# Mesenchymal Stem Cells Cultured in a 3D Microgel Environment Containing Platelet-Rich Plasma Significantly Modify Their Chondrogenesis-Related miRNA Expression

**DOI:** 10.3390/ijms25020937

**Published:** 2024-01-11

**Authors:** Manuel Mata, Rubén Salvador-Clavell, Joaquín Ródenas-Rochina, María Sancho-Tello, Gloria Gallego Ferrer, José Luis Gómez Ribelles

**Affiliations:** 1 Departamento de Patología, Facultad de Medicina y Odontología, Universitat de València, 46010 Valencia, Spain; ruben.salvador@uv.es (R.S.-C.); maria.sancho-tello@uv.es (M.S.-T.); 2 INCLIVA Biomedical Research Institute, 46010 Valencia, Spain; 3 Centro de Investigación Biomédica en Red de Bioingeniería Biomateriales y Nanomedicina, Instituto de Salud Carlos III, 28029 Madrid, Spain; jrodenasr@gmail.com (J.R.-R.); ggallego@ter.upv.es (G.G.F.); jlgomez@ter.upv.es (J.L.G.R.); 4 Centre for Biomaterials and Tissue Engineering (CBIT), Universitat Politècnica de València, 46022 Valencia, Spain

**Keywords:** platelet-rich plasma, mesenchymal stem cells, chondrogenesis, miRNA expression

## Abstract

The aim of this work is to study the effect of platelet factors on the differentiation of mesenchymal stem cells (MSCs) to hyaline cartilage chondrocytes in a three-dimensional environment. MSCs were cultured in a microgel environment with a chondrogenic medium. The microgel consisted of microspheres that combine gelatin and platelet-rich plasma (PRP). The gelatin/PRP microdroplets were produced by emulsion. The gelatin containing the microdroplets was enzymatically gelled, retaining PRP and, just before seeding the cells, platelets were activated by adding calcium chloride so that platelet growth factors were released into the culture media but not before. Platelet activation was analyzed before activation to rule out the possibility that the gelatin cross-linking process itself activated the platelets. The gene expression of characteristic chondrogenic markers and miRNA expression were analyzed in cells cultured in a differentiation medium and significant differences were found between gelation/PRP microgels and those containing only pure gelatin. In summary, the gelatin microspheres effectively encapsulated platelets that secreted and released factors that significantly contributed to cellular chondrogenic differentiation. At the same time, the microgel constituted a 3D medium that provided the cells with adherent surfaces and the possibility of three-dimensional cell–cell contact.

## 1. Introduction

Platelet-rich plasma (PRP) has been proposed as an autologous, easy-to-obtain, and cost-effective vehicle for a cocktail of growth factors, capable of releasing them with a positive effect on tissue regeneration. It is indicated for application through different procedures in various pathologies: wound healing, periodontal surgery or regeneration of bone, articular cartilage, tendon, or intervertebral disc [1,2]. However, both clinical evaluation and studies in animal models are controversial, which some authors justify by the variability in the PRP preparation procedure [2].

PRP is obtained by separating anticoagulated circulating blood by centrifugation. The PRP fraction is located between the bottom layer of red blood cells and the platelet-poor plasma fraction above. Some authors have established that the platelet concentration that can be considered the threshold for designating plasma as PRP is 1.4 × 10^6^ platelets per microliter [3]. Once the platelet-rich fraction is separated, it can be activated with thrombin and/or calcium chloride. Platelets actively release growth factors during the first 10 min after activation, and within 1 h they release more than 95% of the factors they had presynthesized, although they continue to synthesize and release factors after being transplanted [4]. Therefore, it is important to inoculate PRP immediately after its activation, although there are also studies that show its effectiveness even hours after its preparation. For all these facts, some variability can be expected in the effects found with PRP [2].

One of the important problems in the administration of recombinant growth factors in regenerative therapies is related to their rapid dispersion in the tissue and their short life in the organism. These facts mean that the doses that show effectiveness in the delivery of the growth factors are really very high, and there is concern about their systemic distribution throughout the organism [5,6]. These features should also be considered in the supply of platelet growth factors. Thus, it may be interesting to encapsulate PRP in a medium that retains growth factors and delays their release into the tissue, to increase the effectiveness of the treatment. For example, the dispersion of PRP in hydrogels such as polyethylene glycol [7] or gelatin [8,9,10,11,12] have been studied.

Regarding the regeneration of articular cartilage, the effect of PRP on the proliferation and differentiation of chondrocytes and mesenchymal stem cells (MSCs) has been studied [7,13,14,15], and its effect on osteochondral regeneration has been evaluated in animal models [16]. One of the most widespread clinical applications has been the treatment of osteoarthritis through intra-articular injections [17,18,19].

On the other hand, microRNAs (miRNAs) are small non-coding RNAs of approximately 21–22 nucleotides in length, which are key factors in the regulation of several cellular processes such as apoptosis, cell cycle, angiogenesis, aging, proliferation, etc. [20]. In recent years, the regulatory role of miRNAs has been studied in relation to the maintenance, regeneration, and degeneration of cartilage tissue. Thus, there are different studies focused on the role of these molecules in the differentiation of MSCs of diverse origin into chondrocytes, and also human-induced pluripotent stem cells [21]. Furthermore, the regulatory role of miRNAs in degenerative processes, especially osteoarthritis, has also been studied [22]. However, to the best of our knowledge, there are no studies focused on the effect of PRP on miRNAs during chondrogenic differentiation of MSCs.

Regeneration of an articular cartilage lesion requires a source of cells with chondrogenic capacity, as well as a supportive environment at the site where regeneration must take place, as well as the supply of growth factors. The role of the support biomaterial is to transmit to the cells the dynamic stresses to which the articular cartilage is subjected. This local load will be essential in the generation or maintenance of the phenotype of hyaline cartilage cells. The strategy based on the implantation of solid microspheres as mechanical support has the advantage of allowing their displacement while the formation and growth of the newly formed tissue occurs, allowing it to organize itself with the specific structure of the hyaline cartilage. On the other hand, it allows mesenchymal cells to easily migrate from the subchondral bone if the implantation of the support material is combined with an injury to the subchondral bone with techniques such as microfracture. This strategy allowed the formation of a tissue with all the characteristics of hyaline cartilage in a rabbit knee model [23].

In this study we propose the production of microspheres containing PRP for the regeneration of focal lesions of articular cartilage. The matrix used to encapsulate the PRP is gelatin, which has already been used alone [24,25] or in combination with PRP [8,9] for the regeneration of articular cartilage. The goal of our work has been to avoid platelet activation during the preparation process of the microspheres, producing them through an enzymatic process of gelatin cross-linking in an emulsion containing PRP, without platelet activation being involved in the cross-linking. We checked the level of platelet activation and the effect of platelet factors released from the microspheres on chondrocytes differentiation in vitro. To do this, we established an in vitro model of differentiation of porcine MSCs into chondrocytes, cultured in gelatin microgels with or without PRP. It allows us, on the one hand, to evaluate changes in the gene expression of chondrogenic markers such as *COL2A1*, *ACAN, COL1A1*, *COL10A1*, *VEGF*, *MMP13*, *SOX6* and *SOX9* and, on the other hand, to study the changes in the expression profile of miRNAs due to the differentiation process. The results presented here complement those obtained by other authors in relation to the chondrogenic potential of PRP and propose new regulatory molecules involved in the cartilage differentiation process.

## 2. Results

**Figure 1:**
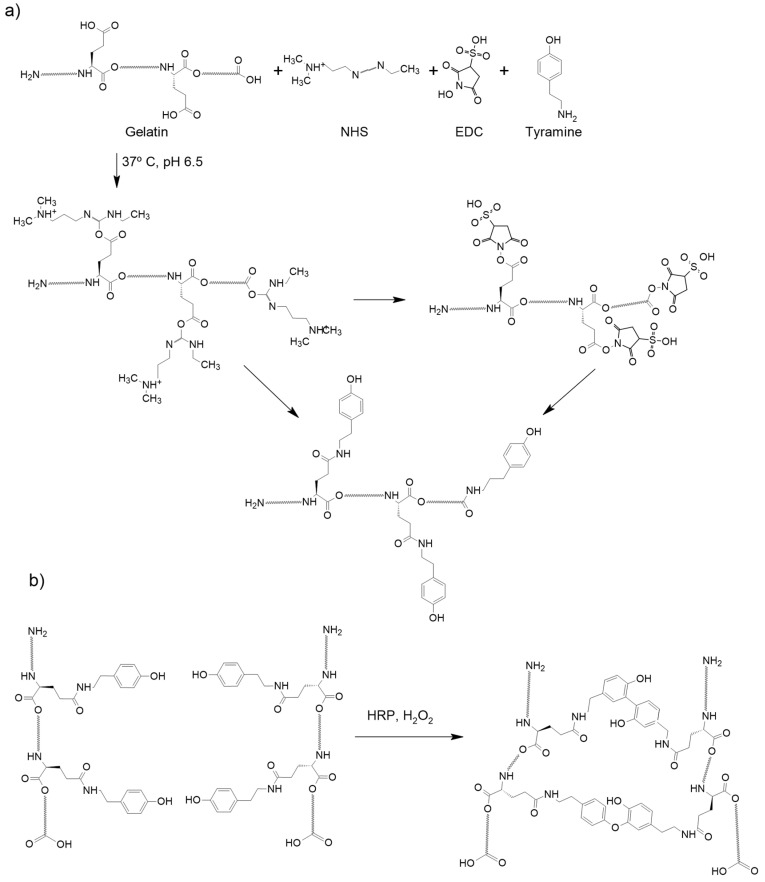
(**a**) Scheme of the modification of gelatin to add phenol groups. (**b**) Scheme of the gelatin network.

The schematic modification of gelatin to add a tyramine and peroxidase cross-linking reaction to produce the gelatin network, catalyzed by hydrogen peroxide, is shown in Figure 1.

**Figure 2:**
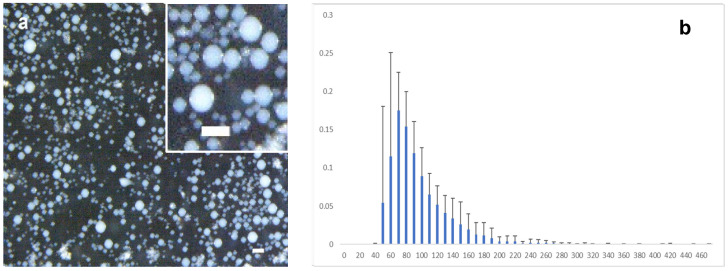
(**a**) Stereoscopic microscope image of the gelatin/PRP microspheres obtained with 12 s of vortex mixing; the size bar corresponds to 200 μm. A representative panoramic view of the size distribution and an inset with a more detailed view are shown. Insert size bar equals to 200 μm. (**b**) Microsphere diameter histogram. Mean ± SD of n = 6 independent experiments are shown.

Microspheres were produced using a water-in-oil emulsion. The aqueous phase consisted of the mixture of PRP with a solution of tyraminated gelatin (G-Tyr), which contained HRP. Different vegetable oils with a wide range of viscosity were tested as the oil medium. The emulsion was formed by adding the required amount of hydrogen peroxide to the aqueous phase, which was immediately added to the Falcon tube containing the oil and mixed by vortex for 12 s. This time is sufficient for the emulsion to form, which remains stable because the enzymatic reaction of gelatin cross-linking begins in seconds when using the composition described in this work [26]. The suspension was kept for 20 min at room temperature without stirring to allow the cross-linking reaction to be completed. The microspheres were then extracted from the oil suspension by centrifugation with the help of a filter that retained the microspheres while allowing the oil to pass through. Figure 2a shows the morphology of the microspheres in a stereoscopic microscopy image. Figure 2b shows the microspheres diameter distribution. The mean diameter was 76.8 ± 24.4 µm (mean ± SD). Shorter stirring times achieved larger particle size and greater size dispersion; thus, stirring for only 6 s produced particles with a size of 95.8 ± 41.2 µm.

**Figure 3:**
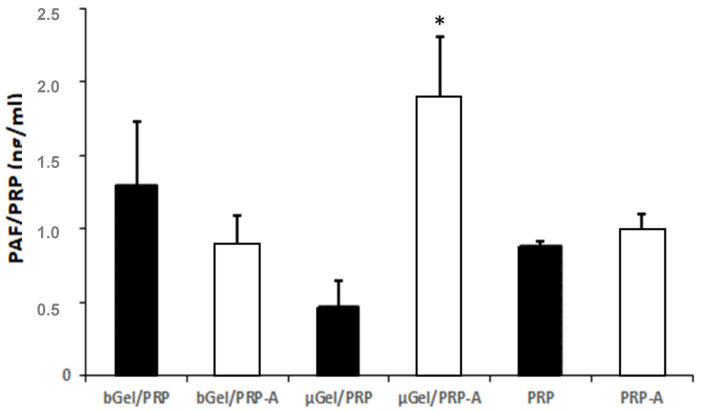
Level of platelet activating factor (PAF) in the different groups studied. The results were normalized according to the PRP content. Immersion in CaCl_2_ activated platelets encapsulated in μGel/PRP microspheres, which showed a much lower level of PAF than without activation by CaCl_2_. The details of the experimental groups are shown in Table 1. The mean ± SD of three different experiments is represented as * *p* < 0.05 compared to the control group (black bars).

The cross-linking of tyramine phenol groups by peroxidase action is highly selective. Thus, the coagulation of the microsphere does not imply, at least intentionally, the activation of the platelets. After this process, the microspheres will be called μGel/PRP. Just before adding the microspheres into the cell culture, a series of microspheres were immersed in calcium chloride to activate the platelets (μGel/PRP-A). Platelet activating factor (PAF) was measured to quantify platelet activation in the microspheres (Figure 3). Pure PRP, activated with CaCl_2_ (PRP-A), and not activated (PRP) were used as controls. Furthermore, bulk gelatin/PRP hydrogels activated with CaCl_2_ (bGel/PRP-A) and not activated (bGel/PRP) were tested for comparison (Table 1).

**Figure 4:**
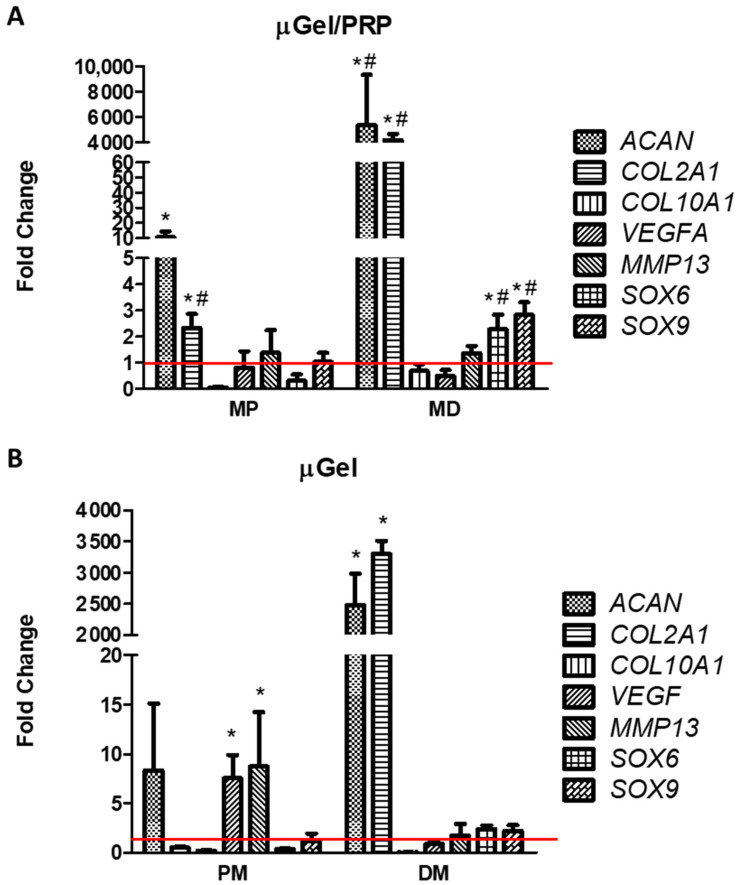
Expression levels of genes related to chondrocytes. pMSCs were cultured for 6 weeks in gelatin microgels containing (**A**) or not containing (**B**) PRP with proliferation (PM) or chondrogenic differentiation media (DM). The relative gene expression levels of *ACAN*, *COL2A1*, *COL10A1*, *VEGF*, *MMP13*, *SOX6* and *SOX9* were evaluated by real-time RT-PCR. *GAPDH* was used as a housekeeping gene. The fold change (2^−∆∆Ct^) was calculated using the relative expression of cells cultured in 2D with PM as a control group (indicated by the red line). The mean ± SD of three different experiments is represented as * *p* < 0.05 compared to the control group (2D cultured cells), and # *p* < 0.05 comparing cells cultured in µgel in the presence or absence of PRP.

Next, a 3D cell culture system was performed to analyze gene expression changes in well-known cartilage-related genes. As described above, pMSCs were isolated and cultured in microgels manufactured from gelatin microparticles containing or not containing PRP at a density of 3 × 10^5^ cells/mL. The cells in microgels were then cultured for 6 weeks with either proliferation (PM) or chondrogenic differentiation culture medium (DM). RT-PCR was used to study changes in the relative expression of cartilage-related genes, which was calculated with respect to cells cultured in 2D with PM. The results obtained are shown in Figure 4.

Our final objective was to study whether PRP could induce changes in the expression of miRNAs during the chondrogenic differentiation process. To this end, we analyzed the miRNAs expression profiles of pMSCs cultured in gelatin microgels, in the presence or absence of PRP, cultured with PM or chondrogenic DM. We used the same total RNAs previously extracted and analyzed in the gene expression studies. Once the miRNA expression data were obtained, we performed the following comparative analyses: (i) pMSCs cultured with PM in gelatin microgels vs. those cultured in gelatin-PRP microgels, and (ii) pMSCs cultured with chondrogenic DM in gelatin microgels vs. those cultured in gelatin-PRP microgels. To detect significant changes, the *t*-test was used after false discovery rate (FDR) *p*-value correction. The results were filtered according to an absolute fold change > 1.7 and an adjusted *p* < 0.05.

**Figure 5:**
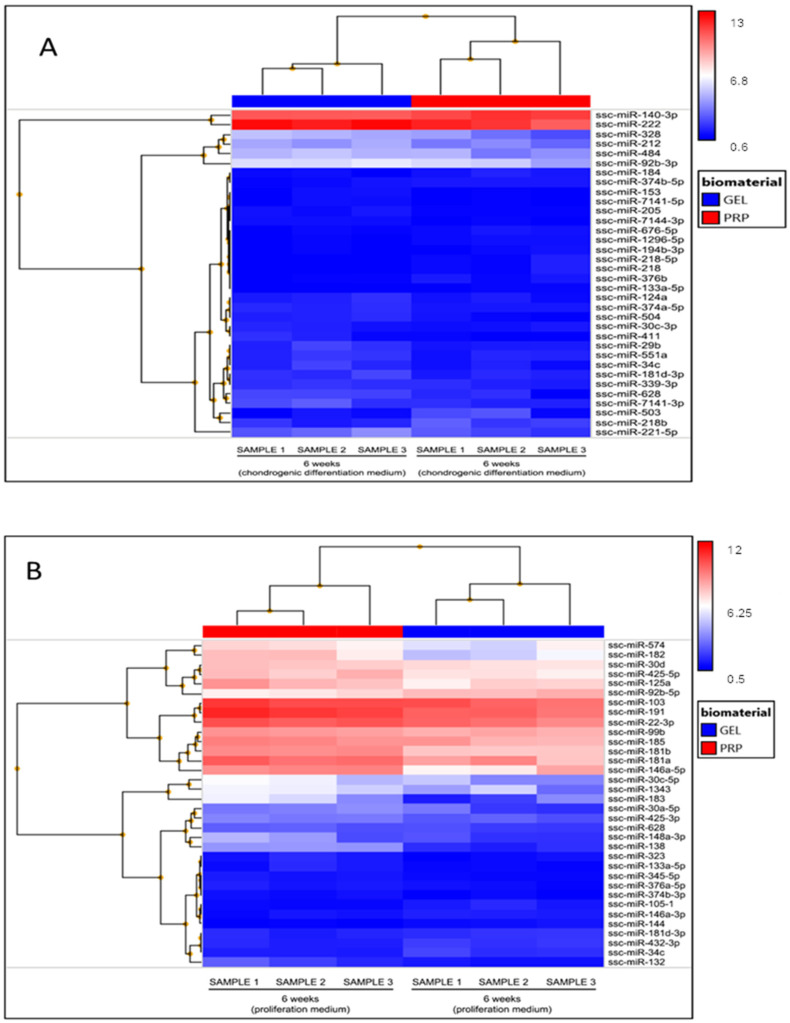
Expression levels of genes related to chondrogenesis. pMSCs were cultured in gelatin microgels containing or not containing PRP, with (**A**) chondrogenic differentiation or (**B**) proliferation culture medium for 6 weeks. Total RNA was extracted and analyzed using Affymetrix miRNA arrays. The *t*-test following the false discovery rate (FDR) *p*-value correction was used to detect significant changes between cells culture in PRP containing microgels compared to non-PRP microgels. Hierarchical clustering was used to analyze the data obtained.

Regarding the cells cultured in PM, we observed 34 miRNAs that passed the filtering criteria. Table 2 shows the miRNA ID, fold change, and adjusted *p*-value. More detailed tables containing the annotation and description of the potential targets of the miRNAs that passed the filters are included as supplementary material (Supplementary Materials Tables S1 and S2, with data from cells cultured in differentiation and proliferation medium, respectively). Hierarchical clustering analysis demonstrated that the expression values of these miRNAs efficiently discriminated between the defined experimental conditions (Figure 5A).

To study whether our scaffold could improve chondrogenesis in a more realistic in vitro model, we compared miRNA expression under the same experimental conditions but using a chondrogenic DM. In this way, we wanted to evaluate the potential of these factors to improve the effect of the biochemical molecules used for the induction of the chondral phenotype from MSCs. The samples were analyzed in the same way as for cells cultured with PM and filtered according to the same parameters. Interestingly, we found variations in the same number of miRNAs, which is nothing more than a coincidence. Therefore, 34 miRNAs passed the filtering criteria. Table 2 shows the miRNA’s ID, fold change, and adjusted *p*-value. Hierarchical clustering demonstrated that the expression values of these miRNAs efficiently discriminated between the defined experimental conditions (Figure 5B).

## 3. Discussion

The results show that gelatin/PRP microspheres, as obtained after enzymatic cross-linking (μGel/PRP), show a much lower degree of activation than when activated with CaCl_2_ (μGel/PRP-A; Figure 3). This result allows us to trust that the microsphere manufacturing process preserves the release of platelet growth factors, which would cause their loss in the successive washes. Therefore, platelets can be activated just before starting a cell culture or before implanting them in the body.

pMSCs cultured in gelatin microgels with PM did not show the acquisition of a chondrogenic phenotype. No significant increase in *COL2A1* expression was observed and, although an increase in *ACAN* expression was detected, it was not significant. On the contrary, the use of chondrogenic DM induced a statistically significant increase in the expression of *COL2A1* and *ACAN*. No induction was detected in the expression of hypertrophy markers such as *COL10A1*, *VEGF*, or *MMP13*. The use of PRP in the scaffold induced an appreciable differentiation of chondrocyte. Even when cultured in PM, a remarkable increase in the expression of *ACAN* and *COL2A1* was detected, which was notably higher in the group in which PRP was used along with DM. These increases were of greater magnitude than in the groups in which gelatin microparticles without PRP were used. Likewise, no changes were observed in the expression of hypertrophy markers. In the experimental group cultured with microparticles and PRP in DM, a significant increase in the expression of *SOX6* and *SOX9* was also detected.

As expected, the use of chondrogenic DM induced the expression of the two main components of the chondral matrix, *COL2A1* and *ACAN* [27]. This increase was notably enhanced by PRP, which agrees with other authors [28,29]. It is noteworthy that PRP alone was able to induce the expression of both *COL2A1* and *ACAN*, demonstrating its potential to stimulate the secretion of certain chondrogenic factors such as TGFβ, which could stimulate their differentiation in a paracrine way [29].

On the other hand, hypertrophy encompasses a set of processes that can lead to cartilage degeneration. Different factors are involved in this process, among which the increased expression of *COL10A1* (related to the acquisition of the hypertrophic phenotype), *VEFG* (related to angiogenesis) or *MMP13* (related to chondral matrix remodeling) are well known [30]. We have not detected the induction of any of these markers, indicating that at least at the times tested, the culture environment developed fits a model of cartilage generation. Moreover, we have detected a significant increase in the expression of *SOX6* and *SOX9*, which are two key factors that control the chondrogenic differentiation from MSCs [31].

When pMSCs were cultured in the microgel in PM, the most upregulated miRNAs were miR-182 and miR-183 (with a 6.46- and 9.24-fold change, respectively). Both miRNAs belong to the same cluster and there is experimental evidence that relates them to the induction of chondrogenesis by strengthening the regulation of the SOX5-SOX6 axis, which leads to type II collagen responses, or the HIF1α-PGF-axis, which leads to various hypoxia responses [32]. A significant induction of miR-183 was observed in the PRP-treated group compared to the untreated group (4.3-fold change). Some authors have previously reported that the level of miR-138 was significantly decreased in the chondral tissues of patients with osteoarthritis (OA) compared to those of control patients. Those authors associated the induction of this miRNA with an inhibition of OA by suppressing the effects mediated by TNF-alpha or IL6, through mechanisms regulated by NF-kB [33].

Overexpression of hsa-miR-148a inhibits hypertrophic chondrocyte differentiation by decreasing *COL10A1*, *MMP13*, and *ADAMTS5*, and increases type II collagen production and deposition by OA chondrocytes, accompanied by increased proteoglycan retention. Therefore, in OA, hsa-miR-148a might be a potential disease-modulatory compound since it promotes the production of hyaline cartilage [34]. We found an induction in miR-148a expression levels of 4.51-fold change in cells exposed to PRP compared to control groups, which confirms the results obtained in real-time RT-PCR experiments. Similarly, miR-30a was shown to promote chondrogenic differentiation with the deposition of characteristic chondral proteins such as type II collagen or *ACAN*, through mechanisms related to the inhibition of the Notch pathway [35]. We found that the expression of this miRNA increased by 3.17-fold. We also found a significant upregulation of miR182a and b, which have been shown to induce chondrogenesis through mechanisms involving downregulation of the Wnt/β-catenin pathway [36], in association with other miRNAs such as miR-185 (whose expression increased 2.08-fold).

Regarding the downregulated miRNAs, we want to highlight the downregulation of miR-92b, a member of the miR-17-92 cluster, whose downregulation has been reported through experimental evidence with the induction of the chondral matrix secretion in vitro [37].

These results, supported by those obtained in gene expression experiments, demonstrate the potential of undifferentiated MSCs to induce the chondrogenic phenotype, because of the release of growth and differentiation factors from PRP.

We observed between 2.86 and −2.88-fold changes in mRNA expression in cells cultured in the chondrogenic differentiation medium. The most upregulated miRNA was miR-503, which is significantly upregulated in MSCs differentiated into mature chondrocytes and has been related to cell cycle arrest in different cell types [38]. Another significantly upregulated miRNA is miR-140, a well-known regulator of chondrogenesis. This transcriptional regulator targets RALA and enhances SOX9 by stimulating chondrogenesis in vitro, upregulating these molecules at the protein level [39]. During chondrogenesis, there is a balance between the characteristic deposition of chondral matrix proteins and the development of the hypertrophic phenotype. In this sense, miRNAs such as miR-218 have been directly associated with targets related to hypertrophy, such as *COL10A1*, *MEF2C*, and *RUNX2*, causing a reduction in the accumulated MEF2C and RUNX2 proteins, with attenuation of *COL10A1* expression and a significant concomitant reduction of alkaline phosphatase (ALP) activity. Additionally, upregulation of miR-218 in human MSCs attenuated hypertrophic markers (*MEF2C*, *RUNX2*, *COL10A1*, *ALPL*), although without an increase in chondrogenic markers (GAG deposition, *COL2A1*) due to activation of WNT/β-catenin signaling [40]. Our data support these findings and are consistent with the real-time gene expression of *COL10A1* detected in our model.

Regarding the downregulated miRNAs, we distinguish miR-212 whose downregulation has been related to an increase in well-known chondrogenesis inducers, such as SOX6 [41]. Also noteworthy are the inhibitory effect of miR-222 and miR-411. The former regulates MMP13 that targets HDAC4 during osteoarthritis and its inhibition has been reported to accelerate the chondrogenic process [42]. The second one is related to the induction of chondrocyte autophagy through HID-1α-dependent mechanisms [43].

Finally, we want to point out that some caution is necessary when interpreting the data presented in this manuscript. On the one hand, in relation to the comparison between MSCs cultured in the PM, the miRNAs detected are directly related and are responsible for the differentiation of the chondral phenotype. In the comparison between MSCs cultured with DM, we are evaluating the modulatory, additive, or synergistic effect of PRP on biochemical elements that are already chondrogenic. To our knowledge, this is the first work that addresses the effects of PRP from this perspective, and reinforces, in line with the extensive existing bibliography, the beneficial effects of PRP for cartilage regeneration. In addition, the scaffold presented here allows a controlled release of the factors contained in the PRP, which increases the novelty of this work.

This is, therefore, a preliminary in vitro study that supports the use of these materials for future tests in experimental animal models more representative of what happens in humans, such as pigs. For this reason, and thinking about these future experiments, we have incorporated pig mesenchymal cells in our experiments.

## 4. Materials and Methods

### 4.1. Materials

The reagents purchased from Sigma (Madrid, Spain) were 4-Morpholineethanesulfonic acid, 2-(*N*-Morpholino)ethanesulfonic acid (MES; M3671), Horseradish peroxidase (HRP; P8375), *N*-Hydroxysuccinimide 98% (130672), Gelatin type A 300 bloom strength from porcine skin (G2500), and Calcium Chloride (223506). Sodium Chloride was purchased from Scharlab (SO0225005P, Barcelona, Spain). N-(3-Dimethylaminopropyl)-*N*-ethylcarbodiimide hydrochloride was purchased from Iris Biotech GmbH (RL-1022.0025, Marktredwitz, Germany), and Dulbecco’s phosphate buffered saline (DPBS) from Gibco (14200-059, Madrid, Spain). Olive oil (Aceites Toledo; Batch 394819135, Madrid, Spain) was sterilized by heating the oil at 150 °C for 90 min. The biocompatibility of all the materials used was verified in previous studies [26,44].

### 4.2. Gelatin Modification to Add Tyramine Groups

The chemical modification of gelatin with tyramine substitution was performed by reacting carbodiimide with carboxyl groups of gelatin, following previously published work [26]. Gelatin was dissolved at 2% *w*/*v* in 20 mL ultrapure water with 0.98% *w*/*v* MES at 60 °C and 0.56% *w*/*v* tyramine, before equilibrating the solution to pH 6. Cross-linking reagents (0.04% *w*/*v* N-Hydroxysuccinimide (NHS) and 0.61% *w*/*v* 1-Ethyl-3-(3-dimethylaminopropyl)carbodiimide (EDC) were added and once dissolved, the sample was incubated at 37 °C and stirred to allow the reaction. The sample was poured onto a cellulose membrane to dialyze and remove small molecules, changing the washing water every 8 h for three days. Finally, the tyramined gelatin was filtered through a filter with 0.22 µm pores and lyophilized. The degree of tyramine substitution was determined by absorbance at 275 nm.

### 4.3. PRP Collection

Sheep blood was donated by Instituto Universitario de Investigación de Ciencia y Tecnología Animal, Universitat Politècnica de València (ICTA; Valencia, Spain). Blood was collected into 5 mL citrate vacuum tubes and gently mixed to prevent blood clotting. The blood was centrifuged for 18 min at 1800 rpm to separate the formed blood elements from the plasma. The plasma fraction was divided into 4 portions, with the platelet-rich plasma (PRP) being the lower portion, in contact with the red fraction. Platelet-poor plasma fractions were discarded and PRP was stored frozen at −80 °C in sterile centrifuge tubes.

### 4.4. Preparation of the Microspheres

Gelatin microgels were obtained using a water-in-oil emulsion. The modified gelatin was dissolved at 4% (*w*/*v*) in calcium-free Krebbs–Ringer buffer (115 mM NaCl, 5 mM KCl, 1 mM KH_2_PO_4_ and 25 mM HEPES at pH 7.4) and sterilized by filtration. The gelatin solution was mixed with PRP and 12.5 U/mL HRP in a 4:4:1 solution. A volume of 180 µL of gelatin/PRP solution was mixed with 20 µL of 22 mM H_2_O_2_, under sterile conditions, poured into a 50 mL tube with 5 mL of sterile olive oil and emulsified for 12 s with a vortex (444–1372 (EU), VWR international, Radnor, PA, USA). The emulsion obtained was kept at room temperature for 20 min to complete the cross-linking reaction before removing the oil by filtration. The microspheres formed were then washed with DPBS, removing any remaining oil in the supernatant. Platelets were activated immediately after being added to the cell culture by immersing the microspheres in a solution of 0.5% CaCl_2_ with 0.9% NaCl. The diameter of the microspheres was determined in optical microscopy pictures by image analysis using Image J software v 1.53t (National Insitute of Health, Bethesda, MD, USA) from 10 pictures/batch from two different batches.

### 4.5. Evaluation of Platelet Activation

The platelet activation factor (PAF) quantification assay was performed on the gelatin/PRP microspheres using pure gelatin microspheres and bulk PRP, gelatin, and gelatin/PRP hydrogels as controls. Pure gelatin microspheres were prepared as described above without the addition of PRP. Control bulk gelatin hydrogels were obtained from a solution containing 4% tyramined gelatin, 12.5 U/mL HRP, and 22 mM H_2_O_2_ in a ratio of 8:1:1. Control bulk gelatin/PRP hydrogels were obtained by mixing 8% *w*/*v* tyramined gelatin, *w*/*v* PRP, 12.5 U/mL HRP and 22 mM H_2_O_2_ in a 4:4:1:1 solution. Both control bulk hydrogels were produced by pouring 200 µL of the corresponding solutions into a silicone mold and allowing them to coagulate at room temperature. Control PRP gels were obtained by mixing 200 µL of PRP with 10 µL of 1% *w*/*v* CaCl_2_ for 40 min at 37 °C in a glass vial. The gels were washed three times with 0.9% *w*/*v* NaCl. The microspheres and bulk hydrogel samples were seeded in a 48-well plate and incubated with 200 µL of 0.9% NaCl (non-activated platelets) or 0.5% CaCl_2_ (to activate platelets) for 30 min at 37 °C.

### 4.6. Cell Isolation and Culture

Pig mesenchymal stem cells (pMSCs) from bone marrow were used to perform all tests. Porcine mesenchymal stem cells were harvested from the femoral bone marrow. The femur bone was cut in the upper part in aseptic conditions and the gelatinous bone marrow was removed. Bone marrow was grinded and sieved with a 40 µm nylon mesh to remove tissue debris. Cells were counted with trypan blue and acetic acid and seeded at 4 × 10^5^ cells/cm in a T75 cm^2^ culture flask. Non-adherent cells were removed by changing the culture medium. Cells at passage three were seeded in culture flasks with a proliferation medium (PM) containing Dulbeccos’s modified minimum essential media (DMEM; Gibco, Waltham, MA, USA) supplemented with 10% fetal bovine serum (FBS; Gibco), 1% L glutamine (Gibco), 1% penicillin/streptomycin (Gibco), and 1% fungizone (Gibco), and were cultured in a humidified atmosphere incubator at 37 °C and 5% CO_2_. Culture media were changed every 2–3 days. Cells were detached from the flasks with a 0.25% (*w*/*v*) trypsin-0.91 mM EDTA solution (Gibco) and cultured at a density of 3 × 10^5^ cells in 30 µL of sample (µGel or µGel/PRP) in 0.5 mL microcentrifuge tubes. After 24 h, the culture media of half of the samples were changed to chondrogenic differentiation medium (DM) containing proliferation medium (1% FBS) supplemented with 1% insulin-transferrin-selenium (ITS; Gibco), 1% non-essential aminoacids (Gibco), 1% ascorbic acid (Gibco), 1% sodium pyruvate (Gibco), and 10 ng/mL transforming growth factor beta 1 (TGFβ1; Gibco).

### 4.7. Analysis of Relative Gene Expression of Chondrocyte-Related Genes

Total RNA was extracted from 3D cultures using Trizol reagent (Thermo Fischer Scientific Inc., Waltham, MA, USA) according to the manufacturer’s instructions. RNA concentration was determined by spectrophotometry using a Nanodrop 2000 spectrophotometer (Fischer Scientific, Madrid, Spain). Only extracts with a 260/280 ratio > 1.8 were used. RNA integrity (RI) was evaluated by capillary electrophoresis using a Bioanalyzer (Agilent Technologies, Santa Clara, CA, USA). Only extracts with an RI number (RIN) of ~10 were used to determine gene expression levels. The expression levels of *COL1A1*, *COL2A1*, *ACAN*, *COL10A1* and *GAPDH* genes were assayed by reverse transcriptase polymerase chain reaction (RT-PCR) using Assays on Demand (Applied Biosystems, Madrid, Spain, ID Ss03373340_m1, Ss03373344_g1, Ss03374823_m1, Ss03391766_m1 and Ss03375629_u1, respectively). The reactions were carried out in a 7900HT real-time Thermocycler (Applied Biosystems, Madrid, Spain). The comparative ΔΔCt method with glyceraldehyde 3 phosphate dehydrogenase (*GAPDH*) was used as an endogenous control to calculate relative gene expression levels [45].

### 4.8. miRNAs Analysis

For analysis of global miRNA expression, Affymetrix miRNA 3.0 arrays (Affymetrix, CA, USA) were used, as previously reported [46]. The miRNA was extracted from cells using Trizol reagent as recommended by the manufacturer (Thermo Fischer Scientific Inc., Waltham, MA, USA). The miRNA extracts were labeled with biotin, using 3DNA Array Detection FlashTag™ Biotin HSR (Genisphere, Hatfield, PA, USA) and hybridized with the microarrays in a 645-hybridization oven (Affymetrix, CA, USA), following standardized protocols provided by Affymetrix. Microarray staining, washing, and scanning were then performed using a Fluidic 450 Station and a GeneChip 3000 7G confocal scanner (Affymetrix, Santa Clara, CA, USA). DAT and CEL files were acquired using GeneChip Command Console Software (version 4.3.2, AGCC, Affymetrix, CA, USA). Then, miRNA expressions were analyzed using Transcriptome Analysis Console (TAC) Software (version 4.0, Applied Biosystems). To do this, quality control of the array was performed, and data were normalized, followed by statistical tests to study the differential expression of these miRNA. Finally, the results were interpreted, and the significant differential expressions were represented using the hierarchical clustering method, comparing samples in different conditions (µGel vs. µGel/PRP, and proliferation samples vs. induced chondrogenic differentiation).

### 4.9. Data Presentation and Statistical Analysis

The experimental samples were replicated three times. The mean ± standard deviation (SD) was used. For statistical analysis, Tukey’s one-way ANOVA test was performed to find differences between samples groups, with a significance threshold of *p*-value of ≤0.05. miRNA arrays were normalized using the quartil method. ANOVA was used to select significant changes. False Discovery Rate (FDR) algorithm was used to correct the *p*-value.

## 5. Conclusions

By means of an emulsion process, cross-linked gelatin microspheres containing PRP can be prepared, avoiding at least in part, the activation of platelets during this process. Subsequent activation of platelets by calcium chloride immediately before cell seeding allows the release of platelet growth factors to be as efficient as possible. The release of these factors during the culture of MSCs in DM significantly induces chondrogenesis, as demonstrated by the expression of genes and miRNAs characteristic of the hyaline cartilage chondrocyte.

## Figures and Tables

**Table 1:** Experimental series.

μGel/PRP	Gelatin/PRP Microspheres
μGel/PRP-A	Gelatin/PRP microspheres activated with CaCl_2_
bGel/PRP	Bulk Gelatin/PRP
bGel/PRP-A	Bulk Gelatin/PRP activated with CaCl_2_
PRP	Pure PRP
PRP-A	Pure PRP activated with CaCl_2_

**Table 2:** Differential expression of miRNAs. pMSCs were cultured in gelatin microgels containing or not PRP, in proliferation or chondrogenic differentiation culture media. Data was filtered according to a fold change > 1.7 and adjusted to *p* < 0.05. Means and adjusted *p*-value of n = 3 replicates are shown.

Proliferation Medium			Chondrogenic Differentiation Medium		
miRNA	Fold Change	Adjusted *p*-Value	miRNA	Fold Change	Adjusted *p*-Value
ssc-miR-374b-3p	1.75	0.0706	ssc-miR-503	3.36	0.1456
ssc-miR-132	1.98	0.0542	ssc-miR-140-3p	2.06	0.0137
ssc-miR-181b	2.55	0.00000402	ssc-miR-218b	1.99	0.1005
ssc-miR-323	1.78	0.0994	ssc-miR-376b	1.87	0.0612
ssc-miR-125a	2.08	0.0653	ssc-miR-133a-5p	1.82	0.0226
ssc-miR-144	−1.94	0.0167	ssc-miR-194b-3p	1.78	0.174
ssc-miR-105-1	−1.96	0.0236	ssc-miR-1296-5p	1.78	0.0269
ssc-miR-432-3p	−2.13	0.0161	ssc-miR-676-5p	1.74	0.0882
ssc-miR-138	4.63	0.0000379	ssc-miR-184	1.73	0.0856
ssc-miR-148a-3p	4.51	0.0889	ssc-miR-218	1.73	0.083
ssc-miR-181a	2.42	0.041	ssc-miR-218-5p	1.73	0.083
ssc-miR-30d	2.07	0.0098	ssc-miR-374b-5p	1.71	0.0364
ssc-miR-103	1.89	0.0903	ssc-miR-124a	−1.71	0.1324
ssc-miR-628	1.73	0.0598	ssc-miR-7141-5p	−1.71	0.1605
ssc-miR-146a-3p	−1.91	0.0282	ssc-miR-205	−1.72	0.0238
ssc-miR-92b-5p	−2.62	0.0076	ssc-miR-7144-3p	−1.72	0.008
ssc-miR-22-3p	2.1	0.0644	ssc-miR-339-3p	−1.74	0.0988
ssc-miR-30c-5p	5.88	0.0861	ssc-miR-374a-5p	−1.76	0.0084
ssc-miR-99b	1.9	0.028	ssc-miR-92b-3p	−1.76	0.1694
ssc-miR-1343	3.73	0.0838	ssc-miR-181d-3p	−1.78	0.1152
ssc-miR-185	2.08	0.058	ssc-miR-30c-3p	−1.78	0.1384
ssc-miR-133a-5p	1.76	0.0941	ssc-miR-153	−1.79	0.1415
ssc-miR-30a-5p	3.17	0.0763	ssc-miR-34c	−1.81	0.1241
ssc-miR-425-3p	2.15	0.0073	ssc-miR-551a	−1.83	0.1258
ssc-miR-183	9.24	0.0538	ssc-miR-29b	−1.91	0.0807
ssc-miR-376a-5p	1.71	0.0444	ssc-miR-504	−1.98	0.03
ssc-miR-425-5p	1.8	0.0188	ssc-miR-628	−1.99	0.0747
ssc-miR-345-5p	1.72	0.0043	ssc-miR-411	−2.07	0.0789
ssc-miR-34c	−1.97	0.0341	ssc-miR-484	-2.22	0.1198
ssc-miR-181d-3p	−1.94	0.0561	ssc-miR-221-5p	−2.27	0.1237
ssc-miR-574	2.59	0.0699	ssc-miR-7141-3p	−2.34	0.0945
ssc-miR-182	6.46	0.0163	ssc-miR-222	−2.51	0.083
ssc-miR-146a-5p	5.3	0.0401	ssc-miR-212	−2.6	0.0398
ssc-miR-191	1.85	0.0115	ssc-miR-328	−3.38	0.0559

## Data Availability

Complete array data have been submitted to the GEO database (accession code ID GSE250185).

## Supplementary Materials

The supporting information can be downloaded at: https://www.mdpi.com/article/10.3390/ijms25020937/s1.
