# Supplementary material for: Mesenchymal Stem Cells Cultured in a 3D Microgel Environment Containing Platelet-Rich Plasma Significantly Modify Their Chondrogenesis-Related miRNA Expression
Source: Int J Mol Sci. 2024 Jan 11;25(2):937. doi: 10.3390/ijms25020937 (PMC10815493; doi:10.3390/ijms25020937)
Supplement: Supplementary file 1 [file ijms-25-00937-s001.zip › Table S1_DIFF.pdf]

Supplementary Table S1. Differential expression of miRNAs. pMSCs were cultured in gelatin microgels containing or not PRP, in chondrogenic differentiation culture media. Data was filtered according to a fold change > 1.7

miRNA	ID	DESCRIPTION	FOLD CHANGE	Adjusted p_value
ssc-miR-503	AC068987.1	hypothetical gene	3.36	0.1456
ssc-miR-503	ACHE	acetylcholinesterase [Source:VGNC Symbol;Acc:VGNC:97029]	3.36	0.1456
ssc-miR-503	ACSL6	acyl-CoA synthetase long chain family member 6 [Source:VGNC Symbol;Acc:VGNC:99581]	3.36	0.1456
ssc-miR-503	ACVR2B	activin A receptor type 2B [Source:VGNC Symbol;Acc:VGNC:108629]	3.36	0.1456
ssc-miR-503	AGO3	argonaute RISC component 1 [Source:NCBI gene (formerly Entrezgene);Acc:100499510]	3.36	0.1456
ssc-miR-503	AIFM1	apoptosis inducing factor mitochondria associated 1 [Source:VGNC Symbol;Acc:VGNC:85198]	3.36	0.1456
ssc-miR-503	AK4	hypothetical gene	3.36	0.1456
ssc-miR-503	AKIRIN1	akirin 1 [Source:VGNC Symbol;Acc:VGNC:85227]	3.36	0.1456
ssc-miR-503	AKT3	AKT serine/threonine kinase 3 [Source:VGNC Symbol;Acc:VGNC:96306]	3.36	0.1456
ssc-miR-503	ANKRD13B	ankyrin repeat domain 13B [Source:VGNC Symbol;Acc:VGNC:85325]	3.36	0.1456
ssc-miR-503	ANKS1A	ankyrin repeat and sterile alpha motif domain containing 1A [Source:VGNC Symbol;Acc:VGNC:85348]	3.36	0.1456
ssc-miR-503	ANLN	anillin actin binding protein [Source:VGNC Symbol;Acc:VGNC:85353]	3.36	0.1456
ssc-miR-503	AP3D1	adaptor related protein complex 3 subunit delta 1 [Source:VGNC Symbol;Acc:VGNC:85389]	3.36	0.1456
ssc-miR-503	APLN	apelin [Source:HGNC Symbol;Acc:HGNC:16665]	3.36	0.1456
ssc-miR-503	AQP11	aquaporin 11 [Source:VGNC Symbol;Acc:VGNC:85430]	3.36	0.1456
ssc-miR-503	ARF3	ADP ribosylation factor 3 [Source:NCBI gene (formerly Entrezgene);Acc:100301570]	3.36	0.1456
ssc-miR-503	ARHGAP20	Rho GTPase activating protein 20 [Source:VGNC Symbol;Acc:VGNC:97884]	3.36	0.1456
ssc-miR-503	ARL2	ADP ribosylation factor like GTPase 2 [Source:VGNC Symbol;Acc:VGNC:97885]	3.36	0.1456
ssc-miR-503	ARPP19	cAMP regulated phosphoprotein 19 [Source:NCBI gene (formerly Entrezgene);Acc:397362]	3.36	0.1456
ssc-miR-503	ASB6	ankyrin repeat and SOCS box containing 6 [Source:VGNC Symbol;Acc:VGNC:85565]	3.36	0.1456
ssc-miR-503	ATOH1	atonal bHLH transcription factor 1 [Source:VGNC Symbol;Acc:VGNC:85631]	3.36	0.1456
ssc-miR-503	ATP5J2-PTCD1	hypothetical gene	3.36	0.1456
ssc-miR-503	B3GALT1	beta-1,3-galactosyltransferase 1 [Source:VGNC Symbol;Acc:VGNC:96494]	3.36	0.1456
ssc-miR-503	BCL2	BCL2 apoptosis regulator [Source:HGNC Symbol;Acc:HGNC:990]	3.36	0.1456
ssc-miR-503	BCL9L	BCL9 like [Source:VGNC Symbol;Acc:VGNC:96568]	3.36	0.1456
ssc-miR-503	BMPR1A	bone morphotic protein receptor type 1A [Source:VGNC Symbol;Acc:VGNC:85846]	3.36	0.1456
ssc-miR-503	BPTF	bromodomain PHD finger transcription factor [Source:VGNC Symbol;Acc:VGNC:97904]	3.36	0.1456
ssc-miR-503	BSN	bassoon presynaptic cytomatrix protein [Source:VGNC Symbol;Acc:VGNC:85893]	3.36	0.1456
ssc-miR-503	BTAF1	B-TFIID TATA-box binding protein associated factor 1 [Source:VGNC Symbol;Acc:VGNC:85899]	3.36	0.1456
ssc-miR-503	BTBD19	BTB domain containing 19 [Source:VGNC Symbol;Acc:VGNC:85905]	3.36	0.1456
ssc-miR-503	BTF3	basic transcription factor 3 [Source:VGNC Symbol;Acc:VGNC:99597]	3.36	0.1456
ssc-miR-503	BTLA	hypothetical gene	3.36	0.1456
ssc-miR-503	BTRC	beta-transducin repeat containing E3 ubiquitin protein ligase [Source:VGNC Symbol;Acc:VGNC:107368]	3.36	0.1456
ssc-miR-503	BZW1	basic leucine zipper and W2 domains 1 [Source:VGNC Symbol;Acc:VGNC:95885]	3.36	0.1456
ssc-miR-503	C17orf59	hypothetical gene	3.36	0.1456
ssc-miR-503	C1orf21	hypothetical gene	3.36	0.1456
ssc-miR-503	C20orf112	hypothetical gene	3.36	0.1456
ssc-miR-503	C20orf194	hypothetical gene	3.36	0.1456
ssc-miR-503	C21orf128	hypothetical gene	3.36	0.1456
ssc-miR-503	C2orf72	chromosome 15 C2orf72 homolog [Source:VGNC Symbol;Acc:VGNC:96157]	3.36	0.1456
ssc-miR-503	CACNA1I	calcium voltage-gated channel subunit alpha1 I [Source:VGNC Symbol;Acc:VGNC:97908]	3.36	0.1456
ssc-miR-503	CACUL1	CDK2 associated cullin domain 1 [Source:VGNC Symbol;Acc:VGNC:86132]	3.36	0.1456
ssc-miR-503	CAPN6	calpain 6 [Source:VGNC Symbol;Acc:VGNC:86176]	3.36	0.1456
ssc-miR-503	CASR	calcium sensing receptor [Source:VGNC Symbol;Acc:VGNC:108635]	3.36	0.1456
ssc-miR-503	CAST	calpastatin [Source:VGNC Symbol;Acc:VGNC:99603]	3.36	0.1456
ssc-miR-503	CBFA2T3	CBFA2/RUNX1 partner transcriptional co-repressor 3 [Source:VGNC Symbol;Acc:VGNC:96574]	3.36	0.1456

ssc-miR-503	CBX2	chromobox 2 [Source:VGNC Symbol;Acc:VGNC:86230]	3.36	0.1456
ssc-miR-503	CBX5	chromobox 5 [Source:VGNC Symbol;Acc:VGNC:86232]	3.36	0.1456
ssc-miR-503	CC2D1B	coiled-coil and C2 domain containing 1B [Source:VGNC Symbol;Acc:VGNC:86236]	3.36	0.1456
ssc-miR-503	CCAR2	cell cycle and apoptosis regulator 2 [Source:VGNC Symbol;Acc:VGNC:86239]	3.36	0.1456
ssc-miR-503	CCDC42B	hypothetical gene	3.36	0.1456
ssc-miR-503	CCDC6	coiled-coil domain containing 6 [Source:VGNC Symbol;Acc:VGNC:86304]	3.36	0.1456
ssc-miR-503	CCND2	cyclin D2 [Source:VGNC Symbol;Acc:VGNC:103222]	3.36	0.1456
ssc-miR-503	CCND3	cyclin D3 [Source:VGNC Symbol;Acc:VGNC:86353]	3.36	0.1456
ssc-miR-503	CCNYL1	hypothetical gene	3.36	0.1456
ssc-miR-503	CD28	CD28 molecule [Source:VGNC Symbol;Acc:VGNC:95813]	3.36	0.1456
ssc-miR-503	CD2AP	CD2 associated protein [Source:VGNC Symbol;Acc:VGNC:86406]	3.36	0.1456
ssc-miR-503	CDAN1	codanin 1 [Source:VGNC Symbol;Acc:VGNC:86441]	3.36	0.1456
ssc-miR-503	CDC25A	cell division cycle 25A [Source:VGNC Symbol;Acc:VGNC:86446]	3.36	0.1456
ssc-miR-503	CDC37L1	cell division cycle 37 like 1 [Source:VGNC Symbol;Acc:VGNC:86451]	3.36	0.1456
ssc-miR-503	CDC42SE2	CDC42 small effector 2 [Source:VGNC Symbol;Acc:VGNC:86461]	3.36	0.1456
ssc-miR-503	CDCA2	cell division cycle associated 2 [Source:VGNC Symbol;Acc:VGNC:86465]	3.36	0.1456
ssc-miR-503	CDCA4	cell division cycle associated 4 [Source:VGNC Symbol;Acc:VGNC:86467]	3.36	0.1456
ssc-miR-503	CDH4	cadherin 4 [Source:VGNC Symbol;Acc:VGNC:95853]	3.36	0.1456
ssc-miR-503	CDK5R1	cyclin dependent kinase 5 regulatory subunit 1 [Source:VGNC Symbol;Acc:VGNC:98980]	3.36	0.1456
ssc-miR-503	CHAC1	ChaC glutathione specific gamma-glutamylcyclotransferase 1 [Source:VGNC Symbol;Acc:VGNC:86619]	3.36	0.1456
ssc-miR-503	CHEK1	checkpoint kinase 1 [Source:VGNC Symbol;Acc:VGNC:86637]	3.36	0.1456
ssc-miR-503	CHPF	chondroitin polymerizing factor [Source:VGNC Symbol;Acc:VGNC:96038]	3.36	0.1456
ssc-miR-503	CLCN5	chloride voltage-gated channel 5 [Source:VGNC Symbol;Acc:VGNC:103925]	3.36	0.1456
ssc-miR-503	CLDN12	hypothetical gene	3.36	0.1456
ssc-miR-503	CMPK1	cytidine/uridine monophosphate kinase 1 [Source:VGNC Symbol;Acc:VGNC:96953]	3.36	0.1456
ssc-miR-503	CNNM2	cyclin and CBS domain divalent metal cation transport mediator 2 [Source:VGNC Symbol;Acc:VGNC:86828]	3.36	0.1456
ssc-miR-503	CREBL2	cAMP responsive element binding protein like 2 [Source:VGNC Symbol;Acc:VGNC:86986]	3.36	0.1456
ssc-miR-503	CRK	CRK proto-onco, adaptor protein [Source:NCBI gene (formerly Entrezgene);Acc:100192444]	3.36	0.1456
ssc-miR-503	CTDSPL	CTD small phosphatase like [Source:VGNC Symbol;Acc:VGNC:107131]	3.36	0.1456
ssc-miR-503	CXCL10	C-X-C motif chemokine ligand 10 [Source:VGNC Symbol;Acc:VGNC:87101]	3.36	0.1456
ssc-miR-503	CYB561D1	cytochrome b561 family member D1 [Source:VGNC Symbol;Acc:VGNC:87121]	3.36	0.1456
ssc-miR-503	CYP26B1	cytochrome P450 family 26 subfamily B member 1 [Source:VGNC Symbol;Acc:VGNC:103374]	3.36	0.1456
ssc-miR-503	CYP4F11	hypothetical gene	3.36	0.1456
ssc-miR-503	DCAF7	DDB1 and CUL4 associated factor 7 [Source:VGNC Symbol;Acc:VGNC:87178]	3.36	0.1456
ssc-miR-503	DERL3	derlin 3 [Source:VGNC Symbol;Acc:VGNC:87262]	3.36	0.1456
ssc-miR-503	DESI1	desumoylating isopeptidase 1 [Source:VGNC Symbol;Acc:VGNC:87263]	3.36	0.1456
ssc-miR-503	DGCR2	DiGeorge syndrome critical region gene 2 [Source:HGNC Symbol;Acc:HGNC:2845]	3.36	0.1456
ssc-miR-503	DIAPH2	diaphanous related formin 2 [Source:VGNC Symbol;Acc:VGNC:87300]	3.36	0.1456
ssc-miR-503	DIXDC1	hypothetical gene	3.36	0.1456
ssc-miR-503	DNAJA2	DnaJ heat shock protein family (Hsp40) member A2 [Source:VGNC Symbol;Acc:VGNC:96621]	3.36	0.1456
ssc-miR-503	DNAJC16	DnaJ heat shock protein family (Hsp40) member C16 [Source:VGNC Symbol;Acc:VGNC:96624]	3.36	0.1456
ssc-miR-503	DPP8	dipeptidyl peptidase 8 [Source:VGNC Symbol;Acc:VGNC:87423]	3.36	0.1456
ssc-miR-503	DYRK1B	dual specificity tyrosine phosphorylation regulated kinase 1B [Source:VGNC Symbol;Acc:VGNC:87506]	3.36	0.1456
ssc-miR-503	E2F3	E2F transcription factor 3 [Source:VGNC Symbol;Acc:VGNC:87514]	3.36	0.1456
ssc-miR-503	EED	embryonic ectoderm development [Source:VGNC Symbol;Acc:VGNC:87552]	3.36	0.1456
ssc-miR-503	EIF4E	eukaryotic translation initiation factor 4E [Source:VGNC Symbol;Acc:VGNC:87626]	3.36	0.1456
ssc-miR-503	EIF4G1	eukaryotic translation initiation factor 4 gamma 1 [Source:VGNC Symbol;Acc:VGNC:87631]	3.36	0.1456
ssc-miR-503	EPB41L4B	erythrocyte membrane protein band 4.1 like 4B [Source:VGNC Symbol;Acc:VGNC:103088]	3.36	0.1456
ssc-miR-503	EPOR	erythropoietin receptor [Source:VGNC Symbol;Acc:VGNC:87746]	3.36	0.1456
ssc-miR-503	ERLIN2	ER lipid raft associated 2 [Source:VGNC Symbol;Acc:VGNC:95586]	3.36	0.1456

ssc-miR-503	ESRP1	epithelial splicing regulatory protein 1 [Source:VGNC Symbol;Acc:VGNC:87791]	3.36	0.1456
ssc-miR-503	EXTL3	exostosin like glycosyltransferase 3 [Source:VGNC Symbol;Acc:VGNC:87850]	3.36	0.1456
ssc-miR-503	FAM110C	family with sequence similarity 110 member C [Source:HGNC Symbol;Acc:HGNC:33340]	3.36	0.1456
ssc-miR-503	FAM122A	hypothetical gene	3.36	0.1456
ssc-miR-503	FAM160B2	hypothetical gene	3.36	0.1456
ssc-miR-503	FAM57B	hypothetical gene	3.36	0.1456
ssc-miR-503	FAM63B	hypothetical gene	3.36	0.1456
ssc-miR-503	FAM73A	hypothetical gene	3.36	0.1456
ssc-miR-503	FAM73B	hypothetical gene	3.36	0.1456
ssc-miR-503	FANCA	FA complementation group A [Source:VGNC Symbol;Acc:VGNC:88001]	3.36	0.1456
ssc-miR-503	FBXO21	F-box protein 21 [Source:VGNC Symbol;Acc:VGNC:88035]	3.36	0.1456
ssc-miR-503	FCHSD2	FCH and double SH3 domains 2 [Source:VGNC Symbol;Acc:VGNC:88068]	3.36	0.1456
ssc-miR-503	FGF2	hypothetical gene	3.36	0.1456
ssc-miR-503	FGF7	fibroblast growth factor 7 [Source:HGNC Symbol;Acc:HGNC:3685]	3.36	0.1456
ssc-miR-503	FHDC1	FH2 domain containing 1 [Source:VGNC Symbol;Acc:VGNC:88126]	3.36	0.1456
ssc-miR-503	FKRP	fukutin related protein [Source:VGNC Symbol;Acc:VGNC:88152]	3.36	0.1456
ssc-miR-503	FOSL1	FOS like 1, AP-1 transcription factor subunit [Source:VGNC Symbol;Acc:VGNC:88191]	3.36	0.1456
ssc-miR-503	FOSL2	FOS like 2, AP-1 transcription factor subunit [Source:VGNC Symbol;Acc:VGNC:88192]	3.36	0.1456
ssc-miR-503	FOXK1	forkhead box K1 [Source:VGNC Symbol;Acc:VGNC:88214]	3.36	0.1456
ssc-miR-503	FP15737	hypothetical gene	3.36	0.1456
ssc-miR-503	FSTL1	follistatin like 1 [Source:VGNC Symbol;Acc:VGNC:88255]	3.36	0.1456
ssc-miR-503	FSTL4	follistatin like 4 [Source:VGNC Symbol;Acc:VGNC:88256]	3.36	0.1456
ssc-miR-503	G2E3	G2/M-phase specific E3 ubiquitin protein ligase [Source:VGNC Symbol;Acc:VGNC:88287]	3.36	0.1456
ssc-miR-503	G3BP1	G3BP stress granule assembly factor 1 [Source:VGNC Symbol;Acc:VGNC:88288]	3.36	0.1456
ssc-miR-503	GAN	gigaxonin [Source:VGNC Symbol;Acc:VGNC:88343]	3.36	0.1456
ssc-miR-503	GAREM	hypothetical gene	3.36	0.1456
ssc-miR-503	GBA2	glucosylceramidase beta 2 [Source:VGNC Symbol;Acc:VGNC:88373]	3.36	0.1456
ssc-miR-503	GHR	growth hormone receptor [Source:NCBI gene (formerly Entrezgene);Acc:397488]	3.36	0.1456
ssc-miR-503	GJA9	gap junction protein alpha 9 [Source:VGNC Symbol;Acc:VGNC:97065]	3.36	0.1456
ssc-miR-503	GLCE	glucuronic acid epimerase [Source:VGNC Symbol;Acc:VGNC:88477]	3.36	0.1456
ssc-miR-503	GNAI3	G protein subunit alpha i3 [Source:VGNC Symbol;Acc:VGNC:88523]	3.36	0.1456
ssc-miR-503	GPR174	G protein-coupled receptor 174 [Source:VGNC Symbol;Acc:VGNC:88617]	3.36	0.1456
ssc-miR-503	GPR63	G protein-coupled receptor 63 [Source:VGNC Symbol;Acc:VGNC:88634]	3.36	0.1456
ssc-miR-503	HECTD1	HECT domain E3 ubiquitin protein ligase 1 [Source:VGNC Symbol;Acc:VGNC:88832]	3.36	0.1456
ssc-miR-503	HECTD4	HECT domain E3 ubiquitin protein ligase 4 [Source:VGNC Symbol;Acc:VGNC:88834]	3.36	0.1456
ssc-miR-503	HELZ	helicase with zinc finger [Source:VGNC Symbol;Acc:VGNC:88840]	3.36	0.1456
ssc-miR-503	HIPK2	homeodomain interacting protein kinase 2 [Source:VGNC Symbol;Acc:VGNC:88888]	3.36	0.1456
ssc-miR-503	HNRNPA1	heteroous nuclear ribonucleoprotein A1 [Source:VGNC Symbol;Acc:VGNC:88918]	3.36	0.1456
ssc-miR-503	HS3ST3B1	heparan sulfate-glucosamine 3-sulfotransferase 3B1 [Source:VGNC Symbol;Acc:VGNC:99003]	3.36	0.1456
ssc-miR-503	HSPA4L	heat shock protein family A (Hsp70) member 4 like [Source:HGNC Symbol;Acc:HGNC:17041]	3.36	0.1456
ssc-miR-503	HSPE1-MOB4	hypothetical gene	3.36	0.1456
ssc-miR-503	IFFO2	intermediate filament family orphan 2 [Source:VGNC Symbol;Acc:VGNC:98475]	3.36	0.1456
ssc-miR-503	IGF1	insulin like growth factor 1 [Source:VGNC Symbol;Acc:VGNC:98044]	3.36	0.1456
ssc-miR-503	IHH	Indian hedgehog signaling molecule [Source:VGNC Symbol;Acc:VGNC:96370]	3.36	0.1456
ssc-miR-503	IKBKB	inhibitor of nuclear factor kappa B kinase subunit beta [Source:VGNC Symbol;Acc:VGNC:107145]	3.36	0.1456
ssc-miR-503	IL7R	interleukin 7 receptor [Source:VGNC Symbol;Acc:VGNC:89114]	3.36	0.1456
ssc-miR-503	IPO7	importin 7 [Source:VGNC Symbol;Acc:VGNC:89180]	3.36	0.1456
ssc-miR-503	IPO9	importin 9 [Source:VGNC Symbol;Acc:VGNC:95913]	3.36	0.1456
ssc-miR-503	IPPK	inositol-pentakisphosphate 2-kinase [Source:VGNC Symbol;Acc:VGNC:89183]	3.36	0.1456
ssc-miR-503	ISLR	immunoglobulin superfamily containing leucine rich repeat [Source:VGNC Symbol;Acc:VGNC:98052]	3.36	0.1456

ssc-miR-503	ISM2	hypothetical gene	3.36	0.1456
ssc-miR-503	JARID2	jumonji and AT-rich interaction domain containing 2 [Source:VGNC Symbol;Acc:VGNC:89279]	3.36	0.1456
ssc-miR-503	JOSD2	Josephin domain containing 2 [Source:NCBI gene (formerly Entrezgene);Acc:100627842]	3.36	0.1456
ssc-miR-503	KCNK10	potassium two pore domain channel subfamily K member 10 [Source:VGNC Symbol;Acc:VGNC:89364]	3.36	0.1456
ssc-miR-503	KDSR	3-ketodihydrosphingosine reductase [Source:VGNC Symbol;Acc:VGNC:108157]	3.36	0.1456
ssc-miR-503	KIAA0226L	hypothetical gene	3.36	0.1456
ssc-miR-503	KIF1C	kinesin family member 1C [Source:VGNC Symbol;Acc:VGNC:89461]	3.36	0.1456
ssc-miR-503	KIF23	kinesin family member 23 [Source:VGNC Symbol;Acc:VGNC:103113]	3.36	0.1456
ssc-miR-503	KIF5A	kinesin family member 5A [Source:VGNC Symbol;Acc:VGNC:89472]	3.36	0.1456
ssc-miR-503	KIF5C	kinesin family member 5C [Source:VGNC Symbol;Acc:VGNC:96394]	3.36	0.1456
ssc-miR-503	KLHL26	kelch like family member 26 [Source:VGNC Symbol;Acc:VGNC:89522]	3.36	0.1456
ssc-miR-503	KLHL3	kelch like family member 3 [Source:VGNC Symbol;Acc:VGNC:89525]	3.36	0.1456
ssc-miR-503	LAMTOR4	hypothetical gene	3.36	0.1456
ssc-miR-503	LARP1	La ribonucleoprotein 1, translational regulator [Source:VGNC Symbol;Acc:VGNC:98067]	3.36	0.1456
ssc-miR-503	LHX4	LIM homeobox 4 [Source:VGNC Symbol;Acc:VGNC:89714]	3.36	0.1456
ssc-miR-503	LONRF3	LON peptidase N-terminal domain and ring finger 3 [Source:VGNC Symbol;Acc:VGNC:89778]	3.36	0.1456
ssc-miR-503	LPHN1	hypothetical gene	3.36	0.1456
ssc-miR-503	LPP	LIM domain containing preferred translocation partner in lipoma [Source:HGNC Symbol;Acc:HGNC:6679]	3.36	0.1456
ssc-miR-503	LRCH4	leucine rich repeats and calponin homology domain containing 4 [Source:VGNC Symbol;Acc:VGNC:89804]	3.36	0.1456
ssc-miR-503	LRRC58	leucine rich repeat containing 58 [Source:VGNC Symbol;Acc:VGNC:98081]	3.36	0.1456
ssc-miR-503	LSM11	LSM11, U7 small nuclear RNA associated [Source:VGNC Symbol;Acc:VGNC:89871]	3.36	0.1456
ssc-miR-503	MAP2K1	mitogen-activated protein kinase kinase 1 [Source:VGNC Symbol;Acc:VGNC:103121]	3.36	0.1456
ssc-miR-503	MAPK8IP2	mitogen-activated protein kinase 8 interacting protein 2 [Source:VGNC Symbol;Acc:VGNC:103299]	3.36	0.1456
ssc-miR-503	MARCH9	hypothetical gene	3.36	0.1456
ssc-miR-503	MFSD2A	major facilitator superfamily domain containing 2A [Source:VGNC Symbol;Acc:VGNC:90188]	3.36	0.1456
ssc-miR-503	MIDN	midnolin [Source:VGNC Symbol;Acc:VGNC:90216]	3.36	0.1456
ssc-miR-503	MIEF1	hypothetical gene	3.36	0.1456
ssc-miR-503	MMAB	metabolism of cobalamin associated B [Source:VGNC Symbol;Acc:VGNC:96743]	3.36	0.1456
ssc-miR-503	MOB3B	MOB kinase activator 3B [Source:VGNC Symbol;Acc:VGNC:96024]	3.36	0.1456
ssc-miR-503	MOB4	hypothetical gene	3.36	0.1456
ssc-miR-503	MRAP2	melanocortin 2 receptor accessory protein 2 [Source:VGNC Symbol;Acc:VGNC:90339]	3.36	0.1456
ssc-miR-503	MTMR11	myotubularin related protein 11 [Source:VGNC Symbol;Acc:VGNC:90458]	3.36	0.1456
ssc-miR-503	MTMR12	myotubularin related protein 12 [Source:VGNC Symbol;Acc:VGNC:90459]	3.36	0.1456
ssc-miR-503	MYLK	myosin light chain kinase [Source:VGNC Symbol;Acc:VGNC:108676]	3.36	0.1456
ssc-miR-503	MYO5A	myosin VA [Source:HGNC Symbol;Acc:HGNC:7602]	3.36	0.1456
ssc-miR-503	N4BP1	NEDD4 binding protein 1 [Source:VGNC Symbol;Acc:VGNC:90553]	3.36	0.1456
ssc-miR-503	NAV1	neuron navigator 1 [Source:VGNC Symbol;Acc:VGNC:95725]	3.36	0.1456
ssc-miR-503	NBEAL1	cytochrome P450 family 20 subfamily A member 1 [Source:VGNC Symbol;Acc:VGNC:103366]	3.36	0.1456
ssc-miR-503	NCKAP1L	NCK associated protein 1 like [Source:VGNC Symbol;Acc:VGNC:90610]	3.36	0.1456
ssc-miR-503	NEK10	NIMA related kinase 10 [Source:VGNC Symbol;Acc:VGNC:90674]	3.36	0.1456
ssc-miR-503	NFE2L1	NFE2 like bZIP transcription factor 1 [Source:VGNC Symbol;Acc:VGNC:90713]	3.36	0.1456
ssc-miR-503	NFYA	nuclear transcription factor Y subunit alpha [Source:VGNC Symbol;Acc:VGNC:90729]	3.36	0.1456
ssc-miR-503	NISCH	nischarin [Source:VGNC Symbol;Acc:VGNC:107159]	3.36	0.1456
ssc-miR-503	NKD1	NKD inhibitor of WNT signaling pathway 1 [Source:VGNC Symbol;Acc:VGNC:90759]	3.36	0.1456
ssc-miR-503	NLRP1	hypothetical gene	3.36	0.1456
ssc-miR-503	NMNAT2	nicotinamide nucleotide adenylyltransferase 2 [Source:VGNC Symbol;Acc:VGNC:90798]	3.36	0.1456
ssc-miR-503	NRP2	neuropilin 2 [Source:VGNC Symbol;Acc:VGNC:96454]	3.36	0.1456
ssc-miR-503	NUAK2	NUAK family kinase 2 [Source:VGNC Symbol;Acc:VGNC:90941]	3.36	0.1456
ssc-miR-503	NUDT4	hypothetical gene	3.36	0.1456
ssc-miR-503	NUFIP2	nuclear FMR1 interacting protein 2 [Source:VGNC Symbol;Acc:VGNC:90967]	3.36	0.1456

ssc-miR-503	NUP50	nucleoporin 50 [Source:VGNC Symbol;Acc:VGNC:90983]	3.36	0.1456
ssc-miR-503	NUTF2	nuclear transport factor 2 [Source:VGNC Symbol;Acc:VGNC:98524]	3.36	0.1456
ssc-miR-503	NYNRIN	NYN domain and retroviral integrase containing [Source:VGNC Symbol;Acc:VGNC:91003]	3.36	0.1456
ssc-miR-503	OCRL	OCRL inositol polyphosphate-5-phosphatase [Source:VGNC Symbol;Acc:VGNC:91014]	3.36	0.1456
ssc-miR-503	OTOF	otoferlin [Source:VGNC Symbol;Acc:VGNC:91089]	3.36	0.1456
ssc-miR-503	OTUD4	OTU deubiquitinase 4 [Source:VGNC Symbol;Acc:VGNC:91100]	3.36	0.1456
ssc-miR-503	PAFAH1B2	platelet activating factor acetylhydrolase 1b catalytic subunit 2 [Source:VGNC Symbol;Acc:VGNC:91151]	3.36	0.1456
ssc-miR-503	PAM	peptidylglycine alpha-amidating monooxygenase [Source:VGNC Symbol;Acc:VGNC:91163]	3.36	0.1456
ssc-miR-503	PAPD4	hypothetical gene	3.36	0.1456
ssc-miR-503	PAPPA	pappalysin 1 [Source:VGNC Symbol;Acc:VGNC:91170]	3.36	0.1456
ssc-miR-503	PARD6B	par-6 family cell polarity regulator beta [Source:VGNC Symbol;Acc:VGNC:98176]	3.36	0.1456
ssc-miR-503	PAX2	paired box 2 [Source:VGNC Symbol;Acc:VGNC:91192]	3.36	0.1456
ssc-miR-503	PCDHB16	hypothetical gene	3.36	0.1456
ssc-miR-503	PDAP1	PDGFA associated protein 1 [Source:VGNC Symbol;Acc:VGNC:91239]	3.36	0.1456
ssc-miR-503	PDCD4	programmed cell death 4 [Source:VGNC Symbol;Acc:VGNC:91244]	3.36	0.1456
ssc-miR-503	PDZD8	hypothetical gene	3.36	0.1456
ssc-miR-503	PEX13	hypothetical gene	3.36	0.1456
ssc-miR-503	PEX19	peroxisomal biosis factor 19 [Source:VGNC Symbol;Acc:VGNC:104022]	3.36	0.1456
ssc-miR-503	PGM2L1	phosphoglucomutase 2 like 1 [Source:VGNC Symbol;Acc:VGNC:91357]	3.36	0.1456
ssc-miR-503	PHACTR2	phosphatase and actin regulator 2 [Source:VGNC Symbol;Acc:VGNC:91366]	3.36	0.1456
ssc-miR-503	PHOX2B	paired like homeobox 2B [Source:VGNC Symbol;Acc:VGNC:91402]	3.36	0.1456
ssc-miR-503	PI4K2B	phosphatidylinositol 4-kinase type 2 beta [Source:VGNC Symbol;Acc:VGNC:98190]	3.36	0.1456
ssc-miR-503	PIK3C2A	phosphatidylinositol-4-phosphate 3-kinase catalytic subunit type 2 alpha [Source:VGNC Symbol;Acc:VGNC:91436]	3.36	0.1456
ssc-miR-503	PIK3R1	phosphoinositide-3-kinase regulatory subunit 1 [Source:VGNC Symbol;Acc:VGNC:91445]	3.36	0.1456
ssc-miR-503	PIM3	Pim-3 proto-onco, serine/threonine kinase [Source:VGNC Symbol;Acc:VGNC:91450]	3.36	0.1456
ssc-miR-503	PLAGL1	PLAG1 like zinc finger 1 [Source:VGNC Symbol;Acc:VGNC:91510]	3.36	0.1456
ssc-miR-503	PNRC2	proline rich nuclear receptor coactivator 2 [Source:VGNC Symbol;Acc:VGNC:91614]	3.36	0.1456
ssc-miR-503	POM121	hypothetical gene	3.36	0.1456
ssc-miR-503	PPFIA3	PTPRF interacting protein alpha 3 [Source:VGNC Symbol;Acc:VGNC:91693]	3.36	0.1456
ssc-miR-503	PPM1D	protein phosphatase, Mg2+/Mn2+ dependent 1D [Source:VGNC Symbol;Acc:VGNC:91704]	3.36	0.1456
ssc-miR-503	PPP1R11	protein phosphatase 1 regulatory inhibitor subunit 11 [Source:VGNC Symbol;Acc:VGNC:91718]	3.36	0.1456
ssc-miR-503	PPP3CB	protein phosphatase 3 catalytic subunit beta [Source:VGNC Symbol;Acc:VGNC:104035]	3.36	0.1456
ssc-miR-503	PRDM4	PR/SET domain 4 [Source:VGNC Symbol;Acc:VGNC:91779]	3.36	0.1456
ssc-miR-503	PRR11	proline rich 11 [Source:VGNC Symbol;Acc:VGNC:91850]	3.36	0.1456
ssc-miR-503	PSKH1	protein serine kinase H1 [Source:VGNC Symbol;Acc:VGNC:91899]	3.36	0.1456
ssc-miR-503	PTCD1	pentatricopeptide repeat domain 1 [Source:HGNC Symbol;Acc:HGNC:22198]	3.36	0.1456
ssc-miR-503	PTGFR	prostaglandin F receptor [Source:VGNC Symbol;Acc:VGNC:91954]	3.36	0.1456
ssc-miR-503	PTGIR	prostaglandin I2 receptor [Source:VGNC Symbol;Acc:VGNC:91956]	3.36	0.1456
ssc-miR-503	PTK7	protein tyrosine kinase 7 (inactive) [Source:HGNC Symbol;Acc:HGNC:9618]	3.36	0.1456
ssc-miR-503	PTPN3	protein tyrosine phosphatase non-receptor type 3 [Source:VGNC Symbol;Acc:VGNC:91980]	3.36	0.1456
ssc-miR-503	PTPN4	protein tyrosine phosphatase non-receptor type 4 [Source:VGNC Symbol;Acc:VGNC:96521]	3.36	0.1456
ssc-miR-503	PVRL1	hypothetical gene	3.36	0.1456
ssc-miR-503	RAB35	RAB35, member RAS onco family [Source:VGNC Symbol;Acc:VGNC:98259]	3.36	0.1456
ssc-miR-503	RAB9B	RAB9B, member RAS onco family [Source:VGNC Symbol;Acc:VGNC:98278]	3.36	0.1456
ssc-miR-503	RAD9A	RAD9 checkpoint clamp component A [Source:VGNC Symbol;Acc:VGNC:92062]	3.36	0.1456
ssc-miR-503	RAF1	Raf-1 proto-onco, serine/threonine kinase [Source:VGNC Symbol;Acc:VGNC:108688]	3.36	0.1456
ssc-miR-503	RASGEF1B	RasGEF domain family member 1B [Source:VGNC Symbol;Acc:VGNC:92109]	3.36	0.1456
ssc-miR-503	RBCK1	RANBP2-type and C3HC4-type zinc finger containing 1 [Source:VGNC Symbol;Acc:VGNC:98284]	3.36	0.1456
ssc-miR-503	RBM23	RNA binding motif protein 23 [Source:HGNC Symbol;Acc:HGNC:20155]	3.36	0.1456
ssc-miR-503	RECK	reversion inducing cysteine rich protein with kazal motifs [Source:VGNC Symbol;Acc:VGNC:92194]	3.36	0.1456

ssc-miR-503	RGP1	RGP1 homolog, RAB6A GEF complex partner 1 [Source:VGNC Symbol;Acc:VGNC:92257]	3.36	0.1456
ssc-miR-503	RICTOR	RPTOR independent companion of MTOR complex 2 [Source:VGNC Symbol;Acc:VGNC:92303]	3.36	0.1456
ssc-miR-503	RIMKLB	ribosomal modification protein rimK like family member B [Source:VGNC Symbol;Acc:VGNC:92308]	3.36	0.1456
ssc-miR-503	RNF138	ring finger protein 138 [Source:VGNC Symbol;Acc:VGNC:92356]	3.36	0.1456
ssc-miR-503	RNF144B	ring finger protein 144B [Source:VGNC Symbol;Acc:VGNC:92359]	3.36	0.1456
ssc-miR-503	RNF41	ring finger protein 41 [Source:VGNC Symbol;Acc:VGNC:92394]	3.36	0.1456
ssc-miR-503	RP4-559A3.7	hypothetical gene	3.36	0.1456
ssc-miR-503	RPS6KA6	ribosomal protein S6 kinase A6 [Source:VGNC Symbol;Acc:VGNC:92445]	3.36	0.1456
ssc-miR-503	RSPO3	R-spondin 3 [Source:VGNC Symbol;Acc:VGNC:92485]	3.36	0.1456
ssc-miR-503	SCAI	suppressor of cancer cell invasion [Source:VGNC Symbol;Acc:VGNC:103170]	3.36	0.1456
ssc-miR-503	SENP5	SUMO specific peptidase 5 [Source:VGNC Symbol;Acc:VGNC:92714]	3.36	0.1456
ssc-miR-503	SESTD1	SEC14 and spectrin domain containing 1 [Source:VGNC Symbol;Acc:VGNC:95884]	3.36	0.1456
ssc-miR-503	SETD1B	SET domain containing 1B, histone lysine methyltransferase [Source:HGNC Symbol;Acc:HGNC:29187]	3.36	0.1456
ssc-miR-503	SETD6	SET domain containing 6, protein lysine methyltransferase [Source:VGNC Symbol;Acc:VGNC:92761]	3.36	0.1456
ssc-miR-503	SIK1	salt inducible kinase 1 [Source:HGNC Symbol;Acc:HGNC:11142]	3.36	0.1456
ssc-miR-503	SKI	SKI proto-onco [Source:VGNC Symbol;Acc:VGNC:92902]	3.36	0.1456
ssc-miR-503	SLC10A6	solute carrier family 10 member 6 [Source:VGNC Symbol;Acc:VGNC:92918]	3.36	0.1456
ssc-miR-503	SLC20A2	solute carrier family 20 member 2 [Source:VGNC Symbol;Acc:VGNC:95498]	3.36	0.1456
ssc-miR-503	SLC25A29	hypothetical gene	3.36	0.1456
ssc-miR-503	SLC25A37	hypothetical gene	3.36	0.1456
ssc-miR-503	SLC2A14	hypothetical gene	3.36	0.1456
ssc-miR-503	SLC2A3	hypothetical gene	3.36	0.1456
ssc-miR-503	SLC35G1	solute carrier family 35 member G1 [Source:VGNC Symbol;Acc:VGNC:93086]	3.36	0.1456
ssc-miR-503	SLC4A4	solute carrier family 4 member 4 [Source:VGNC Symbol;Acc:VGNC:93133]	3.36	0.1456
ssc-miR-503	SLC52A3	solute carrier family 52 member 3 [Source:VGNC Symbol;Acc:VGNC:95777]	3.36	0.1456
ssc-miR-503	SLC6A11	solute carrier family 6 member 11 [Source:VGNC Symbol;Acc:VGNC:93154]	3.36	0.1456
ssc-miR-503	SLIT2	slit guidance ligand 2 [Source:VGNC Symbol;Acc:VGNC:93204]	3.36	0.1456
ssc-miR-503	SMAD7	SMAD family member 7 [Source:VGNC Symbol;Acc:VGNC:93221]	3.36	0.1456
ssc-miR-503	SMURF1	SMAD specific E3 ubiquitin protein ligase 1 [Source:VGNC Symbol;Acc:VGNC:93270]	3.36	0.1456
ssc-miR-503	SMURF2	SMAD specific E3 ubiquitin protein ligase 2 [Source:VGNC Symbol;Acc:VGNC:93271]	3.36	0.1456
ssc-miR-503	SNTB2	syntrophin beta 2 [Source:VGNC Symbol;Acc:VGNC:93298]	3.36	0.1456
ssc-miR-503	SOCS5	suppressor of cytokine signaling 5 [Source:VGNC Symbol;Acc:VGNC:93332]	3.36	0.1456
ssc-miR-503	SOCS6	suppressor of cytokine signaling 6 [Source:VGNC Symbol;Acc:VGNC:93333]	3.36	0.1456
ssc-miR-503	SOWAHB	sosondowah ankyrin repeat domain family member B [Source:VGNC Symbol;Acc:VGNC:93348]	3.36	0.1456
ssc-miR-503	SPRED1	sprouty related EVH1 domain containing 1 [Source:VGNC Symbol;Acc:VGNC:93420]	3.36	0.1456
ssc-miR-503	SPTBN2	spectrin beta, non-erythrocytic 2 [Source:VGNC Symbol;Acc:VGNC:93437]	3.36	0.1456
ssc-miR-503	SRCAP	Snf2 related CREBBP activator protein [Source:HGNC Symbol;Acc:HGNC:16974]	3.36	0.1456
ssc-miR-503	SRPR	hypothetical gene	3.36	0.1456
ssc-miR-503	SRSF10	serine and arginine rich splicing factor 10 [Source:VGNC Symbol;Acc:VGNC:93472]	3.36	0.1456
ssc-miR-503	ST8SIA3	ST8 alpha-N-acetyl-neuraminide alpha-2,8-sialyltransferase 3 [Source:VGNC Symbol;Acc:VGNC:93518]	3.36	0.1456
ssc-miR-503	STEAP3	STEAP3 metalloreductase [Source:VGNC Symbol;Acc:VGNC:96048]	3.36	0.1456
ssc-miR-503	STOX2	storkhead box 2 [Source:VGNC Symbol;Acc:VGNC:96082]	3.36	0.1456
ssc-miR-503	STRN	striatin [Source:VGNC Symbol;Acc:VGNC:93578]	3.36	0.1456
ssc-miR-503	STYX	serine/threonine/tyrosine interacting protein [Source:VGNC Symbol;Acc:VGNC:93602]	3.36	0.1456
ssc-miR-503	SYDE2	synapse defective Rho GTPase homolog 2 [Source:VGNC Symbol;Acc:VGNC:98874]	3.36	0.1456
ssc-miR-503	SYT3	synaptotagmin 3 [Source:VGNC Symbol;Acc:VGNC:93684]	3.36	0.1456
ssc-miR-503	TCTE1	t-complex-associated-testis-expressed 1 [Source:VGNC Symbol;Acc:VGNC:93836]	3.36	0.1456
ssc-miR-503	TERT	telomerase reverse transcriptase [Source:VGNC Symbol;Acc:VGNC:93883]	3.36	0.1456
ssc-miR-503	TFCP2L1	transcription factor CP2 like 1 [Source:HGNC Symbol;Acc:HGNC:17925]	3.36	0.1456
ssc-miR-503	THUMPD1	THUMP domain containing 1 [Source:VGNC Symbol;Acc:VGNC:93970]	3.36	0.1456

ssc-miR-503	TLK1	tousled like kinase 1 [Source:VGNC Symbol;Acc:VGNC:95543]	3.36	0.1456
ssc-miR-503	TLL1	tolloid like 1 [Source:VGNC Symbol;Acc:VGNC:94018]	3.36	0.1456
ssc-miR-503	TMC7	transmembrane channel like 7 [Source:VGNC Symbol;Acc:VGNC:94044]	3.36	0.1456
ssc-miR-503	TMCC1	transmembrane and coiled-coil domain family 1 [Source:VGNC Symbol;Acc:VGNC:94046]	3.36	0.1456
ssc-miR-503	TMEM178B	transmembrane protein 178B [Source:HGNC Symbol;Acc:HGNC:44112]	3.36	0.1456
ssc-miR-503	TMEM55A	hypothetical gene	3.36	0.1456
ssc-miR-503	TMEM55B	hypothetical gene	3.36	0.1456
ssc-miR-503	TMEM74B	transmembrane protein 74B [Source:HGNC Symbol;Acc:HGNC:15893]	3.36	0.1456
ssc-miR-503	TNFSF13B	TNF superfamily member 13b [Source:VGNC Symbol;Acc:VGNC:94269]	3.36	0.1456
ssc-miR-503	TNPO1	transportin 1 [Source:VGNC Symbol;Acc:VGNC:94287]	3.36	0.1456
ssc-miR-503	TPRG1	tumor protein p63 regulated 1 [Source:VGNC Symbol;Acc:VGNC:94348]	3.36	0.1456
ssc-miR-503	TRABD2B	TraB domain containing 2B [Source:VGNC Symbol;Acc:VGNC:94356]	3.36	0.1456
ssc-miR-503	TRAK1	trafficking kinesin protein 1 [Source:VGNC Symbol;Acc:VGNC:94369]	3.36	0.1456
ssc-miR-503	TSC22D2	TSC22 domain family member 2 [Source:VGNC Symbol;Acc:VGNC:94482]	3.36	0.1456
ssc-miR-503	TSPYL2	TSPY like 2 [Source:VGNC Symbol;Acc:VGNC:94519]	3.36	0.1456
ssc-miR-503	UBFD1	ubiquitin family domain containing 1 [Source:VGNC Symbol;Acc:VGNC:94658]	3.36	0.1456
ssc-miR-503	UBN2	ubinuclein 2 [Source:VGNC Symbol;Acc:VGNC:94664]	3.36	0.1456
ssc-miR-503	UHMK1	U2AF homology motif kinase 1 [Source:VGNC Symbol;Acc:VGNC:94688]	3.36	0.1456
ssc-miR-503	UNC13A	hypothetical gene	3.36	0.1456
ssc-miR-503	USP2	ubiquitin specific peptidase 2 [Source:VGNC Symbol;Acc:VGNC:94751]	3.36	0.1456
ssc-miR-503	USP31	ubiquitin specific peptidase 31 [Source:HGNC Symbol;Acc:HGNC:20060]	3.36	0.1456
ssc-miR-503	VPS37C	VPS37C subunit of ESCRT-I [Source:VGNC Symbol;Acc:VGNC:94852]	3.36	0.1456
ssc-miR-503	WBP11	hypothetical gene	3.36	0.1456
ssc-miR-503	WDR55	WD repeat domain 55 [Source:VGNC Symbol;Acc:VGNC:94929]	3.36	0.1456
ssc-miR-503	WEE1	WEE1 G2 checkpoint kinase [Source:VGNC Symbol;Acc:VGNC:104095]	3.36	0.1456
ssc-miR-503	WIPI2	WD repeat domain, phosphoinositide interacting 2 [Source:VGNC Symbol;Acc:VGNC:94960]	3.36	0.1456
ssc-miR-503	WNT2B	Wnt family member 2B [Source:VGNC Symbol;Acc:VGNC:94969]	3.36	0.1456
ssc-miR-503	WNT3A	Wnt family member 3A [Source:VGNC Symbol;Acc:VGNC:94971]	3.36	0.1456
ssc-miR-503	WNT4	hypothetical gene	3.36	0.1456
ssc-miR-503	WNT7A	Wnt family member 7A [Source:VGNC Symbol;Acc:VGNC:94974]	3.36	0.1456
ssc-miR-503	XPO4	exportin 4 [Source:VGNC Symbol;Acc:VGNC:95004]	3.36	0.1456
ssc-miR-503	YOD1	YOD1 deubiquitinase [Source:VGNC Symbol;Acc:VGNC:95035]	3.36	0.1456
ssc-miR-503	YTHDC1	YTH domain containing 1 [Source:VGNC Symbol;Acc:VGNC:95041]	3.36	0.1456
ssc-miR-503	ZADH2	prostaglandin reductase 3 [Source:VGNC Symbol;Acc:VGNC:95049]	3.36	0.1456
ssc-miR-503	ZBTB39	zinc finger and BTB domain containing 39 [Source:VGNC Symbol;Acc:VGNC:95073]	3.36	0.1456
ssc-miR-503	ZC3H11A	hypothetical gene	3.36	0.1456
ssc-miR-503	ZC3H6	zinc finger CCCH-type containing 6 [Source:VGNC Symbol;Acc:VGNC:95098]	3.36	0.1456
ssc-miR-503	ZDHHC18	zinc finger DHHC-type palmitoyltransferase 18 [Source:VGNC Symbol;Acc:VGNC:95120]	3.36	0.1456
ssc-miR-503	ZER1	zyg-11 related cell cycle regulator [Source:VGNC Symbol;Acc:VGNC:95132]	3.36	0.1456
ssc-miR-503	ZHX3	zinc fingers and homeoboxes 3 [Source:VGNC Symbol;Acc:VGNC:95717]	3.36	0.1456
ssc-miR-503	ZMAT3	zinc finger matrin-type 3 [Source:VGNC Symbol;Acc:VGNC:95177]	3.36	0.1456
ssc-miR-503	ZNF217	zinc finger protein 217 [Source:VGNC Symbol;Acc:VGNC:95573]	3.36	0.1456
ssc-miR-503	ZNF423	zinc finger protein 423 [Source:VGNC Symbol;Acc:VGNC:98752]	3.36	0.1456
ssc-miR-503	ZNF449	zinc finger protein 449 [Source:VGNC Symbol;Acc:VGNC:95242]	3.36	0.1456
ssc-miR-503	ZNF622	zinc finger protein 622 [Source:VGNC Symbol;Acc:VGNC:95277]	3.36	0.1456
ssc-miR-503	ZNF629	zinc finger protein 629 [Source:VGNC Symbol;Acc:VGNC:95279]	3.36	0.1456
ssc-miR-503	ZNF697	zinc finger protein 697 [Source:HGNC Symbol;Acc:HGNC:32034]	3.36	0.1456
ssc-miR-503	ZNF74	zinc finger protein 74 [Source:HGNC Symbol;Acc:HGNC:13144]	3.36	0.1456
ssc-miR-503	ZNRF2	zinc and ring finger 2 [Source:VGNC Symbol;Acc:VGNC:95317]	3.36	0.1456
ssc-miR-140-3p	ABHD15	abhydrolase domain containing 15 [Source:VGNC Symbol;Acc:VGNC:84974]	2.06	0.0137

ssc-miR-140-3p	ABHD17C	abhydrolase domain containing 17C, depalmitoylase [Source:VGNC Symbol;Acc:VGNC:84978]	2.06	0.0137
ssc-miR-140-3p	ABHD2	abhydrolase domain containing 2, acylglycerol lipase [Source:VGNC Symbol;Acc:VGNC:84980]	2.06	0.0137
ssc-miR-140-3p	ACER2	alkaline ceramidase 2 [Source:VGNC Symbol;Acc:VGNC:85010]	2.06	0.0137
ssc-miR-140-3p	ACPL2	hypothetical gene	2.06	0.0137
ssc-miR-140-3p	ACVR2B	activin A receptor type 2B [Source:VGNC Symbol;Acc:VGNC:108629]	2.06	0.0137
ssc-miR-140-3p	ADAM10	ADAM metallopeptidase domain 10 [Source:VGNC Symbol;Acc:VGNC:85061]	2.06	0.0137
ssc-miR-140-3p	ADAM17	ADAM metallopeptidase domain 17 [Source:VGNC Symbol;Acc:VGNC:85065]	2.06	0.0137
ssc-miR-140-3p	ADCY9	adenylate cyclase 9 [Source:VGNC Symbol;Acc:VGNC:85113]	2.06	0.0137
ssc-miR-140-3p	AFF4	AF4/FMR2 family member 4 [Source:VGNC Symbol;Acc:VGNC:85169]	2.06	0.0137
ssc-miR-140-3p	AGO2	argonaute RISC catalytic component 2 [Source:VGNC Symbol;Acc:VGNC:97871]	2.06	0.0137
ssc-miR-140-3p	AGO4	hypothetical gene	2.06	0.0137
ssc-miR-140-3p	AHCYL2	adenosylhomocysteinase like 2 [Source:VGNC Symbol;Acc:VGNC:97874]	2.06	0.0137
ssc-miR-140-3p	AHDC1	hypothetical gene	2.06	0.0137
ssc-miR-140-3p	AHRR	aryl-hydrocarbon receptor repressor [Source:VGNC Symbol;Acc:VGNC:103065]	2.06	0.0137
ssc-miR-140-3p	ALDH1A3	aldehyde dehydrogenase 1 family member A3 [Source:VGNC Symbol;Acc:VGNC:97879]	2.06	0.0137
ssc-miR-140-3p	ALX1	ALX homeobox 1 [Source:VGNC Symbol;Acc:VGNC:85271]	2.06	0.0137
ssc-miR-140-3p	ANGEL1	angel homolog 1 [Source:VGNC Symbol;Acc:VGNC:85301]	2.06	0.0137
ssc-miR-140-3p	ANGPTL3	angiopoietin like 3 [Source:VGNC Symbol;Acc:VGNC:85305]	2.06	0.0137
ssc-miR-140-3p	ANK2	hypothetical gene	2.06	0.0137
ssc-miR-140-3p	ANKRD52	ankyrin repeat domain 52 [Source:VGNC Symbol;Acc:VGNC:85342]	2.06	0.0137
ssc-miR-140-3p	ANKS1A	ankyrin repeat and sterile alpha motif domain containing 1A [Source:VGNC Symbol;Acc:VGNC:85348]	2.06	0.0137
ssc-miR-140-3p	AP2A2	adaptor related protein complex 2 subunit alpha 2 [Source:VGNC Symbol;Acc:VGNC:85384]	2.06	0.0137
ssc-miR-140-3p	APC	APC regulator of WNT signaling pathway [Source:VGNC Symbol;Acc:VGNC:99584]	2.06	0.0137
ssc-miR-140-3p	APLP2	amyloid beta like protein 2 [Source:VGNC Symbol;Acc:VGNC:85414]	2.06	0.0137
ssc-miR-140-3p	APOLD1	apolipoprotein L domain containing 1 [Source:VGNC Symbol;Acc:VGNC:103215]	2.06	0.0137
ssc-miR-140-3p	APPBP2	amyloid beta protein binding protein 2 [Source:VGNC Symbol;Acc:VGNC:85425]	2.06	0.0137
ssc-miR-140-3p	AQP10	aquaporin 10 [Source:VGNC Symbol;Acc:VGNC:85429]	2.06	0.0137
ssc-miR-140-3p	ARID1B	AT-rich interaction domain 1B [Source:VGNC Symbol;Acc:VGNC:85506]	2.06	0.0137
ssc-miR-140-3p	ARID2	AT-rich interaction domain 2 [Source:HGNC Symbol;Acc:HGNC:18037]	2.06	0.0137
ssc-miR-140-3p	ARIH1	ariadne RBR E3 ubiquitin protein ligase 1 [Source:HGNC Symbol;Acc:HGNC:689]	2.06	0.0137
ssc-miR-140-3p	ARL4C	ADP ribosylation factor like GTPase 4C [Source:VGNC Symbol;Acc:VGNC:96419]	2.06	0.0137
ssc-miR-140-3p	ARL5A	ADP ribosylation factor like GTPase 5A [Source:VGNC Symbol;Acc:VGNC:96418]	2.06	0.0137
ssc-miR-140-3p	ARMC7	hypothetical gene	2.06	0.0137
ssc-miR-140-3p	ATF7IP	activating transcription factor 7 interacting protein [Source:VGNC Symbol;Acc:VGNC:85611]	2.06	0.0137
ssc-miR-140-3p	ATG4B	autophagy related 4B cysteine peptidase [Source:VGNC Symbol;Acc:VGNC:95947]	2.06	0.0137
ssc-miR-140-3p	ATL2	atlastin GTPase 2 [Source:VGNC Symbol;Acc:VGNC:85628]	2.06	0.0137
ssc-miR-140-3p	ATP1B1	ATPase Na+/K+ transporting subunit beta 1 [Source:VGNC Symbol;Acc:VGNC:85642]	2.06	0.0137
ssc-miR-140-3p	ATP2A2	ATPase sarcoplasmic/endoplasmic reticulum Ca2+ transporting 2 [Source:VGNC Symbol;Acc:VGNC:85647]	2.06	0.0137
ssc-miR-140-3p	ATP2B2	ATPase plasma membrane Ca2+ transporting 2 [Source:VGNC Symbol;Acc:VGNC:85649]	2.06	0.0137
ssc-miR-140-3p	ATXN7L3	ataxin 7 like 3 [Source:VGNC Symbol;Acc:VGNC:85694]	2.06	0.0137
ssc-miR-140-3p	ATXN7L3B	ataxin 7 like 3B [Source:HGNC Symbol;Acc:HGNC:37931]	2.06	0.0137
ssc-miR-140-3p	B3GALNT2	beta-1,3-N-acetylgalactosaminyltransferase 2 [Source:VGNC Symbol;Acc:VGNC:85715]	2.06	0.0137
ssc-miR-140-3p	B3GNT1	hypothetical gene	2.06	0.0137
ssc-miR-140-3p	BACE1	beta-secretase 1 [Source:VGNC Symbol;Acc:VGNC:85740]	2.06	0.0137
ssc-miR-140-3p	BAHD1	bromo adjacent homology domain containing 1 [Source:VGNC Symbol;Acc:VGNC:85747]	2.06	0.0137
ssc-miR-140-3p	BAK1	hypothetical gene	2.06	0.0137
ssc-miR-140-3p	BCL2	BCL2 apoptosis regulator [Source:HGNC Symbol;Acc:HGNC:990]	2.06	0.0137
ssc-miR-140-3p	BCL9	BCL9 transcription coactivator [Source:VGNC Symbol;Acc:VGNC:96567]	2.06	0.0137
ssc-miR-140-3p	BCL9L	BCL9 like [Source:VGNC Symbol;Acc:VGNC:96568]	2.06	0.0137
ssc-miR-140-3p	BDNF	brain derived neurotrophic factor [Source:VGNC Symbol;Acc:VGNC:85795]	2.06	0.0137

ssc-miR-140-3p	BRWD3	bromodomain and WD repeat domain containing 3 [Source:VGNC Symbol;Acc:VGNC:85890]	2.06	0.0137
ssc-miR-140-3p	BSN	bassoon presynaptic cytomatrix protein [Source:VGNC Symbol;Acc:VGNC:85893]	2.06	0.0137
ssc-miR-140-3p	BTG1	BTG anti-proliferation factor 1 [Source:VGNC Symbol;Acc:VGNC:103907]	2.06	0.0137
ssc-miR-140-3p	C10orf76	hypothetical gene	2.06	0.0137
ssc-miR-140-3p	C16orf52	hypothetical gene	2.06	0.0137
ssc-miR-140-3p	C1orf21	hypothetical gene	2.06	0.0137
ssc-miR-140-3p	C2orf91	hypothetical gene	2.06	0.0137
ssc-miR-140-3p	C3orf83	hypothetical gene	2.06	0.0137
ssc-miR-140-3p	CABLES2	Cdk5 and Abl enzyme substrate 2 [Source:VGNC Symbol;Acc:VGNC:95753]	2.06	0.0137
ssc-miR-140-3p	CACUL1	CDK2 associated cullin domain 1 [Source:VGNC Symbol;Acc:VGNC:86132]	2.06	0.0137
ssc-miR-140-3p	CAND1	cullin associated and neddylation dissociated 1 [Source:VGNC Symbol;Acc:VGNC:97912]	2.06	0.0137
ssc-miR-140-3p	CAPRIN1	cell cycle associated protein 1 [Source:VGNC Symbol;Acc:VGNC:86178]	2.06	0.0137
ssc-miR-140-3p	CAPZB	capping actin protein of muscle Z-line subunit beta [Source:VGNC Symbol;Acc:VGNC:86184]	2.06	0.0137
ssc-miR-140-3p	CASD1	CAS1 domain containing 1 [Source:VGNC Symbol;Acc:VGNC:86199]	2.06	0.0137
ssc-miR-140-3p	CBL	Cbl proto-onco [Source:VGNC Symbol;Acc:VGNC:86222]	2.06	0.0137
ssc-miR-140-3p	CBX6	chromobox 6 [Source:VGNC Symbol;Acc:VGNC:97915]	2.06	0.0137
ssc-miR-140-3p	CCAR2	cell cycle and apoptosis regulator 2 [Source:VGNC Symbol;Acc:VGNC:86239]	2.06	0.0137
ssc-miR-140-3p	CCDC171	coiled-coil domain containing 171 [Source:VGNC Symbol;Acc:VGNC:103044]	2.06	0.0137
ssc-miR-140-3p	CCDC179	coiled-coil domain containing 179 [Source:HGNC Symbol;Acc:HGNC:44653]	2.06	0.0137
ssc-miR-140-3p	CCDC25	coiled-coil domain containing 25 [Source:VGNC Symbol;Acc:VGNC:86288]	2.06	0.0137
ssc-miR-140-3p	CCDC85C	coiled-coil domain containing 85C [Source:VGNC Symbol;Acc:VGNC:86322]	2.06	0.0137
ssc-miR-140-3p	CCDC88C	coiled-coil domain containing 88C [Source:VGNC Symbol;Acc:VGNC:97917]	2.06	0.0137
ssc-miR-140-3p	CCNT2	cyclin T2 [Source:VGNC Symbol;Acc:VGNC:103916]	2.06	0.0137
ssc-miR-140-3p	CD274	CD274 molecule [Source:VGNC Symbol;Acc:VGNC:86404]	2.06	0.0137
ssc-miR-140-3p	CDC42EP3	CDC42 effector protein 3 [Source:VGNC Symbol;Acc:VGNC:86457]	2.06	0.0137
ssc-miR-140-3p	CDC5L	cell division cycle 5 like [Source:VGNC Symbol;Acc:VGNC:86463]	2.06	0.0137
ssc-miR-140-3p	CDC7	cell division cycle 7 [Source:VGNC Symbol;Acc:VGNC:97922]	2.06	0.0137
ssc-miR-140-3p	CDH18	cadherin 18 [Source:VGNC Symbol;Acc:VGNC:86481]	2.06	0.0137
ssc-miR-140-3p	CDK6	cyclin dependent kinase 6 [Source:HGNC Symbol;Acc:HGNC:1777]	2.06	0.0137
ssc-miR-140-3p	CDYL2	chromodomain Y like 2 [Source:VGNC Symbol;Acc:VGNC:86529]	2.06	0.0137
ssc-miR-140-3p	CELF1	CUGBP Elav-like family member 1 [Source:VGNC Symbol;Acc:VGNC:86537]	2.06	0.0137
ssc-miR-140-3p	CELF2	hypothetical gene	2.06	0.0137
ssc-miR-140-3p	CELSR3	cadherin EGF LAG seven-pass G-type receptor 3 [Source:VGNC Symbol;Acc:VGNC:86541]	2.06	0.0137
ssc-miR-140-3p	CENPC	centromere protein C [Source:VGNC Symbol;Acc:VGNC:86546]	2.06	0.0137
ssc-miR-140-3p	CHD6	chromodomain helicase DNA binding protein 6 [Source:VGNC Symbol;Acc:VGNC:95903]	2.06	0.0137
ssc-miR-140-3p	CHD7	chromodomain helicase DNA binding protein 7 [Source:VGNC Symbol;Acc:VGNC:86633]	2.06	0.0137
ssc-miR-140-3p	CHIC1	cysteine rich hydrophobic domain 1 [Source:VGNC Symbol;Acc:VGNC:86642]	2.06	0.0137
ssc-miR-140-3p	CHIC2	cysteine rich hydrophobic domain 2 [Source:VGNC Symbol;Acc:VGNC:86643]	2.06	0.0137
ssc-miR-140-3p	CHL1	cell adhesion molecule L1 like [Source:VGNC Symbol;Acc:VGNC:108639]	2.06	0.0137
ssc-miR-140-3p	CHN1	hypothetical gene	2.06	0.0137
ssc-miR-140-3p	CISD1	hypothetical gene	2.06	0.0137
ssc-miR-140-3p	CISD2	CDGSH iron sulfur domain 2 [Source:VGNC Symbol;Acc:VGNC:86707]	2.06	0.0137
ssc-miR-140-3p	CLDN1	claudin 1 [Source:VGNC Symbol;Acc:VGNC:103926]	2.06	0.0137
ssc-miR-140-3p	CLIC5	chloride intracellular channel 5 [Source:VGNC Symbol;Acc:VGNC:86764]	2.06	0.0137
ssc-miR-140-3p	CMTR1	cap methyltransferase 1 [Source:HGNC Symbol;Acc:HGNC:21077]	2.06	0.0137
ssc-miR-140-3p	CNEP1R1	CTD nuclear envelope phosphatase 1 regulatory subunit 1 [Source:VGNC Symbol;Acc:VGNC:86813]	2.06	0.0137
ssc-miR-140-3p	COBLL1	cordon-bleu WH2 repeat protein like 1 [Source:VGNC Symbol;Acc:VGNC:96013]	2.06	0.0137
ssc-miR-140-3p	CPE	carboxypeptidase E [Source:VGNC Symbol;Acc:VGNC:86936]	2.06	0.0137
ssc-miR-140-3p	CPEB1	cytoplasmic polyadenylation element binding protein 1 [Source:NCBI gene (formerly Entrezgene);Acc:100048944]	2.06	0.0137
ssc-miR-140-3p	CREB1	cAMP responsive element binding protein 1 [Source:VGNC Symbol;Acc:VGNC:96004]	2.06	0.0137

ssc-miR-140-3p	CRISPLD2	cysteine rich secretory protein LCCL domain containing 2 [Source:VGNC Symbol;Acc:VGNC:96958]	2.06	0.0137
ssc-miR-140-3p	CTBP1	C-terminal binding protein 1 [Source:VGNC Symbol;Acc:VGNC:87053]	2.06	0.0137
ssc-miR-140-3p	CXADR	CXADR Ig-like cell adhesion molecule [Source:VGNC Symbol;Acc:VGNC:87100]	2.06	0.0137
ssc-miR-140-3p	CYTH3	cytohesin 3 [Source:VGNC Symbol;Acc:VGNC:87136]	2.06	0.0137
ssc-miR-140-3p	DAAM1	dishevelled associated activator of morphosis 1 [Source:VGNC Symbol;Acc:VGNC:87141]	2.06	0.0137
ssc-miR-140-3p	DACH1	dachshund family transcription factor 1 [Source:VGNC Symbol;Acc:VGNC:87146]	2.06	0.0137
ssc-miR-140-3p	DAND5	hypothetical gene	2.06	0.0137
ssc-miR-140-3p	DAZAP2	DAZ associated protein 2 [Source:VGNC Symbol;Acc:VGNC:87162]	2.06	0.0137
ssc-miR-140-3p	DCUN1D1	hypothetical gene	2.06	0.0137
ssc-miR-140-3p	DCUN1D3	defective in cullin neddylation 1 domain containing 3 [Source:VGNC Symbol;Acc:VGNC:87196]	2.06	0.0137
ssc-miR-140-3p	DDN	dendrin [Source:VGNC Symbol;Acc:VGNC:87210]	2.06	0.0137
ssc-miR-140-3p	DDX11	DEAD/H-box helicase 11 [Source:HGNC Symbol;Acc:HGNC:2736]	2.06	0.0137
ssc-miR-140-3p	DDX5	DEAD-box helicase 5 [Source:VGNC Symbol;Acc:VGNC:87226]	2.06	0.0137
ssc-miR-140-3p	DENND6A	DENN domain containing 6A [Source:VGNC Symbol;Acc:VGNC:87256]	2.06	0.0137
ssc-miR-140-3p	DGKE	diacylglycerol kinase epsilon [Source:VGNC Symbol;Acc:VGNC:87271]	2.06	0.0137
ssc-miR-140-3p	DGKG	diacylglycerol kinase gamma [Source:VGNC Symbol;Acc:VGNC:87272]	2.06	0.0137
ssc-miR-140-3p	DIP2A	disco interacting A [Source:VGNC Symbol;Acc:VGNC:95836]	2.06	0.0137
ssc-miR-140-3p	DNAJB12	DnaJ heat shock protein family (Hsp40) member B12 [Source:VGNC Symbol;Acc:VGNC:96679]	2.06	0.0137
ssc-miR-140-3p	DNM1	dynamin 1 [Source:VGNC Symbol;Acc:VGNC:87380]	2.06	0.0137
ssc-miR-140-3p	DOCK7	dedicator of cytokinesis 7 [Source:VGNC Symbol;Acc:VGNC:87397]	2.06	0.0137
ssc-miR-140-3p	DOCK9	dedicator of cytokinesis 9 [Source:VGNC Symbol;Acc:VGNC:87399]	2.06	0.0137
ssc-miR-140-3p	DPYSL3	dihydropyrimidinase like 3 [Source:VGNC Symbol;Acc:VGNC:87431]	2.06	0.0137
ssc-miR-140-3p	DRP2	dystrophin related protein 2 [Source:VGNC Symbol;Acc:VGNC:87451]	2.06	0.0137
ssc-miR-140-3p	DTX4	deltex E3 ubiquitin ligase 4 [Source:VGNC Symbol;Acc:VGNC:87470]	2.06	0.0137
ssc-miR-140-3p	DYNLL2	dynein light chain LC8-type 2 [Source:VGNC Symbol;Acc:VGNC:87501]	2.06	0.0137
ssc-miR-140-3p	DYRK2	dual specificity tyrosine phosphorylation regulated kinase 2 [Source:VGNC Symbol;Acc:VGNC:87507]	2.06	0.0137
ssc-miR-140-3p	E2F7	E2F transcription factor 7 [Source:VGNC Symbol;Acc:VGNC:87518]	2.06	0.0137
ssc-miR-140-3p	EAF1	ELL associated factor 1 [Source:VGNC Symbol;Acc:VGNC:87521]	2.06	0.0137
ssc-miR-140-3p	EGLN3	egl-9 family hypoxia inducible factor 3 [Source:VGNC Symbol;Acc:VGNC:87589]	2.06	0.0137
ssc-miR-140-3p	EIF4EBP2	eukaryotic translation initiation factor 4E binding protein 2 [Source:VGNC Symbol;Acc:VGNC:87629]	2.06	0.0137
ssc-miR-140-3p	EIF5A2	eukaryotic translation initiation factor 5A2 [Source:VGNC Symbol;Acc:VGNC:87633]	2.06	0.0137
ssc-miR-140-3p	ELAVL2	ELAV like RNA binding protein 2 [Source:VGNC Symbol;Acc:VGNC:87639]	2.06	0.0137
ssc-miR-140-3p	ELMO2	engulfment and cell motility 2 [Source:VGNC Symbol;Acc:VGNC:95837]	2.06	0.0137
ssc-miR-140-3p	EN2	engrailed homeobox 2 [Source:VGNC Symbol;Acc:VGNC:87694]	2.06	0.0137
ssc-miR-140-3p	EPHA4	EPH receptor A4 [Source:VGNC Symbol;Acc:VGNC:96280]	2.06	0.0137
ssc-miR-140-3p	EPOR	erythropoietin receptor [Source:VGNC Symbol;Acc:VGNC:87746]	2.06	0.0137
ssc-miR-140-3p	ERLIN2	ER lipid raft associated 2 [Source:VGNC Symbol;Acc:VGNC:95586]	2.06	0.0137
ssc-miR-140-3p	FAF2	Fas associated factor family member 2 [Source:VGNC Symbol;Acc:VGNC:87880]	2.06	0.0137
ssc-miR-140-3p	FAIM	Fas apoptotic inhibitory molecule [Source:VGNC Symbol;Acc:VGNC:87882]	2.06	0.0137
ssc-miR-140-3p	FAM105B	hypothetical gene	2.06	0.0137
ssc-miR-140-3p	FAM169A	family with sequence similarity 169 member A [Source:VGNC Symbol;Acc:VGNC:87927]	2.06	0.0137
ssc-miR-140-3p	FAM179B	hypothetical gene	2.06	0.0137
ssc-miR-140-3p	FAM199X	family with sequence similarity 199, X-linked [Source:HGNC Symbol;Acc:HGNC:25195]	2.06	0.0137
ssc-miR-140-3p	FAM19A1	hypothetical gene	2.06	0.0137
ssc-miR-140-3p	FAM20C	FAM20C golgi associated secretory pathway kinase [Source:VGNC Symbol;Acc:VGNC:87951]	2.06	0.0137
ssc-miR-140-3p	FANCA	FA complementation group A [Source:VGNC Symbol;Acc:VGNC:88001]	2.06	0.0137
ssc-miR-140-3p	FAR1	fatty acyl-CoA reductase 1 [Source:VGNC Symbol;Acc:VGNC:88010]	2.06	0.0137
ssc-miR-140-3p	FARP1	FERM, ARH/RhoGEF and pleckstrin domain protein 1 [Source:VGNC Symbol;Acc:VGNC:88012]	2.06	0.0137
ssc-miR-140-3p	FAT3	hypothetical gene	2.06	0.0137
ssc-miR-140-3p	FBXL20	F-box and leucine rich repeat protein 20 [Source:VGNC Symbol;Acc:VGNC:98006]	2.06	0.0137

ssc-miR-140-3p	FBXO21	F-box protein 21 [Source:VGNC Symbol;Acc:VGNC:88035]	2.06	0.0137
ssc-miR-140-3p	FBXO33	F-box protein 33 [Source:VGNC Symbol;Acc:VGNC:88040]	2.06	0.0137
ssc-miR-140-3p	FBXO44	hypothetical gene	2.06	0.0137
ssc-miR-140-3p	FBXW7	F-box and WD repeat domain containing 7 [Source:VGNC Symbol;Acc:VGNC:98925]	2.06	0.0137
ssc-miR-140-3p	FCRLB	Fc receptor like B [Source:VGNC Symbol;Acc:VGNC:88074]	2.06	0.0137
ssc-miR-140-3p	FGF12	fibroblast growth factor 12 [Source:VGNC Symbol;Acc:VGNC:88102]	2.06	0.0137
ssc-miR-140-3p	FGF2	hypothetical gene	2.06	0.0137
ssc-miR-140-3p	FGF9	fibroblast growth factor 9 [Source:VGNC Symbol;Acc:VGNC:103943]	2.06	0.0137
ssc-miR-140-3p	FHL5	four and a half LIM domains 5 [Source:VGNC Symbol;Acc:VGNC:88130]	2.06	0.0137
ssc-miR-140-3p	FOXG1	forkhead box G1 [Source:VGNC Symbol;Acc:VGNC:88206]	2.06	0.0137
ssc-miR-140-3p	FOXP1	forkhead box P1 [Source:VGNC Symbol;Acc:VGNC:88222]	2.06	0.0137
ssc-miR-140-3p	FRRS1	ferric chelate reductase 1 [Source:VGNC Symbol;Acc:VGNC:88244]	2.06	0.0137
ssc-miR-140-3p	FUT11	fucosyltransferase 11 [Source:VGNC Symbol;Acc:VGNC:88269]	2.06	0.0137
ssc-miR-140-3p	FZD5	frizzled class receptor 5 [Source:VGNC Symbol;Acc:VGNC:96309]	2.06	0.0137
ssc-miR-140-3p	G6PC2	glucose-6-phosphatase catalytic subunit 2 [Source:VGNC Symbol;Acc:VGNC:96189]	2.06	0.0137
ssc-miR-140-3p	GAB1	GRB2 associated binding protein 1 [Source:VGNC Symbol;Acc:VGNC:88294]	2.06	0.0137
ssc-miR-140-3p	GAB2	GRB2 associated binding protein 2 [Source:VGNC Symbol;Acc:VGNC:108586]	2.06	0.0137
ssc-miR-140-3p	GABRB1	gamma-aminobutyric acid type A receptor subunit beta1 [Source:VGNC Symbol;Acc:VGNC:88306]	2.06	0.0137
ssc-miR-140-3p	GABRB2	gamma-aminobutyric acid type A receptor subunit beta2 [Source:VGNC Symbol;Acc:VGNC:88307]	2.06	0.0137
ssc-miR-140-3p	GAD2	glutamate decarboxylase 2 [Source:VGNC Symbol;Acc:VGNC:108273]	2.06	0.0137
ssc-miR-140-3p	GAN	gigaxonin [Source:VGNC Symbol;Acc:VGNC:88343]	2.06	0.0137
ssc-miR-140-3p	GDNF	glial cell derived neurotrophic factor [Source:VGNC Symbol;Acc:VGNC:88406]	2.06	0.0137
ssc-miR-140-3p	GDPD5	glycerophosphodiester phosphodiesterase domain containing 5 [Source:VGNC Symbol;Acc:VGNC:88409]	2.06	0.0137
ssc-miR-140-3p	GLG1	golgi glycoprotein 1 [Source:VGNC Symbol;Acc:VGNC:88481]	2.06	0.0137
ssc-miR-140-3p	GLYCTK	glycerate kinase [Source:VGNC Symbol;Acc:VGNC:88504]	2.06	0.0137
ssc-miR-140-3p	GMFB	glia maturation factor beta [Source:VGNC Symbol;Acc:VGNC:88509]	2.06	0.0137
ssc-miR-140-3p	GNG4	G protein subunit gamma 4 [Source:HGNC Symbol;Acc:HGNC:4407]	2.06	0.0137
ssc-miR-140-3p	GOPC	golgi associated PDZ and coiled-coil motif containing [Source:VGNC Symbol;Acc:VGNC:98024]	2.06	0.0137
ssc-miR-140-3p	GPCPD1	glycerophosphocholine phosphodiesterase 1 [Source:VGNC Symbol;Acc:VGNC:96148]	2.06	0.0137
ssc-miR-140-3p	GPN2	GPN-loop GTPase 2 [Source:HGNC Symbol;Acc:HGNC:25513]	2.06	0.0137
ssc-miR-140-3p	GPR12	G protein-coupled receptor 12 [Source:VGNC Symbol;Acc:VGNC:88598]	2.06	0.0137
ssc-miR-140-3p	GPR158	G protein-coupled receptor 158 [Source:VGNC Symbol;Acc:VGNC:96016]	2.06	0.0137
ssc-miR-140-3p	GRHL2	grainyhead like transcription factor 2 [Source:VGNC Symbol;Acc:VGNC:88667]	2.06	0.0137
ssc-miR-140-3p	GRM2	glutamate metabotropic receptor 2 [Source:VGNC Symbol;Acc:VGNC:88701]	2.06	0.0137
ssc-miR-140-3p	GSG1L	GSG1 like [Source:VGNC Symbol;Acc:VGNC:88722]	2.06	0.0137
ssc-miR-140-3p	HAS2	hyaluronan synthase 2 [Source:HGNC Symbol;Acc:HGNC:4819]	2.06	0.0137
ssc-miR-140-3p	HBP1	HMG-box transcription factor 1 [Source:VGNC Symbol;Acc:VGNC:88794]	2.06	0.0137
ssc-miR-140-3p	HCN1	hyperpolarization activated cyclic nucleotide gated potassium channel 1 [Source:VGNC Symbol;Acc:VGNC:88802]	2.06	0.0137
ssc-miR-140-3p	HDAC4	histone deacetylase 4 [Source:VGNC Symbol;Acc:VGNC:95602]	2.06	0.0137
ssc-miR-140-3p	HDC	histidine decarboxylase [Source:VGNC Symbol;Acc:VGNC:88817]	2.06	0.0137
ssc-miR-140-3p	HELLS	helicase, lymphoid specific [Source:VGNC Symbol;Acc:VGNC:88838]	2.06	0.0137
ssc-miR-140-3p	HGF	hepatocyte growth factor [Source:VGNC Symbol;Acc:VGNC:88869]	2.06	0.0137
ssc-miR-140-3p	HIC1	HIC ZBTB transcriptional repressor 1 [Source:VGNC Symbol;Acc:VGNC:88878]	2.06	0.0137
ssc-miR-140-3p	HIF1AN	hypoxia inducible factor 1 subunit alpha inhibitor [Source:VGNC Symbol;Acc:VGNC:98033]	2.06	0.0137
ssc-miR-140-3p	HIPK3	homeodomain interacting protein kinase 3 [Source:VGNC Symbol;Acc:VGNC:88889]	2.06	0.0137
ssc-miR-140-3p	HLF	HLF transcription factor, PAR bZIP family member [Source:VGNC Symbol;Acc:VGNC:88896]	2.06	0.0137
ssc-miR-140-3p	HMBOX1	homeobox containing 1 [Source:VGNC Symbol;Acc:VGNC:88898]	2.06	0.0137
ssc-miR-140-3p	HMGN3	high mobility group nucleosomal binding domain 3 [Source:VGNC Symbol;Acc:VGNC:88908]	2.06	0.0137
ssc-miR-140-3p	HMGXB4	HMG-box containing 4 [Source:VGNC Symbol;Acc:VGNC:96346]	2.06	0.0137
ssc-miR-140-3p	HNRNPA1	heterous nuclear ribonucleoprotein A1 [Source:VGNC Symbol;Acc:VGNC:88918]	2.06	0.0137

ssc-miR-140-3p	HNRNPA3	hypothetical gene	2.06	0.0137
ssc-miR-140-3p	HNRNPDL	heterogeneous nuclear ribonucleoprotein D like [Source:HGNC Symbol;Acc:HGNC:5037]	2.06	0.0137
ssc-miR-140-3p	HNRNPUL2	heteroous nuclear ribonucleoprotein U like 2 [Source:VGNC Symbol;Acc:VGNC:99758]	2.06	0.0137
ssc-miR-140-3p	HOXA9	homeobox A9 [Source:HGNC Symbol;Acc:HGNC:5109]	2.06	0.0137
ssc-miR-140-3p	HOXB5	homeobox B5 [Source:VGNC Symbol;Acc:VGNC:88945]	2.06	0.0137
ssc-miR-140-3p	HPCAL1	hippocalcin like 1 [Source:VGNC Symbol;Acc:VGNC:88958]	2.06	0.0137
ssc-miR-140-3p	HPCAL4	hippocalcin like 4 [Source:VGNC Symbol;Acc:VGNC:88959]	2.06	0.0137
ssc-miR-140-3p	HR	HR lysine demethylase and nuclear receptor corepressor [Source:VGNC Symbol;Acc:VGNC:88969]	2.06	0.0137
ssc-miR-140-3p	HS2ST1	heparan sulfate 2-O-sulfotransferase 1 [Source:HGNC Symbol;Acc:HGNC:5193]	2.06	0.0137
ssc-miR-140-3p	HS3ST1	heparan sulfate-glucosamine 3-sulfotransferase 1 [Source:VGNC Symbol;Acc:VGNC:88977]	2.06	0.0137
ssc-miR-140-3p	HS3ST5	heparan sulfate-glucosamine 3-sulfotransferase 5 [Source:VGNC Symbol;Acc:VGNC:88979]	2.06	0.0137
ssc-miR-140-3p	HS6ST2	heparan sulfate 6-O-sulfotransferase 2 [Source:HGNC Symbol;Acc:HGNC:19133]	2.06	0.0137
ssc-miR-140-3p	HSPG2	heparan sulfate proteoglycan 2 [Source:VGNC Symbol;Acc:VGNC:97099]	2.06	0.0137
ssc-miR-140-3p	IFRD1	interferon related developmental regulator 1 [Source:NCBI gene (formerly Entrezgene);Acc:493185]	2.06	0.0137
ssc-miR-140-3p	IKZF1	IKAROS family zinc finger 1 [Source:VGNC Symbol;Acc:VGNC:89073]	2.06	0.0137
ssc-miR-140-3p	IKZF3	IKAROS family zinc finger 3 [Source:VGNC Symbol;Acc:VGNC:89074]	2.06	0.0137
ssc-miR-140-3p	IL21R	interleukin 21 receptor [Source:VGNC Symbol;Acc:VGNC:89096]	2.06	0.0137
ssc-miR-140-3p	IL6ST	interleukin 6 cytokine family signal transducer [Source:VGNC Symbol;Acc:VGNC:89113]	2.06	0.0137
ssc-miR-140-3p	INO80	INO80 complex ATPase subunit [Source:VGNC Symbol;Acc:VGNC:89138]	2.06	0.0137
ssc-miR-140-3p	INO80D	INO80 complex subunit D [Source:VGNC Symbol;Acc:VGNC:96113]	2.06	0.0137
ssc-miR-140-3p	IP6K1	inositol hexakisphosphate kinase 1 [Source:VGNC Symbol;Acc:VGNC:89172]	2.06	0.0137
ssc-miR-140-3p	IQCJ-SCHIP1	hypothetical gene	2.06	0.0137
ssc-miR-140-3p	IQGAP2	IQ motif containing GTPase activating protein 2 [Source:VGNC Symbol;Acc:VGNC:89192]	2.06	0.0137
ssc-miR-140-3p	IRGQ	immunity related GTPase Q [Source:VGNC Symbol;Acc:VGNC:89213]	2.06	0.0137
ssc-miR-140-3p	ITK	IL2 inducible T cell kinase [Source:VGNC Symbol;Acc:VGNC:89249]	2.06	0.0137
ssc-miR-140-3p	JAG1	jagged canonical Notch ligand 1 [Source:VGNC Symbol;Acc:VGNC:96385]	2.06	0.0137
ssc-miR-140-3p	KANSL1	KAT8 regulatory NSL complex subunit 1 [Source:VGNC Symbol;Acc:VGNC:89299]	2.06	0.0137
ssc-miR-140-3p	KAT7	lysine acetyltransferase 7 [Source:VGNC Symbol;Acc:VGNC:89307]	2.06	0.0137
ssc-miR-140-3p	KCMF1	potassium channel modulatory factor 1 [Source:VGNC Symbol;Acc:VGNC:89322]	2.06	0.0137
ssc-miR-140-3p	KCNB1	potassium voltage-gated channel subfamily B member 1 [Source:VGNC Symbol;Acc:VGNC:96386]	2.06	0.0137
ssc-miR-140-3p	KCNH6	potassium voltage-gated channel subfamily H member 6 [Source:VGNC Symbol;Acc:VGNC:89346]	2.06	0.0137
ssc-miR-140-3p	KCNK17	potassium two pore domain channel subfamily K member 17 [Source:VGNC Symbol;Acc:VGNC:89368]	2.06	0.0137
ssc-miR-140-3p	KCTD16	potassium channel tetramerization domain containing 16 [Source:VGNC Symbol;Acc:VGNC:89395]	2.06	0.0137
ssc-miR-140-3p	KCTD6	potassium channel tetramerization domain containing 6 [Source:VGNC Symbol;Acc:VGNC:89402]	2.06	0.0137
ssc-miR-140-3p	KDM5A	lysine demethylase 5A [Source:VGNC Symbol;Acc:VGNC:89415]	2.06	0.0137
ssc-miR-140-3p	KHDRBS2	KH RNA binding domain containing, signal transduction associated 2 [Source:VGNC Symbol;Acc:VGNC:89423]	2.06	0.0137
ssc-miR-140-3p	KHDRBS3	KH RNA binding domain containing, signal transduction associated 3 [Source:VGNC Symbol;Acc:VGNC:89424]	2.06	0.0137
ssc-miR-140-3p	KIAA0141	hypothetical gene	2.06	0.0137
ssc-miR-140-3p	KIAA0232	hypothetical gene	2.06	0.0137
ssc-miR-140-3p	KIAA1147	hypothetical gene	2.06	0.0137
ssc-miR-140-3p	KIAA1468	hypothetical gene	2.06	0.0137
ssc-miR-140-3p	KIAA2022	hypothetical gene	2.06	0.0137
ssc-miR-140-3p	KIF1C	kinesin family member 1C [Source:VGNC Symbol;Acc:VGNC:89461]	2.06	0.0137
ssc-miR-140-3p	KIF2A	kinesin family member 2A [Source:VGNC Symbol;Acc:VGNC:89467]	2.06	0.0137
ssc-miR-140-3p	KIF5A	kinesin family member 5A [Source:VGNC Symbol;Acc:VGNC:89472]	2.06	0.0137
ssc-miR-140-3p	KIF6	hypothetical gene	2.06	0.0137
ssc-miR-140-3p	KLF4	Kruppel like factor 4 [Source:VGNC Symbol;Acc:VGNC:98062]	2.06	0.0137
ssc-miR-140-3p	KLF5	Kruppel like factor 5 [Source:VGNC Symbol;Acc:VGNC:89499]	2.06	0.0137
ssc-miR-140-3p	KLHDC9	kelch domain containing 9 [Source:VGNC Symbol;Acc:VGNC:89510]	2.06	0.0137
ssc-miR-140-3p	KPNA1	karyopherin subunit alpha 1 [Source:VGNC Symbol;Acc:VGNC:89560]	2.06	0.0137

ssc-miR-140-3p	KPNA4	karyopherin subunit alpha 4 [Source:VGNC Symbol;Acc:VGNC:89563]	2.06	0.0137
ssc-miR-140-3p	KPNB1	karyopherin subunit beta 1 [Source:VGNC Symbol;Acc:VGNC:89567]	2.06	0.0137
ssc-miR-140-3p	KRT2	keratin 2 [Source:VGNC Symbol;Acc:VGNC:89581]	2.06	0.0137
ssc-miR-140-3p	LARP1	La ribonucleoprotein 1, translational regulator [Source:VGNC Symbol;Acc:VGNC:98067]	2.06	0.0137
ssc-miR-140-3p	LATS2	large tumor suppressor kinase 2 [Source:VGNC Symbol;Acc:VGNC:89649]	2.06	0.0137
ssc-miR-140-3p	LDLRAD4	low density lipoprotein receptor class A domain containing 4 [Source:VGNC Symbol;Acc:VGNC:89673]	2.06	0.0137
ssc-miR-140-3p	LGSN	lengsin, lens protein with glutamine synthetase domain [Source:HGNC Symbol;Acc:HGNC:21016]	2.06	0.0137
ssc-miR-140-3p	LHFPL2	LHFPL tetraspan subfamily member 2 [Source:VGNC Symbol;Acc:VGNC:89705]	2.06	0.0137
ssc-miR-140-3p	LIF	LIF interleukin 6 family cytokine [Source:VGNC Symbol;Acc:VGNC:89719]	2.06	0.0137
ssc-miR-140-3p	LMAN2	lectin, mannose binding 2 [Source:VGNC Symbol;Acc:VGNC:89750]	2.06	0.0137
ssc-miR-140-3p	LPHN2	hypothetical gene	2.06	0.0137
ssc-miR-140-3p	LPP	LIM domain containing preferred translocation partner in lipoma [Source:HGNC Symbol;Acc:HGNC:6679]	2.06	0.0137
ssc-miR-140-3p	LRRC4C	leucine rich repeat containing 4C [Source:VGNC Symbol;Acc:VGNC:89848]	2.06	0.0137
ssc-miR-140-3p	LSM11	LSM11, U7 small nuclear RNA associated [Source:VGNC Symbol;Acc:VGNC:89871]	2.06	0.0137
ssc-miR-140-3p	LUZP1	leucine zipper protein 1 [Source:VGNC Symbol;Acc:VGNC:89897]	2.06	0.0137
ssc-miR-140-3p	LYG2	lysozyme g2 [Source:HGNC Symbol;Acc:HGNC:29615]	2.06	0.0137
ssc-miR-140-3p	MAFG	MAF bZIP transcription factor G [Source:VGNC Symbol;Acc:VGNC:89948]	2.06	0.0137
ssc-miR-140-3p	MAFK	MAF bZIP transcription factor K [Source:HGNC Symbol;Acc:HGNC:6782]	2.06	0.0137
ssc-miR-140-3p	MAGIX	MAGI family member, X-linked [Source:HGNC Symbol;Acc:HGNC:30006]	2.06	0.0137
ssc-miR-140-3p	MAP1LC3A	microtubule associated protein 1 light chain 3 alpha [Source:VGNC Symbol;Acc:VGNC:96408]	2.06	0.0137
ssc-miR-140-3p	MAP2K6	mitogen-activated protein kinase kinase 6 [Source:VGNC Symbol;Acc:VGNC:98102]	2.06	0.0137
ssc-miR-140-3p	MAPK1	mitogen-activated protein kinase 1 [Source:VGNC Symbol;Acc:VGNC:89996]	2.06	0.0137
ssc-miR-140-3p	MAPRE3	microtubule associated protein RP/EB family member 3 [Source:VGNC Symbol;Acc:VGNC:90015]	2.06	0.0137
ssc-miR-140-3p	MARCH6	hypothetical gene	2.06	0.0137
ssc-miR-140-3p	MARCH7	hypothetical gene	2.06	0.0137
ssc-miR-140-3p	MARCKS	myristoylated alanine rich protein kinase C substrate [Source:VGNC Symbol;Acc:VGNC:90024]	2.06	0.0137
ssc-miR-140-3p	MCTS1	MCTS1 re-initiation and release factor [Source:HGNC Symbol;Acc:HGNC:23357]	2.06	0.0137
ssc-miR-140-3p	MECP2	methyl-CpG binding protein 2 [Source:VGNC Symbol;Acc:VGNC:90101]	2.06	0.0137
ssc-miR-140-3p	MED12L	mediator complex subunit 12L [Source:VGNC Symbol;Acc:VGNC:90105]	2.06	0.0137
ssc-miR-140-3p	MED13	mediator complex subunit 13 [Source:VGNC Symbol;Acc:VGNC:90106]	2.06	0.0137
ssc-miR-140-3p	MEIS2	Meis homeobox 2 [Source:VGNC Symbol;Acc:VGNC:90136]	2.06	0.0137
ssc-miR-140-3p	MELK	maternal embryonic leucine zipper kinase [Source:VGNC Symbol;Acc:VGNC:90137]	2.06	0.0137
ssc-miR-140-3p	MEMO1	mediator of cell motility 1 [Source:VGNC Symbol;Acc:VGNC:90139]	2.06	0.0137
ssc-miR-140-3p	METTL21A	methyltransferase 21A, HSPA lysine [Source:VGNC Symbol;Acc:VGNC:96242]	2.06	0.0137
ssc-miR-140-3p	MGAT1	alpha-1,3-mannosyl-glycoprotein 2-beta-N-acetylglucosaminyltransferase [Source:VGNC Symbol;Acc:VGNC:96738]	2.06	0.0137
ssc-miR-140-3p	MIER3	MIER family member 3 [Source:VGNC Symbol;Acc:VGNC:90221]	2.06	0.0137
ssc-miR-140-3p	MINA	hypothetical gene	2.06	0.0137
ssc-miR-140-3p	MLLT3	MLLT3 super elongation complex subunit [Source:VGNC Symbol;Acc:VGNC:90256]	2.06	0.0137
ssc-miR-140-3p	MMP16	matrix metallopeptidase 16 [Source:VGNC Symbol;Acc:VGNC:90271]	2.06	0.0137
ssc-miR-140-3p	MMRN2	multimerin 2 [Source:VGNC Symbol;Acc:VGNC:90282]	2.06	0.0137
ssc-miR-140-3p	MOCS2	molybdenum cofactor synthesis 2 [Source:VGNC Symbol;Acc:VGNC:90295]	2.06	0.0137
ssc-miR-140-3p	MPZL1	myelin protein zero like 1 [Source:VGNC Symbol;Acc:VGNC:90335]	2.06	0.0137
ssc-miR-140-3p	MRFAP1	hypothetical gene	2.06	0.0137
ssc-miR-140-3p	MSH3	mutS homolog 3 [Source:VGNC Symbol;Acc:VGNC:90419]	2.06	0.0137
ssc-miR-140-3p	MSH6	mutS homolog 6 [Source:VGNC Symbol;Acc:VGNC:90420]	2.06	0.0137
ssc-miR-140-3p	MSI2	musashi RNA binding protein 2 [Source:VGNC Symbol;Acc:VGNC:90422]	2.06	0.0137
ssc-miR-140-3p	MTSS1	MTSS I-BAR domain containing 1 [Source:VGNC Symbol;Acc:VGNC:90474]	2.06	0.0137
ssc-miR-140-3p	MVB12B	multivesicular body subunit 12B [Source:VGNC Symbol;Acc:VGNC:90488]	2.06	0.0137
ssc-miR-140-3p	MYB	MYB proto-onco, transcription factor [Source:VGNC Symbol;Acc:VGNC:90496]	2.06	0.0137
ssc-miR-140-3p	MYLK4	hypothetical gene	2.06	0.0137

ssc-miR-140-3p	NAA50	N-alpha-acetyltransferase 50, NatE catalytic subunit [Source:VGNC Symbol;Acc:VGNC:104002]	2.06	0.0137
ssc-miR-140-3p	NCAN	neurocan [Source:VGNC Symbol;Acc:VGNC:90596]	2.06	0.0137
ssc-miR-140-3p	NCL	nucleolin [Source:VGNC Symbol;Acc:VGNC:96433]	2.06	0.0137
ssc-miR-140-3p	NDC1	NDC1 transmembrane nucleoporin [Source:VGNC Symbol;Acc:VGNC:90622]	2.06	0.0137
ssc-miR-140-3p	NDST1	N-deacetylase and N-sulfotransferase 1 [Source:VGNC Symbol;Acc:VGNC:90634]	2.06	0.0137
ssc-miR-140-3p	NETO2	neuropilin and tolloid like 2 [Source:VGNC Symbol;Acc:VGNC:90691]	2.06	0.0137
ssc-miR-140-3p	NFAT5	nuclear factor of activated T cells 5 [Source:VGNC Symbol;Acc:VGNC:90708]	2.06	0.0137
ssc-miR-140-3p	NFATC4	nuclear factor of activated T cells 4 [Source:VGNC Symbol;Acc:VGNC:90711]	2.06	0.0137
ssc-miR-140-3p	NFYA	nuclear transcription factor Y subunit alpha [Source:VGNC Symbol;Acc:VGNC:90729]	2.06	0.0137
ssc-miR-140-3p	NKAIN2	sodium/potassium transporting ATPase interacting 2 [Source:VGNC Symbol;Acc:VGNC:103141]	2.06	0.0137
ssc-miR-140-3p	NKAP	NFKB activating protein [Source:VGNC Symbol;Acc:VGNC:90756]	2.06	0.0137
ssc-miR-140-3p	NKRF	NFKB repressing factor [Source:VGNC Symbol;Acc:VGNC:90764]	2.06	0.0137
ssc-miR-140-3p	NME2	NME/NM23 nucleoside diphosphate kinase 2 [Source:NCBI gene (formerly Entrezgene);Acc:733683]	2.06	0.0137
ssc-miR-140-3p	NNAT	hypothetical gene	2.06	0.0137
ssc-miR-140-3p	NOMO1	hypothetical gene	2.06	0.0137
ssc-miR-140-3p	NOMO3	hypothetical gene	2.06	0.0137
ssc-miR-140-3p	NOVA1	NOVA alternative splicing regulator 1 [Source:VGNC Symbol;Acc:VGNC:90827]	2.06	0.0137
ssc-miR-140-3p	NR1D2	nuclear receptor subfamily 1 group D member 2 [Source:VGNC Symbol;Acc:VGNC:99726]	2.06	0.0137
ssc-miR-140-3p	NR1I2	nuclear receptor subfamily 1 group I member 2 [Source:VGNC Symbol;Acc:VGNC:90873]	2.06	0.0137
ssc-miR-140-3p	NR2F2	nuclear receptor subfamily 2 group F member 2 [Source:VGNC Symbol;Acc:VGNC:90881]	2.06	0.0137
ssc-miR-140-3p	NREP	neuronal regeneration related protein [Source:HGNC Symbol;Acc:HGNC:16834]	2.06	0.0137
ssc-miR-140-3p	NRG3	neuregulin 3 [Source:VGNC Symbol;Acc:VGNC:104011]	2.06	0.0137
ssc-miR-140-3p	NRIP1	nuclear receptor interacting protein 1 [Source:VGNC Symbol;Acc:VGNC:90896]	2.06	0.0137
ssc-miR-140-3p	NSF	N-ethylmaleimide sensitive factor, vesicle fusing ATPase [Source:VGNC Symbol;Acc:VGNC:90906]	2.06	0.0137
ssc-miR-140-3p	NSL1	NSL1 component of MIS12 kinetochore complex [Source:VGNC Symbol;Acc:VGNC:90908]	2.06	0.0137
ssc-miR-140-3p	NUP153	nucleoporin 153 [Source:VGNC Symbol;Acc:VGNC:90973]	2.06	0.0137
ssc-miR-140-3p	NUP188	nucleoporin 188 [Source:VGNC Symbol;Acc:VGNC:98165]	2.06	0.0137
ssc-miR-140-3p	NXN	nucleoredoxin [Source:VGNC Symbol;Acc:VGNC:90994]	2.06	0.0137
ssc-miR-140-3p	OGFRL1	opioid growth factor receptor like 1 [Source:VGNC Symbol;Acc:VGNC:91026]	2.06	0.0137
ssc-miR-140-3p	OLA1	Obg like ATPase 1 [Source:VGNC Symbol;Acc:VGNC:96188]	2.06	0.0137
ssc-miR-140-3p	ONECUT2	one cut homeobox 2 [Source:VGNC Symbol;Acc:VGNC:91043]	2.06	0.0137
ssc-miR-140-3p	ORC6	origin recognition complex subunit 6 [Source:VGNC Symbol;Acc:VGNC:91063]	2.06	0.0137
ssc-miR-140-3p	OTUB2	OTU deubiquitinase, ubiquitin aldehyde binding 2 [Source:VGNC Symbol;Acc:VGNC:91098]	2.06	0.0137
ssc-miR-140-3p	OTUD7B	OTU deubiquitinase 7B [Source:VGNC Symbol;Acc:VGNC:91105]	2.06	0.0137
ssc-miR-140-3p	OTX2	orthodenticle homeobox 2 [Source:VGNC Symbol;Acc:VGNC:98171]	2.06	0.0137
ssc-miR-140-3p	P2RY2	purinergic receptor P2Y2 [Source:VGNC Symbol;Acc:VGNC:98173]	2.06	0.0137
ssc-miR-140-3p	PACSIN1	protein kinase C and casein kinase substrate in neurons 1 [Source:VGNC Symbol;Acc:VGNC:91142]	2.06	0.0137
ssc-miR-140-3p	PAFAH1B2	platelet activating factor acetylhydrolase 1b catalytic subunit 2 [Source:VGNC Symbol;Acc:VGNC:91151]	2.06	0.0137
ssc-miR-140-3p	PAG1	phosphoprotein membrane anchor with glycosphingolipid microdomains 1 [Source:VGNC Symbol;Acc:VGNC:91154]	2.06	0.0137
ssc-miR-140-3p	PAK2	p21 (RAC1) activated kinase 2 [Source:HGNC Symbol;Acc:HGNC:8591]	2.06	0.0137
ssc-miR-140-3p	PANK1	pantothenate kinase 1 [Source:VGNC Symbol;Acc:VGNC:107164]	2.06	0.0137
ssc-miR-140-3p	PAPD7	hypothetical gene	2.06	0.0137
ssc-miR-140-3p	PAPSS2	3'-phosphoadenosine 5'-phosphosulfate synthase 2 [Source:VGNC Symbol;Acc:VGNC:91173]	2.06	0.0137
ssc-miR-140-3p	PARG	poly(ADP-ribose) glycohydrolase [Source:HGNC Symbol;Acc:HGNC:8605]	2.06	0.0137
ssc-miR-140-3p	PARP2	poly(ADP-ribose) polymerase 2 [Source:HGNC Symbol;Acc:HGNC:272]	2.06	0.0137
ssc-miR-140-3p	PCDH10	protocadherin 10 [Source:HGNC Symbol;Acc:HGNC:13404]	2.06	0.0137
ssc-miR-140-3p	PCDH11Y	hypothetical gene	2.06	0.0137
ssc-miR-140-3p	PCDH17	protocadherin 17 [Source:VGNC Symbol;Acc:VGNC:91214]	2.06	0.0137
ssc-miR-140-3p	PCDH7	protocadherin 7 [Source:NCBI gene (formerly Entrezgene);Acc:100520035]	2.06	0.0137
ssc-miR-140-3p	PCDH8	protocadherin 8 [Source:HGNC Symbol;Acc:HGNC:8660]	2.06	0.0137

ssc-miR-140-3p	PCGF5	hypothetical gene	2.06	0.0137
ssc-miR-140-3p	PDE3A	phosphodiesterase 3A [Source:VGNC Symbol;Acc:VGNC:91252]	2.06	0.0137
ssc-miR-140-3p	PDE3B	phosphodiesterase 3B [Source:VGNC Symbol;Acc:VGNC:91253]	2.06	0.0137
ssc-miR-140-3p	PEAK1	pseudopodium enriched atypical kinase 1 [Source:VGNC Symbol;Acc:VGNC:91300]	2.06	0.0137
ssc-miR-140-3p	PGBD5	piggyBac transposable element derived 5 [Source:VGNC Symbol;Acc:VGNC:91346]	2.06	0.0137
ssc-miR-140-3p	PHF12	PHD finger protein 12 [Source:VGNC Symbol;Acc:VGNC:91378]	2.06	0.0137
ssc-miR-140-3p	PHLDA3	pleckstrin homology like domain family A member 3 [Source:HGNC Symbol;Acc:HGNC:8934]	2.06	0.0137
ssc-miR-140-3p	PI4KB	phosphatidylinositol 4-kinase beta [Source:VGNC Symbol;Acc:VGNC:91408]	2.06	0.0137
ssc-miR-140-3p	PIK3CA	phosphatidylinositol-4,5-bisphosphate 3-kinase catalytic subunit alpha [Source:VGNC Symbol;Acc:VGNC:91440]	2.06	0.0137
ssc-miR-140-3p	PIK3R3	phosphoinositide-3-kinase regulatory subunit 3 [Source:HGNC Symbol;Acc:HGNC:8981]	2.06	0.0137
ssc-miR-140-3p	PIP4K2B	phosphatidylinositol-5-phosphate 4-kinase type 2 beta [Source:VGNC Symbol;Acc:VGNC:91454]	2.06	0.0137
ssc-miR-140-3p	PITPNM3	PITPNM family member 3 [Source:HGNC Symbol;Acc:HGNC:21043]	2.06	0.0137
ssc-miR-140-3p	PKD2	polycystin 2, transient receptor potential cation channel [Source:VGNC Symbol;Acc:VGNC:91472]	2.06	0.0137
ssc-miR-140-3p	PLEKHG3	pleckstrin homology and RhoGEF domain containing G3 [Source:VGNC Symbol;Acc:VGNC:91544]	2.06	0.0137
ssc-miR-140-3p	PLXNC1	plexin C1 [Source:VGNC Symbol;Acc:VGNC:91584]	2.06	0.0137
ssc-miR-140-3p	POMGNT1	protein O-linked mannose N-acetylglucosaminyltransferase 1 (beta 1,2-) [Source:VGNC Symbol;Acc:VGNC:98536]	2.06	0.0137
ssc-miR-140-3p	PPAP2B	hypothetical gene	2.06	0.0137
ssc-miR-140-3p	PPARGC1A	PPARG coactivator 1 alpha [Source:VGNC Symbol;Acc:VGNC:91685]	2.06	0.0137
ssc-miR-140-3p	PPIL1	peptidylprolyl isomerase like 1 [Source:VGNC Symbol;Acc:VGNC:104033]	2.06	0.0137
ssc-miR-140-3p	PPM1A	protein phosphatase, Mg2+/Mn2+ dependent 1A [Source:VGNC Symbol;Acc:VGNC:91703]	2.06	0.0137
ssc-miR-140-3p	PPP1CB	protein phosphatase 1 catalytic subunit beta [Source:NCBI gene (formerly Entrezgene);Acc:397378]	2.06	0.0137
ssc-miR-140-3p	PPP2R2C	protein phosphatase 2 regulatory subunit Bgamma [Source:VGNC Symbol;Acc:VGNC:91749]	2.06	0.0137
ssc-miR-140-3p	PRCP	prolylcarboxypeptidase [Source:VGNC Symbol;Acc:VGNC:91771]	2.06	0.0137
ssc-miR-140-3p	PRKG1	protein kinase cGMP-dependent 1 [Source:VGNC Symbol;Acc:VGNC:91816]	2.06	0.0137
ssc-miR-140-3p	PROX1	prospero homeobox 1 [Source:VGNC Symbol;Acc:VGNC:91837]	2.06	0.0137
ssc-miR-140-3p	PRSS42	hypothetical gene	2.06	0.0137
ssc-miR-140-3p	PTAR1	protein prenyltransferase alpha subunit repeat containing 1 [Source:VGNC Symbol;Acc:VGNC:91936]	2.06	0.0137
ssc-miR-140-3p	PTCH1	patched 1 [Source:VGNC Symbol;Acc:VGNC:96513]	2.06	0.0137
ssc-miR-140-3p	PTCHD1	patched domain containing 1 [Source:VGNC Symbol;Acc:VGNC:91942]	2.06	0.0137
ssc-miR-140-3p	PTEN	hypothetical gene	2.06	0.0137
ssc-miR-140-3p	PTPN1	protein tyrosine phosphatase non-receptor type 1 [Source:VGNC Symbol;Acc:VGNC:96519]	2.06	0.0137
ssc-miR-140-3p	PTPN3	protein tyrosine phosphatase non-receptor type 3 [Source:VGNC Symbol;Acc:VGNC:91980]	2.06	0.0137
ssc-miR-140-3p	PURA	purine rich element binding protein A [Source:HGNC Symbol;Acc:HGNC:9701]	2.06	0.0137
ssc-miR-140-3p	PVRL3	hypothetical gene	2.06	0.0137
ssc-miR-140-3p	PYGO1	pygopus family PHD finger 1 [Source:VGNC Symbol;Acc:VGNC:98231]	2.06	0.0137
ssc-miR-140-3p	RAB21	RAB21, member RAS oncogene family [Source:HGNC Symbol;Acc:HGNC:18263]	2.06	0.0137
ssc-miR-140-3p	RAB27B	RAB27B, member RAS onco family [Source:VGNC Symbol;Acc:VGNC:98250]	2.06	0.0137
ssc-miR-140-3p	RAB2A	RAB2A, member RAS onco family [Source:VGNC Symbol;Acc:VGNC:98252]	2.06	0.0137
ssc-miR-140-3p	RAB30	RAB30, member RAS onco family [Source:VGNC Symbol;Acc:VGNC:98254]	2.06	0.0137
ssc-miR-140-3p	RAB3B	RAB3B, member RAS onco family [Source:VGNC Symbol;Acc:VGNC:98265]	2.06	0.0137
ssc-miR-140-3p	RAB8B	RAB8B, member RAS onco family [Source:VGNC Symbol;Acc:VGNC:98276]	2.06	0.0137
ssc-miR-140-3p	RAP1B	RAP1B, member of RAS oncogene family [Source:HGNC Symbol;Acc:HGNC:9857]	2.06	0.0137
ssc-miR-140-3p	RARB	retinoic acid receptor beta [Source:HGNC Symbol;Acc:HGNC:9865]	2.06	0.0137
ssc-miR-140-3p	RASA2	RAS p21 protein activator 2 [Source:VGNC Symbol;Acc:VGNC:92102]	2.06	0.0137
ssc-miR-140-3p	RASGEF1B	RasGEF domain family member 1B [Source:VGNC Symbol;Acc:VGNC:92109]	2.06	0.0137
ssc-miR-140-3p	RASIP1	Ras interacting protein 1 [Source:VGNC Symbol;Acc:VGNC:92116]	2.06	0.0137
ssc-miR-140-3p	RASSF6	Ras association domain family member 6 [Source:VGNC Symbol;Acc:VGNC:92126]	2.06	0.0137
ssc-miR-140-3p	RBFOX2	RNA binding fox-1 homolog 2 [Source:VGNC Symbol;Acc:VGNC:92140]	2.06	0.0137
ssc-miR-140-3p	RBMS1	RNA binding motif single stranded interacting protein 1 [Source:VGNC Symbol;Acc:VGNC:96534]	2.06	0.0137
ssc-miR-140-3p	RC3H1	ring finger and CCCH-type domains 1 [Source:VGNC Symbol;Acc:VGNC:92168]	2.06	0.0137

ssc-miR-140-3p	REEP5	receptor accessory protein 5 [Source:VGNC Symbol;Acc:VGNC:92201]	2.06	0.0137
ssc-miR-140-3p	REL	REL proto-onco, NF-kB subunit [Source:VGNC Symbol;Acc:VGNC:92202]	2.06	0.0137
ssc-miR-140-3p	RELT	RELT TNF receptor [Source:VGNC Symbol;Acc:VGNC:92209]	2.06	0.0137
ssc-miR-140-3p	RFT1	RFT1 homolog [Source:HGNC Symbol;Acc:HGNC:30220]	2.06	0.0137
ssc-miR-140-3p	RFX1	regulatory factor X1 [Source:VGNC Symbol;Acc:VGNC:92243]	2.06	0.0137
ssc-miR-140-3p	RFX7	regulatory factor X7 [Source:VGNC Symbol;Acc:VGNC:92248]	2.06	0.0137
ssc-miR-140-3p	RHOA	ras homolog family member A [Source:VGNC Symbol;Acc:VGNC:92284]	2.06	0.0137
ssc-miR-140-3p	RNF115	ring finger protein 115 [Source:VGNC Symbol;Acc:VGNC:92348]	2.06	0.0137
ssc-miR-140-3p	RNF152	ring finger protein 152 [Source:VGNC Symbol;Acc:VGNC:92364]	2.06	0.0137
ssc-miR-140-3p	RNF165	ring finger protein 165 [Source:VGNC Symbol;Acc:VGNC:92366]	2.06	0.0137
ssc-miR-140-3p	RNF44	ring finger protein 44 [Source:VGNC Symbol;Acc:VGNC:92395]	2.06	0.0137
ssc-miR-140-3p	RNF7	ring finger protein 7 [Source:VGNC Symbol;Acc:VGNC:92398]	2.06	0.0137
ssc-miR-140-3p	ROBO2	roundabout guidance receptor 2 [Source:HGNC Symbol;Acc:HGNC:10250]	2.06	0.0137
ssc-miR-140-3p	RP1-170O19.20	hypothetical gene	2.06	0.0137
ssc-miR-140-3p	RP11-160N1.10	hypothetical gene	2.06	0.0137
ssc-miR-140-3p	RPL11	ribosomal protein L11 [Source:VGNC Symbol;Acc:VGNC:98603]	2.06	0.0137
ssc-miR-140-3p	RPS6KA3	ribosomal protein S6 kinase A3 [Source:VGNC Symbol;Acc:VGNC:92442]	2.06	0.0137
ssc-miR-140-3p	RRAGC	Ras related GTP binding C [Source:HGNC Symbol;Acc:HGNC:19902]	2.06	0.0137
ssc-miR-140-3p	RRAS2	hypothetical gene	2.06	0.0137
ssc-miR-140-3p	RTN3	hypothetical gene	2.06	0.0137
ssc-miR-140-3p	RUNX1T1	RUNX1 partner transcriptional co-repressor 1 [Source:VGNC Symbol;Acc:VGNC:96594]	2.06	0.0137
ssc-miR-140-3p	RUSC1	RUN and SH3 domain containing 1 [Source:VGNC Symbol;Acc:VGNC:92519]	2.06	0.0137
ssc-miR-140-3p	SAMD8	sterile alpha motif domain containing 8 [Source:VGNC Symbol;Acc:VGNC:92571]	2.06	0.0137
ssc-miR-140-3p	SAR1A	secretion associated Ras related GTPase 1A [Source:VGNC Symbol;Acc:VGNC:92579]	2.06	0.0137
ssc-miR-140-3p	SATB2	SATB homeobox 2 [Source:VGNC Symbol;Acc:VGNC:95972]	2.06	0.0137
ssc-miR-140-3p	SBSPON	somatomedin B and thrombospondin type 1 domain containing [Source:VGNC Symbol;Acc:VGNC:92599]	2.06	0.0137
ssc-miR-140-3p	SCHIP1	hypothetical gene	2.06	0.0137
ssc-miR-140-3p	SCN1A	sodium voltage-gated channel alpha subunit 1 [Source:VGNC Symbol;Acc:VGNC:95478]	2.06	0.0137
ssc-miR-140-3p	SCN3A	sodium voltage-gated channel alpha subunit 3 [Source:VGNC Symbol;Acc:VGNC:95479]	2.06	0.0137
ssc-miR-140-3p	SDC4	hypothetical gene	2.06	0.0137
ssc-miR-140-3p	SEPT4	hypothetical gene	2.06	0.0137
ssc-miR-140-3p	SFMBT1	Scm like with four mbt domains 1 [Source:VGNC Symbol;Acc:VGNC:92773]	2.06	0.0137
ssc-miR-140-3p	SGIP1	SH3GL interacting endocytic adaptor 1 [Source:VGNC Symbol;Acc:VGNC:92792]	2.06	0.0137
ssc-miR-140-3p	SH3BGRL2	SH3 domain binding glutamate rich protein like 2 [Source:VGNC Symbol;Acc:VGNC:92816]	2.06	0.0137
ssc-miR-140-3p	SIAH3	siah E3 ubiquitin protein ligase family member 3 [Source:VGNC Symbol;Acc:VGNC:92867]	2.06	0.0137
ssc-miR-140-3p	SIK2	salt inducible kinase 2 [Source:VGNC Symbol;Acc:VGNC:92872]	2.06	0.0137
ssc-miR-140-3p	SIRPA	hypothetical gene	2.06	0.0137
ssc-miR-140-3p	SIRT1	sirtuin 1 [Source:VGNC Symbol;Acc:VGNC:92884]	2.06	0.0137
ssc-miR-140-3p	SKIL	SKI like proto-onco [Source:VGNC Symbol;Acc:VGNC:92903]	2.06	0.0137
ssc-miR-140-3p	SLAIN2	SLAIN motif family member 2 [Source:VGNC Symbol;Acc:VGNC:92910]	2.06	0.0137
ssc-miR-140-3p	SLC10A4	solute carrier family 10 member 4 [Source:VGNC Symbol;Acc:VGNC:92917]	2.06	0.0137
ssc-miR-140-3p	SLC16A3	solute carrier family 16 member 3 [Source:VGNC Symbol;Acc:VGNC:92943]	2.06	0.0137
ssc-miR-140-3p	SLC25A36	solute carrier family 25 member 36 [Source:VGNC Symbol;Acc:VGNC:93012]	2.06	0.0137
ssc-miR-140-3p	SLC30A3	solute carrier family 30 member 3 [Source:VGNC Symbol;Acc:VGNC:93057]	2.06	0.0137
ssc-miR-140-3p	SLC35B2	solute carrier family 35 member B2 [Source:VGNC Symbol;Acc:VGNC:93072]	2.06	0.0137
ssc-miR-140-3p	SLC35F1	solute carrier family 35 member F1 [Source:VGNC Symbol;Acc:VGNC:93081]	2.06	0.0137
ssc-miR-140-3p	SLC5A1	solute carrier family 5 member 1 [Source:VGNC Symbol;Acc:VGNC:93140]	2.06	0.0137
ssc-miR-140-3p	SLC5A3	solute carrier family 5 member 3 [Source:VGNC Symbol;Acc:VGNC:93144]	2.06	0.0137
ssc-miR-140-3p	SLC6A9	solute carrier family 6 member 9 [Source:VGNC Symbol;Acc:VGNC:93168]	2.06	0.0137
ssc-miR-140-3p	SLC7A1	solute carrier family 7 member 1 [Source:VGNC Symbol;Acc:VGNC:93169]	2.06	0.0137

ssc-miR-140-3p	SLC8A1	solute carrier family 8 member A1 [Source:VGNC Symbol;Acc:VGNC:93178]	2.06	0.0137
ssc-miR-140-3p	SLTM	SAFB like transcription modulator [Source:VGNC Symbol;Acc:VGNC:93213]	2.06	0.0137
ssc-miR-140-3p	SMARCA2	hypothetical gene	2.06	0.0137
ssc-miR-140-3p	SMARCC1	SWI/SNF related, matrix associated, actin dependent regulator of chromatin subfamily c member 1 [Source:VGNC Symbol;Acc:VGNC:93231]	2.06	0.0137
ssc-miR-140-3p	SMARCC2	SWI/SNF related, matrix associated, actin dependent regulator of chromatin subfamily c member 2 [Source:VGNC Symbol;Acc:VGNC:93232]	2.06	0.0137
ssc-miR-140-3p	SMCR8	SMCR8-C9orf72 complex subunit [Source:VGNC Symbol;Acc:VGNC:96598]	2.06	0.0137
ssc-miR-140-3p	SMIM13	small integral membrane protein 13 [Source:HGNC Symbol;Acc:HGNC:27356]	2.06	0.0137
ssc-miR-140-3p	SMUG1	single-strand-selective monofunctional uracil-DNA glycosylase 1 [Source:VGNC Symbol;Acc:VGNC:93269]	2.06	0.0137
ssc-miR-140-3p	SMURF1	SMAD specific E3 ubiquitin protein ligase 1 [Source:VGNC Symbol;Acc:VGNC:93270]	2.06	0.0137
ssc-miR-140-3p	SNAP25	synaptosome associated protein 25 [Source:VGNC Symbol;Acc:VGNC:95509]	2.06	0.0137
ssc-miR-140-3p	SNTA1	syntrophin alpha 1 [Source:HGNC Symbol;Acc:HGNC:11167]	2.06	0.0137
ssc-miR-140-3p	SNX29	sorting nexin 29 [Source:VGNC Symbol;Acc:VGNC:98331]	2.06	0.0137
ssc-miR-140-3p	SOBP	sine oculis binding protein homolog [Source:VGNC Symbol;Acc:VGNC:93329]	2.06	0.0137
ssc-miR-140-3p	SOCS4	suppressor of cytokine signaling 4 [Source:VGNC Symbol;Acc:VGNC:93331]	2.06	0.0137
ssc-miR-140-3p	SON	SON DNA and RNA binding protein [Source:VGNC Symbol;Acc:VGNC:93336]	2.06	0.0137
ssc-miR-140-3p	SOS1	SOS Ras/Rac guanine nucleotide exchange factor 1 [Source:HGNC Symbol;Acc:HGNC:11187]	2.06	0.0137
ssc-miR-140-3p	SOX12	SRY-box transcription factor 12 [Source:HGNC Symbol;Acc:HGNC:11198]	2.06	0.0137
ssc-miR-140-3p	SOX4	SRY-box transcription factor 4 [Source:HGNC Symbol;Acc:HGNC:11200]	2.06	0.0137
ssc-miR-140-3p	SP3	Sp3 transcription factor [Source:VGNC Symbol;Acc:VGNC:95511]	2.06	0.0137
ssc-miR-140-3p	SP4	Sp4 transcription factor [Source:VGNC Symbol;Acc:VGNC:93362]	2.06	0.0137
ssc-miR-140-3p	SPPL3	signal peptide peptidase like 3 [Source:VGNC Symbol;Acc:VGNC:98340]	2.06	0.0137
ssc-miR-140-3p	SPRED2	sprouty related EVH1 domain containing 2 [Source:VGNC Symbol;Acc:VGNC:93421]	2.06	0.0137
ssc-miR-140-3p	SPRY4	sprouty RTK signaling antagonist 4 [Source:VGNC Symbol;Acc:VGNC:93427]	2.06	0.0137
ssc-miR-140-3p	SREK1	splicing regulatory glutamic acid and lysine rich protein 1 [Source:VGNC Symbol;Acc:VGNC:93451]	2.06	0.0137
ssc-miR-140-3p	SRGAP1	SLIT-ROBO Rho GTPase activating protein 1 [Source:VGNC Symbol;Acc:VGNC:93454]	2.06	0.0137
ssc-miR-140-3p	SRGAP3	SLIT-ROBO Rho GTPase activating protein 3 [Source:VGNC Symbol;Acc:VGNC:93455]	2.06	0.0137
ssc-miR-140-3p	SRPX2	sushi repeat containing protein X-linked 2 [Source:HGNC Symbol;Acc:HGNC:30668]	2.06	0.0137
ssc-miR-140-3p	SRSF10	serine and arginine rich splicing factor 10 [Source:VGNC Symbol;Acc:VGNC:93472]	2.06	0.0137
ssc-miR-140-3p	SSH3	slingshot protein phosphatase 3 [Source:VGNC Symbol;Acc:VGNC:93487]	2.06	0.0137
ssc-miR-140-3p	SSR2	signal sequence receptor subunit 2 [Source:VGNC Symbol;Acc:VGNC:93491]	2.06	0.0137
ssc-miR-140-3p	SSR3	signal sequence receptor subunit 3 [Source:VGNC Symbol;Acc:VGNC:93492]	2.06	0.0137
ssc-miR-140-3p	ST18	ST18 C2H2C-type zinc finger transcription factor [Source:VGNC Symbol;Acc:VGNC:93501]	2.06	0.0137
ssc-miR-140-3p	STAG2	stromal antigen 2 [Source:VGNC Symbol;Acc:VGNC:93525]	2.06	0.0137
ssc-miR-140-3p	STAT1	signal transducer and activator of transcription 1 [Source:VGNC Symbol;Acc:VGNC:95523]	2.06	0.0137
ssc-miR-140-3p	SUCO	SUN domain containing ossification factor [Source:VGNC Symbol;Acc:VGNC:93606]	2.06	0.0137
ssc-miR-140-3p	SUPT7L	SPT7 like, STAGA complex subunit gamma [Source:VGNC Symbol;Acc:VGNC:93627]	2.06	0.0137
ssc-miR-140-3p	SUV420H1	hypothetical gene	2.06	0.0137
ssc-miR-140-3p	SUZ12	SUZ12 polycomb repressive complex 2 subunit [Source:VGNC Symbol;Acc:VGNC:93638]	2.06	0.0137
ssc-miR-140-3p	SYN1	synapsin I [Source:NCBI gene (formerly Entrezgene);Acc:100216310]	2.06	0.0137
ssc-miR-140-3p	TAB2	TGF-beta activated kinase 1 (MAP3K7) binding protein 2 [Source:VGNC Symbol;Acc:VGNC:103186]	2.06	0.0137
ssc-miR-140-3p	TADA3	transcriptional adaptor 3 [Source:VGNC Symbol;Acc:VGNC:93708]	2.06	0.0137
ssc-miR-140-3p	TAF1D	TATA-box binding protein associated factor, RNA polymerase I subunit D [Source:VGNC Symbol;Acc:VGNC:93714]	2.06	0.0137
ssc-miR-140-3p	TAF2	TATA-box binding protein associated factor 2 [Source:VGNC Symbol;Acc:VGNC:93715]	2.06	0.0137
ssc-miR-140-3p	TBC1D25	TBC1 domain family member 25 [Source:VGNC Symbol;Acc:VGNC:93773]	2.06	0.0137
ssc-miR-140-3p	TCEB3	hypothetical gene	2.06	0.0137
ssc-miR-140-3p	TENM1	teneurin transmembrane protein 1 [Source:VGNC Symbol;Acc:VGNC:98363]	2.06	0.0137
ssc-miR-140-3p	TET2	hypothetical gene	2.06	0.0137
ssc-miR-140-3p	TET3	tet methylcytosine dioxygenase 3 [Source:VGNC Symbol;Acc:VGNC:93890]	2.06	0.0137
ssc-miR-140-3p	TGFA	transforming growth factor alpha [Source:VGNC Symbol;Acc:VGNC:93928]	2.06	0.0137
ssc-miR-140-3p	TGFB3	transforming growth factor beta 3 [Source:VGNC Symbol;Acc:VGNC:98367]	2.06	0.0137

ssc-miR-140-3p	TGIF2	TGFB induced factor homeobox 2 [Source:VGNC Symbol;Acc:VGNC:95656]	2.06	0.0137
ssc-miR-140-3p	TIFAB	TIFA inhibitor [Source:VGNC Symbol;Acc:VGNC:93979]	2.06	0.0137
ssc-miR-140-3p	TLE4	TLE family member 4, transcriptional corepressor [Source:VGNC Symbol;Acc:VGNC:98372]	2.06	0.0137
ssc-miR-140-3p	TLL2	tolloid like 2 [Source:VGNC Symbol;Acc:VGNC:94019]	2.06	0.0137
ssc-miR-140-3p	TMED8	transmembrane p24 trafficking protein family member 8 [Source:VGNC Symbol;Acc:VGNC:94058]	2.06	0.0137
ssc-miR-140-3p	TMEFF2	transmembrane protein with EGF like and two follistatin like domains 2 [Source:HGNC Symbol;Acc:HGNC:11867]	2.06	0.0137
ssc-miR-140-3p	TMEM108	transmembrane protein 108 [Source:VGNC Symbol;Acc:VGNC:94065]	2.06	0.0137
ssc-miR-140-3p	TMEM178B	transmembrane protein 178B [Source:HGNC Symbol;Acc:HGNC:44112]	2.06	0.0137
ssc-miR-140-3p	TNIK	TRAF2 and NCK interacting kinase [Source:VGNC Symbol;Acc:VGNC:98381]	2.06	0.0137
ssc-miR-140-3p	TNKS	tankyrase [Source:VGNC Symbol;Acc:VGNC:95546]	2.06	0.0137
ssc-miR-140-3p	TPRG1	tumor protein p63 regulated 1 [Source:VGNC Symbol;Acc:VGNC:94348]	2.06	0.0137
ssc-miR-140-3p	TRIM33	tripartite motif containing 33 [Source:VGNC Symbol;Acc:VGNC:98881]	2.06	0.0137
ssc-miR-140-3p	TRIM39	tripartite motif containing 39 [Source:NCBI gene (formerly Entrezgene);Acc:100151742]	2.06	0.0137
ssc-miR-140-3p	TRIM71	tripartite motif containing 71 [Source:VGNC Symbol;Acc:VGNC:94431]	2.06	0.0137
ssc-miR-140-3p	TRPM7	transient receptor potential cation channel subfamily M member 7 [Source:VGNC Symbol;Acc:VGNC:94470]	2.06	0.0137
ssc-miR-140-3p	TRPS1	transcriptional repressor GATA binding 1 [Source:VGNC Symbol;Acc:VGNC:94471]	2.06	0.0137
ssc-miR-140-3p	TSPAN3	tetraspanin 3 [Source:VGNC Symbol;Acc:VGNC:94510]	2.06	0.0137
ssc-miR-140-3p	TSTD2	thiosulfate sulfurtransferase like domain containing 2 [Source:VGNC Symbol;Acc:VGNC:94532]	2.06	0.0137
ssc-miR-140-3p	TTC1	tetratricopeptide repeat domain 1 [Source:VGNC Symbol;Acc:VGNC:104087]	2.06	0.0137
ssc-miR-140-3p	TTC39C	tetratricopeptide repeat domain 39C [Source:VGNC Symbol;Acc:VGNC:94553]	2.06	0.0137
ssc-miR-140-3p	TTN	hypothetical gene	2.06	0.0137
ssc-miR-140-3p	TTYH3	tweety family member 3 [Source:VGNC Symbol;Acc:VGNC:94576]	2.06	0.0137
ssc-miR-140-3p	TXLNG	taxilin gamma [Source:HGNC Symbol;Acc:HGNC:18578]	2.06	0.0137
ssc-miR-140-3p	TYRO3	TYRO3 protein tyrosine kinase [Source:VGNC Symbol;Acc:VGNC:94618]	2.06	0.0137
ssc-miR-140-3p	UBAP2L	ubiquitin associated protein 2 like [Source:VGNC Symbol;Acc:VGNC:94635]	2.06	0.0137
ssc-miR-140-3p	UBD	ubiquitin D [Source:NCBI gene (formerly Entrezgene);Acc:100294688]	2.06	0.0137
ssc-miR-140-3p	UBE2E3	ubiquitin conjugating enzyme E2 E3 [Source:VGNC Symbol;Acc:VGNC:106461]	2.06	0.0137
ssc-miR-140-3p	UBE2H	ubiquitin conjugating enzyme E2 H [Source:VGNC Symbol;Acc:VGNC:94643]	2.06	0.0137
ssc-miR-140-3p	UBE2Q1	ubiquitin conjugating enzyme E2 Q1 [Source:VGNC Symbol;Acc:VGNC:98888]	2.06	0.0137
ssc-miR-140-3p	UBE2QL1	ubiquitin conjugating enzyme E2 Q family like 1 [Source:VGNC Symbol;Acc:VGNC:94650]	2.06	0.0137
ssc-miR-140-3p	UBN2	ubinuclein 2 [Source:VGNC Symbol;Acc:VGNC:94664]	2.06	0.0137
ssc-miR-140-3p	UBQLN2	ubiquilin 2 [Source:VGNC Symbol;Acc:VGNC:94665]	2.06	0.0137
ssc-miR-140-3p	UBR1	ubiquitin protein ligase E3 component n-recognin 1 [Source:VGNC Symbol;Acc:VGNC:94668]	2.06	0.0137
ssc-miR-140-3p	UCK2	uridine-cytidine kinase 2 [Source:VGNC Symbol;Acc:VGNC:98399]	2.06	0.0137
ssc-miR-140-3p	UFC1	ubiquitin-fold modifier conjugating enzyme 1 [Source:VGNC Symbol;Acc:VGNC:98892]	2.06	0.0137
ssc-miR-140-3p	UNKL	unk like zinc finger [Source:VGNC Symbol;Acc:VGNC:94716]	2.06	0.0137
ssc-miR-140-3p	USP1	ubiquitin specific peptidase 1 [Source:VGNC Symbol;Acc:VGNC:94742]	2.06	0.0137
ssc-miR-140-3p	USP31	ubiquitin specific peptidase 31 [Source:HGNC Symbol;Acc:HGNC:20060]	2.06	0.0137
ssc-miR-140-3p	USP34	ubiquitin specific peptidase 34 [Source:VGNC Symbol;Acc:VGNC:94759]	2.06	0.0137
ssc-miR-140-3p	USP49	ubiquitin specific peptidase 49 [Source:VGNC Symbol;Acc:VGNC:94771]	2.06	0.0137
ssc-miR-140-3p	USP9X	ubiquitin specific peptidase 9 X-linked [Source:HGNC Symbol;Acc:HGNC:12632]	2.06	0.0137
ssc-miR-140-3p	VANGL2	VANGL planar cell polarity protein 2 [Source:VGNC Symbol;Acc:VGNC:94798]	2.06	0.0137
ssc-miR-140-3p	VCPIP1	valosin containing protein interacting protein 1 [Source:VGNC Symbol;Acc:VGNC:94813]	2.06	0.0137
ssc-miR-140-3p	VGLL2	vestigial like family member 2 [Source:VGNC Symbol;Acc:VGNC:94822]	2.06	0.0137
ssc-miR-140-3p	VKORC1L1	vitamin K epoxide reductase complex subunit 1 like 1 [Source:VGNC Symbol;Acc:VGNC:94831]	2.06	0.0137
ssc-miR-140-3p	VSX2	visual system homeobox 2 [Source:VGNC Symbol;Acc:VGNC:94876]	2.06	0.0137
ssc-miR-140-3p	VTI1A	vesicle transport through interaction with t-SNAREs 1A [Source:VGNC Symbol;Acc:VGNC:94879]	2.06	0.0137
ssc-miR-140-3p	WDFY1	WD repeat and FYVE domain containing 1 [Source:VGNC Symbol;Acc:VGNC:99733]	2.06	0.0137
ssc-miR-140-3p	WDFY2	WD repeat and FYVE domain containing 2 [Source:VGNC Symbol;Acc:VGNC:94902]	2.06	0.0137
ssc-miR-140-3p	WDR13	WD repeat domain 13 [Source:VGNC Symbol;Acc:VGNC:94907]	2.06	0.0137

ssc-miR-140-3p	WDR37	WD repeat domain 37 [Source:VGNC Symbol;Acc:VGNC:96258]	2.06	0.0137
ssc-miR-140-3p	WDR91	WD repeat domain 91 [Source:VGNC Symbol;Acc:VGNC:94949]	2.06	0.0137
ssc-miR-140-3p	WEE1	WEE1 G2 checkpoint kinase [Source:VGNC Symbol;Acc:VGNC:104095]	2.06	0.0137
ssc-miR-140-3p	WNT2B	Wnt family member 2B [Source:VGNC Symbol;Acc:VGNC:94969]	2.06	0.0137
ssc-miR-140-3p	WNT9A	Wnt family member 9A [Source:VGNC Symbol;Acc:VGNC:94978]	2.06	0.0137
ssc-miR-140-3p	XPR1	xenotropic and polytropic retrovirus receptor 1 [Source:VGNC Symbol;Acc:VGNC:108625]	2.06	0.0137
ssc-miR-140-3p	YWHAG	hypothetical gene	2.06	0.0137
ssc-miR-140-3p	YWHAZ	tyrosine 3-monooxygenase/tryptophan 5-monooxygenase activation protein zeta [Source:VGNC Symbol;Acc:VGNC:95047]	2.06	0.0137
ssc-miR-140-3p	ZBTB18	zinc finger and BTB domain containing 18 [Source:HGNC Symbol;Acc:HGNC:13030]	2.06	0.0137
ssc-miR-140-3p	ZBTB20	zinc finger and BTB domain containing 20 [Source:VGNC Symbol;Acc:VGNC:95063]	2.06	0.0137
ssc-miR-140-3p	ZBTB38	zinc finger and BTB domain containing 38 [Source:VGNC Symbol;Acc:VGNC:95072]	2.06	0.0137
ssc-miR-140-3p	ZC3H4	zinc finger CCCH-type containing 4 [Source:VGNC Symbol;Acc:VGNC:95097]	2.06	0.0137
ssc-miR-140-3p	ZC3H6	zinc finger CCCH-type containing 6 [Source:VGNC Symbol;Acc:VGNC:95098]	2.06	0.0137
ssc-miR-140-3p	ZC3H7B	zinc finger CCCH-type containing 7B [Source:VGNC Symbol;Acc:VGNC:95100]	2.06	0.0137
ssc-miR-140-3p	ZCCHC5	hypothetical gene	2.06	0.0137
ssc-miR-140-3p	ZFAND5	zinc finger AN1-type containing 5 [Source:VGNC Symbol;Acc:VGNC:95137]	2.06	0.0137
ssc-miR-140-3p	ZFPM2	zinc finger protein, FOG family member 2 [Source:VGNC Symbol;Acc:VGNC:95153]	2.06	0.0137
ssc-miR-140-3p	ZMAT4	zinc finger matrin-type 4 [Source:VGNC Symbol;Acc:VGNC:96109]	2.06	0.0137
ssc-miR-140-3p	ZMYM4	zinc finger MYM-type containing 4 [Source:VGNC Symbol;Acc:VGNC:95183]	2.06	0.0137
ssc-miR-140-3p	ZNF131	zinc finger protein 131 [Source:HGNC Symbol;Acc:HGNC:12915]	2.06	0.0137
ssc-miR-140-3p	ZNF146	zinc finger protein 146 [Source:HGNC Symbol;Acc:HGNC:12931]	2.06	0.0137
ssc-miR-140-3p	ZNF207	zinc finger protein 207 [Source:VGNC Symbol;Acc:VGNC:95205]	2.06	0.0137
ssc-miR-140-3p	ZNF226	zinc finger protein 226 [Source:HGNC Symbol;Acc:HGNC:13019]	2.06	0.0137
ssc-miR-140-3p	ZNF24	zinc finger protein 24 [Source:VGNC Symbol;Acc:VGNC:98422]	2.06	0.0137
ssc-miR-140-3p	ZNF644	zinc finger protein 644 [Source:VGNC Symbol;Acc:VGNC:95283]	2.06	0.0137
ssc-miR-140-3p	ZNF777	zinc finger protein 777 [Source:HGNC Symbol;Acc:HGNC:22213]	2.06	0.0137
ssc-miR-218b	NA	hypothetical gene	1.99	0.1005
ssc-miR-376b	AAK1	AP2 associated kinase 1 [Source:VGNC Symbol;Acc:VGNC:100379]	1.87	0.0612
ssc-miR-376b	ABCB8	ATP binding cassette subfamily B member 8 [Source:VGNC Symbol;Acc:VGNC:97856]	1.87	0.0612
ssc-miR-376b	ABCD2	ATP binding cassette subfamily D member 2 [Source:VGNC Symbol;Acc:VGNC:84962]	1.87	0.0612
ssc-miR-376b	ABI2	abl interactor 2 [Source:VGNC Symbol;Acc:VGNC:96027]	1.87	0.0612
ssc-miR-376b	ADAT2	adenosine deaminase tRNA specific 2 [Source:VGNC Symbol;Acc:VGNC:85100]	1.87	0.0612
ssc-miR-376b	AGO2	argonaute RISC catalytic component 2 [Source:VGNC Symbol;Acc:VGNC:97871]	1.87	0.0612
ssc-miR-376b	AIFM1	apoptosis inducing factor mitochondria associated 1 [Source:VGNC Symbol;Acc:VGNC:85198]	1.87	0.0612
ssc-miR-376b	AMER2	APC membrane recruitment protein 2 [Source:VGNC Symbol;Acc:VGNC:85277]	1.87	0.0612
ssc-miR-376b	ANK2	hypothetical gene	1.87	0.0612
ssc-miR-376b	ARL5B	ADP ribosylation factor like GTPase 5B [Source:VGNC Symbol;Acc:VGNC:95985]	1.87	0.0612
ssc-miR-376b	ARMC5	armadillo repeat containing 5 [Source:VGNC Symbol;Acc:VGNC:85529]	1.87	0.0612
ssc-miR-376b	ATG2A	autophagy related 2A [Source:VGNC Symbol;Acc:VGNC:85619]	1.87	0.0612
ssc-miR-376b	ATP13A3	ATPase 13A3 [Source:VGNC Symbol;Acc:VGNC:85639]	1.87	0.0612
ssc-miR-376b	ATP5G3	hypothetical gene	1.87	0.0612
ssc-miR-376b	ATP6V1A	ATPase H+ transporting V1 subunit A [Source:VGNC Symbol;Acc:VGNC:85669]	1.87	0.0612
ssc-miR-376b	ATP6V1G1	ATPase H+ transporting V1 subunit G1 [Source:NCBI gene (formerly Entrezgene);Acc:100154379]	1.87	0.0612
ssc-miR-376b	BAG4	BAG cochaperone 4 [Source:VGNC Symbol;Acc:VGNC:96503]	1.87	0.0612
ssc-miR-376b	BCL2L11	BCL2 like 11 [Source:NCBI gene (formerly Entrezgene);Acc:396632]	1.87	0.0612
ssc-miR-376b	BEND4	BEN domain containing 4 [Source:VGNC Symbol;Acc:VGNC:85801]	1.87	0.0612
ssc-miR-376b	BEND6	BEN domain containing 6 [Source:VGNC Symbol;Acc:VGNC:85802]	1.87	0.0612
ssc-miR-376b	BMPR2	bone morphotic protein receptor type 2 [Source:VGNC Symbol;Acc:VGNC:95494]	1.87	0.0612
ssc-miR-376b	BNC1	basonuclin 1 [Source:VGNC Symbol;Acc:VGNC:85851]	1.87	0.0612
ssc-miR-376b	BPTF	bromodomain PHD finger transcription factor [Source:VGNC Symbol;Acc:VGNC:97904]	1.87	0.0612

ssc-miR-376b	BRWD1	bromodomain and WD repeat domain containing 1 [Source:VGNC Symbol;Acc:VGNC:108153]	1.87	0.0612
ssc-miR-376b	BRWD3	bromodomain and WD repeat domain containing 3 [Source:VGNC Symbol;Acc:VGNC:85890]	1.87	0.0612
ssc-miR-376b	BTAF1	B-TFIID TATA-box binding protein associated factor 1 [Source:VGNC Symbol;Acc:VGNC:85899]	1.87	0.0612
ssc-miR-376b	C15orf48	chromosome 1 C15orf48 homolog [Source:VGNC Symbol;Acc:VGNC:85961]	1.87	0.0612
ssc-miR-376b	C1orf52	chromosome 4 C1orf52 homolog [Source:VGNC Symbol;Acc:VGNC:98742]	1.87	0.0612
ssc-miR-376b	C2orf43	hypothetical gene	1.87	0.0612
ssc-miR-376b	C3orf80	chromosome 13 C3orf80 homolog [Source:VGNC Symbol;Acc:VGNC:85938]	1.87	0.0612
ssc-miR-376b	C8orf44-SGK3	hypothetical gene	1.87	0.0612
ssc-miR-376b	CABIN1	calcineurin binding protein 1 [Source:VGNC Symbol;Acc:VGNC:86108]	1.87	0.0612
ssc-miR-376b	CALR	calreticulin [Source:VGNC Symbol;Acc:VGNC:86149]	1.87	0.0612
ssc-miR-376b	CAPZA1	capping actin protein of muscle Z-line subunit alpha 1 [Source:VGNC Symbol;Acc:VGNC:86183]	1.87	0.0612
ssc-miR-376b	CASP8	caspase 8 [Source:VGNC Symbol;Acc:VGNC:95639]	1.87	0.0612
ssc-miR-376b	CCBE1	collagen and calcium binding EGF domains 1 [Source:VGNC Symbol;Acc:VGNC:86240]	1.87	0.0612
ssc-miR-376b	CCDC120	coiled-coil domain containing 120 [Source:VGNC Symbol;Acc:VGNC:86249]	1.87	0.0612
ssc-miR-376b	CCDC144A	hypothetical gene	1.87	0.0612
ssc-miR-376b	CCNJ	cyclin J [Source:VGNC Symbol;Acc:VGNC:86360]	1.87	0.0612
ssc-miR-376b	CCSER1	coiled-coil serine rich protein 1 [Source:VGNC Symbol;Acc:VGNC:86378]	1.87	0.0612
ssc-miR-376b	CDC123	cell division cycle 123 [Source:VGNC Symbol;Acc:VGNC:95819]	1.87	0.0612
ssc-miR-376b	CDH6	cadherin 6 [Source:HGNC Symbol;Acc:HGNC:1765]	1.87	0.0612
ssc-miR-376b	CDK19	cyclin dependent kinase 19 [Source:VGNC Symbol;Acc:VGNC:86502]	1.87	0.0612
ssc-miR-376b	CDK5R1	cyclin dependent kinase 5 regulatory subunit 1 [Source:VGNC Symbol;Acc:VGNC:98980]	1.87	0.0612
ssc-miR-376b	CEBPB	CCAAT enhancer binding protein beta [Source:VGNC Symbol;Acc:VGNC:95883]	1.87	0.0612
ssc-miR-376b	CHD9	chromodomain helicase DNA binding protein 9 [Source:VGNC Symbol;Acc:VGNC:86635]	1.87	0.0612
ssc-miR-376b	CLASP2	cytoplasmic linker associated protein 2 [Source:VGNC Symbol;Acc:VGNC:86720]	1.87	0.0612
ssc-miR-376b	CLVS2	clavesin 2 [Source:VGNC Symbol;Acc:VGNC:86795]	1.87	0.0612
ssc-miR-376b	CNKSR3	CNKSR family member 3 [Source:VGNC Symbol;Acc:VGNC:86823]	1.87	0.0612
ssc-miR-376b	CNOT2	CCR4-NOT transcription complex subunit 2 [Source:VGNC Symbol;Acc:VGNC:86834]	1.87	0.0612
ssc-miR-376b	COPS7A	COP9 signalosome subunit 7A [Source:VGNC Symbol;Acc:VGNC:86902]	1.87	0.0612
ssc-miR-376b	CRIP3	cysteine rich protein 3 [Source:VGNC Symbol;Acc:VGNC:86996]	1.87	0.0612
ssc-miR-376b	CRISPLD1	cysteine rich secretory protein LCCL domain containing 1 [Source:VGNC Symbol;Acc:VGNC:97945]	1.87	0.0612
ssc-miR-376b	CRISPLD2	cysteine rich secretory protein LCCL domain containing 2 [Source:VGNC Symbol;Acc:VGNC:96958]	1.87	0.0612
ssc-miR-376b	CTBP2	C-terminal binding protein 2 [Source:VGNC Symbol;Acc:VGNC:87054]	1.87	0.0612
ssc-miR-376b	CTNNA3	catenin alpha 3 [Source:VGNC Symbol;Acc:VGNC:87064]	1.87	0.0612
ssc-miR-376b	CUX2	cut like homeobox 2 [Source:VGNC Symbol;Acc:VGNC:87094]	1.87	0.0612
ssc-miR-376b	DCAF5	DDB1 and CUL4 associated factor 5 [Source:VGNC Symbol;Acc:VGNC:87176]	1.87	0.0612
ssc-miR-376b	DCAF8	DDB1 and CUL4 associated factor 8 [Source:NCBI gene (formerly Entrezgene);Acc:100153655]	1.87	0.0612
ssc-miR-376b	DCP1A	decapping mRNA 1A [Source:VGNC Symbol;Acc:VGNC:97960]	1.87	0.0612
ssc-miR-376b	DDB2	damage specific DNA binding protein 2 [Source:VGNC Symbol;Acc:VGNC:87203]	1.87	0.0612
ssc-miR-376b	DDHD1	DDHD domain containing 1 [Source:VGNC Symbol;Acc:VGNC:87205]	1.87	0.0612
ssc-miR-376b	DGKI	diacylglycerol kinase iota [Source:VGNC Symbol;Acc:VGNC:87274]	1.87	0.0612
ssc-miR-376b	DKK3	dickkopf WNT signaling pathway inhibitor 3 [Source:VGNC Symbol;Acc:VGNC:87323]	1.87	0.0612
ssc-miR-376b	DLAT	dihydrolipoamide S-acetyltransferase [Source:VGNC Symbol;Acc:VGNC:87325]	1.87	0.0612
ssc-miR-376b	DLX5	distal-less homeobox 5 [Source:VGNC Symbol;Acc:VGNC:87341]	1.87	0.0612
ssc-miR-376b	DOK1	docking protein 1 [Source:VGNC Symbol;Acc:VGNC:87401]	1.87	0.0612
ssc-miR-376b	DSC1	desmocollin 1 [Source:VGNC Symbol;Acc:VGNC:87452]	1.87	0.0612
ssc-miR-376b	DSC3	desmocollin 3 [Source:HGNC Symbol;Acc:HGNC:3037]	1.87	0.0612
ssc-miR-376b	DSCAML1	DS cell adhesion molecule like 1 [Source:VGNC Symbol;Acc:VGNC:97969]	1.87	0.0612
ssc-miR-376b	DUSP18	dual specificity phosphatase 18 [Source:VGNC Symbol;Acc:VGNC:103937]	1.87	0.0612
ssc-miR-376b	DYRK2	dual specificity tyrosine phosphorylation regulated kinase 2 [Source:VGNC Symbol;Acc:VGNC:87507]	1.87	0.0612
ssc-miR-376b	EBF2	EBF transcription factor 2 [Source:VGNC Symbol;Acc:VGNC:87526]	1.87	0.0612

ssc-miR-376b	EID1	EP300 interacting inhibitor of differentiation 1 [Source:HGNC Symbol;Acc:HGNC:1191]	1.87	0.0612
ssc-miR-376b	EIF4B	eukaryotic translation initiation factor 4B [Source:HGNC Symbol;Acc:HGNC:3285]	1.87	0.0612
ssc-miR-376b	ELF1	E74 like ETS transcription factor 1 [Source:VGNC Symbol;Acc:VGNC:87641]	1.87	0.0612
ssc-miR-376b	ELOVL7	ELOVL fatty acid elongase 7 [Source:HGNC Symbol;Acc:HGNC:26292]	1.87	0.0612
ssc-miR-376b	ELP5	elongator acetyltransferase complex subunit 5 [Source:VGNC Symbol;Acc:VGNC:87666]	1.87	0.0612
ssc-miR-376b	EML1	EMAP like 1 [Source:VGNC Symbol;Acc:VGNC:87682]	1.87	0.0612
ssc-miR-376b	EN2	engrailed homeobox 2 [Source:VGNC Symbol;Acc:VGNC:87694]	1.87	0.0612
ssc-miR-376b	ENAH	ENAH actin regulator [Source:VGNC Symbol;Acc:VGNC:108271]	1.87	0.0612
ssc-miR-376b	ENPEP	glutamyl aminopeptidase [Source:VGNC Symbol;Acc:VGNC:98923]	1.87	0.0612
ssc-miR-376b	ENTPD1	ectonucleoside triphosphate diphosphohydrolase 1 [Source:VGNC Symbol;Acc:VGNC:97977]	1.87	0.0612
ssc-miR-376b	EPT1	hypothetical gene	1.87	0.0612
ssc-miR-376b	ERBB4	erb-b2 receptor tyrosine kinase 4 [Source:VGNC Symbol;Acc:VGNC:96284]	1.87	0.0612
ssc-miR-376b	ERCC8	ERCC excision repair 8, CSA ubiquitin ligase complex subunit [Source:VGNC Symbol;Acc:VGNC:87765]	1.87	0.0612
ssc-miR-376b	EVA1C	eva-1 homolog C [Source:VGNC Symbol;Acc:VGNC:87817]	1.87	0.0612
ssc-miR-376b	EVI5	ecotropic viral integration site 5 [Source:VGNC Symbol;Acc:VGNC:98793]	1.87	0.0612
ssc-miR-376b	EXOSC10	exosome component 10 [Source:VGNC Symbol;Acc:VGNC:87837]	1.87	0.0612
ssc-miR-376b	EZR	ezrin [Source:VGNC Symbol;Acc:VGNC:87856]	1.87	0.0612
ssc-miR-376b	FAM126B	family with sequence similarity 126 member B [Source:HGNC Symbol;Acc:HGNC:28593]	1.87	0.0612
ssc-miR-376b	FAM131B	family with sequence similarity 131 member B [Source:VGNC Symbol;Acc:VGNC:87901]	1.87	0.0612
ssc-miR-376b	FAM175B	hypothetical gene	1.87	0.0612
ssc-miR-376b	FAM63B	hypothetical gene	1.87	0.0612
ssc-miR-376b	FANCB	FA complementation group B [Source:VGNC Symbol;Acc:VGNC:88002]	1.87	0.0612
ssc-miR-376b	FBXO11	F-box protein 11 [Source:VGNC Symbol;Acc:VGNC:88032]	1.87	0.0612
ssc-miR-376b	FERMT2	FERM domain containing kindlin 2 [Source:VGNC Symbol;Acc:VGNC:88088]	1.87	0.0612
ssc-miR-376b	FGFR1	fibroblast growth factor receptor 1 [Source:HGNC Symbol;Acc:HGNC:3688]	1.87	0.0612
ssc-miR-376b	FIBIN	fin bud initiation factor homolog [Source:VGNC Symbol;Acc:VGNC:99750]	1.87	0.0612
ssc-miR-376b	FLG2	hypothetical gene	1.87	0.0612
ssc-miR-376b	FOXJ3	forkhead box J3 [Source:VGNC Symbol;Acc:VGNC:88213]	1.87	0.0612
ssc-miR-376b	FTO	FTO alpha-ketoglutarate dependent dioxygenase [Source:VGNC Symbol;Acc:VGNC:88259]	1.87	0.0612
ssc-miR-376b	GAB1	GRB2 associated binding protein 1 [Source:VGNC Symbol;Acc:VGNC:88294]	1.87	0.0612
ssc-miR-376b	GAL3ST4	galactose-3-O-sulfotransferase 4 [Source:VGNC Symbol;Acc:VGNC:88323]	1.87	0.0612
ssc-miR-376b	GALNTL6	polypeptide N-acetylgalactosaminyltransferase like 6 [Source:VGNC Symbol;Acc:VGNC:103947]	1.87	0.0612
ssc-miR-376b	GLIPR1	GLI pathosis related 1 [Source:VGNC Symbol;Acc:VGNC:98020]	1.87	0.0612
ssc-miR-376b	GLRA3	glycine receptor alpha 3 [Source:VGNC Symbol;Acc:VGNC:88493]	1.87	0.0612
ssc-miR-376b	GNB4	G protein subunit beta 4 [Source:VGNC Symbol;Acc:VGNC:88533]	1.87	0.0612
ssc-miR-376b	GPC4	glypican 4 [Source:VGNC Symbol;Acc:VGNC:88581]	1.87	0.0612
ssc-miR-376b	GPC6	glypican 6 [Source:HGNC Symbol;Acc:HGNC:4454]	1.87	0.0612
ssc-miR-376b	GPD1L	glycerol-3-phosphate dehydrogenase 1 like [Source:VGNC Symbol;Acc:VGNC:108661]	1.87	0.0612
ssc-miR-376b	GPR180	G protein-coupled receptor 180 [Source:VGNC Symbol;Acc:VGNC:88621]	1.87	0.0612
ssc-miR-376b	GPR22	G protein-coupled receptor 22 [Source:VGNC Symbol;Acc:VGNC:88625]	1.87	0.0612
ssc-miR-376b	GPX8	glutathione peroxidase 8 (putative) [Source:HGNC Symbol;Acc:HGNC:33100]	1.87	0.0612
ssc-miR-376b	GRAMD3	hypothetical gene	1.87	0.0612
ssc-miR-376b	GRIK2	glutamate ionotropic receptor kainate type subunit 2 [Source:VGNC Symbol;Acc:VGNC:88678]	1.87	0.0612
ssc-miR-376b	GRIK3	glutamate ionotropic receptor kainate type subunit 3 [Source:VGNC Symbol;Acc:VGNC:88679]	1.87	0.0612
ssc-miR-376b	GRIN2A	glutamate ionotropic receptor NMDA type subunit 2A [Source:VGNC Symbol;Acc:VGNC:88683]	1.87	0.0612
ssc-miR-376b	GRIN3A	glutamate ionotropic receptor NMDA type subunit 3A [Source:VGNC Symbol;Acc:VGNC:88687]	1.87	0.0612
ssc-miR-376b	GRM5	glutamate metabotropic receptor 5 [Source:VGNC Symbol;Acc:VGNC:88703]	1.87	0.0612
ssc-miR-376b	HAS2	hyaluronan synthase 2 [Source:HGNC Symbol;Acc:HGNC:4819]	1.87	0.0612
ssc-miR-376b	HAT1	histone acetyltransferase 1 [Source:VGNC Symbol;Acc:VGNC:96340]	1.87	0.0612
ssc-miR-376b	HBEGF	heparin binding EGF like growth factor [Source:VGNC Symbol;Acc:VGNC:88792]	1.87	0.0612

ssc-miR-376b	HCN1	hyperpolarization activated cyclic nucleotide gated potassium channel 1 [Source:VGNC Symbol;Acc:VGNC:88802]	1.87	0.0612
ssc-miR-376b	HDAC9	histone deacetylase 9 [Source:HGNC Symbol;Acc:HGNC:14065]	1.87	0.0612
ssc-miR-376b	HDDC2	HD domain containing 2 [Source:VGNC Symbol;Acc:VGNC:103105]	1.87	0.0612
ssc-miR-376b	HELZ	helicase with zinc finger [Source:VGNC Symbol;Acc:VGNC:88840]	1.87	0.0612
ssc-miR-376b	HERPUD1	homocysteine inducible ER protein with ubiquitin like domain 1 [Source:VGNC Symbol;Acc:VGNC:88852]	1.87	0.0612
ssc-miR-376b	HES5	hes family bHLH transcription factor 5 [Source:VGNC Symbol;Acc:VGNC:88858]	1.87	0.0612
ssc-miR-376b	HIF1A	hypoxia inducible factor 1 subunit alpha [Source:VGNC Symbol;Acc:VGNC:88880]	1.87	0.0612
ssc-miR-376b	HIPK2	homeodomain interacting protein kinase 2 [Source:VGNC Symbol;Acc:VGNC:88888]	1.87	0.0612
ssc-miR-376b	HK2	hexokinase 2 [Source:VGNC Symbol;Acc:VGNC:98034]	1.87	0.0612
ssc-miR-376b	HNRNPA0	hypothetical gene	1.87	0.0612
ssc-miR-376b	HOXC8	homeobox C8 [Source:VGNC Symbol;Acc:VGNC:88954]	1.87	0.0612
ssc-miR-376b	HUNK	hormonally up-regulated Neu-associated kinase [Source:VGNC Symbol;Acc:VGNC:89007]	1.87	0.0612
ssc-miR-376b	IGF1R	insulin like growth factor 1 receptor [Source:NCBI gene (formerly Entrezgene);Acc:397350]	1.87	0.0612
ssc-miR-376b	IGSF3	immunoglobulin superfamily member 3 [Source:VGNC Symbol;Acc:VGNC:89067]	1.87	0.0612
ssc-miR-376b	IL1RAPL1	interleukin 1 receptor accessory protein like 1 [Source:VGNC Symbol;Acc:VGNC:103967]	1.87	0.0612
ssc-miR-376b	IMPG1	interphotoreceptor matrix proteoglycan 1 [Source:VGNC Symbol;Acc:VGNC:89127]	1.87	0.0612
ssc-miR-376b	INSIG2	insulin induced 2 [Source:VGNC Symbol;Acc:VGNC:103970]	1.87	0.0612
ssc-miR-376b	IP6K1	inositol hexakisphosphate kinase 1 [Source:VGNC Symbol;Acc:VGNC:89172]	1.87	0.0612
ssc-miR-376b	IPMK	inositol polyphosphate multikinase [Source:VGNC Symbol;Acc:VGNC:89175]	1.87	0.0612
ssc-miR-376b	IPPK	inositol-pentakisphosphate 2-kinase [Source:VGNC Symbol;Acc:VGNC:89183]	1.87	0.0612
ssc-miR-376b	ITGB8	integrin subunit beta 8 [Source:VGNC Symbol;Acc:VGNC:89246]	1.87	0.0612
ssc-miR-376b	ITPRIP	inositol 1,4,5-trisphosphate receptor interacting protein [Source:VGNC Symbol;Acc:VGNC:89256]	1.87	0.0612
ssc-miR-376b	JAKMIP2	janus kinase and microtubule interacting protein 2 [Source:VGNC Symbol;Acc:VGNC:89274]	1.87	0.0612
ssc-miR-376b	KATNBL1	katanin regulatory subunit B1 like 1 [Source:VGNC Symbol;Acc:VGNC:89313]	1.87	0.0612
ssc-miR-376b	KCTD20	potassium channel tetramerization domain containing 20 [Source:VGNC Symbol;Acc:VGNC:89399]	1.87	0.0612
ssc-miR-376b	KHDRBS3	KH RNA binding domain containing, signal transduction associated 3 [Source:VGNC Symbol;Acc:VGNC:89424]	1.87	0.0612
ssc-miR-376b	KIAA0825	KIAA0825 [Source:VGNC Symbol;Acc:VGNC:99781]	1.87	0.0612
ssc-miR-376b	KIAA1462	hypothetical gene	1.87	0.0612
ssc-miR-376b	KIF24	kinesin family member 24 [Source:VGNC Symbol;Acc:VGNC:95928]	1.87	0.0612
ssc-miR-376b	KLF12	Kruppel like factor 12 [Source:VGNC Symbol;Acc:VGNC:89492]	1.87	0.0612
ssc-miR-376b	KLF15	Kruppel like factor 15 [Source:VGNC Symbol;Acc:VGNC:89495]	1.87	0.0612
ssc-miR-376b	KLHDC9	kelch domain containing 9 [Source:VGNC Symbol;Acc:VGNC:89510]	1.87	0.0612
ssc-miR-376b	KLHL36	kelch like family member 36 [Source:VGNC Symbol;Acc:VGNC:89530]	1.87	0.0612
ssc-miR-376b	KPNA4	karyopherin subunit alpha 4 [Source:VGNC Symbol;Acc:VGNC:89563]	1.87	0.0612
ssc-miR-376b	KPNB1	karyopherin subunit beta 1 [Source:VGNC Symbol;Acc:VGNC:89567]	1.87	0.0612
ssc-miR-376b	LAMC1	laminin subunit gamma 1 [Source:VGNC Symbol;Acc:VGNC:89624]	1.87	0.0612
ssc-miR-376b	LAYN	layilin [Source:VGNC Symbol;Acc:VGNC:89651]	1.87	0.0612
ssc-miR-376b	LIN9	lin-9 DREAM MuvB core complex component [Source:VGNC Symbol;Acc:VGNC:103976]	1.87	0.0612
ssc-miR-376b	LMBR1	limb development membrane protein 1 [Source:VGNC Symbol;Acc:VGNC:89752]	1.87	0.0612
ssc-miR-376b	LNPEP	leucyl and cystinyl aminopeptidase [Source:VGNC Symbol;Acc:VGNC:89772]	1.87	0.0612
ssc-miR-376b	LRIG2	leucine rich repeats and immunoglobulin like domains 2 [Source:VGNC Symbol;Acc:VGNC:89811]	1.87	0.0612
ssc-miR-376b	LRP12	LDL receptor related protein 12 [Source:VGNC Symbol;Acc:VGNC:89818]	1.87	0.0612
ssc-miR-376b	MACROD2	mono-ADP ribosylhydrolase 2 [Source:VGNC Symbol;Acc:VGNC:108725]	1.87	0.0612
ssc-miR-376b	MAN1C1	mannosidase alpha class 1C member 1 [Source:VGNC Symbol;Acc:VGNC:89968]	1.87	0.0612
ssc-miR-376b	MAP2K6	mitogen-activated protein kinase kinase 6 [Source:VGNC Symbol;Acc:VGNC:98102]	1.87	0.0612
ssc-miR-376b	MARCH11	hypothetical gene	1.87	0.0612
ssc-miR-376b	MBD5	methyl-CpG binding domain protein 5 [Source:VGNC Symbol;Acc:VGNC:95940]	1.87	0.0612
ssc-miR-376b	MED1	mediator complex subunit 1 [Source:VGNC Symbol;Acc:VGNC:90102]	1.87	0.0612
ssc-miR-376b	MELK	maternal embryonic leucine zipper kinase [Source:VGNC Symbol;Acc:VGNC:90137]	1.87	0.0612
ssc-miR-376b	METTL21A	methyltransferase 21A, HSPA lysine [Source:VGNC Symbol;Acc:VGNC:96242]	1.87	0.0612

ssc-miR-376b	MFSD6	major facilitator superfamily domain containing 6 [Source:VGNC Symbol;Acc:VGNC:96052]	1.87	0.0612
ssc-miR-376b	MGA	MAX dimerization protein MGA [Source:VGNC Symbol;Acc:VGNC:90195]	1.87	0.0612
ssc-miR-376b	MPP5	hypothetical gene	1.87	0.0612
ssc-miR-376b	MR1	major histocompatibility complex, class I-related [Source:VGNC Symbol;Acc:VGNC:90338]	1.87	0.0612
ssc-miR-376b	MRPS25	mitochondrial ribosomal protein S25 [Source:VGNC Symbol;Acc:VGNC:90391]	1.87	0.0612
ssc-miR-376b	MT1A	metallothionein 1A [Source:NCBI gene (formerly Entrezgene);Acc:397417]	1.87	0.0612
ssc-miR-376b	MUC19	hypothetical gene	1.87	0.0612
ssc-miR-376b	MYH9	myosin heavy chain 9 [Source:VGNC Symbol;Acc:VGNC:90512]	1.87	0.0612
ssc-miR-376b	MYLK	myosin light chain kinase [Source:VGNC Symbol;Acc:VGNC:108676]	1.87	0.0612
ssc-miR-376b	NAA50	N-alpha-acetyltransferase 50, NatE catalytic subunit [Source:VGNC Symbol;Acc:VGNC:104002]	1.87	0.0612
ssc-miR-376b	NABP1	nucleic acid binding protein 1 [Source:VGNC Symbol;Acc:VGNC:96122]	1.87	0.0612
ssc-miR-376b	NDC1	NDC1 transmembrane nucleoporin [Source:VGNC Symbol;Acc:VGNC:90622]	1.87	0.0612
ssc-miR-376b	NDNF	neuron derived neurotrophic factor [Source:VGNC Symbol;Acc:VGNC:90629]	1.87	0.0612
ssc-miR-376b	NDST1	N-deacetylase and N-sulfotransferase 1 [Source:VGNC Symbol;Acc:VGNC:90634]	1.87	0.0612
ssc-miR-376b	NEK10	NIMA related kinase 10 [Source:VGNC Symbol;Acc:VGNC:90674]	1.87	0.0612
ssc-miR-376b	NFKBIZ	NFKB inhibitor zeta [Source:VGNC Symbol;Acc:VGNC:90726]	1.87	0.0612
ssc-miR-376b	NIPAL1	NIPA like domain containing 1 [Source:VGNC Symbol;Acc:VGNC:90748]	1.87	0.0612
ssc-miR-376b	NME2	NME/NM23 nucleoside diphosphate kinase 2 [Source:NCBI gene (formerly Entrezgene);Acc:733683]	1.87	0.0612
ssc-miR-376b	NPNT	nephronectin [Source:VGNC Symbol;Acc:VGNC:90855]	1.87	0.0612
ssc-miR-376b	NRP1	neuropilin 1 [Source:VGNC Symbol;Acc:VGNC:104012]	1.87	0.0612
ssc-miR-376b	NUFIP2	nuclear FMR1 interacting protein 2 [Source:VGNC Symbol;Acc:VGNC:90967]	1.87	0.0612
ssc-miR-376b	OPN5	opsin 5 [Source:VGNC Symbol;Acc:VGNC:91052]	1.87	0.0612
ssc-miR-376b	OTUD3	OTU deubiquitinase 3 [Source:VGNC Symbol;Acc:VGNC:91099]	1.87	0.0612
ssc-miR-376b	PARD6B	par-6 family cell polarity regulator beta [Source:VGNC Symbol;Acc:VGNC:98176]	1.87	0.0612
ssc-miR-376b	PAX6	paired box 6 [Source:VGNC Symbol;Acc:VGNC:91195]	1.87	0.0612
ssc-miR-376b	PCDH12	protocadherin 12 [Source:VGNC Symbol;Acc:VGNC:91212]	1.87	0.0612
ssc-miR-376b	PCDH17	protocadherin 17 [Source:VGNC Symbol;Acc:VGNC:91214]	1.87	0.0612
ssc-miR-376b	PCDH18	protocadherin 18 [Source:VGNC Symbol;Acc:VGNC:91215]	1.87	0.0612
ssc-miR-376b	PDE8A	phosphodiesterase 8A [Source:VGNC Symbol;Acc:VGNC:91263]	1.87	0.0612
ssc-miR-376b	PDIA6	protein disulfide isomerase family A member 6 [Source:VGNC Symbol;Acc:VGNC:91275]	1.87	0.0612
ssc-miR-376b	PDS5A	PDS5 cohesin associated factor A [Source:HGNC Symbol;Acc:HGNC:29088]	1.87	0.0612
ssc-miR-376b	PEX12	peroxisomal biosis factor 12 [Source:VGNC Symbol;Acc:VGNC:91323]	1.87	0.0612
ssc-miR-376b	PEX5L	peroxisomal biosis factor 5 like [Source:VGNC Symbol;Acc:VGNC:91329]	1.87	0.0612
ssc-miR-376b	PGAM5	PGAM family member 5, mitochondrial serine/threonine protein phosphatase [Source:HGNC Symbol;Acc:HGNC:28763]	1.87	0.0612
ssc-miR-376b	PGM2L1	phosphoglucomutase 2 like 1 [Source:VGNC Symbol;Acc:VGNC:91357]	1.87	0.0612
ssc-miR-376b	PGR	progesterone receptor [Source:VGNC Symbol;Acc:VGNC:91362]	1.87	0.0612
ssc-miR-376b	PIGG	phosphatidylinositol glycan anchor biosynthesis class G [Source:VGNC Symbol;Acc:VGNC:91422]	1.87	0.0612
ssc-miR-376b	PIK3CB	phosphatidylinositol-4,5-bisphosphate 3-kinase catalytic subunit beta [Source:VGNC Symbol;Acc:VGNC:91441]	1.87	0.0612
ssc-miR-376b	PIK3R1	phosphoinositide-3-kinase regulatory subunit 1 [Source:VGNC Symbol;Acc:VGNC:91445]	1.87	0.0612
ssc-miR-376b	PITPNM2	phosphatidylinositol transfer protein membrane associated 2 [Source:VGNC Symbol;Acc:VGNC:91466]	1.87	0.0612
ssc-miR-376b	PKNOX1	PBX/knotted 1 homeobox 1 [Source:VGNC Symbol;Acc:VGNC:91484]	1.87	0.0612
ssc-miR-376b	PLAG1	PLAG1 zinc finger [Source:VGNC Symbol;Acc:VGNC:91509]	1.87	0.0612
ssc-miR-376b	PLEKHA5	pleckstrin homology domain containing A5 [Source:VGNC Symbol;Acc:VGNC:91535]	1.87	0.0612
ssc-miR-376b	POLK	DNA polymerase kappa [Source:VGNC Symbol;Acc:VGNC:91637]	1.87	0.0612
ssc-miR-376b	PON2	paraoxonase 2 [Source:VGNC Symbol;Acc:VGNC:91662]	1.87	0.0612
ssc-miR-376b	POP1	POP1 homolog, ribonuclease P/MRP subunit [Source:VGNC Symbol;Acc:VGNC:91663]	1.87	0.0612
ssc-miR-376b	PPP1CC	protein phosphatase 1 catalytic subunit gamma [Source:VGNC Symbol;Acc:VGNC:91716]	1.87	0.0612
ssc-miR-376b	PPP1R10	protein phosphatase 1 regulatory subunit 10 [Source:VGNC Symbol;Acc:VGNC:91717]	1.87	0.0612
ssc-miR-376b	PPP1R3F	protein phosphatase 1 regulatory subunit 3F [Source:VGNC Symbol;Acc:VGNC:91739]	1.87	0.0612
ssc-miR-376b	PRKACB	protein kinase cAMP-activated catalytic subunit beta [Source:VGNC Symbol;Acc:VGNC:91800]	1.87	0.0612

ssc-miR-376b	PRKCE	protein kinase C epsilon [Source:VGNC Symbol;Acc:VGNC:91807]	1.87	0.0612
ssc-miR-376b	PRKD1	protein kinase D1 [Source:VGNC Symbol;Acc:VGNC:91812]	1.87	0.0612
ssc-miR-376b	PTBP3	polypyrimidine tract binding protein 3 [Source:VGNC Symbol;Acc:VGNC:91938]	1.87	0.0612
ssc-miR-376b	PTHLH	parathyroid hormone like hormone [Source:VGNC Symbol;Acc:VGNC:91963]	1.87	0.0612
ssc-miR-376b	PTMA	prothymosin alpha [Source:VGNC Symbol;Acc:VGNC:106455]	1.87	0.0612
ssc-miR-376b	PYGO1	pygopus family PHD finger 1 [Source:VGNC Symbol;Acc:VGNC:98231]	1.87	0.0612
ssc-miR-376b	RAB15	RAB15, member RAS onco family [Source:VGNC Symbol;Acc:VGNC:98239]	1.87	0.0612
ssc-miR-376b	RAD54L2	RAD54 like 2 [Source:VGNC Symbol;Acc:VGNC:92061]	1.87	0.0612
ssc-miR-376b	RAPGEFL1	Rap guanine nucleotide exchange factor like 1 [Source:VGNC Symbol;Acc:VGNC:92095]	1.87	0.0612
ssc-miR-376b	RASA1	RAS p21 protein activator 1 [Source:VGNC Symbol;Acc:VGNC:92101]	1.87	0.0612
ssc-miR-376b	RBM18	RNA binding motif protein 18 [Source:VGNC Symbol;Acc:VGNC:92149]	1.87	0.0612
ssc-miR-376b	RBM41	RNA binding motif protein 41 [Source:VGNC Symbol;Acc:VGNC:92155]	1.87	0.0612
ssc-miR-376b	RBM7	RNA binding motif protein 7 [Source:VGNC Symbol;Acc:VGNC:108612]	1.87	0.0612
ssc-miR-376b	RBMS1	RNA binding motif single stranded interacting protein 1 [Source:VGNC Symbol;Acc:VGNC:96534]	1.87	0.0612
ssc-miR-376b	RHBDL2	rhomboid like 2 [Source:VGNC Symbol;Acc:VGNC:92278]	1.87	0.0612
ssc-miR-376b	RIPK4	receptor interacting serine/threonine kinase 4 [Source:VGNC Symbol;Acc:VGNC:92321]	1.87	0.0612
ssc-miR-376b	RNF20	ring finger protein 20 [Source:VGNC Symbol;Acc:VGNC:92378]	1.87	0.0612
ssc-miR-376b	RP11-766F14.2	hypothetical gene	1.87	0.0612
ssc-miR-376b	RPL5	ribosomal protein L5 [Source:NCBI gene (formerly Entrezgene);Acc:100135671]	1.87	0.0612
ssc-miR-376b	RRAGD	Ras related GTP binding D [Source:VGNC Symbol;Acc:VGNC:92457]	1.87	0.0612
ssc-miR-376b	RUVBL1	RuvB like AAA ATPase 1 [Source:VGNC Symbol;Acc:VGNC:92521]	1.87	0.0612
ssc-miR-376b	RYBP	RING1 and YY1 binding protein [Source:VGNC Symbol;Acc:VGNC:92532]	1.87	0.0612
ssc-miR-376b	SATB1	SATB homeobox 1 [Source:VGNC Symbol;Acc:VGNC:92587]	1.87	0.0612
ssc-miR-376b	SCOC	short coiled-coil protein [Source:VGNC Symbol;Acc:VGNC:98960]	1.87	0.0612
ssc-miR-376b	SEC22A	SEC22 homolog A, vesicle trafficking protein [Source:VGNC Symbol;Acc:VGNC:92673]	1.87	0.0612
ssc-miR-376b	SEMA4C	semaphorin 4C [Source:VGNC Symbol;Acc:VGNC:92702]	1.87	0.0612
ssc-miR-376b	SENP2	SUMO specific peptidase 2 [Source:VGNC Symbol;Acc:VGNC:92713]	1.87	0.0612
ssc-miR-376b	SENP6	SUMO specific peptidase 6 [Source:VGNC Symbol;Acc:VGNC:92715]	1.87	0.0612
ssc-miR-376b	SEPT7	hypothetical gene	1.87	0.0612
ssc-miR-376b	SERINC5	serine incorporator 5 [Source:VGNC Symbol;Acc:VGNC:92730]	1.87	0.0612
ssc-miR-376b	SETD7	SET domain containing 7, histone lysine methyltransferase [Source:VGNC Symbol;Acc:VGNC:92762]	1.87	0.0612
ssc-miR-376b	SGK3	serum/glucocorticoid regulated kinase family member 3 [Source:HGNC Symbol;Acc:HGNC:10812]	1.87	0.0612
ssc-miR-376b	SGMS1	sphingomyelin synthase 1 [Source:VGNC Symbol;Acc:VGNC:92794]	1.87	0.0612
ssc-miR-376b	SIDT2	SID1 transmembrane family member 2 [Source:VGNC Symbol;Acc:VGNC:92869]	1.87	0.0612
ssc-miR-376b	SKAP2	src kinase associated phosphoprotein 2 [Source:VGNC Symbol;Acc:VGNC:92901]	1.87	0.0612
ssc-miR-376b	SLC4A10	solute carrier family 4 member 10 [Source:VGNC Symbol;Acc:VGNC:95592]	1.87	0.0612
ssc-miR-376b	SLC4A4	solute carrier family 4 member 4 [Source:VGNC Symbol;Acc:VGNC:93133]	1.87	0.0612
ssc-miR-376b	SLC6A1	solute carrier family 6 member 1 [Source:VGNC Symbol;Acc:VGNC:93153]	1.87	0.0612
ssc-miR-376b	SLC7A11	solute carrier family 7 member 11 [Source:VGNC Symbol;Acc:VGNC:93171]	1.87	0.0612
ssc-miR-376b	SLIT2	slit guidance ligand 2 [Source:VGNC Symbol;Acc:VGNC:93204]	1.87	0.0612
ssc-miR-376b	SNX24	sorting nexin 24 [Source:VGNC Symbol;Acc:VGNC:93315]	1.87	0.0612
ssc-miR-376b	SNX27	sorting nexin 27 [Source:VGNC Symbol;Acc:VGNC:93316]	1.87	0.0612
ssc-miR-376b	SOCS5	suppressor of cytokine signaling 5 [Source:VGNC Symbol;Acc:VGNC:93332]	1.87	0.0612
ssc-miR-376b	SP1	Sp1 transcription factor [Source:VGNC Symbol;Acc:VGNC:93360]	1.87	0.0612
ssc-miR-376b	SPAG9	sperm associated antigen 9 [Source:VGNC Symbol;Acc:VGNC:93374]	1.87	0.0612
ssc-miR-376b	SPATS2	spermatogenesis associated serine rich 2 [Source:HGNC Symbol;Acc:HGNC:18650]	1.87	0.0612
ssc-miR-376b	SREK1IP1	SREK1 interacting protein 1 [Source:HGNC Symbol;Acc:HGNC:26716]	1.87	0.0612
ssc-miR-376b	SRGAP3	SLIT-ROBO Rho GTPase activating protein 3 [Source:VGNC Symbol;Acc:VGNC:93455]	1.87	0.0612
ssc-miR-376b	SRSF1	serine and arginine rich splicing factor 1 [Source:VGNC Symbol;Acc:VGNC:99084]	1.87	0.0612
ssc-miR-376b	STC1	stanniocalcin 1 [Source:VGNC Symbol;Acc:VGNC:93542]	1.87	0.0612

ssc-miR-376b	STON2	stonin 2 [Source:VGNC Symbol;Acc:VGNC:93568]	1.87	0.0612
ssc-miR-376b	STRN3	striatin 3 [Source:VGNC Symbol;Acc:VGNC:93579]	1.87	0.0612
ssc-miR-376b	SUGT1	SGT1 homolog, MIS12 kinetochore complex assembly cochaperone [Source:VGNC Symbol;Acc:VGNC:93611]	1.87	0.0612
ssc-miR-376b	TBX20	T-box transcription factor 20 [Source:VGNC Symbol;Acc:VGNC:93799]	1.87	0.0612
ssc-miR-376b	TEFM	transcription elongation factor, mitochondrial [Source:VGNC Symbol;Acc:VGNC:93865]	1.87	0.0612
ssc-miR-376b	TET1	tet methylcytosine dioxygenase 1 [Source:VGNC Symbol;Acc:VGNC:93888]	1.87	0.0612
ssc-miR-376b	TFAM	transcription factor A, mitochondrial [Source:NCBI gene (formerly Entrezgene);Acc:397279]	1.87	0.0612
ssc-miR-376b	TFCP2L1	transcription factor CP2 like 1 [Source:HGNC Symbol;Acc:HGNC:17925]	1.87	0.0612
ssc-miR-376b	TIAM1	TIAM Rac1 associated GEF 1 [Source:VGNC Symbol;Acc:VGNC:96601]	1.87	0.0612
ssc-miR-376b	TMED7	transmembrane p24 trafficking protein 7 [Source:NCBI gene (formerly Entrezgene);Acc:100522183]	1.87	0.0612
ssc-miR-376b	TMIGD1	transmembrane and immunoglobulin domain containing 1 [Source:VGNC Symbol;Acc:VGNC:94224]	1.87	0.0612
ssc-miR-376b	TMTC4	transmembrane O-mannosyltransferase targeting cadherins 4 [Source:VGNC Symbol;Acc:VGNC:94244]	1.87	0.0612
ssc-miR-376b	TP53TG5	TP53 target 5 [Source:VGNC Symbol;Acc:VGNC:108740]	1.87	0.0612
ssc-miR-376b	TRAF3IP2	TRAF3 interacting protein 2 [Source:VGNC Symbol;Acc:VGNC:94361]	1.87	0.0612
ssc-miR-376b	TRHDE	thyrotropin releasing hormone degrading enzyme [Source:VGNC Symbol;Acc:VGNC:94390]	1.87	0.0612
ssc-miR-376b	TRIM13	tripartite motif containing 13 [Source:VGNC Symbol;Acc:VGNC:94394]	1.87	0.0612
ssc-miR-376b	TSN	translin [Source:VGNC Symbol;Acc:VGNC:95556]	1.87	0.0612
ssc-miR-376b	TTC33	tetratricopeptide repeat domain 33 [Source:VGNC Symbol;Acc:VGNC:94547]	1.87	0.0612
ssc-miR-376b	UBE2D2	ubiquitin conjugating enzyme E2 D2 [Source:VGNC Symbol;Acc:VGNC:98391]	1.87	0.0612
ssc-miR-376b	UBE2W	ubiquitin conjugating enzyme E2 W [Source:VGNC Symbol;Acc:VGNC:98890]	1.87	0.0612
ssc-miR-376b	UBR4	ubiquitin protein ligase E3 component n-recognin 4 [Source:VGNC Symbol;Acc:VGNC:94670]	1.87	0.0612
ssc-miR-376b	USP49	ubiquitin specific peptidase 49 [Source:VGNC Symbol;Acc:VGNC:94771]	1.87	0.0612
ssc-miR-376b	USP6NL	USP6 N-terminal like [Source:VGNC Symbol;Acc:VGNC:95820]	1.87	0.0612
ssc-miR-376b	UST	uronyl 2-sulfotransferase [Source:VGNC Symbol;Acc:VGNC:94780]	1.87	0.0612
ssc-miR-376b	VASP	vasodilator stimulated phosphoprotein [Source:VGNC Symbol;Acc:VGNC:94804]	1.87	0.0612
ssc-miR-376b	VCPIP1	valosin containing protein interacting protein 1 [Source:VGNC Symbol;Acc:VGNC:94813]	1.87	0.0612
ssc-miR-376b	VPS13B	vacuolar protein sorting 13 homolog B [Source:VGNC Symbol;Acc:VGNC:94839]	1.87	0.0612
ssc-miR-376b	VPS29	VPS29 retromer complex component [Source:VGNC Symbol;Acc:VGNC:94847]	1.87	0.0612
ssc-miR-376b	VPS37B	VPS37B subunit of ESCRT-I [Source:VGNC Symbol;Acc:VGNC:94851]	1.87	0.0612
ssc-miR-376b	VPS54	VPS54 subunit of GARP complex [Source:VGNC Symbol;Acc:VGNC:94862]	1.87	0.0612
ssc-miR-376b	WIPI2	WD repeat domain, phosphoinositide interacting 2 [Source:VGNC Symbol;Acc:VGNC:94960]	1.87	0.0612
ssc-miR-376b	WNT2B	Wnt family member 2B [Source:VGNC Symbol;Acc:VGNC:94969]	1.87	0.0612
ssc-miR-376b	WWTR1	WW domain containing transcription regulator 1 [Source:VGNC Symbol;Acc:VGNC:94991]	1.87	0.0612
ssc-miR-376b	XKR4	XK related 4 [Source:VGNC Symbol;Acc:VGNC:98900]	1.87	0.0612
ssc-miR-376b	XPR1	xenotropic and polytropic retrovirus receptor 1 [Source:VGNC Symbol;Acc:VGNC:108625]	1.87	0.0612
ssc-miR-376b	ZBTB10	zinc finger and BTB domain containing 10 [Source:VGNC Symbol;Acc:VGNC:95058]	1.87	0.0612
ssc-miR-376b	ZBTB7A	zinc finger and BTB domain containing 7A [Source:VGNC Symbol;Acc:VGNC:95081]	1.87	0.0612
ssc-miR-376b	ZBTB8A	zinc finger and BTB domain containing 8A [Source:VGNC Symbol;Acc:VGNC:95084]	1.87	0.0612
ssc-miR-376b	ZC3H4	zinc finger CCCH-type containing 4 [Source:VGNC Symbol;Acc:VGNC:95097]	1.87	0.0612
ssc-miR-376b	ZFHX3	zinc finger homeobox 3 [Source:VGNC Symbol;Acc:VGNC:95141]	1.87	0.0612
ssc-miR-376b	ZFP69B	ZFP69 zinc finger protein B [Source:HGNC Symbol;Acc:HGNC:28053]	1.87	0.0612
ssc-miR-376b	ZNF570	zinc finger protein 570 [Source:VGNC Symbol;Acc:VGNC:95267]	1.87	0.0612
ssc-miR-376b	ZNF80	hypothetical gene	1.87	0.0612
ssc-miR-376b	ZSWIM5	zinc finger SWIM-type containing 5 [Source:VGNC Symbol;Acc:VGNC:95334]	1.87	0.0612
ssc-miR-133a-5p	NA	hypothetical gene	1.82	0.0226
ssc-miR-1296-5p	NA	hypothetical gene	1.78	0.0269
ssc-miR-194b-3p	NA	hypothetical gene	1.78	0.174
ssc-miR-184	AGO2	argonaute RISC catalytic component 2 [Source:VGNC Symbol;Acc:VGNC:97871]	1.74	0.0856
ssc-miR-184	ALDH4A1	aldehyde dehydrogenase 4 family member A1 [Source:VGNC Symbol;Acc:VGNC:85240]	1.74	0.0856
ssc-miR-184	BMPR1A	bone morphotic protein receptor type 1A [Source:VGNC Symbol;Acc:VGNC:85846]	1.74	0.0856

ssc-miR-184	C15orf37	hypothetical gene	1.74	0.0856
ssc-miR-184	C20orf112	hypothetical gene	1.74	0.0856
ssc-miR-184	C9orf114	hypothetical gene	1.74	0.0856
ssc-miR-184	C9orf116	hypothetical gene	1.74	0.0856
ssc-miR-184	CARM1	coactivator associated arginine methyltransferase 1 [Source:VGNC Symbol;Acc:VGNC:86191]	1.74	0.0856
ssc-miR-184	CBX6	chromobox 6 [Source:VGNC Symbol;Acc:VGNC:97915]	1.74	0.0856
ssc-miR-184	CRISPLD2	cysteine rich secretory protein LCCL domain containing 2 [Source:VGNC Symbol;Acc:VGNC:96958]	1.74	0.0856
ssc-miR-184	CRTC1	CREB regulated transcription coactivator 1 [Source:VGNC Symbol;Acc:VGNC:87007]	1.74	0.0856
ssc-miR-184	DBNL	drebrin like [Source:VGNC Symbol;Acc:VGNC:87166]	1.74	0.0856
ssc-miR-184	EPB41L5	erythrocyte membrane protein band 4.1 like 5 [Source:VGNC Symbol;Acc:VGNC:97979]	1.74	0.0856
ssc-miR-184	FBXO28	F-box protein 28 [Source:HGNC Symbol;Acc:HGNC:29046]	1.74	0.0856
ssc-miR-184	FCRLB	Fc receptor like B [Source:VGNC Symbol;Acc:VGNC:88074]	1.74	0.0856
ssc-miR-184	GBX2	gastrulation brain homeobox 2 [Source:VGNC Symbol;Acc:VGNC:96316]	1.74	0.0856
ssc-miR-184	HAND2	heart and neural crest derivatives expressed 2 [Source:VGNC Symbol;Acc:VGNC:88778]	1.74	0.0856
ssc-miR-184	HIF1AN	hypoxia inducible factor 1 subunit alpha inhibitor [Source:VGNC Symbol;Acc:VGNC:98033]	1.74	0.0856
ssc-miR-184	MFRP	membrane frizzled-related protein [Source:VGNC Symbol;Acc:VGNC:90181]	1.74	0.0856
ssc-miR-184	MLEC	malectin [Source:HGNC Symbol;Acc:HGNC:28973]	1.74	0.0856
ssc-miR-184	NCOR2	nuclear receptor corepressor 2 [Source:VGNC Symbol;Acc:VGNC:98148]	1.74	0.0856
ssc-miR-184	NFIC	nuclear factor I C [Source:VGNC Symbol;Acc:VGNC:100313]	1.74	0.0856
ssc-miR-184	NUS1	NUS1 dehydrodolichyl diphosphate synthase subunit [Source:VGNC Symbol;Acc:VGNC:90990]	1.74	0.0856
ssc-miR-184	PPAP2B	hypothetical gene	1.74	0.0856
ssc-miR-184	REEP5	receptor accessory protein 5 [Source:VGNC Symbol;Acc:VGNC:92201]	1.74	0.0856
ssc-miR-184	SF1	splicing factor 1 [Source:HGNC Symbol;Acc:HGNC:12950]	1.74	0.0856
ssc-miR-184	SIDT2	SID1 transmembrane family member 2 [Source:VGNC Symbol;Acc:VGNC:92869]	1.74	0.0856
ssc-miR-184	SLC25A22	solute carrier family 25 member 22 [Source:VGNC Symbol;Acc:VGNC:92999]	1.74	0.0856
ssc-miR-184	SLC35E4	solute carrier family 35 member E4 [Source:VGNC Symbol;Acc:VGNC:93080]	1.74	0.0856
ssc-miR-184	STC2	stanniocalcin 2 [Source:VGNC Symbol;Acc:VGNC:93543]	1.74	0.0856
ssc-miR-184	TNRC6B	trinucleotide repeat containing adaptor 6B [Source:VGNC Symbol;Acc:VGNC:94293]	1.74	0.0856
ssc-miR-184	ZC3H4	zinc finger CCCH-type containing 4 [Source:VGNC Symbol;Acc:VGNC:95097]	1.74	0.0856
ssc-miR-184	ZIC4	Zic family member 4 [Source:VGNC Symbol;Acc:VGNC:95169]	1.74	0.0856
ssc-miR-184	ZNF740	zinc finger protein 740 [Source:VGNC Symbol;Acc:VGNC:95298]	1.74	0.0856
ssc-miR-184	ZNF865	zinc finger protein 865 [Source:VGNC Symbol;Acc:VGNC:95312]	1.74	0.0856
ssc-miR-676-5p	NA	hypothetical gene	1.74	0.0882
ssc-miR-218	ABAT	4-aminobutyrate aminotransferase [Source:VGNC Symbol;Acc:VGNC:96910]	1.73	0.083
ssc-miR-218	ABCD2	ATP binding cassette subfamily D member 2 [Source:VGNC Symbol;Acc:VGNC:84962]	1.73	0.083
ssc-miR-218	ABCG4	ATP binding cassette subfamily G member 4 [Source:HGNC Symbol;Acc:HGNC:13884]	1.73	0.083
ssc-miR-218	ABI2	abl interactor 2 [Source:VGNC Symbol;Acc:VGNC:96027]	1.73	0.083
ssc-miR-218	ABL2	ABL proto-onco 2, non-receptor tyrosine kinase [Source:VGNC Symbol;Acc:VGNC:84985]	1.73	0.083
ssc-miR-218	ABLIM3	actin binding LIM protein family member 3 [Source:VGNC Symbol;Acc:VGNC:84988]	1.73	0.083
ssc-miR-218	AC010441.1	hypothetical gene	1.73	0.083
ssc-miR-218	ACO1	aconitase 1 [Source:VGNC Symbol;Acc:VGNC:95539]	1.73	0.083
ssc-miR-218	ACSL1	acyl-CoA synthetase long chain family member 1 [Source:VGNC Symbol;Acc:VGNC:96294]	1.73	0.083
ssc-miR-218	ACSL6	acyl-CoA synthetase long chain family member 6 [Source:VGNC Symbol;Acc:VGNC:99581]	1.73	0.083
ssc-miR-218	ACTN1	actinin alpha 1 [Source:NCBI gene (formerly Entrezgene);Acc:100513412]	1.73	0.083
ssc-miR-218	ADAM10	ADAM metallopeptidase domain 10 [Source:VGNC Symbol;Acc:VGNC:85061]	1.73	0.083
ssc-miR-218	ADAM23	ADAM metallopeptidase domain 23 [Source:VGNC Symbol;Acc:VGNC:95933]	1.73	0.083
ssc-miR-218	ADAMTS18	ADAM metallopeptidase with thrombospondin type 1 motif 18 [Source:VGNC Symbol;Acc:VGNC:85080]	1.73	0.083
ssc-miR-218	ADARB1	adenosine deaminase RNA specific B1 [Source:VGNC Symbol;Acc:VGNC:85098]	1.73	0.083
ssc-miR-218	ADCY9	adenylate cyclase 9 [Source:VGNC Symbol;Acc:VGNC:85113]	1.73	0.083
ssc-miR-218	ADD2	adducin 2 [Source:VGNC Symbol;Acc:VGNC:85117]	1.73	0.083

ssc-miR-218	ADIPOR2	adiponectin receptor 2 [Source:VGNC Symbol;Acc:VGNC:85141]	1.73	0.083
ssc-miR-218	ADO	2-aminoethanethiol dioxygenase [Source:VGNC Symbol;Acc:VGNC:85146]	1.73	0.083
ssc-miR-218	ADRB1	adrenoceptor beta 1 [Source:VGNC Symbol;Acc:VGNC:107363]	1.73	0.083
ssc-miR-218	ADRBK2	hypothetical gene	1.73	0.083
ssc-miR-218	AFAP1L1	actin filament associated protein 1 like 1 [Source:VGNC Symbol;Acc:VGNC:85166]	1.73	0.083
ssc-miR-218	AFF1	AF4/FMR2 family member 1 [Source:VGNC Symbol;Acc:VGNC:85167]	1.73	0.083
ssc-miR-218	AFF4	AF4/FMR2 family member 4 [Source:VGNC Symbol;Acc:VGNC:85169]	1.73	0.083
ssc-miR-218	AGA	aspartylglucosaminidase [Source:VGNC Symbol;Acc:VGNC:96261]	1.73	0.083
ssc-miR-218	AGAP1	ArfGAP with GTPase domain, ankyrin repeat and PH domain 1 [Source:HGNC Symbol;Acc:HGNC:16922]	1.73	0.083
ssc-miR-218	AGO1	hypothetical gene	1.73	0.083
ssc-miR-218	AGO3	argonaute RISC component 1 [Source:NCBI gene (formerly Entrezgene);Acc:100499510]	1.73	0.083
ssc-miR-218	AGPAT3	1-acylglycerol-3-phosphate O-acyltransferase 3 [Source:VGNC Symbol;Acc:VGNC:85185]	1.73	0.083
ssc-miR-218	AHSA2	hypothetical gene	1.73	0.083
ssc-miR-218	AIF1L	allograft inflammatory factor 1 like [Source:VGNC Symbol;Acc:VGNC:103022]	1.73	0.083
ssc-miR-218	AJAP1	adherens junctions associated protein 1 [Source:VGNC Symbol;Acc:VGNC:85207]	1.73	0.083
ssc-miR-218	AK4	hypothetical gene	1.73	0.083
ssc-miR-218	AKIRIN1	akirin 1 [Source:VGNC Symbol;Acc:VGNC:85227]	1.73	0.083
ssc-miR-218	AL021546.6	hypothetical gene	1.73	0.083
ssc-miR-218	AL626787.1	hypothetical gene	1.73	0.083
ssc-miR-218	ALDH5A1	aldehyde dehydrogenase 5 family member A1 [Source:VGNC Symbol;Acc:VGNC:85241]	1.73	0.083
ssc-miR-218	ALG6	ALG6 alpha-1,3-glucosyltransferase [Source:VGNC Symbol;Acc:VGNC:85253]	1.73	0.083
ssc-miR-218	AMIGO2	adhesion molecule with Ig like domain 2 [Source:VGNC Symbol;Acc:VGNC:85281]	1.73	0.083
ssc-miR-218	AMMECR1	AMMECR nuclear protein 1 [Source:VGNC Symbol;Acc:VGNC:96559]	1.73	0.083
ssc-miR-218	ANK1	ankyrin 1 [Source:VGNC Symbol;Acc:VGNC:96344]	1.73	0.083
ssc-miR-218	ANKRD13B	ankyrin repeat domain 13B [Source:VGNC Symbol;Acc:VGNC:85325]	1.73	0.083
ssc-miR-218	ANKRD27	ankyrin repeat domain 27 [Source:VGNC Symbol;Acc:VGNC:96907]	1.73	0.083
ssc-miR-218	ANKRD34A	ankyrin repeat domain 34A [Source:VGNC Symbol;Acc:VGNC:85335]	1.73	0.083
ssc-miR-218	ANKRD40	ankyrin repeat domain 40 [Source:VGNC Symbol;Acc:VGNC:85338]	1.73	0.083
ssc-miR-218	ANKRD44	ankyrin repeat domain 44 [Source:VGNC Symbol;Acc:VGNC:107123]	1.73	0.083
ssc-miR-218	ANKRD52	ankyrin repeat domain 52 [Source:VGNC Symbol;Acc:VGNC:85342]	1.73	0.083
ssc-miR-218	ANKS1B	ankyrin repeat and sterile alpha motif domain containing 1B [Source:VGNC Symbol;Acc:VGNC:103214]	1.73	0.083
ssc-miR-218	AP1AR	adaptor related protein complex 1 associated regulatory protein [Source:HGNC Symbol;Acc:HGNC:28808]	1.73	0.083
ssc-miR-218	AP1S2	adaptor related protein complex 1 subunit sigma 2 [Source:VGNC Symbol;Acc:VGNC:85382]	1.73	0.083
ssc-miR-218	AP2A1	adaptor related protein complex 2 subunit alpha 1 [Source:VGNC Symbol;Acc:VGNC:85383]	1.73	0.083
ssc-miR-218	AP3M1	adaptor related protein complex 3 subunit mu 1 [Source:VGNC Symbol;Acc:VGNC:85390]	1.73	0.083
ssc-miR-218	APEX1	apurinic/apyrimidinic endodeoxyribonuclease 1 [Source:VGNC Symbol;Acc:VGNC:103896]	1.73	0.083
ssc-miR-218	APH1B	aph-1 homolog B, gamma-secretase subunit [Source:VGNC Symbol;Acc:VGNC:103898]	1.73	0.083
ssc-miR-218	ARAF	A-Raf proto-onco, serine/threonine kinase [Source:VGNC Symbol;Acc:VGNC:85438]	1.73	0.083
ssc-miR-218	ARF6	ADP ribosylation factor 6 [Source:VGNC Symbol;Acc:VGNC:85448]	1.73	0.083
ssc-miR-218	ARHGAP17	Rho GTPase activating protein 17 [Source:VGNC Symbol;Acc:VGNC:85459]	1.73	0.083
ssc-miR-218	ARHGAP32	Rho GTPase activating protein 32 [Source:VGNC Symbol;Acc:VGNC:85471]	1.73	0.083
ssc-miR-218	ARHGAP5	Rho GTPase activating protein 5 [Source:VGNC Symbol;Acc:VGNC:85480]	1.73	0.083
ssc-miR-218	ARHGEF12	Rho guanine nucleotide exchange factor 12 [Source:VGNC Symbol;Acc:VGNC:85488]	1.73	0.083
ssc-miR-218	ARHGEF39	Rho guanine nucleotide exchange factor 39 [Source:VGNC Symbol;Acc:VGNC:85500]	1.73	0.083
ssc-miR-218	ARID4B	AT-rich interaction domain 4B [Source:HGNC Symbol;Acc:HGNC:15550]	1.73	0.083
ssc-miR-218	ARL3	ADP ribosylation factor like GTPase 3 [Source:VGNC Symbol;Acc:VGNC:107366]	1.73	0.083
ssc-miR-218	ARL4C	ADP ribosylation factor like GTPase 4C [Source:VGNC Symbol;Acc:VGNC:96419]	1.73	0.083
ssc-miR-218	ARL5B	ADP ribosylation factor like GTPase 5B [Source:VGNC Symbol;Acc:VGNC:95985]	1.73	0.083
ssc-miR-218	ARPP19	cAMP regulated phosphoprotein 19 [Source:NCBI gene (formerly Entrezgene);Acc:397362]	1.73	0.083
ssc-miR-218	ARRB1	arrestin beta 1 [Source:VGNC Symbol;Acc:VGNC:85542]	1.73	0.083

ssc-miR-218	ASAP1	ArfGAP with SH3 domain, ankyrin repeat and PH domain 1 [Source:VGNC Symbol;Acc:VGNC:98734]	1.73	0.083
ssc-miR-218	ASIC1	acid sensing ion channel subunit 1 [Source:VGNC Symbol;Acc:VGNC:85578]	1.73	0.083
ssc-miR-218	ASXL3	ASXL transcriptional regulator 3 [Source:VGNC Symbol;Acc:VGNC:85596]	1.73	0.083
ssc-miR-218	ATP8B4	ATPase phospholipid transporting 8B4 (putative) [Source:VGNC Symbol;Acc:VGNC:103036]	1.73	0.083
ssc-miR-218	ATRN	attractin [Source:VGNC Symbol;Acc:VGNC:96479]	1.73	0.083
ssc-miR-218	ATRX	ATRX chromatin remodeler [Source:VGNC Symbol;Acc:VGNC:85686]	1.73	0.083
ssc-miR-218	ATXN1	ataxin 1 [Source:VGNC Symbol;Acc:VGNC:85687]	1.73	0.083
ssc-miR-218	ATXN2	ataxin 2 [Source:VGNC Symbol;Acc:VGNC:85690]	1.73	0.083
ssc-miR-218	AURKA	hypothetical gene	1.73	0.083
ssc-miR-218	AZIN1	antizyme inhibitor 1 [Source:VGNC Symbol;Acc:VGNC:85712]	1.73	0.083
ssc-miR-218	B3GAT1	beta-1,3-glucuronyltransferase 1 [Source:VGNC Symbol;Acc:VGNC:85719]	1.73	0.083
ssc-miR-218	B3GAT2	beta-1,3-glucuronyltransferase 2 [Source:VGNC Symbol;Acc:VGNC:85720]	1.73	0.083
ssc-miR-218	BAHD1	bromo adjacent homology domain containing 1 [Source:VGNC Symbol;Acc:VGNC:85747]	1.73	0.083
ssc-miR-218	BAI3	hypothetical gene	1.73	0.083
ssc-miR-218	BAZ2B	bromodomain adjacent to zinc finger domain 2B [Source:HGNC Symbol;Acc:HGNC:963]	1.73	0.083
ssc-miR-218	BBX	BBX high mobility group box domain containing [Source:VGNC Symbol;Acc:VGNC:85766]	1.73	0.083
ssc-miR-218	BCAT1	branched chain amino acid transaminase 1 [Source:VGNC Symbol;Acc:VGNC:85773]	1.73	0.083
ssc-miR-218	BCL11B	BAF chromatin remodeling complex subunit BCL11B [Source:VGNC Symbol;Acc:VGNC:96563]	1.73	0.083
ssc-miR-218	BCL9	BCL9 transcription coactivator [Source:VGNC Symbol;Acc:VGNC:96567]	1.73	0.083
ssc-miR-218	BDP1	B double prime 1, subunit of RNA polymerase III transcription initiation factor IIIB [Source:VGNC Symbol;Acc:VGNC:85796]	1.73	0.083
ssc-miR-218	BEND3	BEN domain containing 3 [Source:VGNC Symbol;Acc:VGNC:85800]	1.73	0.083
ssc-miR-218	BICD1	BICD cargo adaptor 1 [Source:VGNC Symbol;Acc:VGNC:85818]	1.73	0.083
ssc-miR-218	BIRC6	baculoviral IAP repeat containing 6 [Source:VGNC Symbol;Acc:VGNC:97038]	1.73	0.083
ssc-miR-218	BIVM	basic, immunoglobulin-like variable motif containing [Source:NCBI gene (formerly Entrezgene);Acc:100512068]	1.73	0.083
ssc-miR-218	BMI1	BMI1 proto-onco, polycomb ring finger [Source:VGNC Symbol;Acc:VGNC:108265]	1.73	0.083
ssc-miR-218	BMP5	bone morphotic protein 5 [Source:VGNC Symbol;Acc:VGNC:85844]	1.73	0.083
ssc-miR-218	BMPR2	bone morphotic protein receptor type 2 [Source:VGNC Symbol;Acc:VGNC:95494]	1.73	0.083
ssc-miR-218	BPHL	biphenyl hydrolase like [Source:VGNC Symbol;Acc:VGNC:85864]	1.73	0.083
ssc-miR-218	BRCC3	BRCA1/BRCA2-containing complex subunit 3 [Source:VGNC Symbol;Acc:VGNC:85870]	1.73	0.083
ssc-miR-218	BRPF3	bromodomain and PHD finger containing 3 [Source:VGNC Symbol;Acc:VGNC:85886]	1.73	0.083
ssc-miR-218	BSN	bassoon presynaptic cytomatrix protein [Source:VGNC Symbol;Acc:VGNC:85893]	1.73	0.083
ssc-miR-218	BTG2	BTG anti-proliferation factor 2 [Source:NCBI gene (formerly Entrezgene);Acc:100048932]	1.73	0.083
ssc-miR-218	BVES	blood vessel epicardial substance [Source:VGNC Symbol;Acc:VGNC:103040]	1.73	0.083
ssc-miR-218	C10orf35	hypothetical gene	1.73	0.083
ssc-miR-218	C11orf1	hypothetical gene	1.73	0.083
ssc-miR-218	C11orf87	chromosome 11 open reading frame 87 [Source:HGNC Symbol;Acc:HGNC:33788]	1.73	0.083
ssc-miR-218	C16orf52	hypothetical gene	1.73	0.083
ssc-miR-218	C16orf70	hypothetical gene	1.73	0.083
ssc-miR-218	C17orf70	hypothetical gene	1.73	0.083
ssc-miR-218	C18orf25	chromosome 1 C18orf25 homolog [Source:VGNC Symbol;Acc:VGNC:85964]	1.73	0.083
ssc-miR-218	C1orf21	hypothetical gene	1.73	0.083
ssc-miR-218	C1orf27	hypothetical gene	1.73	0.083
ssc-miR-218	C1orf35	chromosome 2 C1orf35 homolog [Source:VGNC Symbol;Acc:VGNC:86008]	1.73	0.083
ssc-miR-218	C20orf112	hypothetical gene	1.73	0.083
ssc-miR-218	C20orf194	hypothetical gene	1.73	0.083
ssc-miR-218	C2orf88	chromosome 15 C2orf88 homolog [Source:VGNC Symbol;Acc:VGNC:96170]	1.73	0.083
ssc-miR-218	C3orf70	chromosome 13 C3orf70 homolog [Source:VGNC Symbol;Acc:VGNC:85937]	1.73	0.083
ssc-miR-218	C5orf15	chromosome 2 C5orf15 homolog [Source:VGNC Symbol;Acc:VGNC:86009]	1.73	0.083
ssc-miR-218	C6orf120	chromosome 1 C6orf120 homolog [Source:VGNC Symbol;Acc:VGNC:85967]	1.73	0.083
ssc-miR-218	C6orf47	hypothetical gene	1.73	0.083

ssc-miR-218	C6orf62	chromosome 7 C6orf62 homolog [Source:VGNC Symbol;Acc:VGNC:86074]	1.73	0.083
ssc-miR-218	C7orf25	chromosome 18 C7orf25 homolog [Source:VGNC Symbol;Acc:VGNC:85955]	1.73	0.083
ssc-miR-218	C7orf41	hypothetical gene	1.73	0.083
ssc-miR-218	C7orf60	hypothetical gene	1.73	0.083
ssc-miR-218	C8orf46	hypothetical gene	1.73	0.083
ssc-miR-218	C9orf156	hypothetical gene	1.73	0.083
ssc-miR-218	C9orf47	hypothetical gene	1.73	0.083
ssc-miR-218	CA2	carbonic anhydrase 2 [Source:VGNC Symbol;Acc:VGNC:98746]	1.73	0.083
ssc-miR-218	CAB39L	calcium binding protein 39 like [Source:VGNC Symbol;Acc:VGNC:86106]	1.73	0.083
ssc-miR-218	CABLES1	Cdk5 and Abl enzyme substrate 1 [Source:VGNC Symbol;Acc:VGNC:86109]	1.73	0.083
ssc-miR-218	CACNA1G	calcium voltage-gated channel subunit alpha1 G [Source:VGNC Symbol;Acc:VGNC:86119]	1.73	0.083
ssc-miR-218	CACNA1I	calcium voltage-gated channel subunit alpha1 I [Source:VGNC Symbol;Acc:VGNC:97908]	1.73	0.083
ssc-miR-218	CACNB4	calcium voltage-gated channel auxiliary subunit beta 4 [Source:VGNC Symbol;Acc:VGNC:108507]	1.73	0.083
ssc-miR-218	CACUL1	CDK2 associated cullin domain 1 [Source:VGNC Symbol;Acc:VGNC:86132]	1.73	0.083
ssc-miR-218	CADM2	cell adhesion molecule 2 [Source:VGNC Symbol;Acc:VGNC:97910]	1.73	0.083
ssc-miR-218	CALCOCO2	calcium binding and coiled-coil domain 2 [Source:VGNC Symbol;Acc:VGNC:86141]	1.73	0.083
ssc-miR-218	CALM1	calmodulin 1 [Source:NCBI gene (formerly Entrezgene);Acc:100154056]	1.73	0.083
ssc-miR-218	CALN1	hypothetical gene	1.73	0.083
ssc-miR-218	CAMK4	calcium/calmodulin dependent protein kinase IV [Source:VGNC Symbol;Acc:VGNC:99602]	1.73	0.083
ssc-miR-218	CAMKK2	calcium/calmodulin dependent protein kinase kinase 2 [Source:VGNC Symbol;Acc:VGNC:86158]	1.73	0.083
ssc-miR-218	CASK	calcium/calmodulin dependent serine protein kinase [Source:HGNC Symbol;Acc:HGNC:1497]	1.73	0.083
ssc-miR-218	CASKIN1	CASK interacting protein 1 [Source:VGNC Symbol;Acc:VGNC:86200]	1.73	0.083
ssc-miR-218	CBL	Cbl proto-onco [Source:VGNC Symbol;Acc:VGNC:86222]	1.73	0.083
ssc-miR-218	CBLB	Cbl proto-onco B [Source:VGNC Symbol;Acc:VGNC:86223]	1.73	0.083
ssc-miR-218	CBX3	chromobox 3 [Source:HGNC Symbol;Acc:HGNC:1553]	1.73	0.083
ssc-miR-218	CBX5	chromobox 5 [Source:VGNC Symbol;Acc:VGNC:86232]	1.73	0.083
ssc-miR-218	CBX7	chromobox 7 [Source:HGNC Symbol;Acc:HGNC:1557]	1.73	0.083
ssc-miR-218	CBX8	chromobox 8 [Source:VGNC Symbol;Acc:VGNC:86233]	1.73	0.083
ssc-miR-218	CC2D1B	coiled-coil and C2 domain containing 1B [Source:VGNC Symbol;Acc:VGNC:86236]	1.73	0.083
ssc-miR-218	CCDC113	coiled-coil domain containing 113 [Source:VGNC Symbol;Acc:VGNC:86247]	1.73	0.083
ssc-miR-218	CCDC144A	hypothetical gene	1.73	0.083
ssc-miR-218	CCDC176	hypothetical gene	1.73	0.083
ssc-miR-218	CCDC177	coiled-coil domain containing 177 [Source:VGNC Symbol;Acc:VGNC:86275]	1.73	0.083
ssc-miR-218	CCDC24	coiled-coil domain containing 24 [Source:VGNC Symbol;Acc:VGNC:86287]	1.73	0.083
ssc-miR-218	CCDC6	coiled-coil domain containing 6 [Source:VGNC Symbol;Acc:VGNC:86304]	1.73	0.083
ssc-miR-218	CCDC64	hypothetical gene	1.73	0.083
ssc-miR-218	CCDC88A	coiled-coil domain containing 88A [Source:VGNC Symbol;Acc:VGNC:86325]	1.73	0.083
ssc-miR-218	CCDC89	coiled-coil domain containing 89 [Source:HGNC Symbol;Acc:HGNC:26762]	1.73	0.083
ssc-miR-218	CCNK	cyclin K [Source:VGNC Symbol;Acc:VGNC:86362]	1.73	0.083
ssc-miR-218	CCP110	centriolar coiled-coil protein 110 [Source:VGNC Symbol;Acc:VGNC:86367]	1.73	0.083
ssc-miR-218	CDC20B	cell division cycle 20B [Source:HGNC Symbol;Acc:HGNC:24222]	1.73	0.083
ssc-miR-218	CDC42BPA	CDC42 binding protein kinase alpha [Source:VGNC Symbol;Acc:VGNC:95847]	1.73	0.083
ssc-miR-218	CDC42SE2	CDC42 small effector 2 [Source:VGNC Symbol;Acc:VGNC:86461]	1.73	0.083
ssc-miR-218	CDC73	cell division cycle 73 [Source:VGNC Symbol;Acc:VGNC:95817]	1.73	0.083
ssc-miR-218	CDCA7L	cell division cycle associated 7 like [Source:VGNC Symbol;Acc:VGNC:86469]	1.73	0.083
ssc-miR-218	CDH2	cadherin 2 [Source:VGNC Symbol;Acc:VGNC:86483]	1.73	0.083
ssc-miR-218	CDH4	cadherin 4 [Source:VGNC Symbol;Acc:VGNC:95853]	1.73	0.083
ssc-miR-218	CDK6	cyclin dependent kinase 6 [Source:HGNC Symbol;Acc:HGNC:1777]	1.73	0.083
ssc-miR-218	CDK8	cyclin dependent kinase 8 [Source:VGNC Symbol;Acc:VGNC:86507]	1.73	0.083
ssc-miR-218	CDR1	hypothetical gene	1.73	0.083

ssc-miR-218	CDS1	CDP-diacylglycerol synthase 1 [Source:VGNC Symbol;Acc:VGNC:86523]	1.73	0.083
ssc-miR-218	CDX2	caudal type homeobox 2 [Source:VGNC Symbol;Acc:VGNC:86527]	1.73	0.083
ssc-miR-218	CEBPA	CCAAT enhancer binding protein alpha [Source:VGNC Symbol;Acc:VGNC:86531]	1.73	0.083
ssc-miR-218	CELF1	CUGBP Elav-like family member 1 [Source:VGNC Symbol;Acc:VGNC:86537]	1.73	0.083
ssc-miR-218	CELF5	CUGBP Elav-like family member 5 [Source:VGNC Symbol;Acc:VGNC:86540]	1.73	0.083
ssc-miR-218	CELF6	CUGBP Elav-like family member 6 [Source:HGNC Symbol;Acc:HGNC:14059]	1.73	0.083
ssc-miR-218	CENPB	centromere protein B [Source:HGNC Symbol;Acc:HGNC:1852]	1.73	0.083
ssc-miR-218	CEP55	centrosomal protein 55 [Source:VGNC Symbol;Acc:VGNC:86572]	1.73	0.083
ssc-miR-218	CEP85L	centrosomal protein 85 like [Source:VGNC Symbol;Acc:VGNC:86580]	1.73	0.083
ssc-miR-218	CEP97	centrosomal protein 97 [Source:VGNC Symbol;Acc:VGNC:108638]	1.73	0.083
ssc-miR-218	CGGBP1	CGG triplet repeat binding protein 1 [Source:VGNC Symbol;Acc:VGNC:86615]	1.73	0.083
ssc-miR-218	CHD6	chromodomain helicase DNA binding protein 6 [Source:VGNC Symbol;Acc:VGNC:95903]	1.73	0.083
ssc-miR-218	CHFR	checkpoint with forkhead and ring finger domains [Source:HGNC Symbol;Acc:HGNC:20455]	1.73	0.083
ssc-miR-218	CHL1	cell adhesion molecule L1 like [Source:VGNC Symbol;Acc:VGNC:108639]	1.73	0.083
ssc-miR-218	CHM	CHM Rab escort protein [Source:VGNC Symbol;Acc:VGNC:86647]	1.73	0.083
ssc-miR-218	CHRM2	cholinergic receptor muscarinic 2 [Source:VGNC Symbol;Acc:VGNC:103922]	1.73	0.083
ssc-miR-218	CHST8	carbohydrate sulfotransferase 8 [Source:VGNC Symbol;Acc:VGNC:86682]	1.73	0.083
ssc-miR-218	CISD2	CDGSH iron sulfur domain 2 [Source:VGNC Symbol;Acc:VGNC:86707]	1.73	0.083
ssc-miR-218	CLASP2	cytoplasmic linker associated protein 2 [Source:VGNC Symbol;Acc:VGNC:86720]	1.73	0.083
ssc-miR-218	CLCN3	chloride voltage-gated channel 3 [Source:VGNC Symbol;Acc:VGNC:86727]	1.73	0.083
ssc-miR-218	CLCN5	chloride voltage-gated channel 5 [Source:VGNC Symbol;Acc:VGNC:103925]	1.73	0.083
ssc-miR-218	CLCN6	chloride voltage-gated channel 6 [Source:VGNC Symbol;Acc:VGNC:86729]	1.73	0.083
ssc-miR-218	CLDN2	claudin 2 [Source:VGNC Symbol;Acc:VGNC:86737]	1.73	0.083
ssc-miR-218	CLK3	CDC like kinase 3 [Source:VGNC Symbol;Acc:VGNC:107129]	1.73	0.083
ssc-miR-218	CLNK	cytokine dependent hematopoietic cell linker [Source:VGNC Symbol;Acc:VGNC:86772]	1.73	0.083
ssc-miR-218	CLVS1	clavesin 1 [Source:VGNC Symbol;Acc:VGNC:86794]	1.73	0.083
ssc-miR-218	CNN1	calponin 1 [Source:VGNC Symbol;Acc:VGNC:86825]	1.73	0.083
ssc-miR-218	CNNM2	cyclin and CBS domain divalent metal cation transport mediator 2 [Source:VGNC Symbol;Acc:VGNC:86828]	1.73	0.083
ssc-miR-218	CNOT2	CCR4-NOT transcription complex subunit 2 [Source:VGNC Symbol;Acc:VGNC:86834]	1.73	0.083
ssc-miR-218	CNOT6L	CCR4-NOT transcription complex subunit 6 like [Source:VGNC Symbol;Acc:VGNC:86838]	1.73	0.083
ssc-miR-218	CNOT7	CCR4-NOT transcription complex subunit 7 [Source:VGNC Symbol;Acc:VGNC:95604]	1.73	0.083
ssc-miR-218	CNTN1	contactin 1 [Source:VGNC Symbol;Acc:VGNC:86848]	1.73	0.083
ssc-miR-218	CNTNAP2	hypothetical gene	1.73	0.083
ssc-miR-218	CNTNAP5	contactin associated protein family member 5 [Source:VGNC Symbol;Acc:VGNC:95459]	1.73	0.083
ssc-miR-218	COA1	hypothetical gene	1.73	0.083
ssc-miR-218	COA5	cytochrome c oxidase assembly factor 5 [Source:HGNC Symbol;Acc:HGNC:33848]	1.73	0.083
ssc-miR-218	COL1A1	collagen type I alpha 1 chain [Source:VGNC Symbol;Acc:VGNC:86870]	1.73	0.083
ssc-miR-218	COL4A3BP	hypothetical gene	1.73	0.083
ssc-miR-218	COMMD3-BMI1	hypothetical gene	1.73	0.083
ssc-miR-218	COMMD8	COMM domain containing 8 [Source:VGNC Symbol;Acc:VGNC:86890]	1.73	0.083
ssc-miR-218	COPS7B	COP9 signalosome subunit 7B [Source:HGNC Symbol;Acc:HGNC:16760]	1.73	0.083
ssc-miR-218	CPEB2	cytoplasmic polyadenylation element binding protein 2 [Source:VGNC Symbol;Acc:VGNC:86937]	1.73	0.083
ssc-miR-218	CPNE8	copine 8 [Source:VGNC Symbol;Acc:VGNC:86955]	1.73	0.083
ssc-miR-218	CREB1	cAMP responsive element binding protein 1 [Source:VGNC Symbol;Acc:VGNC:96004]	1.73	0.083
ssc-miR-218	CREBZF	CREB/ATF bZIP transcription factor [Source:VGNC Symbol;Acc:VGNC:86988]	1.73	0.083
ssc-miR-218	CRKL	CRK like proto-onco, adaptor protein [Source:VGNC Symbol;Acc:VGNC:86997]	1.73	0.083
ssc-miR-218	CRTAP	cartilage associated protein [Source:HGNC Symbol;Acc:HGNC:2379]	1.73	0.083
ssc-miR-218	CRTC3	CREB regulated transcription coactivator 3 [Source:VGNC Symbol;Acc:VGNC:87009]	1.73	0.083
ssc-miR-218	CSMD3	CUB and Sushi multiple domains 3 [Source:VGNC Symbol;Acc:VGNC:98765]	1.73	0.083
ssc-miR-218	CSNK2A1	hypothetical gene	1.73	0.083

ssc-miR-218	CTC-360G5.1	hypothetical gene	1.73	0.083
ssc-miR-218	CTD-2207O23.12	hypothetical gene	1.73	0.083
ssc-miR-218	CTDSPL	CTD small phosphatase like [Source:VGNC Symbol;Acc:VGNC:107131]	1.73	0.083
ssc-miR-218	CTGF	hypothetical gene	1.73	0.083
ssc-miR-218	CTNNA2	catenin alpha 2 [Source:HGNC Symbol;Acc:HGNC:2510]	1.73	0.083
ssc-miR-218	CTNND2	catenin delta 2 [Source:VGNC Symbol;Acc:VGNC:87068]	1.73	0.083
ssc-miR-218	CTTNBP2NL	CTTNBP2 N-terminal like [Source:VGNC Symbol;Acc:VGNC:87082]	1.73	0.083
ssc-miR-218	CUL3	cullin 3 [Source:VGNC Symbol;Acc:VGNC:96091]	1.73	0.083
ssc-miR-218	CXorf23	hypothetical gene	1.73	0.083
ssc-miR-218	CYB5D1	cytochrome b5 domain containing 1 [Source:HGNC Symbol;Acc:HGNC:26516]	1.73	0.083
ssc-miR-218	CYP7B1	cytochrome P450 family 7 subfamily B member 1 [Source:VGNC Symbol;Acc:VGNC:103362]	1.73	0.083
ssc-miR-218	DAAM1	dishevelled associated activator of morphosis 1 [Source:VGNC Symbol;Acc:VGNC:87141]	1.73	0.083
ssc-miR-218	DACH1	dachshund family transcription factor 1 [Source:VGNC Symbol;Acc:VGNC:87146]	1.73	0.083
ssc-miR-218	DAGLA	diacylglycerol lipase alpha [Source:VGNC Symbol;Acc:VGNC:87152]	1.73	0.083
ssc-miR-218	DAZAP1	DAZ associated protein 1 [Source:NCBI gene (formerly Entrezgene);Acc:100625005]	1.73	0.083
ssc-miR-218	DBN1	drebrin 1 [Source:VGNC Symbol;Acc:VGNC:87164]	1.73	0.083
ssc-miR-218	DBNDD1	dysbindin domain containing 1 [Source:VGNC Symbol;Acc:VGNC:87165]	1.73	0.083
ssc-miR-218	DCBLD2	discoidin, CUB and LCCL domain containing 2 [Source:VGNC Symbol;Acc:VGNC:87180]	1.73	0.083
ssc-miR-218	DCP2	decapping mRNA 2 [Source:VGNC Symbol;Acc:VGNC:87188]	1.73	0.083
ssc-miR-218	DCUN1D1	hypothetical gene	1.73	0.083
ssc-miR-218	DCUN1D4	defective in cullin neddylation 1 domain containing 4 [Source:VGNC Symbol;Acc:VGNC:87197]	1.73	0.083
ssc-miR-218	DCUN1D5	defective in cullin neddylation 1 domain containing 5 [Source:VGNC Symbol;Acc:VGNC:87198]	1.73	0.083
ssc-miR-218	DCX	doublecortin [Source:HGNC Symbol;Acc:HGNC:2714]	1.73	0.083
ssc-miR-218	DDX21	DExD-box helicase 21 [Source:VGNC Symbol;Acc:VGNC:87215]	1.73	0.083
ssc-miR-218	DDX41	DEAD-box helicase 41 [Source:VGNC Symbol;Acc:VGNC:87221]	1.73	0.083
ssc-miR-218	DDX5	DEAD-box helicase 5 [Source:VGNC Symbol;Acc:VGNC:87226]	1.73	0.083
ssc-miR-218	DDX6	DEAD-box helicase 6 [Source:VGNC Symbol;Acc:VGNC:87233]	1.73	0.083
ssc-miR-218	DERL2	derlin 2 [Source:VGNC Symbol;Acc:VGNC:98986]	1.73	0.083
ssc-miR-218	DES	desmin [Source:VGNC Symbol;Acc:VGNC:96159]	1.73	0.083
ssc-miR-218	DESI2	desumoylating isopeptidase 2 [Source:VGNC Symbol;Acc:VGNC:96037]	1.73	0.083
ssc-miR-218	DFNB59	hypothetical gene	1.73	0.083
ssc-miR-218	DHX29	DExH-box helicase 29 [Source:VGNC Symbol;Acc:VGNC:87288]	1.73	0.083
ssc-miR-218	DIP2A	disco interacting A [Source:VGNC Symbol;Acc:VGNC:95836]	1.73	0.083
ssc-miR-218	DIRAS1	DIRAS family GTPase 1 [Source:VGNC Symbol;Acc:VGNC:87312]	1.73	0.083
ssc-miR-218	DIRAS2	DIRAS family GTPase 2 [Source:VGNC Symbol;Acc:VGNC:87313]	1.73	0.083
ssc-miR-218	DKK2	dickkopf WNT signaling pathway inhibitor 2 [Source:VGNC Symbol;Acc:VGNC:87322]	1.73	0.083
ssc-miR-218	DKK3	dickkopf WNT signaling pathway inhibitor 3 [Source:VGNC Symbol;Acc:VGNC:87323]	1.73	0.083
ssc-miR-218	DLG2	discs large MAGUK scaffold protein 2 [Source:VGNC Symbol;Acc:VGNC:108581]	1.73	0.083
ssc-miR-218	DLST	dihydrolipoamide S-succinyltransferase [Source:VGNC Symbol;Acc:VGNC:87338]	1.73	0.083
ssc-miR-218	DNAJA4	DnaJ heat shock protein family (Hsp40) member A4 [Source:NCBI gene (formerly Entrezgene);Acc:397613]	1.73	0.083
ssc-miR-218	DNAJB13	DnaJ heat shock protein family (Hsp40) member B13 [Source:VGNC Symbol;Acc:VGNC:108582]	1.73	0.083
ssc-miR-218	DNAJC13	DnaJ heat shock protein family (Hsp40) member C13 [Source:VGNC Symbol;Acc:VGNC:108650]	1.73	0.083
ssc-miR-218	DNAJC27	DnaJ heat shock protein family (Hsp40) member C27 [Source:VGNC Symbol;Acc:VGNC:102563]	1.73	0.083
ssc-miR-218	DNAJC3	DnaJ heat shock protein family (Hsp40) member C3 [Source:NCBI gene (formerly Entrezgene);Acc:100154166]	1.73	0.083
ssc-miR-218	DNAL1	dynein axonemal light chain 1 [Source:VGNC Symbol;Acc:VGNC:87373]	1.73	0.083
ssc-miR-218	DNMT3A	DNA methyltransferase 3 alpha [Source:VGNC Symbol;Acc:VGNC:87384]	1.73	0.083
ssc-miR-218	DOCK7	dedicator of cytokinesis 7 [Source:VGNC Symbol;Acc:VGNC:87397]	1.73	0.083
ssc-miR-218	DOCK9	dedicator of cytokinesis 9 [Source:VGNC Symbol;Acc:VGNC:87399]	1.73	0.083
ssc-miR-218	DOLPP1	dolichyldiphosphatase 1 [Source:VGNC Symbol;Acc:VGNC:87407]	1.73	0.083
ssc-miR-218	DPF1	double PHD fingers 1 [Source:VGNC Symbol;Acc:VGNC:87412]	1.73	0.083

ssc-miR-218	DPF3	double PHD fingers 3 [Source:VGNC Symbol;Acc:VGNC:87414]	1.73	0.083
ssc-miR-218	DPP6	dipeptidyl peptidase like 6 [Source:VGNC Symbol;Acc:VGNC:87421]	1.73	0.083
ssc-miR-218	DPP9	dipeptidyl peptidase 9 [Source:VGNC Symbol;Acc:VGNC:87424]	1.73	0.083
ssc-miR-218	DPY19L3	dpy-19 like C-mannosyltransferase 3 [Source:VGNC Symbol;Acc:VGNC:87426]	1.73	0.083
ssc-miR-218	DST	dystonin [Source:HGNC Symbol;Acc:HGNC:1090]	1.73	0.083
ssc-miR-218	DUSP18	dual specificity phosphatase 18 [Source:VGNC Symbol;Acc:VGNC:103937]	1.73	0.083
ssc-miR-218	DUSP5	dual specificity phosphatase 5 [Source:VGNC Symbol;Acc:VGNC:87487]	1.73	0.083
ssc-miR-218	DYNC1I2	dynein cytoplasmic 1 intermediate chain 2 [Source:VGNC Symbol;Acc:VGNC:96224]	1.73	0.083
ssc-miR-218	EBF1	EBF transcription factor 1 [Source:VGNC Symbol;Acc:VGNC:87525]	1.73	0.083
ssc-miR-218	EBF3	EBF transcription factor 3 [Source:VGNC Symbol;Acc:VGNC:87527]	1.73	0.083
ssc-miR-218	EDEM1	ER degradation enhancing alpha-mannosidase like protein 1 [Source:VGNC Symbol;Acc:VGNC:87545]	1.73	0.083
ssc-miR-218	EFNB2	ephrin B2 [Source:VGNC Symbol;Acc:VGNC:87577]	1.73	0.083
ssc-miR-218	EGLN3	egl-9 family hypoxia inducible factor 3 [Source:VGNC Symbol;Acc:VGNC:87589]	1.73	0.083
ssc-miR-218	EI24	EI24 autophagy associated transmembrane protein [Source:VGNC Symbol;Acc:VGNC:87602]	1.73	0.083
ssc-miR-218	EIF3J	eukaryotic translation initiation factor 3 subunit J [Source:VGNC Symbol;Acc:VGNC:87621]	1.73	0.083
ssc-miR-218	EIF5A2	eukaryotic translation initiation factor 5A2 [Source:VGNC Symbol;Acc:VGNC:87633]	1.73	0.083
ssc-miR-218	ELFN2	extracellular leucine rich repeat and fibronectin type III domain containing 2 [Source:VGNC Symbol;Acc:VGNC:103232]	1.73	0.083
ssc-miR-218	ELK4	ETS transcription factor ELK4 [Source:VGNC Symbol;Acc:VGNC:87647]	1.73	0.083
ssc-miR-218	ELL2	elongation factor for RNA polymerase II 2 [Source:VGNC Symbol;Acc:VGNC:87649]	1.73	0.083
ssc-miR-218	ELMO1	engulfment and cell motility 1 [Source:HGNC Symbol;Acc:HGNC:16286]	1.73	0.083
ssc-miR-218	ELMSAN1	hypothetical gene	1.73	0.083
ssc-miR-218	ELOVL5	ELOVL fatty acid elongase 5 [Source:VGNC Symbol;Acc:VGNC:87661]	1.73	0.083
ssc-miR-218	EPB41L1	hypothetical gene	1.73	0.083
ssc-miR-218	EPHA5	EPH receptor A5 [Source:VGNC Symbol;Acc:VGNC:87733]	1.73	0.083
ssc-miR-218	EPHA7	EPH receptor A7 [Source:VGNC Symbol;Acc:VGNC:87734]	1.73	0.083
ssc-miR-218	EPHA8	EPH receptor A8 [Source:VGNC Symbol;Acc:VGNC:87735]	1.73	0.083
ssc-miR-218	EPS8	epidermal growth factor receptor pathway substrate 8 [Source:VGNC Symbol;Acc:VGNC:87749]	1.73	0.083
ssc-miR-218	ERBB4	erb-b2 receptor tyrosine kinase 4 [Source:VGNC Symbol;Acc:VGNC:96284]	1.73	0.083
ssc-miR-218	ERC2	ELKS/RAB6-interacting/CAST family member 2 [Source:VGNC Symbol;Acc:VGNC:87762]	1.73	0.083
ssc-miR-218	ERGIC2	ERGIC and golgi 2 [Source:VGNC Symbol;Acc:VGNC:87771]	1.73	0.083
ssc-miR-218	ERMP1	endoplasmic reticulum metallopeptidase 1 [Source:VGNC Symbol;Acc:VGNC:87777]	1.73	0.083
ssc-miR-218	ERN1	endoplasmic reticulum to nucleus signaling 1 [Source:HGNC Symbol;Acc:HGNC:3449]	1.73	0.083
ssc-miR-218	ERRFI1	ERBB receptor feedback inhibitor 1 [Source:VGNC Symbol;Acc:VGNC:87784]	1.73	0.083
ssc-miR-218	ETNK1	ethanolamine kinase 1 [Source:VGNC Symbol;Acc:VGNC:87806]	1.73	0.083
ssc-miR-218	EXD2	exonuclease 3'-5' domain containing 2 [Source:VGNC Symbol;Acc:VGNC:97990]	1.73	0.083
ssc-miR-218	EXOC5	exocyst complex component 5 [Source:VGNC Symbol;Acc:VGNC:87831]	1.73	0.083
ssc-miR-218	EXTL3	exostosin like glycosyltransferase 3 [Source:VGNC Symbol;Acc:VGNC:87850]	1.73	0.083
ssc-miR-218	FAM109A	hypothetical gene	1.73	0.083
ssc-miR-218	FAM126A	family with sequence similarity 126 member A [Source:VGNC Symbol;Acc:VGNC:87899]	1.73	0.083
ssc-miR-218	FAM126B	family with sequence similarity 126 member B [Source:HGNC Symbol;Acc:HGNC:28593]	1.73	0.083
ssc-miR-218	FAM13A	family with sequence similarity 13 member A [Source:VGNC Symbol;Acc:VGNC:98924]	1.73	0.083
ssc-miR-218	FAM162B	family with sequence similarity 162 member B [Source:VGNC Symbol;Acc:VGNC:87919]	1.73	0.083
ssc-miR-218	FAM171B	family with sequence similarity 171 member B [Source:VGNC Symbol;Acc:VGNC:96218]	1.73	0.083
ssc-miR-218	FAM172A	family with sequence similarity 172 member A [Source:VGNC Symbol;Acc:VGNC:87930]	1.73	0.083
ssc-miR-218	FAM175B	hypothetical gene	1.73	0.083
ssc-miR-218	FAM178A	hypothetical gene	1.73	0.083
ssc-miR-218	FAM196A	hypothetical gene	1.73	0.083
ssc-miR-218	FAM196B	hypothetical gene	1.73	0.083
ssc-miR-218	FAM20B	FAM20B glycosaminoglycan xylosylkinase [Source:VGNC Symbol;Acc:VGNC:87950]	1.73	0.083
ssc-miR-218	FAM214A	family with sequence similarity 214 member A [Source:VGNC Symbol;Acc:VGNC:87952]	1.73	0.083

ssc-miR-218	FAM217B	family with sequence similarity 217 member B [Source:VGNC Symbol;Acc:VGNC:95767]	1.73	0.083
ssc-miR-218	FAM3C	FAM3 metabolism regulating signaling molecule C [Source:VGNC Symbol;Acc:VGNC:87969]	1.73	0.083
ssc-miR-218	FAM63B	hypothetical gene	1.73	0.083
ssc-miR-218	FAM65B	hypothetical gene	1.73	0.083
ssc-miR-218	FAM78A	family with sequence similarity 78 member A [Source:VGNC Symbol;Acc:VGNC:87984]	1.73	0.083
ssc-miR-218	FAM81A	family with sequence similarity 81 member A [Source:VGNC Symbol;Acc:VGNC:87986]	1.73	0.083
ssc-miR-218	FAT3	hypothetical gene	1.73	0.083
ssc-miR-218	FBN2	fibrillin 2 [Source:VGNC Symbol;Acc:VGNC:88025]	1.73	0.083
ssc-miR-218	FBRSL1	hypothetical gene	1.73	0.083
ssc-miR-218	FBXL2	F-box and leucine rich repeat protein 2 [Source:VGNC Symbol;Acc:VGNC:98005]	1.73	0.083
ssc-miR-218	FBXL20	F-box and leucine rich repeat protein 20 [Source:VGNC Symbol;Acc:VGNC:98006]	1.73	0.083
ssc-miR-218	FBXO28	F-box protein 28 [Source:HGNC Symbol;Acc:HGNC:29046]	1.73	0.083
ssc-miR-218	FBXO36	F-box protein 36 [Source:HGNC Symbol;Acc:HGNC:27020]	1.73	0.083
ssc-miR-218	FBXO41	F-box protein 41 [Source:VGNC Symbol;Acc:VGNC:88046]	1.73	0.083
ssc-miR-218	FCHO2	FCH and mu domain containing endocytic adaptor 2 [Source:VGNC Symbol;Acc:VGNC:88066]	1.73	0.083
ssc-miR-218	FCHSD2	FCH and double SH3 domains 2 [Source:VGNC Symbol;Acc:VGNC:88068]	1.73	0.083
ssc-miR-218	FGD4	FYVE, RhoGEF and PH domain containing 4 [Source:VGNC Symbol;Acc:VGNC:88097]	1.73	0.083
ssc-miR-218	FGD6	FYVE, RhoGEF and PH domain containing 6 [Source:VGNC Symbol;Acc:VGNC:88099]	1.73	0.083
ssc-miR-218	FGF12	fibroblast growth factor 12 [Source:VGNC Symbol;Acc:VGNC:88102]	1.73	0.083
ssc-miR-218	FGFR2	fibroblast growth factor receptor 2 [Source:NCBI gene (formerly Entrezgene);Acc:396762]	1.73	0.083
ssc-miR-218	FHL5	four and a half LIM domains 5 [Source:VGNC Symbol;Acc:VGNC:88130]	1.73	0.083
ssc-miR-218	FHOD3	formin homology 2 domain containing 3 [Source:VGNC Symbol;Acc:VGNC:88132]	1.73	0.083
ssc-miR-218	FIGN	fidgetin, microtubule severing factor [Source:VGNC Symbol;Acc:VGNC:95580]	1.73	0.083
ssc-miR-218	FLNC	filamin C [Source:VGNC Symbol;Acc:VGNC:88157]	1.73	0.083
ssc-miR-218	FLRT2	fibronectin leucine rich transmembrane protein 2 [Source:VGNC Symbol;Acc:VGNC:88161]	1.73	0.083
ssc-miR-218	FLRT3	fibronectin leucine rich transmembrane protein 3 [Source:VGNC Symbol;Acc:VGNC:96304]	1.73	0.083
ssc-miR-218	FOXN2	forkhead box N2 [Source:VGNC Symbol;Acc:VGNC:88219]	1.73	0.083
ssc-miR-218	FOXN3	forkhead box N3 [Source:VGNC Symbol;Acc:VGNC:88220]	1.73	0.083
ssc-miR-218	FOXP2	forkhead box P2 [Source:VGNC Symbol;Acc:VGNC:98014]	1.73	0.083
ssc-miR-218	FRMD4A	FERM domain containing 4A [Source:VGNC Symbol;Acc:VGNC:96088]	1.73	0.083
ssc-miR-218	FRMD4B	FERM domain containing 4B [Source:VGNC Symbol;Acc:VGNC:88237]	1.73	0.083
ssc-miR-218	FRMD6	FERM domain containing 6 [Source:VGNC Symbol;Acc:VGNC:88239]	1.73	0.083
ssc-miR-218	FRMPD4	FERM and PDZ domain containing 4 [Source:VGNC Symbol;Acc:VGNC:88243]	1.73	0.083
ssc-miR-218	FRZB	frizzled related protein [Source:VGNC Symbol;Acc:VGNC:96308]	1.73	0.083
ssc-miR-218	FSBP	hypothetical gene	1.73	0.083
ssc-miR-218	FUBP1	far upstream element binding protein 1 [Source:VGNC Symbol;Acc:VGNC:96781]	1.73	0.083
ssc-miR-218	FUT9	fucosyltransferase 9 [Source:VGNC Symbol;Acc:VGNC:88271]	1.73	0.083
ssc-miR-218	FYCO1	hypothetical gene	1.73	0.083
ssc-miR-218	FZD4	frizzled class receptor 4 [Source:VGNC Symbol;Acc:VGNC:88282]	1.73	0.083
ssc-miR-218	GAB2	GRB2 associated binding protein 2 [Source:VGNC Symbol;Acc:VGNC:108586]	1.73	0.083
ssc-miR-218	GABPA	GA binding protein transcription factor subunit alpha [Source:VGNC Symbol;Acc:VGNC:103945]	1.73	0.083
ssc-miR-218	GABRB2	gamma-aminobutyric acid type A receptor subunit beta2 [Source:VGNC Symbol;Acc:VGNC:88307]	1.73	0.083
ssc-miR-218	GABRB3	gamma-aminobutyric acid type A receptor subunit beta3 [Source:VGNC Symbol;Acc:VGNC:88308]	1.73	0.083
ssc-miR-218	GALNT1	polypeptide N-acetylgalactosaminyltransferase 1 [Source:VGNC Symbol;Acc:VGNC:97062]	1.73	0.083
ssc-miR-218	GALNT13	polypeptide N-acetylgalactosaminyltransferase 13 [Source:VGNC Symbol;Acc:VGNC:95992]	1.73	0.083
ssc-miR-218	GALNT3	polypeptide N-acetylgalactosaminyltransferase 3 [Source:VGNC Symbol;Acc:VGNC:96313]	1.73	0.083
ssc-miR-218	GALNT4	polypeptide N-acetylgalactosaminyltransferase 4 [Source:HGNC Symbol;Acc:HGNC:4126]	1.73	0.083
ssc-miR-218	GAN	gigaxonin [Source:VGNC Symbol;Acc:VGNC:88343]	1.73	0.083
ssc-miR-218	GAPVD1	GTPase activating protein and VPS9 domains 1 [Source:VGNC Symbol;Acc:VGNC:88347]	1.73	0.083
ssc-miR-218	GAREML	hypothetical gene	1.73	0.083

ssc-miR-218	GATC	glutamyl-tRNA amidotransferase subunit C [Source:VGNC Symbol;Acc:VGNC:103948]	1.73	0.083
ssc-miR-218	GDAP2	ganglioside induced differentiation associated protein 2 [Source:VGNC Symbol;Acc:VGNC:88395]	1.73	0.083
ssc-miR-218	GDE1	glycerophosphodiester phosphodiesterase 1 [Source:VGNC Symbol;Acc:VGNC:88396]	1.73	0.083
ssc-miR-218	GDI1	GDP dissociation inhibitor 1 [Source:VGNC Symbol;Acc:VGNC:88405]	1.73	0.083
ssc-miR-218	GDPD5	glycerophosphodiester phosphodiesterase domain containing 5 [Source:VGNC Symbol;Acc:VGNC:88409]	1.73	0.083
ssc-miR-218	GET4	hypothetical gene	1.73	0.083
ssc-miR-218	GFPT1	glutamine--fructose-6-phosphate transaminase 1 [Source:VGNC Symbol;Acc:VGNC:88426]	1.73	0.083
ssc-miR-218	GFRA1	GDNF family receptor alpha 1 [Source:VGNC Symbol;Acc:VGNC:88428]	1.73	0.083
ssc-miR-218	GHITM	growth hormone inducible transmembrane protein [Source:VGNC Symbol;Acc:VGNC:88442]	1.73	0.083
ssc-miR-218	GINS2	GINS complex subunit 2 [Source:HGNC Symbol;Acc:HGNC:24575]	1.73	0.083
ssc-miR-218	GJA1	gap junction protein alpha 1 [Source:VGNC Symbol;Acc:VGNC:103098]	1.73	0.083
ssc-miR-218	GLCE	glucuronic acid epimerase [Source:VGNC Symbol;Acc:VGNC:88477]	1.73	0.083
ssc-miR-218	GLIPR2	GLI pathosis related 2 [Source:VGNC Symbol;Acc:VGNC:98023]	1.73	0.083
ssc-miR-218	GMEB1	glucocorticoid modulatory element binding protein 1 [Source:VGNC Symbol;Acc:VGNC:88508]	1.73	0.083
ssc-miR-218	GNA13	G protein subunit alpha 13 [Source:VGNC Symbol;Acc:VGNC:98997]	1.73	0.083
ssc-miR-218	GNAI2	G protein subunit alpha i2 [Source:VGNC Symbol;Acc:VGNC:88522]	1.73	0.083
ssc-miR-218	GNAI3	G protein subunit alpha i3 [Source:VGNC Symbol;Acc:VGNC:88523]	1.73	0.083
ssc-miR-218	GNAO1	hypothetical gene	1.73	0.083
ssc-miR-218	GNAS	hypothetical gene	1.73	0.083
ssc-miR-218	GNB1	G protein subunit beta 1 [Source:VGNC Symbol;Acc:VGNC:88529]	1.73	0.083
ssc-miR-218	GNG3	hypothetical gene	1.73	0.083
ssc-miR-218	GNG4	G protein subunit gamma 4 [Source:HGNC Symbol;Acc:HGNC:4407]	1.73	0.083
ssc-miR-218	GNPTG	N-acetylglucosamine-1-phosphate transferase subunit gamma [Source:VGNC Symbol;Acc:VGNC:88547]	1.73	0.083
ssc-miR-218	GOLGA7	golgin A7 [Source:VGNC Symbol;Acc:VGNC:96059]	1.73	0.083
ssc-miR-218	GOLGA7B	golgin A7 family member B [Source:VGNC Symbol;Acc:VGNC:88553]	1.73	0.083
ssc-miR-218	GOLT1B	golgi transport 1B [Source:VGNC Symbol;Acc:VGNC:88557]	1.73	0.083
ssc-miR-218	GPAM	glycerol-3-phosphate acyltransferase, mitochondrial [Source:VGNC Symbol;Acc:VGNC:88570]	1.73	0.083
ssc-miR-218	GPD1L	glycerol-3-phosphate dehydrogenase 1 like [Source:VGNC Symbol;Acc:VGNC:108661]	1.73	0.083
ssc-miR-218	GPLD1	glycosylphosphatidylinositol specific phospholipase D1 [Source:VGNC Symbol;Acc:VGNC:88590]	1.73	0.083
ssc-miR-218	GPR126	hypothetical gene	1.73	0.083
ssc-miR-218	GPR137C	G protein-coupled receptor 137C [Source:VGNC Symbol;Acc:VGNC:88602]	1.73	0.083
ssc-miR-218	GPR153	G protein-coupled receptor 153 [Source:VGNC Symbol;Acc:VGNC:88609]	1.73	0.083
ssc-miR-218	GPR161	G protein-coupled receptor 161 [Source:VGNC Symbol;Acc:VGNC:88613]	1.73	0.083
ssc-miR-218	GPR45	G protein-coupled receptor 45 [Source:VGNC Symbol;Acc:VGNC:88632]	1.73	0.083
ssc-miR-218	GPR63	G protein-coupled receptor 63 [Source:VGNC Symbol;Acc:VGNC:88634]	1.73	0.083
ssc-miR-218	GPR83	G protein-coupled receptor 83 [Source:VGNC Symbol;Acc:VGNC:88637]	1.73	0.083
ssc-miR-218	GPR85	G protein-coupled receptor 85 [Source:VGNC Symbol;Acc:VGNC:88638]	1.73	0.083
ssc-miR-218	GPRC5B	G protein-coupled receptor class C group 5 member B [Source:VGNC Symbol;Acc:VGNC:88643]	1.73	0.083
ssc-miR-218	GRAMD4	GRAM domain containing 4 [Source:VGNC Symbol;Acc:VGNC:88659]	1.73	0.083
ssc-miR-218	GREM1	gremlin 1, DAN family BMP antagonist [Source:VGNC Symbol;Acc:VGNC:88665]	1.73	0.083
ssc-miR-218	GRIA2	glutamate ionotropic receptor AMPA type subunit 2 [Source:VGNC Symbol;Acc:VGNC:88671]	1.73	0.083
ssc-miR-218	GRIK2	glutamate ionotropic receptor kainate type subunit 2 [Source:VGNC Symbol;Acc:VGNC:88678]	1.73	0.083
ssc-miR-218	GRIK3	glutamate ionotropic receptor kainate type subunit 3 [Source:VGNC Symbol;Acc:VGNC:88679]	1.73	0.083
ssc-miR-218	GRIN2B	glutamate ionotropic receptor NMDA type subunit 2B [Source:VGNC Symbol;Acc:VGNC:88684]	1.73	0.083
ssc-miR-218	GRIP1	glutamate receptor interacting protein 1 [Source:VGNC Symbol;Acc:VGNC:88690]	1.73	0.083
ssc-miR-218	GRM1	glutamate metabotropic receptor 1 [Source:VGNC Symbol;Acc:VGNC:88700]	1.73	0.083
ssc-miR-218	GRM3	glutamate metabotropic receptor 3 [Source:VGNC Symbol;Acc:VGNC:88702]	1.73	0.083
ssc-miR-218	GSK3B	glycogen synthase kinase 3 beta [Source:VGNC Symbol;Acc:VGNC:88723]	1.73	0.083
ssc-miR-218	GSKIP	GSK3B interacting protein [Source:VGNC Symbol;Acc:VGNC:103956]	1.73	0.083
ssc-miR-218	GSPT1	G1 to S phase transition 1 [Source:VGNC Symbol;Acc:VGNC:88724]	1.73	0.083

ssc-miR-218	GSX2	GS homeobox 2 [Source:VGNC Symbol;Acc:VGNC:88729]	1.73	0.083
ssc-miR-218	GTF2H3	ral transcription factor IIH subunit 3 [Source:VGNC Symbol;Acc:VGNC:88735]	1.73	0.083
ssc-miR-218	GUCY1A2	guanylate cyclase 1 soluble subunit alpha 2 [Source:VGNC Symbol;Acc:VGNC:88751]	1.73	0.083
ssc-miR-218	HAPLN1	hyaluronan and proteoglycan link protein 1 [Source:VGNC Symbol;Acc:VGNC:88780]	1.73	0.083
ssc-miR-218	HCFC1	host cell factor C1 [Source:HGNC Symbol;Acc:HGNC:4839]	1.73	0.083
ssc-miR-218	HCN3	hyperpolarization activated cyclic nucleotide gated potassium channel 3 [Source:VGNC Symbol;Acc:VGNC:88804]	1.73	0.083
ssc-miR-218	HCN4	hyperpolarization activated cyclic nucleotide gated potassium channel 4 [Source:VGNC Symbol;Acc:VGNC:88805]	1.73	0.083
ssc-miR-218	HDAC7	histone deacetylase 7 [Source:VGNC Symbol;Acc:VGNC:103286]	1.73	0.083
ssc-miR-218	HECTD2	HECT domain E3 ubiquitin protein ligase 2 [Source:VGNC Symbol;Acc:VGNC:88833]	1.73	0.083
ssc-miR-218	HECTD3	HECT domain E3 ubiquitin protein ligase 3 [Source:VGNC Symbol;Acc:VGNC:97096]	1.73	0.083
ssc-miR-218	HECW1	HECT, C2 and WW domain containing E3 ubiquitin protein ligase 1 [Source:VGNC Symbol;Acc:VGNC:88835]	1.73	0.083
ssc-miR-218	HERPUD2	HERPUD family member 2 [Source:VGNC Symbol;Acc:VGNC:88853]	1.73	0.083
ssc-miR-218	HIC2	HIC ZBTB transcriptional repressor 2 [Source:HGNC Symbol;Acc:HGNC:18595]	1.73	0.083
ssc-miR-218	HIVEP1	HIVEP zinc finger 1 [Source:VGNC Symbol;Acc:VGNC:96585]	1.73	0.083
ssc-miR-218	HIVEP2	HIVEP zinc finger 2 [Source:VGNC Symbol;Acc:VGNC:96586]	1.73	0.083
ssc-miR-218	HIVEP3	hypothetical gene	1.73	0.083
ssc-miR-218	HLF	HLF transcription factor, PAR bZIP family member [Source:VGNC Symbol;Acc:VGNC:88896]	1.73	0.083
ssc-miR-218	HMGB1	hypothetical gene	1.73	0.083
ssc-miR-218	HNRNPA1	heterous nuclear ribonucleoprotein A1 [Source:VGNC Symbol;Acc:VGNC:88918]	1.73	0.083
ssc-miR-218	HNRNPA3	hypothetical gene	1.73	0.083
ssc-miR-218	HNRNPC	hypothetical gene	1.73	0.083
ssc-miR-218	HOOK1	hypothetical gene	1.73	0.083
ssc-miR-218	HOXA1	homeobox A1 [Source:VGNC Symbol;Acc:VGNC:88933]	1.73	0.083
ssc-miR-218	HOXA10	homeobox A10 [Source:VGNC Symbol;Acc:VGNC:88934]	1.73	0.083
ssc-miR-218	HOXB3	homeobox B3 [Source:VGNC Symbol;Acc:VGNC:88944]	1.73	0.083
ssc-miR-218	HOXD10	homeobox D10 [Source:VGNC Symbol;Acc:VGNC:96351]	1.73	0.083
ssc-miR-218	HOXD4	homeobox D4 [Source:HGNC Symbol;Acc:HGNC:5138]	1.73	0.083
ssc-miR-218	HOXD8	homeobox D8 [Source:VGNC Symbol;Acc:VGNC:96356]	1.73	0.083
ssc-miR-218	HP1BP3	heterochromatin protein 1 binding protein 3 [Source:VGNC Symbol;Acc:VGNC:88956]	1.73	0.083
ssc-miR-218	HPGD	15-hydroxyprostaglandin dehydrogenase [Source:VGNC Symbol;Acc:VGNC:88961]	1.73	0.083
ssc-miR-218	HRAS	HRas proto-onco, GTPase [Source:VGNC Symbol;Acc:VGNC:88970]	1.73	0.083
ssc-miR-218	HS3ST2	heparan sulfate-glucosamine 3-sulfotransferase 2 [Source:VGNC Symbol;Acc:VGNC:88978]	1.73	0.083
ssc-miR-218	HS3ST3B1	heparan sulfate-glucosamine 3-sulfotransferase 3B1 [Source:VGNC Symbol;Acc:VGNC:99003]	1.73	0.083
ssc-miR-218	HS6ST3	heparan sulfate 6-O-sulfotransferase 3 [Source:HGNC Symbol;Acc:HGNC:19134]	1.73	0.083
ssc-miR-218	ICK	hypothetical gene	1.73	0.083
ssc-miR-218	IDH3A	isocitrate dehydrogenase (NAD(+)) 3 catalytic subunit alpha [Source:HGNC Symbol;Acc:HGNC:5384]	1.73	0.083
ssc-miR-218	IGSF11	immunoglobulin superfamily member 11 [Source:VGNC Symbol;Acc:VGNC:89064]	1.73	0.083
ssc-miR-218	IKZF1	IKAROS family zinc finger 1 [Source:VGNC Symbol;Acc:VGNC:89073]	1.73	0.083
ssc-miR-218	IMPAD1	hypothetical gene	1.73	0.083
ssc-miR-218	IMPG2	interphotoreceptor matrix proteoglycan 2 [Source:VGNC Symbol;Acc:VGNC:98048]	1.73	0.083
ssc-miR-218	INHBB	inhibin subunit beta B [Source:VGNC Symbol;Acc:VGNC:103969]	1.73	0.083
ssc-miR-218	INSIG1	insulin induced 1 [Source:VGNC Symbol;Acc:VGNC:89150]	1.73	0.083
ssc-miR-218	INTS6	integrator complex subunit 6 [Source:VGNC Symbol;Acc:VGNC:89166]	1.73	0.083
ssc-miR-218	IPMK	inositol polyphosphate multikinase [Source:VGNC Symbol;Acc:VGNC:89175]	1.73	0.083
ssc-miR-218	ISCA2	iron-sulfur cluster assembly 2 [Source:VGNC Symbol;Acc:VGNC:89221]	1.73	0.083
ssc-miR-218	ISLR2	immunoglobulin superfamily containing leucine rich repeat 2 [Source:VGNC Symbol;Acc:VGNC:89227]	1.73	0.083
ssc-miR-218	ITM2C	integral membrane protein 2C [Source:VGNC Symbol;Acc:VGNC:96383]	1.73	0.083
ssc-miR-218	ITSN1	intersectin 1 [Source:VGNC Symbol;Acc:VGNC:108669]	1.73	0.083
ssc-miR-218	JAG1	jagged canonical Notch ligand 1 [Source:VGNC Symbol;Acc:VGNC:96385]	1.73	0.083
ssc-miR-218	JAKMIP2	janus kinase and microtubule interacting protein 2 [Source:VGNC Symbol;Acc:VGNC:89274]	1.73	0.083

ssc-miR-218	JAKMIP3	Janus kinase and microtubule interacting protein 3 [Source:VGNC Symbol;Acc:VGNC:89275]	1.73	0.083
ssc-miR-218	JDP2	Jun dimerization protein 2 [Source:VGNC Symbol;Acc:VGNC:89282]	1.73	0.083
ssc-miR-218	JMY	junction mediating and regulatory protein, p53 cofactor [Source:VGNC Symbol;Acc:VGNC:89289]	1.73	0.083
ssc-miR-218	KANSL1	KAT8 regulatory NSL complex subunit 1 [Source:VGNC Symbol;Acc:VGNC:89299]	1.73	0.083
ssc-miR-218	KAT8	lysine acetyltransferase 8 [Source:VGNC Symbol;Acc:VGNC:89308]	1.73	0.083
ssc-miR-218	KCNA1	potassium voltage-gated channel subfamily A member 1 [Source:VGNC Symbol;Acc:VGNC:89323]	1.73	0.083
ssc-miR-218	KCNAB1	potassium voltage-gated channel subfamily A regulatory beta subunit 1 [Source:VGNC Symbol;Acc:VGNC:89328]	1.73	0.083
ssc-miR-218	KCNB1	potassium voltage-gated channel subfamily B member 1 [Source:VGNC Symbol;Acc:VGNC:96386]	1.73	0.083
ssc-miR-218	KCNC2	potassium voltage-gated channel subfamily C member 2 [Source:VGNC Symbol;Acc:VGNC:89333]	1.73	0.083
ssc-miR-218	KCND1	potassium voltage-gated channel subfamily D member 1 [Source:VGNC Symbol;Acc:VGNC:89336]	1.73	0.083
ssc-miR-218	KCND2	potassium voltage-gated channel subfamily D member 2 [Source:HGNC Symbol;Acc:HGNC:6238]	1.73	0.083
ssc-miR-218	KCNE1	potassium voltage-gated channel subfamily E regulatory subunit 1 [Source:NCBI gene (formerly Entrezgene);Acc:100621502]	1.73	0.083
ssc-miR-218	KCNH1	potassium voltage-gated channel subfamily H member 1 [Source:VGNC Symbol;Acc:VGNC:108599]	1.73	0.083
ssc-miR-218	KCNH5	potassium voltage-gated channel subfamily H member 5 [Source:VGNC Symbol;Acc:VGNC:89345]	1.73	0.083
ssc-miR-218	KCNIP3	potassium voltage-gated channel interacting protein 3 [Source:VGNC Symbol;Acc:VGNC:89350]	1.73	0.083
ssc-miR-218	KCNJ6	potassium inwardly rectifying channel subfamily J member 6 [Source:VGNC Symbol;Acc:VGNC:89360]	1.73	0.083
ssc-miR-218	KCNK1	potassium two pore domain channel subfamily K member 1 [Source:VGNC Symbol;Acc:VGNC:89363]	1.73	0.083
ssc-miR-218	KCNK12	potassium two pore domain channel subfamily K member 12 [Source:HGNC Symbol;Acc:HGNC:6274]	1.73	0.083
ssc-miR-218	KCNK15	potassium two pore domain channel subfamily K member 15 [Source:VGNC Symbol;Acc:VGNC:95593]	1.73	0.083
ssc-miR-218	KCNK2	potassium two pore domain channel subfamily K member 2 [Source:VGNC Symbol;Acc:VGNC:89369]	1.73	0.083
ssc-miR-218	KCNMB4	potassium calcium-activated channel subfamily M regulatory beta subunit 4 [Source:VGNC Symbol;Acc:VGNC:89378]	1.73	0.083
ssc-miR-218	KCNN3	potassium calcium-activated channel subfamily N member 3 [Source:VGNC Symbol;Acc:VGNC:98056]	1.73	0.083
ssc-miR-218	KCNQ3	potassium voltage-gated channel subfamily Q member 3 [Source:VGNC Symbol;Acc:VGNC:89382]	1.73	0.083
ssc-miR-218	KCNQ4	potassium voltage-gated channel subfamily Q member 4 [Source:VGNC Symbol;Acc:VGNC:89383]	1.73	0.083
ssc-miR-218	KCNQ5	potassium voltage-gated channel subfamily Q member 5 [Source:VGNC Symbol;Acc:VGNC:89384]	1.73	0.083
ssc-miR-218	KCNT1	potassium sodium-activated channel subfamily T member 1 [Source:VGNC Symbol;Acc:VGNC:89388]	1.73	0.083
ssc-miR-218	KCTD16	potassium channel tetramerization domain containing 16 [Source:VGNC Symbol;Acc:VGNC:89395]	1.73	0.083
ssc-miR-218	KCTD9	potassium channel tetramerization domain containing 9 [Source:VGNC Symbol;Acc:VGNC:89404]	1.73	0.083
ssc-miR-218	KDM2A	lysine demethylase 2A [Source:VGNC Symbol;Acc:VGNC:89410]	1.73	0.083
ssc-miR-218	KDM3B	lysine demethylase 3B [Source:VGNC Symbol;Acc:VGNC:89412]	1.73	0.083
ssc-miR-218	KDM5A	lysine demethylase 5A [Source:VGNC Symbol;Acc:VGNC:89415]	1.73	0.083
ssc-miR-218	KHNYN	KH and NYN domain containing [Source:VGNC Symbol;Acc:VGNC:89426]	1.73	0.083
ssc-miR-218	KIAA0040	KIAA0040 [Source:VGNC Symbol;Acc:VGNC:98059]	1.73	0.083
ssc-miR-218	KIAA1024	hypothetical gene	1.73	0.083
ssc-miR-218	KIAA1045	hypothetical gene	1.73	0.083
ssc-miR-218	KIAA1161	hypothetical gene	1.73	0.083
ssc-miR-218	KIAA1324	hypothetical gene	1.73	0.083
ssc-miR-218	KIAA1429	hypothetical gene	1.73	0.083
ssc-miR-218	KIAA1456	hypothetical gene	1.73	0.083
ssc-miR-218	KIAA1522	KIAA1522 [Source:VGNC Symbol;Acc:VGNC:89444]	1.73	0.083
ssc-miR-218	KIAA1549	KIAA1549 [Source:VGNC Symbol;Acc:VGNC:99719]	1.73	0.083
ssc-miR-218	KIAA1549L	KIAA1549 like [Source:VGNC Symbol;Acc:VGNC:89445]	1.73	0.083
ssc-miR-218	KIAA2022	hypothetical gene	1.73	0.083
ssc-miR-218	KIF13A	kinesin family member 13A [Source:VGNC Symbol;Acc:VGNC:89454]	1.73	0.083
ssc-miR-218	KIF21B	kinesin family member 21B [Source:VGNC Symbol;Acc:VGNC:96219]	1.73	0.083
ssc-miR-218	KIF2A	kinesin family member 2A [Source:VGNC Symbol;Acc:VGNC:89467]	1.73	0.083
ssc-miR-218	KIF3C	kinesin family member 3C [Source:VGNC Symbol;Acc:VGNC:89471]	1.73	0.083
ssc-miR-218	KIRREL3	kirre like nephrin family adhesion molecule 3 [Source:VGNC Symbol;Acc:VGNC:89482]	1.73	0.083
ssc-miR-218	KIT	KIT proto-onco, receptor tyrosine kinase [Source:VGNC Symbol;Acc:VGNC:98060]	1.73	0.083
ssc-miR-218	KLF12	Kruppel like factor 12 [Source:VGNC Symbol;Acc:VGNC:89492]	1.73	0.083

ssc-miR-218	KLF3	Kruppel like factor 3 [Source:VGNC Symbol;Acc:VGNC:89498]	1.73	0.083
ssc-miR-218	KLF8	Kruppel like factor 8 [Source:VGNC Symbol;Acc:VGNC:89500]	1.73	0.083
ssc-miR-218	KLF9	Kruppel like factor 9 [Source:VGNC Symbol;Acc:VGNC:103114]	1.73	0.083
ssc-miR-218	KLHDC10	kelch domain containing 10 [Source:VGNC Symbol;Acc:VGNC:89502]	1.73	0.083
ssc-miR-218	KLHDC8A	kelch domain containing 8A [Source:VGNC Symbol;Acc:VGNC:89508]	1.73	0.083
ssc-miR-218	KLHL11	kelch like family member 11 [Source:VGNC Symbol;Acc:VGNC:89513]	1.73	0.083
ssc-miR-218	KLHL13	kelch like family member 13 [Source:VGNC Symbol;Acc:VGNC:89514]	1.73	0.083
ssc-miR-218	KLHL18	kelch like family member 18 [Source:VGNC Symbol;Acc:VGNC:89517]	1.73	0.083
ssc-miR-218	KLHL23	kelch like family member 23 [Source:VGNC Symbol;Acc:VGNC:96158]	1.73	0.083
ssc-miR-218	KLHL25	kelch like family member 25 [Source:HGNC Symbol;Acc:HGNC:25732]	1.73	0.083
ssc-miR-218	KLHL29	kelch like family member 29 [Source:VGNC Symbol;Acc:VGNC:89524]	1.73	0.083
ssc-miR-218	KMT2A	lysine methyltransferase 2A [Source:VGNC Symbol;Acc:VGNC:108600]	1.73	0.083
ssc-miR-218	KPNA1	karyopherin subunit alpha 1 [Source:VGNC Symbol;Acc:VGNC:89560]	1.73	0.083
ssc-miR-218	KPNA4	karyopherin subunit alpha 4 [Source:VGNC Symbol;Acc:VGNC:89563]	1.73	0.083
ssc-miR-218	KRIT1	KRIT1 ankyrin repeat containing [Source:HGNC Symbol;Acc:HGNC:1573]	1.73	0.083
ssc-miR-218	KRTAP3-3	hypothetical gene	1.73	0.083
ssc-miR-218	L3MBTL3	L3MBTL histone methyl-lysine binding protein 3 [Source:VGNC Symbol;Acc:VGNC:98066]	1.73	0.083
ssc-miR-218	L3MBTL4	hypothetical gene	1.73	0.083
ssc-miR-218	LAMP2	lysosomal associated membrane protein 2 [Source:VGNC Symbol;Acc:VGNC:89628]	1.73	0.083
ssc-miR-218	LARP4B	hypothetical gene	1.73	0.083
ssc-miR-218	LASP1	LIM and SH3 protein 1 [Source:VGNC Symbol;Acc:VGNC:89646]	1.73	0.083
ssc-miR-218	LCORL	ligand dependent nuclear receptor corepressor like [Source:VGNC Symbol;Acc:VGNC:98937]	1.73	0.083
ssc-miR-218	LDLRAD4	low density lipoprotein receptor class A domain containing 4 [Source:VGNC Symbol;Acc:VGNC:89673]	1.73	0.083
ssc-miR-218	LGALSL	galectin like [Source:VGNC Symbol;Acc:VGNC:89698]	1.73	0.083
ssc-miR-218	LGR4	leucine rich repeat containing G protein-coupled receptor 4 [Source:VGNC Symbol;Acc:VGNC:89703]	1.73	0.083
ssc-miR-218	LHFP	hypothetical gene	1.73	0.083
ssc-miR-218	LHX1	LIM homeobox 1 [Source:VGNC Symbol;Acc:VGNC:89711]	1.73	0.083
ssc-miR-218	LHX4	LIM homeobox 4 [Source:VGNC Symbol;Acc:VGNC:89714]	1.73	0.083
ssc-miR-218	LHX9	LIM homeobox 9 [Source:VGNC Symbol;Acc:VGNC:95608]	1.73	0.083
ssc-miR-218	LIFR	LIF receptor subunit alpha [Source:HGNC Symbol;Acc:HGNC:6597]	1.73	0.083
ssc-miR-218	LIMD1	LIM domain containing 1 [Source:VGNC Symbol;Acc:VGNC:89725]	1.73	0.083
ssc-miR-218	LIN7A	lin-7 homolog A, crumbs cell polarity complex component [Source:VGNC Symbol;Acc:VGNC:89730]	1.73	0.083
ssc-miR-218	LIPG	lipase G, endothelial type [Source:VGNC Symbol;Acc:VGNC:89739]	1.73	0.083
ssc-miR-218	LMLN	leishmanolysin like peptidase [Source:VGNC Symbol;Acc:VGNC:89759]	1.73	0.083
ssc-miR-218	LMNB1	lamin B1 [Source:VGNC Symbol;Acc:VGNC:89760]	1.73	0.083
ssc-miR-218	LMO3	hypothetical gene	1.73	0.083
ssc-miR-218	LMO7	LIM domain 7 [Source:VGNC Symbol;Acc:VGNC:89767]	1.73	0.083
ssc-miR-218	LNPEP	leucyl and cystinyl aminopeptidase [Source:VGNC Symbol;Acc:VGNC:89772]	1.73	0.083
ssc-miR-218	LPCAT1	lysophosphatidylcholine acyltransferase 1 [Source:VGNC Symbol;Acc:VGNC:89788]	1.73	0.083
ssc-miR-218	LPHN1	hypothetical gene	1.73	0.083
ssc-miR-218	LPHN3	hypothetical gene	1.73	0.083
ssc-miR-218	LPP	LIM domain containing preferred translocation partner in lipoma [Source:HGNC Symbol;Acc:HGNC:6679]	1.73	0.083
ssc-miR-218	LPPR4	hypothetical gene	1.73	0.083
ssc-miR-218	LRAT	lecithin retinol acyltransferase [Source:VGNC Symbol;Acc:VGNC:89798]	1.73	0.083
ssc-miR-218	LRCH1	leucine rich repeats and calponin homology domain containing 1 [Source:VGNC Symbol;Acc:VGNC:89801]	1.73	0.083
ssc-miR-218	LRIG1	leucine rich repeats and immunoglobulin like domains 1 [Source:VGNC Symbol;Acc:VGNC:89810]	1.73	0.083
ssc-miR-218	LRIG3	leucine rich repeats and immunoglobulin like domains 3 [Source:VGNC Symbol;Acc:VGNC:89812]	1.73	0.083
ssc-miR-218	LRP1B	LDL receptor related protein 1B [Source:HGNC Symbol;Acc:HGNC:6693]	1.73	0.083
ssc-miR-218	LRRC1	leucine rich repeat containing 1 [Source:VGNC Symbol;Acc:VGNC:103977]	1.73	0.083
ssc-miR-218	LRRC16A	hypothetical gene	1.73	0.083

ssc-miR-218	LRRC55	leucine rich repeat containing 55 [Source:VGNC Symbol;Acc:VGNC:89850]	1.73	0.083
ssc-miR-218	LRRC7	leucine rich repeat containing 7 [Source:HGNC Symbol;Acc:HGNC:18531]	1.73	0.083
ssc-miR-218	LRRFIP1	hypothetical gene	1.73	0.083
ssc-miR-218	LRRN1	leucine rich repeat neuronal 1 [Source:VGNC Symbol;Acc:VGNC:89860]	1.73	0.083
ssc-miR-218	LYPD6B	LY6/PLAUR domain containing 6B [Source:VGNC Symbol;Acc:VGNC:96150]	1.73	0.083
ssc-miR-218	LYSMD3	LysM domain containing 3 [Source:VGNC Symbol;Acc:VGNC:89919]	1.73	0.083
ssc-miR-218	LYVE1	lymphatic vessel endothelial hyaluronan receptor 1 [Source:VGNC Symbol;Acc:VGNC:89922]	1.73	0.083
ssc-miR-218	MAFG	MAF bZIP transcription factor G [Source:VGNC Symbol;Acc:VGNC:89948]	1.73	0.083
ssc-miR-218	MAGI2	membrane associated guanylate kinase, WW and PDZ domain containing 2 [Source:VGNC Symbol;Acc:VGNC:89955]	1.73	0.083
ssc-miR-218	MALSU1	mitochondrial assembly of ribosomal large subunit 1 [Source:VGNC Symbol;Acc:VGNC:89960]	1.73	0.083
ssc-miR-218	MAN2A1	mannosidase alpha class 2A member 1 [Source:VGNC Symbol;Acc:VGNC:98098]	1.73	0.083
ssc-miR-218	MAP3K2	mitogen-activated protein kinase kinase kinase 2 [Source:VGNC Symbol;Acc:VGNC:98107]	1.73	0.083
ssc-miR-218	MAP7D2	MAP7 domain containing 2 [Source:VGNC Symbol;Acc:VGNC:89994]	1.73	0.083
ssc-miR-218	MAPK1IP1L	mitogen-activated protein kinase 1 interacting protein 1 like [Source:VGNC Symbol;Acc:VGNC:90002]	1.73	0.083
ssc-miR-218	MAPK8IP3	mitogen-activated protein kinase 8 interacting protein 3 [Source:VGNC Symbol;Acc:VGNC:90008]	1.73	0.083
ssc-miR-218	MARCH4	hypothetical gene	1.73	0.083
ssc-miR-218	MARCKS	myristoylated alanine rich protein kinase C substrate [Source:VGNC Symbol;Acc:VGNC:90024]	1.73	0.083
ssc-miR-218	MARK1	microtubule affinity regulating kinase 1 [Source:VGNC Symbol;Acc:VGNC:96411]	1.73	0.083
ssc-miR-218	MARK2	microtubule affinity regulating kinase 2 [Source:VGNC Symbol;Acc:VGNC:98118]	1.73	0.083
ssc-miR-218	MBD5	methyl-CpG binding domain protein 5 [Source:VGNC Symbol;Acc:VGNC:95940]	1.73	0.083
ssc-miR-218	MBD6	methyl-CpG binding domain protein 6 [Source:VGNC Symbol;Acc:VGNC:90051]	1.73	0.083
ssc-miR-218	MBLAC2	metallo-beta-lactamase domain containing 2 [Source:VGNC Symbol;Acc:VGNC:90053]	1.73	0.083
ssc-miR-218	MBNL1	muscleblind like splicing regulator 1 [Source:VGNC Symbol;Acc:VGNC:90054]	1.73	0.083
ssc-miR-218	MBNL2	muscleblind like splicing regulator 2 [Source:VGNC Symbol;Acc:VGNC:90055]	1.73	0.083
ssc-miR-218	MCC	MCC regulator of WNT signaling pathway [Source:VGNC Symbol;Acc:VGNC:96588]	1.73	0.083
ssc-miR-218	MCF2	MCF.2 cell line derived transforming sequence [Source:VGNC Symbol;Acc:VGNC:90067]	1.73	0.083
ssc-miR-218	MDGA1	MAM domain containing glycosylphosphatidylinositol anchor 1 [Source:VGNC Symbol;Acc:VGNC:90091]	1.73	0.083
ssc-miR-218	MDGA2	MAM domain containing glycosylphosphatidylinositol anchor 2 [Source:VGNC Symbol;Acc:VGNC:90092]	1.73	0.083
ssc-miR-218	MECP2	methyl-CpG binding protein 2 [Source:VGNC Symbol;Acc:VGNC:90101]	1.73	0.083
ssc-miR-218	MED1	mediator complex subunit 1 [Source:VGNC Symbol;Acc:VGNC:90102]	1.73	0.083
ssc-miR-218	MED12L	mediator complex subunit 12L [Source:VGNC Symbol;Acc:VGNC:90105]	1.73	0.083
ssc-miR-218	MED17	mediator complex subunit 17 [Source:HGNC Symbol;Acc:HGNC:2375]	1.73	0.083
ssc-miR-218	MEF2C	myocyte enhancer factor 2C [Source:VGNC Symbol;Acc:VGNC:90127]	1.73	0.083
ssc-miR-218	MEF2D	myocyte enhancer factor 2D [Source:VGNC Symbol;Acc:VGNC:90128]	1.73	0.083
ssc-miR-218	MEGF9	multiple EGF like domains 9 [Source:VGNC Symbol;Acc:VGNC:90130]	1.73	0.083
ssc-miR-218	MEMO1	mediator of cell motility 1 [Source:VGNC Symbol;Acc:VGNC:90139]	1.73	0.083
ssc-miR-218	MEPCE	methylphosphate capping enzyme [Source:VGNC Symbol;Acc:VGNC:90144]	1.73	0.083
ssc-miR-218	METTL20	hypothetical gene	1.73	0.083
ssc-miR-218	METTL21A	methyltransferase 21A, HSPA lysine [Source:VGNC Symbol;Acc:VGNC:96242]	1.73	0.083
ssc-miR-218	METTL24	methyltransferase like 24 [Source:VGNC Symbol;Acc:VGNC:90161]	1.73	0.083
ssc-miR-218	MFHAS1	multifunctional ROCO family signaling regulator 1 [Source:VGNC Symbol;Acc:VGNC:107361]	1.73	0.083
ssc-miR-218	MID2	midline 2 [Source:VGNC Symbol;Acc:VGNC:98127]	1.73	0.083
ssc-miR-218	MIER3	MIER family member 3 [Source:VGNC Symbol;Acc:VGNC:90221]	1.73	0.083
ssc-miR-218	MITF	melanocyte inducing transcription factor [Source:VGNC Symbol;Acc:VGNC:90243]	1.73	0.083
ssc-miR-218	MLLT3	MLLT3 super elongation complex subunit [Source:VGNC Symbol;Acc:VGNC:90256]	1.73	0.083
ssc-miR-218	MLXIP	MLX interacting protein [Source:VGNC Symbol;Acc:VGNC:90262]	1.73	0.083
ssc-miR-218	MME	membrane metalloendopeptidase [Source:VGNC Symbol;Acc:VGNC:90265]	1.73	0.083
ssc-miR-218	MOB1B	hypothetical gene	1.73	0.083
ssc-miR-218	MOGAT3	hypothetical gene	1.73	0.083
ssc-miR-218	MOSPD1	motile sperm domain containing 1 [Source:VGNC Symbol;Acc:VGNC:103990]	1.73	0.083

ssc-miR-218	MPP6	hypothetical gene	1.73	0.083
ssc-miR-218	MPPED2	metallophosphoesterase domain containing 2 [Source:VGNC Symbol;Acc:VGNC:90331]	1.73	0.083
ssc-miR-218	MPRIP	hypothetical gene	1.73	0.083
ssc-miR-218	MPZL1	myelin protein zero like 1 [Source:VGNC Symbol;Acc:VGNC:90335]	1.73	0.083
ssc-miR-218	MRGBP	MRG domain binding protein [Source:HGNC Symbol;Acc:HGNC:15866]	1.73	0.083
ssc-miR-218	MRPS36	mitochondrial ribosomal protein S36 [Source:VGNC Symbol;Acc:VGNC:90397]	1.73	0.083
ssc-miR-218	MRVI1	hypothetical gene	1.73	0.083
ssc-miR-218	MSI2	musashi RNA binding protein 2 [Source:VGNC Symbol;Acc:VGNC:90422]	1.73	0.083
ssc-miR-218	MSL2	MSL complex subunit 2 [Source:VGNC Symbol;Acc:VGNC:90424]	1.73	0.083
ssc-miR-218	MTF2	metal response element binding transcription factor 2 [Source:VGNC Symbol;Acc:VGNC:90444]	1.73	0.083
ssc-miR-218	MTHFD2	methylenetetrahydrofolate dehydrogenase (NADP+ dependent) 2, methenyltetrahydrofolate cyclohydrolase [Source:HGNC Symbol;Acc:HGNC:7434]	1.73	0.083
ssc-miR-218	MTHFSD	methenyltetrahydrofolate synthetase domain containing [Source:VGNC Symbol;Acc:VGNC:90452]	1.73	0.083
ssc-miR-218	MTMR1	myotubularin related protein 1 [Source:VGNC Symbol;Acc:VGNC:90456]	1.73	0.083
ssc-miR-218	MTMR12	myotubularin related protein 12 [Source:VGNC Symbol;Acc:VGNC:90459]	1.73	0.083
ssc-miR-218	MUL1	mitochondrial E3 ubiquitin protein ligase 1 [Source:VGNC Symbol;Acc:VGNC:90484]	1.73	0.083
ssc-miR-218	MVB12B	multivesicular body subunit 12B [Source:VGNC Symbol;Acc:VGNC:90488]	1.73	0.083
ssc-miR-218	MYO16	hypothetical gene	1.73	0.083
ssc-miR-218	MYPN	myopalladin [Source:VGNC Symbol;Acc:VGNC:107412]	1.73	0.083
ssc-miR-218	MYSM1	Myb like, SWIRM and MPN domains 1 [Source:VGNC Symbol;Acc:VGNC:90548]	1.73	0.083
ssc-miR-218	MYT1L	myelin transcription factor 1 like [Source:VGNC Symbol;Acc:VGNC:90549]	1.73	0.083
ssc-miR-218	NAA15	N-alpha-acetyltransferase 15, NatA auxiliary subunit [Source:VGNC Symbol;Acc:VGNC:96747]	1.73	0.083
ssc-miR-218	NAB1	NGFI-A binding protein 1 [Source:VGNC Symbol;Acc:VGNC:96431]	1.73	0.083
ssc-miR-218	NACC1	nucleus accumbens associated 1 [Source:VGNC Symbol;Acc:VGNC:90563]	1.73	0.083
ssc-miR-218	NACC2	NACC family member 2 [Source:VGNC Symbol;Acc:VGNC:90564]	1.73	0.083
ssc-miR-218	NAGS	N-acetylglutamate synthase [Source:VGNC Symbol;Acc:VGNC:90571]	1.73	0.083
ssc-miR-218	NAPEPLD	N-acyl phosphatidylethanolamine phospholipase D [Source:VGNC Symbol;Acc:VGNC:90579]	1.73	0.083
ssc-miR-218	NAT8L	N-acetyltransferase 8 like [Source:VGNC Symbol;Acc:VGNC:90586]	1.73	0.083
ssc-miR-218	NAV1	neuron navigator 1 [Source:VGNC Symbol;Acc:VGNC:95725]	1.73	0.083
ssc-miR-218	NAV3	neuron navigator 3 [Source:VGNC Symbol;Acc:VGNC:90589]	1.73	0.083
ssc-miR-218	NBEA	neurobeachin [Source:VGNC Symbol;Acc:VGNC:90590]	1.73	0.083
ssc-miR-218	NBR1	NBR1 autophagy cargo receptor [Source:VGNC Symbol;Acc:VGNC:90593]	1.73	0.083
ssc-miR-218	NCAN	neurocan [Source:VGNC Symbol;Acc:VGNC:90596]	1.73	0.083
ssc-miR-218	NCMAP	hypothetical gene	1.73	0.083
ssc-miR-218	NDRG4	NDRG family member 4 [Source:VGNC Symbol;Acc:VGNC:90633]	1.73	0.083
ssc-miR-218	NEBL	nebulette [Source:VGNC Symbol;Acc:VGNC:95824]	1.73	0.083
ssc-miR-218	NECAB1	N-terminal EF-hand calcium binding protein 1 [Source:VGNC Symbol;Acc:VGNC:90659]	1.73	0.083
ssc-miR-218	NEURL4	neuralized E3 ubiquitin protein ligase 4 [Source:VGNC Symbol;Acc:VGNC:99022]	1.73	0.083
ssc-miR-218	NEUROD4	neuronal differentiation 4 [Source:VGNC Symbol;Acc:VGNC:90698]	1.73	0.083
ssc-miR-218	NF2	NF2, moesin-ezrin-radixin like (MERLIN) tumor suppressor [Source:VGNC Symbol;Acc:VGNC:90705]	1.73	0.083
ssc-miR-218	NFAT5	nuclear factor of activated T cells 5 [Source:VGNC Symbol;Acc:VGNC:90708]	1.73	0.083
ssc-miR-218	NFATC1	nuclear factor of activated T cells 1 [Source:VGNC Symbol;Acc:VGNC:103140]	1.73	0.083
ssc-miR-218	NFATC3	nuclear factor of activated T cells 3 [Source:VGNC Symbol;Acc:VGNC:90710]	1.73	0.083
ssc-miR-218	NFE2L1	NFE2 like bZIP transcription factor 1 [Source:VGNC Symbol;Acc:VGNC:90713]	1.73	0.083
ssc-miR-218	NFIA	nuclear factor I A [Source:VGNC Symbol;Acc:VGNC:90715]	1.73	0.083
ssc-miR-218	NFIX	nuclear factor I X [Source:VGNC Symbol;Acc:VGNC:90718]	1.73	0.083
ssc-miR-218	NHLRC3	NHL repeat containing 3 [Source:HGNC Symbol;Acc:HGNC:33751]	1.73	0.083
ssc-miR-218	NMNAT3	nicotinamide nucleotide adenylyltransferase 3 [Source:VGNC Symbol;Acc:VGNC:108679]	1.73	0.083
ssc-miR-218	NMT2	N-myristoyltransferase 2 [Source:VGNC Symbol;Acc:VGNC:96448]	1.73	0.083
ssc-miR-218	NPAS2	neuronal PAS domain protein 2 [Source:VGNC Symbol;Acc:VGNC:90837]	1.73	0.083
ssc-miR-218	NPY1R	neuropeptide Y receptor Y1 [Source:VGNC Symbol;Acc:VGNC:90865]	1.73	0.083

ssc-miR-218	NR1D2	nuclear receptor subfamily 1 group D member 2 [Source:VGNC Symbol;Acc:VGNC:99726]	1.73	0.083
ssc-miR-218	NRAS	NRAS proto-onco, GTPase [Source:VGNC Symbol;Acc:VGNC:98827]	1.73	0.083
ssc-miR-218	NRXN1	neurexin 1 [Source:HGNC Symbol;Acc:HGNC:8008]	1.73	0.083
ssc-miR-218	NRXN3	neurexin 3 [Source:HGNC Symbol;Acc:HGNC:8010]	1.73	0.083
ssc-miR-218	NSRP1	nuclear speckle splicing regulatory protein 1 [Source:VGNC Symbol;Acc:VGNC:90913]	1.73	0.083
ssc-miR-218	NUDT10	hypothetical gene	1.73	0.083
ssc-miR-218	NUFIP2	nuclear FMR1 interacting protein 2 [Source:VGNC Symbol;Acc:VGNC:90967]	1.73	0.083
ssc-miR-218	NUMB	NUMB endocytic adaptor protein [Source:VGNC Symbol;Acc:VGNC:90970]	1.73	0.083
ssc-miR-218	NUMBL	NUMB like endocytic adaptor protein [Source:HGNC Symbol;Acc:HGNC:8061]	1.73	0.083
ssc-miR-218	NUP50	nucleoporin 50 [Source:VGNC Symbol;Acc:VGNC:90983]	1.73	0.083
ssc-miR-218	NUPL2	hypothetical gene	1.73	0.083
ssc-miR-218	NXF1	nuclear RNA export factor 1 [Source:VGNC Symbol;Acc:VGNC:90993]	1.73	0.083
ssc-miR-218	NXT2	nuclear transport factor 2 like export factor 2 [Source:VGNC Symbol;Acc:VGNC:91001]	1.73	0.083
ssc-miR-218	OCIAD2	OCIA domain containing 2 [Source:VGNC Symbol;Acc:VGNC:91012]	1.73	0.083
ssc-miR-218	ONECUT2	one cut homeobox 2 [Source:VGNC Symbol;Acc:VGNC:91043]	1.73	0.083
ssc-miR-218	ONECUT3	one cut homeobox 3 [Source:VGNC Symbol;Acc:VGNC:91044]	1.73	0.083
ssc-miR-218	ORC2	origin recognition complex subunit 2 [Source:VGNC Symbol;Acc:VGNC:96464]	1.73	0.083
ssc-miR-218	ORC5	origin recognition complex subunit 5 [Source:VGNC Symbol;Acc:VGNC:91062]	1.73	0.083
ssc-miR-218	ORC6	origin recognition complex subunit 6 [Source:VGNC Symbol;Acc:VGNC:91063]	1.73	0.083
ssc-miR-218	OTP	orthopedia homeobox [Source:VGNC Symbol;Acc:VGNC:91096]	1.73	0.083
ssc-miR-218	OTUB2	OTU deubiquitinase, ubiquitin aldehyde binding 2 [Source:VGNC Symbol;Acc:VGNC:91098]	1.73	0.083
ssc-miR-218	OTUD7B	OTU deubiquitinase 7B [Source:VGNC Symbol;Acc:VGNC:91105]	1.73	0.083
ssc-miR-218	OTX2	orthodenticle homeobox 2 [Source:VGNC Symbol;Acc:VGNC:98171]	1.73	0.083
ssc-miR-218	OXSR1	hypothetical gene	1.73	0.083
ssc-miR-218	P2RY2	purinergic receptor P2Y2 [Source:VGNC Symbol;Acc:VGNC:98173]	1.73	0.083
ssc-miR-218	PAG1	phosphoprotein membrane anchor with glycosphingolipid microdomains 1 [Source:VGNC Symbol;Acc:VGNC:91154]	1.73	0.083
ssc-miR-218	PAIP2	poly(A) binding protein interacting protein 2 [Source:VGNC Symbol;Acc:VGNC:96618]	1.73	0.083
ssc-miR-218	PAK3	p21 (RAC1) activated kinase 3 [Source:HGNC Symbol;Acc:HGNC:8592]	1.73	0.083
ssc-miR-218	PALM2	hypothetical gene	1.73	0.083
ssc-miR-218	PAN3	poly(A) specific ribonuclease subunit PAN3 [Source:HGNC Symbol;Acc:HGNC:29991]	1.73	0.083
ssc-miR-218	PANK2	pantothenate kinase 2 [Source:VGNC Symbol;Acc:VGNC:95695]	1.73	0.083
ssc-miR-218	PARP8	poly(ADP-ribose) polymerase family member 8 [Source:HGNC Symbol;Acc:HGNC:26124]	1.73	0.083
ssc-miR-218	PAX2	paired box 2 [Source:VGNC Symbol;Acc:VGNC:91192]	1.73	0.083
ssc-miR-218	PAX5	paired box 5 [Source:VGNC Symbol;Acc:VGNC:91194]	1.73	0.083
ssc-miR-218	PBX2	PBX homeobox 2 [Source:VGNC Symbol;Acc:VGNC:91204]	1.73	0.083
ssc-miR-218	PCDH1	protocadherin 1 [Source:VGNC Symbol;Acc:VGNC:91211]	1.73	0.083
ssc-miR-218	PCDH17	protocadherin 17 [Source:VGNC Symbol;Acc:VGNC:91214]	1.73	0.083
ssc-miR-218	PCDH8	protocadherin 8 [Source:HGNC Symbol;Acc:HGNC:8660]	1.73	0.083
ssc-miR-218	PCDH9	protocadherin 9 [Source:VGNC Symbol;Acc:VGNC:91217]	1.73	0.083
ssc-miR-218	PCDHA1	hypothetical gene	1.73	0.083
ssc-miR-218	PCDHA10	hypothetical gene	1.73	0.083
ssc-miR-218	PCDHA11	hypothetical gene	1.73	0.083
ssc-miR-218	PCDHA12	hypothetical gene	1.73	0.083
ssc-miR-218	PCDHA13	protocadherin alpha 13 [Source:HGNC Symbol;Acc:HGNC:8667]	1.73	0.083
ssc-miR-218	PCDHA2	hypothetical gene	1.73	0.083
ssc-miR-218	PCDHA3	protocadherin alpha 3 [Source:HGNC Symbol;Acc:HGNC:8669]	1.73	0.083
ssc-miR-218	PCDHA4	hypothetical gene	1.73	0.083
ssc-miR-218	PCDHA5	hypothetical gene	1.73	0.083
ssc-miR-218	PCDHA6	hypothetical gene	1.73	0.083
ssc-miR-218	PCDHA7	hypothetical gene	1.73	0.083

ssc-miR-218	PCDHA8	hypothetical gene	1.73	0.083
ssc-miR-218	PCDHA9	hypothetical gene	1.73	0.083
ssc-miR-218	PCDHAC1	hypothetical gene	1.73	0.083
ssc-miR-218	PCDHAC2	protocadherin alpha subfamily C, 2 [Source:HGNC Symbol;Acc:HGNC:8677]	1.73	0.083
ssc-miR-218	PCGF2	polycomb group ring finger 2 [Source:VGNC Symbol;Acc:VGNC:91219]	1.73	0.083
ssc-miR-218	PCGF5	hypothetical gene	1.73	0.083
ssc-miR-218	PCLO	hypothetical gene	1.73	0.083
ssc-miR-218	PCMTD2	protein-L-isoaspartate (D-aspartate) O-methyltransferase domain containing 2 [Source:VGNC Symbol;Acc:VGNC:108730]	1.73	0.083
ssc-miR-218	PCNP	PEST proteolytic signal containing nuclear protein [Source:VGNC Symbol;Acc:VGNC:104018]	1.73	0.083
ssc-miR-218	PDE12	phosphodiesterase 12 [Source:VGNC Symbol;Acc:VGNC:104020]	1.73	0.083
ssc-miR-218	PDE3A	phosphodiesterase 3A [Source:VGNC Symbol;Acc:VGNC:91252]	1.73	0.083
ssc-miR-218	PDE7A	phosphodiesterase 7A [Source:VGNC Symbol;Acc:VGNC:91261]	1.73	0.083
ssc-miR-218	PDE8B	phosphodiesterase 8B [Source:VGNC Symbol;Acc:VGNC:100321]	1.73	0.083
ssc-miR-218	PDGFRA	platelet derived growth factor receptor alpha [Source:VGNC Symbol;Acc:VGNC:98179]	1.73	0.083
ssc-miR-218	PDYN	prodynorphin [Source:VGNC Symbol;Acc:VGNC:96477]	1.73	0.083
ssc-miR-218	PDZD4	PDZ domain containing 4 [Source:VGNC Symbol;Acc:VGNC:91296]	1.73	0.083
ssc-miR-218	PEX5L	peroxisomal biosis factor 5 like [Source:VGNC Symbol;Acc:VGNC:91329]	1.73	0.083
ssc-miR-218	PGM2L1	phosphoglucomutase 2 like 1 [Source:VGNC Symbol;Acc:VGNC:91357]	1.73	0.083
ssc-miR-218	PHC3	polyhomeotic homolog 3 [Source:VGNC Symbol;Acc:VGNC:91372]	1.73	0.083
ssc-miR-218	PHF15	hypothetical gene	1.73	0.083
ssc-miR-218	PHF17	hypothetical gene	1.73	0.083
ssc-miR-218	PHF20	PHD finger protein 20 [Source:HGNC Symbol;Acc:HGNC:16098]	1.73	0.083
ssc-miR-218	PHF6	PHD finger protein 6 [Source:VGNC Symbol;Acc:VGNC:91389]	1.73	0.083
ssc-miR-218	PHYH	phytanoyl-CoA 2-hydroxylase [Source:VGNC Symbol;Acc:VGNC:96483]	1.73	0.083
ssc-miR-218	PI4K2A	phosphatidylinositol 4-kinase type 2 alpha [Source:HGNC Symbol;Acc:HGNC:30031]	1.73	0.083
ssc-miR-218	PIAS1	protein inhibitor of activated STAT 1 [Source:VGNC Symbol;Acc:VGNC:91410]	1.73	0.083
ssc-miR-218	PIEZO2	piezo type mechanosensitive ion channel component 2 [Source:HGNC Symbol;Acc:HGNC:26270]	1.73	0.083
ssc-miR-218	PIGH	phosphatidylinositol glycan anchor biosynthesis class H [Source:VGNC Symbol;Acc:VGNC:91423]	1.73	0.083
ssc-miR-218	PIK3C2A	phosphatidylinositol-4-phosphate 3-kinase catalytic subunit type 2 alpha [Source:VGNC Symbol;Acc:VGNC:91436]	1.73	0.083
ssc-miR-218	PIK3R1	phosphoinositide-3-kinase regulatory subunit 1 [Source:VGNC Symbol;Acc:VGNC:91445]	1.73	0.083
ssc-miR-218	PKP4	plakophilin 4 [Source:VGNC Symbol;Acc:VGNC:96488]	1.73	0.083
ssc-miR-218	PLCE1	phospholipase C epsilon 1 [Source:VGNC Symbol;Acc:VGNC:91519]	1.73	0.083
ssc-miR-218	PLCG1	phospholipase C gamma 1 [Source:VGNC Symbol;Acc:VGNC:96492]	1.73	0.083
ssc-miR-218	PLCL2	phospholipase C like 2 [Source:VGNC Symbol;Acc:VGNC:91523]	1.73	0.083
ssc-miR-218	PLCXD3	phosphatidylinositol specific phospholipase C X domain containing 3 [Source:VGNC Symbol;Acc:VGNC:91524]	1.73	0.083
ssc-miR-218	PLD5	phospholipase D family member 5 [Source:VGNC Symbol;Acc:VGNC:96145]	1.73	0.083
ssc-miR-218	PLEKHA3	pleckstrin homology domain containing A3 [Source:NCBI gene (formerly Entrezgene);Acc:100154010]	1.73	0.083
ssc-miR-218	PLEKHG1	pleckstrin homology and RhoGEF domain containing G1 [Source:VGNC Symbol;Acc:VGNC:91542]	1.73	0.083
ssc-miR-218	PLEKHG3	pleckstrin homology and RhoGEF domain containing G3 [Source:VGNC Symbol;Acc:VGNC:91544]	1.73	0.083
ssc-miR-218	PLGRKT	plasminogen receptor with a C-terminal lysine [Source:VGNC Symbol;Acc:VGNC:91556]	1.73	0.083
ssc-miR-218	PLXDC2	plexin domain containing 2 [Source:VGNC Symbol;Acc:VGNC:95958]	1.73	0.083
ssc-miR-218	PLXNA2	plexin A2 [Source:VGNC Symbol;Acc:VGNC:91580]	1.73	0.083
ssc-miR-218	PLXNA4	plexin A4 [Source:VGNC Symbol;Acc:VGNC:98204]	1.73	0.083
ssc-miR-218	PLXNC1	plexin C1 [Source:VGNC Symbol;Acc:VGNC:91584]	1.73	0.083
ssc-miR-218	PLXND1	plexin D1 [Source:VGNC Symbol;Acc:VGNC:91585]	1.73	0.083
ssc-miR-218	POC1B-GALNT4	hypothetical gene	1.73	0.083
ssc-miR-218	POLD3	DNA polymerase delta 3, accessory subunit [Source:VGNC Symbol;Acc:VGNC:99727]	1.73	0.083
ssc-miR-218	POLH	DNA polymerase eta [Source:VGNC Symbol;Acc:VGNC:91635]	1.73	0.083
ssc-miR-218	POLR2C	RNA polymerase II subunit C [Source:VGNC Symbol;Acc:VGNC:91648]	1.73	0.083
ssc-miR-218	POMGNT1	protein O-linked mannose N-acetylglucosaminyltransferase 1 (beta 1,2-) [Source:VGNC Symbol;Acc:VGNC:98536]	1.73	0.083

ssc-miR-218	POU2F1	POU class 2 homeobox 1 [Source:VGNC Symbol;Acc:VGNC:91672]	1.73	0.083
ssc-miR-218	POU3F2	POU class 3 homeobox 2 [Source:HGNC Symbol;Acc:HGNC:9215]	1.73	0.083
ssc-miR-218	POU3F3	POU class 3 homeobox 3 [Source:VGNC Symbol;Acc:VGNC:91676]	1.73	0.083
ssc-miR-218	PPAP2B	hypothetical gene	1.73	0.083
ssc-miR-218	PPAPDC2	hypothetical gene	1.73	0.083
ssc-miR-218	PPARGC1A	PPARG coactivator 1 alpha [Source:VGNC Symbol;Acc:VGNC:91685]	1.73	0.083
ssc-miR-218	PPIG	peptidylprolyl isomerase G [Source:VGNC Symbol;Acc:VGNC:98214]	1.73	0.083
ssc-miR-218	PPM1K	protein phosphatase, Mg2+/Mn2+ dependent 1K [Source:VGNC Symbol;Acc:VGNC:91710]	1.73	0.083
ssc-miR-218	PPME1	protein phosphatase methylesterase 1 [Source:VGNC Symbol;Acc:VGNC:91713]	1.73	0.083
ssc-miR-218	PPP1CB	protein phosphatase 1 catalytic subunit beta [Source:NCBI gene (formerly Entrezgene);Acc:397378]	1.73	0.083
ssc-miR-218	PPP1CC	protein phosphatase 1 catalytic subunit gamma [Source:VGNC Symbol;Acc:VGNC:91716]	1.73	0.083
ssc-miR-218	PPP1R26	protein phosphatase 1 regulatory subunit 26 [Source:VGNC Symbol;Acc:VGNC:91730]	1.73	0.083
ssc-miR-218	PPP2R2A	protein phosphatase 2 regulatory subunit Balpha [Source:VGNC Symbol;Acc:VGNC:91748]	1.73	0.083
ssc-miR-218	PPP2R2C	protein phosphatase 2 regulatory subunit Bgamma [Source:VGNC Symbol;Acc:VGNC:91749]	1.73	0.083
ssc-miR-218	PPP2R4	hypothetical gene	1.73	0.083
ssc-miR-218	PPP2R5A	protein phosphatase 2 regulatory subunit B'alpha [Source:VGNC Symbol;Acc:VGNC:91751]	1.73	0.083
ssc-miR-218	PPP4R1L	hypothetical gene	1.73	0.083
ssc-miR-218	PPP4R2	protein phosphatase 4 regulatory subunit 2 [Source:VGNC Symbol;Acc:VGNC:91756]	1.73	0.083
ssc-miR-218	PPP6C	protein phosphatase 6 catalytic subunit [Source:VGNC Symbol;Acc:VGNC:98219]	1.73	0.083
ssc-miR-218	PRKAR2B	protein kinase cAMP-dependent type II regulatory subunit beta [Source:VGNC Symbol;Acc:VGNC:91805]	1.73	0.083
ssc-miR-218	PRKCE	protein kinase C epsilon [Source:VGNC Symbol;Acc:VGNC:91807]	1.73	0.083
ssc-miR-218	PRKG1	protein kinase cGMP-dependent 1 [Source:VGNC Symbol;Acc:VGNC:91816]	1.73	0.083
ssc-miR-218	PRLR	prolactin receptor [Source:VGNC Symbol;Acc:VGNC:91819]	1.73	0.083
ssc-miR-218	PROSC	hypothetical gene	1.73	0.083
ssc-miR-218	PROX1	prospero homeobox 1 [Source:VGNC Symbol;Acc:VGNC:91837]	1.73	0.083
ssc-miR-218	PRRG3	proline rich and Gla domain 3 [Source:VGNC Symbol;Acc:VGNC:91869]	1.73	0.083
ssc-miR-218	PSD2	pleckstrin and Sec7 domain containing 2 [Source:VGNC Symbol;Acc:VGNC:91896]	1.73	0.083
ssc-miR-218	PSD3	pleckstrin and Sec7 domain containing 3 [Source:VGNC Symbol;Acc:VGNC:107166]	1.73	0.083
ssc-miR-218	PSMB8	proteasome 20S subunit beta 8 [Source:VGNC Symbol;Acc:VGNC:91910]	1.73	0.083
ssc-miR-218	PSMC2	proteasome 26S subunit, ATPase 2 [Source:VGNC Symbol;Acc:VGNC:91913]	1.73	0.083
ssc-miR-218	PSME4	proteasome activator subunit 4 [Source:VGNC Symbol;Acc:VGNC:91928]	1.73	0.083
ssc-miR-218	PTCHD1	patched domain containing 1 [Source:VGNC Symbol;Acc:VGNC:91942]	1.73	0.083
ssc-miR-218	PTP4A1	protein tyrosine phosphatase 4A1 [Source:HGNC Symbol;Acc:HGNC:9634]	1.73	0.083
ssc-miR-218	PTPN11	protein tyrosine phosphatase non-receptor type 11 [Source:VGNC Symbol;Acc:VGNC:91972]	1.73	0.083
ssc-miR-218	PTPRA	protein tyrosine phosphatase receptor type A [Source:VGNC Symbol;Acc:VGNC:96522]	1.73	0.083
ssc-miR-218	PTPRG	protein tyrosine phosphatase receptor type G [Source:VGNC Symbol;Acc:VGNC:91988]	1.73	0.083
ssc-miR-218	PTPRK	protein tyrosine phosphatase receptor type K [Source:VGNC Symbol;Acc:VGNC:91991]	1.73	0.083
ssc-miR-218	PTPRM	protein tyrosine phosphatase receptor type M [Source:VGNC Symbol;Acc:VGNC:91992]	1.73	0.083
ssc-miR-218	PTPRN2	protein tyrosine phosphatase receptor type N2 [Source:VGNC Symbol;Acc:VGNC:91993]	1.73	0.083
ssc-miR-218	PTPRR	protein tyrosine phosphatase receptor type R [Source:HGNC Symbol;Acc:HGNC:9680]	1.73	0.083
ssc-miR-218	PTPRT	protein tyrosine phosphatase receptor type T [Source:VGNC Symbol;Acc:VGNC:96524]	1.73	0.083
ssc-miR-218	PTRH2	peptidyl-tRNA hydrolase 2 [Source:VGNC Symbol;Acc:VGNC:91998]	1.73	0.083
ssc-miR-218	PTS	6-pyruvoyltetrahydropterin synthase [Source:VGNC Symbol;Acc:VGNC:104039]	1.73	0.083
ssc-miR-218	PUM1	pumilio RNA binding family member 1 [Source:VGNC Symbol;Acc:VGNC:92001]	1.73	0.083
ssc-miR-218	PUM2	pumilio RNA binding family member 2 [Source:VGNC Symbol;Acc:VGNC:92002]	1.73	0.083
ssc-miR-218	PURA	purine rich element binding protein A [Source:HGNC Symbol;Acc:HGNC:9701]	1.73	0.083
ssc-miR-218	PURB	purine rich element binding protein B [Source:VGNC Symbol;Acc:VGNC:92004]	1.73	0.083
ssc-miR-218	PVRL1	hypothetical gene	1.73	0.083
ssc-miR-218	PXN	paxillin [Source:HGNC Symbol;Acc:HGNC:9718]	1.73	0.083
ssc-miR-218	QSER1	glutamine and serine rich 1 [Source:VGNC Symbol;Acc:VGNC:92032]	1.73	0.083

ssc-miR-218	RAB1A	RAB1A, member RAS onco family [Source:VGNC Symbol;Acc:VGNC:104042]	1.73	0.083
ssc-miR-218	RAB30	RAB30, member RAS onco family [Source:VGNC Symbol;Acc:VGNC:98254]	1.73	0.083
ssc-miR-218	RAB33B	RAB33B, member RAS onco family [Source:VGNC Symbol;Acc:VGNC:98257]	1.73	0.083
ssc-miR-218	RAB6A	RAB6A, member RAS onco family [Source:VGNC Symbol;Acc:VGNC:108610]	1.73	0.083
ssc-miR-218	RAB8A	RAB8A, member RAS onco family [Source:VGNC Symbol;Acc:VGNC:98275]	1.73	0.083
ssc-miR-218	RAB8B	RAB8B, member RAS onco family [Source:VGNC Symbol;Acc:VGNC:98276]	1.73	0.083
ssc-miR-218	RABEP1	rabaptin, RAB GTPase binding effector protein 1 [Source:VGNC Symbol;Acc:VGNC:99032]	1.73	0.083
ssc-miR-218	RABGAP1L	RAB GTPase activating protein 1 like [Source:VGNC Symbol;Acc:VGNC:108611]	1.73	0.083
ssc-miR-218	RAD51L3-RFFL	hypothetical gene	1.73	0.083
ssc-miR-218	RAD52	RAD52 homolog, DNA repair protein [Source:VGNC Symbol;Acc:VGNC:92060]	1.73	0.083
ssc-miR-218	RALGAPA2	Ral GTPase activating protein catalytic subunit alpha 2 [Source:VGNC Symbol;Acc:VGNC:95454]	1.73	0.083
ssc-miR-218	RANBP10	RAN binding protein 10 [Source:VGNC Symbol;Acc:VGNC:92077]	1.73	0.083
ssc-miR-218	RAP1GAP	RAP1 GTPase activating protein [Source:VGNC Symbol;Acc:VGNC:92085]	1.73	0.083
ssc-miR-218	RAP1GDS1	Rap1 GTPase-GDP dissociation stimulator 1 [Source:VGNC Symbol;Acc:VGNC:92087]	1.73	0.083
ssc-miR-218	RAPGEF2	Rap guanine nucleotide exchange factor 2 [Source:VGNC Symbol;Acc:VGNC:92092]	1.73	0.083
ssc-miR-218	RAPGEF4	Rap guanine nucleotide exchange factor 4 [Source:VGNC Symbol;Acc:VGNC:95814]	1.73	0.083
ssc-miR-218	RARA	retinoic acid receptor alpha [Source:VGNC Symbol;Acc:VGNC:92096]	1.73	0.083
ssc-miR-218	RASAL2	RAS protein activator like 2 [Source:VGNC Symbol;Acc:VGNC:92105]	1.73	0.083
ssc-miR-218	RASGEF1A	RasGEF domain family member 1A [Source:VGNC Symbol;Acc:VGNC:92108]	1.73	0.083
ssc-miR-218	RASSF2	Ras association domain family member 2 [Source:VGNC Symbol;Acc:VGNC:96531]	1.73	0.083
ssc-miR-218	RASSF5	Ras association domain family member 5 [Source:VGNC Symbol;Acc:VGNC:92125]	1.73	0.083
ssc-miR-218	RBBP7	RB binding protein 7, chromatin remodeling factor [Source:VGNC Symbol;Acc:VGNC:92137]	1.73	0.083
ssc-miR-218	RBM18	RNA binding motif protein 18 [Source:VGNC Symbol;Acc:VGNC:92149]	1.73	0.083
ssc-miR-218	RBM47	RNA binding motif protein 47 [Source:VGNC Symbol;Acc:VGNC:92158]	1.73	0.083
ssc-miR-218	RBPJ	recombination signal binding protein for immunoglobulin kappa J region [Source:VGNC Symbol;Acc:VGNC:98956]	1.73	0.083
ssc-miR-218	RCBTB1	RCC1 and BTB domain containing protein 1 [Source:VGNC Symbol;Acc:VGNC:92173]	1.73	0.083
ssc-miR-218	RCC1	regulator of chromosome condensation 1 [Source:VGNC Symbol;Acc:VGNC:92175]	1.73	0.083
ssc-miR-218	RCOR1	REST corepressor 1 [Source:VGNC Symbol;Acc:VGNC:92183]	1.73	0.083
ssc-miR-218	RELN	reelin [Source:VGNC Symbol;Acc:VGNC:92208]	1.73	0.083
ssc-miR-218	REPS1	RALBP1 associated Eps domain containing 1 [Source:VGNC Symbol;Acc:VGNC:92213]	1.73	0.083
ssc-miR-218	REPS2	RALBP1 associated Eps domain containing 2 [Source:VGNC Symbol;Acc:VGNC:92214]	1.73	0.083
ssc-miR-218	RER1	retention in endoplasmic reticulum sorting receptor 1 [Source:VGNC Symbol;Acc:VGNC:92215]	1.73	0.083
ssc-miR-218	RET	ret proto-onco [Source:VGNC Symbol;Acc:VGNC:92220]	1.73	0.083
ssc-miR-218	RFFL	ring finger and FYVE like domain containing E3 ubiquitin protein ligase [Source:VGNC Symbol;Acc:VGNC:98289]	1.73	0.083
ssc-miR-218	RFX3	regulatory factor X3 [Source:VGNC Symbol;Acc:VGNC:92245]	1.73	0.083
ssc-miR-218	RGS17	regulator of G protein signaling 17 [Source:VGNC Symbol;Acc:VGNC:92263]	1.73	0.083
ssc-miR-218	RGS20	regulator of G protein signaling 20 [Source:VGNC Symbol;Acc:VGNC:92264]	1.73	0.083
ssc-miR-218	RHOBTB1	Rho related BTB domain containing 1 [Source:VGNC Symbol;Acc:VGNC:92285]	1.73	0.083
ssc-miR-218	RHOQ	ras homolog family member Q [Source:VGNC Symbol;Acc:VGNC:92292]	1.73	0.083
ssc-miR-218	RIC3	RIC3 acetylcholine receptor chaperone [Source:VGNC Symbol;Acc:VGNC:92300]	1.73	0.083
ssc-miR-218	RICTOR	RPTOR independent companion of MTOR complex 2 [Source:VGNC Symbol;Acc:VGNC:92303]	1.73	0.083
ssc-miR-218	RIMBP2	RIMS binding protein 2 [Source:VGNC Symbol;Acc:VGNC:92306]	1.73	0.083
ssc-miR-218	RIMS1	regulating synaptic membrane exocytosis 1 [Source:VGNC Symbol;Acc:VGNC:92309]	1.73	0.083
ssc-miR-218	RIMS3	regulating synaptic membrane exocytosis 3 [Source:VGNC Symbol;Acc:VGNC:92310]	1.73	0.083
ssc-miR-218	RIMS4	regulating synaptic membrane exocytosis 4 [Source:VGNC Symbol;Acc:VGNC:95775]	1.73	0.083
ssc-miR-218	RLIM	ring finger protein, LIM domain interacting [Source:HGNC Symbol;Acc:HGNC:13429]	1.73	0.083
ssc-miR-218	RNF103	ring finger protein 103 [Source:VGNC Symbol;Acc:VGNC:98291]	1.73	0.083
ssc-miR-218	RNF114	ring finger protein 114 [Source:VGNC Symbol;Acc:VGNC:108733]	1.73	0.083
ssc-miR-218	RNF139	ring finger protein 139 [Source:HGNC Symbol;Acc:HGNC:17023]	1.73	0.083
ssc-miR-218	RNF149	ring finger protein 149 [Source:VGNC Symbol;Acc:VGNC:92362]	1.73	0.083

ssc-miR-218	RNF152	ring finger protein 152 [Source:VGNC Symbol;Acc:VGNC:92364]	1.73	0.083
ssc-miR-218	RNF165	ring finger protein 165 [Source:VGNC Symbol;Acc:VGNC:92366]	1.73	0.083
ssc-miR-218	RNF19B	ring finger protein 19B [Source:VGNC Symbol;Acc:VGNC:92376]	1.73	0.083
ssc-miR-218	RNF219	hypothetical gene	1.73	0.083
ssc-miR-218	RNF220	ring finger protein 220 [Source:VGNC Symbol;Acc:VGNC:98602]	1.73	0.083
ssc-miR-218	RNF38	ring finger protein 38 [Source:VGNC Symbol;Acc:VGNC:92390]	1.73	0.083
ssc-miR-218	RNF41	ring finger protein 41 [Source:VGNC Symbol;Acc:VGNC:92394]	1.73	0.083
ssc-miR-218	ROBO1	roundabout guidance receptor 1 [Source:HGNC Symbol;Acc:HGNC:10249]	1.73	0.083
ssc-miR-218	ROBO2	roundabout guidance receptor 2 [Source:HGNC Symbol;Acc:HGNC:10250]	1.73	0.083
ssc-miR-218	ROCK1	Rho associated coiled-coil containing protein kinase 1 [Source:VGNC Symbol;Acc:VGNC:98294]	1.73	0.083
ssc-miR-218	RORA	RAR related orphan receptor A [Source:VGNC Symbol;Acc:VGNC:92408]	1.73	0.083
ssc-miR-218	RORB	RAR related orphan receptor B [Source:VGNC Symbol;Acc:VGNC:92409]	1.73	0.083
ssc-miR-218	RP11-302B13.5	hypothetical gene	1.73	0.083
ssc-miR-218	RP11-315D16.2	hypothetical gene	1.73	0.083
ssc-miR-218	RP11-47I22.4	hypothetical gene	1.73	0.083
ssc-miR-218	RP11-770J1.4	hypothetical gene	1.73	0.083
ssc-miR-218	RPA3	replication protein A3 [Source:VGNC Symbol;Acc:VGNC:92415]	1.73	0.083
ssc-miR-218	RPP14	hypothetical gene	1.73	0.083
ssc-miR-218	RPP25	ribonuclease P and MRP subunit p25 [Source:VGNC Symbol;Acc:VGNC:92432]	1.73	0.083
ssc-miR-218	RPS6KA3	ribosomal protein S6 kinase A3 [Source:VGNC Symbol;Acc:VGNC:92442]	1.73	0.083
ssc-miR-218	RPS6KA6	ribosomal protein S6 kinase A6 [Source:VGNC Symbol;Acc:VGNC:92445]	1.73	0.083
ssc-miR-218	RPS6KB1	ribosomal protein S6 kinase B1 [Source:VGNC Symbol;Acc:VGNC:99037]	1.73	0.083
ssc-miR-218	RPS9	ribosomal protein S9 [Source:VGNC Symbol;Acc:VGNC:92449]	1.73	0.083
ssc-miR-218	RRP1B	ribosomal RNA processing 1B [Source:VGNC Symbol;Acc:VGNC:92466]	1.73	0.083
ssc-miR-218	RRP8	ribosomal RNA processing 8 [Source:VGNC Symbol;Acc:VGNC:92467]	1.73	0.083
ssc-miR-218	RSBN1	round spermatid basic protein 1 [Source:VGNC Symbol;Acc:VGNC:92473]	1.73	0.083
ssc-miR-218	RSRC2	arginine and serine rich coiled-coil 2 [Source:VGNC Symbol;Acc:VGNC:92488]	1.73	0.083
ssc-miR-218	RTN3	hypothetical gene	1.73	0.083
ssc-miR-218	RUNX1T1	RUNX1 partner transcriptional co-repressor 1 [Source:VGNC Symbol;Acc:VGNC:96594]	1.73	0.083
ssc-miR-218	RUNX2	RUNX family transcription factor 2 [Source:VGNC Symbol;Acc:VGNC:92517]	1.73	0.083
ssc-miR-218	RUNX3	RUNX family transcription factor 3 [Source:VGNC Symbol;Acc:VGNC:92518]	1.73	0.083
ssc-miR-218	RYBP	RING1 and YY1 binding protein [Source:VGNC Symbol;Acc:VGNC:92532]	1.73	0.083
ssc-miR-218	RYK	receptor like tyrosine kinase [Source:VGNC Symbol;Acc:VGNC:92533]	1.73	0.083
ssc-miR-218	SALL3	spalt like transcription factor 3 [Source:VGNC Symbol;Acc:VGNC:92563]	1.73	0.083
ssc-miR-218	SAMD12	sterile alpha motif domain containing 12 [Source:VGNC Symbol;Acc:VGNC:92565]	1.73	0.083
ssc-miR-218	SAMD8	sterile alpha motif domain containing 8 [Source:VGNC Symbol;Acc:VGNC:92571]	1.73	0.083
ssc-miR-218	SASH1	SAM and SH3 domain containing 1 [Source:VGNC Symbol;Acc:VGNC:92583]	1.73	0.083
ssc-miR-218	SATB2	SATB homeobox 2 [Source:VGNC Symbol;Acc:VGNC:95972]	1.73	0.083
ssc-miR-218	SCAI	suppressor of cancer cell invasion [Source:VGNC Symbol;Acc:VGNC:103170]	1.73	0.083
ssc-miR-218	SCAMP5	secretory carrier membrane protein 5 [Source:VGNC Symbol;Acc:VGNC:92609]	1.73	0.083
ssc-miR-218	SCD	stearoyl-CoA desaturase [Source:NCBI gene (formerly Entrezgene);Acc:396670]	1.73	0.083
ssc-miR-218	SCN1A	sodium voltage-gated channel alpha subunit 1 [Source:VGNC Symbol;Acc:VGNC:95478]	1.73	0.083
ssc-miR-218	SCN2B	sodium voltage-gated channel beta subunit 2 [Source:VGNC Symbol;Acc:VGNC:92633]	1.73	0.083
ssc-miR-218	SCN4B	hypothetical gene	1.73	0.083
ssc-miR-218	SCN8A	sodium voltage-gated channel alpha subunit 8 [Source:VGNC Symbol;Acc:VGNC:92638]	1.73	0.083
ssc-miR-218	SCRT1	scratch family transcriptional repressor 1 [Source:VGNC Symbol;Acc:VGNC:92649]	1.73	0.083
ssc-miR-218	SCRT2	scratch family transcriptional repressor 2 [Source:VGNC Symbol;Acc:VGNC:95721]	1.73	0.083
ssc-miR-218	SDC2	syndecan 2 [Source:VGNC Symbol;Acc:VGNC:92655]	1.73	0.083
ssc-miR-218	SEC14L1	SEC14 like lipid binding 1 [Source:VGNC Symbol;Acc:VGNC:92669]	1.73	0.083
ssc-miR-218	SEC14L2	SEC14 like lipid binding 2 [Source:NCBI gene (formerly Entrezgene);Acc:100152451]	1.73	0.083

ssc-miR-218	SEC61A1	SEC61 translocon subunit alpha 1 [Source:VGNC Symbol;Acc:VGNC:92682]	1.73	0.083
ssc-miR-218	SEMA5A	semaphorin 5A [Source:VGNC Symbol;Acc:VGNC:92706]	1.73	0.083
ssc-miR-218	SEMA6A	semaphorin 6A [Source:VGNC Symbol;Acc:VGNC:92708]	1.73	0.083
ssc-miR-218	SEMA6B	semaphorin 6B [Source:VGNC Symbol;Acc:VGNC:92709]	1.73	0.083
ssc-miR-218	SENP1	SUMO specific peptidase 1 [Source:VGNC Symbol;Acc:VGNC:92712]	1.73	0.083
ssc-miR-218	SENP2	SUMO specific peptidase 2 [Source:VGNC Symbol;Acc:VGNC:92713]	1.73	0.083
ssc-miR-218	SENP5	SUMO specific peptidase 5 [Source:VGNC Symbol;Acc:VGNC:92714]	1.73	0.083
ssc-miR-218	SEPHS1	selenophosphate synthetase 1 [Source:VGNC Symbol;Acc:VGNC:104053]	1.73	0.083
ssc-miR-218	SERBP1	SERPINE1 mRNA binding protein 1 [Source:VGNC Symbol;Acc:VGNC:92727]	1.73	0.083
ssc-miR-218	SERINC5	serine incorporator 5 [Source:VGNC Symbol;Acc:VGNC:92730]	1.73	0.083
ssc-miR-218	SERP1	stress associated endoplasmic reticulum protein 1 [Source:NCBI gene (formerly Entrezgene);Acc:100156392]	1.73	0.083
ssc-miR-218	SERPINI1	serpin family I member 1 [Source:VGNC Symbol;Acc:VGNC:92747]	1.73	0.083
ssc-miR-218	SERTAD2	SERTA domain containing 2 [Source:VGNC Symbol;Acc:VGNC:92750]	1.73	0.083
ssc-miR-218	SERTAD4	SERTA domain containing 4 [Source:VGNC Symbol;Acc:VGNC:92752]	1.73	0.083
ssc-miR-218	SESN3	sestrin 3 [Source:VGNC Symbol;Acc:VGNC:92755]	1.73	0.083
ssc-miR-218	SETBP1	SET binding protein 1 [Source:VGNC Symbol;Acc:VGNC:92756]	1.73	0.083
ssc-miR-218	SETD7	SET domain containing 7, histone lysine methyltransferase [Source:VGNC Symbol;Acc:VGNC:92762]	1.73	0.083
ssc-miR-218	SFMBT1	Scm like with four mbt domains 1 [Source:VGNC Symbol;Acc:VGNC:92773]	1.73	0.083
ssc-miR-218	SFMBT2	Scm like with four mbt domains 2 [Source:VGNC Symbol;Acc:VGNC:95935]	1.73	0.083
ssc-miR-218	SFT2D3	SFT2 domain containing 3 [Source:VGNC Symbol;Acc:VGNC:96186]	1.73	0.083
ssc-miR-218	SFXN5	sideroflexin 5 [Source:VGNC Symbol;Acc:VGNC:92787]	1.73	0.083
ssc-miR-218	SGCD	sarcoglycan delta [Source:NCBI gene (formerly Entrezgene);Acc:100240724]	1.73	0.083
ssc-miR-218	SGCZ	sarcoglycan zeta [Source:VGNC Symbol;Acc:VGNC:104055]	1.73	0.083
ssc-miR-218	SGMS2	sphingomyelin synthase 2 [Source:VGNC Symbol;Acc:VGNC:92795]	1.73	0.083
ssc-miR-218	SGSM1	small G protein signaling modulator 1 [Source:VGNC Symbol;Acc:VGNC:92800]	1.73	0.083
ssc-miR-218	SH3BP2	SH3 domain binding protein 2 [Source:VGNC Symbol;Acc:VGNC:92818]	1.73	0.083
ssc-miR-218	SH3BP4	SH3 domain binding protein 4 [Source:VGNC Symbol;Acc:VGNC:95495]	1.73	0.083
ssc-miR-218	SH3D19	SH3 domain containing 19 [Source:VGNC Symbol;Acc:VGNC:92821]	1.73	0.083
ssc-miR-218	SH3GL1	SH3 domain containing GRB2 like 1, endophilin A2 [Source:VGNC Symbol;Acc:VGNC:92823]	1.73	0.083
ssc-miR-218	SH3KBP1	SH3 domain containing kinase binding protein 1 [Source:VGNC Symbol;Acc:VGNC:92828]	1.73	0.083
ssc-miR-218	SH3RF1	SH3 domain containing ring finger 1 [Source:VGNC Symbol;Acc:VGNC:98316]	1.73	0.083
ssc-miR-218	SHANK2	SH3 and multiple ankyrin repeat domains 2 [Source:VGNC Symbol;Acc:VGNC:92835]	1.73	0.083
ssc-miR-218	SHC1	SHC adaptor protein 1 [Source:VGNC Symbol;Acc:VGNC:92837]	1.73	0.083
ssc-miR-218	SHC3	SHC adaptor protein 3 [Source:VGNC Symbol;Acc:VGNC:92838]	1.73	0.083
ssc-miR-218	SHC4	SHC adaptor protein 4 [Source:HGNC Symbol;Acc:HGNC:16743]	1.73	0.083
ssc-miR-218	SHISA6	hypothetical gene	1.73	0.083
ssc-miR-218	SHISA7	shisa family member 7 [Source:VGNC Symbol;Acc:VGNC:92848]	1.73	0.083
ssc-miR-218	SHISA9	shisa family member 9 [Source:HGNC Symbol;Acc:HGNC:37231]	1.73	0.083
ssc-miR-218	SHMT1	serine hydroxymethyltransferase 1 [Source:VGNC Symbol;Acc:VGNC:92855]	1.73	0.083
ssc-miR-218	SHOC2	SHOC2 leucine rich repeat scaffold protein [Source:VGNC Symbol;Acc:VGNC:98318]	1.73	0.083
ssc-miR-218	SIAE	sialic acid acetylesterase [Source:VGNC Symbol;Acc:VGNC:92864]	1.73	0.083
ssc-miR-218	SIK2	salt inducible kinase 2 [Source:VGNC Symbol;Acc:VGNC:92872]	1.73	0.083
ssc-miR-218	SIN3A	SIN3 transcription regulator family member A [Source:VGNC Symbol;Acc:VGNC:92879]	1.73	0.083
ssc-miR-218	SKA2	spindle and kinetochore associated complex subunit 2 [Source:VGNC Symbol;Acc:VGNC:92898]	1.73	0.083
ssc-miR-218	SKAP1	src kinase associated phosphoprotein 1 [Source:VGNC Symbol;Acc:VGNC:92900]	1.73	0.083
ssc-miR-218	SKI	SKI proto-onco [Source:VGNC Symbol;Acc:VGNC:92902]	1.73	0.083
ssc-miR-218	SKIL	SKI like proto-onco [Source:VGNC Symbol;Acc:VGNC:92903]	1.73	0.083
ssc-miR-218	SLAIN1	SLAIN motif family member 1 [Source:VGNC Symbol;Acc:VGNC:92909]	1.73	0.083
ssc-miR-218	SLAIN2	SLAIN motif family member 2 [Source:VGNC Symbol;Acc:VGNC:92910]	1.73	0.083
ssc-miR-218	SLC12A2	solute carrier family 12 member 2 [Source:VGNC Symbol;Acc:VGNC:92922]	1.73	0.083

ssc-miR-218	SLC16A10	solute carrier family 16 member 10 [Source:VGNC Symbol;Acc:VGNC:103178]	1.73	0.083
ssc-miR-218	SLC16A14	solute carrier family 16 member 14 [Source:VGNC Symbol;Acc:VGNC:96129]	1.73	0.083
ssc-miR-218	SLC17A6	solute carrier family 17 member 6 [Source:VGNC Symbol;Acc:VGNC:92954]	1.73	0.083
ssc-miR-218	SLC18A2	solute carrier family 18 member A2 [Source:VGNC Symbol;Acc:VGNC:92958]	1.73	0.083
ssc-miR-218	SLC22A23	solute carrier family 22 member 23 [Source:HGNC Symbol;Acc:HGNC:21106]	1.73	0.083
ssc-miR-218	SLC24A4	solute carrier family 24 member 4 [Source:VGNC Symbol;Acc:VGNC:92988]	1.73	0.083
ssc-miR-218	SLC25A16	solute carrier family 25 member 16 [Source:VGNC Symbol;Acc:VGNC:92995]	1.73	0.083
ssc-miR-218	SLC25A51	hypothetical gene	1.73	0.083
ssc-miR-218	SLC35B2	solute carrier family 35 member B2 [Source:VGNC Symbol;Acc:VGNC:93072]	1.73	0.083
ssc-miR-218	SLC35F1	solute carrier family 35 member F1 [Source:VGNC Symbol;Acc:VGNC:93081]	1.73	0.083
ssc-miR-218	SLC38A1	solute carrier family 38 member 1 [Source:HGNC Symbol;Acc:HGNC:13447]	1.73	0.083
ssc-miR-218	SLC38A9	solute carrier family 38 member 9 [Source:VGNC Symbol;Acc:VGNC:93100]	1.73	0.083
ssc-miR-218	SLC39A1	solute carrier family 39 member 1 [Source:VGNC Symbol;Acc:VGNC:93101]	1.73	0.083
ssc-miR-218	SLC39A14	solute carrier family 39 member 14 [Source:HGNC Symbol;Acc:HGNC:20858]	1.73	0.083
ssc-miR-218	SLC45A3	solute carrier family 45 member 3 [Source:VGNC Symbol;Acc:VGNC:93124]	1.73	0.083
ssc-miR-218	SLC5A3	solute carrier family 5 member 3 [Source:VGNC Symbol;Acc:VGNC:93144]	1.73	0.083
ssc-miR-218	SLC6A1	solute carrier family 6 member 1 [Source:VGNC Symbol;Acc:VGNC:93153]	1.73	0.083
ssc-miR-218	SLC6A17	solute carrier family 6 member 17 [Source:VGNC Symbol;Acc:VGNC:93158]	1.73	0.083
ssc-miR-218	SLC6A6	solute carrier family 6 member 6 [Source:VGNC Symbol;Acc:VGNC:93166]	1.73	0.083
ssc-miR-218	SLCO5A1	solute carrier organic anion transporter family member 5A1 [Source:HGNC Symbol;Acc:HGNC:19046]	1.73	0.083
ssc-miR-218	SLIT2	slit guidance ligand 2 [Source:VGNC Symbol;Acc:VGNC:93204]	1.73	0.083
ssc-miR-218	SLITRK6	SLIT and NTRK like family member 6 [Source:VGNC Symbol;Acc:VGNC:93211]	1.73	0.083
ssc-miR-218	SLK	STE20 like kinase [Source:VGNC Symbol;Acc:VGNC:98328]	1.73	0.083
ssc-miR-218	SMCHD1	structural maintenance of chromosomes flexible hinge domain containing 1 [Source:VGNC Symbol;Acc:VGNC:93242]	1.73	0.083
ssc-miR-218	SMEK2	hypothetical gene	1.73	0.083
ssc-miR-218	SMG7	SMG7 nonsense mediated mRNA decay factor [Source:VGNC Symbol;Acc:VGNC:93250]	1.73	0.083
ssc-miR-218	SMPD3	sphingomyelin phosphodiesterase 3 [Source:VGNC Symbol;Acc:VGNC:93263]	1.73	0.083
ssc-miR-218	SMUG1	single-strand-selective monofunctional uracil-DNA glycosylase 1 [Source:VGNC Symbol;Acc:VGNC:93269]	1.73	0.083
ssc-miR-218	SMYD1	SET and MYND domain containing 1 [Source:VGNC Symbol;Acc:VGNC:93272]	1.73	0.083
ssc-miR-218	SNAPC5	small nuclear RNA activating complex polypeptide 5 [Source:VGNC Symbol;Acc:VGNC:93283]	1.73	0.083
ssc-miR-218	SNCB	synuclein beta [Source:VGNC Symbol;Acc:VGNC:93286]	1.73	0.083
ssc-miR-218	SNTB2	syntrophin beta 2 [Source:VGNC Symbol;Acc:VGNC:93298]	1.73	0.083
ssc-miR-218	SNTG1	syntrophin gamma 1 [Source:VGNC Symbol;Acc:VGNC:93299]	1.73	0.083
ssc-miR-218	SNX12	sorting nexin 12 [Source:VGNC Symbol;Acc:VGNC:93305]	1.73	0.083
ssc-miR-218	SNX13	sorting nexin 13 [Source:VGNC Symbol;Acc:VGNC:93306]	1.73	0.083
ssc-miR-218	SNX18	sorting nexin 18 [Source:VGNC Symbol;Acc:VGNC:93310]	1.73	0.083
ssc-miR-218	SNX4	sorting nexin 4 [Source:VGNC Symbol;Acc:VGNC:93322]	1.73	0.083
ssc-miR-218	SOCS3	suppressor of cytokine signaling 3 [Source:VGNC Symbol;Acc:VGNC:99052]	1.73	0.083
ssc-miR-218	SOCS4	suppressor of cytokine signaling 4 [Source:VGNC Symbol;Acc:VGNC:93331]	1.73	0.083
ssc-miR-218	SOCS5	suppressor of cytokine signaling 5 [Source:VGNC Symbol;Acc:VGNC:93332]	1.73	0.083
ssc-miR-218	SOCS6	suppressor of cytokine signaling 6 [Source:VGNC Symbol;Acc:VGNC:93333]	1.73	0.083
ssc-miR-218	SOCS7	suppressor of cytokine signaling 7 [Source:VGNC Symbol;Acc:VGNC:93334]	1.73	0.083
ssc-miR-218	SORBS1	sorbin and SH3 domain containing 1 [Source:VGNC Symbol;Acc:VGNC:93337]	1.73	0.083
ssc-miR-218	SORCS1	sortilin related VPS10 domain containing receptor 1 [Source:VGNC Symbol;Acc:VGNC:93339]	1.73	0.083
ssc-miR-218	SOST	sclerostin [Source:VGNC Symbol;Acc:VGNC:93345]	1.73	0.083
ssc-miR-218	SOX11	SRY-box transcription factor 11 [Source:VGNC Symbol;Acc:VGNC:93351]	1.73	0.083
ssc-miR-218	SOX5	SRY-box transcription factor 5 [Source:VGNC Symbol;Acc:VGNC:93357]	1.73	0.083
ssc-miR-218	SOX6	SRY-box transcription factor 6 [Source:VGNC Symbol;Acc:VGNC:93358]	1.73	0.083
ssc-miR-218	SP1	Sp1 transcription factor [Source:VGNC Symbol;Acc:VGNC:93360]	1.73	0.083
ssc-miR-218	SPAG9	sperm associated antigen 9 [Source:VGNC Symbol;Acc:VGNC:93374]	1.73	0.083

ssc-miR-218	SPAST	spastin [Source:VGNC Symbol;Acc:VGNC:98333]	1.73	0.083
ssc-miR-218	SPECC1L	sperm antigen with calponin homology and coiled-coil domains 1 like [Source:HGNC Symbol;Acc:HGNC:29022]	1.73	0.083
ssc-miR-218	SPHKAP	SPHK1 interactor, AKAP domain containing [Source:VGNC Symbol;Acc:VGNC:96245]	1.73	0.083
ssc-miR-218	SPI1	Spi-1 proto-onco [Source:VGNC Symbol;Acc:VGNC:98335]	1.73	0.083
ssc-miR-218	SPOCK3	SPARC (osteonectin), cwcv and kazal like domains proteoglycan 3 [Source:HGNC Symbol;Acc:HGNC:13565]	1.73	0.083
ssc-miR-218	SPRED1	sprouty related EVH1 domain containing 1 [Source:VGNC Symbol;Acc:VGNC:93420]	1.73	0.083
ssc-miR-218	SPRED2	sprouty related EVH1 domain containing 2 [Source:VGNC Symbol;Acc:VGNC:93421]	1.73	0.083
ssc-miR-218	SPSB1	splA/ryanodine receptor domain and SOCS box containing 1 [Source:VGNC Symbol;Acc:VGNC:93430]	1.73	0.083
ssc-miR-218	SRCIN1	SRC kinase signaling inhibitor 1 [Source:VGNC Symbol;Acc:VGNC:93448]	1.73	0.083
ssc-miR-218	SREK1	splicing regulatory glutamic acid and lysine rich protein 1 [Source:VGNC Symbol;Acc:VGNC:93451]	1.73	0.083
ssc-miR-218	SREK1IP1	SREK1 interacting protein 1 [Source:HGNC Symbol;Acc:HGNC:26716]	1.73	0.083
ssc-miR-218	SRGAP2	hypothetical gene	1.73	0.083
ssc-miR-218	SRP72	signal recognition particle 72 [Source:VGNC Symbol;Acc:VGNC:93461]	1.73	0.083
ssc-miR-218	SRPX2	sushi repeat containing protein X-linked 2 [Source:HGNC Symbol;Acc:HGNC:30668]	1.73	0.083
ssc-miR-218	SRSF10	serine and arginine rich splicing factor 10 [Source:VGNC Symbol;Acc:VGNC:93472]	1.73	0.083
ssc-miR-218	SSH1	slingshot protein phosphatase 1 [Source:VGNC Symbol;Acc:VGNC:93485]	1.73	0.083
ssc-miR-218	SSR1	signal sequence receptor subunit 1 [Source:VGNC Symbol;Acc:VGNC:93490]	1.73	0.083
ssc-miR-218	SSR3	signal sequence receptor subunit 3 [Source:VGNC Symbol;Acc:VGNC:93492]	1.73	0.083
ssc-miR-218	ST18	ST18 C2H2C-type zinc finger transcription factor [Source:VGNC Symbol;Acc:VGNC:93501]	1.73	0.083
ssc-miR-218	ST8SIA1	ST8 alpha-N-acetyl-neuraminide alpha-2,8-sialyltransferase 1 [Source:VGNC Symbol;Acc:VGNC:93516]	1.73	0.083
ssc-miR-218	ST8SIA3	ST8 alpha-N-acetyl-neuraminide alpha-2,8-sialyltransferase 3 [Source:VGNC Symbol;Acc:VGNC:93518]	1.73	0.083
ssc-miR-218	ST8SIA4	ST8 alpha-N-acetyl-neuraminide alpha-2,8-sialyltransferase 4 [Source:VGNC Symbol;Acc:VGNC:93519]	1.73	0.083
ssc-miR-218	ST8SIA5	ST8 alpha-N-acetyl-neuraminide alpha-2,8-sialyltransferase 5 [Source:VGNC Symbol;Acc:VGNC:93520]	1.73	0.083
ssc-miR-218	STAM2	signal transducing adaptor molecule 2 [Source:VGNC Symbol;Acc:VGNC:95521]	1.73	0.083
ssc-miR-218	STEAP3	STEAP3 metalloreductase [Source:VGNC Symbol;Acc:VGNC:96048]	1.73	0.083
ssc-miR-218	STK16	serine/threonine kinase 16 [Source:VGNC Symbol;Acc:VGNC:95526]	1.73	0.083
ssc-miR-218	STK38	serine/threonine kinase 38 [Source:VGNC Symbol;Acc:VGNC:93556]	1.73	0.083
ssc-miR-218	STX1B	syntaxin 1B [Source:VGNC Symbol;Acc:VGNC:98352]	1.73	0.083
ssc-miR-218	STXBP1	syntaxin binding protein 1 [Source:VGNC Symbol;Acc:VGNC:93596]	1.73	0.083
ssc-miR-218	STXBP4	syntaxin binding protein 4 [Source:VGNC Symbol;Acc:VGNC:93598]	1.73	0.083
ssc-miR-218	STXBP5	syntaxin binding protein 5 [Source:VGNC Symbol;Acc:VGNC:93599]	1.73	0.083
ssc-miR-218	STYX	serine/threonine/tyrosine interacting protein [Source:VGNC Symbol;Acc:VGNC:93602]	1.73	0.083
ssc-miR-218	SUPT3H	SPT3 homolog, SAGA and STAGA complex component [Source:VGNC Symbol;Acc:VGNC:93623]	1.73	0.083
ssc-miR-218	SV2A	synaptic vesicle glycoprotein 2A [Source:VGNC Symbol;Acc:VGNC:93639]	1.73	0.083
ssc-miR-218	SV2B	synaptic vesicle glycoprotein 2B [Source:VGNC Symbol;Acc:VGNC:93640]	1.73	0.083
ssc-miR-218	SYCE3	synaptonemal complex central element protein 3 [Source:VGNC Symbol;Acc:VGNC:93652]	1.73	0.083
ssc-miR-218	SYNE1	hypothetical gene	1.73	0.083
ssc-miR-218	SYNGR1	synaptogyrin 1 [Source:VGNC Symbol;Acc:VGNC:93665]	1.73	0.083
ssc-miR-218	SYS1	SYS1 golgi trafficking protein [Source:VGNC Symbol;Acc:VGNC:98354]	1.73	0.083
ssc-miR-218	SYT1	synaptotagmin 1 [Source:VGNC Symbol;Acc:VGNC:93678]	1.73	0.083
ssc-miR-218	SYT13	synaptotagmin 13 [Source:VGNC Symbol;Acc:VGNC:93682]	1.73	0.083
ssc-miR-218	TAC1	tachykinin 1 [Source:VGNC Symbol;Acc:VGNC:93697]	1.73	0.083
ssc-miR-218	TACC1	hypothetical gene	1.73	0.083
ssc-miR-218	TAF11	TATA-box binding protein associated factor 11 [Source:NCBI gene (formerly Entrezgene);Acc:100151814]	1.73	0.083
ssc-miR-218	TANC2	tetratricopeptide repeat, ankyrin repeat and coiled-coil containing 2 [Source:VGNC Symbol;Acc:VGNC:93733]	1.73	0.083
ssc-miR-218	TAOK3	TAO kinase 3 [Source:VGNC Symbol;Acc:VGNC:98357]	1.73	0.083
ssc-miR-218	TBC1D12	TBC1 domain family member 12 [Source:VGNC Symbol;Acc:VGNC:93762]	1.73	0.083
ssc-miR-218	TBC1D8B	TBC1 domain family member 8B [Source:VGNC Symbol;Acc:VGNC:93780]	1.73	0.083
ssc-miR-218	TBC1D9B	TBC1 domain family member 9B [Source:VGNC Symbol;Acc:VGNC:93782]	1.73	0.083
ssc-miR-218	TBX15	hypothetical gene	1.73	0.083

ssc-miR-218	TBX20	T-box transcription factor 20 [Source:VGNC Symbol;Acc:VGNC:93799]	1.73	0.083
ssc-miR-218	TCF12	transcription factor 12 [Source:VGNC Symbol;Acc:VGNC:93817]	1.73	0.083
ssc-miR-218	TCF19	transcription factor 19 [Source:VGNC Symbol;Acc:VGNC:93818]	1.73	0.083
ssc-miR-218	TCF20	transcription factor 20 [Source:HGNC Symbol;Acc:HGNC:11631]	1.73	0.083
ssc-miR-218	TCF4	transcription factor 4 [Source:VGNC Symbol;Acc:VGNC:93823]	1.73	0.083
ssc-miR-218	TDP1	tyrosyl-DNA phosphodiesterase 1 [Source:VGNC Symbol;Acc:VGNC:93844]	1.73	0.083
ssc-miR-218	TEAD1	TEA domain transcription factor 1 [Source:VGNC Symbol;Acc:VGNC:93853]	1.73	0.083
ssc-miR-218	TENM3	teneurin transmembrane protein 3 [Source:VGNC Symbol;Acc:VGNC:96233]	1.73	0.083
ssc-miR-218	TFAP2E	transcription factor AP-2 epsilon [Source:VGNC Symbol;Acc:VGNC:93911]	1.73	0.083
ssc-miR-218	TGIF2	TGFB induced factor homeobox 2 [Source:VGNC Symbol;Acc:VGNC:95656]	1.73	0.083
ssc-miR-218	THOC2	THO complex 2 [Source:VGNC Symbol;Acc:VGNC:93957]	1.73	0.083
ssc-miR-218	THRB	thyroid hormone receptor beta [Source:VGNC Symbol;Acc:VGNC:93965]	1.73	0.083
ssc-miR-218	THSD7A	thrombospondin type 1 domain containing 7A [Source:VGNC Symbol;Acc:VGNC:93968]	1.73	0.083
ssc-miR-218	THTPA	thiamine triphosphatase [Source:VGNC Symbol;Acc:VGNC:93969]	1.73	0.083
ssc-miR-218	TLK2	tousled like kinase 2 [Source:VGNC Symbol;Acc:VGNC:94017]	1.73	0.083
ssc-miR-218	TLL1	tolloid like 1 [Source:VGNC Symbol;Acc:VGNC:94018]	1.73	0.083
ssc-miR-218	TMED7-TICAM2	hypothetical gene	1.73	0.083
ssc-miR-218	TMED8	transmembrane p24 trafficking protein family member 8 [Source:VGNC Symbol;Acc:VGNC:94058]	1.73	0.083
ssc-miR-218	TMEM123	hypothetical gene	1.73	0.083
ssc-miR-218	TMEM127	transmembrane protein 127 [Source:HGNC Symbol;Acc:HGNC:26038]	1.73	0.083
ssc-miR-218	TMEM132B	transmembrane protein 132B [Source:HGNC Symbol;Acc:HGNC:29397]	1.73	0.083
ssc-miR-218	TMEM150C	transmembrane protein 150C [Source:VGNC Symbol;Acc:VGNC:94096]	1.73	0.083
ssc-miR-218	TMEM151A	transmembrane protein 151A [Source:VGNC Symbol;Acc:VGNC:94097]	1.73	0.083
ssc-miR-218	TMEM156	transmembrane protein 156 [Source:VGNC Symbol;Acc:VGNC:94099]	1.73	0.083
ssc-miR-218	TMEM163	transmembrane protein 163 [Source:VGNC Symbol;Acc:VGNC:96078]	1.73	0.083
ssc-miR-218	TMEM178A	transmembrane protein 178A [Source:VGNC Symbol;Acc:VGNC:94114]	1.73	0.083
ssc-miR-218	TMEM184A	transmembrane protein 184A [Source:VGNC Symbol;Acc:VGNC:94119]	1.73	0.083
ssc-miR-218	TMEM185B	transmembrane protein 185B [Source:VGNC Symbol;Acc:VGNC:95900]	1.73	0.083
ssc-miR-218	TMEM229B	hypothetical gene	1.73	0.083
ssc-miR-218	TMEM25	transmembrane protein 25 [Source:VGNC Symbol;Acc:VGNC:94157]	1.73	0.083
ssc-miR-218	TMEM33	transmembrane protein 33 [Source:VGNC Symbol;Acc:VGNC:94171]	1.73	0.083
ssc-miR-218	TMEM68	transmembrane protein 68 [Source:VGNC Symbol;Acc:VGNC:94202]	1.73	0.083
ssc-miR-218	TMTC2	transmembrane O-mannosyltransferase targeting cadherins 2 [Source:VGNC Symbol;Acc:VGNC:94242]	1.73	0.083
ssc-miR-218	TMUB2	transmembrane and ubiquitin like domain containing 2 [Source:VGNC Symbol;Acc:VGNC:94246]	1.73	0.083
ssc-miR-218	TMX1	thioredoxin related transmembrane protein 1 [Source:VGNC Symbol;Acc:VGNC:94247]	1.73	0.083
ssc-miR-218	TNC	tenascin C [Source:VGNC Symbol;Acc:VGNC:103192]	1.73	0.083
ssc-miR-218	TNFAIP1	TNF alpha induced protein 1 [Source:VGNC Symbol;Acc:VGNC:94250]	1.73	0.083
ssc-miR-218	TNIK	TRAF2 and NCK interacting kinase [Source:VGNC Symbol;Acc:VGNC:98381]	1.73	0.083
ssc-miR-218	TOB1	transducer of ERBB2, 1 [Source:NCBI gene (formerly Entrezgene);Acc:100144440]	1.73	0.083
ssc-miR-218	TOX3	TOX high mobility group box family member 3 [Source:VGNC Symbol;Acc:VGNC:94323]	1.73	0.083
ssc-miR-218	TP53INP1	tumor protein p53 inducible nuclear protein 1 [Source:VGNC Symbol;Acc:VGNC:94329]	1.73	0.083
ssc-miR-218	TP53INP2	tumor protein p53 inducible nuclear protein 2 [Source:VGNC Symbol;Acc:VGNC:95737]	1.73	0.083
ssc-miR-218	TPBGL	hypothetical gene	1.73	0.083
ssc-miR-218	TPCN1	two pore segment channel 1 [Source:VGNC Symbol;Acc:VGNC:94333]	1.73	0.083
ssc-miR-218	TPD52	tumor protein D52 [Source:VGNC Symbol;Acc:VGNC:98383]	1.73	0.083
ssc-miR-218	TPM3	tropomyosin 3 [Source:VGNC Symbol;Acc:VGNC:98877]	1.73	0.083
ssc-miR-218	TPRG1	tumor protein p63 regulated 1 [Source:VGNC Symbol;Acc:VGNC:94348]	1.73	0.083
ssc-miR-218	TRHDE	thyrotropin releasing hormone degrading enzyme [Source:VGNC Symbol;Acc:VGNC:94390]	1.73	0.083
ssc-miR-218	TRIM52	tripartite motif containing 52 [Source:HGNC Symbol;Acc:HGNC:19024]	1.73	0.083
ssc-miR-218	TRIM71	tripartite motif containing 71 [Source:VGNC Symbol;Acc:VGNC:94431]	1.73	0.083

ssc-miR-218	TRIM9	tripartite motif containing 9 [Source:VGNC Symbol;Acc:VGNC:94434]	1.73	0.083
ssc-miR-218	TRIO	trio Rho guanine nucleotide exchange factor [Source:VGNC Symbol;Acc:VGNC:94435]	1.73	0.083
ssc-miR-218	TRIP12	thyroid hormone receptor interactor 12 [Source:VGNC Symbol;Acc:VGNC:95555]	1.73	0.083
ssc-miR-218	TRPC3	transient receptor potential cation channel subfamily C member 3 [Source:VGNC Symbol;Acc:VGNC:94461]	1.73	0.083
ssc-miR-218	TRPM3	transient receptor potential cation channel subfamily M member 3 [Source:VGNC Symbol;Acc:VGNC:98384]	1.73	0.083
ssc-miR-218	TSHZ2	teashirt zinc finger homeobox 2 [Source:NCBI gene (formerly Entrezgene);Acc:100136903]	1.73	0.083
ssc-miR-218	TSPAN3	tetraspanin 3 [Source:VGNC Symbol;Acc:VGNC:94510]	1.73	0.083
ssc-miR-218	TSPAN5	tetraspanin 5 [Source:VGNC Symbol;Acc:VGNC:94514]	1.73	0.083
ssc-miR-218	TSPAN9	tetraspanin 9 [Source:VGNC Symbol;Acc:VGNC:94516]	1.73	0.083
ssc-miR-218	TSR1	TSR1 ribosome maturation factor [Source:VGNC Symbol;Acc:VGNC:94521]	1.73	0.083
ssc-miR-218	TTC39C	tetratricopeptide repeat domain 39C [Source:VGNC Symbol;Acc:VGNC:94553]	1.73	0.083
ssc-miR-218	TTYH3	tweety family member 3 [Source:VGNC Symbol;Acc:VGNC:94576]	1.73	0.083
ssc-miR-218	TUB	TUB bipartite transcription factor [Source:VGNC Symbol;Acc:VGNC:94577]	1.73	0.083
ssc-miR-218	TUSC5	hypothetical gene	1.73	0.083
ssc-miR-218	TXLNG	taxilin gamma [Source:HGNC Symbol;Acc:HGNC:18578]	1.73	0.083
ssc-miR-218	TXNDC5	thioredoxin domain containing 5 [Source:VGNC Symbol;Acc:VGNC:94608]	1.73	0.083
ssc-miR-218	TXNIP	thioredoxin interacting protein [Source:VGNC Symbol;Acc:VGNC:94610]	1.73	0.083
ssc-miR-218	UBASH3A	ubiquitin associated and SH3 domain containing A [Source:VGNC Symbol;Acc:VGNC:94636]	1.73	0.083
ssc-miR-218	UBE2E2	ubiquitin conjugating enzyme E2 E2 [Source:NCBI gene (formerly Entrezgene);Acc:780417]	1.73	0.083
ssc-miR-218	UBE2G1	ubiquitin conjugating enzyme E2 G1 [Source:HGNC Symbol;Acc:HGNC:12482]	1.73	0.083
ssc-miR-218	UBE2H	ubiquitin conjugating enzyme E2 H [Source:VGNC Symbol;Acc:VGNC:94643]	1.73	0.083
ssc-miR-218	UBE2J1	ubiquitin conjugating enzyme E2 J1 [Source:VGNC Symbol;Acc:VGNC:94644]	1.73	0.083
ssc-miR-218	UBE2V2	ubiquitin conjugating enzyme E2 V2 [Source:VGNC Symbol;Acc:VGNC:98394]	1.73	0.083
ssc-miR-218	UBE3A	ubiquitin protein ligase E3A [Source:VGNC Symbol;Acc:VGNC:94652]	1.73	0.083
ssc-miR-218	UBN2	ubinuclein 2 [Source:VGNC Symbol;Acc:VGNC:94664]	1.73	0.083
ssc-miR-218	UBQLN2	ubiquilin 2 [Source:VGNC Symbol;Acc:VGNC:94665]	1.73	0.083
ssc-miR-218	UBR3	ubiquitin protein ligase E3 component n-recognin 3 [Source:VGNC Symbol;Acc:VGNC:96771]	1.73	0.083
ssc-miR-218	UBTD1	ubiquitin domain containing 1 [Source:VGNC Symbol;Acc:VGNC:94672]	1.73	0.083
ssc-miR-218	UGT8	UDP glycosyltransferase 8 [Source:VGNC Symbol;Acc:VGNC:98401]	1.73	0.083
ssc-miR-218	UHRF1BP1L	UHRF1 binding protein 1 like [Source:VGNC Symbol;Acc:VGNC:94691]	1.73	0.083
ssc-miR-218	UNC13A	hypothetical gene	1.73	0.083
ssc-miR-218	UQCR10	ubiquinol-cytochrome c reductase, complex III subunit X [Source:VGNC Symbol;Acc:VGNC:94727]	1.73	0.083
ssc-miR-218	USP31	ubiquitin specific peptidase 31 [Source:HGNC Symbol;Acc:HGNC:20060]	1.73	0.083
ssc-miR-218	USP32	hypothetical gene	1.73	0.083
ssc-miR-218	USP34	ubiquitin specific peptidase 34 [Source:VGNC Symbol;Acc:VGNC:94759]	1.73	0.083
ssc-miR-218	USP45	ubiquitin specific peptidase 45 [Source:VGNC Symbol;Acc:VGNC:94767]	1.73	0.083
ssc-miR-218	USP46	ubiquitin specific peptidase 46 [Source:VGNC Symbol;Acc:VGNC:94768]	1.73	0.083
ssc-miR-218	USP49	ubiquitin specific peptidase 49 [Source:VGNC Symbol;Acc:VGNC:94771]	1.73	0.083
ssc-miR-218	USP6	hypothetical gene	1.73	0.083
ssc-miR-218	USP9X	ubiquitin specific peptidase 9 X-linked [Source:HGNC Symbol;Acc:HGNC:12632]	1.73	0.083
ssc-miR-218	VAMP7	vesicle associated membrane protein 7 [Source:VGNC Symbol;Acc:VGNC:94796]	1.73	0.083
ssc-miR-218	VASH2	vasohibin 2 [Source:VGNC Symbol;Acc:VGNC:94802]	1.73	0.083
ssc-miR-218	VAT1	vesicle amine transport 1 [Source:VGNC Symbol;Acc:VGNC:94805]	1.73	0.083
ssc-miR-218	VAV3	hypothetical gene	1.73	0.083
ssc-miR-218	VOPP1	VOPP1 WW domain binding protein [Source:VGNC Symbol;Acc:VGNC:94835]	1.73	0.083
ssc-miR-218	VPS13B	vacuolar protein sorting 13 homolog B [Source:VGNC Symbol;Acc:VGNC:94839]	1.73	0.083
ssc-miR-218	VPS37A	VPS37A subunit of ESCRT-I [Source:VGNC Symbol;Acc:VGNC:104092]	1.73	0.083
ssc-miR-218	VPS4A	vacuolar protein sorting 4 homolog A [Source:NCBI gene (formerly Entrezgene);Acc:100627841]	1.73	0.083
ssc-miR-218	VPS52	VPS52 subunit of GARP complex [Source:VGNC Symbol;Acc:VGNC:94860]	1.73	0.083
ssc-miR-218	VSIG10	V-set and immunoglobulin domain containing 10 [Source:HGNC Symbol;Acc:HGNC:26078]	1.73	0.083

ssc-miR-218	VSIG8	V-set and immunoglobulin domain containing 8 [Source:VGNC Symbol;Acc:VGNC:98894]	1.73	0.083
ssc-miR-218	VWC2	von Willebrand factor C domain containing 2 [Source:VGNC Symbol;Acc:VGNC:94887]	1.73	0.083
ssc-miR-218	WASF3	WASP family member 3 [Source:VGNC Symbol;Acc:VGNC:94894]	1.73	0.083
ssc-miR-218	WASL	WASP like actin nucleation promoting factor [Source:VGNC Symbol;Acc:VGNC:96604]	1.73	0.083
ssc-miR-218	WDR26	WD repeat domain 26 [Source:VGNC Symbol;Acc:VGNC:95960]	1.73	0.083
ssc-miR-218	WDR44	WD repeat domain 44 [Source:VGNC Symbol;Acc:VGNC:94921]	1.73	0.083
ssc-miR-218	WDR47	WD repeat domain 47 [Source:VGNC Symbol;Acc:VGNC:94924]	1.73	0.083
ssc-miR-218	WDR48	WD repeat domain 48 [Source:VGNC Symbol;Acc:VGNC:94925]	1.73	0.083
ssc-miR-218	WDR55	WD repeat domain 55 [Source:VGNC Symbol;Acc:VGNC:94929]	1.73	0.083
ssc-miR-218	WIPF2	WAS/WASL interacting protein family member 2 [Source:VGNC Symbol;Acc:VGNC:99106]	1.73	0.083
ssc-miR-218	WIPI2	WD repeat domain, phosphoinositide interacting 2 [Source:VGNC Symbol;Acc:VGNC:94960]	1.73	0.083
ssc-miR-218	WNT2B	Wnt family member 2B [Source:VGNC Symbol;Acc:VGNC:94969]	1.73	0.083
ssc-miR-218	XKR4	XK related 4 [Source:VGNC Symbol;Acc:VGNC:98900]	1.73	0.083
ssc-miR-218	XKR7	XK related 7 [Source:VGNC Symbol;Acc:VGNC:95987]	1.73	0.083
ssc-miR-218	XPO5	exportin 5 [Source:VGNC Symbol;Acc:VGNC:95005]	1.73	0.083
ssc-miR-218	XRCC5	X-ray repair cross complementing 5 [Source:VGNC Symbol;Acc:VGNC:95570]	1.73	0.083
ssc-miR-218	YEATS4	YEATS domain containing 4 [Source:HGNC Symbol;Acc:HGNC:24859]	1.73	0.083
ssc-miR-218	YIPF6	Yip1 domain family member 6 [Source:VGNC Symbol;Acc:VGNC:95032]	1.73	0.083
ssc-miR-218	YPEL2	yippee like 2 [Source:VGNC Symbol;Acc:VGNC:95036]	1.73	0.083
ssc-miR-218	YWHAZ	tyrosine 3-monooxygenase/tryptophan 5-monooxygenase activation protein zeta [Source:VGNC Symbol;Acc:VGNC:95047]	1.73	0.083
ssc-miR-218	YY1	YY1 transcription factor [Source:HGNC Symbol;Acc:HGNC:12856]	1.73	0.083
ssc-miR-218	ZBTB10	zinc finger and BTB domain containing 10 [Source:VGNC Symbol;Acc:VGNC:95058]	1.73	0.083
ssc-miR-218	ZBTB11	zinc finger and BTB domain containing 11 [Source:VGNC Symbol;Acc:VGNC:95059]	1.73	0.083
ssc-miR-218	ZBTB16	zinc finger and BTB domain containing 16 [Source:VGNC Symbol;Acc:VGNC:108626]	1.73	0.083
ssc-miR-218	ZBTB20	zinc finger and BTB domain containing 20 [Source:VGNC Symbol;Acc:VGNC:95063]	1.73	0.083
ssc-miR-218	ZBTB34	zinc finger and BTB domain containing 34 [Source:VGNC Symbol;Acc:VGNC:95070]	1.73	0.083
ssc-miR-218	ZBTB39	zinc finger and BTB domain containing 39 [Source:VGNC Symbol;Acc:VGNC:95073]	1.73	0.083
ssc-miR-218	ZBTB41	zinc finger and BTB domain containing 41 [Source:VGNC Symbol;Acc:VGNC:108284]	1.73	0.083
ssc-miR-218	ZC3H12B	zinc finger CCCH-type containing 12B [Source:VGNC Symbol;Acc:VGNC:95090]	1.73	0.083
ssc-miR-218	ZC3H12D	zinc finger CCCH-type containing 12D [Source:VGNC Symbol;Acc:VGNC:95092]	1.73	0.083
ssc-miR-218	ZC4H2	zinc finger C4H2-type containing [Source:VGNC Symbol;Acc:VGNC:95105]	1.73	0.083
ssc-miR-218	ZDHHC18	zinc finger DHHC-type palmitoyltransferase 18 [Source:VGNC Symbol;Acc:VGNC:95120]	1.73	0.083
ssc-miR-218	ZDHHC2	zinc finger DHHC-type palmitoyltransferase 2 [Source:VGNC Symbol;Acc:VGNC:95886]	1.73	0.083
ssc-miR-218	ZDHHC23	zinc finger DHHC-type palmitoyltransferase 23 [Source:VGNC Symbol;Acc:VGNC:95125]	1.73	0.083
ssc-miR-218	ZDHHC8	zinc finger DHHC-type palmitoyltransferase 8 [Source:VGNC Symbol;Acc:VGNC:95130]	1.73	0.083
ssc-miR-218	ZEB2	hypothetical gene	1.73	0.083
ssc-miR-218	ZFHX2	zinc finger homeobox 2 [Source:VGNC Symbol;Acc:VGNC:95140]	1.73	0.083
ssc-miR-218	ZFP91	ZFP91 zinc finger protein, atypical E3 ubiquitin ligase [Source:HGNC Symbol;Acc:HGNC:14983]	1.73	0.083
ssc-miR-218	ZFX	hypothetical gene	1.73	0.083
ssc-miR-218	ZFY	zinc finger protein Y-linked [Source:HGNC Symbol;Acc:HGNC:12870]	1.73	0.083
ssc-miR-218	ZFYVE26	zinc finger FYVE-type containing 26 [Source:VGNC Symbol;Acc:VGNC:95160]	1.73	0.083
ssc-miR-218	ZFYVE28	zinc finger FYVE-type containing 28 [Source:VGNC Symbol;Acc:VGNC:95162]	1.73	0.083
ssc-miR-218	ZIC5	Zic family member 5 [Source:VGNC Symbol;Acc:VGNC:95170]	1.73	0.083
ssc-miR-218	ZKSCAN1	zinc finger with KRAB and SCAN domains 1 [Source:VGNC Symbol;Acc:VGNC:95171]	1.73	0.083
ssc-miR-218	ZMAT3	zinc finger matrin-type 3 [Source:VGNC Symbol;Acc:VGNC:95177]	1.73	0.083
ssc-miR-218	ZMAT4	zinc finger matrin-type 4 [Source:VGNC Symbol;Acc:VGNC:96109]	1.73	0.083
ssc-miR-218	ZMIZ1	zinc finger MIZ-type containing 1 [Source:VGNC Symbol;Acc:VGNC:95178]	1.73	0.083
ssc-miR-218	ZMIZ2	zinc finger MIZ-type containing 2 [Source:VGNC Symbol;Acc:VGNC:95179]	1.73	0.083
ssc-miR-218	ZNF202	zinc finger protein 202 [Source:HGNC Symbol;Acc:HGNC:12994]	1.73	0.083
ssc-miR-218	ZNF236	zinc finger protein 236 [Source:VGNC Symbol;Acc:VGNC:95209]	1.73	0.083

ssc-miR-218	ZNF280C	zinc finger protein 280C [Source:VGNC Symbol;Acc:VGNC:95216]	1.73	0.083
ssc-miR-218	ZNF385A	zinc finger protein 385A [Source:VGNC Symbol;Acc:VGNC:95233]	1.73	0.083
ssc-miR-218	ZNF462	zinc finger protein 462 [Source:VGNC Symbol;Acc:VGNC:95244]	1.73	0.083
ssc-miR-218	ZNF518B	zinc finger protein 518B [Source:VGNC Symbol;Acc:VGNC:95258]	1.73	0.083
ssc-miR-218	ZNF583	zinc finger protein 583 [Source:VGNC Symbol;Acc:VGNC:98731]	1.73	0.083
ssc-miR-218	ZNF609	zinc finger protein 609 [Source:VGNC Symbol;Acc:VGNC:95274]	1.73	0.083
ssc-miR-218	ZNF638	zinc finger protein 638 [Source:VGNC Symbol;Acc:VGNC:95281]	1.73	0.083
ssc-miR-218	ZNF654	zinc finger protein 654 [Source:VGNC Symbol;Acc:VGNC:95285]	1.73	0.083
ssc-miR-218	ZNF697	zinc finger protein 697 [Source:HGNC Symbol;Acc:HGNC:32034]	1.73	0.083
ssc-miR-218	ZNF831	zinc finger protein 831 [Source:VGNC Symbol;Acc:VGNC:95765]	1.73	0.083
ssc-miR-218	ZNFX1	zinc finger NFX1-type containing 1 [Source:VGNC Symbol;Acc:VGNC:96210]	1.73	0.083
ssc-miR-218	ZRANB1	zinc finger RANBP2-type containing 1 [Source:VGNC Symbol;Acc:VGNC:95322]	1.73	0.083
ssc-miR-218	ZWILCH	zwilch kinetochore protein [Source:VGNC Symbol;Acc:VGNC:95341]	1.73	0.083
ssc-miR-218	ZXDA	hypothetical gene	1.73	0.083
ssc-miR-218-5p	ABAT	4-aminobutyrate aminotransferase [Source:VGNC Symbol;Acc:VGNC:96910]	1.73	0.083
ssc-miR-218-5p	ABCD2	ATP binding cassette subfamily D member 2 [Source:VGNC Symbol;Acc:VGNC:84962]	1.73	0.083
ssc-miR-218-5p	ABCG4	ATP binding cassette subfamily G member 4 [Source:HGNC Symbol;Acc:HGNC:13884]	1.73	0.083
ssc-miR-218-5p	ABI2	abl interactor 2 [Source:VGNC Symbol;Acc:VGNC:96027]	1.73	0.083
ssc-miR-218-5p	ABL2	ABL proto-onco 2, non-receptor tyrosine kinase [Source:VGNC Symbol;Acc:VGNC:84985]	1.73	0.083
ssc-miR-218-5p	ABLIM3	actin binding LIM protein family member 3 [Source:VGNC Symbol;Acc:VGNC:84988]	1.73	0.083
ssc-miR-218-5p	AC010441.1	hypothetical gene	1.73	0.083
ssc-miR-218-5p	ACO1	aconitase 1 [Source:VGNC Symbol;Acc:VGNC:95539]	1.73	0.083
ssc-miR-218-5p	ACSL1	acyl-CoA synthetase long chain family member 1 [Source:VGNC Symbol;Acc:VGNC:96294]	1.73	0.083
ssc-miR-218-5p	ACSL6	acyl-CoA synthetase long chain family member 6 [Source:VGNC Symbol;Acc:VGNC:99581]	1.73	0.083
ssc-miR-218-5p	ACTN1	actinin alpha 1 [Source:NCBI gene (formerly Entrezgene);Acc:100513412]	1.73	0.083
ssc-miR-218-5p	ADAM10	ADAM metallopeptidase domain 10 [Source:VGNC Symbol;Acc:VGNC:85061]	1.73	0.083
ssc-miR-218-5p	ADAM23	ADAM metallopeptidase domain 23 [Source:VGNC Symbol;Acc:VGNC:95933]	1.73	0.083
ssc-miR-218-5p	ADAMTS18	ADAM metallopeptidase with thrombospondin type 1 motif 18 [Source:VGNC Symbol;Acc:VGNC:85080]	1.73	0.083
ssc-miR-218-5p	ADARB1	adenosine deaminase RNA specific B1 [Source:VGNC Symbol;Acc:VGNC:85098]	1.73	0.083
ssc-miR-218-5p	ADCY9	adenylate cyclase 9 [Source:VGNC Symbol;Acc:VGNC:85113]	1.73	0.083
ssc-miR-218-5p	ADD2	adducin 2 [Source:VGNC Symbol;Acc:VGNC:85117]	1.73	0.083
ssc-miR-218-5p	ADIPOR2	adiponectin receptor 2 [Source:VGNC Symbol;Acc:VGNC:85141]	1.73	0.083
ssc-miR-218-5p	ADO	2-aminoethanethiol dioxygenase [Source:VGNC Symbol;Acc:VGNC:85146]	1.73	0.083
ssc-miR-218-5p	ADRB1	adrenoceptor beta 1 [Source:VGNC Symbol;Acc:VGNC:107363]	1.73	0.083
ssc-miR-218-5p	ADRBK2	hypothetical gene	1.73	0.083
ssc-miR-218-5p	AFAP1L1	actin filament associated protein 1 like 1 [Source:VGNC Symbol;Acc:VGNC:85166]	1.73	0.083
ssc-miR-218-5p	AFF1	AF4/FMR2 family member 1 [Source:VGNC Symbol;Acc:VGNC:85167]	1.73	0.083
ssc-miR-218-5p	AFF4	AF4/FMR2 family member 4 [Source:VGNC Symbol;Acc:VGNC:85169]	1.73	0.083
ssc-miR-218-5p	AGAP1	ArfGAP with GTPase domain, ankyrin repeat and PH domain 1 [Source:HGNC Symbol;Acc:HGNC:16922]	1.73	0.083
ssc-miR-218-5p	AGO1	hypothetical gene	1.73	0.083
ssc-miR-218-5p	AGO3	argonaute RISC component 1 [Source:NCBI gene (formerly Entrezgene);Acc:100499510]	1.73	0.083
ssc-miR-218-5p	AGPAT3	1-acylglycerol-3-phosphate O-acyltransferase 3 [Source:VGNC Symbol;Acc:VGNC:85185]	1.73	0.083
ssc-miR-218-5p	AHSA2	hypothetical gene	1.73	0.083
ssc-miR-218-5p	AIF1L	allograft inflammatory factor 1 like [Source:VGNC Symbol;Acc:VGNC:103022]	1.73	0.083
ssc-miR-218-5p	AIMP1	aminoacyl tRNA synthetase complex interacting multifunctional protein 1 [Source:VGNC Symbol;Acc:VGNC:85202]	1.73	0.083
ssc-miR-218-5p	AJAP1	adherens junctions associated protein 1 [Source:VGNC Symbol;Acc:VGNC:85207]	1.73	0.083
ssc-miR-218-5p	AK4	hypothetical gene	1.73	0.083
ssc-miR-218-5p	AKIRIN1	akirin 1 [Source:VGNC Symbol;Acc:VGNC:85227]	1.73	0.083
ssc-miR-218-5p	AL021546.6	hypothetical gene	1.73	0.083
ssc-miR-218-5p	AL626787.1	hypothetical gene	1.73	0.083

ssc-miR-218-5p	ALG6	ALG6 alpha-1,3-glucosyltransferase [Source:VGNC Symbol;Acc:VGNC:85253]	1.73	0.083
ssc-miR-218-5p	AMMECR1	AMMECR nuclear protein 1 [Source:VGNC Symbol;Acc:VGNC:96559]	1.73	0.083
ssc-miR-218-5p	ANK1	ankyrin 1 [Source:VGNC Symbol;Acc:VGNC:96344]	1.73	0.083
ssc-miR-218-5p	ANKRD13B	ankyrin repeat domain 13B [Source:VGNC Symbol;Acc:VGNC:85325]	1.73	0.083
ssc-miR-218-5p	ANKRD27	ankyrin repeat domain 27 [Source:VGNC Symbol;Acc:VGNC:96907]	1.73	0.083
ssc-miR-218-5p	ANKRD34A	ankyrin repeat domain 34A [Source:VGNC Symbol;Acc:VGNC:85335]	1.73	0.083
ssc-miR-218-5p	ANKRD40	ankyrin repeat domain 40 [Source:VGNC Symbol;Acc:VGNC:85338]	1.73	0.083
ssc-miR-218-5p	ANKRD44	ankyrin repeat domain 44 [Source:VGNC Symbol;Acc:VGNC:107123]	1.73	0.083
ssc-miR-218-5p	ANKRD52	ankyrin repeat domain 52 [Source:VGNC Symbol;Acc:VGNC:85342]	1.73	0.083
ssc-miR-218-5p	ANKS1B	ankyrin repeat and sterile alpha motif domain containing 1B [Source:VGNC Symbol;Acc:VGNC:103214]	1.73	0.083
ssc-miR-218-5p	AP1AR	adaptor related protein complex 1 associated regulatory protein [Source:HGNC Symbol;Acc:HGNC:28808]	1.73	0.083
ssc-miR-218-5p	AP1S2	adaptor related protein complex 1 subunit sigma 2 [Source:VGNC Symbol;Acc:VGNC:85382]	1.73	0.083
ssc-miR-218-5p	AP2A1	adaptor related protein complex 2 subunit alpha 1 [Source:VGNC Symbol;Acc:VGNC:85383]	1.73	0.083
ssc-miR-218-5p	AP3M1	adaptor related protein complex 3 subunit mu 1 [Source:VGNC Symbol;Acc:VGNC:85390]	1.73	0.083
ssc-miR-218-5p	APEX1	apurinic/apyrimidinic endodeoxyribonuclease 1 [Source:VGNC Symbol;Acc:VGNC:103896]	1.73	0.083
ssc-miR-218-5p	APH1B	aph-1 homolog B, gamma-secretase subunit [Source:VGNC Symbol;Acc:VGNC:103898]	1.73	0.083
ssc-miR-218-5p	APPBP2	amyloid beta protein binding protein 2 [Source:VGNC Symbol;Acc:VGNC:85425]	1.73	0.083
ssc-miR-218-5p	ARAF	A-Raf proto-onco, serine/threonine kinase [Source:VGNC Symbol;Acc:VGNC:85438]	1.73	0.083
ssc-miR-218-5p	ARF6	ADP ribosylation factor 6 [Source:VGNC Symbol;Acc:VGNC:85448]	1.73	0.083
ssc-miR-218-5p	ARHGAP17	Rho GTPase activating protein 17 [Source:VGNC Symbol;Acc:VGNC:85459]	1.73	0.083
ssc-miR-218-5p	ARHGAP32	Rho GTPase activating protein 32 [Source:VGNC Symbol;Acc:VGNC:85471]	1.73	0.083
ssc-miR-218-5p	ARHGAP5	Rho GTPase activating protein 5 [Source:VGNC Symbol;Acc:VGNC:85480]	1.73	0.083
ssc-miR-218-5p	ARHGEF12	Rho guanine nucleotide exchange factor 12 [Source:VGNC Symbol;Acc:VGNC:85488]	1.73	0.083
ssc-miR-218-5p	ARHGEF39	Rho guanine nucleotide exchange factor 39 [Source:VGNC Symbol;Acc:VGNC:85500]	1.73	0.083
ssc-miR-218-5p	ARID4B	AT-rich interaction domain 4B [Source:HGNC Symbol;Acc:HGNC:15550]	1.73	0.083
ssc-miR-218-5p	ARL3	ADP ribosylation factor like GTPase 3 [Source:VGNC Symbol;Acc:VGNC:107366]	1.73	0.083
ssc-miR-218-5p	ARL4C	ADP ribosylation factor like GTPase 4C [Source:VGNC Symbol;Acc:VGNC:96419]	1.73	0.083
ssc-miR-218-5p	ARL5B	ADP ribosylation factor like GTPase 5B [Source:VGNC Symbol;Acc:VGNC:95985]	1.73	0.083
ssc-miR-218-5p	ARPP19	cAMP regulated phosphoprotein 19 [Source:NCBI gene (formerly Entrezgene);Acc:397362]	1.73	0.083
ssc-miR-218-5p	ARRB1	arrestin beta 1 [Source:VGNC Symbol;Acc:VGNC:85542]	1.73	0.083
ssc-miR-218-5p	ASAP1	ArfGAP with SH3 domain, ankyrin repeat and PH domain 1 [Source:VGNC Symbol;Acc:VGNC:98734]	1.73	0.083
ssc-miR-218-5p	ASIC1	acid sensing ion channel subunit 1 [Source:VGNC Symbol;Acc:VGNC:85578]	1.73	0.083
ssc-miR-218-5p	ASXL3	ASXL transcriptional regulator 3 [Source:VGNC Symbol;Acc:VGNC:85596]	1.73	0.083
ssc-miR-218-5p	ATP8B4	ATPase phospholipid transporting 8B4 (putative) [Source:VGNC Symbol;Acc:VGNC:103036]	1.73	0.083
ssc-miR-218-5p	ATRN	attractin [Source:VGNC Symbol;Acc:VGNC:96479]	1.73	0.083
ssc-miR-218-5p	ATRX	ATRX chromatin remodeler [Source:VGNC Symbol;Acc:VGNC:85686]	1.73	0.083
ssc-miR-218-5p	ATXN1	ataxin 1 [Source:VGNC Symbol;Acc:VGNC:85687]	1.73	0.083
ssc-miR-218-5p	ATXN2	ataxin 2 [Source:VGNC Symbol;Acc:VGNC:85690]	1.73	0.083
ssc-miR-218-5p	AZIN1	antizyme inhibitor 1 [Source:VGNC Symbol;Acc:VGNC:85712]	1.73	0.083
ssc-miR-218-5p	B3GAT1	beta-1,3-glucuronyltransferase 1 [Source:VGNC Symbol;Acc:VGNC:85719]	1.73	0.083
ssc-miR-218-5p	B3GAT2	beta-1,3-glucuronyltransferase 2 [Source:VGNC Symbol;Acc:VGNC:85720]	1.73	0.083
ssc-miR-218-5p	BAHD1	bromo adjacent homology domain containing 1 [Source:VGNC Symbol;Acc:VGNC:85747]	1.73	0.083
ssc-miR-218-5p	BAI3	hypothetical gene	1.73	0.083
ssc-miR-218-5p	BAZ2B	bromodomain adjacent to zinc finger domain 2B [Source:HGNC Symbol;Acc:HGNC:963]	1.73	0.083
ssc-miR-218-5p	BBX	BBX high mobility group box domain containing [Source:VGNC Symbol;Acc:VGNC:85766]	1.73	0.083
ssc-miR-218-5p	BCAT1	branched chain amino acid transaminase 1 [Source:VGNC Symbol;Acc:VGNC:85773]	1.73	0.083
ssc-miR-218-5p	BCL11B	BAF chromatin remodeling complex subunit BCL11B [Source:VGNC Symbol;Acc:VGNC:96563]	1.73	0.083
ssc-miR-218-5p	BCL9	BCL9 transcription coactivator [Source:VGNC Symbol;Acc:VGNC:96567]	1.73	0.083
ssc-miR-218-5p	BDP1	B double prime 1, subunit of RNA polymerase III transcription initiation factor IIIB [Source:VGNC Symbol;Acc:VGNC:85796]	1.73	0.083
ssc-miR-218-5p	BEND3	BEN domain containing 3 [Source:VGNC Symbol;Acc:VGNC:85800]	1.73	0.083

ssc-miR-218-5p	BICD1	BICD cargo adaptor 1 [Source:VGNC Symbol;Acc:VGNC:85818]	1.73	0.083
ssc-miR-218-5p	BIRC6	baculoviral IAP repeat containing 6 [Source:VGNC Symbol;Acc:VGNC:97038]	1.73	0.083
ssc-miR-218-5p	BIVM	basic, immunoglobulin-like variable motif containing [Source:NCBI gene (formerly Entrezgene);Acc:100512068]	1.73	0.083
ssc-miR-218-5p	BMI1	BMI1 proto-onco, polycomb ring finger [Source:VGNC Symbol;Acc:VGNC:108265]	1.73	0.083
ssc-miR-218-5p	BMP5	bone morphotic protein 5 [Source:VGNC Symbol;Acc:VGNC:85844]	1.73	0.083
ssc-miR-218-5p	BMPR2	bone morphotic protein receptor type 2 [Source:VGNC Symbol;Acc:VGNC:95494]	1.73	0.083
ssc-miR-218-5p	BPHL	biphenyl hydrolase like [Source:VGNC Symbol;Acc:VGNC:85864]	1.73	0.083
ssc-miR-218-5p	BRCC3	BRCA1/BRCA2-containing complex subunit 3 [Source:VGNC Symbol;Acc:VGNC:85870]	1.73	0.083
ssc-miR-218-5p	BRPF3	bromodomain and PHD finger containing 3 [Source:VGNC Symbol;Acc:VGNC:85886]	1.73	0.083
ssc-miR-218-5p	BSN	bassoon presynaptic cytomatrix protein [Source:VGNC Symbol;Acc:VGNC:85893]	1.73	0.083
ssc-miR-218-5p	BTG2	BTG anti-proliferation factor 2 [Source:NCBI gene (formerly Entrezgene);Acc:100048932]	1.73	0.083
ssc-miR-218-5p	BVES	blood vessel epicardial substance [Source:VGNC Symbol;Acc:VGNC:103040]	1.73	0.083
ssc-miR-218-5p	C10orf35	hypothetical gene	1.73	0.083
ssc-miR-218-5p	C11orf87	chromosome 11 open reading frame 87 [Source:HGNC Symbol;Acc:HGNC:33788]	1.73	0.083
ssc-miR-218-5p	C16orf52	hypothetical gene	1.73	0.083
ssc-miR-218-5p	C16orf70	hypothetical gene	1.73	0.083
ssc-miR-218-5p	C17orf70	hypothetical gene	1.73	0.083
ssc-miR-218-5p	C18orf25	chromosome 1 C18orf25 homolog [Source:VGNC Symbol;Acc:VGNC:85964]	1.73	0.083
ssc-miR-218-5p	C1orf115	chromosome 10 C1orf115 homolog [Source:VGNC Symbol;Acc:VGNC:96111]	1.73	0.083
ssc-miR-218-5p	C1orf21	hypothetical gene	1.73	0.083
ssc-miR-218-5p	C1orf35	chromosome 2 C1orf35 homolog [Source:VGNC Symbol;Acc:VGNC:86008]	1.73	0.083
ssc-miR-218-5p	C20orf112	hypothetical gene	1.73	0.083
ssc-miR-218-5p	C20orf194	hypothetical gene	1.73	0.083
ssc-miR-218-5p	C2orf88	chromosome 15 C2orf88 homolog [Source:VGNC Symbol;Acc:VGNC:96170]	1.73	0.083
ssc-miR-218-5p	C3orf70	chromosome 13 C3orf70 homolog [Source:VGNC Symbol;Acc:VGNC:85937]	1.73	0.083
ssc-miR-218-5p	C5orf15	chromosome 2 C5orf15 homolog [Source:VGNC Symbol;Acc:VGNC:86009]	1.73	0.083
ssc-miR-218-5p	C6orf120	chromosome 1 C6orf120 homolog [Source:VGNC Symbol;Acc:VGNC:85967]	1.73	0.083
ssc-miR-218-5p	C6orf47	hypothetical gene	1.73	0.083
ssc-miR-218-5p	C6orf62	chromosome 7 C6orf62 homolog [Source:VGNC Symbol;Acc:VGNC:86074]	1.73	0.083
ssc-miR-218-5p	C7orf25	chromosome 18 C7orf25 homolog [Source:VGNC Symbol;Acc:VGNC:85955]	1.73	0.083
ssc-miR-218-5p	C7orf41	hypothetical gene	1.73	0.083
ssc-miR-218-5p	C7orf60	hypothetical gene	1.73	0.083
ssc-miR-218-5p	C8orf46	hypothetical gene	1.73	0.083
ssc-miR-218-5p	C9orf156	hypothetical gene	1.73	0.083
ssc-miR-218-5p	C9orf47	hypothetical gene	1.73	0.083
ssc-miR-218-5p	CA2	carbonic anhydrase 2 [Source:VGNC Symbol;Acc:VGNC:98746]	1.73	0.083
ssc-miR-218-5p	CAB39L	calcium binding protein 39 like [Source:VGNC Symbol;Acc:VGNC:86106]	1.73	0.083
ssc-miR-218-5p	CABLES1	Cdk5 and Abl enzyme substrate 1 [Source:VGNC Symbol;Acc:VGNC:86109]	1.73	0.083
ssc-miR-218-5p	CACNA1G	calcium voltage-gated channel subunit alpha1 G [Source:VGNC Symbol;Acc:VGNC:86119]	1.73	0.083
ssc-miR-218-5p	CACNA1I	calcium voltage-gated channel subunit alpha1 I [Source:VGNC Symbol;Acc:VGNC:97908]	1.73	0.083
ssc-miR-218-5p	CACNB4	calcium voltage-gated channel auxiliary subunit beta 4 [Source:VGNC Symbol;Acc:VGNC:108507]	1.73	0.083
ssc-miR-218-5p	CACUL1	CDK2 associated cullin domain 1 [Source:VGNC Symbol;Acc:VGNC:86132]	1.73	0.083
ssc-miR-218-5p	CADM2	cell adhesion molecule 2 [Source:VGNC Symbol;Acc:VGNC:97910]	1.73	0.083
ssc-miR-218-5p	CALCOCO2	calcium binding and coiled-coil domain 2 [Source:VGNC Symbol;Acc:VGNC:86141]	1.73	0.083
ssc-miR-218-5p	CALM1	calmodulin 1 [Source:NCBI gene (formerly Entrezgene);Acc:100154056]	1.73	0.083
ssc-miR-218-5p	CALN1	hypothetical gene	1.73	0.083
ssc-miR-218-5p	CAMK4	calcium/calmodulin dependent protein kinase IV [Source:VGNC Symbol;Acc:VGNC:99602]	1.73	0.083
ssc-miR-218-5p	CAMKK2	calcium/calmodulin dependent protein kinase kinase 2 [Source:VGNC Symbol;Acc:VGNC:86158]	1.73	0.083
ssc-miR-218-5p	CASK	calcium/calmodulin dependent serine protein kinase [Source:HGNC Symbol;Acc:HGNC:1497]	1.73	0.083
ssc-miR-218-5p	CASKIN1	CASK interacting protein 1 [Source:VGNC Symbol;Acc:VGNC:86200]	1.73	0.083

ssc-miR-218-5p	CBL	Cbl proto-onco [Source:VGNC Symbol;Acc:VGNC:86222]	1.73	0.083
ssc-miR-218-5p	CBLB	Cbl proto-onco B [Source:VGNC Symbol;Acc:VGNC:86223]	1.73	0.083
ssc-miR-218-5p	CBX3	chromobox 3 [Source:HGNC Symbol;Acc:HGNC:1553]	1.73	0.083
ssc-miR-218-5p	CBX5	chromobox 5 [Source:VGNC Symbol;Acc:VGNC:86232]	1.73	0.083
ssc-miR-218-5p	CBX7	chromobox 7 [Source:HGNC Symbol;Acc:HGNC:1557]	1.73	0.083
ssc-miR-218-5p	CBX8	chromobox 8 [Source:VGNC Symbol;Acc:VGNC:86233]	1.73	0.083
ssc-miR-218-5p	CC2D1B	coiled-coil and C2 domain containing 1B [Source:VGNC Symbol;Acc:VGNC:86236]	1.73	0.083
ssc-miR-218-5p	CCDC144A	hypothetical gene	1.73	0.083
ssc-miR-218-5p	CCDC176	hypothetical gene	1.73	0.083
ssc-miR-218-5p	CCDC177	coiled-coil domain containing 177 [Source:VGNC Symbol;Acc:VGNC:86275]	1.73	0.083
ssc-miR-218-5p	CCDC24	coiled-coil domain containing 24 [Source:VGNC Symbol;Acc:VGNC:86287]	1.73	0.083
ssc-miR-218-5p	CCDC6	coiled-coil domain containing 6 [Source:VGNC Symbol;Acc:VGNC:86304]	1.73	0.083
ssc-miR-218-5p	CCDC64	hypothetical gene	1.73	0.083
ssc-miR-218-5p	CCDC88A	coiled-coil domain containing 88A [Source:VGNC Symbol;Acc:VGNC:86325]	1.73	0.083
ssc-miR-218-5p	CCDC89	coiled-coil domain containing 89 [Source:HGNC Symbol;Acc:HGNC:26762]	1.73	0.083
ssc-miR-218-5p	CCNK	cyclin K [Source:VGNC Symbol;Acc:VGNC:86362]	1.73	0.083
ssc-miR-218-5p	CCP110	centriolar coiled-coil protein 110 [Source:VGNC Symbol;Acc:VGNC:86367]	1.73	0.083
ssc-miR-218-5p	CDC20B	cell division cycle 20B [Source:HGNC Symbol;Acc:HGNC:24222]	1.73	0.083
ssc-miR-218-5p	CDC42BPA	CDC42 binding protein kinase alpha [Source:VGNC Symbol;Acc:VGNC:95847]	1.73	0.083
ssc-miR-218-5p	CDC42SE2	CDC42 small effector 2 [Source:VGNC Symbol;Acc:VGNC:86461]	1.73	0.083
ssc-miR-218-5p	CDC73	cell division cycle 73 [Source:VGNC Symbol;Acc:VGNC:95817]	1.73	0.083
ssc-miR-218-5p	CDCA7L	cell division cycle associated 7 like [Source:VGNC Symbol;Acc:VGNC:86469]	1.73	0.083
ssc-miR-218-5p	CDH2	cadherin 2 [Source:VGNC Symbol;Acc:VGNC:86483]	1.73	0.083
ssc-miR-218-5p	CDH4	cadherin 4 [Source:VGNC Symbol;Acc:VGNC:95853]	1.73	0.083
ssc-miR-218-5p	CDK6	cyclin dependent kinase 6 [Source:HGNC Symbol;Acc:HGNC:1777]	1.73	0.083
ssc-miR-218-5p	CDK8	cyclin dependent kinase 8 [Source:VGNC Symbol;Acc:VGNC:86507]	1.73	0.083
ssc-miR-218-5p	CDR1	hypothetical gene	1.73	0.083
ssc-miR-218-5p	CDS1	CDP-diacylglycerol synthase 1 [Source:VGNC Symbol;Acc:VGNC:86523]	1.73	0.083
ssc-miR-218-5p	CDX2	caudal type homeobox 2 [Source:VGNC Symbol;Acc:VGNC:86527]	1.73	0.083
ssc-miR-218-5p	CEBPA	CCAAT enhancer binding protein alpha [Source:VGNC Symbol;Acc:VGNC:86531]	1.73	0.083
ssc-miR-218-5p	CELF1	CUGBP Elav-like family member 1 [Source:VGNC Symbol;Acc:VGNC:86537]	1.73	0.083
ssc-miR-218-5p	CELF5	CUGBP Elav-like family member 5 [Source:VGNC Symbol;Acc:VGNC:86540]	1.73	0.083
ssc-miR-218-5p	CELF6	CUGBP Elav-like family member 6 [Source:HGNC Symbol;Acc:HGNC:14059]	1.73	0.083
ssc-miR-218-5p	CENPB	centromere protein B [Source:HGNC Symbol;Acc:HGNC:1852]	1.73	0.083
ssc-miR-218-5p	CEP55	centrosomal protein 55 [Source:VGNC Symbol;Acc:VGNC:86572]	1.73	0.083
ssc-miR-218-5p	CEP85L	centrosomal protein 85 like [Source:VGNC Symbol;Acc:VGNC:86580]	1.73	0.083
ssc-miR-218-5p	CEP97	centrosomal protein 97 [Source:VGNC Symbol;Acc:VGNC:108638]	1.73	0.083
ssc-miR-218-5p	CGGBP1	CGG triplet repeat binding protein 1 [Source:VGNC Symbol;Acc:VGNC:86615]	1.73	0.083
ssc-miR-218-5p	CHD6	chromodomain helicase DNA binding protein 6 [Source:VGNC Symbol;Acc:VGNC:95903]	1.73	0.083
ssc-miR-218-5p	CHFR	checkpoint with forkhead and ring finger domains [Source:HGNC Symbol;Acc:HGNC:20455]	1.73	0.083
ssc-miR-218-5p	CHL1	cell adhesion molecule L1 like [Source:VGNC Symbol;Acc:VGNC:108639]	1.73	0.083
ssc-miR-218-5p	CHM	CHM Rab escort protein [Source:VGNC Symbol;Acc:VGNC:86647]	1.73	0.083
ssc-miR-218-5p	CHST8	carbohydrate sulfotransferase 8 [Source:VGNC Symbol;Acc:VGNC:86682]	1.73	0.083
ssc-miR-218-5p	CHSY1	chondroitin sulfate synthase 1 [Source:VGNC Symbol;Acc:VGNC:86684]	1.73	0.083
ssc-miR-218-5p	CLASP2	cytoplasmic linker associated protein 2 [Source:VGNC Symbol;Acc:VGNC:86720]	1.73	0.083
ssc-miR-218-5p	CLCN3	chloride voltage-gated channel 3 [Source:VGNC Symbol;Acc:VGNC:86727]	1.73	0.083
ssc-miR-218-5p	CLCN5	chloride voltage-gated channel 5 [Source:VGNC Symbol;Acc:VGNC:103925]	1.73	0.083
ssc-miR-218-5p	CLCN6	chloride voltage-gated channel 6 [Source:VGNC Symbol;Acc:VGNC:86729]	1.73	0.083
ssc-miR-218-5p	CLDN2	claudin 2 [Source:VGNC Symbol;Acc:VGNC:86737]	1.73	0.083
ssc-miR-218-5p	CLK3	CDC like kinase 3 [Source:VGNC Symbol;Acc:VGNC:107129]	1.73	0.083

ssc-miR-218-5p	CLNK	cytokine dependent hematopoietic cell linker [Source:VGNC Symbol;Acc:VGNC:86772]	1.73	0.083
ssc-miR-218-5p	CLVS1	clavesin 1 [Source:VGNC Symbol;Acc:VGNC:86794]	1.73	0.083
ssc-miR-218-5p	CNN1	calponin 1 [Source:VGNC Symbol;Acc:VGNC:86825]	1.73	0.083
ssc-miR-218-5p	CNNM2	cyclin and CBS domain divalent metal cation transport mediator 2 [Source:VGNC Symbol;Acc:VGNC:86828]	1.73	0.083
ssc-miR-218-5p	CNOT2	CCR4-NOT transcription complex subunit 2 [Source:VGNC Symbol;Acc:VGNC:86834]	1.73	0.083
ssc-miR-218-5p	CNOT6L	CCR4-NOT transcription complex subunit 6 like [Source:VGNC Symbol;Acc:VGNC:86838]	1.73	0.083
ssc-miR-218-5p	CNOT7	CCR4-NOT transcription complex subunit 7 [Source:VGNC Symbol;Acc:VGNC:95604]	1.73	0.083
ssc-miR-218-5p	CNTN1	contactin 1 [Source:VGNC Symbol;Acc:VGNC:86848]	1.73	0.083
ssc-miR-218-5p	CNTNAP2	hypothetical gene	1.73	0.083
ssc-miR-218-5p	CNTNAP5	contactin associated protein family member 5 [Source:VGNC Symbol;Acc:VGNC:95459]	1.73	0.083
ssc-miR-218-5p	COA1	hypothetical gene	1.73	0.083
ssc-miR-218-5p	COA5	cytochrome c oxidase assembly factor 5 [Source:HGNC Symbol;Acc:HGNC:33848]	1.73	0.083
ssc-miR-218-5p	COL1A1	collagen type I alpha 1 chain [Source:VGNC Symbol;Acc:VGNC:86870]	1.73	0.083
ssc-miR-218-5p	COL4A3BP	hypothetical gene	1.73	0.083
ssc-miR-218-5p	COMMD3-BMI1	hypothetical gene	1.73	0.083
ssc-miR-218-5p	COMMD8	COMM domain containing 8 [Source:VGNC Symbol;Acc:VGNC:86890]	1.73	0.083
ssc-miR-218-5p	COPS7B	COP9 signalosome subunit 7B [Source:HGNC Symbol;Acc:HGNC:16760]	1.73	0.083
ssc-miR-218-5p	CPEB2	cytoplasmic polyadenylation element binding protein 2 [Source:VGNC Symbol;Acc:VGNC:86937]	1.73	0.083
ssc-miR-218-5p	CPNE8	copine 8 [Source:VGNC Symbol;Acc:VGNC:86955]	1.73	0.083
ssc-miR-218-5p	CREB1	cAMP responsive element binding protein 1 [Source:VGNC Symbol;Acc:VGNC:96004]	1.73	0.083
ssc-miR-218-5p	CREBZF	CREB/ATF bZIP transcription factor [Source:VGNC Symbol;Acc:VGNC:86988]	1.73	0.083
ssc-miR-218-5p	CRKL	CRK like proto-onco, adaptor protein [Source:VGNC Symbol;Acc:VGNC:86997]	1.73	0.083
ssc-miR-218-5p	CRTAP	cartilage associated protein [Source:HGNC Symbol;Acc:HGNC:2379]	1.73	0.083
ssc-miR-218-5p	CRTC3	CREB regulated transcription coactivator 3 [Source:VGNC Symbol;Acc:VGNC:87009]	1.73	0.083
ssc-miR-218-5p	CSMD3	CUB and Sushi multiple domains 3 [Source:VGNC Symbol;Acc:VGNC:98765]	1.73	0.083
ssc-miR-218-5p	CSNK2A1	hypothetical gene	1.73	0.083
ssc-miR-218-5p	CTC-360G5.1	hypothetical gene	1.73	0.083
ssc-miR-218-5p	CTD-2207O23.12	hypothetical gene	1.73	0.083
ssc-miR-218-5p	CTDSPL	CTD small phosphatase like [Source:VGNC Symbol;Acc:VGNC:107131]	1.73	0.083
ssc-miR-218-5p	CTGF	hypothetical gene	1.73	0.083
ssc-miR-218-5p	CTNNA2	catenin alpha 2 [Source:HGNC Symbol;Acc:HGNC:2510]	1.73	0.083
ssc-miR-218-5p	CTNND2	catenin delta 2 [Source:VGNC Symbol;Acc:VGNC:87068]	1.73	0.083
ssc-miR-218-5p	CTTNBP2NL	CTTNBP2 N-terminal like [Source:VGNC Symbol;Acc:VGNC:87082]	1.73	0.083
ssc-miR-218-5p	CUL3	cullin 3 [Source:VGNC Symbol;Acc:VGNC:96091]	1.73	0.083
ssc-miR-218-5p	CXorf23	hypothetical gene	1.73	0.083
ssc-miR-218-5p	CYB5D1	cytochrome b5 domain containing 1 [Source:HGNC Symbol;Acc:HGNC:26516]	1.73	0.083
ssc-miR-218-5p	CYP7B1	cytochrome P450 family 7 subfamily B member 1 [Source:VGNC Symbol;Acc:VGNC:103362]	1.73	0.083
ssc-miR-218-5p	DAAM1	dishevelled associated activator of morphosis 1 [Source:VGNC Symbol;Acc:VGNC:87141]	1.73	0.083
ssc-miR-218-5p	DACH1	dachshund family transcription factor 1 [Source:VGNC Symbol;Acc:VGNC:87146]	1.73	0.083
ssc-miR-218-5p	DAGLA	diacylglycerol lipase alpha [Source:VGNC Symbol;Acc:VGNC:87152]	1.73	0.083
ssc-miR-218-5p	DAZAP1	DAZ associated protein 1 [Source:NCBI gene (formerly Entrezgene);Acc:100625005]	1.73	0.083
ssc-miR-218-5p	DBN1	drebrin 1 [Source:VGNC Symbol;Acc:VGNC:87164]	1.73	0.083
ssc-miR-218-5p	DBNDD1	dysbindin domain containing 1 [Source:VGNC Symbol;Acc:VGNC:87165]	1.73	0.083
ssc-miR-218-5p	DCBLD2	discoidin, CUB and LCCL domain containing 2 [Source:VGNC Symbol;Acc:VGNC:87180]	1.73	0.083
ssc-miR-218-5p	DCP2	decapping mRNA 2 [Source:VGNC Symbol;Acc:VGNC:87188]	1.73	0.083
ssc-miR-218-5p	DCUN1D1	hypothetical gene	1.73	0.083
ssc-miR-218-5p	DCUN1D4	defective in cullin neddylation 1 domain containing 4 [Source:VGNC Symbol;Acc:VGNC:87197]	1.73	0.083
ssc-miR-218-5p	DCUN1D5	defective in cullin neddylation 1 domain containing 5 [Source:VGNC Symbol;Acc:VGNC:87198]	1.73	0.083
ssc-miR-218-5p	DCX	doublecortin [Source:HGNC Symbol;Acc:HGNC:2714]	1.73	0.083
ssc-miR-218-5p	DDX21	DExD-box helicase 21 [Source:VGNC Symbol;Acc:VGNC:87215]	1.73	0.083

ssc-miR-218-5p	DDX41	DEAD-box helicase 41 [Source:VGNC Symbol;Acc:VGNC:87221]	1.73	0.083
ssc-miR-218-5p	DDX5	DEAD-box helicase 5 [Source:VGNC Symbol;Acc:VGNC:87226]	1.73	0.083
ssc-miR-218-5p	DDX6	DEAD-box helicase 6 [Source:VGNC Symbol;Acc:VGNC:87233]	1.73	0.083
ssc-miR-218-5p	DERL2	derlin 2 [Source:VGNC Symbol;Acc:VGNC:98986]	1.73	0.083
ssc-miR-218-5p	DES	desmin [Source:VGNC Symbol;Acc:VGNC:96159]	1.73	0.083
ssc-miR-218-5p	DESI2	desumoylating isopeptidase 2 [Source:VGNC Symbol;Acc:VGNC:96037]	1.73	0.083
ssc-miR-218-5p	DFNB59	hypothetical gene	1.73	0.083
ssc-miR-218-5p	DHX29	DExH-box helicase 29 [Source:VGNC Symbol;Acc:VGNC:87288]	1.73	0.083
ssc-miR-218-5p	DIP2A	disco interacting A [Source:VGNC Symbol;Acc:VGNC:95836]	1.73	0.083
ssc-miR-218-5p	DIRAS1	DIRAS family GTPase 1 [Source:VGNC Symbol;Acc:VGNC:87312]	1.73	0.083
ssc-miR-218-5p	DIRAS2	DIRAS family GTPase 2 [Source:VGNC Symbol;Acc:VGNC:87313]	1.73	0.083
ssc-miR-218-5p	DKK2	dickkopf WNT signaling pathway inhibitor 2 [Source:VGNC Symbol;Acc:VGNC:87322]	1.73	0.083
ssc-miR-218-5p	DKK3	dickkopf WNT signaling pathway inhibitor 3 [Source:VGNC Symbol;Acc:VGNC:87323]	1.73	0.083
ssc-miR-218-5p	DLG2	discs large MAGUK scaffold protein 2 [Source:VGNC Symbol;Acc:VGNC:108581]	1.73	0.083
ssc-miR-218-5p	DLST	dihydrolipoamide S-succinyltransferase [Source:VGNC Symbol;Acc:VGNC:87338]	1.73	0.083
ssc-miR-218-5p	DNAJA4	DnaJ heat shock protein family (Hsp40) member A4 [Source:NCBI gene (formerly Entrezgene);Acc:397613]	1.73	0.083
ssc-miR-218-5p	DNAJB13	DnaJ heat shock protein family (Hsp40) member B13 [Source:VGNC Symbol;Acc:VGNC:108582]	1.73	0.083
ssc-miR-218-5p	DNAJC13	DnaJ heat shock protein family (Hsp40) member C13 [Source:VGNC Symbol;Acc:VGNC:108650]	1.73	0.083
ssc-miR-218-5p	DNAJC27	DnaJ heat shock protein family (Hsp40) member C27 [Source:VGNC Symbol;Acc:VGNC:102563]	1.73	0.083
ssc-miR-218-5p	DNAJC3	DnaJ heat shock protein family (Hsp40) member C3 [Source:NCBI gene (formerly Entrezgene);Acc:100154166]	1.73	0.083
ssc-miR-218-5p	DNAL1	dynein axonemal light chain 1 [Source:VGNC Symbol;Acc:VGNC:87373]	1.73	0.083
ssc-miR-218-5p	DNMT3A	DNA methyltransferase 3 alpha [Source:VGNC Symbol;Acc:VGNC:87384]	1.73	0.083
ssc-miR-218-5p	DOCK7	dedicator of cytokinesis 7 [Source:VGNC Symbol;Acc:VGNC:87397]	1.73	0.083
ssc-miR-218-5p	DOCK9	dedicator of cytokinesis 9 [Source:VGNC Symbol;Acc:VGNC:87399]	1.73	0.083
ssc-miR-218-5p	DOK6	docking protein 6 [Source:VGNC Symbol;Acc:VGNC:87404]	1.73	0.083
ssc-miR-218-5p	DOLPP1	dolichyldiphosphatase 1 [Source:VGNC Symbol;Acc:VGNC:87407]	1.73	0.083
ssc-miR-218-5p	DPF1	double PHD fingers 1 [Source:VGNC Symbol;Acc:VGNC:87412]	1.73	0.083
ssc-miR-218-5p	DPF3	double PHD fingers 3 [Source:VGNC Symbol;Acc:VGNC:87414]	1.73	0.083
ssc-miR-218-5p	DPP6	dipeptidyl peptidase like 6 [Source:VGNC Symbol;Acc:VGNC:87421]	1.73	0.083
ssc-miR-218-5p	DPP9	dipeptidyl peptidase 9 [Source:VGNC Symbol;Acc:VGNC:87424]	1.73	0.083
ssc-miR-218-5p	DPY19L3	dpy-19 like C-mannosyltransferase 3 [Source:VGNC Symbol;Acc:VGNC:87426]	1.73	0.083
ssc-miR-218-5p	DR1	down-regulator of transcription 1 [Source:VGNC Symbol;Acc:VGNC:87435]	1.73	0.083
ssc-miR-218-5p	DST	dystonin [Source:HGNC Symbol;Acc:HGNC:1090]	1.73	0.083
ssc-miR-218-5p	DUSP18	dual specificity phosphatase 18 [Source:VGNC Symbol;Acc:VGNC:103937]	1.73	0.083
ssc-miR-218-5p	DUSP5	dual specificity phosphatase 5 [Source:VGNC Symbol;Acc:VGNC:87487]	1.73	0.083
ssc-miR-218-5p	DYNC1I2	dynein cytoplasmic 1 intermediate chain 2 [Source:VGNC Symbol;Acc:VGNC:96224]	1.73	0.083
ssc-miR-218-5p	EBF1	EBF transcription factor 1 [Source:VGNC Symbol;Acc:VGNC:87525]	1.73	0.083
ssc-miR-218-5p	EBF3	EBF transcription factor 3 [Source:VGNC Symbol;Acc:VGNC:87527]	1.73	0.083
ssc-miR-218-5p	EDEM1	ER degradation enhancing alpha-mannosidase like protein 1 [Source:VGNC Symbol;Acc:VGNC:87545]	1.73	0.083
ssc-miR-218-5p	EFNB2	ephrin B2 [Source:VGNC Symbol;Acc:VGNC:87577]	1.73	0.083
ssc-miR-218-5p	EGLN3	egl-9 family hypoxia inducible factor 3 [Source:VGNC Symbol;Acc:VGNC:87589]	1.73	0.083
ssc-miR-218-5p	EI24	EI24 autophagy associated transmembrane protein [Source:VGNC Symbol;Acc:VGNC:87602]	1.73	0.083
ssc-miR-218-5p	EIF3J	eukaryotic translation initiation factor 3 subunit J [Source:VGNC Symbol;Acc:VGNC:87621]	1.73	0.083
ssc-miR-218-5p	EIF5A2	eukaryotic translation initiation factor 5A2 [Source:VGNC Symbol;Acc:VGNC:87633]	1.73	0.083
ssc-miR-218-5p	ELFN2	extracellular leucine rich repeat and fibronectin type III domain containing 2 [Source:VGNC Symbol;Acc:VGNC:103232]	1.73	0.083
ssc-miR-218-5p	ELK4	ETS transcription factor ELK4 [Source:VGNC Symbol;Acc:VGNC:87647]	1.73	0.083
ssc-miR-218-5p	ELL2	elongation factor for RNA polymerase II 2 [Source:VGNC Symbol;Acc:VGNC:87649]	1.73	0.083
ssc-miR-218-5p	ELMO1	engulfment and cell motility 1 [Source:HGNC Symbol;Acc:HGNC:16286]	1.73	0.083
ssc-miR-218-5p	ELMSAN1	hypothetical gene	1.73	0.083
ssc-miR-218-5p	ELOVL5	ELOVL fatty acid elongase 5 [Source:VGNC Symbol;Acc:VGNC:87661]	1.73	0.083

ssc-miR-218-5p	EPB41L1	hypothetical gene	1.73	0.083
ssc-miR-218-5p	EPHA5	EPH receptor A5 [Source:VGNC Symbol;Acc:VGNC:87733]	1.73	0.083
ssc-miR-218-5p	EPHA7	EPH receptor A7 [Source:VGNC Symbol;Acc:VGNC:87734]	1.73	0.083
ssc-miR-218-5p	EPHA8	EPH receptor A8 [Source:VGNC Symbol;Acc:VGNC:87735]	1.73	0.083
ssc-miR-218-5p	EPS8	epidermal growth factor receptor pathway substrate 8 [Source:VGNC Symbol;Acc:VGNC:87749]	1.73	0.083
ssc-miR-218-5p	ERBB4	erb-b2 receptor tyrosine kinase 4 [Source:VGNC Symbol;Acc:VGNC:96284]	1.73	0.083
ssc-miR-218-5p	ERC2	ELKS/RAB6-interacting/CAST family member 2 [Source:VGNC Symbol;Acc:VGNC:87762]	1.73	0.083
ssc-miR-218-5p	ERGIC2	ERGIC and golgi 2 [Source:VGNC Symbol;Acc:VGNC:87771]	1.73	0.083
ssc-miR-218-5p	ERMP1	endoplasmic reticulum metallopeptidase 1 [Source:VGNC Symbol;Acc:VGNC:87777]	1.73	0.083
ssc-miR-218-5p	ERN1	endoplasmic reticulum to nucleus signaling 1 [Source:HGNC Symbol;Acc:HGNC:3449]	1.73	0.083
ssc-miR-218-5p	ETNK1	ethanolamine kinase 1 [Source:VGNC Symbol;Acc:VGNC:87806]	1.73	0.083
ssc-miR-218-5p	EXD2	exonuclease 3'-5' domain containing 2 [Source:VGNC Symbol;Acc:VGNC:97990]	1.73	0.083
ssc-miR-218-5p	EXOC5	exocyst complex component 5 [Source:VGNC Symbol;Acc:VGNC:87831]	1.73	0.083
ssc-miR-218-5p	EXTL3	exostosin like glycosyltransferase 3 [Source:VGNC Symbol;Acc:VGNC:87850]	1.73	0.083
ssc-miR-218-5p	FAM109A	hypothetical gene	1.73	0.083
ssc-miR-218-5p	FAM126A	family with sequence similarity 126 member A [Source:VGNC Symbol;Acc:VGNC:87899]	1.73	0.083
ssc-miR-218-5p	FAM126B	family with sequence similarity 126 member B [Source:HGNC Symbol;Acc:HGNC:28593]	1.73	0.083
ssc-miR-218-5p	FAM13A	family with sequence similarity 13 member A [Source:VGNC Symbol;Acc:VGNC:98924]	1.73	0.083
ssc-miR-218-5p	FAM162B	family with sequence similarity 162 member B [Source:VGNC Symbol;Acc:VGNC:87919]	1.73	0.083
ssc-miR-218-5p	FAM171B	family with sequence similarity 171 member B [Source:VGNC Symbol;Acc:VGNC:96218]	1.73	0.083
ssc-miR-218-5p	FAM175B	hypothetical gene	1.73	0.083
ssc-miR-218-5p	FAM178A	hypothetical gene	1.73	0.083
ssc-miR-218-5p	FAM196A	hypothetical gene	1.73	0.083
ssc-miR-218-5p	FAM196B	hypothetical gene	1.73	0.083
ssc-miR-218-5p	FAM20B	FAM20B glycosaminoglycan xylosylkinase [Source:VGNC Symbol;Acc:VGNC:87950]	1.73	0.083
ssc-miR-218-5p	FAM214A	family with sequence similarity 214 member A [Source:VGNC Symbol;Acc:VGNC:87952]	1.73	0.083
ssc-miR-218-5p	FAM217B	family with sequence similarity 217 member B [Source:VGNC Symbol;Acc:VGNC:95767]	1.73	0.083
ssc-miR-218-5p	FAM3C	FAM3 metabolism regulating signaling molecule C [Source:VGNC Symbol;Acc:VGNC:87969]	1.73	0.083
ssc-miR-218-5p	FAM63B	hypothetical gene	1.73	0.083
ssc-miR-218-5p	FAM65B	hypothetical gene	1.73	0.083
ssc-miR-218-5p	FAM78A	family with sequence similarity 78 member A [Source:VGNC Symbol;Acc:VGNC:87984]	1.73	0.083
ssc-miR-218-5p	FAM81A	family with sequence similarity 81 member A [Source:VGNC Symbol;Acc:VGNC:87986]	1.73	0.083
ssc-miR-218-5p	FAT3	hypothetical gene	1.73	0.083
ssc-miR-218-5p	FBN2	fibrillin 2 [Source:VGNC Symbol;Acc:VGNC:88025]	1.73	0.083
ssc-miR-218-5p	FBRSL1	hypothetical gene	1.73	0.083
ssc-miR-218-5p	FBXL2	F-box and leucine rich repeat protein 2 [Source:VGNC Symbol;Acc:VGNC:98005]	1.73	0.083
ssc-miR-218-5p	FBXL20	F-box and leucine rich repeat protein 20 [Source:VGNC Symbol;Acc:VGNC:98006]	1.73	0.083
ssc-miR-218-5p	FBXO28	F-box protein 28 [Source:HGNC Symbol;Acc:HGNC:29046]	1.73	0.083
ssc-miR-218-5p	FBXO41	F-box protein 41 [Source:VGNC Symbol;Acc:VGNC:88046]	1.73	0.083
ssc-miR-218-5p	FCHO2	FCH and mu domain containing endocytic adaptor 2 [Source:VGNC Symbol;Acc:VGNC:88066]	1.73	0.083
ssc-miR-218-5p	FCHSD2	FCH and double SH3 domains 2 [Source:VGNC Symbol;Acc:VGNC:88068]	1.73	0.083
ssc-miR-218-5p	FGD4	FYVE, RhoGEF and PH domain containing 4 [Source:VGNC Symbol;Acc:VGNC:88097]	1.73	0.083
ssc-miR-218-5p	FGD6	FYVE, RhoGEF and PH domain containing 6 [Source:VGNC Symbol;Acc:VGNC:88099]	1.73	0.083
ssc-miR-218-5p	FGF12	fibroblast growth factor 12 [Source:VGNC Symbol;Acc:VGNC:88102]	1.73	0.083
ssc-miR-218-5p	FGFR2	fibroblast growth factor receptor 2 [Source:NCBI gene (formerly Entrezgene);Acc:396762]	1.73	0.083
ssc-miR-218-5p	FHL5	four and a half LIM domains 5 [Source:VGNC Symbol;Acc:VGNC:88130]	1.73	0.083
ssc-miR-218-5p	FHOD3	formin homology 2 domain containing 3 [Source:VGNC Symbol;Acc:VGNC:88132]	1.73	0.083
ssc-miR-218-5p	FIGN	fidgetin, microtubule severing factor [Source:VGNC Symbol;Acc:VGNC:95580]	1.73	0.083
ssc-miR-218-5p	FLNC	filamin C [Source:VGNC Symbol;Acc:VGNC:88157]	1.73	0.083
ssc-miR-218-5p	FLRT2	fibronectin leucine rich transmembrane protein 2 [Source:VGNC Symbol;Acc:VGNC:88161]	1.73	0.083

ssc-miR-218-5p	FLRT3	fibronectin leucine rich transmembrane protein 3 [Source:VGNC Symbol;Acc:VGNC:96304]	1.73	0.083
ssc-miR-218-5p	FOSL2	FOS like 2, AP-1 transcription factor subunit [Source:VGNC Symbol;Acc:VGNC:88192]	1.73	0.083
ssc-miR-218-5p	FOXN2	forkhead box N2 [Source:VGNC Symbol;Acc:VGNC:88219]	1.73	0.083
ssc-miR-218-5p	FOXN3	forkhead box N3 [Source:VGNC Symbol;Acc:VGNC:88220]	1.73	0.083
ssc-miR-218-5p	FOXP2	forkhead box P2 [Source:VGNC Symbol;Acc:VGNC:98014]	1.73	0.083
ssc-miR-218-5p	FRMD4A	FERM domain containing 4A [Source:VGNC Symbol;Acc:VGNC:96088]	1.73	0.083
ssc-miR-218-5p	FRMD4B	FERM domain containing 4B [Source:VGNC Symbol;Acc:VGNC:88237]	1.73	0.083
ssc-miR-218-5p	FRMD6	FERM domain containing 6 [Source:VGNC Symbol;Acc:VGNC:88239]	1.73	0.083
ssc-miR-218-5p	FRMPD4	FERM and PDZ domain containing 4 [Source:VGNC Symbol;Acc:VGNC:88243]	1.73	0.083
ssc-miR-218-5p	FRZB	frizzled related protein [Source:VGNC Symbol;Acc:VGNC:96308]	1.73	0.083
ssc-miR-218-5p	FSBP	hypothetical gene	1.73	0.083
ssc-miR-218-5p	FUBP1	far upstream element binding protein 1 [Source:VGNC Symbol;Acc:VGNC:96781]	1.73	0.083
ssc-miR-218-5p	FUT9	fucosyltransferase 9 [Source:VGNC Symbol;Acc:VGNC:88271]	1.73	0.083
ssc-miR-218-5p	FYCO1	hypothetical gene	1.73	0.083
ssc-miR-218-5p	FZD4	frizzled class receptor 4 [Source:VGNC Symbol;Acc:VGNC:88282]	1.73	0.083
ssc-miR-218-5p	GAB2	GRB2 associated binding protein 2 [Source:VGNC Symbol;Acc:VGNC:108586]	1.73	0.083
ssc-miR-218-5p	GABPA	GA binding protein transcription factor subunit alpha [Source:VGNC Symbol;Acc:VGNC:103945]	1.73	0.083
ssc-miR-218-5p	GABRB2	gamma-aminobutyric acid type A receptor subunit beta2 [Source:VGNC Symbol;Acc:VGNC:88307]	1.73	0.083
ssc-miR-218-5p	GABRB3	gamma-aminobutyric acid type A receptor subunit beta3 [Source:VGNC Symbol;Acc:VGNC:88308]	1.73	0.083
ssc-miR-218-5p	GALNT1	polypeptide N-acetylgalactosaminyltransferase 1 [Source:VGNC Symbol;Acc:VGNC:97062]	1.73	0.083
ssc-miR-218-5p	GALNT13	polypeptide N-acetylgalactosaminyltransferase 13 [Source:VGNC Symbol;Acc:VGNC:95992]	1.73	0.083
ssc-miR-218-5p	GALNT3	polypeptide N-acetylgalactosaminyltransferase 3 [Source:VGNC Symbol;Acc:VGNC:96313]	1.73	0.083
ssc-miR-218-5p	GALNT4	polypeptide N-acetylgalactosaminyltransferase 4 [Source:HGNC Symbol;Acc:HGNC:4126]	1.73	0.083
ssc-miR-218-5p	GALR1	galanin receptor 1 [Source:VGNC Symbol;Acc:VGNC:88339]	1.73	0.083
ssc-miR-218-5p	GAN	gigaxonin [Source:VGNC Symbol;Acc:VGNC:88343]	1.73	0.083
ssc-miR-218-5p	GAPVD1	GTPase activating protein and VPS9 domains 1 [Source:VGNC Symbol;Acc:VGNC:88347]	1.73	0.083
ssc-miR-218-5p	GAREML	hypothetical gene	1.73	0.083
ssc-miR-218-5p	GATC	glutamyl-tRNA amidotransferase subunit C [Source:VGNC Symbol;Acc:VGNC:103948]	1.73	0.083
ssc-miR-218-5p	GDAP2	ganglioside induced differentiation associated protein 2 [Source:VGNC Symbol;Acc:VGNC:88395]	1.73	0.083
ssc-miR-218-5p	GDE1	glycerophosphodiester phosphodiesterase 1 [Source:VGNC Symbol;Acc:VGNC:88396]	1.73	0.083
ssc-miR-218-5p	GDI1	GDP dissociation inhibitor 1 [Source:VGNC Symbol;Acc:VGNC:88405]	1.73	0.083
ssc-miR-218-5p	GDPD5	glycerophosphodiester phosphodiesterase domain containing 5 [Source:VGNC Symbol;Acc:VGNC:88409]	1.73	0.083
ssc-miR-218-5p	GET4	hypothetical gene	1.73	0.083
ssc-miR-218-5p	GFPT1	glutamine--fructose-6-phosphate transaminase 1 [Source:VGNC Symbol;Acc:VGNC:88426]	1.73	0.083
ssc-miR-218-5p	GFRA1	GDNF family receptor alpha 1 [Source:VGNC Symbol;Acc:VGNC:88428]	1.73	0.083
ssc-miR-218-5p	GHITM	growth hormone inducible transmembrane protein [Source:VGNC Symbol;Acc:VGNC:88442]	1.73	0.083
ssc-miR-218-5p	GINS2	GINS complex subunit 2 [Source:HGNC Symbol;Acc:HGNC:24575]	1.73	0.083
ssc-miR-218-5p	GJA1	gap junction protein alpha 1 [Source:VGNC Symbol;Acc:VGNC:103098]	1.73	0.083
ssc-miR-218-5p	GLCE	glucuronic acid epimerase [Source:VGNC Symbol;Acc:VGNC:88477]	1.73	0.083
ssc-miR-218-5p	GLIPR2	GLI pathosis related 2 [Source:VGNC Symbol;Acc:VGNC:98023]	1.73	0.083
ssc-miR-218-5p	GMEB1	glucocorticoid modulatory element binding protein 1 [Source:VGNC Symbol;Acc:VGNC:88508]	1.73	0.083
ssc-miR-218-5p	GNA13	G protein subunit alpha 13 [Source:VGNC Symbol;Acc:VGNC:98997]	1.73	0.083
ssc-miR-218-5p	GNAI2	G protein subunit alpha i2 [Source:VGNC Symbol;Acc:VGNC:88522]	1.73	0.083
ssc-miR-218-5p	GNAI3	G protein subunit alpha i3 [Source:VGNC Symbol;Acc:VGNC:88523]	1.73	0.083
ssc-miR-218-5p	GNAO1	hypothetical gene	1.73	0.083
ssc-miR-218-5p	GNAS	hypothetical gene	1.73	0.083
ssc-miR-218-5p	GNB1	G protein subunit beta 1 [Source:VGNC Symbol;Acc:VGNC:88529]	1.73	0.083
ssc-miR-218-5p	GNG3	hypothetical gene	1.73	0.083
ssc-miR-218-5p	GNG4	G protein subunit gamma 4 [Source:HGNC Symbol;Acc:HGNC:4407]	1.73	0.083
ssc-miR-218-5p	GNPTG	N-acetylglucosamine-1-phosphate transferase subunit gamma [Source:VGNC Symbol;Acc:VGNC:88547]	1.73	0.083

ssc-miR-218-5p	GOLGA7	golgin A7 [Source:VGNC Symbol;Acc:VGNC:96059]	1.73	0.083
ssc-miR-218-5p	GOLT1B	golgi transport 1B [Source:VGNC Symbol;Acc:VGNC:88557]	1.73	0.083
ssc-miR-218-5p	GPAM	glycerol-3-phosphate acyltransferase, mitochondrial [Source:VGNC Symbol;Acc:VGNC:88570]	1.73	0.083
ssc-miR-218-5p	GPD1L	glycerol-3-phosphate dehydrogenase 1 like [Source:VGNC Symbol;Acc:VGNC:108661]	1.73	0.083
ssc-miR-218-5p	GPR126	hypothetical gene	1.73	0.083
ssc-miR-218-5p	GPR137C	G protein-coupled receptor 137C [Source:VGNC Symbol;Acc:VGNC:88602]	1.73	0.083
ssc-miR-218-5p	GPR153	G protein-coupled receptor 153 [Source:VGNC Symbol;Acc:VGNC:88609]	1.73	0.083
ssc-miR-218-5p	GPR161	G protein-coupled receptor 161 [Source:VGNC Symbol;Acc:VGNC:88613]	1.73	0.083
ssc-miR-218-5p	GPR45	G protein-coupled receptor 45 [Source:VGNC Symbol;Acc:VGNC:88632]	1.73	0.083
ssc-miR-218-5p	GPR63	G protein-coupled receptor 63 [Source:VGNC Symbol;Acc:VGNC:88634]	1.73	0.083
ssc-miR-218-5p	GPR83	G protein-coupled receptor 83 [Source:VGNC Symbol;Acc:VGNC:88637]	1.73	0.083
ssc-miR-218-5p	GPR85	G protein-coupled receptor 85 [Source:VGNC Symbol;Acc:VGNC:88638]	1.73	0.083
ssc-miR-218-5p	GPRC5B	G protein-coupled receptor class C group 5 member B [Source:VGNC Symbol;Acc:VGNC:88643]	1.73	0.083
ssc-miR-218-5p	GRAMD4	GRAM domain containing 4 [Source:VGNC Symbol;Acc:VGNC:88659]	1.73	0.083
ssc-miR-218-5p	GREM1	gremlin 1, DAN family BMP antagonist [Source:VGNC Symbol;Acc:VGNC:88665]	1.73	0.083
ssc-miR-218-5p	GRIA2	glutamate ionotropic receptor AMPA type subunit 2 [Source:VGNC Symbol;Acc:VGNC:88671]	1.73	0.083
ssc-miR-218-5p	GRIK2	glutamate ionotropic receptor kainate type subunit 2 [Source:VGNC Symbol;Acc:VGNC:88678]	1.73	0.083
ssc-miR-218-5p	GRIK3	glutamate ionotropic receptor kainate type subunit 3 [Source:VGNC Symbol;Acc:VGNC:88679]	1.73	0.083
ssc-miR-218-5p	GRIN2B	glutamate ionotropic receptor NMDA type subunit 2B [Source:VGNC Symbol;Acc:VGNC:88684]	1.73	0.083
ssc-miR-218-5p	GRIP1	glutamate receptor interacting protein 1 [Source:VGNC Symbol;Acc:VGNC:88690]	1.73	0.083
ssc-miR-218-5p	GRM1	glutamate metabotropic receptor 1 [Source:VGNC Symbol;Acc:VGNC:88700]	1.73	0.083
ssc-miR-218-5p	GRM3	glutamate metabotropic receptor 3 [Source:VGNC Symbol;Acc:VGNC:88702]	1.73	0.083
ssc-miR-218-5p	GSK3B	glycogen synthase kinase 3 beta [Source:VGNC Symbol;Acc:VGNC:88723]	1.73	0.083
ssc-miR-218-5p	GSKIP	GSK3B interacting protein [Source:VGNC Symbol;Acc:VGNC:103956]	1.73	0.083
ssc-miR-218-5p	GSPT1	G1 to S phase transition 1 [Source:VGNC Symbol;Acc:VGNC:88724]	1.73	0.083
ssc-miR-218-5p	GSX2	GS homeobox 2 [Source:VGNC Symbol;Acc:VGNC:88729]	1.73	0.083
ssc-miR-218-5p	GTF2H3	ral transcription factor IIH subunit 3 [Source:VGNC Symbol;Acc:VGNC:88735]	1.73	0.083
ssc-miR-218-5p	GUCY1A2	guanylate cyclase 1 soluble subunit alpha 2 [Source:VGNC Symbol;Acc:VGNC:88751]	1.73	0.083
ssc-miR-218-5p	HAPLN1	hyaluronan and proteoglycan link protein 1 [Source:VGNC Symbol;Acc:VGNC:88780]	1.73	0.083
ssc-miR-218-5p	HCFC1	host cell factor C1 [Source:HGNC Symbol;Acc:HGNC:4839]	1.73	0.083
ssc-miR-218-5p	HCN3	hyperpolarization activated cyclic nucleotide gated potassium channel 3 [Source:VGNC Symbol;Acc:VGNC:88804]	1.73	0.083
ssc-miR-218-5p	HCN4	hyperpolarization activated cyclic nucleotide gated potassium channel 4 [Source:VGNC Symbol;Acc:VGNC:88805]	1.73	0.083
ssc-miR-218-5p	HDAC7	histone deacetylase 7 [Source:VGNC Symbol;Acc:VGNC:103286]	1.73	0.083
ssc-miR-218-5p	HECTD2	HECT domain E3 ubiquitin protein ligase 2 [Source:VGNC Symbol;Acc:VGNC:88833]	1.73	0.083
ssc-miR-218-5p	HECTD3	HECT domain E3 ubiquitin protein ligase 3 [Source:VGNC Symbol;Acc:VGNC:97096]	1.73	0.083
ssc-miR-218-5p	HECW1	HECT, C2 and WW domain containing E3 ubiquitin protein ligase 1 [Source:VGNC Symbol;Acc:VGNC:88835]	1.73	0.083
ssc-miR-218-5p	HERPUD2	HERPUD family member 2 [Source:VGNC Symbol;Acc:VGNC:88853]	1.73	0.083
ssc-miR-218-5p	HIC2	HIC ZBTB transcriptional repressor 2 [Source:HGNC Symbol;Acc:HGNC:18595]	1.73	0.083
ssc-miR-218-5p	HIVEP1	HIVEP zinc finger 1 [Source:VGNC Symbol;Acc:VGNC:96585]	1.73	0.083
ssc-miR-218-5p	HIVEP2	HIVEP zinc finger 2 [Source:VGNC Symbol;Acc:VGNC:96586]	1.73	0.083
ssc-miR-218-5p	HIVEP3	hypothetical gene	1.73	0.083
ssc-miR-218-5p	HLF	HLF transcription factor, PAR bZIP family member [Source:VGNC Symbol;Acc:VGNC:88896]	1.73	0.083
ssc-miR-218-5p	HMGB1	hypothetical gene	1.73	0.083
ssc-miR-218-5p	HNRNPA1	heterous nuclear ribonucleoprotein A1 [Source:VGNC Symbol;Acc:VGNC:88918]	1.73	0.083
ssc-miR-218-5p	HNRNPA3	hypothetical gene	1.73	0.083
ssc-miR-218-5p	HNRNPC	hypothetical gene	1.73	0.083
ssc-miR-218-5p	HOOK1	hypothetical gene	1.73	0.083
ssc-miR-218-5p	HOXA1	homeobox A1 [Source:VGNC Symbol;Acc:VGNC:88933]	1.73	0.083
ssc-miR-218-5p	HOXA10	homeobox A10 [Source:VGNC Symbol;Acc:VGNC:88934]	1.73	0.083
ssc-miR-218-5p	HOXB3	homeobox B3 [Source:VGNC Symbol;Acc:VGNC:88944]	1.73	0.083

ssc-miR-218-5p	HOXD10	homeobox D10 [Source:VGNC Symbol;Acc:VGNC:96351]	1.73	0.083
ssc-miR-218-5p	HOXD4	homeobox D4 [Source:HGNC Symbol;Acc:HGNC:5138]	1.73	0.083
ssc-miR-218-5p	HOXD8	homeobox D8 [Source:VGNC Symbol;Acc:VGNC:96356]	1.73	0.083
ssc-miR-218-5p	HP1BP3	heterochromatin protein 1 binding protein 3 [Source:VGNC Symbol;Acc:VGNC:88956]	1.73	0.083
ssc-miR-218-5p	HPGD	15-hydroxyprostaglandin dehydrogenase [Source:VGNC Symbol;Acc:VGNC:88961]	1.73	0.083
ssc-miR-218-5p	HRAS	HRas proto-onco, GTPase [Source:VGNC Symbol;Acc:VGNC:88970]	1.73	0.083
ssc-miR-218-5p	HS3ST2	heparan sulfate-glucosamine 3-sulfotransferase 2 [Source:VGNC Symbol;Acc:VGNC:88978]	1.73	0.083
ssc-miR-218-5p	HS3ST3B1	heparan sulfate-glucosamine 3-sulfotransferase 3B1 [Source:VGNC Symbol;Acc:VGNC:99003]	1.73	0.083
ssc-miR-218-5p	HS6ST3	heparan sulfate 6-O-sulfotransferase 3 [Source:HGNC Symbol;Acc:HGNC:19134]	1.73	0.083
ssc-miR-218-5p	ICK	hypothetical gene	1.73	0.083
ssc-miR-218-5p	IDH3A	isocitrate dehydrogenase (NAD(+)) 3 catalytic subunit alpha [Source:HGNC Symbol;Acc:HGNC:5384]	1.73	0.083
ssc-miR-218-5p	IGSF11	immunoglobulin superfamily member 11 [Source:VGNC Symbol;Acc:VGNC:89064]	1.73	0.083
ssc-miR-218-5p	IKZF1	IKAROS family zinc finger 1 [Source:VGNC Symbol;Acc:VGNC:89073]	1.73	0.083
ssc-miR-218-5p	IMPAD1	hypothetical gene	1.73	0.083
ssc-miR-218-5p	IMPG2	interphotoreceptor matrix proteoglycan 2 [Source:VGNC Symbol;Acc:VGNC:98048]	1.73	0.083
ssc-miR-218-5p	INHBB	inhibin subunit beta B [Source:VGNC Symbol;Acc:VGNC:103969]	1.73	0.083
ssc-miR-218-5p	INSIG1	insulin induced 1 [Source:VGNC Symbol;Acc:VGNC:89150]	1.73	0.083
ssc-miR-218-5p	INTS6	integrator complex subunit 6 [Source:VGNC Symbol;Acc:VGNC:89166]	1.73	0.083
ssc-miR-218-5p	IPMK	inositol polyphosphate multikinase [Source:VGNC Symbol;Acc:VGNC:89175]	1.73	0.083
ssc-miR-218-5p	ISCA2	iron-sulfur cluster assembly 2 [Source:VGNC Symbol;Acc:VGNC:89221]	1.73	0.083
ssc-miR-218-5p	ISLR2	immunoglobulin superfamily containing leucine rich repeat 2 [Source:VGNC Symbol;Acc:VGNC:89227]	1.73	0.083
ssc-miR-218-5p	ITGBL1	integrin subunit beta like 1 [Source:VGNC Symbol;Acc:VGNC:89247]	1.73	0.083
ssc-miR-218-5p	ITM2C	integral membrane protein 2C [Source:VGNC Symbol;Acc:VGNC:96383]	1.73	0.083
ssc-miR-218-5p	ITSN1	intersectin 1 [Source:VGNC Symbol;Acc:VGNC:108669]	1.73	0.083
ssc-miR-218-5p	JAG1	jagged canonical Notch ligand 1 [Source:VGNC Symbol;Acc:VGNC:96385]	1.73	0.083
ssc-miR-218-5p	JAKMIP2	janus kinase and microtubule interacting protein 2 [Source:VGNC Symbol;Acc:VGNC:89274]	1.73	0.083
ssc-miR-218-5p	JAKMIP3	Janus kinase and microtubule interacting protein 3 [Source:VGNC Symbol;Acc:VGNC:89275]	1.73	0.083
ssc-miR-218-5p	JDP2	Jun dimerization protein 2 [Source:VGNC Symbol;Acc:VGNC:89282]	1.73	0.083
ssc-miR-218-5p	JMY	junction mediating and regulatory protein, p53 cofactor [Source:VGNC Symbol;Acc:VGNC:89289]	1.73	0.083
ssc-miR-218-5p	KANSL1	KAT8 regulatory NSL complex subunit 1 [Source:VGNC Symbol;Acc:VGNC:89299]	1.73	0.083
ssc-miR-218-5p	KAT8	lysine acetyltransferase 8 [Source:VGNC Symbol;Acc:VGNC:89308]	1.73	0.083
ssc-miR-218-5p	KCNA1	potassium voltage-gated channel subfamily A member 1 [Source:VGNC Symbol;Acc:VGNC:89323]	1.73	0.083
ssc-miR-218-5p	KCNAB1	potassium voltage-gated channel subfamily A regulatory beta subunit 1 [Source:VGNC Symbol;Acc:VGNC:89328]	1.73	0.083
ssc-miR-218-5p	KCNB1	potassium voltage-gated channel subfamily B member 1 [Source:VGNC Symbol;Acc:VGNC:96386]	1.73	0.083
ssc-miR-218-5p	KCNC2	potassium voltage-gated channel subfamily C member 2 [Source:VGNC Symbol;Acc:VGNC:89333]	1.73	0.083
ssc-miR-218-5p	KCND1	potassium voltage-gated channel subfamily D member 1 [Source:VGNC Symbol;Acc:VGNC:89336]	1.73	0.083
ssc-miR-218-5p	KCND2	potassium voltage-gated channel subfamily D member 2 [Source:HGNC Symbol;Acc:HGNC:6238]	1.73	0.083
ssc-miR-218-5p	KCNE1	potassium voltage-gated channel subfamily E regulatory subunit 1 [Source:NCBI gene (formerly Entrezgene);Acc:100621502]	1.73	0.083
ssc-miR-218-5p	KCNH1	potassium voltage-gated channel subfamily H member 1 [Source:VGNC Symbol;Acc:VGNC:108599]	1.73	0.083
ssc-miR-218-5p	KCNH5	potassium voltage-gated channel subfamily H member 5 [Source:VGNC Symbol;Acc:VGNC:89345]	1.73	0.083
ssc-miR-218-5p	KCNIP3	potassium voltage-gated channel interacting protein 3 [Source:VGNC Symbol;Acc:VGNC:89350]	1.73	0.083
ssc-miR-218-5p	KCNJ6	potassium inwardly rectifying channel subfamily J member 6 [Source:VGNC Symbol;Acc:VGNC:89360]	1.73	0.083
ssc-miR-218-5p	KCNK1	potassium two pore domain channel subfamily K member 1 [Source:VGNC Symbol;Acc:VGNC:89363]	1.73	0.083
ssc-miR-218-5p	KCNK12	potassium two pore domain channel subfamily K member 12 [Source:HGNC Symbol;Acc:HGNC:6274]	1.73	0.083
ssc-miR-218-5p	KCNK15	potassium two pore domain channel subfamily K member 15 [Source:VGNC Symbol;Acc:VGNC:95593]	1.73	0.083
ssc-miR-218-5p	KCNK2	potassium two pore domain channel subfamily K member 2 [Source:VGNC Symbol;Acc:VGNC:89369]	1.73	0.083
ssc-miR-218-5p	KCNMB4	potassium calcium-activated channel subfamily M regulatory beta subunit 4 [Source:VGNC Symbol;Acc:VGNC:89378]	1.73	0.083
ssc-miR-218-5p	KCNN3	potassium calcium-activated channel subfamily N member 3 [Source:VGNC Symbol;Acc:VGNC:98056]	1.73	0.083
ssc-miR-218-5p	KCNQ3	potassium voltage-gated channel subfamily Q member 3 [Source:VGNC Symbol;Acc:VGNC:89382]	1.73	0.083
ssc-miR-218-5p	KCNQ4	potassium voltage-gated channel subfamily Q member 4 [Source:VGNC Symbol;Acc:VGNC:89383]	1.73	0.083

ssc-miR-218-5p	KCNQ5	potassium voltage-gated channel subfamily Q member 5 [Source:VGNC Symbol;Acc:VGNC:89384]	1.73	0.083
ssc-miR-218-5p	KCNT1	potassium sodium-activated channel subfamily T member 1 [Source:VGNC Symbol;Acc:VGNC:89388]	1.73	0.083
ssc-miR-218-5p	KCTD16	potassium channel tetramerization domain containing 16 [Source:VGNC Symbol;Acc:VGNC:89395]	1.73	0.083
ssc-miR-218-5p	KCTD9	potassium channel tetramerization domain containing 9 [Source:VGNC Symbol;Acc:VGNC:89404]	1.73	0.083
ssc-miR-218-5p	KDM2A	lysine demethylase 2A [Source:VGNC Symbol;Acc:VGNC:89410]	1.73	0.083
ssc-miR-218-5p	KDM3B	lysine demethylase 3B [Source:VGNC Symbol;Acc:VGNC:89412]	1.73	0.083
ssc-miR-218-5p	KDM5A	lysine demethylase 5A [Source:VGNC Symbol;Acc:VGNC:89415]	1.73	0.083
ssc-miR-218-5p	KHNYN	KH and NYN domain containing [Source:VGNC Symbol;Acc:VGNC:89426]	1.73	0.083
ssc-miR-218-5p	KIAA0040	KIAA0040 [Source:VGNC Symbol;Acc:VGNC:98059]	1.73	0.083
ssc-miR-218-5p	KIAA1024	hypothetical gene	1.73	0.083
ssc-miR-218-5p	KIAA1045	hypothetical gene	1.73	0.083
ssc-miR-218-5p	KIAA1161	hypothetical gene	1.73	0.083
ssc-miR-218-5p	KIAA1324	hypothetical gene	1.73	0.083
ssc-miR-218-5p	KIAA1429	hypothetical gene	1.73	0.083
ssc-miR-218-5p	KIAA1456	hypothetical gene	1.73	0.083
ssc-miR-218-5p	KIAA1522	KIAA1522 [Source:VGNC Symbol;Acc:VGNC:89444]	1.73	0.083
ssc-miR-218-5p	KIAA1549	KIAA1549 [Source:VGNC Symbol;Acc:VGNC:99719]	1.73	0.083
ssc-miR-218-5p	KIAA1549L	KIAA1549 like [Source:VGNC Symbol;Acc:VGNC:89445]	1.73	0.083
ssc-miR-218-5p	KIAA2022	hypothetical gene	1.73	0.083
ssc-miR-218-5p	KIF13A	kinesin family member 13A [Source:VGNC Symbol;Acc:VGNC:89454]	1.73	0.083
ssc-miR-218-5p	KIF21B	kinesin family member 21B [Source:VGNC Symbol;Acc:VGNC:96219]	1.73	0.083
ssc-miR-218-5p	KIF2A	kinesin family member 2A [Source:VGNC Symbol;Acc:VGNC:89467]	1.73	0.083
ssc-miR-218-5p	KIF3C	kinesin family member 3C [Source:VGNC Symbol;Acc:VGNC:89471]	1.73	0.083
ssc-miR-218-5p	KIRREL3	kirre like nephrin family adhesion molecule 3 [Source:VGNC Symbol;Acc:VGNC:89482]	1.73	0.083
ssc-miR-218-5p	KIT	KIT proto-onco, receptor tyrosine kinase [Source:VGNC Symbol;Acc:VGNC:98060]	1.73	0.083
ssc-miR-218-5p	KLF12	Kruppel like factor 12 [Source:VGNC Symbol;Acc:VGNC:89492]	1.73	0.083
ssc-miR-218-5p	KLF3	Kruppel like factor 3 [Source:VGNC Symbol;Acc:VGNC:89498]	1.73	0.083
ssc-miR-218-5p	KLF8	Kruppel like factor 8 [Source:VGNC Symbol;Acc:VGNC:89500]	1.73	0.083
ssc-miR-218-5p	KLF9	Kruppel like factor 9 [Source:VGNC Symbol;Acc:VGNC:103114]	1.73	0.083
ssc-miR-218-5p	KLHDC10	kelch domain containing 10 [Source:VGNC Symbol;Acc:VGNC:89502]	1.73	0.083
ssc-miR-218-5p	KLHDC8A	kelch domain containing 8A [Source:VGNC Symbol;Acc:VGNC:89508]	1.73	0.083
ssc-miR-218-5p	KLHL11	kelch like family member 11 [Source:VGNC Symbol;Acc:VGNC:89513]	1.73	0.083
ssc-miR-218-5p	KLHL13	kelch like family member 13 [Source:VGNC Symbol;Acc:VGNC:89514]	1.73	0.083
ssc-miR-218-5p	KLHL18	kelch like family member 18 [Source:VGNC Symbol;Acc:VGNC:89517]	1.73	0.083
ssc-miR-218-5p	KLHL23	kelch like family member 23 [Source:VGNC Symbol;Acc:VGNC:96158]	1.73	0.083
ssc-miR-218-5p	KLHL25	kelch like family member 25 [Source:HGNC Symbol;Acc:HGNC:25732]	1.73	0.083
ssc-miR-218-5p	KLHL29	kelch like family member 29 [Source:VGNC Symbol;Acc:VGNC:89524]	1.73	0.083
ssc-miR-218-5p	KMO	kynurenine 3-monooxygenase [Source:VGNC Symbol;Acc:VGNC:96398]	1.73	0.083
ssc-miR-218-5p	KMT2A	lysine methyltransferase 2A [Source:VGNC Symbol;Acc:VGNC:108600]	1.73	0.083
ssc-miR-218-5p	KPNA1	karyopherin subunit alpha 1 [Source:VGNC Symbol;Acc:VGNC:89560]	1.73	0.083
ssc-miR-218-5p	KPNA4	karyopherin subunit alpha 4 [Source:VGNC Symbol;Acc:VGNC:89563]	1.73	0.083
ssc-miR-218-5p	KRIT1	KRIT1 ankyrin repeat containing [Source:HGNC Symbol;Acc:HGNC:1573]	1.73	0.083
ssc-miR-218-5p	KRTAP3-3	hypothetical gene	1.73	0.083
ssc-miR-218-5p	L3MBTL3	L3MBTL histone methyl-lysine binding protein 3 [Source:VGNC Symbol;Acc:VGNC:98066]	1.73	0.083
ssc-miR-218-5p	L3MBTL4	hypothetical gene	1.73	0.083
ssc-miR-218-5p	LAMP2	lysosomal associated membrane protein 2 [Source:VGNC Symbol;Acc:VGNC:89628]	1.73	0.083
ssc-miR-218-5p	LARP4B	hypothetical gene	1.73	0.083
ssc-miR-218-5p	LASP1	LIM and SH3 protein 1 [Source:VGNC Symbol;Acc:VGNC:89646]	1.73	0.083
ssc-miR-218-5p	LCORL	ligand dependent nuclear receptor corepressor like [Source:VGNC Symbol;Acc:VGNC:98937]	1.73	0.083
ssc-miR-218-5p	LDLRAD4	low density lipoprotein receptor class A domain containing 4 [Source:VGNC Symbol;Acc:VGNC:89673]	1.73	0.083

ssc-miR-218-5p	LGALSL	galectin like [Source:VGNC Symbol;Acc:VGNC:89698]	1.73	0.083
ssc-miR-218-5p	LGR4	leucine rich repeat containing G protein-coupled receptor 4 [Source:VGNC Symbol;Acc:VGNC:89703]	1.73	0.083
ssc-miR-218-5p	LHFP	hypothetical gene	1.73	0.083
ssc-miR-218-5p	LHX1	LIM homeobox 1 [Source:VGNC Symbol;Acc:VGNC:89711]	1.73	0.083
ssc-miR-218-5p	LHX4	LIM homeobox 4 [Source:VGNC Symbol;Acc:VGNC:89714]	1.73	0.083
ssc-miR-218-5p	LHX9	LIM homeobox 9 [Source:VGNC Symbol;Acc:VGNC:95608]	1.73	0.083
ssc-miR-218-5p	LIFR	LIF receptor subunit alpha [Source:HGNC Symbol;Acc:HGNC:6597]	1.73	0.083
ssc-miR-218-5p	LIMD1	LIM domain containing 1 [Source:VGNC Symbol;Acc:VGNC:89725]	1.73	0.083
ssc-miR-218-5p	LIN7A	lin-7 homolog A, crumbs cell polarity complex component [Source:VGNC Symbol;Acc:VGNC:89730]	1.73	0.083
ssc-miR-218-5p	LIPG	lipase G, endothelial type [Source:VGNC Symbol;Acc:VGNC:89739]	1.73	0.083
ssc-miR-218-5p	LMLN	leishmanolysin like peptidase [Source:VGNC Symbol;Acc:VGNC:89759]	1.73	0.083
ssc-miR-218-5p	LMNB1	lamin B1 [Source:VGNC Symbol;Acc:VGNC:89760]	1.73	0.083
ssc-miR-218-5p	LMO3	hypothetical gene	1.73	0.083
ssc-miR-218-5p	LMO7	LIM domain 7 [Source:VGNC Symbol;Acc:VGNC:89767]	1.73	0.083
ssc-miR-218-5p	LNPEP	leucyl and cystinyl aminopeptidase [Source:VGNC Symbol;Acc:VGNC:89772]	1.73	0.083
ssc-miR-218-5p	LPCAT1	lysophosphatidylcholine acyltransferase 1 [Source:VGNC Symbol;Acc:VGNC:89788]	1.73	0.083
ssc-miR-218-5p	LPHN1	hypothetical gene	1.73	0.083
ssc-miR-218-5p	LPHN3	hypothetical gene	1.73	0.083
ssc-miR-218-5p	LPP	LIM domain containing preferred translocation partner in lipoma [Source:HGNC Symbol;Acc:HGNC:6679]	1.73	0.083
ssc-miR-218-5p	LPPR4	hypothetical gene	1.73	0.083
ssc-miR-218-5p	LRAT	lecithin retinol acyltransferase [Source:VGNC Symbol;Acc:VGNC:89798]	1.73	0.083
ssc-miR-218-5p	LRCH1	leucine rich repeats and calponin homology domain containing 1 [Source:VGNC Symbol;Acc:VGNC:89801]	1.73	0.083
ssc-miR-218-5p	LRIG1	leucine rich repeats and immunoglobulin like domains 1 [Source:VGNC Symbol;Acc:VGNC:89810]	1.73	0.083
ssc-miR-218-5p	LRIG3	leucine rich repeats and immunoglobulin like domains 3 [Source:VGNC Symbol;Acc:VGNC:89812]	1.73	0.083
ssc-miR-218-5p	LRP1B	LDL receptor related protein 1B [Source:HGNC Symbol;Acc:HGNC:6693]	1.73	0.083
ssc-miR-218-5p	LRRC1	leucine rich repeat containing 1 [Source:VGNC Symbol;Acc:VGNC:103977]	1.73	0.083
ssc-miR-218-5p	LRRC16A	hypothetical gene	1.73	0.083
ssc-miR-218-5p	LRRC19	leucine rich repeat containing 19 [Source:VGNC Symbol;Acc:VGNC:89829]	1.73	0.083
ssc-miR-218-5p	LRRC55	leucine rich repeat containing 55 [Source:VGNC Symbol;Acc:VGNC:89850]	1.73	0.083
ssc-miR-218-5p	LRRC7	leucine rich repeat containing 7 [Source:HGNC Symbol;Acc:HGNC:18531]	1.73	0.083
ssc-miR-218-5p	LRRFIP1	hypothetical gene	1.73	0.083
ssc-miR-218-5p	LRRN1	leucine rich repeat neuronal 1 [Source:VGNC Symbol;Acc:VGNC:89860]	1.73	0.083
ssc-miR-218-5p	LYPD6B	LY6/PLAUR domain containing 6B [Source:VGNC Symbol;Acc:VGNC:96150]	1.73	0.083
ssc-miR-218-5p	LYSMD3	LysM domain containing 3 [Source:VGNC Symbol;Acc:VGNC:89919]	1.73	0.083
ssc-miR-218-5p	LYVE1	lymphatic vessel endothelial hyaluronan receptor 1 [Source:VGNC Symbol;Acc:VGNC:89922]	1.73	0.083
ssc-miR-218-5p	MAFG	MAF bZIP transcription factor G [Source:VGNC Symbol;Acc:VGNC:89948]	1.73	0.083
ssc-miR-218-5p	MAGI2	membrane associated guanylate kinase, WW and PDZ domain containing 2 [Source:VGNC Symbol;Acc:VGNC:89955]	1.73	0.083
ssc-miR-218-5p	MALSU1	mitochondrial assembly of ribosomal large subunit 1 [Source:VGNC Symbol;Acc:VGNC:89960]	1.73	0.083
ssc-miR-218-5p	MAN2A1	mannosidase alpha class 2A member 1 [Source:VGNC Symbol;Acc:VGNC:98098]	1.73	0.083
ssc-miR-218-5p	MAP3K2	mitogen-activated protein kinase kinase kinase 2 [Source:VGNC Symbol;Acc:VGNC:98107]	1.73	0.083
ssc-miR-218-5p	MAP7D2	MAP7 domain containing 2 [Source:VGNC Symbol;Acc:VGNC:89994]	1.73	0.083
ssc-miR-218-5p	MAPK1IP1L	mitogen-activated protein kinase 1 interacting protein 1 like [Source:VGNC Symbol;Acc:VGNC:90002]	1.73	0.083
ssc-miR-218-5p	MAPK8IP3	mitogen-activated protein kinase 8 interacting protein 3 [Source:VGNC Symbol;Acc:VGNC:90008]	1.73	0.083
ssc-miR-218-5p	MARCH4	hypothetical gene	1.73	0.083
ssc-miR-218-5p	MARCKS	myristoylated alanine rich protein kinase C substrate [Source:VGNC Symbol;Acc:VGNC:90024]	1.73	0.083
ssc-miR-218-5p	MARK1	microtubule affinity regulating kinase 1 [Source:VGNC Symbol;Acc:VGNC:96411]	1.73	0.083
ssc-miR-218-5p	MARK2	microtubule affinity regulating kinase 2 [Source:VGNC Symbol;Acc:VGNC:98118]	1.73	0.083
ssc-miR-218-5p	MBD5	methyl-CpG binding domain protein 5 [Source:VGNC Symbol;Acc:VGNC:95940]	1.73	0.083
ssc-miR-218-5p	MBD6	methyl-CpG binding domain protein 6 [Source:VGNC Symbol;Acc:VGNC:90051]	1.73	0.083
ssc-miR-218-5p	MBLAC2	metallo-beta-lactamase domain containing 2 [Source:VGNC Symbol;Acc:VGNC:90053]	1.73	0.083

ssc-miR-218-5p	MBNL1	muscleblind like splicing regulator 1 [Source:VGNC Symbol;Acc:VGNC:90054]	1.73	0.083
ssc-miR-218-5p	MBNL2	muscleblind like splicing regulator 2 [Source:VGNC Symbol;Acc:VGNC:90055]	1.73	0.083
ssc-miR-218-5p	MCAT	malonyl-CoA-acyl carrier protein transacylase [Source:VGNC Symbol;Acc:VGNC:90062]	1.73	0.083
ssc-miR-218-5p	MCC	MCC regulator of WNT signaling pathway [Source:VGNC Symbol;Acc:VGNC:96588]	1.73	0.083
ssc-miR-218-5p	MCF2	MCF.2 cell line derived transforming sequence [Source:VGNC Symbol;Acc:VGNC:90067]	1.73	0.083
ssc-miR-218-5p	MDGA1	MAM domain containing glycosylphosphatidylinositol anchor 1 [Source:VGNC Symbol;Acc:VGNC:90091]	1.73	0.083
ssc-miR-218-5p	MDGA2	MAM domain containing glycosylphosphatidylinositol anchor 2 [Source:VGNC Symbol;Acc:VGNC:90092]	1.73	0.083
ssc-miR-218-5p	MECP2	methyl-CpG binding protein 2 [Source:VGNC Symbol;Acc:VGNC:90101]	1.73	0.083
ssc-miR-218-5p	MED1	mediator complex subunit 1 [Source:VGNC Symbol;Acc:VGNC:90102]	1.73	0.083
ssc-miR-218-5p	MED12L	mediator complex subunit 12L [Source:VGNC Symbol;Acc:VGNC:90105]	1.73	0.083
ssc-miR-218-5p	MED17	mediator complex subunit 17 [Source:HGNC Symbol;Acc:HGNC:2375]	1.73	0.083
ssc-miR-218-5p	MEF2C	myocyte enhancer factor 2C [Source:VGNC Symbol;Acc:VGNC:90127]	1.73	0.083
ssc-miR-218-5p	MEF2D	myocyte enhancer factor 2D [Source:VGNC Symbol;Acc:VGNC:90128]	1.73	0.083
ssc-miR-218-5p	MEGF9	multiple EGF like domains 9 [Source:VGNC Symbol;Acc:VGNC:90130]	1.73	0.083
ssc-miR-218-5p	MEMO1	mediator of cell motility 1 [Source:VGNC Symbol;Acc:VGNC:90139]	1.73	0.083
ssc-miR-218-5p	MEPCE	methylphosphate capping enzyme [Source:VGNC Symbol;Acc:VGNC:90144]	1.73	0.083
ssc-miR-218-5p	METTL20	hypothetical gene	1.73	0.083
ssc-miR-218-5p	METTL21A	methyltransferase 21A, HSPA lysine [Source:VGNC Symbol;Acc:VGNC:96242]	1.73	0.083
ssc-miR-218-5p	METTL24	methyltransferase like 24 [Source:VGNC Symbol;Acc:VGNC:90161]	1.73	0.083
ssc-miR-218-5p	MFHAS1	multifunctional ROCO family signaling regulator 1 [Source:VGNC Symbol;Acc:VGNC:107361]	1.73	0.083
ssc-miR-218-5p	MID2	midline 2 [Source:VGNC Symbol;Acc:VGNC:98127]	1.73	0.083
ssc-miR-218-5p	MIER3	MIER family member 3 [Source:VGNC Symbol;Acc:VGNC:90221]	1.73	0.083
ssc-miR-218-5p	MITF	melanocyte inducing transcription factor [Source:VGNC Symbol;Acc:VGNC:90243]	1.73	0.083
ssc-miR-218-5p	MLLT3	MLLT3 super elongation complex subunit [Source:VGNC Symbol;Acc:VGNC:90256]	1.73	0.083
ssc-miR-218-5p	MME	membrane metalloendopeptidase [Source:VGNC Symbol;Acc:VGNC:90265]	1.73	0.083
ssc-miR-218-5p	MOB1B	hypothetical gene	1.73	0.083
ssc-miR-218-5p	MOGAT3	hypothetical gene	1.73	0.083
ssc-miR-218-5p	MOSPD1	motile sperm domain containing 1 [Source:VGNC Symbol;Acc:VGNC:103990]	1.73	0.083
ssc-miR-218-5p	MPP6	hypothetical gene	1.73	0.083
ssc-miR-218-5p	MPPED2	metallophosphoesterase domain containing 2 [Source:VGNC Symbol;Acc:VGNC:90331]	1.73	0.083
ssc-miR-218-5p	MPRIP	hypothetical gene	1.73	0.083
ssc-miR-218-5p	MPZL1	myelin protein zero like 1 [Source:VGNC Symbol;Acc:VGNC:90335]	1.73	0.083
ssc-miR-218-5p	MRGBP	MRG domain binding protein [Source:HGNC Symbol;Acc:HGNC:15866]	1.73	0.083
ssc-miR-218-5p	MRPS36	mitochondrial ribosomal protein S36 [Source:VGNC Symbol;Acc:VGNC:90397]	1.73	0.083
ssc-miR-218-5p	MRVI1	hypothetical gene	1.73	0.083
ssc-miR-218-5p	MSI2	musashi RNA binding protein 2 [Source:VGNC Symbol;Acc:VGNC:90422]	1.73	0.083
ssc-miR-218-5p	MSL2	MSL complex subunit 2 [Source:VGNC Symbol;Acc:VGNC:90424]	1.73	0.083
ssc-miR-218-5p	MTF2	metal response element binding transcription factor 2 [Source:VGNC Symbol;Acc:VGNC:90444]	1.73	0.083
ssc-miR-218-5p	MTHFSD	methenyltetrahydrofolate synthetase domain containing [Source:VGNC Symbol;Acc:VGNC:90452]	1.73	0.083
ssc-miR-218-5p	MTMR1	myotubularin related protein 1 [Source:VGNC Symbol;Acc:VGNC:90456]	1.73	0.083
ssc-miR-218-5p	MTMR12	myotubularin related protein 12 [Source:VGNC Symbol;Acc:VGNC:90459]	1.73	0.083
ssc-miR-218-5p	MUL1	mitochondrial E3 ubiquitin protein ligase 1 [Source:VGNC Symbol;Acc:VGNC:90484]	1.73	0.083
ssc-miR-218-5p	MVB12B	multivesicular body subunit 12B [Source:VGNC Symbol;Acc:VGNC:90488]	1.73	0.083
ssc-miR-218-5p	MYO16	hypothetical gene	1.73	0.083
ssc-miR-218-5p	MYO5A	myosin VA [Source:HGNC Symbol;Acc:HGNC:7602]	1.73	0.083
ssc-miR-218-5p	MYPN	myopalladin [Source:VGNC Symbol;Acc:VGNC:107412]	1.73	0.083
ssc-miR-218-5p	MYSM1	Myb like, SWIRM and MPN domains 1 [Source:VGNC Symbol;Acc:VGNC:90548]	1.73	0.083
ssc-miR-218-5p	MYT1L	myelin transcription factor 1 like [Source:VGNC Symbol;Acc:VGNC:90549]	1.73	0.083
ssc-miR-218-5p	NAA15	N-alpha-acetyltransferase 15, NatA auxiliary subunit [Source:VGNC Symbol;Acc:VGNC:96747]	1.73	0.083
ssc-miR-218-5p	NAB1	NGFI-A binding protein 1 [Source:VGNC Symbol;Acc:VGNC:96431]	1.73	0.083

ssc-miR-218-5p	NACC1	nucleus accumbens associated 1 [Source:VGNC Symbol;Acc:VGNC:90563]	1.73	0.083
ssc-miR-218-5p	NACC2	NACC family member 2 [Source:VGNC Symbol;Acc:VGNC:90564]	1.73	0.083
ssc-miR-218-5p	NAGS	N-acetylglutamate synthase [Source:VGNC Symbol;Acc:VGNC:90571]	1.73	0.083
ssc-miR-218-5p	NAPEPLD	N-acyl phosphatidylethanolamine phospholipase D [Source:VGNC Symbol;Acc:VGNC:90579]	1.73	0.083
ssc-miR-218-5p	NAT8L	N-acetyltransferase 8 like [Source:VGNC Symbol;Acc:VGNC:90586]	1.73	0.083
ssc-miR-218-5p	NAV1	neuron navigator 1 [Source:VGNC Symbol;Acc:VGNC:95725]	1.73	0.083
ssc-miR-218-5p	NAV3	neuron navigator 3 [Source:VGNC Symbol;Acc:VGNC:90589]	1.73	0.083
ssc-miR-218-5p	NBEA	neurobeachin [Source:VGNC Symbol;Acc:VGNC:90590]	1.73	0.083
ssc-miR-218-5p	NBR1	NBR1 autophagy cargo receptor [Source:VGNC Symbol;Acc:VGNC:90593]	1.73	0.083
ssc-miR-218-5p	NCAN	neurocan [Source:VGNC Symbol;Acc:VGNC:90596]	1.73	0.083
ssc-miR-218-5p	NDRG4	NDRG family member 4 [Source:VGNC Symbol;Acc:VGNC:90633]	1.73	0.083
ssc-miR-218-5p	NEBL	nebulette [Source:VGNC Symbol;Acc:VGNC:95824]	1.73	0.083
ssc-miR-218-5p	NECAB1	N-terminal EF-hand calcium binding protein 1 [Source:VGNC Symbol;Acc:VGNC:90659]	1.73	0.083
ssc-miR-218-5p	NEURL4	neuralized E3 ubiquitin protein ligase 4 [Source:VGNC Symbol;Acc:VGNC:99022]	1.73	0.083
ssc-miR-218-5p	NEUROD4	neuronal differentiation 4 [Source:VGNC Symbol;Acc:VGNC:90698]	1.73	0.083
ssc-miR-218-5p	NF2	NF2, moesin-ezrin-radixin like (MERLIN) tumor suppressor [Source:VGNC Symbol;Acc:VGNC:90705]	1.73	0.083
ssc-miR-218-5p	NFATC1	nuclear factor of activated T cells 1 [Source:VGNC Symbol;Acc:VGNC:103140]	1.73	0.083
ssc-miR-218-5p	NFATC3	nuclear factor of activated T cells 3 [Source:VGNC Symbol;Acc:VGNC:90710]	1.73	0.083
ssc-miR-218-5p	NFE2L1	NFE2 like bZIP transcription factor 1 [Source:VGNC Symbol;Acc:VGNC:90713]	1.73	0.083
ssc-miR-218-5p	NFIA	nuclear factor I A [Source:VGNC Symbol;Acc:VGNC:90715]	1.73	0.083
ssc-miR-218-5p	NFIX	nuclear factor I X [Source:VGNC Symbol;Acc:VGNC:90718]	1.73	0.083
ssc-miR-218-5p	NHLRC3	NHL repeat containing 3 [Source:HGNC Symbol;Acc:HGNC:33751]	1.73	0.083
ssc-miR-218-5p	NMNAT3	nicotinamide nucleotide adenylyltransferase 3 [Source:VGNC Symbol;Acc:VGNC:108679]	1.73	0.083
ssc-miR-218-5p	NMT2	N-myristoyltransferase 2 [Source:VGNC Symbol;Acc:VGNC:96448]	1.73	0.083
ssc-miR-218-5p	NPAS2	neuronal PAS domain protein 2 [Source:VGNC Symbol;Acc:VGNC:90837]	1.73	0.083
ssc-miR-218-5p	NPY1R	neuropeptide Y receptor Y1 [Source:VGNC Symbol;Acc:VGNC:90865]	1.73	0.083
ssc-miR-218-5p	NR1D2	nuclear receptor subfamily 1 group D member 2 [Source:VGNC Symbol;Acc:VGNC:99726]	1.73	0.083
ssc-miR-218-5p	NRAS	NRAS proto-onco, GTPase [Source:VGNC Symbol;Acc:VGNC:98827]	1.73	0.083
ssc-miR-218-5p	NRXN1	neurexin 1 [Source:HGNC Symbol;Acc:HGNC:8008]	1.73	0.083
ssc-miR-218-5p	NRXN3	neurexin 3 [Source:HGNC Symbol;Acc:HGNC:8010]	1.73	0.083
ssc-miR-218-5p	NSRP1	nuclear speckle splicing regulatory protein 1 [Source:VGNC Symbol;Acc:VGNC:90913]	1.73	0.083
ssc-miR-218-5p	NUDT10	hypothetical gene	1.73	0.083
ssc-miR-218-5p	NUFIP2	nuclear FMR1 interacting protein 2 [Source:VGNC Symbol;Acc:VGNC:90967]	1.73	0.083
ssc-miR-218-5p	NUMB	NUMB endocytic adaptor protein [Source:VGNC Symbol;Acc:VGNC:90970]	1.73	0.083
ssc-miR-218-5p	NUMBL	NUMB like endocytic adaptor protein [Source:HGNC Symbol;Acc:HGNC:8061]	1.73	0.083
ssc-miR-218-5p	NUP50	nucleoporin 50 [Source:VGNC Symbol;Acc:VGNC:90983]	1.73	0.083
ssc-miR-218-5p	NUPL2	hypothetical gene	1.73	0.083
ssc-miR-218-5p	NXF1	nuclear RNA export factor 1 [Source:VGNC Symbol;Acc:VGNC:90993]	1.73	0.083
ssc-miR-218-5p	NXT2	nuclear transport factor 2 like export factor 2 [Source:VGNC Symbol;Acc:VGNC:91001]	1.73	0.083
ssc-miR-218-5p	ONECUT2	one cut homeobox 2 [Source:VGNC Symbol;Acc:VGNC:91043]	1.73	0.083
ssc-miR-218-5p	ONECUT3	one cut homeobox 3 [Source:VGNC Symbol;Acc:VGNC:91044]	1.73	0.083
ssc-miR-218-5p	ORC2	origin recognition complex subunit 2 [Source:VGNC Symbol;Acc:VGNC:96464]	1.73	0.083
ssc-miR-218-5p	ORC5	origin recognition complex subunit 5 [Source:VGNC Symbol;Acc:VGNC:91062]	1.73	0.083
ssc-miR-218-5p	ORC6	origin recognition complex subunit 6 [Source:VGNC Symbol;Acc:VGNC:91063]	1.73	0.083
ssc-miR-218-5p	OTP	orthopedia homeobox [Source:VGNC Symbol;Acc:VGNC:91096]	1.73	0.083
ssc-miR-218-5p	OTUB2	OTU deubiquitinase, ubiquitin aldehyde binding 2 [Source:VGNC Symbol;Acc:VGNC:91098]	1.73	0.083
ssc-miR-218-5p	OTUD7B	OTU deubiquitinase 7B [Source:VGNC Symbol;Acc:VGNC:91105]	1.73	0.083
ssc-miR-218-5p	OTX2	orthodenticle homeobox 2 [Source:VGNC Symbol;Acc:VGNC:98171]	1.73	0.083
ssc-miR-218-5p	OXSR1	hypothetical gene	1.73	0.083
ssc-miR-218-5p	P2RY2	purinergic receptor P2Y2 [Source:VGNC Symbol;Acc:VGNC:98173]	1.73	0.083

ssc-miR-218-5p	PAG1	phosphoprotein membrane anchor with glycosphingolipid microdomains 1 [Source:VGNC Symbol;Acc:VGNC:91154]	1.73	0.083
ssc-miR-218-5p	PAIP2	poly(A) binding protein interacting protein 2 [Source:VGNC Symbol;Acc:VGNC:96618]	1.73	0.083
ssc-miR-218-5p	PAK3	p21 (RAC1) activated kinase 3 [Source:HGNC Symbol;Acc:HGNC:8592]	1.73	0.083
ssc-miR-218-5p	PALM2	hypothetical gene	1.73	0.083
ssc-miR-218-5p	PAN3	poly(A) specific ribonuclease subunit PAN3 [Source:HGNC Symbol;Acc:HGNC:29991]	1.73	0.083
ssc-miR-218-5p	PARP8	poly(ADP-ribose) polymerase family member 8 [Source:HGNC Symbol;Acc:HGNC:26124]	1.73	0.083
ssc-miR-218-5p	PAX2	paired box 2 [Source:VGNC Symbol;Acc:VGNC:91192]	1.73	0.083
ssc-miR-218-5p	PAX5	paired box 5 [Source:VGNC Symbol;Acc:VGNC:91194]	1.73	0.083
ssc-miR-218-5p	PBX2	PBX homeobox 2 [Source:VGNC Symbol;Acc:VGNC:91204]	1.73	0.083
ssc-miR-218-5p	PCDH1	protocadherin 1 [Source:VGNC Symbol;Acc:VGNC:91211]	1.73	0.083
ssc-miR-218-5p	PCDH17	protocadherin 17 [Source:VGNC Symbol;Acc:VGNC:91214]	1.73	0.083
ssc-miR-218-5p	PCDH8	protocadherin 8 [Source:HGNC Symbol;Acc:HGNC:8660]	1.73	0.083
ssc-miR-218-5p	PCDH9	protocadherin 9 [Source:VGNC Symbol;Acc:VGNC:91217]	1.73	0.083
ssc-miR-218-5p	PCDHA1	hypothetical gene	1.73	0.083
ssc-miR-218-5p	PCDHA10	hypothetical gene	1.73	0.083
ssc-miR-218-5p	PCDHA11	hypothetical gene	1.73	0.083
ssc-miR-218-5p	PCDHA12	hypothetical gene	1.73	0.083
ssc-miR-218-5p	PCDHA13	protocadherin alpha 13 [Source:HGNC Symbol;Acc:HGNC:8667]	1.73	0.083
ssc-miR-218-5p	PCDHA2	hypothetical gene	1.73	0.083
ssc-miR-218-5p	PCDHA3	protocadherin alpha 3 [Source:HGNC Symbol;Acc:HGNC:8669]	1.73	0.083
ssc-miR-218-5p	PCDHA4	hypothetical gene	1.73	0.083
ssc-miR-218-5p	PCDHA5	hypothetical gene	1.73	0.083
ssc-miR-218-5p	PCDHA6	hypothetical gene	1.73	0.083
ssc-miR-218-5p	PCDHA7	hypothetical gene	1.73	0.083
ssc-miR-218-5p	PCDHA8	hypothetical gene	1.73	0.083
ssc-miR-218-5p	PCDHA9	hypothetical gene	1.73	0.083
ssc-miR-218-5p	PCDHAC1	hypothetical gene	1.73	0.083
ssc-miR-218-5p	PCDHAC2	protocadherin alpha subfamily C, 2 [Source:HGNC Symbol;Acc:HGNC:8677]	1.73	0.083
ssc-miR-218-5p	PCDHB16	hypothetical gene	1.73	0.083
ssc-miR-218-5p	PCGF2	polycomb group ring finger 2 [Source:VGNC Symbol;Acc:VGNC:91219]	1.73	0.083
ssc-miR-218-5p	PCGF5	hypothetical gene	1.73	0.083
ssc-miR-218-5p	PCLO	hypothetical gene	1.73	0.083
ssc-miR-218-5p	PCMTD2	protein-L-isoaspartate (D-aspartate) O-methyltransferase domain containing 2 [Source:VGNC Symbol;Acc:VGNC:108730]	1.73	0.083
ssc-miR-218-5p	PCNP	PEST proteolytic signal containing nuclear protein [Source:VGNC Symbol;Acc:VGNC:104018]	1.73	0.083
ssc-miR-218-5p	PDE12	phosphodiesterase 12 [Source:VGNC Symbol;Acc:VGNC:104020]	1.73	0.083
ssc-miR-218-5p	PDE3A	phosphodiesterase 3A [Source:VGNC Symbol;Acc:VGNC:91252]	1.73	0.083
ssc-miR-218-5p	PDE7A	phosphodiesterase 7A [Source:VGNC Symbol;Acc:VGNC:91261]	1.73	0.083
ssc-miR-218-5p	PDE8B	phosphodiesterase 8B [Source:VGNC Symbol;Acc:VGNC:100321]	1.73	0.083
ssc-miR-218-5p	PDGFRA	platelet derived growth factor receptor alpha [Source:VGNC Symbol;Acc:VGNC:98179]	1.73	0.083
ssc-miR-218-5p	PDYN	prodynorphin [Source:VGNC Symbol;Acc:VGNC:96477]	1.73	0.083
ssc-miR-218-5p	PDZD4	PDZ domain containing 4 [Source:VGNC Symbol;Acc:VGNC:91296]	1.73	0.083
ssc-miR-218-5p	PEX5L	peroxisomal biosis factor 5 like [Source:VGNC Symbol;Acc:VGNC:91329]	1.73	0.083
ssc-miR-218-5p	PGM2L1	phosphoglucomutase 2 like 1 [Source:VGNC Symbol;Acc:VGNC:91357]	1.73	0.083
ssc-miR-218-5p	PHC3	polyhomeotic homolog 3 [Source:VGNC Symbol;Acc:VGNC:91372]	1.73	0.083
ssc-miR-218-5p	PHF15	hypothetical gene	1.73	0.083
ssc-miR-218-5p	PHF17	hypothetical gene	1.73	0.083
ssc-miR-218-5p	PHF20	PHD finger protein 20 [Source:HGNC Symbol;Acc:HGNC:16098]	1.73	0.083
ssc-miR-218-5p	PHF6	PHD finger protein 6 [Source:VGNC Symbol;Acc:VGNC:91389]	1.73	0.083
ssc-miR-218-5p	PHYH	phytanoyl-CoA 2-hydroxylase [Source:VGNC Symbol;Acc:VGNC:96483]	1.73	0.083
ssc-miR-218-5p	PI4K2A	phosphatidylinositol 4-kinase type 2 alpha [Source:HGNC Symbol;Acc:HGNC:30031]	1.73	0.083

ssc-miR-218-5p	PIAS1	protein inhibitor of activated STAT 1 [Source:VGNC Symbol;Acc:VGNC:91410]	1.73	0.083
ssc-miR-218-5p	PIEZO2	piezo type mechanosensitive ion channel component 2 [Source:HGNC Symbol;Acc:HGNC:26270]	1.73	0.083
ssc-miR-218-5p	PIGH	phosphatidylinositol glycan anchor biosynthesis class H [Source:VGNC Symbol;Acc:VGNC:91423]	1.73	0.083
ssc-miR-218-5p	PIK3C2A	phosphatidylinositol-4-phosphate 3-kinase catalytic subunit type 2 alpha [Source:VGNC Symbol;Acc:VGNC:91436]	1.73	0.083
ssc-miR-218-5p	PIK3R1	phosphoinositide-3-kinase regulatory subunit 1 [Source:VGNC Symbol;Acc:VGNC:91445]	1.73	0.083
ssc-miR-218-5p	PKP4	plakophilin 4 [Source:VGNC Symbol;Acc:VGNC:96488]	1.73	0.083
ssc-miR-218-5p	PLCE1	phospholipase C epsilon 1 [Source:VGNC Symbol;Acc:VGNC:91519]	1.73	0.083
ssc-miR-218-5p	PLCG1	phospholipase C gamma 1 [Source:VGNC Symbol;Acc:VGNC:96492]	1.73	0.083
ssc-miR-218-5p	PLCL2	phospholipase C like 2 [Source:VGNC Symbol;Acc:VGNC:91523]	1.73	0.083
ssc-miR-218-5p	PLCXD3	phosphatidylinositol specific phospholipase C X domain containing 3 [Source:VGNC Symbol;Acc:VGNC:91524]	1.73	0.083
ssc-miR-218-5p	PLD5	phospholipase D family member 5 [Source:VGNC Symbol;Acc:VGNC:96145]	1.73	0.083
ssc-miR-218-5p	PLEKHA3	pleckstrin homology domain containing A3 [Source:NCBI gene (formerly Entrezgene);Acc:100154010]	1.73	0.083
ssc-miR-218-5p	PLEKHG1	pleckstrin homology and RhoGEF domain containing G1 [Source:VGNC Symbol;Acc:VGNC:91542]	1.73	0.083
ssc-miR-218-5p	PLEKHG3	pleckstrin homology and RhoGEF domain containing G3 [Source:VGNC Symbol;Acc:VGNC:91544]	1.73	0.083
ssc-miR-218-5p	PLGRKT	plasminogen receptor with a C-terminal lysine [Source:VGNC Symbol;Acc:VGNC:91556]	1.73	0.083
ssc-miR-218-5p	PLXDC2	plexin domain containing 2 [Source:VGNC Symbol;Acc:VGNC:95958]	1.73	0.083
ssc-miR-218-5p	PLXNA2	plexin A2 [Source:VGNC Symbol;Acc:VGNC:91580]	1.73	0.083
ssc-miR-218-5p	PLXNA4	plexin A4 [Source:VGNC Symbol;Acc:VGNC:98204]	1.73	0.083
ssc-miR-218-5p	PLXNC1	plexin C1 [Source:VGNC Symbol;Acc:VGNC:91584]	1.73	0.083
ssc-miR-218-5p	PLXND1	plexin D1 [Source:VGNC Symbol;Acc:VGNC:91585]	1.73	0.083
ssc-miR-218-5p	POC1B-GALNT4	hypothetical gene	1.73	0.083
ssc-miR-218-5p	POLD3	DNA polymerase delta 3, accessory subunit [Source:VGNC Symbol;Acc:VGNC:99727]	1.73	0.083
ssc-miR-218-5p	POLH	DNA polymerase eta [Source:VGNC Symbol;Acc:VGNC:91635]	1.73	0.083
ssc-miR-218-5p	POLR2C	RNA polymerase II subunit C [Source:VGNC Symbol;Acc:VGNC:91648]	1.73	0.083
ssc-miR-218-5p	POMGNT1	protein O-linked mannose N-acetylglucosaminyltransferase 1 (beta 1,2-) [Source:VGNC Symbol;Acc:VGNC:98536]	1.73	0.083
ssc-miR-218-5p	POTEG	hypothetical gene	1.73	0.083
ssc-miR-218-5p	POTEM	hypothetical gene	1.73	0.083
ssc-miR-218-5p	POU2F1	POU class 2 homeobox 1 [Source:VGNC Symbol;Acc:VGNC:91672]	1.73	0.083
ssc-miR-218-5p	POU3F2	POU class 3 homeobox 2 [Source:HGNC Symbol;Acc:HGNC:9215]	1.73	0.083
ssc-miR-218-5p	POU3F3	POU class 3 homeobox 3 [Source:VGNC Symbol;Acc:VGNC:91676]	1.73	0.083
ssc-miR-218-5p	PPAP2B	hypothetical gene	1.73	0.083
ssc-miR-218-5p	PPARGC1A	PPARG coactivator 1 alpha [Source:VGNC Symbol;Acc:VGNC:91685]	1.73	0.083
ssc-miR-218-5p	PPIG	peptidylprolyl isomerase G [Source:VGNC Symbol;Acc:VGNC:98214]	1.73	0.083
ssc-miR-218-5p	PPME1	protein phosphatase methylesterase 1 [Source:VGNC Symbol;Acc:VGNC:91713]	1.73	0.083
ssc-miR-218-5p	PPP1CB	protein phosphatase 1 catalytic subunit beta [Source:NCBI gene (formerly Entrezgene);Acc:397378]	1.73	0.083
ssc-miR-218-5p	PPP1CC	protein phosphatase 1 catalytic subunit gamma [Source:VGNC Symbol;Acc:VGNC:91716]	1.73	0.083
ssc-miR-218-5p	PPP1R26	protein phosphatase 1 regulatory subunit 26 [Source:VGNC Symbol;Acc:VGNC:91730]	1.73	0.083
ssc-miR-218-5p	PPP2R2A	protein phosphatase 2 regulatory subunit Balpha [Source:VGNC Symbol;Acc:VGNC:91748]	1.73	0.083
ssc-miR-218-5p	PPP2R2C	protein phosphatase 2 regulatory subunit Bgamma [Source:VGNC Symbol;Acc:VGNC:91749]	1.73	0.083
ssc-miR-218-5p	PPP2R4	hypothetical gene	1.73	0.083
ssc-miR-218-5p	PPP2R5A	protein phosphatase 2 regulatory subunit B'alpha [Source:VGNC Symbol;Acc:VGNC:91751]	1.73	0.083
ssc-miR-218-5p	PPP4R1L	hypothetical gene	1.73	0.083
ssc-miR-218-5p	PPP4R2	protein phosphatase 4 regulatory subunit 2 [Source:VGNC Symbol;Acc:VGNC:91756]	1.73	0.083
ssc-miR-218-5p	PPP6C	protein phosphatase 6 catalytic subunit [Source:VGNC Symbol;Acc:VGNC:98219]	1.73	0.083
ssc-miR-218-5p	PQLC3	hypothetical gene	1.73	0.083
ssc-miR-218-5p	PRKAR2B	protein kinase cAMP-dependent type II regulatory subunit beta [Source:VGNC Symbol;Acc:VGNC:91805]	1.73	0.083
ssc-miR-218-5p	PRKCE	protein kinase C epsilon [Source:VGNC Symbol;Acc:VGNC:91807]	1.73	0.083
ssc-miR-218-5p	PRKG1	protein kinase cGMP-dependent 1 [Source:VGNC Symbol;Acc:VGNC:91816]	1.73	0.083
ssc-miR-218-5p	PRKX	protein kinase X-linked [Source:HGNC Symbol;Acc:HGNC:9441]	1.73	0.083
ssc-miR-218-5p	PRLR	prolactin receptor [Source:VGNC Symbol;Acc:VGNC:91819]	1.73	0.083

ssc-miR-218-5p	PROSC	hypothetical gene	1.73	0.083
ssc-miR-218-5p	PROX1	prospero homeobox 1 [Source:VGNC Symbol;Acc:VGNC:91837]	1.73	0.083
ssc-miR-218-5p	PRRG3	proline rich and Gla domain 3 [Source:VGNC Symbol;Acc:VGNC:91869]	1.73	0.083
ssc-miR-218-5p	PSD2	pleckstrin and Sec7 domain containing 2 [Source:VGNC Symbol;Acc:VGNC:91896]	1.73	0.083
ssc-miR-218-5p	PSD3	pleckstrin and Sec7 domain containing 3 [Source:VGNC Symbol;Acc:VGNC:107166]	1.73	0.083
ssc-miR-218-5p	PSMC2	proteasome 26S subunit, ATPase 2 [Source:VGNC Symbol;Acc:VGNC:91913]	1.73	0.083
ssc-miR-218-5p	PSME4	proteasome activator subunit 4 [Source:VGNC Symbol;Acc:VGNC:91928]	1.73	0.083
ssc-miR-218-5p	PTCHD1	patched domain containing 1 [Source:VGNC Symbol;Acc:VGNC:91942]	1.73	0.083
ssc-miR-218-5p	PTP4A1	protein tyrosine phosphatase 4A1 [Source:HGNC Symbol;Acc:HGNC:9634]	1.73	0.083
ssc-miR-218-5p	PTPN11	protein tyrosine phosphatase non-receptor type 11 [Source:VGNC Symbol;Acc:VGNC:91972]	1.73	0.083
ssc-miR-218-5p	PTPRA	protein tyrosine phosphatase receptor type A [Source:VGNC Symbol;Acc:VGNC:96522]	1.73	0.083
ssc-miR-218-5p	PTPRG	protein tyrosine phosphatase receptor type G [Source:VGNC Symbol;Acc:VGNC:91988]	1.73	0.083
ssc-miR-218-5p	PTPRK	protein tyrosine phosphatase receptor type K [Source:VGNC Symbol;Acc:VGNC:91991]	1.73	0.083
ssc-miR-218-5p	PTPRM	protein tyrosine phosphatase receptor type M [Source:VGNC Symbol;Acc:VGNC:91992]	1.73	0.083
ssc-miR-218-5p	PTPRN2	protein tyrosine phosphatase receptor type N2 [Source:VGNC Symbol;Acc:VGNC:91993]	1.73	0.083
ssc-miR-218-5p	PTPRR	protein tyrosine phosphatase receptor type R [Source:HGNC Symbol;Acc:HGNC:9680]	1.73	0.083
ssc-miR-218-5p	PTPRT	protein tyrosine phosphatase receptor type T [Source:VGNC Symbol;Acc:VGNC:96524]	1.73	0.083
ssc-miR-218-5p	PTRH2	peptidyl-tRNA hydrolase 2 [Source:VGNC Symbol;Acc:VGNC:91998]	1.73	0.083
ssc-miR-218-5p	PTS	6-pyruvoyltetrahydropterin synthase [Source:VGNC Symbol;Acc:VGNC:104039]	1.73	0.083
ssc-miR-218-5p	PUM1	pumilio RNA binding family member 1 [Source:VGNC Symbol;Acc:VGNC:92001]	1.73	0.083
ssc-miR-218-5p	PUM2	pumilio RNA binding family member 2 [Source:VGNC Symbol;Acc:VGNC:92002]	1.73	0.083
ssc-miR-218-5p	PURA	purine rich element binding protein A [Source:HGNC Symbol;Acc:HGNC:9701]	1.73	0.083
ssc-miR-218-5p	PURB	purine rich element binding protein B [Source:VGNC Symbol;Acc:VGNC:92004]	1.73	0.083
ssc-miR-218-5p	PVRL1	hypothetical gene	1.73	0.083
ssc-miR-218-5p	PXN	paxillin [Source:HGNC Symbol;Acc:HGNC:9718]	1.73	0.083
ssc-miR-218-5p	QSER1	glutamine and serine rich 1 [Source:VGNC Symbol;Acc:VGNC:92032]	1.73	0.083
ssc-miR-218-5p	RAB1A	RAB1A, member RAS onco family [Source:VGNC Symbol;Acc:VGNC:104042]	1.73	0.083
ssc-miR-218-5p	RAB30	RAB30, member RAS onco family [Source:VGNC Symbol;Acc:VGNC:98254]	1.73	0.083
ssc-miR-218-5p	RAB33B	RAB33B, member RAS onco family [Source:VGNC Symbol;Acc:VGNC:98257]	1.73	0.083
ssc-miR-218-5p	RAB6A	RAB6A, member RAS onco family [Source:VGNC Symbol;Acc:VGNC:108610]	1.73	0.083
ssc-miR-218-5p	RAB6C	hypothetical gene	1.73	0.083
ssc-miR-218-5p	RAB8A	RAB8A, member RAS onco family [Source:VGNC Symbol;Acc:VGNC:98275]	1.73	0.083
ssc-miR-218-5p	RAB8B	RAB8B, member RAS onco family [Source:VGNC Symbol;Acc:VGNC:98276]	1.73	0.083
ssc-miR-218-5p	RABEP1	rabaptin, RAB GTPase binding effector protein 1 [Source:VGNC Symbol;Acc:VGNC:99032]	1.73	0.083
ssc-miR-218-5p	RABGAP1L	RAB GTPase activating protein 1 like [Source:VGNC Symbol;Acc:VGNC:108611]	1.73	0.083
ssc-miR-218-5p	RAD51L3-RFFL	hypothetical gene	1.73	0.083
ssc-miR-218-5p	RAD52	RAD52 homolog, DNA repair protein [Source:VGNC Symbol;Acc:VGNC:92060]	1.73	0.083
ssc-miR-218-5p	RALGAPA2	Ral GTPase activating protein catalytic subunit alpha 2 [Source:VGNC Symbol;Acc:VGNC:95454]	1.73	0.083
ssc-miR-218-5p	RANBP10	RAN binding protein 10 [Source:VGNC Symbol;Acc:VGNC:92077]	1.73	0.083
ssc-miR-218-5p	RAP1GAP	RAP1 GTPase activating protein [Source:VGNC Symbol;Acc:VGNC:92085]	1.73	0.083
ssc-miR-218-5p	RAP1GDS1	Rap1 GTPase-GDP dissociation stimulator 1 [Source:VGNC Symbol;Acc:VGNC:92087]	1.73	0.083
ssc-miR-218-5p	RAPGEF2	Rap guanine nucleotide exchange factor 2 [Source:VGNC Symbol;Acc:VGNC:92092]	1.73	0.083
ssc-miR-218-5p	RAPGEF4	Rap guanine nucleotide exchange factor 4 [Source:VGNC Symbol;Acc:VGNC:95814]	1.73	0.083
ssc-miR-218-5p	RARA	retinoic acid receptor alpha [Source:VGNC Symbol;Acc:VGNC:92096]	1.73	0.083
ssc-miR-218-5p	RASAL2	RAS protein activator like 2 [Source:VGNC Symbol;Acc:VGNC:92105]	1.73	0.083
ssc-miR-218-5p	RASGEF1A	RasGEF domain family member 1A [Source:VGNC Symbol;Acc:VGNC:92108]	1.73	0.083
ssc-miR-218-5p	RASSF2	Ras association domain family member 2 [Source:VGNC Symbol;Acc:VGNC:96531]	1.73	0.083
ssc-miR-218-5p	RASSF5	Ras association domain family member 5 [Source:VGNC Symbol;Acc:VGNC:92125]	1.73	0.083
ssc-miR-218-5p	RBBP7	RB binding protein 7, chromatin remodeling factor [Source:VGNC Symbol;Acc:VGNC:92137]	1.73	0.083
ssc-miR-218-5p	RBM18	RNA binding motif protein 18 [Source:VGNC Symbol;Acc:VGNC:92149]	1.73	0.083

ssc-miR-218-5p	RBM47	RNA binding motif protein 47 [Source:VGNC Symbol;Acc:VGNC:92158]	1.73	0.083
ssc-miR-218-5p	RBPJ	recombination signal binding protein for immunoglobulin kappa J region [Source:VGNC Symbol;Acc:VGNC:98956]	1.73	0.083
ssc-miR-218-5p	RCBTB1	RCC1 and BTB domain containing protein 1 [Source:VGNC Symbol;Acc:VGNC:92173]	1.73	0.083
ssc-miR-218-5p	RCC1	regulator of chromosome condensation 1 [Source:VGNC Symbol;Acc:VGNC:92175]	1.73	0.083
ssc-miR-218-5p	RCOR1	REST corepressor 1 [Source:VGNC Symbol;Acc:VGNC:92183]	1.73	0.083
ssc-miR-218-5p	RELN	reelin [Source:VGNC Symbol;Acc:VGNC:92208]	1.73	0.083
ssc-miR-218-5p	REPS1	RALBP1 associated Eps domain containing 1 [Source:VGNC Symbol;Acc:VGNC:92213]	1.73	0.083
ssc-miR-218-5p	REPS2	RALBP1 associated Eps domain containing 2 [Source:VGNC Symbol;Acc:VGNC:92214]	1.73	0.083
ssc-miR-218-5p	RER1	retention in endoplasmic reticulum sorting receptor 1 [Source:VGNC Symbol;Acc:VGNC:92215]	1.73	0.083
ssc-miR-218-5p	RET	ret proto-onco [Source:VGNC Symbol;Acc:VGNC:92220]	1.73	0.083
ssc-miR-218-5p	RFFL	ring finger and FYVE like domain containing E3 ubiquitin protein ligase [Source:VGNC Symbol;Acc:VGNC:98289]	1.73	0.083
ssc-miR-218-5p	RFX3	regulatory factor X3 [Source:VGNC Symbol;Acc:VGNC:92245]	1.73	0.083
ssc-miR-218-5p	RGS17	regulator of G protein signaling 17 [Source:VGNC Symbol;Acc:VGNC:92263]	1.73	0.083
ssc-miR-218-5p	RGS20	regulator of G protein signaling 20 [Source:VGNC Symbol;Acc:VGNC:92264]	1.73	0.083
ssc-miR-218-5p	RHOBTB1	Rho related BTB domain containing 1 [Source:VGNC Symbol;Acc:VGNC:92285]	1.73	0.083
ssc-miR-218-5p	RHOQ	ras homolog family member Q [Source:VGNC Symbol;Acc:VGNC:92292]	1.73	0.083
ssc-miR-218-5p	RIC3	RIC3 acetylcholine receptor chaperone [Source:VGNC Symbol;Acc:VGNC:92300]	1.73	0.083
ssc-miR-218-5p	RICTOR	RPTOR independent companion of MTOR complex 2 [Source:VGNC Symbol;Acc:VGNC:92303]	1.73	0.083
ssc-miR-218-5p	RIMBP2	RIMS binding protein 2 [Source:VGNC Symbol;Acc:VGNC:92306]	1.73	0.083
ssc-miR-218-5p	RIMS1	regulating synaptic membrane exocytosis 1 [Source:VGNC Symbol;Acc:VGNC:92309]	1.73	0.083
ssc-miR-218-5p	RIMS3	regulating synaptic membrane exocytosis 3 [Source:VGNC Symbol;Acc:VGNC:92310]	1.73	0.083
ssc-miR-218-5p	RIMS4	regulating synaptic membrane exocytosis 4 [Source:VGNC Symbol;Acc:VGNC:95775]	1.73	0.083
ssc-miR-218-5p	RLIM	ring finger protein, LIM domain interacting [Source:HGNC Symbol;Acc:HGNC:13429]	1.73	0.083
ssc-miR-218-5p	RNF103	ring finger protein 103 [Source:VGNC Symbol;Acc:VGNC:98291]	1.73	0.083
ssc-miR-218-5p	RNF114	ring finger protein 114 [Source:VGNC Symbol;Acc:VGNC:108733]	1.73	0.083
ssc-miR-218-5p	RNF139	ring finger protein 139 [Source:HGNC Symbol;Acc:HGNC:17023]	1.73	0.083
ssc-miR-218-5p	RNF149	ring finger protein 149 [Source:VGNC Symbol;Acc:VGNC:92362]	1.73	0.083
ssc-miR-218-5p	RNF152	ring finger protein 152 [Source:VGNC Symbol;Acc:VGNC:92364]	1.73	0.083
ssc-miR-218-5p	RNF165	ring finger protein 165 [Source:VGNC Symbol;Acc:VGNC:92366]	1.73	0.083
ssc-miR-218-5p	RNF19B	ring finger protein 19B [Source:VGNC Symbol;Acc:VGNC:92376]	1.73	0.083
ssc-miR-218-5p	RNF219	hypothetical gene	1.73	0.083
ssc-miR-218-5p	RNF220	ring finger protein 220 [Source:VGNC Symbol;Acc:VGNC:98602]	1.73	0.083
ssc-miR-218-5p	RNF38	ring finger protein 38 [Source:VGNC Symbol;Acc:VGNC:92390]	1.73	0.083
ssc-miR-218-5p	RNF41	ring finger protein 41 [Source:VGNC Symbol;Acc:VGNC:92394]	1.73	0.083
ssc-miR-218-5p	ROBO1	roundabout guidance receptor 1 [Source:HGNC Symbol;Acc:HGNC:10249]	1.73	0.083
ssc-miR-218-5p	ROBO2	roundabout guidance receptor 2 [Source:HGNC Symbol;Acc:HGNC:10250]	1.73	0.083
ssc-miR-218-5p	ROCK1	Rho associated coiled-coil containing protein kinase 1 [Source:VGNC Symbol;Acc:VGNC:98294]	1.73	0.083
ssc-miR-218-5p	RORA	RAR related orphan receptor A [Source:VGNC Symbol;Acc:VGNC:92408]	1.73	0.083
ssc-miR-218-5p	RORB	RAR related orphan receptor B [Source:VGNC Symbol;Acc:VGNC:92409]	1.73	0.083
ssc-miR-218-5p	RP11-302B13.5	hypothetical gene	1.73	0.083
ssc-miR-218-5p	RP11-315D16.2	hypothetical gene	1.73	0.083
ssc-miR-218-5p	RP11-47I22.4	hypothetical gene	1.73	0.083
ssc-miR-218-5p	RP11-770J1.4	hypothetical gene	1.73	0.083
ssc-miR-218-5p	RPA3	replication protein A3 [Source:VGNC Symbol;Acc:VGNC:92415]	1.73	0.083
ssc-miR-218-5p	RPL31	hypothetical gene	1.73	0.083
ssc-miR-218-5p	RPP14	hypothetical gene	1.73	0.083
ssc-miR-218-5p	RPP25	ribonuclease P and MRP subunit p25 [Source:VGNC Symbol;Acc:VGNC:92432]	1.73	0.083
ssc-miR-218-5p	RPS6KA3	ribosomal protein S6 kinase A3 [Source:VGNC Symbol;Acc:VGNC:92442]	1.73	0.083
ssc-miR-218-5p	RPS6KA6	ribosomal protein S6 kinase A6 [Source:VGNC Symbol;Acc:VGNC:92445]	1.73	0.083
ssc-miR-218-5p	RPS6KB1	ribosomal protein S6 kinase B1 [Source:VGNC Symbol;Acc:VGNC:99037]	1.73	0.083

ssc-miR-218-5p	RPS9	ribosomal protein S9 [Source:VGNC Symbol;Acc:VGNC:92449]	1.73	0.083
ssc-miR-218-5p	RRP1B	ribosomal RNA processing 1B [Source:VGNC Symbol;Acc:VGNC:92466]	1.73	0.083
ssc-miR-218-5p	RRP8	ribosomal RNA processing 8 [Source:VGNC Symbol;Acc:VGNC:92467]	1.73	0.083
ssc-miR-218-5p	RSBN1	round spermatid basic protein 1 [Source:VGNC Symbol;Acc:VGNC:92473]	1.73	0.083
ssc-miR-218-5p	RSRC2	arginine and serine rich coiled-coil 2 [Source:VGNC Symbol;Acc:VGNC:92488]	1.73	0.083
ssc-miR-218-5p	RTN3	hypothetical gene	1.73	0.083
ssc-miR-218-5p	RUNX1T1	RUNX1 partner transcriptional co-repressor 1 [Source:VGNC Symbol;Acc:VGNC:96594]	1.73	0.083
ssc-miR-218-5p	RUNX2	RUNX family transcription factor 2 [Source:VGNC Symbol;Acc:VGNC:92517]	1.73	0.083
ssc-miR-218-5p	RUNX3	RUNX family transcription factor 3 [Source:VGNC Symbol;Acc:VGNC:92518]	1.73	0.083
ssc-miR-218-5p	RYBP	RING1 and YY1 binding protein [Source:VGNC Symbol;Acc:VGNC:92532]	1.73	0.083
ssc-miR-218-5p	RYK	receptor like tyrosine kinase [Source:VGNC Symbol;Acc:VGNC:92533]	1.73	0.083
ssc-miR-218-5p	SALL3	spalt like transcription factor 3 [Source:VGNC Symbol;Acc:VGNC:92563]	1.73	0.083
ssc-miR-218-5p	SAMD12	sterile alpha motif domain containing 12 [Source:VGNC Symbol;Acc:VGNC:92565]	1.73	0.083
ssc-miR-218-5p	SAMD8	sterile alpha motif domain containing 8 [Source:VGNC Symbol;Acc:VGNC:92571]	1.73	0.083
ssc-miR-218-5p	SASH1	SAM and SH3 domain containing 1 [Source:VGNC Symbol;Acc:VGNC:92583]	1.73	0.083
ssc-miR-218-5p	SATB2	SATB homeobox 2 [Source:VGNC Symbol;Acc:VGNC:95972]	1.73	0.083
ssc-miR-218-5p	SCAI	suppressor of cancer cell invasion [Source:VGNC Symbol;Acc:VGNC:103170]	1.73	0.083
ssc-miR-218-5p	SCAMP5	secretory carrier membrane protein 5 [Source:VGNC Symbol;Acc:VGNC:92609]	1.73	0.083
ssc-miR-218-5p	SCN1A	sodium voltage-gated channel alpha subunit 1 [Source:VGNC Symbol;Acc:VGNC:95478]	1.73	0.083
ssc-miR-218-5p	SCN2B	sodium voltage-gated channel beta subunit 2 [Source:VGNC Symbol;Acc:VGNC:92633]	1.73	0.083
ssc-miR-218-5p	SCN4B	hypothetical gene	1.73	0.083
ssc-miR-218-5p	SCN8A	sodium voltage-gated channel alpha subunit 8 [Source:VGNC Symbol;Acc:VGNC:92638]	1.73	0.083
ssc-miR-218-5p	SCRT1	scratch family transcriptional repressor 1 [Source:VGNC Symbol;Acc:VGNC:92649]	1.73	0.083
ssc-miR-218-5p	SCRT2	scratch family transcriptional repressor 2 [Source:VGNC Symbol;Acc:VGNC:95721]	1.73	0.083
ssc-miR-218-5p	SDC2	syndecan 2 [Source:VGNC Symbol;Acc:VGNC:92655]	1.73	0.083
ssc-miR-218-5p	SEC14L1	SEC14 like lipid binding 1 [Source:VGNC Symbol;Acc:VGNC:92669]	1.73	0.083
ssc-miR-218-5p	SEC14L2	SEC14 like lipid binding 2 [Source:NCBI gene (formerly Entrezgene);Acc:100152451]	1.73	0.083
ssc-miR-218-5p	SEC22C	SEC22 homolog C, vesicle trafficking protein [Source:VGNC Symbol;Acc:VGNC:92674]	1.73	0.083
ssc-miR-218-5p	SEC61A1	SEC61 translocon subunit alpha 1 [Source:VGNC Symbol;Acc:VGNC:92682]	1.73	0.083
ssc-miR-218-5p	SEMA5A	semaphorin 5A [Source:VGNC Symbol;Acc:VGNC:92706]	1.73	0.083
ssc-miR-218-5p	SEMA6A	semaphorin 6A [Source:VGNC Symbol;Acc:VGNC:92708]	1.73	0.083
ssc-miR-218-5p	SEMA6B	semaphorin 6B [Source:VGNC Symbol;Acc:VGNC:92709]	1.73	0.083
ssc-miR-218-5p	SENP1	SUMO specific peptidase 1 [Source:VGNC Symbol;Acc:VGNC:92712]	1.73	0.083
ssc-miR-218-5p	SENP2	SUMO specific peptidase 2 [Source:VGNC Symbol;Acc:VGNC:92713]	1.73	0.083
ssc-miR-218-5p	SENP5	SUMO specific peptidase 5 [Source:VGNC Symbol;Acc:VGNC:92714]	1.73	0.083
ssc-miR-218-5p	SEPHS1	selenophosphate synthetase 1 [Source:VGNC Symbol;Acc:VGNC:104053]	1.73	0.083
ssc-miR-218-5p	SERBP1	SERPINE1 mRNA binding protein 1 [Source:VGNC Symbol;Acc:VGNC:92727]	1.73	0.083
ssc-miR-218-5p	SERINC5	serine incorporator 5 [Source:VGNC Symbol;Acc:VGNC:92730]	1.73	0.083
ssc-miR-218-5p	SERP1	stress associated endoplasmic reticulum protein 1 [Source:NCBI gene (formerly Entrezgene);Acc:100156392]	1.73	0.083
ssc-miR-218-5p	SERPINI1	serpin family I member 1 [Source:VGNC Symbol;Acc:VGNC:92747]	1.73	0.083
ssc-miR-218-5p	SERTAD2	SERTA domain containing 2 [Source:VGNC Symbol;Acc:VGNC:92750]	1.73	0.083
ssc-miR-218-5p	SERTAD4	SERTA domain containing 4 [Source:VGNC Symbol;Acc:VGNC:92752]	1.73	0.083
ssc-miR-218-5p	SESN3	sestrin 3 [Source:VGNC Symbol;Acc:VGNC:92755]	1.73	0.083
ssc-miR-218-5p	SETBP1	SET binding protein 1 [Source:VGNC Symbol;Acc:VGNC:92756]	1.73	0.083
ssc-miR-218-5p	SETD7	SET domain containing 7, histone lysine methyltransferase [Source:VGNC Symbol;Acc:VGNC:92762]	1.73	0.083
ssc-miR-218-5p	SFMBT1	Scm like with four mbt domains 1 [Source:VGNC Symbol;Acc:VGNC:92773]	1.73	0.083
ssc-miR-218-5p	SFMBT2	Scm like with four mbt domains 2 [Source:VGNC Symbol;Acc:VGNC:95935]	1.73	0.083
ssc-miR-218-5p	SFXN5	sideroflexin 5 [Source:VGNC Symbol;Acc:VGNC:92787]	1.73	0.083
ssc-miR-218-5p	SGCD	sarcoglycan delta [Source:NCBI gene (formerly Entrezgene);Acc:100240724]	1.73	0.083
ssc-miR-218-5p	SGCZ	sarcoglycan zeta [Source:VGNC Symbol;Acc:VGNC:104055]	1.73	0.083

ssc-miR-218-5p	SGMS2	sphingomyelin synthase 2 [Source:VGNC Symbol;Acc:VGNC:92795]	1.73	0.083
ssc-miR-218-5p	SGSM1	small G protein signaling modulator 1 [Source:VGNC Symbol;Acc:VGNC:92800]	1.73	0.083
ssc-miR-218-5p	SH3BP4	SH3 domain binding protein 4 [Source:VGNC Symbol;Acc:VGNC:95495]	1.73	0.083
ssc-miR-218-5p	SH3D19	SH3 domain containing 19 [Source:VGNC Symbol;Acc:VGNC:92821]	1.73	0.083
ssc-miR-218-5p	SH3GL1	SH3 domain containing GRB2 like 1, endophilin A2 [Source:VGNC Symbol;Acc:VGNC:92823]	1.73	0.083
ssc-miR-218-5p	SH3KBP1	SH3 domain containing kinase binding protein 1 [Source:VGNC Symbol;Acc:VGNC:92828]	1.73	0.083
ssc-miR-218-5p	SH3RF1	SH3 domain containing ring finger 1 [Source:VGNC Symbol;Acc:VGNC:98316]	1.73	0.083
ssc-miR-218-5p	SHANK2	SH3 and multiple ankyrin repeat domains 2 [Source:VGNC Symbol;Acc:VGNC:92835]	1.73	0.083
ssc-miR-218-5p	SHC1	SHC adaptor protein 1 [Source:VGNC Symbol;Acc:VGNC:92837]	1.73	0.083
ssc-miR-218-5p	SHC3	SHC adaptor protein 3 [Source:VGNC Symbol;Acc:VGNC:92838]	1.73	0.083
ssc-miR-218-5p	SHC4	SHC adaptor protein 4 [Source:HGNC Symbol;Acc:HGNC:16743]	1.73	0.083
ssc-miR-218-5p	SHISA6	hypothetical gene	1.73	0.083
ssc-miR-218-5p	SHISA7	shisa family member 7 [Source:VGNC Symbol;Acc:VGNC:92848]	1.73	0.083
ssc-miR-218-5p	SHISA9	shisa family member 9 [Source:HGNC Symbol;Acc:HGNC:37231]	1.73	0.083
ssc-miR-218-5p	SHMT1	serine hydroxymethyltransferase 1 [Source:VGNC Symbol;Acc:VGNC:92855]	1.73	0.083
ssc-miR-218-5p	SHOC2	SHOC2 leucine rich repeat scaffold protein [Source:VGNC Symbol;Acc:VGNC:98318]	1.73	0.083
ssc-miR-218-5p	SIAE	sialic acid acetylesterase [Source:VGNC Symbol;Acc:VGNC:92864]	1.73	0.083
ssc-miR-218-5p	SIK2	salt inducible kinase 2 [Source:VGNC Symbol;Acc:VGNC:92872]	1.73	0.083
ssc-miR-218-5p	SIN3A	SIN3 transcription regulator family member A [Source:VGNC Symbol;Acc:VGNC:92879]	1.73	0.083
ssc-miR-218-5p	SKAP1	src kinase associated phosphoprotein 1 [Source:VGNC Symbol;Acc:VGNC:92900]	1.73	0.083
ssc-miR-218-5p	SKI	SKI proto-onco [Source:VGNC Symbol;Acc:VGNC:92902]	1.73	0.083
ssc-miR-218-5p	SKIL	SKI like proto-onco [Source:VGNC Symbol;Acc:VGNC:92903]	1.73	0.083
ssc-miR-218-5p	SLAIN1	SLAIN motif family member 1 [Source:VGNC Symbol;Acc:VGNC:92909]	1.73	0.083
ssc-miR-218-5p	SLAIN2	SLAIN motif family member 2 [Source:VGNC Symbol;Acc:VGNC:92910]	1.73	0.083
ssc-miR-218-5p	SLC12A2	solute carrier family 12 member 2 [Source:VGNC Symbol;Acc:VGNC:92922]	1.73	0.083
ssc-miR-218-5p	SLC16A10	solute carrier family 16 member 10 [Source:VGNC Symbol;Acc:VGNC:103178]	1.73	0.083
ssc-miR-218-5p	SLC16A14	solute carrier family 16 member 14 [Source:VGNC Symbol;Acc:VGNC:96129]	1.73	0.083
ssc-miR-218-5p	SLC17A6	solute carrier family 17 member 6 [Source:VGNC Symbol;Acc:VGNC:92954]	1.73	0.083
ssc-miR-218-5p	SLC18A2	solute carrier family 18 member A2 [Source:VGNC Symbol;Acc:VGNC:92958]	1.73	0.083
ssc-miR-218-5p	SLC22A23	solute carrier family 22 member 23 [Source:HGNC Symbol;Acc:HGNC:21106]	1.73	0.083
ssc-miR-218-5p	SLC24A4	solute carrier family 24 member 4 [Source:VGNC Symbol;Acc:VGNC:92988]	1.73	0.083
ssc-miR-218-5p	SLC25A16	solute carrier family 25 member 16 [Source:VGNC Symbol;Acc:VGNC:92995]	1.73	0.083
ssc-miR-218-5p	SLC35B2	solute carrier family 35 member B2 [Source:VGNC Symbol;Acc:VGNC:93072]	1.73	0.083
ssc-miR-218-5p	SLC35F1	solute carrier family 35 member F1 [Source:VGNC Symbol;Acc:VGNC:93081]	1.73	0.083
ssc-miR-218-5p	SLC38A1	solute carrier family 38 member 1 [Source:HGNC Symbol;Acc:HGNC:13447]	1.73	0.083
ssc-miR-218-5p	SLC38A9	solute carrier family 38 member 9 [Source:VGNC Symbol;Acc:VGNC:93100]	1.73	0.083
ssc-miR-218-5p	SLC39A1	solute carrier family 39 member 1 [Source:VGNC Symbol;Acc:VGNC:93101]	1.73	0.083
ssc-miR-218-5p	SLC39A14	solute carrier family 39 member 14 [Source:HGNC Symbol;Acc:HGNC:20858]	1.73	0.083
ssc-miR-218-5p	SLC45A3	solute carrier family 45 member 3 [Source:VGNC Symbol;Acc:VGNC:93124]	1.73	0.083
ssc-miR-218-5p	SLC5A3	solute carrier family 5 member 3 [Source:VGNC Symbol;Acc:VGNC:93144]	1.73	0.083
ssc-miR-218-5p	SLC6A1	solute carrier family 6 member 1 [Source:VGNC Symbol;Acc:VGNC:93153]	1.73	0.083
ssc-miR-218-5p	SLC6A17	solute carrier family 6 member 17 [Source:VGNC Symbol;Acc:VGNC:93158]	1.73	0.083
ssc-miR-218-5p	SLC6A6	solute carrier family 6 member 6 [Source:VGNC Symbol;Acc:VGNC:93166]	1.73	0.083
ssc-miR-218-5p	SLCO5A1	solute carrier organic anion transporter family member 5A1 [Source:HGNC Symbol;Acc:HGNC:19046]	1.73	0.083
ssc-miR-218-5p	SLIT2	slit guidance ligand 2 [Source:VGNC Symbol;Acc:VGNC:93204]	1.73	0.083
ssc-miR-218-5p	SLITRK6	SLIT and NTRK like family member 6 [Source:VGNC Symbol;Acc:VGNC:93211]	1.73	0.083
ssc-miR-218-5p	SLK	STE20 like kinase [Source:VGNC Symbol;Acc:VGNC:98328]	1.73	0.083
ssc-miR-218-5p	SMCHD1	structural maintenance of chromosomes flexible hinge domain containing 1 [Source:VGNC Symbol;Acc:VGNC:93242]	1.73	0.083
ssc-miR-218-5p	SMEK2	hypothetical gene	1.73	0.083
ssc-miR-218-5p	SMG7	SMG7 nonsense mediated mRNA decay factor [Source:VGNC Symbol;Acc:VGNC:93250]	1.73	0.083

ssc-miR-218-5p	SMIM17	small integral membrane protein 17 [Source:VGNC Symbol;Acc:VGNC:98615]	1.73	0.083
ssc-miR-218-5p	SMPD3	sphingomyelin phosphodiesterase 3 [Source:VGNC Symbol;Acc:VGNC:93263]	1.73	0.083
ssc-miR-218-5p	SMUG1	single-strand-selective monofunctional uracil-DNA glycosylase 1 [Source:VGNC Symbol;Acc:VGNC:93269]	1.73	0.083
ssc-miR-218-5p	SMYD1	SET and MYND domain containing 1 [Source:VGNC Symbol;Acc:VGNC:93272]	1.73	0.083
ssc-miR-218-5p	SNAPC5	small nuclear RNA activating complex polypeptide 5 [Source:VGNC Symbol;Acc:VGNC:93283]	1.73	0.083
ssc-miR-218-5p	SNCB	synuclein beta [Source:VGNC Symbol;Acc:VGNC:93286]	1.73	0.083
ssc-miR-218-5p	SNTB2	syntrophin beta 2 [Source:VGNC Symbol;Acc:VGNC:93298]	1.73	0.083
ssc-miR-218-5p	SNTG1	syntrophin gamma 1 [Source:VGNC Symbol;Acc:VGNC:93299]	1.73	0.083
ssc-miR-218-5p	SNX12	sorting nexin 12 [Source:VGNC Symbol;Acc:VGNC:93305]	1.73	0.083
ssc-miR-218-5p	SNX13	sorting nexin 13 [Source:VGNC Symbol;Acc:VGNC:93306]	1.73	0.083
ssc-miR-218-5p	SNX18	sorting nexin 18 [Source:VGNC Symbol;Acc:VGNC:93310]	1.73	0.083
ssc-miR-218-5p	SNX4	sorting nexin 4 [Source:VGNC Symbol;Acc:VGNC:93322]	1.73	0.083
ssc-miR-218-5p	SOCS3	suppressor of cytokine signaling 3 [Source:VGNC Symbol;Acc:VGNC:99052]	1.73	0.083
ssc-miR-218-5p	SOCS4	suppressor of cytokine signaling 4 [Source:VGNC Symbol;Acc:VGNC:93331]	1.73	0.083
ssc-miR-218-5p	SOCS5	suppressor of cytokine signaling 5 [Source:VGNC Symbol;Acc:VGNC:93332]	1.73	0.083
ssc-miR-218-5p	SOCS6	suppressor of cytokine signaling 6 [Source:VGNC Symbol;Acc:VGNC:93333]	1.73	0.083
ssc-miR-218-5p	SOCS7	suppressor of cytokine signaling 7 [Source:VGNC Symbol;Acc:VGNC:93334]	1.73	0.083
ssc-miR-218-5p	SORBS1	sorbin and SH3 domain containing 1 [Source:VGNC Symbol;Acc:VGNC:93337]	1.73	0.083
ssc-miR-218-5p	SORCS1	sortilin related VPS10 domain containing receptor 1 [Source:VGNC Symbol;Acc:VGNC:93339]	1.73	0.083
ssc-miR-218-5p	SOST	sclerostin [Source:VGNC Symbol;Acc:VGNC:93345]	1.73	0.083
ssc-miR-218-5p	SOX11	SRY-box transcription factor 11 [Source:VGNC Symbol;Acc:VGNC:93351]	1.73	0.083
ssc-miR-218-5p	SOX5	SRY-box transcription factor 5 [Source:VGNC Symbol;Acc:VGNC:93357]	1.73	0.083
ssc-miR-218-5p	SOX6	SRY-box transcription factor 6 [Source:VGNC Symbol;Acc:VGNC:93358]	1.73	0.083
ssc-miR-218-5p	SP1	Sp1 transcription factor [Source:VGNC Symbol;Acc:VGNC:93360]	1.73	0.083
ssc-miR-218-5p	SPAG9	sperm associated antigen 9 [Source:VGNC Symbol;Acc:VGNC:93374]	1.73	0.083
ssc-miR-218-5p	SPAST	spastin [Source:VGNC Symbol;Acc:VGNC:98333]	1.73	0.083
ssc-miR-218-5p	SPATS2L	spermatosis associated serine rich 2 like [Source:VGNC Symbol;Acc:VGNC:96047]	1.73	0.083
ssc-miR-218-5p	SPECC1L	sperm antigen with calponin homology and coiled-coil domains 1 like [Source:HGNC Symbol;Acc:HGNC:29022]	1.73	0.083
ssc-miR-218-5p	SPHKAP	SPHK1 interactor, AKAP domain containing [Source:VGNC Symbol;Acc:VGNC:96245]	1.73	0.083
ssc-miR-218-5p	SPI1	Spi-1 proto-onco [Source:VGNC Symbol;Acc:VGNC:98335]	1.73	0.083
ssc-miR-218-5p	SPOCK3	SPARC (osteonectin), cwcv and kazal like domains proteoglycan 3 [Source:HGNC Symbol;Acc:HGNC:13565]	1.73	0.083
ssc-miR-218-5p	SPRED1	sprouty related EVH1 domain containing 1 [Source:VGNC Symbol;Acc:VGNC:93420]	1.73	0.083
ssc-miR-218-5p	SPRED2	sprouty related EVH1 domain containing 2 [Source:VGNC Symbol;Acc:VGNC:93421]	1.73	0.083
ssc-miR-218-5p	SPSB1	splA/ryanodine receptor domain and SOCS box containing 1 [Source:VGNC Symbol;Acc:VGNC:93430]	1.73	0.083
ssc-miR-218-5p	SRCIN1	SRC kinase signaling inhibitor 1 [Source:VGNC Symbol;Acc:VGNC:93448]	1.73	0.083
ssc-miR-218-5p	SREK1	splicing regulatory glutamic acid and lysine rich protein 1 [Source:VGNC Symbol;Acc:VGNC:93451]	1.73	0.083
ssc-miR-218-5p	SREK1IP1	SREK1 interacting protein 1 [Source:HGNC Symbol;Acc:HGNC:26716]	1.73	0.083
ssc-miR-218-5p	SRGAP2	hypothetical gene	1.73	0.083
ssc-miR-218-5p	SRP72	signal recognition particle 72 [Source:VGNC Symbol;Acc:VGNC:93461]	1.73	0.083
ssc-miR-218-5p	SRPX2	sushi repeat containing protein X-linked 2 [Source:HGNC Symbol;Acc:HGNC:30668]	1.73	0.083
ssc-miR-218-5p	SRSF10	serine and arginine rich splicing factor 10 [Source:VGNC Symbol;Acc:VGNC:93472]	1.73	0.083
ssc-miR-218-5p	SSH1	slingshot protein phosphatase 1 [Source:VGNC Symbol;Acc:VGNC:93485]	1.73	0.083
ssc-miR-218-5p	SSR1	signal sequence receptor subunit 1 [Source:VGNC Symbol;Acc:VGNC:93490]	1.73	0.083
ssc-miR-218-5p	SSR3	signal sequence receptor subunit 3 [Source:VGNC Symbol;Acc:VGNC:93492]	1.73	0.083
ssc-miR-218-5p	ST18	ST18 C2H2C-type zinc finger transcription factor [Source:VGNC Symbol;Acc:VGNC:93501]	1.73	0.083
ssc-miR-218-5p	ST8SIA1	ST8 alpha-N-acetyl-neuraminide alpha-2,8-sialyltransferase 1 [Source:VGNC Symbol;Acc:VGNC:93516]	1.73	0.083
ssc-miR-218-5p	ST8SIA3	ST8 alpha-N-acetyl-neuraminide alpha-2,8-sialyltransferase 3 [Source:VGNC Symbol;Acc:VGNC:93518]	1.73	0.083
ssc-miR-218-5p	ST8SIA4	ST8 alpha-N-acetyl-neuraminide alpha-2,8-sialyltransferase 4 [Source:VGNC Symbol;Acc:VGNC:93519]	1.73	0.083
ssc-miR-218-5p	ST8SIA5	ST8 alpha-N-acetyl-neuraminide alpha-2,8-sialyltransferase 5 [Source:VGNC Symbol;Acc:VGNC:93520]	1.73	0.083
ssc-miR-218-5p	STAM2	signal transducing adaptor molecule 2 [Source:VGNC Symbol;Acc:VGNC:95521]	1.73	0.083

ssc-miR-218-5p	STEAP3	STEAP3 metalloreductase [Source:VGNC Symbol;Acc:VGNC:96048]	1.73	0.083
ssc-miR-218-5p	STK16	serine/threonine kinase 16 [Source:VGNC Symbol;Acc:VGNC:95526]	1.73	0.083
ssc-miR-218-5p	STK38	serine/threonine kinase 38 [Source:VGNC Symbol;Acc:VGNC:93556]	1.73	0.083
ssc-miR-218-5p	STX1B	syntaxin 1B [Source:VGNC Symbol;Acc:VGNC:98352]	1.73	0.083
ssc-miR-218-5p	STXBP1	syntaxin binding protein 1 [Source:VGNC Symbol;Acc:VGNC:93596]	1.73	0.083
ssc-miR-218-5p	STXBP4	syntaxin binding protein 4 [Source:VGNC Symbol;Acc:VGNC:93598]	1.73	0.083
ssc-miR-218-5p	STXBP5	syntaxin binding protein 5 [Source:VGNC Symbol;Acc:VGNC:93599]	1.73	0.083
ssc-miR-218-5p	STYX	serine/threonine/tyrosine interacting protein [Source:VGNC Symbol;Acc:VGNC:93602]	1.73	0.083
ssc-miR-218-5p	SUPT3H	SPT3 homolog, SAGA and STAGA complex component [Source:VGNC Symbol;Acc:VGNC:93623]	1.73	0.083
ssc-miR-218-5p	SV2A	synaptic vesicle glycoprotein 2A [Source:VGNC Symbol;Acc:VGNC:93639]	1.73	0.083
ssc-miR-218-5p	SV2B	synaptic vesicle glycoprotein 2B [Source:VGNC Symbol;Acc:VGNC:93640]	1.73	0.083
ssc-miR-218-5p	SYCE3	synaptonemal complex central element protein 3 [Source:VGNC Symbol;Acc:VGNC:93652]	1.73	0.083
ssc-miR-218-5p	SYNE1	hypothetical gene	1.73	0.083
ssc-miR-218-5p	SYNGR1	synaptogyrin 1 [Source:VGNC Symbol;Acc:VGNC:93665]	1.73	0.083
ssc-miR-218-5p	SYS1	SYS1 golgi trafficking protein [Source:VGNC Symbol;Acc:VGNC:98354]	1.73	0.083
ssc-miR-218-5p	SYT1	synaptotagmin 1 [Source:VGNC Symbol;Acc:VGNC:93678]	1.73	0.083
ssc-miR-218-5p	SYT13	synaptotagmin 13 [Source:VGNC Symbol;Acc:VGNC:93682]	1.73	0.083
ssc-miR-218-5p	TAC1	tachykinin 1 [Source:VGNC Symbol;Acc:VGNC:93697]	1.73	0.083
ssc-miR-218-5p	TACC1	hypothetical gene	1.73	0.083
ssc-miR-218-5p	TAF11	TATA-box binding protein associated factor 11 [Source:NCBI gene (formerly Entrezgene);Acc:100151814]	1.73	0.083
ssc-miR-218-5p	TANC2	tetratricopeptide repeat, ankyrin repeat and coiled-coil containing 2 [Source:VGNC Symbol;Acc:VGNC:93733]	1.73	0.083
ssc-miR-218-5p	TAOK3	TAO kinase 3 [Source:VGNC Symbol;Acc:VGNC:98357]	1.73	0.083
ssc-miR-218-5p	TBC1D12	TBC1 domain family member 12 [Source:VGNC Symbol;Acc:VGNC:93762]	1.73	0.083
ssc-miR-218-5p	TBC1D8B	TBC1 domain family member 8B [Source:VGNC Symbol;Acc:VGNC:93780]	1.73	0.083
ssc-miR-218-5p	TBC1D9B	TBC1 domain family member 9B [Source:VGNC Symbol;Acc:VGNC:93782]	1.73	0.083
ssc-miR-218-5p	TBX15	hypothetical gene	1.73	0.083
ssc-miR-218-5p	TBX20	T-box transcription factor 20 [Source:VGNC Symbol;Acc:VGNC:93799]	1.73	0.083
ssc-miR-218-5p	TCF12	transcription factor 12 [Source:VGNC Symbol;Acc:VGNC:93817]	1.73	0.083
ssc-miR-218-5p	TCF19	transcription factor 19 [Source:VGNC Symbol;Acc:VGNC:93818]	1.73	0.083
ssc-miR-218-5p	TCF20	transcription factor 20 [Source:HGNC Symbol;Acc:HGNC:11631]	1.73	0.083
ssc-miR-218-5p	TCF4	transcription factor 4 [Source:VGNC Symbol;Acc:VGNC:93823]	1.73	0.083
ssc-miR-218-5p	TDP1	tyrosyl-DNA phosphodiesterase 1 [Source:VGNC Symbol;Acc:VGNC:93844]	1.73	0.083
ssc-miR-218-5p	TEAD1	TEA domain transcription factor 1 [Source:VGNC Symbol;Acc:VGNC:93853]	1.73	0.083
ssc-miR-218-5p	TENM3	teneurin transmembrane protein 3 [Source:VGNC Symbol;Acc:VGNC:96233]	1.73	0.083
ssc-miR-218-5p	TFAP2E	transcription factor AP-2 epsilon [Source:VGNC Symbol;Acc:VGNC:93911]	1.73	0.083
ssc-miR-218-5p	TGIF2	TGFB induced factor homeobox 2 [Source:VGNC Symbol;Acc:VGNC:95656]	1.73	0.083
ssc-miR-218-5p	THOC2	THO complex 2 [Source:VGNC Symbol;Acc:VGNC:93957]	1.73	0.083
ssc-miR-218-5p	THRB	thyroid hormone receptor beta [Source:VGNC Symbol;Acc:VGNC:93965]	1.73	0.083
ssc-miR-218-5p	THSD7A	thrombospondin type 1 domain containing 7A [Source:VGNC Symbol;Acc:VGNC:93968]	1.73	0.083
ssc-miR-218-5p	THTPA	thiamine triphosphatase [Source:VGNC Symbol;Acc:VGNC:93969]	1.73	0.083
ssc-miR-218-5p	TLK2	tousled like kinase 2 [Source:VGNC Symbol;Acc:VGNC:94017]	1.73	0.083
ssc-miR-218-5p	TLL1	tolloid like 1 [Source:VGNC Symbol;Acc:VGNC:94018]	1.73	0.083
ssc-miR-218-5p	TMED7-TICAM2	hypothetical gene	1.73	0.083
ssc-miR-218-5p	TMED8	transmembrane p24 trafficking protein family member 8 [Source:VGNC Symbol;Acc:VGNC:94058]	1.73	0.083
ssc-miR-218-5p	TMEM123	hypothetical gene	1.73	0.083
ssc-miR-218-5p	TMEM127	transmembrane protein 127 [Source:HGNC Symbol;Acc:HGNC:26038]	1.73	0.083
ssc-miR-218-5p	TMEM132B	transmembrane protein 132B [Source:HGNC Symbol;Acc:HGNC:29397]	1.73	0.083
ssc-miR-218-5p	TMEM150C	transmembrane protein 150C [Source:VGNC Symbol;Acc:VGNC:94096]	1.73	0.083
ssc-miR-218-5p	TMEM151A	transmembrane protein 151A [Source:VGNC Symbol;Acc:VGNC:94097]	1.73	0.083
ssc-miR-218-5p	TMEM156	transmembrane protein 156 [Source:VGNC Symbol;Acc:VGNC:94099]	1.73	0.083

ssc-miR-218-5p	TMEM163	transmembrane protein 163 [Source:VGNC Symbol;Acc:VGNC:96078]	1.73	0.083
ssc-miR-218-5p	TMEM178A	transmembrane protein 178A [Source:VGNC Symbol;Acc:VGNC:94114]	1.73	0.083
ssc-miR-218-5p	TMEM184A	transmembrane protein 184A [Source:VGNC Symbol;Acc:VGNC:94119]	1.73	0.083
ssc-miR-218-5p	TMEM185B	transmembrane protein 185B [Source:VGNC Symbol;Acc:VGNC:95900]	1.73	0.083
ssc-miR-218-5p	TMEM229B	hypothetical gene	1.73	0.083
ssc-miR-218-5p	TMEM231	transmembrane protein 231 [Source:VGNC Symbol;Acc:VGNC:98377]	1.73	0.083
ssc-miR-218-5p	TMEM25	transmembrane protein 25 [Source:VGNC Symbol;Acc:VGNC:94157]	1.73	0.083
ssc-miR-218-5p	TMEM33	transmembrane protein 33 [Source:VGNC Symbol;Acc:VGNC:94171]	1.73	0.083
ssc-miR-218-5p	TMEM68	transmembrane protein 68 [Source:VGNC Symbol;Acc:VGNC:94202]	1.73	0.083
ssc-miR-218-5p	TMTC2	transmembrane O-mannosyltransferase targeting cadherins 2 [Source:VGNC Symbol;Acc:VGNC:94242]	1.73	0.083
ssc-miR-218-5p	TMUB2	transmembrane and ubiquitin like domain containing 2 [Source:VGNC Symbol;Acc:VGNC:94246]	1.73	0.083
ssc-miR-218-5p	TMX1	thioredoxin related transmembrane protein 1 [Source:VGNC Symbol;Acc:VGNC:94247]	1.73	0.083
ssc-miR-218-5p	TNC	tenascin C [Source:VGNC Symbol;Acc:VGNC:103192]	1.73	0.083
ssc-miR-218-5p	TNFAIP1	TNF alpha induced protein 1 [Source:VGNC Symbol;Acc:VGNC:94250]	1.73	0.083
ssc-miR-218-5p	TNIK	TRAF2 and NCK interacting kinase [Source:VGNC Symbol;Acc:VGNC:98381]	1.73	0.083
ssc-miR-218-5p	TOB1	transducer of ERBB2, 1 [Source:NCBI gene (formerly Entrezgene);Acc:100144440]	1.73	0.083
ssc-miR-218-5p	TOX3	TOX high mobility group box family member 3 [Source:VGNC Symbol;Acc:VGNC:94323]	1.73	0.083
ssc-miR-218-5p	TP53INP1	tumor protein p53 inducible nuclear protein 1 [Source:VGNC Symbol;Acc:VGNC:94329]	1.73	0.083
ssc-miR-218-5p	TP53INP2	tumor protein p53 inducible nuclear protein 2 [Source:VGNC Symbol;Acc:VGNC:95737]	1.73	0.083
ssc-miR-218-5p	TP73	tumor protein p73 [Source:VGNC Symbol;Acc:VGNC:94331]	1.73	0.083
ssc-miR-218-5p	TPBGL	hypothetical gene	1.73	0.083
ssc-miR-218-5p	TPCN1	two pore segment channel 1 [Source:VGNC Symbol;Acc:VGNC:94333]	1.73	0.083
ssc-miR-218-5p	TPD52	tumor protein D52 [Source:VGNC Symbol;Acc:VGNC:98383]	1.73	0.083
ssc-miR-218-5p	TPM3	tropomyosin 3 [Source:VGNC Symbol;Acc:VGNC:98877]	1.73	0.083
ssc-miR-218-5p	TPRG1	tumor protein p63 regulated 1 [Source:VGNC Symbol;Acc:VGNC:94348]	1.73	0.083
ssc-miR-218-5p	TRHDE	thyrotropin releasing hormone degrading enzyme [Source:VGNC Symbol;Acc:VGNC:94390]	1.73	0.083
ssc-miR-218-5p	TRIM71	tripartite motif containing 71 [Source:VGNC Symbol;Acc:VGNC:94431]	1.73	0.083
ssc-miR-218-5p	TRIM9	tripartite motif containing 9 [Source:VGNC Symbol;Acc:VGNC:94434]	1.73	0.083
ssc-miR-218-5p	TRIO	trio Rho guanine nucleotide exchange factor [Source:VGNC Symbol;Acc:VGNC:94435]	1.73	0.083
ssc-miR-218-5p	TRIP12	thyroid hormone receptor interactor 12 [Source:VGNC Symbol;Acc:VGNC:95555]	1.73	0.083
ssc-miR-218-5p	TRPC3	transient receptor potential cation channel subfamily C member 3 [Source:VGNC Symbol;Acc:VGNC:94461]	1.73	0.083
ssc-miR-218-5p	TRPM3	transient receptor potential cation channel subfamily M member 3 [Source:VGNC Symbol;Acc:VGNC:98384]	1.73	0.083
ssc-miR-218-5p	TSHZ2	teashirt zinc finger homeobox 2 [Source:NCBI gene (formerly Entrezgene);Acc:100136903]	1.73	0.083
ssc-miR-218-5p	TSPAN3	tetraspanin 3 [Source:VGNC Symbol;Acc:VGNC:94510]	1.73	0.083
ssc-miR-218-5p	TSPAN5	tetraspanin 5 [Source:VGNC Symbol;Acc:VGNC:94514]	1.73	0.083
ssc-miR-218-5p	TSPAN9	tetraspanin 9 [Source:VGNC Symbol;Acc:VGNC:94516]	1.73	0.083
ssc-miR-218-5p	TSR1	TSR1 ribosome maturation factor [Source:VGNC Symbol;Acc:VGNC:94521]	1.73	0.083
ssc-miR-218-5p	TTC39C	tetratricopeptide repeat domain 39C [Source:VGNC Symbol;Acc:VGNC:94553]	1.73	0.083
ssc-miR-218-5p	TTYH3	tweety family member 3 [Source:VGNC Symbol;Acc:VGNC:94576]	1.73	0.083
ssc-miR-218-5p	TUB	TUB bipartite transcription factor [Source:VGNC Symbol;Acc:VGNC:94577]	1.73	0.083
ssc-miR-218-5p	TUSC5	hypothetical gene	1.73	0.083
ssc-miR-218-5p	TXLNG	taxilin gamma [Source:HGNC Symbol;Acc:HGNC:18578]	1.73	0.083
ssc-miR-218-5p	TXNDC5	thioredoxin domain containing 5 [Source:VGNC Symbol;Acc:VGNC:94608]	1.73	0.083
ssc-miR-218-5p	UBASH3A	ubiquitin associated and SH3 domain containing A [Source:VGNC Symbol;Acc:VGNC:94636]	1.73	0.083
ssc-miR-218-5p	UBE2E2	ubiquitin conjugating enzyme E2 E2 [Source:NCBI gene (formerly Entrezgene);Acc:780417]	1.73	0.083
ssc-miR-218-5p	UBE2G1	ubiquitin conjugating enzyme E2 G1 [Source:HGNC Symbol;Acc:HGNC:12482]	1.73	0.083
ssc-miR-218-5p	UBE2H	ubiquitin conjugating enzyme E2 H [Source:VGNC Symbol;Acc:VGNC:94643]	1.73	0.083
ssc-miR-218-5p	UBE2J1	ubiquitin conjugating enzyme E2 J1 [Source:VGNC Symbol;Acc:VGNC:94644]	1.73	0.083
ssc-miR-218-5p	UBE2V2	ubiquitin conjugating enzyme E2 V2 [Source:VGNC Symbol;Acc:VGNC:98394]	1.73	0.083
ssc-miR-218-5p	UBE3A	ubiquitin protein ligase E3A [Source:VGNC Symbol;Acc:VGNC:94652]	1.73	0.083

ssc-miR-218-5p	UBN2	ubinuclein 2 [Source:VGNC Symbol;Acc:VGNC:94664]	1.73	0.083
ssc-miR-218-5p	UBQLN2	ubiquilin 2 [Source:VGNC Symbol;Acc:VGNC:94665]	1.73	0.083
ssc-miR-218-5p	UBR3	ubiquitin protein ligase E3 component n-recognin 3 [Source:VGNC Symbol;Acc:VGNC:96771]	1.73	0.083
ssc-miR-218-5p	UBTD1	ubiquitin domain containing 1 [Source:VGNC Symbol;Acc:VGNC:94672]	1.73	0.083
ssc-miR-218-5p	UGT8	UDP glycosyltransferase 8 [Source:VGNC Symbol;Acc:VGNC:98401]	1.73	0.083
ssc-miR-218-5p	UHRF1BP1L	UHRF1 binding protein 1 like [Source:VGNC Symbol;Acc:VGNC:94691]	1.73	0.083
ssc-miR-218-5p	UNC13A	hypothetical gene	1.73	0.083
ssc-miR-218-5p	UQCR10	ubiquinol-cytochrome c reductase, complex III subunit X [Source:VGNC Symbol;Acc:VGNC:94727]	1.73	0.083
ssc-miR-218-5p	USP31	ubiquitin specific peptidase 31 [Source:HGNC Symbol;Acc:HGNC:20060]	1.73	0.083
ssc-miR-218-5p	USP32	hypothetical gene	1.73	0.083
ssc-miR-218-5p	USP34	ubiquitin specific peptidase 34 [Source:VGNC Symbol;Acc:VGNC:94759]	1.73	0.083
ssc-miR-218-5p	USP45	ubiquitin specific peptidase 45 [Source:VGNC Symbol;Acc:VGNC:94767]	1.73	0.083
ssc-miR-218-5p	USP46	ubiquitin specific peptidase 46 [Source:VGNC Symbol;Acc:VGNC:94768]	1.73	0.083
ssc-miR-218-5p	USP49	ubiquitin specific peptidase 49 [Source:VGNC Symbol;Acc:VGNC:94771]	1.73	0.083
ssc-miR-218-5p	USP6	hypothetical gene	1.73	0.083
ssc-miR-218-5p	USP9X	ubiquitin specific peptidase 9 X-linked [Source:HGNC Symbol;Acc:HGNC:12632]	1.73	0.083
ssc-miR-218-5p	VAMP7	vesicle associated membrane protein 7 [Source:VGNC Symbol;Acc:VGNC:94796]	1.73	0.083
ssc-miR-218-5p	VASH2	vasohibin 2 [Source:VGNC Symbol;Acc:VGNC:94802]	1.73	0.083
ssc-miR-218-5p	VAT1	vesicle amine transport 1 [Source:VGNC Symbol;Acc:VGNC:94805]	1.73	0.083
ssc-miR-218-5p	VAV3	hypothetical gene	1.73	0.083
ssc-miR-218-5p	VOPP1	VOPP1 WW domain binding protein [Source:VGNC Symbol;Acc:VGNC:94835]	1.73	0.083
ssc-miR-218-5p	VPS13B	vacuolar protein sorting 13 homolog B [Source:VGNC Symbol;Acc:VGNC:94839]	1.73	0.083
ssc-miR-218-5p	VPS37A	VPS37A subunit of ESCRT-I [Source:VGNC Symbol;Acc:VGNC:104092]	1.73	0.083
ssc-miR-218-5p	VSIG10	V-set and immunoglobulin domain containing 10 [Source:HGNC Symbol;Acc:HGNC:26078]	1.73	0.083
ssc-miR-218-5p	VSIG8	V-set and immunoglobulin domain containing 8 [Source:VGNC Symbol;Acc:VGNC:98894]	1.73	0.083
ssc-miR-218-5p	VWC2	von Willebrand factor C domain containing 2 [Source:VGNC Symbol;Acc:VGNC:94887]	1.73	0.083
ssc-miR-218-5p	WASF3	WASP family member 3 [Source:VGNC Symbol;Acc:VGNC:94894]	1.73	0.083
ssc-miR-218-5p	WASL	WASP like actin nucleation promoting factor [Source:VGNC Symbol;Acc:VGNC:96604]	1.73	0.083
ssc-miR-218-5p	WBP1L	WW domain binding protein 1 like [Source:VGNC Symbol;Acc:VGNC:94898]	1.73	0.083
ssc-miR-218-5p	WDR26	WD repeat domain 26 [Source:VGNC Symbol;Acc:VGNC:95960]	1.73	0.083
ssc-miR-218-5p	WDR44	WD repeat domain 44 [Source:VGNC Symbol;Acc:VGNC:94921]	1.73	0.083
ssc-miR-218-5p	WDR47	WD repeat domain 47 [Source:VGNC Symbol;Acc:VGNC:94924]	1.73	0.083
ssc-miR-218-5p	WDR48	WD repeat domain 48 [Source:VGNC Symbol;Acc:VGNC:94925]	1.73	0.083
ssc-miR-218-5p	WDR55	WD repeat domain 55 [Source:VGNC Symbol;Acc:VGNC:94929]	1.73	0.083
ssc-miR-218-5p	WIPF1	hypothetical gene	1.73	0.083
ssc-miR-218-5p	WIPF2	WAS/WASL interacting protein family member 2 [Source:VGNC Symbol;Acc:VGNC:99106]	1.73	0.083
ssc-miR-218-5p	WIPI2	WD repeat domain, phosphoinositide interacting 2 [Source:VGNC Symbol;Acc:VGNC:94960]	1.73	0.083
ssc-miR-218-5p	WNT2B	Wnt family member 2B [Source:VGNC Symbol;Acc:VGNC:94969]	1.73	0.083
ssc-miR-218-5p	XKR4	XK related 4 [Source:VGNC Symbol;Acc:VGNC:98900]	1.73	0.083
ssc-miR-218-5p	XKR7	XK related 7 [Source:VGNC Symbol;Acc:VGNC:95987]	1.73	0.083
ssc-miR-218-5p	XPO5	exportin 5 [Source:VGNC Symbol;Acc:VGNC:95005]	1.73	0.083
ssc-miR-218-5p	XRCC5	X-ray repair cross complementing 5 [Source:VGNC Symbol;Acc:VGNC:95570]	1.73	0.083
ssc-miR-218-5p	YEATS4	YEATS domain containing 4 [Source:HGNC Symbol;Acc:HGNC:24859]	1.73	0.083
ssc-miR-218-5p	YIPF6	Yip1 domain family member 6 [Source:VGNC Symbol;Acc:VGNC:95032]	1.73	0.083
ssc-miR-218-5p	YPEL2	yippee like 2 [Source:VGNC Symbol;Acc:VGNC:95036]	1.73	0.083
ssc-miR-218-5p	YWHAZ	tyrosine 3-monooxygenase/tryptophan 5-monooxygenase activation protein zeta [Source:VGNC Symbol;Acc:VGNC:95047]	1.73	0.083
ssc-miR-218-5p	YY1	YY1 transcription factor [Source:HGNC Symbol;Acc:HGNC:12856]	1.73	0.083
ssc-miR-218-5p	ZBTB10	zinc finger and BTB domain containing 10 [Source:VGNC Symbol;Acc:VGNC:95058]	1.73	0.083
ssc-miR-218-5p	ZBTB11	zinc finger and BTB domain containing 11 [Source:VGNC Symbol;Acc:VGNC:95059]	1.73	0.083
ssc-miR-218-5p	ZBTB16	zinc finger and BTB domain containing 16 [Source:VGNC Symbol;Acc:VGNC:108626]	1.73	0.083

ssc-miR-218-5p	ZBTB20	zinc finger and BTB domain containing 20 [Source:VGNC Symbol;Acc:VGNC:95063]	1.73	0.083
ssc-miR-218-5p	ZBTB34	zinc finger and BTB domain containing 34 [Source:VGNC Symbol;Acc:VGNC:95070]	1.73	0.083
ssc-miR-218-5p	ZBTB39	zinc finger and BTB domain containing 39 [Source:VGNC Symbol;Acc:VGNC:95073]	1.73	0.083
ssc-miR-218-5p	ZBTB41	zinc finger and BTB domain containing 41 [Source:VGNC Symbol;Acc:VGNC:108284]	1.73	0.083
ssc-miR-218-5p	ZC3H12B	zinc finger CCCH-type containing 12B [Source:VGNC Symbol;Acc:VGNC:95090]	1.73	0.083
ssc-miR-218-5p	ZC4H2	zinc finger C4H2-type containing [Source:VGNC Symbol;Acc:VGNC:95105]	1.73	0.083
ssc-miR-218-5p	ZDHHC18	zinc finger DHHC-type palmitoyltransferase 18 [Source:VGNC Symbol;Acc:VGNC:95120]	1.73	0.083
ssc-miR-218-5p	ZDHHC2	zinc finger DHHC-type palmitoyltransferase 2 [Source:VGNC Symbol;Acc:VGNC:95886]	1.73	0.083
ssc-miR-218-5p	ZDHHC23	zinc finger DHHC-type palmitoyltransferase 23 [Source:VGNC Symbol;Acc:VGNC:95125]	1.73	0.083
ssc-miR-218-5p	ZDHHC8	zinc finger DHHC-type palmitoyltransferase 8 [Source:VGNC Symbol;Acc:VGNC:95130]	1.73	0.083
ssc-miR-218-5p	ZEB2	hypothetical gene	1.73	0.083
ssc-miR-218-5p	ZFAND4	zinc finger AN1-type containing 4 [Source:VGNC Symbol;Acc:VGNC:95136]	1.73	0.083
ssc-miR-218-5p	ZFHX2	zinc finger homeobox 2 [Source:VGNC Symbol;Acc:VGNC:95140]	1.73	0.083
ssc-miR-218-5p	ZFP91	ZFP91 zinc finger protein, atypical E3 ubiquitin ligase [Source:HGNC Symbol;Acc:HGNC:14983]	1.73	0.083
ssc-miR-218-5p	ZFX	hypothetical gene	1.73	0.083
ssc-miR-218-5p	ZFY	zinc finger protein Y-linked [Source:HGNC Symbol;Acc:HGNC:12870]	1.73	0.083
ssc-miR-218-5p	ZFYVE26	zinc finger FYVE-type containing 26 [Source:VGNC Symbol;Acc:VGNC:95160]	1.73	0.083
ssc-miR-218-5p	ZFYVE28	zinc finger FYVE-type containing 28 [Source:VGNC Symbol;Acc:VGNC:95162]	1.73	0.083
ssc-miR-218-5p	ZIC5	Zic family member 5 [Source:VGNC Symbol;Acc:VGNC:95170]	1.73	0.083
ssc-miR-218-5p	ZKSCAN1	zinc finger with KRAB and SCAN domains 1 [Source:VGNC Symbol;Acc:VGNC:95171]	1.73	0.083
ssc-miR-218-5p	ZMAT4	zinc finger matrin-type 4 [Source:VGNC Symbol;Acc:VGNC:96109]	1.73	0.083
ssc-miR-218-5p	ZMIZ1	zinc finger MIZ-type containing 1 [Source:VGNC Symbol;Acc:VGNC:95178]	1.73	0.083
ssc-miR-218-5p	ZMIZ2	zinc finger MIZ-type containing 2 [Source:VGNC Symbol;Acc:VGNC:95179]	1.73	0.083
ssc-miR-218-5p	ZNF202	zinc finger protein 202 [Source:HGNC Symbol;Acc:HGNC:12994]	1.73	0.083
ssc-miR-218-5p	ZNF236	zinc finger protein 236 [Source:VGNC Symbol;Acc:VGNC:95209]	1.73	0.083
ssc-miR-218-5p	ZNF280C	zinc finger protein 280C [Source:VGNC Symbol;Acc:VGNC:95216]	1.73	0.083
ssc-miR-218-5p	ZNF385A	zinc finger protein 385A [Source:VGNC Symbol;Acc:VGNC:95233]	1.73	0.083
ssc-miR-218-5p	ZNF462	zinc finger protein 462 [Source:VGNC Symbol;Acc:VGNC:95244]	1.73	0.083
ssc-miR-218-5p	ZNF518B	zinc finger protein 518B [Source:VGNC Symbol;Acc:VGNC:95258]	1.73	0.083
ssc-miR-218-5p	ZNF583	zinc finger protein 583 [Source:VGNC Symbol;Acc:VGNC:98731]	1.73	0.083
ssc-miR-218-5p	ZNF609	zinc finger protein 609 [Source:VGNC Symbol;Acc:VGNC:95274]	1.73	0.083
ssc-miR-218-5p	ZNF638	zinc finger protein 638 [Source:VGNC Symbol;Acc:VGNC:95281]	1.73	0.083
ssc-miR-218-5p	ZNF654	zinc finger protein 654 [Source:VGNC Symbol;Acc:VGNC:95285]	1.73	0.083
ssc-miR-218-5p	ZNF697	zinc finger protein 697 [Source:HGNC Symbol;Acc:HGNC:32034]	1.73	0.083
ssc-miR-218-5p	ZNF831	zinc finger protein 831 [Source:VGNC Symbol;Acc:VGNC:95765]	1.73	0.083
ssc-miR-218-5p	ZNFX1	zinc finger NFX1-type containing 1 [Source:VGNC Symbol;Acc:VGNC:96210]	1.73	0.083
ssc-miR-218-5p	ZRANB1	zinc finger RANBP2-type containing 1 [Source:VGNC Symbol;Acc:VGNC:95322]	1.73	0.083
ssc-miR-218-5p	ZWILCH	zwilch kinetochore protein [Source:VGNC Symbol;Acc:VGNC:95341]	1.73	0.083
ssc-miR-218-5p	ZXDA	hypothetical gene	1.73	0.083
ssc-miR-374b-5p	ABAT	4-aminobutyrate aminotransferase [Source:VGNC Symbol;Acc:VGNC:96910]	1.71	0.0364
ssc-miR-374b-5p	ABCA8	ATP binding cassette subfamily A member 8 [Source:HGNC Symbol;Acc:HGNC:38]	1.71	0.0364
ssc-miR-374b-5p	ABCB11	ATP binding cassette subfamily B member 11 [Source:VGNC Symbol;Acc:VGNC:97853]	1.71	0.0364
ssc-miR-374b-5p	ABCE1	ATP binding cassette subfamily E member 1 [Source:VGNC Symbol;Acc:VGNC:84965]	1.71	0.0364
ssc-miR-374b-5p	ABCG2	ATP-binding cassette, sub-family G (WHITE), member 2 [Source:NCBI gene (formerly Entrezgene);Acc:397073]	1.71	0.0364
ssc-miR-374b-5p	ABR	ABR activator of RhoGEF and GTPase [Source:VGNC Symbol;Acc:VGNC:84989]	1.71	0.0364
ssc-miR-374b-5p	AC006946.15	hypothetical gene	1.71	0.0364
ssc-miR-374b-5p	ACSL6	acyl-CoA synthetase long chain family member 6 [Source:VGNC Symbol;Acc:VGNC:99581]	1.71	0.0364
ssc-miR-374b-5p	ACTN1	actinin alpha 1 [Source:NCBI gene (formerly Entrezgene);Acc:100513412]	1.71	0.0364
ssc-miR-374b-5p	ACTN4	actinin alpha 4 [Source:VGNC Symbol;Acc:VGNC:85047]	1.71	0.0364
ssc-miR-374b-5p	ACVR2B	activin A receptor type 2B [Source:VGNC Symbol;Acc:VGNC:108629]	1.71	0.0364

ssc-miR-374b-5p	ADAM10	ADAM metallopeptidase domain 10 [Source:VGNC Symbol;Acc:VGNC:85061]	1.71	0.0364
ssc-miR-374b-5p	ADAM12	ADAM metallopeptidase domain 12 [Source:VGNC Symbol;Acc:VGNC:85063]	1.71	0.0364
ssc-miR-374b-5p	ADAMTS5	ADAM metallopeptidase with thrombospondin type 1 motif 5 [Source:VGNC Symbol;Acc:VGNC:85085]	1.71	0.0364
ssc-miR-374b-5p	ADARB2	adenosine deaminase RNA specific B2 (inactive) [Source:HGNC Symbol;Acc:HGNC:227]	1.71	0.0364
ssc-miR-374b-5p	ADNP	activity dependent neuroprotector homeobox [Source:VGNC Symbol;Acc:VGNC:95657]	1.71	0.0364
ssc-miR-374b-5p	ADORA2B	adenosine A2b receptor [Source:VGNC Symbol;Acc:VGNC:85149]	1.71	0.0364
ssc-miR-374b-5p	ADRB1	adrenoceptor beta 1 [Source:VGNC Symbol;Acc:VGNC:107363]	1.71	0.0364
ssc-miR-374b-5p	AFF1	AF4/FMR2 family member 1 [Source:VGNC Symbol;Acc:VGNC:85167]	1.71	0.0364
ssc-miR-374b-5p	AFF3	AF4/FMR2 family member 3 [Source:HGNC Symbol;Acc:HGNC:6473]	1.71	0.0364
ssc-miR-374b-5p	AFF4	AF4/FMR2 family member 4 [Source:VGNC Symbol;Acc:VGNC:85169]	1.71	0.0364
ssc-miR-374b-5p	AGFG2	ArfGAP with FG repeats 2 [Source:VGNC Symbol;Acc:VGNC:85179]	1.71	0.0364
ssc-miR-374b-5p	AHCY	adenosylhomocysteinase [Source:VGNC Symbol;Acc:VGNC:108708]	1.71	0.0364
ssc-miR-374b-5p	AHDC1	hypothetical gene	1.71	0.0364
ssc-miR-374b-5p	AHSA2	hypothetical gene	1.71	0.0364
ssc-miR-374b-5p	AK4	hypothetical gene	1.71	0.0364
ssc-miR-374b-5p	AKAP6	A-kinase anchoring protein 6 [Source:VGNC Symbol;Acc:VGNC:85221]	1.71	0.0364
ssc-miR-374b-5p	AKT1	AKT serine/threonine kinase 1 [Source:VGNC Symbol;Acc:VGNC:96930]	1.71	0.0364
ssc-miR-374b-5p	AKT3	AKT serine/threonine kinase 3 [Source:VGNC Symbol;Acc:VGNC:96306]	1.71	0.0364
ssc-miR-374b-5p	AMD1	hypothetical gene	1.71	0.0364
ssc-miR-374b-5p	AMN	amnion associated transmembrane protein [Source:VGNC Symbol;Acc:VGNC:85282]	1.71	0.0364
ssc-miR-374b-5p	ANAPC16	anaphase promoting complex subunit 16 [Source:NCBI gene (formerly Entrezgene);Acc:100155019]	1.71	0.0364
ssc-miR-374b-5p	ANK3	hypothetical gene	1.71	0.0364
ssc-miR-374b-5p	ANKRD12	ankyrin repeat domain 12 [Source:VGNC Symbol;Acc:VGNC:96906]	1.71	0.0364
ssc-miR-374b-5p	ANKRD13C	ankyrin repeat domain 13C [Source:VGNC Symbol;Acc:VGNC:85326]	1.71	0.0364
ssc-miR-374b-5p	ANKRD50	ankyrin repeat domain containing 50 [Source:VGNC Symbol;Acc:VGNC:85341]	1.71	0.0364
ssc-miR-374b-5p	ANLN	anillin actin binding protein [Source:VGNC Symbol;Acc:VGNC:85353]	1.71	0.0364
ssc-miR-374b-5p	ANTXR2	ANTXR cell adhesion molecule 2 [Source:VGNC Symbol;Acc:VGNC:85365]	1.71	0.0364
ssc-miR-374b-5p	AP005482.1	hypothetical gene	1.71	0.0364
ssc-miR-374b-5p	APC	APC regulator of WNT signaling pathway [Source:VGNC Symbol;Acc:VGNC:99584]	1.71	0.0364
ssc-miR-374b-5p	APOPT1	hypothetical gene	1.71	0.0364
ssc-miR-374b-5p	ARCN1	archain 1 [Source:VGNC Symbol;Acc:VGNC:85443]	1.71	0.0364
ssc-miR-374b-5p	ARHGAP20	Rho GTPase activating protein 20 [Source:VGNC Symbol;Acc:VGNC:97884]	1.71	0.0364
ssc-miR-374b-5p	ARHGAP23	Rho GTPase activating protein 23 [Source:VGNC Symbol;Acc:VGNC:85462]	1.71	0.0364
ssc-miR-374b-5p	ARHGAP5	Rho GTPase activating protein 5 [Source:VGNC Symbol;Acc:VGNC:85480]	1.71	0.0364
ssc-miR-374b-5p	ARHGEF40	Rho guanine nucleotide exchange factor 40 [Source:VGNC Symbol;Acc:VGNC:85501]	1.71	0.0364
ssc-miR-374b-5p	ARID2	AT-rich interaction domain 2 [Source:HGNC Symbol;Acc:HGNC:18037]	1.71	0.0364
ssc-miR-374b-5p	ARIH2	ariadne RBR E3 ubiquitin protein ligase 2 [Source:VGNC Symbol;Acc:VGNC:85512]	1.71	0.0364
ssc-miR-374b-5p	ARL15	ADP ribosylation factor like GTPase 15 [Source:VGNC Symbol;Acc:VGNC:85520]	1.71	0.0364
ssc-miR-374b-5p	ARL2BP	ADP ribosylation factor like GTPase 2 binding protein [Source:VGNC Symbol;Acc:VGNC:85521]	1.71	0.0364
ssc-miR-374b-5p	ARL5A	ADP ribosylation factor like GTPase 5A [Source:VGNC Symbol;Acc:VGNC:96418]	1.71	0.0364
ssc-miR-374b-5p	ARMCX5	armadillo repeat containing X-linked 5 [Source:HGNC Symbol;Acc:HGNC:25772]	1.71	0.0364
ssc-miR-374b-5p	ASB4	ankyrin repeat and SOCS box containing 4 [Source:VGNC Symbol;Acc:VGNC:97891]	1.71	0.0364
ssc-miR-374b-5p	ASCL1	achaete-scute family bHLH transcription factor 1 [Source:HGNC Symbol;Acc:HGNC:738]	1.71	0.0364
ssc-miR-374b-5p	ASPN	asporin [Source:VGNC Symbol;Acc:VGNC:85586]	1.71	0.0364
ssc-miR-374b-5p	ATAD1	ATPase family AAA domain containing 1 [Source:HGNC Symbol;Acc:HGNC:25903]	1.71	0.0364
ssc-miR-374b-5p	ATAD2	ATPase family AAA domain containing 2 [Source:VGNC Symbol;Acc:VGNC:85599]	1.71	0.0364
ssc-miR-374b-5p	ATE1	arginyltransferase 1 [Source:VGNC Symbol;Acc:VGNC:85604]	1.71	0.0364
ssc-miR-374b-5p	ATF2	activating transcription factor 2 [Source:VGNC Symbol;Acc:VGNC:96446]	1.71	0.0364
ssc-miR-374b-5p	ATMIN	hypothetical gene	1.71	0.0364
ssc-miR-374b-5p	ATP1A1	ATPase Na+/K+ transporting subunit alpha 1 [Source:VGNC Symbol;Acc:VGNC:98735]	1.71	0.0364

ssc-miR-374b-5p	ATP1B4	ATPase Na+/K+ transporting family member beta 4 [Source:VGNC Symbol;Acc:VGNC:85644]	1.71	0.0364
ssc-miR-374b-5p	ATP2B2	ATPase plasma membrane Ca2+ transporting 2 [Source:VGNC Symbol;Acc:VGNC:85649]	1.71	0.0364
ssc-miR-374b-5p	ATP8B2	ATPase phospholipid transporting 8B2 [Source:VGNC Symbol;Acc:VGNC:85680]	1.71	0.0364
ssc-miR-374b-5p	ATXN1	ataxin 1 [Source:VGNC Symbol;Acc:VGNC:85687]	1.71	0.0364
ssc-miR-374b-5p	ATXN7	ataxin 7 [Source:VGNC Symbol;Acc:VGNC:99704]	1.71	0.0364
ssc-miR-374b-5p	BACE1	beta-secretase 1 [Source:VGNC Symbol;Acc:VGNC:85740]	1.71	0.0364
ssc-miR-374b-5p	BARHL2	BarH like homeobox 2 [Source:VGNC Symbol;Acc:VGNC:85756]	1.71	0.0364
ssc-miR-374b-5p	BCAT1	branched chain amino acid transaminase 1 [Source:VGNC Symbol;Acc:VGNC:85773]	1.71	0.0364
ssc-miR-374b-5p	BCL10	BCL10 immune signaling adaptor [Source:VGNC Symbol;Acc:VGNC:96561]	1.71	0.0364
ssc-miR-374b-5p	BCL11B	BAF chromatin remodeling complex subunit BCL11B [Source:VGNC Symbol;Acc:VGNC:96563]	1.71	0.0364
ssc-miR-374b-5p	BEND4	BEN domain containing 4 [Source:VGNC Symbol;Acc:VGNC:85801]	1.71	0.0364
ssc-miR-374b-5p	BHLHE40	basic helix-loop-helix family member e40 [Source:VGNC Symbol;Acc:VGNC:85814]	1.71	0.0364
ssc-miR-374b-5p	BICC1	BicC family RNA binding protein 1 [Source:VGNC Symbol;Acc:VGNC:85817]	1.71	0.0364
ssc-miR-374b-5p	BIRC3	baculoviral IAP repeat containing 3 [Source:HGNC Symbol;Acc:HGNC:591]	1.71	0.0364
ssc-miR-374b-5p	BLOC1S6	biosis of lysosomal organelles complex 1 subunit 6 [Source:VGNC Symbol;Acc:VGNC:85832]	1.71	0.0364
ssc-miR-374b-5p	BMP2	bone morphotic protein 2 [Source:VGNC Symbol;Acc:VGNC:95488]	1.71	0.0364
ssc-miR-374b-5p	BMP3	bone morphotic protein 3 [Source:VGNC Symbol;Acc:VGNC:85842]	1.71	0.0364
ssc-miR-374b-5p	BMPER	BMP binding endothelial regulator [Source:VGNC Symbol;Acc:VGNC:97902]	1.71	0.0364
ssc-miR-374b-5p	BOLA3	hypothetical gene	1.71	0.0364
ssc-miR-374b-5p	BRINP3	BMP/retinoic acid inducible neural specific 3 [Source:VGNC Symbol;Acc:VGNC:95983]	1.71	0.0364
ssc-miR-374b-5p	BSDC1	BSD domain containing 1 [Source:VGNC Symbol;Acc:VGNC:85892]	1.71	0.0364
ssc-miR-374b-5p	BTBD7	BTB domain containing 7 [Source:VGNC Symbol;Acc:VGNC:85908]	1.71	0.0364
ssc-miR-374b-5p	BTBD9	BTB domain containing 9 [Source:VGNC Symbol;Acc:VGNC:85909]	1.71	0.0364
ssc-miR-374b-5p	BZRAP1	hypothetical gene	1.71	0.0364
ssc-miR-374b-5p	BZW1	basic leucine zipper and W2 domains 1 [Source:VGNC Symbol;Acc:VGNC:95885]	1.71	0.0364
ssc-miR-374b-5p	C10orf12	hypothetical gene	1.71	0.0364
ssc-miR-374b-5p	C11orf58	chromosome 2 C11orf58 homolog [Source:VGNC Symbol;Acc:VGNC:85998]	1.71	0.0364
ssc-miR-374b-5p	C11orf87	chromosome 11 open reading frame 87 [Source:HGNC Symbol;Acc:HGNC:33788]	1.71	0.0364
ssc-miR-374b-5p	C16orf72	chromosome 3 C16orf72 homolog [Source:VGNC Symbol;Acc:VGNC:86016]	1.71	0.0364
ssc-miR-374b-5p	C19orf81	chromosome 6 C19orf81 homolog [Source:VGNC Symbol;Acc:VGNC:86058]	1.71	0.0364
ssc-miR-374b-5p	C1orf95	hypothetical gene	1.71	0.0364
ssc-miR-374b-5p	C1QL3	complement C1q like 3 [Source:VGNC Symbol;Acc:VGNC:95910]	1.71	0.0364
ssc-miR-374b-5p	C20orf194	hypothetical gene	1.71	0.0364
ssc-miR-374b-5p	C3orf58	hypothetical gene	1.71	0.0364
ssc-miR-374b-5p	C3orf70	chromosome 13 C3orf70 homolog [Source:VGNC Symbol;Acc:VGNC:85937]	1.71	0.0364
ssc-miR-374b-5p	C5orf28	hypothetical gene	1.71	0.0364
ssc-miR-374b-5p	C5orf47	chromosome 16 C5orf47 homolog [Source:VGNC Symbol;Acc:VGNC:85952]	1.71	0.0364
ssc-miR-374b-5p	C5orf51	hypothetical gene	1.71	0.0364
ssc-miR-374b-5p	C8orf44-SGK3	hypothetical gene	1.71	0.0364
ssc-miR-374b-5p	CA12	carbonic anhydrase 12 [Source:VGNC Symbol;Acc:VGNC:86097]	1.71	0.0364
ssc-miR-374b-5p	CADM1	cell adhesion molecule 1 [Source:VGNC Symbol;Acc:VGNC:86134]	1.71	0.0364
ssc-miR-374b-5p	CADM2	cell adhesion molecule 2 [Source:VGNC Symbol;Acc:VGNC:97910]	1.71	0.0364
ssc-miR-374b-5p	CALCOCO1	calcium binding and coiled-coil domain 1 [Source:VGNC Symbol;Acc:VGNC:86140]	1.71	0.0364
ssc-miR-374b-5p	CALD1	caldesmon 1 [Source:VGNC Symbol;Acc:VGNC:86143]	1.71	0.0364
ssc-miR-374b-5p	CAMK4	calcium/calmodulin dependent protein kinase IV [Source:VGNC Symbol;Acc:VGNC:99602]	1.71	0.0364
ssc-miR-374b-5p	CAMSAP2	calmodulin regulated spectrin associated protein family member 2 [Source:VGNC Symbol;Acc:VGNC:96205]	1.71	0.0364
ssc-miR-374b-5p	CAND1	cullin associated and neddylation dissociated 1 [Source:VGNC Symbol;Acc:VGNC:97912]	1.71	0.0364
ssc-miR-374b-5p	CAPZA2	capping actin protein of muscle Z-line subunit alpha 2 [Source:VGNC Symbol;Acc:VGNC:103910]	1.71	0.0364
ssc-miR-374b-5p	CCDC148	hypothetical gene	1.71	0.0364
ssc-miR-374b-5p	CCDC89	coiled-coil domain containing 89 [Source:HGNC Symbol;Acc:HGNC:26762]	1.71	0.0364

ssc-miR-374b-5p	CCL2	chemokine (C-C motif) ligand 2 [Source:NCBI gene (formerly Entrezgene);Acc:397422]	1.71	0.0364
ssc-miR-374b-5p	CCL22	C-C motif chemokine ligand 22 [Source:NCBI gene (formerly Entrezgene);Acc:100516016]	1.71	0.0364
ssc-miR-374b-5p	CCL8	chemokine (C-C motif) ligand 8 [Source:NCBI gene (formerly Entrezgene);Acc:100302703]	1.71	0.0364
ssc-miR-374b-5p	CCNE2	cyclin E2 [Source:VGNC Symbol;Acc:VGNC:86355]	1.71	0.0364
ssc-miR-374b-5p	CCNG2	cyclin G2 [Source:VGNC Symbol;Acc:VGNC:86358]	1.71	0.0364
ssc-miR-374b-5p	CCNL1	cyclin L1 [Source:VGNC Symbol;Acc:VGNC:86363]	1.71	0.0364
ssc-miR-374b-5p	CD40LG	CD40 ligand [Source:VGNC Symbol;Acc:VGNC:86418]	1.71	0.0364
ssc-miR-374b-5p	CD47	CD47 molecule [Source:VGNC Symbol;Acc:VGNC:108637]	1.71	0.0364
ssc-miR-374b-5p	CDA	cytidine deaminase [Source:VGNC Symbol;Acc:VGNC:86439]	1.71	0.0364
ssc-miR-374b-5p	CDC42BPG	CDC42 binding protein kinase gamma [Source:VGNC Symbol;Acc:VGNC:86454]	1.71	0.0364
ssc-miR-374b-5p	CDC42EP3	CDC42 effector protein 3 [Source:VGNC Symbol;Acc:VGNC:86457]	1.71	0.0364
ssc-miR-374b-5p	CDC73	cell division cycle 73 [Source:VGNC Symbol;Acc:VGNC:95817]	1.71	0.0364
ssc-miR-374b-5p	CDK19	cyclin dependent kinase 19 [Source:VGNC Symbol;Acc:VGNC:86502]	1.71	0.0364
ssc-miR-374b-5p	CDYL2	chromodomain Y like 2 [Source:VGNC Symbol;Acc:VGNC:86529]	1.71	0.0364
ssc-miR-374b-5p	CEBPA	CCAAT enhancer binding protein alpha [Source:VGNC Symbol;Acc:VGNC:86531]	1.71	0.0364
ssc-miR-374b-5p	CEBPB	CCAAT enhancer binding protein beta [Source:VGNC Symbol;Acc:VGNC:95883]	1.71	0.0364
ssc-miR-374b-5p	CELF2	hypothetical gene	1.71	0.0364
ssc-miR-374b-5p	CEP350	centrosomal protein 350 [Source:HGNC Symbol;Acc:HGNC:24238]	1.71	0.0364
ssc-miR-374b-5p	CERS3	ceramide synthase 3 [Source:VGNC Symbol;Acc:VGNC:86588]	1.71	0.0364
ssc-miR-374b-5p	CFL2	cofilin 2 [Source:VGNC Symbol;Acc:VGNC:86611]	1.71	0.0364
ssc-miR-374b-5p	CHD7	chromodomain helicase DNA binding protein 7 [Source:VGNC Symbol;Acc:VGNC:86633]	1.71	0.0364
ssc-miR-374b-5p	CHRM2	cholinergic receptor muscarinic 2 [Source:VGNC Symbol;Acc:VGNC:103922]	1.71	0.0364
ssc-miR-374b-5p	CHST2	carbohydrate sulfotransferase 2 [Source:VGNC Symbol;Acc:VGNC:86678]	1.71	0.0364
ssc-miR-374b-5p	CLDN14	claudin 14 [Source:VGNC Symbol;Acc:VGNC:86731]	1.71	0.0364
ssc-miR-374b-5p	CLIP4	CAP-Gly domain containing linker protein family member 4 [Source:VGNC Symbol;Acc:VGNC:97932]	1.71	0.0364
ssc-miR-374b-5p	CLSTN3	calsyntenin 3 [Source:VGNC Symbol;Acc:VGNC:86786]	1.71	0.0364
ssc-miR-374b-5p	CNIH1	cornichon family AMPA receptor auxiliary protein 1 [Source:VGNC Symbol;Acc:VGNC:86820]	1.71	0.0364
ssc-miR-374b-5p	CNOT2	CCR4-NOT transcription complex subunit 2 [Source:VGNC Symbol;Acc:VGNC:86834]	1.71	0.0364
ssc-miR-374b-5p	CNOT6	CCR4-NOT transcription complex subunit 6 [Source:VGNC Symbol;Acc:VGNC:86837]	1.71	0.0364
ssc-miR-374b-5p	CNOT8	CCR4-NOT transcription complex subunit 8 [Source:VGNC Symbol;Acc:VGNC:86839]	1.71	0.0364
ssc-miR-374b-5p	CNST	consortin, connexin sorting protein [Source:VGNC Symbol;Acc:VGNC:96131]	1.71	0.0364
ssc-miR-374b-5p	CNTN4	contactin 4 [Source:VGNC Symbol;Acc:VGNC:97934]	1.71	0.0364
ssc-miR-374b-5p	COA6	hypothetical gene	1.71	0.0364
ssc-miR-374b-5p	COG3	component of oligomeric golgi complex 3 [Source:HGNC Symbol;Acc:HGNC:18619]	1.71	0.0364
ssc-miR-374b-5p	COPS2	COP9 signalosome subunit 2 [Source:VGNC Symbol;Acc:VGNC:86899]	1.71	0.0364
ssc-miR-374b-5p	CORO1C	coronin 1C [Source:VGNC Symbol;Acc:VGNC:86914]	1.71	0.0364
ssc-miR-374b-5p	CPEB4	cytoplasmic polyadenylation element binding protein 4 [Source:VGNC Symbol;Acc:VGNC:86939]	1.71	0.0364
ssc-miR-374b-5p	CRAMP1L	hypothetical gene	1.71	0.0364
ssc-miR-374b-5p	CREB1	cAMP responsive element binding protein 1 [Source:VGNC Symbol;Acc:VGNC:96004]	1.71	0.0364
ssc-miR-374b-5p	CREB3L2	cAMP responsive element binding protein 3 like 2 [Source:VGNC Symbol;Acc:VGNC:86981]	1.71	0.0364
ssc-miR-374b-5p	CRIM1	cysteine rich transmembrane BMP regulator 1 [Source:VGNC Symbol;Acc:VGNC:86995]	1.71	0.0364
ssc-miR-374b-5p	CRK	CRK proto-onco, adaptor protein [Source:NCBI gene (formerly Entrezgene);Acc:100192444]	1.71	0.0364
ssc-miR-374b-5p	CRNKL1	crooked neck pre-mRNA splicing factor 1 [Source:VGNC Symbol;Acc:VGNC:95654]	1.71	0.0364
ssc-miR-374b-5p	CRTC2	CREB regulated transcription coactivator 2 [Source:VGNC Symbol;Acc:VGNC:87008]	1.71	0.0364
ssc-miR-374b-5p	CSMD1	CUB and Sushi multiple domains 1 [Source:VGNC Symbol;Acc:VGNC:95600]	1.71	0.0364
ssc-miR-374b-5p	CSMD2	hypothetical gene	1.71	0.0364
ssc-miR-374b-5p	CSNK1G1	casein kinase 1 gamma 1 [Source:VGNC Symbol;Acc:VGNC:97949]	1.71	0.0364
ssc-miR-374b-5p	CSRNP3	cysteine and serine rich nuclear protein 3 [Source:VGNC Symbol;Acc:VGNC:96249]	1.71	0.0364
ssc-miR-374b-5p	CTAGE5	hypothetical gene	1.71	0.0364
ssc-miR-374b-5p	CTBS	chitobiase [Source:VGNC Symbol;Acc:VGNC:87055]	1.71	0.0364

ssc-miR-374b-5p	CTDNEP1	CTD nuclear envelope phosphatase 1 [Source:HGNC Symbol;Acc:HGNC:19085]	1.71	0.0364
ssc-miR-374b-5p	CTDSPL2	CTD small phosphatase like 2 [Source:VGNC Symbol;Acc:VGNC:87060]	1.71	0.0364
ssc-miR-374b-5p	CTTNBP2NL	CTTNBP2 N-terminal like [Source:VGNC Symbol;Acc:VGNC:87082]	1.71	0.0364
ssc-miR-374b-5p	CYBRD1	cytochrome b reductase 1 [Source:VGNC Symbol;Acc:VGNC:95948]	1.71	0.0364
ssc-miR-374b-5p	CYFIP1	cytoplasmic FMR1 interacting protein 1 [Source:HGNC Symbol;Acc:HGNC:13759]	1.71	0.0364
ssc-miR-374b-5p	CYP26A1	cytochrome P450 family 26 subfamily A member 1 [Source:VGNC Symbol;Acc:VGNC:103370]	1.71	0.0364
ssc-miR-374b-5p	CYP26B1	cytochrome P450 family 26 subfamily B member 1 [Source:VGNC Symbol;Acc:VGNC:103374]	1.71	0.0364
ssc-miR-374b-5p	CYP7B1	cytochrome P450 family 7 subfamily B member 1 [Source:VGNC Symbol;Acc:VGNC:103362]	1.71	0.0364
ssc-miR-374b-5p	CYTH1	cytohesin 1 [Source:VGNC Symbol;Acc:VGNC:87135]	1.71	0.0364
ssc-miR-374b-5p	CYTH3	cytohesin 3 [Source:VGNC Symbol;Acc:VGNC:87136]	1.71	0.0364
ssc-miR-374b-5p	DACH1	dachshund family transcription factor 1 [Source:VGNC Symbol;Acc:VGNC:87146]	1.71	0.0364
ssc-miR-374b-5p	DBR1	debranching RNA lariats 1 [Source:VGNC Symbol;Acc:VGNC:87168]	1.71	0.0364
ssc-miR-374b-5p	DCLK1	doublecortin like kinase 1 [Source:HGNC Symbol;Acc:HGNC:2700]	1.71	0.0364
ssc-miR-374b-5p	DCP2	decapping mRNA 2 [Source:VGNC Symbol;Acc:VGNC:87188]	1.71	0.0364
ssc-miR-374b-5p	DCUN1D1	hypothetical gene	1.71	0.0364
ssc-miR-374b-5p	DDX53	hypothetical gene	1.71	0.0364
ssc-miR-374b-5p	DENND4A	DENN domain containing 4A [Source:VGNC Symbol;Acc:VGNC:87251]	1.71	0.0364
ssc-miR-374b-5p	DESI1	desumoylating isopeptidase 1 [Source:VGNC Symbol;Acc:VGNC:87263]	1.71	0.0364
ssc-miR-374b-5p	DGAT2	diacylglycerol O-acyltransferase 2 [Source:NCBI gene (formerly Entrezgene);Acc:100294675]	1.71	0.0364
ssc-miR-374b-5p	DLG3	discs large MAGUK scaffold protein 3 [Source:VGNC Symbol;Acc:VGNC:87327]	1.71	0.0364
ssc-miR-374b-5p	DMD	dystrophin [Source:NCBI gene (formerly Entrezgene);Acc:497636]	1.71	0.0364
ssc-miR-374b-5p	DMXL1	Dmx like 1 [Source:VGNC Symbol;Acc:VGNC:87359]	1.71	0.0364
ssc-miR-374b-5p	DNAJB1	DnaJ heat shock protein family (Hsp40) member B1 [Source:VGNC Symbol;Acc:VGNC:96614]	1.71	0.0364
ssc-miR-374b-5p	DNAJC14	DnaJ heat shock protein family (Hsp40) member C14 [Source:NCBI gene (formerly Entrezgene);Acc:100151876]	1.71	0.0364
ssc-miR-374b-5p	DNAJC21	hypothetical gene	1.71	0.0364
ssc-miR-374b-5p	DNAJC22	DnaJ heat shock protein family (Hsp40) member C22 [Source:VGNC Symbol;Acc:VGNC:103231]	1.71	0.0364
ssc-miR-374b-5p	DNAJC6	DnaJ heat shock protein family (Hsp40) member C6 [Source:VGNC Symbol;Acc:VGNC:96625]	1.71	0.0364
ssc-miR-374b-5p	DNMT3A	DNA methyltransferase 3 alpha [Source:VGNC Symbol;Acc:VGNC:87384]	1.71	0.0364
ssc-miR-374b-5p	DOK6	docking protein 6 [Source:VGNC Symbol;Acc:VGNC:87404]	1.71	0.0364
ssc-miR-374b-5p	DR1	down-regulator of transcription 1 [Source:VGNC Symbol;Acc:VGNC:87435]	1.71	0.0364
ssc-miR-374b-5p	DSEL	dermatan sulfate epimerase like [Source:VGNC Symbol;Acc:VGNC:87454]	1.71	0.0364
ssc-miR-374b-5p	DSG3	desmoglein 3 [Source:VGNC Symbol;Acc:VGNC:87457]	1.71	0.0364
ssc-miR-374b-5p	DUSP19	dual specificity phosphatase 19 [Source:VGNC Symbol;Acc:VGNC:95899]	1.71	0.0364
ssc-miR-374b-5p	DUSP6	dual specificity phosphatase 6 [Source:VGNC Symbol;Acc:VGNC:87488]	1.71	0.0364
ssc-miR-374b-5p	DYRK1A	dual specificity tyrosine phosphorylation regulated kinase 1A [Source:VGNC Symbol;Acc:VGNC:87505]	1.71	0.0364
ssc-miR-374b-5p	DZIP1	DAZ interacting zinc finger protein 1 [Source:VGNC Symbol;Acc:VGNC:87510]	1.71	0.0364
ssc-miR-374b-5p	DZIP1L	DAZ interacting zinc finger protein 1 like [Source:VGNC Symbol;Acc:VGNC:87511]	1.71	0.0364
ssc-miR-374b-5p	EDAR	ectodysplasin A receptor [Source:VGNC Symbol;Acc:VGNC:87541]	1.71	0.0364
ssc-miR-374b-5p	EDEM3	ER degradation enhancing alpha-mannosidase like protein 3 [Source:VGNC Symbol;Acc:VGNC:87546]	1.71	0.0364
ssc-miR-374b-5p	EDIL3	EGF like repeats and discoidin domains 3 [Source:VGNC Symbol;Acc:VGNC:99642]	1.71	0.0364
ssc-miR-374b-5p	EFNA2	ephrin A2 [Source:VGNC Symbol;Acc:VGNC:87573]	1.71	0.0364
ssc-miR-374b-5p	EFNB2	ephrin B2 [Source:VGNC Symbol;Acc:VGNC:87577]	1.71	0.0364
ssc-miR-374b-5p	EGR2	early growth response 2 [Source:VGNC Symbol;Acc:VGNC:103939]	1.71	0.0364
ssc-miR-374b-5p	EIF2AK4	eukaryotic translation initiation factor 2 alpha kinase 4 [Source:VGNC Symbol;Acc:VGNC:87609]	1.71	0.0364
ssc-miR-374b-5p	EIF2S2	eukaryotic translation initiation factor 2 subunit beta [Source:VGNC Symbol;Acc:VGNC:96265]	1.71	0.0364
ssc-miR-374b-5p	EIF3A	eukaryotic translation initiation factor 3 subunit A [Source:VGNC Symbol;Acc:VGNC:87615]	1.71	0.0364
ssc-miR-374b-5p	EIF4E	eukaryotic translation initiation factor 4E [Source:VGNC Symbol;Acc:VGNC:87626]	1.71	0.0364
ssc-miR-374b-5p	EIF4G1	eukaryotic translation initiation factor 4 gamma 1 [Source:VGNC Symbol;Acc:VGNC:87631]	1.71	0.0364
ssc-miR-374b-5p	EIF4G2	eukaryotic translation initiation factor 4 gamma 2 [Source:VGNC Symbol;Acc:VGNC:99645]	1.71	0.0364
ssc-miR-374b-5p	EIF5A2	eukaryotic translation initiation factor 5A2 [Source:VGNC Symbol;Acc:VGNC:87633]	1.71	0.0364

ssc-miR-374b-5p	ELAC2	elaC ribonuclease Z 2 [Source:VGNC Symbol;Acc:VGNC:87636]	1.71	0.0364
ssc-miR-374b-5p	ELAVL4	ELAV like RNA binding protein 4 [Source:VGNC Symbol;Acc:VGNC:97047]	1.71	0.0364
ssc-miR-374b-5p	ELOVL7	ELOVL fatty acid elongase 7 [Source:HGNC Symbol;Acc:HGNC:26292]	1.71	0.0364
ssc-miR-374b-5p	EN1	engrailed homeobox 1 [Source:VGNC Symbol;Acc:VGNC:96273]	1.71	0.0364
ssc-miR-374b-5p	EN2	engrailed homeobox 2 [Source:VGNC Symbol;Acc:VGNC:87694]	1.71	0.0364
ssc-miR-374b-5p	EPB41L1	hypothetical gene	1.71	0.0364
ssc-miR-374b-5p	EPHA4	EPH receptor A4 [Source:VGNC Symbol;Acc:VGNC:96280]	1.71	0.0364
ssc-miR-374b-5p	EPHA8	EPH receptor A8 [Source:VGNC Symbol;Acc:VGNC:87735]	1.71	0.0364
ssc-miR-374b-5p	EPS15	epidermal growth factor receptor pathway substrate 15 [Source:VGNC Symbol;Acc:VGNC:87747]	1.71	0.0364
ssc-miR-374b-5p	ERC1	ELKS/RAB6-interacting/CAST family member 1 [Source:VGNC Symbol;Acc:VGNC:87761]	1.71	0.0364
ssc-miR-374b-5p	ESRRG	estrogen related receptor gamma [Source:VGNC Symbol;Acc:VGNC:96289]	1.71	0.0364
ssc-miR-374b-5p	ETNK1	ethanolamine kinase 1 [Source:VGNC Symbol;Acc:VGNC:87806]	1.71	0.0364
ssc-miR-374b-5p	ETV3	ETS variant transcription factor 3 [Source:VGNC Symbol;Acc:VGNC:87812]	1.71	0.0364
ssc-miR-374b-5p	ETV5	ETS variant transcription factor 5 [Source:VGNC Symbol;Acc:VGNC:87814]	1.71	0.0364
ssc-miR-374b-5p	ETV6	ETS variant transcription factor 6 [Source:VGNC Symbol;Acc:VGNC:87815]	1.71	0.0364
ssc-miR-374b-5p	FABP2	fatty acid binding protein 2 [Source:VGNC Symbol;Acc:VGNC:87871]	1.71	0.0364
ssc-miR-374b-5p	FAF2	Fas associated factor family member 2 [Source:VGNC Symbol;Acc:VGNC:87880]	1.71	0.0364
ssc-miR-374b-5p	FAM131B	family with sequence similarity 131 member B [Source:VGNC Symbol;Acc:VGNC:87901]	1.71	0.0364
ssc-miR-374b-5p	FAM13C	family with sequence similarity 13 member C [Source:VGNC Symbol;Acc:VGNC:87907]	1.71	0.0364
ssc-miR-374b-5p	FAM169A	family with sequence similarity 169 member A [Source:VGNC Symbol;Acc:VGNC:87927]	1.71	0.0364
ssc-miR-374b-5p	FAM19A1	hypothetical gene	1.71	0.0364
ssc-miR-374b-5p	FAM19A2	hypothetical gene	1.71	0.0364
ssc-miR-374b-5p	FAM216A	family with sequence similarity 216 member A [Source:VGNC Symbol;Acc:VGNC:87954]	1.71	0.0364
ssc-miR-374b-5p	FAM222B	family with sequence similarity 222 member B [Source:VGNC Symbol;Acc:VGNC:87960]	1.71	0.0364
ssc-miR-374b-5p	FAM63B	hypothetical gene	1.71	0.0364
ssc-miR-374b-5p	FAM65B	hypothetical gene	1.71	0.0364
ssc-miR-374b-5p	FANCM	FA complementation group M [Source:VGNC Symbol;Acc:VGNC:88008]	1.71	0.0364
ssc-miR-374b-5p	FBXL3	F-box and leucine rich repeat protein 3 [Source:VGNC Symbol;Acc:VGNC:88030]	1.71	0.0364
ssc-miR-374b-5p	FBXO32	F-box protein 32 [Source:VGNC Symbol;Acc:VGNC:88039]	1.71	0.0364
ssc-miR-374b-5p	FBXO42	F-box protein 42 [Source:VGNC Symbol;Acc:VGNC:88047]	1.71	0.0364
ssc-miR-374b-5p	FECH	ferrochelatase [Source:VGNC Symbol;Acc:VGNC:88081]	1.71	0.0364
ssc-miR-374b-5p	FGF12	fibroblast growth factor 12 [Source:VGNC Symbol;Acc:VGNC:88102]	1.71	0.0364
ssc-miR-374b-5p	FGF18	fibroblast growth factor 18 [Source:VGNC Symbol;Acc:VGNC:88107]	1.71	0.0364
ssc-miR-374b-5p	FGF5	fibroblast growth factor 5 [Source:HGNC Symbol;Acc:HGNC:3683]	1.71	0.0364
ssc-miR-374b-5p	FGF9	fibroblast growth factor 9 [Source:VGNC Symbol;Acc:VGNC:103943]	1.71	0.0364
ssc-miR-374b-5p	FGFR2	fibroblast growth factor receptor 2 [Source:NCBI gene (formerly Entrezgene);Acc:396762]	1.71	0.0364
ssc-miR-374b-5p	FNIP1	folliculin interacting protein 1 [Source:VGNC Symbol;Acc:VGNC:88187]	1.71	0.0364
ssc-miR-374b-5p	FOSB	FosB proto-onco, AP-1 transcription factor subunit [Source:VGNC Symbol;Acc:VGNC:88190]	1.71	0.0364
ssc-miR-374b-5p	FOXD2	forkhead box D2 [Source:VGNC Symbol;Acc:VGNC:88200]	1.71	0.0364
ssc-miR-374b-5p	FOXD3	forkhead box D3 [Source:VGNC Symbol;Acc:VGNC:88201]	1.71	0.0364
ssc-miR-374b-5p	FOXG1	forkhead box G1 [Source:VGNC Symbol;Acc:VGNC:88206]	1.71	0.0364
ssc-miR-374b-5p	FOXO1	forkhead box O1 [Source:VGNC Symbol;Acc:VGNC:98013]	1.71	0.0364
ssc-miR-374b-5p	FOXP1	forkhead box P1 [Source:VGNC Symbol;Acc:VGNC:88222]	1.71	0.0364
ssc-miR-374b-5p	FRMPD4	FERM and PDZ domain containing 4 [Source:VGNC Symbol;Acc:VGNC:88243]	1.71	0.0364
ssc-miR-374b-5p	FRS2	fibroblast growth factor receptor substrate 2 [Source:VGNC Symbol;Acc:VGNC:88246]	1.71	0.0364
ssc-miR-374b-5p	FUT9	fucosyltransferase 9 [Source:VGNC Symbol;Acc:VGNC:88271]	1.71	0.0364
ssc-miR-374b-5p	FZD5	frizzled class receptor 5 [Source:VGNC Symbol;Acc:VGNC:96309]	1.71	0.0364
ssc-miR-374b-5p	FZD8	frizzled class receptor 8 [Source:VGNC Symbol;Acc:VGNC:96311]	1.71	0.0364
ssc-miR-374b-5p	GABPA	GA binding protein transcription factor subunit alpha [Source:VGNC Symbol;Acc:VGNC:103945]	1.71	0.0364
ssc-miR-374b-5p	GABPB2	GA binding protein transcription factor subunit beta 2 [Source:VGNC Symbol;Acc:VGNC:98796]	1.71	0.0364

ssc-miR-374b-5p	GABRA1	gamma-aminobutyric acid type A receptor subunit alpha1 [Source:VGNC Symbol;Acc:VGNC:88300]	1.71	0.0364
ssc-miR-374b-5p	GADD45A	growth arrest and DNA damage inducible alpha [Source:VGNC Symbol;Acc:VGNC:88316]	1.71	0.0364
ssc-miR-374b-5p	GAS7	growth arrest specific 7 [Source:VGNC Symbol;Acc:VGNC:88359]	1.71	0.0364
ssc-miR-374b-5p	GATA3	GATA binding protein 3 [Source:VGNC Symbol;Acc:VGNC:96315]	1.71	0.0364
ssc-miR-374b-5p	GBX2	gastrulation brain homeobox 2 [Source:VGNC Symbol;Acc:VGNC:96316]	1.71	0.0364
ssc-miR-374b-5p	GLG1	golgi glycoprotein 1 [Source:VGNC Symbol;Acc:VGNC:88481]	1.71	0.0364
ssc-miR-374b-5p	GLIS3	GLIS family zinc finger 3 [Source:VGNC Symbol;Acc:VGNC:88485]	1.71	0.0364
ssc-miR-374b-5p	GMEB1	glucocorticoid modulatory element binding protein 1 [Source:VGNC Symbol;Acc:VGNC:88508]	1.71	0.0364
ssc-miR-374b-5p	GNB2	G protein subunit beta 2 [Source:VGNC Symbol;Acc:VGNC:88531]	1.71	0.0364
ssc-miR-374b-5p	GNPNAT1	glucosamine-phosphate N-acetyltransferase 1 [Source:VGNC Symbol;Acc:VGNC:103102]	1.71	0.0364
ssc-miR-374b-5p	GNPTG	N-acetylglucosamine-1-phosphate transferase subunit gamma [Source:VGNC Symbol;Acc:VGNC:88547]	1.71	0.0364
ssc-miR-374b-5p	GOLGA1	golgin A1 [Source:VGNC Symbol;Acc:VGNC:88549]	1.71	0.0364
ssc-miR-374b-5p	GOPC	golgi associated PDZ and coiled-coil motif containing [Source:VGNC Symbol;Acc:VGNC:98024]	1.71	0.0364
ssc-miR-374b-5p	GPC6	glypican 6 [Source:HGNC Symbol;Acc:HGNC:4454]	1.71	0.0364
ssc-miR-374b-5p	GPR161	G protein-coupled receptor 161 [Source:VGNC Symbol;Acc:VGNC:88613]	1.71	0.0364
ssc-miR-374b-5p	GPR50	G protein-coupled receptor 50 [Source:VGNC Symbol;Acc:VGNC:88633]	1.71	0.0364
ssc-miR-374b-5p	GPR75	G protein-coupled receptor 75 [Source:VGNC Symbol;Acc:VGNC:88636]	1.71	0.0364
ssc-miR-374b-5p	GPT2	glutamic--pyruvic transaminase 2 [Source:VGNC Symbol;Acc:VGNC:88651]	1.71	0.0364
ssc-miR-374b-5p	GRAMD1B	GRAM domain containing 1B [Source:VGNC Symbol;Acc:VGNC:88656]	1.71	0.0364
ssc-miR-374b-5p	GRAMD4	GRAM domain containing 4 [Source:VGNC Symbol;Acc:VGNC:88659]	1.71	0.0364
ssc-miR-374b-5p	GRIN2B	glutamate ionotropic receptor NMDA type subunit 2B [Source:VGNC Symbol;Acc:VGNC:88684]	1.71	0.0364
ssc-miR-374b-5p	GTDC1	glycosyltransferase like domain containing 1 [Source:VGNC Symbol;Acc:VGNC:95955]	1.71	0.0364
ssc-miR-374b-5p	GTF2A2	general transcription factor IIA subunit 2 [Source:HGNC Symbol;Acc:HGNC:4647]	1.71	0.0364
ssc-miR-374b-5p	GTPBP1	GTP binding protein 1 [Source:VGNC Symbol;Acc:VGNC:88742]	1.71	0.0364
ssc-miR-374b-5p	GTPBP10	GTP binding protein 10 [Source:VGNC Symbol;Acc:VGNC:103959]	1.71	0.0364
ssc-miR-374b-5p	H3F3B	hypothetical gene	1.71	0.0364
ssc-miR-374b-5p	HAPLN1	hyaluronan and proteoglycan link protein 1 [Source:VGNC Symbol;Acc:VGNC:88780]	1.71	0.0364
ssc-miR-374b-5p	HCFC1	host cell factor C1 [Source:HGNC Symbol;Acc:HGNC:4839]	1.71	0.0364
ssc-miR-374b-5p	HDAC9	histone deacetylase 9 [Source:HGNC Symbol;Acc:HGNC:14065]	1.71	0.0364
ssc-miR-374b-5p	HECTD1	HECT domain E3 ubiquitin protein ligase 1 [Source:VGNC Symbol;Acc:VGNC:88832]	1.71	0.0364
ssc-miR-374b-5p	HECTD4	HECT domain E3 ubiquitin protein ligase 4 [Source:VGNC Symbol;Acc:VGNC:88834]	1.71	0.0364
ssc-miR-374b-5p	HEG1	heart development protein with EGF like domains 1 [Source:VGNC Symbol;Acc:VGNC:88836]	1.71	0.0364
ssc-miR-374b-5p	HELZ2	helicase with zinc finger 2 [Source:VGNC Symbol;Acc:VGNC:88841]	1.71	0.0364
ssc-miR-374b-5p	HERC2	HECT and RLD domain containing E3 ubiquitin protein ligase 2 [Source:VGNC Symbol;Acc:VGNC:99716]	1.71	0.0364
ssc-miR-374b-5p	HES1	hes family bHLH transcription factor 1 [Source:VGNC Symbol;Acc:VGNC:88854]	1.71	0.0364
ssc-miR-374b-5p	HGF	hepatocyte growth factor [Source:VGNC Symbol;Acc:VGNC:88869]	1.71	0.0364
ssc-miR-374b-5p	HIAT1	hypothetical gene	1.71	0.0364
ssc-miR-374b-5p	HIPK2	homeodomain interacting protein kinase 2 [Source:VGNC Symbol;Acc:VGNC:88888]	1.71	0.0364
ssc-miR-374b-5p	HLF	HLF transcription factor, PAR bZIP family member [Source:VGNC Symbol;Acc:VGNC:88896]	1.71	0.0364
ssc-miR-374b-5p	HOXA1	homeobox A1 [Source:VGNC Symbol;Acc:VGNC:88933]	1.71	0.0364
ssc-miR-374b-5p	HOXA10	homeobox A10 [Source:VGNC Symbol;Acc:VGNC:88934]	1.71	0.0364
ssc-miR-374b-5p	HOXA11	homeobox A11 [Source:VGNC Symbol;Acc:VGNC:88935]	1.71	0.0364
ssc-miR-374b-5p	HOXB2	homeobox B2 [Source:VGNC Symbol;Acc:VGNC:88943]	1.71	0.0364
ssc-miR-374b-5p	HOXD11	homeobox D11 [Source:VGNC Symbol;Acc:VGNC:96352]	1.71	0.0364
ssc-miR-374b-5p	HOXD13	homeobox D13 [Source:HGNC Symbol;Acc:HGNC:5136]	1.71	0.0364
ssc-miR-374b-5p	HR	HR lysine demethylase and nuclear receptor corepressor [Source:VGNC Symbol;Acc:VGNC:88969]	1.71	0.0364
ssc-miR-374b-5p	HSBP1	heat shock factor binding protein 1 [Source:NCBI gene (formerly Entrezgene);Acc:100579143]	1.71	0.0364
ssc-miR-374b-5p	HSD11B1	hydroxysteroid 11-beta dehydrogenase 1 [Source:VGNC Symbol;Acc:VGNC:88983]	1.71	0.0364
ssc-miR-374b-5p	HSDL2	hydroxysteroid dehydrogenase like 2 [Source:VGNC Symbol;Acc:VGNC:88992]	1.71	0.0364
ssc-miR-374b-5p	HSPA4	heat shock protein family A (Hsp70) member 4 [Source:HGNC Symbol;Acc:HGNC:5237]	1.71	0.0364

ssc-miR-374b-5p	HSPA9	heat shock protein family A (Hsp70) member 9 [Source:VGNC Symbol;Acc:VGNC:99761]	1.71	0.0364
ssc-miR-374b-5p	HSPE1-MOB4	hypothetical gene	1.71	0.0364
ssc-miR-374b-5p	HSPH1	heat shock protein family H (Hsp110) member 1 [Source:NCBI gene (formerly Entrezgene);Acc:100048931]	1.71	0.0364
ssc-miR-374b-5p	HUNK	hormonally up-regulated Neu-associated kinase [Source:VGNC Symbol;Acc:VGNC:89007]	1.71	0.0364
ssc-miR-374b-5p	ICK	hypothetical gene	1.71	0.0364
ssc-miR-374b-5p	IFIT5	interferon induced protein with tetratricopeptide repeats 5 [Source:VGNC Symbol;Acc:VGNC:107402]	1.71	0.0364
ssc-miR-374b-5p	IGSF10	immunoglobulin superfamily member 10 [Source:VGNC Symbol;Acc:VGNC:89063]	1.71	0.0364
ssc-miR-374b-5p	IKZF2	IKAROS family zinc finger 2 [Source:VGNC Symbol;Acc:VGNC:95576]	1.71	0.0364
ssc-miR-374b-5p	IL10	interleukin 10 [Source:VGNC Symbol;Acc:VGNC:108593]	1.71	0.0364
ssc-miR-374b-5p	IL1A	interleukin 1 alpha [Source:VGNC Symbol;Acc:VGNC:89091]	1.71	0.0364
ssc-miR-374b-5p	IL1RAPL1	interleukin 1 receptor accessory protein like 1 [Source:VGNC Symbol;Acc:VGNC:103967]	1.71	0.0364
ssc-miR-374b-5p	ING1	inhibitor of growth family member 1 [Source:VGNC Symbol;Acc:VGNC:89131]	1.71	0.0364
ssc-miR-374b-5p	INHBA	inhibin subunit beta A [Source:VGNC Symbol;Acc:VGNC:89133]	1.71	0.0364
ssc-miR-374b-5p	INHBB	inhibin subunit beta B [Source:VGNC Symbol;Acc:VGNC:103969]	1.71	0.0364
ssc-miR-374b-5p	IQCK	IQ motif containing K [Source:VGNC Symbol;Acc:VGNC:89191]	1.71	0.0364
ssc-miR-374b-5p	IQSEC2	IQ motif and Sec7 domain ArfGEF 2 [Source:VGNC Symbol;Acc:VGNC:89195]	1.71	0.0364
ssc-miR-374b-5p	IRF2BP2	interferon regulatory factor 2 binding protein 2 [Source:VGNC Symbol;Acc:VGNC:89205]	1.71	0.0364
ssc-miR-374b-5p	IRS4	insulin receptor substrate 4 [Source:HGNC Symbol;Acc:HGNC:6128]	1.71	0.0364
ssc-miR-374b-5p	ITGB1BP1	integrin subunit beta 1 binding protein 1 [Source:VGNC Symbol;Acc:VGNC:89241]	1.71	0.0364
ssc-miR-374b-5p	JAM2	junctional adhesion molecule 2 [Source:VGNC Symbol;Acc:VGNC:89276]	1.71	0.0364
ssc-miR-374b-5p	JHDM1D	hypothetical gene	1.71	0.0364
ssc-miR-374b-5p	JMY	junction mediating and regulatory protein, p53 cofactor [Source:VGNC Symbol;Acc:VGNC:89289]	1.71	0.0364
ssc-miR-374b-5p	KANSL1L	KAT8 regulatory NSL complex subunit 1 like [Source:HGNC Symbol;Acc:HGNC:26310]	1.71	0.0364
ssc-miR-374b-5p	KAT6A	hypothetical gene	1.71	0.0364
ssc-miR-374b-5p	KCNJ6	potassium inwardly rectifying channel subfamily J member 6 [Source:VGNC Symbol;Acc:VGNC:89360]	1.71	0.0364
ssc-miR-374b-5p	KCNN3	potassium calcium-activated channel subfamily N member 3 [Source:VGNC Symbol;Acc:VGNC:98056]	1.71	0.0364
ssc-miR-374b-5p	KCTD19	potassium channel tetramerization domain containing 19 [Source:VGNC Symbol;Acc:VGNC:89397]	1.71	0.0364
ssc-miR-374b-5p	KDELC2	hypothetical gene	1.71	0.0364
ssc-miR-374b-5p	KERA	keratocan [Source:VGNC Symbol;Acc:VGNC:89420]	1.71	0.0364
ssc-miR-374b-5p	KIAA0408	KIAA0408 [Source:VGNC Symbol;Acc:VGNC:89432]	1.71	0.0364
ssc-miR-374b-5p	KIAA1024	hypothetical gene	1.71	0.0364
ssc-miR-374b-5p	KIAA1161	hypothetical gene	1.71	0.0364
ssc-miR-374b-5p	KIAA1199	hypothetical gene	1.71	0.0364
ssc-miR-374b-5p	KIAA1432	hypothetical gene	1.71	0.0364
ssc-miR-374b-5p	KIAA1468	hypothetical gene	1.71	0.0364
ssc-miR-374b-5p	KIF20A	kinesin family member 20A [Source:VGNC Symbol;Acc:VGNC:89462]	1.71	0.0364
ssc-miR-374b-5p	KIRREL	hypothetical gene	1.71	0.0364
ssc-miR-374b-5p	KLF3	Kruppel like factor 3 [Source:VGNC Symbol;Acc:VGNC:89498]	1.71	0.0364
ssc-miR-374b-5p	KLF7	Kruppel like factor 7 [Source:VGNC Symbol;Acc:VGNC:96396]	1.71	0.0364
ssc-miR-374b-5p	KLF8	Kruppel like factor 8 [Source:VGNC Symbol;Acc:VGNC:89500]	1.71	0.0364
ssc-miR-374b-5p	KLHL18	kelch like family member 18 [Source:VGNC Symbol;Acc:VGNC:89517]	1.71	0.0364
ssc-miR-374b-5p	KLHL29	kelch like family member 29 [Source:VGNC Symbol;Acc:VGNC:89524]	1.71	0.0364
ssc-miR-374b-5p	KRR1	KRR1 small subunit processome component homolog [Source:VGNC Symbol;Acc:VGNC:89575]	1.71	0.0364
ssc-miR-374b-5p	KTN1	kinectin 1 [Source:VGNC Symbol;Acc:VGNC:89608]	1.71	0.0364
ssc-miR-374b-5p	L1CAM	L1 cell adhesion molecule [Source:HGNC Symbol;Acc:HGNC:6470]	1.71	0.0364
ssc-miR-374b-5p	L3HYPDH	trans-L-3-hydroxyproline dehydratase [Source:VGNC Symbol;Acc:VGNC:89612]	1.71	0.0364
ssc-miR-374b-5p	LARP1	La ribonucleoprotein 1, translational regulator [Source:VGNC Symbol;Acc:VGNC:98067]	1.71	0.0364
ssc-miR-374b-5p	LARP4	La ribonucleoprotein 4 [Source:VGNC Symbol;Acc:VGNC:89641]	1.71	0.0364
ssc-miR-374b-5p	LARP4B	hypothetical gene	1.71	0.0364
ssc-miR-374b-5p	LCA5	lebercilin LCA5 [Source:VGNC Symbol;Acc:VGNC:89654]	1.71	0.0364

ssc-miR-374b-5p	LDLRAD4	low density lipoprotein receptor class A domain containing 4 [Source:VGNC Symbol;Acc:VGNC:89673]	1.71	0.0364
ssc-miR-374b-5p	LEMD3	LEM domain containing 3 [Source:VGNC Symbol;Acc:VGNC:89679]	1.71	0.0364
ssc-miR-374b-5p	LHFPL2	LHFPL tetraspan subfamily member 2 [Source:VGNC Symbol;Acc:VGNC:89705]	1.71	0.0364
ssc-miR-374b-5p	LHX5	LIM homeobox 5 [Source:VGNC Symbol;Acc:VGNC:89715]	1.71	0.0364
ssc-miR-374b-5p	LIG3	DNA ligase 3 [Source:VGNC Symbol;Acc:VGNC:89721]	1.71	0.0364
ssc-miR-374b-5p	LIN9	lin-9 DREAM MuvB core complex component [Source:VGNC Symbol;Acc:VGNC:103976]	1.71	0.0364
ssc-miR-374b-5p	LMLN	leishmanolysin like peptidase [Source:VGNC Symbol;Acc:VGNC:89759]	1.71	0.0364
ssc-miR-374b-5p	LPAR1	hypothetical gene	1.71	0.0364
ssc-miR-374b-5p	LPAR3	lysophosphatidic acid receptor 3 [Source:VGNC Symbol;Acc:VGNC:89785]	1.71	0.0364
ssc-miR-374b-5p	LPHN2	hypothetical gene	1.71	0.0364
ssc-miR-374b-5p	LPP	LIM domain containing preferred translocation partner in lipoma [Source:HGNC Symbol;Acc:HGNC:6679]	1.71	0.0364
ssc-miR-374b-5p	LRCH1	leucine rich repeats and calponin homology domain containing 1 [Source:VGNC Symbol;Acc:VGNC:89801]	1.71	0.0364
ssc-miR-374b-5p	LRIG1	leucine rich repeats and immunoglobulin like domains 1 [Source:VGNC Symbol;Acc:VGNC:89810]	1.71	0.0364
ssc-miR-374b-5p	LRP6	LDL receptor related protein 6 [Source:VGNC Symbol;Acc:VGNC:89821]	1.71	0.0364
ssc-miR-374b-5p	LSAMP	limbic system associated membrane protein [Source:VGNC Symbol;Acc:VGNC:89868]	1.71	0.0364
ssc-miR-374b-5p	MAP2	microtubule associated protein 2 [Source:VGNC Symbol;Acc:VGNC:96409]	1.71	0.0364
ssc-miR-374b-5p	MAP2K4	mitogen-activated protein kinase kinase 4 [Source:VGNC Symbol;Acc:VGNC:98101]	1.71	0.0364
ssc-miR-374b-5p	MAP2K6	mitogen-activated protein kinase kinase 6 [Source:VGNC Symbol;Acc:VGNC:98102]	1.71	0.0364
ssc-miR-374b-5p	MAP3K2	mitogen-activated protein kinase kinase kinase 2 [Source:VGNC Symbol;Acc:VGNC:98107]	1.71	0.0364
ssc-miR-374b-5p	MAPK1IP1L	mitogen-activated protein kinase 1 interacting protein 1 like [Source:VGNC Symbol;Acc:VGNC:90002]	1.71	0.0364
ssc-miR-374b-5p	MAPK6	mitogen-activated protein kinase 6 [Source:VGNC Symbol;Acc:VGNC:103123]	1.71	0.0364
ssc-miR-374b-5p	MAPRE2	microtubule associated protein RP/EB family member 2 [Source:VGNC Symbol;Acc:VGNC:98117]	1.71	0.0364
ssc-miR-374b-5p	MBD5	methyl-CpG binding domain protein 5 [Source:VGNC Symbol;Acc:VGNC:95940]	1.71	0.0364
ssc-miR-374b-5p	MBNL1	muscleblind like splicing regulator 1 [Source:VGNC Symbol;Acc:VGNC:90054]	1.71	0.0364
ssc-miR-374b-5p	MBNL2	muscleblind like splicing regulator 2 [Source:VGNC Symbol;Acc:VGNC:90055]	1.71	0.0364
ssc-miR-374b-5p	MCFD2	multiple coagulation factor deficiency 2, ER cargo receptor complex subunit [Source:VGNC Symbol;Acc:VGNC:103980]	1.71	0.0364
ssc-miR-374b-5p	MECP2	methyl-CpG binding protein 2 [Source:VGNC Symbol;Acc:VGNC:90101]	1.71	0.0364
ssc-miR-374b-5p	MED12L	mediator complex subunit 12L [Source:VGNC Symbol;Acc:VGNC:90105]	1.71	0.0364
ssc-miR-374b-5p	MED13	mediator complex subunit 13 [Source:VGNC Symbol;Acc:VGNC:90106]	1.71	0.0364
ssc-miR-374b-5p	MEF2D	myocyte enhancer factor 2D [Source:VGNC Symbol;Acc:VGNC:90128]	1.71	0.0364
ssc-miR-374b-5p	MEIS1	Meis homeobox 1 [Source:VGNC Symbol;Acc:VGNC:90135]	1.71	0.0364
ssc-miR-374b-5p	MET	MET proto-onco, receptor tyrosine kinase [Source:NCBI gene (formerly Entrezgene);Acc:654328]	1.71	0.0364
ssc-miR-374b-5p	METRNL	meteorin like, glial cell differentiation regulator [Source:VGNC Symbol;Acc:VGNC:90153]	1.71	0.0364
ssc-miR-374b-5p	METTL21A	methyltransferase 21A, HSPA lysine [Source:VGNC Symbol;Acc:VGNC:96242]	1.71	0.0364
ssc-miR-374b-5p	MEX3A	mex-3 RNA binding family member A [Source:VGNC Symbol;Acc:VGNC:90168]	1.71	0.0364
ssc-miR-374b-5p	MEX3B	mex-3 RNA binding family member B [Source:VGNC Symbol;Acc:VGNC:90169]	1.71	0.0364
ssc-miR-374b-5p	MFAP3L	microfibril associated protein 3 like [Source:VGNC Symbol;Acc:VGNC:90175]	1.71	0.0364
ssc-miR-374b-5p	MGAT4A	alpha-1,3-mannosyl-glycoprotein 4-beta-N-acetylglucosaminyltransferase A [Source:VGNC Symbol;Acc:VGNC:108158]	1.71	0.0364
ssc-miR-374b-5p	MICALCL	hypothetical gene	1.71	0.0364
ssc-miR-374b-5p	MIEF1	hypothetical gene	1.71	0.0364
ssc-miR-374b-5p	MIER1	MIER1 transcriptional regulator [Source:VGNC Symbol;Acc:VGNC:90219]	1.71	0.0364
ssc-miR-374b-5p	MIPOL1	mirror-image polydactyly 1 [Source:VGNC Symbol;Acc:VGNC:90237]	1.71	0.0364
ssc-miR-374b-5p	MKX	mohawk homeobox [Source:VGNC Symbol;Acc:VGNC:96020]	1.71	0.0364
ssc-miR-374b-5p	MLX	MAX dimerization protein MLX [Source:VGNC Symbol;Acc:VGNC:90261]	1.71	0.0364
ssc-miR-374b-5p	MME	membrane metalloendopeptidase [Source:VGNC Symbol;Acc:VGNC:90265]	1.71	0.0364
ssc-miR-374b-5p	MMP14	matrix metallopeptidase 14 [Source:NCBI gene (formerly Entrezgene);Acc:397471]	1.71	0.0364
ssc-miR-374b-5p	MOB4	hypothetical gene	1.71	0.0364
ssc-miR-374b-5p	MON2	MON2 homolog, regulator of endosome-to-Golgi trafficking [Source:VGNC Symbol;Acc:VGNC:90301]	1.71	0.0364
ssc-miR-374b-5p	MPP2	MAGUK p55 scaffold protein 2 [Source:VGNC Symbol;Acc:VGNC:98133]	1.71	0.0364
ssc-miR-374b-5p	MPP6	hypothetical gene	1.71	0.0364

ssc-miR-374b-5p	MSH3	mutS homolog 3 [Source:VGNC Symbol;Acc:VGNC:90419]	1.71	0.0364
ssc-miR-374b-5p	MSI2	musashi RNA binding protein 2 [Source:VGNC Symbol;Acc:VGNC:90422]	1.71	0.0364
ssc-miR-374b-5p	MSN	moesin [Source:VGNC Symbol;Acc:VGNC:90426]	1.71	0.0364
ssc-miR-374b-5p	MSX1	msh homeobox 1 [Source:VGNC Symbol;Acc:VGNC:90431]	1.71	0.0364
ssc-miR-374b-5p	MTMR12	myotubularin related protein 12 [Source:VGNC Symbol;Acc:VGNC:90459]	1.71	0.0364
ssc-miR-374b-5p	MUM1L1	hypothetical gene	1.71	0.0364
ssc-miR-374b-5p	MYLK	myosin light chain kinase [Source:VGNC Symbol;Acc:VGNC:108676]	1.71	0.0364
ssc-miR-374b-5p	MYO10	myosin X [Source:VGNC Symbol;Acc:VGNC:90522]	1.71	0.0364
ssc-miR-374b-5p	MYO9A	myosin IXA [Source:VGNC Symbol;Acc:VGNC:103138]	1.71	0.0364
ssc-miR-374b-5p	MYT1L	myelin transcription factor 1 like [Source:VGNC Symbol;Acc:VGNC:90549]	1.71	0.0364
ssc-miR-374b-5p	MYZAP	myocardial zonula adherens protein [Source:HGNC Symbol;Acc:HGNC:43444]	1.71	0.0364
ssc-miR-374b-5p	MZT1	mitotic spindle organizing protein 1 [Source:VGNC Symbol;Acc:VGNC:90552]	1.71	0.0364
ssc-miR-374b-5p	N4BP2	NEDD4 binding protein 2 [Source:VGNC Symbol;Acc:VGNC:90554]	1.71	0.0364
ssc-miR-374b-5p	NAA35	N-alpha-acetyltransferase 35, NatC auxiliary subunit [Source:VGNC Symbol;Acc:VGNC:96752]	1.71	0.0364
ssc-miR-374b-5p	NAPEPLD	N-acyl phosphatidylethanolamine phospholipase D [Source:VGNC Symbol;Acc:VGNC:90579]	1.71	0.0364
ssc-miR-374b-5p	NCBP1	nuclear cap binding protein subunit 1 [Source:VGNC Symbol;Acc:VGNC:90603]	1.71	0.0364
ssc-miR-374b-5p	NCK1	NCK adaptor protein 1 [Source:VGNC Symbol;Acc:VGNC:90609]	1.71	0.0364
ssc-miR-374b-5p	NCKAP1	NCK associated protein 1 [Source:VGNC Symbol;Acc:VGNC:96432]	1.71	0.0364
ssc-miR-374b-5p	NCOA1	nuclear receptor coactivator 1 [Source:VGNC Symbol;Acc:VGNC:90615]	1.71	0.0364
ssc-miR-374b-5p	NCOA2	nuclear receptor coactivator 2 [Source:VGNC Symbol;Acc:VGNC:90616]	1.71	0.0364
ssc-miR-374b-5p	NDST1	N-deacetylase and N-sulfotransferase 1 [Source:VGNC Symbol;Acc:VGNC:90634]	1.71	0.0364
ssc-miR-374b-5p	NEDD4L	NEDD4 like E3 ubiquitin protein ligase [Source:VGNC Symbol;Acc:VGNC:90667]	1.71	0.0364
ssc-miR-374b-5p	NEO1	neogenin 1 [Source:HGNC Symbol;Acc:HGNC:7754]	1.71	0.0364
ssc-miR-374b-5p	NEUROD1	neuronal differentiation 1 [Source:VGNC Symbol;Acc:VGNC:96439]	1.71	0.0364
ssc-miR-374b-5p	NEUROG2	neurogenin 2 [Source:VGNC Symbol;Acc:VGNC:90701]	1.71	0.0364
ssc-miR-374b-5p	NFIA	nuclear factor I A [Source:VGNC Symbol;Acc:VGNC:90715]	1.71	0.0364
ssc-miR-374b-5p	NFIB	nuclear factor I B [Source:VGNC Symbol;Acc:VGNC:90716]	1.71	0.0364
ssc-miR-374b-5p	NFIL3	nuclear factor, interleukin 3 regulated [Source:VGNC Symbol;Acc:VGNC:90717]	1.71	0.0364
ssc-miR-374b-5p	NFIX	nuclear factor I X [Source:VGNC Symbol;Acc:VGNC:90718]	1.71	0.0364
ssc-miR-374b-5p	NFKBIZ	NFKB inhibitor zeta [Source:VGNC Symbol;Acc:VGNC:90726]	1.71	0.0364
ssc-miR-374b-5p	NFYB	nuclear transcription factor Y subunit beta [Source:VGNC Symbol;Acc:VGNC:90730]	1.71	0.0364
ssc-miR-374b-5p	NHLRC2	NHL repeat containing 2 [Source:VGNC Symbol;Acc:VGNC:90737]	1.71	0.0364
ssc-miR-374b-5p	NIPBL	NIPBL cohesin loading factor [Source:VGNC Symbol;Acc:VGNC:90752]	1.71	0.0364
ssc-miR-374b-5p	NKX2-2	NK2 homeobox 2 [Source:VGNC Symbol;Acc:VGNC:96444]	1.71	0.0364
ssc-miR-374b-5p	NLN	neurolysin [Source:VGNC Symbol;Acc:VGNC:90780]	1.71	0.0364
ssc-miR-374b-5p	NMT1	N-myristoyltransferase 1 [Source:VGNC Symbol;Acc:VGNC:90802]	1.71	0.0364
ssc-miR-374b-5p	NOG	noggin [Source:VGNC Symbol;Acc:VGNC:90812]	1.71	0.0364
ssc-miR-374b-5p	NOVA1	NOVA alternative splicing regulator 1 [Source:VGNC Symbol;Acc:VGNC:90827]	1.71	0.0364
ssc-miR-374b-5p	NPPC	natriuretic peptide C [Source:VGNC Symbol;Acc:VGNC:96450]	1.71	0.0364
ssc-miR-374b-5p	NPTX2	neuronal pentraxin 2 [Source:VGNC Symbol;Acc:VGNC:90863]	1.71	0.0364
ssc-miR-374b-5p	NR2F2	nuclear receptor subfamily 2 group F member 2 [Source:VGNC Symbol;Acc:VGNC:90881]	1.71	0.0364
ssc-miR-374b-5p	NR3C1	nuclear receptor subfamily 3 group C member 1 [Source:VGNC Symbol;Acc:VGNC:90883]	1.71	0.0364
ssc-miR-374b-5p	NR4A2	nuclear receptor subfamily 4 group A member 2 [Source:VGNC Symbol;Acc:VGNC:96451]	1.71	0.0364
ssc-miR-374b-5p	NR4A3	nuclear receptor subfamily 4 group A member 3 [Source:VGNC Symbol;Acc:VGNC:90885]	1.71	0.0364
ssc-miR-374b-5p	NRDE2	NRDE-2, necessary for RNA interference, domain containing [Source:VGNC Symbol;Acc:VGNC:90894]	1.71	0.0364
ssc-miR-374b-5p	NRG2	neuregulin 2 [Source:VGNC Symbol;Acc:VGNC:98160]	1.71	0.0364
ssc-miR-374b-5p	NRK	Nik related kinase [Source:VGNC Symbol;Acc:VGNC:98161]	1.71	0.0364
ssc-miR-374b-5p	NRN1	neuritin 1 [Source:VGNC Symbol;Acc:VGNC:90900]	1.71	0.0364
ssc-miR-374b-5p	NSF	N-ethylmaleimide sensitive factor, vesicle fusing ATPase [Source:VGNC Symbol;Acc:VGNC:90906]	1.71	0.0364
ssc-miR-374b-5p	NTF3	neurotrophin 3 [Source:VGNC Symbol;Acc:VGNC:90928]	1.71	0.0364

ssc-miR-374b-5p	NTN1	netrin 1 [Source:VGNC Symbol;Acc:VGNC:90931]	1.71	0.0364
ssc-miR-374b-5p	NUAK1	NUAK family kinase 1 [Source:VGNC Symbol;Acc:VGNC:90940]	1.71	0.0364
ssc-miR-374b-5p	NUMB	NUMB endocytic adaptor protein [Source:VGNC Symbol;Acc:VGNC:90970]	1.71	0.0364
ssc-miR-374b-5p	NUMBL	NUMB like endocytic adaptor protein [Source:HGNC Symbol;Acc:HGNC:8061]	1.71	0.0364
ssc-miR-374b-5p	NUP107	nucleoporin 107 [Source:VGNC Symbol;Acc:VGNC:90971]	1.71	0.0364
ssc-miR-374b-5p	NUP35	nucleoporin 35 [Source:VGNC Symbol;Acc:VGNC:96229]	1.71	0.0364
ssc-miR-374b-5p	NUP85	nucleoporin 85 [Source:VGNC Symbol;Acc:VGNC:90985]	1.71	0.0364
ssc-miR-374b-5p	NXPE3	neurexophilin and PC-esterase domain family member 3 [Source:VGNC Symbol;Acc:VGNC:90997]	1.71	0.0364
ssc-miR-374b-5p	OCRL	OCRL inositol polyphosphate-5-phosphatase [Source:VGNC Symbol;Acc:VGNC:91014]	1.71	0.0364
ssc-miR-374b-5p	OGT	O-linked N-acetylglucosamine (GlcNAc) transferase [Source:NCBI gene (formerly Entrezgene);Acc:664652]	1.71	0.0364
ssc-miR-374b-5p	ONECUT2	one cut homeobox 2 [Source:VGNC Symbol;Acc:VGNC:91043]	1.71	0.0364
ssc-miR-374b-5p	ONECUT3	one cut homeobox 3 [Source:VGNC Symbol;Acc:VGNC:91044]	1.71	0.0364
ssc-miR-374b-5p	OSBP	oxysterol binding protein [Source:VGNC Symbol;Acc:VGNC:91066]	1.71	0.0364
ssc-miR-374b-5p	OSTF1	osteoclast stimulating factor 1 [Source:VGNC Symbol;Acc:VGNC:91085]	1.71	0.0364
ssc-miR-374b-5p	PABPC1	poly(A) binding protein cytoplasmic 1 [Source:VGNC Symbol;Acc:VGNC:98829]	1.71	0.0364
ssc-miR-374b-5p	PAPD4	hypothetical gene	1.71	0.0364
ssc-miR-374b-5p	PAPLN	papilin, proteoglycan like sulfated glycoprotein [Source:VGNC Symbol;Acc:VGNC:91169]	1.71	0.0364
ssc-miR-374b-5p	PAPPA	pappalysin 1 [Source:VGNC Symbol;Acc:VGNC:91170]	1.71	0.0364
ssc-miR-374b-5p	PAPPA2	pappalysin 2 [Source:VGNC Symbol;Acc:VGNC:91171]	1.71	0.0364
ssc-miR-374b-5p	PAQR3	progestin and adipoQ receptor family member 3 [Source:VGNC Symbol;Acc:VGNC:91174]	1.71	0.0364
ssc-miR-374b-5p	PARD6B	par-6 family cell polarity regulator beta [Source:VGNC Symbol;Acc:VGNC:98176]	1.71	0.0364
ssc-miR-374b-5p	PARP8	poly(ADP-ribose) polymerase family member 8 [Source:HGNC Symbol;Acc:HGNC:26124]	1.71	0.0364
ssc-miR-374b-5p	PAX6	paired box 6 [Source:VGNC Symbol;Acc:VGNC:91195]	1.71	0.0364
ssc-miR-374b-5p	PCDH10	protocadherin 10 [Source:HGNC Symbol;Acc:HGNC:13404]	1.71	0.0364
ssc-miR-374b-5p	PCDHA1	hypothetical gene	1.71	0.0364
ssc-miR-374b-5p	PCDHA10	hypothetical gene	1.71	0.0364
ssc-miR-374b-5p	PCDHA11	hypothetical gene	1.71	0.0364
ssc-miR-374b-5p	PCDHA12	hypothetical gene	1.71	0.0364
ssc-miR-374b-5p	PCDHA13	protocadherin alpha 13 [Source:HGNC Symbol;Acc:HGNC:8667]	1.71	0.0364
ssc-miR-374b-5p	PCDHA2	hypothetical gene	1.71	0.0364
ssc-miR-374b-5p	PCDHA3	protocadherin alpha 3 [Source:HGNC Symbol;Acc:HGNC:8669]	1.71	0.0364
ssc-miR-374b-5p	PCDHA4	hypothetical gene	1.71	0.0364
ssc-miR-374b-5p	PCDHA5	hypothetical gene	1.71	0.0364
ssc-miR-374b-5p	PCDHA6	hypothetical gene	1.71	0.0364
ssc-miR-374b-5p	PCDHA7	hypothetical gene	1.71	0.0364
ssc-miR-374b-5p	PCDHA8	hypothetical gene	1.71	0.0364
ssc-miR-374b-5p	PCDHA9	hypothetical gene	1.71	0.0364
ssc-miR-374b-5p	PCDHAC1	hypothetical gene	1.71	0.0364
ssc-miR-374b-5p	PCDHAC2	protocadherin alpha subfamily C, 2 [Source:HGNC Symbol;Acc:HGNC:8677]	1.71	0.0364
ssc-miR-374b-5p	PCF11	PCF11 cleavage and polyadenylation factor subunit [Source:VGNC Symbol;Acc:VGNC:91218]	1.71	0.0364
ssc-miR-374b-5p	PCGF3	polycomb group ring finger 3 [Source:VGNC Symbol;Acc:VGNC:91220]	1.71	0.0364
ssc-miR-374b-5p	PCNX	hypothetical gene	1.71	0.0364
ssc-miR-374b-5p	PDE10A	phosphodiesterase 10A [Source:VGNC Symbol;Acc:VGNC:91248]	1.71	0.0364
ssc-miR-374b-5p	PDE3A	phosphodiesterase 3A [Source:VGNC Symbol;Acc:VGNC:91252]	1.71	0.0364
ssc-miR-374b-5p	PDE4A	phosphodiesterase 4A [Source:VGNC Symbol;Acc:VGNC:91254]	1.71	0.0364
ssc-miR-374b-5p	PDE4D	phosphodiesterase 4D [Source:VGNC Symbol;Acc:VGNC:91256]	1.71	0.0364
ssc-miR-374b-5p	PDE6A	phosphodiesterase 6A [Source:VGNC Symbol;Acc:VGNC:91258]	1.71	0.0364
ssc-miR-374b-5p	PDE7B	phosphodiesterase 7B [Source:VGNC Symbol;Acc:VGNC:91262]	1.71	0.0364
ssc-miR-374b-5p	PDE8B	phosphodiesterase 8B [Source:VGNC Symbol;Acc:VGNC:100321]	1.71	0.0364
ssc-miR-374b-5p	PDK4	pyruvate dehydrogenase kinase 4 [Source:VGNC Symbol;Acc:VGNC:91280]	1.71	0.0364

ssc-miR-374b-5p	PDP2	pyruvate dehyrogenase phosphatase catalytic subunit 2 [Source:VGNC Symbol;Acc:VGNC:91287]	1.71	0.0364
ssc-miR-374b-5p	PDPK1	3-phosphoinositide dependent protein kinase 1 [Source:HGNC Symbol;Acc:HGNC:8816]	1.71	0.0364
ssc-miR-374b-5p	PDZD2	hypothetical gene	1.71	0.0364
ssc-miR-374b-5p	PELI1	pellino E3 ubiquitin protein ligase 1 [Source:VGNC Symbol;Acc:VGNC:91306]	1.71	0.0364
ssc-miR-374b-5p	PGR	progesterone receptor [Source:VGNC Symbol;Acc:VGNC:91362]	1.71	0.0364
ssc-miR-374b-5p	PHACTR2	phosphatase and actin regulator 2 [Source:VGNC Symbol;Acc:VGNC:91366]	1.71	0.0364
ssc-miR-374b-5p	PHF21A	PHD finger protein 21A [Source:VGNC Symbol;Acc:VGNC:91384]	1.71	0.0364
ssc-miR-374b-5p	PHRF1	PHD and ring finger domains 1 [Source:VGNC Symbol;Acc:VGNC:91403]	1.71	0.0364
ssc-miR-374b-5p	PI15	peptidase inhibitor 15 [Source:VGNC Symbol;Acc:VGNC:98188]	1.71	0.0364
ssc-miR-374b-5p	PIGW	phosphatidylinositol glycan anchor biosynthesis class W [Source:VGNC Symbol;Acc:VGNC:91431]	1.71	0.0364
ssc-miR-374b-5p	PIK3CA	phosphatidylinositol-4,5-bisphosphate 3-kinase catalytic subunit alpha [Source:VGNC Symbol;Acc:VGNC:91440]	1.71	0.0364
ssc-miR-374b-5p	PIKFYVE	phosphoinositide kinase, FYVE-type zinc finger containing [Source:VGNC Symbol;Acc:VGNC:96023]	1.71	0.0364
ssc-miR-374b-5p	PITPNC1	phosphatidylinositol transfer protein cytoplasmic 1 [Source:NCBI gene (formerly Entrezgene);Acc:100233199]	1.71	0.0364
ssc-miR-374b-5p	PITX1	paired like homeodomain 1 [Source:NCBI gene (formerly Entrezgene);Acc:100689266]	1.71	0.0364
ssc-miR-374b-5p	PITX2	paired like homeodomain 2 [Source:VGNC Symbol;Acc:VGNC:91467]	1.71	0.0364
ssc-miR-374b-5p	PKIA	cAMP-dependent protein kinase inhibitor alpha [Source:VGNC Symbol;Acc:VGNC:91477]	1.71	0.0364
ssc-miR-374b-5p	PKN2	protein kinase N2 [Source:VGNC Symbol;Acc:VGNC:91482]	1.71	0.0364
ssc-miR-374b-5p	PKNOX1	PBX/knotted 1 homeobox 1 [Source:VGNC Symbol;Acc:VGNC:91484]	1.71	0.0364
ssc-miR-374b-5p	PLAT	plasminogen activator, tissue type [Source:VGNC Symbol;Acc:VGNC:98201]	1.71	0.0364
ssc-miR-374b-5p	PLCE1	phospholipase C epsilon 1 [Source:VGNC Symbol;Acc:VGNC:91519]	1.71	0.0364
ssc-miR-374b-5p	PLD5	phospholipase D family member 5 [Source:VGNC Symbol;Acc:VGNC:96145]	1.71	0.0364
ssc-miR-374b-5p	PLEKHM3	pleckstrin homology domain containing M3 [Source:VGNC Symbol;Acc:VGNC:96281]	1.71	0.0364
ssc-miR-374b-5p	PLXNA2	plexin A2 [Source:VGNC Symbol;Acc:VGNC:91580]	1.71	0.0364
ssc-miR-374b-5p	PLXNC1	plexin C1 [Source:VGNC Symbol;Acc:VGNC:91584]	1.71	0.0364
ssc-miR-374b-5p	PMEPA1	prostate transmembrane protein, androgen induced 1 [Source:VGNC Symbol;Acc:VGNC:95606]	1.71	0.0364
ssc-miR-374b-5p	PMPCB	peptidase, mitochondrial processing subunit beta [Source:VGNC Symbol;Acc:VGNC:91592]	1.71	0.0364
ssc-miR-374b-5p	PNPT1	polyribonucleotide nucleotidyltransferase 1 [Source:VGNC Symbol;Acc:VGNC:91612]	1.71	0.0364
ssc-miR-374b-5p	PNRC1	proline rich nuclear receptor coactivator 1 [Source:VGNC Symbol;Acc:VGNC:91613]	1.71	0.0364
ssc-miR-374b-5p	POC1B-GALNT4	hypothetical gene	1.71	0.0364
ssc-miR-374b-5p	POGZ	pogo transposable element derived with ZNF domain [Source:VGNC Symbol;Acc:VGNC:91625]	1.71	0.0364
ssc-miR-374b-5p	POMP	proteasome maturation protein [Source:HGNC Symbol;Acc:HGNC:20330]	1.71	0.0364
ssc-miR-374b-5p	POU4F1	POU class 4 homeobox 1 [Source:VGNC Symbol;Acc:VGNC:91678]	1.71	0.0364
ssc-miR-374b-5p	PPAP2B	hypothetical gene	1.71	0.0364
ssc-miR-374b-5p	PPARGC1A	PPARG coactivator 1 alpha [Source:VGNC Symbol;Acc:VGNC:91685]	1.71	0.0364
ssc-miR-374b-5p	PPARGC1B	PPARG coactivator 1 beta [Source:VGNC Symbol;Acc:VGNC:91686]	1.71	0.0364
ssc-miR-374b-5p	PPIG	peptidylprolyl isomerase G [Source:VGNC Symbol;Acc:VGNC:98214]	1.71	0.0364
ssc-miR-374b-5p	PPM1D	protein phosphatase, Mg2+/Mn2+ dependent 1D [Source:VGNC Symbol;Acc:VGNC:91704]	1.71	0.0364
ssc-miR-374b-5p	PPM1H	protein phosphatase, Mg2+/Mn2+ dependent 1H [Source:VGNC Symbol;Acc:VGNC:91708]	1.71	0.0364
ssc-miR-374b-5p	PPM1K	protein phosphatase, Mg2+/Mn2+ dependent 1K [Source:VGNC Symbol;Acc:VGNC:91710]	1.71	0.0364
ssc-miR-374b-5p	PPP1R3F	protein phosphatase 1 regulatory subunit 3F [Source:VGNC Symbol;Acc:VGNC:91739]	1.71	0.0364
ssc-miR-374b-5p	PPP2R2C	protein phosphatase 2 regulatory subunit Bgamma [Source:VGNC Symbol;Acc:VGNC:91749]	1.71	0.0364
ssc-miR-374b-5p	PPP6R1	protein phosphatase 6 regulatory subunit 1 [Source:VGNC Symbol;Acc:VGNC:91760]	1.71	0.0364
ssc-miR-374b-5p	PPTC7	protein phosphatase targeting COQ7 [Source:VGNC Symbol;Acc:VGNC:91765]	1.71	0.0364
ssc-miR-374b-5p	PRIM1	DNA primase subunit 1 [Source:VGNC Symbol;Acc:VGNC:103313]	1.71	0.0364
ssc-miR-374b-5p	PRIMA1	proline rich membrane anchor 1 [Source:HGNC Symbol;Acc:HGNC:18319]	1.71	0.0364
ssc-miR-374b-5p	PRKCA	protein kinase C alpha [Source:VGNC Symbol;Acc:VGNC:99028]	1.71	0.0364
ssc-miR-374b-5p	PRKCE	protein kinase C epsilon [Source:VGNC Symbol;Acc:VGNC:91807]	1.71	0.0364
ssc-miR-374b-5p	PRKG1	protein kinase cGMP-dependent 1 [Source:VGNC Symbol;Acc:VGNC:91816]	1.71	0.0364
ssc-miR-374b-5p	PROK2	prokineticin 2 [Source:VGNC Symbol;Acc:VGNC:91831]	1.71	0.0364
ssc-miR-374b-5p	PRPF38B	hypothetical gene	1.71	0.0364

ssc-miR-374b-5p	PRPF40A	pre-mRNA processing factor 40 homolog A [Source:VGNC Symbol;Acc:VGNC:98222]	1.71	0.0364
ssc-miR-374b-5p	PRR16	proline rich 16 [Source:VGNC Symbol;Acc:VGNC:91855]	1.71	0.0364
ssc-miR-374b-5p	PRRG1	proline rich and Gla domain 1 [Source:VGNC Symbol;Acc:VGNC:101494]	1.71	0.0364
ssc-miR-374b-5p	PSD2	pleckstrin and Sec7 domain containing 2 [Source:VGNC Symbol;Acc:VGNC:91896]	1.71	0.0364
ssc-miR-374b-5p	PSME3	proteasome activator subunit 3 [Source:VGNC Symbol;Acc:VGNC:91926]	1.71	0.0364
ssc-miR-374b-5p	PSMF1	proteasome inhibitor subunit 1 [Source:VGNC Symbol;Acc:VGNC:96512]	1.71	0.0364
ssc-miR-374b-5p	PTEN	hypothetical gene	1.71	0.0364
ssc-miR-374b-5p	PTGES3L	prostaglandin E synthase 3 like [Source:VGNC Symbol;Acc:VGNC:91953]	1.71	0.0364
ssc-miR-374b-5p	PTPN11	protein tyrosine phosphatase non-receptor type 11 [Source:VGNC Symbol;Acc:VGNC:91972]	1.71	0.0364
ssc-miR-374b-5p	PTPRG	protein tyrosine phosphatase receptor type G [Source:VGNC Symbol;Acc:VGNC:91988]	1.71	0.0364
ssc-miR-374b-5p	PUM2	pumilio RNA binding family member 2 [Source:VGNC Symbol;Acc:VGNC:92002]	1.71	0.0364
ssc-miR-374b-5p	PURA	purine rich element binding protein A [Source:HGNC Symbol;Acc:HGNC:9701]	1.71	0.0364
ssc-miR-374b-5p	PURG	purine rich element binding protein G [Source:VGNC Symbol;Acc:VGNC:95865]	1.71	0.0364
ssc-miR-374b-5p	QKI	QKI, KH domain containing RNA binding [Source:VGNC Symbol;Acc:VGNC:92025]	1.71	0.0364
ssc-miR-374b-5p	RAB10	RAB10, member RAS onco family [Source:VGNC Symbol;Acc:VGNC:98235]	1.71	0.0364
ssc-miR-374b-5p	RAB1A	RAB1A, member RAS onco family [Source:VGNC Symbol;Acc:VGNC:104042]	1.71	0.0364
ssc-miR-374b-5p	RAB21	RAB21, member RAS oncogene family [Source:HGNC Symbol;Acc:HGNC:18263]	1.71	0.0364
ssc-miR-374b-5p	RAB22A	RAB22A, member RAS onco family [Source:VGNC Symbol;Acc:VGNC:98244]	1.71	0.0364
ssc-miR-374b-5p	RAB2A	RAB2A, member RAS onco family [Source:VGNC Symbol;Acc:VGNC:98252]	1.71	0.0364
ssc-miR-374b-5p	RAB38	RAB38, member RAS onco family [Source:VGNC Symbol;Acc:VGNC:98261]	1.71	0.0364
ssc-miR-374b-5p	RAB5B	RAB5B, member RAS oncogene family [Source:HGNC Symbol;Acc:HGNC:9784]	1.71	0.0364
ssc-miR-374b-5p	RAD21	RAD21 cohesin complex component [Source:VGNC Symbol;Acc:VGNC:92054]	1.71	0.0364
ssc-miR-374b-5p	RAI1	retinoic acid induced 1 [Source:VGNC Symbol;Acc:VGNC:92067]	1.71	0.0364
ssc-miR-374b-5p	RALA	RAS like proto-oncogene A [Source:HGNC Symbol;Acc:HGNC:9839]	1.71	0.0364
ssc-miR-374b-5p	RALGDS	ral guanine nucleotide dissociation stimulator [Source:HGNC Symbol;Acc:HGNC:9842]	1.71	0.0364
ssc-miR-374b-5p	RANBP9	RAN binding protein 9 [Source:VGNC Symbol;Acc:VGNC:92082]	1.71	0.0364
ssc-miR-374b-5p	RASA1	RAS p21 protein activator 1 [Source:VGNC Symbol;Acc:VGNC:92101]	1.71	0.0364
ssc-miR-374b-5p	RASA2	RAS p21 protein activator 2 [Source:VGNC Symbol;Acc:VGNC:92102]	1.71	0.0364
ssc-miR-374b-5p	RASSF8	Ras association domain family member 8 [Source:VGNC Symbol;Acc:VGNC:92128]	1.71	0.0364
ssc-miR-374b-5p	RBFOX2	RNA binding fox-1 homolog 2 [Source:VGNC Symbol;Acc:VGNC:92140]	1.71	0.0364
ssc-miR-374b-5p	RBM27	RNA binding motif protein 27 [Source:HGNC Symbol;Acc:HGNC:29243]	1.71	0.0364
ssc-miR-374b-5p	RBM38	RNA binding motif protein 38 [Source:VGNC Symbol;Acc:VGNC:95666]	1.71	0.0364
ssc-miR-374b-5p	RC3H1	ring finger and CCCH-type domains 1 [Source:VGNC Symbol;Acc:VGNC:92168]	1.71	0.0364
ssc-miR-374b-5p	RCAN1	regulator of calcineurin 1 [Source:VGNC Symbol;Acc:VGNC:92170]	1.71	0.0364
ssc-miR-374b-5p	RER1	retention in endoplasmic reticulum sorting receptor 1 [Source:VGNC Symbol;Acc:VGNC:92215]	1.71	0.0364
ssc-miR-374b-5p	RFESD	Rieske Fe-S domain containing [Source:VGNC Symbol;Acc:VGNC:92236]	1.71	0.0364
ssc-miR-374b-5p	RFTN2	raftlin family member 2 [Source:VGNC Symbol;Acc:VGNC:96128]	1.71	0.0364
ssc-miR-374b-5p	RFX3	regulatory factor X3 [Source:VGNC Symbol;Acc:VGNC:92245]	1.71	0.0364
ssc-miR-374b-5p	RFX4	regulatory factor X4 [Source:HGNC Symbol;Acc:HGNC:9985]	1.71	0.0364
ssc-miR-374b-5p	RGS14	regulator of G protein signaling 14 [Source:VGNC Symbol;Acc:VGNC:92261]	1.71	0.0364
ssc-miR-374b-5p	RGS7BP	regulator of G protein signaling 7 binding protein [Source:VGNC Symbol;Acc:VGNC:92268]	1.71	0.0364
ssc-miR-374b-5p	RHOQ	ras homolog family member Q [Source:VGNC Symbol;Acc:VGNC:92292]	1.71	0.0364
ssc-miR-374b-5p	RMND5A	required for meiotic nuclear division 5 homolog A [Source:VGNC Symbol;Acc:VGNC:92336]	1.71	0.0364
ssc-miR-374b-5p	RNF11	ring finger protein 11 [Source:VGNC Symbol;Acc:VGNC:98601]	1.71	0.0364
ssc-miR-374b-5p	RNF115	ring finger protein 115 [Source:VGNC Symbol;Acc:VGNC:92348]	1.71	0.0364
ssc-miR-374b-5p	RNF14	ring finger protein 14 [Source:VGNC Symbol;Acc:VGNC:92357]	1.71	0.0364
ssc-miR-374b-5p	RNF214	ring finger protein 214 [Source:VGNC Symbol;Acc:VGNC:92382]	1.71	0.0364
ssc-miR-374b-5p	RNF222	ring finger protein 222 [Source:VGNC Symbol;Acc:VGNC:92384]	1.71	0.0364
ssc-miR-374b-5p	RNF38	ring finger protein 38 [Source:VGNC Symbol;Acc:VGNC:92390]	1.71	0.0364
ssc-miR-374b-5p	RNF44	ring finger protein 44 [Source:VGNC Symbol;Acc:VGNC:92395]	1.71	0.0364

ssc-miR-374b-5p	RORA	RAR related orphan receptor A [Source:VGNC Symbol;Acc:VGNC:92408]	1.71	0.0364
ssc-miR-374b-5p	RORB	RAR related orphan receptor B [Source:VGNC Symbol;Acc:VGNC:92409]	1.71	0.0364
ssc-miR-374b-5p	RP11-407N17.3	hypothetical gene	1.71	0.0364
ssc-miR-374b-5p	RPS6KA3	ribosomal protein S6 kinase A3 [Source:VGNC Symbol;Acc:VGNC:92442]	1.71	0.0364
ssc-miR-374b-5p	RRP15	ribosomal RNA processing 15 homolog [Source:VGNC Symbol;Acc:VGNC:96036]	1.71	0.0364
ssc-miR-374b-5p	RRP1B	ribosomal RNA processing 1B [Source:VGNC Symbol;Acc:VGNC:92466]	1.71	0.0364
ssc-miR-374b-5p	RSF1	remodeling and spacing factor 1 [Source:VGNC Symbol;Acc:VGNC:92476]	1.71	0.0364
ssc-miR-374b-5p	RSRC2	arginine and serine rich coiled-coil 2 [Source:VGNC Symbol;Acc:VGNC:92488]	1.71	0.0364
ssc-miR-374b-5p	RTKN2	rhotekin 2 [Source:VGNC Symbol;Acc:VGNC:92495]	1.71	0.0364
ssc-miR-374b-5p	RYBP	RING1 and YY1 binding protein [Source:VGNC Symbol;Acc:VGNC:92532]	1.71	0.0364
ssc-miR-374b-5p	SAP30	Sin3A associated protein 30 [Source:VGNC Symbol;Acc:VGNC:92575]	1.71	0.0364
ssc-miR-374b-5p	SATB1	SATB homeobox 1 [Source:VGNC Symbol;Acc:VGNC:92587]	1.71	0.0364
ssc-miR-374b-5p	SCAI	suppressor of cancer cell invasion [Source:VGNC Symbol;Acc:VGNC:103170]	1.71	0.0364
ssc-miR-374b-5p	SCIN	scinderin [Source:NCBI gene (formerly Entrezgene);Acc:100512981]	1.71	0.0364
ssc-miR-374b-5p	SCN1A	sodium voltage-gated channel alpha subunit 1 [Source:VGNC Symbol;Acc:VGNC:95478]	1.71	0.0364
ssc-miR-374b-5p	SCN2B	sodium voltage-gated channel beta subunit 2 [Source:VGNC Symbol;Acc:VGNC:92633]	1.71	0.0364
ssc-miR-374b-5p	SCN5A	sodium voltage-gated channel alpha subunit 5 [Source:VGNC Symbol;Acc:VGNC:92637]	1.71	0.0364
ssc-miR-374b-5p	SCN7A	sodium voltage-gated channel alpha subunit 7 [Source:VGNC Symbol;Acc:VGNC:95480]	1.71	0.0364
ssc-miR-374b-5p	SCOC	short coiled-coil protein [Source:VGNC Symbol;Acc:VGNC:98960]	1.71	0.0364
ssc-miR-374b-5p	SCRN3	secernin 3 [Source:VGNC Symbol;Acc:VGNC:96241]	1.71	0.0364
ssc-miR-374b-5p	SEC23B	SEC23 homolog B, COPII coat complex component [Source:VGNC Symbol;Acc:VGNC:95490]	1.71	0.0364
ssc-miR-374b-5p	SEC23IP	SEC23 interacting protein [Source:VGNC Symbol;Acc:VGNC:92675]	1.71	0.0364
ssc-miR-374b-5p	SEMA3D	semaphorin 3D [Source:VGNC Symbol;Acc:VGNC:92696]	1.71	0.0364
ssc-miR-374b-5p	SEMA5A	semaphorin 5A [Source:VGNC Symbol;Acc:VGNC:92706]	1.71	0.0364
ssc-miR-374b-5p	SEPT7	hypothetical gene	1.71	0.0364
ssc-miR-374b-5p	SERTAD2	SERTA domain containing 2 [Source:VGNC Symbol;Acc:VGNC:92750]	1.71	0.0364
ssc-miR-374b-5p	SERTAD4	SERTA domain containing 4 [Source:VGNC Symbol;Acc:VGNC:92752]	1.71	0.0364
ssc-miR-374b-5p	SETBP1	SET binding protein 1 [Source:VGNC Symbol;Acc:VGNC:92756]	1.71	0.0364
ssc-miR-374b-5p	SETD1B	SET domain containing 1B, histone lysine methyltransferase [Source:HGNC Symbol;Acc:HGNC:29187]	1.71	0.0364
ssc-miR-374b-5p	SETD2	SET domain containing 2, histone lysine methyltransferase [Source:VGNC Symbol;Acc:VGNC:92757]	1.71	0.0364
ssc-miR-374b-5p	SETD7	SET domain containing 7, histone lysine methyltransferase [Source:VGNC Symbol;Acc:VGNC:92762]	1.71	0.0364
ssc-miR-374b-5p	SEZ6	seizure related 6 homolog [Source:VGNC Symbol;Acc:VGNC:92767]	1.71	0.0364
ssc-miR-374b-5p	SF1	splicing factor 1 [Source:HGNC Symbol;Acc:HGNC:12950]	1.71	0.0364
ssc-miR-374b-5p	SGIP1	SH3GL interacting endocytic adaptor 1 [Source:VGNC Symbol;Acc:VGNC:92792]	1.71	0.0364
ssc-miR-374b-5p	SGK3	serum/glucocorticoid regulated kinase family member 3 [Source:HGNC Symbol;Acc:HGNC:10812]	1.71	0.0364
ssc-miR-374b-5p	SH3BP5	SH3 domain binding protein 5 [Source:VGNC Symbol;Acc:VGNC:92819]	1.71	0.0364
ssc-miR-374b-5p	SHANK3	hypothetical gene	1.71	0.0364
ssc-miR-374b-5p	SHISA6	hypothetical gene	1.71	0.0364
ssc-miR-374b-5p	SHOC2	SHOC2 leucine rich repeat scaffold protein [Source:VGNC Symbol;Acc:VGNC:98318]	1.71	0.0364
ssc-miR-374b-5p	SIKE1	suppressor of IKBKE 1 [Source:VGNC Symbol;Acc:VGNC:92874]	1.71	0.0364
ssc-miR-374b-5p	SIX6	SIX homeobox 6 [Source:VGNC Symbol;Acc:VGNC:92897]	1.71	0.0364
ssc-miR-374b-5p	SKI	SKI proto-onco [Source:VGNC Symbol;Acc:VGNC:92902]	1.71	0.0364
ssc-miR-374b-5p	SLC10A7	solute carrier family 10 member 7 [Source:VGNC Symbol;Acc:VGNC:92919]	1.71	0.0364
ssc-miR-374b-5p	SLC15A4	solute carrier family 15 member 4 [Source:VGNC Symbol;Acc:VGNC:92937]	1.71	0.0364
ssc-miR-374b-5p	SLC16A1	hypothetical gene	1.71	0.0364
ssc-miR-374b-5p	SLC18A2	solute carrier family 18 member A2 [Source:VGNC Symbol;Acc:VGNC:92958]	1.71	0.0364
ssc-miR-374b-5p	SLC24A2	solute carrier family 24 member 2 [Source:VGNC Symbol;Acc:VGNC:92987]	1.71	0.0364
ssc-miR-374b-5p	SLC25A16	solute carrier family 25 member 16 [Source:VGNC Symbol;Acc:VGNC:92995]	1.71	0.0364
ssc-miR-374b-5p	SLC25A33	solute carrier family 25 member 33 [Source:VGNC Symbol;Acc:VGNC:93009]	1.71	0.0364
ssc-miR-374b-5p	SLC2A2	solute carrier family 2 member 2 [Source:VGNC Symbol;Acc:VGNC:93048]	1.71	0.0364

ssc-miR-374b-5p	SLC30A1	solute carrier family 30 member 1 [Source:VGNC Symbol;Acc:VGNC:93055]	1.71	0.0364
ssc-miR-374b-5p	SLC30A10	hypothetical gene	1.71	0.0364
ssc-miR-374b-5p	SLC30A5	solute carrier family 30 member 5 [Source:VGNC Symbol;Acc:VGNC:93059]	1.71	0.0364
ssc-miR-374b-5p	SLC35A3	solute carrier family 35 member A3 [Source:VGNC Symbol;Acc:VGNC:98860]	1.71	0.0364
ssc-miR-374b-5p	SLC35B3	solute carrier family 35 member B3 [Source:VGNC Symbol;Acc:VGNC:93073]	1.71	0.0364
ssc-miR-374b-5p	SLC6A14	solute carrier family 6 member 14 [Source:NCBI gene (formerly Entrezgene);Acc:431671]	1.71	0.0364
ssc-miR-374b-5p	SLC6A17	solute carrier family 6 member 17 [Source:VGNC Symbol;Acc:VGNC:93158]	1.71	0.0364
ssc-miR-374b-5p	SLC6A5	solute carrier family 6 member 5 [Source:VGNC Symbol;Acc:VGNC:93165]	1.71	0.0364
ssc-miR-374b-5p	SLC7A14	solute carrier family 7 member 14 [Source:VGNC Symbol;Acc:VGNC:93173]	1.71	0.0364
ssc-miR-374b-5p	SLCO2A1	solute carrier organic anion transporter family member 2A1 [Source:VGNC Symbol;Acc:VGNC:93195]	1.71	0.0364
ssc-miR-374b-5p	SLIT3	slit guidance ligand 3 [Source:VGNC Symbol;Acc:VGNC:93205]	1.71	0.0364
ssc-miR-374b-5p	SMAD6	SMAD family member 6 [Source:VGNC Symbol;Acc:VGNC:93220]	1.71	0.0364
ssc-miR-374b-5p	SMARCA5	SWI/SNF related, matrix associated, actin dependent regulator of chromatin, subfamily a, member 5 [Source:VGNC Symbol;Acc:VGNC:93228]	1.71	0.0364
ssc-miR-374b-5p	SMARCAD1	SWI/SNF-related, matrix-associated actin-dependent regulator of chromatin, subfamily a, containing DEAD/H box 1 [Source:VGNC Symbol;Acc:VGNC:93229]	1.71	0.0364
ssc-miR-374b-5p	SMYD4	SET and MYND domain containing 4 [Source:VGNC Symbol;Acc:VGNC:93274]	1.71	0.0364
ssc-miR-374b-5p	SNRPC	small nuclear ribonucleoprotein polypeptide C [Source:VGNC Symbol;Acc:VGNC:93295]	1.71	0.0364
ssc-miR-374b-5p	SNTB1	syntrophin beta 1 [Source:VGNC Symbol;Acc:VGNC:93297]	1.71	0.0364
ssc-miR-374b-5p	SNTB2	syntrophin beta 2 [Source:VGNC Symbol;Acc:VGNC:93298]	1.71	0.0364
ssc-miR-374b-5p	SNTG1	syntrophin gamma 1 [Source:VGNC Symbol;Acc:VGNC:93299]	1.71	0.0364
ssc-miR-374b-5p	SNX17	sorting nexin 17 [Source:VGNC Symbol;Acc:VGNC:93309]	1.71	0.0364
ssc-miR-374b-5p	SOCS4	suppressor of cytokine signaling 4 [Source:VGNC Symbol;Acc:VGNC:93331]	1.71	0.0364
ssc-miR-374b-5p	SOGA3	SOGA family member 3 [Source:HGNC Symbol;Acc:HGNC:21494]	1.71	0.0364
ssc-miR-374b-5p	SOX4	SRY-box transcription factor 4 [Source:HGNC Symbol;Acc:HGNC:11200]	1.71	0.0364
ssc-miR-374b-5p	SP1	Sp1 transcription factor [Source:VGNC Symbol;Acc:VGNC:93360]	1.71	0.0364
ssc-miR-374b-5p	SP3	Sp3 transcription factor [Source:VGNC Symbol;Acc:VGNC:95511]	1.71	0.0364
ssc-miR-374b-5p	SP4	Sp4 transcription factor [Source:VGNC Symbol;Acc:VGNC:93362]	1.71	0.0364
ssc-miR-374b-5p	SPACA1	sperm acrosome associated 1 [Source:VGNC Symbol;Acc:VGNC:93367]	1.71	0.0364
ssc-miR-374b-5p	SPAG9	sperm associated antigen 9 [Source:VGNC Symbol;Acc:VGNC:93374]	1.71	0.0364
ssc-miR-374b-5p	SPAST	spastin [Source:VGNC Symbol;Acc:VGNC:98333]	1.71	0.0364
ssc-miR-374b-5p	SPICE1	spindle and centriole associated protein 1 [Source:HGNC Symbol;Acc:HGNC:25083]	1.71	0.0364
ssc-miR-374b-5p	SPOPL	speckle type BTB/POZ protein like [Source:VGNC Symbol;Acc:VGNC:96161]	1.71	0.0364
ssc-miR-374b-5p	SPPL2A	signal peptide peptidase like 2A [Source:VGNC Symbol;Acc:VGNC:98337]	1.71	0.0364
ssc-miR-374b-5p	SPPL3	signal peptide peptidase like 3 [Source:VGNC Symbol;Acc:VGNC:98340]	1.71	0.0364
ssc-miR-374b-5p	SPRY2	sprouty RTK signaling antagonist 2 [Source:VGNC Symbol;Acc:VGNC:93425]	1.71	0.0364
ssc-miR-374b-5p	SPTLC1	serine palmitoyltransferase long chain base subunit 1 [Source:VGNC Symbol;Acc:VGNC:93439]	1.71	0.0364
ssc-miR-374b-5p	SRCIN1	SRC kinase signaling inhibitor 1 [Source:VGNC Symbol;Acc:VGNC:93448]	1.71	0.0364
ssc-miR-374b-5p	SRFBP1	serum response factor binding protein 1 [Source:VGNC Symbol;Acc:VGNC:93453]	1.71	0.0364
ssc-miR-374b-5p	SRGAP1	SLIT-ROBO Rho GTPase activating protein 1 [Source:VGNC Symbol;Acc:VGNC:93454]	1.71	0.0364
ssc-miR-374b-5p	SRPK2	SRSF protein kinase 2 [Source:VGNC Symbol;Acc:VGNC:93463]	1.71	0.0364
ssc-miR-374b-5p	SRSF10	serine and arginine rich splicing factor 10 [Source:VGNC Symbol;Acc:VGNC:93472]	1.71	0.0364
ssc-miR-374b-5p	SRSF7	serine and arginine rich splicing factor 7 [Source:VGNC Symbol;Acc:VGNC:93477]	1.71	0.0364
ssc-miR-374b-5p	SSH1	slingshot protein phosphatase 1 [Source:VGNC Symbol;Acc:VGNC:93485]	1.71	0.0364
ssc-miR-374b-5p	SSX2IP	SSX family member 2 interacting protein [Source:VGNC Symbol;Acc:VGNC:93499]	1.71	0.0364
ssc-miR-374b-5p	ST18	ST18 C2H2C-type zinc finger transcription factor [Source:VGNC Symbol;Acc:VGNC:93501]	1.71	0.0364
ssc-miR-374b-5p	ST6GALNAC3	ST6 N-acetylgalactosaminide alpha-2,6-sialyltransferase 3 [Source:VGNC Symbol;Acc:VGNC:93511]	1.71	0.0364
ssc-miR-374b-5p	ST8SIA2	ST8 alpha-N-acetyl-neuraminide alpha-2,8-sialyltransferase 2 [Source:VGNC Symbol;Acc:VGNC:93517]	1.71	0.0364
ssc-miR-374b-5p	ST8SIA4	ST8 alpha-N-acetyl-neuraminide alpha-2,8-sialyltransferase 4 [Source:VGNC Symbol;Acc:VGNC:93519]	1.71	0.0364
ssc-miR-374b-5p	STIM2	stromal interaction molecule 2 [Source:VGNC Symbol;Acc:VGNC:98967]	1.71	0.0364
ssc-miR-374b-5p	STK38L	serine/threonine kinase 38 like [Source:VGNC Symbol;Acc:VGNC:93557]	1.71	0.0364
ssc-miR-374b-5p	STK4	serine/threonine kinase 4 [Source:VGNC Symbol;Acc:VGNC:98349]	1.71	0.0364

ssc-miR-374b-5p	STMN2	stathmin 2 [Source:VGNC Symbol;Acc:VGNC:93561]	1.71	0.0364
ssc-miR-374b-5p	STRN	striatin [Source:VGNC Symbol;Acc:VGNC:93578]	1.71	0.0364
ssc-miR-374b-5p	STX17	syntaxin 17 [Source:VGNC Symbol;Acc:VGNC:93587]	1.71	0.0364
ssc-miR-374b-5p	STX7	syntaxin 7 [Source:VGNC Symbol;Acc:VGNC:93595]	1.71	0.0364
ssc-miR-374b-5p	STXBP5	syntaxin binding protein 5 [Source:VGNC Symbol;Acc:VGNC:93599]	1.71	0.0364
ssc-miR-374b-5p	STYX	serine/threonine/tyrosine interacting protein [Source:VGNC Symbol;Acc:VGNC:93602]	1.71	0.0364
ssc-miR-374b-5p	SUCNR1	succinate receptor 1 [Source:VGNC Symbol;Acc:VGNC:93605]	1.71	0.0364
ssc-miR-374b-5p	SUPT3H	SPT3 homolog, SAGA and STAGA complex component [Source:VGNC Symbol;Acc:VGNC:93623]	1.71	0.0364
ssc-miR-374b-5p	SWT1	SWT1 RNA endoribonuclease homolog [Source:VGNC Symbol;Acc:VGNC:93647]	1.71	0.0364
ssc-miR-374b-5p	SYDE2	synapse defective Rho GTPase homolog 2 [Source:VGNC Symbol;Acc:VGNC:98874]	1.71	0.0364
ssc-miR-374b-5p	SYNE1	hypothetical gene	1.71	0.0364
ssc-miR-374b-5p	SYP	synaptophysin [Source:VGNC Symbol;Acc:VGNC:93676]	1.71	0.0364
ssc-miR-374b-5p	SYT14	synaptotagmin 14 [Source:VGNC Symbol;Acc:VGNC:108619]	1.71	0.0364
ssc-miR-374b-5p	TACC1	hypothetical gene	1.71	0.0364
ssc-miR-374b-5p	TAF4B	TATA-box binding protein associated factor 4b [Source:VGNC Symbol;Acc:VGNC:93716]	1.71	0.0364
ssc-miR-374b-5p	TAF5L	TATA-box binding protein associated factor 5 like [Source:VGNC Symbol;Acc:VGNC:93718]	1.71	0.0364
ssc-miR-374b-5p	TAOK1	TAO kinase 1 [Source:VGNC Symbol;Acc:VGNC:98355]	1.71	0.0364
ssc-miR-374b-5p	TAT	tyrosine aminotransferase [Source:VGNC Symbol;Acc:VGNC:93752]	1.71	0.0364
ssc-miR-374b-5p	TBC1D9	TBC1 domain family member 9 [Source:VGNC Symbol;Acc:VGNC:93781]	1.71	0.0364
ssc-miR-374b-5p	TBX4	T-box transcription factor 4 [Source:VGNC Symbol;Acc:VGNC:93803]	1.71	0.0364
ssc-miR-374b-5p	TCERG1	transcription elongation regulator 1 [Source:VGNC Symbol;Acc:VGNC:93815]	1.71	0.0364
ssc-miR-374b-5p	TCF21	transcription factor 21 [Source:VGNC Symbol;Acc:VGNC:93819]	1.71	0.0364
ssc-miR-374b-5p	TCF4	transcription factor 4 [Source:VGNC Symbol;Acc:VGNC:93823]	1.71	0.0364
ssc-miR-374b-5p	TEAD3	TEA domain transcription factor 3 [Source:VGNC Symbol;Acc:VGNC:93855]	1.71	0.0364
ssc-miR-374b-5p	TENM1	teneurin transmembrane protein 1 [Source:VGNC Symbol;Acc:VGNC:98363]	1.71	0.0364
ssc-miR-374b-5p	TET2	hypothetical gene	1.71	0.0364
ssc-miR-374b-5p	TET3	tet methylcytosine dioxygenase 3 [Source:VGNC Symbol;Acc:VGNC:93890]	1.71	0.0364
ssc-miR-374b-5p	TFAP4	transcription factor AP-4 [Source:VGNC Symbol;Acc:VGNC:93912]	1.71	0.0364
ssc-miR-374b-5p	TFDP1	transcription factor Dp-1 [Source:HGNC Symbol;Acc:HGNC:11749]	1.71	0.0364
ssc-miR-374b-5p	TGFA	transforming growth factor alpha [Source:VGNC Symbol;Acc:VGNC:93928]	1.71	0.0364
ssc-miR-374b-5p	TLE4	TLE family member 4, transcriptional corepressor [Source:VGNC Symbol;Acc:VGNC:98372]	1.71	0.0364
ssc-miR-374b-5p	TLR4	toll like receptor 4 [Source:VGNC Symbol;Acc:VGNC:94025]	1.71	0.0364
ssc-miR-374b-5p	TMC7	transmembrane channel like 7 [Source:VGNC Symbol;Acc:VGNC:94044]	1.71	0.0364
ssc-miR-374b-5p	TMEM108	transmembrane protein 108 [Source:VGNC Symbol;Acc:VGNC:94065]	1.71	0.0364
ssc-miR-374b-5p	TMEM123	hypothetical gene	1.71	0.0364
ssc-miR-374b-5p	TMEM161B	transmembrane protein 161B [Source:VGNC Symbol;Acc:VGNC:94102]	1.71	0.0364
ssc-miR-374b-5p	TMEM184C	transmembrane protein 184C [Source:VGNC Symbol;Acc:VGNC:94121]	1.71	0.0364
ssc-miR-374b-5p	TMEM185A	transmembrane protein 185A [Source:VGNC Symbol;Acc:VGNC:94122]	1.71	0.0364
ssc-miR-374b-5p	TMEM38B	transmembrane protein 38B [Source:VGNC Symbol;Acc:VGNC:94175]	1.71	0.0364
ssc-miR-374b-5p	TMEM56	hypothetical gene	1.71	0.0364
ssc-miR-374b-5p	TMSB10	thymosin beta 10 [Source:NCBI gene (formerly Entrezgene);Acc:100037998]	1.71	0.0364
ssc-miR-374b-5p	TNKS2	tankyrase 2 [Source:VGNC Symbol;Acc:VGNC:94280]	1.71	0.0364
ssc-miR-374b-5p	TNRC18	trinucleotide repeat containing 18 [Source:VGNC Symbol;Acc:VGNC:94291]	1.71	0.0364
ssc-miR-374b-5p	TNRC6A	trinucleotide repeat containing adaptor 6A [Source:VGNC Symbol;Acc:VGNC:94292]	1.71	0.0364
ssc-miR-374b-5p	TOP1	hypothetical gene	1.71	0.0364
ssc-miR-374b-5p	TOR1AIP1	torsin 1A interacting protein 1 [Source:VGNC Symbol;Acc:VGNC:94317]	1.71	0.0364
ssc-miR-374b-5p	TPRG1	tumor protein p63 regulated 1 [Source:VGNC Symbol;Acc:VGNC:94348]	1.71	0.0364
ssc-miR-374b-5p	TRAFD1	TRAF-type zinc finger domain containing 1 [Source:VGNC Symbol;Acc:VGNC:94367]	1.71	0.0364
ssc-miR-374b-5p	TRAK2	trafficking kinesin protein 2 [Source:VGNC Symbol;Acc:VGNC:95577]	1.71	0.0364
ssc-miR-374b-5p	TRAPPC13	trafficking protein particle complex subunit 13 [Source:VGNC Symbol;Acc:VGNC:104085]	1.71	0.0364

ssc-miR-374b-5p	TRAPPC8	trafficking protein particle complex subunit 8 [Source:VGNC Symbol;Acc:VGNC:94379]	1.71	0.0364
ssc-miR-374b-5p	TRERF1	transcriptional regulating factor 1 [Source:VGNC Symbol;Acc:VGNC:94387]	1.71	0.0364
ssc-miR-374b-5p	TRPM3	transient receptor potential cation channel subfamily M member 3 [Source:VGNC Symbol;Acc:VGNC:98384]	1.71	0.0364
ssc-miR-374b-5p	TRPM7	transient receptor potential cation channel subfamily M member 7 [Source:VGNC Symbol;Acc:VGNC:94470]	1.71	0.0364
ssc-miR-374b-5p	TRPV3	transient receptor potential cation channel subfamily V member 3 [Source:VGNC Symbol;Acc:VGNC:94475]	1.71	0.0364
ssc-miR-374b-5p	TTBK2	tau tubulin kinase 2 [Source:VGNC Symbol;Acc:VGNC:98387]	1.71	0.0364
ssc-miR-374b-5p	TTC30A	hypothetical gene	1.71	0.0364
ssc-miR-374b-5p	TTC37	hypothetical gene	1.71	0.0364
ssc-miR-374b-5p	TTLL7	tubulin tyrosine ligase like 7 [Source:VGNC Symbol;Acc:VGNC:94571]	1.71	0.0364
ssc-miR-374b-5p	TXLNB	taxilin beta [Source:VGNC Symbol;Acc:VGNC:94601]	1.71	0.0364
ssc-miR-374b-5p	TXNL1	thioredoxin like 1 [Source:VGNC Symbol;Acc:VGNC:94611]	1.71	0.0364
ssc-miR-374b-5p	UBA6	ubiquitin like modifier activating enzyme 6 [Source:VGNC Symbol;Acc:VGNC:94630]	1.71	0.0364
ssc-miR-374b-5p	UBE2B	ubiquitin conjugating enzyme E2 B [Source:VGNC Symbol;Acc:VGNC:94640]	1.71	0.0364
ssc-miR-374b-5p	UBE2E3	ubiquitin conjugating enzyme E2 E3 [Source:VGNC Symbol;Acc:VGNC:106461]	1.71	0.0364
ssc-miR-374b-5p	UBE2H	ubiquitin conjugating enzyme E2 H [Source:VGNC Symbol;Acc:VGNC:94643]	1.71	0.0364
ssc-miR-374b-5p	UBE2I	hypothetical gene	1.71	0.0364
ssc-miR-374b-5p	UBE2J1	ubiquitin conjugating enzyme E2 J1 [Source:VGNC Symbol;Acc:VGNC:94644]	1.71	0.0364
ssc-miR-374b-5p	UBE2W	ubiquitin conjugating enzyme E2 W [Source:VGNC Symbol;Acc:VGNC:98890]	1.71	0.0364
ssc-miR-374b-5p	UBXN4	UBX domain protein 4 [Source:VGNC Symbol;Acc:VGNC:98397]	1.71	0.0364
ssc-miR-374b-5p	UGCG	UDP-glucose ceramide glucosyltransferase [Source:VGNC Symbol;Acc:VGNC:94685]	1.71	0.0364
ssc-miR-374b-5p	UHMK1	U2AF homology motif kinase 1 [Source:VGNC Symbol;Acc:VGNC:94688]	1.71	0.0364
ssc-miR-374b-5p	USP15	ubiquitin specific peptidase 15 [Source:VGNC Symbol;Acc:VGNC:94748]	1.71	0.0364
ssc-miR-374b-5p	USP30	ubiquitin specific peptidase 30 [Source:VGNC Symbol;Acc:VGNC:94758]	1.71	0.0364
ssc-miR-374b-5p	USP38	ubiquitin specific peptidase 38 [Source:VGNC Symbol;Acc:VGNC:94762]	1.71	0.0364
ssc-miR-374b-5p	USP47	ubiquitin specific peptidase 47 [Source:VGNC Symbol;Acc:VGNC:94769]	1.71	0.0364
ssc-miR-374b-5p	UST	uronyl 2-sulfotransferase [Source:VGNC Symbol;Acc:VGNC:94780]	1.71	0.0364
ssc-miR-374b-5p	VAMP2	vesicle associated membrane protein 2 [Source:HGNC Symbol;Acc:HGNC:12643]	1.71	0.0364
ssc-miR-374b-5p	VCAM1	vascular cell adhesion molecule 1 [Source:VGNC Symbol;Acc:VGNC:94811]	1.71	0.0364
ssc-miR-374b-5p	VCAN	versican [Source:VGNC Symbol;Acc:VGNC:108163]	1.71	0.0364
ssc-miR-374b-5p	VCPIP1	valosin containing protein interacting protein 1 [Source:VGNC Symbol;Acc:VGNC:94813]	1.71	0.0364
ssc-miR-374b-5p	VEGFC	vascular endothelial growth factor C [Source:VGNC Symbol;Acc:VGNC:95563]	1.71	0.0364
ssc-miR-374b-5p	VHL	hypothetical gene	1.71	0.0364
ssc-miR-374b-5p	VPRBP	hypothetical gene	1.71	0.0364
ssc-miR-374b-5p	VSNL1	visinin like 1 [Source:VGNC Symbol;Acc:VGNC:94871]	1.71	0.0364
ssc-miR-374b-5p	VWC2	von Willebrand factor C domain containing 2 [Source:VGNC Symbol;Acc:VGNC:94887]	1.71	0.0364
ssc-miR-374b-5p	WDR11	WD repeat domain 11 [Source:VGNC Symbol;Acc:VGNC:94906]	1.71	0.0364
ssc-miR-374b-5p	WDR37	WD repeat domain 37 [Source:VGNC Symbol;Acc:VGNC:96258]	1.71	0.0364
ssc-miR-374b-5p	WDR52	hypothetical gene	1.71	0.0364
ssc-miR-374b-5p	WDR72	WD repeat domain 72 [Source:VGNC Symbol;Acc:VGNC:94938]	1.71	0.0364
ssc-miR-374b-5p	WISP3	hypothetical gene	1.71	0.0364
ssc-miR-374b-5p	WNT16	Wnt family member 16 [Source:VGNC Symbol;Acc:VGNC:94967]	1.71	0.0364
ssc-miR-374b-5p	WNT2B	Wnt family member 2B [Source:VGNC Symbol;Acc:VGNC:94969]	1.71	0.0364
ssc-miR-374b-5p	WNT3	Wnt family member 3 [Source:VGNC Symbol;Acc:VGNC:94970]	1.71	0.0364
ssc-miR-374b-5p	WNT5A	Wnt family member 5A [Source:VGNC Symbol;Acc:VGNC:94973]	1.71	0.0364
ssc-miR-374b-5p	WRN	WRN RecQ like helicase [Source:VGNC Symbol;Acc:VGNC:96605]	1.71	0.0364
ssc-miR-374b-5p	WWC2	WW and C2 domain containing 2 [Source:VGNC Symbol;Acc:VGNC:96033]	1.71	0.0364
ssc-miR-374b-5p	XIAP	X-linked inhibitor of apoptosis [Source:NCBI gene (formerly Entrezgene);Acc:100037300]	1.71	0.0364
ssc-miR-374b-5p	XK	X-linked Kx blood group [Source:HGNC Symbol;Acc:HGNC:12811]	1.71	0.0364
ssc-miR-374b-5p	XPOT	exportin for tRNA [Source:VGNC Symbol;Acc:VGNC:95008]	1.71	0.0364
ssc-miR-374b-5p	YOD1	YOD1 deubiquitinase [Source:VGNC Symbol;Acc:VGNC:95035]	1.71	0.0364

ssc-miR-374b-5p	ZBTB10	zinc finger and BTB domain containing 10 [Source:VGNC Symbol;Acc:VGNC:95058]	1.71	0.0364
ssc-miR-374b-5p	ZBTB20	zinc finger and BTB domain containing 20 [Source:VGNC Symbol;Acc:VGNC:95063]	1.71	0.0364
ssc-miR-374b-5p	ZBTB34	zinc finger and BTB domain containing 34 [Source:VGNC Symbol;Acc:VGNC:95070]	1.71	0.0364
ssc-miR-374b-5p	ZBTB43	zinc finger and BTB domain containing 43 [Source:VGNC Symbol;Acc:VGNC:95075]	1.71	0.0364
ssc-miR-374b-5p	ZBTB46	zinc finger and BTB domain containing 46 [Source:VGNC Symbol;Acc:VGNC:95733]	1.71	0.0364
ssc-miR-374b-5p	ZC3H6	zinc finger CCCH-type containing 6 [Source:VGNC Symbol;Acc:VGNC:95098]	1.71	0.0364
ssc-miR-374b-5p	ZC3H7B	zinc finger CCCH-type containing 7B [Source:VGNC Symbol;Acc:VGNC:95100]	1.71	0.0364
ssc-miR-374b-5p	ZC3H8	zinc finger CCCH-type containing 8 [Source:VGNC Symbol;Acc:VGNC:95101]	1.71	0.0364
ssc-miR-374b-5p	ZCCHC14	zinc finger CCHC-type containing 14 [Source:VGNC Symbol;Acc:VGNC:95107]	1.71	0.0364
ssc-miR-374b-5p	ZCCHC24	zinc finger CCHC-type containing 24 [Source:HGNC Symbol;Acc:HGNC:26911]	1.71	0.0364
ssc-miR-374b-5p	ZCWPW2	zinc finger CW-type and PWWP domain containing 2 [Source:VGNC Symbol;Acc:VGNC:108706]	1.71	0.0364
ssc-miR-374b-5p	ZDHHC20	zinc finger DHHC-type palmitoyltransferase 20 [Source:VGNC Symbol;Acc:VGNC:95122]	1.71	0.0364
ssc-miR-374b-5p	ZDHHC23	zinc finger DHHC-type palmitoyltransferase 23 [Source:VGNC Symbol;Acc:VGNC:95125]	1.71	0.0364
ssc-miR-374b-5p	ZDHHC3	zinc finger DHHC-type palmitoyltransferase 3 [Source:VGNC Symbol;Acc:VGNC:95126]	1.71	0.0364
ssc-miR-374b-5p	ZDHHC5	zinc finger DHHC-type palmitoyltransferase 5 [Source:VGNC Symbol;Acc:VGNC:95127]	1.71	0.0364
ssc-miR-374b-5p	ZDHHC9	zinc finger DHHC-type palmitoyltransferase 9 [Source:VGNC Symbol;Acc:VGNC:95131]	1.71	0.0364
ssc-miR-374b-5p	ZEB2	hypothetical gene	1.71	0.0364
ssc-miR-374b-5p	ZER1	zyg-11 related cell cycle regulator [Source:VGNC Symbol;Acc:VGNC:95132]	1.71	0.0364
ssc-miR-374b-5p	ZFAND4	zinc finger AN1-type containing 4 [Source:VGNC Symbol;Acc:VGNC:95136]	1.71	0.0364
ssc-miR-374b-5p	ZFP91	ZFP91 zinc finger protein, atypical E3 ubiquitin ligase [Source:HGNC Symbol;Acc:HGNC:14983]	1.71	0.0364
ssc-miR-374b-5p	ZHX1	zinc fingers and homeoboxes 1 [Source:VGNC Symbol;Acc:VGNC:95164]	1.71	0.0364
ssc-miR-374b-5p	ZMAT3	zinc finger matrin-type 3 [Source:VGNC Symbol;Acc:VGNC:95177]	1.71	0.0364
ssc-miR-374b-5p	ZNF189	zinc finger protein 189 [Source:VGNC Symbol;Acc:VGNC:103205]	1.71	0.0364
ssc-miR-374b-5p	ZNF207	zinc finger protein 207 [Source:VGNC Symbol;Acc:VGNC:95205]	1.71	0.0364
ssc-miR-374b-5p	ZNF236	zinc finger protein 236 [Source:VGNC Symbol;Acc:VGNC:95209]	1.71	0.0364
ssc-miR-374b-5p	ZNF281	zinc finger protein 281 [Source:VGNC Symbol;Acc:VGNC:108285]	1.71	0.0364
ssc-miR-374b-5p	ZNF423	zinc finger protein 423 [Source:VGNC Symbol;Acc:VGNC:98752]	1.71	0.0364
ssc-miR-374b-5p	ZNF462	zinc finger protein 462 [Source:VGNC Symbol;Acc:VGNC:95244]	1.71	0.0364
ssc-miR-374b-5p	ZNF516	zinc finger protein 516 [Source:VGNC Symbol;Acc:VGNC:95256]	1.71	0.0364
ssc-miR-374b-5p	ZNF644	zinc finger protein 644 [Source:VGNC Symbol;Acc:VGNC:95283]	1.71	0.0364
ssc-miR-374b-5p	ZNF704	zinc finger protein 704 [Source:VGNC Symbol;Acc:VGNC:95296]	1.71	0.0364
ssc-miR-374b-5p	ZNF710	zinc finger protein 710 [Source:VGNC Symbol;Acc:VGNC:107180]	1.71	0.0364
ssc-miR-374b-5p	ZNF771	zinc finger protein 771 [Source:HGNC Symbol;Acc:HGNC:29653]	1.71	0.0364
ssc-miR-374b-5p	ZNF81	zinc finger protein 81 [Source:VGNC Symbol;Acc:VGNC:95308]	1.71	0.0364
ssc-miR-374b-5p	ZNHIT6	zinc finger HIT-type containing 6 [Source:VGNC Symbol;Acc:VGNC:98909]	1.71	0.0364
ssc-miR-374b-5p	ZSWIM6	zinc finger SWIM-type containing 6 [Source:VGNC Symbol;Acc:VGNC:95335]	1.71	0.0364
ssc-miR-374b-5p	ZZZ3	zinc finger ZZ-type containing 3 [Source:VGNC Symbol;Acc:VGNC:95348]	1.71	0.0364
ssc-miR-124a	AAK1	AP2 associated kinase 1 [Source:VGNC Symbol;Acc:VGNC:100379]	-1.71	0.1324
ssc-miR-124a	ABCA1	ATP binding cassette subfamily A member 1 [Source:VGNC Symbol;Acc:VGNC:84947]	-1.71	0.1324
ssc-miR-124a	ABCA2	ATP binding cassette subfamily A member 2 [Source:HGNC Symbol;Acc:HGNC:32]	-1.71	0.1324
ssc-miR-124a	ABCC4	hypothetical gene	-1.71	0.1324
ssc-miR-124a	ABCD2	ATP binding cassette subfamily D member 2 [Source:VGNC Symbol;Acc:VGNC:84962]	-1.71	0.1324
ssc-miR-124a	ABHD17C	abhydrolase domain containing 17C, depalmitoylase [Source:VGNC Symbol;Acc:VGNC:84978]	-1.71	0.1324
ssc-miR-124a	ABHD2	abhydrolase domain containing 2, acylglycerol lipase [Source:VGNC Symbol;Acc:VGNC:84980]	-1.71	0.1324
ssc-miR-124a	ABHD3	abhydrolase domain containing 3, phospholipase [Source:VGNC Symbol;Acc:VGNC:84981]	-1.71	0.1324
ssc-miR-124a	ABHD4	abhydrolase domain containing 4, N-acyl phospholipase B [Source:VGNC Symbol;Acc:VGNC:97859]	-1.71	0.1324
ssc-miR-124a	ABHD5	abhydrolase domain containing 5, lysophosphatidic acid acyltransferase [Source:VGNC Symbol;Acc:VGNC:97860]	-1.71	0.1324
ssc-miR-124a	ABR	ABR activator of RhoGEF and GTPase [Source:VGNC Symbol;Acc:VGNC:84989]	-1.71	0.1324
ssc-miR-124a	ABTB2	ankyrin repeat and BTB domain containing 2 [Source:VGNC Symbol;Acc:VGNC:84994]	-1.71	0.1324
ssc-miR-124a	AC012215.1	hypothetical gene	-1.71	0.1324

ssc-miR-124a	AC090616.2	hypothetical gene	-1.71	0.1324
ssc-miR-124a	AC129492.6	hypothetical gene	-1.71	0.1324
ssc-miR-124a	ACAA2	acetyl-CoA acyltransferase 2 [Source:VGNC Symbol;Acc:VGNC:84996]	-1.71	0.1324
ssc-miR-124a	ACADM	acyl-CoA dehydrogenase medium chain [Source:VGNC Symbol;Acc:VGNC:85000]	-1.71	0.1324
ssc-miR-124a	ACADSB	acyl-CoA dehydrogenase short/branched chain [Source:VGNC Symbol;Acc:VGNC:97863]	-1.71	0.1324
ssc-miR-124a	ACADVL	acyl-CoA dehydrogenase very long chain [Source:VGNC Symbol;Acc:VGNC:97864]	-1.71	0.1324
ssc-miR-124a	ACAN	aggrecan [Source:NCBI gene (formerly Entrezgene);Acc:397255]	-1.71	0.1324
ssc-miR-124a	ACER3	alkaline ceramidase 3 [Source:VGNC Symbol;Acc:VGNC:103893]	-1.71	0.1324
ssc-miR-124a	ACHE	acetylcholinesterase [Source:VGNC Symbol;Acc:VGNC:97029]	-1.71	0.1324
ssc-miR-124a	ACOX1	acyl-CoA oxidase 1 [Source:VGNC Symbol;Acc:VGNC:85020]	-1.71	0.1324
ssc-miR-124a	ACSL1	acyl-CoA synthetase long chain family member 1 [Source:VGNC Symbol;Acc:VGNC:96294]	-1.71	0.1324
ssc-miR-124a	ACSS1	acyl-CoA synthetase short chain family member 1 [Source:VGNC Symbol;Acc:VGNC:95732]	-1.71	0.1324
ssc-miR-124a	ACTB	actin beta [Source:VGNC Symbol;Acc:VGNC:96915]	-1.71	0.1324
ssc-miR-124a	ACVR2A	activin A receptor type 2A [Source:VGNC Symbol;Acc:VGNC:95843]	-1.71	0.1324
ssc-miR-124a	ACVR2B	activin A receptor type 2B [Source:VGNC Symbol;Acc:VGNC:108629]	-1.71	0.1324
ssc-miR-124a	ADAM19	ADAM metallopeptidase domain 19 [Source:VGNC Symbol;Acc:VGNC:85066]	-1.71	0.1324
ssc-miR-124a	ADAM22	ADAM metallopeptidase domain 22 [Source:VGNC Symbol;Acc:VGNC:85067]	-1.71	0.1324
ssc-miR-124a	ADAM23	ADAM metallopeptidase domain 23 [Source:VGNC Symbol;Acc:VGNC:95933]	-1.71	0.1324
ssc-miR-124a	ADAMTS14	ADAM metallopeptidase with thrombospondin type 1 motif 14 [Source:VGNC Symbol;Acc:VGNC:85076]	-1.71	0.1324
ssc-miR-124a	ADAMTS20	ADAM metallopeptidase with thrombospondin type 1 motif 20 [Source:VGNC Symbol;Acc:VGNC:85082]	-1.71	0.1324
ssc-miR-124a	ADAMTS9	ADAM metallopeptidase with thrombospondin type 1 motif 9 [Source:VGNC Symbol;Acc:VGNC:85089]	-1.71	0.1324
ssc-miR-124a	ADCY9	adenylate cyclase 9 [Source:VGNC Symbol;Acc:VGNC:85113]	-1.71	0.1324
ssc-miR-124a	ADCYAP1	adenylate cyclase activating polypeptide 1 [Source:VGNC Symbol;Acc:VGNC:85114]	-1.71	0.1324
ssc-miR-124a	ADI1	acireductone dioxygenase 1 [Source:VGNC Symbol;Acc:VGNC:85139]	-1.71	0.1324
ssc-miR-124a	ADIPOR2	adiponectin receptor 2 [Source:VGNC Symbol;Acc:VGNC:85141]	-1.71	0.1324
ssc-miR-124a	ADNP2	ADNP homeobox 2 [Source:VGNC Symbol;Acc:VGNC:85145]	-1.71	0.1324
ssc-miR-124a	AES	hypothetical gene	-1.71	0.1324
ssc-miR-124a	AFF1	AF4/FMR2 family member 1 [Source:VGNC Symbol;Acc:VGNC:85167]	-1.71	0.1324
ssc-miR-124a	AFF3	AF4/FMR2 family member 3 [Source:HGNC Symbol;Acc:HGNC:6473]	-1.71	0.1324
ssc-miR-124a	AFF4	AF4/FMR2 family member 4 [Source:VGNC Symbol;Acc:VGNC:85169]	-1.71	0.1324
ssc-miR-124a	AGBL4	hypothetical gene	-1.71	0.1324
ssc-miR-124a	AGFG1	ArfGAP with FG repeats 1 [Source:VGNC Symbol;Acc:VGNC:96359]	-1.71	0.1324
ssc-miR-124a	AGO1	hypothetical gene	-1.71	0.1324
ssc-miR-124a	AGO3	argonaute RISC component 1 [Source:NCBI gene (formerly Entrezgene);Acc:100499510]	-1.71	0.1324
ssc-miR-124a	AGPAT5	1-acylglycerol-3-phosphate O-acyltransferase 5 [Source:VGNC Symbol;Acc:VGNC:95954]	-1.71	0.1324
ssc-miR-124a	AGPAT9	hypothetical gene	-1.71	0.1324
ssc-miR-124a	AGPS	alkylglycerone phosphate synthase [Source:VGNC Symbol;Acc:VGNC:96267]	-1.71	0.1324
ssc-miR-124a	AGRN	agrin [Source:VGNC Symbol;Acc:VGNC:85188]	-1.71	0.1324
ssc-miR-124a	AHCYL1	adenosylhomocysteinase like 1 [Source:VGNC Symbol;Acc:VGNC:97873]	-1.71	0.1324
ssc-miR-124a	AHNAK	hypothetical gene	-1.71	0.1324
ssc-miR-124a	AHR	aryl hydrocarbon receptor [Source:NCBI gene (formerly Entrezgene);Acc:396654]	-1.71	0.1324
ssc-miR-124a	AHRR	aryl-hydrocarbon receptor repressor [Source:VGNC Symbol;Acc:VGNC:103065]	-1.71	0.1324
ssc-miR-124a	AIDA	axin interactor, dorsalization associated [Source:VGNC Symbol;Acc:VGNC:96103]	-1.71	0.1324
ssc-miR-124a	AIF1L	allograft inflammatory factor 1 like [Source:VGNC Symbol;Acc:VGNC:103022]	-1.71	0.1324
ssc-miR-124a	AK2	hypothetical gene	-1.71	0.1324
ssc-miR-124a	AK3	adenylate kinase 3 [Source:VGNC Symbol;Acc:VGNC:97876]	-1.71	0.1324
ssc-miR-124a	AK4	hypothetical gene	-1.71	0.1324
ssc-miR-124a	AKAP13	hypothetical gene	-1.71	0.1324
ssc-miR-124a	AKAP5	A-kinase anchoring protein 5 [Source:VGNC Symbol;Acc:VGNC:85220]	-1.71	0.1324
ssc-miR-124a	AKAP6	A-kinase anchoring protein 6 [Source:VGNC Symbol;Acc:VGNC:85221]	-1.71	0.1324

ssc-miR-124a	AKIRIN1	akirin 1 [Source:VGNC Symbol;Acc:VGNC:85227]	-1.71	0.1324
ssc-miR-124a	AKR7A2	aldo-keto reductase family 7 member A2 [Source:NCBI gene (formerly Entrezgene);Acc:100511331]	-1.71	0.1324
ssc-miR-124a	AKT1S1	AKT1 substrate 1 [Source:VGNC Symbol;Acc:VGNC:85231]	-1.71	0.1324
ssc-miR-124a	AKT2	AKT serine/threonine kinase 2 [Source:VGNC Symbol;Acc:VGNC:85232]	-1.71	0.1324
ssc-miR-124a	AKT3	AKT serine/threonine kinase 3 [Source:VGNC Symbol;Acc:VGNC:96306]	-1.71	0.1324
ssc-miR-124a	AL626787.1	hypothetical gene	-1.71	0.1324
ssc-miR-124a	ALCAM	activated leukocyte cell adhesion molecule [Source:VGNC Symbol;Acc:VGNC:85236]	-1.71	0.1324
ssc-miR-124a	ALDH4A1	aldehyde dehydrogenase 4 family member A1 [Source:VGNC Symbol;Acc:VGNC:85240]	-1.71	0.1324
ssc-miR-124a	ALDH6A1	aldehyde dehydrogenase 6 family member A1 [Source:VGNC Symbol;Acc:VGNC:85242]	-1.71	0.1324
ssc-miR-124a	ALG2	ALG2 alpha-1,3/1,6-mannosyltransferase [Source:VGNC Symbol;Acc:VGNC:85250]	-1.71	0.1324
ssc-miR-124a	ALG9	ALG9 alpha-1,2-mannosyltransferase [Source:NCBI gene (formerly Entrezgene);Acc:100519965]	-1.71	0.1324
ssc-miR-124a	ALS2	alsin Rho guanine nucleotide exchange factor ALS2 [Source:VGNC Symbol;Acc:VGNC:96327]	-1.71	0.1324
ssc-miR-124a	AMER2	APC membrane recruitment protein 2 [Source:VGNC Symbol;Acc:VGNC:85277]	-1.71	0.1324
ssc-miR-124a	AMMECR1	AMMECR nuclear protein 1 [Source:VGNC Symbol;Acc:VGNC:96559]	-1.71	0.1324
ssc-miR-124a	AMMECR1L	AMMECR1 like [Source:VGNC Symbol;Acc:VGNC:103895]	-1.71	0.1324
ssc-miR-124a	AMOT	angiomotin [Source:VGNC Symbol;Acc:VGNC:85283]	-1.71	0.1324
ssc-miR-124a	AMOTL1	angiomotin like 1 [Source:VGNC Symbol;Acc:VGNC:85284]	-1.71	0.1324
ssc-miR-124a	AMPD3	adenosine monophosphate deaminase 3 [Source:VGNC Symbol;Acc:VGNC:85288]	-1.71	0.1324
ssc-miR-124a	ANAPC7	anaphase promoting complex subunit 7 [Source:VGNC Symbol;Acc:VGNC:85300]	-1.71	0.1324
ssc-miR-124a	ANGEL1	angel homolog 1 [Source:VGNC Symbol;Acc:VGNC:85301]	-1.71	0.1324
ssc-miR-124a	ANK1	ankyrin 1 [Source:VGNC Symbol;Acc:VGNC:96344]	-1.71	0.1324
ssc-miR-124a	ANKFY1	ankyrin repeat and FYVE domain containing 1 [Source:VGNC Symbol;Acc:VGNC:85314]	-1.71	0.1324
ssc-miR-124a	ANKIB1	ankyrin repeat and IBR domain containing 1 [Source:VGNC Symbol;Acc:VGNC:85316]	-1.71	0.1324
ssc-miR-124a	ANKLE2	ankyrin repeat and LEM domain containing 2 [Source:VGNC Symbol;Acc:VGNC:85318]	-1.71	0.1324
ssc-miR-124a	ANKRD13A	ankyrin repeat domain 13A [Source:VGNC Symbol;Acc:VGNC:85324]	-1.71	0.1324
ssc-miR-124a	ANKRD13C	ankyrin repeat domain 13C [Source:VGNC Symbol;Acc:VGNC:85326]	-1.71	0.1324
ssc-miR-124a	ANKRD27	ankyrin repeat domain 27 [Source:VGNC Symbol;Acc:VGNC:96907]	-1.71	0.1324
ssc-miR-124a	ANKRD44	ankyrin repeat domain 44 [Source:VGNC Symbol;Acc:VGNC:107123]	-1.71	0.1324
ssc-miR-124a	ANKRD50	ankyrin repeat domain containing 50 [Source:VGNC Symbol;Acc:VGNC:85341]	-1.71	0.1324
ssc-miR-124a	ANKS1B	ankyrin repeat and sterile alpha motif domain containing 1B [Source:VGNC Symbol;Acc:VGNC:103214]	-1.71	0.1324
ssc-miR-124a	ANO5	anoctamin 5 [Source:VGNC Symbol;Acc:VGNC:85358]	-1.71	0.1324
ssc-miR-124a	ANP32E	acidic nuclear phosphoprotein 32 family member E [Source:HGNC Symbol;Acc:HGNC:16673]	-1.71	0.1324
ssc-miR-124a	ANTXR2	ANTXR cell adhesion molecule 2 [Source:VGNC Symbol;Acc:VGNC:85365]	-1.71	0.1324
ssc-miR-124a	ANXA11	annexin A11 [Source:VGNC Symbol;Acc:VGNC:85367]	-1.71	0.1324
ssc-miR-124a	ANXA5	annexin A5 [Source:VGNC Symbol;Acc:VGNC:85370]	-1.71	0.1324
ssc-miR-124a	ANXA7	annexin A7 [Source:VGNC Symbol;Acc:VGNC:85372]	-1.71	0.1324
ssc-miR-124a	AP1G1	adaptor related protein complex 1 subunit gamma 1 [Source:VGNC Symbol;Acc:VGNC:85378]	-1.71	0.1324
ssc-miR-124a	AP1M2	adaptor related protein complex 1 subunit mu 2 [Source:VGNC Symbol;Acc:VGNC:85381]	-1.71	0.1324
ssc-miR-124a	AP3M1	adaptor related protein complex 3 subunit mu 1 [Source:VGNC Symbol;Acc:VGNC:85390]	-1.71	0.1324
ssc-miR-124a	APBA3	amyloid beta protein binding family A member 3 [Source:VGNC Symbol;Acc:VGNC:85401]	-1.71	0.1324
ssc-miR-124a	APBB2	amyloid beta protein binding family B member 2 [Source:VGNC Symbol;Acc:VGNC:85403]	-1.71	0.1324
ssc-miR-124a	APEX2	apurinic/apyrimidinic endodeoxyribonuclease 2 [Source:VGNC Symbol;Acc:VGNC:103897]	-1.71	0.1324
ssc-miR-124a	APH1B	aph-1 homolog B, gamma-secretase subunit [Source:VGNC Symbol;Acc:VGNC:103898]	-1.71	0.1324
ssc-miR-124a	APLN	apelin [Source:HGNC Symbol;Acc:HGNC:16665]	-1.71	0.1324
ssc-miR-124a	AR	androgen receptor [Source:VGNC Symbol;Acc:VGNC:103903]	-1.71	0.1324
ssc-miR-124a	ARF6	ADP ribosylation factor 6 [Source:VGNC Symbol;Acc:VGNC:85448]	-1.71	0.1324
ssc-miR-124a	ARFGEF2	ADP ribosylation factor guanine nucleotide exchange factor 2 [Source:VGNC Symbol;Acc:VGNC:95672]	-1.71	0.1324
ssc-miR-124a	ARFIP1	ADP ribosylation factor interacting protein 1 [Source:VGNC Symbol;Acc:VGNC:85453]	-1.71	0.1324
ssc-miR-124a	ARG2	arginase 2 [Source:VGNC Symbol;Acc:VGNC:85455]	-1.71	0.1324
ssc-miR-124a	ARGLU1	arginine and glutamate rich 1 [Source:VGNC Symbol;Acc:VGNC:85456]	-1.71	0.1324

ssc-miR-124a	ARHGAP17	Rho GTPase activating protein 17 [Source:VGNC Symbol;Acc:VGNC:85459]	-1.71	0.1324
ssc-miR-124a	ARHGAP31	Rho GTPase activating protein 31 [Source:VGNC Symbol;Acc:VGNC:85470]	-1.71	0.1324
ssc-miR-124a	ARHGAP32	Rho GTPase activating protein 32 [Source:VGNC Symbol;Acc:VGNC:85471]	-1.71	0.1324
ssc-miR-124a	ARHGAP39	Rho GTPase activating protein 39 [Source:VGNC Symbol;Acc:VGNC:85475]	-1.71	0.1324
ssc-miR-124a	ARHGDIA	Rho GDP dissociation inhibitor alpha [Source:VGNC Symbol;Acc:VGNC:85483]	-1.71	0.1324
ssc-miR-124a	ARHGEF1	Rho guanine nucleotide exchange factor 1 [Source:VGNC Symbol;Acc:VGNC:96922]	-1.71	0.1324
ssc-miR-124a	ARHGEF17	hypothetical gene	-1.71	0.1324
ssc-miR-124a	ARHGEF3	Rho guanine nucleotide exchange factor 3 [Source:VGNC Symbol;Acc:VGNC:85496]	-1.71	0.1324
ssc-miR-124a	ARHGEF37	Rho guanine nucleotide exchange factor 37 [Source:VGNC Symbol;Acc:VGNC:85498]	-1.71	0.1324
ssc-miR-124a	ARHGEF4	hypothetical gene	-1.71	0.1324
ssc-miR-124a	ARHGEF40	Rho guanine nucleotide exchange factor 40 [Source:VGNC Symbol;Acc:VGNC:85501]	-1.71	0.1324
ssc-miR-124a	ARHGEF7	Rho guanine nucleotide exchange factor 7 [Source:VGNC Symbol;Acc:VGNC:85503]	-1.71	0.1324
ssc-miR-124a	ARID5B	AT-rich interaction domain 5B [Source:VGNC Symbol;Acc:VGNC:85511]	-1.71	0.1324
ssc-miR-124a	ARIH1	ariadne RBR E3 ubiquitin protein ligase 1 [Source:HGNC Symbol;Acc:HGNC:689]	-1.71	0.1324
ssc-miR-124a	ARL10	ADP ribosylation factor like GTPase 10 [Source:VGNC Symbol;Acc:VGNC:85514]	-1.71	0.1324
ssc-miR-124a	ARL5A	ADP ribosylation factor like GTPase 5A [Source:VGNC Symbol;Acc:VGNC:96418]	-1.71	0.1324
ssc-miR-124a	ARL5B	ADP ribosylation factor like GTPase 5B [Source:VGNC Symbol;Acc:VGNC:95985]	-1.71	0.1324
ssc-miR-124a	ARPP19	cAMP regulated phosphoprotein 19 [Source:NCBI gene (formerly Entrezgene);Acc:397362]	-1.71	0.1324
ssc-miR-124a	ARRB1	arrestin beta 1 [Source:VGNC Symbol;Acc:VGNC:85542]	-1.71	0.1324
ssc-miR-124a	ARRDC1	arrestin domain containing 1 [Source:VGNC Symbol;Acc:VGNC:85544]	-1.71	0.1324
ssc-miR-124a	ASB1	ankyrin repeat and SOCS box containing 1 [Source:VGNC Symbol;Acc:VGNC:95728]	-1.71	0.1324
ssc-miR-124a	ASCC2	activating signal cointegrator 1 complex subunit 2 [Source:VGNC Symbol;Acc:VGNC:85570]	-1.71	0.1324
ssc-miR-124a	ASF1A	anti-silencing function 1A histone chaperone [Source:VGNC Symbol;Acc:VGNC:85573]	-1.71	0.1324
ssc-miR-124a	ASIC1	acid sensing ion channel subunit 1 [Source:VGNC Symbol;Acc:VGNC:85578]	-1.71	0.1324
ssc-miR-124a	ASPA	aspartoacylase [Source:VGNC Symbol;Acc:VGNC:85582]	-1.71	0.1324
ssc-miR-124a	ASXL2	ASXL transcriptional regulator 2 [Source:VGNC Symbol;Acc:VGNC:85595]	-1.71	0.1324
ssc-miR-124a	ATAD2B	ATPase family AAA domain containing 2B [Source:VGNC Symbol;Acc:VGNC:85600]	-1.71	0.1324
ssc-miR-124a	ATCAY	ATCAY kinesin light chain interacting caytaxin [Source:VGNC Symbol;Acc:VGNC:85603]	-1.71	0.1324
ssc-miR-124a	ATF7IP	activating transcription factor 7 interacting protein [Source:VGNC Symbol;Acc:VGNC:85611]	-1.71	0.1324
ssc-miR-124a	ATL3	atlastin GTPase 3 [Source:VGNC Symbol;Acc:VGNC:85629]	-1.71	0.1324
ssc-miR-124a	ATMIN	hypothetical gene	-1.71	0.1324
ssc-miR-124a	ATP11A	ATPase phospholipid transporting 11A [Source:VGNC Symbol;Acc:VGNC:85634]	-1.71	0.1324
ssc-miR-124a	ATP1A1	ATPase Na+/K+ transporting subunit alpha 1 [Source:VGNC Symbol;Acc:VGNC:98735]	-1.71	0.1324
ssc-miR-124a	ATP2B4	ATPase plasma membrane Ca2+ transporting 4 [Source:VGNC Symbol;Acc:VGNC:85651]	-1.71	0.1324
ssc-miR-124a	ATP6V0A2	ATPase H+ transporting V0 subunit a2 [Source:VGNC Symbol;Acc:VGNC:85664]	-1.71	0.1324
ssc-miR-124a	ATP6V0E1	hypothetical gene	-1.71	0.1324
ssc-miR-124a	ATP6V1G1	ATPase H+ transporting V1 subunit G1 [Source:NCBI gene (formerly Entrezgene);Acc:100154379]	-1.71	0.1324
ssc-miR-124a	ATP7A	ATPase copper transporting alpha [Source:VGNC Symbol;Acc:VGNC:85678]	-1.71	0.1324
ssc-miR-124a	ATPAF1	ATP synthase mitochondrial F1 complex assembly factor 1 [Source:VGNC Symbol;Acc:VGNC:96924]	-1.71	0.1324
ssc-miR-124a	ATRX	ATRX chromatin remodeler [Source:VGNC Symbol;Acc:VGNC:85686]	-1.71	0.1324
ssc-miR-124a	ATXN1L	ataxin 1 like [Source:VGNC Symbol;Acc:VGNC:85689]	-1.71	0.1324
ssc-miR-124a	AXIN1	axin 1 [Source:VGNC Symbol;Acc:VGNC:85707]	-1.71	0.1324
ssc-miR-124a	B3GALT1	beta-1,3-galactosyltransferase 1 [Source:VGNC Symbol;Acc:VGNC:96494]	-1.71	0.1324
ssc-miR-124a	B3GALTL	hypothetical gene	-1.71	0.1324
ssc-miR-124a	B4GALNT3	beta-1,4-N-acetyl-galactosaminyltransferase 3 [Source:VGNC Symbol;Acc:VGNC:85731]	-1.71	0.1324
ssc-miR-124a	B4GALT1	beta-1,4-galactosyltransferase 1 [Source:VGNC Symbol;Acc:VGNC:96497]	-1.71	0.1324
ssc-miR-124a	B4GALT6	beta-1,4-galactosyltransferase 6 [Source:VGNC Symbol;Acc:VGNC:85734]	-1.71	0.1324
ssc-miR-124a	BACE1	beta-secretase 1 [Source:VGNC Symbol;Acc:VGNC:85740]	-1.71	0.1324
ssc-miR-124a	BACH2	BTB domain and CNC homolog 2 [Source:VGNC Symbol;Acc:VGNC:85742]	-1.71	0.1324
ssc-miR-124a	BAG5	BAG cochaperone 5 [Source:VGNC Symbol;Acc:VGNC:85746]	-1.71	0.1324

ssc-miR-124a	BAHD1	bromo adjacent homology domain containing 1 [Source:VGNC Symbol;Acc:VGNC:85747]	-1.71	0.1324
ssc-miR-124a	BARX2	BARX homeobox 2 [Source:VGNC Symbol;Acc:VGNC:85758]	-1.71	0.1324
ssc-miR-124a	BAZ2B	bromodomain adjacent to zinc finger domain 2B [Source:HGNC Symbol;Acc:HGNC:963]	-1.71	0.1324
ssc-miR-124a	BCAT1	branched chain amino acid transaminase 1 [Source:VGNC Symbol;Acc:VGNC:85773]	-1.71	0.1324
ssc-miR-124a	BCL11B	BAF chromatin remodeling complex subunit BCL11B [Source:VGNC Symbol;Acc:VGNC:96563]	-1.71	0.1324
ssc-miR-124a	BCL2L11	BCL2 like 11 [Source:NCBI gene (formerly Entrezgene);Acc:396632]	-1.71	0.1324
ssc-miR-124a	BCL6	BCL6 transcription repressor [Source:VGNC Symbol;Acc:VGNC:96565]	-1.71	0.1324
ssc-miR-124a	BCL7A	BAF chromatin remodeling complex subunit BCL7A [Source:VGNC Symbol;Acc:VGNC:85784]	-1.71	0.1324
ssc-miR-124a	BCL9L	BCL9 like [Source:VGNC Symbol;Acc:VGNC:96568]	-1.71	0.1324
ssc-miR-124a	BECN1	beclin 1 [Source:VGNC Symbol;Acc:VGNC:85798]	-1.71	0.1324
ssc-miR-124a	BEND3	BEN domain containing 3 [Source:VGNC Symbol;Acc:VGNC:85800]	-1.71	0.1324
ssc-miR-124a	BICC1	BicC family RNA binding protein 1 [Source:VGNC Symbol;Acc:VGNC:85817]	-1.71	0.1324
ssc-miR-124a	BICD1	BICD cargo adaptor 1 [Source:VGNC Symbol;Acc:VGNC:85818]	-1.71	0.1324
ssc-miR-124a	BICD2	BICD cargo adaptor 2 [Source:VGNC Symbol;Acc:VGNC:85819]	-1.71	0.1324
ssc-miR-124a	BID	BH3 interacting domain death agonist [Source:VGNC Symbol;Acc:VGNC:85824]	-1.71	0.1324
ssc-miR-124a	BLOC1S6	biosis of lysosomal organelles complex 1 subunit 6 [Source:VGNC Symbol;Acc:VGNC:85832]	-1.71	0.1324
ssc-miR-124a	BMF	Bcl2 modifying factor [Source:VGNC Symbol;Acc:VGNC:85837]	-1.71	0.1324
ssc-miR-124a	BMP3	bone morphotic protein 3 [Source:VGNC Symbol;Acc:VGNC:85842]	-1.71	0.1324
ssc-miR-124a	BMP6	bone morphotic protein 6 [Source:VGNC Symbol;Acc:VGNC:85845]	-1.71	0.1324
ssc-miR-124a	BMPR1A	bone morphotic protein receptor type 1A [Source:VGNC Symbol;Acc:VGNC:85846]	-1.71	0.1324
ssc-miR-124a	BMPR1B	bone morphotic protein receptor type 1B [Source:VGNC Symbol;Acc:VGNC:85847]	-1.71	0.1324
ssc-miR-124a	BMS1	BMS1 ribosome biosis factor [Source:VGNC Symbol;Acc:VGNC:85848]	-1.71	0.1324
ssc-miR-124a	BRD4	bromodomain containing 4 [Source:VGNC Symbol;Acc:VGNC:85873]	-1.71	0.1324
ssc-miR-124a	BRWD1	bromodomain and WD repeat domain containing 1 [Source:VGNC Symbol;Acc:VGNC:108153]	-1.71	0.1324
ssc-miR-124a	BRWD3	bromodomain and WD repeat domain containing 3 [Source:VGNC Symbol;Acc:VGNC:85890]	-1.71	0.1324
ssc-miR-124a	BTAF1	B-TFIID TATA-box binding protein associated factor 1 [Source:VGNC Symbol;Acc:VGNC:85899]	-1.71	0.1324
ssc-miR-124a	BTBD10	hypothetical gene	-1.71	0.1324
ssc-miR-124a	BTBD7	BTB domain containing 7 [Source:VGNC Symbol;Acc:VGNC:85908]	-1.71	0.1324
ssc-miR-124a	BTG2	BTG anti-proliferation factor 2 [Source:NCBI gene (formerly Entrezgene);Acc:100048932]	-1.71	0.1324
ssc-miR-124a	BZRAP1	hypothetical gene	-1.71	0.1324
ssc-miR-124a	C10orf12	hypothetical gene	-1.71	0.1324
ssc-miR-124a	C10orf137	hypothetical gene	-1.71	0.1324
ssc-miR-124a	C11orf57	hypothetical gene	-1.71	0.1324
ssc-miR-124a	C11orf84	hypothetical gene	-1.71	0.1324
ssc-miR-124a	C11orf87	chromosome 11 open reading frame 87 [Source:HGNC Symbol;Acc:HGNC:33788]	-1.71	0.1324
ssc-miR-124a	C12orf23	hypothetical gene	-1.71	0.1324
ssc-miR-124a	C12orf66	hypothetical gene	-1.71	0.1324
ssc-miR-124a	C16orf72	chromosome 3 C16orf72 homolog [Source:VGNC Symbol;Acc:VGNC:86016]	-1.71	0.1324
ssc-miR-124a	C17orf103	hypothetical gene	-1.71	0.1324
ssc-miR-124a	C1GALT1	hypothetical gene	-1.71	0.1324
ssc-miR-124a	C1orf198	chromosome 14 C1orf198 homolog [Source:VGNC Symbol;Acc:VGNC:85947]	-1.71	0.1324
ssc-miR-124a	C1orf21	hypothetical gene	-1.71	0.1324
ssc-miR-124a	C1orf233	hypothetical gene	-1.71	0.1324
ssc-miR-124a	C1QL3	complement C1q like 3 [Source:VGNC Symbol;Acc:VGNC:95910]	-1.71	0.1324
ssc-miR-124a	C20orf194	hypothetical gene	-1.71	0.1324
ssc-miR-124a	C21orf91	chromosome 13 C21orf91 homolog [Source:VGNC Symbol;Acc:VGNC:85930]	-1.71	0.1324
ssc-miR-124a	C2CD3	C2 domain containing 3 centriole elongation regulator [Source:VGNC Symbol;Acc:VGNC:85992]	-1.71	0.1324
ssc-miR-124a	C2orf68	chromosome 3 C2orf68 homolog [Source:VGNC Symbol;Acc:VGNC:86023]	-1.71	0.1324
ssc-miR-124a	C2orf71	hypothetical gene	-1.71	0.1324
ssc-miR-124a	C2orf88	chromosome 15 C2orf88 homolog [Source:VGNC Symbol;Acc:VGNC:96170]	-1.71	0.1324

ssc-miR-124a	C3orf17	hypothetical gene	-1.71	0.1324
ssc-miR-124a	C3orf38	chromosome 13 C3orf38 homolog [Source:VGNC Symbol;Acc:VGNC:85934]	-1.71	0.1324
ssc-miR-124a	C3orf58	hypothetical gene	-1.71	0.1324
ssc-miR-124a	C3orf70	chromosome 13 C3orf70 homolog [Source:VGNC Symbol;Acc:VGNC:85937]	-1.71	0.1324
ssc-miR-124a	C4orf46	chromosome 8 C4orf46 homolog [Source:VGNC Symbol;Acc:VGNC:86081]	-1.71	0.1324
ssc-miR-124a	C5orf28	hypothetical gene	-1.71	0.1324
ssc-miR-124a	C7orf55-LUC7L2	hypothetical gene	-1.71	0.1324
ssc-miR-124a	C9orf41	hypothetical gene	-1.71	0.1324
ssc-miR-124a	C9orf72	chromosome 10 C9orf72 homolog [Source:VGNC Symbol;Acc:VGNC:96173]	-1.71	0.1324
ssc-miR-124a	CA5B	hypothetical gene	-1.71	0.1324
ssc-miR-124a	CACFD1	calcium channel flower domain containing 1 [Source:VGNC Symbol;Acc:VGNC:86115]	-1.71	0.1324
ssc-miR-124a	CACNA2D1	calcium voltage-gated channel auxiliary subunit alpha2delta 1 [Source:VGNC Symbol;Acc:VGNC:86120]	-1.71	0.1324
ssc-miR-124a	CACNA2D2	calcium voltage-gated channel auxiliary subunit alpha2delta 2 [Source:VGNC Symbol;Acc:VGNC:86121]	-1.71	0.1324
ssc-miR-124a	CACNB2	calcium voltage-gated channel auxiliary subunit beta 2 [Source:VGNC Symbol;Acc:VGNC:95599]	-1.71	0.1324
ssc-miR-124a	CACNB4	calcium voltage-gated channel auxiliary subunit beta 4 [Source:VGNC Symbol;Acc:VGNC:108507]	-1.71	0.1324
ssc-miR-124a	CACUL1	CDK2 associated cullin domain 1 [Source:VGNC Symbol;Acc:VGNC:86132]	-1.71	0.1324
ssc-miR-124a	CADM1	cell adhesion molecule 1 [Source:VGNC Symbol;Acc:VGNC:86134]	-1.71	0.1324
ssc-miR-124a	CADM2	cell adhesion molecule 2 [Source:VGNC Symbol;Acc:VGNC:97910]	-1.71	0.1324
ssc-miR-124a	CADPS	hypothetical gene	-1.71	0.1324
ssc-miR-124a	CALCOCO1	calcium binding and coiled-coil domain 1 [Source:VGNC Symbol;Acc:VGNC:86140]	-1.71	0.1324
ssc-miR-124a	CALU	calumenin [Source:VGNC Symbol;Acc:VGNC:86151]	-1.71	0.1324
ssc-miR-124a	CAMTA2	calmodulin binding transcription activator 2 [Source:VGNC Symbol;Acc:VGNC:86165]	-1.71	0.1324
ssc-miR-124a	CAPN1	calpain 1 [Source:VGNC Symbol;Acc:VGNC:86170]	-1.71	0.1324
ssc-miR-124a	CAPN2	calpain 2 [Source:VGNC Symbol;Acc:VGNC:95630]	-1.71	0.1324
ssc-miR-124a	CAPN6	calpain 6 [Source:VGNC Symbol;Acc:VGNC:86176]	-1.71	0.1324
ssc-miR-124a	CAPNS1	calpain small subunit 1 [Source:VGNC Symbol;Acc:VGNC:96937]	-1.71	0.1324
ssc-miR-124a	CAPZA1	capping actin protein of muscle Z-line subunit alpha 1 [Source:VGNC Symbol;Acc:VGNC:86183]	-1.71	0.1324
ssc-miR-124a	CASC3	CASC3 exon junction complex subunit [Source:VGNC Symbol;Acc:VGNC:96572]	-1.71	0.1324
ssc-miR-124a	CASC4	hypothetical gene	-1.71	0.1324
ssc-miR-124a	CASK	calcium/calmodulin dependent serine protein kinase [Source:HGNC Symbol;Acc:HGNC:1497]	-1.71	0.1324
ssc-miR-124a	CASKIN1	CASK interacting protein 1 [Source:VGNC Symbol;Acc:VGNC:86200]	-1.71	0.1324
ssc-miR-124a	CAV1	caveolin 1 [Source:NCBI gene (formerly Entrezgene);Acc:404693]	-1.71	0.1324
ssc-miR-124a	CBFB	core-binding factor subunit beta [Source:VGNC Symbol;Acc:VGNC:86221]	-1.71	0.1324
ssc-miR-124a	CBL	Cbl proto-onco [Source:VGNC Symbol;Acc:VGNC:86222]	-1.71	0.1324
ssc-miR-124a	CBLL1	Cbl proto-onco like 1 [Source:VGNC Symbol;Acc:VGNC:108573]	-1.71	0.1324
ssc-miR-124a	CBLN2	cerebellin 2 [Source:VGNC Symbol;Acc:VGNC:86226]	-1.71	0.1324
ssc-miR-124a	CBLN4	cerebellin 4 [Source:VGNC Symbol;Acc:VGNC:95793]	-1.71	0.1324
ssc-miR-124a	CBX2	chromobox 2 [Source:VGNC Symbol;Acc:VGNC:86230]	-1.71	0.1324
ssc-miR-124a	CBX5	chromobox 5 [Source:VGNC Symbol;Acc:VGNC:86232]	-1.71	0.1324
ssc-miR-124a	CC2D1B	coiled-coil and C2 domain containing 1B [Source:VGNC Symbol;Acc:VGNC:86236]	-1.71	0.1324
ssc-miR-124a	CCAR1	cell division cycle and apoptosis regulator 1 [Source:VGNC Symbol;Acc:VGNC:86238]	-1.71	0.1324
ssc-miR-124a	CCDC117	coiled-coil domain containing 117 [Source:VGNC Symbol;Acc:VGNC:86248]	-1.71	0.1324
ssc-miR-124a	CCDC130	hypothetical gene	-1.71	0.1324
ssc-miR-124a	CCDC171	coiled-coil domain containing 171 [Source:VGNC Symbol;Acc:VGNC:103044]	-1.71	0.1324
ssc-miR-124a	CCDC177	coiled-coil domain containing 177 [Source:VGNC Symbol;Acc:VGNC:86275]	-1.71	0.1324
ssc-miR-124a	CCDC28A	coiled-coil domain containing 28A [Source:VGNC Symbol;Acc:VGNC:86289]	-1.71	0.1324
ssc-miR-124a	CCDC50	coiled-coil domain containing 50 [Source:VGNC Symbol;Acc:VGNC:86299]	-1.71	0.1324
ssc-miR-124a	CCDC6	coiled-coil domain containing 6 [Source:VGNC Symbol;Acc:VGNC:86304]	-1.71	0.1324
ssc-miR-124a	CCDC71L	coiled-coil domain containing 71 like [Source:HGNC Symbol;Acc:HGNC:26685]	-1.71	0.1324
ssc-miR-124a	CCDC86	coiled-coil domain containing 86 [Source:VGNC Symbol;Acc:VGNC:86323]	-1.71	0.1324

ssc-miR-124a	CCDC88A	coiled-coil domain containing 88A [Source:VGNC Symbol;Acc:VGNC:86325]	-1.71	0.1324
ssc-miR-124a	CCDC89	coiled-coil domain containing 89 [Source:HGNC Symbol;Acc:HGNC:26762]	-1.71	0.1324
ssc-miR-124a	CCL2	chemokine (C-C motif) ligand 2 [Source:NCBI gene (formerly Entrezgene);Acc:397422]	-1.71	0.1324
ssc-miR-124a	CCND2	cyclin D2 [Source:VGNC Symbol;Acc:VGNC:103222]	-1.71	0.1324
ssc-miR-124a	CCNT2	cyclin T2 [Source:VGNC Symbol;Acc:VGNC:103916]	-1.71	0.1324
ssc-miR-124a	CCT5	chaperonin containing TCP1 subunit 5 [Source:VGNC Symbol;Acc:VGNC:86383]	-1.71	0.1324
ssc-miR-124a	CD151	CD151 molecule [Source:VGNC Symbol;Acc:VGNC:96613]	-1.71	0.1324
ssc-miR-124a	CD164	CD164 molecule [Source:HGNC Symbol;Acc:HGNC:1632]	-1.71	0.1324
ssc-miR-124a	CD276	CD276 molecule [Source:VGNC Symbol;Acc:VGNC:86405]	-1.71	0.1324
ssc-miR-124a	CD82	CD82 molecule [Source:VGNC Symbol;Acc:VGNC:86432]	-1.71	0.1324
ssc-miR-124a	CDC14A	cell division cycle 14A [Source:VGNC Symbol;Acc:VGNC:86442]	-1.71	0.1324
ssc-miR-124a	CDC27	cell division cycle 27 [Source:VGNC Symbol;Acc:VGNC:86448]	-1.71	0.1324
ssc-miR-124a	CDC42BPA	CDC42 binding protein kinase alpha [Source:VGNC Symbol;Acc:VGNC:95847]	-1.71	0.1324
ssc-miR-124a	CDC42EP3	CDC42 effector protein 3 [Source:VGNC Symbol;Acc:VGNC:86457]	-1.71	0.1324
ssc-miR-124a	CDC42SE2	CDC42 small effector 2 [Source:VGNC Symbol;Acc:VGNC:86461]	-1.71	0.1324
ssc-miR-124a	CDC6	cell division cycle 6 [Source:VGNC Symbol;Acc:VGNC:86464]	-1.71	0.1324
ssc-miR-124a	CDC73	cell division cycle 73 [Source:VGNC Symbol;Acc:VGNC:95817]	-1.71	0.1324
ssc-miR-124a	CDCA7	cell division cycle associated 7 [Source:VGNC Symbol;Acc:VGNC:95623]	-1.71	0.1324
ssc-miR-124a	CDH11	cadherin 11 [Source:VGNC Symbol;Acc:VGNC:86475]	-1.71	0.1324
ssc-miR-124a	CDH2	cadherin 2 [Source:VGNC Symbol;Acc:VGNC:86483]	-1.71	0.1324
ssc-miR-124a	CDH4	cadherin 4 [Source:VGNC Symbol;Acc:VGNC:95853]	-1.71	0.1324
ssc-miR-124a	CDH9	cadherin 9 [Source:VGNC Symbol;Acc:VGNC:86488]	-1.71	0.1324
ssc-miR-124a	CDK13	cyclin dependent kinase 13 [Source:VGNC Symbol;Acc:VGNC:86497]	-1.71	0.1324
ssc-miR-124a	CDK18	cyclin dependent kinase 18 [Source:VGNC Symbol;Acc:VGNC:86501]	-1.71	0.1324
ssc-miR-124a	CDK4	cyclin dependent kinase 4 [Source:VGNC Symbol;Acc:VGNC:86504]	-1.71	0.1324
ssc-miR-124a	CDK6	cyclin dependent kinase 6 [Source:HGNC Symbol;Acc:HGNC:1777]	-1.71	0.1324
ssc-miR-124a	CDKL2	cyclin dependent kinase like 2 [Source:VGNC Symbol;Acc:VGNC:86511]	-1.71	0.1324
ssc-miR-124a	CDR2L	cerebellar deration related protein 2 like [Source:VGNC Symbol;Acc:VGNC:86522]	-1.71	0.1324
ssc-miR-124a	CDYL2	chromodomain Y like 2 [Source:VGNC Symbol;Acc:VGNC:86529]	-1.71	0.1324
ssc-miR-124a	CEBPA	CCAAT enhancer binding protein alpha [Source:VGNC Symbol;Acc:VGNC:86531]	-1.71	0.1324
ssc-miR-124a	CEBPG	CCAAT enhancer binding protein gamma [Source:VGNC Symbol;Acc:VGNC:86534]	-1.71	0.1324
ssc-miR-124a	CELSR1	cadherin EGF LAG seven-pass G-type receptor 1 [Source:HGNC Symbol;Acc:HGNC:1850]	-1.71	0.1324
ssc-miR-124a	CELSR3	cadherin EGF LAG seven-pass G-type receptor 3 [Source:VGNC Symbol;Acc:VGNC:86541]	-1.71	0.1324
ssc-miR-124a	CENPL	centromere protein L [Source:VGNC Symbol;Acc:VGNC:86552]	-1.71	0.1324
ssc-miR-124a	CEP152	centrosomal protein 152 [Source:HGNC Symbol;Acc:HGNC:29298]	-1.71	0.1324
ssc-miR-124a	CEP350	centrosomal protein 350 [Source:HGNC Symbol;Acc:HGNC:24238]	-1.71	0.1324
ssc-miR-124a	CEP41	centrosomal protein 41 [Source:VGNC Symbol;Acc:VGNC:86569]	-1.71	0.1324
ssc-miR-124a	CEP68	centrosomal protein 68 [Source:VGNC Symbol;Acc:VGNC:86575]	-1.71	0.1324
ssc-miR-124a	CEP85L	centrosomal protein 85 like [Source:VGNC Symbol;Acc:VGNC:86580]	-1.71	0.1324
ssc-miR-124a	CERS2	ceramide synthase 2 [Source:VGNC Symbol;Acc:VGNC:86587]	-1.71	0.1324
ssc-miR-124a	CFL2	cofilin 2 [Source:VGNC Symbol;Acc:VGNC:86611]	-1.71	0.1324
ssc-miR-124a	CGN	hypothetical gene	-1.71	0.1324
ssc-miR-124a	CGRRF1	cell growth regulator with ring finger domain 1 [Source:VGNC Symbol;Acc:VGNC:86617]	-1.71	0.1324
ssc-miR-124a	CHD1	chromodomain helicase DNA binding protein 1 [Source:VGNC Symbol;Acc:VGNC:86629]	-1.71	0.1324
ssc-miR-124a	CHIC1	cysteine rich hydrophobic domain 1 [Source:VGNC Symbol;Acc:VGNC:86642]	-1.71	0.1324
ssc-miR-124a	CHIC2	cysteine rich hydrophobic domain 2 [Source:VGNC Symbol;Acc:VGNC:86643]	-1.71	0.1324
ssc-miR-124a	CHMP2B	charged multivesicular body protein 2B [Source:VGNC Symbol;Acc:VGNC:86650]	-1.71	0.1324
ssc-miR-124a	CHODL	chondrolectin [Source:VGNC Symbol;Acc:VGNC:86655]	-1.71	0.1324
ssc-miR-124a	CHP1	calcineurin like EF-hand protein 1 [Source:VGNC Symbol;Acc:VGNC:86657]	-1.71	0.1324
ssc-miR-124a	CHRD	chordin [Source:VGNC Symbol;Acc:VGNC:86661]	-1.71	0.1324

ssc-miR-124a	CHST1	carbohydrate sulfotransferase 1 [Source:VGNC Symbol;Acc:VGNC:86673]	-1.71	0.1324
ssc-miR-124a	CHST14	carbohydrate sulfotransferase 14 [Source:HGNC Symbol;Acc:HGNC:24464]	-1.71	0.1324
ssc-miR-124a	CHSY1	chondroitin sulfate synthase 1 [Source:VGNC Symbol;Acc:VGNC:86684]	-1.71	0.1324
ssc-miR-124a	CIRH1A	hypothetical gene	-1.71	0.1324
ssc-miR-124a	CISD1	hypothetical gene	-1.71	0.1324
ssc-miR-124a	CISD2	CDGSH iron sulfur domain 2 [Source:VGNC Symbol;Acc:VGNC:86707]	-1.71	0.1324
ssc-miR-124a	CLCN6	chloride voltage-gated channel 6 [Source:VGNC Symbol;Acc:VGNC:86729]	-1.71	0.1324
ssc-miR-124a	CLDN11	claudin 11 [Source:NCBI gene (formerly Entrezgene);Acc:100302016]	-1.71	0.1324
ssc-miR-124a	CLDND1	claudin domain containing 1 [Source:VGNC Symbol;Acc:VGNC:86742]	-1.71	0.1324
ssc-miR-124a	CLIC5	chloride intracellular channel 5 [Source:VGNC Symbol;Acc:VGNC:86764]	-1.71	0.1324
ssc-miR-124a	CLIC6	chloride intracellular channel 6 [Source:VGNC Symbol;Acc:VGNC:108640]	-1.71	0.1324
ssc-miR-124a	CLIP1	CAP-Gly domain containing linker protein 1 [Source:VGNC Symbol;Acc:VGNC:86766]	-1.71	0.1324
ssc-miR-124a	CLIP3	CAP-Gly domain containing linker protein 3 [Source:VGNC Symbol;Acc:VGNC:97931]	-1.71	0.1324
ssc-miR-124a	CLMN	calmin [Source:VGNC Symbol;Acc:VGNC:86769]	-1.71	0.1324
ssc-miR-124a	CLMP	CXADR like membrane protein [Source:VGNC Symbol;Acc:VGNC:86770]	-1.71	0.1324
ssc-miR-124a	CLOCK	clock circadian regulator [Source:VGNC Symbol;Acc:VGNC:86774]	-1.71	0.1324
ssc-miR-124a	CLVS2	clavesin 2 [Source:VGNC Symbol;Acc:VGNC:86795]	-1.71	0.1324
ssc-miR-124a	CMPK1	cytidine/uridine monophosphate kinase 1 [Source:VGNC Symbol;Acc:VGNC:96953]	-1.71	0.1324
ssc-miR-124a	CMTM4	CKLF like MARVEL transmembrane domain containing 4 [Source:VGNC Symbol;Acc:VGNC:86804]	-1.71	0.1324
ssc-miR-124a	CMTM6	hypothetical gene	-1.71	0.1324
ssc-miR-124a	CMTR2	cap methyltransferase 2 [Source:VGNC Symbol;Acc:VGNC:86808]	-1.71	0.1324
ssc-miR-124a	CNEP1R1	CTD nuclear envelope phosphatase 1 regulatory subunit 1 [Source:VGNC Symbol;Acc:VGNC:86813]	-1.71	0.1324
ssc-miR-124a	CNGA3	cyclic nucleotide gated channel subunit alpha 3 [Source:VGNC Symbol;Acc:VGNC:86817]	-1.71	0.1324
ssc-miR-124a	CNIH4	cornichon family AMPA receptor auxiliary protein 4 [Source:VGNC Symbol;Acc:VGNC:96060]	-1.71	0.1324
ssc-miR-124a	CNKSR3	CNKSR family member 3 [Source:VGNC Symbol;Acc:VGNC:86823]	-1.71	0.1324
ssc-miR-124a	CNN3	calponin 3 [Source:VGNC Symbol;Acc:VGNC:86826]	-1.71	0.1324
ssc-miR-124a	CNOT1	CCR4-NOT transcription complex subunit 1 [Source:VGNC Symbol;Acc:VGNC:86831]	-1.71	0.1324
ssc-miR-124a	CNOT11	CCR4-NOT transcription complex subunit 11 [Source:VGNC Symbol;Acc:VGNC:86833]	-1.71	0.1324
ssc-miR-124a	CNOT2	CCR4-NOT transcription complex subunit 2 [Source:VGNC Symbol;Acc:VGNC:86834]	-1.71	0.1324
ssc-miR-124a	CNOT7	CCR4-NOT transcription complex subunit 7 [Source:VGNC Symbol;Acc:VGNC:95604]	-1.71	0.1324
ssc-miR-124a	CNTN1	contactin 1 [Source:VGNC Symbol;Acc:VGNC:86848]	-1.71	0.1324
ssc-miR-124a	CNTN3	contactin 3 [Source:VGNC Symbol;Acc:VGNC:108642]	-1.71	0.1324
ssc-miR-124a	CNTN4	contactin 4 [Source:VGNC Symbol;Acc:VGNC:97934]	-1.71	0.1324
ssc-miR-124a	CNTNAP5	contactin associated protein family member 5 [Source:VGNC Symbol;Acc:VGNC:95459]	-1.71	0.1324
ssc-miR-124a	COBLL1	cordon-bleu WH2 repeat protein like 1 [Source:VGNC Symbol;Acc:VGNC:96013]	-1.71	0.1324
ssc-miR-124a	COL12A1	collagen type XII alpha 1 chain [Source:VGNC Symbol;Acc:VGNC:86864]	-1.71	0.1324
ssc-miR-124a	COL23A1	collagen type XXIII alpha 1 chain [Source:VGNC Symbol;Acc:VGNC:99620]	-1.71	0.1324
ssc-miR-124a	COL4A1	collagen type IV alpha 1 chain [Source:HGNC Symbol;Acc:HGNC:2202]	-1.71	0.1324
ssc-miR-124a	COL4A3BP	hypothetical gene	-1.71	0.1324
ssc-miR-124a	COLGALT1	collagen beta(1-O)galactosyltransferase 1 [Source:HGNC Symbol;Acc:HGNC:26182]	-1.71	0.1324
ssc-miR-124a	COPZ1	COPI coat complex subunit zeta 1 [Source:VGNC Symbol;Acc:VGNC:86903]	-1.71	0.1324
ssc-miR-124a	COQ10A	coenzyme Q10A [Source:VGNC Symbol;Acc:VGNC:86905]	-1.71	0.1324
ssc-miR-124a	CORO2A	coronin 2A [Source:VGNC Symbol;Acc:VGNC:86915]	-1.71	0.1324
ssc-miR-124a	COTL1	coactosin like F-actin binding protein 1 [Source:HGNC Symbol;Acc:HGNC:18304]	-1.71	0.1324
ssc-miR-124a	CPD	carboxypeptidase D [Source:VGNC Symbol;Acc:VGNC:86935]	-1.71	0.1324
ssc-miR-124a	CPEB1	cytoplasmic polyadenylation element binding protein 1 [Source:NCBI gene (formerly Entrezgene);Acc:100048944]	-1.71	0.1324
ssc-miR-124a	CPNE3	copine 3 [Source:VGNC Symbol;Acc:VGNC:86951]	-1.71	0.1324
ssc-miR-124a	CPNE5	copine 5 [Source:VGNC Symbol;Acc:VGNC:86953]	-1.71	0.1324
ssc-miR-124a	CPNE8	copine 8 [Source:VGNC Symbol;Acc:VGNC:86955]	-1.71	0.1324
ssc-miR-124a	CPOX	coproporphyrinogen oxidase [Source:VGNC Symbol;Acc:VGNC:86956]	-1.71	0.1324

ssc-miR-124a	CPPED1	calcineurin like phosphoesterase domain containing 1 [Source:VGNC Symbol;Acc:VGNC:86957]	-1.71	0.1324
ssc-miR-124a	CPS1	carbamoyl-phosphate synthase 1 [Source:VGNC Symbol;Acc:VGNC:95991]	-1.71	0.1324
ssc-miR-124a	CPT1A	carnitine palmitoyltransferase 1A [Source:VGNC Symbol;Acc:VGNC:86964]	-1.71	0.1324
ssc-miR-124a	CRAT	carnitine O-acetyltransferase [Source:VGNC Symbol;Acc:VGNC:86976]	-1.71	0.1324
ssc-miR-124a	CREB1	cAMP responsive element binding protein 1 [Source:VGNC Symbol;Acc:VGNC:96004]	-1.71	0.1324
ssc-miR-124a	CREB3L2	cAMP responsive element binding protein 3 like 2 [Source:VGNC Symbol;Acc:VGNC:86981]	-1.71	0.1324
ssc-miR-124a	CREBRF	CREB3 regulatory factor [Source:VGNC Symbol;Acc:VGNC:86987]	-1.71	0.1324
ssc-miR-124a	CRKL	CRK like proto-onco, adaptor protein [Source:VGNC Symbol;Acc:VGNC:86997]	-1.71	0.1324
ssc-miR-124a	CRTC1	CREB regulated transcription coactivator 1 [Source:VGNC Symbol;Acc:VGNC:87007]	-1.71	0.1324
ssc-miR-124a	CRTC3	CREB regulated transcription coactivator 3 [Source:VGNC Symbol;Acc:VGNC:87009]	-1.71	0.1324
ssc-miR-124a	CRYBG3	crystallin beta-gamma domain containing 3 [Source:VGNC Symbol;Acc:VGNC:87020]	-1.71	0.1324
ssc-miR-124a	CSDE1	cold shock domain containing E1 [Source:VGNC Symbol;Acc:VGNC:87029]	-1.71	0.1324
ssc-miR-124a	CSH1	hypothetical gene	-1.71	0.1324
ssc-miR-124a	CSH2	hypothetical gene	-1.71	0.1324
ssc-miR-124a	CSHL1	hypothetical gene	-1.71	0.1324
ssc-miR-124a	CSNK1G1	casein kinase 1 gamma 1 [Source:VGNC Symbol;Acc:VGNC:97949]	-1.71	0.1324
ssc-miR-124a	CSNK1G2	casein kinase 1 gamma 2 [Source:VGNC Symbol;Acc:VGNC:97950]	-1.71	0.1324
ssc-miR-124a	CSPP1	centrosome and spindle pole associated protein 1 [Source:VGNC Symbol;Acc:VGNC:87045]	-1.71	0.1324
ssc-miR-124a	CSTF3	cleavage stimulation factor subunit 3 [Source:VGNC Symbol;Acc:VGNC:87052]	-1.71	0.1324
ssc-miR-124a	CTC1	CST telomere replication complex component 1 [Source:VGNC Symbol;Acc:VGNC:87056]	-1.71	0.1324
ssc-miR-124a	CTD-2228K2.5	hypothetical gene	-1.71	0.1324
ssc-miR-124a	CTDSP1	CTD small phosphatase 1 [Source:VGNC Symbol;Acc:VGNC:95971]	-1.71	0.1324
ssc-miR-124a	CTDSP2	CTD small phosphatase 2 [Source:VGNC Symbol;Acc:VGNC:87059]	-1.71	0.1324
ssc-miR-124a	CTDSPL	CTD small phosphatase like [Source:VGNC Symbol;Acc:VGNC:107131]	-1.71	0.1324
ssc-miR-124a	CTNND1	catenin delta 1 [Source:VGNC Symbol;Acc:VGNC:87067]	-1.71	0.1324
ssc-miR-124a	CTNS	cystinosin, lysosomal cystine transporter [Source:VGNC Symbol;Acc:VGNC:87069]	-1.71	0.1324
ssc-miR-124a	CTSH	cathepsin H [Source:VGNC Symbol;Acc:VGNC:87077]	-1.71	0.1324
ssc-miR-124a	CTXN1	cortexin 1 [Source:VGNC Symbol;Acc:VGNC:87084]	-1.71	0.1324
ssc-miR-124a	CUL4A	cullin 4A [Source:VGNC Symbol;Acc:VGNC:87088]	-1.71	0.1324
ssc-miR-124a	CUL5	cullin 5 [Source:VGNC Symbol;Acc:VGNC:87089]	-1.71	0.1324
ssc-miR-124a	CWC22	CWC22 spliceosome associated protein homolog [Source:VGNC Symbol;Acc:VGNC:96215]	-1.71	0.1324
ssc-miR-124a	CXADR	CXADR Ig-like cell adhesion molecule [Source:VGNC Symbol;Acc:VGNC:87100]	-1.71	0.1324
ssc-miR-124a	CXorf24	hypothetical gene	-1.71	0.1324
ssc-miR-124a	CYB561D1	cytochrome b561 family member D1 [Source:VGNC Symbol;Acc:VGNC:87121]	-1.71	0.1324
ssc-miR-124a	CYB5A	cytochrome b5 type A [Source:VGNC Symbol;Acc:VGNC:96727]	-1.71	0.1324
ssc-miR-124a	CYBRD1	cytochrome b reductase 1 [Source:VGNC Symbol;Acc:VGNC:95948]	-1.71	0.1324
ssc-miR-124a	CYLD	CYLD lysine 63 deubiquitinase [Source:VGNC Symbol;Acc:VGNC:87130]	-1.71	0.1324
ssc-miR-124a	CYP2U1	cytochrome P450 family 2 subfamily U member 1 [Source:VGNC Symbol;Acc:VGNC:103357]	-1.71	0.1324
ssc-miR-124a	DAAM2	dishevelled associated activator of morphosis 2 [Source:VGNC Symbol;Acc:VGNC:87142]	-1.71	0.1324
ssc-miR-124a	DAB2	DAB adaptor protein 2 [Source:VGNC Symbol;Acc:VGNC:87144]	-1.71	0.1324
ssc-miR-124a	DACT1	dishevelled binding antagonist of beta catenin 1 [Source:VGNC Symbol;Acc:VGNC:87147]	-1.71	0.1324
ssc-miR-124a	DAP	death associated protein [Source:VGNC Symbol;Acc:VGNC:106447]	-1.71	0.1324
ssc-miR-124a	DAPK1	death associated protein kinase 1 [Source:HGNC Symbol;Acc:HGNC:2674]	-1.71	0.1324
ssc-miR-124a	DCAF12L1	hypothetical gene	-1.71	0.1324
ssc-miR-124a	DCAF16	DDB1 and CUL4 associated factor 16 [Source:HGNC Symbol;Acc:HGNC:25987]	-1.71	0.1324
ssc-miR-124a	DCAF5	DDB1 and CUL4 associated factor 5 [Source:VGNC Symbol;Acc:VGNC:87176]	-1.71	0.1324
ssc-miR-124a	DCAF7	DDB1 and CUL4 associated factor 7 [Source:VGNC Symbol;Acc:VGNC:87178]	-1.71	0.1324
ssc-miR-124a	DCAKD	dephospho-CoA kinase domain containing [Source:VGNC Symbol;Acc:VGNC:97958]	-1.71	0.1324
ssc-miR-124a	DCHS1	dachsous cadherin-related 1 [Source:VGNC Symbol;Acc:VGNC:97959]	-1.71	0.1324
ssc-miR-124a	DCLRE1C	DNA cross-link repair 1C [Source:VGNC Symbol;Acc:VGNC:108268]	-1.71	0.1324

ssc-miR-124a	DCP1A	decapping mRNA 1A [Source:VGNC Symbol;Acc:VGNC:97960]	-1.71	0.1324
ssc-miR-124a	DCTN4	dynactin subunit 4 [Source:VGNC Symbol;Acc:VGNC:99635]	-1.71	0.1324
ssc-miR-124a	DCUN1D1	hypothetical gene	-1.71	0.1324
ssc-miR-124a	DCUN1D3	defective in cullin neddylation 1 domain containing 3 [Source:VGNC Symbol;Acc:VGNC:87196]	-1.71	0.1324
ssc-miR-124a	DCUN1D5	defective in cullin neddylation 1 domain containing 5 [Source:VGNC Symbol;Acc:VGNC:87198]	-1.71	0.1324
ssc-miR-124a	DDA1	DET1 and DDB1 associated 1 [Source:VGNC Symbol;Acc:VGNC:87199]	-1.71	0.1324
ssc-miR-124a	DDHD1	DDHD domain containing 1 [Source:VGNC Symbol;Acc:VGNC:87205]	-1.71	0.1324
ssc-miR-124a	DDI2	hypothetical gene	-1.71	0.1324
ssc-miR-124a	DDX3X	DEAD-box helicase 3 X-linked [Source:NCBI gene (formerly Entrezgene);Acc:100515940]	-1.71	0.1324
ssc-miR-124a	DDX3Y	hypothetical gene	-1.71	0.1324
ssc-miR-124a	DDX5	DEAD-box helicase 5 [Source:VGNC Symbol;Acc:VGNC:87226]	-1.71	0.1324
ssc-miR-124a	DDX6	DEAD-box helicase 6 [Source:VGNC Symbol;Acc:VGNC:87233]	-1.71	0.1324
ssc-miR-124a	DEF6	DEF6 guanine nucleotide exchange factor [Source:VGNC Symbol;Acc:VGNC:87238]	-1.71	0.1324
ssc-miR-124a	DENND4C	DENN domain containing 4C [Source:VGNC Symbol;Acc:VGNC:87253]	-1.71	0.1324
ssc-miR-124a	DENR	density regulated re-initiation and release factor [Source:VGNC Symbol;Acc:VGNC:97963]	-1.71	0.1324
ssc-miR-124a	DEPDC1	DEP domain containing 1 [Source:VGNC Symbol;Acc:VGNC:87258]	-1.71	0.1324
ssc-miR-124a	DEPTOR	DEP domain containing MTOR interacting protein [Source:HGNC Symbol;Acc:HGNC:22953]	-1.71	0.1324
ssc-miR-124a	DESI2	desumoylating isopeptidase 2 [Source:VGNC Symbol;Acc:VGNC:96037]	-1.71	0.1324
ssc-miR-124a	DFFA	DNA fragmentation factor subunit alpha [Source:VGNC Symbol;Acc:VGNC:87265]	-1.71	0.1324
ssc-miR-124a	DGAT2	diacylglycerol O-acyltransferase 2 [Source:NCBI gene (formerly Entrezgene);Acc:100294675]	-1.71	0.1324
ssc-miR-124a	DGKH	diacylglycerol kinase eta [Source:VGNC Symbol;Acc:VGNC:87273]	-1.71	0.1324
ssc-miR-124a	DGKI	diacylglycerol kinase iota [Source:VGNC Symbol;Acc:VGNC:87274]	-1.71	0.1324
ssc-miR-124a	DHCR24	24-dehydrocholesterol reductase [Source:NCBI gene (formerly Entrezgene);Acc:100628197]	-1.71	0.1324
ssc-miR-124a	DHDDS	dehydrodolichyl diphosphate synthase subunit [Source:VGNC Symbol;Acc:VGNC:87279]	-1.71	0.1324
ssc-miR-124a	DHX36	DEAH-box helicase 36 [Source:VGNC Symbol;Acc:VGNC:87292]	-1.71	0.1324
ssc-miR-124a	DHX40	DEAH-box helicase 40 [Source:VGNC Symbol;Acc:VGNC:87295]	-1.71	0.1324
ssc-miR-124a	DIAPH1	diaphanous related formin 1 [Source:VGNC Symbol;Acc:VGNC:99632]	-1.71	0.1324
ssc-miR-124a	DIAPH2	diaphanous related formin 2 [Source:VGNC Symbol;Acc:VGNC:87300]	-1.71	0.1324
ssc-miR-124a	DICER1	dicer 1, ribonuclease III [Source:VGNC Symbol;Acc:VGNC:87302]	-1.71	0.1324
ssc-miR-124a	DIP2B	disco interacting B [Source:VGNC Symbol;Acc:VGNC:87306]	-1.71	0.1324
ssc-miR-124a	DIXDC1	hypothetical gene	-1.71	0.1324
ssc-miR-124a	DLGAP2	DLG associated protein 2 [Source:VGNC Symbol;Acc:VGNC:99711]	-1.71	0.1324
ssc-miR-124a	DLL4	delta like canonical Notch ligand 4 [Source:VGNC Symbol;Acc:VGNC:87337]	-1.71	0.1324
ssc-miR-124a	DLX2	distal-less homeobox 2 [Source:VGNC Symbol;Acc:VGNC:96200]	-1.71	0.1324
ssc-miR-124a	DLX5	distal-less homeobox 5 [Source:VGNC Symbol;Acc:VGNC:87341]	-1.71	0.1324
ssc-miR-124a	DMD	dystrophin [Source:NCBI gene (formerly Entrezgene);Acc:497636]	-1.71	0.1324
ssc-miR-124a	DMRTA1	DMRT like family A1 [Source:VGNC Symbol;Acc:VGNC:87353]	-1.71	0.1324
ssc-miR-124a	DMXL1	Dmx like 1 [Source:VGNC Symbol;Acc:VGNC:87359]	-1.71	0.1324
ssc-miR-124a	DNAJB12	DnaJ heat shock protein family (Hsp40) member B12 [Source:VGNC Symbol;Acc:VGNC:96679]	-1.71	0.1324
ssc-miR-124a	DNAJB14	DnaJ heat shock protein family (Hsp40) member B14 [Source:VGNC Symbol;Acc:VGNC:98919]	-1.71	0.1324
ssc-miR-124a	DNAJC1	DnaJ heat shock protein family (Hsp40) member C1 [Source:VGNC Symbol;Acc:VGNC:96674]	-1.71	0.1324
ssc-miR-124a	DNAJC25	DnaJ heat shock protein family (Hsp40) member C25 [Source:VGNC Symbol;Acc:VGNC:103083]	-1.71	0.1324
ssc-miR-124a	DNAJC25-GNG10	hypothetical gene	-1.71	0.1324
ssc-miR-124a	DNASE2	deoxyribonuclease 2, lysosomal [Source:VGNC Symbol;Acc:VGNC:87377]	-1.71	0.1324
ssc-miR-124a	DNER	delta/notch like EGF repeat containing [Source:VGNC Symbol;Acc:VGNC:96041]	-1.71	0.1324
ssc-miR-124a	DNM2	dynamin 2 [Source:VGNC Symbol;Acc:VGNC:87381]	-1.71	0.1324
ssc-miR-124a	DNMT3A	DNA methyltransferase 3 alpha [Source:VGNC Symbol;Acc:VGNC:87384]	-1.71	0.1324
ssc-miR-124a	DNMT3B	DNA methyltransferase 3 beta [Source:VGNC Symbol;Acc:VGNC:96228]	-1.71	0.1324
ssc-miR-124a	DONSON	DNA replication fork stabilization factor DONSON [Source:VGNC Symbol;Acc:VGNC:96920]	-1.71	0.1324
ssc-miR-124a	DPH3	diphthamide biosynthesis 3 [Source:NCBI gene (formerly Entrezgene);Acc:100156362]	-1.71	0.1324

ssc-miR-124a	DPY19L1	dpy-19 like C-mannosyltransferase 1 [Source:VGNC Symbol;Acc:VGNC:99712]	-1.71	0.1324
ssc-miR-124a	DPY19L3	dpy-19 like C-mannosyltransferase 3 [Source:VGNC Symbol;Acc:VGNC:87426]	-1.71	0.1324
ssc-miR-124a	DPYSL5	dihydropyrimidinase like 5 [Source:VGNC Symbol;Acc:VGNC:87433]	-1.71	0.1324
ssc-miR-124a	DRAM1	DNA damage regulated autophagy modulator 1 [Source:VGNC Symbol;Acc:VGNC:87436]	-1.71	0.1324
ssc-miR-124a	DRD2	dopamine receptor D2 [Source:VGNC Symbol;Acc:VGNC:87444]	-1.71	0.1324
ssc-miR-124a	DRP2	dystrophin related protein 2 [Source:VGNC Symbol;Acc:VGNC:87451]	-1.71	0.1324
ssc-miR-124a	DSC1	desmocollin 1 [Source:VGNC Symbol;Acc:VGNC:87452]	-1.71	0.1324
ssc-miR-124a	DSG2	desmoglein 2 [Source:VGNC Symbol;Acc:VGNC:87456]	-1.71	0.1324
ssc-miR-124a	DTNA	dystrobrevin alpha [Source:VGNC Symbol;Acc:VGNC:87462]	-1.71	0.1324
ssc-miR-124a	DUSP15	dual specificity phosphatase 15 [Source:HGNC Symbol;Acc:HGNC:16236]	-1.71	0.1324
ssc-miR-124a	DUSP18	dual specificity phosphatase 18 [Source:VGNC Symbol;Acc:VGNC:103937]	-1.71	0.1324
ssc-miR-124a	DUSP3	dual specificity phosphatase 3 [Source:VGNC Symbol;Acc:VGNC:87486]	-1.71	0.1324
ssc-miR-124a	DUSP6	dual specificity phosphatase 6 [Source:VGNC Symbol;Acc:VGNC:87488]	-1.71	0.1324
ssc-miR-124a	DYM	dymeclin [Source:VGNC Symbol;Acc:VGNC:87495]	-1.71	0.1324
ssc-miR-124a	DYNC1LI1	dynein cytoplasmic 1 light intermediate chain 1 [Source:VGNC Symbol;Acc:VGNC:87497]	-1.71	0.1324
ssc-miR-124a	DYNC1LI2	dynein cytoplasmic 1 light intermediate chain 2 [Source:VGNC Symbol;Acc:VGNC:87498]	-1.71	0.1324
ssc-miR-124a	DYRK1A	dual specificity tyrosine phosphorylation regulated kinase 1A [Source:VGNC Symbol;Acc:VGNC:87505]	-1.71	0.1324
ssc-miR-124a	DYRK2	dual specificity tyrosine phosphorylation regulated kinase 2 [Source:VGNC Symbol;Acc:VGNC:87507]	-1.71	0.1324
ssc-miR-124a	DZIP1	DAZ interacting zinc finger protein 1 [Source:VGNC Symbol;Acc:VGNC:87510]	-1.71	0.1324
ssc-miR-124a	E2F2	E2F transcription factor 2 [Source:VGNC Symbol;Acc:VGNC:87513]	-1.71	0.1324
ssc-miR-124a	E2F3	E2F transcription factor 3 [Source:VGNC Symbol;Acc:VGNC:87514]	-1.71	0.1324
ssc-miR-124a	E2F5	E2F transcription factor 5 [Source:VGNC Symbol;Acc:VGNC:87516]	-1.71	0.1324
ssc-miR-124a	E2F6	E2F transcription factor 6 [Source:VGNC Symbol;Acc:VGNC:87517]	-1.71	0.1324
ssc-miR-124a	EAF1	ELL associated factor 1 [Source:VGNC Symbol;Acc:VGNC:87521]	-1.71	0.1324
ssc-miR-124a	EBF1	EBF transcription factor 1 [Source:VGNC Symbol;Acc:VGNC:87525]	-1.71	0.1324
ssc-miR-124a	EBF2	EBF transcription factor 2 [Source:VGNC Symbol;Acc:VGNC:87526]	-1.71	0.1324
ssc-miR-124a	EBF3	EBF transcription factor 3 [Source:VGNC Symbol;Acc:VGNC:87527]	-1.71	0.1324
ssc-miR-124a	ECE1	endothelin converting enzyme 1 [Source:VGNC Symbol;Acc:VGNC:87531]	-1.71	0.1324
ssc-miR-124a	ECI2	enoyl-CoA delta isomerase 2 [Source:VGNC Symbol;Acc:VGNC:87532]	-1.71	0.1324
ssc-miR-124a	EDC3	enhancer of mRNA decapping 3 [Source:VGNC Symbol;Acc:VGNC:87543]	-1.71	0.1324
ssc-miR-124a	EDC4	enhancer of mRNA decapping 4 [Source:VGNC Symbol;Acc:VGNC:87544]	-1.71	0.1324
ssc-miR-124a	EDEM1	ER degradation enhancing alpha-mannosidase like protein 1 [Source:VGNC Symbol;Acc:VGNC:87545]	-1.71	0.1324
ssc-miR-124a	EDNRB	endothelin receptor type B [Source:VGNC Symbol;Acc:VGNC:87550]	-1.71	0.1324
ssc-miR-124a	EEA1	early endosome antigen 1 [Source:HGNC Symbol;Acc:HGNC:3185]	-1.71	0.1324
ssc-miR-124a	EFCAB14	EF-hand calcium binding domain 14 [Source:VGNC Symbol;Acc:VGNC:87563]	-1.71	0.1324
ssc-miR-124a	EFHC1	EF-hand domain containing 1 [Source:VGNC Symbol;Acc:VGNC:87570]	-1.71	0.1324
ssc-miR-124a	EFHD2	EF-hand domain family member D2 [Source:HGNC Symbol;Acc:HGNC:28670]	-1.71	0.1324
ssc-miR-124a	EFNA5	ephrin A5 [Source:VGNC Symbol;Acc:VGNC:87575]	-1.71	0.1324
ssc-miR-124a	EFNB1	ephrin B1 [Source:VGNC Symbol;Acc:VGNC:87576]	-1.71	0.1324
ssc-miR-124a	EFNB2	ephrin B2 [Source:VGNC Symbol;Acc:VGNC:87577]	-1.71	0.1324
ssc-miR-124a	EFNB3	ephrin B3 [Source:VGNC Symbol;Acc:VGNC:87578]	-1.71	0.1324
ssc-miR-124a	EGR1	early growth response 1 [Source:VGNC Symbol;Acc:VGNC:87590]	-1.71	0.1324
ssc-miR-124a	EGR2	early growth response 2 [Source:VGNC Symbol;Acc:VGNC:103939]	-1.71	0.1324
ssc-miR-124a	EHD3	EH domain containing 3 [Source:VGNC Symbol;Acc:VGNC:87597]	-1.71	0.1324
ssc-miR-124a	EIF2S1	eukaryotic translation initiation factor 2 subunit alpha [Source:VGNC Symbol;Acc:VGNC:87614]	-1.71	0.1324
ssc-miR-124a	EIF3B	eukaryotic translation initiation factor 3 subunit B [Source:HGNC Symbol;Acc:HGNC:3280]	-1.71	0.1324
ssc-miR-124a	EIF3J	eukaryotic translation initiation factor 3 subunit J [Source:VGNC Symbol;Acc:VGNC:87621]	-1.71	0.1324
ssc-miR-124a	EIF4EBP2	eukaryotic translation initiation factor 4E binding protein 2 [Source:VGNC Symbol;Acc:VGNC:87629]	-1.71	0.1324
ssc-miR-124a	ELF3	E74 like ETS transcription factor 3 [Source:VGNC Symbol;Acc:VGNC:96271]	-1.71	0.1324
ssc-miR-124a	ELK3	ETS transcription factor ELK3 [Source:VGNC Symbol;Acc:VGNC:87646]	-1.71	0.1324

ssc-miR-124a	ELK4	ETS transcription factor ELK4 [Source:VGNC Symbol;Acc:VGNC:87647]	-1.71	0.1324
ssc-miR-124a	ELL2	elongation factor for RNA polymerase II 2 [Source:VGNC Symbol;Acc:VGNC:87649]	-1.71	0.1324
ssc-miR-124a	ELMO2	engulfment and cell motility 2 [Source:VGNC Symbol;Acc:VGNC:95837]	-1.71	0.1324
ssc-miR-124a	ELMSAN1	hypothetical gene	-1.71	0.1324
ssc-miR-124a	ELOVL1	ELOVL fatty acid elongase 1 [Source:VGNC Symbol;Acc:VGNC:87657]	-1.71	0.1324
ssc-miR-124a	ELOVL5	ELOVL fatty acid elongase 5 [Source:VGNC Symbol;Acc:VGNC:87661]	-1.71	0.1324
ssc-miR-124a	EMC10	ER membrane protein complex subunit 10 [Source:VGNC Symbol;Acc:VGNC:87669]	-1.71	0.1324
ssc-miR-124a	EMD	emerin [Source:VGNC Symbol;Acc:VGNC:87675]	-1.71	0.1324
ssc-miR-124a	EML5	hypothetical gene	-1.71	0.1324
ssc-miR-124a	EML6	EMAP like 6 [Source:VGNC Symbol;Acc:VGNC:87687]	-1.71	0.1324
ssc-miR-124a	EMP2	epithelial membrane protein 2 [Source:VGNC Symbol;Acc:VGNC:87689]	-1.71	0.1324
ssc-miR-124a	EMX1	empty spiracles homeobox 1 [Source:VGNC Symbol;Acc:VGNC:87692]	-1.71	0.1324
ssc-miR-124a	EN2	engrailed homeobox 2 [Source:VGNC Symbol;Acc:VGNC:87694]	-1.71	0.1324
ssc-miR-124a	ENAH	ENAH actin regulator [Source:VGNC Symbol;Acc:VGNC:108271]	-1.71	0.1324
ssc-miR-124a	ENDOD1	endonuclease domain containing 1 [Source:VGNC Symbol;Acc:VGNC:87697]	-1.71	0.1324
ssc-miR-124a	ENOX1	ecto-NOX disulfide-thiol exchanger 1 [Source:VGNC Symbol;Acc:VGNC:87706]	-1.71	0.1324
ssc-miR-124a	ENPP4	ectonucleotide pyrophosphatase/phosphodiesterase 4 [Source:VGNC Symbol;Acc:VGNC:87711]	-1.71	0.1324
ssc-miR-124a	ENTPD5	ectonucleoside triphosphate diphosphohydrolase 5 (inactive) [Source:HGNC Symbol;Acc:HGNC:3367]	-1.71	0.1324
ssc-miR-124a	ENTPD7	ectonucleoside triphosphate diphosphohydrolase 7 [Source:VGNC Symbol;Acc:VGNC:87716]	-1.71	0.1324
ssc-miR-124a	ENY2	ENY2 transcription and export complex 2 subunit [Source:VGNC Symbol;Acc:VGNC:98792]	-1.71	0.1324
ssc-miR-124a	EPB41L3	erythrocyte membrane protein band 4.1 like 3 [Source:VGNC Symbol;Acc:VGNC:87724]	-1.71	0.1324
ssc-miR-124a	EPC1	enhancer of polycomb homolog 1 [Source:VGNC Symbol;Acc:VGNC:95923]	-1.71	0.1324
ssc-miR-124a	EPHA10	EPH receptor A10 [Source:VGNC Symbol;Acc:VGNC:87730]	-1.71	0.1324
ssc-miR-124a	EPHA3	EPH receptor A3 [Source:VGNC Symbol;Acc:VGNC:87732]	-1.71	0.1324
ssc-miR-124a	EPHA7	EPH receptor A7 [Source:VGNC Symbol;Acc:VGNC:87734]	-1.71	0.1324
ssc-miR-124a	EPHB4	EPH receptor B4 [Source:VGNC Symbol;Acc:VGNC:87739]	-1.71	0.1324
ssc-miR-124a	EPHX4	epoxide hydrolase 4 [Source:VGNC Symbol;Acc:VGNC:97981]	-1.71	0.1324
ssc-miR-124a	EPN2	epsin 2 [Source:VGNC Symbol;Acc:VGNC:97982]	-1.71	0.1324
ssc-miR-124a	EPS8	epidermal growth factor receptor pathway substrate 8 [Source:VGNC Symbol;Acc:VGNC:87749]	-1.71	0.1324
ssc-miR-124a	EPT1	hypothetical gene	-1.71	0.1324
ssc-miR-124a	ERC2	ELKS/RAB6-interacting/CAST family member 2 [Source:VGNC Symbol;Acc:VGNC:87762]	-1.71	0.1324
ssc-miR-124a	ERF	ETS2 repressor factor [Source:VGNC Symbol;Acc:VGNC:87767]	-1.71	0.1324
ssc-miR-124a	ERMP1	endoplasmic reticulum metallopeptidase 1 [Source:VGNC Symbol;Acc:VGNC:87777]	-1.71	0.1324
ssc-miR-124a	ERN1	endoplasmic reticulum to nucleus signaling 1 [Source:HGNC Symbol;Acc:HGNC:3449]	-1.71	0.1324
ssc-miR-124a	ESRP1	epithelial splicing regulatory protein 1 [Source:VGNC Symbol;Acc:VGNC:87791]	-1.71	0.1324
ssc-miR-124a	ESYT2	extended synaptotagmin 2 [Source:VGNC Symbol;Acc:VGNC:87797]	-1.71	0.1324
ssc-miR-124a	ETF1	eukaryotic translation termination factor 1 [Source:VGNC Symbol;Acc:VGNC:87800]	-1.71	0.1324
ssc-miR-124a	ETNK1	ethanolamine kinase 1 [Source:VGNC Symbol;Acc:VGNC:87806]	-1.71	0.1324
ssc-miR-124a	ETNK2	ethanolamine kinase 2 [Source:VGNC Symbol;Acc:VGNC:87807]	-1.71	0.1324
ssc-miR-124a	ETS1	ETS proto-onco 1, transcription factor [Source:VGNC Symbol;Acc:VGNC:87808]	-1.71	0.1324
ssc-miR-124a	ETV1	ETS variant transcription factor 1 [Source:VGNC Symbol;Acc:VGNC:87810]	-1.71	0.1324
ssc-miR-124a	ETV3	ETS variant transcription factor 3 [Source:VGNC Symbol;Acc:VGNC:87812]	-1.71	0.1324
ssc-miR-124a	EVI5	ecotropic viral integration site 5 [Source:VGNC Symbol;Acc:VGNC:98793]	-1.71	0.1324
ssc-miR-124a	EXOSC2	exosome component 2 [Source:VGNC Symbol;Acc:VGNC:87838]	-1.71	0.1324
ssc-miR-124a	EXTL2	exostosin like glycosyltransferase 2 [Source:VGNC Symbol;Acc:VGNC:87849]	-1.71	0.1324
ssc-miR-124a	EXTL3	exostosin like glycosyltransferase 3 [Source:VGNC Symbol;Acc:VGNC:87850]	-1.71	0.1324
ssc-miR-124a	EYA1	EYA transcriptional coactivator and phosphatase 1 [Source:VGNC Symbol;Acc:VGNC:87851]	-1.71	0.1324
ssc-miR-124a	EYA2	EYA transcriptional coactivator and phosphatase 2 [Source:VGNC Symbol;Acc:VGNC:96292]	-1.71	0.1324
ssc-miR-124a	EYA3	EYA transcriptional coactivator and phosphatase 3 [Source:VGNC Symbol;Acc:VGNC:87852]	-1.71	0.1324
ssc-miR-124a	EYA4	EYA transcriptional coactivator and phosphatase 4 [Source:VGNC Symbol;Acc:VGNC:87853]	-1.71	0.1324

ssc-miR-124a	EZH2	enhancer of zeste 2 polycomb repressive complex 2 subunit [Source:VGNC Symbol;Acc:VGNC:87855]	-1.71	0.1324
ssc-miR-124a	F11R	F11 receptor [Source:VGNC Symbol;Acc:VGNC:87857]	-1.71	0.1324
ssc-miR-124a	FA2H	fatty acid 2-hydroxylase [Source:VGNC Symbol;Acc:VGNC:87868]	-1.71	0.1324
ssc-miR-124a	FAF2	Fas associated factor family member 2 [Source:VGNC Symbol;Acc:VGNC:87880]	-1.71	0.1324
ssc-miR-124a	FAM104A	family with sequence similarity 104 member A [Source:VGNC Symbol;Acc:VGNC:98990]	-1.71	0.1324
ssc-miR-124a	FAM107B	hypothetical gene	-1.71	0.1324
ssc-miR-124a	FAM117A	family with sequence similarity 117 member A [Source:VGNC Symbol;Acc:VGNC:87892]	-1.71	0.1324
ssc-miR-124a	FAM122A	hypothetical gene	-1.71	0.1324
ssc-miR-124a	FAM122B	hypothetical gene	-1.71	0.1324
ssc-miR-124a	FAM127A	hypothetical gene	-1.71	0.1324
ssc-miR-124a	FAM127B	hypothetical gene	-1.71	0.1324
ssc-miR-124a	FAM127C	hypothetical gene	-1.71	0.1324
ssc-miR-124a	FAM129B	hypothetical gene	-1.71	0.1324
ssc-miR-124a	FAM133A	family with sequence similarity 133 member A [Source:VGNC Symbol;Acc:VGNC:87903]	-1.71	0.1324
ssc-miR-124a	FAM133B	family with sequence similarity 133 member B [Source:HGNC Symbol;Acc:HGNC:28629]	-1.71	0.1324
ssc-miR-124a	FAM134B	hypothetical gene	-1.71	0.1324
ssc-miR-124a	FAM135B	family with sequence similarity 135 member B [Source:VGNC Symbol;Acc:VGNC:87905]	-1.71	0.1324
ssc-miR-124a	FAM150B	hypothetical gene	-1.71	0.1324
ssc-miR-124a	FAM160A1	hypothetical gene	-1.71	0.1324
ssc-miR-124a	FAM171A1	family with sequence similarity 171 member A1 [Source:VGNC Symbol;Acc:VGNC:96011]	-1.71	0.1324
ssc-miR-124a	FAM171B	family with sequence similarity 171 member B [Source:VGNC Symbol;Acc:VGNC:96218]	-1.71	0.1324
ssc-miR-124a	FAM172A	family with sequence similarity 172 member A [Source:VGNC Symbol;Acc:VGNC:87930]	-1.71	0.1324
ssc-miR-124a	FAM174B	family with sequence similarity 174 member B [Source:VGNC Symbol;Acc:VGNC:87932]	-1.71	0.1324
ssc-miR-124a	FAM177A1	family with sequence similarity 177 member A1 [Source:VGNC Symbol;Acc:VGNC:87933]	-1.71	0.1324
ssc-miR-124a	FAM178A	hypothetical gene	-1.71	0.1324
ssc-miR-124a	FAM199X	family with sequence similarity 199, X-linked [Source:HGNC Symbol;Acc:HGNC:25195]	-1.71	0.1324
ssc-miR-124a	FAM19A2	hypothetical gene	-1.71	0.1324
ssc-miR-124a	FAM204A	family with sequence similarity 204 member A [Source:VGNC Symbol;Acc:VGNC:107389]	-1.71	0.1324
ssc-miR-124a	FAM20B	FAM20B glycosaminoglycan xylosylkinase [Source:VGNC Symbol;Acc:VGNC:87950]	-1.71	0.1324
ssc-miR-124a	FAM211A	hypothetical gene	-1.71	0.1324
ssc-miR-124a	FAM219A	family with sequence similarity 219 member A [Source:HGNC Symbol;Acc:HGNC:19920]	-1.71	0.1324
ssc-miR-124a	FAM219B	family with sequence similarity 219 member B [Source:VGNC Symbol;Acc:VGNC:87956]	-1.71	0.1324
ssc-miR-124a	FAM222B	family with sequence similarity 222 member B [Source:VGNC Symbol;Acc:VGNC:87960]	-1.71	0.1324
ssc-miR-124a	FAM53B	family with sequence similarity 53 member B [Source:VGNC Symbol;Acc:VGNC:87974]	-1.71	0.1324
ssc-miR-124a	FAM73A	hypothetical gene	-1.71	0.1324
ssc-miR-124a	FAM73B	hypothetical gene	-1.71	0.1324
ssc-miR-124a	FAM76A	family with sequence similarity 76 member A [Source:VGNC Symbol;Acc:VGNC:87982]	-1.71	0.1324
ssc-miR-124a	FAM76B	family with sequence similarity 76 member B [Source:VGNC Symbol;Acc:VGNC:87983]	-1.71	0.1324
ssc-miR-124a	FAM78A	family with sequence similarity 78 member A [Source:VGNC Symbol;Acc:VGNC:87984]	-1.71	0.1324
ssc-miR-124a	FAM81A	family with sequence similarity 81 member A [Source:VGNC Symbol;Acc:VGNC:87986]	-1.71	0.1324
ssc-miR-124a	FAM83F	family with sequence similarity 83 member F [Source:VGNC Symbol;Acc:VGNC:87991]	-1.71	0.1324
ssc-miR-124a	FAM89A	family with sequence similarity 89 member A [Source:VGNC Symbol;Acc:VGNC:87994]	-1.71	0.1324
ssc-miR-124a	FAM92B	hypothetical gene	-1.71	0.1324
ssc-miR-124a	FANCD2	FA complementation group D2 [Source:VGNC Symbol;Acc:VGNC:88003]	-1.71	0.1324
ssc-miR-124a	FAR1	fatty acyl-CoA reductase 1 [Source:VGNC Symbol;Acc:VGNC:88010]	-1.71	0.1324
ssc-miR-124a	FARP1	FERM, ARH/RhoGEF and pleckstrin domain protein 1 [Source:VGNC Symbol;Acc:VGNC:88012]	-1.71	0.1324
ssc-miR-124a	FARSA	phenylalanyl-tRNA synthetase subunit alpha [Source:VGNC Symbol;Acc:VGNC:88014]	-1.71	0.1324
ssc-miR-124a	FBRSL1	hypothetical gene	-1.71	0.1324
ssc-miR-124a	FBXL4	F-box and leucine rich repeat protein 4 [Source:VGNC Symbol;Acc:VGNC:98007]	-1.71	0.1324
ssc-miR-124a	FBXL7	F-box and leucine rich repeat protein 7 [Source:VGNC Symbol;Acc:VGNC:98009]	-1.71	0.1324

ssc-miR-124a	FBXO10	F-box protein 10 [Source:VGNC Symbol;Acc:VGNC:103092]	-1.71	0.1324
ssc-miR-124a	FBXO28	F-box protein 28 [Source:HGNC Symbol;Acc:HGNC:29046]	-1.71	0.1324
ssc-miR-124a	FBXO30	F-box protein 30 [Source:VGNC Symbol;Acc:VGNC:108155]	-1.71	0.1324
ssc-miR-124a	FBXO31	F-box protein 31 [Source:VGNC Symbol;Acc:VGNC:88038]	-1.71	0.1324
ssc-miR-124a	FBXO38	F-box protein 38 [Source:VGNC Symbol;Acc:VGNC:88042]	-1.71	0.1324
ssc-miR-124a	FBXO42	F-box protein 42 [Source:VGNC Symbol;Acc:VGNC:88047]	-1.71	0.1324
ssc-miR-124a	FCHO2	FCH and mu domain containing endocytic adaptor 2 [Source:VGNC Symbol;Acc:VGNC:88066]	-1.71	0.1324
ssc-miR-124a	FCHSD2	FCH and double SH3 domains 2 [Source:VGNC Symbol;Acc:VGNC:88068]	-1.71	0.1324
ssc-miR-124a	FEM1B	fem-1 homolog B [Source:VGNC Symbol;Acc:VGNC:88083]	-1.71	0.1324
ssc-miR-124a	FGD5	FYVE, RhoGEF and PH domain containing 5 [Source:VGNC Symbol;Acc:VGNC:88098]	-1.71	0.1324
ssc-miR-124a	FGF14	fibroblast growth factor 14 [Source:VGNC Symbol;Acc:VGNC:88104]	-1.71	0.1324
ssc-miR-124a	FGFR1OP	hypothetical gene	-1.71	0.1324
ssc-miR-124a	FGFR2	fibroblast growth factor receptor 2 [Source:NCBI gene (formerly Entrezgene);Acc:396762]	-1.71	0.1324
ssc-miR-124a	FGL2	fibrinogen like 2 [Source:VGNC Symbol;Acc:VGNC:88123]	-1.71	0.1324
ssc-miR-124a	FIGN	fidgetin, microtubule severing factor [Source:VGNC Symbol;Acc:VGNC:95580]	-1.71	0.1324
ssc-miR-124a	FKBP15	FKBP prolyl isomerase family member 15 [Source:VGNC Symbol;Acc:VGNC:88145]	-1.71	0.1324
ssc-miR-124a	FKBP1B	FKBP prolyl isomerase 1B [Source:VGNC Symbol;Acc:VGNC:106579]	-1.71	0.1324
ssc-miR-124a	FLI1	Fli-1 proto-onco, ETS transcription factor [Source:VGNC Symbol;Acc:VGNC:88153]	-1.71	0.1324
ssc-miR-124a	FLNB	filamin B [Source:VGNC Symbol;Acc:VGNC:88156]	-1.71	0.1324
ssc-miR-124a	FLOT1	flotillin 1 [Source:VGNC Symbol;Acc:VGNC:88158]	-1.71	0.1324
ssc-miR-124a	FLOT2	flotillin 2 [Source:VGNC Symbol;Acc:VGNC:88159]	-1.71	0.1324
ssc-miR-124a	FLRT3	fibronectin leucine rich transmembrane protein 3 [Source:VGNC Symbol;Acc:VGNC:96304]	-1.71	0.1324
ssc-miR-124a	FMNL2	formin like 2 [Source:VGNC Symbol;Acc:VGNC:95879]	-1.71	0.1324
ssc-miR-124a	FMR1	FMRP translational regulator 1 [Source:VGNC Symbol;Acc:VGNC:88175]	-1.71	0.1324
ssc-miR-124a	FNBP1L	formin binding protein 1 like [Source:VGNC Symbol;Acc:VGNC:88179]	-1.71	0.1324
ssc-miR-124a	FNIP2	folliculin interacting protein 2 [Source:VGNC Symbol;Acc:VGNC:88188]	-1.71	0.1324
ssc-miR-124a	FOXK2	forkhead box K2 [Source:VGNC Symbol;Acc:VGNC:88215]	-1.71	0.1324
ssc-miR-124a	FOXQ1	forkhead box Q1 [Source:VGNC Symbol;Acc:VGNC:88225]	-1.71	0.1324
ssc-miR-124a	FRMD4A	FERM domain containing 4A [Source:VGNC Symbol;Acc:VGNC:96088]	-1.71	0.1324
ssc-miR-124a	FRMD4B	FERM domain containing 4B [Source:VGNC Symbol;Acc:VGNC:88237]	-1.71	0.1324
ssc-miR-124a	FRMD8	FERM domain containing 8 [Source:VGNC Symbol;Acc:VGNC:99653]	-1.71	0.1324
ssc-miR-124a	FRRS1L	ferric chelate reductase 1 like [Source:VGNC Symbol;Acc:VGNC:88245]	-1.71	0.1324
ssc-miR-124a	FRS2	fibroblast growth factor receptor substrate 2 [Source:VGNC Symbol;Acc:VGNC:88246]	-1.71	0.1324
ssc-miR-124a	FSBP	hypothetical gene	-1.71	0.1324
ssc-miR-124a	FSD1	fibronectin type III and SPRY domain containing 1 [Source:VGNC Symbol;Acc:VGNC:88251]	-1.71	0.1324
ssc-miR-124a	FSD1L	fibronectin type III and SPRY domain containing 1 like [Source:HGNC Symbol;Acc:HGNC:13753]	-1.71	0.1324
ssc-miR-124a	FSTL1	follistatin like 1 [Source:VGNC Symbol;Acc:VGNC:88255]	-1.71	0.1324
ssc-miR-124a	FSTL3	follistatin like 3 [Source:VGNC Symbol;Acc:VGNC:99655]	-1.71	0.1324
ssc-miR-124a	FSTL4	follistatin like 4 [Source:VGNC Symbol;Acc:VGNC:88256]	-1.71	0.1324
ssc-miR-124a	FUBP1	far upstream element binding protein 1 [Source:VGNC Symbol;Acc:VGNC:96781]	-1.71	0.1324
ssc-miR-124a	FURIN	furin, paired basic amino acid cleaving enzyme [Source:VGNC Symbol;Acc:VGNC:88266]	-1.71	0.1324
ssc-miR-124a	FUT10	fucosyltransferase 10 [Source:VGNC Symbol;Acc:VGNC:88268]	-1.71	0.1324
ssc-miR-124a	FXR1	FMR1 autosomal homolog 1 [Source:VGNC Symbol;Acc:VGNC:108659]	-1.71	0.1324
ssc-miR-124a	FXR2	FMR1 autosomal homolog 2 [Source:HGNC Symbol;Acc:HGNC:4024]	-1.71	0.1324
ssc-miR-124a	FZD4	frizzled class receptor 4 [Source:VGNC Symbol;Acc:VGNC:88282]	-1.71	0.1324
ssc-miR-124a	FZD5	frizzled class receptor 5 [Source:VGNC Symbol;Acc:VGNC:96309]	-1.71	0.1324
ssc-miR-124a	FZD8	frizzled class receptor 8 [Source:VGNC Symbol;Acc:VGNC:96311]	-1.71	0.1324
ssc-miR-124a	G3BP1	G3BP stress granule assembly factor 1 [Source:VGNC Symbol;Acc:VGNC:88288]	-1.71	0.1324
ssc-miR-124a	G3BP2	G3BP stress granule assembly factor 2 [Source:VGNC Symbol;Acc:VGNC:88289]	-1.71	0.1324
ssc-miR-124a	GABPB2	GA binding protein transcription factor subunit beta 2 [Source:VGNC Symbol;Acc:VGNC:98796]	-1.71	0.1324

ssc-miR-124a	GABRA6	gamma-aminobutyric acid type A receptor subunit alpha6 [Source:VGNC Symbol;Acc:VGNC:88305]	-1.71	0.1324
ssc-miR-124a	GABRB1	gamma-aminobutyric acid type A receptor subunit beta1 [Source:VGNC Symbol;Acc:VGNC:88306]	-1.71	0.1324
ssc-miR-124a	GABRB3	gamma-aminobutyric acid type A receptor subunit beta3 [Source:VGNC Symbol;Acc:VGNC:88308]	-1.71	0.1324
ssc-miR-124a	GABRE	gamma-aminobutyric acid type A receptor subunit epsilon [Source:VGNC Symbol;Acc:VGNC:88310]	-1.71	0.1324
ssc-miR-124a	GABRQ	gamma-aminobutyric acid type A receptor subunit theta [Source:VGNC Symbol;Acc:VGNC:88315]	-1.71	0.1324
ssc-miR-124a	GALNT1	polypeptide N-acetylgalactosaminyltransferase 1 [Source:VGNC Symbol;Acc:VGNC:97062]	-1.71	0.1324
ssc-miR-124a	GALNT10	polypeptide N-acetylgalactosaminyltransferase 10 [Source:VGNC Symbol;Acc:VGNC:88327]	-1.71	0.1324
ssc-miR-124a	GALNT13	polypeptide N-acetylgalactosaminyltransferase 13 [Source:VGNC Symbol;Acc:VGNC:95992]	-1.71	0.1324
ssc-miR-124a	GALNT4	polypeptide N-acetylgalactosaminyltransferase 4 [Source:HGNC Symbol;Acc:HGNC:4126]	-1.71	0.1324
ssc-miR-124a	GALNT9	polypeptide N-acetylgalactosaminyltransferase 9 [Source:VGNC Symbol;Acc:VGNC:88336]	-1.71	0.1324
ssc-miR-124a	GAPVD1	GTPase activating protein and VPS9 domains 1 [Source:VGNC Symbol;Acc:VGNC:88347]	-1.71	0.1324
ssc-miR-124a	GAREM	hypothetical gene	-1.71	0.1324
ssc-miR-124a	GAREML	hypothetical gene	-1.71	0.1324
ssc-miR-124a	GAS1	growth arrest specific 1 [Source:HGNC Symbol;Acc:HGNC:4165]	-1.71	0.1324
ssc-miR-124a	GAS2	growth arrest specific 2 [Source:VGNC Symbol;Acc:VGNC:88354]	-1.71	0.1324
ssc-miR-124a	GATA6	GATA binding protein 6 [Source:VGNC Symbol;Acc:VGNC:88366]	-1.71	0.1324
ssc-miR-124a	GATAD2A	GATA zinc finger domain containing 2A [Source:VGNC Symbol;Acc:VGNC:88368]	-1.71	0.1324
ssc-miR-124a	GATSL2	hypothetical gene	-1.71	0.1324
ssc-miR-124a	GCC1	GRIP and coiled-coil domain containing 1 [Source:VGNC Symbol;Acc:VGNC:88379]	-1.71	0.1324
ssc-miR-124a	GCDH	glutaryl-CoA dehydrogenase [Source:VGNC Symbol;Acc:VGNC:88381]	-1.71	0.1324
ssc-miR-124a	GCH1	GTP cyclohydrolase 1 [Source:VGNC Symbol;Acc:VGNC:88384]	-1.71	0.1324
ssc-miR-124a	GCN1L1	hypothetical gene	-1.71	0.1324
ssc-miR-124a	GCNT4	glucosaminyl (N-acetyl) transferase 4 [Source:VGNC Symbol;Acc:VGNC:96616]	-1.71	0.1324
ssc-miR-124a	GDAP1L1	ganglioside induced differentiation associated protein 1 like 1 [Source:VGNC Symbol;Acc:VGNC:96318]	-1.71	0.1324
ssc-miR-124a	GDAP2	ganglioside induced differentiation associated protein 2 [Source:VGNC Symbol;Acc:VGNC:88395]	-1.71	0.1324
ssc-miR-124a	GDPD5	glycerophosphodiester phosphodiesterase domain containing 5 [Source:VGNC Symbol;Acc:VGNC:88409]	-1.71	0.1324
ssc-miR-124a	GFPT2	glutamine-fructose-6-phosphate transaminase 2 [Source:VGNC Symbol;Acc:VGNC:88427]	-1.71	0.1324
ssc-miR-124a	GGA1	golgi associated, gamma adaptin ear containing, ARF binding protein 1 [Source:VGNC Symbol;Acc:VGNC:88431]	-1.71	0.1324
ssc-miR-124a	GGA2	golgi associated, gamma adaptin ear containing, ARF binding protein 2 [Source:VGNC Symbol;Acc:VGNC:88432]	-1.71	0.1324
ssc-miR-124a	GGPS1	geranylgeranyl diphosphate synthase 1 [Source:VGNC Symbol;Acc:VGNC:88438]	-1.71	0.1324
ssc-miR-124a	GH2	hypothetical gene	-1.71	0.1324
ssc-miR-124a	GID8	GID complex subunit 8 homolog [Source:VGNC Symbol;Acc:VGNC:95674]	-1.71	0.1324
ssc-miR-124a	GIGYF1	GRB10 interacting GYF protein 1 [Source:VGNC Symbol;Acc:VGNC:88444]	-1.71	0.1324
ssc-miR-124a	GINM1	glycoprotein integral membrane 1 [Source:VGNC Symbol;Acc:VGNC:88450]	-1.71	0.1324
ssc-miR-124a	GIT2	GIT ArfGAP 2 [Source:VGNC Symbol;Acc:VGNC:88458]	-1.71	0.1324
ssc-miR-124a	GJA5	gap junction protein alpha 5 [Source:VGNC Symbol;Acc:VGNC:88460]	-1.71	0.1324
ssc-miR-124a	GJA9	gap junction protein alpha 9 [Source:VGNC Symbol;Acc:VGNC:97065]	-1.71	0.1324
ssc-miR-124a	GJC1	gap junction protein gamma 1 [Source:VGNC Symbol;Acc:VGNC:88468]	-1.71	0.1324
ssc-miR-124a	GK5	glycerol kinase 5 [Source:VGNC Symbol;Acc:VGNC:96734]	-1.71	0.1324
ssc-miR-124a	GLCE	glucuronic acid epimerase [Source:VGNC Symbol;Acc:VGNC:88477]	-1.71	0.1324
ssc-miR-124a	GLI3	GLI family zinc finger 3 [Source:VGNC Symbol;Acc:VGNC:88483]	-1.71	0.1324
ssc-miR-124a	GLIS2	GLIS family zinc finger 2 [Source:VGNC Symbol;Acc:VGNC:88484]	-1.71	0.1324
ssc-miR-124a	GLRB	glycine receptor beta [Source:VGNC Symbol;Acc:VGNC:88494]	-1.71	0.1324
ssc-miR-124a	GLT8D1	glycosyltransferase 8 domain containing 1 [Source:VGNC Symbol;Acc:VGNC:88500]	-1.71	0.1324
ssc-miR-124a	GLTP	glycolipid transfer protein [Source:VGNC Symbol;Acc:VGNC:107395]	-1.71	0.1324
ssc-miR-124a	GMCL1	hypothetical gene	-1.71	0.1324
ssc-miR-124a	GMFB	glia maturation factor beta [Source:VGNC Symbol;Acc:VGNC:88509]	-1.71	0.1324
ssc-miR-124a	GNA13	G protein subunit alpha 13 [Source:VGNC Symbol;Acc:VGNC:98997]	-1.71	0.1324
ssc-miR-124a	GNAI1	G protein subunit alpha i1 [Source:VGNC Symbol;Acc:VGNC:88521]	-1.71	0.1324
ssc-miR-124a	GNAI2	G protein subunit alpha i2 [Source:VGNC Symbol;Acc:VGNC:88522]	-1.71	0.1324

ssc-miR-124a	GNAI3	G protein subunit alpha i3 [Source:VGNC Symbol;Acc:VGNC:88523]	-1.71	0.1324
ssc-miR-124a	GNAL	G protein subunit alpha L [Source:VGNC Symbol;Acc:VGNC:88524]	-1.71	0.1324
ssc-miR-124a	GNAQ	G protein subunit alpha q [Source:VGNC Symbol;Acc:VGNC:103100]	-1.71	0.1324
ssc-miR-124a	GNG10	G protein subunit gamma 10 [Source:HGNC Symbol;Acc:HGNC:4402]	-1.71	0.1324
ssc-miR-124a	GNL1	G protein nucleolar 1 (putative) [Source:NCBI gene (formerly Entrezgene);Acc:100151741]	-1.71	0.1324
ssc-miR-124a	GNMT	glycine N-methyltransferase [Source:VGNC Symbol;Acc:VGNC:88542]	-1.71	0.1324
ssc-miR-124a	GNS	glucosamine (N-acetyl)-6-sulfatase [Source:VGNC Symbol;Acc:VGNC:103284]	-1.71	0.1324
ssc-miR-124a	GOLGA8A	hypothetical gene	-1.71	0.1324
ssc-miR-124a	GOLGA8B	hypothetical gene	-1.71	0.1324
ssc-miR-124a	GOLGA8H	hypothetical gene	-1.71	0.1324
ssc-miR-124a	GOLGA8I	hypothetical gene	-1.71	0.1324
ssc-miR-124a	GOLGA8N	hypothetical gene	-1.71	0.1324
ssc-miR-124a	GOLT1B	golgi transport 1B [Source:VGNC Symbol;Acc:VGNC:88557]	-1.71	0.1324
ssc-miR-124a	GPAM	glycerol-3-phosphate acyltransferase, mitochondrial [Source:VGNC Symbol;Acc:VGNC:88570]	-1.71	0.1324
ssc-miR-124a	GPATCH8	G-patch domain containing 8 [Source:HGNC Symbol;Acc:HGNC:29066]	-1.71	0.1324
ssc-miR-124a	GPC4	glypican 4 [Source:VGNC Symbol;Acc:VGNC:88581]	-1.71	0.1324
ssc-miR-124a	GPD2	glycerol-3-phosphate dehydrogenase 2 [Source:VGNC Symbol;Acc:VGNC:96330]	-1.71	0.1324
ssc-miR-124a	GPM6B	hypothetical gene	-1.71	0.1324
ssc-miR-124a	GPR124	hypothetical gene	-1.71	0.1324
ssc-miR-124a	GPR153	G protein-coupled receptor 153 [Source:VGNC Symbol;Acc:VGNC:88609]	-1.71	0.1324
ssc-miR-124a	GPR173	G protein-coupled receptor 173 [Source:VGNC Symbol;Acc:VGNC:88616]	-1.71	0.1324
ssc-miR-124a	GPR22	G protein-coupled receptor 22 [Source:VGNC Symbol;Acc:VGNC:88625]	-1.71	0.1324
ssc-miR-124a	GPR37	G protein-coupled receptor 37 [Source:VGNC Symbol;Acc:VGNC:88630]	-1.71	0.1324
ssc-miR-124a	GPR50	G protein-coupled receptor 50 [Source:VGNC Symbol;Acc:VGNC:88633]	-1.71	0.1324
ssc-miR-124a	GPR85	G protein-coupled receptor 85 [Source:VGNC Symbol;Acc:VGNC:88638]	-1.71	0.1324
ssc-miR-124a	GPRIN3	hypothetical gene	-1.71	0.1324
ssc-miR-124a	GPT2	glutamic--pyruvic transaminase 2 [Source:VGNC Symbol;Acc:VGNC:88651]	-1.71	0.1324
ssc-miR-124a	GRASP	hypothetical gene	-1.71	0.1324
ssc-miR-124a	GRB2	growth factor receptor bound protein 2 [Source:VGNC Symbol;Acc:VGNC:88663]	-1.71	0.1324
ssc-miR-124a	GREB1	growth regulating estrogen receptor binding 1 [Source:VGNC Symbol;Acc:VGNC:96583]	-1.71	0.1324
ssc-miR-124a	GREB1L	GREB1 like retinoic acid receptor coactivator [Source:VGNC Symbol;Acc:VGNC:96584]	-1.71	0.1324
ssc-miR-124a	GRIA2	glutamate ionotropic receptor AMPA type subunit 2 [Source:VGNC Symbol;Acc:VGNC:88671]	-1.71	0.1324
ssc-miR-124a	GRID1	glutamate ionotropic receptor delta type subunit 1 [Source:VGNC Symbol;Acc:VGNC:88674]	-1.71	0.1324
ssc-miR-124a	GRID2	glutamate ionotropic receptor delta type subunit 2 [Source:VGNC Symbol;Acc:VGNC:98932]	-1.71	0.1324
ssc-miR-124a	GRIN2A	glutamate ionotropic receptor NMDA type subunit 2A [Source:VGNC Symbol;Acc:VGNC:88683]	-1.71	0.1324
ssc-miR-124a	GRIN3A	glutamate ionotropic receptor NMDA type subunit 3A [Source:VGNC Symbol;Acc:VGNC:88687]	-1.71	0.1324
ssc-miR-124a	GRK5	G protein-coupled receptor kinase 5 [Source:VGNC Symbol;Acc:VGNC:88697]	-1.71	0.1324
ssc-miR-124a	GRSF1	G-rich RNA sequence binding factor 1 [Source:VGNC Symbol;Acc:VGNC:88710]	-1.71	0.1324
ssc-miR-124a	GSK3B	glycogen synthase kinase 3 beta [Source:VGNC Symbol;Acc:VGNC:88723]	-1.71	0.1324
ssc-miR-124a	GSN	gelsolin [Source:HGNC Symbol;Acc:HGNC:4620]	-1.71	0.1324
ssc-miR-124a	GSS	glutathione synthetase [Source:VGNC Symbol;Acc:VGNC:96338]	-1.71	0.1324
ssc-miR-124a	GSTO2	glutathione S-transferase omega 2 [Source:VGNC Symbol;Acc:VGNC:107396]	-1.71	0.1324
ssc-miR-124a	GTF2A1	ral transcription factor IIA subunit 1 [Source:VGNC Symbol;Acc:VGNC:88730]	-1.71	0.1324
ssc-miR-124a	GUCD1	guanylyl cyclase domain containing 1 [Source:VGNC Symbol;Acc:VGNC:88749]	-1.71	0.1324
ssc-miR-124a	GXYLT1	glucoside xylosyltransferase 1 [Source:VGNC Symbol;Acc:VGNC:88756]	-1.71	0.1324
ssc-miR-124a	GZF1	GDNF inducible zinc finger protein 1 [Source:VGNC Symbol;Acc:VGNC:108722]	-1.71	0.1324
ssc-miR-124a	HADH	hydroxyacyl-CoA dehydrogenase [Source:VGNC Symbol;Acc:VGNC:88771]	-1.71	0.1324
ssc-miR-124a	HADHA	hydroxyacyl-CoA dehydrogenase trifunctional multienzyme complex subunit alpha [Source:VGNC Symbol;Acc:VGNC:88772]	-1.71	0.1324
ssc-miR-124a	HBP1	HMG-box transcription factor 1 [Source:VGNC Symbol;Acc:VGNC:88794]	-1.71	0.1324
ssc-miR-124a	HDAC4	histone deacetylase 4 [Source:VGNC Symbol;Acc:VGNC:95602]	-1.71	0.1324

ssc-miR-124a	HDAC5	histone deacetylase 5 [Source:VGNC Symbol;Acc:VGNC:88815]	-1.71	0.1324
ssc-miR-124a	HDLBP	high density lipoprotein binding protein [Source:HGNC Symbol;Acc:HGNC:4857]	-1.71	0.1324
ssc-miR-124a	HEATR1	HEAT repeat containing 1 [Source:VGNC Symbol;Acc:VGNC:88825]	-1.71	0.1324
ssc-miR-124a	HEATR6	HEAT repeat containing 6 [Source:VGNC Symbol;Acc:VGNC:88828]	-1.71	0.1324
ssc-miR-124a	HEBP2	hypothetical gene	-1.71	0.1324
ssc-miR-124a	HECTD2	HECT domain E3 ubiquitin protein ligase 2 [Source:VGNC Symbol;Acc:VGNC:88833]	-1.71	0.1324
ssc-miR-124a	HELZ	helicase with zinc finger [Source:VGNC Symbol;Acc:VGNC:88840]	-1.71	0.1324
ssc-miR-124a	HEPACAM	hepatic and glial cell adhesion molecule [Source:VGNC Symbol;Acc:VGNC:88845]	-1.71	0.1324
ssc-miR-124a	HEXIM1	HEXIM P-TEFb complex subunit 1 [Source:HGNC Symbol;Acc:HGNC:24953]	-1.71	0.1324
ssc-miR-124a	HIAT1	hypothetical gene	-1.71	0.1324
ssc-miR-124a	HIATL1	hypothetical gene	-1.71	0.1324
ssc-miR-124a	HIC1	HIC ZBTB transcriptional repressor 1 [Source:VGNC Symbol;Acc:VGNC:88878]	-1.71	0.1324
ssc-miR-124a	HIF1AN	hypoxia inducible factor 1 subunit alpha inhibitor [Source:VGNC Symbol;Acc:VGNC:98033]	-1.71	0.1324
ssc-miR-124a	HIP1	huntingtin interacting protein 1 [Source:VGNC Symbol;Acc:VGNC:88885]	-1.71	0.1324
ssc-miR-124a	HIPK1	homeodomain interacting protein kinase 1 [Source:VGNC Symbol;Acc:VGNC:88887]	-1.71	0.1324
ssc-miR-124a	HIPK2	homeodomain interacting protein kinase 2 [Source:VGNC Symbol;Acc:VGNC:88888]	-1.71	0.1324
ssc-miR-124a	HIPK3	homeodomain interacting protein kinase 3 [Source:VGNC Symbol;Acc:VGNC:88889]	-1.71	0.1324
ssc-miR-124a	HIVEP1	HIVEP zinc finger 1 [Source:VGNC Symbol;Acc:VGNC:96585]	-1.71	0.1324
ssc-miR-124a	HIVEP2	HIVEP zinc finger 2 [Source:VGNC Symbol;Acc:VGNC:96586]	-1.71	0.1324
ssc-miR-124a	HIVEP3	hypothetical gene	-1.71	0.1324
ssc-miR-124a	HLF	HLF transcription factor, PAR bZIP family member [Source:VGNC Symbol;Acc:VGNC:88896]	-1.71	0.1324
ssc-miR-124a	HMGA1	high mobility group AT-hook 1 [Source:VGNC Symbol;Acc:VGNC:103070]	-1.71	0.1324
ssc-miR-124a	HMGXB4	HMG-box containing 4 [Source:VGNC Symbol;Acc:VGNC:96346]	-1.71	0.1324
ssc-miR-124a	HN1L	hypothetical gene	-1.71	0.1324
ssc-miR-124a	HOOK3	hook microtubule tethering protein 3 [Source:VGNC Symbol;Acc:VGNC:98037]	-1.71	0.1324
ssc-miR-124a	HOXA13	homeobox A13 [Source:NCBI gene (formerly Entrezgene);Acc:100359352]	-1.71	0.1324
ssc-miR-124a	HOXA5	homeobox A5 [Source:VGNC Symbol;Acc:VGNC:88939]	-1.71	0.1324
ssc-miR-124a	HOXB1	homeobox B1 [Source:VGNC Symbol;Acc:VGNC:88941]	-1.71	0.1324
ssc-miR-124a	HOXD11	homeobox D11 [Source:VGNC Symbol;Acc:VGNC:96352]	-1.71	0.1324
ssc-miR-124a	HRCT1	histidine rich carboxyl terminus 1 [Source:HGNC Symbol;Acc:HGNC:33872]	-1.71	0.1324
ssc-miR-124a	HRH1	histamine receptor H1 [Source:VGNC Symbol;Acc:VGNC:88973]	-1.71	0.1324
ssc-miR-124a	HS1BP3	HCLS1 binding protein 3 [Source:VGNC Symbol;Acc:VGNC:88976]	-1.71	0.1324
ssc-miR-124a	HS2ST1	heparan sulfate 2-O-sulfotransferase 1 [Source:HGNC Symbol;Acc:HGNC:5193]	-1.71	0.1324
ssc-miR-124a	HS3ST3B1	heparan sulfate-glucosamine 3-sulfotransferase 3B1 [Source:VGNC Symbol;Acc:VGNC:99003]	-1.71	0.1324
ssc-miR-124a	HSPA2	hypothetical gene	-1.71	0.1324
ssc-miR-124a	HTR2C	5-hydroxytryptamine receptor 2C [Source:VGNC Symbol;Acc:VGNC:96737]	-1.71	0.1324
ssc-miR-124a	IAPP	hypothetical gene	-1.71	0.1324
ssc-miR-124a	ICMT	isoprenylcysteine carboxyl methyltransferase [Source:VGNC Symbol;Acc:VGNC:89022]	-1.71	0.1324
ssc-miR-124a	IDE	insulin degrading enzyme [Source:VGNC Symbol;Acc:VGNC:89025]	-1.71	0.1324
ssc-miR-124a	IFFO2	intermediate filament family orphan 2 [Source:VGNC Symbol;Acc:VGNC:98475]	-1.71	0.1324
ssc-miR-124a	IFRG15	hypothetical gene	-1.71	0.1324
ssc-miR-124a	IGDCC4	immunoglobulin superfamily DCC subclass member 4 [Source:VGNC Symbol;Acc:VGNC:89054]	-1.71	0.1324
ssc-miR-124a	IGF2BP1	insulin like growth factor 2 mRNA binding protein 1 [Source:VGNC Symbol;Acc:VGNC:99006]	-1.71	0.1324
ssc-miR-124a	IGF2R	insulin like growth factor 2 receptor [Source:VGNC Symbol;Acc:VGNC:89057]	-1.71	0.1324
ssc-miR-124a	IKZF2	IKAROS family zinc finger 2 [Source:VGNC Symbol;Acc:VGNC:95576]	-1.71	0.1324
ssc-miR-124a	IL11	interleukin 11 [Source:VGNC Symbol;Acc:VGNC:89078]	-1.71	0.1324
ssc-miR-124a	IL17RD	interleukin 17 receptor D [Source:VGNC Symbol;Acc:VGNC:89087]	-1.71	0.1324
ssc-miR-124a	IL6R	interleukin 6 receptor [Source:VGNC Symbol;Acc:VGNC:89112]	-1.71	0.1324
ssc-miR-124a	ILDR2	immunoglobulin like domain containing receptor 2 [Source:VGNC Symbol;Acc:VGNC:89118]	-1.71	0.1324
ssc-miR-124a	IMPACT	impact RWD domain protein [Source:HGNC Symbol;Acc:HGNC:20387]	-1.71	0.1324

ssc-miR-124a	IMPAD1	hypothetical gene	-1.71	0.1324
ssc-miR-124a	INF2	hypothetical gene	-1.71	0.1324
ssc-miR-124a	ING1	inhibitor of growth family member 1 [Source:VGNC Symbol;Acc:VGNC:89131]	-1.71	0.1324
ssc-miR-124a	INO80C	INO80 complex subunit C [Source:VGNC Symbol;Acc:VGNC:98049]	-1.71	0.1324
ssc-miR-124a	INO80D	INO80 complex subunit D [Source:VGNC Symbol;Acc:VGNC:96113]	-1.71	0.1324
ssc-miR-124a	INSIG2	insulin induced 2 [Source:VGNC Symbol;Acc:VGNC:103970]	-1.71	0.1324
ssc-miR-124a	INSM1	INSM transcriptional repressor 1 [Source:HGNC Symbol;Acc:HGNC:6090]	-1.71	0.1324
ssc-miR-124a	INTS12	integrator complex subunit 12 [Source:VGNC Symbol;Acc:VGNC:98051]	-1.71	0.1324
ssc-miR-124a	INTU	inturned planar cell polarity protein [Source:VGNC Symbol;Acc:VGNC:89170]	-1.71	0.1324
ssc-miR-124a	IPCEF1	interaction protein for cytohesin exchange factors 1 [Source:HGNC Symbol;Acc:HGNC:21204]	-1.71	0.1324
ssc-miR-124a	IPMK	inositol polyphosphate multikinase [Source:VGNC Symbol;Acc:VGNC:89175]	-1.71	0.1324
ssc-miR-124a	IPO11	importin 11 [Source:VGNC Symbol;Acc:VGNC:89176]	-1.71	0.1324
ssc-miR-124a	IPO4	importin 4 [Source:VGNC Symbol;Acc:VGNC:89178]	-1.71	0.1324
ssc-miR-124a	IPO8	importin 8 [Source:VGNC Symbol;Acc:VGNC:89181]	-1.71	0.1324
ssc-miR-124a	IPP	intracisternal A particle-promoted polypeptide [Source:VGNC Symbol;Acc:VGNC:89182]	-1.71	0.1324
ssc-miR-124a	IQGAP1	IQ motif containing GTPase activating protein 1 [Source:HGNC Symbol;Acc:HGNC:6110]	-1.71	0.1324
ssc-miR-124a	IQGAP2	IQ motif containing GTPase activating protein 2 [Source:VGNC Symbol;Acc:VGNC:89192]	-1.71	0.1324
ssc-miR-124a	IRF1	interferon regulatory factor 1 [Source:VGNC Symbol;Acc:VGNC:89203]	-1.71	0.1324
ssc-miR-124a	IRF2BP2	interferon regulatory factor 2 binding protein 2 [Source:VGNC Symbol;Acc:VGNC:89205]	-1.71	0.1324
ssc-miR-124a	IRF2BPL	interferon regulatory factor 2 binding protein like [Source:HGNC Symbol;Acc:HGNC:14282]	-1.71	0.1324
ssc-miR-124a	IRGQ	immunity related GTPase Q [Source:VGNC Symbol;Acc:VGNC:89213]	-1.71	0.1324
ssc-miR-124a	ISG20L2	interferon stimulated exonuclease 20 like 2 [Source:VGNC Symbol;Acc:VGNC:89224]	-1.71	0.1324
ssc-miR-124a	IST1	IST1 factor associated with ESCRT-III [Source:VGNC Symbol;Acc:VGNC:98480]	-1.71	0.1324
ssc-miR-124a	ISY1-RAB43	hypothetical gene	-1.71	0.1324
ssc-miR-124a	ITFG1	integrin alpha FG-GAP repeat containing 1 [Source:VGNC Symbol;Acc:VGNC:89229]	-1.71	0.1324
ssc-miR-124a	ITFG3	hypothetical gene	-1.71	0.1324
ssc-miR-124a	ITGA11	integrin subunit alpha 11 [Source:VGNC Symbol;Acc:VGNC:89233]	-1.71	0.1324
ssc-miR-124a	ITGA3	integrin subunit alpha 3 [Source:VGNC Symbol;Acc:VGNC:89235]	-1.71	0.1324
ssc-miR-124a	ITGA6	integrin subunit alpha 6 [Source:VGNC Symbol;Acc:VGNC:96378]	-1.71	0.1324
ssc-miR-124a	ITGA7	integrin subunit alpha 7 [Source:VGNC Symbol;Acc:VGNC:89237]	-1.71	0.1324
ssc-miR-124a	ITGAE	integrin subunit alpha E [Source:VGNC Symbol;Acc:VGNC:89239]	-1.71	0.1324
ssc-miR-124a	ITGB1	integrin subunit beta 1 [Source:VGNC Symbol;Acc:VGNC:96381]	-1.71	0.1324
ssc-miR-124a	ITPKB	hypothetical gene	-1.71	0.1324
ssc-miR-124a	ITPR3	inositol 1,4,5-trisphosphate receptor type 3 [Source:VGNC Symbol;Acc:VGNC:89254]	-1.71	0.1324
ssc-miR-124a	ITPRIP	inositol 1,4,5-trisphosphate receptor interacting protein [Source:VGNC Symbol;Acc:VGNC:89256]	-1.71	0.1324
ssc-miR-124a	ITPRIPL2	ITPRIP like 2 [Source:VGNC Symbol;Acc:VGNC:89258]	-1.71	0.1324
ssc-miR-124a	ITSN2	intersectin 2 [Source:VGNC Symbol;Acc:VGNC:89259]	-1.71	0.1324
ssc-miR-124a	JAG1	jagged canonical Notch ligand 1 [Source:VGNC Symbol;Acc:VGNC:96385]	-1.71	0.1324
ssc-miR-124a	JAG2	jagged canonical Notch ligand 2 [Source:VGNC Symbol;Acc:VGNC:89269]	-1.71	0.1324
ssc-miR-124a	JAGN1	hypothetical gene	-1.71	0.1324
ssc-miR-124a	JAKMIP3	Janus kinase and microtubule interacting protein 3 [Source:VGNC Symbol;Acc:VGNC:89275]	-1.71	0.1324
ssc-miR-124a	JAM2	junctional adhesion molecule 2 [Source:VGNC Symbol;Acc:VGNC:89276]	-1.71	0.1324
ssc-miR-124a	JAZF1	JAZF zinc finger 1 [Source:VGNC Symbol;Acc:VGNC:89280]	-1.71	0.1324
ssc-miR-124a	JPH1	junctophilin 1 [Source:VGNC Symbol;Acc:VGNC:89291]	-1.71	0.1324
ssc-miR-124a	KANK1	KN motif and ankyrin repeat domains 1 [Source:VGNC Symbol;Acc:VGNC:103112]	-1.71	0.1324
ssc-miR-124a	KANK2	KN motif and ankyrin repeat domains 2 [Source:VGNC Symbol;Acc:VGNC:89296]	-1.71	0.1324
ssc-miR-124a	KANK4	KN motif and ankyrin repeat domains 4 [Source:VGNC Symbol;Acc:VGNC:89298]	-1.71	0.1324
ssc-miR-124a	KAT7	lysine acetyltransferase 7 [Source:VGNC Symbol;Acc:VGNC:89307]	-1.71	0.1324
ssc-miR-124a	KATNA1	katanin catalytic subunit A1 [Source:VGNC Symbol;Acc:VGNC:89309]	-1.71	0.1324
ssc-miR-124a	KATNBL1	katanin regulatory subunit B1 like 1 [Source:VGNC Symbol;Acc:VGNC:89313]	-1.71	0.1324

ssc-miR-124a	KCNA1	potassium voltage-gated channel subfamily A member 1 [Source:VGNC Symbol;Acc:VGNC:89323]	-1.71	0.1324
ssc-miR-124a	KCNA6	potassium voltage-gated channel subfamily A member 6 [Source:HGNC Symbol;Acc:HGNC:6225]	-1.71	0.1324
ssc-miR-124a	KCNB1	potassium voltage-gated channel subfamily B member 1 [Source:VGNC Symbol;Acc:VGNC:96386]	-1.71	0.1324
ssc-miR-124a	KCNE1L	hypothetical gene	-1.71	0.1324
ssc-miR-124a	KCNH1	potassium voltage-gated channel subfamily H member 1 [Source:VGNC Symbol;Acc:VGNC:108599]	-1.71	0.1324
ssc-miR-124a	KCNJ12	hypothetical gene	-1.71	0.1324
ssc-miR-124a	KCNJ2	potassium inwardly rectifying channel subfamily J member 2 [Source:VGNC Symbol;Acc:VGNC:89357]	-1.71	0.1324
ssc-miR-124a	KCNJ3	potassium inwardly rectifying channel subfamily J member 3 [Source:HGNC Symbol;Acc:HGNC:6264]	-1.71	0.1324
ssc-miR-124a	KCNJ5	potassium inwardly rectifying channel subfamily J member 5 [Source:VGNC Symbol;Acc:VGNC:89359]	-1.71	0.1324
ssc-miR-124a	KCNJ6	potassium inwardly rectifying channel subfamily J member 6 [Source:VGNC Symbol;Acc:VGNC:89360]	-1.71	0.1324
ssc-miR-124a	KCNK10	potassium two pore domain channel subfamily K member 10 [Source:VGNC Symbol;Acc:VGNC:89364]	-1.71	0.1324
ssc-miR-124a	KCNK2	potassium two pore domain channel subfamily K member 2 [Source:VGNC Symbol;Acc:VGNC:89369]	-1.71	0.1324
ssc-miR-124a	KCNK3	potassium two pore domain channel subfamily K member 3 [Source:VGNC Symbol;Acc:VGNC:89370]	-1.71	0.1324
ssc-miR-124a	KCNMB4	potassium calcium-activated channel subfamily M regulatory beta subunit 4 [Source:VGNC Symbol;Acc:VGNC:89378]	-1.71	0.1324
ssc-miR-124a	KCNN3	potassium calcium-activated channel subfamily N member 3 [Source:VGNC Symbol;Acc:VGNC:98056]	-1.71	0.1324
ssc-miR-124a	KCNQ3	potassium voltage-gated channel subfamily Q member 3 [Source:VGNC Symbol;Acc:VGNC:89382]	-1.71	0.1324
ssc-miR-124a	KCNQ5	potassium voltage-gated channel subfamily Q member 5 [Source:VGNC Symbol;Acc:VGNC:89384]	-1.71	0.1324
ssc-miR-124a	KCNS2	potassium voltage-gated channel modifier subfamily S member 2 [Source:VGNC Symbol;Acc:VGNC:89386]	-1.71	0.1324
ssc-miR-124a	KCNV1	potassium voltage-gated channel modifier subfamily V member 1 [Source:VGNC Symbol;Acc:VGNC:89390]	-1.71	0.1324
ssc-miR-124a	KCTD12	potassium channel tetramerization domain containing 12 [Source:HGNC Symbol;Acc:HGNC:14678]	-1.71	0.1324
ssc-miR-124a	KCTD16	potassium channel tetramerization domain containing 16 [Source:VGNC Symbol;Acc:VGNC:89395]	-1.71	0.1324
ssc-miR-124a	KCTD5	hypothetical gene	-1.71	0.1324
ssc-miR-124a	KCTD7	potassium channel tetramerization domain containing 7 [Source:VGNC Symbol;Acc:VGNC:89403]	-1.71	0.1324
ssc-miR-124a	KCTD8	potassium channel tetramerization domain containing 8 [Source:HGNC Symbol;Acc:HGNC:22394]	-1.71	0.1324
ssc-miR-124a	KDELC2	hypothetical gene	-1.71	0.1324
ssc-miR-124a	KDELR2	KDEL endoplasmic reticulum protein retention receptor 2 [Source:VGNC Symbol;Acc:VGNC:89405]	-1.71	0.1324
ssc-miR-124a	KDM1B	lysine demethylase 1B [Source:VGNC Symbol;Acc:VGNC:89409]	-1.71	0.1324
ssc-miR-124a	KDM5A	lysine demethylase 5A [Source:VGNC Symbol;Acc:VGNC:89415]	-1.71	0.1324
ssc-miR-124a	KIAA0226	hypothetical gene	-1.71	0.1324
ssc-miR-124a	KIAA0247	hypothetical gene	-1.71	0.1324
ssc-miR-124a	KIAA0368	hypothetical gene	-1.71	0.1324
ssc-miR-124a	KIAA0408	KIAA0408 [Source:VGNC Symbol;Acc:VGNC:89432]	-1.71	0.1324
ssc-miR-124a	KIAA0556	hypothetical gene	-1.71	0.1324
ssc-miR-124a	KIAA0895	KIAA0895 [Source:VGNC Symbol;Acc:VGNC:89436]	-1.71	0.1324
ssc-miR-124a	KIAA0947	hypothetical gene	-1.71	0.1324
ssc-miR-124a	KIAA1024	hypothetical gene	-1.71	0.1324
ssc-miR-124a	KIAA1147	hypothetical gene	-1.71	0.1324
ssc-miR-124a	KIAA1161	hypothetical gene	-1.71	0.1324
ssc-miR-124a	KIAA1211	hypothetical gene	-1.71	0.1324
ssc-miR-124a	KIAA1239	hypothetical gene	-1.71	0.1324
ssc-miR-124a	KIAA1244	hypothetical gene	-1.71	0.1324
ssc-miR-124a	KIAA1324L	hypothetical gene	-1.71	0.1324
ssc-miR-124a	KIAA1467	hypothetical gene	-1.71	0.1324
ssc-miR-124a	KIAA1468	hypothetical gene	-1.71	0.1324
ssc-miR-124a	KIAA1522	KIAA1522 [Source:VGNC Symbol;Acc:VGNC:89444]	-1.71	0.1324
ssc-miR-124a	KIAA1644	hypothetical gene	-1.71	0.1324
ssc-miR-124a	KIAA1671	hypothetical gene	-1.71	0.1324
ssc-miR-124a	KIAA1958	KIAA1958 [Source:VGNC Symbol;Acc:VGNC:89448]	-1.71	0.1324
ssc-miR-124a	KIAA2013	KIAA2013 [Source:VGNC Symbol;Acc:VGNC:89449]	-1.71	0.1324
ssc-miR-124a	KIAA2018	hypothetical gene	-1.71	0.1324

ssc-miR-124a	KIF13B	kinesin family member 13B [Source:VGNC Symbol;Acc:VGNC:89455]	-1.71	0.1324
ssc-miR-124a	KIF16B	kinesin family member 16B [Source:VGNC Symbol;Acc:VGNC:106450]	-1.71	0.1324
ssc-miR-124a	KIF21B	kinesin family member 21B [Source:VGNC Symbol;Acc:VGNC:96219]	-1.71	0.1324
ssc-miR-124a	KIF26A	kinesin family member 26A [Source:VGNC Symbol;Acc:VGNC:89466]	-1.71	0.1324
ssc-miR-124a	KIF26B	kinesin family member 26B [Source:VGNC Symbol;Acc:VGNC:96087]	-1.71	0.1324
ssc-miR-124a	KIF2A	kinesin family member 2A [Source:VGNC Symbol;Acc:VGNC:89467]	-1.71	0.1324
ssc-miR-124a	KIF3A	kinesin family member 3A [Source:VGNC Symbol;Acc:VGNC:89470]	-1.71	0.1324
ssc-miR-124a	KIF5A	kinesin family member 5A [Source:VGNC Symbol;Acc:VGNC:89472]	-1.71	0.1324
ssc-miR-124a	KIRREL	hypothetical gene	-1.71	0.1324
ssc-miR-124a	KLC2	kinesin light chain 2 [Source:VGNC Symbol;Acc:VGNC:89486]	-1.71	0.1324
ssc-miR-124a	KLF13	Kruppel like factor 13 [Source:VGNC Symbol;Acc:VGNC:89493]	-1.71	0.1324
ssc-miR-124a	KLF16	Kruppel like factor 16 [Source:VGNC Symbol;Acc:VGNC:89496]	-1.71	0.1324
ssc-miR-124a	KLF4	Kruppel like factor 4 [Source:VGNC Symbol;Acc:VGNC:98062]	-1.71	0.1324
ssc-miR-124a	KLF6	Kruppel like factor 6 [Source:VGNC Symbol;Acc:VGNC:98063]	-1.71	0.1324
ssc-miR-124a	KLHDC10	kelch domain containing 10 [Source:VGNC Symbol;Acc:VGNC:89502]	-1.71	0.1324
ssc-miR-124a	KLHL17	pleckstrin homology domain containing N1 [Source:VGNC Symbol;Acc:VGNC:98535]	-1.71	0.1324
ssc-miR-124a	KLHL21	kelch like family member 21 [Source:VGNC Symbol;Acc:VGNC:89520]	-1.71	0.1324
ssc-miR-124a	KLHL24	kelch like family member 24 [Source:VGNC Symbol;Acc:VGNC:89521]	-1.71	0.1324
ssc-miR-124a	KLHL28	kelch like family member 28 [Source:VGNC Symbol;Acc:VGNC:89523]	-1.71	0.1324
ssc-miR-124a	KLHL42	kelch like family member 42 [Source:VGNC Symbol;Acc:VGNC:89534]	-1.71	0.1324
ssc-miR-124a	KLLN	hypothetical gene	-1.71	0.1324
ssc-miR-124a	KPNA1	karyopherin subunit alpha 1 [Source:VGNC Symbol;Acc:VGNC:89560]	-1.71	0.1324
ssc-miR-124a	KPNA3	karyopherin subunit alpha 3 [Source:VGNC Symbol;Acc:VGNC:89562]	-1.71	0.1324
ssc-miR-124a	KPNA4	karyopherin subunit alpha 4 [Source:VGNC Symbol;Acc:VGNC:89563]	-1.71	0.1324
ssc-miR-124a	KPNB1	karyopherin subunit beta 1 [Source:VGNC Symbol;Acc:VGNC:89567]	-1.71	0.1324
ssc-miR-124a	KRAS	KRAS proto-onco, GTPase [Source:VGNC Symbol;Acc:VGNC:89569]	-1.71	0.1324
ssc-miR-124a	KRR1	KRR1 small subunit processome component homolog [Source:VGNC Symbol;Acc:VGNC:89575]	-1.71	0.1324
ssc-miR-124a	KRT85	keratin 85 [Source:HGNC Symbol;Acc:HGNC:6462]	-1.71	0.1324
ssc-miR-124a	L2HGDH	L-2-hydroxyglutarate dehydrogenase [Source:VGNC Symbol;Acc:VGNC:89611]	-1.71	0.1324
ssc-miR-124a	LAMC1	laminin subunit gamma 1 [Source:VGNC Symbol;Acc:VGNC:89624]	-1.71	0.1324
ssc-miR-124a	LANCL3	LanC like 3 [Source:VGNC Symbol;Acc:VGNC:89634]	-1.71	0.1324
ssc-miR-124a	LARP1	La ribonucleoprotein 1, translational regulator [Source:VGNC Symbol;Acc:VGNC:98067]	-1.71	0.1324
ssc-miR-124a	LARP4B	hypothetical gene	-1.71	0.1324
ssc-miR-124a	LCLAT1	lysocardiolipin acyltransferase 1 [Source:VGNC Symbol;Acc:VGNC:89658]	-1.71	0.1324
ssc-miR-124a	LCP1	lymphocyte cytosolic protein 1 [Source:VGNC Symbol;Acc:VGNC:89663]	-1.71	0.1324
ssc-miR-124a	LDLRAD4	low density lipoprotein receptor class A domain containing 4 [Source:VGNC Symbol;Acc:VGNC:89673]	-1.71	0.1324
ssc-miR-124a	LEMD2	LEM domain nuclear envelope protein 2 [Source:VGNC Symbol;Acc:VGNC:89678]	-1.71	0.1324
ssc-miR-124a	LEMD3	LEM domain containing 3 [Source:VGNC Symbol;Acc:VGNC:89679]	-1.71	0.1324
ssc-miR-124a	LHX2	LIM homeobox 2 [Source:VGNC Symbol;Acc:VGNC:89712]	-1.71	0.1324
ssc-miR-124a	LHX4	LIM homeobox 4 [Source:VGNC Symbol;Acc:VGNC:89714]	-1.71	0.1324
ssc-miR-124a	LHX9	LIM homeobox 9 [Source:VGNC Symbol;Acc:VGNC:95608]	-1.71	0.1324
ssc-miR-124a	LIF	LIF interleukin 6 family cytokine [Source:VGNC Symbol;Acc:VGNC:89719]	-1.71	0.1324
ssc-miR-124a	LIMA1	LIM domain and actin binding 1 [Source:VGNC Symbol;Acc:VGNC:98070]	-1.71	0.1324
ssc-miR-124a	LIMCH1	LIM and calponin homology domains 1 [Source:VGNC Symbol;Acc:VGNC:89724]	-1.71	0.1324
ssc-miR-124a	LIMD1	LIM domain containing 1 [Source:VGNC Symbol;Acc:VGNC:89725]	-1.71	0.1324
ssc-miR-124a	LIMD2	LIM domain containing 2 [Source:VGNC Symbol;Acc:VGNC:89726]	-1.71	0.1324
ssc-miR-124a	LIMS1	hypothetical gene	-1.71	0.1324
ssc-miR-124a	LIN28B	lin-28 homolog B [Source:VGNC Symbol;Acc:VGNC:89729]	-1.71	0.1324
ssc-miR-124a	LIPA	lipase A, lysosomal acid type [Source:VGNC Symbol;Acc:VGNC:89737]	-1.71	0.1324
ssc-miR-124a	LIPE	lipase E, hormone sensitive type [Source:VGNC Symbol;Acc:VGNC:98493]	-1.71	0.1324

ssc-miR-124a	LITAF	hypothetical gene	-1.71	0.1324
ssc-miR-124a	LIX1	limb and CNS expressed 1 [Source:VGNC Symbol;Acc:VGNC:89743]	-1.71	0.1324
ssc-miR-124a	LMAN1	lectin, mannose binding 1 [Source:VGNC Symbol;Acc:VGNC:89748]	-1.71	0.1324
ssc-miR-124a	LMAN2L	lectin, mannose binding 2 like [Source:VGNC Symbol;Acc:VGNC:89751]	-1.71	0.1324
ssc-miR-124a	LMBR1	limb development membrane protein 1 [Source:VGNC Symbol;Acc:VGNC:89752]	-1.71	0.1324
ssc-miR-124a	LMBRD2	LMBR1 domain containing 2 [Source:VGNC Symbol;Acc:VGNC:89755]	-1.71	0.1324
ssc-miR-124a	LMF2	lipase maturation factor 2 [Source:VGNC Symbol;Acc:VGNC:89758]	-1.71	0.1324
ssc-miR-124a	LMNB1	lamin B1 [Source:VGNC Symbol;Acc:VGNC:89760]	-1.71	0.1324
ssc-miR-124a	LMO3	hypothetical gene	-1.71	0.1324
ssc-miR-124a	LMO4	hypothetical gene	-1.71	0.1324
ssc-miR-124a	LMTK2	lemur tyrosine kinase 2 [Source:HGNC Symbol;Acc:HGNC:17880]	-1.71	0.1324
ssc-miR-124a	LNX2	ligand of numb-protein X 2 [Source:VGNC Symbol;Acc:VGNC:89774]	-1.71	0.1324
ssc-miR-124a	LONRF1	LON peptidase N-terminal domain and ring finger 1 [Source:VGNC Symbol;Acc:VGNC:96123]	-1.71	0.1324
ssc-miR-124a	LOX	lysyl oxidase [Source:VGNC Symbol;Acc:VGNC:99785]	-1.71	0.1324
ssc-miR-124a	LPCAT2	lysophosphatidylcholine acyltransferase 2 [Source:VGNC Symbol;Acc:VGNC:89789]	-1.71	0.1324
ssc-miR-124a	LPCAT3	lysophosphatidylcholine acyltransferase 3 [Source:VGNC Symbol;Acc:VGNC:89790]	-1.71	0.1324
ssc-miR-124a	LPHN1	hypothetical gene	-1.71	0.1324
ssc-miR-124a	LPIN1	lipin 1 [Source:VGNC Symbol;Acc:VGNC:89793]	-1.71	0.1324
ssc-miR-124a	LPP	LIM domain containing preferred translocation partner in lipoma [Source:HGNC Symbol;Acc:HGNC:6679]	-1.71	0.1324
ssc-miR-124a	LRCH4	leucine rich repeats and calponin homology domain containing 4 [Source:VGNC Symbol;Acc:VGNC:89804]	-1.71	0.1324
ssc-miR-124a	LRFN1	leucine rich repeat and fibronectin type III domain containing 1 [Source:HGNC Symbol;Acc:HGNC:29290]	-1.71	0.1324
ssc-miR-124a	LRGUK	leucine rich repeats and guanylate kinase domain containing [Source:VGNC Symbol;Acc:VGNC:89808]	-1.71	0.1324
ssc-miR-124a	LRIG1	leucine rich repeats and immunoglobulin like domains 1 [Source:VGNC Symbol;Acc:VGNC:89810]	-1.71	0.1324
ssc-miR-124a	LRP10	LDL receptor related protein 10 [Source:VGNC Symbol;Acc:VGNC:89816]	-1.71	0.1324
ssc-miR-124a	LRP6	LDL receptor related protein 6 [Source:VGNC Symbol;Acc:VGNC:89821]	-1.71	0.1324
ssc-miR-124a	LRRC1	leucine rich repeat containing 1 [Source:VGNC Symbol;Acc:VGNC:103977]	-1.71	0.1324
ssc-miR-124a	LRRC3	leucine rich repeat containing 3 [Source:HGNC Symbol;Acc:HGNC:14965]	-1.71	0.1324
ssc-miR-124a	LRRC40	hypothetical gene	-1.71	0.1324
ssc-miR-124a	LRRC57	leucine rich repeat containing 57 [Source:VGNC Symbol;Acc:VGNC:103119]	-1.71	0.1324
ssc-miR-124a	LRRC58	leucine rich repeat containing 58 [Source:VGNC Symbol;Acc:VGNC:98081]	-1.71	0.1324
ssc-miR-124a	LRRC7	leucine rich repeat containing 7 [Source:HGNC Symbol;Acc:HGNC:18531]	-1.71	0.1324
ssc-miR-124a	LRRFIP2	LRR binding FLII interacting protein 2 [Source:VGNC Symbol;Acc:VGNC:89858]	-1.71	0.1324
ssc-miR-124a	LUC7L2	LUC7 like 2, pre-mRNA splicing factor [Source:HGNC Symbol;Acc:HGNC:21608]	-1.71	0.1324
ssc-miR-124a	LYPD6	LY6/PLAUR domain containing 6 [Source:HGNC Symbol;Acc:HGNC:28751]	-1.71	0.1324
ssc-miR-124a	LYSMD3	LysM domain containing 3 [Source:VGNC Symbol;Acc:VGNC:89919]	-1.71	0.1324
ssc-miR-124a	LZIC	leucine zipper and CTNNBIP1 domain containing [Source:VGNC Symbol;Acc:VGNC:89925]	-1.71	0.1324
ssc-miR-124a	LZTS1	leucine zipper tumor suppressor 1 [Source:VGNC Symbol;Acc:VGNC:89928]	-1.71	0.1324
ssc-miR-124a	MACF1	microtubule actin crosslinking factor 1 [Source:VGNC Symbol;Acc:VGNC:98496]	-1.71	0.1324
ssc-miR-124a	MACROD2	mono-ADP ribosylhydrolase 2 [Source:VGNC Symbol;Acc:VGNC:108725]	-1.71	0.1324
ssc-miR-124a	MAGI2	membrane associated guanylate kinase, WW and PDZ domain containing 2 [Source:VGNC Symbol;Acc:VGNC:89955]	-1.71	0.1324
ssc-miR-124a	MAGT1	magnesium transporter 1 [Source:VGNC Symbol;Acc:VGNC:89957]	-1.71	0.1324
ssc-miR-124a	MAML1	mastermind like transcriptional coactivator 1 [Source:VGNC Symbol;Acc:VGNC:89963]	-1.71	0.1324
ssc-miR-124a	MAN2A1	mannosidase alpha class 2A member 1 [Source:VGNC Symbol;Acc:VGNC:98098]	-1.71	0.1324
ssc-miR-124a	MAP1B	microtubule associated protein 1B [Source:VGNC Symbol;Acc:VGNC:89979]	-1.71	0.1324
ssc-miR-124a	MAP2K4	mitogen-activated protein kinase kinase 4 [Source:VGNC Symbol;Acc:VGNC:98101]	-1.71	0.1324
ssc-miR-124a	MAP3K1	mitogen-activated protein kinase kinase kinase 1 [Source:VGNC Symbol;Acc:VGNC:98104]	-1.71	0.1324
ssc-miR-124a	MAP3K11	mitogen-activated protein kinase kinase kinase 11 [Source:VGNC Symbol;Acc:VGNC:89982]	-1.71	0.1324
ssc-miR-124a	MAP3K2	mitogen-activated protein kinase kinase kinase 2 [Source:VGNC Symbol;Acc:VGNC:98107]	-1.71	0.1324
ssc-miR-124a	MAP3K3	mitogen-activated protein kinase kinase kinase 3 [Source:VGNC Symbol;Acc:VGNC:98108]	-1.71	0.1324
ssc-miR-124a	MAP7	microtubule associated protein 7 [Source:VGNC Symbol;Acc:VGNC:89992]	-1.71	0.1324

ssc-miR-124a	MAP7D3	hypothetical gene	-1.71	0.1324
ssc-miR-124a	MAPK1	mitogen-activated protein kinase 1 [Source:VGNC Symbol;Acc:VGNC:89996]	-1.71	0.1324
ssc-miR-124a	MAPK10	mitogen-activated protein kinase 10 [Source:VGNC Symbol;Acc:VGNC:98116]	-1.71	0.1324
ssc-miR-124a	MAPK14	mitogen-activated protein kinase 14 [Source:VGNC Symbol;Acc:VGNC:90000]	-1.71	0.1324
ssc-miR-124a	MAPK4	hypothetical gene	-1.71	0.1324
ssc-miR-124a	MAPK7	mitogen-activated protein kinase 7 [Source:VGNC Symbol;Acc:VGNC:90005]	-1.71	0.1324
ssc-miR-124a	MAPKAPK2	MAPK activated protein kinase 2 [Source:VGNC Symbol;Acc:VGNC:90011]	-1.71	0.1324
ssc-miR-124a	MAPRE1	microtubule associated protein RP/EB family member 1 [Source:VGNC Symbol;Acc:VGNC:96410]	-1.71	0.1324
ssc-miR-124a	MARCH6	hypothetical gene	-1.71	0.1324
ssc-miR-124a	MARCH7	hypothetical gene	-1.71	0.1324
ssc-miR-124a	MARCH8	hypothetical gene	-1.71	0.1324
ssc-miR-124a	MARK1	microtubule affinity regulating kinase 1 [Source:VGNC Symbol;Acc:VGNC:96411]	-1.71	0.1324
ssc-miR-124a	MARVELD1	MARVEL domain containing 1 [Source:HGNC Symbol;Acc:HGNC:28674]	-1.71	0.1324
ssc-miR-124a	MAT2A	methionine adenosyltransferase 2A [Source:VGNC Symbol;Acc:VGNC:90039]	-1.71	0.1324
ssc-miR-124a	MATN2	matrilin 2 [Source:VGNC Symbol;Acc:VGNC:90043]	-1.71	0.1324
ssc-miR-124a	MAU2	MAU2 sister chromatid cohesion factor [Source:VGNC Symbol;Acc:VGNC:90045]	-1.71	0.1324
ssc-miR-124a	MBLAC2	metallo-beta-lactamase domain containing 2 [Source:VGNC Symbol;Acc:VGNC:90053]	-1.71	0.1324
ssc-miR-124a	MBNL3	muscleblind like splicing regulator 3 [Source:VGNC Symbol;Acc:VGNC:90056]	-1.71	0.1324
ssc-miR-124a	MBOAT2	membrane bound O-acyltransferase domain containing 2 [Source:VGNC Symbol;Acc:VGNC:90058]	-1.71	0.1324
ssc-miR-124a	MCCC2	methylcrotonyl-CoA carboxylase subunit 2 [Source:VGNC Symbol;Acc:VGNC:90064]	-1.71	0.1324
ssc-miR-124a	MCF2	MCF.2 cell line derived transforming sequence [Source:VGNC Symbol;Acc:VGNC:90067]	-1.71	0.1324
ssc-miR-124a	MCM10	minichromosome maintenance 10 replication initiation factor [Source:VGNC Symbol;Acc:VGNC:95871]	-1.71	0.1324
ssc-miR-124a	MCU	mitochondrial calcium uniporter [Source:VGNC Symbol;Acc:VGNC:90085]	-1.71	0.1324
ssc-miR-124a	MCUR1	mitochondrial calcium uniporter regulator 1 [Source:HGNC Symbol;Acc:HGNC:21097]	-1.71	0.1324
ssc-miR-124a	MDC1	mediator of DNA damage checkpoint 1 [Source:NCBI gene (formerly Entrezgene);Acc:100144453]	-1.71	0.1324
ssc-miR-124a	MDGA1	MAM domain containing glycosylphosphatidylinositol anchor 1 [Source:VGNC Symbol;Acc:VGNC:90091]	-1.71	0.1324
ssc-miR-124a	MDGA2	MAM domain containing glycosylphosphatidylinositol anchor 2 [Source:VGNC Symbol;Acc:VGNC:90092]	-1.71	0.1324
ssc-miR-124a	MDK	midkine [Source:VGNC Symbol;Acc:VGNC:100167]	-1.71	0.1324
ssc-miR-124a	MECOM	MDS1 and EVI1 complex locus [Source:VGNC Symbol;Acc:VGNC:90100]	-1.71	0.1324
ssc-miR-124a	MECP2	methyl-CpG binding protein 2 [Source:VGNC Symbol;Acc:VGNC:90101]	-1.71	0.1324
ssc-miR-124a	MED12L	mediator complex subunit 12L [Source:VGNC Symbol;Acc:VGNC:90105]	-1.71	0.1324
ssc-miR-124a	MED26	mediator complex subunit 26 [Source:VGNC Symbol;Acc:VGNC:100168]	-1.71	0.1324
ssc-miR-124a	MEF2A	myocyte enhancer factor 2A [Source:VGNC Symbol;Acc:VGNC:98123]	-1.71	0.1324
ssc-miR-124a	MEMO1	mediator of cell motility 1 [Source:VGNC Symbol;Acc:VGNC:90139]	-1.71	0.1324
ssc-miR-124a	METAP2	methionyl aminopeptidase 2 [Source:VGNC Symbol;Acc:VGNC:90152]	-1.71	0.1324
ssc-miR-124a	METTL13	methyltransferase 13, eEF1A lysine and N-terminal methyltransferase [Source:VGNC Symbol;Acc:VGNC:87554]	-1.71	0.1324
ssc-miR-124a	METTL8	methyltransferase 8, methylcytidine [Source:VGNC Symbol;Acc:VGNC:96110]	-1.71	0.1324
ssc-miR-124a	MFSD6	major facilitator superfamily domain containing 6 [Source:VGNC Symbol;Acc:VGNC:96052]	-1.71	0.1324
ssc-miR-124a	MGA	MAX dimerization protein MGA [Source:VGNC Symbol;Acc:VGNC:90195]	-1.71	0.1324
ssc-miR-124a	MGAT4A	alpha-1,3-mannosyl-glycoprotein 4-beta-N-acetylglucosaminyltransferase A [Source:VGNC Symbol;Acc:VGNC:108158]	-1.71	0.1324
ssc-miR-124a	MGAT5	alpha-1,6-mannosylglycoprotein 6-beta-N-acetylglucosaminyltransferase [Source:VGNC Symbol;Acc:VGNC:96741]	-1.71	0.1324
ssc-miR-124a	MIB1	MIB E3 ubiquitin protein ligase 1 [Source:VGNC Symbol;Acc:VGNC:90205]	-1.71	0.1324
ssc-miR-124a	MICALCL	hypothetical gene	-1.71	0.1324
ssc-miR-124a	MID1	midline 1 [Source:VGNC Symbol;Acc:VGNC:90213]	-1.71	0.1324
ssc-miR-124a	MID1IP1	MID1 interacting protein 1 [Source:VGNC Symbol;Acc:VGNC:90214]	-1.71	0.1324
ssc-miR-124a	MIPOL1	mirror-image polydactyly 1 [Source:VGNC Symbol;Acc:VGNC:90237]	-1.71	0.1324
ssc-miR-124a	MITF	melanocyte inducing transcription factor [Source:VGNC Symbol;Acc:VGNC:90243]	-1.71	0.1324
ssc-miR-124a	MKX	mohawk homeobox [Source:VGNC Symbol;Acc:VGNC:96020]	-1.71	0.1324
ssc-miR-124a	MLANA	melan-A [Source:VGNC Symbol;Acc:VGNC:90250]	-1.71	0.1324
ssc-miR-124a	MLEC	malectin [Source:HGNC Symbol;Acc:HGNC:28973]	-1.71	0.1324

ssc-miR-124a	MLLT1	MLLT1 super elongation complex subunit [Source:VGNC Symbol;Acc:VGNC:90255]	-1.71	0.1324
ssc-miR-124a	MLLT3	MLLT3 super elongation complex subunit [Source:VGNC Symbol;Acc:VGNC:90256]	-1.71	0.1324
ssc-miR-124a	MLXIP	MLX interacting protein [Source:VGNC Symbol;Acc:VGNC:90262]	-1.71	0.1324
ssc-miR-124a	MMD2	monocyte to macrophage differentiation associated 2 [Source:HGNC Symbol;Acc:HGNC:30133]	-1.71	0.1324
ssc-miR-124a	MMP16	matrix metallopeptidase 16 [Source:VGNC Symbol;Acc:VGNC:90271]	-1.71	0.1324
ssc-miR-124a	MMP2	matrix metallopeptidase 2 [Source:VGNC Symbol;Acc:VGNC:90274]	-1.71	0.1324
ssc-miR-124a	MN1	MN1 proto-onco, transcriptional regulator [Source:VGNC Symbol;Acc:VGNC:90285]	-1.71	0.1324
ssc-miR-124a	MOB1B	hypothetical gene	-1.71	0.1324
ssc-miR-124a	MOCS1	molybdenum cofactor synthesis 1 [Source:VGNC Symbol;Acc:VGNC:107153]	-1.71	0.1324
ssc-miR-124a	MON2	MON2 homolog, regulator of endosome-to-Golgi trafficking [Source:VGNC Symbol;Acc:VGNC:90301]	-1.71	0.1324
ssc-miR-124a	MORC4	MORC family CW-type zinc finger 4 [Source:VGNC Symbol;Acc:VGNC:90305]	-1.71	0.1324
ssc-miR-124a	MPC1	hypothetical gene	-1.71	0.1324
ssc-miR-124a	MPP5	hypothetical gene	-1.71	0.1324
ssc-miR-124a	MPP6	hypothetical gene	-1.71	0.1324
ssc-miR-124a	MPZL1	myelin protein zero like 1 [Source:VGNC Symbol;Acc:VGNC:90335]	-1.71	0.1324
ssc-miR-124a	MRE11A	hypothetical gene	-1.71	0.1324
ssc-miR-124a	MSL2	MSL complex subunit 2 [Source:VGNC Symbol;Acc:VGNC:90424]	-1.71	0.1324
ssc-miR-124a	MSRA	methionine sulfoxide reductase A [Source:HGNC Symbol;Acc:HGNC:7377]	-1.71	0.1324
ssc-miR-124a	MSRB1	methionine sulfoxide reductase B1 [Source:VGNC Symbol;Acc:VGNC:107154]	-1.71	0.1324
ssc-miR-124a	MST4	hypothetical gene	-1.71	0.1324
ssc-miR-124a	MTDH	metadherin [Source:VGNC Symbol;Acc:VGNC:90439]	-1.71	0.1324
ssc-miR-124a	MTF1	metal regulatory transcription factor 1 [Source:VGNC Symbol;Acc:VGNC:90443]	-1.71	0.1324
ssc-miR-124a	MTF2	metal response element binding transcription factor 2 [Source:VGNC Symbol;Acc:VGNC:90444]	-1.71	0.1324
ssc-miR-124a	MTM1	myotubularin 1 [Source:VGNC Symbol;Acc:VGNC:90455]	-1.71	0.1324
ssc-miR-124a	MTMR10	myotubularin related protein 10 [Source:VGNC Symbol;Acc:VGNC:90457]	-1.71	0.1324
ssc-miR-124a	MTMR12	myotubularin related protein 12 [Source:VGNC Symbol;Acc:VGNC:90459]	-1.71	0.1324
ssc-miR-124a	MTMR2	myotubularin related protein 2 [Source:VGNC Symbol;Acc:VGNC:90460]	-1.71	0.1324
ssc-miR-124a	MTMR3	myotubularin related protein 3 [Source:VGNC Symbol;Acc:VGNC:90461]	-1.71	0.1324
ssc-miR-124a	MTMR6	myotubularin related protein 6 [Source:HGNC Symbol;Acc:HGNC:7453]	-1.71	0.1324
ssc-miR-124a	MTPN	myotrophin [Source:VGNC Symbol;Acc:VGNC:90467]	-1.71	0.1324
ssc-miR-124a	MTX3	metaxin 3 [Source:VGNC Symbol;Acc:VGNC:90479]	-1.71	0.1324
ssc-miR-124a	MXD1	MAX dimerization protein 1 [Source:VGNC Symbol;Acc:VGNC:90492]	-1.71	0.1324
ssc-miR-124a	MXD4	MAX dimerization protein 4 [Source:VGNC Symbol;Acc:VGNC:90493]	-1.71	0.1324
ssc-miR-124a	MYADM	myeloid associated differentiation marker [Source:VGNC Symbol;Acc:VGNC:98508]	-1.71	0.1324
ssc-miR-124a	MYB	MYB proto-onco, transcription factor [Source:VGNC Symbol;Acc:VGNC:90496]	-1.71	0.1324
ssc-miR-124a	MYCBP	MYC binding protein [Source:NCBI gene (formerly Entrezgene);Acc:100513990]	-1.71	0.1324
ssc-miR-124a	MYH10	hypothetical gene	-1.71	0.1324
ssc-miR-124a	MYH11	myosin heavy chain 11 [Source:VGNC Symbol;Acc:VGNC:90510]	-1.71	0.1324
ssc-miR-124a	MYH9	myosin heavy chain 9 [Source:VGNC Symbol;Acc:VGNC:90512]	-1.71	0.1324
ssc-miR-124a	MYLIP	myosin regulatory light chain interacting protein [Source:VGNC Symbol;Acc:VGNC:103999]	-1.71	0.1324
ssc-miR-124a	MYNN	myoneurin [Source:VGNC Symbol;Acc:VGNC:90521]	-1.71	0.1324
ssc-miR-124a	MYO10	myosin X [Source:VGNC Symbol;Acc:VGNC:90522]	-1.71	0.1324
ssc-miR-124a	MYO1C	myosin IC [Source:VGNC Symbol;Acc:VGNC:90526]	-1.71	0.1324
ssc-miR-124a	MYO1E	myosin IE [Source:VGNC Symbol;Acc:VGNC:90528]	-1.71	0.1324
ssc-miR-124a	MYO5A	myosin VA [Source:HGNC Symbol;Acc:HGNC:7602]	-1.71	0.1324
ssc-miR-124a	MYO5B	myosin VB [Source:VGNC Symbol;Acc:VGNC:103131]	-1.71	0.1324
ssc-miR-124a	MYO9A	myosin IXA [Source:VGNC Symbol;Acc:VGNC:103138]	-1.71	0.1324
ssc-miR-124a	MYO9B	myosin IXB [Source:VGNC Symbol;Acc:VGNC:90534]	-1.71	0.1324
ssc-miR-124a	MYPN	myopalladin [Source:VGNC Symbol;Acc:VGNC:107412]	-1.71	0.1324
ssc-miR-124a	MYRF	hypothetical gene	-1.71	0.1324

ssc-miR-124a	MYRIP	myosin VIIA and Rab interacting protein [Source:NCBI gene (formerly Entrezgene);Acc:100156583]	-1.71	0.1324
ssc-miR-124a	MYZAP	myocardial zonula adherens protein [Source:HGNC Symbol;Acc:HGNC:43444]	-1.71	0.1324
ssc-miR-124a	N4BP1	NEDD4 binding protein 1 [Source:VGNC Symbol;Acc:VGNC:90553]	-1.71	0.1324
ssc-miR-124a	NAA15	N-alpha-acetyltransferase 15, NatA auxiliary subunit [Source:VGNC Symbol;Acc:VGNC:96747]	-1.71	0.1324
ssc-miR-124a	NAB1	NGFI-A binding protein 1 [Source:VGNC Symbol;Acc:VGNC:96431]	-1.71	0.1324
ssc-miR-124a	NACC1	nucleus accumbens associated 1 [Source:VGNC Symbol;Acc:VGNC:90563]	-1.71	0.1324
ssc-miR-124a	NACC2	NACC family member 2 [Source:VGNC Symbol;Acc:VGNC:90564]	-1.71	0.1324
ssc-miR-124a	NAMPT	nicotinamide phosphoribosyltransferase [Source:VGNC Symbol;Acc:VGNC:90573]	-1.71	0.1324
ssc-miR-124a	NANP	N-acetylneuraminic acid phosphatase [Source:HGNC Symbol;Acc:HGNC:16140]	-1.71	0.1324
ssc-miR-124a	NAP1L1	nucleosome assembly protein 1 like 1 [Source:VGNC Symbol;Acc:VGNC:90576]	-1.71	0.1324
ssc-miR-124a	NAP1L5	nucleosome assembly protein 1 like 5 [Source:NCBI gene (formerly Entrezgene);Acc:100616076]	-1.71	0.1324
ssc-miR-124a	NAPEPLD	N-acyl phosphatidylethanolamine phospholipase D [Source:VGNC Symbol;Acc:VGNC:90579]	-1.71	0.1324
ssc-miR-124a	NAT8L	N-acetyltransferase 8 like [Source:VGNC Symbol;Acc:VGNC:90586]	-1.71	0.1324
ssc-miR-124a	NAV1	neuron navigator 1 [Source:VGNC Symbol;Acc:VGNC:95725]	-1.71	0.1324
ssc-miR-124a	NAV2	neuron navigator 2 [Source:VGNC Symbol;Acc:VGNC:90588]	-1.71	0.1324
ssc-miR-124a	NAV3	neuron navigator 3 [Source:VGNC Symbol;Acc:VGNC:90589]	-1.71	0.1324
ssc-miR-124a	NCAM2	neural cell adhesion molecule 2 [Source:VGNC Symbol;Acc:VGNC:90595]	-1.71	0.1324
ssc-miR-124a	NCKIPSD	NCK interacting protein with SH3 domain [Source:VGNC Symbol;Acc:VGNC:90612]	-1.71	0.1324
ssc-miR-124a	NCMAP	hypothetical gene	-1.71	0.1324
ssc-miR-124a	NCOA2	nuclear receptor coactivator 2 [Source:VGNC Symbol;Acc:VGNC:90616]	-1.71	0.1324
ssc-miR-124a	NCOA4	nuclear receptor coactivator 4 [Source:VGNC Symbol;Acc:VGNC:90617]	-1.71	0.1324
ssc-miR-124a	NCOR2	nuclear receptor corepressor 2 [Source:VGNC Symbol;Acc:VGNC:98148]	-1.71	0.1324
ssc-miR-124a	NDFIP1	Nedd4 family interacting protein 1 [Source:VGNC Symbol;Acc:VGNC:90626]	-1.71	0.1324
ssc-miR-124a	NDFIP2	Nedd4 family interacting protein 2 [Source:VGNC Symbol;Acc:VGNC:90627]	-1.71	0.1324
ssc-miR-124a	NDST1	N-deacetylase and N-sulfotransferase 1 [Source:VGNC Symbol;Acc:VGNC:90634]	-1.71	0.1324
ssc-miR-124a	NEBL	nebulette [Source:VGNC Symbol;Acc:VGNC:95824]	-1.71	0.1324
ssc-miR-124a	NECAP2	NECAP endocytosis associated 2 [Source:VGNC Symbol;Acc:VGNC:90661]	-1.71	0.1324
ssc-miR-124a	NEDD4L	NEDD4 like E3 ubiquitin protein ligase [Source:VGNC Symbol;Acc:VGNC:90667]	-1.71	0.1324
ssc-miR-124a	NEFM	hypothetical gene	-1.71	0.1324
ssc-miR-124a	NEGR1	neuronal growth regulator 1 [Source:VGNC Symbol;Acc:VGNC:90671]	-1.71	0.1324
ssc-miR-124a	NEK6	NIMA related kinase 6 [Source:VGNC Symbol;Acc:VGNC:90678]	-1.71	0.1324
ssc-miR-124a	NEK9	NIMA related kinase 9 [Source:VGNC Symbol;Acc:VGNC:90679]	-1.71	0.1324
ssc-miR-124a	NEURL1B	neuralized E3 ubiquitin protein ligase 1B [Source:VGNC Symbol;Acc:VGNC:90695]	-1.71	0.1324
ssc-miR-124a	NEUROD1	neuronal differentiation 1 [Source:VGNC Symbol;Acc:VGNC:96439]	-1.71	0.1324
ssc-miR-124a	NEUROG3	neurogenin 3 [Source:VGNC Symbol;Acc:VGNC:90702]	-1.71	0.1324
ssc-miR-124a	NFASC	neurofascin [Source:VGNC Symbol;Acc:VGNC:90707]	-1.71	0.1324
ssc-miR-124a	NFAT5	nuclear factor of activated T cells 5 [Source:VGNC Symbol;Acc:VGNC:90708]	-1.71	0.1324
ssc-miR-124a	NFATC1	nuclear factor of activated T cells 1 [Source:VGNC Symbol;Acc:VGNC:103140]	-1.71	0.1324
ssc-miR-124a	NFATC2	nuclear factor of activated T cells 2 [Source:VGNC Symbol;Acc:VGNC:96440]	-1.71	0.1324
ssc-miR-124a	NFIA	nuclear factor I A [Source:VGNC Symbol;Acc:VGNC:90715]	-1.71	0.1324
ssc-miR-124a	NFIB	nuclear factor I B [Source:VGNC Symbol;Acc:VGNC:90716]	-1.71	0.1324
ssc-miR-124a	NFIC	nuclear factor I C [Source:VGNC Symbol;Acc:VGNC:100313]	-1.71	0.1324
ssc-miR-124a	NFIX	nuclear factor I X [Source:VGNC Symbol;Acc:VGNC:90718]	-1.71	0.1324
ssc-miR-124a	NHLH1	nescient helix-loop-helix 1 [Source:VGNC Symbol;Acc:VGNC:90735]	-1.71	0.1324
ssc-miR-124a	NHLRC3	NHL repeat containing 3 [Source:HGNC Symbol;Acc:HGNC:33751]	-1.71	0.1324
ssc-miR-124a	NHSL2	NHS like 2 [Source:VGNC Symbol;Acc:VGNC:98155]	-1.71	0.1324
ssc-miR-124a	NID1	nidogen 1 [Source:VGNC Symbol;Acc:VGNC:90742]	-1.71	0.1324
ssc-miR-124a	NIPA1	NIPA magnesium transporter 1 [Source:VGNC Symbol;Acc:VGNC:95455]	-1.71	0.1324
ssc-miR-124a	NIPBL	NIPBL cohesin loading factor [Source:VGNC Symbol;Acc:VGNC:90752]	-1.71	0.1324
ssc-miR-124a	NKAIN2	sodium/potassium transporting ATPase interacting 2 [Source:VGNC Symbol;Acc:VGNC:103141]	-1.71	0.1324

ssc-miR-124a	NKAP	NFKB activating protein [Source:VGNC Symbol;Acc:VGNC:90756]	-1.71	0.1324
ssc-miR-124a	NKRF	NFKB repressing factor [Source:VGNC Symbol;Acc:VGNC:90764]	-1.71	0.1324
ssc-miR-124a	NLGN4X	hypothetical gene	-1.71	0.1324
ssc-miR-124a	NME4	NME/NM23 nucleoside diphosphate kinase 4 [Source:VGNC Symbol;Acc:VGNC:90792]	-1.71	0.1324
ssc-miR-124a	NMT1	N-myristoyltransferase 1 [Source:VGNC Symbol;Acc:VGNC:90802]	-1.71	0.1324
ssc-miR-124a	NODAL	nodal growth differentiation factor [Source:VGNC Symbol;Acc:VGNC:90811]	-1.71	0.1324
ssc-miR-124a	NOL4	nucleolar protein 4 [Source:VGNC Symbol;Acc:VGNC:90814]	-1.71	0.1324
ssc-miR-124a	NPAT	nuclear protein, coactivator of histone transcription [Source:VGNC Symbol;Acc:VGNC:90840]	-1.71	0.1324
ssc-miR-124a	NPLOC4	NPL4 homolog, ubiquitin recognition factor [Source:VGNC Symbol;Acc:VGNC:90852]	-1.71	0.1324
ssc-miR-124a	NPTN	neuroplastin [Source:VGNC Symbol;Acc:VGNC:90861]	-1.71	0.1324
ssc-miR-124a	NPTX1	neuronal pentraxin 1 [Source:VGNC Symbol;Acc:VGNC:90862]	-1.71	0.1324
ssc-miR-124a	NR1D2	nuclear receptor subfamily 1 group D member 2 [Source:VGNC Symbol;Acc:VGNC:99726]	-1.71	0.1324
ssc-miR-124a	NR2C2	nuclear receptor subfamily 2 group C member 2 [Source:VGNC Symbol;Acc:VGNC:90876]	-1.71	0.1324
ssc-miR-124a	NR3C1	nuclear receptor subfamily 3 group C member 1 [Source:VGNC Symbol;Acc:VGNC:90883]	-1.71	0.1324
ssc-miR-124a	NR3C2	nuclear receptor subfamily 3 group C member 2 [Source:VGNC Symbol;Acc:VGNC:90884]	-1.71	0.1324
ssc-miR-124a	NR4A1	nuclear receptor subfamily 4 group A member 1 [Source:HGNC Symbol;Acc:HGNC:7980]	-1.71	0.1324
ssc-miR-124a	NR4A3	nuclear receptor subfamily 4 group A member 3 [Source:VGNC Symbol;Acc:VGNC:90885]	-1.71	0.1324
ssc-miR-124a	NR5A2	nuclear receptor subfamily 5 group A member 2 [Source:VGNC Symbol;Acc:VGNC:96452]	-1.71	0.1324
ssc-miR-124a	NRAS	NRAS proto-onco, GTPase [Source:VGNC Symbol;Acc:VGNC:98827]	-1.71	0.1324
ssc-miR-124a	NRCAM	neuronal cell adhesion molecule [Source:HGNC Symbol;Acc:HGNC:7994]	-1.71	0.1324
ssc-miR-124a	NRG1	neuregulin 1 [Source:VGNC Symbol;Acc:VGNC:96453]	-1.71	0.1324
ssc-miR-124a	NRG2	neuregulin 2 [Source:VGNC Symbol;Acc:VGNC:98160]	-1.71	0.1324
ssc-miR-124a	NRP1	neuropilin 1 [Source:VGNC Symbol;Acc:VGNC:104012]	-1.71	0.1324
ssc-miR-124a	NRP2	neuropilin 2 [Source:VGNC Symbol;Acc:VGNC:96454]	-1.71	0.1324
ssc-miR-124a	NSUN2	NOP2/Sun RNA methyltransferase 2 [Source:VGNC Symbol;Acc:VGNC:90914]	-1.71	0.1324
ssc-miR-124a	NSUN3	NOP2/Sun RNA methyltransferase 3 [Source:VGNC Symbol;Acc:VGNC:90915]	-1.71	0.1324
ssc-miR-124a	NT5M	5',3'-nucleotidase, mitochondrial [Source:VGNC Symbol;Acc:VGNC:90926]	-1.71	0.1324
ssc-miR-124a	NTHL1	nth like DNA glycosylase 1 [Source:VGNC Symbol;Acc:VGNC:90929]	-1.71	0.1324
ssc-miR-124a	NTNG2	netrin G2 [Source:VGNC Symbol;Acc:VGNC:90936]	-1.71	0.1324
ssc-miR-124a	NUDCD2	NudC domain containing 2 [Source:VGNC Symbol;Acc:VGNC:90950]	-1.71	0.1324
ssc-miR-124a	NUDT3	nudix hydrolase 3 [Source:NCBI gene (formerly Entrezgene);Acc:100737442]	-1.71	0.1324
ssc-miR-124a	NUDT4	hypothetical gene	-1.71	0.1324
ssc-miR-124a	NUFIP2	nuclear FMR1 interacting protein 2 [Source:VGNC Symbol;Acc:VGNC:90967]	-1.71	0.1324
ssc-miR-124a	NUMA1	nuclear mitotic apparatus protein 1 [Source:VGNC Symbol;Acc:VGNC:90969]	-1.71	0.1324
ssc-miR-124a	NUP210	nucleoporin 210 [Source:VGNC Symbol;Acc:VGNC:90977]	-1.71	0.1324
ssc-miR-124a	NUPL1	hypothetical gene	-1.71	0.1324
ssc-miR-124a	NXN	nucleoredoxin [Source:VGNC Symbol;Acc:VGNC:90994]	-1.71	0.1324
ssc-miR-124a	NXT2	nuclear transport factor 2 like export factor 2 [Source:VGNC Symbol;Acc:VGNC:91001]	-1.71	0.1324
ssc-miR-124a	NYNRIN	NYN domain and retroviral integrase containing [Source:VGNC Symbol;Acc:VGNC:91003]	-1.71	0.1324
ssc-miR-124a	OAF	out at first homolog [Source:VGNC Symbol;Acc:VGNC:91005]	-1.71	0.1324
ssc-miR-124a	OGFOD3	2-oxoglutarate and iron dependent oxygenase domain containing 3 [Source:VGNC Symbol;Acc:VGNC:91025]	-1.71	0.1324
ssc-miR-124a	OGFRL1	opioid growth factor receptor like 1 [Source:VGNC Symbol;Acc:VGNC:91026]	-1.71	0.1324
ssc-miR-124a	OIP5	Opa interacting protein 5 [Source:VGNC Symbol;Acc:VGNC:91029]	-1.71	0.1324
ssc-miR-124a	OLFM3	olfactomedin 3 [Source:VGNC Symbol;Acc:VGNC:91033]	-1.71	0.1324
ssc-miR-124a	ONECUT1	one cut homeobox 1 [Source:VGNC Symbol;Acc:VGNC:91042]	-1.71	0.1324
ssc-miR-124a	ONECUT2	one cut homeobox 2 [Source:VGNC Symbol;Acc:VGNC:91043]	-1.71	0.1324
ssc-miR-124a	ONECUT3	one cut homeobox 3 [Source:VGNC Symbol;Acc:VGNC:91044]	-1.71	0.1324
ssc-miR-124a	OPRK1	opioid receptor kappa 1 [Source:VGNC Symbol;Acc:VGNC:91054]	-1.71	0.1324
ssc-miR-124a	ORC2	origin recognition complex subunit 2 [Source:VGNC Symbol;Acc:VGNC:96464]	-1.71	0.1324
ssc-miR-124a	OSBP	oxysterol binding protein [Source:VGNC Symbol;Acc:VGNC:91066]	-1.71	0.1324

ssc-miR-124a	OSBP2	oxysterol binding protein 2 [Source:VGNC Symbol;Acc:VGNC:91067]	-1.71	0.1324
ssc-miR-124a	OSBPL10	oxysterol binding protein like 10 [Source:VGNC Symbol;Acc:VGNC:91068]	-1.71	0.1324
ssc-miR-124a	OSBPL11	oxysterol binding protein like 11 [Source:VGNC Symbol;Acc:VGNC:91069]	-1.71	0.1324
ssc-miR-124a	OSBPL3	oxysterol binding protein like 3 [Source:VGNC Symbol;Acc:VGNC:91071]	-1.71	0.1324
ssc-miR-124a	OSBPL5	oxysterol binding protein like 5 [Source:VGNC Symbol;Acc:VGNC:91072]	-1.71	0.1324
ssc-miR-124a	OSBPL6	oxysterol binding protein like 6 [Source:VGNC Symbol;Acc:VGNC:95807]	-1.71	0.1324
ssc-miR-124a	OSBPL7	oxysterol binding protein like 7 [Source:VGNC Symbol;Acc:VGNC:91073]	-1.71	0.1324
ssc-miR-124a	OSBPL8	oxysterol binding protein like 8 [Source:VGNC Symbol;Acc:VGNC:91074]	-1.71	0.1324
ssc-miR-124a	OTUD1	OTU deubiquitinase 1 [Source:VGNC Symbol;Acc:VGNC:96156]	-1.71	0.1324
ssc-miR-124a	OTUD3	OTU deubiquitinase 3 [Source:VGNC Symbol;Acc:VGNC:91099]	-1.71	0.1324
ssc-miR-124a	OTUD4	OTU deubiquitinase 4 [Source:VGNC Symbol;Acc:VGNC:91100]	-1.71	0.1324
ssc-miR-124a	OVOL2	ovo like zinc finger 2 [Source:VGNC Symbol;Acc:VGNC:95663]	-1.71	0.1324
ssc-miR-124a	OXSR1	hypothetical gene	-1.71	0.1324
ssc-miR-124a	P2RX7	purinergic receptor P2X 7 [Source:HGNC Symbol;Acc:HGNC:8537]	-1.71	0.1324
ssc-miR-124a	P4HA1	prolyl 4-hydroxylase subunit alpha 1 [Source:VGNC Symbol;Acc:VGNC:91132]	-1.71	0.1324
ssc-miR-124a	P4HA2	prolyl 4-hydroxylase subunit alpha 2 [Source:VGNC Symbol;Acc:VGNC:91133]	-1.71	0.1324
ssc-miR-124a	PABPC4L	poly(A) binding protein cytoplasmic 4 like [Source:VGNC Symbol;Acc:VGNC:108517]	-1.71	0.1324
ssc-miR-124a	PADI2	peptidyl arginine deiminase 2 [Source:VGNC Symbol;Acc:VGNC:91146]	-1.71	0.1324
ssc-miR-124a	PAFAH1B1	platelet activating factor acetylhydrolase 1b regulatory subunit 1 [Source:VGNC Symbol;Acc:VGNC:99026]	-1.71	0.1324
ssc-miR-124a	PAG1	phosphoprotein membrane anchor with glycosphingolipid microdomains 1 [Source:VGNC Symbol;Acc:VGNC:91154]	-1.71	0.1324
ssc-miR-124a	PAK2	p21 (RAC1) activated kinase 2 [Source:HGNC Symbol;Acc:HGNC:8591]	-1.71	0.1324
ssc-miR-124a	PALLD	palladin, cytoskeletal associated protein [Source:HGNC Symbol;Acc:HGNC:17068]	-1.71	0.1324
ssc-miR-124a	PALM	paralemmin [Source:VGNC Symbol;Acc:VGNC:91160]	-1.71	0.1324
ssc-miR-124a	PALM2	hypothetical gene	-1.71	0.1324
ssc-miR-124a	PAM	peptidylglycine alpha-amidating monooxygenase [Source:VGNC Symbol;Acc:VGNC:91163]	-1.71	0.1324
ssc-miR-124a	PAN3	poly(A) specific ribonuclease subunit PAN3 [Source:HGNC Symbol;Acc:HGNC:29991]	-1.71	0.1324
ssc-miR-124a	PANK3	pantothenate kinase 3 [Source:VGNC Symbol;Acc:VGNC:91165]	-1.71	0.1324
ssc-miR-124a	PAPD4	hypothetical gene	-1.71	0.1324
ssc-miR-124a	PAPD5	hypothetical gene	-1.71	0.1324
ssc-miR-124a	PAPOLG	poly(A) polymerase gamma [Source:HGNC Symbol;Acc:HGNC:14982]	-1.71	0.1324
ssc-miR-124a	PAPPA	pappalysin 1 [Source:VGNC Symbol;Acc:VGNC:91170]	-1.71	0.1324
ssc-miR-124a	PAPSS2	3'-phosphoadenosine 5'-phosphosulfate synthase 2 [Source:VGNC Symbol;Acc:VGNC:91173]	-1.71	0.1324
ssc-miR-124a	PAQR8	progestin and adipoQ receptor family member 8 [Source:VGNC Symbol;Acc:VGNC:91178]	-1.71	0.1324
ssc-miR-124a	PAQR9	progestin and adipoQ receptor family member 9 [Source:HGNC Symbol;Acc:HGNC:30131]	-1.71	0.1324
ssc-miR-124a	PARP16	poly(ADP-ribose) polymerase family member 16 [Source:VGNC Symbol;Acc:VGNC:103145]	-1.71	0.1324
ssc-miR-124a	PARP8	poly(ADP-ribose) polymerase family member 8 [Source:HGNC Symbol;Acc:HGNC:26124]	-1.71	0.1324
ssc-miR-124a	PARP9	poly(ADP-ribose) polymerase family member 9 [Source:VGNC Symbol;Acc:VGNC:108682]	-1.71	0.1324
ssc-miR-124a	PAWR	pro-apoptotic WT1 regulator [Source:VGNC Symbol;Acc:VGNC:91191]	-1.71	0.1324
ssc-miR-124a	PAX3	paired box 3 [Source:VGNC Symbol;Acc:VGNC:96468]	-1.71	0.1324
ssc-miR-124a	PAXBP1	PAX3 and PAX7 binding protein 1 [Source:VGNC Symbol;Acc:VGNC:91197]	-1.71	0.1324
ssc-miR-124a	PBX3	PBX homeobox 3 [Source:VGNC Symbol;Acc:VGNC:91205]	-1.71	0.1324
ssc-miR-124a	PCCB	propionyl-CoA carboxylase subunit beta [Source:VGNC Symbol;Acc:VGNC:91210]	-1.71	0.1324
ssc-miR-124a	PCDH1	protocadherin 1 [Source:VGNC Symbol;Acc:VGNC:91211]	-1.71	0.1324
ssc-miR-124a	PCDH17	protocadherin 17 [Source:VGNC Symbol;Acc:VGNC:91214]	-1.71	0.1324
ssc-miR-124a	PCDH19	protocadherin 19 [Source:VGNC Symbol;Acc:VGNC:91216]	-1.71	0.1324
ssc-miR-124a	PCDH7	protocadherin 7 [Source:NCBI gene (formerly Entrezgene);Acc:100520035]	-1.71	0.1324
ssc-miR-124a	PCDH8	protocadherin 8 [Source:HGNC Symbol;Acc:HGNC:8660]	-1.71	0.1324
ssc-miR-124a	PCDHA1	hypothetical gene	-1.71	0.1324
ssc-miR-124a	PCDHA10	hypothetical gene	-1.71	0.1324
ssc-miR-124a	PCDHA11	hypothetical gene	-1.71	0.1324

ssc-miR-124a	PCDHA12	hypothetical gene	-1.71	0.1324
ssc-miR-124a	PCDHA13	protocadherin alpha 13 [Source:HGNC Symbol;Acc:HGNC:8667]	-1.71	0.1324
ssc-miR-124a	PCDHA2	hypothetical gene	-1.71	0.1324
ssc-miR-124a	PCDHA3	protocadherin alpha 3 [Source:HGNC Symbol;Acc:HGNC:8669]	-1.71	0.1324
ssc-miR-124a	PCDHA4	hypothetical gene	-1.71	0.1324
ssc-miR-124a	PCDHA5	hypothetical gene	-1.71	0.1324
ssc-miR-124a	PCDHA6	hypothetical gene	-1.71	0.1324
ssc-miR-124a	PCDHA7	hypothetical gene	-1.71	0.1324
ssc-miR-124a	PCDHA8	hypothetical gene	-1.71	0.1324
ssc-miR-124a	PCDHA9	hypothetical gene	-1.71	0.1324
ssc-miR-124a	PCDHAC1	hypothetical gene	-1.71	0.1324
ssc-miR-124a	PCDHAC2	protocadherin alpha subfamily C, 2 [Source:HGNC Symbol;Acc:HGNC:8677]	-1.71	0.1324
ssc-miR-124a	PCGF5	hypothetical gene	-1.71	0.1324
ssc-miR-124a	PCSK6	proprotein convertase subtilisin/kexin type 6 [Source:VGNC Symbol;Acc:VGNC:103148]	-1.71	0.1324
ssc-miR-124a	PCYOX1	prenylcysteine oxidase 1 [Source:VGNC Symbol;Acc:VGNC:91235]	-1.71	0.1324
ssc-miR-124a	PDCD10	programmed cell death 10 [Source:VGNC Symbol;Acc:VGNC:91241]	-1.71	0.1324
ssc-miR-124a	PDCD6	programmed cell death 6 [Source:VGNC Symbol;Acc:VGNC:98178]	-1.71	0.1324
ssc-miR-124a	PDE10A	phosphodiesterase 10A [Source:VGNC Symbol;Acc:VGNC:91248]	-1.71	0.1324
ssc-miR-124a	PDE12	phosphodiesterase 12 [Source:VGNC Symbol;Acc:VGNC:104020]	-1.71	0.1324
ssc-miR-124a	PDE2A	phosphodiesterase 2A [Source:VGNC Symbol;Acc:VGNC:91251]	-1.71	0.1324
ssc-miR-124a	PDE3B	phosphodiesterase 3B [Source:VGNC Symbol;Acc:VGNC:91253]	-1.71	0.1324
ssc-miR-124a	PDE4A	phosphodiesterase 4A [Source:VGNC Symbol;Acc:VGNC:91254]	-1.71	0.1324
ssc-miR-124a	PDE4B	phosphodiesterase 4B [Source:VGNC Symbol;Acc:VGNC:91255]	-1.71	0.1324
ssc-miR-124a	PDE4D	phosphodiesterase 4D [Source:VGNC Symbol;Acc:VGNC:91256]	-1.71	0.1324
ssc-miR-124a	PDE5A	phosphodiesterase 5A [Source:VGNC Symbol;Acc:VGNC:91257]	-1.71	0.1324
ssc-miR-124a	PDE7A	phosphodiesterase 7A [Source:VGNC Symbol;Acc:VGNC:91261]	-1.71	0.1324
ssc-miR-124a	PDE7B	phosphodiesterase 7B [Source:VGNC Symbol;Acc:VGNC:91262]	-1.71	0.1324
ssc-miR-124a	PDE8A	phosphodiesterase 8A [Source:VGNC Symbol;Acc:VGNC:91263]	-1.71	0.1324
ssc-miR-124a	PDGFA	platelet derived growth factor subunit A [Source:VGNC Symbol;Acc:VGNC:91265]	-1.71	0.1324
ssc-miR-124a	PDIK1L	PDLIM1 interacting kinase 1 like [Source:VGNC Symbol;Acc:VGNC:91276]	-1.71	0.1324
ssc-miR-124a	PDK2	pyruvate dehydrogenase kinase 2 [Source:VGNC Symbol;Acc:VGNC:91278]	-1.71	0.1324
ssc-miR-124a	PDPK1	3-phosphoinositide dependent protein kinase 1 [Source:HGNC Symbol;Acc:HGNC:8816]	-1.71	0.1324
ssc-miR-124a	PDXK	pyridoxal kinase [Source:VGNC Symbol;Acc:VGNC:108684]	-1.71	0.1324
ssc-miR-124a	PDZD2	hypothetical gene	-1.71	0.1324
ssc-miR-124a	PEA15	proliferation and apoptosis adaptor protein 15 [Source:VGNC Symbol;Acc:VGNC:98181]	-1.71	0.1324
ssc-miR-124a	PEAK1	pseudopodium enriched atypical kinase 1 [Source:VGNC Symbol;Acc:VGNC:91300]	-1.71	0.1324
ssc-miR-124a	PECR	peroxisomal trans-2-enoyl-CoA reductase [Source:VGNC Symbol;Acc:VGNC:95880]	-1.71	0.1324
ssc-miR-124a	PEG10	paternally expressed 10 [Source:NCBI gene (formerly Entrezgene);Acc:654416]	-1.71	0.1324
ssc-miR-124a	PEG3	paternally expressed 3 [Source:VGNC Symbol;Acc:VGNC:98530]	-1.71	0.1324
ssc-miR-124a	PEMT	hypothetical gene	-1.71	0.1324
ssc-miR-124a	PER3	hypothetical gene	-1.71	0.1324
ssc-miR-124a	PEX19	peroxisomal biosis factor 19 [Source:VGNC Symbol;Acc:VGNC:104022]	-1.71	0.1324
ssc-miR-124a	PGAP1	post-GPI attachment to proteins inositol deacylase 1 [Source:VGNC Symbol;Acc:VGNC:96099]	-1.71	0.1324
ssc-miR-124a	PGF	placental growth factor [Source:HGNC Symbol;Acc:HGNC:8893]	-1.71	0.1324
ssc-miR-124a	PGM1	hypothetical gene	-1.71	0.1324
ssc-miR-124a	PGM2	phosphoglucomutase 2 [Source:VGNC Symbol;Acc:VGNC:91356]	-1.71	0.1324
ssc-miR-124a	PGM2L1	phosphoglucomutase 2 like 1 [Source:VGNC Symbol;Acc:VGNC:91357]	-1.71	0.1324
ssc-miR-124a	PGPEP1	pyroglutamyl-peptidase I [Source:VGNC Symbol;Acc:VGNC:91360]	-1.71	0.1324
ssc-miR-124a	PGRMC2	progesterone receptor membrane component 2 [Source:VGNC Symbol;Acc:VGNC:98952]	-1.71	0.1324
ssc-miR-124a	PHACTR2	phosphatase and actin regulator 2 [Source:VGNC Symbol;Acc:VGNC:91366]	-1.71	0.1324

ssc-miR-124a	PHC2	polyhomeotic homolog 2 [Source:VGNC Symbol;Acc:VGNC:91371]	-1.71	0.1324
ssc-miR-124a	PHEX	phosphate regulating endopeptidase homolog X-linked [Source:NCBI gene (formerly Entrezgene);Acc:100154258]	-1.71	0.1324
ssc-miR-124a	PHF12	PHD finger protein 12 [Source:VGNC Symbol;Acc:VGNC:91378]	-1.71	0.1324
ssc-miR-124a	PHF17	hypothetical gene	-1.71	0.1324
ssc-miR-124a	PHF19	PHD finger protein 19 [Source:VGNC Symbol;Acc:VGNC:91381]	-1.71	0.1324
ssc-miR-124a	PHF6	PHD finger protein 6 [Source:VGNC Symbol;Acc:VGNC:91389]	-1.71	0.1324
ssc-miR-124a	PHIP	pleckstrin homology domain interacting protein [Source:VGNC Symbol;Acc:VGNC:91392]	-1.71	0.1324
ssc-miR-124a	PHKA1	phosphorylase kinase regulatory subunit alpha 1 [Source:VGNC Symbol;Acc:VGNC:91393]	-1.71	0.1324
ssc-miR-124a	PHLDB1	pleckstrin homology like domain family B member 1 [Source:VGNC Symbol;Acc:VGNC:91398]	-1.71	0.1324
ssc-miR-124a	PHTF2	putative homeodomain transcription factor 2 [Source:VGNC Symbol;Acc:VGNC:91405]	-1.71	0.1324
ssc-miR-124a	PI4K2B	phosphatidylinositol 4-kinase type 2 beta [Source:VGNC Symbol;Acc:VGNC:98190]	-1.71	0.1324
ssc-miR-124a	PIAS1	protein inhibitor of activated STAT 1 [Source:VGNC Symbol;Acc:VGNC:91410]	-1.71	0.1324
ssc-miR-124a	PID1	phosphotyrosine interaction domain containing 1 [Source:VGNC Symbol;Acc:VGNC:96114]	-1.71	0.1324
ssc-miR-124a	PIEZO2	piezo type mechanosensitive ion channel component 2 [Source:HGNC Symbol;Acc:HGNC:26270]	-1.71	0.1324
ssc-miR-124a	PIGM	hypothetical gene	-1.71	0.1324
ssc-miR-124a	PIK3C2A	phosphatidylinositol-4-phosphate 3-kinase catalytic subunit type 2 alpha [Source:VGNC Symbol;Acc:VGNC:91436]	-1.71	0.1324
ssc-miR-124a	PIK3CA	phosphatidylinositol-4,5-bisphosphate 3-kinase catalytic subunit alpha [Source:VGNC Symbol;Acc:VGNC:91440]	-1.71	0.1324
ssc-miR-124a	PIK3IP1	phosphoinositide-3-kinase interacting protein 1 [Source:VGNC Symbol;Acc:VGNC:91444]	-1.71	0.1324
ssc-miR-124a	PIKFYVE	phosphoinositide kinase, FYVE-type zinc finger containing [Source:VGNC Symbol;Acc:VGNC:96023]	-1.71	0.1324
ssc-miR-124a	PIM1	Pim-1 proto-oncogene, serine/threonine kinase [Source:HGNC Symbol;Acc:HGNC:8986]	-1.71	0.1324
ssc-miR-124a	PIM3	Pim-3 proto-onco, serine/threonine kinase [Source:VGNC Symbol;Acc:VGNC:91450]	-1.71	0.1324
ssc-miR-124a	PIP4K2A	phosphatidylinositol-5-phosphate 4-kinase type 2 alpha [Source:VGNC Symbol;Acc:VGNC:96485]	-1.71	0.1324
ssc-miR-124a	PIP4K2C	phosphatidylinositol-5-phosphate 4-kinase type 2 gamma [Source:VGNC Symbol;Acc:VGNC:91455]	-1.71	0.1324
ssc-miR-124a	PIRT	phosphoinositide interacting regulator of transient receptor potential channels [Source:VGNC Symbol;Acc:VGNC:91461]	-1.71	0.1324
ssc-miR-124a	PITPNA	phosphatidylinositol transfer protein alpha [Source:VGNC Symbol;Acc:VGNC:91464]	-1.71	0.1324
ssc-miR-124a	PITPNM3	PITPNM family member 3 [Source:HGNC Symbol;Acc:HGNC:21043]	-1.71	0.1324
ssc-miR-124a	PJA2	praja ring finger ubiquitin ligase 2 [Source:VGNC Symbol;Acc:VGNC:91470]	-1.71	0.1324
ssc-miR-124a	PKN2	protein kinase N2 [Source:VGNC Symbol;Acc:VGNC:91482]	-1.71	0.1324
ssc-miR-124a	PLA2G12A	phospholipase A2 group XIIA [Source:HGNC Symbol;Acc:HGNC:18554]	-1.71	0.1324
ssc-miR-124a	PLAG1	PLAG1 zinc finger [Source:VGNC Symbol;Acc:VGNC:91509]	-1.71	0.1324
ssc-miR-124a	PLAGL2	PLAG1 like zinc finger 2 [Source:VGNC Symbol;Acc:VGNC:96490]	-1.71	0.1324
ssc-miR-124a	PLCB1	phospholipase C beta 1 [Source:VGNC Symbol;Acc:VGNC:95706]	-1.71	0.1324
ssc-miR-124a	PLCXD3	phosphatidylinositol specific phospholipase C X domain containing 3 [Source:VGNC Symbol;Acc:VGNC:91524]	-1.71	0.1324
ssc-miR-124a	PLD1	phospholipase D1 [Source:VGNC Symbol;Acc:VGNC:91525]	-1.71	0.1324
ssc-miR-124a	PLD5	phospholipase D family member 5 [Source:VGNC Symbol;Acc:VGNC:96145]	-1.71	0.1324
ssc-miR-124a	PLEC	plectin [Source:VGNC Symbol;Acc:VGNC:91530]	-1.71	0.1324
ssc-miR-124a	PLEKHA6	pleckstrin homology domain containing A6 [Source:VGNC Symbol;Acc:VGNC:91536]	-1.71	0.1324
ssc-miR-124a	PLEKHF2	pleckstrin homology and FYVE domain containing 2 [Source:VGNC Symbol;Acc:VGNC:91541]	-1.71	0.1324
ssc-miR-124a	PLEKHG3	pleckstrin homology and RhoGEF domain containing G3 [Source:VGNC Symbol;Acc:VGNC:91544]	-1.71	0.1324
ssc-miR-124a	PLEKHH1	pleckstrin homology, MyTH4 and FERM domain containing H1 [Source:HGNC Symbol;Acc:HGNC:17733]	-1.71	0.1324
ssc-miR-124a	PLEKHM3	pleckstrin homology domain containing M3 [Source:VGNC Symbol;Acc:VGNC:96281]	-1.71	0.1324
ssc-miR-124a	PLIN3	perilipin 3 [Source:VGNC Symbol;Acc:VGNC:91559]	-1.71	0.1324
ssc-miR-124a	PLOD2	procollagen-lysine,2-oxoglutarate 5-dioxygenase 2 [Source:VGNC Symbol;Acc:VGNC:91567]	-1.71	0.1324
ssc-miR-124a	PLOD3	procollagen-lysine,2-oxoglutarate 5-dioxygenase 3 [Source:VGNC Symbol;Acc:VGNC:91568]	-1.71	0.1324
ssc-miR-124a	PLP2	proteolipid protein 2 [Source:NCBI gene (formerly Entrezgene);Acc:100512511]	-1.71	0.1324
ssc-miR-124a	PLXNA2	plexin A2 [Source:VGNC Symbol;Acc:VGNC:91580]	-1.71	0.1324
ssc-miR-124a	PLXNA3	plexin A3 [Source:VGNC Symbol;Acc:VGNC:91581]	-1.71	0.1324
ssc-miR-124a	PLXNB2	plexin B2 [Source:HGNC Symbol;Acc:HGNC:9104]	-1.71	0.1324
ssc-miR-124a	PMEPA1	prostate transmembrane protein, androgen induced 1 [Source:VGNC Symbol;Acc:VGNC:95606]	-1.71	0.1324
ssc-miR-124a	PML	PML nuclear body scaffold [Source:VGNC Symbol;Acc:VGNC:99296]	-1.71	0.1324

ssc-miR-124a	PNN	pinin, desmosome associated protein [Source:VGNC Symbol;Acc:VGNC:91603]	-1.71	0.1324
ssc-miR-124a	PNPLA2	patatin like phospholipase domain containing 2 [Source:VGNC Symbol;Acc:VGNC:91607]	-1.71	0.1324
ssc-miR-124a	POC1B	POC1 centriolar protein B [Source:VGNC Symbol;Acc:VGNC:91616]	-1.71	0.1324
ssc-miR-124a	POC1B-GALNT4	hypothetical gene	-1.71	0.1324
ssc-miR-124a	PODXL	hypothetical gene	-1.71	0.1324
ssc-miR-124a	POGK	pogo transposable element derived with KRAB domain [Source:VGNC Symbol;Acc:VGNC:91622]	-1.71	0.1324
ssc-miR-124a	POGLUT1	protein O-glucosyltransferase 1 [Source:VGNC Symbol;Acc:VGNC:91623]	-1.71	0.1324
ssc-miR-124a	POLH	DNA polymerase eta [Source:VGNC Symbol;Acc:VGNC:91635]	-1.71	0.1324
ssc-miR-124a	POLR3G	hypothetical gene	-1.71	0.1324
ssc-miR-124a	POTEI	hypothetical gene	-1.71	0.1324
ssc-miR-124a	POU2F1	POU class 2 homeobox 1 [Source:VGNC Symbol;Acc:VGNC:91672]	-1.71	0.1324
ssc-miR-124a	PPARA	peroxisome proliferator activated receptor alpha [Source:VGNC Symbol;Acc:VGNC:91682]	-1.71	0.1324
ssc-miR-124a	PPARGC1B	PPARG coactivator 1 beta [Source:VGNC Symbol;Acc:VGNC:91686]	-1.71	0.1324
ssc-miR-124a	PPDPF	hypothetical gene	-1.71	0.1324
ssc-miR-124a	PPFIBP2	PPFIA binding protein 2 [Source:VGNC Symbol;Acc:VGNC:91696]	-1.71	0.1324
ssc-miR-124a	PPM1A	protein phosphatase, Mg2+/Mn2+ dependent 1A [Source:VGNC Symbol;Acc:VGNC:91703]	-1.71	0.1324
ssc-miR-124a	PPM1F	protein phosphatase, Mg2+/Mn2+ dependent 1F [Source:VGNC Symbol;Acc:VGNC:91706]	-1.71	0.1324
ssc-miR-124a	PPM1L	protein phosphatase, Mg2+/Mn2+ dependent 1L [Source:VGNC Symbol;Acc:VGNC:91711]	-1.71	0.1324
ssc-miR-124a	PPP1R13L	protein phosphatase 1 regulatory subunit 13 like [Source:VGNC Symbol;Acc:VGNC:91722]	-1.71	0.1324
ssc-miR-124a	PPP1R3B	protein phosphatase 1 regulatory subunit 3B [Source:VGNC Symbol;Acc:VGNC:95636]	-1.71	0.1324
ssc-miR-124a	PPP1R3D	protein phosphatase 1 regulatory subunit 3D [Source:VGNC Symbol;Acc:VGNC:96500]	-1.71	0.1324
ssc-miR-124a	PPP1R9A	protein phosphatase 1 regulatory subunit 9A [Source:VGNC Symbol;Acc:VGNC:91743]	-1.71	0.1324
ssc-miR-124a	PPP2R5E	protein phosphatase 2 regulatory subunit B'epsilon [Source:VGNC Symbol;Acc:VGNC:91754]	-1.71	0.1324
ssc-miR-124a	PPP4R1	protein phosphatase 4 regulatory subunit 1 [Source:VGNC Symbol;Acc:VGNC:98540]	-1.71	0.1324
ssc-miR-124a	PPP4R1L	hypothetical gene	-1.71	0.1324
ssc-miR-124a	PPP6R2	protein phosphatase 6 regulatory subunit 2 [Source:VGNC Symbol;Acc:VGNC:91761]	-1.71	0.1324
ssc-miR-124a	PPTC7	protein phosphatase targeting COQ7 [Source:VGNC Symbol;Acc:VGNC:91765]	-1.71	0.1324
ssc-miR-124a	PQLC3	hypothetical gene	-1.71	0.1324
ssc-miR-124a	PRDM13	PR/SET domain 13 [Source:VGNC Symbol;Acc:VGNC:91775]	-1.71	0.1324
ssc-miR-124a	PRDM2	PR/SET domain 2 [Source:VGNC Symbol;Acc:VGNC:91778]	-1.71	0.1324
ssc-miR-124a	PRDM4	PR/SET domain 4 [Source:VGNC Symbol;Acc:VGNC:91779]	-1.71	0.1324
ssc-miR-124a	PRDX6	peroxiredoxin 6 [Source:VGNC Symbol;Acc:VGNC:91785]	-1.71	0.1324
ssc-miR-124a	PRICKLE2	prickle planar cell polarity protein 2 [Source:VGNC Symbol;Acc:VGNC:91794]	-1.71	0.1324
ssc-miR-124a	PRKAA2	protein kinase AMP-activated catalytic subunit alpha 2 [Source:VGNC Symbol;Acc:VGNC:91798]	-1.71	0.1324
ssc-miR-124a	PRKAG2	protein kinase AMP-activated non-catalytic subunit gamma 2 [Source:VGNC Symbol;Acc:VGNC:91801]	-1.71	0.1324
ssc-miR-124a	PRKD1	protein kinase D1 [Source:VGNC Symbol;Acc:VGNC:91812]	-1.71	0.1324
ssc-miR-124a	PRKG1	protein kinase cGMP-dependent 1 [Source:VGNC Symbol;Acc:VGNC:91816]	-1.71	0.1324
ssc-miR-124a	PRKX	protein kinase X-linked [Source:HGNC Symbol;Acc:HGNC:9441]	-1.71	0.1324
ssc-miR-124a	PRLR	prolactin receptor [Source:VGNC Symbol;Acc:VGNC:91819]	-1.71	0.1324
ssc-miR-124a	PROX1	prospero homeobox 1 [Source:VGNC Symbol;Acc:VGNC:91837]	-1.71	0.1324
ssc-miR-124a	PRPF38B	hypothetical gene	-1.71	0.1324
ssc-miR-124a	PRPF40A	pre-mRNA processing factor 40 homolog A [Source:VGNC Symbol;Acc:VGNC:98222]	-1.71	0.1324
ssc-miR-124a	PRPS1	hypothetical gene	-1.71	0.1324
ssc-miR-124a	PRR14L	proline rich 14 like [Source:VGNC Symbol;Acc:VGNC:91853]	-1.71	0.1324
ssc-miR-124a	PRR18	hypothetical gene	-1.71	0.1324
ssc-miR-124a	PRRC1	proline rich coiled-coil 1 [Source:VGNC Symbol;Acc:VGNC:91864]	-1.71	0.1324
ssc-miR-124a	PRRX1	paired related homeobox 1 [Source:VGNC Symbol;Acc:VGNC:91875]	-1.71	0.1324
ssc-miR-124a	PSEN1	presenilin 1 [Source:VGNC Symbol;Acc:VGNC:91897]	-1.71	0.1324
ssc-miR-124a	PSKH1	protein serine kinase H1 [Source:VGNC Symbol;Acc:VGNC:91899]	-1.71	0.1324
ssc-miR-124a	PSMB5	hypothetical gene	-1.71	0.1324

ssc-miR-124a	PSMD5	proteasome 26S subunit, non-ATPase 5 [Source:VGNC Symbol;Acc:VGNC:91923]	-1.71	0.1324
ssc-miR-124a	PTAR1	protein prenyltransferase alpha subunit repeat containing 1 [Source:VGNC Symbol;Acc:VGNC:91936]	-1.71	0.1324
ssc-miR-124a	PTBP1	polypyrimidine tract binding protein 1 [Source:VGNC Symbol;Acc:VGNC:100330]	-1.71	0.1324
ssc-miR-124a	PTBP2	polypyrimidine tract binding protein 2 [Source:VGNC Symbol;Acc:VGNC:91937]	-1.71	0.1324
ssc-miR-124a	PTBP3	polypyrimidine tract binding protein 3 [Source:VGNC Symbol;Acc:VGNC:91938]	-1.71	0.1324
ssc-miR-124a	PTDSS1	phosphatidylserine synthase 1 [Source:VGNC Symbol;Acc:VGNC:91945]	-1.71	0.1324
ssc-miR-124a	PTER	phosphotriesterase related [Source:VGNC Symbol;Acc:VGNC:96514]	-1.71	0.1324
ssc-miR-124a	PTGFRN	prostaglandin F2 receptor inhibitor [Source:VGNC Symbol;Acc:VGNC:91955]	-1.71	0.1324
ssc-miR-124a	PTPN1	protein tyrosine phosphatase non-receptor type 1 [Source:VGNC Symbol;Acc:VGNC:96519]	-1.71	0.1324
ssc-miR-124a	PTPN11	protein tyrosine phosphatase non-receptor type 11 [Source:VGNC Symbol;Acc:VGNC:91972]	-1.71	0.1324
ssc-miR-124a	PTPN12	protein tyrosine phosphatase non-receptor type 12 [Source:VGNC Symbol;Acc:VGNC:91973]	-1.71	0.1324
ssc-miR-124a	PTPN4	protein tyrosine phosphatase non-receptor type 4 [Source:VGNC Symbol;Acc:VGNC:96521]	-1.71	0.1324
ssc-miR-124a	PTPN9	protein tyrosine phosphatase non-receptor type 9 [Source:VGNC Symbol;Acc:VGNC:91983]	-1.71	0.1324
ssc-miR-124a	PTPRD	protein tyrosine phosphatase receptor type D [Source:HGNC Symbol;Acc:HGNC:9668]	-1.71	0.1324
ssc-miR-124a	PTPRJ	protein tyrosine phosphatase receptor type J [Source:VGNC Symbol;Acc:VGNC:91990]	-1.71	0.1324
ssc-miR-124a	PTPRQ	protein tyrosine phosphatase receptor type Q [Source:HGNC Symbol;Acc:HGNC:9679]	-1.71	0.1324
ssc-miR-124a	PTPRZ1	protein tyrosine phosphatase receptor type Z1 [Source:VGNC Symbol;Acc:VGNC:91996]	-1.71	0.1324
ssc-miR-124a	PTTG1IP	PTTG1 interacting protein [Source:VGNC Symbol;Acc:VGNC:91999]	-1.71	0.1324
ssc-miR-124a	PURA	purine rich element binding protein A [Source:HGNC Symbol;Acc:HGNC:9701]	-1.71	0.1324
ssc-miR-124a	PURB	purine rich element binding protein B [Source:VGNC Symbol;Acc:VGNC:92004]	-1.71	0.1324
ssc-miR-124a	PUS7	pseudouridine synthase 7 [Source:VGNC Symbol;Acc:VGNC:108161]	-1.71	0.1324
ssc-miR-124a	PXMP4	hypothetical gene	-1.71	0.1324
ssc-miR-124a	QDPR	quinoid dihydropteridine reductase [Source:VGNC Symbol;Acc:VGNC:98232]	-1.71	0.1324
ssc-miR-124a	QKI	QKI, KH domain containing RNA binding [Source:VGNC Symbol;Acc:VGNC:92025]	-1.71	0.1324
ssc-miR-124a	QRICH1	hypothetical gene	-1.71	0.1324
ssc-miR-124a	QSER1	glutamine and serine rich 1 [Source:VGNC Symbol;Acc:VGNC:92032]	-1.71	0.1324
ssc-miR-124a	RAB10	RAB10, member RAS onco family [Source:VGNC Symbol;Acc:VGNC:98235]	-1.71	0.1324
ssc-miR-124a	RAB11A	RAB11A, member RAS onco family [Source:VGNC Symbol;Acc:VGNC:98236]	-1.71	0.1324
ssc-miR-124a	RAB11FIP1	hypothetical gene	-1.71	0.1324
ssc-miR-124a	RAB11FIP4	RAB11 family interacting protein 4 [Source:VGNC Symbol;Acc:VGNC:92041]	-1.71	0.1324
ssc-miR-124a	RAB11FIP5	RAB11 family interacting protein 5 [Source:HGNC Symbol;Acc:HGNC:24845]	-1.71	0.1324
ssc-miR-124a	RAB14	RAB14, member RAS onco family [Source:VGNC Symbol;Acc:VGNC:98238]	-1.71	0.1324
ssc-miR-124a	RAB22A	RAB22A, member RAS onco family [Source:VGNC Symbol;Acc:VGNC:98244]	-1.71	0.1324
ssc-miR-124a	RAB27A	RAB27A, member RAS onco family [Source:VGNC Symbol;Acc:VGNC:98249]	-1.71	0.1324
ssc-miR-124a	RAB2A	RAB2A, member RAS onco family [Source:VGNC Symbol;Acc:VGNC:98252]	-1.71	0.1324
ssc-miR-124a	RAB32	RAB32, member RAS onco family [Source:NCBI gene (formerly Entrezgene);Acc:100144496]	-1.71	0.1324
ssc-miR-124a	RAB34	RAB34, member RAS onco family [Source:VGNC Symbol;Acc:VGNC:98258]	-1.71	0.1324
ssc-miR-124a	RAB38	RAB38, member RAS onco family [Source:VGNC Symbol;Acc:VGNC:98261]	-1.71	0.1324
ssc-miR-124a	RAB3D	RAB3D, member RAS onco family [Source:VGNC Symbol;Acc:VGNC:98266]	-1.71	0.1324
ssc-miR-124a	RAB43	RAB43, member RAS onco family [Source:VGNC Symbol;Acc:VGNC:98268]	-1.71	0.1324
ssc-miR-124a	RAB4A	RAB4A, member RAS onco family [Source:VGNC Symbol;Acc:VGNC:98270]	-1.71	0.1324
ssc-miR-124a	RAB9B	RAB9B, member RAS onco family [Source:VGNC Symbol;Acc:VGNC:98278]	-1.71	0.1324
ssc-miR-124a	RAD17	RAD17 checkpoint clamp loader component [Source:VGNC Symbol;Acc:VGNC:92052]	-1.71	0.1324
ssc-miR-124a	RAD51L3-RFFL	hypothetical gene	-1.71	0.1324
ssc-miR-124a	RAD54B	fibrinogen silencer binding protein [Source:VGNC Symbol;Acc:VGNC:98795]	-1.71	0.1324
ssc-miR-124a	RAD54L2	RAD54 like 2 [Source:VGNC Symbol;Acc:VGNC:92061]	-1.71	0.1324
ssc-miR-124a	RAF1	Raf-1 proto-onco, serine/threonine kinase [Source:VGNC Symbol;Acc:VGNC:108688]	-1.71	0.1324
ssc-miR-124a	RAI14	retinoic acid induced 14 [Source:VGNC Symbol;Acc:VGNC:92068]	-1.71	0.1324
ssc-miR-124a	RALA	RAS like proto-oncogene A [Source:HGNC Symbol;Acc:HGNC:9839]	-1.71	0.1324
ssc-miR-124a	RALBP1	ralA binding protein 1 [Source:VGNC Symbol;Acc:VGNC:92070]	-1.71	0.1324

ssc-miR-124a	RALGDS	ral guanine nucleotide dissociation stimulator [Source:HGNC Symbol;Acc:HGNC:9842]	-1.71	0.1324
ssc-miR-124a	RALGPS1	Ral GEF with PH domain and SH3 binding motif 1 [Source:VGNC Symbol;Acc:VGNC:92072]	-1.71	0.1324
ssc-miR-124a	RALGPS2	Ral GEF with PH domain and SH3 binding motif 2 [Source:VGNC Symbol;Acc:VGNC:92073]	-1.71	0.1324
ssc-miR-124a	RANBP10	RAN binding protein 10 [Source:VGNC Symbol;Acc:VGNC:92077]	-1.71	0.1324
ssc-miR-124a	RAP1A	RAP1A, member of RAS onco family [Source:VGNC Symbol;Acc:VGNC:92084]	-1.71	0.1324
ssc-miR-124a	RAP1B	RAP1B, member of RAS oncogene family [Source:HGNC Symbol;Acc:HGNC:9857]	-1.71	0.1324
ssc-miR-124a	RAP1GAP2	RAP1 GTPase activating protein 2 [Source:VGNC Symbol;Acc:VGNC:92086]	-1.71	0.1324
ssc-miR-124a	RAP2A	RAP2A, member of RAS onco family [Source:VGNC Symbol;Acc:VGNC:92088]	-1.71	0.1324
ssc-miR-124a	RAP2B	RAP2B, member of RAS onco family [Source:VGNC Symbol;Acc:VGNC:92089]	-1.71	0.1324
ssc-miR-124a	RAP2C	RAP2C, member of RAS onco family [Source:VGNC Symbol;Acc:VGNC:92090]	-1.71	0.1324
ssc-miR-124a	RAPGEF1	Rap guanine nucleotide exchange factor 1 [Source:VGNC Symbol;Acc:VGNC:92091]	-1.71	0.1324
ssc-miR-124a	RAPGEF2	Rap guanine nucleotide exchange factor 2 [Source:VGNC Symbol;Acc:VGNC:92092]	-1.71	0.1324
ssc-miR-124a	RAPH1	hypothetical gene	-1.71	0.1324
ssc-miR-124a	RARG	retinoic acid receptor gamma [Source:HGNC Symbol;Acc:HGNC:9866]	-1.71	0.1324
ssc-miR-124a	RASAL2	RAS protein activator like 2 [Source:VGNC Symbol;Acc:VGNC:92105]	-1.71	0.1324
ssc-miR-124a	RASGEF1A	RasGEF domain family member 1A [Source:VGNC Symbol;Acc:VGNC:92108]	-1.71	0.1324
ssc-miR-124a	RASL10B	RAS like family 10 member B [Source:VGNC Symbol;Acc:VGNC:92118]	-1.71	0.1324
ssc-miR-124a	RASSF3	Ras association domain family member 3 [Source:VGNC Symbol;Acc:VGNC:92124]	-1.71	0.1324
ssc-miR-124a	RASSF5	Ras association domain family member 5 [Source:VGNC Symbol;Acc:VGNC:92125]	-1.71	0.1324
ssc-miR-124a	RASSF8	Ras association domain family member 8 [Source:VGNC Symbol;Acc:VGNC:92128]	-1.71	0.1324
ssc-miR-124a	RAVER1	ribonucleoprotein, PTB binding 1 [Source:HGNC Symbol;Acc:HGNC:30296]	-1.71	0.1324
ssc-miR-124a	RAVER2	ribonucleoprotein, PTB binding 2 [Source:VGNC Symbol;Acc:VGNC:98283]	-1.71	0.1324
ssc-miR-124a	RB1CC1	RB1 inducible coiled-coil 1 [Source:VGNC Symbol;Acc:VGNC:92133]	-1.71	0.1324
ssc-miR-124a	RBCK1	RANBP2-type and C3HC4-type zinc finger containing 1 [Source:VGNC Symbol;Acc:VGNC:98284]	-1.71	0.1324
ssc-miR-124a	RBM12	RNA binding motif protein 12 [Source:HGNC Symbol;Acc:HGNC:9898]	-1.71	0.1324
ssc-miR-124a	RBM20	RNA binding motif protein 20 [Source:HGNC Symbol;Acc:HGNC:27424]	-1.71	0.1324
ssc-miR-124a	RBM24	hypothetical gene	-1.71	0.1324
ssc-miR-124a	RBM33	hypothetical gene	-1.71	0.1324
ssc-miR-124a	RBM47	RNA binding motif protein 47 [Source:VGNC Symbol;Acc:VGNC:92158]	-1.71	0.1324
ssc-miR-124a	RBMS1	RNA binding motif single stranded interacting protein 1 [Source:VGNC Symbol;Acc:VGNC:96534]	-1.71	0.1324
ssc-miR-124a	RBMS2	RNA binding motif single stranded interacting protein 2 [Source:VGNC Symbol;Acc:VGNC:98286]	-1.71	0.1324
ssc-miR-124a	RBMS3	RNA binding motif single stranded interacting protein 3 [Source:VGNC Symbol;Acc:VGNC:92163]	-1.71	0.1324
ssc-miR-124a	RCAN1	regulator of calcineurin 1 [Source:VGNC Symbol;Acc:VGNC:92170]	-1.71	0.1324
ssc-miR-124a	RCC2	regulator of chromosome condensation 2 [Source:VGNC Symbol;Acc:VGNC:98547]	-1.71	0.1324
ssc-miR-124a	RCOR1	REST corepressor 1 [Source:VGNC Symbol;Acc:VGNC:92183]	-1.71	0.1324
ssc-miR-124a	RDH10	retinol dehydrogenase 10 [Source:VGNC Symbol;Acc:VGNC:92190]	-1.71	0.1324
ssc-miR-124a	REEP1	receptor accessory protein 1 [Source:VGNC Symbol;Acc:VGNC:92197]	-1.71	0.1324
ssc-miR-124a	REEP2	receptor accessory protein 2 [Source:VGNC Symbol;Acc:VGNC:92198]	-1.71	0.1324
ssc-miR-124a	REEP3	receptor accessory protein 3 [Source:VGNC Symbol;Acc:VGNC:92199]	-1.71	0.1324
ssc-miR-124a	RELA	RELA proto-onco, NF-kB subunit [Source:VGNC Symbol;Acc:VGNC:92203]	-1.71	0.1324
ssc-miR-124a	RERE	hypothetical gene	-1.71	0.1324
ssc-miR-124a	RFFL	ring finger and FYVE like domain containing E3 ubiquitin protein ligase [Source:VGNC Symbol;Acc:VGNC:98289]	-1.71	0.1324
ssc-miR-124a	RFX1	regulatory factor X1 [Source:VGNC Symbol;Acc:VGNC:92243]	-1.71	0.1324
ssc-miR-124a	RFX3	regulatory factor X3 [Source:VGNC Symbol;Acc:VGNC:92245]	-1.71	0.1324
ssc-miR-124a	RFX4	regulatory factor X4 [Source:HGNC Symbol;Acc:HGNC:9985]	-1.71	0.1324
ssc-miR-124a	RGS9	regulator of G protein signaling 9 [Source:VGNC Symbol;Acc:VGNC:92270]	-1.71	0.1324
ssc-miR-124a	RHBDD1	rhomboid domain containing 1 [Source:VGNC Symbol;Acc:VGNC:95989]	-1.71	0.1324
ssc-miR-124a	RHBDL3	rhomboid like 3 [Source:VGNC Symbol;Acc:VGNC:92279]	-1.71	0.1324
ssc-miR-124a	RHOA	ras homolog family member A [Source:VGNC Symbol;Acc:VGNC:92284]	-1.71	0.1324
ssc-miR-124a	RHOG	hypothetical gene	-1.71	0.1324

ssc-miR-124a	RHOQ	ras homolog family member Q [Source:VGNC Symbol;Acc:VGNC:92292]	-1.71	0.1324
ssc-miR-124a	RHOU	ras homolog family member U [Source:VGNC Symbol;Acc:VGNC:92294]	-1.71	0.1324
ssc-miR-124a	RICTOR	RPTOR independent companion of MTOR complex 2 [Source:VGNC Symbol;Acc:VGNC:92303]	-1.71	0.1324
ssc-miR-124a	RILPL1	Rab interacting lysosomal protein like 1 [Source:VGNC Symbol;Acc:VGNC:92304]	-1.71	0.1324
ssc-miR-124a	RIMBP2	RIMS binding protein 2 [Source:VGNC Symbol;Acc:VGNC:92306]	-1.71	0.1324
ssc-miR-124a	RLIM	ring finger protein, LIM domain interacting [Source:HGNC Symbol;Acc:HGNC:13429]	-1.71	0.1324
ssc-miR-124a	RNF11	ring finger protein 11 [Source:VGNC Symbol;Acc:VGNC:98601]	-1.71	0.1324
ssc-miR-124a	RNF114	ring finger protein 114 [Source:VGNC Symbol;Acc:VGNC:108733]	-1.71	0.1324
ssc-miR-124a	RNF125	ring finger protein 125 [Source:VGNC Symbol;Acc:VGNC:92352]	-1.71	0.1324
ssc-miR-124a	RNF128	ring finger protein 128 [Source:VGNC Symbol;Acc:VGNC:92354]	-1.71	0.1324
ssc-miR-124a	RNF135	ring finger protein 135 [Source:HGNC Symbol;Acc:HGNC:21158]	-1.71	0.1324
ssc-miR-124a	RNF141	ring finger protein 141 [Source:VGNC Symbol;Acc:VGNC:98292]	-1.71	0.1324
ssc-miR-124a	RNF144A	ring finger protein 144A [Source:VGNC Symbol;Acc:VGNC:92358]	-1.71	0.1324
ssc-miR-124a	RNF144B	ring finger protein 144B [Source:VGNC Symbol;Acc:VGNC:92359]	-1.71	0.1324
ssc-miR-124a	RNF165	ring finger protein 165 [Source:VGNC Symbol;Acc:VGNC:92366]	-1.71	0.1324
ssc-miR-124a	RNF19A	ring finger protein 19A, RBR E3 ubiquitin protein ligase [Source:VGNC Symbol;Acc:VGNC:92375]	-1.71	0.1324
ssc-miR-124a	RNF213	hypothetical gene	-1.71	0.1324
ssc-miR-124a	RNF216	ring finger protein 216 [Source:VGNC Symbol;Acc:VGNC:92383]	-1.71	0.1324
ssc-miR-124a	RNF217	ring finger protein 217 [Source:VGNC Symbol;Acc:VGNC:103162]	-1.71	0.1324
ssc-miR-124a	RNFT1	ring finger protein, transmembrane 1 [Source:VGNC Symbol;Acc:VGNC:104046]	-1.71	0.1324
ssc-miR-124a	RNPEPL1	arginyl aminopeptidase like 1 [Source:VGNC Symbol;Acc:VGNC:95470]	-1.71	0.1324
ssc-miR-124a	ROCK1	Rho associated coiled-coil containing protein kinase 1 [Source:VGNC Symbol;Acc:VGNC:98294]	-1.71	0.1324
ssc-miR-124a	ROCK2	Rho associated coiled-coil containing protein kinase 2 [Source:HGNC Symbol;Acc:HGNC:10252]	-1.71	0.1324
ssc-miR-124a	ROR2	receptor tyrosine kinase like orphan receptor 2 [Source:VGNC Symbol;Acc:VGNC:92407]	-1.71	0.1324
ssc-miR-124a	RP11-10A14.4	hypothetical gene	-1.71	0.1324
ssc-miR-124a	RP11-382J12.1	hypothetical gene	-1.71	0.1324
ssc-miR-124a	RP11-766F14.2	hypothetical gene	-1.71	0.1324
ssc-miR-124a	RPH3AL	rabphilin 3A like (without C2 domains) [Source:HGNC Symbol;Acc:HGNC:10296]	-1.71	0.1324
ssc-miR-124a	RPIA	ribose 5-phosphate isomerase A [Source:NCBI gene (formerly Entrezgene);Acc:100038014]	-1.71	0.1324
ssc-miR-124a	RPS6KA1	ribosomal protein S6 kinase A1 [Source:VGNC Symbol;Acc:VGNC:92440]	-1.71	0.1324
ssc-miR-124a	RPS6KA4	ribosomal protein S6 kinase A4 [Source:VGNC Symbol;Acc:VGNC:92443]	-1.71	0.1324
ssc-miR-124a	RPS6KB1	ribosomal protein S6 kinase B1 [Source:VGNC Symbol;Acc:VGNC:99037]	-1.71	0.1324
ssc-miR-124a	RPS6KL1	ribosomal protein S6 kinase like 1 [Source:VGNC Symbol;Acc:VGNC:92448]	-1.71	0.1324
ssc-miR-124a	RRAGD	Ras related GTP binding D [Source:VGNC Symbol;Acc:VGNC:92457]	-1.71	0.1324
ssc-miR-124a	RRAS	RAS related [Source:VGNC Symbol;Acc:VGNC:92458]	-1.71	0.1324
ssc-miR-124a	RRBP1	ribosome binding protein 1 [Source:VGNC Symbol;Acc:VGNC:95474]	-1.71	0.1324
ssc-miR-124a	RREB1	hypothetical gene	-1.71	0.1324
ssc-miR-124a	RRM2B	ribonucleotide reductase regulatory TP53 inducible subunit M2B [Source:VGNC Symbol;Acc:VGNC:92462]	-1.71	0.1324
ssc-miR-124a	RRP36	ribosomal RNA processing 36 [Source:NCBI gene (formerly Entrezgene);Acc:100141407]	-1.71	0.1324
ssc-miR-124a	RSBN1L	round spermatid basic protein 1 like [Source:VGNC Symbol;Acc:VGNC:92474]	-1.71	0.1324
ssc-miR-124a	RSRC2	arginine and serine rich coiled-coil 2 [Source:VGNC Symbol;Acc:VGNC:92488]	-1.71	0.1324
ssc-miR-124a	RTCB	RNA 2',3'-cyclic phosphate and 5'-OH ligase [Source:VGNC Symbol;Acc:VGNC:92492]	-1.71	0.1324
ssc-miR-124a	RTN3	hypothetical gene	-1.71	0.1324
ssc-miR-124a	RUFY2	RUN and FYVE domain containing 2 [Source:VGNC Symbol;Acc:VGNC:92512]	-1.71	0.1324
ssc-miR-124a	RWDD4	RWD domain containing 4 [Source:VGNC Symbol;Acc:VGNC:96022]	-1.71	0.1324
ssc-miR-124a	RXRA	retinoid X receptor alpha [Source:VGNC Symbol;Acc:VGNC:92528]	-1.71	0.1324
ssc-miR-124a	RYBP	RING1 and YY1 binding protein [Source:VGNC Symbol;Acc:VGNC:92532]	-1.71	0.1324
ssc-miR-124a	RYK	receptor like tyrosine kinase [Source:VGNC Symbol;Acc:VGNC:92533]	-1.71	0.1324
ssc-miR-124a	RYR1	ryanodine receptor 1 [Source:NCBI gene (formerly Entrezgene);Acc:396718]	-1.71	0.1324
ssc-miR-124a	RYR2	ryanodine receptor 2 [Source:VGNC Symbol;Acc:VGNC:92534]	-1.71	0.1324

ssc-miR-124a	RYR3	ryanodine receptor 3 [Source:HGNC Symbol;Acc:HGNC:10485]	-1.71	0.1324
ssc-miR-124a	SALL4	spalt like transcription factor 4 [Source:NCBI gene (formerly Entrezgene);Acc:100136902]	-1.71	0.1324
ssc-miR-124a	SAMD12	sterile alpha motif domain containing 12 [Source:VGNC Symbol;Acc:VGNC:92565]	-1.71	0.1324
ssc-miR-124a	SAMD4A	sterile alpha motif domain containing 4A [Source:VGNC Symbol;Acc:VGNC:92569]	-1.71	0.1324
ssc-miR-124a	SAMD4B	sterile alpha motif domain containing 4B [Source:VGNC Symbol;Acc:VGNC:98610]	-1.71	0.1324
ssc-miR-124a	SAP30L	SAP30 like [Source:VGNC Symbol;Acc:VGNC:92577]	-1.71	0.1324
ssc-miR-124a	SAR1B	secretion associated Ras related GTPase 1B [Source:VGNC Symbol;Acc:VGNC:92580]	-1.71	0.1324
ssc-miR-124a	SART3	spliceosome associated factor 3, U4/U6 recycling protein [Source:VGNC Symbol;Acc:VGNC:96595]	-1.71	0.1324
ssc-miR-124a	SASH1	SAM and SH3 domain containing 1 [Source:VGNC Symbol;Acc:VGNC:92583]	-1.71	0.1324
ssc-miR-124a	SBK1	SH3 domain binding kinase 1 [Source:HGNC Symbol;Acc:HGNC:17699]	-1.71	0.1324
ssc-miR-124a	SBNO2	strawberry notch homolog 2 [Source:VGNC Symbol;Acc:VGNC:92597]	-1.71	0.1324
ssc-miR-124a	SC5D	sterol-C5-desaturase [Source:VGNC Symbol;Acc:VGNC:92600]	-1.71	0.1324
ssc-miR-124a	SCAMP2	secretory carrier membrane protein 2 [Source:VGNC Symbol;Acc:VGNC:92606]	-1.71	0.1324
ssc-miR-124a	SCD	stearoyl-CoA desaturase [Source:NCBI gene (formerly Entrezgene);Acc:396670]	-1.71	0.1324
ssc-miR-124a	SCML4	Scm polycomb group protein like 4 [Source:VGNC Symbol;Acc:VGNC:92629]	-1.71	0.1324
ssc-miR-124a	SCN2B	sodium voltage-gated channel beta subunit 2 [Source:VGNC Symbol;Acc:VGNC:92633]	-1.71	0.1324
ssc-miR-124a	SCN4B	hypothetical gene	-1.71	0.1324
ssc-miR-124a	SDAD1	SDA1 domain containing 1 [Source:VGNC Symbol;Acc:VGNC:98961]	-1.71	0.1324
ssc-miR-124a	SDC2	syndecan 2 [Source:VGNC Symbol;Acc:VGNC:92655]	-1.71	0.1324
ssc-miR-124a	SDC4	hypothetical gene	-1.71	0.1324
ssc-miR-124a	SDCBP	syndecan binding protein [Source:VGNC Symbol;Acc:VGNC:92657]	-1.71	0.1324
ssc-miR-124a	SDF2L1	stromal cell derived factor 2 like 1 [Source:HGNC Symbol;Acc:HGNC:10676]	-1.71	0.1324
ssc-miR-124a	SDK1	sidekick cell adhesion molecule 1 [Source:VGNC Symbol;Acc:VGNC:92660]	-1.71	0.1324
ssc-miR-124a	SEC13	SEC13 homolog, nuclear pore and COPII coat complex component [Source:VGNC Symbol;Acc:VGNC:92668]	-1.71	0.1324
ssc-miR-124a	SEC22C	SEC22 homolog C, vesicle trafficking protein [Source:VGNC Symbol;Acc:VGNC:92674]	-1.71	0.1324
ssc-miR-124a	SEC61A2	SEC61 translocon subunit alpha 2 [Source:VGNC Symbol;Acc:VGNC:95457]	-1.71	0.1324
ssc-miR-124a	SEC62	SEC62 homolog, preprotein translocation factor [Source:VGNC Symbol;Acc:VGNC:108691]	-1.71	0.1324
ssc-miR-124a	SEC63	SEC63 homolog, protein translocation regulator [Source:VGNC Symbol;Acc:VGNC:103172]	-1.71	0.1324
ssc-miR-124a	SEMA4F	ssemaphorin 4F [Source:VGNC Symbol;Acc:VGNC:92704]	-1.71	0.1324
ssc-miR-124a	SEMA5A	semaphorin 5A [Source:VGNC Symbol;Acc:VGNC:92706]	-1.71	0.1324
ssc-miR-124a	SEMA6A	semaphorin 6A [Source:VGNC Symbol;Acc:VGNC:92708]	-1.71	0.1324
ssc-miR-124a	SEMA6C	semaphorin 6C [Source:VGNC Symbol;Acc:VGNC:92710]	-1.71	0.1324
ssc-miR-124a	SEMA6D	semaphorin 6D [Source:VGNC Symbol;Acc:VGNC:92711]	-1.71	0.1324
ssc-miR-124a	SEPT10	hypothetical gene	-1.71	0.1324
ssc-miR-124a	SEPT11	hypothetical gene	-1.71	0.1324
ssc-miR-124a	SEPT9	hypothetical gene	-1.71	0.1324
ssc-miR-124a	SERINC2	serine incorporator 2 [Source:VGNC Symbol;Acc:VGNC:92729]	-1.71	0.1324
ssc-miR-124a	SERP1	stress associated endoplasmic reticulum protein 1 [Source:NCBI gene (formerly Entrezgene);Acc:100156392]	-1.71	0.1324
ssc-miR-124a	SERTAD2	SERTA domain containing 2 [Source:VGNC Symbol;Acc:VGNC:92750]	-1.71	0.1324
ssc-miR-124a	SERTAD3	SERTA domain containing 3 [Source:VGNC Symbol;Acc:VGNC:92751]	-1.71	0.1324
ssc-miR-124a	SERTAD4	SERTA domain containing 4 [Source:VGNC Symbol;Acc:VGNC:92752]	-1.71	0.1324
ssc-miR-124a	SESN3	sestrin 3 [Source:VGNC Symbol;Acc:VGNC:92755]	-1.71	0.1324
ssc-miR-124a	SESTD1	SEC14 and spectrin domain containing 1 [Source:VGNC Symbol;Acc:VGNC:95884]	-1.71	0.1324
ssc-miR-124a	SETBP1	SET binding protein 1 [Source:VGNC Symbol;Acc:VGNC:92756]	-1.71	0.1324
ssc-miR-124a	SFMBT2	Scm like with four mbt domains 2 [Source:VGNC Symbol;Acc:VGNC:95935]	-1.71	0.1324
ssc-miR-124a	SFT2D2	SFT2 domain containing 2 [Source:VGNC Symbol;Acc:VGNC:98856]	-1.71	0.1324
ssc-miR-124a	SFXN2	sideroflexin 2 [Source:VGNC Symbol;Acc:VGNC:92784]	-1.71	0.1324
ssc-miR-124a	SGCD	sarcoglycan delta [Source:NCBI gene (formerly Entrezgene);Acc:100240724]	-1.71	0.1324
ssc-miR-124a	SGCZ	sarcoglycan zeta [Source:VGNC Symbol;Acc:VGNC:104055]	-1.71	0.1324
ssc-miR-124a	SGIP1	SH3GL interacting endocytic adaptor 1 [Source:VGNC Symbol;Acc:VGNC:92792]	-1.71	0.1324

ssc-miR-124a	SGK1	serum/glucocorticoid regulated kinase 1 [Source:VGNC Symbol;Acc:VGNC:92793]	-1.71	0.1324
ssc-miR-124a	SGMS1	sphingomyelin synthase 1 [Source:VGNC Symbol;Acc:VGNC:92794]	-1.71	0.1324
ssc-miR-124a	SGMS2	sphingomyelin synthase 2 [Source:VGNC Symbol;Acc:VGNC:92795]	-1.71	0.1324
ssc-miR-124a	SGPL1	sphingosine-1-phosphate lyase 1 [Source:VGNC Symbol;Acc:VGNC:92797]	-1.71	0.1324
ssc-miR-124a	SGPP1	sphingosine-1-phosphate phosphatase 1 [Source:VGNC Symbol;Acc:VGNC:92798]	-1.71	0.1324
ssc-miR-124a	SGSM2	small G protein signaling modulator 2 [Source:VGNC Symbol;Acc:VGNC:92801]	-1.71	0.1324
ssc-miR-124a	SH2B3	SH2B adaptor protein 3 [Source:VGNC Symbol;Acc:VGNC:92807]	-1.71	0.1324
ssc-miR-124a	SH2D5	SH2 domain containing 5 [Source:VGNC Symbol;Acc:VGNC:92812]	-1.71	0.1324
ssc-miR-124a	SH3BP5L	SH3 binding domain protein 5 like [Source:VGNC Symbol;Acc:VGNC:92820]	-1.71	0.1324
ssc-miR-124a	SH3GL3	SH3 domain containing GRB2 like 3, endophilin A3 [Source:VGNC Symbol;Acc:VGNC:92825]	-1.71	0.1324
ssc-miR-124a	SH3KBP1	SH3 domain containing kinase binding protein 1 [Source:VGNC Symbol;Acc:VGNC:92828]	-1.71	0.1324
ssc-miR-124a	SH3PXD2A	SH3 and PX domains 2A [Source:VGNC Symbol;Acc:VGNC:92829]	-1.71	0.1324
ssc-miR-124a	SH3PXD2B	SH3 and PX domains 2B [Source:VGNC Symbol;Acc:VGNC:92830]	-1.71	0.1324
ssc-miR-124a	SHANK2	SH3 and multiple ankyrin repeat domains 2 [Source:VGNC Symbol;Acc:VGNC:92835]	-1.71	0.1324
ssc-miR-124a	SHC1	SHC adaptor protein 1 [Source:VGNC Symbol;Acc:VGNC:92837]	-1.71	0.1324
ssc-miR-124a	SHC3	SHC adaptor protein 3 [Source:VGNC Symbol;Acc:VGNC:92838]	-1.71	0.1324
ssc-miR-124a	SHC4	SHC adaptor protein 4 [Source:HGNC Symbol;Acc:HGNC:16743]	-1.71	0.1324
ssc-miR-124a	SHH	sonic hedgehog signaling molecule [Source:VGNC Symbol;Acc:VGNC:92844]	-1.71	0.1324
ssc-miR-124a	SHISA9	shisa family member 9 [Source:HGNC Symbol;Acc:HGNC:37231]	-1.71	0.1324
ssc-miR-124a	SHKBP1	SH3KBP1 binding protein 1 [Source:VGNC Symbol;Acc:VGNC:92853]	-1.71	0.1324
ssc-miR-124a	SHPK	sedoheptulokinase [Source:HGNC Symbol;Acc:HGNC:1492]	-1.71	0.1324
ssc-miR-124a	SHROOM3	hypothetical gene	-1.71	0.1324
ssc-miR-124a	SHROOM4	shroom family member 4 [Source:VGNC Symbol;Acc:VGNC:92861]	-1.71	0.1324
ssc-miR-124a	SIGMAR1	sigma non-opioid intracellular receptor 1 [Source:VGNC Symbol;Acc:VGNC:96462]	-1.71	0.1324
ssc-miR-124a	SIK1	salt inducible kinase 1 [Source:HGNC Symbol;Acc:HGNC:11142]	-1.71	0.1324
ssc-miR-124a	SIK2	salt inducible kinase 2 [Source:VGNC Symbol;Acc:VGNC:92872]	-1.71	0.1324
ssc-miR-124a	SIRT1	sirtuin 1 [Source:VGNC Symbol;Acc:VGNC:92884]	-1.71	0.1324
ssc-miR-124a	SIX4	SIX homeobox 4 [Source:VGNC Symbol;Acc:VGNC:92895]	-1.71	0.1324
ssc-miR-124a	SKI	SKI proto-onco [Source:VGNC Symbol;Acc:VGNC:92902]	-1.71	0.1324
ssc-miR-124a	SLAIN2	SLAIN motif family member 2 [Source:VGNC Symbol;Acc:VGNC:92910]	-1.71	0.1324
ssc-miR-124a	SLBP	stem-loop binding protein [Source:NCBI gene (formerly Entrezgene);Acc:100513605]	-1.71	0.1324
ssc-miR-124a	SLC10A7	solute carrier family 10 member 7 [Source:VGNC Symbol;Acc:VGNC:92919]	-1.71	0.1324
ssc-miR-124a	SLC12A2	solute carrier family 12 member 2 [Source:VGNC Symbol;Acc:VGNC:92922]	-1.71	0.1324
ssc-miR-124a	SLC12A9	solute carrier family 12 member 9 [Source:VGNC Symbol;Acc:VGNC:92928]	-1.71	0.1324
ssc-miR-124a	SLC15A4	solute carrier family 15 member 4 [Source:VGNC Symbol;Acc:VGNC:92937]	-1.71	0.1324
ssc-miR-124a	SLC16A1	hypothetical gene	-1.71	0.1324
ssc-miR-124a	SLC16A10	solute carrier family 16 member 10 [Source:VGNC Symbol;Acc:VGNC:103178]	-1.71	0.1324
ssc-miR-124a	SLC16A13	solute carrier family 16 member 13 [Source:VGNC Symbol;Acc:VGNC:92941]	-1.71	0.1324
ssc-miR-124a	SLC16A14	solute carrier family 16 member 14 [Source:VGNC Symbol;Acc:VGNC:96129]	-1.71	0.1324
ssc-miR-124a	SLC17A5	solute carrier family 17 member 5 [Source:VGNC Symbol;Acc:VGNC:92953]	-1.71	0.1324
ssc-miR-124a	SLC18A2	solute carrier family 18 member A2 [Source:VGNC Symbol;Acc:VGNC:92958]	-1.71	0.1324
ssc-miR-124a	SLC19A2	solute carrier family 19 member 2 [Source:VGNC Symbol;Acc:VGNC:92962]	-1.71	0.1324
ssc-miR-124a	SLC1A4	solute carrier family 1 member 4 [Source:VGNC Symbol;Acc:VGNC:92966]	-1.71	0.1324
ssc-miR-124a	SLC22A5	solute carrier family 22 member 5 [Source:HGNC Symbol;Acc:HGNC:10969]	-1.71	0.1324
ssc-miR-124a	SLC25A1	solute carrier family 25 member 1 [Source:VGNC Symbol;Acc:VGNC:92990]	-1.71	0.1324
ssc-miR-124a	SLC25A20	solute carrier family 25 member 20 [Source:VGNC Symbol;Acc:VGNC:92998]	-1.71	0.1324
ssc-miR-124a	SLC25A25	solute carrier family 25 member 25 [Source:VGNC Symbol;Acc:VGNC:93002]	-1.71	0.1324
ssc-miR-124a	SLC25A37	hypothetical gene	-1.71	0.1324
ssc-miR-124a	SLC25A39	solute carrier family 25 member 39 [Source:VGNC Symbol;Acc:VGNC:93014]	-1.71	0.1324
ssc-miR-124a	SLC26A2	solute carrier family 26 member 2 [Source:VGNC Symbol;Acc:VGNC:93027]	-1.71	0.1324

ssc-miR-124a	SLC27A1	solute carrier family 27 member 1 [Source:VGNC Symbol;Acc:VGNC:93035]	-1.71	0.1324
ssc-miR-124a	SLC29A1	solute carrier family 29 member 1 (Augustine blood group) [Source:HGNC Symbol;Acc:HGNC:11003]	-1.71	0.1324
ssc-miR-124a	SLC2A12	solute carrier family 2 member 12 [Source:VGNC Symbol;Acc:VGNC:93046]	-1.71	0.1324
ssc-miR-124a	SLC2A13	solute carrier family 2 member 13 [Source:VGNC Symbol;Acc:VGNC:93047]	-1.71	0.1324
ssc-miR-124a	SLC30A2	solute carrier family 30 member 2 [Source:VGNC Symbol;Acc:VGNC:93056]	-1.71	0.1324
ssc-miR-124a	SLC30A4	solute carrier family 30 member 4 [Source:VGNC Symbol;Acc:VGNC:93058]	-1.71	0.1324
ssc-miR-124a	SLC30A7	solute carrier family 30 member 7 [Source:VGNC Symbol;Acc:VGNC:93061]	-1.71	0.1324
ssc-miR-124a	SLC31A1	solute carrier family 31 member 1 [Source:VGNC Symbol;Acc:VGNC:103180]	-1.71	0.1324
ssc-miR-124a	SLC31A2	solute carrier family 31 member 2 [Source:VGNC Symbol;Acc:VGNC:93063]	-1.71	0.1324
ssc-miR-124a	SLC33A1	solute carrier family 33 member 1 [Source:VGNC Symbol;Acc:VGNC:103074]	-1.71	0.1324
ssc-miR-124a	SLC34A2	solute carrier family 34 member 2 [Source:VGNC Symbol;Acc:VGNC:93065]	-1.71	0.1324
ssc-miR-124a	SLC35A1	solute carrier family 35 member A1 [Source:VGNC Symbol;Acc:VGNC:93067]	-1.71	0.1324
ssc-miR-124a	SLC35A3	solute carrier family 35 member A3 [Source:VGNC Symbol;Acc:VGNC:98860]	-1.71	0.1324
ssc-miR-124a	SLC35A4	solute carrier family 35 member A4 [Source:VGNC Symbol;Acc:VGNC:93069]	-1.71	0.1324
ssc-miR-124a	SLC35B3	solute carrier family 35 member B3 [Source:VGNC Symbol;Acc:VGNC:93073]	-1.71	0.1324
ssc-miR-124a	SLC35F3	solute carrier family 35 member F3 [Source:VGNC Symbol;Acc:VGNC:93083]	-1.71	0.1324
ssc-miR-124a	SLC35F5	solute carrier family 35 member F5 [Source:VGNC Symbol;Acc:VGNC:96015]	-1.71	0.1324
ssc-miR-124a	SLC35G1	solute carrier family 35 member G1 [Source:VGNC Symbol;Acc:VGNC:93086]	-1.71	0.1324
ssc-miR-124a	SLC36A1	solute carrier family 36 member 1 [Source:VGNC Symbol;Acc:VGNC:93088]	-1.71	0.1324
ssc-miR-124a	SLC39A9	solute carrier family 39 member 9 [Source:VGNC Symbol;Acc:VGNC:93110]	-1.71	0.1324
ssc-miR-124a	SLC40A1	solute carrier family 40 member 1 [Source:VGNC Symbol;Acc:VGNC:95497]	-1.71	0.1324
ssc-miR-124a	SLC41A2	solute carrier family 41 member 2 [Source:VGNC Symbol;Acc:VGNC:93114]	-1.71	0.1324
ssc-miR-124a	SLC43A2	solute carrier family 43 member 2 [Source:VGNC Symbol;Acc:VGNC:93117]	-1.71	0.1324
ssc-miR-124a	SLC44A1	solute carrier family 44 member 1 [Source:VGNC Symbol;Acc:VGNC:93118]	-1.71	0.1324
ssc-miR-124a	SLC44A5	solute carrier family 44 member 5 [Source:VGNC Symbol;Acc:VGNC:93121]	-1.71	0.1324
ssc-miR-124a	SLC48A1	solute carrier family 48 member 1 [Source:VGNC Symbol;Acc:VGNC:93129]	-1.71	0.1324
ssc-miR-124a	SLC4A7	solute carrier family 4 member 7 [Source:VGNC Symbol;Acc:VGNC:93135]	-1.71	0.1324
ssc-miR-124a	SLC4A8	solute carrier family 4 member 8 [Source:VGNC Symbol;Acc:VGNC:98326]	-1.71	0.1324
ssc-miR-124a	SLC50A1	solute carrier family 50 member 1 [Source:VGNC Symbol;Acc:VGNC:93137]	-1.71	0.1324
ssc-miR-124a	SLC5A3	solute carrier family 5 member 3 [Source:VGNC Symbol;Acc:VGNC:93144]	-1.71	0.1324
ssc-miR-124a	SLC6A2	solute carrier family 6 member 2 [Source:VGNC Symbol;Acc:VGNC:93161]	-1.71	0.1324
ssc-miR-124a	SLC6A5	solute carrier family 6 member 5 [Source:VGNC Symbol;Acc:VGNC:93165]	-1.71	0.1324
ssc-miR-124a	SLC7A1	solute carrier family 7 member 1 [Source:VGNC Symbol;Acc:VGNC:93169]	-1.71	0.1324
ssc-miR-124a	SLC7A14	solute carrier family 7 member 14 [Source:VGNC Symbol;Acc:VGNC:93173]	-1.71	0.1324
ssc-miR-124a	SLC7A8	solute carrier family 7 member 8 [Source:VGNC Symbol;Acc:VGNC:93176]	-1.71	0.1324
ssc-miR-124a	SLC8A1	solute carrier family 8 member A1 [Source:VGNC Symbol;Acc:VGNC:93178]	-1.71	0.1324
ssc-miR-124a	SLC9A2	solute carrier family 9 member A2 [Source:VGNC Symbol;Acc:VGNC:93182]	-1.71	0.1324
ssc-miR-124a	SLC9A6	solute carrier family 9 member A6 [Source:VGNC Symbol;Acc:VGNC:93188]	-1.71	0.1324
ssc-miR-124a	SLC9A9	solute carrier family 9 member A9 [Source:VGNC Symbol;Acc:VGNC:93190]	-1.71	0.1324
ssc-miR-124a	SLCO5A1	solute carrier organic anion transporter family member 5A1 [Source:HGNC Symbol;Acc:HGNC:19046]	-1.71	0.1324
ssc-miR-124a	SLIT3	slit guidance ligand 3 [Source:VGNC Symbol;Acc:VGNC:93205]	-1.71	0.1324
ssc-miR-124a	SLITRK2	SLIT and NTRK like family member 2 [Source:VGNC Symbol;Acc:VGNC:93207]	-1.71	0.1324
ssc-miR-124a	SLITRK3	SLIT and NTRK like family member 3 [Source:VGNC Symbol;Acc:VGNC:93208]	-1.71	0.1324
ssc-miR-124a	SLITRK4	SLIT and NTRK like family member 4 [Source:VGNC Symbol;Acc:VGNC:93209]	-1.71	0.1324
ssc-miR-124a	SLITRK5	SLIT and NTRK like family member 5 [Source:VGNC Symbol;Acc:VGNC:93210]	-1.71	0.1324
ssc-miR-124a	SLITRK6	SLIT and NTRK like family member 6 [Source:VGNC Symbol;Acc:VGNC:93211]	-1.71	0.1324
ssc-miR-124a	SLK	STE20 like kinase [Source:VGNC Symbol;Acc:VGNC:98328]	-1.71	0.1324
ssc-miR-124a	SLMAP	sarcolemma associated protein [Source:VGNC Symbol;Acc:VGNC:93212]	-1.71	0.1324
ssc-miR-124a	SMAD2	SMAD family member 2 [Source:VGNC Symbol;Acc:VGNC:98329]	-1.71	0.1324
ssc-miR-124a	SMARCAD1	SWI/SNF-related, matrix-associated actin-dependent regulator of chromatin, subfamily a, containing DEAD/H box 1 [Source:VGNC Symbol;Acc:VGNC:93229]	-1.71	0.1324

ssc-miR-124a	SMARCC1	SWI/SNF related, matrix associated, actin dependent regulator of chromatin subfamily c member 1 [Source:VGNC Symbol;Acc:VGNC:93231]	-1.71	0.1324
ssc-miR-124a	SMCHD1	structural maintenance of chromosomes flexible hinge domain containing 1 [Source:VGNC Symbol;Acc:VGNC:93242]	-1.71	0.1324
ssc-miR-124a	SMCO4	single-pass membrane protein with coiled-coil domains 4 [Source:HGNC Symbol;Acc:HGNC:24810]	-1.71	0.1324
ssc-miR-124a	SMCR8	SMCR8-C9orf72 complex subunit [Source:VGNC Symbol;Acc:VGNC:96598]	-1.71	0.1324
ssc-miR-124a	SMOX	spermine oxidase [Source:VGNC Symbol;Acc:VGNC:95679]	-1.71	0.1324
ssc-miR-124a	SMPD3	sphingomyelin phosphodiesterase 3 [Source:VGNC Symbol;Acc:VGNC:93263]	-1.71	0.1324
ssc-miR-124a	SMURF2	SMAD specific E3 ubiquitin protein ligase 2 [Source:VGNC Symbol;Acc:VGNC:93271]	-1.71	0.1324
ssc-miR-124a	SMYD5	SMYD family member 5 [Source:VGNC Symbol;Acc:VGNC:93275]	-1.71	0.1324
ssc-miR-124a	SNAI2	snail family transcriptional repressor 2 [Source:VGNC Symbol;Acc:VGNC:98330]	-1.71	0.1324
ssc-miR-124a	SNAP29	synaptosome associated protein 29 [Source:VGNC Symbol;Acc:VGNC:93278]	-1.71	0.1324
ssc-miR-124a	SNCAIP	hypothetical gene	-1.71	0.1324
ssc-miR-124a	SNF8	SNF8 subunit of ESCRT-II [Source:VGNC Symbol;Acc:VGNC:93288]	-1.71	0.1324
ssc-miR-124a	SNIP1	Smad nuclear interacting protein 1 [Source:VGNC Symbol;Acc:VGNC:93289]	-1.71	0.1324
ssc-miR-124a	SNRPB2	hypothetical gene	-1.71	0.1324
ssc-miR-124a	SNTA1	syntrophin alpha 1 [Source:HGNC Symbol;Acc:HGNC:11167]	-1.71	0.1324
ssc-miR-124a	SNTB2	syntrophin beta 2 [Source:VGNC Symbol;Acc:VGNC:93298]	-1.71	0.1324
ssc-miR-124a	SNTG1	syntrophin gamma 1 [Source:VGNC Symbol;Acc:VGNC:93299]	-1.71	0.1324
ssc-miR-124a	SNX16	sorting nexin 16 [Source:VGNC Symbol;Acc:VGNC:93308]	-1.71	0.1324
ssc-miR-124a	SNX18	sorting nexin 18 [Source:VGNC Symbol;Acc:VGNC:93310]	-1.71	0.1324
ssc-miR-124a	SNX2	sorting nexin 2 [Source:VGNC Symbol;Acc:VGNC:93312]	-1.71	0.1324
ssc-miR-124a	SNX30	sorting nexin family member 30 [Source:VGNC Symbol;Acc:VGNC:93318]	-1.71	0.1324
ssc-miR-124a	SNX6	sorting nexin 6 [Source:VGNC Symbol;Acc:VGNC:93323]	-1.71	0.1324
ssc-miR-124a	SNX9	sorting nexin 9 [Source:VGNC Symbol;Acc:VGNC:93326]	-1.71	0.1324
ssc-miR-124a	SOCS3	suppressor of cytokine signaling 3 [Source:VGNC Symbol;Acc:VGNC:99052]	-1.71	0.1324
ssc-miR-124a	SOCS5	suppressor of cytokine signaling 5 [Source:VGNC Symbol;Acc:VGNC:93332]	-1.71	0.1324
ssc-miR-124a	SOGA3	SOGA family member 3 [Source:HGNC Symbol;Acc:HGNC:21494]	-1.71	0.1324
ssc-miR-124a	SORCS1	sortilin related VPS10 domain containing receptor 1 [Source:VGNC Symbol;Acc:VGNC:93339]	-1.71	0.1324
ssc-miR-124a	SORD	sorbitol dehydrogenase [Source:VGNC Symbol;Acc:VGNC:93342]	-1.71	0.1324
ssc-miR-124a	SORL1	sortilin related receptor 1 [Source:VGNC Symbol;Acc:VGNC:108617]	-1.71	0.1324
ssc-miR-124a	SOS1	SOS Ras/Rac guanine nucleotide exchange factor 1 [Source:HGNC Symbol;Acc:HGNC:11187]	-1.71	0.1324
ssc-miR-124a	SOS2	SOS Ras/Rho guanine nucleotide exchange factor 2 [Source:VGNC Symbol;Acc:VGNC:93344]	-1.71	0.1324
ssc-miR-124a	SOX12	SRY-box transcription factor 12 [Source:HGNC Symbol;Acc:HGNC:11198]	-1.71	0.1324
ssc-miR-124a	SOX8	SRY-box transcription factor 8 [Source:VGNC Symbol;Acc:VGNC:93359]	-1.71	0.1324
ssc-miR-124a	SP1	Sp1 transcription factor [Source:VGNC Symbol;Acc:VGNC:93360]	-1.71	0.1324
ssc-miR-124a	SP2	Sp2 transcription factor [Source:VGNC Symbol;Acc:VGNC:93361]	-1.71	0.1324
ssc-miR-124a	SP3	Sp3 transcription factor [Source:VGNC Symbol;Acc:VGNC:95511]	-1.71	0.1324
ssc-miR-124a	SP7	Sp7 transcription factor [Source:HGNC Symbol;Acc:HGNC:17321]	-1.71	0.1324
ssc-miR-124a	SP9	Sp9 transcription factor [Source:VGNC Symbol;Acc:VGNC:96247]	-1.71	0.1324
ssc-miR-124a	SPAG9	sperm associated antigen 9 [Source:VGNC Symbol;Acc:VGNC:93374]	-1.71	0.1324
ssc-miR-124a	SPATA13	hypothetical gene	-1.71	0.1324
ssc-miR-124a	SPECC1L	sperm antigen with calponin homology and coiled-coil domains 1 like [Source:HGNC Symbol;Acc:HGNC:29022]	-1.71	0.1324
ssc-miR-124a	SPOCK1	SPARC (osteonectin), cwcv and kazal like domains proteoglycan 1 [Source:VGNC Symbol;Acc:VGNC:96619]	-1.71	0.1324
ssc-miR-124a	SPOCK2	SPARC (osteonectin), cwcv and kazal like domains proteoglycan 2 [Source:VGNC Symbol;Acc:VGNC:107362]	-1.71	0.1324
ssc-miR-124a	SPOPL	speckle type BTB/POZ protein like [Source:VGNC Symbol;Acc:VGNC:96161]	-1.71	0.1324
ssc-miR-124a	SPPL2A	signal peptide peptidase like 2A [Source:VGNC Symbol;Acc:VGNC:98337]	-1.71	0.1324
ssc-miR-124a	SPRED1	sprouty related EVH1 domain containing 1 [Source:VGNC Symbol;Acc:VGNC:93420]	-1.71	0.1324
ssc-miR-124a	SPRED2	sprouty related EVH1 domain containing 2 [Source:VGNC Symbol;Acc:VGNC:93421]	-1.71	0.1324
ssc-miR-124a	SPRTN	SprT-like N-terminal domain [Source:VGNC Symbol;Acc:VGNC:93423]	-1.71	0.1324
ssc-miR-124a	SPRY1	sprouty RTK signaling antagonist 1 [Source:VGNC Symbol;Acc:VGNC:93424]	-1.71	0.1324
ssc-miR-124a	SPRY2	sprouty RTK signaling antagonist 2 [Source:VGNC Symbol;Acc:VGNC:93425]	-1.71	0.1324

ssc-miR-124a	SPRY3	sprouty RTK signaling antagonist 3 [Source:VGNC Symbol;Acc:VGNC:93426]	-1.71	0.1324
ssc-miR-124a	SPTLC1	serine palmitoyltransferase long chain base subunit 1 [Source:VGNC Symbol;Acc:VGNC:93439]	-1.71	0.1324
ssc-miR-124a	SPTLC2	serine palmitoyltransferase long chain base subunit 2 [Source:VGNC Symbol;Acc:VGNC:93440]	-1.71	0.1324
ssc-miR-124a	SPTY2D1	SPT2 chromatin protein domain containing 1 [Source:VGNC Symbol;Acc:VGNC:100867]	-1.71	0.1324
ssc-miR-124a	SREK1	splicing regulatory glutamic acid and lysine rich protein 1 [Source:VGNC Symbol;Acc:VGNC:93451]	-1.71	0.1324
ssc-miR-124a	SREK1IP1	SREK1 interacting protein 1 [Source:HGNC Symbol;Acc:HGNC:26716]	-1.71	0.1324
ssc-miR-124a	SRGAP1	SLIT-ROBO Rho GTPase activating protein 1 [Source:VGNC Symbol;Acc:VGNC:93454]	-1.71	0.1324
ssc-miR-124a	SRGAP2	hypothetical gene	-1.71	0.1324
ssc-miR-124a	SRGAP3	SLIT-ROBO Rho GTPase activating protein 3 [Source:VGNC Symbol;Acc:VGNC:93455]	-1.71	0.1324
ssc-miR-124a	SRI	sorcin [Source:VGNC Symbol;Acc:VGNC:93457]	-1.71	0.1324
ssc-miR-124a	SRPK3	hypothetical gene	-1.71	0.1324
ssc-miR-124a	SRSF6	serine and arginine rich splicing factor 6 [Source:NCBI gene (formerly Entrezgene);Acc:768102]	-1.71	0.1324
ssc-miR-124a	SRSF7	serine and arginine rich splicing factor 7 [Source:VGNC Symbol;Acc:VGNC:93477]	-1.71	0.1324
ssc-miR-124a	SRSF9	serine and arginine rich splicing factor 9 [Source:VGNC Symbol;Acc:VGNC:104063]	-1.71	0.1324
ssc-miR-124a	SSFA2	hypothetical gene	-1.71	0.1324
ssc-miR-124a	SSH1	slingshot protein phosphatase 1 [Source:VGNC Symbol;Acc:VGNC:93485]	-1.71	0.1324
ssc-miR-124a	SSH3	slingshot protein phosphatase 3 [Source:VGNC Symbol;Acc:VGNC:93487]	-1.71	0.1324
ssc-miR-124a	SSR1	signal sequence receptor subunit 1 [Source:VGNC Symbol;Acc:VGNC:93490]	-1.71	0.1324
ssc-miR-124a	ST3GAL1	ST3 beta-galactoside alpha-2,3-sialyltransferase 1 [Source:VGNC Symbol;Acc:VGNC:98863]	-1.71	0.1324
ssc-miR-124a	ST5	hypothetical gene	-1.71	0.1324
ssc-miR-124a	ST8SIA1	ST8 alpha-N-acetyl-neuraminide alpha-2,8-sialyltransferase 1 [Source:VGNC Symbol;Acc:VGNC:93516]	-1.71	0.1324
ssc-miR-124a	ST8SIA2	ST8 alpha-N-acetyl-neuraminide alpha-2,8-sialyltransferase 2 [Source:VGNC Symbol;Acc:VGNC:93517]	-1.71	0.1324
ssc-miR-124a	ST8SIA4	ST8 alpha-N-acetyl-neuraminide alpha-2,8-sialyltransferase 4 [Source:VGNC Symbol;Acc:VGNC:93519]	-1.71	0.1324
ssc-miR-124a	STAC	SH3 and cysteine rich domain [Source:HGNC Symbol;Acc:HGNC:11353]	-1.71	0.1324
ssc-miR-124a	STAG2	stromal antigen 2 [Source:VGNC Symbol;Acc:VGNC:93525]	-1.71	0.1324
ssc-miR-124a	STARD4	StAR related lipid transfer domain containing 4 [Source:VGNC Symbol;Acc:VGNC:93534]	-1.71	0.1324
ssc-miR-124a	STARD7	StAR related lipid transfer domain containing 7 [Source:VGNC Symbol;Acc:VGNC:93537]	-1.71	0.1324
ssc-miR-124a	STAT3	signal transducer and activator of transcription 3 [Source:VGNC Symbol;Acc:VGNC:93540]	-1.71	0.1324
ssc-miR-124a	STEAP2	STEAP2 metalloreductase [Source:HGNC Symbol;Acc:HGNC:17885]	-1.71	0.1324
ssc-miR-124a	STEAP3	STEAP3 metalloreductase [Source:VGNC Symbol;Acc:VGNC:96048]	-1.71	0.1324
ssc-miR-124a	STK35	serine/threonine kinase 35 [Source:VGNC Symbol;Acc:VGNC:95802]	-1.71	0.1324
ssc-miR-124a	STK36	serine/threonine kinase 36 [Source:VGNC Symbol;Acc:VGNC:95835]	-1.71	0.1324
ssc-miR-124a	STK38	serine/threonine kinase 38 [Source:VGNC Symbol;Acc:VGNC:93556]	-1.71	0.1324
ssc-miR-124a	STK39	serine/threonine kinase 39 [Source:VGNC Symbol;Acc:VGNC:98348]	-1.71	0.1324
ssc-miR-124a	STK4	serine/threonine kinase 4 [Source:VGNC Symbol;Acc:VGNC:98349]	-1.71	0.1324
ssc-miR-124a	STOM	stomatin [Source:VGNC Symbol;Acc:VGNC:93565]	-1.71	0.1324
ssc-miR-124a	STON2	stonin 2 [Source:VGNC Symbol;Acc:VGNC:93568]	-1.71	0.1324
ssc-miR-124a	STOX2	storkhead box 2 [Source:VGNC Symbol;Acc:VGNC:96082]	-1.71	0.1324
ssc-miR-124a	STRN	striatin [Source:VGNC Symbol;Acc:VGNC:93578]	-1.71	0.1324
ssc-miR-124a	STRN3	striatin 3 [Source:VGNC Symbol;Acc:VGNC:93579]	-1.71	0.1324
ssc-miR-124a	STT3A	STT3 oligosaccharyltransferase complex catalytic subunit A [Source:VGNC Symbol;Acc:VGNC:93581]	-1.71	0.1324
ssc-miR-124a	STX10	syntaxin 10 [Source:VGNC Symbol;Acc:VGNC:93584]	-1.71	0.1324
ssc-miR-124a	STX17	syntaxin 17 [Source:VGNC Symbol;Acc:VGNC:93587]	-1.71	0.1324
ssc-miR-124a	STX7	syntaxin 7 [Source:VGNC Symbol;Acc:VGNC:93595]	-1.71	0.1324
ssc-miR-124a	STYX	serine/threonine/tyrosine interacting protein [Source:VGNC Symbol;Acc:VGNC:93602]	-1.71	0.1324
ssc-miR-124a	SUB1	SUB1 regulator of transcription [Source:HGNC Symbol;Acc:HGNC:19985]	-1.71	0.1324
ssc-miR-124a	SUCLG2	succinate-CoA ligase GDP-forming subunit beta [Source:VGNC Symbol;Acc:VGNC:93604]	-1.71	0.1324
ssc-miR-124a	SUCO	SUN domain containing ossification factor [Source:VGNC Symbol;Acc:VGNC:93606]	-1.71	0.1324
ssc-miR-124a	SUGT1	SGT1 homolog, MIS12 kinetochore complex assembly cochaperone [Source:VGNC Symbol;Acc:VGNC:93611]	-1.71	0.1324
ssc-miR-124a	SUMF1	sulfatase modifying factor 1 [Source:VGNC Symbol;Acc:VGNC:93617]	-1.71	0.1324

ssc-miR-124a	SUPT7L	SPT7 like, STAGA complex subunit gamma [Source:VGNC Symbol;Acc:VGNC:93627]	-1.71	0.1324
ssc-miR-124a	SURF4	surfeit 4 [Source:VGNC Symbol;Acc:VGNC:93631]	-1.71	0.1324
ssc-miR-124a	SVIL	supervillin [Source:VGNC Symbol;Acc:VGNC:95528]	-1.71	0.1324
ssc-miR-124a	SYMPK	symplekin scaffold protein [Source:VGNC Symbol;Acc:VGNC:93658]	-1.71	0.1324
ssc-miR-124a	SYNC	syncoilin, intermediate filament protein [Source:VGNC Symbol;Acc:VGNC:93660]	-1.71	0.1324
ssc-miR-124a	SYNCRIP	synaptotagmin binding cytoplasmic RNA interacting protein [Source:VGNC Symbol;Acc:VGNC:103185]	-1.71	0.1324
ssc-miR-124a	SYNGR2	synaptogyrin 2 [Source:HGNC Symbol;Acc:HGNC:11499]	-1.71	0.1324
ssc-miR-124a	SYT11	synaptotagmin 11 [Source:VGNC Symbol;Acc:VGNC:93680]	-1.71	0.1324
ssc-miR-124a	SYT14	synaptotagmin 14 [Source:VGNC Symbol;Acc:VGNC:108619]	-1.71	0.1324
ssc-miR-124a	SYT9	synaptotagmin 9 [Source:VGNC Symbol;Acc:VGNC:93689]	-1.71	0.1324
ssc-miR-124a	TAB2	TGF-beta activated kinase 1 (MAP3K7) binding protein 2 [Source:VGNC Symbol;Acc:VGNC:103186]	-1.71	0.1324
ssc-miR-124a	TACC1	hypothetical gene	-1.71	0.1324
ssc-miR-124a	TACC2	transforming acidic coiled-coil containing protein 2 [Source:NCBI gene (formerly Entrezgene);Acc:100152804]	-1.71	0.1324
ssc-miR-124a	TADA2B	transcriptional adaptor 2B [Source:VGNC Symbol;Acc:VGNC:93707]	-1.71	0.1324
ssc-miR-124a	TAL1	TAL bHLH transcription factor 1, erythroid differentiation factor [Source:HGNC Symbol;Acc:HGNC:11556]	-1.71	0.1324
ssc-miR-124a	TANC2	tetratricopeptide repeat, ankyrin repeat and coiled-coil containing 2 [Source:VGNC Symbol;Acc:VGNC:93733]	-1.71	0.1324
ssc-miR-124a	TAOK1	TAO kinase 1 [Source:VGNC Symbol;Acc:VGNC:98355]	-1.71	0.1324
ssc-miR-124a	TARBP1	TAR (HIV-1) RNA binding protein 1 [Source:HGNC Symbol;Acc:HGNC:11568]	-1.71	0.1324
ssc-miR-124a	TATDN2	TatD DNase domain containing 2 [Source:HGNC Symbol;Acc:HGNC:28988]	-1.71	0.1324
ssc-miR-124a	TBC1D1	TBC1 domain family member 1 [Source:VGNC Symbol;Acc:VGNC:93758]	-1.71	0.1324
ssc-miR-124a	TBC1D13	TBC1 domain family member 13 [Source:VGNC Symbol;Acc:VGNC:93763]	-1.71	0.1324
ssc-miR-124a	TBC1D14	hypothetical gene	-1.71	0.1324
ssc-miR-124a	TBC1D16	hypothetical gene	-1.71	0.1324
ssc-miR-124a	TBC1D20	TBC1 domain family member 20 [Source:VGNC Symbol;Acc:VGNC:95752]	-1.71	0.1324
ssc-miR-124a	TBC1D5	TBC1 domain family member 5 [Source:VGNC Symbol;Acc:VGNC:98360]	-1.71	0.1324
ssc-miR-124a	TBC1D9B	TBC1 domain family member 9B [Source:VGNC Symbol;Acc:VGNC:93782]	-1.71	0.1324
ssc-miR-124a	TBR1	T-box brain transcription factor 1 [Source:VGNC Symbol;Acc:VGNC:95533]	-1.71	0.1324
ssc-miR-124a	TCEB1	hypothetical gene	-1.71	0.1324
ssc-miR-124a	TCF20	transcription factor 20 [Source:HGNC Symbol;Acc:HGNC:11631]	-1.71	0.1324
ssc-miR-124a	TCF3	transcription factor 3 [Source:VGNC Symbol;Acc:VGNC:93822]	-1.71	0.1324
ssc-miR-124a	TCF4	transcription factor 4 [Source:VGNC Symbol;Acc:VGNC:93823]	-1.71	0.1324
ssc-miR-124a	TCTA	T cell leukemia translocation altered [Source:HGNC Symbol;Acc:HGNC:11692]	-1.71	0.1324
ssc-miR-124a	TDG	thymine DNA glycosylase [Source:VGNC Symbol;Acc:VGNC:93842]	-1.71	0.1324
ssc-miR-124a	TDRP	testis development related protein [Source:VGNC Symbol;Acc:VGNC:104069]	-1.71	0.1324
ssc-miR-124a	TEAD1	TEA domain transcription factor 1 [Source:VGNC Symbol;Acc:VGNC:93853]	-1.71	0.1324
ssc-miR-124a	TECPR2	tectonin beta-propeller repeat containing 2 [Source:VGNC Symbol;Acc:VGNC:99731]	-1.71	0.1324
ssc-miR-124a	TECTB	tectorin beta [Source:VGNC Symbol;Acc:VGNC:93860]	-1.71	0.1324
ssc-miR-124a	TENM1	teneurin transmembrane protein 1 [Source:VGNC Symbol;Acc:VGNC:98363]	-1.71	0.1324
ssc-miR-124a	TET1	tet methylcytosine dioxygenase 1 [Source:VGNC Symbol;Acc:VGNC:93888]	-1.71	0.1324
ssc-miR-124a	TET3	tet methylcytosine dioxygenase 3 [Source:VGNC Symbol;Acc:VGNC:93890]	-1.71	0.1324
ssc-miR-124a	TEX261	testis expressed 261 [Source:HGNC Symbol;Acc:HGNC:30712]	-1.71	0.1324
ssc-miR-124a	TFAP4	transcription factor AP-4 [Source:VGNC Symbol;Acc:VGNC:93912]	-1.71	0.1324
ssc-miR-124a	TFCP2L1	transcription factor CP2 like 1 [Source:HGNC Symbol;Acc:HGNC:17925]	-1.71	0.1324
ssc-miR-124a	TFDP2	transcription factor Dp-2 [Source:VGNC Symbol;Acc:VGNC:93915]	-1.71	0.1324
ssc-miR-124a	TFE3	transcription factor binding to IGHM enhancer 3 [Source:VGNC Symbol;Acc:VGNC:93916]	-1.71	0.1324
ssc-miR-124a	TFEB	transcription factor EB [Source:VGNC Symbol;Acc:VGNC:93917]	-1.71	0.1324
ssc-miR-124a	TFRC	transferrin receptor [Source:VGNC Symbol;Acc:VGNC:93925]	-1.71	0.1324
ssc-miR-124a	TGFBR1	transforming growth factor beta receptor 1 [Source:VGNC Symbol;Acc:VGNC:98368]	-1.71	0.1324
ssc-miR-124a	TGFBR3	transforming growth factor beta receptor 3 [Source:VGNC Symbol;Acc:VGNC:93932]	-1.71	0.1324
ssc-miR-124a	THAP2	THAP domain containing 2 [Source:VGNC Symbol;Acc:VGNC:104071]	-1.71	0.1324

ssc-miR-124a	THBS2	thrombospondin 2 [Source:VGNC Symbol;Acc:VGNC:93947]	-1.71	0.1324
ssc-miR-124a	THEM6	thioesterase superfamily member 6 [Source:VGNC Symbol;Acc:VGNC:93953]	-1.71	0.1324
ssc-miR-124a	THOP1	thimet oligopeptidase 1 [Source:VGNC Symbol;Acc:VGNC:93961]	-1.71	0.1324
ssc-miR-124a	THRB	thyroid hormone receptor beta [Source:VGNC Symbol;Acc:VGNC:93965]	-1.71	0.1324
ssc-miR-124a	THSD4	thrombospondin type 1 domain containing 4 [Source:VGNC Symbol;Acc:VGNC:93967]	-1.71	0.1324
ssc-miR-124a	THSD7B	thrombospondin type 1 domain containing 7B [Source:VGNC Symbol;Acc:VGNC:96216]	-1.71	0.1324
ssc-miR-124a	THUMPD1	THUMP domain containing 1 [Source:VGNC Symbol;Acc:VGNC:93970]	-1.71	0.1324
ssc-miR-124a	THUMPD3	THUMP domain containing 3 [Source:VGNC Symbol;Acc:VGNC:93972]	-1.71	0.1324
ssc-miR-124a	TIA1	TIA1 cytotoxic granule associated RNA binding protein [Source:VGNC Symbol;Acc:VGNC:93975]	-1.71	0.1324
ssc-miR-124a	TIAL1	TIA1 cytotoxic granule associated RNA binding protein like 1 [Source:VGNC Symbol;Acc:VGNC:104073]	-1.71	0.1324
ssc-miR-124a	TIGD5	tigger transposable element derived 5 [Source:VGNC Symbol;Acc:VGNC:98876]	-1.71	0.1324
ssc-miR-124a	TIPRL	TOR signaling pathway regulator [Source:VGNC Symbol;Acc:VGNC:94002]	-1.71	0.1324
ssc-miR-124a	TJP1	tight junction protein 1 [Source:VGNC Symbol;Acc:VGNC:94005]	-1.71	0.1324
ssc-miR-124a	TJP2	tight junction protein 2 [Source:VGNC Symbol;Acc:VGNC:103188]	-1.71	0.1324
ssc-miR-124a	TLE4	TLE family member 4, transcriptional corepressor [Source:VGNC Symbol;Acc:VGNC:98372]	-1.71	0.1324
ssc-miR-124a	TLL1	tolloid like 1 [Source:VGNC Symbol;Acc:VGNC:94018]	-1.71	0.1324
ssc-miR-124a	TLL2	tolloid like 2 [Source:VGNC Symbol;Acc:VGNC:94019]	-1.71	0.1324
ssc-miR-124a	TLN1	talin 1 [Source:VGNC Symbol;Acc:VGNC:94020]	-1.71	0.1324
ssc-miR-124a	TLR4	toll like receptor 4 [Source:VGNC Symbol;Acc:VGNC:94025]	-1.71	0.1324
ssc-miR-124a	TMCC3	transmembrane and coiled-coil domain family 3 [Source:VGNC Symbol;Acc:VGNC:98375]	-1.71	0.1324
ssc-miR-124a	TMCO3	transmembrane and coiled-coil domains 3 [Source:VGNC Symbol;Acc:VGNC:94050]	-1.71	0.1324
ssc-miR-124a	TMED1	transmembrane p24 trafficking protein 1 [Source:VGNC Symbol;Acc:VGNC:94053]	-1.71	0.1324
ssc-miR-124a	TMED7	transmembrane p24 trafficking protein 7 [Source:NCBI gene (formerly Entrezgene);Acc:100522183]	-1.71	0.1324
ssc-miR-124a	TMED8	transmembrane p24 trafficking protein family member 8 [Source:VGNC Symbol;Acc:VGNC:94058]	-1.71	0.1324
ssc-miR-124a	TMEM104	transmembrane protein 104 [Source:VGNC Symbol;Acc:VGNC:94062]	-1.71	0.1324
ssc-miR-124a	TMEM109	transmembrane protein 109 [Source:VGNC Symbol;Acc:VGNC:94066]	-1.71	0.1324
ssc-miR-124a	TMEM110	hypothetical gene	-1.71	0.1324
ssc-miR-124a	TMEM117	transmembrane protein 117 [Source:VGNC Symbol;Acc:VGNC:94070]	-1.71	0.1324
ssc-miR-124a	TMEM128	transmembrane protein 128 [Source:VGNC Symbol;Acc:VGNC:94075]	-1.71	0.1324
ssc-miR-124a	TMEM129	transmembrane protein 129, E3 ubiquitin ligase [Source:VGNC Symbol;Acc:VGNC:94076]	-1.71	0.1324
ssc-miR-124a	TMEM134	transmembrane protein 134 [Source:VGNC Symbol;Acc:VGNC:94084]	-1.71	0.1324
ssc-miR-124a	TMEM143	transmembrane protein 143 [Source:VGNC Symbol;Acc:VGNC:94090]	-1.71	0.1324
ssc-miR-124a	TMEM150A	transmembrane protein 150A [Source:VGNC Symbol;Acc:VGNC:94095]	-1.71	0.1324
ssc-miR-124a	TMEM168	transmembrane protein 168 [Source:VGNC Symbol;Acc:VGNC:94106]	-1.71	0.1324
ssc-miR-124a	TMEM170B	transmembrane protein 170B [Source:VGNC Symbol;Acc:VGNC:94108]	-1.71	0.1324
ssc-miR-124a	TMEM178A	transmembrane protein 178A [Source:VGNC Symbol;Acc:VGNC:94114]	-1.71	0.1324
ssc-miR-124a	TMEM178B	transmembrane protein 178B [Source:HGNC Symbol;Acc:HGNC:44112]	-1.71	0.1324
ssc-miR-124a	TMEM181	transmembrane protein 181 [Source:VGNC Symbol;Acc:VGNC:94117]	-1.71	0.1324
ssc-miR-124a	TMEM184B	transmembrane protein 184B [Source:VGNC Symbol;Acc:VGNC:94120]	-1.71	0.1324
ssc-miR-124a	TMEM194A	hypothetical gene	-1.71	0.1324
ssc-miR-124a	TMEM198	transmembrane protein 198 [Source:VGNC Symbol;Acc:VGNC:96274]	-1.71	0.1324
ssc-miR-124a	TMEM229A	transmembrane protein 229A [Source:VGNC Symbol;Acc:VGNC:94146]	-1.71	0.1324
ssc-miR-124a	TMEM245	transmembrane protein 245 [Source:HGNC Symbol;Acc:HGNC:1363]	-1.71	0.1324
ssc-miR-124a	TMEM248	transmembrane protein 248 [Source:VGNC Symbol;Acc:VGNC:94155]	-1.71	0.1324
ssc-miR-124a	TMEM256-PLSCR3	hypothetical gene	-1.71	0.1324
ssc-miR-124a	TMEM257	hypothetical gene	-1.71	0.1324
ssc-miR-124a	TMEM259	transmembrane protein 259 [Source:VGNC Symbol;Acc:VGNC:94162]	-1.71	0.1324
ssc-miR-124a	TMEM26	transmembrane protein 26 [Source:VGNC Symbol;Acc:VGNC:94163]	-1.71	0.1324
ssc-miR-124a	TMEM260	transmembrane protein 260 [Source:VGNC Symbol;Acc:VGNC:94164]	-1.71	0.1324
ssc-miR-124a	TMEM38A	transmembrane protein 38A [Source:VGNC Symbol;Acc:VGNC:94174]	-1.71	0.1324

ssc-miR-124a	TMEM41A	transmembrane protein 41A [Source:VGNC Symbol;Acc:VGNC:104080]	-1.71	0.1324
ssc-miR-124a	TMEM45A	transmembrane protein 45A [Source:VGNC Symbol;Acc:VGNC:94182]	-1.71	0.1324
ssc-miR-124a	TMEM50B	transmembrane protein 50B [Source:VGNC Symbol;Acc:VGNC:94186]	-1.71	0.1324
ssc-miR-124a	TMEM57	hypothetical gene	-1.71	0.1324
ssc-miR-124a	TMEM63C	transmembrane protein 63C [Source:VGNC Symbol;Acc:VGNC:94198]	-1.71	0.1324
ssc-miR-124a	TMEM87B	transmembrane protein 87B [Source:VGNC Symbol;Acc:VGNC:94213]	-1.71	0.1324
ssc-miR-124a	TMOD1	tropomodulin 1 [Source:HGNC Symbol;Acc:HGNC:11871]	-1.71	0.1324
ssc-miR-124a	TMOD2	tropomodulin 2 [Source:VGNC Symbol;Acc:VGNC:94227]	-1.71	0.1324
ssc-miR-124a	TNFSF11	TNF superfamily member 11 [Source:HGNC Symbol;Acc:HGNC:11926]	-1.71	0.1324
ssc-miR-124a	TNKS	tankyrase [Source:VGNC Symbol;Acc:VGNC:95546]	-1.71	0.1324
ssc-miR-124a	TNRC6B	trinucleotide repeat containing adaptor 6B [Source:VGNC Symbol;Acc:VGNC:94293]	-1.71	0.1324
ssc-miR-124a	TNS3	tensin 3 [Source:VGNC Symbol;Acc:VGNC:94296]	-1.71	0.1324
ssc-miR-124a	TOB2	transducer of ERBB2, 2 [Source:VGNC Symbol;Acc:VGNC:94298]	-1.71	0.1324
ssc-miR-124a	TOM1	target of myb1 membrane trafficking protein [Source:VGNC Symbol;Acc:VGNC:95552]	-1.71	0.1324
ssc-miR-124a	TOMM34	translocase of outer mitochondrial membrane 34 [Source:VGNC Symbol;Acc:VGNC:95647]	-1.71	0.1324
ssc-miR-124a	TOR1AIP2	torsin 1A interacting protein 2 [Source:VGNC Symbol;Acc:VGNC:94318]	-1.71	0.1324
ssc-miR-124a	TOR1B	torsin family 1 member B [Source:HGNC Symbol;Acc:HGNC:11995]	-1.71	0.1324
ssc-miR-124a	TOR3A	torsin family 3 member A [Source:VGNC Symbol;Acc:VGNC:94320]	-1.71	0.1324
ssc-miR-124a	TOX	thymocyte selection associated high mobility group box [Source:VGNC Symbol;Acc:VGNC:94322]	-1.71	0.1324
ssc-miR-124a	TP53INP2	tumor protein p53 inducible nuclear protein 2 [Source:VGNC Symbol;Acc:VGNC:95737]	-1.71	0.1324
ssc-miR-124a	TPCN1	two pore segment channel 1 [Source:VGNC Symbol;Acc:VGNC:94333]	-1.71	0.1324
ssc-miR-124a	TPCN2	two pore segment channel 2 [Source:VGNC Symbol;Acc:VGNC:100876]	-1.71	0.1324
ssc-miR-124a	TPD52L2	TPD52 like 2 [Source:VGNC Symbol;Acc:VGNC:95554]	-1.71	0.1324
ssc-miR-124a	TPP2	tripeptidyl peptidase 2 [Source:VGNC Symbol;Acc:VGNC:94343]	-1.71	0.1324
ssc-miR-124a	TPST2	tyrosylprotein sulfotransferase 2 [Source:NCBI gene (formerly Entrezgene);Acc:100154448]	-1.71	0.1324
ssc-miR-124a	TRABD	TraB domain containing [Source:VGNC Symbol;Acc:VGNC:94355]	-1.71	0.1324
ssc-miR-124a	TRAF3	TNF receptor associated factor 3 [Source:VGNC Symbol;Acc:VGNC:94360]	-1.71	0.1324
ssc-miR-124a	TRAF6	TNF receptor associated factor 6 [Source:VGNC Symbol;Acc:VGNC:94365]	-1.71	0.1324
ssc-miR-124a	TRAM2	translocation associated membrane protein 2 [Source:HGNC Symbol;Acc:HGNC:16855]	-1.71	0.1324
ssc-miR-124a	TRAPPC10	trafficking protein particle complex subunit 10 [Source:VGNC Symbol;Acc:VGNC:101497]	-1.71	0.1324
ssc-miR-124a	TRIB3	tribbles pseudokinase 3 [Source:VGNC Symbol;Acc:VGNC:95791]	-1.71	0.1324
ssc-miR-124a	TRIM14	tripartite motif containing 14 [Source:VGNC Symbol;Acc:VGNC:103196]	-1.71	0.1324
ssc-miR-124a	TRIM2	tripartite motif containing 2 [Source:VGNC Symbol;Acc:VGNC:94397]	-1.71	0.1324
ssc-miR-124a	TRIM39	tripartite motif containing 39 [Source:NCBI gene (formerly Entrezgene);Acc:100151742]	-1.71	0.1324
ssc-miR-124a	TRIM45	tripartite motif containing 45 [Source:VGNC Symbol;Acc:VGNC:94417]	-1.71	0.1324
ssc-miR-124a	TRIM71	tripartite motif containing 71 [Source:VGNC Symbol;Acc:VGNC:94431]	-1.71	0.1324
ssc-miR-124a	TRIM9	tripartite motif containing 9 [Source:VGNC Symbol;Acc:VGNC:94434]	-1.71	0.1324
ssc-miR-124a	TRIP10	thyroid hormone receptor interactor 10 [Source:VGNC Symbol;Acc:VGNC:94436]	-1.71	0.1324
ssc-miR-124a	TRIP12	thyroid hormone receptor interactor 12 [Source:VGNC Symbol;Acc:VGNC:95555]	-1.71	0.1324
ssc-miR-124a	TRMT10A	tRNA methyltransferase 10A [Source:VGNC Symbol;Acc:VGNC:94442]	-1.71	0.1324
ssc-miR-124a	TRMT6	tRNA methyltransferase 6 non-catalytic subunit [Source:VGNC Symbol;Acc:VGNC:95956]	-1.71	0.1324
ssc-miR-124a	TRPS1	transcriptional repressor GATA binding 1 [Source:VGNC Symbol;Acc:VGNC:94471]	-1.71	0.1324
ssc-miR-124a	TRUB1	TruB pseudouridine synthase family member 1 [Source:VGNC Symbol;Acc:VGNC:94478]	-1.71	0.1324
ssc-miR-124a	TSC22D1	hypothetical gene	-1.71	0.1324
ssc-miR-124a	TSC22D4	TSC22 domain family member 4 [Source:HGNC Symbol;Acc:HGNC:21696]	-1.71	0.1324
ssc-miR-124a	TSEN54	tRNA splicing endonuclease subunit 54 [Source:VGNC Symbol;Acc:VGNC:94485]	-1.71	0.1324
ssc-miR-124a	TSHZ1	teashirt zinc finger homeobox 1 [Source:VGNC Symbol;Acc:VGNC:94492]	-1.71	0.1324
ssc-miR-124a	TSHZ2	teashirt zinc finger homeobox 2 [Source:NCBI gene (formerly Entrezgene);Acc:100136903]	-1.71	0.1324
ssc-miR-124a	TSKU	tsukushi, small leucine rich proteoglycan [Source:VGNC Symbol;Acc:VGNC:94495]	-1.71	0.1324
ssc-miR-124a	TSN	translin [Source:VGNC Symbol;Acc:VGNC:95556]	-1.71	0.1324

ssc-miR-124a	TSPAN15	tetraspanin 15 [Source:VGNC Symbol;Acc:VGNC:94504]	-1.71	0.1324
ssc-miR-124a	TTC26	tetratricopeptide repeat domain 26 [Source:VGNC Symbol;Acc:VGNC:94541]	-1.71	0.1324
ssc-miR-124a	TTC3	tetratricopeptide repeat domain 3 [Source:VGNC Symbol;Acc:VGNC:94545]	-1.71	0.1324
ssc-miR-124a	TTC37	hypothetical gene	-1.71	0.1324
ssc-miR-124a	TTC7A	tetratricopeptide repeat domain 7A [Source:VGNC Symbol;Acc:VGNC:94557]	-1.71	0.1324
ssc-miR-124a	TTC9	tetratricopeptide repeat domain 9 [Source:VGNC Symbol;Acc:VGNC:94560]	-1.71	0.1324
ssc-miR-124a	TTL	hypothetical gene	-1.71	0.1324
ssc-miR-124a	TTPA	alpha tocopherol transfer protein [Source:HGNC Symbol;Acc:HGNC:12404]	-1.71	0.1324
ssc-miR-124a	TUB	TUB bipartite transcription factor [Source:VGNC Symbol;Acc:VGNC:94577]	-1.71	0.1324
ssc-miR-124a	TUBG1	tubulin gamma 1 [Source:HGNC Symbol;Acc:HGNC:12417]	-1.71	0.1324
ssc-miR-124a	TWSG1	twisted gastrulation BMP signaling modulator 1 [Source:VGNC Symbol;Acc:VGNC:94598]	-1.71	0.1324
ssc-miR-124a	TXLNA	taxilin alpha [Source:VGNC Symbol;Acc:VGNC:94600]	-1.71	0.1324
ssc-miR-124a	TXNDC5	thioredoxin domain containing 5 [Source:VGNC Symbol;Acc:VGNC:94608]	-1.71	0.1324
ssc-miR-124a	TXNRD1	thioredoxin reductase 1 [Source:NCBI gene (formerly Entrezgene);Acc:396681]	-1.71	0.1324
ssc-miR-124a	TYK2	tyrosine kinase 2 [Source:VGNC Symbol;Acc:VGNC:94615]	-1.71	0.1324
ssc-miR-124a	UACA	uveal autoantigen with coiled-coil domains and ankyrin repeats [Source:VGNC Symbol;Acc:VGNC:94625]	-1.71	0.1324
ssc-miR-124a	UBA6	ubiquitin like modifier activating enzyme 6 [Source:VGNC Symbol;Acc:VGNC:94630]	-1.71	0.1324
ssc-miR-124a	UBE2B	ubiquitin conjugating enzyme E2 B [Source:VGNC Symbol;Acc:VGNC:94640]	-1.71	0.1324
ssc-miR-124a	UBE2G1	ubiquitin conjugating enzyme E2 G1 [Source:HGNC Symbol;Acc:HGNC:12482]	-1.71	0.1324
ssc-miR-124a	UBE2G2	ubiquitin conjugating enzyme E2 G2 [Source:VGNC Symbol;Acc:VGNC:94642]	-1.71	0.1324
ssc-miR-124a	UBE2O	ubiquitin conjugating enzyme E2 O [Source:VGNC Symbol;Acc:VGNC:94649]	-1.71	0.1324
ssc-miR-124a	UBE2Q2	ubiquitin conjugating enzyme E2 Q2 [Source:HGNC Symbol;Acc:HGNC:19248]	-1.71	0.1324
ssc-miR-124a	UBE2V2	ubiquitin conjugating enzyme E2 V2 [Source:VGNC Symbol;Acc:VGNC:98394]	-1.71	0.1324
ssc-miR-124a	UBE2W	ubiquitin conjugating enzyme E2 W [Source:VGNC Symbol;Acc:VGNC:98890]	-1.71	0.1324
ssc-miR-124a	UBE4A	ubiquitination factor E4A [Source:VGNC Symbol;Acc:VGNC:94656]	-1.71	0.1324
ssc-miR-124a	UBL3	hypothetical gene	-1.71	0.1324
ssc-miR-124a	UBN1	ubinuclein 1 [Source:VGNC Symbol;Acc:VGNC:94663]	-1.71	0.1324
ssc-miR-124a	UBN2	ubinuclein 2 [Source:VGNC Symbol;Acc:VGNC:94664]	-1.71	0.1324
ssc-miR-124a	UBOX5	U-box domain containing 5 [Source:VGNC Symbol;Acc:VGNC:95860]	-1.71	0.1324
ssc-miR-124a	UBR1	ubiquitin protein ligase E3 component n-recognin 1 [Source:VGNC Symbol;Acc:VGNC:94668]	-1.71	0.1324
ssc-miR-124a	UBR2	ubiquitin protein ligase E3 component n-recognin 2 [Source:VGNC Symbol;Acc:VGNC:94669]	-1.71	0.1324
ssc-miR-124a	UBR3	ubiquitin protein ligase E3 component n-recognin 3 [Source:VGNC Symbol;Acc:VGNC:96771]	-1.71	0.1324
ssc-miR-124a	UBR7	ubiquitin protein ligase E3 component n-recognin 7 [Source:HGNC Symbol;Acc:HGNC:20344]	-1.71	0.1324
ssc-miR-124a	UBTD2	ubiquitin domain containing 2 [Source:VGNC Symbol;Acc:VGNC:94673]	-1.71	0.1324
ssc-miR-124a	UBXN1	UBX domain protein 1 [Source:VGNC Symbol;Acc:VGNC:94674]	-1.71	0.1324
ssc-miR-124a	UBXN2A	UBX domain protein 2A [Source:VGNC Symbol;Acc:VGNC:94677]	-1.71	0.1324
ssc-miR-124a	UCK2	uridine-cytidine kinase 2 [Source:VGNC Symbol;Acc:VGNC:98399]	-1.71	0.1324
ssc-miR-124a	UGCG	UDP-glucose ceramide glucosyltransferase [Source:VGNC Symbol;Acc:VGNC:94685]	-1.71	0.1324
ssc-miR-124a	UGGT1	UDP-glucose glycoprotein glucosyltransferase 1 [Source:VGNC Symbol;Acc:VGNC:95646]	-1.71	0.1324
ssc-miR-124a	UGT8	UDP glycosyltransferase 8 [Source:VGNC Symbol;Acc:VGNC:98401]	-1.71	0.1324
ssc-miR-124a	UHMK1	U2AF homology motif kinase 1 [Source:VGNC Symbol;Acc:VGNC:94688]	-1.71	0.1324
ssc-miR-124a	ULK2	unc-51 like autophagy activating kinase 2 [Source:VGNC Symbol;Acc:VGNC:94695]	-1.71	0.1324
ssc-miR-124a	UNC119B	unc-119 lipid binding chaperone B [Source:HGNC Symbol;Acc:HGNC:16488]	-1.71	0.1324
ssc-miR-124a	UNC5D	unc-5 netrin receptor D [Source:VGNC Symbol;Acc:VGNC:95892]	-1.71	0.1324
ssc-miR-124a	UPF3B	UPF3B regulator of nonsense mediated mRNA decay [Source:VGNC Symbol;Acc:VGNC:94720]	-1.71	0.1324
ssc-miR-124a	URGCP	upregulator of cell proliferation [Source:HGNC Symbol;Acc:HGNC:30890]	-1.71	0.1324
ssc-miR-124a	USO1	USO1 vesicle transport factor [Source:VGNC Symbol;Acc:VGNC:94741]	-1.71	0.1324
ssc-miR-124a	USP1	ubiquitin specific peptidase 1 [Source:VGNC Symbol;Acc:VGNC:94742]	-1.71	0.1324
ssc-miR-124a	USP14	ubiquitin specific peptidase 14 [Source:VGNC Symbol;Acc:VGNC:94747]	-1.71	0.1324
ssc-miR-124a	USP2	ubiquitin specific peptidase 2 [Source:VGNC Symbol;Acc:VGNC:94751]	-1.71	0.1324

ssc-miR-124a	USP24	ubiquitin specific peptidase 24 [Source:VGNC Symbol;Acc:VGNC:94754]	-1.71	0.1324
ssc-miR-124a	USP30	ubiquitin specific peptidase 30 [Source:VGNC Symbol;Acc:VGNC:94758]	-1.71	0.1324
ssc-miR-124a	USP37	ubiquitin specific peptidase 37 [Source:VGNC Symbol;Acc:VGNC:98404]	-1.71	0.1324
ssc-miR-124a	USP38	ubiquitin specific peptidase 38 [Source:VGNC Symbol;Acc:VGNC:94762]	-1.71	0.1324
ssc-miR-124a	USP45	ubiquitin specific peptidase 45 [Source:VGNC Symbol;Acc:VGNC:94767]	-1.71	0.1324
ssc-miR-124a	USP47	ubiquitin specific peptidase 47 [Source:VGNC Symbol;Acc:VGNC:94769]	-1.71	0.1324
ssc-miR-124a	USP48	ubiquitin specific peptidase 48 [Source:VGNC Symbol;Acc:VGNC:94770]	-1.71	0.1324
ssc-miR-124a	USP49	ubiquitin specific peptidase 49 [Source:VGNC Symbol;Acc:VGNC:94771]	-1.71	0.1324
ssc-miR-124a	UST	uronyl 2-sulfotransferase [Source:VGNC Symbol;Acc:VGNC:94780]	-1.71	0.1324
ssc-miR-124a	VAMP3	hypothetical gene	-1.71	0.1324
ssc-miR-124a	VAMP4	vesicle associated membrane protein 4 [Source:VGNC Symbol;Acc:VGNC:94794]	-1.71	0.1324
ssc-miR-124a	VAMP7	vesicle associated membrane protein 7 [Source:VGNC Symbol;Acc:VGNC:94796]	-1.71	0.1324
ssc-miR-124a	VANGL1	VANGL planar cell polarity protein 1 [Source:VGNC Symbol;Acc:VGNC:94797]	-1.71	0.1324
ssc-miR-124a	VANGL2	VANGL planar cell polarity protein 2 [Source:VGNC Symbol;Acc:VGNC:94798]	-1.71	0.1324
ssc-miR-124a	VAT1	vesicle amine transport 1 [Source:VGNC Symbol;Acc:VGNC:94805]	-1.71	0.1324
ssc-miR-124a	VAT1L	vesicle amine transport 1 like [Source:VGNC Symbol;Acc:VGNC:94806]	-1.71	0.1324
ssc-miR-124a	VCAN	versican [Source:VGNC Symbol;Acc:VGNC:108163]	-1.71	0.1324
ssc-miR-124a	VDAC2	hypothetical gene	-1.71	0.1324
ssc-miR-124a	VDAC3	voltage dependent anion channel 3 [Source:VGNC Symbol;Acc:VGNC:95562]	-1.71	0.1324
ssc-miR-124a	VDR	vitamin D receptor [Source:VGNC Symbol;Acc:VGNC:94814]	-1.71	0.1324
ssc-miR-124a	VEZF1	vascular endothelial zinc finger 1 [Source:VGNC Symbol;Acc:VGNC:94818]	-1.71	0.1324
ssc-miR-124a	VGLL4	vestigial like family member 4 [Source:VGNC Symbol;Acc:VGNC:94824]	-1.71	0.1324
ssc-miR-124a	VIM	vimentin [Source:VGNC Symbol;Acc:VGNC:95565]	-1.71	0.1324
ssc-miR-124a	VKORC1L1	vitamin K epoxide reductase complex subunit 1 like 1 [Source:VGNC Symbol;Acc:VGNC:94831]	-1.71	0.1324
ssc-miR-124a	VLDLR	very low density lipoprotein receptor [Source:VGNC Symbol;Acc:VGNC:94832]	-1.71	0.1324
ssc-miR-124a	VMP1	vacuole membrane protein 1 [Source:HGNC Symbol;Acc:HGNC:29559]	-1.71	0.1324
ssc-miR-124a	VPS13A	vacuolar protein sorting 13 homolog A [Source:VGNC Symbol;Acc:VGNC:94838]	-1.71	0.1324
ssc-miR-124a	VPS35	VPS35 retromer complex component [Source:VGNC Symbol;Acc:VGNC:94848]	-1.71	0.1324
ssc-miR-124a	VPS37B	VPS37B subunit of ESCRT-I [Source:VGNC Symbol;Acc:VGNC:94851]	-1.71	0.1324
ssc-miR-124a	VPS37C	VPS37C subunit of ESCRT-I [Source:VGNC Symbol;Acc:VGNC:94852]	-1.71	0.1324
ssc-miR-124a	VPS4B	vacuolar protein sorting 4 homolog B [Source:VGNC Symbol;Acc:VGNC:94857]	-1.71	0.1324
ssc-miR-124a	VSIG10	V-set and immunoglobulin domain containing 10 [Source:HGNC Symbol;Acc:HGNC:26078]	-1.71	0.1324
ssc-miR-124a	VSNL1	visinin like 1 [Source:VGNC Symbol;Acc:VGNC:94871]	-1.71	0.1324
ssc-miR-124a	VSTM4	V-set and transmembrane domain containing 4 [Source:VGNC Symbol;Acc:VGNC:94874]	-1.71	0.1324
ssc-miR-124a	WAPAL	hypothetical gene	-1.71	0.1324
ssc-miR-124a	WASF1	hypothetical gene	-1.71	0.1324
ssc-miR-124a	WASF2	WASP family member 2 [Source:VGNC Symbol;Acc:VGNC:94893]	-1.71	0.1324
ssc-miR-124a	WDFY1	WD repeat and FYVE domain containing 1 [Source:VGNC Symbol;Acc:VGNC:99733]	-1.71	0.1324
ssc-miR-124a	WDFY3	WD repeat and FYVE domain containing 3 [Source:VGNC Symbol;Acc:VGNC:94903]	-1.71	0.1324
ssc-miR-124a	WDR33	WD repeat domain 33 [Source:VGNC Symbol;Acc:VGNC:96097]	-1.71	0.1324
ssc-miR-124a	WDR44	WD repeat domain 44 [Source:VGNC Symbol;Acc:VGNC:94921]	-1.71	0.1324
ssc-miR-124a	WDR45B	WD repeat domain 45B [Source:NCBI gene (formerly Entrezgene);Acc:100462760]	-1.71	0.1324
ssc-miR-124a	WDR48	WD repeat domain 48 [Source:VGNC Symbol;Acc:VGNC:94925]	-1.71	0.1324
ssc-miR-124a	WDR81	WD repeat domain 81 [Source:VGNC Symbol;Acc:VGNC:99104]	-1.71	0.1324
ssc-miR-124a	WIPF1	hypothetical gene	-1.71	0.1324
ssc-miR-124a	WIPF2	WAS/WASL interacting protein family member 2 [Source:VGNC Symbol;Acc:VGNC:99106]	-1.71	0.1324
ssc-miR-124a	WIPF3	WAS/WASL interacting protein family member 3 [Source:VGNC Symbol;Acc:VGNC:94958]	-1.71	0.1324
ssc-miR-124a	WNK1	WNK lysine deficient protein kinase 1 [Source:VGNC Symbol;Acc:VGNC:94962]	-1.71	0.1324
ssc-miR-124a	WNK3	WNK lysine deficient protein kinase 3 [Source:VGNC Symbol;Acc:VGNC:94964]	-1.71	0.1324
ssc-miR-124a	WSB2	WD repeat and SOCS box containing 2 [Source:VGNC Symbol;Acc:VGNC:94982]	-1.71	0.1324

ssc-miR-124a	WWC3	WWC family member 3 [Source:VGNC Symbol;Acc:VGNC:94987]	-1.71	0.1324
ssc-miR-124a	XKR4	XK related 4 [Source:VGNC Symbol;Acc:VGNC:98900]	-1.71	0.1324
ssc-miR-124a	XKR6	XK related 6 [Source:HGNC Symbol;Acc:HGNC:27806]	-1.71	0.1324
ssc-miR-124a	XKR7	XK related 7 [Source:VGNC Symbol;Acc:VGNC:95987]	-1.71	0.1324
ssc-miR-124a	XKR8	XK related 8 [Source:VGNC Symbol;Acc:VGNC:94996]	-1.71	0.1324
ssc-miR-124a	XPO4	exportin 4 [Source:VGNC Symbol;Acc:VGNC:95004]	-1.71	0.1324
ssc-miR-124a	XPO5	exportin 5 [Source:VGNC Symbol;Acc:VGNC:95005]	-1.71	0.1324
ssc-miR-124a	XRN1	5'-3' exoribonuclease 1 [Source:VGNC Symbol;Acc:VGNC:108705]	-1.71	0.1324
ssc-miR-124a	XYLT1	xylosyltransferase 1 [Source:VGNC Symbol;Acc:VGNC:95015]	-1.71	0.1324
ssc-miR-124a	YEATS2	YEATS domain containing 2 [Source:VGNC Symbol;Acc:VGNC:95023]	-1.71	0.1324
ssc-miR-124a	YIPF6	Yip1 domain family member 6 [Source:VGNC Symbol;Acc:VGNC:95032]	-1.71	0.1324
ssc-miR-124a	YME1L1	hypothetical gene	-1.71	0.1324
ssc-miR-124a	YOD1	YOD1 deubiquitinase [Source:VGNC Symbol;Acc:VGNC:95035]	-1.71	0.1324
ssc-miR-124a	YTHDF1	YTH N6-methyladenosine RNA binding protein 1 [Source:VGNC Symbol;Acc:VGNC:95681]	-1.71	0.1324
ssc-miR-124a	YTHDF3	YTH N6-methyladenosine RNA binding protein 3 [Source:VGNC Symbol;Acc:VGNC:95044]	-1.71	0.1324
ssc-miR-124a	YY1	YY1 transcription factor [Source:HGNC Symbol;Acc:HGNC:12856]	-1.71	0.1324
ssc-miR-124a	ZADH2	prostaglandin reductase 3 [Source:VGNC Symbol;Acc:VGNC:95049]	-1.71	0.1324
ssc-miR-124a	ZBED4	hypothetical gene	-1.71	0.1324
ssc-miR-124a	ZBTB11	zinc finger and BTB domain containing 11 [Source:VGNC Symbol;Acc:VGNC:95059]	-1.71	0.1324
ssc-miR-124a	ZBTB16	zinc finger and BTB domain containing 16 [Source:VGNC Symbol;Acc:VGNC:108626]	-1.71	0.1324
ssc-miR-124a	ZBTB17	zinc finger and BTB domain containing 17 [Source:VGNC Symbol;Acc:VGNC:98682]	-1.71	0.1324
ssc-miR-124a	ZBTB18	zinc finger and BTB domain containing 18 [Source:HGNC Symbol;Acc:HGNC:13030]	-1.71	0.1324
ssc-miR-124a	ZBTB20	zinc finger and BTB domain containing 20 [Source:VGNC Symbol;Acc:VGNC:95063]	-1.71	0.1324
ssc-miR-124a	ZBTB34	zinc finger and BTB domain containing 34 [Source:VGNC Symbol;Acc:VGNC:95070]	-1.71	0.1324
ssc-miR-124a	ZBTB38	zinc finger and BTB domain containing 38 [Source:VGNC Symbol;Acc:VGNC:95072]	-1.71	0.1324
ssc-miR-124a	ZBTB39	zinc finger and BTB domain containing 39 [Source:VGNC Symbol;Acc:VGNC:95073]	-1.71	0.1324
ssc-miR-124a	ZBTB41	zinc finger and BTB domain containing 41 [Source:VGNC Symbol;Acc:VGNC:108284]	-1.71	0.1324
ssc-miR-124a	ZBTB5	zinc finger and BTB domain containing 5 [Source:VGNC Symbol;Acc:VGNC:103204]	-1.71	0.1324
ssc-miR-124a	ZBTB6	zinc finger and BTB domain containing 6 [Source:VGNC Symbol;Acc:VGNC:95080]	-1.71	0.1324
ssc-miR-124a	ZBTB7A	zinc finger and BTB domain containing 7A [Source:VGNC Symbol;Acc:VGNC:95081]	-1.71	0.1324
ssc-miR-124a	ZBTB7B	zinc finger and BTB domain containing 7B [Source:VGNC Symbol;Acc:VGNC:95082]	-1.71	0.1324
ssc-miR-124a	ZBTB7C	zinc finger and BTB domain containing 7C [Source:VGNC Symbol;Acc:VGNC:95083]	-1.71	0.1324
ssc-miR-124a	ZC3H10	zinc finger CCCH-type containing 10 [Source:VGNC Symbol;Acc:VGNC:95088]	-1.71	0.1324
ssc-miR-124a	ZC4H2	zinc finger C4H2-type containing [Source:VGNC Symbol;Acc:VGNC:95105]	-1.71	0.1324
ssc-miR-124a	ZCCHC14	zinc finger CCHC-type containing 14 [Source:VGNC Symbol;Acc:VGNC:95107]	-1.71	0.1324
ssc-miR-124a	ZCCHC24	zinc finger CCHC-type containing 24 [Source:HGNC Symbol;Acc:HGNC:26911]	-1.71	0.1324
ssc-miR-124a	ZCWPW2	zinc finger CW-type and PWWP domain containing 2 [Source:VGNC Symbol;Acc:VGNC:108706]	-1.71	0.1324
ssc-miR-124a	ZDHHC14	zinc finger DHHC-type palmitoyltransferase 14 [Source:VGNC Symbol;Acc:VGNC:95117]	-1.71	0.1324
ssc-miR-124a	ZDHHC17	zinc finger DHHC-type palmitoyltransferase 17 [Source:VGNC Symbol;Acc:VGNC:95119]	-1.71	0.1324
ssc-miR-124a	ZDHHC2	zinc finger DHHC-type palmitoyltransferase 2 [Source:VGNC Symbol;Acc:VGNC:95886]	-1.71	0.1324
ssc-miR-124a	ZDHHC20	zinc finger DHHC-type palmitoyltransferase 20 [Source:VGNC Symbol;Acc:VGNC:95122]	-1.71	0.1324
ssc-miR-124a	ZDHHC3	zinc finger DHHC-type palmitoyltransferase 3 [Source:VGNC Symbol;Acc:VGNC:95126]	-1.71	0.1324
ssc-miR-124a	ZDHHC7	zinc finger DHHC-type palmitoyltransferase 7 [Source:VGNC Symbol;Acc:VGNC:95129]	-1.71	0.1324
ssc-miR-124a	ZDHHC8	zinc finger DHHC-type palmitoyltransferase 8 [Source:VGNC Symbol;Acc:VGNC:95130]	-1.71	0.1324
ssc-miR-124a	ZEB1	zinc finger E-box binding homeobox 1 [Source:VGNC Symbol;Acc:VGNC:95536]	-1.71	0.1324
ssc-miR-124a	ZFP3	hypothetical gene	-1.71	0.1324
ssc-miR-124a	ZFP36L1	ZFP36 ring finger protein like 1 [Source:VGNC Symbol;Acc:VGNC:95146]	-1.71	0.1324
ssc-miR-124a	ZFP36L2	ZFP36 ring finger protein like 2 [Source:VGNC Symbol;Acc:VGNC:95147]	-1.71	0.1324
ssc-miR-124a	ZFPM2	zinc finger protein, FOG family member 2 [Source:VGNC Symbol;Acc:VGNC:95153]	-1.71	0.1324
ssc-miR-124a	ZFR2	zinc finger RNA binding protein 2 [Source:VGNC Symbol;Acc:VGNC:95155]	-1.71	0.1324

ssc-miR-124a	ZFYVE21	zinc finger FYVE-type containing 21 [Source:VGNC Symbol;Acc:VGNC:95159]	-1.71	0.1324
ssc-miR-124a	ZHX3	zinc fingers and homeoboxes 3 [Source:VGNC Symbol;Acc:VGNC:95717]	-1.71	0.1324
ssc-miR-124a	ZKSCAN3	hypothetical gene	-1.71	0.1324
ssc-miR-124a	ZKSCAN4	zinc finger with KRAB and SCAN domains 4 [Source:VGNC Symbol;Acc:VGNC:95173]	-1.71	0.1324
ssc-miR-124a	ZKSCAN8	zinc finger with KRAB and SCAN domains 8 [Source:HGNC Symbol;Acc:HGNC:12983]	-1.71	0.1324
ssc-miR-124a	ZMAT3	zinc finger matrin-type 3 [Source:VGNC Symbol;Acc:VGNC:95177]	-1.71	0.1324
ssc-miR-124a	ZNF131	zinc finger protein 131 [Source:HGNC Symbol;Acc:HGNC:12915]	-1.71	0.1324
ssc-miR-124a	ZNF148	zinc finger protein 148 [Source:VGNC Symbol;Acc:VGNC:95192]	-1.71	0.1324
ssc-miR-124a	ZNF189	zinc finger protein 189 [Source:VGNC Symbol;Acc:VGNC:103205]	-1.71	0.1324
ssc-miR-124a	ZNF2	zinc finger protein 2 [Source:VGNC Symbol;Acc:VGNC:98421]	-1.71	0.1324
ssc-miR-124a	ZNF219	zinc finger protein 219 [Source:VGNC Symbol;Acc:VGNC:95207]	-1.71	0.1324
ssc-miR-124a	ZNF25	zinc finger protein 25 [Source:VGNC Symbol;Acc:VGNC:98423]	-1.71	0.1324
ssc-miR-124a	ZNF275	hypothetical gene	-1.71	0.1324
ssc-miR-124a	ZNF287	zinc finger protein 287 [Source:VGNC Symbol;Acc:VGNC:99107]	-1.71	0.1324
ssc-miR-124a	ZNF292	zinc finger protein 292 [Source:VGNC Symbol;Acc:VGNC:95218]	-1.71	0.1324
ssc-miR-124a	ZNF318	zinc finger protein 318 [Source:VGNC Symbol;Acc:VGNC:95222]	-1.71	0.1324
ssc-miR-124a	ZNF326	zinc finger protein 326 [Source:HGNC Symbol;Acc:HGNC:14104]	-1.71	0.1324
ssc-miR-124a	ZNF362	zinc finger protein 362 [Source:VGNC Symbol;Acc:VGNC:95229]	-1.71	0.1324
ssc-miR-124a	ZNF391	zinc finger protein 391 [Source:HGNC Symbol;Acc:HGNC:18779]	-1.71	0.1324
ssc-miR-124a	ZNF397	zinc finger protein 397 [Source:VGNC Symbol;Acc:VGNC:95236]	-1.71	0.1324
ssc-miR-124a	ZNF432	hypothetical gene	-1.71	0.1324
ssc-miR-124a	ZNF449	zinc finger protein 449 [Source:VGNC Symbol;Acc:VGNC:95242]	-1.71	0.1324
ssc-miR-124a	ZNF451	zinc finger protein 451 [Source:HGNC Symbol;Acc:HGNC:21091]	-1.71	0.1324
ssc-miR-124a	ZNF503	zinc finger protein 503 [Source:VGNC Symbol;Acc:VGNC:95252]	-1.71	0.1324
ssc-miR-124a	ZNF507	zinc finger protein 507 [Source:VGNC Symbol;Acc:VGNC:95253]	-1.71	0.1324
ssc-miR-124a	ZNF518B	zinc finger protein 518B [Source:VGNC Symbol;Acc:VGNC:95258]	-1.71	0.1324
ssc-miR-124a	ZNF546	zinc finger protein 546 [Source:VGNC Symbol;Acc:VGNC:98716]	-1.71	0.1324
ssc-miR-124a	ZNF605	zinc finger protein 605 [Source:HGNC Symbol;Acc:HGNC:28068]	-1.71	0.1324
ssc-miR-124a	ZNF608	hypothetical gene	-1.71	0.1324
ssc-miR-124a	ZNF609	zinc finger protein 609 [Source:VGNC Symbol;Acc:VGNC:95274]	-1.71	0.1324
ssc-miR-124a	ZNF618	zinc finger protein 618 [Source:VGNC Symbol;Acc:VGNC:95276]	-1.71	0.1324
ssc-miR-124a	ZNF652	zinc finger protein 652 [Source:VGNC Symbol;Acc:VGNC:99108]	-1.71	0.1324
ssc-miR-124a	ZNF654	zinc finger protein 654 [Source:VGNC Symbol;Acc:VGNC:95285]	-1.71	0.1324
ssc-miR-124a	ZNF687	zinc finger protein 687 [Source:VGNC Symbol;Acc:VGNC:95292]	-1.71	0.1324
ssc-miR-124a	ZNF704	zinc finger protein 704 [Source:VGNC Symbol;Acc:VGNC:95296]	-1.71	0.1324
ssc-miR-124a	ZNF706	hypothetical gene	-1.71	0.1324
ssc-miR-124a	ZNF710	zinc finger protein 710 [Source:VGNC Symbol;Acc:VGNC:107180]	-1.71	0.1324
ssc-miR-124a	ZNF740	zinc finger protein 740 [Source:VGNC Symbol;Acc:VGNC:95298]	-1.71	0.1324
ssc-miR-124a	ZNF772	zinc finger protein 772 [Source:HGNC Symbol;Acc:HGNC:33106]	-1.71	0.1324
ssc-miR-124a	ZNF784	zinc finger protein 784 [Source:VGNC Symbol;Acc:VGNC:95301]	-1.71	0.1324
ssc-miR-124a	ZNF831	zinc finger protein 831 [Source:VGNC Symbol;Acc:VGNC:95765]	-1.71	0.1324
ssc-miR-124a	ZNHIT6	zinc finger HIT-type containing 6 [Source:VGNC Symbol;Acc:VGNC:98909]	-1.71	0.1324
ssc-miR-124a	ZNRF3	zinc and ring finger 3 [Source:VGNC Symbol;Acc:VGNC:95318]	-1.71	0.1324
ssc-miR-124a	ZRANB2	zinc finger RANBP2-type containing 2 [Source:VGNC Symbol;Acc:VGNC:95323]	-1.71	0.1324
ssc-miR-124a	ZXDA	hypothetical gene	-1.71	0.1324
ssc-miR-124a	ZXDB	hypothetical gene	-1.71	0.1324
ssc-miR-7141-5p	NA	hypothetical gene	-1.71	0.1605
ssc-miR-205	AAK1	AP2 associated kinase 1 [Source:VGNC Symbol;Acc:VGNC:100379]	-1.72	0.0238
ssc-miR-205	ABCA9	ATP binding cassette subfamily A member 9 [Source:HGNC Symbol;Acc:HGNC:39]	-1.72	0.0238
ssc-miR-205	ABCD2	ATP binding cassette subfamily D member 2 [Source:VGNC Symbol;Acc:VGNC:84962]	-1.72	0.0238

ssc-miR-205	ABHD10	abhydrolase domain containing 10, depalmitoylase [Source:VGNC Symbol;Acc:VGNC:84969]	-1.72	0.0238
ssc-miR-205	ABHD17B	abhydrolase domain containing 17B, depalmitoylase [Source:VGNC Symbol;Acc:VGNC:84977]	-1.72	0.0238
ssc-miR-205	ABI2	abl interactor 2 [Source:VGNC Symbol;Acc:VGNC:96027]	-1.72	0.0238
ssc-miR-205	AC010336.1	hypothetical gene	-1.72	0.0238
ssc-miR-205	AC012215.1	hypothetical gene	-1.72	0.0238
ssc-miR-205	AC104841.2	hypothetical gene	-1.72	0.0238
ssc-miR-205	AC129492.6	hypothetical gene	-1.72	0.0238
ssc-miR-205	ACBD5	acyl-CoA binding domain containing 5 [Source:VGNC Symbol;Acc:VGNC:95997]	-1.72	0.0238
ssc-miR-205	ACSL1	acyl-CoA synthetase long chain family member 1 [Source:VGNC Symbol;Acc:VGNC:96294]	-1.72	0.0238
ssc-miR-205	ACTB	actin beta [Source:VGNC Symbol;Acc:VGNC:96915]	-1.72	0.0238
ssc-miR-205	ADAMTS9	ADAM metallopeptidase with thrombospondin type 1 motif 9 [Source:VGNC Symbol;Acc:VGNC:85089]	-1.72	0.0238
ssc-miR-205	AEBP2	AE binding protein 2 [Source:VGNC Symbol;Acc:VGNC:85163]	-1.72	0.0238
ssc-miR-205	AFF1	AF4/FMR2 family member 1 [Source:VGNC Symbol;Acc:VGNC:85167]	-1.72	0.0238
ssc-miR-205	AFF3	AF4/FMR2 family member 3 [Source:HGNC Symbol;Acc:HGNC:6473]	-1.72	0.0238
ssc-miR-205	AFF4	AF4/FMR2 family member 4 [Source:VGNC Symbol;Acc:VGNC:85169]	-1.72	0.0238
ssc-miR-205	AGO1	hypothetical gene	-1.72	0.0238
ssc-miR-205	AGPAT6	hypothetical gene	-1.72	0.0238
ssc-miR-205	AL031666.2	hypothetical gene	-1.72	0.0238
ssc-miR-205	AL626787.1	hypothetical gene	-1.72	0.0238
ssc-miR-205	ALDH6A1	aldehyde dehydrogenase 6 family member A1 [Source:VGNC Symbol;Acc:VGNC:85242]	-1.72	0.0238
ssc-miR-205	ALDOB	aldolase, fructose-bisphosphate B [Source:VGNC Symbol;Acc:VGNC:85245]	-1.72	0.0238
ssc-miR-205	ALG11	ALG11 alpha-1,2-mannosyltransferase [Source:VGNC Symbol;Acc:VGNC:85247]	-1.72	0.0238
ssc-miR-205	ALX4	ALX homeobox 4 [Source:VGNC Symbol;Acc:VGNC:96791]	-1.72	0.0238
ssc-miR-205	AMOT	angiomotin [Source:VGNC Symbol;Acc:VGNC:85283]	-1.72	0.0238
ssc-miR-205	ANGPT2	angiopoietin 2 [Source:NCBI gene (formerly Entrezgene);Acc:396730]	-1.72	0.0238
ssc-miR-205	ANK2	hypothetical gene	-1.72	0.0238
ssc-miR-205	ANKRD50	ankyrin repeat domain containing 50 [Source:VGNC Symbol;Acc:VGNC:85341]	-1.72	0.0238
ssc-miR-205	ANKRD53	ankyrin repeat domain 53 [Source:HGNC Symbol;Acc:HGNC:25691]	-1.72	0.0238
ssc-miR-205	ANO10	anoctamin 10 [Source:VGNC Symbol;Acc:VGNC:85355]	-1.72	0.0238
ssc-miR-205	AP1AR	adaptor related protein complex 1 associated regulatory protein [Source:HGNC Symbol;Acc:HGNC:28808]	-1.72	0.0238
ssc-miR-205	AP1G1	adaptor related protein complex 1 subunit gamma 1 [Source:VGNC Symbol;Acc:VGNC:85378]	-1.72	0.0238
ssc-miR-205	APBB2	amyloid beta protein binding family B member 2 [Source:VGNC Symbol;Acc:VGNC:85403]	-1.72	0.0238
ssc-miR-205	ARFGEF1	ADP ribosylation factor guanine nucleotide exchange factor 1 [Source:VGNC Symbol;Acc:VGNC:85451]	-1.72	0.0238
ssc-miR-205	ARHGAP17	Rho GTPase activating protein 17 [Source:VGNC Symbol;Acc:VGNC:85459]	-1.72	0.0238
ssc-miR-205	ARHGAP19-SLIT1	hypothetical gene	-1.72	0.0238
ssc-miR-205	ARHGAP24	Rho GTPase activating protein 24 [Source:VGNC Symbol;Acc:VGNC:85463]	-1.72	0.0238
ssc-miR-205	ARID1A	AT-rich interaction domain 1A [Source:VGNC Symbol;Acc:VGNC:85505]	-1.72	0.0238
ssc-miR-205	ARMC8	armadillo repeat containing 8 [Source:VGNC Symbol;Acc:VGNC:85531]	-1.72	0.0238
ssc-miR-205	ARRB2	arrestin beta 2 [Source:VGNC Symbol;Acc:VGNC:85543]	-1.72	0.0238
ssc-miR-205	ATP7A	ATPase copper transporting alpha [Source:VGNC Symbol;Acc:VGNC:85678]	-1.72	0.0238
ssc-miR-205	ATP8A2	ATPase phospholipid transporting 8A2 [Source:HGNC Symbol;Acc:HGNC:13533]	-1.72	0.0238
ssc-miR-205	AXIN2	axin 2 [Source:VGNC Symbol;Acc:VGNC:85708]	-1.72	0.0238
ssc-miR-205	B3GNT5	UDP-GlcNAc:betaGal beta-1,3-N-acetylglucosaminyltransferase 5 [Source:VGNC Symbol;Acc:VGNC:85726]	-1.72	0.0238
ssc-miR-205	B4GALT4	beta-1,4-galactosyltransferase 4 [Source:HGNC Symbol;Acc:HGNC:927]	-1.72	0.0238
ssc-miR-205	B4GALT5	beta-1,4-galactosyltransferase 5 [Source:VGNC Symbol;Acc:VGNC:96499]	-1.72	0.0238
ssc-miR-205	B4GALT6	beta-1,4-galactosyltransferase 6 [Source:VGNC Symbol;Acc:VGNC:85734]	-1.72	0.0238
ssc-miR-205	BAMBI	BMP and activin membrane bound inhibitor [Source:VGNC Symbol;Acc:VGNC:96238]	-1.72	0.0238
ssc-miR-205	BCAS3	BCAS3 microtubule associated cell migration factor [Source:VGNC Symbol;Acc:VGNC:85772]	-1.72	0.0238
ssc-miR-205	BCAS4	breast carcinoma amplified sequence 4 [Source:NCBI gene (formerly Entrezgene);Acc:100141422]	-1.72	0.0238
ssc-miR-205	BCL6	BCL6 transcription repressor [Source:VGNC Symbol;Acc:VGNC:96565]	-1.72	0.0238

ssc-miR-205	BEAN1	brain expressed associated with NEDD4 1 [Source:VGNC Symbol;Acc:VGNC:85797]	-1.72	0.0238
ssc-miR-205	BEND4	BEN domain containing 4 [Source:VGNC Symbol;Acc:VGNC:85801]	-1.72	0.0238
ssc-miR-205	BHLHE40	basic helix-loop-helix family member e40 [Source:VGNC Symbol;Acc:VGNC:85814]	-1.72	0.0238
ssc-miR-205	BICC1	BicC family RNA binding protein 1 [Source:VGNC Symbol;Acc:VGNC:85817]	-1.72	0.0238
ssc-miR-205	BMF	Bcl2 modifying factor [Source:VGNC Symbol;Acc:VGNC:85837]	-1.72	0.0238
ssc-miR-205	BMPER	BMP binding endothelial regulator [Source:VGNC Symbol;Acc:VGNC:97902]	-1.72	0.0238
ssc-miR-205	BROX	BRO1 domain and CAAX motif containing [Source:VGNC Symbol;Acc:VGNC:96132]	-1.72	0.0238
ssc-miR-205	BTBD3	BTB domain containing 3 [Source:VGNC Symbol;Acc:VGNC:95673]	-1.72	0.0238
ssc-miR-205	C10orf131	hypothetical gene	-1.72	0.0238
ssc-miR-205	C11orf34	hypothetical gene	-1.72	0.0238
ssc-miR-205	C11orf57	hypothetical gene	-1.72	0.0238
ssc-miR-205	C11orf86	chromosome 2 C11orf86 homolog [Source:VGNC Symbol;Acc:VGNC:86000]	-1.72	0.0238
ssc-miR-205	C12orf29	chromosome 5 C12orf29 homolog [Source:VGNC Symbol;Acc:VGNC:86040]	-1.72	0.0238
ssc-miR-205	C15orf61	chromosome 1 C15orf61 homolog [Source:VGNC Symbol;Acc:VGNC:85962]	-1.72	0.0238
ssc-miR-205	C16orf52	hypothetical gene	-1.72	0.0238
ssc-miR-205	C17orf97	hypothetical gene	-1.72	0.0238
ssc-miR-205	C20orf194	hypothetical gene	-1.72	0.0238
ssc-miR-205	C3orf80	chromosome 13 C3orf80 homolog [Source:VGNC Symbol;Acc:VGNC:85938]	-1.72	0.0238
ssc-miR-205	C4orf36	chromosome 8 C4orf36 homolog [Source:VGNC Symbol;Acc:VGNC:98914]	-1.72	0.0238
ssc-miR-205	CADM1	cell adhesion molecule 1 [Source:VGNC Symbol;Acc:VGNC:86134]	-1.72	0.0238
ssc-miR-205	CALCOCO1	calcium binding and coiled-coil domain 1 [Source:VGNC Symbol;Acc:VGNC:86140]	-1.72	0.0238
ssc-miR-205	CALCRL	calcitonin receptor like receptor [Source:NCBI gene (formerly Entrezgene);Acc:397172]	-1.72	0.0238
ssc-miR-205	CALM1	calmodulin 1 [Source:NCBI gene (formerly Entrezgene);Acc:100154056]	-1.72	0.0238
ssc-miR-205	CALU	calumenin [Source:VGNC Symbol;Acc:VGNC:86151]	-1.72	0.0238
ssc-miR-205	CAMK2N1	calcium/calmodulin dependent protein kinase II inhibitor 1 [Source:VGNC Symbol;Acc:VGNC:96935]	-1.72	0.0238
ssc-miR-205	CAMKK1	calcium/calmodulin dependent protein kinase kinase 1 [Source:VGNC Symbol;Acc:VGNC:98977]	-1.72	0.0238
ssc-miR-205	CAND1	cullin associated and neddylation dissociated 1 [Source:VGNC Symbol;Acc:VGNC:97912]	-1.72	0.0238
ssc-miR-205	CASC4	hypothetical gene	-1.72	0.0238
ssc-miR-205	CASD1	CAS1 domain containing 1 [Source:VGNC Symbol;Acc:VGNC:86199]	-1.72	0.0238
ssc-miR-205	CBX1	chromobox 1 [Source:VGNC Symbol;Acc:VGNC:97914]	-1.72	0.0238
ssc-miR-205	CCDC149	coiled-coil domain containing 149 [Source:VGNC Symbol;Acc:VGNC:86262]	-1.72	0.0238
ssc-miR-205	CCDC176	hypothetical gene	-1.72	0.0238
ssc-miR-205	CCDC43	coiled-coil domain containing 43 [Source:VGNC Symbol;Acc:VGNC:98979]	-1.72	0.0238
ssc-miR-205	CCDC85C	coiled-coil domain containing 85C [Source:VGNC Symbol;Acc:VGNC:86322]	-1.72	0.0238
ssc-miR-205	CCDC93	coiled-coil domain containing 93 [Source:VGNC Symbol;Acc:VGNC:96095]	-1.72	0.0238
ssc-miR-205	CCNJ	cyclin J [Source:VGNC Symbol;Acc:VGNC:86360]	-1.72	0.0238
ssc-miR-205	CCNT1	cyclin T1 [Source:VGNC Symbol;Acc:VGNC:86366]	-1.72	0.0238
ssc-miR-205	CDC20B	cell division cycle 20B [Source:HGNC Symbol;Acc:HGNC:24222]	-1.72	0.0238
ssc-miR-205	CDC27	cell division cycle 27 [Source:VGNC Symbol;Acc:VGNC:86448]	-1.72	0.0238
ssc-miR-205	CDC42BPB	CDC42 binding protein kinase beta [Source:VGNC Symbol;Acc:VGNC:86453]	-1.72	0.0238
ssc-miR-205	CDH11	cadherin 11 [Source:VGNC Symbol;Acc:VGNC:86475]	-1.72	0.0238
ssc-miR-205	CDH19	cadherin 19 [Source:VGNC Symbol;Acc:VGNC:86482]	-1.72	0.0238
ssc-miR-205	CDH7	cadherin 7 [Source:VGNC Symbol;Acc:VGNC:97923]	-1.72	0.0238
ssc-miR-205	CDK14	cyclin dependent kinase 14 [Source:VGNC Symbol;Acc:VGNC:86498]	-1.72	0.0238
ssc-miR-205	CDK19	cyclin dependent kinase 19 [Source:VGNC Symbol;Acc:VGNC:86502]	-1.72	0.0238
ssc-miR-205	CENPE	centromere protein E [Source:VGNC Symbol;Acc:VGNC:86547]	-1.72	0.0238
ssc-miR-205	CENPF	centromere protein F [Source:VGNC Symbol;Acc:VGNC:86548]	-1.72	0.0238
ssc-miR-205	CENPO	centromere protein O [Source:HGNC Symbol;Acc:HGNC:28152]	-1.72	0.0238
ssc-miR-205	CEP128	centrosomal protein 128 [Source:VGNC Symbol;Acc:VGNC:86560]	-1.72	0.0238
ssc-miR-205	CEP63	centrosomal protein 63 [Source:VGNC Symbol;Acc:VGNC:86574]	-1.72	0.0238

ssc-miR-205	CFL2	cofilin 2 [Source:VGNC Symbol;Acc:VGNC:86611]	-1.72	0.0238
ssc-miR-205	CHIC1	cysteine rich hydrophobic domain 1 [Source:VGNC Symbol;Acc:VGNC:86642]	-1.72	0.0238
ssc-miR-205	CHN1	hypothetical gene	-1.72	0.0238
ssc-miR-205	CHST7	carbohydrate sulfotransferase 7 [Source:VGNC Symbol;Acc:VGNC:86681]	-1.72	0.0238
ssc-miR-205	CLCN6	chloride voltage-gated channel 6 [Source:VGNC Symbol;Acc:VGNC:86729]	-1.72	0.0238
ssc-miR-205	CLDN11	claudin 11 [Source:NCBI gene (formerly Entrezgene);Acc:100302016]	-1.72	0.0238
ssc-miR-205	CLDN8	claudin 8 [Source:VGNC Symbol;Acc:VGNC:86740]	-1.72	0.0238
ssc-miR-205	CLINT1	clathrin interactor 1 [Source:VGNC Symbol;Acc:VGNC:86765]	-1.72	0.0238
ssc-miR-205	CLK3	CDC like kinase 3 [Source:VGNC Symbol;Acc:VGNC:107129]	-1.72	0.0238
ssc-miR-205	CLTC	clathrin heavy chain [Source:VGNC Symbol;Acc:VGNC:86789]	-1.72	0.0238
ssc-miR-205	CMTM4	CKLF like MARVEL transmembrane domain containing 4 [Source:VGNC Symbol;Acc:VGNC:86804]	-1.72	0.0238
ssc-miR-205	CNIH1	cornichon family AMPA receptor auxiliary protein 1 [Source:VGNC Symbol;Acc:VGNC:86820]	-1.72	0.0238
ssc-miR-205	CNNM1	cyclin and CBS domain divalent metal cation transport mediator 1 [Source:VGNC Symbol;Acc:VGNC:86827]	-1.72	0.0238
ssc-miR-205	CNP	2',3'-cyclic nucleotide 3' phosphodiesterase [Source:VGNC Symbol;Acc:VGNC:86840]	-1.72	0.0238
ssc-miR-205	CNR2	cannabinoid receptor 2 [Source:VGNC Symbol;Acc:VGNC:86844]	-1.72	0.0238
ssc-miR-205	COL16A1	collagen type XVI alpha 1 chain [Source:VGNC Symbol;Acc:VGNC:86867]	-1.72	0.0238
ssc-miR-205	COMMD10	COMM domain containing 10 [Source:VGNC Symbol;Acc:VGNC:86886]	-1.72	0.0238
ssc-miR-205	COQ10B	coenzyme Q10B [Source:VGNC Symbol;Acc:VGNC:96107]	-1.72	0.0238
ssc-miR-205	COX4I1	cytochrome c oxidase subunit 4I1 [Source:VGNC Symbol;Acc:VGNC:96957]	-1.72	0.0238
ssc-miR-205	CPEB2	cytoplasmic polyadenylation element binding protein 2 [Source:VGNC Symbol;Acc:VGNC:86937]	-1.72	0.0238
ssc-miR-205	CREB1	cAMP responsive element binding protein 1 [Source:VGNC Symbol;Acc:VGNC:96004]	-1.72	0.0238
ssc-miR-205	CREBRF	CREB3 regulatory factor [Source:VGNC Symbol;Acc:VGNC:86987]	-1.72	0.0238
ssc-miR-205	CRMP1	collapsin response mediator protein 1 [Source:VGNC Symbol;Acc:VGNC:87000]	-1.72	0.0238
ssc-miR-205	CSF1	colony stimulating factor 1 [Source:VGNC Symbol;Acc:VGNC:87030]	-1.72	0.0238
ssc-miR-205	CSNK1A1	casein kinase 1 alpha 1 [Source:VGNC Symbol;Acc:VGNC:99630]	-1.72	0.0238
ssc-miR-205	CSNK2A1	hypothetical gene	-1.72	0.0238
ssc-miR-205	CSPP1	centrosome and spindle pole associated protein 1 [Source:VGNC Symbol;Acc:VGNC:87045]	-1.72	0.0238
ssc-miR-205	CTH	cystathionine gamma-lyase [Source:VGNC Symbol;Acc:VGNC:96961]	-1.72	0.0238
ssc-miR-205	CTNNA1	catenin alpha 1 [Source:VGNC Symbol;Acc:VGNC:87063]	-1.72	0.0238
ssc-miR-205	CTPS2	CTP synthase 2 [Source:VGNC Symbol;Acc:VGNC:87071]	-1.72	0.0238
ssc-miR-205	CUX2	cut like homeobox 2 [Source:VGNC Symbol;Acc:VGNC:87094]	-1.72	0.0238
ssc-miR-205	CXorf21	hypothetical gene	-1.72	0.0238
ssc-miR-205	DAB2	DAB adaptor protein 2 [Source:VGNC Symbol;Acc:VGNC:87144]	-1.72	0.0238
ssc-miR-205	DAW1	dynein assembly factor with WD repeats 1 [Source:HGNC Symbol;Acc:HGNC:26383]	-1.72	0.0238
ssc-miR-205	DBF4	hypothetical gene	-1.72	0.0238
ssc-miR-205	DCDC2	doublecortin domain containing 2 [Source:VGNC Symbol;Acc:VGNC:87181]	-1.72	0.0238
ssc-miR-205	DCHS1	dachsous cadherin-related 1 [Source:VGNC Symbol;Acc:VGNC:97959]	-1.72	0.0238
ssc-miR-205	DDI2	hypothetical gene	-1.72	0.0238
ssc-miR-205	DDR1	discoidin domain receptor tyrosine kinase 1 [Source:VGNC Symbol;Acc:VGNC:107135]	-1.72	0.0238
ssc-miR-205	DDX11	DEAD/H-box helicase 11 [Source:HGNC Symbol;Acc:HGNC:2736]	-1.72	0.0238
ssc-miR-205	DDX5	DEAD-box helicase 5 [Source:VGNC Symbol;Acc:VGNC:87226]	-1.72	0.0238
ssc-miR-205	DDX52	DExD-box helicase 52 [Source:VGNC Symbol;Acc:VGNC:87229]	-1.72	0.0238
ssc-miR-205	DDX6	DEAD-box helicase 6 [Source:VGNC Symbol;Acc:VGNC:87233]	-1.72	0.0238
ssc-miR-205	DGCR14	hypothetical gene	-1.72	0.0238
ssc-miR-205	DGCR2	DiGeorge syndrome critical region gene 2 [Source:HGNC Symbol;Acc:HGNC:2845]	-1.72	0.0238
ssc-miR-205	DGCR8	DGCR8 microprocessor complex subunit [Source:VGNC Symbol;Acc:VGNC:87268]	-1.72	0.0238
ssc-miR-205	DGKI	diacylglycerol kinase iota [Source:VGNC Symbol;Acc:VGNC:87274]	-1.72	0.0238
ssc-miR-205	DHCR24	24-dehydrocholesterol reductase [Source:NCBI gene (formerly Entrezgene);Acc:100628197]	-1.72	0.0238
ssc-miR-205	DLEU1	hypothetical gene	-1.72	0.0238
ssc-miR-205	DLG2	discs large MAGUK scaffold protein 2 [Source:VGNC Symbol;Acc:VGNC:108581]	-1.72	0.0238

ssc-miR-205	DMXL1	Dmx like 1 [Source:VGNC Symbol;Acc:VGNC:87359]	-1.72	0.0238
ssc-miR-205	DNAJA1	hypothetical gene	-1.72	0.0238
ssc-miR-205	DNAJC8	DnaJ heat shock protein family (Hsp40) member C8 [Source:VGNC Symbol;Acc:VGNC:96626]	-1.72	0.0238
ssc-miR-205	DNM3	dynamin 3 [Source:VGNC Symbol;Acc:VGNC:87382]	-1.72	0.0238
ssc-miR-205	DOCK11	dedicator of cytokinesis 11 [Source:VGNC Symbol;Acc:VGNC:87391]	-1.72	0.0238
ssc-miR-205	DOCK3	dedicator of cytokinesis 3 [Source:VGNC Symbol;Acc:VGNC:87393]	-1.72	0.0238
ssc-miR-205	DOK4	docking protein 4 [Source:VGNC Symbol;Acc:VGNC:87403]	-1.72	0.0238
ssc-miR-205	DSC1	desmocollin 1 [Source:VGNC Symbol;Acc:VGNC:87452]	-1.72	0.0238
ssc-miR-205	DSC2	desmocollin 2 [Source:HGNC Symbol;Acc:HGNC:3036]	-1.72	0.0238
ssc-miR-205	DSC3	desmocollin 3 [Source:HGNC Symbol;Acc:HGNC:3037]	-1.72	0.0238
ssc-miR-205	DST	dystonin [Source:HGNC Symbol;Acc:HGNC:1090]	-1.72	0.0238
ssc-miR-205	DUSP19	dual specificity phosphatase 19 [Source:VGNC Symbol;Acc:VGNC:95899]	-1.72	0.0238
ssc-miR-205	DUSP7	dual specificity phosphatase 7 [Source:HGNC Symbol;Acc:HGNC:3073]	-1.72	0.0238
ssc-miR-205	E2F1	E2F transcription factor 1 [Source:VGNC Symbol;Acc:VGNC:96256]	-1.72	0.0238
ssc-miR-205	EAF1	ELL associated factor 1 [Source:VGNC Symbol;Acc:VGNC:87521]	-1.72	0.0238
ssc-miR-205	EFCAB4A	hypothetical gene	-1.72	0.0238
ssc-miR-205	EIF4E	eukaryotic translation initiation factor 4E [Source:VGNC Symbol;Acc:VGNC:87626]	-1.72	0.0238
ssc-miR-205	ELAVL4	ELAV like RNA binding protein 4 [Source:VGNC Symbol;Acc:VGNC:97047]	-1.72	0.0238
ssc-miR-205	ELF1	E74 like ETS transcription factor 1 [Source:VGNC Symbol;Acc:VGNC:87641]	-1.72	0.0238
ssc-miR-205	ELK3	ETS transcription factor ELK3 [Source:VGNC Symbol;Acc:VGNC:87646]	-1.72	0.0238
ssc-miR-205	ELMSAN1	hypothetical gene	-1.72	0.0238
ssc-miR-205	EMP2	epithelial membrane protein 2 [Source:VGNC Symbol;Acc:VGNC:87689]	-1.72	0.0238
ssc-miR-205	ENC1	ectodermal-neural cortex 1 [Source:VGNC Symbol;Acc:VGNC:87696]	-1.72	0.0238
ssc-miR-205	ENDOD1	endonuclease domain containing 1 [Source:VGNC Symbol;Acc:VGNC:87697]	-1.72	0.0238
ssc-miR-205	ENPEP	glutamyl aminopeptidase [Source:VGNC Symbol;Acc:VGNC:98923]	-1.72	0.0238
ssc-miR-205	ENPP4	ectonucleotide pyrophosphatase/phosphodiesterase 4 [Source:VGNC Symbol;Acc:VGNC:87711]	-1.72	0.0238
ssc-miR-205	ENTPD1	ectonucleoside triphosphate diphosphohydrolase 1 [Source:VGNC Symbol;Acc:VGNC:97977]	-1.72	0.0238
ssc-miR-205	EPB41	erythrocyte membrane protein band 4.1 [Source:VGNC Symbol;Acc:VGNC:87722]	-1.72	0.0238
ssc-miR-205	EPB41L1	hypothetical gene	-1.72	0.0238
ssc-miR-205	EPB41L4B	erythrocyte membrane protein band 4.1 like 4B [Source:VGNC Symbol;Acc:VGNC:103088]	-1.72	0.0238
ssc-miR-205	EPS15	epidermal growth factor receptor pathway substrate 15 [Source:VGNC Symbol;Acc:VGNC:87747]	-1.72	0.0238
ssc-miR-205	EPS8	epidermal growth factor receptor pathway substrate 8 [Source:VGNC Symbol;Acc:VGNC:87749]	-1.72	0.0238
ssc-miR-205	ERBB3	erb-b2 receptor tyrosine kinase 3 [Source:VGNC Symbol;Acc:VGNC:87760]	-1.72	0.0238
ssc-miR-205	ERBB4	erb-b2 receptor tyrosine kinase 4 [Source:VGNC Symbol;Acc:VGNC:96284]	-1.72	0.0238
ssc-miR-205	ERCC6L2	ERCC excision repair 6 like 2 [Source:VGNC Symbol;Acc:VGNC:107140]	-1.72	0.0238
ssc-miR-205	EREG	epiregulin [Source:VGNC Symbol;Acc:VGNC:87766]	-1.72	0.0238
ssc-miR-205	ERMP1	endoplasmic reticulum metallopeptidase 1 [Source:VGNC Symbol;Acc:VGNC:87777]	-1.72	0.0238
ssc-miR-205	ERP29	endoplasmic reticulum protein 29 [Source:VGNC Symbol;Acc:VGNC:87782]	-1.72	0.0238
ssc-miR-205	ERRFI1	ERBB receptor feedback inhibitor 1 [Source:VGNC Symbol;Acc:VGNC:87784]	-1.72	0.0238
ssc-miR-205	ESRRG	estrogen related receptor gamma [Source:VGNC Symbol;Acc:VGNC:96289]	-1.72	0.0238
ssc-miR-205	ETF1	eukaryotic translation termination factor 1 [Source:VGNC Symbol;Acc:VGNC:87800]	-1.72	0.0238
ssc-miR-205	ETNK1	ethanolamine kinase 1 [Source:VGNC Symbol;Acc:VGNC:87806]	-1.72	0.0238
ssc-miR-205	EVA1A	eva-1 homolog A, regulator of programmed cell death [Source:VGNC Symbol;Acc:VGNC:97988]	-1.72	0.0238
ssc-miR-205	EVA1C	eva-1 homolog C [Source:VGNC Symbol;Acc:VGNC:87817]	-1.72	0.0238
ssc-miR-205	EVX1	even-skipped homeobox 1 [Source:VGNC Symbol;Acc:VGNC:87821]	-1.72	0.0238
ssc-miR-205	EXOC8	exocyst complex component 8 [Source:VGNC Symbol;Acc:VGNC:87834]	-1.72	0.0238
ssc-miR-205	EXT1	exostosin glycosyltransferase 1 [Source:VGNC Symbol;Acc:VGNC:87846]	-1.72	0.0238
ssc-miR-205	EZR	ezrin [Source:VGNC Symbol;Acc:VGNC:87856]	-1.72	0.0238
ssc-miR-205	FA2H	fatty acid 2-hydroxylase [Source:VGNC Symbol;Acc:VGNC:87868]	-1.72	0.0238
ssc-miR-205	FAM104A	family with sequence similarity 104 member A [Source:VGNC Symbol;Acc:VGNC:98990]	-1.72	0.0238

ssc-miR-205	FAM104B	hypothetical gene	-1.72	0.0238
ssc-miR-205	FAM118B	family with sequence similarity 118 member B [Source:VGNC Symbol;Acc:VGNC:87894]	-1.72	0.0238
ssc-miR-205	FAM120A	family with sequence similarity 120A [Source:VGNC Symbol;Acc:VGNC:99713]	-1.72	0.0238
ssc-miR-205	FAM126A	family with sequence similarity 126 member A [Source:VGNC Symbol;Acc:VGNC:87899]	-1.72	0.0238
ssc-miR-205	FAM136A	hypothetical gene	-1.72	0.0238
ssc-miR-205	FAM155A	hypothetical gene	-1.72	0.0238
ssc-miR-205	FAM155B	hypothetical gene	-1.72	0.0238
ssc-miR-205	FAM168A	family with sequence similarity 168 member A [Source:VGNC Symbol;Acc:VGNC:87926]	-1.72	0.0238
ssc-miR-205	FAM175B	hypothetical gene	-1.72	0.0238
ssc-miR-205	FAM179A	hypothetical gene	-1.72	0.0238
ssc-miR-205	FAM208A	hypothetical gene	-1.72	0.0238
ssc-miR-205	FAM216B	family with sequence similarity 216 member B [Source:VGNC Symbol;Acc:VGNC:87955]	-1.72	0.0238
ssc-miR-205	FAM63B	hypothetical gene	-1.72	0.0238
ssc-miR-205	FAM73B	hypothetical gene	-1.72	0.0238
ssc-miR-205	FAM84B	hypothetical gene	-1.72	0.0238
ssc-miR-205	FAT3	hypothetical gene	-1.72	0.0238
ssc-miR-205	FBXL3	F-box and leucine rich repeat protein 3 [Source:VGNC Symbol;Acc:VGNC:88030]	-1.72	0.0238
ssc-miR-205	FBXO22	F-box protein 22 [Source:HGNC Symbol;Acc:HGNC:13593]	-1.72	0.0238
ssc-miR-205	FBXO24	F-box protein 24 [Source:VGNC Symbol;Acc:VGNC:88036]	-1.72	0.0238
ssc-miR-205	FBXO38	F-box protein 38 [Source:VGNC Symbol;Acc:VGNC:88042]	-1.72	0.0238
ssc-miR-205	FGF14	fibroblast growth factor 14 [Source:VGNC Symbol;Acc:VGNC:88104]	-1.72	0.0238
ssc-miR-205	FGFRL1	fibroblast growth factor receptor like 1 [Source:VGNC Symbol;Acc:VGNC:88121]	-1.72	0.0238
ssc-miR-205	FNBP1L	formin binding protein 1 like [Source:VGNC Symbol;Acc:VGNC:88179]	-1.72	0.0238
ssc-miR-205	FOXF1	forkhead box F1 [Source:VGNC Symbol;Acc:VGNC:88204]	-1.72	0.0238
ssc-miR-205	FOXP2	forkhead box P2 [Source:VGNC Symbol;Acc:VGNC:98014]	-1.72	0.0238
ssc-miR-205	FRK	fyn related Src family tyrosine kinase [Source:VGNC Symbol;Acc:VGNC:88235]	-1.72	0.0238
ssc-miR-205	FSD1L	fibronectin type III and SPRY domain containing 1 like [Source:HGNC Symbol;Acc:HGNC:13753]	-1.72	0.0238
ssc-miR-205	FUT8	fucosyltransferase 8 [Source:NCBI gene (formerly Entrezgene);Acc:396933]	-1.72	0.0238
ssc-miR-205	FZD3	frizzled class receptor 3 [Source:VGNC Symbol;Acc:VGNC:88281]	-1.72	0.0238
ssc-miR-205	GALNT3	polypeptide N-acetylgalactosaminyltransferase 3 [Source:VGNC Symbol;Acc:VGNC:96313]	-1.72	0.0238
ssc-miR-205	GATA3	GATA binding protein 3 [Source:VGNC Symbol;Acc:VGNC:96315]	-1.72	0.0238
ssc-miR-205	GBA	glucosylceramidase beta [Source:VGNC Symbol;Acc:VGNC:98802]	-1.72	0.0238
ssc-miR-205	GCC2	GRIP and coiled-coil domain containing 2 [Source:VGNC Symbol;Acc:VGNC:88380]	-1.72	0.0238
ssc-miR-205	GDPD1	hypothetical gene	-1.72	0.0238
ssc-miR-205	GDPD5	glycerophosphodiester phosphodiesterase domain containing 5 [Source:VGNC Symbol;Acc:VGNC:88409]	-1.72	0.0238
ssc-miR-205	GEMIN6	gem nuclear organelle associated protein 6 [Source:VGNC Symbol;Acc:VGNC:88414]	-1.72	0.0238
ssc-miR-205	GGCX	gamma-glutamyl carboxylase [Source:VGNC Symbol;Acc:VGNC:88435]	-1.72	0.0238
ssc-miR-205	GINS1	GINS complex subunit 1 [Source:VGNC Symbol;Acc:VGNC:103950]	-1.72	0.0238
ssc-miR-205	GJC1	gap junction protein gamma 1 [Source:VGNC Symbol;Acc:VGNC:88468]	-1.72	0.0238
ssc-miR-205	GLIS3	GLIS family zinc finger 3 [Source:VGNC Symbol;Acc:VGNC:88485]	-1.72	0.0238
ssc-miR-205	GLRB	glycine receptor beta [Source:VGNC Symbol;Acc:VGNC:88494]	-1.72	0.0238
ssc-miR-205	GMFB	glia maturation factor beta [Source:VGNC Symbol;Acc:VGNC:88509]	-1.72	0.0238
ssc-miR-205	GORASP1	golgi reassembly stacking protein 1 [Source:VGNC Symbol;Acc:VGNC:88559]	-1.72	0.0238
ssc-miR-205	GPATCH2L	G-patch domain containing 2 like [Source:VGNC Symbol;Acc:VGNC:88575]	-1.72	0.0238
ssc-miR-205	GPR111	hypothetical gene	-1.72	0.0238
ssc-miR-205	GPR135	G protein-coupled receptor 135 [Source:VGNC Symbol;Acc:VGNC:88599]	-1.72	0.0238
ssc-miR-205	GPR146	hypothetical gene	-1.72	0.0238
ssc-miR-205	GPR180	G protein-coupled receptor 180 [Source:VGNC Symbol;Acc:VGNC:88621]	-1.72	0.0238
ssc-miR-205	GPR61	G protein-coupled receptor 61 [Source:HGNC Symbol;Acc:HGNC:13300]	-1.72	0.0238
ssc-miR-205	GPX3	glutathione peroxidase 3 [Source:VGNC Symbol;Acc:VGNC:98026]	-1.72	0.0238

ssc-miR-205	GRAMD1C	GRAM domain containing 1C [Source:VGNC Symbol;Acc:VGNC:88657]	-1.72	0.0238
ssc-miR-205	GRAMD2	hypothetical gene	-1.72	0.0238
ssc-miR-205	GRB10	growth factor receptor bound protein 10 [Source:VGNC Symbol;Acc:VGNC:88662]	-1.72	0.0238
ssc-miR-205	GRSF1	G-rich RNA sequence binding factor 1 [Source:VGNC Symbol;Acc:VGNC:88710]	-1.72	0.0238
ssc-miR-205	GTDC1	glycosyltransferase like domain containing 1 [Source:VGNC Symbol;Acc:VGNC:95955]	-1.72	0.0238
ssc-miR-205	GTF3C2	ral transcription factor IIIC subunit 2 [Source:VGNC Symbol;Acc:VGNC:88739]	-1.72	0.0238
ssc-miR-205	GXYLT1	glucoside xylosyltransferase 1 [Source:VGNC Symbol;Acc:VGNC:88756]	-1.72	0.0238
ssc-miR-205	H2AFJ	hypothetical gene	-1.72	0.0238
ssc-miR-205	HCN1	hyperpolarization activated cyclic nucleotide gated potassium channel 1 [Source:VGNC Symbol;Acc:VGNC:88802]	-1.72	0.0238
ssc-miR-205	HDX	highly divergent homeobox [Source:VGNC Symbol;Acc:VGNC:88824]	-1.72	0.0238
ssc-miR-205	HELLS	helicase, lymphoid specific [Source:VGNC Symbol;Acc:VGNC:88838]	-1.72	0.0238
ssc-miR-205	HEPACAM2	HEPACAM family member 2 [Source:VGNC Symbol;Acc:VGNC:88846]	-1.72	0.0238
ssc-miR-205	HERC3	HECT and RLD domain containing E3 ubiquitin protein ligase 3 [Source:VGNC Symbol;Acc:VGNC:98934]	-1.72	0.0238
ssc-miR-205	HIATL1	hypothetical gene	-1.72	0.0238
ssc-miR-205	HIF1AN	hypoxia inducible factor 1 subunit alpha inhibitor [Source:VGNC Symbol;Acc:VGNC:98033]	-1.72	0.0238
ssc-miR-205	HIPK3	homeodomain interacting protein kinase 3 [Source:VGNC Symbol;Acc:VGNC:88889]	-1.72	0.0238
ssc-miR-205	HMGB1	hypothetical gene	-1.72	0.0238
ssc-miR-205	HMX3	H6 family homeobox 3 [Source:VGNC Symbol;Acc:VGNC:88915]	-1.72	0.0238
ssc-miR-205	HNRNPH3	heteroous nuclear ribonucleoprotein H3 [Source:VGNC Symbol;Acc:VGNC:88922]	-1.72	0.0238
ssc-miR-205	HNRNPK	heteroous nuclear ribonucleoprotein K [Source:VGNC Symbol;Acc:VGNC:103963]	-1.72	0.0238
ssc-miR-205	HNRNPR	heteroous nuclear ribonucleoprotein R [Source:VGNC Symbol;Acc:VGNC:88925]	-1.72	0.0238
ssc-miR-205	HOOK3	hook microtubule tethering protein 3 [Source:VGNC Symbol;Acc:VGNC:98037]	-1.72	0.0238
ssc-miR-205	HOXC12	homeobox C12 [Source:VGNC Symbol;Acc:VGNC:88950]	-1.72	0.0238
ssc-miR-205	HOXD13	homeobox D13 [Source:HGNC Symbol;Acc:HGNC:5136]	-1.72	0.0238
ssc-miR-205	HOXD9	homeobox D9 [Source:VGNC Symbol;Acc:VGNC:96357]	-1.72	0.0238
ssc-miR-205	HS3ST1	heparan sulfate-glucosamine 3-sulfotransferase 1 [Source:VGNC Symbol;Acc:VGNC:88977]	-1.72	0.0238
ssc-miR-205	HSD17B11	hydroxysteroid 17-beta dehydrogenase 11 [Source:HGNC Symbol;Acc:HGNC:22960]	-1.72	0.0238
ssc-miR-205	HSPA13	heat shock protein family A (Hsp70) member 13 [Source:VGNC Symbol;Acc:VGNC:108663]	-1.72	0.0238
ssc-miR-205	IFRG15	hypothetical gene	-1.72	0.0238
ssc-miR-205	IKZF4	IKAROS family zinc finger 4 [Source:VGNC Symbol;Acc:VGNC:89075]	-1.72	0.0238
ssc-miR-205	IL1R1	interleukin 1 receptor type 1 [Source:VGNC Symbol;Acc:VGNC:89092]	-1.72	0.0238
ssc-miR-205	IL1RAPL1	interleukin 1 receptor accessory protein like 1 [Source:VGNC Symbol;Acc:VGNC:103967]	-1.72	0.0238
ssc-miR-205	IL6R	interleukin 6 receptor [Source:VGNC Symbol;Acc:VGNC:89112]	-1.72	0.0238
ssc-miR-205	IL6ST	interleukin 6 cytokine family signal transducer [Source:VGNC Symbol;Acc:VGNC:89113]	-1.72	0.0238
ssc-miR-205	IMMT	inner membrane mitochondrial protein [Source:VGNC Symbol;Acc:VGNC:89124]	-1.72	0.0238
ssc-miR-205	INHBA	inhibin subunit beta A [Source:VGNC Symbol;Acc:VGNC:89133]	-1.72	0.0238
ssc-miR-205	INPPL1	inositol polyphosphate phosphatase like 1 [Source:VGNC Symbol;Acc:VGNC:89148]	-1.72	0.0238
ssc-miR-205	INSR	insulin receptor [Source:VGNC Symbol;Acc:VGNC:89153]	-1.72	0.0238
ssc-miR-205	IPO7	importin 7 [Source:VGNC Symbol;Acc:VGNC:89180]	-1.72	0.0238
ssc-miR-205	IPPK	inositol-pentakisphosphate 2-kinase [Source:VGNC Symbol;Acc:VGNC:89183]	-1.72	0.0238
ssc-miR-205	IRF1	interferon regulatory factor 1 [Source:VGNC Symbol;Acc:VGNC:89203]	-1.72	0.0238
ssc-miR-205	IRF2BPL	interferon regulatory factor 2 binding protein like [Source:HGNC Symbol;Acc:HGNC:14282]	-1.72	0.0238
ssc-miR-205	ITGA5	integrin subunit alpha 5 [Source:VGNC Symbol;Acc:VGNC:89236]	-1.72	0.0238
ssc-miR-205	ITGB3BP	hypothetical gene	-1.72	0.0238
ssc-miR-205	ITIH5	inter-alpha-trypsin inhibitor heavy chain 5 [Source:VGNC Symbol;Acc:VGNC:95966]	-1.72	0.0238
ssc-miR-205	IVNS1ABP	influenza virus NS1A binding protein [Source:NCBI gene (formerly Entrezgene);Acc:100302027]	-1.72	0.0238
ssc-miR-205	JAKMIP3	Janus kinase and microtubule interacting protein 3 [Source:VGNC Symbol;Acc:VGNC:89275]	-1.72	0.0238
ssc-miR-205	JPH4	junctophilin 4 [Source:VGNC Symbol;Acc:VGNC:89292]	-1.72	0.0238
ssc-miR-205	KAT7	lysine acetyltransferase 7 [Source:VGNC Symbol;Acc:VGNC:89307]	-1.72	0.0238
ssc-miR-205	KAZN	kazrin, periplakin interacting protein [Source:HGNC Symbol;Acc:HGNC:29173]	-1.72	0.0238

ssc-miR-205	KCNB1	potassium voltage-gated channel subfamily B member 1 [Source:VGNC Symbol;Acc:VGNC:96386]	-1.72	0.0238
ssc-miR-205	KCND3	potassium voltage-gated channel subfamily D member 3 [Source:VGNC Symbol;Acc:VGNC:98814]	-1.72	0.0238
ssc-miR-205	KCNMB4	potassium calcium-activated channel subfamily M regulatory beta subunit 4 [Source:VGNC Symbol;Acc:VGNC:89378]	-1.72	0.0238
ssc-miR-205	KHNYN	KH and NYN domain containing [Source:VGNC Symbol;Acc:VGNC:89426]	-1.72	0.0238
ssc-miR-205	KIF1B	kinesin family member 1B [Source:VGNC Symbol;Acc:VGNC:89460]	-1.72	0.0238
ssc-miR-205	KIF1C	kinesin family member 1C [Source:VGNC Symbol;Acc:VGNC:89461]	-1.72	0.0238
ssc-miR-205	KIF26B	kinesin family member 26B [Source:VGNC Symbol;Acc:VGNC:96087]	-1.72	0.0238
ssc-miR-205	KLF12	Kruppel like factor 12 [Source:VGNC Symbol;Acc:VGNC:89492]	-1.72	0.0238
ssc-miR-205	KLF3	Kruppel like factor 3 [Source:VGNC Symbol;Acc:VGNC:89498]	-1.72	0.0238
ssc-miR-205	KLF7	Kruppel like factor 7 [Source:VGNC Symbol;Acc:VGNC:96396]	-1.72	0.0238
ssc-miR-205	KLHL15	kelch like family member 15 [Source:VGNC Symbol;Acc:VGNC:89516]	-1.72	0.0238
ssc-miR-205	KLHL31	kelch like family member 31 [Source:VGNC Symbol;Acc:VGNC:89526]	-1.72	0.0238
ssc-miR-205	KMT2A	lysine methyltransferase 2A [Source:VGNC Symbol;Acc:VGNC:108600]	-1.72	0.0238
ssc-miR-205	KNTC1	kinetochore associated 1 [Source:VGNC Symbol;Acc:VGNC:89559]	-1.72	0.0238
ssc-miR-205	KPNA1	karyopherin subunit alpha 1 [Source:VGNC Symbol;Acc:VGNC:89560]	-1.72	0.0238
ssc-miR-205	KRBA2	hypothetical gene	-1.72	0.0238
ssc-miR-205	KSR1	kinase suppressor of ras 1 [Source:VGNC Symbol;Acc:VGNC:89606]	-1.72	0.0238
ssc-miR-205	LAMC1	laminin subunit gamma 1 [Source:VGNC Symbol;Acc:VGNC:89624]	-1.72	0.0238
ssc-miR-205	LANCL3	LanC like 3 [Source:VGNC Symbol;Acc:VGNC:89634]	-1.72	0.0238
ssc-miR-205	LBH	LBH regulator of WNT signaling pathway [Source:HGNC Symbol;Acc:HGNC:29532]	-1.72	0.0238
ssc-miR-205	LCOR	ligand dependent nuclear receptor corepressor [Source:HGNC Symbol;Acc:HGNC:29503]	-1.72	0.0238
ssc-miR-205	LDB3	hypothetical gene	-1.72	0.0238
ssc-miR-205	LDLRAD3	low density lipoprotein receptor class A domain containing 3 [Source:VGNC Symbol;Acc:VGNC:89672]	-1.72	0.0238
ssc-miR-205	LENG9	leukocyte receptor cluster member 9 [Source:VGNC Symbol;Acc:VGNC:89682]	-1.72	0.0238
ssc-miR-205	LHFPL2	LHFPL tetraspan subfamily member 2 [Source:VGNC Symbol;Acc:VGNC:89705]	-1.72	0.0238
ssc-miR-205	LIMS2	LIM zinc finger domain containing 2 [Source:VGNC Symbol;Acc:VGNC:95731]	-1.72	0.0238
ssc-miR-205	LIN9	lin-9 DREAM MuvB core complex component [Source:VGNC Symbol;Acc:VGNC:103976]	-1.72	0.0238
ssc-miR-205	LMNB1	lamin B1 [Source:VGNC Symbol;Acc:VGNC:89760]	-1.72	0.0238
ssc-miR-205	LONRF3	LON peptidase N-terminal domain and ring finger 3 [Source:VGNC Symbol;Acc:VGNC:89778]	-1.72	0.0238
ssc-miR-205	LPAR1	hypothetical gene	-1.72	0.0238
ssc-miR-205	LPCAT1	lysophosphatidylcholine acyltransferase 1 [Source:VGNC Symbol;Acc:VGNC:89788]	-1.72	0.0238
ssc-miR-205	LPHN3	hypothetical gene	-1.72	0.0238
ssc-miR-205	LRCH3	leucine rich repeats and calponin homology domain containing 3 [Source:VGNC Symbol;Acc:VGNC:89803]	-1.72	0.0238
ssc-miR-205	LRP1	hypothetical gene	-1.72	0.0238
ssc-miR-205	LRP4	LDL receptor related protein 4 [Source:VGNC Symbol;Acc:VGNC:89820]	-1.72	0.0238
ssc-miR-205	LRP6	LDL receptor related protein 6 [Source:VGNC Symbol;Acc:VGNC:89821]	-1.72	0.0238
ssc-miR-205	LRRK2	leucine rich repeat kinase 2 [Source:VGNC Symbol;Acc:VGNC:98094]	-1.72	0.0238
ssc-miR-205	LSAMP	limbic system associated membrane protein [Source:VGNC Symbol;Acc:VGNC:89868]	-1.72	0.0238
ssc-miR-205	LUC7L3	hypothetical gene	-1.72	0.0238
ssc-miR-205	LYN	LYN proto-onco, Src family tyrosine kinase [Source:VGNC Symbol;Acc:VGNC:89910]	-1.72	0.0238
ssc-miR-205	LYPD6	LY6/PLAUR domain containing 6 [Source:HGNC Symbol;Acc:HGNC:28751]	-1.72	0.0238
ssc-miR-205	LYSMD3	LysM domain containing 3 [Source:VGNC Symbol;Acc:VGNC:89919]	-1.72	0.0238
ssc-miR-205	LYSMD4	LysM domain containing 4 [Source:VGNC Symbol;Acc:VGNC:89920]	-1.72	0.0238
ssc-miR-205	MAGI1	membrane associated guanylate kinase, WW and PDZ domain containing 1 [Source:VGNC Symbol;Acc:VGNC:98097]	-1.72	0.0238
ssc-miR-205	MAGI2	membrane associated guanylate kinase, WW and PDZ domain containing 2 [Source:VGNC Symbol;Acc:VGNC:89955]	-1.72	0.0238
ssc-miR-205	MAGI3	membrane associated guanylate kinase, WW and PDZ domain containing 3 [Source:VGNC Symbol;Acc:VGNC:89956]	-1.72	0.0238
ssc-miR-205	MAP3K9	mitogen-activated protein kinase kinase kinase 9 [Source:VGNC Symbol;Acc:VGNC:89989]	-1.72	0.0238
ssc-miR-205	MAPK3	mitogen-activated protein kinase 3 [Source:VGNC Symbol;Acc:VGNC:90003]	-1.72	0.0238
ssc-miR-205	MARCKS	myristoylated alanine rich protein kinase C substrate [Source:VGNC Symbol;Acc:VGNC:90024]	-1.72	0.0238
ssc-miR-205	MBNL3	muscleblind like splicing regulator 3 [Source:VGNC Symbol;Acc:VGNC:90056]	-1.72	0.0238

ssc-miR-205	MDM4	MDM4 regulator of p53 [Source:VGNC Symbol;Acc:VGNC:90094]	-1.72	0.0238
ssc-miR-205	MECP2	methyl-CpG binding protein 2 [Source:VGNC Symbol;Acc:VGNC:90101]	-1.72	0.0238
ssc-miR-205	MED1	mediator complex subunit 1 [Source:VGNC Symbol;Acc:VGNC:90102]	-1.72	0.0238
ssc-miR-205	MED13L	mediator complex subunit 13L [Source:VGNC Symbol;Acc:VGNC:90107]	-1.72	0.0238
ssc-miR-205	MELK	maternal embryonic leucine zipper kinase [Source:VGNC Symbol;Acc:VGNC:90137]	-1.72	0.0238
ssc-miR-205	METTL21B	hypothetical gene	-1.72	0.0238
ssc-miR-205	MFNG	MFNG O-fucosylpeptide 3-beta-N-acetylglucosaminyltransferase [Source:VGNC Symbol;Acc:VGNC:90180]	-1.72	0.0238
ssc-miR-205	MGA	MAX dimerization protein MGA [Source:VGNC Symbol;Acc:VGNC:90195]	-1.72	0.0238
ssc-miR-205	MGAT4A	alpha-1,3-mannosyl-glycoprotein 4-beta-N-acetylglucosaminyltransferase A [Source:VGNC Symbol;Acc:VGNC:108158]	-1.72	0.0238
ssc-miR-205	MGRN1	mahogunin ring finger 1 [Source:VGNC Symbol;Acc:VGNC:90202]	-1.72	0.0238
ssc-miR-205	MICAL2	hypothetical gene	-1.72	0.0238
ssc-miR-205	MICU3	mitochondrial calcium uptake family member 3 [Source:VGNC Symbol;Acc:VGNC:96160]	-1.72	0.0238
ssc-miR-205	MID1IP1	MID1 interacting protein 1 [Source:VGNC Symbol;Acc:VGNC:90214]	-1.72	0.0238
ssc-miR-205	MIER3	MIER family member 3 [Source:VGNC Symbol;Acc:VGNC:90221]	-1.72	0.0238
ssc-miR-205	MKRN2	makorin ring finger protein 2 [Source:VGNC Symbol;Acc:VGNC:90248]	-1.72	0.0238
ssc-miR-205	MLLT4	hypothetical gene	-1.72	0.0238
ssc-miR-205	MMAB	metabolism of cobalamin associated B [Source:VGNC Symbol;Acc:VGNC:96743]	-1.72	0.0238
ssc-miR-205	MMD	monocyte to macrophage differentiation associated [Source:VGNC Symbol;Acc:VGNC:90264]	-1.72	0.0238
ssc-miR-205	MORN4	MORN repeat containing 4 [Source:VGNC Symbol;Acc:VGNC:90310]	-1.72	0.0238
ssc-miR-205	MPPED1	metallophosphoesterase domain containing 1 [Source:VGNC Symbol;Acc:VGNC:90330]	-1.72	0.0238
ssc-miR-205	MPRIP	hypothetical gene	-1.72	0.0238
ssc-miR-205	MRPS18A	mitochondrial ribosomal protein S18A [Source:VGNC Symbol;Acc:VGNC:90384]	-1.72	0.0238
ssc-miR-205	MSI2	musashi RNA binding protein 2 [Source:VGNC Symbol;Acc:VGNC:90422]	-1.72	0.0238
ssc-miR-205	MSL2	MSL complex subunit 2 [Source:VGNC Symbol;Acc:VGNC:90424]	-1.72	0.0238
ssc-miR-205	MTF1	metal regulatory transcription factor 1 [Source:VGNC Symbol;Acc:VGNC:90443]	-1.72	0.0238
ssc-miR-205	MTFR1L	mitochondrial fission regulator 1 like [Source:VGNC Symbol;Acc:VGNC:90448]	-1.72	0.0238
ssc-miR-205	MTMR4	myotubularin related protein 4 [Source:VGNC Symbol;Acc:VGNC:90462]	-1.72	0.0238
ssc-miR-205	MTRF1L	mitochondrial translation release factor 1 like [Source:VGNC Symbol;Acc:VGNC:90472]	-1.72	0.0238
ssc-miR-205	MXD1	MAX dimerization protein 1 [Source:VGNC Symbol;Acc:VGNC:90492]	-1.72	0.0238
ssc-miR-205	MXI1	hypothetical gene	-1.72	0.0238
ssc-miR-205	MYLK4	hypothetical gene	-1.72	0.0238
ssc-miR-205	MYO5B	myosin VB [Source:VGNC Symbol;Acc:VGNC:103131]	-1.72	0.0238
ssc-miR-205	NAA10	N-alpha-acetyltransferase 10, NatA catalytic subunit [Source:VGNC Symbol;Acc:VGNC:96746]	-1.72	0.0238
ssc-miR-205	NAA11	N-alpha-acetyltransferase 11, NatA catalytic subunit [Source:VGNC Symbol;Acc:VGNC:98943]	-1.72	0.0238
ssc-miR-205	NAA25	N-alpha-acetyltransferase 25, NatB auxiliary subunit [Source:VGNC Symbol;Acc:VGNC:96750]	-1.72	0.0238
ssc-miR-205	NAA35	N-alpha-acetyltransferase 35, NatC auxiliary subunit [Source:VGNC Symbol;Acc:VGNC:96752]	-1.72	0.0238
ssc-miR-205	NACC2	NACC family member 2 [Source:VGNC Symbol;Acc:VGNC:90564]	-1.72	0.0238
ssc-miR-205	NCEH1	neutral cholesterol ester hydrolase 1 [Source:VGNC Symbol;Acc:VGNC:98147]	-1.72	0.0238
ssc-miR-205	NCOA1	nuclear receptor coactivator 1 [Source:VGNC Symbol;Acc:VGNC:90615]	-1.72	0.0238
ssc-miR-205	NDNF	neuron derived neurotrophic factor [Source:VGNC Symbol;Acc:VGNC:90629]	-1.72	0.0238
ssc-miR-205	NDUFA4	NDUFA4 mitochondrial complex associated [Source:NCBI gene (formerly Entrezgene);Acc:100579141]	-1.72	0.0238
ssc-miR-205	NECAP1	NECAP endocytosis associated 1 [Source:VGNC Symbol;Acc:VGNC:90660]	-1.72	0.0238
ssc-miR-205	NEK6	NIMA related kinase 6 [Source:VGNC Symbol;Acc:VGNC:90678]	-1.72	0.0238
ssc-miR-205	NEU1	neuraminidase 1 [Source:VGNC Symbol;Acc:VGNC:90692]	-1.72	0.0238
ssc-miR-205	NFAT5	nuclear factor of activated T cells 5 [Source:VGNC Symbol;Acc:VGNC:90708]	-1.72	0.0238
ssc-miR-205	NFIA	nuclear factor I A [Source:VGNC Symbol;Acc:VGNC:90715]	-1.72	0.0238
ssc-miR-205	NFIB	nuclear factor I B [Source:VGNC Symbol;Acc:VGNC:90716]	-1.72	0.0238
ssc-miR-205	NFIX	nuclear factor I X [Source:VGNC Symbol;Acc:VGNC:90718]	-1.72	0.0238
ssc-miR-205	NHP2	hypothetical gene	-1.72	0.0238
ssc-miR-205	NHP2L1	hypothetical gene	-1.72	0.0238

ssc-miR-205	NHS	NHS actin remodeling regulator [Source:VGNC Symbol;Acc:VGNC:90738]	-1.72	0.0238
ssc-miR-205	NHSL2	NHS like 2 [Source:VGNC Symbol;Acc:VGNC:98155]	-1.72	0.0238
ssc-miR-205	NKAP	NFKB activating protein [Source:VGNC Symbol;Acc:VGNC:90756]	-1.72	0.0238
ssc-miR-205	NKD1	NKD inhibitor of WNT signaling pathway 1 [Source:VGNC Symbol;Acc:VGNC:90759]	-1.72	0.0238
ssc-miR-205	NKX2-3	NK2 homeobox 3 [Source:VGNC Symbol;Acc:VGNC:90768]	-1.72	0.0238
ssc-miR-205	NMT2	N-myristoyltransferase 2 [Source:VGNC Symbol;Acc:VGNC:96448]	-1.72	0.0238
ssc-miR-205	NOP9	NOP9 nucleolar protein [Source:VGNC Symbol;Acc:VGNC:90822]	-1.72	0.0238
ssc-miR-205	NOTCH2	notch receptor 2 [Source:HGNC Symbol;Acc:HGNC:7882]	-1.72	0.0238
ssc-miR-205	NPEPPS	aminopeptidase puromycin sensitive [Source:VGNC Symbol;Acc:VGNC:98157]	-1.72	0.0238
ssc-miR-205	NR3C2	nuclear receptor subfamily 3 group C member 2 [Source:VGNC Symbol;Acc:VGNC:90884]	-1.72	0.0238
ssc-miR-205	NR6A1	nuclear receptor subfamily 6 group A member 1 [Source:VGNC Symbol;Acc:VGNC:90887]	-1.72	0.0238
ssc-miR-205	NRCAM	neuronal cell adhesion molecule [Source:HGNC Symbol;Acc:HGNC:7994]	-1.72	0.0238
ssc-miR-205	NSF	N-ethylmaleimide sensitive factor, vesicle fusing ATPase [Source:VGNC Symbol;Acc:VGNC:90906]	-1.72	0.0238
ssc-miR-205	NSFL1C	NSFL1 cofactor [Source:VGNC Symbol;Acc:VGNC:95704]	-1.72	0.0238
ssc-miR-205	NSUN5	NOP2/Sun RNA methyltransferase 5 [Source:VGNC Symbol;Acc:VGNC:90916]	-1.72	0.0238
ssc-miR-205	NTNG1	netrin G1 [Source:VGNC Symbol;Acc:VGNC:90935]	-1.72	0.0238
ssc-miR-205	NUDT18	nudix hydrolase 18 [Source:VGNC Symbol;Acc:VGNC:90960]	-1.72	0.0238
ssc-miR-205	NUFIP2	nuclear FMR1 interacting protein 2 [Source:VGNC Symbol;Acc:VGNC:90967]	-1.72	0.0238
ssc-miR-205	NUP35	nucleoporin 35 [Source:VGNC Symbol;Acc:VGNC:96229]	-1.72	0.0238
ssc-miR-205	NUP62CL	nucleoporin 62 C-terminal like [Source:VGNC Symbol;Acc:VGNC:104013]	-1.72	0.0238
ssc-miR-205	NUTF2	nuclear transport factor 2 [Source:VGNC Symbol;Acc:VGNC:98524]	-1.72	0.0238
ssc-miR-205	OAF	out at first homolog [Source:VGNC Symbol;Acc:VGNC:91005]	-1.72	0.0238
ssc-miR-205	OCIAD1	OCIA domain containing 1 [Source:HGNC Symbol;Acc:HGNC:16074]	-1.72	0.0238
ssc-miR-205	ONECUT1	one cut homeobox 1 [Source:VGNC Symbol;Acc:VGNC:91042]	-1.72	0.0238
ssc-miR-205	OSER1	oxidative stress responsive serine rich 1 [Source:VGNC Symbol;Acc:VGNC:95738]	-1.72	0.0238
ssc-miR-205	P2RY1	purinergic receptor P2Y1 [Source:HGNC Symbol;Acc:HGNC:8539]	-1.72	0.0238
ssc-miR-205	PACS1	phosphofurin acidic cluster sorting protein 1 [Source:VGNC Symbol;Acc:VGNC:91140]	-1.72	0.0238
ssc-miR-205	PAFAH1B1	platelet activating factor acetylhydrolase 1b regulatory subunit 1 [Source:VGNC Symbol;Acc:VGNC:99026]	-1.72	0.0238
ssc-miR-205	PAPD5	hypothetical gene	-1.72	0.0238
ssc-miR-205	PAPLN	papilin, proteoglycan like sulfated glycoprotein [Source:VGNC Symbol;Acc:VGNC:91169]	-1.72	0.0238
ssc-miR-205	PAQR5	progestin and adipoQ receptor family member 5 [Source:VGNC Symbol;Acc:VGNC:91175]	-1.72	0.0238
ssc-miR-205	PARD6B	par-6 family cell polarity regulator beta [Source:VGNC Symbol;Acc:VGNC:98176]	-1.72	0.0238
ssc-miR-205	PAX9	paired box 9 [Source:HGNC Symbol;Acc:HGNC:8623]	-1.72	0.0238
ssc-miR-205	PCDH20	protocadherin 20 [Source:HGNC Symbol;Acc:HGNC:14257]	-1.72	0.0238
ssc-miR-205	PCNX	hypothetical gene	-1.72	0.0238
ssc-miR-205	PDE3A	phosphodiesterase 3A [Source:VGNC Symbol;Acc:VGNC:91252]	-1.72	0.0238
ssc-miR-205	PDE3B	phosphodiesterase 3B [Source:VGNC Symbol;Acc:VGNC:91253]	-1.72	0.0238
ssc-miR-205	PDE7A	phosphodiesterase 7A [Source:VGNC Symbol;Acc:VGNC:91261]	-1.72	0.0238
ssc-miR-205	PDS5A	PDS5 cohesin associated factor A [Source:HGNC Symbol;Acc:HGNC:29088]	-1.72	0.0238
ssc-miR-205	PET117	PET117 cytochrome c oxidase chaperone [Source:HGNC Symbol;Acc:HGNC:40045]	-1.72	0.0238
ssc-miR-205	PFDN1	prefoldin subunit 1 [Source:VGNC Symbol;Acc:VGNC:91332]	-1.72	0.0238
ssc-miR-205	PFKFB3	6-phosphofructo-2-kinase/fructose-2,6-biphosphatase 3 [Source:VGNC Symbol;Acc:VGNC:96480]	-1.72	0.0238
ssc-miR-205	PGAP3	post-GPI attachment to proteins phospholipase 3 [Source:VGNC Symbol;Acc:VGNC:91343]	-1.72	0.0238
ssc-miR-205	PHB	hypothetical gene	-1.72	0.0238
ssc-miR-205	PHC2	polyhomeotic homolog 2 [Source:VGNC Symbol;Acc:VGNC:91371]	-1.72	0.0238
ssc-miR-205	PHF16	hypothetical gene	-1.72	0.0238
ssc-miR-205	PHF17	hypothetical gene	-1.72	0.0238
ssc-miR-205	PHLDA3	pleckstrin homology like domain family A member 3 [Source:HGNC Symbol;Acc:HGNC:8934]	-1.72	0.0238
ssc-miR-205	PHYHIPL	phytanoyl-CoA 2-hydroxylase interacting protein like [Source:VGNC Symbol;Acc:VGNC:91407]	-1.72	0.0238
ssc-miR-205	PI16	peptidase inhibitor 16 [Source:VGNC Symbol;Acc:VGNC:98189]	-1.72	0.0238

ssc-miR-205	PIAS1	protein inhibitor of activated STAT 1 [Source:VGNC Symbol;Acc:VGNC:91410]	-1.72	0.0238
ssc-miR-205	PIAS2	protein inhibitor of activated STAT 2 [Source:VGNC Symbol;Acc:VGNC:91411]	-1.72	0.0238
ssc-miR-205	PICALM	phosphatidylinositol binding clathrin assembly protein [Source:VGNC Symbol;Acc:VGNC:91415]	-1.72	0.0238
ssc-miR-205	PIEZO1	piezo type mechanosensitive ion channel component 1 [Source:VGNC Symbol;Acc:VGNC:91417]	-1.72	0.0238
ssc-miR-205	PIGV	phosphatidylinositol glycan anchor biosynthesis class V [Source:VGNC Symbol;Acc:VGNC:91430]	-1.72	0.0238
ssc-miR-205	PJA2	praja ring finger ubiquitin ligase 2 [Source:VGNC Symbol;Acc:VGNC:91470]	-1.72	0.0238
ssc-miR-205	PLAT	plasminogen activator, tissue type [Source:VGNC Symbol;Acc:VGNC:98201]	-1.72	0.0238
ssc-miR-205	PLCB1	phospholipase C beta 1 [Source:VGNC Symbol;Acc:VGNC:95706]	-1.72	0.0238
ssc-miR-205	PLCL1	phospholipase C like 1 (inactive) [Source:HGNC Symbol;Acc:HGNC:9063]	-1.72	0.0238
ssc-miR-205	PLCXD3	phosphatidylinositol specific phospholipase C X domain containing 3 [Source:VGNC Symbol;Acc:VGNC:91524]	-1.72	0.0238
ssc-miR-205	PLD1	phospholipase D1 [Source:VGNC Symbol;Acc:VGNC:91525]	-1.72	0.0238
ssc-miR-205	PMEPA1	prostate transmembrane protein, androgen induced 1 [Source:VGNC Symbol;Acc:VGNC:95606]	-1.72	0.0238
ssc-miR-205	PNN	pinin, desmosome associated protein [Source:VGNC Symbol;Acc:VGNC:91603]	-1.72	0.0238
ssc-miR-205	POTEG	hypothetical gene	-1.72	0.0238
ssc-miR-205	POTEI	hypothetical gene	-1.72	0.0238
ssc-miR-205	POTEM	hypothetical gene	-1.72	0.0238
ssc-miR-205	POU2F1	POU class 2 homeobox 1 [Source:VGNC Symbol;Acc:VGNC:91672]	-1.72	0.0238
ssc-miR-205	PPM1H	protein phosphatase, Mg2+/Mn2+ dependent 1H [Source:VGNC Symbol;Acc:VGNC:91708]	-1.72	0.0238
ssc-miR-205	PPP1R13B	protein phosphatase 1 regulatory subunit 13B [Source:VGNC Symbol;Acc:VGNC:91721]	-1.72	0.0238
ssc-miR-205	PPP1R15B	protein phosphatase 1 regulatory subunit 15B [Source:VGNC Symbol;Acc:VGNC:91726]	-1.72	0.0238
ssc-miR-205	PPP1R1C	protein phosphatase 1 regulatory inhibitor subunit 1C [Source:HGNC Symbol;Acc:HGNC:14940]	-1.72	0.0238
ssc-miR-205	PPP1R3A	protein phosphatase 1 regulatory subunit 3A [Source:VGNC Symbol;Acc:VGNC:91736]	-1.72	0.0238
ssc-miR-205	PPP1R8	protein phosphatase 1 regulatory subunit 8 [Source:VGNC Symbol;Acc:VGNC:91742]	-1.72	0.0238
ssc-miR-205	PPP1R9A	protein phosphatase 1 regulatory subunit 9A [Source:VGNC Symbol;Acc:VGNC:91743]	-1.72	0.0238
ssc-miR-205	PPP3R1	protein phosphatase 3 regulatory subunit B, alpha [Source:VGNC Symbol;Acc:VGNC:106453]	-1.72	0.0238
ssc-miR-205	PRDM16	PR/SET domain 16 [Source:VGNC Symbol;Acc:VGNC:91777]	-1.72	0.0238
ssc-miR-205	PREPL	prolyl endopeptidase like [Source:VGNC Symbol;Acc:VGNC:91790]	-1.72	0.0238
ssc-miR-205	PRKCA	protein kinase C alpha [Source:VGNC Symbol;Acc:VGNC:99028]	-1.72	0.0238
ssc-miR-205	PRKCE	protein kinase C epsilon [Source:VGNC Symbol;Acc:VGNC:91807]	-1.72	0.0238
ssc-miR-205	PRMT6	protein arginine methyltransferase 6 [Source:VGNC Symbol;Acc:VGNC:91823]	-1.72	0.0238
ssc-miR-205	PROX1	prospero homeobox 1 [Source:VGNC Symbol;Acc:VGNC:91837]	-1.72	0.0238
ssc-miR-205	PRPF38B	hypothetical gene	-1.72	0.0238
ssc-miR-205	PSD3	pleckstrin and Sec7 domain containing 3 [Source:VGNC Symbol;Acc:VGNC:107166]	-1.72	0.0238
ssc-miR-205	PSMA4	proteasome 20S subunit alpha 4 [Source:VGNC Symbol;Acc:VGNC:91901]	-1.72	0.0238
ssc-miR-205	PSMB5	hypothetical gene	-1.72	0.0238
ssc-miR-205	PSME3	proteasome activator subunit 3 [Source:VGNC Symbol;Acc:VGNC:91926]	-1.72	0.0238
ssc-miR-205	PSTK	phosphoseryl-tRNA kinase [Source:VGNC Symbol;Acc:VGNC:91934]	-1.72	0.0238
ssc-miR-205	PTCHD1	patched domain containing 1 [Source:VGNC Symbol;Acc:VGNC:91942]	-1.72	0.0238
ssc-miR-205	PTEN	hypothetical gene	-1.72	0.0238
ssc-miR-205	PTK7	protein tyrosine kinase 7 (inactive) [Source:HGNC Symbol;Acc:HGNC:9618]	-1.72	0.0238
ssc-miR-205	PTP4A1	protein tyrosine phosphatase 4A1 [Source:HGNC Symbol;Acc:HGNC:9634]	-1.72	0.0238
ssc-miR-205	PTPRD	protein tyrosine phosphatase receptor type D [Source:HGNC Symbol;Acc:HGNC:9668]	-1.72	0.0238
ssc-miR-205	PTPRG	protein tyrosine phosphatase receptor type G [Source:VGNC Symbol;Acc:VGNC:91988]	-1.72	0.0238
ssc-miR-205	PTPRJ	protein tyrosine phosphatase receptor type J [Source:VGNC Symbol;Acc:VGNC:91990]	-1.72	0.0238
ssc-miR-205	PTPRM	protein tyrosine phosphatase receptor type M [Source:VGNC Symbol;Acc:VGNC:91992]	-1.72	0.0238
ssc-miR-205	PURG	purine rich element binding protein G [Source:VGNC Symbol;Acc:VGNC:95865]	-1.72	0.0238
ssc-miR-205	QKI	QKI, KH domain containing RNA binding [Source:VGNC Symbol;Acc:VGNC:92025]	-1.72	0.0238
ssc-miR-205	RAB11FIP1	hypothetical gene	-1.72	0.0238
ssc-miR-205	RAB11FIP2	RAB11 family interacting protein 2 [Source:VGNC Symbol;Acc:VGNC:92039]	-1.72	0.0238
ssc-miR-205	RAB14	RAB14, member RAS onco family [Source:VGNC Symbol;Acc:VGNC:98238]	-1.72	0.0238

ssc-miR-205	RAB23	RAB23, member RAS onco family [Source:VGNC Symbol;Acc:VGNC:98245]	-1.72	0.0238
ssc-miR-205	RAB30	RAB30, member RAS onco family [Source:VGNC Symbol;Acc:VGNC:98254]	-1.72	0.0238
ssc-miR-205	RAB9B	RAB9B, member RAS onco family [Source:VGNC Symbol;Acc:VGNC:98278]	-1.72	0.0238
ssc-miR-205	RALGAPB	Ral GTPase activating protein non-catalytic subunit beta [Source:VGNC Symbol;Acc:VGNC:95463]	-1.72	0.0238
ssc-miR-205	RAN	hypothetical gene	-1.72	0.0238
ssc-miR-205	RANBP2	RAN binding protein 2 [Source:HGNC Symbol;Acc:HGNC:9848]	-1.72	0.0238
ssc-miR-205	RAP2B	RAP2B, member of RAS onco family [Source:VGNC Symbol;Acc:VGNC:92089]	-1.72	0.0238
ssc-miR-205	RARA	retinoic acid receptor alpha [Source:VGNC Symbol;Acc:VGNC:92096]	-1.72	0.0238
ssc-miR-205	RASSF2	Ras association domain family member 2 [Source:VGNC Symbol;Acc:VGNC:96531]	-1.72	0.0238
ssc-miR-205	RBFOX3	RNA binding fox-1 homolog 3 [Source:VGNC Symbol;Acc:VGNC:92141]	-1.72	0.0238
ssc-miR-205	RBM12	RNA binding motif protein 12 [Source:HGNC Symbol;Acc:HGNC:9898]	-1.72	0.0238
ssc-miR-205	RBM47	RNA binding motif protein 47 [Source:VGNC Symbol;Acc:VGNC:92158]	-1.72	0.0238
ssc-miR-205	RBM8A	RNA binding motif protein 8A [Source:VGNC Symbol;Acc:VGNC:92162]	-1.72	0.0238
ssc-miR-205	RBMS1	RNA binding motif single stranded interacting protein 1 [Source:VGNC Symbol;Acc:VGNC:96534]	-1.72	0.0238
ssc-miR-205	RBMS2	RNA binding motif single stranded interacting protein 2 [Source:VGNC Symbol;Acc:VGNC:98286]	-1.72	0.0238
ssc-miR-205	RBMX	hypothetical gene	-1.72	0.0238
ssc-miR-205	RBPMS2	RNA binding protein, mRNA processing factor 2 [Source:VGNC Symbol;Acc:VGNC:92166]	-1.72	0.0238
ssc-miR-205	RCBTB1	RCC1 and BTB domain containing protein 1 [Source:VGNC Symbol;Acc:VGNC:92173]	-1.72	0.0238
ssc-miR-205	REEP5	receptor accessory protein 5 [Source:VGNC Symbol;Acc:VGNC:92201]	-1.72	0.0238
ssc-miR-205	RGPD4	hypothetical gene	-1.72	0.0238
ssc-miR-205	RGPD6	hypothetical gene	-1.72	0.0238
ssc-miR-205	RGS18	regulator of G protein signaling 18 [Source:VGNC Symbol;Acc:VGNC:95609]	-1.72	0.0238
ssc-miR-205	RND3	Rho family GTPase 3 [Source:VGNC Symbol;Acc:VGNC:96407]	-1.72	0.0238
ssc-miR-205	RNF11	ring finger protein 11 [Source:VGNC Symbol;Acc:VGNC:98601]	-1.72	0.0238
ssc-miR-205	RNF146	ring finger protein 146 [Source:VGNC Symbol;Acc:VGNC:92361]	-1.72	0.0238
ssc-miR-205	RNF157	ring finger protein 157 [Source:VGNC Symbol;Acc:VGNC:92365]	-1.72	0.0238
ssc-miR-205	RNF19A	ring finger protein 19A, RBR E3 ubiquitin protein ligase [Source:VGNC Symbol;Acc:VGNC:92375]	-1.72	0.0238
ssc-miR-205	RNF19B	ring finger protein 19B [Source:VGNC Symbol;Acc:VGNC:92376]	-1.72	0.0238
ssc-miR-205	RNF207	ring finger protein 207 [Source:VGNC Symbol;Acc:VGNC:92379]	-1.72	0.0238
ssc-miR-205	RNF213	hypothetical gene	-1.72	0.0238
ssc-miR-205	RNF217	ring finger protein 217 [Source:VGNC Symbol;Acc:VGNC:103162]	-1.72	0.0238
ssc-miR-205	RNF4	ring finger protein 4 [Source:VGNC Symbol;Acc:VGNC:92392]	-1.72	0.0238
ssc-miR-205	ROCK2	Rho associated coiled-coil containing protein kinase 2 [Source:HGNC Symbol;Acc:HGNC:10252]	-1.72	0.0238
ssc-miR-205	RORA	RAR related orphan receptor A [Source:VGNC Symbol;Acc:VGNC:92408]	-1.72	0.0238
ssc-miR-205	RP11-156E8.1	hypothetical gene	-1.72	0.0238
ssc-miR-205	RP11-17M16.1	hypothetical gene	-1.72	0.0238
ssc-miR-205	RP11-210M15.2	hypothetical gene	-1.72	0.0238
ssc-miR-205	RPS6KA3	ribosomal protein S6 kinase A3 [Source:VGNC Symbol;Acc:VGNC:92442]	-1.72	0.0238
ssc-miR-205	RTN3	hypothetical gene	-1.72	0.0238
ssc-miR-205	RTTN	rotatin [Source:VGNC Symbol;Acc:VGNC:92508]	-1.72	0.0238
ssc-miR-205	RUNX1	RUNX family transcription factor 1 [Source:VGNC Symbol;Acc:VGNC:92516]	-1.72	0.0238
ssc-miR-205	RUNX2	RUNX family transcription factor 2 [Source:VGNC Symbol;Acc:VGNC:92517]	-1.72	0.0238
ssc-miR-205	RUNX3	RUNX family transcription factor 3 [Source:VGNC Symbol;Acc:VGNC:92518]	-1.72	0.0238
ssc-miR-205	S1PR1	sphingosine-1-phosphate receptor 1 [Source:VGNC Symbol;Acc:VGNC:92551]	-1.72	0.0238
ssc-miR-205	SAE1	SUMO1 activating enzyme subunit 1 [Source:VGNC Symbol;Acc:VGNC:92558]	-1.72	0.0238
ssc-miR-205	SALL4	spalt like transcription factor 4 [Source:NCBI gene (formerly Entrezgene);Acc:100136902]	-1.72	0.0238
ssc-miR-205	SATB2	SATB homeobox 2 [Source:VGNC Symbol;Acc:VGNC:95972]	-1.72	0.0238
ssc-miR-205	SBF2	SET binding factor 2 [Source:VGNC Symbol;Acc:VGNC:92594]	-1.72	0.0238
ssc-miR-205	SCD5	stearoyl-CoA desaturase 5 [Source:VGNC Symbol;Acc:VGNC:92617]	-1.72	0.0238
ssc-miR-205	SCMH1	Scm polycomb group protein homolog 1 [Source:VGNC Symbol;Acc:VGNC:92627]	-1.72	0.0238

ssc-miR-205	SCN2A	hypothetical gene	-1.72	0.0238
ssc-miR-205	SCPEP1	serine carboxypeptidase 1 [Source:VGNC Symbol;Acc:VGNC:92645]	-1.72	0.0238
ssc-miR-205	SECISBP2L	SECIS binding protein 2 like [Source:VGNC Symbol;Acc:VGNC:92686]	-1.72	0.0238
ssc-miR-205	SEH1L	centrosomal protein 192 [Source:VGNC Symbol;Acc:VGNC:96946]	-1.72	0.0238
ssc-miR-205	SELT	hypothetical gene	-1.72	0.0238
ssc-miR-205	SEMA4C	semaphorin 4C [Source:VGNC Symbol;Acc:VGNC:92702]	-1.72	0.0238
ssc-miR-205	SEMA7A	semaphorin 7A (John Milton Hagen blood group) [Source:HGNC Symbol;Acc:HGNC:10741]	-1.72	0.0238
ssc-miR-205	SEPT11	hypothetical gene	-1.72	0.0238
ssc-miR-205	SEPT3	hypothetical gene	-1.72	0.0238
ssc-miR-205	SEPT4	hypothetical gene	-1.72	0.0238
ssc-miR-205	SEPT5	hypothetical gene	-1.72	0.0238
ssc-miR-205	SERTAD2	SERTA domain containing 2 [Source:VGNC Symbol;Acc:VGNC:92750]	-1.72	0.0238
ssc-miR-205	SGMS1	sphingomyelin synthase 1 [Source:VGNC Symbol;Acc:VGNC:92794]	-1.72	0.0238
ssc-miR-205	SH3BGRL3	SH3 domain binding glutamate rich protein like 3 [Source:VGNC Symbol;Acc:VGNC:92817]	-1.72	0.0238
ssc-miR-205	SH3GL3	SH3 domain containing GRB2 like 3, endophilin A3 [Source:VGNC Symbol;Acc:VGNC:92825]	-1.72	0.0238
ssc-miR-205	SHROOM3	hypothetical gene	-1.72	0.0238
ssc-miR-205	SIAH1	siah E3 ubiquitin protein ligase 1 [Source:VGNC Symbol;Acc:VGNC:92865]	-1.72	0.0238
ssc-miR-205	SIK2	salt inducible kinase 2 [Source:VGNC Symbol;Acc:VGNC:92872]	-1.72	0.0238
ssc-miR-205	SIPA1L1	signal induced proliferation associated 1 like 1 [Source:VGNC Symbol;Acc:VGNC:92882]	-1.72	0.0238
ssc-miR-205	SLC16A13	solute carrier family 16 member 13 [Source:VGNC Symbol;Acc:VGNC:92941]	-1.72	0.0238
ssc-miR-205	SLC16A2	solute carrier family 16 member 2 [Source:VGNC Symbol;Acc:VGNC:92942]	-1.72	0.0238
ssc-miR-205	SLC19A2	solute carrier family 19 member 2 [Source:VGNC Symbol;Acc:VGNC:92962]	-1.72	0.0238
ssc-miR-205	SLC24A2	solute carrier family 24 member 2 [Source:VGNC Symbol;Acc:VGNC:92987]	-1.72	0.0238
ssc-miR-205	SLC26A2	solute carrier family 26 member 2 [Source:VGNC Symbol;Acc:VGNC:93027]	-1.72	0.0238
ssc-miR-205	SLC30A7	solute carrier family 30 member 7 [Source:VGNC Symbol;Acc:VGNC:93061]	-1.72	0.0238
ssc-miR-205	SLC35A1	solute carrier family 35 member A1 [Source:VGNC Symbol;Acc:VGNC:93067]	-1.72	0.0238
ssc-miR-205	SLC35B3	solute carrier family 35 member B3 [Source:VGNC Symbol;Acc:VGNC:93073]	-1.72	0.0238
ssc-miR-205	SLC4A4	solute carrier family 4 member 4 [Source:VGNC Symbol;Acc:VGNC:93133]	-1.72	0.0238
ssc-miR-205	SLC5A3	solute carrier family 5 member 3 [Source:VGNC Symbol;Acc:VGNC:93144]	-1.72	0.0238
ssc-miR-205	SLC8A1	solute carrier family 8 member A1 [Source:VGNC Symbol;Acc:VGNC:93178]	-1.72	0.0238
ssc-miR-205	SLK	STE20 like kinase [Source:VGNC Symbol;Acc:VGNC:98328]	-1.72	0.0238
ssc-miR-205	SMAD1	SMAD family member 1 [Source:VGNC Symbol;Acc:VGNC:93216]	-1.72	0.0238
ssc-miR-205	SMAD4	SMAD family member 4 [Source:VGNC Symbol;Acc:VGNC:93218]	-1.72	0.0238
ssc-miR-205	SMAD5	SMAD family member 5 [Source:VGNC Symbol;Acc:VGNC:93219]	-1.72	0.0238
ssc-miR-205	SMARCD1	SWI/SNF related, matrix associated, actin dependent regulator of chromatin, subfamily d, member 1 [Source:VGNC Symbol;Acc:VGNC:93233]	-1.72	0.0238
ssc-miR-205	SMIM14	small integral membrane protein 14 [Source:VGNC Symbol;Acc:VGNC:93252]	-1.72	0.0238
ssc-miR-205	SNN	stannin [Source:HGNC Symbol;Acc:HGNC:11149]	-1.72	0.0238
ssc-miR-205	SNX24	sorting nexin 24 [Source:VGNC Symbol;Acc:VGNC:93315]	-1.72	0.0238
ssc-miR-205	SNX27	sorting nexin 27 [Source:VGNC Symbol;Acc:VGNC:93316]	-1.72	0.0238
ssc-miR-205	SOGA3	SOGA family member 3 [Source:HGNC Symbol;Acc:HGNC:21494]	-1.72	0.0238
ssc-miR-205	SORBS1	sorbin and SH3 domain containing 1 [Source:VGNC Symbol;Acc:VGNC:93337]	-1.72	0.0238
ssc-miR-205	SOX7	SRY-box transcription factor 7 [Source:HGNC Symbol;Acc:HGNC:18196]	-1.72	0.0238
ssc-miR-205	SP4	Sp4 transcription factor [Source:VGNC Symbol;Acc:VGNC:93362]	-1.72	0.0238
ssc-miR-205	SP6	Sp6 transcription factor [Source:VGNC Symbol;Acc:VGNC:93363]	-1.72	0.0238
ssc-miR-205	SPATA13	hypothetical gene	-1.72	0.0238
ssc-miR-205	SPG11	SPG11 vesicle trafficking associated, spatacsin [Source:VGNC Symbol;Acc:VGNC:93402]	-1.72	0.0238
ssc-miR-205	SPOPL	speckle type BTB/POZ protein like [Source:VGNC Symbol;Acc:VGNC:96161]	-1.72	0.0238
ssc-miR-205	SPRY1	sprouty RTK signaling antagonist 1 [Source:VGNC Symbol;Acc:VGNC:93424]	-1.72	0.0238
ssc-miR-205	SREK1	splicing regulatory glutamic acid and lysine rich protein 1 [Source:VGNC Symbol;Acc:VGNC:93451]	-1.72	0.0238
ssc-miR-205	SRGAP1	SLIT-ROBO Rho GTPase activating protein 1 [Source:VGNC Symbol;Acc:VGNC:93454]	-1.72	0.0238

ssc-miR-205	SRSF10	serine and arginine rich splicing factor 10 [Source:VGNC Symbol;Acc:VGNC:93472]	-1.72	0.0238
ssc-miR-205	SS18	SS18 subunit of BAF chromatin remodeling complex [Source:VGNC Symbol;Acc:VGNC:93478]	-1.72	0.0238
ssc-miR-205	SSBP3	single stranded DNA binding protein 3 [Source:VGNC Symbol;Acc:VGNC:93481]	-1.72	0.0238
ssc-miR-205	STIM2	stromal interaction molecule 2 [Source:VGNC Symbol;Acc:VGNC:98967]	-1.72	0.0238
ssc-miR-205	STK3	serine/threonine kinase 3 [Source:VGNC Symbol;Acc:VGNC:98347]	-1.72	0.0238
ssc-miR-205	STON2	stonin 2 [Source:VGNC Symbol;Acc:VGNC:93568]	-1.72	0.0238
ssc-miR-205	STRBP	spermatid perinuclear RNA binding protein [Source:VGNC Symbol;Acc:VGNC:93575]	-1.72	0.0238
ssc-miR-205	STRN	striatin [Source:VGNC Symbol;Acc:VGNC:93578]	-1.72	0.0238
ssc-miR-205	STS	hypothetical gene	-1.72	0.0238
ssc-miR-205	STXBP6	syntaxin binding protein 6 [Source:NCBI gene (formerly Entrezgene);Acc:100514852]	-1.72	0.0238
ssc-miR-205	SUGT1	SGT1 homolog, MIS12 kinetochore complex assembly cochaperone [Source:VGNC Symbol;Acc:VGNC:93611]	-1.72	0.0238
ssc-miR-205	SULF1	sulfatase 1 [Source:VGNC Symbol;Acc:VGNC:93612]	-1.72	0.0238
ssc-miR-205	SUSD1	sushi domain containing 1 [Source:VGNC Symbol;Acc:VGNC:93633]	-1.72	0.0238
ssc-miR-205	SV2B	synaptic vesicle glycoprotein 2B [Source:VGNC Symbol;Acc:VGNC:93640]	-1.72	0.0238
ssc-miR-205	SVIP	small VCP interacting protein [Source:HGNC Symbol;Acc:HGNC:25238]	-1.72	0.0238
ssc-miR-205	SYNJ2BP	synaptojanin 2 binding protein [Source:NCBI gene (formerly Entrezgene);Acc:100514718]	-1.72	0.0238
ssc-miR-205	SYPL2	synaptophysin like 2 [Source:VGNC Symbol;Acc:VGNC:93677]	-1.72	0.0238
ssc-miR-205	SYT14	synaptotagmin 14 [Source:VGNC Symbol;Acc:VGNC:108619]	-1.72	0.0238
ssc-miR-205	TACO1	translational activator of cytochrome c oxidase I [Source:VGNC Symbol;Acc:VGNC:93700]	-1.72	0.0238
ssc-miR-205	TAOK1	TAO kinase 1 [Source:VGNC Symbol;Acc:VGNC:98355]	-1.72	0.0238
ssc-miR-205	TAPT1	transmembrane anterior posterior transformation 1 [Source:VGNC Symbol;Acc:VGNC:93736]	-1.72	0.0238
ssc-miR-205	TBC1D13	TBC1 domain family member 13 [Source:VGNC Symbol;Acc:VGNC:93763]	-1.72	0.0238
ssc-miR-205	TBCEL	tubulin folding cofactor E like [Source:HGNC Symbol;Acc:HGNC:28115]	-1.72	0.0238
ssc-miR-205	TBX18	T-box transcription factor 18 [Source:VGNC Symbol;Acc:VGNC:103187]	-1.72	0.0238
ssc-miR-205	TBX3	T-box transcription factor 3 [Source:VGNC Symbol;Acc:VGNC:93802]	-1.72	0.0238
ssc-miR-205	TCEA2	transcription elongation factor A2 [Source:VGNC Symbol;Acc:VGNC:95534]	-1.72	0.0238
ssc-miR-205	TEAD1	TEA domain transcription factor 1 [Source:VGNC Symbol;Acc:VGNC:93853]	-1.72	0.0238
ssc-miR-205	TESK2	testis associated actin remodelling kinase 2 [Source:VGNC Symbol;Acc:VGNC:93886]	-1.72	0.0238
ssc-miR-205	TET1	tet methylcytosine dioxygenase 1 [Source:VGNC Symbol;Acc:VGNC:93888]	-1.72	0.0238
ssc-miR-205	TFDP2	transcription factor Dp-2 [Source:VGNC Symbol;Acc:VGNC:93915]	-1.72	0.0238
ssc-miR-205	TFE3	transcription factor binding to IGHM enhancer 3 [Source:VGNC Symbol;Acc:VGNC:93916]	-1.72	0.0238
ssc-miR-205	TGFA	transforming growth factor alpha [Source:VGNC Symbol;Acc:VGNC:93928]	-1.72	0.0238
ssc-miR-205	THAP8	THAP domain containing 8 [Source:HGNC Symbol;Acc:HGNC:23191]	-1.72	0.0238
ssc-miR-205	THBS1	thrombospondin 1 [Source:VGNC Symbol;Acc:VGNC:93946]	-1.72	0.0238
ssc-miR-205	TIAL1	TIA1 cytotoxic granule associated RNA binding protein like 1 [Source:VGNC Symbol;Acc:VGNC:104073]	-1.72	0.0238
ssc-miR-205	TIFA	TRAF interacting protein with forkhead associated domain [Source:VGNC Symbol;Acc:VGNC:93978]	-1.72	0.0238
ssc-miR-205	TIMM17A	translocase of inner mitochondrial membrane 17A [Source:VGNC Symbol;Acc:VGNC:104074]	-1.72	0.0238
ssc-miR-205	TLK1	tousled like kinase 1 [Source:VGNC Symbol;Acc:VGNC:95543]	-1.72	0.0238
ssc-miR-205	TM2D3	TM2 domain containing 3 [Source:VGNC Symbol;Acc:VGNC:94029]	-1.72	0.0238
ssc-miR-205	TM9SF2	transmembrane 9 superfamily member 2 [Source:VGNC Symbol;Acc:VGNC:94036]	-1.72	0.0238
ssc-miR-205	TM9SF3	transmembrane 9 superfamily member 3 [Source:VGNC Symbol;Acc:VGNC:94037]	-1.72	0.0238
ssc-miR-205	TMEM136	hypothetical gene	-1.72	0.0238
ssc-miR-205	TMEM144	transmembrane protein 144 [Source:VGNC Symbol;Acc:VGNC:94091]	-1.72	0.0238
ssc-miR-205	TMEM236	transmembrane protein 236 [Source:VGNC Symbol;Acc:VGNC:96006]	-1.72	0.0238
ssc-miR-205	TMEM255A	transmembrane protein 255A [Source:VGNC Symbol;Acc:VGNC:104079]	-1.72	0.0238
ssc-miR-205	TMEM260	transmembrane protein 260 [Source:VGNC Symbol;Acc:VGNC:94164]	-1.72	0.0238
ssc-miR-205	TNFAIP8	TNF alpha induced protein 8 [Source:VGNC Symbol;Acc:VGNC:94253]	-1.72	0.0238
ssc-miR-205	TNFSF15	TNF superfamily member 15 [Source:NCBI gene (formerly Entrezgene);Acc:100624969]	-1.72	0.0238
ssc-miR-205	TNPO1	transportin 1 [Source:VGNC Symbol;Acc:VGNC:94287]	-1.72	0.0238
ssc-miR-205	TNR	tenascin R [Source:VGNC Symbol;Acc:VGNC:94290]	-1.72	0.0238

ssc-miR-205	TNRC6B	trinucleotide repeat containing adaptor 6B [Source:VGNC Symbol;Acc:VGNC:94293]	-1.72	0.0238
ssc-miR-205	TNRC6C	trinucleotide repeat containing adaptor 6C [Source:VGNC Symbol;Acc:VGNC:94294]	-1.72	0.0238
ssc-miR-205	TNS1	tensin 1 [Source:VGNC Symbol;Acc:VGNC:104082]	-1.72	0.0238
ssc-miR-205	TOB2	transducer of ERBB2, 2 [Source:VGNC Symbol;Acc:VGNC:94298]	-1.72	0.0238
ssc-miR-205	TOR1AIP2	torsin 1A interacting protein 2 [Source:VGNC Symbol;Acc:VGNC:94318]	-1.72	0.0238
ssc-miR-205	TP53BP2	tumor protein p53 binding protein 2 [Source:VGNC Symbol;Acc:VGNC:95553]	-1.72	0.0238
ssc-miR-205	TP53INP1	tumor protein p53 inducible nuclear protein 1 [Source:VGNC Symbol;Acc:VGNC:94329]	-1.72	0.0238
ssc-miR-205	TPP2	tripeptidyl peptidase 2 [Source:VGNC Symbol;Acc:VGNC:94343]	-1.72	0.0238
ssc-miR-205	TRAK2	trafficking kinesin protein 2 [Source:VGNC Symbol;Acc:VGNC:95577]	-1.72	0.0238
ssc-miR-205	TRAM2	translocation associated membrane protein 2 [Source:HGNC Symbol;Acc:HGNC:16855]	-1.72	0.0238
ssc-miR-205	TRPS1	transcriptional repressor GATA binding 1 [Source:VGNC Symbol;Acc:VGNC:94471]	-1.72	0.0238
ssc-miR-205	TSC22D1	hypothetical gene	-1.72	0.0238
ssc-miR-205	TSHR	thyroid stimulating hormone receptor [Source:VGNC Symbol;Acc:VGNC:94491]	-1.72	0.0238
ssc-miR-205	TSHZ3	teashirt zinc finger homeobox 3 [Source:VGNC Symbol;Acc:VGNC:94493]	-1.72	0.0238
ssc-miR-205	TTC19	tetratricopeptide repeat domain 19 [Source:VGNC Symbol;Acc:VGNC:99101]	-1.72	0.0238
ssc-miR-205	TTI1	TELO2 interacting protein 1 [Source:VGNC Symbol;Acc:VGNC:96194]	-1.72	0.0238
ssc-miR-205	TTLL7	tubulin tyrosine ligase like 7 [Source:VGNC Symbol;Acc:VGNC:94571]	-1.72	0.0238
ssc-miR-205	TUBB6	tubulin beta 6 class V [Source:VGNC Symbol;Acc:VGNC:94581]	-1.72	0.0238
ssc-miR-205	TXNRD1	thioredoxin reductase 1 [Source:NCBI gene (formerly Entrezgene);Acc:396681]	-1.72	0.0238
ssc-miR-205	UBE2E3	ubiquitin conjugating enzyme E2 E3 [Source:VGNC Symbol;Acc:VGNC:106461]	-1.72	0.0238
ssc-miR-205	UBE2G1	ubiquitin conjugating enzyme E2 G1 [Source:HGNC Symbol;Acc:HGNC:12482]	-1.72	0.0238
ssc-miR-205	UBE2N	hypothetical gene	-1.72	0.0238
ssc-miR-205	UBE2NL	hypothetical gene	-1.72	0.0238
ssc-miR-205	UBFD1	ubiquitin family domain containing 1 [Source:VGNC Symbol;Acc:VGNC:94658]	-1.72	0.0238
ssc-miR-205	UBIAD1	UbiA prenyltransferase domain containing 1 [Source:VGNC Symbol;Acc:VGNC:94659]	-1.72	0.0238
ssc-miR-205	UBQLN2	ubiquilin 2 [Source:VGNC Symbol;Acc:VGNC:94665]	-1.72	0.0238
ssc-miR-205	UBR1	ubiquitin protein ligase E3 component n-recognin 1 [Source:VGNC Symbol;Acc:VGNC:94668]	-1.72	0.0238
ssc-miR-205	UNC5D	unc-5 netrin receptor D [Source:VGNC Symbol;Acc:VGNC:95892]	-1.72	0.0238
ssc-miR-205	USP13	ubiquitin specific peptidase 13 [Source:VGNC Symbol;Acc:VGNC:94746]	-1.72	0.0238
ssc-miR-205	USP38	ubiquitin specific peptidase 38 [Source:VGNC Symbol;Acc:VGNC:94762]	-1.72	0.0238
ssc-miR-205	USP7	ubiquitin specific peptidase 7 [Source:VGNC Symbol;Acc:VGNC:94777]	-1.72	0.0238
ssc-miR-205	VASN	vasorin [Source:VGNC Symbol;Acc:VGNC:94803]	-1.72	0.0238
ssc-miR-205	VEGFA	vascular endothelial growth factor A [Source:VGNC Symbol;Acc:VGNC:94815]	-1.72	0.0238
ssc-miR-205	VEZT	vezatin, adherens junctions transmembrane protein [Source:VGNC Symbol;Acc:VGNC:94819]	-1.72	0.0238
ssc-miR-205	VPS13B	vacuolar protein sorting 13 homolog B [Source:VGNC Symbol;Acc:VGNC:94839]	-1.72	0.0238
ssc-miR-205	VPS52	VPS52 subunit of GARP complex [Source:VGNC Symbol;Acc:VGNC:94860]	-1.72	0.0238
ssc-miR-205	VTI1B	vesicle transport through interaction with t-SNAREs 1B [Source:VGNC Symbol;Acc:VGNC:94880]	-1.72	0.0238
ssc-miR-205	VWA7	von Willebrand factor A domain containing 7 [Source:VGNC Symbol;Acc:VGNC:94885]	-1.72	0.0238
ssc-miR-205	WAC	WW domain containing adaptor with coiled-coil [Source:VGNC Symbol;Acc:VGNC:95845]	-1.72	0.0238
ssc-miR-205	WDTC1	WD and tetratricopeptide repeats 1 [Source:VGNC Symbol;Acc:VGNC:94952]	-1.72	0.0238
ssc-miR-205	WNK2	WNK lysine deficient protein kinase 2 [Source:VGNC Symbol;Acc:VGNC:94963]	-1.72	0.0238
ssc-miR-205	WWC1	WW and C2 domain containing 1 [Source:VGNC Symbol;Acc:VGNC:94986]	-1.72	0.0238
ssc-miR-205	WWC2	WW and C2 domain containing 2 [Source:VGNC Symbol;Acc:VGNC:96033]	-1.72	0.0238
ssc-miR-205	WWC3	WWC family member 3 [Source:VGNC Symbol;Acc:VGNC:94987]	-1.72	0.0238
ssc-miR-205	XIRP1	xin actin binding repeat containing 1 [Source:NCBI gene (formerly Entrezgene);Acc:100134978]	-1.72	0.0238
ssc-miR-205	XIRP2	xin actin binding repeat containing 2 [Source:HGNC Symbol;Acc:HGNC:14303]	-1.72	0.0238
ssc-miR-205	XPO4	exportin 4 [Source:VGNC Symbol;Acc:VGNC:95004]	-1.72	0.0238
ssc-miR-205	XPR1	xenotropic and polytropic retrovirus receptor 1 [Source:VGNC Symbol;Acc:VGNC:108625]	-1.72	0.0238
ssc-miR-205	XRN1	5'-3' exoribonuclease 1 [Source:VGNC Symbol;Acc:VGNC:108705]	-1.72	0.0238
ssc-miR-205	XRRA1	X-ray radiation resistance associated 1 [Source:VGNC Symbol;Acc:VGNC:95012]	-1.72	0.0238

ssc-miR-205	XYLT1	xylosyltransferase 1 [Source:VGNC Symbol;Acc:VGNC:95015]	-1.72	0.0238
ssc-miR-205	YAP1	Yes1 associated transcriptional regulator [Source:VGNC Symbol;Acc:VGNC:95019]	-1.72	0.0238
ssc-miR-205	YES1	YES proto-onco 1, Src family tyrosine kinase [Source:VGNC Symbol;Acc:VGNC:95024]	-1.72	0.0238
ssc-miR-205	ZBTB20	zinc finger and BTB domain containing 20 [Source:VGNC Symbol;Acc:VGNC:95063]	-1.72	0.0238
ssc-miR-205	ZBTB38	zinc finger and BTB domain containing 38 [Source:VGNC Symbol;Acc:VGNC:95072]	-1.72	0.0238
ssc-miR-205	ZCCHC14	zinc finger CCHC-type containing 14 [Source:VGNC Symbol;Acc:VGNC:95107]	-1.72	0.0238
ssc-miR-205	ZDHHC3	zinc finger DHHC-type palmitoyltransferase 3 [Source:VGNC Symbol;Acc:VGNC:95126]	-1.72	0.0238
ssc-miR-205	ZDHHC9	zinc finger DHHC-type palmitoyltransferase 9 [Source:VGNC Symbol;Acc:VGNC:95131]	-1.72	0.0238
ssc-miR-205	ZEB1	zinc finger E-box binding homeobox 1 [Source:VGNC Symbol;Acc:VGNC:95536]	-1.72	0.0238
ssc-miR-205	ZEB2	hypothetical gene	-1.72	0.0238
ssc-miR-205	ZER1	zyg-11 related cell cycle regulator [Source:VGNC Symbol;Acc:VGNC:95132]	-1.72	0.0238
ssc-miR-205	ZFP57	ZFP57 zinc finger protein [Source:NCBI gene (formerly Entrezgene);Acc:100141415]	-1.72	0.0238
ssc-miR-205	ZFP62	ZFP62 zinc finger protein [Source:VGNC Symbol;Acc:VGNC:95148]	-1.72	0.0238
ssc-miR-205	ZFYVE16	zinc finger FYVE-type containing 16 [Source:VGNC Symbol;Acc:VGNC:95157]	-1.72	0.0238
ssc-miR-205	ZHX3	zinc fingers and homeoboxes 3 [Source:VGNC Symbol;Acc:VGNC:95717]	-1.72	0.0238
ssc-miR-205	ZKSCAN5	zinc finger with KRAB and SCAN domains 5 [Source:VGNC Symbol;Acc:VGNC:95174]	-1.72	0.0238
ssc-miR-205	ZNF148	zinc finger protein 148 [Source:VGNC Symbol;Acc:VGNC:95192]	-1.72	0.0238
ssc-miR-205	ZNF436	zinc finger protein 436 [Source:VGNC Symbol;Acc:VGNC:95239]	-1.72	0.0238
ssc-miR-205	ZNF518B	zinc finger protein 518B [Source:VGNC Symbol;Acc:VGNC:95258]	-1.72	0.0238
ssc-miR-205	ZNF536	zinc finger protein 536 [Source:VGNC Symbol;Acc:VGNC:95262]	-1.72	0.0238
ssc-miR-205	ZNF597	hypothetical gene	-1.72	0.0238
ssc-miR-205	ZNF609	zinc finger protein 609 [Source:VGNC Symbol;Acc:VGNC:95274]	-1.72	0.0238
ssc-miR-205	ZNF638	zinc finger protein 638 [Source:VGNC Symbol;Acc:VGNC:95281]	-1.72	0.0238
ssc-miR-205	ZNF652	zinc finger protein 652 [Source:VGNC Symbol;Acc:VGNC:99108]	-1.72	0.0238
ssc-miR-205	ZNF800	zinc finger protein 800 [Source:VGNC Symbol;Acc:VGNC:95306]	-1.72	0.0238
ssc-miR-205	ZSWIM4	zinc finger SWIM-type containing 4 [Source:VGNC Symbol;Acc:VGNC:95333]	-1.72	0.0238
ssc-miR-7144-3p	NA	hypothetical gene	-1.72	0.008
ssc-miR-339-3p	NA	hypothetical gene	-1.74	0.0988
ssc-miR-374a-5p	ABAT	4-aminobutyrate aminotransferase [Source:VGNC Symbol;Acc:VGNC:96910]	-1.76	0.0084
ssc-miR-374a-5p	ABCA8	ATP binding cassette subfamily A member 8 [Source:HGNC Symbol;Acc:HGNC:38]	-1.76	0.0084
ssc-miR-374a-5p	ABCB11	ATP binding cassette subfamily B member 11 [Source:VGNC Symbol;Acc:VGNC:97853]	-1.76	0.0084
ssc-miR-374a-5p	ABCE1	ATP binding cassette subfamily E member 1 [Source:VGNC Symbol;Acc:VGNC:84965]	-1.76	0.0084
ssc-miR-374a-5p	ABCG2	ATP-binding cassette, sub-family G (WHITE), member 2 [Source:NCBI gene (formerly Entrezgene);Acc:397073]	-1.76	0.0084
ssc-miR-374a-5p	ABR	ABR activator of RhoGEF and GTPase [Source:VGNC Symbol;Acc:VGNC:84989]	-1.76	0.0084
ssc-miR-374a-5p	AC006946.15	hypothetical gene	-1.76	0.0084
ssc-miR-374a-5p	ACSL6	acyl-CoA synthetase long chain family member 6 [Source:VGNC Symbol;Acc:VGNC:99581]	-1.76	0.0084
ssc-miR-374a-5p	ACTN1	actinin alpha 1 [Source:NCBI gene (formerly Entrezgene);Acc:100513412]	-1.76	0.0084
ssc-miR-374a-5p	ACTN4	actinin alpha 4 [Source:VGNC Symbol;Acc:VGNC:85047]	-1.76	0.0084
ssc-miR-374a-5p	ACVR2B	activin A receptor type 2B [Source:VGNC Symbol;Acc:VGNC:108629]	-1.76	0.0084
ssc-miR-374a-5p	ADAM10	ADAM metallopeptidase domain 10 [Source:VGNC Symbol;Acc:VGNC:85061]	-1.76	0.0084
ssc-miR-374a-5p	ADAMTS5	ADAM metallopeptidase with thrombospondin type 1 motif 5 [Source:VGNC Symbol;Acc:VGNC:85085]	-1.76	0.0084
ssc-miR-374a-5p	ADNP	activity dependent neuroprotector homeobox [Source:VGNC Symbol;Acc:VGNC:95657]	-1.76	0.0084
ssc-miR-374a-5p	ADORA2B	adenosine A2b receptor [Source:VGNC Symbol;Acc:VGNC:85149]	-1.76	0.0084
ssc-miR-374a-5p	ADRB1	adrenoceptor beta 1 [Source:VGNC Symbol;Acc:VGNC:107363]	-1.76	0.0084
ssc-miR-374a-5p	AFF1	AF4/FMR2 family member 1 [Source:VGNC Symbol;Acc:VGNC:85167]	-1.76	0.0084
ssc-miR-374a-5p	AFF3	AF4/FMR2 family member 3 [Source:HGNC Symbol;Acc:HGNC:6473]	-1.76	0.0084
ssc-miR-374a-5p	AFF4	AF4/FMR2 family member 4 [Source:VGNC Symbol;Acc:VGNC:85169]	-1.76	0.0084
ssc-miR-374a-5p	AGFG2	ArfGAP with FG repeats 2 [Source:VGNC Symbol;Acc:VGNC:85179]	-1.76	0.0084
ssc-miR-374a-5p	AHDC1	hypothetical gene	-1.76	0.0084
ssc-miR-374a-5p	AHSA2	hypothetical gene	-1.76	0.0084

ssc-miR-374a-5p	AK4	hypothetical gene	-1.76	0.0084
ssc-miR-374a-5p	AKAP6	A-kinase anchoring protein 6 [Source:VGNC Symbol;Acc:VGNC:85221]	-1.76	0.0084
ssc-miR-374a-5p	AKT1	AKT serine/threonine kinase 1 [Source:VGNC Symbol;Acc:VGNC:96930]	-1.76	0.0084
ssc-miR-374a-5p	AKT3	AKT serine/threonine kinase 3 [Source:VGNC Symbol;Acc:VGNC:96306]	-1.76	0.0084
ssc-miR-374a-5p	AMD1	hypothetical gene	-1.76	0.0084
ssc-miR-374a-5p	AMN	amnion associated transmembrane protein [Source:VGNC Symbol;Acc:VGNC:85282]	-1.76	0.0084
ssc-miR-374a-5p	ANAPC16	anaphase promoting complex subunit 16 [Source:NCBI gene (formerly Entrezgene);Acc:100155019]	-1.76	0.0084
ssc-miR-374a-5p	ANK3	hypothetical gene	-1.76	0.0084
ssc-miR-374a-5p	ANKRD12	ankyrin repeat domain 12 [Source:VGNC Symbol;Acc:VGNC:96906]	-1.76	0.0084
ssc-miR-374a-5p	ANKRD13C	ankyrin repeat domain 13C [Source:VGNC Symbol;Acc:VGNC:85326]	-1.76	0.0084
ssc-miR-374a-5p	ANLN	anillin actin binding protein [Source:VGNC Symbol;Acc:VGNC:85353]	-1.76	0.0084
ssc-miR-374a-5p	ANTXR2	ANTXR cell adhesion molecule 2 [Source:VGNC Symbol;Acc:VGNC:85365]	-1.76	0.0084
ssc-miR-374a-5p	AP005482.1	hypothetical gene	-1.76	0.0084
ssc-miR-374a-5p	APC	APC regulator of WNT signaling pathway [Source:VGNC Symbol;Acc:VGNC:99584]	-1.76	0.0084
ssc-miR-374a-5p	APOPT1	hypothetical gene	-1.76	0.0084
ssc-miR-374a-5p	ARCN1	archain 1 [Source:VGNC Symbol;Acc:VGNC:85443]	-1.76	0.0084
ssc-miR-374a-5p	ARHGAP20	Rho GTPase activating protein 20 [Source:VGNC Symbol;Acc:VGNC:97884]	-1.76	0.0084
ssc-miR-374a-5p	ARHGAP23	Rho GTPase activating protein 23 [Source:VGNC Symbol;Acc:VGNC:85462]	-1.76	0.0084
ssc-miR-374a-5p	ARHGAP5	Rho GTPase activating protein 5 [Source:VGNC Symbol;Acc:VGNC:85480]	-1.76	0.0084
ssc-miR-374a-5p	ARHGEF40	Rho guanine nucleotide exchange factor 40 [Source:VGNC Symbol;Acc:VGNC:85501]	-1.76	0.0084
ssc-miR-374a-5p	ARID2	AT-rich interaction domain 2 [Source:HGNC Symbol;Acc:HGNC:18037]	-1.76	0.0084
ssc-miR-374a-5p	ARIH2	ariadne RBR E3 ubiquitin protein ligase 2 [Source:VGNC Symbol;Acc:VGNC:85512]	-1.76	0.0084
ssc-miR-374a-5p	ARL15	ADP ribosylation factor like GTPase 15 [Source:VGNC Symbol;Acc:VGNC:85520]	-1.76	0.0084
ssc-miR-374a-5p	ARL2BP	ADP ribosylation factor like GTPase 2 binding protein [Source:VGNC Symbol;Acc:VGNC:85521]	-1.76	0.0084
ssc-miR-374a-5p	ARL5A	ADP ribosylation factor like GTPase 5A [Source:VGNC Symbol;Acc:VGNC:96418]	-1.76	0.0084
ssc-miR-374a-5p	ARMCX5	armadillo repeat containing X-linked 5 [Source:HGNC Symbol;Acc:HGNC:25772]	-1.76	0.0084
ssc-miR-374a-5p	ASB4	ankyrin repeat and SOCS box containing 4 [Source:VGNC Symbol;Acc:VGNC:97891]	-1.76	0.0084
ssc-miR-374a-5p	ASCL1	achaete-scute family bHLH transcription factor 1 [Source:HGNC Symbol;Acc:HGNC:738]	-1.76	0.0084
ssc-miR-374a-5p	ASPN	asporin [Source:VGNC Symbol;Acc:VGNC:85586]	-1.76	0.0084
ssc-miR-374a-5p	ATAD1	ATPase family AAA domain containing 1 [Source:HGNC Symbol;Acc:HGNC:25903]	-1.76	0.0084
ssc-miR-374a-5p	ATAD2	ATPase family AAA domain containing 2 [Source:VGNC Symbol;Acc:VGNC:85599]	-1.76	0.0084
ssc-miR-374a-5p	ATE1	arginyltransferase 1 [Source:VGNC Symbol;Acc:VGNC:85604]	-1.76	0.0084
ssc-miR-374a-5p	ATF2	activating transcription factor 2 [Source:VGNC Symbol;Acc:VGNC:96446]	-1.76	0.0084
ssc-miR-374a-5p	ATMIN	hypothetical gene	-1.76	0.0084
ssc-miR-374a-5p	ATP1A1	ATPase Na+/K+ transporting subunit alpha 1 [Source:VGNC Symbol;Acc:VGNC:98735]	-1.76	0.0084
ssc-miR-374a-5p	ATP2B2	ATPase plasma membrane Ca2+ transporting 2 [Source:VGNC Symbol;Acc:VGNC:85649]	-1.76	0.0084
ssc-miR-374a-5p	ATP8B2	ATPase phospholipid transporting 8B2 [Source:VGNC Symbol;Acc:VGNC:85680]	-1.76	0.0084
ssc-miR-374a-5p	ATXN1	ataxin 1 [Source:VGNC Symbol;Acc:VGNC:85687]	-1.76	0.0084
ssc-miR-374a-5p	ATXN7	ataxin 7 [Source:VGNC Symbol;Acc:VGNC:99704]	-1.76	0.0084
ssc-miR-374a-5p	BACE1	beta-secretase 1 [Source:VGNC Symbol;Acc:VGNC:85740]	-1.76	0.0084
ssc-miR-374a-5p	BARHL2	BarH like homeobox 2 [Source:VGNC Symbol;Acc:VGNC:85756]	-1.76	0.0084
ssc-miR-374a-5p	BCAT1	branched chain amino acid transaminase 1 [Source:VGNC Symbol;Acc:VGNC:85773]	-1.76	0.0084
ssc-miR-374a-5p	BCL11B	BAF chromatin remodeling complex subunit BCL11B [Source:VGNC Symbol;Acc:VGNC:96563]	-1.76	0.0084
ssc-miR-374a-5p	BEND4	BEN domain containing 4 [Source:VGNC Symbol;Acc:VGNC:85801]	-1.76	0.0084
ssc-miR-374a-5p	BHLHE40	basic helix-loop-helix family member e40 [Source:VGNC Symbol;Acc:VGNC:85814]	-1.76	0.0084
ssc-miR-374a-5p	BICC1	BicC family RNA binding protein 1 [Source:VGNC Symbol;Acc:VGNC:85817]	-1.76	0.0084
ssc-miR-374a-5p	BIRC3	baculoviral IAP repeat containing 3 [Source:HGNC Symbol;Acc:HGNC:591]	-1.76	0.0084
ssc-miR-374a-5p	BLOC1S6	biosis of lysosomal organelles complex 1 subunit 6 [Source:VGNC Symbol;Acc:VGNC:85832]	-1.76	0.0084
ssc-miR-374a-5p	BMP2	bone morphotic protein 2 [Source:VGNC Symbol;Acc:VGNC:95488]	-1.76	0.0084
ssc-miR-374a-5p	BMP3	bone morphotic protein 3 [Source:VGNC Symbol;Acc:VGNC:85842]	-1.76	0.0084

ssc-miR-374a-5p	BMPER	BMP binding endothelial regulator [Source:VGNC Symbol;Acc:VGNC:97902]	-1.76	0.0084
ssc-miR-374a-5p	BOLA3	hypothetical gene	-1.76	0.0084
ssc-miR-374a-5p	BRINP3	BMP/retinoic acid inducible neural specific 3 [Source:VGNC Symbol;Acc:VGNC:95983]	-1.76	0.0084
ssc-miR-374a-5p	BSDC1	BSD domain containing 1 [Source:VGNC Symbol;Acc:VGNC:85892]	-1.76	0.0084
ssc-miR-374a-5p	BTBD7	BTB domain containing 7 [Source:VGNC Symbol;Acc:VGNC:85908]	-1.76	0.0084
ssc-miR-374a-5p	BTBD9	BTB domain containing 9 [Source:VGNC Symbol;Acc:VGNC:85909]	-1.76	0.0084
ssc-miR-374a-5p	BZRAP1	hypothetical gene	-1.76	0.0084
ssc-miR-374a-5p	BZW1	basic leucine zipper and W2 domains 1 [Source:VGNC Symbol;Acc:VGNC:95885]	-1.76	0.0084
ssc-miR-374a-5p	C10orf12	hypothetical gene	-1.76	0.0084
ssc-miR-374a-5p	C11orf58	chromosome 2 C11orf58 homolog [Source:VGNC Symbol;Acc:VGNC:85998]	-1.76	0.0084
ssc-miR-374a-5p	C11orf87	chromosome 11 open reading frame 87 [Source:HGNC Symbol;Acc:HGNC:33788]	-1.76	0.0084
ssc-miR-374a-5p	C16orf72	chromosome 3 C16orf72 homolog [Source:VGNC Symbol;Acc:VGNC:86016]	-1.76	0.0084
ssc-miR-374a-5p	C19orf81	chromosome 6 C19orf81 homolog [Source:VGNC Symbol;Acc:VGNC:86058]	-1.76	0.0084
ssc-miR-374a-5p	C1orf95	hypothetical gene	-1.76	0.0084
ssc-miR-374a-5p	C1QL3	complement C1q like 3 [Source:VGNC Symbol;Acc:VGNC:95910]	-1.76	0.0084
ssc-miR-374a-5p	C3orf58	hypothetical gene	-1.76	0.0084
ssc-miR-374a-5p	C5orf28	hypothetical gene	-1.76	0.0084
ssc-miR-374a-5p	C5orf47	chromosome 16 C5orf47 homolog [Source:VGNC Symbol;Acc:VGNC:85952]	-1.76	0.0084
ssc-miR-374a-5p	C5orf51	hypothetical gene	-1.76	0.0084
ssc-miR-374a-5p	C8orf44-SGK3	hypothetical gene	-1.76	0.0084
ssc-miR-374a-5p	CA12	carbonic anhydrase 12 [Source:VGNC Symbol;Acc:VGNC:86097]	-1.76	0.0084
ssc-miR-374a-5p	CADM2	cell adhesion molecule 2 [Source:VGNC Symbol;Acc:VGNC:97910]	-1.76	0.0084
ssc-miR-374a-5p	CALCOCO1	calcium binding and coiled-coil domain 1 [Source:VGNC Symbol;Acc:VGNC:86140]	-1.76	0.0084
ssc-miR-374a-5p	CALD1	caldesmon 1 [Source:VGNC Symbol;Acc:VGNC:86143]	-1.76	0.0084
ssc-miR-374a-5p	CAMK4	calcium/calmodulin dependent protein kinase IV [Source:VGNC Symbol;Acc:VGNC:99602]	-1.76	0.0084
ssc-miR-374a-5p	CAMSAP2	calmodulin regulated spectrin associated protein family member 2 [Source:VGNC Symbol;Acc:VGNC:96205]	-1.76	0.0084
ssc-miR-374a-5p	CAND1	cullin associated and neddylation dissociated 1 [Source:VGNC Symbol;Acc:VGNC:97912]	-1.76	0.0084
ssc-miR-374a-5p	CAPZA2	capping actin protein of muscle Z-line subunit alpha 2 [Source:VGNC Symbol;Acc:VGNC:103910]	-1.76	0.0084
ssc-miR-374a-5p	CCDC148	hypothetical gene	-1.76	0.0084
ssc-miR-374a-5p	CCDC89	coiled-coil domain containing 89 [Source:HGNC Symbol;Acc:HGNC:26762]	-1.76	0.0084
ssc-miR-374a-5p	CCL2	chemokine (C-C motif) ligand 2 [Source:NCBI gene (formerly Entrezgene);Acc:397422]	-1.76	0.0084
ssc-miR-374a-5p	CCL22	C-C motif chemokine ligand 22 [Source:NCBI gene (formerly Entrezgene);Acc:100516016]	-1.76	0.0084
ssc-miR-374a-5p	CCL8	chemokine (C-C motif) ligand 8 [Source:NCBI gene (formerly Entrezgene);Acc:100302703]	-1.76	0.0084
ssc-miR-374a-5p	CCNE2	cyclin E2 [Source:VGNC Symbol;Acc:VGNC:86355]	-1.76	0.0084
ssc-miR-374a-5p	CCNL1	cyclin L1 [Source:VGNC Symbol;Acc:VGNC:86363]	-1.76	0.0084
ssc-miR-374a-5p	CD40LG	CD40 ligand [Source:VGNC Symbol;Acc:VGNC:86418]	-1.76	0.0084
ssc-miR-374a-5p	CD47	CD47 molecule [Source:VGNC Symbol;Acc:VGNC:108637]	-1.76	0.0084
ssc-miR-374a-5p	CDA	cytidine deaminase [Source:VGNC Symbol;Acc:VGNC:86439]	-1.76	0.0084
ssc-miR-374a-5p	CDC42BPG	CDC42 binding protein kinase gamma [Source:VGNC Symbol;Acc:VGNC:86454]	-1.76	0.0084
ssc-miR-374a-5p	CDC42EP3	CDC42 effector protein 3 [Source:VGNC Symbol;Acc:VGNC:86457]	-1.76	0.0084
ssc-miR-374a-5p	CDC73	cell division cycle 73 [Source:VGNC Symbol;Acc:VGNC:95817]	-1.76	0.0084
ssc-miR-374a-5p	CDK19	cyclin dependent kinase 19 [Source:VGNC Symbol;Acc:VGNC:86502]	-1.76	0.0084
ssc-miR-374a-5p	CDYL2	chromodomain Y like 2 [Source:VGNC Symbol;Acc:VGNC:86529]	-1.76	0.0084
ssc-miR-374a-5p	CEBPA	CCAAT enhancer binding protein alpha [Source:VGNC Symbol;Acc:VGNC:86531]	-1.76	0.0084
ssc-miR-374a-5p	CEBPB	CCAAT enhancer binding protein beta [Source:VGNC Symbol;Acc:VGNC:95883]	-1.76	0.0084
ssc-miR-374a-5p	CELF2	hypothetical gene	-1.76	0.0084
ssc-miR-374a-5p	CEP350	centrosomal protein 350 [Source:HGNC Symbol;Acc:HGNC:24238]	-1.76	0.0084
ssc-miR-374a-5p	CERS3	ceramide synthase 3 [Source:VGNC Symbol;Acc:VGNC:86588]	-1.76	0.0084
ssc-miR-374a-5p	CHD7	chromodomain helicase DNA binding protein 7 [Source:VGNC Symbol;Acc:VGNC:86633]	-1.76	0.0084
ssc-miR-374a-5p	CHRM2	cholinergic receptor muscarinic 2 [Source:VGNC Symbol;Acc:VGNC:103922]	-1.76	0.0084

ssc-miR-374a-5p	CHST2	carbohydrate sulfotransferase 2 [Source:VGNC Symbol;Acc:VGNC:86678]	-1.76	0.0084
ssc-miR-374a-5p	CLDN14	claudin 14 [Source:VGNC Symbol;Acc:VGNC:86731]	-1.76	0.0084
ssc-miR-374a-5p	CLIP4	CAP-Gly domain containing linker protein family member 4 [Source:VGNC Symbol;Acc:VGNC:97932]	-1.76	0.0084
ssc-miR-374a-5p	CNIH1	cornichon family AMPA receptor auxiliary protein 1 [Source:VGNC Symbol;Acc:VGNC:86820]	-1.76	0.0084
ssc-miR-374a-5p	CNOT2	CCR4-NOT transcription complex subunit 2 [Source:VGNC Symbol;Acc:VGNC:86834]	-1.76	0.0084
ssc-miR-374a-5p	CNOT6	CCR4-NOT transcription complex subunit 6 [Source:VGNC Symbol;Acc:VGNC:86837]	-1.76	0.0084
ssc-miR-374a-5p	CNOT8	CCR4-NOT transcription complex subunit 8 [Source:VGNC Symbol;Acc:VGNC:86839]	-1.76	0.0084
ssc-miR-374a-5p	CNST	consortin, connexin sorting protein [Source:VGNC Symbol;Acc:VGNC:96131]	-1.76	0.0084
ssc-miR-374a-5p	CNTN4	contactin 4 [Source:VGNC Symbol;Acc:VGNC:97934]	-1.76	0.0084
ssc-miR-374a-5p	COG3	component of oligomeric golgi complex 3 [Source:HGNC Symbol;Acc:HGNC:18619]	-1.76	0.0084
ssc-miR-374a-5p	COPS2	COP9 signalosome subunit 2 [Source:VGNC Symbol;Acc:VGNC:86899]	-1.76	0.0084
ssc-miR-374a-5p	CORO1C	coronin 1C [Source:VGNC Symbol;Acc:VGNC:86914]	-1.76	0.0084
ssc-miR-374a-5p	CPEB4	cytoplasmic polyadenylation element binding protein 4 [Source:VGNC Symbol;Acc:VGNC:86939]	-1.76	0.0084
ssc-miR-374a-5p	CRAMP1L	hypothetical gene	-1.76	0.0084
ssc-miR-374a-5p	CREB1	cAMP responsive element binding protein 1 [Source:VGNC Symbol;Acc:VGNC:96004]	-1.76	0.0084
ssc-miR-374a-5p	CREB3L2	cAMP responsive element binding protein 3 like 2 [Source:VGNC Symbol;Acc:VGNC:86981]	-1.76	0.0084
ssc-miR-374a-5p	CRIM1	cysteine rich transmembrane BMP regulator 1 [Source:VGNC Symbol;Acc:VGNC:86995]	-1.76	0.0084
ssc-miR-374a-5p	CRK	CRK proto-onco, adaptor protein [Source:NCBI gene (formerly Entrezgene);Acc:100192444]	-1.76	0.0084
ssc-miR-374a-5p	CRNKL1	crooked neck pre-mRNA splicing factor 1 [Source:VGNC Symbol;Acc:VGNC:95654]	-1.76	0.0084
ssc-miR-374a-5p	CRTC2	CREB regulated transcription coactivator 2 [Source:VGNC Symbol;Acc:VGNC:87008]	-1.76	0.0084
ssc-miR-374a-5p	CSMD1	CUB and Sushi multiple domains 1 [Source:VGNC Symbol;Acc:VGNC:95600]	-1.76	0.0084
ssc-miR-374a-5p	CSMD2	hypothetical gene	-1.76	0.0084
ssc-miR-374a-5p	CSNK1G1	casein kinase 1 gamma 1 [Source:VGNC Symbol;Acc:VGNC:97949]	-1.76	0.0084
ssc-miR-374a-5p	CSRNP3	cysteine and serine rich nuclear protein 3 [Source:VGNC Symbol;Acc:VGNC:96249]	-1.76	0.0084
ssc-miR-374a-5p	CTAGE5	hypothetical gene	-1.76	0.0084
ssc-miR-374a-5p	CTDNEP1	CTD nuclear envelope phosphatase 1 [Source:HGNC Symbol;Acc:HGNC:19085]	-1.76	0.0084
ssc-miR-374a-5p	CTDSPL2	CTD small phosphatase like 2 [Source:VGNC Symbol;Acc:VGNC:87060]	-1.76	0.0084
ssc-miR-374a-5p	CTTNBP2NL	CTTNBP2 N-terminal like [Source:VGNC Symbol;Acc:VGNC:87082]	-1.76	0.0084
ssc-miR-374a-5p	CYBRD1	cytochrome b reductase 1 [Source:VGNC Symbol;Acc:VGNC:95948]	-1.76	0.0084
ssc-miR-374a-5p	CYFIP1	cytoplasmic FMR1 interacting protein 1 [Source:HGNC Symbol;Acc:HGNC:13759]	-1.76	0.0084
ssc-miR-374a-5p	CYP26A1	cytochrome P450 family 26 subfamily A member 1 [Source:VGNC Symbol;Acc:VGNC:103370]	-1.76	0.0084
ssc-miR-374a-5p	CYP26B1	cytochrome P450 family 26 subfamily B member 1 [Source:VGNC Symbol;Acc:VGNC:103374]	-1.76	0.0084
ssc-miR-374a-5p	CYP7B1	cytochrome P450 family 7 subfamily B member 1 [Source:VGNC Symbol;Acc:VGNC:103362]	-1.76	0.0084
ssc-miR-374a-5p	CYTH1	cytohesin 1 [Source:VGNC Symbol;Acc:VGNC:87135]	-1.76	0.0084
ssc-miR-374a-5p	CYTH3	cytohesin 3 [Source:VGNC Symbol;Acc:VGNC:87136]	-1.76	0.0084
ssc-miR-374a-5p	DACH1	dachshund family transcription factor 1 [Source:VGNC Symbol;Acc:VGNC:87146]	-1.76	0.0084
ssc-miR-374a-5p	DBR1	debranching RNA lariats 1 [Source:VGNC Symbol;Acc:VGNC:87168]	-1.76	0.0084
ssc-miR-374a-5p	DCLK1	doublecortin like kinase 1 [Source:HGNC Symbol;Acc:HGNC:2700]	-1.76	0.0084
ssc-miR-374a-5p	DCP2	decapping mRNA 2 [Source:VGNC Symbol;Acc:VGNC:87188]	-1.76	0.0084
ssc-miR-374a-5p	DCUN1D1	hypothetical gene	-1.76	0.0084
ssc-miR-374a-5p	DDX53	hypothetical gene	-1.76	0.0084
ssc-miR-374a-5p	DENND4A	DENN domain containing 4A [Source:VGNC Symbol;Acc:VGNC:87251]	-1.76	0.0084
ssc-miR-374a-5p	DESI1	desumoylating isopeptidase 1 [Source:VGNC Symbol;Acc:VGNC:87263]	-1.76	0.0084
ssc-miR-374a-5p	DGAT2	diacylglycerol O-acyltransferase 2 [Source:NCBI gene (formerly Entrezgene);Acc:100294675]	-1.76	0.0084
ssc-miR-374a-5p	DLG3	discs large MAGUK scaffold protein 3 [Source:VGNC Symbol;Acc:VGNC:87327]	-1.76	0.0084
ssc-miR-374a-5p	DMD	dystrophin [Source:NCBI gene (formerly Entrezgene);Acc:497636]	-1.76	0.0084
ssc-miR-374a-5p	DMXL1	Dmx like 1 [Source:VGNC Symbol;Acc:VGNC:87359]	-1.76	0.0084
ssc-miR-374a-5p	DNAJB1	DnaJ heat shock protein family (Hsp40) member B1 [Source:VGNC Symbol;Acc:VGNC:96614]	-1.76	0.0084
ssc-miR-374a-5p	DNAJC14	DnaJ heat shock protein family (Hsp40) member C14 [Source:NCBI gene (formerly Entrezgene);Acc:100151876]	-1.76	0.0084
ssc-miR-374a-5p	DNAJC22	DnaJ heat shock protein family (Hsp40) member C22 [Source:VGNC Symbol;Acc:VGNC:103231]	-1.76	0.0084

ssc-miR-374a-5p	DNAJC6	DnaJ heat shock protein family (Hsp40) member C6 [Source:VGNC Symbol;Acc:VGNC:96625]	-1.76	0.0084
ssc-miR-374a-5p	DNMT3A	DNA methyltransferase 3 alpha [Source:VGNC Symbol;Acc:VGNC:87384]	-1.76	0.0084
ssc-miR-374a-5p	DOK6	docking protein 6 [Source:VGNC Symbol;Acc:VGNC:87404]	-1.76	0.0084
ssc-miR-374a-5p	DR1	down-regulator of transcription 1 [Source:VGNC Symbol;Acc:VGNC:87435]	-1.76	0.0084
ssc-miR-374a-5p	DSEL	dermatan sulfate epimerase like [Source:VGNC Symbol;Acc:VGNC:87454]	-1.76	0.0084
ssc-miR-374a-5p	DSG3	desmoglein 3 [Source:VGNC Symbol;Acc:VGNC:87457]	-1.76	0.0084
ssc-miR-374a-5p	DUSP19	dual specificity phosphatase 19 [Source:VGNC Symbol;Acc:VGNC:95899]	-1.76	0.0084
ssc-miR-374a-5p	DUSP6	dual specificity phosphatase 6 [Source:VGNC Symbol;Acc:VGNC:87488]	-1.76	0.0084
ssc-miR-374a-5p	DYRK1A	dual specificity tyrosine phosphorylation regulated kinase 1A [Source:VGNC Symbol;Acc:VGNC:87505]	-1.76	0.0084
ssc-miR-374a-5p	DZIP1	DAZ interacting zinc finger protein 1 [Source:VGNC Symbol;Acc:VGNC:87510]	-1.76	0.0084
ssc-miR-374a-5p	DZIP1L	DAZ interacting zinc finger protein 1 like [Source:VGNC Symbol;Acc:VGNC:87511]	-1.76	0.0084
ssc-miR-374a-5p	EDAR	ectodysplasin A receptor [Source:VGNC Symbol;Acc:VGNC:87541]	-1.76	0.0084
ssc-miR-374a-5p	EDEM3	ER degradation enhancing alpha-mannosidase like protein 3 [Source:VGNC Symbol;Acc:VGNC:87546]	-1.76	0.0084
ssc-miR-374a-5p	EDIL3	EGF like repeats and discoidin domains 3 [Source:VGNC Symbol;Acc:VGNC:99642]	-1.76	0.0084
ssc-miR-374a-5p	EFNA2	ephrin A2 [Source:VGNC Symbol;Acc:VGNC:87573]	-1.76	0.0084
ssc-miR-374a-5p	EFNB2	ephrin B2 [Source:VGNC Symbol;Acc:VGNC:87577]	-1.76	0.0084
ssc-miR-374a-5p	EGR2	early growth response 2 [Source:VGNC Symbol;Acc:VGNC:103939]	-1.76	0.0084
ssc-miR-374a-5p	EIF2AK4	eukaryotic translation initiation factor 2 alpha kinase 4 [Source:VGNC Symbol;Acc:VGNC:87609]	-1.76	0.0084
ssc-miR-374a-5p	EIF2S2	eukaryotic translation initiation factor 2 subunit beta [Source:VGNC Symbol;Acc:VGNC:96265]	-1.76	0.0084
ssc-miR-374a-5p	EIF3A	eukaryotic translation initiation factor 3 subunit A [Source:VGNC Symbol;Acc:VGNC:87615]	-1.76	0.0084
ssc-miR-374a-5p	EIF4E	eukaryotic translation initiation factor 4E [Source:VGNC Symbol;Acc:VGNC:87626]	-1.76	0.0084
ssc-miR-374a-5p	EIF4G1	eukaryotic translation initiation factor 4 gamma 1 [Source:VGNC Symbol;Acc:VGNC:87631]	-1.76	0.0084
ssc-miR-374a-5p	EIF4G2	eukaryotic translation initiation factor 4 gamma 2 [Source:VGNC Symbol;Acc:VGNC:99645]	-1.76	0.0084
ssc-miR-374a-5p	ELAC2	elaC ribonuclease Z 2 [Source:VGNC Symbol;Acc:VGNC:87636]	-1.76	0.0084
ssc-miR-374a-5p	ELAVL4	ELAV like RNA binding protein 4 [Source:VGNC Symbol;Acc:VGNC:97047]	-1.76	0.0084
ssc-miR-374a-5p	ELOVL7	ELOVL fatty acid elongase 7 [Source:HGNC Symbol;Acc:HGNC:26292]	-1.76	0.0084
ssc-miR-374a-5p	EN1	engrailed homeobox 1 [Source:VGNC Symbol;Acc:VGNC:96273]	-1.76	0.0084
ssc-miR-374a-5p	EN2	engrailed homeobox 2 [Source:VGNC Symbol;Acc:VGNC:87694]	-1.76	0.0084
ssc-miR-374a-5p	EPB41L1	hypothetical gene	-1.76	0.0084
ssc-miR-374a-5p	EPHA4	EPH receptor A4 [Source:VGNC Symbol;Acc:VGNC:96280]	-1.76	0.0084
ssc-miR-374a-5p	EPHA8	EPH receptor A8 [Source:VGNC Symbol;Acc:VGNC:87735]	-1.76	0.0084
ssc-miR-374a-5p	EPS15	epidermal growth factor receptor pathway substrate 15 [Source:VGNC Symbol;Acc:VGNC:87747]	-1.76	0.0084
ssc-miR-374a-5p	ERC1	ELKS/RAB6-interacting/CAST family member 1 [Source:VGNC Symbol;Acc:VGNC:87761]	-1.76	0.0084
ssc-miR-374a-5p	ESRRG	estrogen related receptor gamma [Source:VGNC Symbol;Acc:VGNC:96289]	-1.76	0.0084
ssc-miR-374a-5p	ETNK1	ethanolamine kinase 1 [Source:VGNC Symbol;Acc:VGNC:87806]	-1.76	0.0084
ssc-miR-374a-5p	ETV3	ETS variant transcription factor 3 [Source:VGNC Symbol;Acc:VGNC:87812]	-1.76	0.0084
ssc-miR-374a-5p	ETV5	ETS variant transcription factor 5 [Source:VGNC Symbol;Acc:VGNC:87814]	-1.76	0.0084
ssc-miR-374a-5p	ETV6	ETS variant transcription factor 6 [Source:VGNC Symbol;Acc:VGNC:87815]	-1.76	0.0084
ssc-miR-374a-5p	FABP2	fatty acid binding protein 2 [Source:VGNC Symbol;Acc:VGNC:87871]	-1.76	0.0084
ssc-miR-374a-5p	FAF2	Fas associated factor family member 2 [Source:VGNC Symbol;Acc:VGNC:87880]	-1.76	0.0084
ssc-miR-374a-5p	FAM131B	family with sequence similarity 131 member B [Source:VGNC Symbol;Acc:VGNC:87901]	-1.76	0.0084
ssc-miR-374a-5p	FAM13C	family with sequence similarity 13 member C [Source:VGNC Symbol;Acc:VGNC:87907]	-1.76	0.0084
ssc-miR-374a-5p	FAM169A	family with sequence similarity 169 member A [Source:VGNC Symbol;Acc:VGNC:87927]	-1.76	0.0084
ssc-miR-374a-5p	FAM19A2	hypothetical gene	-1.76	0.0084
ssc-miR-374a-5p	FAM216A	family with sequence similarity 216 member A [Source:VGNC Symbol;Acc:VGNC:87954]	-1.76	0.0084
ssc-miR-374a-5p	FAM222B	family with sequence similarity 222 member B [Source:VGNC Symbol;Acc:VGNC:87960]	-1.76	0.0084
ssc-miR-374a-5p	FAM63B	hypothetical gene	-1.76	0.0084
ssc-miR-374a-5p	FAM65B	hypothetical gene	-1.76	0.0084
ssc-miR-374a-5p	FANCM	FA complementation group M [Source:VGNC Symbol;Acc:VGNC:88008]	-1.76	0.0084
ssc-miR-374a-5p	FBXL3	F-box and leucine rich repeat protein 3 [Source:VGNC Symbol;Acc:VGNC:88030]	-1.76	0.0084

ssc-miR-374a-5p	FBXO32	F-box protein 32 [Source:VGNC Symbol;Acc:VGNC:88039]	-1.76	0.0084
ssc-miR-374a-5p	FBXO42	F-box protein 42 [Source:VGNC Symbol;Acc:VGNC:88047]	-1.76	0.0084
ssc-miR-374a-5p	FECH	ferrochelatase [Source:VGNC Symbol;Acc:VGNC:88081]	-1.76	0.0084
ssc-miR-374a-5p	FGF12	fibroblast growth factor 12 [Source:VGNC Symbol;Acc:VGNC:88102]	-1.76	0.0084
ssc-miR-374a-5p	FGF18	fibroblast growth factor 18 [Source:VGNC Symbol;Acc:VGNC:88107]	-1.76	0.0084
ssc-miR-374a-5p	FGF5	fibroblast growth factor 5 [Source:HGNC Symbol;Acc:HGNC:3683]	-1.76	0.0084
ssc-miR-374a-5p	FGF9	fibroblast growth factor 9 [Source:VGNC Symbol;Acc:VGNC:103943]	-1.76	0.0084
ssc-miR-374a-5p	FGFR2	fibroblast growth factor receptor 2 [Source:NCBI gene (formerly Entrezgene);Acc:396762]	-1.76	0.0084
ssc-miR-374a-5p	FNIP1	folliculin interacting protein 1 [Source:VGNC Symbol;Acc:VGNC:88187]	-1.76	0.0084
ssc-miR-374a-5p	FOSB	FosB proto-onco, AP-1 transcription factor subunit [Source:VGNC Symbol;Acc:VGNC:88190]	-1.76	0.0084
ssc-miR-374a-5p	FOXD2	forkhead box D2 [Source:VGNC Symbol;Acc:VGNC:88200]	-1.76	0.0084
ssc-miR-374a-5p	FOXD3	forkhead box D3 [Source:VGNC Symbol;Acc:VGNC:88201]	-1.76	0.0084
ssc-miR-374a-5p	FOXG1	forkhead box G1 [Source:VGNC Symbol;Acc:VGNC:88206]	-1.76	0.0084
ssc-miR-374a-5p	FOXO1	forkhead box O1 [Source:VGNC Symbol;Acc:VGNC:98013]	-1.76	0.0084
ssc-miR-374a-5p	FOXP1	forkhead box P1 [Source:VGNC Symbol;Acc:VGNC:88222]	-1.76	0.0084
ssc-miR-374a-5p	FRMPD4	FERM and PDZ domain containing 4 [Source:VGNC Symbol;Acc:VGNC:88243]	-1.76	0.0084
ssc-miR-374a-5p	FRS2	fibroblast growth factor receptor substrate 2 [Source:VGNC Symbol;Acc:VGNC:88246]	-1.76	0.0084
ssc-miR-374a-5p	FUT9	fucosyltransferase 9 [Source:VGNC Symbol;Acc:VGNC:88271]	-1.76	0.0084
ssc-miR-374a-5p	FZD5	frizzled class receptor 5 [Source:VGNC Symbol;Acc:VGNC:96309]	-1.76	0.0084
ssc-miR-374a-5p	FZD8	frizzled class receptor 8 [Source:VGNC Symbol;Acc:VGNC:96311]	-1.76	0.0084
ssc-miR-374a-5p	GABPA	GA binding protein transcription factor subunit alpha [Source:VGNC Symbol;Acc:VGNC:103945]	-1.76	0.0084
ssc-miR-374a-5p	GABPB2	GA binding protein transcription factor subunit beta 2 [Source:VGNC Symbol;Acc:VGNC:98796]	-1.76	0.0084
ssc-miR-374a-5p	GADD45A	growth arrest and DNA damage inducible alpha [Source:VGNC Symbol;Acc:VGNC:88316]	-1.76	0.0084
ssc-miR-374a-5p	GAS7	growth arrest specific 7 [Source:VGNC Symbol;Acc:VGNC:88359]	-1.76	0.0084
ssc-miR-374a-5p	GATA3	GATA binding protein 3 [Source:VGNC Symbol;Acc:VGNC:96315]	-1.76	0.0084
ssc-miR-374a-5p	GBX2	gastrulation brain homeobox 2 [Source:VGNC Symbol;Acc:VGNC:96316]	-1.76	0.0084
ssc-miR-374a-5p	GLG1	golgi glycoprotein 1 [Source:VGNC Symbol;Acc:VGNC:88481]	-1.76	0.0084
ssc-miR-374a-5p	GLIS3	GLIS family zinc finger 3 [Source:VGNC Symbol;Acc:VGNC:88485]	-1.76	0.0084
ssc-miR-374a-5p	GMEB1	glucocorticoid modulatory element binding protein 1 [Source:VGNC Symbol;Acc:VGNC:88508]	-1.76	0.0084
ssc-miR-374a-5p	GNB2	G protein subunit beta 2 [Source:VGNC Symbol;Acc:VGNC:88531]	-1.76	0.0084
ssc-miR-374a-5p	GNPNAT1	glucosamine-phosphate N-acetyltransferase 1 [Source:VGNC Symbol;Acc:VGNC:103102]	-1.76	0.0084
ssc-miR-374a-5p	GNPTG	N-acetylglucosamine-1-phosphate transferase subunit gamma [Source:VGNC Symbol;Acc:VGNC:88547]	-1.76	0.0084
ssc-miR-374a-5p	GOLGA1	golgin A1 [Source:VGNC Symbol;Acc:VGNC:88549]	-1.76	0.0084
ssc-miR-374a-5p	GOPC	golgi associated PDZ and coiled-coil motif containing [Source:VGNC Symbol;Acc:VGNC:98024]	-1.76	0.0084
ssc-miR-374a-5p	GPC6	glypican 6 [Source:HGNC Symbol;Acc:HGNC:4454]	-1.76	0.0084
ssc-miR-374a-5p	GPR161	G protein-coupled receptor 161 [Source:VGNC Symbol;Acc:VGNC:88613]	-1.76	0.0084
ssc-miR-374a-5p	GPR50	G protein-coupled receptor 50 [Source:VGNC Symbol;Acc:VGNC:88633]	-1.76	0.0084
ssc-miR-374a-5p	GPR75	G protein-coupled receptor 75 [Source:VGNC Symbol;Acc:VGNC:88636]	-1.76	0.0084
ssc-miR-374a-5p	GPT2	glutamic--pyruvic transaminase 2 [Source:VGNC Symbol;Acc:VGNC:88651]	-1.76	0.0084
ssc-miR-374a-5p	GRAMD1B	GRAM domain containing 1B [Source:VGNC Symbol;Acc:VGNC:88656]	-1.76	0.0084
ssc-miR-374a-5p	GRAMD4	GRAM domain containing 4 [Source:VGNC Symbol;Acc:VGNC:88659]	-1.76	0.0084
ssc-miR-374a-5p	GRIN2B	glutamate ionotropic receptor NMDA type subunit 2B [Source:VGNC Symbol;Acc:VGNC:88684]	-1.76	0.0084
ssc-miR-374a-5p	GTDC1	glycosyltransferase like domain containing 1 [Source:VGNC Symbol;Acc:VGNC:95955]	-1.76	0.0084
ssc-miR-374a-5p	GTF2A2	general transcription factor IIA subunit 2 [Source:HGNC Symbol;Acc:HGNC:4647]	-1.76	0.0084
ssc-miR-374a-5p	GTPBP1	GTP binding protein 1 [Source:VGNC Symbol;Acc:VGNC:88742]	-1.76	0.0084
ssc-miR-374a-5p	GTPBP10	GTP binding protein 10 [Source:VGNC Symbol;Acc:VGNC:103959]	-1.76	0.0084
ssc-miR-374a-5p	H3F3B	hypothetical gene	-1.76	0.0084
ssc-miR-374a-5p	HAPLN1	hyaluronan and proteoglycan link protein 1 [Source:VGNC Symbol;Acc:VGNC:88780]	-1.76	0.0084
ssc-miR-374a-5p	HCFC1	host cell factor C1 [Source:HGNC Symbol;Acc:HGNC:4839]	-1.76	0.0084
ssc-miR-374a-5p	HDAC9	histone deacetylase 9 [Source:HGNC Symbol;Acc:HGNC:14065]	-1.76	0.0084

ssc-miR-374a-5p	HECTD1	HECT domain E3 ubiquitin protein ligase 1 [Source:VGNC Symbol;Acc:VGNC:88832]	-1.76	0.0084
ssc-miR-374a-5p	HECTD4	HECT domain E3 ubiquitin protein ligase 4 [Source:VGNC Symbol;Acc:VGNC:88834]	-1.76	0.0084
ssc-miR-374a-5p	HEG1	heart development protein with EGF like domains 1 [Source:VGNC Symbol;Acc:VGNC:88836]	-1.76	0.0084
ssc-miR-374a-5p	HERC2	HECT and RLD domain containing E3 ubiquitin protein ligase 2 [Source:VGNC Symbol;Acc:VGNC:99716]	-1.76	0.0084
ssc-miR-374a-5p	HES1	hes family bHLH transcription factor 1 [Source:VGNC Symbol;Acc:VGNC:88854]	-1.76	0.0084
ssc-miR-374a-5p	HGF	hepatocyte growth factor [Source:VGNC Symbol;Acc:VGNC:88869]	-1.76	0.0084
ssc-miR-374a-5p	HIAT1	hypothetical gene	-1.76	0.0084
ssc-miR-374a-5p	HIPK2	homeodomain interacting protein kinase 2 [Source:VGNC Symbol;Acc:VGNC:88888]	-1.76	0.0084
ssc-miR-374a-5p	HLF	HLF transcription factor, PAR bZIP family member [Source:VGNC Symbol;Acc:VGNC:88896]	-1.76	0.0084
ssc-miR-374a-5p	HOXA1	homeobox A1 [Source:VGNC Symbol;Acc:VGNC:88933]	-1.76	0.0084
ssc-miR-374a-5p	HOXA10	homeobox A10 [Source:VGNC Symbol;Acc:VGNC:88934]	-1.76	0.0084
ssc-miR-374a-5p	HOXA11	homeobox A11 [Source:VGNC Symbol;Acc:VGNC:88935]	-1.76	0.0084
ssc-miR-374a-5p	HOXD11	homeobox D11 [Source:VGNC Symbol;Acc:VGNC:96352]	-1.76	0.0084
ssc-miR-374a-5p	HR	HR lysine demethylase and nuclear receptor corepressor [Source:VGNC Symbol;Acc:VGNC:88969]	-1.76	0.0084
ssc-miR-374a-5p	HSBP1	heat shock factor binding protein 1 [Source:NCBI gene (formerly Entrezgene);Acc:100579143]	-1.76	0.0084
ssc-miR-374a-5p	HSD11B1	hydroxysteroid 11-beta dehydrogenase 1 [Source:VGNC Symbol;Acc:VGNC:88983]	-1.76	0.0084
ssc-miR-374a-5p	HSDL2	hydroxysteroid dehydrogenase like 2 [Source:VGNC Symbol;Acc:VGNC:88992]	-1.76	0.0084
ssc-miR-374a-5p	HSPA4	heat shock protein family A (Hsp70) member 4 [Source:HGNC Symbol;Acc:HGNC:5237]	-1.76	0.0084
ssc-miR-374a-5p	HSPA9	heat shock protein family A (Hsp70) member 9 [Source:VGNC Symbol;Acc:VGNC:99761]	-1.76	0.0084
ssc-miR-374a-5p	HSPE1-MOB4	hypothetical gene	-1.76	0.0084
ssc-miR-374a-5p	HSPH1	heat shock protein family H (Hsp110) member 1 [Source:NCBI gene (formerly Entrezgene);Acc:100048931]	-1.76	0.0084
ssc-miR-374a-5p	HUNK	hormonally up-regulated Neu-associated kinase [Source:VGNC Symbol;Acc:VGNC:89007]	-1.76	0.0084
ssc-miR-374a-5p	ICK	hypothetical gene	-1.76	0.0084
ssc-miR-374a-5p	IFIT5	interferon induced protein with tetratricopeptide repeats 5 [Source:VGNC Symbol;Acc:VGNC:107402]	-1.76	0.0084
ssc-miR-374a-5p	IGSF10	immunoglobulin superfamily member 10 [Source:VGNC Symbol;Acc:VGNC:89063]	-1.76	0.0084
ssc-miR-374a-5p	IKZF2	IKAROS family zinc finger 2 [Source:VGNC Symbol;Acc:VGNC:95576]	-1.76	0.0084
ssc-miR-374a-5p	IL10	interleukin 10 [Source:VGNC Symbol;Acc:VGNC:108593]	-1.76	0.0084
ssc-miR-374a-5p	IL1A	interleukin 1 alpha [Source:VGNC Symbol;Acc:VGNC:89091]	-1.76	0.0084
ssc-miR-374a-5p	IL1RAPL1	interleukin 1 receptor accessory protein like 1 [Source:VGNC Symbol;Acc:VGNC:103967]	-1.76	0.0084
ssc-miR-374a-5p	ING1	inhibitor of growth family member 1 [Source:VGNC Symbol;Acc:VGNC:89131]	-1.76	0.0084
ssc-miR-374a-5p	INHBA	inhibin subunit beta A [Source:VGNC Symbol;Acc:VGNC:89133]	-1.76	0.0084
ssc-miR-374a-5p	INHBB	inhibin subunit beta B [Source:VGNC Symbol;Acc:VGNC:103969]	-1.76	0.0084
ssc-miR-374a-5p	IQCK	IQ motif containing K [Source:VGNC Symbol;Acc:VGNC:89191]	-1.76	0.0084
ssc-miR-374a-5p	IQSEC2	IQ motif and Sec7 domain ArfGEF 2 [Source:VGNC Symbol;Acc:VGNC:89195]	-1.76	0.0084
ssc-miR-374a-5p	IRF2BP2	interferon regulatory factor 2 binding protein 2 [Source:VGNC Symbol;Acc:VGNC:89205]	-1.76	0.0084
ssc-miR-374a-5p	IRS4	insulin receptor substrate 4 [Source:HGNC Symbol;Acc:HGNC:6128]	-1.76	0.0084
ssc-miR-374a-5p	ITGB1BP1	integrin subunit beta 1 binding protein 1 [Source:VGNC Symbol;Acc:VGNC:89241]	-1.76	0.0084
ssc-miR-374a-5p	JAM2	junctional adhesion molecule 2 [Source:VGNC Symbol;Acc:VGNC:89276]	-1.76	0.0084
ssc-miR-374a-5p	JHDM1D	hypothetical gene	-1.76	0.0084
ssc-miR-374a-5p	JMY	junction mediating and regulatory protein, p53 cofactor [Source:VGNC Symbol;Acc:VGNC:89289]	-1.76	0.0084
ssc-miR-374a-5p	KANSL1L	KAT8 regulatory NSL complex subunit 1 like [Source:HGNC Symbol;Acc:HGNC:26310]	-1.76	0.0084
ssc-miR-374a-5p	KAT6A	hypothetical gene	-1.76	0.0084
ssc-miR-374a-5p	KCNJ6	potassium inwardly rectifying channel subfamily J member 6 [Source:VGNC Symbol;Acc:VGNC:89360]	-1.76	0.0084
ssc-miR-374a-5p	KCNN3	potassium calcium-activated channel subfamily N member 3 [Source:VGNC Symbol;Acc:VGNC:98056]	-1.76	0.0084
ssc-miR-374a-5p	KCTD19	potassium channel tetramerization domain containing 19 [Source:VGNC Symbol;Acc:VGNC:89397]	-1.76	0.0084
ssc-miR-374a-5p	KERA	keratocan [Source:VGNC Symbol;Acc:VGNC:89420]	-1.76	0.0084
ssc-miR-374a-5p	KIAA0408	KIAA0408 [Source:VGNC Symbol;Acc:VGNC:89432]	-1.76	0.0084
ssc-miR-374a-5p	KIAA1024	hypothetical gene	-1.76	0.0084
ssc-miR-374a-5p	KIAA1161	hypothetical gene	-1.76	0.0084
ssc-miR-374a-5p	KIAA1199	hypothetical gene	-1.76	0.0084

ssc-miR-374a-5p	KIAA1432	hypothetical gene	-1.76	0.0084
ssc-miR-374a-5p	KIAA1468	hypothetical gene	-1.76	0.0084
ssc-miR-374a-5p	KIF20A	kinesin family member 20A [Source:VGNC Symbol;Acc:VGNC:89462]	-1.76	0.0084
ssc-miR-374a-5p	KIRREL	hypothetical gene	-1.76	0.0084
ssc-miR-374a-5p	KLF3	Kruppel like factor 3 [Source:VGNC Symbol;Acc:VGNC:89498]	-1.76	0.0084
ssc-miR-374a-5p	KLF7	Kruppel like factor 7 [Source:VGNC Symbol;Acc:VGNC:96396]	-1.76	0.0084
ssc-miR-374a-5p	KLF8	Kruppel like factor 8 [Source:VGNC Symbol;Acc:VGNC:89500]	-1.76	0.0084
ssc-miR-374a-5p	KLHL18	kelch like family member 18 [Source:VGNC Symbol;Acc:VGNC:89517]	-1.76	0.0084
ssc-miR-374a-5p	KLHL29	kelch like family member 29 [Source:VGNC Symbol;Acc:VGNC:89524]	-1.76	0.0084
ssc-miR-374a-5p	KRR1	KRR1 small subunit processome component homolog [Source:VGNC Symbol;Acc:VGNC:89575]	-1.76	0.0084
ssc-miR-374a-5p	KTN1	kinectin 1 [Source:VGNC Symbol;Acc:VGNC:89608]	-1.76	0.0084
ssc-miR-374a-5p	L1CAM	L1 cell adhesion molecule [Source:HGNC Symbol;Acc:HGNC:6470]	-1.76	0.0084
ssc-miR-374a-5p	L3HYPDH	trans-L-3-hydroxyproline dehydratase [Source:VGNC Symbol;Acc:VGNC:89612]	-1.76	0.0084
ssc-miR-374a-5p	LARP1	La ribonucleoprotein 1, translational regulator [Source:VGNC Symbol;Acc:VGNC:98067]	-1.76	0.0084
ssc-miR-374a-5p	LARP4	La ribonucleoprotein 4 [Source:VGNC Symbol;Acc:VGNC:89641]	-1.76	0.0084
ssc-miR-374a-5p	LARP4B	hypothetical gene	-1.76	0.0084
ssc-miR-374a-5p	LCA5	lebercilin LCA5 [Source:VGNC Symbol;Acc:VGNC:89654]	-1.76	0.0084
ssc-miR-374a-5p	LDLRAD4	low density lipoprotein receptor class A domain containing 4 [Source:VGNC Symbol;Acc:VGNC:89673]	-1.76	0.0084
ssc-miR-374a-5p	LEMD3	LEM domain containing 3 [Source:VGNC Symbol;Acc:VGNC:89679]	-1.76	0.0084
ssc-miR-374a-5p	LHFPL2	LHFPL tetraspan subfamily member 2 [Source:VGNC Symbol;Acc:VGNC:89705]	-1.76	0.0084
ssc-miR-374a-5p	LHX5	LIM homeobox 5 [Source:VGNC Symbol;Acc:VGNC:89715]	-1.76	0.0084
ssc-miR-374a-5p	LIG3	DNA ligase 3 [Source:VGNC Symbol;Acc:VGNC:89721]	-1.76	0.0084
ssc-miR-374a-5p	LIN9	lin-9 DREAM MuvB core complex component [Source:VGNC Symbol;Acc:VGNC:103976]	-1.76	0.0084
ssc-miR-374a-5p	LMLN	leishmanolysin like peptidase [Source:VGNC Symbol;Acc:VGNC:89759]	-1.76	0.0084
ssc-miR-374a-5p	LPAR1	hypothetical gene	-1.76	0.0084
ssc-miR-374a-5p	LPAR3	lysophosphatidic acid receptor 3 [Source:VGNC Symbol;Acc:VGNC:89785]	-1.76	0.0084
ssc-miR-374a-5p	LPHN2	hypothetical gene	-1.76	0.0084
ssc-miR-374a-5p	LPP	LIM domain containing preferred translocation partner in lipoma [Source:HGNC Symbol;Acc:HGNC:6679]	-1.76	0.0084
ssc-miR-374a-5p	LRCH1	leucine rich repeats and calponin homology domain containing 1 [Source:VGNC Symbol;Acc:VGNC:89801]	-1.76	0.0084
ssc-miR-374a-5p	LRP6	LDL receptor related protein 6 [Source:VGNC Symbol;Acc:VGNC:89821]	-1.76	0.0084
ssc-miR-374a-5p	LSAMP	limbic system associated membrane protein [Source:VGNC Symbol;Acc:VGNC:89868]	-1.76	0.0084
ssc-miR-374a-5p	MAP2	microtubule associated protein 2 [Source:VGNC Symbol;Acc:VGNC:96409]	-1.76	0.0084
ssc-miR-374a-5p	MAP2K4	mitogen-activated protein kinase kinase 4 [Source:VGNC Symbol;Acc:VGNC:98101]	-1.76	0.0084
ssc-miR-374a-5p	MAP2K6	mitogen-activated protein kinase kinase 6 [Source:VGNC Symbol;Acc:VGNC:98102]	-1.76	0.0084
ssc-miR-374a-5p	MAP3K2	mitogen-activated protein kinase kinase kinase 2 [Source:VGNC Symbol;Acc:VGNC:98107]	-1.76	0.0084
ssc-miR-374a-5p	MAPK1IP1L	mitogen-activated protein kinase 1 interacting protein 1 like [Source:VGNC Symbol;Acc:VGNC:90002]	-1.76	0.0084
ssc-miR-374a-5p	MAPK6	mitogen-activated protein kinase 6 [Source:VGNC Symbol;Acc:VGNC:103123]	-1.76	0.0084
ssc-miR-374a-5p	MAPRE2	microtubule associated protein RP/EB family member 2 [Source:VGNC Symbol;Acc:VGNC:98117]	-1.76	0.0084
ssc-miR-374a-5p	MBD5	methyl-CpG binding domain protein 5 [Source:VGNC Symbol;Acc:VGNC:95940]	-1.76	0.0084
ssc-miR-374a-5p	MBNL1	muscleblind like splicing regulator 1 [Source:VGNC Symbol;Acc:VGNC:90054]	-1.76	0.0084
ssc-miR-374a-5p	MBNL2	muscleblind like splicing regulator 2 [Source:VGNC Symbol;Acc:VGNC:90055]	-1.76	0.0084
ssc-miR-374a-5p	MCFD2	multiple coagulation factor deficiency 2, ER cargo receptor complex subunit [Source:VGNC Symbol;Acc:VGNC:103980]	-1.76	0.0084
ssc-miR-374a-5p	MECP2	methyl-CpG binding protein 2 [Source:VGNC Symbol;Acc:VGNC:90101]	-1.76	0.0084
ssc-miR-374a-5p	MED12L	mediator complex subunit 12L [Source:VGNC Symbol;Acc:VGNC:90105]	-1.76	0.0084
ssc-miR-374a-5p	MED13	mediator complex subunit 13 [Source:VGNC Symbol;Acc:VGNC:90106]	-1.76	0.0084
ssc-miR-374a-5p	MEF2D	myocyte enhancer factor 2D [Source:VGNC Symbol;Acc:VGNC:90128]	-1.76	0.0084
ssc-miR-374a-5p	MEIS1	Meis homeobox 1 [Source:VGNC Symbol;Acc:VGNC:90135]	-1.76	0.0084
ssc-miR-374a-5p	METRNL	meteorin like, glial cell differentiation regulator [Source:VGNC Symbol;Acc:VGNC:90153]	-1.76	0.0084
ssc-miR-374a-5p	METTL21A	methyltransferase 21A, HSPA lysine [Source:VGNC Symbol;Acc:VGNC:96242]	-1.76	0.0084
ssc-miR-374a-5p	MEX3A	mex-3 RNA binding family member A [Source:VGNC Symbol;Acc:VGNC:90168]	-1.76	0.0084

ssc-miR-374a-5p	MEX3B	mex-3 RNA binding family member B [Source:VGNC Symbol;Acc:VGNC:90169]	-1.76	0.0084
ssc-miR-374a-5p	MFAP3L	microfibril associated protein 3 like [Source:VGNC Symbol;Acc:VGNC:90175]	-1.76	0.0084
ssc-miR-374a-5p	MGAT4A	alpha-1,3-mannosyl-glycoprotein 4-beta-N-acetylglucosaminyltransferase A [Source:VGNC Symbol;Acc:VGNC:108158]	-1.76	0.0084
ssc-miR-374a-5p	MICALCL	hypothetical gene	-1.76	0.0084
ssc-miR-374a-5p	MIER1	MIER1 transcriptional regulator [Source:VGNC Symbol;Acc:VGNC:90219]	-1.76	0.0084
ssc-miR-374a-5p	MIPOL1	mirror-image polydactyly 1 [Source:VGNC Symbol;Acc:VGNC:90237]	-1.76	0.0084
ssc-miR-374a-5p	MKX	mohawk homeobox [Source:VGNC Symbol;Acc:VGNC:96020]	-1.76	0.0084
ssc-miR-374a-5p	MLX	MAX dimerization protein MLX [Source:VGNC Symbol;Acc:VGNC:90261]	-1.76	0.0084
ssc-miR-374a-5p	MME	membrane metalloendopeptidase [Source:VGNC Symbol;Acc:VGNC:90265]	-1.76	0.0084
ssc-miR-374a-5p	MMP14	matrix metallopeptidase 14 [Source:NCBI gene (formerly Entrezgene);Acc:397471]	-1.76	0.0084
ssc-miR-374a-5p	MOB4	hypothetical gene	-1.76	0.0084
ssc-miR-374a-5p	MON2	MON2 homolog, regulator of endosome-to-Golgi trafficking [Source:VGNC Symbol;Acc:VGNC:90301]	-1.76	0.0084
ssc-miR-374a-5p	MPP6	hypothetical gene	-1.76	0.0084
ssc-miR-374a-5p	MSH3	mutS homolog 3 [Source:VGNC Symbol;Acc:VGNC:90419]	-1.76	0.0084
ssc-miR-374a-5p	MSI2	musashi RNA binding protein 2 [Source:VGNC Symbol;Acc:VGNC:90422]	-1.76	0.0084
ssc-miR-374a-5p	MSX1	msh homeobox 1 [Source:VGNC Symbol;Acc:VGNC:90431]	-1.76	0.0084
ssc-miR-374a-5p	MUM1L1	hypothetical gene	-1.76	0.0084
ssc-miR-374a-5p	MYLK	myosin light chain kinase [Source:VGNC Symbol;Acc:VGNC:108676]	-1.76	0.0084
ssc-miR-374a-5p	MYO10	myosin X [Source:VGNC Symbol;Acc:VGNC:90522]	-1.76	0.0084
ssc-miR-374a-5p	MYO9A	myosin IXA [Source:VGNC Symbol;Acc:VGNC:103138]	-1.76	0.0084
ssc-miR-374a-5p	MYT1L	myelin transcription factor 1 like [Source:VGNC Symbol;Acc:VGNC:90549]	-1.76	0.0084
ssc-miR-374a-5p	MYZAP	myocardial zonula adherens protein [Source:HGNC Symbol;Acc:HGNC:43444]	-1.76	0.0084
ssc-miR-374a-5p	MZT1	mitotic spindle organizing protein 1 [Source:VGNC Symbol;Acc:VGNC:90552]	-1.76	0.0084
ssc-miR-374a-5p	N4BP2	NEDD4 binding protein 2 [Source:VGNC Symbol;Acc:VGNC:90554]	-1.76	0.0084
ssc-miR-374a-5p	NAA35	N-alpha-acetyltransferase 35, NatC auxiliary subunit [Source:VGNC Symbol;Acc:VGNC:96752]	-1.76	0.0084
ssc-miR-374a-5p	NAPEPLD	N-acyl phosphatidylethanolamine phospholipase D [Source:VGNC Symbol;Acc:VGNC:90579]	-1.76	0.0084
ssc-miR-374a-5p	NCBP1	nuclear cap binding protein subunit 1 [Source:VGNC Symbol;Acc:VGNC:90603]	-1.76	0.0084
ssc-miR-374a-5p	NCK1	NCK adaptor protein 1 [Source:VGNC Symbol;Acc:VGNC:90609]	-1.76	0.0084
ssc-miR-374a-5p	NCKAP1	NCK associated protein 1 [Source:VGNC Symbol;Acc:VGNC:96432]	-1.76	0.0084
ssc-miR-374a-5p	NCOA1	nuclear receptor coactivator 1 [Source:VGNC Symbol;Acc:VGNC:90615]	-1.76	0.0084
ssc-miR-374a-5p	NCOA2	nuclear receptor coactivator 2 [Source:VGNC Symbol;Acc:VGNC:90616]	-1.76	0.0084
ssc-miR-374a-5p	NEDD4L	NEDD4 like E3 ubiquitin protein ligase [Source:VGNC Symbol;Acc:VGNC:90667]	-1.76	0.0084
ssc-miR-374a-5p	NEO1	neogenin 1 [Source:HGNC Symbol;Acc:HGNC:7754]	-1.76	0.0084
ssc-miR-374a-5p	NEUROD1	neuronal differentiation 1 [Source:VGNC Symbol;Acc:VGNC:96439]	-1.76	0.0084
ssc-miR-374a-5p	NEUROG2	neurogenin 2 [Source:VGNC Symbol;Acc:VGNC:90701]	-1.76	0.0084
ssc-miR-374a-5p	NFIA	nuclear factor I A [Source:VGNC Symbol;Acc:VGNC:90715]	-1.76	0.0084
ssc-miR-374a-5p	NFIB	nuclear factor I B [Source:VGNC Symbol;Acc:VGNC:90716]	-1.76	0.0084
ssc-miR-374a-5p	NFIL3	nuclear factor, interleukin 3 regulated [Source:VGNC Symbol;Acc:VGNC:90717]	-1.76	0.0084
ssc-miR-374a-5p	NFIX	nuclear factor I X [Source:VGNC Symbol;Acc:VGNC:90718]	-1.76	0.0084
ssc-miR-374a-5p	NFKBIZ	NFKB inhibitor zeta [Source:VGNC Symbol;Acc:VGNC:90726]	-1.76	0.0084
ssc-miR-374a-5p	NFYB	nuclear transcription factor Y subunit beta [Source:VGNC Symbol;Acc:VGNC:90730]	-1.76	0.0084
ssc-miR-374a-5p	NHLRC2	NHL repeat containing 2 [Source:VGNC Symbol;Acc:VGNC:90737]	-1.76	0.0084
ssc-miR-374a-5p	NIPBL	NIPBL cohesin loading factor [Source:VGNC Symbol;Acc:VGNC:90752]	-1.76	0.0084
ssc-miR-374a-5p	NKX2-2	NK2 homeobox 2 [Source:VGNC Symbol;Acc:VGNC:96444]	-1.76	0.0084
ssc-miR-374a-5p	NLN	neurolysin [Source:VGNC Symbol;Acc:VGNC:90780]	-1.76	0.0084
ssc-miR-374a-5p	NMT1	N-myristoyltransferase 1 [Source:VGNC Symbol;Acc:VGNC:90802]	-1.76	0.0084
ssc-miR-374a-5p	NOG	noggin [Source:VGNC Symbol;Acc:VGNC:90812]	-1.76	0.0084
ssc-miR-374a-5p	NOVA1	NOVA alternative splicing regulator 1 [Source:VGNC Symbol;Acc:VGNC:90827]	-1.76	0.0084
ssc-miR-374a-5p	NPPC	natriuretic peptide C [Source:VGNC Symbol;Acc:VGNC:96450]	-1.76	0.0084
ssc-miR-374a-5p	NPTX2	neuronal pentraxin 2 [Source:VGNC Symbol;Acc:VGNC:90863]	-1.76	0.0084

ssc-miR-374a-5p	NR2F2	nuclear receptor subfamily 2 group F member 2 [Source:VGNC Symbol;Acc:VGNC:90881]	-1.76	0.0084
ssc-miR-374a-5p	NR3C1	nuclear receptor subfamily 3 group C member 1 [Source:VGNC Symbol;Acc:VGNC:90883]	-1.76	0.0084
ssc-miR-374a-5p	NR4A2	nuclear receptor subfamily 4 group A member 2 [Source:VGNC Symbol;Acc:VGNC:96451]	-1.76	0.0084
ssc-miR-374a-5p	NR4A3	nuclear receptor subfamily 4 group A member 3 [Source:VGNC Symbol;Acc:VGNC:90885]	-1.76	0.0084
ssc-miR-374a-5p	NRDE2	NRDE-2, necessary for RNA interference, domain containing [Source:VGNC Symbol;Acc:VGNC:90894]	-1.76	0.0084
ssc-miR-374a-5p	NRG2	neuregulin 2 [Source:VGNC Symbol;Acc:VGNC:98160]	-1.76	0.0084
ssc-miR-374a-5p	NRK	Nik related kinase [Source:VGNC Symbol;Acc:VGNC:98161]	-1.76	0.0084
ssc-miR-374a-5p	NRN1	neuritin 1 [Source:VGNC Symbol;Acc:VGNC:90900]	-1.76	0.0084
ssc-miR-374a-5p	NTF3	neurotrophin 3 [Source:VGNC Symbol;Acc:VGNC:90928]	-1.76	0.0084
ssc-miR-374a-5p	NTN1	netrin 1 [Source:VGNC Symbol;Acc:VGNC:90931]	-1.76	0.0084
ssc-miR-374a-5p	NUAK1	NUAK family kinase 1 [Source:VGNC Symbol;Acc:VGNC:90940]	-1.76	0.0084
ssc-miR-374a-5p	NUMBL	NUMB like endocytic adaptor protein [Source:HGNC Symbol;Acc:HGNC:8061]	-1.76	0.0084
ssc-miR-374a-5p	NUP35	nucleoporin 35 [Source:VGNC Symbol;Acc:VGNC:96229]	-1.76	0.0084
ssc-miR-374a-5p	NUP85	nucleoporin 85 [Source:VGNC Symbol;Acc:VGNC:90985]	-1.76	0.0084
ssc-miR-374a-5p	NXPE3	neurexophilin and PC-esterase domain family member 3 [Source:VGNC Symbol;Acc:VGNC:90997]	-1.76	0.0084
ssc-miR-374a-5p	OCRL	OCRL inositol polyphosphate-5-phosphatase [Source:VGNC Symbol;Acc:VGNC:91014]	-1.76	0.0084
ssc-miR-374a-5p	ONECUT2	one cut homeobox 2 [Source:VGNC Symbol;Acc:VGNC:91043]	-1.76	0.0084
ssc-miR-374a-5p	ONECUT3	one cut homeobox 3 [Source:VGNC Symbol;Acc:VGNC:91044]	-1.76	0.0084
ssc-miR-374a-5p	OSBP	oxysterol binding protein [Source:VGNC Symbol;Acc:VGNC:91066]	-1.76	0.0084
ssc-miR-374a-5p	OSTF1	osteoclast stimulating factor 1 [Source:VGNC Symbol;Acc:VGNC:91085]	-1.76	0.0084
ssc-miR-374a-5p	PABPC1	poly(A) binding protein cytoplasmic 1 [Source:VGNC Symbol;Acc:VGNC:98829]	-1.76	0.0084
ssc-miR-374a-5p	PAPD4	hypothetical gene	-1.76	0.0084
ssc-miR-374a-5p	PAPLN	papilin, proteoglycan like sulfated glycoprotein [Source:VGNC Symbol;Acc:VGNC:91169]	-1.76	0.0084
ssc-miR-374a-5p	PAPPA	pappalysin 1 [Source:VGNC Symbol;Acc:VGNC:91170]	-1.76	0.0084
ssc-miR-374a-5p	PAPPA2	pappalysin 2 [Source:VGNC Symbol;Acc:VGNC:91171]	-1.76	0.0084
ssc-miR-374a-5p	PAQR3	progestin and adipoQ receptor family member 3 [Source:VGNC Symbol;Acc:VGNC:91174]	-1.76	0.0084
ssc-miR-374a-5p	PARD6B	par-6 family cell polarity regulator beta [Source:VGNC Symbol;Acc:VGNC:98176]	-1.76	0.0084
ssc-miR-374a-5p	PARP8	poly(ADP-ribose) polymerase family member 8 [Source:HGNC Symbol;Acc:HGNC:26124]	-1.76	0.0084
ssc-miR-374a-5p	PAX6	paired box 6 [Source:VGNC Symbol;Acc:VGNC:91195]	-1.76	0.0084
ssc-miR-374a-5p	PCDH10	protocadherin 10 [Source:HGNC Symbol;Acc:HGNC:13404]	-1.76	0.0084
ssc-miR-374a-5p	PCDHA1	hypothetical gene	-1.76	0.0084
ssc-miR-374a-5p	PCDHA10	hypothetical gene	-1.76	0.0084
ssc-miR-374a-5p	PCDHA11	hypothetical gene	-1.76	0.0084
ssc-miR-374a-5p	PCDHA12	hypothetical gene	-1.76	0.0084
ssc-miR-374a-5p	PCDHA13	protocadherin alpha 13 [Source:HGNC Symbol;Acc:HGNC:8667]	-1.76	0.0084
ssc-miR-374a-5p	PCDHA2	hypothetical gene	-1.76	0.0084
ssc-miR-374a-5p	PCDHA3	protocadherin alpha 3 [Source:HGNC Symbol;Acc:HGNC:8669]	-1.76	0.0084
ssc-miR-374a-5p	PCDHA4	hypothetical gene	-1.76	0.0084
ssc-miR-374a-5p	PCDHA5	hypothetical gene	-1.76	0.0084
ssc-miR-374a-5p	PCDHA6	hypothetical gene	-1.76	0.0084
ssc-miR-374a-5p	PCDHA7	hypothetical gene	-1.76	0.0084
ssc-miR-374a-5p	PCDHA8	hypothetical gene	-1.76	0.0084
ssc-miR-374a-5p	PCDHA9	hypothetical gene	-1.76	0.0084
ssc-miR-374a-5p	PCDHAC1	hypothetical gene	-1.76	0.0084
ssc-miR-374a-5p	PCDHAC2	protocadherin alpha subfamily C, 2 [Source:HGNC Symbol;Acc:HGNC:8677]	-1.76	0.0084
ssc-miR-374a-5p	PCF11	PCF11 cleavage and polyadenylation factor subunit [Source:VGNC Symbol;Acc:VGNC:91218]	-1.76	0.0084
ssc-miR-374a-5p	PCGF3	polycomb group ring finger 3 [Source:VGNC Symbol;Acc:VGNC:91220]	-1.76	0.0084
ssc-miR-374a-5p	PCNX	hypothetical gene	-1.76	0.0084
ssc-miR-374a-5p	PDE10A	phosphodiesterase 10A [Source:VGNC Symbol;Acc:VGNC:91248]	-1.76	0.0084
ssc-miR-374a-5p	PDE3A	phosphodiesterase 3A [Source:VGNC Symbol;Acc:VGNC:91252]	-1.76	0.0084

ssc-miR-374a-5p	PDE4A	phosphodiesterase 4A [Source:VGNC Symbol;Acc:VGNC:91254]	-1.76	0.0084
ssc-miR-374a-5p	PDE4D	phosphodiesterase 4D [Source:VGNC Symbol;Acc:VGNC:91256]	-1.76	0.0084
ssc-miR-374a-5p	PDE7B	phosphodiesterase 7B [Source:VGNC Symbol;Acc:VGNC:91262]	-1.76	0.0084
ssc-miR-374a-5p	PDE8B	phosphodiesterase 8B [Source:VGNC Symbol;Acc:VGNC:100321]	-1.76	0.0084
ssc-miR-374a-5p	PDP2	pyruvate dehyrogenase phosphatase catalytic subunit 2 [Source:VGNC Symbol;Acc:VGNC:91287]	-1.76	0.0084
ssc-miR-374a-5p	PDPK1	3-phosphoinositide dependent protein kinase 1 [Source:HGNC Symbol;Acc:HGNC:8816]	-1.76	0.0084
ssc-miR-374a-5p	PDZD2	hypothetical gene	-1.76	0.0084
ssc-miR-374a-5p	PELI1	pellino E3 ubiquitin protein ligase 1 [Source:VGNC Symbol;Acc:VGNC:91306]	-1.76	0.0084
ssc-miR-374a-5p	PGR	progesterone receptor [Source:VGNC Symbol;Acc:VGNC:91362]	-1.76	0.0084
ssc-miR-374a-5p	PHACTR2	phosphatase and actin regulator 2 [Source:VGNC Symbol;Acc:VGNC:91366]	-1.76	0.0084
ssc-miR-374a-5p	PHF21A	PHD finger protein 21A [Source:VGNC Symbol;Acc:VGNC:91384]	-1.76	0.0084
ssc-miR-374a-5p	PHRF1	PHD and ring finger domains 1 [Source:VGNC Symbol;Acc:VGNC:91403]	-1.76	0.0084
ssc-miR-374a-5p	PI15	peptidase inhibitor 15 [Source:VGNC Symbol;Acc:VGNC:98188]	-1.76	0.0084
ssc-miR-374a-5p	PIGW	phosphatidylinositol glycan anchor biosynthesis class W [Source:VGNC Symbol;Acc:VGNC:91431]	-1.76	0.0084
ssc-miR-374a-5p	PIK3CA	phosphatidylinositol-4,5-bisphosphate 3-kinase catalytic subunit alpha [Source:VGNC Symbol;Acc:VGNC:91440]	-1.76	0.0084
ssc-miR-374a-5p	PIKFYVE	phosphoinositide kinase, FYVE-type zinc finger containing [Source:VGNC Symbol;Acc:VGNC:96023]	-1.76	0.0084
ssc-miR-374a-5p	PITPNC1	phosphatidylinositol transfer protein cytoplasmic 1 [Source:NCBI gene (formerly Entrezgene);Acc:100233199]	-1.76	0.0084
ssc-miR-374a-5p	PITX1	paired like homeodomain 1 [Source:NCBI gene (formerly Entrezgene);Acc:100689266]	-1.76	0.0084
ssc-miR-374a-5p	PITX2	paired like homeodomain 2 [Source:VGNC Symbol;Acc:VGNC:91467]	-1.76	0.0084
ssc-miR-374a-5p	PKIA	cAMP-dependent protein kinase inhibitor alpha [Source:VGNC Symbol;Acc:VGNC:91477]	-1.76	0.0084
ssc-miR-374a-5p	PKNOX1	PBX/knotted 1 homeobox 1 [Source:VGNC Symbol;Acc:VGNC:91484]	-1.76	0.0084
ssc-miR-374a-5p	PLCE1	phospholipase C epsilon 1 [Source:VGNC Symbol;Acc:VGNC:91519]	-1.76	0.0084
ssc-miR-374a-5p	PLD5	phospholipase D family member 5 [Source:VGNC Symbol;Acc:VGNC:96145]	-1.76	0.0084
ssc-miR-374a-5p	PLEKHM3	pleckstrin homology domain containing M3 [Source:VGNC Symbol;Acc:VGNC:96281]	-1.76	0.0084
ssc-miR-374a-5p	PLXNA2	plexin A2 [Source:VGNC Symbol;Acc:VGNC:91580]	-1.76	0.0084
ssc-miR-374a-5p	PLXNC1	plexin C1 [Source:VGNC Symbol;Acc:VGNC:91584]	-1.76	0.0084
ssc-miR-374a-5p	PMEPA1	prostate transmembrane protein, androgen induced 1 [Source:VGNC Symbol;Acc:VGNC:95606]	-1.76	0.0084
ssc-miR-374a-5p	PMPCB	peptidase, mitochondrial processing subunit beta [Source:VGNC Symbol;Acc:VGNC:91592]	-1.76	0.0084
ssc-miR-374a-5p	PNPT1	polyribonucleotide nucleotidyltransferase 1 [Source:VGNC Symbol;Acc:VGNC:91612]	-1.76	0.0084
ssc-miR-374a-5p	PNRC1	proline rich nuclear receptor coactivator 1 [Source:VGNC Symbol;Acc:VGNC:91613]	-1.76	0.0084
ssc-miR-374a-5p	POC1B-GALNT4	hypothetical gene	-1.76	0.0084
ssc-miR-374a-5p	POGZ	pogo transposable element derived with ZNF domain [Source:VGNC Symbol;Acc:VGNC:91625]	-1.76	0.0084
ssc-miR-374a-5p	POMP	proteasome maturation protein [Source:HGNC Symbol;Acc:HGNC:20330]	-1.76	0.0084
ssc-miR-374a-5p	POU4F1	POU class 4 homeobox 1 [Source:VGNC Symbol;Acc:VGNC:91678]	-1.76	0.0084
ssc-miR-374a-5p	PPAP2B	hypothetical gene	-1.76	0.0084
ssc-miR-374a-5p	PPARGC1A	PPARG coactivator 1 alpha [Source:VGNC Symbol;Acc:VGNC:91685]	-1.76	0.0084
ssc-miR-374a-5p	PPARGC1B	PPARG coactivator 1 beta [Source:VGNC Symbol;Acc:VGNC:91686]	-1.76	0.0084
ssc-miR-374a-5p	PPIG	peptidylprolyl isomerase G [Source:VGNC Symbol;Acc:VGNC:98214]	-1.76	0.0084
ssc-miR-374a-5p	PPM1D	protein phosphatase, Mg2+/Mn2+ dependent 1D [Source:VGNC Symbol;Acc:VGNC:91704]	-1.76	0.0084
ssc-miR-374a-5p	PPM1H	protein phosphatase, Mg2+/Mn2+ dependent 1H [Source:VGNC Symbol;Acc:VGNC:91708]	-1.76	0.0084
ssc-miR-374a-5p	PPM1K	protein phosphatase, Mg2+/Mn2+ dependent 1K [Source:VGNC Symbol;Acc:VGNC:91710]	-1.76	0.0084
ssc-miR-374a-5p	PPP1R3F	protein phosphatase 1 regulatory subunit 3F [Source:VGNC Symbol;Acc:VGNC:91739]	-1.76	0.0084
ssc-miR-374a-5p	PPP2R2C	protein phosphatase 2 regulatory subunit Bgamma [Source:VGNC Symbol;Acc:VGNC:91749]	-1.76	0.0084
ssc-miR-374a-5p	PPP6R1	protein phosphatase 6 regulatory subunit 1 [Source:VGNC Symbol;Acc:VGNC:91760]	-1.76	0.0084
ssc-miR-374a-5p	PPTC7	protein phosphatase targeting COQ7 [Source:VGNC Symbol;Acc:VGNC:91765]	-1.76	0.0084
ssc-miR-374a-5p	PRIM1	DNA primase subunit 1 [Source:VGNC Symbol;Acc:VGNC:103313]	-1.76	0.0084
ssc-miR-374a-5p	PRIMA1	proline rich membrane anchor 1 [Source:HGNC Symbol;Acc:HGNC:18319]	-1.76	0.0084
ssc-miR-374a-5p	PRKCA	protein kinase C alpha [Source:VGNC Symbol;Acc:VGNC:99028]	-1.76	0.0084
ssc-miR-374a-5p	PRKCE	protein kinase C epsilon [Source:VGNC Symbol;Acc:VGNC:91807]	-1.76	0.0084
ssc-miR-374a-5p	PRKG1	protein kinase cGMP-dependent 1 [Source:VGNC Symbol;Acc:VGNC:91816]	-1.76	0.0084

ssc-miR-374a-5p	PROK2	prokineticin 2 [Source:VGNC Symbol;Acc:VGNC:91831]	-1.76	0.0084
ssc-miR-374a-5p	PRPF38B	hypothetical gene	-1.76	0.0084
ssc-miR-374a-5p	PRPF40A	pre-mRNA processing factor 40 homolog A [Source:VGNC Symbol;Acc:VGNC:98222]	-1.76	0.0084
ssc-miR-374a-5p	PRR16	proline rich 16 [Source:VGNC Symbol;Acc:VGNC:91855]	-1.76	0.0084
ssc-miR-374a-5p	PRRG1	proline rich and Gla domain 1 [Source:VGNC Symbol;Acc:VGNC:101494]	-1.76	0.0084
ssc-miR-374a-5p	PSD2	pleckstrin and Sec7 domain containing 2 [Source:VGNC Symbol;Acc:VGNC:91896]	-1.76	0.0084
ssc-miR-374a-5p	PSME3	proteasome activator subunit 3 [Source:VGNC Symbol;Acc:VGNC:91926]	-1.76	0.0084
ssc-miR-374a-5p	PSMF1	proteasome inhibitor subunit 1 [Source:VGNC Symbol;Acc:VGNC:96512]	-1.76	0.0084
ssc-miR-374a-5p	PTEN	hypothetical gene	-1.76	0.0084
ssc-miR-374a-5p	PTGES3L	prostaglandin E synthase 3 like [Source:VGNC Symbol;Acc:VGNC:91953]	-1.76	0.0084
ssc-miR-374a-5p	PTPN11	protein tyrosine phosphatase non-receptor type 11 [Source:VGNC Symbol;Acc:VGNC:91972]	-1.76	0.0084
ssc-miR-374a-5p	PTPRG	protein tyrosine phosphatase receptor type G [Source:VGNC Symbol;Acc:VGNC:91988]	-1.76	0.0084
ssc-miR-374a-5p	PUM2	pumilio RNA binding family member 2 [Source:VGNC Symbol;Acc:VGNC:92002]	-1.76	0.0084
ssc-miR-374a-5p	PURA	purine rich element binding protein A [Source:HGNC Symbol;Acc:HGNC:9701]	-1.76	0.0084
ssc-miR-374a-5p	PURG	purine rich element binding protein G [Source:VGNC Symbol;Acc:VGNC:95865]	-1.76	0.0084
ssc-miR-374a-5p	QKI	QKI, KH domain containing RNA binding [Source:VGNC Symbol;Acc:VGNC:92025]	-1.76	0.0084
ssc-miR-374a-5p	RAB10	RAB10, member RAS onco family [Source:VGNC Symbol;Acc:VGNC:98235]	-1.76	0.0084
ssc-miR-374a-5p	RAB1A	RAB1A, member RAS onco family [Source:VGNC Symbol;Acc:VGNC:104042]	-1.76	0.0084
ssc-miR-374a-5p	RAB21	RAB21, member RAS oncogene family [Source:HGNC Symbol;Acc:HGNC:18263]	-1.76	0.0084
ssc-miR-374a-5p	RAB22A	RAB22A, member RAS onco family [Source:VGNC Symbol;Acc:VGNC:98244]	-1.76	0.0084
ssc-miR-374a-5p	RAB2A	RAB2A, member RAS onco family [Source:VGNC Symbol;Acc:VGNC:98252]	-1.76	0.0084
ssc-miR-374a-5p	RAB38	RAB38, member RAS onco family [Source:VGNC Symbol;Acc:VGNC:98261]	-1.76	0.0084
ssc-miR-374a-5p	RAB5B	RAB5B, member RAS oncogene family [Source:HGNC Symbol;Acc:HGNC:9784]	-1.76	0.0084
ssc-miR-374a-5p	RAD21	RAD21 cohesin complex component [Source:VGNC Symbol;Acc:VGNC:92054]	-1.76	0.0084
ssc-miR-374a-5p	RAI1	retinoic acid induced 1 [Source:VGNC Symbol;Acc:VGNC:92067]	-1.76	0.0084
ssc-miR-374a-5p	RALA	RAS like proto-oncogene A [Source:HGNC Symbol;Acc:HGNC:9839]	-1.76	0.0084
ssc-miR-374a-5p	RALGDS	ral guanine nucleotide dissociation stimulator [Source:HGNC Symbol;Acc:HGNC:9842]	-1.76	0.0084
ssc-miR-374a-5p	RANBP9	RAN binding protein 9 [Source:VGNC Symbol;Acc:VGNC:92082]	-1.76	0.0084
ssc-miR-374a-5p	RASA1	RAS p21 protein activator 1 [Source:VGNC Symbol;Acc:VGNC:92101]	-1.76	0.0084
ssc-miR-374a-5p	RASA2	RAS p21 protein activator 2 [Source:VGNC Symbol;Acc:VGNC:92102]	-1.76	0.0084
ssc-miR-374a-5p	RASSF8	Ras association domain family member 8 [Source:VGNC Symbol;Acc:VGNC:92128]	-1.76	0.0084
ssc-miR-374a-5p	RBFOX2	RNA binding fox-1 homolog 2 [Source:VGNC Symbol;Acc:VGNC:92140]	-1.76	0.0084
ssc-miR-374a-5p	RBM27	RNA binding motif protein 27 [Source:HGNC Symbol;Acc:HGNC:29243]	-1.76	0.0084
ssc-miR-374a-5p	RC3H1	ring finger and CCCH-type domains 1 [Source:VGNC Symbol;Acc:VGNC:92168]	-1.76	0.0084
ssc-miR-374a-5p	RCAN1	regulator of calcineurin 1 [Source:VGNC Symbol;Acc:VGNC:92170]	-1.76	0.0084
ssc-miR-374a-5p	RER1	retention in endoplasmic reticulum sorting receptor 1 [Source:VGNC Symbol;Acc:VGNC:92215]	-1.76	0.0084
ssc-miR-374a-5p	RFESD	Rieske Fe-S domain containing [Source:VGNC Symbol;Acc:VGNC:92236]	-1.76	0.0084
ssc-miR-374a-5p	RFTN2	raftlin family member 2 [Source:VGNC Symbol;Acc:VGNC:96128]	-1.76	0.0084
ssc-miR-374a-5p	RFX3	regulatory factor X3 [Source:VGNC Symbol;Acc:VGNC:92245]	-1.76	0.0084
ssc-miR-374a-5p	RFX4	regulatory factor X4 [Source:HGNC Symbol;Acc:HGNC:9985]	-1.76	0.0084
ssc-miR-374a-5p	RGS14	regulator of G protein signaling 14 [Source:VGNC Symbol;Acc:VGNC:92261]	-1.76	0.0084
ssc-miR-374a-5p	RGS7BP	regulator of G protein signaling 7 binding protein [Source:VGNC Symbol;Acc:VGNC:92268]	-1.76	0.0084
ssc-miR-374a-5p	RHOQ	ras homolog family member Q [Source:VGNC Symbol;Acc:VGNC:92292]	-1.76	0.0084
ssc-miR-374a-5p	RMND5A	required for meiotic nuclear division 5 homolog A [Source:VGNC Symbol;Acc:VGNC:92336]	-1.76	0.0084
ssc-miR-374a-5p	RNF11	ring finger protein 11 [Source:VGNC Symbol;Acc:VGNC:98601]	-1.76	0.0084
ssc-miR-374a-5p	RNF115	ring finger protein 115 [Source:VGNC Symbol;Acc:VGNC:92348]	-1.76	0.0084
ssc-miR-374a-5p	RNF14	ring finger protein 14 [Source:VGNC Symbol;Acc:VGNC:92357]	-1.76	0.0084
ssc-miR-374a-5p	RNF214	ring finger protein 214 [Source:VGNC Symbol;Acc:VGNC:92382]	-1.76	0.0084
ssc-miR-374a-5p	RNF222	ring finger protein 222 [Source:VGNC Symbol;Acc:VGNC:92384]	-1.76	0.0084
ssc-miR-374a-5p	RNF38	ring finger protein 38 [Source:VGNC Symbol;Acc:VGNC:92390]	-1.76	0.0084

ssc-miR-374a-5p	RNF44	ring finger protein 44 [Source:VGNC Symbol;Acc:VGNC:92395]	-1.76	0.0084
ssc-miR-374a-5p	RORA	RAR related orphan receptor A [Source:VGNC Symbol;Acc:VGNC:92408]	-1.76	0.0084
ssc-miR-374a-5p	RORB	RAR related orphan receptor B [Source:VGNC Symbol;Acc:VGNC:92409]	-1.76	0.0084
ssc-miR-374a-5p	RP11-407N17.3	hypothetical gene	-1.76	0.0084
ssc-miR-374a-5p	RPS6KA3	ribosomal protein S6 kinase A3 [Source:VGNC Symbol;Acc:VGNC:92442]	-1.76	0.0084
ssc-miR-374a-5p	RRP15	ribosomal RNA processing 15 homolog [Source:VGNC Symbol;Acc:VGNC:96036]	-1.76	0.0084
ssc-miR-374a-5p	RSF1	remodeling and spacing factor 1 [Source:VGNC Symbol;Acc:VGNC:92476]	-1.76	0.0084
ssc-miR-374a-5p	RSRC2	arginine and serine rich coiled-coil 2 [Source:VGNC Symbol;Acc:VGNC:92488]	-1.76	0.0084
ssc-miR-374a-5p	RTKN2	rhotekin 2 [Source:VGNC Symbol;Acc:VGNC:92495]	-1.76	0.0084
ssc-miR-374a-5p	RYBP	RING1 and YY1 binding protein [Source:VGNC Symbol;Acc:VGNC:92532]	-1.76	0.0084
ssc-miR-374a-5p	SAP30	Sin3A associated protein 30 [Source:VGNC Symbol;Acc:VGNC:92575]	-1.76	0.0084
ssc-miR-374a-5p	SATB1	SATB homeobox 1 [Source:VGNC Symbol;Acc:VGNC:92587]	-1.76	0.0084
ssc-miR-374a-5p	SCIN	scinderin [Source:NCBI gene (formerly Entrezgene);Acc:100512981]	-1.76	0.0084
ssc-miR-374a-5p	SCN1A	sodium voltage-gated channel alpha subunit 1 [Source:VGNC Symbol;Acc:VGNC:95478]	-1.76	0.0084
ssc-miR-374a-5p	SCN2B	sodium voltage-gated channel beta subunit 2 [Source:VGNC Symbol;Acc:VGNC:92633]	-1.76	0.0084
ssc-miR-374a-5p	SCN5A	sodium voltage-gated channel alpha subunit 5 [Source:VGNC Symbol;Acc:VGNC:92637]	-1.76	0.0084
ssc-miR-374a-5p	SCN7A	sodium voltage-gated channel alpha subunit 7 [Source:VGNC Symbol;Acc:VGNC:95480]	-1.76	0.0084
ssc-miR-374a-5p	SCOC	short coiled-coil protein [Source:VGNC Symbol;Acc:VGNC:98960]	-1.76	0.0084
ssc-miR-374a-5p	SCRN3	secernin 3 [Source:VGNC Symbol;Acc:VGNC:96241]	-1.76	0.0084
ssc-miR-374a-5p	SEC23B	SEC23 homolog B, COPII coat complex component [Source:VGNC Symbol;Acc:VGNC:95490]	-1.76	0.0084
ssc-miR-374a-5p	SEC23IP	SEC23 interacting protein [Source:VGNC Symbol;Acc:VGNC:92675]	-1.76	0.0084
ssc-miR-374a-5p	SEMA3D	semaphorin 3D [Source:VGNC Symbol;Acc:VGNC:92696]	-1.76	0.0084
ssc-miR-374a-5p	SEMA5A	semaphorin 5A [Source:VGNC Symbol;Acc:VGNC:92706]	-1.76	0.0084
ssc-miR-374a-5p	SERTAD2	SERTA domain containing 2 [Source:VGNC Symbol;Acc:VGNC:92750]	-1.76	0.0084
ssc-miR-374a-5p	SERTAD4	SERTA domain containing 4 [Source:VGNC Symbol;Acc:VGNC:92752]	-1.76	0.0084
ssc-miR-374a-5p	SETBP1	SET binding protein 1 [Source:VGNC Symbol;Acc:VGNC:92756]	-1.76	0.0084
ssc-miR-374a-5p	SETD1B	SET domain containing 1B, histone lysine methyltransferase [Source:HGNC Symbol;Acc:HGNC:29187]	-1.76	0.0084
ssc-miR-374a-5p	SETD2	SET domain containing 2, histone lysine methyltransferase [Source:VGNC Symbol;Acc:VGNC:92757]	-1.76	0.0084
ssc-miR-374a-5p	SETD7	SET domain containing 7, histone lysine methyltransferase [Source:VGNC Symbol;Acc:VGNC:92762]	-1.76	0.0084
ssc-miR-374a-5p	SEZ6	seizure related 6 homolog [Source:VGNC Symbol;Acc:VGNC:92767]	-1.76	0.0084
ssc-miR-374a-5p	SF1	splicing factor 1 [Source:HGNC Symbol;Acc:HGNC:12950]	-1.76	0.0084
ssc-miR-374a-5p	SGIP1	SH3GL interacting endocytic adaptor 1 [Source:VGNC Symbol;Acc:VGNC:92792]	-1.76	0.0084
ssc-miR-374a-5p	SGK3	serum/glucocorticoid regulated kinase family member 3 [Source:HGNC Symbol;Acc:HGNC:10812]	-1.76	0.0084
ssc-miR-374a-5p	SH3BP5	SH3 domain binding protein 5 [Source:VGNC Symbol;Acc:VGNC:92819]	-1.76	0.0084
ssc-miR-374a-5p	SHANK3	hypothetical gene	-1.76	0.0084
ssc-miR-374a-5p	SHISA6	hypothetical gene	-1.76	0.0084
ssc-miR-374a-5p	SHOC2	SHOC2 leucine rich repeat scaffold protein [Source:VGNC Symbol;Acc:VGNC:98318]	-1.76	0.0084
ssc-miR-374a-5p	SIX6	SIX homeobox 6 [Source:VGNC Symbol;Acc:VGNC:92897]	-1.76	0.0084
ssc-miR-374a-5p	SKI	SKI proto-onco [Source:VGNC Symbol;Acc:VGNC:92902]	-1.76	0.0084
ssc-miR-374a-5p	SLC10A7	solute carrier family 10 member 7 [Source:VGNC Symbol;Acc:VGNC:92919]	-1.76	0.0084
ssc-miR-374a-5p	SLC15A4	solute carrier family 15 member 4 [Source:VGNC Symbol;Acc:VGNC:92937]	-1.76	0.0084
ssc-miR-374a-5p	SLC16A1	hypothetical gene	-1.76	0.0084
ssc-miR-374a-5p	SLC24A2	solute carrier family 24 member 2 [Source:VGNC Symbol;Acc:VGNC:92987]	-1.76	0.0084
ssc-miR-374a-5p	SLC25A16	solute carrier family 25 member 16 [Source:VGNC Symbol;Acc:VGNC:92995]	-1.76	0.0084
ssc-miR-374a-5p	SLC25A33	solute carrier family 25 member 33 [Source:VGNC Symbol;Acc:VGNC:93009]	-1.76	0.0084
ssc-miR-374a-5p	SLC2A2	solute carrier family 2 member 2 [Source:VGNC Symbol;Acc:VGNC:93048]	-1.76	0.0084
ssc-miR-374a-5p	SLC30A1	solute carrier family 30 member 1 [Source:VGNC Symbol;Acc:VGNC:93055]	-1.76	0.0084
ssc-miR-374a-5p	SLC30A10	hypothetical gene	-1.76	0.0084
ssc-miR-374a-5p	SLC30A5	solute carrier family 30 member 5 [Source:VGNC Symbol;Acc:VGNC:93059]	-1.76	0.0084
ssc-miR-374a-5p	SLC35A3	solute carrier family 35 member A3 [Source:VGNC Symbol;Acc:VGNC:98860]	-1.76	0.0084

ssc-miR-374a-5p	SLC35B3	solute carrier family 35 member B3 [Source:VGNC Symbol;Acc:VGNC:93073]	-1.76	0.0084
ssc-miR-374a-5p	SLC6A14	solute carrier family 6 member 14 [Source:NCBI gene (formerly Entrezgene);Acc:431671]	-1.76	0.0084
ssc-miR-374a-5p	SLC6A17	solute carrier family 6 member 17 [Source:VGNC Symbol;Acc:VGNC:93158]	-1.76	0.0084
ssc-miR-374a-5p	SLC6A5	solute carrier family 6 member 5 [Source:VGNC Symbol;Acc:VGNC:93165]	-1.76	0.0084
ssc-miR-374a-5p	SLC7A14	solute carrier family 7 member 14 [Source:VGNC Symbol;Acc:VGNC:93173]	-1.76	0.0084
ssc-miR-374a-5p	SLIT3	slit guidance ligand 3 [Source:VGNC Symbol;Acc:VGNC:93205]	-1.76	0.0084
ssc-miR-374a-5p	SMAD6	SMAD family member 6 [Source:VGNC Symbol;Acc:VGNC:93220]	-1.76	0.0084
ssc-miR-374a-5p	SMARCAD1	SWI/SNF-related, matrix-associated actin-dependent regulator of chromatin, subfamily a, containing DEAD/H box 1 [Source:VGNC Symbol;Acc:VGNC:93229]	-1.76	0.0084
ssc-miR-374a-5p	SMYD4	SET and MYND domain containing 4 [Source:VGNC Symbol;Acc:VGNC:93274]	-1.76	0.0084
ssc-miR-374a-5p	SNRPC	small nuclear ribonucleoprotein polypeptide C [Source:VGNC Symbol;Acc:VGNC:93295]	-1.76	0.0084
ssc-miR-374a-5p	SNTB1	syntrophin beta 1 [Source:VGNC Symbol;Acc:VGNC:93297]	-1.76	0.0084
ssc-miR-374a-5p	SNTB2	syntrophin beta 2 [Source:VGNC Symbol;Acc:VGNC:93298]	-1.76	0.0084
ssc-miR-374a-5p	SNTG1	syntrophin gamma 1 [Source:VGNC Symbol;Acc:VGNC:93299]	-1.76	0.0084
ssc-miR-374a-5p	SNX17	sorting nexin 17 [Source:VGNC Symbol;Acc:VGNC:93309]	-1.76	0.0084
ssc-miR-374a-5p	SOCS4	suppressor of cytokine signaling 4 [Source:VGNC Symbol;Acc:VGNC:93331]	-1.76	0.0084
ssc-miR-374a-5p	SOGA3	SOGA family member 3 [Source:HGNC Symbol;Acc:HGNC:21494]	-1.76	0.0084
ssc-miR-374a-5p	SOX4	SRY-box transcription factor 4 [Source:HGNC Symbol;Acc:HGNC:11200]	-1.76	0.0084
ssc-miR-374a-5p	SP1	Sp1 transcription factor [Source:VGNC Symbol;Acc:VGNC:93360]	-1.76	0.0084
ssc-miR-374a-5p	SP3	Sp3 transcription factor [Source:VGNC Symbol;Acc:VGNC:95511]	-1.76	0.0084
ssc-miR-374a-5p	SP4	Sp4 transcription factor [Source:VGNC Symbol;Acc:VGNC:93362]	-1.76	0.0084
ssc-miR-374a-5p	SPACA1	sperm acrosome associated 1 [Source:VGNC Symbol;Acc:VGNC:93367]	-1.76	0.0084
ssc-miR-374a-5p	SPICE1	spindle and centriole associated protein 1 [Source:HGNC Symbol;Acc:HGNC:25083]	-1.76	0.0084
ssc-miR-374a-5p	SPOPL	speckle type BTB/POZ protein like [Source:VGNC Symbol;Acc:VGNC:96161]	-1.76	0.0084
ssc-miR-374a-5p	SPPL2A	signal peptide peptidase like 2A [Source:VGNC Symbol;Acc:VGNC:98337]	-1.76	0.0084
ssc-miR-374a-5p	SPPL3	signal peptide peptidase like 3 [Source:VGNC Symbol;Acc:VGNC:98340]	-1.76	0.0084
ssc-miR-374a-5p	SPRY2	sprouty RTK signaling antagonist 2 [Source:VGNC Symbol;Acc:VGNC:93425]	-1.76	0.0084
ssc-miR-374a-5p	SPTLC1	serine palmitoyltransferase long chain base subunit 1 [Source:VGNC Symbol;Acc:VGNC:93439]	-1.76	0.0084
ssc-miR-374a-5p	SRCIN1	SRC kinase signaling inhibitor 1 [Source:VGNC Symbol;Acc:VGNC:93448]	-1.76	0.0084
ssc-miR-374a-5p	SRFBP1	serum response factor binding protein 1 [Source:VGNC Symbol;Acc:VGNC:93453]	-1.76	0.0084
ssc-miR-374a-5p	SRGAP1	SLIT-ROBO Rho GTPase activating protein 1 [Source:VGNC Symbol;Acc:VGNC:93454]	-1.76	0.0084
ssc-miR-374a-5p	SRPK2	SRSF protein kinase 2 [Source:VGNC Symbol;Acc:VGNC:93463]	-1.76	0.0084
ssc-miR-374a-5p	SRSF7	serine and arginine rich splicing factor 7 [Source:VGNC Symbol;Acc:VGNC:93477]	-1.76	0.0084
ssc-miR-374a-5p	SSX2IP	SSX family member 2 interacting protein [Source:VGNC Symbol;Acc:VGNC:93499]	-1.76	0.0084
ssc-miR-374a-5p	ST18	ST18 C2H2C-type zinc finger transcription factor [Source:VGNC Symbol;Acc:VGNC:93501]	-1.76	0.0084
ssc-miR-374a-5p	ST6GALNAC3	ST6 N-acetylgalactosaminide alpha-2,6-sialyltransferase 3 [Source:VGNC Symbol;Acc:VGNC:93511]	-1.76	0.0084
ssc-miR-374a-5p	ST8SIA2	ST8 alpha-N-acetyl-neuraminide alpha-2,8-sialyltransferase 2 [Source:VGNC Symbol;Acc:VGNC:93517]	-1.76	0.0084
ssc-miR-374a-5p	ST8SIA4	ST8 alpha-N-acetyl-neuraminide alpha-2,8-sialyltransferase 4 [Source:VGNC Symbol;Acc:VGNC:93519]	-1.76	0.0084
ssc-miR-374a-5p	STK38L	serine/threonine kinase 38 like [Source:VGNC Symbol;Acc:VGNC:93557]	-1.76	0.0084
ssc-miR-374a-5p	STK4	serine/threonine kinase 4 [Source:VGNC Symbol;Acc:VGNC:98349]	-1.76	0.0084
ssc-miR-374a-5p	STMN2	stathmin 2 [Source:VGNC Symbol;Acc:VGNC:93561]	-1.76	0.0084
ssc-miR-374a-5p	STRN	striatin [Source:VGNC Symbol;Acc:VGNC:93578]	-1.76	0.0084
ssc-miR-374a-5p	STX17	syntaxin 17 [Source:VGNC Symbol;Acc:VGNC:93587]	-1.76	0.0084
ssc-miR-374a-5p	STX7	syntaxin 7 [Source:VGNC Symbol;Acc:VGNC:93595]	-1.76	0.0084
ssc-miR-374a-5p	STXBP5	syntaxin binding protein 5 [Source:VGNC Symbol;Acc:VGNC:93599]	-1.76	0.0084
ssc-miR-374a-5p	STYX	serine/threonine/tyrosine interacting protein [Source:VGNC Symbol;Acc:VGNC:93602]	-1.76	0.0084
ssc-miR-374a-5p	SUCNR1	succinate receptor 1 [Source:VGNC Symbol;Acc:VGNC:93605]	-1.76	0.0084
ssc-miR-374a-5p	SUPT3H	SPT3 homolog, SAGA and STAGA complex component [Source:VGNC Symbol;Acc:VGNC:93623]	-1.76	0.0084
ssc-miR-374a-5p	SWT1	SWT1 RNA endoribonuclease homolog [Source:VGNC Symbol;Acc:VGNC:93647]	-1.76	0.0084
ssc-miR-374a-5p	SYDE2	synapse defective Rho GTPase homolog 2 [Source:VGNC Symbol;Acc:VGNC:98874]	-1.76	0.0084
ssc-miR-374a-5p	SYNE1	hypothetical gene	-1.76	0.0084

ssc-miR-374a-5p	SYP	synaptophysin [Source:VGNC Symbol;Acc:VGNC:93676]	-1.76	0.0084
ssc-miR-374a-5p	SYT14	synaptotagmin 14 [Source:VGNC Symbol;Acc:VGNC:108619]	-1.76	0.0084
ssc-miR-374a-5p	TACC1	hypothetical gene	-1.76	0.0084
ssc-miR-374a-5p	TAF4B	TATA-box binding protein associated factor 4b [Source:VGNC Symbol;Acc:VGNC:93716]	-1.76	0.0084
ssc-miR-374a-5p	TAF5L	TATA-box binding protein associated factor 5 like [Source:VGNC Symbol;Acc:VGNC:93718]	-1.76	0.0084
ssc-miR-374a-5p	TAOK1	TAO kinase 1 [Source:VGNC Symbol;Acc:VGNC:98355]	-1.76	0.0084
ssc-miR-374a-5p	TAT	tyrosine aminotransferase [Source:VGNC Symbol;Acc:VGNC:93752]	-1.76	0.0084
ssc-miR-374a-5p	TBC1D9	TBC1 domain family member 9 [Source:VGNC Symbol;Acc:VGNC:93781]	-1.76	0.0084
ssc-miR-374a-5p	TBX4	T-box transcription factor 4 [Source:VGNC Symbol;Acc:VGNC:93803]	-1.76	0.0084
ssc-miR-374a-5p	TCERG1	transcription elongation regulator 1 [Source:VGNC Symbol;Acc:VGNC:93815]	-1.76	0.0084
ssc-miR-374a-5p	TCF21	transcription factor 21 [Source:VGNC Symbol;Acc:VGNC:93819]	-1.76	0.0084
ssc-miR-374a-5p	TCF4	transcription factor 4 [Source:VGNC Symbol;Acc:VGNC:93823]	-1.76	0.0084
ssc-miR-374a-5p	TEAD3	TEA domain transcription factor 3 [Source:VGNC Symbol;Acc:VGNC:93855]	-1.76	0.0084
ssc-miR-374a-5p	TENM1	teneurin transmembrane protein 1 [Source:VGNC Symbol;Acc:VGNC:98363]	-1.76	0.0084
ssc-miR-374a-5p	TET2	hypothetical gene	-1.76	0.0084
ssc-miR-374a-5p	TET3	tet methylcytosine dioxygenase 3 [Source:VGNC Symbol;Acc:VGNC:93890]	-1.76	0.0084
ssc-miR-374a-5p	TFAP4	transcription factor AP-4 [Source:VGNC Symbol;Acc:VGNC:93912]	-1.76	0.0084
ssc-miR-374a-5p	TFDP1	transcription factor Dp-1 [Source:HGNC Symbol;Acc:HGNC:11749]	-1.76	0.0084
ssc-miR-374a-5p	TGFA	transforming growth factor alpha [Source:VGNC Symbol;Acc:VGNC:93928]	-1.76	0.0084
ssc-miR-374a-5p	TLE4	TLE family member 4, transcriptional corepressor [Source:VGNC Symbol;Acc:VGNC:98372]	-1.76	0.0084
ssc-miR-374a-5p	TLR4	toll like receptor 4 [Source:VGNC Symbol;Acc:VGNC:94025]	-1.76	0.0084
ssc-miR-374a-5p	TMC7	transmembrane channel like 7 [Source:VGNC Symbol;Acc:VGNC:94044]	-1.76	0.0084
ssc-miR-374a-5p	TMEM108	transmembrane protein 108 [Source:VGNC Symbol;Acc:VGNC:94065]	-1.76	0.0084
ssc-miR-374a-5p	TMEM123	hypothetical gene	-1.76	0.0084
ssc-miR-374a-5p	TMEM161B	transmembrane protein 161B [Source:VGNC Symbol;Acc:VGNC:94102]	-1.76	0.0084
ssc-miR-374a-5p	TMEM184C	transmembrane protein 184C [Source:VGNC Symbol;Acc:VGNC:94121]	-1.76	0.0084
ssc-miR-374a-5p	TMEM185A	transmembrane protein 185A [Source:VGNC Symbol;Acc:VGNC:94122]	-1.76	0.0084
ssc-miR-374a-5p	TMEM38B	transmembrane protein 38B [Source:VGNC Symbol;Acc:VGNC:94175]	-1.76	0.0084
ssc-miR-374a-5p	TMSB10	thymosin beta 10 [Source:NCBI gene (formerly Entrezgene);Acc:100037998]	-1.76	0.0084
ssc-miR-374a-5p	TNKS2	tankyrase 2 [Source:VGNC Symbol;Acc:VGNC:94280]	-1.76	0.0084
ssc-miR-374a-5p	TNRC18	trinucleotide repeat containing 18 [Source:VGNC Symbol;Acc:VGNC:94291]	-1.76	0.0084
ssc-miR-374a-5p	TNRC6A	trinucleotide repeat containing adaptor 6A [Source:VGNC Symbol;Acc:VGNC:94292]	-1.76	0.0084
ssc-miR-374a-5p	TOP1	hypothetical gene	-1.76	0.0084
ssc-miR-374a-5p	TOR1AIP1	torsin 1A interacting protein 1 [Source:VGNC Symbol;Acc:VGNC:94317]	-1.76	0.0084
ssc-miR-374a-5p	TPRG1	tumor protein p63 regulated 1 [Source:VGNC Symbol;Acc:VGNC:94348]	-1.76	0.0084
ssc-miR-374a-5p	TRAFD1	TRAF-type zinc finger domain containing 1 [Source:VGNC Symbol;Acc:VGNC:94367]	-1.76	0.0084
ssc-miR-374a-5p	TRAK2	trafficking kinesin protein 2 [Source:VGNC Symbol;Acc:VGNC:95577]	-1.76	0.0084
ssc-miR-374a-5p	TRAPPC13	trafficking protein particle complex subunit 13 [Source:VGNC Symbol;Acc:VGNC:104085]	-1.76	0.0084
ssc-miR-374a-5p	TRAPPC8	trafficking protein particle complex subunit 8 [Source:VGNC Symbol;Acc:VGNC:94379]	-1.76	0.0084
ssc-miR-374a-5p	TRERF1	transcriptional regulating factor 1 [Source:VGNC Symbol;Acc:VGNC:94387]	-1.76	0.0084
ssc-miR-374a-5p	TRPM3	transient receptor potential cation channel subfamily M member 3 [Source:VGNC Symbol;Acc:VGNC:98384]	-1.76	0.0084
ssc-miR-374a-5p	TRPM7	transient receptor potential cation channel subfamily M member 7 [Source:VGNC Symbol;Acc:VGNC:94470]	-1.76	0.0084
ssc-miR-374a-5p	TRPV3	transient receptor potential cation channel subfamily V member 3 [Source:VGNC Symbol;Acc:VGNC:94475]	-1.76	0.0084
ssc-miR-374a-5p	TTBK2	tau tubulin kinase 2 [Source:VGNC Symbol;Acc:VGNC:98387]	-1.76	0.0084
ssc-miR-374a-5p	TTC37	hypothetical gene	-1.76	0.0084
ssc-miR-374a-5p	TTLL7	tubulin tyrosine ligase like 7 [Source:VGNC Symbol;Acc:VGNC:94571]	-1.76	0.0084
ssc-miR-374a-5p	TXLNB	taxilin beta [Source:VGNC Symbol;Acc:VGNC:94601]	-1.76	0.0084
ssc-miR-374a-5p	TXNL1	thioredoxin like 1 [Source:VGNC Symbol;Acc:VGNC:94611]	-1.76	0.0084
ssc-miR-374a-5p	UBA6	ubiquitin like modifier activating enzyme 6 [Source:VGNC Symbol;Acc:VGNC:94630]	-1.76	0.0084
ssc-miR-374a-5p	UBE2B	ubiquitin conjugating enzyme E2 B [Source:VGNC Symbol;Acc:VGNC:94640]	-1.76	0.0084

ssc-miR-374a-5p	UBE2E3	ubiquitin conjugating enzyme E2 E3 [Source:VGNC Symbol;Acc:VGNC:106461]	-1.76	0.0084
ssc-miR-374a-5p	UBE2H	ubiquitin conjugating enzyme E2 H [Source:VGNC Symbol;Acc:VGNC:94643]	-1.76	0.0084
ssc-miR-374a-5p	UBE2I	hypothetical gene	-1.76	0.0084
ssc-miR-374a-5p	UBE2J1	ubiquitin conjugating enzyme E2 J1 [Source:VGNC Symbol;Acc:VGNC:94644]	-1.76	0.0084
ssc-miR-374a-5p	UBXN4	UBX domain protein 4 [Source:VGNC Symbol;Acc:VGNC:98397]	-1.76	0.0084
ssc-miR-374a-5p	UGCG	UDP-glucose ceramide glucosyltransferase [Source:VGNC Symbol;Acc:VGNC:94685]	-1.76	0.0084
ssc-miR-374a-5p	UHMK1	U2AF homology motif kinase 1 [Source:VGNC Symbol;Acc:VGNC:94688]	-1.76	0.0084
ssc-miR-374a-5p	USP15	ubiquitin specific peptidase 15 [Source:VGNC Symbol;Acc:VGNC:94748]	-1.76	0.0084
ssc-miR-374a-5p	USP30	ubiquitin specific peptidase 30 [Source:VGNC Symbol;Acc:VGNC:94758]	-1.76	0.0084
ssc-miR-374a-5p	USP38	ubiquitin specific peptidase 38 [Source:VGNC Symbol;Acc:VGNC:94762]	-1.76	0.0084
ssc-miR-374a-5p	USP47	ubiquitin specific peptidase 47 [Source:VGNC Symbol;Acc:VGNC:94769]	-1.76	0.0084
ssc-miR-374a-5p	UST	uronyl 2-sulfotransferase [Source:VGNC Symbol;Acc:VGNC:94780]	-1.76	0.0084
ssc-miR-374a-5p	VAMP2	vesicle associated membrane protein 2 [Source:HGNC Symbol;Acc:HGNC:12643]	-1.76	0.0084
ssc-miR-374a-5p	VCAM1	vascular cell adhesion molecule 1 [Source:VGNC Symbol;Acc:VGNC:94811]	-1.76	0.0084
ssc-miR-374a-5p	VCPIP1	valosin containing protein interacting protein 1 [Source:VGNC Symbol;Acc:VGNC:94813]	-1.76	0.0084
ssc-miR-374a-5p	VEGFC	vascular endothelial growth factor C [Source:VGNC Symbol;Acc:VGNC:95563]	-1.76	0.0084
ssc-miR-374a-5p	VHL	hypothetical gene	-1.76	0.0084
ssc-miR-374a-5p	VPRBP	hypothetical gene	-1.76	0.0084
ssc-miR-374a-5p	VWC2	von Willebrand factor C domain containing 2 [Source:VGNC Symbol;Acc:VGNC:94887]	-1.76	0.0084
ssc-miR-374a-5p	WDR11	WD repeat domain 11 [Source:VGNC Symbol;Acc:VGNC:94906]	-1.76	0.0084
ssc-miR-374a-5p	WDR37	WD repeat domain 37 [Source:VGNC Symbol;Acc:VGNC:96258]	-1.76	0.0084
ssc-miR-374a-5p	WDR52	hypothetical gene	-1.76	0.0084
ssc-miR-374a-5p	WISP3	hypothetical gene	-1.76	0.0084
ssc-miR-374a-5p	WNT16	Wnt family member 16 [Source:VGNC Symbol;Acc:VGNC:94967]	-1.76	0.0084
ssc-miR-374a-5p	WNT2B	Wnt family member 2B [Source:VGNC Symbol;Acc:VGNC:94969]	-1.76	0.0084
ssc-miR-374a-5p	WNT3	Wnt family member 3 [Source:VGNC Symbol;Acc:VGNC:94970]	-1.76	0.0084
ssc-miR-374a-5p	WNT5A	Wnt family member 5A [Source:VGNC Symbol;Acc:VGNC:94973]	-1.76	0.0084
ssc-miR-374a-5p	WRN	WRN RecQ like helicase [Source:VGNC Symbol;Acc:VGNC:96605]	-1.76	0.0084
ssc-miR-374a-5p	WWC2	WW and C2 domain containing 2 [Source:VGNC Symbol;Acc:VGNC:96033]	-1.76	0.0084
ssc-miR-374a-5p	XIAP	X-linked inhibitor of apoptosis [Source:NCBI gene (formerly Entrezgene);Acc:100037300]	-1.76	0.0084
ssc-miR-374a-5p	XK	X-linked Kx blood group [Source:HGNC Symbol;Acc:HGNC:12811]	-1.76	0.0084
ssc-miR-374a-5p	XPOT	exportin for tRNA [Source:VGNC Symbol;Acc:VGNC:95008]	-1.76	0.0084
ssc-miR-374a-5p	YOD1	YOD1 deubiquitinase [Source:VGNC Symbol;Acc:VGNC:95035]	-1.76	0.0084
ssc-miR-374a-5p	ZBTB10	zinc finger and BTB domain containing 10 [Source:VGNC Symbol;Acc:VGNC:95058]	-1.76	0.0084
ssc-miR-374a-5p	ZBTB20	zinc finger and BTB domain containing 20 [Source:VGNC Symbol;Acc:VGNC:95063]	-1.76	0.0084
ssc-miR-374a-5p	ZBTB34	zinc finger and BTB domain containing 34 [Source:VGNC Symbol;Acc:VGNC:95070]	-1.76	0.0084
ssc-miR-374a-5p	ZBTB43	zinc finger and BTB domain containing 43 [Source:VGNC Symbol;Acc:VGNC:95075]	-1.76	0.0084
ssc-miR-374a-5p	ZBTB46	zinc finger and BTB domain containing 46 [Source:VGNC Symbol;Acc:VGNC:95733]	-1.76	0.0084
ssc-miR-374a-5p	ZC3H6	zinc finger CCCH-type containing 6 [Source:VGNC Symbol;Acc:VGNC:95098]	-1.76	0.0084
ssc-miR-374a-5p	ZC3H7B	zinc finger CCCH-type containing 7B [Source:VGNC Symbol;Acc:VGNC:95100]	-1.76	0.0084
ssc-miR-374a-5p	ZC3H8	zinc finger CCCH-type containing 8 [Source:VGNC Symbol;Acc:VGNC:95101]	-1.76	0.0084
ssc-miR-374a-5p	ZCCHC14	zinc finger CCHC-type containing 14 [Source:VGNC Symbol;Acc:VGNC:95107]	-1.76	0.0084
ssc-miR-374a-5p	ZCCHC24	zinc finger CCHC-type containing 24 [Source:HGNC Symbol;Acc:HGNC:26911]	-1.76	0.0084
ssc-miR-374a-5p	ZCWPW2	zinc finger CW-type and PWWP domain containing 2 [Source:VGNC Symbol;Acc:VGNC:108706]	-1.76	0.0084
ssc-miR-374a-5p	ZDHHC20	zinc finger DHHC-type palmitoyltransferase 20 [Source:VGNC Symbol;Acc:VGNC:95122]	-1.76	0.0084
ssc-miR-374a-5p	ZDHHC23	zinc finger DHHC-type palmitoyltransferase 23 [Source:VGNC Symbol;Acc:VGNC:95125]	-1.76	0.0084
ssc-miR-374a-5p	ZDHHC3	zinc finger DHHC-type palmitoyltransferase 3 [Source:VGNC Symbol;Acc:VGNC:95126]	-1.76	0.0084
ssc-miR-374a-5p	ZDHHC5	zinc finger DHHC-type palmitoyltransferase 5 [Source:VGNC Symbol;Acc:VGNC:95127]	-1.76	0.0084
ssc-miR-374a-5p	ZDHHC9	zinc finger DHHC-type palmitoyltransferase 9 [Source:VGNC Symbol;Acc:VGNC:95131]	-1.76	0.0084
ssc-miR-374a-5p	ZEB2	hypothetical gene	-1.76	0.0084

ssc-miR-374a-5p	ZER1	zyg-11 related cell cycle regulator [Source:VGNC Symbol;Acc:VGNC:95132]	-1.76	0.0084
ssc-miR-374a-5p	ZFAND4	zinc finger AN1-type containing 4 [Source:VGNC Symbol;Acc:VGNC:95136]	-1.76	0.0084
ssc-miR-374a-5p	ZFP91	ZFP91 zinc finger protein, atypical E3 ubiquitin ligase [Source:HGNC Symbol;Acc:HGNC:14983]	-1.76	0.0084
ssc-miR-374a-5p	ZMAT3	zinc finger matrin-type 3 [Source:VGNC Symbol;Acc:VGNC:95177]	-1.76	0.0084
ssc-miR-374a-5p	ZNF189	zinc finger protein 189 [Source:VGNC Symbol;Acc:VGNC:103205]	-1.76	0.0084
ssc-miR-374a-5p	ZNF207	zinc finger protein 207 [Source:VGNC Symbol;Acc:VGNC:95205]	-1.76	0.0084
ssc-miR-374a-5p	ZNF236	zinc finger protein 236 [Source:VGNC Symbol;Acc:VGNC:95209]	-1.76	0.0084
ssc-miR-374a-5p	ZNF281	zinc finger protein 281 [Source:VGNC Symbol;Acc:VGNC:108285]	-1.76	0.0084
ssc-miR-374a-5p	ZNF423	zinc finger protein 423 [Source:VGNC Symbol;Acc:VGNC:98752]	-1.76	0.0084
ssc-miR-374a-5p	ZNF462	zinc finger protein 462 [Source:VGNC Symbol;Acc:VGNC:95244]	-1.76	0.0084
ssc-miR-374a-5p	ZNF516	zinc finger protein 516 [Source:VGNC Symbol;Acc:VGNC:95256]	-1.76	0.0084
ssc-miR-374a-5p	ZNF644	zinc finger protein 644 [Source:VGNC Symbol;Acc:VGNC:95283]	-1.76	0.0084
ssc-miR-374a-5p	ZNF704	zinc finger protein 704 [Source:VGNC Symbol;Acc:VGNC:95296]	-1.76	0.0084
ssc-miR-374a-5p	ZNF710	zinc finger protein 710 [Source:VGNC Symbol;Acc:VGNC:107180]	-1.76	0.0084
ssc-miR-374a-5p	ZNF771	zinc finger protein 771 [Source:HGNC Symbol;Acc:HGNC:29653]	-1.76	0.0084
ssc-miR-374a-5p	ZNF81	zinc finger protein 81 [Source:VGNC Symbol;Acc:VGNC:95308]	-1.76	0.0084
ssc-miR-374a-5p	ZNHIT6	zinc finger HIT-type containing 6 [Source:VGNC Symbol;Acc:VGNC:98909]	-1.76	0.0084
ssc-miR-374a-5p	ZSWIM6	zinc finger SWIM-type containing 6 [Source:VGNC Symbol;Acc:VGNC:95335]	-1.76	0.0084
ssc-miR-374a-5p	ZZZ3	zinc finger ZZ-type containing 3 [Source:VGNC Symbol;Acc:VGNC:95348]	-1.76	0.0084
ssc-miR-92b-3p	AARS	hypothetical gene	-1.76	0.1694
ssc-miR-92b-3p	AATK	apoptosis associated tyrosine kinase [Source:VGNC Symbol;Acc:VGNC:84946]	-1.76	0.1694
ssc-miR-92b-3p	ABHD13	abhydrolase domain containing 13 [Source:VGNC Symbol;Acc:VGNC:84972]	-1.76	0.1694
ssc-miR-92b-3p	ABI2	abl interactor 2 [Source:VGNC Symbol;Acc:VGNC:96027]	-1.76	0.1694
ssc-miR-92b-3p	ABL2	ABL proto-onco 2, non-receptor tyrosine kinase [Source:VGNC Symbol;Acc:VGNC:84985]	-1.76	0.1694
ssc-miR-92b-3p	AC010327.2	hypothetical gene	-1.76	0.1694
ssc-miR-92b-3p	AC012123.1	hypothetical gene	-1.76	0.1694
ssc-miR-92b-3p	AC068987.1	hypothetical gene	-1.76	0.1694
ssc-miR-92b-3p	ACAN	aggrecan [Source:NCBI gene (formerly Entrezgene);Acc:397255]	-1.76	0.1694
ssc-miR-92b-3p	ACOX1	acyl-CoA oxidase 1 [Source:VGNC Symbol;Acc:VGNC:85020]	-1.76	0.1694
ssc-miR-92b-3p	ACTC1	actin alpha cardiac muscle 1 [Source:VGNC Symbol;Acc:VGNC:85040]	-1.76	0.1694
ssc-miR-92b-3p	ACTR3	actin related protein 3 [Source:VGNC Symbol;Acc:VGNC:103894]	-1.76	0.1694
ssc-miR-92b-3p	ADAM10	ADAM metallopeptidase domain 10 [Source:VGNC Symbol;Acc:VGNC:85061]	-1.76	0.1694
ssc-miR-92b-3p	ADAM19	ADAM metallopeptidase domain 19 [Source:VGNC Symbol;Acc:VGNC:85066]	-1.76	0.1694
ssc-miR-92b-3p	ADAM23	ADAM metallopeptidase domain 23 [Source:VGNC Symbol;Acc:VGNC:95933]	-1.76	0.1694
ssc-miR-92b-3p	ADAMTS9	ADAM metallopeptidase with thrombospondin type 1 motif 9 [Source:VGNC Symbol;Acc:VGNC:85089]	-1.76	0.1694
ssc-miR-92b-3p	ADAMTSL1	ADAMTS like 1 [Source:VGNC Symbol;Acc:VGNC:85090]	-1.76	0.1694
ssc-miR-92b-3p	ADAMTSL3	ADAMTS like 3 [Source:VGNC Symbol;Acc:VGNC:85092]	-1.76	0.1694
ssc-miR-92b-3p	ADCY3	adenylate cyclase 3 [Source:VGNC Symbol;Acc:VGNC:85107]	-1.76	0.1694
ssc-miR-92b-3p	ADM	adrenomedullin [Source:VGNC Symbol;Acc:VGNC:85144]	-1.76	0.1694
ssc-miR-92b-3p	ADO	2-aminoethanethiol dioxygenase [Source:VGNC Symbol;Acc:VGNC:85146]	-1.76	0.1694
ssc-miR-92b-3p	ADRB1	adrenoceptor beta 1 [Source:VGNC Symbol;Acc:VGNC:107363]	-1.76	0.1694
ssc-miR-92b-3p	AFF1	AF4/FMR2 family member 1 [Source:VGNC Symbol;Acc:VGNC:85167]	-1.76	0.1694
ssc-miR-92b-3p	AFF3	AF4/FMR2 family member 3 [Source:HGNC Symbol;Acc:HGNC:6473]	-1.76	0.1694
ssc-miR-92b-3p	AFF4	AF4/FMR2 family member 4 [Source:VGNC Symbol;Acc:VGNC:85169]	-1.76	0.1694
ssc-miR-92b-3p	AGGF1	angiogenic factor with G-patch and FHA domains 1 [Source:VGNC Symbol;Acc:VGNC:96789]	-1.76	0.1694
ssc-miR-92b-3p	AGO3	argonaute RISC component 1 [Source:NCBI gene (formerly Entrezgene);Acc:100499510]	-1.76	0.1694
ssc-miR-92b-3p	AHCYL1	adenosylhomocysteinase like 1 [Source:VGNC Symbol;Acc:VGNC:97873]	-1.76	0.1694
ssc-miR-92b-3p	AIDA	axin interactor, dorsalization associated [Source:VGNC Symbol;Acc:VGNC:96103]	-1.76	0.1694
ssc-miR-92b-3p	AIFM1	apoptosis inducing factor mitochondria associated 1 [Source:VGNC Symbol;Acc:VGNC:85198]	-1.76	0.1694
ssc-miR-92b-3p	AIFM2	apoptosis inducing factor mitochondria associated 2 [Source:VGNC Symbol;Acc:VGNC:85199]	-1.76	0.1694

ssc-miR-92b-3p	AKAP1	A-kinase anchoring protein 1 [Source:VGNC Symbol;Acc:VGNC:85214]	-1.76	0.1694
ssc-miR-92b-3p	AKAP10	A-kinase anchoring protein 10 [Source:VGNC Symbol;Acc:VGNC:85215]	-1.76	0.1694
ssc-miR-92b-3p	AKT1	AKT serine/threonine kinase 1 [Source:VGNC Symbol;Acc:VGNC:96930]	-1.76	0.1694
ssc-miR-92b-3p	ALDH1A2	aldehyde dehydrogenase 1 family member A2 [Source:VGNC Symbol;Acc:VGNC:97878]	-1.76	0.1694
ssc-miR-92b-3p	ALPK3	alpha kinase 3 [Source:HGNC Symbol;Acc:HGNC:17574]	-1.76	0.1694
ssc-miR-92b-3p	ALX4	ALX homeobox 4 [Source:VGNC Symbol;Acc:VGNC:96791]	-1.76	0.1694
ssc-miR-92b-3p	ANGPTL2	angiopoietin like 2 [Source:VGNC Symbol;Acc:VGNC:85304]	-1.76	0.1694
ssc-miR-92b-3p	ANK3	hypothetical gene	-1.76	0.1694
ssc-miR-92b-3p	ANKIB1	ankyrin repeat and IBR domain containing 1 [Source:VGNC Symbol;Acc:VGNC:85316]	-1.76	0.1694
ssc-miR-92b-3p	ANKRD13C	ankyrin repeat domain 13C [Source:VGNC Symbol;Acc:VGNC:85326]	-1.76	0.1694
ssc-miR-92b-3p	ANKRD44	ankyrin repeat domain 44 [Source:VGNC Symbol;Acc:VGNC:107123]	-1.76	0.1694
ssc-miR-92b-3p	ANO4	anoctamin 4 [Source:VGNC Symbol;Acc:VGNC:85357]	-1.76	0.1694
ssc-miR-92b-3p	ANO8	anoctamin 8 [Source:VGNC Symbol;Acc:VGNC:85360]	-1.76	0.1694
ssc-miR-92b-3p	ANP32E	acidic nuclear phosphoprotein 32 family member E [Source:HGNC Symbol;Acc:HGNC:16673]	-1.76	0.1694
ssc-miR-92b-3p	AP1AR	adaptor related protein complex 1 associated regulatory protein [Source:HGNC Symbol;Acc:HGNC:28808]	-1.76	0.1694
ssc-miR-92b-3p	AP5M1	adaptor related protein complex 5 subunit mu 1 [Source:VGNC Symbol;Acc:VGNC:85396]	-1.76	0.1694
ssc-miR-92b-3p	APPL1	adaptor protein, phosphotyrosine interacting with PH domain and leucine zipper 1 [Source:VGNC Symbol;Acc:VGNC:85426]	-1.76	0.1694
ssc-miR-92b-3p	AQP2	aquaporin 2 [Source:VGNC Symbol;Acc:VGNC:85431]	-1.76	0.1694
ssc-miR-92b-3p	ARF1	ADP ribosylation factor 1 [Source:VGNC Symbol;Acc:VGNC:96793]	-1.76	0.1694
ssc-miR-92b-3p	ARFGEF1	ADP ribosylation factor guanine nucleotide exchange factor 1 [Source:VGNC Symbol;Acc:VGNC:85451]	-1.76	0.1694
ssc-miR-92b-3p	ARFGEF2	ADP ribosylation factor guanine nucleotide exchange factor 2 [Source:VGNC Symbol;Acc:VGNC:95672]	-1.76	0.1694
ssc-miR-92b-3p	ARHGAP29	Rho GTPase activating protein 29 [Source:VGNC Symbol;Acc:VGNC:85468]	-1.76	0.1694
ssc-miR-92b-3p	ARHGEF10	Rho guanine nucleotide exchange factor 10 [Source:VGNC Symbol;Acc:VGNC:95605]	-1.76	0.1694
ssc-miR-92b-3p	ARHGEF17	hypothetical gene	-1.76	0.1694
ssc-miR-92b-3p	ARID1B	AT-rich interaction domain 1B [Source:VGNC Symbol;Acc:VGNC:85506]	-1.76	0.1694
ssc-miR-92b-3p	ARID5B	AT-rich interaction domain 5B [Source:VGNC Symbol;Acc:VGNC:85511]	-1.76	0.1694
ssc-miR-92b-3p	ARL5B	ADP ribosylation factor like GTPase 5B [Source:VGNC Symbol;Acc:VGNC:95985]	-1.76	0.1694
ssc-miR-92b-3p	ARMC1	armadillo repeat containing 1 [Source:VGNC Symbol;Acc:VGNC:85526]	-1.76	0.1694
ssc-miR-92b-3p	ARPC2	actin related protein 2/3 complex subunit 2 [Source:VGNC Symbol;Acc:VGNC:96420]	-1.76	0.1694
ssc-miR-92b-3p	ARPP19	cAMP regulated phosphoprotein 19 [Source:NCBI gene (formerly Entrezgene);Acc:397362]	-1.76	0.1694
ssc-miR-92b-3p	ARRDC3	arrestin domain containing 3 [Source:VGNC Symbol;Acc:VGNC:85546]	-1.76	0.1694
ssc-miR-92b-3p	ARRDC4	arrestin domain containing 4 [Source:VGNC Symbol;Acc:VGNC:85547]	-1.76	0.1694
ssc-miR-92b-3p	ASB7	ankyrin repeat and SOCS box containing 7 [Source:VGNC Symbol;Acc:VGNC:85566]	-1.76	0.1694
ssc-miR-92b-3p	ASPH	aspartate beta-hydroxylase [Source:VGNC Symbol;Acc:VGNC:85584]	-1.76	0.1694
ssc-miR-92b-3p	ASPN	asporin [Source:VGNC Symbol;Acc:VGNC:85586]	-1.76	0.1694
ssc-miR-92b-3p	ASTN1	astrotactin 1 [Source:VGNC Symbol;Acc:VGNC:85593]	-1.76	0.1694
ssc-miR-92b-3p	ASXL2	ASXL transcriptional regulator 2 [Source:VGNC Symbol;Acc:VGNC:85595]	-1.76	0.1694
ssc-miR-92b-3p	ATG14	autophagy related 14 [Source:VGNC Symbol;Acc:VGNC:85617]	-1.76	0.1694
ssc-miR-92b-3p	ATL3	atlastin GTPase 3 [Source:VGNC Symbol;Acc:VGNC:85629]	-1.76	0.1694
ssc-miR-92b-3p	ATMIN	hypothetical gene	-1.76	0.1694
ssc-miR-92b-3p	ATP11A	ATPase phospholipid transporting 11A [Source:VGNC Symbol;Acc:VGNC:85634]	-1.76	0.1694
ssc-miR-92b-3p	ATP2A2	ATPase sarcoplasmic/endoplasmic reticulum Ca2+ transporting 2 [Source:VGNC Symbol;Acc:VGNC:85647]	-1.76	0.1694
ssc-miR-92b-3p	ATP2B4	ATPase plasma membrane Ca2+ transporting 4 [Source:VGNC Symbol;Acc:VGNC:85651]	-1.76	0.1694
ssc-miR-92b-3p	ATP6V1B2	ATPase H+ transporting V1 subunit B2 [Source:VGNC Symbol;Acc:VGNC:85671]	-1.76	0.1694
ssc-miR-92b-3p	ATP7A	ATPase copper transporting alpha [Source:VGNC Symbol;Acc:VGNC:85678]	-1.76	0.1694
ssc-miR-92b-3p	ATP8B1	ATPase phospholipid transporting 8B1 [Source:VGNC Symbol;Acc:VGNC:85679]	-1.76	0.1694
ssc-miR-92b-3p	ATRX	ATRX chromatin remodeler [Source:VGNC Symbol;Acc:VGNC:85686]	-1.76	0.1694
ssc-miR-92b-3p	ATXN1	ataxin 1 [Source:VGNC Symbol;Acc:VGNC:85687]	-1.76	0.1694
ssc-miR-92b-3p	ATXN1L	ataxin 1 like [Source:VGNC Symbol;Acc:VGNC:85689]	-1.76	0.1694
ssc-miR-92b-3p	ATXN3	hypothetical gene	-1.76	0.1694

ssc-miR-92b-3p	ATXN7	ataxin 7 [Source:VGNC Symbol;Acc:VGNC:99704]	-1.76	0.1694
ssc-miR-92b-3p	AURKA	hypothetical gene	-1.76	0.1694
ssc-miR-92b-3p	AVL9	AVL9 cell migration associated [Source:VGNC Symbol;Acc:VGNC:85701]	-1.76	0.1694
ssc-miR-92b-3p	AXL	AXL receptor tyrosine kinase [Source:VGNC Symbol;Acc:VGNC:85709]	-1.76	0.1694
ssc-miR-92b-3p	AZIN1	antizyme inhibitor 1 [Source:VGNC Symbol;Acc:VGNC:85712]	-1.76	0.1694
ssc-miR-92b-3p	B3GALT2	beta-1,3-galactosyltransferase 2 [Source:VGNC Symbol;Acc:VGNC:96495]	-1.76	0.1694
ssc-miR-92b-3p	BACH1	BTB domain and CNC homolog 1 [Source:VGNC Symbol;Acc:VGNC:85741]	-1.76	0.1694
ssc-miR-92b-3p	BAI3	hypothetical gene	-1.76	0.1694
ssc-miR-92b-3p	BAK1	hypothetical gene	-1.76	0.1694
ssc-miR-92b-3p	BAZ2A	bromodomain adjacent to zinc finger domain 2A [Source:VGNC Symbol;Acc:VGNC:85763]	-1.76	0.1694
ssc-miR-92b-3p	BAZ2B	bromodomain adjacent to zinc finger domain 2B [Source:HGNC Symbol;Acc:HGNC:963]	-1.76	0.1694
ssc-miR-92b-3p	BCAT2	branched chain amino acid transaminase 2 [Source:VGNC Symbol;Acc:VGNC:85774]	-1.76	0.1694
ssc-miR-92b-3p	BCL11B	BAF chromatin remodeling complex subunit BCL11B [Source:VGNC Symbol;Acc:VGNC:96563]	-1.76	0.1694
ssc-miR-92b-3p	BCL2L11	BCL2 like 11 [Source:NCBI gene (formerly Entrezgene);Acc:396632]	-1.76	0.1694
ssc-miR-92b-3p	BCL2L14	BCL2 like 14 [Source:VGNC Symbol;Acc:VGNC:85782]	-1.76	0.1694
ssc-miR-92b-3p	BCL9	BCL9 transcription coactivator [Source:VGNC Symbol;Acc:VGNC:96567]	-1.76	0.1694
ssc-miR-92b-3p	BGN	biglycan [Source:VGNC Symbol;Acc:VGNC:85811]	-1.76	0.1694
ssc-miR-92b-3p	BLOC1S5	biosis of lysosomal organelles complex 1 subunit 5 [Source:NCBI gene (formerly Entrezgene);Acc:100049677]	-1.76	0.1694
ssc-miR-92b-3p	BMP2K	BMP2 inducible kinase [Source:VGNC Symbol;Acc:VGNC:85841]	-1.76	0.1694
ssc-miR-92b-3p	BMP7	bone morphotic protein 7 [Source:VGNC Symbol;Acc:VGNC:95491]	-1.76	0.1694
ssc-miR-92b-3p	BMPR1A	bone morphotic protein receptor type 1A [Source:VGNC Symbol;Acc:VGNC:85846]	-1.76	0.1694
ssc-miR-92b-3p	BMPR2	bone morphotic protein receptor type 2 [Source:VGNC Symbol;Acc:VGNC:95494]	-1.76	0.1694
ssc-miR-92b-3p	BRMS1L	BRMS1 like transcriptional repressor [Source:VGNC Symbol;Acc:VGNC:96571]	-1.76	0.1694
ssc-miR-92b-3p	BRWD1	bromodomain and WD repeat domain containing 1 [Source:VGNC Symbol;Acc:VGNC:108153]	-1.76	0.1694
ssc-miR-92b-3p	BSDC1	BSD domain containing 1 [Source:VGNC Symbol;Acc:VGNC:85892]	-1.76	0.1694
ssc-miR-92b-3p	BSN	bassoon presynaptic cytomatrix protein [Source:VGNC Symbol;Acc:VGNC:85893]	-1.76	0.1694
ssc-miR-92b-3p	BTG2	BTG anti-proliferation factor 2 [Source:NCBI gene (formerly Entrezgene);Acc:100048932]	-1.76	0.1694
ssc-miR-92b-3p	BTLA	hypothetical gene	-1.76	0.1694
ssc-miR-92b-3p	C10orf118	hypothetical gene	-1.76	0.1694
ssc-miR-92b-3p	C11orf24	hypothetical gene	-1.76	0.1694
ssc-miR-92b-3p	C11orf87	chromosome 11 open reading frame 87 [Source:HGNC Symbol;Acc:HGNC:33788]	-1.76	0.1694
ssc-miR-92b-3p	C16orf52	hypothetical gene	-1.76	0.1694
ssc-miR-92b-3p	C16orf87	chromosome 6 C16orf87 homolog [Source:VGNC Symbol;Acc:VGNC:96927]	-1.76	0.1694
ssc-miR-92b-3p	C18orf25	chromosome 1 C18orf25 homolog [Source:VGNC Symbol;Acc:VGNC:85964]	-1.76	0.1694
ssc-miR-92b-3p	C20orf112	hypothetical gene	-1.76	0.1694
ssc-miR-92b-3p	C20orf194	hypothetical gene	-1.76	0.1694
ssc-miR-92b-3p	C21orf91	chromosome 13 C21orf91 homolog [Source:VGNC Symbol;Acc:VGNC:85930]	-1.76	0.1694
ssc-miR-92b-3p	C2CD4C	C2 calcium dependent domain containing 4C [Source:HGNC Symbol;Acc:HGNC:29417]	-1.76	0.1694
ssc-miR-92b-3p	C2orf69	chromosome 2 open reading frame 69 [Source:HGNC Symbol;Acc:HGNC:26799]	-1.76	0.1694
ssc-miR-92b-3p	C5orf24	chromosome 2 C5orf24 homolog [Source:VGNC Symbol;Acc:VGNC:86010]	-1.76	0.1694
ssc-miR-92b-3p	C5orf30	hypothetical gene	-1.76	0.1694
ssc-miR-92b-3p	C6orf62	chromosome 7 C6orf62 homolog [Source:VGNC Symbol;Acc:VGNC:86074]	-1.76	0.1694
ssc-miR-92b-3p	C8orf44-SGK3	hypothetical gene	-1.76	0.1694
ssc-miR-92b-3p	CACNA1C	calcium voltage-gated channel subunit alpha1 C [Source:HGNC Symbol;Acc:HGNC:1390]	-1.76	0.1694
ssc-miR-92b-3p	CACNA1H	calcium voltage-gated channel subunit alpha1 H [Source:VGNC Symbol;Acc:VGNC:97041]	-1.76	0.1694
ssc-miR-92b-3p	CACNA1I	calcium voltage-gated channel subunit alpha1 I [Source:VGNC Symbol;Acc:VGNC:97908]	-1.76	0.1694
ssc-miR-92b-3p	CACNA2D1	calcium voltage-gated channel auxiliary subunit alpha2delta 1 [Source:VGNC Symbol;Acc:VGNC:86120]	-1.76	0.1694
ssc-miR-92b-3p	CADM2	cell adhesion molecule 2 [Source:VGNC Symbol;Acc:VGNC:97910]	-1.76	0.1694
ssc-miR-92b-3p	CALD1	caldesmon 1 [Source:VGNC Symbol;Acc:VGNC:86143]	-1.76	0.1694
ssc-miR-92b-3p	CALM3	calmodulin 3 [Source:NCBI gene (formerly Entrezgene);Acc:396838]	-1.76	0.1694

ssc-miR-92b-3p	CALN1	hypothetical gene	-1.76	0.1694
ssc-miR-92b-3p	CAMK2A	calcium/calmodulin dependent protein kinase II alpha [Source:VGNC Symbol;Acc:VGNC:86155]	-1.76	0.1694
ssc-miR-92b-3p	CAND1	cullin associated and neddylation dissociated 1 [Source:VGNC Symbol;Acc:VGNC:97912]	-1.76	0.1694
ssc-miR-92b-3p	CASD1	CAS1 domain containing 1 [Source:VGNC Symbol;Acc:VGNC:86199]	-1.76	0.1694
ssc-miR-92b-3p	CASK	calcium/calmodulin dependent serine protein kinase [Source:HGNC Symbol;Acc:HGNC:1497]	-1.76	0.1694
ssc-miR-92b-3p	CASKIN1	CASK interacting protein 1 [Source:VGNC Symbol;Acc:VGNC:86200]	-1.76	0.1694
ssc-miR-92b-3p	CASZ1	castor zinc finger 1 [Source:VGNC Symbol;Acc:VGNC:86207]	-1.76	0.1694
ssc-miR-92b-3p	CBFA2T3	CBFA2/RUNX1 partner transcriptional co-repressor 3 [Source:VGNC Symbol;Acc:VGNC:96574]	-1.76	0.1694
ssc-miR-92b-3p	CBLN4	cerebellin 4 [Source:VGNC Symbol;Acc:VGNC:95793]	-1.76	0.1694
ssc-miR-92b-3p	CCDC171	coiled-coil domain containing 171 [Source:VGNC Symbol;Acc:VGNC:103044]	-1.76	0.1694
ssc-miR-92b-3p	CCDC71L	coiled-coil domain containing 71 like [Source:HGNC Symbol;Acc:HGNC:26685]	-1.76	0.1694
ssc-miR-92b-3p	CCNC	cyclin C [Source:VGNC Symbol;Acc:VGNC:103046]	-1.76	0.1694
ssc-miR-92b-3p	CCNE2	cyclin E2 [Source:VGNC Symbol;Acc:VGNC:86355]	-1.76	0.1694
ssc-miR-92b-3p	CCNJ	cyclin J [Source:VGNC Symbol;Acc:VGNC:86360]	-1.76	0.1694
ssc-miR-92b-3p	CCNJL	cyclin J like [Source:VGNC Symbol;Acc:VGNC:86361]	-1.76	0.1694
ssc-miR-92b-3p	CCNL2	cyclin L2 [Source:VGNC Symbol;Acc:VGNC:86364]	-1.76	0.1694
ssc-miR-92b-3p	CCSER2	coiled-coil serine rich protein 2 [Source:VGNC Symbol;Acc:VGNC:86379]	-1.76	0.1694
ssc-miR-92b-3p	CCT6A	chaperonin containing TCP1 subunit 6A [Source:VGNC Symbol;Acc:VGNC:106569]	-1.76	0.1694
ssc-miR-92b-3p	CD2AP	CD2 associated protein [Source:VGNC Symbol;Acc:VGNC:86406]	-1.76	0.1694
ssc-miR-92b-3p	CD69	CD69 molecule [Source:VGNC Symbol;Acc:VGNC:103223]	-1.76	0.1694
ssc-miR-92b-3p	CDC27	cell division cycle 27 [Source:VGNC Symbol;Acc:VGNC:86448]	-1.76	0.1694
ssc-miR-92b-3p	CDC42	cell division cycle 42 [Source:NCBI gene (formerly Entrezgene);Acc:780428]	-1.76	0.1694
ssc-miR-92b-3p	CDC42BPA	CDC42 binding protein kinase alpha [Source:VGNC Symbol;Acc:VGNC:95847]	-1.76	0.1694
ssc-miR-92b-3p	CDC42EP2	CDC42 effector protein 2 [Source:VGNC Symbol;Acc:VGNC:86456]	-1.76	0.1694
ssc-miR-92b-3p	CDCA7L	cell division cycle associated 7 like [Source:VGNC Symbol;Acc:VGNC:86469]	-1.76	0.1694
ssc-miR-92b-3p	CDH19	cadherin 19 [Source:VGNC Symbol;Acc:VGNC:86482]	-1.76	0.1694
ssc-miR-92b-3p	CDK16	cyclin dependent kinase 16 [Source:VGNC Symbol;Acc:VGNC:86499]	-1.76	0.1694
ssc-miR-92b-3p	CDK5R1	cyclin dependent kinase 5 regulatory subunit 1 [Source:VGNC Symbol;Acc:VGNC:98980]	-1.76	0.1694
ssc-miR-92b-3p	CDKN1C	cyclin dependent kinase inhibitor 1C [Source:VGNC Symbol;Acc:VGNC:86516]	-1.76	0.1694
ssc-miR-92b-3p	CEBPA	CCAAT enhancer binding protein alpha [Source:VGNC Symbol;Acc:VGNC:86531]	-1.76	0.1694
ssc-miR-92b-3p	CELF2	hypothetical gene	-1.76	0.1694
ssc-miR-92b-3p	CEND1	cell cycle exit and neuronal differentiation 1 [Source:VGNC Symbol;Acc:VGNC:86544]	-1.76	0.1694
ssc-miR-92b-3p	CENPF	centromere protein F [Source:VGNC Symbol;Acc:VGNC:86548]	-1.76	0.1694
ssc-miR-92b-3p	CENPP	centromere protein P [Source:VGNC Symbol;Acc:VGNC:98865]	-1.76	0.1694
ssc-miR-92b-3p	CEP350	centrosomal protein 350 [Source:HGNC Symbol;Acc:HGNC:24238]	-1.76	0.1694
ssc-miR-92b-3p	CEP41	centrosomal protein 41 [Source:VGNC Symbol;Acc:VGNC:86569]	-1.76	0.1694
ssc-miR-92b-3p	CERK	ceramide kinase [Source:VGNC Symbol;Acc:VGNC:86586]	-1.76	0.1694
ssc-miR-92b-3p	CFL2	cofilin 2 [Source:VGNC Symbol;Acc:VGNC:86611]	-1.76	0.1694
ssc-miR-92b-3p	CHCHD10	coiled-coil-helix-coiled-coil-helix domain containing 10 [Source:VGNC Symbol;Acc:VGNC:86626]	-1.76	0.1694
ssc-miR-92b-3p	CHD9	chromodomain helicase DNA binding protein 9 [Source:VGNC Symbol;Acc:VGNC:86635]	-1.76	0.1694
ssc-miR-92b-3p	CHGA	chromogranin A [Source:VGNC Symbol;Acc:VGNC:103919]	-1.76	0.1694
ssc-miR-92b-3p	CHKA	choline kinase alpha [Source:VGNC Symbol;Acc:VGNC:86646]	-1.76	0.1694
ssc-miR-92b-3p	CHMP7	charged multivesicular body protein 7 [Source:VGNC Symbol;Acc:VGNC:86654]	-1.76	0.1694
ssc-miR-92b-3p	CHRM2	cholinergic receptor muscarinic 2 [Source:VGNC Symbol;Acc:VGNC:103922]	-1.76	0.1694
ssc-miR-92b-3p	CHRM5	cholinergic receptor muscarinic 5 [Source:VGNC Symbol;Acc:VGNC:86666]	-1.76	0.1694
ssc-miR-92b-3p	CHST1	carbohydrate sulfotransferase 1 [Source:VGNC Symbol;Acc:VGNC:86673]	-1.76	0.1694
ssc-miR-92b-3p	CHST11	carbohydrate sulfotransferase 11 [Source:VGNC Symbol;Acc:VGNC:86675]	-1.76	0.1694
ssc-miR-92b-3p	CHST7	carbohydrate sulfotransferase 7 [Source:VGNC Symbol;Acc:VGNC:86681]	-1.76	0.1694
ssc-miR-92b-3p	CHSY3	chondroitin sulfate synthase 3 [Source:VGNC Symbol;Acc:VGNC:86685]	-1.76	0.1694
ssc-miR-92b-3p	CIC	capicua transcriptional repressor [Source:VGNC Symbol;Acc:VGNC:96950]	-1.76	0.1694

ssc-miR-92b-3p	CLCF1	cardiotrophin like cytokine factor 1 [Source:VGNC Symbol;Acc:VGNC:86725]	-1.76	0.1694
ssc-miR-92b-3p	CLDN11	claudin 11 [Source:NCBI gene (formerly Entrezgene);Acc:100302016]	-1.76	0.1694
ssc-miR-92b-3p	CLEC16A	C-type lectin domain containing 16A [Source:VGNC Symbol;Acc:VGNC:86748]	-1.76	0.1694
ssc-miR-92b-3p	CLGN	calmegin [Source:VGNC Symbol;Acc:VGNC:86759]	-1.76	0.1694
ssc-miR-92b-3p	CLIP4	CAP-Gly domain containing linker protein family member 4 [Source:VGNC Symbol;Acc:VGNC:97932]	-1.76	0.1694
ssc-miR-92b-3p	CLK3	CDC like kinase 3 [Source:VGNC Symbol;Acc:VGNC:107129]	-1.76	0.1694
ssc-miR-92b-3p	CLPX	caseinolytic mitochondrial matrix peptidase chaperone subunit X [Source:VGNC Symbol;Acc:VGNC:86780]	-1.76	0.1694
ssc-miR-92b-3p	CNEP1R1	CTD nuclear envelope phosphatase 1 regulatory subunit 1 [Source:VGNC Symbol;Acc:VGNC:86813]	-1.76	0.1694
ssc-miR-92b-3p	CNIH1	cornichon family AMPA receptor auxiliary protein 1 [Source:VGNC Symbol;Acc:VGNC:86820]	-1.76	0.1694
ssc-miR-92b-3p	CNNM4	cyclin and CBS domain divalent metal cation transport mediator 4 [Source:VGNC Symbol;Acc:VGNC:86830]	-1.76	0.1694
ssc-miR-92b-3p	CNOT2	CCR4-NOT transcription complex subunit 2 [Source:VGNC Symbol;Acc:VGNC:86834]	-1.76	0.1694
ssc-miR-92b-3p	CNST	consortin, connexin sorting protein [Source:VGNC Symbol;Acc:VGNC:96131]	-1.76	0.1694
ssc-miR-92b-3p	CNTN1	contactin 1 [Source:VGNC Symbol;Acc:VGNC:86848]	-1.76	0.1694
ssc-miR-92b-3p	CNTN4	contactin 4 [Source:VGNC Symbol;Acc:VGNC:97934]	-1.76	0.1694
ssc-miR-92b-3p	COG1	component of oligomeric golgi complex 1 [Source:VGNC Symbol;Acc:VGNC:86854]	-1.76	0.1694
ssc-miR-92b-3p	COG3	component of oligomeric golgi complex 3 [Source:HGNC Symbol;Acc:HGNC:18619]	-1.76	0.1694
ssc-miR-92b-3p	COG5	component of oligomeric golgi complex 5 [Source:VGNC Symbol;Acc:VGNC:86857]	-1.76	0.1694
ssc-miR-92b-3p	COL19A1	collagen type XIX alpha 1 chain [Source:HGNC Symbol;Acc:HGNC:2196]	-1.76	0.1694
ssc-miR-92b-3p	COL1A2	collagen type I alpha 2 chain [Source:VGNC Symbol;Acc:VGNC:86871]	-1.76	0.1694
ssc-miR-92b-3p	COL24A1	collagen type XXIV alpha 1 chain [Source:HGNC Symbol;Acc:HGNC:20821]	-1.76	0.1694
ssc-miR-92b-3p	COL27A1	collagen type XXVII alpha 1 chain [Source:VGNC Symbol;Acc:VGNC:86872]	-1.76	0.1694
ssc-miR-92b-3p	COL5A1	collagen type V alpha 1 chain [Source:VGNC Symbol;Acc:VGNC:86877]	-1.76	0.1694
ssc-miR-92b-3p	COPS2	COP9 signalosome subunit 2 [Source:VGNC Symbol;Acc:VGNC:86899]	-1.76	0.1694
ssc-miR-92b-3p	CORO2A	coronin 2A [Source:VGNC Symbol;Acc:VGNC:86915]	-1.76	0.1694
ssc-miR-92b-3p	COX10	cytochrome c oxidase assembly factor heme A:farnesyltransferase COX10 [Source:VGNC Symbol;Acc:VGNC:96724]	-1.76	0.1694
ssc-miR-92b-3p	CPEB1	cytoplasmic polyadenylation element binding protein 1 [Source:NCBI gene (formerly Entrezgene);Acc:100048944]	-1.76	0.1694
ssc-miR-92b-3p	CPEB2	cytoplasmic polyadenylation element binding protein 2 [Source:VGNC Symbol;Acc:VGNC:86937]	-1.76	0.1694
ssc-miR-92b-3p	CPEB3	cytoplasmic polyadenylation element binding protein 3 [Source:VGNC Symbol;Acc:VGNC:86938]	-1.76	0.1694
ssc-miR-92b-3p	CPEB4	cytoplasmic polyadenylation element binding protein 4 [Source:VGNC Symbol;Acc:VGNC:86939]	-1.76	0.1694
ssc-miR-92b-3p	CPLX1	complexin 1 [Source:VGNC Symbol;Acc:VGNC:86943]	-1.76	0.1694
ssc-miR-92b-3p	CPLX2	complexin 2 [Source:VGNC Symbol;Acc:VGNC:86944]	-1.76	0.1694
ssc-miR-92b-3p	CREB1	cAMP responsive element binding protein 1 [Source:VGNC Symbol;Acc:VGNC:96004]	-1.76	0.1694
ssc-miR-92b-3p	CREB3L2	cAMP responsive element binding protein 3 like 2 [Source:VGNC Symbol;Acc:VGNC:86981]	-1.76	0.1694
ssc-miR-92b-3p	CRTAM	cytotoxic and regulatory T cell molecule [Source:VGNC Symbol;Acc:VGNC:87005]	-1.76	0.1694
ssc-miR-92b-3p	CSMD1	CUB and Sushi multiple domains 1 [Source:VGNC Symbol;Acc:VGNC:95600]	-1.76	0.1694
ssc-miR-92b-3p	CSMD3	CUB and Sushi multiple domains 3 [Source:VGNC Symbol;Acc:VGNC:98765]	-1.76	0.1694
ssc-miR-92b-3p	CSRNP3	cysteine and serine rich nuclear protein 3 [Source:VGNC Symbol;Acc:VGNC:96249]	-1.76	0.1694
ssc-miR-92b-3p	CTNNBIP1	catenin beta interacting protein 1 [Source:VGNC Symbol;Acc:VGNC:87066]	-1.76	0.1694
ssc-miR-92b-3p	CTTNBP2	cortactin binding protein 2 [Source:HGNC Symbol;Acc:HGNC:15679]	-1.76	0.1694
ssc-miR-92b-3p	CUL3	cullin 3 [Source:VGNC Symbol;Acc:VGNC:96091]	-1.76	0.1694
ssc-miR-92b-3p	CXCL5	hypothetical gene	-1.76	0.1694
ssc-miR-92b-3p	CXXC4	CXXC finger protein 4 [Source:HGNC Symbol;Acc:HGNC:24593]	-1.76	0.1694
ssc-miR-92b-3p	CXXC5	CXXC finger protein 5 [Source:VGNC Symbol;Acc:VGNC:87118]	-1.76	0.1694
ssc-miR-92b-3p	CYBB	hypothetical gene	-1.76	0.1694
ssc-miR-92b-3p	CYFIP1	cytoplasmic FMR1 interacting protein 1 [Source:HGNC Symbol;Acc:HGNC:13759]	-1.76	0.1694
ssc-miR-92b-3p	CYP2S1	cytochrome P450 family 2 subfamily S member 1 [Source:VGNC Symbol;Acc:VGNC:103351]	-1.76	0.1694
ssc-miR-92b-3p	DAAM1	dishevelled associated activator of morphosis 1 [Source:VGNC Symbol;Acc:VGNC:87141]	-1.76	0.1694
ssc-miR-92b-3p	DAB2IP	DAB2 interacting protein [Source:VGNC Symbol;Acc:VGNC:87145]	-1.76	0.1694
ssc-miR-92b-3p	DACT1	dishevelled binding antagonist of beta catenin 1 [Source:VGNC Symbol;Acc:VGNC:87147]	-1.76	0.1694
ssc-miR-92b-3p	DAG1	dystroglycan 1 [Source:VGNC Symbol;Acc:VGNC:87151]	-1.76	0.1694

ssc-miR-92b-3p	DARS	hypothetical gene	-1.76	0.1694
ssc-miR-92b-3p	DBT	dihydrolipoamide branched chain transacylase E2 [Source:VGNC Symbol;Acc:VGNC:87169]	-1.76	0.1694
ssc-miR-92b-3p	DCAF10	DDB1 and CUL4 associated factor 10 [Source:VGNC Symbol;Acc:VGNC:87173]	-1.76	0.1694
ssc-miR-92b-3p	DCAF6	DDB1 and CUL4 associated factor 6 [Source:VGNC Symbol;Acc:VGNC:87177]	-1.76	0.1694
ssc-miR-92b-3p	DCAF8	DDB1 and CUL4 associated factor 8 [Source:NCBI gene (formerly Entrezgene);Acc:100153655]	-1.76	0.1694
ssc-miR-92b-3p	DCBLD1	discoidin, CUB and LCCL domain containing 1 [Source:VGNC Symbol;Acc:VGNC:87179]	-1.76	0.1694
ssc-miR-92b-3p	DCC	DCC netrin 1 receptor [Source:VGNC Symbol;Acc:VGNC:103078]	-1.76	0.1694
ssc-miR-92b-3p	DCHS2	dachsous cadherin-related 2 [Source:HGNC Symbol;Acc:HGNC:23111]	-1.76	0.1694
ssc-miR-92b-3p	DCLK2	doublecortin like kinase 2 [Source:VGNC Symbol;Acc:VGNC:98918]	-1.76	0.1694
ssc-miR-92b-3p	DCP1A	decapping mRNA 1A [Source:VGNC Symbol;Acc:VGNC:97960]	-1.76	0.1694
ssc-miR-92b-3p	DCP2	decapping mRNA 2 [Source:VGNC Symbol;Acc:VGNC:87188]	-1.76	0.1694
ssc-miR-92b-3p	DCX	doublecortin [Source:HGNC Symbol;Acc:HGNC:2714]	-1.76	0.1694
ssc-miR-92b-3p	DDHD2	DDHD domain containing 2 [Source:VGNC Symbol;Acc:VGNC:96197]	-1.76	0.1694
ssc-miR-92b-3p	DDI2	hypothetical gene	-1.76	0.1694
ssc-miR-92b-3p	DDIT4	DNA damage inducible transcript 4 [Source:VGNC Symbol;Acc:VGNC:87208]	-1.76	0.1694
ssc-miR-92b-3p	DDX3X	DEAD-box helicase 3 X-linked [Source:NCBI gene (formerly Entrezgene);Acc:100515940]	-1.76	0.1694
ssc-miR-92b-3p	DDX3Y	hypothetical gene	-1.76	0.1694
ssc-miR-92b-3p	DENND4B	DENN domain containing 4B [Source:VGNC Symbol;Acc:VGNC:87252]	-1.76	0.1694
ssc-miR-92b-3p	DENND4C	DENN domain containing 4C [Source:VGNC Symbol;Acc:VGNC:87253]	-1.76	0.1694
ssc-miR-92b-3p	DESI1	desumoylating isopeptidase 1 [Source:VGNC Symbol;Acc:VGNC:87263]	-1.76	0.1694
ssc-miR-92b-3p	DHX32	DEAH-box helicase 32 (putative) [Source:HGNC Symbol;Acc:HGNC:16717]	-1.76	0.1694
ssc-miR-92b-3p	DIRAS1	DIRAS family GTPase 1 [Source:VGNC Symbol;Acc:VGNC:87312]	-1.76	0.1694
ssc-miR-92b-3p	DKK3	dickkopf WNT signaling pathway inhibitor 3 [Source:VGNC Symbol;Acc:VGNC:87323]	-1.76	0.1694
ssc-miR-92b-3p	DLG5	discs large MAGUK scaffold protein 5 [Source:VGNC Symbol;Acc:VGNC:87329]	-1.76	0.1694
ssc-miR-92b-3p	DLGAP2	DLG associated protein 2 [Source:VGNC Symbol;Acc:VGNC:99711]	-1.76	0.1694
ssc-miR-92b-3p	DMP1	dentin matrix acidic phosphoprotein 1 [Source:VGNC Symbol;Acc:VGNC:87348]	-1.76	0.1694
ssc-miR-92b-3p	DMXL1	Dmx like 1 [Source:VGNC Symbol;Acc:VGNC:87359]	-1.76	0.1694
ssc-miR-92b-3p	DNAJB12	DnaJ heat shock protein family (Hsp40) member B12 [Source:VGNC Symbol;Acc:VGNC:96679]	-1.76	0.1694
ssc-miR-92b-3p	DNAJB9	DnaJ heat shock protein family (Hsp40) member B9 [Source:VGNC Symbol;Acc:VGNC:96687]	-1.76	0.1694
ssc-miR-92b-3p	DNAJC27	DnaJ heat shock protein family (Hsp40) member C27 [Source:VGNC Symbol;Acc:VGNC:102563]	-1.76	0.1694
ssc-miR-92b-3p	DNAJC30	DnaJ heat shock protein family (Hsp40) member C30 [Source:HGNC Symbol;Acc:HGNC:16410]	-1.76	0.1694
ssc-miR-92b-3p	DNAJC4	DnaJ heat shock protein family (Hsp40) member C4 [Source:HGNC Symbol;Acc:HGNC:5271]	-1.76	0.1694
ssc-miR-92b-3p	DNMT3A	DNA methyltransferase 3 alpha [Source:VGNC Symbol;Acc:VGNC:87384]	-1.76	0.1694
ssc-miR-92b-3p	DOCK1	dedicator of cytokinesis 1 [Source:VGNC Symbol;Acc:VGNC:87390]	-1.76	0.1694
ssc-miR-92b-3p	DOCK4	dedicator of cytokinesis 4 [Source:VGNC Symbol;Acc:VGNC:87394]	-1.76	0.1694
ssc-miR-92b-3p	DOCK5	dedicator of cytokinesis 5 [Source:VGNC Symbol;Acc:VGNC:87395]	-1.76	0.1694
ssc-miR-92b-3p	DOCK9	dedicator of cytokinesis 9 [Source:VGNC Symbol;Acc:VGNC:87399]	-1.76	0.1694
ssc-miR-92b-3p	DOK5	docking protein 5 [Source:VGNC Symbol;Acc:VGNC:95768]	-1.76	0.1694
ssc-miR-92b-3p	DPP10	dipeptidyl peptidase like 10 [Source:VGNC Symbol;Acc:VGNC:95949]	-1.76	0.1694
ssc-miR-92b-3p	DPP8	dipeptidyl peptidase 8 [Source:VGNC Symbol;Acc:VGNC:87423]	-1.76	0.1694
ssc-miR-92b-3p	DPY30	dpy-30 histone methyltransferase complex regulatory subunit [Source:VGNC Symbol;Acc:VGNC:87428]	-1.76	0.1694
ssc-miR-92b-3p	DRGX	dorsal root ganglia homeobox [Source:VGNC Symbol;Acc:VGNC:97968]	-1.76	0.1694
ssc-miR-92b-3p	DSC2	desmocollin 2 [Source:HGNC Symbol;Acc:HGNC:3036]	-1.76	0.1694
ssc-miR-92b-3p	DSCAML1	DS cell adhesion molecule like 1 [Source:VGNC Symbol;Acc:VGNC:97969]	-1.76	0.1694
ssc-miR-92b-3p	DSTYK	dual serine/threonine and tyrosine protein kinase [Source:VGNC Symbol;Acc:VGNC:87460]	-1.76	0.1694
ssc-miR-92b-3p	DTX2	deltex E3 ubiquitin ligase 2 [Source:VGNC Symbol;Acc:VGNC:87467]	-1.76	0.1694
ssc-miR-92b-3p	DUS2	dihydrouridine synthase 2 [Source:HGNC Symbol;Acc:HGNC:26014]	-1.76	0.1694
ssc-miR-92b-3p	DUSP1	dual specificity phosphatase 1 [Source:VGNC Symbol;Acc:VGNC:87476]	-1.76	0.1694
ssc-miR-92b-3p	DUSP10	dual specificity phosphatase 10 [Source:VGNC Symbol;Acc:VGNC:96246]	-1.76	0.1694
ssc-miR-92b-3p	DUSP4	dual specificity phosphatase 4 [Source:VGNC Symbol;Acc:VGNC:96248]	-1.76	0.1694

ssc-miR-92b-3p	DUSP5	dual specificity phosphatase 5 [Source:VGNC Symbol;Acc:VGNC:87487]	-1.76	0.1694
ssc-miR-92b-3p	DUSP6	dual specificity phosphatase 6 [Source:VGNC Symbol;Acc:VGNC:87488]	-1.76	0.1694
ssc-miR-92b-3p	DYRK2	dual specificity tyrosine phosphorylation regulated kinase 2 [Source:VGNC Symbol;Acc:VGNC:87507]	-1.76	0.1694
ssc-miR-92b-3p	E2F3	E2F transcription factor 3 [Source:VGNC Symbol;Acc:VGNC:87514]	-1.76	0.1694
ssc-miR-92b-3p	EBAG9	estrogen receptor binding site associated antigen 9 [Source:VGNC Symbol;Acc:VGNC:98788]	-1.76	0.1694
ssc-miR-92b-3p	EDEM1	ER degradation enhancing alpha-mannosidase like protein 1 [Source:VGNC Symbol;Acc:VGNC:87545]	-1.76	0.1694
ssc-miR-92b-3p	EFR3A	EFR3 homolog A [Source:VGNC Symbol;Acc:VGNC:87579]	-1.76	0.1694
ssc-miR-92b-3p	EFR3B	EFR3 homolog B [Source:VGNC Symbol;Acc:VGNC:87580]	-1.76	0.1694
ssc-miR-92b-3p	EGR2	early growth response 2 [Source:VGNC Symbol;Acc:VGNC:103939]	-1.76	0.1694
ssc-miR-92b-3p	EIF1	eukaryotic translation initiation factor 1 [Source:VGNC Symbol;Acc:VGNC:98988]	-1.76	0.1694
ssc-miR-92b-3p	EIF4G2	eukaryotic translation initiation factor 4 gamma 2 [Source:VGNC Symbol;Acc:VGNC:99645]	-1.76	0.1694
ssc-miR-92b-3p	EIF5A2	eukaryotic translation initiation factor 5A2 [Source:VGNC Symbol;Acc:VGNC:87633]	-1.76	0.1694
ssc-miR-92b-3p	EIF5B	eukaryotic translation initiation factor 5B [Source:VGNC Symbol;Acc:VGNC:87634]	-1.76	0.1694
ssc-miR-92b-3p	ELFN2	extracellular leucine rich repeat and fibronectin type III domain containing 2 [Source:VGNC Symbol;Acc:VGNC:103232]	-1.76	0.1694
ssc-miR-92b-3p	ELK4	ETS transcription factor ELK4 [Source:VGNC Symbol;Acc:VGNC:87647]	-1.76	0.1694
ssc-miR-92b-3p	ELOVL4	ELOVL fatty acid elongase 4 [Source:VGNC Symbol;Acc:VGNC:87660]	-1.76	0.1694
ssc-miR-92b-3p	ELOVL6	ELOVL fatty acid elongase 6 [Source:VGNC Symbol;Acc:VGNC:87662]	-1.76	0.1694
ssc-miR-92b-3p	EN2	engrailed homeobox 2 [Source:VGNC Symbol;Acc:VGNC:87694]	-1.76	0.1694
ssc-miR-92b-3p	ENTPD4	ectonucleoside triphosphate diphosphohydrolase 4 [Source:VGNC Symbol;Acc:VGNC:107386]	-1.76	0.1694
ssc-miR-92b-3p	ENTPD5	ectonucleoside triphosphate diphosphohydrolase 5 (inactive) [Source:HGNC Symbol;Acc:HGNC:3367]	-1.76	0.1694
ssc-miR-92b-3p	EOMES	eomesodermin [Source:VGNC Symbol;Acc:VGNC:87719]	-1.76	0.1694
ssc-miR-92b-3p	EPB41L4A	erythrocyte membrane protein band 4.1 like 4A [Source:HGNC Symbol;Acc:HGNC:13278]	-1.76	0.1694
ssc-miR-92b-3p	EPB41L4A-AS2	hypothetical gene	-1.76	0.1694
ssc-miR-92b-3p	EPC2	enhancer of polycomb homolog 2 [Source:VGNC Symbol;Acc:VGNC:96045]	-1.76	0.1694
ssc-miR-92b-3p	EPG5	ectopic P-granules 5 autophagy tethering factor [Source:VGNC Symbol;Acc:VGNC:87728]	-1.76	0.1694
ssc-miR-92b-3p	EPHA3	EPH receptor A3 [Source:VGNC Symbol;Acc:VGNC:87732]	-1.76	0.1694
ssc-miR-92b-3p	EPHA8	EPH receptor A8 [Source:VGNC Symbol;Acc:VGNC:87735]	-1.76	0.1694
ssc-miR-92b-3p	EPS8	epidermal growth factor receptor pathway substrate 8 [Source:VGNC Symbol;Acc:VGNC:87749]	-1.76	0.1694
ssc-miR-92b-3p	ERBB2IP	hypothetical gene	-1.76	0.1694
ssc-miR-92b-3p	ERC2	ELKS/RAB6-interacting/CAST family member 2 [Source:VGNC Symbol;Acc:VGNC:87762]	-1.76	0.1694
ssc-miR-92b-3p	ERGIC2	ERGIC and golgi 2 [Source:VGNC Symbol;Acc:VGNC:87771]	-1.76	0.1694
ssc-miR-92b-3p	ESRP1	epithelial splicing regulatory protein 1 [Source:VGNC Symbol;Acc:VGNC:87791]	-1.76	0.1694
ssc-miR-92b-3p	ESRRG	estrogen related receptor gamma [Source:VGNC Symbol;Acc:VGNC:96289]	-1.76	0.1694
ssc-miR-92b-3p	ESYT2	extended synaptotagmin 2 [Source:VGNC Symbol;Acc:VGNC:87797]	-1.76	0.1694
ssc-miR-92b-3p	EVI5	ecotropic viral integration site 5 [Source:VGNC Symbol;Acc:VGNC:98793]	-1.76	0.1694
ssc-miR-92b-3p	EVX2	even-skipped homeobox 2 [Source:VGNC Symbol;Acc:VGNC:96290]	-1.76	0.1694
ssc-miR-92b-3p	EXOC5	exocyst complex component 5 [Source:VGNC Symbol;Acc:VGNC:87831]	-1.76	0.1694
ssc-miR-92b-3p	EXT1	exostosin glycosyltransferase 1 [Source:VGNC Symbol;Acc:VGNC:87846]	-1.76	0.1694
ssc-miR-92b-3p	EZH2	enhancer of zeste 2 polycomb repressive complex 2 subunit [Source:VGNC Symbol;Acc:VGNC:87855]	-1.76	0.1694
ssc-miR-92b-3p	FAM110B	family with sequence similarity 110 member B [Source:VGNC Symbol;Acc:VGNC:87885]	-1.76	0.1694
ssc-miR-92b-3p	FAM110C	family with sequence similarity 110 member C [Source:HGNC Symbol;Acc:HGNC:33340]	-1.76	0.1694
ssc-miR-92b-3p	FAM117B	family with sequence similarity 117 member B [Source:VGNC Symbol;Acc:VGNC:95616]	-1.76	0.1694
ssc-miR-92b-3p	FAM126B	family with sequence similarity 126 member B [Source:HGNC Symbol;Acc:HGNC:28593]	-1.76	0.1694
ssc-miR-92b-3p	FAM133B	family with sequence similarity 133 member B [Source:HGNC Symbol;Acc:HGNC:28629]	-1.76	0.1694
ssc-miR-92b-3p	FAM135A	family with sequence similarity 135 member A [Source:VGNC Symbol;Acc:VGNC:87904]	-1.76	0.1694
ssc-miR-92b-3p	FAM150B	hypothetical gene	-1.76	0.1694
ssc-miR-92b-3p	FAM160A2	hypothetical gene	-1.76	0.1694
ssc-miR-92b-3p	FAM160B1	hypothetical gene	-1.76	0.1694
ssc-miR-92b-3p	FAM179A	hypothetical gene	-1.76	0.1694
ssc-miR-92b-3p	FAM196A	hypothetical gene	-1.76	0.1694

ssc-miR-92b-3p	FAM196B	hypothetical gene	-1.76	0.1694
ssc-miR-92b-3p	FAM19A1	hypothetical gene	-1.76	0.1694
ssc-miR-92b-3p	FAM20C	FAM20C golgi associated secretory pathway kinase [Source:VGNC Symbol;Acc:VGNC:87951]	-1.76	0.1694
ssc-miR-92b-3p	FAM214A	family with sequence similarity 214 member A [Source:VGNC Symbol;Acc:VGNC:87952]	-1.76	0.1694
ssc-miR-92b-3p	FAM46A	hypothetical gene	-1.76	0.1694
ssc-miR-92b-3p	FAM76B	family with sequence similarity 76 member B [Source:VGNC Symbol;Acc:VGNC:87983]	-1.76	0.1694
ssc-miR-92b-3p	FAM81A	family with sequence similarity 81 member A [Source:VGNC Symbol;Acc:VGNC:87986]	-1.76	0.1694
ssc-miR-92b-3p	FAM84A	hypothetical gene	-1.76	0.1694
ssc-miR-92b-3p	FAR1	fatty acyl-CoA reductase 1 [Source:VGNC Symbol;Acc:VGNC:88010]	-1.76	0.1694
ssc-miR-92b-3p	FARP1	FERM, ARH/RhoGEF and pleckstrin domain protein 1 [Source:VGNC Symbol;Acc:VGNC:88012]	-1.76	0.1694
ssc-miR-92b-3p	FASLG	Fas ligand [Source:VGNC Symbol;Acc:VGNC:103942]	-1.76	0.1694
ssc-miR-92b-3p	FBN1	fibrillin 1 [Source:VGNC Symbol;Acc:VGNC:103090]	-1.76	0.1694
ssc-miR-92b-3p	FBN2	fibrillin 2 [Source:VGNC Symbol;Acc:VGNC:88025]	-1.76	0.1694
ssc-miR-92b-3p	FBXL17	F-box and leucine rich repeat protein 17 [Source:VGNC Symbol;Acc:VGNC:99650]	-1.76	0.1694
ssc-miR-92b-3p	FBXO21	F-box protein 21 [Source:VGNC Symbol;Acc:VGNC:88035]	-1.76	0.1694
ssc-miR-92b-3p	FBXO28	F-box protein 28 [Source:HGNC Symbol;Acc:HGNC:29046]	-1.76	0.1694
ssc-miR-92b-3p	FBXO32	F-box protein 32 [Source:VGNC Symbol;Acc:VGNC:88039]	-1.76	0.1694
ssc-miR-92b-3p	FBXO33	F-box protein 33 [Source:VGNC Symbol;Acc:VGNC:88040]	-1.76	0.1694
ssc-miR-92b-3p	FBXO41	F-box protein 41 [Source:VGNC Symbol;Acc:VGNC:88046]	-1.76	0.1694
ssc-miR-92b-3p	FBXW7	F-box and WD repeat domain containing 7 [Source:VGNC Symbol;Acc:VGNC:98925]	-1.76	0.1694
ssc-miR-92b-3p	FCHO2	FCH and mu domain containing endocytic adaptor 2 [Source:VGNC Symbol;Acc:VGNC:88066]	-1.76	0.1694
ssc-miR-92b-3p	FGB	fibrinogen beta chain [Source:VGNC Symbol;Acc:VGNC:98927]	-1.76	0.1694
ssc-miR-92b-3p	FHL2	hypothetical gene	-1.76	0.1694
ssc-miR-92b-3p	FHL3	four and a half LIM domains 3 [Source:VGNC Symbol;Acc:VGNC:88129]	-1.76	0.1694
ssc-miR-92b-3p	FKBP14	FKBP prolyl isomerase 14 [Source:VGNC Symbol;Acc:VGNC:88144]	-1.76	0.1694
ssc-miR-92b-3p	FKBP1A	hypothetical gene	-1.76	0.1694
ssc-miR-92b-3p	FMN2	hypothetical gene	-1.76	0.1694
ssc-miR-92b-3p	FMR1	FMRP translational regulator 1 [Source:VGNC Symbol;Acc:VGNC:88175]	-1.76	0.1694
ssc-miR-92b-3p	FNBP4	formin binding protein 4 [Source:VGNC Symbol;Acc:VGNC:88180]	-1.76	0.1694
ssc-miR-92b-3p	FNDC3B	fibronectin type III domain containing 3B [Source:VGNC Symbol;Acc:VGNC:88183]	-1.76	0.1694
ssc-miR-92b-3p	FNIP1	folliculin interacting protein 1 [Source:VGNC Symbol;Acc:VGNC:88187]	-1.76	0.1694
ssc-miR-92b-3p	FNIP2	folliculin interacting protein 2 [Source:VGNC Symbol;Acc:VGNC:88188]	-1.76	0.1694
ssc-miR-92b-3p	FOSL2	FOS like 2, AP-1 transcription factor subunit [Source:VGNC Symbol;Acc:VGNC:88192]	-1.76	0.1694
ssc-miR-92b-3p	FOXG1	forkhead box G1 [Source:VGNC Symbol;Acc:VGNC:88206]	-1.76	0.1694
ssc-miR-92b-3p	FOXN2	forkhead box N2 [Source:VGNC Symbol;Acc:VGNC:88219]	-1.76	0.1694
ssc-miR-92b-3p	FOXN3	forkhead box N3 [Source:VGNC Symbol;Acc:VGNC:88220]	-1.76	0.1694
ssc-miR-92b-3p	FOXP1	forkhead box P1 [Source:VGNC Symbol;Acc:VGNC:88222]	-1.76	0.1694
ssc-miR-92b-3p	FOXP2	forkhead box P2 [Source:VGNC Symbol;Acc:VGNC:98014]	-1.76	0.1694
ssc-miR-92b-3p	FOXP4	forkhead box P4 [Source:VGNC Symbol;Acc:VGNC:88224]	-1.76	0.1694
ssc-miR-92b-3p	FREM2	FRAS1 related extracellular matrix 2 [Source:VGNC Symbol;Acc:VGNC:88234]	-1.76	0.1694
ssc-miR-92b-3p	FRMPD3	FERM and PDZ domain containing 3 [Source:VGNC Symbol;Acc:VGNC:88242]	-1.76	0.1694
ssc-miR-92b-3p	FRS2	fibroblast growth factor receptor substrate 2 [Source:VGNC Symbol;Acc:VGNC:88246]	-1.76	0.1694
ssc-miR-92b-3p	FRY	hypothetical gene	-1.76	0.1694
ssc-miR-92b-3p	FRYL	FRY like transcription coactivator [Source:VGNC Symbol;Acc:VGNC:98015]	-1.76	0.1694
ssc-miR-92b-3p	FST	follistatin [Source:NCBI gene (formerly Entrezgene);Acc:445002]	-1.76	0.1694
ssc-miR-92b-3p	FSTL1	follistatin like 1 [Source:VGNC Symbol;Acc:VGNC:88255]	-1.76	0.1694
ssc-miR-92b-3p	FUBP1	far upstream element binding protein 1 [Source:VGNC Symbol;Acc:VGNC:96781]	-1.76	0.1694
ssc-miR-92b-3p	FUT11	fucosyltransferase 11 [Source:VGNC Symbol;Acc:VGNC:88269]	-1.76	0.1694
ssc-miR-92b-3p	FXR1	FMR1 autosomal homolog 1 [Source:VGNC Symbol;Acc:VGNC:108659]	-1.76	0.1694
ssc-miR-92b-3p	FZD10	frizzled class receptor 10 [Source:VGNC Symbol;Acc:VGNC:88279]	-1.76	0.1694

ssc-miR-92b-3p	G3BP2	G3BP stress granule assembly factor 2 [Source:VGNC Symbol;Acc:VGNC:88289]	-1.76	0.1694
ssc-miR-92b-3p	GAA	alpha glucosidase [Source:VGNC Symbol;Acc:VGNC:88293]	-1.76	0.1694
ssc-miR-92b-3p	GABRA3	gamma-aminobutyric acid type A receptor subunit alpha3 [Source:VGNC Symbol;Acc:VGNC:88302]	-1.76	0.1694
ssc-miR-92b-3p	GALNT7	polypeptide N-acetylgalactosaminyltransferase 7 [Source:VGNC Symbol;Acc:VGNC:103946]	-1.76	0.1694
ssc-miR-92b-3p	GAN	gigaxonin [Source:VGNC Symbol;Acc:VGNC:88343]	-1.76	0.1694
ssc-miR-92b-3p	GAP43	growth associated protein 43 [Source:VGNC Symbol;Acc:VGNC:98018]	-1.76	0.1694
ssc-miR-92b-3p	GAS2L3	growth arrest specific 2 like 3 [Source:VGNC Symbol;Acc:VGNC:88357]	-1.76	0.1694
ssc-miR-92b-3p	GATA2	GATA binding protein 2 [Source:VGNC Symbol;Acc:VGNC:88364]	-1.76	0.1694
ssc-miR-92b-3p	GATA6	GATA binding protein 6 [Source:VGNC Symbol;Acc:VGNC:88366]	-1.76	0.1694
ssc-miR-92b-3p	GATAD2B	GATA zinc finger domain containing 2B [Source:VGNC Symbol;Acc:VGNC:88369]	-1.76	0.1694
ssc-miR-92b-3p	GCLM	glutamate-cysteine ligase modifier subunit [Source:VGNC Symbol;Acc:VGNC:88387]	-1.76	0.1694
ssc-miR-92b-3p	GDF11	growth differentiation factor 11 [Source:VGNC Symbol;Acc:VGNC:88398]	-1.76	0.1694
ssc-miR-92b-3p	GFPT2	glutamine-fructose-6-phosphate transaminase 2 [Source:VGNC Symbol;Acc:VGNC:88427]	-1.76	0.1694
ssc-miR-92b-3p	GID4	GID complex subunit 4 homolog [Source:VGNC Symbol;Acc:VGNC:98996]	-1.76	0.1694
ssc-miR-92b-3p	GIGYF2	GRB10 interacting GYF protein 2 [Source:VGNC Symbol;Acc:VGNC:95551]	-1.76	0.1694
ssc-miR-92b-3p	GIT2	GIT ArfGAP 2 [Source:VGNC Symbol;Acc:VGNC:88458]	-1.76	0.1694
ssc-miR-92b-3p	GJA3	gap junction protein alpha 3 [Source:HGNC Symbol;Acc:HGNC:4277]	-1.76	0.1694
ssc-miR-92b-3p	GLB1L2	galactosidase beta 1 like 2 [Source:HGNC Symbol;Acc:HGNC:25129]	-1.76	0.1694
ssc-miR-92b-3p	GLCE	glucuronic acid epimerase [Source:VGNC Symbol;Acc:VGNC:88477]	-1.76	0.1694
ssc-miR-92b-3p	GLRA1	glycine receptor alpha 1 [Source:VGNC Symbol;Acc:VGNC:88491]	-1.76	0.1694
ssc-miR-92b-3p	GLTSCR1L	hypothetical gene	-1.76	0.1694
ssc-miR-92b-3p	GLUL	hypothetical gene	-1.76	0.1694
ssc-miR-92b-3p	GLYR1	glyoxylate reductase 1 homolog [Source:VGNC Symbol;Acc:VGNC:88505]	-1.76	0.1694
ssc-miR-92b-3p	GNAL	G protein subunit alpha L [Source:VGNC Symbol;Acc:VGNC:88524]	-1.76	0.1694
ssc-miR-92b-3p	GNAQ	G protein subunit alpha q [Source:VGNC Symbol;Acc:VGNC:103100]	-1.76	0.1694
ssc-miR-92b-3p	GNS	glucosamine (N-acetyl)-6-sulfatase [Source:VGNC Symbol;Acc:VGNC:103284]	-1.76	0.1694
ssc-miR-92b-3p	GOLGA1	golgin A1 [Source:VGNC Symbol;Acc:VGNC:88549]	-1.76	0.1694
ssc-miR-92b-3p	GOLGA3	golgin A3 [Source:VGNC Symbol;Acc:VGNC:88550]	-1.76	0.1694
ssc-miR-92b-3p	GOLGA4	hypothetical gene	-1.76	0.1694
ssc-miR-92b-3p	GOLGA7	golgin A7 [Source:VGNC Symbol;Acc:VGNC:96059]	-1.76	0.1694
ssc-miR-92b-3p	GOLGA8A	hypothetical gene	-1.76	0.1694
ssc-miR-92b-3p	GOLGA8B	hypothetical gene	-1.76	0.1694
ssc-miR-92b-3p	GOLGA8I	hypothetical gene	-1.76	0.1694
ssc-miR-92b-3p	GOLGA8J	hypothetical gene	-1.76	0.1694
ssc-miR-92b-3p	GOLGA8M	hypothetical gene	-1.76	0.1694
ssc-miR-92b-3p	GOLGA8O	hypothetical gene	-1.76	0.1694
ssc-miR-92b-3p	GOLGA8R	hypothetical gene	-1.76	0.1694
ssc-miR-92b-3p	GPATCH8	G-patch domain containing 8 [Source:HGNC Symbol;Acc:HGNC:29066]	-1.76	0.1694
ssc-miR-92b-3p	GPC6	glypican 6 [Source:HGNC Symbol;Acc:HGNC:4454]	-1.76	0.1694
ssc-miR-92b-3p	GPM6A	glycoprotein M6A [Source:VGNC Symbol;Acc:VGNC:98025]	-1.76	0.1694
ssc-miR-92b-3p	GPR12	G protein-coupled receptor 12 [Source:VGNC Symbol;Acc:VGNC:88598]	-1.76	0.1694
ssc-miR-92b-3p	GPR124	hypothetical gene	-1.76	0.1694
ssc-miR-92b-3p	GPR137C	G protein-coupled receptor 137C [Source:VGNC Symbol;Acc:VGNC:88602]	-1.76	0.1694
ssc-miR-92b-3p	GPR158	G protein-coupled receptor 158 [Source:VGNC Symbol;Acc:VGNC:96016]	-1.76	0.1694
ssc-miR-92b-3p	GPR173	G protein-coupled receptor 173 [Source:VGNC Symbol;Acc:VGNC:88616]	-1.76	0.1694
ssc-miR-92b-3p	GPR180	G protein-coupled receptor 180 [Source:VGNC Symbol;Acc:VGNC:88621]	-1.76	0.1694
ssc-miR-92b-3p	GPRC5A	G protein-coupled receptor class C group 5 member A [Source:VGNC Symbol;Acc:VGNC:88642]	-1.76	0.1694
ssc-miR-92b-3p	GRAMD1B	GRAM domain containing 1B [Source:VGNC Symbol;Acc:VGNC:88656]	-1.76	0.1694
ssc-miR-92b-3p	GRAMD3	hypothetical gene	-1.76	0.1694
ssc-miR-92b-3p	GRAMD4	GRAM domain containing 4 [Source:VGNC Symbol;Acc:VGNC:88659]	-1.76	0.1694

ssc-miR-92b-3p	GREB1L	GREB1 like retinoic acid receptor coactivator [Source:VGNC Symbol;Acc:VGNC:96584]	-1.76	0.1694
ssc-miR-92b-3p	GRHL1	grainyhead like transcription factor 1 [Source:VGNC Symbol;Acc:VGNC:88666]	-1.76	0.1694
ssc-miR-92b-3p	GRHL2	grainyhead like transcription factor 2 [Source:VGNC Symbol;Acc:VGNC:88667]	-1.76	0.1694
ssc-miR-92b-3p	GRIA1	glutamate ionotropic receptor AMPA type subunit 1 [Source:VGNC Symbol;Acc:VGNC:88670]	-1.76	0.1694
ssc-miR-92b-3p	GRID2	glutamate ionotropic receptor delta type subunit 2 [Source:VGNC Symbol;Acc:VGNC:98932]	-1.76	0.1694
ssc-miR-92b-3p	GRIK3	glutamate ionotropic receptor kainate type subunit 3 [Source:VGNC Symbol;Acc:VGNC:88679]	-1.76	0.1694
ssc-miR-92b-3p	GRK5	G protein-coupled receptor kinase 5 [Source:VGNC Symbol;Acc:VGNC:88697]	-1.76	0.1694
ssc-miR-92b-3p	GRM2	glutamate metabotropic receptor 2 [Source:VGNC Symbol;Acc:VGNC:88701]	-1.76	0.1694
ssc-miR-92b-3p	GRM7	glutamate metabotropic receptor 7 [Source:VGNC Symbol;Acc:VGNC:98028]	-1.76	0.1694
ssc-miR-92b-3p	GXYLT1	glucoside xylosyltransferase 1 [Source:VGNC Symbol;Acc:VGNC:88756]	-1.76	0.1694
ssc-miR-92b-3p	H3F3B	hypothetical gene	-1.76	0.1694
ssc-miR-92b-3p	H3F3C	hypothetical gene	-1.76	0.1694
ssc-miR-92b-3p	HAND1	heart and neural crest derivatives expressed 1 [Source:VGNC Symbol;Acc:VGNC:88777]	-1.76	0.1694
ssc-miR-92b-3p	HAND2	heart and neural crest derivatives expressed 2 [Source:VGNC Symbol;Acc:VGNC:88778]	-1.76	0.1694
ssc-miR-92b-3p	HAPLN1	hyaluronan and proteoglycan link protein 1 [Source:VGNC Symbol;Acc:VGNC:88780]	-1.76	0.1694
ssc-miR-92b-3p	HAS2	hyaluronan synthase 2 [Source:HGNC Symbol;Acc:HGNC:4819]	-1.76	0.1694
ssc-miR-92b-3p	HAS3	hyaluronan synthase 3 [Source:NCBI gene (formerly Entrezgene);Acc:408053]	-1.76	0.1694
ssc-miR-92b-3p	HCN2	hyperpolarization activated cyclic nucleotide gated potassium and sodium channel 2 [Source:VGNC Symbol;Acc:VGNC:88803]	-1.76	0.1694
ssc-miR-92b-3p	HECW1	HECT, C2 and WW domain containing E3 ubiquitin protein ligase 1 [Source:VGNC Symbol;Acc:VGNC:88835]	-1.76	0.1694
ssc-miR-92b-3p	HECW2	HECT, C2 and WW domain containing E3 ubiquitin protein ligase 2 [Source:NCBI gene (formerly Entrezgene);Acc:100155879]	-1.76	0.1694
ssc-miR-92b-3p	HEG1	heart development protein with EGF like domains 1 [Source:VGNC Symbol;Acc:VGNC:88836]	-1.76	0.1694
ssc-miR-92b-3p	HERC2	HECT and RLD domain containing E3 ubiquitin protein ligase 2 [Source:VGNC Symbol;Acc:VGNC:99716]	-1.76	0.1694
ssc-miR-92b-3p	HERC3	HECT and RLD domain containing E3 ubiquitin protein ligase 3 [Source:VGNC Symbol;Acc:VGNC:98934]	-1.76	0.1694
ssc-miR-92b-3p	HERPUD2	HERPUD family member 2 [Source:VGNC Symbol;Acc:VGNC:88853]	-1.76	0.1694
ssc-miR-92b-3p	HHIP	hedgehog interacting protein [Source:VGNC Symbol;Acc:VGNC:88875]	-1.76	0.1694
ssc-miR-92b-3p	HIF1A	hypoxia inducible factor 1 subunit alpha [Source:VGNC Symbol;Acc:VGNC:88880]	-1.76	0.1694
ssc-miR-92b-3p	HIPK1	homeodomain interacting protein kinase 1 [Source:VGNC Symbol;Acc:VGNC:88887]	-1.76	0.1694
ssc-miR-92b-3p	HIPK3	homeodomain interacting protein kinase 3 [Source:VGNC Symbol;Acc:VGNC:88889]	-1.76	0.1694
ssc-miR-92b-3p	HIVEP1	HIVEP zinc finger 1 [Source:VGNC Symbol;Acc:VGNC:96585]	-1.76	0.1694
ssc-miR-92b-3p	HMGA2	hypothetical gene	-1.76	0.1694
ssc-miR-92b-3p	HMX3	H6 family homeobox 3 [Source:VGNC Symbol;Acc:VGNC:88915]	-1.76	0.1694
ssc-miR-92b-3p	HNF1B	HNF1 homeobox B [Source:NCBI gene (formerly Entrezgene);Acc:397002]	-1.76	0.1694
ssc-miR-92b-3p	HNRNPA0	hypothetical gene	-1.76	0.1694
ssc-miR-92b-3p	HNRNPU	heterogeneous nuclear ribonucleoprotein U [Source:HGNC Symbol;Acc:HGNC:5048]	-1.76	0.1694
ssc-miR-92b-3p	HOXA3	homeobox A3 [Source:VGNC Symbol;Acc:VGNC:88938]	-1.76	0.1694
ssc-miR-92b-3p	HOXB8	homeobox B8 [Source:VGNC Symbol;Acc:VGNC:88948]	-1.76	0.1694
ssc-miR-92b-3p	HOXC11	homeobox C11 [Source:VGNC Symbol;Acc:VGNC:103287]	-1.76	0.1694
ssc-miR-92b-3p	HOXC12	homeobox C12 [Source:VGNC Symbol;Acc:VGNC:88950]	-1.76	0.1694
ssc-miR-92b-3p	HOXC4	homeobox C4 [Source:VGNC Symbol;Acc:VGNC:88952]	-1.76	0.1694
ssc-miR-92b-3p	HOXC8	homeobox C8 [Source:VGNC Symbol;Acc:VGNC:88954]	-1.76	0.1694
ssc-miR-92b-3p	HOXD10	homeobox D10 [Source:VGNC Symbol;Acc:VGNC:96351]	-1.76	0.1694
ssc-miR-92b-3p	HOXD11	homeobox D11 [Source:VGNC Symbol;Acc:VGNC:96352]	-1.76	0.1694
ssc-miR-92b-3p	HPS6	HPS6 biosis of lysosomal organelles complex 2 subunit 3 [Source:NCBI gene (formerly Entrezgene);Acc:100049675]	-1.76	0.1694
ssc-miR-92b-3p	HS6ST3	heparan sulfate 6-O-sulfotransferase 3 [Source:HGNC Symbol;Acc:HGNC:19134]	-1.76	0.1694
ssc-miR-92b-3p	HSPE1-MOB4	hypothetical gene	-1.76	0.1694
ssc-miR-92b-3p	HYPK	huntingtin interacting protein K [Source:HGNC Symbol;Acc:HGNC:18418]	-1.76	0.1694
ssc-miR-92b-3p	IBSP	integrin binding sialoprotein [Source:VGNC Symbol;Acc:VGNC:98040]	-1.76	0.1694
ssc-miR-92b-3p	IBTK	inhibitor of Bruton tyrosine kinase [Source:VGNC Symbol;Acc:VGNC:89017]	-1.76	0.1694
ssc-miR-92b-3p	ICK	hypothetical gene	-1.76	0.1694
ssc-miR-92b-3p	IDH1	isocitrate dehydrogenase (NADP(+)) 1 [Source:HGNC Symbol;Acc:HGNC:5382]	-1.76	0.1694

ssc-miR-92b-3p	IGFBP7	insulin like growth factor binding protein 7 [Source:VGNC Symbol;Acc:VGNC:89060]	-1.76	0.1694
ssc-miR-92b-3p	IKZF2	IKAROS family zinc finger 2 [Source:VGNC Symbol;Acc:VGNC:95576]	-1.76	0.1694
ssc-miR-92b-3p	IKZF4	IKAROS family zinc finger 4 [Source:VGNC Symbol;Acc:VGNC:89075]	-1.76	0.1694
ssc-miR-92b-3p	IMPA2	inositol monophosphatase 2 [Source:HGNC Symbol;Acc:HGNC:6051]	-1.76	0.1694
ssc-miR-92b-3p	ING2	inhibitor of growth family member 2 [Source:VGNC Symbol;Acc:VGNC:99717]	-1.76	0.1694
ssc-miR-92b-3p	ING5	inhibitor of growth family member 5 [Source:VGNC Symbol;Acc:VGNC:95912]	-1.76	0.1694
ssc-miR-92b-3p	INSIG1	insulin induced 1 [Source:VGNC Symbol;Acc:VGNC:89150]	-1.76	0.1694
ssc-miR-92b-3p	INSL5	hypothetical gene	-1.76	0.1694
ssc-miR-92b-3p	IPO5	importin 5 [Source:VGNC Symbol;Acc:VGNC:89179]	-1.76	0.1694
ssc-miR-92b-3p	IQGAP2	IQ motif containing GTPase activating protein 2 [Source:VGNC Symbol;Acc:VGNC:89192]	-1.76	0.1694
ssc-miR-92b-3p	IRS2	insulin receptor substrate 2 [Source:VGNC Symbol;Acc:VGNC:89214]	-1.76	0.1694
ssc-miR-92b-3p	ISCA1	iron-sulfur cluster assembly 1 [Source:HGNC Symbol;Acc:HGNC:28660]	-1.76	0.1694
ssc-miR-92b-3p	ITGA5	integrin subunit alpha 5 [Source:VGNC Symbol;Acc:VGNC:89236]	-1.76	0.1694
ssc-miR-92b-3p	ITGA6	integrin subunit alpha 6 [Source:VGNC Symbol;Acc:VGNC:96378]	-1.76	0.1694
ssc-miR-92b-3p	ITGA8	integrin subunit alpha 8 [Source:VGNC Symbol;Acc:VGNC:96379]	-1.76	0.1694
ssc-miR-92b-3p	ITGAV	integrin subunit alpha V [Source:VGNC Symbol;Acc:VGNC:96380]	-1.76	0.1694
ssc-miR-92b-3p	ITM2B	integral membrane protein 2B [Source:VGNC Symbol;Acc:VGNC:103971]	-1.76	0.1694
ssc-miR-92b-3p	ITPR1	inositol 1,4,5-trisphosphate receptor type 1 [Source:VGNC Symbol;Acc:VGNC:89253]	-1.76	0.1694
ssc-miR-92b-3p	ITPRIPL2	ITPRIP like 2 [Source:VGNC Symbol;Acc:VGNC:89258]	-1.76	0.1694
ssc-miR-92b-3p	JARID2	jumonji and AT-rich interaction domain containing 2 [Source:VGNC Symbol;Acc:VGNC:89279]	-1.76	0.1694
ssc-miR-92b-3p	JMY	junction mediating and regulatory protein, p53 cofactor [Source:VGNC Symbol;Acc:VGNC:89289]	-1.76	0.1694
ssc-miR-92b-3p	JOSD1	Josephin domain containing 1 [Source:VGNC Symbol;Acc:VGNC:89290]	-1.76	0.1694
ssc-miR-92b-3p	JPH2	hypothetical gene	-1.76	0.1694
ssc-miR-92b-3p	KALRN	hypothetical gene	-1.76	0.1694
ssc-miR-92b-3p	KAT2B	lysine acetyltransferase 2B [Source:VGNC Symbol;Acc:VGNC:89304]	-1.76	0.1694
ssc-miR-92b-3p	KAT7	lysine acetyltransferase 7 [Source:VGNC Symbol;Acc:VGNC:89307]	-1.76	0.1694
ssc-miR-92b-3p	KBTBD8	kelch repeat and BTB domain containing 8 [Source:VGNC Symbol;Acc:VGNC:89321]	-1.76	0.1694
ssc-miR-92b-3p	KCNA1	potassium voltage-gated channel subfamily A member 1 [Source:VGNC Symbol;Acc:VGNC:89323]	-1.76	0.1694
ssc-miR-92b-3p	KCNC4	potassium voltage-gated channel subfamily C member 4 [Source:VGNC Symbol;Acc:VGNC:89335]	-1.76	0.1694
ssc-miR-92b-3p	KCND2	potassium voltage-gated channel subfamily D member 2 [Source:HGNC Symbol;Acc:HGNC:6238]	-1.76	0.1694
ssc-miR-92b-3p	KCNH1	potassium voltage-gated channel subfamily H member 1 [Source:VGNC Symbol;Acc:VGNC:108599]	-1.76	0.1694
ssc-miR-92b-3p	KCNJ3	potassium inwardly rectifying channel subfamily J member 3 [Source:HGNC Symbol;Acc:HGNC:6264]	-1.76	0.1694
ssc-miR-92b-3p	KCNK10	potassium two pore domain channel subfamily K member 10 [Source:VGNC Symbol;Acc:VGNC:89364]	-1.76	0.1694
ssc-miR-92b-3p	KCNK3	potassium two pore domain channel subfamily K member 3 [Source:VGNC Symbol;Acc:VGNC:89370]	-1.76	0.1694
ssc-miR-92b-3p	KCNN3	potassium calcium-activated channel subfamily N member 3 [Source:VGNC Symbol;Acc:VGNC:98056]	-1.76	0.1694
ssc-miR-92b-3p	KCTD8	potassium channel tetramerization domain containing 8 [Source:HGNC Symbol;Acc:HGNC:22394]	-1.76	0.1694
ssc-miR-92b-3p	KDM2A	lysine demethylase 2A [Source:VGNC Symbol;Acc:VGNC:89410]	-1.76	0.1694
ssc-miR-92b-3p	KIAA1024	hypothetical gene	-1.76	0.1694
ssc-miR-92b-3p	KIAA1109	KIAA1109 [Source:VGNC Symbol;Acc:VGNC:89439]	-1.76	0.1694
ssc-miR-92b-3p	KIAA1211	hypothetical gene	-1.76	0.1694
ssc-miR-92b-3p	KIAA1279	hypothetical gene	-1.76	0.1694
ssc-miR-92b-3p	KIAA1432	hypothetical gene	-1.76	0.1694
ssc-miR-92b-3p	KIAA1549L	KIAA1549 like [Source:VGNC Symbol;Acc:VGNC:89445]	-1.76	0.1694
ssc-miR-92b-3p	KIAA1644	hypothetical gene	-1.76	0.1694
ssc-miR-92b-3p	KIF1B	kinesin family member 1B [Source:VGNC Symbol;Acc:VGNC:89460]	-1.76	0.1694
ssc-miR-92b-3p	KIF2A	kinesin family member 2A [Source:VGNC Symbol;Acc:VGNC:89467]	-1.76	0.1694
ssc-miR-92b-3p	KIF3B	kinesin family member 3B [Source:VGNC Symbol;Acc:VGNC:96392]	-1.76	0.1694
ssc-miR-92b-3p	KIF5B	kinesin family member 5B [Source:VGNC Symbol;Acc:VGNC:96393]	-1.76	0.1694
ssc-miR-92b-3p	KLF2	Kruppel like factor 2 [Source:VGNC Symbol;Acc:VGNC:89497]	-1.76	0.1694
ssc-miR-92b-3p	KLF3	Kruppel like factor 3 [Source:VGNC Symbol;Acc:VGNC:89498]	-1.76	0.1694

ssc-miR-92b-3p	KLF4	Kruppel like factor 4 [Source:VGNC Symbol;Acc:VGNC:98062]	-1.76	0.1694
ssc-miR-92b-3p	KLF6	Kruppel like factor 6 [Source:VGNC Symbol;Acc:VGNC:98063]	-1.76	0.1694
ssc-miR-92b-3p	KLHDC10	kelch domain containing 10 [Source:VGNC Symbol;Acc:VGNC:89502]	-1.76	0.1694
ssc-miR-92b-3p	KLHL11	kelch like family member 11 [Source:VGNC Symbol;Acc:VGNC:89513]	-1.76	0.1694
ssc-miR-92b-3p	KLHL14	kelch like family member 14 [Source:VGNC Symbol;Acc:VGNC:89515]	-1.76	0.1694
ssc-miR-92b-3p	KLHL15	kelch like family member 15 [Source:VGNC Symbol;Acc:VGNC:89516]	-1.76	0.1694
ssc-miR-92b-3p	KLHL18	kelch like family member 18 [Source:VGNC Symbol;Acc:VGNC:89517]	-1.76	0.1694
ssc-miR-92b-3p	KLHL29	kelch like family member 29 [Source:VGNC Symbol;Acc:VGNC:89524]	-1.76	0.1694
ssc-miR-92b-3p	KLHL3	kelch like family member 3 [Source:VGNC Symbol;Acc:VGNC:89525]	-1.76	0.1694
ssc-miR-92b-3p	KLHL31	kelch like family member 31 [Source:VGNC Symbol;Acc:VGNC:89526]	-1.76	0.1694
ssc-miR-92b-3p	KMT2E	lysine methyltransferase 2E (inactive) [Source:VGNC Symbol;Acc:VGNC:89551]	-1.76	0.1694
ssc-miR-92b-3p	KPNA5	karyopherin subunit alpha 5 [Source:VGNC Symbol;Acc:VGNC:89564]	-1.76	0.1694
ssc-miR-92b-3p	KRT1	keratin 1 [Source:VGNC Symbol;Acc:VGNC:89576]	-1.76	0.1694
ssc-miR-92b-3p	LATS2	large tumor suppressor kinase 2 [Source:VGNC Symbol;Acc:VGNC:89649]	-1.76	0.1694
ssc-miR-92b-3p	LBX1	ladybird homeobox 1 [Source:VGNC Symbol;Acc:VGNC:89652]	-1.76	0.1694
ssc-miR-92b-3p	LCOR	ligand dependent nuclear receptor corepressor [Source:HGNC Symbol;Acc:HGNC:29503]	-1.76	0.1694
ssc-miR-92b-3p	LDLRAD4	low density lipoprotein receptor class A domain containing 4 [Source:VGNC Symbol;Acc:VGNC:89673]	-1.76	0.1694
ssc-miR-92b-3p	LEPREL2	hypothetical gene	-1.76	0.1694
ssc-miR-92b-3p	LGALSL	galectin like [Source:VGNC Symbol;Acc:VGNC:89698]	-1.76	0.1694
ssc-miR-92b-3p	LHFPL2	LHFPL tetraspan subfamily member 2 [Source:VGNC Symbol;Acc:VGNC:89705]	-1.76	0.1694
ssc-miR-92b-3p	LIN28A	lin-28 homolog A [Source:VGNC Symbol;Acc:VGNC:98492]	-1.76	0.1694
ssc-miR-92b-3p	LIN54	lin-54 DREAM MuvB core complex component [Source:VGNC Symbol;Acc:VGNC:98072]	-1.76	0.1694
ssc-miR-92b-3p	LMBR1L	limb development membrane protein 1 like [Source:VGNC Symbol;Acc:VGNC:89753]	-1.76	0.1694
ssc-miR-92b-3p	LMO2	LIM domain only 2 [Source:VGNC Symbol;Acc:VGNC:89765]	-1.76	0.1694
ssc-miR-92b-3p	LNX2	ligand of numb-protein X 2 [Source:VGNC Symbol;Acc:VGNC:89774]	-1.76	0.1694
ssc-miR-92b-3p	LONRF3	LON peptidase N-terminal domain and ring finger 3 [Source:VGNC Symbol;Acc:VGNC:89778]	-1.76	0.1694
ssc-miR-92b-3p	LPIN1	lipin 1 [Source:VGNC Symbol;Acc:VGNC:89793]	-1.76	0.1694
ssc-miR-92b-3p	LPIN2	lipin 2 [Source:VGNC Symbol;Acc:VGNC:89794]	-1.76	0.1694
ssc-miR-92b-3p	LPP	LIM domain containing preferred translocation partner in lipoma [Source:HGNC Symbol;Acc:HGNC:6679]	-1.76	0.1694
ssc-miR-92b-3p	LRCH1	leucine rich repeats and calponin homology domain containing 1 [Source:VGNC Symbol;Acc:VGNC:89801]	-1.76	0.1694
ssc-miR-92b-3p	LRP1B	LDL receptor related protein 1B [Source:HGNC Symbol;Acc:HGNC:6693]	-1.76	0.1694
ssc-miR-92b-3p	LRRC1	leucine rich repeat containing 1 [Source:VGNC Symbol;Acc:VGNC:103977]	-1.76	0.1694
ssc-miR-92b-3p	LRRC20	leucine rich repeat containing 20 [Source:VGNC Symbol;Acc:VGNC:89830]	-1.76	0.1694
ssc-miR-92b-3p	LRRC4	leucine rich repeat containing 4 [Source:VGNC Symbol;Acc:VGNC:89839]	-1.76	0.1694
ssc-miR-92b-3p	LRRC8B	leucine rich repeat containing 8 VRAC subunit B [Source:VGNC Symbol;Acc:VGNC:98086]	-1.76	0.1694
ssc-miR-92b-3p	LRRFIP2	LRR binding FLII interacting protein 2 [Source:VGNC Symbol;Acc:VGNC:89858]	-1.76	0.1694
ssc-miR-92b-3p	LURAP1L	leucine rich adaptor protein 1 like [Source:VGNC Symbol;Acc:VGNC:89896]	-1.76	0.1694
ssc-miR-92b-3p	LUZP1	leucine zipper protein 1 [Source:VGNC Symbol;Acc:VGNC:89897]	-1.76	0.1694
ssc-miR-92b-3p	LYST	lysosomal trafficking regulator [Source:VGNC Symbol;Acc:VGNC:89921]	-1.76	0.1694
ssc-miR-92b-3p	MAFK	MAF bZIP transcription factor K [Source:HGNC Symbol;Acc:HGNC:6782]	-1.76	0.1694
ssc-miR-92b-3p	MAN2A1	mannosidase alpha class 2A member 1 [Source:VGNC Symbol;Acc:VGNC:98098]	-1.76	0.1694
ssc-miR-92b-3p	MAP1B	microtubule associated protein 1B [Source:VGNC Symbol;Acc:VGNC:89979]	-1.76	0.1694
ssc-miR-92b-3p	MAP2K4	mitogen-activated protein kinase kinase 4 [Source:VGNC Symbol;Acc:VGNC:98101]	-1.76	0.1694
ssc-miR-92b-3p	MAP4K2	mitogen-activated protein kinase kinase kinase kinase 2 [Source:VGNC Symbol;Acc:VGNC:98112]	-1.76	0.1694
ssc-miR-92b-3p	MAPK8	mitogen-activated protein kinase 8 [Source:VGNC Symbol;Acc:VGNC:90006]	-1.76	0.1694
ssc-miR-92b-3p	MARCH1	hypothetical gene	-1.76	0.1694
ssc-miR-92b-3p	MARCH3	hypothetical gene	-1.76	0.1694
ssc-miR-92b-3p	MARCH4	hypothetical gene	-1.76	0.1694
ssc-miR-92b-3p	MARCH8	hypothetical gene	-1.76	0.1694
ssc-miR-92b-3p	MARK1	microtubule affinity regulating kinase 1 [Source:VGNC Symbol;Acc:VGNC:96411]	-1.76	0.1694

ssc-miR-92b-3p	MAT2B	methionine adenosyltransferase 2B [Source:VGNC Symbol;Acc:VGNC:90040]	-1.76	0.1694
ssc-miR-92b-3p	MBD2	methyl-CpG binding domain protein 2 [Source:VGNC Symbol;Acc:VGNC:90048]	-1.76	0.1694
ssc-miR-92b-3p	MBNL3	muscleblind like splicing regulator 3 [Source:VGNC Symbol;Acc:VGNC:90056]	-1.76	0.1694
ssc-miR-92b-3p	MBOAT2	membrane bound O-acyltransferase domain containing 2 [Source:VGNC Symbol;Acc:VGNC:90058]	-1.76	0.1694
ssc-miR-92b-3p	MCFD2	multiple coagulation factor deficiency 2, ER cargo receptor complex subunit [Source:VGNC Symbol;Acc:VGNC:103980]	-1.76	0.1694
ssc-miR-92b-3p	MCOLN2	mucolipin TRP cation channel 2 [Source:VGNC Symbol;Acc:VGNC:90080]	-1.76	0.1694
ssc-miR-92b-3p	MED29	hypothetical gene	-1.76	0.1694
ssc-miR-92b-3p	MEF2D	myocyte enhancer factor 2D [Source:VGNC Symbol;Acc:VGNC:90128]	-1.76	0.1694
ssc-miR-92b-3p	METAP1	methionyl aminopeptidase 1 [Source:VGNC Symbol;Acc:VGNC:90151]	-1.76	0.1694
ssc-miR-92b-3p	MFHAS1	multifunctional ROCO family signaling regulator 1 [Source:VGNC Symbol;Acc:VGNC:107361]	-1.76	0.1694
ssc-miR-92b-3p	MGLL	monoglyceride lipase [Source:VGNC Symbol;Acc:VGNC:90199]	-1.76	0.1694
ssc-miR-92b-3p	MIA3	hypothetical gene	-1.76	0.1694
ssc-miR-92b-3p	MIER3	MIER family member 3 [Source:VGNC Symbol;Acc:VGNC:90221]	-1.76	0.1694
ssc-miR-92b-3p	MITF	melanocyte inducing transcription factor [Source:VGNC Symbol;Acc:VGNC:90243]	-1.76	0.1694
ssc-miR-92b-3p	MLLT10	MLLT10 histone lysine methyltransferase DOT1L cofactor [Source:VGNC Symbol;Acc:VGNC:96590]	-1.76	0.1694
ssc-miR-92b-3p	MLLT3	MLLT3 super elongation complex subunit [Source:VGNC Symbol;Acc:VGNC:90256]	-1.76	0.1694
ssc-miR-92b-3p	MMD	monocyte to macrophage differentiation associated [Source:VGNC Symbol;Acc:VGNC:90264]	-1.76	0.1694
ssc-miR-92b-3p	MMP16	matrix metallopeptidase 16 [Source:VGNC Symbol;Acc:VGNC:90271]	-1.76	0.1694
ssc-miR-92b-3p	MOAP1	hypothetical gene	-1.76	0.1694
ssc-miR-92b-3p	MOB4	hypothetical gene	-1.76	0.1694
ssc-miR-92b-3p	MOCS2	molybdenum cofactor synthesis 2 [Source:VGNC Symbol;Acc:VGNC:90295]	-1.76	0.1694
ssc-miR-92b-3p	MORC3	MORC family CW-type zinc finger 3 [Source:VGNC Symbol;Acc:VGNC:90304]	-1.76	0.1694
ssc-miR-92b-3p	MPP1	hypothetical gene	-1.76	0.1694
ssc-miR-92b-3p	MRPS25	mitochondrial ribosomal protein S25 [Source:VGNC Symbol;Acc:VGNC:90391]	-1.76	0.1694
ssc-miR-92b-3p	MRS2	magnesium transporter MRS2 [Source:VGNC Symbol;Acc:VGNC:90403]	-1.76	0.1694
ssc-miR-92b-3p	MS4A2	membrane spanning 4-domains A2 [Source:VGNC Symbol;Acc:VGNC:90411]	-1.76	0.1694
ssc-miR-92b-3p	MSRB3	methionine sulfoxide reductase B3 [Source:VGNC Symbol;Acc:VGNC:90427]	-1.76	0.1694
ssc-miR-92b-3p	MTDH	metadherin [Source:VGNC Symbol;Acc:VGNC:90439]	-1.76	0.1694
ssc-miR-92b-3p	MTF1	metal regulatory transcription factor 1 [Source:VGNC Symbol;Acc:VGNC:90443]	-1.76	0.1694
ssc-miR-92b-3p	MTF2	metal response element binding transcription factor 2 [Source:VGNC Symbol;Acc:VGNC:90444]	-1.76	0.1694
ssc-miR-92b-3p	MTHFD2	methylenetetrahydrofolate dehydrogenase (NADP+ dependent) 2, methenyltetrahydrofolate cyclohydrolase [Source:HGNC Symbol;Acc:HGNC:7434]	-1.76	0.1694
ssc-miR-92b-3p	MTMR7	myotubularin related protein 7 [Source:VGNC Symbol;Acc:VGNC:108726]	-1.76	0.1694
ssc-miR-92b-3p	MTMR9	myotubularin related protein 9 [Source:VGNC Symbol;Acc:VGNC:90464]	-1.76	0.1694
ssc-miR-92b-3p	MYCBP2	MYC binding protein 2 [Source:VGNC Symbol;Acc:VGNC:90502]	-1.76	0.1694
ssc-miR-92b-3p	MYH9	myosin heavy chain 9 [Source:VGNC Symbol;Acc:VGNC:90512]	-1.76	0.1694
ssc-miR-92b-3p	MYLIP	myosin regulatory light chain interacting protein [Source:VGNC Symbol;Acc:VGNC:103999]	-1.76	0.1694
ssc-miR-92b-3p	MYLK	myosin light chain kinase [Source:VGNC Symbol;Acc:VGNC:108676]	-1.76	0.1694
ssc-miR-92b-3p	MYO10	myosin X [Source:VGNC Symbol;Acc:VGNC:90522]	-1.76	0.1694
ssc-miR-92b-3p	MYO18A	myosin XVIIIA [Source:HGNC Symbol;Acc:HGNC:31104]	-1.76	0.1694
ssc-miR-92b-3p	MYO1B	myosin IB [Source:VGNC Symbol;Acc:VGNC:96429]	-1.76	0.1694
ssc-miR-92b-3p	MYO5A	myosin VA [Source:HGNC Symbol;Acc:HGNC:7602]	-1.76	0.1694
ssc-miR-92b-3p	MYT1L	myelin transcription factor 1 like [Source:VGNC Symbol;Acc:VGNC:90549]	-1.76	0.1694
ssc-miR-92b-3p	N4BP2	NEDD4 binding protein 2 [Source:VGNC Symbol;Acc:VGNC:90554]	-1.76	0.1694
ssc-miR-92b-3p	NAA15	N-alpha-acetyltransferase 15, NatA auxiliary subunit [Source:VGNC Symbol;Acc:VGNC:96747]	-1.76	0.1694
ssc-miR-92b-3p	NAA50	N-alpha-acetyltransferase 50, NatE catalytic subunit [Source:VGNC Symbol;Acc:VGNC:104002]	-1.76	0.1694
ssc-miR-92b-3p	NACC2	NACC family member 2 [Source:VGNC Symbol;Acc:VGNC:90564]	-1.76	0.1694
ssc-miR-92b-3p	NAV3	neuron navigator 3 [Source:VGNC Symbol;Acc:VGNC:90589]	-1.76	0.1694
ssc-miR-92b-3p	NBEAL1	cytochrome P450 family 20 subfamily A member 1 [Source:VGNC Symbol;Acc:VGNC:103366]	-1.76	0.1694
ssc-miR-92b-3p	NCKAP5	NCK associated protein 5 [Source:HGNC Symbol;Acc:HGNC:29847]	-1.76	0.1694
ssc-miR-92b-3p	NCOA1	nuclear receptor coactivator 1 [Source:VGNC Symbol;Acc:VGNC:90615]	-1.76	0.1694

ssc-miR-92b-3p	NCOA3	nuclear receptor coactivator 3 [Source:VGNC Symbol;Acc:VGNC:96434]	-1.76	0.1694
ssc-miR-92b-3p	NECAP1	NECAP endocytosis associated 1 [Source:VGNC Symbol;Acc:VGNC:90660]	-1.76	0.1694
ssc-miR-92b-3p	NEDD4L	NEDD4 like E3 ubiquitin protein ligase [Source:VGNC Symbol;Acc:VGNC:90667]	-1.76	0.1694
ssc-miR-92b-3p	NEFH	neurofilament heavy chain [Source:VGNC Symbol;Acc:VGNC:90669]	-1.76	0.1694
ssc-miR-92b-3p	NEFM	hypothetical gene	-1.76	0.1694
ssc-miR-92b-3p	NEGR1	neuronal growth regulator 1 [Source:VGNC Symbol;Acc:VGNC:90671]	-1.76	0.1694
ssc-miR-92b-3p	NEO1	neogenin 1 [Source:HGNC Symbol;Acc:HGNC:7754]	-1.76	0.1694
ssc-miR-92b-3p	NETO1	neuropilin and tolloid like 1 [Source:VGNC Symbol;Acc:VGNC:90690]	-1.76	0.1694
ssc-miR-92b-3p	NEURL1B	neuralized E3 ubiquitin protein ligase 1B [Source:VGNC Symbol;Acc:VGNC:90695]	-1.76	0.1694
ssc-miR-92b-3p	NF1	neurofibromin 1 [Source:VGNC Symbol;Acc:VGNC:90704]	-1.76	0.1694
ssc-miR-92b-3p	NF2	NF2, moesin-ezrin-radixin like (MERLIN) tumor suppressor [Source:VGNC Symbol;Acc:VGNC:90705]	-1.76	0.1694
ssc-miR-92b-3p	NFAT5	nuclear factor of activated T cells 5 [Source:VGNC Symbol;Acc:VGNC:90708]	-1.76	0.1694
ssc-miR-92b-3p	NFATC2IP	nuclear factor of activated T cells 2 interacting protein [Source:VGNC Symbol;Acc:VGNC:90709]	-1.76	0.1694
ssc-miR-92b-3p	NFE2L1	NFE2 like bZIP transcription factor 1 [Source:VGNC Symbol;Acc:VGNC:90713]	-1.76	0.1694
ssc-miR-92b-3p	NFIA	nuclear factor I A [Source:VGNC Symbol;Acc:VGNC:90715]	-1.76	0.1694
ssc-miR-92b-3p	NFIB	nuclear factor I B [Source:VGNC Symbol;Acc:VGNC:90716]	-1.76	0.1694
ssc-miR-92b-3p	NFIC	nuclear factor I C [Source:VGNC Symbol;Acc:VGNC:100313]	-1.76	0.1694
ssc-miR-92b-3p	NFIX	nuclear factor I X [Source:VGNC Symbol;Acc:VGNC:90718]	-1.76	0.1694
ssc-miR-92b-3p	NFYB	nuclear transcription factor Y subunit beta [Source:VGNC Symbol;Acc:VGNC:90730]	-1.76	0.1694
ssc-miR-92b-3p	NHLH2	nescient helix-loop-helix 2 [Source:VGNC Symbol;Acc:VGNC:90736]	-1.76	0.1694
ssc-miR-92b-3p	NIPAL1	NIPA like domain containing 1 [Source:VGNC Symbol;Acc:VGNC:90748]	-1.76	0.1694
ssc-miR-92b-3p	NIPBL	NIPBL cohesin loading factor [Source:VGNC Symbol;Acc:VGNC:90752]	-1.76	0.1694
ssc-miR-92b-3p	NKPD1	NTPase KAP family P-loop domain containing 1 [Source:VGNC Symbol;Acc:VGNC:98518]	-1.76	0.1694
ssc-miR-92b-3p	NKX2-3	NK2 homeobox 3 [Source:VGNC Symbol;Acc:VGNC:90768]	-1.76	0.1694
ssc-miR-92b-3p	NKX2-4	NK2 homeobox 4 [Source:VGNC Symbol;Acc:VGNC:96445]	-1.76	0.1694
ssc-miR-92b-3p	NLK	nemo like kinase [Source:VGNC Symbol;Acc:VGNC:90779]	-1.76	0.1694
ssc-miR-92b-3p	NOL4	nucleolar protein 4 [Source:VGNC Symbol;Acc:VGNC:90814]	-1.76	0.1694
ssc-miR-92b-3p	NOL7	nucleolar protein 7 [Source:VGNC Symbol;Acc:VGNC:90815]	-1.76	0.1694
ssc-miR-92b-3p	NOMO1	hypothetical gene	-1.76	0.1694
ssc-miR-92b-3p	NOMO2	hypothetical gene	-1.76	0.1694
ssc-miR-92b-3p	NOTCH1	notch receptor 1 [Source:HGNC Symbol;Acc:HGNC:7881]	-1.76	0.1694
ssc-miR-92b-3p	NOV	hypothetical gene	-1.76	0.1694
ssc-miR-92b-3p	NOVA1	NOVA alternative splicing regulator 1 [Source:VGNC Symbol;Acc:VGNC:90827]	-1.76	0.1694
ssc-miR-92b-3p	NOVA2	NOVA alternative splicing regulator 2 [Source:VGNC Symbol;Acc:VGNC:90828]	-1.76	0.1694
ssc-miR-92b-3p	NPAS3	neuronal PAS domain protein 3 [Source:VGNC Symbol;Acc:VGNC:90838]	-1.76	0.1694
ssc-miR-92b-3p	NPC1	NPC intracellular cholesterol transporter 1 [Source:VGNC Symbol;Acc:VGNC:98521]	-1.76	0.1694
ssc-miR-92b-3p	NPNT	nephronectin [Source:VGNC Symbol;Acc:VGNC:90855]	-1.76	0.1694
ssc-miR-92b-3p	NPTN	neuroplastin [Source:VGNC Symbol;Acc:VGNC:90861]	-1.76	0.1694
ssc-miR-92b-3p	NR4A3	nuclear receptor subfamily 4 group A member 3 [Source:VGNC Symbol;Acc:VGNC:90885]	-1.76	0.1694
ssc-miR-92b-3p	NRAS	NRAS proto-onco, GTPase [Source:VGNC Symbol;Acc:VGNC:98827]	-1.76	0.1694
ssc-miR-92b-3p	NRF1	nuclear respiratory factor 1 [Source:VGNC Symbol;Acc:VGNC:90895]	-1.76	0.1694
ssc-miR-92b-3p	NRG1	neuregulin 1 [Source:VGNC Symbol;Acc:VGNC:96453]	-1.76	0.1694
ssc-miR-92b-3p	NRK	Nik related kinase [Source:VGNC Symbol;Acc:VGNC:98161]	-1.76	0.1694
ssc-miR-92b-3p	NRP1	neuropilin 1 [Source:VGNC Symbol;Acc:VGNC:104012]	-1.76	0.1694
ssc-miR-92b-3p	NSF	N-ethylmaleimide sensitive factor, vesicle fusing ATPase [Source:VGNC Symbol;Acc:VGNC:90906]	-1.76	0.1694
ssc-miR-92b-3p	NSG1	neuronal vesicle trafficking associated 1 [Source:VGNC Symbol;Acc:VGNC:90907]	-1.76	0.1694
ssc-miR-92b-3p	NSMAF	neutral sphingomyelinase activation associated factor [Source:VGNC Symbol;Acc:VGNC:90909]	-1.76	0.1694
ssc-miR-92b-3p	NSMF	NMDA receptor synaptonuclear signaling and neuronal migration factor [Source:VGNC Symbol;Acc:VGNC:90912]	-1.76	0.1694
ssc-miR-92b-3p	NSUN3	NOP2/Sun RNA methyltransferase 3 [Source:VGNC Symbol;Acc:VGNC:90915]	-1.76	0.1694
ssc-miR-92b-3p	NUCKS1	nuclear casein kinase and cyclin dependent kinase substrate 1 [Source:VGNC Symbol;Acc:VGNC:90947]	-1.76	0.1694

ssc-miR-92b-3p	NUDCD3	NudC domain containing 3 [Source:VGNC Symbol;Acc:VGNC:90951]	-1.76	0.1694
ssc-miR-92b-3p	NUFIP2	nuclear FMR1 interacting protein 2 [Source:VGNC Symbol;Acc:VGNC:90967]	-1.76	0.1694
ssc-miR-92b-3p	NUP43	nucleoporin 43 [Source:VGNC Symbol;Acc:VGNC:90982]	-1.76	0.1694
ssc-miR-92b-3p	NUTF2	nuclear transport factor 2 [Source:VGNC Symbol;Acc:VGNC:98524]	-1.76	0.1694
ssc-miR-92b-3p	NXPE3	neurexophilin and PC-esterase domain family member 3 [Source:VGNC Symbol;Acc:VGNC:90997]	-1.76	0.1694
ssc-miR-92b-3p	OAZ3	ornithine decarboxylase antizyme 3 [Source:VGNC Symbol;Acc:VGNC:91010]	-1.76	0.1694
ssc-miR-92b-3p	OLFM3	olfactomedin 3 [Source:VGNC Symbol;Acc:VGNC:91033]	-1.76	0.1694
ssc-miR-92b-3p	OPCML	opioid binding protein/cell adhesion molecule like [Source:VGNC Symbol;Acc:VGNC:91047]	-1.76	0.1694
ssc-miR-92b-3p	OSBPL5	oxysterol binding protein like 5 [Source:VGNC Symbol;Acc:VGNC:91072]	-1.76	0.1694
ssc-miR-92b-3p	OSBPL8	oxysterol binding protein like 8 [Source:VGNC Symbol;Acc:VGNC:91074]	-1.76	0.1694
ssc-miR-92b-3p	OSER1	oxidative stress responsive serine rich 1 [Source:VGNC Symbol;Acc:VGNC:95738]	-1.76	0.1694
ssc-miR-92b-3p	OTUD3	OTU deubiquitinase 3 [Source:VGNC Symbol;Acc:VGNC:91099]	-1.76	0.1694
ssc-miR-92b-3p	OTUD4	OTU deubiquitinase 4 [Source:VGNC Symbol;Acc:VGNC:91100]	-1.76	0.1694
ssc-miR-92b-3p	OXSR1	hypothetical gene	-1.76	0.1694
ssc-miR-92b-3p	PAFAH1B1	platelet activating factor acetylhydrolase 1b regulatory subunit 1 [Source:VGNC Symbol;Acc:VGNC:99026]	-1.76	0.1694
ssc-miR-92b-3p	PAIP1	poly(A) binding protein interacting protein 1 [Source:HGNC Symbol;Acc:HGNC:16945]	-1.76	0.1694
ssc-miR-92b-3p	PALLD	palladin, cytoskeletal associated protein [Source:HGNC Symbol;Acc:HGNC:17068]	-1.76	0.1694
ssc-miR-92b-3p	PALM2	hypothetical gene	-1.76	0.1694
ssc-miR-92b-3p	PANK3	pantothenate kinase 3 [Source:VGNC Symbol;Acc:VGNC:91165]	-1.76	0.1694
ssc-miR-92b-3p	PANX1	pannexin 1 [Source:VGNC Symbol;Acc:VGNC:91166]	-1.76	0.1694
ssc-miR-92b-3p	PAPD4	hypothetical gene	-1.76	0.1694
ssc-miR-92b-3p	PAPD5	hypothetical gene	-1.76	0.1694
ssc-miR-92b-3p	PAPD7	hypothetical gene	-1.76	0.1694
ssc-miR-92b-3p	PAPOLA	poly(A) polymerase alpha [Source:HGNC Symbol;Acc:HGNC:14981]	-1.76	0.1694
ssc-miR-92b-3p	PAQR3	progestin and adipoQ receptor family member 3 [Source:VGNC Symbol;Acc:VGNC:91174]	-1.76	0.1694
ssc-miR-92b-3p	PARP8	poly(ADP-ribose) polymerase family member 8 [Source:HGNC Symbol;Acc:HGNC:26124]	-1.76	0.1694
ssc-miR-92b-3p	PAWR	pro-apoptotic WT1 regulator [Source:VGNC Symbol;Acc:VGNC:91191]	-1.76	0.1694
ssc-miR-92b-3p	PAX3	paired box 3 [Source:VGNC Symbol;Acc:VGNC:96468]	-1.76	0.1694
ssc-miR-92b-3p	PAX5	paired box 5 [Source:VGNC Symbol;Acc:VGNC:91194]	-1.76	0.1694
ssc-miR-92b-3p	PAX9	paired box 9 [Source:HGNC Symbol;Acc:HGNC:8623]	-1.76	0.1694
ssc-miR-92b-3p	PAXBP1	PAX3 and PAX7 binding protein 1 [Source:VGNC Symbol;Acc:VGNC:91197]	-1.76	0.1694
ssc-miR-92b-3p	PCDH1	protocadherin 1 [Source:VGNC Symbol;Acc:VGNC:91211]	-1.76	0.1694
ssc-miR-92b-3p	PCDH10	protocadherin 10 [Source:HGNC Symbol;Acc:HGNC:13404]	-1.76	0.1694
ssc-miR-92b-3p	PCDH11X	hypothetical gene	-1.76	0.1694
ssc-miR-92b-3p	PCDH11Y	hypothetical gene	-1.76	0.1694
ssc-miR-92b-3p	PCDH20	protocadherin 20 [Source:HGNC Symbol;Acc:HGNC:14257]	-1.76	0.1694
ssc-miR-92b-3p	PCDH7	protocadherin 7 [Source:NCBI gene (formerly Entrezgene);Acc:100520035]	-1.76	0.1694
ssc-miR-92b-3p	PCGF3	polycomb group ring finger 3 [Source:VGNC Symbol;Acc:VGNC:91220]	-1.76	0.1694
ssc-miR-92b-3p	PCMTD1	protein-L-isoaspartate (D-aspartate) O-methyltransferase domain containing 1 [Source:VGNC Symbol;Acc:VGNC:98830]	-1.76	0.1694
ssc-miR-92b-3p	PCOLCE2	procollagen C-endopeptidase enhancer 2 [Source:HGNC Symbol;Acc:HGNC:8739]	-1.76	0.1694
ssc-miR-92b-3p	PCYT1B	phosphate cytidylyltransferase 1B, choline [Source:VGNC Symbol;Acc:VGNC:91237]	-1.76	0.1694
ssc-miR-92b-3p	PDCD6IP	hypothetical gene	-1.76	0.1694
ssc-miR-92b-3p	PDE10A	phosphodiesterase 10A [Source:VGNC Symbol;Acc:VGNC:91248]	-1.76	0.1694
ssc-miR-92b-3p	PDE3B	phosphodiesterase 3B [Source:VGNC Symbol;Acc:VGNC:91253]	-1.76	0.1694
ssc-miR-92b-3p	PDE8A	phosphodiesterase 8A [Source:VGNC Symbol;Acc:VGNC:91263]	-1.76	0.1694
ssc-miR-92b-3p	PDHB	pyruvate dehydrogenase E1 subunit beta [Source:VGNC Symbol;Acc:VGNC:91269]	-1.76	0.1694
ssc-miR-92b-3p	PDP2	pyruvate dehyrogenase phosphatase catalytic subunit 2 [Source:VGNC Symbol;Acc:VGNC:91287]	-1.76	0.1694
ssc-miR-92b-3p	PDS5A	PDS5 cohesin associated factor A [Source:HGNC Symbol;Acc:HGNC:29088]	-1.76	0.1694
ssc-miR-92b-3p	PDS5B	PDS5 cohesin associated factor B [Source:VGNC Symbol;Acc:VGNC:91290]	-1.76	0.1694
ssc-miR-92b-3p	PDXDC1	pyridoxal dependent decarboxylase domain containing 1 [Source:HGNC Symbol;Acc:HGNC:28995]	-1.76	0.1694

ssc-miR-92b-3p	PDZD2	hypothetical gene	-1.76	0.1694
ssc-miR-92b-3p	PDZD8	hypothetical gene	-1.76	0.1694
ssc-miR-92b-3p	PEAK1	pseudopodium enriched atypical kinase 1 [Source:VGNC Symbol;Acc:VGNC:91300]	-1.76	0.1694
ssc-miR-92b-3p	PER2	period circadian regulator 2 [Source:VGNC Symbol;Acc:VGNC:96478]	-1.76	0.1694
ssc-miR-92b-3p	PEX3	peroxisomal biosis factor 3 [Source:VGNC Symbol;Acc:VGNC:91327]	-1.76	0.1694
ssc-miR-92b-3p	PEX5L	peroxisomal biosis factor 5 like [Source:VGNC Symbol;Acc:VGNC:91329]	-1.76	0.1694
ssc-miR-92b-3p	PGAM1	phosphoglycerate mutase 1 [Source:HGNC Symbol;Acc:HGNC:8888]	-1.76	0.1694
ssc-miR-92b-3p	PHF15	hypothetical gene	-1.76	0.1694
ssc-miR-92b-3p	PHF3	PHD finger protein 3 [Source:VGNC Symbol;Acc:VGNC:91387]	-1.76	0.1694
ssc-miR-92b-3p	PHLDA1	pleckstrin homology like domain family A member 1 [Source:HGNC Symbol;Acc:HGNC:8933]	-1.76	0.1694
ssc-miR-92b-3p	PHLDB2	pleckstrin homology like domain family B member 2 [Source:VGNC Symbol;Acc:VGNC:91399]	-1.76	0.1694
ssc-miR-92b-3p	PHLPP2	PH domain and leucine rich repeat protein phosphatase 2 [Source:VGNC Symbol;Acc:VGNC:98186]	-1.76	0.1694
ssc-miR-92b-3p	PHTF2	putative homeodomain transcription factor 2 [Source:VGNC Symbol;Acc:VGNC:91405]	-1.76	0.1694
ssc-miR-92b-3p	PIAS4	protein inhibitor of activated STAT 4 [Source:VGNC Symbol;Acc:VGNC:91413]	-1.76	0.1694
ssc-miR-92b-3p	PIK3AP1	phosphoinositide-3-kinase adaptor protein 1 [Source:VGNC Symbol;Acc:VGNC:91435]	-1.76	0.1694
ssc-miR-92b-3p	PIK3CA	phosphatidylinositol-4,5-bisphosphate 3-kinase catalytic subunit alpha [Source:VGNC Symbol;Acc:VGNC:91440]	-1.76	0.1694
ssc-miR-92b-3p	PIK3CB	phosphatidylinositol-4,5-bisphosphate 3-kinase catalytic subunit beta [Source:VGNC Symbol;Acc:VGNC:91441]	-1.76	0.1694
ssc-miR-92b-3p	PIK3R3	phosphoinositide-3-kinase regulatory subunit 3 [Source:HGNC Symbol;Acc:HGNC:8981]	-1.76	0.1694
ssc-miR-92b-3p	PIKFYVE	phosphoinositide kinase, FYVE-type zinc finger containing [Source:VGNC Symbol;Acc:VGNC:96023]	-1.76	0.1694
ssc-miR-92b-3p	PIP4K2C	phosphatidylinositol-5-phosphate 4-kinase type 2 gamma [Source:VGNC Symbol;Acc:VGNC:91455]	-1.76	0.1694
ssc-miR-92b-3p	PIP5K1C	phosphatidylinositol-4-phosphate 5-kinase type 1 gamma [Source:VGNC Symbol;Acc:VGNC:91459]	-1.76	0.1694
ssc-miR-92b-3p	PITPNA	phosphatidylinositol transfer protein alpha [Source:VGNC Symbol;Acc:VGNC:91464]	-1.76	0.1694
ssc-miR-92b-3p	PITPNC1	phosphatidylinositol transfer protein cytoplasmic 1 [Source:NCBI gene (formerly Entrezgene);Acc:100233199]	-1.76	0.1694
ssc-miR-92b-3p	PITPNM2	phosphatidylinositol transfer protein membrane associated 2 [Source:VGNC Symbol;Acc:VGNC:91466]	-1.76	0.1694
ssc-miR-92b-3p	PKDCC	protein kinase domain containing, cytoplasmic [Source:VGNC Symbol;Acc:VGNC:91475]	-1.76	0.1694
ssc-miR-92b-3p	PLD1	phospholipase D1 [Source:VGNC Symbol;Acc:VGNC:91525]	-1.76	0.1694
ssc-miR-92b-3p	PLEKHA1	pleckstrin homology domain containing A1 [Source:VGNC Symbol;Acc:VGNC:91533]	-1.76	0.1694
ssc-miR-92b-3p	PLEKHA6	pleckstrin homology domain containing A6 [Source:VGNC Symbol;Acc:VGNC:91536]	-1.76	0.1694
ssc-miR-92b-3p	PLEKHB2	pleckstrin homology domain containing B2 [Source:VGNC Symbol;Acc:VGNC:95907]	-1.76	0.1694
ssc-miR-92b-3p	PLEKHG3	pleckstrin homology and RhoGEF domain containing G3 [Source:VGNC Symbol;Acc:VGNC:91544]	-1.76	0.1694
ssc-miR-92b-3p	PLEKHM1	pleckstrin homology and RUN domain containing M1 [Source:VGNC Symbol;Acc:VGNC:91552]	-1.76	0.1694
ssc-miR-92b-3p	PLXDC2	plexin domain containing 2 [Source:VGNC Symbol;Acc:VGNC:95958]	-1.76	0.1694
ssc-miR-92b-3p	PMEPA1	prostate transmembrane protein, androgen induced 1 [Source:VGNC Symbol;Acc:VGNC:95606]	-1.76	0.1694
ssc-miR-92b-3p	PNISR	PNN interacting serine and arginine rich protein [Source:VGNC Symbol;Acc:VGNC:91594]	-1.76	0.1694
ssc-miR-92b-3p	POFUT2	hypothetical gene	-1.76	0.1694
ssc-miR-92b-3p	POLK	DNA polymerase kappa [Source:VGNC Symbol;Acc:VGNC:91637]	-1.76	0.1694
ssc-miR-92b-3p	PPAP2B	hypothetical gene	-1.76	0.1694
ssc-miR-92b-3p	PPARGC1B	PPARG coactivator 1 beta [Source:VGNC Symbol;Acc:VGNC:91686]	-1.76	0.1694
ssc-miR-92b-3p	PPCS	phosphopantothenoylcysteine synthetase [Source:HGNC Symbol;Acc:HGNC:25686]	-1.76	0.1694
ssc-miR-92b-3p	PPIP5K2	diphosphoinositol pentakisphosphate kinase 2 [Source:VGNC Symbol;Acc:VGNC:91701]	-1.76	0.1694
ssc-miR-92b-3p	PPM1A	protein phosphatase, Mg2+/Mn2+ dependent 1A [Source:VGNC Symbol;Acc:VGNC:91703]	-1.76	0.1694
ssc-miR-92b-3p	PPM1H	protein phosphatase, Mg2+/Mn2+ dependent 1H [Source:VGNC Symbol;Acc:VGNC:91708]	-1.76	0.1694
ssc-miR-92b-3p	PPP1R12A	protein phosphatase 1 regulatory subunit 12A [Source:VGNC Symbol;Acc:VGNC:91719]	-1.76	0.1694
ssc-miR-92b-3p	PPP1R12C	protein phosphatase 1 regulatory subunit 12C [Source:VGNC Symbol;Acc:VGNC:91720]	-1.76	0.1694
ssc-miR-92b-3p	PPP1R37	protein phosphatase 1 regulatory subunit 37 [Source:VGNC Symbol;Acc:VGNC:91735]	-1.76	0.1694
ssc-miR-92b-3p	PPP1R3D	protein phosphatase 1 regulatory subunit 3D [Source:VGNC Symbol;Acc:VGNC:96500]	-1.76	0.1694
ssc-miR-92b-3p	PPP1R9A	protein phosphatase 1 regulatory subunit 9A [Source:VGNC Symbol;Acc:VGNC:91743]	-1.76	0.1694
ssc-miR-92b-3p	PPP6C	protein phosphatase 6 catalytic subunit [Source:VGNC Symbol;Acc:VGNC:98219]	-1.76	0.1694
ssc-miR-92b-3p	PRC1	protein regulator of cytokinesis 1 [Source:VGNC Symbol;Acc:VGNC:104036]	-1.76	0.1694
ssc-miR-92b-3p	PRDM13	PR/SET domain 13 [Source:VGNC Symbol;Acc:VGNC:91775]	-1.76	0.1694

ssc-miR-92b-3p	PRDM15	PR/SET domain 15 [Source:VGNC Symbol;Acc:VGNC:98220]	-1.76	0.1694
ssc-miR-92b-3p	PRDM16	PR/SET domain 16 [Source:VGNC Symbol;Acc:VGNC:91777]	-1.76	0.1694
ssc-miR-92b-3p	PRKAA1	protein kinase AMP-activated catalytic subunit alpha 1 [Source:VGNC Symbol;Acc:VGNC:91797]	-1.76	0.1694
ssc-miR-92b-3p	PRKAA2	protein kinase AMP-activated catalytic subunit alpha 2 [Source:VGNC Symbol;Acc:VGNC:91798]	-1.76	0.1694
ssc-miR-92b-3p	PRKAB2	protein kinase AMP-activated non-catalytic subunit beta 2 [Source:VGNC Symbol;Acc:VGNC:91799]	-1.76	0.1694
ssc-miR-92b-3p	PRKAR1A	protein kinase cAMP-dependent type I regulatory subunit alpha [Source:VGNC Symbol;Acc:VGNC:91802]	-1.76	0.1694
ssc-miR-92b-3p	PRKAR1B	protein kinase cAMP-dependent type I regulatory subunit beta [Source:VGNC Symbol;Acc:VGNC:91803]	-1.76	0.1694
ssc-miR-92b-3p	PRKAR2B	protein kinase cAMP-dependent type II regulatory subunit beta [Source:VGNC Symbol;Acc:VGNC:91805]	-1.76	0.1694
ssc-miR-92b-3p	PRKCE	protein kinase C epsilon [Source:VGNC Symbol;Acc:VGNC:91807]	-1.76	0.1694
ssc-miR-92b-3p	PROB1	proline rich basic protein 1 [Source:VGNC Symbol;Acc:VGNC:91827]	-1.76	0.1694
ssc-miR-92b-3p	PROX1	prospero homeobox 1 [Source:VGNC Symbol;Acc:VGNC:91837]	-1.76	0.1694
ssc-miR-92b-3p	PRPF40A	pre-mRNA processing factor 40 homolog A [Source:VGNC Symbol;Acc:VGNC:98222]	-1.76	0.1694
ssc-miR-92b-3p	PRR24	hypothetical gene	-1.76	0.1694
ssc-miR-92b-3p	PRRC2B	proline rich coiled-coil 2B [Source:VGNC Symbol;Acc:VGNC:91866]	-1.76	0.1694
ssc-miR-92b-3p	PRSS12	serine protease 12 [Source:VGNC Symbol;Acc:VGNC:91877]	-1.76	0.1694
ssc-miR-92b-3p	PSMD14	proteasome 26S subunit, non-ATPase 14 [Source:VGNC Symbol;Acc:VGNC:95823]	-1.76	0.1694
ssc-miR-92b-3p	PTAR1	protein prenyltransferase alpha subunit repeat containing 1 [Source:VGNC Symbol;Acc:VGNC:91936]	-1.76	0.1694
ssc-miR-92b-3p	PTEN	hypothetical gene	-1.76	0.1694
ssc-miR-92b-3p	PTF1A	pancreas associated transcription factor 1a [Source:VGNC Symbol;Acc:VGNC:96021]	-1.76	0.1694
ssc-miR-92b-3p	PTGER4	prostaglandin E receptor 4 [Source:VGNC Symbol;Acc:VGNC:91949]	-1.76	0.1694
ssc-miR-92b-3p	PTPRD	protein tyrosine phosphatase receptor type D [Source:HGNC Symbol;Acc:HGNC:9668]	-1.76	0.1694
ssc-miR-92b-3p	PTPRG	protein tyrosine phosphatase receptor type G [Source:VGNC Symbol;Acc:VGNC:91988]	-1.76	0.1694
ssc-miR-92b-3p	PTPRJ	protein tyrosine phosphatase receptor type J [Source:VGNC Symbol;Acc:VGNC:91990]	-1.76	0.1694
ssc-miR-92b-3p	PTPRK	protein tyrosine phosphatase receptor type K [Source:VGNC Symbol;Acc:VGNC:91991]	-1.76	0.1694
ssc-miR-92b-3p	PURG	purine rich element binding protein G [Source:VGNC Symbol;Acc:VGNC:95865]	-1.76	0.1694
ssc-miR-92b-3p	PVRL1	hypothetical gene	-1.76	0.1694
ssc-miR-92b-3p	PWWP2A	hypothetical gene	-1.76	0.1694
ssc-miR-92b-3p	QKI	QKI, KH domain containing RNA binding [Source:VGNC Symbol;Acc:VGNC:92025]	-1.76	0.1694
ssc-miR-92b-3p	QPCT	glutaminyl-peptide cyclotransferase [Source:VGNC Symbol;Acc:VGNC:92026]	-1.76	0.1694
ssc-miR-92b-3p	QSER1	glutamine and serine rich 1 [Source:VGNC Symbol;Acc:VGNC:92032]	-1.76	0.1694
ssc-miR-92b-3p	RAB11FIP2	RAB11 family interacting protein 2 [Source:VGNC Symbol;Acc:VGNC:92039]	-1.76	0.1694
ssc-miR-92b-3p	RAB14	RAB14, member RAS onco family [Source:VGNC Symbol;Acc:VGNC:98238]	-1.76	0.1694
ssc-miR-92b-3p	RAB23	RAB23, member RAS onco family [Source:VGNC Symbol;Acc:VGNC:98245]	-1.76	0.1694
ssc-miR-92b-3p	RAB3B	RAB3B, member RAS onco family [Source:VGNC Symbol;Acc:VGNC:98265]	-1.76	0.1694
ssc-miR-92b-3p	RAB3C	RAB3C, member RAS onco family [Source:VGNC Symbol;Acc:VGNC:104043]	-1.76	0.1694
ssc-miR-92b-3p	RAB8B	RAB8B, member RAS onco family [Source:VGNC Symbol;Acc:VGNC:98276]	-1.76	0.1694
ssc-miR-92b-3p	RABGAP1L	RAB GTPase activating protein 1 like [Source:VGNC Symbol;Acc:VGNC:108611]	-1.76	0.1694
ssc-miR-92b-3p	RAD21	RAD21 cohesin complex component [Source:VGNC Symbol;Acc:VGNC:92054]	-1.76	0.1694
ssc-miR-92b-3p	RAD51D	RAD51 paralog D [Source:HGNC Symbol;Acc:HGNC:9823]	-1.76	0.1694
ssc-miR-92b-3p	RAG1	recombination activating 1 [Source:VGNC Symbol;Acc:VGNC:92065]	-1.76	0.1694
ssc-miR-92b-3p	RANBP9	RAN binding protein 9 [Source:VGNC Symbol;Acc:VGNC:92082]	-1.76	0.1694
ssc-miR-92b-3p	RAP1A	RAP1A, member of RAS onco family [Source:VGNC Symbol;Acc:VGNC:92084]	-1.76	0.1694
ssc-miR-92b-3p	RAP1B	RAP1B, member of RAS oncogene family [Source:HGNC Symbol;Acc:HGNC:9857]	-1.76	0.1694
ssc-miR-92b-3p	RASAL2	RAS protein activator like 2 [Source:VGNC Symbol;Acc:VGNC:92105]	-1.76	0.1694
ssc-miR-92b-3p	RASSF2	Ras association domain family member 2 [Source:VGNC Symbol;Acc:VGNC:96531]	-1.76	0.1694
ssc-miR-92b-3p	RASSF3	Ras association domain family member 3 [Source:VGNC Symbol;Acc:VGNC:92124]	-1.76	0.1694
ssc-miR-92b-3p	RBFOX1	RNA binding fox-1 homolog 1 [Source:VGNC Symbol;Acc:VGNC:92139]	-1.76	0.1694
ssc-miR-92b-3p	RBFOX2	RNA binding fox-1 homolog 2 [Source:VGNC Symbol;Acc:VGNC:92140]	-1.76	0.1694
ssc-miR-92b-3p	RBL2	RB transcriptional corepressor like 2 [Source:VGNC Symbol;Acc:VGNC:92143]	-1.76	0.1694
ssc-miR-92b-3p	RBM15B	RNA binding motif protein 15B [Source:VGNC Symbol;Acc:VGNC:98285]	-1.76	0.1694

ssc-miR-92b-3p	RBM27	RNA binding motif protein 27 [Source:HGNC Symbol;Acc:HGNC:29243]	-1.76	0.1694
ssc-miR-92b-3p	RBM47	RNA binding motif protein 47 [Source:VGNC Symbol;Acc:VGNC:92158]	-1.76	0.1694
ssc-miR-92b-3p	RBPJ	recombination signal binding protein for immunoglobulin kappa J region [Source:VGNC Symbol;Acc:VGNC:98956]	-1.76	0.1694
ssc-miR-92b-3p	RBPMS2	RNA binding protein, mRNA processing factor 2 [Source:VGNC Symbol;Acc:VGNC:92166]	-1.76	0.1694
ssc-miR-92b-3p	RCOR1	REST corepressor 1 [Source:VGNC Symbol;Acc:VGNC:92183]	-1.76	0.1694
ssc-miR-92b-3p	REST	hypothetical gene	-1.76	0.1694
ssc-miR-92b-3p	REV3L	REV3 like, DNA directed polymerase zeta catalytic subunit [Source:VGNC Symbol;Acc:VGNC:92226]	-1.76	0.1694
ssc-miR-92b-3p	REXO1	RNA exonuclease 1 homolog [Source:VGNC Symbol;Acc:VGNC:100851]	-1.76	0.1694
ssc-miR-92b-3p	RFX1	regulatory factor X1 [Source:VGNC Symbol;Acc:VGNC:92243]	-1.76	0.1694
ssc-miR-92b-3p	RFX3	regulatory factor X3 [Source:VGNC Symbol;Acc:VGNC:92245]	-1.76	0.1694
ssc-miR-92b-3p	RFX7	regulatory factor X7 [Source:VGNC Symbol;Acc:VGNC:92248]	-1.76	0.1694
ssc-miR-92b-3p	RGL1	ral guanine nucleotide dissociation stimulator like 1 [Source:VGNC Symbol;Acc:VGNC:92252]	-1.76	0.1694
ssc-miR-92b-3p	RGS17	regulator of G protein signaling 17 [Source:VGNC Symbol;Acc:VGNC:92263]	-1.76	0.1694
ssc-miR-92b-3p	RGS3	hypothetical gene	-1.76	0.1694
ssc-miR-92b-3p	RHPN2	rhophilin Rho GTPase binding protein 2 [Source:VGNC Symbol;Acc:VGNC:98548]	-1.76	0.1694
ssc-miR-92b-3p	RIMS2	regulating synaptic membrane exocytosis 2 [Source:HGNC Symbol;Acc:HGNC:17283]	-1.76	0.1694
ssc-miR-92b-3p	RNF10	ring finger protein 10 [Source:VGNC Symbol;Acc:VGNC:92345]	-1.76	0.1694
ssc-miR-92b-3p	RNF11	ring finger protein 11 [Source:VGNC Symbol;Acc:VGNC:98601]	-1.76	0.1694
ssc-miR-92b-3p	RNF141	ring finger protein 141 [Source:VGNC Symbol;Acc:VGNC:98292]	-1.76	0.1694
ssc-miR-92b-3p	RNF150	ring finger protein 150 [Source:VGNC Symbol;Acc:VGNC:98293]	-1.76	0.1694
ssc-miR-92b-3p	RNF157	ring finger protein 157 [Source:VGNC Symbol;Acc:VGNC:92365]	-1.76	0.1694
ssc-miR-92b-3p	RNF166	ring finger protein 166 [Source:VGNC Symbol;Acc:VGNC:92367]	-1.76	0.1694
ssc-miR-92b-3p	RNF180	ring finger protein 180 [Source:VGNC Symbol;Acc:VGNC:92370]	-1.76	0.1694
ssc-miR-92b-3p	RNF38	ring finger protein 38 [Source:VGNC Symbol;Acc:VGNC:92390]	-1.76	0.1694
ssc-miR-92b-3p	RNF4	ring finger protein 4 [Source:VGNC Symbol;Acc:VGNC:92392]	-1.76	0.1694
ssc-miR-92b-3p	RNF44	ring finger protein 44 [Source:VGNC Symbol;Acc:VGNC:92395]	-1.76	0.1694
ssc-miR-92b-3p	ROBO1	roundabout guidance receptor 1 [Source:HGNC Symbol;Acc:HGNC:10249]	-1.76	0.1694
ssc-miR-92b-3p	ROBO2	roundabout guidance receptor 2 [Source:HGNC Symbol;Acc:HGNC:10250]	-1.76	0.1694
ssc-miR-92b-3p	RORA	RAR related orphan receptor A [Source:VGNC Symbol;Acc:VGNC:92408]	-1.76	0.1694
ssc-miR-92b-3p	RP1-170O19.20	hypothetical gene	-1.76	0.1694
ssc-miR-92b-3p	RP11-1055B8.7	hypothetical gene	-1.76	0.1694
ssc-miR-92b-3p	RP11-766F14.2	hypothetical gene	-1.76	0.1694
ssc-miR-92b-3p	RP11-770J1.4	hypothetical gene	-1.76	0.1694
ssc-miR-92b-3p	RPL15	ribosomal protein L15 [Source:NCBI gene (formerly Entrezgene);Acc:100620094]	-1.76	0.1694
ssc-miR-92b-3p	RPS6KA4	ribosomal protein S6 kinase A4 [Source:VGNC Symbol;Acc:VGNC:92443]	-1.76	0.1694
ssc-miR-92b-3p	RPS6KB1	ribosomal protein S6 kinase B1 [Source:VGNC Symbol;Acc:VGNC:99037]	-1.76	0.1694
ssc-miR-92b-3p	RRBP1	ribosome binding protein 1 [Source:VGNC Symbol;Acc:VGNC:95474]	-1.76	0.1694
ssc-miR-92b-3p	RRN3	RRN3 homolog, RNA polymerase I transcription factor [Source:HGNC Symbol;Acc:HGNC:30346]	-1.76	0.1694
ssc-miR-92b-3p	RSBN1	round spermatid basic protein 1 [Source:VGNC Symbol;Acc:VGNC:92473]	-1.76	0.1694
ssc-miR-92b-3p	RUNX1T1	RUNX1 partner transcriptional co-repressor 1 [Source:VGNC Symbol;Acc:VGNC:96594]	-1.76	0.1694
ssc-miR-92b-3p	RYR3	ryanodine receptor 3 [Source:HGNC Symbol;Acc:HGNC:10485]	-1.76	0.1694
ssc-miR-92b-3p	S100A2	S100 calcium binding protein A2 [Source:HGNC Symbol;Acc:HGNC:10492]	-1.76	0.1694
ssc-miR-92b-3p	S1PR1	sphingosine-1-phosphate receptor 1 [Source:VGNC Symbol;Acc:VGNC:92551]	-1.76	0.1694
ssc-miR-92b-3p	SAP18	Sin3A associated protein 18 [Source:VGNC Symbol;Acc:VGNC:92574]	-1.76	0.1694
ssc-miR-92b-3p	SAR1B	secretion associated Ras related GTPase 1B [Source:VGNC Symbol;Acc:VGNC:92580]	-1.76	0.1694
ssc-miR-92b-3p	SATB2	SATB homeobox 2 [Source:VGNC Symbol;Acc:VGNC:95972]	-1.76	0.1694
ssc-miR-92b-3p	SBK1	SH3 domain binding kinase 1 [Source:HGNC Symbol;Acc:HGNC:17699]	-1.76	0.1694
ssc-miR-92b-3p	SBNO1	strawberry notch homolog 1 [Source:VGNC Symbol;Acc:VGNC:92596]	-1.76	0.1694
ssc-miR-92b-3p	SCN8A	sodium voltage-gated channel alpha subunit 8 [Source:VGNC Symbol;Acc:VGNC:92638]	-1.76	0.1694
ssc-miR-92b-3p	SCRG1	stimulator of chondrosis 1 [Source:VGNC Symbol;Acc:VGNC:92646]	-1.76	0.1694

ssc-miR-92b-3p	SCUBE3	signal peptide, CUB domain and EGF like domain containing 3 [Source:VGNC Symbol;Acc:VGNC:92651]	-1.76	0.1694
ssc-miR-92b-3p	SDC2	syndecan 2 [Source:VGNC Symbol;Acc:VGNC:92655]	-1.76	0.1694
ssc-miR-92b-3p	SDC3	syndecan 3 [Source:VGNC Symbol;Acc:VGNC:92656]	-1.76	0.1694
ssc-miR-92b-3p	SDK1	sidekick cell adhesion molecule 1 [Source:VGNC Symbol;Acc:VGNC:92660]	-1.76	0.1694
ssc-miR-92b-3p	SEC16A	SEC16 homolog A, endoplasmic reticulum export factor [Source:VGNC Symbol;Acc:VGNC:92671]	-1.76	0.1694
ssc-miR-92b-3p	SEC31B	SEC31 homolog B, COPII coat complex component [Source:VGNC Symbol;Acc:VGNC:92681]	-1.76	0.1694
ssc-miR-92b-3p	SEL1L3	SEL1L family member 3 [Source:VGNC Symbol;Acc:VGNC:92688]	-1.76	0.1694
ssc-miR-92b-3p	SELT	hypothetical gene	-1.76	0.1694
ssc-miR-92b-3p	SEMA3A	semaphorin 3A [Source:VGNC Symbol;Acc:VGNC:92693]	-1.76	0.1694
ssc-miR-92b-3p	SEMA6D	semaphorin 6D [Source:VGNC Symbol;Acc:VGNC:92711]	-1.76	0.1694
ssc-miR-92b-3p	SERPINF1	serpin family F member 1 [Source:VGNC Symbol;Acc:VGNC:92743]	-1.76	0.1694
ssc-miR-92b-3p	SERTAD2	SERTA domain containing 2 [Source:VGNC Symbol;Acc:VGNC:92750]	-1.76	0.1694
ssc-miR-92b-3p	SERTAD3	SERTA domain containing 3 [Source:VGNC Symbol;Acc:VGNC:92751]	-1.76	0.1694
ssc-miR-92b-3p	SESN3	sestrin 3 [Source:VGNC Symbol;Acc:VGNC:92755]	-1.76	0.1694
ssc-miR-92b-3p	SESTD1	SEC14 and spectrin domain containing 1 [Source:VGNC Symbol;Acc:VGNC:95884]	-1.76	0.1694
ssc-miR-92b-3p	SETD1B	SET domain containing 1B, histone lysine methyltransferase [Source:HGNC Symbol;Acc:HGNC:29187]	-1.76	0.1694
ssc-miR-92b-3p	SETD5	SET domain containing 5 [Source:VGNC Symbol;Acc:VGNC:92760]	-1.76	0.1694
ssc-miR-92b-3p	SETD7	SET domain containing 7, histone lysine methyltransferase [Source:VGNC Symbol;Acc:VGNC:92762]	-1.76	0.1694
ssc-miR-92b-3p	SFMBT2	Scm like with four mbt domains 2 [Source:VGNC Symbol;Acc:VGNC:95935]	-1.76	0.1694
ssc-miR-92b-3p	SFXN1	sideroflexin 1 [Source:VGNC Symbol;Acc:VGNC:92783]	-1.76	0.1694
ssc-miR-92b-3p	SGCD	sarcoglycan delta [Source:NCBI gene (formerly Entrezgene);Acc:100240724]	-1.76	0.1694
ssc-miR-92b-3p	SGK3	serum/glucocorticoid regulated kinase family member 3 [Source:HGNC Symbol;Acc:HGNC:10812]	-1.76	0.1694
ssc-miR-92b-3p	SGPP1	sphingosine-1-phosphate phosphatase 1 [Source:VGNC Symbol;Acc:VGNC:92798]	-1.76	0.1694
ssc-miR-92b-3p	SH3D19	SH3 domain containing 19 [Source:VGNC Symbol;Acc:VGNC:92821]	-1.76	0.1694
ssc-miR-92b-3p	SH3PXD2A	SH3 and PX domains 2A [Source:VGNC Symbol;Acc:VGNC:92829]	-1.76	0.1694
ssc-miR-92b-3p	SHOC2	SHOC2 leucine rich repeat scaffold protein [Source:VGNC Symbol;Acc:VGNC:98318]	-1.76	0.1694
ssc-miR-92b-3p	SHOX	hypothetical gene	-1.76	0.1694
ssc-miR-92b-3p	SIK1	salt inducible kinase 1 [Source:HGNC Symbol;Acc:HGNC:11142]	-1.76	0.1694
ssc-miR-92b-3p	SIK2	salt inducible kinase 2 [Source:VGNC Symbol;Acc:VGNC:92872]	-1.76	0.1694
ssc-miR-92b-3p	SIM2	SIM bHLH transcription factor 2 [Source:VGNC Symbol;Acc:VGNC:92877]	-1.76	0.1694
ssc-miR-92b-3p	SIRPA	hypothetical gene	-1.76	0.1694
ssc-miR-92b-3p	SKI	SKI proto-onco [Source:VGNC Symbol;Acc:VGNC:92902]	-1.76	0.1694
ssc-miR-92b-3p	SKOR1	SKI family transcriptional corepressor 1 [Source:VGNC Symbol;Acc:VGNC:92905]	-1.76	0.1694
ssc-miR-92b-3p	SLC12A2	solute carrier family 12 member 2 [Source:VGNC Symbol;Acc:VGNC:92922]	-1.76	0.1694
ssc-miR-92b-3p	SLC12A5	solute carrier family 12 member 5 [Source:VGNC Symbol;Acc:VGNC:95594]	-1.76	0.1694
ssc-miR-92b-3p	SLC16A10	solute carrier family 16 member 10 [Source:VGNC Symbol;Acc:VGNC:103178]	-1.76	0.1694
ssc-miR-92b-3p	SLC17A6	solute carrier family 17 member 6 [Source:VGNC Symbol;Acc:VGNC:92954]	-1.76	0.1694
ssc-miR-92b-3p	SLC1A1	solute carrier family 1 member 1 [Source:VGNC Symbol;Acc:VGNC:92963]	-1.76	0.1694
ssc-miR-92b-3p	SLC24A3	solute carrier family 24 member 3 [Source:VGNC Symbol;Acc:VGNC:95501]	-1.76	0.1694
ssc-miR-92b-3p	SLC24A4	solute carrier family 24 member 4 [Source:VGNC Symbol;Acc:VGNC:92988]	-1.76	0.1694
ssc-miR-92b-3p	SLC25A16	solute carrier family 25 member 16 [Source:VGNC Symbol;Acc:VGNC:92995]	-1.76	0.1694
ssc-miR-92b-3p	SLC25A32	solute carrier family 25 member 32 [Source:VGNC Symbol;Acc:VGNC:98859]	-1.76	0.1694
ssc-miR-92b-3p	SLC25A36	solute carrier family 25 member 36 [Source:VGNC Symbol;Acc:VGNC:93012]	-1.76	0.1694
ssc-miR-92b-3p	SLC25A51	hypothetical gene	-1.76	0.1694
ssc-miR-92b-3p	SLC2A14	hypothetical gene	-1.76	0.1694
ssc-miR-92b-3p	SLC2A3	hypothetical gene	-1.76	0.1694
ssc-miR-92b-3p	SLC30A10	hypothetical gene	-1.76	0.1694
ssc-miR-92b-3p	SLC30A7	solute carrier family 30 member 7 [Source:VGNC Symbol;Acc:VGNC:93061]	-1.76	0.1694
ssc-miR-92b-3p	SLC32A1	solute carrier family 32 member 1 [Source:VGNC Symbol;Acc:VGNC:95504]	-1.76	0.1694
ssc-miR-92b-3p	SLC37A3	solute carrier family 37 member 3 [Source:VGNC Symbol;Acc:VGNC:98324]	-1.76	0.1694

ssc-miR-92b-3p	SLC38A2	solute carrier family 38 member 2 [Source:VGNC Symbol;Acc:VGNC:93094]	-1.76	0.1694
ssc-miR-92b-3p	SLC39A6	solute carrier family 39 member 6 [Source:VGNC Symbol;Acc:VGNC:93107]	-1.76	0.1694
ssc-miR-92b-3p	SLC41A1	solute carrier family 41 member 1 [Source:VGNC Symbol;Acc:VGNC:93113]	-1.76	0.1694
ssc-miR-92b-3p	SLC44A1	solute carrier family 44 member 1 [Source:VGNC Symbol;Acc:VGNC:93118]	-1.76	0.1694
ssc-miR-92b-3p	SLC45A3	solute carrier family 45 member 3 [Source:VGNC Symbol;Acc:VGNC:93124]	-1.76	0.1694
ssc-miR-92b-3p	SLC45A4	solute carrier family 45 member 4 [Source:VGNC Symbol;Acc:VGNC:93125]	-1.76	0.1694
ssc-miR-92b-3p	SLC4A10	solute carrier family 4 member 10 [Source:VGNC Symbol;Acc:VGNC:95592]	-1.76	0.1694
ssc-miR-92b-3p	SLC4A8	solute carrier family 4 member 8 [Source:VGNC Symbol;Acc:VGNC:98326]	-1.76	0.1694
ssc-miR-92b-3p	SLC6A1	solute carrier family 6 member 1 [Source:VGNC Symbol;Acc:VGNC:93153]	-1.76	0.1694
ssc-miR-92b-3p	SLC7A11	solute carrier family 7 member 11 [Source:VGNC Symbol;Acc:VGNC:93171]	-1.76	0.1694
ssc-miR-92b-3p	SLC8A1	solute carrier family 8 member A1 [Source:VGNC Symbol;Acc:VGNC:93178]	-1.76	0.1694
ssc-miR-92b-3p	SLC9A1	solute carrier family 9 member A1 [Source:NCBI gene (formerly Entrezgene);Acc:397458]	-1.76	0.1694
ssc-miR-92b-3p	SLC9A2	solute carrier family 9 member A2 [Source:VGNC Symbol;Acc:VGNC:93182]	-1.76	0.1694
ssc-miR-92b-3p	SLC9A7	solute carrier family 9 member A7 [Source:VGNC Symbol;Acc:VGNC:93189]	-1.76	0.1694
ssc-miR-92b-3p	SLX4	SLX4 structure-specific endonuclease subunit [Source:VGNC Symbol;Acc:VGNC:93215]	-1.76	0.1694
ssc-miR-92b-3p	SMAD6	SMAD family member 6 [Source:VGNC Symbol;Acc:VGNC:93220]	-1.76	0.1694
ssc-miR-92b-3p	SMAD7	SMAD family member 7 [Source:VGNC Symbol;Acc:VGNC:93221]	-1.76	0.1694
ssc-miR-92b-3p	SMARCA5	SWI/SNF related, matrix associated, actin dependent regulator of chromatin, subfamily a, member 5 [Source:VGNC Symbol;Acc:VGNC:93228]	-1.76	0.1694
ssc-miR-92b-3p	SMCHD1	structural maintenance of chromosomes flexible hinge domain containing 1 [Source:VGNC Symbol;Acc:VGNC:93242]	-1.76	0.1694
ssc-miR-92b-3p	SMG1	SMG1 nonsense mediated mRNA decay associated PI3K related kinase [Source:VGNC Symbol;Acc:VGNC:93248]	-1.76	0.1694
ssc-miR-92b-3p	SMIM14	small integral membrane protein 14 [Source:VGNC Symbol;Acc:VGNC:93252]	-1.76	0.1694
ssc-miR-92b-3p	SMURF1	SMAD specific E3 ubiquitin protein ligase 1 [Source:VGNC Symbol;Acc:VGNC:93270]	-1.76	0.1694
ssc-miR-92b-3p	SNAI1	snail family transcriptional repressor 1 [Source:VGNC Symbol;Acc:VGNC:95508]	-1.76	0.1694
ssc-miR-92b-3p	SNAP91	synaptosome associated protein 91 [Source:VGNC Symbol;Acc:VGNC:93279]	-1.76	0.1694
ssc-miR-92b-3p	SNAPC1	small nuclear RNA activating complex polypeptide 1 [Source:VGNC Symbol;Acc:VGNC:93280]	-1.76	0.1694
ssc-miR-92b-3p	SNN	stannin [Source:HGNC Symbol;Acc:HGNC:11149]	-1.76	0.1694
ssc-miR-92b-3p	SNX13	sorting nexin 13 [Source:VGNC Symbol;Acc:VGNC:93306]	-1.76	0.1694
ssc-miR-92b-3p	SNX2	sorting nexin 2 [Source:VGNC Symbol;Acc:VGNC:93312]	-1.76	0.1694
ssc-miR-92b-3p	SNX25	sorting nexin 25 [Source:VGNC Symbol;Acc:VGNC:95978]	-1.76	0.1694
ssc-miR-92b-3p	SNX30	sorting nexin family member 30 [Source:VGNC Symbol;Acc:VGNC:93318]	-1.76	0.1694
ssc-miR-92b-3p	SOBP	sine oculis binding protein homolog [Source:VGNC Symbol;Acc:VGNC:93329]	-1.76	0.1694
ssc-miR-92b-3p	SOCS5	suppressor of cytokine signaling 5 [Source:VGNC Symbol;Acc:VGNC:93332]	-1.76	0.1694
ssc-miR-92b-3p	SOCS6	suppressor of cytokine signaling 6 [Source:VGNC Symbol;Acc:VGNC:93333]	-1.76	0.1694
ssc-miR-92b-3p	SORCS3	sortilin related VPS10 domain containing receptor 3 [Source:VGNC Symbol;Acc:VGNC:93341]	-1.76	0.1694
ssc-miR-92b-3p	SORL1	sortilin related receptor 1 [Source:VGNC Symbol;Acc:VGNC:108617]	-1.76	0.1694
ssc-miR-92b-3p	SORT1	sortilin 1 [Source:VGNC Symbol;Acc:VGNC:93343]	-1.76	0.1694
ssc-miR-92b-3p	SOSTDC1	sclerostin domain containing 1 [Source:VGNC Symbol;Acc:VGNC:93346]	-1.76	0.1694
ssc-miR-92b-3p	SOX11	SRY-box transcription factor 11 [Source:VGNC Symbol;Acc:VGNC:93351]	-1.76	0.1694
ssc-miR-92b-3p	SOX4	SRY-box transcription factor 4 [Source:HGNC Symbol;Acc:HGNC:11200]	-1.76	0.1694
ssc-miR-92b-3p	SP1	Sp1 transcription factor [Source:VGNC Symbol;Acc:VGNC:93360]	-1.76	0.1694
ssc-miR-92b-3p	SP4	Sp4 transcription factor [Source:VGNC Symbol;Acc:VGNC:93362]	-1.76	0.1694
ssc-miR-92b-3p	SPHK2	sphingosine kinase 2 [Source:VGNC Symbol;Acc:VGNC:93405]	-1.76	0.1694
ssc-miR-92b-3p	SPOCK2	SPARC (osteonectin), cwcv and kazal like domains proteoglycan 2 [Source:VGNC Symbol;Acc:VGNC:107362]	-1.76	0.1694
ssc-miR-92b-3p	SPRYD4	SPRY domain containing 4 [Source:HGNC Symbol;Acc:HGNC:27468]	-1.76	0.1694
ssc-miR-92b-3p	SPSB1	splA/ryanodine receptor domain and SOCS box containing 1 [Source:VGNC Symbol;Acc:VGNC:93430]	-1.76	0.1694
ssc-miR-92b-3p	SPTBN4	spectrin beta, non-erythrocytic 4 [Source:VGNC Symbol;Acc:VGNC:93438]	-1.76	0.1694
ssc-miR-92b-3p	SREK1IP1	SREK1 interacting protein 1 [Source:HGNC Symbol;Acc:HGNC:26716]	-1.76	0.1694
ssc-miR-92b-3p	SRPK2	SRSF protein kinase 2 [Source:VGNC Symbol;Acc:VGNC:93463]	-1.76	0.1694
ssc-miR-92b-3p	SRPR	hypothetical gene	-1.76	0.1694
ssc-miR-92b-3p	SSBP2	single stranded DNA binding protein 2 [Source:VGNC Symbol;Acc:VGNC:93480]	-1.76	0.1694

ssc-miR-92b-3p	SSBP3	single stranded DNA binding protein 3 [Source:VGNC Symbol;Acc:VGNC:93481]	-1.76	0.1694
ssc-miR-92b-3p	SSFA2	hypothetical gene	-1.76	0.1694
ssc-miR-92b-3p	ST13	ST13 Hsp70 interacting protein [Source:HGNC Symbol;Acc:HGNC:11343]	-1.76	0.1694
ssc-miR-92b-3p	ST6GAL2	ST6 beta-galactoside alpha-2,6-sialyltransferase 2 [Source:VGNC Symbol;Acc:VGNC:93508]	-1.76	0.1694
ssc-miR-92b-3p	STAG2	stromal antigen 2 [Source:VGNC Symbol;Acc:VGNC:93525]	-1.76	0.1694
ssc-miR-92b-3p	STARD13	hypothetical gene	-1.76	0.1694
ssc-miR-92b-3p	STAU2	staufen double-stranded RNA binding protein 2 [Source:VGNC Symbol;Acc:VGNC:98873]	-1.76	0.1694
ssc-miR-92b-3p	STK39	serine/threonine kinase 39 [Source:VGNC Symbol;Acc:VGNC:98348]	-1.76	0.1694
ssc-miR-92b-3p	STRN	striatin [Source:VGNC Symbol;Acc:VGNC:93578]	-1.76	0.1694
ssc-miR-92b-3p	STRN3	striatin 3 [Source:VGNC Symbol;Acc:VGNC:93579]	-1.76	0.1694
ssc-miR-92b-3p	STX17	syntaxin 17 [Source:VGNC Symbol;Acc:VGNC:93587]	-1.76	0.1694
ssc-miR-92b-3p	STX7	syntaxin 7 [Source:VGNC Symbol;Acc:VGNC:93595]	-1.76	0.1694
ssc-miR-92b-3p	STYX	serine/threonine/tyrosine interacting protein [Source:VGNC Symbol;Acc:VGNC:93602]	-1.76	0.1694
ssc-miR-92b-3p	SUN1	Sad1 and UNC84 domain containing 1 [Source:VGNC Symbol;Acc:VGNC:93618]	-1.76	0.1694
ssc-miR-92b-3p	SUV420H1	hypothetical gene	-1.76	0.1694
ssc-miR-92b-3p	SYNDIG1	synapse differentiation inducing 1 [Source:VGNC Symbol;Acc:VGNC:95689]	-1.76	0.1694
ssc-miR-92b-3p	SYNJ1	synaptojanin 1 [Source:VGNC Symbol;Acc:VGNC:93668]	-1.76	0.1694
ssc-miR-92b-3p	SYT1	synaptotagmin 1 [Source:VGNC Symbol;Acc:VGNC:93678]	-1.76	0.1694
ssc-miR-92b-3p	SYT2	synaptotagmin 2 [Source:VGNC Symbol;Acc:VGNC:95530]	-1.76	0.1694
ssc-miR-92b-3p	TACC2	transforming acidic coiled-coil containing protein 2 [Source:NCBI gene (formerly Entrezgene);Acc:100152804]	-1.76	0.1694
ssc-miR-92b-3p	TAF1	hypothetical gene	-1.76	0.1694
ssc-miR-92b-3p	TAGAP	T cell activation RhoGTPase activating protein [Source:VGNC Symbol;Acc:VGNC:93729]	-1.76	0.1694
ssc-miR-92b-3p	TBC1D12	TBC1 domain family member 12 [Source:VGNC Symbol;Acc:VGNC:93762]	-1.76	0.1694
ssc-miR-92b-3p	TBC1D19	TBC1 domain family member 19 [Source:VGNC Symbol;Acc:VGNC:93767]	-1.76	0.1694
ssc-miR-92b-3p	TBC1D30	TBC1 domain family member 30 [Source:VGNC Symbol;Acc:VGNC:93775]	-1.76	0.1694
ssc-miR-92b-3p	TBC1D8	TBC1 domain family member 8 [Source:VGNC Symbol;Acc:VGNC:93779]	-1.76	0.1694
ssc-miR-92b-3p	TBL1XR1	TBL1X receptor 1 [Source:VGNC Symbol;Acc:VGNC:96600]	-1.76	0.1694
ssc-miR-92b-3p	TBX20	T-box transcription factor 20 [Source:VGNC Symbol;Acc:VGNC:93799]	-1.76	0.1694
ssc-miR-92b-3p	TBX3	T-box transcription factor 3 [Source:VGNC Symbol;Acc:VGNC:93802]	-1.76	0.1694
ssc-miR-92b-3p	TCEB3	hypothetical gene	-1.76	0.1694
ssc-miR-92b-3p	TCF21	transcription factor 21 [Source:VGNC Symbol;Acc:VGNC:93819]	-1.76	0.1694
ssc-miR-92b-3p	TCF4	transcription factor 4 [Source:VGNC Symbol;Acc:VGNC:93823]	-1.76	0.1694
ssc-miR-92b-3p	TCHP	hypothetical gene	-1.76	0.1694
ssc-miR-92b-3p	TEAD1	TEA domain transcription factor 1 [Source:VGNC Symbol;Acc:VGNC:93853]	-1.76	0.1694
ssc-miR-92b-3p	TECPR2	tectonin beta-propeller repeat containing 2 [Source:VGNC Symbol;Acc:VGNC:99731]	-1.76	0.1694
ssc-miR-92b-3p	TEF	TEF transcription factor, PAR bZIP family member [Source:VGNC Symbol;Acc:VGNC:93864]	-1.76	0.1694
ssc-miR-92b-3p	TENM1	teneurin transmembrane protein 1 [Source:VGNC Symbol;Acc:VGNC:98363]	-1.76	0.1694
ssc-miR-92b-3p	TET2	hypothetical gene	-1.76	0.1694
ssc-miR-92b-3p	TEX2	testis expressed 2 [Source:VGNC Symbol;Acc:VGNC:93894]	-1.76	0.1694
ssc-miR-92b-3p	TFAP2A	transcription factor AP-2 alpha [Source:VGNC Symbol;Acc:VGNC:93908]	-1.76	0.1694
ssc-miR-92b-3p	TFAP2E	transcription factor AP-2 epsilon [Source:VGNC Symbol;Acc:VGNC:93911]	-1.76	0.1694
ssc-miR-92b-3p	TFDP2	transcription factor Dp-2 [Source:VGNC Symbol;Acc:VGNC:93915]	-1.76	0.1694
ssc-miR-92b-3p	TGFB2	transforming growth factor beta 2 [Source:VGNC Symbol;Acc:VGNC:95541]	-1.76	0.1694
ssc-miR-92b-3p	TGIF1	TGFB induced factor homeobox 1 [Source:VGNC Symbol;Acc:VGNC:93935]	-1.76	0.1694
ssc-miR-92b-3p	TMCC1	transmembrane and coiled-coil domain family 1 [Source:VGNC Symbol;Acc:VGNC:94046]	-1.76	0.1694
ssc-miR-92b-3p	TMCC3	transmembrane and coiled-coil domain family 3 [Source:VGNC Symbol;Acc:VGNC:98375]	-1.76	0.1694
ssc-miR-92b-3p	TMED5	transmembrane p24 trafficking protein 5 [Source:VGNC Symbol;Acc:VGNC:94057]	-1.76	0.1694
ssc-miR-92b-3p	TMEM125	transmembrane protein 125 [Source:VGNC Symbol;Acc:VGNC:94073]	-1.76	0.1694
ssc-miR-92b-3p	TMEM143	transmembrane protein 143 [Source:VGNC Symbol;Acc:VGNC:94090]	-1.76	0.1694
ssc-miR-92b-3p	TMEM184B	transmembrane protein 184B [Source:VGNC Symbol;Acc:VGNC:94120]	-1.76	0.1694

ssc-miR-92b-3p	TMEM200B	transmembrane protein 200B [Source:HGNC Symbol;Acc:HGNC:33785]	-1.76	0.1694
ssc-miR-92b-3p	TMEM229A	transmembrane protein 229A [Source:VGNC Symbol;Acc:VGNC:94146]	-1.76	0.1694
ssc-miR-92b-3p	TMEM255A	transmembrane protein 255A [Source:VGNC Symbol;Acc:VGNC:104079]	-1.76	0.1694
ssc-miR-92b-3p	TMEM50B	transmembrane protein 50B [Source:VGNC Symbol;Acc:VGNC:94186]	-1.76	0.1694
ssc-miR-92b-3p	TMEM87A	transmembrane protein 87A [Source:VGNC Symbol;Acc:VGNC:94212]	-1.76	0.1694
ssc-miR-92b-3p	TMF1	TATA element modulatory factor 1 [Source:VGNC Symbol;Acc:VGNC:94223]	-1.76	0.1694
ssc-miR-92b-3p	TNPO1	transportin 1 [Source:VGNC Symbol;Acc:VGNC:94287]	-1.76	0.1694
ssc-miR-92b-3p	TNRC6B	trinucleotide repeat containing adaptor 6B [Source:VGNC Symbol;Acc:VGNC:94293]	-1.76	0.1694
ssc-miR-92b-3p	TOB1	transducer of ERBB2, 1 [Source:NCBI gene (formerly Entrezgene);Acc:100144440]	-1.76	0.1694
ssc-miR-92b-3p	TOB2	transducer of ERBB2, 2 [Source:VGNC Symbol;Acc:VGNC:94298]	-1.76	0.1694
ssc-miR-92b-3p	TOP1	hypothetical gene	-1.76	0.1694
ssc-miR-92b-3p	TOR1AIP2	torsin 1A interacting protein 2 [Source:VGNC Symbol;Acc:VGNC:94318]	-1.76	0.1694
ssc-miR-92b-3p	TOR1B	torsin family 1 member B [Source:HGNC Symbol;Acc:HGNC:11995]	-1.76	0.1694
ssc-miR-92b-3p	TPBGL	hypothetical gene	-1.76	0.1694
ssc-miR-92b-3p	TPCN1	two pore segment channel 1 [Source:VGNC Symbol;Acc:VGNC:94333]	-1.76	0.1694
ssc-miR-92b-3p	TRAF3	TNF receptor associated factor 3 [Source:VGNC Symbol;Acc:VGNC:94360]	-1.76	0.1694
ssc-miR-92b-3p	TRAK2	trafficking kinesin protein 2 [Source:VGNC Symbol;Acc:VGNC:95577]	-1.76	0.1694
ssc-miR-92b-3p	TRAM2	translocation associated membrane protein 2 [Source:HGNC Symbol;Acc:HGNC:16855]	-1.76	0.1694
ssc-miR-92b-3p	TRHDE	thyrotropin releasing hormone degrading enzyme [Source:VGNC Symbol;Acc:VGNC:94390]	-1.76	0.1694
ssc-miR-92b-3p	TRIM33	tripartite motif containing 33 [Source:VGNC Symbol;Acc:VGNC:98881]	-1.76	0.1694
ssc-miR-92b-3p	TRIM36	tripartite motif containing 36 [Source:VGNC Symbol;Acc:VGNC:94409]	-1.76	0.1694
ssc-miR-92b-3p	TRIO	trio Rho guanine nucleotide exchange factor [Source:VGNC Symbol;Acc:VGNC:94435]	-1.76	0.1694
ssc-miR-92b-3p	TRIP12	thyroid hormone receptor interactor 12 [Source:VGNC Symbol;Acc:VGNC:95555]	-1.76	0.1694
ssc-miR-92b-3p	TRPC4	transient receptor potential cation channel subfamily C member 4 [Source:VGNC Symbol;Acc:VGNC:94462]	-1.76	0.1694
ssc-miR-92b-3p	TRPM3	transient receptor potential cation channel subfamily M member 3 [Source:VGNC Symbol;Acc:VGNC:98384]	-1.76	0.1694
ssc-miR-92b-3p	TRPM7	transient receptor potential cation channel subfamily M member 7 [Source:VGNC Symbol;Acc:VGNC:94470]	-1.76	0.1694
ssc-miR-92b-3p	TSC1	TSC complex subunit 1 [Source:HGNC Symbol;Acc:HGNC:12362]	-1.76	0.1694
ssc-miR-92b-3p	TSSK6	testis specific serine kinase 6 [Source:VGNC Symbol;Acc:VGNC:94528]	-1.76	0.1694
ssc-miR-92b-3p	TTBK2	tau tubulin kinase 2 [Source:VGNC Symbol;Acc:VGNC:98387]	-1.76	0.1694
ssc-miR-92b-3p	TTC14	tetratricopeptide repeat domain 14 [Source:VGNC Symbol;Acc:VGNC:94535]	-1.76	0.1694
ssc-miR-92b-3p	TTC28	tetratricopeptide repeat domain 28 [Source:VGNC Symbol;Acc:VGNC:94543]	-1.76	0.1694
ssc-miR-92b-3p	TTC9	tetratricopeptide repeat domain 9 [Source:VGNC Symbol;Acc:VGNC:94560]	-1.76	0.1694
ssc-miR-92b-3p	TTLL7	tubulin tyrosine ligase like 7 [Source:VGNC Symbol;Acc:VGNC:94571]	-1.76	0.1694
ssc-miR-92b-3p	TTPAL	alpha tocopherol transfer protein like [Source:VGNC Symbol;Acc:VGNC:95742]	-1.76	0.1694
ssc-miR-92b-3p	TUB	TUB bipartite transcription factor [Source:VGNC Symbol;Acc:VGNC:94577]	-1.76	0.1694
ssc-miR-92b-3p	TULP4	hypothetical gene	-1.76	0.1694
ssc-miR-92b-3p	TWF1	twinfilin actin binding protein 1 [Source:VGNC Symbol;Acc:VGNC:94595]	-1.76	0.1694
ssc-miR-92b-3p	TWIST1	twist family bHLH transcription factor 1 [Source:HGNC Symbol;Acc:HGNC:12428]	-1.76	0.1694
ssc-miR-92b-3p	UBASH3B	ubiquitin associated and SH3 domain containing B [Source:VGNC Symbol;Acc:VGNC:94637]	-1.76	0.1694
ssc-miR-92b-3p	UBE2G1	ubiquitin conjugating enzyme E2 G1 [Source:HGNC Symbol;Acc:HGNC:12482]	-1.76	0.1694
ssc-miR-92b-3p	UBE2W	ubiquitin conjugating enzyme E2 W [Source:VGNC Symbol;Acc:VGNC:98890]	-1.76	0.1694
ssc-miR-92b-3p	UBE2Z	ubiquitin conjugating enzyme E2 Z [Source:VGNC Symbol;Acc:VGNC:98395]	-1.76	0.1694
ssc-miR-92b-3p	UBR1	ubiquitin protein ligase E3 component n-recognin 1 [Source:VGNC Symbol;Acc:VGNC:94668]	-1.76	0.1694
ssc-miR-92b-3p	UBXN4	UBX domain protein 4 [Source:VGNC Symbol;Acc:VGNC:98397]	-1.76	0.1694
ssc-miR-92b-3p	UGP2	UDP-glucose pyrophosphorylase 2 [Source:VGNC Symbol;Acc:VGNC:98400]	-1.76	0.1694
ssc-miR-92b-3p	UNC79	unc-79 homolog, NALCN channel complex subunit [Source:HGNC Symbol;Acc:HGNC:19966]	-1.76	0.1694
ssc-miR-92b-3p	UPF2	UPF2 regulator of nonsense mediated mRNA decay [Source:VGNC Symbol;Acc:VGNC:98402]	-1.76	0.1694
ssc-miR-92b-3p	USF2	upstream transcription factor 2, c-fos interacting [Source:VGNC Symbol;Acc:VGNC:96633]	-1.76	0.1694
ssc-miR-92b-3p	USP28	ubiquitin specific peptidase 28 [Source:VGNC Symbol;Acc:VGNC:94756]	-1.76	0.1694
ssc-miR-92b-3p	USP31	ubiquitin specific peptidase 31 [Source:HGNC Symbol;Acc:HGNC:20060]	-1.76	0.1694

ssc-miR-92b-3p	USP36	ubiquitin specific peptidase 36 [Source:VGNC Symbol;Acc:VGNC:94761]	-1.76	0.1694
ssc-miR-92b-3p	USP44	ubiquitin specific peptidase 44 [Source:VGNC Symbol;Acc:VGNC:94766]	-1.76	0.1694
ssc-miR-92b-3p	USP45	ubiquitin specific peptidase 45 [Source:VGNC Symbol;Acc:VGNC:94767]	-1.76	0.1694
ssc-miR-92b-3p	UVRAG	UV radiation resistance associated [Source:VGNC Symbol;Acc:VGNC:94789]	-1.76	0.1694
ssc-miR-92b-3p	VCL	vinculin [Source:VGNC Symbol;Acc:VGNC:94812]	-1.76	0.1694
ssc-miR-92b-3p	VPS13B	vacuolar protein sorting 13 homolog B [Source:VGNC Symbol;Acc:VGNC:94839]	-1.76	0.1694
ssc-miR-92b-3p	VPS4B	vacuolar protein sorting 4 homolog B [Source:VGNC Symbol;Acc:VGNC:94857]	-1.76	0.1694
ssc-miR-92b-3p	VPS54	VPS54 subunit of GARP complex [Source:VGNC Symbol;Acc:VGNC:94862]	-1.76	0.1694
ssc-miR-92b-3p	VWA5B2	von Willebrand factor A domain containing 5B2 [Source:HGNC Symbol;Acc:HGNC:25144]	-1.76	0.1694
ssc-miR-92b-3p	WASL	WASP like actin nucleation promoting factor [Source:VGNC Symbol;Acc:VGNC:96604]	-1.76	0.1694
ssc-miR-92b-3p	WDFY3	WD repeat and FYVE domain containing 3 [Source:VGNC Symbol;Acc:VGNC:94903]	-1.76	0.1694
ssc-miR-92b-3p	WDR37	WD repeat domain 37 [Source:VGNC Symbol;Acc:VGNC:96258]	-1.76	0.1694
ssc-miR-92b-3p	WDR81	WD repeat domain 81 [Source:VGNC Symbol;Acc:VGNC:99104]	-1.76	0.1694
ssc-miR-92b-3p	WHSC1L1	hypothetical gene	-1.76	0.1694
ssc-miR-92b-3p	WRNIP1	WRN helicase interacting protein 1 [Source:VGNC Symbol;Acc:VGNC:104096]	-1.76	0.1694
ssc-miR-92b-3p	WWC1	WW and C2 domain containing 1 [Source:VGNC Symbol;Acc:VGNC:94986]	-1.76	0.1694
ssc-miR-92b-3p	WWP2	WW domain containing E3 ubiquitin protein ligase 2 [Source:VGNC Symbol;Acc:VGNC:94990]	-1.76	0.1694
ssc-miR-92b-3p	XBP1	X-box binding protein 1 [Source:VGNC Symbol;Acc:VGNC:94993]	-1.76	0.1694
ssc-miR-92b-3p	XKR7	XK related 7 [Source:VGNC Symbol;Acc:VGNC:95987]	-1.76	0.1694
ssc-miR-92b-3p	XPR1	xenotropic and polytropic retrovirus receptor 1 [Source:VGNC Symbol;Acc:VGNC:108625]	-1.76	0.1694
ssc-miR-92b-3p	XRN1	5'-3' exoribonuclease 1 [Source:VGNC Symbol;Acc:VGNC:108705]	-1.76	0.1694
ssc-miR-92b-3p	XYLT2	xylosyltransferase 2 [Source:VGNC Symbol;Acc:VGNC:95016]	-1.76	0.1694
ssc-miR-92b-3p	YIPF4	Yip1 domain family member 4 [Source:VGNC Symbol;Acc:VGNC:95030]	-1.76	0.1694
ssc-miR-92b-3p	YWHAZ	tyrosine 3-monooxygenase/tryptophan 5-monooxygenase activation protein zeta [Source:VGNC Symbol;Acc:VGNC:95047]	-1.76	0.1694
ssc-miR-92b-3p	ZBTB10	zinc finger and BTB domain containing 10 [Source:VGNC Symbol;Acc:VGNC:95058]	-1.76	0.1694
ssc-miR-92b-3p	ZBTB18	zinc finger and BTB domain containing 18 [Source:HGNC Symbol;Acc:HGNC:13030]	-1.76	0.1694
ssc-miR-92b-3p	ZBTB20	zinc finger and BTB domain containing 20 [Source:VGNC Symbol;Acc:VGNC:95063]	-1.76	0.1694
ssc-miR-92b-3p	ZBTB34	zinc finger and BTB domain containing 34 [Source:VGNC Symbol;Acc:VGNC:95070]	-1.76	0.1694
ssc-miR-92b-3p	ZBTB40	zinc finger and BTB domain containing 40 [Source:VGNC Symbol;Acc:VGNC:95074]	-1.76	0.1694
ssc-miR-92b-3p	ZBTB46	zinc finger and BTB domain containing 46 [Source:VGNC Symbol;Acc:VGNC:95733]	-1.76	0.1694
ssc-miR-92b-3p	ZC2HC1A	zinc finger C2HC-type containing 1A [Source:VGNC Symbol;Acc:VGNC:95085]	-1.76	0.1694
ssc-miR-92b-3p	ZC3H12B	zinc finger CCCH-type containing 12B [Source:VGNC Symbol;Acc:VGNC:95090]	-1.76	0.1694
ssc-miR-92b-3p	ZCCHC2	zinc finger CCHC-type containing 2 [Source:VGNC Symbol;Acc:VGNC:95109]	-1.76	0.1694
ssc-miR-92b-3p	ZDHHC21	zinc finger DHHC-type palmitoyltransferase 21 [Source:VGNC Symbol;Acc:VGNC:95123]	-1.76	0.1694
ssc-miR-92b-3p	ZDHHC23	zinc finger DHHC-type palmitoyltransferase 23 [Source:VGNC Symbol;Acc:VGNC:95125]	-1.76	0.1694
ssc-miR-92b-3p	ZDHHC3	zinc finger DHHC-type palmitoyltransferase 3 [Source:VGNC Symbol;Acc:VGNC:95126]	-1.76	0.1694
ssc-miR-92b-3p	ZDHHC5	zinc finger DHHC-type palmitoyltransferase 5 [Source:VGNC Symbol;Acc:VGNC:95127]	-1.76	0.1694
ssc-miR-92b-3p	ZEB2	hypothetical gene	-1.76	0.1694
ssc-miR-92b-3p	ZFC3H1	zinc finger C3H1-type containing [Source:VGNC Symbol;Acc:VGNC:95139]	-1.76	0.1694
ssc-miR-92b-3p	ZFHX3	zinc finger homeobox 3 [Source:VGNC Symbol;Acc:VGNC:95141]	-1.76	0.1694
ssc-miR-92b-3p	ZFHX4	zinc finger homeobox 4 [Source:VGNC Symbol;Acc:VGNC:95142]	-1.76	0.1694
ssc-miR-92b-3p	ZFP91	ZFP91 zinc finger protein, atypical E3 ubiquitin ligase [Source:HGNC Symbol;Acc:HGNC:14983]	-1.76	0.1694
ssc-miR-92b-3p	ZFPM2	zinc finger protein, FOG family member 2 [Source:VGNC Symbol;Acc:VGNC:95153]	-1.76	0.1694
ssc-miR-92b-3p	ZFYVE21	zinc finger FYVE-type containing 21 [Source:VGNC Symbol;Acc:VGNC:95159]	-1.76	0.1694
ssc-miR-92b-3p	ZNF148	zinc finger protein 148 [Source:VGNC Symbol;Acc:VGNC:95192]	-1.76	0.1694
ssc-miR-92b-3p	ZNF287	zinc finger protein 287 [Source:VGNC Symbol;Acc:VGNC:99107]	-1.76	0.1694
ssc-miR-92b-3p	ZNF385B	zinc finger protein 385B [Source:VGNC Symbol;Acc:VGNC:98425]	-1.76	0.1694
ssc-miR-92b-3p	ZNF385D	zinc finger protein 385D [Source:VGNC Symbol;Acc:VGNC:95234]	-1.76	0.1694
ssc-miR-92b-3p	ZNF451	zinc finger protein 451 [Source:HGNC Symbol;Acc:HGNC:21091]	-1.76	0.1694
ssc-miR-92b-3p	ZNF469	hypothetical gene	-1.76	0.1694

ssc-miR-92b-3p	ZNF496	zinc finger protein 496 [Source:VGNC Symbol;Acc:VGNC:95251]	-1.76	0.1694
ssc-miR-92b-3p	ZNF512B	zinc finger protein 512B [Source:HGNC Symbol;Acc:HGNC:29212]	-1.76	0.1694
ssc-miR-92b-3p	ZNF521	zinc finger protein 521 [Source:VGNC Symbol;Acc:VGNC:98715]	-1.76	0.1694
ssc-miR-92b-3p	ZNF532	zinc finger protein 532 [Source:HGNC Symbol;Acc:HGNC:30940]	-1.76	0.1694
ssc-miR-92b-3p	ZNF597	hypothetical gene	-1.76	0.1694
ssc-miR-92b-3p	ZNF652	zinc finger protein 652 [Source:VGNC Symbol;Acc:VGNC:99108]	-1.76	0.1694
ssc-miR-92b-3p	ZNF654	zinc finger protein 654 [Source:VGNC Symbol;Acc:VGNC:95285]	-1.76	0.1694
ssc-miR-92b-3p	ZNF804A	zinc finger protein 804A [Source:VGNC Symbol;Acc:VGNC:95976]	-1.76	0.1694
ssc-miR-92b-3p	ZNF804B	zinc finger protein 804B [Source:VGNC Symbol;Acc:VGNC:95307]	-1.76	0.1694
ssc-miR-92b-3p	ZNF827	zinc finger protein 827 [Source:VGNC Symbol;Acc:VGNC:98970]	-1.76	0.1694
ssc-miR-181d-3p	NA	hypothetical gene	-1.78	0.1152
ssc-miR-30c-3p	NA	hypothetical gene	-1.78	0.1384
ssc-miR-153	A1CF	APOBEC1 complementation factor [Source:VGNC Symbol;Acc:VGNC:84933]	-1.79	0.1415
ssc-miR-153	AAK1	AP2 associated kinase 1 [Source:VGNC Symbol;Acc:VGNC:100379]	-1.79	0.1415
ssc-miR-153	ABAT	4-aminobutyrate aminotransferase [Source:VGNC Symbol;Acc:VGNC:96910]	-1.79	0.1415
ssc-miR-153	ABCC12	ATP binding cassette subfamily C member 12 [Source:VGNC Symbol;Acc:VGNC:84955]	-1.79	0.1415
ssc-miR-153	ABHD13	abhydrolase domain containing 13 [Source:VGNC Symbol;Acc:VGNC:84972]	-1.79	0.1415
ssc-miR-153	AC002451.1	hypothetical gene	-1.79	0.1415
ssc-miR-153	AC012123.1	hypothetical gene	-1.79	0.1415
ssc-miR-153	ACAP3	ArfGAP with coiled-coil, ankyrin repeat and PH domains 3 [Source:VGNC Symbol;Acc:VGNC:85003]	-1.79	0.1415
ssc-miR-153	ACOT13	acyl-CoA thioesterase 13 [Source:VGNC Symbol;Acc:VGNC:85018]	-1.79	0.1415
ssc-miR-153	ACTN4	actinin alpha 4 [Source:VGNC Symbol;Acc:VGNC:85047]	-1.79	0.1415
ssc-miR-153	ACVR1B	activin A receptor type 1B [Source:VGNC Symbol;Acc:VGNC:85056]	-1.79	0.1415
ssc-miR-153	ACVR2A	activin A receptor type 2A [Source:VGNC Symbol;Acc:VGNC:95843]	-1.79	0.1415
ssc-miR-153	ADAM10	ADAM metallopeptidase domain 10 [Source:VGNC Symbol;Acc:VGNC:85061]	-1.79	0.1415
ssc-miR-153	ADAM19	ADAM metallopeptidase domain 19 [Source:VGNC Symbol;Acc:VGNC:85066]	-1.79	0.1415
ssc-miR-153	ADAM23	ADAM metallopeptidase domain 23 [Source:VGNC Symbol;Acc:VGNC:95933]	-1.79	0.1415
ssc-miR-153	ADAMTS8	ADAM metallopeptidase with thrombospondin type 1 motif 8 [Source:VGNC Symbol;Acc:VGNC:85088]	-1.79	0.1415
ssc-miR-153	ADC	hypothetical gene	-1.79	0.1415
ssc-miR-153	ADD1	adducin 1 [Source:VGNC Symbol;Acc:VGNC:85116]	-1.79	0.1415
ssc-miR-153	ADO	2-aminoethanethiol dioxygenase [Source:VGNC Symbol;Acc:VGNC:85146]	-1.79	0.1415
ssc-miR-153	AFF1	AF4/FMR2 family member 1 [Source:VGNC Symbol;Acc:VGNC:85167]	-1.79	0.1415
ssc-miR-153	AFF4	AF4/FMR2 family member 4 [Source:VGNC Symbol;Acc:VGNC:85169]	-1.79	0.1415
ssc-miR-153	AGO1	hypothetical gene	-1.79	0.1415
ssc-miR-153	AGO3	argonaute RISC component 1 [Source:NCBI gene (formerly Entrezgene);Acc:100499510]	-1.79	0.1415
ssc-miR-153	AIDA	axin interactor, dorsalization associated [Source:VGNC Symbol;Acc:VGNC:96103]	-1.79	0.1415
ssc-miR-153	AKAP6	A-kinase anchoring protein 6 [Source:VGNC Symbol;Acc:VGNC:85221]	-1.79	0.1415
ssc-miR-153	AKT3	AKT serine/threonine kinase 3 [Source:VGNC Symbol;Acc:VGNC:96306]	-1.79	0.1415
ssc-miR-153	AL590483.1	hypothetical gene	-1.79	0.1415
ssc-miR-153	AMOT	angiomotin [Source:VGNC Symbol;Acc:VGNC:85283]	-1.79	0.1415
ssc-miR-153	ANGPT1	angiopoietin 1 [Source:VGNC Symbol;Acc:VGNC:98732]	-1.79	0.1415
ssc-miR-153	ANK1	ankyrin 1 [Source:VGNC Symbol;Acc:VGNC:96344]	-1.79	0.1415
ssc-miR-153	ANK3	hypothetical gene	-1.79	0.1415
ssc-miR-153	ANKRD10	ankyrin repeat domain 10 [Source:VGNC Symbol;Acc:VGNC:85322]	-1.79	0.1415
ssc-miR-153	ANKRD29	ankyrin repeat domain 29 [Source:VGNC Symbol;Acc:VGNC:85332]	-1.79	0.1415
ssc-miR-153	ANKRD40	ankyrin repeat domain 40 [Source:VGNC Symbol;Acc:VGNC:85338]	-1.79	0.1415
ssc-miR-153	ANKRD50	ankyrin repeat domain containing 50 [Source:VGNC Symbol;Acc:VGNC:85341]	-1.79	0.1415
ssc-miR-153	ANKRD52	ankyrin repeat domain 52 [Source:VGNC Symbol;Acc:VGNC:85342]	-1.79	0.1415
ssc-miR-153	ANO3	anoctamin 3 [Source:VGNC Symbol;Acc:VGNC:85356]	-1.79	0.1415
ssc-miR-153	AP3B1	adaptor related protein complex 3 subunit beta 1 [Source:VGNC Symbol;Acc:VGNC:85387]	-1.79	0.1415

ssc-miR-153	AP5M1	adaptor related protein complex 5 subunit mu 1 [Source:VGNC Symbol;Acc:VGNC:85396]	-1.79	0.1415
ssc-miR-153	APBB2	amyloid beta protein binding family B member 2 [Source:VGNC Symbol;Acc:VGNC:85403]	-1.79	0.1415
ssc-miR-153	APC	APC regulator of WNT signaling pathway [Source:VGNC Symbol;Acc:VGNC:99584]	-1.79	0.1415
ssc-miR-153	APLF	aprataxin and PNKP like factor [Source:VGNC Symbol;Acc:VGNC:97033]	-1.79	0.1415
ssc-miR-153	APLP2	amyloid beta like protein 2 [Source:VGNC Symbol;Acc:VGNC:85414]	-1.79	0.1415
ssc-miR-153	APP	amyloid beta protein [Source:VGNC Symbol;Acc:VGNC:103902]	-1.79	0.1415
ssc-miR-153	APPL1	adaptor protein, phosphotyrosine interacting with PH domain and leucine zipper 1 [Source:VGNC Symbol;Acc:VGNC:85426]	-1.79	0.1415
ssc-miR-153	ARF1	ADP ribosylation factor 1 [Source:VGNC Symbol;Acc:VGNC:96793]	-1.79	0.1415
ssc-miR-153	ARHGAP36	hypothetical gene	-1.79	0.1415
ssc-miR-153	ARHGEF40	Rho guanine nucleotide exchange factor 40 [Source:VGNC Symbol;Acc:VGNC:85501]	-1.79	0.1415
ssc-miR-153	ARID1A	AT-rich interaction domain 1A [Source:VGNC Symbol;Acc:VGNC:85505]	-1.79	0.1415
ssc-miR-153	ARID3A	AT-rich interaction domain 3A [Source:VGNC Symbol;Acc:VGNC:85507]	-1.79	0.1415
ssc-miR-153	ARID4A	AT-rich interaction domain 4A [Source:VGNC Symbol;Acc:VGNC:85509]	-1.79	0.1415
ssc-miR-153	ARL4A	ADP ribosylation factor like GTPase 4A [Source:VGNC Symbol;Acc:VGNC:85522]	-1.79	0.1415
ssc-miR-153	ARNT	aryl hydrocarbon receptor nuclear translocator [Source:VGNC Symbol;Acc:VGNC:98733]	-1.79	0.1415
ssc-miR-153	ARNTL	aryl hydrocarbon receptor nuclear translocator like [Source:VGNC Symbol;Acc:VGNC:96796]	-1.79	0.1415
ssc-miR-153	ARPP19	cAMP regulated phosphoprotein 19 [Source:NCBI gene (formerly Entrezgene);Acc:397362]	-1.79	0.1415
ssc-miR-153	ASB7	ankyrin repeat and SOCS box containing 7 [Source:VGNC Symbol;Acc:VGNC:85566]	-1.79	0.1415
ssc-miR-153	ASIC2	acid sensing ion channel subunit 2 [Source:VGNC Symbol;Acc:VGNC:97893]	-1.79	0.1415
ssc-miR-153	ASXL1	ASXL transcriptional regulator 1 [Source:VGNC Symbol;Acc:VGNC:95881]	-1.79	0.1415
ssc-miR-153	ATAD2B	ATPase family AAA domain containing 2B [Source:VGNC Symbol;Acc:VGNC:85600]	-1.79	0.1415
ssc-miR-153	ATF6	activating transcription factor 6 [Source:VGNC Symbol;Acc:VGNC:85609]	-1.79	0.1415
ssc-miR-153	ATG5	autophagy related 5 [Source:VGNC Symbol;Acc:VGNC:103029]	-1.79	0.1415
ssc-miR-153	ATP13A3	ATPase 13A3 [Source:VGNC Symbol;Acc:VGNC:85639]	-1.79	0.1415
ssc-miR-153	ATP2A2	ATPase sarcoplasmic/endoplasmic reticulum Ca2+ transporting 2 [Source:VGNC Symbol;Acc:VGNC:85647]	-1.79	0.1415
ssc-miR-153	ATP2B2	ATPase plasma membrane Ca2+ transporting 2 [Source:VGNC Symbol;Acc:VGNC:85649]	-1.79	0.1415
ssc-miR-153	ATP2B4	ATPase plasma membrane Ca2+ transporting 4 [Source:VGNC Symbol;Acc:VGNC:85651]	-1.79	0.1415
ssc-miR-153	ATP2C1	ATPase secretory pathway Ca2+ transporting 1 [Source:VGNC Symbol;Acc:VGNC:85652]	-1.79	0.1415
ssc-miR-153	ATP8A1	ATPase phospholipid transporting 8A1 [Source:VGNC Symbol;Acc:VGNC:97894]	-1.79	0.1415
ssc-miR-153	ATXN1L	ataxin 1 like [Source:VGNC Symbol;Acc:VGNC:85689]	-1.79	0.1415
ssc-miR-153	ATXN3	hypothetical gene	-1.79	0.1415
ssc-miR-153	ATXN7	ataxin 7 [Source:VGNC Symbol;Acc:VGNC:99704]	-1.79	0.1415
ssc-miR-153	ATXN7L3B	ataxin 7 like 3B [Source:HGNC Symbol;Acc:HGNC:37931]	-1.79	0.1415
ssc-miR-153	AUTS2	activator of transcription and developmental regulator AUTS2 [Source:VGNC Symbol;Acc:VGNC:97897]	-1.79	0.1415
ssc-miR-153	AZIN1	antizyme inhibitor 1 [Source:VGNC Symbol;Acc:VGNC:85712]	-1.79	0.1415
ssc-miR-153	B3GALNT2	beta-1,3-N-acetylgalactosaminyltransferase 2 [Source:VGNC Symbol;Acc:VGNC:85715]	-1.79	0.1415
ssc-miR-153	B3GAT2	beta-1,3-glucuronyltransferase 2 [Source:VGNC Symbol;Acc:VGNC:85720]	-1.79	0.1415
ssc-miR-153	B4GALT1	beta-1,4-galactosyltransferase 1 [Source:VGNC Symbol;Acc:VGNC:96497]	-1.79	0.1415
ssc-miR-153	BACH2	BTB domain and CNC homolog 2 [Source:VGNC Symbol;Acc:VGNC:85742]	-1.79	0.1415
ssc-miR-153	BAG4	BAG cochaperone 4 [Source:VGNC Symbol;Acc:VGNC:96503]	-1.79	0.1415
ssc-miR-153	BBX	BBX high mobility group box domain containing [Source:VGNC Symbol;Acc:VGNC:85766]	-1.79	0.1415
ssc-miR-153	BCL2	BCL2 apoptosis regulator [Source:HGNC Symbol;Acc:HGNC:990]	-1.79	0.1415
ssc-miR-153	BEND4	BEN domain containing 4 [Source:VGNC Symbol;Acc:VGNC:85801]	-1.79	0.1415
ssc-miR-153	BMPR1A	bone morphotic protein receptor type 1A [Source:VGNC Symbol;Acc:VGNC:85846]	-1.79	0.1415
ssc-miR-153	BMPR2	bone morphotic protein receptor type 2 [Source:VGNC Symbol;Acc:VGNC:95494]	-1.79	0.1415
ssc-miR-153	BOD1L1	biorientation of chromosomes in cell division 1 like 1 [Source:VGNC Symbol;Acc:VGNC:97903]	-1.79	0.1415
ssc-miR-153	BPTF	bromodomain PHD finger transcription factor [Source:VGNC Symbol;Acc:VGNC:97904]	-1.79	0.1415
ssc-miR-153	BRMS1L	BRMS1 like transcriptional repressor [Source:VGNC Symbol;Acc:VGNC:96571]	-1.79	0.1415
ssc-miR-153	BSN	bassoon presynaptic cytomatrix protein [Source:VGNC Symbol;Acc:VGNC:85893]	-1.79	0.1415
ssc-miR-153	BTBD7	BTB domain containing 7 [Source:VGNC Symbol;Acc:VGNC:85908]	-1.79	0.1415

ssc-miR-153	C14orf28	chromosome 1 C14orf28 homolog [Source:VGNC Symbol;Acc:VGNC:85958]	-1.79	0.1415
ssc-miR-153	C16orf52	hypothetical gene	-1.79	0.1415
ssc-miR-153	C17orf75	chromosome 12 C17orf75 homolog [Source:VGNC Symbol;Acc:VGNC:85925]	-1.79	0.1415
ssc-miR-153	C20orf112	hypothetical gene	-1.79	0.1415
ssc-miR-153	C21orf91	chromosome 13 C21orf91 homolog [Source:VGNC Symbol;Acc:VGNC:85930]	-1.79	0.1415
ssc-miR-153	C2CD2	C2 calcium dependent domain containing 2 [Source:VGNC Symbol;Acc:VGNC:85990]	-1.79	0.1415
ssc-miR-153	C5orf24	chromosome 2 C5orf24 homolog [Source:VGNC Symbol;Acc:VGNC:86010]	-1.79	0.1415
ssc-miR-153	C6orf106	hypothetical gene	-1.79	0.1415
ssc-miR-153	C6orf120	chromosome 1 C6orf120 homolog [Source:VGNC Symbol;Acc:VGNC:85967]	-1.79	0.1415
ssc-miR-153	C6orf62	chromosome 7 C6orf62 homolog [Source:VGNC Symbol;Acc:VGNC:86074]	-1.79	0.1415
ssc-miR-153	C8orf44-SGK3	hypothetical gene	-1.79	0.1415
ssc-miR-153	C9orf40	chromosome 1 C9orf40 homolog [Source:VGNC Symbol;Acc:VGNC:85973]	-1.79	0.1415
ssc-miR-153	CABP7	calcium binding protein 7 [Source:VGNC Symbol;Acc:VGNC:107372]	-1.79	0.1415
ssc-miR-153	CACNA1B	calcium voltage-gated channel subunit alpha1 B [Source:HGNC Symbol;Acc:HGNC:1389]	-1.79	0.1415
ssc-miR-153	CACNA1C	calcium voltage-gated channel subunit alpha1 C [Source:HGNC Symbol;Acc:HGNC:1390]	-1.79	0.1415
ssc-miR-153	CACNB4	calcium voltage-gated channel auxiliary subunit beta 4 [Source:VGNC Symbol;Acc:VGNC:108507]	-1.79	0.1415
ssc-miR-153	CADM2	cell adhesion molecule 2 [Source:VGNC Symbol;Acc:VGNC:97910]	-1.79	0.1415
ssc-miR-153	CADM3	cell adhesion molecule 3 [Source:VGNC Symbol;Acc:VGNC:86135]	-1.79	0.1415
ssc-miR-153	CALN1	hypothetical gene	-1.79	0.1415
ssc-miR-153	CAMK1D	calcium/calmodulin dependent protein kinase ID [Source:VGNC Symbol;Acc:VGNC:95909]	-1.79	0.1415
ssc-miR-153	CAMK2G	calcium/calmodulin dependent protein kinase II gamma [Source:VGNC Symbol;Acc:VGNC:86157]	-1.79	0.1415
ssc-miR-153	CAMKK2	calcium/calmodulin dependent protein kinase kinase 2 [Source:VGNC Symbol;Acc:VGNC:86158]	-1.79	0.1415
ssc-miR-153	CAND1	cullin associated and neddylation dissociated 1 [Source:VGNC Symbol;Acc:VGNC:97912]	-1.79	0.1415
ssc-miR-153	CANX	calnexin [Source:VGNC Symbol;Acc:VGNC:86167]	-1.79	0.1415
ssc-miR-153	CAP1	cyclase associated actin cytoskeleton regulatory protein 1 [Source:VGNC Symbol;Acc:VGNC:96936]	-1.79	0.1415
ssc-miR-153	CAPN15	calpain 15 [Source:VGNC Symbol;Acc:VGNC:86174]	-1.79	0.1415
ssc-miR-153	CASK	calcium/calmodulin dependent serine protein kinase [Source:HGNC Symbol;Acc:HGNC:1497]	-1.79	0.1415
ssc-miR-153	CBFB	core-binding factor subunit beta [Source:VGNC Symbol;Acc:VGNC:86221]	-1.79	0.1415
ssc-miR-153	CBX5	chromobox 5 [Source:VGNC Symbol;Acc:VGNC:86232]	-1.79	0.1415
ssc-miR-153	CCDC144A	hypothetical gene	-1.79	0.1415
ssc-miR-153	CCDC50	coiled-coil domain containing 50 [Source:VGNC Symbol;Acc:VGNC:86299]	-1.79	0.1415
ssc-miR-153	CCND2	cyclin D2 [Source:VGNC Symbol;Acc:VGNC:103222]	-1.79	0.1415
ssc-miR-153	CCNJ	cyclin J [Source:VGNC Symbol;Acc:VGNC:86360]	-1.79	0.1415
ssc-miR-153	CCNT1	cyclin T1 [Source:VGNC Symbol;Acc:VGNC:86366]	-1.79	0.1415
ssc-miR-153	CCSAP	centriole, cilia and spindle associated protein [Source:VGNC Symbol;Acc:VGNC:86377]	-1.79	0.1415
ssc-miR-153	CCSER2	coiled-coil serine rich protein 2 [Source:VGNC Symbol;Acc:VGNC:86379]	-1.79	0.1415
ssc-miR-153	CDH4	cadherin 4 [Source:VGNC Symbol;Acc:VGNC:95853]	-1.79	0.1415
ssc-miR-153	CDH6	cadherin 6 [Source:HGNC Symbol;Acc:HGNC:1765]	-1.79	0.1415
ssc-miR-153	CDK13	cyclin dependent kinase 13 [Source:VGNC Symbol;Acc:VGNC:86497]	-1.79	0.1415
ssc-miR-153	CDK8	cyclin dependent kinase 8 [Source:VGNC Symbol;Acc:VGNC:86507]	-1.79	0.1415
ssc-miR-153	CDON	cell adhesion associated, onco regulated [Source:VGNC Symbol;Acc:VGNC:86519]	-1.79	0.1415
ssc-miR-153	CELF2	hypothetical gene	-1.79	0.1415
ssc-miR-153	CELF6	CUGBP Elav-like family member 6 [Source:HGNC Symbol;Acc:HGNC:14059]	-1.79	0.1415
ssc-miR-153	CEP350	centrosomal protein 350 [Source:HGNC Symbol;Acc:HGNC:24238]	-1.79	0.1415
ssc-miR-153	CFL2	cofilin 2 [Source:VGNC Symbol;Acc:VGNC:86611]	-1.79	0.1415
ssc-miR-153	CHD1	chromodomain helicase DNA binding protein 1 [Source:VGNC Symbol;Acc:VGNC:86629]	-1.79	0.1415
ssc-miR-153	CHM	CHM Rab escort protein [Source:VGNC Symbol;Acc:VGNC:86647]	-1.79	0.1415
ssc-miR-153	CHRM2	cholinergic receptor muscarinic 2 [Source:VGNC Symbol;Acc:VGNC:103922]	-1.79	0.1415
ssc-miR-153	CHST15	carbohydrate sulfotransferase 15 [Source:VGNC Symbol;Acc:VGNC:86677]	-1.79	0.1415
ssc-miR-153	CIB2	calcium and integrin binding family member 2 [Source:VGNC Symbol;Acc:VGNC:103923]	-1.79	0.1415

ssc-miR-153	CIC	capicua transcriptional repressor [Source:VGNC Symbol;Acc:VGNC:96950]	-1.79	0.1415
ssc-miR-153	CITED2	Cbp/p300 interacting transactivator with Glu/Asp rich carboxy-terminal domain 2 [Source:VGNC Symbol;Acc:VGNC:86712]	-1.79	0.1415
ssc-miR-153	CKAP4	cytoskeleton associated protein 4 [Source:VGNC Symbol;Acc:VGNC:86716]	-1.79	0.1415
ssc-miR-153	CLASP2	cytoplasmic linker associated protein 2 [Source:VGNC Symbol;Acc:VGNC:86720]	-1.79	0.1415
ssc-miR-153	CLCN3	chloride voltage-gated channel 3 [Source:VGNC Symbol;Acc:VGNC:86727]	-1.79	0.1415
ssc-miR-153	CLCN4	chloride voltage-gated channel 4 [Source:VGNC Symbol;Acc:VGNC:86728]	-1.79	0.1415
ssc-miR-153	CLCN5	chloride voltage-gated channel 5 [Source:VGNC Symbol;Acc:VGNC:103925]	-1.79	0.1415
ssc-miR-153	CLMP	CXADR like membrane protein [Source:VGNC Symbol;Acc:VGNC:86770]	-1.79	0.1415
ssc-miR-153	CLTC	clathrin heavy chain [Source:VGNC Symbol;Acc:VGNC:86789]	-1.79	0.1415
ssc-miR-153	CMC1	C-X9-C motif containing 1 [Source:VGNC Symbol;Acc:VGNC:108641]	-1.79	0.1415
ssc-miR-153	CMIP	c-Maf inducing protein [Source:VGNC Symbol;Acc:VGNC:86799]	-1.79	0.1415
ssc-miR-153	CNN3	calponin 3 [Source:VGNC Symbol;Acc:VGNC:86826]	-1.79	0.1415
ssc-miR-153	CPEB2	cytoplasmic polyadenylation element binding protein 2 [Source:VGNC Symbol;Acc:VGNC:86937]	-1.79	0.1415
ssc-miR-153	CPEB4	cytoplasmic polyadenylation element binding protein 4 [Source:VGNC Symbol;Acc:VGNC:86939]	-1.79	0.1415
ssc-miR-153	CREB5	cAMP responsive element binding protein 5 [Source:VGNC Symbol;Acc:VGNC:86984]	-1.79	0.1415
ssc-miR-153	CREBBP	CREB binding protein [Source:VGNC Symbol;Acc:VGNC:86985]	-1.79	0.1415
ssc-miR-153	CREBRF	CREB3 regulatory factor [Source:VGNC Symbol;Acc:VGNC:86987]	-1.79	0.1415
ssc-miR-153	CREM	hypothetical gene	-1.79	0.1415
ssc-miR-153	CRMP1	collapsin response mediator protein 1 [Source:VGNC Symbol;Acc:VGNC:87000]	-1.79	0.1415
ssc-miR-153	CROT	carnitine O-octanoyltransferase [Source:VGNC Symbol;Acc:VGNC:87002]	-1.79	0.1415
ssc-miR-153	CRTC1	CREB regulated transcription coactivator 1 [Source:VGNC Symbol;Acc:VGNC:87007]	-1.79	0.1415
ssc-miR-153	CSNK1A1	casein kinase 1 alpha 1 [Source:VGNC Symbol;Acc:VGNC:99630]	-1.79	0.1415
ssc-miR-153	CTDSPL2	CTD small phosphatase like 2 [Source:VGNC Symbol;Acc:VGNC:87060]	-1.79	0.1415
ssc-miR-153	CUX2	cut like homeobox 2 [Source:VGNC Symbol;Acc:VGNC:87094]	-1.79	0.1415
ssc-miR-153	CYFIP2	cytoplasmic FMR1 interacting protein 2 [Source:VGNC Symbol;Acc:VGNC:87127]	-1.79	0.1415
ssc-miR-153	CYTH3	cytohesin 3 [Source:VGNC Symbol;Acc:VGNC:87136]	-1.79	0.1415
ssc-miR-153	DAAM1	dishevelled associated activator of morphosis 1 [Source:VGNC Symbol;Acc:VGNC:87141]	-1.79	0.1415
ssc-miR-153	DAB1	DAB adaptor protein 1 [Source:VGNC Symbol;Acc:VGNC:87143]	-1.79	0.1415
ssc-miR-153	DAB2	DAB adaptor protein 2 [Source:VGNC Symbol;Acc:VGNC:87144]	-1.79	0.1415
ssc-miR-153	DACH1	dachshund family transcription factor 1 [Source:VGNC Symbol;Acc:VGNC:87146]	-1.79	0.1415
ssc-miR-153	DAGLA	diacylglycerol lipase alpha [Source:VGNC Symbol;Acc:VGNC:87152]	-1.79	0.1415
ssc-miR-153	DARS	hypothetical gene	-1.79	0.1415
ssc-miR-153	DCAF10	DDB1 and CUL4 associated factor 10 [Source:VGNC Symbol;Acc:VGNC:87173]	-1.79	0.1415
ssc-miR-153	DCAF5	DDB1 and CUL4 associated factor 5 [Source:VGNC Symbol;Acc:VGNC:87176]	-1.79	0.1415
ssc-miR-153	DCLK1	doublecortin like kinase 1 [Source:HGNC Symbol;Acc:HGNC:2700]	-1.79	0.1415
ssc-miR-153	DCP1A	decapping mRNA 1A [Source:VGNC Symbol;Acc:VGNC:97960]	-1.79	0.1415
ssc-miR-153	DCP2	decapping mRNA 2 [Source:VGNC Symbol;Acc:VGNC:87188]	-1.79	0.1415
ssc-miR-153	DCT	dopachrome tautomerase [Source:VGNC Symbol;Acc:VGNC:87192]	-1.79	0.1415
ssc-miR-153	DDI2	hypothetical gene	-1.79	0.1415
ssc-miR-153	DDIT4	DNA damage inducible transcript 4 [Source:VGNC Symbol;Acc:VGNC:87208]	-1.79	0.1415
ssc-miR-153	DESI2	desumoylating isopeptidase 2 [Source:VGNC Symbol;Acc:VGNC:96037]	-1.79	0.1415
ssc-miR-153	DEXI	Dexi homolog [Source:HGNC Symbol;Acc:HGNC:13267]	-1.79	0.1415
ssc-miR-153	DKK2	dickkopf WNT signaling pathway inhibitor 2 [Source:VGNC Symbol;Acc:VGNC:87322]	-1.79	0.1415
ssc-miR-153	DLG2	discs large MAGUK scaffold protein 2 [Source:VGNC Symbol;Acc:VGNC:108581]	-1.79	0.1415
ssc-miR-153	DLGAP2	DLG associated protein 2 [Source:VGNC Symbol;Acc:VGNC:99711]	-1.79	0.1415
ssc-miR-153	DLL4	delta like canonical Notch ligand 4 [Source:VGNC Symbol;Acc:VGNC:87337]	-1.79	0.1415
ssc-miR-153	DLX1	distal-less homeobox 1 [Source:VGNC Symbol;Acc:VGNC:96199]	-1.79	0.1415
ssc-miR-153	DMD	dystrophin [Source:NCBI gene (formerly Entrezgene);Acc:497636]	-1.79	0.1415
ssc-miR-153	DMXL2	Dmx like 2 [Source:VGNC Symbol;Acc:VGNC:87360]	-1.79	0.1415
ssc-miR-153	DNAJB12	DnaJ heat shock protein family (Hsp40) member B12 [Source:VGNC Symbol;Acc:VGNC:96679]	-1.79	0.1415

ssc-miR-153	DNAJB4	DnaJ heat shock protein family (Hsp40) member B4 [Source:VGNC Symbol;Acc:VGNC:96623]	-1.79	0.1415
ssc-miR-153	DNM3	dynamin 3 [Source:VGNC Symbol;Acc:VGNC:87382]	-1.79	0.1415
ssc-miR-153	DOC2A	double C2 domain alpha [Source:VGNC Symbol;Acc:VGNC:87388]	-1.79	0.1415
ssc-miR-153	DOT1L	DOT1 like histone lysine methyltransferase [Source:VGNC Symbol;Acc:VGNC:87410]	-1.79	0.1415
ssc-miR-153	DPP6	dipeptidyl peptidase like 6 [Source:VGNC Symbol;Acc:VGNC:87421]	-1.79	0.1415
ssc-miR-153	DPY19L1	dpy-19 like C-mannosyltransferase 1 [Source:VGNC Symbol;Acc:VGNC:99712]	-1.79	0.1415
ssc-miR-153	DPYSL5	dihydropyrimidinase like 5 [Source:VGNC Symbol;Acc:VGNC:87433]	-1.79	0.1415
ssc-miR-153	DSE	dermatan sulfate epimerase [Source:VGNC Symbol;Acc:VGNC:103084]	-1.79	0.1415
ssc-miR-153	DTX3	deltex E3 ubiquitin ligase 3 [Source:VGNC Symbol;Acc:VGNC:87468]	-1.79	0.1415
ssc-miR-153	DUSP3	dual specificity phosphatase 3 [Source:VGNC Symbol;Acc:VGNC:87486]	-1.79	0.1415
ssc-miR-153	DVL3	dishevelled segment polarity protein 3 [Source:VGNC Symbol;Acc:VGNC:87492]	-1.79	0.1415
ssc-miR-153	DYRK1A	dual specificity tyrosine phosphorylation regulated kinase 1A [Source:VGNC Symbol;Acc:VGNC:87505]	-1.79	0.1415
ssc-miR-153	E2F3	E2F transcription factor 3 [Source:VGNC Symbol;Acc:VGNC:87514]	-1.79	0.1415
ssc-miR-153	EBF2	EBF transcription factor 2 [Source:VGNC Symbol;Acc:VGNC:87526]	-1.79	0.1415
ssc-miR-153	EDEM1	ER degradation enhancing alpha-mannosidase like protein 1 [Source:VGNC Symbol;Acc:VGNC:87545]	-1.79	0.1415
ssc-miR-153	EFHD1	EF-hand domain family member D1 [Source:VGNC Symbol;Acc:VGNC:96222]	-1.79	0.1415
ssc-miR-153	EFHD2	EF-hand domain family member D2 [Source:HGNC Symbol;Acc:HGNC:28670]	-1.79	0.1415
ssc-miR-153	EFNA3	ephrin A3 [Source:VGNC Symbol;Acc:VGNC:98790]	-1.79	0.1415
ssc-miR-153	EFNB2	ephrin B2 [Source:VGNC Symbol;Acc:VGNC:87577]	-1.79	0.1415
ssc-miR-153	EIF1AX	eukaryotic translation initiation factor 1A X-linked [Source:NCBI gene (formerly Entrezgene);Acc:100522912]	-1.79	0.1415
ssc-miR-153	ELAVL2	ELAV like RNA binding protein 2 [Source:VGNC Symbol;Acc:VGNC:87639]	-1.79	0.1415
ssc-miR-153	ELAVL4	ELAV like RNA binding protein 4 [Source:VGNC Symbol;Acc:VGNC:97047]	-1.79	0.1415
ssc-miR-153	ELK4	ETS transcription factor ELK4 [Source:VGNC Symbol;Acc:VGNC:87647]	-1.79	0.1415
ssc-miR-153	ELL2	elongation factor for RNA polymerase II 2 [Source:VGNC Symbol;Acc:VGNC:87649]	-1.79	0.1415
ssc-miR-153	ELMSAN1	hypothetical gene	-1.79	0.1415
ssc-miR-153	EML6	EMAP like 6 [Source:VGNC Symbol;Acc:VGNC:87687]	-1.79	0.1415
ssc-miR-153	EPC1	enhancer of polycomb homolog 1 [Source:VGNC Symbol;Acc:VGNC:95923]	-1.79	0.1415
ssc-miR-153	EPDR1	ependymin related 1 [Source:VGNC Symbol;Acc:VGNC:87727]	-1.79	0.1415
ssc-miR-153	EPHA4	EPH receptor A4 [Source:VGNC Symbol;Acc:VGNC:96280]	-1.79	0.1415
ssc-miR-153	ERI2	ERI1 exoribonuclease family member 2 [Source:VGNC Symbol;Acc:VGNC:106577]	-1.79	0.1415
ssc-miR-153	ERO1LB	hypothetical gene	-1.79	0.1415
ssc-miR-153	ETS2	ETS proto-onco 2, transcription factor [Source:VGNC Symbol;Acc:VGNC:87809]	-1.79	0.1415
ssc-miR-153	EXOC8	exocyst complex component 8 [Source:VGNC Symbol;Acc:VGNC:87834]	-1.79	0.1415
ssc-miR-153	EXT1	exostosin glycosyltransferase 1 [Source:VGNC Symbol;Acc:VGNC:87846]	-1.79	0.1415
ssc-miR-153	FAF1	Fas associated factor 1 [Source:VGNC Symbol;Acc:VGNC:87879]	-1.79	0.1415
ssc-miR-153	FAM102A	family with sequence similarity 102 member A [Source:HGNC Symbol;Acc:HGNC:31419]	-1.79	0.1415
ssc-miR-153	FAM105B	hypothetical gene	-1.79	0.1415
ssc-miR-153	FAM124A	family with sequence similarity 124 member A [Source:VGNC Symbol;Acc:VGNC:87898]	-1.79	0.1415
ssc-miR-153	FAM129B	hypothetical gene	-1.79	0.1415
ssc-miR-153	FAM135B	family with sequence similarity 135 member B [Source:VGNC Symbol;Acc:VGNC:87905]	-1.79	0.1415
ssc-miR-153	FAM160A1	hypothetical gene	-1.79	0.1415
ssc-miR-153	FAM168A	family with sequence similarity 168 member A [Source:VGNC Symbol;Acc:VGNC:87926]	-1.79	0.1415
ssc-miR-153	FAM168B	family with sequence similarity 168 member B [Source:VGNC Symbol;Acc:VGNC:95462]	-1.79	0.1415
ssc-miR-153	FAM171A1	family with sequence similarity 171 member A1 [Source:VGNC Symbol;Acc:VGNC:96011]	-1.79	0.1415
ssc-miR-153	FAM177A1	family with sequence similarity 177 member A1 [Source:VGNC Symbol;Acc:VGNC:87933]	-1.79	0.1415
ssc-miR-153	FAM184B	family with sequence similarity 184 member B [Source:VGNC Symbol;Acc:VGNC:87938]	-1.79	0.1415
ssc-miR-153	FAM210B	family with sequence similarity 210 member B [Source:VGNC Symbol;Acc:VGNC:95736]	-1.79	0.1415
ssc-miR-153	FAM214A	family with sequence similarity 214 member A [Source:VGNC Symbol;Acc:VGNC:87952]	-1.79	0.1415
ssc-miR-153	FAR1	fatty acyl-CoA reductase 1 [Source:VGNC Symbol;Acc:VGNC:88010]	-1.79	0.1415
ssc-miR-153	FAT2	FAT atypical cadherin 2 [Source:VGNC Symbol;Acc:VGNC:88019]	-1.79	0.1415

ssc-miR-153	FAT3	hypothetical gene	-1.79	0.1415
ssc-miR-153	FBRSL1	hypothetical gene	-1.79	0.1415
ssc-miR-153	FBXL3	F-box and leucine rich repeat protein 3 [Source:VGNC Symbol;Acc:VGNC:88030]	-1.79	0.1415
ssc-miR-153	FBXL4	F-box and leucine rich repeat protein 4 [Source:VGNC Symbol;Acc:VGNC:98007]	-1.79	0.1415
ssc-miR-153	FBXO32	F-box protein 32 [Source:VGNC Symbol;Acc:VGNC:88039]	-1.79	0.1415
ssc-miR-153	FBXO33	F-box protein 33 [Source:VGNC Symbol;Acc:VGNC:88040]	-1.79	0.1415
ssc-miR-153	FBXO8	F-box protein 8 [Source:VGNC Symbol;Acc:VGNC:98010]	-1.79	0.1415
ssc-miR-153	FEM1C	fem-1 homolog C [Source:VGNC Symbol;Acc:VGNC:88084]	-1.79	0.1415
ssc-miR-153	FERMT2	FERM domain containing kindlin 2 [Source:VGNC Symbol;Acc:VGNC:88088]	-1.79	0.1415
ssc-miR-153	FFAR4	free fatty acid receptor 4 [Source:VGNC Symbol;Acc:VGNC:107392]	-1.79	0.1415
ssc-miR-153	FGFR2	fibroblast growth factor receptor 2 [Source:NCBI gene (formerly Entrezgene);Acc:396762]	-1.79	0.1415
ssc-miR-153	FIGN	fidgetin, microtubule severing factor [Source:VGNC Symbol;Acc:VGNC:95580]	-1.79	0.1415
ssc-miR-153	FLI1	Fli-1 proto-onco, ETS transcription factor [Source:VGNC Symbol;Acc:VGNC:88153]	-1.79	0.1415
ssc-miR-153	FLRT2	fibronectin leucine rich transmembrane protein 2 [Source:VGNC Symbol;Acc:VGNC:88161]	-1.79	0.1415
ssc-miR-153	FMR1	FMRP translational regulator 1 [Source:VGNC Symbol;Acc:VGNC:88175]	-1.79	0.1415
ssc-miR-153	FOXO1	forkhead box O1 [Source:VGNC Symbol;Acc:VGNC:98013]	-1.79	0.1415
ssc-miR-153	FOXO3	forkhead box O3 [Source:VGNC Symbol;Acc:VGNC:99715]	-1.79	0.1415
ssc-miR-153	FRK	fyn related Src family tyrosine kinase [Source:VGNC Symbol;Acc:VGNC:88235]	-1.79	0.1415
ssc-miR-153	FRMD5	FERM domain containing 5 [Source:VGNC Symbol;Acc:VGNC:88238]	-1.79	0.1415
ssc-miR-153	FRMPD4	FERM and PDZ domain containing 4 [Source:VGNC Symbol;Acc:VGNC:88243]	-1.79	0.1415
ssc-miR-153	FRS2	fibroblast growth factor receptor substrate 2 [Source:VGNC Symbol;Acc:VGNC:88246]	-1.79	0.1415
ssc-miR-153	FSD1L	fibronectin type III and SPRY domain containing 1 like [Source:HGNC Symbol;Acc:HGNC:13753]	-1.79	0.1415
ssc-miR-153	FURIN	furin, paired basic amino acid cleaving enzyme [Source:VGNC Symbol;Acc:VGNC:88266]	-1.79	0.1415
ssc-miR-153	FXR1	FMR1 autosomal homolog 1 [Source:VGNC Symbol;Acc:VGNC:108659]	-1.79	0.1415
ssc-miR-153	FYN	FYN proto-onco, Src family tyrosine kinase [Source:VGNC Symbol;Acc:VGNC:88276]	-1.79	0.1415
ssc-miR-153	FYTTD1	forty-two-three domain containing 1 [Source:VGNC Symbol;Acc:VGNC:88277]	-1.79	0.1415
ssc-miR-153	FZD3	frizzled class receptor 3 [Source:VGNC Symbol;Acc:VGNC:88281]	-1.79	0.1415
ssc-miR-153	FZD4	frizzled class receptor 4 [Source:VGNC Symbol;Acc:VGNC:88282]	-1.79	0.1415
ssc-miR-153	GAB1	GRB2 associated binding protein 1 [Source:VGNC Symbol;Acc:VGNC:88294]	-1.79	0.1415
ssc-miR-153	GABPB2	GA binding protein transcription factor subunit beta 2 [Source:VGNC Symbol;Acc:VGNC:98796]	-1.79	0.1415
ssc-miR-153	GABRA1	gamma-aminobutyric acid type A receptor subunit alpha1 [Source:VGNC Symbol;Acc:VGNC:88300]	-1.79	0.1415
ssc-miR-153	GALNT3	polypeptide N-acetylgalactosaminyltransferase 3 [Source:VGNC Symbol;Acc:VGNC:96313]	-1.79	0.1415
ssc-miR-153	GALNT7	polypeptide N-acetylgalactosaminyltransferase 7 [Source:VGNC Symbol;Acc:VGNC:103946]	-1.79	0.1415
ssc-miR-153	GAN	gigaxonin [Source:VGNC Symbol;Acc:VGNC:88343]	-1.79	0.1415
ssc-miR-153	GAREM	hypothetical gene	-1.79	0.1415
ssc-miR-153	GATAD2A	GATA zinc finger domain containing 2A [Source:VGNC Symbol;Acc:VGNC:88368]	-1.79	0.1415
ssc-miR-153	GFPT2	glutamine-fructose-6-phosphate transaminase 2 [Source:VGNC Symbol;Acc:VGNC:88427]	-1.79	0.1415
ssc-miR-153	GGCX	gamma-glutamyl carboxylase [Source:VGNC Symbol;Acc:VGNC:88435]	-1.79	0.1415
ssc-miR-153	GJA9	gap junction protein alpha 9 [Source:VGNC Symbol;Acc:VGNC:97065]	-1.79	0.1415
ssc-miR-153	GJD2	gap junction protein delta 2 [Source:VGNC Symbol;Acc:VGNC:88471]	-1.79	0.1415
ssc-miR-153	GKAP1	G kinase anchoring protein 1 [Source:VGNC Symbol;Acc:VGNC:95850]	-1.79	0.1415
ssc-miR-153	GLCCI1	glucocorticoid induced 1 [Source:VGNC Symbol;Acc:VGNC:88476]	-1.79	0.1415
ssc-miR-153	GLCE	glucuronic acid epimerase [Source:VGNC Symbol;Acc:VGNC:88477]	-1.79	0.1415
ssc-miR-153	GLRA3	glycine receptor alpha 3 [Source:VGNC Symbol;Acc:VGNC:88493]	-1.79	0.1415
ssc-miR-153	GMCL1	hypothetical gene	-1.79	0.1415
ssc-miR-153	GNAI3	G protein subunit alpha i3 [Source:VGNC Symbol;Acc:VGNC:88523]	-1.79	0.1415
ssc-miR-153	GOLGA8N	hypothetical gene	-1.79	0.1415
ssc-miR-153	GOLPH3	hypothetical gene	-1.79	0.1415
ssc-miR-153	GPCPD1	glycerophosphocholine phosphodiesterase 1 [Source:VGNC Symbol;Acc:VGNC:96148]	-1.79	0.1415
ssc-miR-153	GPHN	gephyrin [Source:VGNC Symbol;Acc:VGNC:88587]	-1.79	0.1415

ssc-miR-153	GPR124	hypothetical gene	-1.79	0.1415
ssc-miR-153	GPR155	G protein-coupled receptor 155 [Source:VGNC Symbol;Acc:VGNC:95984]	-1.79	0.1415
ssc-miR-153	GPR158	G protein-coupled receptor 158 [Source:VGNC Symbol;Acc:VGNC:96016]	-1.79	0.1415
ssc-miR-153	GPR37	G protein-coupled receptor 37 [Source:VGNC Symbol;Acc:VGNC:88630]	-1.79	0.1415
ssc-miR-153	GPR4	G protein-coupled receptor 4 [Source:VGNC Symbol;Acc:VGNC:88631]	-1.79	0.1415
ssc-miR-153	GRB2	growth factor receptor bound protein 2 [Source:VGNC Symbol;Acc:VGNC:88663]	-1.79	0.1415
ssc-miR-153	GRHL3	grainyhead like transcription factor 3 [Source:VGNC Symbol;Acc:VGNC:88668]	-1.79	0.1415
ssc-miR-153	GRIK2	glutamate ionotropic receptor kainate type subunit 2 [Source:VGNC Symbol;Acc:VGNC:88678]	-1.79	0.1415
ssc-miR-153	GRIK3	glutamate ionotropic receptor kainate type subunit 3 [Source:VGNC Symbol;Acc:VGNC:88679]	-1.79	0.1415
ssc-miR-153	GRIK4	glutamate ionotropic receptor kainate type subunit 4 [Source:VGNC Symbol;Acc:VGNC:88680]	-1.79	0.1415
ssc-miR-153	GRIP1	glutamate receptor interacting protein 1 [Source:VGNC Symbol;Acc:VGNC:88690]	-1.79	0.1415
ssc-miR-153	GXYLT1	glucoside xylosyltransferase 1 [Source:VGNC Symbol;Acc:VGNC:88756]	-1.79	0.1415
ssc-miR-153	GXYLT2	glucoside xylosyltransferase 2 [Source:VGNC Symbol;Acc:VGNC:88757]	-1.79	0.1415
ssc-miR-153	HAUS3	hypothetical gene	-1.79	0.1415
ssc-miR-153	HCFC2	host cell factor C2 [Source:VGNC Symbol;Acc:VGNC:88800]	-1.79	0.1415
ssc-miR-153	HCN1	hyperpolarization activated cyclic nucleotide gated potassium channel 1 [Source:VGNC Symbol;Acc:VGNC:88802]	-1.79	0.1415
ssc-miR-153	HDLBP	high density lipoprotein binding protein [Source:HGNC Symbol;Acc:HGNC:4857]	-1.79	0.1415
ssc-miR-153	HECW1	HECT, C2 and WW domain containing E3 ubiquitin protein ligase 1 [Source:VGNC Symbol;Acc:VGNC:88835]	-1.79	0.1415
ssc-miR-153	HECW2	HECT, C2 and WW domain containing E3 ubiquitin protein ligase 2 [Source:NCBI gene (formerly Entrezgene);Acc:100155879]	-1.79	0.1415
ssc-miR-153	HELZ	helicase with zinc finger [Source:VGNC Symbol;Acc:VGNC:88840]	-1.79	0.1415
ssc-miR-153	HERC3	HECT and RLD domain containing E3 ubiquitin protein ligase 3 [Source:VGNC Symbol;Acc:VGNC:98934]	-1.79	0.1415
ssc-miR-153	HEY2	hes related family bHLH transcription factor with YRPW motif 2 [Source:VGNC Symbol;Acc:VGNC:88864]	-1.79	0.1415
ssc-miR-153	HIPK3	homeodomain interacting protein kinase 3 [Source:VGNC Symbol;Acc:VGNC:88889]	-1.79	0.1415
ssc-miR-153	HLCS	holocarboxylase synthetase [Source:VGNC Symbol;Acc:VGNC:88895]	-1.79	0.1415
ssc-miR-153	HMCES	5-hydroxymethylcytosine binding, ES cell specific [Source:VGNC Symbol;Acc:VGNC:96736]	-1.79	0.1415
ssc-miR-153	HNRNPA0	hypothetical gene	-1.79	0.1415
ssc-miR-153	HNRNPA1	heteroous nuclear ribonucleoprotein A1 [Source:VGNC Symbol;Acc:VGNC:88918]	-1.79	0.1415
ssc-miR-153	HNRNPK	heteroous nuclear ribonucleoprotein K [Source:VGNC Symbol;Acc:VGNC:103963]	-1.79	0.1415
ssc-miR-153	HOXC11	homeobox C11 [Source:VGNC Symbol;Acc:VGNC:103287]	-1.79	0.1415
ssc-miR-153	HOXC12	homeobox C12 [Source:VGNC Symbol;Acc:VGNC:88950]	-1.79	0.1415
ssc-miR-153	HOXC8	homeobox C8 [Source:VGNC Symbol;Acc:VGNC:88954]	-1.79	0.1415
ssc-miR-153	HS6ST1	heparan sulfate 6-O-sulfotransferase 1 [Source:VGNC Symbol;Acc:VGNC:96362]	-1.79	0.1415
ssc-miR-153	HTR1F	5-hydroxytryptamine receptor 1F [Source:VGNC Symbol;Acc:VGNC:88999]	-1.79	0.1415
ssc-miR-153	IARS	hypothetical gene	-1.79	0.1415
ssc-miR-153	IDE	insulin degrading enzyme [Source:VGNC Symbol;Acc:VGNC:89025]	-1.79	0.1415
ssc-miR-153	IFFO2	intermediate filament family orphan 2 [Source:VGNC Symbol;Acc:VGNC:98475]	-1.79	0.1415
ssc-miR-153	IFIT5	interferon induced protein with tetratricopeptide repeats 5 [Source:VGNC Symbol;Acc:VGNC:107402]	-1.79	0.1415
ssc-miR-153	IFNAR2	interferon alpha and beta receptor subunit 2 [Source:NCBI gene (formerly Entrezgene);Acc:100533555]	-1.79	0.1415
ssc-miR-153	IGF1R	insulin like growth factor 1 receptor [Source:NCBI gene (formerly Entrezgene);Acc:397350]	-1.79	0.1415
ssc-miR-153	IGSF10	immunoglobulin superfamily member 10 [Source:VGNC Symbol;Acc:VGNC:89063]	-1.79	0.1415
ssc-miR-153	IKZF1	IKAROS family zinc finger 1 [Source:VGNC Symbol;Acc:VGNC:89073]	-1.79	0.1415
ssc-miR-153	IKZF2	IKAROS family zinc finger 2 [Source:VGNC Symbol;Acc:VGNC:95576]	-1.79	0.1415
ssc-miR-153	IKZF5	IKAROS family zinc finger 5 [Source:VGNC Symbol;Acc:VGNC:89076]	-1.79	0.1415
ssc-miR-153	IMPAD1	hypothetical gene	-1.79	0.1415
ssc-miR-153	ING2	inhibitor of growth family member 2 [Source:VGNC Symbol;Acc:VGNC:99717]	-1.79	0.1415
ssc-miR-153	INHBA	inhibin subunit beta A [Source:VGNC Symbol;Acc:VGNC:89133]	-1.79	0.1415
ssc-miR-153	INHBB	inhibin subunit beta B [Source:VGNC Symbol;Acc:VGNC:103969]	-1.79	0.1415
ssc-miR-153	INO80D	INO80 complex subunit D [Source:VGNC Symbol;Acc:VGNC:96113]	-1.79	0.1415
ssc-miR-153	IPO7	importin 7 [Source:VGNC Symbol;Acc:VGNC:89180]	-1.79	0.1415
ssc-miR-153	IQCK	IQ motif containing K [Source:VGNC Symbol;Acc:VGNC:89191]	-1.79	0.1415

ssc-miR-153	IRF2	interferon regulatory factor 2 [Source:VGNC Symbol;Acc:VGNC:96375]	-1.79	0.1415
ssc-miR-153	IRS2	insulin receptor substrate 2 [Source:VGNC Symbol;Acc:VGNC:89214]	-1.79	0.1415
ssc-miR-153	IRX2	iroquois homeobox 2 [Source:VGNC Symbol;Acc:VGNC:89216]	-1.79	0.1415
ssc-miR-153	ITPR1	inositol 1,4,5-trisphosphate receptor type 1 [Source:VGNC Symbol;Acc:VGNC:89253]	-1.79	0.1415
ssc-miR-153	ITSN2	intersectin 2 [Source:VGNC Symbol;Acc:VGNC:89259]	-1.79	0.1415
ssc-miR-153	JAG1	jagged canonical Notch ligand 1 [Source:VGNC Symbol;Acc:VGNC:96385]	-1.79	0.1415
ssc-miR-153	JAKMIP3	Janus kinase and microtubule interacting protein 3 [Source:VGNC Symbol;Acc:VGNC:89275]	-1.79	0.1415
ssc-miR-153	JARID2	jumonji and AT-rich interaction domain containing 2 [Source:VGNC Symbol;Acc:VGNC:89279]	-1.79	0.1415
ssc-miR-153	JHDM1D	hypothetical gene	-1.79	0.1415
ssc-miR-153	JOSD1	Josephin domain containing 1 [Source:VGNC Symbol;Acc:VGNC:89290]	-1.79	0.1415
ssc-miR-153	KANSL1	KAT8 regulatory NSL complex subunit 1 [Source:VGNC Symbol;Acc:VGNC:89299]	-1.79	0.1415
ssc-miR-153	KBTBD8	kelch repeat and BTB domain containing 8 [Source:VGNC Symbol;Acc:VGNC:89321]	-1.79	0.1415
ssc-miR-153	KCNA1	potassium voltage-gated channel subfamily A member 1 [Source:VGNC Symbol;Acc:VGNC:89323]	-1.79	0.1415
ssc-miR-153	KCNA4	potassium voltage-gated channel subfamily A member 4 [Source:VGNC Symbol;Acc:VGNC:89326]	-1.79	0.1415
ssc-miR-153	KCNA6	potassium voltage-gated channel subfamily A member 6 [Source:HGNC Symbol;Acc:HGNC:6225]	-1.79	0.1415
ssc-miR-153	KCNB1	potassium voltage-gated channel subfamily B member 1 [Source:VGNC Symbol;Acc:VGNC:96386]	-1.79	0.1415
ssc-miR-153	KCNC3	potassium voltage-gated channel subfamily C member 3 [Source:VGNC Symbol;Acc:VGNC:89334]	-1.79	0.1415
ssc-miR-153	KCND1	potassium voltage-gated channel subfamily D member 1 [Source:VGNC Symbol;Acc:VGNC:89336]	-1.79	0.1415
ssc-miR-153	KCNH5	potassium voltage-gated channel subfamily H member 5 [Source:VGNC Symbol;Acc:VGNC:89345]	-1.79	0.1415
ssc-miR-153	KCNH7	potassium voltage-gated channel subfamily H member 7 [Source:VGNC Symbol;Acc:VGNC:95897]	-1.79	0.1415
ssc-miR-153	KCNIP2	potassium voltage-gated channel interacting protein 2 [Source:VGNC Symbol;Acc:VGNC:89349]	-1.79	0.1415
ssc-miR-153	KCNIP4	potassium voltage-gated channel interacting protein 4 [Source:VGNC Symbol;Acc:VGNC:89351]	-1.79	0.1415
ssc-miR-153	KCNK10	potassium two pore domain channel subfamily K member 10 [Source:VGNC Symbol;Acc:VGNC:89364]	-1.79	0.1415
ssc-miR-153	KCNK12	potassium two pore domain channel subfamily K member 12 [Source:HGNC Symbol;Acc:HGNC:6274]	-1.79	0.1415
ssc-miR-153	KCNK3	potassium two pore domain channel subfamily K member 3 [Source:VGNC Symbol;Acc:VGNC:89370]	-1.79	0.1415
ssc-miR-153	KCNMB2	potassium calcium-activated channel subfamily M regulatory beta subunit 2 [Source:HGNC Symbol;Acc:HGNC:6286]	-1.79	0.1415
ssc-miR-153	KCNQ3	potassium voltage-gated channel subfamily Q member 3 [Source:VGNC Symbol;Acc:VGNC:89382]	-1.79	0.1415
ssc-miR-153	KCNQ4	potassium voltage-gated channel subfamily Q member 4 [Source:VGNC Symbol;Acc:VGNC:89383]	-1.79	0.1415
ssc-miR-153	KCTD16	potassium channel tetramerization domain containing 16 [Source:VGNC Symbol;Acc:VGNC:89395]	-1.79	0.1415
ssc-miR-153	KCTD5	hypothetical gene	-1.79	0.1415
ssc-miR-153	KCTD6	potassium channel tetramerization domain containing 6 [Source:VGNC Symbol;Acc:VGNC:89402]	-1.79	0.1415
ssc-miR-153	KCTD9	potassium channel tetramerization domain containing 9 [Source:VGNC Symbol;Acc:VGNC:89404]	-1.79	0.1415
ssc-miR-153	KDM2B	lysine demethylase 2B [Source:VGNC Symbol;Acc:VGNC:98057]	-1.79	0.1415
ssc-miR-153	KDM5A	lysine demethylase 5A [Source:VGNC Symbol;Acc:VGNC:89415]	-1.79	0.1415
ssc-miR-153	KDM5B	lysine demethylase 5B [Source:VGNC Symbol;Acc:VGNC:95870]	-1.79	0.1415
ssc-miR-153	KDM6A	lysine demethylase 6A [Source:HGNC Symbol;Acc:HGNC:12637]	-1.79	0.1415
ssc-miR-153	KDSR	3-ketodihydrosphingosine reductase [Source:VGNC Symbol;Acc:VGNC:108157]	-1.79	0.1415
ssc-miR-153	KIAA0247	hypothetical gene	-1.79	0.1415
ssc-miR-153	KIAA0556	hypothetical gene	-1.79	0.1415
ssc-miR-153	KIAA1429	hypothetical gene	-1.79	0.1415
ssc-miR-153	KIAA1456	hypothetical gene	-1.79	0.1415
ssc-miR-153	KIAA1522	KIAA1522 [Source:VGNC Symbol;Acc:VGNC:89444]	-1.79	0.1415
ssc-miR-153	KIAA1549	KIAA1549 [Source:VGNC Symbol;Acc:VGNC:99719]	-1.79	0.1415
ssc-miR-153	KIF20A	kinesin family member 20A [Source:VGNC Symbol;Acc:VGNC:89462]	-1.79	0.1415
ssc-miR-153	KIF26B	kinesin family member 26B [Source:VGNC Symbol;Acc:VGNC:96087]	-1.79	0.1415
ssc-miR-153	KLF13	Kruppel like factor 13 [Source:VGNC Symbol;Acc:VGNC:89493]	-1.79	0.1415
ssc-miR-153	KLF3	Kruppel like factor 3 [Source:VGNC Symbol;Acc:VGNC:89498]	-1.79	0.1415
ssc-miR-153	KLF5	Kruppel like factor 5 [Source:VGNC Symbol;Acc:VGNC:89499]	-1.79	0.1415
ssc-miR-153	KLF6	Kruppel like factor 6 [Source:VGNC Symbol;Acc:VGNC:98063]	-1.79	0.1415
ssc-miR-153	KLF7	Kruppel like factor 7 [Source:VGNC Symbol;Acc:VGNC:96396]	-1.79	0.1415

ssc-miR-153	KLHL3	kelch like family member 3 [Source:VGNC Symbol;Acc:VGNC:89525]		-1.79	0.1415
ssc-miR-153	KLHL5	kelch like family member 5 [Source:VGNC Symbol;Acc:VGNC:89535]		-1.79	0.1415
ssc-miR-153	KMT2A	lysine methyltransferase 2A [Source:VGNC Symbol;Acc:VGNC:108600]		-1.79	0.1415
ssc-miR-153	KMT2B	lysine methyltransferase 2B [Source:VGNC Symbol;Acc:VGNC:98489]		-1.79	0.1415
ssc-miR-153	KPNA4	karyopherin subunit alpha 4 [Source:VGNC Symbol;Acc:VGNC:89563]		-1.79	0.1415
ssc-miR-153	KPNA5	karyopherin subunit alpha 5 [Source:VGNC Symbol;Acc:VGNC:89564]		-1.79	0.1415
ssc-miR-153	KREMEN1	kringle containing transmembrane protein 1 [Source:VGNC Symbol;Acc:VGNC:89572]		-1.79	0.1415
ssc-miR-153	KRT222	keratin 222 [Source:HGNC Symbol;Acc:HGNC:28695]		-1.79	0.1415
ssc-miR-153	LAMC1	laminin subunit gamma 1 [Source:VGNC Symbol;Acc:VGNC:89624]		-1.79	0.1415
ssc-miR-153	LAMP1	lysosomal associated membrane protein 1 [Source:VGNC Symbol;Acc:VGNC:89627]		-1.79	0.1415
ssc-miR-153	LAMP5	lysosomal associated membrane protein family member 5 [Source:VGNC Symbol;Acc:VGNC:95735]		-1.79	0.1415
ssc-miR-153	LARP1	La ribonucleoprotein 1, translational regulator [Source:VGNC Symbol;Acc:VGNC:98067]		-1.79	0.1415
ssc-miR-153	LCOR	ligand dependent nuclear receptor corepressor [Source:HGNC Symbol;Acc:HGNC:29503]		-1.79	0.1415
ssc-miR-153	LDLRAD4	low density lipoprotein receptor class A domain containing 4 [Source:VGNC Symbol;Acc:VGNC:89673]		-1.79	0.1415
ssc-miR-153	LGI2	leucine rich repeat LGI family member 2 [Source:VGNC Symbol;Acc:VGNC:89699]		-1.79	0.1415
ssc-miR-153	LHX9	LIM homeobox 9 [Source:VGNC Symbol;Acc:VGNC:95608]		-1.79	0.1415
ssc-miR-153	LIMS1	hypothetical gene		-1.79	0.1415
ssc-miR-153	LINGO3	leucine rich repeat and Ig domain containing 3 [Source:VGNC Symbol;Acc:VGNC:89734]		-1.79	0.1415
ssc-miR-153	LMAN1	lectin, mannose binding 1 [Source:VGNC Symbol;Acc:VGNC:89748]		-1.79	0.1415
ssc-miR-153	LNPEP	leucyl and cystinyl aminopeptidase [Source:VGNC Symbol;Acc:VGNC:89772]		-1.79	0.1415
ssc-miR-153	LPGAT1	lysophosphatidylglycerol acyltransferase 1 [Source:VGNC Symbol;Acc:VGNC:89792]		-1.79	0.1415
ssc-miR-153	LPP	LIM domain containing preferred translocation partner in lipoma [Source:HGNC Symbol;Acc:HGNC:6679]		-1.79	0.1415
ssc-miR-153	LPPR4	hypothetical gene		-1.79	0.1415
ssc-miR-153	LRCH1	leucine rich repeats and calponin homology domain containing 1 [Source:VGNC Symbol;Acc:VGNC:89801]		-1.79	0.1415
ssc-miR-153	LRIG3	leucine rich repeats and immunoglobulin like domains 3 [Source:VGNC Symbol;Acc:VGNC:89812]		-1.79	0.1415
ssc-miR-153	LRP12	LDL receptor related protein 12 [Source:VGNC Symbol;Acc:VGNC:89818]		-1.79	0.1415
ssc-miR-153	LRRC40	hypothetical gene		-1.79	0.1415
ssc-miR-153	LRRC57	leucine rich repeat containing 57 [Source:VGNC Symbol;Acc:VGNC:103119]		-1.79	0.1415
ssc-miR-153	LRRC58	leucine rich repeat containing 58 [Source:VGNC Symbol;Acc:VGNC:98081]		-1.79	0.1415
ssc-miR-153	LZIC	leucine zipper and CTNNBIP1 domain containing [Source:VGNC Symbol;Acc:VGNC:89925]		-1.79	0.1415
ssc-miR-153	MAFA	MAF bZIP transcription factor A [Source:VGNC Symbol;Acc:VGNC:89946]		-1.79	0.1415
ssc-miR-153	MAGI1	membrane associated guanylate kinase, WW and PDZ domain containing 1 [Source:VGNC Symbol;Acc:VGNC:98097]		-1.79	0.1415
ssc-miR-153	MAP2K6	mitogen-activated protein kinase kinase 6 [Source:VGNC Symbol;Acc:VGNC:98102]		-1.79	0.1415
ssc-miR-153	MAP3K1	mitogen-activated protein kinase kinase kinase 1 [Source:VGNC Symbol;Acc:VGNC:98104]		-1.79	0.1415
ssc-miR-153	MAP4K5	mitogen-activated protein kinase kinase kinase kinase 5 [Source:VGNC Symbol;Acc:VGNC:98115]		-1.79	0.1415
ssc-miR-153	MAPK1IP1L	mitogen-activated protein kinase 1 interacting protein 1 like [Source:VGNC Symbol;Acc:VGNC:90002]		-1.79	0.1415
ssc-miR-153	MAPK4	hypothetical gene		-1.79	0.1415
ssc-miR-153	MAT2A	methionine adenosyltransferase 2A [Source:VGNC Symbol;Acc:VGNC:90039]		-1.79	0.1415
ssc-miR-153	MATN2	matrilin 2 [Source:VGNC Symbol;Acc:VGNC:90043]		-1.79	0.1415
ssc-miR-153	MBD6	methyl-CpG binding domain protein 6 [Source:VGNC Symbol;Acc:VGNC:90051]		-1.79	0.1415
ssc-miR-153	MBNL3	muscleblind like splicing regulator 3 [Source:VGNC Symbol;Acc:VGNC:90056]		-1.79	0.1415
ssc-miR-153	MBTD1	mbt domain containing 1 [Source:VGNC Symbol;Acc:VGNC:90060]		-1.79	0.1415
ssc-miR-153	MCFD2	multiple coagulation factor deficiency 2, ER cargo receptor complex subunit [Source:VGNC Symbol;Acc:VGNC:103980]		-1.79	0.1415
ssc-miR-153	MCL1	MCL1 apoptosis regulator, BCL2 family member [Source:VGNC Symbol;Acc:VGNC:90072]		-1.79	0.1415
ssc-miR-153	MDGA2	MAM domain containing glycosylphosphatidylinositol anchor 2 [Source:VGNC Symbol;Acc:VGNC:90092]		-1.79	0.1415
ssc-miR-153	MDN1	midasin AAA ATPase 1 [Source:VGNC Symbol;Acc:VGNC:98121]		-1.79	0.1415
ssc-miR-153	ME1	malic enzyme 1 [Source:VGNC Symbol;Acc:VGNC:90095]		-1.79	0.1415
ssc-miR-153	MEF2A	myocyte enhancer factor 2A [Source:VGNC Symbol;Acc:VGNC:98123]		-1.79	0.1415
ssc-miR-153	MEF2C	myocyte enhancer factor 2C [Source:VGNC Symbol;Acc:VGNC:90127]		-1.79	0.1415
ssc-miR-153	METAP1	methionyl aminopeptidase 1 [Source:VGNC Symbol;Acc:VGNC:90151]		-1.79	0.1415

ssc-miR-153	MFAP3L	microfibril associated protein 3 like [Source:VGNC Symbol;Acc:VGNC:90175]	-1.79	0.1415
ssc-miR-153	MFHAS1	multifunctional ROCO family signaling regulator 1 [Source:VGNC Symbol;Acc:VGNC:107361]	-1.79	0.1415
ssc-miR-153	MFN1	mitofusin 1 [Source:VGNC Symbol;Acc:VGNC:90178]	-1.79	0.1415
ssc-miR-153	MGAT5	alpha-1,6-mannosylglycoprotein 6-beta-N-acetylglucosaminyltransferase [Source:VGNC Symbol;Acc:VGNC:96741]	-1.79	0.1415
ssc-miR-153	MIER3	MIER family member 3 [Source:VGNC Symbol;Acc:VGNC:90221]	-1.79	0.1415
ssc-miR-153	MIOS	meiosis regulator for oocyte development [Source:VGNC Symbol;Acc:VGNC:90234]	-1.79	0.1415
ssc-miR-153	MITF	melanocyte inducing transcription factor [Source:VGNC Symbol;Acc:VGNC:90243]	-1.79	0.1415
ssc-miR-153	MKNK2	MAPK interacting serine/threonine kinase 2 [Source:VGNC Symbol;Acc:VGNC:90247]	-1.79	0.1415
ssc-miR-153	MOB1B	hypothetical gene	-1.79	0.1415
ssc-miR-153	MON2	MON2 homolog, regulator of endosome-to-Golgi trafficking [Source:VGNC Symbol;Acc:VGNC:90301]	-1.79	0.1415
ssc-miR-153	MORC3	MORC family CW-type zinc finger 3 [Source:VGNC Symbol;Acc:VGNC:90304]	-1.79	0.1415
ssc-miR-153	MOV10	Mov10 RISC complex RNA helicase [Source:VGNC Symbol;Acc:VGNC:90316]	-1.79	0.1415
ssc-miR-153	MPP2	MAGUK p55 scaffold protein 2 [Source:VGNC Symbol;Acc:VGNC:98133]	-1.79	0.1415
ssc-miR-153	MPPED2	metallophosphoesterase domain containing 2 [Source:VGNC Symbol;Acc:VGNC:90331]	-1.79	0.1415
ssc-miR-153	MRE11A	hypothetical gene	-1.79	0.1415
ssc-miR-153	MSI2	musashi RNA binding protein 2 [Source:VGNC Symbol;Acc:VGNC:90422]	-1.79	0.1415
ssc-miR-153	MTDH	metadherin [Source:VGNC Symbol;Acc:VGNC:90439]	-1.79	0.1415
ssc-miR-153	MTF1	metal regulatory transcription factor 1 [Source:VGNC Symbol;Acc:VGNC:90443]	-1.79	0.1415
ssc-miR-153	MTF2	metal response element binding transcription factor 2 [Source:VGNC Symbol;Acc:VGNC:90444]	-1.79	0.1415
ssc-miR-153	MTHFD2	methylenetetrahydrofolate dehydrogenase (NADP+ dependent) 2, methenyltetrahydrofolate cyclohydrolase [Source:HGNC Symbol;Acc:HGNC:7434]	-1.79	0.1415
ssc-miR-153	MTMR12	myotubularin related protein 12 [Source:VGNC Symbol;Acc:VGNC:90459]	-1.79	0.1415
ssc-miR-153	MTRF1L	mitochondrial translation release factor 1 like [Source:VGNC Symbol;Acc:VGNC:90472]	-1.79	0.1415
ssc-miR-153	MYB	MYB proto-onco, transcription factor [Source:VGNC Symbol;Acc:VGNC:90496]	-1.79	0.1415
ssc-miR-153	MYCBP	MYC binding protein [Source:NCBI gene (formerly Entrezgene);Acc:100513990]	-1.79	0.1415
ssc-miR-153	MYCL	hypothetical gene	-1.79	0.1415
ssc-miR-153	MYO5A	myosin VA [Source:HGNC Symbol;Acc:HGNC:7602]	-1.79	0.1415
ssc-miR-153	NAA15	N-alpha-acetyltransferase 15, NatA auxiliary subunit [Source:VGNC Symbol;Acc:VGNC:96747]	-1.79	0.1415
ssc-miR-153	NAA50	N-alpha-acetyltransferase 50, NatE catalytic subunit [Source:VGNC Symbol;Acc:VGNC:104002]	-1.79	0.1415
ssc-miR-153	NAV1	neuron navigator 1 [Source:VGNC Symbol;Acc:VGNC:95725]	-1.79	0.1415
ssc-miR-153	NAV2	neuron navigator 2 [Source:VGNC Symbol;Acc:VGNC:90588]	-1.79	0.1415
ssc-miR-153	NAV3	neuron navigator 3 [Source:VGNC Symbol;Acc:VGNC:90589]	-1.79	0.1415
ssc-miR-153	NBEA	neurobeachin [Source:VGNC Symbol;Acc:VGNC:90590]	-1.79	0.1415
ssc-miR-153	NCAM2	neural cell adhesion molecule 2 [Source:VGNC Symbol;Acc:VGNC:90595]	-1.79	0.1415
ssc-miR-153	NCOA2	nuclear receptor coactivator 2 [Source:VGNC Symbol;Acc:VGNC:90616]	-1.79	0.1415
ssc-miR-153	NDUFB9	NADH:ubiquinone oxidoreductase subunit B9 [Source:VGNC Symbol;Acc:VGNC:90649]	-1.79	0.1415
ssc-miR-153	NEBL	nebulette [Source:VGNC Symbol;Acc:VGNC:95824]	-1.79	0.1415
ssc-miR-153	NEFM	hypothetical gene	-1.79	0.1415
ssc-miR-153	NEK9	NIMA related kinase 9 [Source:VGNC Symbol;Acc:VGNC:90679]	-1.79	0.1415
ssc-miR-153	NEUROD1	neuronal differentiation 1 [Source:VGNC Symbol;Acc:VGNC:96439]	-1.79	0.1415
ssc-miR-153	NEUROD2	neuronal differentiation 2 [Source:VGNC Symbol;Acc:VGNC:90697]	-1.79	0.1415
ssc-miR-153	NEUROD4	neuronal differentiation 4 [Source:VGNC Symbol;Acc:VGNC:90698]	-1.79	0.1415
ssc-miR-153	NEUROD6	neuronal differentiation 6 [Source:VGNC Symbol;Acc:VGNC:90699]	-1.79	0.1415
ssc-miR-153	NF1	neurofibromin 1 [Source:VGNC Symbol;Acc:VGNC:90704]	-1.79	0.1415
ssc-miR-153	NFASC	neurofascin [Source:VGNC Symbol;Acc:VGNC:90707]	-1.79	0.1415
ssc-miR-153	NFATC2	nuclear factor of activated T cells 2 [Source:VGNC Symbol;Acc:VGNC:96440]	-1.79	0.1415
ssc-miR-153	NFATC3	nuclear factor of activated T cells 3 [Source:VGNC Symbol;Acc:VGNC:90710]	-1.79	0.1415
ssc-miR-153	NFE2L2	NFE2 like bZIP transcription factor 2 [Source:VGNC Symbol;Acc:VGNC:96441]	-1.79	0.1415
ssc-miR-153	NFIA	nuclear factor I A [Source:VGNC Symbol;Acc:VGNC:90715]	-1.79	0.1415
ssc-miR-153	NFIB	nuclear factor I B [Source:VGNC Symbol;Acc:VGNC:90716]	-1.79	0.1415
ssc-miR-153	NFIC	nuclear factor I C [Source:VGNC Symbol;Acc:VGNC:100313]	-1.79	0.1415

ssc-miR-153	NFYA	nuclear transcription factor Y subunit alpha [Source:VGNC Symbol;Acc:VGNC:90729]	-1.79	0.1415
ssc-miR-153	NLGN4X	hypothetical gene	-1.79	0.1415
ssc-miR-153	NMNAT2	nicotinamide nucleotide adenylyltransferase 2 [Source:VGNC Symbol;Acc:VGNC:90798]	-1.79	0.1415
ssc-miR-153	NMT2	N-myristoyltransferase 2 [Source:VGNC Symbol;Acc:VGNC:96448]	-1.79	0.1415
ssc-miR-153	NOVA1	NOVA alternative splicing regulator 1 [Source:VGNC Symbol;Acc:VGNC:90827]	-1.79	0.1415
ssc-miR-153	NPTN	neuroplastin [Source:VGNC Symbol;Acc:VGNC:90861]	-1.79	0.1415
ssc-miR-153	NR3C2	nuclear receptor subfamily 3 group C member 2 [Source:VGNC Symbol;Acc:VGNC:90884]	-1.79	0.1415
ssc-miR-153	NR5A2	nuclear receptor subfamily 5 group A member 2 [Source:VGNC Symbol;Acc:VGNC:96452]	-1.79	0.1415
ssc-miR-153	NRARP	NOTCH regulated ankyrin repeat protein [Source:VGNC Symbol;Acc:VGNC:90889]	-1.79	0.1415
ssc-miR-153	NREP	neuronal regeneration related protein [Source:HGNC Symbol;Acc:HGNC:16834]	-1.79	0.1415
ssc-miR-153	NUDT4	hypothetical gene	-1.79	0.1415
ssc-miR-153	NUFIP1	nuclear FMR1 interacting protein 1 [Source:VGNC Symbol;Acc:VGNC:90966]	-1.79	0.1415
ssc-miR-153	NUFIP2	nuclear FMR1 interacting protein 2 [Source:VGNC Symbol;Acc:VGNC:90967]	-1.79	0.1415
ssc-miR-153	NUP43	nucleoporin 43 [Source:VGNC Symbol;Acc:VGNC:90982]	-1.79	0.1415
ssc-miR-153	NUPL1	hypothetical gene	-1.79	0.1415
ssc-miR-153	NXF1	nuclear RNA export factor 1 [Source:VGNC Symbol;Acc:VGNC:90993]	-1.79	0.1415
ssc-miR-153	NXNL2	nucleoredoxin like 2 [Source:VGNC Symbol;Acc:VGNC:90996]	-1.79	0.1415
ssc-miR-153	NXPE3	neurexophilin and PC-esterase domain family member 3 [Source:VGNC Symbol;Acc:VGNC:90997]	-1.79	0.1415
ssc-miR-153	ONECUT1	one cut homeobox 1 [Source:VGNC Symbol;Acc:VGNC:91042]	-1.79	0.1415
ssc-miR-153	ONECUT2	one cut homeobox 2 [Source:VGNC Symbol;Acc:VGNC:91043]	-1.79	0.1415
ssc-miR-153	ORC2	origin recognition complex subunit 2 [Source:VGNC Symbol;Acc:VGNC:96464]	-1.79	0.1415
ssc-miR-153	ORC4	origin recognition complex subunit 4 [Source:VGNC Symbol;Acc:VGNC:96465]	-1.79	0.1415
ssc-miR-153	OSBPL6	oxysterol binding protein like 6 [Source:VGNC Symbol;Acc:VGNC:95807]	-1.79	0.1415
ssc-miR-153	OTUD4	OTU deubiquitinase 4 [Source:VGNC Symbol;Acc:VGNC:91100]	-1.79	0.1415
ssc-miR-153	OTUD7A	OTU deubiquitinase 7A [Source:VGNC Symbol;Acc:VGNC:91104]	-1.79	0.1415
ssc-miR-153	OTUD7B	OTU deubiquitinase 7B [Source:VGNC Symbol;Acc:VGNC:91105]	-1.79	0.1415
ssc-miR-153	OTX2	orthodenticle homeobox 2 [Source:VGNC Symbol;Acc:VGNC:98171]	-1.79	0.1415
ssc-miR-153	OXR1	oxidation resistance 1 [Source:VGNC Symbol;Acc:VGNC:91116]	-1.79	0.1415
ssc-miR-153	PALM2	hypothetical gene	-1.79	0.1415
ssc-miR-153	PAPD7	hypothetical gene	-1.79	0.1415
ssc-miR-153	PAPOLG	poly(A) polymerase gamma [Source:HGNC Symbol;Acc:HGNC:14982]	-1.79	0.1415
ssc-miR-153	PARD6B	par-6 family cell polarity regulator beta [Source:VGNC Symbol;Acc:VGNC:98176]	-1.79	0.1415
ssc-miR-153	PAX2	paired box 2 [Source:VGNC Symbol;Acc:VGNC:91192]	-1.79	0.1415
ssc-miR-153	PAXBP1	PAX3 and PAX7 binding protein 1 [Source:VGNC Symbol;Acc:VGNC:91197]	-1.79	0.1415
ssc-miR-153	PBXIP1	PBX homeobox interacting protein 1 [Source:HGNC Symbol;Acc:HGNC:21199]	-1.79	0.1415
ssc-miR-153	PCDH7	protocadherin 7 [Source:NCBI gene (formerly Entrezgene);Acc:100520035]	-1.79	0.1415
ssc-miR-153	PCDH8	protocadherin 8 [Source:HGNC Symbol;Acc:HGNC:8660]	-1.79	0.1415
ssc-miR-153	PCDHA1	hypothetical gene	-1.79	0.1415
ssc-miR-153	PCDHA10	hypothetical gene	-1.79	0.1415
ssc-miR-153	PCDHA11	hypothetical gene	-1.79	0.1415
ssc-miR-153	PCDHA12	hypothetical gene	-1.79	0.1415
ssc-miR-153	PCDHA13	protocadherin alpha 13 [Source:HGNC Symbol;Acc:HGNC:8667]	-1.79	0.1415
ssc-miR-153	PCDHA2	hypothetical gene	-1.79	0.1415
ssc-miR-153	PCDHA3	protocadherin alpha 3 [Source:HGNC Symbol;Acc:HGNC:8669]	-1.79	0.1415
ssc-miR-153	PCDHA4	hypothetical gene	-1.79	0.1415
ssc-miR-153	PCDHA5	hypothetical gene	-1.79	0.1415
ssc-miR-153	PCDHA6	hypothetical gene	-1.79	0.1415
ssc-miR-153	PCDHA7	hypothetical gene	-1.79	0.1415
ssc-miR-153	PCDHA8	hypothetical gene	-1.79	0.1415
ssc-miR-153	PCDHA9	hypothetical gene	-1.79	0.1415

ssc-miR-153	PCDHAC1	hypothetical gene	-1.79	0.1415
ssc-miR-153	PCDHAC2	protocadherin alpha subfamily C, 2 [Source:HGNC Symbol;Acc:HGNC:8677]	-1.79	0.1415
ssc-miR-153	PCDHGA1	hypothetical gene	-1.79	0.1415
ssc-miR-153	PCDHGA10	hypothetical gene	-1.79	0.1415
ssc-miR-153	PCDHGA11	hypothetical gene	-1.79	0.1415
ssc-miR-153	PCDHGA12	hypothetical gene	-1.79	0.1415
ssc-miR-153	PCDHGA2	hypothetical gene	-1.79	0.1415
ssc-miR-153	PCDHGA3	hypothetical gene	-1.79	0.1415
ssc-miR-153	PCDHGA4	protocadherin gamma subfamily A, 4 [Source:HGNC Symbol;Acc:HGNC:8702]	-1.79	0.1415
ssc-miR-153	PCDHGA5	hypothetical gene	-1.79	0.1415
ssc-miR-153	PCDHGA6	protocadherin gamma subfamily A, 6 [Source:HGNC Symbol;Acc:HGNC:8704]	-1.79	0.1415
ssc-miR-153	PCDHGA7	hypothetical gene	-1.79	0.1415
ssc-miR-153	PCDHGA8	hypothetical gene	-1.79	0.1415
ssc-miR-153	PCDHGA9	hypothetical gene	-1.79	0.1415
ssc-miR-153	PCDHGB1	protocadherin gamma subfamily B, 1 [Source:HGNC Symbol;Acc:HGNC:8708]	-1.79	0.1415
ssc-miR-153	PCDHGB2	hypothetical gene	-1.79	0.1415
ssc-miR-153	PCDHGB3	hypothetical gene	-1.79	0.1415
ssc-miR-153	PCDHGB4	hypothetical gene	-1.79	0.1415
ssc-miR-153	PCDHGB6	hypothetical gene	-1.79	0.1415
ssc-miR-153	PCDHGB7	hypothetical gene	-1.79	0.1415
ssc-miR-153	PCDHGC3	hypothetical gene	-1.79	0.1415
ssc-miR-153	PCDHGC4	hypothetical gene	-1.79	0.1415
ssc-miR-153	PCDHGC5	hypothetical gene	-1.79	0.1415
ssc-miR-153	PCLO	hypothetical gene	-1.79	0.1415
ssc-miR-153	PCNX	hypothetical gene	-1.79	0.1415
ssc-miR-153	PCYT1B	phosphate cytidylyltransferase 1B, choline [Source:VGNC Symbol;Acc:VGNC:91237]	-1.79	0.1415
ssc-miR-153	PDE3B	phosphodiesterase 3B [Source:VGNC Symbol;Acc:VGNC:91253]	-1.79	0.1415
ssc-miR-153	PDE4D	phosphodiesterase 4D [Source:VGNC Symbol;Acc:VGNC:91256]	-1.79	0.1415
ssc-miR-153	PDE7A	phosphodiesterase 7A [Source:VGNC Symbol;Acc:VGNC:91261]	-1.79	0.1415
ssc-miR-153	PDGFA	platelet derived growth factor subunit A [Source:VGNC Symbol;Acc:VGNC:91265]	-1.79	0.1415
ssc-miR-153	PDP1	pyruvate dehydrogenase phosphatase catalytic subunit 1 [Source:VGNC Symbol;Acc:VGNC:91286]	-1.79	0.1415
ssc-miR-153	PDP2	pyruvate dehyrogenase phosphatase catalytic subunit 2 [Source:VGNC Symbol;Acc:VGNC:91287]	-1.79	0.1415
ssc-miR-153	PDS5A	PDS5 cohesin associated factor A [Source:HGNC Symbol;Acc:HGNC:29088]	-1.79	0.1415
ssc-miR-153	PEA15	proliferation and apoptosis adaptor protein 15 [Source:VGNC Symbol;Acc:VGNC:98181]	-1.79	0.1415
ssc-miR-153	PELI1	pellino E3 ubiquitin protein ligase 1 [Source:VGNC Symbol;Acc:VGNC:91306]	-1.79	0.1415
ssc-miR-153	PHF13	PHD finger protein 13 [Source:VGNC Symbol;Acc:VGNC:91379]	-1.79	0.1415
ssc-miR-153	PHF17	hypothetical gene	-1.79	0.1415
ssc-miR-153	PHF21A	PHD finger protein 21A [Source:VGNC Symbol;Acc:VGNC:91384]	-1.79	0.1415
ssc-miR-153	PHF3	PHD finger protein 3 [Source:VGNC Symbol;Acc:VGNC:91387]	-1.79	0.1415
ssc-miR-153	PHF7	PHD finger protein 7 [Source:VGNC Symbol;Acc:VGNC:91390]	-1.79	0.1415
ssc-miR-153	PHIP	pleckstrin homology domain interacting protein [Source:VGNC Symbol;Acc:VGNC:91392]	-1.79	0.1415
ssc-miR-153	PHOSPHO1	hypothetical gene	-1.79	0.1415
ssc-miR-153	PIAS2	protein inhibitor of activated STAT 2 [Source:VGNC Symbol;Acc:VGNC:91411]	-1.79	0.1415
ssc-miR-153	PIGA	phosphatidylinositol glycan anchor biosynthesis class A [Source:VGNC Symbol;Acc:VGNC:91419]	-1.79	0.1415
ssc-miR-153	PIK3R1	phosphoinositide-3-kinase regulatory subunit 1 [Source:VGNC Symbol;Acc:VGNC:91445]	-1.79	0.1415
ssc-miR-153	PITPNB	phosphatidylinositol transfer protein beta [Source:VGNC Symbol;Acc:VGNC:104026]	-1.79	0.1415
ssc-miR-153	PKHD1	PKHD1 ciliary IPT domain containing fibrocystin/polyductin [Source:VGNC Symbol;Acc:VGNC:91476]	-1.79	0.1415
ssc-miR-153	PKIG	cAMP-dependent protein kinase inhibitor gamma [Source:VGNC Symbol;Acc:VGNC:96486]	-1.79	0.1415
ssc-miR-153	PKN2	protein kinase N2 [Source:VGNC Symbol;Acc:VGNC:91482]	-1.79	0.1415
ssc-miR-153	PKNOX2	PBX/knotted 1 homeobox 2 [Source:VGNC Symbol;Acc:VGNC:91485]	-1.79	0.1415

ssc-miR-153	PKP4	plakophilin 4 [Source:VGNC Symbol;Acc:VGNC:96488]	-1.79	0.1415
ssc-miR-153	PLCB1	phospholipase C beta 1 [Source:VGNC Symbol;Acc:VGNC:95706]	-1.79	0.1415
ssc-miR-153	PLEKHA3	pleckstrin homology domain containing A3 [Source:NCBI gene (formerly Entrezgene);Acc:100154010]	-1.79	0.1415
ssc-miR-153	PLEKHM1	pleckstrin homology and RUN domain containing M1 [Source:VGNC Symbol;Acc:VGNC:91552]	-1.79	0.1415
ssc-miR-153	PLXNA4	plexin A4 [Source:VGNC Symbol;Acc:VGNC:98204]	-1.79	0.1415
ssc-miR-153	POFUT1	protein O-fucosyltransferase 1 [Source:VGNC Symbol;Acc:VGNC:95637]	-1.79	0.1415
ssc-miR-153	POLH	DNA polymerase eta [Source:VGNC Symbol;Acc:VGNC:91635]	-1.79	0.1415
ssc-miR-153	POU2F1	POU class 2 homeobox 1 [Source:VGNC Symbol;Acc:VGNC:91672]	-1.79	0.1415
ssc-miR-153	POU4F1	POU class 4 homeobox 1 [Source:VGNC Symbol;Acc:VGNC:91678]	-1.79	0.1415
ssc-miR-153	PPARGC1A	PPARG coactivator 1 alpha [Source:VGNC Symbol;Acc:VGNC:91685]	-1.79	0.1415
ssc-miR-153	PPARGC1B	PPARG coactivator 1 beta [Source:VGNC Symbol;Acc:VGNC:91686]	-1.79	0.1415
ssc-miR-153	PPM1D	protein phosphatase, Mg2+/Mn2+ dependent 1D [Source:VGNC Symbol;Acc:VGNC:91704]	-1.79	0.1415
ssc-miR-153	PPM1H	protein phosphatase, Mg2+/Mn2+ dependent 1H [Source:VGNC Symbol;Acc:VGNC:91708]	-1.79	0.1415
ssc-miR-153	PPM1K	protein phosphatase, Mg2+/Mn2+ dependent 1K [Source:VGNC Symbol;Acc:VGNC:91710]	-1.79	0.1415
ssc-miR-153	PPP1R12A	protein phosphatase 1 regulatory subunit 12A [Source:VGNC Symbol;Acc:VGNC:91719]	-1.79	0.1415
ssc-miR-153	PPP1R12B	protein phosphatase 1 regulatory subunit 12B [Source:VGNC Symbol;Acc:VGNC:96430]	-1.79	0.1415
ssc-miR-153	PPP2R2B	protein phosphatase 2 regulatory subunit Bbeta [Source:VGNC Symbol;Acc:VGNC:100336]	-1.79	0.1415
ssc-miR-153	PPP2R2D	hypothetical gene	-1.79	0.1415
ssc-miR-153	PPP3CA	protein phosphatase 3 catalytic subunit alpha [Source:VGNC Symbol;Acc:VGNC:98218]	-1.79	0.1415
ssc-miR-153	PPRC1	PPARG related coactivator 1 [Source:VGNC Symbol;Acc:VGNC:91763]	-1.79	0.1415
ssc-miR-153	PRDM16	PR/SET domain 16 [Source:VGNC Symbol;Acc:VGNC:91777]	-1.79	0.1415
ssc-miR-153	PRDM2	PR/SET domain 2 [Source:VGNC Symbol;Acc:VGNC:91778]	-1.79	0.1415
ssc-miR-153	PRKAB2	protein kinase AMP-activated non-catalytic subunit beta 2 [Source:VGNC Symbol;Acc:VGNC:91799]	-1.79	0.1415
ssc-miR-153	PRKACB	protein kinase cAMP-activated catalytic subunit beta [Source:VGNC Symbol;Acc:VGNC:91800]	-1.79	0.1415
ssc-miR-153	PRKAR2B	protein kinase cAMP-dependent type II regulatory subunit beta [Source:VGNC Symbol;Acc:VGNC:91805]	-1.79	0.1415
ssc-miR-153	PRKG1	protein kinase cGMP-dependent 1 [Source:VGNC Symbol;Acc:VGNC:91816]	-1.79	0.1415
ssc-miR-153	PROSC	hypothetical gene	-1.79	0.1415
ssc-miR-153	PROX1	prospero homeobox 1 [Source:VGNC Symbol;Acc:VGNC:91837]	-1.79	0.1415
ssc-miR-153	PRR16	proline rich 16 [Source:VGNC Symbol;Acc:VGNC:91855]	-1.79	0.1415
ssc-miR-153	PRRC1	proline rich coiled-coil 1 [Source:VGNC Symbol;Acc:VGNC:91864]	-1.79	0.1415
ssc-miR-153	PTAR1	protein prenyltransferase alpha subunit repeat containing 1 [Source:VGNC Symbol;Acc:VGNC:91936]	-1.79	0.1415
ssc-miR-153	PTBP1	polypyrimidine tract binding protein 1 [Source:VGNC Symbol;Acc:VGNC:100330]	-1.79	0.1415
ssc-miR-153	PTCH1	patched 1 [Source:VGNC Symbol;Acc:VGNC:96513]	-1.79	0.1415
ssc-miR-153	PTCHD3	patched domain containing 3 (gene/pseudogene) [Source:HGNC Symbol;Acc:HGNC:24776]	-1.79	0.1415
ssc-miR-153	PTCHD4	patched domain containing 4 [Source:VGNC Symbol;Acc:VGNC:91943]	-1.79	0.1415
ssc-miR-153	PTEN	hypothetical gene	-1.79	0.1415
ssc-miR-153	PTF1A	pancreas associated transcription factor 1a [Source:VGNC Symbol;Acc:VGNC:96021]	-1.79	0.1415
ssc-miR-153	PTGFRN	prostaglandin F2 receptor inhibitor [Source:VGNC Symbol;Acc:VGNC:91955]	-1.79	0.1415
ssc-miR-153	PTPLA	hypothetical gene	-1.79	0.1415
ssc-miR-153	PTPLB	hypothetical gene	-1.79	0.1415
ssc-miR-153	PTPN14	protein tyrosine phosphatase non-receptor type 14 [Source:VGNC Symbol;Acc:VGNC:91975]	-1.79	0.1415
ssc-miR-153	PTPN3	protein tyrosine phosphatase non-receptor type 3 [Source:VGNC Symbol;Acc:VGNC:91980]	-1.79	0.1415
ssc-miR-153	PTPRD	protein tyrosine phosphatase receptor type D [Source:HGNC Symbol;Acc:HGNC:9668]	-1.79	0.1415
ssc-miR-153	PURA	purine rich element binding protein A [Source:HGNC Symbol;Acc:HGNC:9701]	-1.79	0.1415
ssc-miR-153	PVRL3	hypothetical gene	-1.79	0.1415
ssc-miR-153	PXDN	peroxidasin [Source:VGNC Symbol;Acc:VGNC:92014]	-1.79	0.1415
ssc-miR-153	PXK	PX domain containing serine/threonine kinase like [Source:VGNC Symbol;Acc:VGNC:92015]	-1.79	0.1415
ssc-miR-153	PXMP4	hypothetical gene	-1.79	0.1415
ssc-miR-153	QKI	QKI, KH domain containing RNA binding [Source:VGNC Symbol;Acc:VGNC:92025]	-1.79	0.1415
ssc-miR-153	RAB11FIP2	RAB11 family interacting protein 2 [Source:VGNC Symbol;Acc:VGNC:92039]	-1.79	0.1415

ssc-miR-153	RAB7A	RAB7A, member RAS onco family [Source:VGNC Symbol;Acc:VGNC:98273]	-1.79	0.1415
ssc-miR-153	RABGAP1	RAB GTPase activating protein 1 [Source:HGNC Symbol;Acc:HGNC:17155]	-1.79	0.1415
ssc-miR-153	RABL3	RAB, member of RAS onco family like 3 [Source:VGNC Symbol;Acc:VGNC:98279]	-1.79	0.1415
ssc-miR-153	RAD51L3-RFFL	hypothetical gene	-1.79	0.1415
ssc-miR-153	RAD54L2	RAD54 like 2 [Source:VGNC Symbol;Acc:VGNC:92061]	-1.79	0.1415
ssc-miR-153	RAI14	retinoic acid induced 14 [Source:VGNC Symbol;Acc:VGNC:92068]	-1.79	0.1415
ssc-miR-153	RALA	RAS like proto-oncogene A [Source:HGNC Symbol;Acc:HGNC:9839]	-1.79	0.1415
ssc-miR-153	RALGPS2	Ral GEF with PH domain and SH3 binding motif 2 [Source:VGNC Symbol;Acc:VGNC:92073]	-1.79	0.1415
ssc-miR-153	RANBP2	RAN binding protein 2 [Source:HGNC Symbol;Acc:HGNC:9848]	-1.79	0.1415
ssc-miR-153	RAP2B	RAP2B, member of RAS onco family [Source:VGNC Symbol;Acc:VGNC:92089]	-1.79	0.1415
ssc-miR-153	RAP2C	RAP2C, member of RAS onco family [Source:VGNC Symbol;Acc:VGNC:92090]	-1.79	0.1415
ssc-miR-153	RASA1	RAS p21 protein activator 1 [Source:VGNC Symbol;Acc:VGNC:92101]	-1.79	0.1415
ssc-miR-153	RASGRF2	Ras protein specific guanine nucleotide releasing factor 2 [Source:VGNC Symbol;Acc:VGNC:92112]	-1.79	0.1415
ssc-miR-153	RASL12	RAS like family 12 [Source:VGNC Symbol;Acc:VGNC:92121]	-1.79	0.1415
ssc-miR-153	RASSF4	Ras association domain family member 4 [Source:NCBI gene (formerly Entrezgene);Acc:100152580]	-1.79	0.1415
ssc-miR-153	RBAK	RB associated KRAB zinc finger [Source:VGNC Symbol;Acc:VGNC:92134]	-1.79	0.1415
ssc-miR-153	RBBP5	RB binding protein 5, histone lysine methyltransferase complex subunit [Source:VGNC Symbol;Acc:VGNC:92135]	-1.79	0.1415
ssc-miR-153	RBM26	RNA binding motif protein 26 [Source:VGNC Symbol;Acc:VGNC:92153]	-1.79	0.1415
ssc-miR-153	RBM7	RNA binding motif protein 7 [Source:VGNC Symbol;Acc:VGNC:108612]	-1.79	0.1415
ssc-miR-153	RCAN3	RCAN family member 3 [Source:VGNC Symbol;Acc:VGNC:92172]	-1.79	0.1415
ssc-miR-153	RCHY1	ring finger and CHY zinc finger domain containing 1 [Source:VGNC Symbol;Acc:VGNC:92179]	-1.79	0.1415
ssc-miR-153	RCOR1	REST corepressor 1 [Source:VGNC Symbol;Acc:VGNC:92183]	-1.79	0.1415
ssc-miR-153	REST	hypothetical gene	-1.79	0.1415
ssc-miR-153	RFFL	ring finger and FYVE like domain containing E3 ubiquitin protein ligase [Source:VGNC Symbol;Acc:VGNC:98289]	-1.79	0.1415
ssc-miR-153	RGS17	regulator of G protein signaling 17 [Source:VGNC Symbol;Acc:VGNC:92263]	-1.79	0.1415
ssc-miR-153	RGS7BP	regulator of G protein signaling 7 binding protein [Source:VGNC Symbol;Acc:VGNC:92268]	-1.79	0.1415
ssc-miR-153	RHOU	ras homolog family member U [Source:VGNC Symbol;Acc:VGNC:92294]	-1.79	0.1415
ssc-miR-153	RIC8B	RIC8 guanine nucleotide exchange factor B [Source:VGNC Symbol;Acc:VGNC:92302]	-1.79	0.1415
ssc-miR-153	RICTOR	RPTOR independent companion of MTOR complex 2 [Source:VGNC Symbol;Acc:VGNC:92303]	-1.79	0.1415
ssc-miR-153	RNF125	ring finger protein 125 [Source:VGNC Symbol;Acc:VGNC:92352]	-1.79	0.1415
ssc-miR-153	RNF152	ring finger protein 152 [Source:VGNC Symbol;Acc:VGNC:92364]	-1.79	0.1415
ssc-miR-153	RNF165	ring finger protein 165 [Source:VGNC Symbol;Acc:VGNC:92366]	-1.79	0.1415
ssc-miR-153	RNF169	ring finger protein 169 [Source:VGNC Symbol;Acc:VGNC:92368]	-1.79	0.1415
ssc-miR-153	RNF213	hypothetical gene	-1.79	0.1415
ssc-miR-153	RNF26	ring finger protein 26 [Source:VGNC Symbol;Acc:VGNC:92387]	-1.79	0.1415
ssc-miR-153	RNGTT	RNA guanylyltransferase and 5'-phosphatase [Source:VGNC Symbol;Acc:VGNC:92400]	-1.79	0.1415
ssc-miR-153	RNPEP	arginyl aminopeptidase [Source:VGNC Symbol;Acc:VGNC:95469]	-1.79	0.1415
ssc-miR-153	ROBO2	roundabout guidance receptor 2 [Source:HGNC Symbol;Acc:HGNC:10250]	-1.79	0.1415
ssc-miR-153	ROCK1	Rho associated coiled-coil containing protein kinase 1 [Source:VGNC Symbol;Acc:VGNC:98294]	-1.79	0.1415
ssc-miR-153	ROCK2	Rho associated coiled-coil containing protein kinase 2 [Source:HGNC Symbol;Acc:HGNC:10252]	-1.79	0.1415
ssc-miR-153	ROR1	receptor tyrosine kinase like orphan receptor 1 [Source:VGNC Symbol;Acc:VGNC:98295]	-1.79	0.1415
ssc-miR-153	RP11-1055B8.7	hypothetical gene	-1.79	0.1415
ssc-miR-153	RP5-850E9.3	hypothetical gene	-1.79	0.1415
ssc-miR-153	RP6-24A23.6	hypothetical gene	-1.79	0.1415
ssc-miR-153	RPL22	ribosomal protein L22 [Source:NCBI gene (formerly Entrezgene);Acc:397047]	-1.79	0.1415
ssc-miR-153	RPL36A-HNRNPH2	hypothetical gene	-1.79	0.1415
ssc-miR-153	RPS6KA5	ribosomal protein S6 kinase A5 [Source:VGNC Symbol;Acc:VGNC:92444]	-1.79	0.1415
ssc-miR-153	RPS6KB1	ribosomal protein S6 kinase B1 [Source:VGNC Symbol;Acc:VGNC:99037]	-1.79	0.1415
ssc-miR-153	RSBN1	round spermatid basic protein 1 [Source:VGNC Symbol;Acc:VGNC:92473]	-1.79	0.1415
ssc-miR-153	RSF1	remodeling and spacing factor 1 [Source:VGNC Symbol;Acc:VGNC:92476]	-1.79	0.1415

ssc-miR-153	RTN4RL1	reticulon 4 receptor like 1 [Source:VGNC Symbol;Acc:VGNC:92504]	-1.79	0.1415
ssc-miR-153	RTTN	rotatin [Source:VGNC Symbol;Acc:VGNC:92508]	-1.79	0.1415
ssc-miR-153	RUNX1T1	RUNX1 partner transcriptional co-repressor 1 [Source:VGNC Symbol;Acc:VGNC:96594]	-1.79	0.1415
ssc-miR-153	RUNX2	RUNX family transcription factor 2 [Source:VGNC Symbol;Acc:VGNC:92517]	-1.79	0.1415
ssc-miR-153	RYK	receptor like tyrosine kinase [Source:VGNC Symbol;Acc:VGNC:92533]	-1.79	0.1415
ssc-miR-153	RYR2	ryanodine receptor 2 [Source:VGNC Symbol;Acc:VGNC:92534]	-1.79	0.1415
ssc-miR-153	RYR3	ryanodine receptor 3 [Source:HGNC Symbol;Acc:HGNC:10485]	-1.79	0.1415
ssc-miR-153	SAMD12	sterile alpha motif domain containing 12 [Source:VGNC Symbol;Acc:VGNC:92565]	-1.79	0.1415
ssc-miR-153	SAR1B	secretion associated Ras related GTPase 1B [Source:VGNC Symbol;Acc:VGNC:92580]	-1.79	0.1415
ssc-miR-153	SATB1	SATB homeobox 1 [Source:VGNC Symbol;Acc:VGNC:92587]	-1.79	0.1415
ssc-miR-153	SBNO2	strawberry notch homolog 2 [Source:VGNC Symbol;Acc:VGNC:92597]	-1.79	0.1415
ssc-miR-153	SCAMP5	secretory carrier membrane protein 5 [Source:VGNC Symbol;Acc:VGNC:92609]	-1.79	0.1415
ssc-miR-153	SCML1	hypothetical gene	-1.79	0.1415
ssc-miR-153	SCML2	Scm polycomb group protein like 2 [Source:VGNC Symbol;Acc:VGNC:92628]	-1.79	0.1415
ssc-miR-153	SCN3A	sodium voltage-gated channel alpha subunit 3 [Source:VGNC Symbol;Acc:VGNC:95479]	-1.79	0.1415
ssc-miR-153	SCN5A	sodium voltage-gated channel alpha subunit 5 [Source:VGNC Symbol;Acc:VGNC:92637]	-1.79	0.1415
ssc-miR-153	SCN8A	sodium voltage-gated channel alpha subunit 8 [Source:VGNC Symbol;Acc:VGNC:92638]	-1.79	0.1415
ssc-miR-153	SCRT2	scratch family transcriptional repressor 2 [Source:VGNC Symbol;Acc:VGNC:95721]	-1.79	0.1415
ssc-miR-153	SEC22A	SEC22 homolog A, vesicle trafficking protein [Source:VGNC Symbol;Acc:VGNC:92673]	-1.79	0.1415
ssc-miR-153	SEMA4F	ssemaphorin 4F [Source:VGNC Symbol;Acc:VGNC:92704]	-1.79	0.1415
ssc-miR-153	SEMA4G	semaphorin 4G [Source:VGNC Symbol;Acc:VGNC:92705]	-1.79	0.1415
ssc-miR-153	SENP7	SUMO specific peptidase 7 [Source:VGNC Symbol;Acc:VGNC:92716]	-1.79	0.1415
ssc-miR-153	SEPT11	hypothetical gene	-1.79	0.1415
ssc-miR-153	SEPT8	hypothetical gene	-1.79	0.1415
ssc-miR-153	SERTAD2	SERTA domain containing 2 [Source:VGNC Symbol;Acc:VGNC:92750]	-1.79	0.1415
ssc-miR-153	SESN3	sestrin 3 [Source:VGNC Symbol;Acc:VGNC:92755]	-1.79	0.1415
ssc-miR-153	SESTD1	SEC14 and spectrin domain containing 1 [Source:VGNC Symbol;Acc:VGNC:95884]	-1.79	0.1415
ssc-miR-153	SETD7	SET domain containing 7, histone lysine methyltransferase [Source:VGNC Symbol;Acc:VGNC:92762]	-1.79	0.1415
ssc-miR-153	SGCD	sarcoglycan delta [Source:NCBI gene (formerly Entrezgene);Acc:100240724]	-1.79	0.1415
ssc-miR-153	SGIP1	SH3GL interacting endocytic adaptor 1 [Source:VGNC Symbol;Acc:VGNC:92792]	-1.79	0.1415
ssc-miR-153	SGK3	serum/glucocorticoid regulated kinase family member 3 [Source:HGNC Symbol;Acc:HGNC:10812]	-1.79	0.1415
ssc-miR-153	SGMS2	sphingomyelin synthase 2 [Source:VGNC Symbol;Acc:VGNC:92795]	-1.79	0.1415
ssc-miR-153	SH2B3	SH2B adaptor protein 3 [Source:VGNC Symbol;Acc:VGNC:92807]	-1.79	0.1415
ssc-miR-153	SH3BP4	SH3 domain binding protein 4 [Source:VGNC Symbol;Acc:VGNC:95495]	-1.79	0.1415
ssc-miR-153	SH3KBP1	SH3 domain containing kinase binding protein 1 [Source:VGNC Symbol;Acc:VGNC:92828]	-1.79	0.1415
ssc-miR-153	SHC1	SHC adaptor protein 1 [Source:VGNC Symbol;Acc:VGNC:92837]	-1.79	0.1415
ssc-miR-153	SIGMAR1	sigma non-opioid intracellular receptor 1 [Source:VGNC Symbol;Acc:VGNC:96462]	-1.79	0.1415
ssc-miR-153	SIX3	SIX homeobox 3 [Source:VGNC Symbol;Acc:VGNC:92894]	-1.79	0.1415
ssc-miR-153	SKI	SKI proto-onco [Source:VGNC Symbol;Acc:VGNC:92902]	-1.79	0.1415
ssc-miR-153	SLAIN2	SLAIN motif family member 2 [Source:VGNC Symbol;Acc:VGNC:92910]	-1.79	0.1415
ssc-miR-153	SLC10A3	solute carrier family 10 member 3 [Source:VGNC Symbol;Acc:VGNC:92916]	-1.79	0.1415
ssc-miR-153	SLC16A7	solute carrier family 16 member 7 [Source:VGNC Symbol;Acc:VGNC:92947]	-1.79	0.1415
ssc-miR-153	SLC24A4	solute carrier family 24 member 4 [Source:VGNC Symbol;Acc:VGNC:92988]	-1.79	0.1415
ssc-miR-153	SLC25A12	solute carrier family 25 member 12 [Source:VGNC Symbol;Acc:VGNC:95502]	-1.79	0.1415
ssc-miR-153	SLC25A53	solute carrier family 25 member 53 [Source:VGNC Symbol;Acc:VGNC:93023]	-1.79	0.1415
ssc-miR-153	SLC30A3	solute carrier family 30 member 3 [Source:VGNC Symbol;Acc:VGNC:93057]	-1.79	0.1415
ssc-miR-153	SLC35A3	solute carrier family 35 member A3 [Source:VGNC Symbol;Acc:VGNC:98860]	-1.79	0.1415
ssc-miR-153	SLC35B4	solute carrier family 35 member B4 [Source:VGNC Symbol;Acc:VGNC:93074]	-1.79	0.1415
ssc-miR-153	SLC36A2	solute carrier family 36 member 2 [Source:VGNC Symbol;Acc:VGNC:93089]	-1.79	0.1415
ssc-miR-153	SLC38A1	solute carrier family 38 member 1 [Source:HGNC Symbol;Acc:HGNC:13447]	-1.79	0.1415

ssc-miR-153	SLC44A1	solute carrier family 44 member 1 [Source:VGNC Symbol;Acc:VGNC:93118]	-1.79	0.1415
ssc-miR-153	SLC4A4	solute carrier family 4 member 4 [Source:VGNC Symbol;Acc:VGNC:93133]	-1.79	0.1415
ssc-miR-153	SLC6A15	solute carrier family 6 member 15 [Source:VGNC Symbol;Acc:VGNC:93157]	-1.79	0.1415
ssc-miR-153	SLC9A6	solute carrier family 9 member A6 [Source:VGNC Symbol;Acc:VGNC:93188]	-1.79	0.1415
ssc-miR-153	SLC9A9	solute carrier family 9 member A9 [Source:VGNC Symbol;Acc:VGNC:93190]	-1.79	0.1415
ssc-miR-153	SLCO5A1	solute carrier organic anion transporter family member 5A1 [Source:HGNC Symbol;Acc:HGNC:19046]	-1.79	0.1415
ssc-miR-153	SMAD5	SMAD family member 5 [Source:VGNC Symbol;Acc:VGNC:93219]	-1.79	0.1415
ssc-miR-153	SMARCA5	SWI/SNF related, matrix associated, actin dependent regulator of chromatin, subfamily a, member 5 [Source:VGNC Symbol;Acc:VGNC:93228]	-1.79	0.1415
ssc-miR-153	SMARCC1	SWI/SNF related, matrix associated, actin dependent regulator of chromatin subfamily c member 1 [Source:VGNC Symbol;Acc:VGNC:93231]	-1.79	0.1415
ssc-miR-153	SMARCD2	SWI/SNF related, matrix associated, actin dependent regulator of chromatin, subfamily d, member 2 [Source:NCBI gene (formerly Entrezgene);Acc:100512367]	-1.79	0.1415
ssc-miR-153	SMIM13	small integral membrane protein 13 [Source:HGNC Symbol;Acc:HGNC:27356]	-1.79	0.1415
ssc-miR-153	SMIM14	small integral membrane protein 14 [Source:VGNC Symbol;Acc:VGNC:93252]	-1.79	0.1415
ssc-miR-153	SMURF1	SMAD specific E3 ubiquitin protein ligase 1 [Source:VGNC Symbol;Acc:VGNC:93270]	-1.79	0.1415
ssc-miR-153	SNAI1	snail family transcriptional repressor 1 [Source:VGNC Symbol;Acc:VGNC:95508]	-1.79	0.1415
ssc-miR-153	SNAP25	synaptosome associated protein 25 [Source:VGNC Symbol;Acc:VGNC:95509]	-1.79	0.1415
ssc-miR-153	SNCA	synuclein alpha [Source:VGNC Symbol;Acc:VGNC:93285]	-1.79	0.1415
ssc-miR-153	SNPH	syntaphilin [Source:VGNC Symbol;Acc:VGNC:95716]	-1.79	0.1415
ssc-miR-153	SNTB1	syntrophin beta 1 [Source:VGNC Symbol;Acc:VGNC:93297]	-1.79	0.1415
ssc-miR-153	SNTB2	syntrophin beta 2 [Source:VGNC Symbol;Acc:VGNC:93298]	-1.79	0.1415
ssc-miR-153	SOCS2	suppressor of cytokine signaling 2 [Source:NCBI gene (formerly Entrezgene);Acc:100037966]	-1.79	0.1415
ssc-miR-153	SOCS5	suppressor of cytokine signaling 5 [Source:VGNC Symbol;Acc:VGNC:93332]	-1.79	0.1415
ssc-miR-153	SORL1	sortilin related receptor 1 [Source:VGNC Symbol;Acc:VGNC:108617]	-1.79	0.1415
ssc-miR-153	SOST	sclerostin [Source:VGNC Symbol;Acc:VGNC:93345]	-1.79	0.1415
ssc-miR-153	SOX11	SRY-box transcription factor 11 [Source:VGNC Symbol;Acc:VGNC:93351]	-1.79	0.1415
ssc-miR-153	SOX6	SRY-box transcription factor 6 [Source:VGNC Symbol;Acc:VGNC:93358]	-1.79	0.1415
ssc-miR-153	SOX8	SRY-box transcription factor 8 [Source:VGNC Symbol;Acc:VGNC:93359]	-1.79	0.1415
ssc-miR-153	SP3	Sp3 transcription factor [Source:VGNC Symbol;Acc:VGNC:95511]	-1.79	0.1415
ssc-miR-153	SP4	Sp4 transcription factor [Source:VGNC Symbol;Acc:VGNC:93362]	-1.79	0.1415
ssc-miR-153	SP8	Sp8 transcription factor [Source:VGNC Symbol;Acc:VGNC:93365]	-1.79	0.1415
ssc-miR-153	SP9	Sp9 transcription factor [Source:VGNC Symbol;Acc:VGNC:96247]	-1.79	0.1415
ssc-miR-153	SPHK2	sphingosine kinase 2 [Source:VGNC Symbol;Acc:VGNC:93405]	-1.79	0.1415
ssc-miR-153	SPIC	Spi-C transcription factor [Source:VGNC Symbol;Acc:VGNC:104061]	-1.79	0.1415
ssc-miR-153	SPOPL	speckle type BTB/POZ protein like [Source:VGNC Symbol;Acc:VGNC:96161]	-1.79	0.1415
ssc-miR-153	SPRED1	sprouty related EVH1 domain containing 1 [Source:VGNC Symbol;Acc:VGNC:93420]	-1.79	0.1415
ssc-miR-153	SPRY2	sprouty RTK signaling antagonist 2 [Source:VGNC Symbol;Acc:VGNC:93425]	-1.79	0.1415
ssc-miR-153	SPTBN1	spectrin beta, non-erythrocytic 1 [Source:VGNC Symbol;Acc:VGNC:93436]	-1.79	0.1415
ssc-miR-153	SPTBN4	spectrin beta, non-erythrocytic 4 [Source:VGNC Symbol;Acc:VGNC:93438]	-1.79	0.1415
ssc-miR-153	SPTSSB	serine palmitoyltransferase small subunit B [Source:VGNC Symbol;Acc:VGNC:93441]	-1.79	0.1415
ssc-miR-153	SPTY2D1	SPT2 chromatin protein domain containing 1 [Source:VGNC Symbol;Acc:VGNC:100867]	-1.79	0.1415
ssc-miR-153	SRC	SRC proto-onco, non-receptor tyrosine kinase [Source:VGNC Symbol;Acc:VGNC:95516]	-1.79	0.1415
ssc-miR-153	SRPK1	SRSF protein kinase 1 [Source:VGNC Symbol;Acc:VGNC:93462]	-1.79	0.1415
ssc-miR-153	SRSF1	serine and arginine rich splicing factor 1 [Source:VGNC Symbol;Acc:VGNC:99084]	-1.79	0.1415
ssc-miR-153	SRSF10	serine and arginine rich splicing factor 10 [Source:VGNC Symbol;Acc:VGNC:93472]	-1.79	0.1415
ssc-miR-153	SS18	SS18 subunit of BAF chromatin remodeling complex [Source:VGNC Symbol;Acc:VGNC:93478]	-1.79	0.1415
ssc-miR-153	SSBP3	single stranded DNA binding protein 3 [Source:VGNC Symbol;Acc:VGNC:93481]	-1.79	0.1415
ssc-miR-153	STAU2	staufen double-stranded RNA binding protein 2 [Source:VGNC Symbol;Acc:VGNC:98873]	-1.79	0.1415
ssc-miR-153	STEAP2	STEAP2 metalloreductase [Source:HGNC Symbol;Acc:HGNC:17885]	-1.79	0.1415
ssc-miR-153	STIM2	stromal interaction molecule 2 [Source:VGNC Symbol;Acc:VGNC:98967]	-1.79	0.1415
ssc-miR-153	STK16	serine/threonine kinase 16 [Source:VGNC Symbol;Acc:VGNC:95526]	-1.79	0.1415
ssc-miR-153	STK24	serine/threonine kinase 24 [Source:VGNC Symbol;Acc:VGNC:98344]	-1.79	0.1415

ssc-miR-153	STMN2	stathmin 2 [Source:VGNC Symbol;Acc:VGNC:93561]	-1.79	0.1415
ssc-miR-153	STON2	stonin 2 [Source:VGNC Symbol;Acc:VGNC:93568]	-1.79	0.1415
ssc-miR-153	STRN	striatin [Source:VGNC Symbol;Acc:VGNC:93578]	-1.79	0.1415
ssc-miR-153	STX1B	syntaxin 1B [Source:VGNC Symbol;Acc:VGNC:98352]	-1.79	0.1415
ssc-miR-153	STYX	serine/threonine/tyrosine interacting protein [Source:VGNC Symbol;Acc:VGNC:93602]	-1.79	0.1415
ssc-miR-153	SUN1	Sad1 and UNC84 domain containing 1 [Source:VGNC Symbol;Acc:VGNC:93618]	-1.79	0.1415
ssc-miR-153	SUN2	Sad1 and UNC84 domain containing 2 [Source:VGNC Symbol;Acc:VGNC:93619]	-1.79	0.1415
ssc-miR-153	SUV420H1	hypothetical gene	-1.79	0.1415
ssc-miR-153	SYDE2	synapse defective Rho GTPase homolog 2 [Source:VGNC Symbol;Acc:VGNC:98874]	-1.79	0.1415
ssc-miR-153	SYN1	synapsin I [Source:NCBI gene (formerly Entrezgene);Acc:100216310]	-1.79	0.1415
ssc-miR-153	SYT1	synaptotagmin 1 [Source:VGNC Symbol;Acc:VGNC:93678]	-1.79	0.1415
ssc-miR-153	SYT10	synaptotagmin 10 [Source:VGNC Symbol;Acc:VGNC:93679]	-1.79	0.1415
ssc-miR-153	SYT4	synaptotagmin 4 [Source:VGNC Symbol;Acc:VGNC:93685]	-1.79	0.1415
ssc-miR-153	TAB2	TGF-beta activated kinase 1 (MAP3K7) binding protein 2 [Source:VGNC Symbol;Acc:VGNC:103186]	-1.79	0.1415
ssc-miR-153	TAB3	TGF-beta activated kinase 1 (MAP3K7) binding protein 3 [Source:HGNC Symbol;Acc:HGNC:30681]	-1.79	0.1415
ssc-miR-153	TAF4	TATA-box binding protein associated factor 4 [Source:VGNC Symbol;Acc:VGNC:95532]	-1.79	0.1415
ssc-miR-153	TAF5	TATA-box binding protein associated factor 5 [Source:VGNC Symbol;Acc:VGNC:93717]	-1.79	0.1415
ssc-miR-153	TAGLN3	transgelin 3 [Source:VGNC Symbol;Acc:VGNC:108697]	-1.79	0.1415
ssc-miR-153	TBC1D19	TBC1 domain family member 19 [Source:VGNC Symbol;Acc:VGNC:93767]	-1.79	0.1415
ssc-miR-153	TBC1D30	TBC1 domain family member 30 [Source:VGNC Symbol;Acc:VGNC:93775]	-1.79	0.1415
ssc-miR-153	TBPL1	TATA-box binding protein like 1 [Source:VGNC Symbol;Acc:VGNC:93792]	-1.79	0.1415
ssc-miR-153	TBX2	T-box transcription factor 2 [Source:VGNC Symbol;Acc:VGNC:93798]	-1.79	0.1415
ssc-miR-153	TBX3	T-box transcription factor 3 [Source:VGNC Symbol;Acc:VGNC:93802]	-1.79	0.1415
ssc-miR-153	TCEB1	hypothetical gene	-1.79	0.1415
ssc-miR-153	TCERG1L	transcription elongation regulator 1 like [Source:VGNC Symbol;Acc:VGNC:93816]	-1.79	0.1415
ssc-miR-153	TCF4	transcription factor 4 [Source:VGNC Symbol;Acc:VGNC:93823]	-1.79	0.1415
ssc-miR-153	TDGF1	hypothetical gene	-1.79	0.1415
ssc-miR-153	TES	testin LIM domain protein [Source:VGNC Symbol;Acc:VGNC:93884]	-1.79	0.1415
ssc-miR-153	TESK2	testis associated actin remodelling kinase 2 [Source:VGNC Symbol;Acc:VGNC:93886]	-1.79	0.1415
ssc-miR-153	TET2	hypothetical gene	-1.79	0.1415
ssc-miR-153	TET3	tet methylcytosine dioxygenase 3 [Source:VGNC Symbol;Acc:VGNC:93890]	-1.79	0.1415
ssc-miR-153	TFAM	transcription factor A, mitochondrial [Source:NCBI gene (formerly Entrezgene);Acc:397279]	-1.79	0.1415
ssc-miR-153	TFDP2	transcription factor Dp-2 [Source:VGNC Symbol;Acc:VGNC:93915]	-1.79	0.1415
ssc-miR-153	TFEC	transcription factor EC [Source:VGNC Symbol;Acc:VGNC:93918]	-1.79	0.1415
ssc-miR-153	TGFB2	transforming growth factor beta 2 [Source:VGNC Symbol;Acc:VGNC:95541]	-1.79	0.1415
ssc-miR-153	TGFBR1	transforming growth factor beta receptor 1 [Source:VGNC Symbol;Acc:VGNC:98368]	-1.79	0.1415
ssc-miR-153	TGFBR2	transforming growth factor beta receptor 2 [Source:VGNC Symbol;Acc:VGNC:93931]	-1.79	0.1415
ssc-miR-153	THAP2	THAP domain containing 2 [Source:VGNC Symbol;Acc:VGNC:104071]	-1.79	0.1415
ssc-miR-153	TIA1	TIA1 cytotoxic granule associated RNA binding protein [Source:VGNC Symbol;Acc:VGNC:93975]	-1.79	0.1415
ssc-miR-153	TJP1	tight junction protein 1 [Source:VGNC Symbol;Acc:VGNC:94005]	-1.79	0.1415
ssc-miR-153	TLCD2	TLC domain containing 2 [Source:VGNC Symbol;Acc:VGNC:94011]	-1.79	0.1415
ssc-miR-153	TLK1	tousled like kinase 1 [Source:VGNC Symbol;Acc:VGNC:95543]	-1.79	0.1415
ssc-miR-153	TM9SF3	transmembrane 9 superfamily member 3 [Source:VGNC Symbol;Acc:VGNC:94037]	-1.79	0.1415
ssc-miR-153	TMED8	transmembrane p24 trafficking protein family member 8 [Source:VGNC Symbol;Acc:VGNC:94058]	-1.79	0.1415
ssc-miR-153	TMEM110	hypothetical gene	-1.79	0.1415
ssc-miR-153	TMEM151B	transmembrane protein 151B [Source:VGNC Symbol;Acc:VGNC:98376]	-1.79	0.1415
ssc-miR-153	TMEM167A	hypothetical gene	-1.79	0.1415
ssc-miR-153	TMEM168	transmembrane protein 168 [Source:VGNC Symbol;Acc:VGNC:94106]	-1.79	0.1415
ssc-miR-153	TMEM170B	transmembrane protein 170B [Source:VGNC Symbol;Acc:VGNC:94108]	-1.79	0.1415
ssc-miR-153	TMEM178A	transmembrane protein 178A [Source:VGNC Symbol;Acc:VGNC:94114]	-1.79	0.1415

ssc-miR-153	TMEM181	transmembrane protein 181 [Source:VGNC Symbol;Acc:VGNC:94117]	-1.79	0.1415
ssc-miR-153	TMEM192	transmembrane protein 192 [Source:VGNC Symbol;Acc:VGNC:94126]	-1.79	0.1415
ssc-miR-153	TMEM233	transmembrane protein 233 [Source:HGNC Symbol;Acc:HGNC:37219]	-1.79	0.1415
ssc-miR-153	TMEM25	transmembrane protein 25 [Source:VGNC Symbol;Acc:VGNC:94157]	-1.79	0.1415
ssc-miR-153	TMEM55A	hypothetical gene	-1.79	0.1415
ssc-miR-153	TMEM56	hypothetical gene	-1.79	0.1415
ssc-miR-153	TMEM64	transmembrane protein 64 [Source:VGNC Symbol;Acc:VGNC:94199]	-1.79	0.1415
ssc-miR-153	TMSB4X	thymosin beta 4 X-linked [Source:NCBI gene (formerly Entrezgene);Acc:733606]	-1.79	0.1415
ssc-miR-153	TMSB4Y	hypothetical gene	-1.79	0.1415
ssc-miR-153	TMX1	thioredoxin related transmembrane protein 1 [Source:VGNC Symbol;Acc:VGNC:94247]	-1.79	0.1415
ssc-miR-153	TNIK	TRAF2 and NCK interacting kinase [Source:VGNC Symbol;Acc:VGNC:98381]	-1.79	0.1415
ssc-miR-153	TNKS	tankyrase [Source:VGNC Symbol;Acc:VGNC:95546]	-1.79	0.1415
ssc-miR-153	TNP2	transition protein 2 [Source:VGNC Symbol;Acc:VGNC:94286]	-1.79	0.1415
ssc-miR-153	TNRC6A	trinucleotide repeat containing adaptor 6A [Source:VGNC Symbol;Acc:VGNC:94292]	-1.79	0.1415
ssc-miR-153	TNRC6B	trinucleotide repeat containing adaptor 6B [Source:VGNC Symbol;Acc:VGNC:94293]	-1.79	0.1415
ssc-miR-153	TNRC6C	trinucleotide repeat containing adaptor 6C [Source:VGNC Symbol;Acc:VGNC:94294]	-1.79	0.1415
ssc-miR-153	TOR1AIP1	torsin 1A interacting protein 1 [Source:VGNC Symbol;Acc:VGNC:94317]	-1.79	0.1415
ssc-miR-153	TP53INP1	tumor protein p53 inducible nuclear protein 1 [Source:VGNC Symbol;Acc:VGNC:94329]	-1.79	0.1415
ssc-miR-153	TP53INP2	tumor protein p53 inducible nuclear protein 2 [Source:VGNC Symbol;Acc:VGNC:95737]	-1.79	0.1415
ssc-miR-153	TPBG	trophoblast glycoprotein [Source:VGNC Symbol;Acc:VGNC:94332]	-1.79	0.1415
ssc-miR-153	TPCN1	two pore segment channel 1 [Source:VGNC Symbol;Acc:VGNC:94333]	-1.79	0.1415
ssc-miR-153	TPPP	tubulin polymerization promoting protein [Source:VGNC Symbol;Acc:VGNC:94344]	-1.79	0.1415
ssc-miR-153	TRAF3	TNF receptor associated factor 3 [Source:VGNC Symbol;Acc:VGNC:94360]	-1.79	0.1415
ssc-miR-153	TRAK2	trafficking kinesin protein 2 [Source:VGNC Symbol;Acc:VGNC:95577]	-1.79	0.1415
ssc-miR-153	TRHDE	thyrotropin releasing hormone degrading enzyme [Source:VGNC Symbol;Acc:VGNC:94390]	-1.79	0.1415
ssc-miR-153	TRIM71	tripartite motif containing 71 [Source:VGNC Symbol;Acc:VGNC:94431]	-1.79	0.1415
ssc-miR-153	TRIO	trio Rho guanine nucleotide exchange factor [Source:VGNC Symbol;Acc:VGNC:94435]	-1.79	0.1415
ssc-miR-153	TRIP11	thyroid hormone receptor interactor 11 [Source:VGNC Symbol;Acc:VGNC:94437]	-1.79	0.1415
ssc-miR-153	TROVE2	hypothetical gene	-1.79	0.1415
ssc-miR-153	TRPC4	transient receptor potential cation channel subfamily C member 4 [Source:VGNC Symbol;Acc:VGNC:94462]	-1.79	0.1415
ssc-miR-153	TRPM3	transient receptor potential cation channel subfamily M member 3 [Source:VGNC Symbol;Acc:VGNC:98384]	-1.79	0.1415
ssc-miR-153	TRPS1	transcriptional repressor GATA binding 1 [Source:VGNC Symbol;Acc:VGNC:94471]	-1.79	0.1415
ssc-miR-153	TSC22D4	TSC22 domain family member 4 [Source:HGNC Symbol;Acc:HGNC:21696]	-1.79	0.1415
ssc-miR-153	TSHZ1	teashirt zinc finger homeobox 1 [Source:VGNC Symbol;Acc:VGNC:94492]	-1.79	0.1415
ssc-miR-153	TSPYL2	TSPY like 2 [Source:VGNC Symbol;Acc:VGNC:94519]	-1.79	0.1415
ssc-miR-153	TTC28	tetratricopeptide repeat domain 28 [Source:VGNC Symbol;Acc:VGNC:94543]	-1.79	0.1415
ssc-miR-153	TTYH3	tweety family member 3 [Source:VGNC Symbol;Acc:VGNC:94576]	-1.79	0.1415
ssc-miR-153	TXLNG	taxilin gamma [Source:HGNC Symbol;Acc:HGNC:18578]	-1.79	0.1415
ssc-miR-153	TXNDC11	thioredoxin domain containing 11 [Source:VGNC Symbol;Acc:VGNC:94603]	-1.79	0.1415
ssc-miR-153	UBA2	hypothetical gene	-1.79	0.1415
ssc-miR-153	UBE2G1	ubiquitin conjugating enzyme E2 G1 [Source:HGNC Symbol;Acc:HGNC:12482]	-1.79	0.1415
ssc-miR-153	UBE2K	ubiquitin conjugating enzyme E2 K [Source:VGNC Symbol;Acc:VGNC:94646]	-1.79	0.1415
ssc-miR-153	UBE2W	ubiquitin conjugating enzyme E2 W [Source:VGNC Symbol;Acc:VGNC:98890]	-1.79	0.1415
ssc-miR-153	UBE3B	ubiquitin protein ligase E3B [Source:VGNC Symbol;Acc:VGNC:94653]	-1.79	0.1415
ssc-miR-153	UBFD1	ubiquitin family domain containing 1 [Source:VGNC Symbol;Acc:VGNC:94658]	-1.79	0.1415
ssc-miR-153	UBN2	ubinuclein 2 [Source:VGNC Symbol;Acc:VGNC:94664]	-1.79	0.1415
ssc-miR-153	UBR1	ubiquitin protein ligase E3 component n-recognin 1 [Source:VGNC Symbol;Acc:VGNC:94668]	-1.79	0.1415
ssc-miR-153	UBR7	ubiquitin protein ligase E3 component n-recognin 7 [Source:HGNC Symbol;Acc:HGNC:20344]	-1.79	0.1415
ssc-miR-153	UBXN2A	UBX domain protein 2A [Source:VGNC Symbol;Acc:VGNC:94677]	-1.79	0.1415
ssc-miR-153	UHRF2	ubiquitin like with PHD and ring finger domains 2 [Source:VGNC Symbol;Acc:VGNC:94692]	-1.79	0.1415

ssc-miR-153	UNC5C	unc-5 netrin receptor C [Source:VGNC Symbol;Acc:VGNC:94709]	-1.79	0.1415
ssc-miR-153	UNKL	unk like zinc finger [Source:VGNC Symbol;Acc:VGNC:94716]	-1.79	0.1415
ssc-miR-153	USP28	ubiquitin specific peptidase 28 [Source:VGNC Symbol;Acc:VGNC:94756]	-1.79	0.1415
ssc-miR-153	USP31	ubiquitin specific peptidase 31 [Source:HGNC Symbol;Acc:HGNC:20060]	-1.79	0.1415
ssc-miR-153	USP44	ubiquitin specific peptidase 44 [Source:VGNC Symbol;Acc:VGNC:94766]	-1.79	0.1415
ssc-miR-153	USP49	ubiquitin specific peptidase 49 [Source:VGNC Symbol;Acc:VGNC:94771]	-1.79	0.1415
ssc-miR-153	USP6	hypothetical gene	-1.79	0.1415
ssc-miR-153	USP9X	ubiquitin specific peptidase 9 X-linked [Source:HGNC Symbol;Acc:HGNC:12632]	-1.79	0.1415
ssc-miR-153	USP9Y	hypothetical gene	-1.79	0.1415
ssc-miR-153	UTRN	utrophin [Source:VGNC Symbol;Acc:VGNC:103200]	-1.79	0.1415
ssc-miR-153	UTY	hypothetical gene	-1.79	0.1415
ssc-miR-153	UXS1	UDP-glucuronate decarboxylase 1 [Source:VGNC Symbol;Acc:VGNC:94791]	-1.79	0.1415
ssc-miR-153	VAMP2	vesicle associated membrane protein 2 [Source:HGNC Symbol;Acc:HGNC:12643]	-1.79	0.1415
ssc-miR-153	VASH2	vasohibin 2 [Source:VGNC Symbol;Acc:VGNC:94802]	-1.79	0.1415
ssc-miR-153	VCL	vinculin [Source:VGNC Symbol;Acc:VGNC:94812]	-1.79	0.1415
ssc-miR-153	VEZF1	vascular endothelial zinc finger 1 [Source:VGNC Symbol;Acc:VGNC:94818]	-1.79	0.1415
ssc-miR-153	VIL1	villin 1 [Source:VGNC Symbol;Acc:VGNC:95564]	-1.79	0.1415
ssc-miR-153	VPS37A	VPS37A subunit of ESCRT-I [Source:VGNC Symbol;Acc:VGNC:104092]	-1.79	0.1415
ssc-miR-153	VSTM2A	V-set and transmembrane domain containing 2A [Source:VGNC Symbol;Acc:VGNC:94872]	-1.79	0.1415
ssc-miR-153	VWA5B2	von Willebrand factor A domain containing 5B2 [Source:HGNC Symbol;Acc:HGNC:25144]	-1.79	0.1415
ssc-miR-153	WASF2	WASP family member 2 [Source:VGNC Symbol;Acc:VGNC:94893]	-1.79	0.1415
ssc-miR-153	WDR26	WD repeat domain 26 [Source:VGNC Symbol;Acc:VGNC:95960]	-1.79	0.1415
ssc-miR-153	WDR47	WD repeat domain 47 [Source:VGNC Symbol;Acc:VGNC:94924]	-1.79	0.1415
ssc-miR-153	WIPF1	hypothetical gene	-1.79	0.1415
ssc-miR-153	WIPF2	WAS/WASL interacting protein family member 2 [Source:VGNC Symbol;Acc:VGNC:99106]	-1.79	0.1415
ssc-miR-153	WNK3	WNK lysine deficient protein kinase 3 [Source:VGNC Symbol;Acc:VGNC:94964]	-1.79	0.1415
ssc-miR-153	WRNIP1	WRN helicase interacting protein 1 [Source:VGNC Symbol;Acc:VGNC:104096]	-1.79	0.1415
ssc-miR-153	WWC2	WW and C2 domain containing 2 [Source:VGNC Symbol;Acc:VGNC:96033]	-1.79	0.1415
ssc-miR-153	WWP1	WW domain containing E3 ubiquitin protein ligase 1 [Source:VGNC Symbol;Acc:VGNC:94989]	-1.79	0.1415
ssc-miR-153	XKR4	XK related 4 [Source:VGNC Symbol;Acc:VGNC:98900]	-1.79	0.1415
ssc-miR-153	XKR7	XK related 7 [Source:VGNC Symbol;Acc:VGNC:95987]	-1.79	0.1415
ssc-miR-153	XYLT1	xylosyltransferase 1 [Source:VGNC Symbol;Acc:VGNC:95015]	-1.79	0.1415
ssc-miR-153	XYLT2	xylosyltransferase 2 [Source:VGNC Symbol;Acc:VGNC:95016]	-1.79	0.1415
ssc-miR-153	YBX1	hypothetical gene	-1.79	0.1415
ssc-miR-153	YIPF2	Yip1 domain family member 2 [Source:VGNC Symbol;Acc:VGNC:95028]	-1.79	0.1415
ssc-miR-153	YTHDC1	YTH domain containing 1 [Source:VGNC Symbol;Acc:VGNC:95041]	-1.79	0.1415
ssc-miR-153	ZBTB10	zinc finger and BTB domain containing 10 [Source:VGNC Symbol;Acc:VGNC:95058]	-1.79	0.1415
ssc-miR-153	ZBTB2	zinc finger and BTB domain containing 2 [Source:VGNC Symbol;Acc:VGNC:95062]	-1.79	0.1415
ssc-miR-153	ZBTB20	zinc finger and BTB domain containing 20 [Source:VGNC Symbol;Acc:VGNC:95063]	-1.79	0.1415
ssc-miR-153	ZBTB21	zinc finger and BTB domain containing 21 [Source:HGNC Symbol;Acc:HGNC:13083]	-1.79	0.1415
ssc-miR-153	ZBTB26	zinc finger and BTB domain containing 26 [Source:VGNC Symbol;Acc:VGNC:95066]	-1.79	0.1415
ssc-miR-153	ZBTB34	zinc finger and BTB domain containing 34 [Source:VGNC Symbol;Acc:VGNC:95070]	-1.79	0.1415
ssc-miR-153	ZBTB43	zinc finger and BTB domain containing 43 [Source:VGNC Symbol;Acc:VGNC:95075]	-1.79	0.1415
ssc-miR-153	ZBTB7B	zinc finger and BTB domain containing 7B [Source:VGNC Symbol;Acc:VGNC:95082]	-1.79	0.1415
ssc-miR-153	ZC2HC1A	zinc finger C2HC-type containing 1A [Source:VGNC Symbol;Acc:VGNC:95085]	-1.79	0.1415
ssc-miR-153	ZC3H12C	zinc finger CCCH-type containing 12C [Source:VGNC Symbol;Acc:VGNC:95091]	-1.79	0.1415
ssc-miR-153	ZC3HAV1	zinc finger CCCH-type containing, antiviral 1 [Source:VGNC Symbol;Acc:VGNC:95102]	-1.79	0.1415
ssc-miR-153	ZCCHC14	zinc finger CCHC-type containing 14 [Source:VGNC Symbol;Acc:VGNC:95107]	-1.79	0.1415
ssc-miR-153	ZCCHC2	zinc finger CCHC-type containing 2 [Source:VGNC Symbol;Acc:VGNC:95109]	-1.79	0.1415
ssc-miR-153	ZCCHC24	zinc finger CCHC-type containing 24 [Source:HGNC Symbol;Acc:HGNC:26911]	-1.79	0.1415

ssc-miR-153	ZDHHC1	zinc finger DHHC-type containing 1 [Source:VGNC Symbol;Acc:VGNC:95115]	-1.79	0.1415
ssc-miR-153	ZDHHC2	zinc finger DHHC-type palmitoyltransferase 2 [Source:VGNC Symbol;Acc:VGNC:95886]	-1.79	0.1415
ssc-miR-153	ZDHHC20	zinc finger DHHC-type palmitoyltransferase 20 [Source:VGNC Symbol;Acc:VGNC:95122]	-1.79	0.1415
ssc-miR-153	ZDHHC23	zinc finger DHHC-type palmitoyltransferase 23 [Source:VGNC Symbol;Acc:VGNC:95125]	-1.79	0.1415
ssc-miR-153	ZDHHC6	zinc finger DHHC-type palmitoyltransferase 6 [Source:VGNC Symbol;Acc:VGNC:95128]	-1.79	0.1415
ssc-miR-153	ZEB2	hypothetical gene	-1.79	0.1415
ssc-miR-153	ZFPM2	zinc finger protein, FOG family member 2 [Source:VGNC Symbol;Acc:VGNC:95153]	-1.79	0.1415
ssc-miR-153	ZFYVE9	zinc finger FYVE-type containing 9 [Source:VGNC Symbol;Acc:VGNC:95163]	-1.79	0.1415
ssc-miR-153	ZMAT3	zinc finger matrin-type 3 [Source:VGNC Symbol;Acc:VGNC:95177]	-1.79	0.1415
ssc-miR-153	ZMIZ1	zinc finger MIZ-type containing 1 [Source:VGNC Symbol;Acc:VGNC:95178]	-1.79	0.1415
ssc-miR-153	ZMYND11	zinc finger MYND-type containing 11 [Source:VGNC Symbol;Acc:VGNC:95827]	-1.79	0.1415
ssc-miR-153	ZNF146	zinc finger protein 146 [Source:HGNC Symbol;Acc:HGNC:12931]	-1.79	0.1415
ssc-miR-153	ZNF148	zinc finger protein 148 [Source:VGNC Symbol;Acc:VGNC:95192]	-1.79	0.1415
ssc-miR-153	ZNF275	hypothetical gene	-1.79	0.1415
ssc-miR-153	ZNF35	zinc finger protein 35 [Source:VGNC Symbol;Acc:VGNC:95228]	-1.79	0.1415
ssc-miR-153	ZNF385D	zinc finger protein 385D [Source:VGNC Symbol;Acc:VGNC:95234]	-1.79	0.1415
ssc-miR-153	ZNF449	zinc finger protein 449 [Source:VGNC Symbol;Acc:VGNC:95242]	-1.79	0.1415
ssc-miR-153	ZNF462	zinc finger protein 462 [Source:VGNC Symbol;Acc:VGNC:95244]	-1.79	0.1415
ssc-miR-153	ZNF521	zinc finger protein 521 [Source:VGNC Symbol;Acc:VGNC:98715]	-1.79	0.1415
ssc-miR-153	ZNF592	zinc finger protein 592 [Source:HGNC Symbol;Acc:HGNC:28986]	-1.79	0.1415
ssc-miR-153	ZNF609	zinc finger protein 609 [Source:VGNC Symbol;Acc:VGNC:95274]	-1.79	0.1415
ssc-miR-153	ZNF621	zinc finger protein 621 [Source:HGNC Symbol;Acc:HGNC:24787]	-1.79	0.1415
ssc-miR-153	ZNF652	zinc finger protein 652 [Source:VGNC Symbol;Acc:VGNC:99108]	-1.79	0.1415
ssc-miR-153	ZNF654	zinc finger protein 654 [Source:VGNC Symbol;Acc:VGNC:95285]	-1.79	0.1415
ssc-miR-153	ZNF703	zinc finger protein 703 [Source:VGNC Symbol;Acc:VGNC:96112]	-1.79	0.1415
ssc-miR-153	ZNF704	zinc finger protein 704 [Source:VGNC Symbol;Acc:VGNC:95296]	-1.79	0.1415
ssc-miR-153	ZNF827	zinc finger protein 827 [Source:VGNC Symbol;Acc:VGNC:98970]	-1.79	0.1415
ssc-miR-153	ZNFX1	zinc finger NFX1-type containing 1 [Source:VGNC Symbol;Acc:VGNC:96210]	-1.79	0.1415
ssc-miR-153	ZNRF2	zinc and ring finger 2 [Source:VGNC Symbol;Acc:VGNC:95317]	-1.79	0.1415
ssc-miR-153	ZSWIM5	zinc finger SWIM-type containing 5 [Source:VGNC Symbol;Acc:VGNC:95334]	-1.79	0.1415
ssc-miR-153	ZSWIM6	zinc finger SWIM-type containing 6 [Source:VGNC Symbol;Acc:VGNC:95335]	-1.79	0.1415
ssc-miR-153	ZZEF1	zinc finger ZZ-type and EF-hand domain containing 1 [Source:VGNC Symbol;Acc:VGNC:95347]	-1.79	0.1415
ssc-miR-34c	AAGAB	alpha and gamma adaptin binding protein [Source:VGNC Symbol;Acc:VGNC:84939]	-1.81	0.1241
ssc-miR-34c	ABHD4	abhydrolase domain containing 4, N-acyl phospholipase B [Source:VGNC Symbol;Acc:VGNC:97859]	-1.81	0.1241
ssc-miR-34c	ABR	ABR activator of RhoGEF and GTPase [Source:VGNC Symbol;Acc:VGNC:84989]	-1.81	0.1241
ssc-miR-34c	ACBD3	acyl-CoA binding domain containing 3 [Source:VGNC Symbol;Acc:VGNC:95641]	-1.81	0.1241
ssc-miR-34c	ACOT11	acyl-CoA thioesterase 11 [Source:VGNC Symbol;Acc:VGNC:85016]	-1.81	0.1241
ssc-miR-34c	ACSL1	acyl-CoA synthetase long chain family member 1 [Source:VGNC Symbol;Acc:VGNC:96294]	-1.81	0.1241
ssc-miR-34c	ACSL4	acyl-CoA synthetase long chain family member 4 [Source:VGNC Symbol;Acc:VGNC:85033]	-1.81	0.1241
ssc-miR-34c	ACTR1A	actin related protein 1A [Source:VGNC Symbol;Acc:VGNC:85049]	-1.81	0.1241
ssc-miR-34c	ACVR2B	activin A receptor type 2B [Source:VGNC Symbol;Acc:VGNC:108629]	-1.81	0.1241
ssc-miR-34c	ACVRL1	activin A receptor like type 1 [Source:VGNC Symbol;Acc:VGNC:85057]	-1.81	0.1241
ssc-miR-34c	ADAM10	ADAM metallopeptidase domain 10 [Source:VGNC Symbol;Acc:VGNC:85061]	-1.81	0.1241
ssc-miR-34c	ADAM11	ADAM metallopeptidase domain 11 [Source:VGNC Symbol;Acc:VGNC:85062]	-1.81	0.1241
ssc-miR-34c	ADAM22	ADAM metallopeptidase domain 22 [Source:VGNC Symbol;Acc:VGNC:85067]	-1.81	0.1241
ssc-miR-34c	ADAMTS5	ADAM metallopeptidase with thrombospondin type 1 motif 5 [Source:VGNC Symbol;Acc:VGNC:85085]	-1.81	0.1241
ssc-miR-34c	ADAT2	adenosine deaminase tRNA specific 2 [Source:VGNC Symbol;Acc:VGNC:85100]	-1.81	0.1241
ssc-miR-34c	ADCY5	adenylate cyclase 5 [Source:VGNC Symbol;Acc:VGNC:85109]	-1.81	0.1241
ssc-miR-34c	ADD2	adducin 2 [Source:VGNC Symbol;Acc:VGNC:85117]	-1.81	0.1241
ssc-miR-34c	ADIPOR2	adiponectin receptor 2 [Source:VGNC Symbol;Acc:VGNC:85141]	-1.81	0.1241

ssc-miR-34c	ADK	adenosine kinase [Source:VGNC Symbol;Acc:VGNC:85143]	-1.81	0.1241
ssc-miR-34c	ADO	2-aminoethanethiol dioxygenase [Source:VGNC Symbol;Acc:VGNC:85146]	-1.81	0.1241
ssc-miR-34c	AFAP1	actin filament associated protein 1 [Source:VGNC Symbol;Acc:VGNC:85165]	-1.81	0.1241
ssc-miR-34c	AFF3	AF4/FMR2 family member 3 [Source:HGNC Symbol;Acc:HGNC:6473]	-1.81	0.1241
ssc-miR-34c	AFF4	AF4/FMR2 family member 4 [Source:VGNC Symbol;Acc:VGNC:85169]	-1.81	0.1241
ssc-miR-34c	AGAP2	ArfGAP with GTPase domain, ankyrin repeat and PH domain 2 [Source:VGNC Symbol;Acc:VGNC:85174]	-1.81	0.1241
ssc-miR-34c	AGO1	hypothetical gene	-1.81	0.1241
ssc-miR-34c	AGO4	hypothetical gene	-1.81	0.1241
ssc-miR-34c	AGTRAP	hypothetical gene	-1.81	0.1241
ssc-miR-34c	AHCYL2	adenosylhomocysteinase like 2 [Source:VGNC Symbol;Acc:VGNC:97874]	-1.81	0.1241
ssc-miR-34c	AHSA2	hypothetical gene	-1.81	0.1241
ssc-miR-34c	AIDA	axin interactor, dorsalization associated [Source:VGNC Symbol;Acc:VGNC:96103]	-1.81	0.1241
ssc-miR-34c	AK2	hypothetical gene	-1.81	0.1241
ssc-miR-34c	AL117190.3	hypothetical gene	-1.81	0.1241
ssc-miR-34c	AL163636.6	hypothetical gene	-1.81	0.1241
ssc-miR-34c	ALCAM	activated leukocyte cell adhesion molecule [Source:VGNC Symbol;Acc:VGNC:85236]	-1.81	0.1241
ssc-miR-34c	ALDOA	aldolase, fructose-bisphosphate A [Source:VGNC Symbol;Acc:VGNC:102549]	-1.81	0.1241
ssc-miR-34c	AMER1	APC membrane recruitment protein 1 [Source:VGNC Symbol;Acc:VGNC:85276]	-1.81	0.1241
ssc-miR-34c	AMER2	APC membrane recruitment protein 2 [Source:VGNC Symbol;Acc:VGNC:85277]	-1.81	0.1241
ssc-miR-34c	ANK2	hypothetical gene	-1.81	0.1241
ssc-miR-34c	ANK3	hypothetical gene	-1.81	0.1241
ssc-miR-34c	ANKFY1	ankyrin repeat and FYVE domain containing 1 [Source:VGNC Symbol;Acc:VGNC:85314]	-1.81	0.1241
ssc-miR-34c	ANKRD52	ankyrin repeat domain 52 [Source:VGNC Symbol;Acc:VGNC:85342]	-1.81	0.1241
ssc-miR-34c	ANKS1A	ankyrin repeat and sterile alpha motif domain containing 1A [Source:VGNC Symbol;Acc:VGNC:85348]	-1.81	0.1241
ssc-miR-34c	ANO6	anoctamin 6 [Source:VGNC Symbol;Acc:VGNC:85359]	-1.81	0.1241
ssc-miR-34c	ANP32B	acidic nuclear phosphoprotein 32 family member B [Source:HGNC Symbol;Acc:HGNC:16677]	-1.81	0.1241
ssc-miR-34c	AP1B1	adaptor related protein complex 1 subunit beta 1 [Source:VGNC Symbol;Acc:VGNC:85377]	-1.81	0.1241
ssc-miR-34c	AP1S2	adaptor related protein complex 1 subunit sigma 2 [Source:VGNC Symbol;Acc:VGNC:85382]	-1.81	0.1241
ssc-miR-34c	AP3S2	adaptor-related protein complex 3, sigma 2 subunit [Source:NCBI gene (formerly Entrezgene);Acc:100153862]	-1.81	0.1241
ssc-miR-34c	APH1A	aph-1 homolog A, gamma-secretase subunit [Source:VGNC Symbol;Acc:VGNC:85409]	-1.81	0.1241
ssc-miR-34c	AREG	amphiregulin [Source:VGNC Symbol;Acc:VGNC:85444]	-1.81	0.1241
ssc-miR-34c	AREGB	hypothetical gene	-1.81	0.1241
ssc-miR-34c	ARHGAP1	Rho GTPase activating protein 1 [Source:VGNC Symbol;Acc:VGNC:85457]	-1.81	0.1241
ssc-miR-34c	ARHGAP18	Rho GTPase activating protein 18 [Source:VGNC Symbol;Acc:VGNC:85460]	-1.81	0.1241
ssc-miR-34c	ARHGAP19-SLIT1	hypothetical gene	-1.81	0.1241
ssc-miR-34c	ARHGAP26	Rho GTPase activating protein 26 [Source:VGNC Symbol;Acc:VGNC:85465]	-1.81	0.1241
ssc-miR-34c	ARHGAP36	hypothetical gene	-1.81	0.1241
ssc-miR-34c	ARHGAP39	Rho GTPase activating protein 39 [Source:VGNC Symbol;Acc:VGNC:85475]	-1.81	0.1241
ssc-miR-34c	ARID4A	AT-rich interaction domain 4A [Source:VGNC Symbol;Acc:VGNC:85509]	-1.81	0.1241
ssc-miR-34c	ARID4B	AT-rich interaction domain 4B [Source:HGNC Symbol;Acc:HGNC:15550]	-1.81	0.1241
ssc-miR-34c	ARMC7	hypothetical gene	-1.81	0.1241
ssc-miR-34c	ARPC4-TTLL3	hypothetical gene	-1.81	0.1241
ssc-miR-34c	ARPP19	cAMP regulated phosphoprotein 19 [Source:NCBI gene (formerly Entrezgene);Acc:397362]	-1.81	0.1241
ssc-miR-34c	ARSJ	arylsulfatase family member J [Source:VGNC Symbol;Acc:VGNC:85549]	-1.81	0.1241
ssc-miR-34c	ASB1	ankyrin repeat and SOCS box containing 1 [Source:VGNC Symbol;Acc:VGNC:95728]	-1.81	0.1241
ssc-miR-34c	ASB6	ankyrin repeat and SOCS box containing 6 [Source:VGNC Symbol;Acc:VGNC:85565]	-1.81	0.1241
ssc-miR-34c	ASCL1	achaete-scute family bHLH transcription factor 1 [Source:HGNC Symbol;Acc:HGNC:738]	-1.81	0.1241
ssc-miR-34c	ASIC2	acid sensing ion channel subunit 2 [Source:VGNC Symbol;Acc:VGNC:97893]	-1.81	0.1241
ssc-miR-34c	ASPHD2	aspartate beta-hydroxylase domain containing 2 [Source:VGNC Symbol;Acc:VGNC:85585]	-1.81	0.1241
ssc-miR-34c	ATG4B	autophagy related 4B cysteine peptidase [Source:VGNC Symbol;Acc:VGNC:95947]	-1.81	0.1241

ssc-miR-34c	ATG5	autophagy related 5 [Source:VGNC Symbol;Acc:VGNC:103029]	-1.81	0.1241
ssc-miR-34c	ATG9A	autophagy related 9A [Source:VGNC Symbol;Acc:VGNC:99703]	-1.81	0.1241
ssc-miR-34c	ATMIN	hypothetical gene	-1.81	0.1241
ssc-miR-34c	ATOH1	atonal bHLH transcription factor 1 [Source:VGNC Symbol;Acc:VGNC:85631]	-1.81	0.1241
ssc-miR-34c	ATP1A2	ATPase Na+/K+ transporting subunit alpha 2 [Source:NCBI gene (formerly Entrezgene);Acc:396828]	-1.81	0.1241
ssc-miR-34c	ATP2B4	ATPase plasma membrane Ca2+ transporting 4 [Source:VGNC Symbol;Acc:VGNC:85651]	-1.81	0.1241
ssc-miR-34c	ATP5S	hypothetical gene	-1.81	0.1241
ssc-miR-34c	ATRN	attractin [Source:VGNC Symbol;Acc:VGNC:96479]	-1.81	0.1241
ssc-miR-34c	ATXN7	ataxin 7 [Source:VGNC Symbol;Acc:VGNC:99704]	-1.81	0.1241
ssc-miR-34c	ATXN7L1	ataxin 7 like 1 [Source:VGNC Symbol;Acc:VGNC:85692]	-1.81	0.1241
ssc-miR-34c	ATXN7L3	ataxin 7 like 3 [Source:VGNC Symbol;Acc:VGNC:85694]	-1.81	0.1241
ssc-miR-34c	ATXN7L3B	ataxin 7 like 3B [Source:HGNC Symbol;Acc:HGNC:37931]	-1.81	0.1241
ssc-miR-34c	AXL	AXL receptor tyrosine kinase [Source:VGNC Symbol;Acc:VGNC:85709]	-1.81	0.1241
ssc-miR-34c	B4GALT2	beta-1,4-galactosyltransferase 2 [Source:VGNC Symbol;Acc:VGNC:85733]	-1.81	0.1241
ssc-miR-34c	BAG2	BAG cochaperone 2 [Source:VGNC Symbol;Acc:VGNC:85744]	-1.81	0.1241
ssc-miR-34c	BAZ2A	bromodomain adjacent to zinc finger domain 2A [Source:VGNC Symbol;Acc:VGNC:85763]	-1.81	0.1241
ssc-miR-34c	BCL11B	BAF chromatin remodeling complex subunit BCL11B [Source:VGNC Symbol;Acc:VGNC:96563]	-1.81	0.1241
ssc-miR-34c	BCL2	BCL2 apoptosis regulator [Source:HGNC Symbol;Acc:HGNC:990]	-1.81	0.1241
ssc-miR-34c	BCL2L13	BCL2 like 13 [Source:VGNC Symbol;Acc:VGNC:85781]	-1.81	0.1241
ssc-miR-34c	BCL9L	BCL9 like [Source:VGNC Symbol;Acc:VGNC:96568]	-1.81	0.1241
ssc-miR-34c	BMP3	bone morphotic protein 3 [Source:VGNC Symbol;Acc:VGNC:85842]	-1.81	0.1241
ssc-miR-34c	BMP8B	hypothetical gene	-1.81	0.1241
ssc-miR-34c	BMPR2	bone morphotic protein receptor type 2 [Source:VGNC Symbol;Acc:VGNC:95494]	-1.81	0.1241
ssc-miR-34c	BNC2	basonuclin 2 [Source:VGNC Symbol;Acc:VGNC:85852]	-1.81	0.1241
ssc-miR-34c	BRINP1	BMP/retinoic acid inducible neural specific 1 [Source:VGNC Symbol;Acc:VGNC:85881]	-1.81	0.1241
ssc-miR-34c	BRPF3	bromodomain and PHD finger containing 3 [Source:VGNC Symbol;Acc:VGNC:85886]	-1.81	0.1241
ssc-miR-34c	BTBD11	BTB domain containing 11 [Source:VGNC Symbol;Acc:VGNC:85901]	-1.81	0.1241
ssc-miR-34c	C11orf49	hypothetical gene	-1.81	0.1241
ssc-miR-34c	C12orf73	chromosome 12 open reading frame 73 [Source:HGNC Symbol;Acc:HGNC:34450]	-1.81	0.1241
ssc-miR-34c	C14orf28	chromosome 1 C14orf28 homolog [Source:VGNC Symbol;Acc:VGNC:85958]	-1.81	0.1241
ssc-miR-34c	C15orf38-AP3S2	hypothetical gene	-1.81	0.1241
ssc-miR-34c	C15orf39	chromosome 7 C15orf39 homolog [Source:VGNC Symbol;Acc:VGNC:86070]	-1.81	0.1241
ssc-miR-34c	C15orf53	hypothetical gene	-1.81	0.1241
ssc-miR-34c	C17orf75	chromosome 12 C17orf75 homolog [Source:VGNC Symbol;Acc:VGNC:85925]	-1.81	0.1241
ssc-miR-34c	C19orf52	hypothetical gene	-1.81	0.1241
ssc-miR-34c	C1QL3	complement C1q like 3 [Source:VGNC Symbol;Acc:VGNC:95910]	-1.81	0.1241
ssc-miR-34c	C22orf23	chromosome 5 C22orf23 homolog [Source:VGNC Symbol;Acc:VGNC:86049]	-1.81	0.1241
ssc-miR-34c	C2CD4A	hypothetical gene	-1.81	0.1241
ssc-miR-34c	C2orf49	chromosome 3 C2orf49 homolog [Source:VGNC Symbol;Acc:VGNC:86022]	-1.81	0.1241
ssc-miR-34c	C2orf68	chromosome 3 C2orf68 homolog [Source:VGNC Symbol;Acc:VGNC:86023]	-1.81	0.1241
ssc-miR-34c	C3orf58	hypothetical gene	-1.81	0.1241
ssc-miR-34c	C3orf70	chromosome 13 C3orf70 homolog [Source:VGNC Symbol;Acc:VGNC:85937]	-1.81	0.1241
ssc-miR-34c	C5orf51	hypothetical gene	-1.81	0.1241
ssc-miR-34c	C6orf106	hypothetical gene	-1.81	0.1241
ssc-miR-34c	C6orf120	chromosome 1 C6orf120 homolog [Source:VGNC Symbol;Acc:VGNC:85967]	-1.81	0.1241
ssc-miR-34c	C8orf37	hypothetical gene	-1.81	0.1241
ssc-miR-34c	C8orf44-SGK3	hypothetical gene	-1.81	0.1241
ssc-miR-34c	C8orf58	chromosome 14 C8orf58 homolog [Source:VGNC Symbol;Acc:VGNC:107371]	-1.81	0.1241
ssc-miR-34c	C9orf64	chromosome 10 C9orf64 homolog [Source:VGNC Symbol;Acc:VGNC:96167]	-1.81	0.1241
ssc-miR-34c	C9orf69	hypothetical gene	-1.81	0.1241

ssc-miR-34c	CA7	carbonic anhydrase 7 [Source:VGNC Symbol;Acc:VGNC:86104]	-1.81	0.1241
ssc-miR-34c	CACNA1E	calcium voltage-gated channel subunit alpha1 E [Source:VGNC Symbol;Acc:VGNC:86118]	-1.81	0.1241
ssc-miR-34c	CACNA2D1	calcium voltage-gated channel auxiliary subunit alpha2delta 1 [Source:VGNC Symbol;Acc:VGNC:86120]	-1.81	0.1241
ssc-miR-34c	CACNA2D2	calcium voltage-gated channel auxiliary subunit alpha2delta 2 [Source:VGNC Symbol;Acc:VGNC:86121]	-1.81	0.1241
ssc-miR-34c	CACNB1	calcium voltage-gated channel auxiliary subunit beta 1 [Source:VGNC Symbol;Acc:VGNC:97909]	-1.81	0.1241
ssc-miR-34c	CACNB3	calcium voltage-gated channel auxiliary subunit beta 3 [Source:VGNC Symbol;Acc:VGNC:103221]	-1.81	0.1241
ssc-miR-34c	CACNG2	calcium voltage-gated channel auxiliary subunit gamma 2 [Source:VGNC Symbol;Acc:VGNC:86125]	-1.81	0.1241
ssc-miR-34c	CADM4	cell adhesion molecule 4 [Source:VGNC Symbol;Acc:VGNC:86136]	-1.81	0.1241
ssc-miR-34c	CALCR	calcitonin receptor [Source:VGNC Symbol;Acc:VGNC:86142]	-1.81	0.1241
ssc-miR-34c	CALN1	hypothetical gene	-1.81	0.1241
ssc-miR-34c	CAMK4	calcium/calmodulin dependent protein kinase IV [Source:VGNC Symbol;Acc:VGNC:99602]	-1.81	0.1241
ssc-miR-34c	CAMSAP1	calmodulin regulated spectrin associated protein 1 [Source:VGNC Symbol;Acc:VGNC:86162]	-1.81	0.1241
ssc-miR-34c	CAND1	cullin associated and neddylation dissociated 1 [Source:VGNC Symbol;Acc:VGNC:97912]	-1.81	0.1241
ssc-miR-34c	CANT1	calcium activated nucleotidase 1 [Source:VGNC Symbol;Acc:VGNC:86166]	-1.81	0.1241
ssc-miR-34c	CAPN5	calpain 5 [Source:VGNC Symbol;Acc:VGNC:86175]	-1.81	0.1241
ssc-miR-34c	CAPN6	calpain 6 [Source:VGNC Symbol;Acc:VGNC:86176]	-1.81	0.1241
ssc-miR-34c	CASP2	caspase 2 [Source:HGNC Symbol;Acc:HGNC:1503]	-1.81	0.1241
ssc-miR-34c	CBFA2T3	CBFA2/RUNX1 partner transcriptional co-repressor 3 [Source:VGNC Symbol;Acc:VGNC:96574]	-1.81	0.1241
ssc-miR-34c	CBFB	core-binding factor subunit beta [Source:VGNC Symbol;Acc:VGNC:86221]	-1.81	0.1241
ssc-miR-34c	CCDC135	hypothetical gene	-1.81	0.1241
ssc-miR-34c	CCDC80	coiled-coil domain containing 80 [Source:VGNC Symbol;Acc:VGNC:86315]	-1.81	0.1241
ssc-miR-34c	CCDC85A	coiled-coil domain containing 85A [Source:VGNC Symbol;Acc:VGNC:86320]	-1.81	0.1241
ssc-miR-34c	CCDC88A	coiled-coil domain containing 88A [Source:VGNC Symbol;Acc:VGNC:86325]	-1.81	0.1241
ssc-miR-34c	CCND1	cyclin D1 [Source:VGNC Symbol;Acc:VGNC:86352]	-1.81	0.1241
ssc-miR-34c	CCNE2	cyclin E2 [Source:VGNC Symbol;Acc:VGNC:86355]	-1.81	0.1241
ssc-miR-34c	CD47	CD47 molecule [Source:VGNC Symbol;Acc:VGNC:108637]	-1.81	0.1241
ssc-miR-34c	CD82	CD82 molecule [Source:VGNC Symbol;Acc:VGNC:86432]	-1.81	0.1241
ssc-miR-34c	CDC25A	cell division cycle 25A [Source:VGNC Symbol;Acc:VGNC:86446]	-1.81	0.1241
ssc-miR-34c	CDC37	cell division cycle 37, HSP90 cochaperone [Source:VGNC Symbol;Acc:VGNC:86450]	-1.81	0.1241
ssc-miR-34c	CDC40	cell division cycle 40 [Source:VGNC Symbol;Acc:VGNC:86452]	-1.81	0.1241
ssc-miR-34c	CDH4	cadherin 4 [Source:VGNC Symbol;Acc:VGNC:95853]	-1.81	0.1241
ssc-miR-34c	CDIP1	cell death inducing p53 target 1 [Source:VGNC Symbol;Acc:VGNC:86493]	-1.81	0.1241
ssc-miR-34c	CDK6	cyclin dependent kinase 6 [Source:HGNC Symbol;Acc:HGNC:1777]	-1.81	0.1241
ssc-miR-34c	CDKN1C	cyclin dependent kinase inhibitor 1C [Source:VGNC Symbol;Acc:VGNC:86516]	-1.81	0.1241
ssc-miR-34c	CELF3	CUGBP Elav-like family member 3 [Source:VGNC Symbol;Acc:VGNC:86538]	-1.81	0.1241
ssc-miR-34c	CEP170B	centrosomal protein 170B [Source:VGNC Symbol;Acc:VGNC:86564]	-1.81	0.1241
ssc-miR-34c	CERK	ceramide kinase [Source:VGNC Symbol;Acc:VGNC:86586]	-1.81	0.1241
ssc-miR-34c	CERS6	ceramide synthase 6 [Source:VGNC Symbol;Acc:VGNC:96025]	-1.81	0.1241
ssc-miR-34c	CES4A	hypothetical gene	-1.81	0.1241
ssc-miR-34c	CHD1	chromodomain helicase DNA binding protein 1 [Source:VGNC Symbol;Acc:VGNC:86629]	-1.81	0.1241
ssc-miR-34c	CHL1	cell adhesion molecule L1 like [Source:VGNC Symbol;Acc:VGNC:108639]	-1.81	0.1241
ssc-miR-34c	CHMP7	charged multivesicular body protein 7 [Source:VGNC Symbol;Acc:VGNC:86654]	-1.81	0.1241
ssc-miR-34c	CHST12	carbohydrate sulfotransferase 12 [Source:HGNC Symbol;Acc:HGNC:17423]	-1.81	0.1241
ssc-miR-34c	CLCN3	chloride voltage-gated channel 3 [Source:VGNC Symbol;Acc:VGNC:86727]	-1.81	0.1241
ssc-miR-34c	CLLU1	hypothetical gene	-1.81	0.1241
ssc-miR-34c	CLOCK	clock circadian regulator [Source:VGNC Symbol;Acc:VGNC:86774]	-1.81	0.1241
ssc-miR-34c	CLPB	caseinolytic mitochondrial matrix peptidase chaperone subunit B [Source:VGNC Symbol;Acc:VGNC:86776]	-1.81	0.1241
ssc-miR-34c	CMKLR1	chemerin chemokine-like receptor 1 [Source:VGNC Symbol;Acc:VGNC:86800]	-1.81	0.1241
ssc-miR-34c	CNOT6	CCR4-NOT transcription complex subunit 6 [Source:VGNC Symbol;Acc:VGNC:86837]	-1.81	0.1241
ssc-miR-34c	CNOT6L	CCR4-NOT transcription complex subunit 6 like [Source:VGNC Symbol;Acc:VGNC:86838]	-1.81	0.1241

ssc-miR-34c	CNTD1	cyclin N-terminal domain containing 1 [Source:VGNC Symbol;Acc:VGNC:86846]	-1.81	0.1241
ssc-miR-34c	CNTN2	contactin 2 [Source:VGNC Symbol;Acc:VGNC:108577]	-1.81	0.1241
ssc-miR-34c	CNTNAP1	contactin associated protein 1 [Source:VGNC Symbol;Acc:VGNC:86849]	-1.81	0.1241
ssc-miR-34c	CNTNAP2	hypothetical gene	-1.81	0.1241
ssc-miR-34c	COL12A1	collagen type XII alpha 1 chain [Source:VGNC Symbol;Acc:VGNC:86864]	-1.81	0.1241
ssc-miR-34c	COPS7B	COP9 signalosome subunit 7B [Source:HGNC Symbol;Acc:HGNC:16760]	-1.81	0.1241
ssc-miR-34c	CORO1C	coronin 1C [Source:VGNC Symbol;Acc:VGNC:86914]	-1.81	0.1241
ssc-miR-34c	CPD	carboxypeptidase D [Source:VGNC Symbol;Acc:VGNC:86935]	-1.81	0.1241
ssc-miR-34c	CPEB2	cytoplasmic polyadenylation element binding protein 2 [Source:VGNC Symbol;Acc:VGNC:86937]	-1.81	0.1241
ssc-miR-34c	CPLX2	complexin 2 [Source:VGNC Symbol;Acc:VGNC:86944]	-1.81	0.1241
ssc-miR-34c	CREB3L1	cAMP responsive element binding protein 3 like 1 [Source:VGNC Symbol;Acc:VGNC:86980]	-1.81	0.1241
ssc-miR-34c	CREB5	cAMP responsive element binding protein 5 [Source:VGNC Symbol;Acc:VGNC:86984]	-1.81	0.1241
ssc-miR-34c	CREBRF	CREB3 regulatory factor [Source:VGNC Symbol;Acc:VGNC:86987]	-1.81	0.1241
ssc-miR-34c	CRHR1	corticotropin releasing hormone receptor 1 [Source:VGNC Symbol;Acc:VGNC:98983]	-1.81	0.1241
ssc-miR-34c	CRTC1	CREB regulated transcription coactivator 1 [Source:VGNC Symbol;Acc:VGNC:87007]	-1.81	0.1241
ssc-miR-34c	CSF1R	colony stimulating factor 1 receptor [Source:VGNC Symbol;Acc:VGNC:87031]	-1.81	0.1241
ssc-miR-34c	CSNK1G1	casein kinase 1 gamma 1 [Source:VGNC Symbol;Acc:VGNC:97949]	-1.81	0.1241
ssc-miR-34c	CSNK2B-LY6G5B-1181	hypothetical gene	-1.81	0.1241
ssc-miR-34c	CTDSP2	CTD small phosphatase 2 [Source:VGNC Symbol;Acc:VGNC:87059]	-1.81	0.1241
ssc-miR-34c	CTDSPL	CTD small phosphatase like [Source:VGNC Symbol;Acc:VGNC:107131]	-1.81	0.1241
ssc-miR-34c	CTNND1	catenin delta 1 [Source:VGNC Symbol;Acc:VGNC:87067]	-1.81	0.1241
ssc-miR-34c	CTNND2	catenin delta 2 [Source:VGNC Symbol;Acc:VGNC:87068]	-1.81	0.1241
ssc-miR-34c	CTRC	chymotrypsin C [Source:NCBI gene (formerly Entrezgene);Acc:100520911]	-1.81	0.1241
ssc-miR-34c	CTTNBP2NL	CTTNBP2 N-terminal like [Source:VGNC Symbol;Acc:VGNC:87082]	-1.81	0.1241
ssc-miR-34c	CUEDC1	CUE domain containing 1 [Source:VGNC Symbol;Acc:VGNC:87085]	-1.81	0.1241
ssc-miR-34c	CYB561D1	cytochrome b561 family member D1 [Source:VGNC Symbol;Acc:VGNC:87121]	-1.81	0.1241
ssc-miR-34c	CYBRD1	cytochrome b reductase 1 [Source:VGNC Symbol;Acc:VGNC:95948]	-1.81	0.1241
ssc-miR-34c	DAAM1	dishevelled associated activator of morphosis 1 [Source:VGNC Symbol;Acc:VGNC:87141]	-1.81	0.1241
ssc-miR-34c	DAB2IP	DAB2 interacting protein [Source:VGNC Symbol;Acc:VGNC:87145]	-1.81	0.1241
ssc-miR-34c	DAGLA	diacylglycerol lipase alpha [Source:VGNC Symbol;Acc:VGNC:87152]	-1.81	0.1241
ssc-miR-34c	DBNL	drebrin like [Source:VGNC Symbol;Acc:VGNC:87166]	-1.81	0.1241
ssc-miR-34c	DCAF11	DDB1 and CUL4 associated factor 11 [Source:VGNC Symbol;Acc:VGNC:97957]	-1.81	0.1241
ssc-miR-34c	DCAF7	DDB1 and CUL4 associated factor 7 [Source:VGNC Symbol;Acc:VGNC:87178]	-1.81	0.1241
ssc-miR-34c	DCP1A	decapping mRNA 1A [Source:VGNC Symbol;Acc:VGNC:97960]	-1.81	0.1241
ssc-miR-34c	DCX	doublecortin [Source:HGNC Symbol;Acc:HGNC:2714]	-1.81	0.1241
ssc-miR-34c	DDX17	hypothetical gene	-1.81	0.1241
ssc-miR-34c	DGKH	diacylglycerol kinase eta [Source:VGNC Symbol;Acc:VGNC:87273]	-1.81	0.1241
ssc-miR-34c	DGKI	diacylglycerol kinase iota [Source:VGNC Symbol;Acc:VGNC:87274]	-1.81	0.1241
ssc-miR-34c	DGKZ	diacylglycerol kinase zeta [Source:VGNC Symbol;Acc:VGNC:87277]	-1.81	0.1241
ssc-miR-34c	DIXDC1	hypothetical gene	-1.81	0.1241
ssc-miR-34c	DLL1	delta like canonical Notch ligand 1 [Source:VGNC Symbol;Acc:VGNC:87335]	-1.81	0.1241
ssc-miR-34c	DMWD	DM1 locus, WD repeat containing [Source:VGNC Symbol;Acc:VGNC:96978]	-1.81	0.1241
ssc-miR-34c	DMXL1	Dmx like 1 [Source:VGNC Symbol;Acc:VGNC:87359]	-1.81	0.1241
ssc-miR-34c	DNAH10OS	hypothetical gene	-1.81	0.1241
ssc-miR-34c	DNAH17-AS1	hypothetical gene	-1.81	0.1241
ssc-miR-34c	DNAJB1	DnaJ heat shock protein family (Hsp40) member B1 [Source:VGNC Symbol;Acc:VGNC:96614]	-1.81	0.1241
ssc-miR-34c	DNAJC16	DnaJ heat shock protein family (Hsp40) member C16 [Source:VGNC Symbol;Acc:VGNC:96624]	-1.81	0.1241
ssc-miR-34c	DPYSL4	dihydropyrimidinase like 4 [Source:VGNC Symbol;Acc:VGNC:87432]	-1.81	0.1241
ssc-miR-34c	DSEL	dermatan sulfate epimerase like [Source:VGNC Symbol;Acc:VGNC:87454]	-1.81	0.1241
ssc-miR-34c	E2F3	E2F transcription factor 3 [Source:VGNC Symbol;Acc:VGNC:87514]	-1.81	0.1241

ssc-miR-34c	E2F5	E2F transcription factor 5 [Source:VGNC Symbol;Acc:VGNC:87516]	-1.81	0.1241
ssc-miR-34c	EBF4	EBF family member 4 [Source:VGNC Symbol;Acc:VGNC:96211]	-1.81	0.1241
ssc-miR-34c	EEA1	early endosome antigen 1 [Source:HGNC Symbol;Acc:HGNC:3185]	-1.81	0.1241
ssc-miR-34c	EHD4	EH domain containing 4 [Source:VGNC Symbol;Acc:VGNC:87598]	-1.81	0.1241
ssc-miR-34c	EIF2S2	eukaryotic translation initiation factor 2 subunit beta [Source:VGNC Symbol;Acc:VGNC:96265]	-1.81	0.1241
ssc-miR-34c	ELL2	elongation factor for RNA polymerase II 2 [Source:VGNC Symbol;Acc:VGNC:87649]	-1.81	0.1241
ssc-miR-34c	ELMOD1	ELMO domain containing 1 [Source:VGNC Symbol;Acc:VGNC:87652]	-1.81	0.1241
ssc-miR-34c	ELMSAN1	hypothetical gene	-1.81	0.1241
ssc-miR-34c	ELOVL6	ELOVL fatty acid elongase 6 [Source:VGNC Symbol;Acc:VGNC:87662]	-1.81	0.1241
ssc-miR-34c	EMC3	ER membrane protein complex subunit 3 [Source:VGNC Symbol;Acc:VGNC:87671]	-1.81	0.1241
ssc-miR-34c	EML5	hypothetical gene	-1.81	0.1241
ssc-miR-34c	EMX1	empty spiracles homeobox 1 [Source:VGNC Symbol;Acc:VGNC:87692]	-1.81	0.1241
ssc-miR-34c	EPN2	epsin 2 [Source:VGNC Symbol;Acc:VGNC:97982]	-1.81	0.1241
ssc-miR-34c	ERC1	ELKS/RAB6-interacting/CAST family member 1 [Source:VGNC Symbol;Acc:VGNC:87761]	-1.81	0.1241
ssc-miR-34c	ERGIC1	endoplasmic reticulum-golgi intermediate compartment 1 [Source:VGNC Symbol;Acc:VGNC:87770]	-1.81	0.1241
ssc-miR-34c	ERN1	endoplasmic reticulum to nucleus signaling 1 [Source:HGNC Symbol;Acc:HGNC:3449]	-1.81	0.1241
ssc-miR-34c	ERP44	endoplasmic reticulum protein 44 [Source:VGNC Symbol;Acc:VGNC:87783]	-1.81	0.1241
ssc-miR-34c	ESRRA	estrogen related receptor alpha [Source:VGNC Symbol;Acc:VGNC:87793]	-1.81	0.1241
ssc-miR-34c	EVI5L	ecotropic viral integration site 5 like [Source:VGNC Symbol;Acc:VGNC:99646]	-1.81	0.1241
ssc-miR-34c	EVX2	even-skipped homeobox 2 [Source:VGNC Symbol;Acc:VGNC:96290]	-1.81	0.1241
ssc-miR-34c	F2RL2	coagulation factor II thrombin receptor like 2 [Source:VGNC Symbol;Acc:VGNC:87862]	-1.81	0.1241
ssc-miR-34c	FAM117B	family with sequence similarity 117 member B [Source:VGNC Symbol;Acc:VGNC:95616]	-1.81	0.1241
ssc-miR-34c	FAM126B	family with sequence similarity 126 member B [Source:HGNC Symbol;Acc:HGNC:28593]	-1.81	0.1241
ssc-miR-34c	FAM131B	family with sequence similarity 131 member B [Source:VGNC Symbol;Acc:VGNC:87901]	-1.81	0.1241
ssc-miR-34c	FAM133A	family with sequence similarity 133 member A [Source:VGNC Symbol;Acc:VGNC:87903]	-1.81	0.1241
ssc-miR-34c	FAM167A	family with sequence similarity 167 member A [Source:VGNC Symbol;Acc:VGNC:87924]	-1.81	0.1241
ssc-miR-34c	FAM175B	hypothetical gene	-1.81	0.1241
ssc-miR-34c	FAM208A	hypothetical gene	-1.81	0.1241
ssc-miR-34c	FAM46A	hypothetical gene	-1.81	0.1241
ssc-miR-34c	FAM63B	hypothetical gene	-1.81	0.1241
ssc-miR-34c	FAM73B	hypothetical gene	-1.81	0.1241
ssc-miR-34c	FAM76A	family with sequence similarity 76 member A [Source:VGNC Symbol;Acc:VGNC:87982]	-1.81	0.1241
ssc-miR-34c	FAM81A	family with sequence similarity 81 member A [Source:VGNC Symbol;Acc:VGNC:87986]	-1.81	0.1241
ssc-miR-34c	FAM83H	family with sequence similarity 83 member H [Source:VGNC Symbol;Acc:VGNC:87993]	-1.81	0.1241
ssc-miR-34c	FAT3	hypothetical gene	-1.81	0.1241
ssc-miR-34c	FBLIM1	filamin binding LIM protein 1 [Source:VGNC Symbol;Acc:VGNC:88023]	-1.81	0.1241
ssc-miR-34c	FBXO10	F-box protein 10 [Source:VGNC Symbol;Acc:VGNC:103092]	-1.81	0.1241
ssc-miR-34c	FBXO41	F-box protein 41 [Source:VGNC Symbol;Acc:VGNC:88046]	-1.81	0.1241
ssc-miR-34c	FBXW8	F-box and WD repeat domain containing 8 [Source:VGNC Symbol;Acc:VGNC:107391]	-1.81	0.1241
ssc-miR-34c	FGD6	FYVE, RhoGEF and PH domain containing 6 [Source:VGNC Symbol;Acc:VGNC:88099]	-1.81	0.1241
ssc-miR-34c	FGFRL1	fibroblast growth factor receptor like 1 [Source:VGNC Symbol;Acc:VGNC:88121]	-1.81	0.1241
ssc-miR-34c	FKBP1B	FKBP prolyl isomerase 1B [Source:VGNC Symbol;Acc:VGNC:106579]	-1.81	0.1241
ssc-miR-34c	FLOT2	flotillin 2 [Source:VGNC Symbol;Acc:VGNC:88159]	-1.81	0.1241
ssc-miR-34c	FNBP1	formin binding protein 1 [Source:VGNC Symbol;Acc:VGNC:88178]	-1.81	0.1241
ssc-miR-34c	FNDC3A	fibronectin type III domain containing 3A [Source:VGNC Symbol;Acc:VGNC:88182]	-1.81	0.1241
ssc-miR-34c	FNDC3B	fibronectin type III domain containing 3B [Source:VGNC Symbol;Acc:VGNC:88183]	-1.81	0.1241
ssc-miR-34c	FNDC8	fibronectin type III domain containing 8 [Source:VGNC Symbol;Acc:VGNC:88186]	-1.81	0.1241
ssc-miR-34c	FOSL1	FOS like 1, AP-1 transcription factor subunit [Source:VGNC Symbol;Acc:VGNC:88191]	-1.81	0.1241
ssc-miR-34c	FOSL2	FOS like 2, AP-1 transcription factor subunit [Source:VGNC Symbol;Acc:VGNC:88192]	-1.81	0.1241
ssc-miR-34c	FOXG1	forkhead box G1 [Source:VGNC Symbol;Acc:VGNC:88206]	-1.81	0.1241

ssc-miR-34c	FOXJ2	forkhead box J2 [Source:VGNC Symbol;Acc:VGNC:88212]	-1.81	0.1241
ssc-miR-34c	FOXN2	forkhead box N2 [Source:VGNC Symbol;Acc:VGNC:88219]	-1.81	0.1241
ssc-miR-34c	FOXN3	forkhead box N3 [Source:VGNC Symbol;Acc:VGNC:88220]	-1.81	0.1241
ssc-miR-34c	FOXP1	forkhead box P1 [Source:VGNC Symbol;Acc:VGNC:88222]	-1.81	0.1241
ssc-miR-34c	FOXP2	forkhead box P2 [Source:VGNC Symbol;Acc:VGNC:98014]	-1.81	0.1241
ssc-miR-34c	FOXQ1	forkhead box Q1 [Source:VGNC Symbol;Acc:VGNC:88225]	-1.81	0.1241
ssc-miR-34c	FRK	fyn related Src family tyrosine kinase [Source:VGNC Symbol;Acc:VGNC:88235]	-1.81	0.1241
ssc-miR-34c	FRMD4A	FERM domain containing 4A [Source:VGNC Symbol;Acc:VGNC:96088]	-1.81	0.1241
ssc-miR-34c	FRMD5	FERM domain containing 5 [Source:VGNC Symbol;Acc:VGNC:88238]	-1.81	0.1241
ssc-miR-34c	FUT11	fucosyltransferase 11 [Source:VGNC Symbol;Acc:VGNC:88269]	-1.81	0.1241
ssc-miR-34c	FUT8	fucosyltransferase 8 [Source:NCBI gene (formerly Entrezgene);Acc:396933]	-1.81	0.1241
ssc-miR-34c	FUT9	fucosyltransferase 9 [Source:VGNC Symbol;Acc:VGNC:88271]	-1.81	0.1241
ssc-miR-34c	FXYD2	hypothetical gene	-1.81	0.1241
ssc-miR-34c	G3BP1	G3BP stress granule assembly factor 1 [Source:VGNC Symbol;Acc:VGNC:88288]	-1.81	0.1241
ssc-miR-34c	G6PC3	glucose-6-phosphatase catalytic subunit 3 [Source:VGNC Symbol;Acc:VGNC:88291]	-1.81	0.1241
ssc-miR-34c	GABBR1	gamma-aminobutyric acid type B receptor subunit 1 [Source:VGNC Symbol;Acc:VGNC:88297]	-1.81	0.1241
ssc-miR-34c	GABBR2	gamma-aminobutyric acid type B receptor subunit 2 [Source:VGNC Symbol;Acc:VGNC:88298]	-1.81	0.1241
ssc-miR-34c	GABRA3	gamma-aminobutyric acid type A receptor subunit alpha3 [Source:VGNC Symbol;Acc:VGNC:88302]	-1.81	0.1241
ssc-miR-34c	GABRA4	gamma-aminobutyric acid type A receptor subunit alpha4 [Source:VGNC Symbol;Acc:VGNC:88303]	-1.81	0.1241
ssc-miR-34c	GABRB1	gamma-aminobutyric acid type A receptor subunit beta1 [Source:VGNC Symbol;Acc:VGNC:88306]	-1.81	0.1241
ssc-miR-34c	GALNT7	polypeptide N-acetylgalactosaminyltransferase 7 [Source:VGNC Symbol;Acc:VGNC:103946]	-1.81	0.1241
ssc-miR-34c	GAS1	growth arrest specific 1 [Source:HGNC Symbol;Acc:HGNC:4165]	-1.81	0.1241
ssc-miR-34c	GATAD2B	GATA zinc finger domain containing 2B [Source:VGNC Symbol;Acc:VGNC:88369]	-1.81	0.1241
ssc-miR-34c	GATS	hypothetical gene	-1.81	0.1241
ssc-miR-34c	GATSL2	hypothetical gene	-1.81	0.1241
ssc-miR-34c	GCH1	GTP cyclohydrolase 1 [Source:VGNC Symbol;Acc:VGNC:88384]	-1.81	0.1241
ssc-miR-34c	GDAP1L1	ganglioside induced differentiation associated protein 1 like 1 [Source:VGNC Symbol;Acc:VGNC:96318]	-1.81	0.1241
ssc-miR-34c	GFRA1	GDNF family receptor alpha 1 [Source:VGNC Symbol;Acc:VGNC:88428]	-1.81	0.1241
ssc-miR-34c	GHDC	GH3 domain containing [Source:VGNC Symbol;Acc:VGNC:88441]	-1.81	0.1241
ssc-miR-34c	GIGYF1	GRB10 interacting GYF protein 1 [Source:VGNC Symbol;Acc:VGNC:88444]	-1.81	0.1241
ssc-miR-34c	GJB4	gap junction protein beta 4 [Source:VGNC Symbol;Acc:VGNC:88464]	-1.81	0.1241
ssc-miR-34c	GK5	glycerol kinase 5 [Source:VGNC Symbol;Acc:VGNC:96734]	-1.81	0.1241
ssc-miR-34c	GLCE	glucuronic acid epimerase [Source:VGNC Symbol;Acc:VGNC:88477]	-1.81	0.1241
ssc-miR-34c	GLRA3	glycine receptor alpha 3 [Source:VGNC Symbol;Acc:VGNC:88493]	-1.81	0.1241
ssc-miR-34c	GMFB	glia maturation factor beta [Source:VGNC Symbol;Acc:VGNC:88509]	-1.81	0.1241
ssc-miR-34c	GMNC	geminin coiled-coil domain containing [Source:VGNC Symbol;Acc:VGNC:88512]	-1.81	0.1241
ssc-miR-34c	GNAI2	G protein subunit alpha i2 [Source:VGNC Symbol;Acc:VGNC:88522]	-1.81	0.1241
ssc-miR-34c	GNAO1	hypothetical gene	-1.81	0.1241
ssc-miR-34c	GNAQ	G protein subunit alpha q [Source:VGNC Symbol;Acc:VGNC:103100]	-1.81	0.1241
ssc-miR-34c	GORASP2	golgi reassembly stacking protein 2 [Source:VGNC Symbol;Acc:VGNC:95851]	-1.81	0.1241
ssc-miR-34c	GPC6	glypican 6 [Source:HGNC Symbol;Acc:HGNC:4454]	-1.81	0.1241
ssc-miR-34c	GPR12	G protein-coupled receptor 12 [Source:VGNC Symbol;Acc:VGNC:88598]	-1.81	0.1241
ssc-miR-34c	GPR158	G protein-coupled receptor 158 [Source:VGNC Symbol;Acc:VGNC:96016]	-1.81	0.1241
ssc-miR-34c	GPR22	G protein-coupled receptor 22 [Source:VGNC Symbol;Acc:VGNC:88625]	-1.81	0.1241
ssc-miR-34c	GPR26	G protein-coupled receptor 26 [Source:VGNC Symbol;Acc:VGNC:88626]	-1.81	0.1241
ssc-miR-34c	GPR64	hypothetical gene	-1.81	0.1241
ssc-miR-34c	GPR85	G protein-coupled receptor 85 [Source:VGNC Symbol;Acc:VGNC:88638]	-1.81	0.1241
ssc-miR-34c	GPX8	glutathione peroxidase 8 (putative) [Source:HGNC Symbol;Acc:HGNC:33100]	-1.81	0.1241
ssc-miR-34c	GRID1	glutamate ionotropic receptor delta type subunit 1 [Source:VGNC Symbol;Acc:VGNC:88674]	-1.81	0.1241
ssc-miR-34c	GRIN2C	glutamate ionotropic receptor NMDA type subunit 2C [Source:VGNC Symbol;Acc:VGNC:88685]	-1.81	0.1241

ssc-miR-34c	GRM7	glutamate metabotropic receptor 7 [Source:VGNC Symbol;Acc:VGNC:98028]	-1.81	0.1241
ssc-miR-34c	GSG1	germ cell associated 1 [Source:VGNC Symbol;Acc:VGNC:88721]	-1.81	0.1241
ssc-miR-34c	HCFC2	host cell factor C2 [Source:VGNC Symbol;Acc:VGNC:88800]	-1.81	0.1241
ssc-miR-34c	HCN3	hyperpolarization activated cyclic nucleotide gated potassium channel 3 [Source:VGNC Symbol;Acc:VGNC:88804]	-1.81	0.1241
ssc-miR-34c	HDAC1	histone deacetylase 1 [Source:VGNC Symbol;Acc:VGNC:88810]	-1.81	0.1241
ssc-miR-34c	HEBP1	heme binding protein 1 [Source:VGNC Symbol;Acc:VGNC:88830]	-1.81	0.1241
ssc-miR-34c	HECW2	HECT, C2 and WW domain containing E3 ubiquitin protein ligase 2 [Source:NCBI gene (formerly Entrezgene);Acc:100155879]	-1.81	0.1241
ssc-miR-34c	HIP1	huntingtin interacting protein 1 [Source:VGNC Symbol;Acc:VGNC:88885]	-1.81	0.1241
ssc-miR-34c	HK1	hexokinase 1 [Source:VGNC Symbol;Acc:VGNC:88892]	-1.81	0.1241
ssc-miR-34c	HM13	histocompatibility minor 13 [Source:VGNC Symbol;Acc:VGNC:98035]	-1.81	0.1241
ssc-miR-34c	HMGB1	hypothetical gene	-1.81	0.1241
ssc-miR-34c	HNF4A	hepatocyte nuclear factor 4 alpha [Source:VGNC Symbol;Acc:VGNC:96348]	-1.81	0.1241
ssc-miR-34c	HNF4G	hepatocyte nuclear factor 4 gamma [Source:VGNC Symbol;Acc:VGNC:88917]	-1.81	0.1241
ssc-miR-34c	HNRNPA1	heteroous nuclear ribonucleoprotein A1 [Source:VGNC Symbol;Acc:VGNC:88918]	-1.81	0.1241
ssc-miR-34c	HOOK3	hook microtubule tethering protein 3 [Source:VGNC Symbol;Acc:VGNC:98037]	-1.81	0.1241
ssc-miR-34c	HOXA13	homeobox A13 [Source:NCBI gene (formerly Entrezgene);Acc:100359352]	-1.81	0.1241
ssc-miR-34c	HS2ST1	heparan sulfate 2-O-sulfotransferase 1 [Source:HGNC Symbol;Acc:HGNC:5193]	-1.81	0.1241
ssc-miR-34c	HSBP1	heat shock factor binding protein 1 [Source:NCBI gene (formerly Entrezgene);Acc:100579143]	-1.81	0.1241
ssc-miR-34c	HSD17B8	hydroxysteroid 17-beta dehydrogenase 8 [Source:VGNC Symbol;Acc:VGNC:88989]	-1.81	0.1241
ssc-miR-34c	HSPA1B	hypothetical gene	-1.81	0.1241
ssc-miR-34c	HTR2C	5-hydroxytryptamine receptor 2C [Source:VGNC Symbol;Acc:VGNC:96737]	-1.81	0.1241
ssc-miR-34c	IGFBP3	insulin like growth factor binding protein 3 [Source:NCBI gene (formerly Entrezgene);Acc:448812]	-1.81	0.1241
ssc-miR-34c	IGFBP5	insulin like growth factor binding protein 5 [Source:VGNC Symbol;Acc:VGNC:96368]	-1.81	0.1241
ssc-miR-34c	IGSF1	immunoglobulin superfamily member 1 [Source:VGNC Symbol;Acc:VGNC:89062]	-1.81	0.1241
ssc-miR-34c	IL6R	interleukin 6 receptor [Source:VGNC Symbol;Acc:VGNC:89112]	-1.81	0.1241
ssc-miR-34c	INA	internexin neuronal intermediate filament protein alpha [Source:VGNC Symbol;Acc:VGNC:89128]	-1.81	0.1241
ssc-miR-34c	ING5	inhibitor of growth family member 5 [Source:VGNC Symbol;Acc:VGNC:95912]	-1.81	0.1241
ssc-miR-34c	INHBB	inhibin subunit beta B [Source:VGNC Symbol;Acc:VGNC:103969]	-1.81	0.1241
ssc-miR-34c	INO80	INO80 complex ATPase subunit [Source:VGNC Symbol;Acc:VGNC:89138]	-1.81	0.1241
ssc-miR-34c	INPP5K	inositol polyphosphate-5-phosphatase K [Source:VGNC Symbol;Acc:VGNC:89147]	-1.81	0.1241
ssc-miR-34c	IPO11	importin 11 [Source:VGNC Symbol;Acc:VGNC:89176]	-1.81	0.1241
ssc-miR-34c	IQGAP3	IQ motif containing GTPase activating protein 3 [Source:VGNC Symbol;Acc:VGNC:89193]	-1.81	0.1241
ssc-miR-34c	IRF2BP2	interferon regulatory factor 2 binding protein 2 [Source:VGNC Symbol;Acc:VGNC:89205]	-1.81	0.1241
ssc-miR-34c	ISY1-RAB43	hypothetical gene	-1.81	0.1241
ssc-miR-34c	ITCH	itchy E3 ubiquitin protein ligase [Source:HGNC Symbol;Acc:HGNC:13890]	-1.81	0.1241
ssc-miR-34c	ITGB8	integrin subunit beta 8 [Source:VGNC Symbol;Acc:VGNC:89246]	-1.81	0.1241
ssc-miR-34c	ITK	IL2 inducible T cell kinase [Source:VGNC Symbol;Acc:VGNC:89249]	-1.81	0.1241
ssc-miR-34c	ITSN1	intersectin 1 [Source:VGNC Symbol;Acc:VGNC:108669]	-1.81	0.1241
ssc-miR-34c	JAG1	jagged canonical Notch ligand 1 [Source:VGNC Symbol;Acc:VGNC:96385]	-1.81	0.1241
ssc-miR-34c	JHDM1D	hypothetical gene	-1.81	0.1241
ssc-miR-34c	JMJD1C	jumonji domain containing 1C [Source:VGNC Symbol;Acc:VGNC:89285]	-1.81	0.1241
ssc-miR-34c	JPH3	hypothetical gene	-1.81	0.1241
ssc-miR-34c	KCND3	potassium voltage-gated channel subfamily D member 3 [Source:VGNC Symbol;Acc:VGNC:98814]	-1.81	0.1241
ssc-miR-34c	KCNH7	potassium voltage-gated channel subfamily H member 7 [Source:VGNC Symbol;Acc:VGNC:95897]	-1.81	0.1241
ssc-miR-34c	KCNK3	potassium two pore domain channel subfamily K member 3 [Source:VGNC Symbol;Acc:VGNC:89370]	-1.81	0.1241
ssc-miR-34c	KCNQ3	potassium voltage-gated channel subfamily Q member 3 [Source:VGNC Symbol;Acc:VGNC:89382]	-1.81	0.1241
ssc-miR-34c	KCTD16	potassium channel tetramerization domain containing 16 [Source:VGNC Symbol;Acc:VGNC:89395]	-1.81	0.1241
ssc-miR-34c	KIAA0100	KIAA0100 [Source:VGNC Symbol;Acc:VGNC:89428]	-1.81	0.1241
ssc-miR-34c	KIAA0753	hypothetical gene	-1.81	0.1241
ssc-miR-34c	KIAA1024	hypothetical gene	-1.81	0.1241

ssc-miR-34c	KIAA1045	hypothetical gene	-1.81	0.1241
ssc-miR-34c	KIAA1210	hypothetical gene	-1.81	0.1241
ssc-miR-34c	KIAA1217	KIAA1217 [Source:VGNC Symbol;Acc:VGNC:96079]	-1.81	0.1241
ssc-miR-34c	KIAA1462	hypothetical gene	-1.81	0.1241
ssc-miR-34c	KITLG	KIT ligand [Source:VGNC Symbol;Acc:VGNC:98061]	-1.81	0.1241
ssc-miR-34c	KLC2	kinesin light chain 2 [Source:VGNC Symbol;Acc:VGNC:89486]	-1.81	0.1241
ssc-miR-34c	KLF4	Kruppel like factor 4 [Source:VGNC Symbol;Acc:VGNC:98062]	-1.81	0.1241
ssc-miR-34c	KLF7	Kruppel like factor 7 [Source:VGNC Symbol;Acc:VGNC:96396]	-1.81	0.1241
ssc-miR-34c	KLHDC8A	kelch domain containing 8A [Source:VGNC Symbol;Acc:VGNC:89508]	-1.81	0.1241
ssc-miR-34c	KLHL3	kelch like family member 3 [Source:VGNC Symbol;Acc:VGNC:89525]	-1.81	0.1241
ssc-miR-34c	KMT2D	hypothetical gene	-1.81	0.1241
ssc-miR-34c	LDHA	hypothetical gene	-1.81	0.1241
ssc-miR-34c	LEF1	lymphoid enhancer binding factor 1 [Source:VGNC Symbol;Acc:VGNC:89676]	-1.81	0.1241
ssc-miR-34c	LGI1	leucine rich glioma inactivated 1 [Source:HGNC Symbol;Acc:HGNC:6572]	-1.81	0.1241
ssc-miR-34c	LGR4	leucine rich repeat containing G protein-coupled receptor 4 [Source:VGNC Symbol;Acc:VGNC:89703]	-1.81	0.1241
ssc-miR-34c	LHX2	LIM homeobox 2 [Source:VGNC Symbol;Acc:VGNC:89712]	-1.81	0.1241
ssc-miR-34c	LHX9	LIM homeobox 9 [Source:VGNC Symbol;Acc:VGNC:95608]	-1.81	0.1241
ssc-miR-34c	LIMD2	LIM domain containing 2 [Source:VGNC Symbol;Acc:VGNC:89726]	-1.81	0.1241
ssc-miR-34c	LIN28A	lin-28 homolog A [Source:VGNC Symbol;Acc:VGNC:98492]	-1.81	0.1241
ssc-miR-34c	LIN54	lin-54 DREAM MuvB core complex component [Source:VGNC Symbol;Acc:VGNC:98072]	-1.81	0.1241
ssc-miR-34c	LINC00998	hypothetical gene	-1.81	0.1241
ssc-miR-34c	LITAF	hypothetical gene	-1.81	0.1241
ssc-miR-34c	LMAN1	lectin, mannose binding 1 [Source:VGNC Symbol;Acc:VGNC:89748]	-1.81	0.1241
ssc-miR-34c	LMAN2L	lectin, mannose binding 2 like [Source:VGNC Symbol;Acc:VGNC:89751]	-1.81	0.1241
ssc-miR-34c	LMBR1L	limb development membrane protein 1 like [Source:VGNC Symbol;Acc:VGNC:89753]	-1.81	0.1241
ssc-miR-34c	LMNB2	lamin B2 [Source:VGNC Symbol;Acc:VGNC:89761]	-1.81	0.1241
ssc-miR-34c	LMTK3	lemur tyrosine kinase 3 [Source:VGNC Symbol;Acc:VGNC:89769]	-1.81	0.1241
ssc-miR-34c	LMX1B	LIM homeobox transcription factor 1 beta [Source:VGNC Symbol;Acc:VGNC:89771]	-1.81	0.1241
ssc-miR-34c	LNX1	ligand of numb-protein X 1 [Source:VGNC Symbol;Acc:VGNC:89773]	-1.81	0.1241
ssc-miR-34c	LOXL3	lysyl oxidase like 3 [Source:VGNC Symbol;Acc:VGNC:89782]	-1.81	0.1241
ssc-miR-34c	LPAR2	lysophosphatidic acid receptor 2 [Source:VGNC Symbol;Acc:VGNC:89784]	-1.81	0.1241
ssc-miR-34c	LPHN1	hypothetical gene	-1.81	0.1241
ssc-miR-34c	LRRC55	leucine rich repeat containing 55 [Source:VGNC Symbol;Acc:VGNC:89850]	-1.81	0.1241
ssc-miR-34c	LRRC7	leucine rich repeat containing 7 [Source:HGNC Symbol;Acc:HGNC:18531]	-1.81	0.1241
ssc-miR-34c	LRRTM2	leucine rich repeat transmembrane neuronal 2 [Source:VGNC Symbol;Acc:VGNC:99788]	-1.81	0.1241
ssc-miR-34c	LRTM2	leucine rich repeats and transmembrane domains 2 [Source:VGNC Symbol;Acc:VGNC:89866]	-1.81	0.1241
ssc-miR-34c	LTBP2	latent transforming growth factor beta binding protein 2 [Source:VGNC Symbol;Acc:VGNC:89886]	-1.81	0.1241
ssc-miR-34c	LYPLAL1	lysophospholipase like 1 [Source:VGNC Symbol;Acc:VGNC:95939]	-1.81	0.1241
ssc-miR-34c	LYST	lysosomal trafficking regulator [Source:VGNC Symbol;Acc:VGNC:89921]	-1.81	0.1241
ssc-miR-34c	LZTS3	leucine zipper tumor suppressor family member 3 [Source:VGNC Symbol;Acc:VGNC:96237]	-1.81	0.1241
ssc-miR-34c	MAGI2	membrane associated guanylate kinase, WW and PDZ domain containing 2 [Source:VGNC Symbol;Acc:VGNC:89955]	-1.81	0.1241
ssc-miR-34c	MAGT1	magnesium transporter 1 [Source:VGNC Symbol;Acc:VGNC:89957]	-1.81	0.1241
ssc-miR-34c	MAP1A	microtubule associated protein 1A [Source:VGNC Symbol;Acc:VGNC:89978]	-1.81	0.1241
ssc-miR-34c	MAP2K1	mitogen-activated protein kinase kinase 1 [Source:VGNC Symbol;Acc:VGNC:103121]	-1.81	0.1241
ssc-miR-34c	MAP3K7CL	MAP3K7 C-terminal like [Source:VGNC Symbol;Acc:VGNC:89988]	-1.81	0.1241
ssc-miR-34c	MAP4K4	mitogen-activated protein kinase kinase kinase kinase 4 [Source:VGNC Symbol;Acc:VGNC:98114]	-1.81	0.1241
ssc-miR-34c	MAPK1	mitogen-activated protein kinase 1 [Source:VGNC Symbol;Acc:VGNC:89996]	-1.81	0.1241
ssc-miR-34c	MAPT	microtubule associated protein tau [Source:VGNC Symbol;Acc:VGNC:90016]	-1.81	0.1241
ssc-miR-34c	MARCH8	hypothetical gene	-1.81	0.1241
ssc-miR-34c	MAZ	MYC associated zinc finger protein [Source:VGNC Symbol;Acc:VGNC:107150]	-1.81	0.1241

ssc-miR-34c	MBD6	methyl-CpG binding domain protein 6 [Source:VGNC Symbol;Acc:VGNC:90051]	-1.81	0.1241
ssc-miR-34c	MCFD2	multiple coagulation factor deficiency 2, ER cargo receptor complex subunit [Source:VGNC Symbol;Acc:VGNC:103980]	-1.81	0.1241
ssc-miR-34c	MCIDAS	multiciliate differentiation and DNA synthesis associated cell cycle protein [Source:VGNC Symbol;Acc:VGNC:90071]	-1.81	0.1241
ssc-miR-34c	MCTP1	multiple C2 and transmembrane domain containing 1 [Source:VGNC Symbol;Acc:VGNC:90083]	-1.81	0.1241
ssc-miR-34c	MDM4	MDM4 regulator of p53 [Source:VGNC Symbol;Acc:VGNC:90094]	-1.81	0.1241
ssc-miR-34c	MED29	hypothetical gene	-1.81	0.1241
ssc-miR-34c	MET	MET proto-onco, receptor tyrosine kinase [Source:NCBI gene (formerly Entrezgene);Acc:654328]	-1.81	0.1241
ssc-miR-34c	METAP1	methionyl aminopeptidase 1 [Source:VGNC Symbol;Acc:VGNC:90151]	-1.81	0.1241
ssc-miR-34c	MGAT4A	alpha-1,3-mannosyl-glycoprotein 4-beta-N-acetylglucosaminyltransferase A [Source:VGNC Symbol;Acc:VGNC:108158]	-1.81	0.1241
ssc-miR-34c	MGAT5B	alpha-1,6-mannosylglycoprotein 6-beta-N-acetylglucosaminyltransferase B [Source:VGNC Symbol;Acc:VGNC:96742]	-1.81	0.1241
ssc-miR-34c	MLEC	malectin [Source:HGNC Symbol;Acc:HGNC:28973]	-1.81	0.1241
ssc-miR-34c	MLK4	hypothetical gene	-1.81	0.1241
ssc-miR-34c	MLLT1	MLLT1 super elongation complex subunit [Source:VGNC Symbol;Acc:VGNC:90255]	-1.81	0.1241
ssc-miR-34c	MLLT3	MLLT3 super elongation complex subunit [Source:VGNC Symbol;Acc:VGNC:90256]	-1.81	0.1241
ssc-miR-34c	MPP2	MAGUK p55 scaffold protein 2 [Source:VGNC Symbol;Acc:VGNC:98133]	-1.81	0.1241
ssc-miR-34c	MPPED2	metallophosphoesterase domain containing 2 [Source:VGNC Symbol;Acc:VGNC:90331]	-1.81	0.1241
ssc-miR-34c	MRAS	muscle RAS onco homolog [Source:VGNC Symbol;Acc:VGNC:90340]	-1.81	0.1241
ssc-miR-34c	MRPL52	mitochondrial ribosomal protein L52 [Source:VGNC Symbol;Acc:VGNC:90375]	-1.81	0.1241
ssc-miR-34c	MSANTD3-TMEFF1	hypothetical gene	-1.81	0.1241
ssc-miR-34c	MSL2	MSL complex subunit 2 [Source:VGNC Symbol;Acc:VGNC:90424]	-1.81	0.1241
ssc-miR-34c	MTA2	metastasis associated 1 family member 2 [Source:VGNC Symbol;Acc:VGNC:90435]	-1.81	0.1241
ssc-miR-34c	MTA3	metastasis associated 1 family member 3 [Source:VGNC Symbol;Acc:VGNC:90436]	-1.81	0.1241
ssc-miR-34c	MTMR14	myotubularin related protein 14 [Source:HGNC Symbol;Acc:HGNC:26190]	-1.81	0.1241
ssc-miR-34c	MTMR9	myotubularin related protein 9 [Source:VGNC Symbol;Acc:VGNC:90464]	-1.81	0.1241
ssc-miR-34c	MTUS1	microtubule associated scaffold protein 1 [Source:VGNC Symbol;Acc:VGNC:107157]	-1.81	0.1241
ssc-miR-34c	MYADM	myeloid associated differentiation marker [Source:VGNC Symbol;Acc:VGNC:98508]	-1.81	0.1241
ssc-miR-34c	MYCN	MYCN proto-onco, bHLH transcription factor [Source:VGNC Symbol;Acc:VGNC:90504]	-1.81	0.1241
ssc-miR-34c	MYH9	myosin heavy chain 9 [Source:VGNC Symbol;Acc:VGNC:90512]	-1.81	0.1241
ssc-miR-34c	MYL9	myosin light chain 9 [Source:VGNC Symbol;Acc:VGNC:95651]	-1.81	0.1241
ssc-miR-34c	MYO18A	myosin XVIIIA [Source:HGNC Symbol;Acc:HGNC:31104]	-1.81	0.1241
ssc-miR-34c	MYO1C	myosin IC [Source:VGNC Symbol;Acc:VGNC:90526]	-1.81	0.1241
ssc-miR-34c	MYOCD	myocardin [Source:VGNC Symbol;Acc:VGNC:90536]	-1.81	0.1241
ssc-miR-34c	MYRIP	myosin VIIA and Rab interacting protein [Source:NCBI gene (formerly Entrezgene);Acc:100156583]	-1.81	0.1241
ssc-miR-34c	MYSM1	Myb like, SWIRM and MPN domains 1 [Source:VGNC Symbol;Acc:VGNC:90548]	-1.81	0.1241
ssc-miR-34c	NAA50	N-alpha-acetyltransferase 50, NatE catalytic subunit [Source:VGNC Symbol;Acc:VGNC:104002]	-1.81	0.1241
ssc-miR-34c	NABP1	nucleic acid binding protein 1 [Source:VGNC Symbol;Acc:VGNC:96122]	-1.81	0.1241
ssc-miR-34c	NAPEPLD	N-acyl phosphatidylethanolamine phospholipase D [Source:VGNC Symbol;Acc:VGNC:90579]	-1.81	0.1241
ssc-miR-34c	NAV1	neuron navigator 1 [Source:VGNC Symbol;Acc:VGNC:95725]	-1.81	0.1241
ssc-miR-34c	NAV3	neuron navigator 3 [Source:VGNC Symbol;Acc:VGNC:90589]	-1.81	0.1241
ssc-miR-34c	NCEH1	neutral cholesterol ester hydrolase 1 [Source:VGNC Symbol;Acc:VGNC:98147]	-1.81	0.1241
ssc-miR-34c	NCOA1	nuclear receptor coactivator 1 [Source:VGNC Symbol;Acc:VGNC:90615]	-1.81	0.1241
ssc-miR-34c	NDC1	NDC1 transmembrane nucleoporin [Source:VGNC Symbol;Acc:VGNC:90622]	-1.81	0.1241
ssc-miR-34c	NDNF	neuron derived neurotrophic factor [Source:VGNC Symbol;Acc:VGNC:90629]	-1.81	0.1241
ssc-miR-34c	NDST1	N-deacetylase and N-sulfotransferase 1 [Source:VGNC Symbol;Acc:VGNC:90634]	-1.81	0.1241
ssc-miR-34c	NEDD4L	NEDD4 like E3 ubiquitin protein ligase [Source:VGNC Symbol;Acc:VGNC:90667]	-1.81	0.1241
ssc-miR-34c	NEK9	NIMA related kinase 9 [Source:VGNC Symbol;Acc:VGNC:90679]	-1.81	0.1241
ssc-miR-34c	NETO1	neuropilin and tolloid like 1 [Source:VGNC Symbol;Acc:VGNC:90690]	-1.81	0.1241
ssc-miR-34c	NEUROD2	neuronal differentiation 2 [Source:VGNC Symbol;Acc:VGNC:90697]	-1.81	0.1241
ssc-miR-34c	NFE2L1	NFE2 like bZIP transcription factor 1 [Source:VGNC Symbol;Acc:VGNC:90713]	-1.81	0.1241
ssc-miR-34c	NME6	NME/NM23 nucleoside diphosphate kinase 6 [Source:VGNC Symbol;Acc:VGNC:104009]	-1.81	0.1241

ssc-miR-34c	NMT2	N-myristoyltransferase 2 [Source:VGNC Symbol;Acc:VGNC:96448]	-1.81	0.1241
ssc-miR-34c	NONO	non-POU domain containing octamer binding [Source:HGNC Symbol;Acc:HGNC:7871]	-1.81	0.1241
ssc-miR-34c	NOS1AP	hypothetical gene	-1.81	0.1241
ssc-miR-34c	NOTCH1	notch receptor 1 [Source:HGNC Symbol;Acc:HGNC:7881]	-1.81	0.1241
ssc-miR-34c	NOTCH2	notch receptor 2 [Source:HGNC Symbol;Acc:HGNC:7882]	-1.81	0.1241
ssc-miR-34c	NPNT	nephronectin [Source:VGNC Symbol;Acc:VGNC:90855]	-1.81	0.1241
ssc-miR-34c	NR4A2	nuclear receptor subfamily 4 group A member 2 [Source:VGNC Symbol;Acc:VGNC:96451]	-1.81	0.1241
ssc-miR-34c	NRIP3	nuclear receptor interacting protein 3 [Source:HGNC Symbol;Acc:HGNC:1167]	-1.81	0.1241
ssc-miR-34c	NRN1	neuritin 1 [Source:VGNC Symbol;Acc:VGNC:90900]	-1.81	0.1241
ssc-miR-34c	NRXN2	hypothetical gene	-1.81	0.1241
ssc-miR-34c	NSD1	nuclear receptor binding SET domain protein 1 [Source:VGNC Symbol;Acc:VGNC:90904]	-1.81	0.1241
ssc-miR-34c	NSMCE4A	NSE4 homolog A, SMC5-SMC6 complex component [Source:VGNC Symbol;Acc:VGNC:90911]	-1.81	0.1241
ssc-miR-34c	NUMBL	NUMB like endocytic adaptor protein [Source:HGNC Symbol;Acc:HGNC:8061]	-1.81	0.1241
ssc-miR-34c	ONECUT2	one cut homeobox 2 [Source:VGNC Symbol;Acc:VGNC:91043]	-1.81	0.1241
ssc-miR-34c	ORAI2	ORAI calcium release-activated calcium modulator 2 [Source:VGNC Symbol;Acc:VGNC:91058]	-1.81	0.1241
ssc-miR-34c	ORAI3	ORAI calcium release-activated calcium modulator 3 [Source:VGNC Symbol;Acc:VGNC:91059]	-1.81	0.1241
ssc-miR-34c	OSGIN2	oxidative stress induced growth inhibitor family member 2 [Source:VGNC Symbol;Acc:VGNC:91080]	-1.81	0.1241
ssc-miR-34c	OTUD3	OTU deubiquitinase 3 [Source:VGNC Symbol;Acc:VGNC:91099]	-1.81	0.1241
ssc-miR-34c	OTUD7A	OTU deubiquitinase 7A [Source:VGNC Symbol;Acc:VGNC:91104]	-1.81	0.1241
ssc-miR-34c	OXSR1	hypothetical gene	-1.81	0.1241
ssc-miR-34c	PACS1	phosphofurin acidic cluster sorting protein 1 [Source:VGNC Symbol;Acc:VGNC:91140]	-1.81	0.1241
ssc-miR-34c	PAG1	phosphoprotein membrane anchor with glycosphingolipid microdomains 1 [Source:VGNC Symbol;Acc:VGNC:91154]	-1.81	0.1241
ssc-miR-34c	PALLD	palladin, cytoskeletal associated protein [Source:HGNC Symbol;Acc:HGNC:17068]	-1.81	0.1241
ssc-miR-34c	PALM2	hypothetical gene	-1.81	0.1241
ssc-miR-34c	PAN3	poly(A) specific ribonuclease subunit PAN3 [Source:HGNC Symbol;Acc:HGNC:29991]	-1.81	0.1241
ssc-miR-34c	PAOX	polyamine oxidase [Source:HGNC Symbol;Acc:HGNC:20837]	-1.81	0.1241
ssc-miR-34c	PARD6B	par-6 family cell polarity regulator beta [Source:VGNC Symbol;Acc:VGNC:98176]	-1.81	0.1241
ssc-miR-34c	PARM1	prostate androgen-regulated mucin-like protein 1 [Source:VGNC Symbol;Acc:VGNC:91182]	-1.81	0.1241
ssc-miR-34c	PARP15	hypothetical gene	-1.81	0.1241
ssc-miR-34c	PARP8	poly(ADP-ribose) polymerase family member 8 [Source:HGNC Symbol;Acc:HGNC:26124]	-1.81	0.1241
ssc-miR-34c	PCDH1	protocadherin 1 [Source:VGNC Symbol;Acc:VGNC:91211]	-1.81	0.1241
ssc-miR-34c	PCNX	hypothetical gene	-1.81	0.1241
ssc-miR-34c	PDCD4	programmed cell death 4 [Source:VGNC Symbol;Acc:VGNC:91244]	-1.81	0.1241
ssc-miR-34c	PDE4B	phosphodiesterase 4B [Source:VGNC Symbol;Acc:VGNC:91255]	-1.81	0.1241
ssc-miR-34c	PDE7A	phosphodiesterase 7A [Source:VGNC Symbol;Acc:VGNC:91261]	-1.81	0.1241
ssc-miR-34c	PDE7B	phosphodiesterase 7B [Source:VGNC Symbol;Acc:VGNC:91262]	-1.81	0.1241
ssc-miR-34c	PDGFRA	platelet derived growth factor receptor alpha [Source:VGNC Symbol;Acc:VGNC:98179]	-1.81	0.1241
ssc-miR-34c	PDK3	pyruvate dehydrogenase kinase 3 [Source:VGNC Symbol;Acc:VGNC:91279]	-1.81	0.1241
ssc-miR-34c	PDSS1	decaprenyl diphosphate synthase subunit 1 [Source:VGNC Symbol;Acc:VGNC:95858]	-1.81	0.1241
ssc-miR-34c	PDXK	pyridoxal kinase [Source:VGNC Symbol;Acc:VGNC:108684]	-1.81	0.1241
ssc-miR-34c	PEA15	proliferation and apoptosis adaptor protein 15 [Source:VGNC Symbol;Acc:VGNC:98181]	-1.81	0.1241
ssc-miR-34c	PEAK1	pseudopodium enriched atypical kinase 1 [Source:VGNC Symbol;Acc:VGNC:91300]	-1.81	0.1241
ssc-miR-34c	PEG10	paternally expressed 10 [Source:NCBI gene (formerly Entrezgene);Acc:654416]	-1.81	0.1241
ssc-miR-34c	PEX5L	peroxisomal biosis factor 5 like [Source:VGNC Symbol;Acc:VGNC:91329]	-1.81	0.1241
ssc-miR-34c	PGAP2	post-GPI attachment to proteins 2 [Source:VGNC Symbol;Acc:VGNC:91342]	-1.81	0.1241
ssc-miR-34c	PGF	placental growth factor [Source:HGNC Symbol;Acc:HGNC:8893]	-1.81	0.1241
ssc-miR-34c	PGRMC2	progesterone receptor membrane component 2 [Source:VGNC Symbol;Acc:VGNC:98952]	-1.81	0.1241
ssc-miR-34c	PHF15	hypothetical gene	-1.81	0.1241
ssc-miR-34c	PHF19	PHD finger protein 19 [Source:VGNC Symbol;Acc:VGNC:91381]	-1.81	0.1241
ssc-miR-34c	PIP5K1A	phosphatidylinositol-4-phosphate 5-kinase type 1 alpha [Source:VGNC Symbol;Acc:VGNC:98834]	-1.81	0.1241

ssc-miR-34c	PITPNC1	phosphatidylinositol transfer protein cytoplasmic 1 [Source:NCBI gene (formerly Entrezgene);Acc:100233199]	-1.81	0.1241
ssc-miR-34c	PITPNM2	phosphatidylinositol transfer protein membrane associated 2 [Source:VGNC Symbol;Acc:VGNC:91466]	-1.81	0.1241
ssc-miR-34c	PKIA	cAMP-dependent protein kinase inhibitor alpha [Source:VGNC Symbol;Acc:VGNC:91477]	-1.81	0.1241
ssc-miR-34c	PKP4	plakophilin 4 [Source:VGNC Symbol;Acc:VGNC:96488]	-1.81	0.1241
ssc-miR-34c	PLA2G15	phospholipase A2 group XV [Source:HGNC Symbol;Acc:HGNC:17163]	-1.81	0.1241
ssc-miR-34c	PLAG1	PLAG1 zinc finger [Source:VGNC Symbol;Acc:VGNC:91509]	-1.81	0.1241
ssc-miR-34c	PLCG1	phospholipase C gamma 1 [Source:VGNC Symbol;Acc:VGNC:96492]	-1.81	0.1241
ssc-miR-34c	PLEKHG3	pleckstrin homology and RhoGEF domain containing G3 [Source:VGNC Symbol;Acc:VGNC:91544]	-1.81	0.1241
ssc-miR-34c	PLOD1	procollagen-lysine,2-oxoglutarate 5-dioxygenase 1 [Source:VGNC Symbol;Acc:VGNC:91566]	-1.81	0.1241
ssc-miR-34c	PNOC	prepronociceptin [Source:VGNC Symbol;Acc:VGNC:91604]	-1.81	0.1241
ssc-miR-34c	PNRC2	proline rich nuclear receptor coactivator 2 [Source:VGNC Symbol;Acc:VGNC:91614]	-1.81	0.1241
ssc-miR-34c	PODXL	hypothetical gene	-1.81	0.1241
ssc-miR-34c	POFUT1	protein O-fucosyltransferase 1 [Source:VGNC Symbol;Acc:VGNC:95637]	-1.81	0.1241
ssc-miR-34c	POGZ	pogo transposable element derived with ZNF domain [Source:VGNC Symbol;Acc:VGNC:91625]	-1.81	0.1241
ssc-miR-34c	POLR3H	RNA polymerase III subunit H [Source:VGNC Symbol;Acc:VGNC:91658]	-1.81	0.1241
ssc-miR-34c	POMGNT1	protein O-linked mannose N-acetylglucosaminyltransferase 1 (beta 1,2-) [Source:VGNC Symbol;Acc:VGNC:98536]	-1.81	0.1241
ssc-miR-34c	POU2F1	POU class 2 homeobox 1 [Source:VGNC Symbol;Acc:VGNC:91672]	-1.81	0.1241
ssc-miR-34c	POU3F3	POU class 3 homeobox 3 [Source:VGNC Symbol;Acc:VGNC:91676]	-1.81	0.1241
ssc-miR-34c	PPARGC1B	PPARG coactivator 1 beta [Source:VGNC Symbol;Acc:VGNC:91686]	-1.81	0.1241
ssc-miR-34c	PPFIA1	PTPRF interacting protein alpha 1 [Source:VGNC Symbol;Acc:VGNC:91691]	-1.81	0.1241
ssc-miR-34c	PPM1L	protein phosphatase, Mg2+/Mn2+ dependent 1L [Source:VGNC Symbol;Acc:VGNC:91711]	-1.81	0.1241
ssc-miR-34c	PPP1R10	protein phosphatase 1 regulatory subunit 10 [Source:VGNC Symbol;Acc:VGNC:91717]	-1.81	0.1241
ssc-miR-34c	PPP1R11	protein phosphatase 1 regulatory inhibitor subunit 11 [Source:VGNC Symbol;Acc:VGNC:91718]	-1.81	0.1241
ssc-miR-34c	PPP1R16B	protein phosphatase 1 regulatory subunit 16B [Source:VGNC Symbol;Acc:VGNC:95669]	-1.81	0.1241
ssc-miR-34c	PPP2R3A	protein phosphatase 2 regulatory subunit B''alpha [Source:VGNC Symbol;Acc:VGNC:108686]	-1.81	0.1241
ssc-miR-34c	PPP4R1L	hypothetical gene	-1.81	0.1241
ssc-miR-34c	PPP4R2	protein phosphatase 4 regulatory subunit 2 [Source:VGNC Symbol;Acc:VGNC:91756]	-1.81	0.1241
ssc-miR-34c	PPP6C	protein phosphatase 6 catalytic subunit [Source:VGNC Symbol;Acc:VGNC:98219]	-1.81	0.1241
ssc-miR-34c	PPT2	palmitoyl-protein thioesterase 2 [Source:NCBI gene (formerly Entrezgene);Acc:100144537]	-1.81	0.1241
ssc-miR-34c	PPWD1	peptidylprolyl isomerase domain and WD repeat containing 1 [Source:VGNC Symbol;Acc:VGNC:91766]	-1.81	0.1241
ssc-miR-34c	PREB	prolactin regulatory element binding [Source:VGNC Symbol;Acc:VGNC:91786]	-1.81	0.1241
ssc-miR-34c	PRKACB	protein kinase cAMP-activated catalytic subunit beta [Source:VGNC Symbol;Acc:VGNC:91800]	-1.81	0.1241
ssc-miR-34c	PRKD1	protein kinase D1 [Source:VGNC Symbol;Acc:VGNC:91812]	-1.81	0.1241
ssc-miR-34c	PROX1	prospero homeobox 1 [Source:VGNC Symbol;Acc:VGNC:91837]	-1.81	0.1241
ssc-miR-34c	PRPF38B	hypothetical gene	-1.81	0.1241
ssc-miR-34c	PRRG3	proline rich and Gla domain 3 [Source:VGNC Symbol;Acc:VGNC:91869]	-1.81	0.1241
ssc-miR-34c	PTCHD1	patched domain containing 1 [Source:VGNC Symbol;Acc:VGNC:91942]	-1.81	0.1241
ssc-miR-34c	PTPN4	protein tyrosine phosphatase non-receptor type 4 [Source:VGNC Symbol;Acc:VGNC:96521]	-1.81	0.1241
ssc-miR-34c	PTPRD	protein tyrosine phosphatase receptor type D [Source:HGNC Symbol;Acc:HGNC:9668]	-1.81	0.1241
ssc-miR-34c	PTPRM	protein tyrosine phosphatase receptor type M [Source:VGNC Symbol;Acc:VGNC:91992]	-1.81	0.1241
ssc-miR-34c	PURB	purine rich element binding protein B [Source:VGNC Symbol;Acc:VGNC:92004]	-1.81	0.1241
ssc-miR-34c	PVRL1	hypothetical gene	-1.81	0.1241
ssc-miR-34c	RAB11FIP4	RAB11 family interacting protein 4 [Source:VGNC Symbol;Acc:VGNC:92041]	-1.81	0.1241
ssc-miR-34c	RAB21	RAB21, member RAS oncogene family [Source:HGNC Symbol;Acc:HGNC:18263]	-1.81	0.1241
ssc-miR-34c	RAB36	RAB36, member RAS oncogene family [Source:HGNC Symbol;Acc:HGNC:9775]	-1.81	0.1241
ssc-miR-34c	RAB43	RAB43, member RAS onco family [Source:VGNC Symbol;Acc:VGNC:98268]	-1.81	0.1241
ssc-miR-34c	RAB4A	RAB4A, member RAS onco family [Source:VGNC Symbol;Acc:VGNC:98270]	-1.81	0.1241
ssc-miR-34c	RAD51D	RAD51 paralog D [Source:HGNC Symbol;Acc:HGNC:9823]	-1.81	0.1241
ssc-miR-34c	RAE1	ribonucleic acid export 1 [Source:VGNC Symbol;Acc:VGNC:96528]	-1.81	0.1241
ssc-miR-34c	RAI14	retinoic acid induced 14 [Source:VGNC Symbol;Acc:VGNC:92068]	-1.81	0.1241

ssc-miR-34c	RALGDS	ral guanine nucleotide dissociation stimulator [Source:HGNC Symbol;Acc:HGNC:9842]	-1.81	0.1241
ssc-miR-34c	RALGPS1	Ral GEF with PH domain and SH3 binding motif 1 [Source:VGNC Symbol;Acc:VGNC:92072]	-1.81	0.1241
ssc-miR-34c	RALGPS2	Ral GEF with PH domain and SH3 binding motif 2 [Source:VGNC Symbol;Acc:VGNC:92073]	-1.81	0.1241
ssc-miR-34c	RALY	RALY heterous nuclear ribonucleoprotein [Source:VGNC Symbol;Acc:VGNC:95709]	-1.81	0.1241
ssc-miR-34c	RANBP10	RAN binding protein 10 [Source:VGNC Symbol;Acc:VGNC:92077]	-1.81	0.1241
ssc-miR-34c	RAP1GDS1	Rap1 GTPase-GDP dissociation stimulator 1 [Source:VGNC Symbol;Acc:VGNC:92087]	-1.81	0.1241
ssc-miR-34c	RARB	retinoic acid receptor beta [Source:HGNC Symbol;Acc:HGNC:9865]	-1.81	0.1241
ssc-miR-34c	RASGRP4	RAS guanyl releasing protein 4 [Source:VGNC Symbol;Acc:VGNC:98282]	-1.81	0.1241
ssc-miR-34c	RBCK1	RANBP2-type and C3HC4-type zinc finger containing 1 [Source:VGNC Symbol;Acc:VGNC:98284]	-1.81	0.1241
ssc-miR-34c	RCAN1	regulator of calcineurin 1 [Source:VGNC Symbol;Acc:VGNC:92170]	-1.81	0.1241
ssc-miR-34c	RDH11	retinol dehydrogenase 11 [Source:HGNC Symbol;Acc:HGNC:17964]	-1.81	0.1241
ssc-miR-34c	RELN	reelin [Source:VGNC Symbol;Acc:VGNC:92208]	-1.81	0.1241
ssc-miR-34c	REPS2	RALBP1 associated Eps domain containing 2 [Source:VGNC Symbol;Acc:VGNC:92214]	-1.81	0.1241
ssc-miR-34c	RET	ret proto-onco [Source:VGNC Symbol;Acc:VGNC:92220]	-1.81	0.1241
ssc-miR-34c	RFX3	regulatory factor X3 [Source:VGNC Symbol;Acc:VGNC:92245]	-1.81	0.1241
ssc-miR-34c	RGMB	repulsive guidance molecule BMP co-receptor b [Source:VGNC Symbol;Acc:VGNC:92255]	-1.81	0.1241
ssc-miR-34c	RGS17	regulator of G protein signaling 17 [Source:VGNC Symbol;Acc:VGNC:92263]	-1.81	0.1241
ssc-miR-34c	RGS7BP	regulator of G protein signaling 7 binding protein [Source:VGNC Symbol;Acc:VGNC:92268]	-1.81	0.1241
ssc-miR-34c	RHOV	ras homolog family member V [Source:VGNC Symbol;Acc:VGNC:92295]	-1.81	0.1241
ssc-miR-34c	RIC8B	RIC8 guanine nucleotide exchange factor B [Source:VGNC Symbol;Acc:VGNC:92302]	-1.81	0.1241
ssc-miR-34c	RIMS3	regulating synaptic membrane exocytosis 3 [Source:VGNC Symbol;Acc:VGNC:92310]	-1.81	0.1241
ssc-miR-34c	RNF152	ring finger protein 152 [Source:VGNC Symbol;Acc:VGNC:92364]	-1.81	0.1241
ssc-miR-34c	RNF213	hypothetical gene	-1.81	0.1241
ssc-miR-34c	RNF34	ring finger protein 34 [Source:VGNC Symbol;Acc:VGNC:92389]	-1.81	0.1241
ssc-miR-34c	RNF44	ring finger protein 44 [Source:VGNC Symbol;Acc:VGNC:92395]	-1.81	0.1241
ssc-miR-34c	ROCK1	Rho associated coiled-coil containing protein kinase 1 [Source:VGNC Symbol;Acc:VGNC:98294]	-1.81	0.1241
ssc-miR-34c	RORA	RAR related orphan receptor A [Source:VGNC Symbol;Acc:VGNC:92408]	-1.81	0.1241
ssc-miR-34c	RP11-192H23.4	hypothetical gene	-1.81	0.1241
ssc-miR-34c	RPS6KA4	ribosomal protein S6 kinase A4 [Source:VGNC Symbol;Acc:VGNC:92443]	-1.81	0.1241
ssc-miR-34c	RRAGC	Ras related GTP binding C [Source:HGNC Symbol;Acc:HGNC:19902]	-1.81	0.1241
ssc-miR-34c	RRAGD	Ras related GTP binding D [Source:VGNC Symbol;Acc:VGNC:92457]	-1.81	0.1241
ssc-miR-34c	RRAS	RAS related [Source:VGNC Symbol;Acc:VGNC:92458]	-1.81	0.1241
ssc-miR-34c	RRAS2	hypothetical gene	-1.81	0.1241
ssc-miR-34c	RSPO4	R-spondin 4 [Source:VGNC Symbol;Acc:VGNC:95769]	-1.81	0.1241
ssc-miR-34c	RTF1	RTF1 homolog, Paf1/RNA polymerase II complex component [Source:VGNC Symbol;Acc:VGNC:92493]	-1.81	0.1241
ssc-miR-34c	RTN4RL1	reticulon 4 receptor like 1 [Source:VGNC Symbol;Acc:VGNC:92504]	-1.81	0.1241
ssc-miR-34c	RTTN	rotatin [Source:VGNC Symbol;Acc:VGNC:92508]	-1.81	0.1241
ssc-miR-34c	RWDD1	RWD domain containing 1 [Source:VGNC Symbol;Acc:VGNC:104051]	-1.81	0.1241
ssc-miR-34c	SAMD12	sterile alpha motif domain containing 12 [Source:VGNC Symbol;Acc:VGNC:92565]	-1.81	0.1241
ssc-miR-34c	SAR1A	secretion associated Ras related GTPase 1A [Source:VGNC Symbol;Acc:VGNC:92579]	-1.81	0.1241
ssc-miR-34c	SATB1	SATB homeobox 1 [Source:VGNC Symbol;Acc:VGNC:92587]	-1.81	0.1241
ssc-miR-34c	SATB2	SATB homeobox 2 [Source:VGNC Symbol;Acc:VGNC:95972]	-1.81	0.1241
ssc-miR-34c	SBK1	SH3 domain binding kinase 1 [Source:HGNC Symbol;Acc:HGNC:17699]	-1.81	0.1241
ssc-miR-34c	SCARA3	scavenger receptor class A member 3 [Source:HGNC Symbol;Acc:HGNC:19000]	-1.81	0.1241
ssc-miR-34c	SCN1A	sodium voltage-gated channel alpha subunit 1 [Source:VGNC Symbol;Acc:VGNC:95478]	-1.81	0.1241
ssc-miR-34c	SCN2B	sodium voltage-gated channel beta subunit 2 [Source:VGNC Symbol;Acc:VGNC:92633]	-1.81	0.1241
ssc-miR-34c	SDHC	succinate dehydrogenase complex subunit C [Source:VGNC Symbol;Acc:VGNC:98852]	-1.81	0.1241
ssc-miR-34c	SDK2	hypothetical gene	-1.81	0.1241
ssc-miR-34c	SEC22A	SEC22 homolog A, vesicle trafficking protein [Source:VGNC Symbol;Acc:VGNC:92673]	-1.81	0.1241
ssc-miR-34c	SEH1L	centrosomal protein 192 [Source:VGNC Symbol;Acc:VGNC:96946]	-1.81	0.1241

ssc-miR-34c	SELRC1	hypothetical gene	-1.81	0.1241
ssc-miR-34c	SEMA4B	semaphorin 4B [Source:VGNC Symbol;Acc:VGNC:92701]	-1.81	0.1241
ssc-miR-34c	SEMA4C	semaphorin 4C [Source:VGNC Symbol;Acc:VGNC:92702]	-1.81	0.1241
ssc-miR-34c	SEMA4F	ssemaphorin 4F [Source:VGNC Symbol;Acc:VGNC:92704]	-1.81	0.1241
ssc-miR-34c	SEMA5B	semaphorin 5B [Source:VGNC Symbol;Acc:VGNC:92707]	-1.81	0.1241
ssc-miR-34c	SEPT3	hypothetical gene	-1.81	0.1241
ssc-miR-34c	SEPT7	hypothetical gene	-1.81	0.1241
ssc-miR-34c	SERPINE1	serpin family E member 1 [Source:VGNC Symbol;Acc:VGNC:98310]	-1.81	0.1241
ssc-miR-34c	SERPINF2	serpin family F member 2 [Source:VGNC Symbol;Acc:VGNC:92744]	-1.81	0.1241
ssc-miR-34c	SESTD1	SEC14 and spectrin domain containing 1 [Source:VGNC Symbol;Acc:VGNC:95884]	-1.81	0.1241
ssc-miR-34c	SETD3	SET domain containing 3, actin histidine methyltransferase [Source:VGNC Symbol;Acc:VGNC:92758]	-1.81	0.1241
ssc-miR-34c	SGK3	serum/glucocorticoid regulated kinase family member 3 [Source:HGNC Symbol;Acc:HGNC:10812]	-1.81	0.1241
ssc-miR-34c	SGK494	hypothetical gene	-1.81	0.1241
ssc-miR-34c	SGPP1	sphingosine-1-phosphate phosphatase 1 [Source:VGNC Symbol;Acc:VGNC:92798]	-1.81	0.1241
ssc-miR-34c	SGSM2	small G protein signaling modulator 2 [Source:VGNC Symbol;Acc:VGNC:92801]	-1.81	0.1241
ssc-miR-34c	SGTA	small glutamine rich tetratricopeptide repeat co-chaperone alpha [Source:VGNC Symbol;Acc:VGNC:92803]	-1.81	0.1241
ssc-miR-34c	SHANK3	hypothetical gene	-1.81	0.1241
ssc-miR-34c	SHE	Src homology 2 domain containing E [Source:VGNC Symbol;Acc:VGNC:92842]	-1.81	0.1241
ssc-miR-34c	SHISA7	shisa family member 7 [Source:VGNC Symbol;Acc:VGNC:92848]	-1.81	0.1241
ssc-miR-34c	SHKBP1	SH3KBP1 binding protein 1 [Source:VGNC Symbol;Acc:VGNC:92853]	-1.81	0.1241
ssc-miR-34c	SHOC2	SHOC2 leucine rich repeat scaffold protein [Source:VGNC Symbol;Acc:VGNC:98318]	-1.81	0.1241
ssc-miR-34c	SHOX	hypothetical gene	-1.81	0.1241
ssc-miR-34c	SIDT1	SID1 transmembrane family member 1 [Source:VGNC Symbol;Acc:VGNC:92868]	-1.81	0.1241
ssc-miR-34c	SIDT2	SID1 transmembrane family member 2 [Source:VGNC Symbol;Acc:VGNC:92869]	-1.81	0.1241
ssc-miR-34c	SIGMAR1	sigma non-opioid intracellular receptor 1 [Source:VGNC Symbol;Acc:VGNC:96462]	-1.81	0.1241
ssc-miR-34c	SIPA1	signal-induced proliferation-associated 1 [Source:VGNC Symbol;Acc:VGNC:92881]	-1.81	0.1241
ssc-miR-34c	SIX3	SIX homeobox 3 [Source:VGNC Symbol;Acc:VGNC:92894]	-1.81	0.1241
ssc-miR-34c	SLC12A2	solute carrier family 12 member 2 [Source:VGNC Symbol;Acc:VGNC:92922]	-1.81	0.1241
ssc-miR-34c	SLC25A23	solute carrier family 25 member 23 [Source:VGNC Symbol;Acc:VGNC:93000]	-1.81	0.1241
ssc-miR-34c	SLC25A27	solute carrier family 25 member 27 [Source:VGNC Symbol;Acc:VGNC:93004]	-1.81	0.1241
ssc-miR-34c	SLC27A4	solute carrier family 27 member 4 [Source:VGNC Symbol;Acc:VGNC:93038]	-1.81	0.1241
ssc-miR-34c	SLC2A13	solute carrier family 2 member 13 [Source:VGNC Symbol;Acc:VGNC:93047]	-1.81	0.1241
ssc-miR-34c	SLC2A4RG	SLC2A4 regulator [Source:VGNC Symbol;Acc:VGNC:95715]	-1.81	0.1241
ssc-miR-34c	SLC30A3	solute carrier family 30 member 3 [Source:VGNC Symbol;Acc:VGNC:93057]	-1.81	0.1241
ssc-miR-34c	SLC35G2	solute carrier family 35 member G2 [Source:VGNC Symbol;Acc:VGNC:93087]	-1.81	0.1241
ssc-miR-34c	SLC43A2	solute carrier family 43 member 2 [Source:VGNC Symbol;Acc:VGNC:93117]	-1.81	0.1241
ssc-miR-34c	SLC44A2	solute carrier family 44 member 2 [Source:VGNC Symbol;Acc:VGNC:100862]	-1.81	0.1241
ssc-miR-34c	SLC4A5	solute carrier family 4 member 5 [Source:VGNC Symbol;Acc:VGNC:93134]	-1.81	0.1241
ssc-miR-34c	SLC4A7	solute carrier family 4 member 7 [Source:VGNC Symbol;Acc:VGNC:93135]	-1.81	0.1241
ssc-miR-34c	SLC5A3	solute carrier family 5 member 3 [Source:VGNC Symbol;Acc:VGNC:93144]	-1.81	0.1241
ssc-miR-34c	SLC6A1	solute carrier family 6 member 1 [Source:VGNC Symbol;Acc:VGNC:93153]	-1.81	0.1241
ssc-miR-34c	SLC6A17	solute carrier family 6 member 17 [Source:VGNC Symbol;Acc:VGNC:93158]	-1.81	0.1241
ssc-miR-34c	SLC7A11	solute carrier family 7 member 11 [Source:VGNC Symbol;Acc:VGNC:93171]	-1.81	0.1241
ssc-miR-34c	SLC7A2	solute carrier family 7 member 2 [Source:VGNC Symbol;Acc:VGNC:95506]	-1.81	0.1241
ssc-miR-34c	SLC7A6	hypothetical gene	-1.81	0.1241
ssc-miR-34c	SLC8A1	solute carrier family 8 member A1 [Source:VGNC Symbol;Acc:VGNC:93178]	-1.81	0.1241
ssc-miR-34c	SLCO3A1	solute carrier organic anion transporter family member 3A1 [Source:VGNC Symbol;Acc:VGNC:93197]	-1.81	0.1241
ssc-miR-34c	SMAD4	SMAD family member 4 [Source:VGNC Symbol;Acc:VGNC:93218]	-1.81	0.1241
ssc-miR-34c	SMC6	structural maintenance of chromosomes 6 [Source:VGNC Symbol;Acc:VGNC:93241]	-1.81	0.1241
ssc-miR-34c	SMIM15	hypothetical gene	-1.81	0.1241

ssc-miR-34c	SNAP25	synaptosome associated protein 25 [Source:VGNC Symbol;Acc:VGNC:95509]	-1.81	0.1241
ssc-miR-34c	SNAP29	synaptosome associated protein 29 [Source:VGNC Symbol;Acc:VGNC:93278]	-1.81	0.1241
ssc-miR-34c	SNTB2	syntrophin beta 2 [Source:VGNC Symbol;Acc:VGNC:93298]	-1.81	0.1241
ssc-miR-34c	SNX12	sorting nexin 12 [Source:VGNC Symbol;Acc:VGNC:93305]	-1.81	0.1241
ssc-miR-34c	SNX15	sorting nexin 15 [Source:VGNC Symbol;Acc:VGNC:100864]	-1.81	0.1241
ssc-miR-34c	SNX30	sorting nexin family member 30 [Source:VGNC Symbol;Acc:VGNC:93318]	-1.81	0.1241
ssc-miR-34c	SOCS4	suppressor of cytokine signaling 4 [Source:VGNC Symbol;Acc:VGNC:93331]	-1.81	0.1241
ssc-miR-34c	SOGA2	hypothetical gene	-1.81	0.1241
ssc-miR-34c	SOX12	SRY-box transcription factor 12 [Source:HGNC Symbol;Acc:HGNC:11198]	-1.81	0.1241
ssc-miR-34c	SOX4	SRY-box transcription factor 4 [Source:HGNC Symbol;Acc:HGNC:11200]	-1.81	0.1241
ssc-miR-34c	SP2	Sp2 transcription factor [Source:VGNC Symbol;Acc:VGNC:93361]	-1.81	0.1241
ssc-miR-34c	SPCS2	signal peptidase complex subunit 2 [Source:VGNC Symbol;Acc:VGNC:108618]	-1.81	0.1241
ssc-miR-34c	SPEN	hypothetical gene	-1.81	0.1241
ssc-miR-34c	SPRED1	sprouty related EVH1 domain containing 1 [Source:VGNC Symbol;Acc:VGNC:93420]	-1.81	0.1241
ssc-miR-34c	SPRN	shadow of prion protein [Source:HGNC Symbol;Acc:HGNC:16871]	-1.81	0.1241
ssc-miR-34c	SPRY1	sprouty RTK signaling antagonist 1 [Source:VGNC Symbol;Acc:VGNC:93424]	-1.81	0.1241
ssc-miR-34c	SPRY3	sprouty RTK signaling antagonist 3 [Source:VGNC Symbol;Acc:VGNC:93426]	-1.81	0.1241
ssc-miR-34c	SPTBN2	spectrin beta, non-erythrocytic 2 [Source:VGNC Symbol;Acc:VGNC:93437]	-1.81	0.1241
ssc-miR-34c	SRPR	hypothetical gene	-1.81	0.1241
ssc-miR-34c	SRR	serine racemase [Source:HGNC Symbol;Acc:HGNC:14398]	-1.81	0.1241
ssc-miR-34c	ST8SIA3	ST8 alpha-N-acetyl-neuraminide alpha-2,8-sialyltransferase 3 [Source:VGNC Symbol;Acc:VGNC:93518]	-1.81	0.1241
ssc-miR-34c	STAC2	SH3 and cysteine rich domain 2 [Source:VGNC Symbol;Acc:VGNC:93522]	-1.81	0.1241
ssc-miR-34c	STAT6	signal transducer and activator of transcription 6 [Source:VGNC Symbol;Acc:VGNC:93541]	-1.81	0.1241
ssc-miR-34c	STK38L	serine/threonine kinase 38 like [Source:VGNC Symbol;Acc:VGNC:93557]	-1.81	0.1241
ssc-miR-34c	STRN3	striatin 3 [Source:VGNC Symbol;Acc:VGNC:93579]	-1.81	0.1241
ssc-miR-34c	STX17	syntaxin 17 [Source:VGNC Symbol;Acc:VGNC:93587]	-1.81	0.1241
ssc-miR-34c	STX1A	syntaxin 1A [Source:VGNC Symbol;Acc:VGNC:93589]	-1.81	0.1241
ssc-miR-34c	SUPT6H	SPT6 homolog, histone chaperone and transcription elongation factor [Source:VGNC Symbol;Acc:VGNC:93626]	-1.81	0.1241
ssc-miR-34c	SURF4	surfeit 4 [Source:VGNC Symbol;Acc:VGNC:93631]	-1.81	0.1241
ssc-miR-34c	SVIP	small VCP interacting protein [Source:HGNC Symbol;Acc:HGNC:25238]	-1.81	0.1241
ssc-miR-34c	SVOP	SV2 related protein [Source:VGNC Symbol;Acc:VGNC:93643]	-1.81	0.1241
ssc-miR-34c	SYNJ1	synaptojanin 1 [Source:VGNC Symbol;Acc:VGNC:93668]	-1.81	0.1241
ssc-miR-34c	SYT1	synaptotagmin 1 [Source:VGNC Symbol;Acc:VGNC:93678]	-1.81	0.1241
ssc-miR-34c	SYT9	synaptotagmin 9 [Source:VGNC Symbol;Acc:VGNC:93689]	-1.81	0.1241
ssc-miR-34c	SYVN1	synoviolin 1 [Source:HGNC Symbol;Acc:HGNC:20738]	-1.81	0.1241
ssc-miR-34c	SZRD1	hypothetical gene	-1.81	0.1241
ssc-miR-34c	TAF4B	TATA-box binding protein associated factor 4b [Source:VGNC Symbol;Acc:VGNC:93716]	-1.81	0.1241
ssc-miR-34c	TAF5	TATA-box binding protein associated factor 5 [Source:VGNC Symbol;Acc:VGNC:93717]	-1.81	0.1241
ssc-miR-34c	TANC2	tetratricopeptide repeat, ankyrin repeat and coiled-coil containing 2 [Source:VGNC Symbol;Acc:VGNC:93733]	-1.81	0.1241
ssc-miR-34c	TBC1D13	TBC1 domain family member 13 [Source:VGNC Symbol;Acc:VGNC:93763]	-1.81	0.1241
ssc-miR-34c	TBC1D25	TBC1 domain family member 25 [Source:VGNC Symbol;Acc:VGNC:93773]	-1.81	0.1241
ssc-miR-34c	TBC1D30	TBC1 domain family member 30 [Source:VGNC Symbol;Acc:VGNC:93775]	-1.81	0.1241
ssc-miR-34c	TBL1XR1	TBL1X receptor 1 [Source:VGNC Symbol;Acc:VGNC:96600]	-1.81	0.1241
ssc-miR-34c	TCF12	transcription factor 12 [Source:VGNC Symbol;Acc:VGNC:93817]	-1.81	0.1241
ssc-miR-34c	TERT	telomerase reverse transcriptase [Source:VGNC Symbol;Acc:VGNC:93883]	-1.81	0.1241
ssc-miR-34c	TFCP2L1	transcription factor CP2 like 1 [Source:HGNC Symbol;Acc:HGNC:17925]	-1.81	0.1241
ssc-miR-34c	TFDP2	transcription factor Dp-2 [Source:VGNC Symbol;Acc:VGNC:93915]	-1.81	0.1241
ssc-miR-34c	TFRC	transferrin receptor [Source:VGNC Symbol;Acc:VGNC:93925]	-1.81	0.1241
ssc-miR-34c	TGFBRAP1	transforming growth factor beta receptor associated protein 1 [Source:VGNC Symbol;Acc:VGNC:93934]	-1.81	0.1241
ssc-miR-34c	TGIF2	TGFB induced factor homeobox 2 [Source:VGNC Symbol;Acc:VGNC:95656]	-1.81	0.1241

ssc-miR-34c	THAP1	THAP domain containing 1 [Source:VGNC Symbol;Acc:VGNC:95950]	-1.81	0.1241
ssc-miR-34c	THRA	thyroid hormone receptor alpha [Source:VGNC Symbol;Acc:VGNC:93963]	-1.81	0.1241
ssc-miR-34c	THSD4	thrombospondin type 1 domain containing 4 [Source:VGNC Symbol;Acc:VGNC:93967]	-1.81	0.1241
ssc-miR-34c	TM9SF3	transmembrane 9 superfamily member 3 [Source:VGNC Symbol;Acc:VGNC:94037]	-1.81	0.1241
ssc-miR-34c	TMCC3	transmembrane and coiled-coil domain family 3 [Source:VGNC Symbol;Acc:VGNC:98375]	-1.81	0.1241
ssc-miR-34c	TMED8	transmembrane p24 trafficking protein family member 8 [Source:VGNC Symbol;Acc:VGNC:94058]	-1.81	0.1241
ssc-miR-34c	TMEFF1	transmembrane protein with EGF like and two follistatin like domains 1 [Source:HGNC Symbol;Acc:HGNC:11866]	-1.81	0.1241
ssc-miR-34c	TMEM104	transmembrane protein 104 [Source:VGNC Symbol;Acc:VGNC:94062]	-1.81	0.1241
ssc-miR-34c	TMEM109	transmembrane protein 109 [Source:VGNC Symbol;Acc:VGNC:94066]	-1.81	0.1241
ssc-miR-34c	TMEM126B	transmembrane protein 126B [Source:VGNC Symbol;Acc:VGNC:104078]	-1.81	0.1241
ssc-miR-34c	TMEM134	transmembrane protein 134 [Source:VGNC Symbol;Acc:VGNC:94084]	-1.81	0.1241
ssc-miR-34c	TMEM164	transmembrane protein 164 [Source:VGNC Symbol;Acc:VGNC:94103]	-1.81	0.1241
ssc-miR-34c	TMEM184B	transmembrane protein 184B [Source:VGNC Symbol;Acc:VGNC:94120]	-1.81	0.1241
ssc-miR-34c	TMEM185B	transmembrane protein 185B [Source:VGNC Symbol;Acc:VGNC:95900]	-1.81	0.1241
ssc-miR-34c	TMEM200B	transmembrane protein 200B [Source:HGNC Symbol;Acc:HGNC:33785]	-1.81	0.1241
ssc-miR-34c	TMEM222	transmembrane protein 222 [Source:VGNC Symbol;Acc:VGNC:94143]	-1.81	0.1241
ssc-miR-34c	TMEM246	hypothetical gene	-1.81	0.1241
ssc-miR-34c	TMEM251	transmembrane protein 251 [Source:VGNC Symbol;Acc:VGNC:98378]	-1.81	0.1241
ssc-miR-34c	TMEM255A	transmembrane protein 255A [Source:VGNC Symbol;Acc:VGNC:104079]	-1.81	0.1241
ssc-miR-34c	TMEM52B	transmembrane protein 52B [Source:VGNC Symbol;Acc:VGNC:94189]	-1.81	0.1241
ssc-miR-34c	TMEM55A	hypothetical gene	-1.81	0.1241
ssc-miR-34c	TMEM97	transmembrane protein 97 [Source:VGNC Symbol;Acc:VGNC:94220]	-1.81	0.1241
ssc-miR-34c	TMOD2	tropomodulin 2 [Source:VGNC Symbol;Acc:VGNC:94227]	-1.81	0.1241
ssc-miR-34c	TMUB2	transmembrane and ubiquitin like domain containing 2 [Source:VGNC Symbol;Acc:VGNC:94246]	-1.81	0.1241
ssc-miR-34c	TNKS	tankyrase [Source:VGNC Symbol;Acc:VGNC:95546]	-1.81	0.1241
ssc-miR-34c	TNPO3	transportin 3 [Source:VGNC Symbol;Acc:VGNC:94289]	-1.81	0.1241
ssc-miR-34c	TNRC18	trinucleotide repeat containing 18 [Source:VGNC Symbol;Acc:VGNC:94291]	-1.81	0.1241
ssc-miR-34c	TNRC6B	trinucleotide repeat containing adaptor 6B [Source:VGNC Symbol;Acc:VGNC:94293]	-1.81	0.1241
ssc-miR-34c	TOB2	transducer of ERBB2, 2 [Source:VGNC Symbol;Acc:VGNC:94298]	-1.81	0.1241
ssc-miR-34c	TOM1	target of myb1 membrane trafficking protein [Source:VGNC Symbol;Acc:VGNC:95552]	-1.81	0.1241
ssc-miR-34c	TOX	thymocyte selection associated high mobility group box [Source:VGNC Symbol;Acc:VGNC:94322]	-1.81	0.1241
ssc-miR-34c	TP73	tumor protein p73 [Source:VGNC Symbol;Acc:VGNC:94331]	-1.81	0.1241
ssc-miR-34c	TPD52	tumor protein D52 [Source:VGNC Symbol;Acc:VGNC:98383]	-1.81	0.1241
ssc-miR-34c	TRAFD1	TRAF-type zinc finger domain containing 1 [Source:VGNC Symbol;Acc:VGNC:94367]	-1.81	0.1241
ssc-miR-34c	TRANK1	tetratricopeptide repeat and ankyrin repeat containing 1 [Source:VGNC Symbol;Acc:VGNC:94370]	-1.81	0.1241
ssc-miR-34c	TRIM13	tripartite motif containing 13 [Source:VGNC Symbol;Acc:VGNC:94394]	-1.81	0.1241
ssc-miR-34c	TRIM41	hypothetical gene	-1.81	0.1241
ssc-miR-34c	TRIM67	tripartite motif containing 67 [Source:VGNC Symbol;Acc:VGNC:94427]	-1.81	0.1241
ssc-miR-34c	TRIM9	tripartite motif containing 9 [Source:VGNC Symbol;Acc:VGNC:94434]	-1.81	0.1241
ssc-miR-34c	TRPM3	transient receptor potential cation channel subfamily M member 3 [Source:VGNC Symbol;Acc:VGNC:98384]	-1.81	0.1241
ssc-miR-34c	TSN	translin [Source:VGNC Symbol;Acc:VGNC:95556]	-1.81	0.1241
ssc-miR-34c	TSPAN14	tetraspanin 14 [Source:VGNC Symbol;Acc:VGNC:94503]	-1.81	0.1241
ssc-miR-34c	TSPAN18	tetraspanin 18 [Source:VGNC Symbol;Acc:VGNC:94507]	-1.81	0.1241
ssc-miR-34c	TTC19	tetratricopeptide repeat domain 19 [Source:VGNC Symbol;Acc:VGNC:99101]	-1.81	0.1241
ssc-miR-34c	TTPAL	alpha tocopherol transfer protein like [Source:VGNC Symbol;Acc:VGNC:95742]	-1.81	0.1241
ssc-miR-34c	TUSC5	hypothetical gene	-1.81	0.1241
ssc-miR-34c	UBE2G1	ubiquitin conjugating enzyme E2 G1 [Source:HGNC Symbol;Acc:HGNC:12482]	-1.81	0.1241
ssc-miR-34c	UBN2	ubinuclein 2 [Source:VGNC Symbol;Acc:VGNC:94664]	-1.81	0.1241
ssc-miR-34c	UBP1	upstream binding protein 1 [Source:VGNC Symbol;Acc:VGNC:108701]	-1.81	0.1241
ssc-miR-34c	UBXN2B	UBX domain protein 2B [Source:VGNC Symbol;Acc:VGNC:94678]	-1.81	0.1241

ssc-miR-34c	UCK2	uridine-cytidine kinase 2 [Source:VGNC Symbol;Acc:VGNC:98399]	-1.81	0.1241
ssc-miR-34c	UCN2	urocortin 2 [Source:VGNC Symbol;Acc:VGNC:94682]	-1.81	0.1241
ssc-miR-34c	UHRF2	ubiquitin like with PHD and ring finger domains 2 [Source:VGNC Symbol;Acc:VGNC:94692]	-1.81	0.1241
ssc-miR-34c	UNC13C	unc-13 homolog C [Source:VGNC Symbol;Acc:VGNC:94702]	-1.81	0.1241
ssc-miR-34c	UNC45A	unc-45 myosin chaperone A [Source:VGNC Symbol;Acc:VGNC:94704]	-1.81	0.1241
ssc-miR-34c	USF1	upstream transcription factor 1 [Source:VGNC Symbol;Acc:VGNC:98893]	-1.81	0.1241
ssc-miR-34c	USH1G	USH1 protein network component sans [Source:VGNC Symbol;Acc:VGNC:94739]	-1.81	0.1241
ssc-miR-34c	USP24	ubiquitin specific peptidase 24 [Source:VGNC Symbol;Acc:VGNC:94754]	-1.81	0.1241
ssc-miR-34c	USP31	ubiquitin specific peptidase 31 [Source:HGNC Symbol;Acc:HGNC:20060]	-1.81	0.1241
ssc-miR-34c	USP54	ubiquitin specific peptidase 54 [Source:VGNC Symbol;Acc:VGNC:94776]	-1.81	0.1241
ssc-miR-34c	VAMP2	vesicle associated membrane protein 2 [Source:HGNC Symbol;Acc:HGNC:12643]	-1.81	0.1241
ssc-miR-34c	VAT1	vesicle amine transport 1 [Source:VGNC Symbol;Acc:VGNC:94805]	-1.81	0.1241
ssc-miR-34c	VCAM1	vascular cell adhesion molecule 1 [Source:VGNC Symbol;Acc:VGNC:94811]	-1.81	0.1241
ssc-miR-34c	VCL	vinculin [Source:VGNC Symbol;Acc:VGNC:94812]	-1.81	0.1241
ssc-miR-34c	VPS13C	vacuolar protein sorting 13 homolog C [Source:VGNC Symbol;Acc:VGNC:94840]	-1.81	0.1241
ssc-miR-34c	VPS13D	vacuolar protein sorting 13 homolog D [Source:VGNC Symbol;Acc:VGNC:94841]	-1.81	0.1241
ssc-miR-34c	VPS4A	vacuolar protein sorting 4 homolog A [Source:NCBI gene (formerly Entrezgene);Acc:100627841]	-1.81	0.1241
ssc-miR-34c	VTI1B	vesicle transport through interaction with t-SNAREs 1B [Source:VGNC Symbol;Acc:VGNC:94880]	-1.81	0.1241
ssc-miR-34c	VWA5B2	von Willebrand factor A domain containing 5B2 [Source:HGNC Symbol;Acc:HGNC:25144]	-1.81	0.1241
ssc-miR-34c	WAC	WW domain containing adaptor with coiled-coil [Source:VGNC Symbol;Acc:VGNC:95845]	-1.81	0.1241
ssc-miR-34c	WASF1	hypothetical gene	-1.81	0.1241
ssc-miR-34c	WDR91	WD repeat domain 91 [Source:VGNC Symbol;Acc:VGNC:94949]	-1.81	0.1241
ssc-miR-34c	WHAMM	WASP homolog associated with actin, golgi membranes and microtubules [Source:VGNC Symbol;Acc:VGNC:98414]	-1.81	0.1241
ssc-miR-34c	WNK3	WNK lysine deficient protein kinase 3 [Source:VGNC Symbol;Acc:VGNC:94964]	-1.81	0.1241
ssc-miR-34c	WNT1	Wnt family member 1 [Source:HGNC Symbol;Acc:HGNC:12774]	-1.81	0.1241
ssc-miR-34c	WNT2B	Wnt family member 2B [Source:VGNC Symbol;Acc:VGNC:94969]	-1.81	0.1241
ssc-miR-34c	WSCD2	WSC domain containing 2 [Source:HGNC Symbol;Acc:HGNC:29117]	-1.81	0.1241
ssc-miR-34c	WTAP	WT1 associated protein [Source:VGNC Symbol;Acc:VGNC:94984]	-1.81	0.1241
ssc-miR-34c	XPO5	exportin 5 [Source:VGNC Symbol;Acc:VGNC:95005]	-1.81	0.1241
ssc-miR-34c	XYLT1	xylosyltransferase 1 [Source:VGNC Symbol;Acc:VGNC:95015]	-1.81	0.1241
ssc-miR-34c	YTHDC1	YTH domain containing 1 [Source:VGNC Symbol;Acc:VGNC:95041]	-1.81	0.1241
ssc-miR-34c	YY1	YY1 transcription factor [Source:HGNC Symbol;Acc:HGNC:12856]	-1.81	0.1241
ssc-miR-34c	YY2	hypothetical gene	-1.81	0.1241
ssc-miR-34c	ZBTB20	zinc finger and BTB domain containing 20 [Source:VGNC Symbol;Acc:VGNC:95063]	-1.81	0.1241
ssc-miR-34c	ZBTB46	zinc finger and BTB domain containing 46 [Source:VGNC Symbol;Acc:VGNC:95733]	-1.81	0.1241
ssc-miR-34c	ZC3H12B	zinc finger CCCH-type containing 12B [Source:VGNC Symbol;Acc:VGNC:95090]	-1.81	0.1241
ssc-miR-34c	ZC3H4	zinc finger CCCH-type containing 4 [Source:VGNC Symbol;Acc:VGNC:95097]	-1.81	0.1241
ssc-miR-34c	ZCCHC17	zinc finger CCHC-type containing 17 [Source:VGNC Symbol;Acc:VGNC:95108]	-1.81	0.1241
ssc-miR-34c	ZDHHC16	zinc finger DHHC-type palmitoyltransferase 16 [Source:VGNC Symbol;Acc:VGNC:104101]	-1.81	0.1241
ssc-miR-34c	ZDHHC17	zinc finger DHHC-type palmitoyltransferase 17 [Source:VGNC Symbol;Acc:VGNC:95119]	-1.81	0.1241
ssc-miR-34c	ZDHHC23	zinc finger DHHC-type palmitoyltransferase 23 [Source:VGNC Symbol;Acc:VGNC:95125]	-1.81	0.1241
ssc-miR-34c	ZER1	zyg-11 related cell cycle regulator [Source:VGNC Symbol;Acc:VGNC:95132]	-1.81	0.1241
ssc-miR-34c	ZFHX4	zinc finger homeobox 4 [Source:VGNC Symbol;Acc:VGNC:95142]	-1.81	0.1241
ssc-miR-34c	ZIC5	Zic family member 5 [Source:VGNC Symbol;Acc:VGNC:95170]	-1.81	0.1241
ssc-miR-34c	ZMYM4	zinc finger MYM-type containing 4 [Source:VGNC Symbol;Acc:VGNC:95183]	-1.81	0.1241
ssc-miR-34c	ZNF207	zinc finger protein 207 [Source:VGNC Symbol;Acc:VGNC:95205]	-1.81	0.1241
ssc-miR-34c	ZNF281	zinc finger protein 281 [Source:VGNC Symbol;Acc:VGNC:108285]	-1.81	0.1241
ssc-miR-34c	ZNF282	hypothetical gene	-1.81	0.1241
ssc-miR-34c	ZNF304	zinc finger protein 304 [Source:HGNC Symbol;Acc:HGNC:13505]	-1.81	0.1241
ssc-miR-34c	ZNF423	zinc finger protein 423 [Source:VGNC Symbol;Acc:VGNC:98752]	-1.81	0.1241

ssc-miR-34c	ZNF48	zinc finger protein 48 [Source:VGNC Symbol;Acc:VGNC:95247]	-1.81	0.1241
ssc-miR-34c	ZNF579	zinc finger protein 579 [Source:VGNC Symbol;Acc:VGNC:98729]	-1.81	0.1241
ssc-miR-34c	ZNF593	zinc finger protein 593 [Source:HGNC Symbol;Acc:HGNC:30943]	-1.81	0.1241
ssc-miR-34c	ZNF644	zinc finger protein 644 [Source:VGNC Symbol;Acc:VGNC:95283]	-1.81	0.1241
ssc-miR-34c	ZNF772	zinc finger protein 772 [Source:HGNC Symbol;Acc:HGNC:33106]	-1.81	0.1241
ssc-miR-551a	CTIF	cap binding complex dependent translation initiation factor [Source:VGNC Symbol;Acc:VGNC:87062]	-1.83	0.1258
ssc-miR-551a	ERBB4	erb-b2 receptor tyrosine kinase 4 [Source:VGNC Symbol;Acc:VGNC:96284]	-1.83	0.1258
ssc-miR-551a	GALNTL6	polypeptide N-acetylgalactosaminyltransferase like 6 [Source:VGNC Symbol;Acc:VGNC:103947]	-1.83	0.1258
ssc-miR-551a	HEYL	hes related family bHLH transcription factor with YRPW motif like [Source:VGNC Symbol;Acc:VGNC:88865]	-1.83	0.1258
ssc-miR-551a	LPHN1	hypothetical gene	-1.83	0.1258
ssc-miR-551a	MEF2C	myocyte enhancer factor 2C [Source:VGNC Symbol;Acc:VGNC:90127]	-1.83	0.1258
ssc-miR-551a	NFIB	nuclear factor I B [Source:VGNC Symbol;Acc:VGNC:90716]	-1.83	0.1258
ssc-miR-551a	OSBPL6	oxysterol binding protein like 6 [Source:VGNC Symbol;Acc:VGNC:95807]	-1.83	0.1258
ssc-miR-551a	RP11-644F5.10	hypothetical gene	-1.83	0.1258
ssc-miR-29b	AAR2	AAR2 splicing factor [Source:VGNC Symbol;Acc:VGNC:95690]	-1.91	0.0807
ssc-miR-29b	ABCB6	ATP binding cassette subfamily B member 6 (Langereis blood group) [Source:HGNC Symbol;Acc:HGNC:47]	-1.91	0.0807
ssc-miR-29b	ABCE1	ATP binding cassette subfamily E member 1 [Source:VGNC Symbol;Acc:VGNC:84965]	-1.91	0.0807
ssc-miR-29b	ABHD4	abhydrolase domain containing 4, N-acyl phospholipase B [Source:VGNC Symbol;Acc:VGNC:97859]	-1.91	0.0807
ssc-miR-29b	ABHD5	abhydrolase domain containing 5, lysophosphatidic acid acyltransferase [Source:VGNC Symbol;Acc:VGNC:97860]	-1.91	0.0807
ssc-miR-29b	ACER3	alkaline ceramidase 3 [Source:VGNC Symbol;Acc:VGNC:103893]	-1.91	0.0807
ssc-miR-29b	ACOXL	acyl-CoA oxidase like [Source:VGNC Symbol;Acc:VGNC:85023]	-1.91	0.0807
ssc-miR-29b	ACPL2	hypothetical gene	-1.91	0.0807
ssc-miR-29b	ACTN2	actinin alpha 2 [Source:VGNC Symbol;Acc:VGNC:85046]	-1.91	0.0807
ssc-miR-29b	ACVR2A	activin A receptor type 2A [Source:VGNC Symbol;Acc:VGNC:95843]	-1.91	0.0807
ssc-miR-29b	ADA	adenosine deaminase [Source:VGNC Symbol;Acc:VGNC:95889]	-1.91	0.0807
ssc-miR-29b	ADAM12	ADAM metallopeptidase domain 12 [Source:VGNC Symbol;Acc:VGNC:85063]	-1.91	0.0807
ssc-miR-29b	ADAM19	ADAM metallopeptidase domain 19 [Source:VGNC Symbol;Acc:VGNC:85066]	-1.91	0.0807
ssc-miR-29b	ADAMTS10	ADAM metallopeptidase with thrombospondin type 1 motif 10 [Source:VGNC Symbol;Acc:VGNC:85073]	-1.91	0.0807
ssc-miR-29b	ADAMTS14	ADAM metallopeptidase with thrombospondin type 1 motif 14 [Source:VGNC Symbol;Acc:VGNC:85076]	-1.91	0.0807
ssc-miR-29b	ADAMTS15	ADAM metallopeptidase with thrombospondin type 1 motif 15 [Source:VGNC Symbol;Acc:VGNC:85077]	-1.91	0.0807
ssc-miR-29b	ADAMTS16	ADAM metallopeptidase with thrombospondin type 1 motif 16 [Source:VGNC Symbol;Acc:VGNC:85078]	-1.91	0.0807
ssc-miR-29b	ADAMTS17	ADAM metallopeptidase with thrombospondin type 1 motif 17 [Source:VGNC Symbol;Acc:VGNC:85079]	-1.91	0.0807
ssc-miR-29b	ADAMTS18	ADAM metallopeptidase with thrombospondin type 1 motif 18 [Source:VGNC Symbol;Acc:VGNC:85080]	-1.91	0.0807
ssc-miR-29b	ADAMTS2	ADAM metallopeptidase with thrombospondin type 1 motif 2 [Source:VGNC Symbol;Acc:VGNC:85081]	-1.91	0.0807
ssc-miR-29b	ADAMTS20	ADAM metallopeptidase with thrombospondin type 1 motif 20 [Source:VGNC Symbol;Acc:VGNC:85082]	-1.91	0.0807
ssc-miR-29b	ADAMTS5	ADAM metallopeptidase with thrombospondin type 1 motif 5 [Source:VGNC Symbol;Acc:VGNC:85085]	-1.91	0.0807
ssc-miR-29b	ADAMTS7	ADAM metallopeptidase with thrombospondin type 1 motif 7 [Source:VGNC Symbol;Acc:VGNC:85087]	-1.91	0.0807
ssc-miR-29b	ADAMTS9	ADAM metallopeptidase with thrombospondin type 1 motif 9 [Source:VGNC Symbol;Acc:VGNC:85089]	-1.91	0.0807
ssc-miR-29b	ADCYAP1R1	ADCYAP receptor type I [Source:VGNC Symbol;Acc:VGNC:85115]	-1.91	0.0807
ssc-miR-29b	AFF4	AF4/FMR2 family member 4 [Source:VGNC Symbol;Acc:VGNC:85169]	-1.91	0.0807
ssc-miR-29b	AGO1	hypothetical gene	-1.91	0.0807
ssc-miR-29b	AGO3	argonaute RISC component 1 [Source:NCBI gene (formerly Entrezgene);Acc:100499510]	-1.91	0.0807
ssc-miR-29b	AGPAT4	1-acylglycerol-3-phosphate O-acyltransferase 4 [Source:VGNC Symbol;Acc:VGNC:85186]	-1.91	0.0807
ssc-miR-29b	AGTR2	angiotensin II receptor type 2 [Source:VGNC Symbol;Acc:VGNC:85192]	-1.91	0.0807
ssc-miR-29b	AHR	aryl hydrocarbon receptor [Source:NCBI gene (formerly Entrezgene);Acc:396654]	-1.91	0.0807
ssc-miR-29b	AIM1	hypothetical gene	-1.91	0.0807
ssc-miR-29b	AK1	adenylate kinase 1 [Source:VGNC Symbol;Acc:VGNC:97875]	-1.91	0.0807
ssc-miR-29b	AK3	adenylate kinase 3 [Source:VGNC Symbol;Acc:VGNC:97876]	-1.91	0.0807
ssc-miR-29b	AKAP13	hypothetical gene	-1.91	0.0807
ssc-miR-29b	AKAP2	hypothetical gene	-1.91	0.0807

ssc-miR-29b	AKAP5	A-kinase anchoring protein 5 [Source:VGNC Symbol;Acc:VGNC:85220]	-1.91	0.0807
ssc-miR-29b	AKT3	AKT serine/threonine kinase 3 [Source:VGNC Symbol;Acc:VGNC:96306]	-1.91	0.0807
ssc-miR-29b	AL021546.6	hypothetical gene	-1.91	0.0807
ssc-miR-29b	AL626787.1	hypothetical gene	-1.91	0.0807
ssc-miR-29b	ALKBH6	alkB homolog 6 [Source:VGNC Symbol;Acc:VGNC:85261]	-1.91	0.0807
ssc-miR-29b	ALS2	alsin Rho guanine nucleotide exchange factor ALS2 [Source:VGNC Symbol;Acc:VGNC:96327]	-1.91	0.0807
ssc-miR-29b	AMER1	APC membrane recruitment protein 1 [Source:VGNC Symbol;Acc:VGNC:85276]	-1.91	0.0807
ssc-miR-29b	AMFR	autocrine motility factor receptor [Source:VGNC Symbol;Acc:VGNC:85278]	-1.91	0.0807
ssc-miR-29b	AMMECR1	AMMECR nuclear protein 1 [Source:VGNC Symbol;Acc:VGNC:96559]	-1.91	0.0807
ssc-miR-29b	AMMECR1L	AMMECR1 like [Source:VGNC Symbol;Acc:VGNC:103895]	-1.91	0.0807
ssc-miR-29b	AMOT	angiomotin [Source:VGNC Symbol;Acc:VGNC:85283]	-1.91	0.0807
ssc-miR-29b	ANK1	ankyrin 1 [Source:VGNC Symbol;Acc:VGNC:96344]	-1.91	0.0807
ssc-miR-29b	ANK3	hypothetical gene	-1.91	0.0807
ssc-miR-29b	ANKFY1	ankyrin repeat and FYVE domain containing 1 [Source:VGNC Symbol;Acc:VGNC:85314]	-1.91	0.0807
ssc-miR-29b	ANKRD13B	ankyrin repeat domain 13B [Source:VGNC Symbol;Acc:VGNC:85325]	-1.91	0.0807
ssc-miR-29b	ANKRD13C	ankyrin repeat domain 13C [Source:VGNC Symbol;Acc:VGNC:85326]	-1.91	0.0807
ssc-miR-29b	ANKRD27	ankyrin repeat domain 27 [Source:VGNC Symbol;Acc:VGNC:96907]	-1.91	0.0807
ssc-miR-29b	ANKRD52	ankyrin repeat domain 52 [Source:VGNC Symbol;Acc:VGNC:85342]	-1.91	0.0807
ssc-miR-29b	ANTXR2	ANTXR cell adhesion molecule 2 [Source:VGNC Symbol;Acc:VGNC:85365]	-1.91	0.0807
ssc-miR-29b	AP1G1	adaptor related protein complex 1 subunit gamma 1 [Source:VGNC Symbol;Acc:VGNC:85378]	-1.91	0.0807
ssc-miR-29b	AP1S1	adaptor related protein complex 1 subunit sigma 1 [Source:VGNC Symbol;Acc:VGNC:97032]	-1.91	0.0807
ssc-miR-29b	AP4E1	adaptor related protein complex 4 subunit epsilon 1 [Source:VGNC Symbol;Acc:VGNC:85392]	-1.91	0.0807
ssc-miR-29b	AP5S1	adaptor related protein complex 5 subunit sigma 1 [Source:VGNC Symbol;Acc:VGNC:95686]	-1.91	0.0807
ssc-miR-29b	APC	APC regulator of WNT signaling pathway [Source:VGNC Symbol;Acc:VGNC:99584]	-1.91	0.0807
ssc-miR-29b	APLNR	apelin receptor [Source:VGNC Symbol;Acc:VGNC:85412]	-1.91	0.0807
ssc-miR-29b	APPBP2	amyloid beta protein binding protein 2 [Source:VGNC Symbol;Acc:VGNC:85425]	-1.91	0.0807
ssc-miR-29b	AQP4	aquaporin 4 [Source:VGNC Symbol;Acc:VGNC:85432]	-1.91	0.0807
ssc-miR-29b	ARF3	ADP ribosylation factor 3 [Source:NCBI gene (formerly Entrezgene);Acc:100301570]	-1.91	0.0807
ssc-miR-29b	ARF5	ADP ribosylation factor 5 [Source:VGNC Symbol;Acc:VGNC:85447]	-1.91	0.0807
ssc-miR-29b	ARFGEF2	ADP ribosylation factor guanine nucleotide exchange factor 2 [Source:VGNC Symbol;Acc:VGNC:95672]	-1.91	0.0807
ssc-miR-29b	ARHGAP10	Rho GTPase activating protein 10 [Source:VGNC Symbol;Acc:VGNC:85458]	-1.91	0.0807
ssc-miR-29b	ARHGAP28	Rho GTPase activating protein 28 [Source:VGNC Symbol;Acc:VGNC:85467]	-1.91	0.0807
ssc-miR-29b	ARHGAP31	Rho GTPase activating protein 31 [Source:VGNC Symbol;Acc:VGNC:85470]	-1.91	0.0807
ssc-miR-29b	ARHGAP36	hypothetical gene	-1.91	0.0807
ssc-miR-29b	ARHGEF10	Rho guanine nucleotide exchange factor 10 [Source:VGNC Symbol;Acc:VGNC:95605]	-1.91	0.0807
ssc-miR-29b	ARHGEF4	hypothetical gene	-1.91	0.0807
ssc-miR-29b	ARID1B	AT-rich interaction domain 1B [Source:VGNC Symbol;Acc:VGNC:85506]	-1.91	0.0807
ssc-miR-29b	ARL4A	ADP ribosylation factor like GTPase 4A [Source:VGNC Symbol;Acc:VGNC:85522]	-1.91	0.0807
ssc-miR-29b	ARL5B	ADP ribosylation factor like GTPase 5B [Source:VGNC Symbol;Acc:VGNC:95985]	-1.91	0.0807
ssc-miR-29b	ARMC8	armadillo repeat containing 8 [Source:VGNC Symbol;Acc:VGNC:85531]	-1.91	0.0807
ssc-miR-29b	ARNT	aryl hydrocarbon receptor nuclear translocator [Source:VGNC Symbol;Acc:VGNC:98733]	-1.91	0.0807
ssc-miR-29b	ARPC3	actin related protein 2/3 complex subunit 3 [Source:VGNC Symbol;Acc:VGNC:85538]	-1.91	0.0807
ssc-miR-29b	ARPP19	cAMP regulated phosphoprotein 19 [Source:NCBI gene (formerly Entrezgene);Acc:397362]	-1.91	0.0807
ssc-miR-29b	ARRB1	arrestin beta 1 [Source:VGNC Symbol;Acc:VGNC:85542]	-1.91	0.0807
ssc-miR-29b	ARRDC3	arrestin domain containing 3 [Source:VGNC Symbol;Acc:VGNC:85546]	-1.91	0.0807
ssc-miR-29b	ARRDC4	arrestin domain containing 4 [Source:VGNC Symbol;Acc:VGNC:85547]	-1.91	0.0807
ssc-miR-29b	ARSB	arylsulfatase B [Source:VGNC Symbol;Acc:VGNC:96794]	-1.91	0.0807
ssc-miR-29b	ARVCF	ARVCF delta catenin family member [Source:VGNC Symbol;Acc:VGNC:85555]	-1.91	0.0807
ssc-miR-29b	ASAP2	ArfGAP with SH3 domain, ankyrin repeat and PH domain 2 [Source:VGNC Symbol;Acc:VGNC:85558]	-1.91	0.0807
ssc-miR-29b	ASH2L	ASH2 like, histone lysine methyltransferase complex subunit [Source:VGNC Symbol;Acc:VGNC:96426]	-1.91	0.0807

ssc-miR-29b	ASIC1	acid sensing ion channel subunit 1 [Source:VGNC Symbol;Acc:VGNC:85578]	-1.91	0.0807
ssc-miR-29b	ASXL3	ASXL transcriptional regulator 3 [Source:VGNC Symbol;Acc:VGNC:85596]	-1.91	0.0807
ssc-miR-29b	ATAD2B	ATPase family AAA domain containing 2B [Source:VGNC Symbol;Acc:VGNC:85600]	-1.91	0.0807
ssc-miR-29b	ATG14	autophagy related 14 [Source:VGNC Symbol;Acc:VGNC:85617]	-1.91	0.0807
ssc-miR-29b	ATG9A	autophagy related 9A [Source:VGNC Symbol;Acc:VGNC:99703]	-1.91	0.0807
ssc-miR-29b	ATP1B1	ATPase Na+/K+ transporting subunit beta 1 [Source:VGNC Symbol;Acc:VGNC:85642]	-1.91	0.0807
ssc-miR-29b	ATP1B4	ATPase Na+/K+ transporting family member beta 4 [Source:VGNC Symbol;Acc:VGNC:85644]	-1.91	0.0807
ssc-miR-29b	ATP2B2	ATPase plasma membrane Ca2+ transporting 2 [Source:VGNC Symbol;Acc:VGNC:85649]	-1.91	0.0807
ssc-miR-29b	ATP2B4	ATPase plasma membrane Ca2+ transporting 4 [Source:VGNC Symbol;Acc:VGNC:85651]	-1.91	0.0807
ssc-miR-29b	ATP5G3	hypothetical gene	-1.91	0.0807
ssc-miR-29b	ATP6V1A	ATPase H+ transporting V1 subunit A [Source:VGNC Symbol;Acc:VGNC:85669]	-1.91	0.0807
ssc-miR-29b	ATRN	attractin [Source:VGNC Symbol;Acc:VGNC:96479]	-1.91	0.0807
ssc-miR-29b	ATXN1	ataxin 1 [Source:VGNC Symbol;Acc:VGNC:85687]	-1.91	0.0807
ssc-miR-29b	B3GALT1	beta-1,3-galactosyltransferase 1 [Source:VGNC Symbol;Acc:VGNC:96494]	-1.91	0.0807
ssc-miR-29b	B3GNT5	UDP-GlcNAc:betaGal beta-1,3-N-acetylglucosaminyltransferase 5 [Source:VGNC Symbol;Acc:VGNC:85726]	-1.91	0.0807
ssc-miR-29b	BACE1	beta-secretase 1 [Source:VGNC Symbol;Acc:VGNC:85740]	-1.91	0.0807
ssc-miR-29b	BACH2	BTB domain and CNC homolog 2 [Source:VGNC Symbol;Acc:VGNC:85742]	-1.91	0.0807
ssc-miR-29b	BAHD1	bromo adjacent homology domain containing 1 [Source:VGNC Symbol;Acc:VGNC:85747]	-1.91	0.0807
ssc-miR-29b	BAK1	hypothetical gene	-1.91	0.0807
ssc-miR-29b	BBC3	BCL2 binding component 3 [Source:HGNC Symbol;Acc:HGNC:17868]	-1.91	0.0807
ssc-miR-29b	BBS1	Bardet-Biedl syndrome 1 [Source:HGNC Symbol;Acc:HGNC:966]	-1.91	0.0807
ssc-miR-29b	BCL11B	BAF chromatin remodeling complex subunit BCL11B [Source:VGNC Symbol;Acc:VGNC:96563]	-1.91	0.0807
ssc-miR-29b	BCL2L2	BCL2 like 2 [Source:HGNC Symbol;Acc:HGNC:995]	-1.91	0.0807
ssc-miR-29b	BCL7A	BAF chromatin remodeling complex subunit BCL7A [Source:VGNC Symbol;Acc:VGNC:85784]	-1.91	0.0807
ssc-miR-29b	BCL9L	BCL9 like [Source:VGNC Symbol;Acc:VGNC:96568]	-1.91	0.0807
ssc-miR-29b	BCORL1	BCL6 corepressor like 1 [Source:VGNC Symbol;Acc:VGNC:85791]	-1.91	0.0807
ssc-miR-29b	BDKRB2	bradykinin receptor B2 [Source:VGNC Symbol;Acc:VGNC:85794]	-1.91	0.0807
ssc-miR-29b	BEAN1	brain expressed associated with NEDD4 1 [Source:VGNC Symbol;Acc:VGNC:85797]	-1.91	0.0807
ssc-miR-29b	BICD1	BICD cargo adaptor 1 [Source:VGNC Symbol;Acc:VGNC:85818]	-1.91	0.0807
ssc-miR-29b	BLMH	bleomycin hydrolase [Source:HGNC Symbol;Acc:HGNC:1059]	-1.91	0.0807
ssc-miR-29b	BMF	Bcl2 modifying factor [Source:VGNC Symbol;Acc:VGNC:85837]	-1.91	0.0807
ssc-miR-29b	BMP1	bone morphotic protein 1 [Source:VGNC Symbol;Acc:VGNC:85838]	-1.91	0.0807
ssc-miR-29b	BMPR1A	bone morphotic protein receptor type 1A [Source:VGNC Symbol;Acc:VGNC:85846]	-1.91	0.0807
ssc-miR-29b	BRMS1L	BRMS1 like transcriptional repressor [Source:VGNC Symbol;Acc:VGNC:96571]	-1.91	0.0807
ssc-miR-29b	BRWD1	bromodomain and WD repeat domain containing 1 [Source:VGNC Symbol;Acc:VGNC:108153]	-1.91	0.0807
ssc-miR-29b	BRWD3	bromodomain and WD repeat domain containing 3 [Source:VGNC Symbol;Acc:VGNC:85890]	-1.91	0.0807
ssc-miR-29b	BSDC1	BSD domain containing 1 [Source:VGNC Symbol;Acc:VGNC:85892]	-1.91	0.0807
ssc-miR-29b	BSND	barttin CLCNK type accessory subunit beta [Source:VGNC Symbol;Acc:VGNC:85894]	-1.91	0.0807
ssc-miR-29b	BTBD7	BTB domain containing 7 [Source:VGNC Symbol;Acc:VGNC:85908]	-1.91	0.0807
ssc-miR-29b	BTG2	BTG anti-proliferation factor 2 [Source:NCBI gene (formerly Entrezgene);Acc:100048932]	-1.91	0.0807
ssc-miR-29b	BTLA	hypothetical gene	-1.91	0.0807
ssc-miR-29b	BVES	blood vessel epicardial substance [Source:VGNC Symbol;Acc:VGNC:103040]	-1.91	0.0807
ssc-miR-29b	C11orf30	hypothetical gene	-1.91	0.0807
ssc-miR-29b	C11orf57	hypothetical gene	-1.91	0.0807
ssc-miR-29b	C11orf87	chromosome 11 open reading frame 87 [Source:HGNC Symbol;Acc:HGNC:33788]	-1.91	0.0807
ssc-miR-29b	C16orf72	chromosome 3 C16orf72 homolog [Source:VGNC Symbol;Acc:VGNC:86016]	-1.91	0.0807
ssc-miR-29b	C16orf87	chromosome 6 C16orf87 homolog [Source:VGNC Symbol;Acc:VGNC:96927]	-1.91	0.0807
ssc-miR-29b	C17orf103	hypothetical gene	-1.91	0.0807
ssc-miR-29b	C18orf42	hypothetical gene	-1.91	0.0807
ssc-miR-29b	C1orf21	hypothetical gene	-1.91	0.0807

ssc-miR-29b	C1orf226	hypothetical gene	-1.91	0.0807
ssc-miR-29b	C1QTNF6	C1q and TNF related 6 [Source:NCBI gene (formerly Entrezgene);Acc:100217379]	-1.91	0.0807
ssc-miR-29b	C20orf112	hypothetical gene	-1.91	0.0807
ssc-miR-29b	C21orf91	chromosome 13 C21orf91 homolog [Source:VGNC Symbol;Acc:VGNC:85930]	-1.91	0.0807
ssc-miR-29b	C2orf42	chromosome 3 C2orf42 homolog [Source:VGNC Symbol;Acc:VGNC:86021]	-1.91	0.0807
ssc-miR-29b	C2orf88	chromosome 15 C2orf88 homolog [Source:VGNC Symbol;Acc:VGNC:96170]	-1.91	0.0807
ssc-miR-29b	C4orf29	hypothetical gene	-1.91	0.0807
ssc-miR-29b	C4orf32	hypothetical gene	-1.91	0.0807
ssc-miR-29b	C5orf15	chromosome 2 C5orf15 homolog [Source:VGNC Symbol;Acc:VGNC:86009]	-1.91	0.0807
ssc-miR-29b	C5orf24	chromosome 2 C5orf24 homolog [Source:VGNC Symbol;Acc:VGNC:86010]	-1.91	0.0807
ssc-miR-29b	C7orf60	hypothetical gene	-1.91	0.0807
ssc-miR-29b	C7orf73	hypothetical gene	-1.91	0.0807
ssc-miR-29b	C8orf47	hypothetical gene	-1.91	0.0807
ssc-miR-29b	C9orf69	hypothetical gene	-1.91	0.0807
ssc-miR-29b	C9orf72	chromosome 10 C9orf72 homolog [Source:VGNC Symbol;Acc:VGNC:96173]	-1.91	0.0807
ssc-miR-29b	CA3	carbonic anhydrase 3 [Source:VGNC Symbol;Acc:VGNC:86099]	-1.91	0.0807
ssc-miR-29b	CACFD1	calcium channel flower domain containing 1 [Source:VGNC Symbol;Acc:VGNC:86115]	-1.91	0.0807
ssc-miR-29b	CACNA1A	calcium voltage-gated channel subunit alpha1 A [Source:VGNC Symbol;Acc:VGNC:99705]	-1.91	0.0807
ssc-miR-29b	CACNA1B	calcium voltage-gated channel subunit alpha1 B [Source:HGNC Symbol;Acc:HGNC:1389]	-1.91	0.0807
ssc-miR-29b	CACNA1C	calcium voltage-gated channel subunit alpha1 C [Source:HGNC Symbol;Acc:HGNC:1390]	-1.91	0.0807
ssc-miR-29b	CACNG4	calcium voltage-gated channel auxiliary subunit gamma 4 [Source:VGNC Symbol;Acc:VGNC:86127]	-1.91	0.0807
ssc-miR-29b	CACUL1	CDK2 associated cullin domain 1 [Source:VGNC Symbol;Acc:VGNC:86132]	-1.91	0.0807
ssc-miR-29b	CALCR	calcitonin receptor [Source:VGNC Symbol;Acc:VGNC:86142]	-1.91	0.0807
ssc-miR-29b	CALM3	calmodulin 3 [Source:NCBI gene (formerly Entrezgene);Acc:396838]	-1.91	0.0807
ssc-miR-29b	CALU	calumenin [Source:VGNC Symbol;Acc:VGNC:86151]	-1.91	0.0807
ssc-miR-29b	CAMK1D	calcium/calmodulin dependent protein kinase ID [Source:VGNC Symbol;Acc:VGNC:95909]	-1.91	0.0807
ssc-miR-29b	CAMK2G	calcium/calmodulin dependent protein kinase II gamma [Source:VGNC Symbol;Acc:VGNC:86157]	-1.91	0.0807
ssc-miR-29b	CAMK4	calcium/calmodulin dependent protein kinase IV [Source:VGNC Symbol;Acc:VGNC:99602]	-1.91	0.0807
ssc-miR-29b	CAMKK2	calcium/calmodulin dependent protein kinase kinase 2 [Source:VGNC Symbol;Acc:VGNC:86158]	-1.91	0.0807
ssc-miR-29b	CAMSAP2	calmodulin regulated spectrin associated protein family member 2 [Source:VGNC Symbol;Acc:VGNC:96205]	-1.91	0.0807
ssc-miR-29b	CAND1	cullin associated and neddylation dissociated 1 [Source:VGNC Symbol;Acc:VGNC:97912]	-1.91	0.0807
ssc-miR-29b	CAPN7	calpain 7 [Source:NCBI gene (formerly Entrezgene);Acc:100037936]	-1.91	0.0807
ssc-miR-29b	CASP7	caspase 7 [Source:VGNC Symbol;Acc:VGNC:86203]	-1.91	0.0807
ssc-miR-29b	CAV2	caveolin 2 [Source:VGNC Symbol;Acc:VGNC:103914]	-1.91	0.0807
ssc-miR-29b	CBX3	chromobox 3 [Source:HGNC Symbol;Acc:HGNC:1553]	-1.91	0.0807
ssc-miR-29b	CBX5	chromobox 5 [Source:VGNC Symbol;Acc:VGNC:86232]	-1.91	0.0807
ssc-miR-29b	CBX6	chromobox 6 [Source:VGNC Symbol;Acc:VGNC:97915]	-1.91	0.0807
ssc-miR-29b	CCDC117	coiled-coil domain containing 117 [Source:VGNC Symbol;Acc:VGNC:86248]	-1.91	0.0807
ssc-miR-29b	CCDC28B	coiled-coil domain containing 28B [Source:VGNC Symbol;Acc:VGNC:86290]	-1.91	0.0807
ssc-miR-29b	CCDC50	coiled-coil domain containing 50 [Source:VGNC Symbol;Acc:VGNC:86299]	-1.91	0.0807
ssc-miR-29b	CCDC80	coiled-coil domain containing 80 [Source:VGNC Symbol;Acc:VGNC:86315]	-1.91	0.0807
ssc-miR-29b	CCDC85A	coiled-coil domain containing 85A [Source:VGNC Symbol;Acc:VGNC:86320]	-1.91	0.0807
ssc-miR-29b	CCDC88A	coiled-coil domain containing 88A [Source:VGNC Symbol;Acc:VGNC:86325]	-1.91	0.0807
ssc-miR-29b	CCNA2	cyclin A2 [Source:NCBI gene (formerly Entrezgene);Acc:100415929]	-1.91	0.0807
ssc-miR-29b	CCND2	cyclin D2 [Source:VGNC Symbol;Acc:VGNC:103222]	-1.91	0.0807
ssc-miR-29b	CCNJ	cyclin J [Source:VGNC Symbol;Acc:VGNC:86360]	-1.91	0.0807
ssc-miR-29b	CCNL2	cyclin L2 [Source:VGNC Symbol;Acc:VGNC:86364]	-1.91	0.0807
ssc-miR-29b	CCNT2	cyclin T2 [Source:VGNC Symbol;Acc:VGNC:103916]	-1.91	0.0807
ssc-miR-29b	CCNYL1	hypothetical gene	-1.91	0.0807
ssc-miR-29b	CCSAP	centriole, cilia and spindle associated protein [Source:VGNC Symbol;Acc:VGNC:86377]	-1.91	0.0807

ssc-miR-29b	CCSER2	coiled-coil serine rich protein 2 [Source:VGNC Symbol;Acc:VGNC:86379]	-1.91	0.0807
ssc-miR-29b	CD276	CD276 molecule [Source:VGNC Symbol;Acc:VGNC:86405]	-1.91	0.0807
ssc-miR-29b	CDC42	cell division cycle 42 [Source:NCBI gene (formerly Entrezgene);Acc:780428]	-1.91	0.0807
ssc-miR-29b	CDC42BPA	CDC42 binding protein kinase alpha [Source:VGNC Symbol;Acc:VGNC:95847]	-1.91	0.0807
ssc-miR-29b	CDC42SE1	CDC42 small effector 1 [Source:VGNC Symbol;Acc:VGNC:86460]	-1.91	0.0807
ssc-miR-29b	CDC7	cell division cycle 7 [Source:VGNC Symbol;Acc:VGNC:97922]	-1.91	0.0807
ssc-miR-29b	CDCA4	cell division cycle associated 4 [Source:VGNC Symbol;Acc:VGNC:86467]	-1.91	0.0807
ssc-miR-29b	CDK16	cyclin dependent kinase 16 [Source:VGNC Symbol;Acc:VGNC:86499]	-1.91	0.0807
ssc-miR-29b	CDK6	cyclin dependent kinase 6 [Source:HGNC Symbol;Acc:HGNC:1777]	-1.91	0.0807
ssc-miR-29b	CDKL2	cyclin dependent kinase like 2 [Source:VGNC Symbol;Acc:VGNC:86511]	-1.91	0.0807
ssc-miR-29b	CEACAM1	hypothetical gene	-1.91	0.0807
ssc-miR-29b	CELF2	hypothetical gene	-1.91	0.0807
ssc-miR-29b	CELF6	CUGBP Elav-like family member 6 [Source:HGNC Symbol;Acc:HGNC:14059]	-1.91	0.0807
ssc-miR-29b	CEP41	centrosomal protein 41 [Source:VGNC Symbol;Acc:VGNC:86569]	-1.91	0.0807
ssc-miR-29b	CEP85L	centrosomal protein 85 like [Source:VGNC Symbol;Acc:VGNC:86580]	-1.91	0.0807
ssc-miR-29b	CEP97	centrosomal protein 97 [Source:VGNC Symbol;Acc:VGNC:108638]	-1.91	0.0807
ssc-miR-29b	CERS1	growth differentiation factor 1 [Source:VGNC Symbol;Acc:VGNC:103069]	-1.91	0.0807
ssc-miR-29b	CFL2	cofilin 2 [Source:VGNC Symbol;Acc:VGNC:86611]	-1.91	0.0807
ssc-miR-29b	CHFR	checkpoint with forkhead and ring finger domains [Source:HGNC Symbol;Acc:HGNC:20455]	-1.91	0.0807
ssc-miR-29b	CHIC2	cysteine rich hydrophobic domain 2 [Source:VGNC Symbol;Acc:VGNC:86643]	-1.91	0.0807
ssc-miR-29b	CHMP6	charged multivesicular body protein 6 [Source:VGNC Symbol;Acc:VGNC:86653]	-1.91	0.0807
ssc-miR-29b	CHSY1	chondroitin sulfate synthase 1 [Source:VGNC Symbol;Acc:VGNC:86684]	-1.91	0.0807
ssc-miR-29b	CILP2	cartilage intermediate layer protein 2 [Source:VGNC Symbol;Acc:VGNC:86704]	-1.91	0.0807
ssc-miR-29b	CLCN5	chloride voltage-gated channel 5 [Source:VGNC Symbol;Acc:VGNC:103925]	-1.91	0.0807
ssc-miR-29b	CLDN1	claudin 1 [Source:VGNC Symbol;Acc:VGNC:103926]	-1.91	0.0807
ssc-miR-29b	CLEC2L	C-type lectin domain family 2 member L [Source:VGNC Symbol;Acc:VGNC:86752]	-1.91	0.0807
ssc-miR-29b	CLK2	CDC like kinase 2 [Source:VGNC Symbol;Acc:VGNC:86767]	-1.91	0.0807
ssc-miR-29b	CLMN	calmin [Source:VGNC Symbol;Acc:VGNC:86769]	-1.91	0.0807
ssc-miR-29b	CLPX	caseinolytic mitochondrial matrix peptidase chaperone subunit X [Source:VGNC Symbol;Acc:VGNC:86780]	-1.91	0.0807
ssc-miR-29b	CMPK1	cytidine/uridine monophosphate kinase 1 [Source:VGNC Symbol;Acc:VGNC:96953]	-1.91	0.0807
ssc-miR-29b	CNIH1	cornichon family AMPA receptor auxiliary protein 1 [Source:VGNC Symbol;Acc:VGNC:86820]	-1.91	0.0807
ssc-miR-29b	CNNM2	cyclin and CBS domain divalent metal cation transport mediator 2 [Source:VGNC Symbol;Acc:VGNC:86828]	-1.91	0.0807
ssc-miR-29b	CNOT6	CCR4-NOT transcription complex subunit 6 [Source:VGNC Symbol;Acc:VGNC:86837]	-1.91	0.0807
ssc-miR-29b	CNOT8	CCR4-NOT transcription complex subunit 8 [Source:VGNC Symbol;Acc:VGNC:86839]	-1.91	0.0807
ssc-miR-29b	CNR1	cannabinoid receptor 1 [Source:VGNC Symbol;Acc:VGNC:86843]	-1.91	0.0807
ssc-miR-29b	COL11A1	collagen type XI alpha 1 chain [Source:VGNC Symbol;Acc:VGNC:86862]	-1.91	0.0807
ssc-miR-29b	COL15A1	collagen type XV alpha 1 chain [Source:VGNC Symbol;Acc:VGNC:86866]	-1.91	0.0807
ssc-miR-29b	COL16A1	collagen type XVI alpha 1 chain [Source:VGNC Symbol;Acc:VGNC:86867]	-1.91	0.0807
ssc-miR-29b	COL19A1	collagen type XIX alpha 1 chain [Source:HGNC Symbol;Acc:HGNC:2196]	-1.91	0.0807
ssc-miR-29b	COL1A1	collagen type I alpha 1 chain [Source:VGNC Symbol;Acc:VGNC:86870]	-1.91	0.0807
ssc-miR-29b	COL1A2	collagen type I alpha 2 chain [Source:VGNC Symbol;Acc:VGNC:86871]	-1.91	0.0807
ssc-miR-29b	COL21A1	collagen type XXI alpha 1 chain [Source:HGNC Symbol;Acc:HGNC:17025]	-1.91	0.0807
ssc-miR-29b	COL22A1	collagen type XXII alpha 1 chain [Source:VGNC Symbol;Acc:VGNC:97937]	-1.91	0.0807
ssc-miR-29b	COL24A1	collagen type XXIV alpha 1 chain [Source:HGNC Symbol;Acc:HGNC:20821]	-1.91	0.0807
ssc-miR-29b	COL25A1	collagen type XXV alpha 1 chain [Source:HGNC Symbol;Acc:HGNC:18603]	-1.91	0.0807
ssc-miR-29b	COL26A1	collagen type XXVI alpha 1 chain [Source:HGNC Symbol;Acc:HGNC:18038]	-1.91	0.0807
ssc-miR-29b	COL27A1	collagen type XXVII alpha 1 chain [Source:VGNC Symbol;Acc:VGNC:86872]	-1.91	0.0807
ssc-miR-29b	COL2A1	collagen type II alpha 1 chain [Source:VGNC Symbol;Acc:VGNC:86874]	-1.91	0.0807
ssc-miR-29b	COL3A1	collagen type III alpha 1 chain [Source:VGNC Symbol;Acc:VGNC:95979]	-1.91	0.0807
ssc-miR-29b	COL4A1	collagen type IV alpha 1 chain [Source:HGNC Symbol;Acc:HGNC:2202]	-1.91	0.0807

ssc-miR-29b	COL4A2	collagen type IV alpha 2 chain [Source:VGNC Symbol;Acc:VGNC:86875]	-1.91	0.0807
ssc-miR-29b	COL4A3	collagen type IV alpha 3 chain [Source:HGNC Symbol;Acc:HGNC:2204]	-1.91	0.0807
ssc-miR-29b	COL4A4	collagen type IV alpha 4 chain [Source:VGNC Symbol;Acc:VGNC:95980]	-1.91	0.0807
ssc-miR-29b	COL4A5	collagen type IV alpha 5 chain [Source:VGNC Symbol;Acc:VGNC:86876]	-1.91	0.0807
ssc-miR-29b	COL4A6	collagen type IV alpha 6 chain [Source:HGNC Symbol;Acc:HGNC:2208]	-1.91	0.0807
ssc-miR-29b	COL5A1	collagen type V alpha 1 chain [Source:VGNC Symbol;Acc:VGNC:86877]	-1.91	0.0807
ssc-miR-29b	COL5A2	collagen type V alpha 2 chain [Source:VGNC Symbol;Acc:VGNC:95981]	-1.91	0.0807
ssc-miR-29b	COL5A3	collagen type V alpha 3 chain [Source:VGNC Symbol;Acc:VGNC:86878]	-1.91	0.0807
ssc-miR-29b	COL6A2	collagen type VI alpha 2 chain [Source:HGNC Symbol;Acc:HGNC:2212]	-1.91	0.0807
ssc-miR-29b	COL6A3	collagen type VI alpha 3 chain [Source:VGNC Symbol;Acc:VGNC:95982]	-1.91	0.0807
ssc-miR-29b	COL6A6	collagen type VI alpha 6 chain [Source:VGNC Symbol;Acc:VGNC:108644]	-1.91	0.0807
ssc-miR-29b	COL7A1	hypothetical gene	-1.91	0.0807
ssc-miR-29b	COL8A1	collagen type VIII alpha 1 chain [Source:VGNC Symbol;Acc:VGNC:86880]	-1.91	0.0807
ssc-miR-29b	COL9A1	collagen type IX alpha 1 chain [Source:HGNC Symbol;Acc:HGNC:2217]	-1.91	0.0807
ssc-miR-29b	COMMD2	COMM domain containing 2 [Source:VGNC Symbol;Acc:VGNC:86887]	-1.91	0.0807
ssc-miR-29b	COX10	cytochrome c oxidase assembly factor heme A:farnesyltransferase COX10 [Source:VGNC Symbol;Acc:VGNC:96724]	-1.91	0.0807
ssc-miR-29b	CPEB3	cytoplasmic polyadenylation element binding protein 3 [Source:VGNC Symbol;Acc:VGNC:86938]	-1.91	0.0807
ssc-miR-29b	CPM	carboxypeptidase M [Source:VGNC Symbol;Acc:VGNC:86947]	-1.91	0.0807
ssc-miR-29b	CPS1	carbamoyl-phosphate synthase 1 [Source:VGNC Symbol;Acc:VGNC:95991]	-1.91	0.0807
ssc-miR-29b	CPSF7	cleavage and polyadenylation specific factor 7 [Source:VGNC Symbol;Acc:VGNC:86963]	-1.91	0.0807
ssc-miR-29b	CREB5	cAMP responsive element binding protein 5 [Source:VGNC Symbol;Acc:VGNC:86984]	-1.91	0.0807
ssc-miR-29b	CRELD1	cysteine rich with EGF like domains 1 [Source:VGNC Symbol;Acc:VGNC:86991]	-1.91	0.0807
ssc-miR-29b	CRISPLD1	cysteine rich secretory protein LCCL domain containing 1 [Source:VGNC Symbol;Acc:VGNC:97945]	-1.91	0.0807
ssc-miR-29b	CSE1L	chromosome segregation 1 like [Source:VGNC Symbol;Acc:VGNC:96039]	-1.91	0.0807
ssc-miR-29b	CSGALNACT2	chondroitin sulfate N-acetylgalactosaminyltransferase 2 [Source:VGNC Symbol;Acc:VGNC:87036]	-1.91	0.0807
ssc-miR-29b	CSPG4	chondroitin sulfate proteoglycan 4 [Source:VGNC Symbol;Acc:VGNC:87043]	-1.91	0.0807
ssc-miR-29b	CSRNP2	cysteine and serine rich nuclear protein 2 [Source:VGNC Symbol;Acc:VGNC:87047]	-1.91	0.0807
ssc-miR-29b	CTC1	CST telomere replication complex component 1 [Source:VGNC Symbol;Acc:VGNC:87056]	-1.91	0.0807
ssc-miR-29b	CTDSPL2	CTD small phosphatase like 2 [Source:VGNC Symbol;Acc:VGNC:87060]	-1.91	0.0807
ssc-miR-29b	CTNNBIP1	catenin beta interacting protein 1 [Source:VGNC Symbol;Acc:VGNC:87066]	-1.91	0.0807
ssc-miR-29b	CTNND1	catenin delta 1 [Source:VGNC Symbol;Acc:VGNC:87067]	-1.91	0.0807
ssc-miR-29b	CTPS1	CTP synthase 1 [Source:VGNC Symbol;Acc:VGNC:87070]	-1.91	0.0807
ssc-miR-29b	CTSF	cathepsin F [Source:VGNC Symbol;Acc:VGNC:87076]	-1.91	0.0807
ssc-miR-29b	CUEDC1	CUE domain containing 1 [Source:VGNC Symbol;Acc:VGNC:87085]	-1.91	0.0807
ssc-miR-29b	CX3CL1	C-X3-C motif chemokine ligand 1 [Source:HGNC Symbol;Acc:HGNC:10647]	-1.91	0.0807
ssc-miR-29b	CYB561D1	cytochrome b561 family member D1 [Source:VGNC Symbol;Acc:VGNC:87121]	-1.91	0.0807
ssc-miR-29b	CYCS	cytochrome c, somatic [Source:NCBI gene (formerly Entrezgene);Acc:100170131]	-1.91	0.0807
ssc-miR-29b	CYTH3	cytohesin 3 [Source:VGNC Symbol;Acc:VGNC:87136]	-1.91	0.0807
ssc-miR-29b	DAAM1	dishevelled associated activator of morphosis 1 [Source:VGNC Symbol;Acc:VGNC:87141]	-1.91	0.0807
ssc-miR-29b	DAAM2	dishevelled associated activator of morphosis 2 [Source:VGNC Symbol;Acc:VGNC:87142]	-1.91	0.0807
ssc-miR-29b	DAB1	DAB adaptor protein 1 [Source:VGNC Symbol;Acc:VGNC:87143]	-1.91	0.0807
ssc-miR-29b	DAB2IP	DAB2 interacting protein [Source:VGNC Symbol;Acc:VGNC:87145]	-1.91	0.0807
ssc-miR-29b	DCAF12	DDB1 and CUL4 associated factor 12 [Source:VGNC Symbol;Acc:VGNC:95927]	-1.91	0.0807
ssc-miR-29b	DCAF12L1	hypothetical gene	-1.91	0.0807
ssc-miR-29b	DCAF7	DDB1 and CUL4 associated factor 7 [Source:VGNC Symbol;Acc:VGNC:87178]	-1.91	0.0807
ssc-miR-29b	DCAKD	dephospho-CoA kinase domain containing [Source:VGNC Symbol;Acc:VGNC:97958]	-1.91	0.0807
ssc-miR-29b	DCLK3	doublecortin like kinase 3 [Source:VGNC Symbol;Acc:VGNC:87184]	-1.91	0.0807
ssc-miR-29b	DCP2	decapping mRNA 2 [Source:VGNC Symbol;Acc:VGNC:87188]	-1.91	0.0807
ssc-miR-29b	DCUN1D4	defective in cullin neddylation 1 domain containing 4 [Source:VGNC Symbol;Acc:VGNC:87197]	-1.91	0.0807
ssc-miR-29b	DCUN1D5	defective in cullin neddylation 1 domain containing 5 [Source:VGNC Symbol;Acc:VGNC:87198]	-1.91	0.0807

ssc-miR-29b	DCX	doublecortin [Source:HGNC Symbol;Acc:HGNC:2714]	-1.91	0.0807
ssc-miR-29b	DDI2	hypothetical gene	-1.91	0.0807
ssc-miR-29b	DDX11	DEAD/H-box helicase 11 [Source:HGNC Symbol;Acc:HGNC:2736]	-1.91	0.0807
ssc-miR-29b	DDX3X	DEAD-box helicase 3 X-linked [Source:NCBI gene (formerly Entrezgene);Acc:100515940]	-1.91	0.0807
ssc-miR-29b	DDX3Y	hypothetical gene	-1.91	0.0807
ssc-miR-29b	DDX46	DEAD-box helicase 46 [Source:VGNC Symbol;Acc:VGNC:87223]	-1.91	0.0807
ssc-miR-29b	DDX6	DEAD-box helicase 6 [Source:VGNC Symbol;Acc:VGNC:87233]	-1.91	0.0807
ssc-miR-29b	DEDD	death effector domain containing [Source:VGNC Symbol;Acc:VGNC:98777]	-1.91	0.0807
ssc-miR-29b	DENND2C	DENN domain containing 2C [Source:HGNC Symbol;Acc:HGNC:24748]	-1.91	0.0807
ssc-miR-29b	DENND5B	DENN domain containing 5B [Source:VGNC Symbol;Acc:VGNC:87255]	-1.91	0.0807
ssc-miR-29b	DENND6A	DENN domain containing 6A [Source:VGNC Symbol;Acc:VGNC:87256]	-1.91	0.0807
ssc-miR-29b	DENR	density regulated re-initiation and release factor [Source:VGNC Symbol;Acc:VGNC:97963]	-1.91	0.0807
ssc-miR-29b	DGKD	diacylglycerol kinase delta [Source:VGNC Symbol;Acc:VGNC:96180]	-1.91	0.0807
ssc-miR-29b	DGKH	diacylglycerol kinase eta [Source:VGNC Symbol;Acc:VGNC:87273]	-1.91	0.0807
ssc-miR-29b	DHTKD1	dehydrogenase E1 and transketolase domain containing 1 [Source:VGNC Symbol;Acc:VGNC:97966]	-1.91	0.0807
ssc-miR-29b	DIABLO	diablo IAP-binding mitochondrial protein [Source:VGNC Symbol;Acc:VGNC:101487]	-1.91	0.0807
ssc-miR-29b	DIAPH2	diaphanous related formin 2 [Source:VGNC Symbol;Acc:VGNC:87300]	-1.91	0.0807
ssc-miR-29b	DICER1	dicer 1, ribonuclease III [Source:VGNC Symbol;Acc:VGNC:87302]	-1.91	0.0807
ssc-miR-29b	DIO2	iodothyronine deiodinase 2 [Source:VGNC Symbol;Acc:VGNC:103935]	-1.91	0.0807
ssc-miR-29b	DIP2B	disco interacting B [Source:VGNC Symbol;Acc:VGNC:87306]	-1.91	0.0807
ssc-miR-29b	DIP2C	disco interacting C [Source:VGNC Symbol;Acc:VGNC:96201]	-1.91	0.0807
ssc-miR-29b	DIXDC1	hypothetical gene	-1.91	0.0807
ssc-miR-29b	DLG2	discs large MAGUK scaffold protein 2 [Source:VGNC Symbol;Acc:VGNC:108581]	-1.91	0.0807
ssc-miR-29b	DLGAP2	DLG associated protein 2 [Source:VGNC Symbol;Acc:VGNC:99711]	-1.91	0.0807
ssc-miR-29b	DMBX1	diencephalon/mesencephalon homeobox 1 [Source:VGNC Symbol;Acc:VGNC:87345]	-1.91	0.0807
ssc-miR-29b	DNAJB1	DnaJ heat shock protein family (Hsp40) member B1 [Source:VGNC Symbol;Acc:VGNC:96614]	-1.91	0.0807
ssc-miR-29b	DNAJB11	DnaJ heat shock protein family (Hsp40) member B11 [Source:VGNC Symbol;Acc:VGNC:108648]	-1.91	0.0807
ssc-miR-29b	DNAJB14	DnaJ heat shock protein family (Hsp40) member B14 [Source:VGNC Symbol;Acc:VGNC:98919]	-1.91	0.0807
ssc-miR-29b	DNAL1	dynein axonemal light chain 1 [Source:VGNC Symbol;Acc:VGNC:87373]	-1.91	0.0807
ssc-miR-29b	DNM3	dynamin 3 [Source:VGNC Symbol;Acc:VGNC:87382]	-1.91	0.0807
ssc-miR-29b	DNMT3A	DNA methyltransferase 3 alpha [Source:VGNC Symbol;Acc:VGNC:87384]	-1.91	0.0807
ssc-miR-29b	DNMT3B	DNA methyltransferase 3 beta [Source:VGNC Symbol;Acc:VGNC:96228]	-1.91	0.0807
ssc-miR-29b	DOLPP1	dolichyldiphosphatase 1 [Source:VGNC Symbol;Acc:VGNC:87407]	-1.91	0.0807
ssc-miR-29b	DOT1L	DOT1 like histone lysine methyltransferase [Source:VGNC Symbol;Acc:VGNC:87410]	-1.91	0.0807
ssc-miR-29b	DPP3	dipeptidyl peptidase 3 [Source:VGNC Symbol;Acc:VGNC:87420]	-1.91	0.0807
ssc-miR-29b	DPP4	dipeptidyl peptidase 4 [Source:VGNC Symbol;Acc:VGNC:96236]	-1.91	0.0807
ssc-miR-29b	DPYSL2	dihydropyrimidinase like 2 [Source:VGNC Symbol;Acc:VGNC:87430]	-1.91	0.0807
ssc-miR-29b	DPYSL3	dihydropyrimidinase like 3 [Source:VGNC Symbol;Acc:VGNC:87431]	-1.91	0.0807
ssc-miR-29b	DPYSL5	dihydropyrimidinase like 5 [Source:VGNC Symbol;Acc:VGNC:87433]	-1.91	0.0807
ssc-miR-29b	DRP2	dystrophin related protein 2 [Source:VGNC Symbol;Acc:VGNC:87451]	-1.91	0.0807
ssc-miR-29b	DSC2	desmocollin 2 [Source:HGNC Symbol;Acc:HGNC:3036]	-1.91	0.0807
ssc-miR-29b	DSG3	desmoglein 3 [Source:VGNC Symbol;Acc:VGNC:87457]	-1.91	0.0807
ssc-miR-29b	DTWD2	DTW domain containing 2 [Source:VGNC Symbol;Acc:VGNC:87465]	-1.91	0.0807
ssc-miR-29b	DTX4	deltex E3 ubiquitin ligase 4 [Source:VGNC Symbol;Acc:VGNC:87470]	-1.91	0.0807
ssc-miR-29b	DUSP2	dual specificity phosphatase 2 [Source:VGNC Symbol;Acc:VGNC:87481]	-1.91	0.0807
ssc-miR-29b	DUSP22	dual specificity phosphatase 22 [Source:VGNC Symbol;Acc:VGNC:87482]	-1.91	0.0807
ssc-miR-29b	DVL3	dishevelled segment polarity protein 3 [Source:VGNC Symbol;Acc:VGNC:87492]	-1.91	0.0807
ssc-miR-29b	DYNLT1	dynein light chain Tctex-type 1 [Source:VGNC Symbol;Acc:VGNC:87503]	-1.91	0.0807
ssc-miR-29b	E2F7	E2F transcription factor 7 [Source:VGNC Symbol;Acc:VGNC:87518]	-1.91	0.0807
ssc-miR-29b	EDARADD	EDAR associated death domain [Source:VGNC Symbol;Acc:VGNC:87542]	-1.91	0.0807

ssc-miR-29b	EDC3	enhancer of mRNA decapping 3 [Source:VGNC Symbol;Acc:VGNC:87543]	-1.91	0.0807
ssc-miR-29b	EED	embryonic ectoderm development [Source:VGNC Symbol;Acc:VGNC:87552]	-1.91	0.0807
ssc-miR-29b	EFNA2	ephrin A2 [Source:VGNC Symbol;Acc:VGNC:87573]	-1.91	0.0807
ssc-miR-29b	EFNA5	ephrin A5 [Source:VGNC Symbol;Acc:VGNC:87575]	-1.91	0.0807
ssc-miR-29b	EHD1	EH domain containing 1 [Source:VGNC Symbol;Acc:VGNC:87595]	-1.91	0.0807
ssc-miR-29b	EHD2	EH domain containing 2 [Source:VGNC Symbol;Acc:VGNC:87596]	-1.91	0.0807
ssc-miR-29b	EIF2S1	eukaryotic translation initiation factor 2 subunit alpha [Source:VGNC Symbol;Acc:VGNC:87614]	-1.91	0.0807
ssc-miR-29b	EIF2S2	eukaryotic translation initiation factor 2 subunit beta [Source:VGNC Symbol;Acc:VGNC:96265]	-1.91	0.0807
ssc-miR-29b	EIF3J	eukaryotic translation initiation factor 3 subunit J [Source:VGNC Symbol;Acc:VGNC:87621]	-1.91	0.0807
ssc-miR-29b	EIF4E2	eukaryotic translation initiation factor 4E family member 2 [Source:VGNC Symbol;Acc:VGNC:96269]	-1.91	0.0807
ssc-miR-29b	EIF4E3	eukaryotic translation initiation factor 4E family member 3 [Source:VGNC Symbol;Acc:VGNC:87628]	-1.91	0.0807
ssc-miR-29b	ELAVL4	ELAV like RNA binding protein 4 [Source:VGNC Symbol;Acc:VGNC:97047]	-1.91	0.0807
ssc-miR-29b	ELF2	E74 like ETS transcription factor 2 [Source:VGNC Symbol;Acc:VGNC:87642]	-1.91	0.0807
ssc-miR-29b	ELMO2	engulfment and cell motility 2 [Source:VGNC Symbol;Acc:VGNC:95837]	-1.91	0.0807
ssc-miR-29b	ELMSAN1	hypothetical gene	-1.91	0.0807
ssc-miR-29b	ELN	elastin [Source:VGNC Symbol;Acc:VGNC:87655]	-1.91	0.0807
ssc-miR-29b	ELOVL4	ELOVL fatty acid elongase 4 [Source:VGNC Symbol;Acc:VGNC:87660]	-1.91	0.0807
ssc-miR-29b	EMB	embigin [Source:VGNC Symbol;Acc:VGNC:87668]	-1.91	0.0807
ssc-miR-29b	EMID1	EMI domain containing 1 [Source:VGNC Symbol;Acc:VGNC:87679]	-1.91	0.0807
ssc-miR-29b	EML4	EMAP like 4 [Source:VGNC Symbol;Acc:VGNC:87685]	-1.91	0.0807
ssc-miR-29b	EML5	hypothetical gene	-1.91	0.0807
ssc-miR-29b	EML6	EMAP like 6 [Source:VGNC Symbol;Acc:VGNC:87687]	-1.91	0.0807
ssc-miR-29b	EMP1	epithelial membrane protein 1 [Source:VGNC Symbol;Acc:VGNC:87688]	-1.91	0.0807
ssc-miR-29b	EMP2	epithelial membrane protein 2 [Source:VGNC Symbol;Acc:VGNC:87689]	-1.91	0.0807
ssc-miR-29b	EN1	engrailed homeobox 1 [Source:VGNC Symbol;Acc:VGNC:96273]	-1.91	0.0807
ssc-miR-29b	ENAH	ENAH actin regulator [Source:VGNC Symbol;Acc:VGNC:108271]	-1.91	0.0807
ssc-miR-29b	ENHO	energy homeostasis associated [Source:VGNC Symbol;Acc:VGNC:96058]	-1.91	0.0807
ssc-miR-29b	ENPP2	ectonucleotide pyrophosphatase/phosphodiesterase 2 [Source:VGNC Symbol;Acc:VGNC:87709]	-1.91	0.0807
ssc-miR-29b	ENPP3	ectonucleotide pyrophosphatase/phosphodiesterase 3 [Source:VGNC Symbol;Acc:VGNC:87710]	-1.91	0.0807
ssc-miR-29b	ENTPD7	ectonucleoside triphosphate diphosphohydrolase 7 [Source:VGNC Symbol;Acc:VGNC:87716]	-1.91	0.0807
ssc-miR-29b	EOMES	eomesodermin [Source:VGNC Symbol;Acc:VGNC:87719]	-1.91	0.0807
ssc-miR-29b	EPB41L1	hypothetical gene	-1.91	0.0807
ssc-miR-29b	EPB41L4B	erythrocyte membrane protein band 4.1 like 4B [Source:VGNC Symbol;Acc:VGNC:103088]	-1.91	0.0807
ssc-miR-29b	EPC1	enhancer of polycomb homolog 1 [Source:VGNC Symbol;Acc:VGNC:95923]	-1.91	0.0807
ssc-miR-29b	EPHA1	EPH receptor A1 [Source:VGNC Symbol;Acc:VGNC:87729]	-1.91	0.0807
ssc-miR-29b	EPHB3	EPH receptor B3 [Source:VGNC Symbol;Acc:VGNC:87738]	-1.91	0.0807
ssc-miR-29b	EPOR	erythropoietin receptor [Source:VGNC Symbol;Acc:VGNC:87746]	-1.91	0.0807
ssc-miR-29b	EPS15	epidermal growth factor receptor pathway substrate 15 [Source:VGNC Symbol;Acc:VGNC:87747]	-1.91	0.0807
ssc-miR-29b	ERBB2IP	hypothetical gene	-1.91	0.0807
ssc-miR-29b	ERC1	ELKS/RAB6-interacting/CAST family member 1 [Source:VGNC Symbol;Acc:VGNC:87761]	-1.91	0.0807
ssc-miR-29b	ERCC6	hypothetical gene	-1.91	0.0807
ssc-miR-29b	ERLIN2	ER lipid raft associated 2 [Source:VGNC Symbol;Acc:VGNC:95586]	-1.91	0.0807
ssc-miR-29b	ERP29	endoplasmic reticulum protein 29 [Source:VGNC Symbol;Acc:VGNC:87782]	-1.91	0.0807
ssc-miR-29b	ERP44	endoplasmic reticulum protein 44 [Source:VGNC Symbol;Acc:VGNC:87783]	-1.91	0.0807
ssc-miR-29b	ETV4	ETS variant transcription factor 4 [Source:VGNC Symbol;Acc:VGNC:87813]	-1.91	0.0807
ssc-miR-29b	ETV6	ETS variant transcription factor 6 [Source:VGNC Symbol;Acc:VGNC:87815]	-1.91	0.0807
ssc-miR-29b	EXOC7	exocyst complex component 7 [Source:VGNC Symbol;Acc:VGNC:87833]	-1.91	0.0807
ssc-miR-29b	EXT1	exostosin glycosyltransferase 1 [Source:VGNC Symbol;Acc:VGNC:87846]	-1.91	0.0807
ssc-miR-29b	F11R	F11 receptor [Source:VGNC Symbol;Acc:VGNC:87857]	-1.91	0.0807
ssc-miR-29b	FAF2	Fas associated factor family member 2 [Source:VGNC Symbol;Acc:VGNC:87880]	-1.91	0.0807

ssc-miR-29b	FAM101B	hypothetical gene	-1.91	0.0807
ssc-miR-29b	FAM105B	hypothetical gene	-1.91	0.0807
ssc-miR-29b	FAM109B	hypothetical gene	-1.91	0.0807
ssc-miR-29b	FAM124A	family with sequence similarity 124 member A [Source:VGNC Symbol;Acc:VGNC:87898]	-1.91	0.0807
ssc-miR-29b	FAM126B	family with sequence similarity 126 member B [Source:HGNC Symbol;Acc:HGNC:28593]	-1.91	0.0807
ssc-miR-29b	FAM131B	family with sequence similarity 131 member B [Source:VGNC Symbol;Acc:VGNC:87901]	-1.91	0.0807
ssc-miR-29b	FAM134C	hypothetical gene	-1.91	0.0807
ssc-miR-29b	FAM136A	hypothetical gene	-1.91	0.0807
ssc-miR-29b	FAM13B	family with sequence similarity 13 member B [Source:VGNC Symbol;Acc:VGNC:87906]	-1.91	0.0807
ssc-miR-29b	FAM167A	family with sequence similarity 167 member A [Source:VGNC Symbol;Acc:VGNC:87924]	-1.91	0.0807
ssc-miR-29b	FAM168B	family with sequence similarity 168 member B [Source:VGNC Symbol;Acc:VGNC:95462]	-1.91	0.0807
ssc-miR-29b	FAM175B	hypothetical gene	-1.91	0.0807
ssc-miR-29b	FAM184B	family with sequence similarity 184 member B [Source:VGNC Symbol;Acc:VGNC:87938]	-1.91	0.0807
ssc-miR-29b	FAM193B	family with sequence similarity 193 member B [Source:VGNC Symbol;Acc:VGNC:87947]	-1.91	0.0807
ssc-miR-29b	FAM219B	family with sequence similarity 219 member B [Source:VGNC Symbol;Acc:VGNC:87956]	-1.91	0.0807
ssc-miR-29b	FAM3C	FAM3 metabolism regulating signaling molecule C [Source:VGNC Symbol;Acc:VGNC:87969]	-1.91	0.0807
ssc-miR-29b	FAM53C	family with sequence similarity 53 member C [Source:VGNC Symbol;Acc:VGNC:87975]	-1.91	0.0807
ssc-miR-29b	FAM57B	hypothetical gene	-1.91	0.0807
ssc-miR-29b	FAM65B	hypothetical gene	-1.91	0.0807
ssc-miR-29b	FAM73B	hypothetical gene	-1.91	0.0807
ssc-miR-29b	FAM76B	family with sequence similarity 76 member B [Source:VGNC Symbol;Acc:VGNC:87983]	-1.91	0.0807
ssc-miR-29b	FAM83F	family with sequence similarity 83 member F [Source:VGNC Symbol;Acc:VGNC:87991]	-1.91	0.0807
ssc-miR-29b	FAM84B	hypothetical gene	-1.91	0.0807
ssc-miR-29b	FASLG	Fas ligand [Source:VGNC Symbol;Acc:VGNC:103942]	-1.91	0.0807
ssc-miR-29b	FASTK	Fas activated serine/threonine kinase [Source:VGNC Symbol;Acc:VGNC:88017]	-1.91	0.0807
ssc-miR-29b	FAXC	hypothetical gene	-1.91	0.0807
ssc-miR-29b	FBN1	fibrillin 1 [Source:VGNC Symbol;Acc:VGNC:103090]	-1.91	0.0807
ssc-miR-29b	FBN2	fibrillin 2 [Source:VGNC Symbol;Acc:VGNC:88025]	-1.91	0.0807
ssc-miR-29b	FBRS	fibrosin [Source:VGNC Symbol;Acc:VGNC:88027]	-1.91	0.0807
ssc-miR-29b	FBXL18	F-box and leucine rich repeat protein 18 [Source:VGNC Symbol;Acc:VGNC:88029]	-1.91	0.0807
ssc-miR-29b	FBXL20	F-box and leucine rich repeat protein 20 [Source:VGNC Symbol;Acc:VGNC:98006]	-1.91	0.0807
ssc-miR-29b	FBXO28	F-box protein 28 [Source:HGNC Symbol;Acc:HGNC:29046]	-1.91	0.0807
ssc-miR-29b	FBXO42	F-box protein 42 [Source:VGNC Symbol;Acc:VGNC:88047]	-1.91	0.0807
ssc-miR-29b	FBXW2	hypothetical gene	-1.91	0.0807
ssc-miR-29b	FBXW7	F-box and WD repeat domain containing 7 [Source:VGNC Symbol;Acc:VGNC:98925]	-1.91	0.0807
ssc-miR-29b	FBXW9	F-box and WD repeat domain containing 9 [Source:VGNC Symbol;Acc:VGNC:88057]	-1.91	0.0807
ssc-miR-29b	FCHO1	FCH and mu domain containing endocytic adaptor 1 [Source:VGNC Symbol;Acc:VGNC:88065]	-1.91	0.0807
ssc-miR-29b	FEM1A	fem-1 homolog A [Source:VGNC Symbol;Acc:VGNC:88082]	-1.91	0.0807
ssc-miR-29b	FEM1B	fem-1 homolog B [Source:VGNC Symbol;Acc:VGNC:88083]	-1.91	0.0807
ssc-miR-29b	FERMT2	FERM domain containing kindlin 2 [Source:VGNC Symbol;Acc:VGNC:88088]	-1.91	0.0807
ssc-miR-29b	FGD4	FYVE, RhoGEF and PH domain containing 4 [Source:VGNC Symbol;Acc:VGNC:88097]	-1.91	0.0807
ssc-miR-29b	FGF12	fibroblast growth factor 12 [Source:VGNC Symbol;Acc:VGNC:88102]	-1.91	0.0807
ssc-miR-29b	FIGN	fidgetin, microtubule severing factor [Source:VGNC Symbol;Acc:VGNC:95580]	-1.91	0.0807
ssc-miR-29b	FKBP14	FKBP prolyl isomerase 14 [Source:VGNC Symbol;Acc:VGNC:88144]	-1.91	0.0807
ssc-miR-29b	FKBP4	FKBP prolyl isomerase 4 [Source:VGNC Symbol;Acc:VGNC:88147]	-1.91	0.0807
ssc-miR-29b	FMN1	formin 1 [Source:HGNC Symbol;Acc:HGNC:3768]	-1.91	0.0807
ssc-miR-29b	FMNL3	formin like 3 [Source:VGNC Symbol;Acc:VGNC:88169]	-1.91	0.0807
ssc-miR-29b	FNDC5	fibronectin type III domain containing 5 [Source:VGNC Symbol;Acc:VGNC:88185]	-1.91	0.0807
ssc-miR-29b	FOS	Fos proto-onco, AP-1 transcription factor subunit [Source:NCBI gene (formerly Entrezgene);Acc:100144486]	-1.91	0.0807
ssc-miR-29b	FOSL1	FOS like 1, AP-1 transcription factor subunit [Source:VGNC Symbol;Acc:VGNC:88191]	-1.91	0.0807

ssc-miR-29b	FOXJ2	forkhead box J2 [Source:VGNC Symbol;Acc:VGNC:88212]	-1.91	0.0807
ssc-miR-29b	FOXN2	forkhead box N2 [Source:VGNC Symbol;Acc:VGNC:88219]	-1.91	0.0807
ssc-miR-29b	FOXN3	forkhead box N3 [Source:VGNC Symbol;Acc:VGNC:88220]	-1.91	0.0807
ssc-miR-29b	FOXO3	forkhead box O3 [Source:VGNC Symbol;Acc:VGNC:99715]	-1.91	0.0807
ssc-miR-29b	FOXP1	forkhead box P1 [Source:VGNC Symbol;Acc:VGNC:88222]	-1.91	0.0807
ssc-miR-29b	FRAS1	Fraser extracellular matrix complex subunit 1 [Source:VGNC Symbol;Acc:VGNC:88232]	-1.91	0.0807
ssc-miR-29b	FRAT2	FRAT regulator of WNT signaling pathway 2 [Source:VGNC Symbol;Acc:VGNC:107394]	-1.91	0.0807
ssc-miR-29b	FREM1	FRAS1 related extracellular matrix 1 [Source:VGNC Symbol;Acc:VGNC:88233]	-1.91	0.0807
ssc-miR-29b	FREM2	FRAS1 related extracellular matrix 2 [Source:VGNC Symbol;Acc:VGNC:88234]	-1.91	0.0807
ssc-miR-29b	FRMD4A	FERM domain containing 4A [Source:VGNC Symbol;Acc:VGNC:96088]	-1.91	0.0807
ssc-miR-29b	FRS2	fibroblast growth factor receptor substrate 2 [Source:VGNC Symbol;Acc:VGNC:88246]	-1.91	0.0807
ssc-miR-29b	FSTL1	follistatin like 1 [Source:VGNC Symbol;Acc:VGNC:88255]	-1.91	0.0807
ssc-miR-29b	FTCD	formimidoyltransferase cyclodeaminase [Source:NCBI gene (formerly Entrezgene);Acc:397517]	-1.91	0.0807
ssc-miR-29b	FUT11	fucosyltransferase 11 [Source:VGNC Symbol;Acc:VGNC:88269]	-1.91	0.0807
ssc-miR-29b	FZD4	frizzled class receptor 4 [Source:VGNC Symbol;Acc:VGNC:88282]	-1.91	0.0807
ssc-miR-29b	FZD5	frizzled class receptor 5 [Source:VGNC Symbol;Acc:VGNC:96309]	-1.91	0.0807
ssc-miR-29b	G3BP1	G3BP stress granule assembly factor 1 [Source:VGNC Symbol;Acc:VGNC:88288]	-1.91	0.0807
ssc-miR-29b	G6PC	hypothetical gene	-1.91	0.0807
ssc-miR-29b	GAB1	GRB2 associated binding protein 1 [Source:VGNC Symbol;Acc:VGNC:88294]	-1.91	0.0807
ssc-miR-29b	GAB2	GRB2 associated binding protein 2 [Source:VGNC Symbol;Acc:VGNC:108586]	-1.91	0.0807
ssc-miR-29b	GABPB2	GA binding protein transcription factor subunit beta 2 [Source:VGNC Symbol;Acc:VGNC:98796]	-1.91	0.0807
ssc-miR-29b	GABRB1	gamma-aminobutyric acid type A receptor subunit beta1 [Source:VGNC Symbol;Acc:VGNC:88306]	-1.91	0.0807
ssc-miR-29b	GAN	gigaxonin [Source:VGNC Symbol;Acc:VGNC:88343]	-1.91	0.0807
ssc-miR-29b	GAS2L3	growth arrest specific 2 like 3 [Source:VGNC Symbol;Acc:VGNC:88357]	-1.91	0.0807
ssc-miR-29b	GAS7	growth arrest specific 7 [Source:VGNC Symbol;Acc:VGNC:88359]	-1.91	0.0807
ssc-miR-29b	GATC	glutamyl-tRNA amidotransferase subunit C [Source:VGNC Symbol;Acc:VGNC:103948]	-1.91	0.0807
ssc-miR-29b	GEMIN2	gem nuclear organelle associated protein 2 [Source:VGNC Symbol;Acc:VGNC:88411]	-1.91	0.0807
ssc-miR-29b	GGA1	golgi associated, gamma adaptin ear containing, ARF binding protein 1 [Source:VGNC Symbol;Acc:VGNC:88431]	-1.91	0.0807
ssc-miR-29b	GGCT	gamma-glutamylcyclotransferase [Source:HGNC Symbol;Acc:HGNC:21705]	-1.91	0.0807
ssc-miR-29b	GID4	GID complex subunit 4 homolog [Source:VGNC Symbol;Acc:VGNC:98996]	-1.91	0.0807
ssc-miR-29b	GID8	GID complex subunit 8 homolog [Source:VGNC Symbol;Acc:VGNC:95674]	-1.91	0.0807
ssc-miR-29b	GJD2	gap junction protein delta 2 [Source:VGNC Symbol;Acc:VGNC:88471]	-1.91	0.0807
ssc-miR-29b	GLB1L	galactosidase beta 1 like [Source:VGNC Symbol;Acc:VGNC:96165]	-1.91	0.0807
ssc-miR-29b	GLIS2	GLIS family zinc finger 2 [Source:VGNC Symbol;Acc:VGNC:88484]	-1.91	0.0807
ssc-miR-29b	GLIS3	GLIS family zinc finger 3 [Source:VGNC Symbol;Acc:VGNC:88485]	-1.91	0.0807
ssc-miR-29b	GLYCTK	glycerate kinase [Source:VGNC Symbol;Acc:VGNC:88504]	-1.91	0.0807
ssc-miR-29b	GMEB1	glucocorticoid modulatory element binding protein 1 [Source:VGNC Symbol;Acc:VGNC:88508]	-1.91	0.0807
ssc-miR-29b	GMFB	glia maturation factor beta [Source:VGNC Symbol;Acc:VGNC:88509]	-1.91	0.0807
ssc-miR-29b	GMPPB	GDP-mannose pyrophosphorylase B [Source:NCBI gene (formerly Entrezgene);Acc:100513376]	-1.91	0.0807
ssc-miR-29b	GNA13	G protein subunit alpha 13 [Source:VGNC Symbol;Acc:VGNC:98997]	-1.91	0.0807
ssc-miR-29b	GNB4	G protein subunit beta 4 [Source:VGNC Symbol;Acc:VGNC:88533]	-1.91	0.0807
ssc-miR-29b	GNG12	G protein subunit gamma 12 [Source:NCBI gene (formerly Entrezgene);Acc:100270820]	-1.91	0.0807
ssc-miR-29b	GNG2	hypothetical gene	-1.91	0.0807
ssc-miR-29b	GNS	glucosamine (N-acetyl)-6-sulfatase [Source:VGNC Symbol;Acc:VGNC:103284]	-1.91	0.0807
ssc-miR-29b	GOLGA7	golgin A7 [Source:VGNC Symbol;Acc:VGNC:96059]	-1.91	0.0807
ssc-miR-29b	GOLGA7B	golgin A7 family member B [Source:VGNC Symbol;Acc:VGNC:88553]	-1.91	0.0807
ssc-miR-29b	GOPC	golgi associated PDZ and coiled-coil motif containing [Source:VGNC Symbol;Acc:VGNC:98024]	-1.91	0.0807
ssc-miR-29b	GPAM	glycerol-3-phosphate acyltransferase, mitochondrial [Source:VGNC Symbol;Acc:VGNC:88570]	-1.91	0.0807
ssc-miR-29b	GPATCH2	G-patch domain containing 2 [Source:VGNC Symbol;Acc:VGNC:96089]	-1.91	0.0807
ssc-miR-29b	GPCPD1	glycerophosphocholine phosphodiesterase 1 [Source:VGNC Symbol;Acc:VGNC:96148]	-1.91	0.0807

ssc-miR-29b	GPR111	hypothetical gene	-1.91	0.0807
ssc-miR-29b	GPR156	G protein-coupled receptor 156 [Source:VGNC Symbol;Acc:VGNC:88610]	-1.91	0.0807
ssc-miR-29b	GPR37	G protein-coupled receptor 37 [Source:VGNC Symbol;Acc:VGNC:88630]	-1.91	0.0807
ssc-miR-29b	GPR50	G protein-coupled receptor 50 [Source:VGNC Symbol;Acc:VGNC:88633]	-1.91	0.0807
ssc-miR-29b	GPR85	G protein-coupled receptor 85 [Source:VGNC Symbol;Acc:VGNC:88638]	-1.91	0.0807
ssc-miR-29b	GPRIN2	G protein regulated inducer of neurite outgrowth 2 [Source:NCBI gene (formerly Entrezgene);Acc:100568453]	-1.91	0.0807
ssc-miR-29b	GPX7	glutathione peroxidase 7 [Source:VGNC Symbol;Acc:VGNC:97069]	-1.91	0.0807
ssc-miR-29b	GRAMD1B	GRAM domain containing 1B [Source:VGNC Symbol;Acc:VGNC:88656]	-1.91	0.0807
ssc-miR-29b	GRAP2	GRB2 related adaptor protein 2 [Source:VGNC Symbol;Acc:VGNC:88660]	-1.91	0.0807
ssc-miR-29b	GRIN2B	glutamate ionotropic receptor NMDA type subunit 2B [Source:VGNC Symbol;Acc:VGNC:88684]	-1.91	0.0807
ssc-miR-29b	GRIP1	glutamate receptor interacting protein 1 [Source:VGNC Symbol;Acc:VGNC:88690]	-1.91	0.0807
ssc-miR-29b	GRM4	glutamate metabotropic receptor 4 [Source:VGNC Symbol;Acc:VGNC:98027]	-1.91	0.0807
ssc-miR-29b	GRPEL2	GrpE like 2, mitochondrial [Source:VGNC Symbol;Acc:VGNC:88708]	-1.91	0.0807
ssc-miR-29b	GSK3B	glycogen synthase kinase 3 beta [Source:VGNC Symbol;Acc:VGNC:88723]	-1.91	0.0807
ssc-miR-29b	GSTA4	glutathione S-transferase alpha 4 [Source:NCBI gene (formerly Entrezgene);Acc:100152951]	-1.91	0.0807
ssc-miR-29b	GTDC1	glycosyltransferase like domain containing 1 [Source:VGNC Symbol;Acc:VGNC:95955]	-1.91	0.0807
ssc-miR-29b	GTPBP2	GTP binding protein 2 [Source:VGNC Symbol;Acc:VGNC:88743]	-1.91	0.0807
ssc-miR-29b	GXYLT2	glucoside xylosyltransferase 2 [Source:VGNC Symbol;Acc:VGNC:88757]	-1.91	0.0807
ssc-miR-29b	H2AFY	hypothetical gene	-1.91	0.0807
ssc-miR-29b	HAPLN1	hyaluronan and proteoglycan link protein 1 [Source:VGNC Symbol;Acc:VGNC:88780]	-1.91	0.0807
ssc-miR-29b	HAPLN3	hyaluronan and proteoglycan link protein 3 [Source:VGNC Symbol;Acc:VGNC:88782]	-1.91	0.0807
ssc-miR-29b	HAS2	hyaluronan synthase 2 [Source:HGNC Symbol;Acc:HGNC:4819]	-1.91	0.0807
ssc-miR-29b	HAS3	hyaluronan synthase 3 [Source:NCBI gene (formerly Entrezgene);Acc:408053]	-1.91	0.0807
ssc-miR-29b	HBEGF	heparin binding EGF like growth factor [Source:VGNC Symbol;Acc:VGNC:88792]	-1.91	0.0807
ssc-miR-29b	HBP1	HMG-box transcription factor 1 [Source:VGNC Symbol;Acc:VGNC:88794]	-1.91	0.0807
ssc-miR-29b	HCN1	hyperpolarization activated cyclic nucleotide gated potassium channel 1 [Source:VGNC Symbol;Acc:VGNC:88802]	-1.91	0.0807
ssc-miR-29b	HDAC4	histone deacetylase 4 [Source:VGNC Symbol;Acc:VGNC:95602]	-1.91	0.0807
ssc-miR-29b	HDGF	heparin binding growth factor [Source:VGNC Symbol;Acc:VGNC:88819]	-1.91	0.0807
ssc-miR-29b	HECW1	HECT, C2 and WW domain containing E3 ubiquitin protein ligase 1 [Source:VGNC Symbol;Acc:VGNC:88835]	-1.91	0.0807
ssc-miR-29b	HELZ	helicase with zinc finger [Source:VGNC Symbol;Acc:VGNC:88840]	-1.91	0.0807
ssc-miR-29b	HEPACAM	hepatic and glial cell adhesion molecule [Source:VGNC Symbol;Acc:VGNC:88845]	-1.91	0.0807
ssc-miR-29b	HEY2	hes related family bHLH transcription factor with YRPW motif 2 [Source:VGNC Symbol;Acc:VGNC:88864]	-1.91	0.0807
ssc-miR-29b	HEYL	hes related family bHLH transcription factor with YRPW motif like [Source:VGNC Symbol;Acc:VGNC:88865]	-1.91	0.0807
ssc-miR-29b	HHAT	hedgehog acyltransferase [Source:VGNC Symbol;Acc:VGNC:88873]	-1.91	0.0807
ssc-miR-29b	HIF3A	hypoxia inducible factor 3 subunit alpha [Source:VGNC Symbol;Acc:VGNC:88881]	-1.91	0.0807
ssc-miR-29b	HIP1	huntingtin interacting protein 1 [Source:VGNC Symbol;Acc:VGNC:88885]	-1.91	0.0807
ssc-miR-29b	HIST1H2BF	hypothetical gene	-1.91	0.0807
ssc-miR-29b	HLF	HLF transcription factor, PAR bZIP family member [Source:VGNC Symbol;Acc:VGNC:88896]	-1.91	0.0807
ssc-miR-29b	HMCN1	hemicentin 1 [Source:VGNC Symbol;Acc:VGNC:108590]	-1.91	0.0807
ssc-miR-29b	HMGCR	3-hydroxy-3-methylglutaryl-CoA reductase [Source:VGNC Symbol;Acc:VGNC:88906]	-1.91	0.0807
ssc-miR-29b	HMGCS1	3-hydroxy-3-methylglutaryl-CoA synthase 1 [Source:VGNC Symbol;Acc:VGNC:88907]	-1.91	0.0807
ssc-miR-29b	HMGN3	high mobility group nucleosomal binding domain 3 [Source:VGNC Symbol;Acc:VGNC:88908]	-1.91	0.0807
ssc-miR-29b	HN1	hypothetical gene	-1.91	0.0807
ssc-miR-29b	HNF4G	hepatocyte nuclear factor 4 gamma [Source:VGNC Symbol;Acc:VGNC:88917]	-1.91	0.0807
ssc-miR-29b	HNRNPF	hypothetical gene	-1.91	0.0807
ssc-miR-29b	HNRNPUL1	heteroous nuclear ribonucleoprotein U like 1 [Source:VGNC Symbol;Acc:VGNC:88926]	-1.91	0.0807
ssc-miR-29b	HOMER1	homer scaffold protein 1 [Source:VGNC Symbol;Acc:VGNC:88928]	-1.91	0.0807
ssc-miR-29b	HOOK3	hook microtubule tethering protein 3 [Source:VGNC Symbol;Acc:VGNC:98037]	-1.91	0.0807
ssc-miR-29b	HOXC13	homeobox C13 [Source:VGNC Symbol;Acc:VGNC:88951]	-1.91	0.0807
ssc-miR-29b	HOXD12	homeobox D12 [Source:VGNC Symbol;Acc:VGNC:96353]	-1.91	0.0807

ssc-miR-29b	HPCAL4	hippocalcin like 4 [Source:VGNC Symbol;Acc:VGNC:88959]	-1.91	0.0807
ssc-miR-29b	HRK	harakiri, BCL2 interacting protein [Source:HGNC Symbol;Acc:HGNC:5185]	-1.91	0.0807
ssc-miR-29b	HS3ST3B1	heparan sulfate-glucosamine 3-sulfotransferase 3B1 [Source:VGNC Symbol;Acc:VGNC:99003]	-1.91	0.0807
ssc-miR-29b	HSPA4L	heat shock protein family A (Hsp70) member 4 like [Source:HGNC Symbol;Acc:HGNC:17041]	-1.91	0.0807
ssc-miR-29b	HTR6	hypothetical gene	-1.91	0.0807
ssc-miR-29b	HTR7	5-hydroxytryptamine receptor 7 [Source:VGNC Symbol;Acc:VGNC:89003]	-1.91	0.0807
ssc-miR-29b	IBSP	integrin binding sialoprotein [Source:VGNC Symbol;Acc:VGNC:98040]	-1.91	0.0807
ssc-miR-29b	ICOS	inducible T cell costimulator [Source:VGNC Symbol;Acc:VGNC:103966]	-1.91	0.0807
ssc-miR-29b	ID1	inhibitor of DNA binding 1, HLH protein [Source:VGNC Symbol;Acc:VGNC:96366]	-1.91	0.0807
ssc-miR-29b	IDE	insulin degrading enzyme [Source:VGNC Symbol;Acc:VGNC:89025]	-1.91	0.0807
ssc-miR-29b	IFFO1	intermediate filament family orphan 1 [Source:VGNC Symbol;Acc:VGNC:89030]	-1.91	0.0807
ssc-miR-29b	IFFO2	intermediate filament family orphan 2 [Source:VGNC Symbol;Acc:VGNC:98475]	-1.91	0.0807
ssc-miR-29b	IFI30	IFI30 lysosomal thiol reductase [Source:VGNC Symbol;Acc:VGNC:89031]	-1.91	0.0807
ssc-miR-29b	IFNG	interferon gamma [Source:VGNC Symbol;Acc:VGNC:89040]	-1.91	0.0807
ssc-miR-29b	IGF1	insulin like growth factor 1 [Source:VGNC Symbol;Acc:VGNC:98044]	-1.91	0.0807
ssc-miR-29b	IGF2BP1	insulin like growth factor 2 mRNA binding protein 1 [Source:VGNC Symbol;Acc:VGNC:99006]	-1.91	0.0807
ssc-miR-29b	IGSF1	immunoglobulin superfamily member 1 [Source:VGNC Symbol;Acc:VGNC:89062]	-1.91	0.0807
ssc-miR-29b	IL10RB	interleukin 10 receptor subunit beta [Source:NCBI gene (formerly Entrezgene);Acc:396657]	-1.91	0.0807
ssc-miR-29b	IL17RD	interleukin 17 receptor D [Source:VGNC Symbol;Acc:VGNC:89087]	-1.91	0.0807
ssc-miR-29b	IL1RAP	interleukin 1 receptor accessory protein [Source:VGNC Symbol;Acc:VGNC:98046]	-1.91	0.0807
ssc-miR-29b	IL1RAPL1	interleukin 1 receptor accessory protein like 1 [Source:VGNC Symbol;Acc:VGNC:103967]	-1.91	0.0807
ssc-miR-29b	IL5RA	interleukin 5 receptor subunit alpha [Source:VGNC Symbol;Acc:VGNC:89111]	-1.91	0.0807
ssc-miR-29b	ILDR2	immunoglobulin like domain containing receptor 2 [Source:VGNC Symbol;Acc:VGNC:89118]	-1.91	0.0807
ssc-miR-29b	IMPDH1	inosine monophosphate dehydrogenase 1 [Source:VGNC Symbol;Acc:VGNC:89126]	-1.91	0.0807
ssc-miR-29b	INA	internexin neuronal intermediate filament protein alpha [Source:VGNC Symbol;Acc:VGNC:89128]	-1.91	0.0807
ssc-miR-29b	ING2	inhibitor of growth family member 2 [Source:VGNC Symbol;Acc:VGNC:99717]	-1.91	0.0807
ssc-miR-29b	ING4	inhibitor of growth family member 4 [Source:HGNC Symbol;Acc:HGNC:19423]	-1.91	0.0807
ssc-miR-29b	INO80D	INO80 complex subunit D [Source:VGNC Symbol;Acc:VGNC:96113]	-1.91	0.0807
ssc-miR-29b	INSIG1	insulin induced 1 [Source:VGNC Symbol;Acc:VGNC:89150]	-1.91	0.0807
ssc-miR-29b	INSRR	insulin receptor related receptor [Source:VGNC Symbol;Acc:VGNC:89154]	-1.91	0.0807
ssc-miR-29b	IQCJ-SCHIP1	hypothetical gene	-1.91	0.0807
ssc-miR-29b	IREB2	iron responsive element binding protein 2 [Source:NCBI gene (formerly Entrezgene);Acc:100153993]	-1.91	0.0807
ssc-miR-29b	IRGQ	immunity related GTPase Q [Source:VGNC Symbol;Acc:VGNC:89213]	-1.91	0.0807
ssc-miR-29b	IRS1	insulin receptor substrate 1 [Source:VGNC Symbol;Acc:VGNC:96376]	-1.91	0.0807
ssc-miR-29b	ISG20L2	interferon stimulated exonuclease 20 like 2 [Source:VGNC Symbol;Acc:VGNC:89224]	-1.91	0.0807
ssc-miR-29b	ISL1	ISL LIM homeobox 1 [Source:VGNC Symbol;Acc:VGNC:89225]	-1.91	0.0807
ssc-miR-29b	ISLR2	immunoglobulin superfamily containing leucine rich repeat 2 [Source:VGNC Symbol;Acc:VGNC:89227]	-1.91	0.0807
ssc-miR-29b	ITFG3	hypothetical gene	-1.91	0.0807
ssc-miR-29b	ITGA11	integrin subunit alpha 11 [Source:VGNC Symbol;Acc:VGNC:89233]	-1.91	0.0807
ssc-miR-29b	ITGA6	integrin subunit alpha 6 [Source:VGNC Symbol;Acc:VGNC:96378]	-1.91	0.0807
ssc-miR-29b	ITGB1	integrin subunit beta 1 [Source:VGNC Symbol;Acc:VGNC:96381]	-1.91	0.0807
ssc-miR-29b	JARID2	jumonji and AT-rich interaction domain containing 2 [Source:VGNC Symbol;Acc:VGNC:89279]	-1.91	0.0807
ssc-miR-29b	JAZF1	JAZF zinc finger 1 [Source:VGNC Symbol;Acc:VGNC:89280]	-1.91	0.0807
ssc-miR-29b	JMJD1C	jumonji domain containing 1C [Source:VGNC Symbol;Acc:VGNC:89285]	-1.91	0.0807
ssc-miR-29b	JMY	junction mediating and regulatory protein, p53 cofactor [Source:VGNC Symbol;Acc:VGNC:89289]	-1.91	0.0807
ssc-miR-29b	JOSD1	Josephin domain containing 1 [Source:VGNC Symbol;Acc:VGNC:89290]	-1.91	0.0807
ssc-miR-29b	KATNBL1	katanin regulatory subunit B1 like 1 [Source:VGNC Symbol;Acc:VGNC:89313]	-1.91	0.0807
ssc-miR-29b	KBTBD8	kelch repeat and BTB domain containing 8 [Source:VGNC Symbol;Acc:VGNC:89321]	-1.91	0.0807
ssc-miR-29b	KCNC2	potassium voltage-gated channel subfamily C member 2 [Source:VGNC Symbol;Acc:VGNC:89333]	-1.91	0.0807
ssc-miR-29b	KCNC3	potassium voltage-gated channel subfamily C member 3 [Source:VGNC Symbol;Acc:VGNC:89334]	-1.91	0.0807

ssc-miR-29b	KCNIP2	potassium voltage-gated channel interacting protein 2 [Source:VGNC Symbol;Acc:VGNC:89349]	-1.91	0.0807
ssc-miR-29b	KCNJ12	hypothetical gene	-1.91	0.0807
ssc-miR-29b	KCNJ6	potassium inwardly rectifying channel subfamily J member 6 [Source:VGNC Symbol;Acc:VGNC:89360]	-1.91	0.0807
ssc-miR-29b	KCNK10	potassium two pore domain channel subfamily K member 10 [Source:VGNC Symbol;Acc:VGNC:89364]	-1.91	0.0807
ssc-miR-29b	KCNRG	potassium channel regulator [Source:VGNC Symbol;Acc:VGNC:89385]	-1.91	0.0807
ssc-miR-29b	KCNV1	potassium voltage-gated channel modifier subfamily V member 1 [Source:VGNC Symbol;Acc:VGNC:89390]	-1.91	0.0807
ssc-miR-29b	KCTD1	potassium channel tetramerization domain containing 1 [Source:VGNC Symbol;Acc:VGNC:98482]	-1.91	0.0807
ssc-miR-29b	KCTD15	potassium channel tetramerization domain containing 15 [Source:VGNC Symbol;Acc:VGNC:89394]	-1.91	0.0807
ssc-miR-29b	KCTD20	potassium channel tetramerization domain containing 20 [Source:VGNC Symbol;Acc:VGNC:89399]	-1.91	0.0807
ssc-miR-29b	KCTD21	potassium channel tetramerization domain containing 21 [Source:VGNC Symbol;Acc:VGNC:89400]	-1.91	0.0807
ssc-miR-29b	KCTD3	potassium channel tetramerization domain containing 3 [Source:VGNC Symbol;Acc:VGNC:95963]	-1.91	0.0807
ssc-miR-29b	KCTD5	hypothetical gene	-1.91	0.0807
ssc-miR-29b	KDELC1	hypothetical gene	-1.91	0.0807
ssc-miR-29b	KDM2A	lysine demethylase 2A [Source:VGNC Symbol;Acc:VGNC:89410]	-1.91	0.0807
ssc-miR-29b	KDM4A	lysine demethylase 4A [Source:VGNC Symbol;Acc:VGNC:98484]	-1.91	0.0807
ssc-miR-29b	KDM4B	lysine demethylase 4B [Source:VGNC Symbol;Acc:VGNC:89413]	-1.91	0.0807
ssc-miR-29b	KDM5A	lysine demethylase 5A [Source:VGNC Symbol;Acc:VGNC:89415]	-1.91	0.0807
ssc-miR-29b	KDM5B	lysine demethylase 5B [Source:VGNC Symbol;Acc:VGNC:95870]	-1.91	0.0807
ssc-miR-29b	KDM5C	lysine demethylase 5C [Source:NCBI gene (formerly Entrezgene);Acc:100037295]	-1.91	0.0807
ssc-miR-29b	KDM6B	lysine demethylase 6B [Source:VGNC Symbol;Acc:VGNC:89416]	-1.91	0.0807
ssc-miR-29b	KIAA0087	hypothetical gene	-1.91	0.0807
ssc-miR-29b	KIAA0355	hypothetical gene	-1.91	0.0807
ssc-miR-29b	KIAA0895	KIAA0895 [Source:VGNC Symbol;Acc:VGNC:89436]	-1.91	0.0807
ssc-miR-29b	KIAA0895L	KIAA0895 like [Source:VGNC Symbol;Acc:VGNC:89437]	-1.91	0.0807
ssc-miR-29b	KIAA0907	hypothetical gene	-1.91	0.0807
ssc-miR-29b	KIAA1024	hypothetical gene	-1.91	0.0807
ssc-miR-29b	KIAA1199	hypothetical gene	-1.91	0.0807
ssc-miR-29b	KIAA1432	hypothetical gene	-1.91	0.0807
ssc-miR-29b	KIAA1549	KIAA1549 [Source:VGNC Symbol;Acc:VGNC:99719]	-1.91	0.0807
ssc-miR-29b	KIAA1549L	KIAA1549 like [Source:VGNC Symbol;Acc:VGNC:89445]	-1.91	0.0807
ssc-miR-29b	KIAA1644	hypothetical gene	-1.91	0.0807
ssc-miR-29b	KIAA2018	hypothetical gene	-1.91	0.0807
ssc-miR-29b	KIAA2022	hypothetical gene	-1.91	0.0807
ssc-miR-29b	KIF26A	kinesin family member 26A [Source:VGNC Symbol;Acc:VGNC:89466]	-1.91	0.0807
ssc-miR-29b	KIF26B	kinesin family member 26B [Source:VGNC Symbol;Acc:VGNC:96087]	-1.91	0.0807
ssc-miR-29b	KIF3B	kinesin family member 3B [Source:VGNC Symbol;Acc:VGNC:96392]	-1.91	0.0807
ssc-miR-29b	KIF5A	kinesin family member 5A [Source:VGNC Symbol;Acc:VGNC:89472]	-1.91	0.0807
ssc-miR-29b	KIF5B	kinesin family member 5B [Source:VGNC Symbol;Acc:VGNC:96393]	-1.91	0.0807
ssc-miR-29b	KIRREL	hypothetical gene	-1.91	0.0807
ssc-miR-29b	KLF12	Kruppel like factor 12 [Source:VGNC Symbol;Acc:VGNC:89492]	-1.91	0.0807
ssc-miR-29b	KLF13	Kruppel like factor 13 [Source:VGNC Symbol;Acc:VGNC:89493]	-1.91	0.0807
ssc-miR-29b	KLF4	Kruppel like factor 4 [Source:VGNC Symbol;Acc:VGNC:98062]	-1.91	0.0807
ssc-miR-29b	KLHDC10	kelch domain containing 10 [Source:VGNC Symbol;Acc:VGNC:89502]	-1.91	0.0807
ssc-miR-29b	KLHDC3	kelch domain containing 3 [Source:VGNC Symbol;Acc:VGNC:89504]	-1.91	0.0807
ssc-miR-29b	KLHL25	kelch like family member 25 [Source:HGNC Symbol;Acc:HGNC:25732]	-1.91	0.0807
ssc-miR-29b	KLHL28	kelch like family member 28 [Source:VGNC Symbol;Acc:VGNC:89523]	-1.91	0.0807
ssc-miR-29b	KLHL42	kelch like family member 42 [Source:VGNC Symbol;Acc:VGNC:89534]	-1.91	0.0807
ssc-miR-29b	KLHL8	kelch like family member 8 [Source:VGNC Symbol;Acc:VGNC:89536]	-1.91	0.0807
ssc-miR-29b	KLHL9	kelch like family member 9 [Source:VGNC Symbol;Acc:VGNC:89537]	-1.91	0.0807
ssc-miR-29b	KNOP1	lysine rich nucleolar protein 1 [Source:VGNC Symbol;Acc:VGNC:89558]	-1.91	0.0807

ssc-miR-29b	KPNA1	karyopherin subunit alpha 1 [Source:VGNC Symbol;Acc:VGNC:89560]	-1.91	0.0807
ssc-miR-29b	KPNA4	karyopherin subunit alpha 4 [Source:VGNC Symbol;Acc:VGNC:89563]	-1.91	0.0807
ssc-miR-29b	KPNB1	karyopherin subunit beta 1 [Source:VGNC Symbol;Acc:VGNC:89567]	-1.91	0.0807
ssc-miR-29b	L3MBTL4	hypothetical gene	-1.91	0.0807
ssc-miR-29b	LAMA2	hypothetical gene	-1.91	0.0807
ssc-miR-29b	LAMC1	laminin subunit gamma 1 [Source:VGNC Symbol;Acc:VGNC:89624]	-1.91	0.0807
ssc-miR-29b	LAMTOR1	late endosomal/lysosomal adaptor, MAPK and MTOR activator 1 [Source:VGNC Symbol;Acc:VGNC:89629]	-1.91	0.0807
ssc-miR-29b	LARP4	La ribonucleoprotein 4 [Source:VGNC Symbol;Acc:VGNC:89641]	-1.91	0.0807
ssc-miR-29b	LARP4B	hypothetical gene	-1.91	0.0807
ssc-miR-29b	LASP1	LIM and SH3 protein 1 [Source:VGNC Symbol;Acc:VGNC:89646]	-1.91	0.0807
ssc-miR-29b	LDLRAD3	low density lipoprotein receptor class A domain containing 3 [Source:VGNC Symbol;Acc:VGNC:89672]	-1.91	0.0807
ssc-miR-29b	LDLRAD4	low density lipoprotein receptor class A domain containing 4 [Source:VGNC Symbol;Acc:VGNC:89673]	-1.91	0.0807
ssc-miR-29b	LDLRAP1	low density lipoprotein receptor adaptor protein 1 [Source:VGNC Symbol;Acc:VGNC:89674]	-1.91	0.0807
ssc-miR-29b	LDOC1L	hypothetical gene	-1.91	0.0807
ssc-miR-29b	LEP	leptin [Source:VGNC Symbol;Acc:VGNC:89684]	-1.91	0.0807
ssc-miR-29b	LEPRE1	hypothetical gene	-1.91	0.0807
ssc-miR-29b	LGI3	leucine rich repeat LGI family member 3 [Source:VGNC Symbol;Acc:VGNC:89700]	-1.91	0.0807
ssc-miR-29b	LGSN	lengsin, lens protein with glutamine synthetase domain [Source:HGNC Symbol;Acc:HGNC:21016]	-1.91	0.0807
ssc-miR-29b	LIF	LIF interleukin 6 family cytokine [Source:VGNC Symbol;Acc:VGNC:89719]	-1.91	0.0807
ssc-miR-29b	LIG3	DNA ligase 3 [Source:VGNC Symbol;Acc:VGNC:89721]	-1.91	0.0807
ssc-miR-29b	LIMS1	hypothetical gene	-1.91	0.0807
ssc-miR-29b	LIN7A	lin-7 homolog A, crumbs cell polarity complex component [Source:VGNC Symbol;Acc:VGNC:89730]	-1.91	0.0807
ssc-miR-29b	LMLN	leishmanolysin like peptidase [Source:VGNC Symbol;Acc:VGNC:89759]	-1.91	0.0807
ssc-miR-29b	LMTK2	lemur tyrosine kinase 2 [Source:HGNC Symbol;Acc:HGNC:17880]	-1.91	0.0807
ssc-miR-29b	LMX1A	LIM homeobox transcription factor 1 alpha [Source:VGNC Symbol;Acc:VGNC:89770]	-1.91	0.0807
ssc-miR-29b	LNPEP	leucyl and cystinyl aminopeptidase [Source:VGNC Symbol;Acc:VGNC:89772]	-1.91	0.0807
ssc-miR-29b	LOX	lysyl oxidase [Source:VGNC Symbol;Acc:VGNC:99785]	-1.91	0.0807
ssc-miR-29b	LOXL2	lysyl oxidase like 2 [Source:VGNC Symbol;Acc:VGNC:89781]	-1.91	0.0807
ssc-miR-29b	LOXL4	lysyl oxidase like 4 [Source:VGNC Symbol;Acc:VGNC:89783]	-1.91	0.0807
ssc-miR-29b	LPGAT1	lysophosphatidylglycerol acyltransferase 1 [Source:VGNC Symbol;Acc:VGNC:89792]	-1.91	0.0807
ssc-miR-29b	LPL	lipoprotein lipase [Source:VGNC Symbol;Acc:VGNC:89795]	-1.91	0.0807
ssc-miR-29b	LPPR4	hypothetical gene	-1.91	0.0807
ssc-miR-29b	LPPR5	hypothetical gene	-1.91	0.0807
ssc-miR-29b	LRP6	LDL receptor related protein 6 [Source:VGNC Symbol;Acc:VGNC:89821]	-1.91	0.0807
ssc-miR-29b	LRRC28	hypothetical gene	-1.91	0.0807
ssc-miR-29b	LRRC58	leucine rich repeat containing 58 [Source:VGNC Symbol;Acc:VGNC:98081]	-1.91	0.0807
ssc-miR-29b	LRRC59	leucine rich repeat containing 59 [Source:VGNC Symbol;Acc:VGNC:98082]	-1.91	0.0807
ssc-miR-29b	LSM11	LSM11, U7 small nuclear RNA associated [Source:VGNC Symbol;Acc:VGNC:89871]	-1.91	0.0807
ssc-miR-29b	LSM14B	LSM family member 14B [Source:VGNC Symbol;Acc:VGNC:95691]	-1.91	0.0807
ssc-miR-29b	LTBR	lymphotoxin beta receptor [Source:VGNC Symbol;Acc:VGNC:89889]	-1.91	0.0807
ssc-miR-29b	LUZP1	leucine zipper protein 1 [Source:VGNC Symbol;Acc:VGNC:89897]	-1.91	0.0807
ssc-miR-29b	LYSMD1	LysM domain containing 1 [Source:VGNC Symbol;Acc:VGNC:89917]	-1.91	0.0807
ssc-miR-29b	MAFB	MAF bZIP transcription factor B [Source:HGNC Symbol;Acc:HGNC:6408]	-1.91	0.0807
ssc-miR-29b	MAFG	MAF bZIP transcription factor G [Source:VGNC Symbol;Acc:VGNC:89948]	-1.91	0.0807
ssc-miR-29b	MAFK	MAF bZIP transcription factor K [Source:HGNC Symbol;Acc:HGNC:6782]	-1.91	0.0807
ssc-miR-29b	MANEAL	mannosidase endo-alpha like [Source:VGNC Symbol;Acc:VGNC:89972]	-1.91	0.0807
ssc-miR-29b	MAP2K6	mitogen-activated protein kinase kinase 6 [Source:VGNC Symbol;Acc:VGNC:98102]	-1.91	0.0807
ssc-miR-29b	MAP3K2	mitogen-activated protein kinase kinase kinase 2 [Source:VGNC Symbol;Acc:VGNC:98107]	-1.91	0.0807
ssc-miR-29b	MAP4K4	mitogen-activated protein kinase kinase kinase kinase 4 [Source:VGNC Symbol;Acc:VGNC:98114]	-1.91	0.0807
ssc-miR-29b	MAP6D1	MAP6 domain containing 1 [Source:VGNC Symbol;Acc:VGNC:89991]	-1.91	0.0807

ssc-miR-29b	MAPK10	mitogen-activated protein kinase 10 [Source:VGNC Symbol;Acc:VGNC:98116]	-1.91	0.0807
ssc-miR-29b	MAPK8	mitogen-activated protein kinase 8 [Source:VGNC Symbol;Acc:VGNC:90006]	-1.91	0.0807
ssc-miR-29b	MAPKBP1	mitogen-activated protein kinase binding protein 1 [Source:VGNC Symbol;Acc:VGNC:90014]	-1.91	0.0807
ssc-miR-29b	MAPRE1	microtubule associated protein RP/EB family member 1 [Source:VGNC Symbol;Acc:VGNC:96410]	-1.91	0.0807
ssc-miR-29b	MAPRE2	microtubule associated protein RP/EB family member 2 [Source:VGNC Symbol;Acc:VGNC:98117]	-1.91	0.0807
ssc-miR-29b	MARCH1	hypothetical gene	-1.91	0.0807
ssc-miR-29b	MARCH7	hypothetical gene	-1.91	0.0807
ssc-miR-29b	MARK3	microtubule affinity regulating kinase 3 [Source:VGNC Symbol;Acc:VGNC:90026]	-1.91	0.0807
ssc-miR-29b	MARVELD2	MARVEL domain containing 2 [Source:VGNC Symbol;Acc:VGNC:90029]	-1.91	0.0807
ssc-miR-29b	MAT1A	methionine adenosyltransferase 1A [Source:VGNC Symbol;Acc:VGNC:90038]	-1.91	0.0807
ssc-miR-29b	MAT2A	methionine adenosyltransferase 2A [Source:VGNC Symbol;Acc:VGNC:90039]	-1.91	0.0807
ssc-miR-29b	MAU2	MAU2 sister chromatid cohesion factor [Source:VGNC Symbol;Acc:VGNC:90045]	-1.91	0.0807
ssc-miR-29b	MAZ	MYC associated zinc finger protein [Source:VGNC Symbol;Acc:VGNC:107150]	-1.91	0.0807
ssc-miR-29b	MBD5	methyl-CpG binding domain protein 5 [Source:VGNC Symbol;Acc:VGNC:95940]	-1.91	0.0807
ssc-miR-29b	MBLAC2	metallo-beta-lactamase domain containing 2 [Source:VGNC Symbol;Acc:VGNC:90053]	-1.91	0.0807
ssc-miR-29b	MBTD1	mbt domain containing 1 [Source:VGNC Symbol;Acc:VGNC:90060]	-1.91	0.0807
ssc-miR-29b	MCL1	MCL1 apoptosis regulator, BCL2 family member [Source:VGNC Symbol;Acc:VGNC:90072]	-1.91	0.0807
ssc-miR-29b	MCMBP	minichromosome maintenance complex binding protein [Source:VGNC Symbol;Acc:VGNC:90077]	-1.91	0.0807
ssc-miR-29b	MED12L	mediator complex subunit 12L [Source:VGNC Symbol;Acc:VGNC:90105]	-1.91	0.0807
ssc-miR-29b	MED26	mediator complex subunit 26 [Source:VGNC Symbol;Acc:VGNC:100168]	-1.91	0.0807
ssc-miR-29b	MED28	mediator complex subunit 28 [Source:VGNC Symbol;Acc:VGNC:90118]	-1.91	0.0807
ssc-miR-29b	MEGF6	multiple EGF like domains 6 [Source:VGNC Symbol;Acc:VGNC:98499]	-1.91	0.0807
ssc-miR-29b	MEST	mesoderm specific transcript [Source:VGNC Symbol;Acc:VGNC:90150]	-1.91	0.0807
ssc-miR-29b	METAP2	methionyl aminopeptidase 2 [Source:VGNC Symbol;Acc:VGNC:90152]	-1.91	0.0807
ssc-miR-29b	METTL21B	hypothetical gene	-1.91	0.0807
ssc-miR-29b	MEX3B	mex-3 RNA binding family member B [Source:VGNC Symbol;Acc:VGNC:90169]	-1.91	0.0807
ssc-miR-29b	MFAP2	microfibril associated protein 2 [Source:VGNC Symbol;Acc:VGNC:90173]	-1.91	0.0807
ssc-miR-29b	MFAP3	microfibril associated protein 3 [Source:VGNC Symbol;Acc:VGNC:90174]	-1.91	0.0807
ssc-miR-29b	MGA	MAX dimerization protein MGA [Source:VGNC Symbol;Acc:VGNC:90195]	-1.91	0.0807
ssc-miR-29b	MGAT4B	alpha-1,3-mannosyl-glycoprotein 4-beta-N-acetylglucosaminyltransferase B [Source:VGNC Symbol;Acc:VGNC:96740]	-1.91	0.0807
ssc-miR-29b	MIB1	MIB E3 ubiquitin protein ligase 1 [Source:VGNC Symbol;Acc:VGNC:90205]	-1.91	0.0807
ssc-miR-29b	MICALL1	MICAL like 1 [Source:VGNC Symbol;Acc:VGNC:90209]	-1.91	0.0807
ssc-miR-29b	MIEF1	hypothetical gene	-1.91	0.0807
ssc-miR-29b	MIER3	MIER family member 3 [Source:VGNC Symbol;Acc:VGNC:90221]	-1.91	0.0807
ssc-miR-29b	MIF4GD	MIF4G domain containing [Source:VGNC Symbol;Acc:VGNC:90223]	-1.91	0.0807
ssc-miR-29b	MLF1	myeloid leukemia factor 1 [Source:HGNC Symbol;Acc:HGNC:7125]	-1.91	0.0807
ssc-miR-29b	MLIP	muscular LMNA interacting protein [Source:VGNC Symbol;Acc:VGNC:90253]	-1.91	0.0807
ssc-miR-29b	MLLT11	MLLT11 transcription factor 7 cofactor [Source:VGNC Symbol;Acc:VGNC:96591]	-1.91	0.0807
ssc-miR-29b	MLXIP	MLX interacting protein [Source:VGNC Symbol;Acc:VGNC:90262]	-1.91	0.0807
ssc-miR-29b	MMD2	monocyte to macrophage differentiation associated 2 [Source:HGNC Symbol;Acc:HGNC:30133]	-1.91	0.0807
ssc-miR-29b	MMP16	matrix metallopeptidase 16 [Source:VGNC Symbol;Acc:VGNC:90271]	-1.91	0.0807
ssc-miR-29b	MMP2	matrix metallopeptidase 2 [Source:VGNC Symbol;Acc:VGNC:90274]	-1.91	0.0807
ssc-miR-29b	MMP24	matrix metallopeptidase 24 [Source:VGNC Symbol;Acc:VGNC:96421]	-1.91	0.0807
ssc-miR-29b	MOB1A	MOB kinase activator 1A [Source:VGNC Symbol;Acc:VGNC:90290]	-1.91	0.0807
ssc-miR-29b	MOB1B	hypothetical gene	-1.91	0.0807
ssc-miR-29b	MOG	myelin oligodendrocyte glycoprotein [Source:VGNC Symbol;Acc:VGNC:90296]	-1.91	0.0807
ssc-miR-29b	MORF4L1	mortality factor 4 like 1 [Source:VGNC Symbol;Acc:VGNC:90306]	-1.91	0.0807
ssc-miR-29b	MOSPD2	motile sperm domain containing 2 [Source:VGNC Symbol;Acc:VGNC:90314]	-1.91	0.0807
ssc-miR-29b	MPZL3	myelin protein zero like 3 [Source:VGNC Symbol;Acc:VGNC:90337]	-1.91	0.0807
ssc-miR-29b	MRE11A	hypothetical gene	-1.91	0.0807

ssc-miR-29b	MRTO4	MRT4 homolog, ribosome maturation factor [Source:VGNC Symbol;Acc:VGNC:90405]	-1.91	0.0807
ssc-miR-29b	MSL1	MSL complex subunit 1 [Source:VGNC Symbol;Acc:VGNC:90423]	-1.91	0.0807
ssc-miR-29b	MSTN	myostatin [Source:VGNC Symbol;Acc:VGNC:96320]	-1.91	0.0807
ssc-miR-29b	MTMR11	myotubularin related protein 11 [Source:VGNC Symbol;Acc:VGNC:90458]	-1.91	0.0807
ssc-miR-29b	MTMR4	myotubularin related protein 4 [Source:VGNC Symbol;Acc:VGNC:90462]	-1.91	0.0807
ssc-miR-29b	MTMR9	myotubularin related protein 9 [Source:VGNC Symbol;Acc:VGNC:90464]	-1.91	0.0807
ssc-miR-29b	MTSS1L	hypothetical gene	-1.91	0.0807
ssc-miR-29b	MTX3	metaxin 3 [Source:VGNC Symbol;Acc:VGNC:90479]	-1.91	0.0807
ssc-miR-29b	MXD1	MAX dimerization protein 1 [Source:VGNC Symbol;Acc:VGNC:90492]	-1.91	0.0807
ssc-miR-29b	MXD4	MAX dimerization protein 4 [Source:VGNC Symbol;Acc:VGNC:90493]	-1.91	0.0807
ssc-miR-29b	MXI1	hypothetical gene	-1.91	0.0807
ssc-miR-29b	MYBL2	MYB proto-onco like 2 [Source:VGNC Symbol;Acc:VGNC:103997]	-1.91	0.0807
ssc-miR-29b	MYCN	MYCN proto-onco, bHLH transcription factor [Source:VGNC Symbol;Acc:VGNC:90504]	-1.91	0.0807
ssc-miR-29b	MYL6	myosin light chain 6 [Source:NCBI gene (formerly Entrezgene);Acc:396807]	-1.91	0.0807
ssc-miR-29b	MYO16	hypothetical gene	-1.91	0.0807
ssc-miR-29b	MYO18A	myosin XVIIIA [Source:HGNC Symbol;Acc:HGNC:31104]	-1.91	0.0807
ssc-miR-29b	N4BP2	NEDD4 binding protein 2 [Source:VGNC Symbol;Acc:VGNC:90554]	-1.91	0.0807
ssc-miR-29b	N4BP2L1	NEDD4 binding protein 2 like 1 [Source:HGNC Symbol;Acc:HGNC:25037]	-1.91	0.0807
ssc-miR-29b	NAA10	N-alpha-acetyltransferase 10, NatA catalytic subunit [Source:VGNC Symbol;Acc:VGNC:96746]	-1.91	0.0807
ssc-miR-29b	NAA40	N-alpha-acetyltransferase 40, NatD catalytic subunit [Source:VGNC Symbol;Acc:VGNC:96753]	-1.91	0.0807
ssc-miR-29b	NAA60	N-alpha-acetyltransferase 60, NatF catalytic subunit [Source:VGNC Symbol;Acc:VGNC:96754]	-1.91	0.0807
ssc-miR-29b	NAIF1	hypothetical gene	-1.91	0.0807
ssc-miR-29b	NANOS1	nanos C2HC-type zinc finger 1 [Source:VGNC Symbol;Acc:VGNC:90574]	-1.91	0.0807
ssc-miR-29b	NANP	N-acetylneuraminic acid phosphatase [Source:HGNC Symbol;Acc:HGNC:16140]	-1.91	0.0807
ssc-miR-29b	NAPB	NSF attachment protein beta [Source:VGNC Symbol;Acc:VGNC:95650]	-1.91	0.0807
ssc-miR-29b	NARF	nuclear prelamin A recognition factor [Source:VGNC Symbol;Acc:VGNC:90582]	-1.91	0.0807
ssc-miR-29b	NASP	nuclear autoantigenic sperm protein [Source:VGNC Symbol;Acc:VGNC:90584]	-1.91	0.0807
ssc-miR-29b	NAV1	neuron navigator 1 [Source:VGNC Symbol;Acc:VGNC:95725]	-1.91	0.0807
ssc-miR-29b	NAV2	neuron navigator 2 [Source:VGNC Symbol;Acc:VGNC:90588]	-1.91	0.0807
ssc-miR-29b	NAV3	neuron navigator 3 [Source:VGNC Symbol;Acc:VGNC:90589]	-1.91	0.0807
ssc-miR-29b	NCKAP5	NCK associated protein 5 [Source:HGNC Symbol;Acc:HGNC:29847]	-1.91	0.0807
ssc-miR-29b	NCKAP5L	NCK associated protein 5 like [Source:VGNC Symbol;Acc:VGNC:90611]	-1.91	0.0807
ssc-miR-29b	NCMAP	hypothetical gene	-1.91	0.0807
ssc-miR-29b	NCOA3	nuclear receptor coactivator 3 [Source:VGNC Symbol;Acc:VGNC:96434]	-1.91	0.0807
ssc-miR-29b	NCOA4	nuclear receptor coactivator 4 [Source:VGNC Symbol;Acc:VGNC:90617]	-1.91	0.0807
ssc-miR-29b	NCOR2	nuclear receptor corepressor 2 [Source:VGNC Symbol;Acc:VGNC:98148]	-1.91	0.0807
ssc-miR-29b	NDN	necdin, MAGE family member [Source:VGNC Symbol;Acc:VGNC:90628]	-1.91	0.0807
ssc-miR-29b	NDST1	N-deacetylase and N-sulfotransferase 1 [Source:VGNC Symbol;Acc:VGNC:90634]	-1.91	0.0807
ssc-miR-29b	NEBL	nebulette [Source:VGNC Symbol;Acc:VGNC:95824]	-1.91	0.0807
ssc-miR-29b	NEGR1	neuronal growth regulator 1 [Source:VGNC Symbol;Acc:VGNC:90671]	-1.91	0.0807
ssc-miR-29b	NFAT5	nuclear factor of activated T cells 5 [Source:VGNC Symbol;Acc:VGNC:90708]	-1.91	0.0807
ssc-miR-29b	NFATC3	nuclear factor of activated T cells 3 [Source:VGNC Symbol;Acc:VGNC:90710]	-1.91	0.0807
ssc-miR-29b	NFATC4	nuclear factor of activated T cells 4 [Source:VGNC Symbol;Acc:VGNC:90711]	-1.91	0.0807
ssc-miR-29b	NFE2L1	NFE2 like bZIP transcription factor 1 [Source:VGNC Symbol;Acc:VGNC:90713]	-1.91	0.0807
ssc-miR-29b	NFIA	nuclear factor I A [Source:VGNC Symbol;Acc:VGNC:90715]	-1.91	0.0807
ssc-miR-29b	NFIB	nuclear factor I B [Source:VGNC Symbol;Acc:VGNC:90716]	-1.91	0.0807
ssc-miR-29b	NFIC	nuclear factor I C [Source:VGNC Symbol;Acc:VGNC:100313]	-1.91	0.0807
ssc-miR-29b	NFIX	nuclear factor I X [Source:VGNC Symbol;Acc:VGNC:90718]	-1.91	0.0807
ssc-miR-29b	NGRN	neugrin, neurite outgrowth associated [Source:HGNC Symbol;Acc:HGNC:18077]	-1.91	0.0807
ssc-miR-29b	NHLRC2	NHL repeat containing 2 [Source:VGNC Symbol;Acc:VGNC:90737]	-1.91	0.0807

ssc-miR-29b	NID1	nidogen 1 [Source:VGNC Symbol;Acc:VGNC:90742]	-1.91	0.0807
ssc-miR-29b	NIPSNAP3A	nipsnap homolog 3A [Source:HGNC Symbol;Acc:HGNC:23619]	-1.91	0.0807
ssc-miR-29b	NIPSNAP3B	hypothetical gene	-1.91	0.0807
ssc-miR-29b	NIT2	nitrilase family member 2 [Source:VGNC Symbol;Acc:VGNC:108678]	-1.91	0.0807
ssc-miR-29b	NKAIN1	sodium/potassium transporting ATPase interacting 1 [Source:VGNC Symbol;Acc:VGNC:90754]	-1.91	0.0807
ssc-miR-29b	NKAPL	NFKB activating protein like [Source:VGNC Symbol;Acc:VGNC:90758]	-1.91	0.0807
ssc-miR-29b	NKIRAS2	NFKB inhibitor interacting Ras like 2 [Source:VGNC Symbol;Acc:VGNC:90763]	-1.91	0.0807
ssc-miR-29b	NKRF	NFKB repressing factor [Source:VGNC Symbol;Acc:VGNC:90764]	-1.91	0.0807
ssc-miR-29b	NLGN3	neuroligin 3 [Source:VGNC Symbol;Acc:VGNC:90778]	-1.91	0.0807
ssc-miR-29b	NLRX1	NLR family member X1 [Source:NCBI gene (formerly Entrezgene);Acc:100518678]	-1.91	0.0807
ssc-miR-29b	NOTCH2	notch receptor 2 [Source:HGNC Symbol;Acc:HGNC:7882]	-1.91	0.0807
ssc-miR-29b	NPAS3	neuronal PAS domain protein 3 [Source:VGNC Symbol;Acc:VGNC:90838]	-1.91	0.0807
ssc-miR-29b	NPAS4	neuronal PAS domain protein 4 [Source:VGNC Symbol;Acc:VGNC:90839]	-1.91	0.0807
ssc-miR-29b	NPFFR1	neuropeptide FF receptor 1 [Source:VGNC Symbol;Acc:VGNC:90846]	-1.91	0.0807
ssc-miR-29b	NPM3	nucleophosmin/nucleoplasmin 3 [Source:VGNC Symbol;Acc:VGNC:90854]	-1.91	0.0807
ssc-miR-29b	NRAS	NRAS proto-onco, GTPase [Source:VGNC Symbol;Acc:VGNC:98827]	-1.91	0.0807
ssc-miR-29b	NREP	neuronal regeneration related protein [Source:HGNC Symbol;Acc:HGNC:16834]	-1.91	0.0807
ssc-miR-29b	NRSN1	neurensin 1 [Source:VGNC Symbol;Acc:VGNC:90902]	-1.91	0.0807
ssc-miR-29b	NSD1	nuclear receptor binding SET domain protein 1 [Source:VGNC Symbol;Acc:VGNC:90904]	-1.91	0.0807
ssc-miR-29b	NSUN4	NOP2/Sun RNA methyltransferase 4 [Source:VGNC Symbol;Acc:VGNC:98522]	-1.91	0.0807
ssc-miR-29b	NTNG2	netrin G2 [Source:VGNC Symbol;Acc:VGNC:90936]	-1.91	0.0807
ssc-miR-29b	NUCKS1	nuclear casein kinase and cyclin dependent kinase substrate 1 [Source:VGNC Symbol;Acc:VGNC:90947]	-1.91	0.0807
ssc-miR-29b	NUDT11	hypothetical gene	-1.91	0.0807
ssc-miR-29b	NUFIP2	nuclear FMR1 interacting protein 2 [Source:VGNC Symbol;Acc:VGNC:90967]	-1.91	0.0807
ssc-miR-29b	NUP160	nucleoporin 160 [Source:VGNC Symbol;Acc:VGNC:90975]	-1.91	0.0807
ssc-miR-29b	ONECUT1	one cut homeobox 1 [Source:VGNC Symbol;Acc:VGNC:91042]	-1.91	0.0807
ssc-miR-29b	ONECUT2	one cut homeobox 2 [Source:VGNC Symbol;Acc:VGNC:91043]	-1.91	0.0807
ssc-miR-29b	ORAI2	ORAI calcium release-activated calcium modulator 2 [Source:VGNC Symbol;Acc:VGNC:91058]	-1.91	0.0807
ssc-miR-29b	ORAI3	ORAI calcium release-activated calcium modulator 3 [Source:VGNC Symbol;Acc:VGNC:91059]	-1.91	0.0807
ssc-miR-29b	OSBP	oxysterol binding protein [Source:VGNC Symbol;Acc:VGNC:91066]	-1.91	0.0807
ssc-miR-29b	OSBPL3	oxysterol binding protein like 3 [Source:VGNC Symbol;Acc:VGNC:91071]	-1.91	0.0807
ssc-miR-29b	OSBPL6	oxysterol binding protein like 6 [Source:VGNC Symbol;Acc:VGNC:95807]	-1.91	0.0807
ssc-miR-29b	OSTC	hypothetical gene	-1.91	0.0807
ssc-miR-29b	OTUB2	OTU deubiquitinase, ubiquitin aldehyde binding 2 [Source:VGNC Symbol;Acc:VGNC:91098]	-1.91	0.0807
ssc-miR-29b	OTUD4	OTU deubiquitinase 4 [Source:VGNC Symbol;Acc:VGNC:91100]	-1.91	0.0807
ssc-miR-29b	OXCT1	3-oxoacid CoA-transferase 1 [Source:VGNC Symbol;Acc:VGNC:91113]	-1.91	0.0807
ssc-miR-29b	OXTR	oxytocin receptor [Source:VGNC Symbol;Acc:VGNC:108052]	-1.91	0.0807
ssc-miR-29b	P2RX5	purinergic receptor P2X 5 [Source:HGNC Symbol;Acc:HGNC:8536]	-1.91	0.0807
ssc-miR-29b	PAG1	phosphoprotein membrane anchor with glycosphingolipid microdomains 1 [Source:VGNC Symbol;Acc:VGNC:91154]	-1.91	0.0807
ssc-miR-29b	PAIP2	poly(A) binding protein interacting protein 2 [Source:VGNC Symbol;Acc:VGNC:96618]	-1.91	0.0807
ssc-miR-29b	PALM	paralemmin [Source:VGNC Symbol;Acc:VGNC:91160]	-1.91	0.0807
ssc-miR-29b	PALM2	hypothetical gene	-1.91	0.0807
ssc-miR-29b	PALM2-AKAP2	hypothetical gene	-1.91	0.0807
ssc-miR-29b	PAN2	poly(A) specific ribonuclease subunit PAN2 [Source:HGNC Symbol;Acc:HGNC:20074]	-1.91	0.0807
ssc-miR-29b	PAPOLG	poly(A) polymerase gamma [Source:HGNC Symbol;Acc:HGNC:14982]	-1.91	0.0807
ssc-miR-29b	PAQR3	progestin and adipoQ receptor family member 3 [Source:VGNC Symbol;Acc:VGNC:91174]	-1.91	0.0807
ssc-miR-29b	PAQR6	progestin and adipoQ receptor family member 6 [Source:VGNC Symbol;Acc:VGNC:91176]	-1.91	0.0807
ssc-miR-29b	PARG	poly(ADP-ribose) glycohydrolase [Source:HGNC Symbol;Acc:HGNC:8605]	-1.91	0.0807
ssc-miR-29b	PCDH17	protocadherin 17 [Source:VGNC Symbol;Acc:VGNC:91214]	-1.91	0.0807
ssc-miR-29b	PCDH9	protocadherin 9 [Source:VGNC Symbol;Acc:VGNC:91217]	-1.91	0.0807

ssc-miR-29b	PCDHA1	hypothetical gene	-1.91	0.0807
ssc-miR-29b	PCDHA10	hypothetical gene	-1.91	0.0807
ssc-miR-29b	PCDHA11	hypothetical gene	-1.91	0.0807
ssc-miR-29b	PCDHA12	hypothetical gene	-1.91	0.0807
ssc-miR-29b	PCDHA13	protocadherin alpha 13 [Source:HGNC Symbol;Acc:HGNC:8667]	-1.91	0.0807
ssc-miR-29b	PCDHA2	hypothetical gene	-1.91	0.0807
ssc-miR-29b	PCDHA3	protocadherin alpha 3 [Source:HGNC Symbol;Acc:HGNC:8669]	-1.91	0.0807
ssc-miR-29b	PCDHA4	hypothetical gene	-1.91	0.0807
ssc-miR-29b	PCDHA5	hypothetical gene	-1.91	0.0807
ssc-miR-29b	PCDHA6	hypothetical gene	-1.91	0.0807
ssc-miR-29b	PCDHA7	hypothetical gene	-1.91	0.0807
ssc-miR-29b	PCDHA8	hypothetical gene	-1.91	0.0807
ssc-miR-29b	PCDHA9	hypothetical gene	-1.91	0.0807
ssc-miR-29b	PCDHAC1	hypothetical gene	-1.91	0.0807
ssc-miR-29b	PCDHAC2	protocadherin alpha subfamily C, 2 [Source:HGNC Symbol;Acc:HGNC:8677]	-1.91	0.0807
ssc-miR-29b	PCDHB16	hypothetical gene	-1.91	0.0807
ssc-miR-29b	PCGF3	polycomb group ring finger 3 [Source:VGNC Symbol;Acc:VGNC:91220]	-1.91	0.0807
ssc-miR-29b	PCSK5	proprotein convertase subtilisin/kexin type 5 [Source:VGNC Symbol;Acc:VGNC:103147]	-1.91	0.0807
ssc-miR-29b	PCYT1B	phosphate cytidylyltransferase 1B, choline [Source:VGNC Symbol;Acc:VGNC:91237]	-1.91	0.0807
ssc-miR-29b	PDE3A	phosphodiesterase 3A [Source:VGNC Symbol;Acc:VGNC:91252]	-1.91	0.0807
ssc-miR-29b	PDE3B	phosphodiesterase 3B [Source:VGNC Symbol;Acc:VGNC:91253]	-1.91	0.0807
ssc-miR-29b	PDE7A	phosphodiesterase 7A [Source:VGNC Symbol;Acc:VGNC:91261]	-1.91	0.0807
ssc-miR-29b	PDGFA	platelet derived growth factor subunit A [Source:VGNC Symbol;Acc:VGNC:91265]	-1.91	0.0807
ssc-miR-29b	PDGFB	platelet derived growth factor subunit B [Source:HGNC Symbol;Acc:HGNC:8800]	-1.91	0.0807
ssc-miR-29b	PDGFC	platelet derived growth factor C [Source:VGNC Symbol;Acc:VGNC:91266]	-1.91	0.0807
ssc-miR-29b	PDGFRB	platelet derived growth factor receptor beta [Source:VGNC Symbol;Acc:VGNC:91268]	-1.91	0.0807
ssc-miR-29b	PDHX	pyruvate dehydrogenase complex component X [Source:VGNC Symbol;Acc:VGNC:91270]	-1.91	0.0807
ssc-miR-29b	PDIK1L	PDLIM1 interacting kinase 1 like [Source:VGNC Symbol;Acc:VGNC:91276]	-1.91	0.0807
ssc-miR-29b	PDK4	pyruvate dehydrogenase kinase 4 [Source:VGNC Symbol;Acc:VGNC:91280]	-1.91	0.0807
ssc-miR-29b	PDP2	pyruvate dehyrogenase phosphatase catalytic subunit 2 [Source:VGNC Symbol;Acc:VGNC:91287]	-1.91	0.0807
ssc-miR-29b	PEAK1	pseudopodium enriched atypical kinase 1 [Source:VGNC Symbol;Acc:VGNC:91300]	-1.91	0.0807
ssc-miR-29b	PEG10	paternally expressed 10 [Source:NCBI gene (formerly Entrezgene);Acc:654416]	-1.91	0.0807
ssc-miR-29b	PER1	period circadian regulator 1 [Source:VGNC Symbol;Acc:VGNC:91314]	-1.91	0.0807
ssc-miR-29b	PER3	hypothetical gene	-1.91	0.0807
ssc-miR-29b	PEX5	peroxisomal biosis factor 5 [Source:VGNC Symbol;Acc:VGNC:91328]	-1.91	0.0807
ssc-miR-29b	PGAP1	post-GPI attachment to proteins inositol deacylase 1 [Source:VGNC Symbol;Acc:VGNC:96099]	-1.91	0.0807
ssc-miR-29b	PGAP2	post-GPI attachment to proteins 2 [Source:VGNC Symbol;Acc:VGNC:91342]	-1.91	0.0807
ssc-miR-29b	PGM3	phosphoglucomutase 3 [Source:VGNC Symbol;Acc:VGNC:103150]	-1.91	0.0807
ssc-miR-29b	PGP	phosphoglycolate phosphatase [Source:VGNC Symbol;Acc:VGNC:91359]	-1.91	0.0807
ssc-miR-29b	PHACTR2	phosphatase and actin regulator 2 [Source:VGNC Symbol;Acc:VGNC:91366]	-1.91	0.0807
ssc-miR-29b	PHC1	polyhomeotic homolog 1 [Source:VGNC Symbol;Acc:VGNC:91370]	-1.91	0.0807
ssc-miR-29b	PHF21A	PHD finger protein 21A [Source:VGNC Symbol;Acc:VGNC:91384]	-1.91	0.0807
ssc-miR-29b	PHLDB3	pleckstrin homology like domain family B member 3 [Source:VGNC Symbol;Acc:VGNC:91400]	-1.91	0.0807
ssc-miR-29b	PHLPP2	PH domain and leucine rich repeat protein phosphatase 2 [Source:VGNC Symbol;Acc:VGNC:98186]	-1.91	0.0807
ssc-miR-29b	PI15	peptidase inhibitor 15 [Source:VGNC Symbol;Acc:VGNC:98188]	-1.91	0.0807
ssc-miR-29b	PIAS2	protein inhibitor of activated STAT 2 [Source:VGNC Symbol;Acc:VGNC:91411]	-1.91	0.0807
ssc-miR-29b	PIAS4	protein inhibitor of activated STAT 4 [Source:VGNC Symbol;Acc:VGNC:91413]	-1.91	0.0807
ssc-miR-29b	PIK3CA	phosphatidylinositol-4,5-bisphosphate 3-kinase catalytic subunit alpha [Source:VGNC Symbol;Acc:VGNC:91440]	-1.91	0.0807
ssc-miR-29b	PIK3CB	phosphatidylinositol-4,5-bisphosphate 3-kinase catalytic subunit beta [Source:VGNC Symbol;Acc:VGNC:91441]	-1.91	0.0807
ssc-miR-29b	PIK3R1	phosphoinositide-3-kinase regulatory subunit 1 [Source:VGNC Symbol;Acc:VGNC:91445]	-1.91	0.0807

ssc-miR-29b	PIK3R3	phosphoinositide-3-kinase regulatory subunit 3 [Source:HGNC Symbol;Acc:HGNC:8981]	-1.91	0.0807
ssc-miR-29b	PIP5KL1	phosphatidylinositol-4-phosphate 5-kinase like 1 [Source:VGNC Symbol;Acc:VGNC:91460]	-1.91	0.0807
ssc-miR-29b	PITPNA	phosphatidylinositol transfer protein alpha [Source:VGNC Symbol;Acc:VGNC:91464]	-1.91	0.0807
ssc-miR-29b	PITPNM2	phosphatidylinositol transfer protein membrane associated 2 [Source:VGNC Symbol;Acc:VGNC:91466]	-1.91	0.0807
ssc-miR-29b	PITPNM3	PITPNM family member 3 [Source:HGNC Symbol;Acc:HGNC:21043]	-1.91	0.0807
ssc-miR-29b	PLAG1	PLAG1 zinc finger [Source:VGNC Symbol;Acc:VGNC:91509]	-1.91	0.0807
ssc-miR-29b	PLCG2	phospholipase C gamma 2 [Source:VGNC Symbol;Acc:VGNC:91520]	-1.91	0.0807
ssc-miR-29b	PLEK2	pleckstrin 2 [Source:VGNC Symbol;Acc:VGNC:91532]	-1.91	0.0807
ssc-miR-29b	PLEKHA1	pleckstrin homology domain containing A1 [Source:VGNC Symbol;Acc:VGNC:91533]	-1.91	0.0807
ssc-miR-29b	PLEKHA3	pleckstrin homology domain containing A3 [Source:NCBI gene (formerly Entrezgene);Acc:100154010]	-1.91	0.0807
ssc-miR-29b	PLEKHA8	pleckstrin homology domain containing A8 [Source:HGNC Symbol;Acc:HGNC:30037]	-1.91	0.0807
ssc-miR-29b	PLEKHF2	pleckstrin homology and FYVE domain containing 2 [Source:VGNC Symbol;Acc:VGNC:91541]	-1.91	0.0807
ssc-miR-29b	PLGLB1	hypothetical gene	-1.91	0.0807
ssc-miR-29b	PLGLB2	hypothetical gene	-1.91	0.0807
ssc-miR-29b	PLP1	proteolipid protein 1 [Source:VGNC Symbol;Acc:VGNC:91569]	-1.91	0.0807
ssc-miR-29b	PLXDC2	plexin domain containing 2 [Source:VGNC Symbol;Acc:VGNC:95958]	-1.91	0.0807
ssc-miR-29b	PLXNA1	plexin A1 [Source:VGNC Symbol;Acc:VGNC:91579]	-1.91	0.0807
ssc-miR-29b	PLXNC1	plexin C1 [Source:VGNC Symbol;Acc:VGNC:91584]	-1.91	0.0807
ssc-miR-29b	PMP22	peripheral myelin protein 22 [Source:VGNC Symbol;Acc:VGNC:91590]	-1.91	0.0807
ssc-miR-29b	POC1B-GALNT4	hypothetical gene	-1.91	0.0807
ssc-miR-29b	POLE3	DNA polymerase epsilon 3, accessory subunit [Source:VGNC Symbol;Acc:VGNC:103154]	-1.91	0.0807
ssc-miR-29b	POLR1D	RNA polymerase I and III subunit D [Source:VGNC Symbol;Acc:VGNC:104031]	-1.91	0.0807
ssc-miR-29b	POLR3E	RNA polymerase III subunit E [Source:VGNC Symbol;Acc:VGNC:91657]	-1.91	0.0807
ssc-miR-29b	PPARD	peroxisome proliferator activated receptor delta [Source:VGNC Symbol;Acc:VGNC:91683]	-1.91	0.0807
ssc-miR-29b	PPARGC1A	PPARG coactivator 1 alpha [Source:VGNC Symbol;Acc:VGNC:91685]	-1.91	0.0807
ssc-miR-29b	PPIC	peptidylprolyl isomerase C [Source:VGNC Symbol;Acc:VGNC:98211]	-1.91	0.0807
ssc-miR-29b	PPIP5K2	diphosphoinositol pentakisphosphate kinase 2 [Source:VGNC Symbol;Acc:VGNC:91701]	-1.91	0.0807
ssc-miR-29b	PPM1D	protein phosphatase, Mg2+/Mn2+ dependent 1D [Source:VGNC Symbol;Acc:VGNC:91704]	-1.91	0.0807
ssc-miR-29b	PPM1E	protein phosphatase, Mg2+/Mn2+ dependent 1E [Source:VGNC Symbol;Acc:VGNC:91705]	-1.91	0.0807
ssc-miR-29b	PPP1R13B	protein phosphatase 1 regulatory subunit 13B [Source:VGNC Symbol;Acc:VGNC:91721]	-1.91	0.0807
ssc-miR-29b	PPP1R15B	protein phosphatase 1 regulatory subunit 15B [Source:VGNC Symbol;Acc:VGNC:91726]	-1.91	0.0807
ssc-miR-29b	PPP1R3D	protein phosphatase 1 regulatory subunit 3D [Source:VGNC Symbol;Acc:VGNC:96500]	-1.91	0.0807
ssc-miR-29b	PPP1R9A	protein phosphatase 1 regulatory subunit 9A [Source:VGNC Symbol;Acc:VGNC:91743]	-1.91	0.0807
ssc-miR-29b	PPP2CA	protein phosphatase 2 catalytic subunit alpha [Source:VGNC Symbol;Acc:VGNC:91745]	-1.91	0.0807
ssc-miR-29b	PRELP	proline and arginine rich end leucine rich repeat protein [Source:VGNC Symbol;Acc:VGNC:91789]	-1.91	0.0807
ssc-miR-29b	PRICKLE2	prickle planar cell polarity protein 2 [Source:VGNC Symbol;Acc:VGNC:91794]	-1.91	0.0807
ssc-miR-29b	PRKAB2	protein kinase AMP-activated non-catalytic subunit beta 2 [Source:VGNC Symbol;Acc:VGNC:91799]	-1.91	0.0807
ssc-miR-29b	PRKG1	protein kinase cGMP-dependent 1 [Source:VGNC Symbol;Acc:VGNC:91816]	-1.91	0.0807
ssc-miR-29b	PRKRA	protein activator of interferon induced protein kinase EIF2AK2 [Source:VGNC Symbol;Acc:VGNC:96505]	-1.91	0.0807
ssc-miR-29b	PRMT6	protein arginine methyltransferase 6 [Source:VGNC Symbol;Acc:VGNC:91823]	-1.91	0.0807
ssc-miR-29b	PROM1	prominin 1 [Source:VGNC Symbol;Acc:VGNC:91832]	-1.91	0.0807
ssc-miR-29b	PROSER1	proline and serine rich 1 [Source:VGNC Symbol;Acc:VGNC:91836]	-1.91	0.0807
ssc-miR-29b	PRPF38B	hypothetical gene	-1.91	0.0807
ssc-miR-29b	PRPF40A	pre-mRNA processing factor 40 homolog A [Source:VGNC Symbol;Acc:VGNC:98222]	-1.91	0.0807
ssc-miR-29b	PRPH2	peripherin 2 [Source:VGNC Symbol;Acc:VGNC:91848]	-1.91	0.0807
ssc-miR-29b	PRR14L	proline rich 14 like [Source:VGNC Symbol;Acc:VGNC:91853]	-1.91	0.0807
ssc-miR-29b	PRR3	proline rich 3 [Source:HGNC Symbol;Acc:HGNC:21149]	-1.91	0.0807
ssc-miR-29b	PRRC2C	proline rich coiled-coil 2C [Source:VGNC Symbol;Acc:VGNC:91867]	-1.91	0.0807
ssc-miR-29b	PRRT4	proline rich transmembrane protein 4 [Source:VGNC Symbol;Acc:VGNC:91874]	-1.91	0.0807
ssc-miR-29b	PRSS12	serine protease 12 [Source:VGNC Symbol;Acc:VGNC:91877]	-1.91	0.0807

ssc-miR-29b	PSD2	pleckstrin and Sec7 domain containing 2 [Source:VGNC Symbol;Acc:VGNC:91896]	-1.91	0.0807
ssc-miR-29b	PSEN2	presenilin 2 [Source:VGNC Symbol;Acc:VGNC:96508]	-1.91	0.0807
ssc-miR-29b	PTBP3	polypyrimidine tract binding protein 3 [Source:VGNC Symbol;Acc:VGNC:91938]	-1.91	0.0807
ssc-miR-29b	PTEN	hypothetical gene	-1.91	0.0807
ssc-miR-29b	PTHLH	parathyroid hormone like hormone [Source:VGNC Symbol;Acc:VGNC:91963]	-1.91	0.0807
ssc-miR-29b	PTP4A1	protein tyrosine phosphatase 4A1 [Source:HGNC Symbol;Acc:HGNC:9634]	-1.91	0.0807
ssc-miR-29b	PTPN1	protein tyrosine phosphatase non-receptor type 1 [Source:VGNC Symbol;Acc:VGNC:96519]	-1.91	0.0807
ssc-miR-29b	PTPN4	protein tyrosine phosphatase non-receptor type 4 [Source:VGNC Symbol;Acc:VGNC:96521]	-1.91	0.0807
ssc-miR-29b	PTPRD	protein tyrosine phosphatase receptor type D [Source:HGNC Symbol;Acc:HGNC:9668]	-1.91	0.0807
ssc-miR-29b	PTPRK	protein tyrosine phosphatase receptor type K [Source:VGNC Symbol;Acc:VGNC:91991]	-1.91	0.0807
ssc-miR-29b	PTRF	hypothetical gene	-1.91	0.0807
ssc-miR-29b	PTRH2	peptidyl-tRNA hydrolase 2 [Source:VGNC Symbol;Acc:VGNC:91998]	-1.91	0.0807
ssc-miR-29b	PURA	purine rich element binding protein A [Source:HGNC Symbol;Acc:HGNC:9701]	-1.91	0.0807
ssc-miR-29b	PVRL1	hypothetical gene	-1.91	0.0807
ssc-miR-29b	PXDN	peroxidasin [Source:VGNC Symbol;Acc:VGNC:92014]	-1.91	0.0807
ssc-miR-29b	PXMP4	hypothetical gene	-1.91	0.0807
ssc-miR-29b	QKI	QKI, KH domain containing RNA binding [Source:VGNC Symbol;Acc:VGNC:92025]	-1.91	0.0807
ssc-miR-29b	R3HDM4	R3H domain containing 4 [Source:VGNC Symbol;Acc:VGNC:92038]	-1.91	0.0807
ssc-miR-29b	RAB12	RAB12, member RAS onco family [Source:VGNC Symbol;Acc:VGNC:98237]	-1.91	0.0807
ssc-miR-29b	RAB15	RAB15, member RAS onco family [Source:VGNC Symbol;Acc:VGNC:98239]	-1.91	0.0807
ssc-miR-29b	RAB27A	RAB27A, member RAS onco family [Source:VGNC Symbol;Acc:VGNC:98249]	-1.91	0.0807
ssc-miR-29b	RAB30	RAB30, member RAS onco family [Source:VGNC Symbol;Acc:VGNC:98254]	-1.91	0.0807
ssc-miR-29b	RAB3C	RAB3C, member RAS onco family [Source:VGNC Symbol;Acc:VGNC:104043]	-1.91	0.0807
ssc-miR-29b	RAB40C	RAB40C, member RAS onco family [Source:VGNC Symbol;Acc:VGNC:104044]	-1.91	0.0807
ssc-miR-29b	RAB6B	RAB6B, member RAS onco family [Source:VGNC Symbol;Acc:VGNC:98272]	-1.91	0.0807
ssc-miR-29b	RABGEF1	RAB guanine nucleotide exchange factor 1 [Source:VGNC Symbol;Acc:VGNC:108510]	-1.91	0.0807
ssc-miR-29b	RAET1L	hypothetical gene	-1.91	0.0807
ssc-miR-29b	RALGPS1	Ral GEF with PH domain and SH3 binding motif 1 [Source:VGNC Symbol;Acc:VGNC:92072]	-1.91	0.0807
ssc-miR-29b	RAP1A	RAP1A, member of RAS onco family [Source:VGNC Symbol;Acc:VGNC:92084]	-1.91	0.0807
ssc-miR-29b	RAP1GDS1	Rap1 GTPase-GDP dissociation stimulator 1 [Source:VGNC Symbol;Acc:VGNC:92087]	-1.91	0.0807
ssc-miR-29b	RAPGEFL1	Rap guanine nucleotide exchange factor like 1 [Source:VGNC Symbol;Acc:VGNC:92095]	-1.91	0.0807
ssc-miR-29b	RARB	retinoic acid receptor beta [Source:HGNC Symbol;Acc:HGNC:9865]	-1.91	0.0807
ssc-miR-29b	RASAL2	RAS protein activator like 2 [Source:VGNC Symbol;Acc:VGNC:92105]	-1.91	0.0807
ssc-miR-29b	RBAK	RB associated KRAB zinc finger [Source:VGNC Symbol;Acc:VGNC:92134]	-1.91	0.0807
ssc-miR-29b	RBFOX2	RNA binding fox-1 homolog 2 [Source:VGNC Symbol;Acc:VGNC:92140]	-1.91	0.0807
ssc-miR-29b	RBMS2	RNA binding motif single stranded interacting protein 2 [Source:VGNC Symbol;Acc:VGNC:98286]	-1.91	0.0807
ssc-miR-29b	RCC2	regulator of chromosome condensation 2 [Source:VGNC Symbol;Acc:VGNC:98547]	-1.91	0.0807
ssc-miR-29b	RCOR1	REST corepressor 1 [Source:VGNC Symbol;Acc:VGNC:92183]	-1.91	0.0807
ssc-miR-29b	REL	REL proto-onco, NF-kB subunit [Source:VGNC Symbol;Acc:VGNC:92202]	-1.91	0.0807
ssc-miR-29b	REPS2	RALBP1 associated Eps domain containing 2 [Source:VGNC Symbol;Acc:VGNC:92214]	-1.91	0.0807
ssc-miR-29b	RERE	hypothetical gene	-1.91	0.0807
ssc-miR-29b	REST	hypothetical gene	-1.91	0.0807
ssc-miR-29b	REV1	REV1 DNA directed polymerase [Source:HGNC Symbol;Acc:HGNC:14060]	-1.91	0.0807
ssc-miR-29b	REV3L	REV3 like, DNA directed polymerase zeta catalytic subunit [Source:VGNC Symbol;Acc:VGNC:92226]	-1.91	0.0807
ssc-miR-29b	RFESD	Rieske Fe-S domain containing [Source:VGNC Symbol;Acc:VGNC:92236]	-1.91	0.0807
ssc-miR-29b	RFX7	regulatory factor X7 [Source:VGNC Symbol;Acc:VGNC:92248]	-1.91	0.0807
ssc-miR-29b	RGAG4	hypothetical gene	-1.91	0.0807
ssc-miR-29b	RHBDD1	rhomboid domain containing 1 [Source:VGNC Symbol;Acc:VGNC:95989]	-1.91	0.0807
ssc-miR-29b	RHOBTB1	Rho related BTB domain containing 1 [Source:VGNC Symbol;Acc:VGNC:92285]	-1.91	0.0807
ssc-miR-29b	RIC3	RIC3 acetylcholine receptor chaperone [Source:VGNC Symbol;Acc:VGNC:92300]	-1.91	0.0807

ssc-miR-29b	RIC8A	RIC8 guanine nucleotide exchange factor A [Source:VGNC Symbol;Acc:VGNC:92301]	-1.91	0.0807
ssc-miR-29b	RIOK3	RIO kinase 3 [Source:VGNC Symbol;Acc:VGNC:92316]	-1.91	0.0807
ssc-miR-29b	RIT1	Ras like without CAAX 1 [Source:VGNC Symbol;Acc:VGNC:92325]	-1.91	0.0807
ssc-miR-29b	RLF	RLF zinc finger [Source:VGNC Symbol;Acc:VGNC:92328]	-1.91	0.0807
ssc-miR-29b	RLIM	ring finger protein, LIM domain interacting [Source:HGNC Symbol;Acc:HGNC:13429]	-1.91	0.0807
ssc-miR-29b	RMND5A	required for meiotic nuclear division 5 homolog A [Source:VGNC Symbol;Acc:VGNC:92336]	-1.91	0.0807
ssc-miR-29b	RNF122	ring finger protein 122 [Source:VGNC Symbol;Acc:VGNC:92350]	-1.91	0.0807
ssc-miR-29b	RNF125	ring finger protein 125 [Source:VGNC Symbol;Acc:VGNC:92352]	-1.91	0.0807
ssc-miR-29b	RNF138	ring finger protein 138 [Source:VGNC Symbol;Acc:VGNC:92356]	-1.91	0.0807
ssc-miR-29b	RNF141	ring finger protein 141 [Source:VGNC Symbol;Acc:VGNC:98292]	-1.91	0.0807
ssc-miR-29b	RNF144B	ring finger protein 144B [Source:VGNC Symbol;Acc:VGNC:92359]	-1.91	0.0807
ssc-miR-29b	RNF150	ring finger protein 150 [Source:VGNC Symbol;Acc:VGNC:98293]	-1.91	0.0807
ssc-miR-29b	RNF152	ring finger protein 152 [Source:VGNC Symbol;Acc:VGNC:92364]	-1.91	0.0807
ssc-miR-29b	RNF165	ring finger protein 165 [Source:VGNC Symbol;Acc:VGNC:92366]	-1.91	0.0807
ssc-miR-29b	RNF169	ring finger protein 169 [Source:VGNC Symbol;Acc:VGNC:92368]	-1.91	0.0807
ssc-miR-29b	RNF19A	ring finger protein 19A, RBR E3 ubiquitin protein ligase [Source:VGNC Symbol;Acc:VGNC:92375]	-1.91	0.0807
ssc-miR-29b	RNF214	ring finger protein 214 [Source:VGNC Symbol;Acc:VGNC:92382]	-1.91	0.0807
ssc-miR-29b	RNF217	ring finger protein 217 [Source:VGNC Symbol;Acc:VGNC:103162]	-1.91	0.0807
ssc-miR-29b	RNF39	ring finger protein 39 [Source:VGNC Symbol;Acc:VGNC:92391]	-1.91	0.0807
ssc-miR-29b	RNPEPL1	arginyl aminopeptidase like 1 [Source:VGNC Symbol;Acc:VGNC:95470]	-1.91	0.0807
ssc-miR-29b	ROBO1	roundabout guidance receptor 1 [Source:HGNC Symbol;Acc:HGNC:10249]	-1.91	0.0807
ssc-miR-29b	RORA	RAR related orphan receptor A [Source:VGNC Symbol;Acc:VGNC:92408]	-1.91	0.0807
ssc-miR-29b	RP11-192H23.4	hypothetical gene	-1.91	0.0807
ssc-miR-29b	RP11-766F14.2	hypothetical gene	-1.91	0.0807
ssc-miR-29b	RPS15A	hypothetical gene	-1.91	0.0807
ssc-miR-29b	RPS6KA3	ribosomal protein S6 kinase A3 [Source:VGNC Symbol;Acc:VGNC:92442]	-1.91	0.0807
ssc-miR-29b	RRAS2	hypothetical gene	-1.91	0.0807
ssc-miR-29b	RTKN2	rhotekin 2 [Source:VGNC Symbol;Acc:VGNC:92495]	-1.91	0.0807
ssc-miR-29b	RUNX1T1	RUNX1 partner transcriptional co-repressor 1 [Source:VGNC Symbol;Acc:VGNC:96594]	-1.91	0.0807
ssc-miR-29b	RYBP	RING1 and YY1 binding protein [Source:VGNC Symbol;Acc:VGNC:92532]	-1.91	0.0807
ssc-miR-29b	SAMD10	sterile alpha motif domain containing 10 [Source:VGNC Symbol;Acc:VGNC:95750]	-1.91	0.0807
ssc-miR-29b	SAMD4A	sterile alpha motif domain containing 4A [Source:VGNC Symbol;Acc:VGNC:92569]	-1.91	0.0807
ssc-miR-29b	SAP30L	SAP30 like [Source:VGNC Symbol;Acc:VGNC:92577]	-1.91	0.0807
ssc-miR-29b	SARM1	hypothetical gene	-1.91	0.0807
ssc-miR-29b	SBF2	SET binding factor 2 [Source:VGNC Symbol;Acc:VGNC:92594]	-1.91	0.0807
ssc-miR-29b	SCAI	suppressor of cancer cell invasion [Source:VGNC Symbol;Acc:VGNC:103170]	-1.91	0.0807
ssc-miR-29b	SCAMP5	secretory carrier membrane protein 5 [Source:VGNC Symbol;Acc:VGNC:92609]	-1.91	0.0807
ssc-miR-29b	SCHIP1	hypothetical gene	-1.91	0.0807
ssc-miR-29b	SCML2	Scm polycomb group protein like 2 [Source:VGNC Symbol;Acc:VGNC:92628]	-1.91	0.0807
ssc-miR-29b	SCN3B	sodium voltage-gated channel beta subunit 3 [Source:HGNC Symbol;Acc:HGNC:20665]	-1.91	0.0807
ssc-miR-29b	SCN7A	sodium voltage-gated channel alpha subunit 7 [Source:VGNC Symbol;Acc:VGNC:95480]	-1.91	0.0807
ssc-miR-29b	SCYL2	SCY1 like pseudokinase 2 [Source:VGNC Symbol;Acc:VGNC:98305]	-1.91	0.0807
ssc-miR-29b	SDK1	sidekick cell adhesion molecule 1 [Source:VGNC Symbol;Acc:VGNC:92660]	-1.91	0.0807
ssc-miR-29b	SEMA5B	semaphorin 5B [Source:VGNC Symbol;Acc:VGNC:92707]	-1.91	0.0807
ssc-miR-29b	SENP1	SUMO specific peptidase 1 [Source:VGNC Symbol;Acc:VGNC:92712]	-1.91	0.0807
ssc-miR-29b	SENP2	SUMO specific peptidase 2 [Source:VGNC Symbol;Acc:VGNC:92713]	-1.91	0.0807
ssc-miR-29b	SERBP1	SERPINE1 mRNA binding protein 1 [Source:VGNC Symbol;Acc:VGNC:92727]	-1.91	0.0807
ssc-miR-29b	SERINC5	serine incorporator 5 [Source:VGNC Symbol;Acc:VGNC:92730]	-1.91	0.0807
ssc-miR-29b	SERPINH1	serpin family H member 1 [Source:VGNC Symbol;Acc:VGNC:92746]	-1.91	0.0807
ssc-miR-29b	SESN1	sestrin 1 [Source:VGNC Symbol;Acc:VGNC:92753]	-1.91	0.0807

ssc-miR-29b	SESTD1	SEC14 and spectrin domain containing 1 [Source:VGNC Symbol;Acc:VGNC:95884]	-1.91	0.0807
ssc-miR-29b	SETD5	SET domain containing 5 [Source:VGNC Symbol;Acc:VGNC:92760]	-1.91	0.0807
ssc-miR-29b	SETD8	hypothetical gene	-1.91	0.0807
ssc-miR-29b	SETDB1	SET domain bifurcated histone lysine methyltransferase 1 [Source:VGNC Symbol;Acc:VGNC:92764]	-1.91	0.0807
ssc-miR-29b	SETDB2	SET domain bifurcated histone lysine methyltransferase 2 [Source:VGNC Symbol;Acc:VGNC:92765]	-1.91	0.0807
ssc-miR-29b	SFTA3	hypothetical gene	-1.91	0.0807
ssc-miR-29b	SFXN3	sideroflexin 3 [Source:VGNC Symbol;Acc:VGNC:92785]	-1.91	0.0807
ssc-miR-29b	SGCB	sarcoglycan beta [Source:NCBI gene (formerly Entrezgene);Acc:100135674]	-1.91	0.0807
ssc-miR-29b	SGK1	serum/glucocorticoid regulated kinase 1 [Source:VGNC Symbol;Acc:VGNC:92793]	-1.91	0.0807
ssc-miR-29b	SGK494	hypothetical gene	-1.91	0.0807
ssc-miR-29b	SGMS2	sphingomyelin synthase 2 [Source:VGNC Symbol;Acc:VGNC:92795]	-1.91	0.0807
ssc-miR-29b	SH3BP5L	SH3 binding domain protein 5 like [Source:VGNC Symbol;Acc:VGNC:92820]	-1.91	0.0807
ssc-miR-29b	SH3GLB1	SH3 domain containing GRB2 like, endophilin B1 [Source:VGNC Symbol;Acc:VGNC:92826]	-1.91	0.0807
ssc-miR-29b	SH3GLB2	SH3 domain containing GRB2 like, endophilin B2 [Source:VGNC Symbol;Acc:VGNC:92827]	-1.91	0.0807
ssc-miR-29b	SH3PXD2A	SH3 and PX domains 2A [Source:VGNC Symbol;Acc:VGNC:92829]	-1.91	0.0807
ssc-miR-29b	SH3PXD2B	SH3 and PX domains 2B [Source:VGNC Symbol;Acc:VGNC:92830]	-1.91	0.0807
ssc-miR-29b	SH3RF3	SH3 domain containing ring finger 3 [Source:VGNC Symbol;Acc:VGNC:92832]	-1.91	0.0807
ssc-miR-29b	SHB	SH2 domain containing adaptor protein B [Source:VGNC Symbol;Acc:VGNC:103174]	-1.91	0.0807
ssc-miR-29b	SHPRH	SNF2 histone linker PHD RING helicase [Source:VGNC Symbol;Acc:VGNC:92858]	-1.91	0.0807
ssc-miR-29b	SHROOM2	shroom family member 2 [Source:HGNC Symbol;Acc:HGNC:630]	-1.91	0.0807
ssc-miR-29b	SHROOM4	shroom family member 4 [Source:VGNC Symbol;Acc:VGNC:92861]	-1.91	0.0807
ssc-miR-29b	SIAH3	siah E3 ubiquitin protein ligase family member 3 [Source:VGNC Symbol;Acc:VGNC:92867]	-1.91	0.0807
ssc-miR-29b	SIDT1	SID1 transmembrane family member 1 [Source:VGNC Symbol;Acc:VGNC:92868]	-1.91	0.0807
ssc-miR-29b	SIDT2	SID1 transmembrane family member 2 [Source:VGNC Symbol;Acc:VGNC:92869]	-1.91	0.0807
ssc-miR-29b	SIKE1	suppressor of IKBKE 1 [Source:VGNC Symbol;Acc:VGNC:92874]	-1.91	0.0807
ssc-miR-29b	SIM1	SIM bHLH transcription factor 1 [Source:VGNC Symbol;Acc:VGNC:92876]	-1.91	0.0807
ssc-miR-29b	SIRT1	sirtuin 1 [Source:VGNC Symbol;Acc:VGNC:92884]	-1.91	0.0807
ssc-miR-29b	SIX5	SIX homeobox 5 [Source:VGNC Symbol;Acc:VGNC:92896]	-1.91	0.0807
ssc-miR-29b	SKI	SKI proto-onco [Source:VGNC Symbol;Acc:VGNC:92902]	-1.91	0.0807
ssc-miR-29b	SLC10A7	solute carrier family 10 member 7 [Source:VGNC Symbol;Acc:VGNC:92919]	-1.91	0.0807
ssc-miR-29b	SLC16A1	hypothetical gene	-1.91	0.0807
ssc-miR-29b	SLC16A10	solute carrier family 16 member 10 [Source:VGNC Symbol;Acc:VGNC:103178]	-1.91	0.0807
ssc-miR-29b	SLC16A14	solute carrier family 16 member 14 [Source:VGNC Symbol;Acc:VGNC:96129]	-1.91	0.0807
ssc-miR-29b	SLC16A2	solute carrier family 16 member 2 [Source:VGNC Symbol;Acc:VGNC:92942]	-1.91	0.0807
ssc-miR-29b	SLC16A7	solute carrier family 16 member 7 [Source:VGNC Symbol;Acc:VGNC:92947]	-1.91	0.0807
ssc-miR-29b	SLC22A7	solute carrier family 22 member 7 [Source:VGNC Symbol;Acc:VGNC:92983]	-1.91	0.0807
ssc-miR-29b	SLC24A4	solute carrier family 24 member 4 [Source:VGNC Symbol;Acc:VGNC:92988]	-1.91	0.0807
ssc-miR-29b	SLC25A12	solute carrier family 25 member 12 [Source:VGNC Symbol;Acc:VGNC:95502]	-1.91	0.0807
ssc-miR-29b	SLC25A15	solute carrier family 25 member 15 [Source:VGNC Symbol;Acc:VGNC:92994]	-1.91	0.0807
ssc-miR-29b	SLC25A22	solute carrier family 25 member 22 [Source:VGNC Symbol;Acc:VGNC:92999]	-1.91	0.0807
ssc-miR-29b	SLC25A29	hypothetical gene	-1.91	0.0807
ssc-miR-29b	SLC25A44	solute carrier family 25 member 44 [Source:VGNC Symbol;Acc:VGNC:93017]	-1.91	0.0807
ssc-miR-29b	SLC25A45	solute carrier family 25 member 45 [Source:VGNC Symbol;Acc:VGNC:93018]	-1.91	0.0807
ssc-miR-29b	SLC2A14	hypothetical gene	-1.91	0.0807
ssc-miR-29b	SLC2A3	hypothetical gene	-1.91	0.0807
ssc-miR-29b	SLC30A10	hypothetical gene	-1.91	0.0807
ssc-miR-29b	SLC30A3	solute carrier family 30 member 3 [Source:VGNC Symbol;Acc:VGNC:93057]	-1.91	0.0807
ssc-miR-29b	SLC31A1	solute carrier family 31 member 1 [Source:VGNC Symbol;Acc:VGNC:103180]	-1.91	0.0807
ssc-miR-29b	SLC35B4	solute carrier family 35 member B4 [Source:VGNC Symbol;Acc:VGNC:93074]	-1.91	0.0807
ssc-miR-29b	SLC36A1	solute carrier family 36 member 1 [Source:VGNC Symbol;Acc:VGNC:93088]	-1.91	0.0807

ssc-miR-29b	SLC39A13	solute carrier family 39 member 13 [Source:VGNC Symbol;Acc:VGNC:93103]	-1.91	0.0807
ssc-miR-29b	SLC39A9	solute carrier family 39 member 9 [Source:VGNC Symbol;Acc:VGNC:93110]	-1.91	0.0807
ssc-miR-29b	SLC3A1	solute carrier family 3 member 1 [Source:VGNC Symbol;Acc:VGNC:93111]	-1.91	0.0807
ssc-miR-29b	SLC43A1	solute carrier family 43 member 1 [Source:VGNC Symbol;Acc:VGNC:93116]	-1.91	0.0807
ssc-miR-29b	SLC43A2	solute carrier family 43 member 2 [Source:VGNC Symbol;Acc:VGNC:93117]	-1.91	0.0807
ssc-miR-29b	SLC45A4	solute carrier family 45 member 4 [Source:VGNC Symbol;Acc:VGNC:93125]	-1.91	0.0807
ssc-miR-29b	SLC5A8	solute carrier family 5 member 8 [Source:VGNC Symbol;Acc:VGNC:93148]	-1.91	0.0807
ssc-miR-29b	SLC6A14	solute carrier family 6 member 14 [Source:NCBI gene (formerly Entrezgene);Acc:431671]	-1.91	0.0807
ssc-miR-29b	SLC6A17	solute carrier family 6 member 17 [Source:VGNC Symbol;Acc:VGNC:93158]	-1.91	0.0807
ssc-miR-29b	SLC7A1	solute carrier family 7 member 1 [Source:VGNC Symbol;Acc:VGNC:93169]	-1.91	0.0807
ssc-miR-29b	SLC7A6	hypothetical gene	-1.91	0.0807
ssc-miR-29b	SLCO5A1	solute carrier organic anion transporter family member 5A1 [Source:HGNC Symbol;Acc:HGNC:19046]	-1.91	0.0807
ssc-miR-29b	SLITRK3	SLIT and NTRK like family member 3 [Source:VGNC Symbol;Acc:VGNC:93208]	-1.91	0.0807
ssc-miR-29b	SLK	STE20 like kinase [Source:VGNC Symbol;Acc:VGNC:98328]	-1.91	0.0807
ssc-miR-29b	SMARCA2	hypothetical gene	-1.91	0.0807
ssc-miR-29b	SMARCC1	SWI/SNF related, matrix associated, actin dependent regulator of chromatin subfamily c member 1 [Source:VGNC Symbol;Acc:VGNC:93231]	-1.91	0.0807
ssc-miR-29b	SMARCE1	SWI/SNF related, matrix associated, actin dependent regulator of chromatin, subfamily e, member 1 [Source:VGNC Symbol;Acc:VGNC:93235]	-1.91	0.0807
ssc-miR-29b	SMCO3	single-pass membrane protein with coiled-coil domains 3 [Source:VGNC Symbol;Acc:VGNC:93245]	-1.91	0.0807
ssc-miR-29b	SMEK2	hypothetical gene	-1.91	0.0807
ssc-miR-29b	SMIM12	small integral membrane protein 12 [Source:HGNC Symbol;Acc:HGNC:25154]	-1.91	0.0807
ssc-miR-29b	SMIM17	small integral membrane protein 17 [Source:VGNC Symbol;Acc:VGNC:98615]	-1.91	0.0807
ssc-miR-29b	SMPD3	sphingomyelin phosphodiesterase 3 [Source:VGNC Symbol;Acc:VGNC:93263]	-1.91	0.0807
ssc-miR-29b	SMS	spermine synthase [Source:VGNC Symbol;Acc:VGNC:104059]	-1.91	0.0807
ssc-miR-29b	SMTNL2	smoothelin like 2 [Source:VGNC Symbol;Acc:VGNC:93268]	-1.91	0.0807
ssc-miR-29b	SMURF2	SMAD specific E3 ubiquitin protein ligase 2 [Source:VGNC Symbol;Acc:VGNC:93271]	-1.91	0.0807
ssc-miR-29b	SNIP1	Smad nuclear interacting protein 1 [Source:VGNC Symbol;Acc:VGNC:93289]	-1.91	0.0807
ssc-miR-29b	SNRK	SNF related kinase [Source:VGNC Symbol;Acc:VGNC:93290]	-1.91	0.0807
ssc-miR-29b	SNRPC	small nuclear ribonucleoprotein polypeptide C [Source:VGNC Symbol;Acc:VGNC:93295]	-1.91	0.0807
ssc-miR-29b	SNTB2	syntrophin beta 2 [Source:VGNC Symbol;Acc:VGNC:93298]	-1.91	0.0807
ssc-miR-29b	SNX1	sorting nexin 1 [Source:HGNC Symbol;Acc:HGNC:11172]	-1.91	0.0807
ssc-miR-29b	SNX22	sorting nexin 22 [Source:VGNC Symbol;Acc:VGNC:93314]	-1.91	0.0807
ssc-miR-29b	SNX24	sorting nexin 24 [Source:VGNC Symbol;Acc:VGNC:93315]	-1.91	0.0807
ssc-miR-29b	SOCS7	suppressor of cytokine signaling 7 [Source:VGNC Symbol;Acc:VGNC:93334]	-1.91	0.0807
ssc-miR-29b	SOWAHC	hypothetical gene	-1.91	0.0807
ssc-miR-29b	SOX12	SRY-box transcription factor 12 [Source:HGNC Symbol;Acc:HGNC:11198]	-1.91	0.0807
ssc-miR-29b	SOX6	SRY-box transcription factor 6 [Source:VGNC Symbol;Acc:VGNC:93358]	-1.91	0.0807
ssc-miR-29b	SP1	Sp1 transcription factor [Source:VGNC Symbol;Acc:VGNC:93360]	-1.91	0.0807
ssc-miR-29b	SPARC	secreted protein acidic and cysteine rich [Source:VGNC Symbol;Acc:VGNC:98332]	-1.91	0.0807
ssc-miR-29b	SPAST	spastin [Source:VGNC Symbol;Acc:VGNC:98333]	-1.91	0.0807
ssc-miR-29b	SPATA2L	spermatosis associated 2 like [Source:VGNC Symbol;Acc:VGNC:93382]	-1.91	0.0807
ssc-miR-29b	SPEN	hypothetical gene	-1.91	0.0807
ssc-miR-29b	SPNS1	sphingolipid transporter 1 (putative) [Source:NCBI gene (formerly Entrezgene);Acc:100517643]	-1.91	0.0807
ssc-miR-29b	SPRY1	sprouty RTK signaling antagonist 1 [Source:VGNC Symbol;Acc:VGNC:93424]	-1.91	0.0807
ssc-miR-29b	SPRY4	sprouty RTK signaling antagonist 4 [Source:VGNC Symbol;Acc:VGNC:93427]	-1.91	0.0807
ssc-miR-29b	SPSB4	splA/ryanodine receptor domain and SOCS box containing 4 [Source:VGNC Symbol;Acc:VGNC:93433]	-1.91	0.0807
ssc-miR-29b	SPTAN1	spectrin alpha, non-erythrocytic 1 [Source:VGNC Symbol;Acc:VGNC:93434]	-1.91	0.0807
ssc-miR-29b	SPTY2D1	SPT2 chromatin protein domain containing 1 [Source:VGNC Symbol;Acc:VGNC:100867]	-1.91	0.0807
ssc-miR-29b	SRCRB4D	hypothetical gene	-1.91	0.0807
ssc-miR-29b	SRGAP2	hypothetical gene	-1.91	0.0807
ssc-miR-29b	SRSF10	serine and arginine rich splicing factor 10 [Source:VGNC Symbol;Acc:VGNC:93472]	-1.91	0.0807

ssc-miR-29b	SS18L1	SS18L1 subunit of BAF chromatin remodeling complex [Source:VGNC Symbol;Acc:VGNC:98342]	-1.91	0.0807
ssc-miR-29b	STAG2	stromal antigen 2 [Source:VGNC Symbol;Acc:VGNC:93525]	-1.91	0.0807
ssc-miR-29b	STARD8	StAR related lipid transfer domain containing 8 [Source:VGNC Symbol;Acc:VGNC:93538]	-1.91	0.0807
ssc-miR-29b	STARD9	hypothetical gene	-1.91	0.0807
ssc-miR-29b	STAT3	signal transducer and activator of transcription 3 [Source:VGNC Symbol;Acc:VGNC:93540]	-1.91	0.0807
ssc-miR-29b	STC1	stanniocalcin 1 [Source:VGNC Symbol;Acc:VGNC:93542]	-1.91	0.0807
ssc-miR-29b	STEAP3	STEAP3 metalloreductase [Source:VGNC Symbol;Acc:VGNC:96048]	-1.91	0.0807
ssc-miR-29b	STK35	serine/threonine kinase 35 [Source:VGNC Symbol;Acc:VGNC:95802]	-1.91	0.0807
ssc-miR-29b	STK38L	serine/threonine kinase 38 like [Source:VGNC Symbol;Acc:VGNC:93557]	-1.91	0.0807
ssc-miR-29b	STMN2	stathmin 2 [Source:VGNC Symbol;Acc:VGNC:93561]	-1.91	0.0807
ssc-miR-29b	STRIP2	striatin interacting protein 2 [Source:VGNC Symbol;Acc:VGNC:93577]	-1.91	0.0807
ssc-miR-29b	STRN	striatin [Source:VGNC Symbol;Acc:VGNC:93578]	-1.91	0.0807
ssc-miR-29b	STRN3	striatin 3 [Source:VGNC Symbol;Acc:VGNC:93579]	-1.91	0.0807
ssc-miR-29b	STRN4	striatin 4 [Source:VGNC Symbol;Acc:VGNC:93580]	-1.91	0.0807
ssc-miR-29b	STT3A	STT3 oligosaccharyltransferase complex catalytic subunit A [Source:VGNC Symbol;Acc:VGNC:93581]	-1.91	0.0807
ssc-miR-29b	STX16	syntaxin 16 [Source:NCBI gene (formerly Entrezgene);Acc:100144526]	-1.91	0.0807
ssc-miR-29b	STX17	syntaxin 17 [Source:VGNC Symbol;Acc:VGNC:93587]	-1.91	0.0807
ssc-miR-29b	SUB1	SUB1 regulator of transcription [Source:HGNC Symbol;Acc:HGNC:19985]	-1.91	0.0807
ssc-miR-29b	SUN1	Sad1 and UNC84 domain containing 1 [Source:VGNC Symbol;Acc:VGNC:93618]	-1.91	0.0807
ssc-miR-29b	SUV420H1	hypothetical gene	-1.91	0.0807
ssc-miR-29b	SUV420H2	hypothetical gene	-1.91	0.0807
ssc-miR-29b	SVEP1	sushi, von Willebrand factor type A, EGF and pentraxin domain containing 1 [Source:VGNC Symbol;Acc:VGNC:93642]	-1.91	0.0807
ssc-miR-29b	SVIL	supervillin [Source:VGNC Symbol;Acc:VGNC:95528]	-1.91	0.0807
ssc-miR-29b	SYNCRIP	synaptotagmin binding cytoplasmic RNA interacting protein [Source:VGNC Symbol;Acc:VGNC:103185]	-1.91	0.0807
ssc-miR-29b	SYNGAP1	synaptic Ras GTPase activating protein 1 [Source:VGNC Symbol;Acc:VGNC:93664]	-1.91	0.0807
ssc-miR-29b	SYPL2	synaptophysin like 2 [Source:VGNC Symbol;Acc:VGNC:93677]	-1.91	0.0807
ssc-miR-29b	SYT2	synaptotagmin 2 [Source:VGNC Symbol;Acc:VGNC:95530]	-1.91	0.0807
ssc-miR-29b	SYT7	synaptotagmin 7 [Source:VGNC Symbol;Acc:VGNC:100868]	-1.91	0.0807
ssc-miR-29b	SYT9	synaptotagmin 9 [Source:VGNC Symbol;Acc:VGNC:93689]	-1.91	0.0807
ssc-miR-29b	TACC1	hypothetical gene	-1.91	0.0807
ssc-miR-29b	TADA2B	transcriptional adaptor 2B [Source:VGNC Symbol;Acc:VGNC:93707]	-1.91	0.0807
ssc-miR-29b	TAF11	TATA-box binding protein associated factor 11 [Source:NCBI gene (formerly Entrezgene);Acc:100151814]	-1.91	0.0807
ssc-miR-29b	TAF5	TATA-box binding protein associated factor 5 [Source:VGNC Symbol;Acc:VGNC:93717]	-1.91	0.0807
ssc-miR-29b	TANC1	tetratricopeptide repeat, ankyrin repeat and coiled-coil containing 1 [Source:VGNC Symbol;Acc:VGNC:99730]	-1.91	0.0807
ssc-miR-29b	TANGO6	transport and golgi organization 6 homolog [Source:HGNC Symbol;Acc:HGNC:25749]	-1.91	0.0807
ssc-miR-29b	TBC1D13	TBC1 domain family member 13 [Source:VGNC Symbol;Acc:VGNC:93763]	-1.91	0.0807
ssc-miR-29b	TBC1D7	TBC1 domain family member 7 [Source:VGNC Symbol;Acc:VGNC:93778]	-1.91	0.0807
ssc-miR-29b	TBCEL	tubulin folding cofactor E like [Source:HGNC Symbol;Acc:HGNC:28115]	-1.91	0.0807
ssc-miR-29b	TBX21	T-box transcription factor 21 [Source:VGNC Symbol;Acc:VGNC:93800]	-1.91	0.0807
ssc-miR-29b	TCF4	transcription factor 4 [Source:VGNC Symbol;Acc:VGNC:93823]	-1.91	0.0807
ssc-miR-29b	TDG	thymine DNA glycosylase [Source:VGNC Symbol;Acc:VGNC:93842]	-1.91	0.0807
ssc-miR-29b	TECTB	tectorin beta [Source:VGNC Symbol;Acc:VGNC:93860]	-1.91	0.0807
ssc-miR-29b	TENM3	teneurin transmembrane protein 3 [Source:VGNC Symbol;Acc:VGNC:96233]	-1.91	0.0807
ssc-miR-29b	TET1	tet methylcytosine dioxygenase 1 [Source:VGNC Symbol;Acc:VGNC:93888]	-1.91	0.0807
ssc-miR-29b	TET2	hypothetical gene	-1.91	0.0807
ssc-miR-29b	TET3	tet methylcytosine dioxygenase 3 [Source:VGNC Symbol;Acc:VGNC:93890]	-1.91	0.0807
ssc-miR-29b	TFAP2C	transcription factor AP-2 gamma [Source:VGNC Symbol;Acc:VGNC:95540]	-1.91	0.0807
ssc-miR-29b	TFEB	transcription factor EB [Source:VGNC Symbol;Acc:VGNC:93917]	-1.91	0.0807
ssc-miR-29b	TFEC	transcription factor EC [Source:VGNC Symbol;Acc:VGNC:93918]	-1.91	0.0807
ssc-miR-29b	TGFB2	transforming growth factor beta 2 [Source:VGNC Symbol;Acc:VGNC:95541]	-1.91	0.0807

ssc-miR-29b	TGIF2	TGFB induced factor homeobox 2 [Source:VGNC Symbol;Acc:VGNC:95656]	-1.91	0.0807
ssc-miR-29b	THAP8	THAP domain containing 8 [Source:HGNC Symbol;Acc:HGNC:23191]	-1.91	0.0807
ssc-miR-29b	THOC1	THO complex 1 [Source:VGNC Symbol;Acc:VGNC:93956]	-1.91	0.0807
ssc-miR-29b	THPO	thrombopoietin [Source:VGNC Symbol;Acc:VGNC:93962]	-1.91	0.0807
ssc-miR-29b	THRA	thyroid hormone receptor alpha [Source:VGNC Symbol;Acc:VGNC:93963]	-1.91	0.0807
ssc-miR-29b	TIAF1	hypothetical gene	-1.91	0.0807
ssc-miR-29b	TIAM1	TIAM Rac1 associated GEF 1 [Source:VGNC Symbol;Acc:VGNC:96601]	-1.91	0.0807
ssc-miR-29b	TIGD5	tigger transposable element derived 5 [Source:VGNC Symbol;Acc:VGNC:98876]	-1.91	0.0807
ssc-miR-29b	TIMM8B	translocase of inner mitochondrial membrane 8 homolog B [Source:VGNC Symbol;Acc:VGNC:93993]	-1.91	0.0807
ssc-miR-29b	TLL1	tolloid like 1 [Source:VGNC Symbol;Acc:VGNC:94018]	-1.91	0.0807
ssc-miR-29b	TLR6	toll like receptor 6 [Source:VGNC Symbol;Acc:VGNC:94026]	-1.91	0.0807
ssc-miR-29b	TMC5	transmembrane channel like 5 [Source:VGNC Symbol;Acc:VGNC:94042]	-1.91	0.0807
ssc-miR-29b	TMED9	transmembrane p24 trafficking protein 9 [Source:VGNC Symbol;Acc:VGNC:100875]	-1.91	0.0807
ssc-miR-29b	TMEM127	transmembrane protein 127 [Source:HGNC Symbol;Acc:HGNC:26038]	-1.91	0.0807
ssc-miR-29b	TMEM132A	transmembrane protein 132A [Source:VGNC Symbol;Acc:VGNC:94080]	-1.91	0.0807
ssc-miR-29b	TMEM136	hypothetical gene	-1.91	0.0807
ssc-miR-29b	TMEM151B	transmembrane protein 151B [Source:VGNC Symbol;Acc:VGNC:98376]	-1.91	0.0807
ssc-miR-29b	TMEM164	transmembrane protein 164 [Source:VGNC Symbol;Acc:VGNC:94103]	-1.91	0.0807
ssc-miR-29b	TMEM169	transmembrane protein 169 [Source:HGNC Symbol;Acc:HGNC:25130]	-1.91	0.0807
ssc-miR-29b	TMEM178B	transmembrane protein 178B [Source:HGNC Symbol;Acc:HGNC:44112]	-1.91	0.0807
ssc-miR-29b	TMEM179B	transmembrane protein 179B [Source:VGNC Symbol;Acc:VGNC:94116]	-1.91	0.0807
ssc-miR-29b	TMEM183A	transmembrane protein 183A [Source:NCBI gene (formerly Entrezgene);Acc:100512006]	-1.91	0.0807
ssc-miR-29b	TMEM229B	hypothetical gene	-1.91	0.0807
ssc-miR-29b	TMEM234	transmembrane protein 234 [Source:VGNC Symbol;Acc:VGNC:94148]	-1.91	0.0807
ssc-miR-29b	TMEM236	transmembrane protein 236 [Source:VGNC Symbol;Acc:VGNC:96006]	-1.91	0.0807
ssc-miR-29b	TMEM255A	transmembrane protein 255A [Source:VGNC Symbol;Acc:VGNC:104079]	-1.91	0.0807
ssc-miR-29b	TMEM256-PLSCR3	hypothetical gene	-1.91	0.0807
ssc-miR-29b	TMEM259	transmembrane protein 259 [Source:VGNC Symbol;Acc:VGNC:94162]	-1.91	0.0807
ssc-miR-29b	TMEM33	transmembrane protein 33 [Source:VGNC Symbol;Acc:VGNC:94171]	-1.91	0.0807
ssc-miR-29b	TMEM44	transmembrane protein 44 [Source:VGNC Symbol;Acc:VGNC:94181]	-1.91	0.0807
ssc-miR-29b	TMEM65	transmembrane protein 65 [Source:VGNC Symbol;Acc:VGNC:94200]	-1.91	0.0807
ssc-miR-29b	TMEM86A	transmembrane protein 86A [Source:VGNC Symbol;Acc:VGNC:94210]	-1.91	0.0807
ssc-miR-29b	TMOD3	tropomodulin 3 [Source:VGNC Symbol;Acc:VGNC:94228]	-1.91	0.0807
ssc-miR-29b	TMPRSS3	transmembrane serine protease 3 [Source:VGNC Symbol;Acc:VGNC:94235]	-1.91	0.0807
ssc-miR-29b	TMTC3	transmembrane O-mannosyltransferase targeting cadherins 3 [Source:VGNC Symbol;Acc:VGNC:94243]	-1.91	0.0807
ssc-miR-29b	TMUB2	transmembrane and ubiquitin like domain containing 2 [Source:VGNC Symbol;Acc:VGNC:94246]	-1.91	0.0807
ssc-miR-29b	TNFAIP1	TNF alpha induced protein 1 [Source:VGNC Symbol;Acc:VGNC:94250]	-1.91	0.0807
ssc-miR-29b	TNFAIP3	TNF alpha induced protein 3 [Source:VGNC Symbol;Acc:VGNC:94252]	-1.91	0.0807
ssc-miR-29b	TNFRSF11A	TNF receptor superfamily member 11a [Source:VGNC Symbol;Acc:VGNC:94257]	-1.91	0.0807
ssc-miR-29b	TNFRSF1A	TNF receptor superfamily member 1A [Source:NCBI gene (formerly Entrezgene);Acc:397020]	-1.91	0.0807
ssc-miR-29b	TNRC18	trinucleotide repeat containing 18 [Source:VGNC Symbol;Acc:VGNC:94291]	-1.91	0.0807
ssc-miR-29b	TNRC6B	trinucleotide repeat containing adaptor 6B [Source:VGNC Symbol;Acc:VGNC:94293]	-1.91	0.0807
ssc-miR-29b	TP53INP1	tumor protein p53 inducible nuclear protein 1 [Source:VGNC Symbol;Acc:VGNC:94329]	-1.91	0.0807
ssc-miR-29b	TP53INP2	tumor protein p53 inducible nuclear protein 2 [Source:VGNC Symbol;Acc:VGNC:95737]	-1.91	0.0807
ssc-miR-29b	TPK1	thiamin pyrophosphokinase 1 [Source:VGNC Symbol;Acc:VGNC:94337]	-1.91	0.0807
ssc-miR-29b	TPM1	tropomyosin 1 [Source:VGNC Symbol;Acc:VGNC:103194]	-1.91	0.0807
ssc-miR-29b	TRAF3	TNF receptor associated factor 3 [Source:VGNC Symbol;Acc:VGNC:94360]	-1.91	0.0807
ssc-miR-29b	TRAF4	TNF receptor associated factor 4 [Source:VGNC Symbol;Acc:VGNC:94363]	-1.91	0.0807
ssc-miR-29b	TRAF5	TNF receptor associated factor 5 [Source:VGNC Symbol;Acc:VGNC:94364]	-1.91	0.0807
ssc-miR-29b	TRAFD1	TRAF-type zinc finger domain containing 1 [Source:VGNC Symbol;Acc:VGNC:94367]	-1.91	0.0807

ssc-miR-29b	TRAK2	trafficking kinesin protein 2 [Source:VGNC Symbol;Acc:VGNC:95577]	-1.91	0.0807
ssc-miR-29b	TRAM2	translocation associated membrane protein 2 [Source:HGNC Symbol;Acc:HGNC:16855]	-1.91	0.0807
ssc-miR-29b	TRIB2	tribbles pseudokinase 2 [Source:VGNC Symbol;Acc:VGNC:94392]	-1.91	0.0807
ssc-miR-29b	TRIM24	tripartite motif containing 24 [Source:VGNC Symbol;Acc:VGNC:94400]	-1.91	0.0807
ssc-miR-29b	TRIM63	tripartite motif containing 63 [Source:VGNC Symbol;Acc:VGNC:94424]	-1.91	0.0807
ssc-miR-29b	TRIM66	tripartite motif containing 66 [Source:VGNC Symbol;Acc:VGNC:94426]	-1.91	0.0807
ssc-miR-29b	TRIM9	tripartite motif containing 9 [Source:VGNC Symbol;Acc:VGNC:94434]	-1.91	0.0807
ssc-miR-29b	TRPC6	transient receptor potential cation channel subfamily C member 6 [Source:VGNC Symbol;Acc:VGNC:94464]	-1.91	0.0807
ssc-miR-29b	TSC22D3	hypothetical gene	-1.91	0.0807
ssc-miR-29b	TSFM	Ts translation elongation factor, mitochondrial [Source:VGNC Symbol;Acc:VGNC:94486]	-1.91	0.0807
ssc-miR-29b	TSPAN14	tetraspanin 14 [Source:VGNC Symbol;Acc:VGNC:94503]	-1.91	0.0807
ssc-miR-29b	TSPAN4	tetraspanin 4 [Source:VGNC Symbol;Acc:VGNC:94513]	-1.91	0.0807
ssc-miR-29b	TSPAN9	tetraspanin 9 [Source:VGNC Symbol;Acc:VGNC:94516]	-1.91	0.0807
ssc-miR-29b	TTC14	tetratricopeptide repeat domain 14 [Source:VGNC Symbol;Acc:VGNC:94535]	-1.91	0.0807
ssc-miR-29b	TTC30B	hypothetical gene	-1.91	0.0807
ssc-miR-29b	TTC9	tetratricopeptide repeat domain 9 [Source:VGNC Symbol;Acc:VGNC:94560]	-1.91	0.0807
ssc-miR-29b	TTYH2	tweety family member 2 [Source:VGNC Symbol;Acc:VGNC:94575]	-1.91	0.0807
ssc-miR-29b	TUBB2A	tubulin beta 2A class IIa [Source:HGNC Symbol;Acc:HGNC:12412]	-1.91	0.0807
ssc-miR-29b	TUBB2B	tubulin, beta 2B class IIb [Source:NCBI gene (formerly Entrezgene);Acc:100153507]	-1.91	0.0807
ssc-miR-29b	TUBD1	tubulin delta 1 [Source:VGNC Symbol;Acc:VGNC:94582]	-1.91	0.0807
ssc-miR-29b	TXLNA	taxilin alpha [Source:VGNC Symbol;Acc:VGNC:94600]	-1.91	0.0807
ssc-miR-29b	U2SURP	U2 snRNP associated SURP domain containing [Source:VGNC Symbol;Acc:VGNC:94624]	-1.91	0.0807
ssc-miR-29b	UACA	uveal autoantigen with coiled-coil domains and ankyrin repeats [Source:VGNC Symbol;Acc:VGNC:94625]	-1.91	0.0807
ssc-miR-29b	UBE2E3	ubiquitin conjugating enzyme E2 E3 [Source:VGNC Symbol;Acc:VGNC:106461]	-1.91	0.0807
ssc-miR-29b	UBE2H	ubiquitin conjugating enzyme E2 H [Source:VGNC Symbol;Acc:VGNC:94643]	-1.91	0.0807
ssc-miR-29b	UBE2K	ubiquitin conjugating enzyme E2 K [Source:VGNC Symbol;Acc:VGNC:94646]	-1.91	0.0807
ssc-miR-29b	UBFD1	ubiquitin family domain containing 1 [Source:VGNC Symbol;Acc:VGNC:94658]	-1.91	0.0807
ssc-miR-29b	UBIAD1	UbiA prenyltransferase domain containing 1 [Source:VGNC Symbol;Acc:VGNC:94659]	-1.91	0.0807
ssc-miR-29b	UBN1	ubinuclein 1 [Source:VGNC Symbol;Acc:VGNC:94663]	-1.91	0.0807
ssc-miR-29b	UBN2	ubinuclein 2 [Source:VGNC Symbol;Acc:VGNC:94664]	-1.91	0.0807
ssc-miR-29b	UBTD1	ubiquitin domain containing 1 [Source:VGNC Symbol;Acc:VGNC:94672]	-1.91	0.0807
ssc-miR-29b	UBTD2	ubiquitin domain containing 2 [Source:VGNC Symbol;Acc:VGNC:94673]	-1.91	0.0807
ssc-miR-29b	UBTF	upstream binding transcription factor [Source:VGNC Symbol;Acc:VGNC:108185]	-1.91	0.0807
ssc-miR-29b	UBXN7	hypothetical gene	-1.91	0.0807
ssc-miR-29b	UCK2	uridine-cytidine kinase 2 [Source:VGNC Symbol;Acc:VGNC:98399]	-1.91	0.0807
ssc-miR-29b	UHRF1BP1L	UHRF1 binding protein 1 like [Source:VGNC Symbol;Acc:VGNC:94691]	-1.91	0.0807
ssc-miR-29b	UNC13B	unc-13 homolog B [Source:VGNC Symbol;Acc:VGNC:103199]	-1.91	0.0807
ssc-miR-29b	UNK	unk zinc finger [Source:VGNC Symbol;Acc:VGNC:94715]	-1.91	0.0807
ssc-miR-29b	UPK1B	uroplakin 1B [Source:VGNC Symbol;Acc:VGNC:94722]	-1.91	0.0807
ssc-miR-29b	URB1	URB1 ribosome biosis homolog [Source:VGNC Symbol;Acc:VGNC:99817]	-1.91	0.0807
ssc-miR-29b	URM1	ubiquitin related modifier 1 [Source:VGNC Symbol;Acc:VGNC:94732]	-1.91	0.0807
ssc-miR-29b	USP15	ubiquitin specific peptidase 15 [Source:VGNC Symbol;Acc:VGNC:94748]	-1.91	0.0807
ssc-miR-29b	USP2	ubiquitin specific peptidase 2 [Source:VGNC Symbol;Acc:VGNC:94751]	-1.91	0.0807
ssc-miR-29b	USP27X	ubiquitin specific peptidase 27 X-linked [Source:HGNC Symbol;Acc:HGNC:13486]	-1.91	0.0807
ssc-miR-29b	USP31	ubiquitin specific peptidase 31 [Source:HGNC Symbol;Acc:HGNC:20060]	-1.91	0.0807
ssc-miR-29b	USP34	ubiquitin specific peptidase 34 [Source:VGNC Symbol;Acc:VGNC:94759]	-1.91	0.0807
ssc-miR-29b	USP37	ubiquitin specific peptidase 37 [Source:VGNC Symbol;Acc:VGNC:98404]	-1.91	0.0807
ssc-miR-29b	USP42	ubiquitin specific peptidase 42 [Source:HGNC Symbol;Acc:HGNC:20068]	-1.91	0.0807
ssc-miR-29b	USP6NL	USP6 N-terminal like [Source:VGNC Symbol;Acc:VGNC:95820]	-1.91	0.0807
ssc-miR-29b	VAMP3	hypothetical gene	-1.91	0.0807

ssc-miR-29b	VAMP7	vesicle associated membrane protein 7 [Source:VGNC Symbol;Acc:VGNC:94796]	-1.91	0.0807
ssc-miR-29b	VANGL1	VANGL planar cell polarity protein 1 [Source:VGNC Symbol;Acc:VGNC:94797]	-1.91	0.0807
ssc-miR-29b	VAPB	VAMP associated protein B and C [Source:VGNC Symbol;Acc:VGNC:108743]	-1.91	0.0807
ssc-miR-29b	VASH1	vasohibin 1 [Source:VGNC Symbol;Acc:VGNC:94801]	-1.91	0.0807
ssc-miR-29b	VASH2	vasohibin 2 [Source:VGNC Symbol;Acc:VGNC:94802]	-1.91	0.0807
ssc-miR-29b	VCL	vinculin [Source:VGNC Symbol;Acc:VGNC:94812]	-1.91	0.0807
ssc-miR-29b	VCPKMT	valosin containing protein lysine methyltransferase [Source:VGNC Symbol;Acc:VGNC:98406]	-1.91	0.0807
ssc-miR-29b	VEGFA	vascular endothelial growth factor A [Source:VGNC Symbol;Acc:VGNC:94815]	-1.91	0.0807
ssc-miR-29b	VHL	hypothetical gene	-1.91	0.0807
ssc-miR-29b	VIPAS39	VPS33B interacting protein, apical-basolateral polarity regulator, spe-39 homolog [Source:VGNC Symbol;Acc:VGNC:94826]	-1.91	0.0807
ssc-miR-29b	VPS13A	vacuolar protein sorting 13 homolog A [Source:VGNC Symbol;Acc:VGNC:94838]	-1.91	0.0807
ssc-miR-29b	VPS13B	vacuolar protein sorting 13 homolog B [Source:VGNC Symbol;Acc:VGNC:94839]	-1.91	0.0807
ssc-miR-29b	VPS25	vacuolar protein sorting 25 homolog [Source:VGNC Symbol;Acc:VGNC:94843]	-1.91	0.0807
ssc-miR-29b	VPS36	vacuolar protein sorting 36 homolog [Source:VGNC Symbol;Acc:VGNC:94850]	-1.91	0.0807
ssc-miR-29b	VPS37C	VPS37C subunit of ESCRT-I [Source:VGNC Symbol;Acc:VGNC:94852]	-1.91	0.0807
ssc-miR-29b	VTA1	vesicle trafficking 1 [Source:VGNC Symbol;Acc:VGNC:94877]	-1.91	0.0807
ssc-miR-29b	WAC	WW domain containing adaptor with coiled-coil [Source:VGNC Symbol;Acc:VGNC:95845]	-1.91	0.0807
ssc-miR-29b	WASF1	hypothetical gene	-1.91	0.0807
ssc-miR-29b	WASF2	WASP family member 2 [Source:VGNC Symbol;Acc:VGNC:94893]	-1.91	0.0807
ssc-miR-29b	WBP1L	WW domain binding protein 1 like [Source:VGNC Symbol;Acc:VGNC:94898]	-1.91	0.0807
ssc-miR-29b	WDFY1	WD repeat and FYVE domain containing 1 [Source:VGNC Symbol;Acc:VGNC:99733]	-1.91	0.0807
ssc-miR-29b	WDFY3	WD repeat and FYVE domain containing 3 [Source:VGNC Symbol;Acc:VGNC:94903]	-1.91	0.0807
ssc-miR-29b	WDR26	WD repeat domain 26 [Source:VGNC Symbol;Acc:VGNC:95960]	-1.91	0.0807
ssc-miR-29b	WDR31	WD repeat domain 31 [Source:VGNC Symbol;Acc:VGNC:94914]	-1.91	0.0807
ssc-miR-29b	WDR37	WD repeat domain 37 [Source:VGNC Symbol;Acc:VGNC:96258]	-1.91	0.0807
ssc-miR-29b	WDR81	WD repeat domain 81 [Source:VGNC Symbol;Acc:VGNC:99104]	-1.91	0.0807
ssc-miR-29b	WISP1	hypothetical gene	-1.91	0.0807
ssc-miR-29b	WNT10B	Wnt family member 10B [Source:VGNC Symbol;Acc:VGNC:94965]	-1.91	0.0807
ssc-miR-29b	WNT8B	Wnt family member 8B [Source:VGNC Symbol;Acc:VGNC:94977]	-1.91	0.0807
ssc-miR-29b	WSCD2	WSC domain containing 2 [Source:HGNC Symbol;Acc:HGNC:29117]	-1.91	0.0807
ssc-miR-29b	XKR4	XK related 4 [Source:VGNC Symbol;Acc:VGNC:98900]	-1.91	0.0807
ssc-miR-29b	XKR6	XK related 6 [Source:HGNC Symbol;Acc:HGNC:27806]	-1.91	0.0807
ssc-miR-29b	XKR7	XK related 7 [Source:VGNC Symbol;Acc:VGNC:95987]	-1.91	0.0807
ssc-miR-29b	XKRX	XK related X-linked [Source:HGNC Symbol;Acc:HGNC:29845]	-1.91	0.0807
ssc-miR-29b	XPNPEP1	X-prolyl aminopeptidase 1 [Source:VGNC Symbol;Acc:VGNC:95000]	-1.91	0.0807
ssc-miR-29b	XPO4	exportin 4 [Source:VGNC Symbol;Acc:VGNC:95004]	-1.91	0.0807
ssc-miR-29b	XPO5	exportin 5 [Source:VGNC Symbol;Acc:VGNC:95005]	-1.91	0.0807
ssc-miR-29b	XRN1	5'-3' exoribonuclease 1 [Source:VGNC Symbol;Acc:VGNC:108705]	-1.91	0.0807
ssc-miR-29b	XXYLT1	xyloside xylosyltransferase 1 [Source:VGNC Symbol;Acc:VGNC:95013]	-1.91	0.0807
ssc-miR-29b	XYLT1	xylosyltransferase 1 [Source:VGNC Symbol;Acc:VGNC:95015]	-1.91	0.0807
ssc-miR-29b	YBX3	Y-box binding protein 3 [Source:VGNC Symbol;Acc:VGNC:95021]	-1.91	0.0807
ssc-miR-29b	YPEL2	yippee like 2 [Source:VGNC Symbol;Acc:VGNC:95036]	-1.91	0.0807
ssc-miR-29b	YRDC	yrdC N6-threonylcarbamoyltransferase domain containing [Source:VGNC Symbol;Acc:VGNC:95040]	-1.91	0.0807
ssc-miR-29b	YWHAE	tyrosine 3-monooxygenase/tryptophan 5-monooxygenase activation protein epsilon [Source:HGNC Symbol;Acc:HGNC:12851]	-1.91	0.0807
ssc-miR-29b	YY1	YY1 transcription factor [Source:HGNC Symbol;Acc:HGNC:12856]	-1.91	0.0807
ssc-miR-29b	YY2	hypothetical gene	-1.91	0.0807
ssc-miR-29b	ZBTB10	zinc finger and BTB domain containing 10 [Source:VGNC Symbol;Acc:VGNC:95058]	-1.91	0.0807
ssc-miR-29b	ZBTB20	zinc finger and BTB domain containing 20 [Source:VGNC Symbol;Acc:VGNC:95063]	-1.91	0.0807
ssc-miR-29b	ZBTB21	zinc finger and BTB domain containing 21 [Source:HGNC Symbol;Acc:HGNC:13083]	-1.91	0.0807
ssc-miR-29b	ZBTB34	zinc finger and BTB domain containing 34 [Source:VGNC Symbol;Acc:VGNC:95070]	-1.91	0.0807

ssc-miR-29b	ZBTB37	zinc finger and BTB domain containing 37 [Source:VGNC Symbol;Acc:VGNC:95071]	-1.91	0.0807
ssc-miR-29b	ZBTB40	zinc finger and BTB domain containing 40 [Source:VGNC Symbol;Acc:VGNC:95074]	-1.91	0.0807
ssc-miR-29b	ZBTB41	zinc finger and BTB domain containing 41 [Source:VGNC Symbol;Acc:VGNC:108284]	-1.91	0.0807
ssc-miR-29b	ZBTB43	zinc finger and BTB domain containing 43 [Source:VGNC Symbol;Acc:VGNC:95075]	-1.91	0.0807
ssc-miR-29b	ZBTB46	zinc finger and BTB domain containing 46 [Source:VGNC Symbol;Acc:VGNC:95733]	-1.91	0.0807
ssc-miR-29b	ZBTB47	zinc finger and BTB domain containing 47 [Source:VGNC Symbol;Acc:VGNC:95078]	-1.91	0.0807
ssc-miR-29b	ZBTB5	zinc finger and BTB domain containing 5 [Source:VGNC Symbol;Acc:VGNC:103204]	-1.91	0.0807
ssc-miR-29b	ZBTB8B	zinc finger and BTB domain containing 8B [Source:HGNC Symbol;Acc:HGNC:37057]	-1.91	0.0807
ssc-miR-29b	ZC3HAV1L	zinc finger CCCH-type containing, antiviral 1 like [Source:VGNC Symbol;Acc:VGNC:95103]	-1.91	0.0807
ssc-miR-29b	ZC4H2	zinc finger C4H2-type containing [Source:VGNC Symbol;Acc:VGNC:95105]	-1.91	0.0807
ssc-miR-29b	ZCCHC17	zinc finger CCHC-type containing 17 [Source:VGNC Symbol;Acc:VGNC:95108]	-1.91	0.0807
ssc-miR-29b	ZDHHC21	zinc finger DHHC-type palmitoyltransferase 21 [Source:VGNC Symbol;Acc:VGNC:95123]	-1.91	0.0807
ssc-miR-29b	ZDHHC5	zinc finger DHHC-type palmitoyltransferase 5 [Source:VGNC Symbol;Acc:VGNC:95127]	-1.91	0.0807
ssc-miR-29b	ZER1	zyg-11 related cell cycle regulator [Source:VGNC Symbol;Acc:VGNC:95132]	-1.91	0.0807
ssc-miR-29b	ZFP36	ZFP36 ring finger protein [Source:VGNC Symbol;Acc:VGNC:95145]	-1.91	0.0807
ssc-miR-29b	ZFP36L1	ZFP36 ring finger protein like 1 [Source:VGNC Symbol;Acc:VGNC:95146]	-1.91	0.0807
ssc-miR-29b	ZFP91	ZFP91 zinc finger protein, atypical E3 ubiquitin ligase [Source:HGNC Symbol;Acc:HGNC:14983]	-1.91	0.0807
ssc-miR-29b	ZFX	hypothetical gene	-1.91	0.0807
ssc-miR-29b	ZFY	zinc finger protein Y-linked [Source:HGNC Symbol;Acc:HGNC:12870]	-1.91	0.0807
ssc-miR-29b	ZFYVE26	zinc finger FYVE-type containing 26 [Source:VGNC Symbol;Acc:VGNC:95160]	-1.91	0.0807
ssc-miR-29b	ZHX3	zinc fingers and homeoboxes 3 [Source:VGNC Symbol;Acc:VGNC:95717]	-1.91	0.0807
ssc-miR-29b	ZIC5	Zic family member 5 [Source:VGNC Symbol;Acc:VGNC:95170]	-1.91	0.0807
ssc-miR-29b	ZKSCAN3	hypothetical gene	-1.91	0.0807
ssc-miR-29b	ZKSCAN4	zinc finger with KRAB and SCAN domains 4 [Source:VGNC Symbol;Acc:VGNC:95173]	-1.91	0.0807
ssc-miR-29b	ZMIZ1	zinc finger MIZ-type containing 1 [Source:VGNC Symbol;Acc:VGNC:95178]	-1.91	0.0807
ssc-miR-29b	ZMYM2	zinc finger MYM-type containing 2 [Source:VGNC Symbol;Acc:VGNC:95182]	-1.91	0.0807
ssc-miR-29b	ZMYM3	hypothetical gene	-1.91	0.0807
ssc-miR-29b	ZNF24	zinc finger protein 24 [Source:VGNC Symbol;Acc:VGNC:98422]	-1.91	0.0807
ssc-miR-29b	ZNF282	hypothetical gene	-1.91	0.0807
ssc-miR-29b	ZNF362	zinc finger protein 362 [Source:VGNC Symbol;Acc:VGNC:95229]	-1.91	0.0807
ssc-miR-29b	ZNF366	zinc finger protein 366 [Source:VGNC Symbol;Acc:VGNC:95231]	-1.91	0.0807
ssc-miR-29b	ZNF384	zinc finger protein 384 [Source:HGNC Symbol;Acc:HGNC:11955]	-1.91	0.0807
ssc-miR-29b	ZNF469	hypothetical gene	-1.91	0.0807
ssc-miR-29b	ZNF507	zinc finger protein 507 [Source:VGNC Symbol;Acc:VGNC:95253]	-1.91	0.0807
ssc-miR-29b	ZNF512B	zinc finger protein 512B [Source:HGNC Symbol;Acc:HGNC:29212]	-1.91	0.0807
ssc-miR-29b	ZNF518B	zinc finger protein 518B [Source:VGNC Symbol;Acc:VGNC:95258]	-1.91	0.0807
ssc-miR-29b	ZNF532	zinc finger protein 532 [Source:HGNC Symbol;Acc:HGNC:30940]	-1.91	0.0807
ssc-miR-29b	ZNF609	zinc finger protein 609 [Source:VGNC Symbol;Acc:VGNC:95274]	-1.91	0.0807
ssc-miR-29b	ZNF641	zinc finger protein 641 [Source:HGNC Symbol;Acc:HGNC:31834]	-1.91	0.0807
ssc-miR-29b	ZNF652	zinc finger protein 652 [Source:VGNC Symbol;Acc:VGNC:99108]	-1.91	0.0807
ssc-miR-29b	ZNF704	zinc finger protein 704 [Source:VGNC Symbol;Acc:VGNC:95296]	-1.91	0.0807
ssc-miR-29b	ZNF852	zinc finger protein 852 [Source:HGNC Symbol;Acc:HGNC:27713]	-1.91	0.0807
ssc-miR-29b	ZSCAN25	zinc finger and SCAN domain containing 25 [Source:VGNC Symbol;Acc:VGNC:95329]	-1.91	0.0807
ssc-miR-504	NA	hypothetical gene	-1.98	0.03
ssc-miR-628	NA	hypothetical gene	-1.99	0.0747
ssc-miR-411	AC005003.1	hypothetical gene	-2.07	0.0789
ssc-miR-411	ADAMTS20	ADAM metallopeptidase with thrombospondin type 1 motif 20 [Source:VGNC Symbol;Acc:VGNC:85082]	-2.07	0.0789
ssc-miR-411	ADAMTS3	ADAM metallopeptidase with thrombospondin type 1 motif 3 [Source:VGNC Symbol;Acc:VGNC:85083]	-2.07	0.0789
ssc-miR-411	ADNP	activity dependent neuroprotector homeobox [Source:VGNC Symbol;Acc:VGNC:95657]	-2.07	0.0789
ssc-miR-411	ADORA1	adenosine A1 receptor [Source:VGNC Symbol;Acc:VGNC:85147]	-2.07	0.0789

ssc-miR-411	ADPGK	ADP dependent glucokinase [Source:VGNC Symbol;Acc:VGNC:85150]	-2.07	0.0789
ssc-miR-411	AGO1	hypothetical gene	-2.07	0.0789
ssc-miR-411	AKAP5	A-kinase anchoring protein 5 [Source:VGNC Symbol;Acc:VGNC:85220]	-2.07	0.0789
ssc-miR-411	ALDOA	aldolase, fructose-bisphosphate A [Source:VGNC Symbol;Acc:VGNC:102549]	-2.07	0.0789
ssc-miR-411	AMD1	hypothetical gene	-2.07	0.0789
ssc-miR-411	ANTXR2	ANTXR cell adhesion molecule 2 [Source:VGNC Symbol;Acc:VGNC:85365]	-2.07	0.0789
ssc-miR-411	APOLD1	apolipoprotein L domain containing 1 [Source:VGNC Symbol;Acc:VGNC:103215]	-2.07	0.0789
ssc-miR-411	ARHGAP5	Rho GTPase activating protein 5 [Source:VGNC Symbol;Acc:VGNC:85480]	-2.07	0.0789
ssc-miR-411	ARIH1	ariadne RBR E3 ubiquitin protein ligase 1 [Source:HGNC Symbol;Acc:HGNC:689]	-2.07	0.0789
ssc-miR-411	ARRDC3	arrestin domain containing 3 [Source:VGNC Symbol;Acc:VGNC:85546]	-2.07	0.0789
ssc-miR-411	ASB14	ankyrin repeat and SOCS box containing 14 [Source:VGNC Symbol;Acc:VGNC:85562]	-2.07	0.0789
ssc-miR-411	ATP2B4	ATPase plasma membrane Ca2+ transporting 4 [Source:VGNC Symbol;Acc:VGNC:85651]	-2.07	0.0789
ssc-miR-411	ATP5F1	hypothetical gene	-2.07	0.0789
ssc-miR-411	BNIP3	BCL2 interacting protein 3 [Source:VGNC Symbol;Acc:VGNC:85855]	-2.07	0.0789
ssc-miR-411	C16orf52	hypothetical gene	-2.07	0.0789
ssc-miR-411	C21orf91	chromosome 13 C21orf91 homolog [Source:VGNC Symbol;Acc:VGNC:85930]	-2.07	0.0789
ssc-miR-411	C4orf32	hypothetical gene	-2.07	0.0789
ssc-miR-411	CACHD1	cache domain containing 1 [Source:VGNC Symbol;Acc:VGNC:86116]	-2.07	0.0789
ssc-miR-411	CACNB4	calcium voltage-gated channel auxiliary subunit beta 4 [Source:VGNC Symbol;Acc:VGNC:108507]	-2.07	0.0789
ssc-miR-411	CALML4	calmodulin like 4 [Source:VGNC Symbol;Acc:VGNC:86148]	-2.07	0.0789
ssc-miR-411	CAMKK2	calcium/calmodulin dependent protein kinase kinase 2 [Source:VGNC Symbol;Acc:VGNC:86158]	-2.07	0.0789
ssc-miR-411	CAMLG	calcium modulating ligand [Source:VGNC Symbol;Acc:VGNC:86161]	-2.07	0.0789
ssc-miR-411	CAMSAP1	calmodulin regulated spectrin associated protein 1 [Source:VGNC Symbol;Acc:VGNC:86162]	-2.07	0.0789
ssc-miR-411	CASP10	caspase 10 [Source:VGNC Symbol;Acc:VGNC:95638]	-2.07	0.0789
ssc-miR-411	CATSPERG	cation channel sperm associated auxiliary subunit gamma [Source:VGNC Symbol;Acc:VGNC:86215]	-2.07	0.0789
ssc-miR-411	CCDC144A	hypothetical gene	-2.07	0.0789
ssc-miR-411	CCDC85C	coiled-coil domain containing 85C [Source:VGNC Symbol;Acc:VGNC:86322]	-2.07	0.0789
ssc-miR-411	CD200	CD200 molecule [Source:VGNC Symbol;Acc:VGNC:86396]	-2.07	0.0789
ssc-miR-411	CDC37L1	cell division cycle 37 like 1 [Source:VGNC Symbol;Acc:VGNC:86451]	-2.07	0.0789
ssc-miR-411	CDH2	cadherin 2 [Source:VGNC Symbol;Acc:VGNC:86483]	-2.07	0.0789
ssc-miR-411	CDH6	cadherin 6 [Source:HGNC Symbol;Acc:HGNC:1765]	-2.07	0.0789
ssc-miR-411	CELF1	CUGBP Elav-like family member 1 [Source:VGNC Symbol;Acc:VGNC:86537]	-2.07	0.0789
ssc-miR-411	CHURC1-FNTB	hypothetical gene	-2.07	0.0789
ssc-miR-411	CIB1	calcium and integrin binding 1 [Source:VGNC Symbol;Acc:VGNC:86695]	-2.07	0.0789
ssc-miR-411	CLCN5	chloride voltage-gated channel 5 [Source:VGNC Symbol;Acc:VGNC:103925]	-2.07	0.0789
ssc-miR-411	CLEC11A	C-type lectin domain containing 11A [Source:VGNC Symbol;Acc:VGNC:86744]	-2.07	0.0789
ssc-miR-411	CLIP2	hypothetical gene	-2.07	0.0789
ssc-miR-411	CLLU1	hypothetical gene	-2.07	0.0789
ssc-miR-411	CNNM2	cyclin and CBS domain divalent metal cation transport mediator 2 [Source:VGNC Symbol;Acc:VGNC:86828]	-2.07	0.0789
ssc-miR-411	CPEB4	cytoplasmic polyadenylation element binding protein 4 [Source:VGNC Symbol;Acc:VGNC:86939]	-2.07	0.0789
ssc-miR-411	CPT1A	carnitine palmitoyltransferase 1A [Source:VGNC Symbol;Acc:VGNC:86964]	-2.07	0.0789
ssc-miR-411	CREBL2	cAMP responsive element binding protein like 2 [Source:VGNC Symbol;Acc:VGNC:86986]	-2.07	0.0789
ssc-miR-411	CSNK1G1	casein kinase 1 gamma 1 [Source:VGNC Symbol;Acc:VGNC:97949]	-2.07	0.0789
ssc-miR-411	CSRNP3	cysteine and serine rich nuclear protein 3 [Source:VGNC Symbol;Acc:VGNC:96249]	-2.07	0.0789
ssc-miR-411	CTC-432M15.3	hypothetical gene	-2.07	0.0789
ssc-miR-411	CTDP1	CTD phosphatase subunit 1 [Source:VGNC Symbol;Acc:VGNC:87058]	-2.07	0.0789
ssc-miR-411	CTNND1	catenin delta 1 [Source:VGNC Symbol;Acc:VGNC:87067]	-2.07	0.0789
ssc-miR-411	CYLD	CYLD lysine 63 deubiquitinase [Source:VGNC Symbol;Acc:VGNC:87130]	-2.07	0.0789
ssc-miR-411	DGKH	diacylglycerol kinase eta [Source:VGNC Symbol;Acc:VGNC:87273]	-2.07	0.0789
ssc-miR-411	DUSP1	dual specificity phosphatase 1 [Source:VGNC Symbol;Acc:VGNC:87476]	-2.07	0.0789

ssc-miR-411	DUSP13	hypothetical gene	-2.07	0.0789
ssc-miR-411	DVL3	dishevelled segment polarity protein 3 [Source:VGNC Symbol;Acc:VGNC:87492]	-2.07	0.0789
ssc-miR-411	EBF1	EBF transcription factor 1 [Source:VGNC Symbol;Acc:VGNC:87525]	-2.07	0.0789
ssc-miR-411	EBF2	EBF transcription factor 2 [Source:VGNC Symbol;Acc:VGNC:87526]	-2.07	0.0789
ssc-miR-411	EIF3J	eukaryotic translation initiation factor 3 subunit J [Source:VGNC Symbol;Acc:VGNC:87621]	-2.07	0.0789
ssc-miR-411	EIF4G2	eukaryotic translation initiation factor 4 gamma 2 [Source:VGNC Symbol;Acc:VGNC:99645]	-2.07	0.0789
ssc-miR-411	EIF5	hypothetical gene	-2.07	0.0789
ssc-miR-411	ELFN1	extracellular leucine rich repeat and fibronectin type III domain containing 1 [Source:VGNC Symbol;Acc:VGNC:87645]	-2.07	0.0789
ssc-miR-411	EPC1	enhancer of polycomb homolog 1 [Source:VGNC Symbol;Acc:VGNC:95923]	-2.07	0.0789
ssc-miR-411	ERBB4	erb-b2 receptor tyrosine kinase 4 [Source:VGNC Symbol;Acc:VGNC:96284]	-2.07	0.0789
ssc-miR-411	EVI2A	ecotropic viral integration site 2A [Source:HGNC Symbol;Acc:HGNC:3499]	-2.07	0.0789
ssc-miR-411	FAM175B	hypothetical gene	-2.07	0.0789
ssc-miR-411	FAM204A	family with sequence similarity 204 member A [Source:VGNC Symbol;Acc:VGNC:107389]	-2.07	0.0789
ssc-miR-411	FAM53C	family with sequence similarity 53 member C [Source:VGNC Symbol;Acc:VGNC:87975]	-2.07	0.0789
ssc-miR-411	FBXL3	F-box and leucine rich repeat protein 3 [Source:VGNC Symbol;Acc:VGNC:88030]	-2.07	0.0789
ssc-miR-411	FBXL5	F-box and leucine rich repeat protein 5 [Source:VGNC Symbol;Acc:VGNC:98008]	-2.07	0.0789
ssc-miR-411	FNIP1	folliculin interacting protein 1 [Source:VGNC Symbol;Acc:VGNC:88187]	-2.07	0.0789
ssc-miR-411	FNTB	farnesyltransferase, CAAX box, beta [Source:NCBI gene (formerly Entrezgene);Acc:100512293]	-2.07	0.0789
ssc-miR-411	FRMD4A	FERM domain containing 4A [Source:VGNC Symbol;Acc:VGNC:96088]	-2.07	0.0789
ssc-miR-411	FZD4	frizzled class receptor 4 [Source:VGNC Symbol;Acc:VGNC:88282]	-2.07	0.0789
ssc-miR-411	GALNT13	polypeptide N-acetylgalactosaminyltransferase 13 [Source:VGNC Symbol;Acc:VGNC:95992]	-2.07	0.0789
ssc-miR-411	GATAD2B	GATA zinc finger domain containing 2B [Source:VGNC Symbol;Acc:VGNC:88369]	-2.07	0.0789
ssc-miR-411	GK	hypothetical gene	-2.07	0.0789
ssc-miR-411	GNAI3	G protein subunit alpha i3 [Source:VGNC Symbol;Acc:VGNC:88523]	-2.07	0.0789
ssc-miR-411	GNAZ	G protein subunit alpha z [Source:VGNC Symbol;Acc:VGNC:88528]	-2.07	0.0789
ssc-miR-411	GNG11	G protein subunit gamma 11 [Source:HGNC Symbol;Acc:HGNC:4403]	-2.07	0.0789
ssc-miR-411	GPR173	G protein-coupled receptor 173 [Source:VGNC Symbol;Acc:VGNC:88616]	-2.07	0.0789
ssc-miR-411	GRID2	glutamate ionotropic receptor delta type subunit 2 [Source:VGNC Symbol;Acc:VGNC:98932]	-2.07	0.0789
ssc-miR-411	GTF2I	ral transcription factor IIi [Source:VGNC Symbol;Acc:VGNC:88736]	-2.07	0.0789
ssc-miR-411	HERPUD2	HERPUD family member 2 [Source:VGNC Symbol;Acc:VGNC:88853]	-2.07	0.0789
ssc-miR-411	HIPK2	homeodomain interacting protein kinase 2 [Source:VGNC Symbol;Acc:VGNC:88888]	-2.07	0.0789
ssc-miR-411	HLF	HLF transcription factor, PAR bZIP family member [Source:VGNC Symbol;Acc:VGNC:88896]	-2.07	0.0789
ssc-miR-411	HMGN3	high mobility group nucleosomal binding domain 3 [Source:VGNC Symbol;Acc:VGNC:88908]	-2.07	0.0789
ssc-miR-411	HNRNPA1	heteroous nuclear ribonucleoprotein A1 [Source:VGNC Symbol;Acc:VGNC:88918]	-2.07	0.0789
ssc-miR-411	HNRNPH3	heteroous nuclear ribonucleoprotein H3 [Source:VGNC Symbol;Acc:VGNC:88922]	-2.07	0.0789
ssc-miR-411	HNRNPUL1	heteroous nuclear ribonucleoprotein U like 1 [Source:VGNC Symbol;Acc:VGNC:88926]	-2.07	0.0789
ssc-miR-411	IDH2	isocitrate dehydrogenase (NADP(+)) 2 [Source:NCBI gene (formerly Entrezgene);Acc:397603]	-2.07	0.0789
ssc-miR-411	IL17RB	interleukin 17 receptor B [Source:VGNC Symbol;Acc:VGNC:89086]	-2.07	0.0789
ssc-miR-411	IQGAP2	IQ motif containing GTPase activating protein 2 [Source:VGNC Symbol;Acc:VGNC:89192]	-2.07	0.0789
ssc-miR-411	ISM1	isthmin 1 [Source:VGNC Symbol;Acc:VGNC:95787]	-2.07	0.0789
ssc-miR-411	ITGB8	integrin subunit beta 8 [Source:VGNC Symbol;Acc:VGNC:89246]	-2.07	0.0789
ssc-miR-411	ITPK1	inositol-tetrakisphosphate 1-kinase [Source:VGNC Symbol;Acc:VGNC:89251]	-2.07	0.0789
ssc-miR-411	KDM6A	lysine demethylase 6A [Source:HGNC Symbol;Acc:HGNC:12637]	-2.07	0.0789
ssc-miR-411	KIAA1432	hypothetical gene	-2.07	0.0789
ssc-miR-411	KIAA1549L	KIAA1549 like [Source:VGNC Symbol;Acc:VGNC:89445]	-2.07	0.0789
ssc-miR-411	KLHL3	kelch like family member 3 [Source:VGNC Symbol;Acc:VGNC:89525]	-2.07	0.0789
ssc-miR-411	KMT2D	hypothetical gene	-2.07	0.0789
ssc-miR-411	KPNA2	karyopherin subunit alpha 2 [Source:VGNC Symbol;Acc:VGNC:89561]	-2.07	0.0789
ssc-miR-411	KRT13	hypothetical gene	-2.07	0.0789
ssc-miR-411	LAMTOR1	late endosomal/lysosomal adaptor, MAPK and MTOR activator 1 [Source:VGNC Symbol;Acc:VGNC:89629]	-2.07	0.0789

ssc-miR-411	LNX1	ligand of numb-protein X 1 [Source:VGNC Symbol;Acc:VGNC:89773]	-2.07	0.0789
ssc-miR-411	LPGAT1	lysophosphatidylglycerol acyltransferase 1 [Source:VGNC Symbol;Acc:VGNC:89792]	-2.07	0.0789
ssc-miR-411	LRP12	LDL receptor related protein 12 [Source:VGNC Symbol;Acc:VGNC:89818]	-2.07	0.0789
ssc-miR-411	LRRC57	leucine rich repeat containing 57 [Source:VGNC Symbol;Acc:VGNC:103119]	-2.07	0.0789
ssc-miR-411	LRRC59	leucine rich repeat containing 59 [Source:VGNC Symbol;Acc:VGNC:98082]	-2.07	0.0789
ssc-miR-411	LZIC	leucine zipper and CTNNBIP1 domain containing [Source:VGNC Symbol;Acc:VGNC:89925]	-2.07	0.0789
ssc-miR-411	MAML2	mastermind like transcriptional coactivator 2 [Source:VGNC Symbol;Acc:VGNC:89964]	-2.07	0.0789
ssc-miR-411	MAML3	mastermind like transcriptional coactivator 3 [Source:VGNC Symbol;Acc:VGNC:98938]	-2.07	0.0789
ssc-miR-411	MAP2K1	mitogen-activated protein kinase kinase 1 [Source:VGNC Symbol;Acc:VGNC:103121]	-2.07	0.0789
ssc-miR-411	MAP3K1	mitogen-activated protein kinase kinase kinase 1 [Source:VGNC Symbol;Acc:VGNC:98104]	-2.07	0.0789
ssc-miR-411	MATR3	matrin 3 [Source:HGNC Symbol;Acc:HGNC:6912]	-2.07	0.0789
ssc-miR-411	MBNL2	muscleblind like splicing regulator 2 [Source:VGNC Symbol;Acc:VGNC:90055]	-2.07	0.0789
ssc-miR-411	MBNL3	muscleblind like splicing regulator 3 [Source:VGNC Symbol;Acc:VGNC:90056]	-2.07	0.0789
ssc-miR-411	METTL20	hypothetical gene	-2.07	0.0789
ssc-miR-411	MFRP	membrane frizzled-related protein [Source:VGNC Symbol;Acc:VGNC:90181]	-2.07	0.0789
ssc-miR-411	MIB1	MIB E3 ubiquitin protein ligase 1 [Source:VGNC Symbol;Acc:VGNC:90205]	-2.07	0.0789
ssc-miR-411	MMP19	matrix metallopeptidase 19 [Source:VGNC Symbol;Acc:VGNC:90273]	-2.07	0.0789
ssc-miR-411	MPHOSPH8	M-phase phosphoprotein 8 [Source:HGNC Symbol;Acc:HGNC:29810]	-2.07	0.0789
ssc-miR-411	MSH6	mutS homolog 6 [Source:VGNC Symbol;Acc:VGNC:90420]	-2.07	0.0789
ssc-miR-411	MSL2	MSL complex subunit 2 [Source:VGNC Symbol;Acc:VGNC:90424]	-2.07	0.0789
ssc-miR-411	MTA3	metastasis associated 1 family member 3 [Source:VGNC Symbol;Acc:VGNC:90436]	-2.07	0.0789
ssc-miR-411	MTHFD1	methylenetetrahydrofolate dehydrogenase, cyclohydrolase and formyltetrahydrofolate synthetase 1 [Source:VGNC Symbol;Acc:VGNC:90450]	-2.07	0.0789
ssc-miR-411	MTPAP	mitochondrial poly(A) polymerase [Source:VGNC Symbol;Acc:VGNC:96676]	-2.07	0.0789
ssc-miR-411	MYPN	myopalladin [Source:VGNC Symbol;Acc:VGNC:107412]	-2.07	0.0789
ssc-miR-411	NAV3	neuron navigator 3 [Source:VGNC Symbol;Acc:VGNC:90589]	-2.07	0.0789
ssc-miR-411	NFATC2IP	nuclear factor of activated T cells 2 interacting protein [Source:VGNC Symbol;Acc:VGNC:90709]	-2.07	0.0789
ssc-miR-411	NFE2L3	NFE2 like bZIP transcription factor 3 [Source:VGNC Symbol;Acc:VGNC:90714]	-2.07	0.0789
ssc-miR-411	NIPAL2	NIPA like domain containing 2 [Source:VGNC Symbol;Acc:VGNC:90749]	-2.07	0.0789
ssc-miR-411	NKAIN2	sodium/potassium transporting ATPase interacting 2 [Source:VGNC Symbol;Acc:VGNC:103141]	-2.07	0.0789
ssc-miR-411	NR2C2	nuclear receptor subfamily 2 group C member 2 [Source:VGNC Symbol;Acc:VGNC:90876]	-2.07	0.0789
ssc-miR-411	NRF1	nuclear respiratory factor 1 [Source:VGNC Symbol;Acc:VGNC:90895]	-2.07	0.0789
ssc-miR-411	NUDT4	hypothetical gene	-2.07	0.0789
ssc-miR-411	NUDT5	nudix hydrolase 5 [Source:VGNC Symbol;Acc:VGNC:96458]	-2.07	0.0789
ssc-miR-411	ODC1	ornithine decarboxylase 1 [Source:VGNC Symbol;Acc:VGNC:104014]	-2.07	0.0789
ssc-miR-411	OSBP	oxysterol binding protein [Source:VGNC Symbol;Acc:VGNC:91066]	-2.07	0.0789
ssc-miR-411	PA2G4	hypothetical gene	-2.07	0.0789
ssc-miR-411	PAPPA2	pappalysin 2 [Source:VGNC Symbol;Acc:VGNC:91171]	-2.07	0.0789
ssc-miR-411	PDS5A	PDS5 cohesin associated factor A [Source:HGNC Symbol;Acc:HGNC:29088]	-2.07	0.0789
ssc-miR-411	PFN1	profilin 1 [Source:NCBI gene (formerly Entrezgene);Acc:100512494]	-2.07	0.0789
ssc-miR-411	PLA2G15	phospholipase A2 group XV [Source:HGNC Symbol;Acc:HGNC:17163]	-2.07	0.0789
ssc-miR-411	POU4F1	POU class 4 homeobox 1 [Source:VGNC Symbol;Acc:VGNC:91678]	-2.07	0.0789
ssc-miR-411	PPP1R9A	protein phosphatase 1 regulatory subunit 9A [Source:VGNC Symbol;Acc:VGNC:91743]	-2.07	0.0789
ssc-miR-411	PPTC7	protein phosphatase targeting COQ7 [Source:VGNC Symbol;Acc:VGNC:91765]	-2.07	0.0789
ssc-miR-411	PREX1	phosphatidylinositol-3,4,5-trisphosphate dependent Rac exchange factor 1 [Source:VGNC Symbol;Acc:VGNC:96264]	-2.07	0.0789
ssc-miR-411	PREX2	phosphatidylinositol-3,4,5-trisphosphate dependent Rac exchange factor 2 [Source:VGNC Symbol;Acc:VGNC:91791]	-2.07	0.0789
ssc-miR-411	PRPF40A	pre-mRNA processing factor 40 homolog A [Source:VGNC Symbol;Acc:VGNC:98222]	-2.07	0.0789
ssc-miR-411	PSMD12	proteasome 26S subunit, non-ATPase 12 [Source:VGNC Symbol;Acc:VGNC:91920]	-2.07	0.0789
ssc-miR-411	PUM1	pumilio RNA binding family member 1 [Source:VGNC Symbol;Acc:VGNC:92001]	-2.07	0.0789
ssc-miR-411	PXDC1	PX domain containing 1 [Source:VGNC Symbol;Acc:VGNC:92013]	-2.07	0.0789
ssc-miR-411	RAB10	RAB10, member RAS onco family [Source:VGNC Symbol;Acc:VGNC:98235]	-2.07	0.0789

ssc-miR-411	RAB21	RAB21, member RAS oncogene family [Source:HGNC Symbol;Acc:HGNC:18263]	-2.07	0.0789
ssc-miR-411	RAB3C	RAB3C, member RAS onco family [Source:VGNC Symbol;Acc:VGNC:104043]	-2.07	0.0789
ssc-miR-411	RANBP2	RAN binding protein 2 [Source:HGNC Symbol;Acc:HGNC:9848]	-2.07	0.0789
ssc-miR-411	RAPGEF2	Rap guanine nucleotide exchange factor 2 [Source:VGNC Symbol;Acc:VGNC:92092]	-2.07	0.0789
ssc-miR-411	RAPGEF6	Rap guanine nucleotide exchange factor 6 [Source:HGNC Symbol;Acc:HGNC:20655]	-2.07	0.0789
ssc-miR-411	RC3H1	ring finger and CCCH-type domains 1 [Source:VGNC Symbol;Acc:VGNC:92168]	-2.07	0.0789
ssc-miR-411	RHOB	ras homolog family member B [Source:NCBI gene (formerly Entrezgene);Acc:100144503]	-2.07	0.0789
ssc-miR-411	RMDN2	hypothetical gene	-2.07	0.0789
ssc-miR-411	RNF149	ring finger protein 149 [Source:VGNC Symbol;Acc:VGNC:92362]	-2.07	0.0789
ssc-miR-411	RORB	RAR related orphan receptor B [Source:VGNC Symbol;Acc:VGNC:92409]	-2.07	0.0789
ssc-miR-411	RP11-210M15.2	hypothetical gene	-2.07	0.0789
ssc-miR-411	RP11-315D16.2	hypothetical gene	-2.07	0.0789
ssc-miR-411	RP11-644F5.10	hypothetical gene	-2.07	0.0789
ssc-miR-411	RP2	RP2 activator of ARL3 GTPase [Source:VGNC Symbol;Acc:VGNC:92412]	-2.07	0.0789
ssc-miR-411	RPS6KA3	ribosomal protein S6 kinase A3 [Source:VGNC Symbol;Acc:VGNC:92442]	-2.07	0.0789
ssc-miR-411	RUNX1T1	RUNX1 partner transcriptional co-repressor 1 [Source:VGNC Symbol;Acc:VGNC:96594]	-2.07	0.0789
ssc-miR-411	RYBP	RING1 and YY1 binding protein [Source:VGNC Symbol;Acc:VGNC:92532]	-2.07	0.0789
ssc-miR-411	SCD5	stearoyl-CoA desaturase 5 [Source:VGNC Symbol;Acc:VGNC:92617]	-2.07	0.0789
ssc-miR-411	SEC22C	SEC22 homolog C, vesicle trafficking protein [Source:VGNC Symbol;Acc:VGNC:92674]	-2.07	0.0789
ssc-miR-411	SEMA3A	semaphorin 3A [Source:VGNC Symbol;Acc:VGNC:92693]	-2.07	0.0789
ssc-miR-411	SF3B3	splicing factor 3b subunit 3 [Source:VGNC Symbol;Acc:VGNC:92772]	-2.07	0.0789
ssc-miR-411	SHANK2	SH3 and multiple ankyrin repeat domains 2 [Source:VGNC Symbol;Acc:VGNC:92835]	-2.07	0.0789
ssc-miR-411	SHROOM4	shroom family member 4 [Source:VGNC Symbol;Acc:VGNC:92861]	-2.07	0.0789
ssc-miR-411	SLC16A9	solute carrier family 16 member 9 [Source:VGNC Symbol;Acc:VGNC:92949]	-2.07	0.0789
ssc-miR-411	SLC25A29	hypothetical gene	-2.07	0.0789
ssc-miR-411	SLC6A4	solute carrier family 6 member 4 [Source:VGNC Symbol;Acc:VGNC:93164]	-2.07	0.0789
ssc-miR-411	SLCO3A1	solute carrier organic anion transporter family member 3A1 [Source:VGNC Symbol;Acc:VGNC:93197]	-2.07	0.0789
ssc-miR-411	SMAD4	SMAD family member 4 [Source:VGNC Symbol;Acc:VGNC:93218]	-2.07	0.0789
ssc-miR-411	SNAP29	synaptosome associated protein 29 [Source:VGNC Symbol;Acc:VGNC:93278]	-2.07	0.0789
ssc-miR-411	SNX18	sorting nexin 18 [Source:VGNC Symbol;Acc:VGNC:93310]	-2.07	0.0789
ssc-miR-411	SP1	Sp1 transcription factor [Source:VGNC Symbol;Acc:VGNC:93360]	-2.07	0.0789
ssc-miR-411	SP2	Sp2 transcription factor [Source:VGNC Symbol;Acc:VGNC:93361]	-2.07	0.0789
ssc-miR-411	SPRY4	sprouty RTK signaling antagonist 4 [Source:VGNC Symbol;Acc:VGNC:93427]	-2.07	0.0789
ssc-miR-411	SRGAP3	SLIT-ROBO Rho GTPase activating protein 3 [Source:VGNC Symbol;Acc:VGNC:93455]	-2.07	0.0789
ssc-miR-411	SRSF10	serine and arginine rich splicing factor 10 [Source:VGNC Symbol;Acc:VGNC:93472]	-2.07	0.0789
ssc-miR-411	SSR3	signal sequence receptor subunit 3 [Source:VGNC Symbol;Acc:VGNC:93492]	-2.07	0.0789
ssc-miR-411	SSUH2	ssu-2 homolog [Source:VGNC Symbol;Acc:VGNC:96764]	-2.07	0.0789
ssc-miR-411	STIM2	stromal interaction molecule 2 [Source:VGNC Symbol;Acc:VGNC:98967]	-2.07	0.0789
ssc-miR-411	STX18	syntaxin 18 [Source:VGNC Symbol;Acc:VGNC:93588]	-2.07	0.0789
ssc-miR-411	TAOK1	TAO kinase 1 [Source:VGNC Symbol;Acc:VGNC:98355]	-2.07	0.0789
ssc-miR-411	TECPR2	tectonin beta-propeller repeat containing 2 [Source:VGNC Symbol;Acc:VGNC:99731]	-2.07	0.0789
ssc-miR-411	TGFBR2	transforming growth factor beta receptor 2 [Source:VGNC Symbol;Acc:VGNC:93931]	-2.07	0.0789
ssc-miR-411	TLE2	TLE family member 2, transcriptional corepressor [Source:VGNC Symbol;Acc:VGNC:94015]	-2.07	0.0789
ssc-miR-411	TNIK	TRAF2 and NCK interacting kinase [Source:VGNC Symbol;Acc:VGNC:98381]	-2.07	0.0789
ssc-miR-411	TNRC6B	trinucleotide repeat containing adaptor 6B [Source:VGNC Symbol;Acc:VGNC:94293]	-2.07	0.0789
ssc-miR-411	TPPP	tubulin polymerization promoting protein [Source:VGNC Symbol;Acc:VGNC:94344]	-2.07	0.0789
ssc-miR-411	TRIM24	tripartite motif containing 24 [Source:VGNC Symbol;Acc:VGNC:94400]	-2.07	0.0789
ssc-miR-411	UBE2D1	ubiquitin conjugating enzyme E2 D1 [Source:VGNC Symbol;Acc:VGNC:94641]	-2.07	0.0789
ssc-miR-411	UBE2R2	ubiquitin conjugating enzyme E2 R2 [Source:VGNC Symbol;Acc:VGNC:95924]	-2.07	0.0789
ssc-miR-411	UBE2W	ubiquitin conjugating enzyme E2 W [Source:VGNC Symbol;Acc:VGNC:98890]	-2.07	0.0789

ssc-miR-411	USP25	ubiquitin specific peptidase 25 [Source:VGNC Symbol;Acc:VGNC:94755]	-2.07	0.0789
ssc-miR-411	USP32	hypothetical gene	-2.07	0.0789
ssc-miR-411	USP49	ubiquitin specific peptidase 49 [Source:VGNC Symbol;Acc:VGNC:94771]	-2.07	0.0789
ssc-miR-411	USP6	hypothetical gene	-2.07	0.0789
ssc-miR-411	VAMP1	hypothetical gene	-2.07	0.0789
ssc-miR-411	VAMP7	vesicle associated membrane protein 7 [Source:VGNC Symbol;Acc:VGNC:94796]	-2.07	0.0789
ssc-miR-411	VGLL3	vestigial like family member 3 [Source:VGNC Symbol;Acc:VGNC:94823]	-2.07	0.0789
ssc-miR-411	VPS35	VPS35 retromer complex component [Source:VGNC Symbol;Acc:VGNC:94848]	-2.07	0.0789
ssc-miR-411	VPS53	VPS53 subunit of GARP complex [Source:VGNC Symbol;Acc:VGNC:94861]	-2.07	0.0789
ssc-miR-411	WNT10B	Wnt family member 10B [Source:VGNC Symbol;Acc:VGNC:94965]	-2.07	0.0789
ssc-miR-411	WNT7A	Wnt family member 7A [Source:VGNC Symbol;Acc:VGNC:94974]	-2.07	0.0789
ssc-miR-411	YAF2	YY1 associated factor 2 [Source:VGNC Symbol;Acc:VGNC:95018]	-2.07	0.0789
ssc-miR-411	ZBTB16	zinc finger and BTB domain containing 16 [Source:VGNC Symbol;Acc:VGNC:108626]	-2.07	0.0789
ssc-miR-411	ZBTB34	zinc finger and BTB domain containing 34 [Source:VGNC Symbol;Acc:VGNC:95070]	-2.07	0.0789
ssc-miR-411	ZDHHC3	zinc finger DHHC-type palmitoyltransferase 3 [Source:VGNC Symbol;Acc:VGNC:95126]	-2.07	0.0789
ssc-miR-411	ZFAND5	zinc finger AN1-type containing 5 [Source:VGNC Symbol;Acc:VGNC:95137]	-2.07	0.0789
ssc-miR-411	ZNF664	zinc finger protein 664 [Source:VGNC Symbol;Acc:VGNC:95288]	-2.07	0.0789
ssc-miR-411	ZNRF3	zinc and ring finger 3 [Source:VGNC Symbol;Acc:VGNC:95318]	-2.07	0.0789
ssc-miR-484	NA	hypothetical gene	-2.22	0.1198
ssc-miR-221-5p	NA	hypothetical gene	-2.27	0.1237
ssc-miR-7141-3p	NA	hypothetical gene	-2.34	0.0945
ssc-miR-222	AAK1	AP2 associated kinase 1 [Source:VGNC Symbol;Acc:VGNC:100379]	-2.51	0.083
ssc-miR-222	ABCB11	ATP binding cassette subfamily B member 11 [Source:VGNC Symbol;Acc:VGNC:97853]	-2.51	0.083
ssc-miR-222	AC010336.1	hypothetical gene	-2.51	0.083
ssc-miR-222	ACSL6	acyl-CoA synthetase long chain family member 6 [Source:VGNC Symbol;Acc:VGNC:99581]	-2.51	0.083
ssc-miR-222	ACVR2B	activin A receptor type 2B [Source:VGNC Symbol;Acc:VGNC:108629]	-2.51	0.083
ssc-miR-222	ADAM11	ADAM metallopeptidase domain 11 [Source:VGNC Symbol;Acc:VGNC:85062]	-2.51	0.083
ssc-miR-222	ADAM22	ADAM metallopeptidase domain 22 [Source:VGNC Symbol;Acc:VGNC:85067]	-2.51	0.083
ssc-miR-222	AEBP2	AE binding protein 2 [Source:VGNC Symbol;Acc:VGNC:85163]	-2.51	0.083
ssc-miR-222	AGFG1	ArfGAP with FG repeats 1 [Source:VGNC Symbol;Acc:VGNC:96359]	-2.51	0.083
ssc-miR-222	AGFG2	ArfGAP with FG repeats 2 [Source:VGNC Symbol;Acc:VGNC:85179]	-2.51	0.083
ssc-miR-222	AGO4	hypothetical gene	-2.51	0.083
ssc-miR-222	AGPS	alkylglycerone phosphate synthase [Source:VGNC Symbol;Acc:VGNC:96267]	-2.51	0.083
ssc-miR-222	AIDA	axin interactor, dorsalization associated [Source:VGNC Symbol;Acc:VGNC:96103]	-2.51	0.083
ssc-miR-222	AJAP1	adherens junctions associated protein 1 [Source:VGNC Symbol;Acc:VGNC:85207]	-2.51	0.083
ssc-miR-222	AKAP13	hypothetical gene	-2.51	0.083
ssc-miR-222	AKAP5	A-kinase anchoring protein 5 [Source:VGNC Symbol;Acc:VGNC:85220]	-2.51	0.083
ssc-miR-222	ALG14	ALG14 UDP-N-acetylglucosaminyltransferase subunit [Source:VGNC Symbol;Acc:VGNC:85249]	-2.51	0.083
ssc-miR-222	ALG6	ALG6 alpha-1,3-glucosyltransferase [Source:VGNC Symbol;Acc:VGNC:85253]	-2.51	0.083
ssc-miR-222	AMIGO1	adhesion molecule with Ig like domain 1 [Source:VGNC Symbol;Acc:VGNC:85280]	-2.51	0.083
ssc-miR-222	AMMECR1	AMMECR nuclear protein 1 [Source:VGNC Symbol;Acc:VGNC:96559]	-2.51	0.083
ssc-miR-222	ANAPC16	anaphase promoting complex subunit 16 [Source:NCBI gene (formerly Entrezgene);Acc:100155019]	-2.51	0.083
ssc-miR-222	ANGPTL2	angiopoietin like 2 [Source:VGNC Symbol;Acc:VGNC:85304]	-2.51	0.083
ssc-miR-222	ANKHD1	ankyrin repeat and KH domain containing 1 [Source:NCBI gene (formerly Entrezgene);Acc:100512270]	-2.51	0.083
ssc-miR-222	ANKIB1	ankyrin repeat and IBR domain containing 1 [Source:VGNC Symbol;Acc:VGNC:85316]	-2.51	0.083
ssc-miR-222	ANKRD52	ankyrin repeat domain 52 [Source:VGNC Symbol;Acc:VGNC:85342]	-2.51	0.083
ssc-miR-222	AP3M1	adaptor related protein complex 3 subunit mu 1 [Source:VGNC Symbol;Acc:VGNC:85390]	-2.51	0.083
ssc-miR-222	APPBP2	amyloid beta protein binding protein 2 [Source:VGNC Symbol;Acc:VGNC:85425]	-2.51	0.083
ssc-miR-222	ARF4	ADP ribosylation factor 4 [Source:VGNC Symbol;Acc:VGNC:85446]	-2.51	0.083
ssc-miR-222	ARHGAP28	Rho GTPase activating protein 28 [Source:VGNC Symbol;Acc:VGNC:85467]	-2.51	0.083

ssc-miR-222	ARHGAP42	Rho GTPase activating protein 42 [Source:VGNC Symbol;Acc:VGNC:85477]	-2.51	0.083
ssc-miR-222	ARHGEF7	Rho guanine nucleotide exchange factor 7 [Source:VGNC Symbol;Acc:VGNC:85503]	-2.51	0.083
ssc-miR-222	ARID1A	AT-rich interaction domain 1A [Source:VGNC Symbol;Acc:VGNC:85505]	-2.51	0.083
ssc-miR-222	ARID3B	AT-rich interaction domain 3B [Source:VGNC Symbol;Acc:VGNC:85508]	-2.51	0.083
ssc-miR-222	ARNT	aryl hydrocarbon receptor nuclear translocator [Source:VGNC Symbol;Acc:VGNC:98733]	-2.51	0.083
ssc-miR-222	ASPH	aspartate beta-hydroxylase [Source:VGNC Symbol;Acc:VGNC:85584]	-2.51	0.083
ssc-miR-222	ATAD2B	ATPase family AAA domain containing 2B [Source:VGNC Symbol;Acc:VGNC:85600]	-2.51	0.083
ssc-miR-222	ATG7	autophagy related 7 [Source:VGNC Symbol;Acc:VGNC:85625]	-2.51	0.083
ssc-miR-222	ATP11B	hypothetical gene	-2.51	0.083
ssc-miR-222	ATP1B1	ATPase Na+/K+ transporting subunit beta 1 [Source:VGNC Symbol;Acc:VGNC:85642]	-2.51	0.083
ssc-miR-222	ATP8A2	ATPase phospholipid transporting 8A2 [Source:HGNC Symbol;Acc:HGNC:13533]	-2.51	0.083
ssc-miR-222	ATPAF1	ATP synthase mitochondrial F1 complex assembly factor 1 [Source:VGNC Symbol;Acc:VGNC:96924]	-2.51	0.083
ssc-miR-222	ATXN1	ataxin 1 [Source:VGNC Symbol;Acc:VGNC:85687]	-2.51	0.083
ssc-miR-222	ATXN7	ataxin 7 [Source:VGNC Symbol;Acc:VGNC:99704]	-2.51	0.083
ssc-miR-222	AXIN2	axin 2 [Source:VGNC Symbol;Acc:VGNC:85708]	-2.51	0.083
ssc-miR-222	AZI2	5-azacytidine induced 2 [Source:VGNC Symbol;Acc:VGNC:85711]	-2.51	0.083
ssc-miR-222	BACH1	BTB domain and CNC homolog 1 [Source:VGNC Symbol;Acc:VGNC:85741]	-2.51	0.083
ssc-miR-222	BAZ2B	bromodomain adjacent to zinc finger domain 2B [Source:HGNC Symbol;Acc:HGNC:963]	-2.51	0.083
ssc-miR-222	BBC3	BCL2 binding component 3 [Source:HGNC Symbol;Acc:HGNC:17868]	-2.51	0.083
ssc-miR-222	BCL11B	BAF chromatin remodeling complex subunit BCL11B [Source:VGNC Symbol;Acc:VGNC:96563]	-2.51	0.083
ssc-miR-222	BCL2L11	BCL2 like 11 [Source:NCBI gene (formerly Entrezgene);Acc:396632]	-2.51	0.083
ssc-miR-222	BEAN1	brain expressed associated with NEDD4 1 [Source:VGNC Symbol;Acc:VGNC:85797]	-2.51	0.083
ssc-miR-222	BEND4	BEN domain containing 4 [Source:VGNC Symbol;Acc:VGNC:85801]	-2.51	0.083
ssc-miR-222	BMF	Bcl2 modifying factor [Source:VGNC Symbol;Acc:VGNC:85837]	-2.51	0.083
ssc-miR-222	BMI1	BMI1 proto-onco, polycomb ring finger [Source:VGNC Symbol;Acc:VGNC:108265]	-2.51	0.083
ssc-miR-222	BRWD1	bromodomain and WD repeat domain containing 1 [Source:VGNC Symbol;Acc:VGNC:108153]	-2.51	0.083
ssc-miR-222	BRWD3	bromodomain and WD repeat domain containing 3 [Source:VGNC Symbol;Acc:VGNC:85890]	-2.51	0.083
ssc-miR-222	BTG2	BTG anti-proliferation factor 2 [Source:NCBI gene (formerly Entrezgene);Acc:100048932]	-2.51	0.083
ssc-miR-222	BYSL	bystin like [Source:NCBI gene (formerly Entrezgene);Acc:100156292]	-2.51	0.083
ssc-miR-222	BZW1	basic leucine zipper and W2 domains 1 [Source:VGNC Symbol;Acc:VGNC:95885]	-2.51	0.083
ssc-miR-222	C11orf87	chromosome 11 open reading frame 87 [Source:HGNC Symbol;Acc:HGNC:33788]	-2.51	0.083
ssc-miR-222	C11orf88	hypothetical gene	-2.51	0.083
ssc-miR-222	C16orf45	hypothetical gene	-2.51	0.083
ssc-miR-222	C16orf52	hypothetical gene	-2.51	0.083
ssc-miR-222	C16orf92	chromosome 16 open reading frame 92 [Source:HGNC Symbol;Acc:HGNC:26346]	-2.51	0.083
ssc-miR-222	C18orf25	chromosome 1 C18orf25 homolog [Source:VGNC Symbol;Acc:VGNC:85964]	-2.51	0.083
ssc-miR-222	C2CD2	C2 calcium dependent domain containing 2 [Source:VGNC Symbol;Acc:VGNC:85990]	-2.51	0.083
ssc-miR-222	C2orf91	hypothetical gene	-2.51	0.083
ssc-miR-222	C3orf70	chromosome 13 C3orf70 homolog [Source:VGNC Symbol;Acc:VGNC:85937]	-2.51	0.083
ssc-miR-222	C6orf120	chromosome 1 C6orf120 homolog [Source:VGNC Symbol;Acc:VGNC:85967]	-2.51	0.083
ssc-miR-222	CABP7	calcium binding protein 7 [Source:VGNC Symbol;Acc:VGNC:107372]	-2.51	0.083
ssc-miR-222	CACNB4	calcium voltage-gated channel auxiliary subunit beta 4 [Source:VGNC Symbol;Acc:VGNC:108507]	-2.51	0.083
ssc-miR-222	CAMK1D	calcium/calmodulin dependent protein kinase ID [Source:VGNC Symbol;Acc:VGNC:95909]	-2.51	0.083
ssc-miR-222	CAMKK1	calcium/calmodulin dependent protein kinase kinase 1 [Source:VGNC Symbol;Acc:VGNC:98977]	-2.51	0.083
ssc-miR-222	CAND1	cullin associated and neddylation dissociated 1 [Source:VGNC Symbol;Acc:VGNC:97912]	-2.51	0.083
ssc-miR-222	CANX	calnexin [Source:VGNC Symbol;Acc:VGNC:86167]	-2.51	0.083
ssc-miR-222	CAPRIN2	caprin family member 2 [Source:VGNC Symbol;Acc:VGNC:86179]	-2.51	0.083
ssc-miR-222	CASP10	caspase 10 [Source:VGNC Symbol;Acc:VGNC:95638]	-2.51	0.083
ssc-miR-222	CASZ1	castor zinc finger 1 [Source:VGNC Symbol;Acc:VGNC:86207]	-2.51	0.083
ssc-miR-222	CBFB	core-binding factor subunit beta [Source:VGNC Symbol;Acc:VGNC:86221]	-2.51	0.083

ssc-miR-222	CBL	Cbl proto-onco [Source:VGNC Symbol;Acc:VGNC:86222]		-2.51	0.083
ssc-miR-222	CC2D1B	coiled-coil and C2 domain containing 1B [Source:VGNC Symbol;Acc:VGNC:86236]		-2.51	0.083
ssc-miR-222	CCDC171	coiled-coil domain containing 171 [Source:VGNC Symbol;Acc:VGNC:103044]		-2.51	0.083
ssc-miR-222	CCDC50	coiled-coil domain containing 50 [Source:VGNC Symbol;Acc:VGNC:86299]		-2.51	0.083
ssc-miR-222	CCDC64	hypothetical gene		-2.51	0.083
ssc-miR-222	CCDC88A	coiled-coil domain containing 88A [Source:VGNC Symbol;Acc:VGNC:86325]		-2.51	0.083
ssc-miR-222	CCNYL1	hypothetical gene		-2.51	0.083
ssc-miR-222	CCT4	chaperonin containing TCP1 subunit 4 [Source:VGNC Symbol;Acc:VGNC:86382]		-2.51	0.083
ssc-miR-222	CD2AP	CD2 associated protein [Source:VGNC Symbol;Acc:VGNC:86406]		-2.51	0.083
ssc-miR-222	CD4	CD4 molecule [Source:VGNC Symbol;Acc:VGNC:86417]		-2.51	0.083
ssc-miR-222	CD47	CD47 molecule [Source:VGNC Symbol;Acc:VGNC:108637]		-2.51	0.083
ssc-miR-222	CDC5L	cell division cycle 5 like [Source:VGNC Symbol;Acc:VGNC:86463]		-2.51	0.083
ssc-miR-222	CDH2	cadherin 2 [Source:VGNC Symbol;Acc:VGNC:86483]		-2.51	0.083
ssc-miR-222	CDH4	cadherin 4 [Source:VGNC Symbol;Acc:VGNC:95853]		-2.51	0.083
ssc-miR-222	CDK8	cyclin dependent kinase 8 [Source:VGNC Symbol;Acc:VGNC:86507]		-2.51	0.083
ssc-miR-222	CDKN1B	cyclin dependent kinase inhibitor 1B [Source:VGNC Symbol;Acc:VGNC:86515]		-2.51	0.083
ssc-miR-222	CDKN1C	cyclin dependent kinase inhibitor 1C [Source:VGNC Symbol;Acc:VGNC:86516]		-2.51	0.083
ssc-miR-222	CDON	cell adhesion associated, onco regulated [Source:VGNC Symbol;Acc:VGNC:86519]		-2.51	0.083
ssc-miR-222	CENPP	centromere protein P [Source:VGNC Symbol;Acc:VGNC:98865]		-2.51	0.083
ssc-miR-222	CEP97	centrosomal protein 97 [Source:VGNC Symbol;Acc:VGNC:108638]		-2.51	0.083
ssc-miR-222	CHCHD4	coiled-coil-helix-coiled-coil-helix domain containing 4 [Source:NCBI gene (formerly Entrezgene);Acc:100154663]		-2.51	0.083
ssc-miR-222	CHD2	chromodomain helicase DNA binding protein 2 [Source:HGNC Symbol;Acc:HGNC:1917]		-2.51	0.083
ssc-miR-222	CHD7	chromodomain helicase DNA binding protein 7 [Source:VGNC Symbol;Acc:VGNC:86633]		-2.51	0.083
ssc-miR-222	CHD8	chromodomain helicase DNA binding protein 8 [Source:VGNC Symbol;Acc:VGNC:86634]		-2.51	0.083
ssc-miR-222	CHSY1	chondroitin sulfate synthase 1 [Source:VGNC Symbol;Acc:VGNC:86684]		-2.51	0.083
ssc-miR-222	CLVS2	clavesin 2 [Source:VGNC Symbol;Acc:VGNC:86795]		-2.51	0.083
ssc-miR-222	CMTM4	CKLF like MARVEL transmembrane domain containing 4 [Source:VGNC Symbol;Acc:VGNC:86804]		-2.51	0.083
ssc-miR-222	CNOT2	CCR4-NOT transcription complex subunit 2 [Source:VGNC Symbol;Acc:VGNC:86834]		-2.51	0.083
ssc-miR-222	CNOT6	CCR4-NOT transcription complex subunit 6 [Source:VGNC Symbol;Acc:VGNC:86837]		-2.51	0.083
ssc-miR-222	COL4A4	collagen type IV alpha 4 chain [Source:VGNC Symbol;Acc:VGNC:95980]		-2.51	0.083
ssc-miR-222	COMMD3-BMI1	hypothetical gene		-2.51	0.083
ssc-miR-222	CPEB3	cytoplasmic polyadenylation element binding protein 3 [Source:VGNC Symbol;Acc:VGNC:86938]		-2.51	0.083
ssc-miR-222	CPNE8	copine 8 [Source:VGNC Symbol;Acc:VGNC:86955]		-2.51	0.083
ssc-miR-222	CPPED1	calcineurin like phosphoesterase domain containing 1 [Source:VGNC Symbol;Acc:VGNC:86957]		-2.51	0.083
ssc-miR-222	CREB1	cAMP responsive element binding protein 1 [Source:VGNC Symbol;Acc:VGNC:96004]		-2.51	0.083
ssc-miR-222	CREBZF	CREB/ATF bZIP transcription factor [Source:VGNC Symbol;Acc:VGNC:86988]		-2.51	0.083
ssc-miR-222	CRK	CRK proto-onco, adaptor protein [Source:NCBI gene (formerly Entrezgene);Acc:100192444]		-2.51	0.083
ssc-miR-222	CRKL	CRK like proto-onco, adaptor protein [Source:VGNC Symbol;Acc:VGNC:86997]		-2.51	0.083
ssc-miR-222	CTCF	CCCTC-binding factor [Source:VGNC Symbol;Acc:VGNC:87057]		-2.51	0.083
ssc-miR-222	CTDSPL2	CTD small phosphatase like 2 [Source:VGNC Symbol;Acc:VGNC:87060]		-2.51	0.083
ssc-miR-222	CTIF	cap binding complex dependent translation initiation factor [Source:VGNC Symbol;Acc:VGNC:87062]		-2.51	0.083
ssc-miR-222	CTTN	cortactin [Source:VGNC Symbol;Acc:VGNC:87081]		-2.51	0.083
ssc-miR-222	CUX2	cut like homeobox 2 [Source:VGNC Symbol;Acc:VGNC:87094]		-2.51	0.083
ssc-miR-222	CXCR5	C-X-C motif chemokine receptor 5 [Source:VGNC Symbol;Acc:VGNC:87109]		-2.51	0.083
ssc-miR-222	CYP7B1	cytochrome P450 family 7 subfamily B member 1 [Source:VGNC Symbol;Acc:VGNC:103362]		-2.51	0.083
ssc-miR-222	CYR61	hypothetical gene		-2.51	0.083
ssc-miR-222	DBNL	drebrin like [Source:VGNC Symbol;Acc:VGNC:87166]		-2.51	0.083
ssc-miR-222	DCAF12	DDB1 and CUL4 associated factor 12 [Source:VGNC Symbol;Acc:VGNC:95927]		-2.51	0.083
ssc-miR-222	DCAF7	DDB1 and CUL4 associated factor 7 [Source:VGNC Symbol;Acc:VGNC:87178]		-2.51	0.083
ssc-miR-222	DCUN1D1	hypothetical gene		-2.51	0.083

ssc-miR-222	DCUN1D4	defective in cullin neddylation 1 domain containing 4 [Source:VGNC Symbol;Acc:VGNC:87197]	-2.51	0.083
ssc-miR-222	DCX	doublecortin [Source:HGNC Symbol;Acc:HGNC:2714]	-2.51	0.083
ssc-miR-222	DDIT4	DNA damage inducible transcript 4 [Source:VGNC Symbol;Acc:VGNC:87208]	-2.51	0.083
ssc-miR-222	DGKG	diacylglycerol kinase gamma [Source:VGNC Symbol;Acc:VGNC:87272]	-2.51	0.083
ssc-miR-222	DGKH	diacylglycerol kinase eta [Source:VGNC Symbol;Acc:VGNC:87273]	-2.51	0.083
ssc-miR-222	DHX36	DEAH-box helicase 36 [Source:VGNC Symbol;Acc:VGNC:87292]	-2.51	0.083
ssc-miR-222	DICER1	dicer 1, ribonuclease III [Source:VGNC Symbol;Acc:VGNC:87302]	-2.51	0.083
ssc-miR-222	DKK2	dickkopf WNT signaling pathway inhibitor 2 [Source:VGNC Symbol;Acc:VGNC:87322]	-2.51	0.083
ssc-miR-222	DLG2	discs large MAGUK scaffold protein 2 [Source:VGNC Symbol;Acc:VGNC:108581]	-2.51	0.083
ssc-miR-222	DMRT3	doublesex and mab-3 related transcription factor 3 [Source:VGNC Symbol;Acc:VGNC:87352]	-2.51	0.083
ssc-miR-222	DNAJA2	DnaJ heat shock protein family (Hsp40) member A2 [Source:VGNC Symbol;Acc:VGNC:96621]	-2.51	0.083
ssc-miR-222	DNAJC14	DnaJ heat shock protein family (Hsp40) member C14 [Source:NCBI gene (formerly Entrezgene);Acc:100151876]	-2.51	0.083
ssc-miR-222	DNAL1	dynein axonemal light chain 1 [Source:VGNC Symbol;Acc:VGNC:87373]	-2.51	0.083
ssc-miR-222	DPP8	dipeptidyl peptidase 8 [Source:VGNC Symbol;Acc:VGNC:87423]	-2.51	0.083
ssc-miR-222	DYNC1LI2	dynein cytoplasmic 1 light intermediate chain 2 [Source:VGNC Symbol;Acc:VGNC:87498]	-2.51	0.083
ssc-miR-222	DYRK1A	dual specificity tyrosine phosphorylation regulated kinase 1A [Source:VGNC Symbol;Acc:VGNC:87505]	-2.51	0.083
ssc-miR-222	DZIP1	DAZ interacting zinc finger protein 1 [Source:VGNC Symbol;Acc:VGNC:87510]	-2.51	0.083
ssc-miR-222	E2F2	E2F transcription factor 2 [Source:VGNC Symbol;Acc:VGNC:87513]	-2.51	0.083
ssc-miR-222	EFNB2	ephrin B2 [Source:VGNC Symbol;Acc:VGNC:87577]	-2.51	0.083
ssc-miR-222	EGFR	epidermal growth factor receptor [Source:VGNC Symbol;Acc:VGNC:87587]	-2.51	0.083
ssc-miR-222	EIF1	eukaryotic translation initiation factor 1 [Source:VGNC Symbol;Acc:VGNC:98988]	-2.51	0.083
ssc-miR-222	EIF3J	eukaryotic translation initiation factor 3 subunit J [Source:VGNC Symbol;Acc:VGNC:87621]	-2.51	0.083
ssc-miR-222	EIF4E3	eukaryotic translation initiation factor 4E family member 3 [Source:VGNC Symbol;Acc:VGNC:87628]	-2.51	0.083
ssc-miR-222	EIF5A2	eukaryotic translation initiation factor 5A2 [Source:VGNC Symbol;Acc:VGNC:87633]	-2.51	0.083
ssc-miR-222	ELAVL2	ELAV like RNA binding protein 2 [Source:VGNC Symbol;Acc:VGNC:87639]	-2.51	0.083
ssc-miR-222	ELK4	ETS transcription factor ELK4 [Source:VGNC Symbol;Acc:VGNC:87647]	-2.51	0.083
ssc-miR-222	ELP4	elongator acetyltransferase complex subunit 4 [Source:VGNC Symbol;Acc:VGNC:87665]	-2.51	0.083
ssc-miR-222	EML6	EMAP like 6 [Source:VGNC Symbol;Acc:VGNC:87687]	-2.51	0.083
ssc-miR-222	EMX2	empty spiracles homeobox 2 [Source:VGNC Symbol;Acc:VGNC:87693]	-2.51	0.083
ssc-miR-222	ERBB3	erb-b2 receptor tyrosine kinase 3 [Source:VGNC Symbol;Acc:VGNC:87760]	-2.51	0.083
ssc-miR-222	ERBB4	erb-b2 receptor tyrosine kinase 4 [Source:VGNC Symbol;Acc:VGNC:96284]	-2.51	0.083
ssc-miR-222	ESR1	estrogen receptor 1 [Source:VGNC Symbol;Acc:VGNC:103089]	-2.51	0.083
ssc-miR-222	ETS1	ETS proto-onco 1, transcription factor [Source:VGNC Symbol;Acc:VGNC:87808]	-2.51	0.083
ssc-miR-222	ETS2	ETS proto-onco 2, transcription factor [Source:VGNC Symbol;Acc:VGNC:87809]	-2.51	0.083
ssc-miR-222	ETV3	ETS variant transcription factor 3 [Source:VGNC Symbol;Acc:VGNC:87812]	-2.51	0.083
ssc-miR-222	FAM110B	family with sequence similarity 110 member B [Source:VGNC Symbol;Acc:VGNC:87885]	-2.51	0.083
ssc-miR-222	FAM120A	family with sequence similarity 120A [Source:VGNC Symbol;Acc:VGNC:99713]	-2.51	0.083
ssc-miR-222	FAM167A	family with sequence similarity 167 member A [Source:VGNC Symbol;Acc:VGNC:87924]	-2.51	0.083
ssc-miR-222	FAM178A	hypothetical gene	-2.51	0.083
ssc-miR-222	FAM196B	hypothetical gene	-2.51	0.083
ssc-miR-222	FAM199X	family with sequence similarity 199, X-linked [Source:HGNC Symbol;Acc:HGNC:25195]	-2.51	0.083
ssc-miR-222	FAM208A	hypothetical gene	-2.51	0.083
ssc-miR-222	FAM214A	family with sequence similarity 214 member A [Source:VGNC Symbol;Acc:VGNC:87952]	-2.51	0.083
ssc-miR-222	FAM46A	hypothetical gene	-2.51	0.083
ssc-miR-222	FAM53B	family with sequence similarity 53 member B [Source:VGNC Symbol;Acc:VGNC:87974]	-2.51	0.083
ssc-miR-222	FAT2	FAT atypical cadherin 2 [Source:VGNC Symbol;Acc:VGNC:88019]	-2.51	0.083
ssc-miR-222	FBN2	fibrillin 2 [Source:VGNC Symbol;Acc:VGNC:88025]	-2.51	0.083
ssc-miR-222	FBXO28	F-box protein 28 [Source:HGNC Symbol;Acc:HGNC:29046]	-2.51	0.083
ssc-miR-222	FBXO3	F-box protein 3 [Source:VGNC Symbol;Acc:VGNC:88037]	-2.51	0.083
ssc-miR-222	FGF14	fibroblast growth factor 14 [Source:VGNC Symbol;Acc:VGNC:88104]	-2.51	0.083

ssc-miR-222	FIGN	fidgetin, microtubule severing factor [Source:VGNC Symbol;Acc:VGNC:95580]	-2.51	0.083
ssc-miR-222	FKBP9	FKBP prolyl isomerase 9 [Source:VGNC Symbol;Acc:VGNC:88151]	-2.51	0.083
ssc-miR-222	FLJ00388	hypothetical gene	-2.51	0.083
ssc-miR-222	FMR1	FMRP translational regulator 1 [Source:VGNC Symbol;Acc:VGNC:88175]	-2.51	0.083
ssc-miR-222	FNDC3A	fibronectin type III domain containing 3A [Source:VGNC Symbol;Acc:VGNC:88182]	-2.51	0.083
ssc-miR-222	FNDC3B	fibronectin type III domain containing 3B [Source:VGNC Symbol;Acc:VGNC:88183]	-2.51	0.083
ssc-miR-222	FNIP2	folliculin interacting protein 2 [Source:VGNC Symbol;Acc:VGNC:88188]	-2.51	0.083
ssc-miR-222	FOS	Fos proto-onco, AP-1 transcription factor subunit [Source:NCBI gene (formerly Entrezgene);Acc:100144486]	-2.51	0.083
ssc-miR-222	FOXN2	forkhead box N2 [Source:VGNC Symbol;Acc:VGNC:88219]	-2.51	0.083
ssc-miR-222	FOXP1	forkhead box P1 [Source:VGNC Symbol;Acc:VGNC:88222]	-2.51	0.083
ssc-miR-222	FOXP2	forkhead box P2 [Source:VGNC Symbol;Acc:VGNC:98014]	-2.51	0.083
ssc-miR-222	FRAT2	FRAT regulator of WNT signaling pathway 2 [Source:VGNC Symbol;Acc:VGNC:107394]	-2.51	0.083
ssc-miR-222	FRK	fyn related Src family tyrosine kinase [Source:VGNC Symbol;Acc:VGNC:88235]	-2.51	0.083
ssc-miR-222	FRRS1L	ferric chelate reductase 1 like [Source:VGNC Symbol;Acc:VGNC:88245]	-2.51	0.083
ssc-miR-222	FRS2	fibroblast growth factor receptor substrate 2 [Source:VGNC Symbol;Acc:VGNC:88246]	-2.51	0.083
ssc-miR-222	FRY	hypothetical gene	-2.51	0.083
ssc-miR-222	GAB1	GRB2 associated binding protein 1 [Source:VGNC Symbol;Acc:VGNC:88294]	-2.51	0.083
ssc-miR-222	GABRA1	gamma-aminobutyric acid type A receptor subunit alpha1 [Source:VGNC Symbol;Acc:VGNC:88300]	-2.51	0.083
ssc-miR-222	GALNT18	polypeptide N-acetylgalactosaminyltransferase 18 [Source:VGNC Symbol;Acc:VGNC:88333]	-2.51	0.083
ssc-miR-222	GATA4	GATA binding protein 4 [Source:VGNC Symbol;Acc:VGNC:88365]	-2.51	0.083
ssc-miR-222	GBX2	gastrulation brain homeobox 2 [Source:VGNC Symbol;Acc:VGNC:96316]	-2.51	0.083
ssc-miR-222	GFPT1	glutamine--fructose-6-phosphate transaminase 1 [Source:VGNC Symbol;Acc:VGNC:88426]	-2.51	0.083
ssc-miR-222	GGA2	golgi associated, gamma adaptin ear containing, ARF binding protein 2 [Source:VGNC Symbol;Acc:VGNC:88432]	-2.51	0.083
ssc-miR-222	GHR	growth hormone receptor [Source:NCBI gene (formerly Entrezgene);Acc:397488]	-2.51	0.083
ssc-miR-222	GNAI2	G protein subunit alpha i2 [Source:VGNC Symbol;Acc:VGNC:88522]	-2.51	0.083
ssc-miR-222	GNAI3	G protein subunit alpha i3 [Source:VGNC Symbol;Acc:VGNC:88523]	-2.51	0.083
ssc-miR-222	GNAL	G protein subunit alpha L [Source:VGNC Symbol;Acc:VGNC:88524]	-2.51	0.083
ssc-miR-222	GPBP1	GC-rich promoter binding protein 1 [Source:VGNC Symbol;Acc:VGNC:88578]	-2.51	0.083
ssc-miR-222	GPM6A	glycoprotein M6A [Source:VGNC Symbol;Acc:VGNC:98025]	-2.51	0.083
ssc-miR-222	GPR12	G protein-coupled receptor 12 [Source:VGNC Symbol;Acc:VGNC:88598]	-2.51	0.083
ssc-miR-222	GPR22	G protein-coupled receptor 22 [Source:VGNC Symbol;Acc:VGNC:88625]	-2.51	0.083
ssc-miR-222	GPR68	G protein-coupled receptor 68 [Source:VGNC Symbol;Acc:VGNC:88635]	-2.51	0.083
ssc-miR-222	GRB10	growth factor receptor bound protein 10 [Source:VGNC Symbol;Acc:VGNC:88662]	-2.51	0.083
ssc-miR-222	GRIN2B	glutamate ionotropic receptor NMDA type subunit 2B [Source:VGNC Symbol;Acc:VGNC:88684]	-2.51	0.083
ssc-miR-222	GRM1	glutamate metabotropic receptor 1 [Source:VGNC Symbol;Acc:VGNC:88700]	-2.51	0.083
ssc-miR-222	GTF2B	ral transcription factor IIB [Source:VGNC Symbol;Acc:VGNC:88731]	-2.51	0.083
ssc-miR-222	GTF2E1	general transcription factor IIE subunit 1 [Source:HGNC Symbol;Acc:HGNC:4650]	-2.51	0.083
ssc-miR-222	H3F3A	hypothetical gene	-2.51	0.083
ssc-miR-222	HCN4	hyperpolarization activated cyclic nucleotide gated potassium channel 4 [Source:VGNC Symbol;Acc:VGNC:88805]	-2.51	0.083
ssc-miR-222	HECTD2	HECT domain E3 ubiquitin protein ligase 2 [Source:VGNC Symbol;Acc:VGNC:88833]	-2.51	0.083
ssc-miR-222	HECW1	HECT, C2 and WW domain containing E3 ubiquitin protein ligase 1 [Source:VGNC Symbol;Acc:VGNC:88835]	-2.51	0.083
ssc-miR-222	HEG1	heart development protein with EGF like domains 1 [Source:VGNC Symbol;Acc:VGNC:88836]	-2.51	0.083
ssc-miR-222	HIP1	huntingtin interacting protein 1 [Source:VGNC Symbol;Acc:VGNC:88885]	-2.51	0.083
ssc-miR-222	HIPK1	homeodomain interacting protein kinase 1 [Source:VGNC Symbol;Acc:VGNC:88887]	-2.51	0.083
ssc-miR-222	HIPK2	homeodomain interacting protein kinase 2 [Source:VGNC Symbol;Acc:VGNC:88888]	-2.51	0.083
ssc-miR-222	HIPK3	homeodomain interacting protein kinase 3 [Source:VGNC Symbol;Acc:VGNC:88889]	-2.51	0.083
ssc-miR-222	HMBOX1	homeobox containing 1 [Source:VGNC Symbol;Acc:VGNC:88898]	-2.51	0.083
ssc-miR-222	HMGB3	hypothetical gene	-2.51	0.083
ssc-miR-222	HNRNPA0	hypothetical gene	-2.51	0.083
ssc-miR-222	HNRNPA3	hypothetical gene	-2.51	0.083

ssc-miR-222	HNRNPH3	heteroous nuclear ribonucleoprotein H3 [Source:VGNC Symbol;Acc:VGNC:88922]	-2.51	0.083
ssc-miR-222	HOOK3	hook microtubule tethering protein 3 [Source:VGNC Symbol;Acc:VGNC:98037]	-2.51	0.083
ssc-miR-222	HOXA7	homeobox A7 [Source:VGNC Symbol;Acc:VGNC:88940]	-2.51	0.083
ssc-miR-222	HOXC8	homeobox C8 [Source:VGNC Symbol;Acc:VGNC:88954]	-2.51	0.083
ssc-miR-222	HOXD11	homeobox D11 [Source:VGNC Symbol;Acc:VGNC:96352]	-2.51	0.083
ssc-miR-222	IGF1	insulin like growth factor 1 [Source:VGNC Symbol;Acc:VGNC:98044]	-2.51	0.083
ssc-miR-222	IGF2BP2	insulin like growth factor 2 mRNA binding protein 2 [Source:VGNC Symbol;Acc:VGNC:89055]	-2.51	0.083
ssc-miR-222	IGSF3	immunoglobulin superfamily member 3 [Source:VGNC Symbol;Acc:VGNC:89067]	-2.51	0.083
ssc-miR-222	IKZF3	IKAROS family zinc finger 3 [Source:VGNC Symbol;Acc:VGNC:89074]	-2.51	0.083
ssc-miR-222	IKZF4	IKAROS family zinc finger 4 [Source:VGNC Symbol;Acc:VGNC:89075]	-2.51	0.083
ssc-miR-222	IL1RAPL1	interleukin 1 receptor accessory protein like 1 [Source:VGNC Symbol;Acc:VGNC:103967]	-2.51	0.083
ssc-miR-222	INSIG1	insulin induced 1 [Source:VGNC Symbol;Acc:VGNC:89150]	-2.51	0.083
ssc-miR-222	IPO7	importin 7 [Source:VGNC Symbol;Acc:VGNC:89180]	-2.51	0.083
ssc-miR-222	IRF2	interferon regulatory factor 2 [Source:VGNC Symbol;Acc:VGNC:96375]	-2.51	0.083
ssc-miR-222	IRX5	iroquois homeobox 5 [Source:VGNC Symbol;Acc:VGNC:89219]	-2.51	0.083
ssc-miR-222	ITGA2	integrin subunit alpha 2 [Source:VGNC Symbol;Acc:VGNC:89234]	-2.51	0.083
ssc-miR-222	ITGB3	integrin subunit beta 3 [Source:NCBI gene (formerly Entrezgene);Acc:397063]	-2.51	0.083
ssc-miR-222	ITGB8	integrin subunit beta 8 [Source:VGNC Symbol;Acc:VGNC:89246]	-2.51	0.083
ssc-miR-222	KANSL1	KAT8 regulatory NSL complex subunit 1 [Source:VGNC Symbol;Acc:VGNC:89299]	-2.51	0.083
ssc-miR-222	KBTBD8	kelch repeat and BTB domain containing 8 [Source:VGNC Symbol;Acc:VGNC:89321]	-2.51	0.083
ssc-miR-222	KCNK2	potassium two pore domain channel subfamily K member 2 [Source:VGNC Symbol;Acc:VGNC:89369]	-2.51	0.083
ssc-miR-222	KCNQ3	potassium voltage-gated channel subfamily Q member 3 [Source:VGNC Symbol;Acc:VGNC:89382]	-2.51	0.083
ssc-miR-222	KDM5B	lysine demethylase 5B [Source:VGNC Symbol;Acc:VGNC:95870]	-2.51	0.083
ssc-miR-222	KDR	kinase insert domain receptor [Source:VGNC Symbol;Acc:VGNC:89418]	-2.51	0.083
ssc-miR-222	KDSR	3-ketodihydrosphingosine reductase [Source:VGNC Symbol;Acc:VGNC:108157]	-2.51	0.083
ssc-miR-222	KHDRBS2	KH RNA binding domain containing, signal transduction associated 2 [Source:VGNC Symbol;Acc:VGNC:89423]	-2.51	0.083
ssc-miR-222	KIAA0368	hypothetical gene	-2.51	0.083
ssc-miR-222	KIAA0430	hypothetical gene	-2.51	0.083
ssc-miR-222	KIAA1598	hypothetical gene	-2.51	0.083
ssc-miR-222	KIF16B	kinesin family member 16B [Source:VGNC Symbol;Acc:VGNC:106450]	-2.51	0.083
ssc-miR-222	KIF26A	kinesin family member 26A [Source:VGNC Symbol;Acc:VGNC:89466]	-2.51	0.083
ssc-miR-222	KIT	KIT proto-onco, receptor tyrosine kinase [Source:VGNC Symbol;Acc:VGNC:98060]	-2.51	0.083
ssc-miR-222	KLF7	Kruppel like factor 7 [Source:VGNC Symbol;Acc:VGNC:96396]	-2.51	0.083
ssc-miR-222	KLHDC10	kelch domain containing 10 [Source:VGNC Symbol;Acc:VGNC:89502]	-2.51	0.083
ssc-miR-222	KLHL18	kelch like family member 18 [Source:VGNC Symbol;Acc:VGNC:89517]	-2.51	0.083
ssc-miR-222	KLHL24	kelch like family member 24 [Source:VGNC Symbol;Acc:VGNC:89521]	-2.51	0.083
ssc-miR-222	KMT2A	lysine methyltransferase 2A [Source:VGNC Symbol;Acc:VGNC:108600]	-2.51	0.083
ssc-miR-222	KMT2C	lysine methyltransferase 2C [Source:VGNC Symbol;Acc:VGNC:89550]	-2.51	0.083
ssc-miR-222	KPNA1	karyopherin subunit alpha 1 [Source:VGNC Symbol;Acc:VGNC:89560]	-2.51	0.083
ssc-miR-222	KPNA2	karyopherin subunit alpha 2 [Source:VGNC Symbol;Acc:VGNC:89561]	-2.51	0.083
ssc-miR-222	KRAS	KRAS proto-onco, GTPase [Source:VGNC Symbol;Acc:VGNC:89569]	-2.51	0.083
ssc-miR-222	KSR1	kinase suppressor of ras 1 [Source:VGNC Symbol;Acc:VGNC:89606]	-2.51	0.083
ssc-miR-222	LCOR	ligand dependent nuclear receptor corepressor [Source:HGNC Symbol;Acc:HGNC:29503]	-2.51	0.083
ssc-miR-222	LGALSL	galectin like [Source:VGNC Symbol;Acc:VGNC:89698]	-2.51	0.083
ssc-miR-222	LHFPL2	LHFPL tetraspan subfamily member 2 [Source:VGNC Symbol;Acc:VGNC:89705]	-2.51	0.083
ssc-miR-222	LHX8	LIM homeobox 8 [Source:VGNC Symbol;Acc:VGNC:89717]	-2.51	0.083
ssc-miR-222	LIFR	LIF receptor subunit alpha [Source:HGNC Symbol;Acc:HGNC:6597]	-2.51	0.083
ssc-miR-222	LLPH	LLP homolog, long-term synaptic facilitation factor [Source:VGNC Symbol;Acc:VGNC:103297]	-2.51	0.083
ssc-miR-222	LMBR1	limb development membrane protein 1 [Source:VGNC Symbol;Acc:VGNC:89752]	-2.51	0.083
ssc-miR-222	LMX1A	LIM homeobox transcription factor 1 alpha [Source:VGNC Symbol;Acc:VGNC:89770]	-2.51	0.083

ssc-miR-222	LPPR1	hypothetical gene	-2.51	0.083
ssc-miR-222	LRFN2	leucine rich repeat and fibronectin type III domain containing 2 [Source:VGNC Symbol;Acc:VGNC:89805]	-2.51	0.083
ssc-miR-222	LRRC14	leucine rich repeat containing 14 [Source:VGNC Symbol;Acc:VGNC:89826]	-2.51	0.083
ssc-miR-222	LRRC34	leucine rich repeat containing 34 [Source:VGNC Symbol;Acc:VGNC:89835]	-2.51	0.083
ssc-miR-222	LUC7L3	hypothetical gene	-2.51	0.083
ssc-miR-222	LYSMD1	LysM domain containing 1 [Source:VGNC Symbol;Acc:VGNC:89917]	-2.51	0.083
ssc-miR-222	MAGI1	membrane associated guanylate kinase, WW and PDZ domain containing 1 [Source:VGNC Symbol;Acc:VGNC:98097]	-2.51	0.083
ssc-miR-222	MALSU1	mitochondrial assembly of ribosomal large subunit 1 [Source:VGNC Symbol;Acc:VGNC:89960]	-2.51	0.083
ssc-miR-222	MAP3K2	mitogen-activated protein kinase kinase kinase 2 [Source:VGNC Symbol;Acc:VGNC:98107]	-2.51	0.083
ssc-miR-222	MAPK10	mitogen-activated protein kinase 10 [Source:VGNC Symbol;Acc:VGNC:98116]	-2.51	0.083
ssc-miR-222	MAPK6	mitogen-activated protein kinase 6 [Source:VGNC Symbol;Acc:VGNC:103123]	-2.51	0.083
ssc-miR-222	MARCKS	myristoylated alanine rich protein kinase C substrate [Source:VGNC Symbol;Acc:VGNC:90024]	-2.51	0.083
ssc-miR-222	MARK1	microtubule affinity regulating kinase 1 [Source:VGNC Symbol;Acc:VGNC:96411]	-2.51	0.083
ssc-miR-222	MBD2	methyl-CpG binding domain protein 2 [Source:VGNC Symbol;Acc:VGNC:90048]	-2.51	0.083
ssc-miR-222	MBD5	methyl-CpG binding domain protein 5 [Source:VGNC Symbol;Acc:VGNC:95940]	-2.51	0.083
ssc-miR-222	MCFD2	multiple coagulation factor deficiency 2, ER cargo receptor complex subunit [Source:VGNC Symbol;Acc:VGNC:103980]	-2.51	0.083
ssc-miR-222	MCTS1	MCTS1 re-initiation and release factor [Source:HGNC Symbol;Acc:HGNC:23357]	-2.51	0.083
ssc-miR-222	MDM4	MDM4 regulator of p53 [Source:VGNC Symbol;Acc:VGNC:90094]	-2.51	0.083
ssc-miR-222	MED1	mediator complex subunit 1 [Source:VGNC Symbol;Acc:VGNC:90102]	-2.51	0.083
ssc-miR-222	MEGF9	multiple EGF like domains 9 [Source:VGNC Symbol;Acc:VGNC:90130]	-2.51	0.083
ssc-miR-222	MEIS1	Meis homeobox 1 [Source:VGNC Symbol;Acc:VGNC:90135]	-2.51	0.083
ssc-miR-222	MESDC1	hypothetical gene	-2.51	0.083
ssc-miR-222	MEX3A	mex-3 RNA binding family member A [Source:VGNC Symbol;Acc:VGNC:90168]	-2.51	0.083
ssc-miR-222	MFN1	mitofusin 1 [Source:VGNC Symbol;Acc:VGNC:90178]	-2.51	0.083
ssc-miR-222	MIA3	hypothetical gene	-2.51	0.083
ssc-miR-222	MIDN	midnolin [Source:VGNC Symbol;Acc:VGNC:90216]	-2.51	0.083
ssc-miR-222	MIER3	MIER family member 3 [Source:VGNC Symbol;Acc:VGNC:90221]	-2.51	0.083
ssc-miR-222	MITF	melanocyte inducing transcription factor [Source:VGNC Symbol;Acc:VGNC:90243]	-2.51	0.083
ssc-miR-222	MLLT6	MLLT6, PHD finger containing [Source:VGNC Symbol;Acc:VGNC:90257]	-2.51	0.083
ssc-miR-222	MLXIP	MLX interacting protein [Source:VGNC Symbol;Acc:VGNC:90262]	-2.51	0.083
ssc-miR-222	MON2	MON2 homolog, regulator of endosome-to-Golgi trafficking [Source:VGNC Symbol;Acc:VGNC:90301]	-2.51	0.083
ssc-miR-222	MPRIP	hypothetical gene	-2.51	0.083
ssc-miR-222	MSANTD4	Myb/SANT DNA binding domain containing 4 with coiled-coils [Source:VGNC Symbol;Acc:VGNC:90415]	-2.51	0.083
ssc-miR-222	MSL1	MSL complex subunit 1 [Source:VGNC Symbol;Acc:VGNC:90423]	-2.51	0.083
ssc-miR-222	MSL2	MSL complex subunit 2 [Source:VGNC Symbol;Acc:VGNC:90424]	-2.51	0.083
ssc-miR-222	MST4	hypothetical gene	-2.51	0.083
ssc-miR-222	MTERF	hypothetical gene	-2.51	0.083
ssc-miR-222	MTMR2	myotubularin related protein 2 [Source:VGNC Symbol;Acc:VGNC:90460]	-2.51	0.083
ssc-miR-222	MXD1	MAX dimerization protein 1 [Source:VGNC Symbol;Acc:VGNC:90492]	-2.51	0.083
ssc-miR-222	MYBL1	MYB proto-onco like 1 [Source:VGNC Symbol;Acc:VGNC:90498]	-2.51	0.083
ssc-miR-222	MYEF2	myelin expression factor 2 [Source:VGNC Symbol;Acc:VGNC:90507]	-2.51	0.083
ssc-miR-222	MYLIP	myosin regulatory light chain interacting protein [Source:VGNC Symbol;Acc:VGNC:103999]	-2.51	0.083
ssc-miR-222	MYO10	myosin X [Source:VGNC Symbol;Acc:VGNC:90522]	-2.51	0.083
ssc-miR-222	MYT1L	myelin transcription factor 1 like [Source:VGNC Symbol;Acc:VGNC:90549]	-2.51	0.083
ssc-miR-222	NAA25	N-alpha-acetyltransferase 25, NatB auxiliary subunit [Source:VGNC Symbol;Acc:VGNC:96750]	-2.51	0.083
ssc-miR-222	NANOS1	nanos C2HC-type zinc finger 1 [Source:VGNC Symbol;Acc:VGNC:90574]	-2.51	0.083
ssc-miR-222	NAP1L1	nucleosome assembly protein 1 like 1 [Source:VGNC Symbol;Acc:VGNC:90576]	-2.51	0.083
ssc-miR-222	NAP1L2	nucleosome assembly protein 1 like 2 [Source:HGNC Symbol;Acc:HGNC:7638]	-2.51	0.083
ssc-miR-222	NAP1L5	nucleosome assembly protein 1 like 5 [Source:NCBI gene (formerly Entrezgene);Acc:100616076]	-2.51	0.083
ssc-miR-222	NBEAL1	cytochrome P450 family 20 subfamily A member 1 [Source:VGNC Symbol;Acc:VGNC:103366]	-2.51	0.083

ssc-miR-222	NDFIP1	Nedd4 family interacting protein 1 [Source:VGNC Symbol;Acc:VGNC:90626]	-2.51	0.083
ssc-miR-222	NDST3	N-deacetylase and N-sulfotransferase 3 [Source:VGNC Symbol;Acc:VGNC:90635]	-2.51	0.083
ssc-miR-222	NFAT5	nuclear factor of activated T cells 5 [Source:VGNC Symbol;Acc:VGNC:90708]	-2.51	0.083
ssc-miR-222	NFATC2	nuclear factor of activated T cells 2 [Source:VGNC Symbol;Acc:VGNC:96440]	-2.51	0.083
ssc-miR-222	NFATC3	nuclear factor of activated T cells 3 [Source:VGNC Symbol;Acc:VGNC:90710]	-2.51	0.083
ssc-miR-222	NFYB	nuclear transcription factor Y subunit beta [Source:VGNC Symbol;Acc:VGNC:90730]	-2.51	0.083
ssc-miR-222	NGRN	neugrin, neurite outgrowth associated [Source:HGNC Symbol;Acc:HGNC:18077]	-2.51	0.083
ssc-miR-222	NIPAL4	NIPA like domain containing 4 [Source:VGNC Symbol;Acc:VGNC:90751]	-2.51	0.083
ssc-miR-222	NIPBL	NIPBL cohesin loading factor [Source:VGNC Symbol;Acc:VGNC:90752]	-2.51	0.083
ssc-miR-222	NLK	nemo like kinase [Source:VGNC Symbol;Acc:VGNC:90779]	-2.51	0.083
ssc-miR-222	NNT	nicotinamide nucleotide transhydrogenase [Source:HGNC Symbol;Acc:HGNC:7863]	-2.51	0.083
ssc-miR-222	NOP9	NOP9 nucleolar protein [Source:VGNC Symbol;Acc:VGNC:90822]	-2.51	0.083
ssc-miR-222	NOVA1	NOVA alternative splicing regulator 1 [Source:VGNC Symbol;Acc:VGNC:90827]	-2.51	0.083
ssc-miR-222	NR2C1	nuclear receptor subfamily 2 group C member 1 [Source:VGNC Symbol;Acc:VGNC:90875]	-2.51	0.083
ssc-miR-222	NR4A1	nuclear receptor subfamily 4 group A member 1 [Source:HGNC Symbol;Acc:HGNC:7980]	-2.51	0.083
ssc-miR-222	NRG1	neuregulin 1 [Source:VGNC Symbol;Acc:VGNC:96453]	-2.51	0.083
ssc-miR-222	NRK	Nik related kinase [Source:VGNC Symbol;Acc:VGNC:98161]	-2.51	0.083
ssc-miR-222	NSMCE4A	NSE4 homolog A, SMC5-SMC6 complex component [Source:VGNC Symbol;Acc:VGNC:90911]	-2.51	0.083
ssc-miR-222	NSUN4	NOP2/Sun RNA methyltransferase 4 [Source:VGNC Symbol;Acc:VGNC:98522]	-2.51	0.083
ssc-miR-222	NTF3	neurotrophin 3 [Source:VGNC Symbol;Acc:VGNC:90928]	-2.51	0.083
ssc-miR-222	NUFIP2	nuclear FMR1 interacting protein 2 [Source:VGNC Symbol;Acc:VGNC:90967]	-2.51	0.083
ssc-miR-222	NXPH1	neurexophilin 1 [Source:VGNC Symbol;Acc:VGNC:90998]	-2.51	0.083
ssc-miR-222	OIP5	Opa interacting protein 5 [Source:VGNC Symbol;Acc:VGNC:91029]	-2.51	0.083
ssc-miR-222	OSBPL3	oxysterol binding protein like 3 [Source:VGNC Symbol;Acc:VGNC:91071]	-2.51	0.083
ssc-miR-222	OSTM1	osteoclastosis associated transmembrane protein 1 [Source:VGNC Symbol;Acc:VGNC:91086]	-2.51	0.083
ssc-miR-222	OXA1L	OXA1L mitochondrial inner membrane protein [Source:VGNC Symbol;Acc:VGNC:91112]	-2.51	0.083
ssc-miR-222	PACS2	phosphofurin acidic cluster sorting protein 2 [Source:VGNC Symbol;Acc:VGNC:91141]	-2.51	0.083
ssc-miR-222	PAFAH1B1	platelet activating factor acetylhydrolase 1b regulatory subunit 1 [Source:VGNC Symbol;Acc:VGNC:99026]	-2.51	0.083
ssc-miR-222	PAIP1	poly(A) binding protein interacting protein 1 [Source:HGNC Symbol;Acc:HGNC:16945]	-2.51	0.083
ssc-miR-222	PAIP2	poly(A) binding protein interacting protein 2 [Source:VGNC Symbol;Acc:VGNC:96618]	-2.51	0.083
ssc-miR-222	PAK1	p21 (RAC1) activated kinase 1 [Source:HGNC Symbol;Acc:HGNC:8590]	-2.51	0.083
ssc-miR-222	PAM	peptidylglycine alpha-amidating monooxygenase [Source:VGNC Symbol;Acc:VGNC:91163]	-2.51	0.083
ssc-miR-222	PANK3	pantothenate kinase 3 [Source:VGNC Symbol;Acc:VGNC:91165]	-2.51	0.083
ssc-miR-222	PAPD4	hypothetical gene	-2.51	0.083
ssc-miR-222	PAPOLG	poly(A) polymerase gamma [Source:HGNC Symbol;Acc:HGNC:14982]	-2.51	0.083
ssc-miR-222	PBX3	PBX homeobox 3 [Source:VGNC Symbol;Acc:VGNC:91205]	-2.51	0.083
ssc-miR-222	PCDHA1	hypothetical gene	-2.51	0.083
ssc-miR-222	PCDHA10	hypothetical gene	-2.51	0.083
ssc-miR-222	PCDHA11	hypothetical gene	-2.51	0.083
ssc-miR-222	PCDHA12	hypothetical gene	-2.51	0.083
ssc-miR-222	PCDHA13	protocadherin alpha 13 [Source:HGNC Symbol;Acc:HGNC:8667]	-2.51	0.083
ssc-miR-222	PCDHA2	hypothetical gene	-2.51	0.083
ssc-miR-222	PCDHA3	protocadherin alpha 3 [Source:HGNC Symbol;Acc:HGNC:8669]	-2.51	0.083
ssc-miR-222	PCDHA4	hypothetical gene	-2.51	0.083
ssc-miR-222	PCDHA5	hypothetical gene	-2.51	0.083
ssc-miR-222	PCDHA6	hypothetical gene	-2.51	0.083
ssc-miR-222	PCDHA7	hypothetical gene	-2.51	0.083
ssc-miR-222	PCDHA8	hypothetical gene	-2.51	0.083
ssc-miR-222	PCDHA9	hypothetical gene	-2.51	0.083
ssc-miR-222	PCDHAC1	hypothetical gene	-2.51	0.083

ssc-miR-222	PCDHAC2	protocadherin alpha subfamily C, 2 [Source:HGNC Symbol;Acc:HGNC:8677]	-2.51	0.083
ssc-miR-222	PCGF3	polycomb group ring finger 3 [Source:VGNC Symbol;Acc:VGNC:91220]	-2.51	0.083
ssc-miR-222	PCMTD1	protein-L-isoaspartate (D-aspartate) O-methyltransferase domain containing 1 [Source:VGNC Symbol;Acc:VGNC:98830]	-2.51	0.083
ssc-miR-222	PDCD10	programmed cell death 10 [Source:VGNC Symbol;Acc:VGNC:91241]	-2.51	0.083
ssc-miR-222	PDE3A	phosphodiesterase 3A [Source:VGNC Symbol;Acc:VGNC:91252]	-2.51	0.083
ssc-miR-222	PDGFA	platelet derived growth factor subunit A [Source:VGNC Symbol;Acc:VGNC:91265]	-2.51	0.083
ssc-miR-222	PDIK1L	PDLIM1 interacting kinase 1 like [Source:VGNC Symbol;Acc:VGNC:91276]	-2.51	0.083
ssc-miR-222	PDZRN4	PDZ domain containing ring finger 4 [Source:VGNC Symbol;Acc:VGNC:91299]	-2.51	0.083
ssc-miR-222	PEG3	paternally expressed 3 [Source:VGNC Symbol;Acc:VGNC:98530]	-2.51	0.083
ssc-miR-222	PGAP1	post-GPI attachment to proteins inositol deacylase 1 [Source:VGNC Symbol;Acc:VGNC:96099]	-2.51	0.083
ssc-miR-222	PHACTR4	phosphatase and actin regulator 4 [Source:VGNC Symbol;Acc:VGNC:98531]	-2.51	0.083
ssc-miR-222	PHF12	PHD finger protein 12 [Source:VGNC Symbol;Acc:VGNC:91378]	-2.51	0.083
ssc-miR-222	PHF2	PHD finger protein 2 [Source:VGNC Symbol;Acc:VGNC:91382]	-2.51	0.083
ssc-miR-222	PIEZO2	piezo type mechanosensitive ion channel component 2 [Source:HGNC Symbol;Acc:HGNC:26270]	-2.51	0.083
ssc-miR-222	PIK3R1	phosphoinositide-3-kinase regulatory subunit 1 [Source:VGNC Symbol;Acc:VGNC:91445]	-2.51	0.083
ssc-miR-222	PITPNM2	phosphatidylinositol transfer protein membrane associated 2 [Source:VGNC Symbol;Acc:VGNC:91466]	-2.51	0.083
ssc-miR-222	PKDCC	protein kinase domain containing, cytoplasmic [Source:VGNC Symbol;Acc:VGNC:91475]	-2.51	0.083
ssc-miR-222	PKIA	cAMP-dependent protein kinase inhibitor alpha [Source:VGNC Symbol;Acc:VGNC:91477]	-2.51	0.083
ssc-miR-222	PLCL2	phospholipase C like 2 [Source:VGNC Symbol;Acc:VGNC:91523]	-2.51	0.083
ssc-miR-222	PLCXD3	phosphatidylinositol specific phospholipase C X domain containing 3 [Source:VGNC Symbol;Acc:VGNC:91524]	-2.51	0.083
ssc-miR-222	PLD5	phospholipase D family member 5 [Source:VGNC Symbol;Acc:VGNC:96145]	-2.51	0.083
ssc-miR-222	PLEKHA2	pleckstrin homology domain containing A2 [Source:VGNC Symbol;Acc:VGNC:95611]	-2.51	0.083
ssc-miR-222	PLEKHA6	pleckstrin homology domain containing A6 [Source:VGNC Symbol;Acc:VGNC:91536]	-2.51	0.083
ssc-miR-222	PLXNC1	plexin C1 [Source:VGNC Symbol;Acc:VGNC:91584]	-2.51	0.083
ssc-miR-222	PNO1	partner of NOB1 homolog [Source:VGNC Symbol;Acc:VGNC:104030]	-2.51	0.083
ssc-miR-222	PNRC2	proline rich nuclear receptor coactivator 2 [Source:VGNC Symbol;Acc:VGNC:91614]	-2.51	0.083
ssc-miR-222	POGZ	pogo transposable element derived with ZNF domain [Source:VGNC Symbol;Acc:VGNC:91625]	-2.51	0.083
ssc-miR-222	POLR3E	RNA polymerase III subunit E [Source:VGNC Symbol;Acc:VGNC:91657]	-2.51	0.083
ssc-miR-222	PPAP2B	hypothetical gene	-2.51	0.083
ssc-miR-222	PPP1R15B	protein phosphatase 1 regulatory subunit 15B [Source:VGNC Symbol;Acc:VGNC:91726]	-2.51	0.083
ssc-miR-222	PPP2R2A	protein phosphatase 2 regulatory subunit Balpha [Source:VGNC Symbol;Acc:VGNC:91748]	-2.51	0.083
ssc-miR-222	PPP2R5E	protein phosphatase 2 regulatory subunit B'epsilon [Source:VGNC Symbol;Acc:VGNC:91754]	-2.51	0.083
ssc-miR-222	PPP3R1	protein phosphatase 3 regulatory subunit B, alpha [Source:VGNC Symbol;Acc:VGNC:106453]	-2.51	0.083
ssc-miR-222	PPP6C	protein phosphatase 6 catalytic subunit [Source:VGNC Symbol;Acc:VGNC:98219]	-2.51	0.083
ssc-miR-222	PRDM1	PR/SET domain 1 [Source:VGNC Symbol;Acc:VGNC:91772]	-2.51	0.083
ssc-miR-222	PRKACB	protein kinase cAMP-activated catalytic subunit beta [Source:VGNC Symbol;Acc:VGNC:91800]	-2.51	0.083
ssc-miR-222	PRR14L	proline rich 14 like [Source:VGNC Symbol;Acc:VGNC:91853]	-2.51	0.083
ssc-miR-222	PRRG3	proline rich and Gla domain 3 [Source:VGNC Symbol;Acc:VGNC:91869]	-2.51	0.083
ssc-miR-222	PRUNE	hypothetical gene	-2.51	0.083
ssc-miR-222	PSMB5	hypothetical gene	-2.51	0.083
ssc-miR-222	PTBP2	polypyrimidine tract binding protein 2 [Source:VGNC Symbol;Acc:VGNC:91937]	-2.51	0.083
ssc-miR-222	PTBP3	polypyrimidine tract binding protein 3 [Source:VGNC Symbol;Acc:VGNC:91938]	-2.51	0.083
ssc-miR-222	PTCHD1	patched domain containing 1 [Source:VGNC Symbol;Acc:VGNC:91942]	-2.51	0.083
ssc-miR-222	PTPMT1	protein tyrosine phosphatase mitochondrial 1 [Source:VGNC Symbol;Acc:VGNC:91971]	-2.51	0.083
ssc-miR-222	PTPN3	protein tyrosine phosphatase non-receptor type 3 [Source:VGNC Symbol;Acc:VGNC:91980]	-2.51	0.083
ssc-miR-222	PTS	6-pyruvoyltetrahydropterin synthase [Source:VGNC Symbol;Acc:VGNC:104039]	-2.51	0.083
ssc-miR-222	PURA	purine rich element binding protein A [Source:HGNC Symbol;Acc:HGNC:9701]	-2.51	0.083
ssc-miR-222	PVRL1	hypothetical gene	-2.51	0.083
ssc-miR-222	QKI	QKI, KH domain containing RNA binding [Source:VGNC Symbol;Acc:VGNC:92025]	-2.51	0.083
ssc-miR-222	RAB1A	RAB1A, member RAS onco family [Source:VGNC Symbol;Acc:VGNC:104042]	-2.51	0.083

ssc-miR-222	RAB3B	RAB3B, member RAS onco family [Source:VGNC Symbol;Acc:VGNC:98265]	-2.51	0.083
ssc-miR-222	RAB3GAP2	RAB3 GTPase activating non-catalytic protein subunit 2 [Source:VGNC Symbol;Acc:VGNC:95833]	-2.51	0.083
ssc-miR-222	RALGAPA1	Ral GTPase activating protein catalytic subunit alpha 1 [Source:VGNC Symbol;Acc:VGNC:92071]	-2.51	0.083
ssc-miR-222	RANBP10	RAN binding protein 10 [Source:VGNC Symbol;Acc:VGNC:92077]	-2.51	0.083
ssc-miR-222	RANBP2	RAN binding protein 2 [Source:HGNC Symbol;Acc:HGNC:9848]	-2.51	0.083
ssc-miR-222	RBL1	RB transcriptional corepressor like 1 [Source:VGNC Symbol;Acc:VGNC:96533]	-2.51	0.083
ssc-miR-222	RBM24	hypothetical gene	-2.51	0.083
ssc-miR-222	RECK	reversion inducing cysteine rich protein with kazal motifs [Source:VGNC Symbol;Acc:VGNC:92194]	-2.51	0.083
ssc-miR-222	REV1	REV1 DNA directed polymerase [Source:HGNC Symbol;Acc:HGNC:14060]	-2.51	0.083
ssc-miR-222	REV3L	REV3 like, DNA directed polymerase zeta catalytic subunit [Source:VGNC Symbol;Acc:VGNC:92226]	-2.51	0.083
ssc-miR-222	RFX3	regulatory factor X3 [Source:VGNC Symbol;Acc:VGNC:92245]	-2.51	0.083
ssc-miR-222	RFX7	regulatory factor X7 [Source:VGNC Symbol;Acc:VGNC:92248]	-2.51	0.083
ssc-miR-222	RGS17	regulator of G protein signaling 17 [Source:VGNC Symbol;Acc:VGNC:92263]	-2.51	0.083
ssc-miR-222	RIMS3	regulating synaptic membrane exocytosis 3 [Source:VGNC Symbol;Acc:VGNC:92310]	-2.51	0.083
ssc-miR-222	RIMS4	regulating synaptic membrane exocytosis 4 [Source:VGNC Symbol;Acc:VGNC:95775]	-2.51	0.083
ssc-miR-222	RND2	Rho family GTPase 2 [Source:VGNC Symbol;Acc:VGNC:92344]	-2.51	0.083
ssc-miR-222	RNF217	ring finger protein 217 [Source:VGNC Symbol;Acc:VGNC:103162]	-2.51	0.083
ssc-miR-222	RNF4	ring finger protein 4 [Source:VGNC Symbol;Acc:VGNC:92392]	-2.51	0.083
ssc-miR-222	RNF44	ring finger protein 44 [Source:VGNC Symbol;Acc:VGNC:92395]	-2.51	0.083
ssc-miR-222	RNPS1	RNA binding protein with serine rich domain 1 [Source:VGNC Symbol;Acc:VGNC:92403]	-2.51	0.083
ssc-miR-222	RP11-766F14.2	hypothetical gene	-2.51	0.083
ssc-miR-222	RPH3A	rabphilin 3A [Source:VGNC Symbol;Acc:VGNC:92425]	-2.51	0.083
ssc-miR-222	RREB1	hypothetical gene	-2.51	0.083
ssc-miR-222	RSBN1L	round spermatid basic protein 1 like [Source:VGNC Symbol;Acc:VGNC:92474]	-2.51	0.083
ssc-miR-222	RSRC2	arginine and serine rich coiled-coil 2 [Source:VGNC Symbol;Acc:VGNC:92488]	-2.51	0.083
ssc-miR-222	RUNX1T1	RUNX1 partner transcriptional co-repressor 1 [Source:VGNC Symbol;Acc:VGNC:96594]	-2.51	0.083
ssc-miR-222	RUNX2	RUNX family transcription factor 2 [Source:VGNC Symbol;Acc:VGNC:92517]	-2.51	0.083
ssc-miR-222	S100PBP	S100P binding protein [Source:VGNC Symbol;Acc:VGNC:92549]	-2.51	0.083
ssc-miR-222	SBK1	SH3 domain binding kinase 1 [Source:HGNC Symbol;Acc:HGNC:17699]	-2.51	0.083
ssc-miR-222	SCD5	stearoyl-CoA desaturase 5 [Source:VGNC Symbol;Acc:VGNC:92617]	-2.51	0.083
ssc-miR-222	SEC23IP	SEC23 interacting protein [Source:VGNC Symbol;Acc:VGNC:92675]	-2.51	0.083
ssc-miR-222	SEC62	SEC62 homolog, preprotein translocation factor [Source:VGNC Symbol;Acc:VGNC:108691]	-2.51	0.083
ssc-miR-222	SEMA6D	semaphorin 6D [Source:VGNC Symbol;Acc:VGNC:92711]	-2.51	0.083
ssc-miR-222	SESN3	sestrin 3 [Source:VGNC Symbol;Acc:VGNC:92755]	-2.51	0.083
ssc-miR-222	SH3BP4	SH3 domain binding protein 4 [Source:VGNC Symbol;Acc:VGNC:95495]	-2.51	0.083
ssc-miR-222	SH3PXD2B	SH3 and PX domains 2B [Source:VGNC Symbol;Acc:VGNC:92830]	-2.51	0.083
ssc-miR-222	SHANK2	SH3 and multiple ankyrin repeat domains 2 [Source:VGNC Symbol;Acc:VGNC:92835]	-2.51	0.083
ssc-miR-222	SHC3	SHC adaptor protein 3 [Source:VGNC Symbol;Acc:VGNC:92838]	-2.51	0.083
ssc-miR-222	SHISA9	shisa family member 9 [Source:HGNC Symbol;Acc:HGNC:37231]	-2.51	0.083
ssc-miR-222	SKOR1	SKI family transcriptional corepressor 1 [Source:VGNC Symbol;Acc:VGNC:92905]	-2.51	0.083
ssc-miR-222	SKP1	hypothetical gene	-2.51	0.083
ssc-miR-222	SLAIN2	SLAIN motif family member 2 [Source:VGNC Symbol;Acc:VGNC:92910]	-2.51	0.083
ssc-miR-222	SLC16A6	solute carrier family 16 member 6 [Source:VGNC Symbol;Acc:VGNC:92946]	-2.51	0.083
ssc-miR-222	SLC17A6	solute carrier family 17 member 6 [Source:VGNC Symbol;Acc:VGNC:92954]	-2.51	0.083
ssc-miR-222	SLC25A37	hypothetical gene	-2.51	0.083
ssc-miR-222	SLC2A13	solute carrier family 2 member 13 [Source:VGNC Symbol;Acc:VGNC:93047]	-2.51	0.083
ssc-miR-222	SLC35A3	solute carrier family 35 member A3 [Source:VGNC Symbol;Acc:VGNC:98860]	-2.51	0.083
ssc-miR-222	SLC40A1	solute carrier family 40 member 1 [Source:VGNC Symbol;Acc:VGNC:95497]	-2.51	0.083
ssc-miR-222	SLC4A4	solute carrier family 4 member 4 [Source:VGNC Symbol;Acc:VGNC:93133]	-2.51	0.083
ssc-miR-222	SLC6A1	solute carrier family 6 member 1 [Source:VGNC Symbol;Acc:VGNC:93153]	-2.51	0.083

ssc-miR-222	SLCO1A2	solute carrier organic anion transporter family member 1A2 [Source:VGNC Symbol;Acc:VGNC:93193]	-2.51	0.083
ssc-miR-222	SLITRK5	SLIT and NTRK like family member 5 [Source:VGNC Symbol;Acc:VGNC:93210]	-2.51	0.083
ssc-miR-222	SMARCA5	SWI/SNF related, matrix associated, actin dependent regulator of chromatin, subfamily a, member 5 [Source:VGNC Symbol;Acc:VGNC:93228]	-2.51	0.083
ssc-miR-222	SMC1A	structural maintenance of chromosomes 1A [Source:VGNC Symbol;Acc:VGNC:93236]	-2.51	0.083
ssc-miR-222	SMNDC1	survival motor neuron domain containing 1 [Source:VGNC Symbol;Acc:VGNC:93257]	-2.51	0.083
ssc-miR-222	SMURF1	SMAD specific E3 ubiquitin protein ligase 1 [Source:VGNC Symbol;Acc:VGNC:93270]	-2.51	0.083
ssc-miR-222	SNAP29	synaptosome associated protein 29 [Source:VGNC Symbol;Acc:VGNC:93278]	-2.51	0.083
ssc-miR-222	SNAP91	synaptosome associated protein 91 [Source:VGNC Symbol;Acc:VGNC:93279]	-2.51	0.083
ssc-miR-222	SNCB	synuclein beta [Source:VGNC Symbol;Acc:VGNC:93286]	-2.51	0.083
ssc-miR-222	SNX24	sorting nexin 24 [Source:VGNC Symbol;Acc:VGNC:93315]	-2.51	0.083
ssc-miR-222	SNX29	sorting nexin 29 [Source:VGNC Symbol;Acc:VGNC:98331]	-2.51	0.083
ssc-miR-222	SNX4	sorting nexin 4 [Source:VGNC Symbol;Acc:VGNC:93322]	-2.51	0.083
ssc-miR-222	SOCS1	suppressor of cytokine signaling 1 [Source:VGNC Symbol;Acc:VGNC:93330]	-2.51	0.083
ssc-miR-222	SOCS3	suppressor of cytokine signaling 3 [Source:VGNC Symbol;Acc:VGNC:99052]	-2.51	0.083
ssc-miR-222	SOCS5	suppressor of cytokine signaling 5 [Source:VGNC Symbol;Acc:VGNC:93332]	-2.51	0.083
ssc-miR-222	SOCS7	suppressor of cytokine signaling 7 [Source:VGNC Symbol;Acc:VGNC:93334]	-2.51	0.083
ssc-miR-222	SOX1	SRY-box transcription factor 1 [Source:HGNC Symbol;Acc:HGNC:11189]	-2.51	0.083
ssc-miR-222	SOX10	SRY-box transcription factor 10 [Source:VGNC Symbol;Acc:VGNC:93350]	-2.51	0.083
ssc-miR-222	SOX11	SRY-box transcription factor 11 [Source:VGNC Symbol;Acc:VGNC:93351]	-2.51	0.083
ssc-miR-222	SOX18	SRY-box transcription factor 18 [Source:VGNC Symbol;Acc:VGNC:95510]	-2.51	0.083
ssc-miR-222	SPATA2	spermatosis associated 2 [Source:VGNC Symbol;Acc:VGNC:95628]	-2.51	0.083
ssc-miR-222	SPATS2L	spermatosis associated serine rich 2 like [Source:VGNC Symbol;Acc:VGNC:96047]	-2.51	0.083
ssc-miR-222	SPPL3	signal peptide peptidase like 3 [Source:VGNC Symbol;Acc:VGNC:98340]	-2.51	0.083
ssc-miR-222	SPRED1	sprouty related EVH1 domain containing 1 [Source:VGNC Symbol;Acc:VGNC:93420]	-2.51	0.083
ssc-miR-222	SPRED2	sprouty related EVH1 domain containing 2 [Source:VGNC Symbol;Acc:VGNC:93421]	-2.51	0.083
ssc-miR-222	SPTSSA	serine palmitoyltransferase small subunit A [Source:HGNC Symbol;Acc:HGNC:20361]	-2.51	0.083
ssc-miR-222	SRCIN1	SRC kinase signaling inhibitor 1 [Source:VGNC Symbol;Acc:VGNC:93448]	-2.51	0.083
ssc-miR-222	SREK1IP1	SREK1 interacting protein 1 [Source:HGNC Symbol;Acc:HGNC:26716]	-2.51	0.083
ssc-miR-222	SRPK2	SRSF protein kinase 2 [Source:VGNC Symbol;Acc:VGNC:93463]	-2.51	0.083
ssc-miR-222	SRSF10	serine and arginine rich splicing factor 10 [Source:VGNC Symbol;Acc:VGNC:93472]	-2.51	0.083
ssc-miR-222	SRSF2	serine and arginine rich splicing factor 2 [Source:VGNC Symbol;Acc:VGNC:99085]	-2.51	0.083
ssc-miR-222	SRSF7	serine and arginine rich splicing factor 7 [Source:VGNC Symbol;Acc:VGNC:93477]	-2.51	0.083
ssc-miR-222	ST8SIA1	ST8 alpha-N-acetyl-neuraminide alpha-2,8-sialyltransferase 1 [Source:VGNC Symbol;Acc:VGNC:93516]	-2.51	0.083
ssc-miR-222	STMN1	stathmin 1 [Source:VGNC Symbol;Acc:VGNC:93560]	-2.51	0.083
ssc-miR-222	STOX2	storkhead box 2 [Source:VGNC Symbol;Acc:VGNC:96082]	-2.51	0.083
ssc-miR-222	STX1B	syntaxin 1B [Source:VGNC Symbol;Acc:VGNC:98352]	-2.51	0.083
ssc-miR-222	STYX	serine/threonine/tyrosine interacting protein [Source:VGNC Symbol;Acc:VGNC:93602]	-2.51	0.083
ssc-miR-222	SUGT1	SGT1 homolog, MIS12 kinetochore complex assembly cochaperone [Source:VGNC Symbol;Acc:VGNC:93611]	-2.51	0.083
ssc-miR-222	SUN2	Sad1 and UNC84 domain containing 2 [Source:VGNC Symbol;Acc:VGNC:93619]	-2.51	0.083
ssc-miR-222	SYNCRIP	synaptotagmin binding cytoplasmic RNA interacting protein [Source:VGNC Symbol;Acc:VGNC:103185]	-2.51	0.083
ssc-miR-222	SYT10	synaptotagmin 10 [Source:VGNC Symbol;Acc:VGNC:93679]	-2.51	0.083
ssc-miR-222	TAB2	TGF-beta activated kinase 1 (MAP3K7) binding protein 2 [Source:VGNC Symbol;Acc:VGNC:103186]	-2.51	0.083
ssc-miR-222	TAOK1	TAO kinase 1 [Source:VGNC Symbol;Acc:VGNC:98355]	-2.51	0.083
ssc-miR-222	TBC1D19	TBC1 domain family member 19 [Source:VGNC Symbol;Acc:VGNC:93767]	-2.51	0.083
ssc-miR-222	TBC1D22B	TBC1 domain family member 22B [Source:VGNC Symbol;Acc:VGNC:93771]	-2.51	0.083
ssc-miR-222	TBX15	hypothetical gene	-2.51	0.083
ssc-miR-222	TCF12	transcription factor 12 [Source:VGNC Symbol;Acc:VGNC:93817]	-2.51	0.083
ssc-miR-222	TCF24	transcription factor 24 [Source:HGNC Symbol;Acc:HGNC:32275]	-2.51	0.083
ssc-miR-222	TCF3	transcription factor 3 [Source:VGNC Symbol;Acc:VGNC:93822]	-2.51	0.083
ssc-miR-222	TCF4	transcription factor 4 [Source:VGNC Symbol;Acc:VGNC:93823]	-2.51	0.083

ssc-miR-222	TCF7L2	transcription factor 7 like 2 [Source:VGNC Symbol;Acc:VGNC:93825]	-2.51	0.083
ssc-miR-222	TERF2	telomeric repeat binding factor 2 [Source:VGNC Symbol;Acc:VGNC:93882]	-2.51	0.083
ssc-miR-222	TFAP2A	transcription factor AP-2 alpha [Source:VGNC Symbol;Acc:VGNC:93908]	-2.51	0.083
ssc-miR-222	TFG	trafficking from ER to golgi regulator [Source:VGNC Symbol;Acc:VGNC:93921]	-2.51	0.083
ssc-miR-222	THBS1	thrombospondin 1 [Source:VGNC Symbol;Acc:VGNC:93946]	-2.51	0.083
ssc-miR-222	THRB	thyroid hormone receptor beta [Source:VGNC Symbol;Acc:VGNC:93965]	-2.51	0.083
ssc-miR-222	TIMP2	hypothetical gene	-2.51	0.083
ssc-miR-222	TIMP3	TIMP metallopeptidase inhibitor 3 [Source:VGNC Symbol;Acc:VGNC:93996]	-2.51	0.083
ssc-miR-222	TIPARP	TCDD inducible poly(ADP-ribose) polymerase [Source:VGNC Symbol;Acc:VGNC:108699]	-2.51	0.083
ssc-miR-222	TLE3	TLE family member 3, transcriptional corepressor [Source:VGNC Symbol;Acc:VGNC:94016]	-2.51	0.083
ssc-miR-222	TLK1	tousled like kinase 1 [Source:VGNC Symbol;Acc:VGNC:95543]	-2.51	0.083
ssc-miR-222	TMCC1	transmembrane and coiled-coil domain family 1 [Source:VGNC Symbol;Acc:VGNC:94046]	-2.51	0.083
ssc-miR-222	TMEM132B	transmembrane protein 132B [Source:HGNC Symbol;Acc:HGNC:29397]	-2.51	0.083
ssc-miR-222	TMF1	TATA element modulatory factor 1 [Source:VGNC Symbol;Acc:VGNC:94223]	-2.51	0.083
ssc-miR-222	TMSB15B	hypothetical gene	-2.51	0.083
ssc-miR-222	TNRC6C	trinucleotide repeat containing adaptor 6C [Source:VGNC Symbol;Acc:VGNC:94294]	-2.51	0.083
ssc-miR-222	TOR1AIP1	torsin 1A interacting protein 1 [Source:VGNC Symbol;Acc:VGNC:94317]	-2.51	0.083
ssc-miR-222	TOX	thymocyte selection associated high mobility group box [Source:VGNC Symbol;Acc:VGNC:94322]	-2.51	0.083
ssc-miR-222	TP53BP2	tumor protein p53 binding protein 2 [Source:VGNC Symbol;Acc:VGNC:95553]	-2.51	0.083
ssc-miR-222	TP53INP2	tumor protein p53 inducible nuclear protein 2 [Source:VGNC Symbol;Acc:VGNC:95737]	-2.51	0.083
ssc-miR-222	TPM3	tropomyosin 3 [Source:VGNC Symbol;Acc:VGNC:98877]	-2.51	0.083
ssc-miR-222	TRABD2B	TraB domain containing 2B [Source:VGNC Symbol;Acc:VGNC:94356]	-2.51	0.083
ssc-miR-222	TRAM2	translocation associated membrane protein 2 [Source:HGNC Symbol;Acc:HGNC:16855]	-2.51	0.083
ssc-miR-222	TRIB2	tribbles pseudokinase 2 [Source:VGNC Symbol;Acc:VGNC:94392]	-2.51	0.083
ssc-miR-222	TRIM71	tripartite motif containing 71 [Source:VGNC Symbol;Acc:VGNC:94431]	-2.51	0.083
ssc-miR-222	TRPC3	transient receptor potential cation channel subfamily C member 3 [Source:VGNC Symbol;Acc:VGNC:94461]	-2.51	0.083
ssc-miR-222	TRPM3	transient receptor potential cation channel subfamily M member 3 [Source:VGNC Symbol;Acc:VGNC:98384]	-2.51	0.083
ssc-miR-222	TRPS1	transcriptional repressor GATA binding 1 [Source:VGNC Symbol;Acc:VGNC:94471]	-2.51	0.083
ssc-miR-222	TSC22D3	hypothetical gene	-2.51	0.083
ssc-miR-222	TTBK2	tau tubulin kinase 2 [Source:VGNC Symbol;Acc:VGNC:98387]	-2.51	0.083
ssc-miR-222	TUBA1A	tubulin alpha 1a [Source:NCBI gene (formerly Entrezgene);Acc:100135051]	-2.51	0.083
ssc-miR-222	UBE2G1	ubiquitin conjugating enzyme E2 G1 [Source:HGNC Symbol;Acc:HGNC:12482]	-2.51	0.083
ssc-miR-222	UBE2J1	ubiquitin conjugating enzyme E2 J1 [Source:VGNC Symbol;Acc:VGNC:94644]	-2.51	0.083
ssc-miR-222	UBE2N	hypothetical gene	-2.51	0.083
ssc-miR-222	UBL4A	hypothetical gene	-2.51	0.083
ssc-miR-222	UBN2	ubinuclein 2 [Source:VGNC Symbol;Acc:VGNC:94664]	-2.51	0.083
ssc-miR-222	ULK2	unc-51 like autophagy activating kinase 2 [Source:VGNC Symbol;Acc:VGNC:94695]	-2.51	0.083
ssc-miR-222	URI1	URI1 prefoldin like chaperone [Source:VGNC Symbol;Acc:VGNC:94731]	-2.51	0.083
ssc-miR-222	USP49	ubiquitin specific peptidase 49 [Source:VGNC Symbol;Acc:VGNC:94771]	-2.51	0.083
ssc-miR-222	USP6NL	USP6 N-terminal like [Source:VGNC Symbol;Acc:VGNC:95820]	-2.51	0.083
ssc-miR-222	VAPB	VAMP associated protein B and C [Source:VGNC Symbol;Acc:VGNC:108743]	-2.51	0.083
ssc-miR-222	VASH1	vasohibin 1 [Source:VGNC Symbol;Acc:VGNC:94801]	-2.51	0.083
ssc-miR-222	VEZF1	vascular endothelial zinc finger 1 [Source:VGNC Symbol;Acc:VGNC:94818]	-2.51	0.083
ssc-miR-222	VGLL3	vestigial like family member 3 [Source:VGNC Symbol;Acc:VGNC:94823]	-2.51	0.083
ssc-miR-222	VGLL4	vestigial like family member 4 [Source:VGNC Symbol;Acc:VGNC:94824]	-2.51	0.083
ssc-miR-222	VTI1A	vesicle transport through interaction with t-SNAREs 1A [Source:VGNC Symbol;Acc:VGNC:94879]	-2.51	0.083
ssc-miR-222	VWC2L	von Willebrand factor C domain containing 2 like [Source:VGNC Symbol;Acc:VGNC:96301]	-2.51	0.083
ssc-miR-222	WASF2	WASP family member 2 [Source:VGNC Symbol;Acc:VGNC:94893]	-2.51	0.083
ssc-miR-222	WDR37	WD repeat domain 37 [Source:VGNC Symbol;Acc:VGNC:96258]	-2.51	0.083
ssc-miR-222	WDR47	WD repeat domain 47 [Source:VGNC Symbol;Acc:VGNC:94924]	-2.51	0.083

ssc-miR-222	WEE1	WEE1 G2 checkpoint kinase [Source:VGNC Symbol;Acc:VGNC:104095]	-2.51	0.083
ssc-miR-222	WLS	Wnt ligand secretion mediator [Source:VGNC Symbol;Acc:VGNC:94961]	-2.51	0.083
ssc-miR-222	WNK3	WNK lysine deficient protein kinase 3 [Source:VGNC Symbol;Acc:VGNC:94964]	-2.51	0.083
ssc-miR-222	WRN	WRN RecQ like helicase [Source:VGNC Symbol;Acc:VGNC:96605]	-2.51	0.083
ssc-miR-222	WSB2	WD repeat and SOCS box containing 2 [Source:VGNC Symbol;Acc:VGNC:94982]	-2.51	0.083
ssc-miR-222	XKR4	XK related 4 [Source:VGNC Symbol;Acc:VGNC:98900]	-2.51	0.083
ssc-miR-222	YOD1	YOD1 deubiquitinase [Source:VGNC Symbol;Acc:VGNC:95035]	-2.51	0.083
ssc-miR-222	YTHDC1	YTH domain containing 1 [Source:VGNC Symbol;Acc:VGNC:95041]	-2.51	0.083
ssc-miR-222	YWHAG	hypothetical gene	-2.51	0.083
ssc-miR-222	ZADH2	prostaglandin reductase 3 [Source:VGNC Symbol;Acc:VGNC:95049]	-2.51	0.083
ssc-miR-222	ZBTB37	zinc finger and BTB domain containing 37 [Source:VGNC Symbol;Acc:VGNC:95071]	-2.51	0.083
ssc-miR-222	ZBTB39	zinc finger and BTB domain containing 39 [Source:VGNC Symbol;Acc:VGNC:95073]	-2.51	0.083
ssc-miR-222	ZC3H6	zinc finger CCCH-type containing 6 [Source:VGNC Symbol;Acc:VGNC:95098]	-2.51	0.083
ssc-miR-222	ZDHHC17	zinc finger DHHC-type palmitoyltransferase 17 [Source:VGNC Symbol;Acc:VGNC:95119]	-2.51	0.083
ssc-miR-222	ZEB2	hypothetical gene	-2.51	0.083
ssc-miR-222	ZFAND5	zinc finger AN1-type containing 5 [Source:VGNC Symbol;Acc:VGNC:95137]	-2.51	0.083
ssc-miR-222	ZFHX3	zinc finger homeobox 3 [Source:VGNC Symbol;Acc:VGNC:95141]	-2.51	0.083
ssc-miR-222	ZFP36	ZFP36 ring finger protein [Source:VGNC Symbol;Acc:VGNC:95145]	-2.51	0.083
ssc-miR-222	ZFP36L2	ZFP36 ring finger protein like 2 [Source:VGNC Symbol;Acc:VGNC:95147]	-2.51	0.083
ssc-miR-222	ZFPM2	zinc finger protein, FOG family member 2 [Source:VGNC Symbol;Acc:VGNC:95153]	-2.51	0.083
ssc-miR-222	ZFYVE16	zinc finger FYVE-type containing 16 [Source:VGNC Symbol;Acc:VGNC:95157]	-2.51	0.083
ssc-miR-222	ZMYM2	zinc finger MYM-type containing 2 [Source:VGNC Symbol;Acc:VGNC:95182]	-2.51	0.083
ssc-miR-222	ZNF385A	zinc finger protein 385A [Source:VGNC Symbol;Acc:VGNC:95233]	-2.51	0.083
ssc-miR-222	ZNF518B	zinc finger protein 518B [Source:VGNC Symbol;Acc:VGNC:95258]	-2.51	0.083
ssc-miR-222	ZNF618	zinc finger protein 618 [Source:VGNC Symbol;Acc:VGNC:95276]	-2.51	0.083
ssc-miR-222	ZNF629	zinc finger protein 629 [Source:VGNC Symbol;Acc:VGNC:95279]	-2.51	0.083
ssc-miR-222	ZNF652	zinc finger protein 652 [Source:VGNC Symbol;Acc:VGNC:99108]	-2.51	0.083
ssc-miR-222	ZSWIM4	zinc finger SWIM-type containing 4 [Source:VGNC Symbol;Acc:VGNC:95333]	-2.51	0.083
ssc-miR-212	AAK1	AP2 associated kinase 1 [Source:VGNC Symbol;Acc:VGNC:100379]	-2.6	0.0398
ssc-miR-212	ABCA9	ATP binding cassette subfamily A member 9 [Source:HGNC Symbol;Acc:HGNC:39]	-2.6	0.0398
ssc-miR-212	ABCE1	ATP binding cassette subfamily E member 1 [Source:VGNC Symbol;Acc:VGNC:84965]	-2.6	0.0398
ssc-miR-212	ABHD14A-ACY1	hypothetical gene	-2.6	0.0398
ssc-miR-212	ABL2	ABL proto-onco 2, non-receptor tyrosine kinase [Source:VGNC Symbol;Acc:VGNC:84985]	-2.6	0.0398
ssc-miR-212	AC008443.1	hypothetical gene	-2.6	0.0398
ssc-miR-212	AC074091.13	hypothetical gene	-2.6	0.0398
ssc-miR-212	ACVR1	activin A receptor type 1 [Source:VGNC Symbol;Acc:VGNC:95830]	-2.6	0.0398
ssc-miR-212	ACVR2B	activin A receptor type 2B [Source:VGNC Symbol;Acc:VGNC:108629]	-2.6	0.0398
ssc-miR-212	ACY1	aminoacylase 1 [Source:NCBI gene (formerly Entrezgene);Acc:396930]	-2.6	0.0398
ssc-miR-212	ADD2	adducin 2 [Source:VGNC Symbol;Acc:VGNC:85117]	-2.6	0.0398
ssc-miR-212	AGPAT4	1-acylglycerol-3-phosphate O-acyltransferase 4 [Source:VGNC Symbol;Acc:VGNC:85186]	-2.6	0.0398
ssc-miR-212	AIRE	autoimmune regulator [Source:VGNC Symbol;Acc:VGNC:85206]	-2.6	0.0398
ssc-miR-212	AK1	adenylate kinase 1 [Source:VGNC Symbol;Acc:VGNC:97875]	-2.6	0.0398
ssc-miR-212	AKT3	AKT serine/threonine kinase 3 [Source:VGNC Symbol;Acc:VGNC:96306]	-2.6	0.0398
ssc-miR-212	ALDH1L1	aldehyde dehydrogenase 1 family member L1 [Source:VGNC Symbol;Acc:VGNC:97881]	-2.6	0.0398
ssc-miR-212	ALDOA	aldolase, fructose-bisphosphate A [Source:VGNC Symbol;Acc:VGNC:102549]	-2.6	0.0398
ssc-miR-212	AMER1	APC membrane recruitment protein 1 [Source:VGNC Symbol;Acc:VGNC:85276]	-2.6	0.0398
ssc-miR-212	AMER2	APC membrane recruitment protein 2 [Source:VGNC Symbol;Acc:VGNC:85277]	-2.6	0.0398
ssc-miR-212	AMER3	APC membrane recruitment protein 3 [Source:VGNC Symbol;Acc:VGNC:96139]	-2.6	0.0398
ssc-miR-212	AMMECR1	AMMECR nuclear protein 1 [Source:VGNC Symbol;Acc:VGNC:96559]	-2.6	0.0398
ssc-miR-212	ANKHD1-EIF4EBP3	hypothetical gene	-2.6	0.0398

ssc-miR-212	ANKRD40	ankyrin repeat domain 40 [Source:VGNC Symbol;Acc:VGNC:85338]	-2.6	0.0398
ssc-miR-212	ANP32A	hypothetical gene	-2.6	0.0398
ssc-miR-212	APH1B	aph-1 homolog B, gamma-secretase subunit [Source:VGNC Symbol;Acc:VGNC:103898]	-2.6	0.0398
ssc-miR-212	APPL1	adaptor protein, phosphotyrosine interacting with PH domain and leucine zipper 1 [Source:VGNC Symbol;Acc:VGNC:85426]	-2.6	0.0398
ssc-miR-212	AQP2	aquaporin 2 [Source:VGNC Symbol;Acc:VGNC:85431]	-2.6	0.0398
ssc-miR-212	AQR	aquarius intron-binding spliceosomal factor [Source:VGNC Symbol;Acc:VGNC:85437]	-2.6	0.0398
ssc-miR-212	ARHGAP17	Rho GTPase activating protein 17 [Source:VGNC Symbol;Acc:VGNC:85459]	-2.6	0.0398
ssc-miR-212	ARHGAP19-SLIT1	hypothetical gene	-2.6	0.0398
ssc-miR-212	ARHGAP32	Rho GTPase activating protein 32 [Source:VGNC Symbol;Acc:VGNC:85471]	-2.6	0.0398
ssc-miR-212	ARHGEF12	Rho guanine nucleotide exchange factor 12 [Source:VGNC Symbol;Acc:VGNC:85488]	-2.6	0.0398
ssc-miR-212	ARID2	AT-rich interaction domain 2 [Source:HGNC Symbol;Acc:HGNC:18037]	-2.6	0.0398
ssc-miR-212	ARID3A	AT-rich interaction domain 3A [Source:VGNC Symbol;Acc:VGNC:85507]	-2.6	0.0398
ssc-miR-212	ARMC7	hypothetical gene	-2.6	0.0398
ssc-miR-212	ARPC4-TTLL3	hypothetical gene	-2.6	0.0398
ssc-miR-212	ARPP19	cAMP regulated phosphoprotein 19 [Source:NCBI gene (formerly Entrezgene);Acc:397362]	-2.6	0.0398
ssc-miR-212	ARX	aristaless related homeobox [Source:VGNC Symbol;Acc:VGNC:85556]	-2.6	0.0398
ssc-miR-212	ATG12	autophagy related 12 [Source:VGNC Symbol;Acc:VGNC:85615]	-2.6	0.0398
ssc-miR-212	ATG9A	autophagy related 9A [Source:VGNC Symbol;Acc:VGNC:99703]	-2.6	0.0398
ssc-miR-212	ATOH8	atonal bHLH transcription factor 8 [Source:VGNC Symbol;Acc:VGNC:85633]	-2.6	0.0398
ssc-miR-212	ATP7A	ATPase copper transporting alpha [Source:VGNC Symbol;Acc:VGNC:85678]	-2.6	0.0398
ssc-miR-212	ATPAF1	ATP synthase mitochondrial F1 complex assembly factor 1 [Source:VGNC Symbol;Acc:VGNC:96924]	-2.6	0.0398
ssc-miR-212	ATXN1	ataxin 1 [Source:VGNC Symbol;Acc:VGNC:85687]	-2.6	0.0398
ssc-miR-212	ATXN7	ataxin 7 [Source:VGNC Symbol;Acc:VGNC:99704]	-2.6	0.0398
ssc-miR-212	ATXN7L3B	ataxin 7 like 3B [Source:HGNC Symbol;Acc:HGNC:37931]	-2.6	0.0398
ssc-miR-212	AVPR2	vasopressin receptor 2 [Source:VGNC Symbol;Acc:VGNC:97898]	-2.6	0.0398
ssc-miR-212	B3GALT1	beta-1,3-galactosyltransferase 1 [Source:VGNC Symbol;Acc:VGNC:96494]	-2.6	0.0398
ssc-miR-212	B3GALT5	hypothetical gene	-2.6	0.0398
ssc-miR-212	BACE1	beta-secretase 1 [Source:VGNC Symbol;Acc:VGNC:85740]	-2.6	0.0398
ssc-miR-212	BACH2	BTB domain and CNC homolog 2 [Source:VGNC Symbol;Acc:VGNC:85742]	-2.6	0.0398
ssc-miR-212	BAHD1	bromo adjacent homology domain containing 1 [Source:VGNC Symbol;Acc:VGNC:85747]	-2.6	0.0398
ssc-miR-212	BASP1	hypothetical gene	-2.6	0.0398
ssc-miR-212	BBX	BBX high mobility group box domain containing [Source:VGNC Symbol;Acc:VGNC:85766]	-2.6	0.0398
ssc-miR-212	BCKDHB	branched chain keto acid dehydrogenase E1 subunit beta [Source:VGNC Symbol;Acc:VGNC:85777]	-2.6	0.0398
ssc-miR-212	BCKDK	branched chain keto acid dehydrogenase kinase [Source:VGNC Symbol;Acc:VGNC:85778]	-2.6	0.0398
ssc-miR-212	BCL2L2	BCL2 like 2 [Source:HGNC Symbol;Acc:HGNC:995]	-2.6	0.0398
ssc-miR-212	BHLHE41	basic helix-loop-helix family member e41 [Source:VGNC Symbol;Acc:VGNC:85815]	-2.6	0.0398
ssc-miR-212	BICD2	BICD cargo adaptor 2 [Source:VGNC Symbol;Acc:VGNC:85819]	-2.6	0.0398
ssc-miR-212	BID	BH3 interacting domain death agonist [Source:VGNC Symbol;Acc:VGNC:85824]	-2.6	0.0398
ssc-miR-212	BLOC1S5	biosis of lysosomal organelles complex 1 subunit 5 [Source:NCBI gene (formerly Entrezgene);Acc:100049677]	-2.6	0.0398
ssc-miR-212	BNC2	basonuclin 2 [Source:VGNC Symbol;Acc:VGNC:85852]	-2.6	0.0398
ssc-miR-212	BTBD11	BTB domain containing 11 [Source:VGNC Symbol;Acc:VGNC:85901]	-2.6	0.0398
ssc-miR-212	BTBD7	BTB domain containing 7 [Source:VGNC Symbol;Acc:VGNC:85908]	-2.6	0.0398
ssc-miR-212	BTBD9	BTB domain containing 9 [Source:VGNC Symbol;Acc:VGNC:85909]	-2.6	0.0398
ssc-miR-212	BTK	Bruton tyrosine kinase [Source:VGNC Symbol;Acc:VGNC:85914]	-2.6	0.0398
ssc-miR-212	C12orf79	hypothetical gene	-2.6	0.0398
ssc-miR-212	C14orf28	chromosome 1 C14orf28 homolog [Source:VGNC Symbol;Acc:VGNC:85958]	-2.6	0.0398
ssc-miR-212	C16orf54	hypothetical gene	-2.6	0.0398
ssc-miR-212	C16orf87	chromosome 6 C16orf87 homolog [Source:VGNC Symbol;Acc:VGNC:96927]	-2.6	0.0398
ssc-miR-212	C22orf26	hypothetical gene	-2.6	0.0398
ssc-miR-212	C4orf36	chromosome 8 C4orf36 homolog [Source:VGNC Symbol;Acc:VGNC:98914]	-2.6	0.0398

ssc-miR-212	C7orf41	hypothetical gene	-2.6	0.0398
ssc-miR-212	CALCOCO1	calcium binding and coiled-coil domain 1 [Source:VGNC Symbol;Acc:VGNC:86140]	-2.6	0.0398
ssc-miR-212	CALM1	calmodulin 1 [Source:NCBI gene (formerly Entrezgene);Acc:100154056]	-2.6	0.0398
ssc-miR-212	CAMKV	CaM kinase like vesicle associated [Source:VGNC Symbol;Acc:VGNC:86160]	-2.6	0.0398
ssc-miR-212	CASP10	caspase 10 [Source:VGNC Symbol;Acc:VGNC:95638]	-2.6	0.0398
ssc-miR-212	CASP7	caspase 7 [Source:VGNC Symbol;Acc:VGNC:86203]	-2.6	0.0398
ssc-miR-212	CASP8	caspase 8 [Source:VGNC Symbol;Acc:VGNC:95639]	-2.6	0.0398
ssc-miR-212	CAV2	caveolin 2 [Source:VGNC Symbol;Acc:VGNC:103914]	-2.6	0.0398
ssc-miR-212	CBFA2T3	CBFA2/RUNX1 partner transcriptional co-repressor 3 [Source:VGNC Symbol;Acc:VGNC:96574]	-2.6	0.0398
ssc-miR-212	CBLN2	cerebellin 2 [Source:VGNC Symbol;Acc:VGNC:86226]	-2.6	0.0398
ssc-miR-212	CBX5	chromobox 5 [Source:VGNC Symbol;Acc:VGNC:86232]	-2.6	0.0398
ssc-miR-212	CCDC117	coiled-coil domain containing 117 [Source:VGNC Symbol;Acc:VGNC:86248]	-2.6	0.0398
ssc-miR-212	CCDC171	coiled-coil domain containing 171 [Source:VGNC Symbol;Acc:VGNC:103044]	-2.6	0.0398
ssc-miR-212	CCND3	cyclin D3 [Source:VGNC Symbol;Acc:VGNC:86353]	-2.6	0.0398
ssc-miR-212	CCNT2	cyclin T2 [Source:VGNC Symbol;Acc:VGNC:103916]	-2.6	0.0398
ssc-miR-212	CDC20B	cell division cycle 20B [Source:HGNC Symbol;Acc:HGNC:24222]	-2.6	0.0398
ssc-miR-212	CDC27	cell division cycle 27 [Source:VGNC Symbol;Acc:VGNC:86448]	-2.6	0.0398
ssc-miR-212	CDC42EP1	CDC42 effector protein 1 [Source:VGNC Symbol;Acc:VGNC:86455]	-2.6	0.0398
ssc-miR-212	CDIP1	cell death inducing p53 target 1 [Source:VGNC Symbol;Acc:VGNC:86493]	-2.6	0.0398
ssc-miR-212	CDYL2	chromodomain Y like 2 [Source:VGNC Symbol;Acc:VGNC:86529]	-2.6	0.0398
ssc-miR-212	CERS2	ceramide synthase 2 [Source:VGNC Symbol;Acc:VGNC:86587]	-2.6	0.0398
ssc-miR-212	CFL2	cofilin 2 [Source:VGNC Symbol;Acc:VGNC:86611]	-2.6	0.0398
ssc-miR-212	CHD1	chromodomain helicase DNA binding protein 1 [Source:VGNC Symbol;Acc:VGNC:86629]	-2.6	0.0398
ssc-miR-212	CHD2	chromodomain helicase DNA binding protein 2 [Source:HGNC Symbol;Acc:HGNC:1917]	-2.6	0.0398
ssc-miR-212	CHDH	choline dehydrogenase [Source:VGNC Symbol;Acc:VGNC:86636]	-2.6	0.0398
ssc-miR-212	CHRM3	cholinergic receptor muscarinic 3 [Source:VGNC Symbol;Acc:VGNC:86664]	-2.6	0.0398
ssc-miR-212	CHST14	carbohydrate sulfotransferase 14 [Source:HGNC Symbol;Acc:HGNC:24464]	-2.6	0.0398
ssc-miR-212	CLCN5	chloride voltage-gated channel 5 [Source:VGNC Symbol;Acc:VGNC:103925]	-2.6	0.0398
ssc-miR-212	CLDN7	claudin 7 [Source:VGNC Symbol;Acc:VGNC:97930]	-2.6	0.0398
ssc-miR-212	CLIC5	chloride intracellular channel 5 [Source:VGNC Symbol;Acc:VGNC:86764]	-2.6	0.0398
ssc-miR-212	CLMN	calmin [Source:VGNC Symbol;Acc:VGNC:86769]	-2.6	0.0398
ssc-miR-212	CNKSR3	CNKSR family member 3 [Source:VGNC Symbol;Acc:VGNC:86823]	-2.6	0.0398
ssc-miR-212	CNOT2	CCR4-NOT transcription complex subunit 2 [Source:VGNC Symbol;Acc:VGNC:86834]	-2.6	0.0398
ssc-miR-212	CNPY3	canopy FGF signaling regulator 3 [Source:VGNC Symbol;Acc:VGNC:86841]	-2.6	0.0398
ssc-miR-212	CNTN2	contactin 2 [Source:VGNC Symbol;Acc:VGNC:108577]	-2.6	0.0398
ssc-miR-212	COBL	hypothetical gene	-2.6	0.0398
ssc-miR-212	COLQ	collagen like tail subunit of asymmetric acetylcholinesterase [Source:VGNC Symbol;Acc:VGNC:86884]	-2.6	0.0398
ssc-miR-212	CPLX2	complexin 2 [Source:VGNC Symbol;Acc:VGNC:86944]	-2.6	0.0398
ssc-miR-212	CPLX3	complexin 3 [Source:VGNC Symbol;Acc:VGNC:86945]	-2.6	0.0398
ssc-miR-212	CRB3	crumbs cell polarity complex component 3 [Source:HGNC Symbol;Acc:HGNC:20237]	-2.6	0.0398
ssc-miR-212	CRELD1	cysteine rich with EGF like domains 1 [Source:VGNC Symbol;Acc:VGNC:86991]	-2.6	0.0398
ssc-miR-212	CSNK1E	casein kinase 1 epsilon [Source:HGNC Symbol;Acc:HGNC:2453]	-2.6	0.0398
ssc-miR-212	CSNK1G3	casein kinase 1 gamma 3 [Source:VGNC Symbol;Acc:VGNC:97951]	-2.6	0.0398
ssc-miR-212	CTC-432M15.3	hypothetical gene	-2.6	0.0398
ssc-miR-212	CTC1	CST telomere replication complex component 1 [Source:VGNC Symbol;Acc:VGNC:87056]	-2.6	0.0398
ssc-miR-212	CTD-3214H19.16	hypothetical gene	-2.6	0.0398
ssc-miR-212	CTDSP1	CTD small phosphatase 1 [Source:VGNC Symbol;Acc:VGNC:95971]	-2.6	0.0398
ssc-miR-212	CTDSP2	CTD small phosphatase 2 [Source:VGNC Symbol;Acc:VGNC:87059]	-2.6	0.0398
ssc-miR-212	CTGF	hypothetical gene	-2.6	0.0398
ssc-miR-212	CTNND1	catenin delta 1 [Source:VGNC Symbol;Acc:VGNC:87067]	-2.6	0.0398

ssc-miR-212	CUL3	cullin 3 [Source:VGNC Symbol;Acc:VGNC:96091]	-2.6	0.0398
ssc-miR-212	CYB561D1	cytochrome b561 family member D1 [Source:VGNC Symbol;Acc:VGNC:87121]	-2.6	0.0398
ssc-miR-212	CYGB	hypothetical gene	-2.6	0.0398
ssc-miR-212	CYLD	CYLD lysine 63 deubiquitinase [Source:VGNC Symbol;Acc:VGNC:87130]	-2.6	0.0398
ssc-miR-212	CYTH1	cytohesin 1 [Source:VGNC Symbol;Acc:VGNC:87135]	-2.6	0.0398
ssc-miR-212	DAGLA	diacylglycerol lipase alpha [Source:VGNC Symbol;Acc:VGNC:87152]	-2.6	0.0398
ssc-miR-212	DCC	DCC netrin 1 receptor [Source:VGNC Symbol;Acc:VGNC:103078]	-2.6	0.0398
ssc-miR-212	DDR1	discoidin domain receptor tyrosine kinase 1 [Source:VGNC Symbol;Acc:VGNC:107135]	-2.6	0.0398
ssc-miR-212	DDX3X	DEAD-box helicase 3 X-linked [Source:NCBI gene (formerly Entrezgene);Acc:100515940]	-2.6	0.0398
ssc-miR-212	DDX3Y	hypothetical gene	-2.6	0.0398
ssc-miR-212	DERL1	derlin 1 [Source:NCBI gene (formerly Entrezgene);Acc:100626802]	-2.6	0.0398
ssc-miR-212	DGCR2	DiGeorge syndrome critical region gene 2 [Source:HGNC Symbol;Acc:HGNC:2845]	-2.6	0.0398
ssc-miR-212	DIAPH1	diaphanous related formin 1 [Source:VGNC Symbol;Acc:VGNC:99632]	-2.6	0.0398
ssc-miR-212	DLG3	discs large MAGUK scaffold protein 3 [Source:VGNC Symbol;Acc:VGNC:87327]	-2.6	0.0398
ssc-miR-212	DNAJA2	DnaJ heat shock protein family (Hsp40) member A2 [Source:VGNC Symbol;Acc:VGNC:96621]	-2.6	0.0398
ssc-miR-212	DNAJB12	DnaJ heat shock protein family (Hsp40) member B12 [Source:VGNC Symbol;Acc:VGNC:96679]	-2.6	0.0398
ssc-miR-212	DNAJC11	DnaJ heat shock protein family (Hsp40) member C11 [Source:VGNC Symbol;Acc:VGNC:96622]	-2.6	0.0398
ssc-miR-212	DNMT3A	DNA methyltransferase 3 alpha [Source:VGNC Symbol;Acc:VGNC:87384]	-2.6	0.0398
ssc-miR-212	DPYSL3	dihydropyrimidinase like 3 [Source:VGNC Symbol;Acc:VGNC:87431]	-2.6	0.0398
ssc-miR-212	DVL3	dishevelled segment polarity protein 3 [Source:VGNC Symbol;Acc:VGNC:87492]	-2.6	0.0398
ssc-miR-212	EBF3	EBF transcription factor 3 [Source:VGNC Symbol;Acc:VGNC:87527]	-2.6	0.0398
ssc-miR-212	EBF4	EBF family member 4 [Source:VGNC Symbol;Acc:VGNC:96211]	-2.6	0.0398
ssc-miR-212	EEF1E1	eukaryotic translation elongation factor 1 epsilon 1 [Source:NCBI gene (formerly Entrezgene);Acc:100155141]	-2.6	0.0398
ssc-miR-212	EFNA5	ephrin A5 [Source:VGNC Symbol;Acc:VGNC:87575]	-2.6	0.0398
ssc-miR-212	EGR1	early growth response 1 [Source:VGNC Symbol;Acc:VGNC:87590]	-2.6	0.0398
ssc-miR-212	EGR3	early growth response 3 [Source:VGNC Symbol;Acc:VGNC:87591]	-2.6	0.0398
ssc-miR-212	EHD2	EH domain containing 2 [Source:VGNC Symbol;Acc:VGNC:87596]	-2.6	0.0398
ssc-miR-212	EI24	EI24 autophagy associated transmembrane protein [Source:VGNC Symbol;Acc:VGNC:87602]	-2.6	0.0398
ssc-miR-212	EIF3J	eukaryotic translation initiation factor 3 subunit J [Source:VGNC Symbol;Acc:VGNC:87621]	-2.6	0.0398
ssc-miR-212	EIF4A1	hypothetical gene	-2.6	0.0398
ssc-miR-212	EIF4A2	eukaryotic translation initiation factor 4A2 [Source:VGNC Symbol;Acc:VGNC:87624]	-2.6	0.0398
ssc-miR-212	EIF4EBP2	eukaryotic translation initiation factor 4E binding protein 2 [Source:VGNC Symbol;Acc:VGNC:87629]	-2.6	0.0398
ssc-miR-212	EIF4EBP3	eukaryotic translation initiation factor 4E binding protein 3 [Source:NCBI gene (formerly Entrezgene);Acc:100529254]	-2.6	0.0398
ssc-miR-212	ELMOD3	ELMO domain containing 3 [Source:VGNC Symbol;Acc:VGNC:87654]	-2.6	0.0398
ssc-miR-212	EPC1	enhancer of polycomb homolog 1 [Source:VGNC Symbol;Acc:VGNC:95923]	-2.6	0.0398
ssc-miR-212	ERGIC1	endoplasmic reticulum-golgi intermediate compartment 1 [Source:VGNC Symbol;Acc:VGNC:87770]	-2.6	0.0398
ssc-miR-212	ERLIN2	ER lipid raft associated 2 [Source:VGNC Symbol;Acc:VGNC:95586]	-2.6	0.0398
ssc-miR-212	ETF1	eukaryotic translation termination factor 1 [Source:VGNC Symbol;Acc:VGNC:87800]	-2.6	0.0398
ssc-miR-212	FADS3	fatty acid desaturase 3 [Source:VGNC Symbol;Acc:VGNC:87877]	-2.6	0.0398
ssc-miR-212	FAF2	Fas associated factor family member 2 [Source:VGNC Symbol;Acc:VGNC:87880]	-2.6	0.0398
ssc-miR-212	FAM104A	family with sequence similarity 104 member A [Source:VGNC Symbol;Acc:VGNC:98990]	-2.6	0.0398
ssc-miR-212	FAM104B	hypothetical gene	-2.6	0.0398
ssc-miR-212	FAM126A	family with sequence similarity 126 member A [Source:VGNC Symbol;Acc:VGNC:87899]	-2.6	0.0398
ssc-miR-212	FAM126B	family with sequence similarity 126 member B [Source:HGNC Symbol;Acc:HGNC:28593]	-2.6	0.0398
ssc-miR-212	FAM188B	hypothetical gene	-2.6	0.0398
ssc-miR-212	FAM20B	FAM20B glycosaminoglycan xylosylkinase [Source:VGNC Symbol;Acc:VGNC:87950]	-2.6	0.0398
ssc-miR-212	FAM214B	family with sequence similarity 214 member B [Source:VGNC Symbol;Acc:VGNC:87953]	-2.6	0.0398
ssc-miR-212	FAM221B	family with sequence similarity 221 member B [Source:VGNC Symbol;Acc:VGNC:87958]	-2.6	0.0398
ssc-miR-212	FAM63A	hypothetical gene	-2.6	0.0398
ssc-miR-212	FAM81A	family with sequence similarity 81 member A [Source:VGNC Symbol;Acc:VGNC:87986]	-2.6	0.0398

ssc-miR-212	FANCI	FA complementation group I [Source:VGNC Symbol;Acc:VGNC:88006]	-2.6	0.0398
ssc-miR-212	FAXDC2	fatty acid hydroxylase domain containing 2 [Source:VGNC Symbol;Acc:VGNC:88021]	-2.6	0.0398
ssc-miR-212	FBLN7	fibulin 7 [Source:VGNC Symbol;Acc:VGNC:97999]	-2.6	0.0398
ssc-miR-212	FBN2	fibrillin 2 [Source:VGNC Symbol;Acc:VGNC:88025]	-2.6	0.0398
ssc-miR-212	FBXL4	F-box and leucine rich repeat protein 4 [Source:VGNC Symbol;Acc:VGNC:98007]	-2.6	0.0398
ssc-miR-212	FBXO28	F-box protein 28 [Source:HGNC Symbol;Acc:HGNC:29046]	-2.6	0.0398
ssc-miR-212	FBXW11	F-box and WD repeat domain containing 11 [Source:HGNC Symbol;Acc:HGNC:13607]	-2.6	0.0398
ssc-miR-212	FBXW7	F-box and WD repeat domain containing 7 [Source:VGNC Symbol;Acc:VGNC:98925]	-2.6	0.0398
ssc-miR-212	FEM1C	fem-1 homolog C [Source:VGNC Symbol;Acc:VGNC:88084]	-2.6	0.0398
ssc-miR-212	FGF23	fibroblast growth factor 23 [Source:HGNC Symbol;Acc:HGNC:3680]	-2.6	0.0398
ssc-miR-212	FLNB	filamin B [Source:VGNC Symbol;Acc:VGNC:88156]	-2.6	0.0398
ssc-miR-212	FMNL3	formin like 3 [Source:VGNC Symbol;Acc:VGNC:88169]	-2.6	0.0398
ssc-miR-212	FNDC5	fibronectin type III domain containing 5 [Source:VGNC Symbol;Acc:VGNC:88185]	-2.6	0.0398
ssc-miR-212	FOXN3	forkhead box N3 [Source:VGNC Symbol;Acc:VGNC:88220]	-2.6	0.0398
ssc-miR-212	FOXO3	forkhead box O3 [Source:VGNC Symbol;Acc:VGNC:99715]	-2.6	0.0398
ssc-miR-212	FOXP2	forkhead box P2 [Source:VGNC Symbol;Acc:VGNC:98014]	-2.6	0.0398
ssc-miR-212	FOXP4	forkhead box P4 [Source:VGNC Symbol;Acc:VGNC:88224]	-2.6	0.0398
ssc-miR-212	FPGS	folylpolyglutamate synthase [Source:VGNC Symbol;Acc:VGNC:88230]	-2.6	0.0398
ssc-miR-212	FRYL	FRY like transcription coactivator [Source:VGNC Symbol;Acc:VGNC:98015]	-2.6	0.0398
ssc-miR-212	FTO	FTO alpha-ketoglutarate dependent dioxygenase [Source:VGNC Symbol;Acc:VGNC:88259]	-2.6	0.0398
ssc-miR-212	FURIN	furin, paired basic amino acid cleaving enzyme [Source:VGNC Symbol;Acc:VGNC:88266]	-2.6	0.0398
ssc-miR-212	FXR1	FMR1 autosomal homolog 1 [Source:VGNC Symbol;Acc:VGNC:108659]	-2.6	0.0398
ssc-miR-212	FYTTD1	forty-two-three domain containing 1 [Source:VGNC Symbol;Acc:VGNC:88277]	-2.6	0.0398
ssc-miR-212	FZD5	frizzled class receptor 5 [Source:VGNC Symbol;Acc:VGNC:96309]	-2.6	0.0398
ssc-miR-212	G6PC3	glucose-6-phosphatase catalytic subunit 3 [Source:VGNC Symbol;Acc:VGNC:88291]	-2.6	0.0398
ssc-miR-212	GABARAPL1	GABA type A receptor associated protein like 1 [Source:VGNC Symbol;Acc:VGNC:103282]	-2.6	0.0398
ssc-miR-212	GABBR1	gamma-aminobutyric acid type B receptor subunit 1 [Source:VGNC Symbol;Acc:VGNC:88297]	-2.6	0.0398
ssc-miR-212	GABPB2	GA binding protein transcription factor subunit beta 2 [Source:VGNC Symbol;Acc:VGNC:98796]	-2.6	0.0398
ssc-miR-212	GABRA5	gamma-aminobutyric acid type A receptor subunit alpha5 [Source:VGNC Symbol;Acc:VGNC:88304]	-2.6	0.0398
ssc-miR-212	GALNTL6	polypeptide N-acetylgalactosaminyltransferase like 6 [Source:VGNC Symbol;Acc:VGNC:103947]	-2.6	0.0398
ssc-miR-212	GAN	gigaxonin [Source:VGNC Symbol;Acc:VGNC:88343]	-2.6	0.0398
ssc-miR-212	GAPVD1	GTPase activating protein and VPS9 domains 1 [Source:VGNC Symbol;Acc:VGNC:88347]	-2.6	0.0398
ssc-miR-212	GATSL2	hypothetical gene	-2.6	0.0398
ssc-miR-212	GDF11	growth differentiation factor 11 [Source:VGNC Symbol;Acc:VGNC:88398]	-2.6	0.0398
ssc-miR-212	GDF6	growth differentiation factor 6 [Source:VGNC Symbol;Acc:VGNC:88402]	-2.6	0.0398
ssc-miR-212	GDNF	glial cell derived neurotrophic factor [Source:VGNC Symbol;Acc:VGNC:88406]	-2.6	0.0398
ssc-miR-212	GDPD5	glycerophosphodiester phosphodiesterase domain containing 5 [Source:VGNC Symbol;Acc:VGNC:88409]	-2.6	0.0398
ssc-miR-212	GGCX	gamma-glutamyl carboxylase [Source:VGNC Symbol;Acc:VGNC:88435]	-2.6	0.0398
ssc-miR-212	GIT1	GIT ArfGAP 1 [Source:VGNC Symbol;Acc:VGNC:88457]	-2.6	0.0398
ssc-miR-212	GLRB	glycine receptor beta [Source:VGNC Symbol;Acc:VGNC:88494]	-2.6	0.0398
ssc-miR-212	GMPR	guanosine monophosphate reductase [Source:VGNC Symbol;Acc:VGNC:88514]	-2.6	0.0398
ssc-miR-212	GNAZ	G protein subunit alpha z [Source:VGNC Symbol;Acc:VGNC:88528]	-2.6	0.0398
ssc-miR-212	GNG2	hypothetical gene	-2.6	0.0398
ssc-miR-212	GPM6B	hypothetical gene	-2.6	0.0398
ssc-miR-212	GPR180	G protein-coupled receptor 180 [Source:VGNC Symbol;Acc:VGNC:88621]	-2.6	0.0398
ssc-miR-212	GRIK3	glutamate ionotropic receptor kainate type subunit 3 [Source:VGNC Symbol;Acc:VGNC:88679]	-2.6	0.0398
ssc-miR-212	GRIN1	glutamate ionotropic receptor NMDA type subunit 1 [Source:VGNC Symbol;Acc:VGNC:88682]	-2.6	0.0398
ssc-miR-212	GRIN2A	glutamate ionotropic receptor NMDA type subunit 2A [Source:VGNC Symbol;Acc:VGNC:88683]	-2.6	0.0398
ssc-miR-212	GRM3	glutamate metabotropic receptor 3 [Source:VGNC Symbol;Acc:VGNC:88702]	-2.6	0.0398
ssc-miR-212	GTDC1	glycosyltransferase like domain containing 1 [Source:VGNC Symbol;Acc:VGNC:95955]	-2.6	0.0398

ssc-miR-212	GXYLT2	glucoside xylosyltransferase 2 [Source:VGNC Symbol;Acc:VGNC:88757]	-2.6	0.0398
ssc-miR-212	HAGHL	hydroxyacylglutathione hydrolase like [Source:VGNC Symbol;Acc:VGNC:88774]	-2.6	0.0398
ssc-miR-212	HDX	highly divergent homeobox [Source:VGNC Symbol;Acc:VGNC:88824]	-2.6	0.0398
ssc-miR-212	HEMK1	HemK methyltransferase family member 1 [Source:VGNC Symbol;Acc:VGNC:88843]	-2.6	0.0398
ssc-miR-212	HEYL	hes related family bHLH transcription factor with YRPW motif like [Source:VGNC Symbol;Acc:VGNC:88865]	-2.6	0.0398
ssc-miR-212	HIC2	HIC ZBTB transcriptional repressor 2 [Source:HGNC Symbol;Acc:HGNC:18595]	-2.6	0.0398
ssc-miR-212	HIP1	huntingtin interacting protein 1 [Source:VGNC Symbol;Acc:VGNC:88885]	-2.6	0.0398
ssc-miR-212	HIPK2	homeodomain interacting protein kinase 2 [Source:VGNC Symbol;Acc:VGNC:88888]	-2.6	0.0398
ssc-miR-212	HIVEP1	HIVEP zinc finger 1 [Source:VGNC Symbol;Acc:VGNC:96585]	-2.6	0.0398
ssc-miR-212	HNF4G	hepatocyte nuclear factor 4 gamma [Source:VGNC Symbol;Acc:VGNC:88917]	-2.6	0.0398
ssc-miR-212	HNRNPU	heterogeneous nuclear ribonucleoprotein U [Source:HGNC Symbol;Acc:HGNC:5048]	-2.6	0.0398
ssc-miR-212	HOXD9	homeobox D9 [Source:VGNC Symbol;Acc:VGNC:96357]	-2.6	0.0398
ssc-miR-212	HRK	harakiri, BCL2 interacting protein [Source:HGNC Symbol;Acc:HGNC:5185]	-2.6	0.0398
ssc-miR-212	HSCB	HscB mitochondrial iron-sulfur cluster cochaperone [Source:VGNC Symbol;Acc:VGNC:88982]	-2.6	0.0398
ssc-miR-212	HSF1	heat shock transcription factor 1 [Source:VGNC Symbol;Acc:VGNC:88993]	-2.6	0.0398
ssc-miR-212	HSPH1	heat shock protein family H (Hsp110) member 1 [Source:NCBI gene (formerly Entrezgene);Acc:100048931]	-2.6	0.0398
ssc-miR-212	HUNK	hormonally up-regulated Neu-associated kinase [Source:VGNC Symbol;Acc:VGNC:89007]	-2.6	0.0398
ssc-miR-212	IARS	hypothetical gene	-2.6	0.0398
ssc-miR-212	ID3	inhibitor of DNA binding 3, HLH protein [Source:VGNC Symbol;Acc:VGNC:89023]	-2.6	0.0398
ssc-miR-212	IFFO2	intermediate filament family orphan 2 [Source:VGNC Symbol;Acc:VGNC:98475]	-2.6	0.0398
ssc-miR-212	IGF2BP1	insulin like growth factor 2 mRNA binding protein 1 [Source:VGNC Symbol;Acc:VGNC:99006]	-2.6	0.0398
ssc-miR-212	IGFBP3	insulin like growth factor binding protein 3 [Source:NCBI gene (formerly Entrezgene);Acc:448812]	-2.6	0.0398
ssc-miR-212	IGFBP4	insulin like growth factor binding protein 4 [Source:VGNC Symbol;Acc:VGNC:98045]	-2.6	0.0398
ssc-miR-212	IGFBP5	insulin like growth factor binding protein 5 [Source:VGNC Symbol;Acc:VGNC:96368]	-2.6	0.0398
ssc-miR-212	IGSF11	immunoglobulin superfamily member 11 [Source:VGNC Symbol;Acc:VGNC:89064]	-2.6	0.0398
ssc-miR-212	IL17RE	hypothetical gene	-2.6	0.0398
ssc-miR-212	INTU	inturned planar cell polarity protein [Source:VGNC Symbol;Acc:VGNC:89170]	-2.6	0.0398
ssc-miR-212	IQSEC1	IQ motif and Sec7 domain ArfGEF 1 [Source:VGNC Symbol;Acc:VGNC:89194]	-2.6	0.0398
ssc-miR-212	IQSEC2	IQ motif and Sec7 domain ArfGEF 2 [Source:VGNC Symbol;Acc:VGNC:89195]	-2.6	0.0398
ssc-miR-212	IRF2BPL	interferon regulatory factor 2 binding protein like [Source:HGNC Symbol;Acc:HGNC:14282]	-2.6	0.0398
ssc-miR-212	ISL1	ISL LIM homeobox 1 [Source:VGNC Symbol;Acc:VGNC:89225]	-2.6	0.0398
ssc-miR-212	ISM2	hypothetical gene	-2.6	0.0398
ssc-miR-212	ISY1-RAB43	hypothetical gene	-2.6	0.0398
ssc-miR-212	ITPK1	inositol-tetrakisphosphate 1-kinase [Source:VGNC Symbol;Acc:VGNC:89251]	-2.6	0.0398
ssc-miR-212	JMJD6	jumonji domain containing 6, arginine demethylase and lysine hydroxylase [Source:VGNC Symbol;Acc:VGNC:89287]	-2.6	0.0398
ssc-miR-212	JPH2	hypothetical gene	-2.6	0.0398
ssc-miR-212	KAT7	lysine acetyltransferase 7 [Source:VGNC Symbol;Acc:VGNC:89307]	-2.6	0.0398
ssc-miR-212	KCMF1	potassium channel modulatory factor 1 [Source:VGNC Symbol;Acc:VGNC:89322]	-2.6	0.0398
ssc-miR-212	KCNC2	potassium voltage-gated channel subfamily C member 2 [Source:VGNC Symbol;Acc:VGNC:89333]	-2.6	0.0398
ssc-miR-212	KCNIP3	potassium voltage-gated channel interacting protein 3 [Source:VGNC Symbol;Acc:VGNC:89350]	-2.6	0.0398
ssc-miR-212	KCNK2	potassium two pore domain channel subfamily K member 2 [Source:VGNC Symbol;Acc:VGNC:89369]	-2.6	0.0398
ssc-miR-212	KCTD16	potassium channel tetramerization domain containing 16 [Source:VGNC Symbol;Acc:VGNC:89395]	-2.6	0.0398
ssc-miR-212	KDM2B	lysine demethylase 2B [Source:VGNC Symbol;Acc:VGNC:98057]	-2.6	0.0398
ssc-miR-212	KHDRBS2	KH RNA binding domain containing, signal transduction associated 2 [Source:VGNC Symbol;Acc:VGNC:89423]	-2.6	0.0398
ssc-miR-212	KIAA0226L	hypothetical gene	-2.6	0.0398
ssc-miR-212	KIAA0232	hypothetical gene	-2.6	0.0398
ssc-miR-212	KIAA0430	hypothetical gene	-2.6	0.0398
ssc-miR-212	KIAA1522	KIAA1522 [Source:VGNC Symbol;Acc:VGNC:89444]	-2.6	0.0398
ssc-miR-212	KIAA1614	KIAA1614 [Source:VGNC Symbol;Acc:VGNC:89446]	-2.6	0.0398
ssc-miR-212	KIF21B	kinesin family member 21B [Source:VGNC Symbol;Acc:VGNC:96219]	-2.6	0.0398

ssc-miR-212	KLF4	Kruppel like factor 4 [Source:VGNC Symbol;Acc:VGNC:98062]	-2.6	0.0398
ssc-miR-212	KLF7	Kruppel like factor 7 [Source:VGNC Symbol;Acc:VGNC:96396]	-2.6	0.0398
ssc-miR-212	KPNA3	karyopherin subunit alpha 3 [Source:VGNC Symbol;Acc:VGNC:89562]	-2.6	0.0398
ssc-miR-212	LARP4	La ribonucleoprotein 4 [Source:VGNC Symbol;Acc:VGNC:89641]	-2.6	0.0398
ssc-miR-212	LDB1	LIM domain binding 1 [Source:VGNC Symbol;Acc:VGNC:89667]	-2.6	0.0398
ssc-miR-212	LDLRAD4	low density lipoprotein receptor class A domain containing 4 [Source:VGNC Symbol;Acc:VGNC:89673]	-2.6	0.0398
ssc-miR-212	LEF1	lymphoid enhancer binding factor 1 [Source:VGNC Symbol;Acc:VGNC:89676]	-2.6	0.0398
ssc-miR-212	LEMD3	LEM domain containing 3 [Source:VGNC Symbol;Acc:VGNC:89679]	-2.6	0.0398
ssc-miR-212	LHFPL4	LHFPL tetraspan subfamily member 4 [Source:VGNC Symbol;Acc:VGNC:89707]	-2.6	0.0398
ssc-miR-212	LIMD2	LIM domain containing 2 [Source:VGNC Symbol;Acc:VGNC:89726]	-2.6	0.0398
ssc-miR-212	LRRC8C	leucine rich repeat containing 8 VRAC subunit C [Source:VGNC Symbol;Acc:VGNC:98087]	-2.6	0.0398
ssc-miR-212	LRRC8E	leucine rich repeat containing 8 VRAC subunit E [Source:VGNC Symbol;Acc:VGNC:98089]	-2.6	0.0398
ssc-miR-212	LRRTM3	leucine rich repeat transmembrane neuronal 3 [Source:VGNC Symbol;Acc:VGNC:107409]	-2.6	0.0398
ssc-miR-212	LSAMP	limbic system associated membrane protein [Source:VGNC Symbol;Acc:VGNC:89868]	-2.6	0.0398
ssc-miR-212	LUZP1	leucine zipper protein 1 [Source:VGNC Symbol;Acc:VGNC:89897]	-2.6	0.0398
ssc-miR-212	LYPD6	LY6/PLAUR domain containing 6 [Source:HGNC Symbol;Acc:HGNC:28751]	-2.6	0.0398
ssc-miR-212	LYPLA2	lysophospholipase 2 [Source:VGNC Symbol;Acc:VGNC:89914]	-2.6	0.0398
ssc-miR-212	MAN2A1	mannosidase alpha class 2A member 1 [Source:VGNC Symbol;Acc:VGNC:98098]	-2.6	0.0398
ssc-miR-212	MAP1LC3B	microtubule associated protein 1 light chain 3 beta [Source:NCBI gene (formerly Entrezgene);Acc:100462754]	-2.6	0.0398
ssc-miR-212	MAPK1	mitogen-activated protein kinase 1 [Source:VGNC Symbol;Acc:VGNC:89996]	-2.6	0.0398
ssc-miR-212	MAPK3	mitogen-activated protein kinase 3 [Source:VGNC Symbol;Acc:VGNC:90003]	-2.6	0.0398
ssc-miR-212	MARK3	microtubule affinity regulating kinase 3 [Source:VGNC Symbol;Acc:VGNC:90026]	-2.6	0.0398
ssc-miR-212	MAT2A	methionine adenosyltransferase 2A [Source:VGNC Symbol;Acc:VGNC:90039]	-2.6	0.0398
ssc-miR-212	MED13	mediator complex subunit 13 [Source:VGNC Symbol;Acc:VGNC:90106]	-2.6	0.0398
ssc-miR-212	MEIS1	Meis homeobox 1 [Source:VGNC Symbol;Acc:VGNC:90135]	-2.6	0.0398
ssc-miR-212	MET	MET proto-onco, receptor tyrosine kinase [Source:NCBI gene (formerly Entrezgene);Acc:654328]	-2.6	0.0398
ssc-miR-212	METAP2	methionyl aminopeptidase 2 [Source:VGNC Symbol;Acc:VGNC:90152]	-2.6	0.0398
ssc-miR-212	METTL17	methyltransferase like 17 [Source:VGNC Symbol;Acc:VGNC:103982]	-2.6	0.0398
ssc-miR-212	MFHAS1	multifunctional ROCO family signaling regulator 1 [Source:VGNC Symbol;Acc:VGNC:107361]	-2.6	0.0398
ssc-miR-212	MFSD2A	major facilitator superfamily domain containing 2A [Source:VGNC Symbol;Acc:VGNC:90188]	-2.6	0.0398
ssc-miR-212	MGA	MAX dimerization protein MGA [Source:VGNC Symbol;Acc:VGNC:90195]	-2.6	0.0398
ssc-miR-212	MITF	melanocyte inducing transcription factor [Source:VGNC Symbol;Acc:VGNC:90243]	-2.6	0.0398
ssc-miR-212	MLLT3	MLLT3 super elongation complex subunit [Source:VGNC Symbol;Acc:VGNC:90256]	-2.6	0.0398
ssc-miR-212	MMP15	matrix metallopeptidase 15 [Source:VGNC Symbol;Acc:VGNC:90270]	-2.6	0.0398
ssc-miR-212	MMP16	matrix metallopeptidase 16 [Source:VGNC Symbol;Acc:VGNC:90271]	-2.6	0.0398
ssc-miR-212	MMP24	matrix metallopeptidase 24 [Source:VGNC Symbol;Acc:VGNC:96421]	-2.6	0.0398
ssc-miR-212	MOB3B	MOB kinase activator 3B [Source:VGNC Symbol;Acc:VGNC:96024]	-2.6	0.0398
ssc-miR-212	MOCS2	molybdenum cofactor synthesis 2 [Source:VGNC Symbol;Acc:VGNC:90295]	-2.6	0.0398
ssc-miR-212	MPPED2	metallophosphoesterase domain containing 2 [Source:VGNC Symbol;Acc:VGNC:90331]	-2.6	0.0398
ssc-miR-212	MRVI1	hypothetical gene	-2.6	0.0398
ssc-miR-212	MSL1	MSL complex subunit 1 [Source:VGNC Symbol;Acc:VGNC:90423]	-2.6	0.0398
ssc-miR-212	MTMR10	myotubularin related protein 10 [Source:VGNC Symbol;Acc:VGNC:90457]	-2.6	0.0398
ssc-miR-212	MXI1	hypothetical gene	-2.6	0.0398
ssc-miR-212	MYCBP2	MYC binding protein 2 [Source:VGNC Symbol;Acc:VGNC:90502]	-2.6	0.0398
ssc-miR-212	MYD88	MYD88 innate immune signal transduction adaptor [Source:NCBI gene (formerly Entrezgene);Acc:396646]	-2.6	0.0398
ssc-miR-212	MYO1E	myosin IE [Source:VGNC Symbol;Acc:VGNC:90528]	-2.6	0.0398
ssc-miR-212	NABP1	nucleic acid binding protein 1 [Source:VGNC Symbol;Acc:VGNC:96122]	-2.6	0.0398
ssc-miR-212	NACC2	NACC family member 2 [Source:VGNC Symbol;Acc:VGNC:90564]	-2.6	0.0398
ssc-miR-212	NAT10	N-acetyltransferase 10 [Source:VGNC Symbol;Acc:VGNC:90585]	-2.6	0.0398
ssc-miR-212	NECAB3	N-terminal EF-hand calcium binding protein 3 [Source:VGNC Symbol;Acc:VGNC:95670]	-2.6	0.0398

ssc-miR-212	NFIA	nuclear factor I A [Source:VGNC Symbol;Acc:VGNC:90715]	-2.6	0.0398
ssc-miR-212	NFIB	nuclear factor I B [Source:VGNC Symbol;Acc:VGNC:90716]	-2.6	0.0398
ssc-miR-212	NFYC	nuclear transcription factor Y subunit gamma [Source:VGNC Symbol;Acc:VGNC:90731]	-2.6	0.0398
ssc-miR-212	NGFR	nerve growth factor receptor [Source:VGNC Symbol;Acc:VGNC:90733]	-2.6	0.0398
ssc-miR-212	NHP2	hypothetical gene	-2.6	0.0398
ssc-miR-212	NIPBL	NIPBL cohesin loading factor [Source:VGNC Symbol;Acc:VGNC:90752]	-2.6	0.0398
ssc-miR-212	NLGN2	hypothetical gene	-2.6	0.0398
ssc-miR-212	NNAT	hypothetical gene	-2.6	0.0398
ssc-miR-212	NPEPPS	aminopeptidase puromycin sensitive [Source:VGNC Symbol;Acc:VGNC:98157]	-2.6	0.0398
ssc-miR-212	NPFFR1	neuropeptide FF receptor 1 [Source:VGNC Symbol;Acc:VGNC:90846]	-2.6	0.0398
ssc-miR-212	NR2C1	nuclear receptor subfamily 2 group C member 1 [Source:VGNC Symbol;Acc:VGNC:90875]	-2.6	0.0398
ssc-miR-212	NR2F2	nuclear receptor subfamily 2 group F member 2 [Source:VGNC Symbol;Acc:VGNC:90881]	-2.6	0.0398
ssc-miR-212	NR5A1	nuclear receptor subfamily 5 group A member 1 [Source:VGNC Symbol;Acc:VGNC:90886]	-2.6	0.0398
ssc-miR-212	NR6A1	nuclear receptor subfamily 6 group A member 1 [Source:VGNC Symbol;Acc:VGNC:90887]	-2.6	0.0398
ssc-miR-212	NREP	neuronal regeneration related protein [Source:HGNC Symbol;Acc:HGNC:16834]	-2.6	0.0398
ssc-miR-212	NRG2	neuregulin 2 [Source:VGNC Symbol;Acc:VGNC:98160]	-2.6	0.0398
ssc-miR-212	NRP1	neuropilin 1 [Source:VGNC Symbol;Acc:VGNC:104012]	-2.6	0.0398
ssc-miR-212	NRXN1	neurexin 1 [Source:HGNC Symbol;Acc:HGNC:8008]	-2.6	0.0398
ssc-miR-212	NSD1	nuclear receptor binding SET domain protein 1 [Source:VGNC Symbol;Acc:VGNC:90904]	-2.6	0.0398
ssc-miR-212	NTNG1	netrin G1 [Source:VGNC Symbol;Acc:VGNC:90935]	-2.6	0.0398
ssc-miR-212	NUP43	nucleoporin 43 [Source:VGNC Symbol;Acc:VGNC:90982]	-2.6	0.0398
ssc-miR-212	NUP98	nucleoporin 98 and 96 [Source:VGNC Symbol;Acc:VGNC:90988]	-2.6	0.0398
ssc-miR-212	OBFC1	hypothetical gene	-2.6	0.0398
ssc-miR-212	OPALIN	oligodendrocytic myelin paranodal and inner loop protein [Source:VGNC Symbol;Acc:VGNC:91046]	-2.6	0.0398
ssc-miR-212	OSBP2	oxysterol binding protein 2 [Source:VGNC Symbol;Acc:VGNC:91067]	-2.6	0.0398
ssc-miR-212	OSBPL8	oxysterol binding protein like 8 [Source:VGNC Symbol;Acc:VGNC:91074]	-2.6	0.0398
ssc-miR-212	OSER1	oxidative stress responsive serine rich 1 [Source:VGNC Symbol;Acc:VGNC:95738]	-2.6	0.0398
ssc-miR-212	OTUD7A	OTU deubiquitinase 7A [Source:VGNC Symbol;Acc:VGNC:91104]	-2.6	0.0398
ssc-miR-212	OVOL1	ovo like transcriptional repressor 1 [Source:VGNC Symbol;Acc:VGNC:91111]	-2.6	0.0398
ssc-miR-212	PACS2	phosphofurin acidic cluster sorting protein 2 [Source:VGNC Symbol;Acc:VGNC:91141]	-2.6	0.0398
ssc-miR-212	PAFAH1B2	platelet activating factor acetylhydrolase 1b catalytic subunit 2 [Source:VGNC Symbol;Acc:VGNC:91151]	-2.6	0.0398
ssc-miR-212	PAG1	phosphoprotein membrane anchor with glycosphingolipid microdomains 1 [Source:VGNC Symbol;Acc:VGNC:91154]	-2.6	0.0398
ssc-miR-212	PAN3	poly(A) specific ribonuclease subunit PAN3 [Source:HGNC Symbol;Acc:HGNC:29991]	-2.6	0.0398
ssc-miR-212	PAX5	paired box 5 [Source:VGNC Symbol;Acc:VGNC:91194]	-2.6	0.0398
ssc-miR-212	PCBD2	hypothetical gene	-2.6	0.0398
ssc-miR-212	PCDH10	protocadherin 10 [Source:HGNC Symbol;Acc:HGNC:13404]	-2.6	0.0398
ssc-miR-212	PCK2	phosphoenolpyruvate carboxykinase 2, mitochondrial [Source:VGNC Symbol;Acc:VGNC:91223]	-2.6	0.0398
ssc-miR-212	PCP4L1	Purkinje cell protein 4 like 1 [Source:VGNC Symbol;Acc:VGNC:98831]	-2.6	0.0398
ssc-miR-212	PCSK7	proprotein convertase subtilisin/kexin type 7 [Source:VGNC Symbol;Acc:VGNC:91233]	-2.6	0.0398
ssc-miR-212	PDE7A	phosphodiesterase 7A [Source:VGNC Symbol;Acc:VGNC:91261]	-2.6	0.0398
ssc-miR-212	PDE7B	phosphodiesterase 7B [Source:VGNC Symbol;Acc:VGNC:91262]	-2.6	0.0398
ssc-miR-212	PDLIM4	PDZ and LIM domain 4 [Source:VGNC Symbol;Acc:VGNC:91283]	-2.6	0.0398
ssc-miR-212	PER2	period circadian regulator 2 [Source:VGNC Symbol;Acc:VGNC:96478]	-2.6	0.0398
ssc-miR-212	PGP	phosphoglycolate phosphatase [Source:VGNC Symbol;Acc:VGNC:91359]	-2.6	0.0398
ssc-miR-212	PIANP	PILR alpha associated neural protein [Source:VGNC Symbol;Acc:VGNC:91409]	-2.6	0.0398
ssc-miR-212	PIGU	phosphatidylinositol glycan anchor biosynthesis class U [Source:VGNC Symbol;Acc:VGNC:95660]	-2.6	0.0398
ssc-miR-212	PIK3CB	phosphatidylinositol-4,5-bisphosphate 3-kinase catalytic subunit beta [Source:VGNC Symbol;Acc:VGNC:91441]	-2.6	0.0398
ssc-miR-212	PISD	phosphatidylserine decarboxylase [Source:VGNC Symbol;Acc:VGNC:91462]	-2.6	0.0398
ssc-miR-212	PITPNM2	phosphatidylinositol transfer protein membrane associated 2 [Source:VGNC Symbol;Acc:VGNC:91466]	-2.6	0.0398
ssc-miR-212	PKP4	plakophilin 4 [Source:VGNC Symbol;Acc:VGNC:96488]	-2.6	0.0398

ssc-miR-212	PLCD4	phospholipase C delta 4 [Source:NCBI gene (formerly Entrezgene);Acc:397119]	-2.6	0.0398
ssc-miR-212	PLXNA2	plexin A2 [Source:VGNC Symbol;Acc:VGNC:91580]	-2.6	0.0398
ssc-miR-212	PLXND1	plexin D1 [Source:VGNC Symbol;Acc:VGNC:91585]	-2.6	0.0398
ssc-miR-212	POGZ	pogo transposable element derived with ZNF domain [Source:VGNC Symbol;Acc:VGNC:91625]	-2.6	0.0398
ssc-miR-212	POU4F1	POU class 4 homeobox 1 [Source:VGNC Symbol;Acc:VGNC:91678]	-2.6	0.0398
ssc-miR-212	POU5F1B	hypothetical gene	-2.6	0.0398
ssc-miR-212	PPM1F	protein phosphatase, Mg2+/Mn2+ dependent 1F [Source:VGNC Symbol;Acc:VGNC:91706]	-2.6	0.0398
ssc-miR-212	PPP1R16B	protein phosphatase 1 regulatory subunit 16B [Source:VGNC Symbol;Acc:VGNC:95669]	-2.6	0.0398
ssc-miR-212	PPP2CB	protein phosphatase 2 catalytic subunit beta [Source:VGNC Symbol;Acc:VGNC:96501]	-2.6	0.0398
ssc-miR-212	PPP2R5E	protein phosphatase 2 regulatory subunit B'epsilon [Source:VGNC Symbol;Acc:VGNC:91754]	-2.6	0.0398
ssc-miR-212	PPP3CA	protein phosphatase 3 catalytic subunit alpha [Source:VGNC Symbol;Acc:VGNC:98218]	-2.6	0.0398
ssc-miR-212	PQLC1	hypothetical gene	-2.6	0.0398
ssc-miR-212	PRCD	photoreceptor disc component [Source:VGNC Symbol;Acc:VGNC:91770]	-2.6	0.0398
ssc-miR-212	PRDM16	PR/SET domain 16 [Source:VGNC Symbol;Acc:VGNC:91777]	-2.6	0.0398
ssc-miR-212	PRIM2	DNA primase subunit 2 [Source:VGNC Symbol;Acc:VGNC:91796]	-2.6	0.0398
ssc-miR-212	PROX1	prospero homeobox 1 [Source:VGNC Symbol;Acc:VGNC:91837]	-2.6	0.0398
ssc-miR-212	PRR14L	proline rich 14 like [Source:VGNC Symbol;Acc:VGNC:91853]	-2.6	0.0398
ssc-miR-212	PRR9	proline rich 9 [Source:HGNC Symbol;Acc:HGNC:32057]	-2.6	0.0398
ssc-miR-212	PRRX1	paired related homeobox 1 [Source:VGNC Symbol;Acc:VGNC:91875]	-2.6	0.0398
ssc-miR-212	PRRX2	paired related homeobox 2 [Source:VGNC Symbol;Acc:VGNC:91876]	-2.6	0.0398
ssc-miR-212	PSD2	pleckstrin and Sec7 domain containing 2 [Source:VGNC Symbol;Acc:VGNC:91896]	-2.6	0.0398
ssc-miR-212	PTBP2	polypyrimidine tract binding protein 2 [Source:VGNC Symbol;Acc:VGNC:91937]	-2.6	0.0398
ssc-miR-212	PTEN	hypothetical gene	-2.6	0.0398
ssc-miR-212	PTPMT1	protein tyrosine phosphatase mitochondrial 1 [Source:VGNC Symbol;Acc:VGNC:91971]	-2.6	0.0398
ssc-miR-212	PTPN14	protein tyrosine phosphatase non-receptor type 14 [Source:VGNC Symbol;Acc:VGNC:91975]	-2.6	0.0398
ssc-miR-212	PTPN22	protein tyrosine phosphatase non-receptor type 22 [Source:VGNC Symbol;Acc:VGNC:91978]	-2.6	0.0398
ssc-miR-212	PTPRD	protein tyrosine phosphatase receptor type D [Source:HGNC Symbol;Acc:HGNC:9668]	-2.6	0.0398
ssc-miR-212	PTPRU	protein tyrosine phosphatase receptor type U [Source:VGNC Symbol;Acc:VGNC:91995]	-2.6	0.0398
ssc-miR-212	PVRL1	hypothetical gene	-2.6	0.0398
ssc-miR-212	QKI	QKI, KH domain containing RNA binding [Source:VGNC Symbol;Acc:VGNC:92025]	-2.6	0.0398
ssc-miR-212	QSER1	glutamine and serine rich 1 [Source:VGNC Symbol;Acc:VGNC:92032]	-2.6	0.0398
ssc-miR-212	RAB3IP	RAB3A interacting protein [Source:HGNC Symbol;Acc:HGNC:16508]	-2.6	0.0398
ssc-miR-212	RAB43	RAB43, member RAS onco family [Source:VGNC Symbol;Acc:VGNC:98268]	-2.6	0.0398
ssc-miR-212	RAD54B	fibrinogen silencer binding protein [Source:VGNC Symbol;Acc:VGNC:98795]	-2.6	0.0398
ssc-miR-212	RALGDS	ral guanine nucleotide dissociation stimulator [Source:HGNC Symbol;Acc:HGNC:9842]	-2.6	0.0398
ssc-miR-212	RAP1GAP2	RAP1 GTPase activating protein 2 [Source:VGNC Symbol;Acc:VGNC:92086]	-2.6	0.0398
ssc-miR-212	RAPGEF3	Rap guanine nucleotide exchange factor 3 [Source:VGNC Symbol;Acc:VGNC:92093]	-2.6	0.0398
ssc-miR-212	RASA1	RAS p21 protein activator 1 [Source:VGNC Symbol;Acc:VGNC:92101]	-2.6	0.0398
ssc-miR-212	RASA4	hypothetical gene	-2.6	0.0398
ssc-miR-212	RASD1	ras related dexamethasone induced 1 [Source:VGNC Symbol;Acc:VGNC:92107]	-2.6	0.0398
ssc-miR-212	RASSF3	Ras association domain family member 3 [Source:VGNC Symbol;Acc:VGNC:92124]	-2.6	0.0398
ssc-miR-212	RAX	retina and anterior neural fold homeobox [Source:VGNC Symbol;Acc:VGNC:92130]	-2.6	0.0398
ssc-miR-212	RBM8A	RNA binding motif protein 8A [Source:VGNC Symbol;Acc:VGNC:92162]	-2.6	0.0398
ssc-miR-212	RBPJ	recombination signal binding protein for immunoglobulin kappa J region [Source:VGNC Symbol;Acc:VGNC:98956]	-2.6	0.0398
ssc-miR-212	REL	REL proto-onco, NF-kB subunit [Source:VGNC Symbol;Acc:VGNC:92202]	-2.6	0.0398
ssc-miR-212	RFX3	regulatory factor X3 [Source:VGNC Symbol;Acc:VGNC:92245]	-2.6	0.0398
ssc-miR-212	RHOQ	ras homolog family member Q [Source:VGNC Symbol;Acc:VGNC:92292]	-2.6	0.0398
ssc-miR-212	RHPN1	rhophilin Rho GTPase binding protein 1 [Source:VGNC Symbol;Acc:VGNC:92296]	-2.6	0.0398
ssc-miR-212	RIMKLA	ribosomal modification protein rimK like family member A [Source:HGNC Symbol;Acc:HGNC:28725]	-2.6	0.0398
ssc-miR-212	RNF10	ring finger protein 10 [Source:VGNC Symbol;Acc:VGNC:92345]	-2.6	0.0398

ssc-miR-212	RNF115	ring finger protein 115 [Source:VGNC Symbol;Acc:VGNC:92348]	-2.6	0.0398
ssc-miR-212	RNF207	ring finger protein 207 [Source:VGNC Symbol;Acc:VGNC:92379]	-2.6	0.0398
ssc-miR-212	ROBO1	roundabout guidance receptor 1 [Source:HGNC Symbol;Acc:HGNC:10249]	-2.6	0.0398
ssc-miR-212	ROBO2	roundabout guidance receptor 2 [Source:HGNC Symbol;Acc:HGNC:10250]	-2.6	0.0398
ssc-miR-212	RP11-366L20.2	hypothetical gene	-2.6	0.0398
ssc-miR-212	RP1L1	hypothetical gene	-2.6	0.0398
ssc-miR-212	RPRD2	regulation of nuclear pre-mRNA domain containing 2 [Source:VGNC Symbol;Acc:VGNC:92436]	-2.6	0.0398
ssc-miR-212	RPS21	ribosomal protein S21 [Source:VGNC Symbol;Acc:VGNC:108734]	-2.6	0.0398
ssc-miR-212	RSBN1L	round spermatid basic protein 1 like [Source:VGNC Symbol;Acc:VGNC:92474]	-2.6	0.0398
ssc-miR-212	RTFDC1	hypothetical gene	-2.6	0.0398
ssc-miR-212	RUFY2	RUN and FYVE domain containing 2 [Source:VGNC Symbol;Acc:VGNC:92512]	-2.6	0.0398
ssc-miR-212	RUNX1T1	RUNX1 partner transcriptional co-repressor 1 [Source:VGNC Symbol;Acc:VGNC:96594]	-2.6	0.0398
ssc-miR-212	RYBP	RING1 and YY1 binding protein [Source:VGNC Symbol;Acc:VGNC:92532]	-2.6	0.0398
ssc-miR-212	S1PR2	sphingosine-1-phosphate receptor 2 [Source:VGNC Symbol;Acc:VGNC:92552]	-2.6	0.0398
ssc-miR-212	SALL2	spalt like transcription factor 2 [Source:VGNC Symbol;Acc:VGNC:92562]	-2.6	0.0398
ssc-miR-212	SALL3	spalt like transcription factor 3 [Source:VGNC Symbol;Acc:VGNC:92563]	-2.6	0.0398
ssc-miR-212	SAMD12	sterile alpha motif domain containing 12 [Source:VGNC Symbol;Acc:VGNC:92565]	-2.6	0.0398
ssc-miR-212	SAP30L	SAP30 like [Source:VGNC Symbol;Acc:VGNC:92577]	-2.6	0.0398
ssc-miR-212	SCAMP3	secretory carrier membrane protein 3 [Source:VGNC Symbol;Acc:VGNC:92607]	-2.6	0.0398
ssc-miR-212	SCNM1	sodium channel modifier 1 [Source:VGNC Symbol;Acc:VGNC:92639]	-2.6	0.0398
ssc-miR-212	SCYL1	SCY1 like pseudokinase 1 [Source:VGNC Symbol;Acc:VGNC:100856]	-2.6	0.0398
ssc-miR-212	SDC1	syndecan 1 [Source:VGNC Symbol;Acc:VGNC:92654]	-2.6	0.0398
ssc-miR-212	SEMA6A	semaphorin 6A [Source:VGNC Symbol;Acc:VGNC:92708]	-2.6	0.0398
ssc-miR-212	SESN2	sestrin 2 [Source:VGNC Symbol;Acc:VGNC:92754]	-2.6	0.0398
ssc-miR-212	SETBP1	SET binding protein 1 [Source:VGNC Symbol;Acc:VGNC:92756]	-2.6	0.0398
ssc-miR-212	SETD5	SET domain containing 5 [Source:VGNC Symbol;Acc:VGNC:92760]	-2.6	0.0398
ssc-miR-212	SF1	splicing factor 1 [Source:HGNC Symbol;Acc:HGNC:12950]	-2.6	0.0398
ssc-miR-212	SF3B2	splicing factor 3b subunit 2 [Source:VGNC Symbol;Acc:VGNC:92771]	-2.6	0.0398
ssc-miR-212	SH3BP5	SH3 domain binding protein 5 [Source:VGNC Symbol;Acc:VGNC:92819]	-2.6	0.0398
ssc-miR-212	SH3BP5L	SH3 binding domain protein 5 like [Source:VGNC Symbol;Acc:VGNC:92820]	-2.6	0.0398
ssc-miR-212	SHANK2	SH3 and multiple ankyrin repeat domains 2 [Source:VGNC Symbol;Acc:VGNC:92835]	-2.6	0.0398
ssc-miR-212	SHANK3	hypothetical gene	-2.6	0.0398
ssc-miR-212	SHF	Src homology 2 domain containing F [Source:VGNC Symbol;Acc:VGNC:103175]	-2.6	0.0398
ssc-miR-212	SHH	sonic hedgehog signaling molecule [Source:VGNC Symbol;Acc:VGNC:92844]	-2.6	0.0398
ssc-miR-212	SHISA5	shisa family member 5 [Source:VGNC Symbol;Acc:VGNC:92847]	-2.6	0.0398
ssc-miR-212	SHISA9	shisa family member 9 [Source:HGNC Symbol;Acc:HGNC:37231]	-2.6	0.0398
ssc-miR-212	SHROOM4	shroom family member 4 [Source:VGNC Symbol;Acc:VGNC:92861]	-2.6	0.0398
ssc-miR-212	SIDT2	SID1 transmembrane family member 2 [Source:VGNC Symbol;Acc:VGNC:92869]	-2.6	0.0398
ssc-miR-212	SIRPA	hypothetical gene	-2.6	0.0398
ssc-miR-212	SIRT2	sirtuin 2 [Source:VGNC Symbol;Acc:VGNC:92885]	-2.6	0.0398
ssc-miR-212	SLC10A7	solute carrier family 10 member 7 [Source:VGNC Symbol;Acc:VGNC:92919]	-2.6	0.0398
ssc-miR-212	SLC31A1	solute carrier family 31 member 1 [Source:VGNC Symbol;Acc:VGNC:103180]	-2.6	0.0398
ssc-miR-212	SLC35B2	solute carrier family 35 member B2 [Source:VGNC Symbol;Acc:VGNC:93072]	-2.6	0.0398
ssc-miR-212	SLC37A2	solute carrier family 37 member 2 [Source:VGNC Symbol;Acc:VGNC:98323]	-2.6	0.0398
ssc-miR-212	SLC6A8	solute carrier family 6 member 8 [Source:NCBI gene (formerly Entrezgene);Acc:100302506]	-2.6	0.0398
ssc-miR-212	SLC8A1	solute carrier family 8 member A1 [Source:VGNC Symbol;Acc:VGNC:93178]	-2.6	0.0398
ssc-miR-212	SMG1	SMG1 nonsense mediated mRNA decay associated PI3K related kinase [Source:VGNC Symbol;Acc:VGNC:93248]	-2.6	0.0398
ssc-miR-212	SMTNL2	smoothelin like 2 [Source:VGNC Symbol;Acc:VGNC:93268]	-2.6	0.0398
ssc-miR-212	SMUG1	single-strand-selective monofunctional uracil-DNA glycosylase 1 [Source:VGNC Symbol;Acc:VGNC:93269]	-2.6	0.0398
ssc-miR-212	SND1	staphylococcal nuclease and tudor domain containing 1 [Source:VGNC Symbol;Acc:VGNC:93287]	-2.6	0.0398

ssc-miR-212	SOCS2	suppressor of cytokine signaling 2 [Source:NCBI gene (formerly Entrezgene);Acc:100037966]	-2.6	0.0398
ssc-miR-212	SOCS5	suppressor of cytokine signaling 5 [Source:VGNC Symbol;Acc:VGNC:93332]	-2.6	0.0398
ssc-miR-212	SORCS1	sortilin related VPS10 domain containing receptor 1 [Source:VGNC Symbol;Acc:VGNC:93339]	-2.6	0.0398
ssc-miR-212	SOX4	SRY-box transcription factor 4 [Source:HGNC Symbol;Acc:HGNC:11200]	-2.6	0.0398
ssc-miR-212	SOX5	SRY-box transcription factor 5 [Source:VGNC Symbol;Acc:VGNC:93357]	-2.6	0.0398
ssc-miR-212	SPG20	hypothetical gene	-2.6	0.0398
ssc-miR-212	SPOCD1	SPOC domain containing 1 [Source:VGNC Symbol;Acc:VGNC:93414]	-2.6	0.0398
ssc-miR-212	SPTBN2	spectrin beta, non-erythrocytic 2 [Source:VGNC Symbol;Acc:VGNC:93437]	-2.6	0.0398
ssc-miR-212	SRGAP1	SLIT-ROBO Rho GTPase activating protein 1 [Source:VGNC Symbol;Acc:VGNC:93454]	-2.6	0.0398
ssc-miR-212	SRP9	signal recognition particle 9 [Source:VGNC Symbol;Acc:VGNC:108282]	-2.6	0.0398
ssc-miR-212	SRPK1	SRSF protein kinase 1 [Source:VGNC Symbol;Acc:VGNC:93462]	-2.6	0.0398
ssc-miR-212	SRRM4	serine/arginine repetitive matrix 4 [Source:VGNC Symbol;Acc:VGNC:93470]	-2.6	0.0398
ssc-miR-212	SSR3	signal sequence receptor subunit 3 [Source:VGNC Symbol;Acc:VGNC:93492]	-2.6	0.0398
ssc-miR-212	ST3GAL3	ST3 beta-galactoside alpha-2,3-sialyltransferase 3 [Source:VGNC Symbol;Acc:VGNC:93503]	-2.6	0.0398
ssc-miR-212	STARD7	StAR related lipid transfer domain containing 7 [Source:VGNC Symbol;Acc:VGNC:93537]	-2.6	0.0398
ssc-miR-212	STK38	serine/threonine kinase 38 [Source:VGNC Symbol;Acc:VGNC:93556]	-2.6	0.0398
ssc-miR-212	STON2	stonin 2 [Source:VGNC Symbol;Acc:VGNC:93568]	-2.6	0.0398
ssc-miR-212	STX17	syntaxin 17 [Source:VGNC Symbol;Acc:VGNC:93587]	-2.6	0.0398
ssc-miR-212	STX7	syntaxin 7 [Source:VGNC Symbol;Acc:VGNC:93595]	-2.6	0.0398
ssc-miR-212	SUMO2	small ubiquitin like modifier 2 [Source:NCBI gene (formerly Entrezgene);Acc:397044]	-2.6	0.0398
ssc-miR-212	SV2A	synaptic vesicle glycoprotein 2A [Source:VGNC Symbol;Acc:VGNC:93639]	-2.6	0.0398
ssc-miR-212	SYPL1	synaptophysin like 1 [Source:HGNC Symbol;Acc:HGNC:11507]	-2.6	0.0398
ssc-miR-212	SYT9	synaptotagmin 9 [Source:VGNC Symbol;Acc:VGNC:93689]	-2.6	0.0398
ssc-miR-212	TADA2B	transcriptional adaptor 2B [Source:VGNC Symbol;Acc:VGNC:93707]	-2.6	0.0398
ssc-miR-212	TANC2	tetratricopeptide repeat, ankyrin repeat and coiled-coil containing 2 [Source:VGNC Symbol;Acc:VGNC:93733]	-2.6	0.0398
ssc-miR-212	TAOK3	TAO kinase 3 [Source:VGNC Symbol;Acc:VGNC:98357]	-2.6	0.0398
ssc-miR-212	TAT	tyrosine aminotransferase [Source:VGNC Symbol;Acc:VGNC:93752]	-2.6	0.0398
ssc-miR-212	TBC1D22B	TBC1 domain family member 22B [Source:VGNC Symbol;Acc:VGNC:93771]	-2.6	0.0398
ssc-miR-212	TBX15	hypothetical gene	-2.6	0.0398
ssc-miR-212	TCEA2	transcription elongation factor A2 [Source:VGNC Symbol;Acc:VGNC:95534]	-2.6	0.0398
ssc-miR-212	TCEANC2	transcription elongation factor A N-terminal and central domain containing 2 [Source:HGNC Symbol;Acc:HGNC:26494]	-2.6	0.0398
ssc-miR-212	TCF19	transcription factor 19 [Source:VGNC Symbol;Acc:VGNC:93818]	-2.6	0.0398
ssc-miR-212	TCF23	transcription factor 23 [Source:VGNC Symbol;Acc:VGNC:93820]	-2.6	0.0398
ssc-miR-212	TCF7L2	transcription factor 7 like 2 [Source:VGNC Symbol;Acc:VGNC:93825]	-2.6	0.0398
ssc-miR-212	TERT	telomerase reverse transcriptase [Source:VGNC Symbol;Acc:VGNC:93883]	-2.6	0.0398
ssc-miR-212	TFAP2B	transcription factor AP-2 beta [Source:VGNC Symbol;Acc:VGNC:93909]	-2.6	0.0398
ssc-miR-212	TFDP1	transcription factor Dp-1 [Source:HGNC Symbol;Acc:HGNC:11749]	-2.6	0.0398
ssc-miR-212	TGFBR2	transforming growth factor beta receptor 2 [Source:VGNC Symbol;Acc:VGNC:93931]	-2.6	0.0398
ssc-miR-212	TGOLN2	hypothetical gene	-2.6	0.0398
ssc-miR-212	TMEM115	transmembrane protein 115 [Source:HGNC Symbol;Acc:HGNC:30055]	-2.6	0.0398
ssc-miR-212	TMEM127	transmembrane protein 127 [Source:HGNC Symbol;Acc:HGNC:26038]	-2.6	0.0398
ssc-miR-212	TMEM164	transmembrane protein 164 [Source:VGNC Symbol;Acc:VGNC:94103]	-2.6	0.0398
ssc-miR-212	TMEM178B	transmembrane protein 178B [Source:HGNC Symbol;Acc:HGNC:44112]	-2.6	0.0398
ssc-miR-212	TMEM2	hypothetical gene	-2.6	0.0398
ssc-miR-212	TMEM57	hypothetical gene	-2.6	0.0398
ssc-miR-212	TMEM91	transmembrane protein 91 [Source:HGNC Symbol;Acc:HGNC:32393]	-2.6	0.0398
ssc-miR-212	TPD52	tumor protein D52 [Source:VGNC Symbol;Acc:VGNC:98383]	-2.6	0.0398
ssc-miR-212	TPM2	tropomyosin 2 [Source:VGNC Symbol;Acc:VGNC:94338]	-2.6	0.0398
ssc-miR-212	TPRG1	tumor protein p63 regulated 1 [Source:VGNC Symbol;Acc:VGNC:94348]	-2.6	0.0398
ssc-miR-212	TRAF3	TNF receptor associated factor 3 [Source:VGNC Symbol;Acc:VGNC:94360]	-2.6	0.0398

ssc-miR-212	TRAK2	trafficking kinesin protein 2 [Source:VGNC Symbol;Acc:VGNC:95577]	-2.6	0.0398
ssc-miR-212	TRIB2	tribbles pseudokinase 2 [Source:VGNC Symbol;Acc:VGNC:94392]	-2.6	0.0398
ssc-miR-212	TRIM14	tripartite motif containing 14 [Source:VGNC Symbol;Acc:VGNC:103196]	-2.6	0.0398
ssc-miR-212	TRIM45	tripartite motif containing 45 [Source:VGNC Symbol;Acc:VGNC:94417]	-2.6	0.0398
ssc-miR-212	TRIM71	tripartite motif containing 71 [Source:VGNC Symbol;Acc:VGNC:94431]	-2.6	0.0398
ssc-miR-212	TRIM73	hypothetical gene	-2.6	0.0398
ssc-miR-212	TTBK2	tau tubulin kinase 2 [Source:VGNC Symbol;Acc:VGNC:98387]	-2.6	0.0398
ssc-miR-212	TTC28	tetratricopeptide repeat domain 28 [Source:VGNC Symbol;Acc:VGNC:94543]	-2.6	0.0398
ssc-miR-212	TTC39A	tetratricopeptide repeat domain 39A [Source:VGNC Symbol;Acc:VGNC:94551]	-2.6	0.0398
ssc-miR-212	TTLL3	actin related protein 2/3 complex subunit 4 [Source:NCBI gene (formerly Entrezgene);Acc:100155875]	-2.6	0.0398
ssc-miR-212	TTPAL	alpha tocopherol transfer protein like [Source:VGNC Symbol;Acc:VGNC:95742]	-2.6	0.0398
ssc-miR-212	UAP1	UDP-N-acetylglucosamine pyrophosphorylase 1 [Source:VGNC Symbol;Acc:VGNC:94626]	-2.6	0.0398
ssc-miR-212	UBE2QL1	ubiquitin conjugating enzyme E2 Q family like 1 [Source:VGNC Symbol;Acc:VGNC:94650]	-2.6	0.0398
ssc-miR-212	UBE2R2	ubiquitin conjugating enzyme E2 R2 [Source:VGNC Symbol;Acc:VGNC:95924]	-2.6	0.0398
ssc-miR-212	UHRF1BP1	UHRF1 binding protein 1 [Source:VGNC Symbol;Acc:VGNC:94690]	-2.6	0.0398
ssc-miR-212	UNC13C	unc-13 homolog C [Source:VGNC Symbol;Acc:VGNC:94702]	-2.6	0.0398
ssc-miR-212	USB1	U6 snRNA biosis phosphodiesterase 1 [Source:VGNC Symbol;Acc:VGNC:94736]	-2.6	0.0398
ssc-miR-212	USP13	ubiquitin specific peptidase 13 [Source:VGNC Symbol;Acc:VGNC:94746]	-2.6	0.0398
ssc-miR-212	USP46	ubiquitin specific peptidase 46 [Source:VGNC Symbol;Acc:VGNC:94768]	-2.6	0.0398
ssc-miR-212	USP9X	ubiquitin specific peptidase 9 X-linked [Source:HGNC Symbol;Acc:HGNC:12632]	-2.6	0.0398
ssc-miR-212	USP9Y	hypothetical gene	-2.6	0.0398
ssc-miR-212	UVRAG	UV radiation resistance associated [Source:VGNC Symbol;Acc:VGNC:94789]	-2.6	0.0398
ssc-miR-212	VAMP4	vesicle associated membrane protein 4 [Source:VGNC Symbol;Acc:VGNC:94794]	-2.6	0.0398
ssc-miR-212	VAT1	vesicle amine transport 1 [Source:VGNC Symbol;Acc:VGNC:94805]	-2.6	0.0398
ssc-miR-212	VGLL4	vestigial like family member 4 [Source:VGNC Symbol;Acc:VGNC:94824]	-2.6	0.0398
ssc-miR-212	VMP1	vacuole membrane protein 1 [Source:HGNC Symbol;Acc:HGNC:29559]	-2.6	0.0398
ssc-miR-212	VPS13B	vacuolar protein sorting 13 homolog B [Source:VGNC Symbol;Acc:VGNC:94839]	-2.6	0.0398
ssc-miR-212	VPS53	VPS53 subunit of GARP complex [Source:VGNC Symbol;Acc:VGNC:94861]	-2.6	0.0398
ssc-miR-212	VWA1	von Willebrand factor A domain containing 1 [Source:VGNC Symbol;Acc:VGNC:98679]	-2.6	0.0398
ssc-miR-212	VWC2L	von Willebrand factor C domain containing 2 like [Source:VGNC Symbol;Acc:VGNC:96301]	-2.6	0.0398
ssc-miR-212	WSCD1	WSC domain containing 1 [Source:VGNC Symbol;Acc:VGNC:94983]	-2.6	0.0398
ssc-miR-212	XIAP	X-linked inhibitor of apoptosis [Source:NCBI gene (formerly Entrezgene);Acc:100037300]	-2.6	0.0398
ssc-miR-212	XKR4	XK related 4 [Source:VGNC Symbol;Acc:VGNC:98900]	-2.6	0.0398
ssc-miR-212	XPO6	exportin 6 [Source:VGNC Symbol;Acc:VGNC:95006]	-2.6	0.0398
ssc-miR-212	YWHAG	hypothetical gene	-2.6	0.0398
ssc-miR-212	YWHAZ	tyrosine 3-monooxygenase/tryptophan 5-monooxygenase activation protein zeta [Source:VGNC Symbol;Acc:VGNC:95047]	-2.6	0.0398
ssc-miR-212	ZBED4	hypothetical gene	-2.6	0.0398
ssc-miR-212	ZBTB20	zinc finger and BTB domain containing 20 [Source:VGNC Symbol;Acc:VGNC:95063]	-2.6	0.0398
ssc-miR-212	ZC2HC1C	zinc finger C2HC-type containing 1C [Source:VGNC Symbol;Acc:VGNC:95087]	-2.6	0.0398
ssc-miR-212	ZEB2	hypothetical gene	-2.6	0.0398
ssc-miR-212	ZFHX4	zinc finger homeobox 4 [Source:VGNC Symbol;Acc:VGNC:95142]	-2.6	0.0398
ssc-miR-212	ZNF189	zinc finger protein 189 [Source:VGNC Symbol;Acc:VGNC:103205]	-2.6	0.0398
ssc-miR-212	ZNF280C	zinc finger protein 280C [Source:VGNC Symbol;Acc:VGNC:95216]	-2.6	0.0398
ssc-miR-212	ZNF516	zinc finger protein 516 [Source:VGNC Symbol;Acc:VGNC:95256]	-2.6	0.0398
ssc-miR-212	ZNF521	zinc finger protein 521 [Source:VGNC Symbol;Acc:VGNC:98715]	-2.6	0.0398
ssc-miR-212	ZNF526	zinc finger protein 526 [Source:VGNC Symbol;Acc:VGNC:95260]	-2.6	0.0398
ssc-miR-212	ZNF618	zinc finger protein 618 [Source:VGNC Symbol;Acc:VGNC:95276]	-2.6	0.0398
ssc-miR-212	ZNF644	zinc finger protein 644 [Source:VGNC Symbol;Acc:VGNC:95283]	-2.6	0.0398
ssc-miR-212	ZNF710	zinc finger protein 710 [Source:VGNC Symbol;Acc:VGNC:107180]	-2.6	0.0398
ssc-miR-212	ZNF792	zinc finger protein 792 [Source:VGNC Symbol;Acc:VGNC:95304]	-2.6	0.0398

ssc-miR-212	ZNF827	zinc finger protein 827 [Source:VGNC Symbol;Acc:VGNC:98970]	-2.6	0.0398
ssc-miR-212	ZSWIM5	zinc finger SWIM-type containing 5 [Source:VGNC Symbol;Acc:VGNC:95334]	-2.6	0.0398
ssc-miR-212	ZSWIM6	zinc finger SWIM-type containing 6 [Source:VGNC Symbol;Acc:VGNC:95335]	-2.6	0.0398
ssc-miR-328	ACSS2	acyl-CoA synthetase short chain family member 2 [Source:VGNC Symbol;Acc:VGNC:95665]	-3.38	0.0559
ssc-miR-328	ADD2	adducin 2 [Source:VGNC Symbol;Acc:VGNC:85117]	-3.38	0.0559
ssc-miR-328	ADNP	activity dependent neuroprotector homeobox [Source:VGNC Symbol;Acc:VGNC:95657]	-3.38	0.0559
ssc-miR-328	AGO1	hypothetical gene	-3.38	0.0559
ssc-miR-328	AHSA2	hypothetical gene	-3.38	0.0559
ssc-miR-328	AJUBA	ajuba LIM protein [Source:VGNC Symbol;Acc:VGNC:85208]	-3.38	0.0559
ssc-miR-328	ALG6	ALG6 alpha-1,3-glucosyltransferase [Source:VGNC Symbol;Acc:VGNC:85253]	-3.38	0.0559
ssc-miR-328	ANKFY1	ankyrin repeat and FYVE domain containing 1 [Source:VGNC Symbol;Acc:VGNC:85314]	-3.38	0.0559
ssc-miR-328	ANKRD40	ankyrin repeat domain 40 [Source:VGNC Symbol;Acc:VGNC:85338]	-3.38	0.0559
ssc-miR-328	AP5M1	adaptor related protein complex 5 subunit mu 1 [Source:VGNC Symbol;Acc:VGNC:85396]	-3.38	0.0559
ssc-miR-328	ARHGEF17	hypothetical gene	-3.38	0.0559
ssc-miR-328	ARPP19	cAMP regulated phosphoprotein 19 [Source:NCBI gene (formerly Entrezgene);Acc:397362]	-3.38	0.0559
ssc-miR-328	ASIC1	acid sensing ion channel subunit 1 [Source:VGNC Symbol;Acc:VGNC:85578]	-3.38	0.0559
ssc-miR-328	ATG2A	autophagy related 2A [Source:VGNC Symbol;Acc:VGNC:85619]	-3.38	0.0559
ssc-miR-328	ATPAF1	ATP synthase mitochondrial F1 complex assembly factor 1 [Source:VGNC Symbol;Acc:VGNC:96924]	-3.38	0.0559
ssc-miR-328	AXIN1	axin 1 [Source:VGNC Symbol;Acc:VGNC:85707]	-3.38	0.0559
ssc-miR-328	BACE1	beta-secretase 1 [Source:VGNC Symbol;Acc:VGNC:85740]	-3.38	0.0559
ssc-miR-328	BCL9L	BCL9 like [Source:VGNC Symbol;Acc:VGNC:96568]	-3.38	0.0559
ssc-miR-328	BEND4	BEN domain containing 4 [Source:VGNC Symbol;Acc:VGNC:85801]	-3.38	0.0559
ssc-miR-328	BYSL	bystin like [Source:NCBI gene (formerly Entrezgene);Acc:100156292]	-3.38	0.0559
ssc-miR-328	C10orf113	hypothetical gene	-3.38	0.0559
ssc-miR-328	C15orf37	hypothetical gene	-3.38	0.0559
ssc-miR-328	C16orf87	chromosome 6 C16orf87 homolog [Source:VGNC Symbol;Acc:VGNC:96927]	-3.38	0.0559
ssc-miR-328	C19orf43	hypothetical gene	-3.38	0.0559
ssc-miR-328	C1orf226	hypothetical gene	-3.38	0.0559
ssc-miR-328	C20orf112	hypothetical gene	-3.38	0.0559
ssc-miR-328	C6orf223	hypothetical gene	-3.38	0.0559
ssc-miR-328	CA3	carbonic anhydrase 3 [Source:VGNC Symbol;Acc:VGNC:86099]	-3.38	0.0559
ssc-miR-328	CACNG2	calcium voltage-gated channel auxiliary subunit gamma 2 [Source:VGNC Symbol;Acc:VGNC:86125]	-3.38	0.0559
ssc-miR-328	CBL	Cbl proto-onco [Source:VGNC Symbol;Acc:VGNC:86222]	-3.38	0.0559
ssc-miR-328	CBX8	chromobox 8 [Source:VGNC Symbol;Acc:VGNC:86233]	-3.38	0.0559
ssc-miR-328	CCDC88A	coiled-coil domain containing 88A [Source:VGNC Symbol;Acc:VGNC:86325]	-3.38	0.0559
ssc-miR-328	CCDC9	coiled-coil domain containing 9 [Source:VGNC Symbol;Acc:VGNC:86327]	-3.38	0.0559
ssc-miR-328	CELSR2	cadherin EGF LAG seven-pass G-type receptor 2 [Source:VGNC Symbol;Acc:VGNC:98748]	-3.38	0.0559
ssc-miR-328	CELSR3	cadherin EGF LAG seven-pass G-type receptor 3 [Source:VGNC Symbol;Acc:VGNC:86541]	-3.38	0.0559
ssc-miR-328	CENPO	centromere protein O [Source:HGNC Symbol;Acc:HGNC:28152]	-3.38	0.0559
ssc-miR-328	CHP2	calcineurin like EF-hand protein 2 [Source:VGNC Symbol;Acc:VGNC:86658]	-3.38	0.0559
ssc-miR-328	CHST7	carbohydrate sulfotransferase 7 [Source:VGNC Symbol;Acc:VGNC:86681]	-3.38	0.0559
ssc-miR-328	CHTF8	hypothetical gene	-3.38	0.0559
ssc-miR-328	CNIH4	cornichon family AMPA receptor auxiliary protein 4 [Source:VGNC Symbol;Acc:VGNC:96060]	-3.38	0.0559
ssc-miR-328	CNNM2	cyclin and CBS domain divalent metal cation transport mediator 2 [Source:VGNC Symbol;Acc:VGNC:86828]	-3.38	0.0559
ssc-miR-328	CNOT2	CCR4-NOT transcription complex subunit 2 [Source:VGNC Symbol;Acc:VGNC:86834]	-3.38	0.0559
ssc-miR-328	COL23A1	collagen type XXIII alpha 1 chain [Source:VGNC Symbol;Acc:VGNC:99620]	-3.38	0.0559
ssc-miR-328	CPT1A	carnitine palmitoyltransferase 1A [Source:VGNC Symbol;Acc:VGNC:86964]	-3.38	0.0559
ssc-miR-328	CREB1	cAMP responsive element binding protein 1 [Source:VGNC Symbol;Acc:VGNC:96004]	-3.38	0.0559
ssc-miR-328	CRK	CRK proto-onco, adaptor protein [Source:NCBI gene (formerly Entrezgene);Acc:100192444]	-3.38	0.0559
ssc-miR-328	CRP	C-reactive protein, pentraxin-related [Source:NCBI gene (formerly Entrezgene);Acc:396842]	-3.38	0.0559

ssc-miR-328	CTIF	cap binding complex dependent translation initiation factor [Source:VGNC Symbol;Acc:VGNC:87062]	-3.38	0.0559
ssc-miR-328	CTNNBIP1	catenin beta interacting protein 1 [Source:VGNC Symbol;Acc:VGNC:87066]	-3.38	0.0559
ssc-miR-328	CTNS	cystinosin, lysosomal cystine transporter [Source:VGNC Symbol;Acc:VGNC:87069]	-3.38	0.0559
ssc-miR-328	CYP39A1	cytochrome P450 family 39 subfamily A member 1 [Source:VGNC Symbol;Acc:VGNC:103375]	-3.38	0.0559
ssc-miR-328	DDI2	hypothetical gene	-3.38	0.0559
ssc-miR-328	DERL2	derlin 2 [Source:VGNC Symbol;Acc:VGNC:98986]	-3.38	0.0559
ssc-miR-328	DIEXF	hypothetical gene	-3.38	0.0559
ssc-miR-328	DKK3	dickkopf WNT signaling pathway inhibitor 3 [Source:VGNC Symbol;Acc:VGNC:87323]	-3.38	0.0559
ssc-miR-328	DNAH10OS	hypothetical gene	-3.38	0.0559
ssc-miR-328	DNAJB14	DnaJ heat shock protein family (Hsp40) member B14 [Source:VGNC Symbol;Acc:VGNC:98919]	-3.38	0.0559
ssc-miR-328	DNAJC10	DnaJ heat shock protein family (Hsp40) member C10 [Source:HGNC Symbol;Acc:HGNC:24637]	-3.38	0.0559
ssc-miR-328	DNAJC18	DnaJ heat shock protein family (Hsp40) member C18 [Source:VGNC Symbol;Acc:VGNC:96615]	-3.38	0.0559
ssc-miR-328	DNAJC4	DnaJ heat shock protein family (Hsp40) member C4 [Source:HGNC Symbol;Acc:HGNC:5271]	-3.38	0.0559
ssc-miR-328	DRGX	dorsal root ganglia homeobox [Source:VGNC Symbol;Acc:VGNC:97968]	-3.38	0.0559
ssc-miR-328	DUSP16	dual specificity phosphatase 16 [Source:VGNC Symbol;Acc:VGNC:87480]	-3.38	0.0559
ssc-miR-328	DYNC1I1	dynein cytoplasmic 1 intermediate chain 1 [Source:VGNC Symbol;Acc:VGNC:87496]	-3.38	0.0559
ssc-miR-328	DYNLL2	dynein light chain LC8-type 2 [Source:VGNC Symbol;Acc:VGNC:87501]	-3.38	0.0559
ssc-miR-328	EBF4	EBF family member 4 [Source:VGNC Symbol;Acc:VGNC:96211]	-3.38	0.0559
ssc-miR-328	EDARADD	EDAR associated death domain [Source:VGNC Symbol;Acc:VGNC:87542]	-3.38	0.0559
ssc-miR-328	EEF2K	eukaryotic elongation factor 2 kinase [Source:VGNC Symbol;Acc:VGNC:87557]	-3.38	0.0559
ssc-miR-328	EIF4EBP1	eukaryotic translation initiation factor 4E binding protein 1 [Source:VGNC Symbol;Acc:VGNC:96268]	-3.38	0.0559
ssc-miR-328	EMC3	ER membrane protein complex subunit 3 [Source:VGNC Symbol;Acc:VGNC:87671]	-3.38	0.0559
ssc-miR-328	EN2	engrailed homeobox 2 [Source:VGNC Symbol;Acc:VGNC:87694]	-3.38	0.0559
ssc-miR-328	ENTPD1	ectonucleoside triphosphate diphosphohydrolase 1 [Source:VGNC Symbol;Acc:VGNC:97977]	-3.38	0.0559
ssc-miR-328	ENTPD7	ectonucleoside triphosphate diphosphohydrolase 7 [Source:VGNC Symbol;Acc:VGNC:87716]	-3.38	0.0559
ssc-miR-328	EPB41L1	hypothetical gene	-3.38	0.0559
ssc-miR-328	ERMP1	endoplasmic reticulum metallopeptidase 1 [Source:VGNC Symbol;Acc:VGNC:87777]	-3.38	0.0559
ssc-miR-328	ESCO1	establishment of sister chromatid cohesion N-acetyltransferase 1 [Source:VGNC Symbol;Acc:VGNC:87786]	-3.38	0.0559
ssc-miR-328	ESRP1	epithelial splicing regulatory protein 1 [Source:VGNC Symbol;Acc:VGNC:87791]	-3.38	0.0559
ssc-miR-328	ESRRA	estrogen related receptor alpha [Source:VGNC Symbol;Acc:VGNC:87793]	-3.38	0.0559
ssc-miR-328	ETV6	ETS variant transcription factor 6 [Source:VGNC Symbol;Acc:VGNC:87815]	-3.38	0.0559
ssc-miR-328	FAM155B	hypothetical gene	-3.38	0.0559
ssc-miR-328	FAM199X	family with sequence similarity 199, X-linked [Source:HGNC Symbol;Acc:HGNC:25195]	-3.38	0.0559
ssc-miR-328	FAM211A	hypothetical gene	-3.38	0.0559
ssc-miR-328	FAM214B	family with sequence similarity 214 member B [Source:VGNC Symbol;Acc:VGNC:87953]	-3.38	0.0559
ssc-miR-328	FAXDC2	fatty acid hydroxylase domain containing 2 [Source:VGNC Symbol;Acc:VGNC:88021]	-3.38	0.0559
ssc-miR-328	FBXL5	F-box and leucine rich repeat protein 5 [Source:VGNC Symbol;Acc:VGNC:98008]	-3.38	0.0559
ssc-miR-328	FBXO41	F-box protein 41 [Source:VGNC Symbol;Acc:VGNC:88046]	-3.38	0.0559
ssc-miR-328	FGD1	FYVE, RhoGEF and PH domain containing 1 [Source:VGNC Symbol;Acc:VGNC:88094]	-3.38	0.0559
ssc-miR-328	FKBP15	FKBP prolyl isomerase family member 15 [Source:VGNC Symbol;Acc:VGNC:88145]	-3.38	0.0559
ssc-miR-328	FMNL3	formin like 3 [Source:VGNC Symbol;Acc:VGNC:88169]	-3.38	0.0559
ssc-miR-328	FMO2	flavin containing dimethylaniline monoxygenase 2 [Source:VGNC Symbol;Acc:VGNC:88171]	-3.38	0.0559
ssc-miR-328	FOXO4	forkhead box O4 [Source:VGNC Symbol;Acc:VGNC:88221]	-3.38	0.0559
ssc-miR-328	FOXS1	forkhead box S1 [Source:VGNC Symbol;Acc:VGNC:96303]	-3.38	0.0559
ssc-miR-328	FUBP1	far upstream element binding protein 1 [Source:VGNC Symbol;Acc:VGNC:96781]	-3.38	0.0559
ssc-miR-328	GBA	glucosylceramidase beta [Source:VGNC Symbol;Acc:VGNC:98802]	-3.38	0.0559
ssc-miR-328	GBX2	gastrulation brain homeobox 2 [Source:VGNC Symbol;Acc:VGNC:96316]	-3.38	0.0559
ssc-miR-328	GGA2	golgi associated, gamma adaptin ear containing, ARF binding protein 2 [Source:VGNC Symbol;Acc:VGNC:88432]	-3.38	0.0559
ssc-miR-328	GM2A	GM2 ganglioside activator [Source:VGNC Symbol;Acc:VGNC:88506]	-3.38	0.0559
ssc-miR-328	GNG11	G protein subunit gamma 11 [Source:HGNC Symbol;Acc:HGNC:4403]	-3.38	0.0559

ssc-miR-328	GOSR1	golgi SNAP receptor complex member 1 [Source:VGNC Symbol;Acc:VGNC:88560]	-3.38	0.0559
ssc-miR-328	GPR111	hypothetical gene	-3.38	0.0559
ssc-miR-328	GRAMD1B	GRAM domain containing 1B [Source:VGNC Symbol;Acc:VGNC:88656]	-3.38	0.0559
ssc-miR-328	GRIN2B	glutamate ionotropic receptor NMDA type subunit 2B [Source:VGNC Symbol;Acc:VGNC:88684]	-3.38	0.0559
ssc-miR-328	GRM4	glutamate metabotropic receptor 4 [Source:VGNC Symbol;Acc:VGNC:98027]	-3.38	0.0559
ssc-miR-328	GSG1L	GSG1 like [Source:VGNC Symbol;Acc:VGNC:88722]	-3.38	0.0559
ssc-miR-328	GTDC1	glycosyltransferase like domain containing 1 [Source:VGNC Symbol;Acc:VGNC:95955]	-3.38	0.0559
ssc-miR-328	H2AFX	hypothetical gene	-3.38	0.0559
ssc-miR-328	HAP1	huntingtin associated protein 1 [Source:VGNC Symbol;Acc:VGNC:88779]	-3.38	0.0559
ssc-miR-328	HEYL	hes related family bHLH transcription factor with YRPW motif like [Source:VGNC Symbol;Acc:VGNC:88865]	-3.38	0.0559
ssc-miR-328	HIC1	HIC ZBTB transcriptional repressor 1 [Source:VGNC Symbol;Acc:VGNC:88878]	-3.38	0.0559
ssc-miR-328	HIF1AN	hypoxia inducible factor 1 subunit alpha inhibitor [Source:VGNC Symbol;Acc:VGNC:98033]	-3.38	0.0559
ssc-miR-328	HIST1H4D	hypothetical gene	-3.38	0.0559
ssc-miR-328	HIST1H4G	hypothetical gene	-3.38	0.0559
ssc-miR-328	HIST1H4L	hypothetical gene	-3.38	0.0559
ssc-miR-328	HIST2H4A	hypothetical gene	-3.38	0.0559
ssc-miR-328	HIST2H4B	hypothetical gene	-3.38	0.0559
ssc-miR-328	HM13	histocompatibility minor 13 [Source:VGNC Symbol;Acc:VGNC:98035]	-3.38	0.0559
ssc-miR-328	HPCAL4	hippocalcin like 4 [Source:VGNC Symbol;Acc:VGNC:88959]	-3.38	0.0559
ssc-miR-328	IRAK1BP1	interleukin 1 receptor associated kinase 1 binding protein 1 [Source:VGNC Symbol;Acc:VGNC:89199]	-3.38	0.0559
ssc-miR-328	ITGA5	integrin subunit alpha 5 [Source:VGNC Symbol;Acc:VGNC:89236]	-3.38	0.0559
ssc-miR-328	ITGA9	integrin subunit alpha 9 [Source:VGNC Symbol;Acc:VGNC:89238]	-3.38	0.0559
ssc-miR-328	KAT6B	lysine acetyltransferase 6B [Source:VGNC Symbol;Acc:VGNC:89306]	-3.38	0.0559
ssc-miR-328	KCTD16	potassium channel tetramerization domain containing 16 [Source:VGNC Symbol;Acc:VGNC:89395]	-3.38	0.0559
ssc-miR-328	KIAA0247	hypothetical gene	-3.38	0.0559
ssc-miR-328	KIAA1549	KIAA1549 [Source:VGNC Symbol;Acc:VGNC:99719]	-3.38	0.0559
ssc-miR-328	KIAA1614	KIAA1614 [Source:VGNC Symbol;Acc:VGNC:89446]	-3.38	0.0559
ssc-miR-328	KIF2A	kinesin family member 2A [Source:VGNC Symbol;Acc:VGNC:89467]	-3.38	0.0559
ssc-miR-328	KLHL42	kelch like family member 42 [Source:VGNC Symbol;Acc:VGNC:89534]	-3.38	0.0559
ssc-miR-328	LEPROTL1	leptin receptor overlapping transcript like 1 [Source:VGNC Symbol;Acc:VGNC:96404]	-3.38	0.0559
ssc-miR-328	LGR4	leucine rich repeat containing G protein-coupled receptor 4 [Source:VGNC Symbol;Acc:VGNC:89703]	-3.38	0.0559
ssc-miR-328	LHFPL2	LHFPL tetraspan subfamily member 2 [Source:VGNC Symbol;Acc:VGNC:89705]	-3.38	0.0559
ssc-miR-328	LIMD1	LIM domain containing 1 [Source:VGNC Symbol;Acc:VGNC:89725]	-3.38	0.0559
ssc-miR-328	LMOD1	leiomodin 1 [Source:VGNC Symbol;Acc:VGNC:96405]	-3.38	0.0559
ssc-miR-328	LNX2	ligand of numb-protein X 2 [Source:VGNC Symbol;Acc:VGNC:89774]	-3.38	0.0559
ssc-miR-328	LPP	LIM domain containing preferred translocation partner in lipoma [Source:HGNC Symbol;Acc:HGNC:6679]	-3.38	0.0559
ssc-miR-328	LRRC40	hypothetical gene	-3.38	0.0559
ssc-miR-328	LSM14B	LSM family member 14B [Source:VGNC Symbol;Acc:VGNC:95691]	-3.38	0.0559
ssc-miR-328	LURAP1	leucine rich adaptor protein 1 [Source:VGNC Symbol;Acc:VGNC:89895]	-3.38	0.0559
ssc-miR-328	LYVE1	lymphatic vessel endothelial hyaluronan receptor 1 [Source:VGNC Symbol;Acc:VGNC:89922]	-3.38	0.0559
ssc-miR-328	MAN1C1	mannosidase alpha class 1C member 1 [Source:VGNC Symbol;Acc:VGNC:89968]	-3.38	0.0559
ssc-miR-328	MAP3K9	mitogen-activated protein kinase kinase kinase 9 [Source:VGNC Symbol;Acc:VGNC:89989]	-3.38	0.0559
ssc-miR-328	MBNL3	muscleblind like splicing regulator 3 [Source:VGNC Symbol;Acc:VGNC:90056]	-3.38	0.0559
ssc-miR-328	MECP2	methyl-CpG binding protein 2 [Source:VGNC Symbol;Acc:VGNC:90101]	-3.38	0.0559
ssc-miR-328	METTL21B	hypothetical gene	-3.38	0.0559
ssc-miR-328	MFSD2A	major facilitator superfamily domain containing 2A [Source:VGNC Symbol;Acc:VGNC:90188]	-3.38	0.0559
ssc-miR-328	MIB1	MIB E3 ubiquitin protein ligase 1 [Source:VGNC Symbol;Acc:VGNC:90205]	-3.38	0.0559
ssc-miR-328	MICALL1	MICAL like 1 [Source:VGNC Symbol;Acc:VGNC:90209]	-3.38	0.0559
ssc-miR-328	MMP16	matrix metallopeptidase 16 [Source:VGNC Symbol;Acc:VGNC:90271]	-3.38	0.0559
ssc-miR-328	MN1	MN1 proto-onco, transcriptional regulator [Source:VGNC Symbol;Acc:VGNC:90285]	-3.38	0.0559

ssc-miR-328	MPI	mannose phosphate isomerase [Source:VGNC Symbol;Acc:VGNC:90324]	-3.38	0.0559
ssc-miR-328	MPV17	mitochondrial inner membrane protein MPV17 [Source:VGNC Symbol;Acc:VGNC:103991]	-3.38	0.0559
ssc-miR-328	MRPS18B	mitochondrial ribosomal protein S18B [Source:VGNC Symbol;Acc:VGNC:90385]	-3.38	0.0559
ssc-miR-328	MTMR3	myotubularin related protein 3 [Source:VGNC Symbol;Acc:VGNC:90461]	-3.38	0.0559
ssc-miR-328	MXI1	hypothetical gene	-3.38	0.0559
ssc-miR-328	NDOR1	NADPH dependent diflavin oxidoreductase 1 [Source:VGNC Symbol;Acc:VGNC:90630]	-3.38	0.0559
ssc-miR-328	NF2	NF2, moesin-ezrin-radixin like (MERLIN) tumor suppressor [Source:VGNC Symbol;Acc:VGNC:90705]	-3.38	0.0559
ssc-miR-328	NHSL2	NHS like 2 [Source:VGNC Symbol;Acc:VGNC:98155]	-3.38	0.0559
ssc-miR-328	NIT1	nitrilase 1 [Source:VGNC Symbol;Acc:VGNC:90753]	-3.38	0.0559
ssc-miR-328	NKIRAS2	NFKB inhibitor interacting Ras like 2 [Source:VGNC Symbol;Acc:VGNC:90763]	-3.38	0.0559
ssc-miR-328	NKPD1	NTPase KAP family P-loop domain containing 1 [Source:VGNC Symbol;Acc:VGNC:98518]	-3.38	0.0559
ssc-miR-328	NPFFR1	neuropeptide FF receptor 1 [Source:VGNC Symbol;Acc:VGNC:90846]	-3.38	0.0559
ssc-miR-328	NR3C1	nuclear receptor subfamily 3 group C member 1 [Source:VGNC Symbol;Acc:VGNC:90883]	-3.38	0.0559
ssc-miR-328	NR6A1	nuclear receptor subfamily 6 group A member 1 [Source:VGNC Symbol;Acc:VGNC:90887]	-3.38	0.0559
ssc-miR-328	NRG2	neuregulin 2 [Source:VGNC Symbol;Acc:VGNC:98160]	-3.38	0.0559
ssc-miR-328	NRIP1	nuclear receptor interacting protein 1 [Source:VGNC Symbol;Acc:VGNC:90896]	-3.38	0.0559
ssc-miR-328	NUFIP2	nuclear FMR1 interacting protein 2 [Source:VGNC Symbol;Acc:VGNC:90967]	-3.38	0.0559
ssc-miR-328	NXF1	nuclear RNA export factor 1 [Source:VGNC Symbol;Acc:VGNC:90993]	-3.38	0.0559
ssc-miR-328	NXPH3	neurexophilin 3 [Source:VGNC Symbol;Acc:VGNC:90999]	-3.38	0.0559
ssc-miR-328	OSTM1	osteoclastosis associated transmembrane protein 1 [Source:VGNC Symbol;Acc:VGNC:91086]	-3.38	0.0559
ssc-miR-328	OTUB2	OTU deubiquitinase, ubiquitin aldehyde binding 2 [Source:VGNC Symbol;Acc:VGNC:91098]	-3.38	0.0559
ssc-miR-328	PADI2	peptidyl arginine deiminase 2 [Source:VGNC Symbol;Acc:VGNC:91146]	-3.38	0.0559
ssc-miR-328	PARP16	poly(ADP-ribose) polymerase family member 16 [Source:VGNC Symbol;Acc:VGNC:103145]	-3.38	0.0559
ssc-miR-328	PCNXL4	hypothetical gene	-3.38	0.0559
ssc-miR-328	PEF1	penta-EF-hand domain containing 1 [Source:VGNC Symbol;Acc:VGNC:91305]	-3.38	0.0559
ssc-miR-328	PGM2	phosphoglucomutase 2 [Source:VGNC Symbol;Acc:VGNC:91356]	-3.38	0.0559
ssc-miR-328	PHF15	hypothetical gene	-3.38	0.0559
ssc-miR-328	PIGA	phosphatidylinositol glycan anchor biosynthesis class A [Source:VGNC Symbol;Acc:VGNC:91419]	-3.38	0.0559
ssc-miR-328	PIM1	Pim-1 proto-oncogene, serine/threonine kinase [Source:HGNC Symbol;Acc:HGNC:8986]	-3.38	0.0559
ssc-miR-328	PLA2G12A	phospholipase A2 group XIIA [Source:HGNC Symbol;Acc:HGNC:18554]	-3.38	0.0559
ssc-miR-328	PLCE1	phospholipase C epsilon 1 [Source:VGNC Symbol;Acc:VGNC:91519]	-3.38	0.0559
ssc-miR-328	PLCH2	phospholipase C eta 2 [Source:VGNC Symbol;Acc:VGNC:91522]	-3.38	0.0559
ssc-miR-328	PLEKHA6	pleckstrin homology domain containing A6 [Source:VGNC Symbol;Acc:VGNC:91536]	-3.38	0.0559
ssc-miR-328	PLEKHG3	pleckstrin homology and RhoGEF domain containing G3 [Source:VGNC Symbol;Acc:VGNC:91544]	-3.38	0.0559
ssc-miR-328	PLXNC1	plexin C1 [Source:VGNC Symbol;Acc:VGNC:91584]	-3.38	0.0559
ssc-miR-328	PODNL1	podocan like 1 [Source:VGNC Symbol;Acc:VGNC:91618]	-3.38	0.0559
ssc-miR-328	POLR3H	RNA polymerase III subunit H [Source:VGNC Symbol;Acc:VGNC:91658]	-3.38	0.0559
ssc-miR-328	PPIP5K1	diphosphoinositol pentakisphosphate kinase 1 [Source:VGNC Symbol;Acc:VGNC:91700]	-3.38	0.0559
ssc-miR-328	PPP2R5D	protein phosphatase 2 regulatory subunit B'delta [Source:VGNC Symbol;Acc:VGNC:98217]	-3.38	0.0559
ssc-miR-328	PRDM16	PR/SET domain 16 [Source:VGNC Symbol;Acc:VGNC:91777]	-3.38	0.0559
ssc-miR-328	PRNT	hypothetical gene	-3.38	0.0559
ssc-miR-328	PSME3	proteasome activator subunit 3 [Source:VGNC Symbol;Acc:VGNC:91926]	-3.38	0.0559
ssc-miR-328	PTEN	hypothetical gene	-3.38	0.0559
ssc-miR-328	PTPN9	protein tyrosine phosphatase non-receptor type 9 [Source:VGNC Symbol;Acc:VGNC:91983]	-3.38	0.0559
ssc-miR-328	PTPRF	protein tyrosine phosphatase receptor type F [Source:VGNC Symbol;Acc:VGNC:91987]	-3.38	0.0559
ssc-miR-328	PTPRU	protein tyrosine phosphatase receptor type U [Source:VGNC Symbol;Acc:VGNC:91995]	-3.38	0.0559
ssc-miR-328	RAB3C	RAB3C, member RAS onco family [Source:VGNC Symbol;Acc:VGNC:104043]	-3.38	0.0559
ssc-miR-328	RAD23B	RAD23 homolog B, nucleotide excision repair protein [Source:VGNC Symbol;Acc:VGNC:103160]	-3.38	0.0559
ssc-miR-328	RANBP10	RAN binding protein 10 [Source:VGNC Symbol;Acc:VGNC:92077]	-3.38	0.0559
ssc-miR-328	RAP1GAP2	RAP1 GTPase activating protein 2 [Source:VGNC Symbol;Acc:VGNC:92086]	-3.38	0.0559

ssc-miR-328	RBM8A	RNA binding motif protein 8A [Source:VGNC Symbol;Acc:VGNC:92162]	-3.38	0.0559
ssc-miR-328	RBP2	retinol binding protein 2 [Source:NCBI gene (formerly Entrezgene);Acc:407611]	-3.38	0.0559
ssc-miR-328	RGL2	ral guanine nucleotide dissociation stimulator like 2 [Source:VGNC Symbol;Acc:VGNC:92253]	-3.38	0.0559
ssc-miR-328	RGMA	repulsive guidance molecule BMP co-receptor a [Source:VGNC Symbol;Acc:VGNC:92254]	-3.38	0.0559
ssc-miR-328	RGP1	RGP1 homolog, RAB6A GEF complex partner 1 [Source:VGNC Symbol;Acc:VGNC:92257]	-3.38	0.0559
ssc-miR-328	RGS9BP	regulator of G protein signaling 9 binding protein [Source:HGNC Symbol;Acc:HGNC:30304]	-3.38	0.0559
ssc-miR-328	RNF152	ring finger protein 152 [Source:VGNC Symbol;Acc:VGNC:92364]	-3.38	0.0559
ssc-miR-328	RP11-366L20.2	hypothetical gene	-3.38	0.0559
ssc-miR-328	RPP14	hypothetical gene	-3.38	0.0559
ssc-miR-328	RPS3	ribosomal protein S3 [Source:VGNC Symbol;Acc:VGNC:104049]	-3.38	0.0559
ssc-miR-328	RPS9	ribosomal protein S9 [Source:VGNC Symbol;Acc:VGNC:92449]	-3.38	0.0559
ssc-miR-328	RS1	retinoschisin 1 [Source:VGNC Symbol;Acc:VGNC:92470]	-3.38	0.0559
ssc-miR-328	RSBN1L	round spermatid basic protein 1 like [Source:VGNC Symbol;Acc:VGNC:92474]	-3.38	0.0559
ssc-miR-328	RWDD2B	RWD domain containing 2B [Source:HGNC Symbol;Acc:HGNC:1302]	-3.38	0.0559
ssc-miR-328	SCN2B	sodium voltage-gated channel beta subunit 2 [Source:VGNC Symbol;Acc:VGNC:92633]	-3.38	0.0559
ssc-miR-328	SDK2	hypothetical gene	-3.38	0.0559
ssc-miR-328	SF1	splicing factor 1 [Source:HGNC Symbol;Acc:HGNC:12950]	-3.38	0.0559
ssc-miR-328	SF3B3	splicing factor 3b subunit 3 [Source:VGNC Symbol;Acc:VGNC:92772]	-3.38	0.0559
ssc-miR-328	SHISA5	shisa family member 5 [Source:VGNC Symbol;Acc:VGNC:92847]	-3.38	0.0559
ssc-miR-328	SHISA6	hypothetical gene	-3.38	0.0559
ssc-miR-328	SHKBP1	SH3KBP1 binding protein 1 [Source:VGNC Symbol;Acc:VGNC:92853]	-3.38	0.0559
ssc-miR-328	SIX1	SIX homeobox 1 [Source:VGNC Symbol;Acc:VGNC:92892]	-3.38	0.0559
ssc-miR-328	SLC27A4	solute carrier family 27 member 4 [Source:VGNC Symbol;Acc:VGNC:93038]	-3.38	0.0559
ssc-miR-328	SLC2A1	solute carrier family 2 member 1 [Source:HGNC Symbol;Acc:HGNC:11005]	-3.38	0.0559
ssc-miR-328	SLC30A8	solute carrier family 30 member 8 [Source:VGNC Symbol;Acc:VGNC:93062]	-3.38	0.0559
ssc-miR-328	SLC7A6	hypothetical gene	-3.38	0.0559
ssc-miR-328	SLC7A7	solute carrier family 7 member 7 [Source:VGNC Symbol;Acc:VGNC:93175]	-3.38	0.0559
ssc-miR-328	SLC8A1	solute carrier family 8 member A1 [Source:VGNC Symbol;Acc:VGNC:93178]	-3.38	0.0559
ssc-miR-328	SLITRK3	SLIT and NTRK like family member 3 [Source:VGNC Symbol;Acc:VGNC:93208]	-3.38	0.0559
ssc-miR-328	SMC1A	structural maintenance of chromosomes 1A [Source:VGNC Symbol;Acc:VGNC:93236]	-3.38	0.0559
ssc-miR-328	SNRK	SNF related kinase [Source:VGNC Symbol;Acc:VGNC:93290]	-3.38	0.0559
ssc-miR-328	SNX21	sorting nexin family member 21 [Source:VGNC Symbol;Acc:VGNC:95758]	-3.38	0.0559
ssc-miR-328	SOGA1	suppressor of glucose, autophagy associated 1 [Source:VGNC Symbol;Acc:VGNC:95740]	-3.38	0.0559
ssc-miR-328	SORL1	sortilin related receptor 1 [Source:VGNC Symbol;Acc:VGNC:108617]	-3.38	0.0559
ssc-miR-328	SOX11	SRY-box transcription factor 11 [Source:VGNC Symbol;Acc:VGNC:93351]	-3.38	0.0559
ssc-miR-328	SPECC1L	sperm antigen with calponin homology and coiled-coil domains 1 like [Source:HGNC Symbol;Acc:HGNC:29022]	-3.38	0.0559
ssc-miR-328	SRSF9	serine and arginine rich splicing factor 9 [Source:VGNC Symbol;Acc:VGNC:104063]	-3.38	0.0559
ssc-miR-328	STT3A	STT3 oligosaccharyltransferase complex catalytic subunit A [Source:VGNC Symbol;Acc:VGNC:93581]	-3.38	0.0559
ssc-miR-328	TCF7L2	transcription factor 7 like 2 [Source:VGNC Symbol;Acc:VGNC:93825]	-3.38	0.0559
ssc-miR-328	TESK2	testis associated actin remodelling kinase 2 [Source:VGNC Symbol;Acc:VGNC:93886]	-3.38	0.0559
ssc-miR-328	TGFB2	transforming growth factor beta 2 [Source:VGNC Symbol;Acc:VGNC:95541]	-3.38	0.0559
ssc-miR-328	TMEM106B	transmembrane protein 106B [Source:VGNC Symbol;Acc:VGNC:104075]	-3.38	0.0559
ssc-miR-328	TMEM132B	transmembrane protein 132B [Source:HGNC Symbol;Acc:HGNC:29397]	-3.38	0.0559
ssc-miR-328	TMEM229A	transmembrane protein 229A [Source:VGNC Symbol;Acc:VGNC:94146]	-3.38	0.0559
ssc-miR-328	TMEM248	transmembrane protein 248 [Source:VGNC Symbol;Acc:VGNC:94155]	-3.38	0.0559
ssc-miR-328	TMEM64	transmembrane protein 64 [Source:VGNC Symbol;Acc:VGNC:94199]	-3.38	0.0559
ssc-miR-328	TNPO3	transportin 3 [Source:VGNC Symbol;Acc:VGNC:94289]	-3.38	0.0559
ssc-miR-328	TOM1	target of myb1 membrane trafficking protein [Source:VGNC Symbol;Acc:VGNC:95552]	-3.38	0.0559
ssc-miR-328	TOMM40L	translocase of outer mitochondrial membrane 40 like [Source:VGNC Symbol;Acc:VGNC:94306]	-3.38	0.0559
ssc-miR-328	TOX4	TOX high mobility group box family member 4 [Source:VGNC Symbol;Acc:VGNC:98382]	-3.38	0.0559

ssc-miR-328	TP73	tumor protein p73 [Source:VGNC Symbol;Acc:VGNC:94331]	-3.38	0.0559
ssc-miR-328	TRABD2B	TraB domain containing 2B [Source:VGNC Symbol;Acc:VGNC:94356]	-3.38	0.0559
ssc-miR-328	TRAPPC6B	trafficking protein particle complex subunit 6B [Source:VGNC Symbol;Acc:VGNC:94378]	-3.38	0.0559
ssc-miR-328	TRIM71	tripartite motif containing 71 [Source:VGNC Symbol;Acc:VGNC:94431]	-3.38	0.0559
ssc-miR-328	TRMT1L	tRNA methyltransferase 1 like [Source:VGNC Symbol;Acc:VGNC:94447]	-3.38	0.0559
ssc-miR-328	TSC1	TSC complex subunit 1 [Source:HGNC Symbol;Acc:HGNC:12362]	-3.38	0.0559
ssc-miR-328	TTC26	tetratricopeptide repeat domain 26 [Source:VGNC Symbol;Acc:VGNC:94541]	-3.38	0.0559
ssc-miR-328	TTC38	tetratricopeptide repeat domain 38 [Source:VGNC Symbol;Acc:VGNC:94550]	-3.38	0.0559
ssc-miR-328	UBD	ubiquitin D [Source:NCBI gene (formerly Entrezgene);Acc:100294688]	-3.38	0.0559
ssc-miR-328	UBFD1	ubiquitin family domain containing 1 [Source:VGNC Symbol;Acc:VGNC:94658]	-3.38	0.0559
ssc-miR-328	ULK2	unc-51 like autophagy activating kinase 2 [Source:VGNC Symbol;Acc:VGNC:94695]	-3.38	0.0559
ssc-miR-328	USB1	U6 snRNA biosis phosphodiesterase 1 [Source:VGNC Symbol;Acc:VGNC:94736]	-3.38	0.0559
ssc-miR-328	USP37	ubiquitin specific peptidase 37 [Source:VGNC Symbol;Acc:VGNC:98404]	-3.38	0.0559
ssc-miR-328	USP54	ubiquitin specific peptidase 54 [Source:VGNC Symbol;Acc:VGNC:94776]	-3.38	0.0559
ssc-miR-328	USP8	ubiquitin specific peptidase 8 [Source:VGNC Symbol;Acc:VGNC:94778]	-3.38	0.0559
ssc-miR-328	VAMP2	vesicle associated membrane protein 2 [Source:HGNC Symbol;Acc:HGNC:12643]	-3.38	0.0559
ssc-miR-328	WASF2	WASP family member 2 [Source:VGNC Symbol;Acc:VGNC:94893]	-3.38	0.0559
ssc-miR-328	WDTC1	WD and tetratricopeptide repeats 1 [Source:VGNC Symbol;Acc:VGNC:94952]	-3.38	0.0559
ssc-miR-328	XKR7	XK related 7 [Source:VGNC Symbol;Acc:VGNC:95987]	-3.38	0.0559
ssc-miR-328	YBX2	Y-box binding protein 2 [Source:VGNC Symbol;Acc:VGNC:95020]	-3.38	0.0559
ssc-miR-328	YIPF5	Yip1 domain family member 5 [Source:VGNC Symbol;Acc:VGNC:95031]	-3.38	0.0559
ssc-miR-328	YWHAZ	tyrosine 3-monooxygenase/tryptophan 5-monooxygenase activation protein zeta [Source:VGNC Symbol;Acc:VGNC:95047]	-3.38	0.0559
ssc-miR-328	ZC3H12B	zinc finger CCCH-type containing 12B [Source:VGNC Symbol;Acc:VGNC:95090]	-3.38	0.0559
ssc-miR-328	ZNF280B	zinc finger protein 280B [Source:HGNC Symbol;Acc:HGNC:23022]	-3.38	0.0559
ssc-miR-328	ZNF423	zinc finger protein 423 [Source:VGNC Symbol;Acc:VGNC:98752]	-3.38	0.0559
ssc-miR-328	ZNF436	zinc finger protein 436 [Source:VGNC Symbol;Acc:VGNC:95239]	-3.38	0.0559
ssc-miR-328	ZNF618	zinc finger protein 618 [Source:VGNC Symbol;Acc:VGNC:95276]	-3.38	0.0559
ssc-miR-328	ZNF697	zinc finger protein 697 [Source:HGNC Symbol;Acc:HGNC:32034]	-3.38	0.0559
ssc-miR-328	ZZEF1	zinc finger ZZ-type and EF-hand domain containing 1 [Source:VGNC Symbol;Acc:VGNC:95347]	-3.38	0.0559
