# Supplementary material for: Mesenchymal Stem Cells Cultured in a 3D Microgel Environment Containing Platelet-Rich Plasma Significantly Modify Their Chondrogenesis-Related miRNA Expression
Source: Int J Mol Sci. 2024 Jan 11;25(2):937. doi: 10.3390/ijms25020937 (PMC10815493; doi:10.3390/ijms25020937)
Supplement: Supplementary file 1 [file ijms-25-00937-s001.zip › Table S2_PROL.pdf]

Supplementary Table S2. Differential expression of miRNAs. pMSCs were cultured in gelatin microgels containing or not PRP, in chondrogenic proliferation culture media. Data was filtered according to a fold change > 1.7

miRNA	GENE	DESCRIPTION	FOLD CHANGE	adjusted p_value
ssc-miR-183	AAK1	AP2 associated kinase 1 [Source:VGNC Symbol;Acc:VGNC:100379]	9.24	0.0538
ssc-miR-183	ABCA1	ATP binding cassette subfamily A member 1 [Source:VGNC Symbol;Acc:VGNC:84947]	9.24	0.0538
ssc-miR-183	ABCB10	ATP binding cassette subfamily B member 10 [Source:VGNC Symbol;Acc:VGNC:103892]	9.24	0.0538
ssc-miR-183	ABHD13	abhydrolase domain containing 13 [Source:VGNC Symbol;Acc:VGNC:84972]	9.24	0.0538
ssc-miR-183	ABI2	abl interactor 2 [Source:VGNC Symbol;Acc:VGNC:96027]	9.24	0.0538
ssc-miR-183	ACER3	alkaline ceramidase 3 [Source:VGNC Symbol;Acc:VGNC:103893]	9.24	0.0538
ssc-miR-183	ACTR1A	actin related protein 1A [Source:VGNC Symbol;Acc:VGNC:85049]	9.24	0.0538
ssc-miR-183	ACVR2A	activin A receptor type 2A [Source:VGNC Symbol;Acc:VGNC:95843]	9.24	0.0538
ssc-miR-183	ACVR2B	activin A receptor type 2B [Source:VGNC Symbol;Acc:VGNC:108629]	9.24	0.0538
ssc-miR-183	ADCY6	adenylate cyclase 6 [Source:VGNC Symbol;Acc:VGNC:85110]	9.24	0.0538
ssc-miR-183	AFF4	AF4/FMR2 family member 4 [Source:VGNC Symbol;Acc:VGNC:85169]	9.24	0.0538
ssc-miR-183	AGO2	argonaute RISC catalytic component 2 [Source:VGNC Symbol;Acc:VGNC:97871]	9.24	0.0538
ssc-miR-183	AGPAT5	1-acylglycerol-3-phosphate O-acyltransferase 5 [Source:VGNC Symbol;Acc:VGNC:95954]	9.24	0.0538
ssc-miR-183	AJAP1	adherens junctions associated protein 1 [Source:VGNC Symbol;Acc:VGNC:85207]	9.24	0.0538
ssc-miR-183	AKAP12	A-kinase anchoring protein 12 [Source:VGNC Symbol;Acc:VGNC:85216]	9.24	0.0538
ssc-miR-183	AKTIP	AKT interacting protein [Source:VGNC Symbol;Acc:VGNC:85233]	9.24	0.0538
ssc-miR-183	AL590483.1	hypothetical gene	9.24	0.0538
ssc-miR-183	AMD1	hypothetical gene	9.24	0.0538
ssc-miR-183	AMMECR1L	AMMECR1 like [Source:VGNC Symbol;Acc:VGNC:103895]	9.24	0.0538
ssc-miR-183	ANGEL2	angel homolog 2 [Source:VGNC Symbol;Acc:VGNC:85302]	9.24	0.0538
ssc-miR-183	ANKMY2	ankyrin repeat and MYND domain containing 2 [Source:VGNC Symbol;Acc:VGNC:85319]	9.24	0.0538
ssc-miR-183	ANKRD13C	ankyrin repeat domain 13C [Source:VGNC Symbol;Acc:VGNC:85326]	9.24	0.0538
ssc-miR-183	ANKRD28	ankyrin repeat domain 28 [Source:VGNC Symbol;Acc:VGNC:85331]	9.24	0.0538
ssc-miR-183	ANKRD50	ankyrin repeat domain containing 50 [Source:VGNC Symbol;Acc:VGNC:85341]	9.24	0.0538
ssc-miR-183	AP3M1	adaptor related protein complex 3 subunit mu 1 [Source:VGNC Symbol;Acc:VGNC:85390]	9.24	0.0538
ssc-miR-183	AP3M2	adaptor related protein complex 3 subunit mu 2 [Source:VGNC Symbol;Acc:VGNC:96369]	9.24	0.0538
ssc-miR-183	APPBP2	amyloid beta protein binding protein 2 [Source:VGNC Symbol;Acc:VGNC:85425]	9.24	0.0538
ssc-miR-183	ARFGAP2	ADP ribosylation factor GTPase activating protein 2 [Source:VGNC Symbol;Acc:VGNC:85449]	9.24	0.0538
ssc-miR-183	ARHGAP12	Rho GTPase activating protein 12 [Source:VGNC Symbol;Acc:VGNC:95806]	9.24	0.0538
ssc-miR-183	ARHGAP18	Rho GTPase activating protein 18 [Source:VGNC Symbol;Acc:VGNC:85460]	9.24	0.0538
ssc-miR-183	ARHGAP21	Rho GTPase activating protein 21 [Source:VGNC Symbol;Acc:VGNC:96018]	9.24	0.0538
ssc-miR-183	ARHGAP26	Rho GTPase activating protein 26 [Source:VGNC Symbol;Acc:VGNC:85465]	9.24	0.0538
ssc-miR-183	ARHGAP29	Rho GTPase activating protein 29 [Source:VGNC Symbol;Acc:VGNC:85468]	9.24	0.0538
ssc-miR-183	ARHGAP6	hypothetical gene	9.24	0.0538
ssc-miR-183	ARHGEF18	Rho/Rac guanine nucleotide exchange factor 18 [Source:VGNC Symbol;Acc:VGNC:106443]	9.24	0.0538
ssc-miR-183	ARHGEF37	Rho guanine nucleotide exchange factor 37 [Source:VGNC Symbol;Acc:VGNC:85498]	9.24	0.0538
ssc-miR-183	ARID4B	AT-rich interaction domain 4B [Source:HGNC Symbol;Acc:HGNC:15550]	9.24	0.0538
ssc-miR-183	ARPP19	cAMP regulated phosphoprotein 19 [Source:NCBI gene (formerly Entrezgene);Acc:397362]	9.24	0.0538
ssc-miR-183	ASH2L	ASH2 like, histone lysine methyltransferase complex subunit [Source:VGNC Symbol;Acc:VGNC:96426]	9.24	0.0538
ssc-miR-183	ATF2	activating transcription factor 2 [Source:VGNC Symbol;Acc:VGNC:96446]	9.24	0.0538
ssc-miR-183	ATP13A3	ATPase 13A3 [Source:VGNC Symbol;Acc:VGNC:85639]	9.24	0.0538
ssc-miR-183	ATP2B4	ATPase plasma membrane Ca2+ transporting 4 [Source:VGNC Symbol;Acc:VGNC:85651]	9.24	0.0538
ssc-miR-183	ATP2C1	ATPase secretory pathway Ca2+ transporting 1 [Source:VGNC Symbol;Acc:VGNC:85652]	9.24	0.0538
ssc-miR-183	ATRN	attractin [Source:VGNC Symbol;Acc:VGNC:96479]	9.24	0.0538
ssc-miR-183	AUTS2	activator of transcription and developmental regulator AUTS2 [Source:VGNC Symbol;Acc:VGNC:97897]	9.24	0.0538

ssc-miR-183	AZIN1	antizyme inhibitor 1 [Source:VGNC Symbol;Acc:VGNC:85712]	9.24	0.0538
ssc-miR-183	B3GAT2	beta-1,3-glucuronyltransferase 2 [Source:VGNC Symbol;Acc:VGNC:85720]	9.24	0.0538
ssc-miR-183	BACH2	BTB domain and CNC homolog 2 [Source:VGNC Symbol;Acc:VGNC:85742]	9.24	0.0538
ssc-miR-183	BAZ1B	bromodomain adjacent to zinc finger domain 1B [Source:VGNC Symbol;Acc:VGNC:85762]	9.24	0.0538
ssc-miR-183	BCL11B	BAF chromatin remodeling complex subunit BCL11B [Source:VGNC Symbol;Acc:VGNC:96563]	9.24	0.0538
ssc-miR-183	BEND3	BEN domain containing 3 [Source:VGNC Symbol;Acc:VGNC:85800]	9.24	0.0538
ssc-miR-183	BMI1	BMI1 proto-onco, polycomb ring finger [Source:VGNC Symbol;Acc:VGNC:108265]	9.24	0.0538
ssc-miR-183	BMP2K	BMP2 inducible kinase [Source:VGNC Symbol;Acc:VGNC:85841]	9.24	0.0538
ssc-miR-183	BNC2	basonuclin 2 [Source:VGNC Symbol;Acc:VGNC:85852]	9.24	0.0538
ssc-miR-183	BNIP3L	BCL2 interacting protein 3 like [Source:VGNC Symbol;Acc:VGNC:85856]	9.24	0.0538
ssc-miR-183	BRD4	bromodomain containing 4 [Source:VGNC Symbol;Acc:VGNC:85873]	9.24	0.0538
ssc-miR-183	BRMS1L	BRMS1 like transcriptional repressor [Source:VGNC Symbol;Acc:VGNC:96571]	9.24	0.0538
ssc-miR-183	BTG1	BTG anti-proliferation factor 1 [Source:VGNC Symbol;Acc:VGNC:103907]	9.24	0.0538
ssc-miR-183	BVES	blood vessel epicardial substance [Source:VGNC Symbol;Acc:VGNC:103040]	9.24	0.0538
ssc-miR-183	BZW1	basic leucine zipper and W2 domains 1 [Source:VGNC Symbol;Acc:VGNC:95885]	9.24	0.0538
ssc-miR-183	C11orf57	hypothetical gene	9.24	0.0538
ssc-miR-183	C16orf72	chromosome 3 C16orf72 homolog [Source:VGNC Symbol;Acc:VGNC:86016]	9.24	0.0538
ssc-miR-183	C1orf50	hypothetical gene	9.24	0.0538
ssc-miR-183	C2orf44	hypothetical gene	9.24	0.0538
ssc-miR-183	C5orf24	chromosome 2 C5orf24 homolog [Source:VGNC Symbol;Acc:VGNC:86010]	9.24	0.0538
ssc-miR-183	C8orf47	hypothetical gene	9.24	0.0538
ssc-miR-183	CAB39	calcium binding protein 39 [Source:VGNC Symbol;Acc:VGNC:95936]	9.24	0.0538
ssc-miR-183	CACNA1E	calcium voltage-gated channel subunit alpha1 E [Source:VGNC Symbol;Acc:VGNC:86118]	9.24	0.0538
ssc-miR-183	CALN1	hypothetical gene	9.24	0.0538
ssc-miR-183	CAMK2D	calcium/calmodulin dependent protein kinase II delta [Source:VGNC Symbol;Acc:VGNC:97911]	9.24	0.0538
ssc-miR-183	CAND1	cullin associated and neddylation dissociated 1 [Source:VGNC Symbol;Acc:VGNC:97912]	9.24	0.0538
ssc-miR-183	CBL	Cbl proto-onco [Source:VGNC Symbol;Acc:VGNC:86222]	9.24	0.0538
ssc-miR-183	CC2D2B	coiled-coil and C2 domain containing 2B [Source:HGNC Symbol;Acc:HGNC:31666]	9.24	0.0538
ssc-miR-183	CCDC144A	hypothetical gene	9.24	0.0538
ssc-miR-183	CCDC176	hypothetical gene	9.24	0.0538
ssc-miR-183	CCDC43	coiled-coil domain containing 43 [Source:VGNC Symbol;Acc:VGNC:98979]	9.24	0.0538
ssc-miR-183	CCDC50	coiled-coil domain containing 50 [Source:VGNC Symbol;Acc:VGNC:86299]	9.24	0.0538
ssc-miR-183	CCNB1	cyclin B1 [Source:VGNC Symbol;Acc:VGNC:86349]	9.24	0.0538
ssc-miR-183	CCND2	cyclin D2 [Source:VGNC Symbol;Acc:VGNC:103222]	9.24	0.0538
ssc-miR-183	CDC73	cell division cycle 73 [Source:VGNC Symbol;Acc:VGNC:95817]	9.24	0.0538
ssc-miR-183	CDH9	cadherin 9 [Source:VGNC Symbol;Acc:VGNC:86488]	9.24	0.0538
ssc-miR-183	CDK5R1	cyclin dependent kinase 5 regulatory subunit 1 [Source:VGNC Symbol;Acc:VGNC:98980]	9.24	0.0538
ssc-miR-183	CDYL2	chromodomain Y like 2 [Source:VGNC Symbol;Acc:VGNC:86529]	9.24	0.0538
ssc-miR-183	CELF1	CUGBP Elav-like family member 1 [Source:VGNC Symbol;Acc:VGNC:86537]	9.24	0.0538
ssc-miR-183	CELF2	hypothetical gene	9.24	0.0538
ssc-miR-183	CELF6	CUGBP Elav-like family member 6 [Source:HGNC Symbol;Acc:HGNC:14059]	9.24	0.0538
ssc-miR-183	CELSR3	cadherin EGF LAG seven-pass G-type receptor 3 [Source:VGNC Symbol;Acc:VGNC:86541]	9.24	0.0538
ssc-miR-183	CEP170B	centrosomal protein 170B [Source:VGNC Symbol;Acc:VGNC:86564]	9.24	0.0538
ssc-miR-183	CEP97	centrosomal protein 97 [Source:VGNC Symbol;Acc:VGNC:108638]	9.24	0.0538
ssc-miR-183	CERS6	ceramide synthase 6 [Source:VGNC Symbol;Acc:VGNC:96025]	9.24	0.0538
ssc-miR-183	CHD2	chromodomain helicase DNA binding protein 2 [Source:HGNC Symbol;Acc:HGNC:1917]	9.24	0.0538
ssc-miR-183	CLCN3	chloride voltage-gated channel 3 [Source:VGNC Symbol;Acc:VGNC:86727]	9.24	0.0538
ssc-miR-183	CLIC4	chloride intracellular channel 4 [Source:VGNC Symbol;Acc:VGNC:86763]	9.24	0.0538
ssc-miR-183	CLVS2	clavesin 2 [Source:VGNC Symbol;Acc:VGNC:86795]	9.24	0.0538

ssc-miR-183	CNBP	hypothetical gene	9.24	0.0538
ssc-miR-183	CNOT6L	CCR4-NOT transcription complex subunit 6 like [Source:VGNC Symbol;Acc:VGNC:86838]	9.24	0.0538
ssc-miR-183	COL25A1	collagen type XXV alpha 1 chain [Source:HGNC Symbol;Acc:HGNC:18603]	9.24	0.0538
ssc-miR-183	COMMD3-BMI1	hypothetical gene	9.24	0.0538
ssc-miR-183	CPEB1	cytoplasmic polyadenylation element binding protein 1 [Source:NCBI gene (formerly Entrezgene);Acc:100048944]	9.24	0.0538
ssc-miR-183	CPEB2	cytoplasmic polyadenylation element binding protein 2 [Source:VGNC Symbol;Acc:VGNC:86937]	9.24	0.0538
ssc-miR-183	CREBRF	CREB3 regulatory factor [Source:VGNC Symbol;Acc:VGNC:86987]	9.24	0.0538
ssc-miR-183	CRK	CRK proto-onco, adaptor protein [Source:NCBI gene (formerly Entrezgene);Acc:100192444]	9.24	0.0538
ssc-miR-183	CSMD1	CUB and Sushi multiple domains 1 [Source:VGNC Symbol;Acc:VGNC:95600]	9.24	0.0538
ssc-miR-183	CSNK1G1	casein kinase 1 gamma 1 [Source:VGNC Symbol;Acc:VGNC:97949]	9.24	0.0538
ssc-miR-183	CSNK1G3	casein kinase 1 gamma 3 [Source:VGNC Symbol;Acc:VGNC:97951]	9.24	0.0538
ssc-miR-183	CSNK2A1	hypothetical gene	9.24	0.0538
ssc-miR-183	CSTF1	cleavage stimulation factor subunit 1 [Source:VGNC Symbol;Acc:VGNC:96057]	9.24	0.0538
ssc-miR-183	CTDSP1	CTD small phosphatase 1 [Source:VGNC Symbol;Acc:VGNC:95971]	9.24	0.0538
ssc-miR-183	CTDSPL	CTD small phosphatase like [Source:VGNC Symbol;Acc:VGNC:107131]	9.24	0.0538
ssc-miR-183	CTNNA2	catenin alpha 2 [Source:HGNC Symbol;Acc:HGNC:2510]	9.24	0.0538
ssc-miR-183	CX3CL1	C-X3-C motif chemokine ligand 1 [Source:HGNC Symbol;Acc:HGNC:10647]	9.24	0.0538
ssc-miR-183	CYYR1	cysteine and tyrosine rich 1 [Source:VGNC Symbol;Acc:VGNC:87139]	9.24	0.0538
ssc-miR-183	DAAM1	dishevelled associated activator of morphosis 1 [Source:VGNC Symbol;Acc:VGNC:87141]	9.24	0.0538
ssc-miR-183	DAB1	DAB adaptor protein 1 [Source:VGNC Symbol;Acc:VGNC:87143]	9.24	0.0538
ssc-miR-183	DAGLA	diacylglycerol lipase alpha [Source:VGNC Symbol;Acc:VGNC:87152]	9.24	0.0538
ssc-miR-183	DAP	death associated protein [Source:VGNC Symbol;Acc:VGNC:106447]	9.24	0.0538
ssc-miR-183	DCX	doublecortin [Source:HGNC Symbol;Acc:HGNC:2714]	9.24	0.0538
ssc-miR-183	DDI2	hypothetical gene	9.24	0.0538
ssc-miR-183	DDIT4	DNA damage inducible transcript 4 [Source:VGNC Symbol;Acc:VGNC:87208]	9.24	0.0538
ssc-miR-183	DDX3X	DEAD-box helicase 3 X-linked [Source:NCBI gene (formerly Entrezgene);Acc:100515940]	9.24	0.0538
ssc-miR-183	DDX3Y	hypothetical gene	9.24	0.0538
ssc-miR-183	DGCR2	DiGeorge syndrome critical region gene 2 [Source:HGNC Symbol;Acc:HGNC:2845]	9.24	0.0538
ssc-miR-183	DGKH	diacylglycerol kinase eta [Source:VGNC Symbol;Acc:VGNC:87273]	9.24	0.0538
ssc-miR-183	DIP2A	disco interacting A [Source:VGNC Symbol;Acc:VGNC:95836]	9.24	0.0538
ssc-miR-183	DKFZP761J1410	hypothetical gene	9.24	0.0538
ssc-miR-183	DLGAP2	DLG associated protein 2 [Source:VGNC Symbol;Acc:VGNC:99711]	9.24	0.0538
ssc-miR-183	DMXL1	Dmx like 1 [Source:VGNC Symbol;Acc:VGNC:87359]	9.24	0.0538
ssc-miR-183	DNAJA1	hypothetical gene	9.24	0.0538
ssc-miR-183	DNAJB4	DnaJ heat shock protein family (Hsp40) member B4 [Source:VGNC Symbol;Acc:VGNC:96623]	9.24	0.0538
ssc-miR-183	DOCK4	dedicator of cytokinesis 4 [Source:VGNC Symbol;Acc:VGNC:87394]	9.24	0.0538
ssc-miR-183	DPP8	dipeptidyl peptidase 8 [Source:VGNC Symbol;Acc:VGNC:87423]	9.24	0.0538
ssc-miR-183	DST	dystonin [Source:HGNC Symbol;Acc:HGNC:1090]	9.24	0.0538
ssc-miR-183	DTNA	dystrobrevin alpha [Source:VGNC Symbol;Acc:VGNC:87462]	9.24	0.0538
ssc-miR-183	DUSP10	dual specificity phosphatase 10 [Source:VGNC Symbol;Acc:VGNC:96246]	9.24	0.0538
ssc-miR-183	DUSP13	hypothetical gene	9.24	0.0538
ssc-miR-183	EEA1	early endosome antigen 1 [Source:HGNC Symbol;Acc:HGNC:3185]	9.24	0.0538
ssc-miR-183	EEF2	eukaryotic translation elongation factor 2 [Source:VGNC Symbol;Acc:VGNC:87556]	9.24	0.0538
ssc-miR-183	EGR1	early growth response 1 [Source:VGNC Symbol;Acc:VGNC:87590]	9.24	0.0538
ssc-miR-183	EI24	EI24 autophagy associated transmembrane protein [Source:VGNC Symbol;Acc:VGNC:87602]	9.24	0.0538
ssc-miR-183	EIF4A2	eukaryotic translation initiation factor 4A2 [Source:VGNC Symbol;Acc:VGNC:87624]	9.24	0.0538
ssc-miR-183	EIF5B	eukaryotic translation initiation factor 5B [Source:VGNC Symbol;Acc:VGNC:87634]	9.24	0.0538
ssc-miR-183	EMC3	ER membrane protein complex subunit 3 [Source:VGNC Symbol;Acc:VGNC:87671]	9.24	0.0538
ssc-miR-183	EML4	EMAP like 4 [Source:VGNC Symbol;Acc:VGNC:87685]	9.24	0.0538

ssc-miR-183	ENAH	ENAH actin regulator [Source:VGNC Symbol;Acc:VGNC:108271]	9.24	0.0538
ssc-miR-183	ENY2	ENY2 transcription and export complex 2 subunit [Source:VGNC Symbol;Acc:VGNC:98792]	9.24	0.0538
ssc-miR-183	EPHA4	EPH receptor A4 [Source:VGNC Symbol;Acc:VGNC:96280]	9.24	0.0538
ssc-miR-183	EPHA7	EPH receptor A7 [Source:VGNC Symbol;Acc:VGNC:87734]	9.24	0.0538
ssc-miR-183	EPHX4	epoxide hydrolase 4 [Source:VGNC Symbol;Acc:VGNC:97981]	9.24	0.0538
ssc-miR-183	ERBB2IP	hypothetical gene	9.24	0.0538
ssc-miR-183	ERBB3	erb-b2 receptor tyrosine kinase 3 [Source:VGNC Symbol;Acc:VGNC:87760]	9.24	0.0538
ssc-miR-183	ERN1	endoplasmic reticulum to nucleus signaling 1 [Source:HGNC Symbol;Acc:HGNC:3449]	9.24	0.0538
ssc-miR-183	ERP44	endoplasmic reticulum protein 44 [Source:VGNC Symbol;Acc:VGNC:87783]	9.24	0.0538
ssc-miR-183	EVI5	ecotropic viral integration site 5 [Source:VGNC Symbol;Acc:VGNC:98793]	9.24	0.0538
ssc-miR-183	EXOC8	exocyst complex component 8 [Source:VGNC Symbol;Acc:VGNC:87834]	9.24	0.0538
ssc-miR-183	EZR	ezrin [Source:VGNC Symbol;Acc:VGNC:87856]	9.24	0.0538
ssc-miR-183	FAM168A	family with sequence similarity 168 member A [Source:VGNC Symbol;Acc:VGNC:87926]	9.24	0.0538
ssc-miR-183	FAM169A	family with sequence similarity 169 member A [Source:VGNC Symbol;Acc:VGNC:87927]	9.24	0.0538
ssc-miR-183	FAM175B	hypothetical gene	9.24	0.0538
ssc-miR-183	FAM217B	family with sequence similarity 217 member B [Source:VGNC Symbol;Acc:VGNC:95767]	9.24	0.0538
ssc-miR-183	FAM81A	family with sequence similarity 81 member A [Source:VGNC Symbol;Acc:VGNC:87986]	9.24	0.0538
ssc-miR-183	FAT1	FAT atypical cadherin 1 [Source:NCBI gene (formerly Entrezgene);Acc:100154043]	9.24	0.0538
ssc-miR-183	FBN2	fibrillin 2 [Source:VGNC Symbol;Acc:VGNC:88025]	9.24	0.0538
ssc-miR-183	FCHO2	FCH and mu domain containing endocytic adaptor 2 [Source:VGNC Symbol;Acc:VGNC:88066]	9.24	0.0538
ssc-miR-183	FGF9	fibroblast growth factor 9 [Source:VGNC Symbol;Acc:VGNC:103943]	9.24	0.0538
ssc-miR-183	FHDC1	FH2 domain containing 1 [Source:VGNC Symbol;Acc:VGNC:88126]	9.24	0.0538
ssc-miR-183	FLNB	filamin B [Source:VGNC Symbol;Acc:VGNC:88156]	9.24	0.0538
ssc-miR-183	FLRT3	fibronectin leucine rich transmembrane protein 3 [Source:VGNC Symbol;Acc:VGNC:96304]	9.24	0.0538
ssc-miR-183	FN1	fibronectin 1 [Source:VGNC Symbol;Acc:VGNC:96305]	9.24	0.0538
ssc-miR-183	FNDC3B	fibronectin type III domain containing 3B [Source:VGNC Symbol;Acc:VGNC:88183]	9.24	0.0538
ssc-miR-183	FOXN2	forkhead box N2 [Source:VGNC Symbol;Acc:VGNC:88219]	9.24	0.0538
ssc-miR-183	FOXO1	forkhead box O1 [Source:VGNC Symbol;Acc:VGNC:98013]	9.24	0.0538
ssc-miR-183	FOXP1	forkhead box P1 [Source:VGNC Symbol;Acc:VGNC:88222]	9.24	0.0538
ssc-miR-183	FRMD5	FERM domain containing 5 [Source:VGNC Symbol;Acc:VGNC:88238]	9.24	0.0538
ssc-miR-183	FRMD6	FERM domain containing 6 [Source:VGNC Symbol;Acc:VGNC:88239]	9.24	0.0538
ssc-miR-183	FRS2	fibroblast growth factor receptor substrate 2 [Source:VGNC Symbol;Acc:VGNC:88246]	9.24	0.0538
ssc-miR-183	FSBP	hypothetical gene	9.24	0.0538
ssc-miR-183	FUNDC2	FUN14 domain containing 2 [Source:VGNC Symbol;Acc:VGNC:103944]	9.24	0.0538
ssc-miR-183	G3BP1	G3BP stress granule assembly factor 1 [Source:VGNC Symbol;Acc:VGNC:88288]	9.24	0.0538
ssc-miR-183	GABPB2	GA binding protein transcription factor subunit beta 2 [Source:VGNC Symbol;Acc:VGNC:98796]	9.24	0.0538
ssc-miR-183	GALNTL6	polypeptide N-acetylgalactosaminyltransferase like 6 [Source:VGNC Symbol;Acc:VGNC:103947]	9.24	0.0538
ssc-miR-183	GAN	gigaxonin [Source:VGNC Symbol;Acc:VGNC:88343]	9.24	0.0538
ssc-miR-183	GATAD2B	GATA zinc finger domain containing 2B [Source:VGNC Symbol;Acc:VGNC:88369]	9.24	0.0538
ssc-miR-183	GCLM	glutamate-cysteine ligase modifier subunit [Source:VGNC Symbol;Acc:VGNC:88387]	9.24	0.0538
ssc-miR-183	GDI2	GDP dissociation inhibitor 2 [Source:VGNC Symbol;Acc:VGNC:98019]	9.24	0.0538
ssc-miR-183	GJD2	gap junction protein delta 2 [Source:VGNC Symbol;Acc:VGNC:88471]	9.24	0.0538
ssc-miR-183	GLRA3	glycine receptor alpha 3 [Source:VGNC Symbol;Acc:VGNC:88493]	9.24	0.0538
ssc-miR-183	GMFB	glia maturation factor beta [Source:VGNC Symbol;Acc:VGNC:88509]	9.24	0.0538
ssc-miR-183	GNAQ	G protein subunit alpha q [Source:VGNC Symbol;Acc:VGNC:103100]	9.24	0.0538
ssc-miR-183	GNB1	G protein subunit beta 1 [Source:VGNC Symbol;Acc:VGNC:88529]	9.24	0.0538
ssc-miR-183	GNG12	G protein subunit gamma 12 [Source:NCBI gene (formerly Entrezgene);Acc:100270820]	9.24	0.0538
ssc-miR-183	GNG5	hypothetical gene	9.24	0.0538
ssc-miR-183	GOLGA7	golgin A7 [Source:VGNC Symbol;Acc:VGNC:96059]	9.24	0.0538

ssc-miR-183	GOLPH3	hypothetical gene	9.24	0.0538
ssc-miR-183	GPAM	glycerol-3-phosphate acyltransferase, mitochondrial [Source:VGNC Symbol;Acc:VGNC:88570]	9.24	0.0538
ssc-miR-183	GPCPD1	glycerophosphocholine phosphodiesterase 1 [Source:VGNC Symbol;Acc:VGNC:96148]	9.24	0.0538
ssc-miR-183	GPR3	G protein-coupled receptor 3 [Source:VGNC Symbol;Acc:VGNC:88628]	9.24	0.0538
ssc-miR-183	GPR85	G protein-coupled receptor 85 [Source:VGNC Symbol;Acc:VGNC:88638]	9.24	0.0538
ssc-miR-183	GRAMD1B	GRAM domain containing 1B [Source:VGNC Symbol;Acc:VGNC:88656]	9.24	0.0538
ssc-miR-183	GREM2	gremlin 2, DAN family BMP antagonist [Source:VGNC Symbol;Acc:VGNC:95854]	9.24	0.0538
ssc-miR-183	GSE1	Gse1 coiled-coil protein [Source:VGNC Symbol;Acc:VGNC:88720]	9.24	0.0538
ssc-miR-183	GTDC1	glycosyltransferase like domain containing 1 [Source:VGNC Symbol;Acc:VGNC:95955]	9.24	0.0538
ssc-miR-183	GTF2H1	ral transcription factor IIH subunit 1 [Source:VGNC Symbol;Acc:VGNC:88734]	9.24	0.0538
ssc-miR-183	H3F3B	hypothetical gene	9.24	0.0538
ssc-miR-183	HBEGF	heparin binding EGF like growth factor [Source:VGNC Symbol;Acc:VGNC:88792]	9.24	0.0538
ssc-miR-183	HCN1	hyperpolarization activated cyclic nucleotide gated potassium channel 1 [Source:VGNC Symbol;Acc:VGNC:88802]	9.24	0.0538
ssc-miR-183	HEATR1	HEAT repeat containing 1 [Source:VGNC Symbol;Acc:VGNC:88825]	9.24	0.0538
ssc-miR-183	HECTD2	HECT domain E3 ubiquitin protein ligase 2 [Source:VGNC Symbol;Acc:VGNC:88833]	9.24	0.0538
ssc-miR-183	HECW2	HECT, C2 and WW domain containing E3 ubiquitin protein ligase 2 [Source:NCBI gene (formerly Entrezgene);Acc:100155879]	9.24	0.0538
ssc-miR-183	HLF	HLF transcription factor, PAR bZIP family member [Source:VGNC Symbol;Acc:VGNC:88896]	9.24	0.0538
ssc-miR-183	HLTF	helicase like transcription factor [Source:VGNC Symbol;Acc:VGNC:88897]	9.24	0.0538
ssc-miR-183	HN1	hypothetical gene	9.24	0.0538
ssc-miR-183	HOXA9	homeobox A9 [Source:HGNC Symbol;Acc:HGNC:5109]	9.24	0.0538
ssc-miR-183	HOXD8	homeobox D8 [Source:VGNC Symbol;Acc:VGNC:96356]	9.24	0.0538
ssc-miR-183	HS3ST1	heparan sulfate-glucosamine 3-sulfotransferase 1 [Source:VGNC Symbol;Acc:VGNC:88977]	9.24	0.0538
ssc-miR-183	HSPA9	heat shock protein family A (Hsp70) member 9 [Source:VGNC Symbol;Acc:VGNC:99761]	9.24	0.0538
ssc-miR-183	HSPH1	heat shock protein family H (Hsp110) member 1 [Source:NCBI gene (formerly Entrezgene);Acc:100048931]	9.24	0.0538
ssc-miR-183	HTR2A	5-hydroxytryptamine receptor 2A [Source:VGNC Symbol;Acc:VGNC:103964]	9.24	0.0538
ssc-miR-183	ICA1	islet cell autoantigen 1 [Source:VGNC Symbol;Acc:VGNC:89018]	9.24	0.0538
ssc-miR-183	ICK	hypothetical gene	9.24	0.0538
ssc-miR-183	IDH2	isocitrate dehydrogenase (NADP(+)) 2 [Source:NCBI gene (formerly Entrezgene);Acc:397603]	9.24	0.0538
ssc-miR-183	IGFBP5	insulin like growth factor binding protein 5 [Source:VGNC Symbol;Acc:VGNC:96368]	9.24	0.0538
ssc-miR-183	IMPG1	interphotoreceptor matrix proteoglycan 1 [Source:VGNC Symbol;Acc:VGNC:89127]	9.24	0.0538
ssc-miR-183	INSIG1	insulin induced 1 [Source:VGNC Symbol;Acc:VGNC:89150]	9.24	0.0538
ssc-miR-183	INSR	insulin receptor [Source:VGNC Symbol;Acc:VGNC:89153]	9.24	0.0538
ssc-miR-183	IPPK	inositol-pentakisphosphate 2-kinase [Source:VGNC Symbol;Acc:VGNC:89183]	9.24	0.0538
ssc-miR-183	IRS1	insulin receptor substrate 1 [Source:VGNC Symbol;Acc:VGNC:96376]	9.24	0.0538
ssc-miR-183	ISY1-RAB43	hypothetical gene	9.24	0.0538
ssc-miR-183	ITGB1	integrin subunit beta 1 [Source:VGNC Symbol;Acc:VGNC:96381]	9.24	0.0538
ssc-miR-183	ITGB8	integrin subunit beta 8 [Source:VGNC Symbol;Acc:VGNC:89246]	9.24	0.0538
ssc-miR-183	KANK1	KN motif and ankyrin repeat domains 1 [Source:VGNC Symbol;Acc:VGNC:103112]	9.24	0.0538
ssc-miR-183	KAZALD1	Kazal type serine peptidase inhibitor domain 1 [Source:VGNC Symbol;Acc:VGNC:89314]	9.24	0.0538
ssc-miR-183	KCNJ12	hypothetical gene	9.24	0.0538
ssc-miR-183	KCNJ14	potassium inwardly rectifying channel subfamily J member 14 [Source:VGNC Symbol;Acc:VGNC:89354]	9.24	0.0538
ssc-miR-183	KCNK10	potassium two pore domain channel subfamily K member 10 [Source:VGNC Symbol;Acc:VGNC:89364]	9.24	0.0538
ssc-miR-183	KCNK2	potassium two pore domain channel subfamily K member 2 [Source:VGNC Symbol;Acc:VGNC:89369]	9.24	0.0538
ssc-miR-183	KCTD15	potassium channel tetramerization domain containing 15 [Source:VGNC Symbol;Acc:VGNC:89394]	9.24	0.0538
ssc-miR-183	KCTD2	potassium channel tetramerization domain containing 2 [Source:VGNC Symbol;Acc:VGNC:89398]	9.24	0.0538
ssc-miR-183	KDM2B	lysine demethylase 2B [Source:VGNC Symbol;Acc:VGNC:98057]	9.24	0.0538
ssc-miR-183	KHDRBS2	KH RNA binding domain containing, signal transduction associated 2 [Source:VGNC Symbol;Acc:VGNC:89423]	9.24	0.0538
ssc-miR-183	KIAA0101	hypothetical gene	9.24	0.0538
ssc-miR-183	KIAA0355	hypothetical gene	9.24	0.0538

ssc-miR-183	KIAA0368	hypothetical gene	9.24	0.0538
ssc-miR-183	KIAA1324L	hypothetical gene	9.24	0.0538
ssc-miR-183	KIAA2018	hypothetical gene	9.24	0.0538
ssc-miR-183	KIF13A	kinesin family member 13A [Source:VGNC Symbol;Acc:VGNC:89454]	9.24	0.0538
ssc-miR-183	KIF26B	kinesin family member 26B [Source:VGNC Symbol;Acc:VGNC:96087]	9.24	0.0538
ssc-miR-183	KIF2A	kinesin family member 2A [Source:VGNC Symbol;Acc:VGNC:89467]	9.24	0.0538
ssc-miR-183	KLF7	Kruppel like factor 7 [Source:VGNC Symbol;Acc:VGNC:96396]	9.24	0.0538
ssc-miR-183	KLHL23	kelch like family member 23 [Source:VGNC Symbol;Acc:VGNC:96158]	9.24	0.0538
ssc-miR-183	KLHL24	kelch like family member 24 [Source:VGNC Symbol;Acc:VGNC:89521]	9.24	0.0538
ssc-miR-183	KLHL28	kelch like family member 28 [Source:VGNC Symbol;Acc:VGNC:89523]	9.24	0.0538
ssc-miR-183	KLHL3	kelch like family member 3 [Source:VGNC Symbol;Acc:VGNC:89525]	9.24	0.0538
ssc-miR-183	KPNA4	karyopherin subunit alpha 4 [Source:VGNC Symbol;Acc:VGNC:89563]	9.24	0.0538
ssc-miR-183	L3MBTL3	L3MBTL histone methyl-lysine binding protein 3 [Source:VGNC Symbol;Acc:VGNC:98066]	9.24	0.0538
ssc-miR-183	LCMT2	hypothetical gene	9.24	0.0538
ssc-miR-183	LHFPL2	LHFPL tetraspan subfamily member 2 [Source:VGNC Symbol;Acc:VGNC:89705]	9.24	0.0538
ssc-miR-183	LIN52	lin-52 DREAM MuvB core complex component [Source:NCBI gene (formerly Entrezgene);Acc:100157325]	9.24	0.0538
ssc-miR-183	LIN54	lin-54 DREAM MuvB core complex component [Source:VGNC Symbol;Acc:VGNC:98072]	9.24	0.0538
ssc-miR-183	LMO3	hypothetical gene	9.24	0.0538
ssc-miR-183	LNPEP	leucyl and cystinyl aminopeptidase [Source:VGNC Symbol;Acc:VGNC:89772]	9.24	0.0538
ssc-miR-183	LPHN1	hypothetical gene	9.24	0.0538
ssc-miR-183	LPHN2	hypothetical gene	9.24	0.0538
ssc-miR-183	LRP1B	LDL receptor related protein 1B [Source:HGNC Symbol;Acc:HGNC:6693]	9.24	0.0538
ssc-miR-183	LRP6	LDL receptor related protein 6 [Source:VGNC Symbol;Acc:VGNC:89821]	9.24	0.0538
ssc-miR-183	LRRC1	leucine rich repeat containing 1 [Source:VGNC Symbol;Acc:VGNC:103977]	9.24	0.0538
ssc-miR-183	LRRC4C	leucine rich repeat containing 4C [Source:VGNC Symbol;Acc:VGNC:89848]	9.24	0.0538
ssc-miR-183	LSAMP	limbic system associated membrane protein [Source:VGNC Symbol;Acc:VGNC:89868]	9.24	0.0538
ssc-miR-183	LSM11	LSM11, U7 small nuclear RNA associated [Source:VGNC Symbol;Acc:VGNC:89871]	9.24	0.0538
ssc-miR-183	LYPLA2	lysophospholipase 2 [Source:VGNC Symbol;Acc:VGNC:89914]	9.24	0.0538
ssc-miR-183	MACROD2	mono-ADP ribosylhydrolase 2 [Source:VGNC Symbol;Acc:VGNC:108725]	9.24	0.0538
ssc-miR-183	MAL2	mal, T cell differentiation protein 2 [Source:VGNC Symbol;Acc:VGNC:98818]	9.24	0.0538
ssc-miR-183	MAP3K2	mitogen-activated protein kinase kinase kinase 2 [Source:VGNC Symbol;Acc:VGNC:98107]	9.24	0.0538
ssc-miR-183	MAP3K4	mitogen-activated protein kinase kinase kinase 4 [Source:VGNC Symbol;Acc:VGNC:98109]	9.24	0.0538
ssc-miR-183	MAP4K5	mitogen-activated protein kinase kinase kinase kinase 5 [Source:VGNC Symbol;Acc:VGNC:98115]	9.24	0.0538
ssc-miR-183	MAP7D2	MAP7 domain containing 2 [Source:VGNC Symbol;Acc:VGNC:89994]	9.24	0.0538
ssc-miR-183	MAPK1IP1L	mitogen-activated protein kinase 1 interacting protein 1 like [Source:VGNC Symbol;Acc:VGNC:90002]	9.24	0.0538
ssc-miR-183	MAPK4	hypothetical gene	9.24	0.0538
ssc-miR-183	MAPK8IP1	mitogen-activated protein kinase 8 interacting protein 1 [Source:VGNC Symbol;Acc:VGNC:90007]	9.24	0.0538
ssc-miR-183	MARK2	microtubule affinity regulating kinase 2 [Source:VGNC Symbol;Acc:VGNC:98118]	9.24	0.0538
ssc-miR-183	MBNL1	muscleblind like splicing regulator 1 [Source:VGNC Symbol;Acc:VGNC:90054]	9.24	0.0538
ssc-miR-183	MBNL3	muscleblind like splicing regulator 3 [Source:VGNC Symbol;Acc:VGNC:90056]	9.24	0.0538
ssc-miR-183	MDM4	MDM4 regulator of p53 [Source:VGNC Symbol;Acc:VGNC:90094]	9.24	0.0538
ssc-miR-183	MECOM	MDS1 and EVI1 complex locus [Source:VGNC Symbol;Acc:VGNC:90100]	9.24	0.0538
ssc-miR-183	MED1	mediator complex subunit 1 [Source:VGNC Symbol;Acc:VGNC:90102]	9.24	0.0538
ssc-miR-183	MED12L	mediator complex subunit 12L [Source:VGNC Symbol;Acc:VGNC:90105]	9.24	0.0538
ssc-miR-183	MED13	mediator complex subunit 13 [Source:VGNC Symbol;Acc:VGNC:90106]	9.24	0.0538
ssc-miR-183	MEF2C	myocyte enhancer factor 2C [Source:VGNC Symbol;Acc:VGNC:90127]	9.24	0.0538
ssc-miR-183	MFAP3	microfibril associated protein 3 [Source:VGNC Symbol;Acc:VGNC:90174]	9.24	0.0538
ssc-miR-183	MFF	mitochondrial fission factor [Source:VGNC Symbol;Acc:VGNC:103985]	9.24	0.0538
ssc-miR-183	MIB1	MIB E3 ubiquitin protein ligase 1 [Source:VGNC Symbol;Acc:VGNC:90205]	9.24	0.0538

ssc-miR-183	MKL2	hypothetical gene	9.24	0.0538
ssc-miR-183	MORC3	MORC family CW-type zinc finger 3 [Source:VGNC Symbol;Acc:VGNC:90304]	9.24	0.0538
ssc-miR-183	MRPL22	mitochondrial ribosomal protein L22 [Source:VGNC Symbol;Acc:VGNC:103993]	9.24	0.0538
ssc-miR-183	MRVI1	hypothetical gene	9.24	0.0538
ssc-miR-183	MSANTD3	Myb/SANT DNA binding domain containing 3 [Source:HGNC Symbol;Acc:HGNC:23370]	9.24	0.0538
ssc-miR-183	MSI2	musashi RNA binding protein 2 [Source:VGNC Symbol;Acc:VGNC:90422]	9.24	0.0538
ssc-miR-183	MTA1	hypothetical gene	9.24	0.0538
ssc-miR-183	MTDH	metadherin [Source:VGNC Symbol;Acc:VGNC:90439]	9.24	0.0538
ssc-miR-183	MTMR6	myotubularin related protein 6 [Source:HGNC Symbol;Acc:HGNC:7453]	9.24	0.0538
ssc-miR-183	MUC15	mucin 15, cell surface associated [Source:VGNC Symbol;Acc:VGNC:90481]	9.24	0.0538
ssc-miR-183	MXD4	MAX dimerization protein 4 [Source:VGNC Symbol;Acc:VGNC:90493]	9.24	0.0538
ssc-miR-183	MYO5B	myosin VB [Source:VGNC Symbol;Acc:VGNC:103131]	9.24	0.0538
ssc-miR-183	MYT1L	myelin transcription factor 1 like [Source:VGNC Symbol;Acc:VGNC:90549]	9.24	0.0538
ssc-miR-183	NAA30	N-alpha-acetyltransferase 30, NatC catalytic subunit [Source:VGNC Symbol;Acc:VGNC:96751]	9.24	0.0538
ssc-miR-183	NABP1	nucleic acid binding protein 1 [Source:VGNC Symbol;Acc:VGNC:96122]	9.24	0.0538
ssc-miR-183	NADK2	NAD kinase 2, mitochondrial [Source:VGNC Symbol;Acc:VGNC:90566]	9.24	0.0538
ssc-miR-183	NAPEPLD	N-acyl phosphatidylethanolamine phospholipase D [Source:VGNC Symbol;Acc:VGNC:90579]	9.24	0.0538
ssc-miR-183	NCK2	NCK adaptor protein 2 [Source:NCBI gene (formerly Entrezgene);Acc:100192439]	9.24	0.0538
ssc-miR-183	NCKAP1	NCK associated protein 1 [Source:VGNC Symbol;Acc:VGNC:96432]	9.24	0.0538
ssc-miR-183	NCS1	neuronal calcium sensor 1 [Source:VGNC Symbol;Acc:VGNC:90620]	9.24	0.0538
ssc-miR-183	NFE2L1	NFE2 like bZIP transcription factor 1 [Source:VGNC Symbol;Acc:VGNC:90713]	9.24	0.0538
ssc-miR-183	NLGN4X	hypothetical gene	9.24	0.0538
ssc-miR-183	NPAS3	neuronal PAS domain protein 3 [Source:VGNC Symbol;Acc:VGNC:90838]	9.24	0.0538
ssc-miR-183	NR3C1	nuclear receptor subfamily 3 group C member 1 [Source:VGNC Symbol;Acc:VGNC:90883]	9.24	0.0538
ssc-miR-183	NR4A2	nuclear receptor subfamily 4 group A member 2 [Source:VGNC Symbol;Acc:VGNC:96451]	9.24	0.0538
ssc-miR-183	NR4A3	nuclear receptor subfamily 4 group A member 3 [Source:VGNC Symbol;Acc:VGNC:90885]	9.24	0.0538
ssc-miR-183	NRAS	NRAS proto-onco, GTPase [Source:VGNC Symbol;Acc:VGNC:98827]	9.24	0.0538
ssc-miR-183	NRBF2	nuclear receptor binding factor 2 [Source:VGNC Symbol;Acc:VGNC:90890]	9.24	0.0538
ssc-miR-183	NRG1	neuregulin 1 [Source:VGNC Symbol;Acc:VGNC:96453]	9.24	0.0538
ssc-miR-183	NRP1	neuropilin 1 [Source:VGNC Symbol;Acc:VGNC:104012]	9.24	0.0538
ssc-miR-183	NRP2	neuropilin 2 [Source:VGNC Symbol;Acc:VGNC:96454]	9.24	0.0538
ssc-miR-183	NRXN3	neurexin 3 [Source:HGNC Symbol;Acc:HGNC:8010]	9.24	0.0538
ssc-miR-183	NTN4	netrin 4 [Source:VGNC Symbol;Acc:VGNC:90933]	9.24	0.0538
ssc-miR-183	NTNG1	netrin G1 [Source:VGNC Symbol;Acc:VGNC:90935]	9.24	0.0538
ssc-miR-183	NUDT4	hypothetical gene	9.24	0.0538
ssc-miR-183	NUFIP2	nuclear FMR1 interacting protein 2 [Source:VGNC Symbol;Acc:VGNC:90967]	9.24	0.0538
ssc-miR-183	NXF1	nuclear RNA export factor 1 [Source:VGNC Symbol;Acc:VGNC:90993]	9.24	0.0538
ssc-miR-183	OCLN	occludin [Source:VGNC Symbol;Acc:VGNC:91013]	9.24	0.0538
ssc-miR-183	OSBPL8	oxysterol binding protein like 8 [Source:VGNC Symbol;Acc:VGNC:91074]	9.24	0.0538
ssc-miR-183	OTOP3	otopetrin 3 [Source:VGNC Symbol;Acc:VGNC:91095]	9.24	0.0538
ssc-miR-183	P2RY1	purinergic receptor P2Y1 [Source:HGNC Symbol;Acc:HGNC:8539]	9.24	0.0538
ssc-miR-183	PAIP2	poly(A) binding protein interacting protein 2 [Source:VGNC Symbol;Acc:VGNC:96618]	9.24	0.0538
ssc-miR-183	PALM2	hypothetical gene	9.24	0.0538
ssc-miR-183	PAM	peptidylglycine alpha-amidating monooxygenase [Source:VGNC Symbol;Acc:VGNC:91163]	9.24	0.0538
ssc-miR-183	PANK3	pantothenate kinase 3 [Source:VGNC Symbol;Acc:VGNC:91165]	9.24	0.0538
ssc-miR-183	PAX5	paired box 5 [Source:VGNC Symbol;Acc:VGNC:91194]	9.24	0.0538
ssc-miR-183	PCDHGA1	hypothetical gene	9.24	0.0538
ssc-miR-183	PCDHGA10	hypothetical gene	9.24	0.0538
ssc-miR-183	PCDHGA11	hypothetical gene	9.24	0.0538

ssc-miR-183	PCDHGA12	hypothetical gene	9.24	0.0538
ssc-miR-183	PCDHGA2	hypothetical gene	9.24	0.0538
ssc-miR-183	PCDHGA3	hypothetical gene	9.24	0.0538
ssc-miR-183	PCDHGA4	protocadherin gamma subfamily A, 4 [Source:HGNC Symbol;Acc:HGNC:8702]	9.24	0.0538
ssc-miR-183	PCDHGA5	hypothetical gene	9.24	0.0538
ssc-miR-183	PCDHGA6	protocadherin gamma subfamily A, 6 [Source:HGNC Symbol;Acc:HGNC:8704]	9.24	0.0538
ssc-miR-183	PCDHGA7	hypothetical gene	9.24	0.0538
ssc-miR-183	PCDHGA8	hypothetical gene	9.24	0.0538
ssc-miR-183	PCDHGA9	hypothetical gene	9.24	0.0538
ssc-miR-183	PCDHGB1	protocadherin gamma subfamily B, 1 [Source:HGNC Symbol;Acc:HGNC:8708]	9.24	0.0538
ssc-miR-183	PCDHGB2	hypothetical gene	9.24	0.0538
ssc-miR-183	PCDHGB3	hypothetical gene	9.24	0.0538
ssc-miR-183	PCDHGB4	hypothetical gene	9.24	0.0538
ssc-miR-183	PCDHGB6	hypothetical gene	9.24	0.0538
ssc-miR-183	PCDHGB7	hypothetical gene	9.24	0.0538
ssc-miR-183	PCDHGC3	hypothetical gene	9.24	0.0538
ssc-miR-183	PCDHGC4	hypothetical gene	9.24	0.0538
ssc-miR-183	PCDHGC5	hypothetical gene	9.24	0.0538
ssc-miR-183	PCGF5	hypothetical gene	9.24	0.0538
ssc-miR-183	PCYOX1	prenylcysteine oxidase 1 [Source:VGNC Symbol;Acc:VGNC:91235]	9.24	0.0538
ssc-miR-183	PDCD4	programmed cell death 4 [Source:VGNC Symbol;Acc:VGNC:91244]	9.24	0.0538
ssc-miR-183	PDCD6	programmed cell death 6 [Source:VGNC Symbol;Acc:VGNC:98178]	9.24	0.0538
ssc-miR-183	PDE3A	phosphodiesterase 3A [Source:VGNC Symbol;Acc:VGNC:91252]	9.24	0.0538
ssc-miR-183	PDE4D	phosphodiesterase 4D [Source:VGNC Symbol;Acc:VGNC:91256]	9.24	0.0538
ssc-miR-183	PDE7A	phosphodiesterase 7A [Source:VGNC Symbol;Acc:VGNC:91261]	9.24	0.0538
ssc-miR-183	PDGFD	platelet derived growth factor D [Source:VGNC Symbol;Acc:VGNC:91267]	9.24	0.0538
ssc-miR-183	PEX19	peroxisomal biosis factor 19 [Source:VGNC Symbol;Acc:VGNC:104022]	9.24	0.0538
ssc-miR-183	PEX6	peroxisomal biosis factor 6 [Source:VGNC Symbol;Acc:VGNC:91330]	9.24	0.0538
ssc-miR-183	PFN2	hypothetical gene	9.24	0.0538
ssc-miR-183	PGRMC2	progesterone receptor membrane component 2 [Source:VGNC Symbol;Acc:VGNC:98952]	9.24	0.0538
ssc-miR-183	PHB2	prohibitin 2 [Source:VGNC Symbol;Acc:VGNC:91369]	9.24	0.0538
ssc-miR-183	PHF15	hypothetical gene	9.24	0.0538
ssc-miR-183	PHF3	PHD finger protein 3 [Source:VGNC Symbol;Acc:VGNC:91387]	9.24	0.0538
ssc-miR-183	PHF6	PHD finger protein 6 [Source:VGNC Symbol;Acc:VGNC:91389]	9.24	0.0538
ssc-miR-183	PIGA	phosphatidylinositol glycan anchor biosynthesis class A [Source:VGNC Symbol;Acc:VGNC:91419]	9.24	0.0538
ssc-miR-183	PIGX	phosphatidylinositol glycan anchor biosynthesis class X [Source:VGNC Symbol;Acc:VGNC:91432]	9.24	0.0538
ssc-miR-183	PIP4K2B	phosphatidylinositol-5-phosphate 4-kinase type 2 beta [Source:VGNC Symbol;Acc:VGNC:91454]	9.24	0.0538
ssc-miR-183	PKD2	polycystin 2, transient receptor potential cation channel [Source:VGNC Symbol;Acc:VGNC:91472]	9.24	0.0538
ssc-miR-183	PKNOX2	PBX/knotted 1 homeobox 2 [Source:VGNC Symbol;Acc:VGNC:91485]	9.24	0.0538
ssc-miR-183	PKP4	plakophilin 4 [Source:VGNC Symbol;Acc:VGNC:96488]	9.24	0.0538
ssc-miR-183	PLAG1	PLAG1 zinc finger [Source:VGNC Symbol;Acc:VGNC:91509]	9.24	0.0538
ssc-miR-183	PLAGL2	PLAG1 like zinc finger 2 [Source:VGNC Symbol;Acc:VGNC:96490]	9.24	0.0538
ssc-miR-183	PLCB4	phospholipase C beta 4 [Source:VGNC Symbol;Acc:VGNC:96491]	9.24	0.0538
ssc-miR-183	PLCXD3	phosphatidylinositol specific phospholipase C X domain containing 3 [Source:VGNC Symbol;Acc:VGNC:91524]	9.24	0.0538
ssc-miR-183	PLEKHA3	pleckstrin homology domain containing A3 [Source:NCBI gene (formerly Entrezgene);Acc:100154010]	9.24	0.0538
ssc-miR-183	PLEKHA5	pleckstrin homology domain containing A5 [Source:VGNC Symbol;Acc:VGNC:91535]	9.24	0.0538
ssc-miR-183	PLXNA4	plexin A4 [Source:VGNC Symbol;Acc:VGNC:98204]	9.24	0.0538
ssc-miR-183	PNPT1	polyribonucleotide nucleotidyltransferase 1 [Source:VGNC Symbol;Acc:VGNC:91612]	9.24	0.0538
ssc-miR-183	PODXL	hypothetical gene	9.24	0.0538

ssc-miR-183	PPM1E	protein phosphatase, Mg2+/Mn2+ dependent 1E [Source:VGNC Symbol;Acc:VGNC:91705]	9.24	0.0538
ssc-miR-183	PPM1L	protein phosphatase, Mg2+/Mn2+ dependent 1L [Source:VGNC Symbol;Acc:VGNC:91711]	9.24	0.0538
ssc-miR-183	PPP1R3A	protein phosphatase 1 regulatory subunit 3A [Source:VGNC Symbol;Acc:VGNC:91736]	9.24	0.0538
ssc-miR-183	PPP1R9A	protein phosphatase 1 regulatory subunit 9A [Source:VGNC Symbol;Acc:VGNC:91743]	9.24	0.0538
ssc-miR-183	PPP2CA	protein phosphatase 2 catalytic subunit alpha [Source:VGNC Symbol;Acc:VGNC:91745]	9.24	0.0538
ssc-miR-183	PPP2CB	protein phosphatase 2 catalytic subunit beta [Source:VGNC Symbol;Acc:VGNC:96501]	9.24	0.0538
ssc-miR-183	PPP2R2A	protein phosphatase 2 regulatory subunit Balpha [Source:VGNC Symbol;Acc:VGNC:91748]	9.24	0.0538
ssc-miR-183	PPP2R5C	protein phosphatase 2 regulatory subunit B'gamma [Source:VGNC Symbol;Acc:VGNC:91753]	9.24	0.0538
ssc-miR-183	PPP6C	protein phosphatase 6 catalytic subunit [Source:VGNC Symbol;Acc:VGNC:98219]	9.24	0.0538
ssc-miR-183	PRDM5	PR/SET domain 5 [Source:VGNC Symbol;Acc:VGNC:98953]	9.24	0.0538
ssc-miR-183	PRICKLE1	prickle planar cell polarity protein 1 [Source:VGNC Symbol;Acc:VGNC:91793]	9.24	0.0538
ssc-miR-183	PRKACB	protein kinase cAMP-activated catalytic subunit beta [Source:VGNC Symbol;Acc:VGNC:91800]	9.24	0.0538
ssc-miR-183	PRKAR2A	protein kinase cAMP-dependent type II regulatory subunit alpha [Source:VGNC Symbol;Acc:VGNC:91804]	9.24	0.0538
ssc-miR-183	PRKCA	protein kinase C alpha [Source:VGNC Symbol;Acc:VGNC:99028]	9.24	0.0538
ssc-miR-183	PRKCI	protein kinase C iota [Source:VGNC Symbol;Acc:VGNC:91809]	9.24	0.0538
ssc-miR-183	PRR13	proline rich 13 [Source:NCBI gene (formerly Entrezgene);Acc:100517160]	9.24	0.0538
ssc-miR-183	PRRC1	proline rich coiled-coil 1 [Source:VGNC Symbol;Acc:VGNC:91864]	9.24	0.0538
ssc-miR-183	PRUNE2	hypothetical gene	9.24	0.0538
ssc-miR-183	PSD3	pleckstrin and Sec7 domain containing 3 [Source:VGNC Symbol;Acc:VGNC:107166]	9.24	0.0538
ssc-miR-183	PSEN2	presenilin 2 [Source:VGNC Symbol;Acc:VGNC:96508]	9.24	0.0538
ssc-miR-183	PTCH1	patched 1 [Source:VGNC Symbol;Acc:VGNC:96513]	9.24	0.0538
ssc-miR-183	PTDSS1	phosphatidylserine synthase 1 [Source:VGNC Symbol;Acc:VGNC:91945]	9.24	0.0538
ssc-miR-183	PTPDC1	protein tyrosine phosphatase domain containing 1 [Source:VGNC Symbol;Acc:VGNC:91970]	9.24	0.0538
ssc-miR-183	PTPN4	protein tyrosine phosphatase non-receptor type 4 [Source:VGNC Symbol;Acc:VGNC:96521]	9.24	0.0538
ssc-miR-183	PTS	6-pyruvoyltetrahydropterin synthase [Source:VGNC Symbol;Acc:VGNC:104039]	9.24	0.0538
ssc-miR-183	QKI	QKI, KH domain containing RNA binding [Source:VGNC Symbol;Acc:VGNC:92025]	9.24	0.0538
ssc-miR-183	RAB11FIP4	RAB11 family interacting protein 4 [Source:VGNC Symbol;Acc:VGNC:92041]	9.24	0.0538
ssc-miR-183	RAB15	RAB15, member RAS onco family [Source:VGNC Symbol;Acc:VGNC:98239]	9.24	0.0538
ssc-miR-183	RAB21	RAB21, member RAS oncogene family [Source:HGNC Symbol;Acc:HGNC:18263]	9.24	0.0538
ssc-miR-183	RAB30	RAB30, member RAS onco family [Source:VGNC Symbol;Acc:VGNC:98254]	9.24	0.0538
ssc-miR-183	RAB43	RAB43, member RAS onco family [Source:VGNC Symbol;Acc:VGNC:98268]	9.24	0.0538
ssc-miR-183	RAB8B	RAB8B, member RAS onco family [Source:VGNC Symbol;Acc:VGNC:98276]	9.24	0.0538
ssc-miR-183	RABGAP1L	RAB GTPase activating protein 1 like [Source:VGNC Symbol;Acc:VGNC:108611]	9.24	0.0538
ssc-miR-183	RALA	RAS like proto-oncogene A [Source:HGNC Symbol;Acc:HGNC:9839]	9.24	0.0538
ssc-miR-183	RAP2C	RAP2C, member of RAS onco family [Source:VGNC Symbol;Acc:VGNC:92090]	9.24	0.0538
ssc-miR-183	RAPGEF4	Rap guanine nucleotide exchange factor 4 [Source:VGNC Symbol;Acc:VGNC:95814]	9.24	0.0538
ssc-miR-183	RBMS1	RNA binding motif single stranded interacting protein 1 [Source:VGNC Symbol;Acc:VGNC:96534]	9.24	0.0538
ssc-miR-183	RBMX	hypothetical gene	9.24	0.0538
ssc-miR-183	RCAN3	RCAN family member 3 [Source:VGNC Symbol;Acc:VGNC:92172]	9.24	0.0538
ssc-miR-183	RCN2	reticulocalbin 2 [Source:VGNC Symbol;Acc:VGNC:92181]	9.24	0.0538
ssc-miR-183	RDX	radixin [Source:NCBI gene (formerly Entrezgene);Acc:494457]	9.24	0.0538
ssc-miR-183	REPS2	RALBP1 associated Eps domain containing 2 [Source:VGNC Symbol;Acc:VGNC:92214]	9.24	0.0538
ssc-miR-183	REV1	REV1 DNA directed polymerase [Source:HGNC Symbol;Acc:HGNC:14060]	9.24	0.0538
ssc-miR-183	RFX3	regulatory factor X3 [Source:VGNC Symbol;Acc:VGNC:92245]	9.24	0.0538
ssc-miR-183	RGS17	regulator of G protein signaling 17 [Source:VGNC Symbol;Acc:VGNC:92263]	9.24	0.0538
ssc-miR-183	RHOBTB1	Rho related BTB domain containing 1 [Source:VGNC Symbol;Acc:VGNC:92285]	9.24	0.0538
ssc-miR-183	RHPN2	rhophilin Rho GTPase binding protein 2 [Source:VGNC Symbol;Acc:VGNC:98548]	9.24	0.0538
ssc-miR-183	RIMBP2	RIMS binding protein 2 [Source:VGNC Symbol;Acc:VGNC:92306]	9.24	0.0538
ssc-miR-183	RNF138	ring finger protein 138 [Source:VGNC Symbol;Acc:VGNC:92356]	9.24	0.0538

ssc-miR-183	RNF146	ring finger protein 146 [Source:VGNC Symbol;Acc:VGNC:92361]	9.24	0.0538
ssc-miR-183	RNF157	ring finger protein 157 [Source:VGNC Symbol;Acc:VGNC:92365]	9.24	0.0538
ssc-miR-183	RNF168	hypothetical gene	9.24	0.0538
ssc-miR-183	RNF2	ring finger protein 2 [Source:VGNC Symbol;Acc:VGNC:92377]	9.24	0.0538
ssc-miR-183	RNF217	ring finger protein 217 [Source:VGNC Symbol;Acc:VGNC:103162]	9.24	0.0538
ssc-miR-183	ROBO2	roundabout guidance receptor 2 [Source:HGNC Symbol;Acc:HGNC:10250]	9.24	0.0538
ssc-miR-183	RORA	RAR related orphan receptor A [Source:VGNC Symbol;Acc:VGNC:92408]	9.24	0.0538
ssc-miR-183	RP1-170O19.20	hypothetical gene	9.24	0.0538
ssc-miR-183	RP11-113D6.10	hypothetical gene	9.24	0.0538
ssc-miR-183	RPA3-AS1	hypothetical gene	9.24	0.0538
ssc-miR-183	RPE	hypothetical gene	9.24	0.0538
ssc-miR-183	RPL35	ribosomal protein L35 [Source:VGNC Symbol;Acc:VGNC:103164]	9.24	0.0538
ssc-miR-183	RPS6KA3	ribosomal protein S6 kinase A3 [Source:VGNC Symbol;Acc:VGNC:92442]	9.24	0.0538
ssc-miR-183	RREB1	hypothetical gene	9.24	0.0538
ssc-miR-183	RSF1	remodeling and spacing factor 1 [Source:VGNC Symbol;Acc:VGNC:92476]	9.24	0.0538
ssc-miR-183	RUNX1T1	RUNX1 partner transcriptional co-repressor 1 [Source:VGNC Symbol;Acc:VGNC:96594]	9.24	0.0538
ssc-miR-183	RYBP	RING1 and YY1 binding protein [Source:VGNC Symbol;Acc:VGNC:92532]	9.24	0.0538
ssc-miR-183	SACS	sacsin molecular chaperone [Source:HGNC Symbol;Acc:HGNC:10519]	9.24	0.0538
ssc-miR-183	SAMD4A	sterile alpha motif domain containing 4A [Source:VGNC Symbol;Acc:VGNC:92569]	9.24	0.0538
ssc-miR-183	SAR1A	secretion associated Ras related GTPase 1A [Source:VGNC Symbol;Acc:VGNC:92579]	9.24	0.0538
ssc-miR-183	SCARF1	scavenger receptor class F member 1 [Source:VGNC Symbol;Acc:VGNC:92615]	9.24	0.0538
ssc-miR-183	SCN2A	hypothetical gene	9.24	0.0538
ssc-miR-183	SCN3A	sodium voltage-gated channel alpha subunit 3 [Source:VGNC Symbol;Acc:VGNC:95479]	9.24	0.0538
ssc-miR-183	SCP2	hypothetical gene	9.24	0.0538
ssc-miR-183	SCYL3	SCY1 like pseudokinase 3 [Source:VGNC Symbol;Acc:VGNC:92653]	9.24	0.0538
ssc-miR-183	SEC24D	SEC24 homolog D, COPII coat complex component [Source:VGNC Symbol;Acc:VGNC:92679]	9.24	0.0538
ssc-miR-183	SEL1L	SEL1L adaptor subunit of ERAD E3 ubiquitin ligase [Source:VGNC Symbol;Acc:VGNC:92687]	9.24	0.0538
ssc-miR-183	SEMA3E	semaphorin 3E [Source:VGNC Symbol;Acc:VGNC:92697]	9.24	0.0538
ssc-miR-183	SEMA6D	semaphorin 6D [Source:VGNC Symbol;Acc:VGNC:92711]	9.24	0.0538
ssc-miR-183	SEPT7	hypothetical gene	9.24	0.0538
ssc-miR-183	SERP1	stress associated endoplasmic reticulum protein 1 [Source:NCBI gene (formerly Entrezgene);Acc:100156392]	9.24	0.0538
ssc-miR-183	SESN1	sestrin 1 [Source:VGNC Symbol;Acc:VGNC:92753]	9.24	0.0538
ssc-miR-183	SGMS1	sphingomyelin synthase 1 [Source:VGNC Symbol;Acc:VGNC:92794]	9.24	0.0538
ssc-miR-183	SH3BGRL2	SH3 domain binding glutamate rich protein like 2 [Source:VGNC Symbol;Acc:VGNC:92816]	9.24	0.0538
ssc-miR-183	SHH	sonic hedgehog signaling molecule [Source:VGNC Symbol;Acc:VGNC:92844]	9.24	0.0538
ssc-miR-183	SHOX	hypothetical gene	9.24	0.0538
ssc-miR-183	SIDT2	SID1 transmembrane family member 2 [Source:VGNC Symbol;Acc:VGNC:92869]	9.24	0.0538
ssc-miR-183	SIK3	SIK family kinase 3 [Source:VGNC Symbol;Acc:VGNC:92873]	9.24	0.0538
ssc-miR-183	SIN3A	SIN3 transcription regulator family member A [Source:VGNC Symbol;Acc:VGNC:92879]	9.24	0.0538
ssc-miR-183	SIRPA	hypothetical gene	9.24	0.0538
ssc-miR-183	SKIL	SKI like proto-onco [Source:VGNC Symbol;Acc:VGNC:92903]	9.24	0.0538
ssc-miR-183	SLAIN1	SLAIN motif family member 1 [Source:VGNC Symbol;Acc:VGNC:92909]	9.24	0.0538
ssc-miR-183	SLC1A2	solute carrier family 1 member 2 [Source:VGNC Symbol;Acc:VGNC:92964]	9.24	0.0538
ssc-miR-183	SLC22A23	solute carrier family 22 member 23 [Source:HGNC Symbol;Acc:HGNC:21106]	9.24	0.0538
ssc-miR-183	SLC25A15	solute carrier family 25 member 15 [Source:VGNC Symbol;Acc:VGNC:92994]	9.24	0.0538
ssc-miR-183	SLC25A20	solute carrier family 25 member 20 [Source:VGNC Symbol;Acc:VGNC:92998]	9.24	0.0538
ssc-miR-183	SLC25A36	solute carrier family 25 member 36 [Source:VGNC Symbol;Acc:VGNC:93012]	9.24	0.0538
ssc-miR-183	SLC30A6	solute carrier family 30 member 6 [Source:VGNC Symbol;Acc:VGNC:93060]	9.24	0.0538
ssc-miR-183	SLC30A8	solute carrier family 30 member 8 [Source:VGNC Symbol;Acc:VGNC:93062]	9.24	0.0538

ssc-miR-183	SLC30A9	solute carrier family 30 member 9 [Source:VGNC Symbol;Acc:VGNC:98964]	9.24	0.0538
ssc-miR-183	SLC35A1	solute carrier family 35 member A1 [Source:VGNC Symbol;Acc:VGNC:93067]	9.24	0.0538
ssc-miR-183	SLC35E3	solute carrier family 35 member E3 [Source:VGNC Symbol;Acc:VGNC:93079]	9.24	0.0538
ssc-miR-183	SLC44A1	solute carrier family 44 member 1 [Source:VGNC Symbol;Acc:VGNC:93118]	9.24	0.0538
ssc-miR-183	SLC6A6	solute carrier family 6 member 6 [Source:VGNC Symbol;Acc:VGNC:93166]	9.24	0.0538
ssc-miR-183	SLC8A2	solute carrier family 8 member A2 [Source:VGNC Symbol;Acc:VGNC:93179]	9.24	0.0538
ssc-miR-183	SLITRK1	SLIT and NTRK like family member 1 [Source:VGNC Symbol;Acc:VGNC:93206]	9.24	0.0538
ssc-miR-183	SLITRK3	SLIT and NTRK like family member 3 [Source:VGNC Symbol;Acc:VGNC:93208]	9.24	0.0538
ssc-miR-183	SMAD4	SMAD family member 4 [Source:VGNC Symbol;Acc:VGNC:93218]	9.24	0.0538
ssc-miR-183	SMAD5	SMAD family member 5 [Source:VGNC Symbol;Acc:VGNC:93219]	9.24	0.0538
ssc-miR-183	SMCO4	single-pass membrane protein with coiled-coil domains 4 [Source:HGNC Symbol;Acc:HGNC:24810]	9.24	0.0538
ssc-miR-183	SMIM14	small integral membrane protein 14 [Source:VGNC Symbol;Acc:VGNC:93252]	9.24	0.0538
ssc-miR-183	SMLR1	small leucine rich protein 1 [Source:HGNC Symbol;Acc:HGNC:44670]	9.24	0.0538
ssc-miR-183	SMPD3	sphingomyelin phosphodiesterase 3 [Source:VGNC Symbol;Acc:VGNC:93263]	9.24	0.0538
ssc-miR-183	SNCB	synuclein beta [Source:VGNC Symbol;Acc:VGNC:93286]	9.24	0.0538
ssc-miR-183	SNX1	sorting nexin 1 [Source:HGNC Symbol;Acc:HGNC:11172]	9.24	0.0538
ssc-miR-183	SNX30	sorting nexin family member 30 [Source:VGNC Symbol;Acc:VGNC:93318]	9.24	0.0538
ssc-miR-183	SOBP	sine oculis binding protein homolog [Source:VGNC Symbol;Acc:VGNC:93329]	9.24	0.0538
ssc-miR-183	SOCS6	suppressor of cytokine signaling 6 [Source:VGNC Symbol;Acc:VGNC:93333]	9.24	0.0538
ssc-miR-183	SOX6	SRY-box transcription factor 6 [Source:VGNC Symbol;Acc:VGNC:93358]	9.24	0.0538
ssc-miR-183	SP2	Sp2 transcription factor [Source:VGNC Symbol;Acc:VGNC:93361]	9.24	0.0538
ssc-miR-183	SPATA17	spermatosis associated 17 [Source:VGNC Symbol;Acc:VGNC:96065]	9.24	0.0538
ssc-miR-183	SPATS2	spermatogenesis associated serine rich 2 [Source:HGNC Symbol;Acc:HGNC:18650]	9.24	0.0538
ssc-miR-183	SPCS2	signal peptidase complex subunit 2 [Source:VGNC Symbol;Acc:VGNC:108618]	9.24	0.0538
ssc-miR-183	SPECC1	sperm antigen with calponin homology and coiled-coil domains 1 [Source:VGNC Symbol;Acc:VGNC:93398]	9.24	0.0538
ssc-miR-183	SPOPL	speckle type BTB/POZ protein like [Source:VGNC Symbol;Acc:VGNC:96161]	9.24	0.0538
ssc-miR-183	SPPL2A	signal peptide peptidase like 2A [Source:VGNC Symbol;Acc:VGNC:98337]	9.24	0.0538
ssc-miR-183	SPRED1	sprouty related EVH1 domain containing 1 [Source:VGNC Symbol;Acc:VGNC:93420]	9.24	0.0538
ssc-miR-183	SPRY2	sprouty RTK signaling antagonist 2 [Source:VGNC Symbol;Acc:VGNC:93425]	9.24	0.0538
ssc-miR-183	SPRY3	sprouty RTK signaling antagonist 3 [Source:VGNC Symbol;Acc:VGNC:93426]	9.24	0.0538
ssc-miR-183	SREK1IP1	SREK1 interacting protein 1 [Source:HGNC Symbol;Acc:HGNC:26716]	9.24	0.0538
ssc-miR-183	SRSF10	serine and arginine rich splicing factor 10 [Source:VGNC Symbol;Acc:VGNC:93472]	9.24	0.0538
ssc-miR-183	SRSF11	serine and arginine rich splicing factor 11 [Source:VGNC Symbol;Acc:VGNC:93473]	9.24	0.0538
ssc-miR-183	SRSF2	serine and arginine rich splicing factor 2 [Source:VGNC Symbol;Acc:VGNC:99085]	9.24	0.0538
ssc-miR-183	SSBP2	single stranded DNA binding protein 2 [Source:VGNC Symbol;Acc:VGNC:93480]	9.24	0.0538
ssc-miR-183	STC1	stanniocalcin 1 [Source:VGNC Symbol;Acc:VGNC:93542]	9.24	0.0538
ssc-miR-183	STIM2	stromal interaction molecule 2 [Source:VGNC Symbol;Acc:VGNC:98967]	9.24	0.0538
ssc-miR-183	STK38L	serine/threonine kinase 38 like [Source:VGNC Symbol;Acc:VGNC:93557]	9.24	0.0538
ssc-miR-183	STX10	syntaxin 10 [Source:VGNC Symbol;Acc:VGNC:93584]	9.24	0.0538
ssc-miR-183	STX7	syntaxin 7 [Source:VGNC Symbol;Acc:VGNC:93595]	9.24	0.0538
ssc-miR-183	STYX	serine/threonine/tyrosine interacting protein [Source:VGNC Symbol;Acc:VGNC:93602]	9.24	0.0538
ssc-miR-183	SUCO	SUN domain containing ossification factor [Source:VGNC Symbol;Acc:VGNC:93606]	9.24	0.0538
ssc-miR-183	SVEP1	sushi, von Willebrand factor type A, EGF and pentraxin domain containing 1 [Source:VGNC Symbol;Acc:VGNC:93642]	9.24	0.0538
ssc-miR-183	SYT1	synaptotagmin 1 [Source:VGNC Symbol;Acc:VGNC:93678]	9.24	0.0538
ssc-miR-183	SYT17	synaptotagmin 17 [Source:HGNC Symbol;Acc:HGNC:24119]	9.24	0.0538
ssc-miR-183	TAB3	TGF-beta activated kinase 1 (MAP3K7) binding protein 3 [Source:HGNC Symbol;Acc:HGNC:30681]	9.24	0.0538
ssc-miR-183	TAF13	TATA-box binding protein associated factor 13 [Source:VGNC Symbol;Acc:VGNC:93710]	9.24	0.0538
ssc-miR-183	TAOK1	TAO kinase 1 [Source:VGNC Symbol;Acc:VGNC:98355]	9.24	0.0538
ssc-miR-183	TCEANC2	transcription elongation factor A N-terminal and central domain containing 2 [Source:HGNC Symbol;Acc:HGNC:26494]	9.24	0.0538

ssc-miR-183	TCF12	transcription factor 12 [Source:VGNC Symbol;Acc:VGNC:93817]	9.24	0.0538
ssc-miR-183	TCF4	transcription factor 4 [Source:VGNC Symbol;Acc:VGNC:93823]	9.24	0.0538
ssc-miR-183	TCF7L2	transcription factor 7 like 2 [Source:VGNC Symbol;Acc:VGNC:93825]	9.24	0.0538
ssc-miR-183	TEAD1	TEA domain transcription factor 1 [Source:VGNC Symbol;Acc:VGNC:93853]	9.24	0.0538
ssc-miR-183	TET1	tet methylcytosine dioxygenase 1 [Source:VGNC Symbol;Acc:VGNC:93888]	9.24	0.0538
ssc-miR-183	TET3	tet methylcytosine dioxygenase 3 [Source:VGNC Symbol;Acc:VGNC:93890]	9.24	0.0538
ssc-miR-183	THSD7A	thrombospondin type 1 domain containing 7A [Source:VGNC Symbol;Acc:VGNC:93968]	9.24	0.0538
ssc-miR-183	THSD7B	thrombospondin type 1 domain containing 7B [Source:VGNC Symbol;Acc:VGNC:96216]	9.24	0.0538
ssc-miR-183	TIAL1	TIA1 cytotoxic granule associated RNA binding protein like 1 [Source:VGNC Symbol;Acc:VGNC:104073]	9.24	0.0538
ssc-miR-183	TIAM1	TIAM Rac1 associated GEF 1 [Source:VGNC Symbol;Acc:VGNC:96601]	9.24	0.0538
ssc-miR-183	TIMP2	hypothetical gene	9.24	0.0538
ssc-miR-183	TLE4	TLE family member 4, transcriptional corepressor [Source:VGNC Symbol;Acc:VGNC:98372]	9.24	0.0538
ssc-miR-183	TMCC3	transmembrane and coiled-coil domain family 3 [Source:VGNC Symbol;Acc:VGNC:98375]	9.24	0.0538
ssc-miR-183	TMCO1	transmembrane and coiled-coil domains 1 [Source:VGNC Symbol;Acc:VGNC:94048]	9.24	0.0538
ssc-miR-183	TMED7	transmembrane p24 trafficking protein 7 [Source:NCBI gene (formerly Entrezgene);Acc:100522183]	9.24	0.0538
ssc-miR-183	TMED8	transmembrane p24 trafficking protein family member 8 [Source:VGNC Symbol;Acc:VGNC:94058]	9.24	0.0538
ssc-miR-183	TMEM150A	transmembrane protein 150A [Source:VGNC Symbol;Acc:VGNC:94095]	9.24	0.0538
ssc-miR-183	TMEM170A	transmembrane protein 170A [Source:VGNC Symbol;Acc:VGNC:98659]	9.24	0.0538
ssc-miR-183	TMEM184C	transmembrane protein 184C [Source:VGNC Symbol;Acc:VGNC:94121]	9.24	0.0538
ssc-miR-183	TMSB4X	thymosin beta 4 X-linked [Source:NCBI gene (formerly Entrezgene);Acc:733606]	9.24	0.0538
ssc-miR-183	TMSB4Y	hypothetical gene	9.24	0.0538
ssc-miR-183	TNIK	TRAF2 and NCK interacting kinase [Source:VGNC Symbol;Acc:VGNC:98381]	9.24	0.0538
ssc-miR-183	TNRC6B	trinucleotide repeat containing adaptor 6B [Source:VGNC Symbol;Acc:VGNC:94293]	9.24	0.0538
ssc-miR-183	TOMM70A	hypothetical gene	9.24	0.0538
ssc-miR-183	TOX4	TOX high mobility group box family member 4 [Source:VGNC Symbol;Acc:VGNC:98382]	9.24	0.0538
ssc-miR-183	TPM1	tropomyosin 1 [Source:VGNC Symbol;Acc:VGNC:103194]	9.24	0.0538
ssc-miR-183	TPM3	tropomyosin 3 [Source:VGNC Symbol;Acc:VGNC:98877]	9.24	0.0538
ssc-miR-183	TRHDE	thyrotropin releasing hormone degrading enzyme [Source:VGNC Symbol;Acc:VGNC:94390]	9.24	0.0538
ssc-miR-183	TRIM2	tripartite motif containing 2 [Source:VGNC Symbol;Acc:VGNC:94397]	9.24	0.0538
ssc-miR-183	TRIM24	tripartite motif containing 24 [Source:VGNC Symbol;Acc:VGNC:94400]	9.24	0.0538
ssc-miR-183	TSC1	TSC complex subunit 1 [Source:HGNC Symbol;Acc:HGNC:12362]	9.24	0.0538
ssc-miR-183	TSC22D2	TSC22 domain family member 2 [Source:VGNC Symbol;Acc:VGNC:94482]	9.24	0.0538
ssc-miR-183	TTC7B	tetratricopeptide repeat domain 7B [Source:VGNC Symbol;Acc:VGNC:94558]	9.24	0.0538
ssc-miR-183	TUB	TUB bipartite transcription factor [Source:VGNC Symbol;Acc:VGNC:94577]	9.24	0.0538
ssc-miR-183	TXNDC5	thioredoxin domain containing 5 [Source:VGNC Symbol;Acc:VGNC:94608]	9.24	0.0538
ssc-miR-183	UBA3	ubiquitin like modifier activating enzyme 3 [Source:VGNC Symbol;Acc:VGNC:104088]	9.24	0.0538
ssc-miR-183	UBE2I	hypothetical gene	9.24	0.0538
ssc-miR-183	UBE2V2	ubiquitin conjugating enzyme E2 V2 [Source:VGNC Symbol;Acc:VGNC:98394]	9.24	0.0538
ssc-miR-183	UBFD1	ubiquitin family domain containing 1 [Source:VGNC Symbol;Acc:VGNC:94658]	9.24	0.0538
ssc-miR-183	UBP1	upstream binding protein 1 [Source:VGNC Symbol;Acc:VGNC:108701]	9.24	0.0538
ssc-miR-183	UBXN10	UBX domain protein 10 [Source:VGNC Symbol;Acc:VGNC:94675]	9.24	0.0538
ssc-miR-183	UBXN2B	UBX domain protein 2B [Source:VGNC Symbol;Acc:VGNC:94678]	9.24	0.0538
ssc-miR-183	UCHL3	ubiquitin C-terminal hydrolase L3 [Source:VGNC Symbol;Acc:VGNC:98398]	9.24	0.0538
ssc-miR-183	UNC13B	unc-13 homolog B [Source:VGNC Symbol;Acc:VGNC:103199]	9.24	0.0538
ssc-miR-183	UNC5C	unc-5 netrin receptor C [Source:VGNC Symbol;Acc:VGNC:94709]	9.24	0.0538
ssc-miR-183	UQCRB	ubiquinol-cytochrome c reductase binding protein [Source:NCBI gene (formerly Entrezgene);Acc:100157621]	9.24	0.0538
ssc-miR-183	USP47	ubiquitin specific peptidase 47 [Source:VGNC Symbol;Acc:VGNC:94769]	9.24	0.0538
ssc-miR-183	USP6	hypothetical gene	9.24	0.0538
ssc-miR-183	VANGL1	VANGL planar cell polarity protein 1 [Source:VGNC Symbol;Acc:VGNC:94797]	9.24	0.0538

ssc-miR-183	VAPB	VAMP associated protein B and C [Source:VGNC Symbol;Acc:VGNC:108743]	9.24	0.0538
ssc-miR-183	VAT1L	vesicle amine transport 1 like [Source:VGNC Symbol;Acc:VGNC:94806]	9.24	0.0538
ssc-miR-183	VPS13B	vacuolar protein sorting 13 homolog B [Source:VGNC Symbol;Acc:VGNC:94839]	9.24	0.0538
ssc-miR-183	VPS37C	VPS37C subunit of ESCRT-I [Source:VGNC Symbol;Acc:VGNC:94852]	9.24	0.0538
ssc-miR-183	VSX2	visual system homeobox 2 [Source:VGNC Symbol;Acc:VGNC:94876]	9.24	0.0538
ssc-miR-183	XK	X-linked Kx blood group [Source:HGNC Symbol;Acc:HGNC:12811]	9.24	0.0538
ssc-miR-183	XKR7	XK related 7 [Source:VGNC Symbol;Acc:VGNC:95987]	9.24	0.0538
ssc-miR-183	XPOT	exportin for tRNA [Source:VGNC Symbol;Acc:VGNC:95008]	9.24	0.0538
ssc-miR-183	XPR1	xenotropic and polytropic retrovirus receptor 1 [Source:VGNC Symbol;Acc:VGNC:108625]	9.24	0.0538
ssc-miR-183	YAF2	YY1 associated factor 2 [Source:VGNC Symbol;Acc:VGNC:95018]	9.24	0.0538
ssc-miR-183	YOD1	YOD1 deubiquitinase [Source:VGNC Symbol;Acc:VGNC:95035]	9.24	0.0538
ssc-miR-183	YPEL5	yippee like 5 [Source:VGNC Symbol;Acc:VGNC:95039]	9.24	0.0538
ssc-miR-183	YTHDF3	YTH N6-methyladenosine RNA binding protein 3 [Source:VGNC Symbol;Acc:VGNC:95044]	9.24	0.0538
ssc-miR-183	ZBTB34	zinc finger and BTB domain containing 34 [Source:VGNC Symbol;Acc:VGNC:95070]	9.24	0.0538
ssc-miR-183	ZC2HC1A	zinc finger C2HC-type containing 1A [Source:VGNC Symbol;Acc:VGNC:95085]	9.24	0.0538
ssc-miR-183	ZDHHC14	zinc finger DHHC-type palmitoyltransferase 14 [Source:VGNC Symbol;Acc:VGNC:95117]	9.24	0.0538
ssc-miR-183	ZDHHC21	zinc finger DHHC-type palmitoyltransferase 21 [Source:VGNC Symbol;Acc:VGNC:95123]	9.24	0.0538
ssc-miR-183	ZDHHC6	zinc finger DHHC-type palmitoyltransferase 6 [Source:VGNC Symbol;Acc:VGNC:95128]	9.24	0.0538
ssc-miR-183	ZEB1	zinc finger E-box binding homeobox 1 [Source:VGNC Symbol;Acc:VGNC:95536]	9.24	0.0538
ssc-miR-183	ZEB2	hypothetical gene	9.24	0.0538
ssc-miR-183	ZFAND5	zinc finger AN1-type containing 5 [Source:VGNC Symbol;Acc:VGNC:95137]	9.24	0.0538
ssc-miR-183	ZFPM2	zinc finger protein, FOG family member 2 [Source:VGNC Symbol;Acc:VGNC:95153]	9.24	0.0538
ssc-miR-183	ZFYVE26	zinc finger FYVE-type containing 26 [Source:VGNC Symbol;Acc:VGNC:95160]	9.24	0.0538
ssc-miR-183	ZHX3	zinc fingers and homeoboxes 3 [Source:VGNC Symbol;Acc:VGNC:95717]	9.24	0.0538
ssc-miR-183	ZIC3	Zic family member 3 [Source:VGNC Symbol;Acc:VGNC:95168]	9.24	0.0538
ssc-miR-183	ZMYM2	zinc finger MYM-type containing 2 [Source:VGNC Symbol;Acc:VGNC:95182]	9.24	0.0538
ssc-miR-183	ZNF207	zinc finger protein 207 [Source:VGNC Symbol;Acc:VGNC:95205]	9.24	0.0538
ssc-miR-183	ZNF292	zinc finger protein 292 [Source:VGNC Symbol;Acc:VGNC:95218]	9.24	0.0538
ssc-miR-183	ZNF592	zinc finger protein 592 [Source:HGNC Symbol;Acc:HGNC:28986]	9.24	0.0538
ssc-miR-183	ZNF609	zinc finger protein 609 [Source:VGNC Symbol;Acc:VGNC:95274]	9.24	0.0538
ssc-miR-183	ZNF770	zinc finger protein 770 [Source:VGNC Symbol;Acc:VGNC:95300]	9.24	0.0538
ssc-miR-183	ZNF827	zinc finger protein 827 [Source:VGNC Symbol;Acc:VGNC:98970]	9.24	0.0538
ssc-miR-182	AADAT	aminoadipate aminotransferase [Source:VGNC Symbol;Acc:VGNC:84938]	6.46	0.0163
ssc-miR-182	AAK1	AP2 associated kinase 1 [Source:VGNC Symbol;Acc:VGNC:100379]	6.46	0.0163
ssc-miR-182	AATK	apoptosis associated tyrosine kinase [Source:VGNC Symbol;Acc:VGNC:84946]	6.46	0.0163
ssc-miR-182	ABCA2	ATP binding cassette subfamily A member 2 [Source:HGNC Symbol;Acc:HGNC:32]	6.46	0.0163
ssc-miR-182	ABCD2	ATP binding cassette subfamily D member 2 [Source:VGNC Symbol;Acc:VGNC:84962]	6.46	0.0163
ssc-miR-182	ABCE1	ATP binding cassette subfamily E member 1 [Source:VGNC Symbol;Acc:VGNC:84965]	6.46	0.0163
ssc-miR-182	ABCF1	ATP binding cassette subfamily F member 1 [Source:VGNC Symbol;Acc:VGNC:84966]	6.46	0.0163
ssc-miR-182	ABHD13	abhydrolase domain containing 13 [Source:VGNC Symbol;Acc:VGNC:84972]	6.46	0.0163
ssc-miR-182	ABHD17B	abhydrolase domain containing 17B, depalmitoylase [Source:VGNC Symbol;Acc:VGNC:84977]	6.46	0.0163
ssc-miR-182	ABHD2	abhydrolase domain containing 2, acylglycerol lipase [Source:VGNC Symbol;Acc:VGNC:84980]	6.46	0.0163
ssc-miR-182	ABHD5	abhydrolase domain containing 5, lysophosphatidic acid acyltransferase [Source:VGNC Symbol;Acc:VGNC:97860]	6.46	0.0163
ssc-miR-182	ABL2	ABL proto-onco 2, non-receptor tyrosine kinase [Source:VGNC Symbol;Acc:VGNC:84985]	6.46	0.0163
ssc-miR-182	ABLIM1	actin binding LIM protein 1 [Source:VGNC Symbol;Acc:VGNC:84986]	6.46	0.0163
ssc-miR-182	AC003102.1	hypothetical gene	6.46	0.0163
ssc-miR-182	AC012215.1	hypothetical gene	6.46	0.0163
ssc-miR-182	AC079210.1	hypothetical gene	6.46	0.0163
ssc-miR-182	ACAA2	acetyl-CoA acyltransferase 2 [Source:VGNC Symbol;Acc:VGNC:84996]	6.46	0.0163

ssc-miR-182	ACADSB	acyl-CoA dehydrogenase short/branched chain [Source:VGNC Symbol;Acc:VGNC:97863]	6.46	0.0163
ssc-miR-182	ACER2	alkaline ceramidase 2 [Source:VGNC Symbol;Acc:VGNC:85010]	6.46	0.0163
ssc-miR-182	ACER3	alkaline ceramidase 3 [Source:VGNC Symbol;Acc:VGNC:103893]	6.46	0.0163
ssc-miR-182	ACTR2	actin related protein 2 [Source:VGNC Symbol;Acc:VGNC:85051]	6.46	0.0163
ssc-miR-182	ACVR1	activin A receptor type 1 [Source:VGNC Symbol;Acc:VGNC:95830]	6.46	0.0163
ssc-miR-182	ACVR2A	activin A receptor type 2A [Source:VGNC Symbol;Acc:VGNC:95843]	6.46	0.0163
ssc-miR-182	ADAM10	ADAM metallopeptidase domain 10 [Source:VGNC Symbol;Acc:VGNC:85061]	6.46	0.0163
ssc-miR-182	ADAM19	ADAM metallopeptidase domain 19 [Source:VGNC Symbol;Acc:VGNC:85066]	6.46	0.0163
ssc-miR-182	ADAM22	ADAM metallopeptidase domain 22 [Source:VGNC Symbol;Acc:VGNC:85067]	6.46	0.0163
ssc-miR-182	ADAMTS18	ADAM metallopeptidase with thrombospondin type 1 motif 18 [Source:VGNC Symbol;Acc:VGNC:85080]	6.46	0.0163
ssc-miR-182	ADARB2	adenosine deaminase RNA specific B2 (inactive) [Source:HGNC Symbol;Acc:HGNC:227]	6.46	0.0163
ssc-miR-182	ADAT2	adenosine deaminase tRNA specific 2 [Source:VGNC Symbol;Acc:VGNC:85100]	6.46	0.0163
ssc-miR-182	ADCY2	adenylate cyclase 2 [Source:VGNC Symbol;Acc:VGNC:85106]	6.46	0.0163
ssc-miR-182	ADCY6	adenylate cyclase 6 [Source:VGNC Symbol;Acc:VGNC:85110]	6.46	0.0163
ssc-miR-182	ADD2	adducin 2 [Source:VGNC Symbol;Acc:VGNC:85117]	6.46	0.0163
ssc-miR-182	ADD3	adducin 3 [Source:VGNC Symbol;Acc:VGNC:85118]	6.46	0.0163
ssc-miR-182	ADK	adenosine kinase [Source:VGNC Symbol;Acc:VGNC:85143]	6.46	0.0163
ssc-miR-182	ADRA2C	adrenoceptor alpha 2C [Source:VGNC Symbol;Acc:VGNC:85158]	6.46	0.0163
ssc-miR-182	AEBP2	AE binding protein 2 [Source:VGNC Symbol;Acc:VGNC:85163]	6.46	0.0163
ssc-miR-182	AF196779.12	hypothetical gene	6.46	0.0163
ssc-miR-182	AGMAT	agmatinase [Source:VGNC Symbol;Acc:VGNC:85181]	6.46	0.0163
ssc-miR-182	AGO1	hypothetical gene	6.46	0.0163
ssc-miR-182	AGO2	argonaute RISC catalytic component 2 [Source:VGNC Symbol;Acc:VGNC:97871]	6.46	0.0163
ssc-miR-182	AGO3	argonaute RISC component 1 [Source:NCBI gene (formerly Entrezgene);Acc:100499510]	6.46	0.0163
ssc-miR-182	AGO4	hypothetical gene	6.46	0.0163
ssc-miR-182	AHCYL1	adenosylhomocysteinase like 1 [Source:VGNC Symbol;Acc:VGNC:97873]	6.46	0.0163
ssc-miR-182	AHR	aryl hydrocarbon receptor [Source:NCBI gene (formerly Entrezgene);Acc:396654]	6.46	0.0163
ssc-miR-182	AJUBA	ajuba LIM protein [Source:VGNC Symbol;Acc:VGNC:85208]	6.46	0.0163
ssc-miR-182	AK3	adenylate kinase 3 [Source:VGNC Symbol;Acc:VGNC:97876]	6.46	0.0163
ssc-miR-182	AK4	hypothetical gene	6.46	0.0163
ssc-miR-182	AK5	adenylate kinase 5 [Source:VGNC Symbol;Acc:VGNC:85210]	6.46	0.0163
ssc-miR-182	AKAP1	A-kinase anchoring protein 1 [Source:VGNC Symbol;Acc:VGNC:85214]	6.46	0.0163
ssc-miR-182	AKAP13	hypothetical gene	6.46	0.0163
ssc-miR-182	AL590483.1	hypothetical gene	6.46	0.0163
ssc-miR-182	ALDH1B1	aldehyde dehydrogenase 1 family member B1 [Source:VGNC Symbol;Acc:VGNC:97880]	6.46	0.0163
ssc-miR-182	ALDH6A1	aldehyde dehydrogenase 6 family member A1 [Source:VGNC Symbol;Acc:VGNC:85242]	6.46	0.0163
ssc-miR-182	ALG9	ALG9 alpha-1,2-mannosyltransferase [Source:NCBI gene (formerly Entrezgene);Acc:100519965]	6.46	0.0163
ssc-miR-182	AMMECR1L	AMMECR1 like [Source:VGNC Symbol;Acc:VGNC:103895]	6.46	0.0163
ssc-miR-182	AMN1	antagonist of mitotic exit network 1 homolog [Source:VGNC Symbol;Acc:VGNC:97883]	6.46	0.0163
ssc-miR-182	AMOTL2	angiomotin like 2 [Source:VGNC Symbol;Acc:VGNC:85285]	6.46	0.0163
ssc-miR-182	AMPH	amphiphysin [Source:VGNC Symbol;Acc:VGNC:85289]	6.46	0.0163
ssc-miR-182	ANGPTL1	angiopoietin like 1 [Source:VGNC Symbol;Acc:VGNC:85303]	6.46	0.0163
ssc-miR-182	ANK3	hypothetical gene	6.46	0.0163
ssc-miR-182	ANKHD1-EIF4EBP3	hypothetical gene	6.46	0.0163
ssc-miR-182	ANKIB1	ankyrin repeat and IBR domain containing 1 [Source:VGNC Symbol;Acc:VGNC:85316]	6.46	0.0163
ssc-miR-182	ANKRD27	ankyrin repeat domain 27 [Source:VGNC Symbol;Acc:VGNC:96907]	6.46	0.0163
ssc-miR-182	ANKRD28	ankyrin repeat domain 28 [Source:VGNC Symbol;Acc:VGNC:85331]	6.46	0.0163
ssc-miR-182	ANKRD44	ankyrin repeat domain 44 [Source:VGNC Symbol;Acc:VGNC:107123]	6.46	0.0163
ssc-miR-182	ANKRD52	ankyrin repeat domain 52 [Source:VGNC Symbol;Acc:VGNC:85342]	6.46	0.0163

ssc-miR-182	ANKS6	ankyrin repeat and sterile alpha motif domain containing 6 [Source:VGNC Symbol;Acc:VGNC:85351]	6.46	0.0163
ssc-miR-182	ANLN	anillin actin binding protein [Source:VGNC Symbol;Acc:VGNC:85353]	6.46	0.0163
ssc-miR-182	ANP32B	acidic nuclear phosphoprotein 32 family member B [Source:HGNC Symbol;Acc:HGNC:16677]	6.46	0.0163
ssc-miR-182	ANTXR2	ANTXR cell adhesion molecule 2 [Source:VGNC Symbol;Acc:VGNC:85365]	6.46	0.0163
ssc-miR-182	ANXA11	annexin A11 [Source:VGNC Symbol;Acc:VGNC:85367]	6.46	0.0163
ssc-miR-182	AP3M1	adaptor related protein complex 3 subunit mu 1 [Source:VGNC Symbol;Acc:VGNC:85390]	6.46	0.0163
ssc-miR-182	AP5M1	adaptor related protein complex 5 subunit mu 1 [Source:VGNC Symbol;Acc:VGNC:85396]	6.46	0.0163
ssc-miR-182	APBA2	amyloid beta protein binding family A member 2 [Source:VGNC Symbol;Acc:VGNC:85400]	6.46	0.0163
ssc-miR-182	APLN	apelin [Source:HGNC Symbol;Acc:HGNC:16665]	6.46	0.0163
ssc-miR-182	APOB	apolipoprotein B [Source:VGNC Symbol;Acc:VGNC:85417]	6.46	0.0163
ssc-miR-182	APPL1	adaptor protein, phosphotyrosine interacting with PH domain and leucine zipper 1 [Source:VGNC Symbol;Acc:VGNC:85426]	6.46	0.0163
ssc-miR-182	ARCN1	archain 1 [Source:VGNC Symbol;Acc:VGNC:85443]	6.46	0.0163
ssc-miR-182	AREL1	apoptosis resistant E3 ubiquitin protein ligase 1 [Source:VGNC Symbol;Acc:VGNC:85445]	6.46	0.0163
ssc-miR-182	ARF4	ADP ribosylation factor 4 [Source:VGNC Symbol;Acc:VGNC:85446]	6.46	0.0163
ssc-miR-182	ARHGAP29	Rho GTPase activating protein 29 [Source:VGNC Symbol;Acc:VGNC:85468]	6.46	0.0163
ssc-miR-182	ARHGAP6	hypothetical gene	6.46	0.0163
ssc-miR-182	ARHGDIA	Rho GDP dissociation inhibitor alpha [Source:VGNC Symbol;Acc:VGNC:85483]	6.46	0.0163
ssc-miR-182	ARHGEF12	Rho guanine nucleotide exchange factor 12 [Source:VGNC Symbol;Acc:VGNC:85488]	6.46	0.0163
ssc-miR-182	ARHGEF3	Rho guanine nucleotide exchange factor 3 [Source:VGNC Symbol;Acc:VGNC:85496]	6.46	0.0163
ssc-miR-182	ARID2	AT-rich interaction domain 2 [Source:HGNC Symbol;Acc:HGNC:18037]	6.46	0.0163
ssc-miR-182	ARID3A	AT-rich interaction domain 3A [Source:VGNC Symbol;Acc:VGNC:85507]	6.46	0.0163
ssc-miR-182	ARID4A	AT-rich interaction domain 4A [Source:VGNC Symbol;Acc:VGNC:85509]	6.46	0.0163
ssc-miR-182	ARL14EP	ADP ribosylation factor like GTPase 14 effector protein [Source:VGNC Symbol;Acc:VGNC:85518]	6.46	0.0163
ssc-miR-182	ARL4C	ADP ribosylation factor like GTPase 4C [Source:VGNC Symbol;Acc:VGNC:96419]	6.46	0.0163
ssc-miR-182	ARMC1	armadillo repeat containing 1 [Source:VGNC Symbol;Acc:VGNC:85526]	6.46	0.0163
ssc-miR-182	ARRDC3	arrestin domain containing 3 [Source:VGNC Symbol;Acc:VGNC:85546]	6.46	0.0163
ssc-miR-182	ARSJ	arylsulfatase family member J [Source:VGNC Symbol;Acc:VGNC:85549]	6.46	0.0163
ssc-miR-182	AS3MT	arsenite methyltransferase [Source:VGNC Symbol;Acc:VGNC:85557]	6.46	0.0163
ssc-miR-182	ASAP1	ArfGAP with SH3 domain, ankyrin repeat and PH domain 1 [Source:VGNC Symbol;Acc:VGNC:98734]	6.46	0.0163
ssc-miR-182	ASB6	ankyrin repeat and SOCS box containing 6 [Source:VGNC Symbol;Acc:VGNC:85565]	6.46	0.0163
ssc-miR-182	ASCC1	activating signal cointegrator 1 complex subunit 1 [Source:VGNC Symbol;Acc:VGNC:85569]	6.46	0.0163
ssc-miR-182	ASTN1	astrotactin 1 [Source:VGNC Symbol;Acc:VGNC:85593]	6.46	0.0163
ssc-miR-182	ASUN	hypothetical gene	6.46	0.0163
ssc-miR-182	ASXL3	ASXL transcriptional regulator 3 [Source:VGNC Symbol;Acc:VGNC:85596]	6.46	0.0163
ssc-miR-182	ATAD2B	ATPase family AAA domain containing 2B [Source:VGNC Symbol;Acc:VGNC:85600]	6.46	0.0163
ssc-miR-182	ATF7IP	activating transcription factor 7 interacting protein [Source:VGNC Symbol;Acc:VGNC:85611]	6.46	0.0163
ssc-miR-182	ATG16L1	autophagy related 16 like 1 [Source:VGNC Symbol;Acc:VGNC:95967]	6.46	0.0163
ssc-miR-182	ATG7	autophagy related 7 [Source:VGNC Symbol;Acc:VGNC:85625]	6.46	0.0163
ssc-miR-182	ATOH8	atonal bHLH transcription factor 8 [Source:VGNC Symbol;Acc:VGNC:85633]	6.46	0.0163
ssc-miR-182	ATP1B3	hypothetical gene	6.46	0.0163
ssc-miR-182	ATP5S	hypothetical gene	6.46	0.0163
ssc-miR-182	ATP6V1C1	ATPase H+ transporting V1 subunit C1 [Source:VGNC Symbol;Acc:VGNC:85672]	6.46	0.0163
ssc-miR-182	ATP8A1	ATPase phospholipid transporting 8A1 [Source:VGNC Symbol;Acc:VGNC:97894]	6.46	0.0163
ssc-miR-182	ATP8A2	ATPase phospholipid transporting 8A2 [Source:HGNC Symbol;Acc:HGNC:13533]	6.46	0.0163
ssc-miR-182	ATP9A	ATPase phospholipid transporting 9A (putative) [Source:NCBI gene (formerly Entrezgene);Acc:100136901]	6.46	0.0163
ssc-miR-182	ATXN1	ataxin 1 [Source:VGNC Symbol;Acc:VGNC:85687]	6.46	0.0163
ssc-miR-182	ATXN1L	ataxin 1 like [Source:VGNC Symbol;Acc:VGNC:85689]	6.46	0.0163
ssc-miR-182	ATXN3	hypothetical gene	6.46	0.0163
ssc-miR-182	ATXN7	ataxin 7 [Source:VGNC Symbol;Acc:VGNC:99704]	6.46	0.0163

ssc-miR-182	AVPI1	AVP induced 1 [Source:VGNC Symbol;Acc:VGNC:85702]	6.46	0.0163
ssc-miR-182	AZIN1	antizyme inhibitor 1 [Source:VGNC Symbol;Acc:VGNC:85712]	6.46	0.0163
ssc-miR-182	B3GALT1	beta-1,3-galactosyltransferase 1 [Source:VGNC Symbol;Acc:VGNC:96494]	6.46	0.0163
ssc-miR-182	B4GALNT1	beta-1,4-N-acetyl-galactosaminyltransferase 1 [Source:VGNC Symbol;Acc:VGNC:85730]	6.46	0.0163
ssc-miR-182	B4GALT6	beta-1,4-galactosyltransferase 6 [Source:VGNC Symbol;Acc:VGNC:85734]	6.46	0.0163
ssc-miR-182	BACE2	hypothetical gene	6.46	0.0163
ssc-miR-182	BACH2	BTB domain and CNC homolog 2 [Source:VGNC Symbol;Acc:VGNC:85742]	6.46	0.0163
ssc-miR-182	BAG4	BAG cochaperone 4 [Source:VGNC Symbol;Acc:VGNC:96503]	6.46	0.0163
ssc-miR-182	BAI3	hypothetical gene	6.46	0.0163
ssc-miR-182	BCAT1	branched chain amino acid transaminase 1 [Source:VGNC Symbol;Acc:VGNC:85773]	6.46	0.0163
ssc-miR-182	BCAT2	branched chain amino acid transaminase 2 [Source:VGNC Symbol;Acc:VGNC:85774]	6.46	0.0163
ssc-miR-182	BCL10	BCL10 immune signaling adaptor [Source:VGNC Symbol;Acc:VGNC:96561]	6.46	0.0163
ssc-miR-182	BCL11B	BAF chromatin remodeling complex subunit BCL11B [Source:VGNC Symbol;Acc:VGNC:96563]	6.46	0.0163
ssc-miR-182	BCL2	BCL2 apoptosis regulator [Source:HGNC Symbol;Acc:HGNC:990]	6.46	0.0163
ssc-miR-182	BCL2L12	hypothetical gene	6.46	0.0163
ssc-miR-182	BCL2L13	BCL2 like 13 [Source:VGNC Symbol;Acc:VGNC:85781]	6.46	0.0163
ssc-miR-182	BDNF	brain derived neurotrophic factor [Source:VGNC Symbol;Acc:VGNC:85795]	6.46	0.0163
ssc-miR-182	BEND4	BEN domain containing 4 [Source:VGNC Symbol;Acc:VGNC:85801]	6.46	0.0163
ssc-miR-182	BHLHB9	hypothetical gene	6.46	0.0163
ssc-miR-182	BHLHE22	basic helix-loop-helix family member e22 [Source:HGNC Symbol;Acc:HGNC:11963]	6.46	0.0163
ssc-miR-182	BHLHE41	basic helix-loop-helix family member e41 [Source:VGNC Symbol;Acc:VGNC:85815]	6.46	0.0163
ssc-miR-182	BICD1	BICD cargo adaptor 1 [Source:VGNC Symbol;Acc:VGNC:85818]	6.46	0.0163
ssc-miR-182	BICD2	BICD cargo adaptor 2 [Source:VGNC Symbol;Acc:VGNC:85819]	6.46	0.0163
ssc-miR-182	BMP2	bone morphotic protein 2 [Source:VGNC Symbol;Acc:VGNC:95488]	6.46	0.0163
ssc-miR-182	BMP2K	BMP2 inducible kinase [Source:VGNC Symbol;Acc:VGNC:85841]	6.46	0.0163
ssc-miR-182	BMPER	BMP binding endothelial regulator [Source:VGNC Symbol;Acc:VGNC:97902]	6.46	0.0163
ssc-miR-182	BMPR1B	bone morphotic protein receptor type 1B [Source:VGNC Symbol;Acc:VGNC:85847]	6.46	0.0163
ssc-miR-182	BNC2	basonuclin 2 [Source:VGNC Symbol;Acc:VGNC:85852]	6.46	0.0163
ssc-miR-182	BNIP3	BCL2 interacting protein 3 [Source:VGNC Symbol;Acc:VGNC:85855]	6.46	0.0163
ssc-miR-182	BOD1L1	biorientation of chromosomes in cell division 1 like 1 [Source:VGNC Symbol;Acc:VGNC:97903]	6.46	0.0163
ssc-miR-182	BOD1L2	hypothetical gene	6.46	0.0163
ssc-miR-182	BPTF	bromodomain PHD finger transcription factor [Source:VGNC Symbol;Acc:VGNC:97904]	6.46	0.0163
ssc-miR-182	BRE	hypothetical gene	6.46	0.0163
ssc-miR-182	BRMS1L	BRMS1 like transcriptional repressor [Source:VGNC Symbol;Acc:VGNC:96571]	6.46	0.0163
ssc-miR-182	BRPF3	bromodomain and PHD finger containing 3 [Source:VGNC Symbol;Acc:VGNC:85886]	6.46	0.0163
ssc-miR-182	BRWD1	bromodomain and WD repeat domain containing 1 [Source:VGNC Symbol;Acc:VGNC:108153]	6.46	0.0163
ssc-miR-182	BRWD3	bromodomain and WD repeat domain containing 3 [Source:VGNC Symbol;Acc:VGNC:85890]	6.46	0.0163
ssc-miR-182	BTBD11	BTB domain containing 11 [Source:VGNC Symbol;Acc:VGNC:85901]	6.46	0.0163
ssc-miR-182	BZRAP1	hypothetical gene	6.46	0.0163
ssc-miR-182	C10orf131	hypothetical gene	6.46	0.0163
ssc-miR-182	C10orf32	hypothetical gene	6.46	0.0163
ssc-miR-182	C10orf54	hypothetical gene	6.46	0.0163
ssc-miR-182	C12orf4	chromosome 5 C12orf4 homolog [Source:VGNC Symbol;Acc:VGNC:86041]	6.46	0.0163
ssc-miR-182	C12orf73	chromosome 12 open reading frame 73 [Source:HGNC Symbol;Acc:HGNC:34450]	6.46	0.0163
ssc-miR-182	C12orf79	hypothetical gene	6.46	0.0163
ssc-miR-182	C16orf70	hypothetical gene	6.46	0.0163
ssc-miR-182	C19orf26	hypothetical gene	6.46	0.0163
ssc-miR-182	C1GALT1	hypothetical gene	6.46	0.0163
ssc-miR-182	C1orf106	hypothetical gene	6.46	0.0163

ssc-miR-182	C1orf198	chromosome 14 C1orf198 homolog [Source:VGNC Symbol;Acc:VGNC:85947]	6.46	0.0163
ssc-miR-182	C1orf95	hypothetical gene	6.46	0.0163
ssc-miR-182	C20orf194	hypothetical gene	6.46	0.0163
ssc-miR-182	C2CD2	C2 calcium dependent domain containing 2 [Source:VGNC Symbol;Acc:VGNC:85990]	6.46	0.0163
ssc-miR-182	C2CD5	C2 calcium dependent domain containing 5 [Source:VGNC Symbol;Acc:VGNC:85995]	6.46	0.0163
ssc-miR-182	C2orf68	chromosome 3 C2orf68 homolog [Source:VGNC Symbol;Acc:VGNC:86023]	6.46	0.0163
ssc-miR-182	C2orf69	chromosome 2 open reading frame 69 [Source:HGNC Symbol;Acc:HGNC:26799]	6.46	0.0163
ssc-miR-182	C2orf71	hypothetical gene	6.46	0.0163
ssc-miR-182	C2orf72	chromosome 15 C2orf72 homolog [Source:VGNC Symbol;Acc:VGNC:96157]	6.46	0.0163
ssc-miR-182	C5orf30	hypothetical gene	6.46	0.0163
ssc-miR-182	C5orf47	chromosome 16 C5orf47 homolog [Source:VGNC Symbol;Acc:VGNC:85952]	6.46	0.0163
ssc-miR-182	C6orf106	hypothetical gene	6.46	0.0163
ssc-miR-182	C6orf211	hypothetical gene	6.46	0.0163
ssc-miR-182	C7orf41	hypothetical gene	6.46	0.0163
ssc-miR-182	C7orf60	hypothetical gene	6.46	0.0163
ssc-miR-182	C8orf37	hypothetical gene	6.46	0.0163
ssc-miR-182	C8orf44-SGK3	hypothetical gene	6.46	0.0163
ssc-miR-182	C9	complement C9 [Source:VGNC Symbol;Acc:VGNC:86083]	6.46	0.0163
ssc-miR-182	C9orf114	hypothetical gene	6.46	0.0163
ssc-miR-182	C9orf72	chromosome 10 C9orf72 homolog [Source:VGNC Symbol;Acc:VGNC:96173]	6.46	0.0163
ssc-miR-182	CA10	carbonic anhydrase 10 [Source:VGNC Symbol;Acc:VGNC:86096]	6.46	0.0163
ssc-miR-182	CACNA1B	calcium voltage-gated channel subunit alpha1 B [Source:HGNC Symbol;Acc:HGNC:1389]	6.46	0.0163
ssc-miR-182	CACNA2D1	calcium voltage-gated channel auxiliary subunit alpha2delta 1 [Source:VGNC Symbol;Acc:VGNC:86120]	6.46	0.0163
ssc-miR-182	CACNA2D2	calcium voltage-gated channel auxiliary subunit alpha2delta 2 [Source:VGNC Symbol;Acc:VGNC:86121]	6.46	0.0163
ssc-miR-182	CACNB4	calcium voltage-gated channel auxiliary subunit beta 4 [Source:VGNC Symbol;Acc:VGNC:108507]	6.46	0.0163
ssc-miR-182	CADM1	cell adhesion molecule 1 [Source:VGNC Symbol;Acc:VGNC:86134]	6.46	0.0163
ssc-miR-182	CADM2	cell adhesion molecule 2 [Source:VGNC Symbol;Acc:VGNC:97910]	6.46	0.0163
ssc-miR-182	CALCR	calcitonin receptor [Source:VGNC Symbol;Acc:VGNC:86142]	6.46	0.0163
ssc-miR-182	CALN1	hypothetical gene	6.46	0.0163
ssc-miR-182	CAMK2N1	calcium/calmodulin dependent protein kinase II inhibitor 1 [Source:VGNC Symbol;Acc:VGNC:96935]	6.46	0.0163
ssc-miR-182	CAMKK2	calcium/calmodulin dependent protein kinase kinase 2 [Source:VGNC Symbol;Acc:VGNC:86158]	6.46	0.0163
ssc-miR-182	CAMSAP2	calmodulin regulated spectrin associated protein family member 2 [Source:VGNC Symbol;Acc:VGNC:96205]	6.46	0.0163
ssc-miR-182	CAPN5	calpain 5 [Source:VGNC Symbol;Acc:VGNC:86175]	6.46	0.0163
ssc-miR-182	CAPN7	calpain 7 [Source:NCBI gene (formerly Entrezgene);Acc:100037936]	6.46	0.0163
ssc-miR-182	CASD1	CAS1 domain containing 1 [Source:VGNC Symbol;Acc:VGNC:86199]	6.46	0.0163
ssc-miR-182	CASP2	caspase 2 [Source:HGNC Symbol;Acc:HGNC:1503]	6.46	0.0163
ssc-miR-182	CBFA2T3	CBFA2/RUNX1 partner transcriptional co-repressor 3 [Source:VGNC Symbol;Acc:VGNC:96574]	6.46	0.0163
ssc-miR-182	CBL	Cbl proto-onco [Source:VGNC Symbol;Acc:VGNC:86222]	6.46	0.0163
ssc-miR-182	CBLN4	cerebellin 4 [Source:VGNC Symbol;Acc:VGNC:95793]	6.46	0.0163
ssc-miR-182	CBR4	carbonyl reductase 4 [Source:VGNC Symbol;Acc:VGNC:86229]	6.46	0.0163
ssc-miR-182	CBX2	chromobox 2 [Source:VGNC Symbol;Acc:VGNC:86230]	6.46	0.0163
ssc-miR-182	CBX5	chromobox 5 [Source:VGNC Symbol;Acc:VGNC:86232]	6.46	0.0163
ssc-miR-182	CBX7	chromobox 7 [Source:HGNC Symbol;Acc:HGNC:1557]	6.46	0.0163
ssc-miR-182	CCDC117	coiled-coil domain containing 117 [Source:VGNC Symbol;Acc:VGNC:86248]	6.46	0.0163
ssc-miR-182	CCDC144A	hypothetical gene	6.46	0.0163
ssc-miR-182	CCDC177	coiled-coil domain containing 177 [Source:VGNC Symbol;Acc:VGNC:86275]	6.46	0.0163
ssc-miR-182	CCDC41	hypothetical gene	6.46	0.0163
ssc-miR-182	CCDC50	coiled-coil domain containing 50 [Source:VGNC Symbol;Acc:VGNC:86299]	6.46	0.0163
ssc-miR-182	CCDC71L	coiled-coil domain containing 71 like [Source:HGNC Symbol;Acc:HGNC:26685]	6.46	0.0163

ssc-miR-182	CCDC88A	coiled-coil domain containing 88A [Source:VGNC Symbol;Acc:VGNC:86325]	6.46	0.0163
ssc-miR-182	CCDC92	coiled-coil domain containing 92 [Source:VGNC Symbol;Acc:VGNC:86330]	6.46	0.0163
ssc-miR-182	CCND2	cyclin D2 [Source:VGNC Symbol;Acc:VGNC:103222]	6.46	0.0163
ssc-miR-182	CCND3	cyclin D3 [Source:VGNC Symbol;Acc:VGNC:86353]	6.46	0.0163
ssc-miR-182	CCNJ	cyclin J [Source:VGNC Symbol;Acc:VGNC:86360]	6.46	0.0163
ssc-miR-182	CCNY	cyclin Y [Source:VGNC Symbol;Acc:VGNC:95998]	6.46	0.0163
ssc-miR-182	CCSER1	coiled-coil serine rich protein 1 [Source:VGNC Symbol;Acc:VGNC:86378]	6.46	0.0163
ssc-miR-182	CD164	CD164 molecule [Source:HGNC Symbol;Acc:HGNC:1632]	6.46	0.0163
ssc-miR-182	CD2AP	CD2 associated protein [Source:VGNC Symbol;Acc:VGNC:86406]	6.46	0.0163
ssc-miR-182	CD47	CD47 molecule [Source:VGNC Symbol;Acc:VGNC:108637]	6.46	0.0163
ssc-miR-182	CD96	CD96 molecule [Source:VGNC Symbol;Acc:VGNC:86437]	6.46	0.0163
ssc-miR-182	CDC27	cell division cycle 27 [Source:VGNC Symbol;Acc:VGNC:86448]	6.46	0.0163
ssc-miR-182	CDC37	cell division cycle 37, HSP90 cochaperone [Source:VGNC Symbol;Acc:VGNC:86450]	6.46	0.0163
ssc-miR-182	CDC42BPA	CDC42 binding protein kinase alpha [Source:VGNC Symbol;Acc:VGNC:95847]	6.46	0.0163
ssc-miR-182	CDC42BPG	CDC42 binding protein kinase gamma [Source:VGNC Symbol;Acc:VGNC:86454]	6.46	0.0163
ssc-miR-182	CDC73	cell division cycle 73 [Source:VGNC Symbol;Acc:VGNC:95817]	6.46	0.0163
ssc-miR-182	CDH20	cadherin 20 [Source:VGNC Symbol;Acc:VGNC:86484]	6.46	0.0163
ssc-miR-182	CDH7	cadherin 7 [Source:VGNC Symbol;Acc:VGNC:97923]	6.46	0.0163
ssc-miR-182	CDH8	cadherin 8 [Source:VGNC Symbol;Acc:VGNC:86487]	6.46	0.0163
ssc-miR-182	CDK1	cyclin dependent kinase 1 [Source:VGNC Symbol;Acc:VGNC:103917]	6.46	0.0163
ssc-miR-182	CDK18	cyclin dependent kinase 18 [Source:VGNC Symbol;Acc:VGNC:86501]	6.46	0.0163
ssc-miR-182	CDK6	cyclin dependent kinase 6 [Source:HGNC Symbol;Acc:HGNC:1777]	6.46	0.0163
ssc-miR-182	CDO1	cysteine dioxygenase type 1 [Source:NCBI gene (formerly Entrezgene);Acc:100312964]	6.46	0.0163
ssc-miR-182	CDS2	CDP-diacylglycerol synthase 2 [Source:VGNC Symbol;Acc:VGNC:95869]	6.46	0.0163
ssc-miR-182	CDSN	corneodesmosin [Source:NCBI gene (formerly Entrezgene);Acc:100144482]	6.46	0.0163
ssc-miR-182	CEBPA	CCAAT enhancer binding protein alpha [Source:VGNC Symbol;Acc:VGNC:86531]	6.46	0.0163
ssc-miR-182	CELF1	CUGBP Elav-like family member 1 [Source:VGNC Symbol;Acc:VGNC:86537]	6.46	0.0163
ssc-miR-182	CELF2	hypothetical gene	6.46	0.0163
ssc-miR-182	CELF6	CUGBP Elav-like family member 6 [Source:HGNC Symbol;Acc:HGNC:14059]	6.46	0.0163
ssc-miR-182	CELSR1	cadherin EGF LAG seven-pass G-type receptor 1 [Source:HGNC Symbol;Acc:HGNC:1850]	6.46	0.0163
ssc-miR-182	CELSR3	cadherin EGF LAG seven-pass G-type receptor 3 [Source:VGNC Symbol;Acc:VGNC:86541]	6.46	0.0163
ssc-miR-182	CENPP	centromere protein P [Source:VGNC Symbol;Acc:VGNC:98865]	6.46	0.0163
ssc-miR-182	CEP128	centrosomal protein 128 [Source:VGNC Symbol;Acc:VGNC:86560]	6.46	0.0163
ssc-miR-182	CEP170B	centrosomal protein 170B [Source:VGNC Symbol;Acc:VGNC:86564]	6.46	0.0163
ssc-miR-182	CEP250	centrosomal protein 250 [Source:VGNC Symbol;Acc:VGNC:95888]	6.46	0.0163
ssc-miR-182	CEP41	centrosomal protein 41 [Source:VGNC Symbol;Acc:VGNC:86569]	6.46	0.0163
ssc-miR-182	CEP76	centrosomal protein 76 [Source:VGNC Symbol;Acc:VGNC:96947]	6.46	0.0163
ssc-miR-182	CEP85L	centrosomal protein 85 like [Source:VGNC Symbol;Acc:VGNC:86580]	6.46	0.0163
ssc-miR-182	CERS2	ceramide synthase 2 [Source:VGNC Symbol;Acc:VGNC:86587]	6.46	0.0163
ssc-miR-182	CERS6	ceramide synthase 6 [Source:VGNC Symbol;Acc:VGNC:96025]	6.46	0.0163
ssc-miR-182	CFL1	cofilin 1 [Source:NCBI gene (formerly Entrezgene);Acc:445532]	6.46	0.0163
ssc-miR-182	CGGBP1	CGG triplet repeat binding protein 1 [Source:VGNC Symbol;Acc:VGNC:86615]	6.46	0.0163
ssc-miR-182	CHAMP1	hypothetical gene	6.46	0.0163
ssc-miR-182	CHIC1	cysteine rich hydrophobic domain 1 [Source:VGNC Symbol;Acc:VGNC:86642]	6.46	0.0163
ssc-miR-182	CHL1	cell adhesion molecule L1 like [Source:VGNC Symbol;Acc:VGNC:108639]	6.46	0.0163
ssc-miR-182	CHMP1A	charged multivesicular body protein 1A [Source:VGNC Symbol;Acc:VGNC:86648]	6.46	0.0163
ssc-miR-182	CHMP1B	hypothetical gene	6.46	0.0163
ssc-miR-182	CHMP2B	charged multivesicular body protein 2B [Source:VGNC Symbol;Acc:VGNC:86650]	6.46	0.0163
ssc-miR-182	CHMP7	charged multivesicular body protein 7 [Source:VGNC Symbol;Acc:VGNC:86654]	6.46	0.0163

ssc-miR-182	CHST1	carbohydrate sulfotransferase 1 [Source:VGNC Symbol;Acc:VGNC:86673]	6.46	0.0163
ssc-miR-182	CHST10	carbohydrate sulfotransferase 10 [Source:VGNC Symbol;Acc:VGNC:86674]	6.46	0.0163
ssc-miR-182	CHST11	carbohydrate sulfotransferase 11 [Source:VGNC Symbol;Acc:VGNC:86675]	6.46	0.0163
ssc-miR-182	CIAPIN1	cytokine induced apoptosis inhibitor 1 [Source:VGNC Symbol;Acc:VGNC:86693]	6.46	0.0163
ssc-miR-182	CITED2	Cbp/p300 interacting transactivator with Glu/Asp rich carboxy-terminal domain 2 [Source:VGNC Symbol;Acc:VGNC:86712]	6.46	0.0163
ssc-miR-182	CLCC1	chloride channel CLIC like 1 [Source:VGNC Symbol;Acc:VGNC:86724]	6.46	0.0163
ssc-miR-182	CLCN5	chloride voltage-gated channel 5 [Source:VGNC Symbol;Acc:VGNC:103925]	6.46	0.0163
ssc-miR-182	CLCN6	chloride voltage-gated channel 6 [Source:VGNC Symbol;Acc:VGNC:86729]	6.46	0.0163
ssc-miR-182	CLEC9A	hypothetical gene	6.46	0.0163
ssc-miR-182	CLIC5	chloride intracellular channel 5 [Source:VGNC Symbol;Acc:VGNC:86764]	6.46	0.0163
ssc-miR-182	CLLU1	hypothetical gene	6.46	0.0163
ssc-miR-182	CLOCK	clock circadian regulator [Source:VGNC Symbol;Acc:VGNC:86774]	6.46	0.0163
ssc-miR-182	CLPTM1L	CLPTM1 like [Source:VGNC Symbol;Acc:VGNC:86779]	6.46	0.0163
ssc-miR-182	CLSTN2	calsyntenin 2 [Source:VGNC Symbol;Acc:VGNC:86785]	6.46	0.0163
ssc-miR-182	CLVS1	clavesin 1 [Source:VGNC Symbol;Acc:VGNC:86794]	6.46	0.0163
ssc-miR-182	CMTM7	CKLF like MARVEL transmembrane domain containing 7 [Source:VGNC Symbol;Acc:VGNC:103928]	6.46	0.0163
ssc-miR-182	CNGA3	cyclic nucleotide gated channel subunit alpha 3 [Source:VGNC Symbol;Acc:VGNC:86817]	6.46	0.0163
ssc-miR-182	CNIH4	cornichon family AMPA receptor auxiliary protein 4 [Source:VGNC Symbol;Acc:VGNC:96060]	6.46	0.0163
ssc-miR-182	CNN3	calponin 3 [Source:VGNC Symbol;Acc:VGNC:86826]	6.46	0.0163
ssc-miR-182	CNNM2	cyclin and CBS domain divalent metal cation transport mediator 2 [Source:VGNC Symbol;Acc:VGNC:86828]	6.46	0.0163
ssc-miR-182	CNNM3	cyclin and CBS domain divalent metal cation transport mediator 3 [Source:VGNC Symbol;Acc:VGNC:86829]	6.46	0.0163
ssc-miR-182	CNOT2	CCR4-NOT transcription complex subunit 2 [Source:VGNC Symbol;Acc:VGNC:86834]	6.46	0.0163
ssc-miR-182	CNOT6	CCR4-NOT transcription complex subunit 6 [Source:VGNC Symbol;Acc:VGNC:86837]	6.46	0.0163
ssc-miR-182	CNOT6L	CCR4-NOT transcription complex subunit 6 like [Source:VGNC Symbol;Acc:VGNC:86838]	6.46	0.0163
ssc-miR-182	CNPY3	canopy FGF signaling regulator 3 [Source:VGNC Symbol;Acc:VGNC:86841]	6.46	0.0163
ssc-miR-182	CNR1	cannabinoid receptor 1 [Source:VGNC Symbol;Acc:VGNC:86843]	6.46	0.0163
ssc-miR-182	CNTN1	contactin 1 [Source:VGNC Symbol;Acc:VGNC:86848]	6.46	0.0163
ssc-miR-182	CNTN6	contactin 6 [Source:VGNC Symbol;Acc:VGNC:108643]	6.46	0.0163
ssc-miR-182	COBL	hypothetical gene	6.46	0.0163
ssc-miR-182	COBLL1	cordon-bleu WH2 repeat protein like 1 [Source:VGNC Symbol;Acc:VGNC:96013]	6.46	0.0163
ssc-miR-182	COG3	component of oligomeric golgi complex 3 [Source:HGNC Symbol;Acc:HGNC:18619]	6.46	0.0163
ssc-miR-182	COL13A1	collagen type XIII alpha 1 chain [Source:VGNC Symbol;Acc:VGNC:86865]	6.46	0.0163
ssc-miR-182	COL25A1	collagen type XXV alpha 1 chain [Source:HGNC Symbol;Acc:HGNC:18603]	6.46	0.0163
ssc-miR-182	COL4A4	collagen type IV alpha 4 chain [Source:VGNC Symbol;Acc:VGNC:95980]	6.46	0.0163
ssc-miR-182	COL5A1	collagen type V alpha 1 chain [Source:VGNC Symbol;Acc:VGNC:86877]	6.46	0.0163
ssc-miR-182	COPS5	COP9 signalosome subunit 5 [Source:VGNC Symbol;Acc:VGNC:86901]	6.46	0.0163
ssc-miR-182	CORO1C	coronin 1C [Source:VGNC Symbol;Acc:VGNC:86914]	6.46	0.0163
ssc-miR-182	CPEB1	cytoplasmic polyadenylation element binding protein 1 [Source:NCBI gene (formerly Entrezgene);Acc:100048944]	6.46	0.0163
ssc-miR-182	CREB1	cAMP responsive element binding protein 1 [Source:VGNC Symbol;Acc:VGNC:96004]	6.46	0.0163
ssc-miR-182	CREB3L1	cAMP responsive element binding protein 3 like 1 [Source:VGNC Symbol;Acc:VGNC:86980]	6.46	0.0163
ssc-miR-182	CREB3L2	cAMP responsive element binding protein 3 like 2 [Source:VGNC Symbol;Acc:VGNC:86981]	6.46	0.0163
ssc-miR-182	CREG2	cellular repressor of E1A stimulateds 2 [Source:VGNC Symbol;Acc:VGNC:86990]	6.46	0.0163
ssc-miR-182	CRISP3	cysteine rich secretory protein 3 [Source:HGNC Symbol;Acc:HGNC:16904]	6.46	0.0163
ssc-miR-182	CRISPLD2	cysteine rich secretory protein LCCL domain containing 2 [Source:VGNC Symbol;Acc:VGNC:96958]	6.46	0.0163
ssc-miR-182	CRKL	CRK like proto-onco, adaptor protein [Source:VGNC Symbol;Acc:VGNC:86997]	6.46	0.0163
ssc-miR-182	CROT	carnitine O-octanoyltransferase [Source:VGNC Symbol;Acc:VGNC:87002]	6.46	0.0163
ssc-miR-182	CRTC1	CREB regulated transcription coactivator 1 [Source:VGNC Symbol;Acc:VGNC:87007]	6.46	0.0163
ssc-miR-182	CSE1L	chromosome segregation 1 like [Source:VGNC Symbol;Acc:VGNC:96039]	6.46	0.0163
ssc-miR-182	CSRNP3	cysteine and serine rich nuclear protein 3 [Source:VGNC Symbol;Acc:VGNC:96249]	6.46	0.0163

ssc-miR-182	CTC-432M15.3	hypothetical gene	6.46	0.0163
ssc-miR-182	CTDP1	CTD phosphatase subunit 1 [Source:VGNC Symbol;Acc:VGNC:87058]	6.46	0.0163
ssc-miR-182	CTDSP1	CTD small phosphatase 1 [Source:VGNC Symbol;Acc:VGNC:95971]	6.46	0.0163
ssc-miR-182	CTDSP2	CTD small phosphatase 2 [Source:VGNC Symbol;Acc:VGNC:87059]	6.46	0.0163
ssc-miR-182	CTTN	cortactin [Source:VGNC Symbol;Acc:VGNC:87081]	6.46	0.0163
ssc-miR-182	CUL5	cullin 5 [Source:VGNC Symbol;Acc:VGNC:87089]	6.46	0.0163
ssc-miR-182	CXADR	CXADR Ig-like cell adhesion molecule [Source:VGNC Symbol;Acc:VGNC:87100]	6.46	0.0163
ssc-miR-182	CYB561	cytochrome b561 [Source:VGNC Symbol;Acc:VGNC:87119]	6.46	0.0163
ssc-miR-182	CYB561D1	cytochrome b561 family member D1 [Source:VGNC Symbol;Acc:VGNC:87121]	6.46	0.0163
ssc-miR-182	CYB5B	cytochrome b5 type B [Source:VGNC Symbol;Acc:VGNC:96728]	6.46	0.0163
ssc-miR-182	CYCS	cytochrome c, somatic [Source:NCBI gene (formerly Entrezgene);Acc:100170131]	6.46	0.0163
ssc-miR-182	CYLD	CYLD lysine 63 deubiquitinase [Source:VGNC Symbol;Acc:VGNC:87130]	6.46	0.0163
ssc-miR-182	CYP1B1	cytochrome P450 family 1 subfamily B member 1 [Source:VGNC Symbol;Acc:VGNC:103345]	6.46	0.0163
ssc-miR-182	CYP20A1	cytochrome P450 family 20 subfamily A member 1 [Source:NCBI gene (formerly Entrezgene);Acc:100124408]	6.46	0.0163
ssc-miR-182	CYYR1	cysteine and tyrosine rich 1 [Source:VGNC Symbol;Acc:VGNC:87139]	6.46	0.0163
ssc-miR-182	DAAM1	dishevelled associated activator of morphosis 1 [Source:VGNC Symbol;Acc:VGNC:87141]	6.46	0.0163
ssc-miR-182	DAB1	DAB adaptor protein 1 [Source:VGNC Symbol;Acc:VGNC:87143]	6.46	0.0163
ssc-miR-182	DAB2IP	DAB2 interacting protein [Source:VGNC Symbol;Acc:VGNC:87145]	6.46	0.0163
ssc-miR-182	DAZAP2	DAZ associated protein 2 [Source:VGNC Symbol;Acc:VGNC:87162]	6.46	0.0163
ssc-miR-182	DCAF10	DDB1 and CUL4 associated factor 10 [Source:VGNC Symbol;Acc:VGNC:87173]	6.46	0.0163
ssc-miR-182	DCAF12	DDB1 and CUL4 associated factor 12 [Source:VGNC Symbol;Acc:VGNC:95927]	6.46	0.0163
ssc-miR-182	DCBLD1	discoidin, CUB and LCCL domain containing 1 [Source:VGNC Symbol;Acc:VGNC:87179]	6.46	0.0163
ssc-miR-182	DCP2	decapping mRNA 2 [Source:VGNC Symbol;Acc:VGNC:87188]	6.46	0.0163
ssc-miR-182	DCUN1D1	hypothetical gene	6.46	0.0163
ssc-miR-182	DCUN1D3	defective in cullin neddylation 1 domain containing 3 [Source:VGNC Symbol;Acc:VGNC:87196]	6.46	0.0163
ssc-miR-182	DCX	doublecortin [Source:HGNC Symbol;Acc:HGNC:2714]	6.46	0.0163
ssc-miR-182	DDAH1	dimethylarginine dimethylaminohydrolase 1 [Source:VGNC Symbol;Acc:VGNC:87200]	6.46	0.0163
ssc-miR-182	DDHD1	DDHD domain containing 1 [Source:VGNC Symbol;Acc:VGNC:87205]	6.46	0.0163
ssc-miR-182	DDI2	hypothetical gene	6.46	0.0163
ssc-miR-182	DDTL	hypothetical gene	6.46	0.0163
ssc-miR-182	DENND2C	DENN domain containing 2C [Source:HGNC Symbol;Acc:HGNC:24748]	6.46	0.0163
ssc-miR-182	DENND5B	DENN domain containing 5B [Source:VGNC Symbol;Acc:VGNC:87255]	6.46	0.0163
ssc-miR-182	DENND6A	DENN domain containing 6A [Source:VGNC Symbol;Acc:VGNC:87256]	6.46	0.0163
ssc-miR-182	DENR	density regulated re-initiation and release factor [Source:VGNC Symbol;Acc:VGNC:97963]	6.46	0.0163
ssc-miR-182	DEPDC1	DEP domain containing 1 [Source:VGNC Symbol;Acc:VGNC:87258]	6.46	0.0163
ssc-miR-182	DEPTOR	DEP domain containing MTOR interacting protein [Source:HGNC Symbol;Acc:HGNC:22953]	6.46	0.0163
ssc-miR-182	DERL1	derlin 1 [Source:NCBI gene (formerly Entrezgene);Acc:100626802]	6.46	0.0163
ssc-miR-182	DGCR2	DiGeorge syndrome critical region gene 2 [Source:HGNC Symbol;Acc:HGNC:2845]	6.46	0.0163
ssc-miR-182	DGKH	diacylglycerol kinase eta [Source:VGNC Symbol;Acc:VGNC:87273]	6.46	0.0163
ssc-miR-182	DHCR24	24-dehydrocholesterol reductase [Source:NCBI gene (formerly Entrezgene);Acc:100628197]	6.46	0.0163
ssc-miR-182	DHX36	DEAH-box helicase 36 [Source:VGNC Symbol;Acc:VGNC:87292]	6.46	0.0163
ssc-miR-182	DICER1	dicer 1, ribonuclease III [Source:VGNC Symbol;Acc:VGNC:87302]	6.46	0.0163
ssc-miR-182	DIEXF	hypothetical gene	6.46	0.0163
ssc-miR-182	DIO2	iodothyronine deiodinase 2 [Source:VGNC Symbol;Acc:VGNC:103935]	6.46	0.0163
ssc-miR-182	DIXDC1	hypothetical gene	6.46	0.0163
ssc-miR-182	DKK2	dickkopf WNT signaling pathway inhibitor 2 [Source:VGNC Symbol;Acc:VGNC:87322]	6.46	0.0163
ssc-miR-182	DLEU1	hypothetical gene	6.46	0.0163
ssc-miR-182	DLGAP2	DLG associated protein 2 [Source:VGNC Symbol;Acc:VGNC:99711]	6.46	0.0163
ssc-miR-182	DMRT3	doublesex and mab-3 related transcription factor 3 [Source:VGNC Symbol;Acc:VGNC:87352]	6.46	0.0163

ssc-miR-182	DMXL1	Dmx like 1 [Source:VGNC Symbol;Acc:VGNC:87359]	6.46	0.0163
ssc-miR-182	DNAH10OS	hypothetical gene	6.46	0.0163
ssc-miR-182	DNAJB14	DnaJ heat shock protein family (Hsp40) member B14 [Source:VGNC Symbol;Acc:VGNC:98919]	6.46	0.0163
ssc-miR-182	DNAJB9	DnaJ heat shock protein family (Hsp40) member B9 [Source:VGNC Symbol;Acc:VGNC:96687]	6.46	0.0163
ssc-miR-182	DNAJC30	DnaJ heat shock protein family (Hsp40) member C30 [Source:HGNC Symbol;Acc:HGNC:16410]	6.46	0.0163
ssc-miR-182	DNAL1	dynein axonemal light chain 1 [Source:VGNC Symbol;Acc:VGNC:87373]	6.46	0.0163
ssc-miR-182	DOCK11	dedicator of cytokinesis 11 [Source:VGNC Symbol;Acc:VGNC:87391]	6.46	0.0163
ssc-miR-182	DOCK4	dedicator of cytokinesis 4 [Source:VGNC Symbol;Acc:VGNC:87394]	6.46	0.0163
ssc-miR-182	DOCK9	dedicator of cytokinesis 9 [Source:VGNC Symbol;Acc:VGNC:87399]	6.46	0.0163
ssc-miR-182	DOHH	deoxyhypusine hydroxylase [Source:VGNC Symbol;Acc:VGNC:87400]	6.46	0.0163
ssc-miR-182	DOK4	docking protein 4 [Source:VGNC Symbol;Acc:VGNC:87403]	6.46	0.0163
ssc-miR-182	DONSON	DNA replication fork stabilization factor DONSON [Source:VGNC Symbol;Acc:VGNC:96920]	6.46	0.0163
ssc-miR-182	DPY19L3	dpy-19 like C-mannosyltransferase 3 [Source:VGNC Symbol;Acc:VGNC:87426]	6.46	0.0163
ssc-miR-182	DPYSL5	dihydropyrimidinase like 5 [Source:VGNC Symbol;Acc:VGNC:87433]	6.46	0.0163
ssc-miR-182	DR1	down-regulator of transcription 1 [Source:VGNC Symbol;Acc:VGNC:87435]	6.46	0.0163
ssc-miR-182	DRGX	dorsal root ganglia homeobox [Source:VGNC Symbol;Acc:VGNC:97968]	6.46	0.0163
ssc-miR-182	DSCAM	DS cell adhesion molecule [Source:VGNC Symbol;Acc:VGNC:87453]	6.46	0.0163
ssc-miR-182	DSG2	desmoglein 2 [Source:VGNC Symbol;Acc:VGNC:87456]	6.46	0.0163
ssc-miR-182	DST	dystonin [Source:HGNC Symbol;Acc:HGNC:1090]	6.46	0.0163
ssc-miR-182	DTD2	D-aminoacyl-tRNA deacylase 2 [Source:VGNC Symbol;Acc:VGNC:97970]	6.46	0.0163
ssc-miR-182	DTNBP1	dystrobrevin binding protein 1 [Source:VGNC Symbol;Acc:VGNC:87463]	6.46	0.0163
ssc-miR-182	DUSP1	dual specificity phosphatase 1 [Source:VGNC Symbol;Acc:VGNC:87476]	6.46	0.0163
ssc-miR-182	DUSP22	dual specificity phosphatase 22 [Source:VGNC Symbol;Acc:VGNC:87482]	6.46	0.0163
ssc-miR-182	DUSP4	dual specificity phosphatase 4 [Source:VGNC Symbol;Acc:VGNC:96248]	6.46	0.0163
ssc-miR-182	DYNC1I1	dynein cytoplasmic 1 intermediate chain 1 [Source:VGNC Symbol;Acc:VGNC:87496]	6.46	0.0163
ssc-miR-182	DYNC1LI2	dynein cytoplasmic 1 light intermediate chain 2 [Source:VGNC Symbol;Acc:VGNC:87498]	6.46	0.0163
ssc-miR-182	DYNLL2	dynein light chain LC8-type 2 [Source:VGNC Symbol;Acc:VGNC:87501]	6.46	0.0163
ssc-miR-182	DYRK1A	dual specificity tyrosine phosphorylation regulated kinase 1A [Source:VGNC Symbol;Acc:VGNC:87505]	6.46	0.0163
ssc-miR-182	DYRK2	dual specificity tyrosine phosphorylation regulated kinase 2 [Source:VGNC Symbol;Acc:VGNC:87507]	6.46	0.0163
ssc-miR-182	EBF1	EBF transcription factor 1 [Source:VGNC Symbol;Acc:VGNC:87525]	6.46	0.0163
ssc-miR-182	EBF3	EBF transcription factor 3 [Source:VGNC Symbol;Acc:VGNC:87527]	6.46	0.0163
ssc-miR-182	EBF4	EBF family member 4 [Source:VGNC Symbol;Acc:VGNC:96211]	6.46	0.0163
ssc-miR-182	EDC3	enhancer of mRNA decapping 3 [Source:VGNC Symbol;Acc:VGNC:87543]	6.46	0.0163
ssc-miR-182	EDNRB	endothelin receptor type B [Source:VGNC Symbol;Acc:VGNC:87550]	6.46	0.0163
ssc-miR-182	EEA1	early endosome antigen 1 [Source:HGNC Symbol;Acc:HGNC:3185]	6.46	0.0163
ssc-miR-182	EFNA5	ephrin A5 [Source:VGNC Symbol;Acc:VGNC:87575]	6.46	0.0163
ssc-miR-182	EFNB2	ephrin B2 [Source:VGNC Symbol;Acc:VGNC:87577]	6.46	0.0163
ssc-miR-182	EGLN1	egl-9 family hypoxia inducible factor 1 [Source:VGNC Symbol;Acc:VGNC:87588]	6.46	0.0163
ssc-miR-182	EGR3	early growth response 3 [Source:VGNC Symbol;Acc:VGNC:87591]	6.46	0.0163
ssc-miR-182	EI24	EI24 autophagy associated transmembrane protein [Source:VGNC Symbol;Acc:VGNC:87602]	6.46	0.0163
ssc-miR-182	EIF1AX	eukaryotic translation initiation factor 1A X-linked [Source:NCBI gene (formerly Entrezgene);Acc:100522912]	6.46	0.0163
ssc-miR-182	EIF2AK2	eukaryotic translation initiation factor 2 alpha kinase 2 [Source:VGNC Symbol;Acc:VGNC:87607]	6.46	0.0163
ssc-miR-182	EIF2S1	eukaryotic translation initiation factor 2 subunit alpha [Source:VGNC Symbol;Acc:VGNC:87614]	6.46	0.0163
ssc-miR-182	EIF3A	eukaryotic translation initiation factor 3 subunit A [Source:VGNC Symbol;Acc:VGNC:87615]	6.46	0.0163
ssc-miR-182	EIF3H	eukaryotic translation initiation factor 3 subunit H [Source:VGNC Symbol;Acc:VGNC:87620]	6.46	0.0163
ssc-miR-182	EIF3J	eukaryotic translation initiation factor 3 subunit J [Source:VGNC Symbol;Acc:VGNC:87621]	6.46	0.0163
ssc-miR-182	EIF4EBP2	eukaryotic translation initiation factor 4E binding protein 2 [Source:VGNC Symbol;Acc:VGNC:87629]	6.46	0.0163
ssc-miR-182	EIF4EBP3	eukaryotic translation initiation factor 4E binding protein 3 [Source:NCBI gene (formerly Entrezgene);Acc:100529254]	6.46	0.0163
ssc-miR-182	EIF5	hypothetical gene	6.46	0.0163

ssc-miR-182	EIF5A2	eukaryotic translation initiation factor 5A2 [Source:VGNC Symbol;Acc:VGNC:87633]	6.46	0.0163
ssc-miR-182	ELAVL2	ELAV like RNA binding protein 2 [Source:VGNC Symbol;Acc:VGNC:87639]	6.46	0.0163
ssc-miR-182	ELAVL4	ELAV like RNA binding protein 4 [Source:VGNC Symbol;Acc:VGNC:97047]	6.46	0.0163
ssc-miR-182	ELK4	ETS transcription factor ELK4 [Source:VGNC Symbol;Acc:VGNC:87647]	6.46	0.0163
ssc-miR-182	ELL	elongation factor for RNA polymerase II [Source:VGNC Symbol;Acc:VGNC:87648]	6.46	0.0163
ssc-miR-182	ELL2	elongation factor for RNA polymerase II 2 [Source:VGNC Symbol;Acc:VGNC:87649]	6.46	0.0163
ssc-miR-182	ELMO1	engulfment and cell motility 1 [Source:HGNC Symbol;Acc:HGNC:16286]	6.46	0.0163
ssc-miR-182	ELMSAN1	hypothetical gene	6.46	0.0163
ssc-miR-182	ELOVL2	ELOVL fatty acid elongase 2 [Source:VGNC Symbol;Acc:VGNC:87658]	6.46	0.0163
ssc-miR-182	EML5	hypothetical gene	6.46	0.0163
ssc-miR-182	ENTPD1	ectonucleoside triphosphate diphosphohydrolase 1 [Source:VGNC Symbol;Acc:VGNC:97977]	6.46	0.0163
ssc-miR-182	ENY2	ENY2 transcription and export complex 2 subunit [Source:VGNC Symbol;Acc:VGNC:98792]	6.46	0.0163
ssc-miR-182	EOMES	eomesodermin [Source:VGNC Symbol;Acc:VGNC:87719]	6.46	0.0163
ssc-miR-182	EP300	E1A binding protein p300 [Source:VGNC Symbol;Acc:VGNC:87720]	6.46	0.0163
ssc-miR-182	EPAS1	endothelial PAS domain protein 1 [Source:VGNC Symbol;Acc:VGNC:87721]	6.46	0.0163
ssc-miR-182	EPB41L4B	erythrocyte membrane protein band 4.1 like 4B [Source:VGNC Symbol;Acc:VGNC:103088]	6.46	0.0163
ssc-miR-182	EPHA3	EPH receptor A3 [Source:VGNC Symbol;Acc:VGNC:87732]	6.46	0.0163
ssc-miR-182	EPHA7	EPH receptor A7 [Source:VGNC Symbol;Acc:VGNC:87734]	6.46	0.0163
ssc-miR-182	EPHB1	EPH receptor B1 [Source:VGNC Symbol;Acc:VGNC:87736]	6.46	0.0163
ssc-miR-182	EPHX3	epoxide hydrolase 3 [Source:VGNC Symbol;Acc:VGNC:97980]	6.46	0.0163
ssc-miR-182	EPT1	hypothetical gene	6.46	0.0163
ssc-miR-182	ERBB2	erb-b2 receptor tyrosine kinase 2 [Source:VGNC Symbol;Acc:VGNC:87759]	6.46	0.0163
ssc-miR-182	ERBB4	erb-b2 receptor tyrosine kinase 4 [Source:VGNC Symbol;Acc:VGNC:96284]	6.46	0.0163
ssc-miR-182	ERC1	ELKS/RAB6-interacting/CAST family member 1 [Source:VGNC Symbol;Acc:VGNC:87761]	6.46	0.0163
ssc-miR-182	ERG	ETS transcription factor ERG [Source:VGNC Symbol;Acc:VGNC:87768]	6.46	0.0163
ssc-miR-182	ERLIN1	hypothetical gene	6.46	0.0163
ssc-miR-182	ERO1LB	hypothetical gene	6.46	0.0163
ssc-miR-182	ESCO1	establishment of sister chromatid cohesion N-acetyltransferase 1 [Source:VGNC Symbol;Acc:VGNC:87786]	6.46	0.0163
ssc-miR-182	ESRRG	estrogen related receptor gamma [Source:VGNC Symbol;Acc:VGNC:96289]	6.46	0.0163
ssc-miR-182	ESYT1	extended synaptotagmin 1 [Source:VGNC Symbol;Acc:VGNC:87796]	6.46	0.0163
ssc-miR-182	ETF1	eukaryotic translation termination factor 1 [Source:VGNC Symbol;Acc:VGNC:87800]	6.46	0.0163
ssc-miR-182	ETNK2	ethanolamine kinase 2 [Source:VGNC Symbol;Acc:VGNC:87807]	6.46	0.0163
ssc-miR-182	ETV3	ETS variant transcription factor 3 [Source:VGNC Symbol;Acc:VGNC:87812]	6.46	0.0163
ssc-miR-182	EVI5	ecotropic viral integration site 5 [Source:VGNC Symbol;Acc:VGNC:98793]	6.46	0.0163
ssc-miR-182	EVI5L	ecotropic viral integration site 5 like [Source:VGNC Symbol;Acc:VGNC:99646]	6.46	0.0163
ssc-miR-182	EVX2	even-skipped homeobox 2 [Source:VGNC Symbol;Acc:VGNC:96290]	6.46	0.0163
ssc-miR-182	EXOC4	hypothetical gene	6.46	0.0163
ssc-miR-182	EXT1	exostosin glycosyltransferase 1 [Source:VGNC Symbol;Acc:VGNC:87846]	6.46	0.0163
ssc-miR-182	EYA3	EYA transcriptional coactivator and phosphatase 3 [Source:VGNC Symbol;Acc:VGNC:87852]	6.46	0.0163
ssc-miR-182	EZR	ezrin [Source:VGNC Symbol;Acc:VGNC:87856]	6.46	0.0163
ssc-miR-182	F13A1	coagulation factor XIII A chain [Source:VGNC Symbol;Acc:VGNC:87858]	6.46	0.0163
ssc-miR-182	FABP2	fatty acid binding protein 2 [Source:VGNC Symbol;Acc:VGNC:87871]	6.46	0.0163
ssc-miR-182	FAM107A	hypothetical gene	6.46	0.0163
ssc-miR-182	FAM110B	family with sequence similarity 110 member B [Source:VGNC Symbol;Acc:VGNC:87885]	6.46	0.0163
ssc-miR-182	FAM118A	family with sequence similarity 118 member A [Source:VGNC Symbol;Acc:VGNC:87893]	6.46	0.0163
ssc-miR-182	FAM122A	hypothetical gene	6.46	0.0163
ssc-miR-182	FAM126B	family with sequence similarity 126 member B [Source:HGNC Symbol;Acc:HGNC:28593]	6.46	0.0163
ssc-miR-182	FAM133A	family with sequence similarity 133 member A [Source:VGNC Symbol;Acc:VGNC:87903]	6.46	0.0163
ssc-miR-182	FAM134B	hypothetical gene	6.46	0.0163

ssc-miR-182	FAM13A	family with sequence similarity 13 member A [Source:VGNC Symbol;Acc:VGNC:98924]	6.46	0.0163
ssc-miR-182	FAM149B1	family with sequence similarity 149 member B1 [Source:VGNC Symbol;Acc:VGNC:87908]	6.46	0.0163
ssc-miR-182	FAM151B	family with sequence similarity 151 member B [Source:VGNC Symbol;Acc:VGNC:87910]	6.46	0.0163
ssc-miR-182	FAM160B2	hypothetical gene	6.46	0.0163
ssc-miR-182	FAM167A	family with sequence similarity 167 member A [Source:VGNC Symbol;Acc:VGNC:87924]	6.46	0.0163
ssc-miR-182	FAM168A	family with sequence similarity 168 member A [Source:VGNC Symbol;Acc:VGNC:87926]	6.46	0.0163
ssc-miR-182	FAM171A1	family with sequence similarity 171 member A1 [Source:VGNC Symbol;Acc:VGNC:96011]	6.46	0.0163
ssc-miR-182	FAM188A	hypothetical gene	6.46	0.0163
ssc-miR-182	FAM212B	hypothetical gene	6.46	0.0163
ssc-miR-182	FAM216A	family with sequence similarity 216 member A [Source:VGNC Symbol;Acc:VGNC:87954]	6.46	0.0163
ssc-miR-182	FAM222B	family with sequence similarity 222 member B [Source:VGNC Symbol;Acc:VGNC:87960]	6.46	0.0163
ssc-miR-182	FAM43A	family with sequence similarity 43 member A [Source:VGNC Symbol;Acc:VGNC:87970]	6.46	0.0163
ssc-miR-182	FAM49B	hypothetical gene	6.46	0.0163
ssc-miR-182	FAM53C	family with sequence similarity 53 member C [Source:VGNC Symbol;Acc:VGNC:87975]	6.46	0.0163
ssc-miR-182	FAM60A	hypothetical gene	6.46	0.0163
ssc-miR-182	FAM73A	hypothetical gene	6.46	0.0163
ssc-miR-182	FAM78A	family with sequence similarity 78 member A [Source:VGNC Symbol;Acc:VGNC:87984]	6.46	0.0163
ssc-miR-182	FAM8A1	family with sequence similarity 8 member A1 [Source:VGNC Symbol;Acc:VGNC:87996]	6.46	0.0163
ssc-miR-182	FAM91A1	family with sequence similarity 91 member A1 [Source:VGNC Symbol;Acc:VGNC:97998]	6.46	0.0163
ssc-miR-182	FAM98B	family with sequence similarity 98 member B [Source:VGNC Symbol;Acc:VGNC:87998]	6.46	0.0163
ssc-miR-182	FARP1	FERM, ARH/RhoGEF and pleckstrin domain protein 1 [Source:VGNC Symbol;Acc:VGNC:88012]	6.46	0.0163
ssc-miR-182	FAT3	hypothetical gene	6.46	0.0163
ssc-miR-182	FBN1	fibrillin 1 [Source:VGNC Symbol;Acc:VGNC:103090]	6.46	0.0163
ssc-miR-182	FBXL4	F-box and leucine rich repeat protein 4 [Source:VGNC Symbol;Acc:VGNC:98007]	6.46	0.0163
ssc-miR-182	FBXO27	hypothetical gene	6.46	0.0163
ssc-miR-182	FBXO28	F-box protein 28 [Source:HGNC Symbol;Acc:HGNC:29046]	6.46	0.0163
ssc-miR-182	FBXO33	F-box protein 33 [Source:VGNC Symbol;Acc:VGNC:88040]	6.46	0.0163
ssc-miR-182	FBXO41	F-box protein 41 [Source:VGNC Symbol;Acc:VGNC:88046]	6.46	0.0163
ssc-miR-182	FBXO42	F-box protein 42 [Source:VGNC Symbol;Acc:VGNC:88047]	6.46	0.0163
ssc-miR-182	FBXW11	F-box and WD repeat domain containing 11 [Source:HGNC Symbol;Acc:HGNC:13607]	6.46	0.0163
ssc-miR-182	FBXW7	F-box and WD repeat domain containing 7 [Source:VGNC Symbol;Acc:VGNC:98925]	6.46	0.0163
ssc-miR-182	FDX1	ferredoxin 1 [Source:VGNC Symbol;Acc:VGNC:88078]	6.46	0.0163
ssc-miR-182	FEM1B	fem-1 homolog B [Source:VGNC Symbol;Acc:VGNC:88083]	6.46	0.0163
ssc-miR-182	FEM1C	fem-1 homolog C [Source:VGNC Symbol;Acc:VGNC:88084]	6.46	0.0163
ssc-miR-182	FGD4	FYVE, RhoGEF and PH domain containing 4 [Source:VGNC Symbol;Acc:VGNC:88097]	6.46	0.0163
ssc-miR-182	FGF14	fibroblast growth factor 14 [Source:VGNC Symbol;Acc:VGNC:88104]	6.46	0.0163
ssc-miR-182	FGF9	fibroblast growth factor 9 [Source:VGNC Symbol;Acc:VGNC:103943]	6.46	0.0163
ssc-miR-182	FHL1	four and a half LIM domains 1 [Source:VGNC Symbol;Acc:VGNC:88127]	6.46	0.0163
ssc-miR-182	FICD	FIC domain protein adenylyltransferase [Source:VGNC Symbol;Acc:VGNC:88135]	6.46	0.0163
ssc-miR-182	FIGN	fidgetin, microtubule severing factor [Source:VGNC Symbol;Acc:VGNC:95580]	6.46	0.0163
ssc-miR-182	FKBP15	FKBP prolyl isomerase family member 15 [Source:VGNC Symbol;Acc:VGNC:88145]	6.46	0.0163
ssc-miR-182	FLJ00388	hypothetical gene	6.46	0.0163
ssc-miR-182	FLNB	filamin B [Source:VGNC Symbol;Acc:VGNC:88156]	6.46	0.0163
ssc-miR-182	FLOT1	flotillin 1 [Source:VGNC Symbol;Acc:VGNC:88158]	6.46	0.0163
ssc-miR-182	FMR1	FMRP translational regulator 1 [Source:VGNC Symbol;Acc:VGNC:88175]	6.46	0.0163
ssc-miR-182	FN1	fibronectin 1 [Source:VGNC Symbol;Acc:VGNC:96305]	6.46	0.0163
ssc-miR-182	FNBP1	formin binding protein 1 [Source:VGNC Symbol;Acc:VGNC:88178]	6.46	0.0163
ssc-miR-182	FNBP1L	formin binding protein 1 like [Source:VGNC Symbol;Acc:VGNC:88179]	6.46	0.0163
ssc-miR-182	FNDC3B	fibronectin type III domain containing 3B [Source:VGNC Symbol;Acc:VGNC:88183]	6.46	0.0163

ssc-miR-182	FOCAD	focadhesin [Source:VGNC Symbol;Acc:VGNC:88189]	6.46	0.0163
ssc-miR-182	FOSL2	FOS like 2, AP-1 transcription factor subunit [Source:VGNC Symbol;Acc:VGNC:88192]	6.46	0.0163
ssc-miR-182	FOXF2	forkhead box F2 [Source:VGNC Symbol;Acc:VGNC:88205]	6.46	0.0163
ssc-miR-182	FOXK2	forkhead box K2 [Source:VGNC Symbol;Acc:VGNC:88215]	6.46	0.0163
ssc-miR-182	FOXN2	forkhead box N2 [Source:VGNC Symbol;Acc:VGNC:88219]	6.46	0.0163
ssc-miR-182	FOXN3	forkhead box N3 [Source:VGNC Symbol;Acc:VGNC:88220]	6.46	0.0163
ssc-miR-182	FOXO1	forkhead box O1 [Source:VGNC Symbol;Acc:VGNC:98013]	6.46	0.0163
ssc-miR-182	FOXO3	forkhead box O3 [Source:VGNC Symbol;Acc:VGNC:99715]	6.46	0.0163
ssc-miR-182	FOXP2	forkhead box P2 [Source:VGNC Symbol;Acc:VGNC:98014]	6.46	0.0163
ssc-miR-182	FOXQ1	forkhead box Q1 [Source:VGNC Symbol;Acc:VGNC:88225]	6.46	0.0163
ssc-miR-182	FOXRED2	FAD dependent oxidoreductase domain containing 2 [Source:VGNC Symbol;Acc:VGNC:88229]	6.46	0.0163
ssc-miR-182	FRK	fyn related Src family tyrosine kinase [Source:VGNC Symbol;Acc:VGNC:88235]	6.46	0.0163
ssc-miR-182	FRMD4A	FERM domain containing 4A [Source:VGNC Symbol;Acc:VGNC:96088]	6.46	0.0163
ssc-miR-182	FRMD5	FERM domain containing 5 [Source:VGNC Symbol;Acc:VGNC:88238]	6.46	0.0163
ssc-miR-182	FRS2	fibroblast growth factor receptor substrate 2 [Source:VGNC Symbol;Acc:VGNC:88246]	6.46	0.0163
ssc-miR-182	FST	follistatin [Source:NCBI gene (formerly Entrezgene);Acc:445002]	6.46	0.0163
ssc-miR-182	FTSJ2	hypothetical gene	6.46	0.0163
ssc-miR-182	FUT4	fucosyltransferase 4 [Source:HGNC Symbol;Acc:HGNC:4015]	6.46	0.0163
ssc-miR-182	FUT9	fucosyltransferase 9 [Source:VGNC Symbol;Acc:VGNC:88271]	6.46	0.0163
ssc-miR-182	FXR1	FMR1 autosomal homolog 1 [Source:VGNC Symbol;Acc:VGNC:108659]	6.46	0.0163
ssc-miR-182	FYCO1	hypothetical gene	6.46	0.0163
ssc-miR-182	FYTTD1	forty-two-three domain containing 1 [Source:VGNC Symbol;Acc:VGNC:88277]	6.46	0.0163
ssc-miR-182	FZD3	frizzled class receptor 3 [Source:VGNC Symbol;Acc:VGNC:88281]	6.46	0.0163
ssc-miR-182	GAB2	GRB2 associated binding protein 2 [Source:VGNC Symbol;Acc:VGNC:108586]	6.46	0.0163
ssc-miR-182	GAB4	hypothetical gene	6.46	0.0163
ssc-miR-182	GABBR1	gamma-aminobutyric acid type B receptor subunit 1 [Source:VGNC Symbol;Acc:VGNC:88297]	6.46	0.0163
ssc-miR-182	GABRB1	gamma-aminobutyric acid type A receptor subunit beta1 [Source:VGNC Symbol;Acc:VGNC:88306]	6.46	0.0163
ssc-miR-182	GAD2	glutamate decarboxylase 2 [Source:VGNC Symbol;Acc:VGNC:108273]	6.46	0.0163
ssc-miR-182	GALNT2	polypeptide N-acetylgalactosaminyltransferase 2 [Source:VGNC Symbol;Acc:VGNC:88334]	6.46	0.0163
ssc-miR-182	GALNT5	polypeptide N-acetylgalactosaminyltransferase 5 [Source:VGNC Symbol;Acc:VGNC:96314]	6.46	0.0163
ssc-miR-182	GAN	gigaxonin [Source:VGNC Symbol;Acc:VGNC:88343]	6.46	0.0163
ssc-miR-182	GAPVD1	GTPase activating protein and VPS9 domains 1 [Source:VGNC Symbol;Acc:VGNC:88347]	6.46	0.0163
ssc-miR-182	GATA6	GATA binding protein 6 [Source:VGNC Symbol;Acc:VGNC:88366]	6.46	0.0163
ssc-miR-182	GATSL2	hypothetical gene	6.46	0.0163
ssc-miR-182	GCC1	GRIP and coiled-coil domain containing 1 [Source:VGNC Symbol;Acc:VGNC:88379]	6.46	0.0163
ssc-miR-182	GCNT1	glucosaminyl (N-acetyl) transferase 1 [Source:VGNC Symbol;Acc:VGNC:103096]	6.46	0.0163
ssc-miR-182	GCNT4	glucosaminyl (N-acetyl) transferase 4 [Source:VGNC Symbol;Acc:VGNC:96616]	6.46	0.0163
ssc-miR-182	GDF6	growth differentiation factor 6 [Source:VGNC Symbol;Acc:VGNC:88402]	6.46	0.0163
ssc-miR-182	GDNF	glial cell derived neurotrophic factor [Source:VGNC Symbol;Acc:VGNC:88406]	6.46	0.0163
ssc-miR-182	GID4	GID complex subunit 4 homolog [Source:VGNC Symbol;Acc:VGNC:98996]	6.46	0.0163
ssc-miR-182	GIT1	GIT ArfGAP 1 [Source:VGNC Symbol;Acc:VGNC:88457]	6.46	0.0163
ssc-miR-182	GIT2	GIT ArfGAP 2 [Source:VGNC Symbol;Acc:VGNC:88458]	6.46	0.0163
ssc-miR-182	GJA3	gap junction protein alpha 3 [Source:HGNC Symbol;Acc:HGNC:4277]	6.46	0.0163
ssc-miR-182	GJC1	gap junction protein gamma 1 [Source:VGNC Symbol;Acc:VGNC:88468]	6.46	0.0163
ssc-miR-182	GK	hypothetical gene	6.46	0.0163
ssc-miR-182	GLB1L	galactosidase beta 1 like [Source:VGNC Symbol;Acc:VGNC:96165]	6.46	0.0163
ssc-miR-182	GLIPR2	GLI pathosis related 2 [Source:VGNC Symbol;Acc:VGNC:98023]	6.46	0.0163
ssc-miR-182	GLRA3	glycine receptor alpha 3 [Source:VGNC Symbol;Acc:VGNC:88493]	6.46	0.0163
ssc-miR-182	GLRX2	hypothetical gene	6.46	0.0163

ssc-miR-182	GMFB	glia maturation factor beta [Source:VGNC Symbol;Acc:VGNC:88509]	6.46	0.0163
ssc-miR-182	GMPS	guanine monophosphate synthase [Source:VGNC Symbol;Acc:VGNC:88516]	6.46	0.0163
ssc-miR-182	GNA13	G protein subunit alpha 13 [Source:VGNC Symbol;Acc:VGNC:98997]	6.46	0.0163
ssc-miR-182	GNAI3	G protein subunit alpha i3 [Source:VGNC Symbol;Acc:VGNC:88523]	6.46	0.0163
ssc-miR-182	GNAQ	G protein subunit alpha q [Source:VGNC Symbol;Acc:VGNC:103100]	6.46	0.0163
ssc-miR-182	GNB2L1	hypothetical gene	6.46	0.0163
ssc-miR-182	GNE	glucosamine (UDP-N-acetyl)-2-epimerase/N-acetylmannosamine kinase [Source:VGNC Symbol;Acc:VGNC:103101]	6.46	0.0163
ssc-miR-182	GNG7	G protein subunit gamma 7 [Source:HGNC Symbol;Acc:HGNC:4410]	6.46	0.0163
ssc-miR-182	GPATCH2	G-patch domain containing 2 [Source:VGNC Symbol;Acc:VGNC:96089]	6.46	0.0163
ssc-miR-182	GPC1	glypican 1 [Source:VGNC Symbol;Acc:VGNC:96329]	6.46	0.0163
ssc-miR-182	GPHN	gephyrin [Source:VGNC Symbol;Acc:VGNC:88587]	6.46	0.0163
ssc-miR-182	GPR124	hypothetical gene	6.46	0.0163
ssc-miR-182	GPR135	G protein-coupled receptor 135 [Source:VGNC Symbol;Acc:VGNC:88599]	6.46	0.0163
ssc-miR-182	GPR137B	G protein-coupled receptor 137B [Source:VGNC Symbol;Acc:VGNC:88601]	6.46	0.0163
ssc-miR-182	GPR155	G protein-coupled receptor 155 [Source:VGNC Symbol;Acc:VGNC:95984]	6.46	0.0163
ssc-miR-182	GPR158	G protein-coupled receptor 158 [Source:VGNC Symbol;Acc:VGNC:96016]	6.46	0.0163
ssc-miR-182	GPR173	G protein-coupled receptor 173 [Source:VGNC Symbol;Acc:VGNC:88616]	6.46	0.0163
ssc-miR-182	GPR22	G protein-coupled receptor 22 [Source:VGNC Symbol;Acc:VGNC:88625]	6.46	0.0163
ssc-miR-182	GPR6	hypothetical gene	6.46	0.0163
ssc-miR-182	GPR85	G protein-coupled receptor 85 [Source:VGNC Symbol;Acc:VGNC:88638]	6.46	0.0163
ssc-miR-182	GPRASP1	G protein-coupled receptor associated sorting protein 1 [Source:VGNC Symbol;Acc:VGNC:88640]	6.46	0.0163
ssc-miR-182	GPRASP2	G protein-coupled receptor associated sorting protein 2 [Source:VGNC Symbol;Acc:VGNC:88641]	6.46	0.0163
ssc-miR-182	GPRC5A	G protein-coupled receptor class C group 5 member A [Source:VGNC Symbol;Acc:VGNC:88642]	6.46	0.0163
ssc-miR-182	GPRIN3	hypothetical gene	6.46	0.0163
ssc-miR-182	GRAMD1B	GRAM domain containing 1B [Source:VGNC Symbol;Acc:VGNC:88656]	6.46	0.0163
ssc-miR-182	GRB2	growth factor receptor bound protein 2 [Source:VGNC Symbol;Acc:VGNC:88663]	6.46	0.0163
ssc-miR-182	GREB1	growth regulating estrogen receptor binding 1 [Source:VGNC Symbol;Acc:VGNC:96583]	6.46	0.0163
ssc-miR-182	GRHL2	grainyhead like transcription factor 2 [Source:VGNC Symbol;Acc:VGNC:88667]	6.46	0.0163
ssc-miR-182	GRIA1	glutamate ionotropic receptor AMPA type subunit 1 [Source:VGNC Symbol;Acc:VGNC:88670]	6.46	0.0163
ssc-miR-182	GRID1	glutamate ionotropic receptor delta type subunit 1 [Source:VGNC Symbol;Acc:VGNC:88674]	6.46	0.0163
ssc-miR-182	GRID2	glutamate ionotropic receptor delta type subunit 2 [Source:VGNC Symbol;Acc:VGNC:98932]	6.46	0.0163
ssc-miR-182	GRIK3	glutamate ionotropic receptor kainate type subunit 3 [Source:VGNC Symbol;Acc:VGNC:88679]	6.46	0.0163
ssc-miR-182	GRIK4	glutamate ionotropic receptor kainate type subunit 4 [Source:VGNC Symbol;Acc:VGNC:88680]	6.46	0.0163
ssc-miR-182	GRIN2A	glutamate ionotropic receptor NMDA type subunit 2A [Source:VGNC Symbol;Acc:VGNC:88683]	6.46	0.0163
ssc-miR-182	GRM5	glutamate metabotropic receptor 5 [Source:VGNC Symbol;Acc:VGNC:88703]	6.46	0.0163
ssc-miR-182	GRPEL2	GrpE like 2, mitochondrial [Source:VGNC Symbol;Acc:VGNC:88708]	6.46	0.0163
ssc-miR-182	GSPT1	G1 to S phase transition 1 [Source:VGNC Symbol;Acc:VGNC:88724]	6.46	0.0163
ssc-miR-182	GTPBP1	GTP binding protein 1 [Source:VGNC Symbol;Acc:VGNC:88742]	6.46	0.0163
ssc-miR-182	GTPBP10	GTP binding protein 10 [Source:VGNC Symbol;Acc:VGNC:103959]	6.46	0.0163
ssc-miR-182	GUCY1A2	guanylate cyclase 1 soluble subunit alpha 2 [Source:VGNC Symbol;Acc:VGNC:88751]	6.46	0.0163
ssc-miR-182	GUF1	GTP binding elongation factor GUF1 [Source:VGNC Symbol;Acc:VGNC:88754]	6.46	0.0163
ssc-miR-182	GXYLT1	glucoside xylosyltransferase 1 [Source:VGNC Symbol;Acc:VGNC:88756]	6.46	0.0163
ssc-miR-182	GXYLT2	glucoside xylosyltransferase 2 [Source:VGNC Symbol;Acc:VGNC:88757]	6.46	0.0163
ssc-miR-182	H6PD	hexose-6-phosphate dehydrogenase/glucose 1-dehydrogenase [Source:VGNC Symbol;Acc:VGNC:88764]	6.46	0.0163
ssc-miR-182	HAND1	heart and neural crest derivatives expressed 1 [Source:VGNC Symbol;Acc:VGNC:88777]	6.46	0.0163
ssc-miR-182	HAS2	hyaluronan synthase 2 [Source:HGNC Symbol;Acc:HGNC:4819]	6.46	0.0163
ssc-miR-182	HBEGF	heparin binding EGF like growth factor [Source:VGNC Symbol;Acc:VGNC:88792]	6.46	0.0163
ssc-miR-182	HDAC2	histone deacetylase 2 [Source:VGNC Symbol;Acc:VGNC:88813]	6.46	0.0163
ssc-miR-182	HDAC7	histone deacetylase 7 [Source:VGNC Symbol;Acc:VGNC:103286]	6.46	0.0163

ssc-miR-182	HDAC9	histone deacetylase 9 [Source:HGNC Symbol;Acc:HGNC:14065]	6.46	0.0163
ssc-miR-182	HECW2	HECT, C2 and WW domain containing E3 ubiquitin protein ligase 2 [Source:NCBI gene (formerly Entrezgene);Acc:100155879]	6.46	0.0163
ssc-miR-182	HES1	hes family bHLH transcription factor 1 [Source:VGNC Symbol;Acc:VGNC:88854]	6.46	0.0163
ssc-miR-182	HEY2	hes related family bHLH transcription factor with YRPW motif 2 [Source:VGNC Symbol;Acc:VGNC:88864]	6.46	0.0163
ssc-miR-182	HHIP	hedgehog interacting protein [Source:VGNC Symbol;Acc:VGNC:88875]	6.46	0.0163
ssc-miR-182	HIF1AN	hypoxia inducible factor 1 subunit alpha inhibitor [Source:VGNC Symbol;Acc:VGNC:98033]	6.46	0.0163
ssc-miR-182	HIST1H2BH	hypothetical gene	6.46	0.0163
ssc-miR-182	HMCN1	hemicentin 1 [Source:VGNC Symbol;Acc:VGNC:108590]	6.46	0.0163
ssc-miR-182	HMGCLL1	3-hydroxymethyl-3-methylglutaryl-CoA lyase like 1 [Source:VGNC Symbol;Acc:VGNC:88905]	6.46	0.0163
ssc-miR-182	HNF4A	hepatocyte nuclear factor 4 alpha [Source:VGNC Symbol;Acc:VGNC:96348]	6.46	0.0163
ssc-miR-182	HNF4G	hepatocyte nuclear factor 4 gamma [Source:VGNC Symbol;Acc:VGNC:88917]	6.46	0.0163
ssc-miR-182	HOOK3	hook microtubule tethering protein 3 [Source:VGNC Symbol;Acc:VGNC:98037]	6.46	0.0163
ssc-miR-182	HOXA9	homeobox A9 [Source:HGNC Symbol;Acc:HGNC:5109]	6.46	0.0163
ssc-miR-182	HSBP1	heat shock factor binding protein 1 [Source:NCBI gene (formerly Entrezgene);Acc:100579143]	6.46	0.0163
ssc-miR-182	HSDL1	hydroxysteroid dehydrogenase like 1 [Source:VGNC Symbol;Acc:VGNC:88991]	6.46	0.0163
ssc-miR-182	HSPA13	heat shock protein family A (Hsp70) member 13 [Source:VGNC Symbol;Acc:VGNC:108663]	6.46	0.0163
ssc-miR-182	HUNK	hormonally up-regulated Neu-associated kinase [Source:VGNC Symbol;Acc:VGNC:89007]	6.46	0.0163
ssc-miR-182	IBTK	inhibitor of Bruton tyrosine kinase [Source:VGNC Symbol;Acc:VGNC:89017]	6.46	0.0163
ssc-miR-182	IER5L	immediate early response 5 like [Source:VGNC Symbol;Acc:VGNC:89029]	6.46	0.0163
ssc-miR-182	IFFO2	intermediate filament family orphan 2 [Source:VGNC Symbol;Acc:VGNC:98475]	6.46	0.0163
ssc-miR-182	IGDCC3	immunoglobulin superfamily DCC subclass member 3 [Source:HGNC Symbol;Acc:HGNC:9700]	6.46	0.0163
ssc-miR-182	IGF1R	insulin like growth factor 1 receptor [Source:NCBI gene (formerly Entrezgene);Acc:397350]	6.46	0.0163
ssc-miR-182	IGF2BP1	insulin like growth factor 2 mRNA binding protein 1 [Source:VGNC Symbol;Acc:VGNC:99006]	6.46	0.0163
ssc-miR-182	IGSF10	immunoglobulin superfamily member 10 [Source:VGNC Symbol;Acc:VGNC:89063]	6.46	0.0163
ssc-miR-182	IGSF3	immunoglobulin superfamily member 3 [Source:VGNC Symbol;Acc:VGNC:89067]	6.46	0.0163
ssc-miR-182	IKZF1	IKAROS family zinc finger 1 [Source:VGNC Symbol;Acc:VGNC:89073]	6.46	0.0163
ssc-miR-182	IKZF3	IKAROS family zinc finger 3 [Source:VGNC Symbol;Acc:VGNC:89074]	6.46	0.0163
ssc-miR-182	IL5RA	interleukin 5 receptor subunit alpha [Source:VGNC Symbol;Acc:VGNC:89111]	6.46	0.0163
ssc-miR-182	IMPAD1	hypothetical gene	6.46	0.0163
ssc-miR-182	IMPG2	interphotoreceptor matrix proteoglycan 2 [Source:VGNC Symbol;Acc:VGNC:98048]	6.46	0.0163
ssc-miR-182	INIP	INTS3 and NABP interacting protein [Source:VGNC Symbol;Acc:VGNC:89136]	6.46	0.0163
ssc-miR-182	INO80D	INO80 complex subunit D [Source:VGNC Symbol;Acc:VGNC:96113]	6.46	0.0163
ssc-miR-182	INPP4A	inositol polyphosphate-4-phosphatase type I A [Source:VGNC Symbol;Acc:VGNC:89140]	6.46	0.0163
ssc-miR-182	INPP5A	inositol polyphosphate-5-phosphatase A [Source:VGNC Symbol;Acc:VGNC:89142]	6.46	0.0163
ssc-miR-182	INSIG1	insulin induced 1 [Source:VGNC Symbol;Acc:VGNC:89150]	6.46	0.0163
ssc-miR-182	INSIG2	insulin induced 2 [Source:VGNC Symbol;Acc:VGNC:103970]	6.46	0.0163
ssc-miR-182	INSL5	hypothetical gene	6.46	0.0163
ssc-miR-182	INSM2	INSM transcriptional repressor 2 [Source:VGNC Symbol;Acc:VGNC:89152]	6.46	0.0163
ssc-miR-182	INSR	insulin receptor [Source:VGNC Symbol;Acc:VGNC:89153]	6.46	0.0163
ssc-miR-182	INTS6	integrator complex subunit 6 [Source:VGNC Symbol;Acc:VGNC:89166]	6.46	0.0163
ssc-miR-182	IPO9	importin 9 [Source:VGNC Symbol;Acc:VGNC:95913]	6.46	0.0163
ssc-miR-182	IQSEC2	IQ motif and Sec7 domain ArfGEF 2 [Source:VGNC Symbol;Acc:VGNC:89195]	6.46	0.0163
ssc-miR-182	IRF2BPL	interferon regulatory factor 2 binding protein like [Source:HGNC Symbol;Acc:HGNC:14282]	6.46	0.0163
ssc-miR-182	IRF6	interferon regulatory factor 6 [Source:VGNC Symbol;Acc:VGNC:89209]	6.46	0.0163
ssc-miR-182	IRS4	insulin receptor substrate 4 [Source:HGNC Symbol;Acc:HGNC:6128]	6.46	0.0163
ssc-miR-182	ISL1	ISL LIM homeobox 1 [Source:VGNC Symbol;Acc:VGNC:89225]	6.46	0.0163
ssc-miR-182	ITGB8	integrin subunit beta 8 [Source:VGNC Symbol;Acc:VGNC:89246]	6.46	0.0163
ssc-miR-182	ITPR1	inositol 1,4,5-trisphosphate receptor type 1 [Source:VGNC Symbol;Acc:VGNC:89253]	6.46	0.0163
ssc-miR-182	ITSN2	intersectin 2 [Source:VGNC Symbol;Acc:VGNC:89259]	6.46	0.0163

ssc-miR-182	IVNS1ABP	influenza virus NS1A binding protein [Source:NCBI gene (formerly Entrezgene);Acc:100302027]	6.46	0.0163
ssc-miR-182	JAKMIP2	janus kinase and microtubule interacting protein 2 [Source:VGNC Symbol;Acc:VGNC:89274]	6.46	0.0163
ssc-miR-182	JAZF1	JAZF zinc finger 1 [Source:VGNC Symbol;Acc:VGNC:89280]	6.46	0.0163
ssc-miR-182	JMJD1C	jumonji domain containing 1C [Source:VGNC Symbol;Acc:VGNC:89285]	6.46	0.0163
ssc-miR-182	JMY	junction mediating and regulatory protein, p53 cofactor [Source:VGNC Symbol;Acc:VGNC:89289]	6.46	0.0163
ssc-miR-182	KAT7	lysine acetyltransferase 7 [Source:VGNC Symbol;Acc:VGNC:89307]	6.46	0.0163
ssc-miR-182	KCMF1	potassium channel modulatory factor 1 [Source:VGNC Symbol;Acc:VGNC:89322]	6.46	0.0163
ssc-miR-182	KCNA2	potassium voltage-gated channel subfamily A member 2 [Source:VGNC Symbol;Acc:VGNC:89325]	6.46	0.0163
ssc-miR-182	KCNC3	potassium voltage-gated channel subfamily C member 3 [Source:VGNC Symbol;Acc:VGNC:89334]	6.46	0.0163
ssc-miR-182	KCNH5	potassium voltage-gated channel subfamily H member 5 [Source:VGNC Symbol;Acc:VGNC:89345]	6.46	0.0163
ssc-miR-182	KCNJ13	potassium inwardly rectifying channel subfamily J member 13 [Source:VGNC Symbol;Acc:VGNC:96389]	6.46	0.0163
ssc-miR-182	KCNJ14	potassium inwardly rectifying channel subfamily J member 14 [Source:VGNC Symbol;Acc:VGNC:89354]	6.46	0.0163
ssc-miR-182	KCNJ3	potassium inwardly rectifying channel subfamily J member 3 [Source:HGNC Symbol;Acc:HGNC:6264]	6.46	0.0163
ssc-miR-182	KCNJ5	potassium inwardly rectifying channel subfamily J member 5 [Source:VGNC Symbol;Acc:VGNC:89359]	6.46	0.0163
ssc-miR-182	KCNJ6	potassium inwardly rectifying channel subfamily J member 6 [Source:VGNC Symbol;Acc:VGNC:89360]	6.46	0.0163
ssc-miR-182	KCNK10	potassium two pore domain channel subfamily K member 10 [Source:VGNC Symbol;Acc:VGNC:89364]	6.46	0.0163
ssc-miR-182	KCNMB2	potassium calcium-activated channel subfamily M regulatory beta subunit 2 [Source:HGNC Symbol;Acc:HGNC:6286]	6.46	0.0163
ssc-miR-182	KCNN3	potassium calcium-activated channel subfamily N member 3 [Source:VGNC Symbol;Acc:VGNC:98056]	6.46	0.0163
ssc-miR-182	KCTD16	potassium channel tetramerization domain containing 16 [Source:VGNC Symbol;Acc:VGNC:89395]	6.46	0.0163
ssc-miR-182	KCTD2	potassium channel tetramerization domain containing 2 [Source:VGNC Symbol;Acc:VGNC:89398]	6.46	0.0163
ssc-miR-182	KCTD7	potassium channel tetramerization domain containing 7 [Source:VGNC Symbol;Acc:VGNC:89403]	6.46	0.0163
ssc-miR-182	KDELC2	hypothetical gene	6.46	0.0163
ssc-miR-182	KDELR1	KDEL endoplasmic reticulum protein retention receptor 1 [Source:VGNC Symbol;Acc:VGNC:98483]	6.46	0.0163
ssc-miR-182	KDELR2	KDEL endoplasmic reticulum protein retention receptor 2 [Source:VGNC Symbol;Acc:VGNC:89405]	6.46	0.0163
ssc-miR-182	KDM2B	lysine demethylase 2B [Source:VGNC Symbol;Acc:VGNC:98057]	6.46	0.0163
ssc-miR-182	KDM5A	lysine demethylase 5A [Source:VGNC Symbol;Acc:VGNC:89415]	6.46	0.0163
ssc-miR-182	KDM6A	lysine demethylase 6A [Source:HGNC Symbol;Acc:HGNC:12637]	6.46	0.0163
ssc-miR-182	KIAA0087	hypothetical gene	6.46	0.0163
ssc-miR-182	KIAA0408	KIAA0408 [Source:VGNC Symbol;Acc:VGNC:89432]	6.46	0.0163
ssc-miR-182	KIAA0513	KIAA0513 [Source:VGNC Symbol;Acc:VGNC:89433]	6.46	0.0163
ssc-miR-182	KIAA0907	hypothetical gene	6.46	0.0163
ssc-miR-182	KIAA1210	hypothetical gene	6.46	0.0163
ssc-miR-182	KIAA1211	hypothetical gene	6.46	0.0163
ssc-miR-182	KIAA1217	KIAA1217 [Source:VGNC Symbol;Acc:VGNC:96079]	6.46	0.0163
ssc-miR-182	KIAA1244	hypothetical gene	6.46	0.0163
ssc-miR-182	KIAA1324	hypothetical gene	6.46	0.0163
ssc-miR-182	KIAA1324L	hypothetical gene	6.46	0.0163
ssc-miR-182	KIAA1549	KIAA1549 [Source:VGNC Symbol;Acc:VGNC:99719]	6.46	0.0163
ssc-miR-182	KIAA2022	hypothetical gene	6.46	0.0163
ssc-miR-182	KIF19	kinesin family member 19 [Source:VGNC Symbol;Acc:VGNC:89459]	6.46	0.0163
ssc-miR-182	KIF26A	kinesin family member 26A [Source:VGNC Symbol;Acc:VGNC:89466]	6.46	0.0163
ssc-miR-182	KIF2A	kinesin family member 2A [Source:VGNC Symbol;Acc:VGNC:89467]	6.46	0.0163
ssc-miR-182	KIF3B	kinesin family member 3B [Source:VGNC Symbol;Acc:VGNC:96392]	6.46	0.0163
ssc-miR-182	KIF5A	kinesin family member 5A [Source:VGNC Symbol;Acc:VGNC:89472]	6.46	0.0163
ssc-miR-182	KLF13	Kruppel like factor 13 [Source:VGNC Symbol;Acc:VGNC:89493]	6.46	0.0163
ssc-miR-182	KLF15	Kruppel like factor 15 [Source:VGNC Symbol;Acc:VGNC:89495]	6.46	0.0163
ssc-miR-182	KLF7	Kruppel like factor 7 [Source:VGNC Symbol;Acc:VGNC:96396]	6.46	0.0163
ssc-miR-182	KLHDC10	kelch domain containing 10 [Source:VGNC Symbol;Acc:VGNC:89502]	6.46	0.0163
ssc-miR-182	KLHL13	kelch like family member 13 [Source:VGNC Symbol;Acc:VGNC:89514]	6.46	0.0163

ssc-miR-182	KLHL18	kelch like family member 18 [Source:VGNC Symbol;Acc:VGNC:89517]	6.46	0.0163
ssc-miR-182	KLHL2	kelch like family member 2 [Source:VGNC Symbol;Acc:VGNC:89518]	6.46	0.0163
ssc-miR-182	KLHL28	kelch like family member 28 [Source:VGNC Symbol;Acc:VGNC:89523]	6.46	0.0163
ssc-miR-182	KLHL31	kelch like family member 31 [Source:VGNC Symbol;Acc:VGNC:89526]	6.46	0.0163
ssc-miR-182	KLHL38	kelch like family member 38 [Source:VGNC Symbol;Acc:VGNC:89531]	6.46	0.0163
ssc-miR-182	KLHL7	kelch like family member 7 [Source:VGNC Symbol;Acc:VGNC:103974]	6.46	0.0163
ssc-miR-182	KLHL8	kelch like family member 8 [Source:VGNC Symbol;Acc:VGNC:89536]	6.46	0.0163
ssc-miR-182	KMT2A	lysine methyltransferase 2A [Source:VGNC Symbol;Acc:VGNC:108600]	6.46	0.0163
ssc-miR-182	KPNA1	karyopherin subunit alpha 1 [Source:VGNC Symbol;Acc:VGNC:89560]	6.46	0.0163
ssc-miR-182	KPNA3	karyopherin subunit alpha 3 [Source:VGNC Symbol;Acc:VGNC:89562]	6.46	0.0163
ssc-miR-182	KPNA5	karyopherin subunit alpha 5 [Source:VGNC Symbol;Acc:VGNC:89564]	6.46	0.0163
ssc-miR-182	KPNB1	karyopherin subunit beta 1 [Source:VGNC Symbol;Acc:VGNC:89567]	6.46	0.0163
ssc-miR-182	KTN1	kinectin 1 [Source:VGNC Symbol;Acc:VGNC:89608]	6.46	0.0163
ssc-miR-182	L1CAM	L1 cell adhesion molecule [Source:HGNC Symbol;Acc:HGNC:6470]	6.46	0.0163
ssc-miR-182	LAMC1	laminin subunit gamma 1 [Source:VGNC Symbol;Acc:VGNC:89624]	6.46	0.0163
ssc-miR-182	LARP1	La ribonucleoprotein 1, translational regulator [Source:VGNC Symbol;Acc:VGNC:98067]	6.46	0.0163
ssc-miR-182	LARP4	La ribonucleoprotein 4 [Source:VGNC Symbol;Acc:VGNC:89641]	6.46	0.0163
ssc-miR-182	LARP4B	hypothetical gene	6.46	0.0163
ssc-miR-182	LASP1	LIM and SH3 protein 1 [Source:VGNC Symbol;Acc:VGNC:89646]	6.46	0.0163
ssc-miR-182	LCA5	lebercilin LCA5 [Source:VGNC Symbol;Acc:VGNC:89654]	6.46	0.0163
ssc-miR-182	LCOR	ligand dependent nuclear receptor corepressor [Source:HGNC Symbol;Acc:HGNC:29503]	6.46	0.0163
ssc-miR-182	LDB3	hypothetical gene	6.46	0.0163
ssc-miR-182	LDLRAP1	low density lipoprotein receptor adaptor protein 1 [Source:VGNC Symbol;Acc:VGNC:89674]	6.46	0.0163
ssc-miR-182	LDOC1L	hypothetical gene	6.46	0.0163
ssc-miR-182	LGI1	leucine rich glioma inactivated 1 [Source:HGNC Symbol;Acc:HGNC:6572]	6.46	0.0163
ssc-miR-182	LHFPL2	LHFPL tetraspan subfamily member 2 [Source:VGNC Symbol;Acc:VGNC:89705]	6.46	0.0163
ssc-miR-182	LHX1	LIM homeobox 1 [Source:VGNC Symbol;Acc:VGNC:89711]	6.46	0.0163
ssc-miR-182	LHX3	LIM homeobox 3 [Source:VGNC Symbol;Acc:VGNC:89713]	6.46	0.0163
ssc-miR-182	LIMCH1	LIM and calponin homology domains 1 [Source:VGNC Symbol;Acc:VGNC:89724]	6.46	0.0163
ssc-miR-182	LIMS1	hypothetical gene	6.46	0.0163
ssc-miR-182	LIN28A	lin-28 homolog A [Source:VGNC Symbol;Acc:VGNC:98492]	6.46	0.0163
ssc-miR-182	LIPG	lipase G, endothelial type [Source:VGNC Symbol;Acc:VGNC:89739]	6.46	0.0163
ssc-miR-182	LMCD1	LIM and cysteine rich domains 1 [Source:VGNC Symbol;Acc:VGNC:89756]	6.46	0.0163
ssc-miR-182	LMNB2	lamin B2 [Source:VGNC Symbol;Acc:VGNC:89761]	6.46	0.0163
ssc-miR-182	LMOD1	leiomodin 1 [Source:VGNC Symbol;Acc:VGNC:96405]	6.46	0.0163
ssc-miR-182	LMTK2	lemur tyrosine kinase 2 [Source:HGNC Symbol;Acc:HGNC:17880]	6.46	0.0163
ssc-miR-182	LPHN1	hypothetical gene	6.46	0.0163
ssc-miR-182	LPHN2	hypothetical gene	6.46	0.0163
ssc-miR-182	LPHN3	hypothetical gene	6.46	0.0163
ssc-miR-182	LPP	LIM domain containing preferred translocation partner in lipoma [Source:HGNC Symbol;Acc:HGNC:6679]	6.46	0.0163
ssc-miR-182	LPPR4	hypothetical gene	6.46	0.0163
ssc-miR-182	LRCH2	leucine rich repeats and calponin homology domain containing 2 [Source:VGNC Symbol;Acc:VGNC:89802]	6.46	0.0163
ssc-miR-182	LRIG2	leucine rich repeats and immunoglobulin like domains 2 [Source:VGNC Symbol;Acc:VGNC:89811]	6.46	0.0163
ssc-miR-182	LRP10	LDL receptor related protein 10 [Source:VGNC Symbol;Acc:VGNC:89816]	6.46	0.0163
ssc-miR-182	LRP12	LDL receptor related protein 12 [Source:VGNC Symbol;Acc:VGNC:89818]	6.46	0.0163
ssc-miR-182	LRP1B	LDL receptor related protein 1B [Source:HGNC Symbol;Acc:HGNC:6693]	6.46	0.0163
ssc-miR-182	LRRC4	leucine rich repeat containing 4 [Source:VGNC Symbol;Acc:VGNC:89839]	6.46	0.0163
ssc-miR-182	LRRC7	leucine rich repeat containing 7 [Source:HGNC Symbol;Acc:HGNC:18531]	6.46	0.0163
ssc-miR-182	LRRC8C	leucine rich repeat containing 8 VRAC subunit C [Source:VGNC Symbol;Acc:VGNC:98087]	6.46	0.0163

ssc-miR-182	LSM14A	LSM14A mRNA processing body assembly factor [Source:VGNC Symbol;Acc:VGNC:89872]	6.46	0.0163
ssc-miR-182	LUZP1	leucine zipper protein 1 [Source:VGNC Symbol;Acc:VGNC:89897]	6.46	0.0163
ssc-miR-182	LYVE1	lymphatic vessel endothelial hyaluronan receptor 1 [Source:VGNC Symbol;Acc:VGNC:89922]	6.46	0.0163
ssc-miR-182	MAF	MAF bZIP transcription factor [Source:VGNC Symbol;Acc:VGNC:89945]	6.46	0.0163
ssc-miR-182	MAGEL2	hypothetical gene	6.46	0.0163
ssc-miR-182	MAGI1	membrane associated guanylate kinase, WW and PDZ domain containing 1 [Source:VGNC Symbol;Acc:VGNC:98097]	6.46	0.0163
ssc-miR-182	MAK	male germ cell associated kinase [Source:VGNC Symbol;Acc:VGNC:89959]	6.46	0.0163
ssc-miR-182	MAN1A2	mannosidase alpha class 1A member 2 [Source:VGNC Symbol;Acc:VGNC:89967]	6.46	0.0163
ssc-miR-182	MAP1B	microtubule associated protein 1B [Source:VGNC Symbol;Acc:VGNC:89979]	6.46	0.0163
ssc-miR-182	MAP1LC3B	microtubule associated protein 1 light chain 3 beta [Source:NCBI gene (formerly Entrezgene);Acc:100462754]	6.46	0.0163
ssc-miR-182	MAP2K1	mitogen-activated protein kinase kinase 1 [Source:VGNC Symbol;Acc:VGNC:103121]	6.46	0.0163
ssc-miR-182	MAP2K6	mitogen-activated protein kinase kinase 6 [Source:VGNC Symbol;Acc:VGNC:98102]	6.46	0.0163
ssc-miR-182	MAP3K2	mitogen-activated protein kinase kinase kinase 2 [Source:VGNC Symbol;Acc:VGNC:98107]	6.46	0.0163
ssc-miR-182	MAP3K3	mitogen-activated protein kinase kinase kinase 3 [Source:VGNC Symbol;Acc:VGNC:98108]	6.46	0.0163
ssc-miR-182	MAP4K2	mitogen-activated protein kinase kinase kinase kinase 2 [Source:VGNC Symbol;Acc:VGNC:98112]	6.46	0.0163
ssc-miR-182	MAP9	microtubule associated protein 9 [Source:HGNC Symbol;Acc:HGNC:26118]	6.46	0.0163
ssc-miR-182	MAPK1	mitogen-activated protein kinase 1 [Source:VGNC Symbol;Acc:VGNC:89996]	6.46	0.0163
ssc-miR-182	MAPK1IP1L	mitogen-activated protein kinase 1 interacting protein 1 like [Source:VGNC Symbol;Acc:VGNC:90002]	6.46	0.0163
ssc-miR-182	MAPRE1	microtubule associated protein RP/EB family member 1 [Source:VGNC Symbol;Acc:VGNC:96410]	6.46	0.0163
ssc-miR-182	MAPRE2	microtubule associated protein RP/EB family member 2 [Source:VGNC Symbol;Acc:VGNC:98117]	6.46	0.0163
ssc-miR-182	MARC1	hypothetical gene	6.46	0.0163
ssc-miR-182	MARCH3	hypothetical gene	6.46	0.0163
ssc-miR-182	MARCKS	myristoylated alanine rich protein kinase C substrate [Source:VGNC Symbol;Acc:VGNC:90024]	6.46	0.0163
ssc-miR-182	MBLAC2	metallo-beta-lactamase domain containing 2 [Source:VGNC Symbol;Acc:VGNC:90053]	6.46	0.0163
ssc-miR-182	MBNL2	muscleblind like splicing regulator 2 [Source:VGNC Symbol;Acc:VGNC:90055]	6.46	0.0163
ssc-miR-182	MBOAT2	membrane bound O-acyltransferase domain containing 2 [Source:VGNC Symbol;Acc:VGNC:90058]	6.46	0.0163
ssc-miR-182	MCFD2	multiple coagulation factor deficiency 2, ER cargo receptor complex subunit [Source:VGNC Symbol;Acc:VGNC:103980]	6.46	0.0163
ssc-miR-182	MCM10	minichromosome maintenance 10 replication initiation factor [Source:VGNC Symbol;Acc:VGNC:95871]	6.46	0.0163
ssc-miR-182	MCM9	hypothetical gene	6.46	0.0163
ssc-miR-182	MCMBP	minichromosome maintenance complex binding protein [Source:VGNC Symbol;Acc:VGNC:90077]	6.46	0.0163
ssc-miR-182	MCTP2	multiple C2 and transmembrane domain containing 2 [Source:VGNC Symbol;Acc:VGNC:90084]	6.46	0.0163
ssc-miR-182	MDM4	MDM4 regulator of p53 [Source:VGNC Symbol;Acc:VGNC:90094]	6.46	0.0163
ssc-miR-182	ME2	malic enzyme 2 [Source:VGNC Symbol;Acc:VGNC:90096]	6.46	0.0163
ssc-miR-182	MECOM	MDS1 and EVI1 complex locus [Source:VGNC Symbol;Acc:VGNC:90100]	6.46	0.0163
ssc-miR-182	MED1	mediator complex subunit 1 [Source:VGNC Symbol;Acc:VGNC:90102]	6.46	0.0163
ssc-miR-182	MED12L	mediator complex subunit 12L [Source:VGNC Symbol;Acc:VGNC:90105]	6.46	0.0163
ssc-miR-182	MED14	mediator complex subunit 14 [Source:VGNC Symbol;Acc:VGNC:90108]	6.46	0.0163
ssc-miR-182	MEF2C	myocyte enhancer factor 2C [Source:VGNC Symbol;Acc:VGNC:90127]	6.46	0.0163
ssc-miR-182	MEF2D	myocyte enhancer factor 2D [Source:VGNC Symbol;Acc:VGNC:90128]	6.46	0.0163
ssc-miR-182	MEIS2	Meis homeobox 2 [Source:VGNC Symbol;Acc:VGNC:90136]	6.46	0.0163
ssc-miR-182	MEOX1	mesenchyme homeobox 1 [Source:VGNC Symbol;Acc:VGNC:90141]	6.46	0.0163
ssc-miR-182	MET	MET proto-onco, receptor tyrosine kinase [Source:NCBI gene (formerly Entrezgene);Acc:654328]	6.46	0.0163
ssc-miR-182	METTL8	methyltransferase 8, methylcytidine [Source:VGNC Symbol;Acc:VGNC:96110]	6.46	0.0163
ssc-miR-182	MFAP3	microfibril associated protein 3 [Source:VGNC Symbol;Acc:VGNC:90174]	6.46	0.0163
ssc-miR-182	MGAT2	alpha-1,6-mannosyl-glycoprotein 2-beta-N-acetylglucosaminyltransferase [Source:HGNC Symbol;Acc:HGNC:7045]	6.46	0.0163
ssc-miR-182	MGAT5	alpha-1,6-mannosylglycoprotein 6-beta-N-acetylglucosaminyltransferase [Source:VGNC Symbol;Acc:VGNC:96741]	6.46	0.0163
ssc-miR-182	MISP	mitotic spindle positioning [Source:VGNC Symbol;Acc:VGNC:90241]	6.46	0.0163
ssc-miR-182	MITF	melanocyte inducing transcription factor [Source:VGNC Symbol;Acc:VGNC:90243]	6.46	0.0163
ssc-miR-182	MKL2	hypothetical gene	6.46	0.0163

ssc-miR-182	MLLT1	MLLT1 super elongation complex subunit [Source:VGNC Symbol;Acc:VGNC:90255]	6.46	0.0163
ssc-miR-182	MLX	MAX dimerization protein MLX [Source:VGNC Symbol;Acc:VGNC:90261]	6.46	0.0163
ssc-miR-182	MMD	monocyte to macrophage differentiation associated [Source:VGNC Symbol;Acc:VGNC:90264]	6.46	0.0163
ssc-miR-182	MMP16	matrix metallopeptidase 16 [Source:VGNC Symbol;Acc:VGNC:90271]	6.46	0.0163
ssc-miR-182	MOB1B	hypothetical gene	6.46	0.0163
ssc-miR-182	MOB3B	MOB kinase activator 3B [Source:VGNC Symbol;Acc:VGNC:96024]	6.46	0.0163
ssc-miR-182	MORC3	MORC family CW-type zinc finger 3 [Source:VGNC Symbol;Acc:VGNC:90304]	6.46	0.0163
ssc-miR-182	MORF4L1	mortality factor 4 like 1 [Source:VGNC Symbol;Acc:VGNC:90306]	6.46	0.0163
ssc-miR-182	MPP1	hypothetical gene	6.46	0.0163
ssc-miR-182	MPV17L	MPV17 mitochondrial inner membrane protein like [Source:VGNC Symbol;Acc:VGNC:98139]	6.46	0.0163
ssc-miR-182	MRAS	muscle RAS onco homolog [Source:VGNC Symbol;Acc:VGNC:90340]	6.46	0.0163
ssc-miR-182	MRPL39	mitochondrial ribosomal protein L39 [Source:VGNC Symbol;Acc:VGNC:90364]	6.46	0.0163
ssc-miR-182	MSL2	MSL complex subunit 2 [Source:VGNC Symbol;Acc:VGNC:90424]	6.46	0.0163
ssc-miR-182	MSS51	MSS51 mitochondrial translational activator [Source:VGNC Symbol;Acc:VGNC:90428]	6.46	0.0163
ssc-miR-182	MST4	hypothetical gene	6.46	0.0163
ssc-miR-182	MTCH2	hypothetical gene	6.46	0.0163
ssc-miR-182	MTDH	metadherin [Source:VGNC Symbol;Acc:VGNC:90439]	6.46	0.0163
ssc-miR-182	MTERFD1	hypothetical gene	6.46	0.0163
ssc-miR-182	MTSS1	MTSS I-BAR domain containing 1 [Source:VGNC Symbol;Acc:VGNC:90474]	6.46	0.0163
ssc-miR-182	MYADM	myeloid associated differentiation marker [Source:VGNC Symbol;Acc:VGNC:98508]	6.46	0.0163
ssc-miR-182	MYB	MYB proto-onco, transcription factor [Source:VGNC Symbol;Acc:VGNC:90496]	6.46	0.0163
ssc-miR-182	MYCBP2	MYC binding protein 2 [Source:VGNC Symbol;Acc:VGNC:90502]	6.46	0.0163
ssc-miR-182	MYD88	MYD88 innate immune signal transduction adaptor [Source:NCBI gene (formerly Entrezgene);Acc:396646]	6.46	0.0163
ssc-miR-182	MYO19	myosin XIX [Source:VGNC Symbol;Acc:VGNC:90524]	6.46	0.0163
ssc-miR-182	MYO1C	myosin IC [Source:VGNC Symbol;Acc:VGNC:90526]	6.46	0.0163
ssc-miR-182	MYO1D	myosin ID [Source:VGNC Symbol;Acc:VGNC:90527]	6.46	0.0163
ssc-miR-182	MYO9A	myosin IXA [Source:VGNC Symbol;Acc:VGNC:103138]	6.46	0.0163
ssc-miR-182	MYOZ2	myozenin 2 [Source:NCBI gene (formerly Entrezgene);Acc:733663]	6.46	0.0163
ssc-miR-182	MYRIP	myosin VIIA and Rab interacting protein [Source:NCBI gene (formerly Entrezgene);Acc:100156583]	6.46	0.0163
ssc-miR-182	MYT1L	myelin transcription factor 1 like [Source:VGNC Symbol;Acc:VGNC:90549]	6.46	0.0163
ssc-miR-182	N4BP1	NEDD4 binding protein 1 [Source:VGNC Symbol;Acc:VGNC:90553]	6.46	0.0163
ssc-miR-182	NAA50	N-alpha-acetyltransferase 50, NatE catalytic subunit [Source:VGNC Symbol;Acc:VGNC:104002]	6.46	0.0163
ssc-miR-182	NABP1	nucleic acid binding protein 1 [Source:VGNC Symbol;Acc:VGNC:96122]	6.46	0.0163
ssc-miR-182	NAGPA	N-acetylglucosamine-1-phosphodiester alpha-N-acetylglucosaminidase [Source:NCBI gene (formerly Entrezgene);Acc:100512596]	6.46	0.0163
ssc-miR-182	NAMPT	nicotinamide phosphoribosyltransferase [Source:VGNC Symbol;Acc:VGNC:90573]	6.46	0.0163
ssc-miR-182	NAP1L1	nucleosome assembly protein 1 like 1 [Source:VGNC Symbol;Acc:VGNC:90576]	6.46	0.0163
ssc-miR-182	NAT8L	N-acetyltransferase 8 like [Source:VGNC Symbol;Acc:VGNC:90586]	6.46	0.0163
ssc-miR-182	NBEAL1	cytochrome P450 family 20 subfamily A member 1 [Source:VGNC Symbol;Acc:VGNC:103366]	6.46	0.0163
ssc-miR-182	NCALD	neurocalcin delta [Source:VGNC Symbol;Acc:VGNC:90594]	6.46	0.0163
ssc-miR-182	NCKAP1	NCK associated protein 1 [Source:VGNC Symbol;Acc:VGNC:96432]	6.46	0.0163
ssc-miR-182	NCOA4	nuclear receptor coactivator 4 [Source:VGNC Symbol;Acc:VGNC:90617]	6.46	0.0163
ssc-miR-182	NDNF	neuron derived neurotrophic factor [Source:VGNC Symbol;Acc:VGNC:90629]	6.46	0.0163
ssc-miR-182	NDRG1	N-myc downstream regulated 1 [Source:VGNC Symbol;Acc:VGNC:98825]	6.46	0.0163
ssc-miR-182	NECAB3	N-terminal EF-hand calcium binding protein 3 [Source:VGNC Symbol;Acc:VGNC:95670]	6.46	0.0163
ssc-miR-182	NF1	neurofibromin 1 [Source:VGNC Symbol;Acc:VGNC:90704]	6.46	0.0163
ssc-miR-182	NFASC	neurofascin [Source:VGNC Symbol;Acc:VGNC:90707]	6.46	0.0163
ssc-miR-182	NFAT5	nuclear factor of activated T cells 5 [Source:VGNC Symbol;Acc:VGNC:90708]	6.46	0.0163
ssc-miR-182	NFIC	nuclear factor I C [Source:VGNC Symbol;Acc:VGNC:100313]	6.46	0.0163
ssc-miR-182	NFX1	nuclear transcription factor, X-box binding 1 [Source:VGNC Symbol;Acc:VGNC:96442]	6.46	0.0163

ssc-miR-182	NHLRC3	NHL repeat containing 3 [Source:HGNC Symbol;Acc:HGNC:33751]	6.46	0.0163
ssc-miR-182	NHS	NHS actin remodeling regulator [Source:VGNC Symbol;Acc:VGNC:90738]	6.46	0.0163
ssc-miR-182	NID1	nidogen 1 [Source:VGNC Symbol;Acc:VGNC:90742]	6.46	0.0163
ssc-miR-182	NIPA1	NIPA magnesium transporter 1 [Source:VGNC Symbol;Acc:VGNC:95455]	6.46	0.0163
ssc-miR-182	NIPBL	NIPBL cohesin loading factor [Source:VGNC Symbol;Acc:VGNC:90752]	6.46	0.0163
ssc-miR-182	NKAPL	NFKB activating protein like [Source:VGNC Symbol;Acc:VGNC:90758]	6.46	0.0163
ssc-miR-182	NKX2-2	NK2 homeobox 2 [Source:VGNC Symbol;Acc:VGNC:96444]	6.46	0.0163
ssc-miR-182	NLGN2	hypothetical gene	6.46	0.0163
ssc-miR-182	NMT1	N-myristoyltransferase 1 [Source:VGNC Symbol;Acc:VGNC:90802]	6.46	0.0163
ssc-miR-182	NMT2	N-myristoyltransferase 2 [Source:VGNC Symbol;Acc:VGNC:96448]	6.46	0.0163
ssc-miR-182	NNAT	hypothetical gene	6.46	0.0163
ssc-miR-182	NNMT	nicotinamide N-methyltransferase [Source:VGNC Symbol;Acc:VGNC:90805]	6.46	0.0163
ssc-miR-182	NOP9	NOP9 nucleolar protein [Source:VGNC Symbol;Acc:VGNC:90822]	6.46	0.0163
ssc-miR-182	NOVA2	NOVA alternative splicing regulator 2 [Source:VGNC Symbol;Acc:VGNC:90828]	6.46	0.0163
ssc-miR-182	NPFFR1	neuropeptide FF receptor 1 [Source:VGNC Symbol;Acc:VGNC:90846]	6.46	0.0163
ssc-miR-182	NPR1	natriuretic peptide receptor 1 [Source:VGNC Symbol;Acc:VGNC:90856]	6.46	0.0163
ssc-miR-182	NPTX1	neuronal pentraxin 1 [Source:VGNC Symbol;Acc:VGNC:90862]	6.46	0.0163
ssc-miR-182	NPTX2	neuronal pentraxin 2 [Source:VGNC Symbol;Acc:VGNC:90863]	6.46	0.0163
ssc-miR-182	NPY4R	hypothetical gene	6.46	0.0163
ssc-miR-182	NR4A3	nuclear receptor subfamily 4 group A member 3 [Source:VGNC Symbol;Acc:VGNC:90885]	6.46	0.0163
ssc-miR-182	NRCAM	neuronal cell adhesion molecule [Source:HGNC Symbol;Acc:HGNC:7994]	6.46	0.0163
ssc-miR-182	NRF1	nuclear respiratory factor 1 [Source:VGNC Symbol;Acc:VGNC:90895]	6.46	0.0163
ssc-miR-182	NRN1	neuritin 1 [Source:VGNC Symbol;Acc:VGNC:90900]	6.46	0.0163
ssc-miR-182	NRSN1	neurensin 1 [Source:VGNC Symbol;Acc:VGNC:90902]	6.46	0.0163
ssc-miR-182	NSD1	nuclear receptor binding SET domain protein 1 [Source:VGNC Symbol;Acc:VGNC:90904]	6.46	0.0163
ssc-miR-182	NSG1	neuronal vesicle trafficking associated 1 [Source:VGNC Symbol;Acc:VGNC:90907]	6.46	0.0163
ssc-miR-182	NSG2	neuronal vesicle trafficking associated 2 [Source:HGNC Symbol;Acc:HGNC:24955]	6.46	0.0163
ssc-miR-182	NSUN3	NOP2/Sun RNA methyltransferase 3 [Source:VGNC Symbol;Acc:VGNC:90915]	6.46	0.0163
ssc-miR-182	NT5DC3	5'-nucleotidase domain containing 3 [Source:VGNC Symbol;Acc:VGNC:90924]	6.46	0.0163
ssc-miR-182	NTN4	netrin 4 [Source:VGNC Symbol;Acc:VGNC:90933]	6.46	0.0163
ssc-miR-182	NTNG1	netrin G1 [Source:VGNC Symbol;Acc:VGNC:90935]	6.46	0.0163
ssc-miR-182	NUAK1	NUAK family kinase 1 [Source:VGNC Symbol;Acc:VGNC:90940]	6.46	0.0163
ssc-miR-182	NUDCD2	NudC domain containing 2 [Source:VGNC Symbol;Acc:VGNC:90950]	6.46	0.0163
ssc-miR-182	NUDT21	nudix hydrolase 21 [Source:VGNC Symbol;Acc:VGNC:98523]	6.46	0.0163
ssc-miR-182	NUDT3	nudix hydrolase 3 [Source:NCBI gene (formerly Entrezgene);Acc:100737442]	6.46	0.0163
ssc-miR-182	NUFIP2	nuclear FMR1 interacting protein 2 [Source:VGNC Symbol;Acc:VGNC:90967]	6.46	0.0163
ssc-miR-182	NUMB	NUMB endocytic adaptor protein [Source:VGNC Symbol;Acc:VGNC:90970]	6.46	0.0163
ssc-miR-182	NUP37	nucleoporin 37 [Source:VGNC Symbol;Acc:VGNC:90980]	6.46	0.0163
ssc-miR-182	NUP50	nucleoporin 50 [Source:VGNC Symbol;Acc:VGNC:90983]	6.46	0.0163
ssc-miR-182	NUP62CL	nucleoporin 62 C-terminal like [Source:VGNC Symbol;Acc:VGNC:104013]	6.46	0.0163
ssc-miR-182	NUS1	NUS1 dehydrodolichyl diphosphate synthase subunit [Source:VGNC Symbol;Acc:VGNC:90990]	6.46	0.0163
ssc-miR-182	NXT2	nuclear transport factor 2 like export factor 2 [Source:VGNC Symbol;Acc:VGNC:91001]	6.46	0.0163
ssc-miR-182	NYAP2	neuronal tyrosine-phosphorylated phosphoinositide-3-kinase adaptor 2 [Source:VGNC Symbol;Acc:VGNC:96213]	6.46	0.0163
ssc-miR-182	OAS2	2'-5'-oligoadenylate synthetase 2 [Source:VGNC Symbol;Acc:VGNC:91007]	6.46	0.0163
ssc-miR-182	OCRL	OCRL inositol polyphosphate-5-phosphatase [Source:VGNC Symbol;Acc:VGNC:91014]	6.46	0.0163
ssc-miR-182	OGDH	oxoglutarate dehydrogenase [Source:VGNC Symbol;Acc:VGNC:98168]	6.46	0.0163
ssc-miR-182	OGFRL1	opioid growth factor receptor like 1 [Source:VGNC Symbol;Acc:VGNC:91026]	6.46	0.0163
ssc-miR-182	OGT	O-linked N-acetylglucosamine (GlcNAc) transferase [Source:NCBI gene (formerly Entrezgene);Acc:664652]	6.46	0.0163
ssc-miR-182	OIP5	Opa interacting protein 5 [Source:VGNC Symbol;Acc:VGNC:91029]	6.46	0.0163

ssc-miR-182	ONECUT2	one cut homeobox 2 [Source:VGNC Symbol;Acc:VGNC:91043]	6.46	0.0163
ssc-miR-182	ONECUT3	one cut homeobox 3 [Source:VGNC Symbol;Acc:VGNC:91044]	6.46	0.0163
ssc-miR-182	OPHN1	oligophrenin 1 [Source:VGNC Symbol;Acc:VGNC:91048]	6.46	0.0163
ssc-miR-182	ORC4	origin recognition complex subunit 4 [Source:VGNC Symbol;Acc:VGNC:96465]	6.46	0.0163
ssc-miR-182	ORC5	origin recognition complex subunit 5 [Source:VGNC Symbol;Acc:VGNC:91062]	6.46	0.0163
ssc-miR-182	OSBPL10	oxysterol binding protein like 10 [Source:VGNC Symbol;Acc:VGNC:91068]	6.46	0.0163
ssc-miR-182	OSBPL2	oxysterol binding protein like 2 [Source:VGNC Symbol;Acc:VGNC:95653]	6.46	0.0163
ssc-miR-182	OSTF1	osteoclast stimulating factor 1 [Source:VGNC Symbol;Acc:VGNC:91085]	6.46	0.0163
ssc-miR-182	OSTM1	osteoclastosis associated transmembrane protein 1 [Source:VGNC Symbol;Acc:VGNC:91086]	6.46	0.0163
ssc-miR-182	OTC	ornithine transcarbamylase [Source:NCBI gene (formerly Entrezgene);Acc:397438]	6.46	0.0163
ssc-miR-182	OTUD6A	OTU deubiquitinase 6A [Source:VGNC Symbol;Acc:VGNC:91102]	6.46	0.0163
ssc-miR-182	OTUD6B	OTU deubiquitinase 6B [Source:VGNC Symbol;Acc:VGNC:91103]	6.46	0.0163
ssc-miR-182	OTUD7A	OTU deubiquitinase 7A [Source:VGNC Symbol;Acc:VGNC:91104]	6.46	0.0163
ssc-miR-182	OXCT1	3-oxoacid CoA-transferase 1 [Source:VGNC Symbol;Acc:VGNC:91113]	6.46	0.0163
ssc-miR-182	OXSR1	hypothetical gene	6.46	0.0163
ssc-miR-182	PABPC1L2A	hypothetical gene	6.46	0.0163
ssc-miR-182	PABPC1L2B	hypothetical gene	6.46	0.0163
ssc-miR-182	PACS2	phosphofurin acidic cluster sorting protein 2 [Source:VGNC Symbol;Acc:VGNC:91141]	6.46	0.0163
ssc-miR-182	PAFAH1B1	platelet activating factor acetylhydrolase 1b regulatory subunit 1 [Source:VGNC Symbol;Acc:VGNC:99026]	6.46	0.0163
ssc-miR-182	PAFAH1B2	platelet activating factor acetylhydrolase 1b catalytic subunit 2 [Source:VGNC Symbol;Acc:VGNC:91151]	6.46	0.0163
ssc-miR-182	PAIP1	poly(A) binding protein interacting protein 1 [Source:HGNC Symbol;Acc:HGNC:16945]	6.46	0.0163
ssc-miR-182	PAIP2	poly(A) binding protein interacting protein 2 [Source:VGNC Symbol;Acc:VGNC:96618]	6.46	0.0163
ssc-miR-182	PAK3	p21 (RAC1) activated kinase 3 [Source:HGNC Symbol;Acc:HGNC:8592]	6.46	0.0163
ssc-miR-182	PALLD	palladin, cytoskeletal associated protein [Source:HGNC Symbol;Acc:HGNC:17068]	6.46	0.0163
ssc-miR-182	PAN2	poly(A) specific ribonuclease subunit PAN2 [Source:HGNC Symbol;Acc:HGNC:20074]	6.46	0.0163
ssc-miR-182	PAN3	poly(A) specific ribonuclease subunit PAN3 [Source:HGNC Symbol;Acc:HGNC:29991]	6.46	0.0163
ssc-miR-182	PANK3	pantothenate kinase 3 [Source:VGNC Symbol;Acc:VGNC:91165]	6.46	0.0163
ssc-miR-182	PAPPA	pappalysin 1 [Source:VGNC Symbol;Acc:VGNC:91170]	6.46	0.0163
ssc-miR-182	PAQR5	progestin and adipoQ receptor family member 5 [Source:VGNC Symbol;Acc:VGNC:91175]	6.46	0.0163
ssc-miR-182	PARD3B	par-3 family cell polarity regulator beta [Source:VGNC Symbol;Acc:VGNC:95618]	6.46	0.0163
ssc-miR-182	PARM1	prostate androgen-regulated mucin-like protein 1 [Source:VGNC Symbol;Acc:VGNC:91182]	6.46	0.0163
ssc-miR-182	PARPBP	PARP1 binding protein [Source:VGNC Symbol;Acc:VGNC:91183]	6.46	0.0163
ssc-miR-182	PAX5	paired box 5 [Source:VGNC Symbol;Acc:VGNC:91194]	6.46	0.0163
ssc-miR-182	PAX6	paired box 6 [Source:VGNC Symbol;Acc:VGNC:91195]	6.46	0.0163
ssc-miR-182	PBX2	PBX homeobox 2 [Source:VGNC Symbol;Acc:VGNC:91204]	6.46	0.0163
ssc-miR-182	PBX3	PBX homeobox 3 [Source:VGNC Symbol;Acc:VGNC:91205]	6.46	0.0163
ssc-miR-182	PC	pyruvate carboxylase [Source:VGNC Symbol;Acc:VGNC:100318]	6.46	0.0163
ssc-miR-182	PCDH10	protocadherin 10 [Source:HGNC Symbol;Acc:HGNC:13404]	6.46	0.0163
ssc-miR-182	PCDH18	protocadherin 18 [Source:VGNC Symbol;Acc:VGNC:91215]	6.46	0.0163
ssc-miR-182	PCDH8	protocadherin 8 [Source:HGNC Symbol;Acc:HGNC:8660]	6.46	0.0163
ssc-miR-182	PCGF3	polycomb group ring finger 3 [Source:VGNC Symbol;Acc:VGNC:91220]	6.46	0.0163
ssc-miR-182	PCGF5	hypothetical gene	6.46	0.0163
ssc-miR-182	PCMT1	protein-L-isoaspartate (D-aspartate) O-methyltransferase [Source:VGNC Symbol;Acc:VGNC:103146]	6.46	0.0163
ssc-miR-182	PCMTD1	protein-L-isoaspartate (D-aspartate) O-methyltransferase domain containing 1 [Source:VGNC Symbol;Acc:VGNC:98830]	6.46	0.0163
ssc-miR-182	PCNX	hypothetical gene	6.46	0.0163
ssc-miR-182	PCSK5	proprotein convertase subtilisin/kexin type 5 [Source:VGNC Symbol;Acc:VGNC:103147]	6.46	0.0163
ssc-miR-182	PDCD6	programmed cell death 6 [Source:VGNC Symbol;Acc:VGNC:98178]	6.46	0.0163
ssc-miR-182	PDE11A	phosphodiesterase 11A [Source:HGNC Symbol;Acc:HGNC:8773]	6.46	0.0163
ssc-miR-182	PDE7A	phosphodiesterase 7A [Source:VGNC Symbol;Acc:VGNC:91261]	6.46	0.0163

ssc-miR-182	PDE7B	phosphodiesterase 7B [Source:VGNC Symbol;Acc:VGNC:91262]	6.46	0.0163
ssc-miR-182	PDGFRA	platelet derived growth factor receptor alpha [Source:VGNC Symbol;Acc:VGNC:98179]	6.46	0.0163
ssc-miR-182	PDHB	pyruvate dehydrogenase E1 subunit beta [Source:VGNC Symbol;Acc:VGNC:91269]	6.46	0.0163
ssc-miR-182	PDIA4	protein disulfide isomerase family A member 4 [Source:VGNC Symbol;Acc:VGNC:91273]	6.46	0.0163
ssc-miR-182	PDK3	pyruvate dehydrogenase kinase 3 [Source:VGNC Symbol;Acc:VGNC:91279]	6.46	0.0163
ssc-miR-182	PDK4	pyruvate dehydrogenase kinase 4 [Source:VGNC Symbol;Acc:VGNC:91280]	6.46	0.0163
ssc-miR-182	PDPN	podoplanin [Source:VGNC Symbol;Acc:VGNC:91288]	6.46	0.0163
ssc-miR-182	PDXDC1	pyridoxal dependent decarboxylase domain containing 1 [Source:HGNC Symbol;Acc:HGNC:28995]	6.46	0.0163
ssc-miR-182	PDZD2	hypothetical gene	6.46	0.0163
ssc-miR-182	PDZD4	PDZ domain containing 4 [Source:VGNC Symbol;Acc:VGNC:91296]	6.46	0.0163
ssc-miR-182	PDZD8	hypothetical gene	6.46	0.0163
ssc-miR-182	PEG10	paternally expressed 10 [Source:NCBI gene (formerly Entrezgene);Acc:654416]	6.46	0.0163
ssc-miR-182	PEX5	peroxisomal biosis factor 5 [Source:VGNC Symbol;Acc:VGNC:91328]	6.46	0.0163
ssc-miR-182	PFKM	phosphofructokinase, muscle [Source:VGNC Symbol;Acc:VGNC:91338]	6.46	0.0163
ssc-miR-182	PFN1	profilin 1 [Source:NCBI gene (formerly Entrezgene);Acc:100512494]	6.46	0.0163
ssc-miR-182	PGRMC2	progesterone receptor membrane component 2 [Source:VGNC Symbol;Acc:VGNC:98952]	6.46	0.0163
ssc-miR-182	PHF12	PHD finger protein 12 [Source:VGNC Symbol;Acc:VGNC:91378]	6.46	0.0163
ssc-miR-182	PHF13	PHD finger protein 13 [Source:VGNC Symbol;Acc:VGNC:91379]	6.46	0.0163
ssc-miR-182	PHF15	hypothetical gene	6.46	0.0163
ssc-miR-182	PHF20	PHD finger protein 20 [Source:HGNC Symbol;Acc:HGNC:16098]	6.46	0.0163
ssc-miR-182	PHF20L1	PHD finger protein 20 like 1 [Source:VGNC Symbol;Acc:VGNC:91383]	6.46	0.0163
ssc-miR-182	PHF21A	PHD finger protein 21A [Source:VGNC Symbol;Acc:VGNC:91384]	6.46	0.0163
ssc-miR-182	PHF21B	PHD finger protein 21B [Source:VGNC Symbol;Acc:VGNC:91385]	6.46	0.0163
ssc-miR-182	PHIP	pleckstrin homology domain interacting protein [Source:VGNC Symbol;Acc:VGNC:91392]	6.46	0.0163
ssc-miR-182	PHLDB1	pleckstrin homology like domain family B member 1 [Source:VGNC Symbol;Acc:VGNC:91398]	6.46	0.0163
ssc-miR-182	PHLPP2	PH domain and leucine rich repeat protein phosphatase 2 [Source:VGNC Symbol;Acc:VGNC:98186]	6.46	0.0163
ssc-miR-182	PHYHIPL	phytanoyl-CoA 2-hydroxylase interacting protein like [Source:VGNC Symbol;Acc:VGNC:91407]	6.46	0.0163
ssc-miR-182	PI4K2A	phosphatidylinositol 4-kinase type 2 alpha [Source:HGNC Symbol;Acc:HGNC:30031]	6.46	0.0163
ssc-miR-182	PIAS1	protein inhibitor of activated STAT 1 [Source:VGNC Symbol;Acc:VGNC:91410]	6.46	0.0163
ssc-miR-182	PIK3AP1	phosphoinositide-3-kinase adaptor protein 1 [Source:VGNC Symbol;Acc:VGNC:91435]	6.46	0.0163
ssc-miR-182	PIK3R1	phosphoinositide-3-kinase regulatory subunit 1 [Source:VGNC Symbol;Acc:VGNC:91445]	6.46	0.0163
ssc-miR-182	PIP5K1B	phosphatidylinositol-4-phosphate 5-kinase type 1 beta [Source:VGNC Symbol;Acc:VGNC:91458]	6.46	0.0163
ssc-miR-182	PIRT	phosphoinositide interacting regulator of transient receptor potential channels [Source:VGNC Symbol;Acc:VGNC:91461]	6.46	0.0163
ssc-miR-182	PKN2	protein kinase N2 [Source:VGNC Symbol;Acc:VGNC:91482]	6.46	0.0163
ssc-miR-182	PLAG1	PLAG1 zinc finger [Source:VGNC Symbol;Acc:VGNC:91509]	6.46	0.0163
ssc-miR-182	PLCB4	phospholipase C beta 4 [Source:VGNC Symbol;Acc:VGNC:96491]	6.46	0.0163
ssc-miR-182	PLCE1	phospholipase C epsilon 1 [Source:VGNC Symbol;Acc:VGNC:91519]	6.46	0.0163
ssc-miR-182	PLCG1	phospholipase C gamma 1 [Source:VGNC Symbol;Acc:VGNC:96492]	6.46	0.0163
ssc-miR-182	PLCL1	phospholipase C like 1 (inactive) [Source:HGNC Symbol;Acc:HGNC:9063]	6.46	0.0163
ssc-miR-182	PLD1	phospholipase D1 [Source:VGNC Symbol;Acc:VGNC:91525]	6.46	0.0163
ssc-miR-182	PLEKHA7	pleckstrin homology domain containing A7 [Source:VGNC Symbol;Acc:VGNC:91537]	6.46	0.0163
ssc-miR-182	PLEKHA8	pleckstrin homology domain containing A8 [Source:HGNC Symbol;Acc:HGNC:30037]	6.46	0.0163
ssc-miR-182	PLEKHG3	pleckstrin homology and RhoGEF domain containing G3 [Source:VGNC Symbol;Acc:VGNC:91544]	6.46	0.0163
ssc-miR-182	PLEKHM1	pleckstrin homology and RUN domain containing M1 [Source:VGNC Symbol;Acc:VGNC:91552]	6.46	0.0163
ssc-miR-182	PLOD2	procollagen-lysine,2-oxoglutarate 5-dioxygenase 2 [Source:VGNC Symbol;Acc:VGNC:91567]	6.46	0.0163
ssc-miR-182	PLXDC2	plexin domain containing 2 [Source:VGNC Symbol;Acc:VGNC:95958]	6.46	0.0163
ssc-miR-182	PLXNA3	plexin A3 [Source:VGNC Symbol;Acc:VGNC:91581]	6.46	0.0163
ssc-miR-182	PNISR	PNN interacting serine and arginine rich protein [Source:VGNC Symbol;Acc:VGNC:91594]	6.46	0.0163
ssc-miR-182	PNPLA2	patatin like phospholipase domain containing 2 [Source:VGNC Symbol;Acc:VGNC:91607]	6.46	0.0163

ssc-miR-182	PNPLA4	hypothetical gene	6.46	0.0163
ssc-miR-182	PNPLA8	patatin like phospholipase domain containing 8 [Source:VGNC Symbol;Acc:VGNC:91610]	6.46	0.0163
ssc-miR-182	POLH	DNA polymerase eta [Source:VGNC Symbol;Acc:VGNC:91635]	6.46	0.0163
ssc-miR-182	POLR3G	hypothetical gene	6.46	0.0163
ssc-miR-182	POMGNT1	protein O-linked mannose N-acetylglucosaminyltransferase 1 (beta 1,2-) [Source:VGNC Symbol;Acc:VGNC:98536]	6.46	0.0163
ssc-miR-182	POU2F1	POU class 2 homeobox 1 [Source:VGNC Symbol;Acc:VGNC:91672]	6.46	0.0163
ssc-miR-182	POU3F3	POU class 3 homeobox 3 [Source:VGNC Symbol;Acc:VGNC:91676]	6.46	0.0163
ssc-miR-182	POU4F1	POU class 4 homeobox 1 [Source:VGNC Symbol;Acc:VGNC:91678]	6.46	0.0163
ssc-miR-182	PP13439	hypothetical gene	6.46	0.0163
ssc-miR-182	PPFIBP1	PPFIA binding protein 1 [Source:VGNC Symbol;Acc:VGNC:91695]	6.46	0.0163
ssc-miR-182	PPFIBP2	PPFIA binding protein 2 [Source:VGNC Symbol;Acc:VGNC:91696]	6.46	0.0163
ssc-miR-182	PPIL1	peptidylprolyl isomerase like 1 [Source:VGNC Symbol;Acc:VGNC:104033]	6.46	0.0163
ssc-miR-182	PPIL6	peptidylprolyl isomerase like 6 [Source:VGNC Symbol;Acc:VGNC:98216]	6.46	0.0163
ssc-miR-182	PPM1A	protein phosphatase, Mg2+/Mn2+ dependent 1A [Source:VGNC Symbol;Acc:VGNC:91703]	6.46	0.0163
ssc-miR-182	PPM1F	protein phosphatase, Mg2+/Mn2+ dependent 1F [Source:VGNC Symbol;Acc:VGNC:91706]	6.46	0.0163
ssc-miR-182	PPM1H	protein phosphatase, Mg2+/Mn2+ dependent 1H [Source:VGNC Symbol;Acc:VGNC:91708]	6.46	0.0163
ssc-miR-182	PPM1K	protein phosphatase, Mg2+/Mn2+ dependent 1K [Source:VGNC Symbol;Acc:VGNC:91710]	6.46	0.0163
ssc-miR-182	PPM1L	protein phosphatase, Mg2+/Mn2+ dependent 1L [Source:VGNC Symbol;Acc:VGNC:91711]	6.46	0.0163
ssc-miR-182	PPP1R11	protein phosphatase 1 regulatory inhibitor subunit 11 [Source:VGNC Symbol;Acc:VGNC:91718]	6.46	0.0163
ssc-miR-182	PPP1R12A	protein phosphatase 1 regulatory subunit 12A [Source:VGNC Symbol;Acc:VGNC:91719]	6.46	0.0163
ssc-miR-182	PPP1R12B	protein phosphatase 1 regulatory subunit 12B [Source:VGNC Symbol;Acc:VGNC:96430]	6.46	0.0163
ssc-miR-182	PPP1R13B	protein phosphatase 1 regulatory subunit 13B [Source:VGNC Symbol;Acc:VGNC:91721]	6.46	0.0163
ssc-miR-182	PPP1R13L	protein phosphatase 1 regulatory subunit 13 like [Source:VGNC Symbol;Acc:VGNC:91722]	6.46	0.0163
ssc-miR-182	PPP1R15B	protein phosphatase 1 regulatory subunit 15B [Source:VGNC Symbol;Acc:VGNC:91726]	6.46	0.0163
ssc-miR-182	PPP1R1C	protein phosphatase 1 regulatory inhibitor subunit 1C [Source:HGNC Symbol;Acc:HGNC:14940]	6.46	0.0163
ssc-miR-182	PPP1R2	hypothetical gene	6.46	0.0163
ssc-miR-182	PPP1R3D	protein phosphatase 1 regulatory subunit 3D [Source:VGNC Symbol;Acc:VGNC:96500]	6.46	0.0163
ssc-miR-182	PPP1R9A	protein phosphatase 1 regulatory subunit 9A [Source:VGNC Symbol;Acc:VGNC:91743]	6.46	0.0163
ssc-miR-182	PPP1R9B	protein phosphatase 1 regulatory subunit 9B [Source:VGNC Symbol;Acc:VGNC:91744]	6.46	0.0163
ssc-miR-182	PPP3CA	protein phosphatase 3 catalytic subunit alpha [Source:VGNC Symbol;Acc:VGNC:98218]	6.46	0.0163
ssc-miR-182	PPP3R1	protein phosphatase 3 regulatory subunit B, alpha [Source:VGNC Symbol;Acc:VGNC:106453]	6.46	0.0163
ssc-miR-182	PPP3R2	hypothetical gene	6.46	0.0163
ssc-miR-182	PPP4R1L	hypothetical gene	6.46	0.0163
ssc-miR-182	PPP4R2	protein phosphatase 4 regulatory subunit 2 [Source:VGNC Symbol;Acc:VGNC:91756]	6.46	0.0163
ssc-miR-182	PRAF2	hypothetical gene	6.46	0.0163
ssc-miR-182	PRC1	protein regulator of cytokinesis 1 [Source:VGNC Symbol;Acc:VGNC:104036]	6.46	0.0163
ssc-miR-182	PRDM1	PR/SET domain 1 [Source:VGNC Symbol;Acc:VGNC:91772]	6.46	0.0163
ssc-miR-182	PRDM16	PR/SET domain 16 [Source:VGNC Symbol;Acc:VGNC:91777]	6.46	0.0163
ssc-miR-182	PREPL	prolyl endopeptidase like [Source:VGNC Symbol;Acc:VGNC:91790]	6.46	0.0163
ssc-miR-182	PRKAA2	protein kinase AMP-activated catalytic subunit alpha 2 [Source:VGNC Symbol;Acc:VGNC:91798]	6.46	0.0163
ssc-miR-182	PRKACA	protein kinase cAMP-activated catalytic subunit alpha [Source:VGNC Symbol;Acc:VGNC:100332]	6.46	0.0163
ssc-miR-182	PRKACB	protein kinase cAMP-activated catalytic subunit beta [Source:VGNC Symbol;Acc:VGNC:91800]	6.46	0.0163
ssc-miR-182	PRKAR1A	protein kinase cAMP-dependent type I regulatory subunit alpha [Source:VGNC Symbol;Acc:VGNC:91802]	6.46	0.0163
ssc-miR-182	PRKAR2A	protein kinase cAMP-dependent type II regulatory subunit alpha [Source:VGNC Symbol;Acc:VGNC:91804]	6.46	0.0163
ssc-miR-182	PRKCE	protein kinase C epsilon [Source:VGNC Symbol;Acc:VGNC:91807]	6.46	0.0163
ssc-miR-182	PRKD1	protein kinase D1 [Source:VGNC Symbol;Acc:VGNC:91812]	6.46	0.0163
ssc-miR-182	PRLR	prolactin receptor [Source:VGNC Symbol;Acc:VGNC:91819]	6.46	0.0163
ssc-miR-182	PRM2	protamine 2 [Source:NCBI gene (formerly Entrezgene);Acc:397486]	6.46	0.0163
ssc-miR-182	PRPF4B	pre-mRNA processing factor 4B [Source:VGNC Symbol;Acc:VGNC:91845]	6.46	0.0163

ssc-miR-182	PRPS2	phosphoribosyl pyrophosphate synthetase 2 [Source:NCBI gene (formerly Entrezgene);Acc:100510993]	6.46	0.0163
ssc-miR-182	PRR11	proline rich 11 [Source:VGNC Symbol;Acc:VGNC:91850]	6.46	0.0163
ssc-miR-182	PRR14L	proline rich 14 like [Source:VGNC Symbol;Acc:VGNC:91853]	6.46	0.0163
ssc-miR-182	PRR3	proline rich 3 [Source:HGNC Symbol;Acc:HGNC:21149]	6.46	0.0163
ssc-miR-182	PRRG3	proline rich and Gla domain 3 [Source:VGNC Symbol;Acc:VGNC:91869]	6.46	0.0163
ssc-miR-182	PRRT3	proline rich transmembrane protein 3 [Source:VGNC Symbol;Acc:VGNC:91873]	6.46	0.0163
ssc-miR-182	PRRX1	paired related homeobox 1 [Source:VGNC Symbol;Acc:VGNC:91875]	6.46	0.0163
ssc-miR-182	PRUNE2	hypothetical gene	6.46	0.0163
ssc-miR-182	PSME4	proteasome activator subunit 4 [Source:VGNC Symbol;Acc:VGNC:91928]	6.46	0.0163
ssc-miR-182	PTAR1	protein prenyltransferase alpha subunit repeat containing 1 [Source:VGNC Symbol;Acc:VGNC:91936]	6.46	0.0163
ssc-miR-182	PTCHD1	patched domain containing 1 [Source:VGNC Symbol;Acc:VGNC:91942]	6.46	0.0163
ssc-miR-182	PTGER3	prostaglandin E receptor 3 [Source:VGNC Symbol;Acc:VGNC:98225]	6.46	0.0163
ssc-miR-182	PTP4A1	protein tyrosine phosphatase 4A1 [Source:HGNC Symbol;Acc:HGNC:9634]	6.46	0.0163
ssc-miR-182	PTPN4	protein tyrosine phosphatase non-receptor type 4 [Source:VGNC Symbol;Acc:VGNC:96521]	6.46	0.0163
ssc-miR-182	PTPRG	protein tyrosine phosphatase receptor type G [Source:VGNC Symbol;Acc:VGNC:91988]	6.46	0.0163
ssc-miR-182	PTPRN2	protein tyrosine phosphatase receptor type N2 [Source:VGNC Symbol;Acc:VGNC:91993]	6.46	0.0163
ssc-miR-182	PURA	purine rich element binding protein A [Source:HGNC Symbol;Acc:HGNC:9701]	6.46	0.0163
ssc-miR-182	PVRL3	hypothetical gene	6.46	0.0163
ssc-miR-182	PXMP4	hypothetical gene	6.46	0.0163
ssc-miR-182	PYGO2	hypothetical gene	6.46	0.0163
ssc-miR-182	QKI	QKI, KH domain containing RNA binding [Source:VGNC Symbol;Acc:VGNC:92025]	6.46	0.0163
ssc-miR-182	QSER1	glutamine and serine rich 1 [Source:VGNC Symbol;Acc:VGNC:92032]	6.46	0.0163
ssc-miR-182	RAB10	RAB10, member RAS onco family [Source:VGNC Symbol;Acc:VGNC:98235]	6.46	0.0163
ssc-miR-182	RAB11FIP5	RAB11 family interacting protein 5 [Source:HGNC Symbol;Acc:HGNC:24845]	6.46	0.0163
ssc-miR-182	RAB19	RAB19, member RAS onco family [Source:VGNC Symbol;Acc:VGNC:98242]	6.46	0.0163
ssc-miR-182	RAB21	RAB21, member RAS oncogene family [Source:HGNC Symbol;Acc:HGNC:18263]	6.46	0.0163
ssc-miR-182	RAB22A	RAB22A, member RAS onco family [Source:VGNC Symbol;Acc:VGNC:98244]	6.46	0.0163
ssc-miR-182	RAB23	RAB23, member RAS onco family [Source:VGNC Symbol;Acc:VGNC:98245]	6.46	0.0163
ssc-miR-182	RAB27A	RAB27A, member RAS onco family [Source:VGNC Symbol;Acc:VGNC:98249]	6.46	0.0163
ssc-miR-182	RAB2A	RAB2A, member RAS onco family [Source:VGNC Symbol;Acc:VGNC:98252]	6.46	0.0163
ssc-miR-182	RAB30	RAB30, member RAS onco family [Source:VGNC Symbol;Acc:VGNC:98254]	6.46	0.0163
ssc-miR-182	RAB35	RAB35, member RAS onco family [Source:VGNC Symbol;Acc:VGNC:98259]	6.46	0.0163
ssc-miR-182	RAB3B	RAB3B, member RAS onco family [Source:VGNC Symbol;Acc:VGNC:98265]	6.46	0.0163
ssc-miR-182	RAB3C	RAB3C, member RAS onco family [Source:VGNC Symbol;Acc:VGNC:104043]	6.46	0.0163
ssc-miR-182	RAB3GAP2	RAB3 GTPase activating non-catalytic protein subunit 2 [Source:VGNC Symbol;Acc:VGNC:95833]	6.46	0.0163
ssc-miR-182	RAB6B	RAB6B, member RAS onco family [Source:VGNC Symbol;Acc:VGNC:98272]	6.46	0.0163
ssc-miR-182	RAB9B	RAB9B, member RAS onco family [Source:VGNC Symbol;Acc:VGNC:98278]	6.46	0.0163
ssc-miR-182	RABIF	RAB interacting factor [Source:HGNC Symbol;Acc:HGNC:9797]	6.46	0.0163
ssc-miR-182	RAC1	Rac family small GTPase 1 [Source:VGNC Symbol;Acc:VGNC:92047]	6.46	0.0163
ssc-miR-182	RAD23B	RAD23 homolog B, nucleotide excision repair protein [Source:VGNC Symbol;Acc:VGNC:103160]	6.46	0.0163
ssc-miR-182	RAD51	RAD51 recombinase [Source:VGNC Symbol;Acc:VGNC:92056]	6.46	0.0163
ssc-miR-182	RADIL	Rap associating with DIL domain [Source:VGNC Symbol;Acc:VGNC:92064]	6.46	0.0163
ssc-miR-182	RALB	RAS like proto-onco B [Source:VGNC Symbol;Acc:VGNC:96529]	6.46	0.0163
ssc-miR-182	RALGAPB	Ral GTPase activating protein non-catalytic subunit beta [Source:VGNC Symbol;Acc:VGNC:95463]	6.46	0.0163
ssc-miR-182	RALGDS	ral guanine nucleotide dissociation stimulator [Source:HGNC Symbol;Acc:HGNC:9842]	6.46	0.0163
ssc-miR-182	RALGPS1	Ral GEF with PH domain and SH3 binding motif 1 [Source:VGNC Symbol;Acc:VGNC:92072]	6.46	0.0163
ssc-miR-182	RALGPS2	Ral GEF with PH domain and SH3 binding motif 2 [Source:VGNC Symbol;Acc:VGNC:92073]	6.46	0.0163
ssc-miR-182	RAPGEF4	Rap guanine nucleotide exchange factor 4 [Source:VGNC Symbol;Acc:VGNC:95814]	6.46	0.0163
ssc-miR-182	RAPGEF5	hypothetical gene	6.46	0.0163

ssc-miR-182	RAPGEF6	Rap guanine nucleotide exchange factor 6 [Source:HGNC Symbol;Acc:HGNC:20655]	6.46	0.0163
ssc-miR-182	RARG	retinoic acid receptor gamma [Source:HGNC Symbol;Acc:HGNC:9866]	6.46	0.0163
ssc-miR-182	RASA1	RAS p21 protein activator 1 [Source:VGNC Symbol;Acc:VGNC:92101]	6.46	0.0163
ssc-miR-182	RASA2	RAS p21 protein activator 2 [Source:VGNC Symbol;Acc:VGNC:92102]	6.46	0.0163
ssc-miR-182	RAVER2	ribonucleoprotein, PTB binding 2 [Source:VGNC Symbol;Acc:VGNC:98283]	6.46	0.0163
ssc-miR-182	RBM48	RNA binding motif protein 48 [Source:VGNC Symbol;Acc:VGNC:92159]	6.46	0.0163
ssc-miR-182	RBM5	RNA binding motif protein 5 [Source:VGNC Symbol;Acc:VGNC:92160]	6.46	0.0163
ssc-miR-182	RCAN3	RCAN family member 3 [Source:VGNC Symbol;Acc:VGNC:92172]	6.46	0.0163
ssc-miR-182	RCC2	regulator of chromosome condensation 2 [Source:VGNC Symbol;Acc:VGNC:98547]	6.46	0.0163
ssc-miR-182	RCOR1	REST corepressor 1 [Source:VGNC Symbol;Acc:VGNC:92183]	6.46	0.0163
ssc-miR-182	RD3	RD3 regulator of GUCY2D [Source:VGNC Symbol;Acc:VGNC:92188]	6.46	0.0163
ssc-miR-182	RDX	radixin [Source:NCBI gene (formerly Entrezgene);Acc:494457]	6.46	0.0163
ssc-miR-182	RECK	reversion inducing cysteine rich protein with kazal motifs [Source:VGNC Symbol;Acc:VGNC:92194]	6.46	0.0163
ssc-miR-182	REEP1	receptor accessory protein 1 [Source:VGNC Symbol;Acc:VGNC:92197]	6.46	0.0163
ssc-miR-182	REPS2	RALBP1 associated Eps domain containing 2 [Source:VGNC Symbol;Acc:VGNC:92214]	6.46	0.0163
ssc-miR-182	RERE	hypothetical gene	6.46	0.0163
ssc-miR-182	REV1	REV1 DNA directed polymerase [Source:HGNC Symbol;Acc:HGNC:14060]	6.46	0.0163
ssc-miR-182	RFTN1	raftlin, lipid raft linker 1 [Source:VGNC Symbol;Acc:VGNC:92241]	6.46	0.0163
ssc-miR-182	RGS17	regulator of G protein signaling 17 [Source:VGNC Symbol;Acc:VGNC:92263]	6.46	0.0163
ssc-miR-182	RGS5	regulator of G protein signaling 5 [Source:VGNC Symbol;Acc:VGNC:98844]	6.46	0.0163
ssc-miR-182	RGS7BP	regulator of G protein signaling 7 binding protein [Source:VGNC Symbol;Acc:VGNC:92268]	6.46	0.0163
ssc-miR-182	RHBDD1	rhomboid domain containing 1 [Source:VGNC Symbol;Acc:VGNC:95989]	6.46	0.0163
ssc-miR-182	RHD	hypothetical gene	6.46	0.0163
ssc-miR-182	RHOBTB1	Rho related BTB domain containing 1 [Source:VGNC Symbol;Acc:VGNC:92285]	6.46	0.0163
ssc-miR-182	RHOJ	ras homolog family member J [Source:VGNC Symbol;Acc:VGNC:92291]	6.46	0.0163
ssc-miR-182	RHOQ	ras homolog family member Q [Source:VGNC Symbol;Acc:VGNC:92292]	6.46	0.0163
ssc-miR-182	RHPN2	rhophilin Rho GTPase binding protein 2 [Source:VGNC Symbol;Acc:VGNC:98548]	6.46	0.0163
ssc-miR-182	RIC3	RIC3 acetylcholine receptor chaperone [Source:VGNC Symbol;Acc:VGNC:92300]	6.46	0.0163
ssc-miR-182	RICTOR	RPTOR independent companion of MTOR complex 2 [Source:VGNC Symbol;Acc:VGNC:92303]	6.46	0.0163
ssc-miR-182	RILPL1	Rab interacting lysosomal protein like 1 [Source:VGNC Symbol;Acc:VGNC:92304]	6.46	0.0163
ssc-miR-182	RIMBP2	RIMS binding protein 2 [Source:VGNC Symbol;Acc:VGNC:92306]	6.46	0.0163
ssc-miR-182	RIMKLA	ribosomal modification protein rimK like family member A [Source:HGNC Symbol;Acc:HGNC:28725]	6.46	0.0163
ssc-miR-182	RIMS3	regulating synaptic membrane exocytosis 3 [Source:VGNC Symbol;Acc:VGNC:92310]	6.46	0.0163
ssc-miR-182	RMND5A	required for meiotic nuclear division 5 homolog A [Source:VGNC Symbol;Acc:VGNC:92336]	6.46	0.0163
ssc-miR-182	RND3	Rho family GTPase 3 [Source:VGNC Symbol;Acc:VGNC:96407]	6.46	0.0163
ssc-miR-182	RNF112	ring finger protein 112 [Source:VGNC Symbol;Acc:VGNC:92347]	6.46	0.0163
ssc-miR-182	RNF130	ring finger protein 130 [Source:HGNC Symbol;Acc:HGNC:18280]	6.46	0.0163
ssc-miR-182	RNF144A	ring finger protein 144A [Source:VGNC Symbol;Acc:VGNC:92358]	6.46	0.0163
ssc-miR-182	RNF144B	ring finger protein 144B [Source:VGNC Symbol;Acc:VGNC:92359]	6.46	0.0163
ssc-miR-182	RNF152	ring finger protein 152 [Source:VGNC Symbol;Acc:VGNC:92364]	6.46	0.0163
ssc-miR-182	RNF183	ring finger protein 183 [Source:VGNC Symbol;Acc:VGNC:92373]	6.46	0.0163
ssc-miR-182	RNF207	ring finger protein 207 [Source:VGNC Symbol;Acc:VGNC:92379]	6.46	0.0163
ssc-miR-182	RNF208	ring finger protein 208 [Source:VGNC Symbol;Acc:VGNC:92380]	6.46	0.0163
ssc-miR-182	RNF217	ring finger protein 217 [Source:VGNC Symbol;Acc:VGNC:103162]	6.46	0.0163
ssc-miR-182	RNF222	ring finger protein 222 [Source:VGNC Symbol;Acc:VGNC:92384]	6.46	0.0163
ssc-miR-182	RNF4	ring finger protein 4 [Source:VGNC Symbol;Acc:VGNC:92392]	6.46	0.0163
ssc-miR-182	RNF44	ring finger protein 44 [Source:VGNC Symbol;Acc:VGNC:92395]	6.46	0.0163
ssc-miR-182	ROBO1	roundabout guidance receptor 1 [Source:HGNC Symbol;Acc:HGNC:10249]	6.46	0.0163
ssc-miR-182	ROCK1	Rho associated coiled-coil containing protein kinase 1 [Source:VGNC Symbol;Acc:VGNC:98294]	6.46	0.0163

ssc-miR-182	ROCK2	Rho associated coiled-coil containing protein kinase 2 [Source:HGNC Symbol;Acc:HGNC:10252]	6.46	0.0163
ssc-miR-182	RORB	RAR related orphan receptor B [Source:VGNC Symbol;Acc:VGNC:92409]	6.46	0.0163
ssc-miR-182	RP1-170O19.20	hypothetical gene	6.46	0.0163
ssc-miR-182	RP11-10A14.4	hypothetical gene	6.46	0.0163
ssc-miR-182	RP11-181C3.1	hypothetical gene	6.46	0.0163
ssc-miR-182	RPA3-AS1	hypothetical gene	6.46	0.0163
ssc-miR-182	RPH3A	rabphilin 3A [Source:VGNC Symbol;Acc:VGNC:92425]	6.46	0.0163
ssc-miR-182	RPL36A-HNRNPH2	hypothetical gene	6.46	0.0163
ssc-miR-182	RPRD1B	regulation of nuclear pre-mRNA domain containing 1B [Source:VGNC Symbol;Acc:VGNC:95785]	6.46	0.0163
ssc-miR-182	RPS6KA6	ribosomal protein S6 kinase A6 [Source:VGNC Symbol;Acc:VGNC:92445]	6.46	0.0163
ssc-miR-182	RRAGD	Ras related GTP binding D [Source:VGNC Symbol;Acc:VGNC:92457]	6.46	0.0163
ssc-miR-182	RSF1	remodeling and spacing factor 1 [Source:VGNC Symbol;Acc:VGNC:92476]	6.46	0.0163
ssc-miR-182	RSU1	Ras suppressor protein 1 [Source:VGNC Symbol;Acc:VGNC:98298]	6.46	0.0163
ssc-miR-182	RTN4	reticulon 4 [Source:VGNC Symbol;Acc:VGNC:92502]	6.46	0.0163
ssc-miR-182	RTTN	rotatin [Source:VGNC Symbol;Acc:VGNC:92508]	6.46	0.0163
ssc-miR-182	RUFY2	RUN and FYVE domain containing 2 [Source:VGNC Symbol;Acc:VGNC:92512]	6.46	0.0163
ssc-miR-182	RUNDC3B	RUN domain containing 3B [Source:VGNC Symbol;Acc:VGNC:92515]	6.46	0.0163
ssc-miR-182	RWDD2A	RWD domain containing 2A [Source:VGNC Symbol;Acc:VGNC:92523]	6.46	0.0163
ssc-miR-182	RWDD4	RWD domain containing 4 [Source:VGNC Symbol;Acc:VGNC:96022]	6.46	0.0163
ssc-miR-182	S100PBP	S100P binding protein [Source:VGNC Symbol;Acc:VGNC:92549]	6.46	0.0163
ssc-miR-182	S1PR1	sphingosine-1-phosphate receptor 1 [Source:VGNC Symbol;Acc:VGNC:92551]	6.46	0.0163
ssc-miR-182	S1PR2	sphingosine-1-phosphate receptor 2 [Source:VGNC Symbol;Acc:VGNC:92552]	6.46	0.0163
ssc-miR-182	SALL4	spalt like transcription factor 4 [Source:NCBI gene (formerly Entrezgene);Acc:100136902]	6.46	0.0163
ssc-miR-182	SAMD12	sterile alpha motif domain containing 12 [Source:VGNC Symbol;Acc:VGNC:92565]	6.46	0.0163
ssc-miR-182	SAMD4A	sterile alpha motif domain containing 4A [Source:VGNC Symbol;Acc:VGNC:92569]	6.46	0.0163
ssc-miR-182	SAP30L	SAP30 like [Source:VGNC Symbol;Acc:VGNC:92577]	6.46	0.0163
ssc-miR-182	SAR1B	secretion associated Ras related GTPase 1B [Source:VGNC Symbol;Acc:VGNC:92580]	6.46	0.0163
ssc-miR-182	SATB2	SATB homeobox 2 [Source:VGNC Symbol;Acc:VGNC:95972]	6.46	0.0163
ssc-miR-182	SBDS	SBDS ribosome maturation factor [Source:VGNC Symbol;Acc:VGNC:92592]	6.46	0.0163
ssc-miR-182	SC5D	sterol-C5-desaturase [Source:VGNC Symbol;Acc:VGNC:92600]	6.46	0.0163
ssc-miR-182	SCARB2	scavenger receptor class B member 2 [Source:VGNC Symbol;Acc:VGNC:92614]	6.46	0.0163
ssc-miR-182	SCML4	Scm polycomb group protein like 4 [Source:VGNC Symbol;Acc:VGNC:92629]	6.46	0.0163
ssc-miR-182	SCN1A	sodium voltage-gated channel alpha subunit 1 [Source:VGNC Symbol;Acc:VGNC:95478]	6.46	0.0163
ssc-miR-182	SCN2A	hypothetical gene	6.46	0.0163
ssc-miR-182	SCN3A	sodium voltage-gated channel alpha subunit 3 [Source:VGNC Symbol;Acc:VGNC:95479]	6.46	0.0163
ssc-miR-182	SCN8A	sodium voltage-gated channel alpha subunit 8 [Source:VGNC Symbol;Acc:VGNC:92638]	6.46	0.0163
ssc-miR-182	SCN9A	sodium voltage-gated channel alpha subunit 9 [Source:VGNC Symbol;Acc:VGNC:95481]	6.46	0.0163
ssc-miR-182	SCRT1	scratch family transcriptional repressor 1 [Source:VGNC Symbol;Acc:VGNC:92649]	6.46	0.0163
ssc-miR-182	SCUBE3	signal peptide, CUB domain and EGF like domain containing 3 [Source:VGNC Symbol;Acc:VGNC:92651]	6.46	0.0163
ssc-miR-182	SDC1	syndecan 1 [Source:VGNC Symbol;Acc:VGNC:92654]	6.46	0.0163
ssc-miR-182	SDC2	syndecan 2 [Source:VGNC Symbol;Acc:VGNC:92655]	6.46	0.0163
ssc-miR-182	SEMA5A	semaphorin 5A [Source:VGNC Symbol;Acc:VGNC:92706]	6.46	0.0163
ssc-miR-182	SEMA6D	semaphorin 6D [Source:VGNC Symbol;Acc:VGNC:92711]	6.46	0.0163
ssc-miR-182	SEPHS1	selenophosphate synthetase 1 [Source:VGNC Symbol;Acc:VGNC:104053]	6.46	0.0163
ssc-miR-182	SEPT7	hypothetical gene	6.46	0.0163
ssc-miR-182	SEPT9	hypothetical gene	6.46	0.0163
ssc-miR-182	SERINC1	serine incorporator 1 [Source:VGNC Symbol;Acc:VGNC:92728]	6.46	0.0163
ssc-miR-182	SERTAD4	SERTA domain containing 4 [Source:VGNC Symbol;Acc:VGNC:92752]	6.46	0.0163
ssc-miR-182	SESN2	sestrin 2 [Source:VGNC Symbol;Acc:VGNC:92754]	6.46	0.0163

ssc-miR-182	SESN3	sestrin 3 [Source:VGNC Symbol;Acc:VGNC:92755]	6.46	0.0163
ssc-miR-182	SETD7	SET domain containing 7, histone lysine methyltransferase [Source:VGNC Symbol;Acc:VGNC:92762]	6.46	0.0163
ssc-miR-182	SEZ6L2	seizure related 6 homolog like 2 [Source:VGNC Symbol;Acc:VGNC:98312]	6.46	0.0163
ssc-miR-182	SF3A1	splicing factor 3a subunit 1 [Source:VGNC Symbol;Acc:VGNC:92769]	6.46	0.0163
ssc-miR-182	SF3B3	splicing factor 3b subunit 3 [Source:VGNC Symbol;Acc:VGNC:92772]	6.46	0.0163
ssc-miR-182	SFR1	SWI5 dependent homologous recombination repair protein 1 [Source:VGNC Symbol;Acc:VGNC:92774]	6.46	0.0163
ssc-miR-182	SFXN5	sideroflexin 5 [Source:VGNC Symbol;Acc:VGNC:92787]	6.46	0.0163
ssc-miR-182	SGK3	serum/glucocorticoid regulated kinase family member 3 [Source:HGNC Symbol;Acc:HGNC:10812]	6.46	0.0163
ssc-miR-182	SGMS1	sphingomyelin synthase 1 [Source:VGNC Symbol;Acc:VGNC:92794]	6.46	0.0163
ssc-miR-182	SGMS2	sphingomyelin synthase 2 [Source:VGNC Symbol;Acc:VGNC:92795]	6.46	0.0163
ssc-miR-182	SGOL1	hypothetical gene	6.46	0.0163
ssc-miR-182	SGSM1	small G protein signaling modulator 1 [Source:VGNC Symbol;Acc:VGNC:92800]	6.46	0.0163
ssc-miR-182	SH3BGRL	hypothetical gene	6.46	0.0163
ssc-miR-182	SH3BGRL2	SH3 domain binding glutamate rich protein like 2 [Source:VGNC Symbol;Acc:VGNC:92816]	6.46	0.0163
ssc-miR-182	SH3BGRL3	SH3 domain binding glutamate rich protein like 3 [Source:VGNC Symbol;Acc:VGNC:92817]	6.46	0.0163
ssc-miR-182	SH3BP4	SH3 domain binding protein 4 [Source:VGNC Symbol;Acc:VGNC:95495]	6.46	0.0163
ssc-miR-182	SH3BP5	SH3 domain binding protein 5 [Source:VGNC Symbol;Acc:VGNC:92819]	6.46	0.0163
ssc-miR-182	SH3GLB2	SH3 domain containing GRB2 like, endophilin B2 [Source:VGNC Symbol;Acc:VGNC:92827]	6.46	0.0163
ssc-miR-182	SH3KBP1	SH3 domain containing kinase binding protein 1 [Source:VGNC Symbol;Acc:VGNC:92828]	6.46	0.0163
ssc-miR-182	SH3PXD2B	SH3 and PX domains 2B [Source:VGNC Symbol;Acc:VGNC:92830]	6.46	0.0163
ssc-miR-182	SH3RF2	SH3 domain containing ring finger 2 [Source:VGNC Symbol;Acc:VGNC:92831]	6.46	0.0163
ssc-miR-182	SHANK2	SH3 and multiple ankyrin repeat domains 2 [Source:VGNC Symbol;Acc:VGNC:92835]	6.46	0.0163
ssc-miR-182	SHC4	SHC adaptor protein 4 [Source:HGNC Symbol;Acc:HGNC:16743]	6.46	0.0163
ssc-miR-182	SHOC2	SHOC2 leucine rich repeat scaffold protein [Source:VGNC Symbol;Acc:VGNC:98318]	6.46	0.0163
ssc-miR-182	SHOX	hypothetical gene	6.46	0.0163
ssc-miR-182	SHROOM4	shroom family member 4 [Source:VGNC Symbol;Acc:VGNC:92861]	6.46	0.0163
ssc-miR-182	SIK1	salt inducible kinase 1 [Source:HGNC Symbol;Acc:HGNC:11142]	6.46	0.0163
ssc-miR-182	SIK2	salt inducible kinase 2 [Source:VGNC Symbol;Acc:VGNC:92872]	6.46	0.0163
ssc-miR-182	SIM1	SIM bHLH transcription factor 1 [Source:VGNC Symbol;Acc:VGNC:92876]	6.46	0.0163
ssc-miR-182	SKAP2	src kinase associated phosphoprotein 2 [Source:VGNC Symbol;Acc:VGNC:92901]	6.46	0.0163
ssc-miR-182	SKIL	SKI like proto-onco [Source:VGNC Symbol;Acc:VGNC:92903]	6.46	0.0163
ssc-miR-182	SKP1	hypothetical gene	6.46	0.0163
ssc-miR-182	SLAIN2	SLAIN motif family member 2 [Source:VGNC Symbol;Acc:VGNC:92910]	6.46	0.0163
ssc-miR-182	SLC12A6	solute carrier family 12 member 6 [Source:VGNC Symbol;Acc:VGNC:92925]	6.46	0.0163
ssc-miR-182	SLC16A9	solute carrier family 16 member 9 [Source:VGNC Symbol;Acc:VGNC:92949]	6.46	0.0163
ssc-miR-182	SLC18A2	solute carrier family 18 member A2 [Source:VGNC Symbol;Acc:VGNC:92958]	6.46	0.0163
ssc-miR-182	SLC1A1	solute carrier family 1 member 1 [Source:VGNC Symbol;Acc:VGNC:92963]	6.46	0.0163
ssc-miR-182	SLC1A2	solute carrier family 1 member 2 [Source:VGNC Symbol;Acc:VGNC:92964]	6.46	0.0163
ssc-miR-182	SLC22A23	solute carrier family 22 member 23 [Source:HGNC Symbol;Acc:HGNC:21106]	6.46	0.0163
ssc-miR-182	SLC23A2	solute carrier family 23 member 2 [Source:VGNC Symbol;Acc:VGNC:108737]	6.46	0.0163
ssc-miR-182	SLC24A2	solute carrier family 24 member 2 [Source:VGNC Symbol;Acc:VGNC:92987]	6.46	0.0163
ssc-miR-182	SLC25A10	solute carrier family 25 member 10 [Source:VGNC Symbol;Acc:VGNC:99041]	6.46	0.0163
ssc-miR-182	SLC25A16	solute carrier family 25 member 16 [Source:VGNC Symbol;Acc:VGNC:92995]	6.46	0.0163
ssc-miR-182	SLC25A44	solute carrier family 25 member 44 [Source:VGNC Symbol;Acc:VGNC:93017]	6.46	0.0163
ssc-miR-182	SLC25A51	hypothetical gene	6.46	0.0163
ssc-miR-182	SLC26A9	solute carrier family 26 member 9 [Source:VGNC Symbol;Acc:VGNC:93034]	6.46	0.0163
ssc-miR-182	SLC2A8	solute carrier family 2 member 8 [Source:VGNC Symbol;Acc:VGNC:93053]	6.46	0.0163
ssc-miR-182	SLC30A1	solute carrier family 30 member 1 [Source:VGNC Symbol;Acc:VGNC:93055]	6.46	0.0163
ssc-miR-182	SLC30A10	hypothetical gene	6.46	0.0163

ssc-miR-182	SLC30A4	solute carrier family 30 member 4 [Source:VGNC Symbol;Acc:VGNC:93058]	6.46	0.0163
ssc-miR-182	SLC30A7	solute carrier family 30 member 7 [Source:VGNC Symbol;Acc:VGNC:93061]	6.46	0.0163
ssc-miR-182	SLC30A9	solute carrier family 30 member 9 [Source:VGNC Symbol;Acc:VGNC:98964]	6.46	0.0163
ssc-miR-182	SLC31A1	solute carrier family 31 member 1 [Source:VGNC Symbol;Acc:VGNC:103180]	6.46	0.0163
ssc-miR-182	SLC33A1	solute carrier family 33 member 1 [Source:VGNC Symbol;Acc:VGNC:103074]	6.46	0.0163
ssc-miR-182	SLC35A1	solute carrier family 35 member A1 [Source:VGNC Symbol;Acc:VGNC:93067]	6.46	0.0163
ssc-miR-182	SLC35A2	solute carrier family 35 member A2 [Source:VGNC Symbol;Acc:VGNC:93068]	6.46	0.0163
ssc-miR-182	SLC35B4	solute carrier family 35 member B4 [Source:VGNC Symbol;Acc:VGNC:93074]	6.46	0.0163
ssc-miR-182	SLC35C1	solute carrier family 35 member C1 [Source:VGNC Symbol;Acc:VGNC:93075]	6.46	0.0163
ssc-miR-182	SLC35D1	solute carrier family 35 member D1 [Source:VGNC Symbol;Acc:VGNC:98614]	6.46	0.0163
ssc-miR-182	SLC35G1	solute carrier family 35 member G1 [Source:VGNC Symbol;Acc:VGNC:93086]	6.46	0.0163
ssc-miR-182	SLC39A1	solute carrier family 39 member 1 [Source:VGNC Symbol;Acc:VGNC:93101]	6.46	0.0163
ssc-miR-182	SLC39A13	solute carrier family 39 member 13 [Source:VGNC Symbol;Acc:VGNC:93103]	6.46	0.0163
ssc-miR-182	SLC39A9	solute carrier family 39 member 9 [Source:VGNC Symbol;Acc:VGNC:93110]	6.46	0.0163
ssc-miR-182	SLC43A2	solute carrier family 43 member 2 [Source:VGNC Symbol;Acc:VGNC:93117]	6.46	0.0163
ssc-miR-182	SLC44A2	solute carrier family 44 member 2 [Source:VGNC Symbol;Acc:VGNC:100862]	6.46	0.0163
ssc-miR-182	SLC4A1	solute carrier family 4 member 1 [Source:VGNC Symbol;Acc:VGNC:99051]	6.46	0.0163
ssc-miR-182	SLC4A7	solute carrier family 4 member 7 [Source:VGNC Symbol;Acc:VGNC:93135]	6.46	0.0163
ssc-miR-182	SLC4A8	solute carrier family 4 member 8 [Source:VGNC Symbol;Acc:VGNC:98326]	6.46	0.0163
ssc-miR-182	SLC5A3	solute carrier family 5 member 3 [Source:VGNC Symbol;Acc:VGNC:93144]	6.46	0.0163
ssc-miR-182	SLC6A6	solute carrier family 6 member 6 [Source:VGNC Symbol;Acc:VGNC:93166]	6.46	0.0163
ssc-miR-182	SLC6A9	solute carrier family 6 member 9 [Source:VGNC Symbol;Acc:VGNC:93168]	6.46	0.0163
ssc-miR-182	SLC7A14	solute carrier family 7 member 14 [Source:VGNC Symbol;Acc:VGNC:93173]	6.46	0.0163
ssc-miR-182	SLC7A8	solute carrier family 7 member 8 [Source:VGNC Symbol;Acc:VGNC:93176]	6.46	0.0163
ssc-miR-182	SLC8A1	solute carrier family 8 member A1 [Source:VGNC Symbol;Acc:VGNC:93178]	6.46	0.0163
ssc-miR-182	SLC9A2	solute carrier family 9 member A2 [Source:VGNC Symbol;Acc:VGNC:93182]	6.46	0.0163
ssc-miR-182	SLC9A7	solute carrier family 9 member A7 [Source:VGNC Symbol;Acc:VGNC:93189]	6.46	0.0163
ssc-miR-182	SLCO3A1	solute carrier organic anion transporter family member 3A1 [Source:VGNC Symbol;Acc:VGNC:93197]	6.46	0.0163
ssc-miR-182	SLFN12	hypothetical gene	6.46	0.0163
ssc-miR-182	SLITRK4	SLIT and NTRK like family member 4 [Source:VGNC Symbol;Acc:VGNC:93209]	6.46	0.0163
ssc-miR-182	SLMO2	hypothetical gene	6.46	0.0163
ssc-miR-182	SMAD1	SMAD family member 1 [Source:VGNC Symbol;Acc:VGNC:93216]	6.46	0.0163
ssc-miR-182	SMAD7	SMAD family member 7 [Source:VGNC Symbol;Acc:VGNC:93221]	6.46	0.0163
ssc-miR-182	SMARCA5	SWI/SNF related, matrix associated, actin dependent regulator of chromatin, subfamily a, member 5 [Source:VGNC Symbol;Acc:VGNC:93228]	6.46	0.0163
ssc-miR-182	SMCR8	SMCR8-C9orf72 complex subunit [Source:VGNC Symbol;Acc:VGNC:96598]	6.46	0.0163
ssc-miR-182	SMG5	SMG5 nonsense mediated mRNA decay factor [Source:VGNC Symbol;Acc:VGNC:98861]	6.46	0.0163
ssc-miR-182	SMIM13	small integral membrane protein 13 [Source:HGNC Symbol;Acc:HGNC:27356]	6.46	0.0163
ssc-miR-182	SMIM14	small integral membrane protein 14 [Source:VGNC Symbol;Acc:VGNC:93252]	6.46	0.0163
ssc-miR-182	SNAP23	synaptosome associated protein 23 [Source:VGNC Symbol;Acc:VGNC:93277]	6.46	0.0163
ssc-miR-182	SNX17	sorting nexin 17 [Source:VGNC Symbol;Acc:VGNC:93309]	6.46	0.0163
ssc-miR-182	SNX18	sorting nexin 18 [Source:VGNC Symbol;Acc:VGNC:93310]	6.46	0.0163
ssc-miR-182	SNX22	sorting nexin 22 [Source:VGNC Symbol;Acc:VGNC:93314]	6.46	0.0163
ssc-miR-182	SNX29	sorting nexin 29 [Source:VGNC Symbol;Acc:VGNC:98331]	6.46	0.0163
ssc-miR-182	SNX30	sorting nexin family member 30 [Source:VGNC Symbol;Acc:VGNC:93318]	6.46	0.0163
ssc-miR-182	SNX4	sorting nexin 4 [Source:VGNC Symbol;Acc:VGNC:93322]	6.46	0.0163
ssc-miR-182	SOCS5	suppressor of cytokine signaling 5 [Source:VGNC Symbol;Acc:VGNC:93332]	6.46	0.0163
ssc-miR-182	SOCS7	suppressor of cytokine signaling 7 [Source:VGNC Symbol;Acc:VGNC:93334]	6.46	0.0163
ssc-miR-182	SOGA3	SOGA family member 3 [Source:HGNC Symbol;Acc:HGNC:21494]	6.46	0.0163
ssc-miR-182	SORCS1	sortilin related VPS10 domain containing receptor 1 [Source:VGNC Symbol;Acc:VGNC:93339]	6.46	0.0163

ssc-miR-182	SORT1	sortilin 1 [Source:VGNC Symbol;Acc:VGNC:93343]	6.46	0.0163
ssc-miR-182	SOS1	SOS Ras/Rac guanine nucleotide exchange factor 1 [Source:HGNC Symbol;Acc:HGNC:11187]	6.46	0.0163
ssc-miR-182	SOWAHB	sosondowah ankyrin repeat domain family member B [Source:VGNC Symbol;Acc:VGNC:93348]	6.46	0.0163
ssc-miR-182	SOX11	SRY-box transcription factor 11 [Source:VGNC Symbol;Acc:VGNC:93351]	6.46	0.0163
ssc-miR-182	SOX2	SRY-box transcription factor 2 [Source:NCBI gene (formerly Entrezgene);Acc:407739]	6.46	0.0163
ssc-miR-182	SOX5	SRY-box transcription factor 5 [Source:VGNC Symbol;Acc:VGNC:93357]	6.46	0.0163
ssc-miR-182	SOX6	SRY-box transcription factor 6 [Source:VGNC Symbol;Acc:VGNC:93358]	6.46	0.0163
ssc-miR-182	SP3	Sp3 transcription factor [Source:VGNC Symbol;Acc:VGNC:95511]	6.46	0.0163
ssc-miR-182	SP8	Sp8 transcription factor [Source:VGNC Symbol;Acc:VGNC:93365]	6.46	0.0163
ssc-miR-182	SPAG1	sperm associated antigen 1 [Source:VGNC Symbol;Acc:VGNC:93371]	6.46	0.0163
ssc-miR-182	SPAST	spastin [Source:VGNC Symbol;Acc:VGNC:98333]	6.46	0.0163
ssc-miR-182	SPATA13	hypothetical gene	6.46	0.0163
ssc-miR-182	SPATS2L	spermatosis associated serine rich 2 like [Source:VGNC Symbol;Acc:VGNC:96047]	6.46	0.0163
ssc-miR-182	SPCS1	signal peptidase complex subunit 1 [Source:VGNC Symbol;Acc:VGNC:107173]	6.46	0.0163
ssc-miR-182	SPEN	hypothetical gene	6.46	0.0163
ssc-miR-182	SPIN1	spindlin 1 [Source:VGNC Symbol;Acc:VGNC:101495]	6.46	0.0163
ssc-miR-182	SPIN3	spindlin family member 3 [Source:HGNC Symbol;Acc:HGNC:27272]	6.46	0.0163
ssc-miR-182	SPPL2A	signal peptide peptidase like 2A [Source:VGNC Symbol;Acc:VGNC:98337]	6.46	0.0163
ssc-miR-182	SPRTN	SprT-like N-terminal domain [Source:VGNC Symbol;Acc:VGNC:93423]	6.46	0.0163
ssc-miR-182	SPRY3	sprouty RTK signaling antagonist 3 [Source:VGNC Symbol;Acc:VGNC:93426]	6.46	0.0163
ssc-miR-182	SPRY4	sprouty RTK signaling antagonist 4 [Source:VGNC Symbol;Acc:VGNC:93427]	6.46	0.0163
ssc-miR-182	SPSB4	splA/ryanodine receptor domain and SOCS box containing 4 [Source:VGNC Symbol;Acc:VGNC:93433]	6.46	0.0163
ssc-miR-182	SPTBN2	spectrin beta, non-erythrocytic 2 [Source:VGNC Symbol;Acc:VGNC:93437]	6.46	0.0163
ssc-miR-182	SPTSSB	serine palmitoyltransferase small subunit B [Source:VGNC Symbol;Acc:VGNC:93441]	6.46	0.0163
ssc-miR-182	SREK1	splicing regulatory glutamic acid and lysine rich protein 1 [Source:VGNC Symbol;Acc:VGNC:93451]	6.46	0.0163
ssc-miR-182	SRI	sorcin [Source:VGNC Symbol;Acc:VGNC:93457]	6.46	0.0163
ssc-miR-182	SRSF6	serine and arginine rich splicing factor 6 [Source:NCBI gene (formerly Entrezgene);Acc:768102]	6.46	0.0163
ssc-miR-182	SS18L1	SS18L1 subunit of BAF chromatin remodeling complex [Source:VGNC Symbol;Acc:VGNC:98342]	6.46	0.0163
ssc-miR-182	SSH1	slingshot protein phosphatase 1 [Source:VGNC Symbol;Acc:VGNC:93485]	6.46	0.0163
ssc-miR-182	ST3GAL2	ST3 beta-galactoside alpha-2,3-sialyltransferase 2 [Source:HGNC Symbol;Acc:HGNC:10863]	6.46	0.0163
ssc-miR-182	ST6GALNAC3	ST6 N-acetylgalactosaminide alpha-2,6-sialyltransferase 3 [Source:VGNC Symbol;Acc:VGNC:93511]	6.46	0.0163
ssc-miR-182	ST8SIA1	ST8 alpha-N-acetyl-neuraminide alpha-2,8-sialyltransferase 1 [Source:VGNC Symbol;Acc:VGNC:93516]	6.46	0.0163
ssc-miR-182	ST8SIA3	ST8 alpha-N-acetyl-neuraminide alpha-2,8-sialyltransferase 3 [Source:VGNC Symbol;Acc:VGNC:93518]	6.46	0.0163
ssc-miR-182	STAG1	stromal antigen 1 [Source:VGNC Symbol;Acc:VGNC:93524]	6.46	0.0163
ssc-miR-182	STAM2	signal transducing adaptor molecule 2 [Source:VGNC Symbol;Acc:VGNC:95521]	6.46	0.0163
ssc-miR-182	STARD13	hypothetical gene	6.46	0.0163
ssc-miR-182	STK10	serine/threonine kinase 10 [Source:HGNC Symbol;Acc:HGNC:11388]	6.46	0.0163
ssc-miR-182	STK17A	serine/threonine kinase 17a [Source:VGNC Symbol;Acc:VGNC:104064]	6.46	0.0163
ssc-miR-182	STK17B	serine/threonine kinase 17b [Source:VGNC Symbol;Acc:VGNC:95527]	6.46	0.0163
ssc-miR-182	STK19	serine/threonine kinase 19 [Source:VGNC Symbol;Acc:VGNC:93550]	6.46	0.0163
ssc-miR-182	STK35	serine/threonine kinase 35 [Source:VGNC Symbol;Acc:VGNC:95802]	6.46	0.0163
ssc-miR-182	STK36	serine/threonine kinase 36 [Source:VGNC Symbol;Acc:VGNC:95835]	6.46	0.0163
ssc-miR-182	STK38	serine/threonine kinase 38 [Source:VGNC Symbol;Acc:VGNC:93556]	6.46	0.0163
ssc-miR-182	STMND1	stathmin domain containing 1 [Source:VGNC Symbol;Acc:VGNC:93563]	6.46	0.0163
ssc-miR-182	STOX2	storkhead box 2 [Source:VGNC Symbol;Acc:VGNC:96082]	6.46	0.0163
ssc-miR-182	STT3A	STT3 oligosaccharyltransferase complex catalytic subunit A [Source:VGNC Symbol;Acc:VGNC:93581]	6.46	0.0163
ssc-miR-182	STX7	syntaxin 7 [Source:VGNC Symbol;Acc:VGNC:93595]	6.46	0.0163
ssc-miR-182	SUN2	Sad1 and UNC84 domain containing 2 [Source:VGNC Symbol;Acc:VGNC:93619]	6.46	0.0163
ssc-miR-182	SUPT3H	SPT3 homolog, SAGA and STAGA complex component [Source:VGNC Symbol;Acc:VGNC:93623]	6.46	0.0163

ssc-miR-182	SURF2	surfeit 2 [Source:VGNC Symbol;Acc:VGNC:93630]	6.46	0.0163
ssc-miR-182	SV2C	synaptic vesicle glycoprotein 2C [Source:VGNC Symbol;Acc:VGNC:93641]	6.46	0.0163
ssc-miR-182	SVIP	small VCP interacting protein [Source:HGNC Symbol;Acc:HGNC:25238]	6.46	0.0163
ssc-miR-182	SWAP70	switching B cell complex subunit SWAP70 [Source:VGNC Symbol;Acc:VGNC:93645]	6.46	0.0163
ssc-miR-182	SYNCRIP	synaptotagmin binding cytoplasmic RNA interacting protein [Source:VGNC Symbol;Acc:VGNC:103185]	6.46	0.0163
ssc-miR-182	SYNE2	spectrin repeat containing nuclear envelope protein 2 [Source:VGNC Symbol;Acc:VGNC:93662]	6.46	0.0163
ssc-miR-182	SYNGR2	synaptogyrin 2 [Source:HGNC Symbol;Acc:HGNC:11499]	6.46	0.0163
ssc-miR-182	SYPL1	synaptophysin like 1 [Source:HGNC Symbol;Acc:HGNC:11507]	6.46	0.0163
ssc-miR-182	SYT5	synaptotagmin 5 [Source:VGNC Symbol;Acc:VGNC:93686]	6.46	0.0163
ssc-miR-182	TACC1	hypothetical gene	6.46	0.0163
ssc-miR-182	TAF15	TATA-box binding protein associated factor 15 [Source:VGNC Symbol;Acc:VGNC:93711]	6.46	0.0163
ssc-miR-182	TAF4	TATA-box binding protein associated factor 4 [Source:VGNC Symbol;Acc:VGNC:95532]	6.46	0.0163
ssc-miR-182	TAF4B	TATA-box binding protein associated factor 4b [Source:VGNC Symbol;Acc:VGNC:93716]	6.46	0.0163
ssc-miR-182	TAOK1	TAO kinase 1 [Source:VGNC Symbol;Acc:VGNC:98355]	6.46	0.0163
ssc-miR-182	TAPT1	transmembrane anterior posterior transformation 1 [Source:VGNC Symbol;Acc:VGNC:93736]	6.46	0.0163
ssc-miR-182	TARBP1	TAR (HIV-1) RNA binding protein 1 [Source:HGNC Symbol;Acc:HGNC:11568]	6.46	0.0163
ssc-miR-182	TAT	tyrosine aminotransferase [Source:VGNC Symbol;Acc:VGNC:93752]	6.46	0.0163
ssc-miR-182	TBC1D12	TBC1 domain family member 12 [Source:VGNC Symbol;Acc:VGNC:93762]	6.46	0.0163
ssc-miR-182	TBL1X	hypothetical gene	6.46	0.0163
ssc-miR-182	TBR1	T-box brain transcription factor 1 [Source:VGNC Symbol;Acc:VGNC:95533]	6.46	0.0163
ssc-miR-182	TBX1	T-box transcription factor 1 [Source:VGNC Symbol;Acc:VGNC:93795]	6.46	0.0163
ssc-miR-182	TBXA2R	thromboxane A2 receptor [Source:VGNC Symbol;Acc:VGNC:93806]	6.46	0.0163
ssc-miR-182	TCF19	transcription factor 19 [Source:VGNC Symbol;Acc:VGNC:93818]	6.46	0.0163
ssc-miR-182	TCF7L2	transcription factor 7 like 2 [Source:VGNC Symbol;Acc:VGNC:93825]	6.46	0.0163
ssc-miR-182	TCP11L2	t-complex 11 like 2 [Source:VGNC Symbol;Acc:VGNC:93835]	6.46	0.0163
ssc-miR-182	TEAD1	TEA domain transcription factor 1 [Source:VGNC Symbol;Acc:VGNC:93853]	6.46	0.0163
ssc-miR-182	TECTB	tectorin beta [Source:VGNC Symbol;Acc:VGNC:93860]	6.46	0.0163
ssc-miR-182	TENM4	teneurin transmembrane protein 4 [Source:HGNC Symbol;Acc:HGNC:29945]	6.46	0.0163
ssc-miR-182	TEX2	testis expressed 2 [Source:VGNC Symbol;Acc:VGNC:93894]	6.46	0.0163
ssc-miR-182	TFAP2B	transcription factor AP-2 beta [Source:VGNC Symbol;Acc:VGNC:93909]	6.46	0.0163
ssc-miR-182	TGFBI	transforming growth factor beta induced [Source:VGNC Symbol;Acc:VGNC:93930]	6.46	0.0163
ssc-miR-182	TGFBR2	transforming growth factor beta receptor 2 [Source:VGNC Symbol;Acc:VGNC:93931]	6.46	0.0163
ssc-miR-182	THAP1	THAP domain containing 1 [Source:VGNC Symbol;Acc:VGNC:95950]	6.46	0.0163
ssc-miR-182	THBS1	thrombospondin 1 [Source:VGNC Symbol;Acc:VGNC:93946]	6.46	0.0163
ssc-miR-182	THBS2	thrombospondin 2 [Source:VGNC Symbol;Acc:VGNC:93947]	6.46	0.0163
ssc-miR-182	TIAM1	TIAM Rac1 associated GEF 1 [Source:VGNC Symbol;Acc:VGNC:96601]	6.46	0.0163
ssc-miR-182	TLCD2	TLC domain containing 2 [Source:VGNC Symbol;Acc:VGNC:94011]	6.46	0.0163
ssc-miR-182	TLN1	talin 1 [Source:VGNC Symbol;Acc:VGNC:94020]	6.46	0.0163
ssc-miR-182	TLR4	toll like receptor 4 [Source:VGNC Symbol;Acc:VGNC:94025]	6.46	0.0163
ssc-miR-182	TLR6	toll like receptor 6 [Source:VGNC Symbol;Acc:VGNC:94026]	6.46	0.0163
ssc-miR-182	TMED2	hypothetical gene	6.46	0.0163
ssc-miR-182	TMEM106A	transmembrane protein 106A [Source:VGNC Symbol;Acc:VGNC:94063]	6.46	0.0163
ssc-miR-182	TMEM108	transmembrane protein 108 [Source:VGNC Symbol;Acc:VGNC:94065]	6.46	0.0163
ssc-miR-182	TMEM115	transmembrane protein 115 [Source:HGNC Symbol;Acc:HGNC:30055]	6.46	0.0163
ssc-miR-182	TMEM127	transmembrane protein 127 [Source:HGNC Symbol;Acc:HGNC:26038]	6.46	0.0163
ssc-miR-182	TMEM145	transmembrane protein 145 [Source:VGNC Symbol;Acc:VGNC:94092]	6.46	0.0163
ssc-miR-182	TMEM150C	transmembrane protein 150C [Source:VGNC Symbol;Acc:VGNC:94096]	6.46	0.0163
ssc-miR-182	TMEM170B	transmembrane protein 170B [Source:VGNC Symbol;Acc:VGNC:94108]	6.46	0.0163
ssc-miR-182	TMEM178B	transmembrane protein 178B [Source:HGNC Symbol;Acc:HGNC:44112]	6.46	0.0163

ssc-miR-182	TMEM181	transmembrane protein 181 [Source:VGNC Symbol;Acc:VGNC:94117]	6.46	0.0163
ssc-miR-182	TMEM184A	transmembrane protein 184A [Source:VGNC Symbol;Acc:VGNC:94119]	6.46	0.0163
ssc-miR-182	TMEM198	transmembrane protein 198 [Source:VGNC Symbol;Acc:VGNC:96274]	6.46	0.0163
ssc-miR-182	TMEM200B	transmembrane protein 200B [Source:HGNC Symbol;Acc:HGNC:33785]	6.46	0.0163
ssc-miR-182	TMEM203	transmembrane protein 203 [Source:VGNC Symbol;Acc:VGNC:94132]	6.46	0.0163
ssc-miR-182	TMEM218	transmembrane protein 218 [Source:HGNC Symbol;Acc:HGNC:27344]	6.46	0.0163
ssc-miR-182	TMEM231	transmembrane protein 231 [Source:VGNC Symbol;Acc:VGNC:98377]	6.46	0.0163
ssc-miR-182	TMEM245	transmembrane protein 245 [Source:HGNC Symbol;Acc:HGNC:1363]	6.46	0.0163
ssc-miR-182	TMEM246	hypothetical gene	6.46	0.0163
ssc-miR-182	TMEM41A	transmembrane protein 41A [Source:VGNC Symbol;Acc:VGNC:104080]	6.46	0.0163
ssc-miR-182	TMEM47	transmembrane protein 47 [Source:VGNC Symbol;Acc:VGNC:94184]	6.46	0.0163
ssc-miR-182	TMEM50B	transmembrane protein 50B [Source:VGNC Symbol;Acc:VGNC:94186]	6.46	0.0163
ssc-miR-182	TMEM67	transmembrane protein 67 [Source:VGNC Symbol;Acc:VGNC:94201]	6.46	0.0163
ssc-miR-182	TMEM68	transmembrane protein 68 [Source:VGNC Symbol;Acc:VGNC:94202]	6.46	0.0163
ssc-miR-182	TMEM86A	transmembrane protein 86A [Source:VGNC Symbol;Acc:VGNC:94210]	6.46	0.0163
ssc-miR-182	TMOD1	tropomodulin 1 [Source:HGNC Symbol;Acc:HGNC:11871]	6.46	0.0163
ssc-miR-182	TMOD2	tropomodulin 2 [Source:VGNC Symbol;Acc:VGNC:94227]	6.46	0.0163
ssc-miR-182	TMX4	thioredoxin related transmembrane protein 4 [Source:VGNC Symbol;Acc:VGNC:96072]	6.46	0.0163
ssc-miR-182	TNFAIP8	TNF alpha induced protein 8 [Source:VGNC Symbol;Acc:VGNC:94253]	6.46	0.0163
ssc-miR-182	TNFSF11	TNF superfamily member 11 [Source:HGNC Symbol;Acc:HGNC:11926]	6.46	0.0163
ssc-miR-182	TNFSF13B	TNF superfamily member 13b [Source:VGNC Symbol;Acc:VGNC:94269]	6.46	0.0163
ssc-miR-182	TNK2	tyrosine kinase non receptor 2 [Source:VGNC Symbol;Acc:VGNC:94279]	6.46	0.0163
ssc-miR-182	TNKS2	tankyrase 2 [Source:VGNC Symbol;Acc:VGNC:94280]	6.46	0.0163
ssc-miR-182	TNPO1	transportin 1 [Source:VGNC Symbol;Acc:VGNC:94287]	6.46	0.0163
ssc-miR-182	TNRC6A	trinucleotide repeat containing adaptor 6A [Source:VGNC Symbol;Acc:VGNC:94292]	6.46	0.0163
ssc-miR-182	TNS1	tensin 1 [Source:VGNC Symbol;Acc:VGNC:104082]	6.46	0.0163
ssc-miR-182	TNS3	tensin 3 [Source:VGNC Symbol;Acc:VGNC:94296]	6.46	0.0163
ssc-miR-182	TOB1	transducer of ERBB2, 1 [Source:NCBI gene (formerly Entrezgene);Acc:100144440]	6.46	0.0163
ssc-miR-182	TOPBP1	DNA topoisomerase II binding protein 1 [Source:VGNC Symbol;Acc:VGNC:94315]	6.46	0.0163
ssc-miR-182	TOX	thymocyte selection associated high mobility group box [Source:VGNC Symbol;Acc:VGNC:94322]	6.46	0.0163
ssc-miR-182	TOX2	TOX high mobility group box family member 2 [Source:VGNC Symbol;Acc:VGNC:95734]	6.46	0.0163
ssc-miR-182	TOX3	TOX high mobility group box family member 3 [Source:VGNC Symbol;Acc:VGNC:94323]	6.46	0.0163
ssc-miR-182	TP53INP1	tumor protein p53 inducible nuclear protein 1 [Source:VGNC Symbol;Acc:VGNC:94329]	6.46	0.0163
ssc-miR-182	TP53INP2	tumor protein p53 inducible nuclear protein 2 [Source:VGNC Symbol;Acc:VGNC:95737]	6.46	0.0163
ssc-miR-182	TP73	tumor protein p73 [Source:VGNC Symbol;Acc:VGNC:94331]	6.46	0.0163
ssc-miR-182	TPD52	tumor protein D52 [Source:VGNC Symbol;Acc:VGNC:98383]	6.46	0.0163
ssc-miR-182	TPGS2	tubulin polyglutamylase complex subunit 2 [Source:VGNC Symbol;Acc:VGNC:98661]	6.46	0.0163
ssc-miR-182	TPST2	tyrosylprotein sulfotransferase 2 [Source:NCBI gene (formerly Entrezgene);Acc:100154448]	6.46	0.0163
ssc-miR-182	TRABD2B	TraB domain containing 2B [Source:VGNC Symbol;Acc:VGNC:94356]	6.46	0.0163
ssc-miR-182	TRIB3	tribbles pseudokinase 3 [Source:VGNC Symbol;Acc:VGNC:95791]	6.46	0.0163
ssc-miR-182	TRIM37	tripartite motif containing 37 [Source:VGNC Symbol;Acc:VGNC:94410]	6.46	0.0163
ssc-miR-182	TRIM52	tripartite motif containing 52 [Source:HGNC Symbol;Acc:HGNC:19024]	6.46	0.0163
ssc-miR-182	TRIM66	tripartite motif containing 66 [Source:VGNC Symbol;Acc:VGNC:94426]	6.46	0.0163
ssc-miR-182	TRIM71	tripartite motif containing 71 [Source:VGNC Symbol;Acc:VGNC:94431]	6.46	0.0163
ssc-miR-182	TRIM8	tripartite motif containing 8 [Source:VGNC Symbol;Acc:VGNC:94433]	6.46	0.0163
ssc-miR-182	TRIO	trio Rho guanine nucleotide exchange factor [Source:VGNC Symbol;Acc:VGNC:94435]	6.46	0.0163
ssc-miR-182	TRIQK	triple QxxK/R motif containing [Source:VGNC Symbol;Acc:VGNC:94439]	6.46	0.0163
ssc-miR-182	TRMT1L	tRNA methyltransferase 1 like [Source:VGNC Symbol;Acc:VGNC:94447]	6.46	0.0163
ssc-miR-182	TRPC4AP	transient receptor potential cation channel subfamily C member 4 associated protein [Source:VGNC Symbol;Acc:VGNC:95774]	6.46	0.0163

ssc-miR-182	TSC22D2	TSC22 domain family member 2 [Source:VGNC Symbol;Acc:VGNC:94482]	6.46	0.0163
ssc-miR-182	TSHR	thyroid stimulating hormone receptor [Source:VGNC Symbol;Acc:VGNC:94491]	6.46	0.0163
ssc-miR-182	TSKU	tsukushi, small leucine rich proteoglycan [Source:VGNC Symbol;Acc:VGNC:94495]	6.46	0.0163
ssc-miR-182	TSNAX	translin associated factor X [Source:VGNC Symbol;Acc:VGNC:103075]	6.46	0.0163
ssc-miR-182	TSPAN14	tetraspanin 14 [Source:VGNC Symbol;Acc:VGNC:94503]	6.46	0.0163
ssc-miR-182	TSPAN3	tetraspanin 3 [Source:VGNC Symbol;Acc:VGNC:94510]	6.46	0.0163
ssc-miR-182	TSPAN4	tetraspanin 4 [Source:VGNC Symbol;Acc:VGNC:94513]	6.46	0.0163
ssc-miR-182	TSPAN9	tetraspanin 9 [Source:VGNC Symbol;Acc:VGNC:94516]	6.46	0.0163
ssc-miR-182	TTBK2	tau tubulin kinase 2 [Source:VGNC Symbol;Acc:VGNC:98387]	6.46	0.0163
ssc-miR-182	TTC39A	tetratricopeptide repeat domain 39A [Source:VGNC Symbol;Acc:VGNC:94551]	6.46	0.0163
ssc-miR-182	TTYH3	tweety family member 3 [Source:VGNC Symbol;Acc:VGNC:94576]	6.46	0.0163
ssc-miR-182	TUB	TUB bipartite transcription factor [Source:VGNC Symbol;Acc:VGNC:94577]	6.46	0.0163
ssc-miR-182	TUBD1	tubulin delta 1 [Source:VGNC Symbol;Acc:VGNC:94582]	6.46	0.0163
ssc-miR-182	TWISTNB	hypothetical gene	6.46	0.0163
ssc-miR-182	TXNL1	thioredoxin like 1 [Source:VGNC Symbol;Acc:VGNC:94611]	6.46	0.0163
ssc-miR-182	TXNRD1	thioredoxin reductase 1 [Source:NCBI gene (formerly Entrezgene);Acc:396681]	6.46	0.0163
ssc-miR-182	U2SURP	U2 snRNP associated SURP domain containing [Source:VGNC Symbol;Acc:VGNC:94624]	6.46	0.0163
ssc-miR-182	UBASH3B	ubiquitin associated and SH3 domain containing B [Source:VGNC Symbol;Acc:VGNC:94637]	6.46	0.0163
ssc-miR-182	UBE2D4	ubiquitin conjugating enzyme E2 D4 (putative) [Source:HGNC Symbol;Acc:HGNC:21647]	6.46	0.0163
ssc-miR-182	UBE2F	hypothetical gene	6.46	0.0163
ssc-miR-182	UBE2H	ubiquitin conjugating enzyme E2 H [Source:VGNC Symbol;Acc:VGNC:94643]	6.46	0.0163
ssc-miR-182	UBE2K	ubiquitin conjugating enzyme E2 K [Source:VGNC Symbol;Acc:VGNC:94646]	6.46	0.0163
ssc-miR-182	UBE2L3	hypothetical gene	6.46	0.0163
ssc-miR-182	UBE2Q2	ubiquitin conjugating enzyme E2 Q2 [Source:HGNC Symbol;Acc:HGNC:19248]	6.46	0.0163
ssc-miR-182	UBE2R2	ubiquitin conjugating enzyme E2 R2 [Source:VGNC Symbol;Acc:VGNC:95924]	6.46	0.0163
ssc-miR-182	UBE2W	ubiquitin conjugating enzyme E2 W [Source:VGNC Symbol;Acc:VGNC:98890]	6.46	0.0163
ssc-miR-182	UBE3C	ubiquitin protein ligase E3C [Source:VGNC Symbol;Acc:VGNC:94654]	6.46	0.0163
ssc-miR-182	UBN2	ubinuclein 2 [Source:VGNC Symbol;Acc:VGNC:94664]	6.46	0.0163
ssc-miR-182	UBXN10	UBX domain protein 10 [Source:VGNC Symbol;Acc:VGNC:94675]	6.46	0.0163
ssc-miR-182	UBXN7	hypothetical gene	6.46	0.0163
ssc-miR-182	UCK2	uridine-cytidine kinase 2 [Source:VGNC Symbol;Acc:VGNC:98399]	6.46	0.0163
ssc-miR-182	UNC13A	hypothetical gene	6.46	0.0163
ssc-miR-182	UNC13C	unc-13 homolog C [Source:VGNC Symbol;Acc:VGNC:94702]	6.46	0.0163
ssc-miR-182	UNC5D	unc-5 netrin receptor D [Source:VGNC Symbol;Acc:VGNC:95892]	6.46	0.0163
ssc-miR-182	UNKL	unk like zinc finger [Source:VGNC Symbol;Acc:VGNC:94716]	6.46	0.0163
ssc-miR-182	UPRT	uracil phosphoribosyltransferase homolog [Source:VGNC Symbol;Acc:VGNC:98403]	6.46	0.0163
ssc-miR-182	USP13	ubiquitin specific peptidase 13 [Source:VGNC Symbol;Acc:VGNC:94746]	6.46	0.0163
ssc-miR-182	USP15	ubiquitin specific peptidase 15 [Source:VGNC Symbol;Acc:VGNC:94748]	6.46	0.0163
ssc-miR-182	USP24	ubiquitin specific peptidase 24 [Source:VGNC Symbol;Acc:VGNC:94754]	6.46	0.0163
ssc-miR-182	USP36	ubiquitin specific peptidase 36 [Source:VGNC Symbol;Acc:VGNC:94761]	6.46	0.0163
ssc-miR-182	USP44	ubiquitin specific peptidase 44 [Source:VGNC Symbol;Acc:VGNC:94766]	6.46	0.0163
ssc-miR-182	USP5	ubiquitin specific peptidase 5 [Source:VGNC Symbol;Acc:VGNC:94772]	6.46	0.0163
ssc-miR-182	USP6	hypothetical gene	6.46	0.0163
ssc-miR-182	USP6NL	USP6 N-terminal like [Source:VGNC Symbol;Acc:VGNC:95820]	6.46	0.0163
ssc-miR-182	VAMP3	hypothetical gene	6.46	0.0163
ssc-miR-182	VANGL2	VANGL planar cell polarity protein 2 [Source:VGNC Symbol;Acc:VGNC:94798]	6.46	0.0163
ssc-miR-182	VAT1	vesicle amine transport 1 [Source:VGNC Symbol;Acc:VGNC:94805]	6.46	0.0163
ssc-miR-182	VAT1L	vesicle amine transport 1 like [Source:VGNC Symbol;Acc:VGNC:94806]	6.46	0.0163
ssc-miR-182	VAV2	vav guanine nucleotide exchange factor 2 [Source:VGNC Symbol;Acc:VGNC:94808]	6.46	0.0163

ssc-miR-182	VCL	vinculin [Source:VGNC Symbol;Acc:VGNC:94812]	6.46	0.0163
ssc-miR-182	VEZT	vezatin, adherens junctions transmembrane protein [Source:VGNC Symbol;Acc:VGNC:94819]	6.46	0.0163
ssc-miR-182	VGLL3	vestigial like family member 3 [Source:VGNC Symbol;Acc:VGNC:94823]	6.46	0.0163
ssc-miR-182	VLDLR	very low density lipoprotein receptor [Source:VGNC Symbol;Acc:VGNC:94832]	6.46	0.0163
ssc-miR-182	VPS26B	VPS26, retromer complex component B [Source:NCBI gene (formerly Entrezgene);Acc:100192441]	6.46	0.0163
ssc-miR-182	VPS36	vacuolar protein sorting 36 homolog [Source:VGNC Symbol;Acc:VGNC:94850]	6.46	0.0163
ssc-miR-182	VPS53	VPS53 subunit of GARP complex [Source:VGNC Symbol;Acc:VGNC:94861]	6.46	0.0163
ssc-miR-182	WASF2	WASP family member 2 [Source:VGNC Symbol;Acc:VGNC:94893]	6.46	0.0163
ssc-miR-182	WASL	WASP like actin nucleation promoting factor [Source:VGNC Symbol;Acc:VGNC:96604]	6.46	0.0163
ssc-miR-182	WDR45	WD repeat domain 45 [Source:VGNC Symbol;Acc:VGNC:94922]	6.46	0.0163
ssc-miR-182	WDR47	WD repeat domain 47 [Source:VGNC Symbol;Acc:VGNC:94924]	6.46	0.0163
ssc-miR-182	WDR5	WD repeat domain 5 [Source:VGNC Symbol;Acc:VGNC:98412]	6.46	0.0163
ssc-miR-182	WDR52	hypothetical gene	6.46	0.0163
ssc-miR-182	WDR76	WD repeat domain 76 [Source:VGNC Symbol;Acc:VGNC:94941]	6.46	0.0163
ssc-miR-182	WDR82	WD repeat domain 82 [Source:VGNC Symbol;Acc:VGNC:94944]	6.46	0.0163
ssc-miR-182	WFDC9	hypothetical gene	6.46	0.0163
ssc-miR-182	WHAMM	WASP homolog associated with actin, golgi membranes and microtubules [Source:VGNC Symbol;Acc:VGNC:98414]	6.46	0.0163
ssc-miR-182	WHSC1	hypothetical gene	6.46	0.0163
ssc-miR-182	WHSC1L1	hypothetical gene	6.46	0.0163
ssc-miR-182	WIPF1	hypothetical gene	6.46	0.0163
ssc-miR-182	WIPI2	WD repeat domain, phosphoinositide interacting 2 [Source:VGNC Symbol;Acc:VGNC:94960]	6.46	0.0163
ssc-miR-182	WISP1	hypothetical gene	6.46	0.0163
ssc-miR-182	WNK2	WNK lysine deficient protein kinase 2 [Source:VGNC Symbol;Acc:VGNC:94963]	6.46	0.0163
ssc-miR-182	WNT16	Wnt family member 16 [Source:VGNC Symbol;Acc:VGNC:94967]	6.46	0.0163
ssc-miR-182	WNT2B	Wnt family member 2B [Source:VGNC Symbol;Acc:VGNC:94969]	6.46	0.0163
ssc-miR-182	WSB1	WD repeat and SOCS box containing 1 [Source:VGNC Symbol;Acc:VGNC:94981]	6.46	0.0163
ssc-miR-182	WWC2	WW and C2 domain containing 2 [Source:VGNC Symbol;Acc:VGNC:96033]	6.46	0.0163
ssc-miR-182	WWOX	WW domain containing oxidoreductase [Source:VGNC Symbol;Acc:VGNC:94988]	6.46	0.0163
ssc-miR-182	XKR4	XK related 4 [Source:VGNC Symbol;Acc:VGNC:98900]	6.46	0.0163
ssc-miR-182	XPR1	xenotropic and polytropic retrovirus receptor 1 [Source:VGNC Symbol;Acc:VGNC:108625]	6.46	0.0163
ssc-miR-182	XYLT1	xylosyltransferase 1 [Source:VGNC Symbol;Acc:VGNC:95015]	6.46	0.0163
ssc-miR-182	YAF2	YY1 associated factor 2 [Source:VGNC Symbol;Acc:VGNC:95018]	6.46	0.0163
ssc-miR-182	YES1	YES proto-onco 1, Src family tyrosine kinase [Source:VGNC Symbol;Acc:VGNC:95024]	6.46	0.0163
ssc-miR-182	YIPF4	Yip1 domain family member 4 [Source:VGNC Symbol;Acc:VGNC:95030]	6.46	0.0163
ssc-miR-182	YWHAE	tyrosine 3-monooxygenase/tryptophan 5-monooxygenase activation protein epsilon [Source:HGNC Symbol;Acc:HGNC:12851]	6.46	0.0163
ssc-miR-182	YWHAG	hypothetical gene	6.46	0.0163
ssc-miR-182	ZADH2	prostaglandin reductase 3 [Source:VGNC Symbol;Acc:VGNC:95049]	6.46	0.0163
ssc-miR-182	ZBED3	hypothetical gene	6.46	0.0163
ssc-miR-182	ZBTB16	zinc finger and BTB domain containing 16 [Source:VGNC Symbol;Acc:VGNC:108626]	6.46	0.0163
ssc-miR-182	ZBTB20	zinc finger and BTB domain containing 20 [Source:VGNC Symbol;Acc:VGNC:95063]	6.46	0.0163
ssc-miR-182	ZBTB37	zinc finger and BTB domain containing 37 [Source:VGNC Symbol;Acc:VGNC:95071]	6.46	0.0163
ssc-miR-182	ZBTB40	zinc finger and BTB domain containing 40 [Source:VGNC Symbol;Acc:VGNC:95074]	6.46	0.0163
ssc-miR-182	ZBTB41	zinc finger and BTB domain containing 41 [Source:VGNC Symbol;Acc:VGNC:108284]	6.46	0.0163
ssc-miR-182	ZBTB6	zinc finger and BTB domain containing 6 [Source:VGNC Symbol;Acc:VGNC:95080]	6.46	0.0163
ssc-miR-182	ZBTB7A	zinc finger and BTB domain containing 7A [Source:VGNC Symbol;Acc:VGNC:95081]	6.46	0.0163
ssc-miR-182	ZBTB8A	zinc finger and BTB domain containing 8A [Source:VGNC Symbol;Acc:VGNC:95084]	6.46	0.0163
ssc-miR-182	ZC2HC1C	zinc finger C2HC-type containing 1C [Source:VGNC Symbol;Acc:VGNC:95087]	6.46	0.0163
ssc-miR-182	ZC3H10	zinc finger CCCH-type containing 10 [Source:VGNC Symbol;Acc:VGNC:95088]	6.46	0.0163
ssc-miR-182	ZC3H15	zinc finger CCCH-type containing 15 [Source:HGNC Symbol;Acc:HGNC:29528]	6.46	0.0163

ssc-miR-182	ZC3H6	zinc finger CCCH-type containing 6 [Source:VGNC Symbol;Acc:VGNC:95098]	6.46	0.0163
ssc-miR-182	ZCCHC11	hypothetical gene	6.46	0.0163
ssc-miR-182	ZCCHC14	zinc finger CCHC-type containing 14 [Source:VGNC Symbol;Acc:VGNC:95107]	6.46	0.0163
ssc-miR-182	ZCCHC3	zinc finger CCHC-type containing 3 [Source:HGNC Symbol;Acc:HGNC:16230]	6.46	0.0163
ssc-miR-182	ZDHHC2	zinc finger DHHC-type palmitoyltransferase 2 [Source:VGNC Symbol;Acc:VGNC:95886]	6.46	0.0163
ssc-miR-182	ZDHHC5	zinc finger DHHC-type palmitoyltransferase 5 [Source:VGNC Symbol;Acc:VGNC:95127]	6.46	0.0163
ssc-miR-182	ZEB2	hypothetical gene	6.46	0.0163
ssc-miR-182	ZFAND4	zinc finger AN1-type containing 4 [Source:VGNC Symbol;Acc:VGNC:95136]	6.46	0.0163
ssc-miR-182	ZFAND5	zinc finger AN1-type containing 5 [Source:VGNC Symbol;Acc:VGNC:95137]	6.46	0.0163
ssc-miR-182	ZFC3H1	zinc finger C3H1-type containing [Source:VGNC Symbol;Acc:VGNC:95139]	6.46	0.0163
ssc-miR-182	ZFP3	hypothetical gene	6.46	0.0163
ssc-miR-182	ZFP30	ZFP30 zinc finger protein [Source:VGNC Symbol;Acc:VGNC:98741]	6.46	0.0163
ssc-miR-182	ZFP36L1	ZFP36 ring finger protein like 1 [Source:VGNC Symbol;Acc:VGNC:95146]	6.46	0.0163
ssc-miR-182	ZFP36L2	ZFP36 ring finger protein like 2 [Source:VGNC Symbol;Acc:VGNC:95147]	6.46	0.0163
ssc-miR-182	ZHX1	zinc fingers and homeoboxes 1 [Source:VGNC Symbol;Acc:VGNC:95164]	6.46	0.0163
ssc-miR-182	ZHX2	zinc fingers and homeoboxes 2 [Source:VGNC Symbol;Acc:VGNC:95165]	6.46	0.0163
ssc-miR-182	ZHX3	zinc fingers and homeoboxes 3 [Source:VGNC Symbol;Acc:VGNC:95717]	6.46	0.0163
ssc-miR-182	ZIC2	Zic family member 2 [Source:VGNC Symbol;Acc:VGNC:95167]	6.46	0.0163
ssc-miR-182	ZIC3	Zic family member 3 [Source:VGNC Symbol;Acc:VGNC:95168]	6.46	0.0163
ssc-miR-182	ZMAT4	zinc finger matrin-type 4 [Source:VGNC Symbol;Acc:VGNC:96109]	6.46	0.0163
ssc-miR-182	ZMPSTE24	zinc metallopeptidase STE24 [Source:VGNC Symbol;Acc:VGNC:95180]	6.46	0.0163
ssc-miR-182	ZNF189	zinc finger protein 189 [Source:VGNC Symbol;Acc:VGNC:103205]	6.46	0.0163
ssc-miR-182	ZNF2	zinc finger protein 2 [Source:VGNC Symbol;Acc:VGNC:98421]	6.46	0.0163
ssc-miR-182	ZNF200	zinc finger protein 200 [Source:VGNC Symbol;Acc:VGNC:95203]	6.46	0.0163
ssc-miR-182	ZNF24	zinc finger protein 24 [Source:VGNC Symbol;Acc:VGNC:98422]	6.46	0.0163
ssc-miR-182	ZNF280B	zinc finger protein 280B [Source:HGNC Symbol;Acc:HGNC:23022]	6.46	0.0163
ssc-miR-182	ZNF292	zinc finger protein 292 [Source:VGNC Symbol;Acc:VGNC:95218]	6.46	0.0163
ssc-miR-182	ZNF398	zinc finger protein 398 [Source:HGNC Symbol;Acc:HGNC:18373]	6.46	0.0163
ssc-miR-182	ZNF449	zinc finger protein 449 [Source:VGNC Symbol;Acc:VGNC:95242]	6.46	0.0163
ssc-miR-182	ZNF451	zinc finger protein 451 [Source:HGNC Symbol;Acc:HGNC:21091]	6.46	0.0163
ssc-miR-182	ZNF496	zinc finger protein 496 [Source:VGNC Symbol;Acc:VGNC:95251]	6.46	0.0163
ssc-miR-182	ZNF532	zinc finger protein 532 [Source:HGNC Symbol;Acc:HGNC:30940]	6.46	0.0163
ssc-miR-182	ZNF572	hypothetical gene	6.46	0.0163
ssc-miR-182	ZNF697	zinc finger protein 697 [Source:HGNC Symbol;Acc:HGNC:32034]	6.46	0.0163
ssc-miR-182	ZNF704	zinc finger protein 704 [Source:VGNC Symbol;Acc:VGNC:95296]	6.46	0.0163
ssc-miR-182	ZNF706	hypothetical gene	6.46	0.0163
ssc-miR-182	ZNF827	zinc finger protein 827 [Source:VGNC Symbol;Acc:VGNC:98970]	6.46	0.0163
ssc-miR-182	ZNRF1	zinc and ring finger 1 [Source:HGNC Symbol;Acc:HGNC:18452]	6.46	0.0163
ssc-miR-182	ZNRF2	zinc and ring finger 2 [Source:VGNC Symbol;Acc:VGNC:95317]	6.46	0.0163
ssc-miR-182	ZSWIM5	zinc finger SWIM-type containing 5 [Source:VGNC Symbol;Acc:VGNC:95334]	6.46	0.0163
ssc-miR-182	ZZEF1	zinc finger ZZ-type and EF-hand domain containing 1 [Source:VGNC Symbol;Acc:VGNC:95347]	6.46	0.0163
ssc-miR-30c-5p	A1CF	APOBEC1 complementation factor [Source:VGNC Symbol;Acc:VGNC:84933]	5.88	0.0861
ssc-miR-30c-5p	ABAT	4-aminobutyrate aminotransferase [Source:VGNC Symbol;Acc:VGNC:96910]	5.88	0.0861
ssc-miR-30c-5p	ABCA3	ATP binding cassette subfamily A member 3 [Source:VGNC Symbol;Acc:VGNC:96911]	5.88	0.0861
ssc-miR-30c-5p	ABCB7	ATP binding cassette subfamily B member 7 [Source:VGNC Symbol;Acc:VGNC:97855]	5.88	0.0861
ssc-miR-30c-5p	ABCC9	ATP binding cassette subfamily C member 9 [Source:VGNC Symbol;Acc:VGNC:84960]	5.88	0.0861
ssc-miR-30c-5p	ABCD2	ATP binding cassette subfamily D member 2 [Source:VGNC Symbol;Acc:VGNC:84962]	5.88	0.0861
ssc-miR-30c-5p	ABHD6	abhydrolase domain containing 6, acylglycerol lipase [Source:VGNC Symbol;Acc:VGNC:84982]	5.88	0.0861
ssc-miR-30c-5p	ABI2	abl interactor 2 [Source:VGNC Symbol;Acc:VGNC:96027]	5.88	0.0861

ssc-miR-30c-5p	ABI3BP	ABI family member 3 binding protein [Source:VGNC Symbol;Acc:VGNC:108628]	5.88	0.0861
ssc-miR-30c-5p	ABL1	ABL proto-onco 1, non-receptor tyrosine kinase [Source:VGNC Symbol;Acc:VGNC:84984]	5.88	0.0861
ssc-miR-30c-5p	ABL2	ABL proto-onco 2, non-receptor tyrosine kinase [Source:VGNC Symbol;Acc:VGNC:84985]	5.88	0.0861
ssc-miR-30c-5p	AC012215.1	hypothetical gene	5.88	0.0861
ssc-miR-30c-5p	AC104472.1	hypothetical gene	5.88	0.0861
ssc-miR-30c-5p	ACAP2	ArfGAP with coiled-coil, ankyrin repeat and PH domains 2 [Source:VGNC Symbol;Acc:VGNC:85002]	5.88	0.0861
ssc-miR-30c-5p	ACSBG1	acyl-CoA synthetase bubblegum family member 1 [Source:VGNC Symbol;Acc:VGNC:85030]	5.88	0.0861
ssc-miR-30c-5p	ACTC1	actin alpha cardiac muscle 1 [Source:VGNC Symbol;Acc:VGNC:85040]	5.88	0.0861
ssc-miR-30c-5p	ACTN1	actinin alpha 1 [Source:NCBI gene (formerly Entrezgene);Acc:100513412]	5.88	0.0861
ssc-miR-30c-5p	ACTR1A	actin related protein 1A [Source:VGNC Symbol;Acc:VGNC:85049]	5.88	0.0861
ssc-miR-30c-5p	ACTR3C	hypothetical gene	5.88	0.0861
ssc-miR-30c-5p	ACVR1	activin A receptor type 1 [Source:VGNC Symbol;Acc:VGNC:95830]	5.88	0.0861
ssc-miR-30c-5p	ADAM10	ADAM metallopeptidase domain 10 [Source:VGNC Symbol;Acc:VGNC:85061]	5.88	0.0861
ssc-miR-30c-5p	ADAM11	ADAM metallopeptidase domain 11 [Source:VGNC Symbol;Acc:VGNC:85062]	5.88	0.0861
ssc-miR-30c-5p	ADAM12	ADAM metallopeptidase domain 12 [Source:VGNC Symbol;Acc:VGNC:85063]	5.88	0.0861
ssc-miR-30c-5p	ADAM19	ADAM metallopeptidase domain 19 [Source:VGNC Symbol;Acc:VGNC:85066]	5.88	0.0861
ssc-miR-30c-5p	ADAM22	ADAM metallopeptidase domain 22 [Source:VGNC Symbol;Acc:VGNC:85067]	5.88	0.0861
ssc-miR-30c-5p	ADAM9	ADAM metallopeptidase domain 9 [Source:VGNC Symbol;Acc:VGNC:95969]	5.88	0.0861
ssc-miR-30c-5p	ADAMTS14	ADAM metallopeptidase with thrombospondin type 1 motif 14 [Source:VGNC Symbol;Acc:VGNC:85076]	5.88	0.0861
ssc-miR-30c-5p	ADAMTS3	ADAM metallopeptidase with thrombospondin type 1 motif 3 [Source:VGNC Symbol;Acc:VGNC:85083]	5.88	0.0861
ssc-miR-30c-5p	ADAMTS5	ADAM metallopeptidase with thrombospondin type 1 motif 5 [Source:VGNC Symbol;Acc:VGNC:85085]	5.88	0.0861
ssc-miR-30c-5p	ADAMTS9	ADAM metallopeptidase with thrombospondin type 1 motif 9 [Source:VGNC Symbol;Acc:VGNC:85089]	5.88	0.0861
ssc-miR-30c-5p	ADAMTSL2	ADAMTS like 2 [Source:VGNC Symbol;Acc:VGNC:85091]	5.88	0.0861
ssc-miR-30c-5p	ADARB2	adenosine deaminase RNA specific B2 (inactive) [Source:HGNC Symbol;Acc:HGNC:227]	5.88	0.0861
ssc-miR-30c-5p	ADO	2-aminoethanethiol dioxygenase [Source:VGNC Symbol;Acc:VGNC:85146]	5.88	0.0861
ssc-miR-30c-5p	ADRA1D	adrenoceptor alpha 1D [Source:NCBI gene (formerly Entrezgene);Acc:552898]	5.88	0.0861
ssc-miR-30c-5p	ADRA2A	adrenoceptor alpha 2A [Source:VGNC Symbol;Acc:VGNC:85156]	5.88	0.0861
ssc-miR-30c-5p	ADRA2B	adrenoceptor alpha 2B [Source:VGNC Symbol;Acc:VGNC:85157]	5.88	0.0861
ssc-miR-30c-5p	ADRB1	adrenoceptor beta 1 [Source:VGNC Symbol;Acc:VGNC:107363]	5.88	0.0861
ssc-miR-30c-5p	AFAP1L2	actin filament associated protein 1 like 2 [Source:HGNC Symbol;Acc:HGNC:25901]	5.88	0.0861
ssc-miR-30c-5p	AFF3	AF4/FMR2 family member 3 [Source:HGNC Symbol;Acc:HGNC:6473]	5.88	0.0861
ssc-miR-30c-5p	AFF4	AF4/FMR2 family member 4 [Source:VGNC Symbol;Acc:VGNC:85169]	5.88	0.0861
ssc-miR-30c-5p	AGO1	hypothetical gene	5.88	0.0861
ssc-miR-30c-5p	AGO2	argonaute RISC catalytic component 2 [Source:VGNC Symbol;Acc:VGNC:97871]	5.88	0.0861
ssc-miR-30c-5p	AGO3	argonaute RISC component 1 [Source:NCBI gene (formerly Entrezgene);Acc:100499510]	5.88	0.0861
ssc-miR-30c-5p	AGO4	hypothetical gene	5.88	0.0861
ssc-miR-30c-5p	AGPAT4	1-acylglycerol-3-phosphate O-acyltransferase 4 [Source:VGNC Symbol;Acc:VGNC:85186]	5.88	0.0861
ssc-miR-30c-5p	AHNAK	hypothetical gene	5.88	0.0861
ssc-miR-30c-5p	AIDA	axin interactor, dorsalization associated [Source:VGNC Symbol;Acc:VGNC:96103]	5.88	0.0861
ssc-miR-30c-5p	AJAP1	adherens junctions associated protein 1 [Source:VGNC Symbol;Acc:VGNC:85207]	5.88	0.0861
ssc-miR-30c-5p	AKAP10	A-kinase anchoring protein 10 [Source:VGNC Symbol;Acc:VGNC:85215]	5.88	0.0861
ssc-miR-30c-5p	AKIRIN1	akirin 1 [Source:VGNC Symbol;Acc:VGNC:85227]	5.88	0.0861
ssc-miR-30c-5p	AL139099.1	hypothetical gene	5.88	0.0861
ssc-miR-30c-5p	ALG10	hypothetical gene	5.88	0.0861
ssc-miR-30c-5p	ALG9	ALG9 alpha-1,2-mannosyltransferase [Source:NCBI gene (formerly Entrezgene);Acc:100519965]	5.88	0.0861
ssc-miR-30c-5p	ALKBH1	alkB homolog 1, histone H2A dioxygenase [Source:VGNC Symbol;Acc:VGNC:85257]	5.88	0.0861
ssc-miR-30c-5p	ALPK3	alpha kinase 3 [Source:HGNC Symbol;Acc:HGNC:17574]	5.88	0.0861
ssc-miR-30c-5p	AMER1	APC membrane recruitment protein 1 [Source:VGNC Symbol;Acc:VGNC:85276]	5.88	0.0861
ssc-miR-30c-5p	AMOT	angiomotin [Source:VGNC Symbol;Acc:VGNC:85283]	5.88	0.0861

ssc-miR-30c-5p	AMOTL1	angiomotin like 1 [Source:VGNC Symbol;Acc:VGNC:85284]	5.88	0.0861
ssc-miR-30c-5p	AMOTL2	angiomotin like 2 [Source:VGNC Symbol;Acc:VGNC:85285]	5.88	0.0861
ssc-miR-30c-5p	ANGEL2	angel homolog 2 [Source:VGNC Symbol;Acc:VGNC:85302]	5.88	0.0861
ssc-miR-30c-5p	ANKHD1	ankyrin repeat and KH domain containing 1 [Source:NCBI gene (formerly Entrezgene);Acc:100512270]	5.88	0.0861
ssc-miR-30c-5p	ANKRA2	ankyrin repeat family A member 2 [Source:VGNC Symbol;Acc:VGNC:85320]	5.88	0.0861
ssc-miR-30c-5p	ANKRD17	ankyrin repeat domain 17 [Source:VGNC Symbol;Acc:VGNC:85328]	5.88	0.0861
ssc-miR-30c-5p	ANKRD26	hypothetical gene	5.88	0.0861
ssc-miR-30c-5p	ANKRD52	ankyrin repeat domain 52 [Source:VGNC Symbol;Acc:VGNC:85342]	5.88	0.0861
ssc-miR-30c-5p	ANKS4B	ankyrin repeat and sterile alpha motif domain containing 4B [Source:VGNC Symbol;Acc:VGNC:85350]	5.88	0.0861
ssc-miR-30c-5p	ANO4	anoctamin 4 [Source:VGNC Symbol;Acc:VGNC:85357]	5.88	0.0861
ssc-miR-30c-5p	AP1B1	adaptor related protein complex 1 subunit beta 1 [Source:VGNC Symbol;Acc:VGNC:85377]	5.88	0.0861
ssc-miR-30c-5p	AP1S2	adaptor related protein complex 1 subunit sigma 2 [Source:VGNC Symbol;Acc:VGNC:85382]	5.88	0.0861
ssc-miR-30c-5p	AP2A1	adaptor related protein complex 2 subunit alpha 1 [Source:VGNC Symbol;Acc:VGNC:85383]	5.88	0.0861
ssc-miR-30c-5p	AP3M1	adaptor related protein complex 3 subunit mu 1 [Source:VGNC Symbol;Acc:VGNC:85390]	5.88	0.0861
ssc-miR-30c-5p	AP3S1	hypothetical gene	5.88	0.0861
ssc-miR-30c-5p	AP4E1	adaptor related protein complex 4 subunit epsilon 1 [Source:VGNC Symbol;Acc:VGNC:85392]	5.88	0.0861
ssc-miR-30c-5p	AP5M1	adaptor related protein complex 5 subunit mu 1 [Source:VGNC Symbol;Acc:VGNC:85396]	5.88	0.0861
ssc-miR-30c-5p	APBA1	amyloid beta protein binding family A member 1 [Source:VGNC Symbol;Acc:VGNC:85399]	5.88	0.0861
ssc-miR-30c-5p	AQR	aquarius intron-binding spliceosomal factor [Source:VGNC Symbol;Acc:VGNC:85437]	5.88	0.0861
ssc-miR-30c-5p	ARAP2	ArfGAP with RhoGAP domain, ankyrin repeat and PH domain 2 [Source:VGNC Symbol;Acc:VGNC:85440]	5.88	0.0861
ssc-miR-30c-5p	ARF4	ADP ribosylation factor 4 [Source:VGNC Symbol;Acc:VGNC:85446]	5.88	0.0861
ssc-miR-30c-5p	ARHGAP26	Rho GTPase activating protein 26 [Source:VGNC Symbol;Acc:VGNC:85465]	5.88	0.0861
ssc-miR-30c-5p	ARHGEF3	Rho guanine nucleotide exchange factor 3 [Source:VGNC Symbol;Acc:VGNC:85496]	5.88	0.0861
ssc-miR-30c-5p	ARHGEF6	Rac/Cdc42 guanine nucleotide exchange factor 6 [Source:VGNC Symbol;Acc:VGNC:85502]	5.88	0.0861
ssc-miR-30c-5p	ARID1A	AT-rich interaction domain 1A [Source:VGNC Symbol;Acc:VGNC:85505]	5.88	0.0861
ssc-miR-30c-5p	ARID3A	AT-rich interaction domain 3A [Source:VGNC Symbol;Acc:VGNC:85507]	5.88	0.0861
ssc-miR-30c-5p	ARID4A	AT-rich interaction domain 4A [Source:VGNC Symbol;Acc:VGNC:85509]	5.88	0.0861
ssc-miR-30c-5p	ARID4B	AT-rich interaction domain 4B [Source:HGNC Symbol;Acc:HGNC:15550]	5.88	0.0861
ssc-miR-30c-5p	ARID5B	AT-rich interaction domain 5B [Source:VGNC Symbol;Acc:VGNC:85511]	5.88	0.0861
ssc-miR-30c-5p	ARIH1	ariadne RBR E3 ubiquitin protein ligase 1 [Source:HGNC Symbol;Acc:HGNC:689]	5.88	0.0861
ssc-miR-30c-5p	ARL10	ADP ribosylation factor like GTPase 10 [Source:VGNC Symbol;Acc:VGNC:85514]	5.88	0.0861
ssc-miR-30c-5p	ARL15	ADP ribosylation factor like GTPase 15 [Source:VGNC Symbol;Acc:VGNC:85520]	5.88	0.0861
ssc-miR-30c-5p	ARL4A	ADP ribosylation factor like GTPase 4A [Source:VGNC Symbol;Acc:VGNC:85522]	5.88	0.0861
ssc-miR-30c-5p	ARL4C	ADP ribosylation factor like GTPase 4C [Source:VGNC Symbol;Acc:VGNC:96419]	5.88	0.0861
ssc-miR-30c-5p	ARL6IP5	ADP ribosylation factor like GTPase 6 interacting protein 5 [Source:VGNC Symbol;Acc:VGNC:103904]	5.88	0.0861
ssc-miR-30c-5p	ARL6IP6	ADP ribosylation factor like GTPase 6 interacting protein 6 [Source:VGNC Symbol;Acc:VGNC:96029]	5.88	0.0861
ssc-miR-30c-5p	ASB3	ankyrin repeat and SOCS box containing 3 [Source:VGNC Symbol;Acc:VGNC:97034]	5.88	0.0861
ssc-miR-30c-5p	ASB4	ankyrin repeat and SOCS box containing 4 [Source:VGNC Symbol;Acc:VGNC:97891]	5.88	0.0861
ssc-miR-30c-5p	ASCC1	activating signal cointegrator 1 complex subunit 1 [Source:VGNC Symbol;Acc:VGNC:85569]	5.88	0.0861
ssc-miR-30c-5p	ASCC3	activating signal cointegrator 1 complex subunit 3 [Source:VGNC Symbol;Acc:VGNC:85571]	5.88	0.0861
ssc-miR-30c-5p	ASXL3	ASXL transcriptional regulator 3 [Source:VGNC Symbol;Acc:VGNC:85596]	5.88	0.0861
ssc-miR-30c-5p	ATAD2B	ATPase family AAA domain containing 2B [Source:VGNC Symbol;Acc:VGNC:85600]	5.88	0.0861
ssc-miR-30c-5p	ATAD5	ATPase family AAA domain containing 5 [Source:VGNC Symbol;Acc:VGNC:85601]	5.88	0.0861
ssc-miR-30c-5p	ATF1	activating transcription factor 1 [Source:VGNC Symbol;Acc:VGNC:85605]	5.88	0.0861
ssc-miR-30c-5p	ATF7IP2	activating transcription factor 7 interacting protein 2 [Source:VGNC Symbol;Acc:VGNC:85612]	5.88	0.0861
ssc-miR-30c-5p	ATG5	autophagy related 5 [Source:VGNC Symbol;Acc:VGNC:103029]	5.88	0.0861
ssc-miR-30c-5p	ATL2	atlastin GTPase 2 [Source:VGNC Symbol;Acc:VGNC:85628]	5.88	0.0861
ssc-miR-30c-5p	ATP2A2	ATPase sarcoplasmic/endoplasmic reticulum Ca2+ transporting 2 [Source:VGNC Symbol;Acc:VGNC:85647]	5.88	0.0861
ssc-miR-30c-5p	ATP2B1	ATPase plasma membrane Ca2+ transporting 1 [Source:VGNC Symbol;Acc:VGNC:103219]	5.88	0.0861

ssc-miR-30c-5p	ATP2B2	ATPase plasma membrane Ca2+ transporting 2 [Source:VGNC Symbol;Acc:VGNC:85649]	5.88	0.0861
ssc-miR-30c-5p	ATP6V0A1	ATPase H+ transporting V0 subunit a1 [Source:VGNC Symbol;Acc:VGNC:85663]	5.88	0.0861
ssc-miR-30c-5p	ATP6V0D1	ATPase H+ transporting V0 subunit d1 [Source:VGNC Symbol;Acc:VGNC:85667]	5.88	0.0861
ssc-miR-30c-5p	ATP6V1C1	ATPase H+ transporting V1 subunit C1 [Source:VGNC Symbol;Acc:VGNC:85672]	5.88	0.0861
ssc-miR-30c-5p	ATP8A1	ATPase phospholipid transporting 8A1 [Source:VGNC Symbol;Acc:VGNC:97894]	5.88	0.0861
ssc-miR-30c-5p	ATP8A2	ATPase phospholipid transporting 8A2 [Source:HGNC Symbol;Acc:HGNC:13533]	5.88	0.0861
ssc-miR-30c-5p	ATP8B1	ATPase phospholipid transporting 8B1 [Source:VGNC Symbol;Acc:VGNC:85679]	5.88	0.0861
ssc-miR-30c-5p	ATP8B2	ATPase phospholipid transporting 8B2 [Source:VGNC Symbol;Acc:VGNC:85680]	5.88	0.0861
ssc-miR-30c-5p	ATRN	attractin [Source:VGNC Symbol;Acc:VGNC:96479]	5.88	0.0861
ssc-miR-30c-5p	ATRNL1	attractin like 1 [Source:VGNC Symbol;Acc:VGNC:85685]	5.88	0.0861
ssc-miR-30c-5p	ATXN1	ataxin 1 [Source:VGNC Symbol;Acc:VGNC:85687]	5.88	0.0861
ssc-miR-30c-5p	AVEN	apoptosis and caspase activation inhibitor [Source:HGNC Symbol;Acc:HGNC:13509]	5.88	0.0861
ssc-miR-30c-5p	AVL9	AVL9 cell migration associated [Source:VGNC Symbol;Acc:VGNC:85701]	5.88	0.0861
ssc-miR-30c-5p	AZIN1	antizyme inhibitor 1 [Source:VGNC Symbol;Acc:VGNC:85712]	5.88	0.0861
ssc-miR-30c-5p	B3GALT5	hypothetical gene	5.88	0.0861
ssc-miR-30c-5p	B3GALT6	beta-1,3-galactosyltransferase 6 [Source:VGNC Symbol;Acc:VGNC:85718]	5.88	0.0861
ssc-miR-30c-5p	B3GALTL	hypothetical gene	5.88	0.0861
ssc-miR-30c-5p	B3GAT1	beta-1,3-glucuronyltransferase 1 [Source:VGNC Symbol;Acc:VGNC:85719]	5.88	0.0861
ssc-miR-30c-5p	B3GNT5	UDP-GlcNAc:betaGal beta-1,3-N-acetylglucosaminyltransferase 5 [Source:VGNC Symbol;Acc:VGNC:85726]	5.88	0.0861
ssc-miR-30c-5p	B4GALT5	beta-1,4-galactosyltransferase 5 [Source:VGNC Symbol;Acc:VGNC:96499]	5.88	0.0861
ssc-miR-30c-5p	B4GALT6	beta-1,4-galactosyltransferase 6 [Source:VGNC Symbol;Acc:VGNC:85734]	5.88	0.0861
ssc-miR-30c-5p	BACH1	BTB domain and CNC homolog 1 [Source:VGNC Symbol;Acc:VGNC:85741]	5.88	0.0861
ssc-miR-30c-5p	BAHD1	bromo adjacent homology domain containing 1 [Source:VGNC Symbol;Acc:VGNC:85747]	5.88	0.0861
ssc-miR-30c-5p	BAZ1A	bromodomain adjacent to zinc finger domain 1A [Source:VGNC Symbol;Acc:VGNC:85761]	5.88	0.0861
ssc-miR-30c-5p	BAZ2B	bromodomain adjacent to zinc finger domain 2B [Source:HGNC Symbol;Acc:HGNC:963]	5.88	0.0861
ssc-miR-30c-5p	BBX	BBX high mobility group box domain containing [Source:VGNC Symbol;Acc:VGNC:85766]	5.88	0.0861
ssc-miR-30c-5p	BCL11B	BAF chromatin remodeling complex subunit BCL11B [Source:VGNC Symbol;Acc:VGNC:96563]	5.88	0.0861
ssc-miR-30c-5p	BCL2	BCL2 apoptosis regulator [Source:HGNC Symbol;Acc:HGNC:990]	5.88	0.0861
ssc-miR-30c-5p	BCL2L11	BCL2 like 11 [Source:NCBI gene (formerly Entrezgene);Acc:396632]	5.88	0.0861
ssc-miR-30c-5p	BCL6	BCL6 transcription repressor [Source:VGNC Symbol;Acc:VGNC:96565]	5.88	0.0861
ssc-miR-30c-5p	BCL9	BCL9 transcription coactivator [Source:VGNC Symbol;Acc:VGNC:96567]	5.88	0.0861
ssc-miR-30c-5p	BCLAF1	BCL2 associated transcription factor 1 [Source:VGNC Symbol;Acc:VGNC:85787]	5.88	0.0861
ssc-miR-30c-5p	BCOR	BCL6 corepressor [Source:HGNC Symbol;Acc:HGNC:20893]	5.88	0.0861
ssc-miR-30c-5p	BDNF	brain derived neurotrophic factor [Source:VGNC Symbol;Acc:VGNC:85795]	5.88	0.0861
ssc-miR-30c-5p	BDP1	B double prime 1, subunit of RNA polymerase III transcription initiation factor IIIB [Source:VGNC Symbol;Acc:VGNC:85796]	5.88	0.0861
ssc-miR-30c-5p	BEAN1	brain expressed associated with NEDD4 1 [Source:VGNC Symbol;Acc:VGNC:85797]	5.88	0.0861
ssc-miR-30c-5p	BECN1	beclin 1 [Source:VGNC Symbol;Acc:VGNC:85798]	5.88	0.0861
ssc-miR-30c-5p	BEND3	BEN domain containing 3 [Source:VGNC Symbol;Acc:VGNC:85800]	5.88	0.0861
ssc-miR-30c-5p	BEND4	BEN domain containing 4 [Source:VGNC Symbol;Acc:VGNC:85801]	5.88	0.0861
ssc-miR-30c-5p	BEND7	BEN domain containing 7 [Source:VGNC Symbol;Acc:VGNC:106445]	5.88	0.0861
ssc-miR-30c-5p	BIRC6	baculoviral IAP repeat containing 6 [Source:VGNC Symbol;Acc:VGNC:97038]	5.88	0.0861
ssc-miR-30c-5p	BMP7	bone morphotic protein 7 [Source:VGNC Symbol;Acc:VGNC:95491]	5.88	0.0861
ssc-miR-30c-5p	BMPR2	bone morphotic protein receptor type 2 [Source:VGNC Symbol;Acc:VGNC:95494]	5.88	0.0861
ssc-miR-30c-5p	BNC1	basonuclin 1 [Source:VGNC Symbol;Acc:VGNC:85851]	5.88	0.0861
ssc-miR-30c-5p	BNC2	basonuclin 2 [Source:VGNC Symbol;Acc:VGNC:85852]	5.88	0.0861
ssc-miR-30c-5p	BNIP3L	BCL2 interacting protein 3 like [Source:VGNC Symbol;Acc:VGNC:85856]	5.88	0.0861
ssc-miR-30c-5p	BRAP	BRCA1 associated protein [Source:VGNC Symbol;Acc:VGNC:85867]	5.88	0.0861
ssc-miR-30c-5p	BRD1	bromodomain containing 1 [Source:VGNC Symbol;Acc:VGNC:85871]	5.88	0.0861
ssc-miR-30c-5p	BRWD1	bromodomain and WD repeat domain containing 1 [Source:VGNC Symbol;Acc:VGNC:108153]	5.88	0.0861

ssc-miR-30c-5p	BRWD3	bromodomain and WD repeat domain containing 3 [Source:VGNC Symbol;Acc:VGNC:85890]	5.88	0.0861
ssc-miR-30c-5p	BSN	bassoon presynaptic cytomatrix protein [Source:VGNC Symbol;Acc:VGNC:85893]	5.88	0.0861
ssc-miR-30c-5p	BTAF1	B-TFIID TATA-box binding protein associated factor 1 [Source:VGNC Symbol;Acc:VGNC:85899]	5.88	0.0861
ssc-miR-30c-5p	BTBD10	hypothetical gene	5.88	0.0861
ssc-miR-30c-5p	BTBD7	BTB domain containing 7 [Source:VGNC Symbol;Acc:VGNC:85908]	5.88	0.0861
ssc-miR-30c-5p	BZW1	basic leucine zipper and W2 domains 1 [Source:VGNC Symbol;Acc:VGNC:95885]	5.88	0.0861
ssc-miR-30c-5p	C10orf105	chromosome 14 C10orf105 homolog [Source:VGNC Symbol;Acc:VGNC:85940]	5.88	0.0861
ssc-miR-30c-5p	C10orf11	hypothetical gene	5.88	0.0861
ssc-miR-30c-5p	C10orf118	hypothetical gene	5.88	0.0861
ssc-miR-30c-5p	C10orf25	hypothetical gene	5.88	0.0861
ssc-miR-30c-5p	C10orf76	hypothetical gene	5.88	0.0861
ssc-miR-30c-5p	C11orf84	hypothetical gene	5.88	0.0861
ssc-miR-30c-5p	C14orf28	chromosome 1 C14orf28 homolog [Source:VGNC Symbol;Acc:VGNC:85958]	5.88	0.0861
ssc-miR-30c-5p	C16orf52	hypothetical gene	5.88	0.0861
ssc-miR-30c-5p	C16orf87	chromosome 6 C16orf87 homolog [Source:VGNC Symbol;Acc:VGNC:96927]	5.88	0.0861
ssc-miR-30c-5p	C20orf112	hypothetical gene	5.88	0.0861
ssc-miR-30c-5p	C21orf91	chromosome 13 C21orf91 homolog [Source:VGNC Symbol;Acc:VGNC:85930]	5.88	0.0861
ssc-miR-30c-5p	C3orf18	chromosome 13 C3orf18 homolog [Source:VGNC Symbol;Acc:VGNC:85932]	5.88	0.0861
ssc-miR-30c-5p	C3orf58	hypothetical gene	5.88	0.0861
ssc-miR-30c-5p	C4orf19	chromosome 8 C4orf19 homolog [Source:VGNC Symbol;Acc:VGNC:86080]	5.88	0.0861
ssc-miR-30c-5p	C7orf43	hypothetical gene	5.88	0.0861
ssc-miR-30c-5p	C7orf55-LUC7L2	hypothetical gene	5.88	0.0861
ssc-miR-30c-5p	C7orf60	hypothetical gene	5.88	0.0861
ssc-miR-30c-5p	C8orf4	hypothetical gene	5.88	0.0861
ssc-miR-30c-5p	C8orf44-SGK3	hypothetical gene	5.88	0.0861
ssc-miR-30c-5p	C9orf41	hypothetical gene	5.88	0.0861
ssc-miR-30c-5p	C9orf72	chromosome 10 C9orf72 homolog [Source:VGNC Symbol;Acc:VGNC:96173]	5.88	0.0861
ssc-miR-30c-5p	CA10	carbonic anhydrase 10 [Source:VGNC Symbol;Acc:VGNC:86096]	5.88	0.0861
ssc-miR-30c-5p	CABLES2	Cdk5 and Abl enzyme substrate 2 [Source:VGNC Symbol;Acc:VGNC:95753]	5.88	0.0861
ssc-miR-30c-5p	CACHD1	cache domain containing 1 [Source:VGNC Symbol;Acc:VGNC:86116]	5.88	0.0861
ssc-miR-30c-5p	CACNA1C	calcium voltage-gated channel subunit alpha1 C [Source:HGNC Symbol;Acc:HGNC:1390]	5.88	0.0861
ssc-miR-30c-5p	CACNA1D	calcium voltage-gated channel subunit alpha1 D [Source:VGNC Symbol;Acc:VGNC:86117]	5.88	0.0861
ssc-miR-30c-5p	CACNB2	calcium voltage-gated channel auxiliary subunit beta 2 [Source:VGNC Symbol;Acc:VGNC:95599]	5.88	0.0861
ssc-miR-30c-5p	CACUL1	CDK2 associated cullin domain 1 [Source:VGNC Symbol;Acc:VGNC:86132]	5.88	0.0861
ssc-miR-30c-5p	CACYBP	calcyclin binding protein [Source:VGNC Symbol;Acc:VGNC:86133]	5.88	0.0861
ssc-miR-30c-5p	CADM1	cell adhesion molecule 1 [Source:VGNC Symbol;Acc:VGNC:86134]	5.88	0.0861
ssc-miR-30c-5p	CADM2	cell adhesion molecule 2 [Source:VGNC Symbol;Acc:VGNC:97910]	5.88	0.0861
ssc-miR-30c-5p	CADPS	hypothetical gene	5.88	0.0861
ssc-miR-30c-5p	CALB2	calbindin 2 [Source:VGNC Symbol;Acc:VGNC:86139]	5.88	0.0861
ssc-miR-30c-5p	CALCOCO1	calcium binding and coiled-coil domain 1 [Source:VGNC Symbol;Acc:VGNC:86140]	5.88	0.0861
ssc-miR-30c-5p	CALCR	calcitonin receptor [Source:VGNC Symbol;Acc:VGNC:86142]	5.88	0.0861
ssc-miR-30c-5p	CALD1	caldesmon 1 [Source:VGNC Symbol;Acc:VGNC:86143]	5.88	0.0861
ssc-miR-30c-5p	CALU	calumenin [Source:VGNC Symbol;Acc:VGNC:86151]	5.88	0.0861
ssc-miR-30c-5p	CAMK2D	calcium/calmodulin dependent protein kinase II delta [Source:VGNC Symbol;Acc:VGNC:97911]	5.88	0.0861
ssc-miR-30c-5p	CAMK2N1	calcium/calmodulin dependent protein kinase II inhibitor 1 [Source:VGNC Symbol;Acc:VGNC:96935]	5.88	0.0861
ssc-miR-30c-5p	CAMK2N2	calcium/calmodulin dependent protein kinase II inhibitor 2 [Source:HGNC Symbol;Acc:HGNC:24197]	5.88	0.0861
ssc-miR-30c-5p	CAMK4	calcium/calmodulin dependent protein kinase IV [Source:VGNC Symbol;Acc:VGNC:99602]	5.88	0.0861
ssc-miR-30c-5p	CAMKK2	calcium/calmodulin dependent protein kinase kinase 2 [Source:VGNC Symbol;Acc:VGNC:86158]	5.88	0.0861
ssc-miR-30c-5p	CAND1	cullin associated and neddylation dissociated 1 [Source:VGNC Symbol;Acc:VGNC:97912]	5.88	0.0861

ssc-miR-30c-5p	CANT1	calcium activated nucleotidase 1 [Source:VGNC Symbol;Acc:VGNC:86166]	5.88	0.0861
ssc-miR-30c-5p	CAPN5	calpain 5 [Source:VGNC Symbol;Acc:VGNC:86175]	5.88	0.0861
ssc-miR-30c-5p	CAPN7	calpain 7 [Source:NCBI gene (formerly Entrezgene);Acc:100037936]	5.88	0.0861
ssc-miR-30c-5p	CAPRIN1	cell cycle associated protein 1 [Source:VGNC Symbol;Acc:VGNC:86178]	5.88	0.0861
ssc-miR-30c-5p	CAPZA1	capping actin protein of muscle Z-line subunit alpha 1 [Source:VGNC Symbol;Acc:VGNC:86183]	5.88	0.0861
ssc-miR-30c-5p	CARF	calcium responsive transcription factor [Source:VGNC Symbol;Acc:VGNC:95615]	5.88	0.0861
ssc-miR-30c-5p	CARS	hypothetical gene	5.88	0.0861
ssc-miR-30c-5p	CASD1	CAS1 domain containing 1 [Source:VGNC Symbol;Acc:VGNC:86199]	5.88	0.0861
ssc-miR-30c-5p	CAT	catalase [Source:VGNC Symbol;Acc:VGNC:86208]	5.88	0.0861
ssc-miR-30c-5p	CBFB	core-binding factor subunit beta [Source:VGNC Symbol;Acc:VGNC:86221]	5.88	0.0861
ssc-miR-30c-5p	CBLB	Cbl proto-onco B [Source:VGNC Symbol;Acc:VGNC:86223]	5.88	0.0861
ssc-miR-30c-5p	CBX2	chromobox 2 [Source:VGNC Symbol;Acc:VGNC:86230]	5.88	0.0861
ssc-miR-30c-5p	CBX3	chromobox 3 [Source:HGNC Symbol;Acc:HGNC:1553]	5.88	0.0861
ssc-miR-30c-5p	CBX7	chromobox 7 [Source:HGNC Symbol;Acc:HGNC:1557]	5.88	0.0861
ssc-miR-30c-5p	CBX8	chromobox 8 [Source:VGNC Symbol;Acc:VGNC:86233]	5.88	0.0861
ssc-miR-30c-5p	CCDC117	coiled-coil domain containing 117 [Source:VGNC Symbol;Acc:VGNC:86248]	5.88	0.0861
ssc-miR-30c-5p	CCDC120	coiled-coil domain containing 120 [Source:VGNC Symbol;Acc:VGNC:86249]	5.88	0.0861
ssc-miR-30c-5p	CCDC148	hypothetical gene	5.88	0.0861
ssc-miR-30c-5p	CCDC160	coiled-coil domain containing 160 [Source:VGNC Symbol;Acc:VGNC:86269]	5.88	0.0861
ssc-miR-30c-5p	CCDC178	coiled-coil domain containing 178 [Source:VGNC Symbol;Acc:VGNC:86276]	5.88	0.0861
ssc-miR-30c-5p	CCDC43	coiled-coil domain containing 43 [Source:VGNC Symbol;Acc:VGNC:98979]	5.88	0.0861
ssc-miR-30c-5p	CCDC6	coiled-coil domain containing 6 [Source:VGNC Symbol;Acc:VGNC:86304]	5.88	0.0861
ssc-miR-30c-5p	CCDC71L	coiled-coil domain containing 71 like [Source:HGNC Symbol;Acc:HGNC:26685]	5.88	0.0861
ssc-miR-30c-5p	CCDC97	coiled-coil domain containing 97 [Source:VGNC Symbol;Acc:VGNC:86331]	5.88	0.0861
ssc-miR-30c-5p	CCNE2	cyclin E2 [Source:VGNC Symbol;Acc:VGNC:86355]	5.88	0.0861
ssc-miR-30c-5p	CCNF	cyclin F [Source:VGNC Symbol;Acc:VGNC:86356]	5.88	0.0861
ssc-miR-30c-5p	CCNJL	cyclin J like [Source:VGNC Symbol;Acc:VGNC:86361]	5.88	0.0861
ssc-miR-30c-5p	CCNK	cyclin K [Source:VGNC Symbol;Acc:VGNC:86362]	5.88	0.0861
ssc-miR-30c-5p	CCNT2	cyclin T2 [Source:VGNC Symbol;Acc:VGNC:103916]	5.88	0.0861
ssc-miR-30c-5p	CCNY	cyclin Y [Source:VGNC Symbol;Acc:VGNC:95998]	5.88	0.0861
ssc-miR-30c-5p	CCP110	centriolar coiled-coil protein 110 [Source:VGNC Symbol;Acc:VGNC:86367]	5.88	0.0861
ssc-miR-30c-5p	CCPG1	cell cycle progression 1 [Source:VGNC Symbol;Acc:VGNC:86368]	5.88	0.0861
ssc-miR-30c-5p	CCSER1	coiled-coil serine rich protein 1 [Source:VGNC Symbol;Acc:VGNC:86378]	5.88	0.0861
ssc-miR-30c-5p	CCZ1B	hypothetical gene	5.88	0.0861
ssc-miR-30c-5p	CD226	CD226 molecule [Source:VGNC Symbol;Acc:VGNC:86399]	5.88	0.0861
ssc-miR-30c-5p	CD2AP	CD2 associated protein [Source:VGNC Symbol;Acc:VGNC:86406]	5.88	0.0861
ssc-miR-30c-5p	CD302	CD302 molecule [Source:NCBI gene (formerly Entrezgene);Acc:100126280]	5.88	0.0861
ssc-miR-30c-5p	CDC37L1	cell division cycle 37 like 1 [Source:VGNC Symbol;Acc:VGNC:86451]	5.88	0.0861
ssc-miR-30c-5p	CDC42BPA	CDC42 binding protein kinase alpha [Source:VGNC Symbol;Acc:VGNC:95847]	5.88	0.0861
ssc-miR-30c-5p	CDC7	cell division cycle 7 [Source:VGNC Symbol;Acc:VGNC:97922]	5.88	0.0861
ssc-miR-30c-5p	CDCA7	cell division cycle associated 7 [Source:VGNC Symbol;Acc:VGNC:95623]	5.88	0.0861
ssc-miR-30c-5p	CDH13	cadherin 13 [Source:VGNC Symbol;Acc:VGNC:86477]	5.88	0.0861
ssc-miR-30c-5p	CDH20	cadherin 20 [Source:VGNC Symbol;Acc:VGNC:86484]	5.88	0.0861
ssc-miR-30c-5p	CDK12	cyclin dependent kinase 12 [Source:VGNC Symbol;Acc:VGNC:86496]	5.88	0.0861
ssc-miR-30c-5p	CDYL2	chromodomain Y like 2 [Source:VGNC Symbol;Acc:VGNC:86529]	5.88	0.0861
ssc-miR-30c-5p	CEACAM1	hypothetical gene	5.88	0.0861
ssc-miR-30c-5p	CECR2	CECR2 histone acetyl-lysine reader [Source:VGNC Symbol;Acc:VGNC:96576]	5.88	0.0861
ssc-miR-30c-5p	CECR6	hypothetical gene	5.88	0.0861
ssc-miR-30c-5p	CELF1	CUGBP Elav-like family member 1 [Source:VGNC Symbol;Acc:VGNC:86537]	5.88	0.0861

ssc-miR-30c-5p	CELF2	hypothetical gene	5.88	0.0861
ssc-miR-30c-5p	CELF3	CUGBP Elav-like family member 3 [Source:VGNC Symbol;Acc:VGNC:86538]	5.88	0.0861
ssc-miR-30c-5p	CELF5	CUGBP Elav-like family member 5 [Source:VGNC Symbol;Acc:VGNC:86540]	5.88	0.0861
ssc-miR-30c-5p	CELSR3	cadherin EGF LAG seven-pass G-type receptor 3 [Source:VGNC Symbol;Acc:VGNC:86541]	5.88	0.0861
ssc-miR-30c-5p	CEP170	centrosomal protein 170 [Source:VGNC Symbol;Acc:VGNC:97925]	5.88	0.0861
ssc-miR-30c-5p	CEP170B	centrosomal protein 170B [Source:VGNC Symbol;Acc:VGNC:86564]	5.88	0.0861
ssc-miR-30c-5p	CEP350	centrosomal protein 350 [Source:HGNC Symbol;Acc:HGNC:24238]	5.88	0.0861
ssc-miR-30c-5p	CEP41	centrosomal protein 41 [Source:VGNC Symbol;Acc:VGNC:86569]	5.88	0.0861
ssc-miR-30c-5p	CEP44	centrosomal protein 44 [Source:VGNC Symbol;Acc:VGNC:86571]	5.88	0.0861
ssc-miR-30c-5p	CEP76	centrosomal protein 76 [Source:VGNC Symbol;Acc:VGNC:96947]	5.88	0.0861
ssc-miR-30c-5p	CEP85L	centrosomal protein 85 like [Source:VGNC Symbol;Acc:VGNC:86580]	5.88	0.0861
ssc-miR-30c-5p	CERS5	ceramide synthase 5 [Source:VGNC Symbol;Acc:VGNC:97926]	5.88	0.0861
ssc-miR-30c-5p	CERS6	ceramide synthase 6 [Source:VGNC Symbol;Acc:VGNC:96025]	5.88	0.0861
ssc-miR-30c-5p	CFDP1	craniofacial development protein 1 [Source:VGNC Symbol;Acc:VGNC:86610]	5.88	0.0861
ssc-miR-30c-5p	CFL2	cofilin 2 [Source:VGNC Symbol;Acc:VGNC:86611]	5.88	0.0861
ssc-miR-30c-5p	CHD1	chromodomain helicase DNA binding protein 1 [Source:VGNC Symbol;Acc:VGNC:86629]	5.88	0.0861
ssc-miR-30c-5p	CHD5	chromodomain helicase DNA binding protein 5 [Source:VGNC Symbol;Acc:VGNC:86632]	5.88	0.0861
ssc-miR-30c-5p	CHD7	chromodomain helicase DNA binding protein 7 [Source:VGNC Symbol;Acc:VGNC:86633]	5.88	0.0861
ssc-miR-30c-5p	CHD9	chromodomain helicase DNA binding protein 9 [Source:VGNC Symbol;Acc:VGNC:86635]	5.88	0.0861
ssc-miR-30c-5p	CHFR	checkpoint with forkhead and ring finger domains [Source:HGNC Symbol;Acc:HGNC:20455]	5.88	0.0861
ssc-miR-30c-5p	CHIC1	cysteine rich hydrophobic domain 1 [Source:VGNC Symbol;Acc:VGNC:86642]	5.88	0.0861
ssc-miR-30c-5p	CHKA	choline kinase alpha [Source:VGNC Symbol;Acc:VGNC:86646]	5.88	0.0861
ssc-miR-30c-5p	CHL1	cell adhesion molecule L1 like [Source:VGNC Symbol;Acc:VGNC:108639]	5.88	0.0861
ssc-miR-30c-5p	CHMP2B	charged multivesicular body protein 2B [Source:VGNC Symbol;Acc:VGNC:86650]	5.88	0.0861
ssc-miR-30c-5p	CHRM3	cholinergic receptor muscarinic 3 [Source:VGNC Symbol;Acc:VGNC:86664]	5.88	0.0861
ssc-miR-30c-5p	CHST1	carbohydrate sulfotransferase 1 [Source:VGNC Symbol;Acc:VGNC:86673]	5.88	0.0861
ssc-miR-30c-5p	CHST12	carbohydrate sulfotransferase 12 [Source:HGNC Symbol;Acc:HGNC:17423]	5.88	0.0861
ssc-miR-30c-5p	CHST2	carbohydrate sulfotransferase 2 [Source:VGNC Symbol;Acc:VGNC:86678]	5.88	0.0861
ssc-miR-30c-5p	CLCC1	chloride channel CLIC like 1 [Source:VGNC Symbol;Acc:VGNC:86724]	5.88	0.0861
ssc-miR-30c-5p	CLCF1	cardiotrophin like cytokine factor 1 [Source:VGNC Symbol;Acc:VGNC:86725]	5.88	0.0861
ssc-miR-30c-5p	CLCN3	chloride voltage-gated channel 3 [Source:VGNC Symbol;Acc:VGNC:86727]	5.88	0.0861
ssc-miR-30c-5p	CLDN19	claudin 19 [Source:VGNC Symbol;Acc:VGNC:86736]	5.88	0.0861
ssc-miR-30c-5p	CLEC14A	C-type lectin domain containing 14A [Source:VGNC Symbol;Acc:VGNC:86747]	5.88	0.0861
ssc-miR-30c-5p	CLN8	CLN8 transmembrane ER and ERGIC protein [Source:VGNC Symbol;Acc:VGNC:103067]	5.88	0.0861
ssc-miR-30c-5p	CLOCK	clock circadian regulator [Source:VGNC Symbol;Acc:VGNC:86774]	5.88	0.0861
ssc-miR-30c-5p	CLRN1	clarin 1 [Source:VGNC Symbol;Acc:VGNC:97933]	5.88	0.0861
ssc-miR-30c-5p	CMPK2	cytidine/uridine monophosphate kinase 2 [Source:VGNC Symbol;Acc:VGNC:86801]	5.88	0.0861
ssc-miR-30c-5p	CMTM4	CKLF like MARVEL transmembrane domain containing 4 [Source:VGNC Symbol;Acc:VGNC:86804]	5.88	0.0861
ssc-miR-30c-5p	CNGB3	cyclic nucleotide gated channel subunit beta 3 [Source:VGNC Symbol;Acc:VGNC:86819]	5.88	0.0861
ssc-miR-30c-5p	CNKSR2	connector enhancer of kinase suppressor of Ras 2 [Source:VGNC Symbol;Acc:VGNC:86822]	5.88	0.0861
ssc-miR-30c-5p	CNOT6	CCR4-NOT transcription complex subunit 6 [Source:VGNC Symbol;Acc:VGNC:86837]	5.88	0.0861
ssc-miR-30c-5p	CNTN2	contactin 2 [Source:VGNC Symbol;Acc:VGNC:108577]	5.88	0.0861
ssc-miR-30c-5p	CNTN4	contactin 4 [Source:VGNC Symbol;Acc:VGNC:97934]	5.88	0.0861
ssc-miR-30c-5p	COG3	component of oligomeric golgi complex 3 [Source:HGNC Symbol;Acc:HGNC:18619]	5.88	0.0861
ssc-miR-30c-5p	COG5	component of oligomeric golgi complex 5 [Source:VGNC Symbol;Acc:VGNC:86857]	5.88	0.0861
ssc-miR-30c-5p	COL13A1	collagen type XIII alpha 1 chain [Source:VGNC Symbol;Acc:VGNC:86865]	5.88	0.0861
ssc-miR-30c-5p	COL25A1	collagen type XXV alpha 1 chain [Source:HGNC Symbol;Acc:HGNC:18603]	5.88	0.0861
ssc-miR-30c-5p	COL4A3BP	hypothetical gene	5.88	0.0861
ssc-miR-30c-5p	COL9A3	collagen type IX alpha 3 chain [Source:VGNC Symbol;Acc:VGNC:108713]	5.88	0.0861

ssc-miR-30c-5p	COMT	catechol-O-methyltransferase [Source:VGNC Symbol;Acc:VGNC:107378]	5.88	0.0861
ssc-miR-30c-5p	COPS7B	COP9 signalosome subunit 7B [Source:HGNC Symbol;Acc:HGNC:16760]	5.88	0.0861
ssc-miR-30c-5p	COQ3	coenzyme Q3, methyltransferase [Source:HGNC Symbol;Acc:HGNC:18175]	5.88	0.0861
ssc-miR-30c-5p	CORO2A	coronin 2A [Source:VGNC Symbol;Acc:VGNC:86915]	5.88	0.0861
ssc-miR-30c-5p	COTL1	coactosin like F-actin binding protein 1 [Source:HGNC Symbol;Acc:HGNC:18304]	5.88	0.0861
ssc-miR-30c-5p	CPE	carboxypeptidase E [Source:VGNC Symbol;Acc:VGNC:86936]	5.88	0.0861
ssc-miR-30c-5p	CPEB2	cytoplasmic polyadenylation element binding protein 2 [Source:VGNC Symbol;Acc:VGNC:86937]	5.88	0.0861
ssc-miR-30c-5p	CPEB3	cytoplasmic polyadenylation element binding protein 3 [Source:VGNC Symbol;Acc:VGNC:86938]	5.88	0.0861
ssc-miR-30c-5p	CPEB4	cytoplasmic polyadenylation element binding protein 4 [Source:VGNC Symbol;Acc:VGNC:86939]	5.88	0.0861
ssc-miR-30c-5p	CPNE8	copine 8 [Source:VGNC Symbol;Acc:VGNC:86955]	5.88	0.0861
ssc-miR-30c-5p	CPOX	coproporphyrinogen oxidase [Source:VGNC Symbol;Acc:VGNC:86956]	5.88	0.0861
ssc-miR-30c-5p	CPSF6	cleavage and polyadenylation specific factor 6 [Source:VGNC Symbol;Acc:VGNC:86962]	5.88	0.0861
ssc-miR-30c-5p	CREB1	cAMP responsive element binding protein 1 [Source:VGNC Symbol;Acc:VGNC:96004]	5.88	0.0861
ssc-miR-30c-5p	CRKL	CRK like proto-onco, adaptor protein [Source:VGNC Symbol;Acc:VGNC:86997]	5.88	0.0861
ssc-miR-30c-5p	CRLF3	cytokine receptor like factor 3 [Source:VGNC Symbol;Acc:VGNC:86999]	5.88	0.0861
ssc-miR-30c-5p	CRMP1	collapsin response mediator protein 1 [Source:VGNC Symbol;Acc:VGNC:87000]	5.88	0.0861
ssc-miR-30c-5p	CRY2	cryptochrome circadian regulator 2 [Source:VGNC Symbol;Acc:VGNC:87012]	5.88	0.0861
ssc-miR-30c-5p	CSAD	cysteine sulfinic acid decarboxylase [Source:VGNC Symbol;Acc:VGNC:87027]	5.88	0.0861
ssc-miR-30c-5p	CSGALNACT1	chondroitin sulfate N-acetylgalactosaminyltransferase 1 [Source:HGNC Symbol;Acc:HGNC:24290]	5.88	0.0861
ssc-miR-30c-5p	CSNK1A1	casein kinase 1 alpha 1 [Source:VGNC Symbol;Acc:VGNC:99630]	5.88	0.0861
ssc-miR-30c-5p	CSNK1A1L	hypothetical gene	5.88	0.0861
ssc-miR-30c-5p	CSNK1G1	casein kinase 1 gamma 1 [Source:VGNC Symbol;Acc:VGNC:97949]	5.88	0.0861
ssc-miR-30c-5p	CSNK2A1	hypothetical gene	5.88	0.0861
ssc-miR-30c-5p	CTDSPL2	CTD small phosphatase like 2 [Source:VGNC Symbol;Acc:VGNC:87060]	5.88	0.0861
ssc-miR-30c-5p	CTH	cystathionine gamma-lyase [Source:VGNC Symbol;Acc:VGNC:96961]	5.88	0.0861
ssc-miR-30c-5p	CTHRC1	collagen triple helix repeat containing 1 [Source:VGNC Symbol;Acc:VGNC:87061]	5.88	0.0861
ssc-miR-30c-5p	CTPS1	CTP synthase 1 [Source:VGNC Symbol;Acc:VGNC:87070]	5.88	0.0861
ssc-miR-30c-5p	CTTNBP2NL	CTTNBP2 N-terminal like [Source:VGNC Symbol;Acc:VGNC:87082]	5.88	0.0861
ssc-miR-30c-5p	CUL2	cullin 2 [Source:VGNC Symbol;Acc:VGNC:96090]	5.88	0.0861
ssc-miR-30c-5p	CUL9	cullin 9 [Source:VGNC Symbol;Acc:VGNC:87090]	5.88	0.0861
ssc-miR-30c-5p	CYB561	cytochrome b561 [Source:VGNC Symbol;Acc:VGNC:87119]	5.88	0.0861
ssc-miR-30c-5p	CYB5B	cytochrome b5 type B [Source:VGNC Symbol;Acc:VGNC:96728]	5.88	0.0861
ssc-miR-30c-5p	CYLD	CYLD lysine 63 deubiquitinase [Source:VGNC Symbol;Acc:VGNC:87130]	5.88	0.0861
ssc-miR-30c-5p	CYP24A1	cytochrome P450 family 24 subfamily A member 1 [Source:VGNC Symbol;Acc:VGNC:103371]	5.88	0.0861
ssc-miR-30c-5p	CYSLTR1	cysteinyl leukotriene receptor 1 [Source:VGNC Symbol;Acc:VGNC:87132]	5.88	0.0861
ssc-miR-30c-5p	CYYR1	cysteine and tyrosine rich 1 [Source:VGNC Symbol;Acc:VGNC:87139]	5.88	0.0861
ssc-miR-30c-5p	DAB1	DAB adaptor protein 1 [Source:VGNC Symbol;Acc:VGNC:87143]	5.88	0.0861
ssc-miR-30c-5p	DACH2	dachshund family transcription factor 2 [Source:HGNC Symbol;Acc:HGNC:16814]	5.88	0.0861
ssc-miR-30c-5p	DACT1	dishevelled binding antagonist of beta catenin 1 [Source:VGNC Symbol;Acc:VGNC:87147]	5.88	0.0861
ssc-miR-30c-5p	DAG1	dystroglycan 1 [Source:VGNC Symbol;Acc:VGNC:87151]	5.88	0.0861
ssc-miR-30c-5p	DAGLA	diacylglycerol lipase alpha [Source:VGNC Symbol;Acc:VGNC:87152]	5.88	0.0861
ssc-miR-30c-5p	DBF4	hypothetical gene	5.88	0.0861
ssc-miR-30c-5p	DCAF5	DDB1 and CUL4 associated factor 5 [Source:VGNC Symbol;Acc:VGNC:87176]	5.88	0.0861
ssc-miR-30c-5p	DCBLD1	discoidin, CUB and LCCL domain containing 1 [Source:VGNC Symbol;Acc:VGNC:87179]	5.88	0.0861
ssc-miR-30c-5p	DCP1A	decapping mRNA 1A [Source:VGNC Symbol;Acc:VGNC:97960]	5.88	0.0861
ssc-miR-30c-5p	DCP2	decapping mRNA 2 [Source:VGNC Symbol;Acc:VGNC:87188]	5.88	0.0861
ssc-miR-30c-5p	DCTN4	dynactin subunit 4 [Source:VGNC Symbol;Acc:VGNC:99635]	5.88	0.0861
ssc-miR-30c-5p	DCUN1D1	hypothetical gene	5.88	0.0861
ssc-miR-30c-5p	DCUN1D2	defective in cullin neddylation 1 domain containing 2 [Source:VGNC Symbol;Acc:VGNC:87195]	5.88	0.0861

ssc-miR-30c-5p	DCUN1D3	defective in cullin neddylation 1 domain containing 3 [Source:VGNC Symbol;Acc:VGNC:87196]	5.88	0.0861
ssc-miR-30c-5p	DCX	doublecortin [Source:HGNC Symbol;Acc:HGNC:2714]	5.88	0.0861
ssc-miR-30c-5p	DDAH1	dimethylarginine dimethylaminohydrolase 1 [Source:VGNC Symbol;Acc:VGNC:87200]	5.88	0.0861
ssc-miR-30c-5p	DDI2	hypothetical gene	5.88	0.0861
ssc-miR-30c-5p	DDIT4	DNA damage inducible transcript 4 [Source:VGNC Symbol;Acc:VGNC:87208]	5.88	0.0861
ssc-miR-30c-5p	DDX19A	hypothetical gene	5.88	0.0861
ssc-miR-30c-5p	DDX19B	hypothetical gene	5.88	0.0861
ssc-miR-30c-5p	DDX46	DEAD-box helicase 46 [Source:VGNC Symbol;Acc:VGNC:87223]	5.88	0.0861
ssc-miR-30c-5p	DENND2C	DENN domain containing 2C [Source:HGNC Symbol;Acc:HGNC:24748]	5.88	0.0861
ssc-miR-30c-5p	DENND4A	DENN domain containing 4A [Source:VGNC Symbol;Acc:VGNC:87251]	5.88	0.0861
ssc-miR-30c-5p	DENND5B	DENN domain containing 5B [Source:VGNC Symbol;Acc:VGNC:87255]	5.88	0.0861
ssc-miR-30c-5p	DESI2	desumoylating isopeptidase 2 [Source:VGNC Symbol;Acc:VGNC:96037]	5.88	0.0861
ssc-miR-30c-5p	DEXI	Dexi homolog [Source:HGNC Symbol;Acc:HGNC:13267]	5.88	0.0861
ssc-miR-30c-5p	DGKB	diacylglycerol kinase beta [Source:VGNC Symbol;Acc:VGNC:87270]	5.88	0.0861
ssc-miR-30c-5p	DGKD	diacylglycerol kinase delta [Source:VGNC Symbol;Acc:VGNC:96180]	5.88	0.0861
ssc-miR-30c-5p	DGKE	diacylglycerol kinase epsilon [Source:VGNC Symbol;Acc:VGNC:87271]	5.88	0.0861
ssc-miR-30c-5p	DGKH	diacylglycerol kinase eta [Source:VGNC Symbol;Acc:VGNC:87273]	5.88	0.0861
ssc-miR-30c-5p	DGKI	diacylglycerol kinase iota [Source:VGNC Symbol;Acc:VGNC:87274]	5.88	0.0861
ssc-miR-30c-5p	DGKQ	diacylglycerol kinase theta [Source:VGNC Symbol;Acc:VGNC:87276]	5.88	0.0861
ssc-miR-30c-5p	DGKZ	diacylglycerol kinase zeta [Source:VGNC Symbol;Acc:VGNC:87277]	5.88	0.0861
ssc-miR-30c-5p	DHX36	DEAH-box helicase 36 [Source:VGNC Symbol;Acc:VGNC:87292]	5.88	0.0861
ssc-miR-30c-5p	DHX40	DEAH-box helicase 40 [Source:VGNC Symbol;Acc:VGNC:87295]	5.88	0.0861
ssc-miR-30c-5p	DIO2	iodothyronine deiodinase 2 [Source:VGNC Symbol;Acc:VGNC:103935]	5.88	0.0861
ssc-miR-30c-5p	DIP2B	disco interacting B [Source:VGNC Symbol;Acc:VGNC:87306]	5.88	0.0861
ssc-miR-30c-5p	DIP2C	disco interacting C [Source:VGNC Symbol;Acc:VGNC:96201]	5.88	0.0861
ssc-miR-30c-5p	DLD	dihydrolipoamide dehydrogenase [Source:VGNC Symbol;Acc:VGNC:108580]	5.88	0.0861
ssc-miR-30c-5p	DLG5	discs large MAGUK scaffold protein 5 [Source:VGNC Symbol;Acc:VGNC:87329]	5.88	0.0861
ssc-miR-30c-5p	DLGAP1	DLG associated protein 1 [Source:VGNC Symbol;Acc:VGNC:87330]	5.88	0.0861
ssc-miR-30c-5p	DLGAP2	DLG associated protein 2 [Source:VGNC Symbol;Acc:VGNC:99711]	5.88	0.0861
ssc-miR-30c-5p	DLGAP4	DLG associated protein 4 [Source:VGNC Symbol;Acc:VGNC:96043]	5.88	0.0861
ssc-miR-30c-5p	DLK1	delta like non-canonical Notch ligand 1 [Source:VGNC Symbol;Acc:VGNC:87333]	5.88	0.0861
ssc-miR-30c-5p	DLL4	delta like canonical Notch ligand 4 [Source:VGNC Symbol;Acc:VGNC:87337]	5.88	0.0861
ssc-miR-30c-5p	DMD	dystrophin [Source:NCBI gene (formerly Entrezgene);Acc:497636]	5.88	0.0861
ssc-miR-30c-5p	DNAH17	dynein axonemal heavy chain 17 [Source:VGNC Symbol;Acc:VGNC:87369]	5.88	0.0861
ssc-miR-30c-5p	DNAJC13	DnaJ heat shock protein family (Hsp40) member C13 [Source:VGNC Symbol;Acc:VGNC:108650]	5.88	0.0861
ssc-miR-30c-5p	DNAJC15	DnaJ heat shock protein family (Hsp40) member C15 [Source:HGNC Symbol;Acc:HGNC:20325]	5.88	0.0861
ssc-miR-30c-5p	DNAJC25	DnaJ heat shock protein family (Hsp40) member C25 [Source:VGNC Symbol;Acc:VGNC:103083]	5.88	0.0861
ssc-miR-30c-5p	DNAJC25-GNG10	hypothetical gene	5.88	0.0861
ssc-miR-30c-5p	DNMT3A	DNA methyltransferase 3 alpha [Source:VGNC Symbol;Acc:VGNC:87384]	5.88	0.0861
ssc-miR-30c-5p	DOC2A	double C2 domain alpha [Source:VGNC Symbol;Acc:VGNC:87388]	5.88	0.0861
ssc-miR-30c-5p	DOCK7	dedicator of cytokinesis 7 [Source:VGNC Symbol;Acc:VGNC:87397]	5.88	0.0861
ssc-miR-30c-5p	DOLPP1	dolichyldiphosphatase 1 [Source:VGNC Symbol;Acc:VGNC:87407]	5.88	0.0861
ssc-miR-30c-5p	DPY19L1	dpy-19 like C-mannosyltransferase 1 [Source:VGNC Symbol;Acc:VGNC:99712]	5.88	0.0861
ssc-miR-30c-5p	DPY19L3	dpy-19 like C-mannosyltransferase 3 [Source:VGNC Symbol;Acc:VGNC:87426]	5.88	0.0861
ssc-miR-30c-5p	DPYSL2	dihydropyrimidinase like 2 [Source:VGNC Symbol;Acc:VGNC:87430]	5.88	0.0861
ssc-miR-30c-5p	DRAXIN	dorsal inhibitory axon guidance protein [Source:VGNC Symbol;Acc:VGNC:87439]	5.88	0.0861
ssc-miR-30c-5p	DRD1	dopamine receptor D1 [Source:VGNC Symbol;Acc:VGNC:87443]	5.88	0.0861
ssc-miR-30c-5p	DRP2	dystrophin related protein 2 [Source:VGNC Symbol;Acc:VGNC:87451]	5.88	0.0861
ssc-miR-30c-5p	DSC2	desmocollin 2 [Source:HGNC Symbol;Acc:HGNC:3036]	5.88	0.0861

ssc-miR-30c-5p	DSTYK	dual serine/threonine and tyrosine protein kinase [Source:VGNC Symbol;Acc:VGNC:87460]	5.88	0.0861
ssc-miR-30c-5p	DTNA	dystrobrevin alpha [Source:VGNC Symbol;Acc:VGNC:87462]	5.88	0.0861
ssc-miR-30c-5p	E2F3	E2F transcription factor 3 [Source:VGNC Symbol;Acc:VGNC:87514]	5.88	0.0861
ssc-miR-30c-5p	E2F7	E2F transcription factor 7 [Source:VGNC Symbol;Acc:VGNC:87518]	5.88	0.0861
ssc-miR-30c-5p	EAF1	ELL associated factor 1 [Source:VGNC Symbol;Acc:VGNC:87521]	5.88	0.0861
ssc-miR-30c-5p	EBF2	EBF transcription factor 2 [Source:VGNC Symbol;Acc:VGNC:87526]	5.88	0.0861
ssc-miR-30c-5p	EBF3	EBF transcription factor 3 [Source:VGNC Symbol;Acc:VGNC:87527]	5.88	0.0861
ssc-miR-30c-5p	EDA	ectodysplasin A [Source:VGNC Symbol;Acc:VGNC:87540]	5.88	0.0861
ssc-miR-30c-5p	EDC3	enhancer of mRNA decapping 3 [Source:VGNC Symbol;Acc:VGNC:87543]	5.88	0.0861
ssc-miR-30c-5p	EDEM3	ER degradation enhancing alpha-mannosidase like protein 3 [Source:VGNC Symbol;Acc:VGNC:87546]	5.88	0.0861
ssc-miR-30c-5p	EDNRA	endothelin receptor type A [Source:VGNC Symbol;Acc:VGNC:87549]	5.88	0.0861
ssc-miR-30c-5p	EDNRB	endothelin receptor type B [Source:VGNC Symbol;Acc:VGNC:87550]	5.88	0.0861
ssc-miR-30c-5p	EEA1	early endosome antigen 1 [Source:HGNC Symbol;Acc:HGNC:3185]	5.88	0.0861
ssc-miR-30c-5p	EED	embryonic ectoderm development [Source:VGNC Symbol;Acc:VGNC:87552]	5.88	0.0861
ssc-miR-30c-5p	EEF1E1	eukaryotic translation elongation factor 1 epsilon 1 [Source:NCBI gene (formerly Entrezgene);Acc:100155141]	5.88	0.0861
ssc-miR-30c-5p	EEF2K	eukaryotic elongation factor 2 kinase [Source:VGNC Symbol;Acc:VGNC:87557]	5.88	0.0861
ssc-miR-30c-5p	EFNA3	ephrin A3 [Source:VGNC Symbol;Acc:VGNC:98790]	5.88	0.0861
ssc-miR-30c-5p	EFR3A	EFR3 homolog A [Source:VGNC Symbol;Acc:VGNC:87579]	5.88	0.0861
ssc-miR-30c-5p	EIF3A	eukaryotic translation initiation factor 3 subunit A [Source:VGNC Symbol;Acc:VGNC:87615]	5.88	0.0861
ssc-miR-30c-5p	EIF5A2	eukaryotic translation initiation factor 5A2 [Source:VGNC Symbol;Acc:VGNC:87633]	5.88	0.0861
ssc-miR-30c-5p	ELAVL2	ELAV like RNA binding protein 2 [Source:VGNC Symbol;Acc:VGNC:87639]	5.88	0.0861
ssc-miR-30c-5p	ELAVL3	ELAV like RNA binding protein 3 [Source:VGNC Symbol;Acc:VGNC:87640]	5.88	0.0861
ssc-miR-30c-5p	ELAVL4	ELAV like RNA binding protein 4 [Source:VGNC Symbol;Acc:VGNC:97047]	5.88	0.0861
ssc-miR-30c-5p	ELFN2	extracellular leucine rich repeat and fibronectin type III domain containing 2 [Source:VGNC Symbol;Acc:VGNC:103232]	5.88	0.0861
ssc-miR-30c-5p	ELK3	ETS transcription factor ELK3 [Source:VGNC Symbol;Acc:VGNC:87646]	5.88	0.0861
ssc-miR-30c-5p	ELL	elongation factor for RNA polymerase II [Source:VGNC Symbol;Acc:VGNC:87648]	5.88	0.0861
ssc-miR-30c-5p	ELL2	elongation factor for RNA polymerase II 2 [Source:VGNC Symbol;Acc:VGNC:87649]	5.88	0.0861
ssc-miR-30c-5p	ELMO1	engulfment and cell motility 1 [Source:HGNC Symbol;Acc:HGNC:16286]	5.88	0.0861
ssc-miR-30c-5p	ELMOD2	ELMO domain containing 2 [Source:VGNC Symbol;Acc:VGNC:87653]	5.88	0.0861
ssc-miR-30c-5p	ELMSAN1	hypothetical gene	5.88	0.0861
ssc-miR-30c-5p	ELOVL5	ELOVL fatty acid elongase 5 [Source:VGNC Symbol;Acc:VGNC:87661]	5.88	0.0861
ssc-miR-30c-5p	ELOVL6	ELOVL fatty acid elongase 6 [Source:VGNC Symbol;Acc:VGNC:87662]	5.88	0.0861
ssc-miR-30c-5p	EML1	EMAP like 1 [Source:VGNC Symbol;Acc:VGNC:87682]	5.88	0.0861
ssc-miR-30c-5p	EML4	EMAP like 4 [Source:VGNC Symbol;Acc:VGNC:87685]	5.88	0.0861
ssc-miR-30c-5p	ENAH	ENAH actin regulator [Source:VGNC Symbol;Acc:VGNC:108271]	5.88	0.0861
ssc-miR-30c-5p	ENOX2	ecto-NOX disulfide-thiol exchanger 2 [Source:VGNC Symbol;Acc:VGNC:87707]	5.88	0.0861
ssc-miR-30c-5p	ENTHD2	hypothetical gene	5.88	0.0861
ssc-miR-30c-5p	EOGT	EGF domain specific O-linked N-acetylglucosamine transferase [Source:VGNC Symbol;Acc:VGNC:87718]	5.88	0.0861
ssc-miR-30c-5p	EPB41	erythrocyte membrane protein band 4.1 [Source:VGNC Symbol;Acc:VGNC:87722]	5.88	0.0861
ssc-miR-30c-5p	EPB41L3	erythrocyte membrane protein band 4.1 like 3 [Source:VGNC Symbol;Acc:VGNC:87724]	5.88	0.0861
ssc-miR-30c-5p	EPB41L4B	erythrocyte membrane protein band 4.1 like 4B [Source:VGNC Symbol;Acc:VGNC:103088]	5.88	0.0861
ssc-miR-30c-5p	EPB41L5	erythrocyte membrane protein band 4.1 like 5 [Source:VGNC Symbol;Acc:VGNC:97979]	5.88	0.0861
ssc-miR-30c-5p	EPC1	enhancer of polycomb homolog 1 [Source:VGNC Symbol;Acc:VGNC:95923]	5.88	0.0861
ssc-miR-30c-5p	EPC2	enhancer of polycomb homolog 2 [Source:VGNC Symbol;Acc:VGNC:96045]	5.88	0.0861
ssc-miR-30c-5p	EPDR1	ependymin related 1 [Source:VGNC Symbol;Acc:VGNC:87727]	5.88	0.0861
ssc-miR-30c-5p	EPG5	ectopic P-granules 5 autophagy tethering factor [Source:VGNC Symbol;Acc:VGNC:87728]	5.88	0.0861
ssc-miR-30c-5p	EPHB2	EPH receptor B2 [Source:VGNC Symbol;Acc:VGNC:87737]	5.88	0.0861
ssc-miR-30c-5p	EPN2	epsin 2 [Source:VGNC Symbol;Acc:VGNC:97982]	5.88	0.0861
ssc-miR-30c-5p	ERC2	ELKS/RAB6-interacting/CAST family member 2 [Source:VGNC Symbol;Acc:VGNC:87762]	5.88	0.0861

ssc-miR-30c-5p	ERG	ETS transcription factor ERG [Source:VGNC Symbol;Acc:VGNC:87768]	5.88	0.0861
ssc-miR-30c-5p	ERLIN1	hypothetical gene	5.88	0.0861
ssc-miR-30c-5p	ERMAP	erythroblast membrane associated protein [Source:VGNC Symbol;Acc:VGNC:97053]	5.88	0.0861
ssc-miR-30c-5p	ERRFI1	ERBB receptor feedback inhibitor 1 [Source:VGNC Symbol;Acc:VGNC:87784]	5.88	0.0861
ssc-miR-30c-5p	ESCO1	establishment of sister chromatid cohesion N-acetyltransferase 1 [Source:VGNC Symbol;Acc:VGNC:87786]	5.88	0.0861
ssc-miR-30c-5p	ESPN	espin [Source:VGNC Symbol;Acc:VGNC:97054]	5.88	0.0861
ssc-miR-30c-5p	ESRRG	estrogen related receptor gamma [Source:VGNC Symbol;Acc:VGNC:96289]	5.88	0.0861
ssc-miR-30c-5p	EVA1C	eva-1 homolog C [Source:VGNC Symbol;Acc:VGNC:87817]	5.88	0.0861
ssc-miR-30c-5p	EVI5	ecotropic viral integration site 5 [Source:VGNC Symbol;Acc:VGNC:98793]	5.88	0.0861
ssc-miR-30c-5p	EVI5L	ecotropic viral integration site 5 like [Source:VGNC Symbol;Acc:VGNC:99646]	5.88	0.0861
ssc-miR-30c-5p	EVX2	even-skipped homeobox 2 [Source:VGNC Symbol;Acc:VGNC:96290]	5.88	0.0861
ssc-miR-30c-5p	EXTL2	exostosin like glycosyltransferase 2 [Source:VGNC Symbol;Acc:VGNC:87849]	5.88	0.0861
ssc-miR-30c-5p	EYA2	EYA transcriptional coactivator and phosphatase 2 [Source:VGNC Symbol;Acc:VGNC:96292]	5.88	0.0861
ssc-miR-30c-5p	FA2H	fatty acid 2-hydroxylase [Source:VGNC Symbol;Acc:VGNC:87868]	5.88	0.0861
ssc-miR-30c-5p	FAF2	Fas associated factor family member 2 [Source:VGNC Symbol;Acc:VGNC:87880]	5.88	0.0861
ssc-miR-30c-5p	FAM104A	family with sequence similarity 104 member A [Source:VGNC Symbol;Acc:VGNC:98990]	5.88	0.0861
ssc-miR-30c-5p	FAM104B	hypothetical gene	5.88	0.0861
ssc-miR-30c-5p	FAM105B	hypothetical gene	5.88	0.0861
ssc-miR-30c-5p	FAM109A	hypothetical gene	5.88	0.0861
ssc-miR-30c-5p	FAM109B	hypothetical gene	5.88	0.0861
ssc-miR-30c-5p	FAM110B	family with sequence similarity 110 member B [Source:VGNC Symbol;Acc:VGNC:87885]	5.88	0.0861
ssc-miR-30c-5p	FAM124A	family with sequence similarity 124 member A [Source:VGNC Symbol;Acc:VGNC:87898]	5.88	0.0861
ssc-miR-30c-5p	FAM126A	family with sequence similarity 126 member A [Source:VGNC Symbol;Acc:VGNC:87899]	5.88	0.0861
ssc-miR-30c-5p	FAM126B	family with sequence similarity 126 member B [Source:HGNC Symbol;Acc:HGNC:28593]	5.88	0.0861
ssc-miR-30c-5p	FAM129A	hypothetical gene	5.88	0.0861
ssc-miR-30c-5p	FAM131B	family with sequence similarity 131 member B [Source:VGNC Symbol;Acc:VGNC:87901]	5.88	0.0861
ssc-miR-30c-5p	FAM133A	family with sequence similarity 133 member A [Source:VGNC Symbol;Acc:VGNC:87903]	5.88	0.0861
ssc-miR-30c-5p	FAM13A	family with sequence similarity 13 member A [Source:VGNC Symbol;Acc:VGNC:98924]	5.88	0.0861
ssc-miR-30c-5p	FAM13C	family with sequence similarity 13 member C [Source:VGNC Symbol;Acc:VGNC:87907]	5.88	0.0861
ssc-miR-30c-5p	FAM149B1	family with sequence similarity 149 member B1 [Source:VGNC Symbol;Acc:VGNC:87908]	5.88	0.0861
ssc-miR-30c-5p	FAM155A	hypothetical gene	5.88	0.0861
ssc-miR-30c-5p	FAM160B1	hypothetical gene	5.88	0.0861
ssc-miR-30c-5p	FAM168B	family with sequence similarity 168 member B [Source:VGNC Symbol;Acc:VGNC:95462]	5.88	0.0861
ssc-miR-30c-5p	FAM175B	hypothetical gene	5.88	0.0861
ssc-miR-30c-5p	FAM178A	hypothetical gene	5.88	0.0861
ssc-miR-30c-5p	FAM179B	hypothetical gene	5.88	0.0861
ssc-miR-30c-5p	FAM199X	family with sequence similarity 199, X-linked [Source:HGNC Symbol;Acc:HGNC:25195]	5.88	0.0861
ssc-miR-30c-5p	FAM208B	hypothetical gene	5.88	0.0861
ssc-miR-30c-5p	FAM210B	family with sequence similarity 210 member B [Source:VGNC Symbol;Acc:VGNC:95736]	5.88	0.0861
ssc-miR-30c-5p	FAM214A	family with sequence similarity 214 member A [Source:VGNC Symbol;Acc:VGNC:87952]	5.88	0.0861
ssc-miR-30c-5p	FAM216A	family with sequence similarity 216 member A [Source:VGNC Symbol;Acc:VGNC:87954]	5.88	0.0861
ssc-miR-30c-5p	FAM217B	family with sequence similarity 217 member B [Source:VGNC Symbol;Acc:VGNC:95767]	5.88	0.0861
ssc-miR-30c-5p	FAM219B	family with sequence similarity 219 member B [Source:VGNC Symbol;Acc:VGNC:87956]	5.88	0.0861
ssc-miR-30c-5p	FAM43A	family with sequence similarity 43 member A [Source:VGNC Symbol;Acc:VGNC:87970]	5.88	0.0861
ssc-miR-30c-5p	FAM46A	hypothetical gene	5.88	0.0861
ssc-miR-30c-5p	FAM46C	hypothetical gene	5.88	0.0861
ssc-miR-30c-5p	FAM49A	hypothetical gene	5.88	0.0861
ssc-miR-30c-5p	FAM53B	family with sequence similarity 53 member B [Source:VGNC Symbol;Acc:VGNC:87974]	5.88	0.0861
ssc-miR-30c-5p	FAM72B	hypothetical gene	5.88	0.0861

ssc-miR-30c-5p	FAM72D	hypothetical gene	5.88	0.0861
ssc-miR-30c-5p	FAM73B	hypothetical gene	5.88	0.0861
ssc-miR-30c-5p	FAM81A	family with sequence similarity 81 member A [Source:VGNC Symbol;Acc:VGNC:87986]	5.88	0.0861
ssc-miR-30c-5p	FAM83F	family with sequence similarity 83 member F [Source:VGNC Symbol;Acc:VGNC:87991]	5.88	0.0861
ssc-miR-30c-5p	FAM83G	family with sequence similarity 83 member G [Source:VGNC Symbol;Acc:VGNC:87992]	5.88	0.0861
ssc-miR-30c-5p	FAM91A1	family with sequence similarity 91 member A1 [Source:VGNC Symbol;Acc:VGNC:97998]	5.88	0.0861
ssc-miR-30c-5p	FANCF	FA complementation group F [Source:VGNC Symbol;Acc:VGNC:88004]	5.88	0.0861
ssc-miR-30c-5p	FAP	fibroblast activation protein alpha [Source:VGNC Symbol;Acc:VGNC:99714]	5.88	0.0861
ssc-miR-30c-5p	FBXL14	hypothetical gene	5.88	0.0861
ssc-miR-30c-5p	FBXL17	F-box and leucine rich repeat protein 17 [Source:VGNC Symbol;Acc:VGNC:99650]	5.88	0.0861
ssc-miR-30c-5p	FBXL20	F-box and leucine rich repeat protein 20 [Source:VGNC Symbol;Acc:VGNC:98006]	5.88	0.0861
ssc-miR-30c-5p	FBXO28	F-box protein 28 [Source:HGNC Symbol;Acc:HGNC:29046]	5.88	0.0861
ssc-miR-30c-5p	FBXO32	F-box protein 32 [Source:VGNC Symbol;Acc:VGNC:88039]	5.88	0.0861
ssc-miR-30c-5p	FBXO34	F-box protein 34 [Source:VGNC Symbol;Acc:VGNC:88041]	5.88	0.0861
ssc-miR-30c-5p	FBXO42	F-box protein 42 [Source:VGNC Symbol;Acc:VGNC:88047]	5.88	0.0861
ssc-miR-30c-5p	FBXO45	F-box protein 45 [Source:VGNC Symbol;Acc:VGNC:88048]	5.88	0.0861
ssc-miR-30c-5p	FECH	ferrochelatase [Source:VGNC Symbol;Acc:VGNC:88081]	5.88	0.0861
ssc-miR-30c-5p	FGD6	FYVE, RhoGEF and PH domain containing 6 [Source:VGNC Symbol;Acc:VGNC:88099]	5.88	0.0861
ssc-miR-30c-5p	FGF20	fibroblast growth factor 20 [Source:VGNC Symbol;Acc:VGNC:96298]	5.88	0.0861
ssc-miR-30c-5p	FIGN	fidgetin, microtubule severing factor [Source:VGNC Symbol;Acc:VGNC:95580]	5.88	0.0861
ssc-miR-30c-5p	FKBP14	FKBP prolyl isomerase 14 [Source:VGNC Symbol;Acc:VGNC:88144]	5.88	0.0861
ssc-miR-30c-5p	FKBP3	FKBP prolyl isomerase 3 [Source:VGNC Symbol;Acc:VGNC:88146]	5.88	0.0861
ssc-miR-30c-5p	FLJ20373	hypothetical gene	5.88	0.0861
ssc-miR-30c-5p	FLVCR1	FLVCR heme transporter 1 [Source:VGNC Symbol;Acc:VGNC:96580]	5.88	0.0861
ssc-miR-30c-5p	FNDC3A	fibronectin type III domain containing 3A [Source:VGNC Symbol;Acc:VGNC:88182]	5.88	0.0861
ssc-miR-30c-5p	FNDC3B	fibronectin type III domain containing 3B [Source:VGNC Symbol;Acc:VGNC:88183]	5.88	0.0861
ssc-miR-30c-5p	FNIP2	folliculin interacting protein 2 [Source:VGNC Symbol;Acc:VGNC:88188]	5.88	0.0861
ssc-miR-30c-5p	FOSL2	FOS like 2, AP-1 transcription factor subunit [Source:VGNC Symbol;Acc:VGNC:88192]	5.88	0.0861
ssc-miR-30c-5p	FOXA1	forkhead box A1 [Source:VGNC Symbol;Acc:VGNC:88193]	5.88	0.0861
ssc-miR-30c-5p	FOXB1	forkhead box B1 [Source:VGNC Symbol;Acc:VGNC:88195]	5.88	0.0861
ssc-miR-30c-5p	FOXD1	forkhead box D1 [Source:VGNC Symbol;Acc:VGNC:88199]	5.88	0.0861
ssc-miR-30c-5p	FOXG1	forkhead box G1 [Source:VGNC Symbol;Acc:VGNC:88206]	5.88	0.0861
ssc-miR-30c-5p	FOXN2	forkhead box N2 [Source:VGNC Symbol;Acc:VGNC:88219]	5.88	0.0861
ssc-miR-30c-5p	FOXO3	forkhead box O3 [Source:VGNC Symbol;Acc:VGNC:99715]	5.88	0.0861
ssc-miR-30c-5p	FOXP4	forkhead box P4 [Source:VGNC Symbol;Acc:VGNC:88224]	5.88	0.0861
ssc-miR-30c-5p	FRK	fyn related Src family tyrosine kinase [Source:VGNC Symbol;Acc:VGNC:88235]	5.88	0.0861
ssc-miR-30c-5p	FRMD4A	FERM domain containing 4A [Source:VGNC Symbol;Acc:VGNC:96088]	5.88	0.0861
ssc-miR-30c-5p	FRMD6	FERM domain containing 6 [Source:VGNC Symbol;Acc:VGNC:88239]	5.88	0.0861
ssc-miR-30c-5p	FRMPD1	FERM and PDZ domain containing 1 [Source:VGNC Symbol;Acc:VGNC:88240]	5.88	0.0861
ssc-miR-30c-5p	FRMPD4	FERM and PDZ domain containing 4 [Source:VGNC Symbol;Acc:VGNC:88243]	5.88	0.0861
ssc-miR-30c-5p	FRS2	fibroblast growth factor receptor substrate 2 [Source:VGNC Symbol;Acc:VGNC:88246]	5.88	0.0861
ssc-miR-30c-5p	FRZB	frizzled related protein [Source:VGNC Symbol;Acc:VGNC:96308]	5.88	0.0861
ssc-miR-30c-5p	FST	follistatin [Source:NCBI gene (formerly Entrezgene);Acc:445002]	5.88	0.0861
ssc-miR-30c-5p	FSTL4	follistatin like 4 [Source:VGNC Symbol;Acc:VGNC:88256]	5.88	0.0861
ssc-miR-30c-5p	FTO	FTO alpha-ketoglutarate dependent dioxygenase [Source:VGNC Symbol;Acc:VGNC:88259]	5.88	0.0861
ssc-miR-30c-5p	FUBP1	far upstream element binding protein 1 [Source:VGNC Symbol;Acc:VGNC:96781]	5.88	0.0861
ssc-miR-30c-5p	FUBP3	far upstream element binding protein 3 [Source:VGNC Symbol;Acc:VGNC:103093]	5.88	0.0861
ssc-miR-30c-5p	FUCA1	alpha-L-fucosidase 1 [Source:VGNC Symbol;Acc:VGNC:88262]	5.88	0.0861
ssc-miR-30c-5p	FUT4	fucosyltransferase 4 [Source:HGNC Symbol;Acc:HGNC:4015]	5.88	0.0861

ssc-miR-30c-5p	FXR1	FMR1 autosomal homolog 1 [Source:VGNC Symbol;Acc:VGNC:108659]	5.88	0.0861
ssc-miR-30c-5p	FYCO1	hypothetical gene	5.88	0.0861
ssc-miR-30c-5p	FYN	FYN proto-onco, Src family tyrosine kinase [Source:VGNC Symbol;Acc:VGNC:88276]	5.88	0.0861
ssc-miR-30c-5p	FYTTD1	forty-two-three domain containing 1 [Source:VGNC Symbol;Acc:VGNC:88277]	5.88	0.0861
ssc-miR-30c-5p	FZD2	frizzled class receptor 2 [Source:VGNC Symbol;Acc:VGNC:88280]	5.88	0.0861
ssc-miR-30c-5p	FZD3	frizzled class receptor 3 [Source:VGNC Symbol;Acc:VGNC:88281]	5.88	0.0861
ssc-miR-30c-5p	G3BP1	G3BP stress granule assembly factor 1 [Source:VGNC Symbol;Acc:VGNC:88288]	5.88	0.0861
ssc-miR-30c-5p	G3BP2	G3BP stress granule assembly factor 2 [Source:VGNC Symbol;Acc:VGNC:88289]	5.88	0.0861
ssc-miR-30c-5p	G6PC3	glucose-6-phosphatase catalytic subunit 3 [Source:VGNC Symbol;Acc:VGNC:88291]	5.88	0.0861
ssc-miR-30c-5p	GABRA1	gamma-aminobutyric acid type A receptor subunit alpha1 [Source:VGNC Symbol;Acc:VGNC:88300]	5.88	0.0861
ssc-miR-30c-5p	GABRA5	gamma-aminobutyric acid type A receptor subunit alpha5 [Source:VGNC Symbol;Acc:VGNC:88304]	5.88	0.0861
ssc-miR-30c-5p	GABRB1	gamma-aminobutyric acid type A receptor subunit beta1 [Source:VGNC Symbol;Acc:VGNC:88306]	5.88	0.0861
ssc-miR-30c-5p	GADD45GIP1	GADD45G interacting protein 1 [Source:VGNC Symbol;Acc:VGNC:99751]	5.88	0.0861
ssc-miR-30c-5p	GALNT1	polypeptide N-acetylgalactosaminyltransferase 1 [Source:VGNC Symbol;Acc:VGNC:97062]	5.88	0.0861
ssc-miR-30c-5p	GALNT2	polypeptide N-acetylgalactosaminyltransferase 2 [Source:VGNC Symbol;Acc:VGNC:88334]	5.88	0.0861
ssc-miR-30c-5p	GALNT3	polypeptide N-acetylgalactosaminyltransferase 3 [Source:VGNC Symbol;Acc:VGNC:96313]	5.88	0.0861
ssc-miR-30c-5p	GALNT7	polypeptide N-acetylgalactosaminyltransferase 7 [Source:VGNC Symbol;Acc:VGNC:103946]	5.88	0.0861
ssc-miR-30c-5p	GALR1	galanin receptor 1 [Source:VGNC Symbol;Acc:VGNC:88339]	5.88	0.0861
ssc-miR-30c-5p	GAN	gigaxonin [Source:VGNC Symbol;Acc:VGNC:88343]	5.88	0.0861
ssc-miR-30c-5p	GAREM	hypothetical gene	5.88	0.0861
ssc-miR-30c-5p	GAS2	growth arrest specific 2 [Source:VGNC Symbol;Acc:VGNC:88354]	5.88	0.0861
ssc-miR-30c-5p	GAS7	growth arrest specific 7 [Source:VGNC Symbol;Acc:VGNC:88359]	5.88	0.0861
ssc-miR-30c-5p	GATA5	GATA binding protein 5 [Source:VGNC Symbol;Acc:VGNC:95661]	5.88	0.0861
ssc-miR-30c-5p	GATA6	GATA binding protein 6 [Source:VGNC Symbol;Acc:VGNC:88366]	5.88	0.0861
ssc-miR-30c-5p	GATM	glycine amidinotransferase [Source:VGNC Symbol;Acc:VGNC:88372]	5.88	0.0861
ssc-miR-30c-5p	GBP2	guanylate binding protein 2, interferon-inducible [Source:NCBI gene (formerly Entrezgene);Acc:100153137]	5.88	0.0861
ssc-miR-30c-5p	GCH1	GTP cyclohydrolase 1 [Source:VGNC Symbol;Acc:VGNC:88384]	5.88	0.0861
ssc-miR-30c-5p	GCLC	glutamate-cysteine ligase catalytic subunit [Source:VGNC Symbol;Acc:VGNC:88386]	5.88	0.0861
ssc-miR-30c-5p	GCNT2	glucosaminyl (N-acetyl) transferase 2 (I blood group) [Source:HGNC Symbol;Acc:HGNC:4204]	5.88	0.0861
ssc-miR-30c-5p	GDA	guanine deaminase [Source:VGNC Symbol;Acc:VGNC:88393]	5.88	0.0861
ssc-miR-30c-5p	GDF2	growth differentiation factor 2 [Source:VGNC Symbol;Acc:VGNC:88400]	5.88	0.0861
ssc-miR-30c-5p	GDI2	GDP dissociation inhibitor 2 [Source:VGNC Symbol;Acc:VGNC:98019]	5.88	0.0861
ssc-miR-30c-5p	GDNF	glial cell derived neurotrophic factor [Source:VGNC Symbol;Acc:VGNC:88406]	5.88	0.0861
ssc-miR-30c-5p	GFPT2	glutamine-fructose-6-phosphate transaminase 2 [Source:VGNC Symbol;Acc:VGNC:88427]	5.88	0.0861
ssc-miR-30c-5p	GID4	GID complex subunit 4 homolog [Source:VGNC Symbol;Acc:VGNC:98996]	5.88	0.0861
ssc-miR-30c-5p	GIGYF1	GRB10 interacting GYF protein 1 [Source:VGNC Symbol;Acc:VGNC:88444]	5.88	0.0861
ssc-miR-30c-5p	GIGYF2	GRB10 interacting GYF protein 2 [Source:VGNC Symbol;Acc:VGNC:95551]	5.88	0.0861
ssc-miR-30c-5p	GJA1	gap junction protein alpha 1 [Source:VGNC Symbol;Acc:VGNC:103098]	5.88	0.0861
ssc-miR-30c-5p	GJA3	gap junction protein alpha 3 [Source:HGNC Symbol;Acc:HGNC:4277]	5.88	0.0861
ssc-miR-30c-5p	GJC2	gap junction protein gamma 2 [Source:VGNC Symbol;Acc:VGNC:88469]	5.88	0.0861
ssc-miR-30c-5p	GLCCI1	glucocorticoid induced 1 [Source:VGNC Symbol;Acc:VGNC:88476]	5.88	0.0861
ssc-miR-30c-5p	GLCE	glucuronic acid epimerase [Source:VGNC Symbol;Acc:VGNC:88477]	5.88	0.0861
ssc-miR-30c-5p	GLDC	glycine decarboxylase [Source:VGNC Symbol;Acc:VGNC:88478]	5.88	0.0861
ssc-miR-30c-5p	GLI2	GLI family zinc finger 2 [Source:VGNC Symbol;Acc:VGNC:96324]	5.88	0.0861
ssc-miR-30c-5p	GLUD1	glutamate dehydrogenase 1 [Source:NCBI gene (formerly Entrezgene);Acc:100157162]	5.88	0.0861
ssc-miR-30c-5p	GLUD2	hypothetical gene	5.88	0.0861
ssc-miR-30c-5p	GMEB1	glucocorticoid modulatory element binding protein 1 [Source:VGNC Symbol;Acc:VGNC:88508]	5.88	0.0861
ssc-miR-30c-5p	GMEB2	glucocorticoid modulatory element binding protein 2 [Source:VGNC Symbol;Acc:VGNC:96326]	5.88	0.0861
ssc-miR-30c-5p	GMNC	geminin coiled-coil domain containing [Source:VGNC Symbol;Acc:VGNC:88512]	5.88	0.0861

ssc-miR-30c-5p	GNA13	G protein subunit alpha 13 [Source:VGNC Symbol;Acc:VGNC:98997]	5.88	0.0861
ssc-miR-30c-5p	GNAI2	G protein subunit alpha i2 [Source:VGNC Symbol;Acc:VGNC:88522]	5.88	0.0861
ssc-miR-30c-5p	GNAO1	hypothetical gene	5.88	0.0861
ssc-miR-30c-5p	GNAQ	G protein subunit alpha q [Source:VGNC Symbol;Acc:VGNC:103100]	5.88	0.0861
ssc-miR-30c-5p	GNG10	G protein subunit gamma 10 [Source:HGNC Symbol;Acc:HGNC:4402]	5.88	0.0861
ssc-miR-30c-5p	GNPDA1	glucosamine-6-phosphate deaminase 1 [Source:VGNC Symbol;Acc:VGNC:88544]	5.88	0.0861
ssc-miR-30c-5p	GOLGA1	golgin A1 [Source:VGNC Symbol;Acc:VGNC:88549]	5.88	0.0861
ssc-miR-30c-5p	GOLGA4	hypothetical gene	5.88	0.0861
ssc-miR-30c-5p	GOLGA6A	hypothetical gene	5.88	0.0861
ssc-miR-30c-5p	GOLGA6B	hypothetical gene	5.88	0.0861
ssc-miR-30c-5p	GOLGA6C	hypothetical gene	5.88	0.0861
ssc-miR-30c-5p	GOLGA6D	hypothetical gene	5.88	0.0861
ssc-miR-30c-5p	GOLGA6L10	hypothetical gene	5.88	0.0861
ssc-miR-30c-5p	GOLGA6L4	hypothetical gene	5.88	0.0861
ssc-miR-30c-5p	GOLGA8B	hypothetical gene	5.88	0.0861
ssc-miR-30c-5p	GOT2	glutamic-oxaloacetic transaminase 2 [Source:VGNC Symbol;Acc:VGNC:88562]	5.88	0.0861
ssc-miR-30c-5p	GPCPD1	glycerophosphocholine phosphodiesterase 1 [Source:VGNC Symbol;Acc:VGNC:96148]	5.88	0.0861
ssc-miR-30c-5p	GPD1L	glycerol-3-phosphate dehydrogenase 1 like [Source:VGNC Symbol;Acc:VGNC:108661]	5.88	0.0861
ssc-miR-30c-5p	GPER1	G protein-coupled estrogen receptor 1 [Source:VGNC Symbol;Acc:VGNC:88584]	5.88	0.0861
ssc-miR-30c-5p	GPR108	G protein-coupled receptor 108 [Source:VGNC Symbol;Acc:VGNC:88596]	5.88	0.0861
ssc-miR-30c-5p	GPR124	hypothetical gene	5.88	0.0861
ssc-miR-30c-5p	GPR125	hypothetical gene	5.88	0.0861
ssc-miR-30c-5p	GPR137C	G protein-coupled receptor 137C [Source:VGNC Symbol;Acc:VGNC:88602]	5.88	0.0861
ssc-miR-30c-5p	GPR150	G protein-coupled receptor 150 [Source:VGNC Symbol;Acc:VGNC:88607]	5.88	0.0861
ssc-miR-30c-5p	GPR157	G protein-coupled receptor 157 [Source:VGNC Symbol;Acc:VGNC:88611]	5.88	0.0861
ssc-miR-30c-5p	GPR161	G protein-coupled receptor 161 [Source:VGNC Symbol;Acc:VGNC:88613]	5.88	0.0861
ssc-miR-30c-5p	GPR180	G protein-coupled receptor 180 [Source:VGNC Symbol;Acc:VGNC:88621]	5.88	0.0861
ssc-miR-30c-5p	GPR26	G protein-coupled receptor 26 [Source:VGNC Symbol;Acc:VGNC:88626]	5.88	0.0861
ssc-miR-30c-5p	GPR63	G protein-coupled receptor 63 [Source:VGNC Symbol;Acc:VGNC:88634]	5.88	0.0861
ssc-miR-30c-5p	GPR75-ASB3	hypothetical gene	5.88	0.0861
ssc-miR-30c-5p	GPRIN3	hypothetical gene	5.88	0.0861
ssc-miR-30c-5p	GPT2	glutamic--pyruvic transaminase 2 [Source:VGNC Symbol;Acc:VGNC:88651]	5.88	0.0861
ssc-miR-30c-5p	GRAMD2	hypothetical gene	5.88	0.0861
ssc-miR-30c-5p	GRB10	growth factor receptor bound protein 10 [Source:VGNC Symbol;Acc:VGNC:88662]	5.88	0.0861
ssc-miR-30c-5p	GREB1L	GREB1 like retinoic acid receptor coactivator [Source:VGNC Symbol;Acc:VGNC:96584]	5.88	0.0861
ssc-miR-30c-5p	GRHL1	grainyhead like transcription factor 1 [Source:VGNC Symbol;Acc:VGNC:88666]	5.88	0.0861
ssc-miR-30c-5p	GRHL2	grainyhead like transcription factor 2 [Source:VGNC Symbol;Acc:VGNC:88667]	5.88	0.0861
ssc-miR-30c-5p	GRIA2	glutamate ionotropic receptor AMPA type subunit 2 [Source:VGNC Symbol;Acc:VGNC:88671]	5.88	0.0861
ssc-miR-30c-5p	GRIN2A	glutamate ionotropic receptor NMDA type subunit 2A [Source:VGNC Symbol;Acc:VGNC:88683]	5.88	0.0861
ssc-miR-30c-5p	GRK5	G protein-coupled receptor kinase 5 [Source:VGNC Symbol;Acc:VGNC:88697]	5.88	0.0861
ssc-miR-30c-5p	GRK6	G protein-coupled receptor kinase 6 [Source:VGNC Symbol;Acc:VGNC:88698]	5.88	0.0861
ssc-miR-30c-5p	GRM3	glutamate metabotropic receptor 3 [Source:VGNC Symbol;Acc:VGNC:88702]	5.88	0.0861
ssc-miR-30c-5p	GRM5	glutamate metabotropic receptor 5 [Source:VGNC Symbol;Acc:VGNC:88703]	5.88	0.0861
ssc-miR-30c-5p	GSKIP	GSK3B interacting protein [Source:VGNC Symbol;Acc:VGNC:103956]	5.88	0.0861
ssc-miR-30c-5p	GTDC1	glycosyltransferase like domain containing 1 [Source:VGNC Symbol;Acc:VGNC:95955]	5.88	0.0861
ssc-miR-30c-5p	GTF2H1	ral transcription factor IIH subunit 1 [Source:VGNC Symbol;Acc:VGNC:88734]	5.88	0.0861
ssc-miR-30c-5p	GUF1	GTP binding elongation factor GUF1 [Source:VGNC Symbol;Acc:VGNC:88754]	5.88	0.0861
ssc-miR-30c-5p	GXYLT1	glucoside xylosyltransferase 1 [Source:VGNC Symbol;Acc:VGNC:88756]	5.88	0.0861
ssc-miR-30c-5p	GZF1	GDNF inducible zinc finger protein 1 [Source:VGNC Symbol;Acc:VGNC:108722]	5.88	0.0861

ssc-miR-30c-5p	H2AFY	hypothetical gene	5.88	0.0861
ssc-miR-30c-5p	H6PD	hexose-6-phosphate dehydrogenase/glucose 1-dehydrogenase [Source:VGNC Symbol;Acc:VGNC:88764]	5.88	0.0861
ssc-miR-30c-5p	HACE1	HECT domain and ankyrin repeat containing E3 ubiquitin protein ligase 1 [Source:VGNC Symbol;Acc:VGNC:88769]	5.88	0.0861
ssc-miR-30c-5p	HBS1L	HBS1 like translational GTPase [Source:VGNC Symbol;Acc:VGNC:88796]	5.88	0.0861
ssc-miR-30c-5p	HCAR2	hypothetical gene	5.88	0.0861
ssc-miR-30c-5p	HCAR3	hypothetical gene	5.88	0.0861
ssc-miR-30c-5p	HCFC2	host cell factor C2 [Source:VGNC Symbol;Acc:VGNC:88800]	5.88	0.0861
ssc-miR-30c-5p	HCN1	hyperpolarization activated cyclic nucleotide gated potassium channel 1 [Source:VGNC Symbol;Acc:VGNC:88802]	5.88	0.0861
ssc-miR-30c-5p	HDAC5	histone deacetylase 5 [Source:VGNC Symbol;Acc:VGNC:88815]	5.88	0.0861
ssc-miR-30c-5p	HDAC9	histone deacetylase 9 [Source:HGNC Symbol;Acc:HGNC:14065]	5.88	0.0861
ssc-miR-30c-5p	HECTD4	HECT domain E3 ubiquitin protein ligase 4 [Source:VGNC Symbol;Acc:VGNC:88834]	5.88	0.0861
ssc-miR-30c-5p	HECW1	HECT, C2 and WW domain containing E3 ubiquitin protein ligase 1 [Source:VGNC Symbol;Acc:VGNC:88835]	5.88	0.0861
ssc-miR-30c-5p	HELZ	helicase with zinc finger [Source:VGNC Symbol;Acc:VGNC:88840]	5.88	0.0861
ssc-miR-30c-5p	HEPHL1	hephaestin like 1 [Source:VGNC Symbol;Acc:VGNC:88848]	5.88	0.0861
ssc-miR-30c-5p	HERC2	HECT and RLD domain containing E3 ubiquitin protein ligase 2 [Source:VGNC Symbol;Acc:VGNC:99716]	5.88	0.0861
ssc-miR-30c-5p	HERC3	HECT and RLD domain containing E3 ubiquitin protein ligase 3 [Source:VGNC Symbol;Acc:VGNC:98934]	5.88	0.0861
ssc-miR-30c-5p	HHIPL1	hypothetical gene	5.88	0.0861
ssc-miR-30c-5p	HIAT1	hypothetical gene	5.88	0.0861
ssc-miR-30c-5p	HIC2	HIC ZBTB transcriptional repressor 2 [Source:HGNC Symbol;Acc:HGNC:18595]	5.88	0.0861
ssc-miR-30c-5p	HIPK1	homeodomain interacting protein kinase 1 [Source:VGNC Symbol;Acc:VGNC:88887]	5.88	0.0861
ssc-miR-30c-5p	HIPK2	homeodomain interacting protein kinase 2 [Source:VGNC Symbol;Acc:VGNC:88888]	5.88	0.0861
ssc-miR-30c-5p	HIVEP1	HIVEP zinc finger 1 [Source:VGNC Symbol;Acc:VGNC:96585]	5.88	0.0861
ssc-miR-30c-5p	HLF	HLF transcription factor, PAR bZIP family member [Source:VGNC Symbol;Acc:VGNC:88896]	5.88	0.0861
ssc-miR-30c-5p	HLX	H2.0 like homeobox [Source:VGNC Symbol;Acc:VGNC:96345]	5.88	0.0861
ssc-miR-30c-5p	HMGB3	hypothetical gene	5.88	0.0861
ssc-miR-30c-5p	HNRNPA1	heterous nuclear ribonucleoprotein A1 [Source:VGNC Symbol;Acc:VGNC:88918]	5.88	0.0861
ssc-miR-30c-5p	HNRNPA2B1	heterogeneous nuclear ribonucleoprotein A2/B1 [Source:HGNC Symbol;Acc:HGNC:5033]	5.88	0.0861
ssc-miR-30c-5p	HNRNPA3	hypothetical gene	5.88	0.0861
ssc-miR-30c-5p	HNRNPC	hypothetical gene	5.88	0.0861
ssc-miR-30c-5p	HNRNPUL2	heterous nuclear ribonucleoprotein U like 2 [Source:VGNC Symbol;Acc:VGNC:99758]	5.88	0.0861
ssc-miR-30c-5p	HOOK3	hook microtubule tethering protein 3 [Source:VGNC Symbol;Acc:VGNC:98037]	5.88	0.0861
ssc-miR-30c-5p	HOXA1	homeobox A1 [Source:VGNC Symbol;Acc:VGNC:88933]	5.88	0.0861
ssc-miR-30c-5p	HOXA11	homeobox A11 [Source:VGNC Symbol;Acc:VGNC:88935]	5.88	0.0861
ssc-miR-30c-5p	HOXA2	homeobox A2 [Source:VGNC Symbol;Acc:VGNC:88937]	5.88	0.0861
ssc-miR-30c-5p	HOXA9	homeobox A9 [Source:HGNC Symbol;Acc:HGNC:5109]	5.88	0.0861
ssc-miR-30c-5p	HOXB3	homeobox B3 [Source:VGNC Symbol;Acc:VGNC:88944]	5.88	0.0861
ssc-miR-30c-5p	HOXB4	homeobox B4 [Source:HGNC Symbol;Acc:HGNC:5115]	5.88	0.0861
ssc-miR-30c-5p	HOXB8	homeobox B8 [Source:VGNC Symbol;Acc:VGNC:88948]	5.88	0.0861
ssc-miR-30c-5p	HOXD11	homeobox D11 [Source:VGNC Symbol;Acc:VGNC:96352]	5.88	0.0861
ssc-miR-30c-5p	HPS4	HPS4 biosis of lysosomal organelles complex 3 subunit 2 [Source:VGNC Symbol;Acc:VGNC:88966]	5.88	0.0861
ssc-miR-30c-5p	HSPA2	hypothetical gene	5.88	0.0861
ssc-miR-30c-5p	HSPA4L	heat shock protein family A (Hsp70) member 4 like [Source:HGNC Symbol;Acc:HGNC:17041]	5.88	0.0861
ssc-miR-30c-5p	HSPA5	heat shock protein family A (Hsp70) member 5 [Source:VGNC Symbol;Acc:VGNC:103107]	5.88	0.0861
ssc-miR-30c-5p	HSPD1	heat shock protein family D (Hsp60) member 1 [Source:NCBI gene (formerly Entrezgene);Acc:492279]	5.88	0.0861
ssc-miR-30c-5p	HSPE1-MOB4	hypothetical gene	5.88	0.0861
ssc-miR-30c-5p	HTRA3	HtrA serine peptidase 3 [Source:VGNC Symbol;Acc:VGNC:89005]	5.88	0.0861
ssc-miR-30c-5p	ICK	hypothetical gene	5.88	0.0861
ssc-miR-30c-5p	IDE	insulin degrading enzyme [Source:VGNC Symbol;Acc:VGNC:89025]	5.88	0.0861
ssc-miR-30c-5p	IDH1	isocitrate dehydrogenase (NADP(+)) 1 [Source:HGNC Symbol;Acc:HGNC:5382]	5.88	0.0861

ssc-miR-30c-5p	IER2	immediate early response 2 [Source:HGNC Symbol;Acc:HGNC:28871]	5.88	0.0861
ssc-miR-30c-5p	IER5	immediate early response 5 [Source:VGNC Symbol;Acc:VGNC:89028]	5.88	0.0861
ssc-miR-30c-5p	IFFO2	intermediate filament family orphan 2 [Source:VGNC Symbol;Acc:VGNC:98475]	5.88	0.0861
ssc-miR-30c-5p	IFNAR2	interferon alpha and beta receptor subunit 2 [Source:NCBI gene (formerly Entrezgene);Acc:100533555]	5.88	0.0861
ssc-miR-30c-5p	IFNLR1	interferon lambda receptor 1 [Source:VGNC Symbol;Acc:VGNC:89043]	5.88	0.0861
ssc-miR-30c-5p	IGF1R	insulin like growth factor 1 receptor [Source:NCBI gene (formerly Entrezgene);Acc:397350]	5.88	0.0861
ssc-miR-30c-5p	IGF2R	insulin like growth factor 2 receptor [Source:VGNC Symbol;Acc:VGNC:89057]	5.88	0.0861
ssc-miR-30c-5p	IGSF3	immunoglobulin superfamily member 3 [Source:VGNC Symbol;Acc:VGNC:89067]	5.88	0.0861
ssc-miR-30c-5p	IKZF2	IKAROS family zinc finger 2 [Source:VGNC Symbol;Acc:VGNC:95576]	5.88	0.0861
ssc-miR-30c-5p	IL1A	interleukin 1 alpha [Source:VGNC Symbol;Acc:VGNC:89091]	5.88	0.0861
ssc-miR-30c-5p	IL1RAPL2	interleukin 1 receptor accessory protein like 2 [Source:VGNC Symbol;Acc:VGNC:103968]	5.88	0.0861
ssc-miR-30c-5p	IL2RA	interleukin 2 receptor subunit alpha [Source:VGNC Symbol;Acc:VGNC:108275]	5.88	0.0861
ssc-miR-30c-5p	ILDR2	immunoglobulin like domain containing receptor 2 [Source:VGNC Symbol;Acc:VGNC:89118]	5.88	0.0861
ssc-miR-30c-5p	ING5	inhibitor of growth family member 5 [Source:VGNC Symbol;Acc:VGNC:95912]	5.88	0.0861
ssc-miR-30c-5p	INHBA	inhibin subunit beta A [Source:VGNC Symbol;Acc:VGNC:89133]	5.88	0.0861
ssc-miR-30c-5p	INO80D	INO80 complex subunit D [Source:VGNC Symbol;Acc:VGNC:96113]	5.88	0.0861
ssc-miR-30c-5p	INPP4A	inositol polyphosphate-4-phosphatase type I A [Source:VGNC Symbol;Acc:VGNC:89140]	5.88	0.0861
ssc-miR-30c-5p	INSIG2	insulin induced 2 [Source:VGNC Symbol;Acc:VGNC:103970]	5.88	0.0861
ssc-miR-30c-5p	INTS2	integrator complex subunit 2 [Source:VGNC Symbol;Acc:VGNC:89162]	5.88	0.0861
ssc-miR-30c-5p	IP6K3	inositol hexakisphosphate kinase 3 [Source:VGNC Symbol;Acc:VGNC:89174]	5.88	0.0861
ssc-miR-30c-5p	IPMK	inositol polyphosphate multikinase [Source:VGNC Symbol;Acc:VGNC:89175]	5.88	0.0861
ssc-miR-30c-5p	IQCG	IQ motif containing G [Source:VGNC Symbol;Acc:VGNC:89189]	5.88	0.0861
ssc-miR-30c-5p	IRF2BP2	interferon regulatory factor 2 binding protein 2 [Source:VGNC Symbol;Acc:VGNC:89205]	5.88	0.0861
ssc-miR-30c-5p	IRF4	interferon regulatory factor 4 [Source:VGNC Symbol;Acc:VGNC:89207]	5.88	0.0861
ssc-miR-30c-5p	IRS1	insulin receptor substrate 1 [Source:VGNC Symbol;Acc:VGNC:96376]	5.88	0.0861
ssc-miR-30c-5p	IRS2	insulin receptor substrate 2 [Source:VGNC Symbol;Acc:VGNC:89214]	5.88	0.0861
ssc-miR-30c-5p	IRX4	iroquois homeobox 4 [Source:VGNC Symbol;Acc:VGNC:89218]	5.88	0.0861
ssc-miR-30c-5p	ITGA2	integrin subunit alpha 2 [Source:VGNC Symbol;Acc:VGNC:89234]	5.88	0.0861
ssc-miR-30c-5p	ITGA4	integrin subunit alpha 4 [Source:VGNC Symbol;Acc:VGNC:96377]	5.88	0.0861
ssc-miR-30c-5p	ITGA6	integrin subunit alpha 6 [Source:VGNC Symbol;Acc:VGNC:96378]	5.88	0.0861
ssc-miR-30c-5p	ITGA8	integrin subunit alpha 8 [Source:VGNC Symbol;Acc:VGNC:96379]	5.88	0.0861
ssc-miR-30c-5p	ITGA9	integrin subunit alpha 9 [Source:VGNC Symbol;Acc:VGNC:89238]	5.88	0.0861
ssc-miR-30c-5p	ITGB3	integrin subunit beta 3 [Source:NCBI gene (formerly Entrezgene);Acc:397063]	5.88	0.0861
ssc-miR-30c-5p	ITPK1	inositol-tetrakisphosphate 1-kinase [Source:VGNC Symbol;Acc:VGNC:89251]	5.88	0.0861
ssc-miR-30c-5p	ITSN1	intersectin 1 [Source:VGNC Symbol;Acc:VGNC:108669]	5.88	0.0861
ssc-miR-30c-5p	JAG2	jagged canonical Notch ligand 2 [Source:VGNC Symbol;Acc:VGNC:89269]	5.88	0.0861
ssc-miR-30c-5p	JAK1	Janus kinase 1 [Source:VGNC Symbol;Acc:VGNC:89270]	5.88	0.0861
ssc-miR-30c-5p	JAKMIP2	janus kinase and microtubule interacting protein 2 [Source:VGNC Symbol;Acc:VGNC:89274]	5.88	0.0861
ssc-miR-30c-5p	JAKMIP3	Janus kinase and microtubule interacting protein 3 [Source:VGNC Symbol;Acc:VGNC:89275]	5.88	0.0861
ssc-miR-30c-5p	JAM2	junctional adhesion molecule 2 [Source:VGNC Symbol;Acc:VGNC:89276]	5.88	0.0861
ssc-miR-30c-5p	JARID2	jumonji and AT-rich interaction domain containing 2 [Source:VGNC Symbol;Acc:VGNC:89279]	5.88	0.0861
ssc-miR-30c-5p	JDP2	Jun dimerization protein 2 [Source:VGNC Symbol;Acc:VGNC:89282]	5.88	0.0861
ssc-miR-30c-5p	JOSD1	Josephin domain containing 1 [Source:VGNC Symbol;Acc:VGNC:89290]	5.88	0.0861
ssc-miR-30c-5p	JPH4	junctophilin 4 [Source:VGNC Symbol;Acc:VGNC:89292]	5.88	0.0861
ssc-miR-30c-5p	JUNB	JunB proto-onco, AP-1 transcription factor subunit [Source:VGNC Symbol;Acc:VGNC:89295]	5.88	0.0861
ssc-miR-30c-5p	KATNBL1	katanin regulatory subunit B1 like 1 [Source:VGNC Symbol;Acc:VGNC:89313]	5.88	0.0861
ssc-miR-30c-5p	KCNA4	potassium voltage-gated channel subfamily A member 4 [Source:VGNC Symbol;Acc:VGNC:89326]	5.88	0.0861
ssc-miR-30c-5p	KCNJ12	hypothetical gene	5.88	0.0861
ssc-miR-30c-5p	KCNJ15	potassium inwardly rectifying channel subfamily J member 15 [Source:VGNC Symbol;Acc:VGNC:89355]	5.88	0.0861

ssc-miR-30c-5p	KCNJ3	potassium inwardly rectifying channel subfamily J member 3 [Source:HGNC Symbol;Acc:HGNC:6264]	5.88	0.0861
ssc-miR-30c-5p	KCNJ6	potassium inwardly rectifying channel subfamily J member 6 [Source:VGNC Symbol;Acc:VGNC:89360]	5.88	0.0861
ssc-miR-30c-5p	KCNN3	potassium calcium-activated channel subfamily N member 3 [Source:VGNC Symbol;Acc:VGNC:98056]	5.88	0.0861
ssc-miR-30c-5p	KCTD16	potassium channel tetramerization domain containing 16 [Source:VGNC Symbol;Acc:VGNC:89395]	5.88	0.0861
ssc-miR-30c-5p	KCTD20	potassium channel tetramerization domain containing 20 [Source:VGNC Symbol;Acc:VGNC:89399]	5.88	0.0861
ssc-miR-30c-5p	KCTD3	potassium channel tetramerization domain containing 3 [Source:VGNC Symbol;Acc:VGNC:95963]	5.88	0.0861
ssc-miR-30c-5p	KCTD5	hypothetical gene	5.88	0.0861
ssc-miR-30c-5p	KCTD7	potassium channel tetramerization domain containing 7 [Source:VGNC Symbol;Acc:VGNC:89403]	5.88	0.0861
ssc-miR-30c-5p	KCTD8	potassium channel tetramerization domain containing 8 [Source:HGNC Symbol;Acc:HGNC:22394]	5.88	0.0861
ssc-miR-30c-5p	KDM3A	lysine demethylase 3A [Source:VGNC Symbol;Acc:VGNC:89411]	5.88	0.0861
ssc-miR-30c-5p	KDM5B	lysine demethylase 5B [Source:VGNC Symbol;Acc:VGNC:95870]	5.88	0.0861
ssc-miR-30c-5p	KDSR	3-ketodihydrosphingosine reductase [Source:VGNC Symbol;Acc:VGNC:108157]	5.88	0.0861
ssc-miR-30c-5p	KHNYN	KH and NYN domain containing [Source:VGNC Symbol;Acc:VGNC:89426]	5.88	0.0861
ssc-miR-30c-5p	KIAA0087	hypothetical gene	5.88	0.0861
ssc-miR-30c-5p	KIAA0101	hypothetical gene	5.88	0.0861
ssc-miR-30c-5p	KIAA0226L	hypothetical gene	5.88	0.0861
ssc-miR-30c-5p	KIAA0247	hypothetical gene	5.88	0.0861
ssc-miR-30c-5p	KIAA0355	hypothetical gene	5.88	0.0861
ssc-miR-30c-5p	KIAA0408	KIAA0408 [Source:VGNC Symbol;Acc:VGNC:89432]	5.88	0.0861
ssc-miR-30c-5p	KIAA1024	hypothetical gene	5.88	0.0861
ssc-miR-30c-5p	KIAA1033	hypothetical gene	5.88	0.0861
ssc-miR-30c-5p	KIAA1147	hypothetical gene	5.88	0.0861
ssc-miR-30c-5p	KIAA1211	hypothetical gene	5.88	0.0861
ssc-miR-30c-5p	KIAA1211L	hypothetical gene	5.88	0.0861
ssc-miR-30c-5p	KIAA1244	hypothetical gene	5.88	0.0861
ssc-miR-30c-5p	KIAA1522	KIAA1522 [Source:VGNC Symbol;Acc:VGNC:89444]	5.88	0.0861
ssc-miR-30c-5p	KIAA1549	KIAA1549 [Source:VGNC Symbol;Acc:VGNC:99719]	5.88	0.0861
ssc-miR-30c-5p	KIAA1715	hypothetical gene	5.88	0.0861
ssc-miR-30c-5p	KIAA2026	KIAA2026 [Source:VGNC Symbol;Acc:VGNC:89450]	5.88	0.0861
ssc-miR-30c-5p	KIF16B	kinesin family member 16B [Source:VGNC Symbol;Acc:VGNC:106450]	5.88	0.0861
ssc-miR-30c-5p	KIF1C	kinesin family member 1C [Source:VGNC Symbol;Acc:VGNC:89461]	5.88	0.0861
ssc-miR-30c-5p	KIF21B	kinesin family member 21B [Source:VGNC Symbol;Acc:VGNC:96219]	5.88	0.0861
ssc-miR-30c-5p	KIF3A	kinesin family member 3A [Source:VGNC Symbol;Acc:VGNC:89470]	5.88	0.0861
ssc-miR-30c-5p	KIF3C	kinesin family member 3C [Source:VGNC Symbol;Acc:VGNC:89471]	5.88	0.0861
ssc-miR-30c-5p	KLF10	Kruppel like factor 10 [Source:VGNC Symbol;Acc:VGNC:89490]	5.88	0.0861
ssc-miR-30c-5p	KLF11	Kruppel like factor 11 [Source:VGNC Symbol;Acc:VGNC:89491]	5.88	0.0861
ssc-miR-30c-5p	KLF12	Kruppel like factor 12 [Source:VGNC Symbol;Acc:VGNC:89492]	5.88	0.0861
ssc-miR-30c-5p	KLF13	Kruppel like factor 13 [Source:VGNC Symbol;Acc:VGNC:89493]	5.88	0.0861
ssc-miR-30c-5p	KLF14	Kruppel like factor 14 [Source:VGNC Symbol;Acc:VGNC:89494]	5.88	0.0861
ssc-miR-30c-5p	KLF8	Kruppel like factor 8 [Source:VGNC Symbol;Acc:VGNC:89500]	5.88	0.0861
ssc-miR-30c-5p	KLF9	Kruppel like factor 9 [Source:VGNC Symbol;Acc:VGNC:103114]	5.88	0.0861
ssc-miR-30c-5p	KLHL20	kelch like family member 20 [Source:VGNC Symbol;Acc:VGNC:89519]	5.88	0.0861
ssc-miR-30c-5p	KLHL23	kelch like family member 23 [Source:VGNC Symbol;Acc:VGNC:96158]	5.88	0.0861
ssc-miR-30c-5p	KLHL24	kelch like family member 24 [Source:VGNC Symbol;Acc:VGNC:89521]	5.88	0.0861
ssc-miR-30c-5p	KLHL28	kelch like family member 28 [Source:VGNC Symbol;Acc:VGNC:89523]	5.88	0.0861
ssc-miR-30c-5p	KMT2A	lysine methyltransferase 2A [Source:VGNC Symbol;Acc:VGNC:108600]	5.88	0.0861
ssc-miR-30c-5p	KMT2C	lysine methyltransferase 2C [Source:VGNC Symbol;Acc:VGNC:89550]	5.88	0.0861
ssc-miR-30c-5p	KMT2D	hypothetical gene	5.88	0.0861
ssc-miR-30c-5p	KPNA3	karyopherin subunit alpha 3 [Source:VGNC Symbol;Acc:VGNC:89562]	5.88	0.0861

ssc-miR-30c-5p	KPNA6	karyopherin subunit alpha 6 [Source:VGNC Symbol;Acc:VGNC:89565]	5.88	0.0861
ssc-miR-30c-5p	KRAS	KRAS proto-onco, GTPase [Source:VGNC Symbol;Acc:VGNC:89569]	5.88	0.0861
ssc-miR-30c-5p	KREMEN1	kringle containing transmembrane protein 1 [Source:VGNC Symbol;Acc:VGNC:89572]	5.88	0.0861
ssc-miR-30c-5p	KSR1	kinase suppressor of ras 1 [Source:VGNC Symbol;Acc:VGNC:89606]	5.88	0.0861
ssc-miR-30c-5p	KXD1	KxDL motif containing 1 [Source:HGNC Symbol;Acc:HGNC:28420]	5.88	0.0861
ssc-miR-30c-5p	LARGE	hypothetical gene	5.88	0.0861
ssc-miR-30c-5p	LARP1	La ribonucleoprotein 1, translational regulator [Source:VGNC Symbol;Acc:VGNC:98067]	5.88	0.0861
ssc-miR-30c-5p	LARP1B	La ribonucleoprotein 1B [Source:VGNC Symbol;Acc:VGNC:89640]	5.88	0.0861
ssc-miR-30c-5p	LARP4	La ribonucleoprotein 4 [Source:VGNC Symbol;Acc:VGNC:89641]	5.88	0.0861
ssc-miR-30c-5p	LATS2	large tumor suppressor kinase 2 [Source:VGNC Symbol;Acc:VGNC:89649]	5.88	0.0861
ssc-miR-30c-5p	LAYN	layilin [Source:VGNC Symbol;Acc:VGNC:89651]	5.88	0.0861
ssc-miR-30c-5p	LCLAT1	lysocardiolipin acyltransferase 1 [Source:VGNC Symbol;Acc:VGNC:89658]	5.88	0.0861
ssc-miR-30c-5p	LCOR	ligand dependent nuclear receptor corepressor [Source:HGNC Symbol;Acc:HGNC:29503]	5.88	0.0861
ssc-miR-30c-5p	LDLR	low density lipoprotein receptor [Source:VGNC Symbol;Acc:VGNC:89671]	5.88	0.0861
ssc-miR-30c-5p	LEPR	leptin receptor [Source:VGNC Symbol;Acc:VGNC:89685]	5.88	0.0861
ssc-miR-30c-5p	LGI1	leucine rich glioma inactivated 1 [Source:HGNC Symbol;Acc:HGNC:6572]	5.88	0.0861
ssc-miR-30c-5p	LHFPL2	LHFPL tetraspan subfamily member 2 [Source:VGNC Symbol;Acc:VGNC:89705]	5.88	0.0861
ssc-miR-30c-5p	LHX1	LIM homeobox 1 [Source:VGNC Symbol;Acc:VGNC:89711]	5.88	0.0861
ssc-miR-30c-5p	LHX5	LIM homeobox 5 [Source:VGNC Symbol;Acc:VGNC:89715]	5.88	0.0861
ssc-miR-30c-5p	LHX8	LIM homeobox 8 [Source:VGNC Symbol;Acc:VGNC:89717]	5.88	0.0861
ssc-miR-30c-5p	LHX9	LIM homeobox 9 [Source:VGNC Symbol;Acc:VGNC:95608]	5.88	0.0861
ssc-miR-30c-5p	LIFR	LIF receptor subunit alpha [Source:HGNC Symbol;Acc:HGNC:6597]	5.88	0.0861
ssc-miR-30c-5p	LIMCH1	LIM and calponin homology domains 1 [Source:VGNC Symbol;Acc:VGNC:89724]	5.88	0.0861
ssc-miR-30c-5p	LIN28A	lin-28 homolog A [Source:VGNC Symbol;Acc:VGNC:98492]	5.88	0.0861
ssc-miR-30c-5p	LIN28B	lin-28 homolog B [Source:VGNC Symbol;Acc:VGNC:89729]	5.88	0.0861
ssc-miR-30c-5p	LIN7C	lin-7 homolog C, crumbs cell polarity complex component [Source:VGNC Symbol;Acc:VGNC:89732]	5.88	0.0861
ssc-miR-30c-5p	LMBR1	limb development membrane protein 1 [Source:VGNC Symbol;Acc:VGNC:89752]	5.88	0.0861
ssc-miR-30c-5p	LMBR1L	limb development membrane protein 1 like [Source:VGNC Symbol;Acc:VGNC:89753]	5.88	0.0861
ssc-miR-30c-5p	LMLN	leishmanolysin like peptidase [Source:VGNC Symbol;Acc:VGNC:89759]	5.88	0.0861
ssc-miR-30c-5p	LONRF1	LON peptidase N-terminal domain and ring finger 1 [Source:VGNC Symbol;Acc:VGNC:96123]	5.88	0.0861
ssc-miR-30c-5p	LONRF3	LON peptidase N-terminal domain and ring finger 3 [Source:VGNC Symbol;Acc:VGNC:89778]	5.88	0.0861
ssc-miR-30c-5p	LOX	lysyl oxidase [Source:VGNC Symbol;Acc:VGNC:99785]	5.88	0.0861
ssc-miR-30c-5p	LPAR3	lysophosphatidic acid receptor 3 [Source:VGNC Symbol;Acc:VGNC:89785]	5.88	0.0861
ssc-miR-30c-5p	LPGAT1	lysophosphatidylglycerol acyltransferase 1 [Source:VGNC Symbol;Acc:VGNC:89792]	5.88	0.0861
ssc-miR-30c-5p	LPHN3	hypothetical gene	5.88	0.0861
ssc-miR-30c-5p	LPP	LIM domain containing preferred translocation partner in lipoma [Source:HGNC Symbol;Acc:HGNC:6679]	5.88	0.0861
ssc-miR-30c-5p	LPPR4	hypothetical gene	5.88	0.0861
ssc-miR-30c-5p	LRCH2	leucine rich repeats and calponin homology domain containing 2 [Source:VGNC Symbol;Acc:VGNC:89802]	5.88	0.0861
ssc-miR-30c-5p	LRFN2	leucine rich repeat and fibronectin type III domain containing 2 [Source:VGNC Symbol;Acc:VGNC:89805]	5.88	0.0861
ssc-miR-30c-5p	LRIG2	leucine rich repeats and immunoglobulin like domains 2 [Source:VGNC Symbol;Acc:VGNC:89811]	5.88	0.0861
ssc-miR-30c-5p	LRP6	LDL receptor related protein 6 [Source:VGNC Symbol;Acc:VGNC:89821]	5.88	0.0861
ssc-miR-30c-5p	LRRC17	leucine rich repeat containing 17 [Source:VGNC Symbol;Acc:VGNC:89828]	5.88	0.0861
ssc-miR-30c-5p	LRRC40	hypothetical gene	5.88	0.0861
ssc-miR-30c-5p	LRRC58	leucine rich repeat containing 58 [Source:VGNC Symbol;Acc:VGNC:98081]	5.88	0.0861
ssc-miR-30c-5p	LRRC8C	leucine rich repeat containing 8 VRAC subunit C [Source:VGNC Symbol;Acc:VGNC:98087]	5.88	0.0861
ssc-miR-30c-5p	LRRC8D	leucine rich repeat containing 8 VRAC subunit D [Source:VGNC Symbol;Acc:VGNC:98088]	5.88	0.0861
ssc-miR-30c-5p	LRRK2	leucine rich repeat kinase 2 [Source:VGNC Symbol;Acc:VGNC:98094]	5.88	0.0861
ssc-miR-30c-5p	LSM14B	LSM family member 14B [Source:VGNC Symbol;Acc:VGNC:95691]	5.88	0.0861
ssc-miR-30c-5p	LUC7L2	LUC7 like 2, pre-mRNA splicing factor [Source:HGNC Symbol;Acc:HGNC:21608]	5.88	0.0861

ssc-miR-30c-5p	LY75-CD302	hypothetical gene	5.88	0.0861
ssc-miR-30c-5p	LYN	LYN proto-onco, Src family tyrosine kinase [Source:VGNC Symbol;Acc:VGNC:89910]	5.88	0.0861
ssc-miR-30c-5p	LYSMD3	LysM domain containing 3 [Source:VGNC Symbol;Acc:VGNC:89919]	5.88	0.0861
ssc-miR-30c-5p	LYST	lysosomal trafficking regulator [Source:VGNC Symbol;Acc:VGNC:89921]	5.88	0.0861
ssc-miR-30c-5p	MAB21L1	mab-21 like 1 [Source:VGNC Symbol;Acc:VGNC:89932]	5.88	0.0861
ssc-miR-30c-5p	MAF	MAF bZIP transcription factor [Source:VGNC Symbol;Acc:VGNC:89945]	5.88	0.0861
ssc-miR-30c-5p	MAFG	MAF bZIP transcription factor G [Source:VGNC Symbol;Acc:VGNC:89948]	5.88	0.0861
ssc-miR-30c-5p	MAGI2	membrane associated guanylate kinase, WW and PDZ domain containing 2 [Source:VGNC Symbol;Acc:VGNC:89955]	5.88	0.0861
ssc-miR-30c-5p	MAGI3	membrane associated guanylate kinase, WW and PDZ domain containing 3 [Source:VGNC Symbol;Acc:VGNC:89956]	5.88	0.0861
ssc-miR-30c-5p	MAL	hypothetical gene	5.88	0.0861
ssc-miR-30c-5p	MAML1	mastermind like transcriptional coactivator 1 [Source:VGNC Symbol;Acc:VGNC:89963]	5.88	0.0861
ssc-miR-30c-5p	MAN1A2	mannosidase alpha class 1A member 2 [Source:VGNC Symbol;Acc:VGNC:89967]	5.88	0.0861
ssc-miR-30c-5p	MAN1B1	hypothetical gene	5.88	0.0861
ssc-miR-30c-5p	MAP2K6	mitogen-activated protein kinase kinase 6 [Source:VGNC Symbol;Acc:VGNC:98102]	5.88	0.0861
ssc-miR-30c-5p	MAP3K1	mitogen-activated protein kinase kinase kinase 1 [Source:VGNC Symbol;Acc:VGNC:98104]	5.88	0.0861
ssc-miR-30c-5p	MAP3K12	mitogen-activated protein kinase kinase kinase 12 [Source:VGNC Symbol;Acc:VGNC:89983]	5.88	0.0861
ssc-miR-30c-5p	MAP3K19	mitogen-activated protein kinase kinase kinase 19 [Source:VGNC Symbol;Acc:VGNC:98106]	5.88	0.0861
ssc-miR-30c-5p	MAP3K2	mitogen-activated protein kinase kinase kinase 2 [Source:VGNC Symbol;Acc:VGNC:98107]	5.88	0.0861
ssc-miR-30c-5p	MAP3K5	mitogen-activated protein kinase kinase kinase 5 [Source:VGNC Symbol;Acc:VGNC:89985]	5.88	0.0861
ssc-miR-30c-5p	MAP3K7	mitogen-activated protein kinase kinase kinase 7 [Source:VGNC Symbol;Acc:VGNC:89987]	5.88	0.0861
ssc-miR-30c-5p	MAP4K4	mitogen-activated protein kinase kinase kinase kinase 4 [Source:VGNC Symbol;Acc:VGNC:98114]	5.88	0.0861
ssc-miR-30c-5p	MAPK8	mitogen-activated protein kinase 8 [Source:VGNC Symbol;Acc:VGNC:90006]	5.88	0.0861
ssc-miR-30c-5p	MAPKBP1	mitogen-activated protein kinase binding protein 1 [Source:VGNC Symbol;Acc:VGNC:90014]	5.88	0.0861
ssc-miR-30c-5p	MARCH3	hypothetical gene	5.88	0.0861
ssc-miR-30c-5p	MARCH4	hypothetical gene	5.88	0.0861
ssc-miR-30c-5p	MARCH6	hypothetical gene	5.88	0.0861
ssc-miR-30c-5p	MARCH8	hypothetical gene	5.88	0.0861
ssc-miR-30c-5p	MARCKS	myristoylated alanine rich protein kinase C substrate [Source:VGNC Symbol;Acc:VGNC:90024]	5.88	0.0861
ssc-miR-30c-5p	MARK1	microtubule affinity regulating kinase 1 [Source:VGNC Symbol;Acc:VGNC:96411]	5.88	0.0861
ssc-miR-30c-5p	MARK3	microtubule affinity regulating kinase 3 [Source:VGNC Symbol;Acc:VGNC:90026]	5.88	0.0861
ssc-miR-30c-5p	MAST3	microtubule associated serine/threonine kinase 3 [Source:VGNC Symbol;Acc:VGNC:90036]	5.88	0.0861
ssc-miR-30c-5p	MAT2A	methionine adenosyltransferase 2A [Source:VGNC Symbol;Acc:VGNC:90039]	5.88	0.0861
ssc-miR-30c-5p	MATR3	matrin 3 [Source:HGNC Symbol;Acc:HGNC:6912]	5.88	0.0861
ssc-miR-30c-5p	MAU2	MAU2 sister chromatid cohesion factor [Source:VGNC Symbol;Acc:VGNC:90045]	5.88	0.0861
ssc-miR-30c-5p	MBD6	methyl-CpG binding domain protein 6 [Source:VGNC Symbol;Acc:VGNC:90051]	5.88	0.0861
ssc-miR-30c-5p	MBNL1	muscleblind like splicing regulator 1 [Source:VGNC Symbol;Acc:VGNC:90054]	5.88	0.0861
ssc-miR-30c-5p	MBNL2	muscleblind like splicing regulator 2 [Source:VGNC Symbol;Acc:VGNC:90055]	5.88	0.0861
ssc-miR-30c-5p	MBNL3	muscleblind like splicing regulator 3 [Source:VGNC Symbol;Acc:VGNC:90056]	5.88	0.0861
ssc-miR-30c-5p	MBOAT1	membrane bound O-acyltransferase domain containing 1 [Source:VGNC Symbol;Acc:VGNC:90057]	5.88	0.0861
ssc-miR-30c-5p	MBTPS2	hypothetical gene	5.88	0.0861
ssc-miR-30c-5p	MCF2L	MCF.2 cell line derived transforming sequence like [Source:VGNC Symbol;Acc:VGNC:90068]	5.88	0.0861
ssc-miR-30c-5p	MCTS1	MCTS1 re-initiation and release factor [Source:HGNC Symbol;Acc:HGNC:23357]	5.88	0.0861
ssc-miR-30c-5p	MDM4	MDM4 regulator of p53 [Source:VGNC Symbol;Acc:VGNC:90094]	5.88	0.0861
ssc-miR-30c-5p	ME1	malic enzyme 1 [Source:VGNC Symbol;Acc:VGNC:90095]	5.88	0.0861
ssc-miR-30c-5p	MECP2	methyl-CpG binding protein 2 [Source:VGNC Symbol;Acc:VGNC:90101]	5.88	0.0861
ssc-miR-30c-5p	MED12L	mediator complex subunit 12L [Source:VGNC Symbol;Acc:VGNC:90105]	5.88	0.0861
ssc-miR-30c-5p	MEF2D	myocyte enhancer factor 2D [Source:VGNC Symbol;Acc:VGNC:90128]	5.88	0.0861
ssc-miR-30c-5p	MEIS2	Meis homeobox 2 [Source:VGNC Symbol;Acc:VGNC:90136]	5.88	0.0861
ssc-miR-30c-5p	METAP2	methionyl aminopeptidase 2 [Source:VGNC Symbol;Acc:VGNC:90152]	5.88	0.0861

ssc-miR-30c-5p	METTL1	methyltransferase 1, tRNA methylguanosine [Source:HGNC Symbol;Acc:HGNC:7030]	5.88	0.0861
ssc-miR-30c-5p	MEX3B	mex-3 RNA binding family member B [Source:VGNC Symbol;Acc:VGNC:90169]	5.88	0.0861
ssc-miR-30c-5p	MEX3D	mex-3 RNA binding family member D [Source:VGNC Symbol;Acc:VGNC:90171]	5.88	0.0861
ssc-miR-30c-5p	MFAP3L	microfibril associated protein 3 like [Source:VGNC Symbol;Acc:VGNC:90175]	5.88	0.0861
ssc-miR-30c-5p	MFHAS1	multifunctional ROCO family signaling regulator 1 [Source:VGNC Symbol;Acc:VGNC:107361]	5.88	0.0861
ssc-miR-30c-5p	MFSD11	major facilitator superfamily domain containing 11 [Source:VGNC Symbol;Acc:VGNC:90184]	5.88	0.0861
ssc-miR-30c-5p	MFSD6	major facilitator superfamily domain containing 6 [Source:VGNC Symbol;Acc:VGNC:96052]	5.88	0.0861
ssc-miR-30c-5p	MIA3	hypothetical gene	5.88	0.0861
ssc-miR-30c-5p	MIB1	MIB E3 ubiquitin protein ligase 1 [Source:VGNC Symbol;Acc:VGNC:90205]	5.88	0.0861
ssc-miR-30c-5p	MICAL1	microtubule associated monooxygenase, calponin and LIM domain containing 1 [Source:VGNC Symbol;Acc:VGNC:90207]	5.88	0.0861
ssc-miR-30c-5p	MICU3	mitochondrial calcium uptake family member 3 [Source:VGNC Symbol;Acc:VGNC:96160]	5.88	0.0861
ssc-miR-30c-5p	MID2	midline 2 [Source:VGNC Symbol;Acc:VGNC:98127]	5.88	0.0861
ssc-miR-30c-5p	MIER2	MIER family member 2 [Source:VGNC Symbol;Acc:VGNC:90220]	5.88	0.0861
ssc-miR-30c-5p	MIER3	MIER family member 3 [Source:VGNC Symbol;Acc:VGNC:90221]	5.88	0.0861
ssc-miR-30c-5p	MINOS1	hypothetical gene	5.88	0.0861
ssc-miR-30c-5p	MINPP1	multiple inositol-polyphosphate phosphatase 1 [Source:VGNC Symbol;Acc:VGNC:90233]	5.88	0.0861
ssc-miR-30c-5p	MIS18BP1	MIS18 binding protein 1 [Source:VGNC Symbol;Acc:VGNC:90240]	5.88	0.0861
ssc-miR-30c-5p	MKL2	hypothetical gene	5.88	0.0861
ssc-miR-30c-5p	MKRN3	hypothetical gene	5.88	0.0861
ssc-miR-30c-5p	MLK4	hypothetical gene	5.88	0.0861
ssc-miR-30c-5p	MLXIP	MLX interacting protein [Source:VGNC Symbol;Acc:VGNC:90262]	5.88	0.0861
ssc-miR-30c-5p	MMD	monocyte to macrophage differentiation associated [Source:VGNC Symbol;Acc:VGNC:90264]	5.88	0.0861
ssc-miR-30c-5p	MMP16	matrix metallopeptidase 16 [Source:VGNC Symbol;Acc:VGNC:90271]	5.88	0.0861
ssc-miR-30c-5p	MMP19	matrix metallopeptidase 19 [Source:VGNC Symbol;Acc:VGNC:90273]	5.88	0.0861
ssc-miR-30c-5p	MNT	MAX network transcriptional repressor [Source:VGNC Symbol;Acc:VGNC:90289]	5.88	0.0861
ssc-miR-30c-5p	MOB4	hypothetical gene	5.88	0.0861
ssc-miR-30c-5p	MOV10	Mov10 RISC complex RNA helicase [Source:VGNC Symbol;Acc:VGNC:90316]	5.88	0.0861
ssc-miR-30c-5p	MPPED1	metallophosphoesterase domain containing 1 [Source:VGNC Symbol;Acc:VGNC:90330]	5.88	0.0861
ssc-miR-30c-5p	MSANTD3-TMEFF1	hypothetical gene	5.88	0.0861
ssc-miR-30c-5p	MSANTD4	Myb/SANT DNA binding domain containing 4 with coiled-coils [Source:VGNC Symbol;Acc:VGNC:90415]	5.88	0.0861
ssc-miR-30c-5p	MSI2	musashi RNA binding protein 2 [Source:VGNC Symbol;Acc:VGNC:90422]	5.88	0.0861
ssc-miR-30c-5p	MTA1	hypothetical gene	5.88	0.0861
ssc-miR-30c-5p	MTDH	metadherin [Source:VGNC Symbol;Acc:VGNC:90439]	5.88	0.0861
ssc-miR-30c-5p	MTF1	metal regulatory transcription factor 1 [Source:VGNC Symbol;Acc:VGNC:90443]	5.88	0.0861
ssc-miR-30c-5p	MTF2	metal response element binding transcription factor 2 [Source:VGNC Symbol;Acc:VGNC:90444]	5.88	0.0861
ssc-miR-30c-5p	MTHFD1L	methylenetetrahydrofolate dehydrogenase (NADP+ dependent) 1 like [Source:VGNC Symbol;Acc:VGNC:103129]	5.88	0.0861
ssc-miR-30c-5p	MTTP	microsomal triglyceride transfer protein [Source:VGNC Symbol;Acc:VGNC:98942]	5.88	0.0861
ssc-miR-30c-5p	MXRA5	hypothetical gene	5.88	0.0861
ssc-miR-30c-5p	MYBL2	MYB proto-onco like 2 [Source:VGNC Symbol;Acc:VGNC:103997]	5.88	0.0861
ssc-miR-30c-5p	MYH11	myosin heavy chain 11 [Source:VGNC Symbol;Acc:VGNC:90510]	5.88	0.0861
ssc-miR-30c-5p	MYO5A	myosin VA [Source:HGNC Symbol;Acc:HGNC:7602]	5.88	0.0861
ssc-miR-30c-5p	MYO9B	myosin IXB [Source:VGNC Symbol;Acc:VGNC:90534]	5.88	0.0861
ssc-miR-30c-5p	MYOM2	myomesin 2 [Source:HGNC Symbol;Acc:HGNC:7614]	5.88	0.0861
ssc-miR-30c-5p	MYSM1	Myb like, SWIRM and MPN domains 1 [Source:VGNC Symbol;Acc:VGNC:90548]	5.88	0.0861
ssc-miR-30c-5p	MZT1	mitotic spindle organizing protein 1 [Source:VGNC Symbol;Acc:VGNC:90552]	5.88	0.0861
ssc-miR-30c-5p	N4BP2	NEDD4 binding protein 2 [Source:VGNC Symbol;Acc:VGNC:90554]	5.88	0.0861
ssc-miR-30c-5p	NAA25	N-alpha-acetyltransferase 25, NatB auxiliary subunit [Source:VGNC Symbol;Acc:VGNC:96750]	5.88	0.0861
ssc-miR-30c-5p	NAALADL2	N-acetylated alpha-linked acidic dipeptidase like 2 [Source:VGNC Symbol;Acc:VGNC:90559]	5.88	0.0861
ssc-miR-30c-5p	NACC2	NACC family member 2 [Source:VGNC Symbol;Acc:VGNC:90564]	5.88	0.0861

ssc-miR-30c-5p	NADK	NAD kinase [Source:VGNC Symbol;Acc:VGNC:90565]	5.88	0.0861
ssc-miR-30c-5p	NAGPA	N-acetylglucosamine-1-phosphodiester alpha-N-acetylglucosaminidase [Source:NCBI gene (formerly Entrezgene);Acc:100512596]	5.88	0.0861
ssc-miR-30c-5p	NAP1L1	nucleosome assembly protein 1 like 1 [Source:VGNC Symbol;Acc:VGNC:90576]	5.88	0.0861
ssc-miR-30c-5p	NAP1L5	nucleosome assembly protein 1 like 5 [Source:NCBI gene (formerly Entrezgene);Acc:100616076]	5.88	0.0861
ssc-miR-30c-5p	NAPG	NSF attachment protein gamma [Source:VGNC Symbol;Acc:VGNC:90580]	5.88	0.0861
ssc-miR-30c-5p	NAV1	neuron navigator 1 [Source:VGNC Symbol;Acc:VGNC:95725]	5.88	0.0861
ssc-miR-30c-5p	NAV3	neuron navigator 3 [Source:VGNC Symbol;Acc:VGNC:90589]	5.88	0.0861
ssc-miR-30c-5p	NCALD	neurocalcin delta [Source:VGNC Symbol;Acc:VGNC:90594]	5.88	0.0861
ssc-miR-30c-5p	NCAM1	neural cell adhesion molecule 1 [Source:VGNC Symbol;Acc:VGNC:108603]	5.88	0.0861
ssc-miR-30c-5p	NCOA3	nuclear receptor coactivator 3 [Source:VGNC Symbol;Acc:VGNC:96434]	5.88	0.0861
ssc-miR-30c-5p	NCS1	neuronal calcium sensor 1 [Source:VGNC Symbol;Acc:VGNC:90620]	5.88	0.0861
ssc-miR-30c-5p	NDEL1	nudE neurodevelopment protein 1 like 1 [Source:VGNC Symbol;Acc:VGNC:90625]	5.88	0.0861
ssc-miR-30c-5p	NDNF	neuron derived neurotrophic factor [Source:VGNC Symbol;Acc:VGNC:90629]	5.88	0.0861
ssc-miR-30c-5p	NECAB1	N-terminal EF-hand calcium binding protein 1 [Source:VGNC Symbol;Acc:VGNC:90659]	5.88	0.0861
ssc-miR-30c-5p	NECAP1	NECAP endocytosis associated 1 [Source:VGNC Symbol;Acc:VGNC:90660]	5.88	0.0861
ssc-miR-30c-5p	NEDD4	NEDD4 E3 ubiquitin protein ligase [Source:VGNC Symbol;Acc:VGNC:90666]	5.88	0.0861
ssc-miR-30c-5p	NEDD4L	NEDD4 like E3 ubiquitin protein ligase [Source:VGNC Symbol;Acc:VGNC:90667]	5.88	0.0861
ssc-miR-30c-5p	NEFM	hypothetical gene	5.88	0.0861
ssc-miR-30c-5p	NEGR1	neuronal growth regulator 1 [Source:VGNC Symbol;Acc:VGNC:90671]	5.88	0.0861
ssc-miR-30c-5p	NEURL1B	neuralized E3 ubiquitin protein ligase 1B [Source:VGNC Symbol;Acc:VGNC:90695]	5.88	0.0861
ssc-miR-30c-5p	NEUROD1	neuronal differentiation 1 [Source:VGNC Symbol;Acc:VGNC:96439]	5.88	0.0861
ssc-miR-30c-5p	NEUROD6	neuronal differentiation 6 [Source:VGNC Symbol;Acc:VGNC:90699]	5.88	0.0861
ssc-miR-30c-5p	NF1	neurofibromin 1 [Source:VGNC Symbol;Acc:VGNC:90704]	5.88	0.0861
ssc-miR-30c-5p	NFAT5	nuclear factor of activated T cells 5 [Source:VGNC Symbol;Acc:VGNC:90708]	5.88	0.0861
ssc-miR-30c-5p	NFATC2	nuclear factor of activated T cells 2 [Source:VGNC Symbol;Acc:VGNC:96440]	5.88	0.0861
ssc-miR-30c-5p	NFATC3	nuclear factor of activated T cells 3 [Source:VGNC Symbol;Acc:VGNC:90710]	5.88	0.0861
ssc-miR-30c-5p	NFIA	nuclear factor I A [Source:VGNC Symbol;Acc:VGNC:90715]	5.88	0.0861
ssc-miR-30c-5p	NFIB	nuclear factor I B [Source:VGNC Symbol;Acc:VGNC:90716]	5.88	0.0861
ssc-miR-30c-5p	NFYA	nuclear transcription factor Y subunit alpha [Source:VGNC Symbol;Acc:VGNC:90729]	5.88	0.0861
ssc-miR-30c-5p	NFYB	nuclear transcription factor Y subunit beta [Source:VGNC Symbol;Acc:VGNC:90730]	5.88	0.0861
ssc-miR-30c-5p	NHLH2	nescient helix-loop-helix 2 [Source:VGNC Symbol;Acc:VGNC:90736]	5.88	0.0861
ssc-miR-30c-5p	NHS	NHS actin remodeling regulator [Source:VGNC Symbol;Acc:VGNC:90738]	5.88	0.0861
ssc-miR-30c-5p	NID1	nidogen 1 [Source:VGNC Symbol;Acc:VGNC:90742]	5.88	0.0861
ssc-miR-30c-5p	NIPAL1	NIPA like domain containing 1 [Source:VGNC Symbol;Acc:VGNC:90748]	5.88	0.0861
ssc-miR-30c-5p	NKAIN2	sodium/potassium transporting ATPase interacting 2 [Source:VGNC Symbol;Acc:VGNC:103141]	5.88	0.0861
ssc-miR-30c-5p	NKX2-2	NK2 homeobox 2 [Source:VGNC Symbol;Acc:VGNC:96444]	5.88	0.0861
ssc-miR-30c-5p	NLGN1	neuroligin 1 [Source:VGNC Symbol;Acc:VGNC:90777]	5.88	0.0861
ssc-miR-30c-5p	NLK	nemo like kinase [Source:VGNC Symbol;Acc:VGNC:90779]	5.88	0.0861
ssc-miR-30c-5p	NOTCH1	notch receptor 1 [Source:HGNC Symbol;Acc:HGNC:7881]	5.88	0.0861
ssc-miR-30c-5p	NOVA1	NOVA alternative splicing regulator 1 [Source:VGNC Symbol;Acc:VGNC:90827]	5.88	0.0861
ssc-miR-30c-5p	NPTXR	neuronal pentraxin receptor [Source:VGNC Symbol;Acc:VGNC:98158]	5.88	0.0861
ssc-miR-30c-5p	NR2F2	nuclear receptor subfamily 2 group F member 2 [Source:VGNC Symbol;Acc:VGNC:90881]	5.88	0.0861
ssc-miR-30c-5p	NR3C1	nuclear receptor subfamily 3 group C member 1 [Source:VGNC Symbol;Acc:VGNC:90883]	5.88	0.0861
ssc-miR-30c-5p	NR3C2	nuclear receptor subfamily 3 group C member 2 [Source:VGNC Symbol;Acc:VGNC:90884]	5.88	0.0861
ssc-miR-30c-5p	NR4A2	nuclear receptor subfamily 4 group A member 2 [Source:VGNC Symbol;Acc:VGNC:96451]	5.88	0.0861
ssc-miR-30c-5p	NR5A2	nuclear receptor subfamily 5 group A member 2 [Source:VGNC Symbol;Acc:VGNC:96452]	5.88	0.0861
ssc-miR-30c-5p	NR6A1	nuclear receptor subfamily 6 group A member 1 [Source:VGNC Symbol;Acc:VGNC:90887]	5.88	0.0861
ssc-miR-30c-5p	NRBF2	nuclear receptor binding factor 2 [Source:VGNC Symbol;Acc:VGNC:90890]	5.88	0.0861
ssc-miR-30c-5p	NRBP1	nuclear receptor binding protein 1 [Source:VGNC Symbol;Acc:VGNC:90891]	5.88	0.0861

ssc-miR-30c-5p	NREP	neuronal regeneration related protein [Source:HGNC Symbol;Acc:HGNC:16834]	5.88	0.0861
ssc-miR-30c-5p	NRG2	neuregulin 2 [Source:VGNC Symbol;Acc:VGNC:98160]	5.88	0.0861
ssc-miR-30c-5p	NRG3	neuregulin 3 [Source:VGNC Symbol;Acc:VGNC:104011]	5.88	0.0861
ssc-miR-30c-5p	NRIP1	nuclear receptor interacting protein 1 [Source:VGNC Symbol;Acc:VGNC:90896]	5.88	0.0861
ssc-miR-30c-5p	NRK	Nik related kinase [Source:VGNC Symbol;Acc:VGNC:98161]	5.88	0.0861
ssc-miR-30c-5p	NRP1	neuropilin 1 [Source:VGNC Symbol;Acc:VGNC:104012]	5.88	0.0861
ssc-miR-30c-5p	NRXN3	neurexin 3 [Source:HGNC Symbol;Acc:HGNC:8010]	5.88	0.0861
ssc-miR-30c-5p	NSD1	nuclear receptor binding SET domain protein 1 [Source:VGNC Symbol;Acc:VGNC:90904]	5.88	0.0861
ssc-miR-30c-5p	NSG1	neuronal vesicle trafficking associated 1 [Source:VGNC Symbol;Acc:VGNC:90907]	5.88	0.0861
ssc-miR-30c-5p	NSUN3	NOP2/Sun RNA methyltransferase 3 [Source:VGNC Symbol;Acc:VGNC:90915]	5.88	0.0861
ssc-miR-30c-5p	NT5DC3	5'-nucleotidase domain containing 3 [Source:VGNC Symbol;Acc:VGNC:90924]	5.88	0.0861
ssc-miR-30c-5p	NT5E	5'-nucleotidase ecto [Source:VGNC Symbol;Acc:VGNC:90925]	5.88	0.0861
ssc-miR-30c-5p	NTNG1	netrin G1 [Source:VGNC Symbol;Acc:VGNC:90935]	5.88	0.0861
ssc-miR-30c-5p	NUAK1	NUAK family kinase 1 [Source:VGNC Symbol;Acc:VGNC:90940]	5.88	0.0861
ssc-miR-30c-5p	NUCKS1	nuclear casein kinase and cyclin dependent kinase substrate 1 [Source:VGNC Symbol;Acc:VGNC:90947]	5.88	0.0861
ssc-miR-30c-5p	NUDT5	nudix hydrolase 5 [Source:VGNC Symbol;Acc:VGNC:96458]	5.88	0.0861
ssc-miR-30c-5p	NUFIP2	nuclear FMR1 interacting protein 2 [Source:VGNC Symbol;Acc:VGNC:90967]	5.88	0.0861
ssc-miR-30c-5p	NUP43	nucleoporin 43 [Source:VGNC Symbol;Acc:VGNC:90982]	5.88	0.0861
ssc-miR-30c-5p	NUS1	NUS1 dehydrodolichyl diphosphate synthase subunit [Source:VGNC Symbol;Acc:VGNC:90990]	5.88	0.0861
ssc-miR-30c-5p	OCLN	occludin [Source:VGNC Symbol;Acc:VGNC:91013]	5.88	0.0861
ssc-miR-30c-5p	OMG	oligodendrocyte myelin glycoprotein [Source:VGNC Symbol;Acc:VGNC:91041]	5.88	0.0861
ssc-miR-30c-5p	ONECUT2	one cut homeobox 2 [Source:VGNC Symbol;Acc:VGNC:91043]	5.88	0.0861
ssc-miR-30c-5p	ONECUT3	one cut homeobox 3 [Source:VGNC Symbol;Acc:VGNC:91044]	5.88	0.0861
ssc-miR-30c-5p	ORC2	origin recognition complex subunit 2 [Source:VGNC Symbol;Acc:VGNC:96464]	5.88	0.0861
ssc-miR-30c-5p	OSBPL8	oxysterol binding protein like 8 [Source:VGNC Symbol;Acc:VGNC:91074]	5.88	0.0861
ssc-miR-30c-5p	OSTM1	osteoclastosis associated transmembrane protein 1 [Source:VGNC Symbol;Acc:VGNC:91086]	5.88	0.0861
ssc-miR-30c-5p	OTUD4	OTU deubiquitinase 4 [Source:VGNC Symbol;Acc:VGNC:91100]	5.88	0.0861
ssc-miR-30c-5p	OTUD6B	OTU deubiquitinase 6B [Source:VGNC Symbol;Acc:VGNC:91103]	5.88	0.0861
ssc-miR-30c-5p	OVOL1	ovo like transcriptional repressor 1 [Source:VGNC Symbol;Acc:VGNC:91111]	5.88	0.0861
ssc-miR-30c-5p	OVOL2	ovo like zinc finger 2 [Source:VGNC Symbol;Acc:VGNC:95663]	5.88	0.0861
ssc-miR-30c-5p	OXR1	oxidation resistance 1 [Source:VGNC Symbol;Acc:VGNC:91116]	5.88	0.0861
ssc-miR-30c-5p	OXTR	oxytocin receptor [Source:VGNC Symbol;Acc:VGNC:108052]	5.88	0.0861
ssc-miR-30c-5p	P2RY2	purinergic receptor P2Y2 [Source:VGNC Symbol;Acc:VGNC:98173]	5.88	0.0861
ssc-miR-30c-5p	P4HA1	prolyl 4-hydroxylase subunit alpha 1 [Source:VGNC Symbol;Acc:VGNC:91132]	5.88	0.0861
ssc-miR-30c-5p	P4HA2	prolyl 4-hydroxylase subunit alpha 2 [Source:VGNC Symbol;Acc:VGNC:91133]	5.88	0.0861
ssc-miR-30c-5p	P4HA3	prolyl 4-hydroxylase subunit alpha 3 [Source:VGNC Symbol;Acc:VGNC:91134]	5.88	0.0861
ssc-miR-30c-5p	PAAF1	proteasomal ATPase associated factor 1 [Source:VGNC Symbol;Acc:VGNC:108605]	5.88	0.0861
ssc-miR-30c-5p	PABPC1L2A	hypothetical gene	5.88	0.0861
ssc-miR-30c-5p	PABPC1L2B	hypothetical gene	5.88	0.0861
ssc-miR-30c-5p	PACRGL	parkin coregulated like [Source:VGNC Symbol;Acc:VGNC:91139]	5.88	0.0861
ssc-miR-30c-5p	PAFAH1B2	platelet activating factor acetylhydrolase 1b catalytic subunit 2 [Source:VGNC Symbol;Acc:VGNC:91151]	5.88	0.0861
ssc-miR-30c-5p	PAG1	phosphoprotein membrane anchor with glycosphingolipid microdomains 1 [Source:VGNC Symbol;Acc:VGNC:91154]	5.88	0.0861
ssc-miR-30c-5p	PAK4	p21 (RAC1) activated kinase 4 [Source:VGNC Symbol;Acc:VGNC:98526]	5.88	0.0861
ssc-miR-30c-5p	PALM2	hypothetical gene	5.88	0.0861
ssc-miR-30c-5p	PANK3	pantothenate kinase 3 [Source:VGNC Symbol;Acc:VGNC:91165]	5.88	0.0861
ssc-miR-30c-5p	PANX1	pannexin 1 [Source:VGNC Symbol;Acc:VGNC:91166]	5.88	0.0861
ssc-miR-30c-5p	PAPD4	hypothetical gene	5.88	0.0861
ssc-miR-30c-5p	PAPD5	hypothetical gene	5.88	0.0861
ssc-miR-30c-5p	PAPOLB	hypothetical gene	5.88	0.0861

ssc-miR-30c-5p	PAQR5	progestin and adipoQ receptor family member 5 [Source:VGNC Symbol;Acc:VGNC:91175]	5.88	0.0861
ssc-miR-30c-5p	PARP16	poly(ADP-ribose) polymerase family member 16 [Source:VGNC Symbol;Acc:VGNC:103145]	5.88	0.0861
ssc-miR-30c-5p	PARP8	poly(ADP-ribose) polymerase family member 8 [Source:HGNC Symbol;Acc:HGNC:26124]	5.88	0.0861
ssc-miR-30c-5p	PAWR	pro-apoptotic WT1 regulator [Source:VGNC Symbol;Acc:VGNC:91191]	5.88	0.0861
ssc-miR-30c-5p	PAX3	paired box 3 [Source:VGNC Symbol;Acc:VGNC:96468]	5.88	0.0861
ssc-miR-30c-5p	PAX9	paired box 9 [Source:HGNC Symbol;Acc:HGNC:8623]	5.88	0.0861
ssc-miR-30c-5p	PAXBP1	PAX3 and PAX7 binding protein 1 [Source:VGNC Symbol;Acc:VGNC:91197]	5.88	0.0861
ssc-miR-30c-5p	PBRM1	polybromo 1 [Source:VGNC Symbol;Acc:VGNC:91202]	5.88	0.0861
ssc-miR-30c-5p	PCDH10	protocadherin 10 [Source:HGNC Symbol;Acc:HGNC:13404]	5.88	0.0861
ssc-miR-30c-5p	PCDH17	protocadherin 17 [Source:VGNC Symbol;Acc:VGNC:91214]	5.88	0.0861
ssc-miR-30c-5p	PCDH19	protocadherin 19 [Source:VGNC Symbol;Acc:VGNC:91216]	5.88	0.0861
ssc-miR-30c-5p	PCDH20	protocadherin 20 [Source:HGNC Symbol;Acc:HGNC:14257]	5.88	0.0861
ssc-miR-30c-5p	PCGF3	polycomb group ring finger 3 [Source:VGNC Symbol;Acc:VGNC:91220]	5.88	0.0861
ssc-miR-30c-5p	PCGF5	hypothetical gene	5.88	0.0861
ssc-miR-30c-5p	PCNXL2	hypothetical gene	5.88	0.0861
ssc-miR-30c-5p	PDAP1	PDGFA associated protein 1 [Source:VGNC Symbol;Acc:VGNC:91239]	5.88	0.0861
ssc-miR-30c-5p	PDCD10	programmed cell death 10 [Source:VGNC Symbol;Acc:VGNC:91241]	5.88	0.0861
ssc-miR-30c-5p	PDCL	phosducin like [Source:VGNC Symbol;Acc:VGNC:91245]	5.88	0.0861
ssc-miR-30c-5p	PDE3A	phosphodiesterase 3A [Source:VGNC Symbol;Acc:VGNC:91252]	5.88	0.0861
ssc-miR-30c-5p	PDE4D	phosphodiesterase 4D [Source:VGNC Symbol;Acc:VGNC:91256]	5.88	0.0861
ssc-miR-30c-5p	PDE5A	phosphodiesterase 5A [Source:VGNC Symbol;Acc:VGNC:91257]	5.88	0.0861
ssc-miR-30c-5p	PDE7A	phosphodiesterase 7A [Source:VGNC Symbol;Acc:VGNC:91261]	5.88	0.0861
ssc-miR-30c-5p	PDGFRB	platelet derived growth factor receptor beta [Source:VGNC Symbol;Acc:VGNC:91268]	5.88	0.0861
ssc-miR-30c-5p	PDP2	pyruvate dehyrogenase phosphatase catalytic subunit 2 [Source:VGNC Symbol;Acc:VGNC:91287]	5.88	0.0861
ssc-miR-30c-5p	PDS5B	PDS5 cohesin associated factor B [Source:VGNC Symbol;Acc:VGNC:91290]	5.88	0.0861
ssc-miR-30c-5p	PDSS1	decaprenyl diphosphate synthase subunit 1 [Source:VGNC Symbol;Acc:VGNC:95858]	5.88	0.0861
ssc-miR-30c-5p	PDSS2	decaprenyl diphosphate synthase subunit 2 [Source:VGNC Symbol;Acc:VGNC:91291]	5.88	0.0861
ssc-miR-30c-5p	PEAK1	pseudopodium enriched atypical kinase 1 [Source:VGNC Symbol;Acc:VGNC:91300]	5.88	0.0861
ssc-miR-30c-5p	PELI1	pellino E3 ubiquitin protein ligase 1 [Source:VGNC Symbol;Acc:VGNC:91306]	5.88	0.0861
ssc-miR-30c-5p	PELI2	pellino E3 ubiquitin protein ligase family member 2 [Source:VGNC Symbol;Acc:VGNC:91307]	5.88	0.0861
ssc-miR-30c-5p	PER2	period circadian regulator 2 [Source:VGNC Symbol;Acc:VGNC:96478]	5.88	0.0861
ssc-miR-30c-5p	PER3	hypothetical gene	5.88	0.0861
ssc-miR-30c-5p	PEX26	peroxisomal biogenesis factor 26 [Source:HGNC Symbol;Acc:HGNC:22965]	5.88	0.0861
ssc-miR-30c-5p	PEX5L	peroxisomal biosis factor 5 like [Source:VGNC Symbol;Acc:VGNC:91329]	5.88	0.0861
ssc-miR-30c-5p	PFN2	hypothetical gene	5.88	0.0861
ssc-miR-30c-5p	PGGT1B	protein geranylgeranyltransferase type I subunit beta [Source:VGNC Symbol;Acc:VGNC:91350]	5.88	0.0861
ssc-miR-30c-5p	PGM1	hypothetical gene	5.88	0.0861
ssc-miR-30c-5p	PGM2L1	phosphoglucomutase 2 like 1 [Source:VGNC Symbol;Acc:VGNC:91357]	5.88	0.0861
ssc-miR-30c-5p	PGM3	phosphoglucomutase 3 [Source:VGNC Symbol;Acc:VGNC:103150]	5.88	0.0861
ssc-miR-30c-5p	PGP	phosphoglycolate phosphatase [Source:VGNC Symbol;Acc:VGNC:91359]	5.88	0.0861
ssc-miR-30c-5p	PGR	progesterone receptor [Source:VGNC Symbol;Acc:VGNC:91362]	5.88	0.0861
ssc-miR-30c-5p	PHACTR2	phosphatase and actin regulator 2 [Source:VGNC Symbol;Acc:VGNC:91366]	5.88	0.0861
ssc-miR-30c-5p	PHF13	PHD finger protein 13 [Source:VGNC Symbol;Acc:VGNC:91379]	5.88	0.0861
ssc-miR-30c-5p	PHF16	hypothetical gene	5.88	0.0861
ssc-miR-30c-5p	PHF6	PHD finger protein 6 [Source:VGNC Symbol;Acc:VGNC:91389]	5.88	0.0861
ssc-miR-30c-5p	PHIP	pleckstrin homology domain interacting protein [Source:VGNC Symbol;Acc:VGNC:91392]	5.88	0.0861
ssc-miR-30c-5p	PHKA2	phosphorylase kinase regulatory subunit alpha 2 [Source:VGNC Symbol;Acc:VGNC:91394]	5.88	0.0861
ssc-miR-30c-5p	PHLDB2	pleckstrin homology like domain family B member 2 [Source:VGNC Symbol;Acc:VGNC:91399]	5.88	0.0861
ssc-miR-30c-5p	PHTF2	putative homeodomain transcription factor 2 [Source:VGNC Symbol;Acc:VGNC:91405]	5.88	0.0861

ssc-miR-30c-5p	PI4K2B	phosphatidylinositol 4-kinase type 2 beta [Source:VGNC Symbol;Acc:VGNC:98190]	5.88	0.0861
ssc-miR-30c-5p	PIAS1	protein inhibitor of activated STAT 1 [Source:VGNC Symbol;Acc:VGNC:91410]	5.88	0.0861
ssc-miR-30c-5p	PICALM	phosphatidylinositol binding clathrin assembly protein [Source:VGNC Symbol;Acc:VGNC:91415]	5.88	0.0861
ssc-miR-30c-5p	PIEZO2	piezo type mechanosensitive ion channel component 2 [Source:HGNC Symbol;Acc:HGNC:26270]	5.88	0.0861
ssc-miR-30c-5p	PIGA	phosphatidylinositol glycan anchor biosynthesis class A [Source:VGNC Symbol;Acc:VGNC:91419]	5.88	0.0861
ssc-miR-30c-5p	PIGV	phosphatidylinositol glycan anchor biosynthesis class V [Source:VGNC Symbol;Acc:VGNC:91430]	5.88	0.0861
ssc-miR-30c-5p	PIGX	phosphatidylinositol glycan anchor biosynthesis class X [Source:VGNC Symbol;Acc:VGNC:91432]	5.88	0.0861
ssc-miR-30c-5p	PIK3CD	phosphatidylinositol-4,5-bisphosphate 3-kinase catalytic subunit delta [Source:VGNC Symbol;Acc:VGNC:91442]	5.88	0.0861
ssc-miR-30c-5p	PIK3R2	phosphoinositide-3-kinase regulatory subunit 2 [Source:VGNC Symbol;Acc:VGNC:100324]	5.88	0.0861
ssc-miR-30c-5p	PIN4	peptidylprolyl cis/trans isomerase, NIMA-interacting 4 [Source:VGNC Symbol;Acc:VGNC:91452]	5.88	0.0861
ssc-miR-30c-5p	PIP4K2A	phosphatidylinositol-5-phosphate 4-kinase type 2 alpha [Source:VGNC Symbol;Acc:VGNC:96485]	5.88	0.0861
ssc-miR-30c-5p	PIP4K2B	phosphatidylinositol-5-phosphate 4-kinase type 2 beta [Source:VGNC Symbol;Acc:VGNC:91454]	5.88	0.0861
ssc-miR-30c-5p	PIRT	phosphoinositide interacting regulator of transient receptor potential channels [Source:VGNC Symbol;Acc:VGNC:91461]	5.88	0.0861
ssc-miR-30c-5p	PITPNB	phosphatidylinositol transfer protein beta [Source:VGNC Symbol;Acc:VGNC:104026]	5.88	0.0861
ssc-miR-30c-5p	PITPNC1	phosphatidylinositol transfer protein cytoplasmic 1 [Source:NCBI gene (formerly Entrezgene);Acc:100233199]	5.88	0.0861
ssc-miR-30c-5p	PITPNM2	phosphatidylinositol transfer protein membrane associated 2 [Source:VGNC Symbol;Acc:VGNC:91466]	5.88	0.0861
ssc-miR-30c-5p	PITPNM3	PITPNM family member 3 [Source:HGNC Symbol;Acc:HGNC:21043]	5.88	0.0861
ssc-miR-30c-5p	PITX1	paired like homeodomain 1 [Source:NCBI gene (formerly Entrezgene);Acc:100689266]	5.88	0.0861
ssc-miR-30c-5p	PKHD1	PKHD1 ciliary IPT domain containing fibrocystin/polyductin [Source:VGNC Symbol;Acc:VGNC:91476]	5.88	0.0861
ssc-miR-30c-5p	PKNOX2	PBX/knotted 1 homeobox 2 [Source:VGNC Symbol;Acc:VGNC:91485]	5.88	0.0861
ssc-miR-30c-5p	PLA2G12A	phospholipase A2 group XIIA [Source:HGNC Symbol;Acc:HGNC:18554]	5.88	0.0861
ssc-miR-30c-5p	PLAG1	PLAG1 zinc finger [Source:VGNC Symbol;Acc:VGNC:91509]	5.88	0.0861
ssc-miR-30c-5p	PLAGL2	PLAG1 like zinc finger 2 [Source:VGNC Symbol;Acc:VGNC:96490]	5.88	0.0861
ssc-miR-30c-5p	PLCG1	phospholipase C gamma 1 [Source:VGNC Symbol;Acc:VGNC:96492]	5.88	0.0861
ssc-miR-30c-5p	PLCXD3	phosphatidylinositol specific phospholipase C X domain containing 3 [Source:VGNC Symbol;Acc:VGNC:91524]	5.88	0.0861
ssc-miR-30c-5p	PLEKHA6	pleckstrin homology domain containing A6 [Source:VGNC Symbol;Acc:VGNC:91536]	5.88	0.0861
ssc-miR-30c-5p	PLEKHM3	pleckstrin homology domain containing M3 [Source:VGNC Symbol;Acc:VGNC:96281]	5.88	0.0861
ssc-miR-30c-5p	PLEKHO2	pleckstrin homology domain containing O2 [Source:HGNC Symbol;Acc:HGNC:30026]	5.88	0.0861
ssc-miR-30c-5p	PLS1	plastin 1 [Source:VGNC Symbol;Acc:VGNC:91572]	5.88	0.0861
ssc-miR-30c-5p	PLXNA1	plexin A1 [Source:VGNC Symbol;Acc:VGNC:91579]	5.88	0.0861
ssc-miR-30c-5p	PLXNA2	plexin A2 [Source:VGNC Symbol;Acc:VGNC:91580]	5.88	0.0861
ssc-miR-30c-5p	PLXNC1	plexin C1 [Source:VGNC Symbol;Acc:VGNC:91584]	5.88	0.0861
ssc-miR-30c-5p	PMEPA1	prostate transmembrane protein, androgen induced 1 [Source:VGNC Symbol;Acc:VGNC:95606]	5.88	0.0861
ssc-miR-30c-5p	PNN	pinin, desmosome associated protein [Source:VGNC Symbol;Acc:VGNC:91603]	5.88	0.0861
ssc-miR-30c-5p	PNPLA2	patatin like phospholipase domain containing 2 [Source:VGNC Symbol;Acc:VGNC:91607]	5.88	0.0861
ssc-miR-30c-5p	POC1B	POC1 centriolar protein B [Source:VGNC Symbol;Acc:VGNC:91616]	5.88	0.0861
ssc-miR-30c-5p	POGLUT1	protein O-glucosyltransferase 1 [Source:VGNC Symbol;Acc:VGNC:91623]	5.88	0.0861
ssc-miR-30c-5p	POLE3	DNA polymerase epsilon 3, accessory subunit [Source:VGNC Symbol;Acc:VGNC:103154]	5.88	0.0861
ssc-miR-30c-5p	POLR1D	RNA polymerase I and III subunit D [Source:VGNC Symbol;Acc:VGNC:104031]	5.88	0.0861
ssc-miR-30c-5p	POLR3E	RNA polymerase III subunit E [Source:VGNC Symbol;Acc:VGNC:91657]	5.88	0.0861
ssc-miR-30c-5p	POLR3G	hypothetical gene	5.88	0.0861
ssc-miR-30c-5p	PON2	paraoxonase 2 [Source:VGNC Symbol;Acc:VGNC:91662]	5.88	0.0861
ssc-miR-30c-5p	POP1	POP1 homolog, ribonuclease P/MRP subunit [Source:VGNC Symbol;Acc:VGNC:91663]	5.88	0.0861
ssc-miR-30c-5p	POU3F2	POU class 3 homeobox 2 [Source:HGNC Symbol;Acc:HGNC:9215]	5.88	0.0861
ssc-miR-30c-5p	POU4F2	POU class 4 homeobox 2 [Source:VGNC Symbol;Acc:VGNC:91679]	5.88	0.0861
ssc-miR-30c-5p	PPA1	inorganic pyrophosphatase 1 [Source:VGNC Symbol;Acc:VGNC:96757]	5.88	0.0861
ssc-miR-30c-5p	PPAPDC2	hypothetical gene	5.88	0.0861
ssc-miR-30c-5p	PPARGC1A	PPARG coactivator 1 alpha [Source:VGNC Symbol;Acc:VGNC:91685]	5.88	0.0861
ssc-miR-30c-5p	PPARGC1B	PPARG coactivator 1 beta [Source:VGNC Symbol;Acc:VGNC:91686]	5.88	0.0861

ssc-miR-30c-5p	PPFIA2	PTPRF interacting protein alpha 2 [Source:VGNC Symbol;Acc:VGNC:91692]	5.88	0.0861
ssc-miR-30c-5p	PPID	peptidylprolyl isomerase D [Source:VGNC Symbol;Acc:VGNC:98212]	5.88	0.0861
ssc-miR-30c-5p	PPIL3	peptidylprolyl isomerase like 3 [Source:VGNC Symbol;Acc:VGNC:103073]	5.88	0.0861
ssc-miR-30c-5p	PPIL4	peptidylprolyl isomerase like 4 [Source:VGNC Symbol;Acc:VGNC:91699]	5.88	0.0861
ssc-miR-30c-5p	PPIP5K2	diphosphoinositol pentakisphosphate kinase 2 [Source:VGNC Symbol;Acc:VGNC:91701]	5.88	0.0861
ssc-miR-30c-5p	PPM1A	protein phosphatase, Mg2+/Mn2+ dependent 1A [Source:VGNC Symbol;Acc:VGNC:91703]	5.88	0.0861
ssc-miR-30c-5p	PPM1E	protein phosphatase, Mg2+/Mn2+ dependent 1E [Source:VGNC Symbol;Acc:VGNC:91705]	5.88	0.0861
ssc-miR-30c-5p	PPP1R12A	protein phosphatase 1 regulatory subunit 12A [Source:VGNC Symbol;Acc:VGNC:91719]	5.88	0.0861
ssc-miR-30c-5p	PPP1R14C	protein phosphatase 1 regulatory inhibitor subunit 14C [Source:VGNC Symbol;Acc:VGNC:91724]	5.88	0.0861
ssc-miR-30c-5p	PPP1R18	protein phosphatase 1 regulatory subunit 18 [Source:VGNC Symbol;Acc:VGNC:104034]	5.88	0.0861
ssc-miR-30c-5p	PPP1R2	hypothetical gene	5.88	0.0861
ssc-miR-30c-5p	PPP1R9A	protein phosphatase 1 regulatory subunit 9A [Source:VGNC Symbol;Acc:VGNC:91743]	5.88	0.0861
ssc-miR-30c-5p	PPP2R1B	protein phosphatase 2 scaffold subunit Abeta [Source:VGNC Symbol;Acc:VGNC:91747]	5.88	0.0861
ssc-miR-30c-5p	PPP2R4	hypothetical gene	5.88	0.0861
ssc-miR-30c-5p	PPP2R5E	protein phosphatase 2 regulatory subunit B'epsilon [Source:VGNC Symbol;Acc:VGNC:91754]	5.88	0.0861
ssc-miR-30c-5p	PPP3CA	protein phosphatase 3 catalytic subunit alpha [Source:VGNC Symbol;Acc:VGNC:98218]	5.88	0.0861
ssc-miR-30c-5p	PPP3CB	protein phosphatase 3 catalytic subunit beta [Source:VGNC Symbol;Acc:VGNC:104035]	5.88	0.0861
ssc-miR-30c-5p	PPP3R1	protein phosphatase 3 regulatory subunit B, alpha [Source:VGNC Symbol;Acc:VGNC:106453]	5.88	0.0861
ssc-miR-30c-5p	PPP6C	protein phosphatase 6 catalytic subunit [Source:VGNC Symbol;Acc:VGNC:98219]	5.88	0.0861
ssc-miR-30c-5p	PPTC7	protein phosphatase targeting COQ7 [Source:VGNC Symbol;Acc:VGNC:91765]	5.88	0.0861
ssc-miR-30c-5p	PRCD	photoreceptor disc component [Source:VGNC Symbol;Acc:VGNC:91770]	5.88	0.0861
ssc-miR-30c-5p	PRDM1	PR/SET domain 1 [Source:VGNC Symbol;Acc:VGNC:91772]	5.88	0.0861
ssc-miR-30c-5p	PRDM5	PR/SET domain 5 [Source:VGNC Symbol;Acc:VGNC:98953]	5.88	0.0861
ssc-miR-30c-5p	PRICKLE1	prickle planar cell polarity protein 1 [Source:VGNC Symbol;Acc:VGNC:91793]	5.88	0.0861
ssc-miR-30c-5p	PRKAA2	protein kinase AMP-activated catalytic subunit alpha 2 [Source:VGNC Symbol;Acc:VGNC:91798]	5.88	0.0861
ssc-miR-30c-5p	PRKAR1A	protein kinase cAMP-dependent type I regulatory subunit alpha [Source:VGNC Symbol;Acc:VGNC:91802]	5.88	0.0861
ssc-miR-30c-5p	PRKRIR	hypothetical gene	5.88	0.0861
ssc-miR-30c-5p	PRLR	prolactin receptor [Source:VGNC Symbol;Acc:VGNC:91819]	5.88	0.0861
ssc-miR-30c-5p	PROM1	prominin 1 [Source:VGNC Symbol;Acc:VGNC:91832]	5.88	0.0861
ssc-miR-30c-5p	PROSER1	proline and serine rich 1 [Source:VGNC Symbol;Acc:VGNC:91836]	5.88	0.0861
ssc-miR-30c-5p	PRPF40A	pre-mRNA processing factor 40 homolog A [Source:VGNC Symbol;Acc:VGNC:98222]	5.88	0.0861
ssc-miR-30c-5p	PRR14L	proline rich 14 like [Source:VGNC Symbol;Acc:VGNC:91853]	5.88	0.0861
ssc-miR-30c-5p	PRRG1	proline rich and Gla domain 1 [Source:VGNC Symbol;Acc:VGNC:101494]	5.88	0.0861
ssc-miR-30c-5p	PRRT2	proline rich transmembrane protein 2 [Source:HGNC Symbol;Acc:HGNC:30500]	5.88	0.0861
ssc-miR-30c-5p	PRRX1	paired related homeobox 1 [Source:VGNC Symbol;Acc:VGNC:91875]	5.88	0.0861
ssc-miR-30c-5p	PRUNE2	hypothetical gene	5.88	0.0861
ssc-miR-30c-5p	PSD3	pleckstrin and Sec7 domain containing 3 [Source:VGNC Symbol;Acc:VGNC:107166]	5.88	0.0861
ssc-miR-30c-5p	PSEN2	presenilin 2 [Source:VGNC Symbol;Acc:VGNC:96508]	5.88	0.0861
ssc-miR-30c-5p	PSMD7	proteasome 26S subunit, non-ATPase 7 [Source:VGNC Symbol;Acc:VGNC:91925]	5.88	0.0861
ssc-miR-30c-5p	PSMD9	proteasome 26S subunit, non-ATPase 9 [Source:HGNC Symbol;Acc:HGNC:9567]	5.88	0.0861
ssc-miR-30c-5p	PSME3	proteasome activator subunit 3 [Source:VGNC Symbol;Acc:VGNC:91926]	5.88	0.0861
ssc-miR-30c-5p	PTAFR	platelet activating factor receptor [Source:VGNC Symbol;Acc:VGNC:98224]	5.88	0.0861
ssc-miR-30c-5p	PTBP3	polypyrimidine tract binding protein 3 [Source:VGNC Symbol;Acc:VGNC:91938]	5.88	0.0861
ssc-miR-30c-5p	PTCHD1	patched domain containing 1 [Source:VGNC Symbol;Acc:VGNC:91942]	5.88	0.0861
ssc-miR-30c-5p	PTGDR	prostaglandin D2 receptor [Source:HGNC Symbol;Acc:HGNC:9591]	5.88	0.0861
ssc-miR-30c-5p	PTGFRN	prostaglandin F2 receptor inhibitor [Source:VGNC Symbol;Acc:VGNC:91955]	5.88	0.0861
ssc-miR-30c-5p	PTP4A1	protein tyrosine phosphatase 4A1 [Source:HGNC Symbol;Acc:HGNC:9634]	5.88	0.0861
ssc-miR-30c-5p	PTPDC1	protein tyrosine phosphatase domain containing 1 [Source:VGNC Symbol;Acc:VGNC:91970]	5.88	0.0861
ssc-miR-30c-5p	PTPN13	protein tyrosine phosphatase non-receptor type 13 [Source:VGNC Symbol;Acc:VGNC:91974]	5.88	0.0861

ssc-miR-30c-5p	PTPN21	protein tyrosine phosphatase non-receptor type 21 [Source:VGNC Symbol;Acc:VGNC:91977]	5.88	0.0861
ssc-miR-30c-5p	PTPN4	protein tyrosine phosphatase non-receptor type 4 [Source:VGNC Symbol;Acc:VGNC:96521]	5.88	0.0861
ssc-miR-30c-5p	PTPN9	protein tyrosine phosphatase non-receptor type 9 [Source:VGNC Symbol;Acc:VGNC:91983]	5.88	0.0861
ssc-miR-30c-5p	PTPRK	protein tyrosine phosphatase receptor type K [Source:VGNC Symbol;Acc:VGNC:91991]	5.88	0.0861
ssc-miR-30c-5p	PUM2	pumilio RNA binding family member 2 [Source:VGNC Symbol;Acc:VGNC:92002]	5.88	0.0861
ssc-miR-30c-5p	PVRL3	hypothetical gene	5.88	0.0861
ssc-miR-30c-5p	PXK	PX domain containing serine/threonine kinase like [Source:VGNC Symbol;Acc:VGNC:92015]	5.88	0.0861
ssc-miR-30c-5p	PXN	paxillin [Source:HGNC Symbol;Acc:HGNC:9718]	5.88	0.0861
ssc-miR-30c-5p	PYGO1	pygopus family PHD finger 1 [Source:VGNC Symbol;Acc:VGNC:98231]	5.88	0.0861
ssc-miR-30c-5p	QKI	QKI, KH domain containing RNA binding [Source:VGNC Symbol;Acc:VGNC:92025]	5.88	0.0861
ssc-miR-30c-5p	R3HDM1	R3H domain containing 1 [Source:VGNC Symbol;Acc:VGNC:96526]	5.88	0.0861
ssc-miR-30c-5p	RAB10	RAB10, member RAS onco family [Source:VGNC Symbol;Acc:VGNC:98235]	5.88	0.0861
ssc-miR-30c-5p	RAB11A	RAB11A, member RAS onco family [Source:VGNC Symbol;Acc:VGNC:98236]	5.88	0.0861
ssc-miR-30c-5p	RAB15	RAB15, member RAS onco family [Source:VGNC Symbol;Acc:VGNC:98239]	5.88	0.0861
ssc-miR-30c-5p	RAB22A	RAB22A, member RAS onco family [Source:VGNC Symbol;Acc:VGNC:98244]	5.88	0.0861
ssc-miR-30c-5p	RAB23	RAB23, member RAS onco family [Source:VGNC Symbol;Acc:VGNC:98245]	5.88	0.0861
ssc-miR-30c-5p	RAB27B	RAB27B, member RAS onco family [Source:VGNC Symbol;Acc:VGNC:98250]	5.88	0.0861
ssc-miR-30c-5p	RAB32	RAB32, member RAS onco family [Source:NCBI gene (formerly Entrezgene);Acc:100144496]	5.88	0.0861
ssc-miR-30c-5p	RAB33B	RAB33B, member RAS onco family [Source:VGNC Symbol;Acc:VGNC:98257]	5.88	0.0861
ssc-miR-30c-5p	RAB38	RAB38, member RAS onco family [Source:VGNC Symbol;Acc:VGNC:98261]	5.88	0.0861
ssc-miR-30c-5p	RAB3D	RAB3D, member RAS onco family [Source:VGNC Symbol;Acc:VGNC:98266]	5.88	0.0861
ssc-miR-30c-5p	RAB4A	RAB4A, member RAS onco family [Source:VGNC Symbol;Acc:VGNC:98270]	5.88	0.0861
ssc-miR-30c-5p	RAB4B	RAB4B, member RAS onco family [Source:VGNC Symbol;Acc:VGNC:98597]	5.88	0.0861
ssc-miR-30c-5p	RAB7A	RAB7A, member RAS onco family [Source:VGNC Symbol;Acc:VGNC:98273]	5.88	0.0861
ssc-miR-30c-5p	RAB8A	RAB8A, member RAS onco family [Source:VGNC Symbol;Acc:VGNC:98275]	5.88	0.0861
ssc-miR-30c-5p	RABGAP1L	RAB GTPase activating protein 1 like [Source:VGNC Symbol;Acc:VGNC:108611]	5.88	0.0861
ssc-miR-30c-5p	RAD23B	RAD23 homolog B, nucleotide excision repair protein [Source:VGNC Symbol;Acc:VGNC:103160]	5.88	0.0861
ssc-miR-30c-5p	RAD51L3-RFFL	hypothetical gene	5.88	0.0861
ssc-miR-30c-5p	RAD9B	RAD9 checkpoint clamp component B [Source:VGNC Symbol;Acc:VGNC:92063]	5.88	0.0861
ssc-miR-30c-5p	RAI14	retinoic acid induced 14 [Source:VGNC Symbol;Acc:VGNC:92068]	5.88	0.0861
ssc-miR-30c-5p	RALGDS	ral guanine nucleotide dissociation stimulator [Source:HGNC Symbol;Acc:HGNC:9842]	5.88	0.0861
ssc-miR-30c-5p	RALGPS1	Ral GEF with PH domain and SH3 binding motif 1 [Source:VGNC Symbol;Acc:VGNC:92072]	5.88	0.0861
ssc-miR-30c-5p	RALGPS2	Ral GEF with PH domain and SH3 binding motif 2 [Source:VGNC Symbol;Acc:VGNC:92073]	5.88	0.0861
ssc-miR-30c-5p	RANBP10	RAN binding protein 10 [Source:VGNC Symbol;Acc:VGNC:92077]	5.88	0.0861
ssc-miR-30c-5p	RANBP9	RAN binding protein 9 [Source:VGNC Symbol;Acc:VGNC:92082]	5.88	0.0861
ssc-miR-30c-5p	RAP1B	RAP1B, member of RAS oncogene family [Source:HGNC Symbol;Acc:HGNC:9857]	5.88	0.0861
ssc-miR-30c-5p	RAP2A	RAP2A, member of RAS onco family [Source:VGNC Symbol;Acc:VGNC:92088]	5.88	0.0861
ssc-miR-30c-5p	RAP2B	RAP2B, member of RAS onco family [Source:VGNC Symbol;Acc:VGNC:92089]	5.88	0.0861
ssc-miR-30c-5p	RAP2C	RAP2C, member of RAS onco family [Source:VGNC Symbol;Acc:VGNC:92090]	5.88	0.0861
ssc-miR-30c-5p	RAPGEF2	Rap guanine nucleotide exchange factor 2 [Source:VGNC Symbol;Acc:VGNC:92092]	5.88	0.0861
ssc-miR-30c-5p	RAPGEF4	Rap guanine nucleotide exchange factor 4 [Source:VGNC Symbol;Acc:VGNC:95814]	5.88	0.0861
ssc-miR-30c-5p	RAPH1	hypothetical gene	5.88	0.0861
ssc-miR-30c-5p	RARB	retinoic acid receptor beta [Source:HGNC Symbol;Acc:HGNC:9865]	5.88	0.0861
ssc-miR-30c-5p	RARG	retinoic acid receptor gamma [Source:HGNC Symbol;Acc:HGNC:9866]	5.88	0.0861
ssc-miR-30c-5p	RASA1	RAS p21 protein activator 1 [Source:VGNC Symbol;Acc:VGNC:92101]	5.88	0.0861
ssc-miR-30c-5p	RASA2	RAS p21 protein activator 2 [Source:VGNC Symbol;Acc:VGNC:92102]	5.88	0.0861
ssc-miR-30c-5p	RASAL2	RAS protein activator like 2 [Source:VGNC Symbol;Acc:VGNC:92105]	5.88	0.0861
ssc-miR-30c-5p	RASD1	ras related dexamethasone induced 1 [Source:VGNC Symbol;Acc:VGNC:92107]	5.88	0.0861
ssc-miR-30c-5p	RASEF	hypothetical gene	5.88	0.0861

ssc-miR-30c-5p	RASGEF1A	RasGEF domain family member 1A [Source:VGNC Symbol;Acc:VGNC:92108]	5.88	0.0861
ssc-miR-30c-5p	RASGEF1B	RasGEF domain family member 1B [Source:VGNC Symbol;Acc:VGNC:92109]	5.88	0.0861
ssc-miR-30c-5p	RASGRF2	Ras protein specific guanine nucleotide releasing factor 2 [Source:VGNC Symbol;Acc:VGNC:92112]	5.88	0.0861
ssc-miR-30c-5p	RASGRP3	RAS guanyl releasing protein 3 [Source:VGNC Symbol;Acc:VGNC:92115]	5.88	0.0861
ssc-miR-30c-5p	RASL12	RAS like family 12 [Source:VGNC Symbol;Acc:VGNC:92121]	5.88	0.0861
ssc-miR-30c-5p	RASSF4	Ras association domain family member 4 [Source:NCBI gene (formerly Entrezgene);Acc:100152580]	5.88	0.0861
ssc-miR-30c-5p	RASSF8	Ras association domain family member 8 [Source:VGNC Symbol;Acc:VGNC:92128]	5.88	0.0861
ssc-miR-30c-5p	RAVER2	ribonucleoprotein, PTB binding 2 [Source:VGNC Symbol;Acc:VGNC:98283]	5.88	0.0861
ssc-miR-30c-5p	RBFOX1	RNA binding fox-1 homolog 1 [Source:VGNC Symbol;Acc:VGNC:92139]	5.88	0.0861
ssc-miR-30c-5p	RBM12	RNA binding motif protein 12 [Source:HGNC Symbol;Acc:HGNC:9898]	5.88	0.0861
ssc-miR-30c-5p	RBM15B	RNA binding motif protein 15B [Source:VGNC Symbol;Acc:VGNC:98285]	5.88	0.0861
ssc-miR-30c-5p	RCAN1	regulator of calcineurin 1 [Source:VGNC Symbol;Acc:VGNC:92170]	5.88	0.0861
ssc-miR-30c-5p	RCBTB1	RCC1 and BTB domain containing protein 1 [Source:VGNC Symbol;Acc:VGNC:92173]	5.88	0.0861
ssc-miR-30c-5p	RCC2	regulator of chromosome condensation 2 [Source:VGNC Symbol;Acc:VGNC:98547]	5.88	0.0861
ssc-miR-30c-5p	RCOR1	REST corepressor 1 [Source:VGNC Symbol;Acc:VGNC:92183]	5.88	0.0861
ssc-miR-30c-5p	RECK	reversion inducing cysteine rich protein with kazal motifs [Source:VGNC Symbol;Acc:VGNC:92194]	5.88	0.0861
ssc-miR-30c-5p	REEP1	receptor accessory protein 1 [Source:VGNC Symbol;Acc:VGNC:92197]	5.88	0.0861
ssc-miR-30c-5p	REEP3	receptor accessory protein 3 [Source:VGNC Symbol;Acc:VGNC:92199]	5.88	0.0861
ssc-miR-30c-5p	REV1	REV1 DNA directed polymerase [Source:HGNC Symbol;Acc:HGNC:14060]	5.88	0.0861
ssc-miR-30c-5p	REV3L	REV3 like, DNA directed polymerase zeta catalytic subunit [Source:VGNC Symbol;Acc:VGNC:92226]	5.88	0.0861
ssc-miR-30c-5p	RFFL	ring finger and FYVE like domain containing E3 ubiquitin protein ligase [Source:VGNC Symbol;Acc:VGNC:98289]	5.88	0.0861
ssc-miR-30c-5p	RFTN2	raftlin family member 2 [Source:VGNC Symbol;Acc:VGNC:96128]	5.88	0.0861
ssc-miR-30c-5p	RFX2	regulatory factor X2 [Source:VGNC Symbol;Acc:VGNC:92244]	5.88	0.0861
ssc-miR-30c-5p	RFX3	regulatory factor X3 [Source:VGNC Symbol;Acc:VGNC:92245]	5.88	0.0861
ssc-miR-30c-5p	RFX6	regulatory factor X6 [Source:VGNC Symbol;Acc:VGNC:92247]	5.88	0.0861
ssc-miR-30c-5p	RFX7	regulatory factor X7 [Source:VGNC Symbol;Acc:VGNC:92248]	5.88	0.0861
ssc-miR-30c-5p	RGL1	ral guanine nucleotide dissociation stimulator like 1 [Source:VGNC Symbol;Acc:VGNC:92252]	5.88	0.0861
ssc-miR-30c-5p	RGS17	regulator of G protein signaling 17 [Source:VGNC Symbol;Acc:VGNC:92263]	5.88	0.0861
ssc-miR-30c-5p	RGS9BP	regulator of G protein signaling 9 binding protein [Source:HGNC Symbol;Acc:HGNC:30304]	5.88	0.0861
ssc-miR-30c-5p	RHEBL1	RHEB like 1 [Source:VGNC Symbol;Acc:VGNC:92280]	5.88	0.0861
ssc-miR-30c-5p	RHOB	ras homolog family member B [Source:NCBI gene (formerly Entrezgene);Acc:100144503]	5.88	0.0861
ssc-miR-30c-5p	RIMBP2	RIMS binding protein 2 [Source:VGNC Symbol;Acc:VGNC:92306]	5.88	0.0861
ssc-miR-30c-5p	RIMKLB	ribosomal modification protein rimK like family member B [Source:VGNC Symbol;Acc:VGNC:92308]	5.88	0.0861
ssc-miR-30c-5p	RIMS1	regulating synaptic membrane exocytosis 1 [Source:VGNC Symbol;Acc:VGNC:92309]	5.88	0.0861
ssc-miR-30c-5p	RIOK3	RIO kinase 3 [Source:VGNC Symbol;Acc:VGNC:92316]	5.88	0.0861
ssc-miR-30c-5p	RLIM	ring finger protein, LIM domain interacting [Source:HGNC Symbol;Acc:HGNC:13429]	5.88	0.0861
ssc-miR-30c-5p	RNF115	ring finger protein 115 [Source:VGNC Symbol;Acc:VGNC:92348]	5.88	0.0861
ssc-miR-30c-5p	RNF122	ring finger protein 122 [Source:VGNC Symbol;Acc:VGNC:92350]	5.88	0.0861
ssc-miR-30c-5p	RNF157	ring finger protein 157 [Source:VGNC Symbol;Acc:VGNC:92365]	5.88	0.0861
ssc-miR-30c-5p	RNF165	ring finger protein 165 [Source:VGNC Symbol;Acc:VGNC:92366]	5.88	0.0861
ssc-miR-30c-5p	RNF169	ring finger protein 169 [Source:VGNC Symbol;Acc:VGNC:92368]	5.88	0.0861
ssc-miR-30c-5p	RNF213	hypothetical gene	5.88	0.0861
ssc-miR-30c-5p	RNF217	ring finger protein 217 [Source:VGNC Symbol;Acc:VGNC:103162]	5.88	0.0861
ssc-miR-30c-5p	RNF220	ring finger protein 220 [Source:VGNC Symbol;Acc:VGNC:98602]	5.88	0.0861
ssc-miR-30c-5p	RNF24	ring finger protein 24 [Source:HGNC Symbol;Acc:HGNC:13779]	5.88	0.0861
ssc-miR-30c-5p	RNF34	ring finger protein 34 [Source:VGNC Symbol;Acc:VGNC:92389]	5.88	0.0861
ssc-miR-30c-5p	RNF44	ring finger protein 44 [Source:VGNC Symbol;Acc:VGNC:92395]	5.88	0.0861
ssc-miR-30c-5p	RNF7	ring finger protein 7 [Source:VGNC Symbol;Acc:VGNC:92398]	5.88	0.0861
ssc-miR-30c-5p	RNMT	RNA guanine-7 methyltransferase [Source:VGNC Symbol;Acc:VGNC:92402]	5.88	0.0861

ssc-miR-30c-5p	ROCK2	Rho associated coiled-coil containing protein kinase 2 [Source:HGNC Symbol;Acc:HGNC:10252]	5.88	0.0861
ssc-miR-30c-5p	ROR1	receptor tyrosine kinase like orphan receptor 1 [Source:VGNC Symbol;Acc:VGNC:98295]	5.88	0.0861
ssc-miR-30c-5p	ROR2	receptor tyrosine kinase like orphan receptor 2 [Source:VGNC Symbol;Acc:VGNC:92407]	5.88	0.0861
ssc-miR-30c-5p	RORA	RAR related orphan receptor A [Source:VGNC Symbol;Acc:VGNC:92408]	5.88	0.0861
ssc-miR-30c-5p	RP1-170O19.20	hypothetical gene	5.88	0.0861
ssc-miR-30c-5p	RP11-160N1.10	hypothetical gene	5.88	0.0861
ssc-miR-30c-5p	RP11-497E19.2	hypothetical gene	5.88	0.0861
ssc-miR-30c-5p	RP11-766F14.2	hypothetical gene	5.88	0.0861
ssc-miR-30c-5p	RP13-996F3.4	hypothetical gene	5.88	0.0861
ssc-miR-30c-5p	RP13-996F3.5	hypothetical gene	5.88	0.0861
ssc-miR-30c-5p	RP6-24A23.6	hypothetical gene	5.88	0.0861
ssc-miR-30c-5p	RPA1	replication protein A1 [Source:VGNC Symbol;Acc:VGNC:92413]	5.88	0.0861
ssc-miR-30c-5p	RPL22L1	hypothetical gene	5.88	0.0861
ssc-miR-30c-5p	RPRD1A	regulation of nuclear pre-mRNA domain containing 1A [Source:VGNC Symbol;Acc:VGNC:92435]	5.88	0.0861
ssc-miR-30c-5p	RPRD2	regulation of nuclear pre-mRNA domain containing 2 [Source:VGNC Symbol;Acc:VGNC:92436]	5.88	0.0861
ssc-miR-30c-5p	RPS6KA2	ribosomal protein S6 kinase A2 [Source:VGNC Symbol;Acc:VGNC:92441]	5.88	0.0861
ssc-miR-30c-5p	RPS6KA5	ribosomal protein S6 kinase A5 [Source:VGNC Symbol;Acc:VGNC:92444]	5.88	0.0861
ssc-miR-30c-5p	RRAD	RRAD, Ras related glycolysis inhibitor and calcium channel regulator [Source:VGNC Symbol;Acc:VGNC:92454]	5.88	0.0861
ssc-miR-30c-5p	RSF1	remodeling and spacing factor 1 [Source:VGNC Symbol;Acc:VGNC:92476]	5.88	0.0861
ssc-miR-30c-5p	RSU1	Ras suppressor protein 1 [Source:VGNC Symbol;Acc:VGNC:98298]	5.88	0.0861
ssc-miR-30c-5p	RTCB	RNA 2',3'-cyclic phosphate and 5'-OH ligase [Source:VGNC Symbol;Acc:VGNC:92492]	5.88	0.0861
ssc-miR-30c-5p	RTKN2	rhotekin 2 [Source:VGNC Symbol;Acc:VGNC:92495]	5.88	0.0861
ssc-miR-30c-5p	RTN4R	reticulon 4 receptor [Source:HGNC Symbol;Acc:HGNC:18601]	5.88	0.0861
ssc-miR-30c-5p	RUNDC3B	RUN domain containing 3B [Source:VGNC Symbol;Acc:VGNC:92515]	5.88	0.0861
ssc-miR-30c-5p	RUNX1	RUNX family transcription factor 1 [Source:VGNC Symbol;Acc:VGNC:92516]	5.88	0.0861
ssc-miR-30c-5p	RUNX2	RUNX family transcription factor 2 [Source:VGNC Symbol;Acc:VGNC:92517]	5.88	0.0861
ssc-miR-30c-5p	RWDD4	RWD domain containing 4 [Source:VGNC Symbol;Acc:VGNC:96022]	5.88	0.0861
ssc-miR-30c-5p	S100PBP	S100P binding protein [Source:VGNC Symbol;Acc:VGNC:92549]	5.88	0.0861
ssc-miR-30c-5p	SACS	sacsin molecular chaperone [Source:HGNC Symbol;Acc:HGNC:10519]	5.88	0.0861
ssc-miR-30c-5p	SALL4	spalt like transcription factor 4 [Source:NCBI gene (formerly Entrezgene);Acc:100136902]	5.88	0.0861
ssc-miR-30c-5p	SAMD4A	sterile alpha motif domain containing 4A [Source:VGNC Symbol;Acc:VGNC:92569]	5.88	0.0861
ssc-miR-30c-5p	SAMD8	sterile alpha motif domain containing 8 [Source:VGNC Symbol;Acc:VGNC:92571]	5.88	0.0861
ssc-miR-30c-5p	SAP30	Sin3A associated protein 30 [Source:VGNC Symbol;Acc:VGNC:92575]	5.88	0.0861
ssc-miR-30c-5p	SAP30BP	SAP30 binding protein [Source:VGNC Symbol;Acc:VGNC:92576]	5.88	0.0861
ssc-miR-30c-5p	SAR1B	secretion associated Ras related GTPase 1B [Source:VGNC Symbol;Acc:VGNC:92580]	5.88	0.0861
ssc-miR-30c-5p	SATB1	SATB homeobox 1 [Source:VGNC Symbol;Acc:VGNC:92587]	5.88	0.0861
ssc-miR-30c-5p	SATB2	SATB homeobox 2 [Source:VGNC Symbol;Acc:VGNC:95972]	5.88	0.0861
ssc-miR-30c-5p	SBF1	SET binding factor 1 [Source:VGNC Symbol;Acc:VGNC:92593]	5.88	0.0861
ssc-miR-30c-5p	SBK1	SH3 domain binding kinase 1 [Source:HGNC Symbol;Acc:HGNC:17699]	5.88	0.0861
ssc-miR-30c-5p	SCAF4	SR-related CTD associated factor 4 [Source:VGNC Symbol;Acc:VGNC:92603]	5.88	0.0861
ssc-miR-30c-5p	SCAMP1	secretory carrier membrane protein 1 [Source:VGNC Symbol;Acc:VGNC:92605]	5.88	0.0861
ssc-miR-30c-5p	SCN1A	sodium voltage-gated channel alpha subunit 1 [Source:VGNC Symbol;Acc:VGNC:95478]	5.88	0.0861
ssc-miR-30c-5p	SCN2A	hypothetical gene	5.88	0.0861
ssc-miR-30c-5p	SCN3A	sodium voltage-gated channel alpha subunit 3 [Source:VGNC Symbol;Acc:VGNC:95479]	5.88	0.0861
ssc-miR-30c-5p	SCN8A	sodium voltage-gated channel alpha subunit 8 [Source:VGNC Symbol;Acc:VGNC:92638]	5.88	0.0861
ssc-miR-30c-5p	SCN9A	sodium voltage-gated channel alpha subunit 9 [Source:VGNC Symbol;Acc:VGNC:95481]	5.88	0.0861
ssc-miR-30c-5p	SCYL3	SCY1 like pseudokinase 3 [Source:VGNC Symbol;Acc:VGNC:92653]	5.88	0.0861
ssc-miR-30c-5p	SDAD1	SDA1 domain containing 1 [Source:VGNC Symbol;Acc:VGNC:98961]	5.88	0.0861
ssc-miR-30c-5p	SDC2	syndecan 2 [Source:VGNC Symbol;Acc:VGNC:92655]	5.88	0.0861

ssc-miR-30c-5p	SDCBP	syndecan binding protein [Source:VGNC Symbol;Acc:VGNC:92657]	5.88	0.0861
ssc-miR-30c-5p	SDK2	hypothetical gene	5.88	0.0861
ssc-miR-30c-5p	SEC14L2	SEC14 like lipid binding 2 [Source:NCBI gene (formerly Entrezgene);Acc:100152451]	5.88	0.0861
ssc-miR-30c-5p	SEC22A	SEC22 homolog A, vesicle trafficking protein [Source:VGNC Symbol;Acc:VGNC:92673]	5.88	0.0861
ssc-miR-30c-5p	SEC22C	SEC22 homolog C, vesicle trafficking protein [Source:VGNC Symbol;Acc:VGNC:92674]	5.88	0.0861
ssc-miR-30c-5p	SEC23A	hypothetical gene	5.88	0.0861
ssc-miR-30c-5p	SEC23IP	SEC23 interacting protein [Source:VGNC Symbol;Acc:VGNC:92675]	5.88	0.0861
ssc-miR-30c-5p	SEC24A	SEC24 homolog A, COPII coat complex component [Source:VGNC Symbol;Acc:VGNC:92676]	5.88	0.0861
ssc-miR-30c-5p	SEC61A2	SEC61 translocon subunit alpha 2 [Source:VGNC Symbol;Acc:VGNC:95457]	5.88	0.0861
ssc-miR-30c-5p	SEC62	SEC62 homolog, preprotein translocation factor [Source:VGNC Symbol;Acc:VGNC:108691]	5.88	0.0861
ssc-miR-30c-5p	SEL1L3	SEL1L family member 3 [Source:VGNC Symbol;Acc:VGNC:92688]	5.88	0.0861
ssc-miR-30c-5p	SEMA3A	semaphorin 3A [Source:VGNC Symbol;Acc:VGNC:92693]	5.88	0.0861
ssc-miR-30c-5p	SEMA6B	semaphorin 6B [Source:VGNC Symbol;Acc:VGNC:92709]	5.88	0.0861
ssc-miR-30c-5p	SEMA6D	semaphorin 6D [Source:VGNC Symbol;Acc:VGNC:92711]	5.88	0.0861
ssc-miR-30c-5p	SENP5	SUMO specific peptidase 5 [Source:VGNC Symbol;Acc:VGNC:92714]	5.88	0.0861
ssc-miR-30c-5p	SEPHS1	selenophosphate synthetase 1 [Source:VGNC Symbol;Acc:VGNC:104053]	5.88	0.0861
ssc-miR-30c-5p	SEPT3	hypothetical gene	5.88	0.0861
ssc-miR-30c-5p	SEPT7	hypothetical gene	5.88	0.0861
ssc-miR-30c-5p	SEPT8	hypothetical gene	5.88	0.0861
ssc-miR-30c-5p	SERPINE1	serpin family E member 1 [Source:VGNC Symbol;Acc:VGNC:98310]	5.88	0.0861
ssc-miR-30c-5p	SERPINF2	serpin family F member 2 [Source:VGNC Symbol;Acc:VGNC:92744]	5.88	0.0861
ssc-miR-30c-5p	SETD1B	SET domain containing 1B, histone lysine methyltransferase [Source:HGNC Symbol;Acc:HGNC:29187]	5.88	0.0861
ssc-miR-30c-5p	SETD3	SET domain containing 3, actin histidine methyltransferase [Source:VGNC Symbol;Acc:VGNC:92758]	5.88	0.0861
ssc-miR-30c-5p	SETD5	SET domain containing 5 [Source:VGNC Symbol;Acc:VGNC:92760]	5.88	0.0861
ssc-miR-30c-5p	SETD7	SET domain containing 7, histone lysine methyltransferase [Source:VGNC Symbol;Acc:VGNC:92762]	5.88	0.0861
ssc-miR-30c-5p	SETD9	SET domain containing 9 [Source:VGNC Symbol;Acc:VGNC:92763]	5.88	0.0861
ssc-miR-30c-5p	SFMBT1	Scm like with four mbt domains 1 [Source:VGNC Symbol;Acc:VGNC:92773]	5.88	0.0861
ssc-miR-30c-5p	SFXN1	sideroflexin 1 [Source:VGNC Symbol;Acc:VGNC:92783]	5.88	0.0861
ssc-miR-30c-5p	SGCB	sarcoglycan beta [Source:NCBI gene (formerly Entrezgene);Acc:100135674]	5.88	0.0861
ssc-miR-30c-5p	SGK3	serum/glucocorticoid regulated kinase family member 3 [Source:HGNC Symbol;Acc:HGNC:10812]	5.88	0.0861
ssc-miR-30c-5p	SGMS2	sphingomyelin synthase 2 [Source:VGNC Symbol;Acc:VGNC:92795]	5.88	0.0861
ssc-miR-30c-5p	SGSM1	small G protein signaling modulator 1 [Source:VGNC Symbol;Acc:VGNC:92800]	5.88	0.0861
ssc-miR-30c-5p	SH2B3	SH2B adaptor protein 3 [Source:VGNC Symbol;Acc:VGNC:92807]	5.88	0.0861
ssc-miR-30c-5p	SH3BGRL	hypothetical gene	5.88	0.0861
ssc-miR-30c-5p	SH3GLB1	SH3 domain containing GRB2 like, endophilin B1 [Source:VGNC Symbol;Acc:VGNC:92826]	5.88	0.0861
ssc-miR-30c-5p	SH3KBP1	SH3 domain containing kinase binding protein 1 [Source:VGNC Symbol;Acc:VGNC:92828]	5.88	0.0861
ssc-miR-30c-5p	SH3PXD2A	SH3 and PX domains 2A [Source:VGNC Symbol;Acc:VGNC:92829]	5.88	0.0861
ssc-miR-30c-5p	SH3RF1	SH3 domain containing ring finger 1 [Source:VGNC Symbol;Acc:VGNC:98316]	5.88	0.0861
ssc-miR-30c-5p	SHISA2	shisa family member 2 [Source:VGNC Symbol;Acc:VGNC:92845]	5.88	0.0861
ssc-miR-30c-5p	SHOC2	SHOC2 leucine rich repeat scaffold protein [Source:VGNC Symbol;Acc:VGNC:98318]	5.88	0.0861
ssc-miR-30c-5p	SHROOM1	shroom family member 1 [Source:VGNC Symbol;Acc:VGNC:92860]	5.88	0.0861
ssc-miR-30c-5p	SIAH2	siah E3 ubiquitin protein ligase 2 [Source:VGNC Symbol;Acc:VGNC:92866]	5.88	0.0861
ssc-miR-30c-5p	SIDT2	SID1 transmembrane family member 2 [Source:VGNC Symbol;Acc:VGNC:92869]	5.88	0.0861
ssc-miR-30c-5p	SIK3	SIK family kinase 3 [Source:VGNC Symbol;Acc:VGNC:92873]	5.88	0.0861
ssc-miR-30c-5p	SIM1	SIM bHLH transcription factor 1 [Source:VGNC Symbol;Acc:VGNC:92876]	5.88	0.0861
ssc-miR-30c-5p	SIRT1	sirtuin 1 [Source:VGNC Symbol;Acc:VGNC:92884]	5.88	0.0861
ssc-miR-30c-5p	SIX1	SIX homeobox 1 [Source:VGNC Symbol;Acc:VGNC:92892]	5.88	0.0861
ssc-miR-30c-5p	SIX4	SIX homeobox 4 [Source:VGNC Symbol;Acc:VGNC:92895]	5.88	0.0861
ssc-miR-30c-5p	SKIL	SKI like proto-onco [Source:VGNC Symbol;Acc:VGNC:92903]	5.88	0.0861

ssc-miR-30c-5p	SKP2	S-phase kinase associated protein 2 [Source:VGNC Symbol;Acc:VGNC:92907]	5.88	0.0861
ssc-miR-30c-5p	SLAIN2	SLAIN motif family member 2 [Source:VGNC Symbol;Acc:VGNC:92910]	5.88	0.0861
ssc-miR-30c-5p	SLC12A6	solute carrier family 12 member 6 [Source:VGNC Symbol;Acc:VGNC:92925]	5.88	0.0861
ssc-miR-30c-5p	SLC16A14	solute carrier family 16 member 14 [Source:VGNC Symbol;Acc:VGNC:96129]	5.88	0.0861
ssc-miR-30c-5p	SLC22A23	solute carrier family 22 member 23 [Source:HGNC Symbol;Acc:HGNC:21106]	5.88	0.0861
ssc-miR-30c-5p	SLC22A5	solute carrier family 22 member 5 [Source:HGNC Symbol;Acc:HGNC:10969]	5.88	0.0861
ssc-miR-30c-5p	SLC25A34	solute carrier family 25 member 34 [Source:VGNC Symbol;Acc:VGNC:93010]	5.88	0.0861
ssc-miR-30c-5p	SLC25A36	solute carrier family 25 member 36 [Source:VGNC Symbol;Acc:VGNC:93012]	5.88	0.0861
ssc-miR-30c-5p	SLC29A3	solute carrier family 29 member 3 [Source:VGNC Symbol;Acc:VGNC:93044]	5.88	0.0861
ssc-miR-30c-5p	SLC30A10	hypothetical gene	5.88	0.0861
ssc-miR-30c-5p	SLC30A2	solute carrier family 30 member 2 [Source:VGNC Symbol;Acc:VGNC:93056]	5.88	0.0861
ssc-miR-30c-5p	SLC30A4	solute carrier family 30 member 4 [Source:VGNC Symbol;Acc:VGNC:93058]	5.88	0.0861
ssc-miR-30c-5p	SLC30A5	solute carrier family 30 member 5 [Source:VGNC Symbol;Acc:VGNC:93059]	5.88	0.0861
ssc-miR-30c-5p	SLC35A3	solute carrier family 35 member A3 [Source:VGNC Symbol;Acc:VGNC:98860]	5.88	0.0861
ssc-miR-30c-5p	SLC35A5	solute carrier family 35 member A5 [Source:VGNC Symbol;Acc:VGNC:93070]	5.88	0.0861
ssc-miR-30c-5p	SLC35B4	solute carrier family 35 member B4 [Source:VGNC Symbol;Acc:VGNC:93074]	5.88	0.0861
ssc-miR-30c-5p	SLC35C1	solute carrier family 35 member C1 [Source:VGNC Symbol;Acc:VGNC:93075]	5.88	0.0861
ssc-miR-30c-5p	SLC35D3	solute carrier family 35 member D3 [Source:VGNC Symbol;Acc:VGNC:93076]	5.88	0.0861
ssc-miR-30c-5p	SLC35F1	solute carrier family 35 member F1 [Source:VGNC Symbol;Acc:VGNC:93081]	5.88	0.0861
ssc-miR-30c-5p	SLC35F3	solute carrier family 35 member F3 [Source:VGNC Symbol;Acc:VGNC:93083]	5.88	0.0861
ssc-miR-30c-5p	SLC35F4	solute carrier family 35 member F4 [Source:VGNC Symbol;Acc:VGNC:93084]	5.88	0.0861
ssc-miR-30c-5p	SLC36A1	solute carrier family 36 member 1 [Source:VGNC Symbol;Acc:VGNC:93088]	5.88	0.0861
ssc-miR-30c-5p	SLC38A1	solute carrier family 38 member 1 [Source:HGNC Symbol;Acc:HGNC:13447]	5.88	0.0861
ssc-miR-30c-5p	SLC38A2	solute carrier family 38 member 2 [Source:VGNC Symbol;Acc:VGNC:93094]	5.88	0.0861
ssc-miR-30c-5p	SLC38A4	solute carrier family 38 member 4 [Source:VGNC Symbol;Acc:VGNC:93096]	5.88	0.0861
ssc-miR-30c-5p	SLC38A7	solute carrier family 38 member 7 [Source:HGNC Symbol;Acc:HGNC:25582]	5.88	0.0861
ssc-miR-30c-5p	SLC39A11	solute carrier family 39 member 11 [Source:VGNC Symbol;Acc:VGNC:93102]	5.88	0.0861
ssc-miR-30c-5p	SLC41A2	solute carrier family 41 member 2 [Source:VGNC Symbol;Acc:VGNC:93114]	5.88	0.0861
ssc-miR-30c-5p	SLC4A7	solute carrier family 4 member 7 [Source:VGNC Symbol;Acc:VGNC:93135]	5.88	0.0861
ssc-miR-30c-5p	SLC5A11	solute carrier family 5 member 11 [Source:VGNC Symbol;Acc:VGNC:93141]	5.88	0.0861
ssc-miR-30c-5p	SLC5A3	solute carrier family 5 member 3 [Source:VGNC Symbol;Acc:VGNC:93144]	5.88	0.0861
ssc-miR-30c-5p	SLC6A15	solute carrier family 6 member 15 [Source:VGNC Symbol;Acc:VGNC:93157]	5.88	0.0861
ssc-miR-30c-5p	SLC6A3	solute carrier family 6 member 3 [Source:VGNC Symbol;Acc:VGNC:93163]	5.88	0.0861
ssc-miR-30c-5p	SLC6A6	solute carrier family 6 member 6 [Source:VGNC Symbol;Acc:VGNC:93166]	5.88	0.0861
ssc-miR-30c-5p	SLC6A9	solute carrier family 6 member 9 [Source:VGNC Symbol;Acc:VGNC:93168]	5.88	0.0861
ssc-miR-30c-5p	SLC7A10	solute carrier family 7 member 10 [Source:VGNC Symbol;Acc:VGNC:93170]	5.88	0.0861
ssc-miR-30c-5p	SLC7A11	solute carrier family 7 member 11 [Source:VGNC Symbol;Acc:VGNC:93171]	5.88	0.0861
ssc-miR-30c-5p	SLC7A6	hypothetical gene	5.88	0.0861
ssc-miR-30c-5p	SLC9A4	solute carrier family 9 member A4 [Source:VGNC Symbol;Acc:VGNC:93186]	5.88	0.0861
ssc-miR-30c-5p	SLC9A8	solute carrier family 9 member A8 [Source:VGNC Symbol;Acc:VGNC:95943]	5.88	0.0861
ssc-miR-30c-5p	SMAD1	SMAD family member 1 [Source:VGNC Symbol;Acc:VGNC:93216]	5.88	0.0861
ssc-miR-30c-5p	SMAD2	SMAD family member 2 [Source:VGNC Symbol;Acc:VGNC:98329]	5.88	0.0861
ssc-miR-30c-5p	SMAD5	SMAD family member 5 [Source:VGNC Symbol;Acc:VGNC:93219]	5.88	0.0861
ssc-miR-30c-5p	SMAP1	small ArfGAP 1 [Source:VGNC Symbol;Acc:VGNC:93223]	5.88	0.0861
ssc-miR-30c-5p	SMARCA5	SWI/SNF related, matrix associated, actin dependent regulator of chromatin, subfamily a, member 5 [Source:VGNC Symbol;Acc:VGNC:93228]	5.88	0.0861
ssc-miR-30c-5p	SMARCD2	SWI/SNF related, matrix associated, actin dependent regulator of chromatin, subfamily d, member 2 [Source:NCBI gene (formerly Entrezgene);Acc:100512367]	5.88	0.0861
ssc-miR-30c-5p	SMIM14	small integral membrane protein 14 [Source:VGNC Symbol;Acc:VGNC:93252]	5.88	0.0861
ssc-miR-30c-5p	SNAI1	snail family transcriptional repressor 1 [Source:VGNC Symbol;Acc:VGNC:95508]	5.88	0.0861
ssc-miR-30c-5p	SNAPC3	small nuclear RNA activating complex polypeptide 3 [Source:VGNC Symbol;Acc:VGNC:103182]	5.88	0.0861

ssc-miR-30c-5p	SNAPIN	SNAP associated protein [Source:VGNC Symbol;Acc:VGNC:93284]	5.88	0.0861
ssc-miR-30c-5p	SNTB2	syntrophin beta 2 [Source:VGNC Symbol;Acc:VGNC:93298]	5.88	0.0861
ssc-miR-30c-5p	SNX1	sorting nexin 1 [Source:HGNC Symbol;Acc:HGNC:11172]	5.88	0.0861
ssc-miR-30c-5p	SNX10	sorting nexin 10 [Source:VGNC Symbol;Acc:VGNC:93303]	5.88	0.0861
ssc-miR-30c-5p	SNX16	sorting nexin 16 [Source:VGNC Symbol;Acc:VGNC:93308]	5.88	0.0861
ssc-miR-30c-5p	SNX18	sorting nexin 18 [Source:VGNC Symbol;Acc:VGNC:93310]	5.88	0.0861
ssc-miR-30c-5p	SNX2	sorting nexin 2 [Source:VGNC Symbol;Acc:VGNC:93312]	5.88	0.0861
ssc-miR-30c-5p	SNX27	sorting nexin 27 [Source:VGNC Symbol;Acc:VGNC:93316]	5.88	0.0861
ssc-miR-30c-5p	SNX29	sorting nexin 29 [Source:VGNC Symbol;Acc:VGNC:98331]	5.88	0.0861
ssc-miR-30c-5p	SNX30	sorting nexin family member 30 [Source:VGNC Symbol;Acc:VGNC:93318]	5.88	0.0861
ssc-miR-30c-5p	SNX33	sorting nexin 33 [Source:VGNC Symbol;Acc:VGNC:93321]	5.88	0.0861
ssc-miR-30c-5p	SNX6	sorting nexin 6 [Source:VGNC Symbol;Acc:VGNC:93323]	5.88	0.0861
ssc-miR-30c-5p	SNX8	sorting nexin 8 [Source:VGNC Symbol;Acc:VGNC:93325]	5.88	0.0861
ssc-miR-30c-5p	SOBP	sine oculis binding protein homolog [Source:VGNC Symbol;Acc:VGNC:93329]	5.88	0.0861
ssc-miR-30c-5p	SOCS1	suppressor of cytokine signaling 1 [Source:VGNC Symbol;Acc:VGNC:93330]	5.88	0.0861
ssc-miR-30c-5p	SOCS3	suppressor of cytokine signaling 3 [Source:VGNC Symbol;Acc:VGNC:99052]	5.88	0.0861
ssc-miR-30c-5p	SOCS6	suppressor of cytokine signaling 6 [Source:VGNC Symbol;Acc:VGNC:93333]	5.88	0.0861
ssc-miR-30c-5p	SOGA3	SOGA family member 3 [Source:HGNC Symbol;Acc:HGNC:21494]	5.88	0.0861
ssc-miR-30c-5p	SON	SON DNA and RNA binding protein [Source:VGNC Symbol;Acc:VGNC:93336]	5.88	0.0861
ssc-miR-30c-5p	SORCS3	sortilin related VPS10 domain containing receptor 3 [Source:VGNC Symbol;Acc:VGNC:93341]	5.88	0.0861
ssc-miR-30c-5p	SOS1	SOS Ras/Rac guanine nucleotide exchange factor 1 [Source:HGNC Symbol;Acc:HGNC:11187]	5.88	0.0861
ssc-miR-30c-5p	SOX12	SRY-box transcription factor 12 [Source:HGNC Symbol;Acc:HGNC:11198]	5.88	0.0861
ssc-miR-30c-5p	SOX13	SRY-box transcription factor 13 [Source:VGNC Symbol;Acc:VGNC:93352]	5.88	0.0861
ssc-miR-30c-5p	SOX4	SRY-box transcription factor 4 [Source:HGNC Symbol;Acc:HGNC:11200]	5.88	0.0861
ssc-miR-30c-5p	SOX9	SRY-box transcription factor 9 [Source:VGNC Symbol;Acc:VGNC:99053]	5.88	0.0861
ssc-miR-30c-5p	SP4	Sp4 transcription factor [Source:VGNC Symbol;Acc:VGNC:93362]	5.88	0.0861
ssc-miR-30c-5p	SPAST	spastin [Source:VGNC Symbol;Acc:VGNC:98333]	5.88	0.0861
ssc-miR-30c-5p	SPATS2L	spermatosis associated serine rich 2 like [Source:VGNC Symbol;Acc:VGNC:96047]	5.88	0.0861
ssc-miR-30c-5p	SPCS3	signal peptidase complex subunit 3 [Source:VGNC Symbol;Acc:VGNC:96119]	5.88	0.0861
ssc-miR-30c-5p	SPEN	hypothetical gene	5.88	0.0861
ssc-miR-30c-5p	SPHKAP	SPHK1 interactor, AKAP domain containing [Source:VGNC Symbol;Acc:VGNC:96245]	5.88	0.0861
ssc-miR-30c-5p	SPOPL	speckle type BTB/POZ protein like [Source:VGNC Symbol;Acc:VGNC:96161]	5.88	0.0861
ssc-miR-30c-5p	SPTLC2	serine palmitoyltransferase long chain base subunit 2 [Source:VGNC Symbol;Acc:VGNC:93440]	5.88	0.0861
ssc-miR-30c-5p	SPTSSB	serine palmitoyltransferase small subunit B [Source:VGNC Symbol;Acc:VGNC:93441]	5.88	0.0861
ssc-miR-30c-5p	SRGAP3	SLIT-ROBO Rho GTPase activating protein 3 [Source:VGNC Symbol;Acc:VGNC:93455]	5.88	0.0861
ssc-miR-30c-5p	SRI	sorcin [Source:VGNC Symbol;Acc:VGNC:93457]	5.88	0.0861
ssc-miR-30c-5p	SRSF10	serine and arginine rich splicing factor 10 [Source:VGNC Symbol;Acc:VGNC:93472]	5.88	0.0861
ssc-miR-30c-5p	SRSF7	serine and arginine rich splicing factor 7 [Source:VGNC Symbol;Acc:VGNC:93477]	5.88	0.0861
ssc-miR-30c-5p	SSBP2	single stranded DNA binding protein 2 [Source:VGNC Symbol;Acc:VGNC:93480]	5.88	0.0861
ssc-miR-30c-5p	SSBP3	single stranded DNA binding protein 3 [Source:VGNC Symbol;Acc:VGNC:93481]	5.88	0.0861
ssc-miR-30c-5p	SSH1	slingshot protein phosphatase 1 [Source:VGNC Symbol;Acc:VGNC:93485]	5.88	0.0861
ssc-miR-30c-5p	SSR1	signal sequence receptor subunit 1 [Source:VGNC Symbol;Acc:VGNC:93490]	5.88	0.0861
ssc-miR-30c-5p	SSR3	signal sequence receptor subunit 3 [Source:VGNC Symbol;Acc:VGNC:93492]	5.88	0.0861
ssc-miR-30c-5p	SSX2IP	SSX family member 2 interacting protein [Source:VGNC Symbol;Acc:VGNC:93499]	5.88	0.0861
ssc-miR-30c-5p	ST8SIA1	ST8 alpha-N-acetyl-neuraminide alpha-2,8-sialyltransferase 1 [Source:VGNC Symbol;Acc:VGNC:93516]	5.88	0.0861
ssc-miR-30c-5p	ST8SIA4	ST8 alpha-N-acetyl-neuraminide alpha-2,8-sialyltransferase 4 [Source:VGNC Symbol;Acc:VGNC:93519]	5.88	0.0861
ssc-miR-30c-5p	STAC	SH3 and cysteine rich domain [Source:HGNC Symbol;Acc:HGNC:11353]	5.88	0.0861
ssc-miR-30c-5p	STAG2	stromal antigen 2 [Source:VGNC Symbol;Acc:VGNC:93525]	5.88	0.0861
ssc-miR-30c-5p	STAU1	staufen double-stranded RNA binding protein 1 [Source:VGNC Symbol;Acc:VGNC:95525]	5.88	0.0861

ssc-miR-30c-5p	STC1	stanniocalcin 1 [Source:VGNC Symbol;Acc:VGNC:93542]	5.88	0.0861
ssc-miR-30c-5p	STIM2	stromal interaction molecule 2 [Source:VGNC Symbol;Acc:VGNC:98967]	5.88	0.0861
ssc-miR-30c-5p	STK10	serine/threonine kinase 10 [Source:HGNC Symbol;Acc:HGNC:11388]	5.88	0.0861
ssc-miR-30c-5p	STK17B	serine/threonine kinase 17b [Source:VGNC Symbol;Acc:VGNC:95527]	5.88	0.0861
ssc-miR-30c-5p	STK35	serine/threonine kinase 35 [Source:VGNC Symbol;Acc:VGNC:95802]	5.88	0.0861
ssc-miR-30c-5p	STK38L	serine/threonine kinase 38 like [Source:VGNC Symbol;Acc:VGNC:93557]	5.88	0.0861
ssc-miR-30c-5p	STK39	serine/threonine kinase 39 [Source:VGNC Symbol;Acc:VGNC:98348]	5.88	0.0861
ssc-miR-30c-5p	STK4	serine/threonine kinase 4 [Source:VGNC Symbol;Acc:VGNC:98349]	5.88	0.0861
ssc-miR-30c-5p	STOML1	stomatin like 1 [Source:VGNC Symbol;Acc:VGNC:93566]	5.88	0.0861
ssc-miR-30c-5p	STOX2	storkhead box 2 [Source:VGNC Symbol;Acc:VGNC:96082]	5.88	0.0861
ssc-miR-30c-5p	STRIP1	striatin interacting protein 1 [Source:VGNC Symbol;Acc:VGNC:93576]	5.88	0.0861
ssc-miR-30c-5p	STT3B	STT3 oligosaccharyltransferase complex catalytic subunit B [Source:VGNC Symbol;Acc:VGNC:93582]	5.88	0.0861
ssc-miR-30c-5p	STX16	syntaxin 16 [Source:NCBI gene (formerly Entrezgene);Acc:100144526]	5.88	0.0861
ssc-miR-30c-5p	STX17	syntaxin 17 [Source:VGNC Symbol;Acc:VGNC:93587]	5.88	0.0861
ssc-miR-30c-5p	STX2	syntaxin 2 [Source:VGNC Symbol;Acc:VGNC:93590]	5.88	0.0861
ssc-miR-30c-5p	STX3	syntaxin 3 [Source:VGNC Symbol;Acc:VGNC:93591]	5.88	0.0861
ssc-miR-30c-5p	STX6	syntaxin 6 [Source:VGNC Symbol;Acc:VGNC:93594]	5.88	0.0861
ssc-miR-30c-5p	STX7	syntaxin 7 [Source:VGNC Symbol;Acc:VGNC:93595]	5.88	0.0861
ssc-miR-30c-5p	STXBP1	syntaxin binding protein 1 [Source:VGNC Symbol;Acc:VGNC:93596]	5.88	0.0861
ssc-miR-30c-5p	STXBP5	syntaxin binding protein 5 [Source:VGNC Symbol;Acc:VGNC:93599]	5.88	0.0861
ssc-miR-30c-5p	STYX	serine/threonine/tyrosine interacting protein [Source:VGNC Symbol;Acc:VGNC:93602]	5.88	0.0861
ssc-miR-30c-5p	SUCLG2	succinate-CoA ligase GDP-forming subunit beta [Source:VGNC Symbol;Acc:VGNC:93604]	5.88	0.0861
ssc-miR-30c-5p	SUPT3H	SPT3 homolog, SAGA and STAGA complex component [Source:VGNC Symbol;Acc:VGNC:93623]	5.88	0.0861
ssc-miR-30c-5p	SURF4	surfeit 4 [Source:VGNC Symbol;Acc:VGNC:93631]	5.88	0.0861
ssc-miR-30c-5p	SUV39H2	SUV39H2 histone lysine methyltransferase [Source:VGNC Symbol;Acc:VGNC:95841]	5.88	0.0861
ssc-miR-30c-5p	SV2C	synaptic vesicle glycoprotein 2C [Source:VGNC Symbol;Acc:VGNC:93641]	5.88	0.0861
ssc-miR-30c-5p	SYNGR3	synaptogyrin 3 [Source:VGNC Symbol;Acc:VGNC:93666]	5.88	0.0861
ssc-miR-30c-5p	SYPL1	synaptophysin like 1 [Source:HGNC Symbol;Acc:HGNC:11507]	5.88	0.0861
ssc-miR-30c-5p	TAB3	TGF-beta activated kinase 1 (MAP3K7) binding protein 3 [Source:HGNC Symbol;Acc:HGNC:30681]	5.88	0.0861
ssc-miR-30c-5p	TACC1	hypothetical gene	5.88	0.0861
ssc-miR-30c-5p	TADA2B	transcriptional adaptor 2B [Source:VGNC Symbol;Acc:VGNC:93707]	5.88	0.0861
ssc-miR-30c-5p	TAF4B	TATA-box binding protein associated factor 4b [Source:VGNC Symbol;Acc:VGNC:93716]	5.88	0.0861
ssc-miR-30c-5p	TAOK1	TAO kinase 1 [Source:VGNC Symbol;Acc:VGNC:98355]	5.88	0.0861
ssc-miR-30c-5p	TASP1	taspase 1 [Source:VGNC Symbol;Acc:VGNC:95676]	5.88	0.0861
ssc-miR-30c-5p	TBC1D10B	TBC1 domain family member 10B [Source:VGNC Symbol;Acc:VGNC:93760]	5.88	0.0861
ssc-miR-30c-5p	TBC1D15	TBC1 domain family member 15 [Source:VGNC Symbol;Acc:VGNC:93764]	5.88	0.0861
ssc-miR-30c-5p	TBC1D2B	TBC1 domain family member 2B [Source:VGNC Symbol;Acc:VGNC:93774]	5.88	0.0861
ssc-miR-30c-5p	TBC1D9	TBC1 domain family member 9 [Source:VGNC Symbol;Acc:VGNC:93781]	5.88	0.0861
ssc-miR-30c-5p	TBL1X	hypothetical gene	5.88	0.0861
ssc-miR-30c-5p	TBL1XR1	TBL1X receptor 1 [Source:VGNC Symbol;Acc:VGNC:96600]	5.88	0.0861
ssc-miR-30c-5p	TBPL1	TATA-box binding protein like 1 [Source:VGNC Symbol;Acc:VGNC:93792]	5.88	0.0861
ssc-miR-30c-5p	TCF21	transcription factor 21 [Source:VGNC Symbol;Acc:VGNC:93819]	5.88	0.0861
ssc-miR-30c-5p	TCF7	transcription factor 7 [Source:VGNC Symbol;Acc:VGNC:96767]	5.88	0.0861
ssc-miR-30c-5p	TDG	thymine DNA glycosylase [Source:VGNC Symbol;Acc:VGNC:93842]	5.88	0.0861
ssc-miR-30c-5p	TEAD1	TEA domain transcription factor 1 [Source:VGNC Symbol;Acc:VGNC:93853]	5.88	0.0861
ssc-miR-30c-5p	TECPR1	tectonin beta-propeller repeat containing 1 [Source:VGNC Symbol;Acc:VGNC:93858]	5.88	0.0861
ssc-miR-30c-5p	TENM1	teneurin transmembrane protein 1 [Source:VGNC Symbol;Acc:VGNC:98363]	5.88	0.0861
ssc-miR-30c-5p	TENM3	teneurin transmembrane protein 3 [Source:VGNC Symbol;Acc:VGNC:96233]	5.88	0.0861
ssc-miR-30c-5p	TERT	telomerase reverse transcriptase [Source:VGNC Symbol;Acc:VGNC:93883]	5.88	0.0861

ssc-miR-30c-5p	TET1	tet methylcytosine dioxygenase 1 [Source:VGNC Symbol;Acc:VGNC:93888]	5.88	0.0861
ssc-miR-30c-5p	TET3	tet methylcytosine dioxygenase 3 [Source:VGNC Symbol;Acc:VGNC:93890]	5.88	0.0861
ssc-miR-30c-5p	TEX2	testis expressed 2 [Source:VGNC Symbol;Acc:VGNC:93894]	5.88	0.0861
ssc-miR-30c-5p	TFCP2L1	transcription factor CP2 like 1 [Source:HGNC Symbol;Acc:HGNC:17925]	5.88	0.0861
ssc-miR-30c-5p	TFDP1	transcription factor Dp-1 [Source:HGNC Symbol;Acc:HGNC:11749]	5.88	0.0861
ssc-miR-30c-5p	TGFA	transforming growth factor alpha [Source:VGNC Symbol;Acc:VGNC:93928]	5.88	0.0861
ssc-miR-30c-5p	THBS2	thrombospondin 2 [Source:VGNC Symbol;Acc:VGNC:93947]	5.88	0.0861
ssc-miR-30c-5p	TIA1	TIA1 cytotoxic granule associated RNA binding protein [Source:VGNC Symbol;Acc:VGNC:93975]	5.88	0.0861
ssc-miR-30c-5p	TIMM22	translocase of inner mitochondrial membrane 22 [Source:VGNC Symbol;Acc:VGNC:93988]	5.88	0.0861
ssc-miR-30c-5p	TIMP2	hypothetical gene	5.88	0.0861
ssc-miR-30c-5p	TIMP3	TIMP metallopeptidase inhibitor 3 [Source:VGNC Symbol;Acc:VGNC:93996]	5.88	0.0861
ssc-miR-30c-5p	TLL2	tolloid like 2 [Source:VGNC Symbol;Acc:VGNC:94019]	5.88	0.0861
ssc-miR-30c-5p	TM4SF18	transmembrane 4 L six family member 18 [Source:VGNC Symbol;Acc:VGNC:94031]	5.88	0.0861
ssc-miR-30c-5p	TMCC1	transmembrane and coiled-coil domain family 1 [Source:VGNC Symbol;Acc:VGNC:94046]	5.88	0.0861
ssc-miR-30c-5p	TMED2	hypothetical gene	5.88	0.0861
ssc-miR-30c-5p	TMEFF1	transmembrane protein with EGF like and two follistatin like domains 1 [Source:HGNC Symbol;Acc:HGNC:11866]	5.88	0.0861
ssc-miR-30c-5p	TMEM106B	transmembrane protein 106B [Source:VGNC Symbol;Acc:VGNC:104075]	5.88	0.0861
ssc-miR-30c-5p	TMEM110	hypothetical gene	5.88	0.0861
ssc-miR-30c-5p	TMEM121	transmembrane protein 121 [Source:HGNC Symbol;Acc:HGNC:20511]	5.88	0.0861
ssc-miR-30c-5p	TMEM135	transmembrane protein 135 [Source:VGNC Symbol;Acc:VGNC:94085]	5.88	0.0861
ssc-miR-30c-5p	TMEM154	transmembrane protein 154 [Source:VGNC Symbol;Acc:VGNC:94098]	5.88	0.0861
ssc-miR-30c-5p	TMEM168	transmembrane protein 168 [Source:VGNC Symbol;Acc:VGNC:94106]	5.88	0.0861
ssc-miR-30c-5p	TMEM170B	transmembrane protein 170B [Source:VGNC Symbol;Acc:VGNC:94108]	5.88	0.0861
ssc-miR-30c-5p	TMEM181	transmembrane protein 181 [Source:VGNC Symbol;Acc:VGNC:94117]	5.88	0.0861
ssc-miR-30c-5p	TMEM220	transmembrane protein 220 [Source:VGNC Symbol;Acc:VGNC:94142]	5.88	0.0861
ssc-miR-30c-5p	TMEM229A	transmembrane protein 229A [Source:VGNC Symbol;Acc:VGNC:94146]	5.88	0.0861
ssc-miR-30c-5p	TMEM245	transmembrane protein 245 [Source:HGNC Symbol;Acc:HGNC:1363]	5.88	0.0861
ssc-miR-30c-5p	TMEM252	transmembrane protein 252 [Source:VGNC Symbol;Acc:VGNC:94158]	5.88	0.0861
ssc-miR-30c-5p	TMEM41A	transmembrane protein 41A [Source:VGNC Symbol;Acc:VGNC:104080]	5.88	0.0861
ssc-miR-30c-5p	TMEM55A	hypothetical gene	5.88	0.0861
ssc-miR-30c-5p	TMEM56	hypothetical gene	5.88	0.0861
ssc-miR-30c-5p	TMEM70	transmembrane protein 70 [Source:VGNC Symbol;Acc:VGNC:94204]	5.88	0.0861
ssc-miR-30c-5p	TMEM86A	transmembrane protein 86A [Source:VGNC Symbol;Acc:VGNC:94210]	5.88	0.0861
ssc-miR-30c-5p	TMEM87A	transmembrane protein 87A [Source:VGNC Symbol;Acc:VGNC:94212]	5.88	0.0861
ssc-miR-30c-5p	TMOD2	tropomodulin 2 [Source:VGNC Symbol;Acc:VGNC:94227]	5.88	0.0861
ssc-miR-30c-5p	TMOD3	tropomodulin 3 [Source:VGNC Symbol;Acc:VGNC:94228]	5.88	0.0861
ssc-miR-30c-5p	TMTC3	transmembrane O-mannosyltransferase targeting cadherins 3 [Source:VGNC Symbol;Acc:VGNC:94243]	5.88	0.0861
ssc-miR-30c-5p	TNFRSF8	hypothetical gene	5.88	0.0861
ssc-miR-30c-5p	TNIK	TRAF2 and NCK interacting kinase [Source:VGNC Symbol;Acc:VGNC:98381]	5.88	0.0861
ssc-miR-30c-5p	TNIP1	TNFAIP3 interacting protein 1 [Source:VGNC Symbol;Acc:VGNC:94275]	5.88	0.0861
ssc-miR-30c-5p	TNKS	tankyrase [Source:VGNC Symbol;Acc:VGNC:95546]	5.88	0.0861
ssc-miR-30c-5p	TNPO1	transportin 1 [Source:VGNC Symbol;Acc:VGNC:94287]	5.88	0.0861
ssc-miR-30c-5p	TNPO3	transportin 3 [Source:VGNC Symbol;Acc:VGNC:94289]	5.88	0.0861
ssc-miR-30c-5p	TNRC6A	trinucleotide repeat containing adaptor 6A [Source:VGNC Symbol;Acc:VGNC:94292]	5.88	0.0861
ssc-miR-30c-5p	TNRC6B	trinucleotide repeat containing adaptor 6B [Source:VGNC Symbol;Acc:VGNC:94293]	5.88	0.0861
ssc-miR-30c-5p	TNRC6C	trinucleotide repeat containing adaptor 6C [Source:VGNC Symbol;Acc:VGNC:94294]	5.88	0.0861
ssc-miR-30c-5p	TNXB	tenascin XB [Source:NCBI gene (formerly Entrezgene);Acc:445520]	5.88	0.0861
ssc-miR-30c-5p	TOR1B	torsin family 1 member B [Source:HGNC Symbol;Acc:HGNC:11995]	5.88	0.0861
ssc-miR-30c-5p	TOX	thymocyte selection associated high mobility group box [Source:VGNC Symbol;Acc:VGNC:94322]	5.88	0.0861

ssc-miR-30c-5p	TP53INP1	tumor protein p53 inducible nuclear protein 1 [Source:VGNC Symbol;Acc:VGNC:94329]	5.88	0.0861
ssc-miR-30c-5p	TPM4	hypothetical gene	5.88	0.0861
ssc-miR-30c-5p	TPRG1	tumor protein p63 regulated 1 [Source:VGNC Symbol;Acc:VGNC:94348]	5.88	0.0861
ssc-miR-30c-5p	TPRG1L	tumor protein p63 regulated 1 like [Source:VGNC Symbol;Acc:VGNC:94349]	5.88	0.0861
ssc-miR-30c-5p	TRAF3	TNF receptor associated factor 3 [Source:VGNC Symbol;Acc:VGNC:94360]	5.88	0.0861
ssc-miR-30c-5p	TRAF3IP2	TRAF3 interacting protein 2 [Source:VGNC Symbol;Acc:VGNC:94361]	5.88	0.0861
ssc-miR-30c-5p	TRAM2	translocation associated membrane protein 2 [Source:HGNC Symbol;Acc:HGNC:16855]	5.88	0.0861
ssc-miR-30c-5p	TRDN	triadin [Source:VGNC Symbol;Acc:VGNC:103195]	5.88	0.0861
ssc-miR-30c-5p	TRERF1	transcriptional regulating factor 1 [Source:VGNC Symbol;Acc:VGNC:94387]	5.88	0.0861
ssc-miR-30c-5p	TRIM13	tripartite motif containing 13 [Source:VGNC Symbol;Acc:VGNC:94394]	5.88	0.0861
ssc-miR-30c-5p	TRIM23	tripartite motif containing 23 [Source:VGNC Symbol;Acc:VGNC:94399]	5.88	0.0861
ssc-miR-30c-5p	TRIM24	tripartite motif containing 24 [Source:VGNC Symbol;Acc:VGNC:94400]	5.88	0.0861
ssc-miR-30c-5p	TRIM27	tripartite motif containing 27 [Source:VGNC Symbol;Acc:VGNC:94402]	5.88	0.0861
ssc-miR-30c-5p	TRIM33	tripartite motif containing 33 [Source:VGNC Symbol;Acc:VGNC:98881]	5.88	0.0861
ssc-miR-30c-5p	TRIM36	tripartite motif containing 36 [Source:VGNC Symbol;Acc:VGNC:94409]	5.88	0.0861
ssc-miR-30c-5p	TRIM71	tripartite motif containing 71 [Source:VGNC Symbol;Acc:VGNC:94431]	5.88	0.0861
ssc-miR-30c-5p	TRIM9	tripartite motif containing 9 [Source:VGNC Symbol;Acc:VGNC:94434]	5.88	0.0861
ssc-miR-30c-5p	TRIO	trio Rho guanine nucleotide exchange factor [Source:VGNC Symbol;Acc:VGNC:94435]	5.88	0.0861
ssc-miR-30c-5p	TRIP12	thyroid hormone receptor interactor 12 [Source:VGNC Symbol;Acc:VGNC:95555]	5.88	0.0861
ssc-miR-30c-5p	TRMT5	tRNA methyltransferase 5 [Source:VGNC Symbol;Acc:VGNC:94451]	5.88	0.0861
ssc-miR-30c-5p	TRO	trophinin [Source:NCBI gene (formerly Entrezgene);Acc:396561]	5.88	0.0861
ssc-miR-30c-5p	TRPM3	transient receptor potential cation channel subfamily M member 3 [Source:VGNC Symbol;Acc:VGNC:98384]	5.88	0.0861
ssc-miR-30c-5p	TRPM7	transient receptor potential cation channel subfamily M member 7 [Source:VGNC Symbol;Acc:VGNC:94470]	5.88	0.0861
ssc-miR-30c-5p	TRPS1	transcriptional repressor GATA binding 1 [Source:VGNC Symbol;Acc:VGNC:94471]	5.88	0.0861
ssc-miR-30c-5p	TRRAP	transformation/transcription domain associated protein [Source:VGNC Symbol;Acc:VGNC:94477]	5.88	0.0861
ssc-miR-30c-5p	TSC1	TSC complex subunit 1 [Source:HGNC Symbol;Acc:HGNC:12362]	5.88	0.0861
ssc-miR-30c-5p	TSC22D2	TSC22 domain family member 2 [Source:VGNC Symbol;Acc:VGNC:94482]	5.88	0.0861
ssc-miR-30c-5p	TSPAN2	tetraspanin 2 [Source:VGNC Symbol;Acc:VGNC:94509]	5.88	0.0861
ssc-miR-30c-5p	TSPAN33	tetraspanin 33 [Source:VGNC Symbol;Acc:VGNC:94512]	5.88	0.0861
ssc-miR-30c-5p	TSPYL4	TSPY like 4 [Source:HGNC Symbol;Acc:HGNC:21559]	5.88	0.0861
ssc-miR-30c-5p	TTBK1	tau tubulin kinase 1 [Source:VGNC Symbol;Acc:VGNC:98386]	5.88	0.0861
ssc-miR-30c-5p	TTC28	tetratricopeptide repeat domain 28 [Source:VGNC Symbol;Acc:VGNC:94543]	5.88	0.0861
ssc-miR-30c-5p	TTC30A	hypothetical gene	5.88	0.0861
ssc-miR-30c-5p	TTC39A	tetratricopeptide repeat domain 39A [Source:VGNC Symbol;Acc:VGNC:94551]	5.88	0.0861
ssc-miR-30c-5p	TTLL7	tubulin tyrosine ligase like 7 [Source:VGNC Symbol;Acc:VGNC:94571]	5.88	0.0861
ssc-miR-30c-5p	TTPA	alpha tocopherol transfer protein [Source:HGNC Symbol;Acc:HGNC:12404]	5.88	0.0861
ssc-miR-30c-5p	TUBGCP3	tubulin gamma complex associated protein 3 [Source:VGNC Symbol;Acc:VGNC:94585]	5.88	0.0861
ssc-miR-30c-5p	TULP4	hypothetical gene	5.88	0.0861
ssc-miR-30c-5p	TUSC3	tumor suppressor candidate 3 [Source:VGNC Symbol;Acc:VGNC:108742]	5.88	0.0861
ssc-miR-30c-5p	TVP23B	hypothetical gene	5.88	0.0861
ssc-miR-30c-5p	TWF1	twinfilin actin binding protein 1 [Source:VGNC Symbol;Acc:VGNC:94595]	5.88	0.0861
ssc-miR-30c-5p	TXNDC5	thioredoxin domain containing 5 [Source:VGNC Symbol;Acc:VGNC:94608]	5.88	0.0861
ssc-miR-30c-5p	UACA	uveal autoantigen with coiled-coil domains and ankyrin repeats [Source:VGNC Symbol;Acc:VGNC:94625]	5.88	0.0861
ssc-miR-30c-5p	UBAC1	UBA domain containing 1 [Source:VGNC Symbol;Acc:VGNC:94632]	5.88	0.0861
ssc-miR-30c-5p	UBE2D2	ubiquitin conjugating enzyme E2 D2 [Source:VGNC Symbol;Acc:VGNC:98391]	5.88	0.0861
ssc-miR-30c-5p	UBE2D3	ubiquitin conjugating enzyme E2 D3 [Source:NCBI gene (formerly Entrezgene);Acc:780418]	5.88	0.0861
ssc-miR-30c-5p	UBE2F	hypothetical gene	5.88	0.0861
ssc-miR-30c-5p	UBE2G1	ubiquitin conjugating enzyme E2 G1 [Source:HGNC Symbol;Acc:HGNC:12482]	5.88	0.0861
ssc-miR-30c-5p	UBE2I	hypothetical gene	5.88	0.0861

ssc-miR-30c-5p	UBE2J1	ubiquitin conjugating enzyme E2 J1 [Source:VGNC Symbol;Acc:VGNC:94644]	5.88	0.0861
ssc-miR-30c-5p	UBE2K	ubiquitin conjugating enzyme E2 K [Source:VGNC Symbol;Acc:VGNC:94646]	5.88	0.0861
ssc-miR-30c-5p	UBE2O	ubiquitin conjugating enzyme E2 O [Source:VGNC Symbol;Acc:VGNC:94649]	5.88	0.0861
ssc-miR-30c-5p	UBE2QL1	ubiquitin conjugating enzyme E2 Q family like 1 [Source:VGNC Symbol;Acc:VGNC:94650]	5.88	0.0861
ssc-miR-30c-5p	UBE2R2	ubiquitin conjugating enzyme E2 R2 [Source:VGNC Symbol;Acc:VGNC:95924]	5.88	0.0861
ssc-miR-30c-5p	UBE2V2	ubiquitin conjugating enzyme E2 V2 [Source:VGNC Symbol;Acc:VGNC:98394]	5.88	0.0861
ssc-miR-30c-5p	UBE3C	ubiquitin protein ligase E3C [Source:VGNC Symbol;Acc:VGNC:94654]	5.88	0.0861
ssc-miR-30c-5p	UBN1	ubinuclein 1 [Source:VGNC Symbol;Acc:VGNC:94663]	5.88	0.0861
ssc-miR-30c-5p	UBN2	ubinuclein 2 [Source:VGNC Symbol;Acc:VGNC:94664]	5.88	0.0861
ssc-miR-30c-5p	UBXN2A	UBX domain protein 2A [Source:VGNC Symbol;Acc:VGNC:94677]	5.88	0.0861
ssc-miR-30c-5p	UCP3	uncoupling protein 3 [Source:VGNC Symbol;Acc:VGNC:108624]	5.88	0.0861
ssc-miR-30c-5p	UGT8	UDP glycosyltransferase 8 [Source:VGNC Symbol;Acc:VGNC:98401]	5.88	0.0861
ssc-miR-30c-5p	UHRF1BP1	UHRF1 binding protein 1 [Source:VGNC Symbol;Acc:VGNC:94690]	5.88	0.0861
ssc-miR-30c-5p	UNC5C	unc-5 netrin receptor C [Source:VGNC Symbol;Acc:VGNC:94709]	5.88	0.0861
ssc-miR-30c-5p	UNC5D	unc-5 netrin receptor D [Source:VGNC Symbol;Acc:VGNC:95892]	5.88	0.0861
ssc-miR-30c-5p	UNKL	unk like zinc finger [Source:VGNC Symbol;Acc:VGNC:94716]	5.88	0.0861
ssc-miR-30c-5p	URM1	ubiquitin related modifier 1 [Source:VGNC Symbol;Acc:VGNC:94732]	5.88	0.0861
ssc-miR-30c-5p	USP14	ubiquitin specific peptidase 14 [Source:VGNC Symbol;Acc:VGNC:94747]	5.88	0.0861
ssc-miR-30c-5p	USP15	ubiquitin specific peptidase 15 [Source:VGNC Symbol;Acc:VGNC:94748]	5.88	0.0861
ssc-miR-30c-5p	USP2	ubiquitin specific peptidase 2 [Source:VGNC Symbol;Acc:VGNC:94751]	5.88	0.0861
ssc-miR-30c-5p	USP22	ubiquitin specific peptidase 22 [Source:HGNC Symbol;Acc:HGNC:12621]	5.88	0.0861
ssc-miR-30c-5p	USP24	ubiquitin specific peptidase 24 [Source:VGNC Symbol;Acc:VGNC:94754]	5.88	0.0861
ssc-miR-30c-5p	USP37	ubiquitin specific peptidase 37 [Source:VGNC Symbol;Acc:VGNC:98404]	5.88	0.0861
ssc-miR-30c-5p	USP44	ubiquitin specific peptidase 44 [Source:VGNC Symbol;Acc:VGNC:94766]	5.88	0.0861
ssc-miR-30c-5p	USP45	ubiquitin specific peptidase 45 [Source:VGNC Symbol;Acc:VGNC:94767]	5.88	0.0861
ssc-miR-30c-5p	USP47	ubiquitin specific peptidase 47 [Source:VGNC Symbol;Acc:VGNC:94769]	5.88	0.0861
ssc-miR-30c-5p	USP48	ubiquitin specific peptidase 48 [Source:VGNC Symbol;Acc:VGNC:94770]	5.88	0.0861
ssc-miR-30c-5p	USP49	ubiquitin specific peptidase 49 [Source:VGNC Symbol;Acc:VGNC:94771]	5.88	0.0861
ssc-miR-30c-5p	UST	uronyl 2-sulfotransferase [Source:VGNC Symbol;Acc:VGNC:94780]	5.88	0.0861
ssc-miR-30c-5p	VAMP3	hypothetical gene	5.88	0.0861
ssc-miR-30c-5p	VAPA	VAMP associated protein A [Source:VGNC Symbol;Acc:VGNC:94799]	5.88	0.0861
ssc-miR-30c-5p	VASH1	vasohibin 1 [Source:VGNC Symbol;Acc:VGNC:94801]	5.88	0.0861
ssc-miR-30c-5p	VAT1	vesicle amine transport 1 [Source:VGNC Symbol;Acc:VGNC:94805]	5.88	0.0861
ssc-miR-30c-5p	VAT1L	vesicle amine transport 1 like [Source:VGNC Symbol;Acc:VGNC:94806]	5.88	0.0861
ssc-miR-30c-5p	VAV2	vav guanine nucleotide exchange factor 2 [Source:VGNC Symbol;Acc:VGNC:94808]	5.88	0.0861
ssc-miR-30c-5p	VAV3	hypothetical gene	5.88	0.0861
ssc-miR-30c-5p	VCAN	versican [Source:VGNC Symbol;Acc:VGNC:108163]	5.88	0.0861
ssc-miR-30c-5p	VIM	vimentin [Source:VGNC Symbol;Acc:VGNC:95565]	5.88	0.0861
ssc-miR-30c-5p	VIP	vasoactive intestinal peptide [Source:VGNC Symbol;Acc:VGNC:103201]	5.88	0.0861
ssc-miR-30c-5p	VKORC1L1	vitamin K epoxide reductase complex subunit 1 like 1 [Source:VGNC Symbol;Acc:VGNC:94831]	5.88	0.0861
ssc-miR-30c-5p	VOPP1	VOPP1 WW domain binding protein [Source:VGNC Symbol;Acc:VGNC:94835]	5.88	0.0861
ssc-miR-30c-5p	VPS13C	vacuolar protein sorting 13 homolog C [Source:VGNC Symbol;Acc:VGNC:94840]	5.88	0.0861
ssc-miR-30c-5p	VPS13D	vacuolar protein sorting 13 homolog D [Source:VGNC Symbol;Acc:VGNC:94841]	5.88	0.0861
ssc-miR-30c-5p	VPS26B	VPS26, retromer complex component B [Source:NCBI gene (formerly Entrezgene);Acc:100192441]	5.88	0.0861
ssc-miR-30c-5p	VPS33A	VPS33A core subunit of CORVET and HOPS complexes [Source:NCBI gene (formerly Entrezgene);Acc:100049702]	5.88	0.0861
ssc-miR-30c-5p	VPS53	VPS53 subunit of GARP complex [Source:VGNC Symbol;Acc:VGNC:94861]	5.88	0.0861
ssc-miR-30c-5p	VSTM4	V-set and transmembrane domain containing 4 [Source:VGNC Symbol;Acc:VGNC:94874]	5.88	0.0861
ssc-miR-30c-5p	VWDE	von Willebrand factor D and EGF domains [Source:VGNC Symbol;Acc:VGNC:104093]	5.88	0.0861
ssc-miR-30c-5p	WASL	WASP like actin nucleation promoting factor [Source:VGNC Symbol;Acc:VGNC:96604]	5.88	0.0861

ssc-miR-30c-5p	WBP1L	WW domain binding protein 1 like [Source:VGNC Symbol;Acc:VGNC:94898]	5.88	0.0861
ssc-miR-30c-5p	WDFY3	WD repeat and FYVE domain containing 3 [Source:VGNC Symbol;Acc:VGNC:94903]	5.88	0.0861
ssc-miR-30c-5p	WDPCP	WD repeat containing planar cell polarity effector [Source:HGNC Symbol;Acc:HGNC:28027]	5.88	0.0861
ssc-miR-30c-5p	WDR1	WD repeat domain 1 [Source:VGNC Symbol;Acc:VGNC:94905]	5.88	0.0861
ssc-miR-30c-5p	WDR26	WD repeat domain 26 [Source:VGNC Symbol;Acc:VGNC:95960]	5.88	0.0861
ssc-miR-30c-5p	WDR37	WD repeat domain 37 [Source:VGNC Symbol;Acc:VGNC:96258]	5.88	0.0861
ssc-miR-30c-5p	WDR44	WD repeat domain 44 [Source:VGNC Symbol;Acc:VGNC:94921]	5.88	0.0861
ssc-miR-30c-5p	WDR47	WD repeat domain 47 [Source:VGNC Symbol;Acc:VGNC:94924]	5.88	0.0861
ssc-miR-30c-5p	WDR7	WD repeat domain 7 [Source:VGNC Symbol;Acc:VGNC:94936]	5.88	0.0861
ssc-miR-30c-5p	WDR70	WD repeat domain 70 [Source:VGNC Symbol;Acc:VGNC:94937]	5.88	0.0861
ssc-miR-30c-5p	WDR82	WD repeat domain 82 [Source:VGNC Symbol;Acc:VGNC:94944]	5.88	0.0861
ssc-miR-30c-5p	WIPF1	hypothetical gene	5.88	0.0861
ssc-miR-30c-5p	WIPF3	WAS/WASL interacting protein family member 3 [Source:VGNC Symbol;Acc:VGNC:94958]	5.88	0.0861
ssc-miR-30c-5p	WISP1	hypothetical gene	5.88	0.0861
ssc-miR-30c-5p	WNK3	WNK lysine deficient protein kinase 3 [Source:VGNC Symbol;Acc:VGNC:94964]	5.88	0.0861
ssc-miR-30c-5p	WNT7B	Wnt family member 7B [Source:VGNC Symbol;Acc:VGNC:94975]	5.88	0.0861
ssc-miR-30c-5p	WWP1	WW domain containing E3 ubiquitin protein ligase 1 [Source:VGNC Symbol;Acc:VGNC:94989]	5.88	0.0861
ssc-miR-30c-5p	XKR4	XK related 4 [Source:VGNC Symbol;Acc:VGNC:98900]	5.88	0.0861
ssc-miR-30c-5p	XPO1	exportin 1 [Source:VGNC Symbol;Acc:VGNC:95003]	5.88	0.0861
ssc-miR-30c-5p	XPR1	xenotropic and polytropic retrovirus receptor 1 [Source:VGNC Symbol;Acc:VGNC:108625]	5.88	0.0861
ssc-miR-30c-5p	YAF2	YY1 associated factor 2 [Source:VGNC Symbol;Acc:VGNC:95018]	5.88	0.0861
ssc-miR-30c-5p	YBX1	hypothetical gene	5.88	0.0861
ssc-miR-30c-5p	YES1	YES proto-onco 1, Src family tyrosine kinase [Source:VGNC Symbol;Acc:VGNC:95024]	5.88	0.0861
ssc-miR-30c-5p	YOD1	YOD1 deubiquitinase [Source:VGNC Symbol;Acc:VGNC:95035]	5.88	0.0861
ssc-miR-30c-5p	YPEL2	yippee like 2 [Source:VGNC Symbol;Acc:VGNC:95036]	5.88	0.0861
ssc-miR-30c-5p	YPEL5	yippee like 5 [Source:VGNC Symbol;Acc:VGNC:95039]	5.88	0.0861
ssc-miR-30c-5p	YTHDC1	YTH domain containing 1 [Source:VGNC Symbol;Acc:VGNC:95041]	5.88	0.0861
ssc-miR-30c-5p	YTHDF2	YTH N6-methyladenosine RNA binding protein 2 [Source:VGNC Symbol;Acc:VGNC:95043]	5.88	0.0861
ssc-miR-30c-5p	YTHDF3	YTH N6-methyladenosine RNA binding protein 3 [Source:VGNC Symbol;Acc:VGNC:95044]	5.88	0.0861
ssc-miR-30c-5p	YWHAG	hypothetical gene	5.88	0.0861
ssc-miR-30c-5p	YWHAZ	tyrosine 3-monooxygenase/tryptophan 5-monooxygenase activation protein zeta [Source:VGNC Symbol;Acc:VGNC:95047]	5.88	0.0861
ssc-miR-30c-5p	YY1	YY1 transcription factor [Source:HGNC Symbol;Acc:HGNC:12856]	5.88	0.0861
ssc-miR-30c-5p	ZBED4	hypothetical gene	5.88	0.0861
ssc-miR-30c-5p	ZBTB10	zinc finger and BTB domain containing 10 [Source:VGNC Symbol;Acc:VGNC:95058]	5.88	0.0861
ssc-miR-30c-5p	ZBTB11	zinc finger and BTB domain containing 11 [Source:VGNC Symbol;Acc:VGNC:95059]	5.88	0.0861
ssc-miR-30c-5p	ZBTB18	zinc finger and BTB domain containing 18 [Source:HGNC Symbol;Acc:HGNC:13030]	5.88	0.0861
ssc-miR-30c-5p	ZBTB20	zinc finger and BTB domain containing 20 [Source:VGNC Symbol;Acc:VGNC:95063]	5.88	0.0861
ssc-miR-30c-5p	ZBTB34	zinc finger and BTB domain containing 34 [Source:VGNC Symbol;Acc:VGNC:95070]	5.88	0.0861
ssc-miR-30c-5p	ZBTB39	zinc finger and BTB domain containing 39 [Source:VGNC Symbol;Acc:VGNC:95073]	5.88	0.0861
ssc-miR-30c-5p	ZBTB40	zinc finger and BTB domain containing 40 [Source:VGNC Symbol;Acc:VGNC:95074]	5.88	0.0861
ssc-miR-30c-5p	ZBTB41	zinc finger and BTB domain containing 41 [Source:VGNC Symbol;Acc:VGNC:108284]	5.88	0.0861
ssc-miR-30c-5p	ZBTB44	zinc finger and BTB domain containing 44 [Source:VGNC Symbol;Acc:VGNC:95076]	5.88	0.0861
ssc-miR-30c-5p	ZBTB6	zinc finger and BTB domain containing 6 [Source:VGNC Symbol;Acc:VGNC:95080]	5.88	0.0861
ssc-miR-30c-5p	ZBTB7A	zinc finger and BTB domain containing 7A [Source:VGNC Symbol;Acc:VGNC:95081]	5.88	0.0861
ssc-miR-30c-5p	ZCCHC14	zinc finger CCHC-type containing 14 [Source:VGNC Symbol;Acc:VGNC:95107]	5.88	0.0861
ssc-miR-30c-5p	ZCCHC2	zinc finger CCHC-type containing 2 [Source:VGNC Symbol;Acc:VGNC:95109]	5.88	0.0861
ssc-miR-30c-5p	ZCCHC24	zinc finger CCHC-type containing 24 [Source:HGNC Symbol;Acc:HGNC:26911]	5.88	0.0861
ssc-miR-30c-5p	ZCCHC4	zinc finger CCHC-type containing 4 [Source:VGNC Symbol;Acc:VGNC:95110]	5.88	0.0861
ssc-miR-30c-5p	ZDHHC14	zinc finger DHHC-type palmitoyltransferase 14 [Source:VGNC Symbol;Acc:VGNC:95117]	5.88	0.0861

ssc-miR-30c-5p	ZDHHC17	zinc finger DHHC-type palmitoyltransferase 17 [Source:VGNC Symbol;Acc:VGNC:95119]	5.88	0.0861
ssc-miR-30c-5p	ZDHHC20	zinc finger DHHC-type palmitoyltransferase 20 [Source:VGNC Symbol;Acc:VGNC:95122]	5.88	0.0861
ssc-miR-30c-5p	ZDHHC21	zinc finger DHHC-type palmitoyltransferase 21 [Source:VGNC Symbol;Acc:VGNC:95123]	5.88	0.0861
ssc-miR-30c-5p	ZEB2	hypothetical gene	5.88	0.0861
ssc-miR-30c-5p	ZFAND5	zinc finger AN1-type containing 5 [Source:VGNC Symbol;Acc:VGNC:95137]	5.88	0.0861
ssc-miR-30c-5p	ZFC3H1	zinc finger C3H1-type containing [Source:VGNC Symbol;Acc:VGNC:95139]	5.88	0.0861
ssc-miR-30c-5p	ZFHX3	zinc finger homeobox 3 [Source:VGNC Symbol;Acc:VGNC:95141]	5.88	0.0861
ssc-miR-30c-5p	ZFP36L1	ZFP36 ring finger protein like 1 [Source:VGNC Symbol;Acc:VGNC:95146]	5.88	0.0861
ssc-miR-30c-5p	ZFP36L2	ZFP36 ring finger protein like 2 [Source:VGNC Symbol;Acc:VGNC:95147]	5.88	0.0861
ssc-miR-30c-5p	ZFYVE26	zinc finger FYVE-type containing 26 [Source:VGNC Symbol;Acc:VGNC:95160]	5.88	0.0861
ssc-miR-30c-5p	ZIC2	Zic family member 2 [Source:VGNC Symbol;Acc:VGNC:95167]	5.88	0.0861
ssc-miR-30c-5p	ZMYND8	zinc finger MYND-type containing 8 [Source:VGNC Symbol;Acc:VGNC:108744]	5.88	0.0861
ssc-miR-30c-5p	ZNF148	zinc finger protein 148 [Source:VGNC Symbol;Acc:VGNC:95192]	5.88	0.0861
ssc-miR-30c-5p	ZNF197	zinc finger protein 197 [Source:VGNC Symbol;Acc:VGNC:95202]	5.88	0.0861
ssc-miR-30c-5p	ZNF200	zinc finger protein 200 [Source:VGNC Symbol;Acc:VGNC:95203]	5.88	0.0861
ssc-miR-30c-5p	ZNF264	hypothetical gene	5.88	0.0861
ssc-miR-30c-5p	ZNF275	hypothetical gene	5.88	0.0861
ssc-miR-30c-5p	ZNF280B	zinc finger protein 280B [Source:HGNC Symbol;Acc:HGNC:23022]	5.88	0.0861
ssc-miR-30c-5p	ZNF382	zinc finger protein 382 [Source:VGNC Symbol;Acc:VGNC:95232]	5.88	0.0861
ssc-miR-30c-5p	ZNF507	zinc finger protein 507 [Source:VGNC Symbol;Acc:VGNC:95253]	5.88	0.0861
ssc-miR-30c-5p	ZNF521	zinc finger protein 521 [Source:VGNC Symbol;Acc:VGNC:98715]	5.88	0.0861
ssc-miR-30c-5p	ZNF608	hypothetical gene	5.88	0.0861
ssc-miR-30c-5p	ZNF644	zinc finger protein 644 [Source:VGNC Symbol;Acc:VGNC:95283]	5.88	0.0861
ssc-miR-30c-5p	ZNF652	zinc finger protein 652 [Source:VGNC Symbol;Acc:VGNC:99108]	5.88	0.0861
ssc-miR-30c-5p	ZNF704	zinc finger protein 704 [Source:VGNC Symbol;Acc:VGNC:95296]	5.88	0.0861
ssc-miR-30c-5p	ZNF711	zinc finger protein 711 [Source:HGNC Symbol;Acc:HGNC:13128]	5.88	0.0861
ssc-miR-30c-5p	ZNF746	hypothetical gene	5.88	0.0861
ssc-miR-30c-5p	ZNF770	zinc finger protein 770 [Source:VGNC Symbol;Acc:VGNC:95300]	5.88	0.0861
ssc-miR-30c-5p	ZNF827	zinc finger protein 827 [Source:VGNC Symbol;Acc:VGNC:98970]	5.88	0.0861
ssc-miR-30c-5p	ZNF831	zinc finger protein 831 [Source:VGNC Symbol;Acc:VGNC:95765]	5.88	0.0861
ssc-miR-30c-5p	ZNRF1	zinc and ring finger 1 [Source:HGNC Symbol;Acc:HGNC:18452]	5.88	0.0861
ssc-miR-30c-5p	ZPBP2	zona pellucida binding protein 2 [Source:VGNC Symbol;Acc:VGNC:95320]	5.88	0.0861
ssc-miR-30c-5p	ZRANB3	zinc finger RANBP2-type containing 3 [Source:VGNC Symbol;Acc:VGNC:96073]	5.88	0.0861
ssc-miR-30c-5p	ZSCAN29	zinc finger and SCAN domain containing 29 [Source:HGNC Symbol;Acc:HGNC:26673]	5.88	0.0861
ssc-miR-30c-5p	ZSWIM5	zinc finger SWIM-type containing 5 [Source:VGNC Symbol;Acc:VGNC:95334]	5.88	0.0861
ssc-miR-30c-5p	ZSWIM6	zinc finger SWIM-type containing 6 [Source:VGNC Symbol;Acc:VGNC:95335]	5.88	0.0861
ssc-miR-146a-5p	AAK1	AP2 associated kinase 1 [Source:VGNC Symbol;Acc:VGNC:100379]	5.3	0.0401
ssc-miR-146a-5p	ABL2	ABL proto-onco 2, non-receptor tyrosine kinase [Source:VGNC Symbol;Acc:VGNC:84985]	5.3	0.0401
ssc-miR-146a-5p	AC012215.1	hypothetical gene	5.3	0.0401
ssc-miR-146a-5p	AC137932.1	hypothetical gene	5.3	0.0401
ssc-miR-146a-5p	ACER3	alkaline ceramidase 3 [Source:VGNC Symbol;Acc:VGNC:103893]	5.3	0.0401
ssc-miR-146a-5p	ACKR2	atypical chemokine receptor 2 [Source:VGNC Symbol;Acc:VGNC:85012]	5.3	0.0401
ssc-miR-146a-5p	ACYP2	acylphosphatase 2 [Source:VGNC Symbol;Acc:VGNC:97030]	5.3	0.0401
ssc-miR-146a-5p	ADAM19	ADAM metallopeptidase domain 19 [Source:VGNC Symbol;Acc:VGNC:85066]	5.3	0.0401
ssc-miR-146a-5p	AFAP1L2	actin filament associated protein 1 like 2 [Source:HGNC Symbol;Acc:HGNC:25901]	5.3	0.0401
ssc-miR-146a-5p	AMN	amnion associated transmembrane protein [Source:VGNC Symbol;Acc:VGNC:85282]	5.3	0.0401
ssc-miR-146a-5p	APPL1	adaptor protein, phosphotyrosine interacting with PH domain and leucine zipper 1 [Source:VGNC Symbol;Acc:VGNC:85426]	5.3	0.0401
ssc-miR-146a-5p	AR	androgen receptor [Source:VGNC Symbol;Acc:VGNC:103903]	5.3	0.0401
ssc-miR-146a-5p	ARHGAP29	Rho GTPase activating protein 29 [Source:VGNC Symbol;Acc:VGNC:85468]	5.3	0.0401

ssc-miR-146a-5p	ARMC8	armadillo repeat containing 8 [Source:VGNC Symbol;Acc:VGNC:85531]	5.3	0.0401
ssc-miR-146a-5p	ASB6	ankyrin repeat and SOCS box containing 6 [Source:VGNC Symbol;Acc:VGNC:85565]	5.3	0.0401
ssc-miR-146a-5p	ATG7	autophagy related 7 [Source:VGNC Symbol;Acc:VGNC:85625]	5.3	0.0401
ssc-miR-146a-5p	ATP5J2-PTCD1	hypothetical gene	5.3	0.0401
ssc-miR-146a-5p	BAG1	BAG cochaperone 1 [Source:VGNC Symbol;Acc:VGNC:96502]	5.3	0.0401
ssc-miR-146a-5p	BAIAP2L1	BAR/IMD domain containing adaptor protein 2 like 1 [Source:VGNC Symbol;Acc:VGNC:85749]	5.3	0.0401
ssc-miR-146a-5p	BCORL1	BCL6 corepressor like 1 [Source:VGNC Symbol;Acc:VGNC:85791]	5.3	0.0401
ssc-miR-146a-5p	BEND4	BEN domain containing 4 [Source:VGNC Symbol;Acc:VGNC:85801]	5.3	0.0401
ssc-miR-146a-5p	BMPR1A	bone morphotic protein receptor type 1A [Source:VGNC Symbol;Acc:VGNC:85846]	5.3	0.0401
ssc-miR-146a-5p	BNC1	basonuclin 1 [Source:VGNC Symbol;Acc:VGNC:85851]	5.3	0.0401
ssc-miR-146a-5p	BRD4	bromodomain containing 4 [Source:VGNC Symbol;Acc:VGNC:85873]	5.3	0.0401
ssc-miR-146a-5p	BTG2	BTG anti-proliferation factor 2 [Source:NCBI gene (formerly Entrezgene);Acc:100048932]	5.3	0.0401
ssc-miR-146a-5p	C10orf76	hypothetical gene	5.3	0.0401
ssc-miR-146a-5p	C16orf52	hypothetical gene	5.3	0.0401
ssc-miR-146a-5p	C16orf72	chromosome 3 C16orf72 homolog [Source:VGNC Symbol;Acc:VGNC:86016]	5.3	0.0401
ssc-miR-146a-5p	C17orf75	chromosome 12 C17orf75 homolog [Source:VGNC Symbol;Acc:VGNC:85925]	5.3	0.0401
ssc-miR-146a-5p	C17orf78	chromosome 12 C17orf78 homolog [Source:VGNC Symbol;Acc:VGNC:85926]	5.3	0.0401
ssc-miR-146a-5p	C21orf33	hypothetical gene	5.3	0.0401
ssc-miR-146a-5p	C4orf3	chromosome X C4orf3 homolog [Source:VGNC Symbol;Acc:VGNC:107133]	5.3	0.0401
ssc-miR-146a-5p	C5orf51	hypothetical gene	5.3	0.0401
ssc-miR-146a-5p	C8A	complement C8 alpha chain [Source:VGNC Symbol;Acc:VGNC:86076]	5.3	0.0401
ssc-miR-146a-5p	C9orf40	chromosome 1 C9orf40 homolog [Source:VGNC Symbol;Acc:VGNC:85973]	5.3	0.0401
ssc-miR-146a-5p	CAMSAP1	calmodulin regulated spectrin associated protein 1 [Source:VGNC Symbol;Acc:VGNC:86162]	5.3	0.0401
ssc-miR-146a-5p	CARD10	caspase recruitment domain family member 10 [Source:VGNC Symbol;Acc:VGNC:86185]	5.3	0.0401
ssc-miR-146a-5p	CASK	calcium/calmodulin dependent serine protein kinase [Source:HGNC Symbol;Acc:HGNC:1497]	5.3	0.0401
ssc-miR-146a-5p	CBFA2T2	CBFA2/RUNX1 partner transcriptional co-repressor 2 [Source:VGNC Symbol;Acc:VGNC:96573]	5.3	0.0401
ssc-miR-146a-5p	CCDC6	coiled-coil domain containing 6 [Source:VGNC Symbol;Acc:VGNC:86304]	5.3	0.0401
ssc-miR-146a-5p	CCNJ	cyclin J [Source:VGNC Symbol;Acc:VGNC:86360]	5.3	0.0401
ssc-miR-146a-5p	CD28	CD28 molecule [Source:VGNC Symbol;Acc:VGNC:95813]	5.3	0.0401
ssc-miR-146a-5p	CD3D	CD3 delta subunit of T-cell receptor complex [Source:VGNC Symbol;Acc:VGNC:86413]	5.3	0.0401
ssc-miR-146a-5p	CD80	CD80 molecule [Source:VGNC Symbol;Acc:VGNC:97920]	5.3	0.0401
ssc-miR-146a-5p	CD84	CD84 molecule [Source:VGNC Symbol;Acc:VGNC:86434]	5.3	0.0401
ssc-miR-146a-5p	CDC14A	cell division cycle 14A [Source:VGNC Symbol;Acc:VGNC:86442]	5.3	0.0401
ssc-miR-146a-5p	CDKAL1	CDK5 regulatory subunit associated protein 1 like 1 [Source:VGNC Symbol;Acc:VGNC:86509]	5.3	0.0401
ssc-miR-146a-5p	CDKN2AIP	CDKN2A interacting protein [Source:VGNC Symbol;Acc:VGNC:105247]	5.3	0.0401
ssc-miR-146a-5p	CDON	cell adhesion associated, onco regulated [Source:VGNC Symbol;Acc:VGNC:86519]	5.3	0.0401
ssc-miR-146a-5p	CDS1	CDP-diacylglycerol synthase 1 [Source:VGNC Symbol;Acc:VGNC:86523]	5.3	0.0401
ssc-miR-146a-5p	CELF1	CUGBP Elav-like family member 1 [Source:VGNC Symbol;Acc:VGNC:86537]	5.3	0.0401
ssc-miR-146a-5p	CELF2	hypothetical gene	5.3	0.0401
ssc-miR-146a-5p	CELF3	CUGBP Elav-like family member 3 [Source:VGNC Symbol;Acc:VGNC:86538]	5.3	0.0401
ssc-miR-146a-5p	CLCN4	chloride voltage-gated channel 4 [Source:VGNC Symbol;Acc:VGNC:86728]	5.3	0.0401
ssc-miR-146a-5p	CLCN6	chloride voltage-gated channel 6 [Source:VGNC Symbol;Acc:VGNC:86729]	5.3	0.0401
ssc-miR-146a-5p	CNOT6L	CCR4-NOT transcription complex subunit 6 like [Source:VGNC Symbol;Acc:VGNC:86838]	5.3	0.0401
ssc-miR-146a-5p	CNTFR	ciliary neurotrophic factor receptor [Source:VGNC Symbol;Acc:VGNC:95975]	5.3	0.0401
ssc-miR-146a-5p	COG8	component of oligomeric golgi complex 8 [Source:HGNC Symbol;Acc:HGNC:18623]	5.3	0.0401
ssc-miR-146a-5p	CRB3	crumbs cell polarity complex component 3 [Source:HGNC Symbol;Acc:HGNC:20237]	5.3	0.0401
ssc-miR-146a-5p	CRKL	CRK like proto-onco, adaptor protein [Source:VGNC Symbol;Acc:VGNC:86997]	5.3	0.0401
ssc-miR-146a-5p	CRNKL1	crooked neck pre-mRNA splicing factor 1 [Source:VGNC Symbol;Acc:VGNC:95654]	5.3	0.0401
ssc-miR-146a-5p	CUL4B	cullin 4B [Source:HGNC Symbol;Acc:HGNC:2555]	5.3	0.0401

ssc-miR-146a-5p	DCAF12	DDB1 and CUL4 associated factor 12 [Source:VGNC Symbol;Acc:VGNC:95927]	5.3	0.0401
ssc-miR-146a-5p	DCP1A	decapping mRNA 1A [Source:VGNC Symbol;Acc:VGNC:97960]	5.3	0.0401
ssc-miR-146a-5p	DCX	doublecortin [Source:HGNC Symbol;Acc:HGNC:2714]	5.3	0.0401
ssc-miR-146a-5p	DDHD1	DDHD domain containing 1 [Source:VGNC Symbol;Acc:VGNC:87205]	5.3	0.0401
ssc-miR-146a-5p	DDX49	DEAD-box helicase 49 [Source:VGNC Symbol;Acc:VGNC:87225]	5.3	0.0401
ssc-miR-146a-5p	DEPTOR	DEP domain containing MTOR interacting protein [Source:HGNC Symbol;Acc:HGNC:22953]	5.3	0.0401
ssc-miR-146a-5p	DGCR14	hypothetical gene	5.3	0.0401
ssc-miR-146a-5p	DGKG	diacylglycerol kinase gamma [Source:VGNC Symbol;Acc:VGNC:87272]	5.3	0.0401
ssc-miR-146a-5p	DLGAP2	DLG associated protein 2 [Source:VGNC Symbol;Acc:VGNC:99711]	5.3	0.0401
ssc-miR-146a-5p	DNAJB14	DnaJ heat shock protein family (Hsp40) member B14 [Source:VGNC Symbol;Acc:VGNC:98919]	5.3	0.0401
ssc-miR-146a-5p	DNAJC14	DnaJ heat shock protein family (Hsp40) member C14 [Source:NCBI gene (formerly Entrezgene);Acc:100151876]	5.3	0.0401
ssc-miR-146a-5p	DNAL1	dynein axonemal light chain 1 [Source:VGNC Symbol;Acc:VGNC:87373]	5.3	0.0401
ssc-miR-146a-5p	DNPEP	aspartyl aminopeptidase [Source:VGNC Symbol;Acc:VGNC:96230]	5.3	0.0401
ssc-miR-146a-5p	DOT1L	DOT1 like histone lysine methyltransferase [Source:VGNC Symbol;Acc:VGNC:87410]	5.3	0.0401
ssc-miR-146a-5p	DPP9	dipeptidyl peptidase 9 [Source:VGNC Symbol;Acc:VGNC:87424]	5.3	0.0401
ssc-miR-146a-5p	DTL	denticleless E3 ubiquitin [Source:VGNC Symbol;Acc:VGNC:87461]	5.3	0.0401
ssc-miR-146a-5p	DUSP16	dual specificity phosphatase 16 [Source:VGNC Symbol;Acc:VGNC:87480]	5.3	0.0401
ssc-miR-146a-5p	DYNLL2	dynein light chain LC8-type 2 [Source:VGNC Symbol;Acc:VGNC:87501]	5.3	0.0401
ssc-miR-146a-5p	EDNRB	endothelin receptor type B [Source:VGNC Symbol;Acc:VGNC:87550]	5.3	0.0401
ssc-miR-146a-5p	EHF	ETS homologous factor [Source:VGNC Symbol;Acc:VGNC:87599]	5.3	0.0401
ssc-miR-146a-5p	EIF4G2	eukaryotic translation initiation factor 4 gamma 2 [Source:VGNC Symbol;Acc:VGNC:99645]	5.3	0.0401
ssc-miR-146a-5p	EIF5A2	eukaryotic translation initiation factor 5A2 [Source:VGNC Symbol;Acc:VGNC:87633]	5.3	0.0401
ssc-miR-146a-5p	ELMSAN1	hypothetical gene	5.3	0.0401
ssc-miR-146a-5p	ERBB4	erb-b2 receptor tyrosine kinase 4 [Source:VGNC Symbol;Acc:VGNC:96284]	5.3	0.0401
ssc-miR-146a-5p	ERI2	ERI1 exoribonuclease family member 2 [Source:VGNC Symbol;Acc:VGNC:106577]	5.3	0.0401
ssc-miR-146a-5p	ERLEC1	endoplasmic reticulum lectin 1 [Source:VGNC Symbol;Acc:VGNC:87775]	5.3	0.0401
ssc-miR-146a-5p	ESYT2	extended synaptotagmin 2 [Source:VGNC Symbol;Acc:VGNC:87797]	5.3	0.0401
ssc-miR-146a-5p	FAM169A	family with sequence similarity 169 member A [Source:VGNC Symbol;Acc:VGNC:87927]	5.3	0.0401
ssc-miR-146a-5p	FAM208A	hypothetical gene	5.3	0.0401
ssc-miR-146a-5p	FAM210A	family with sequence similarity 210 member A [Source:HGNC Symbol;Acc:HGNC:28346]	5.3	0.0401
ssc-miR-146a-5p	FAM26E	hypothetical gene	5.3	0.0401
ssc-miR-146a-5p	FAM65B	hypothetical gene	5.3	0.0401
ssc-miR-146a-5p	FAM83F	family with sequence similarity 83 member F [Source:VGNC Symbol;Acc:VGNC:87991]	5.3	0.0401
ssc-miR-146a-5p	FBXL13	F-box and leucine rich repeat protein 13 [Source:VGNC Symbol;Acc:VGNC:98000]	5.3	0.0401
ssc-miR-146a-5p	FBXO28	F-box protein 28 [Source:HGNC Symbol;Acc:HGNC:29046]	5.3	0.0401
ssc-miR-146a-5p	FBXW2	hypothetical gene	5.3	0.0401
ssc-miR-146a-5p	FLOT2	flotillin 2 [Source:VGNC Symbol;Acc:VGNC:88159]	5.3	0.0401
ssc-miR-146a-5p	FMO2	flavin containing dimethylaniline monoxygenase 2 [Source:VGNC Symbol;Acc:VGNC:88171]	5.3	0.0401
ssc-miR-146a-5p	FRYL	FRY like transcription coactivator [Source:VGNC Symbol;Acc:VGNC:98015]	5.3	0.0401
ssc-miR-146a-5p	FUT10	fucosyltransferase 10 [Source:VGNC Symbol;Acc:VGNC:88268]	5.3	0.0401
ssc-miR-146a-5p	FZD1	frizzled class receptor 1 [Source:VGNC Symbol;Acc:VGNC:88278]	5.3	0.0401
ssc-miR-146a-5p	GABPA	GA binding protein transcription factor subunit alpha [Source:VGNC Symbol;Acc:VGNC:103945]	5.3	0.0401
ssc-miR-146a-5p	GALNT10	polypeptide N-acetylgalactosaminyltransferase 10 [Source:VGNC Symbol;Acc:VGNC:88327]	5.3	0.0401
ssc-miR-146a-5p	GATA6	GATA binding protein 6 [Source:VGNC Symbol;Acc:VGNC:88366]	5.3	0.0401
ssc-miR-146a-5p	GDNF	glial cell derived neurotrophic factor [Source:VGNC Symbol;Acc:VGNC:88406]	5.3	0.0401
ssc-miR-146a-5p	GGA2	golgi associated, gamma adaptin ear containing, ARF binding protein 2 [Source:VGNC Symbol;Acc:VGNC:88432]	5.3	0.0401
ssc-miR-146a-5p	GINM1	glycoprotein integral membrane 1 [Source:VGNC Symbol;Acc:VGNC:88450]	5.3	0.0401
ssc-miR-146a-5p	GJC1	gap junction protein gamma 1 [Source:VGNC Symbol;Acc:VGNC:88468]	5.3	0.0401
ssc-miR-146a-5p	GLYATL3	glycine-N-acyltransferase like 3 [Source:VGNC Symbol;Acc:VGNC:88503]	5.3	0.0401

ssc-miR-146a-5p	GMPS	guanine monophosphate synthase [Source:VGNC Symbol;Acc:VGNC:88516]	5.3	0.0401
ssc-miR-146a-5p	GNL1	G protein nucleolar 1 (putative) [Source:NCBI gene (formerly Entrezgene);Acc:100151741]	5.3	0.0401
ssc-miR-146a-5p	GOSR1	golgi SNAP receptor complex member 1 [Source:VGNC Symbol;Acc:VGNC:88560]	5.3	0.0401
ssc-miR-146a-5p	GPBP1	GC-rich promoter binding protein 1 [Source:VGNC Symbol;Acc:VGNC:88578]	5.3	0.0401
ssc-miR-146a-5p	GPR75	G protein-coupled receptor 75 [Source:VGNC Symbol;Acc:VGNC:88636]	5.3	0.0401
ssc-miR-146a-5p	GPX8	glutathione peroxidase 8 (putative) [Source:HGNC Symbol;Acc:HGNC:33100]	5.3	0.0401
ssc-miR-146a-5p	GRID1	glutamate ionotropic receptor delta type subunit 1 [Source:VGNC Symbol;Acc:VGNC:88674]	5.3	0.0401
ssc-miR-146a-5p	GRK5	G protein-coupled receptor kinase 5 [Source:VGNC Symbol;Acc:VGNC:88697]	5.3	0.0401
ssc-miR-146a-5p	GRSF1	G-rich RNA sequence binding factor 1 [Source:VGNC Symbol;Acc:VGNC:88710]	5.3	0.0401
ssc-miR-146a-5p	GSTM4	hypothetical gene	5.3	0.0401
ssc-miR-146a-5p	GSTM5	hypothetical gene	5.3	0.0401
ssc-miR-146a-5p	HEYL	hes related family bHLH transcription factor with YRPW motif like [Source:VGNC Symbol;Acc:VGNC:88865]	5.3	0.0401
ssc-miR-146a-5p	HIC2	HIC ZBTB transcriptional repressor 2 [Source:HGNC Symbol;Acc:HGNC:18595]	5.3	0.0401
ssc-miR-146a-5p	HIPK3	homeodomain interacting protein kinase 3 [Source:VGNC Symbol;Acc:VGNC:88889]	5.3	0.0401
ssc-miR-146a-5p	HNRNPD	heterous nuclear ribonucleoprotein D [Source:VGNC Symbol;Acc:VGNC:88919]	5.3	0.0401
ssc-miR-146a-5p	HOXA13	homeobox A13 [Source:NCBI gene (formerly Entrezgene);Acc:100359352]	5.3	0.0401
ssc-miR-146a-5p	HSPA4L	heat shock protein family A (Hsp70) member 4 like [Source:HGNC Symbol;Acc:HGNC:17041]	5.3	0.0401
ssc-miR-146a-5p	IGF2R	insulin like growth factor 2 receptor [Source:VGNC Symbol;Acc:VGNC:89057]	5.3	0.0401
ssc-miR-146a-5p	IGSF1	immunoglobulin superfamily member 1 [Source:VGNC Symbol;Acc:VGNC:89062]	5.3	0.0401
ssc-miR-146a-5p	IKZF3	IKAROS family zinc finger 3 [Source:VGNC Symbol;Acc:VGNC:89074]	5.3	0.0401
ssc-miR-146a-5p	IMPA2	inositol monophosphatase 2 [Source:HGNC Symbol;Acc:HGNC:6051]	5.3	0.0401
ssc-miR-146a-5p	IMPG1	interphotoreceptor matrix proteoglycan 1 [Source:VGNC Symbol;Acc:VGNC:89127]	5.3	0.0401
ssc-miR-146a-5p	IRAK1	interleukin 1 receptor associated kinase 1 [Source:VGNC Symbol;Acc:VGNC:89198]	5.3	0.0401
ssc-miR-146a-5p	IST1	IST1 factor associated with ESCRT-III [Source:VGNC Symbol;Acc:VGNC:98480]	5.3	0.0401
ssc-miR-146a-5p	ITM2B	integral membrane protein 2B [Source:VGNC Symbol;Acc:VGNC:103971]	5.3	0.0401
ssc-miR-146a-5p	JAZF1	JAZF zinc finger 1 [Source:VGNC Symbol;Acc:VGNC:89280]	5.3	0.0401
ssc-miR-146a-5p	KBTBD4	kelch repeat and BTB domain containing 4 [Source:VGNC Symbol;Acc:VGNC:89319]	5.3	0.0401
ssc-miR-146a-5p	KCMF1	potassium channel modulatory factor 1 [Source:VGNC Symbol;Acc:VGNC:89322]	5.3	0.0401
ssc-miR-146a-5p	KCND3	potassium voltage-gated channel subfamily D member 3 [Source:VGNC Symbol;Acc:VGNC:98814]	5.3	0.0401
ssc-miR-146a-5p	KCNK10	potassium two pore domain channel subfamily K member 10 [Source:VGNC Symbol;Acc:VGNC:89364]	5.3	0.0401
ssc-miR-146a-5p	KCTD15	potassium channel tetramerization domain containing 15 [Source:VGNC Symbol;Acc:VGNC:89394]	5.3	0.0401
ssc-miR-146a-5p	KDM2B	lysine demethylase 2B [Source:VGNC Symbol;Acc:VGNC:98057]	5.3	0.0401
ssc-miR-146a-5p	KIAA0355	hypothetical gene	5.3	0.0401
ssc-miR-146a-5p	KIF24	kinesin family member 24 [Source:VGNC Symbol;Acc:VGNC:95928]	5.3	0.0401
ssc-miR-146a-5p	KIF26B	kinesin family member 26B [Source:VGNC Symbol;Acc:VGNC:96087]	5.3	0.0401
ssc-miR-146a-5p	KLF4	Kruppel like factor 4 [Source:VGNC Symbol;Acc:VGNC:98062]	5.3	0.0401
ssc-miR-146a-5p	KLF7	Kruppel like factor 7 [Source:VGNC Symbol;Acc:VGNC:96396]	5.3	0.0401
ssc-miR-146a-5p	KMT2D	hypothetical gene	5.3	0.0401
ssc-miR-146a-5p	KPNA6	karyopherin subunit alpha 6 [Source:VGNC Symbol;Acc:VGNC:89565]	5.3	0.0401
ssc-miR-146a-5p	LAYN	layilin [Source:VGNC Symbol;Acc:VGNC:89651]	5.3	0.0401
ssc-miR-146a-5p	LCOR	ligand dependent nuclear receptor corepressor [Source:HGNC Symbol;Acc:HGNC:29503]	5.3	0.0401
ssc-miR-146a-5p	LCP2	lymphocyte cytosolic protein 2 [Source:VGNC Symbol;Acc:VGNC:89664]	5.3	0.0401
ssc-miR-146a-5p	LFNG	LFNG O-fucosylpeptide 3-beta-N-acetylglucosaminyltransferase [Source:VGNC Symbol;Acc:VGNC:89690]	5.3	0.0401
ssc-miR-146a-5p	LGALS8	galectin 8 [Source:VGNC Symbol;Acc:VGNC:89697]	5.3	0.0401
ssc-miR-146a-5p	LIN28A	lin-28 homolog A [Source:VGNC Symbol;Acc:VGNC:98492]	5.3	0.0401
ssc-miR-146a-5p	LIN7A	lin-7 homolog A, crumbs cell polarity complex component [Source:VGNC Symbol;Acc:VGNC:89730]	5.3	0.0401
ssc-miR-146a-5p	LIPA	lipase A, lysosomal acid type [Source:VGNC Symbol;Acc:VGNC:89737]	5.3	0.0401
ssc-miR-146a-5p	LRIG2	leucine rich repeats and immunoglobulin like domains 2 [Source:VGNC Symbol;Acc:VGNC:89811]	5.3	0.0401
ssc-miR-146a-5p	LRP2	LDL receptor related protein 2 [Source:HGNC Symbol;Acc:HGNC:6694]	5.3	0.0401

ssc-miR-146a-5p	LRRC15	leucine rich repeat containing 15 [Source:VGNC Symbol;Acc:VGNC:98077]	5.3	0.0401
ssc-miR-146a-5p	LRRC8B	leucine rich repeat containing 8 VRAC subunit B [Source:VGNC Symbol;Acc:VGNC:98086]	5.3	0.0401
ssc-miR-146a-5p	LSM11	LSM11, U7 small nuclear RNA associated [Source:VGNC Symbol;Acc:VGNC:89871]	5.3	0.0401
ssc-miR-146a-5p	LTB	lymphotoxin beta [Source:NCBI gene (formerly Entrezgene);Acc:100155581]	5.3	0.0401
ssc-miR-146a-5p	LYVE1	lymphatic vessel endothelial hyaluronan receptor 1 [Source:VGNC Symbol;Acc:VGNC:89922]	5.3	0.0401
ssc-miR-146a-5p	MAFF	MAF bZIP transcription factor F [Source:VGNC Symbol;Acc:VGNC:89947]	5.3	0.0401
ssc-miR-146a-5p	MAN1C1	mannosidase alpha class 1C member 1 [Source:VGNC Symbol;Acc:VGNC:89968]	5.3	0.0401
ssc-miR-146a-5p	MAPT	microtubule associated protein tau [Source:VGNC Symbol;Acc:VGNC:90016]	5.3	0.0401
ssc-miR-146a-5p	MARCH6	hypothetical gene	5.3	0.0401
ssc-miR-146a-5p	MARK1	microtubule affinity regulating kinase 1 [Source:VGNC Symbol;Acc:VGNC:96411]	5.3	0.0401
ssc-miR-146a-5p	MED1	mediator complex subunit 1 [Source:VGNC Symbol;Acc:VGNC:90102]	5.3	0.0401
ssc-miR-146a-5p	MFHAS1	multifunctional ROCO family signaling regulator 1 [Source:VGNC Symbol;Acc:VGNC:107361]	5.3	0.0401
ssc-miR-146a-5p	MMP16	matrix metallopeptidase 16 [Source:VGNC Symbol;Acc:VGNC:90271]	5.3	0.0401
ssc-miR-146a-5p	MTDH	metadherin [Source:VGNC Symbol;Acc:VGNC:90439]	5.3	0.0401
ssc-miR-146a-5p	MYBL1	MYB proto-onco like 1 [Source:VGNC Symbol;Acc:VGNC:90498]	5.3	0.0401
ssc-miR-146a-5p	MYLK3	myosin light chain kinase 3 [Source:VGNC Symbol;Acc:VGNC:90518]	5.3	0.0401
ssc-miR-146a-5p	MYO5A	myosin VA [Source:HGNC Symbol;Acc:HGNC:7602]	5.3	0.0401
ssc-miR-146a-5p	MYO6	myosin VI [Source:VGNC Symbol;Acc:VGNC:103137]	5.3	0.0401
ssc-miR-146a-5p	MYT1	myelin transcription factor 1 [Source:VGNC Symbol;Acc:VGNC:104000]	5.3	0.0401
ssc-miR-146a-5p	NAIF1	hypothetical gene	5.3	0.0401
ssc-miR-146a-5p	NF2	NF2, moesin-ezrin-radixin like (MERLIN) tumor suppressor [Source:VGNC Symbol;Acc:VGNC:90705]	5.3	0.0401
ssc-miR-146a-5p	NFATC2	nuclear factor of activated T cells 2 [Source:VGNC Symbol;Acc:VGNC:96440]	5.3	0.0401
ssc-miR-146a-5p	NLE1	notchless homolog 1 [Source:VGNC Symbol;Acc:VGNC:90776]	5.3	0.0401
ssc-miR-146a-5p	NLGN1	neuroligin 1 [Source:VGNC Symbol;Acc:VGNC:90777]	5.3	0.0401
ssc-miR-146a-5p	NOTCH1	notch receptor 1 [Source:HGNC Symbol;Acc:HGNC:7881]	5.3	0.0401
ssc-miR-146a-5p	NOVA1	NOVA alternative splicing regulator 1 [Source:VGNC Symbol;Acc:VGNC:90827]	5.3	0.0401
ssc-miR-146a-5p	NPAS4	neuronal PAS domain protein 4 [Source:VGNC Symbol;Acc:VGNC:90839]	5.3	0.0401
ssc-miR-146a-5p	NRAS	NRAS proto-onco, GTPase [Source:VGNC Symbol;Acc:VGNC:98827]	5.3	0.0401
ssc-miR-146a-5p	NRP2	neuropilin 2 [Source:VGNC Symbol;Acc:VGNC:96454]	5.3	0.0401
ssc-miR-146a-5p	NSD1	nuclear receptor binding SET domain protein 1 [Source:VGNC Symbol;Acc:VGNC:90904]	5.3	0.0401
ssc-miR-146a-5p	NUCKS1	nuclear casein kinase and cyclin dependent kinase substrate 1 [Source:VGNC Symbol;Acc:VGNC:90947]	5.3	0.0401
ssc-miR-146a-5p	NUDT17	nudix hydrolase 17 [Source:VGNC Symbol;Acc:VGNC:90959]	5.3	0.0401
ssc-miR-146a-5p	NUDT3	nudix hydrolase 3 [Source:NCBI gene (formerly Entrezgene);Acc:100737442]	5.3	0.0401
ssc-miR-146a-5p	NUFIP2	nuclear FMR1 interacting protein 2 [Source:VGNC Symbol;Acc:VGNC:90967]	5.3	0.0401
ssc-miR-146a-5p	NUMB	NUMB endocytic adaptor protein [Source:VGNC Symbol;Acc:VGNC:90970]	5.3	0.0401
ssc-miR-146a-5p	OSTN	osteocrin [Source:VGNC Symbol;Acc:VGNC:91087]	5.3	0.0401
ssc-miR-146a-5p	PAK3	p21 (RAC1) activated kinase 3 [Source:HGNC Symbol;Acc:HGNC:8592]	5.3	0.0401
ssc-miR-146a-5p	PARD6B	par-6 family cell polarity regulator beta [Source:VGNC Symbol;Acc:VGNC:98176]	5.3	0.0401
ssc-miR-146a-5p	PBX2	PBX homeobox 2 [Source:VGNC Symbol;Acc:VGNC:91204]	5.3	0.0401
ssc-miR-146a-5p	PDE7A	phosphodiesterase 7A [Source:VGNC Symbol;Acc:VGNC:91261]	5.3	0.0401
ssc-miR-146a-5p	PDF	peptide deformylase, mitochondrial [Source:HGNC Symbol;Acc:HGNC:30012]	5.3	0.0401
ssc-miR-146a-5p	PDHB	pyruvate dehydrogenase E1 subunit beta [Source:VGNC Symbol;Acc:VGNC:91269]	5.3	0.0401
ssc-miR-146a-5p	PDIA3	protein disulfide isomerase family A member 3 [Source:VGNC Symbol;Acc:VGNC:91272]	5.3	0.0401
ssc-miR-146a-5p	PDZD8	hypothetical gene	5.3	0.0401
ssc-miR-146a-5p	PER1	period circadian regulator 1 [Source:VGNC Symbol;Acc:VGNC:91314]	5.3	0.0401
ssc-miR-146a-5p	PGK1	phosphoglycerate kinase 1 [Source:VGNC Symbol;Acc:VGNC:104025]	5.3	0.0401
ssc-miR-146a-5p	PGP	phosphoglycolate phosphatase [Source:VGNC Symbol;Acc:VGNC:91359]	5.3	0.0401
ssc-miR-146a-5p	PHOX2B	paired like homeobox 2B [Source:VGNC Symbol;Acc:VGNC:91402]	5.3	0.0401
ssc-miR-146a-5p	PIK3CB	phosphatidylinositol-4,5-bisphosphate 3-kinase catalytic subunit beta [Source:VGNC Symbol;Acc:VGNC:91441]	5.3	0.0401

ssc-miR-146a-5p	PIP5K1B	phosphatidylinositol-4-phosphate 5-kinase type 1 beta [Source:VGNC Symbol;Acc:VGNC:91458]	5.3	0.0401
ssc-miR-146a-5p	PM20D2	peptidase M20 domain containing 2 [Source:VGNC Symbol;Acc:VGNC:91586]	5.3	0.0401
ssc-miR-146a-5p	PNKP	polynucleotide kinase 3'-phosphatase [Source:VGNC Symbol;Acc:VGNC:91595]	5.3	0.0401
ssc-miR-146a-5p	POLH	DNA polymerase eta [Source:VGNC Symbol;Acc:VGNC:91635]	5.3	0.0401
ssc-miR-146a-5p	POLR3H	RNA polymerase III subunit H [Source:VGNC Symbol;Acc:VGNC:91658]	5.3	0.0401
ssc-miR-146a-5p	POU3F2	POU class 3 homeobox 2 [Source:HGNC Symbol;Acc:HGNC:9215]	5.3	0.0401
ssc-miR-146a-5p	PPP1R11	protein phosphatase 1 regulatory inhibitor subunit 11 [Source:VGNC Symbol;Acc:VGNC:91718]	5.3	0.0401
ssc-miR-146a-5p	PPP1R3B	protein phosphatase 1 regulatory subunit 3B [Source:VGNC Symbol;Acc:VGNC:95636]	5.3	0.0401
ssc-miR-146a-5p	PQLC1	hypothetical gene	5.3	0.0401
ssc-miR-146a-5p	PRCP	prolylcarboxypeptidase [Source:VGNC Symbol;Acc:VGNC:91771]	5.3	0.0401
ssc-miR-146a-5p	PRELID2	PRELI domain containing 2 [Source:VGNC Symbol;Acc:VGNC:91787]	5.3	0.0401
ssc-miR-146a-5p	PRKAA2	protein kinase AMP-activated catalytic subunit alpha 2 [Source:VGNC Symbol;Acc:VGNC:91798]	5.3	0.0401
ssc-miR-146a-5p	PRRC1	proline rich coiled-coil 1 [Source:VGNC Symbol;Acc:VGNC:91864]	5.3	0.0401
ssc-miR-146a-5p	PRX	periaxin [Source:VGNC Symbol;Acc:VGNC:91891]	5.3	0.0401
ssc-miR-146a-5p	PSEN1	presenilin 1 [Source:VGNC Symbol;Acc:VGNC:91897]	5.3	0.0401
ssc-miR-146a-5p	PSMA4	proteasome 20S subunit alpha 4 [Source:VGNC Symbol;Acc:VGNC:91901]	5.3	0.0401
ssc-miR-146a-5p	PTCD1	pentatricopeptide repeat domain 1 [Source:HGNC Symbol;Acc:HGNC:22198]	5.3	0.0401
ssc-miR-146a-5p	PTGFRN	prostaglandin F2 receptor inhibitor [Source:VGNC Symbol;Acc:VGNC:91955]	5.3	0.0401
ssc-miR-146a-5p	PTPRA	protein tyrosine phosphatase receptor type A [Source:VGNC Symbol;Acc:VGNC:96522]	5.3	0.0401
ssc-miR-146a-5p	PTRH2	peptidyl-tRNA hydrolase 2 [Source:VGNC Symbol;Acc:VGNC:91998]	5.3	0.0401
ssc-miR-146a-5p	QKI	QKI, KH domain containing RNA binding [Source:VGNC Symbol;Acc:VGNC:92025]	5.3	0.0401
ssc-miR-146a-5p	RABGAP1	RAB GTPase activating protein 1 [Source:HGNC Symbol;Acc:HGNC:17155]	5.3	0.0401
ssc-miR-146a-5p	RARB	retinoic acid receptor beta [Source:HGNC Symbol;Acc:HGNC:9865]	5.3	0.0401
ssc-miR-146a-5p	RBM3	RNA binding motif protein 3 [Source:NCBI gene (formerly Entrezgene);Acc:100627807]	5.3	0.0401
ssc-miR-146a-5p	RCAN1	regulator of calcineurin 1 [Source:VGNC Symbol;Acc:VGNC:92170]	5.3	0.0401
ssc-miR-146a-5p	RCN2	reticulocalbin 2 [Source:VGNC Symbol;Acc:VGNC:92181]	5.3	0.0401
ssc-miR-146a-5p	RCSD1	RCSD domain containing 1 [Source:VGNC Symbol;Acc:VGNC:92186]	5.3	0.0401
ssc-miR-146a-5p	REL	REL proto-onco, NF-kB subunit [Source:VGNC Symbol;Acc:VGNC:92202]	5.3	0.0401
ssc-miR-146a-5p	RFTN2	raftlin family member 2 [Source:VGNC Symbol;Acc:VGNC:96128]	5.3	0.0401
ssc-miR-146a-5p	RFX7	regulatory factor X7 [Source:VGNC Symbol;Acc:VGNC:92248]	5.3	0.0401
ssc-miR-146a-5p	RIMS2	regulating synaptic membrane exocytosis 2 [Source:HGNC Symbol;Acc:HGNC:17283]	5.3	0.0401
ssc-miR-146a-5p	RNASEL	ribonuclease L [Source:VGNC Symbol;Acc:VGNC:92342]	5.3	0.0401
ssc-miR-146a-5p	RNF32	hypothetical gene	5.3	0.0401
ssc-miR-146a-5p	RNF4	ring finger protein 4 [Source:VGNC Symbol;Acc:VGNC:92392]	5.3	0.0401
ssc-miR-146a-5p	ROBO1	roundabout guidance receptor 1 [Source:HGNC Symbol;Acc:HGNC:10249]	5.3	0.0401
ssc-miR-146a-5p	RP11-156E8.1	hypothetical gene	5.3	0.0401
ssc-miR-146a-5p	RP11-343C2.12	hypothetical gene	5.3	0.0401
ssc-miR-146a-5p	RPA3	replication protein A3 [Source:VGNC Symbol;Acc:VGNC:92415]	5.3	0.0401
ssc-miR-146a-5p	RPAP2	RNA polymerase II associated protein 2 [Source:VGNC Symbol;Acc:VGNC:92418]	5.3	0.0401
ssc-miR-146a-5p	RPS15	ribosomal protein S15 [Source:NCBI gene (formerly Entrezgene);Acc:397607]	5.3	0.0401
ssc-miR-146a-5p	RRAGD	Ras related GTP binding D [Source:VGNC Symbol;Acc:VGNC:92457]	5.3	0.0401
ssc-miR-146a-5p	RSPO2	R-spondin 2 [Source:VGNC Symbol;Acc:VGNC:92484]	5.3	0.0401
ssc-miR-146a-5p	RUFY1	RUN and FYVE domain containing 1 [Source:VGNC Symbol;Acc:VGNC:92511]	5.3	0.0401
ssc-miR-146a-5p	RUNX1T1	RUNX1 partner transcriptional co-repressor 1 [Source:VGNC Symbol;Acc:VGNC:96594]	5.3	0.0401
ssc-miR-146a-5p	SAMD10	sterile alpha motif domain containing 10 [Source:VGNC Symbol;Acc:VGNC:95750]	5.3	0.0401
ssc-miR-146a-5p	SAMD8	sterile alpha motif domain containing 8 [Source:VGNC Symbol;Acc:VGNC:92571]	5.3	0.0401
ssc-miR-146a-5p	SART3	spliceosome associated factor 3, U4/U6 recycling protein [Source:VGNC Symbol;Acc:VGNC:96595]	5.3	0.0401
ssc-miR-146a-5p	SBSPON	somatomedin B and thrombospondin type 1 domain containing [Source:VGNC Symbol;Acc:VGNC:92599]	5.3	0.0401
ssc-miR-146a-5p	SCN1A	sodium voltage-gated channel alpha subunit 1 [Source:VGNC Symbol;Acc:VGNC:95478]	5.3	0.0401

ssc-miR-146a-5p	SCN3B	sodium voltage-gated channel beta subunit 3 [Source:HGNC Symbol;Acc:HGNC:20665]	5.3	0.0401
ssc-miR-146a-5p	SEC23IP	SEC23 interacting protein [Source:VGNC Symbol;Acc:VGNC:92675]	5.3	0.0401
ssc-miR-146a-5p	SEMA3G	semaphorin 3G [Source:VGNC Symbol;Acc:VGNC:92699]	5.3	0.0401
ssc-miR-146a-5p	SEPSECS	Sep (O-phosphoserine) tRNA:Sec (selenocysteine) tRNA synthase [Source:VGNC Symbol;Acc:VGNC:98962]	5.3	0.0401
ssc-miR-146a-5p	SETD9	SET domain containing 9 [Source:VGNC Symbol;Acc:VGNC:92763]	5.3	0.0401
ssc-miR-146a-5p	SGIP1	SH3GL interacting endocytic adaptor 1 [Source:VGNC Symbol;Acc:VGNC:92792]	5.3	0.0401
ssc-miR-146a-5p	SH3GL2	SH3 domain containing GRB2 like 2, endophilin A1 [Source:VGNC Symbol;Acc:VGNC:92824]	5.3	0.0401
ssc-miR-146a-5p	SIAH2	siah E3 ubiquitin protein ligase 2 [Source:VGNC Symbol;Acc:VGNC:92866]	5.3	0.0401
ssc-miR-146a-5p	SKAP2	src kinase associated phosphoprotein 2 [Source:VGNC Symbol;Acc:VGNC:92901]	5.3	0.0401
ssc-miR-146a-5p	SLAMF8	SLAM family member 8 [Source:VGNC Symbol;Acc:VGNC:92913]	5.3	0.0401
ssc-miR-146a-5p	SLC10A3	solute carrier family 10 member 3 [Source:VGNC Symbol;Acc:VGNC:92916]	5.3	0.0401
ssc-miR-146a-5p	SLC19A3	solute carrier family 19 member 3 [Source:VGNC Symbol;Acc:VGNC:95805]	5.3	0.0401
ssc-miR-146a-5p	SLC2A14	hypothetical gene	5.3	0.0401
ssc-miR-146a-5p	SLC2A3	hypothetical gene	5.3	0.0401
ssc-miR-146a-5p	SLC39A1	solute carrier family 39 member 1 [Source:VGNC Symbol;Acc:VGNC:93101]	5.3	0.0401
ssc-miR-146a-5p	SLCO3A1	solute carrier organic anion transporter family member 3A1 [Source:VGNC Symbol;Acc:VGNC:93197]	5.3	0.0401
ssc-miR-146a-5p	SLITRK3	SLIT and NTRK like family member 3 [Source:VGNC Symbol;Acc:VGNC:93208]	5.3	0.0401
ssc-miR-146a-5p	SMAD4	SMAD family member 4 [Source:VGNC Symbol;Acc:VGNC:93218]	5.3	0.0401
ssc-miR-146a-5p	SMARCA5	SWI/SNF related, matrix associated, actin dependent regulator of chromatin, subfamily a, member 5 [Source:VGNC Symbol;Acc:VGNC:93228]	5.3	0.0401
ssc-miR-146a-5p	SMTNL2	smoothelin like 2 [Source:VGNC Symbol;Acc:VGNC:93268]	5.3	0.0401
ssc-miR-146a-5p	SNAP25	synaptosome associated protein 25 [Source:VGNC Symbol;Acc:VGNC:95509]	5.3	0.0401
ssc-miR-146a-5p	SNX22	sorting nexin 22 [Source:VGNC Symbol;Acc:VGNC:93314]	5.3	0.0401
ssc-miR-146a-5p	SORT1	sortilin 1 [Source:VGNC Symbol;Acc:VGNC:93343]	5.3	0.0401
ssc-miR-146a-5p	SOX5	SRY-box transcription factor 5 [Source:VGNC Symbol;Acc:VGNC:93357]	5.3	0.0401
ssc-miR-146a-5p	SP8	Sp8 transcription factor [Source:VGNC Symbol;Acc:VGNC:93365]	5.3	0.0401
ssc-miR-146a-5p	SPI1	Spi-1 proto-onco [Source:VGNC Symbol;Acc:VGNC:98335]	5.3	0.0401
ssc-miR-146a-5p	SPRY3	sprouty RTK signaling antagonist 3 [Source:VGNC Symbol;Acc:VGNC:93426]	5.3	0.0401
ssc-miR-146a-5p	SRRD	SRR1 domain containing [Source:HGNC Symbol;Acc:HGNC:33910]	5.3	0.0401
ssc-miR-146a-5p	SRRM4	serine/arginine repetitive matrix 4 [Source:VGNC Symbol;Acc:VGNC:93470]	5.3	0.0401
ssc-miR-146a-5p	SRSF12	serine and arginine rich splicing factor 12 [Source:VGNC Symbol;Acc:VGNC:93474]	5.3	0.0401
ssc-miR-146a-5p	ST5	hypothetical gene	5.3	0.0401
ssc-miR-146a-5p	STAU2	staufen double-stranded RNA binding protein 2 [Source:VGNC Symbol;Acc:VGNC:98873]	5.3	0.0401
ssc-miR-146a-5p	STC1	stanniocalcin 1 [Source:VGNC Symbol;Acc:VGNC:93542]	5.3	0.0401
ssc-miR-146a-5p	STK40	serine/threonine kinase 40 [Source:VGNC Symbol;Acc:VGNC:93558]	5.3	0.0401
ssc-miR-146a-5p	STRA13	hypothetical gene	5.3	0.0401
ssc-miR-146a-5p	STRBP	spermatid perinuclear RNA binding protein [Source:VGNC Symbol;Acc:VGNC:93575]	5.3	0.0401
ssc-miR-146a-5p	STRN	striatin [Source:VGNC Symbol;Acc:VGNC:93578]	5.3	0.0401
ssc-miR-146a-5p	STXBP6	syntaxin binding protein 6 [Source:NCBI gene (formerly Entrezgene);Acc:100514852]	5.3	0.0401
ssc-miR-146a-5p	SYT1	synaptotagmin 1 [Source:VGNC Symbol;Acc:VGNC:93678]	5.3	0.0401
ssc-miR-146a-5p	SYT13	synaptotagmin 13 [Source:VGNC Symbol;Acc:VGNC:93682]	5.3	0.0401
ssc-miR-146a-5p	TAF9B	TATA-box binding protein associated factor 9b [Source:VGNC Symbol;Acc:VGNC:93725]	5.3	0.0401
ssc-miR-146a-5p	TANC2	tetratricopeptide repeat, ankyrin repeat and coiled-coil containing 2 [Source:VGNC Symbol;Acc:VGNC:93733]	5.3	0.0401
ssc-miR-146a-5p	TBC1D20	TBC1 domain family member 20 [Source:VGNC Symbol;Acc:VGNC:95752]	5.3	0.0401
ssc-miR-146a-5p	TCF21	transcription factor 21 [Source:VGNC Symbol;Acc:VGNC:93819]	5.3	0.0401
ssc-miR-146a-5p	TDRKH	tudor and KH domain containing [Source:VGNC Symbol;Acc:VGNC:93852]	5.3	0.0401
ssc-miR-146a-5p	TLCD2	TLC domain containing 2 [Source:VGNC Symbol;Acc:VGNC:94011]	5.3	0.0401
ssc-miR-146a-5p	TLR3	toll like receptor 3 [Source:NCBI gene (formerly Entrezgene);Acc:100037937]	5.3	0.0401
ssc-miR-146a-5p	TM9SF2	transmembrane 9 superfamily member 2 [Source:VGNC Symbol;Acc:VGNC:94036]	5.3	0.0401
ssc-miR-146a-5p	TMEM120B	transmembrane protein 120B [Source:VGNC Symbol;Acc:VGNC:94071]	5.3	0.0401

ssc-miR-146a-5p	TMEM136	hypothetical gene	5.3	0.0401
ssc-miR-146a-5p	TMEM194A	hypothetical gene	5.3	0.0401
ssc-miR-146a-5p	TMEM200C	transmembrane protein 200C [Source:VGNC Symbol;Acc:VGNC:94130]	5.3	0.0401
ssc-miR-146a-5p	TMEM216	transmembrane protein 216 [Source:VGNC Symbol;Acc:VGNC:94140]	5.3	0.0401
ssc-miR-146a-5p	TMEM33	transmembrane protein 33 [Source:VGNC Symbol;Acc:VGNC:94171]	5.3	0.0401
ssc-miR-146a-5p	TMPRSS12	transmembrane serine protease 12 [Source:VGNC Symbol;Acc:VGNC:94231]	5.3	0.0401
ssc-miR-146a-5p	TNIK	TRAF2 and NCK interacting kinase [Source:VGNC Symbol;Acc:VGNC:98381]	5.3	0.0401
ssc-miR-146a-5p	TP53RK	TP53 regulating kinase [Source:VGNC Symbol;Acc:VGNC:95781]	5.3	0.0401
ssc-miR-146a-5p	TRAF6	TNF receptor associated factor 6 [Source:VGNC Symbol;Acc:VGNC:94365]	5.3	0.0401
ssc-miR-146a-5p	TRAT1	T cell receptor associated transmembrane adaptor 1 [Source:VGNC Symbol;Acc:VGNC:94381]	5.3	0.0401
ssc-miR-146a-5p	TRMT6	tRNA methyltransferase 6 non-catalytic subunit [Source:VGNC Symbol;Acc:VGNC:95956]	5.3	0.0401
ssc-miR-146a-5p	TTL	hypothetical gene	5.3	0.0401
ssc-miR-146a-5p	TUBB6	tubulin beta 6 class V [Source:VGNC Symbol;Acc:VGNC:94581]	5.3	0.0401
ssc-miR-146a-5p	UBA6	ubiquitin like modifier activating enzyme 6 [Source:VGNC Symbol;Acc:VGNC:94630]	5.3	0.0401
ssc-miR-146a-5p	UBE2G1	ubiquitin conjugating enzyme E2 G1 [Source:HGNC Symbol;Acc:HGNC:12482]	5.3	0.0401
ssc-miR-146a-5p	UBE2W	ubiquitin conjugating enzyme E2 W [Source:VGNC Symbol;Acc:VGNC:98890]	5.3	0.0401
ssc-miR-146a-5p	UBN2	ubinuclein 2 [Source:VGNC Symbol;Acc:VGNC:94664]	5.3	0.0401
ssc-miR-146a-5p	UBXN7	hypothetical gene	5.3	0.0401
ssc-miR-146a-5p	UNC5D	unc-5 netrin receptor D [Source:VGNC Symbol;Acc:VGNC:95892]	5.3	0.0401
ssc-miR-146a-5p	USP3	ubiquitin specific peptidase 3 [Source:VGNC Symbol;Acc:VGNC:94757]	5.3	0.0401
ssc-miR-146a-5p	USP44	ubiquitin specific peptidase 44 [Source:VGNC Symbol;Acc:VGNC:94766]	5.3	0.0401
ssc-miR-146a-5p	USP47	ubiquitin specific peptidase 47 [Source:VGNC Symbol;Acc:VGNC:94769]	5.3	0.0401
ssc-miR-146a-5p	VASN	vasorin [Source:VGNC Symbol;Acc:VGNC:94803]	5.3	0.0401
ssc-miR-146a-5p	VAT1	vesicle amine transport 1 [Source:VGNC Symbol;Acc:VGNC:94805]	5.3	0.0401
ssc-miR-146a-5p	VPS52	VPS52 subunit of GARP complex [Source:VGNC Symbol;Acc:VGNC:94860]	5.3	0.0401
ssc-miR-146a-5p	VTI1B	vesicle transport through interaction with t-SNAREs 1B [Source:VGNC Symbol;Acc:VGNC:94880]	5.3	0.0401
ssc-miR-146a-5p	WASF2	WASP family member 2 [Source:VGNC Symbol;Acc:VGNC:94893]	5.3	0.0401
ssc-miR-146a-5p	WASF3	WASP family member 3 [Source:VGNC Symbol;Acc:VGNC:94894]	5.3	0.0401
ssc-miR-146a-5p	WDR12	WD repeat domain 12 [Source:VGNC Symbol;Acc:VGNC:95603]	5.3	0.0401
ssc-miR-146a-5p	WDR37	WD repeat domain 37 [Source:VGNC Symbol;Acc:VGNC:96258]	5.3	0.0401
ssc-miR-146a-5p	WHAMM	WASP homolog associated with actin, golgi membranes and microtubules [Source:VGNC Symbol;Acc:VGNC:98414]	5.3	0.0401
ssc-miR-146a-5p	WWC2	WW and C2 domain containing 2 [Source:VGNC Symbol;Acc:VGNC:96033]	5.3	0.0401
ssc-miR-146a-5p	XIAP	X-linked inhibitor of apoptosis [Source:NCBI gene (formerly Entrezgene);Acc:100037300]	5.3	0.0401
ssc-miR-146a-5p	XKR4	XK related 4 [Source:VGNC Symbol;Acc:VGNC:98900]	5.3	0.0401
ssc-miR-146a-5p	ZBTB2	zinc finger and BTB domain containing 2 [Source:VGNC Symbol;Acc:VGNC:95062]	5.3	0.0401
ssc-miR-146a-5p	ZBTB26	zinc finger and BTB domain containing 26 [Source:VGNC Symbol;Acc:VGNC:95066]	5.3	0.0401
ssc-miR-146a-5p	ZBTB8B	zinc finger and BTB domain containing 8B [Source:HGNC Symbol;Acc:HGNC:37057]	5.3	0.0401
ssc-miR-146a-5p	ZC3H6	zinc finger CCCH-type containing 6 [Source:VGNC Symbol;Acc:VGNC:95098]	5.3	0.0401
ssc-miR-146a-5p	ZDHHC13	zinc finger DHHC-type palmitoyltransferase 13 [Source:VGNC Symbol;Acc:VGNC:95116]	5.3	0.0401
ssc-miR-146a-5p	ZFAND2B	zinc finger AN1-type containing 2B [Source:VGNC Symbol;Acc:VGNC:96069]	5.3	0.0401
ssc-miR-146a-5p	ZFP36L2	ZFP36 ring finger protein like 2 [Source:VGNC Symbol;Acc:VGNC:95147]	5.3	0.0401
ssc-miR-146a-5p	ZFYVE1	zinc finger FYVE-type containing 1 [Source:VGNC Symbol;Acc:VGNC:95156]	5.3	0.0401
ssc-miR-146a-5p	ZNF148	zinc finger protein 148 [Source:VGNC Symbol;Acc:VGNC:95192]	5.3	0.0401
ssc-miR-146a-5p	ZNF24	zinc finger protein 24 [Source:VGNC Symbol;Acc:VGNC:98422]	5.3	0.0401
ssc-miR-146a-5p	ZNF367	zinc finger protein 367 [Source:VGNC Symbol;Acc:VGNC:95882]	5.3	0.0401
ssc-miR-146a-5p	ZNF37A	zinc finger protein 37A [Source:HGNC Symbol;Acc:HGNC:13102]	5.3	0.0401
ssc-miR-146a-5p	ZNF512B	zinc finger protein 512B [Source:HGNC Symbol;Acc:HGNC:29212]	5.3	0.0401
ssc-miR-146a-5p	ZNF532	zinc finger protein 532 [Source:HGNC Symbol;Acc:HGNC:30940]	5.3	0.0401
ssc-miR-146a-5p	ZNF652	zinc finger protein 652 [Source:VGNC Symbol;Acc:VGNC:99108]	5.3	0.0401

ssc-miR-146a-5p	ZNF827	zinc finger protein 827 [Source:VGNC Symbol;Acc:VGNC:98970]	5.3	0.0401
ssc-miR-146a-5p	ZNRF2	zinc and ring finger 2 [Source:VGNC Symbol;Acc:VGNC:95317]	5.3	0.0401
ssc-miR-146a-5p	ZNRF3	zinc and ring finger 3 [Source:VGNC Symbol;Acc:VGNC:95318]	5.3	0.0401
ssc-miR-146a-5p	ZRSR1	hypothetical gene	5.3	0.0401
ssc-miR-146a-5p	ZSCAN29	zinc finger and SCAN domain containing 29 [Source:HGNC Symbol;Acc:HGNC:26673]	5.3	0.0401
ssc-miR-138	AAK1	AP2 associated kinase 1 [Source:VGNC Symbol;Acc:VGNC:100379]	4.63	0.000038
ssc-miR-138	ACVR2A	activin A receptor type 2A [Source:VGNC Symbol;Acc:VGNC:95843]	4.63	0.000038
ssc-miR-138	ACVR2B	activin A receptor type 2B [Source:VGNC Symbol;Acc:VGNC:108629]	4.63	0.000038
ssc-miR-138	ADAMTS5	ADAM metallopeptidase with thrombospondin type 1 motif 5 [Source:VGNC Symbol;Acc:VGNC:85085]	4.63	0.000038
ssc-miR-138	ADAMTSL3	ADAMTS like 3 [Source:VGNC Symbol;Acc:VGNC:85092]	4.63	0.000038
ssc-miR-138	ADCK4	hypothetical gene	4.63	0.000038
ssc-miR-138	ADCYAP1R1	ADCYAP receptor type I [Source:VGNC Symbol;Acc:VGNC:85115]	4.63	0.000038
ssc-miR-138	ADRA2A	adrenoceptor alpha 2A [Source:VGNC Symbol;Acc:VGNC:85156]	4.63	0.000038
ssc-miR-138	AEBP2	AE binding protein 2 [Source:VGNC Symbol;Acc:VGNC:85163]	4.63	0.000038
ssc-miR-138	AFF1	AF4/FMR2 family member 1 [Source:VGNC Symbol;Acc:VGNC:85167]	4.63	0.000038
ssc-miR-138	AFF3	AF4/FMR2 family member 3 [Source:HGNC Symbol;Acc:HGNC:6473]	4.63	0.000038
ssc-miR-138	AGO1	hypothetical gene	4.63	0.000038
ssc-miR-138	AGO4	hypothetical gene	4.63	0.000038
ssc-miR-138	AHCYL2	adenosylhomocysteinase like 2 [Source:VGNC Symbol;Acc:VGNC:97874]	4.63	0.000038
ssc-miR-138	AHDC1	hypothetical gene	4.63	0.000038
ssc-miR-138	AKAP11	A-kinase anchoring protein 11 [Source:HGNC Symbol;Acc:HGNC:369]	4.63	0.000038
ssc-miR-138	AKAP13	hypothetical gene	4.63	0.000038
ssc-miR-138	AMER1	APC membrane recruitment protein 1 [Source:VGNC Symbol;Acc:VGNC:85276]	4.63	0.000038
ssc-miR-138	AMIGO1	adhesion molecule with Ig like domain 1 [Source:VGNC Symbol;Acc:VGNC:85280]	4.63	0.000038
ssc-miR-138	AMMECR1	AMMECR nuclear protein 1 [Source:VGNC Symbol;Acc:VGNC:96559]	4.63	0.000038
ssc-miR-138	ANK1	ankyrin 1 [Source:VGNC Symbol;Acc:VGNC:96344]	4.63	0.000038
ssc-miR-138	ANKFY1	ankyrin repeat and FYVE domain containing 1 [Source:VGNC Symbol;Acc:VGNC:85314]	4.63	0.000038
ssc-miR-138	ANKRD13B	ankyrin repeat domain 13B [Source:VGNC Symbol;Acc:VGNC:85325]	4.63	0.000038
ssc-miR-138	ANKRD54	ankyrin repeat domain 54 [Source:VGNC Symbol;Acc:VGNC:85344]	4.63	0.000038
ssc-miR-138	ANKS6	ankyrin repeat and sterile alpha motif domain containing 6 [Source:VGNC Symbol;Acc:VGNC:85351]	4.63	0.000038
ssc-miR-138	ANO10	anoctamin 10 [Source:VGNC Symbol;Acc:VGNC:85355]	4.63	0.000038
ssc-miR-138	APH1A	aph-1 homolog A, gamma-secretase subunit [Source:VGNC Symbol;Acc:VGNC:85409]	4.63	0.000038
ssc-miR-138	APOL4	hypothetical gene	4.63	0.000038
ssc-miR-138	APPBP2	amyloid beta protein binding protein 2 [Source:VGNC Symbol;Acc:VGNC:85425]	4.63	0.000038
ssc-miR-138	ARFGEF2	ADP ribosylation factor guanine nucleotide exchange factor 2 [Source:VGNC Symbol;Acc:VGNC:95672]	4.63	0.000038
ssc-miR-138	ARGLU1	arginine and glutamate rich 1 [Source:VGNC Symbol;Acc:VGNC:85456]	4.63	0.000038
ssc-miR-138	ARHGAP19-SLIT1	hypothetical gene	4.63	0.000038
ssc-miR-138	ARHGAP35	Rho GTPase activating protein 35 [Source:VGNC Symbol;Acc:VGNC:85473]	4.63	0.000038
ssc-miR-138	ARHGAP42	Rho GTPase activating protein 42 [Source:VGNC Symbol;Acc:VGNC:85477]	4.63	0.000038
ssc-miR-138	ARHGEF3	Rho guanine nucleotide exchange factor 3 [Source:VGNC Symbol;Acc:VGNC:85496]	4.63	0.000038
ssc-miR-138	ARHGEF39	Rho guanine nucleotide exchange factor 39 [Source:VGNC Symbol;Acc:VGNC:85500]	4.63	0.000038
ssc-miR-138	ARHGEF40	Rho guanine nucleotide exchange factor 40 [Source:VGNC Symbol;Acc:VGNC:85501]	4.63	0.000038
ssc-miR-138	ARL10	ADP ribosylation factor like GTPase 10 [Source:VGNC Symbol;Acc:VGNC:85514]	4.63	0.000038
ssc-miR-138	ARL5B	ADP ribosylation factor like GTPase 5B [Source:VGNC Symbol;Acc:VGNC:95985]	4.63	0.000038
ssc-miR-138	ARL6IP5	ADP ribosylation factor like GTPase 6 interacting protein 5 [Source:VGNC Symbol;Acc:VGNC:103904]	4.63	0.000038
ssc-miR-138	ARPC2	actin related protein 2/3 complex subunit 2 [Source:VGNC Symbol;Acc:VGNC:96420]	4.63	0.000038
ssc-miR-138	ARRB1	arrestin beta 1 [Source:VGNC Symbol;Acc:VGNC:85542]	4.63	0.000038
ssc-miR-138	ARRDC3	arrestin domain containing 3 [Source:VGNC Symbol;Acc:VGNC:85546]	4.63	0.000038
ssc-miR-138	ASXL2	ASXL transcriptional regulator 2 [Source:VGNC Symbol;Acc:VGNC:85595]	4.63	0.000038

ssc-miR-138	ASXL3	ASXL transcriptional regulator 3 [Source:VGNC Symbol;Acc:VGNC:85596]	4.63	0.000038
ssc-miR-138	ATCAY	ATCAY kinesin light chain interacting caytaxin [Source:VGNC Symbol;Acc:VGNC:85603]	4.63	0.000038
ssc-miR-138	ATG4A	autophagy related 4A cysteine peptidase [Source:VGNC Symbol;Acc:VGNC:85622]	4.63	0.000038
ssc-miR-138	ATG5	autophagy related 5 [Source:VGNC Symbol;Acc:VGNC:103029]	4.63	0.000038
ssc-miR-138	ATG7	autophagy related 7 [Source:VGNC Symbol;Acc:VGNC:85625]	4.63	0.000038
ssc-miR-138	ATG9B	autophagy related 9B [Source:VGNC Symbol;Acc:VGNC:85626]	4.63	0.000038
ssc-miR-138	ATP11C	ATPase phospholipid transporting 11C [Source:VGNC Symbol;Acc:VGNC:85635]	4.63	0.000038
ssc-miR-138	AZGP1	hypothetical gene	4.63	0.000038
ssc-miR-138	B3GNT4	UDP-GlcNAc:betaGal beta-1,3-N-acetylglucosaminyltransferase 4 [Source:VGNC Symbol;Acc:VGNC:85725]	4.63	0.000038
ssc-miR-138	BACE2	hypothetical gene	4.63	0.000038
ssc-miR-138	BAG1	BAG cochaperone 1 [Source:VGNC Symbol;Acc:VGNC:96502]	4.63	0.000038
ssc-miR-138	BAZ1B	bromodomain adjacent to zinc finger domain 1B [Source:VGNC Symbol;Acc:VGNC:85762]	4.63	0.000038
ssc-miR-138	BAZ2B	bromodomain adjacent to zinc finger domain 2B [Source:HGNC Symbol;Acc:HGNC:963]	4.63	0.000038
ssc-miR-138	BCKDK	branched chain keto acid dehydrogenase kinase [Source:VGNC Symbol;Acc:VGNC:85778]	4.63	0.000038
ssc-miR-138	BCL9L	BCL9 like [Source:VGNC Symbol;Acc:VGNC:96568]	4.63	0.000038
ssc-miR-138	BHLHE40	basic helix-loop-helix family member e40 [Source:VGNC Symbol;Acc:VGNC:85814]	4.63	0.000038
ssc-miR-138	BNIP3L	BCL2 interacting protein 3 like [Source:VGNC Symbol;Acc:VGNC:85856]	4.63	0.000038
ssc-miR-138	BRI3BP	BRI3 binding protein [Source:HGNC Symbol;Acc:HGNC:14251]	4.63	0.000038
ssc-miR-138	BRPF3	bromodomain and PHD finger containing 3 [Source:VGNC Symbol;Acc:VGNC:85886]	4.63	0.000038
ssc-miR-138	BTG1	BTG anti-proliferation factor 1 [Source:VGNC Symbol;Acc:VGNC:103907]	4.63	0.000038
ssc-miR-138	BVES	blood vessel epicardial substance [Source:VGNC Symbol;Acc:VGNC:103040]	4.63	0.000038
ssc-miR-138	BYSL	bystin like [Source:NCBI gene (formerly Entrezgene);Acc:100156292]	4.63	0.000038
ssc-miR-138	C10orf118	hypothetical gene	4.63	0.000038
ssc-miR-138	C10orf54	hypothetical gene	4.63	0.000038
ssc-miR-138	C11orf57	hypothetical gene	4.63	0.000038
ssc-miR-138	C12orf43	chromosome 14 C12orf43 homolog [Source:VGNC Symbol;Acc:VGNC:85943]	4.63	0.000038
ssc-miR-138	C15orf37	hypothetical gene	4.63	0.000038
ssc-miR-138	C15orf38	hypothetical gene	4.63	0.000038
ssc-miR-138	C16orf47	hypothetical gene	4.63	0.000038
ssc-miR-138	C17orf103	hypothetical gene	4.63	0.000038
ssc-miR-138	C17orf85	hypothetical gene	4.63	0.000038
ssc-miR-138	C19orf25	chromosome 2 C19orf25 homolog [Source:VGNC Symbol;Acc:VGNC:86002]	4.63	0.000038
ssc-miR-138	C19orf66	hypothetical gene	4.63	0.000038
ssc-miR-138	C1orf27	hypothetical gene	4.63	0.000038
ssc-miR-138	C6orf106	hypothetical gene	4.63	0.000038
ssc-miR-138	C6orf47	hypothetical gene	4.63	0.000038
ssc-miR-138	CABLES2	Cdk5 and Abl enzyme substrate 2 [Source:VGNC Symbol;Acc:VGNC:95753]	4.63	0.000038
ssc-miR-138	CACNA1A	calcium voltage-gated channel subunit alpha1 A [Source:VGNC Symbol;Acc:VGNC:99705]	4.63	0.000038
ssc-miR-138	CACNA2D1	calcium voltage-gated channel auxiliary subunit alpha2delta 1 [Source:VGNC Symbol;Acc:VGNC:86120]	4.63	0.000038
ssc-miR-138	CACUL1	CDK2 associated cullin domain 1 [Source:VGNC Symbol;Acc:VGNC:86132]	4.63	0.000038
ssc-miR-138	CADM1	cell adhesion molecule 1 [Source:VGNC Symbol;Acc:VGNC:86134]	4.63	0.000038
ssc-miR-138	CALML4	calmodulin like 4 [Source:VGNC Symbol;Acc:VGNC:86148]	4.63	0.000038
ssc-miR-138	CALN1	hypothetical gene	4.63	0.000038
ssc-miR-138	CAMK2N1	calcium/calmodulin dependent protein kinase II inhibitor 1 [Source:VGNC Symbol;Acc:VGNC:96935]	4.63	0.000038
ssc-miR-138	CAMTA2	calmodulin binding transcription activator 2 [Source:VGNC Symbol;Acc:VGNC:86165]	4.63	0.000038
ssc-miR-138	CARHSP1	calcium regulated heat stable protein 1 [Source:VGNC Symbol;Acc:VGNC:86190]	4.63	0.000038
ssc-miR-138	CASP3	caspase 3 [Source:VGNC Symbol;Acc:VGNC:103911]	4.63	0.000038
ssc-miR-138	CBFA2T2	CBFA2/RUNX1 partner transcriptional co-repressor 2 [Source:VGNC Symbol;Acc:VGNC:96573]	4.63	0.000038
ssc-miR-138	CBX5	chromobox 5 [Source:VGNC Symbol;Acc:VGNC:86232]	4.63	0.000038

ssc-miR-138	CCAR2	cell cycle and apoptosis regulator 2 [Source:VGNC Symbol;Acc:VGNC:86239]	4.63	0.000038
ssc-miR-138	CCDC103	hypothetical gene	4.63	0.000038
ssc-miR-138	CCDC107	coiled-coil domain containing 107 [Source:VGNC Symbol;Acc:VGNC:86245]	4.63	0.000038
ssc-miR-138	CCDC85C	coiled-coil domain containing 85C [Source:VGNC Symbol;Acc:VGNC:86322]	4.63	0.000038
ssc-miR-138	CCND3	cyclin D3 [Source:VGNC Symbol;Acc:VGNC:86353]	4.63	0.000038
ssc-miR-138	CCNE1	hypothetical gene	4.63	0.000038
ssc-miR-138	CCNL2	cyclin L2 [Source:VGNC Symbol;Acc:VGNC:86364]	4.63	0.000038
ssc-miR-138	CCNYL1	hypothetical gene	4.63	0.000038
ssc-miR-138	CD2AP	CD2 associated protein [Source:VGNC Symbol;Acc:VGNC:86406]	4.63	0.000038
ssc-miR-138	CD3EAP	hypothetical gene	4.63	0.000038
ssc-miR-138	CDK14	cyclin dependent kinase 14 [Source:VGNC Symbol;Acc:VGNC:86498]	4.63	0.000038
ssc-miR-138	CDK19	cyclin dependent kinase 19 [Source:VGNC Symbol;Acc:VGNC:86502]	4.63	0.000038
ssc-miR-138	CDK6	cyclin dependent kinase 6 [Source:HGNC Symbol;Acc:HGNC:1777]	4.63	0.000038
ssc-miR-138	CDK8	cyclin dependent kinase 8 [Source:VGNC Symbol;Acc:VGNC:86507]	4.63	0.000038
ssc-miR-138	CDR2L	cerebellar deration related protein 2 like [Source:VGNC Symbol;Acc:VGNC:86522]	4.63	0.000038
ssc-miR-138	CELF5	CUGBP Elav-like family member 5 [Source:VGNC Symbol;Acc:VGNC:86540]	4.63	0.000038
ssc-miR-138	CEP57L1	centrosomal protein 57 like 1 [Source:VGNC Symbol;Acc:VGNC:86573]	4.63	0.000038
ssc-miR-138	CHD6	chromodomain helicase DNA binding protein 6 [Source:VGNC Symbol;Acc:VGNC:95903]	4.63	0.000038
ssc-miR-138	CHERP	calcium homeostasis endoplasmic reticulum protein [Source:VGNC Symbol;Acc:VGNC:86639]	4.63	0.000038
ssc-miR-138	CHMP7	charged multivesicular body protein 7 [Source:VGNC Symbol;Acc:VGNC:86654]	4.63	0.000038
ssc-miR-138	CHST10	carbohydrate sulfotransferase 10 [Source:VGNC Symbol;Acc:VGNC:86674]	4.63	0.000038
ssc-miR-138	CINP	cyclin dependent kinase 2 interacting protein [Source:VGNC Symbol;Acc:VGNC:86705]	4.63	0.000038
ssc-miR-138	CIZ1	CDKN1A interacting zinc finger protein 1 [Source:VGNC Symbol;Acc:VGNC:86713]	4.63	0.000038
ssc-miR-138	CLCN2	chloride voltage-gated channel 2 [Source:VGNC Symbol;Acc:VGNC:86726]	4.63	0.000038
ssc-miR-138	CLIP1	CAP-Gly domain containing linker protein 1 [Source:VGNC Symbol;Acc:VGNC:86766]	4.63	0.000038
ssc-miR-138	CLK3	CDC like kinase 3 [Source:VGNC Symbol;Acc:VGNC:107129]	4.63	0.000038
ssc-miR-138	CLMP	CXADR like membrane protein [Source:VGNC Symbol;Acc:VGNC:86770]	4.63	0.000038
ssc-miR-138	CLN5	CLN5 intracellular trafficking protein [Source:HGNC Symbol;Acc:HGNC:2076]	4.63	0.000038
ssc-miR-138	CLNS1A	chloride nucleotide-sensitive channel 1A [Source:VGNC Symbol;Acc:VGNC:86773]	4.63	0.000038
ssc-miR-138	CLOCK	clock circadian regulator [Source:VGNC Symbol;Acc:VGNC:86774]	4.63	0.000038
ssc-miR-138	CLVS1	clavesin 1 [Source:VGNC Symbol;Acc:VGNC:86794]	4.63	0.000038
ssc-miR-138	CNOT6L	CCR4-NOT transcription complex subunit 6 like [Source:VGNC Symbol;Acc:VGNC:86838]	4.63	0.000038
ssc-miR-138	CNOT8	CCR4-NOT transcription complex subunit 8 [Source:VGNC Symbol;Acc:VGNC:86839]	4.63	0.000038
ssc-miR-138	COL4A2	collagen type IV alpha 2 chain [Source:VGNC Symbol;Acc:VGNC:86875]	4.63	0.000038
ssc-miR-138	COTL1	coactosin like F-actin binding protein 1 [Source:HGNC Symbol;Acc:HGNC:18304]	4.63	0.000038
ssc-miR-138	CPEB4	cytoplasmic polyadenylation element binding protein 4 [Source:VGNC Symbol;Acc:VGNC:86939]	4.63	0.000038
ssc-miR-138	CREB1	cAMP responsive element binding protein 1 [Source:VGNC Symbol;Acc:VGNC:96004]	4.63	0.000038
ssc-miR-138	CREB3L2	cAMP responsive element binding protein 3 like 2 [Source:VGNC Symbol;Acc:VGNC:86981]	4.63	0.000038
ssc-miR-138	CSGALNACT2	chondroitin sulfate N-acetylgalactosaminyltransferase 2 [Source:VGNC Symbol;Acc:VGNC:87036]	4.63	0.000038
ssc-miR-138	CSRNP1	cysteine and serine rich nuclear protein 1 [Source:VGNC Symbol;Acc:VGNC:87046]	4.63	0.000038
ssc-miR-138	CTD-2207O23.12	hypothetical gene	4.63	0.000038
ssc-miR-138	CTDSPL2	CTD small phosphatase like 2 [Source:VGNC Symbol;Acc:VGNC:87060]	4.63	0.000038
ssc-miR-138	CTIF	cap binding complex dependent translation initiation factor [Source:VGNC Symbol;Acc:VGNC:87062]	4.63	0.000038
ssc-miR-138	CTSH	cathepsin H [Source:VGNC Symbol;Acc:VGNC:87077]	4.63	0.000038
ssc-miR-138	CUL5	cullin 5 [Source:VGNC Symbol;Acc:VGNC:87089]	4.63	0.000038
ssc-miR-138	CXorf24	hypothetical gene	4.63	0.000038
ssc-miR-138	CYB5R1	cytochrome b5 reductase 1 [Source:VGNC Symbol;Acc:VGNC:95584]	4.63	0.000038
ssc-miR-138	CYP3A5	hypothetical gene	4.63	0.000038
ssc-miR-138	DAAM2	dishevelled associated activator of morphosis 2 [Source:VGNC Symbol;Acc:VGNC:87142]	4.63	0.000038

ssc-miR-138	DACH1	dachshund family transcription factor 1 [Source:VGNC Symbol;Acc:VGNC:87146]	4.63	0.000038
ssc-miR-138	DAPK2	death associated protein kinase 2 [Source:VGNC Symbol;Acc:VGNC:87157]	4.63	0.000038
ssc-miR-138	DCBLD2	discoidin, CUB and LCCL domain containing 2 [Source:VGNC Symbol;Acc:VGNC:87180]	4.63	0.000038
ssc-miR-138	DCP1A	decapping mRNA 1A [Source:VGNC Symbol;Acc:VGNC:97960]	4.63	0.000038
ssc-miR-138	DCUN1D2	defective in cullin neddylation 1 domain containing 2 [Source:VGNC Symbol;Acc:VGNC:87195]	4.63	0.000038
ssc-miR-138	DCUN1D4	defective in cullin neddylation 1 domain containing 4 [Source:VGNC Symbol;Acc:VGNC:87197]	4.63	0.000038
ssc-miR-138	DCX	doublecortin [Source:HGNC Symbol;Acc:HGNC:2714]	4.63	0.000038
ssc-miR-138	DEK	hypothetical gene	4.63	0.000038
ssc-miR-138	DENND1A	DENN domain containing 1A [Source:VGNC Symbol;Acc:VGNC:87245]	4.63	0.000038
ssc-miR-138	DENND6A	DENN domain containing 6A [Source:VGNC Symbol;Acc:VGNC:87256]	4.63	0.000038
ssc-miR-138	DEPDC5	DEP domain containing 5, GATOR1 subcomplex subunit [Source:VGNC Symbol;Acc:VGNC:107385]	4.63	0.000038
ssc-miR-138	DESI2	desumoylating isopeptidase 2 [Source:VGNC Symbol;Acc:VGNC:96037]	4.63	0.000038
ssc-miR-138	DGCR14	hypothetical gene	4.63	0.000038
ssc-miR-138	DGKE	diacylglycerol kinase epsilon [Source:VGNC Symbol;Acc:VGNC:87271]	4.63	0.000038
ssc-miR-138	DHCR7	7-dehydrocholesterol reductase [Source:VGNC Symbol;Acc:VGNC:97964]	4.63	0.000038
ssc-miR-138	DHDDS	dehydrodolichyl diphosphate synthase subunit [Source:VGNC Symbol;Acc:VGNC:87279]	4.63	0.000038
ssc-miR-138	DLG3	discs large MAGUK scaffold protein 3 [Source:VGNC Symbol;Acc:VGNC:87327]	4.63	0.000038
ssc-miR-138	DLST	dihydrolipoamide S-succinyltransferase [Source:VGNC Symbol;Acc:VGNC:87338]	4.63	0.000038
ssc-miR-138	DMRT2	doublesex and mab-3 related transcription factor 2 [Source:VGNC Symbol;Acc:VGNC:87351]	4.63	0.000038
ssc-miR-138	DNAJB6	DnaJ heat shock protein family (Hsp40) member B6 [Source:HGNC Symbol;Acc:HGNC:14888]	4.63	0.000038
ssc-miR-138	DNAJC15	DnaJ heat shock protein family (Hsp40) member C15 [Source:HGNC Symbol;Acc:HGNC:20325]	4.63	0.000038
ssc-miR-138	DNMT3A	DNA methyltransferase 3 alpha [Source:VGNC Symbol;Acc:VGNC:87384]	4.63	0.000038
ssc-miR-138	DOT1L	DOT1 like histone lysine methyltransferase [Source:VGNC Symbol;Acc:VGNC:87410]	4.63	0.000038
ssc-miR-138	DPY19L1	dpy-19 like C-mannosyltransferase 1 [Source:VGNC Symbol;Acc:VGNC:99712]	4.63	0.000038
ssc-miR-138	DTX1	deltex E3 ubiquitin ligase 1 [Source:VGNC Symbol;Acc:VGNC:87466]	4.63	0.000038
ssc-miR-138	DTX4	deltex E3 ubiquitin ligase 4 [Source:VGNC Symbol;Acc:VGNC:87470]	4.63	0.000038
ssc-miR-138	DUSP16	dual specificity phosphatase 16 [Source:VGNC Symbol;Acc:VGNC:87480]	4.63	0.000038
ssc-miR-138	DVL2	dishevelled segment polarity protein 2 [Source:VGNC Symbol;Acc:VGNC:87491]	4.63	0.000038
ssc-miR-138	E2F2	E2F transcription factor 2 [Source:VGNC Symbol;Acc:VGNC:87513]	4.63	0.000038
ssc-miR-138	E2F3	E2F transcription factor 3 [Source:VGNC Symbol;Acc:VGNC:87514]	4.63	0.000038
ssc-miR-138	EBF1	EBF transcription factor 1 [Source:VGNC Symbol;Acc:VGNC:87525]	4.63	0.000038
ssc-miR-138	EBF2	EBF transcription factor 2 [Source:VGNC Symbol;Acc:VGNC:87526]	4.63	0.000038
ssc-miR-138	EBPL	EBP like [Source:VGNC Symbol;Acc:VGNC:87529]	4.63	0.000038
ssc-miR-138	EDC3	enhancer of mRNA decapping 3 [Source:VGNC Symbol;Acc:VGNC:87543]	4.63	0.000038
ssc-miR-138	EFNB3	ephrin B3 [Source:VGNC Symbol;Acc:VGNC:87578]	4.63	0.000038
ssc-miR-138	EGFR	epidermal growth factor receptor [Source:VGNC Symbol;Acc:VGNC:87587]	4.63	0.000038
ssc-miR-138	EID1	EP300 interacting inhibitor of differentiation 1 [Source:HGNC Symbol;Acc:HGNC:1191]	4.63	0.000038
ssc-miR-138	EIF3J	eukaryotic translation initiation factor 3 subunit J [Source:VGNC Symbol;Acc:VGNC:87621]	4.63	0.000038
ssc-miR-138	EIF4EBP1	eukaryotic translation initiation factor 4E binding protein 1 [Source:VGNC Symbol;Acc:VGNC:96268]	4.63	0.000038
ssc-miR-138	EIF4EBP2	eukaryotic translation initiation factor 4E binding protein 2 [Source:VGNC Symbol;Acc:VGNC:87629]	4.63	0.000038
ssc-miR-138	ELF2	E74 like ETS transcription factor 2 [Source:VGNC Symbol;Acc:VGNC:87642]	4.63	0.000038
ssc-miR-138	ELMOD3	ELMO domain containing 3 [Source:VGNC Symbol;Acc:VGNC:87654]	4.63	0.000038
ssc-miR-138	EMB	embigin [Source:VGNC Symbol;Acc:VGNC:87668]	4.63	0.000038
ssc-miR-138	EN2	engrailed homeobox 2 [Source:VGNC Symbol;Acc:VGNC:87694]	4.63	0.000038
ssc-miR-138	ENC1	ectodermal-neural cortex 1 [Source:VGNC Symbol;Acc:VGNC:87696]	4.63	0.000038
ssc-miR-138	ENG	endoglin [Source:VGNC Symbol;Acc:VGNC:103087]	4.63	0.000038
ssc-miR-138	ENOX2	ecto-NOX disulfide-thiol exchanger 2 [Source:VGNC Symbol;Acc:VGNC:87707]	4.63	0.000038
ssc-miR-138	ENTPD1	ectonucleoside triphosphate diphosphohydrolase 1 [Source:VGNC Symbol;Acc:VGNC:97977]	4.63	0.000038
ssc-miR-138	EPHA8	EPH receptor A8 [Source:VGNC Symbol;Acc:VGNC:87735]	4.63	0.000038

ssc-miR-138	ERBB4	erb-b2 receptor tyrosine kinase 4 [Source:VGNC Symbol;Acc:VGNC:96284]	4.63	0.000038
ssc-miR-138	ERI1	exoribonuclease 1 [Source:VGNC Symbol;Acc:VGNC:97985]	4.63	0.000038
ssc-miR-138	ERP44	endoplasmic reticulum protein 44 [Source:VGNC Symbol;Acc:VGNC:87783]	4.63	0.000038
ssc-miR-138	EVC	EvC ciliary complex subunit 1 [Source:VGNC Symbol;Acc:VGNC:87818]	4.63	0.000038
ssc-miR-138	EXT1	exostosin glycosyltransferase 1 [Source:VGNC Symbol;Acc:VGNC:87846]	4.63	0.000038
ssc-miR-138	EZH2	enhancer of zeste 2 polycomb repressive complex 2 subunit [Source:VGNC Symbol;Acc:VGNC:87855]	4.63	0.000038
ssc-miR-138	FABP3	fatty acid binding protein 3 [Source:NCBI gene (formerly Entrezgene);Acc:399532]	4.63	0.000038
ssc-miR-138	FADS1	fatty acid desaturase 1 [Source:VGNC Symbol;Acc:VGNC:87875]	4.63	0.000038
ssc-miR-138	FAHD2A	hypothetical gene	4.63	0.000038
ssc-miR-138	FAIM3	hypothetical gene	4.63	0.000038
ssc-miR-138	FAM107B	hypothetical gene	4.63	0.000038
ssc-miR-138	FAM134C	hypothetical gene	4.63	0.000038
ssc-miR-138	FAM169A	family with sequence similarity 169 member A [Source:VGNC Symbol;Acc:VGNC:87927]	4.63	0.000038
ssc-miR-138	FAM178A	hypothetical gene	4.63	0.000038
ssc-miR-138	FAM210B	family with sequence similarity 210 member B [Source:VGNC Symbol;Acc:VGNC:95736]	4.63	0.000038
ssc-miR-138	FAM219B	family with sequence similarity 219 member B [Source:VGNC Symbol;Acc:VGNC:87956]	4.63	0.000038
ssc-miR-138	FAM222B	family with sequence similarity 222 member B [Source:VGNC Symbol;Acc:VGNC:87960]	4.63	0.000038
ssc-miR-138	FAM63B	hypothetical gene	4.63	0.000038
ssc-miR-138	FAM84A	hypothetical gene	4.63	0.000038
ssc-miR-138	FBXO10	F-box protein 10 [Source:VGNC Symbol;Acc:VGNC:103092]	4.63	0.000038
ssc-miR-138	FBXO41	F-box protein 41 [Source:VGNC Symbol;Acc:VGNC:88046]	4.63	0.000038
ssc-miR-138	FBXO46	F-box protein 46 [Source:VGNC Symbol;Acc:VGNC:88049]	4.63	0.000038
ssc-miR-138	FEM1C	fem-1 homolog C [Source:VGNC Symbol;Acc:VGNC:88084]	4.63	0.000038
ssc-miR-138	FERMT2	FERM domain containing kindlin 2 [Source:VGNC Symbol;Acc:VGNC:88088]	4.63	0.000038
ssc-miR-138	FGF10	fibroblast growth factor 10 [Source:VGNC Symbol;Acc:VGNC:88101]	4.63	0.000038
ssc-miR-138	FIGN	fidgetin, microtubule severing factor [Source:VGNC Symbol;Acc:VGNC:95580]	4.63	0.000038
ssc-miR-138	FKBP15	FKBP prolyl isomerase family member 15 [Source:VGNC Symbol;Acc:VGNC:88145]	4.63	0.000038
ssc-miR-138	FLNB	filamin B [Source:VGNC Symbol;Acc:VGNC:88156]	4.63	0.000038
ssc-miR-138	FMNL3	formin like 3 [Source:VGNC Symbol;Acc:VGNC:88169]	4.63	0.000038
ssc-miR-138	FNDC8	fibronectin type III domain containing 8 [Source:VGNC Symbol;Acc:VGNC:88186]	4.63	0.000038
ssc-miR-138	FOSB	FosB proto-onco, AP-1 transcription factor subunit [Source:VGNC Symbol;Acc:VGNC:88190]	4.63	0.000038
ssc-miR-138	FOSL2	FOS like 2, AP-1 transcription factor subunit [Source:VGNC Symbol;Acc:VGNC:88192]	4.63	0.000038
ssc-miR-138	FOXC1	forkhead box C1 [Source:VGNC Symbol;Acc:VGNC:88197]	4.63	0.000038
ssc-miR-138	FOXD1	forkhead box D1 [Source:VGNC Symbol;Acc:VGNC:88199]	4.63	0.000038
ssc-miR-138	FOXE1	forkhead box E1 [Source:VGNC Symbol;Acc:VGNC:88202]	4.63	0.000038
ssc-miR-138	FOXK1	forkhead box K1 [Source:VGNC Symbol;Acc:VGNC:88214]	4.63	0.000038
ssc-miR-138	FOXP4	forkhead box P4 [Source:VGNC Symbol;Acc:VGNC:88224]	4.63	0.000038
ssc-miR-138	FPGS	folylpolyglutamate synthase [Source:VGNC Symbol;Acc:VGNC:88230]	4.63	0.000038
ssc-miR-138	FRMD4A	FERM domain containing 4A [Source:VGNC Symbol;Acc:VGNC:96088]	4.63	0.000038
ssc-miR-138	FRMPD3	FERM and PDZ domain containing 3 [Source:VGNC Symbol;Acc:VGNC:88242]	4.63	0.000038
ssc-miR-138	FRS2	fibroblast growth factor receptor substrate 2 [Source:VGNC Symbol;Acc:VGNC:88246]	4.63	0.000038
ssc-miR-138	FSD1	fibronectin type III and SPRY domain containing 1 [Source:VGNC Symbol;Acc:VGNC:88251]	4.63	0.000038
ssc-miR-138	GAB2	GRB2 associated binding protein 2 [Source:VGNC Symbol;Acc:VGNC:108586]	4.63	0.000038
ssc-miR-138	GABRB1	gamma-aminobutyric acid type A receptor subunit beta1 [Source:VGNC Symbol;Acc:VGNC:88306]	4.63	0.000038
ssc-miR-138	GALNTL6	polypeptide N-acetylgalactosaminyltransferase like 6 [Source:VGNC Symbol;Acc:VGNC:103947]	4.63	0.000038
ssc-miR-138	GAS7	growth arrest specific 7 [Source:VGNC Symbol;Acc:VGNC:88359]	4.63	0.000038
ssc-miR-138	GATAD2A	GATA zinc finger domain containing 2A [Source:VGNC Symbol;Acc:VGNC:88368]	4.63	0.000038
ssc-miR-138	GATAD2B	GATA zinc finger domain containing 2B [Source:VGNC Symbol;Acc:VGNC:88369]	4.63	0.000038
ssc-miR-138	GATSL2	hypothetical gene	4.63	0.000038

ssc-miR-138	GBA	glucosylceramidase beta [Source:VGNC Symbol;Acc:VGNC:98802]	4.63	0.000038
ssc-miR-138	GBX2	gastrulation brain homeobox 2 [Source:VGNC Symbol;Acc:VGNC:96316]	4.63	0.000038
ssc-miR-138	GFRA1	GDNF family receptor alpha 1 [Source:VGNC Symbol;Acc:VGNC:88428]	4.63	0.000038
ssc-miR-138	GGCX	gamma-glutamyl carboxylase [Source:VGNC Symbol;Acc:VGNC:88435]	4.63	0.000038
ssc-miR-138	GID4	GID complex subunit 4 homolog [Source:VGNC Symbol;Acc:VGNC:98996]	4.63	0.000038
ssc-miR-138	GINS2	GINS complex subunit 2 [Source:HGNC Symbol;Acc:HGNC:24575]	4.63	0.000038
ssc-miR-138	GIT1	GIT ArfGAP 1 [Source:VGNC Symbol;Acc:VGNC:88457]	4.63	0.000038
ssc-miR-138	GJA5	gap junction protein alpha 5 [Source:VGNC Symbol;Acc:VGNC:88460]	4.63	0.000038
ssc-miR-138	GK5	glycerol kinase 5 [Source:VGNC Symbol;Acc:VGNC:96734]	4.63	0.000038
ssc-miR-138	GLCCI1	glucocorticoid induced 1 [Source:VGNC Symbol;Acc:VGNC:88476]	4.63	0.000038
ssc-miR-138	GLTP	glycolipid transfer protein [Source:VGNC Symbol;Acc:VGNC:107395]	4.63	0.000038
ssc-miR-138	GM2A	GM2 ganglioside activator [Source:VGNC Symbol;Acc:VGNC:88506]	4.63	0.000038
ssc-miR-138	GMPPB	GDP-mannose pyrophosphorylase B [Source:NCBI gene (formerly Entrezgene);Acc:100513376]	4.63	0.000038
ssc-miR-138	GNAI2	G protein subunit alpha i2 [Source:VGNC Symbol;Acc:VGNC:88522]	4.63	0.000038
ssc-miR-138	GNG2	hypothetical gene	4.63	0.000038
ssc-miR-138	GPHN	gephyrin [Source:VGNC Symbol;Acc:VGNC:88587]	4.63	0.000038
ssc-miR-138	GPR124	hypothetical gene	4.63	0.000038
ssc-miR-138	GPR135	G protein-coupled receptor 135 [Source:VGNC Symbol;Acc:VGNC:88599]	4.63	0.000038
ssc-miR-138	GPR158	G protein-coupled receptor 158 [Source:VGNC Symbol;Acc:VGNC:96016]	4.63	0.000038
ssc-miR-138	GPR3	G protein-coupled receptor 3 [Source:VGNC Symbol;Acc:VGNC:88628]	4.63	0.000038
ssc-miR-138	GPRIN3	hypothetical gene	4.63	0.000038
ssc-miR-138	GRB7	growth factor receptor bound protein 7 [Source:VGNC Symbol;Acc:VGNC:88664]	4.63	0.000038
ssc-miR-138	GRHPR	glyoxylate and hydroxypyruvate reductase [Source:VGNC Symbol;Acc:VGNC:88669]	4.63	0.000038
ssc-miR-138	GRID1	glutamate ionotropic receptor delta type subunit 1 [Source:VGNC Symbol;Acc:VGNC:88674]	4.63	0.000038
ssc-miR-138	GRIK1-AS2	hypothetical gene	4.63	0.000038
ssc-miR-138	GRIN2B	glutamate ionotropic receptor NMDA type subunit 2B [Source:VGNC Symbol;Acc:VGNC:88684]	4.63	0.000038
ssc-miR-138	GTPBP1	GTP binding protein 1 [Source:VGNC Symbol;Acc:VGNC:88742]	4.63	0.000038
ssc-miR-138	H2AFX	hypothetical gene	4.63	0.000038
ssc-miR-138	H3F3B	hypothetical gene	4.63	0.000038
ssc-miR-138	H6PD	hexose-6-phosphate dehydrogenase/glucose 1-dehydrogenase [Source:VGNC Symbol;Acc:VGNC:88764]	4.63	0.000038
ssc-miR-138	HAP1	huntingtin associated protein 1 [Source:VGNC Symbol;Acc:VGNC:88779]	4.63	0.000038
ssc-miR-138	HAUS5	HAUS augmin like complex subunit 5 [Source:VGNC Symbol;Acc:VGNC:88788]	4.63	0.000038
ssc-miR-138	HDAC4	histone deacetylase 4 [Source:VGNC Symbol;Acc:VGNC:95602]	4.63	0.000038
ssc-miR-138	HIF1A	hypoxia inducible factor 1 subunit alpha [Source:VGNC Symbol;Acc:VGNC:88880]	4.63	0.000038
ssc-miR-138	HIF1AN	hypoxia inducible factor 1 subunit alpha inhibitor [Source:VGNC Symbol;Acc:VGNC:98033]	4.63	0.000038
ssc-miR-138	HIPK1	homeodomain interacting protein kinase 1 [Source:VGNC Symbol;Acc:VGNC:88887]	4.63	0.000038
ssc-miR-138	HIPK3	homeodomain interacting protein kinase 3 [Source:VGNC Symbol;Acc:VGNC:88889]	4.63	0.000038
ssc-miR-138	HK1	hexokinase 1 [Source:VGNC Symbol;Acc:VGNC:88892]	4.63	0.000038
ssc-miR-138	HMGA1	high mobility group AT-hook 1 [Source:VGNC Symbol;Acc:VGNC:103070]	4.63	0.000038
ssc-miR-138	HMGXB4	HMG-box containing 4 [Source:VGNC Symbol;Acc:VGNC:96346]	4.63	0.000038
ssc-miR-138	HNF4A	hepatocyte nuclear factor 4 alpha [Source:VGNC Symbol;Acc:VGNC:96348]	4.63	0.000038
ssc-miR-138	HNRNPUL1	heterous nuclear ribonucleoprotein U like 1 [Source:VGNC Symbol;Acc:VGNC:88926]	4.63	0.000038
ssc-miR-138	HOXA13	homeobox A13 [Source:NCBI gene (formerly Entrezgene);Acc:100359352]	4.63	0.000038
ssc-miR-138	HOXD11	homeobox D11 [Source:VGNC Symbol;Acc:VGNC:96352]	4.63	0.000038
ssc-miR-138	HOXD12	homeobox D12 [Source:VGNC Symbol;Acc:VGNC:96353]	4.63	0.000038
ssc-miR-138	HRK	harakiri, BCL2 interacting protein [Source:HGNC Symbol;Acc:HGNC:5185]	4.63	0.000038
ssc-miR-138	HS6ST3	heparan sulfate 6-O-sulfotransferase 3 [Source:HGNC Symbol;Acc:HGNC:19134]	4.63	0.000038
ssc-miR-138	HSPA4L	heat shock protein family A (Hsp70) member 4 like [Source:HGNC Symbol;Acc:HGNC:17041]	4.63	0.000038
ssc-miR-138	HTRA2	HtrA serine peptidase 2 [Source:VGNC Symbol;Acc:VGNC:89004]	4.63	0.000038

ssc-miR-138	ID3	inhibitor of DNA binding 3, HLH protein [Source:VGNC Symbol;Acc:VGNC:89023]	4.63	0.000038
ssc-miR-138	IER5L	immediate early response 5 like [Source:VGNC Symbol;Acc:VGNC:89029]	4.63	0.000038
ssc-miR-138	IGF2BP1	insulin like growth factor 2 mRNA binding protein 1 [Source:VGNC Symbol;Acc:VGNC:99006]	4.63	0.000038
ssc-miR-138	IGLON5	hypothetical gene	4.63	0.000038
ssc-miR-138	ILDR2	immunoglobulin like domain containing receptor 2 [Source:VGNC Symbol;Acc:VGNC:89118]	4.63	0.000038
ssc-miR-138	IMPACT	impact RWD domain protein [Source:HGNC Symbol;Acc:HGNC:20387]	4.63	0.000038
ssc-miR-138	IMPAD1	hypothetical gene	4.63	0.000038
ssc-miR-138	INA	internexin neuronal intermediate filament protein alpha [Source:VGNC Symbol;Acc:VGNC:89128]	4.63	0.000038
ssc-miR-138	ING1	inhibitor of growth family member 1 [Source:VGNC Symbol;Acc:VGNC:89131]	4.63	0.000038
ssc-miR-138	INO80	INO80 complex ATPase subunit [Source:VGNC Symbol;Acc:VGNC:89138]	4.63	0.000038
ssc-miR-138	IP6K1	inositol hexakisphosphate kinase 1 [Source:VGNC Symbol;Acc:VGNC:89172]	4.63	0.000038
ssc-miR-138	IPMK	inositol polyphosphate multikinase [Source:VGNC Symbol;Acc:VGNC:89175]	4.63	0.000038
ssc-miR-138	IPO8	importin 8 [Source:VGNC Symbol;Acc:VGNC:89181]	4.63	0.000038
ssc-miR-138	IQSEC1	IQ motif and Sec7 domain ArfGEF 1 [Source:VGNC Symbol;Acc:VGNC:89194]	4.63	0.000038
ssc-miR-138	IQSEC2	IQ motif and Sec7 domain ArfGEF 2 [Source:VGNC Symbol;Acc:VGNC:89195]	4.63	0.000038
ssc-miR-138	ITM2C	integral membrane protein 2C [Source:VGNC Symbol;Acc:VGNC:96383]	4.63	0.000038
ssc-miR-138	JARID2	jumonji and AT-rich interaction domain containing 2 [Source:VGNC Symbol;Acc:VGNC:89279]	4.63	0.000038
ssc-miR-138	JAZF1	JAZF zinc finger 1 [Source:VGNC Symbol;Acc:VGNC:89280]	4.63	0.000038
ssc-miR-138	JMJD1C	jumonji domain containing 1C [Source:VGNC Symbol;Acc:VGNC:89285]	4.63	0.000038
ssc-miR-138	JMJD8	jumonji domain containing 8 [Source:VGNC Symbol;Acc:VGNC:89288]	4.63	0.000038
ssc-miR-138	JOSD1	Josephin domain containing 1 [Source:VGNC Symbol;Acc:VGNC:89290]	4.63	0.000038
ssc-miR-138	KANK1	KN motif and ankyrin repeat domains 1 [Source:VGNC Symbol;Acc:VGNC:103112]	4.63	0.000038
ssc-miR-138	KAT6A	hypothetical gene	4.63	0.000038
ssc-miR-138	KAT6B	lysine acetyltransferase 6B [Source:VGNC Symbol;Acc:VGNC:89306]	4.63	0.000038
ssc-miR-138	KBTBD4	kelch repeat and BTB domain containing 4 [Source:VGNC Symbol;Acc:VGNC:89319]	4.63	0.000038
ssc-miR-138	KCNH1	potassium voltage-gated channel subfamily H member 1 [Source:VGNC Symbol;Acc:VGNC:108599]	4.63	0.000038
ssc-miR-138	KCNK15	potassium two pore domain channel subfamily K member 15 [Source:VGNC Symbol;Acc:VGNC:95593]	4.63	0.000038
ssc-miR-138	KCNK3	potassium two pore domain channel subfamily K member 3 [Source:VGNC Symbol;Acc:VGNC:89370]	4.63	0.000038
ssc-miR-138	KDELR2	KDEL endoplasmic reticulum protein retention receptor 2 [Source:VGNC Symbol;Acc:VGNC:89405]	4.63	0.000038
ssc-miR-138	KDM5A	lysine demethylase 5A [Source:VGNC Symbol;Acc:VGNC:89415]	4.63	0.000038
ssc-miR-138	KDM5C	lysine demethylase 5C [Source:NCBI gene (formerly Entrezgene);Acc:100037295]	4.63	0.000038
ssc-miR-138	KDM6B	lysine demethylase 6B [Source:VGNC Symbol;Acc:VGNC:89416]	4.63	0.000038
ssc-miR-138	KIAA0226L	hypothetical gene	4.63	0.000038
ssc-miR-138	KIAA0895	KIAA0895 [Source:VGNC Symbol;Acc:VGNC:89436]	4.63	0.000038
ssc-miR-138	KIAA0930	KIAA0930 [Source:VGNC Symbol;Acc:VGNC:89438]	4.63	0.000038
ssc-miR-138	KIAA1217	KIAA1217 [Source:VGNC Symbol;Acc:VGNC:96079]	4.63	0.000038
ssc-miR-138	KIAA1244	hypothetical gene	4.63	0.000038
ssc-miR-138	KIAA1468	hypothetical gene	4.63	0.000038
ssc-miR-138	KIAA1958	KIAA1958 [Source:VGNC Symbol;Acc:VGNC:89448]	4.63	0.000038
ssc-miR-138	KIAA2022	hypothetical gene	4.63	0.000038
ssc-miR-138	KIF13A	kinesin family member 13A [Source:VGNC Symbol;Acc:VGNC:89454]	4.63	0.000038
ssc-miR-138	KIF21B	kinesin family member 21B [Source:VGNC Symbol;Acc:VGNC:96219]	4.63	0.000038
ssc-miR-138	KIF26A	kinesin family member 26A [Source:VGNC Symbol;Acc:VGNC:89466]	4.63	0.000038
ssc-miR-138	KIRREL	hypothetical gene	4.63	0.000038
ssc-miR-138	KLF11	Kruppel like factor 11 [Source:VGNC Symbol;Acc:VGNC:89491]	4.63	0.000038
ssc-miR-138	KLF12	Kruppel like factor 12 [Source:VGNC Symbol;Acc:VGNC:89492]	4.63	0.000038
ssc-miR-138	KLHL18	kelch like family member 18 [Source:VGNC Symbol;Acc:VGNC:89517]	4.63	0.000038
ssc-miR-138	KMT2C	lysine methyltransferase 2C [Source:VGNC Symbol;Acc:VGNC:89550]	4.63	0.000038
ssc-miR-138	KNTC1	kinetochore associated 1 [Source:VGNC Symbol;Acc:VGNC:89559]	4.63	0.000038

ssc-miR-138	KPNA3	karyopherin subunit alpha 3 [Source:VGNC Symbol;Acc:VGNC:89562]	4.63	0.000038
ssc-miR-138	KREMEN1	kringle containing transmembrane protein 1 [Source:VGNC Symbol;Acc:VGNC:89572]	4.63	0.000038
ssc-miR-138	L3MBTL3	L3MBTL histone methyl-lysine binding protein 3 [Source:VGNC Symbol;Acc:VGNC:98066]	4.63	0.000038
ssc-miR-138	LCMT2	hypothetical gene	4.63	0.000038
ssc-miR-138	LCOR	ligand dependent nuclear receptor corepressor [Source:HGNC Symbol;Acc:HGNC:29503]	4.63	0.000038
ssc-miR-138	LDOC1L	hypothetical gene	4.63	0.000038
ssc-miR-138	LENG8	leukocyte receptor cluster member 8 [Source:VGNC Symbol;Acc:VGNC:89681]	4.63	0.000038
ssc-miR-138	LEPROT	leptin receptor overlapping transcript [Source:VGNC Symbol;Acc:VGNC:89686]	4.63	0.000038
ssc-miR-138	LGI4	leucine rich repeat LGI family member 4 [Source:VGNC Symbol;Acc:VGNC:89701]	4.63	0.000038
ssc-miR-138	LHFPL3	LHFPL tetraspan subfamily member 3 [Source:VGNC Symbol;Acc:VGNC:89706]	4.63	0.000038
ssc-miR-138	LHX1	LIM homeobox 1 [Source:VGNC Symbol;Acc:VGNC:89711]	4.63	0.000038
ssc-miR-138	LIMCH1	LIM and calponin homology domains 1 [Source:VGNC Symbol;Acc:VGNC:89724]	4.63	0.000038
ssc-miR-138	LIMK1	LIM domain kinase 1 [Source:VGNC Symbol;Acc:VGNC:89727]	4.63	0.000038
ssc-miR-138	LIMS1	hypothetical gene	4.63	0.000038
ssc-miR-138	LIN52	lin-52 DREAM MuvB core complex component [Source:NCBI gene (formerly Entrezgene);Acc:100157325]	4.63	0.000038
ssc-miR-138	LMAN1	lectin, mannose binding 1 [Source:VGNC Symbol;Acc:VGNC:89748]	4.63	0.000038
ssc-miR-138	LMAN2L	lectin, mannose binding 2 like [Source:VGNC Symbol;Acc:VGNC:89751]	4.63	0.000038
ssc-miR-138	LMTK2	lemur tyrosine kinase 2 [Source:HGNC Symbol;Acc:HGNC:17880]	4.63	0.000038
ssc-miR-138	LNPEP	leucyl and cystinyl aminopeptidase [Source:VGNC Symbol;Acc:VGNC:89772]	4.63	0.000038
ssc-miR-138	LONRF3	LON peptidase N-terminal domain and ring finger 3 [Source:VGNC Symbol;Acc:VGNC:89778]	4.63	0.000038
ssc-miR-138	LPHN1	hypothetical gene	4.63	0.000038
ssc-miR-138	LPHN3	hypothetical gene	4.63	0.000038
ssc-miR-138	LRP5L	hypothetical gene	4.63	0.000038
ssc-miR-138	LRRC14B	leucine rich repeat containing 14B [Source:VGNC Symbol;Acc:VGNC:89827]	4.63	0.000038
ssc-miR-138	LSM14A	LSM14A mRNA processing body assembly factor [Source:VGNC Symbol;Acc:VGNC:89872]	4.63	0.000038
ssc-miR-138	LY6H	lymphocyte antigen 6 family member H [Source:VGNC Symbol;Acc:VGNC:89903]	4.63	0.000038
ssc-miR-138	LYN	LYN proto-onco, Src family tyrosine kinase [Source:VGNC Symbol;Acc:VGNC:89910]	4.63	0.000038
ssc-miR-138	LYPLA1	lysophospholipase 1 [Source:VGNC Symbol;Acc:VGNC:98817]	4.63	0.000038
ssc-miR-138	LZTS1	leucine zipper tumor suppressor 1 [Source:VGNC Symbol;Acc:VGNC:89928]	4.63	0.000038
ssc-miR-138	LZTS3	leucine zipper tumor suppressor family member 3 [Source:VGNC Symbol;Acc:VGNC:96237]	4.63	0.000038
ssc-miR-138	MACF1	microtubule actin crosslinking factor 1 [Source:VGNC Symbol;Acc:VGNC:98496]	4.63	0.000038
ssc-miR-138	MAFK	MAF bZIP transcription factor K [Source:HGNC Symbol;Acc:HGNC:6782]	4.63	0.000038
ssc-miR-138	MAML1	mastermind like transcriptional coactivator 1 [Source:VGNC Symbol;Acc:VGNC:89963]	4.63	0.000038
ssc-miR-138	MANEAL	mannosidase endo-alpha like [Source:VGNC Symbol;Acc:VGNC:89972]	4.63	0.000038
ssc-miR-138	MAP2K7	mitogen-activated protein kinase kinase 7 [Source:VGNC Symbol;Acc:VGNC:98103]	4.63	0.000038
ssc-miR-138	MAP3K11	mitogen-activated protein kinase kinase kinase 11 [Source:VGNC Symbol;Acc:VGNC:89982]	4.63	0.000038
ssc-miR-138	MAPK10	mitogen-activated protein kinase 10 [Source:VGNC Symbol;Acc:VGNC:98116]	4.63	0.000038
ssc-miR-138	MAPK6	mitogen-activated protein kinase 6 [Source:VGNC Symbol;Acc:VGNC:103123]	4.63	0.000038
ssc-miR-138	MAPKBP1	mitogen-activated protein kinase binding protein 1 [Source:VGNC Symbol;Acc:VGNC:90014]	4.63	0.000038
ssc-miR-138	MARCH1	hypothetical gene	4.63	0.000038
ssc-miR-138	MARCKS	myristoylated alanine rich protein kinase C substrate [Source:VGNC Symbol;Acc:VGNC:90024]	4.63	0.000038
ssc-miR-138	MARK2	microtubule affinity regulating kinase 2 [Source:VGNC Symbol;Acc:VGNC:98118]	4.63	0.000038
ssc-miR-138	MBTD1	mbt domain containing 1 [Source:VGNC Symbol;Acc:VGNC:90060]	4.63	0.000038
ssc-miR-138	MBTPS2	hypothetical gene	4.63	0.000038
ssc-miR-138	MCTP1	multiple C2 and transmembrane domain containing 1 [Source:VGNC Symbol;Acc:VGNC:90083]	4.63	0.000038
ssc-miR-138	MCU	mitochondrial calcium uniporter [Source:VGNC Symbol;Acc:VGNC:90085]	4.63	0.000038
ssc-miR-138	MEGF11	multiple EGF like domains 11 [Source:VGNC Symbol;Acc:VGNC:103126]	4.63	0.000038
ssc-miR-138	METTL21B	hypothetical gene	4.63	0.000038
ssc-miR-138	MEX3A	mex-3 RNA binding family member A [Source:VGNC Symbol;Acc:VGNC:90168]	4.63	0.000038

ssc-miR-138	MFAP3	microfibril associated protein 3 [Source:VGNC Symbol;Acc:VGNC:90174]	4.63	0.000038
ssc-miR-138	MFSD6	major facilitator superfamily domain containing 6 [Source:VGNC Symbol;Acc:VGNC:96052]	4.63	0.000038
ssc-miR-138	MGAT5	alpha-1,6-mannosylglycoprotein 6-beta-N-acetylglucosaminyltransferase [Source:VGNC Symbol;Acc:VGNC:96741]	4.63	0.000038
ssc-miR-138	MGAT5B	alpha-1,6-mannosylglycoprotein 6-beta-N-acetylglucosaminyltransferase B [Source:VGNC Symbol;Acc:VGNC:96742]	4.63	0.000038
ssc-miR-138	MICAL3	microtubule associated monooxygenase, calponin and LIM domain containing 3 [Source:VGNC Symbol;Acc:VGNC:90208]	4.63	0.000038
ssc-miR-138	MIEF2	mitochondrial elongation factor 2 [Source:VGNC Symbol;Acc:VGNC:90217]	4.63	0.000038
ssc-miR-138	MIER2	MIER family member 2 [Source:VGNC Symbol;Acc:VGNC:90220]	4.63	0.000038
ssc-miR-138	MIER3	MIER family member 3 [Source:VGNC Symbol;Acc:VGNC:90221]	4.63	0.000038
ssc-miR-138	MINK1	misshapen like kinase 1 [Source:VGNC Symbol;Acc:VGNC:98128]	4.63	0.000038
ssc-miR-138	MLLT6	MLLT6, PHD finger containing [Source:VGNC Symbol;Acc:VGNC:90257]	4.63	0.000038
ssc-miR-138	MLXIP	MLX interacting protein [Source:VGNC Symbol;Acc:VGNC:90262]	4.63	0.000038
ssc-miR-138	MMAB	metabolism of cobalamin associated B [Source:VGNC Symbol;Acc:VGNC:96743]	4.63	0.000038
ssc-miR-138	MOCS1	molybdenum cofactor synthesis 1 [Source:VGNC Symbol;Acc:VGNC:107153]	4.63	0.000038
ssc-miR-138	MOGS	mannosyl-oligosaccharide glucosidase [Source:VGNC Symbol;Acc:VGNC:90297]	4.63	0.000038
ssc-miR-138	MON1B	MON1 homolog B, secretory trafficking associated [Source:VGNC Symbol;Acc:VGNC:90300]	4.63	0.000038
ssc-miR-138	MON2	MON2 homolog, regulator of endosome-to-Golgi trafficking [Source:VGNC Symbol;Acc:VGNC:90301]	4.63	0.000038
ssc-miR-138	MPRIP	hypothetical gene	4.63	0.000038
ssc-miR-138	MRAS	muscle RAS onco homolog [Source:VGNC Symbol;Acc:VGNC:90340]	4.63	0.000038
ssc-miR-138	MRPS18B	mitochondrial ribosomal protein S18B [Source:VGNC Symbol;Acc:VGNC:90385]	4.63	0.000038
ssc-miR-138	MSH6	mutS homolog 6 [Source:VGNC Symbol;Acc:VGNC:90420]	4.63	0.000038
ssc-miR-138	MSI1	musashi RNA binding protein 1 [Source:VGNC Symbol;Acc:VGNC:90421]	4.63	0.000038
ssc-miR-138	MST4	hypothetical gene	4.63	0.000038
ssc-miR-138	MTF2	metal response element binding transcription factor 2 [Source:VGNC Symbol;Acc:VGNC:90444]	4.63	0.000038
ssc-miR-138	MTUS2	microtubule associated scaffold protein 2 [Source:VGNC Symbol;Acc:VGNC:90477]	4.63	0.000038
ssc-miR-138	MXD1	MAX dimerization protein 1 [Source:VGNC Symbol;Acc:VGNC:90492]	4.63	0.000038
ssc-miR-138	MXD4	MAX dimerization protein 4 [Source:VGNC Symbol;Acc:VGNC:90493]	4.63	0.000038
ssc-miR-138	MYEF2	myelin expression factor 2 [Source:VGNC Symbol;Acc:VGNC:90507]	4.63	0.000038
ssc-miR-138	MYO1C	myosin IC [Source:VGNC Symbol;Acc:VGNC:90526]	4.63	0.000038
ssc-miR-138	NAPG	NSF attachment protein gamma [Source:VGNC Symbol;Acc:VGNC:90580]	4.63	0.000038
ssc-miR-138	NBEA	neurobeachin [Source:VGNC Symbol;Acc:VGNC:90590]	4.63	0.000038
ssc-miR-138	NBR1	NBR1 autophagy cargo receptor [Source:VGNC Symbol;Acc:VGNC:90593]	4.63	0.000038
ssc-miR-138	NCOA1	nuclear receptor coactivator 1 [Source:VGNC Symbol;Acc:VGNC:90615]	4.63	0.000038
ssc-miR-138	NCOA5	nuclear receptor coactivator 5 [Source:VGNC Symbol;Acc:VGNC:95702]	4.63	0.000038
ssc-miR-138	NDUFAF2	NADH:ubiquinone oxidoreductase complex assembly factor 2 [Source:HGNC Symbol;Acc:HGNC:28086]	4.63	0.000038
ssc-miR-138	NEBL	nebulette [Source:VGNC Symbol;Acc:VGNC:95824]	4.63	0.000038
ssc-miR-138	NETO1	neuropilin and tolloid like 1 [Source:VGNC Symbol;Acc:VGNC:90690]	4.63	0.000038
ssc-miR-138	NEUROD1	neuronal differentiation 1 [Source:VGNC Symbol;Acc:VGNC:96439]	4.63	0.000038
ssc-miR-138	NFAT5	nuclear factor of activated T cells 5 [Source:VGNC Symbol;Acc:VGNC:90708]	4.63	0.000038
ssc-miR-138	NFE2L3	NFE2 like bZIP transcription factor 3 [Source:VGNC Symbol;Acc:VGNC:90714]	4.63	0.000038
ssc-miR-138	NFIA	nuclear factor I A [Source:VGNC Symbol;Acc:VGNC:90715]	4.63	0.000038
ssc-miR-138	NFIB	nuclear factor I B [Source:VGNC Symbol;Acc:VGNC:90716]	4.63	0.000038
ssc-miR-138	NFIX	nuclear factor I X [Source:VGNC Symbol;Acc:VGNC:90718]	4.63	0.000038
ssc-miR-138	NINJ1	ninjurin 1 [Source:HGNC Symbol;Acc:HGNC:7824]	4.63	0.000038
ssc-miR-138	NIPBL	NIPBL cohesin loading factor [Source:VGNC Symbol;Acc:VGNC:90752]	4.63	0.000038
ssc-miR-138	NKAIN1	sodium/potassium transporting ATPase interacting 1 [Source:VGNC Symbol;Acc:VGNC:90754]	4.63	0.000038
ssc-miR-138	NLGN2	hypothetical gene	4.63	0.000038
ssc-miR-138	NLGN4X	hypothetical gene	4.63	0.000038
ssc-miR-138	NNAT	hypothetical gene	4.63	0.000038
ssc-miR-138	NOP2	NOP2 nucleolar protein [Source:VGNC Symbol;Acc:VGNC:103306]	4.63	0.000038

ssc-miR-138	NOVA1	NOVA alternative splicing regulator 1 [Source:VGNC Symbol;Acc:VGNC:90827]	4.63	0.000038
ssc-miR-138	NOVA2	NOVA alternative splicing regulator 2 [Source:VGNC Symbol;Acc:VGNC:90828]	4.63	0.000038
ssc-miR-138	NPFFR1	neuropeptide FF receptor 1 [Source:VGNC Symbol;Acc:VGNC:90846]	4.63	0.000038
ssc-miR-138	NPHP3	nephrocystin 3 [Source:HGNC Symbol;Acc:HGNC:7907]	4.63	0.000038
ssc-miR-138	NPPC	natriuretic peptide C [Source:VGNC Symbol;Acc:VGNC:96450]	4.63	0.000038
ssc-miR-138	NPTX1	neuronal pentraxin 1 [Source:VGNC Symbol;Acc:VGNC:90862]	4.63	0.000038
ssc-miR-138	NR3C1	nuclear receptor subfamily 3 group C member 1 [Source:VGNC Symbol;Acc:VGNC:90883]	4.63	0.000038
ssc-miR-138	NRG2	neuregulin 2 [Source:VGNC Symbol;Acc:VGNC:98160]	4.63	0.000038
ssc-miR-138	NRM	nurim [Source:VGNC Symbol;Acc:VGNC:90899]	4.63	0.000038
ssc-miR-138	NSFL1C	NSFL1 cofactor [Source:VGNC Symbol;Acc:VGNC:95704]	4.63	0.000038
ssc-miR-138	NSMF	NMDA receptor synaptonuclear signaling and neuronal migration factor [Source:VGNC Symbol;Acc:VGNC:90912]	4.63	0.000038
ssc-miR-138	NSUN3	NOP2/Sun RNA methyltransferase 3 [Source:VGNC Symbol;Acc:VGNC:90915]	4.63	0.000038
ssc-miR-138	NTM	neurotrimin [Source:VGNC Symbol;Acc:VGNC:98164]	4.63	0.000038
ssc-miR-138	NUP188	nucleoporin 188 [Source:VGNC Symbol;Acc:VGNC:98165]	4.63	0.000038
ssc-miR-138	NXN	nucleoredoxin [Source:VGNC Symbol;Acc:VGNC:90994]	4.63	0.000038
ssc-miR-138	ONECUT2	one cut homeobox 2 [Source:VGNC Symbol;Acc:VGNC:91043]	4.63	0.000038
ssc-miR-138	OSBP2	oxysterol binding protein 2 [Source:VGNC Symbol;Acc:VGNC:91067]	4.63	0.000038
ssc-miR-138	OTUD3	OTU deubiquitinase 3 [Source:VGNC Symbol;Acc:VGNC:91099]	4.63	0.000038
ssc-miR-138	PABPC1L2A	hypothetical gene	4.63	0.000038
ssc-miR-138	PABPC1L2B	hypothetical gene	4.63	0.000038
ssc-miR-138	PACS1	phosphofurin acidic cluster sorting protein 1 [Source:VGNC Symbol;Acc:VGNC:91140]	4.63	0.000038
ssc-miR-138	PAN3	poly(A) specific ribonuclease subunit PAN3 [Source:HGNC Symbol;Acc:HGNC:29991]	4.63	0.000038
ssc-miR-138	PAPD5	hypothetical gene	4.63	0.000038
ssc-miR-138	PAPPA	pappalysin 1 [Source:VGNC Symbol;Acc:VGNC:91170]	4.63	0.000038
ssc-miR-138	PAQR3	progestin and adipoQ receptor family member 3 [Source:VGNC Symbol;Acc:VGNC:91174]	4.63	0.000038
ssc-miR-138	PARP2	poly(ADP-ribose) polymerase 2 [Source:HGNC Symbol;Acc:HGNC:272]	4.63	0.000038
ssc-miR-138	PARP8	poly(ADP-ribose) polymerase family member 8 [Source:HGNC Symbol;Acc:HGNC:26124]	4.63	0.000038
ssc-miR-138	PATZ1	POZ/BTB and AT hook containing zinc finger 1 [Source:VGNC Symbol;Acc:VGNC:91190]	4.63	0.000038
ssc-miR-138	PAX5	paired box 5 [Source:VGNC Symbol;Acc:VGNC:91194]	4.63	0.000038
ssc-miR-138	PCGF3	polycomb group ring finger 3 [Source:VGNC Symbol;Acc:VGNC:91220]	4.63	0.000038
ssc-miR-138	PCLO	hypothetical gene	4.63	0.000038
ssc-miR-138	PCMTD1	protein-L-isoaspartate (D-aspartate) O-methyltransferase domain containing 1 [Source:VGNC Symbol;Acc:VGNC:98830]	4.63	0.000038
ssc-miR-138	PCNX	hypothetical gene	4.63	0.000038
ssc-miR-138	PDAP1	PDGFA associated protein 1 [Source:VGNC Symbol;Acc:VGNC:91239]	4.63	0.000038
ssc-miR-138	PDE3A	phosphodiesterase 3A [Source:VGNC Symbol;Acc:VGNC:91252]	4.63	0.000038
ssc-miR-138	PDE7B	phosphodiesterase 7B [Source:VGNC Symbol;Acc:VGNC:91262]	4.63	0.000038
ssc-miR-138	PDIK1L	PDLIM1 interacting kinase 1 like [Source:VGNC Symbol;Acc:VGNC:91276]	4.63	0.000038
ssc-miR-138	PDK1	pyruvate dehydrogenase kinase 1 [Source:VGNC Symbol;Acc:VGNC:98180]	4.63	0.000038
ssc-miR-138	PDP1	pyruvate dehydrogenase phosphatase catalytic subunit 1 [Source:VGNC Symbol;Acc:VGNC:91286]	4.63	0.000038
ssc-miR-138	PEG10	paternally expressed 10 [Source:NCBI gene (formerly Entrezgene);Acc:654416]	4.63	0.000038
ssc-miR-138	PELI3	pellino E3 ubiquitin protein ligase family member 3 [Source:VGNC Symbol;Acc:VGNC:91308]	4.63	0.000038
ssc-miR-138	PER1	period circadian regulator 1 [Source:VGNC Symbol;Acc:VGNC:91314]	4.63	0.000038
ssc-miR-138	PER2	period circadian regulator 2 [Source:VGNC Symbol;Acc:VGNC:96478]	4.63	0.000038
ssc-miR-138	PHACTR4	phosphatase and actin regulator 4 [Source:VGNC Symbol;Acc:VGNC:98531]	4.63	0.000038
ssc-miR-138	PHB2	prohibitin 2 [Source:VGNC Symbol;Acc:VGNC:91369]	4.63	0.000038
ssc-miR-138	PHF20	PHD finger protein 20 [Source:HGNC Symbol;Acc:HGNC:16098]	4.63	0.000038
ssc-miR-138	PHF21A	PHD finger protein 21A [Source:VGNC Symbol;Acc:VGNC:91384]	4.63	0.000038
ssc-miR-138	PHF5A	PHD finger protein 5A [Source:VGNC Symbol;Acc:VGNC:91388]	4.63	0.000038
ssc-miR-138	PHKG2	phosphorylase kinase catalytic subunit gamma 2 [Source:VGNC Symbol;Acc:VGNC:91395]	4.63	0.000038

ssc-miR-138	PHOX2B	paired like homeobox 2B [Source:VGNC Symbol;Acc:VGNC:91402]	4.63	0.000038
ssc-miR-138	PIGG	phosphatidylinositol glycan anchor biosynthesis class G [Source:VGNC Symbol;Acc:VGNC:91422]	4.63	0.000038
ssc-miR-138	PIP4K2C	phosphatidylinositol-5-phosphate 4-kinase type 2 gamma [Source:VGNC Symbol;Acc:VGNC:91455]	4.63	0.000038
ssc-miR-138	PITPNM3	PITPNM family member 3 [Source:HGNC Symbol;Acc:HGNC:21043]	4.63	0.000038
ssc-miR-138	PLAGL2	PLAG1 like zinc finger 2 [Source:VGNC Symbol;Acc:VGNC:96490]	4.63	0.000038
ssc-miR-138	PLCH2	phospholipase C eta 2 [Source:VGNC Symbol;Acc:VGNC:91522]	4.63	0.000038
ssc-miR-138	PLD2	phospholipase D2 [Source:VGNC Symbol;Acc:VGNC:91526]	4.63	0.000038
ssc-miR-138	PLD5	phospholipase D family member 5 [Source:VGNC Symbol;Acc:VGNC:96145]	4.63	0.000038
ssc-miR-138	PLEKHM1	pleckstrin homology and RUN domain containing M1 [Source:VGNC Symbol;Acc:VGNC:91552]	4.63	0.000038
ssc-miR-138	PLEKHO2	pleckstrin homology domain containing O2 [Source:HGNC Symbol;Acc:HGNC:30026]	4.63	0.000038
ssc-miR-138	PLLP	plasmolipin [Source:VGNC Symbol;Acc:VGNC:91565]	4.63	0.000038
ssc-miR-138	PLXNA4	plexin A4 [Source:VGNC Symbol;Acc:VGNC:98204]	4.63	0.000038
ssc-miR-138	PLXNB2	plexin B2 [Source:HGNC Symbol;Acc:HGNC:9104]	4.63	0.000038
ssc-miR-138	PMVK	phosphomevalonate kinase [Source:NCBI gene (formerly Entrezgene);Acc:100152301]	4.63	0.000038
ssc-miR-138	PODNL1	podocan like 1 [Source:VGNC Symbol;Acc:VGNC:91618]	4.63	0.000038
ssc-miR-138	PPARD	peroxisome proliferator activated receptor delta [Source:VGNC Symbol;Acc:VGNC:91683]	4.63	0.000038
ssc-miR-138	PPARGC1A	PPARG coactivator 1 alpha [Source:VGNC Symbol;Acc:VGNC:91685]	4.63	0.000038
ssc-miR-138	PPARGC1B	PPARG coactivator 1 beta [Source:VGNC Symbol;Acc:VGNC:91686]	4.63	0.000038
ssc-miR-138	PPFIA4	PTPRF interacting protein alpha 4 [Source:VGNC Symbol;Acc:VGNC:91694]	4.63	0.000038
ssc-miR-138	PPIP5K1	diphosphoinositol pentakisphosphate kinase 1 [Source:VGNC Symbol;Acc:VGNC:91700]	4.63	0.000038
ssc-miR-138	PPM1A	protein phosphatase, Mg2+/Mn2+ dependent 1A [Source:VGNC Symbol;Acc:VGNC:91703]	4.63	0.000038
ssc-miR-138	PPM1L	protein phosphatase, Mg2+/Mn2+ dependent 1L [Source:VGNC Symbol;Acc:VGNC:91711]	4.63	0.000038
ssc-miR-138	PPP1R3F	protein phosphatase 1 regulatory subunit 3F [Source:VGNC Symbol;Acc:VGNC:91739]	4.63	0.000038
ssc-miR-138	PPP6R3	protein phosphatase 6 regulatory subunit 3 [Source:VGNC Symbol;Acc:VGNC:91762]	4.63	0.000038
ssc-miR-138	PRMT7	protein arginine methyltransferase 7 [Source:VGNC Symbol;Acc:VGNC:91824]	4.63	0.000038
ssc-miR-138	PROX1	prospero homeobox 1 [Source:VGNC Symbol;Acc:VGNC:91837]	4.63	0.000038
ssc-miR-138	PRPF40A	pre-mRNA processing factor 40 homolog A [Source:VGNC Symbol;Acc:VGNC:98222]	4.63	0.000038
ssc-miR-138	PRPF4B	pre-mRNA processing factor 4B [Source:VGNC Symbol;Acc:VGNC:91845]	4.63	0.000038
ssc-miR-138	PRR3	proline rich 3 [Source:HGNC Symbol;Acc:HGNC:21149]	4.63	0.000038
ssc-miR-138	PRRT4	proline rich transmembrane protein 4 [Source:VGNC Symbol;Acc:VGNC:91874]	4.63	0.000038
ssc-miR-138	PRSS46	hypothetical gene	4.63	0.000038
ssc-miR-138	PSD	pleckstrin and Sec7 domain containing [Source:VGNC Symbol;Acc:VGNC:91895]	4.63	0.000038
ssc-miR-138	PSD2	pleckstrin and Sec7 domain containing 2 [Source:VGNC Symbol;Acc:VGNC:91896]	4.63	0.000038
ssc-miR-138	PSEN1	presenilin 1 [Source:VGNC Symbol;Acc:VGNC:91897]	4.63	0.000038
ssc-miR-138	PSME3	proteasome activator subunit 3 [Source:VGNC Symbol;Acc:VGNC:91926]	4.63	0.000038
ssc-miR-138	PSMF1	proteasome inhibitor subunit 1 [Source:VGNC Symbol;Acc:VGNC:96512]	4.63	0.000038
ssc-miR-138	PSTPIP1	proline-serine-threonine phosphatase interacting protein 1 [Source:VGNC Symbol;Acc:VGNC:91935]	4.63	0.000038
ssc-miR-138	PTGFRN	prostaglandin F2 receptor inhibitor [Source:VGNC Symbol;Acc:VGNC:91955]	4.63	0.000038
ssc-miR-138	PTK2	protein tyrosine kinase 2 [Source:VGNC Symbol;Acc:VGNC:98840]	4.63	0.000038
ssc-miR-138	PTP4A1	protein tyrosine phosphatase 4A1 [Source:HGNC Symbol;Acc:HGNC:9634]	4.63	0.000038
ssc-miR-138	PTPMT1	protein tyrosine phosphatase mitochondrial 1 [Source:VGNC Symbol;Acc:VGNC:91971]	4.63	0.000038
ssc-miR-138	PTPN11	protein tyrosine phosphatase non-receptor type 11 [Source:VGNC Symbol;Acc:VGNC:91972]	4.63	0.000038
ssc-miR-138	PTPN4	protein tyrosine phosphatase non-receptor type 4 [Source:VGNC Symbol;Acc:VGNC:96521]	4.63	0.000038
ssc-miR-138	PWWP2A	hypothetical gene	4.63	0.000038
ssc-miR-138	PXK	PX domain containing serine/threonine kinase like [Source:VGNC Symbol;Acc:VGNC:92015]	4.63	0.000038
ssc-miR-138	PXMP4	hypothetical gene	4.63	0.000038
ssc-miR-138	PYURF	hypothetical gene	4.63	0.000038
ssc-miR-138	QSOX1	quiescin sulfhydryl oxidase 1 [Source:VGNC Symbol;Acc:VGNC:92033]	4.63	0.000038
ssc-miR-138	RAB11A	RAB11A, member RAS onco family [Source:VGNC Symbol;Acc:VGNC:98236]	4.63	0.000038

ssc-miR-138	RAB22A	RAB22A, member RAS onco family [Source:VGNC Symbol;Acc:VGNC:98244]	4.63	0.000038
ssc-miR-138	RAB3GAP1	RAB3 GTPase activating protein catalytic subunit 1 [Source:VGNC Symbol;Acc:VGNC:98267]	4.63	0.000038
ssc-miR-138	RAB3IL1	RAB3A interacting protein like 1 [Source:VGNC Symbol;Acc:VGNC:92042]	4.63	0.000038
ssc-miR-138	RABL2B	hypothetical gene	4.63	0.000038
ssc-miR-138	RALGPS1	Ral GEF with PH domain and SH3 binding motif 1 [Source:VGNC Symbol;Acc:VGNC:92072]	4.63	0.000038
ssc-miR-138	RALY	RALY heterogeneous nuclear ribonucleoprotein [Source:VGNC Symbol;Acc:VGNC:95709]	4.63	0.000038
ssc-miR-138	RARA	retinoic acid receptor alpha [Source:VGNC Symbol;Acc:VGNC:92096]	4.63	0.000038
ssc-miR-138	RASSF8	Ras association domain family member 8 [Source:VGNC Symbol;Acc:VGNC:92128]	4.63	0.000038
ssc-miR-138	RAVER1	ribonucleoprotein, PTB binding 1 [Source:HGNC Symbol;Acc:HGNC:30296]	4.63	0.000038
ssc-miR-138	RB1CC1	RB1 inducible coiled-coil 1 [Source:VGNC Symbol;Acc:VGNC:92133]	4.63	0.000038
ssc-miR-138	RBBP4	hypothetical gene	4.63	0.000038
ssc-miR-138	RBM28	RNA binding motif protein 28 [Source:VGNC Symbol;Acc:VGNC:92154]	4.63	0.000038
ssc-miR-138	RBPJ	recombination signal binding protein for immunoglobulin kappa J region [Source:VGNC Symbol;Acc:VGNC:98956]	4.63	0.000038
ssc-miR-138	RCAN2	regulator of calcineurin 2 [Source:VGNC Symbol;Acc:VGNC:92171]	4.63	0.000038
ssc-miR-138	RDH8	retinol dehydrogenase 8 [Source:VGNC Symbol;Acc:VGNC:96762]	4.63	0.000038
ssc-miR-138	RELA	RELA proto-onco, NF-kB subunit [Source:VGNC Symbol;Acc:VGNC:92203]	4.63	0.000038
ssc-miR-138	RELN	reelin [Source:VGNC Symbol;Acc:VGNC:92208]	4.63	0.000038
ssc-miR-138	REPIN1	replication initiator 1 [Source:VGNC Symbol;Acc:VGNC:92212]	4.63	0.000038
ssc-miR-138	RETSAT	retinol saturase [Source:VGNC Symbol;Acc:VGNC:92225]	4.63	0.000038
ssc-miR-138	RGMA	repulsive guidance molecule BMP co-receptor a [Source:VGNC Symbol;Acc:VGNC:92254]	4.63	0.000038
ssc-miR-138	RHBDD1	rhomboid domain containing 1 [Source:VGNC Symbol;Acc:VGNC:95989]	4.63	0.000038
ssc-miR-138	RHBDL3	rhomboid like 3 [Source:VGNC Symbol;Acc:VGNC:92279]	4.63	0.000038
ssc-miR-138	RHOBTB2	Rho related BTB domain containing 2 [Source:HGNC Symbol;Acc:HGNC:18756]	4.63	0.000038
ssc-miR-138	RHOC	ras homolog family member C [Source:VGNC Symbol;Acc:VGNC:98846]	4.63	0.000038
ssc-miR-138	RIBC1	RIB43A domain with coiled-coils 1 [Source:VGNC Symbol;Acc:VGNC:92297]	4.63	0.000038
ssc-miR-138	RIMKLA	ribosomal modification protein rimK like family member A [Source:HGNC Symbol;Acc:HGNC:28725]	4.63	0.000038
ssc-miR-138	RIMS2	regulating synaptic membrane exocytosis 2 [Source:HGNC Symbol;Acc:HGNC:17283]	4.63	0.000038
ssc-miR-138	RIMS3	regulating synaptic membrane exocytosis 3 [Source:VGNC Symbol;Acc:VGNC:92310]	4.63	0.000038
ssc-miR-138	RIMS4	regulating synaptic membrane exocytosis 4 [Source:VGNC Symbol;Acc:VGNC:95775]	4.63	0.000038
ssc-miR-138	RMND5A	required for meiotic nuclear division 5 homolog A [Source:VGNC Symbol;Acc:VGNC:92336]	4.63	0.000038
ssc-miR-138	RNF126	ring finger protein 126 [Source:VGNC Symbol;Acc:VGNC:92353]	4.63	0.000038
ssc-miR-138	RNF150	ring finger protein 150 [Source:VGNC Symbol;Acc:VGNC:98293]	4.63	0.000038
ssc-miR-138	RNF222	ring finger protein 222 [Source:VGNC Symbol;Acc:VGNC:92384]	4.63	0.000038
ssc-miR-138	RNF24	ring finger protein 24 [Source:HGNC Symbol;Acc:HGNC:13779]	4.63	0.000038
ssc-miR-138	RNF38	ring finger protein 38 [Source:VGNC Symbol;Acc:VGNC:92390]	4.63	0.000038
ssc-miR-138	ROBO1	roundabout guidance receptor 1 [Source:HGNC Symbol;Acc:HGNC:10249]	4.63	0.000038
ssc-miR-138	ROCK2	Rho associated coiled-coil containing protein kinase 2 [Source:HGNC Symbol;Acc:HGNC:10252]	4.63	0.000038
ssc-miR-138	RORB	RAR related orphan receptor B [Source:VGNC Symbol;Acc:VGNC:92409]	4.63	0.000038
ssc-miR-138	RP11-181C3.1	hypothetical gene	4.63	0.000038
ssc-miR-138	RP11-315D16.2	hypothetical gene	4.63	0.000038
ssc-miR-138	RP11-644F5.10	hypothetical gene	4.63	0.000038
ssc-miR-138	RP11-766F14.2	hypothetical gene	4.63	0.000038
ssc-miR-138	RP5-850E9.3	hypothetical gene	4.63	0.000038
ssc-miR-138	RPRD1B	regulation of nuclear pre-mRNA domain containing 1B [Source:VGNC Symbol;Acc:VGNC:95785]	4.63	0.000038
ssc-miR-138	RPS6KA1	ribosomal protein S6 kinase A1 [Source:VGNC Symbol;Acc:VGNC:92440]	4.63	0.000038
ssc-miR-138	RPUSD1	RNA pseudouridine synthase domain containing 1 [Source:VGNC Symbol;Acc:VGNC:92451]	4.63	0.000038
ssc-miR-138	RRM1	ribonucleotide reductase catalytic subunit M1 [Source:VGNC Symbol;Acc:VGNC:92461]	4.63	0.000038
ssc-miR-138	RSF1	remodeling and spacing factor 1 [Source:VGNC Symbol;Acc:VGNC:92476]	4.63	0.000038
ssc-miR-138	RSPO4	R-spondin 4 [Source:VGNC Symbol;Acc:VGNC:95769]	4.63	0.000038

ssc-miR-138	RTN4RL2	reticulon 4 receptor like 2 [Source:VGNC Symbol;Acc:VGNC:100855]	4.63	0.000038
ssc-miR-138	RUVBL1	RuvB like AAA ATPase 1 [Source:VGNC Symbol;Acc:VGNC:92521]	4.63	0.000038
ssc-miR-138	SAMD12	sterile alpha motif domain containing 12 [Source:VGNC Symbol;Acc:VGNC:92565]	4.63	0.000038
ssc-miR-138	SATB1	SATB homeobox 1 [Source:VGNC Symbol;Acc:VGNC:92587]	4.63	0.000038
ssc-miR-138	SCAI	suppressor of cancer cell invasion [Source:VGNC Symbol;Acc:VGNC:103170]	4.63	0.000038
ssc-miR-138	SCN1B	sodium voltage-gated channel beta subunit 1 [Source:VGNC Symbol;Acc:VGNC:92632]	4.63	0.000038
ssc-miR-138	SCN3B	sodium voltage-gated channel beta subunit 3 [Source:HGNC Symbol;Acc:HGNC:20665]	4.63	0.000038
ssc-miR-138	SCN8A	sodium voltage-gated channel alpha subunit 8 [Source:VGNC Symbol;Acc:VGNC:92638]	4.63	0.000038
ssc-miR-138	SCRN1	secernin 1 [Source:VGNC Symbol;Acc:VGNC:92647]	4.63	0.000038
ssc-miR-138	SCRT1	scratch family transcriptional repressor 1 [Source:VGNC Symbol;Acc:VGNC:92649]	4.63	0.000038
ssc-miR-138	SCYL2	SCY1 like pseudokinase 2 [Source:VGNC Symbol;Acc:VGNC:98305]	4.63	0.000038
ssc-miR-138	SDC3	syndecan 3 [Source:VGNC Symbol;Acc:VGNC:92656]	4.63	0.000038
ssc-miR-138	SEC22A	SEC22 homolog A, vesicle trafficking protein [Source:VGNC Symbol;Acc:VGNC:92673]	4.63	0.000038
ssc-miR-138	SEH1L	centrosomal protein 192 [Source:VGNC Symbol;Acc:VGNC:96946]	4.63	0.000038
ssc-miR-138	SEMA4C	semaphorin 4C [Source:VGNC Symbol;Acc:VGNC:92702]	4.63	0.000038
ssc-miR-138	SEMA6A	semaphorin 6A [Source:VGNC Symbol;Acc:VGNC:92708]	4.63	0.000038
ssc-miR-138	SENP1	SUMO specific peptidase 1 [Source:VGNC Symbol;Acc:VGNC:92712]	4.63	0.000038
ssc-miR-138	SEPT11	hypothetical gene	4.63	0.000038
ssc-miR-138	SERTAD4	SERTA domain containing 4 [Source:VGNC Symbol;Acc:VGNC:92752]	4.63	0.000038
ssc-miR-138	SESN3	sestrin 3 [Source:VGNC Symbol;Acc:VGNC:92755]	4.63	0.000038
ssc-miR-138	SETD6	SET domain containing 6, protein lysine methyltransferase [Source:VGNC Symbol;Acc:VGNC:92761]	4.63	0.000038
ssc-miR-138	SEZ6L2	seizure related 6 homolog like 2 [Source:VGNC Symbol;Acc:VGNC:98312]	4.63	0.000038
ssc-miR-138	SFMBT1	Scm like with four mbt domains 1 [Source:VGNC Symbol;Acc:VGNC:92773]	4.63	0.000038
ssc-miR-138	SFMBT2	Scm like with four mbt domains 2 [Source:VGNC Symbol;Acc:VGNC:95935]	4.63	0.000038
ssc-miR-138	SFRP1	secreted frizzled related protein 1 [Source:VGNC Symbol;Acc:VGNC:95493]	4.63	0.000038
ssc-miR-138	SFSWAP	splicing factor SWAP [Source:VGNC Symbol;Acc:VGNC:92778]	4.63	0.000038
ssc-miR-138	SFXN2	sideroflexin 2 [Source:VGNC Symbol;Acc:VGNC:92784]	4.63	0.000038
ssc-miR-138	SGSM2	small G protein signaling modulator 2 [Source:VGNC Symbol;Acc:VGNC:92801]	4.63	0.000038
ssc-miR-138	SGTA	small glutamine rich tetratricopeptide repeat co-chaperone alpha [Source:VGNC Symbol;Acc:VGNC:92803]	4.63	0.000038
ssc-miR-138	SH2B3	SH2B adaptor protein 3 [Source:VGNC Symbol;Acc:VGNC:92807]	4.63	0.000038
ssc-miR-138	SH2D1B	hypothetical gene	4.63	0.000038
ssc-miR-138	SH3GL1	SH3 domain containing GRB2 like 1, endophilin A2 [Source:VGNC Symbol;Acc:VGNC:92823]	4.63	0.000038
ssc-miR-138	SH3GL2	SH3 domain containing GRB2 like 2, endophilin A1 [Source:VGNC Symbol;Acc:VGNC:92824]	4.63	0.000038
ssc-miR-138	SHANK2	SH3 and multiple ankyrin repeat domains 2 [Source:VGNC Symbol;Acc:VGNC:92835]	4.63	0.000038
ssc-miR-138	SIGLECL1	hypothetical gene	4.63	0.000038
ssc-miR-138	SIM1	SIM bHLH transcription factor 1 [Source:VGNC Symbol;Acc:VGNC:92876]	4.63	0.000038
ssc-miR-138	SIM2	SIM bHLH transcription factor 2 [Source:VGNC Symbol;Acc:VGNC:92877]	4.63	0.000038
ssc-miR-138	SIN3A	SIN3 transcription regulator family member A [Source:VGNC Symbol;Acc:VGNC:92879]	4.63	0.000038
ssc-miR-138	SIRT1	sirtuin 1 [Source:VGNC Symbol;Acc:VGNC:92884]	4.63	0.000038
ssc-miR-138	SIX5	SIX homeobox 5 [Source:VGNC Symbol;Acc:VGNC:92896]	4.63	0.000038
ssc-miR-138	SLC10A7	solute carrier family 10 member 7 [Source:VGNC Symbol;Acc:VGNC:92919]	4.63	0.000038
ssc-miR-138	SLC16A2	solute carrier family 16 member 2 [Source:VGNC Symbol;Acc:VGNC:92942]	4.63	0.000038
ssc-miR-138	SLC17A7	solute carrier family 17 member 7 [Source:VGNC Symbol;Acc:VGNC:92955]	4.63	0.000038
ssc-miR-138	SLC20A1	solute carrier family 20 member 1 [Source:VGNC Symbol;Acc:VGNC:92970]	4.63	0.000038
ssc-miR-138	SLC22A23	solute carrier family 22 member 23 [Source:HGNC Symbol;Acc:HGNC:21106]	4.63	0.000038
ssc-miR-138	SLC23A2	solute carrier family 23 member 2 [Source:VGNC Symbol;Acc:VGNC:108737]	4.63	0.000038
ssc-miR-138	SLC25A26	solute carrier family 25 member 26 [Source:VGNC Symbol;Acc:VGNC:93003]	4.63	0.000038
ssc-miR-138	SLC30A1	solute carrier family 30 member 1 [Source:VGNC Symbol;Acc:VGNC:93055]	4.63	0.000038
ssc-miR-138	SLC35F1	solute carrier family 35 member F1 [Source:VGNC Symbol;Acc:VGNC:93081]	4.63	0.000038

ssc-miR-138	SLC36A2	solute carrier family 36 member 2 [Source:VGNC Symbol;Acc:VGNC:93089]	4.63	0.000038
ssc-miR-138	SLC38A1	solute carrier family 38 member 1 [Source:HGNC Symbol;Acc:HGNC:13447]	4.63	0.000038
ssc-miR-138	SLC39A9	solute carrier family 39 member 9 [Source:VGNC Symbol;Acc:VGNC:93110]	4.63	0.000038
ssc-miR-138	SLC4A8	solute carrier family 4 member 8 [Source:VGNC Symbol;Acc:VGNC:98326]	4.63	0.000038
ssc-miR-138	SLC6A17	solute carrier family 6 member 17 [Source:VGNC Symbol;Acc:VGNC:93158]	4.63	0.000038
ssc-miR-138	SLC6A8	solute carrier family 6 member 8 [Source:NCBI gene (formerly Entrezgene);Acc:100302506]	4.63	0.000038
ssc-miR-138	SLC9A3R1	SLC9A3 regulator 1 [Source:VGNC Symbol;Acc:VGNC:93184]	4.63	0.000038
ssc-miR-138	SLCO3A1	solute carrier organic anion transporter family member 3A1 [Source:VGNC Symbol;Acc:VGNC:93197]	4.63	0.000038
ssc-miR-138	SLFN12	hypothetical gene	4.63	0.000038
ssc-miR-138	SLFN12L	hypothetical gene	4.63	0.000038
ssc-miR-138	SLX4	SLX4 structure-specific endonuclease subunit [Source:VGNC Symbol;Acc:VGNC:93215]	4.63	0.000038
ssc-miR-138	SMG1	SMG1 nonsense mediated mRNA decay associated PI3K related kinase [Source:VGNC Symbol;Acc:VGNC:93248]	4.63	0.000038
ssc-miR-138	SMURF1	SMAD specific E3 ubiquitin protein ligase 1 [Source:VGNC Symbol;Acc:VGNC:93270]	4.63	0.000038
ssc-miR-138	SNAP25	synaptosome associated protein 25 [Source:VGNC Symbol;Acc:VGNC:95509]	4.63	0.000038
ssc-miR-138	SNCAIP	hypothetical gene	4.63	0.000038
ssc-miR-138	SNCB	synuclein beta [Source:VGNC Symbol;Acc:VGNC:93286]	4.63	0.000038
ssc-miR-138	SNIP1	Smad nuclear interacting protein 1 [Source:VGNC Symbol;Acc:VGNC:93289]	4.63	0.000038
ssc-miR-138	SNTB2	syntrophin beta 2 [Source:VGNC Symbol;Acc:VGNC:93298]	4.63	0.000038
ssc-miR-138	SNX30	sorting nexin family member 30 [Source:VGNC Symbol;Acc:VGNC:93318]	4.63	0.000038
ssc-miR-138	SNX33	sorting nexin 33 [Source:VGNC Symbol;Acc:VGNC:93321]	4.63	0.000038
ssc-miR-138	SOBP	sine oculis binding protein homolog [Source:VGNC Symbol;Acc:VGNC:93329]	4.63	0.000038
ssc-miR-138	SOCS5	suppressor of cytokine signaling 5 [Source:VGNC Symbol;Acc:VGNC:93332]	4.63	0.000038
ssc-miR-138	SOCS6	suppressor of cytokine signaling 6 [Source:VGNC Symbol;Acc:VGNC:93333]	4.63	0.000038
ssc-miR-138	SOGA1	suppressor of glucose, autophagy associated 1 [Source:VGNC Symbol;Acc:VGNC:95740]	4.63	0.000038
ssc-miR-138	SOGA3	SOGA family member 3 [Source:HGNC Symbol;Acc:HGNC:21494]	4.63	0.000038
ssc-miR-138	SORBS2	sorbin and SH3 domain containing 2 [Source:VGNC Symbol;Acc:VGNC:96030]	4.63	0.000038
ssc-miR-138	SOX12	SRY-box transcription factor 12 [Source:HGNC Symbol;Acc:HGNC:11198]	4.63	0.000038
ssc-miR-138	SOX4	SRY-box transcription factor 4 [Source:HGNC Symbol;Acc:HGNC:11200]	4.63	0.000038
ssc-miR-138	SOX5	SRY-box transcription factor 5 [Source:VGNC Symbol;Acc:VGNC:93357]	4.63	0.000038
ssc-miR-138	SOX9	SRY-box transcription factor 9 [Source:VGNC Symbol;Acc:VGNC:99053]	4.63	0.000038
ssc-miR-138	SPEG	hypothetical gene	4.63	0.000038
ssc-miR-138	SPRED1	sprouty related EVH1 domain containing 1 [Source:VGNC Symbol;Acc:VGNC:93420]	4.63	0.000038
ssc-miR-138	SQSTM1	sequestosome 1 [Source:VGNC Symbol;Acc:VGNC:93445]	4.63	0.000038
ssc-miR-138	SRGAP3	SLIT-ROBO Rho GTPase activating protein 3 [Source:VGNC Symbol;Acc:VGNC:93455]	4.63	0.000038
ssc-miR-138	SRRM4	serine/arginine repetitive matrix 4 [Source:VGNC Symbol;Acc:VGNC:93470]	4.63	0.000038
ssc-miR-138	SRSF6	serine and arginine rich splicing factor 6 [Source:NCBI gene (formerly Entrezgene);Acc:768102]	4.63	0.000038
ssc-miR-138	SSSCA1	hypothetical gene	4.63	0.000038
ssc-miR-138	ST3GAL2	ST3 beta-galactoside alpha-2,3-sialyltransferase 2 [Source:HGNC Symbol;Acc:HGNC:10863]	4.63	0.000038
ssc-miR-138	ST3GAL6	ST3 beta-galactoside alpha-2,3-sialyltransferase 6 [Source:VGNC Symbol;Acc:VGNC:93506]	4.63	0.000038
ssc-miR-138	ST6GALNAC4	ST6 N-acetylgalactosaminide alpha-2,6-sialyltransferase 4 [Source:VGNC Symbol;Acc:VGNC:93512]	4.63	0.000038
ssc-miR-138	ST6GALNAC5	ST6 N-acetylgalactosaminide alpha-2,6-sialyltransferase 5 [Source:VGNC Symbol;Acc:VGNC:93513]	4.63	0.000038
ssc-miR-138	ST8SIA3	ST8 alpha-N-acetyl-neuraminide alpha-2,8-sialyltransferase 3 [Source:VGNC Symbol;Acc:VGNC:93518]	4.63	0.000038
ssc-miR-138	STAG1	stromal antigen 1 [Source:VGNC Symbol;Acc:VGNC:93524]	4.63	0.000038
ssc-miR-138	STK16	serine/threonine kinase 16 [Source:VGNC Symbol;Acc:VGNC:95526]	4.63	0.000038
ssc-miR-138	STK4	serine/threonine kinase 4 [Source:VGNC Symbol;Acc:VGNC:98349]	4.63	0.000038
ssc-miR-138	STOX2	storkhead box 2 [Source:VGNC Symbol;Acc:VGNC:96082]	4.63	0.000038
ssc-miR-138	STXBP5	syntaxin binding protein 5 [Source:VGNC Symbol;Acc:VGNC:93599]	4.63	0.000038
ssc-miR-138	STXBP6	syntaxin binding protein 6 [Source:NCBI gene (formerly Entrezgene);Acc:100514852]	4.63	0.000038
ssc-miR-138	SULF2	sulfatase 2 [Source:VGNC Symbol;Acc:VGNC:95938]	4.63	0.000038

ssc-miR-138	SUV420H1	hypothetical gene	4.63	0.000038
ssc-miR-138	SV2A	synaptic vesicle glycoprotein 2A [Source:VGNC Symbol;Acc:VGNC:93639]	4.63	0.000038
ssc-miR-138	SYNGR2	synaptogyrin 2 [Source:HGNC Symbol;Acc:HGNC:11499]	4.63	0.000038
ssc-miR-138	SYNGR3	synaptogyrin 3 [Source:VGNC Symbol;Acc:VGNC:93666]	4.63	0.000038
ssc-miR-138	SYS1	SYS1 golgi trafficking protein [Source:VGNC Symbol;Acc:VGNC:98354]	4.63	0.000038
ssc-miR-138	SYT13	synaptotagmin 13 [Source:VGNC Symbol;Acc:VGNC:93682]	4.63	0.000038
ssc-miR-138	SZRD1	hypothetical gene	4.63	0.000038
ssc-miR-138	TAF10	TATA-box binding protein associated factor 10 [Source:VGNC Symbol;Acc:VGNC:104067]	4.63	0.000038
ssc-miR-138	TAOK1	TAO kinase 1 [Source:VGNC Symbol;Acc:VGNC:98355]	4.63	0.000038
ssc-miR-138	TBC1D4	TBC1 domain family member 4 [Source:HGNC Symbol;Acc:HGNC:19165]	4.63	0.000038
ssc-miR-138	TBL1X	hypothetical gene	4.63	0.000038
ssc-miR-138	TBL1Y	hypothetical gene	4.63	0.000038
ssc-miR-138	TBL3	transducin beta like 3 [Source:VGNC Symbol;Acc:VGNC:93790]	4.63	0.000038
ssc-miR-138	TCEAL3	hypothetical gene	4.63	0.000038
ssc-miR-138	TCEAL5	hypothetical gene	4.63	0.000038
ssc-miR-138	TCF12	transcription factor 12 [Source:VGNC Symbol;Acc:VGNC:93817]	4.63	0.000038
ssc-miR-138	TCF3	transcription factor 3 [Source:VGNC Symbol;Acc:VGNC:93822]	4.63	0.000038
ssc-miR-138	TCF4	transcription factor 4 [Source:VGNC Symbol;Acc:VGNC:93823]	4.63	0.000038
ssc-miR-138	TCF7L1	hypothetical gene	4.63	0.000038
ssc-miR-138	TEAD1	TEA domain transcription factor 1 [Source:VGNC Symbol;Acc:VGNC:93853]	4.63	0.000038
ssc-miR-138	TENM1	teneurin transmembrane protein 1 [Source:VGNC Symbol;Acc:VGNC:98363]	4.63	0.000038
ssc-miR-138	TENM4	teneurin transmembrane protein 4 [Source:HGNC Symbol;Acc:HGNC:29945]	4.63	0.000038
ssc-miR-138	TERF2	telomeric repeat binding factor 2 [Source:VGNC Symbol;Acc:VGNC:93882]	4.63	0.000038
ssc-miR-138	TET2	hypothetical gene	4.63	0.000038
ssc-miR-138	THAP1	THAP domain containing 1 [Source:VGNC Symbol;Acc:VGNC:95950]	4.63	0.000038
ssc-miR-138	THAP11	THAP domain containing 11 [Source:VGNC Symbol;Acc:VGNC:93941]	4.63	0.000038
ssc-miR-138	THRA	thyroid hormone receptor alpha [Source:VGNC Symbol;Acc:VGNC:93963]	4.63	0.000038
ssc-miR-138	THRAP3	thyroid hormone receptor associated protein 3 [Source:VGNC Symbol;Acc:VGNC:93964]	4.63	0.000038
ssc-miR-138	THRB	thyroid hormone receptor beta [Source:VGNC Symbol;Acc:VGNC:93965]	4.63	0.000038
ssc-miR-138	THSD4	thrombospondin type 1 domain containing 4 [Source:VGNC Symbol;Acc:VGNC:93967]	4.63	0.000038
ssc-miR-138	TIMP4	TIMP metallopeptidase inhibitor 4 [Source:VGNC Symbol;Acc:VGNC:93997]	4.63	0.000038
ssc-miR-138	TIPARP	TCDD inducible poly(ADP-ribose) polymerase [Source:VGNC Symbol;Acc:VGNC:108699]	4.63	0.000038
ssc-miR-138	TJP1	tight junction protein 1 [Source:VGNC Symbol;Acc:VGNC:94005]	4.63	0.000038
ssc-miR-138	TLX1	T cell leukemia homeobox 1 [Source:HGNC Symbol;Acc:HGNC:5056]	4.63	0.000038
ssc-miR-138	TMEM132C	transmembrane protein 132C [Source:VGNC Symbol;Acc:VGNC:94081]	4.63	0.000038
ssc-miR-138	TMEM178B	transmembrane protein 178B [Source:HGNC Symbol;Acc:HGNC:44112]	4.63	0.000038
ssc-miR-138	TMEM181	transmembrane protein 181 [Source:VGNC Symbol;Acc:VGNC:94117]	4.63	0.000038
ssc-miR-138	TMEM189	hypothetical gene	4.63	0.000038
ssc-miR-138	TMEM189-UBE2V1	hypothetical gene	4.63	0.000038
ssc-miR-138	TMEM198	transmembrane protein 198 [Source:VGNC Symbol;Acc:VGNC:96274]	4.63	0.000038
ssc-miR-138	TMEM231	transmembrane protein 231 [Source:VGNC Symbol;Acc:VGNC:98377]	4.63	0.000038
ssc-miR-138	TMEM37	hypothetical gene	4.63	0.000038
ssc-miR-138	TMEM38B	transmembrane protein 38B [Source:VGNC Symbol;Acc:VGNC:94175]	4.63	0.000038
ssc-miR-138	TMEM40	transmembrane protein 40 [Source:VGNC Symbol;Acc:VGNC:94178]	4.63	0.000038
ssc-miR-138	TMOD2	tropomodulin 2 [Source:VGNC Symbol;Acc:VGNC:94227]	4.63	0.000038
ssc-miR-138	TMTC4	transmembrane O-mannosyltransferase targeting cadherins 4 [Source:VGNC Symbol;Acc:VGNC:94244]	4.63	0.000038
ssc-miR-138	TNFAIP8	TNF alpha induced protein 8 [Source:VGNC Symbol;Acc:VGNC:94253]	4.63	0.000038
ssc-miR-138	TNFSF4	TNF superfamily member 4 [Source:VGNC Symbol;Acc:VGNC:104081]	4.63	0.000038
ssc-miR-138	TNIK	TRAF2 and NCK interacting kinase [Source:VGNC Symbol;Acc:VGNC:98381]	4.63	0.000038

ssc-miR-138	TNRC6B	trinucleotide repeat containing adaptor 6B [Source:VGNC Symbol;Acc:VGNC:94293]	4.63	0.000038
ssc-miR-138	TOM1	target of myb1 membrane trafficking protein [Source:VGNC Symbol;Acc:VGNC:95552]	4.63	0.000038
ssc-miR-138	TP53BP2	tumor protein p53 binding protein 2 [Source:VGNC Symbol;Acc:VGNC:95553]	4.63	0.000038
ssc-miR-138	TP53INP1	tumor protein p53 inducible nuclear protein 1 [Source:VGNC Symbol;Acc:VGNC:94329]	4.63	0.000038
ssc-miR-138	TP53INP2	tumor protein p53 inducible nuclear protein 2 [Source:VGNC Symbol;Acc:VGNC:95737]	4.63	0.000038
ssc-miR-138	TRAM2	translocation associated membrane protein 2 [Source:HGNC Symbol;Acc:HGNC:16855]	4.63	0.000038
ssc-miR-138	TRIM13	tripartite motif containing 13 [Source:VGNC Symbol;Acc:VGNC:94394]	4.63	0.000038
ssc-miR-138	TRIM62	tripartite motif containing 62 [Source:VGNC Symbol;Acc:VGNC:94423]	4.63	0.000038
ssc-miR-138	TRIM66	tripartite motif containing 66 [Source:VGNC Symbol;Acc:VGNC:94426]	4.63	0.000038
ssc-miR-138	TRIP11	thyroid hormone receptor interactor 11 [Source:VGNC Symbol;Acc:VGNC:94437]	4.63	0.000038
ssc-miR-138	TRPC5	transient receptor potential cation channel subfamily C member 5 [Source:VGNC Symbol;Acc:VGNC:94463]	4.63	0.000038
ssc-miR-138	TRPC7	transient receptor potential cation channel subfamily C member 7 [Source:VGNC Symbol;Acc:VGNC:94465]	4.63	0.000038
ssc-miR-138	TRPS1	transcriptional repressor GATA binding 1 [Source:VGNC Symbol;Acc:VGNC:94471]	4.63	0.000038
ssc-miR-138	TRUB2	TruB pseudouridine synthase family member 2 [Source:VGNC Symbol;Acc:VGNC:94479]	4.63	0.000038
ssc-miR-138	TSPAN31	tetraspanin 31 [Source:VGNC Symbol;Acc:VGNC:94511]	4.63	0.000038
ssc-miR-138	TSR1	TSR1 ribosome maturation factor [Source:VGNC Symbol;Acc:VGNC:94521]	4.63	0.000038
ssc-miR-138	TTC30A	hypothetical gene	4.63	0.000038
ssc-miR-138	TTLL12	tubulin tyrosine ligase like 12 [Source:VGNC Symbol;Acc:VGNC:94568]	4.63	0.000038
ssc-miR-138	TUBB6	tubulin beta 6 class V [Source:VGNC Symbol;Acc:VGNC:94581]	4.63	0.000038
ssc-miR-138	TULP4	hypothetical gene	4.63	0.000038
ssc-miR-138	UBE2Q1	ubiquitin conjugating enzyme E2 Q1 [Source:VGNC Symbol;Acc:VGNC:98888]	4.63	0.000038
ssc-miR-138	UBE2R2	ubiquitin conjugating enzyme E2 R2 [Source:VGNC Symbol;Acc:VGNC:95924]	4.63	0.000038
ssc-miR-138	UBE2V1	ubiquitin conjugating enzyme E2 V1 [Source:VGNC Symbol;Acc:VGNC:98393]	4.63	0.000038
ssc-miR-138	UBP1	upstream binding protein 1 [Source:VGNC Symbol;Acc:VGNC:108701]	4.63	0.000038
ssc-miR-138	UHRF1BP1	UHRF1 binding protein 1 [Source:VGNC Symbol;Acc:VGNC:94690]	4.63	0.000038
ssc-miR-138	UIMC1	ubiquitin interaction motif containing 1 [Source:VGNC Symbol;Acc:VGNC:94693]	4.63	0.000038
ssc-miR-138	UNC5A	unc-5 netrin receptor A [Source:VGNC Symbol;Acc:VGNC:94707]	4.63	0.000038
ssc-miR-138	UNC5D	unc-5 netrin receptor D [Source:VGNC Symbol;Acc:VGNC:95892]	4.63	0.000038
ssc-miR-138	UPF2	UPF2 regulator of nonsense mediated mRNA decay [Source:VGNC Symbol;Acc:VGNC:98402]	4.63	0.000038
ssc-miR-138	USP10	ubiquitin specific peptidase 10 [Source:VGNC Symbol;Acc:VGNC:94743]	4.63	0.000038
ssc-miR-138	USP14	ubiquitin specific peptidase 14 [Source:VGNC Symbol;Acc:VGNC:94747]	4.63	0.000038
ssc-miR-138	USP39	ubiquitin specific peptidase 39 [Source:VGNC Symbol;Acc:VGNC:94763]	4.63	0.000038
ssc-miR-138	USP47	ubiquitin specific peptidase 47 [Source:VGNC Symbol;Acc:VGNC:94769]	4.63	0.000038
ssc-miR-138	USP49	ubiquitin specific peptidase 49 [Source:VGNC Symbol;Acc:VGNC:94771]	4.63	0.000038
ssc-miR-138	VANGL1	VANGL planar cell polarity protein 1 [Source:VGNC Symbol;Acc:VGNC:94797]	4.63	0.000038
ssc-miR-138	VAPB	VAMP associated protein B and C [Source:VGNC Symbol;Acc:VGNC:108743]	4.63	0.000038
ssc-miR-138	VDAC2	hypothetical gene	4.63	0.000038
ssc-miR-138	VEZF1	vascular endothelial zinc finger 1 [Source:VGNC Symbol;Acc:VGNC:94818]	4.63	0.000038
ssc-miR-138	VGLL3	vestigial like family member 3 [Source:VGNC Symbol;Acc:VGNC:94823]	4.63	0.000038
ssc-miR-138	VIM	vimentin [Source:VGNC Symbol;Acc:VGNC:95565]	4.63	0.000038
ssc-miR-138	VPS26A	VPS26, retromer complex component A [Source:VGNC Symbol;Acc:VGNC:94844]	4.63	0.000038
ssc-miR-138	VPS26B	VPS26, retromer complex component B [Source:NCBI gene (formerly Entrezgene);Acc:100192441]	4.63	0.000038
ssc-miR-138	VPS37A	VPS37A subunit of ESCRT-I [Source:VGNC Symbol;Acc:VGNC:104092]	4.63	0.000038
ssc-miR-138	VPS53	VPS53 subunit of GARP complex [Source:VGNC Symbol;Acc:VGNC:94861]	4.63	0.000038
ssc-miR-138	VSTM2L	V-set and transmembrane domain containing 2 like [Source:VGNC Symbol;Acc:VGNC:98410]	4.63	0.000038
ssc-miR-138	VWC2L	von Willebrand factor C domain containing 2 like [Source:VGNC Symbol;Acc:VGNC:96301]	4.63	0.000038
ssc-miR-138	WDR45B	WD repeat domain 45B [Source:NCBI gene (formerly Entrezgene);Acc:100462760]	4.63	0.000038
ssc-miR-138	WDR55	WD repeat domain 55 [Source:VGNC Symbol;Acc:VGNC:94929]	4.63	0.000038
ssc-miR-138	WEE1	WEE1 G2 checkpoint kinase [Source:VGNC Symbol;Acc:VGNC:104095]	4.63	0.000038

ssc-miR-138	WNK2	WNK lysine deficient protein kinase 2 [Source:VGNC Symbol;Acc:VGNC:94963]	4.63	0.000038
ssc-miR-138	WNT9A	Wnt family member 9A [Source:VGNC Symbol;Acc:VGNC:94978]	4.63	0.000038
ssc-miR-138	WWC1	WW and C2 domain containing 1 [Source:VGNC Symbol;Acc:VGNC:94986]	4.63	0.000038
ssc-miR-138	XPR1	xenotropic and polytropic retrovirus receptor 1 [Source:VGNC Symbol;Acc:VGNC:108625]	4.63	0.000038
ssc-miR-138	XRN1	5'-3' exoribonuclease 1 [Source:VGNC Symbol;Acc:VGNC:108705]	4.63	0.000038
ssc-miR-138	YARS2	tyrosyl-tRNA synthetase 2 [Source:VGNC Symbol;Acc:VGNC:104097]	4.63	0.000038
ssc-miR-138	YIPF5	Yip1 domain family member 5 [Source:VGNC Symbol;Acc:VGNC:95031]	4.63	0.000038
ssc-miR-138	YPEL4	yippee like 4 [Source:VGNC Symbol;Acc:VGNC:95038]	4.63	0.000038
ssc-miR-138	YWHAH	tyrosine 3-monooxygenase/tryptophan 5-monooxygenase activation protein eta [Source:VGNC Symbol;Acc:VGNC:95045]	4.63	0.000038
ssc-miR-138	ZBTB20	zinc finger and BTB domain containing 20 [Source:VGNC Symbol;Acc:VGNC:95063]	4.63	0.000038
ssc-miR-138	ZBTB4	hypothetical gene	4.63	0.000038
ssc-miR-138	ZBTB44	zinc finger and BTB domain containing 44 [Source:VGNC Symbol;Acc:VGNC:95076]	4.63	0.000038
ssc-miR-138	ZBTB47	zinc finger and BTB domain containing 47 [Source:VGNC Symbol;Acc:VGNC:95078]	4.63	0.000038
ssc-miR-138	ZBTB8A	zinc finger and BTB domain containing 8A [Source:VGNC Symbol;Acc:VGNC:95084]	4.63	0.000038
ssc-miR-138	ZC3H7B	zinc finger CCCH-type containing 7B [Source:VGNC Symbol;Acc:VGNC:95100]	4.63	0.000038
ssc-miR-138	ZCCHC14	zinc finger CCHC-type containing 14 [Source:VGNC Symbol;Acc:VGNC:95107]	4.63	0.000038
ssc-miR-138	ZEB2	hypothetical gene	4.63	0.000038
ssc-miR-138	ZER1	zyg-11 related cell cycle regulator [Source:VGNC Symbol;Acc:VGNC:95132]	4.63	0.000038
ssc-miR-138	ZFAT	zinc finger and AT-hook domain containing [Source:VGNC Symbol;Acc:VGNC:95138]	4.63	0.000038
ssc-miR-138	ZFP36L2	ZFP36 ring finger protein like 2 [Source:VGNC Symbol;Acc:VGNC:95147]	4.63	0.000038
ssc-miR-138	ZFPM2	zinc finger protein, FOG family member 2 [Source:VGNC Symbol;Acc:VGNC:95153]	4.63	0.000038
ssc-miR-138	ZKSCAN4	zinc finger with KRAB and SCAN domains 4 [Source:VGNC Symbol;Acc:VGNC:95173]	4.63	0.000038
ssc-miR-138	ZMIZ1	zinc finger MIZ-type containing 1 [Source:VGNC Symbol;Acc:VGNC:95178]	4.63	0.000038
ssc-miR-138	ZMYND11	zinc finger MYND-type containing 11 [Source:VGNC Symbol;Acc:VGNC:95827]	4.63	0.000038
ssc-miR-138	ZNF148	zinc finger protein 148 [Source:VGNC Symbol;Acc:VGNC:95192]	4.63	0.000038
ssc-miR-138	ZNF226	zinc finger protein 226 [Source:HGNC Symbol;Acc:HGNC:13019]	4.63	0.000038
ssc-miR-138	ZNF275	hypothetical gene	4.63	0.000038
ssc-miR-138	ZNF282	hypothetical gene	4.63	0.000038
ssc-miR-138	ZNF292	zinc finger protein 292 [Source:VGNC Symbol;Acc:VGNC:95218]	4.63	0.000038
ssc-miR-138	ZNF354C	zinc finger protein 354C [Source:HGNC Symbol;Acc:HGNC:16736]	4.63	0.000038
ssc-miR-138	ZNF385A	zinc finger protein 385A [Source:VGNC Symbol;Acc:VGNC:95233]	4.63	0.000038
ssc-miR-138	ZNF444	zinc finger protein 444 [Source:VGNC Symbol;Acc:VGNC:98426]	4.63	0.000038
ssc-miR-138	ZNF584	zinc finger protein 584 [Source:VGNC Symbol;Acc:VGNC:95271]	4.63	0.000038
ssc-miR-138	ZNF607	hypothetical gene	4.63	0.000038
ssc-miR-138	ZNF609	zinc finger protein 609 [Source:VGNC Symbol;Acc:VGNC:95274]	4.63	0.000038
ssc-miR-138	ZNF704	zinc finger protein 704 [Source:VGNC Symbol;Acc:VGNC:95296]	4.63	0.000038
ssc-miR-138	ZNF74	zinc finger protein 74 [Source:HGNC Symbol;Acc:HGNC:13144]	4.63	0.000038
ssc-miR-138	ZNF746	hypothetical gene	4.63	0.000038
ssc-miR-138	ZNF784	zinc finger protein 784 [Source:VGNC Symbol;Acc:VGNC:95301]	4.63	0.000038
ssc-miR-138	ZRANB1	zinc finger RANBP2-type containing 1 [Source:VGNC Symbol;Acc:VGNC:95322]	4.63	0.000038
ssc-miR-148a-3p	ABCA1	ATP binding cassette subfamily A member 1 [Source:VGNC Symbol;Acc:VGNC:84947]	4.51	0.0889
ssc-miR-148a-3p	ABCB10	ATP binding cassette subfamily B member 10 [Source:VGNC Symbol;Acc:VGNC:103892]	4.51	0.0889
ssc-miR-148a-3p	ABCB7	ATP binding cassette subfamily B member 7 [Source:VGNC Symbol;Acc:VGNC:97855]	4.51	0.0889
ssc-miR-148a-3p	ABCD3	ATP binding cassette subfamily D member 3 [Source:VGNC Symbol;Acc:VGNC:84963]	4.51	0.0889
ssc-miR-148a-3p	ABHD13	abhydrolase domain containing 13 [Source:VGNC Symbol;Acc:VGNC:84972]	4.51	0.0889
ssc-miR-148a-3p	ABL2	ABL proto-onco 2, non-receptor tyrosine kinase [Source:VGNC Symbol;Acc:VGNC:84985]	4.51	0.0889
ssc-miR-148a-3p	AC012215.1	hypothetical gene	4.51	0.0889
ssc-miR-148a-3p	AC068987.1	hypothetical gene	4.51	0.0889
ssc-miR-148a-3p	AC135178.1	hypothetical gene	4.51	0.0889

ssc-miR-148a-3p	AC140061.12	hypothetical gene	4.51	0.0889
ssc-miR-148a-3p	ACHE	acetylcholinesterase [Source:VGNC Symbol;Acc:VGNC:97029]	4.51	0.0889
ssc-miR-148a-3p	ACVR1	activin A receptor type 1 [Source:VGNC Symbol;Acc:VGNC:95830]	4.51	0.0889
ssc-miR-148a-3p	ACVR2B	activin A receptor type 2B [Source:VGNC Symbol;Acc:VGNC:108629]	4.51	0.0889
ssc-miR-148a-3p	ADAM10	ADAM metallopeptidase domain 10 [Source:VGNC Symbol;Acc:VGNC:85061]	4.51	0.0889
ssc-miR-148a-3p	ADAM12	ADAM metallopeptidase domain 12 [Source:VGNC Symbol;Acc:VGNC:85063]	4.51	0.0889
ssc-miR-148a-3p	ADAM17	ADAM metallopeptidase domain 17 [Source:VGNC Symbol;Acc:VGNC:85065]	4.51	0.0889
ssc-miR-148a-3p	ADAM22	ADAM metallopeptidase domain 22 [Source:VGNC Symbol;Acc:VGNC:85067]	4.51	0.0889
ssc-miR-148a-3p	ADAMTS1	ADAM metallopeptidase with thrombospondin type 1 motif 1 [Source:VGNC Symbol;Acc:VGNC:85072]	4.51	0.0889
ssc-miR-148a-3p	ADAMTS15	ADAM metallopeptidase with thrombospondin type 1 motif 15 [Source:VGNC Symbol;Acc:VGNC:85077]	4.51	0.0889
ssc-miR-148a-3p	ADAMTS18	ADAM metallopeptidase with thrombospondin type 1 motif 18 [Source:VGNC Symbol;Acc:VGNC:85080]	4.51	0.0889
ssc-miR-148a-3p	ADAMTS19	ADAM metallopeptidase with thrombospondin type 1 motif 19 [Source:VGNC Symbol;Acc:VGNC:96786]	4.51	0.0889
ssc-miR-148a-3p	ADAMTS5	ADAM metallopeptidase with thrombospondin type 1 motif 5 [Source:VGNC Symbol;Acc:VGNC:85085]	4.51	0.0889
ssc-miR-148a-3p	ADCY2	adenylate cyclase 2 [Source:VGNC Symbol;Acc:VGNC:85106]	4.51	0.0889
ssc-miR-148a-3p	AGFG1	ArfGAP with FG repeats 1 [Source:VGNC Symbol;Acc:VGNC:96359]	4.51	0.0889
ssc-miR-148a-3p	AGO1	hypothetical gene	4.51	0.0889
ssc-miR-148a-3p	AGO3	argonaute RISC component 1 [Source:NCBI gene (formerly Entrezgene);Acc:100499510]	4.51	0.0889
ssc-miR-148a-3p	AGO4	hypothetical gene	4.51	0.0889
ssc-miR-148a-3p	AGPAT5	1-acylglycerol-3-phosphate O-acyltransferase 5 [Source:VGNC Symbol;Acc:VGNC:95954]	4.51	0.0889
ssc-miR-148a-3p	AGXT2	alanine--glyoxylate aminotransferase 2 [Source:VGNC Symbol;Acc:VGNC:97872]	4.51	0.0889
ssc-miR-148a-3p	AHDC1	hypothetical gene	4.51	0.0889
ssc-miR-148a-3p	AHSA2	hypothetical gene	4.51	0.0889
ssc-miR-148a-3p	AJUBA	ajuba LIM protein [Source:VGNC Symbol;Acc:VGNC:85208]	4.51	0.0889
ssc-miR-148a-3p	AKAP1	A-kinase anchoring protein 1 [Source:VGNC Symbol;Acc:VGNC:85214]	4.51	0.0889
ssc-miR-148a-3p	AL020996.1	hypothetical gene	4.51	0.0889
ssc-miR-148a-3p	AL021546.6	hypothetical gene	4.51	0.0889
ssc-miR-148a-3p	ALCAM	activated leukocyte cell adhesion molecule [Source:VGNC Symbol;Acc:VGNC:85236]	4.51	0.0889
ssc-miR-148a-3p	AMER1	APC membrane recruitment protein 1 [Source:VGNC Symbol;Acc:VGNC:85276]	4.51	0.0889
ssc-miR-148a-3p	AMMECR1	AMMECR nuclear protein 1 [Source:VGNC Symbol;Acc:VGNC:96559]	4.51	0.0889
ssc-miR-148a-3p	ANK2	hypothetical gene	4.51	0.0889
ssc-miR-148a-3p	ANKRD13A	ankyrin repeat domain 13A [Source:VGNC Symbol;Acc:VGNC:85324]	4.51	0.0889
ssc-miR-148a-3p	ANKRD32	hypothetical gene	4.51	0.0889
ssc-miR-148a-3p	ANKRD52	ankyrin repeat domain 52 [Source:VGNC Symbol;Acc:VGNC:85342]	4.51	0.0889
ssc-miR-148a-3p	AP1G1	adaptor related protein complex 1 subunit gamma 1 [Source:VGNC Symbol;Acc:VGNC:85378]	4.51	0.0889
ssc-miR-148a-3p	AP4E1	adaptor related protein complex 4 subunit epsilon 1 [Source:VGNC Symbol;Acc:VGNC:85392]	4.51	0.0889
ssc-miR-148a-3p	AQP11	aquaporin 11 [Source:VGNC Symbol;Acc:VGNC:85430]	4.51	0.0889
ssc-miR-148a-3p	ARAP2	ArfGAP with RhoGAP domain, ankyrin repeat and PH domain 2 [Source:VGNC Symbol;Acc:VGNC:85440]	4.51	0.0889
ssc-miR-148a-3p	ARF4	ADP ribosylation factor 4 [Source:VGNC Symbol;Acc:VGNC:85446]	4.51	0.0889
ssc-miR-148a-3p	ARFGEF1	ADP ribosylation factor guanine nucleotide exchange factor 1 [Source:VGNC Symbol;Acc:VGNC:85451]	4.51	0.0889
ssc-miR-148a-3p	ARFIP1	ADP ribosylation factor interacting protein 1 [Source:VGNC Symbol;Acc:VGNC:85453]	4.51	0.0889
ssc-miR-148a-3p	ARHGAP20	Rho GTPase activating protein 20 [Source:VGNC Symbol;Acc:VGNC:97884]	4.51	0.0889
ssc-miR-148a-3p	ARHGAP21	Rho GTPase activating protein 21 [Source:VGNC Symbol;Acc:VGNC:96018]	4.51	0.0889
ssc-miR-148a-3p	ARHGEF12	Rho guanine nucleotide exchange factor 12 [Source:VGNC Symbol;Acc:VGNC:85488]	4.51	0.0889
ssc-miR-148a-3p	ARHGEF17	hypothetical gene	4.51	0.0889
ssc-miR-148a-3p	ARHGEF37	Rho guanine nucleotide exchange factor 37 [Source:VGNC Symbol;Acc:VGNC:85498]	4.51	0.0889
ssc-miR-148a-3p	ARID3A	AT-rich interaction domain 3A [Source:VGNC Symbol;Acc:VGNC:85507]	4.51	0.0889
ssc-miR-148a-3p	ARL6IP1	hypothetical gene	4.51	0.0889
ssc-miR-148a-3p	ARL8B	ADP ribosylation factor like GTPase 8B [Source:VGNC Symbol;Acc:VGNC:103905]	4.51	0.0889
ssc-miR-148a-3p	ARMC5	armadillo repeat containing 5 [Source:VGNC Symbol;Acc:VGNC:85529]	4.51	0.0889

ssc-miR-148a-3p	ARPP19	cAMP regulated phosphoprotein 19 [Source:NCBI gene (formerly Entrezgene);Acc:397362]	4.51	0.0889
ssc-miR-148a-3p	ARRDC3	arrestin domain containing 3 [Source:VGNC Symbol;Acc:VGNC:85546]	4.51	0.0889
ssc-miR-148a-3p	ASPH	aspartate beta-hydroxylase [Source:VGNC Symbol;Acc:VGNC:85584]	4.51	0.0889
ssc-miR-148a-3p	ATG14	autophagy related 14 [Source:VGNC Symbol;Acc:VGNC:85617]	4.51	0.0889
ssc-miR-148a-3p	ATG4D	autophagy related 4D cysteine peptidase [Source:VGNC Symbol;Acc:VGNC:85624]	4.51	0.0889
ssc-miR-148a-3p	ATP11A	ATPase phospholipid transporting 11A [Source:VGNC Symbol;Acc:VGNC:85634]	4.51	0.0889
ssc-miR-148a-3p	ATP2A2	ATPase sarcoplasmic/endoplasmic reticulum Ca2+ transporting 2 [Source:VGNC Symbol;Acc:VGNC:85647]	4.51	0.0889
ssc-miR-148a-3p	ATP2B4	ATPase plasma membrane Ca2+ transporting 4 [Source:VGNC Symbol;Acc:VGNC:85651]	4.51	0.0889
ssc-miR-148a-3p	ATP6AP2	ATPase H+ transporting accessory protein 2 [Source:VGNC Symbol;Acc:VGNC:85662]	4.51	0.0889
ssc-miR-148a-3p	ATP7A	ATPase copper transporting alpha [Source:VGNC Symbol;Acc:VGNC:85678]	4.51	0.0889
ssc-miR-148a-3p	ATP8A1	ATPase phospholipid transporting 8A1 [Source:VGNC Symbol;Acc:VGNC:97894]	4.51	0.0889
ssc-miR-148a-3p	ATXN1	ataxin 1 [Source:VGNC Symbol;Acc:VGNC:85687]	4.51	0.0889
ssc-miR-148a-3p	ATXN3	hypothetical gene	4.51	0.0889
ssc-miR-148a-3p	ATXN7L1	ataxin 7 like 1 [Source:VGNC Symbol;Acc:VGNC:85692]	4.51	0.0889
ssc-miR-148a-3p	B4GALT2	beta-1,4-galactosyltransferase 2 [Source:VGNC Symbol;Acc:VGNC:85733]	4.51	0.0889
ssc-miR-148a-3p	B4GALT5	beta-1,4-galactosyltransferase 5 [Source:VGNC Symbol;Acc:VGNC:96499]	4.51	0.0889
ssc-miR-148a-3p	B4GALT6	beta-1,4-galactosyltransferase 6 [Source:VGNC Symbol;Acc:VGNC:85734]	4.51	0.0889
ssc-miR-148a-3p	BACH2	BTB domain and CNC homolog 2 [Source:VGNC Symbol;Acc:VGNC:85742]	4.51	0.0889
ssc-miR-148a-3p	BAI3	hypothetical gene	4.51	0.0889
ssc-miR-148a-3p	BARHL2	BarH like homeobox 2 [Source:VGNC Symbol;Acc:VGNC:85756]	4.51	0.0889
ssc-miR-148a-3p	BAZ2A	bromodomain adjacent to zinc finger domain 2A [Source:VGNC Symbol;Acc:VGNC:85763]	4.51	0.0889
ssc-miR-148a-3p	BAZ2B	bromodomain adjacent to zinc finger domain 2B [Source:HGNC Symbol;Acc:HGNC:963]	4.51	0.0889
ssc-miR-148a-3p	BBX	BBX high mobility group box domain containing [Source:VGNC Symbol;Acc:VGNC:85766]	4.51	0.0889
ssc-miR-148a-3p	BCL2L11	BCL2 like 11 [Source:NCBI gene (formerly Entrezgene);Acc:396632]	4.51	0.0889
ssc-miR-148a-3p	BCL2L13	BCL2 like 13 [Source:VGNC Symbol;Acc:VGNC:85781]	4.51	0.0889
ssc-miR-148a-3p	BCL2L2	BCL2 like 2 [Source:HGNC Symbol;Acc:HGNC:995]	4.51	0.0889
ssc-miR-148a-3p	BCL7A	BAF chromatin remodeling complex subunit BCL7A [Source:VGNC Symbol;Acc:VGNC:85784]	4.51	0.0889
ssc-miR-148a-3p	BCL7B	BAF chromatin remodeling complex subunit BCL7B [Source:VGNC Symbol;Acc:VGNC:85785]	4.51	0.0889
ssc-miR-148a-3p	BEST1	bestrophin 1 [Source:VGNC Symbol;Acc:VGNC:85803]	4.51	0.0889
ssc-miR-148a-3p	BHLHE41	basic helix-loop-helix family member e41 [Source:VGNC Symbol;Acc:VGNC:85815]	4.51	0.0889
ssc-miR-148a-3p	BICC1	BicC family RNA binding protein 1 [Source:VGNC Symbol;Acc:VGNC:85817]	4.51	0.0889
ssc-miR-148a-3p	BMP3	bone morphotic protein 3 [Source:VGNC Symbol;Acc:VGNC:85842]	4.51	0.0889
ssc-miR-148a-3p	BNIP2	BCL2 interacting protein 2 [Source:VGNC Symbol;Acc:VGNC:85854]	4.51	0.0889
ssc-miR-148a-3p	BRPF1	bromodomain and PHD finger containing 1 [Source:VGNC Symbol;Acc:VGNC:85885]	4.51	0.0889
ssc-miR-148a-3p	BRWD3	bromodomain and WD repeat domain containing 3 [Source:VGNC Symbol;Acc:VGNC:85890]	4.51	0.0889
ssc-miR-148a-3p	BTAF1	B-TFIID TATA-box binding protein associated factor 1 [Source:VGNC Symbol;Acc:VGNC:85899]	4.51	0.0889
ssc-miR-148a-3p	BTBD3	BTB domain containing 3 [Source:VGNC Symbol;Acc:VGNC:95673]	4.51	0.0889
ssc-miR-148a-3p	BZRAP1	hypothetical gene	4.51	0.0889
ssc-miR-148a-3p	C11orf30	hypothetical gene	4.51	0.0889
ssc-miR-148a-3p	C12orf79	hypothetical gene	4.51	0.0889
ssc-miR-148a-3p	C15orf27	hypothetical gene	4.51	0.0889
ssc-miR-148a-3p	C16orf52	hypothetical gene	4.51	0.0889
ssc-miR-148a-3p	C16orf70	hypothetical gene	4.51	0.0889
ssc-miR-148a-3p	C18orf25	chromosome 1 C18orf25 homolog [Source:VGNC Symbol;Acc:VGNC:85964]	4.51	0.0889
ssc-miR-148a-3p	C1GALT1	hypothetical gene	4.51	0.0889
ssc-miR-148a-3p	C1orf35	chromosome 2 C1orf35 homolog [Source:VGNC Symbol;Acc:VGNC:86008]	4.51	0.0889
ssc-miR-148a-3p	C1orf50	hypothetical gene	4.51	0.0889
ssc-miR-148a-3p	C20orf112	hypothetical gene	4.51	0.0889
ssc-miR-148a-3p	C2orf91	hypothetical gene	4.51	0.0889

ssc-miR-148a-3p	C3orf58	hypothetical gene	4.51	0.0889
ssc-miR-148a-3p	C5orf24	chromosome 2 C5orf24 homolog [Source:VGNC Symbol;Acc:VGNC:86010]	4.51	0.0889
ssc-miR-148a-3p	C5orf30	hypothetical gene	4.51	0.0889
ssc-miR-148a-3p	C6orf222	hypothetical gene	4.51	0.0889
ssc-miR-148a-3p	C6orf62	chromosome 7 C6orf62 homolog [Source:VGNC Symbol;Acc:VGNC:86074]	4.51	0.0889
ssc-miR-148a-3p	C7orf60	hypothetical gene	4.51	0.0889
ssc-miR-148a-3p	C7orf71	hypothetical gene	4.51	0.0889
ssc-miR-148a-3p	CABP7	calcium binding protein 7 [Source:VGNC Symbol;Acc:VGNC:107372]	4.51	0.0889
ssc-miR-148a-3p	CADM1	cell adhesion molecule 1 [Source:VGNC Symbol;Acc:VGNC:86134]	4.51	0.0889
ssc-miR-148a-3p	CADM3	cell adhesion molecule 3 [Source:VGNC Symbol;Acc:VGNC:86135]	4.51	0.0889
ssc-miR-148a-3p	CALR	calreticulin [Source:VGNC Symbol;Acc:VGNC:86149]	4.51	0.0889
ssc-miR-148a-3p	CAMK2A	calcium/calmodulin dependent protein kinase II alpha [Source:VGNC Symbol;Acc:VGNC:86155]	4.51	0.0889
ssc-miR-148a-3p	CAMKK1	calcium/calmodulin dependent protein kinase kinase 1 [Source:VGNC Symbol;Acc:VGNC:98977]	4.51	0.0889
ssc-miR-148a-3p	CAMSAP1	calmodulin regulated spectrin associated protein 1 [Source:VGNC Symbol;Acc:VGNC:86162]	4.51	0.0889
ssc-miR-148a-3p	CAMSAP2	calmodulin regulated spectrin associated protein family member 2 [Source:VGNC Symbol;Acc:VGNC:96205]	4.51	0.0889
ssc-miR-148a-3p	CAND1	cullin associated and neddylation dissociated 1 [Source:VGNC Symbol;Acc:VGNC:97912]	4.51	0.0889
ssc-miR-148a-3p	CANX	calnexin [Source:VGNC Symbol;Acc:VGNC:86167]	4.51	0.0889
ssc-miR-148a-3p	CAPRIN1	cell cycle associated protein 1 [Source:VGNC Symbol;Acc:VGNC:86178]	4.51	0.0889
ssc-miR-148a-3p	CAST	calpastatin [Source:VGNC Symbol;Acc:VGNC:99603]	4.51	0.0889
ssc-miR-148a-3p	CASZ1	castor zinc finger 1 [Source:VGNC Symbol;Acc:VGNC:86207]	4.51	0.0889
ssc-miR-148a-3p	CAV2	caveolin 2 [Source:VGNC Symbol;Acc:VGNC:103914]	4.51	0.0889
ssc-miR-148a-3p	CBLB	Cbl proto-onco B [Source:VGNC Symbol;Acc:VGNC:86223]	4.51	0.0889
ssc-miR-148a-3p	CBX5	chromobox 5 [Source:VGNC Symbol;Acc:VGNC:86232]	4.51	0.0889
ssc-miR-148a-3p	CCDC144A	hypothetical gene	4.51	0.0889
ssc-miR-148a-3p	CCDC6	coiled-coil domain containing 6 [Source:VGNC Symbol;Acc:VGNC:86304]	4.51	0.0889
ssc-miR-148a-3p	CCDC71	coiled-coil domain containing 71 [Source:VGNC Symbol;Acc:VGNC:86311]	4.51	0.0889
ssc-miR-148a-3p	CCDC85A	coiled-coil domain containing 85A [Source:VGNC Symbol;Acc:VGNC:86320]	4.51	0.0889
ssc-miR-148a-3p	CCDC88A	coiled-coil domain containing 88A [Source:VGNC Symbol;Acc:VGNC:86325]	4.51	0.0889
ssc-miR-148a-3p	CCNA2	cyclin A2 [Source:NCBI gene (formerly Entrezgene);Acc:100415929]	4.51	0.0889
ssc-miR-148a-3p	CCNF	cyclin F [Source:VGNC Symbol;Acc:VGNC:86356]	4.51	0.0889
ssc-miR-148a-3p	CCNK	cyclin K [Source:VGNC Symbol;Acc:VGNC:86362]	4.51	0.0889
ssc-miR-148a-3p	CCNY	cyclin Y [Source:VGNC Symbol;Acc:VGNC:95998]	4.51	0.0889
ssc-miR-148a-3p	CCT6A	chaperonin containing TCP1 subunit 6A [Source:VGNC Symbol;Acc:VGNC:106569]	4.51	0.0889
ssc-miR-148a-3p	CD22	CD22 molecule [Source:VGNC Symbol;Acc:VGNC:86398]	4.51	0.0889
ssc-miR-148a-3p	CDC14A	cell division cycle 14A [Source:VGNC Symbol;Acc:VGNC:86442]	4.51	0.0889
ssc-miR-148a-3p	CDC25B	cell division cycle 25B [Source:VGNC Symbol;Acc:VGNC:95839]	4.51	0.0889
ssc-miR-148a-3p	CDC42SE2	CDC42 small effector 2 [Source:VGNC Symbol;Acc:VGNC:86461]	4.51	0.0889
ssc-miR-148a-3p	CDH20	cadherin 20 [Source:VGNC Symbol;Acc:VGNC:86484]	4.51	0.0889
ssc-miR-148a-3p	CDH7	cadherin 7 [Source:VGNC Symbol;Acc:VGNC:97923]	4.51	0.0889
ssc-miR-148a-3p	CDK13	cyclin dependent kinase 13 [Source:VGNC Symbol;Acc:VGNC:86497]	4.51	0.0889
ssc-miR-148a-3p	CDK14	cyclin dependent kinase 14 [Source:VGNC Symbol;Acc:VGNC:86498]	4.51	0.0889
ssc-miR-148a-3p	CDK19	cyclin dependent kinase 19 [Source:VGNC Symbol;Acc:VGNC:86502]	4.51	0.0889
ssc-miR-148a-3p	CDK5R1	cyclin dependent kinase 5 regulatory subunit 1 [Source:VGNC Symbol;Acc:VGNC:98980]	4.51	0.0889
ssc-miR-148a-3p	CDK6	cyclin dependent kinase 6 [Source:HGNC Symbol;Acc:HGNC:1777]	4.51	0.0889
ssc-miR-148a-3p	CDK8	cyclin dependent kinase 8 [Source:VGNC Symbol;Acc:VGNC:86507]	4.51	0.0889
ssc-miR-148a-3p	CDKN1B	cyclin dependent kinase inhibitor 1B [Source:VGNC Symbol;Acc:VGNC:86515]	4.51	0.0889
ssc-miR-148a-3p	CEBPG	CCAAT enhancer binding protein gamma [Source:VGNC Symbol;Acc:VGNC:86534]	4.51	0.0889
ssc-miR-148a-3p	CECR6	hypothetical gene	4.51	0.0889
ssc-miR-148a-3p	CENPP	centromere protein P [Source:VGNC Symbol;Acc:VGNC:98865]	4.51	0.0889

ssc-miR-148a-3p	CEP55	centrosomal protein 55 [Source:VGNC Symbol;Acc:VGNC:86572]	4.51	0.0889
ssc-miR-148a-3p	CEP63	centrosomal protein 63 [Source:VGNC Symbol;Acc:VGNC:86574]	4.51	0.0889
ssc-miR-148a-3p	CEP72	hypothetical gene	4.51	0.0889
ssc-miR-148a-3p	CEP97	centrosomal protein 97 [Source:VGNC Symbol;Acc:VGNC:108638]	4.51	0.0889
ssc-miR-148a-3p	CERS5	ceramide synthase 5 [Source:VGNC Symbol;Acc:VGNC:97926]	4.51	0.0889
ssc-miR-148a-3p	CERS6	ceramide synthase 6 [Source:VGNC Symbol;Acc:VGNC:96025]	4.51	0.0889
ssc-miR-148a-3p	CFL2	cofilin 2 [Source:VGNC Symbol;Acc:VGNC:86611]	4.51	0.0889
ssc-miR-148a-3p	CHCHD4	coiled-coil-helix-coiled-coil-helix domain containing 4 [Source:NCBI gene (formerly Entrezgene);Acc:100154663]	4.51	0.0889
ssc-miR-148a-3p	CHD1	chromodomain helicase DNA binding protein 1 [Source:VGNC Symbol;Acc:VGNC:86629]	4.51	0.0889
ssc-miR-148a-3p	CHD7	chromodomain helicase DNA binding protein 7 [Source:VGNC Symbol;Acc:VGNC:86633]	4.51	0.0889
ssc-miR-148a-3p	CHD9	chromodomain helicase DNA binding protein 9 [Source:VGNC Symbol;Acc:VGNC:86635]	4.51	0.0889
ssc-miR-148a-3p	CHMP4B	charged multivesicular body protein 4B [Source:VGNC Symbol;Acc:VGNC:108711]	4.51	0.0889
ssc-miR-148a-3p	CHRNB2	cholinergic receptor nicotinic beta 2 subunit [Source:VGNC Symbol;Acc:VGNC:86672]	4.51	0.0889
ssc-miR-148a-3p	CHST1	carbohydrate sulfotransferase 1 [Source:VGNC Symbol;Acc:VGNC:86673]	4.51	0.0889
ssc-miR-148a-3p	CHUK	component of inhibitor of nuclear factor kappa B kinase complex [Source:VGNC Symbol;Acc:VGNC:86688]	4.51	0.0889
ssc-miR-148a-3p	CLCN3	chloride voltage-gated channel 3 [Source:VGNC Symbol;Acc:VGNC:86727]	4.51	0.0889
ssc-miR-148a-3p	CLCN6	chloride voltage-gated channel 6 [Source:VGNC Symbol;Acc:VGNC:86729]	4.51	0.0889
ssc-miR-148a-3p	CLOCK	clock circadian regulator [Source:VGNC Symbol;Acc:VGNC:86774]	4.51	0.0889
ssc-miR-148a-3p	CLUH	clustered mitochondria homolog [Source:VGNC Symbol;Acc:VGNC:86792]	4.51	0.0889
ssc-miR-148a-3p	CLVS2	clavesin 2 [Source:VGNC Symbol;Acc:VGNC:86795]	4.51	0.0889
ssc-miR-148a-3p	CNIH4	cornichon family AMPA receptor auxiliary protein 4 [Source:VGNC Symbol;Acc:VGNC:96060]	4.51	0.0889
ssc-miR-148a-3p	CNNM2	cyclin and CBS domain divalent metal cation transport mediator 2 [Source:VGNC Symbol;Acc:VGNC:86828]	4.51	0.0889
ssc-miR-148a-3p	CNOT6	CCR4-NOT transcription complex subunit 6 [Source:VGNC Symbol;Acc:VGNC:86837]	4.51	0.0889
ssc-miR-148a-3p	CNOT7	CCR4-NOT transcription complex subunit 7 [Source:VGNC Symbol;Acc:VGNC:95604]	4.51	0.0889
ssc-miR-148a-3p	CNR1	cannabinoid receptor 1 [Source:VGNC Symbol;Acc:VGNC:86843]	4.51	0.0889
ssc-miR-148a-3p	CNTN4	contactin 4 [Source:VGNC Symbol;Acc:VGNC:97934]	4.51	0.0889
ssc-miR-148a-3p	COG5	component of oligomeric golgi complex 5 [Source:VGNC Symbol;Acc:VGNC:86857]	4.51	0.0889
ssc-miR-148a-3p	COL2A1	collagen type II alpha 1 chain [Source:VGNC Symbol;Acc:VGNC:86874]	4.51	0.0889
ssc-miR-148a-3p	COL4A1	collagen type IV alpha 1 chain [Source:HGNC Symbol;Acc:HGNC:2202]	4.51	0.0889
ssc-miR-148a-3p	COL6A3	collagen type VI alpha 3 chain [Source:VGNC Symbol;Acc:VGNC:95982]	4.51	0.0889
ssc-miR-148a-3p	CPD	carboxypeptidase D [Source:VGNC Symbol;Acc:VGNC:86935]	4.51	0.0889
ssc-miR-148a-3p	CPEB4	cytoplasmic polyadenylation element binding protein 4 [Source:VGNC Symbol;Acc:VGNC:86939]	4.51	0.0889
ssc-miR-148a-3p	CPT1A	carnitine palmitoyltransferase 1A [Source:VGNC Symbol;Acc:VGNC:86964]	4.51	0.0889
ssc-miR-148a-3p	CRIM1	cysteine rich transmembrane BMP regulator 1 [Source:VGNC Symbol;Acc:VGNC:86995]	4.51	0.0889
ssc-miR-148a-3p	CS	citrate synthase [Source:VGNC Symbol;Acc:VGNC:103225]	4.51	0.0889
ssc-miR-148a-3p	CSF1	colony stimulating factor 1 [Source:VGNC Symbol;Acc:VGNC:87030]	4.51	0.0889
ssc-miR-148a-3p	CSNK2A1	hypothetical gene	4.51	0.0889
ssc-miR-148a-3p	CTD-2140B24.4	hypothetical gene	4.51	0.0889
ssc-miR-148a-3p	CTSA	cathepsin A [Source:VGNC Symbol;Acc:VGNC:96498]	4.51	0.0889
ssc-miR-148a-3p	CTTNBP2NL	CTTNBP2 N-terminal like [Source:VGNC Symbol;Acc:VGNC:87082]	4.51	0.0889
ssc-miR-148a-3p	CUL4A	cullin 4A [Source:VGNC Symbol;Acc:VGNC:87088]	4.51	0.0889
ssc-miR-148a-3p	CUL5	cullin 5 [Source:VGNC Symbol;Acc:VGNC:87089]	4.51	0.0889
ssc-miR-148a-3p	CXorf23	hypothetical gene	4.51	0.0889
ssc-miR-148a-3p	CYB5R4	cytochrome b5 reductase 4 [Source:HGNC Symbol;Acc:HGNC:20147]	4.51	0.0889
ssc-miR-148a-3p	CYTH3	cytohesin 3 [Source:VGNC Symbol;Acc:VGNC:87136]	4.51	0.0889
ssc-miR-148a-3p	DCP1A	decapping mRNA 1A [Source:VGNC Symbol;Acc:VGNC:97960]	4.51	0.0889
ssc-miR-148a-3p	DCP2	decapping mRNA 2 [Source:VGNC Symbol;Acc:VGNC:87188]	4.51	0.0889
ssc-miR-148a-3p	DCUN1D3	defective in cullin neddylation 1 domain containing 3 [Source:VGNC Symbol;Acc:VGNC:87196]	4.51	0.0889
ssc-miR-148a-3p	DCUN1D4	defective in cullin neddylation 1 domain containing 4 [Source:VGNC Symbol;Acc:VGNC:87197]	4.51	0.0889

ssc-miR-148a-3p	DDAH1	dimethylarginine dimethylaminohydrolase 1 [Source:VGNC Symbol;Acc:VGNC:87200]	4.51	0.0889
ssc-miR-148a-3p	DDX17	hypothetical gene	4.51	0.0889
ssc-miR-148a-3p	DDX6	DEAD-box helicase 6 [Source:VGNC Symbol;Acc:VGNC:87233]	4.51	0.0889
ssc-miR-148a-3p	DEDD	death effector domain containing [Source:VGNC Symbol;Acc:VGNC:98777]	4.51	0.0889
ssc-miR-148a-3p	DENND4C	DENN domain containing 4C [Source:VGNC Symbol;Acc:VGNC:87253]	4.51	0.0889
ssc-miR-148a-3p	DENR	density regulated re-initiation and release factor [Source:VGNC Symbol;Acc:VGNC:97963]	4.51	0.0889
ssc-miR-148a-3p	DGCR8	DGCR8 microprocessor complex subunit [Source:VGNC Symbol;Acc:VGNC:87268]	4.51	0.0889
ssc-miR-148a-3p	DHRS13	dehydrogenase/reductase 13 [Source:VGNC Symbol;Acc:VGNC:97965]	4.51	0.0889
ssc-miR-148a-3p	DICER1	dicer 1, ribonuclease III [Source:VGNC Symbol;Acc:VGNC:87302]	4.51	0.0889
ssc-miR-148a-3p	DLG2	discs large MAGUK scaffold protein 2 [Source:VGNC Symbol;Acc:VGNC:108581]	4.51	0.0889
ssc-miR-148a-3p	DLL1	delta like canonical Notch ligand 1 [Source:VGNC Symbol;Acc:VGNC:87335]	4.51	0.0889
ssc-miR-148a-3p	DLL4	delta like canonical Notch ligand 4 [Source:VGNC Symbol;Acc:VGNC:87337]	4.51	0.0889
ssc-miR-148a-3p	DMPK	DM1 protein kinase [Source:VGNC Symbol;Acc:VGNC:87349]	4.51	0.0889
ssc-miR-148a-3p	DMRTA2	DMRT like family A2 [Source:VGNC Symbol;Acc:VGNC:87354]	4.51	0.0889
ssc-miR-148a-3p	DMXL1	Dmx like 1 [Source:VGNC Symbol;Acc:VGNC:87359]	4.51	0.0889
ssc-miR-148a-3p	DNAJB12	DnaJ heat shock protein family (Hsp40) member B12 [Source:VGNC Symbol;Acc:VGNC:96679]	4.51	0.0889
ssc-miR-148a-3p	DNAJB4	DnaJ heat shock protein family (Hsp40) member B4 [Source:VGNC Symbol;Acc:VGNC:96623]	4.51	0.0889
ssc-miR-148a-3p	DNAJC16	DnaJ heat shock protein family (Hsp40) member C16 [Source:VGNC Symbol;Acc:VGNC:96624]	4.51	0.0889
ssc-miR-148a-3p	DNAJC18	DnaJ heat shock protein family (Hsp40) member C18 [Source:VGNC Symbol;Acc:VGNC:96615]	4.51	0.0889
ssc-miR-148a-3p	DNMT1	DNA methyltransferase 1 [Source:VGNC Symbol;Acc:VGNC:87383]	4.51	0.0889
ssc-miR-148a-3p	DOCK6	dedicator of cytokinesis 6 [Source:VGNC Symbol;Acc:VGNC:87396]	4.51	0.0889
ssc-miR-148a-3p	DPP9	dipeptidyl peptidase 9 [Source:VGNC Symbol;Acc:VGNC:87424]	4.51	0.0889
ssc-miR-148a-3p	DPYSL5	dihydropyrimidinase like 5 [Source:VGNC Symbol;Acc:VGNC:87433]	4.51	0.0889
ssc-miR-148a-3p	DRGX	dorsal root ganglia homeobox [Source:VGNC Symbol;Acc:VGNC:97968]	4.51	0.0889
ssc-miR-148a-3p	DSC2	desmocollin 2 [Source:HGNC Symbol;Acc:HGNC:3036]	4.51	0.0889
ssc-miR-148a-3p	DSTYK	dual serine/threonine and tyrosine protein kinase [Source:VGNC Symbol;Acc:VGNC:87460]	4.51	0.0889
ssc-miR-148a-3p	DUSP1	dual specificity phosphatase 1 [Source:VGNC Symbol;Acc:VGNC:87476]	4.51	0.0889
ssc-miR-148a-3p	DUSP16	dual specificity phosphatase 16 [Source:VGNC Symbol;Acc:VGNC:87480]	4.51	0.0889
ssc-miR-148a-3p	DUSP3	dual specificity phosphatase 3 [Source:VGNC Symbol;Acc:VGNC:87486]	4.51	0.0889
ssc-miR-148a-3p	DUSP8	dual specificity phosphatase 8 [Source:VGNC Symbol;Acc:VGNC:87489]	4.51	0.0889
ssc-miR-148a-3p	DYNLL2	dynein light chain LC8-type 2 [Source:VGNC Symbol;Acc:VGNC:87501]	4.51	0.0889
ssc-miR-148a-3p	DYRK1A	dual specificity tyrosine phosphorylation regulated kinase 1A [Source:VGNC Symbol;Acc:VGNC:87505]	4.51	0.0889
ssc-miR-148a-3p	DYRK1B	dual specificity tyrosine phosphorylation regulated kinase 1B [Source:VGNC Symbol;Acc:VGNC:87506]	4.51	0.0889
ssc-miR-148a-3p	DYRK2	dual specificity tyrosine phosphorylation regulated kinase 2 [Source:VGNC Symbol;Acc:VGNC:87507]	4.51	0.0889
ssc-miR-148a-3p	E2F3	E2F transcription factor 3 [Source:VGNC Symbol;Acc:VGNC:87514]	4.51	0.0889
ssc-miR-148a-3p	E2F7	E2F transcription factor 7 [Source:VGNC Symbol;Acc:VGNC:87518]	4.51	0.0889
ssc-miR-148a-3p	EDARADD	EDAR associated death domain [Source:VGNC Symbol;Acc:VGNC:87542]	4.51	0.0889
ssc-miR-148a-3p	EDNRA	endothelin receptor type A [Source:VGNC Symbol;Acc:VGNC:87549]	4.51	0.0889
ssc-miR-148a-3p	EEA1	early endosome antigen 1 [Source:HGNC Symbol;Acc:HGNC:3185]	4.51	0.0889
ssc-miR-148a-3p	EFNB2	ephrin B2 [Source:VGNC Symbol;Acc:VGNC:87577]	4.51	0.0889
ssc-miR-148a-3p	EGFR	epidermal growth factor receptor [Source:VGNC Symbol;Acc:VGNC:87587]	4.51	0.0889
ssc-miR-148a-3p	EGR2	early growth response 2 [Source:VGNC Symbol;Acc:VGNC:103939]	4.51	0.0889
ssc-miR-148a-3p	EGR3	early growth response 3 [Source:VGNC Symbol;Acc:VGNC:87591]	4.51	0.0889
ssc-miR-148a-3p	EIF4E3	eukaryotic translation initiation factor 4E family member 3 [Source:VGNC Symbol;Acc:VGNC:87628]	4.51	0.0889
ssc-miR-148a-3p	ELAVL2	ELAV like RNA binding protein 2 [Source:VGNC Symbol;Acc:VGNC:87639]	4.51	0.0889
ssc-miR-148a-3p	ELAVL4	ELAV like RNA binding protein 4 [Source:VGNC Symbol;Acc:VGNC:97047]	4.51	0.0889
ssc-miR-148a-3p	ELF5	E74 like ETS transcription factor 5 [Source:VGNC Symbol;Acc:VGNC:87644]	4.51	0.0889
ssc-miR-148a-3p	ELMO1	engulfment and cell motility 1 [Source:HGNC Symbol;Acc:HGNC:16286]	4.51	0.0889
ssc-miR-148a-3p	EML2	EMAP like 2 [Source:VGNC Symbol;Acc:VGNC:87683]	4.51	0.0889

ssc-miR-148a-3p	EML6	EMAP like 6 [Source:VGNC Symbol;Acc:VGNC:87687]	4.51	0.0889
ssc-miR-148a-3p	EMX2	empty spiracles homeobox 2 [Source:VGNC Symbol;Acc:VGNC:87693]	4.51	0.0889
ssc-miR-148a-3p	ENTPD5	ectonucleoside triphosphate diphosphohydrolase 5 (inactive) [Source:HGNC Symbol;Acc:HGNC:3367]	4.51	0.0889
ssc-miR-148a-3p	EOGT	EGF domain specific O-linked N-acetylglucosamine transferase [Source:VGNC Symbol;Acc:VGNC:87718]	4.51	0.0889
ssc-miR-148a-3p	EPAS1	endothelial PAS domain protein 1 [Source:VGNC Symbol;Acc:VGNC:87721]	4.51	0.0889
ssc-miR-148a-3p	EPB41L1	hypothetical gene	4.51	0.0889
ssc-miR-148a-3p	EPHA8	EPH receptor A8 [Source:VGNC Symbol;Acc:VGNC:87735]	4.51	0.0889
ssc-miR-148a-3p	EPN2	epsin 2 [Source:VGNC Symbol;Acc:VGNC:97982]	4.51	0.0889
ssc-miR-148a-3p	EPS15	epidermal growth factor receptor pathway substrate 15 [Source:VGNC Symbol;Acc:VGNC:87747]	4.51	0.0889
ssc-miR-148a-3p	ERBB3	erb-b2 receptor tyrosine kinase 3 [Source:VGNC Symbol;Acc:VGNC:87760]	4.51	0.0889
ssc-miR-148a-3p	ERLIN1	hypothetical gene	4.51	0.0889
ssc-miR-148a-3p	ERRFI1	ERBB receptor feedback inhibitor 1 [Source:VGNC Symbol;Acc:VGNC:87784]	4.51	0.0889
ssc-miR-148a-3p	ESCO1	establishment of sister chromatid cohesion N-acetyltransferase 1 [Source:VGNC Symbol;Acc:VGNC:87786]	4.51	0.0889
ssc-miR-148a-3p	ESR1	estrogen receptor 1 [Source:VGNC Symbol;Acc:VGNC:103089]	4.51	0.0889
ssc-miR-148a-3p	ESRRG	estrogen related receptor gamma [Source:VGNC Symbol;Acc:VGNC:96289]	4.51	0.0889
ssc-miR-148a-3p	ETNK1	ethanolamine kinase 1 [Source:VGNC Symbol;Acc:VGNC:87806]	4.51	0.0889
ssc-miR-148a-3p	ETV1	ETS variant transcription factor 1 [Source:VGNC Symbol;Acc:VGNC:87810]	4.51	0.0889
ssc-miR-148a-3p	ETV3	ETS variant transcription factor 3 [Source:VGNC Symbol;Acc:VGNC:87812]	4.51	0.0889
ssc-miR-148a-3p	ETV5	ETS variant transcription factor 5 [Source:VGNC Symbol;Acc:VGNC:87814]	4.51	0.0889
ssc-miR-148a-3p	EXTL3	exostosin like glycosyltransferase 3 [Source:VGNC Symbol;Acc:VGNC:87850]	4.51	0.0889
ssc-miR-148a-3p	EYA3	EYA transcriptional coactivator and phosphatase 3 [Source:VGNC Symbol;Acc:VGNC:87852]	4.51	0.0889
ssc-miR-148a-3p	F3	coagulation factor III, tissue factor [Source:VGNC Symbol;Acc:VGNC:87864]	4.51	0.0889
ssc-miR-148a-3p	FAF1	Fas associated factor 1 [Source:VGNC Symbol;Acc:VGNC:87879]	4.51	0.0889
ssc-miR-148a-3p	FAM104A	family with sequence similarity 104 member A [Source:VGNC Symbol;Acc:VGNC:98990]	4.51	0.0889
ssc-miR-148a-3p	FAM104B	hypothetical gene	4.51	0.0889
ssc-miR-148a-3p	FAM122A	hypothetical gene	4.51	0.0889
ssc-miR-148a-3p	FAM136A	hypothetical gene	4.51	0.0889
ssc-miR-148a-3p	FAM161A	FAM161 centrosomal protein A [Source:VGNC Symbol;Acc:VGNC:87916]	4.51	0.0889
ssc-miR-148a-3p	FAM168B	family with sequence similarity 168 member B [Source:VGNC Symbol;Acc:VGNC:95462]	4.51	0.0889
ssc-miR-148a-3p	FAM178A	hypothetical gene	4.51	0.0889
ssc-miR-148a-3p	FAM184A	family with sequence similarity 184 member A [Source:VGNC Symbol;Acc:VGNC:87937]	4.51	0.0889
ssc-miR-148a-3p	FAM21A	hypothetical gene	4.51	0.0889
ssc-miR-148a-3p	FAM21B	hypothetical gene	4.51	0.0889
ssc-miR-148a-3p	FAM21C	hypothetical gene	4.51	0.0889
ssc-miR-148a-3p	FAM21D	hypothetical gene	4.51	0.0889
ssc-miR-148a-3p	FAM43A	family with sequence similarity 43 member A [Source:VGNC Symbol;Acc:VGNC:87970]	4.51	0.0889
ssc-miR-148a-3p	FAM46A	hypothetical gene	4.51	0.0889
ssc-miR-148a-3p	FAM73B	hypothetical gene	4.51	0.0889
ssc-miR-148a-3p	FAM81A	family with sequence similarity 81 member A [Source:VGNC Symbol;Acc:VGNC:87986]	4.51	0.0889
ssc-miR-148a-3p	FANCA	FA complementation group A [Source:VGNC Symbol;Acc:VGNC:88001]	4.51	0.0889
ssc-miR-148a-3p	FBN1	fibrillin 1 [Source:VGNC Symbol;Acc:VGNC:103090]	4.51	0.0889
ssc-miR-148a-3p	FBN3	fibrillin 3 [Source:VGNC Symbol;Acc:VGNC:88026]	4.51	0.0889
ssc-miR-148a-3p	FBXL19	F-box and leucine rich repeat protein 19 [Source:VGNC Symbol;Acc:VGNC:98004]	4.51	0.0889
ssc-miR-148a-3p	FBXO11	F-box protein 11 [Source:VGNC Symbol;Acc:VGNC:88032]	4.51	0.0889
ssc-miR-148a-3p	FBXO28	F-box protein 28 [Source:HGNC Symbol;Acc:HGNC:29046]	4.51	0.0889
ssc-miR-148a-3p	FBXO33	F-box protein 33 [Source:VGNC Symbol;Acc:VGNC:88040]	4.51	0.0889
ssc-miR-148a-3p	FCHO2	FCH and mu domain containing endocytic adaptor 2 [Source:VGNC Symbol;Acc:VGNC:88066]	4.51	0.0889
ssc-miR-148a-3p	FCHSD1	FCH and double SH3 domains 1 [Source:VGNC Symbol;Acc:VGNC:88067]	4.51	0.0889
ssc-miR-148a-3p	FEM1C	fem-1 homolog C [Source:VGNC Symbol;Acc:VGNC:88084]	4.51	0.0889

ssc-miR-148a-3p	FEZ2	fasciculation and elongation protein zeta 2 [Source:VGNC Symbol;Acc:VGNC:88093]	4.51	0.0889
ssc-miR-148a-3p	FGF2	hypothetical gene	4.51	0.0889
ssc-miR-148a-3p	FIGN	fidgetin, microtubule severing factor [Source:VGNC Symbol;Acc:VGNC:95580]	4.51	0.0889
ssc-miR-148a-3p	FLJ00418	hypothetical gene	4.51	0.0889
ssc-miR-148a-3p	FLOT2	flotillin 2 [Source:VGNC Symbol;Acc:VGNC:88159]	4.51	0.0889
ssc-miR-148a-3p	FMNL3	formin like 3 [Source:VGNC Symbol;Acc:VGNC:88169]	4.51	0.0889
ssc-miR-148a-3p	FMR1	FMRP translational regulator 1 [Source:VGNC Symbol;Acc:VGNC:88175]	4.51	0.0889
ssc-miR-148a-3p	FNBP1L	formin binding protein 1 like [Source:VGNC Symbol;Acc:VGNC:88179]	4.51	0.0889
ssc-miR-148a-3p	FOSB	FosB proto-onco, AP-1 transcription factor subunit [Source:VGNC Symbol;Acc:VGNC:88190]	4.51	0.0889
ssc-miR-148a-3p	FOXF1	forkhead box F1 [Source:VGNC Symbol;Acc:VGNC:88204]	4.51	0.0889
ssc-miR-148a-3p	FOXK2	forkhead box K2 [Source:VGNC Symbol;Acc:VGNC:88215]	4.51	0.0889
ssc-miR-148a-3p	FRYL	FRY like transcription coactivator [Source:VGNC Symbol;Acc:VGNC:98015]	4.51	0.0889
ssc-miR-148a-3p	FST	follistatin [Source:NCBI gene (formerly Entrezgene);Acc:445002]	4.51	0.0889
ssc-miR-148a-3p	FUBP1	far upstream element binding protein 1 [Source:VGNC Symbol;Acc:VGNC:96781]	4.51	0.0889
ssc-miR-148a-3p	FUT9	fucosyltransferase 9 [Source:VGNC Symbol;Acc:VGNC:88271]	4.51	0.0889
ssc-miR-148a-3p	FXR1	FMR1 autosomal homolog 1 [Source:VGNC Symbol;Acc:VGNC:108659]	4.51	0.0889
ssc-miR-148a-3p	GABRA1	gamma-aminobutyric acid type A receptor subunit alpha1 [Source:VGNC Symbol;Acc:VGNC:88300]	4.51	0.0889
ssc-miR-148a-3p	GADD45A	growth arrest and DNA damage inducible alpha [Source:VGNC Symbol;Acc:VGNC:88316]	4.51	0.0889
ssc-miR-148a-3p	GAP43	growth associated protein 43 [Source:VGNC Symbol;Acc:VGNC:98018]	4.51	0.0889
ssc-miR-148a-3p	GAPVD1	GTPase activating protein and VPS9 domains 1 [Source:VGNC Symbol;Acc:VGNC:88347]	4.51	0.0889
ssc-miR-148a-3p	GAS1	growth arrest specific 1 [Source:HGNC Symbol;Acc:HGNC:4165]	4.51	0.0889
ssc-miR-148a-3p	GATAD2B	GATA zinc finger domain containing 2B [Source:VGNC Symbol;Acc:VGNC:88369]	4.51	0.0889
ssc-miR-148a-3p	GATC	glutamyl-tRNA amidotransferase subunit C [Source:VGNC Symbol;Acc:VGNC:103948]	4.51	0.0889
ssc-miR-148a-3p	GBP2	guanylate binding protein 2, interferon-inducible [Source:NCBI gene (formerly Entrezgene);Acc:100153137]	4.51	0.0889
ssc-miR-148a-3p	GDF6	growth differentiation factor 6 [Source:VGNC Symbol;Acc:VGNC:88402]	4.51	0.0889
ssc-miR-148a-3p	GDI2	GDP dissociation inhibitor 2 [Source:VGNC Symbol;Acc:VGNC:98019]	4.51	0.0889
ssc-miR-148a-3p	GGA2	golgi associated, gamma adaptin ear containing, ARF binding protein 2 [Source:VGNC Symbol;Acc:VGNC:88432]	4.51	0.0889
ssc-miR-148a-3p	GJD2	gap junction protein delta 2 [Source:VGNC Symbol;Acc:VGNC:88471]	4.51	0.0889
ssc-miR-148a-3p	GLRX5	glutaredoxin 5 [Source:VGNC Symbol;Acc:VGNC:88496]	4.51	0.0889
ssc-miR-148a-3p	GMFB	glia maturation factor beta [Source:VGNC Symbol;Acc:VGNC:88509]	4.51	0.0889
ssc-miR-148a-3p	GPATCH8	G-patch domain containing 8 [Source:HGNC Symbol;Acc:HGNC:29066]	4.51	0.0889
ssc-miR-148a-3p	GPCPD1	glycerophosphocholine phosphodiesterase 1 [Source:VGNC Symbol;Acc:VGNC:96148]	4.51	0.0889
ssc-miR-148a-3p	GPD2	glycerol-3-phosphate dehydrogenase 2 [Source:VGNC Symbol;Acc:VGNC:96330]	4.51	0.0889
ssc-miR-148a-3p	GPI	glucose-6-phosphate isomerase [Source:VGNC Symbol;Acc:VGNC:97068]	4.51	0.0889
ssc-miR-148a-3p	GPM6A	glycoprotein M6A [Source:VGNC Symbol;Acc:VGNC:98025]	4.51	0.0889
ssc-miR-148a-3p	GPR116	hypothetical gene	4.51	0.0889
ssc-miR-148a-3p	GPR137C	G protein-coupled receptor 137C [Source:VGNC Symbol;Acc:VGNC:88602]	4.51	0.0889
ssc-miR-148a-3p	GPR180	G protein-coupled receptor 180 [Source:VGNC Symbol;Acc:VGNC:88621]	4.51	0.0889
ssc-miR-148a-3p	GRAMD1B	GRAM domain containing 1B [Source:VGNC Symbol;Acc:VGNC:88656]	4.51	0.0889
ssc-miR-148a-3p	GRID2	glutamate ionotropic receptor delta type subunit 2 [Source:VGNC Symbol;Acc:VGNC:98932]	4.51	0.0889
ssc-miR-148a-3p	GRIN2A	glutamate ionotropic receptor NMDA type subunit 2A [Source:VGNC Symbol;Acc:VGNC:88683]	4.51	0.0889
ssc-miR-148a-3p	GRIN2B	glutamate ionotropic receptor NMDA type subunit 2B [Source:VGNC Symbol;Acc:VGNC:88684]	4.51	0.0889
ssc-miR-148a-3p	GSR	glutathione-disulfide reductase [Source:VGNC Symbol;Acc:VGNC:96337]	4.51	0.0889
ssc-miR-148a-3p	GTF2H1	ral transcription factor IIH subunit 1 [Source:VGNC Symbol;Acc:VGNC:88734]	4.51	0.0889
ssc-miR-148a-3p	H2AFY	hypothetical gene	4.51	0.0889
ssc-miR-148a-3p	HAUS5	HAUS augmin like complex subunit 5 [Source:VGNC Symbol;Acc:VGNC:88788]	4.51	0.0889
ssc-miR-148a-3p	HDGF	heparin binding growth factor [Source:VGNC Symbol;Acc:VGNC:88819]	4.51	0.0889
ssc-miR-148a-3p	HECW2	HECT, C2 and WW domain containing E3 ubiquitin protein ligase 2 [Source:NCBI gene (formerly Entrezgene);Acc:100155879]	4.51	0.0889
ssc-miR-148a-3p	HIATL1	hypothetical gene	4.51	0.0889

ssc-miR-148a-3p	HIF1AN	hypoxia inducible factor 1 subunit alpha inhibitor [Source:VGNC Symbol;Acc:VGNC:98033]	4.51	0.0889
ssc-miR-148a-3p	HIPK2	homeodomain interacting protein kinase 2 [Source:VGNC Symbol;Acc:VGNC:88888]	4.51	0.0889
ssc-miR-148a-3p	HIPK3	homeodomain interacting protein kinase 3 [Source:VGNC Symbol;Acc:VGNC:88889]	4.51	0.0889
ssc-miR-148a-3p	HIVEP1	HIVEP zinc finger 1 [Source:VGNC Symbol;Acc:VGNC:96585]	4.51	0.0889
ssc-miR-148a-3p	HIVEP3	hypothetical gene	4.51	0.0889
ssc-miR-148a-3p	HLA-DQB1	hypothetical gene	4.51	0.0889
ssc-miR-148a-3p	HLA-DQB2	hypothetical gene	4.51	0.0889
ssc-miR-148a-3p	HLF	HLF transcription factor, PAR bZIP family member [Source:VGNC Symbol;Acc:VGNC:88896]	4.51	0.0889
ssc-miR-148a-3p	HMG20A	high mobility group 20A [Source:VGNC Symbol;Acc:VGNC:88900]	4.51	0.0889
ssc-miR-148a-3p	HMGA2	hypothetical gene	4.51	0.0889
ssc-miR-148a-3p	HMGB3	hypothetical gene	4.51	0.0889
ssc-miR-148a-3p	HNF4G	hepatocyte nuclear factor 4 gamma [Source:VGNC Symbol;Acc:VGNC:88917]	4.51	0.0889
ssc-miR-148a-3p	HNRNPR	heterous nuclear ribonucleoprotein R [Source:VGNC Symbol;Acc:VGNC:88925]	4.51	0.0889
ssc-miR-148a-3p	HOMER1	homer scaffold protein 1 [Source:VGNC Symbol;Acc:VGNC:88928]	4.51	0.0889
ssc-miR-148a-3p	HOXC8	homeobox C8 [Source:VGNC Symbol;Acc:VGNC:88954]	4.51	0.0889
ssc-miR-148a-3p	HS2ST1	heparan sulfate 2-O-sulfotransferase 1 [Source:HGNC Symbol;Acc:HGNC:5193]	4.51	0.0889
ssc-miR-148a-3p	HSP90B1	heat shock protein 90 beta family member 1 [Source:VGNC Symbol;Acc:VGNC:103290]	4.51	0.0889
ssc-miR-148a-3p	HSPA4L	heat shock protein family A (Hsp70) member 4 like [Source:HGNC Symbol;Acc:HGNC:17041]	4.51	0.0889
ssc-miR-148a-3p	IGF1	insulin like growth factor 1 [Source:VGNC Symbol;Acc:VGNC:98044]	4.51	0.0889
ssc-miR-148a-3p	IGF2BP3	insulin like growth factor 2 mRNA binding protein 3 [Source:VGNC Symbol;Acc:VGNC:89056]	4.51	0.0889
ssc-miR-148a-3p	IL15	interleukin 15 [Source:VGNC Symbol;Acc:VGNC:89083]	4.51	0.0889
ssc-miR-148a-3p	IL18BP	hypothetical gene	4.51	0.0889
ssc-miR-148a-3p	IL6ST	interleukin 6 cytokine family signal transducer [Source:VGNC Symbol;Acc:VGNC:89113]	4.51	0.0889
ssc-miR-148a-3p	ING2	inhibitor of growth family member 2 [Source:VGNC Symbol;Acc:VGNC:99717]	4.51	0.0889
ssc-miR-148a-3p	INHBB	inhibin subunit beta B [Source:VGNC Symbol;Acc:VGNC:103969]	4.51	0.0889
ssc-miR-148a-3p	INO80	INO80 complex ATPase subunit [Source:VGNC Symbol;Acc:VGNC:89138]	4.51	0.0889
ssc-miR-148a-3p	INSIG2	insulin induced 2 [Source:VGNC Symbol;Acc:VGNC:103970]	4.51	0.0889
ssc-miR-148a-3p	IPO5	importin 5 [Source:VGNC Symbol;Acc:VGNC:89179]	4.51	0.0889
ssc-miR-148a-3p	ISM1	isthmin 1 [Source:VGNC Symbol;Acc:VGNC:95787]	4.51	0.0889
ssc-miR-148a-3p	ITFG3	hypothetical gene	4.51	0.0889
ssc-miR-148a-3p	ITGA1	integrin subunit alpha 1 [Source:VGNC Symbol;Acc:VGNC:89231]	4.51	0.0889
ssc-miR-148a-3p	ITGA11	integrin subunit alpha 11 [Source:VGNC Symbol;Acc:VGNC:89233]	4.51	0.0889
ssc-miR-148a-3p	ITGA5	integrin subunit alpha 5 [Source:VGNC Symbol;Acc:VGNC:89236]	4.51	0.0889
ssc-miR-148a-3p	ITGA9	integrin subunit alpha 9 [Source:VGNC Symbol;Acc:VGNC:89238]	4.51	0.0889
ssc-miR-148a-3p	ITGAV	integrin subunit alpha V [Source:VGNC Symbol;Acc:VGNC:96380]	4.51	0.0889
ssc-miR-148a-3p	ITGB8	integrin subunit beta 8 [Source:VGNC Symbol;Acc:VGNC:89246]	4.51	0.0889
ssc-miR-148a-3p	ITPK1	inositol-tetrakisphosphate 1-kinase [Source:VGNC Symbol;Acc:VGNC:89251]	4.51	0.0889
ssc-miR-148a-3p	ITSN2	intersectin 2 [Source:VGNC Symbol;Acc:VGNC:89259]	4.51	0.0889
ssc-miR-148a-3p	JARID2	jumonji and AT-rich interaction domain containing 2 [Source:VGNC Symbol;Acc:VGNC:89279]	4.51	0.0889
ssc-miR-148a-3p	JHDM1D	hypothetical gene	4.51	0.0889
ssc-miR-148a-3p	JMJD1C	jumonji domain containing 1C [Source:VGNC Symbol;Acc:VGNC:89285]	4.51	0.0889
ssc-miR-148a-3p	JMY	junction mediating and regulatory protein, p53 cofactor [Source:VGNC Symbol;Acc:VGNC:89289]	4.51	0.0889
ssc-miR-148a-3p	JPH3	hypothetical gene	4.51	0.0889
ssc-miR-148a-3p	KANSL1	KAT8 regulatory NSL complex subunit 1 [Source:VGNC Symbol;Acc:VGNC:89299]	4.51	0.0889
ssc-miR-148a-3p	KAT7	lysine acetyltransferase 7 [Source:VGNC Symbol;Acc:VGNC:89307]	4.51	0.0889
ssc-miR-148a-3p	KATNA1	katanin catalytic subunit A1 [Source:VGNC Symbol;Acc:VGNC:89309]	4.51	0.0889
ssc-miR-148a-3p	KCND3	potassium voltage-gated channel subfamily D member 3 [Source:VGNC Symbol;Acc:VGNC:98814]	4.51	0.0889
ssc-miR-148a-3p	KCNH1	potassium voltage-gated channel subfamily H member 1 [Source:VGNC Symbol;Acc:VGNC:108599]	4.51	0.0889
ssc-miR-148a-3p	KCNIP3	potassium voltage-gated channel interacting protein 3 [Source:VGNC Symbol;Acc:VGNC:89350]	4.51	0.0889

ssc-miR-148a-3p	KCNJ6	potassium inwardly rectifying channel subfamily J member 6 [Source:VGNC Symbol;Acc:VGNC:89360]	4.51	0.0889
ssc-miR-148a-3p	KCNN3	potassium calcium-activated channel subfamily N member 3 [Source:VGNC Symbol;Acc:VGNC:98056]	4.51	0.0889
ssc-miR-148a-3p	KCNQ4	potassium voltage-gated channel subfamily Q member 4 [Source:VGNC Symbol;Acc:VGNC:89383]	4.51	0.0889
ssc-miR-148a-3p	KDM2B	lysine demethylase 2B [Source:VGNC Symbol;Acc:VGNC:98057]	4.51	0.0889
ssc-miR-148a-3p	KDM5A	lysine demethylase 5A [Source:VGNC Symbol;Acc:VGNC:89415]	4.51	0.0889
ssc-miR-148a-3p	KDM6B	lysine demethylase 6B [Source:VGNC Symbol;Acc:VGNC:89416]	4.51	0.0889
ssc-miR-148a-3p	KIAA0226	hypothetical gene	4.51	0.0889
ssc-miR-148a-3p	KIAA0232	hypothetical gene	4.51	0.0889
ssc-miR-148a-3p	KIAA1045	hypothetical gene	4.51	0.0889
ssc-miR-148a-3p	KIAA1217	KIAA1217 [Source:VGNC Symbol;Acc:VGNC:96079]	4.51	0.0889
ssc-miR-148a-3p	KIAA1324L	hypothetical gene	4.51	0.0889
ssc-miR-148a-3p	KIAA1468	hypothetical gene	4.51	0.0889
ssc-miR-148a-3p	KIAA1549	KIAA1549 [Source:VGNC Symbol;Acc:VGNC:99719]	4.51	0.0889
ssc-miR-148a-3p	KIT	KIT proto-onco, receptor tyrosine kinase [Source:VGNC Symbol;Acc:VGNC:98060]	4.51	0.0889
ssc-miR-148a-3p	KLC2	kinesin light chain 2 [Source:VGNC Symbol;Acc:VGNC:89486]	4.51	0.0889
ssc-miR-148a-3p	KLF4	Kruppel like factor 4 [Source:VGNC Symbol;Acc:VGNC:98062]	4.51	0.0889
ssc-miR-148a-3p	KLF5	Kruppel like factor 5 [Source:VGNC Symbol;Acc:VGNC:89499]	4.51	0.0889
ssc-miR-148a-3p	KLF6	Kruppel like factor 6 [Source:VGNC Symbol;Acc:VGNC:98063]	4.51	0.0889
ssc-miR-148a-3p	KLHL18	kelch like family member 18 [Source:VGNC Symbol;Acc:VGNC:89517]	4.51	0.0889
ssc-miR-148a-3p	KLHL5	kelch like family member 5 [Source:VGNC Symbol;Acc:VGNC:89535]	4.51	0.0889
ssc-miR-148a-3p	KMT2A	lysine methyltransferase 2A [Source:VGNC Symbol;Acc:VGNC:108600]	4.51	0.0889
ssc-miR-148a-3p	KPNA1	karyopherin subunit alpha 1 [Source:VGNC Symbol;Acc:VGNC:89560]	4.51	0.0889
ssc-miR-148a-3p	KPNA4	karyopherin subunit alpha 4 [Source:VGNC Symbol;Acc:VGNC:89563]	4.51	0.0889
ssc-miR-148a-3p	KPNA6	karyopherin subunit alpha 6 [Source:VGNC Symbol;Acc:VGNC:89565]	4.51	0.0889
ssc-miR-148a-3p	LAMA4	laminin subunit alpha 4 [Source:VGNC Symbol;Acc:VGNC:103116]	4.51	0.0889
ssc-miR-148a-3p	LBR	lamin B receptor [Source:VGNC Symbol;Acc:VGNC:98068]	4.51	0.0889
ssc-miR-148a-3p	LCOR	ligand dependent nuclear receptor corepressor [Source:HGNC Symbol;Acc:HGNC:29503]	4.51	0.0889
ssc-miR-148a-3p	LDLR	low density lipoprotein receptor [Source:VGNC Symbol;Acc:VGNC:89671]	4.51	0.0889
ssc-miR-148a-3p	LEPROTL1	leptin receptor overlapping transcript like 1 [Source:VGNC Symbol;Acc:VGNC:96404]	4.51	0.0889
ssc-miR-148a-3p	LGALS8	galectin 8 [Source:VGNC Symbol;Acc:VGNC:89697]	4.51	0.0889
ssc-miR-148a-3p	LGALSL	galectin like [Source:VGNC Symbol;Acc:VGNC:89698]	4.51	0.0889
ssc-miR-148a-3p	LIMD2	LIM domain containing 2 [Source:VGNC Symbol;Acc:VGNC:89726]	4.51	0.0889
ssc-miR-148a-3p	LIN28A	lin-28 homolog A [Source:VGNC Symbol;Acc:VGNC:98492]	4.51	0.0889
ssc-miR-148a-3p	LIN28B	lin-28 homolog B [Source:VGNC Symbol;Acc:VGNC:89729]	4.51	0.0889
ssc-miR-148a-3p	LIPA	lipase A, lysosomal acid type [Source:VGNC Symbol;Acc:VGNC:89737]	4.51	0.0889
ssc-miR-148a-3p	LMAN1	lectin, mannose binding 1 [Source:VGNC Symbol;Acc:VGNC:89748]	4.51	0.0889
ssc-miR-148a-3p	LMBR1L	limb development membrane protein 1 like [Source:VGNC Symbol;Acc:VGNC:89753]	4.51	0.0889
ssc-miR-148a-3p	LMTK2	lemur tyrosine kinase 2 [Source:HGNC Symbol;Acc:HGNC:17880]	4.51	0.0889
ssc-miR-148a-3p	LNPEP	leucyl and cystinyl aminopeptidase [Source:VGNC Symbol;Acc:VGNC:89772]	4.51	0.0889
ssc-miR-148a-3p	LONRF3	LON peptidase N-terminal domain and ring finger 3 [Source:VGNC Symbol;Acc:VGNC:89778]	4.51	0.0889
ssc-miR-148a-3p	LPPR5	hypothetical gene	4.51	0.0889
ssc-miR-148a-3p	LRCH1	leucine rich repeats and calponin homology domain containing 1 [Source:VGNC Symbol;Acc:VGNC:89801]	4.51	0.0889
ssc-miR-148a-3p	LRFN1	leucine rich repeat and fibronectin type III domain containing 1 [Source:HGNC Symbol;Acc:HGNC:29290]	4.51	0.0889
ssc-miR-148a-3p	LRP2	LDL receptor related protein 2 [Source:HGNC Symbol;Acc:HGNC:6694]	4.51	0.0889
ssc-miR-148a-3p	LRP4	LDL receptor related protein 4 [Source:VGNC Symbol;Acc:VGNC:89820]	4.51	0.0889
ssc-miR-148a-3p	LRP8	LDL receptor related protein 8 [Source:VGNC Symbol;Acc:VGNC:89822]	4.51	0.0889
ssc-miR-148a-3p	LRRC41	leucine rich repeat containing 41 [Source:VGNC Symbol;Acc:VGNC:89840]	4.51	0.0889
ssc-miR-148a-3p	LTBP1	latent transforming growth factor beta binding protein 1 [Source:VGNC Symbol;Acc:VGNC:89885]	4.51	0.0889
ssc-miR-148a-3p	LYPD3	LY6/PLAUR domain containing 3 [Source:VGNC Symbol;Acc:VGNC:89911]	4.51	0.0889

ssc-miR-148a-3p	LYRM2	LYR motif containing 2 [Source:HGNC Symbol;Acc:HGNC:25229]	4.51	0.0889
ssc-miR-148a-3p	LYSMD2	LysM domain containing 2 [Source:VGNC Symbol;Acc:VGNC:89918]	4.51	0.0889
ssc-miR-148a-3p	LYSMD3	LysM domain containing 3 [Source:VGNC Symbol;Acc:VGNC:89919]	4.51	0.0889
ssc-miR-148a-3p	MAF	MAF bZIP transcription factor [Source:VGNC Symbol;Acc:VGNC:89945]	4.51	0.0889
ssc-miR-148a-3p	MAF1	MAF1 homolog, negative regulator of RNA polymerase III [Source:HGNC Symbol;Acc:HGNC:24966]	4.51	0.0889
ssc-miR-148a-3p	MAFB	MAF bZIP transcription factor B [Source:HGNC Symbol;Acc:HGNC:6408]	4.51	0.0889
ssc-miR-148a-3p	MAFG	MAF bZIP transcription factor G [Source:VGNC Symbol;Acc:VGNC:89948]	4.51	0.0889
ssc-miR-148a-3p	MAP1B	microtubule associated protein 1B [Source:VGNC Symbol;Acc:VGNC:89979]	4.51	0.0889
ssc-miR-148a-3p	MAP2K1	mitogen-activated protein kinase kinase 1 [Source:VGNC Symbol;Acc:VGNC:103121]	4.51	0.0889
ssc-miR-148a-3p	MAP3K2	mitogen-activated protein kinase kinase kinase 2 [Source:VGNC Symbol;Acc:VGNC:98107]	4.51	0.0889
ssc-miR-148a-3p	MAP3K4	mitogen-activated protein kinase kinase kinase 4 [Source:VGNC Symbol;Acc:VGNC:98109]	4.51	0.0889
ssc-miR-148a-3p	MAP3K9	mitogen-activated protein kinase kinase kinase 9 [Source:VGNC Symbol;Acc:VGNC:89989]	4.51	0.0889
ssc-miR-148a-3p	MAP4K2	mitogen-activated protein kinase kinase kinase kinase 2 [Source:VGNC Symbol;Acc:VGNC:98112]	4.51	0.0889
ssc-miR-148a-3p	MARCH2	hypothetical gene	4.51	0.0889
ssc-miR-148a-3p	MARCH3	hypothetical gene	4.51	0.0889
ssc-miR-148a-3p	MAX	MYC associated factor X [Source:VGNC Symbol;Acc:VGNC:90046]	4.51	0.0889
ssc-miR-148a-3p	MBOAT2	membrane bound O-acyltransferase domain containing 2 [Source:VGNC Symbol;Acc:VGNC:90058]	4.51	0.0889
ssc-miR-148a-3p	MCM3AP	minichromosome maintenance complex component 3 associated protein [Source:VGNC Symbol;Acc:VGNC:96414]	4.51	0.0889
ssc-miR-148a-3p	MDFIC	MyoD family inhibitor domain containing [Source:VGNC Symbol;Acc:VGNC:90089]	4.51	0.0889
ssc-miR-148a-3p	MDGA2	MAM domain containing glycosylphosphatidylinositol anchor 2 [Source:VGNC Symbol;Acc:VGNC:90092]	4.51	0.0889
ssc-miR-148a-3p	MDM4	MDM4 regulator of p53 [Source:VGNC Symbol;Acc:VGNC:90094]	4.51	0.0889
ssc-miR-148a-3p	MECP2	methyl-CpG binding protein 2 [Source:VGNC Symbol;Acc:VGNC:90101]	4.51	0.0889
ssc-miR-148a-3p	MED1	mediator complex subunit 1 [Source:VGNC Symbol;Acc:VGNC:90102]	4.51	0.0889
ssc-miR-148a-3p	MED12L	mediator complex subunit 12L [Source:VGNC Symbol;Acc:VGNC:90105]	4.51	0.0889
ssc-miR-148a-3p	MEOX2	mesenchyme homeobox 2 [Source:VGNC Symbol;Acc:VGNC:90142]	4.51	0.0889
ssc-miR-148a-3p	MET	MET proto-onco, receptor tyrosine kinase [Source:NCBI gene (formerly Entrezgene);Acc:654328]	4.51	0.0889
ssc-miR-148a-3p	MGAT4A	alpha-1,3-mannosyl-glycoprotein 4-beta-N-acetylglucosaminyltransferase A [Source:VGNC Symbol;Acc:VGNC:108158]	4.51	0.0889
ssc-miR-148a-3p	MGAT5	alpha-1,6-mannosylglycoprotein 6-beta-N-acetylglucosaminyltransferase [Source:VGNC Symbol;Acc:VGNC:96741]	4.51	0.0889
ssc-miR-148a-3p	MGLL	monoglyceride lipase [Source:VGNC Symbol;Acc:VGNC:90199]	4.51	0.0889
ssc-miR-148a-3p	MIER1	MIER1 transcriptional regulator [Source:VGNC Symbol;Acc:VGNC:90219]	4.51	0.0889
ssc-miR-148a-3p	MITF	melanocyte inducing transcription factor [Source:VGNC Symbol;Acc:VGNC:90243]	4.51	0.0889
ssc-miR-148a-3p	MLEC	malectin [Source:HGNC Symbol;Acc:HGNC:28973]	4.51	0.0889
ssc-miR-148a-3p	MLLT10	MLLT10 histone lysine methyltransferase DOT1L cofactor [Source:VGNC Symbol;Acc:VGNC:96590]	4.51	0.0889
ssc-miR-148a-3p	MLLT6	MLLT6, PHD finger containing [Source:VGNC Symbol;Acc:VGNC:90257]	4.51	0.0889
ssc-miR-148a-3p	MMD	monocyte to macrophage differentiation associated [Source:VGNC Symbol;Acc:VGNC:90264]	4.51	0.0889
ssc-miR-148a-3p	MMP15	matrix metallopeptidase 15 [Source:VGNC Symbol;Acc:VGNC:90270]	4.51	0.0889
ssc-miR-148a-3p	MMP19	matrix metallopeptidase 19 [Source:VGNC Symbol;Acc:VGNC:90273]	4.51	0.0889
ssc-miR-148a-3p	MMS19	MMS19 homolog, cytosolic iron-sulfur assembly component [Source:VGNC Symbol;Acc:VGNC:90283]	4.51	0.0889
ssc-miR-148a-3p	MNT	MAX network transcriptional repressor [Source:VGNC Symbol;Acc:VGNC:90289]	4.51	0.0889
ssc-miR-148a-3p	MOB1B	hypothetical gene	4.51	0.0889
ssc-miR-148a-3p	MOCOS	molybdenum cofactor sulfurase [Source:VGNC Symbol;Acc:VGNC:90294]	4.51	0.0889
ssc-miR-148a-3p	MOGS	mannosyl-oligosaccharide glucosidase [Source:VGNC Symbol;Acc:VGNC:90297]	4.51	0.0889
ssc-miR-148a-3p	MOSPD1	motile sperm domain containing 1 [Source:VGNC Symbol;Acc:VGNC:103990]	4.51	0.0889
ssc-miR-148a-3p	MPP2	MAGUK p55 scaffold protein 2 [Source:VGNC Symbol;Acc:VGNC:98133]	4.51	0.0889
ssc-miR-148a-3p	MPPED2	metallophosphoesterase domain containing 2 [Source:VGNC Symbol;Acc:VGNC:90331]	4.51	0.0889
ssc-miR-148a-3p	MRAS	muscle RAS onco homolog [Source:VGNC Symbol;Acc:VGNC:90340]	4.51	0.0889
ssc-miR-148a-3p	MRPS10	mitochondrial ribosomal protein S10 [Source:VGNC Symbol;Acc:VGNC:90381]	4.51	0.0889
ssc-miR-148a-3p	MRPS25	mitochondrial ribosomal protein S25 [Source:VGNC Symbol;Acc:VGNC:90391]	4.51	0.0889
ssc-miR-148a-3p	MSI2	musashi RNA binding protein 2 [Source:VGNC Symbol;Acc:VGNC:90422]	4.51	0.0889

ssc-miR-148a-3p	MTA2	metastasis associated 1 family member 2 [Source:VGNC Symbol;Acc:VGNC:90435]	4.51	0.0889
ssc-miR-148a-3p	MTF1	metal regulatory transcription factor 1 [Source:VGNC Symbol;Acc:VGNC:90443]	4.51	0.0889
ssc-miR-148a-3p	MTMR1	myotubularin related protein 1 [Source:VGNC Symbol;Acc:VGNC:90456]	4.51	0.0889
ssc-miR-148a-3p	MTMR10	myotubularin related protein 10 [Source:VGNC Symbol;Acc:VGNC:90457]	4.51	0.0889
ssc-miR-148a-3p	MTMR12	myotubularin related protein 12 [Source:VGNC Symbol;Acc:VGNC:90459]	4.51	0.0889
ssc-miR-148a-3p	MTMR14	myotubularin related protein 14 [Source:HGNC Symbol;Acc:HGNC:26190]	4.51	0.0889
ssc-miR-148a-3p	MTMR9	myotubularin related protein 9 [Source:VGNC Symbol;Acc:VGNC:90464]	4.51	0.0889
ssc-miR-148a-3p	MTSS1L	hypothetical gene	4.51	0.0889
ssc-miR-148a-3p	MUM1L1	hypothetical gene	4.51	0.0889
ssc-miR-148a-3p	MXD1	MAX dimerization protein 1 [Source:VGNC Symbol;Acc:VGNC:90492]	4.51	0.0889
ssc-miR-148a-3p	MYBL1	MYB proto-onco like 1 [Source:VGNC Symbol;Acc:VGNC:90498]	4.51	0.0889
ssc-miR-148a-3p	MYO9A	myosin IXA [Source:VGNC Symbol;Acc:VGNC:103138]	4.51	0.0889
ssc-miR-148a-3p	NAA15	N-alpha-acetyltransferase 15, NatA auxiliary subunit [Source:VGNC Symbol;Acc:VGNC:96747]	4.51	0.0889
ssc-miR-148a-3p	NACC2	NACC family member 2 [Source:VGNC Symbol;Acc:VGNC:90564]	4.51	0.0889
ssc-miR-148a-3p	NAP1L1	nucleosome assembly protein 1 like 1 [Source:VGNC Symbol;Acc:VGNC:90576]	4.51	0.0889
ssc-miR-148a-3p	NAT14	N-acetyltransferase 14 (putative) [Source:HGNC Symbol;Acc:HGNC:28918]	4.51	0.0889
ssc-miR-148a-3p	NAT8L	N-acetyltransferase 8 like [Source:VGNC Symbol;Acc:VGNC:90586]	4.51	0.0889
ssc-miR-148a-3p	NCAM1	neural cell adhesion molecule 1 [Source:VGNC Symbol;Acc:VGNC:108603]	4.51	0.0889
ssc-miR-148a-3p	NCEH1	neutral cholesterol ester hydrolase 1 [Source:VGNC Symbol;Acc:VGNC:98147]	4.51	0.0889
ssc-miR-148a-3p	NCKAP1	NCK associated protein 1 [Source:VGNC Symbol;Acc:VGNC:96432]	4.51	0.0889
ssc-miR-148a-3p	NCKIPSD	NCK interacting protein with SH3 domain [Source:VGNC Symbol;Acc:VGNC:90612]	4.51	0.0889
ssc-miR-148a-3p	NCOA1	nuclear receptor coactivator 1 [Source:VGNC Symbol;Acc:VGNC:90615]	4.51	0.0889
ssc-miR-148a-3p	NCOR1	nuclear receptor corepressor 1 [Source:VGNC Symbol;Acc:VGNC:99723]	4.51	0.0889
ssc-miR-148a-3p	NCS1	neuronal calcium sensor 1 [Source:VGNC Symbol;Acc:VGNC:90620]	4.51	0.0889
ssc-miR-148a-3p	NDP	norrin cystine knot growth factor NDP [Source:VGNC Symbol;Acc:VGNC:90631]	4.51	0.0889
ssc-miR-148a-3p	NEURL1B	neuralized E3 ubiquitin protein ligase 1B [Source:VGNC Symbol;Acc:VGNC:90695]	4.51	0.0889
ssc-miR-148a-3p	NEURL4	neuralized E3 ubiquitin protein ligase 4 [Source:VGNC Symbol;Acc:VGNC:99022]	4.51	0.0889
ssc-miR-148a-3p	NEUROD1	neuronal differentiation 1 [Source:VGNC Symbol;Acc:VGNC:96439]	4.51	0.0889
ssc-miR-148a-3p	NFAT5	nuclear factor of activated T cells 5 [Source:VGNC Symbol;Acc:VGNC:90708]	4.51	0.0889
ssc-miR-148a-3p	NFE2L1	NFE2 like bZIP transcription factor 1 [Source:VGNC Symbol;Acc:VGNC:90713]	4.51	0.0889
ssc-miR-148a-3p	NFIX	nuclear factor I X [Source:VGNC Symbol;Acc:VGNC:90718]	4.51	0.0889
ssc-miR-148a-3p	NFYA	nuclear transcription factor Y subunit alpha [Source:VGNC Symbol;Acc:VGNC:90729]	4.51	0.0889
ssc-miR-148a-3p	NGLY1	N-glycanase 1 [Source:VGNC Symbol;Acc:VGNC:90734]	4.51	0.0889
ssc-miR-148a-3p	NHS	NHS actin remodeling regulator [Source:VGNC Symbol;Acc:VGNC:90738]	4.51	0.0889
ssc-miR-148a-3p	NLK	nemo like kinase [Source:VGNC Symbol;Acc:VGNC:90779]	4.51	0.0889
ssc-miR-148a-3p	NOG	noggin [Source:VGNC Symbol;Acc:VGNC:90812]	4.51	0.0889
ssc-miR-148a-3p	NOL4	nucleolar protein 4 [Source:VGNC Symbol;Acc:VGNC:90814]	4.51	0.0889
ssc-miR-148a-3p	NOVA1	NOVA alternative splicing regulator 1 [Source:VGNC Symbol;Acc:VGNC:90827]	4.51	0.0889
ssc-miR-148a-3p	NOVA2	NOVA alternative splicing regulator 2 [Source:VGNC Symbol;Acc:VGNC:90828]	4.51	0.0889
ssc-miR-148a-3p	NPAT	nuclear protein, coactivator of histone transcription [Source:VGNC Symbol;Acc:VGNC:90840]	4.51	0.0889
ssc-miR-148a-3p	NPEPL1	aminopeptidase like 1 [Source:VGNC Symbol;Acc:VGNC:95798]	4.51	0.0889
ssc-miR-148a-3p	NPTN	neuroplastin [Source:VGNC Symbol;Acc:VGNC:90861]	4.51	0.0889
ssc-miR-148a-3p	NPTX1	neuronal pentraxin 1 [Source:VGNC Symbol;Acc:VGNC:90862]	4.51	0.0889
ssc-miR-148a-3p	NPTX2	neuronal pentraxin 2 [Source:VGNC Symbol;Acc:VGNC:90863]	4.51	0.0889
ssc-miR-148a-3p	NR2C2AP	nuclear receptor 2C2 associated protein [Source:VGNC Symbol;Acc:VGNC:90877]	4.51	0.0889
ssc-miR-148a-3p	NR2E1	nuclear receptor subfamily 2 group E member 1 [Source:VGNC Symbol;Acc:VGNC:90878]	4.51	0.0889
ssc-miR-148a-3p	NRARP	NOTCH regulated ankyrin repeat protein [Source:VGNC Symbol;Acc:VGNC:90889]	4.51	0.0889
ssc-miR-148a-3p	NRAS	NRAS proto-onco, GTPase [Source:VGNC Symbol;Acc:VGNC:98827]	4.51	0.0889
ssc-miR-148a-3p	NRD1	hypothetical gene	4.51	0.0889

ssc-miR-148a-3p	NRP1	neuropilin 1 [Source:VGNC Symbol;Acc:VGNC:104012]	4.51	0.0889
ssc-miR-148a-3p	NSD1	nuclear receptor binding SET domain protein 1 [Source:VGNC Symbol;Acc:VGNC:90904]	4.51	0.0889
ssc-miR-148a-3p	NSG2	neuronal vesicle trafficking associated 2 [Source:HGNC Symbol;Acc:HGNC:24955]	4.51	0.0889
ssc-miR-148a-3p	NUDT3	nudix hydrolase 3 [Source:NCBI gene (formerly Entrezgene);Acc:100737442]	4.51	0.0889
ssc-miR-148a-3p	NUFIP2	nuclear FMR1 interacting protein 2 [Source:VGNC Symbol;Acc:VGNC:90967]	4.51	0.0889
ssc-miR-148a-3p	NUP43	nucleoporin 43 [Source:VGNC Symbol;Acc:VGNC:90982]	4.51	0.0889
ssc-miR-148a-3p	ORMDL1	ORMDL sphingolipid biosynthesis regulator 1 [Source:VGNC Symbol;Acc:VGNC:104015]	4.51	0.0889
ssc-miR-148a-3p	OSBPL11	oxysterol binding protein like 11 [Source:VGNC Symbol;Acc:VGNC:91069]	4.51	0.0889
ssc-miR-148a-3p	OSTF1	osteoclast stimulating factor 1 [Source:VGNC Symbol;Acc:VGNC:91085]	4.51	0.0889
ssc-miR-148a-3p	OTUD4	OTU deubiquitinase 4 [Source:VGNC Symbol;Acc:VGNC:91100]	4.51	0.0889
ssc-miR-148a-3p	OTX2	orthodenticle homeobox 2 [Source:VGNC Symbol;Acc:VGNC:98171]	4.51	0.0889
ssc-miR-148a-3p	OXSR1	hypothetical gene	4.51	0.0889
ssc-miR-148a-3p	PACS2	phosphofurin acidic cluster sorting protein 2 [Source:VGNC Symbol;Acc:VGNC:91141]	4.51	0.0889
ssc-miR-148a-3p	PAG1	phosphoprotein membrane anchor with glycosphingolipid microdomains 1 [Source:VGNC Symbol;Acc:VGNC:91154]	4.51	0.0889
ssc-miR-148a-3p	PAN3	poly(A) specific ribonuclease subunit PAN3 [Source:HGNC Symbol;Acc:HGNC:29991]	4.51	0.0889
ssc-miR-148a-3p	PAPD4	hypothetical gene	4.51	0.0889
ssc-miR-148a-3p	PAPPA	pappalysin 1 [Source:VGNC Symbol;Acc:VGNC:91170]	4.51	0.0889
ssc-miR-148a-3p	PAQR9	progestin and adipoQ receptor family member 9 [Source:HGNC Symbol;Acc:HGNC:30131]	4.51	0.0889
ssc-miR-148a-3p	PATL1	PAT1 homolog 1, processing body mRNA decay factor [Source:VGNC Symbol;Acc:VGNC:91189]	4.51	0.0889
ssc-miR-148a-3p	PBXIP1	PBX homeobox interacting protein 1 [Source:HGNC Symbol;Acc:HGNC:21199]	4.51	0.0889
ssc-miR-148a-3p	PCYT1B	phosphate cytidylyltransferase 1B, choline [Source:VGNC Symbol;Acc:VGNC:91237]	4.51	0.0889
ssc-miR-148a-3p	PCYT2	phosphate cytidylyltransferase 2, ethanolamine [Source:VGNC Symbol;Acc:VGNC:91238]	4.51	0.0889
ssc-miR-148a-3p	PDE1B	phosphodiesterase 1B [Source:VGNC Symbol;Acc:VGNC:91249]	4.51	0.0889
ssc-miR-148a-3p	PDE4B	phosphodiesterase 4B [Source:VGNC Symbol;Acc:VGNC:91255]	4.51	0.0889
ssc-miR-148a-3p	PDE4D	phosphodiesterase 4D [Source:VGNC Symbol;Acc:VGNC:91256]	4.51	0.0889
ssc-miR-148a-3p	PDE5A	phosphodiesterase 5A [Source:VGNC Symbol;Acc:VGNC:91257]	4.51	0.0889
ssc-miR-148a-3p	PDE7A	phosphodiesterase 7A [Source:VGNC Symbol;Acc:VGNC:91261]	4.51	0.0889
ssc-miR-148a-3p	PDIA3	protein disulfide isomerase family A member 3 [Source:VGNC Symbol;Acc:VGNC:91272]	4.51	0.0889
ssc-miR-148a-3p	PDK4	pyruvate dehydrogenase kinase 4 [Source:VGNC Symbol;Acc:VGNC:91280]	4.51	0.0889
ssc-miR-148a-3p	PEA15	proliferation and apoptosis adaptor protein 15 [Source:VGNC Symbol;Acc:VGNC:98181]	4.51	0.0889
ssc-miR-148a-3p	PEAK1	pseudopodium enriched atypical kinase 1 [Source:VGNC Symbol;Acc:VGNC:91300]	4.51	0.0889
ssc-miR-148a-3p	PHACTR2	phosphatase and actin regulator 2 [Source:VGNC Symbol;Acc:VGNC:91366]	4.51	0.0889
ssc-miR-148a-3p	PHAX	phosphorylated adaptor for RNA export [Source:VGNC Symbol;Acc:VGNC:91367]	4.51	0.0889
ssc-miR-148a-3p	PHF20	PHD finger protein 20 [Source:HGNC Symbol;Acc:HGNC:16098]	4.51	0.0889
ssc-miR-148a-3p	PHF3	PHD finger protein 3 [Source:VGNC Symbol;Acc:VGNC:91387]	4.51	0.0889
ssc-miR-148a-3p	PHIP	pleckstrin homology domain interacting protein [Source:VGNC Symbol;Acc:VGNC:91392]	4.51	0.0889
ssc-miR-148a-3p	PI4K2A	phosphatidylinositol 4-kinase type 2 alpha [Source:HGNC Symbol;Acc:HGNC:30031]	4.51	0.0889
ssc-miR-148a-3p	PI4KA	phosphatidylinositol 4-kinase alpha [Source:VGNC Symbol;Acc:VGNC:98191]	4.51	0.0889
ssc-miR-148a-3p	PICALM	phosphatidylinositol binding clathrin assembly protein [Source:VGNC Symbol;Acc:VGNC:91415]	4.51	0.0889
ssc-miR-148a-3p	PIGA	phosphatidylinositol glycan anchor biosynthesis class A [Source:VGNC Symbol;Acc:VGNC:91419]	4.51	0.0889
ssc-miR-148a-3p	PIGG	phosphatidylinositol glycan anchor biosynthesis class G [Source:VGNC Symbol;Acc:VGNC:91422]	4.51	0.0889
ssc-miR-148a-3p	PIK3C2A	phosphatidylinositol-4-phosphate 3-kinase catalytic subunit type 2 alpha [Source:VGNC Symbol;Acc:VGNC:91436]	4.51	0.0889
ssc-miR-148a-3p	PIK3CA	phosphatidylinositol-4,5-bisphosphate 3-kinase catalytic subunit alpha [Source:VGNC Symbol;Acc:VGNC:91440]	4.51	0.0889
ssc-miR-148a-3p	PIK3R3	phosphoinositide-3-kinase regulatory subunit 3 [Source:HGNC Symbol;Acc:HGNC:8981]	4.51	0.0889
ssc-miR-148a-3p	PITPNM2	phosphatidylinositol transfer protein membrane associated 2 [Source:VGNC Symbol;Acc:VGNC:91466]	4.51	0.0889
ssc-miR-148a-3p	PLA2G15	phospholipase A2 group XV [Source:HGNC Symbol;Acc:HGNC:17163]	4.51	0.0889
ssc-miR-148a-3p	PLAA	phospholipase A2 activating protein [Source:VGNC Symbol;Acc:VGNC:91502]	4.51	0.0889
ssc-miR-148a-3p	PLEKHH1	pleckstrin homology, MyTH4 and FERM domain containing H1 [Source:HGNC Symbol;Acc:HGNC:17733]	4.51	0.0889
ssc-miR-148a-3p	PNPLA2	patatin like phospholipase domain containing 2 [Source:VGNC Symbol;Acc:VGNC:91607]	4.51	0.0889

ssc-miR-148a-3p	PNPLA6	patatin like phospholipase domain containing 6 [Source:VGNC Symbol;Acc:VGNC:91608]	4.51	0.0889
ssc-miR-148a-3p	PNRC1	proline rich nuclear receptor coactivator 1 [Source:VGNC Symbol;Acc:VGNC:91613]	4.51	0.0889
ssc-miR-148a-3p	PNRC2	proline rich nuclear receptor coactivator 2 [Source:VGNC Symbol;Acc:VGNC:91614]	4.51	0.0889
ssc-miR-148a-3p	PODXL	hypothetical gene	4.51	0.0889
ssc-miR-148a-3p	POLH	DNA polymerase eta [Source:VGNC Symbol;Acc:VGNC:91635]	4.51	0.0889
ssc-miR-148a-3p	POU3F2	POU class 3 homeobox 2 [Source:HGNC Symbol;Acc:HGNC:9215]	4.51	0.0889
ssc-miR-148a-3p	POU4F2	POU class 4 homeobox 2 [Source:VGNC Symbol;Acc:VGNC:91679]	4.51	0.0889
ssc-miR-148a-3p	PPAP2B	hypothetical gene	4.51	0.0889
ssc-miR-148a-3p	PPARD	peroxisome proliferator activated receptor delta [Source:VGNC Symbol;Acc:VGNC:91683]	4.51	0.0889
ssc-miR-148a-3p	PPARGC1A	PPARG coactivator 1 alpha [Source:VGNC Symbol;Acc:VGNC:91685]	4.51	0.0889
ssc-miR-148a-3p	PPARGC1B	PPARG coactivator 1 beta [Source:VGNC Symbol;Acc:VGNC:91686]	4.51	0.0889
ssc-miR-148a-3p	PPFIA2	PTPRF interacting protein alpha 2 [Source:VGNC Symbol;Acc:VGNC:91692]	4.51	0.0889
ssc-miR-148a-3p	PPP1CB	protein phosphatase 1 catalytic subunit beta [Source:NCBI gene (formerly Entrezgene);Acc:397378]	4.51	0.0889
ssc-miR-148a-3p	PPP1R10	protein phosphatase 1 regulatory subunit 10 [Source:VGNC Symbol;Acc:VGNC:91717]	4.51	0.0889
ssc-miR-148a-3p	PPP1R12A	protein phosphatase 1 regulatory subunit 12A [Source:VGNC Symbol;Acc:VGNC:91719]	4.51	0.0889
ssc-miR-148a-3p	PPP1R3B	protein phosphatase 1 regulatory subunit 3B [Source:VGNC Symbol;Acc:VGNC:95636]	4.51	0.0889
ssc-miR-148a-3p	PPP1R7	protein phosphatase 1 regulatory subunit 7 [Source:NCBI gene (formerly Entrezgene);Acc:100511842]	4.51	0.0889
ssc-miR-148a-3p	PPP1R9A	protein phosphatase 1 regulatory subunit 9A [Source:VGNC Symbol;Acc:VGNC:91743]	4.51	0.0889
ssc-miR-148a-3p	PPP1R9B	protein phosphatase 1 regulatory subunit 9B [Source:VGNC Symbol;Acc:VGNC:91744]	4.51	0.0889
ssc-miR-148a-3p	PPP2R5E	protein phosphatase 2 regulatory subunit B'epsilon [Source:VGNC Symbol;Acc:VGNC:91754]	4.51	0.0889
ssc-miR-148a-3p	PPP6R1	protein phosphatase 6 regulatory subunit 1 [Source:VGNC Symbol;Acc:VGNC:91760]	4.51	0.0889
ssc-miR-148a-3p	PRDM15	PR/SET domain 15 [Source:VGNC Symbol;Acc:VGNC:98220]	4.51	0.0889
ssc-miR-148a-3p	PREX1	phosphatidylinositol-3,4,5-trisphosphate dependent Rac exchange factor 1 [Source:VGNC Symbol;Acc:VGNC:96264]	4.51	0.0889
ssc-miR-148a-3p	PRICKLE2	prickle planar cell polarity protein 2 [Source:VGNC Symbol;Acc:VGNC:91794]	4.51	0.0889
ssc-miR-148a-3p	PRKAA1	protein kinase AMP-activated catalytic subunit alpha 1 [Source:VGNC Symbol;Acc:VGNC:91797]	4.51	0.0889
ssc-miR-148a-3p	PRKAA2	protein kinase AMP-activated catalytic subunit alpha 2 [Source:VGNC Symbol;Acc:VGNC:91798]	4.51	0.0889
ssc-miR-148a-3p	PRKAG2	protein kinase AMP-activated non-catalytic subunit gamma 2 [Source:VGNC Symbol;Acc:VGNC:91801]	4.51	0.0889
ssc-miR-148a-3p	PRNP	prion protein [Source:NCBI gene (formerly Entrezgene);Acc:494014]	4.51	0.0889
ssc-miR-148a-3p	PRR15	proline rich 15 [Source:VGNC Symbol;Acc:VGNC:91854]	4.51	0.0889
ssc-miR-148a-3p	PRR5L	proline rich 5 like [Source:VGNC Symbol;Acc:VGNC:91862]	4.51	0.0889
ssc-miR-148a-3p	PRRG1	proline rich and Gla domain 1 [Source:VGNC Symbol;Acc:VGNC:101494]	4.51	0.0889
ssc-miR-148a-3p	PRUNE	hypothetical gene	4.51	0.0889
ssc-miR-148a-3p	PSEN1	presenilin 1 [Source:VGNC Symbol;Acc:VGNC:91897]	4.51	0.0889
ssc-miR-148a-3p	PTCD3	pentatricopeptide repeat domain 3 [Source:VGNC Symbol;Acc:VGNC:91940]	4.51	0.0889
ssc-miR-148a-3p	PTEN	hypothetical gene	4.51	0.0889
ssc-miR-148a-3p	PTGES3	prostaglandin E synthase 3 [Source:VGNC Symbol;Acc:VGNC:91952]	4.51	0.0889
ssc-miR-148a-3p	PTPN14	protein tyrosine phosphatase non-receptor type 14 [Source:VGNC Symbol;Acc:VGNC:91975]	4.51	0.0889
ssc-miR-148a-3p	PTPRA	protein tyrosine phosphatase receptor type A [Source:VGNC Symbol;Acc:VGNC:96522]	4.51	0.0889
ssc-miR-148a-3p	PTPRD	protein tyrosine phosphatase receptor type D [Source:HGNC Symbol;Acc:HGNC:9668]	4.51	0.0889
ssc-miR-148a-3p	PTPRM	protein tyrosine phosphatase receptor type M [Source:VGNC Symbol;Acc:VGNC:91992]	4.51	0.0889
ssc-miR-148a-3p	PVRL1	hypothetical gene	4.51	0.0889
ssc-miR-148a-3p	QKI	QKI, KH domain containing RNA binding [Source:VGNC Symbol;Acc:VGNC:92025]	4.51	0.0889
ssc-miR-148a-3p	RAB10	RAB10, member RAS onco family [Source:VGNC Symbol;Acc:VGNC:98235]	4.51	0.0889
ssc-miR-148a-3p	RAB11B	RAB11B, member RAS onco family [Source:VGNC Symbol;Acc:VGNC:100847]	4.51	0.0889
ssc-miR-148a-3p	RAB12	RAB12, member RAS onco family [Source:VGNC Symbol;Acc:VGNC:98237]	4.51	0.0889
ssc-miR-148a-3p	RAB14	RAB14, member RAS onco family [Source:VGNC Symbol;Acc:VGNC:98238]	4.51	0.0889
ssc-miR-148a-3p	RAB24	hypothetical gene	4.51	0.0889
ssc-miR-148a-3p	RAB34	RAB34, member RAS onco family [Source:VGNC Symbol;Acc:VGNC:98258]	4.51	0.0889
ssc-miR-148a-3p	RAB35	RAB35, member RAS onco family [Source:VGNC Symbol;Acc:VGNC:98259]	4.51	0.0889

ssc-miR-148a-3p	RAB39B	RAB39B, member RAS onco family [Source:VGNC Symbol;Acc:VGNC:98263]	4.51	0.0889
ssc-miR-148a-3p	RAI14	retinoic acid induced 14 [Source:VGNC Symbol;Acc:VGNC:92068]	4.51	0.0889
ssc-miR-148a-3p	RALA	RAS like proto-oncogene A [Source:HGNC Symbol;Acc:HGNC:9839]	4.51	0.0889
ssc-miR-148a-3p	RALBP1	ralA binding protein 1 [Source:VGNC Symbol;Acc:VGNC:92070]	4.51	0.0889
ssc-miR-148a-3p	RALGPS1	Ral GEF with PH domain and SH3 binding motif 1 [Source:VGNC Symbol;Acc:VGNC:92072]	4.51	0.0889
ssc-miR-148a-3p	RALY	RALY heteroous nuclear ribonucleoprotein [Source:VGNC Symbol;Acc:VGNC:95709]	4.51	0.0889
ssc-miR-148a-3p	RANBP6	RAN binding protein 6 [Source:VGNC Symbol;Acc:VGNC:92081]	4.51	0.0889
ssc-miR-148a-3p	RASAL1	RAS protein activator like 1 [Source:VGNC Symbol;Acc:VGNC:92104]	4.51	0.0889
ssc-miR-148a-3p	RASSF8	Ras association domain family member 8 [Source:VGNC Symbol;Acc:VGNC:92128]	4.51	0.0889
ssc-miR-148a-3p	RBFOX2	RNA binding fox-1 homolog 2 [Source:VGNC Symbol;Acc:VGNC:92140]	4.51	0.0889
ssc-miR-148a-3p	RBM24	hypothetical gene	4.51	0.0889
ssc-miR-148a-3p	RBM3	RNA binding motif protein 3 [Source:NCBI gene (formerly Entrezgene);Acc:100627807]	4.51	0.0889
ssc-miR-148a-3p	RC3H1	ring finger and CCCH-type domains 1 [Source:VGNC Symbol;Acc:VGNC:92168]	4.51	0.0889
ssc-miR-148a-3p	RCC2	regulator of chromosome condensation 2 [Source:VGNC Symbol;Acc:VGNC:98547]	4.51	0.0889
ssc-miR-148a-3p	RERE	hypothetical gene	4.51	0.0889
ssc-miR-148a-3p	RFX7	regulatory factor X7 [Source:VGNC Symbol;Acc:VGNC:92248]	4.51	0.0889
ssc-miR-148a-3p	RGMA	repulsive guidance molecule BMP co-receptor a [Source:VGNC Symbol;Acc:VGNC:92254]	4.51	0.0889
ssc-miR-148a-3p	RICTOR	RPTOR independent companion of MTOR complex 2 [Source:VGNC Symbol;Acc:VGNC:92303]	4.51	0.0889
ssc-miR-148a-3p	RLIM	ring finger protein, LIM domain interacting [Source:HGNC Symbol;Acc:HGNC:13429]	4.51	0.0889
ssc-miR-148a-3p	RMND5A	required for meiotic nuclear division 5 homolog A [Source:VGNC Symbol;Acc:VGNC:92336]	4.51	0.0889
ssc-miR-148a-3p	RNF149	ring finger protein 149 [Source:VGNC Symbol;Acc:VGNC:92362]	4.51	0.0889
ssc-miR-148a-3p	RNF219	hypothetical gene	4.51	0.0889
ssc-miR-148a-3p	RNF38	ring finger protein 38 [Source:VGNC Symbol;Acc:VGNC:92390]	4.51	0.0889
ssc-miR-148a-3p	RNF44	ring finger protein 44 [Source:VGNC Symbol;Acc:VGNC:92395]	4.51	0.0889
ssc-miR-148a-3p	RNPEPL1	arginyl aminopeptidase like 1 [Source:VGNC Symbol;Acc:VGNC:95470]	4.51	0.0889
ssc-miR-148a-3p	ROBO1	roundabout guidance receptor 1 [Source:HGNC Symbol;Acc:HGNC:10249]	4.51	0.0889
ssc-miR-148a-3p	ROBO2	roundabout guidance receptor 2 [Source:HGNC Symbol;Acc:HGNC:10250]	4.51	0.0889
ssc-miR-148a-3p	ROCK1	Rho associated coiled-coil containing protein kinase 1 [Source:VGNC Symbol;Acc:VGNC:98294]	4.51	0.0889
ssc-miR-148a-3p	RORA	RAR related orphan receptor A [Source:VGNC Symbol;Acc:VGNC:92408]	4.51	0.0889
ssc-miR-148a-3p	RORB	RAR related orphan receptor B [Source:VGNC Symbol;Acc:VGNC:92409]	4.51	0.0889
ssc-miR-148a-3p	RP11-1055B8.7	hypothetical gene	4.51	0.0889
ssc-miR-148a-3p	RPH3AL	rabphilin 3A like (without C2 domains) [Source:HGNC Symbol;Acc:HGNC:10296]	4.51	0.0889
ssc-miR-148a-3p	RPS6KA5	ribosomal protein S6 kinase A5 [Source:VGNC Symbol;Acc:VGNC:92444]	4.51	0.0889
ssc-miR-148a-3p	RPS6KA6	ribosomal protein S6 kinase A6 [Source:VGNC Symbol;Acc:VGNC:92445]	4.51	0.0889
ssc-miR-148a-3p	RPUSD1	RNA pseudouridine synthase domain containing 1 [Source:VGNC Symbol;Acc:VGNC:92451]	4.51	0.0889
ssc-miR-148a-3p	RRAGD	Ras related GTP binding D [Source:VGNC Symbol;Acc:VGNC:92457]	4.51	0.0889
ssc-miR-148a-3p	RSBN1L	round spermatid basic protein 1 like [Source:VGNC Symbol;Acc:VGNC:92474]	4.51	0.0889
ssc-miR-148a-3p	RTN1	reticulon 1 [Source:VGNC Symbol;Acc:VGNC:103169]	4.51	0.0889
ssc-miR-148a-3p	RTN3	hypothetical gene	4.51	0.0889
ssc-miR-148a-3p	RTN4	reticulon 4 [Source:VGNC Symbol;Acc:VGNC:92502]	4.51	0.0889
ssc-miR-148a-3p	RUNX1T1	RUNX1 partner transcriptional co-repressor 1 [Source:VGNC Symbol;Acc:VGNC:96594]	4.51	0.0889
ssc-miR-148a-3p	RXRA	retinoid X receptor alpha [Source:VGNC Symbol;Acc:VGNC:92528]	4.51	0.0889
ssc-miR-148a-3p	RYBP	RING1 and YY1 binding protein [Source:VGNC Symbol;Acc:VGNC:92532]	4.51	0.0889
ssc-miR-148a-3p	S100A7A	hypothetical gene	4.51	0.0889
ssc-miR-148a-3p	S1PR1	sphingosine-1-phosphate receptor 1 [Source:VGNC Symbol;Acc:VGNC:92551]	4.51	0.0889
ssc-miR-148a-3p	SBF2	SET binding factor 2 [Source:VGNC Symbol;Acc:VGNC:92594]	4.51	0.0889
ssc-miR-148a-3p	SBNO1	strawberry notch homolog 1 [Source:VGNC Symbol;Acc:VGNC:92596]	4.51	0.0889
ssc-miR-148a-3p	SCML2	Scm polycomb group protein like 2 [Source:VGNC Symbol;Acc:VGNC:92628]	4.51	0.0889
ssc-miR-148a-3p	SCN8A	sodium voltage-gated channel alpha subunit 8 [Source:VGNC Symbol;Acc:VGNC:92638]	4.51	0.0889

ssc-miR-148a-3p	SEC63	SEC63 homolog, protein translocation regulator [Source:VGNC Symbol;Acc:VGNC:103172]	4.51	0.0889
ssc-miR-148a-3p	SECISBP2L	SECIS binding protein 2 like [Source:VGNC Symbol;Acc:VGNC:92686]	4.51	0.0889
ssc-miR-148a-3p	SEMA4F	ssemaphorin 4F [Source:VGNC Symbol;Acc:VGNC:92704]	4.51	0.0889
ssc-miR-148a-3p	SERBP1	SERPINE1 mRNA binding protein 1 [Source:VGNC Symbol;Acc:VGNC:92727]	4.51	0.0889
ssc-miR-148a-3p	SERPINE1	serpin family E member 1 [Source:VGNC Symbol;Acc:VGNC:98310]	4.51	0.0889
ssc-miR-148a-3p	SESN2	sestrin 2 [Source:VGNC Symbol;Acc:VGNC:92754]	4.51	0.0889
ssc-miR-148a-3p	SESN3	sestrin 3 [Source:VGNC Symbol;Acc:VGNC:92755]	4.51	0.0889
ssc-miR-148a-3p	SESTD1	SEC14 and spectrin domain containing 1 [Source:VGNC Symbol;Acc:VGNC:95884]	4.51	0.0889
ssc-miR-148a-3p	SFMBT1	Scm like with four mbt domains 1 [Source:VGNC Symbol;Acc:VGNC:92773]	4.51	0.0889
ssc-miR-148a-3p	SGCB	sarcoglycan beta [Source:NCBI gene (formerly Entrezgene);Acc:100135674]	4.51	0.0889
ssc-miR-148a-3p	SGMS1	sphingomyelin synthase 1 [Source:VGNC Symbol;Acc:VGNC:92794]	4.51	0.0889
ssc-miR-148a-3p	SH2B3	SH2B adaptor protein 3 [Source:VGNC Symbol;Acc:VGNC:92807]	4.51	0.0889
ssc-miR-148a-3p	SH3BP5	SH3 domain binding protein 5 [Source:VGNC Symbol;Acc:VGNC:92819]	4.51	0.0889
ssc-miR-148a-3p	SH3KBP1	SH3 domain containing kinase binding protein 1 [Source:VGNC Symbol;Acc:VGNC:92828]	4.51	0.0889
ssc-miR-148a-3p	SH3PXD2A	SH3 and PX domains 2A [Source:VGNC Symbol;Acc:VGNC:92829]	4.51	0.0889
ssc-miR-148a-3p	SIK1	salt inducible kinase 1 [Source:HGNC Symbol;Acc:HGNC:11142]	4.51	0.0889
ssc-miR-148a-3p	SIRT7	sirtuin 7 [Source:VGNC Symbol;Acc:VGNC:92890]	4.51	0.0889
ssc-miR-148a-3p	SIX4	SIX homeobox 4 [Source:VGNC Symbol;Acc:VGNC:92895]	4.51	0.0889
ssc-miR-148a-3p	SKIDA1	SKI/DACH domain containing 1 [Source:VGNC Symbol;Acc:VGNC:96266]	4.51	0.0889
ssc-miR-148a-3p	SKP1	hypothetical gene	4.51	0.0889
ssc-miR-148a-3p	SLC13A2	solute carrier family 13 member 2 [Source:VGNC Symbol;Acc:VGNC:92930]	4.51	0.0889
ssc-miR-148a-3p	SLC16A6	solute carrier family 16 member 6 [Source:VGNC Symbol;Acc:VGNC:92946]	4.51	0.0889
ssc-miR-148a-3p	SLC24A2	solute carrier family 24 member 2 [Source:VGNC Symbol;Acc:VGNC:92987]	4.51	0.0889
ssc-miR-148a-3p	SLC24A4	solute carrier family 24 member 4 [Source:VGNC Symbol;Acc:VGNC:92988]	4.51	0.0889
ssc-miR-148a-3p	SLC25A44	solute carrier family 25 member 44 [Source:VGNC Symbol;Acc:VGNC:93017]	4.51	0.0889
ssc-miR-148a-3p	SLC2A1	solute carrier family 2 member 1 [Source:HGNC Symbol;Acc:HGNC:11005]	4.51	0.0889
ssc-miR-148a-3p	SLC2A14	hypothetical gene	4.51	0.0889
ssc-miR-148a-3p	SLC2A3	hypothetical gene	4.51	0.0889
ssc-miR-148a-3p	SLC30A9	solute carrier family 30 member 9 [Source:VGNC Symbol;Acc:VGNC:98964]	4.51	0.0889
ssc-miR-148a-3p	SLC35F1	solute carrier family 35 member F1 [Source:VGNC Symbol;Acc:VGNC:93081]	4.51	0.0889
ssc-miR-148a-3p	SLC43A2	solute carrier family 43 member 2 [Source:VGNC Symbol;Acc:VGNC:93117]	4.51	0.0889
ssc-miR-148a-3p	SLC45A3	solute carrier family 45 member 3 [Source:VGNC Symbol;Acc:VGNC:93124]	4.51	0.0889
ssc-miR-148a-3p	SLC5A3	solute carrier family 5 member 3 [Source:VGNC Symbol;Acc:VGNC:93144]	4.51	0.0889
ssc-miR-148a-3p	SLC6A15	solute carrier family 6 member 15 [Source:VGNC Symbol;Acc:VGNC:93157]	4.51	0.0889
ssc-miR-148a-3p	SLC7A11	solute carrier family 7 member 11 [Source:VGNC Symbol;Acc:VGNC:93171]	4.51	0.0889
ssc-miR-148a-3p	SLC7A14	solute carrier family 7 member 14 [Source:VGNC Symbol;Acc:VGNC:93173]	4.51	0.0889
ssc-miR-148a-3p	SLC7A5	solute carrier family 7 member 5 [Source:HGNC Symbol;Acc:HGNC:11063]	4.51	0.0889
ssc-miR-148a-3p	SLITRK3	SLIT and NTRK like family member 3 [Source:VGNC Symbol;Acc:VGNC:93208]	4.51	0.0889
ssc-miR-148a-3p	SMAD2	SMAD family member 2 [Source:VGNC Symbol;Acc:VGNC:98329]	4.51	0.0889
ssc-miR-148a-3p	SMAD5	SMAD family member 5 [Source:VGNC Symbol;Acc:VGNC:93219]	4.51	0.0889
ssc-miR-148a-3p	SMARCD1	SWI/SNF related, matrix associated, actin dependent regulator of chromatin, subfamily d, member 1 [Source:VGNC Symbol;Acc:VGNC:93233]	4.51	0.0889
ssc-miR-148a-3p	SMIM12	small integral membrane protein 12 [Source:HGNC Symbol;Acc:HGNC:25154]	4.51	0.0889
ssc-miR-148a-3p	SMIM17	small integral membrane protein 17 [Source:VGNC Symbol;Acc:VGNC:98615]	4.51	0.0889
ssc-miR-148a-3p	SMS	spermine synthase [Source:VGNC Symbol;Acc:VGNC:104059]	4.51	0.0889
ssc-miR-148a-3p	SMURF2	SMAD specific E3 ubiquitin protein ligase 2 [Source:VGNC Symbol;Acc:VGNC:93271]	4.51	0.0889
ssc-miR-148a-3p	SNAP91	synaptosome associated protein 91 [Source:VGNC Symbol;Acc:VGNC:93279]	4.51	0.0889
ssc-miR-148a-3p	SNN	stannin [Source:HGNC Symbol;Acc:HGNC:11149]	4.51	0.0889
ssc-miR-148a-3p	SNPH	syntaphilin [Source:VGNC Symbol;Acc:VGNC:95716]	4.51	0.0889
ssc-miR-148a-3p	SNRK	SNF related kinase [Source:VGNC Symbol;Acc:VGNC:93290]	4.51	0.0889

ssc-miR-148a-3p	SNX17	sorting nexin 17 [Source:VGNC Symbol;Acc:VGNC:93309]	4.51	0.0889
ssc-miR-148a-3p	SNX2	sorting nexin 2 [Source:VGNC Symbol;Acc:VGNC:93312]	4.51	0.0889
ssc-miR-148a-3p	SNX21	sorting nexin family member 21 [Source:VGNC Symbol;Acc:VGNC:95758]	4.51	0.0889
ssc-miR-148a-3p	SNX27	sorting nexin 27 [Source:VGNC Symbol;Acc:VGNC:93316]	4.51	0.0889
ssc-miR-148a-3p	SNX3	sorting nexin 3 [Source:VGNC Symbol;Acc:VGNC:93317]	4.51	0.0889
ssc-miR-148a-3p	SOBP	sine oculis binding protein homolog [Source:VGNC Symbol;Acc:VGNC:93329]	4.51	0.0889
ssc-miR-148a-3p	SOCS3	suppressor of cytokine signaling 3 [Source:VGNC Symbol;Acc:VGNC:99052]	4.51	0.0889
ssc-miR-148a-3p	SOGA2	hypothetical gene	4.51	0.0889
ssc-miR-148a-3p	SOS1	SOS Ras/Rac guanine nucleotide exchange factor 1 [Source:HGNC Symbol;Acc:HGNC:11187]	4.51	0.0889
ssc-miR-148a-3p	SOS2	SOS Ras/Rho guanine nucleotide exchange factor 2 [Source:VGNC Symbol;Acc:VGNC:93344]	4.51	0.0889
ssc-miR-148a-3p	SOX11	SRY-box transcription factor 11 [Source:VGNC Symbol;Acc:VGNC:93351]	4.51	0.0889
ssc-miR-148a-3p	SOX5	SRY-box transcription factor 5 [Source:VGNC Symbol;Acc:VGNC:93357]	4.51	0.0889
ssc-miR-148a-3p	SPIN1	spindlin 1 [Source:VGNC Symbol;Acc:VGNC:101495]	4.51	0.0889
ssc-miR-148a-3p	SPIRE1	spire type actin nucleation factor 1 [Source:VGNC Symbol;Acc:VGNC:98336]	4.51	0.0889
ssc-miR-148a-3p	SPRY3	sprouty RTK signaling antagonist 3 [Source:VGNC Symbol;Acc:VGNC:93426]	4.51	0.0889
ssc-miR-148a-3p	SPRYD7	SPRY domain containing 7 [Source:VGNC Symbol;Acc:VGNC:93429]	4.51	0.0889
ssc-miR-148a-3p	SPTY2D1	SPT2 chromatin protein domain containing 1 [Source:VGNC Symbol;Acc:VGNC:100867]	4.51	0.0889
ssc-miR-148a-3p	SRPK2	SRSF protein kinase 2 [Source:VGNC Symbol;Acc:VGNC:93463]	4.51	0.0889
ssc-miR-148a-3p	SRSF11	serine and arginine rich splicing factor 11 [Source:VGNC Symbol;Acc:VGNC:93473]	4.51	0.0889
ssc-miR-148a-3p	SSBP3	single stranded DNA binding protein 3 [Source:VGNC Symbol;Acc:VGNC:93481]	4.51	0.0889
ssc-miR-148a-3p	SSR1	signal sequence receptor subunit 1 [Source:VGNC Symbol;Acc:VGNC:93490]	4.51	0.0889
ssc-miR-148a-3p	ST18	ST18 C2H2C-type zinc finger transcription factor [Source:VGNC Symbol;Acc:VGNC:93501]	4.51	0.0889
ssc-miR-148a-3p	ST8SIA3	ST8 alpha-N-acetyl-neuraminide alpha-2,8-sialyltransferase 3 [Source:VGNC Symbol;Acc:VGNC:93518]	4.51	0.0889
ssc-miR-148a-3p	STAM	signal transducing adaptor molecule [Source:VGNC Symbol;Acc:VGNC:95520]	4.51	0.0889
ssc-miR-148a-3p	STARD13	hypothetical gene	4.51	0.0889
ssc-miR-148a-3p	STK38L	serine/threonine kinase 38 like [Source:VGNC Symbol;Acc:VGNC:93557]	4.51	0.0889
ssc-miR-148a-3p	STOX2	storkhead box 2 [Source:VGNC Symbol;Acc:VGNC:96082]	4.51	0.0889
ssc-miR-148a-3p	STRADB	STE20 related adaptor beta [Source:VGNC Symbol;Acc:VGNC:98351]	4.51	0.0889
ssc-miR-148a-3p	STT3A	STT3 oligosaccharyltransferase complex catalytic subunit A [Source:VGNC Symbol;Acc:VGNC:93581]	4.51	0.0889
ssc-miR-148a-3p	STX11	syntaxin 11 [Source:VGNC Symbol;Acc:VGNC:93585]	4.51	0.0889
ssc-miR-148a-3p	STX3	syntaxin 3 [Source:VGNC Symbol;Acc:VGNC:93591]	4.51	0.0889
ssc-miR-148a-3p	STX6	syntaxin 6 [Source:VGNC Symbol;Acc:VGNC:93594]	4.51	0.0889
ssc-miR-148a-3p	STXBP5	syntaxin binding protein 5 [Source:VGNC Symbol;Acc:VGNC:93599]	4.51	0.0889
ssc-miR-148a-3p	STYX	serine/threonine/tyrosine interacting protein [Source:VGNC Symbol;Acc:VGNC:93602]	4.51	0.0889
ssc-miR-148a-3p	SUGT1	SGT1 homolog, MIS12 kinetochore complex assembly cochaperone [Source:VGNC Symbol;Acc:VGNC:93611]	4.51	0.0889
ssc-miR-148a-3p	SULF1	sulfatase 1 [Source:VGNC Symbol;Acc:VGNC:93612]	4.51	0.0889
ssc-miR-148a-3p	SUN2	Sad1 and UNC84 domain containing 2 [Source:VGNC Symbol;Acc:VGNC:93619]	4.51	0.0889
ssc-miR-148a-3p	SYNCRIP	synaptotagmin binding cytoplasmic RNA interacting protein [Source:VGNC Symbol;Acc:VGNC:103185]	4.51	0.0889
ssc-miR-148a-3p	SYNJ1	synaptojanin 1 [Source:VGNC Symbol;Acc:VGNC:93668]	4.51	0.0889
ssc-miR-148a-3p	SYT1	synaptotagmin 1 [Source:VGNC Symbol;Acc:VGNC:93678]	4.51	0.0889
ssc-miR-148a-3p	SYT2	synaptotagmin 2 [Source:VGNC Symbol;Acc:VGNC:95530]	4.51	0.0889
ssc-miR-148a-3p	SZRD1	hypothetical gene	4.51	0.0889
ssc-miR-148a-3p	TAF11	TATA-box binding protein associated factor 11 [Source:NCBI gene (formerly Entrezgene);Acc:100151814]	4.51	0.0889
ssc-miR-148a-3p	TAF1D	TATA-box binding protein associated factor, RNA polymerase I subunit D [Source:VGNC Symbol;Acc:VGNC:93714]	4.51	0.0889
ssc-miR-148a-3p	TAF4	TATA-box binding protein associated factor 4 [Source:VGNC Symbol;Acc:VGNC:95532]	4.51	0.0889
ssc-miR-148a-3p	TAF4B	TATA-box binding protein associated factor 4b [Source:VGNC Symbol;Acc:VGNC:93716]	4.51	0.0889
ssc-miR-148a-3p	TANC1	tetratricopeptide repeat, ankyrin repeat and coiled-coil containing 1 [Source:VGNC Symbol;Acc:VGNC:99730]	4.51	0.0889
ssc-miR-148a-3p	TBC1D14	hypothetical gene	4.51	0.0889
ssc-miR-148a-3p	TBC1D5	TBC1 domain family member 5 [Source:VGNC Symbol;Acc:VGNC:98360]	4.51	0.0889

ssc-miR-148a-3p	TBC1D8	TBC1 domain family member 8 [Source:VGNC Symbol;Acc:VGNC:93779]	4.51	0.0889
ssc-miR-148a-3p	TBCK	TBC1 domain containing kinase [Source:VGNC Symbol;Acc:VGNC:93786]	4.51	0.0889
ssc-miR-148a-3p	TBL1XR1	TBL1X receptor 1 [Source:VGNC Symbol;Acc:VGNC:96600]	4.51	0.0889
ssc-miR-148a-3p	TBPL1	TATA-box binding protein like 1 [Source:VGNC Symbol;Acc:VGNC:93792]	4.51	0.0889
ssc-miR-148a-3p	TCF4	transcription factor 4 [Source:VGNC Symbol;Acc:VGNC:93823]	4.51	0.0889
ssc-miR-148a-3p	TDRD15	hypothetical gene	4.51	0.0889
ssc-miR-148a-3p	TEAD1	TEA domain transcription factor 1 [Source:VGNC Symbol;Acc:VGNC:93853]	4.51	0.0889
ssc-miR-148a-3p	TEK	TEK receptor tyrosine kinase [Source:VGNC Symbol;Acc:VGNC:93866]	4.51	0.0889
ssc-miR-148a-3p	TENM4	teneurin transmembrane protein 4 [Source:HGNC Symbol;Acc:HGNC:29945]	4.51	0.0889
ssc-miR-148a-3p	TERF2	telomeric repeat binding factor 2 [Source:VGNC Symbol;Acc:VGNC:93882]	4.51	0.0889
ssc-miR-148a-3p	TET2	hypothetical gene	4.51	0.0889
ssc-miR-148a-3p	TFDP2	transcription factor Dp-2 [Source:VGNC Symbol;Acc:VGNC:93915]	4.51	0.0889
ssc-miR-148a-3p	TFRC	transferrin receptor [Source:VGNC Symbol;Acc:VGNC:93925]	4.51	0.0889
ssc-miR-148a-3p	TGFA	transforming growth factor alpha [Source:VGNC Symbol;Acc:VGNC:93928]	4.51	0.0889
ssc-miR-148a-3p	TGFB2	transforming growth factor beta 2 [Source:VGNC Symbol;Acc:VGNC:95541]	4.51	0.0889
ssc-miR-148a-3p	TGFBR1	transforming growth factor beta receptor 1 [Source:VGNC Symbol;Acc:VGNC:98368]	4.51	0.0889
ssc-miR-148a-3p	TGFBRAP1	transforming growth factor beta receptor associated protein 1 [Source:VGNC Symbol;Acc:VGNC:93934]	4.51	0.0889
ssc-miR-148a-3p	TGIF2	TGFB induced factor homeobox 2 [Source:VGNC Symbol;Acc:VGNC:95656]	4.51	0.0889
ssc-miR-148a-3p	TIGD5	tigger transposable element derived 5 [Source:VGNC Symbol;Acc:VGNC:98876]	4.51	0.0889
ssc-miR-148a-3p	TIMM8A	translocase of inner mitochondrial membrane 8A [Source:VGNC Symbol;Acc:VGNC:93992]	4.51	0.0889
ssc-miR-148a-3p	TMED7	transmembrane p24 trafficking protein 7 [Source:NCBI gene (formerly Entrezgene);Acc:100522183]	4.51	0.0889
ssc-miR-148a-3p	TMEM11	transmembrane protein 11 [Source:VGNC Symbol;Acc:VGNC:94067]	4.51	0.0889
ssc-miR-148a-3p	TMEM127	transmembrane protein 127 [Source:HGNC Symbol;Acc:HGNC:26038]	4.51	0.0889
ssc-miR-148a-3p	TMEM164	transmembrane protein 164 [Source:VGNC Symbol;Acc:VGNC:94103]	4.51	0.0889
ssc-miR-148a-3p	TMEM170A	transmembrane protein 170A [Source:VGNC Symbol;Acc:VGNC:98659]	4.51	0.0889
ssc-miR-148a-3p	TMEM170B	transmembrane protein 170B [Source:VGNC Symbol;Acc:VGNC:94108]	4.51	0.0889
ssc-miR-148a-3p	TMEM52B	transmembrane protein 52B [Source:VGNC Symbol;Acc:VGNC:94189]	4.51	0.0889
ssc-miR-148a-3p	TMEM54	transmembrane protein 54 [Source:VGNC Symbol;Acc:VGNC:94191]	4.51	0.0889
ssc-miR-148a-3p	TMEM55A	hypothetical gene	4.51	0.0889
ssc-miR-148a-3p	TMEM63B	transmembrane protein 63B [Source:VGNC Symbol;Acc:VGNC:94197]	4.51	0.0889
ssc-miR-148a-3p	TMEM9B	TMEM9 domain family member B [Source:VGNC Symbol;Acc:VGNC:94222]	4.51	0.0889
ssc-miR-148a-3p	TMSB10	thymosin beta 10 [Source:NCBI gene (formerly Entrezgene);Acc:100037998]	4.51	0.0889
ssc-miR-148a-3p	TMSB4X	thymosin beta 4 X-linked [Source:NCBI gene (formerly Entrezgene);Acc:733606]	4.51	0.0889
ssc-miR-148a-3p	TMSB4Y	hypothetical gene	4.51	0.0889
ssc-miR-148a-3p	TNFRSF1B	TNF receptor superfamily member 1B [Source:VGNC Symbol;Acc:VGNC:94264]	4.51	0.0889
ssc-miR-148a-3p	TNPO1	transportin 1 [Source:VGNC Symbol;Acc:VGNC:94287]	4.51	0.0889
ssc-miR-148a-3p	TNRC6A	trinucleotide repeat containing adaptor 6A [Source:VGNC Symbol;Acc:VGNC:94292]	4.51	0.0889
ssc-miR-148a-3p	TNRC6B	trinucleotide repeat containing adaptor 6B [Source:VGNC Symbol;Acc:VGNC:94293]	4.51	0.0889
ssc-miR-148a-3p	TNRC6C	trinucleotide repeat containing adaptor 6C [Source:VGNC Symbol;Acc:VGNC:94294]	4.51	0.0889
ssc-miR-148a-3p	TOMM70A	hypothetical gene	4.51	0.0889
ssc-miR-148a-3p	TPM3	tropomyosin 3 [Source:VGNC Symbol;Acc:VGNC:98877]	4.51	0.0889
ssc-miR-148a-3p	TRAK2	trafficking kinesin protein 2 [Source:VGNC Symbol;Acc:VGNC:95577]	4.51	0.0889
ssc-miR-148a-3p	TRIP12	thyroid hormone receptor interactor 12 [Source:VGNC Symbol;Acc:VGNC:95555]	4.51	0.0889
ssc-miR-148a-3p	TRPS1	transcriptional repressor GATA binding 1 [Source:VGNC Symbol;Acc:VGNC:94471]	4.51	0.0889
ssc-miR-148a-3p	TSPAN18	tetraspanin 18 [Source:VGNC Symbol;Acc:VGNC:94507]	4.51	0.0889
ssc-miR-148a-3p	TSPAN31	tetraspanin 31 [Source:VGNC Symbol;Acc:VGNC:94511]	4.51	0.0889
ssc-miR-148a-3p	TSPYL1	TSPY like 1 [Source:VGNC Symbol;Acc:VGNC:103197]	4.51	0.0889
ssc-miR-148a-3p	TTBK2	tau tubulin kinase 2 [Source:VGNC Symbol;Acc:VGNC:98387]	4.51	0.0889
ssc-miR-148a-3p	TTC38	tetratricopeptide repeat domain 38 [Source:VGNC Symbol;Acc:VGNC:94550]	4.51	0.0889

ssc-miR-148a-3p	TUBB6	tubulin beta 6 class V [Source:VGNC Symbol;Acc:VGNC:94581]	4.51	0.0889
ssc-miR-148a-3p	TXLNG	taxilin gamma [Source:HGNC Symbol;Acc:HGNC:18578]	4.51	0.0889
ssc-miR-148a-3p	TXNIP	thioredoxin interacting protein [Source:VGNC Symbol;Acc:VGNC:94610]	4.51	0.0889
ssc-miR-148a-3p	UBA6	ubiquitin like modifier activating enzyme 6 [Source:VGNC Symbol;Acc:VGNC:94630]	4.51	0.0889
ssc-miR-148a-3p	UBAP2L	ubiquitin associated protein 2 like [Source:VGNC Symbol;Acc:VGNC:94635]	4.51	0.0889
ssc-miR-148a-3p	UBB	ubiquitin B [Source:VGNC Symbol;Acc:VGNC:98389]	4.51	0.0889
ssc-miR-148a-3p	UBE2D1	ubiquitin conjugating enzyme E2 D1 [Source:VGNC Symbol;Acc:VGNC:94641]	4.51	0.0889
ssc-miR-148a-3p	UBE2D3	ubiquitin conjugating enzyme E2 D3 [Source:NCBI gene (formerly Entrezgene);Acc:780418]	4.51	0.0889
ssc-miR-148a-3p	UBE2K	ubiquitin conjugating enzyme E2 K [Source:VGNC Symbol;Acc:VGNC:94646]	4.51	0.0889
ssc-miR-148a-3p	UBE2W	ubiquitin conjugating enzyme E2 W [Source:VGNC Symbol;Acc:VGNC:98890]	4.51	0.0889
ssc-miR-148a-3p	UBE3B	ubiquitin protein ligase E3B [Source:VGNC Symbol;Acc:VGNC:94653]	4.51	0.0889
ssc-miR-148a-3p	UBE4B	ubiquitination factor E4B [Source:VGNC Symbol;Acc:VGNC:94657]	4.51	0.0889
ssc-miR-148a-3p	UBN2	ubinuclein 2 [Source:VGNC Symbol;Acc:VGNC:94664]	4.51	0.0889
ssc-miR-148a-3p	UBXN1	UBX domain protein 1 [Source:VGNC Symbol;Acc:VGNC:94674]	4.51	0.0889
ssc-miR-148a-3p	UCP3	uncoupling protein 3 [Source:VGNC Symbol;Acc:VGNC:108624]	4.51	0.0889
ssc-miR-148a-3p	UGCG	UDP-glucose ceramide glucosyltransferase [Source:VGNC Symbol;Acc:VGNC:94685]	4.51	0.0889
ssc-miR-148a-3p	UGT8	UDP glycosyltransferase 8 [Source:VGNC Symbol;Acc:VGNC:98401]	4.51	0.0889
ssc-miR-148a-3p	UHMK1	U2AF homology motif kinase 1 [Source:VGNC Symbol;Acc:VGNC:94688]	4.51	0.0889
ssc-miR-148a-3p	ULK3	unc-51 like kinase 3 [Source:VGNC Symbol;Acc:VGNC:94696]	4.51	0.0889
ssc-miR-148a-3p	UNC5D	unc-5 netrin receptor D [Source:VGNC Symbol;Acc:VGNC:95892]	4.51	0.0889
ssc-miR-148a-3p	UNC79	unc-79 homolog, NALCN channel complex subunit [Source:HGNC Symbol;Acc:HGNC:19966]	4.51	0.0889
ssc-miR-148a-3p	USP32	hypothetical gene	4.51	0.0889
ssc-miR-148a-3p	USP33	ubiquitin specific peptidase 33 [Source:HGNC Symbol;Acc:HGNC:20059]	4.51	0.0889
ssc-miR-148a-3p	USP37	ubiquitin specific peptidase 37 [Source:VGNC Symbol;Acc:VGNC:98404]	4.51	0.0889
ssc-miR-148a-3p	USP38	ubiquitin specific peptidase 38 [Source:VGNC Symbol;Acc:VGNC:94762]	4.51	0.0889
ssc-miR-148a-3p	USP4	ubiquitin specific peptidase 4 [Source:VGNC Symbol;Acc:VGNC:94764]	4.51	0.0889
ssc-miR-148a-3p	USP47	ubiquitin specific peptidase 47 [Source:VGNC Symbol;Acc:VGNC:94769]	4.51	0.0889
ssc-miR-148a-3p	USP48	ubiquitin specific peptidase 48 [Source:VGNC Symbol;Acc:VGNC:94770]	4.51	0.0889
ssc-miR-148a-3p	USP6	hypothetical gene	4.51	0.0889
ssc-miR-148a-3p	USP6NL	USP6 N-terminal like [Source:VGNC Symbol;Acc:VGNC:95820]	4.51	0.0889
ssc-miR-148a-3p	VASH2	vasohibin 2 [Source:VGNC Symbol;Acc:VGNC:94802]	4.51	0.0889
ssc-miR-148a-3p	VAV2	vav guanine nucleotide exchange factor 2 [Source:VGNC Symbol;Acc:VGNC:94808]	4.51	0.0889
ssc-miR-148a-3p	VCPIP1	valosin containing protein interacting protein 1 [Source:VGNC Symbol;Acc:VGNC:94813]	4.51	0.0889
ssc-miR-148a-3p	VMP1	vacuole membrane protein 1 [Source:HGNC Symbol;Acc:HGNC:29559]	4.51	0.0889
ssc-miR-148a-3p	VSIG10	V-set and immunoglobulin domain containing 10 [Source:HGNC Symbol;Acc:HGNC:26078]	4.51	0.0889
ssc-miR-148a-3p	VSNL1	visinin like 1 [Source:VGNC Symbol;Acc:VGNC:94871]	4.51	0.0889
ssc-miR-148a-3p	VSTM4	V-set and transmembrane domain containing 4 [Source:VGNC Symbol;Acc:VGNC:94874]	4.51	0.0889
ssc-miR-148a-3p	VTI1A	vesicle transport through interaction with t-SNAREs 1A [Source:VGNC Symbol;Acc:VGNC:94879]	4.51	0.0889
ssc-miR-148a-3p	WAPAL	hypothetical gene	4.51	0.0889
ssc-miR-148a-3p	WASL	WASP like actin nucleation promoting factor [Source:VGNC Symbol;Acc:VGNC:96604]	4.51	0.0889
ssc-miR-148a-3p	WDR20	WD repeat domain 20 [Source:VGNC Symbol;Acc:VGNC:94910]	4.51	0.0889
ssc-miR-148a-3p	WDR47	WD repeat domain 47 [Source:VGNC Symbol;Acc:VGNC:94924]	4.51	0.0889
ssc-miR-148a-3p	WHSC1	hypothetical gene	4.51	0.0889
ssc-miR-148a-3p	WIPI2	WD repeat domain, phosphoinositide interacting 2 [Source:VGNC Symbol;Acc:VGNC:94960]	4.51	0.0889
ssc-miR-148a-3p	WNT1	Wnt family member 1 [Source:HGNC Symbol;Acc:HGNC:12774]	4.51	0.0889
ssc-miR-148a-3p	WNT10B	Wnt family member 10B [Source:VGNC Symbol;Acc:VGNC:94965]	4.51	0.0889
ssc-miR-148a-3p	XPO4	exportin 4 [Source:VGNC Symbol;Acc:VGNC:95004]	4.51	0.0889
ssc-miR-148a-3p	YOD1	YOD1 deubiquitinase [Source:VGNC Symbol;Acc:VGNC:95035]	4.51	0.0889
ssc-miR-148a-3p	YPEL3	yippee like 3 [Source:VGNC Symbol;Acc:VGNC:95037]	4.51	0.0889

ssc-miR-148a-3p	YTHDC2	YTH domain containing 2 [Source:VGNC Symbol;Acc:VGNC:95042]	4.51	0.0889
ssc-miR-148a-3p	YWHAB	tyrosine 3-monooxygenase/tryptophan 5-monooxygenase activation protein beta [Source:VGNC Symbol;Acc:VGNC:98417]	4.51	0.0889
ssc-miR-148a-3p	ZADH2	prostaglandin reductase 3 [Source:VGNC Symbol;Acc:VGNC:95049]	4.51	0.0889
ssc-miR-148a-3p	ZBED4	hypothetical gene	4.51	0.0889
ssc-miR-148a-3p	ZBTB16	zinc finger and BTB domain containing 16 [Source:VGNC Symbol;Acc:VGNC:108626]	4.51	0.0889
ssc-miR-148a-3p	ZBTB18	zinc finger and BTB domain containing 18 [Source:HGNC Symbol;Acc:HGNC:13030]	4.51	0.0889
ssc-miR-148a-3p	ZBTB20	zinc finger and BTB domain containing 20 [Source:VGNC Symbol;Acc:VGNC:95063]	4.51	0.0889
ssc-miR-148a-3p	ZBTB7A	zinc finger and BTB domain containing 7A [Source:VGNC Symbol;Acc:VGNC:95081]	4.51	0.0889
ssc-miR-148a-3p	ZBTB8A	zinc finger and BTB domain containing 8A [Source:VGNC Symbol;Acc:VGNC:95084]	4.51	0.0889
ssc-miR-148a-3p	ZC3H12B	zinc finger CCCH-type containing 12B [Source:VGNC Symbol;Acc:VGNC:95090]	4.51	0.0889
ssc-miR-148a-3p	ZC3H12C	zinc finger CCCH-type containing 12C [Source:VGNC Symbol;Acc:VGNC:95091]	4.51	0.0889
ssc-miR-148a-3p	ZCCHC2	zinc finger CCHC-type containing 2 [Source:VGNC Symbol;Acc:VGNC:95109]	4.51	0.0889
ssc-miR-148a-3p	ZDHHC17	zinc finger DHHC-type palmitoyltransferase 17 [Source:VGNC Symbol;Acc:VGNC:95119]	4.51	0.0889
ssc-miR-148a-3p	ZDHHC2	zinc finger DHHC-type palmitoyltransferase 2 [Source:VGNC Symbol;Acc:VGNC:95886]	4.51	0.0889
ssc-miR-148a-3p	ZDHHC23	zinc finger DHHC-type palmitoyltransferase 23 [Source:VGNC Symbol;Acc:VGNC:95125]	4.51	0.0889
ssc-miR-148a-3p	ZDHHC7	zinc finger DHHC-type palmitoyltransferase 7 [Source:VGNC Symbol;Acc:VGNC:95129]	4.51	0.0889
ssc-miR-148a-3p	ZFHX3	zinc finger homeobox 3 [Source:VGNC Symbol;Acc:VGNC:95141]	4.51	0.0889
ssc-miR-148a-3p	ZFPM2	zinc finger protein, FOG family member 2 [Source:VGNC Symbol;Acc:VGNC:95153]	4.51	0.0889
ssc-miR-148a-3p	ZFYVE26	zinc finger FYVE-type containing 26 [Source:VGNC Symbol;Acc:VGNC:95160]	4.51	0.0889
ssc-miR-148a-3p	ZNF217	zinc finger protein 217 [Source:VGNC Symbol;Acc:VGNC:95573]	4.51	0.0889
ssc-miR-148a-3p	ZNF268	zinc finger protein 268 [Source:HGNC Symbol;Acc:HGNC:13061]	4.51	0.0889
ssc-miR-148a-3p	ZNF274	hypothetical gene	4.51	0.0889
ssc-miR-148a-3p	ZNF469	hypothetical gene	4.51	0.0889
ssc-miR-148a-3p	ZNF618	zinc finger protein 618 [Source:VGNC Symbol;Acc:VGNC:95276]	4.51	0.0889
ssc-miR-148a-3p	ZNF704	zinc finger protein 704 [Source:VGNC Symbol;Acc:VGNC:95296]	4.51	0.0889
ssc-miR-148a-3p	ZNF784	zinc finger protein 784 [Source:VGNC Symbol;Acc:VGNC:95301]	4.51	0.0889
ssc-miR-148a-3p	ZNF804A	zinc finger protein 804A [Source:VGNC Symbol;Acc:VGNC:95976]	4.51	0.0889
ssc-miR-148a-3p	ZNF821	zinc finger protein 821 [Source:VGNC Symbol;Acc:VGNC:95309]	4.51	0.0889
ssc-miR-148a-3p	ZNRF1	zinc and ring finger 1 [Source:HGNC Symbol;Acc:HGNC:18452]	4.51	0.0889
ssc-miR-1343	NA	hypothetical gene	3.73	0.0838
ssc-miR-30a-5p	A1CF	APOBEC1 complementation factor [Source:VGNC Symbol;Acc:VGNC:84933]	3.17	0.0763
ssc-miR-30a-5p	ABAT	4-aminobutyrate aminotransferase [Source:VGNC Symbol;Acc:VGNC:96910]	3.17	0.0763
ssc-miR-30a-5p	ABCA3	ATP binding cassette subfamily A member 3 [Source:VGNC Symbol;Acc:VGNC:96911]	3.17	0.0763
ssc-miR-30a-5p	ABCB7	ATP binding cassette subfamily B member 7 [Source:VGNC Symbol;Acc:VGNC:97855]	3.17	0.0763
ssc-miR-30a-5p	ABCC9	ATP binding cassette subfamily C member 9 [Source:VGNC Symbol;Acc:VGNC:84960]	3.17	0.0763
ssc-miR-30a-5p	ABCD2	ATP binding cassette subfamily D member 2 [Source:VGNC Symbol;Acc:VGNC:84962]	3.17	0.0763
ssc-miR-30a-5p	ABHD6	abhydrolase domain containing 6, acylglycerol lipase [Source:VGNC Symbol;Acc:VGNC:84982]	3.17	0.0763
ssc-miR-30a-5p	ABI2	abl interactor 2 [Source:VGNC Symbol;Acc:VGNC:96027]	3.17	0.0763
ssc-miR-30a-5p	ABI3BP	ABI family member 3 binding protein [Source:VGNC Symbol;Acc:VGNC:108628]	3.17	0.0763
ssc-miR-30a-5p	ABL1	ABL proto-onco 1, non-receptor tyrosine kinase [Source:VGNC Symbol;Acc:VGNC:84984]	3.17	0.0763
ssc-miR-30a-5p	ABL2	ABL proto-onco 2, non-receptor tyrosine kinase [Source:VGNC Symbol;Acc:VGNC:84985]	3.17	0.0763
ssc-miR-30a-5p	AC002451.1	hypothetical gene	3.17	0.0763
ssc-miR-30a-5p	AC012215.1	hypothetical gene	3.17	0.0763
ssc-miR-30a-5p	AC104472.1	hypothetical gene	3.17	0.0763
ssc-miR-30a-5p	ACAP2	ArfGAP with coiled-coil, ankyrin repeat and PH domains 2 [Source:VGNC Symbol;Acc:VGNC:85002]	3.17	0.0763
ssc-miR-30a-5p	ACSBG1	acyl-CoA synthetase bubblegum family member 1 [Source:VGNC Symbol;Acc:VGNC:85030]	3.17	0.0763
ssc-miR-30a-5p	ACTC1	actin alpha cardiac muscle 1 [Source:VGNC Symbol;Acc:VGNC:85040]	3.17	0.0763
ssc-miR-30a-5p	ACTN1	actinin alpha 1 [Source:NCBI gene (formerly Entrezgene);Acc:100513412]	3.17	0.0763
ssc-miR-30a-5p	ACTR1A	actin related protein 1A [Source:VGNC Symbol;Acc:VGNC:85049]	3.17	0.0763

ssc-miR-30a-5p	ACTR3C	hypothetical gene	3.17	0.0763
ssc-miR-30a-5p	ACVR1	activin A receptor type 1 [Source:VGNC Symbol;Acc:VGNC:95830]	3.17	0.0763
ssc-miR-30a-5p	ADAM10	ADAM metallopeptidase domain 10 [Source:VGNC Symbol;Acc:VGNC:85061]	3.17	0.0763
ssc-miR-30a-5p	ADAM11	ADAM metallopeptidase domain 11 [Source:VGNC Symbol;Acc:VGNC:85062]	3.17	0.0763
ssc-miR-30a-5p	ADAM12	ADAM metallopeptidase domain 12 [Source:VGNC Symbol;Acc:VGNC:85063]	3.17	0.0763
ssc-miR-30a-5p	ADAM19	ADAM metallopeptidase domain 19 [Source:VGNC Symbol;Acc:VGNC:85066]	3.17	0.0763
ssc-miR-30a-5p	ADAM22	ADAM metallopeptidase domain 22 [Source:VGNC Symbol;Acc:VGNC:85067]	3.17	0.0763
ssc-miR-30a-5p	ADAM9	ADAM metallopeptidase domain 9 [Source:VGNC Symbol;Acc:VGNC:95969]	3.17	0.0763
ssc-miR-30a-5p	ADAMTS14	ADAM metallopeptidase with thrombospondin type 1 motif 14 [Source:VGNC Symbol;Acc:VGNC:85076]	3.17	0.0763
ssc-miR-30a-5p	ADAMTS3	ADAM metallopeptidase with thrombospondin type 1 motif 3 [Source:VGNC Symbol;Acc:VGNC:85083]	3.17	0.0763
ssc-miR-30a-5p	ADAMTS5	ADAM metallopeptidase with thrombospondin type 1 motif 5 [Source:VGNC Symbol;Acc:VGNC:85085]	3.17	0.0763
ssc-miR-30a-5p	ADAMTS9	ADAM metallopeptidase with thrombospondin type 1 motif 9 [Source:VGNC Symbol;Acc:VGNC:85089]	3.17	0.0763
ssc-miR-30a-5p	ADAMTSL2	ADAMTS like 2 [Source:VGNC Symbol;Acc:VGNC:85091]	3.17	0.0763
ssc-miR-30a-5p	ADARB2	adenosine deaminase RNA specific B2 (inactive) [Source:HGNC Symbol;Acc:HGNC:227]	3.17	0.0763
ssc-miR-30a-5p	ADO	2-aminoethanethiol dioxygenase [Source:VGNC Symbol;Acc:VGNC:85146]	3.17	0.0763
ssc-miR-30a-5p	ADRA1D	adrenoceptor alpha 1D [Source:NCBI gene (formerly Entrezgene);Acc:552898]	3.17	0.0763
ssc-miR-30a-5p	ADRA2A	adrenoceptor alpha 2A [Source:VGNC Symbol;Acc:VGNC:85156]	3.17	0.0763
ssc-miR-30a-5p	ADRA2B	adrenoceptor alpha 2B [Source:VGNC Symbol;Acc:VGNC:85157]	3.17	0.0763
ssc-miR-30a-5p	ADRB1	adrenoceptor beta 1 [Source:VGNC Symbol;Acc:VGNC:107363]	3.17	0.0763
ssc-miR-30a-5p	AFAP1L2	actin filament associated protein 1 like 2 [Source:HGNC Symbol;Acc:HGNC:25901]	3.17	0.0763
ssc-miR-30a-5p	AFF3	AF4/FMR2 family member 3 [Source:HGNC Symbol;Acc:HGNC:6473]	3.17	0.0763
ssc-miR-30a-5p	AFF4	AF4/FMR2 family member 4 [Source:VGNC Symbol;Acc:VGNC:85169]	3.17	0.0763
ssc-miR-30a-5p	AGO1	hypothetical gene	3.17	0.0763
ssc-miR-30a-5p	AGO2	argonaute RISC catalytic component 2 [Source:VGNC Symbol;Acc:VGNC:97871]	3.17	0.0763
ssc-miR-30a-5p	AGO3	argonaute RISC component 1 [Source:NCBI gene (formerly Entrezgene);Acc:100499510]	3.17	0.0763
ssc-miR-30a-5p	AGO4	hypothetical gene	3.17	0.0763
ssc-miR-30a-5p	AGPAT4	1-acylglycerol-3-phosphate O-acyltransferase 4 [Source:VGNC Symbol;Acc:VGNC:85186]	3.17	0.0763
ssc-miR-30a-5p	AHNAK	hypothetical gene	3.17	0.0763
ssc-miR-30a-5p	AIDA	axin interactor, dorsalization associated [Source:VGNC Symbol;Acc:VGNC:96103]	3.17	0.0763
ssc-miR-30a-5p	AJAP1	adherens junctions associated protein 1 [Source:VGNC Symbol;Acc:VGNC:85207]	3.17	0.0763
ssc-miR-30a-5p	AK4	hypothetical gene	3.17	0.0763
ssc-miR-30a-5p	AKAP10	A-kinase anchoring protein 10 [Source:VGNC Symbol;Acc:VGNC:85215]	3.17	0.0763
ssc-miR-30a-5p	AKIRIN1	akirin 1 [Source:VGNC Symbol;Acc:VGNC:85227]	3.17	0.0763
ssc-miR-30a-5p	AL139099.1	hypothetical gene	3.17	0.0763
ssc-miR-30a-5p	ALG10	hypothetical gene	3.17	0.0763
ssc-miR-30a-5p	ALG9	ALG9 alpha-1,2-mannosyltransferase [Source:NCBI gene (formerly Entrezgene);Acc:100519965]	3.17	0.0763
ssc-miR-30a-5p	ALKBH1	alkB homolog 1, histone H2A dioxygenase [Source:VGNC Symbol;Acc:VGNC:85257]	3.17	0.0763
ssc-miR-30a-5p	ALPK3	alpha kinase 3 [Source:HGNC Symbol;Acc:HGNC:17574]	3.17	0.0763
ssc-miR-30a-5p	AMER1	APC membrane recruitment protein 1 [Source:VGNC Symbol;Acc:VGNC:85276]	3.17	0.0763
ssc-miR-30a-5p	AMOT	angiomotin [Source:VGNC Symbol;Acc:VGNC:85283]	3.17	0.0763
ssc-miR-30a-5p	AMOTL1	angiomotin like 1 [Source:VGNC Symbol;Acc:VGNC:85284]	3.17	0.0763
ssc-miR-30a-5p	AMOTL2	angiomotin like 2 [Source:VGNC Symbol;Acc:VGNC:85285]	3.17	0.0763
ssc-miR-30a-5p	ANGEL2	angel homolog 2 [Source:VGNC Symbol;Acc:VGNC:85302]	3.17	0.0763
ssc-miR-30a-5p	ANKHD1	ankyrin repeat and KH domain containing 1 [Source:NCBI gene (formerly Entrezgene);Acc:100512270]	3.17	0.0763
ssc-miR-30a-5p	ANKRA2	ankyrin repeat family A member 2 [Source:VGNC Symbol;Acc:VGNC:85320]	3.17	0.0763
ssc-miR-30a-5p	ANKRD17	ankyrin repeat domain 17 [Source:VGNC Symbol;Acc:VGNC:85328]	3.17	0.0763
ssc-miR-30a-5p	ANKRD26	hypothetical gene	3.17	0.0763
ssc-miR-30a-5p	ANKRD52	ankyrin repeat domain 52 [Source:VGNC Symbol;Acc:VGNC:85342]	3.17	0.0763
ssc-miR-30a-5p	ANKS4B	ankyrin repeat and sterile alpha motif domain containing 4B [Source:VGNC Symbol;Acc:VGNC:85350]	3.17	0.0763

ssc-miR-30a-5p	ANO4	anoctamin 4 [Source:VGNC Symbol;Acc:VGNC:85357]	3.17	0.0763
ssc-miR-30a-5p	AP1B1	adaptor related protein complex 1 subunit beta 1 [Source:VGNC Symbol;Acc:VGNC:85377]	3.17	0.0763
ssc-miR-30a-5p	AP1S2	adaptor related protein complex 1 subunit sigma 2 [Source:VGNC Symbol;Acc:VGNC:85382]	3.17	0.0763
ssc-miR-30a-5p	AP2A1	adaptor related protein complex 2 subunit alpha 1 [Source:VGNC Symbol;Acc:VGNC:85383]	3.17	0.0763
ssc-miR-30a-5p	AP3M1	adaptor related protein complex 3 subunit mu 1 [Source:VGNC Symbol;Acc:VGNC:85390]	3.17	0.0763
ssc-miR-30a-5p	AP3S1	hypothetical gene	3.17	0.0763
ssc-miR-30a-5p	AP4E1	adaptor related protein complex 4 subunit epsilon 1 [Source:VGNC Symbol;Acc:VGNC:85392]	3.17	0.0763
ssc-miR-30a-5p	AP5M1	adaptor related protein complex 5 subunit mu 1 [Source:VGNC Symbol;Acc:VGNC:85396]	3.17	0.0763
ssc-miR-30a-5p	APBA1	amyloid beta protein binding family A member 1 [Source:VGNC Symbol;Acc:VGNC:85399]	3.17	0.0763
ssc-miR-30a-5p	AQR	aquarius intron-binding spliceosomal factor [Source:VGNC Symbol;Acc:VGNC:85437]	3.17	0.0763
ssc-miR-30a-5p	ARAP2	ArfGAP with RhoGAP domain, ankyrin repeat and PH domain 2 [Source:VGNC Symbol;Acc:VGNC:85440]	3.17	0.0763
ssc-miR-30a-5p	ARF4	ADP ribosylation factor 4 [Source:VGNC Symbol;Acc:VGNC:85446]	3.17	0.0763
ssc-miR-30a-5p	ARHGAP26	Rho GTPase activating protein 26 [Source:VGNC Symbol;Acc:VGNC:85465]	3.17	0.0763
ssc-miR-30a-5p	ARHGEF3	Rho guanine nucleotide exchange factor 3 [Source:VGNC Symbol;Acc:VGNC:85496]	3.17	0.0763
ssc-miR-30a-5p	ARHGEF6	Rac/Cdc42 guanine nucleotide exchange factor 6 [Source:VGNC Symbol;Acc:VGNC:85502]	3.17	0.0763
ssc-miR-30a-5p	ARID1A	AT-rich interaction domain 1A [Source:VGNC Symbol;Acc:VGNC:85505]	3.17	0.0763
ssc-miR-30a-5p	ARID3A	AT-rich interaction domain 3A [Source:VGNC Symbol;Acc:VGNC:85507]	3.17	0.0763
ssc-miR-30a-5p	ARID4A	AT-rich interaction domain 4A [Source:VGNC Symbol;Acc:VGNC:85509]	3.17	0.0763
ssc-miR-30a-5p	ARID4B	AT-rich interaction domain 4B [Source:HGNC Symbol;Acc:HGNC:15550]	3.17	0.0763
ssc-miR-30a-5p	ARID5B	AT-rich interaction domain 5B [Source:VGNC Symbol;Acc:VGNC:85511]	3.17	0.0763
ssc-miR-30a-5p	ARIH1	ariadne RBR E3 ubiquitin protein ligase 1 [Source:HGNC Symbol;Acc:HGNC:689]	3.17	0.0763
ssc-miR-30a-5p	ARL10	ADP ribosylation factor like GTPase 10 [Source:VGNC Symbol;Acc:VGNC:85514]	3.17	0.0763
ssc-miR-30a-5p	ARL15	ADP ribosylation factor like GTPase 15 [Source:VGNC Symbol;Acc:VGNC:85520]	3.17	0.0763
ssc-miR-30a-5p	ARL4A	ADP ribosylation factor like GTPase 4A [Source:VGNC Symbol;Acc:VGNC:85522]	3.17	0.0763
ssc-miR-30a-5p	ARL4C	ADP ribosylation factor like GTPase 4C [Source:VGNC Symbol;Acc:VGNC:96419]	3.17	0.0763
ssc-miR-30a-5p	ARL6IP5	ADP ribosylation factor like GTPase 6 interacting protein 5 [Source:VGNC Symbol;Acc:VGNC:103904]	3.17	0.0763
ssc-miR-30a-5p	ARL6IP6	ADP ribosylation factor like GTPase 6 interacting protein 6 [Source:VGNC Symbol;Acc:VGNC:96029]	3.17	0.0763
ssc-miR-30a-5p	ASB14	ankyrin repeat and SOCS box containing 14 [Source:VGNC Symbol;Acc:VGNC:85562]	3.17	0.0763
ssc-miR-30a-5p	ASB3	ankyrin repeat and SOCS box containing 3 [Source:VGNC Symbol;Acc:VGNC:97034]	3.17	0.0763
ssc-miR-30a-5p	ASB4	ankyrin repeat and SOCS box containing 4 [Source:VGNC Symbol;Acc:VGNC:97891]	3.17	0.0763
ssc-miR-30a-5p	ASCC1	activating signal cointegrator 1 complex subunit 1 [Source:VGNC Symbol;Acc:VGNC:85569]	3.17	0.0763
ssc-miR-30a-5p	ASCC3	activating signal cointegrator 1 complex subunit 3 [Source:VGNC Symbol;Acc:VGNC:85571]	3.17	0.0763
ssc-miR-30a-5p	ASXL3	ASXL transcriptional regulator 3 [Source:VGNC Symbol;Acc:VGNC:85596]	3.17	0.0763
ssc-miR-30a-5p	ATAD2B	ATPase family AAA domain containing 2B [Source:VGNC Symbol;Acc:VGNC:85600]	3.17	0.0763
ssc-miR-30a-5p	ATAD5	ATPase family AAA domain containing 5 [Source:VGNC Symbol;Acc:VGNC:85601]	3.17	0.0763
ssc-miR-30a-5p	ATF1	activating transcription factor 1 [Source:VGNC Symbol;Acc:VGNC:85605]	3.17	0.0763
ssc-miR-30a-5p	ATF7IP2	activating transcription factor 7 interacting protein 2 [Source:VGNC Symbol;Acc:VGNC:85612]	3.17	0.0763
ssc-miR-30a-5p	ATG12	autophagy related 12 [Source:VGNC Symbol;Acc:VGNC:85615]	3.17	0.0763
ssc-miR-30a-5p	ATG5	autophagy related 5 [Source:VGNC Symbol;Acc:VGNC:103029]	3.17	0.0763
ssc-miR-30a-5p	ATL2	atlastin GTPase 2 [Source:VGNC Symbol;Acc:VGNC:85628]	3.17	0.0763
ssc-miR-30a-5p	ATP2A2	ATPase sarcoplasmic/endoplasmic reticulum Ca2+ transporting 2 [Source:VGNC Symbol;Acc:VGNC:85647]	3.17	0.0763
ssc-miR-30a-5p	ATP2B1	ATPase plasma membrane Ca2+ transporting 1 [Source:VGNC Symbol;Acc:VGNC:103219]	3.17	0.0763
ssc-miR-30a-5p	ATP2B2	ATPase plasma membrane Ca2+ transporting 2 [Source:VGNC Symbol;Acc:VGNC:85649]	3.17	0.0763
ssc-miR-30a-5p	ATP6V0A1	ATPase H+ transporting V0 subunit a1 [Source:VGNC Symbol;Acc:VGNC:85663]	3.17	0.0763
ssc-miR-30a-5p	ATP6V0D1	ATPase H+ transporting V0 subunit d1 [Source:VGNC Symbol;Acc:VGNC:85667]	3.17	0.0763
ssc-miR-30a-5p	ATP6V1C1	ATPase H+ transporting V1 subunit C1 [Source:VGNC Symbol;Acc:VGNC:85672]	3.17	0.0763
ssc-miR-30a-5p	ATP8A1	ATPase phospholipid transporting 8A1 [Source:VGNC Symbol;Acc:VGNC:97894]	3.17	0.0763
ssc-miR-30a-5p	ATP8A2	ATPase phospholipid transporting 8A2 [Source:HGNC Symbol;Acc:HGNC:13533]	3.17	0.0763
ssc-miR-30a-5p	ATP8B1	ATPase phospholipid transporting 8B1 [Source:VGNC Symbol;Acc:VGNC:85679]	3.17	0.0763

ssc-miR-30a-5p	ATP8B2	ATPase phospholipid transporting 8B2 [Source:VGNC Symbol;Acc:VGNC:85680]	3.17	0.0763
ssc-miR-30a-5p	ATRN	attractin [Source:VGNC Symbol;Acc:VGNC:96479]	3.17	0.0763
ssc-miR-30a-5p	ATRNL1	attractin like 1 [Source:VGNC Symbol;Acc:VGNC:85685]	3.17	0.0763
ssc-miR-30a-5p	ATXN1	ataxin 1 [Source:VGNC Symbol;Acc:VGNC:85687]	3.17	0.0763
ssc-miR-30a-5p	ATXN3	hypothetical gene	3.17	0.0763
ssc-miR-30a-5p	AVEN	apoptosis and caspase activation inhibitor [Source:HGNC Symbol;Acc:HGNC:13509]	3.17	0.0763
ssc-miR-30a-5p	AVL9	AVL9 cell migration associated [Source:VGNC Symbol;Acc:VGNC:85701]	3.17	0.0763
ssc-miR-30a-5p	AZIN1	antizyme inhibitor 1 [Source:VGNC Symbol;Acc:VGNC:85712]	3.17	0.0763
ssc-miR-30a-5p	B3GALT5	hypothetical gene	3.17	0.0763
ssc-miR-30a-5p	B3GALT6	beta-1,3-galactosyltransferase 6 [Source:VGNC Symbol;Acc:VGNC:85718]	3.17	0.0763
ssc-miR-30a-5p	B3GALTL	hypothetical gene	3.17	0.0763
ssc-miR-30a-5p	B3GAT1	beta-1,3-glucuronyltransferase 1 [Source:VGNC Symbol;Acc:VGNC:85719]	3.17	0.0763
ssc-miR-30a-5p	B3GNT5	UDP-GlcNAc:betaGal beta-1,3-N-acetylglucosaminyltransferase 5 [Source:VGNC Symbol;Acc:VGNC:85726]	3.17	0.0763
ssc-miR-30a-5p	B4GALT5	beta-1,4-galactosyltransferase 5 [Source:VGNC Symbol;Acc:VGNC:96499]	3.17	0.0763
ssc-miR-30a-5p	B4GALT6	beta-1,4-galactosyltransferase 6 [Source:VGNC Symbol;Acc:VGNC:85734]	3.17	0.0763
ssc-miR-30a-5p	BACH1	BTB domain and CNC homolog 1 [Source:VGNC Symbol;Acc:VGNC:85741]	3.17	0.0763
ssc-miR-30a-5p	BAHD1	bromo adjacent homology domain containing 1 [Source:VGNC Symbol;Acc:VGNC:85747]	3.17	0.0763
ssc-miR-30a-5p	BAZ1A	bromodomain adjacent to zinc finger domain 1A [Source:VGNC Symbol;Acc:VGNC:85761]	3.17	0.0763
ssc-miR-30a-5p	BAZ2B	bromodomain adjacent to zinc finger domain 2B [Source:HGNC Symbol;Acc:HGNC:963]	3.17	0.0763
ssc-miR-30a-5p	BBX	BBX high mobility group box domain containing [Source:VGNC Symbol;Acc:VGNC:85766]	3.17	0.0763
ssc-miR-30a-5p	BCL11B	BAF chromatin remodeling complex subunit BCL11B [Source:VGNC Symbol;Acc:VGNC:96563]	3.17	0.0763
ssc-miR-30a-5p	BCL2	BCL2 apoptosis regulator [Source:HGNC Symbol;Acc:HGNC:990]	3.17	0.0763
ssc-miR-30a-5p	BCL2L11	BCL2 like 11 [Source:NCBI gene (formerly Entrezgene);Acc:396632]	3.17	0.0763
ssc-miR-30a-5p	BCL6	BCL6 transcription repressor [Source:VGNC Symbol;Acc:VGNC:96565]	3.17	0.0763
ssc-miR-30a-5p	BCL9	BCL9 transcription coactivator [Source:VGNC Symbol;Acc:VGNC:96567]	3.17	0.0763
ssc-miR-30a-5p	BCLAF1	BCL2 associated transcription factor 1 [Source:VGNC Symbol;Acc:VGNC:85787]	3.17	0.0763
ssc-miR-30a-5p	BCOR	BCL6 corepressor [Source:HGNC Symbol;Acc:HGNC:20893]	3.17	0.0763
ssc-miR-30a-5p	BDNF	brain derived neurotrophic factor [Source:VGNC Symbol;Acc:VGNC:85795]	3.17	0.0763
ssc-miR-30a-5p	BDP1	B double prime 1, subunit of RNA polymerase III transcription initiation factor IIIB [Source:VGNC Symbol;Acc:VGNC:85796]	3.17	0.0763
ssc-miR-30a-5p	BEAN1	brain expressed associated with NEDD4 1 [Source:VGNC Symbol;Acc:VGNC:85797]	3.17	0.0763
ssc-miR-30a-5p	BECN1	beclin 1 [Source:VGNC Symbol;Acc:VGNC:85798]	3.17	0.0763
ssc-miR-30a-5p	BEND3	BEN domain containing 3 [Source:VGNC Symbol;Acc:VGNC:85800]	3.17	0.0763
ssc-miR-30a-5p	BEND4	BEN domain containing 4 [Source:VGNC Symbol;Acc:VGNC:85801]	3.17	0.0763
ssc-miR-30a-5p	BEND7	BEN domain containing 7 [Source:VGNC Symbol;Acc:VGNC:106445]	3.17	0.0763
ssc-miR-30a-5p	BIRC6	baculoviral IAP repeat containing 6 [Source:VGNC Symbol;Acc:VGNC:97038]	3.17	0.0763
ssc-miR-30a-5p	BMP7	bone morphotic protein 7 [Source:VGNC Symbol;Acc:VGNC:95491]	3.17	0.0763
ssc-miR-30a-5p	BMPR2	bone morphotic protein receptor type 2 [Source:VGNC Symbol;Acc:VGNC:95494]	3.17	0.0763
ssc-miR-30a-5p	BNC1	basonuclin 1 [Source:VGNC Symbol;Acc:VGNC:85851]	3.17	0.0763
ssc-miR-30a-5p	BNC2	basonuclin 2 [Source:VGNC Symbol;Acc:VGNC:85852]	3.17	0.0763
ssc-miR-30a-5p	BNIP3L	BCL2 interacting protein 3 like [Source:VGNC Symbol;Acc:VGNC:85856]	3.17	0.0763
ssc-miR-30a-5p	BRAP	BRCA1 associated protein [Source:VGNC Symbol;Acc:VGNC:85867]	3.17	0.0763
ssc-miR-30a-5p	BRD1	bromodomain containing 1 [Source:VGNC Symbol;Acc:VGNC:85871]	3.17	0.0763
ssc-miR-30a-5p	BRWD1	bromodomain and WD repeat domain containing 1 [Source:VGNC Symbol;Acc:VGNC:108153]	3.17	0.0763
ssc-miR-30a-5p	BRWD3	bromodomain and WD repeat domain containing 3 [Source:VGNC Symbol;Acc:VGNC:85890]	3.17	0.0763
ssc-miR-30a-5p	BSN	bassoon presynaptic cytomatrix protein [Source:VGNC Symbol;Acc:VGNC:85893]	3.17	0.0763
ssc-miR-30a-5p	BTAF1	B-TFIID TATA-box binding protein associated factor 1 [Source:VGNC Symbol;Acc:VGNC:85899]	3.17	0.0763
ssc-miR-30a-5p	BTBD10	hypothetical gene	3.17	0.0763
ssc-miR-30a-5p	BTBD7	BTB domain containing 7 [Source:VGNC Symbol;Acc:VGNC:85908]	3.17	0.0763
ssc-miR-30a-5p	BZW1	basic leucine zipper and W2 domains 1 [Source:VGNC Symbol;Acc:VGNC:95885]	3.17	0.0763

ssc-miR-30a-5p	C10orf105	chromosome 14 C10orf105 homolog [Source:VGNC Symbol;Acc:VGNC:85940]	3.17	0.0763
ssc-miR-30a-5p	C10orf11	hypothetical gene	3.17	0.0763
ssc-miR-30a-5p	C10orf118	hypothetical gene	3.17	0.0763
ssc-miR-30a-5p	C10orf25	hypothetical gene	3.17	0.0763
ssc-miR-30a-5p	C10orf76	hypothetical gene	3.17	0.0763
ssc-miR-30a-5p	C11orf84	hypothetical gene	3.17	0.0763
ssc-miR-30a-5p	C14orf28	chromosome 1 C14orf28 homolog [Source:VGNC Symbol;Acc:VGNC:85958]	3.17	0.0763
ssc-miR-30a-5p	C16orf52	hypothetical gene	3.17	0.0763
ssc-miR-30a-5p	C16orf87	chromosome 6 C16orf87 homolog [Source:VGNC Symbol;Acc:VGNC:96927]	3.17	0.0763
ssc-miR-30a-5p	C20orf112	hypothetical gene	3.17	0.0763
ssc-miR-30a-5p	C21orf91	chromosome 13 C21orf91 homolog [Source:VGNC Symbol;Acc:VGNC:85930]	3.17	0.0763
ssc-miR-30a-5p	C3orf18	chromosome 13 C3orf18 homolog [Source:VGNC Symbol;Acc:VGNC:85932]	3.17	0.0763
ssc-miR-30a-5p	C3orf58	hypothetical gene	3.17	0.0763
ssc-miR-30a-5p	C4orf19	chromosome 8 C4orf19 homolog [Source:VGNC Symbol;Acc:VGNC:86080]	3.17	0.0763
ssc-miR-30a-5p	C7orf43	hypothetical gene	3.17	0.0763
ssc-miR-30a-5p	C7orf55-LUC7L2	hypothetical gene	3.17	0.0763
ssc-miR-30a-5p	C7orf60	hypothetical gene	3.17	0.0763
ssc-miR-30a-5p	C8orf4	hypothetical gene	3.17	0.0763
ssc-miR-30a-5p	C8orf44-SGK3	hypothetical gene	3.17	0.0763
ssc-miR-30a-5p	C9orf170	hypothetical gene	3.17	0.0763
ssc-miR-30a-5p	C9orf41	hypothetical gene	3.17	0.0763
ssc-miR-30a-5p	C9orf72	chromosome 10 C9orf72 homolog [Source:VGNC Symbol;Acc:VGNC:96173]	3.17	0.0763
ssc-miR-30a-5p	CA10	carbonic anhydrase 10 [Source:VGNC Symbol;Acc:VGNC:86096]	3.17	0.0763
ssc-miR-30a-5p	CABLES2	Cdk5 and Abl enzyme substrate 2 [Source:VGNC Symbol;Acc:VGNC:95753]	3.17	0.0763
ssc-miR-30a-5p	CACHD1	cache domain containing 1 [Source:VGNC Symbol;Acc:VGNC:86116]	3.17	0.0763
ssc-miR-30a-5p	CACNA1C	calcium voltage-gated channel subunit alpha1 C [Source:HGNC Symbol;Acc:HGNC:1390]	3.17	0.0763
ssc-miR-30a-5p	CACNA1D	calcium voltage-gated channel subunit alpha1 D [Source:VGNC Symbol;Acc:VGNC:86117]	3.17	0.0763
ssc-miR-30a-5p	CACNB2	calcium voltage-gated channel auxiliary subunit beta 2 [Source:VGNC Symbol;Acc:VGNC:95599]	3.17	0.0763
ssc-miR-30a-5p	CACUL1	CDK2 associated cullin domain 1 [Source:VGNC Symbol;Acc:VGNC:86132]	3.17	0.0763
ssc-miR-30a-5p	CACYBP	calcyclin binding protein [Source:VGNC Symbol;Acc:VGNC:86133]	3.17	0.0763
ssc-miR-30a-5p	CADM1	cell adhesion molecule 1 [Source:VGNC Symbol;Acc:VGNC:86134]	3.17	0.0763
ssc-miR-30a-5p	CADM2	cell adhesion molecule 2 [Source:VGNC Symbol;Acc:VGNC:97910]	3.17	0.0763
ssc-miR-30a-5p	CADPS	hypothetical gene	3.17	0.0763
ssc-miR-30a-5p	CALB2	calbindin 2 [Source:VGNC Symbol;Acc:VGNC:86139]	3.17	0.0763
ssc-miR-30a-5p	CALCOCO1	calcium binding and coiled-coil domain 1 [Source:VGNC Symbol;Acc:VGNC:86140]	3.17	0.0763
ssc-miR-30a-5p	CALCR	calcitonin receptor [Source:VGNC Symbol;Acc:VGNC:86142]	3.17	0.0763
ssc-miR-30a-5p	CALD1	caldesmon 1 [Source:VGNC Symbol;Acc:VGNC:86143]	3.17	0.0763
ssc-miR-30a-5p	CALU	calumenin [Source:VGNC Symbol;Acc:VGNC:86151]	3.17	0.0763
ssc-miR-30a-5p	CAMK2D	calcium/calmodulin dependent protein kinase II delta [Source:VGNC Symbol;Acc:VGNC:97911]	3.17	0.0763
ssc-miR-30a-5p	CAMK2N1	calcium/calmodulin dependent protein kinase II inhibitor 1 [Source:VGNC Symbol;Acc:VGNC:96935]	3.17	0.0763
ssc-miR-30a-5p	CAMK2N2	calcium/calmodulin dependent protein kinase II inhibitor 2 [Source:HGNC Symbol;Acc:HGNC:24197]	3.17	0.0763
ssc-miR-30a-5p	CAMK4	calcium/calmodulin dependent protein kinase IV [Source:VGNC Symbol;Acc:VGNC:99602]	3.17	0.0763
ssc-miR-30a-5p	CAMKK2	calcium/calmodulin dependent protein kinase kinase 2 [Source:VGNC Symbol;Acc:VGNC:86158]	3.17	0.0763
ssc-miR-30a-5p	CAND1	cullin associated and neddylation dissociated 1 [Source:VGNC Symbol;Acc:VGNC:97912]	3.17	0.0763
ssc-miR-30a-5p	CANT1	calcium activated nucleotidase 1 [Source:VGNC Symbol;Acc:VGNC:86166]	3.17	0.0763
ssc-miR-30a-5p	CAPN5	calpain 5 [Source:VGNC Symbol;Acc:VGNC:86175]	3.17	0.0763
ssc-miR-30a-5p	CAPN7	calpain 7 [Source:NCBI gene (formerly Entrezgene);Acc:100037936]	3.17	0.0763
ssc-miR-30a-5p	CAPRIN1	cell cycle associated protein 1 [Source:VGNC Symbol;Acc:VGNC:86178]	3.17	0.0763
ssc-miR-30a-5p	CAPZA1	capping actin protein of muscle Z-line subunit alpha 1 [Source:VGNC Symbol;Acc:VGNC:86183]	3.17	0.0763

ssc-miR-30a-5p	CARF	calcium responsive transcription factor [Source:VGNC Symbol;Acc:VGNC:95615]	3.17	0.0763
ssc-miR-30a-5p	CARS	hypothetical gene	3.17	0.0763
ssc-miR-30a-5p	CASD1	CAS1 domain containing 1 [Source:VGNC Symbol;Acc:VGNC:86199]	3.17	0.0763
ssc-miR-30a-5p	CAT	catalase [Source:VGNC Symbol;Acc:VGNC:86208]	3.17	0.0763
ssc-miR-30a-5p	CBFB	core-binding factor subunit beta [Source:VGNC Symbol;Acc:VGNC:86221]	3.17	0.0763
ssc-miR-30a-5p	CBLB	Cbl proto-onco B [Source:VGNC Symbol;Acc:VGNC:86223]	3.17	0.0763
ssc-miR-30a-5p	CBX2	chromobox 2 [Source:VGNC Symbol;Acc:VGNC:86230]	3.17	0.0763
ssc-miR-30a-5p	CBX3	chromobox 3 [Source:HGNC Symbol;Acc:HGNC:1553]	3.17	0.0763
ssc-miR-30a-5p	CBX7	chromobox 7 [Source:HGNC Symbol;Acc:HGNC:1557]	3.17	0.0763
ssc-miR-30a-5p	CBX8	chromobox 8 [Source:VGNC Symbol;Acc:VGNC:86233]	3.17	0.0763
ssc-miR-30a-5p	CCDC117	coiled-coil domain containing 117 [Source:VGNC Symbol;Acc:VGNC:86248]	3.17	0.0763
ssc-miR-30a-5p	CCDC120	coiled-coil domain containing 120 [Source:VGNC Symbol;Acc:VGNC:86249]	3.17	0.0763
ssc-miR-30a-5p	CCDC148	hypothetical gene	3.17	0.0763
ssc-miR-30a-5p	CCDC160	coiled-coil domain containing 160 [Source:VGNC Symbol;Acc:VGNC:86269]	3.17	0.0763
ssc-miR-30a-5p	CCDC178	coiled-coil domain containing 178 [Source:VGNC Symbol;Acc:VGNC:86276]	3.17	0.0763
ssc-miR-30a-5p	CCDC43	coiled-coil domain containing 43 [Source:VGNC Symbol;Acc:VGNC:98979]	3.17	0.0763
ssc-miR-30a-5p	CCDC6	coiled-coil domain containing 6 [Source:VGNC Symbol;Acc:VGNC:86304]	3.17	0.0763
ssc-miR-30a-5p	CCDC71L	coiled-coil domain containing 71 like [Source:HGNC Symbol;Acc:HGNC:26685]	3.17	0.0763
ssc-miR-30a-5p	CCDC97	coiled-coil domain containing 97 [Source:VGNC Symbol;Acc:VGNC:86331]	3.17	0.0763
ssc-miR-30a-5p	CCNE2	cyclin E2 [Source:VGNC Symbol;Acc:VGNC:86355]	3.17	0.0763
ssc-miR-30a-5p	CCNF	cyclin F [Source:VGNC Symbol;Acc:VGNC:86356]	3.17	0.0763
ssc-miR-30a-5p	CCNJL	cyclin J like [Source:VGNC Symbol;Acc:VGNC:86361]	3.17	0.0763
ssc-miR-30a-5p	CCNK	cyclin K [Source:VGNC Symbol;Acc:VGNC:86362]	3.17	0.0763
ssc-miR-30a-5p	CCNT2	cyclin T2 [Source:VGNC Symbol;Acc:VGNC:103916]	3.17	0.0763
ssc-miR-30a-5p	CCNY	cyclin Y [Source:VGNC Symbol;Acc:VGNC:95998]	3.17	0.0763
ssc-miR-30a-5p	CCP110	centriolar coiled-coil protein 110 [Source:VGNC Symbol;Acc:VGNC:86367]	3.17	0.0763
ssc-miR-30a-5p	CCPG1	cell cycle progression 1 [Source:VGNC Symbol;Acc:VGNC:86368]	3.17	0.0763
ssc-miR-30a-5p	CCSER1	coiled-coil serine rich protein 1 [Source:VGNC Symbol;Acc:VGNC:86378]	3.17	0.0763
ssc-miR-30a-5p	CCZ1B	hypothetical gene	3.17	0.0763
ssc-miR-30a-5p	CD226	CD226 molecule [Source:VGNC Symbol;Acc:VGNC:86399]	3.17	0.0763
ssc-miR-30a-5p	CD2AP	CD2 associated protein [Source:VGNC Symbol;Acc:VGNC:86406]	3.17	0.0763
ssc-miR-30a-5p	CD302	CD302 molecule [Source:NCBI gene (formerly Entrezgene);Acc:100126280]	3.17	0.0763
ssc-miR-30a-5p	CDC37L1	cell division cycle 37 like 1 [Source:VGNC Symbol;Acc:VGNC:86451]	3.17	0.0763
ssc-miR-30a-5p	CDC42BPA	CDC42 binding protein kinase alpha [Source:VGNC Symbol;Acc:VGNC:95847]	3.17	0.0763
ssc-miR-30a-5p	CDC7	cell division cycle 7 [Source:VGNC Symbol;Acc:VGNC:97922]	3.17	0.0763
ssc-miR-30a-5p	CDCA7	cell division cycle associated 7 [Source:VGNC Symbol;Acc:VGNC:95623]	3.17	0.0763
ssc-miR-30a-5p	CDH13	cadherin 13 [Source:VGNC Symbol;Acc:VGNC:86477]	3.17	0.0763
ssc-miR-30a-5p	CDH20	cadherin 20 [Source:VGNC Symbol;Acc:VGNC:86484]	3.17	0.0763
ssc-miR-30a-5p	CDK12	cyclin dependent kinase 12 [Source:VGNC Symbol;Acc:VGNC:86496]	3.17	0.0763
ssc-miR-30a-5p	CDYL2	chromodomain Y like 2 [Source:VGNC Symbol;Acc:VGNC:86529]	3.17	0.0763
ssc-miR-30a-5p	CEACAM1	hypothetical gene	3.17	0.0763
ssc-miR-30a-5p	CECR2	CECR2 histone acetyl-lysine reader [Source:VGNC Symbol;Acc:VGNC:96576]	3.17	0.0763
ssc-miR-30a-5p	CECR6	hypothetical gene	3.17	0.0763
ssc-miR-30a-5p	CELF1	CUGBP Elav-like family member 1 [Source:VGNC Symbol;Acc:VGNC:86537]	3.17	0.0763
ssc-miR-30a-5p	CELF2	hypothetical gene	3.17	0.0763
ssc-miR-30a-5p	CELF3	CUGBP Elav-like family member 3 [Source:VGNC Symbol;Acc:VGNC:86538]	3.17	0.0763
ssc-miR-30a-5p	CELF5	CUGBP Elav-like family member 5 [Source:VGNC Symbol;Acc:VGNC:86540]	3.17	0.0763
ssc-miR-30a-5p	CELSR3	cadherin EGF LAG seven-pass G-type receptor 3 [Source:VGNC Symbol;Acc:VGNC:86541]	3.17	0.0763
ssc-miR-30a-5p	CEP170	centrosomal protein 170 [Source:VGNC Symbol;Acc:VGNC:97925]	3.17	0.0763

ssc-miR-30a-5p	CEP170B	centrosomal protein 170B [Source:VGNC Symbol;Acc:VGNC:86564]	3.17	0.0763
ssc-miR-30a-5p	CEP350	centrosomal protein 350 [Source:HGNC Symbol;Acc:HGNC:24238]	3.17	0.0763
ssc-miR-30a-5p	CEP41	centrosomal protein 41 [Source:VGNC Symbol;Acc:VGNC:86569]	3.17	0.0763
ssc-miR-30a-5p	CEP44	centrosomal protein 44 [Source:VGNC Symbol;Acc:VGNC:86571]	3.17	0.0763
ssc-miR-30a-5p	CEP76	centrosomal protein 76 [Source:VGNC Symbol;Acc:VGNC:96947]	3.17	0.0763
ssc-miR-30a-5p	CEP85L	centrosomal protein 85 like [Source:VGNC Symbol;Acc:VGNC:86580]	3.17	0.0763
ssc-miR-30a-5p	CERS5	ceramide synthase 5 [Source:VGNC Symbol;Acc:VGNC:97926]	3.17	0.0763
ssc-miR-30a-5p	CERS6	ceramide synthase 6 [Source:VGNC Symbol;Acc:VGNC:96025]	3.17	0.0763
ssc-miR-30a-5p	CFDP1	craniofacial development protein 1 [Source:VGNC Symbol;Acc:VGNC:86610]	3.17	0.0763
ssc-miR-30a-5p	CFL2	cofilin 2 [Source:VGNC Symbol;Acc:VGNC:86611]	3.17	0.0763
ssc-miR-30a-5p	CHD1	chromodomain helicase DNA binding protein 1 [Source:VGNC Symbol;Acc:VGNC:86629]	3.17	0.0763
ssc-miR-30a-5p	CHD5	chromodomain helicase DNA binding protein 5 [Source:VGNC Symbol;Acc:VGNC:86632]	3.17	0.0763
ssc-miR-30a-5p	CHD7	chromodomain helicase DNA binding protein 7 [Source:VGNC Symbol;Acc:VGNC:86633]	3.17	0.0763
ssc-miR-30a-5p	CHD9	chromodomain helicase DNA binding protein 9 [Source:VGNC Symbol;Acc:VGNC:86635]	3.17	0.0763
ssc-miR-30a-5p	CHFR	checkpoint with forkhead and ring finger domains [Source:HGNC Symbol;Acc:HGNC:20455]	3.17	0.0763
ssc-miR-30a-5p	CHIC1	cysteine rich hydrophobic domain 1 [Source:VGNC Symbol;Acc:VGNC:86642]	3.17	0.0763
ssc-miR-30a-5p	CHIC2	cysteine rich hydrophobic domain 2 [Source:VGNC Symbol;Acc:VGNC:86643]	3.17	0.0763
ssc-miR-30a-5p	CHKA	choline kinase alpha [Source:VGNC Symbol;Acc:VGNC:86646]	3.17	0.0763
ssc-miR-30a-5p	CHL1	cell adhesion molecule L1 like [Source:VGNC Symbol;Acc:VGNC:108639]	3.17	0.0763
ssc-miR-30a-5p	CHMP2B	charged multivesicular body protein 2B [Source:VGNC Symbol;Acc:VGNC:86650]	3.17	0.0763
ssc-miR-30a-5p	CHRM3	cholinergic receptor muscarinic 3 [Source:VGNC Symbol;Acc:VGNC:86664]	3.17	0.0763
ssc-miR-30a-5p	CHST1	carbohydrate sulfotransferase 1 [Source:VGNC Symbol;Acc:VGNC:86673]	3.17	0.0763
ssc-miR-30a-5p	CHST12	carbohydrate sulfotransferase 12 [Source:HGNC Symbol;Acc:HGNC:17423]	3.17	0.0763
ssc-miR-30a-5p	CHST2	carbohydrate sulfotransferase 2 [Source:VGNC Symbol;Acc:VGNC:86678]	3.17	0.0763
ssc-miR-30a-5p	CLCC1	chloride channel CLIC like 1 [Source:VGNC Symbol;Acc:VGNC:86724]	3.17	0.0763
ssc-miR-30a-5p	CLCF1	cardiotrophin like cytokine factor 1 [Source:VGNC Symbol;Acc:VGNC:86725]	3.17	0.0763
ssc-miR-30a-5p	CLCN3	chloride voltage-gated channel 3 [Source:VGNC Symbol;Acc:VGNC:86727]	3.17	0.0763
ssc-miR-30a-5p	CLDN19	claudin 19 [Source:VGNC Symbol;Acc:VGNC:86736]	3.17	0.0763
ssc-miR-30a-5p	CLEC14A	C-type lectin domain containing 14A [Source:VGNC Symbol;Acc:VGNC:86747]	3.17	0.0763
ssc-miR-30a-5p	CLN8	CLN8 transmembrane ER and ERGIC protein [Source:VGNC Symbol;Acc:VGNC:103067]	3.17	0.0763
ssc-miR-30a-5p	CLOCK	clock circadian regulator [Source:VGNC Symbol;Acc:VGNC:86774]	3.17	0.0763
ssc-miR-30a-5p	CLRN1	clarin 1 [Source:VGNC Symbol;Acc:VGNC:97933]	3.17	0.0763
ssc-miR-30a-5p	CLSPN	claspin [Source:VGNC Symbol;Acc:VGNC:86783]	3.17	0.0763
ssc-miR-30a-5p	CMPK2	cytidine/uridine monophosphate kinase 2 [Source:VGNC Symbol;Acc:VGNC:86801]	3.17	0.0763
ssc-miR-30a-5p	CMTM4	CKLF like MARVEL transmembrane domain containing 4 [Source:VGNC Symbol;Acc:VGNC:86804]	3.17	0.0763
ssc-miR-30a-5p	CNGB3	cyclic nucleotide gated channel subunit beta 3 [Source:VGNC Symbol;Acc:VGNC:86819]	3.17	0.0763
ssc-miR-30a-5p	CNKSR2	connector enhancer of kinase suppressor of Ras 2 [Source:VGNC Symbol;Acc:VGNC:86822]	3.17	0.0763
ssc-miR-30a-5p	CNOT6	CCR4-NOT transcription complex subunit 6 [Source:VGNC Symbol;Acc:VGNC:86837]	3.17	0.0763
ssc-miR-30a-5p	CNTN2	contactin 2 [Source:VGNC Symbol;Acc:VGNC:108577]	3.17	0.0763
ssc-miR-30a-5p	CNTN4	contactin 4 [Source:VGNC Symbol;Acc:VGNC:97934]	3.17	0.0763
ssc-miR-30a-5p	COG3	component of oligomeric golgi complex 3 [Source:HGNC Symbol;Acc:HGNC:18619]	3.17	0.0763
ssc-miR-30a-5p	COG5	component of oligomeric golgi complex 5 [Source:VGNC Symbol;Acc:VGNC:86857]	3.17	0.0763
ssc-miR-30a-5p	COL13A1	collagen type XIII alpha 1 chain [Source:VGNC Symbol;Acc:VGNC:86865]	3.17	0.0763
ssc-miR-30a-5p	COL25A1	collagen type XXV alpha 1 chain [Source:HGNC Symbol;Acc:HGNC:18603]	3.17	0.0763
ssc-miR-30a-5p	COL4A3BP	hypothetical gene	3.17	0.0763
ssc-miR-30a-5p	COL9A3	collagen type IX alpha 3 chain [Source:VGNC Symbol;Acc:VGNC:108713]	3.17	0.0763
ssc-miR-30a-5p	COMT	catechol-O-methyltransferase [Source:VGNC Symbol;Acc:VGNC:107378]	3.17	0.0763
ssc-miR-30a-5p	COPS7B	COP9 signalosome subunit 7B [Source:HGNC Symbol;Acc:HGNC:16760]	3.17	0.0763
ssc-miR-30a-5p	COQ3	coenzyme Q3, methyltransferase [Source:HGNC Symbol;Acc:HGNC:18175]	3.17	0.0763

ssc-miR-30a-5p	CORO2A	coronin 2A [Source:VGNC Symbol;Acc:VGNC:86915]	3.17	0.0763
ssc-miR-30a-5p	COTL1	coactosin like F-actin binding protein 1 [Source:HGNC Symbol;Acc:HGNC:18304]	3.17	0.0763
ssc-miR-30a-5p	CPE	carboxypeptidase E [Source:VGNC Symbol;Acc:VGNC:86936]	3.17	0.0763
ssc-miR-30a-5p	CPEB2	cytoplasmic polyadenylation element binding protein 2 [Source:VGNC Symbol;Acc:VGNC:86937]	3.17	0.0763
ssc-miR-30a-5p	CPEB3	cytoplasmic polyadenylation element binding protein 3 [Source:VGNC Symbol;Acc:VGNC:86938]	3.17	0.0763
ssc-miR-30a-5p	CPEB4	cytoplasmic polyadenylation element binding protein 4 [Source:VGNC Symbol;Acc:VGNC:86939]	3.17	0.0763
ssc-miR-30a-5p	CPNE8	copine 8 [Source:VGNC Symbol;Acc:VGNC:86955]	3.17	0.0763
ssc-miR-30a-5p	CPOX	coproporphyrinogen oxidase [Source:VGNC Symbol;Acc:VGNC:86956]	3.17	0.0763
ssc-miR-30a-5p	CPSF6	cleavage and polyadenylation specific factor 6 [Source:VGNC Symbol;Acc:VGNC:86962]	3.17	0.0763
ssc-miR-30a-5p	CREB1	cAMP responsive element binding protein 1 [Source:VGNC Symbol;Acc:VGNC:96004]	3.17	0.0763
ssc-miR-30a-5p	CRKL	CRK like proto-onco, adaptor protein [Source:VGNC Symbol;Acc:VGNC:86997]	3.17	0.0763
ssc-miR-30a-5p	CRLF3	cytokine receptor like factor 3 [Source:VGNC Symbol;Acc:VGNC:86999]	3.17	0.0763
ssc-miR-30a-5p	CRMP1	collapsin response mediator protein 1 [Source:VGNC Symbol;Acc:VGNC:87000]	3.17	0.0763
ssc-miR-30a-5p	CRY2	cryptochrome circadian regulator 2 [Source:VGNC Symbol;Acc:VGNC:87012]	3.17	0.0763
ssc-miR-30a-5p	CSAD	cysteine sulfinic acid decarboxylase [Source:VGNC Symbol;Acc:VGNC:87027]	3.17	0.0763
ssc-miR-30a-5p	CSGALNACT1	chondroitin sulfate N-acetylgalactosaminyltransferase 1 [Source:HGNC Symbol;Acc:HGNC:24290]	3.17	0.0763
ssc-miR-30a-5p	CSNK1A1	casein kinase 1 alpha 1 [Source:VGNC Symbol;Acc:VGNC:99630]	3.17	0.0763
ssc-miR-30a-5p	CSNK1A1L	hypothetical gene	3.17	0.0763
ssc-miR-30a-5p	CSNK1G1	casein kinase 1 gamma 1 [Source:VGNC Symbol;Acc:VGNC:97949]	3.17	0.0763
ssc-miR-30a-5p	CSNK2A1	hypothetical gene	3.17	0.0763
ssc-miR-30a-5p	CTDSPL2	CTD small phosphatase like 2 [Source:VGNC Symbol;Acc:VGNC:87060]	3.17	0.0763
ssc-miR-30a-5p	CTH	cystathionine gamma-lyase [Source:VGNC Symbol;Acc:VGNC:96961]	3.17	0.0763
ssc-miR-30a-5p	CTHRC1	collagen triple helix repeat containing 1 [Source:VGNC Symbol;Acc:VGNC:87061]	3.17	0.0763
ssc-miR-30a-5p	CTPS1	CTP synthase 1 [Source:VGNC Symbol;Acc:VGNC:87070]	3.17	0.0763
ssc-miR-30a-5p	CTTNBP2NL	CTTNBP2 N-terminal like [Source:VGNC Symbol;Acc:VGNC:87082]	3.17	0.0763
ssc-miR-30a-5p	CUL2	cullin 2 [Source:VGNC Symbol;Acc:VGNC:96090]	3.17	0.0763
ssc-miR-30a-5p	CUL9	cullin 9 [Source:VGNC Symbol;Acc:VGNC:87090]	3.17	0.0763
ssc-miR-30a-5p	CYB561	cytochrome b561 [Source:VGNC Symbol;Acc:VGNC:87119]	3.17	0.0763
ssc-miR-30a-5p	CYB5B	cytochrome b5 type B [Source:VGNC Symbol;Acc:VGNC:96728]	3.17	0.0763
ssc-miR-30a-5p	CYLD	CYLD lysine 63 deubiquitinase [Source:VGNC Symbol;Acc:VGNC:87130]	3.17	0.0763
ssc-miR-30a-5p	CYP24A1	cytochrome P450 family 24 subfamily A member 1 [Source:VGNC Symbol;Acc:VGNC:103371]	3.17	0.0763
ssc-miR-30a-5p	CYSLTR1	cysteinyl leukotriene receptor 1 [Source:VGNC Symbol;Acc:VGNC:87132]	3.17	0.0763
ssc-miR-30a-5p	CYYR1	cysteine and tyrosine rich 1 [Source:VGNC Symbol;Acc:VGNC:87139]	3.17	0.0763
ssc-miR-30a-5p	DAAM2	dishevelled associated activator of morphosis 2 [Source:VGNC Symbol;Acc:VGNC:87142]	3.17	0.0763
ssc-miR-30a-5p	DAB1	DAB adaptor protein 1 [Source:VGNC Symbol;Acc:VGNC:87143]	3.17	0.0763
ssc-miR-30a-5p	DACH2	dachshund family transcription factor 2 [Source:HGNC Symbol;Acc:HGNC:16814]	3.17	0.0763
ssc-miR-30a-5p	DACT1	dishevelled binding antagonist of beta catenin 1 [Source:VGNC Symbol;Acc:VGNC:87147]	3.17	0.0763
ssc-miR-30a-5p	DAG1	dystroglycan 1 [Source:VGNC Symbol;Acc:VGNC:87151]	3.17	0.0763
ssc-miR-30a-5p	DAGLA	diacylglycerol lipase alpha [Source:VGNC Symbol;Acc:VGNC:87152]	3.17	0.0763
ssc-miR-30a-5p	DBF4	hypothetical gene	3.17	0.0763
ssc-miR-30a-5p	DCAF5	DDB1 and CUL4 associated factor 5 [Source:VGNC Symbol;Acc:VGNC:87176]	3.17	0.0763
ssc-miR-30a-5p	DCBLD1	discoidin, CUB and LCCL domain containing 1 [Source:VGNC Symbol;Acc:VGNC:87179]	3.17	0.0763
ssc-miR-30a-5p	DCP1A	decapping mRNA 1A [Source:VGNC Symbol;Acc:VGNC:97960]	3.17	0.0763
ssc-miR-30a-5p	DCP2	decapping mRNA 2 [Source:VGNC Symbol;Acc:VGNC:87188]	3.17	0.0763
ssc-miR-30a-5p	DCTN4	dynactin subunit 4 [Source:VGNC Symbol;Acc:VGNC:99635]	3.17	0.0763
ssc-miR-30a-5p	DCUN1D1	hypothetical gene	3.17	0.0763
ssc-miR-30a-5p	DCUN1D2	defective in cullin neddylation 1 domain containing 2 [Source:VGNC Symbol;Acc:VGNC:87195]	3.17	0.0763
ssc-miR-30a-5p	DCUN1D3	defective in cullin neddylation 1 domain containing 3 [Source:VGNC Symbol;Acc:VGNC:87196]	3.17	0.0763
ssc-miR-30a-5p	DCX	doublecortin [Source:HGNC Symbol;Acc:HGNC:2714]	3.17	0.0763

ssc-miR-30a-5p	DDAH1	dimethylarginine dimethylaminohydrolase 1 [Source:VGNC Symbol;Acc:VGNC:87200]	3.17	0.0763
ssc-miR-30a-5p	DDI2	hypothetical gene	3.17	0.0763
ssc-miR-30a-5p	DDIT4	DNA damage inducible transcript 4 [Source:VGNC Symbol;Acc:VGNC:87208]	3.17	0.0763
ssc-miR-30a-5p	DDX19A	hypothetical gene	3.17	0.0763
ssc-miR-30a-5p	DDX19B	hypothetical gene	3.17	0.0763
ssc-miR-30a-5p	DDX46	DEAD-box helicase 46 [Source:VGNC Symbol;Acc:VGNC:87223]	3.17	0.0763
ssc-miR-30a-5p	DENND2C	DENN domain containing 2C [Source:HGNC Symbol;Acc:HGNC:24748]	3.17	0.0763
ssc-miR-30a-5p	DENND4A	DENN domain containing 4A [Source:VGNC Symbol;Acc:VGNC:87251]	3.17	0.0763
ssc-miR-30a-5p	DENND5B	DENN domain containing 5B [Source:VGNC Symbol;Acc:VGNC:87255]	3.17	0.0763
ssc-miR-30a-5p	DESI2	desumoylating isopeptidase 2 [Source:VGNC Symbol;Acc:VGNC:96037]	3.17	0.0763
ssc-miR-30a-5p	DEXI	Dexi homolog [Source:HGNC Symbol;Acc:HGNC:13267]	3.17	0.0763
ssc-miR-30a-5p	DGKB	diacylglycerol kinase beta [Source:VGNC Symbol;Acc:VGNC:87270]	3.17	0.0763
ssc-miR-30a-5p	DGKD	diacylglycerol kinase delta [Source:VGNC Symbol;Acc:VGNC:96180]	3.17	0.0763
ssc-miR-30a-5p	DGKE	diacylglycerol kinase epsilon [Source:VGNC Symbol;Acc:VGNC:87271]	3.17	0.0763
ssc-miR-30a-5p	DGKH	diacylglycerol kinase eta [Source:VGNC Symbol;Acc:VGNC:87273]	3.17	0.0763
ssc-miR-30a-5p	DGKI	diacylglycerol kinase iota [Source:VGNC Symbol;Acc:VGNC:87274]	3.17	0.0763
ssc-miR-30a-5p	DGKQ	diacylglycerol kinase theta [Source:VGNC Symbol;Acc:VGNC:87276]	3.17	0.0763
ssc-miR-30a-5p	DGKZ	diacylglycerol kinase zeta [Source:VGNC Symbol;Acc:VGNC:87277]	3.17	0.0763
ssc-miR-30a-5p	DHX36	DEAH-box helicase 36 [Source:VGNC Symbol;Acc:VGNC:87292]	3.17	0.0763
ssc-miR-30a-5p	DHX40	DEAH-box helicase 40 [Source:VGNC Symbol;Acc:VGNC:87295]	3.17	0.0763
ssc-miR-30a-5p	DIO2	iodothyronine deiodinase 2 [Source:VGNC Symbol;Acc:VGNC:103935]	3.17	0.0763
ssc-miR-30a-5p	DIP2B	disco interacting B [Source:VGNC Symbol;Acc:VGNC:87306]	3.17	0.0763
ssc-miR-30a-5p	DIP2C	disco interacting C [Source:VGNC Symbol;Acc:VGNC:96201]	3.17	0.0763
ssc-miR-30a-5p	DLD	dihydrolipoamide dehydrogenase [Source:VGNC Symbol;Acc:VGNC:108580]	3.17	0.0763
ssc-miR-30a-5p	DLG5	discs large MAGUK scaffold protein 5 [Source:VGNC Symbol;Acc:VGNC:87329]	3.17	0.0763
ssc-miR-30a-5p	DLGAP1	DLG associated protein 1 [Source:VGNC Symbol;Acc:VGNC:87330]	3.17	0.0763
ssc-miR-30a-5p	DLGAP2	DLG associated protein 2 [Source:VGNC Symbol;Acc:VGNC:99711]	3.17	0.0763
ssc-miR-30a-5p	DLGAP4	DLG associated protein 4 [Source:VGNC Symbol;Acc:VGNC:96043]	3.17	0.0763
ssc-miR-30a-5p	DLK1	delta like non-canonical Notch ligand 1 [Source:VGNC Symbol;Acc:VGNC:87333]	3.17	0.0763
ssc-miR-30a-5p	DLL4	delta like canonical Notch ligand 4 [Source:VGNC Symbol;Acc:VGNC:87337]	3.17	0.0763
ssc-miR-30a-5p	DMD	dystrophin [Source:NCBI gene (formerly Entrezgene);Acc:497636]	3.17	0.0763
ssc-miR-30a-5p	DNAH17	dynein axonemal heavy chain 17 [Source:VGNC Symbol;Acc:VGNC:87369]	3.17	0.0763
ssc-miR-30a-5p	DNAJC13	DnaJ heat shock protein family (Hsp40) member C13 [Source:VGNC Symbol;Acc:VGNC:108650]	3.17	0.0763
ssc-miR-30a-5p	DNAJC15	DnaJ heat shock protein family (Hsp40) member C15 [Source:HGNC Symbol;Acc:HGNC:20325]	3.17	0.0763
ssc-miR-30a-5p	DNAJC25	DnaJ heat shock protein family (Hsp40) member C25 [Source:VGNC Symbol;Acc:VGNC:103083]	3.17	0.0763
ssc-miR-30a-5p	DNAJC25-GNG10	hypothetical gene	3.17	0.0763
ssc-miR-30a-5p	DNMT3A	DNA methyltransferase 3 alpha [Source:VGNC Symbol;Acc:VGNC:87384]	3.17	0.0763
ssc-miR-30a-5p	DOC2A	double C2 domain alpha [Source:VGNC Symbol;Acc:VGNC:87388]	3.17	0.0763
ssc-miR-30a-5p	DOCK7	dedicator of cytokinesis 7 [Source:VGNC Symbol;Acc:VGNC:87397]	3.17	0.0763
ssc-miR-30a-5p	DOLPP1	dolichyldiphosphatase 1 [Source:VGNC Symbol;Acc:VGNC:87407]	3.17	0.0763
ssc-miR-30a-5p	DPY19L1	dpy-19 like C-mannosyltransferase 1 [Source:VGNC Symbol;Acc:VGNC:99712]	3.17	0.0763
ssc-miR-30a-5p	DPY19L3	dpy-19 like C-mannosyltransferase 3 [Source:VGNC Symbol;Acc:VGNC:87426]	3.17	0.0763
ssc-miR-30a-5p	DPYSL2	dihydropyrimidinase like 2 [Source:VGNC Symbol;Acc:VGNC:87430]	3.17	0.0763
ssc-miR-30a-5p	DRAXIN	dorsal inhibitory axon guidance protein [Source:VGNC Symbol;Acc:VGNC:87439]	3.17	0.0763
ssc-miR-30a-5p	DRD1	dopamine receptor D1 [Source:VGNC Symbol;Acc:VGNC:87443]	3.17	0.0763
ssc-miR-30a-5p	DRP2	dystrophin related protein 2 [Source:VGNC Symbol;Acc:VGNC:87451]	3.17	0.0763
ssc-miR-30a-5p	DSC2	desmocollin 2 [Source:HGNC Symbol;Acc:HGNC:3036]	3.17	0.0763
ssc-miR-30a-5p	DSTYK	dual serine/threonine and tyrosine protein kinase [Source:VGNC Symbol;Acc:VGNC:87460]	3.17	0.0763
ssc-miR-30a-5p	DTNA	dystrobrevin alpha [Source:VGNC Symbol;Acc:VGNC:87462]	3.17	0.0763

ssc-miR-30a-5p	E2F3	E2F transcription factor 3 [Source:VGNC Symbol;Acc:VGNC:87514]	3.17	0.0763
ssc-miR-30a-5p	E2F7	E2F transcription factor 7 [Source:VGNC Symbol;Acc:VGNC:87518]	3.17	0.0763
ssc-miR-30a-5p	EAF1	ELL associated factor 1 [Source:VGNC Symbol;Acc:VGNC:87521]	3.17	0.0763
ssc-miR-30a-5p	EBF2	EBF transcription factor 2 [Source:VGNC Symbol;Acc:VGNC:87526]	3.17	0.0763
ssc-miR-30a-5p	EBF3	EBF transcription factor 3 [Source:VGNC Symbol;Acc:VGNC:87527]	3.17	0.0763
ssc-miR-30a-5p	EDA	ectodysplasin A [Source:VGNC Symbol;Acc:VGNC:87540]	3.17	0.0763
ssc-miR-30a-5p	EDC3	enhancer of mRNA decapping 3 [Source:VGNC Symbol;Acc:VGNC:87543]	3.17	0.0763
ssc-miR-30a-5p	EDEM3	ER degradation enhancing alpha-mannosidase like protein 3 [Source:VGNC Symbol;Acc:VGNC:87546]	3.17	0.0763
ssc-miR-30a-5p	EDNRA	endothelin receptor type A [Source:VGNC Symbol;Acc:VGNC:87549]	3.17	0.0763
ssc-miR-30a-5p	EDNRB	endothelin receptor type B [Source:VGNC Symbol;Acc:VGNC:87550]	3.17	0.0763
ssc-miR-30a-5p	EEA1	early endosome antigen 1 [Source:HGNC Symbol;Acc:HGNC:3185]	3.17	0.0763
ssc-miR-30a-5p	EED	embryonic ectoderm development [Source:VGNC Symbol;Acc:VGNC:87552]	3.17	0.0763
ssc-miR-30a-5p	EEF1E1	eukaryotic translation elongation factor 1 epsilon 1 [Source:NCBI gene (formerly Entrezgene);Acc:100155141]	3.17	0.0763
ssc-miR-30a-5p	EEF2K	eukaryotic elongation factor 2 kinase [Source:VGNC Symbol;Acc:VGNC:87557]	3.17	0.0763
ssc-miR-30a-5p	EFNA3	ephrin A3 [Source:VGNC Symbol;Acc:VGNC:98790]	3.17	0.0763
ssc-miR-30a-5p	EFR3A	EFR3 homolog A [Source:VGNC Symbol;Acc:VGNC:87579]	3.17	0.0763
ssc-miR-30a-5p	EIF3A	eukaryotic translation initiation factor 3 subunit A [Source:VGNC Symbol;Acc:VGNC:87615]	3.17	0.0763
ssc-miR-30a-5p	EIF5A2	eukaryotic translation initiation factor 5A2 [Source:VGNC Symbol;Acc:VGNC:87633]	3.17	0.0763
ssc-miR-30a-5p	ELAVL2	ELAV like RNA binding protein 2 [Source:VGNC Symbol;Acc:VGNC:87639]	3.17	0.0763
ssc-miR-30a-5p	ELAVL3	ELAV like RNA binding protein 3 [Source:VGNC Symbol;Acc:VGNC:87640]	3.17	0.0763
ssc-miR-30a-5p	ELAVL4	ELAV like RNA binding protein 4 [Source:VGNC Symbol;Acc:VGNC:97047]	3.17	0.0763
ssc-miR-30a-5p	ELFN2	extracellular leucine rich repeat and fibronectin type III domain containing 2 [Source:VGNC Symbol;Acc:VGNC:103232]	3.17	0.0763
ssc-miR-30a-5p	ELK3	ETS transcription factor ELK3 [Source:VGNC Symbol;Acc:VGNC:87646]	3.17	0.0763
ssc-miR-30a-5p	ELL	elongation factor for RNA polymerase II [Source:VGNC Symbol;Acc:VGNC:87648]	3.17	0.0763
ssc-miR-30a-5p	ELL2	elongation factor for RNA polymerase II 2 [Source:VGNC Symbol;Acc:VGNC:87649]	3.17	0.0763
ssc-miR-30a-5p	ELMO1	engulfment and cell motility 1 [Source:HGNC Symbol;Acc:HGNC:16286]	3.17	0.0763
ssc-miR-30a-5p	ELMOD2	ELMO domain containing 2 [Source:VGNC Symbol;Acc:VGNC:87653]	3.17	0.0763
ssc-miR-30a-5p	ELMSAN1	hypothetical gene	3.17	0.0763
ssc-miR-30a-5p	ELOVL5	ELOVL fatty acid elongase 5 [Source:VGNC Symbol;Acc:VGNC:87661]	3.17	0.0763
ssc-miR-30a-5p	ELOVL6	ELOVL fatty acid elongase 6 [Source:VGNC Symbol;Acc:VGNC:87662]	3.17	0.0763
ssc-miR-30a-5p	EML1	EMAP like 1 [Source:VGNC Symbol;Acc:VGNC:87682]	3.17	0.0763
ssc-miR-30a-5p	EML4	EMAP like 4 [Source:VGNC Symbol;Acc:VGNC:87685]	3.17	0.0763
ssc-miR-30a-5p	ENAH	ENAH actin regulator [Source:VGNC Symbol;Acc:VGNC:108271]	3.17	0.0763
ssc-miR-30a-5p	ENOX2	ecto-NOX disulfide-thiol exchanger 2 [Source:VGNC Symbol;Acc:VGNC:87707]	3.17	0.0763
ssc-miR-30a-5p	ENTHD2	hypothetical gene	3.17	0.0763
ssc-miR-30a-5p	EOGT	EGF domain specific O-linked N-acetylglucosamine transferase [Source:VGNC Symbol;Acc:VGNC:87718]	3.17	0.0763
ssc-miR-30a-5p	EPB41	erythrocyte membrane protein band 4.1 [Source:VGNC Symbol;Acc:VGNC:87722]	3.17	0.0763
ssc-miR-30a-5p	EPB41L3	erythrocyte membrane protein band 4.1 like 3 [Source:VGNC Symbol;Acc:VGNC:87724]	3.17	0.0763
ssc-miR-30a-5p	EPB41L4B	erythrocyte membrane protein band 4.1 like 4B [Source:VGNC Symbol;Acc:VGNC:103088]	3.17	0.0763
ssc-miR-30a-5p	EPB41L5	erythrocyte membrane protein band 4.1 like 5 [Source:VGNC Symbol;Acc:VGNC:97979]	3.17	0.0763
ssc-miR-30a-5p	EPC1	enhancer of polycomb homolog 1 [Source:VGNC Symbol;Acc:VGNC:95923]	3.17	0.0763
ssc-miR-30a-5p	EPC2	enhancer of polycomb homolog 2 [Source:VGNC Symbol;Acc:VGNC:96045]	3.17	0.0763
ssc-miR-30a-5p	EPDR1	ependymin related 1 [Source:VGNC Symbol;Acc:VGNC:87727]	3.17	0.0763
ssc-miR-30a-5p	EPG5	ectopic P-granules 5 autophagy tethering factor [Source:VGNC Symbol;Acc:VGNC:87728]	3.17	0.0763
ssc-miR-30a-5p	EPHB2	EPH receptor B2 [Source:VGNC Symbol;Acc:VGNC:87737]	3.17	0.0763
ssc-miR-30a-5p	EPN2	epsin 2 [Source:VGNC Symbol;Acc:VGNC:97982]	3.17	0.0763
ssc-miR-30a-5p	ERC2	ELKS/RAB6-interacting/CAST family member 2 [Source:VGNC Symbol;Acc:VGNC:87762]	3.17	0.0763
ssc-miR-30a-5p	ERG	ETS transcription factor ERG [Source:VGNC Symbol;Acc:VGNC:87768]	3.17	0.0763
ssc-miR-30a-5p	ERLIN1	hypothetical gene	3.17	0.0763

ssc-miR-30a-5p	ERMAP	erythroblast membrane associated protein [Source:VGNC Symbol;Acc:VGNC:97053]	3.17	0.0763
ssc-miR-30a-5p	ERRFI1	ERBB receptor feedback inhibitor 1 [Source:VGNC Symbol;Acc:VGNC:87784]	3.17	0.0763
ssc-miR-30a-5p	ESCO1	establishment of sister chromatid cohesion N-acetyltransferase 1 [Source:VGNC Symbol;Acc:VGNC:87786]	3.17	0.0763
ssc-miR-30a-5p	ESPN	espin [Source:VGNC Symbol;Acc:VGNC:97054]	3.17	0.0763
ssc-miR-30a-5p	ESRRG	estrogen related receptor gamma [Source:VGNC Symbol;Acc:VGNC:96289]	3.17	0.0763
ssc-miR-30a-5p	EVA1C	eva-1 homolog C [Source:VGNC Symbol;Acc:VGNC:87817]	3.17	0.0763
ssc-miR-30a-5p	EVI5	ecotropic viral integration site 5 [Source:VGNC Symbol;Acc:VGNC:98793]	3.17	0.0763
ssc-miR-30a-5p	EVI5L	ecotropic viral integration site 5 like [Source:VGNC Symbol;Acc:VGNC:99646]	3.17	0.0763
ssc-miR-30a-5p	EVX2	even-skipped homeobox 2 [Source:VGNC Symbol;Acc:VGNC:96290]	3.17	0.0763
ssc-miR-30a-5p	EXTL2	exostosin like glycosyltransferase 2 [Source:VGNC Symbol;Acc:VGNC:87849]	3.17	0.0763
ssc-miR-30a-5p	EYA2	EYA transcriptional coactivator and phosphatase 2 [Source:VGNC Symbol;Acc:VGNC:96292]	3.17	0.0763
ssc-miR-30a-5p	FA2H	fatty acid 2-hydroxylase [Source:VGNC Symbol;Acc:VGNC:87868]	3.17	0.0763
ssc-miR-30a-5p	FAF2	Fas associated factor family member 2 [Source:VGNC Symbol;Acc:VGNC:87880]	3.17	0.0763
ssc-miR-30a-5p	FAM104A	family with sequence similarity 104 member A [Source:VGNC Symbol;Acc:VGNC:98990]	3.17	0.0763
ssc-miR-30a-5p	FAM104B	hypothetical gene	3.17	0.0763
ssc-miR-30a-5p	FAM105B	hypothetical gene	3.17	0.0763
ssc-miR-30a-5p	FAM109A	hypothetical gene	3.17	0.0763
ssc-miR-30a-5p	FAM109B	hypothetical gene	3.17	0.0763
ssc-miR-30a-5p	FAM110B	family with sequence similarity 110 member B [Source:VGNC Symbol;Acc:VGNC:87885]	3.17	0.0763
ssc-miR-30a-5p	FAM124A	family with sequence similarity 124 member A [Source:VGNC Symbol;Acc:VGNC:87898]	3.17	0.0763
ssc-miR-30a-5p	FAM126A	family with sequence similarity 126 member A [Source:VGNC Symbol;Acc:VGNC:87899]	3.17	0.0763
ssc-miR-30a-5p	FAM126B	family with sequence similarity 126 member B [Source:HGNC Symbol;Acc:HGNC:28593]	3.17	0.0763
ssc-miR-30a-5p	FAM129A	hypothetical gene	3.17	0.0763
ssc-miR-30a-5p	FAM131B	family with sequence similarity 131 member B [Source:VGNC Symbol;Acc:VGNC:87901]	3.17	0.0763
ssc-miR-30a-5p	FAM133A	family with sequence similarity 133 member A [Source:VGNC Symbol;Acc:VGNC:87903]	3.17	0.0763
ssc-miR-30a-5p	FAM13A	family with sequence similarity 13 member A [Source:VGNC Symbol;Acc:VGNC:98924]	3.17	0.0763
ssc-miR-30a-5p	FAM13C	family with sequence similarity 13 member C [Source:VGNC Symbol;Acc:VGNC:87907]	3.17	0.0763
ssc-miR-30a-5p	FAM149B1	family with sequence similarity 149 member B1 [Source:VGNC Symbol;Acc:VGNC:87908]	3.17	0.0763
ssc-miR-30a-5p	FAM155A	hypothetical gene	3.17	0.0763
ssc-miR-30a-5p	FAM160B1	hypothetical gene	3.17	0.0763
ssc-miR-30a-5p	FAM168B	family with sequence similarity 168 member B [Source:VGNC Symbol;Acc:VGNC:95462]	3.17	0.0763
ssc-miR-30a-5p	FAM175B	hypothetical gene	3.17	0.0763
ssc-miR-30a-5p	FAM178A	hypothetical gene	3.17	0.0763
ssc-miR-30a-5p	FAM179B	hypothetical gene	3.17	0.0763
ssc-miR-30a-5p	FAM199X	family with sequence similarity 199, X-linked [Source:HGNC Symbol;Acc:HGNC:25195]	3.17	0.0763
ssc-miR-30a-5p	FAM208B	hypothetical gene	3.17	0.0763
ssc-miR-30a-5p	FAM210B	family with sequence similarity 210 member B [Source:VGNC Symbol;Acc:VGNC:95736]	3.17	0.0763
ssc-miR-30a-5p	FAM214A	family with sequence similarity 214 member A [Source:VGNC Symbol;Acc:VGNC:87952]	3.17	0.0763
ssc-miR-30a-5p	FAM216A	family with sequence similarity 216 member A [Source:VGNC Symbol;Acc:VGNC:87954]	3.17	0.0763
ssc-miR-30a-5p	FAM217B	family with sequence similarity 217 member B [Source:VGNC Symbol;Acc:VGNC:95767]	3.17	0.0763
ssc-miR-30a-5p	FAM219B	family with sequence similarity 219 member B [Source:VGNC Symbol;Acc:VGNC:87956]	3.17	0.0763
ssc-miR-30a-5p	FAM43A	family with sequence similarity 43 member A [Source:VGNC Symbol;Acc:VGNC:87970]	3.17	0.0763
ssc-miR-30a-5p	FAM46A	hypothetical gene	3.17	0.0763
ssc-miR-30a-5p	FAM46C	hypothetical gene	3.17	0.0763
ssc-miR-30a-5p	FAM49A	hypothetical gene	3.17	0.0763
ssc-miR-30a-5p	FAM53B	family with sequence similarity 53 member B [Source:VGNC Symbol;Acc:VGNC:87974]	3.17	0.0763
ssc-miR-30a-5p	FAM72B	hypothetical gene	3.17	0.0763
ssc-miR-30a-5p	FAM72D	hypothetical gene	3.17	0.0763
ssc-miR-30a-5p	FAM73B	hypothetical gene	3.17	0.0763

ssc-miR-30a-5p	FAM81A	family with sequence similarity 81 member A [Source:VGNC Symbol;Acc:VGNC:87986]	3.17	0.0763
ssc-miR-30a-5p	FAM83F	family with sequence similarity 83 member F [Source:VGNC Symbol;Acc:VGNC:87991]	3.17	0.0763
ssc-miR-30a-5p	FAM83G	family with sequence similarity 83 member G [Source:VGNC Symbol;Acc:VGNC:87992]	3.17	0.0763
ssc-miR-30a-5p	FAM91A1	family with sequence similarity 91 member A1 [Source:VGNC Symbol;Acc:VGNC:97998]	3.17	0.0763
ssc-miR-30a-5p	FANCF	FA complementation group F [Source:VGNC Symbol;Acc:VGNC:88004]	3.17	0.0763
ssc-miR-30a-5p	FAP	fibroblast activation protein alpha [Source:VGNC Symbol;Acc:VGNC:99714]	3.17	0.0763
ssc-miR-30a-5p	FBXL14	hypothetical gene	3.17	0.0763
ssc-miR-30a-5p	FBXL17	F-box and leucine rich repeat protein 17 [Source:VGNC Symbol;Acc:VGNC:99650]	3.17	0.0763
ssc-miR-30a-5p	FBXL20	F-box and leucine rich repeat protein 20 [Source:VGNC Symbol;Acc:VGNC:98006]	3.17	0.0763
ssc-miR-30a-5p	FBXO28	F-box protein 28 [Source:HGNC Symbol;Acc:HGNC:29046]	3.17	0.0763
ssc-miR-30a-5p	FBXO32	F-box protein 32 [Source:VGNC Symbol;Acc:VGNC:88039]	3.17	0.0763
ssc-miR-30a-5p	FBXO34	F-box protein 34 [Source:VGNC Symbol;Acc:VGNC:88041]	3.17	0.0763
ssc-miR-30a-5p	FBXO42	F-box protein 42 [Source:VGNC Symbol;Acc:VGNC:88047]	3.17	0.0763
ssc-miR-30a-5p	FBXO45	F-box protein 45 [Source:VGNC Symbol;Acc:VGNC:88048]	3.17	0.0763
ssc-miR-30a-5p	FECH	ferrochelatase [Source:VGNC Symbol;Acc:VGNC:88081]	3.17	0.0763
ssc-miR-30a-5p	FGD6	FYVE, RhoGEF and PH domain containing 6 [Source:VGNC Symbol;Acc:VGNC:88099]	3.17	0.0763
ssc-miR-30a-5p	FGF20	fibroblast growth factor 20 [Source:VGNC Symbol;Acc:VGNC:96298]	3.17	0.0763
ssc-miR-30a-5p	FIGN	fidgetin, microtubule severing factor [Source:VGNC Symbol;Acc:VGNC:95580]	3.17	0.0763
ssc-miR-30a-5p	FKBP14	FKBP prolyl isomerase 14 [Source:VGNC Symbol;Acc:VGNC:88144]	3.17	0.0763
ssc-miR-30a-5p	FKBP3	FKBP prolyl isomerase 3 [Source:VGNC Symbol;Acc:VGNC:88146]	3.17	0.0763
ssc-miR-30a-5p	FLJ20373	hypothetical gene	3.17	0.0763
ssc-miR-30a-5p	FLVCR1	FLVCR heme transporter 1 [Source:VGNC Symbol;Acc:VGNC:96580]	3.17	0.0763
ssc-miR-30a-5p	FNDC3A	fibronectin type III domain containing 3A [Source:VGNC Symbol;Acc:VGNC:88182]	3.17	0.0763
ssc-miR-30a-5p	FNDC3B	fibronectin type III domain containing 3B [Source:VGNC Symbol;Acc:VGNC:88183]	3.17	0.0763
ssc-miR-30a-5p	FNIP2	folliculin interacting protein 2 [Source:VGNC Symbol;Acc:VGNC:88188]	3.17	0.0763
ssc-miR-30a-5p	FOSL2	FOS like 2, AP-1 transcription factor subunit [Source:VGNC Symbol;Acc:VGNC:88192]	3.17	0.0763
ssc-miR-30a-5p	FOXA1	forkhead box A1 [Source:VGNC Symbol;Acc:VGNC:88193]	3.17	0.0763
ssc-miR-30a-5p	FOXB1	forkhead box B1 [Source:VGNC Symbol;Acc:VGNC:88195]	3.17	0.0763
ssc-miR-30a-5p	FOXD1	forkhead box D1 [Source:VGNC Symbol;Acc:VGNC:88199]	3.17	0.0763
ssc-miR-30a-5p	FOXG1	forkhead box G1 [Source:VGNC Symbol;Acc:VGNC:88206]	3.17	0.0763
ssc-miR-30a-5p	FOXN2	forkhead box N2 [Source:VGNC Symbol;Acc:VGNC:88219]	3.17	0.0763
ssc-miR-30a-5p	FOXO3	forkhead box O3 [Source:VGNC Symbol;Acc:VGNC:99715]	3.17	0.0763
ssc-miR-30a-5p	FOXP4	forkhead box P4 [Source:VGNC Symbol;Acc:VGNC:88224]	3.17	0.0763
ssc-miR-30a-5p	FRK	fyn related Src family tyrosine kinase [Source:VGNC Symbol;Acc:VGNC:88235]	3.17	0.0763
ssc-miR-30a-5p	FRMD4A	FERM domain containing 4A [Source:VGNC Symbol;Acc:VGNC:96088]	3.17	0.0763
ssc-miR-30a-5p	FRMD6	FERM domain containing 6 [Source:VGNC Symbol;Acc:VGNC:88239]	3.17	0.0763
ssc-miR-30a-5p	FRMPD1	FERM and PDZ domain containing 1 [Source:VGNC Symbol;Acc:VGNC:88240]	3.17	0.0763
ssc-miR-30a-5p	FRMPD4	FERM and PDZ domain containing 4 [Source:VGNC Symbol;Acc:VGNC:88243]	3.17	0.0763
ssc-miR-30a-5p	FRS2	fibroblast growth factor receptor substrate 2 [Source:VGNC Symbol;Acc:VGNC:88246]	3.17	0.0763
ssc-miR-30a-5p	FRZB	frizzled related protein [Source:VGNC Symbol;Acc:VGNC:96308]	3.17	0.0763
ssc-miR-30a-5p	FST	follistatin [Source:NCBI gene (formerly Entrezgene);Acc:445002]	3.17	0.0763
ssc-miR-30a-5p	FSTL4	follistatin like 4 [Source:VGNC Symbol;Acc:VGNC:88256]	3.17	0.0763
ssc-miR-30a-5p	FTO	FTO alpha-ketoglutarate dependent dioxygenase [Source:VGNC Symbol;Acc:VGNC:88259]	3.17	0.0763
ssc-miR-30a-5p	FUBP1	far upstream element binding protein 1 [Source:VGNC Symbol;Acc:VGNC:96781]	3.17	0.0763
ssc-miR-30a-5p	FUBP3	far upstream element binding protein 3 [Source:VGNC Symbol;Acc:VGNC:103093]	3.17	0.0763
ssc-miR-30a-5p	FUCA1	alpha-L-fucosidase 1 [Source:VGNC Symbol;Acc:VGNC:88262]	3.17	0.0763
ssc-miR-30a-5p	FUT4	fucosyltransferase 4 [Source:HGNC Symbol;Acc:HGNC:4015]	3.17	0.0763
ssc-miR-30a-5p	FXR1	FMR1 autosomal homolog 1 [Source:VGNC Symbol;Acc:VGNC:108659]	3.17	0.0763
ssc-miR-30a-5p	FYCO1	hypothetical gene	3.17	0.0763

ssc-miR-30a-5p	FYN	FYN proto-onco, Src family tyrosine kinase [Source:VGNC Symbol;Acc:VGNC:88276]	3.17	0.0763
ssc-miR-30a-5p	FYTTD1	forty-two-three domain containing 1 [Source:VGNC Symbol;Acc:VGNC:88277]	3.17	0.0763
ssc-miR-30a-5p	FZD2	frizzled class receptor 2 [Source:VGNC Symbol;Acc:VGNC:88280]	3.17	0.0763
ssc-miR-30a-5p	FZD3	frizzled class receptor 3 [Source:VGNC Symbol;Acc:VGNC:88281]	3.17	0.0763
ssc-miR-30a-5p	G3BP1	G3BP stress granule assembly factor 1 [Source:VGNC Symbol;Acc:VGNC:88288]	3.17	0.0763
ssc-miR-30a-5p	G3BP2	G3BP stress granule assembly factor 2 [Source:VGNC Symbol;Acc:VGNC:88289]	3.17	0.0763
ssc-miR-30a-5p	G6PC3	glucose-6-phosphatase catalytic subunit 3 [Source:VGNC Symbol;Acc:VGNC:88291]	3.17	0.0763
ssc-miR-30a-5p	GABRA1	gamma-aminobutyric acid type A receptor subunit alpha1 [Source:VGNC Symbol;Acc:VGNC:88300]	3.17	0.0763
ssc-miR-30a-5p	GABRA5	gamma-aminobutyric acid type A receptor subunit alpha5 [Source:VGNC Symbol;Acc:VGNC:88304]	3.17	0.0763
ssc-miR-30a-5p	GABRB1	gamma-aminobutyric acid type A receptor subunit beta1 [Source:VGNC Symbol;Acc:VGNC:88306]	3.17	0.0763
ssc-miR-30a-5p	GADD45GIP1	GADD45G interacting protein 1 [Source:VGNC Symbol;Acc:VGNC:99751]	3.17	0.0763
ssc-miR-30a-5p	GALNT1	polypeptide N-acetylgalactosaminyltransferase 1 [Source:VGNC Symbol;Acc:VGNC:97062]	3.17	0.0763
ssc-miR-30a-5p	GALNT2	polypeptide N-acetylgalactosaminyltransferase 2 [Source:VGNC Symbol;Acc:VGNC:88334]	3.17	0.0763
ssc-miR-30a-5p	GALNT3	polypeptide N-acetylgalactosaminyltransferase 3 [Source:VGNC Symbol;Acc:VGNC:96313]	3.17	0.0763
ssc-miR-30a-5p	GALNT7	polypeptide N-acetylgalactosaminyltransferase 7 [Source:VGNC Symbol;Acc:VGNC:103946]	3.17	0.0763
ssc-miR-30a-5p	GALR1	galanin receptor 1 [Source:VGNC Symbol;Acc:VGNC:88339]	3.17	0.0763
ssc-miR-30a-5p	GAN	gigaxonin [Source:VGNC Symbol;Acc:VGNC:88343]	3.17	0.0763
ssc-miR-30a-5p	GAREM	hypothetical gene	3.17	0.0763
ssc-miR-30a-5p	GAS2	growth arrest specific 2 [Source:VGNC Symbol;Acc:VGNC:88354]	3.17	0.0763
ssc-miR-30a-5p	GAS7	growth arrest specific 7 [Source:VGNC Symbol;Acc:VGNC:88359]	3.17	0.0763
ssc-miR-30a-5p	GATA5	GATA binding protein 5 [Source:VGNC Symbol;Acc:VGNC:95661]	3.17	0.0763
ssc-miR-30a-5p	GATA6	GATA binding protein 6 [Source:VGNC Symbol;Acc:VGNC:88366]	3.17	0.0763
ssc-miR-30a-5p	GATM	glycine amidinotransferase [Source:VGNC Symbol;Acc:VGNC:88372]	3.17	0.0763
ssc-miR-30a-5p	GBP2	guanylate binding protein 2, interferon-inducible [Source:NCBI gene (formerly Entrezgene);Acc:100153137]	3.17	0.0763
ssc-miR-30a-5p	GCH1	GTP cyclohydrolase 1 [Source:VGNC Symbol;Acc:VGNC:88384]	3.17	0.0763
ssc-miR-30a-5p	GCLC	glutamate-cysteine ligase catalytic subunit [Source:VGNC Symbol;Acc:VGNC:88386]	3.17	0.0763
ssc-miR-30a-5p	GCNT2	glucosaminyl (N-acetyl) transferase 2 (I blood group) [Source:HGNC Symbol;Acc:HGNC:4204]	3.17	0.0763
ssc-miR-30a-5p	GDA	guanine deaminase [Source:VGNC Symbol;Acc:VGNC:88393]	3.17	0.0763
ssc-miR-30a-5p	GDF2	growth differentiation factor 2 [Source:VGNC Symbol;Acc:VGNC:88400]	3.17	0.0763
ssc-miR-30a-5p	GDI2	GDP dissociation inhibitor 2 [Source:VGNC Symbol;Acc:VGNC:98019]	3.17	0.0763
ssc-miR-30a-5p	GDNF	glial cell derived neurotrophic factor [Source:VGNC Symbol;Acc:VGNC:88406]	3.17	0.0763
ssc-miR-30a-5p	GFPT2	glutamine-fructose-6-phosphate transaminase 2 [Source:VGNC Symbol;Acc:VGNC:88427]	3.17	0.0763
ssc-miR-30a-5p	GID4	GID complex subunit 4 homolog [Source:VGNC Symbol;Acc:VGNC:98996]	3.17	0.0763
ssc-miR-30a-5p	GIGYF1	GRB10 interacting GYF protein 1 [Source:VGNC Symbol;Acc:VGNC:88444]	3.17	0.0763
ssc-miR-30a-5p	GIGYF2	GRB10 interacting GYF protein 2 [Source:VGNC Symbol;Acc:VGNC:95551]	3.17	0.0763
ssc-miR-30a-5p	GJA1	gap junction protein alpha 1 [Source:VGNC Symbol;Acc:VGNC:103098]	3.17	0.0763
ssc-miR-30a-5p	GJA3	gap junction protein alpha 3 [Source:HGNC Symbol;Acc:HGNC:4277]	3.17	0.0763
ssc-miR-30a-5p	GJC2	gap junction protein gamma 2 [Source:VGNC Symbol;Acc:VGNC:88469]	3.17	0.0763
ssc-miR-30a-5p	GLCCI1	glucocorticoid induced 1 [Source:VGNC Symbol;Acc:VGNC:88476]	3.17	0.0763
ssc-miR-30a-5p	GLCE	glucuronic acid epimerase [Source:VGNC Symbol;Acc:VGNC:88477]	3.17	0.0763
ssc-miR-30a-5p	GLDC	glycine decarboxylase [Source:VGNC Symbol;Acc:VGNC:88478]	3.17	0.0763
ssc-miR-30a-5p	GLI2	GLI family zinc finger 2 [Source:VGNC Symbol;Acc:VGNC:96324]	3.17	0.0763
ssc-miR-30a-5p	GLUD1	glutamate dehydrogenase 1 [Source:NCBI gene (formerly Entrezgene);Acc:100157162]	3.17	0.0763
ssc-miR-30a-5p	GLUD2	hypothetical gene	3.17	0.0763
ssc-miR-30a-5p	GMEB1	glucocorticoid modulatory element binding protein 1 [Source:VGNC Symbol;Acc:VGNC:88508]	3.17	0.0763
ssc-miR-30a-5p	GMEB2	glucocorticoid modulatory element binding protein 2 [Source:VGNC Symbol;Acc:VGNC:96326]	3.17	0.0763
ssc-miR-30a-5p	GMNC	geminin coiled-coil domain containing [Source:VGNC Symbol;Acc:VGNC:88512]	3.17	0.0763
ssc-miR-30a-5p	GNA13	G protein subunit alpha 13 [Source:VGNC Symbol;Acc:VGNC:98997]	3.17	0.0763
ssc-miR-30a-5p	GNAI2	G protein subunit alpha i2 [Source:VGNC Symbol;Acc:VGNC:88522]	3.17	0.0763

ssc-miR-30a-5p	GNAO1	hypothetical gene	3.17	0.0763
ssc-miR-30a-5p	GNAQ	G protein subunit alpha q [Source:VGNC Symbol;Acc:VGNC:103100]	3.17	0.0763
ssc-miR-30a-5p	GNG10	G protein subunit gamma 10 [Source:HGNC Symbol;Acc:HGNC:4402]	3.17	0.0763
ssc-miR-30a-5p	GNPDA1	glucosamine-6-phosphate deaminase 1 [Source:VGNC Symbol;Acc:VGNC:88544]	3.17	0.0763
ssc-miR-30a-5p	GOLGA1	golgin A1 [Source:VGNC Symbol;Acc:VGNC:88549]	3.17	0.0763
ssc-miR-30a-5p	GOLGA4	hypothetical gene	3.17	0.0763
ssc-miR-30a-5p	GOLGA6A	hypothetical gene	3.17	0.0763
ssc-miR-30a-5p	GOLGA6B	hypothetical gene	3.17	0.0763
ssc-miR-30a-5p	GOLGA6C	hypothetical gene	3.17	0.0763
ssc-miR-30a-5p	GOLGA6D	hypothetical gene	3.17	0.0763
ssc-miR-30a-5p	GOLGA6L10	hypothetical gene	3.17	0.0763
ssc-miR-30a-5p	GOLGA6L4	hypothetical gene	3.17	0.0763
ssc-miR-30a-5p	GOLGA6L9	hypothetical gene	3.17	0.0763
ssc-miR-30a-5p	GOLGA8B	hypothetical gene	3.17	0.0763
ssc-miR-30a-5p	GOT2	glutamic-oxaloacetic transaminase 2 [Source:VGNC Symbol;Acc:VGNC:88562]	3.17	0.0763
ssc-miR-30a-5p	GPCPD1	glycerophosphocholine phosphodiesterase 1 [Source:VGNC Symbol;Acc:VGNC:96148]	3.17	0.0763
ssc-miR-30a-5p	GPD1L	glycerol-3-phosphate dehydrogenase 1 like [Source:VGNC Symbol;Acc:VGNC:108661]	3.17	0.0763
ssc-miR-30a-5p	GPER1	G protein-coupled estrogen receptor 1 [Source:VGNC Symbol;Acc:VGNC:88584]	3.17	0.0763
ssc-miR-30a-5p	GPR108	G protein-coupled receptor 108 [Source:VGNC Symbol;Acc:VGNC:88596]	3.17	0.0763
ssc-miR-30a-5p	GPR124	hypothetical gene	3.17	0.0763
ssc-miR-30a-5p	GPR125	hypothetical gene	3.17	0.0763
ssc-miR-30a-5p	GPR137C	G protein-coupled receptor 137C [Source:VGNC Symbol;Acc:VGNC:88602]	3.17	0.0763
ssc-miR-30a-5p	GPR150	G protein-coupled receptor 150 [Source:VGNC Symbol;Acc:VGNC:88607]	3.17	0.0763
ssc-miR-30a-5p	GPR156	G protein-coupled receptor 156 [Source:VGNC Symbol;Acc:VGNC:88610]	3.17	0.0763
ssc-miR-30a-5p	GPR157	G protein-coupled receptor 157 [Source:VGNC Symbol;Acc:VGNC:88611]	3.17	0.0763
ssc-miR-30a-5p	GPR161	G protein-coupled receptor 161 [Source:VGNC Symbol;Acc:VGNC:88613]	3.17	0.0763
ssc-miR-30a-5p	GPR180	G protein-coupled receptor 180 [Source:VGNC Symbol;Acc:VGNC:88621]	3.17	0.0763
ssc-miR-30a-5p	GPR26	G protein-coupled receptor 26 [Source:VGNC Symbol;Acc:VGNC:88626]	3.17	0.0763
ssc-miR-30a-5p	GPR63	G protein-coupled receptor 63 [Source:VGNC Symbol;Acc:VGNC:88634]	3.17	0.0763
ssc-miR-30a-5p	GPR75-ASB3	hypothetical gene	3.17	0.0763
ssc-miR-30a-5p	GPRIN3	hypothetical gene	3.17	0.0763
ssc-miR-30a-5p	GPT2	glutamic--pyruvic transaminase 2 [Source:VGNC Symbol;Acc:VGNC:88651]	3.17	0.0763
ssc-miR-30a-5p	GRAMD2	hypothetical gene	3.17	0.0763
ssc-miR-30a-5p	GRB10	growth factor receptor bound protein 10 [Source:VGNC Symbol;Acc:VGNC:88662]	3.17	0.0763
ssc-miR-30a-5p	GREB1L	GREB1 like retinoic acid receptor coactivator [Source:VGNC Symbol;Acc:VGNC:96584]	3.17	0.0763
ssc-miR-30a-5p	GRHL1	grainyhead like transcription factor 1 [Source:VGNC Symbol;Acc:VGNC:88666]	3.17	0.0763
ssc-miR-30a-5p	GRHL2	grainyhead like transcription factor 2 [Source:VGNC Symbol;Acc:VGNC:88667]	3.17	0.0763
ssc-miR-30a-5p	GRIA2	glutamate ionotropic receptor AMPA type subunit 2 [Source:VGNC Symbol;Acc:VGNC:88671]	3.17	0.0763
ssc-miR-30a-5p	GRIN2A	glutamate ionotropic receptor NMDA type subunit 2A [Source:VGNC Symbol;Acc:VGNC:88683]	3.17	0.0763
ssc-miR-30a-5p	GRIN3A	glutamate ionotropic receptor NMDA type subunit 3A [Source:VGNC Symbol;Acc:VGNC:88687]	3.17	0.0763
ssc-miR-30a-5p	GRK5	G protein-coupled receptor kinase 5 [Source:VGNC Symbol;Acc:VGNC:88697]	3.17	0.0763
ssc-miR-30a-5p	GRK6	G protein-coupled receptor kinase 6 [Source:VGNC Symbol;Acc:VGNC:88698]	3.17	0.0763
ssc-miR-30a-5p	GRM3	glutamate metabotropic receptor 3 [Source:VGNC Symbol;Acc:VGNC:88702]	3.17	0.0763
ssc-miR-30a-5p	GRM5	glutamate metabotropic receptor 5 [Source:VGNC Symbol;Acc:VGNC:88703]	3.17	0.0763
ssc-miR-30a-5p	GSKIP	GSK3B interacting protein [Source:VGNC Symbol;Acc:VGNC:103956]	3.17	0.0763
ssc-miR-30a-5p	GTDC1	glycosyltransferase like domain containing 1 [Source:VGNC Symbol;Acc:VGNC:95955]	3.17	0.0763
ssc-miR-30a-5p	GTF2H1	ral transcription factor IIH subunit 1 [Source:VGNC Symbol;Acc:VGNC:88734]	3.17	0.0763
ssc-miR-30a-5p	GUF1	GTP binding elongation factor GUF1 [Source:VGNC Symbol;Acc:VGNC:88754]	3.17	0.0763
ssc-miR-30a-5p	GXYLT1	glucoside xylosyltransferase 1 [Source:VGNC Symbol;Acc:VGNC:88756]	3.17	0.0763

ssc-miR-30a-5p	GZF1	GDNF inducible zinc finger protein 1 [Source:VGNC Symbol;Acc:VGNC:108722]	3.17	0.0763
ssc-miR-30a-5p	H2AFY	hypothetical gene	3.17	0.0763
ssc-miR-30a-5p	H6PD	hexose-6-phosphate dehydrogenase/glucose 1-dehydrogenase [Source:VGNC Symbol;Acc:VGNC:88764]	3.17	0.0763
ssc-miR-30a-5p	HACE1	HECT domain and ankyrin repeat containing E3 ubiquitin protein ligase 1 [Source:VGNC Symbol;Acc:VGNC:88769]	3.17	0.0763
ssc-miR-30a-5p	HBS1L	HBS1 like translational GTPase [Source:VGNC Symbol;Acc:VGNC:88796]	3.17	0.0763
ssc-miR-30a-5p	HCAR2	hypothetical gene	3.17	0.0763
ssc-miR-30a-5p	HCAR3	hypothetical gene	3.17	0.0763
ssc-miR-30a-5p	HCFC2	host cell factor C2 [Source:VGNC Symbol;Acc:VGNC:88800]	3.17	0.0763
ssc-miR-30a-5p	HCN1	hyperpolarization activated cyclic nucleotide gated potassium channel 1 [Source:VGNC Symbol;Acc:VGNC:88802]	3.17	0.0763
ssc-miR-30a-5p	HDAC5	histone deacetylase 5 [Source:VGNC Symbol;Acc:VGNC:88815]	3.17	0.0763
ssc-miR-30a-5p	HDAC9	histone deacetylase 9 [Source:HGNC Symbol;Acc:HGNC:14065]	3.17	0.0763
ssc-miR-30a-5p	HECTD4	HECT domain E3 ubiquitin protein ligase 4 [Source:VGNC Symbol;Acc:VGNC:88834]	3.17	0.0763
ssc-miR-30a-5p	HECW1	HECT, C2 and WW domain containing E3 ubiquitin protein ligase 1 [Source:VGNC Symbol;Acc:VGNC:88835]	3.17	0.0763
ssc-miR-30a-5p	HELZ	helicase with zinc finger [Source:VGNC Symbol;Acc:VGNC:88840]	3.17	0.0763
ssc-miR-30a-5p	HEPHL1	hephaestin like 1 [Source:VGNC Symbol;Acc:VGNC:88848]	3.17	0.0763
ssc-miR-30a-5p	HERC2	HECT and RLD domain containing E3 ubiquitin protein ligase 2 [Source:VGNC Symbol;Acc:VGNC:99716]	3.17	0.0763
ssc-miR-30a-5p	HERC3	HECT and RLD domain containing E3 ubiquitin protein ligase 3 [Source:VGNC Symbol;Acc:VGNC:98934]	3.17	0.0763
ssc-miR-30a-5p	HHIPL1	hypothetical gene	3.17	0.0763
ssc-miR-30a-5p	HIAT1	hypothetical gene	3.17	0.0763
ssc-miR-30a-5p	HIC2	HIC ZBTB transcriptional repressor 2 [Source:HGNC Symbol;Acc:HGNC:18595]	3.17	0.0763
ssc-miR-30a-5p	HIPK1	homeodomain interacting protein kinase 1 [Source:VGNC Symbol;Acc:VGNC:88887]	3.17	0.0763
ssc-miR-30a-5p	HIPK2	homeodomain interacting protein kinase 2 [Source:VGNC Symbol;Acc:VGNC:88888]	3.17	0.0763
ssc-miR-30a-5p	HIRIP3	HIRA interacting protein 3 [Source:HGNC Symbol;Acc:HGNC:4917]	3.17	0.0763
ssc-miR-30a-5p	HIVEP1	HIVEP zinc finger 1 [Source:VGNC Symbol;Acc:VGNC:96585]	3.17	0.0763
ssc-miR-30a-5p	HLF	HLF transcription factor, PAR bZIP family member [Source:VGNC Symbol;Acc:VGNC:88896]	3.17	0.0763
ssc-miR-30a-5p	HLX	H2.0 like homeobox [Source:VGNC Symbol;Acc:VGNC:96345]	3.17	0.0763
ssc-miR-30a-5p	HMGB3	hypothetical gene	3.17	0.0763
ssc-miR-30a-5p	HNRNPA1	heterous nuclear ribonucleoprotein A1 [Source:VGNC Symbol;Acc:VGNC:88918]	3.17	0.0763
ssc-miR-30a-5p	HNRNPA2B1	heterogeneous nuclear ribonucleoprotein A2/B1 [Source:HGNC Symbol;Acc:HGNC:5033]	3.17	0.0763
ssc-miR-30a-5p	HNRNPA3	hypothetical gene	3.17	0.0763
ssc-miR-30a-5p	HNRNPC	hypothetical gene	3.17	0.0763
ssc-miR-30a-5p	HNRNPUL2	heterous nuclear ribonucleoprotein U like 2 [Source:VGNC Symbol;Acc:VGNC:99758]	3.17	0.0763
ssc-miR-30a-5p	HOOK3	hook microtubule tethering protein 3 [Source:VGNC Symbol;Acc:VGNC:98037]	3.17	0.0763
ssc-miR-30a-5p	HOXA1	homeobox A1 [Source:VGNC Symbol;Acc:VGNC:88933]	3.17	0.0763
ssc-miR-30a-5p	HOXA11	homeobox A11 [Source:VGNC Symbol;Acc:VGNC:88935]	3.17	0.0763
ssc-miR-30a-5p	HOXA2	homeobox A2 [Source:VGNC Symbol;Acc:VGNC:88937]	3.17	0.0763
ssc-miR-30a-5p	HOXA9	homeobox A9 [Source:HGNC Symbol;Acc:HGNC:5109]	3.17	0.0763
ssc-miR-30a-5p	HOXB3	homeobox B3 [Source:VGNC Symbol;Acc:VGNC:88944]	3.17	0.0763
ssc-miR-30a-5p	HOXB4	homeobox B4 [Source:HGNC Symbol;Acc:HGNC:5115]	3.17	0.0763
ssc-miR-30a-5p	HOXB8	homeobox B8 [Source:VGNC Symbol;Acc:VGNC:88948]	3.17	0.0763
ssc-miR-30a-5p	HOXD11	homeobox D11 [Source:VGNC Symbol;Acc:VGNC:96352]	3.17	0.0763
ssc-miR-30a-5p	HPCAL1	hippocalcin like 1 [Source:VGNC Symbol;Acc:VGNC:88958]	3.17	0.0763
ssc-miR-30a-5p	HPS4	HPS4 biosis of lysosomal organelles complex 3 subunit 2 [Source:VGNC Symbol;Acc:VGNC:88966]	3.17	0.0763
ssc-miR-30a-5p	HSPA2	hypothetical gene	3.17	0.0763
ssc-miR-30a-5p	HSPA4L	heat shock protein family A (Hsp70) member 4 like [Source:HGNC Symbol;Acc:HGNC:17041]	3.17	0.0763
ssc-miR-30a-5p	HSPA5	heat shock protein family A (Hsp70) member 5 [Source:VGNC Symbol;Acc:VGNC:103107]	3.17	0.0763
ssc-miR-30a-5p	HSPD1	heat shock protein family D (Hsp60) member 1 [Source:NCBI gene (formerly Entrezgene);Acc:492279]	3.17	0.0763
ssc-miR-30a-5p	HSPE1-MOB4	hypothetical gene	3.17	0.0763
ssc-miR-30a-5p	HTRA3	HtrA serine peptidase 3 [Source:VGNC Symbol;Acc:VGNC:89005]	3.17	0.0763

ssc-miR-30a-5p	ICK	hypothetical gene	3.17	0.0763
ssc-miR-30a-5p	IDE	insulin degrading enzyme [Source:VGNC Symbol;Acc:VGNC:89025]	3.17	0.0763
ssc-miR-30a-5p	IDH1	isocitrate dehydrogenase (NADP(+)) 1 [Source:HGNC Symbol;Acc:HGNC:5382]	3.17	0.0763
ssc-miR-30a-5p	IER2	immediate early response 2 [Source:HGNC Symbol;Acc:HGNC:28871]	3.17	0.0763
ssc-miR-30a-5p	IER5	immediate early response 5 [Source:VGNC Symbol;Acc:VGNC:89028]	3.17	0.0763
ssc-miR-30a-5p	IFFO2	intermediate filament family orphan 2 [Source:VGNC Symbol;Acc:VGNC:98475]	3.17	0.0763
ssc-miR-30a-5p	IFNAR2	interferon alpha and beta receptor subunit 2 [Source:NCBI gene (formerly Entrezgene);Acc:100533555]	3.17	0.0763
ssc-miR-30a-5p	IFNLR1	interferon lambda receptor 1 [Source:VGNC Symbol;Acc:VGNC:89043]	3.17	0.0763
ssc-miR-30a-5p	IGF1R	insulin like growth factor 1 receptor [Source:NCBI gene (formerly Entrezgene);Acc:397350]	3.17	0.0763
ssc-miR-30a-5p	IGF2R	insulin like growth factor 2 receptor [Source:VGNC Symbol;Acc:VGNC:89057]	3.17	0.0763
ssc-miR-30a-5p	IGSF3	immunoglobulin superfamily member 3 [Source:VGNC Symbol;Acc:VGNC:89067]	3.17	0.0763
ssc-miR-30a-5p	IKZF2	IKAROS family zinc finger 2 [Source:VGNC Symbol;Acc:VGNC:95576]	3.17	0.0763
ssc-miR-30a-5p	IL1A	interleukin 1 alpha [Source:VGNC Symbol;Acc:VGNC:89091]	3.17	0.0763
ssc-miR-30a-5p	IL1RAPL2	interleukin 1 receptor accessory protein like 2 [Source:VGNC Symbol;Acc:VGNC:103968]	3.17	0.0763
ssc-miR-30a-5p	IL2RA	interleukin 2 receptor subunit alpha [Source:VGNC Symbol;Acc:VGNC:108275]	3.17	0.0763
ssc-miR-30a-5p	ILDR2	immunoglobulin like domain containing receptor 2 [Source:VGNC Symbol;Acc:VGNC:89118]	3.17	0.0763
ssc-miR-30a-5p	ING5	inhibitor of growth family member 5 [Source:VGNC Symbol;Acc:VGNC:95912]	3.17	0.0763
ssc-miR-30a-5p	INHBA	inhibin subunit beta A [Source:VGNC Symbol;Acc:VGNC:89133]	3.17	0.0763
ssc-miR-30a-5p	INO80D	INO80 complex subunit D [Source:VGNC Symbol;Acc:VGNC:96113]	3.17	0.0763
ssc-miR-30a-5p	INPP4A	inositol polyphosphate-4-phosphatase type I A [Source:VGNC Symbol;Acc:VGNC:89140]	3.17	0.0763
ssc-miR-30a-5p	INSIG2	insulin induced 2 [Source:VGNC Symbol;Acc:VGNC:103970]	3.17	0.0763
ssc-miR-30a-5p	INTS2	integrator complex subunit 2 [Source:VGNC Symbol;Acc:VGNC:89162]	3.17	0.0763
ssc-miR-30a-5p	IP6K3	inositol hexakisphosphate kinase 3 [Source:VGNC Symbol;Acc:VGNC:89174]	3.17	0.0763
ssc-miR-30a-5p	IPMK	inositol polyphosphate multikinase [Source:VGNC Symbol;Acc:VGNC:89175]	3.17	0.0763
ssc-miR-30a-5p	IQCG	IQ motif containing G [Source:VGNC Symbol;Acc:VGNC:89189]	3.17	0.0763
ssc-miR-30a-5p	IRF2BP2	interferon regulatory factor 2 binding protein 2 [Source:VGNC Symbol;Acc:VGNC:89205]	3.17	0.0763
ssc-miR-30a-5p	IRF4	interferon regulatory factor 4 [Source:VGNC Symbol;Acc:VGNC:89207]	3.17	0.0763
ssc-miR-30a-5p	IRS1	insulin receptor substrate 1 [Source:VGNC Symbol;Acc:VGNC:96376]	3.17	0.0763
ssc-miR-30a-5p	IRS2	insulin receptor substrate 2 [Source:VGNC Symbol;Acc:VGNC:89214]	3.17	0.0763
ssc-miR-30a-5p	IRX4	iroquois homeobox 4 [Source:VGNC Symbol;Acc:VGNC:89218]	3.17	0.0763
ssc-miR-30a-5p	ITGA2	integrin subunit alpha 2 [Source:VGNC Symbol;Acc:VGNC:89234]	3.17	0.0763
ssc-miR-30a-5p	ITGA4	integrin subunit alpha 4 [Source:VGNC Symbol;Acc:VGNC:96377]	3.17	0.0763
ssc-miR-30a-5p	ITGA6	integrin subunit alpha 6 [Source:VGNC Symbol;Acc:VGNC:96378]	3.17	0.0763
ssc-miR-30a-5p	ITGA8	integrin subunit alpha 8 [Source:VGNC Symbol;Acc:VGNC:96379]	3.17	0.0763
ssc-miR-30a-5p	ITGA9	integrin subunit alpha 9 [Source:VGNC Symbol;Acc:VGNC:89238]	3.17	0.0763
ssc-miR-30a-5p	ITGB3	integrin subunit beta 3 [Source:NCBI gene (formerly Entrezgene);Acc:397063]	3.17	0.0763
ssc-miR-30a-5p	ITPK1	inositol-tetrakisphosphate 1-kinase [Source:VGNC Symbol;Acc:VGNC:89251]	3.17	0.0763
ssc-miR-30a-5p	ITSN1	intersectin 1 [Source:VGNC Symbol;Acc:VGNC:108669]	3.17	0.0763
ssc-miR-30a-5p	JAG2	jagged canonical Notch ligand 2 [Source:VGNC Symbol;Acc:VGNC:89269]	3.17	0.0763
ssc-miR-30a-5p	JAK1	Janus kinase 1 [Source:VGNC Symbol;Acc:VGNC:89270]	3.17	0.0763
ssc-miR-30a-5p	JAKMIP2	janus kinase and microtubule interacting protein 2 [Source:VGNC Symbol;Acc:VGNC:89274]	3.17	0.0763
ssc-miR-30a-5p	JAKMIP3	Janus kinase and microtubule interacting protein 3 [Source:VGNC Symbol;Acc:VGNC:89275]	3.17	0.0763
ssc-miR-30a-5p	JAM2	junctional adhesion molecule 2 [Source:VGNC Symbol;Acc:VGNC:89276]	3.17	0.0763
ssc-miR-30a-5p	JARID2	jumonji and AT-rich interaction domain containing 2 [Source:VGNC Symbol;Acc:VGNC:89279]	3.17	0.0763
ssc-miR-30a-5p	JDP2	Jun dimerization protein 2 [Source:VGNC Symbol;Acc:VGNC:89282]	3.17	0.0763
ssc-miR-30a-5p	JMY	junction mediating and regulatory protein, p53 cofactor [Source:VGNC Symbol;Acc:VGNC:89289]	3.17	0.0763
ssc-miR-30a-5p	JOSD1	Josephin domain containing 1 [Source:VGNC Symbol;Acc:VGNC:89290]	3.17	0.0763
ssc-miR-30a-5p	JPH4	junctophilin 4 [Source:VGNC Symbol;Acc:VGNC:89292]	3.17	0.0763
ssc-miR-30a-5p	JUNB	JunB proto-onco, AP-1 transcription factor subunit [Source:VGNC Symbol;Acc:VGNC:89295]	3.17	0.0763

ssc-miR-30a-5p	KATNBL1	katanin regulatory subunit B1 like 1 [Source:VGNC Symbol;Acc:VGNC:89313]	3.17	0.0763
ssc-miR-30a-5p	KCNA4	potassium voltage-gated channel subfamily A member 4 [Source:VGNC Symbol;Acc:VGNC:89326]	3.17	0.0763
ssc-miR-30a-5p	KCNJ12	hypothetical gene	3.17	0.0763
ssc-miR-30a-5p	KCNJ15	potassium inwardly rectifying channel subfamily J member 15 [Source:VGNC Symbol;Acc:VGNC:89355]	3.17	0.0763
ssc-miR-30a-5p	KCNJ3	potassium inwardly rectifying channel subfamily J member 3 [Source:HGNC Symbol;Acc:HGNC:6264]	3.17	0.0763
ssc-miR-30a-5p	KCNJ6	potassium inwardly rectifying channel subfamily J member 6 [Source:VGNC Symbol;Acc:VGNC:89360]	3.17	0.0763
ssc-miR-30a-5p	KCNN3	potassium calcium-activated channel subfamily N member 3 [Source:VGNC Symbol;Acc:VGNC:98056]	3.17	0.0763
ssc-miR-30a-5p	KCTD16	potassium channel tetramerization domain containing 16 [Source:VGNC Symbol;Acc:VGNC:89395]	3.17	0.0763
ssc-miR-30a-5p	KCTD20	potassium channel tetramerization domain containing 20 [Source:VGNC Symbol;Acc:VGNC:89399]	3.17	0.0763
ssc-miR-30a-5p	KCTD3	potassium channel tetramerization domain containing 3 [Source:VGNC Symbol;Acc:VGNC:95963]	3.17	0.0763
ssc-miR-30a-5p	KCTD5	hypothetical gene	3.17	0.0763
ssc-miR-30a-5p	KCTD7	potassium channel tetramerization domain containing 7 [Source:VGNC Symbol;Acc:VGNC:89403]	3.17	0.0763
ssc-miR-30a-5p	KCTD8	potassium channel tetramerization domain containing 8 [Source:HGNC Symbol;Acc:HGNC:22394]	3.17	0.0763
ssc-miR-30a-5p	KDM3A	lysine demethylase 3A [Source:VGNC Symbol;Acc:VGNC:89411]	3.17	0.0763
ssc-miR-30a-5p	KDM5B	lysine demethylase 5B [Source:VGNC Symbol;Acc:VGNC:95870]	3.17	0.0763
ssc-miR-30a-5p	KDSR	3-ketodihydrosphingosine reductase [Source:VGNC Symbol;Acc:VGNC:108157]	3.17	0.0763
ssc-miR-30a-5p	KHNYN	KH and NYN domain containing [Source:VGNC Symbol;Acc:VGNC:89426]	3.17	0.0763
ssc-miR-30a-5p	KIAA0087	hypothetical gene	3.17	0.0763
ssc-miR-30a-5p	KIAA0101	hypothetical gene	3.17	0.0763
ssc-miR-30a-5p	KIAA0226L	hypothetical gene	3.17	0.0763
ssc-miR-30a-5p	KIAA0247	hypothetical gene	3.17	0.0763
ssc-miR-30a-5p	KIAA0355	hypothetical gene	3.17	0.0763
ssc-miR-30a-5p	KIAA0408	KIAA0408 [Source:VGNC Symbol;Acc:VGNC:89432]	3.17	0.0763
ssc-miR-30a-5p	KIAA0930	KIAA0930 [Source:VGNC Symbol;Acc:VGNC:89438]	3.17	0.0763
ssc-miR-30a-5p	KIAA1024	hypothetical gene	3.17	0.0763
ssc-miR-30a-5p	KIAA1033	hypothetical gene	3.17	0.0763
ssc-miR-30a-5p	KIAA1147	hypothetical gene	3.17	0.0763
ssc-miR-30a-5p	KIAA1211	hypothetical gene	3.17	0.0763
ssc-miR-30a-5p	KIAA1211L	hypothetical gene	3.17	0.0763
ssc-miR-30a-5p	KIAA1244	hypothetical gene	3.17	0.0763
ssc-miR-30a-5p	KIAA1522	KIAA1522 [Source:VGNC Symbol;Acc:VGNC:89444]	3.17	0.0763
ssc-miR-30a-5p	KIAA1549	KIAA1549 [Source:VGNC Symbol;Acc:VGNC:99719]	3.17	0.0763
ssc-miR-30a-5p	KIAA1715	hypothetical gene	3.17	0.0763
ssc-miR-30a-5p	KIAA2026	KIAA2026 [Source:VGNC Symbol;Acc:VGNC:89450]	3.17	0.0763
ssc-miR-30a-5p	KIF16B	kinesin family member 16B [Source:VGNC Symbol;Acc:VGNC:106450]	3.17	0.0763
ssc-miR-30a-5p	KIF1C	kinesin family member 1C [Source:VGNC Symbol;Acc:VGNC:89461]	3.17	0.0763
ssc-miR-30a-5p	KIF21B	kinesin family member 21B [Source:VGNC Symbol;Acc:VGNC:96219]	3.17	0.0763
ssc-miR-30a-5p	KIF3A	kinesin family member 3A [Source:VGNC Symbol;Acc:VGNC:89470]	3.17	0.0763
ssc-miR-30a-5p	KIF3C	kinesin family member 3C [Source:VGNC Symbol;Acc:VGNC:89471]	3.17	0.0763
ssc-miR-30a-5p	KLF10	Kruppel like factor 10 [Source:VGNC Symbol;Acc:VGNC:89490]	3.17	0.0763
ssc-miR-30a-5p	KLF11	Kruppel like factor 11 [Source:VGNC Symbol;Acc:VGNC:89491]	3.17	0.0763
ssc-miR-30a-5p	KLF12	Kruppel like factor 12 [Source:VGNC Symbol;Acc:VGNC:89492]	3.17	0.0763
ssc-miR-30a-5p	KLF13	Kruppel like factor 13 [Source:VGNC Symbol;Acc:VGNC:89493]	3.17	0.0763
ssc-miR-30a-5p	KLF14	Kruppel like factor 14 [Source:VGNC Symbol;Acc:VGNC:89494]	3.17	0.0763
ssc-miR-30a-5p	KLF8	Kruppel like factor 8 [Source:VGNC Symbol;Acc:VGNC:89500]	3.17	0.0763
ssc-miR-30a-5p	KLF9	Kruppel like factor 9 [Source:VGNC Symbol;Acc:VGNC:103114]	3.17	0.0763
ssc-miR-30a-5p	KLHL20	kelch like family member 20 [Source:VGNC Symbol;Acc:VGNC:89519]	3.17	0.0763
ssc-miR-30a-5p	KLHL23	kelch like family member 23 [Source:VGNC Symbol;Acc:VGNC:96158]	3.17	0.0763
ssc-miR-30a-5p	KLHL24	kelch like family member 24 [Source:VGNC Symbol;Acc:VGNC:89521]	3.17	0.0763

ssc-miR-30a-5p	KLHL28	kelch like family member 28 [Source:VGNC Symbol;Acc:VGNC:89523]	3.17	0.0763
ssc-miR-30a-5p	KMT2A	lysine methyltransferase 2A [Source:VGNC Symbol;Acc:VGNC:108600]	3.17	0.0763
ssc-miR-30a-5p	KMT2C	lysine methyltransferase 2C [Source:VGNC Symbol;Acc:VGNC:89550]	3.17	0.0763
ssc-miR-30a-5p	KMT2D	hypothetical gene	3.17	0.0763
ssc-miR-30a-5p	KPNA3	karyopherin subunit alpha 3 [Source:VGNC Symbol;Acc:VGNC:89562]	3.17	0.0763
ssc-miR-30a-5p	KPNA6	karyopherin subunit alpha 6 [Source:VGNC Symbol;Acc:VGNC:89565]	3.17	0.0763
ssc-miR-30a-5p	KRAS	KRAS proto-onco, GTPase [Source:VGNC Symbol;Acc:VGNC:89569]	3.17	0.0763
ssc-miR-30a-5p	KREMEN1	kringle containing transmembrane protein 1 [Source:VGNC Symbol;Acc:VGNC:89572]	3.17	0.0763
ssc-miR-30a-5p	KSR1	kinase suppressor of ras 1 [Source:VGNC Symbol;Acc:VGNC:89606]	3.17	0.0763
ssc-miR-30a-5p	KXD1	KxDL motif containing 1 [Source:HGNC Symbol;Acc:HGNC:28420]	3.17	0.0763
ssc-miR-30a-5p	LARGE	hypothetical gene	3.17	0.0763
ssc-miR-30a-5p	LARP1	La ribonucleoprotein 1, translational regulator [Source:VGNC Symbol;Acc:VGNC:98067]	3.17	0.0763
ssc-miR-30a-5p	LARP1B	La ribonucleoprotein 1B [Source:VGNC Symbol;Acc:VGNC:89640]	3.17	0.0763
ssc-miR-30a-5p	LARP4	La ribonucleoprotein 4 [Source:VGNC Symbol;Acc:VGNC:89641]	3.17	0.0763
ssc-miR-30a-5p	LATS2	large tumor suppressor kinase 2 [Source:VGNC Symbol;Acc:VGNC:89649]	3.17	0.0763
ssc-miR-30a-5p	LAYN	layilin [Source:VGNC Symbol;Acc:VGNC:89651]	3.17	0.0763
ssc-miR-30a-5p	LCLAT1	lysocardiolipin acyltransferase 1 [Source:VGNC Symbol;Acc:VGNC:89658]	3.17	0.0763
ssc-miR-30a-5p	LCOR	ligand dependent nuclear receptor corepressor [Source:HGNC Symbol;Acc:HGNC:29503]	3.17	0.0763
ssc-miR-30a-5p	LDLR	low density lipoprotein receptor [Source:VGNC Symbol;Acc:VGNC:89671]	3.17	0.0763
ssc-miR-30a-5p	LEPR	leptin receptor [Source:VGNC Symbol;Acc:VGNC:89685]	3.17	0.0763
ssc-miR-30a-5p	LETMD1	LETM1 domain containing 1 [Source:VGNC Symbol;Acc:VGNC:89688]	3.17	0.0763
ssc-miR-30a-5p	LGI1	leucine rich glioma inactivated 1 [Source:HGNC Symbol;Acc:HGNC:6572]	3.17	0.0763
ssc-miR-30a-5p	LHFPL2	LHFPL tetraspan subfamily member 2 [Source:VGNC Symbol;Acc:VGNC:89705]	3.17	0.0763
ssc-miR-30a-5p	LHX1	LIM homeobox 1 [Source:VGNC Symbol;Acc:VGNC:89711]	3.17	0.0763
ssc-miR-30a-5p	LHX5	LIM homeobox 5 [Source:VGNC Symbol;Acc:VGNC:89715]	3.17	0.0763
ssc-miR-30a-5p	LHX8	LIM homeobox 8 [Source:VGNC Symbol;Acc:VGNC:89717]	3.17	0.0763
ssc-miR-30a-5p	LHX9	LIM homeobox 9 [Source:VGNC Symbol;Acc:VGNC:95608]	3.17	0.0763
ssc-miR-30a-5p	LIFR	LIF receptor subunit alpha [Source:HGNC Symbol;Acc:HGNC:6597]	3.17	0.0763
ssc-miR-30a-5p	LIMCH1	LIM and calponin homology domains 1 [Source:VGNC Symbol;Acc:VGNC:89724]	3.17	0.0763
ssc-miR-30a-5p	LIN28A	lin-28 homolog A [Source:VGNC Symbol;Acc:VGNC:98492]	3.17	0.0763
ssc-miR-30a-5p	LIN28B	lin-28 homolog B [Source:VGNC Symbol;Acc:VGNC:89729]	3.17	0.0763
ssc-miR-30a-5p	LIN7C	lin-7 homolog C, crumbs cell polarity complex component [Source:VGNC Symbol;Acc:VGNC:89732]	3.17	0.0763
ssc-miR-30a-5p	LMBR1	limb development membrane protein 1 [Source:VGNC Symbol;Acc:VGNC:89752]	3.17	0.0763
ssc-miR-30a-5p	LMBR1L	limb development membrane protein 1 like [Source:VGNC Symbol;Acc:VGNC:89753]	3.17	0.0763
ssc-miR-30a-5p	LMLN	leishmanolysin like peptidase [Source:VGNC Symbol;Acc:VGNC:89759]	3.17	0.0763
ssc-miR-30a-5p	LONRF1	LON peptidase N-terminal domain and ring finger 1 [Source:VGNC Symbol;Acc:VGNC:96123]	3.17	0.0763
ssc-miR-30a-5p	LONRF3	LON peptidase N-terminal domain and ring finger 3 [Source:VGNC Symbol;Acc:VGNC:89778]	3.17	0.0763
ssc-miR-30a-5p	LOX	lysyl oxidase [Source:VGNC Symbol;Acc:VGNC:99785]	3.17	0.0763
ssc-miR-30a-5p	LPAR3	lysophosphatidic acid receptor 3 [Source:VGNC Symbol;Acc:VGNC:89785]	3.17	0.0763
ssc-miR-30a-5p	LPGAT1	lysophosphatidylglycerol acyltransferase 1 [Source:VGNC Symbol;Acc:VGNC:89792]	3.17	0.0763
ssc-miR-30a-5p	LPHN3	hypothetical gene	3.17	0.0763
ssc-miR-30a-5p	LPP	LIM domain containing preferred translocation partner in lipoma [Source:HGNC Symbol;Acc:HGNC:6679]	3.17	0.0763
ssc-miR-30a-5p	LPPR4	hypothetical gene	3.17	0.0763
ssc-miR-30a-5p	LRCH2	leucine rich repeats and calponin homology domain containing 2 [Source:VGNC Symbol;Acc:VGNC:89802]	3.17	0.0763
ssc-miR-30a-5p	LRFN2	leucine rich repeat and fibronectin type III domain containing 2 [Source:VGNC Symbol;Acc:VGNC:89805]	3.17	0.0763
ssc-miR-30a-5p	LRIG2	leucine rich repeats and immunoglobulin like domains 2 [Source:VGNC Symbol;Acc:VGNC:89811]	3.17	0.0763
ssc-miR-30a-5p	LRP6	LDL receptor related protein 6 [Source:VGNC Symbol;Acc:VGNC:89821]	3.17	0.0763
ssc-miR-30a-5p	LRRC17	leucine rich repeat containing 17 [Source:VGNC Symbol;Acc:VGNC:89828]	3.17	0.0763
ssc-miR-30a-5p	LRRC40	hypothetical gene	3.17	0.0763

ssc-miR-30a-5p	LRRC58	leucine rich repeat containing 58 [Source:VGNC Symbol;Acc:VGNC:98081]	3.17	0.0763
ssc-miR-30a-5p	LRRC8C	leucine rich repeat containing 8 VRAC subunit C [Source:VGNC Symbol;Acc:VGNC:98087]	3.17	0.0763
ssc-miR-30a-5p	LRRC8D	leucine rich repeat containing 8 VRAC subunit D [Source:VGNC Symbol;Acc:VGNC:98088]	3.17	0.0763
ssc-miR-30a-5p	LRRK2	leucine rich repeat kinase 2 [Source:VGNC Symbol;Acc:VGNC:98094]	3.17	0.0763
ssc-miR-30a-5p	LSM14B	LSM family member 14B [Source:VGNC Symbol;Acc:VGNC:95691]	3.17	0.0763
ssc-miR-30a-5p	LUC7L2	LUC7 like 2, pre-mRNA splicing factor [Source:HGNC Symbol;Acc:HGNC:21608]	3.17	0.0763
ssc-miR-30a-5p	LY75-CD302	hypothetical gene	3.17	0.0763
ssc-miR-30a-5p	LYN	LYN proto-onco, Src family tyrosine kinase [Source:VGNC Symbol;Acc:VGNC:89910]	3.17	0.0763
ssc-miR-30a-5p	LYSMD3	LysM domain containing 3 [Source:VGNC Symbol;Acc:VGNC:89919]	3.17	0.0763
ssc-miR-30a-5p	LYST	lysosomal trafficking regulator [Source:VGNC Symbol;Acc:VGNC:89921]	3.17	0.0763
ssc-miR-30a-5p	MAB21L1	mab-21 like 1 [Source:VGNC Symbol;Acc:VGNC:89932]	3.17	0.0763
ssc-miR-30a-5p	MAF	MAF bZIP transcription factor [Source:VGNC Symbol;Acc:VGNC:89945]	3.17	0.0763
ssc-miR-30a-5p	MAFG	MAF bZIP transcription factor G [Source:VGNC Symbol;Acc:VGNC:89948]	3.17	0.0763
ssc-miR-30a-5p	MAGI2	membrane associated guanylate kinase, WW and PDZ domain containing 2 [Source:VGNC Symbol;Acc:VGNC:89955]	3.17	0.0763
ssc-miR-30a-5p	MAGI3	membrane associated guanylate kinase, WW and PDZ domain containing 3 [Source:VGNC Symbol;Acc:VGNC:89956]	3.17	0.0763
ssc-miR-30a-5p	MAL	hypothetical gene	3.17	0.0763
ssc-miR-30a-5p	MAML1	mastermind like transcriptional coactivator 1 [Source:VGNC Symbol;Acc:VGNC:89963]	3.17	0.0763
ssc-miR-30a-5p	MAN1A2	mannosidase alpha class 1A member 2 [Source:VGNC Symbol;Acc:VGNC:89967]	3.17	0.0763
ssc-miR-30a-5p	MAN1B1	hypothetical gene	3.17	0.0763
ssc-miR-30a-5p	MAP2K6	mitogen-activated protein kinase kinase 6 [Source:VGNC Symbol;Acc:VGNC:98102]	3.17	0.0763
ssc-miR-30a-5p	MAP3K1	mitogen-activated protein kinase kinase kinase 1 [Source:VGNC Symbol;Acc:VGNC:98104]	3.17	0.0763
ssc-miR-30a-5p	MAP3K12	mitogen-activated protein kinase kinase kinase 12 [Source:VGNC Symbol;Acc:VGNC:89983]	3.17	0.0763
ssc-miR-30a-5p	MAP3K19	mitogen-activated protein kinase kinase kinase 19 [Source:VGNC Symbol;Acc:VGNC:98106]	3.17	0.0763
ssc-miR-30a-5p	MAP3K2	mitogen-activated protein kinase kinase kinase 2 [Source:VGNC Symbol;Acc:VGNC:98107]	3.17	0.0763
ssc-miR-30a-5p	MAP3K5	mitogen-activated protein kinase kinase kinase 5 [Source:VGNC Symbol;Acc:VGNC:89985]	3.17	0.0763
ssc-miR-30a-5p	MAP3K7	mitogen-activated protein kinase kinase kinase 7 [Source:VGNC Symbol;Acc:VGNC:89987]	3.17	0.0763
ssc-miR-30a-5p	MAP4K4	mitogen-activated protein kinase kinase kinase kinase 4 [Source:VGNC Symbol;Acc:VGNC:98114]	3.17	0.0763
ssc-miR-30a-5p	MAPK8	mitogen-activated protein kinase 8 [Source:VGNC Symbol;Acc:VGNC:90006]	3.17	0.0763
ssc-miR-30a-5p	MAPKBP1	mitogen-activated protein kinase binding protein 1 [Source:VGNC Symbol;Acc:VGNC:90014]	3.17	0.0763
ssc-miR-30a-5p	MARCH3	hypothetical gene	3.17	0.0763
ssc-miR-30a-5p	MARCH4	hypothetical gene	3.17	0.0763
ssc-miR-30a-5p	MARCH6	hypothetical gene	3.17	0.0763
ssc-miR-30a-5p	MARCH8	hypothetical gene	3.17	0.0763
ssc-miR-30a-5p	MARCKS	myristoylated alanine rich protein kinase C substrate [Source:VGNC Symbol;Acc:VGNC:90024]	3.17	0.0763
ssc-miR-30a-5p	MARK1	microtubule affinity regulating kinase 1 [Source:VGNC Symbol;Acc:VGNC:96411]	3.17	0.0763
ssc-miR-30a-5p	MARK3	microtubule affinity regulating kinase 3 [Source:VGNC Symbol;Acc:VGNC:90026]	3.17	0.0763
ssc-miR-30a-5p	MAST3	microtubule associated serine/threonine kinase 3 [Source:VGNC Symbol;Acc:VGNC:90036]	3.17	0.0763
ssc-miR-30a-5p	MAT2A	methionine adenosyltransferase 2A [Source:VGNC Symbol;Acc:VGNC:90039]	3.17	0.0763
ssc-miR-30a-5p	MATR3	matrin 3 [Source:HGNC Symbol;Acc:HGNC:6912]	3.17	0.0763
ssc-miR-30a-5p	MAU2	MAU2 sister chromatid cohesion factor [Source:VGNC Symbol;Acc:VGNC:90045]	3.17	0.0763
ssc-miR-30a-5p	MBD6	methyl-CpG binding domain protein 6 [Source:VGNC Symbol;Acc:VGNC:90051]	3.17	0.0763
ssc-miR-30a-5p	MBNL1	muscleblind like splicing regulator 1 [Source:VGNC Symbol;Acc:VGNC:90054]	3.17	0.0763
ssc-miR-30a-5p	MBNL2	muscleblind like splicing regulator 2 [Source:VGNC Symbol;Acc:VGNC:90055]	3.17	0.0763
ssc-miR-30a-5p	MBNL3	muscleblind like splicing regulator 3 [Source:VGNC Symbol;Acc:VGNC:90056]	3.17	0.0763
ssc-miR-30a-5p	MBOAT1	membrane bound O-acyltransferase domain containing 1 [Source:VGNC Symbol;Acc:VGNC:90057]	3.17	0.0763
ssc-miR-30a-5p	MBTPS2	hypothetical gene	3.17	0.0763
ssc-miR-30a-5p	MCF2L	MCF.2 cell line derived transforming sequence like [Source:VGNC Symbol;Acc:VGNC:90068]	3.17	0.0763
ssc-miR-30a-5p	MCTS1	MCTS1 re-initiation and release factor [Source:HGNC Symbol;Acc:HGNC:23357]	3.17	0.0763
ssc-miR-30a-5p	MDM4	MDM4 regulator of p53 [Source:VGNC Symbol;Acc:VGNC:90094]	3.17	0.0763

ssc-miR-30a-5p	ME1	malic enzyme 1 [Source:VGNC Symbol;Acc:VGNC:90095]	3.17	0.0763
ssc-miR-30a-5p	MECP2	methyl-CpG binding protein 2 [Source:VGNC Symbol;Acc:VGNC:90101]	3.17	0.0763
ssc-miR-30a-5p	MED12L	mediator complex subunit 12L [Source:VGNC Symbol;Acc:VGNC:90105]	3.17	0.0763
ssc-miR-30a-5p	MEF2D	myocyte enhancer factor 2D [Source:VGNC Symbol;Acc:VGNC:90128]	3.17	0.0763
ssc-miR-30a-5p	MEIS2	Meis homeobox 2 [Source:VGNC Symbol;Acc:VGNC:90136]	3.17	0.0763
ssc-miR-30a-5p	METAP2	methionyl aminopeptidase 2 [Source:VGNC Symbol;Acc:VGNC:90152]	3.17	0.0763
ssc-miR-30a-5p	METTL1	methyltransferase 1, tRNA methylguanosine [Source:HGNC Symbol;Acc:HGNC:7030]	3.17	0.0763
ssc-miR-30a-5p	MEX3B	mex-3 RNA binding family member B [Source:VGNC Symbol;Acc:VGNC:90169]	3.17	0.0763
ssc-miR-30a-5p	MEX3D	mex-3 RNA binding family member D [Source:VGNC Symbol;Acc:VGNC:90171]	3.17	0.0763
ssc-miR-30a-5p	MFAP3L	microfibril associated protein 3 like [Source:VGNC Symbol;Acc:VGNC:90175]	3.17	0.0763
ssc-miR-30a-5p	MFHAS1	multifunctional ROCO family signaling regulator 1 [Source:VGNC Symbol;Acc:VGNC:107361]	3.17	0.0763
ssc-miR-30a-5p	MFSD11	major facilitator superfamily domain containing 11 [Source:VGNC Symbol;Acc:VGNC:90184]	3.17	0.0763
ssc-miR-30a-5p	MFSD6	major facilitator superfamily domain containing 6 [Source:VGNC Symbol;Acc:VGNC:96052]	3.17	0.0763
ssc-miR-30a-5p	MIA3	hypothetical gene	3.17	0.0763
ssc-miR-30a-5p	MIB1	MIB E3 ubiquitin protein ligase 1 [Source:VGNC Symbol;Acc:VGNC:90205]	3.17	0.0763
ssc-miR-30a-5p	MICAL1	microtubule associated monooxygenase, calponin and LIM domain containing 1 [Source:VGNC Symbol;Acc:VGNC:90207]	3.17	0.0763
ssc-miR-30a-5p	MICU3	mitochondrial calcium uptake family member 3 [Source:VGNC Symbol;Acc:VGNC:96160]	3.17	0.0763
ssc-miR-30a-5p	MID2	midline 2 [Source:VGNC Symbol;Acc:VGNC:98127]	3.17	0.0763
ssc-miR-30a-5p	MIER2	MIER family member 2 [Source:VGNC Symbol;Acc:VGNC:90220]	3.17	0.0763
ssc-miR-30a-5p	MIER3	MIER family member 3 [Source:VGNC Symbol;Acc:VGNC:90221]	3.17	0.0763
ssc-miR-30a-5p	MINOS1	hypothetical gene	3.17	0.0763
ssc-miR-30a-5p	MINPP1	multiple inositol-polyphosphate phosphatase 1 [Source:VGNC Symbol;Acc:VGNC:90233]	3.17	0.0763
ssc-miR-30a-5p	MIS12	MIS12 kinetochore complex component [Source:VGNC Symbol;Acc:VGNC:90238]	3.17	0.0763
ssc-miR-30a-5p	MIS18BP1	MIS18 binding protein 1 [Source:VGNC Symbol;Acc:VGNC:90240]	3.17	0.0763
ssc-miR-30a-5p	MKL2	hypothetical gene	3.17	0.0763
ssc-miR-30a-5p	MKRN3	hypothetical gene	3.17	0.0763
ssc-miR-30a-5p	MLK4	hypothetical gene	3.17	0.0763
ssc-miR-30a-5p	MLXIP	MLX interacting protein [Source:VGNC Symbol;Acc:VGNC:90262]	3.17	0.0763
ssc-miR-30a-5p	MMD	monocyte to macrophage differentiation associated [Source:VGNC Symbol;Acc:VGNC:90264]	3.17	0.0763
ssc-miR-30a-5p	MMP16	matrix metallopeptidase 16 [Source:VGNC Symbol;Acc:VGNC:90271]	3.17	0.0763
ssc-miR-30a-5p	MMP19	matrix metallopeptidase 19 [Source:VGNC Symbol;Acc:VGNC:90273]	3.17	0.0763
ssc-miR-30a-5p	MNT	MAX network transcriptional repressor [Source:VGNC Symbol;Acc:VGNC:90289]	3.17	0.0763
ssc-miR-30a-5p	MOB4	hypothetical gene	3.17	0.0763
ssc-miR-30a-5p	MOV10	Mov10 RISC complex RNA helicase [Source:VGNC Symbol;Acc:VGNC:90316]	3.17	0.0763
ssc-miR-30a-5p	MPPED1	metallophosphoesterase domain containing 1 [Source:VGNC Symbol;Acc:VGNC:90330]	3.17	0.0763
ssc-miR-30a-5p	MSANTD3-TMEFF1	hypothetical gene	3.17	0.0763
ssc-miR-30a-5p	MSANTD4	Myb/SANT DNA binding domain containing 4 with coiled-coils [Source:VGNC Symbol;Acc:VGNC:90415]	3.17	0.0763
ssc-miR-30a-5p	MSI2	musashi RNA binding protein 2 [Source:VGNC Symbol;Acc:VGNC:90422]	3.17	0.0763
ssc-miR-30a-5p	MTA1	hypothetical gene	3.17	0.0763
ssc-miR-30a-5p	MTDH	metadherin [Source:VGNC Symbol;Acc:VGNC:90439]	3.17	0.0763
ssc-miR-30a-5p	MTF1	metal regulatory transcription factor 1 [Source:VGNC Symbol;Acc:VGNC:90443]	3.17	0.0763
ssc-miR-30a-5p	MTF2	metal response element binding transcription factor 2 [Source:VGNC Symbol;Acc:VGNC:90444]	3.17	0.0763
ssc-miR-30a-5p	MTHFD1L	methylenetetrahydrofolate dehydrogenase (NADP+ dependent) 1 like [Source:VGNC Symbol;Acc:VGNC:103129]	3.17	0.0763
ssc-miR-30a-5p	MTTP	microsomal triglyceride transfer protein [Source:VGNC Symbol;Acc:VGNC:98942]	3.17	0.0763
ssc-miR-30a-5p	MXRA5	hypothetical gene	3.17	0.0763
ssc-miR-30a-5p	MYBL2	MYB proto-onco like 2 [Source:VGNC Symbol;Acc:VGNC:103997]	3.17	0.0763
ssc-miR-30a-5p	MYH11	myosin heavy chain 11 [Source:VGNC Symbol;Acc:VGNC:90510]	3.17	0.0763
ssc-miR-30a-5p	MYLK	myosin light chain kinase [Source:VGNC Symbol;Acc:VGNC:108676]	3.17	0.0763
ssc-miR-30a-5p	MYO5A	myosin VA [Source:HGNC Symbol;Acc:HGNC:7602]	3.17	0.0763

ssc-miR-30a-5p	MYO9B	myosin IXB [Source:VGNC Symbol;Acc:VGNC:90534]	3.17	0.0763
ssc-miR-30a-5p	MYOM2	myomesin 2 [Source:HGNC Symbol;Acc:HGNC:7614]	3.17	0.0763
ssc-miR-30a-5p	MYSM1	Myb like, SWIRM and MPN domains 1 [Source:VGNC Symbol;Acc:VGNC:90548]	3.17	0.0763
ssc-miR-30a-5p	MZT1	mitotic spindle organizing protein 1 [Source:VGNC Symbol;Acc:VGNC:90552]	3.17	0.0763
ssc-miR-30a-5p	N4BP2	NEDD4 binding protein 2 [Source:VGNC Symbol;Acc:VGNC:90554]	3.17	0.0763
ssc-miR-30a-5p	NAA25	N-alpha-acetyltransferase 25, NatB auxiliary subunit [Source:VGNC Symbol;Acc:VGNC:96750]	3.17	0.0763
ssc-miR-30a-5p	NAALADL2	N-acetylated alpha-linked acidic dipeptidase like 2 [Source:VGNC Symbol;Acc:VGNC:90559]	3.17	0.0763
ssc-miR-30a-5p	NACC2	NACC family member 2 [Source:VGNC Symbol;Acc:VGNC:90564]	3.17	0.0763
ssc-miR-30a-5p	NADK	NAD kinase [Source:VGNC Symbol;Acc:VGNC:90565]	3.17	0.0763
ssc-miR-30a-5p	NAGPA	N-acetylglucosamine-1-phosphodiester alpha-N-acetylglucosaminidase [Source:NCBI gene (formerly Entrezgene);Acc:100512596]	3.17	0.0763
ssc-miR-30a-5p	NAP1L1	nucleosome assembly protein 1 like 1 [Source:VGNC Symbol;Acc:VGNC:90576]	3.17	0.0763
ssc-miR-30a-5p	NAP1L5	nucleosome assembly protein 1 like 5 [Source:NCBI gene (formerly Entrezgene);Acc:100616076]	3.17	0.0763
ssc-miR-30a-5p	NAPG	NSF attachment protein gamma [Source:VGNC Symbol;Acc:VGNC:90580]	3.17	0.0763
ssc-miR-30a-5p	NAV1	neuron navigator 1 [Source:VGNC Symbol;Acc:VGNC:95725]	3.17	0.0763
ssc-miR-30a-5p	NAV3	neuron navigator 3 [Source:VGNC Symbol;Acc:VGNC:90589]	3.17	0.0763
ssc-miR-30a-5p	NCALD	neurocalcin delta [Source:VGNC Symbol;Acc:VGNC:90594]	3.17	0.0763
ssc-miR-30a-5p	NCAM1	neural cell adhesion molecule 1 [Source:VGNC Symbol;Acc:VGNC:108603]	3.17	0.0763
ssc-miR-30a-5p	NCOA3	nuclear receptor coactivator 3 [Source:VGNC Symbol;Acc:VGNC:96434]	3.17	0.0763
ssc-miR-30a-5p	NCS1	neuronal calcium sensor 1 [Source:VGNC Symbol;Acc:VGNC:90620]	3.17	0.0763
ssc-miR-30a-5p	NDEL1	nudE neurodevelopment protein 1 like 1 [Source:VGNC Symbol;Acc:VGNC:90625]	3.17	0.0763
ssc-miR-30a-5p	NDNF	neuron derived neurotrophic factor [Source:VGNC Symbol;Acc:VGNC:90629]	3.17	0.0763
ssc-miR-30a-5p	NECAB1	N-terminal EF-hand calcium binding protein 1 [Source:VGNC Symbol;Acc:VGNC:90659]	3.17	0.0763
ssc-miR-30a-5p	NECAP1	NECAP endocytosis associated 1 [Source:VGNC Symbol;Acc:VGNC:90660]	3.17	0.0763
ssc-miR-30a-5p	NEDD4	NEDD4 E3 ubiquitin protein ligase [Source:VGNC Symbol;Acc:VGNC:90666]	3.17	0.0763
ssc-miR-30a-5p	NEDD4L	NEDD4 like E3 ubiquitin protein ligase [Source:VGNC Symbol;Acc:VGNC:90667]	3.17	0.0763
ssc-miR-30a-5p	NEFM	hypothetical gene	3.17	0.0763
ssc-miR-30a-5p	NEGR1	neuronal growth regulator 1 [Source:VGNC Symbol;Acc:VGNC:90671]	3.17	0.0763
ssc-miR-30a-5p	NEURL1B	neuralized E3 ubiquitin protein ligase 1B [Source:VGNC Symbol;Acc:VGNC:90695]	3.17	0.0763
ssc-miR-30a-5p	NEUROD1	neuronal differentiation 1 [Source:VGNC Symbol;Acc:VGNC:96439]	3.17	0.0763
ssc-miR-30a-5p	NEUROD6	neuronal differentiation 6 [Source:VGNC Symbol;Acc:VGNC:90699]	3.17	0.0763
ssc-miR-30a-5p	NF1	neurofibromin 1 [Source:VGNC Symbol;Acc:VGNC:90704]	3.17	0.0763
ssc-miR-30a-5p	NFAT5	nuclear factor of activated T cells 5 [Source:VGNC Symbol;Acc:VGNC:90708]	3.17	0.0763
ssc-miR-30a-5p	NFATC2	nuclear factor of activated T cells 2 [Source:VGNC Symbol;Acc:VGNC:96440]	3.17	0.0763
ssc-miR-30a-5p	NFATC3	nuclear factor of activated T cells 3 [Source:VGNC Symbol;Acc:VGNC:90710]	3.17	0.0763
ssc-miR-30a-5p	NFIA	nuclear factor I A [Source:VGNC Symbol;Acc:VGNC:90715]	3.17	0.0763
ssc-miR-30a-5p	NFIB	nuclear factor I B [Source:VGNC Symbol;Acc:VGNC:90716]	3.17	0.0763
ssc-miR-30a-5p	NFYA	nuclear transcription factor Y subunit alpha [Source:VGNC Symbol;Acc:VGNC:90729]	3.17	0.0763
ssc-miR-30a-5p	NFYB	nuclear transcription factor Y subunit beta [Source:VGNC Symbol;Acc:VGNC:90730]	3.17	0.0763
ssc-miR-30a-5p	NHLH2	nescient helix-loop-helix 2 [Source:VGNC Symbol;Acc:VGNC:90736]	3.17	0.0763
ssc-miR-30a-5p	NHS	NHS actin remodeling regulator [Source:VGNC Symbol;Acc:VGNC:90738]	3.17	0.0763
ssc-miR-30a-5p	NID1	nidogen 1 [Source:VGNC Symbol;Acc:VGNC:90742]	3.17	0.0763
ssc-miR-30a-5p	NIPAL1	NIPA like domain containing 1 [Source:VGNC Symbol;Acc:VGNC:90748]	3.17	0.0763
ssc-miR-30a-5p	NKAIN2	sodium/potassium transporting ATPase interacting 2 [Source:VGNC Symbol;Acc:VGNC:103141]	3.17	0.0763
ssc-miR-30a-5p	NKX2-2	NK2 homeobox 2 [Source:VGNC Symbol;Acc:VGNC:96444]	3.17	0.0763
ssc-miR-30a-5p	NLGN1	neuroligin 1 [Source:VGNC Symbol;Acc:VGNC:90777]	3.17	0.0763
ssc-miR-30a-5p	NLK	nemo like kinase [Source:VGNC Symbol;Acc:VGNC:90779]	3.17	0.0763
ssc-miR-30a-5p	NOTCH1	notch receptor 1 [Source:HGNC Symbol;Acc:HGNC:7881]	3.17	0.0763
ssc-miR-30a-5p	NOVA1	NOVA alternative splicing regulator 1 [Source:VGNC Symbol;Acc:VGNC:90827]	3.17	0.0763
ssc-miR-30a-5p	NPTXR	neuronal pentraxin receptor [Source:VGNC Symbol;Acc:VGNC:98158]	3.17	0.0763

ssc-miR-30a-5p	NR2F2	nuclear receptor subfamily 2 group F member 2 [Source:VGNC Symbol;Acc:VGNC:90881]	3.17	0.0763
ssc-miR-30a-5p	NR3C1	nuclear receptor subfamily 3 group C member 1 [Source:VGNC Symbol;Acc:VGNC:90883]	3.17	0.0763
ssc-miR-30a-5p	NR3C2	nuclear receptor subfamily 3 group C member 2 [Source:VGNC Symbol;Acc:VGNC:90884]	3.17	0.0763
ssc-miR-30a-5p	NR4A2	nuclear receptor subfamily 4 group A member 2 [Source:VGNC Symbol;Acc:VGNC:96451]	3.17	0.0763
ssc-miR-30a-5p	NR5A2	nuclear receptor subfamily 5 group A member 2 [Source:VGNC Symbol;Acc:VGNC:96452]	3.17	0.0763
ssc-miR-30a-5p	NR6A1	nuclear receptor subfamily 6 group A member 1 [Source:VGNC Symbol;Acc:VGNC:90887]	3.17	0.0763
ssc-miR-30a-5p	NRBF2	nuclear receptor binding factor 2 [Source:VGNC Symbol;Acc:VGNC:90890]	3.17	0.0763
ssc-miR-30a-5p	NRBP1	nuclear receptor binding protein 1 [Source:VGNC Symbol;Acc:VGNC:90891]	3.17	0.0763
ssc-miR-30a-5p	NREP	neuronal regeneration related protein [Source:HGNC Symbol;Acc:HGNC:16834]	3.17	0.0763
ssc-miR-30a-5p	NRG2	neuregulin 2 [Source:VGNC Symbol;Acc:VGNC:98160]	3.17	0.0763
ssc-miR-30a-5p	NRG3	neuregulin 3 [Source:VGNC Symbol;Acc:VGNC:104011]	3.17	0.0763
ssc-miR-30a-5p	NRIP1	nuclear receptor interacting protein 1 [Source:VGNC Symbol;Acc:VGNC:90896]	3.17	0.0763
ssc-miR-30a-5p	NRK	Nik related kinase [Source:VGNC Symbol;Acc:VGNC:98161]	3.17	0.0763
ssc-miR-30a-5p	NRP1	neuropilin 1 [Source:VGNC Symbol;Acc:VGNC:104012]	3.17	0.0763
ssc-miR-30a-5p	NRXN3	neurexin 3 [Source:HGNC Symbol;Acc:HGNC:8010]	3.17	0.0763
ssc-miR-30a-5p	NSD1	nuclear receptor binding SET domain protein 1 [Source:VGNC Symbol;Acc:VGNC:90904]	3.17	0.0763
ssc-miR-30a-5p	NSG1	neuronal vesicle trafficking associated 1 [Source:VGNC Symbol;Acc:VGNC:90907]	3.17	0.0763
ssc-miR-30a-5p	NSUN3	NOP2/Sun RNA methyltransferase 3 [Source:VGNC Symbol;Acc:VGNC:90915]	3.17	0.0763
ssc-miR-30a-5p	NT5DC1	5'-nucleotidase domain containing 1 [Source:VGNC Symbol;Acc:VGNC:90922]	3.17	0.0763
ssc-miR-30a-5p	NT5DC3	5'-nucleotidase domain containing 3 [Source:VGNC Symbol;Acc:VGNC:90924]	3.17	0.0763
ssc-miR-30a-5p	NT5E	5'-nucleotidase ecto [Source:VGNC Symbol;Acc:VGNC:90925]	3.17	0.0763
ssc-miR-30a-5p	NTNG1	netrin G1 [Source:VGNC Symbol;Acc:VGNC:90935]	3.17	0.0763
ssc-miR-30a-5p	NUAK1	NUAK family kinase 1 [Source:VGNC Symbol;Acc:VGNC:90940]	3.17	0.0763
ssc-miR-30a-5p	NUCKS1	nuclear casein kinase and cyclin dependent kinase substrate 1 [Source:VGNC Symbol;Acc:VGNC:90947]	3.17	0.0763
ssc-miR-30a-5p	NUDT5	nudix hydrolase 5 [Source:VGNC Symbol;Acc:VGNC:96458]	3.17	0.0763
ssc-miR-30a-5p	NUFIP2	nuclear FMR1 interacting protein 2 [Source:VGNC Symbol;Acc:VGNC:90967]	3.17	0.0763
ssc-miR-30a-5p	NUP43	nucleoporin 43 [Source:VGNC Symbol;Acc:VGNC:90982]	3.17	0.0763
ssc-miR-30a-5p	NUS1	NUS1 dehydrodolichyl diphosphate synthase subunit [Source:VGNC Symbol;Acc:VGNC:90990]	3.17	0.0763
ssc-miR-30a-5p	OCLN	occludin [Source:VGNC Symbol;Acc:VGNC:91013]	3.17	0.0763
ssc-miR-30a-5p	OMG	oligodendrocyte myelin glycoprotein [Source:VGNC Symbol;Acc:VGNC:91041]	3.17	0.0763
ssc-miR-30a-5p	ONECUT2	one cut homeobox 2 [Source:VGNC Symbol;Acc:VGNC:91043]	3.17	0.0763
ssc-miR-30a-5p	ONECUT3	one cut homeobox 3 [Source:VGNC Symbol;Acc:VGNC:91044]	3.17	0.0763
ssc-miR-30a-5p	ORC2	origin recognition complex subunit 2 [Source:VGNC Symbol;Acc:VGNC:96464]	3.17	0.0763
ssc-miR-30a-5p	OSBPL8	oxysterol binding protein like 8 [Source:VGNC Symbol;Acc:VGNC:91074]	3.17	0.0763
ssc-miR-30a-5p	OSTM1	osteoclastosis associated transmembrane protein 1 [Source:VGNC Symbol;Acc:VGNC:91086]	3.17	0.0763
ssc-miR-30a-5p	OTOR	hypothetical gene	3.17	0.0763
ssc-miR-30a-5p	OTUD4	OTU deubiquitinase 4 [Source:VGNC Symbol;Acc:VGNC:91100]	3.17	0.0763
ssc-miR-30a-5p	OTUD6B	OTU deubiquitinase 6B [Source:VGNC Symbol;Acc:VGNC:91103]	3.17	0.0763
ssc-miR-30a-5p	OVOL1	ovo like transcriptional repressor 1 [Source:VGNC Symbol;Acc:VGNC:91111]	3.17	0.0763
ssc-miR-30a-5p	OVOL2	ovo like zinc finger 2 [Source:VGNC Symbol;Acc:VGNC:95663]	3.17	0.0763
ssc-miR-30a-5p	OXR1	oxidation resistance 1 [Source:VGNC Symbol;Acc:VGNC:91116]	3.17	0.0763
ssc-miR-30a-5p	OXTR	oxytocin receptor [Source:VGNC Symbol;Acc:VGNC:108052]	3.17	0.0763
ssc-miR-30a-5p	P2RY2	purinergic receptor P2Y2 [Source:VGNC Symbol;Acc:VGNC:98173]	3.17	0.0763
ssc-miR-30a-5p	P4HA1	prolyl 4-hydroxylase subunit alpha 1 [Source:VGNC Symbol;Acc:VGNC:91132]	3.17	0.0763
ssc-miR-30a-5p	P4HA2	prolyl 4-hydroxylase subunit alpha 2 [Source:VGNC Symbol;Acc:VGNC:91133]	3.17	0.0763
ssc-miR-30a-5p	P4HA3	prolyl 4-hydroxylase subunit alpha 3 [Source:VGNC Symbol;Acc:VGNC:91134]	3.17	0.0763
ssc-miR-30a-5p	PAAF1	proteasomal ATPase associated factor 1 [Source:VGNC Symbol;Acc:VGNC:108605]	3.17	0.0763
ssc-miR-30a-5p	PABPC1L2A	hypothetical gene	3.17	0.0763
ssc-miR-30a-5p	PABPC1L2B	hypothetical gene	3.17	0.0763

ssc-miR-30a-5p	PACRGL	parkin coregulated like [Source:VGNC Symbol;Acc:VGNC:91139]	3.17	0.0763
ssc-miR-30a-5p	PAFAH1B2	platelet activating factor acetylhydrolase 1b catalytic subunit 2 [Source:VGNC Symbol;Acc:VGNC:91151]	3.17	0.0763
ssc-miR-30a-5p	PAG1	phosphoprotein membrane anchor with glycosphingolipid microdomains 1 [Source:VGNC Symbol;Acc:VGNC:91154]	3.17	0.0763
ssc-miR-30a-5p	PAK4	p21 (RAC1) activated kinase 4 [Source:VGNC Symbol;Acc:VGNC:98526]	3.17	0.0763
ssc-miR-30a-5p	PALM2	hypothetical gene	3.17	0.0763
ssc-miR-30a-5p	PANK3	pantothenate kinase 3 [Source:VGNC Symbol;Acc:VGNC:91165]	3.17	0.0763
ssc-miR-30a-5p	PANX1	pannexin 1 [Source:VGNC Symbol;Acc:VGNC:91166]	3.17	0.0763
ssc-miR-30a-5p	PAPD4	hypothetical gene	3.17	0.0763
ssc-miR-30a-5p	PAPD5	hypothetical gene	3.17	0.0763
ssc-miR-30a-5p	PAPOLB	hypothetical gene	3.17	0.0763
ssc-miR-30a-5p	PAQR5	progestin and adipoQ receptor family member 5 [Source:VGNC Symbol;Acc:VGNC:91175]	3.17	0.0763
ssc-miR-30a-5p	PARP16	poly(ADP-ribose) polymerase family member 16 [Source:VGNC Symbol;Acc:VGNC:103145]	3.17	0.0763
ssc-miR-30a-5p	PARP8	poly(ADP-ribose) polymerase family member 8 [Source:HGNC Symbol;Acc:HGNC:26124]	3.17	0.0763
ssc-miR-30a-5p	PAWR	pro-apoptotic WT1 regulator [Source:VGNC Symbol;Acc:VGNC:91191]	3.17	0.0763
ssc-miR-30a-5p	PAX3	paired box 3 [Source:VGNC Symbol;Acc:VGNC:96468]	3.17	0.0763
ssc-miR-30a-5p	PAX9	paired box 9 [Source:HGNC Symbol;Acc:HGNC:8623]	3.17	0.0763
ssc-miR-30a-5p	PAXBP1	PAX3 and PAX7 binding protein 1 [Source:VGNC Symbol;Acc:VGNC:91197]	3.17	0.0763
ssc-miR-30a-5p	PBRM1	polybromo 1 [Source:VGNC Symbol;Acc:VGNC:91202]	3.17	0.0763
ssc-miR-30a-5p	PCDH10	protocadherin 10 [Source:HGNC Symbol;Acc:HGNC:13404]	3.17	0.0763
ssc-miR-30a-5p	PCDH17	protocadherin 17 [Source:VGNC Symbol;Acc:VGNC:91214]	3.17	0.0763
ssc-miR-30a-5p	PCDH19	protocadherin 19 [Source:VGNC Symbol;Acc:VGNC:91216]	3.17	0.0763
ssc-miR-30a-5p	PCDH20	protocadherin 20 [Source:HGNC Symbol;Acc:HGNC:14257]	3.17	0.0763
ssc-miR-30a-5p	PCGF3	polycomb group ring finger 3 [Source:VGNC Symbol;Acc:VGNC:91220]	3.17	0.0763
ssc-miR-30a-5p	PCGF5	hypothetical gene	3.17	0.0763
ssc-miR-30a-5p	PCNXL2	hypothetical gene	3.17	0.0763
ssc-miR-30a-5p	PDAP1	PDGFA associated protein 1 [Source:VGNC Symbol;Acc:VGNC:91239]	3.17	0.0763
ssc-miR-30a-5p	PDCD10	programmed cell death 10 [Source:VGNC Symbol;Acc:VGNC:91241]	3.17	0.0763
ssc-miR-30a-5p	PDCL	phosducin like [Source:VGNC Symbol;Acc:VGNC:91245]	3.17	0.0763
ssc-miR-30a-5p	PDE3A	phosphodiesterase 3A [Source:VGNC Symbol;Acc:VGNC:91252]	3.17	0.0763
ssc-miR-30a-5p	PDE4D	phosphodiesterase 4D [Source:VGNC Symbol;Acc:VGNC:91256]	3.17	0.0763
ssc-miR-30a-5p	PDE5A	phosphodiesterase 5A [Source:VGNC Symbol;Acc:VGNC:91257]	3.17	0.0763
ssc-miR-30a-5p	PDE7A	phosphodiesterase 7A [Source:VGNC Symbol;Acc:VGNC:91261]	3.17	0.0763
ssc-miR-30a-5p	PDGFRB	platelet derived growth factor receptor beta [Source:VGNC Symbol;Acc:VGNC:91268]	3.17	0.0763
ssc-miR-30a-5p	PDP2	pyruvate dehyrogenase phosphatase catalytic subunit 2 [Source:VGNC Symbol;Acc:VGNC:91287]	3.17	0.0763
ssc-miR-30a-5p	PDS5B	PDS5 cohesin associated factor B [Source:VGNC Symbol;Acc:VGNC:91290]	3.17	0.0763
ssc-miR-30a-5p	PDSS1	decaprenyl diphosphate synthase subunit 1 [Source:VGNC Symbol;Acc:VGNC:95858]	3.17	0.0763
ssc-miR-30a-5p	PDSS2	decaprenyl diphosphate synthase subunit 2 [Source:VGNC Symbol;Acc:VGNC:91291]	3.17	0.0763
ssc-miR-30a-5p	PEAK1	pseudopodium enriched atypical kinase 1 [Source:VGNC Symbol;Acc:VGNC:91300]	3.17	0.0763
ssc-miR-30a-5p	PELI1	pellino E3 ubiquitin protein ligase 1 [Source:VGNC Symbol;Acc:VGNC:91306]	3.17	0.0763
ssc-miR-30a-5p	PELI2	pellino E3 ubiquitin protein ligase family member 2 [Source:VGNC Symbol;Acc:VGNC:91307]	3.17	0.0763
ssc-miR-30a-5p	PER2	period circadian regulator 2 [Source:VGNC Symbol;Acc:VGNC:96478]	3.17	0.0763
ssc-miR-30a-5p	PER3	hypothetical gene	3.17	0.0763
ssc-miR-30a-5p	PEX26	peroxisomal biogenesis factor 26 [Source:HGNC Symbol;Acc:HGNC:22965]	3.17	0.0763
ssc-miR-30a-5p	PEX5L	peroxisomal biosis factor 5 like [Source:VGNC Symbol;Acc:VGNC:91329]	3.17	0.0763
ssc-miR-30a-5p	PFN2	hypothetical gene	3.17	0.0763
ssc-miR-30a-5p	PGGT1B	protein geranylgeranyltransferase type I subunit beta [Source:VGNC Symbol;Acc:VGNC:91350]	3.17	0.0763
ssc-miR-30a-5p	PGM1	hypothetical gene	3.17	0.0763
ssc-miR-30a-5p	PGM2L1	phosphoglucomutase 2 like 1 [Source:VGNC Symbol;Acc:VGNC:91357]	3.17	0.0763
ssc-miR-30a-5p	PGM3	phosphoglucomutase 3 [Source:VGNC Symbol;Acc:VGNC:103150]	3.17	0.0763

ssc-miR-30a-5p	PGP	phosphoglycolate phosphatase [Source:VGNC Symbol;Acc:VGNC:91359]	3.17	0.0763
ssc-miR-30a-5p	PGR	progesterone receptor [Source:VGNC Symbol;Acc:VGNC:91362]	3.17	0.0763
ssc-miR-30a-5p	PHACTR2	phosphatase and actin regulator 2 [Source:VGNC Symbol;Acc:VGNC:91366]	3.17	0.0763
ssc-miR-30a-5p	PHF13	PHD finger protein 13 [Source:VGNC Symbol;Acc:VGNC:91379]	3.17	0.0763
ssc-miR-30a-5p	PHF16	hypothetical gene	3.17	0.0763
ssc-miR-30a-5p	PHF6	PHD finger protein 6 [Source:VGNC Symbol;Acc:VGNC:91389]	3.17	0.0763
ssc-miR-30a-5p	PHIP	pleckstrin homology domain interacting protein [Source:VGNC Symbol;Acc:VGNC:91392]	3.17	0.0763
ssc-miR-30a-5p	PHKA2	phosphorylase kinase regulatory subunit alpha 2 [Source:VGNC Symbol;Acc:VGNC:91394]	3.17	0.0763
ssc-miR-30a-5p	PHLDB2	pleckstrin homology like domain family B member 2 [Source:VGNC Symbol;Acc:VGNC:91399]	3.17	0.0763
ssc-miR-30a-5p	PHTF2	putative homeodomain transcription factor 2 [Source:VGNC Symbol;Acc:VGNC:91405]	3.17	0.0763
ssc-miR-30a-5p	PI4K2B	phosphatidylinositol 4-kinase type 2 beta [Source:VGNC Symbol;Acc:VGNC:98190]	3.17	0.0763
ssc-miR-30a-5p	PIAS1	protein inhibitor of activated STAT 1 [Source:VGNC Symbol;Acc:VGNC:91410]	3.17	0.0763
ssc-miR-30a-5p	PICALM	phosphatidylinositol binding clathrin assembly protein [Source:VGNC Symbol;Acc:VGNC:91415]	3.17	0.0763
ssc-miR-30a-5p	PIEZO2	piezo type mechanosensitive ion channel component 2 [Source:HGNC Symbol;Acc:HGNC:26270]	3.17	0.0763
ssc-miR-30a-5p	PIGA	phosphatidylinositol glycan anchor biosynthesis class A [Source:VGNC Symbol;Acc:VGNC:91419]	3.17	0.0763
ssc-miR-30a-5p	PIGV	phosphatidylinositol glycan anchor biosynthesis class V [Source:VGNC Symbol;Acc:VGNC:91430]	3.17	0.0763
ssc-miR-30a-5p	PIGX	phosphatidylinositol glycan anchor biosynthesis class X [Source:VGNC Symbol;Acc:VGNC:91432]	3.17	0.0763
ssc-miR-30a-5p	PIK3CD	phosphatidylinositol-4,5-bisphosphate 3-kinase catalytic subunit delta [Source:VGNC Symbol;Acc:VGNC:91442]	3.17	0.0763
ssc-miR-30a-5p	PIK3R2	phosphoinositide-3-kinase regulatory subunit 2 [Source:VGNC Symbol;Acc:VGNC:100324]	3.17	0.0763
ssc-miR-30a-5p	PIN4	peptidylprolyl cis/trans isomerase, NIMA-interacting 4 [Source:VGNC Symbol;Acc:VGNC:91452]	3.17	0.0763
ssc-miR-30a-5p	PIP4K2A	phosphatidylinositol-5-phosphate 4-kinase type 2 alpha [Source:VGNC Symbol;Acc:VGNC:96485]	3.17	0.0763
ssc-miR-30a-5p	PIP4K2B	phosphatidylinositol-5-phosphate 4-kinase type 2 beta [Source:VGNC Symbol;Acc:VGNC:91454]	3.17	0.0763
ssc-miR-30a-5p	PIRT	phosphoinositide interacting regulator of transient receptor potential channels [Source:VGNC Symbol;Acc:VGNC:91461]	3.17	0.0763
ssc-miR-30a-5p	PITPNB	phosphatidylinositol transfer protein beta [Source:VGNC Symbol;Acc:VGNC:104026]	3.17	0.0763
ssc-miR-30a-5p	PITPNC1	phosphatidylinositol transfer protein cytoplasmic 1 [Source:NCBI gene (formerly Entrezgene);Acc:100233199]	3.17	0.0763
ssc-miR-30a-5p	PITPNM2	phosphatidylinositol transfer protein membrane associated 2 [Source:VGNC Symbol;Acc:VGNC:91466]	3.17	0.0763
ssc-miR-30a-5p	PITPNM3	PITPNM family member 3 [Source:HGNC Symbol;Acc:HGNC:21043]	3.17	0.0763
ssc-miR-30a-5p	PITX1	paired like homeodomain 1 [Source:NCBI gene (formerly Entrezgene);Acc:100689266]	3.17	0.0763
ssc-miR-30a-5p	PKHD1	PKHD1 ciliary IPT domain containing fibrocystin/polyductin [Source:VGNC Symbol;Acc:VGNC:91476]	3.17	0.0763
ssc-miR-30a-5p	PKNOX2	PBX/knotted 1 homeobox 2 [Source:VGNC Symbol;Acc:VGNC:91485]	3.17	0.0763
ssc-miR-30a-5p	PLA2G12A	phospholipase A2 group XIIA [Source:HGNC Symbol;Acc:HGNC:18554]	3.17	0.0763
ssc-miR-30a-5p	PLAG1	PLAG1 zinc finger [Source:VGNC Symbol;Acc:VGNC:91509]	3.17	0.0763
ssc-miR-30a-5p	PLAGL2	PLAG1 like zinc finger 2 [Source:VGNC Symbol;Acc:VGNC:96490]	3.17	0.0763
ssc-miR-30a-5p	PLCG1	phospholipase C gamma 1 [Source:VGNC Symbol;Acc:VGNC:96492]	3.17	0.0763
ssc-miR-30a-5p	PLCXD3	phosphatidylinositol specific phospholipase C X domain containing 3 [Source:VGNC Symbol;Acc:VGNC:91524]	3.17	0.0763
ssc-miR-30a-5p	PLEKHA6	pleckstrin homology domain containing A6 [Source:VGNC Symbol;Acc:VGNC:91536]	3.17	0.0763
ssc-miR-30a-5p	PLEKHM3	pleckstrin homology domain containing M3 [Source:VGNC Symbol;Acc:VGNC:96281]	3.17	0.0763
ssc-miR-30a-5p	PLEKHO2	pleckstrin homology domain containing O2 [Source:HGNC Symbol;Acc:HGNC:30026]	3.17	0.0763
ssc-miR-30a-5p	PLS1	plastin 1 [Source:VGNC Symbol;Acc:VGNC:91572]	3.17	0.0763
ssc-miR-30a-5p	PLXNA1	plexin A1 [Source:VGNC Symbol;Acc:VGNC:91579]	3.17	0.0763
ssc-miR-30a-5p	PLXNA2	plexin A2 [Source:VGNC Symbol;Acc:VGNC:91580]	3.17	0.0763
ssc-miR-30a-5p	PLXNC1	plexin C1 [Source:VGNC Symbol;Acc:VGNC:91584]	3.17	0.0763
ssc-miR-30a-5p	PMEPA1	prostate transmembrane protein, androgen induced 1 [Source:VGNC Symbol;Acc:VGNC:95606]	3.17	0.0763
ssc-miR-30a-5p	PNN	pinin, desmosome associated protein [Source:VGNC Symbol;Acc:VGNC:91603]	3.17	0.0763
ssc-miR-30a-5p	PNPLA2	patatin like phospholipase domain containing 2 [Source:VGNC Symbol;Acc:VGNC:91607]	3.17	0.0763
ssc-miR-30a-5p	POC1B	POC1 centriolar protein B [Source:VGNC Symbol;Acc:VGNC:91616]	3.17	0.0763
ssc-miR-30a-5p	POGLUT1	protein O-glucosyltransferase 1 [Source:VGNC Symbol;Acc:VGNC:91623]	3.17	0.0763
ssc-miR-30a-5p	POLE3	DNA polymerase epsilon 3, accessory subunit [Source:VGNC Symbol;Acc:VGNC:103154]	3.17	0.0763
ssc-miR-30a-5p	POLR1D	RNA polymerase I and III subunit D [Source:VGNC Symbol;Acc:VGNC:104031]	3.17	0.0763

ssc-miR-30a-5p	POLR3E	RNA polymerase III subunit E [Source:VGNC Symbol;Acc:VGNC:91657]	3.17	0.0763
ssc-miR-30a-5p	POLR3G	hypothetical gene	3.17	0.0763
ssc-miR-30a-5p	PON2	paraoxonase 2 [Source:VGNC Symbol;Acc:VGNC:91662]	3.17	0.0763
ssc-miR-30a-5p	POP1	POP1 homolog, ribonuclease P/MRP subunit [Source:VGNC Symbol;Acc:VGNC:91663]	3.17	0.0763
ssc-miR-30a-5p	POU3F2	POU class 3 homeobox 2 [Source:HGNC Symbol;Acc:HGNC:9215]	3.17	0.0763
ssc-miR-30a-5p	POU4F2	POU class 4 homeobox 2 [Source:VGNC Symbol;Acc:VGNC:91679]	3.17	0.0763
ssc-miR-30a-5p	PPA1	inorganic pyrophosphatase 1 [Source:VGNC Symbol;Acc:VGNC:96757]	3.17	0.0763
ssc-miR-30a-5p	PPAPDC2	hypothetical gene	3.17	0.0763
ssc-miR-30a-5p	PPARGC1A	PPARG coactivator 1 alpha [Source:VGNC Symbol;Acc:VGNC:91685]	3.17	0.0763
ssc-miR-30a-5p	PPARGC1B	PPARG coactivator 1 beta [Source:VGNC Symbol;Acc:VGNC:91686]	3.17	0.0763
ssc-miR-30a-5p	PPFIA2	PTPRF interacting protein alpha 2 [Source:VGNC Symbol;Acc:VGNC:91692]	3.17	0.0763
ssc-miR-30a-5p	PPID	peptidylprolyl isomerase D [Source:VGNC Symbol;Acc:VGNC:98212]	3.17	0.0763
ssc-miR-30a-5p	PPIL3	peptidylprolyl isomerase like 3 [Source:VGNC Symbol;Acc:VGNC:103073]	3.17	0.0763
ssc-miR-30a-5p	PPIL4	peptidylprolyl isomerase like 4 [Source:VGNC Symbol;Acc:VGNC:91699]	3.17	0.0763
ssc-miR-30a-5p	PPIP5K2	diphosphoinositol pentakisphosphate kinase 2 [Source:VGNC Symbol;Acc:VGNC:91701]	3.17	0.0763
ssc-miR-30a-5p	PPM1A	protein phosphatase, Mg2+/Mn2+ dependent 1A [Source:VGNC Symbol;Acc:VGNC:91703]	3.17	0.0763
ssc-miR-30a-5p	PPM1E	protein phosphatase, Mg2+/Mn2+ dependent 1E [Source:VGNC Symbol;Acc:VGNC:91705]	3.17	0.0763
ssc-miR-30a-5p	PPP1R12A	protein phosphatase 1 regulatory subunit 12A [Source:VGNC Symbol;Acc:VGNC:91719]	3.17	0.0763
ssc-miR-30a-5p	PPP1R14C	protein phosphatase 1 regulatory inhibitor subunit 14C [Source:VGNC Symbol;Acc:VGNC:91724]	3.17	0.0763
ssc-miR-30a-5p	PPP1R18	protein phosphatase 1 regulatory subunit 18 [Source:VGNC Symbol;Acc:VGNC:104034]	3.17	0.0763
ssc-miR-30a-5p	PPP1R2	hypothetical gene	3.17	0.0763
ssc-miR-30a-5p	PPP1R9A	protein phosphatase 1 regulatory subunit 9A [Source:VGNC Symbol;Acc:VGNC:91743]	3.17	0.0763
ssc-miR-30a-5p	PPP2R1B	protein phosphatase 2 scaffold subunit Abeta [Source:VGNC Symbol;Acc:VGNC:91747]	3.17	0.0763
ssc-miR-30a-5p	PPP2R4	hypothetical gene	3.17	0.0763
ssc-miR-30a-5p	PPP2R5E	protein phosphatase 2 regulatory subunit B'epsilon [Source:VGNC Symbol;Acc:VGNC:91754]	3.17	0.0763
ssc-miR-30a-5p	PPP3CA	protein phosphatase 3 catalytic subunit alpha [Source:VGNC Symbol;Acc:VGNC:98218]	3.17	0.0763
ssc-miR-30a-5p	PPP3CB	protein phosphatase 3 catalytic subunit beta [Source:VGNC Symbol;Acc:VGNC:104035]	3.17	0.0763
ssc-miR-30a-5p	PPP3R1	protein phosphatase 3 regulatory subunit B, alpha [Source:VGNC Symbol;Acc:VGNC:106453]	3.17	0.0763
ssc-miR-30a-5p	PPP6C	protein phosphatase 6 catalytic subunit [Source:VGNC Symbol;Acc:VGNC:98219]	3.17	0.0763
ssc-miR-30a-5p	PPTC7	protein phosphatase targeting COQ7 [Source:VGNC Symbol;Acc:VGNC:91765]	3.17	0.0763
ssc-miR-30a-5p	PRCD	photoreceptor disc component [Source:VGNC Symbol;Acc:VGNC:91770]	3.17	0.0763
ssc-miR-30a-5p	PRDM1	PR/SET domain 1 [Source:VGNC Symbol;Acc:VGNC:91772]	3.17	0.0763
ssc-miR-30a-5p	PRDM5	PR/SET domain 5 [Source:VGNC Symbol;Acc:VGNC:98953]	3.17	0.0763
ssc-miR-30a-5p	PRICKLE1	prickle planar cell polarity protein 1 [Source:VGNC Symbol;Acc:VGNC:91793]	3.17	0.0763
ssc-miR-30a-5p	PRKAA2	protein kinase AMP-activated catalytic subunit alpha 2 [Source:VGNC Symbol;Acc:VGNC:91798]	3.17	0.0763
ssc-miR-30a-5p	PRKAR1A	protein kinase cAMP-dependent type I regulatory subunit alpha [Source:VGNC Symbol;Acc:VGNC:91802]	3.17	0.0763
ssc-miR-30a-5p	PRKRIR	hypothetical gene	3.17	0.0763
ssc-miR-30a-5p	PRLR	prolactin receptor [Source:VGNC Symbol;Acc:VGNC:91819]	3.17	0.0763
ssc-miR-30a-5p	PROM1	prominin 1 [Source:VGNC Symbol;Acc:VGNC:91832]	3.17	0.0763
ssc-miR-30a-5p	PROSER1	proline and serine rich 1 [Source:VGNC Symbol;Acc:VGNC:91836]	3.17	0.0763
ssc-miR-30a-5p	PRPF40A	pre-mRNA processing factor 40 homolog A [Source:VGNC Symbol;Acc:VGNC:98222]	3.17	0.0763
ssc-miR-30a-5p	PRR14L	proline rich 14 like [Source:VGNC Symbol;Acc:VGNC:91853]	3.17	0.0763
ssc-miR-30a-5p	PRRG1	proline rich and Gla domain 1 [Source:VGNC Symbol;Acc:VGNC:101494]	3.17	0.0763
ssc-miR-30a-5p	PRRT2	proline rich transmembrane protein 2 [Source:HGNC Symbol;Acc:HGNC:30500]	3.17	0.0763
ssc-miR-30a-5p	PRRX1	paired related homeobox 1 [Source:VGNC Symbol;Acc:VGNC:91875]	3.17	0.0763
ssc-miR-30a-5p	PRUNE2	hypothetical gene	3.17	0.0763
ssc-miR-30a-5p	PSD3	pleckstrin and Sec7 domain containing 3 [Source:VGNC Symbol;Acc:VGNC:107166]	3.17	0.0763
ssc-miR-30a-5p	PSEN2	presenilin 2 [Source:VGNC Symbol;Acc:VGNC:96508]	3.17	0.0763
ssc-miR-30a-5p	PSMD7	proteasome 26S subunit, non-ATPase 7 [Source:VGNC Symbol;Acc:VGNC:91925]	3.17	0.0763

ssc-miR-30a-5p	PSMD9	proteasome 26S subunit, non-ATPase 9 [Source:HGNC Symbol;Acc:HGNC:9567]	3.17	0.0763
ssc-miR-30a-5p	PSME3	proteasome activator subunit 3 [Source:VGNC Symbol;Acc:VGNC:91926]	3.17	0.0763
ssc-miR-30a-5p	PTAFR	platelet activating factor receptor [Source:VGNC Symbol;Acc:VGNC:98224]	3.17	0.0763
ssc-miR-30a-5p	PTBP3	polypyrimidine tract binding protein 3 [Source:VGNC Symbol;Acc:VGNC:91938]	3.17	0.0763
ssc-miR-30a-5p	PTCHD1	patched domain containing 1 [Source:VGNC Symbol;Acc:VGNC:91942]	3.17	0.0763
ssc-miR-30a-5p	PTGDR	prostaglandin D2 receptor [Source:HGNC Symbol;Acc:HGNC:9591]	3.17	0.0763
ssc-miR-30a-5p	PTGER3	prostaglandin E receptor 3 [Source:VGNC Symbol;Acc:VGNC:98225]	3.17	0.0763
ssc-miR-30a-5p	PTGFRN	prostaglandin F2 receptor inhibitor [Source:VGNC Symbol;Acc:VGNC:91955]	3.17	0.0763
ssc-miR-30a-5p	PTP4A1	protein tyrosine phosphatase 4A1 [Source:HGNC Symbol;Acc:HGNC:9634]	3.17	0.0763
ssc-miR-30a-5p	PTPDC1	protein tyrosine phosphatase domain containing 1 [Source:VGNC Symbol;Acc:VGNC:91970]	3.17	0.0763
ssc-miR-30a-5p	PTPN13	protein tyrosine phosphatase non-receptor type 13 [Source:VGNC Symbol;Acc:VGNC:91974]	3.17	0.0763
ssc-miR-30a-5p	PTPN21	protein tyrosine phosphatase non-receptor type 21 [Source:VGNC Symbol;Acc:VGNC:91977]	3.17	0.0763
ssc-miR-30a-5p	PTPN4	protein tyrosine phosphatase non-receptor type 4 [Source:VGNC Symbol;Acc:VGNC:96521]	3.17	0.0763
ssc-miR-30a-5p	PTPN9	protein tyrosine phosphatase non-receptor type 9 [Source:VGNC Symbol;Acc:VGNC:91983]	3.17	0.0763
ssc-miR-30a-5p	PTPRK	protein tyrosine phosphatase receptor type K [Source:VGNC Symbol;Acc:VGNC:91991]	3.17	0.0763
ssc-miR-30a-5p	PUM2	pumilio RNA binding family member 2 [Source:VGNC Symbol;Acc:VGNC:92002]	3.17	0.0763
ssc-miR-30a-5p	PVRL3	hypothetical gene	3.17	0.0763
ssc-miR-30a-5p	PXK	PX domain containing serine/threonine kinase like [Source:VGNC Symbol;Acc:VGNC:92015]	3.17	0.0763
ssc-miR-30a-5p	PXN	paxillin [Source:HGNC Symbol;Acc:HGNC:9718]	3.17	0.0763
ssc-miR-30a-5p	PYGO1	pygopus family PHD finger 1 [Source:VGNC Symbol;Acc:VGNC:98231]	3.17	0.0763
ssc-miR-30a-5p	QKI	QKI, KH domain containing RNA binding [Source:VGNC Symbol;Acc:VGNC:92025]	3.17	0.0763
ssc-miR-30a-5p	R3HDM1	R3H domain containing 1 [Source:VGNC Symbol;Acc:VGNC:96526]	3.17	0.0763
ssc-miR-30a-5p	RAB10	RAB10, member RAS onco family [Source:VGNC Symbol;Acc:VGNC:98235]	3.17	0.0763
ssc-miR-30a-5p	RAB11A	RAB11A, member RAS onco family [Source:VGNC Symbol;Acc:VGNC:98236]	3.17	0.0763
ssc-miR-30a-5p	RAB15	RAB15, member RAS onco family [Source:VGNC Symbol;Acc:VGNC:98239]	3.17	0.0763
ssc-miR-30a-5p	RAB22A	RAB22A, member RAS onco family [Source:VGNC Symbol;Acc:VGNC:98244]	3.17	0.0763
ssc-miR-30a-5p	RAB23	RAB23, member RAS onco family [Source:VGNC Symbol;Acc:VGNC:98245]	3.17	0.0763
ssc-miR-30a-5p	RAB27B	RAB27B, member RAS onco family [Source:VGNC Symbol;Acc:VGNC:98250]	3.17	0.0763
ssc-miR-30a-5p	RAB32	RAB32, member RAS onco family [Source:NCBI gene (formerly Entrezgene);Acc:100144496]	3.17	0.0763
ssc-miR-30a-5p	RAB33B	RAB33B, member RAS onco family [Source:VGNC Symbol;Acc:VGNC:98257]	3.17	0.0763
ssc-miR-30a-5p	RAB38	RAB38, member RAS onco family [Source:VGNC Symbol;Acc:VGNC:98261]	3.17	0.0763
ssc-miR-30a-5p	RAB3D	RAB3D, member RAS onco family [Source:VGNC Symbol;Acc:VGNC:98266]	3.17	0.0763
ssc-miR-30a-5p	RAB4A	RAB4A, member RAS onco family [Source:VGNC Symbol;Acc:VGNC:98270]	3.17	0.0763
ssc-miR-30a-5p	RAB4B	RAB4B, member RAS onco family [Source:VGNC Symbol;Acc:VGNC:98597]	3.17	0.0763
ssc-miR-30a-5p	RAB7A	RAB7A, member RAS onco family [Source:VGNC Symbol;Acc:VGNC:98273]	3.17	0.0763
ssc-miR-30a-5p	RAB8A	RAB8A, member RAS onco family [Source:VGNC Symbol;Acc:VGNC:98275]	3.17	0.0763
ssc-miR-30a-5p	RABGAP1L	RAB GTPase activating protein 1 like [Source:VGNC Symbol;Acc:VGNC:108611]	3.17	0.0763
ssc-miR-30a-5p	RAD23B	RAD23 homolog B, nucleotide excision repair protein [Source:VGNC Symbol;Acc:VGNC:103160]	3.17	0.0763
ssc-miR-30a-5p	RAD51L3-RFFL	hypothetical gene	3.17	0.0763
ssc-miR-30a-5p	RAD9B	RAD9 checkpoint clamp component B [Source:VGNC Symbol;Acc:VGNC:92063]	3.17	0.0763
ssc-miR-30a-5p	RAI14	retinoic acid induced 14 [Source:VGNC Symbol;Acc:VGNC:92068]	3.17	0.0763
ssc-miR-30a-5p	RALGDS	ral guanine nucleotide dissociation stimulator [Source:HGNC Symbol;Acc:HGNC:9842]	3.17	0.0763
ssc-miR-30a-5p	RALGPS1	Ral GEF with PH domain and SH3 binding motif 1 [Source:VGNC Symbol;Acc:VGNC:92072]	3.17	0.0763
ssc-miR-30a-5p	RALGPS2	Ral GEF with PH domain and SH3 binding motif 2 [Source:VGNC Symbol;Acc:VGNC:92073]	3.17	0.0763
ssc-miR-30a-5p	RANBP10	RAN binding protein 10 [Source:VGNC Symbol;Acc:VGNC:92077]	3.17	0.0763
ssc-miR-30a-5p	RANBP9	RAN binding protein 9 [Source:VGNC Symbol;Acc:VGNC:92082]	3.17	0.0763
ssc-miR-30a-5p	RAP1B	RAP1B, member of RAS oncogene family [Source:HGNC Symbol;Acc:HGNC:9857]	3.17	0.0763
ssc-miR-30a-5p	RAP2A	RAP2A, member of RAS onco family [Source:VGNC Symbol;Acc:VGNC:92088]	3.17	0.0763
ssc-miR-30a-5p	RAP2B	RAP2B, member of RAS onco family [Source:VGNC Symbol;Acc:VGNC:92089]	3.17	0.0763

ssc-miR-30a-5p	RAP2C	RAP2C, member of RAS onco family [Source:VGNC Symbol;Acc:VGNC:92090]	3.17	0.0763
ssc-miR-30a-5p	RAPGEF2	Rap guanine nucleotide exchange factor 2 [Source:VGNC Symbol;Acc:VGNC:92092]	3.17	0.0763
ssc-miR-30a-5p	RAPGEF4	Rap guanine nucleotide exchange factor 4 [Source:VGNC Symbol;Acc:VGNC:95814]	3.17	0.0763
ssc-miR-30a-5p	RAPH1	hypothetical gene	3.17	0.0763
ssc-miR-30a-5p	RARB	retinoic acid receptor beta [Source:HGNC Symbol;Acc:HGNC:9865]	3.17	0.0763
ssc-miR-30a-5p	RARG	retinoic acid receptor gamma [Source:HGNC Symbol;Acc:HGNC:9866]	3.17	0.0763
ssc-miR-30a-5p	RASA1	RAS p21 protein activator 1 [Source:VGNC Symbol;Acc:VGNC:92101]	3.17	0.0763
ssc-miR-30a-5p	RASA2	RAS p21 protein activator 2 [Source:VGNC Symbol;Acc:VGNC:92102]	3.17	0.0763
ssc-miR-30a-5p	RASAL2	RAS protein activator like 2 [Source:VGNC Symbol;Acc:VGNC:92105]	3.17	0.0763
ssc-miR-30a-5p	RASD1	ras related dexamethasone induced 1 [Source:VGNC Symbol;Acc:VGNC:92107]	3.17	0.0763
ssc-miR-30a-5p	RASEF	hypothetical gene	3.17	0.0763
ssc-miR-30a-5p	RASGEF1A	RasGEF domain family member 1A [Source:VGNC Symbol;Acc:VGNC:92108]	3.17	0.0763
ssc-miR-30a-5p	RASGEF1B	RasGEF domain family member 1B [Source:VGNC Symbol;Acc:VGNC:92109]	3.17	0.0763
ssc-miR-30a-5p	RASGRF2	Ras protein specific guanine nucleotide releasing factor 2 [Source:VGNC Symbol;Acc:VGNC:92112]	3.17	0.0763
ssc-miR-30a-5p	RASGRP3	RAS guanyl releasing protein 3 [Source:VGNC Symbol;Acc:VGNC:92115]	3.17	0.0763
ssc-miR-30a-5p	RASL12	RAS like family 12 [Source:VGNC Symbol;Acc:VGNC:92121]	3.17	0.0763
ssc-miR-30a-5p	RASSF4	Ras association domain family member 4 [Source:NCBI gene (formerly Entrezgene);Acc:100152580]	3.17	0.0763
ssc-miR-30a-5p	RASSF8	Ras association domain family member 8 [Source:VGNC Symbol;Acc:VGNC:92128]	3.17	0.0763
ssc-miR-30a-5p	RAVER2	ribonucleoprotein, PTB binding 2 [Source:VGNC Symbol;Acc:VGNC:98283]	3.17	0.0763
ssc-miR-30a-5p	RBFOX1	RNA binding fox-1 homolog 1 [Source:VGNC Symbol;Acc:VGNC:92139]	3.17	0.0763
ssc-miR-30a-5p	RBM12	RNA binding motif protein 12 [Source:HGNC Symbol;Acc:HGNC:9898]	3.17	0.0763
ssc-miR-30a-5p	RBM15B	RNA binding motif protein 15B [Source:VGNC Symbol;Acc:VGNC:98285]	3.17	0.0763
ssc-miR-30a-5p	RCAN1	regulator of calcineurin 1 [Source:VGNC Symbol;Acc:VGNC:92170]	3.17	0.0763
ssc-miR-30a-5p	RCBTB1	RCC1 and BTB domain containing protein 1 [Source:VGNC Symbol;Acc:VGNC:92173]	3.17	0.0763
ssc-miR-30a-5p	RCC2	regulator of chromosome condensation 2 [Source:VGNC Symbol;Acc:VGNC:98547]	3.17	0.0763
ssc-miR-30a-5p	RCOR1	REST corepressor 1 [Source:VGNC Symbol;Acc:VGNC:92183]	3.17	0.0763
ssc-miR-30a-5p	RECK	reversion inducing cysteine rich protein with kazal motifs [Source:VGNC Symbol;Acc:VGNC:92194]	3.17	0.0763
ssc-miR-30a-5p	REEP1	receptor accessory protein 1 [Source:VGNC Symbol;Acc:VGNC:92197]	3.17	0.0763
ssc-miR-30a-5p	REEP3	receptor accessory protein 3 [Source:VGNC Symbol;Acc:VGNC:92199]	3.17	0.0763
ssc-miR-30a-5p	REV1	REV1 DNA directed polymerase [Source:HGNC Symbol;Acc:HGNC:14060]	3.17	0.0763
ssc-miR-30a-5p	REV3L	REV3 like, DNA directed polymerase zeta catalytic subunit [Source:VGNC Symbol;Acc:VGNC:92226]	3.17	0.0763
ssc-miR-30a-5p	RFFL	ring finger and FYVE like domain containing E3 ubiquitin protein ligase [Source:VGNC Symbol;Acc:VGNC:98289]	3.17	0.0763
ssc-miR-30a-5p	RFTN2	raftlin family member 2 [Source:VGNC Symbol;Acc:VGNC:96128]	3.17	0.0763
ssc-miR-30a-5p	RFX2	regulatory factor X2 [Source:VGNC Symbol;Acc:VGNC:92244]	3.17	0.0763
ssc-miR-30a-5p	RFX3	regulatory factor X3 [Source:VGNC Symbol;Acc:VGNC:92245]	3.17	0.0763
ssc-miR-30a-5p	RFX6	regulatory factor X6 [Source:VGNC Symbol;Acc:VGNC:92247]	3.17	0.0763
ssc-miR-30a-5p	RFX7	regulatory factor X7 [Source:VGNC Symbol;Acc:VGNC:92248]	3.17	0.0763
ssc-miR-30a-5p	RGL1	ral guanine nucleotide dissociation stimulator like 1 [Source:VGNC Symbol;Acc:VGNC:92252]	3.17	0.0763
ssc-miR-30a-5p	RGS17	regulator of G protein signaling 17 [Source:VGNC Symbol;Acc:VGNC:92263]	3.17	0.0763
ssc-miR-30a-5p	RGS9BP	regulator of G protein signaling 9 binding protein [Source:HGNC Symbol;Acc:HGNC:30304]	3.17	0.0763
ssc-miR-30a-5p	RHEBL1	RHEB like 1 [Source:VGNC Symbol;Acc:VGNC:92280]	3.17	0.0763
ssc-miR-30a-5p	RHOB	ras homolog family member B [Source:NCBI gene (formerly Entrezgene);Acc:100144503]	3.17	0.0763
ssc-miR-30a-5p	RHOH	ras homolog family member H [Source:VGNC Symbol;Acc:VGNC:92290]	3.17	0.0763
ssc-miR-30a-5p	RIMBP2	RIMS binding protein 2 [Source:VGNC Symbol;Acc:VGNC:92306]	3.17	0.0763
ssc-miR-30a-5p	RIMKLB	ribosomal modification protein rimK like family member B [Source:VGNC Symbol;Acc:VGNC:92308]	3.17	0.0763
ssc-miR-30a-5p	RIMS1	regulating synaptic membrane exocytosis 1 [Source:VGNC Symbol;Acc:VGNC:92309]	3.17	0.0763
ssc-miR-30a-5p	RIOK3	RIO kinase 3 [Source:VGNC Symbol;Acc:VGNC:92316]	3.17	0.0763
ssc-miR-30a-5p	RLIM	ring finger protein, LIM domain interacting [Source:HGNC Symbol;Acc:HGNC:13429]	3.17	0.0763
ssc-miR-30a-5p	RNF115	ring finger protein 115 [Source:VGNC Symbol;Acc:VGNC:92348]	3.17	0.0763

ssc-miR-30a-5p	RNF122	ring finger protein 122 [Source:VGNC Symbol;Acc:VGNC:92350]	3.17	0.0763
ssc-miR-30a-5p	RNF157	ring finger protein 157 [Source:VGNC Symbol;Acc:VGNC:92365]	3.17	0.0763
ssc-miR-30a-5p	RNF165	ring finger protein 165 [Source:VGNC Symbol;Acc:VGNC:92366]	3.17	0.0763
ssc-miR-30a-5p	RNF169	ring finger protein 169 [Source:VGNC Symbol;Acc:VGNC:92368]	3.17	0.0763
ssc-miR-30a-5p	RNF213	hypothetical gene	3.17	0.0763
ssc-miR-30a-5p	RNF217	ring finger protein 217 [Source:VGNC Symbol;Acc:VGNC:103162]	3.17	0.0763
ssc-miR-30a-5p	RNF220	ring finger protein 220 [Source:VGNC Symbol;Acc:VGNC:98602]	3.17	0.0763
ssc-miR-30a-5p	RNF24	ring finger protein 24 [Source:HGNC Symbol;Acc:HGNC:13779]	3.17	0.0763
ssc-miR-30a-5p	RNF34	ring finger protein 34 [Source:VGNC Symbol;Acc:VGNC:92389]	3.17	0.0763
ssc-miR-30a-5p	RNF44	ring finger protein 44 [Source:VGNC Symbol;Acc:VGNC:92395]	3.17	0.0763
ssc-miR-30a-5p	RNF7	ring finger protein 7 [Source:VGNC Symbol;Acc:VGNC:92398]	3.17	0.0763
ssc-miR-30a-5p	RNMT	RNA guanine-7 methyltransferase [Source:VGNC Symbol;Acc:VGNC:92402]	3.17	0.0763
ssc-miR-30a-5p	ROCK2	Rho associated coiled-coil containing protein kinase 2 [Source:HGNC Symbol;Acc:HGNC:10252]	3.17	0.0763
ssc-miR-30a-5p	ROR1	receptor tyrosine kinase like orphan receptor 1 [Source:VGNC Symbol;Acc:VGNC:98295]	3.17	0.0763
ssc-miR-30a-5p	ROR2	receptor tyrosine kinase like orphan receptor 2 [Source:VGNC Symbol;Acc:VGNC:92407]	3.17	0.0763
ssc-miR-30a-5p	RORA	RAR related orphan receptor A [Source:VGNC Symbol;Acc:VGNC:92408]	3.17	0.0763
ssc-miR-30a-5p	RP1-170O19.20	hypothetical gene	3.17	0.0763
ssc-miR-30a-5p	RP11-160N1.10	hypothetical gene	3.17	0.0763
ssc-miR-30a-5p	RP11-497E19.2	hypothetical gene	3.17	0.0763
ssc-miR-30a-5p	RP11-766F14.2	hypothetical gene	3.17	0.0763
ssc-miR-30a-5p	RP13-996F3.4	hypothetical gene	3.17	0.0763
ssc-miR-30a-5p	RP13-996F3.5	hypothetical gene	3.17	0.0763
ssc-miR-30a-5p	RP6-24A23.6	hypothetical gene	3.17	0.0763
ssc-miR-30a-5p	RPA1	replication protein A1 [Source:VGNC Symbol;Acc:VGNC:92413]	3.17	0.0763
ssc-miR-30a-5p	RPL22L1	hypothetical gene	3.17	0.0763
ssc-miR-30a-5p	RPP14	hypothetical gene	3.17	0.0763
ssc-miR-30a-5p	RPRD1A	regulation of nuclear pre-mRNA domain containing 1A [Source:VGNC Symbol;Acc:VGNC:92435]	3.17	0.0763
ssc-miR-30a-5p	RPRD2	regulation of nuclear pre-mRNA domain containing 2 [Source:VGNC Symbol;Acc:VGNC:92436]	3.17	0.0763
ssc-miR-30a-5p	RPS6KA2	ribosomal protein S6 kinase A2 [Source:VGNC Symbol;Acc:VGNC:92441]	3.17	0.0763
ssc-miR-30a-5p	RPS6KA5	ribosomal protein S6 kinase A5 [Source:VGNC Symbol;Acc:VGNC:92444]	3.17	0.0763
ssc-miR-30a-5p	RRAD	RRAD, Ras related glycolysis inhibitor and calcium channel regulator [Source:VGNC Symbol;Acc:VGNC:92454]	3.17	0.0763
ssc-miR-30a-5p	RSF1	remodeling and spacing factor 1 [Source:VGNC Symbol;Acc:VGNC:92476]	3.17	0.0763
ssc-miR-30a-5p	RSU1	Ras suppressor protein 1 [Source:VGNC Symbol;Acc:VGNC:98298]	3.17	0.0763
ssc-miR-30a-5p	RTCB	RNA 2',3'-cyclic phosphate and 5'-OH ligase [Source:VGNC Symbol;Acc:VGNC:92492]	3.17	0.0763
ssc-miR-30a-5p	RTKN2	rhotekin 2 [Source:VGNC Symbol;Acc:VGNC:92495]	3.17	0.0763
ssc-miR-30a-5p	RTN4R	reticulon 4 receptor [Source:HGNC Symbol;Acc:HGNC:18601]	3.17	0.0763
ssc-miR-30a-5p	RUNDC3B	RUN domain containing 3B [Source:VGNC Symbol;Acc:VGNC:92515]	3.17	0.0763
ssc-miR-30a-5p	RUNX1	RUNX family transcription factor 1 [Source:VGNC Symbol;Acc:VGNC:92516]	3.17	0.0763
ssc-miR-30a-5p	RUNX2	RUNX family transcription factor 2 [Source:VGNC Symbol;Acc:VGNC:92517]	3.17	0.0763
ssc-miR-30a-5p	RWDD4	RWD domain containing 4 [Source:VGNC Symbol;Acc:VGNC:96022]	3.17	0.0763
ssc-miR-30a-5p	S100PBP	S100P binding protein [Source:VGNC Symbol;Acc:VGNC:92549]	3.17	0.0763
ssc-miR-30a-5p	SACS	sacsin molecular chaperone [Source:HGNC Symbol;Acc:HGNC:10519]	3.17	0.0763
ssc-miR-30a-5p	SALL4	spalt like transcription factor 4 [Source:NCBI gene (formerly Entrezgene);Acc:100136902]	3.17	0.0763
ssc-miR-30a-5p	SAMD4A	sterile alpha motif domain containing 4A [Source:VGNC Symbol;Acc:VGNC:92569]	3.17	0.0763
ssc-miR-30a-5p	SAMD8	sterile alpha motif domain containing 8 [Source:VGNC Symbol;Acc:VGNC:92571]	3.17	0.0763
ssc-miR-30a-5p	SAP30	Sin3A associated protein 30 [Source:VGNC Symbol;Acc:VGNC:92575]	3.17	0.0763
ssc-miR-30a-5p	SAP30BP	SAP30 binding protein [Source:VGNC Symbol;Acc:VGNC:92576]	3.17	0.0763
ssc-miR-30a-5p	SAR1B	secretion associated Ras related GTPase 1B [Source:VGNC Symbol;Acc:VGNC:92580]	3.17	0.0763
ssc-miR-30a-5p	SATB1	SATB homeobox 1 [Source:VGNC Symbol;Acc:VGNC:92587]	3.17	0.0763

ssc-miR-30a-5p	SATB2	SATB homeobox 2 [Source:VGNC Symbol;Acc:VGNC:95972]	3.17	0.0763
ssc-miR-30a-5p	SBF1	SET binding factor 1 [Source:VGNC Symbol;Acc:VGNC:92593]	3.17	0.0763
ssc-miR-30a-5p	SBK1	SH3 domain binding kinase 1 [Source:HGNC Symbol;Acc:HGNC:17699]	3.17	0.0763
ssc-miR-30a-5p	SCAF4	SR-related CTD associated factor 4 [Source:VGNC Symbol;Acc:VGNC:92603]	3.17	0.0763
ssc-miR-30a-5p	SCAMP1	secretory carrier membrane protein 1 [Source:VGNC Symbol;Acc:VGNC:92605]	3.17	0.0763
ssc-miR-30a-5p	SCN1A	sodium voltage-gated channel alpha subunit 1 [Source:VGNC Symbol;Acc:VGNC:95478]	3.17	0.0763
ssc-miR-30a-5p	SCN2A	hypothetical gene	3.17	0.0763
ssc-miR-30a-5p	SCN3A	sodium voltage-gated channel alpha subunit 3 [Source:VGNC Symbol;Acc:VGNC:95479]	3.17	0.0763
ssc-miR-30a-5p	SCN8A	sodium voltage-gated channel alpha subunit 8 [Source:VGNC Symbol;Acc:VGNC:92638]	3.17	0.0763
ssc-miR-30a-5p	SCN9A	sodium voltage-gated channel alpha subunit 9 [Source:VGNC Symbol;Acc:VGNC:95481]	3.17	0.0763
ssc-miR-30a-5p	SCYL3	SCY1 like pseudokinase 3 [Source:VGNC Symbol;Acc:VGNC:92653]	3.17	0.0763
ssc-miR-30a-5p	SDAD1	SDA1 domain containing 1 [Source:VGNC Symbol;Acc:VGNC:98961]	3.17	0.0763
ssc-miR-30a-5p	SDC2	syndecan 2 [Source:VGNC Symbol;Acc:VGNC:92655]	3.17	0.0763
ssc-miR-30a-5p	SDCBP	syndecan binding protein [Source:VGNC Symbol;Acc:VGNC:92657]	3.17	0.0763
ssc-miR-30a-5p	SDCCAG3	hypothetical gene	3.17	0.0763
ssc-miR-30a-5p	SDK2	hypothetical gene	3.17	0.0763
ssc-miR-30a-5p	SEC14L2	SEC14 like lipid binding 2 [Source:NCBI gene (formerly Entrezgene);Acc:100152451]	3.17	0.0763
ssc-miR-30a-5p	SEC22A	SEC22 homolog A, vesicle trafficking protein [Source:VGNC Symbol;Acc:VGNC:92673]	3.17	0.0763
ssc-miR-30a-5p	SEC22C	SEC22 homolog C, vesicle trafficking protein [Source:VGNC Symbol;Acc:VGNC:92674]	3.17	0.0763
ssc-miR-30a-5p	SEC23A	hypothetical gene	3.17	0.0763
ssc-miR-30a-5p	SEC23IP	SEC23 interacting protein [Source:VGNC Symbol;Acc:VGNC:92675]	3.17	0.0763
ssc-miR-30a-5p	SEC24A	SEC24 homolog A, COPII coat complex component [Source:VGNC Symbol;Acc:VGNC:92676]	3.17	0.0763
ssc-miR-30a-5p	SEC61A2	SEC61 translocon subunit alpha 2 [Source:VGNC Symbol;Acc:VGNC:95457]	3.17	0.0763
ssc-miR-30a-5p	SEC62	SEC62 homolog, preprotein translocation factor [Source:VGNC Symbol;Acc:VGNC:108691]	3.17	0.0763
ssc-miR-30a-5p	SEL1L3	SEL1L family member 3 [Source:VGNC Symbol;Acc:VGNC:92688]	3.17	0.0763
ssc-miR-30a-5p	SEMA3A	semaphorin 3A [Source:VGNC Symbol;Acc:VGNC:92693]	3.17	0.0763
ssc-miR-30a-5p	SEMA6B	semaphorin 6B [Source:VGNC Symbol;Acc:VGNC:92709]	3.17	0.0763
ssc-miR-30a-5p	SEMA6D	semaphorin 6D [Source:VGNC Symbol;Acc:VGNC:92711]	3.17	0.0763
ssc-miR-30a-5p	SENP5	SUMO specific peptidase 5 [Source:VGNC Symbol;Acc:VGNC:92714]	3.17	0.0763
ssc-miR-30a-5p	SEPHS1	selenophosphate synthetase 1 [Source:VGNC Symbol;Acc:VGNC:104053]	3.17	0.0763
ssc-miR-30a-5p	SEPT3	hypothetical gene	3.17	0.0763
ssc-miR-30a-5p	SEPT7	hypothetical gene	3.17	0.0763
ssc-miR-30a-5p	SEPT8	hypothetical gene	3.17	0.0763
ssc-miR-30a-5p	SERPINE1	serpin family E member 1 [Source:VGNC Symbol;Acc:VGNC:98310]	3.17	0.0763
ssc-miR-30a-5p	SERPINF2	serpin family F member 2 [Source:VGNC Symbol;Acc:VGNC:92744]	3.17	0.0763
ssc-miR-30a-5p	SETD1B	SET domain containing 1B, histone lysine methyltransferase [Source:HGNC Symbol;Acc:HGNC:29187]	3.17	0.0763
ssc-miR-30a-5p	SETD3	SET domain containing 3, actin histidine methyltransferase [Source:VGNC Symbol;Acc:VGNC:92758]	3.17	0.0763
ssc-miR-30a-5p	SETD5	SET domain containing 5 [Source:VGNC Symbol;Acc:VGNC:92760]	3.17	0.0763
ssc-miR-30a-5p	SETD7	SET domain containing 7, histone lysine methyltransferase [Source:VGNC Symbol;Acc:VGNC:92762]	3.17	0.0763
ssc-miR-30a-5p	SETD9	SET domain containing 9 [Source:VGNC Symbol;Acc:VGNC:92763]	3.17	0.0763
ssc-miR-30a-5p	SFMBT1	Scm like with four mbt domains 1 [Source:VGNC Symbol;Acc:VGNC:92773]	3.17	0.0763
ssc-miR-30a-5p	SFXN1	sideroflexin 1 [Source:VGNC Symbol;Acc:VGNC:92783]	3.17	0.0763
ssc-miR-30a-5p	SGCB	sarcoglycan beta [Source:NCBI gene (formerly Entrezgene);Acc:100135674]	3.17	0.0763
ssc-miR-30a-5p	SGK3	serum/glucocorticoid regulated kinase family member 3 [Source:HGNC Symbol;Acc:HGNC:10812]	3.17	0.0763
ssc-miR-30a-5p	SGMS2	sphingomyelin synthase 2 [Source:VGNC Symbol;Acc:VGNC:92795]	3.17	0.0763
ssc-miR-30a-5p	SGSM1	small G protein signaling modulator 1 [Source:VGNC Symbol;Acc:VGNC:92800]	3.17	0.0763
ssc-miR-30a-5p	SH2B3	SH2B adaptor protein 3 [Source:VGNC Symbol;Acc:VGNC:92807]	3.17	0.0763
ssc-miR-30a-5p	SH3BGRL	hypothetical gene	3.17	0.0763
ssc-miR-30a-5p	SH3GLB1	SH3 domain containing GRB2 like, endophilin B1 [Source:VGNC Symbol;Acc:VGNC:92826]	3.17	0.0763

ssc-miR-30a-5p	SH3KBP1	SH3 domain containing kinase binding protein 1 [Source:VGNC Symbol;Acc:VGNC:92828]	3.17	0.0763
ssc-miR-30a-5p	SH3PXD2A	SH3 and PX domains 2A [Source:VGNC Symbol;Acc:VGNC:92829]	3.17	0.0763
ssc-miR-30a-5p	SH3RF1	SH3 domain containing ring finger 1 [Source:VGNC Symbol;Acc:VGNC:98316]	3.17	0.0763
ssc-miR-30a-5p	SHISA2	shisa family member 2 [Source:VGNC Symbol;Acc:VGNC:92845]	3.17	0.0763
ssc-miR-30a-5p	SHOC2	SHOC2 leucine rich repeat scaffold protein [Source:VGNC Symbol;Acc:VGNC:98318]	3.17	0.0763
ssc-miR-30a-5p	SHROOM1	shroom family member 1 [Source:VGNC Symbol;Acc:VGNC:92860]	3.17	0.0763
ssc-miR-30a-5p	SIAH2	siah E3 ubiquitin protein ligase 2 [Source:VGNC Symbol;Acc:VGNC:92866]	3.17	0.0763
ssc-miR-30a-5p	SIDT2	SID1 transmembrane family member 2 [Source:VGNC Symbol;Acc:VGNC:92869]	3.17	0.0763
ssc-miR-30a-5p	SIK3	SIK family kinase 3 [Source:VGNC Symbol;Acc:VGNC:92873]	3.17	0.0763
ssc-miR-30a-5p	SIM1	SIM bHLH transcription factor 1 [Source:VGNC Symbol;Acc:VGNC:92876]	3.17	0.0763
ssc-miR-30a-5p	SIRT1	sirtuin 1 [Source:VGNC Symbol;Acc:VGNC:92884]	3.17	0.0763
ssc-miR-30a-5p	SIX1	SIX homeobox 1 [Source:VGNC Symbol;Acc:VGNC:92892]	3.17	0.0763
ssc-miR-30a-5p	SIX4	SIX homeobox 4 [Source:VGNC Symbol;Acc:VGNC:92895]	3.17	0.0763
ssc-miR-30a-5p	SKIL	SKI like proto-onco [Source:VGNC Symbol;Acc:VGNC:92903]	3.17	0.0763
ssc-miR-30a-5p	SKP2	S-phase kinase associated protein 2 [Source:VGNC Symbol;Acc:VGNC:92907]	3.17	0.0763
ssc-miR-30a-5p	SLAIN2	SLAIN motif family member 2 [Source:VGNC Symbol;Acc:VGNC:92910]	3.17	0.0763
ssc-miR-30a-5p	SLC12A6	solute carrier family 12 member 6 [Source:VGNC Symbol;Acc:VGNC:92925]	3.17	0.0763
ssc-miR-30a-5p	SLC16A14	solute carrier family 16 member 14 [Source:VGNC Symbol;Acc:VGNC:96129]	3.17	0.0763
ssc-miR-30a-5p	SLC18A2	solute carrier family 18 member A2 [Source:VGNC Symbol;Acc:VGNC:92958]	3.17	0.0763
ssc-miR-30a-5p	SLC22A23	solute carrier family 22 member 23 [Source:HGNC Symbol;Acc:HGNC:21106]	3.17	0.0763
ssc-miR-30a-5p	SLC22A5	solute carrier family 22 member 5 [Source:HGNC Symbol;Acc:HGNC:10969]	3.17	0.0763
ssc-miR-30a-5p	SLC25A34	solute carrier family 25 member 34 [Source:VGNC Symbol;Acc:VGNC:93010]	3.17	0.0763
ssc-miR-30a-5p	SLC25A36	solute carrier family 25 member 36 [Source:VGNC Symbol;Acc:VGNC:93012]	3.17	0.0763
ssc-miR-30a-5p	SLC29A3	solute carrier family 29 member 3 [Source:VGNC Symbol;Acc:VGNC:93044]	3.17	0.0763
ssc-miR-30a-5p	SLC30A10	hypothetical gene	3.17	0.0763
ssc-miR-30a-5p	SLC30A2	solute carrier family 30 member 2 [Source:VGNC Symbol;Acc:VGNC:93056]	3.17	0.0763
ssc-miR-30a-5p	SLC30A4	solute carrier family 30 member 4 [Source:VGNC Symbol;Acc:VGNC:93058]	3.17	0.0763
ssc-miR-30a-5p	SLC30A5	solute carrier family 30 member 5 [Source:VGNC Symbol;Acc:VGNC:93059]	3.17	0.0763
ssc-miR-30a-5p	SLC35A3	solute carrier family 35 member A3 [Source:VGNC Symbol;Acc:VGNC:98860]	3.17	0.0763
ssc-miR-30a-5p	SLC35A5	solute carrier family 35 member A5 [Source:VGNC Symbol;Acc:VGNC:93070]	3.17	0.0763
ssc-miR-30a-5p	SLC35B4	solute carrier family 35 member B4 [Source:VGNC Symbol;Acc:VGNC:93074]	3.17	0.0763
ssc-miR-30a-5p	SLC35C1	solute carrier family 35 member C1 [Source:VGNC Symbol;Acc:VGNC:93075]	3.17	0.0763
ssc-miR-30a-5p	SLC35D3	solute carrier family 35 member D3 [Source:VGNC Symbol;Acc:VGNC:93076]	3.17	0.0763
ssc-miR-30a-5p	SLC35F1	solute carrier family 35 member F1 [Source:VGNC Symbol;Acc:VGNC:93081]	3.17	0.0763
ssc-miR-30a-5p	SLC35F3	solute carrier family 35 member F3 [Source:VGNC Symbol;Acc:VGNC:93083]	3.17	0.0763
ssc-miR-30a-5p	SLC35F4	solute carrier family 35 member F4 [Source:VGNC Symbol;Acc:VGNC:93084]	3.17	0.0763
ssc-miR-30a-5p	SLC36A1	solute carrier family 36 member 1 [Source:VGNC Symbol;Acc:VGNC:93088]	3.17	0.0763
ssc-miR-30a-5p	SLC38A1	solute carrier family 38 member 1 [Source:HGNC Symbol;Acc:HGNC:13447]	3.17	0.0763
ssc-miR-30a-5p	SLC38A2	solute carrier family 38 member 2 [Source:VGNC Symbol;Acc:VGNC:93094]	3.17	0.0763
ssc-miR-30a-5p	SLC38A4	solute carrier family 38 member 4 [Source:VGNC Symbol;Acc:VGNC:93096]	3.17	0.0763
ssc-miR-30a-5p	SLC38A7	solute carrier family 38 member 7 [Source:HGNC Symbol;Acc:HGNC:25582]	3.17	0.0763
ssc-miR-30a-5p	SLC39A11	solute carrier family 39 member 11 [Source:VGNC Symbol;Acc:VGNC:93102]	3.17	0.0763
ssc-miR-30a-5p	SLC41A2	solute carrier family 41 member 2 [Source:VGNC Symbol;Acc:VGNC:93114]	3.17	0.0763
ssc-miR-30a-5p	SLC4A7	solute carrier family 4 member 7 [Source:VGNC Symbol;Acc:VGNC:93135]	3.17	0.0763
ssc-miR-30a-5p	SLC5A11	solute carrier family 5 member 11 [Source:VGNC Symbol;Acc:VGNC:93141]	3.17	0.0763
ssc-miR-30a-5p	SLC5A3	solute carrier family 5 member 3 [Source:VGNC Symbol;Acc:VGNC:93144]	3.17	0.0763
ssc-miR-30a-5p	SLC6A15	solute carrier family 6 member 15 [Source:VGNC Symbol;Acc:VGNC:93157]	3.17	0.0763
ssc-miR-30a-5p	SLC6A3	solute carrier family 6 member 3 [Source:VGNC Symbol;Acc:VGNC:93163]	3.17	0.0763
ssc-miR-30a-5p	SLC6A6	solute carrier family 6 member 6 [Source:VGNC Symbol;Acc:VGNC:93166]	3.17	0.0763

ssc-miR-30a-5p	SLC6A9	solute carrier family 6 member 9 [Source:VGNC Symbol;Acc:VGNC:93168]	3.17	0.0763
ssc-miR-30a-5p	SLC7A10	solute carrier family 7 member 10 [Source:VGNC Symbol;Acc:VGNC:93170]	3.17	0.0763
ssc-miR-30a-5p	SLC7A11	solute carrier family 7 member 11 [Source:VGNC Symbol;Acc:VGNC:93171]	3.17	0.0763
ssc-miR-30a-5p	SLC7A6	hypothetical gene	3.17	0.0763
ssc-miR-30a-5p	SLC9A4	solute carrier family 9 member A4 [Source:VGNC Symbol;Acc:VGNC:93186]	3.17	0.0763
ssc-miR-30a-5p	SLC9A8	solute carrier family 9 member A8 [Source:VGNC Symbol;Acc:VGNC:95943]	3.17	0.0763
ssc-miR-30a-5p	SMAD1	SMAD family member 1 [Source:VGNC Symbol;Acc:VGNC:93216]	3.17	0.0763
ssc-miR-30a-5p	SMAD2	SMAD family member 2 [Source:VGNC Symbol;Acc:VGNC:98329]	3.17	0.0763
ssc-miR-30a-5p	SMAD5	SMAD family member 5 [Source:VGNC Symbol;Acc:VGNC:93219]	3.17	0.0763
ssc-miR-30a-5p	SMAP1	small ArfGAP 1 [Source:VGNC Symbol;Acc:VGNC:93223]	3.17	0.0763
ssc-miR-30a-5p	SMARCA5	SWI/SNF related, matrix associated, actin dependent regulator of chromatin, subfamily a, member 5 [Source:VGNC Symbol;Acc:VGNC:93228]	3.17	0.0763
ssc-miR-30a-5p	SMARCD2	SWI/SNF related, matrix associated, actin dependent regulator of chromatin, subfamily d, member 2 [Source:NCBI gene (formerly Entrezgene);Acc:100512367]	3.17	0.0763
ssc-miR-30a-5p	SMIM14	small integral membrane protein 14 [Source:VGNC Symbol;Acc:VGNC:93252]	3.17	0.0763
ssc-miR-30a-5p	SNAI1	snail family transcriptional repressor 1 [Source:VGNC Symbol;Acc:VGNC:95508]	3.17	0.0763
ssc-miR-30a-5p	SNAPC3	small nuclear RNA activating complex polypeptide 3 [Source:VGNC Symbol;Acc:VGNC:103182]	3.17	0.0763
ssc-miR-30a-5p	SNAPIN	SNAP associated protein [Source:VGNC Symbol;Acc:VGNC:93284]	3.17	0.0763
ssc-miR-30a-5p	SNTB2	syntrophin beta 2 [Source:VGNC Symbol;Acc:VGNC:93298]	3.17	0.0763
ssc-miR-30a-5p	SNX1	sorting nexin 1 [Source:HGNC Symbol;Acc:HGNC:11172]	3.17	0.0763
ssc-miR-30a-5p	SNX10	sorting nexin 10 [Source:VGNC Symbol;Acc:VGNC:93303]	3.17	0.0763
ssc-miR-30a-5p	SNX16	sorting nexin 16 [Source:VGNC Symbol;Acc:VGNC:93308]	3.17	0.0763
ssc-miR-30a-5p	SNX18	sorting nexin 18 [Source:VGNC Symbol;Acc:VGNC:93310]	3.17	0.0763
ssc-miR-30a-5p	SNX2	sorting nexin 2 [Source:VGNC Symbol;Acc:VGNC:93312]	3.17	0.0763
ssc-miR-30a-5p	SNX27	sorting nexin 27 [Source:VGNC Symbol;Acc:VGNC:93316]	3.17	0.0763
ssc-miR-30a-5p	SNX29	sorting nexin 29 [Source:VGNC Symbol;Acc:VGNC:98331]	3.17	0.0763
ssc-miR-30a-5p	SNX30	sorting nexin family member 30 [Source:VGNC Symbol;Acc:VGNC:93318]	3.17	0.0763
ssc-miR-30a-5p	SNX33	sorting nexin 33 [Source:VGNC Symbol;Acc:VGNC:93321]	3.17	0.0763
ssc-miR-30a-5p	SNX6	sorting nexin 6 [Source:VGNC Symbol;Acc:VGNC:93323]	3.17	0.0763
ssc-miR-30a-5p	SNX8	sorting nexin 8 [Source:VGNC Symbol;Acc:VGNC:93325]	3.17	0.0763
ssc-miR-30a-5p	SOBP	sine oculis binding protein homolog [Source:VGNC Symbol;Acc:VGNC:93329]	3.17	0.0763
ssc-miR-30a-5p	SOCS1	suppressor of cytokine signaling 1 [Source:VGNC Symbol;Acc:VGNC:93330]	3.17	0.0763
ssc-miR-30a-5p	SOCS3	suppressor of cytokine signaling 3 [Source:VGNC Symbol;Acc:VGNC:99052]	3.17	0.0763
ssc-miR-30a-5p	SOCS6	suppressor of cytokine signaling 6 [Source:VGNC Symbol;Acc:VGNC:93333]	3.17	0.0763
ssc-miR-30a-5p	SOGA3	SOGA family member 3 [Source:HGNC Symbol;Acc:HGNC:21494]	3.17	0.0763
ssc-miR-30a-5p	SON	SON DNA and RNA binding protein [Source:VGNC Symbol;Acc:VGNC:93336]	3.17	0.0763
ssc-miR-30a-5p	SORCS3	sortilin related VPS10 domain containing receptor 3 [Source:VGNC Symbol;Acc:VGNC:93341]	3.17	0.0763
ssc-miR-30a-5p	SOS1	SOS Ras/Rac guanine nucleotide exchange factor 1 [Source:HGNC Symbol;Acc:HGNC:11187]	3.17	0.0763
ssc-miR-30a-5p	SOX12	SRY-box transcription factor 12 [Source:HGNC Symbol;Acc:HGNC:11198]	3.17	0.0763
ssc-miR-30a-5p	SOX13	SRY-box transcription factor 13 [Source:VGNC Symbol;Acc:VGNC:93352]	3.17	0.0763
ssc-miR-30a-5p	SOX4	SRY-box transcription factor 4 [Source:HGNC Symbol;Acc:HGNC:11200]	3.17	0.0763
ssc-miR-30a-5p	SOX9	SRY-box transcription factor 9 [Source:VGNC Symbol;Acc:VGNC:99053]	3.17	0.0763
ssc-miR-30a-5p	SP4	Sp4 transcription factor [Source:VGNC Symbol;Acc:VGNC:93362]	3.17	0.0763
ssc-miR-30a-5p	SPAST	spastin [Source:VGNC Symbol;Acc:VGNC:98333]	3.17	0.0763
ssc-miR-30a-5p	SPATS2L	spermatosis associated serine rich 2 like [Source:VGNC Symbol;Acc:VGNC:96047]	3.17	0.0763
ssc-miR-30a-5p	SPCS3	signal peptidase complex subunit 3 [Source:VGNC Symbol;Acc:VGNC:96119]	3.17	0.0763
ssc-miR-30a-5p	SPEN	hypothetical gene	3.17	0.0763
ssc-miR-30a-5p	SPHKAP	SPHK1 interactor, AKAP domain containing [Source:VGNC Symbol;Acc:VGNC:96245]	3.17	0.0763
ssc-miR-30a-5p	SPIN1	spindlin 1 [Source:VGNC Symbol;Acc:VGNC:101495]	3.17	0.0763
ssc-miR-30a-5p	SPOPL	speckle type BTB/POZ protein like [Source:VGNC Symbol;Acc:VGNC:96161]	3.17	0.0763
ssc-miR-30a-5p	SPRYD7	SPRY domain containing 7 [Source:VGNC Symbol;Acc:VGNC:93429]	3.17	0.0763

ssc-miR-30a-5p	SPTLC2	serine palmitoyltransferase long chain base subunit 2 [Source:VGNC Symbol;Acc:VGNC:93440]	3.17	0.0763
ssc-miR-30a-5p	SPTSSB	serine palmitoyltransferase small subunit B [Source:VGNC Symbol;Acc:VGNC:93441]	3.17	0.0763
ssc-miR-30a-5p	SRGAP3	SLIT-ROBO Rho GTPase activating protein 3 [Source:VGNC Symbol;Acc:VGNC:93455]	3.17	0.0763
ssc-miR-30a-5p	SRI	sorcin [Source:VGNC Symbol;Acc:VGNC:93457]	3.17	0.0763
ssc-miR-30a-5p	SRP14	signal recognition particle 14 [Source:VGNC Symbol;Acc:VGNC:93458]	3.17	0.0763
ssc-miR-30a-5p	SRSF10	serine and arginine rich splicing factor 10 [Source:VGNC Symbol;Acc:VGNC:93472]	3.17	0.0763
ssc-miR-30a-5p	SRSF7	serine and arginine rich splicing factor 7 [Source:VGNC Symbol;Acc:VGNC:93477]	3.17	0.0763
ssc-miR-30a-5p	SSBP2	single stranded DNA binding protein 2 [Source:VGNC Symbol;Acc:VGNC:93480]	3.17	0.0763
ssc-miR-30a-5p	SSBP3	single stranded DNA binding protein 3 [Source:VGNC Symbol;Acc:VGNC:93481]	3.17	0.0763
ssc-miR-30a-5p	SSH1	slingshot protein phosphatase 1 [Source:VGNC Symbol;Acc:VGNC:93485]	3.17	0.0763
ssc-miR-30a-5p	SSR1	signal sequence receptor subunit 1 [Source:VGNC Symbol;Acc:VGNC:93490]	3.17	0.0763
ssc-miR-30a-5p	SSR3	signal sequence receptor subunit 3 [Source:VGNC Symbol;Acc:VGNC:93492]	3.17	0.0763
ssc-miR-30a-5p	SSX2IP	SSX family member 2 interacting protein [Source:VGNC Symbol;Acc:VGNC:93499]	3.17	0.0763
ssc-miR-30a-5p	ST8SIA1	ST8 alpha-N-acetyl-neuraminide alpha-2,8-sialyltransferase 1 [Source:VGNC Symbol;Acc:VGNC:93516]	3.17	0.0763
ssc-miR-30a-5p	ST8SIA4	ST8 alpha-N-acetyl-neuraminide alpha-2,8-sialyltransferase 4 [Source:VGNC Symbol;Acc:VGNC:93519]	3.17	0.0763
ssc-miR-30a-5p	STAC	SH3 and cysteine rich domain [Source:HGNC Symbol;Acc:HGNC:11353]	3.17	0.0763
ssc-miR-30a-5p	STAG2	stromal antigen 2 [Source:VGNC Symbol;Acc:VGNC:93525]	3.17	0.0763
ssc-miR-30a-5p	STAU1	staufen double-stranded RNA binding protein 1 [Source:VGNC Symbol;Acc:VGNC:95525]	3.17	0.0763
ssc-miR-30a-5p	STC1	stanniocalcin 1 [Source:VGNC Symbol;Acc:VGNC:93542]	3.17	0.0763
ssc-miR-30a-5p	STIM2	stromal interaction molecule 2 [Source:VGNC Symbol;Acc:VGNC:98967]	3.17	0.0763
ssc-miR-30a-5p	STK10	serine/threonine kinase 10 [Source:HGNC Symbol;Acc:HGNC:11388]	3.17	0.0763
ssc-miR-30a-5p	STK17B	serine/threonine kinase 17b [Source:VGNC Symbol;Acc:VGNC:95527]	3.17	0.0763
ssc-miR-30a-5p	STK35	serine/threonine kinase 35 [Source:VGNC Symbol;Acc:VGNC:95802]	3.17	0.0763
ssc-miR-30a-5p	STK38L	serine/threonine kinase 38 like [Source:VGNC Symbol;Acc:VGNC:93557]	3.17	0.0763
ssc-miR-30a-5p	STK39	serine/threonine kinase 39 [Source:VGNC Symbol;Acc:VGNC:98348]	3.17	0.0763
ssc-miR-30a-5p	STK4	serine/threonine kinase 4 [Source:VGNC Symbol;Acc:VGNC:98349]	3.17	0.0763
ssc-miR-30a-5p	STOML1	stomatin like 1 [Source:VGNC Symbol;Acc:VGNC:93566]	3.17	0.0763
ssc-miR-30a-5p	STOX2	storkhead box 2 [Source:VGNC Symbol;Acc:VGNC:96082]	3.17	0.0763
ssc-miR-30a-5p	STRIP1	striatin interacting protein 1 [Source:VGNC Symbol;Acc:VGNC:93576]	3.17	0.0763
ssc-miR-30a-5p	STT3B	STT3 oligosaccharyltransferase complex catalytic subunit B [Source:VGNC Symbol;Acc:VGNC:93582]	3.17	0.0763
ssc-miR-30a-5p	STX16	syntaxin 16 [Source:NCBI gene (formerly Entrezgene);Acc:100144526]	3.17	0.0763
ssc-miR-30a-5p	STX17	syntaxin 17 [Source:VGNC Symbol;Acc:VGNC:93587]	3.17	0.0763
ssc-miR-30a-5p	STX2	syntaxin 2 [Source:VGNC Symbol;Acc:VGNC:93590]	3.17	0.0763
ssc-miR-30a-5p	STX3	syntaxin 3 [Source:VGNC Symbol;Acc:VGNC:93591]	3.17	0.0763
ssc-miR-30a-5p	STX6	syntaxin 6 [Source:VGNC Symbol;Acc:VGNC:93594]	3.17	0.0763
ssc-miR-30a-5p	STX7	syntaxin 7 [Source:VGNC Symbol;Acc:VGNC:93595]	3.17	0.0763
ssc-miR-30a-5p	STXBP1	syntaxin binding protein 1 [Source:VGNC Symbol;Acc:VGNC:93596]	3.17	0.0763
ssc-miR-30a-5p	STXBP5	syntaxin binding protein 5 [Source:VGNC Symbol;Acc:VGNC:93599]	3.17	0.0763
ssc-miR-30a-5p	STYX	serine/threonine/tyrosine interacting protein [Source:VGNC Symbol;Acc:VGNC:93602]	3.17	0.0763
ssc-miR-30a-5p	SUCLG2	succinate-CoA ligase GDP-forming subunit beta [Source:VGNC Symbol;Acc:VGNC:93604]	3.17	0.0763
ssc-miR-30a-5p	SUPT3H	SPT3 homolog, SAGA and STAGA complex component [Source:VGNC Symbol;Acc:VGNC:93623]	3.17	0.0763
ssc-miR-30a-5p	SURF4	surfeit 4 [Source:VGNC Symbol;Acc:VGNC:93631]	3.17	0.0763
ssc-miR-30a-5p	SUV39H2	SUV39H2 histone lysine methyltransferase [Source:VGNC Symbol;Acc:VGNC:95841]	3.17	0.0763
ssc-miR-30a-5p	SV2C	synaptic vesicle glycoprotein 2C [Source:VGNC Symbol;Acc:VGNC:93641]	3.17	0.0763
ssc-miR-30a-5p	SYNGR3	synaptogyrin 3 [Source:VGNC Symbol;Acc:VGNC:93666]	3.17	0.0763
ssc-miR-30a-5p	SYPL1	synaptophysin like 1 [Source:HGNC Symbol;Acc:HGNC:11507]	3.17	0.0763
ssc-miR-30a-5p	TAB3	TGF-beta activated kinase 1 (MAP3K7) binding protein 3 [Source:HGNC Symbol;Acc:HGNC:30681]	3.17	0.0763
ssc-miR-30a-5p	TACC1	hypothetical gene	3.17	0.0763
ssc-miR-30a-5p	TADA2B	transcriptional adaptor 2B [Source:VGNC Symbol;Acc:VGNC:93707]	3.17	0.0763

ssc-miR-30a-5p	TAF4B	TATA-box binding protein associated factor 4b [Source:VGNC Symbol;Acc:VGNC:93716]	3.17	0.0763
ssc-miR-30a-5p	TAOK1	TAO kinase 1 [Source:VGNC Symbol;Acc:VGNC:98355]	3.17	0.0763
ssc-miR-30a-5p	TASP1	taspase 1 [Source:VGNC Symbol;Acc:VGNC:95676]	3.17	0.0763
ssc-miR-30a-5p	TBC1D10B	TBC1 domain family member 10B [Source:VGNC Symbol;Acc:VGNC:93760]	3.17	0.0763
ssc-miR-30a-5p	TBC1D15	TBC1 domain family member 15 [Source:VGNC Symbol;Acc:VGNC:93764]	3.17	0.0763
ssc-miR-30a-5p	TBC1D2B	TBC1 domain family member 2B [Source:VGNC Symbol;Acc:VGNC:93774]	3.17	0.0763
ssc-miR-30a-5p	TBC1D9	TBC1 domain family member 9 [Source:VGNC Symbol;Acc:VGNC:93781]	3.17	0.0763
ssc-miR-30a-5p	TBL1X	hypothetical gene	3.17	0.0763
ssc-miR-30a-5p	TBL1XR1	TBL1X receptor 1 [Source:VGNC Symbol;Acc:VGNC:96600]	3.17	0.0763
ssc-miR-30a-5p	TBPL1	TATA-box binding protein like 1 [Source:VGNC Symbol;Acc:VGNC:93792]	3.17	0.0763
ssc-miR-30a-5p	TCERG1	transcription elongation regulator 1 [Source:VGNC Symbol;Acc:VGNC:93815]	3.17	0.0763
ssc-miR-30a-5p	TCF21	transcription factor 21 [Source:VGNC Symbol;Acc:VGNC:93819]	3.17	0.0763
ssc-miR-30a-5p	TCF7	transcription factor 7 [Source:VGNC Symbol;Acc:VGNC:96767]	3.17	0.0763
ssc-miR-30a-5p	TDG	thymine DNA glycosylase [Source:VGNC Symbol;Acc:VGNC:93842]	3.17	0.0763
ssc-miR-30a-5p	TEAD1	TEA domain transcription factor 1 [Source:VGNC Symbol;Acc:VGNC:93853]	3.17	0.0763
ssc-miR-30a-5p	TECPR1	tectonin beta-propeller repeat containing 1 [Source:VGNC Symbol;Acc:VGNC:93858]	3.17	0.0763
ssc-miR-30a-5p	TENM1	teneurin transmembrane protein 1 [Source:VGNC Symbol;Acc:VGNC:98363]	3.17	0.0763
ssc-miR-30a-5p	TENM3	teneurin transmembrane protein 3 [Source:VGNC Symbol;Acc:VGNC:96233]	3.17	0.0763
ssc-miR-30a-5p	TERT	telomerase reverse transcriptase [Source:VGNC Symbol;Acc:VGNC:93883]	3.17	0.0763
ssc-miR-30a-5p	TET1	tet methylcytosine dioxygenase 1 [Source:VGNC Symbol;Acc:VGNC:93888]	3.17	0.0763
ssc-miR-30a-5p	TET3	tet methylcytosine dioxygenase 3 [Source:VGNC Symbol;Acc:VGNC:93890]	3.17	0.0763
ssc-miR-30a-5p	TEX2	testis expressed 2 [Source:VGNC Symbol;Acc:VGNC:93894]	3.17	0.0763
ssc-miR-30a-5p	TFCP2L1	transcription factor CP2 like 1 [Source:HGNC Symbol;Acc:HGNC:17925]	3.17	0.0763
ssc-miR-30a-5p	TFDP1	transcription factor Dp-1 [Source:HGNC Symbol;Acc:HGNC:11749]	3.17	0.0763
ssc-miR-30a-5p	TGFA	transforming growth factor alpha [Source:VGNC Symbol;Acc:VGNC:93928]	3.17	0.0763
ssc-miR-30a-5p	THBS2	thrombospondin 2 [Source:VGNC Symbol;Acc:VGNC:93947]	3.17	0.0763
ssc-miR-30a-5p	TIA1	TIA1 cytotoxic granule associated RNA binding protein [Source:VGNC Symbol;Acc:VGNC:93975]	3.17	0.0763
ssc-miR-30a-5p	TICAM2	hypothetical gene	3.17	0.0763
ssc-miR-30a-5p	TIMM22	translocase of inner mitochondrial membrane 22 [Source:VGNC Symbol;Acc:VGNC:93988]	3.17	0.0763
ssc-miR-30a-5p	TIMP2	hypothetical gene	3.17	0.0763
ssc-miR-30a-5p	TIMP3	TIMP metallopeptidase inhibitor 3 [Source:VGNC Symbol;Acc:VGNC:93996]	3.17	0.0763
ssc-miR-30a-5p	TLL2	tolloid like 2 [Source:VGNC Symbol;Acc:VGNC:94019]	3.17	0.0763
ssc-miR-30a-5p	TM4SF18	transmembrane 4 L six family member 18 [Source:VGNC Symbol;Acc:VGNC:94031]	3.17	0.0763
ssc-miR-30a-5p	TMCC1	transmembrane and coiled-coil domain family 1 [Source:VGNC Symbol;Acc:VGNC:94046]	3.17	0.0763
ssc-miR-30a-5p	TMED2	hypothetical gene	3.17	0.0763
ssc-miR-30a-5p	TMEFF1	transmembrane protein with EGF like and two follistatin like domains 1 [Source:HGNC Symbol;Acc:HGNC:11866]	3.17	0.0763
ssc-miR-30a-5p	TMEM106B	transmembrane protein 106B [Source:VGNC Symbol;Acc:VGNC:104075]	3.17	0.0763
ssc-miR-30a-5p	TMEM110	hypothetical gene	3.17	0.0763
ssc-miR-30a-5p	TMEM121	transmembrane protein 121 [Source:HGNC Symbol;Acc:HGNC:20511]	3.17	0.0763
ssc-miR-30a-5p	TMEM133	hypothetical gene	3.17	0.0763
ssc-miR-30a-5p	TMEM135	transmembrane protein 135 [Source:VGNC Symbol;Acc:VGNC:94085]	3.17	0.0763
ssc-miR-30a-5p	TMEM154	transmembrane protein 154 [Source:VGNC Symbol;Acc:VGNC:94098]	3.17	0.0763
ssc-miR-30a-5p	TMEM168	transmembrane protein 168 [Source:VGNC Symbol;Acc:VGNC:94106]	3.17	0.0763
ssc-miR-30a-5p	TMEM170B	transmembrane protein 170B [Source:VGNC Symbol;Acc:VGNC:94108]	3.17	0.0763
ssc-miR-30a-5p	TMEM181	transmembrane protein 181 [Source:VGNC Symbol;Acc:VGNC:94117]	3.17	0.0763
ssc-miR-30a-5p	TMEM220	transmembrane protein 220 [Source:VGNC Symbol;Acc:VGNC:94142]	3.17	0.0763
ssc-miR-30a-5p	TMEM229A	transmembrane protein 229A [Source:VGNC Symbol;Acc:VGNC:94146]	3.17	0.0763
ssc-miR-30a-5p	TMEM245	transmembrane protein 245 [Source:HGNC Symbol;Acc:HGNC:1363]	3.17	0.0763
ssc-miR-30a-5p	TMEM252	transmembrane protein 252 [Source:VGNC Symbol;Acc:VGNC:94158]	3.17	0.0763

ssc-miR-30a-5p	TMEM41A	transmembrane protein 41A [Source:VGNC Symbol;Acc:VGNC:104080]	3.17	0.0763
ssc-miR-30a-5p	TMEM55A	hypothetical gene	3.17	0.0763
ssc-miR-30a-5p	TMEM56	hypothetical gene	3.17	0.0763
ssc-miR-30a-5p	TMEM70	transmembrane protein 70 [Source:VGNC Symbol;Acc:VGNC:94204]	3.17	0.0763
ssc-miR-30a-5p	TMEM86A	transmembrane protein 86A [Source:VGNC Symbol;Acc:VGNC:94210]	3.17	0.0763
ssc-miR-30a-5p	TMEM87A	transmembrane protein 87A [Source:VGNC Symbol;Acc:VGNC:94212]	3.17	0.0763
ssc-miR-30a-5p	TMOD2	tropomodulin 2 [Source:VGNC Symbol;Acc:VGNC:94227]	3.17	0.0763
ssc-miR-30a-5p	TMOD3	tropomodulin 3 [Source:VGNC Symbol;Acc:VGNC:94228]	3.17	0.0763
ssc-miR-30a-5p	TMTC3	transmembrane O-mannosyltransferase targeting cadherins 3 [Source:VGNC Symbol;Acc:VGNC:94243]	3.17	0.0763
ssc-miR-30a-5p	TNFRSF8	hypothetical gene	3.17	0.0763
ssc-miR-30a-5p	TNIK	TRAF2 and NCK interacting kinase [Source:VGNC Symbol;Acc:VGNC:98381]	3.17	0.0763
ssc-miR-30a-5p	TNIP1	TNFAIP3 interacting protein 1 [Source:VGNC Symbol;Acc:VGNC:94275]	3.17	0.0763
ssc-miR-30a-5p	TNKS	tankyrase [Source:VGNC Symbol;Acc:VGNC:95546]	3.17	0.0763
ssc-miR-30a-5p	TNPO1	transportin 1 [Source:VGNC Symbol;Acc:VGNC:94287]	3.17	0.0763
ssc-miR-30a-5p	TNPO3	transportin 3 [Source:VGNC Symbol;Acc:VGNC:94289]	3.17	0.0763
ssc-miR-30a-5p	TNRC6A	trinucleotide repeat containing adaptor 6A [Source:VGNC Symbol;Acc:VGNC:94292]	3.17	0.0763
ssc-miR-30a-5p	TNRC6B	trinucleotide repeat containing adaptor 6B [Source:VGNC Symbol;Acc:VGNC:94293]	3.17	0.0763
ssc-miR-30a-5p	TNRC6C	trinucleotide repeat containing adaptor 6C [Source:VGNC Symbol;Acc:VGNC:94294]	3.17	0.0763
ssc-miR-30a-5p	TNXB	tenascin XB [Source:NCBI gene (formerly Entrezgene);Acc:445520]	3.17	0.0763
ssc-miR-30a-5p	TOR1B	torsin family 1 member B [Source:HGNC Symbol;Acc:HGNC:11995]	3.17	0.0763
ssc-miR-30a-5p	TOX	thymocyte selection associated high mobility group box [Source:VGNC Symbol;Acc:VGNC:94322]	3.17	0.0763
ssc-miR-30a-5p	TP53INP1	tumor protein p53 inducible nuclear protein 1 [Source:VGNC Symbol;Acc:VGNC:94329]	3.17	0.0763
ssc-miR-30a-5p	TPM4	hypothetical gene	3.17	0.0763
ssc-miR-30a-5p	TPRG1	tumor protein p63 regulated 1 [Source:VGNC Symbol;Acc:VGNC:94348]	3.17	0.0763
ssc-miR-30a-5p	TPRG1L	tumor protein p63 regulated 1 like [Source:VGNC Symbol;Acc:VGNC:94349]	3.17	0.0763
ssc-miR-30a-5p	TRAF3	TNF receptor associated factor 3 [Source:VGNC Symbol;Acc:VGNC:94360]	3.17	0.0763
ssc-miR-30a-5p	TRAF3IP2	TRAF3 interacting protein 2 [Source:VGNC Symbol;Acc:VGNC:94361]	3.17	0.0763
ssc-miR-30a-5p	TRAM2	translocation associated membrane protein 2 [Source:HGNC Symbol;Acc:HGNC:16855]	3.17	0.0763
ssc-miR-30a-5p	TRDN	triadin [Source:VGNC Symbol;Acc:VGNC:103195]	3.17	0.0763
ssc-miR-30a-5p	TRERF1	transcriptional regulating factor 1 [Source:VGNC Symbol;Acc:VGNC:94387]	3.17	0.0763
ssc-miR-30a-5p	TRIM13	tripartite motif containing 13 [Source:VGNC Symbol;Acc:VGNC:94394]	3.17	0.0763
ssc-miR-30a-5p	TRIM23	tripartite motif containing 23 [Source:VGNC Symbol;Acc:VGNC:94399]	3.17	0.0763
ssc-miR-30a-5p	TRIM24	tripartite motif containing 24 [Source:VGNC Symbol;Acc:VGNC:94400]	3.17	0.0763
ssc-miR-30a-5p	TRIM27	tripartite motif containing 27 [Source:VGNC Symbol;Acc:VGNC:94402]	3.17	0.0763
ssc-miR-30a-5p	TRIM33	tripartite motif containing 33 [Source:VGNC Symbol;Acc:VGNC:98881]	3.17	0.0763
ssc-miR-30a-5p	TRIM36	tripartite motif containing 36 [Source:VGNC Symbol;Acc:VGNC:94409]	3.17	0.0763
ssc-miR-30a-5p	TRIM71	tripartite motif containing 71 [Source:VGNC Symbol;Acc:VGNC:94431]	3.17	0.0763
ssc-miR-30a-5p	TRIM9	tripartite motif containing 9 [Source:VGNC Symbol;Acc:VGNC:94434]	3.17	0.0763
ssc-miR-30a-5p	TRIO	trio Rho guanine nucleotide exchange factor [Source:VGNC Symbol;Acc:VGNC:94435]	3.17	0.0763
ssc-miR-30a-5p	TRIP12	thyroid hormone receptor interactor 12 [Source:VGNC Symbol;Acc:VGNC:95555]	3.17	0.0763
ssc-miR-30a-5p	TRMT5	tRNA methyltransferase 5 [Source:VGNC Symbol;Acc:VGNC:94451]	3.17	0.0763
ssc-miR-30a-5p	TRO	trophinin [Source:NCBI gene (formerly Entrezgene);Acc:396561]	3.17	0.0763
ssc-miR-30a-5p	TRPM3	transient receptor potential cation channel subfamily M member 3 [Source:VGNC Symbol;Acc:VGNC:98384]	3.17	0.0763
ssc-miR-30a-5p	TRPM7	transient receptor potential cation channel subfamily M member 7 [Source:VGNC Symbol;Acc:VGNC:94470]	3.17	0.0763
ssc-miR-30a-5p	TRPS1	transcriptional repressor GATA binding 1 [Source:VGNC Symbol;Acc:VGNC:94471]	3.17	0.0763
ssc-miR-30a-5p	TRRAP	transformation/transcription domain associated protein [Source:VGNC Symbol;Acc:VGNC:94477]	3.17	0.0763
ssc-miR-30a-5p	TSC1	TSC complex subunit 1 [Source:HGNC Symbol;Acc:HGNC:12362]	3.17	0.0763
ssc-miR-30a-5p	TSC22D2	TSC22 domain family member 2 [Source:VGNC Symbol;Acc:VGNC:94482]	3.17	0.0763
ssc-miR-30a-5p	TSPAN2	tetraspanin 2 [Source:VGNC Symbol;Acc:VGNC:94509]	3.17	0.0763

ssc-miR-30a-5p	TSPAN33	tetraspanin 33 [Source:VGNC Symbol;Acc:VGNC:94512]	3.17	0.0763
ssc-miR-30a-5p	TSPYL4	TSPY like 4 [Source:HGNC Symbol;Acc:HGNC:21559]	3.17	0.0763
ssc-miR-30a-5p	TTBK1	tau tubulin kinase 1 [Source:VGNC Symbol;Acc:VGNC:98386]	3.17	0.0763
ssc-miR-30a-5p	TTC28	tetratricopeptide repeat domain 28 [Source:VGNC Symbol;Acc:VGNC:94543]	3.17	0.0763
ssc-miR-30a-5p	TTC30A	hypothetical gene	3.17	0.0763
ssc-miR-30a-5p	TTC39A	tetratricopeptide repeat domain 39A [Source:VGNC Symbol;Acc:VGNC:94551]	3.17	0.0763
ssc-miR-30a-5p	TTLL7	tubulin tyrosine ligase like 7 [Source:VGNC Symbol;Acc:VGNC:94571]	3.17	0.0763
ssc-miR-30a-5p	TTPA	alpha tocopherol transfer protein [Source:HGNC Symbol;Acc:HGNC:12404]	3.17	0.0763
ssc-miR-30a-5p	TUBGCP3	tubulin gamma complex associated protein 3 [Source:VGNC Symbol;Acc:VGNC:94585]	3.17	0.0763
ssc-miR-30a-5p	TULP4	hypothetical gene	3.17	0.0763
ssc-miR-30a-5p	TUSC3	tumor suppressor candidate 3 [Source:VGNC Symbol;Acc:VGNC:108742]	3.17	0.0763
ssc-miR-30a-5p	TVP23B	hypothetical gene	3.17	0.0763
ssc-miR-30a-5p	TWF1	twinfilin actin binding protein 1 [Source:VGNC Symbol;Acc:VGNC:94595]	3.17	0.0763
ssc-miR-30a-5p	TXNDC5	thioredoxin domain containing 5 [Source:VGNC Symbol;Acc:VGNC:94608]	3.17	0.0763
ssc-miR-30a-5p	UACA	uveal autoantigen with coiled-coil domains and ankyrin repeats [Source:VGNC Symbol;Acc:VGNC:94625]	3.17	0.0763
ssc-miR-30a-5p	UBAC1	UBA domain containing 1 [Source:VGNC Symbol;Acc:VGNC:94632]	3.17	0.0763
ssc-miR-30a-5p	UBE2D2	ubiquitin conjugating enzyme E2 D2 [Source:VGNC Symbol;Acc:VGNC:98391]	3.17	0.0763
ssc-miR-30a-5p	UBE2D3	ubiquitin conjugating enzyme E2 D3 [Source:NCBI gene (formerly Entrezgene);Acc:780418]	3.17	0.0763
ssc-miR-30a-5p	UBE2F	hypothetical gene	3.17	0.0763
ssc-miR-30a-5p	UBE2G1	ubiquitin conjugating enzyme E2 G1 [Source:HGNC Symbol;Acc:HGNC:12482]	3.17	0.0763
ssc-miR-30a-5p	UBE2I	hypothetical gene	3.17	0.0763
ssc-miR-30a-5p	UBE2J1	ubiquitin conjugating enzyme E2 J1 [Source:VGNC Symbol;Acc:VGNC:94644]	3.17	0.0763
ssc-miR-30a-5p	UBE2K	ubiquitin conjugating enzyme E2 K [Source:VGNC Symbol;Acc:VGNC:94646]	3.17	0.0763
ssc-miR-30a-5p	UBE2O	ubiquitin conjugating enzyme E2 O [Source:VGNC Symbol;Acc:VGNC:94649]	3.17	0.0763
ssc-miR-30a-5p	UBE2QL1	ubiquitin conjugating enzyme E2 Q family like 1 [Source:VGNC Symbol;Acc:VGNC:94650]	3.17	0.0763
ssc-miR-30a-5p	UBE2R2	ubiquitin conjugating enzyme E2 R2 [Source:VGNC Symbol;Acc:VGNC:95924]	3.17	0.0763
ssc-miR-30a-5p	UBE2V2	ubiquitin conjugating enzyme E2 V2 [Source:VGNC Symbol;Acc:VGNC:98394]	3.17	0.0763
ssc-miR-30a-5p	UBE3C	ubiquitin protein ligase E3C [Source:VGNC Symbol;Acc:VGNC:94654]	3.17	0.0763
ssc-miR-30a-5p	UBN1	ubinuclein 1 [Source:VGNC Symbol;Acc:VGNC:94663]	3.17	0.0763
ssc-miR-30a-5p	UBN2	ubinuclein 2 [Source:VGNC Symbol;Acc:VGNC:94664]	3.17	0.0763
ssc-miR-30a-5p	UBR7	ubiquitin protein ligase E3 component n-recognin 7 [Source:HGNC Symbol;Acc:HGNC:20344]	3.17	0.0763
ssc-miR-30a-5p	UBXN2A	UBX domain protein 2A [Source:VGNC Symbol;Acc:VGNC:94677]	3.17	0.0763
ssc-miR-30a-5p	UBXN7	hypothetical gene	3.17	0.0763
ssc-miR-30a-5p	UCP3	uncoupling protein 3 [Source:VGNC Symbol;Acc:VGNC:108624]	3.17	0.0763
ssc-miR-30a-5p	UGT8	UDP glycosyltransferase 8 [Source:VGNC Symbol;Acc:VGNC:98401]	3.17	0.0763
ssc-miR-30a-5p	UHRF1BP1	UHRF1 binding protein 1 [Source:VGNC Symbol;Acc:VGNC:94690]	3.17	0.0763
ssc-miR-30a-5p	UNC5C	unc-5 netrin receptor C [Source:VGNC Symbol;Acc:VGNC:94709]	3.17	0.0763
ssc-miR-30a-5p	UNC5D	unc-5 netrin receptor D [Source:VGNC Symbol;Acc:VGNC:95892]	3.17	0.0763
ssc-miR-30a-5p	UNKL	unk like zinc finger [Source:VGNC Symbol;Acc:VGNC:94716]	3.17	0.0763
ssc-miR-30a-5p	URM1	ubiquitin related modifier 1 [Source:VGNC Symbol;Acc:VGNC:94732]	3.17	0.0763
ssc-miR-30a-5p	USP14	ubiquitin specific peptidase 14 [Source:VGNC Symbol;Acc:VGNC:94747]	3.17	0.0763
ssc-miR-30a-5p	USP15	ubiquitin specific peptidase 15 [Source:VGNC Symbol;Acc:VGNC:94748]	3.17	0.0763
ssc-miR-30a-5p	USP2	ubiquitin specific peptidase 2 [Source:VGNC Symbol;Acc:VGNC:94751]	3.17	0.0763
ssc-miR-30a-5p	USP22	ubiquitin specific peptidase 22 [Source:HGNC Symbol;Acc:HGNC:12621]	3.17	0.0763
ssc-miR-30a-5p	USP24	ubiquitin specific peptidase 24 [Source:VGNC Symbol;Acc:VGNC:94754]	3.17	0.0763
ssc-miR-30a-5p	USP37	ubiquitin specific peptidase 37 [Source:VGNC Symbol;Acc:VGNC:98404]	3.17	0.0763
ssc-miR-30a-5p	USP44	ubiquitin specific peptidase 44 [Source:VGNC Symbol;Acc:VGNC:94766]	3.17	0.0763
ssc-miR-30a-5p	USP45	ubiquitin specific peptidase 45 [Source:VGNC Symbol;Acc:VGNC:94767]	3.17	0.0763
ssc-miR-30a-5p	USP47	ubiquitin specific peptidase 47 [Source:VGNC Symbol;Acc:VGNC:94769]	3.17	0.0763

ssc-miR-30a-5p	USP48	ubiquitin specific peptidase 48 [Source:VGNC Symbol;Acc:VGNC:94770]	3.17	0.0763
ssc-miR-30a-5p	USP49	ubiquitin specific peptidase 49 [Source:VGNC Symbol;Acc:VGNC:94771]	3.17	0.0763
ssc-miR-30a-5p	UST	uronyl 2-sulfotransferase [Source:VGNC Symbol;Acc:VGNC:94780]	3.17	0.0763
ssc-miR-30a-5p	VAMP3	hypothetical gene	3.17	0.0763
ssc-miR-30a-5p	VAPA	VAMP associated protein A [Source:VGNC Symbol;Acc:VGNC:94799]	3.17	0.0763
ssc-miR-30a-5p	VASH1	vasohibin 1 [Source:VGNC Symbol;Acc:VGNC:94801]	3.17	0.0763
ssc-miR-30a-5p	VAT1	vesicle amine transport 1 [Source:VGNC Symbol;Acc:VGNC:94805]	3.17	0.0763
ssc-miR-30a-5p	VAT1L	vesicle amine transport 1 like [Source:VGNC Symbol;Acc:VGNC:94806]	3.17	0.0763
ssc-miR-30a-5p	VAV2	vav guanine nucleotide exchange factor 2 [Source:VGNC Symbol;Acc:VGNC:94808]	3.17	0.0763
ssc-miR-30a-5p	VAV3	hypothetical gene	3.17	0.0763
ssc-miR-30a-5p	VCAN	versican [Source:VGNC Symbol;Acc:VGNC:108163]	3.17	0.0763
ssc-miR-30a-5p	VIM	vimentin [Source:VGNC Symbol;Acc:VGNC:95565]	3.17	0.0763
ssc-miR-30a-5p	VIP	vasoactive intestinal peptide [Source:VGNC Symbol;Acc:VGNC:103201]	3.17	0.0763
ssc-miR-30a-5p	VKORC1L1	vitamin K epoxide reductase complex subunit 1 like 1 [Source:VGNC Symbol;Acc:VGNC:94831]	3.17	0.0763
ssc-miR-30a-5p	VOPP1	VOPP1 WW domain binding protein [Source:VGNC Symbol;Acc:VGNC:94835]	3.17	0.0763
ssc-miR-30a-5p	VPS13C	vacuolar protein sorting 13 homolog C [Source:VGNC Symbol;Acc:VGNC:94840]	3.17	0.0763
ssc-miR-30a-5p	VPS13D	vacuolar protein sorting 13 homolog D [Source:VGNC Symbol;Acc:VGNC:94841]	3.17	0.0763
ssc-miR-30a-5p	VPS26B	VPS26, retromer complex component B [Source:NCBI gene (formerly Entrezgene);Acc:100192441]	3.17	0.0763
ssc-miR-30a-5p	VPS33A	VPS33A core subunit of CORVET and HOPS complexes [Source:NCBI gene (formerly Entrezgene);Acc:100049702]	3.17	0.0763
ssc-miR-30a-5p	VPS53	VPS53 subunit of GARP complex [Source:VGNC Symbol;Acc:VGNC:94861]	3.17	0.0763
ssc-miR-30a-5p	VSTM4	V-set and transmembrane domain containing 4 [Source:VGNC Symbol;Acc:VGNC:94874]	3.17	0.0763
ssc-miR-30a-5p	VWDE	von Willebrand factor D and EGF domains [Source:VGNC Symbol;Acc:VGNC:104093]	3.17	0.0763
ssc-miR-30a-5p	WASL	WASP like actin nucleation promoting factor [Source:VGNC Symbol;Acc:VGNC:96604]	3.17	0.0763
ssc-miR-30a-5p	WBP1L	WW domain binding protein 1 like [Source:VGNC Symbol;Acc:VGNC:94898]	3.17	0.0763
ssc-miR-30a-5p	WDFY3	WD repeat and FYVE domain containing 3 [Source:VGNC Symbol;Acc:VGNC:94903]	3.17	0.0763
ssc-miR-30a-5p	WDPCP	WD repeat containing planar cell polarity effector [Source:HGNC Symbol;Acc:HGNC:28027]	3.17	0.0763
ssc-miR-30a-5p	WDR1	WD repeat domain 1 [Source:VGNC Symbol;Acc:VGNC:94905]	3.17	0.0763
ssc-miR-30a-5p	WDR26	WD repeat domain 26 [Source:VGNC Symbol;Acc:VGNC:95960]	3.17	0.0763
ssc-miR-30a-5p	WDR37	WD repeat domain 37 [Source:VGNC Symbol;Acc:VGNC:96258]	3.17	0.0763
ssc-miR-30a-5p	WDR44	WD repeat domain 44 [Source:VGNC Symbol;Acc:VGNC:94921]	3.17	0.0763
ssc-miR-30a-5p	WDR47	WD repeat domain 47 [Source:VGNC Symbol;Acc:VGNC:94924]	3.17	0.0763
ssc-miR-30a-5p	WDR7	WD repeat domain 7 [Source:VGNC Symbol;Acc:VGNC:94936]	3.17	0.0763
ssc-miR-30a-5p	WDR70	WD repeat domain 70 [Source:VGNC Symbol;Acc:VGNC:94937]	3.17	0.0763
ssc-miR-30a-5p	WDR82	WD repeat domain 82 [Source:VGNC Symbol;Acc:VGNC:94944]	3.17	0.0763
ssc-miR-30a-5p	WIPF1	hypothetical gene	3.17	0.0763
ssc-miR-30a-5p	WIPF3	WAS/WASL interacting protein family member 3 [Source:VGNC Symbol;Acc:VGNC:94958]	3.17	0.0763
ssc-miR-30a-5p	WISP1	hypothetical gene	3.17	0.0763
ssc-miR-30a-5p	WNK3	WNK lysine deficient protein kinase 3 [Source:VGNC Symbol;Acc:VGNC:94964]	3.17	0.0763
ssc-miR-30a-5p	WNT7B	Wnt family member 7B [Source:VGNC Symbol;Acc:VGNC:94975]	3.17	0.0763
ssc-miR-30a-5p	WWP1	WW domain containing E3 ubiquitin protein ligase 1 [Source:VGNC Symbol;Acc:VGNC:94989]	3.17	0.0763
ssc-miR-30a-5p	XKR4	XK related 4 [Source:VGNC Symbol;Acc:VGNC:98900]	3.17	0.0763
ssc-miR-30a-5p	XPO1	exportin 1 [Source:VGNC Symbol;Acc:VGNC:95003]	3.17	0.0763
ssc-miR-30a-5p	XPR1	xenotropic and polytropic retrovirus receptor 1 [Source:VGNC Symbol;Acc:VGNC:108625]	3.17	0.0763
ssc-miR-30a-5p	YAF2	YY1 associated factor 2 [Source:VGNC Symbol;Acc:VGNC:95018]	3.17	0.0763
ssc-miR-30a-5p	YBX1	hypothetical gene	3.17	0.0763
ssc-miR-30a-5p	YES1	YES proto-onco 1, Src family tyrosine kinase [Source:VGNC Symbol;Acc:VGNC:95024]	3.17	0.0763
ssc-miR-30a-5p	YOD1	YOD1 deubiquitinase [Source:VGNC Symbol;Acc:VGNC:95035]	3.17	0.0763
ssc-miR-30a-5p	YPEL2	yippee like 2 [Source:VGNC Symbol;Acc:VGNC:95036]	3.17	0.0763
ssc-miR-30a-5p	YPEL5	yippee like 5 [Source:VGNC Symbol;Acc:VGNC:95039]	3.17	0.0763

ssc-miR-30a-5p	YTHDC1	YTH domain containing 1 [Source:VGNC Symbol;Acc:VGNC:95041]	3.17	0.0763
ssc-miR-30a-5p	YTHDF2	YTH N6-methyladenosine RNA binding protein 2 [Source:VGNC Symbol;Acc:VGNC:95043]	3.17	0.0763
ssc-miR-30a-5p	YTHDF3	YTH N6-methyladenosine RNA binding protein 3 [Source:VGNC Symbol;Acc:VGNC:95044]	3.17	0.0763
ssc-miR-30a-5p	YWHAG	hypothetical gene	3.17	0.0763
ssc-miR-30a-5p	YWHAZ	tyrosine 3-monooxygenase/tryptophan 5-monooxygenase activation protein zeta [Source:VGNC Symbol;Acc:VGNC:95047]	3.17	0.0763
ssc-miR-30a-5p	YY1	YY1 transcription factor [Source:HGNC Symbol;Acc:HGNC:12856]	3.17	0.0763
ssc-miR-30a-5p	ZBED4	hypothetical gene	3.17	0.0763
ssc-miR-30a-5p	ZBTB10	zinc finger and BTB domain containing 10 [Source:VGNC Symbol;Acc:VGNC:95058]	3.17	0.0763
ssc-miR-30a-5p	ZBTB11	zinc finger and BTB domain containing 11 [Source:VGNC Symbol;Acc:VGNC:95059]	3.17	0.0763
ssc-miR-30a-5p	ZBTB18	zinc finger and BTB domain containing 18 [Source:HGNC Symbol;Acc:HGNC:13030]	3.17	0.0763
ssc-miR-30a-5p	ZBTB20	zinc finger and BTB domain containing 20 [Source:VGNC Symbol;Acc:VGNC:95063]	3.17	0.0763
ssc-miR-30a-5p	ZBTB34	zinc finger and BTB domain containing 34 [Source:VGNC Symbol;Acc:VGNC:95070]	3.17	0.0763
ssc-miR-30a-5p	ZBTB39	zinc finger and BTB domain containing 39 [Source:VGNC Symbol;Acc:VGNC:95073]	3.17	0.0763
ssc-miR-30a-5p	ZBTB40	zinc finger and BTB domain containing 40 [Source:VGNC Symbol;Acc:VGNC:95074]	3.17	0.0763
ssc-miR-30a-5p	ZBTB41	zinc finger and BTB domain containing 41 [Source:VGNC Symbol;Acc:VGNC:108284]	3.17	0.0763
ssc-miR-30a-5p	ZBTB44	zinc finger and BTB domain containing 44 [Source:VGNC Symbol;Acc:VGNC:95076]	3.17	0.0763
ssc-miR-30a-5p	ZBTB6	zinc finger and BTB domain containing 6 [Source:VGNC Symbol;Acc:VGNC:95080]	3.17	0.0763
ssc-miR-30a-5p	ZBTB7A	zinc finger and BTB domain containing 7A [Source:VGNC Symbol;Acc:VGNC:95081]	3.17	0.0763
ssc-miR-30a-5p	ZCCHC14	zinc finger CCHC-type containing 14 [Source:VGNC Symbol;Acc:VGNC:95107]	3.17	0.0763
ssc-miR-30a-5p	ZCCHC2	zinc finger CCHC-type containing 2 [Source:VGNC Symbol;Acc:VGNC:95109]	3.17	0.0763
ssc-miR-30a-5p	ZCCHC24	zinc finger CCHC-type containing 24 [Source:HGNC Symbol;Acc:HGNC:26911]	3.17	0.0763
ssc-miR-30a-5p	ZCCHC4	zinc finger CCHC-type containing 4 [Source:VGNC Symbol;Acc:VGNC:95110]	3.17	0.0763
ssc-miR-30a-5p	ZDHHC14	zinc finger DHHC-type palmitoyltransferase 14 [Source:VGNC Symbol;Acc:VGNC:95117]	3.17	0.0763
ssc-miR-30a-5p	ZDHHC17	zinc finger DHHC-type palmitoyltransferase 17 [Source:VGNC Symbol;Acc:VGNC:95119]	3.17	0.0763
ssc-miR-30a-5p	ZDHHC20	zinc finger DHHC-type palmitoyltransferase 20 [Source:VGNC Symbol;Acc:VGNC:95122]	3.17	0.0763
ssc-miR-30a-5p	ZDHHC21	zinc finger DHHC-type palmitoyltransferase 21 [Source:VGNC Symbol;Acc:VGNC:95123]	3.17	0.0763
ssc-miR-30a-5p	ZEB2	hypothetical gene	3.17	0.0763
ssc-miR-30a-5p	ZFAND5	zinc finger AN1-type containing 5 [Source:VGNC Symbol;Acc:VGNC:95137]	3.17	0.0763
ssc-miR-30a-5p	ZFC3H1	zinc finger C3H1-type containing [Source:VGNC Symbol;Acc:VGNC:95139]	3.17	0.0763
ssc-miR-30a-5p	ZFHX3	zinc finger homeobox 3 [Source:VGNC Symbol;Acc:VGNC:95141]	3.17	0.0763
ssc-miR-30a-5p	ZFP36L1	ZFP36 ring finger protein like 1 [Source:VGNC Symbol;Acc:VGNC:95146]	3.17	0.0763
ssc-miR-30a-5p	ZFP36L2	ZFP36 ring finger protein like 2 [Source:VGNC Symbol;Acc:VGNC:95147]	3.17	0.0763
ssc-miR-30a-5p	ZFP90	ZFP90 zinc finger protein [Source:VGNC Symbol;Acc:VGNC:95149]	3.17	0.0763
ssc-miR-30a-5p	ZFYVE26	zinc finger FYVE-type containing 26 [Source:VGNC Symbol;Acc:VGNC:95160]	3.17	0.0763
ssc-miR-30a-5p	ZG16	hypothetical gene	3.17	0.0763
ssc-miR-30a-5p	ZIC2	Zic family member 2 [Source:VGNC Symbol;Acc:VGNC:95167]	3.17	0.0763
ssc-miR-30a-5p	ZMYND8	zinc finger MYND-type containing 8 [Source:VGNC Symbol;Acc:VGNC:108744]	3.17	0.0763
ssc-miR-30a-5p	ZNF148	zinc finger protein 148 [Source:VGNC Symbol;Acc:VGNC:95192]	3.17	0.0763
ssc-miR-30a-5p	ZNF197	zinc finger protein 197 [Source:VGNC Symbol;Acc:VGNC:95202]	3.17	0.0763
ssc-miR-30a-5p	ZNF200	zinc finger protein 200 [Source:VGNC Symbol;Acc:VGNC:95203]	3.17	0.0763
ssc-miR-30a-5p	ZNF264	hypothetical gene	3.17	0.0763
ssc-miR-30a-5p	ZNF275	hypothetical gene	3.17	0.0763
ssc-miR-30a-5p	ZNF280B	zinc finger protein 280B [Source:HGNC Symbol;Acc:HGNC:23022]	3.17	0.0763
ssc-miR-30a-5p	ZNF382	zinc finger protein 382 [Source:VGNC Symbol;Acc:VGNC:95232]	3.17	0.0763
ssc-miR-30a-5p	ZNF507	zinc finger protein 507 [Source:VGNC Symbol;Acc:VGNC:95253]	3.17	0.0763
ssc-miR-30a-5p	ZNF521	zinc finger protein 521 [Source:VGNC Symbol;Acc:VGNC:98715]	3.17	0.0763
ssc-miR-30a-5p	ZNF608	hypothetical gene	3.17	0.0763
ssc-miR-30a-5p	ZNF644	zinc finger protein 644 [Source:VGNC Symbol;Acc:VGNC:95283]	3.17	0.0763
ssc-miR-30a-5p	ZNF652	zinc finger protein 652 [Source:VGNC Symbol;Acc:VGNC:99108]	3.17	0.0763

ssc-miR-30a-5p	ZNF704	zinc finger protein 704 [Source:VGNC Symbol;Acc:VGNC:95296]	3.17	0.0763
ssc-miR-30a-5p	ZNF711	zinc finger protein 711 [Source:HGNC Symbol;Acc:HGNC:13128]	3.17	0.0763
ssc-miR-30a-5p	ZNF746	hypothetical gene	3.17	0.0763
ssc-miR-30a-5p	ZNF770	zinc finger protein 770 [Source:VGNC Symbol;Acc:VGNC:95300]	3.17	0.0763
ssc-miR-30a-5p	ZNF827	zinc finger protein 827 [Source:VGNC Symbol;Acc:VGNC:98970]	3.17	0.0763
ssc-miR-30a-5p	ZNF831	zinc finger protein 831 [Source:VGNC Symbol;Acc:VGNC:95765]	3.17	0.0763
ssc-miR-30a-5p	ZNRF1	zinc and ring finger 1 [Source:HGNC Symbol;Acc:HGNC:18452]	3.17	0.0763
ssc-miR-30a-5p	ZPBP2	zona pellucida binding protein 2 [Source:VGNC Symbol;Acc:VGNC:95320]	3.17	0.0763
ssc-miR-30a-5p	ZRANB3	zinc finger RANBP2-type containing 3 [Source:VGNC Symbol;Acc:VGNC:96073]	3.17	0.0763
ssc-miR-30a-5p	ZSCAN29	zinc finger and SCAN domain containing 29 [Source:HGNC Symbol;Acc:HGNC:26673]	3.17	0.0763
ssc-miR-30a-5p	ZSWIM5	zinc finger SWIM-type containing 5 [Source:VGNC Symbol;Acc:VGNC:95334]	3.17	0.0763
ssc-miR-30a-5p	ZSWIM6	zinc finger SWIM-type containing 6 [Source:VGNC Symbol;Acc:VGNC:95335]	3.17	0.0763
ssc-miR-574	NA	hypothetical gene	2.59	0.0699
ssc-miR-181b	AAK1	AP2 associated kinase 1 [Source:VGNC Symbol;Acc:VGNC:100379]	2.55	0.000004
ssc-miR-181b	AASDHPPT	aminoadipate-semialdehyde dehydrogenase-phosphopantetheinyl transferase [Source:VGNC Symbol;Acc:VGNC:84943]	2.55	0.000004
ssc-miR-181b	ABHD13	abhydrolase domain containing 13 [Source:VGNC Symbol;Acc:VGNC:84972]	2.55	0.000004
ssc-miR-181b	ABI1	abl interactor 1 [Source:VGNC Symbol;Acc:VGNC:95519]	2.55	0.000004
ssc-miR-181b	ABI3BP	ABI family member 3 binding protein [Source:VGNC Symbol;Acc:VGNC:108628]	2.55	0.000004
ssc-miR-181b	ABTB2	ankyrin repeat and BTB domain containing 2 [Source:VGNC Symbol;Acc:VGNC:84994]	2.55	0.000004
ssc-miR-181b	AC012215.1	hypothetical gene	2.55	0.000004
ssc-miR-181b	AC068987.1	hypothetical gene	2.55	0.000004
ssc-miR-181b	ACAN	aggrecan [Source:NCBI gene (formerly Entrezgene);Acc:397255]	2.55	0.000004
ssc-miR-181b	ACAP2	ArfGAP with coiled-coil, ankyrin repeat and PH domains 2 [Source:VGNC Symbol;Acc:VGNC:85002]	2.55	0.000004
ssc-miR-181b	ACER3	alkaline ceramidase 3 [Source:VGNC Symbol;Acc:VGNC:103893]	2.55	0.000004
ssc-miR-181b	ACSL6	acyl-CoA synthetase long chain family member 6 [Source:VGNC Symbol;Acc:VGNC:99581]	2.55	0.000004
ssc-miR-181b	ACTA2	actin alpha 2, smooth muscle [Source:VGNC Symbol;Acc:VGNC:85038]	2.55	0.000004
ssc-miR-181b	ACTR3	actin related protein 3 [Source:VGNC Symbol;Acc:VGNC:103894]	2.55	0.000004
ssc-miR-181b	ACTRT3	actin related protein T3 [Source:VGNC Symbol;Acc:VGNC:85055]	2.55	0.000004
ssc-miR-181b	ACVR1C	activin A receptor type 1C [Source:VGNC Symbol;Acc:VGNC:95873]	2.55	0.000004
ssc-miR-181b	ACVR2A	activin A receptor type 2A [Source:VGNC Symbol;Acc:VGNC:95843]	2.55	0.000004
ssc-miR-181b	ACVR2B	activin A receptor type 2B [Source:VGNC Symbol;Acc:VGNC:108629]	2.55	0.000004
ssc-miR-181b	ADAM11	ADAM metallopeptidase domain 11 [Source:VGNC Symbol;Acc:VGNC:85062]	2.55	0.000004
ssc-miR-181b	ADAMTS1	ADAM metallopeptidase with thrombospondin type 1 motif 1 [Source:VGNC Symbol;Acc:VGNC:85072]	2.55	0.000004
ssc-miR-181b	ADAMTS18	ADAM metallopeptidase with thrombospondin type 1 motif 18 [Source:VGNC Symbol;Acc:VGNC:85080]	2.55	0.000004
ssc-miR-181b	ADAMTS5	ADAM metallopeptidase with thrombospondin type 1 motif 5 [Source:VGNC Symbol;Acc:VGNC:85085]	2.55	0.000004
ssc-miR-181b	ADAMTSL1	ADAMTS like 1 [Source:VGNC Symbol;Acc:VGNC:85090]	2.55	0.000004
ssc-miR-181b	ADARB1	adenosine deaminase RNA specific B1 [Source:VGNC Symbol;Acc:VGNC:85098]	2.55	0.000004
ssc-miR-181b	ADARB2	adenosine deaminase RNA specific B2 (inactive) [Source:HGNC Symbol;Acc:HGNC:227]	2.55	0.000004
ssc-miR-181b	ADAT2	adenosine deaminase tRNA specific 2 [Source:VGNC Symbol;Acc:VGNC:85100]	2.55	0.000004
ssc-miR-181b	ADCY9	adenylate cyclase 9 [Source:VGNC Symbol;Acc:VGNC:85113]	2.55	0.000004
ssc-miR-181b	ADHFE1	alcohol dehydrogenase iron containing 1 [Source:VGNC Symbol;Acc:VGNC:98730]	2.55	0.000004
ssc-miR-181b	ADO	2-aminoethanethiol dioxygenase [Source:VGNC Symbol;Acc:VGNC:85146]	2.55	0.000004
ssc-miR-181b	ADRBK1	hypothetical gene	2.55	0.000004
ssc-miR-181b	AFAP1	actin filament associated protein 1 [Source:VGNC Symbol;Acc:VGNC:85165]	2.55	0.000004
ssc-miR-181b	AFF1	AF4/FMR2 family member 1 [Source:VGNC Symbol;Acc:VGNC:85167]	2.55	0.000004
ssc-miR-181b	AFG3L2	AFG3 like matrix AAA peptidase subunit 2 [Source:HGNC Symbol;Acc:HGNC:315]	2.55	0.000004
ssc-miR-181b	AFTPH	aftiphilin [Source:VGNC Symbol;Acc:VGNC:85173]	2.55	0.000004
ssc-miR-181b	AGFG1	ArfGAP with FG repeats 1 [Source:VGNC Symbol;Acc:VGNC:96359]	2.55	0.000004
ssc-miR-181b	AGO4	hypothetical gene	2.55	0.000004

ssc-miR-181b	AHNAK	hypothetical gene	2.55	0.000004
ssc-miR-181b	AHSA2	hypothetical gene	2.55	0.000004
ssc-miR-181b	AK3	adenylate kinase 3 [Source:VGNC Symbol;Acc:VGNC:97876]	2.55	0.000004
ssc-miR-181b	AKAP5	A-kinase anchoring protein 5 [Source:VGNC Symbol;Acc:VGNC:85220]	2.55	0.000004
ssc-miR-181b	AKIRIN1	akirin 1 [Source:VGNC Symbol;Acc:VGNC:85227]	2.55	0.000004
ssc-miR-181b	AKT3	AKT serine/threonine kinase 3 [Source:VGNC Symbol;Acc:VGNC:96306]	2.55	0.000004
ssc-miR-181b	AL021546.6	hypothetical gene	2.55	0.000004
ssc-miR-181b	AL590483.1	hypothetical gene	2.55	0.000004
ssc-miR-181b	ALDH1A3	aldehyde dehydrogenase 1 family member A3 [Source:VGNC Symbol;Acc:VGNC:97879]	2.55	0.000004
ssc-miR-181b	AMER2	APC membrane recruitment protein 2 [Source:VGNC Symbol;Acc:VGNC:85277]	2.55	0.000004
ssc-miR-181b	ANAPC16	anaphase promoting complex subunit 16 [Source:NCBI gene (formerly Entrezgene);Acc:100155019]	2.55	0.000004
ssc-miR-181b	ANGPTL3	angiopoietin like 3 [Source:VGNC Symbol;Acc:VGNC:85305]	2.55	0.000004
ssc-miR-181b	ANK1	ankyrin 1 [Source:VGNC Symbol;Acc:VGNC:96344]	2.55	0.000004
ssc-miR-181b	ANKFY1	ankyrin repeat and FYVE domain containing 1 [Source:VGNC Symbol;Acc:VGNC:85314]	2.55	0.000004
ssc-miR-181b	ANKRD13C	ankyrin repeat domain 13C [Source:VGNC Symbol;Acc:VGNC:85326]	2.55	0.000004
ssc-miR-181b	ANKRD33B	ankyrin repeat domain 33B [Source:VGNC Symbol;Acc:VGNC:85334]	2.55	0.000004
ssc-miR-181b	ANKRD44	ankyrin repeat domain 44 [Source:VGNC Symbol;Acc:VGNC:107123]	2.55	0.000004
ssc-miR-181b	ANKRD50	ankyrin repeat domain containing 50 [Source:VGNC Symbol;Acc:VGNC:85341]	2.55	0.000004
ssc-miR-181b	ANKRD52	ankyrin repeat domain 52 [Source:VGNC Symbol;Acc:VGNC:85342]	2.55	0.000004
ssc-miR-181b	ANP32A	hypothetical gene	2.55	0.000004
ssc-miR-181b	ANXA11	annexin A11 [Source:VGNC Symbol;Acc:VGNC:85367]	2.55	0.000004
ssc-miR-181b	AP1AR	adaptor related protein complex 1 associated regulatory protein [Source:HGNC Symbol;Acc:HGNC:28808]	2.55	0.000004
ssc-miR-181b	AP1G1	adaptor related protein complex 1 subunit gamma 1 [Source:VGNC Symbol;Acc:VGNC:85378]	2.55	0.000004
ssc-miR-181b	AP1S3	adaptor related protein complex 1 subunit sigma 3 [Source:VGNC Symbol;Acc:VGNC:95901]	2.55	0.000004
ssc-miR-181b	AP5M1	adaptor related protein complex 5 subunit mu 1 [Source:VGNC Symbol;Acc:VGNC:85396]	2.55	0.000004
ssc-miR-181b	APBA1	amyloid beta protein binding family A member 1 [Source:VGNC Symbol;Acc:VGNC:85399]	2.55	0.000004
ssc-miR-181b	APLP2	amyloid beta like protein 2 [Source:VGNC Symbol;Acc:VGNC:85414]	2.55	0.000004
ssc-miR-181b	APOO	apolipoprotein O [Source:VGNC Symbol;Acc:VGNC:103900]	2.55	0.000004
ssc-miR-181b	APP	amyloid beta protein [Source:VGNC Symbol;Acc:VGNC:103902]	2.55	0.000004
ssc-miR-181b	ARF3	ADP ribosylation factor 3 [Source:NCBI gene (formerly Entrezgene);Acc:100301570]	2.55	0.000004
ssc-miR-181b	ARF6	ADP ribosylation factor 6 [Source:VGNC Symbol;Acc:VGNC:85448]	2.55	0.000004
ssc-miR-181b	ARHGEF3	Rho guanine nucleotide exchange factor 3 [Source:VGNC Symbol;Acc:VGNC:85496]	2.55	0.000004
ssc-miR-181b	ARHGEF37	Rho guanine nucleotide exchange factor 37 [Source:VGNC Symbol;Acc:VGNC:85498]	2.55	0.000004
ssc-miR-181b	ARID2	AT-rich interaction domain 2 [Source:HGNC Symbol;Acc:HGNC:18037]	2.55	0.000004
ssc-miR-181b	ARIH1	ariadne RBR E3 ubiquitin protein ligase 1 [Source:HGNC Symbol;Acc:HGNC:689]	2.55	0.000004
ssc-miR-181b	ARL1	ADP ribosylation factor like GTPase 1 [Source:VGNC Symbol;Acc:VGNC:85513]	2.55	0.000004
ssc-miR-181b	ARL3	ADP ribosylation factor like GTPase 3 [Source:VGNC Symbol;Acc:VGNC:107366]	2.55	0.000004
ssc-miR-181b	ARL5A	ADP ribosylation factor like GTPase 5A [Source:VGNC Symbol;Acc:VGNC:96418]	2.55	0.000004
ssc-miR-181b	ARNT2	aryl hydrocarbon receptor nuclear translocator 2 [Source:HGNC Symbol;Acc:HGNC:16876]	2.55	0.000004
ssc-miR-181b	ARRDC3	arrestin domain containing 3 [Source:VGNC Symbol;Acc:VGNC:85546]	2.55	0.000004
ssc-miR-181b	ARSJ	arylsulfatase family member J [Source:VGNC Symbol;Acc:VGNC:85549]	2.55	0.000004
ssc-miR-181b	ASAH2	hypothetical gene	2.55	0.000004
ssc-miR-181b	ASAH2B	hypothetical gene	2.55	0.000004
ssc-miR-181b	ASAH2C	hypothetical gene	2.55	0.000004
ssc-miR-181b	ASIC1	acid sensing ion channel subunit 1 [Source:VGNC Symbol;Acc:VGNC:85578]	2.55	0.000004
ssc-miR-181b	ASPHD2	aspartate beta-hydroxylase domain containing 2 [Source:VGNC Symbol;Acc:VGNC:85585]	2.55	0.000004
ssc-miR-181b	ASTN1	astrotactin 1 [Source:VGNC Symbol;Acc:VGNC:85593]	2.55	0.000004
ssc-miR-181b	ASXL2	ASXL transcriptional regulator 2 [Source:VGNC Symbol;Acc:VGNC:85595]	2.55	0.000004
ssc-miR-181b	ASXL3	ASXL transcriptional regulator 3 [Source:VGNC Symbol;Acc:VGNC:85596]	2.55	0.000004

ssc-miR-181b	ATF2	activating transcription factor 2 [Source:VGNC Symbol;Acc:VGNC:96446]	2.55	0.000004
ssc-miR-181b	ATG2B	autophagy related 2B [Source:VGNC Symbol;Acc:VGNC:85620]	2.55	0.000004
ssc-miR-181b	ATG7	autophagy related 7 [Source:VGNC Symbol;Acc:VGNC:85625]	2.55	0.000004
ssc-miR-181b	ATL3	atlastin GTPase 3 [Source:VGNC Symbol;Acc:VGNC:85629]	2.55	0.000004
ssc-miR-181b	ATM	ATM serine/threonine kinase [Source:VGNC Symbol;Acc:VGNC:108572]	2.55	0.000004
ssc-miR-181b	ATMIN	hypothetical gene	2.55	0.000004
ssc-miR-181b	ATP11A	ATPase phospholipid transporting 11A [Source:VGNC Symbol;Acc:VGNC:85634]	2.55	0.000004
ssc-miR-181b	ATP11C	ATPase phospholipid transporting 11C [Source:VGNC Symbol;Acc:VGNC:85635]	2.55	0.000004
ssc-miR-181b	ATP1B1	ATPase Na+/K+ transporting subunit beta 1 [Source:VGNC Symbol;Acc:VGNC:85642]	2.55	0.000004
ssc-miR-181b	ATP2A2	ATPase sarcoplasmic/endoplasmic reticulum Ca2+ transporting 2 [Source:VGNC Symbol;Acc:VGNC:85647]	2.55	0.000004
ssc-miR-181b	ATP2B1	ATPase plasma membrane Ca2+ transporting 1 [Source:VGNC Symbol;Acc:VGNC:103219]	2.55	0.000004
ssc-miR-181b	ATP2B2	ATPase plasma membrane Ca2+ transporting 2 [Source:VGNC Symbol;Acc:VGNC:85649]	2.55	0.000004
ssc-miR-181b	ATP6V1A	ATPase H+ transporting V1 subunit A [Source:VGNC Symbol;Acc:VGNC:85669]	2.55	0.000004
ssc-miR-181b	ATP6V1C1	ATPase H+ transporting V1 subunit C1 [Source:VGNC Symbol;Acc:VGNC:85672]	2.55	0.000004
ssc-miR-181b	ATP8A1	ATPase phospholipid transporting 8A1 [Source:VGNC Symbol;Acc:VGNC:97894]	2.55	0.000004
ssc-miR-181b	ATP8B1	ATPase phospholipid transporting 8B1 [Source:VGNC Symbol;Acc:VGNC:85679]	2.55	0.000004
ssc-miR-181b	ATP8B2	ATPase phospholipid transporting 8B2 [Source:VGNC Symbol;Acc:VGNC:85680]	2.55	0.000004
ssc-miR-181b	ATP9A	ATPase phospholipid transporting 9A (putative) [Source:NCBI gene (formerly Entrezgene);Acc:100136901]	2.55	0.000004
ssc-miR-181b	ATRNL1	attractin like 1 [Source:VGNC Symbol;Acc:VGNC:85685]	2.55	0.000004
ssc-miR-181b	ATXN1	ataxin 1 [Source:VGNC Symbol;Acc:VGNC:85687]	2.55	0.000004
ssc-miR-181b	ATXN3	hypothetical gene	2.55	0.000004
ssc-miR-181b	ATXN7	ataxin 7 [Source:VGNC Symbol;Acc:VGNC:99704]	2.55	0.000004
ssc-miR-181b	AVL9	AVL9 cell migration associated [Source:VGNC Symbol;Acc:VGNC:85701]	2.55	0.000004
ssc-miR-181b	B3GALT1	beta-1,3-galactosyltransferase 1 [Source:VGNC Symbol;Acc:VGNC:96494]	2.55	0.000004
ssc-miR-181b	B3GNT4	UDP-GlcNAc:betaGal beta-1,3-N-acetylglucosaminyltransferase 4 [Source:VGNC Symbol;Acc:VGNC:85725]	2.55	0.000004
ssc-miR-181b	B4GALT1	beta-1,4-galactosyltransferase 1 [Source:VGNC Symbol;Acc:VGNC:96497]	2.55	0.000004
ssc-miR-181b	BAAT	bile acid-CoA:amino acid N-acyltransferase [Source:VGNC Symbol;Acc:VGNC:103038]	2.55	0.000004
ssc-miR-181b	BACH2	BTB domain and CNC homolog 2 [Source:VGNC Symbol;Acc:VGNC:85742]	2.55	0.000004
ssc-miR-181b	BAG4	BAG cochaperone 4 [Source:VGNC Symbol;Acc:VGNC:96503]	2.55	0.000004
ssc-miR-181b	BAI3	hypothetical gene	2.55	0.000004
ssc-miR-181b	BAZ1A	bromodomain adjacent to zinc finger domain 1A [Source:VGNC Symbol;Acc:VGNC:85761]	2.55	0.000004
ssc-miR-181b	BAZ2A	bromodomain adjacent to zinc finger domain 2A [Source:VGNC Symbol;Acc:VGNC:85763]	2.55	0.000004
ssc-miR-181b	BAZ2B	bromodomain adjacent to zinc finger domain 2B [Source:HGNC Symbol;Acc:HGNC:963]	2.55	0.000004
ssc-miR-181b	BCL2	BCL2 apoptosis regulator [Source:HGNC Symbol;Acc:HGNC:990]	2.55	0.000004
ssc-miR-181b	BCL2L11	BCL2 like 11 [Source:NCBI gene (formerly Entrezgene);Acc:396632]	2.55	0.000004
ssc-miR-181b	BCL2L13	BCL2 like 13 [Source:VGNC Symbol;Acc:VGNC:85781]	2.55	0.000004
ssc-miR-181b	BCL6	BCL6 transcription repressor [Source:VGNC Symbol;Acc:VGNC:96565]	2.55	0.000004
ssc-miR-181b	BCL9	BCL9 transcription coactivator [Source:VGNC Symbol;Acc:VGNC:96567]	2.55	0.000004
ssc-miR-181b	BCLAF1	BCL2 associated transcription factor 1 [Source:VGNC Symbol;Acc:VGNC:85787]	2.55	0.000004
ssc-miR-181b	BCR	BCR activator of RhoGEF and GTPase [Source:HGNC Symbol;Acc:HGNC:1014]	2.55	0.000004
ssc-miR-181b	BEND3	BEN domain containing 3 [Source:VGNC Symbol;Acc:VGNC:85800]	2.55	0.000004
ssc-miR-181b	BEND4	BEN domain containing 4 [Source:VGNC Symbol;Acc:VGNC:85801]	2.55	0.000004
ssc-miR-181b	BHLHE40	basic helix-loop-helix family member e40 [Source:VGNC Symbol;Acc:VGNC:85814]	2.55	0.000004
ssc-miR-181b	BHLHE41	basic helix-loop-helix family member e41 [Source:VGNC Symbol;Acc:VGNC:85815]	2.55	0.000004
ssc-miR-181b	BIRC6	baculoviral IAP repeat containing 6 [Source:VGNC Symbol;Acc:VGNC:97038]	2.55	0.000004
ssc-miR-181b	BLOC1S6	biosis of lysosomal organelles complex 1 subunit 6 [Source:VGNC Symbol;Acc:VGNC:85832]	2.55	0.000004
ssc-miR-181b	BMF	Bcl2 modifying factor [Source:VGNC Symbol;Acc:VGNC:85837]	2.55	0.000004
ssc-miR-181b	BMP3	bone morphotic protein 3 [Source:VGNC Symbol;Acc:VGNC:85842]	2.55	0.000004
ssc-miR-181b	BMPR2	bone morphotic protein receptor type 2 [Source:VGNC Symbol;Acc:VGNC:95494]	2.55	0.000004

ssc-miR-181b	BNC2	basonuclin 2 [Source:VGNC Symbol;Acc:VGNC:85852]	2.55	0.000004
ssc-miR-181b	BPTF	bromodomain PHD finger transcription factor [Source:VGNC Symbol;Acc:VGNC:97904]	2.55	0.000004
ssc-miR-181b	BRAP	BRCA1 associated protein [Source:VGNC Symbol;Acc:VGNC:85867]	2.55	0.000004
ssc-miR-181b	BRD1	bromodomain containing 1 [Source:VGNC Symbol;Acc:VGNC:85871]	2.55	0.000004
ssc-miR-181b	BRWD1	bromodomain and WD repeat domain containing 1 [Source:VGNC Symbol;Acc:VGNC:108153]	2.55	0.000004
ssc-miR-181b	BTBD3	BTB domain containing 3 [Source:VGNC Symbol;Acc:VGNC:95673]	2.55	0.000004
ssc-miR-181b	BTG1	BTG anti-proliferation factor 1 [Source:VGNC Symbol;Acc:VGNC:103907]	2.55	0.000004
ssc-miR-181b	BTG2	BTG anti-proliferation factor 2 [Source:NCBI gene (formerly Entrezgene);Acc:100048932]	2.55	0.000004
ssc-miR-181b	BTLA	hypothetical gene	2.55	0.000004
ssc-miR-181b	C11orf30	hypothetical gene	2.55	0.000004
ssc-miR-181b	C11orf73	hypothetical gene	2.55	0.000004
ssc-miR-181b	C14orf28	chromosome 1 C14orf28 homolog [Source:VGNC Symbol;Acc:VGNC:85958]	2.55	0.000004
ssc-miR-181b	C15ORF37	hypothetical gene	2.55	0.000004
ssc-miR-181b	C16orf87	chromosome 6 C16orf87 homolog [Source:VGNC Symbol;Acc:VGNC:96927]	2.55	0.000004
ssc-miR-181b	C1GALT1	hypothetical gene	2.55	0.000004
ssc-miR-181b	C1orf50	hypothetical gene	2.55	0.000004
ssc-miR-181b	C1QTNF3	C1q and TNF related 3 [Source:NCBI gene (formerly Entrezgene);Acc:100270814]	2.55	0.000004
ssc-miR-181b	C2CD5	C2 calcium dependent domain containing 5 [Source:VGNC Symbol;Acc:VGNC:85995]	2.55	0.000004
ssc-miR-181b	C2orf69	chromosome 2 open reading frame 69 [Source:HGNC Symbol;Acc:HGNC:26799]	2.55	0.000004
ssc-miR-181b	C4orf29	hypothetical gene	2.55	0.000004
ssc-miR-181b	C6orf62	chromosome 7 C6orf62 homolog [Source:VGNC Symbol;Acc:VGNC:86074]	2.55	0.000004
ssc-miR-181b	C6orf89	chromosome 7 C6orf89 homolog [Source:VGNC Symbol;Acc:VGNC:86075]	2.55	0.000004
ssc-miR-181b	C7orf41	hypothetical gene	2.55	0.000004
ssc-miR-181b	C8orf4	hypothetical gene	2.55	0.000004
ssc-miR-181b	C8orf44-SGK3	hypothetical gene	2.55	0.000004
ssc-miR-181b	CACNA2D1	calcium voltage-gated channel auxiliary subunit alpha2delta 1 [Source:VGNC Symbol;Acc:VGNC:86120]	2.55	0.000004
ssc-miR-181b	CACNA2D2	calcium voltage-gated channel auxiliary subunit alpha2delta 2 [Source:VGNC Symbol;Acc:VGNC:86121]	2.55	0.000004
ssc-miR-181b	CALCR	calcitonin receptor [Source:VGNC Symbol;Acc:VGNC:86142]	2.55	0.000004
ssc-miR-181b	CALM1	calmodulin 1 [Source:NCBI gene (formerly Entrezgene);Acc:100154056]	2.55	0.000004
ssc-miR-181b	CALR	calreticulin [Source:VGNC Symbol;Acc:VGNC:86149]	2.55	0.000004
ssc-miR-181b	CAMK2D	calcium/calmodulin dependent protein kinase II delta [Source:VGNC Symbol;Acc:VGNC:97911]	2.55	0.000004
ssc-miR-181b	CAMK2G	calcium/calmodulin dependent protein kinase II gamma [Source:VGNC Symbol;Acc:VGNC:86157]	2.55	0.000004
ssc-miR-181b	CAMKK1	calcium/calmodulin dependent protein kinase kinase 1 [Source:VGNC Symbol;Acc:VGNC:98977]	2.55	0.000004
ssc-miR-181b	CAMSAP1	calmodulin regulated spectrin associated protein 1 [Source:VGNC Symbol;Acc:VGNC:86162]	2.55	0.000004
ssc-miR-181b	CAMSAP2	calmodulin regulated spectrin associated protein family member 2 [Source:VGNC Symbol;Acc:VGNC:96205]	2.55	0.000004
ssc-miR-181b	CAMTA2	calmodulin binding transcription activator 2 [Source:VGNC Symbol;Acc:VGNC:86165]	2.55	0.000004
ssc-miR-181b	CAND1	cullin associated and neddylation dissociated 1 [Source:VGNC Symbol;Acc:VGNC:97912]	2.55	0.000004
ssc-miR-181b	CAPRIN2	caprin family member 2 [Source:VGNC Symbol;Acc:VGNC:86179]	2.55	0.000004
ssc-miR-181b	CARD10	caspase recruitment domain family member 10 [Source:VGNC Symbol;Acc:VGNC:86185]	2.55	0.000004
ssc-miR-181b	CARD11	caspase recruitment domain family member 11 [Source:VGNC Symbol;Acc:VGNC:86186]	2.55	0.000004
ssc-miR-181b	CARM1	coactivator associated arginine methyltransferase 1 [Source:VGNC Symbol;Acc:VGNC:86191]	2.55	0.000004
ssc-miR-181b	CASP10	caspase 10 [Source:VGNC Symbol;Acc:VGNC:95638]	2.55	0.000004
ssc-miR-181b	CBFA2T2	CBFA2/RUNX1 partner transcriptional co-repressor 2 [Source:VGNC Symbol;Acc:VGNC:96573]	2.55	0.000004
ssc-miR-181b	CBFA2T3	CBFA2/RUNX1 partner transcriptional co-repressor 3 [Source:VGNC Symbol;Acc:VGNC:96574]	2.55	0.000004
ssc-miR-181b	CBLB	Cbl proto-onco B [Source:VGNC Symbol;Acc:VGNC:86223]	2.55	0.000004
ssc-miR-181b	CBX4	chromobox 4 [Source:VGNC Symbol;Acc:VGNC:86231]	2.55	0.000004
ssc-miR-181b	CBX5	chromobox 5 [Source:VGNC Symbol;Acc:VGNC:86232]	2.55	0.000004
ssc-miR-181b	CBX7	chromobox 7 [Source:HGNC Symbol;Acc:HGNC:1557]	2.55	0.000004
ssc-miR-181b	CBX8	chromobox 8 [Source:VGNC Symbol;Acc:VGNC:86233]	2.55	0.000004

ssc-miR-181b	CCAR1	cell division cycle and apoptosis regulator 1 [Source:VGNC Symbol;Acc:VGNC:86238]	2.55	0.000004
ssc-miR-181b	CCDC117	coiled-coil domain containing 117 [Source:VGNC Symbol;Acc:VGNC:86248]	2.55	0.000004
ssc-miR-181b	CCDC14	coiled-coil domain containing 14 [Source:VGNC Symbol;Acc:VGNC:86260]	2.55	0.000004
ssc-miR-181b	CCDC6	coiled-coil domain containing 6 [Source:VGNC Symbol;Acc:VGNC:86304]	2.55	0.000004
ssc-miR-181b	CCDC64	hypothetical gene	2.55	0.000004
ssc-miR-181b	CCDC85A	coiled-coil domain containing 85A [Source:VGNC Symbol;Acc:VGNC:86320]	2.55	0.000004
ssc-miR-181b	CCDC92	coiled-coil domain containing 92 [Source:VGNC Symbol;Acc:VGNC:86330]	2.55	0.000004
ssc-miR-181b	CCL8	chemokine (C-C motif) ligand 8 [Source:NCBI gene (formerly Entrezgene);Acc:100302703]	2.55	0.000004
ssc-miR-181b	CCNB1	cyclin B1 [Source:VGNC Symbol;Acc:VGNC:86349]	2.55	0.000004
ssc-miR-181b	CCNDBP1	cyclin D1 binding protein 1 [Source:VGNC Symbol;Acc:VGNC:86354]	2.55	0.000004
ssc-miR-181b	CCNG1	cyclin G1 [Source:VGNC Symbol;Acc:VGNC:86357]	2.55	0.000004
ssc-miR-181b	CCNJ	cyclin J [Source:VGNC Symbol;Acc:VGNC:86360]	2.55	0.000004
ssc-miR-181b	CCNK	cyclin K [Source:VGNC Symbol;Acc:VGNC:86362]	2.55	0.000004
ssc-miR-181b	CCNT1	cyclin T1 [Source:VGNC Symbol;Acc:VGNC:86366]	2.55	0.000004
ssc-miR-181b	CCP110	centriolar coiled-coil protein 110 [Source:VGNC Symbol;Acc:VGNC:86367]	2.55	0.000004
ssc-miR-181b	CD163	CD163 molecule [Source:NCBI gene (formerly Entrezgene);Acc:397031]	2.55	0.000004
ssc-miR-181b	CD2AP	CD2 associated protein [Source:VGNC Symbol;Acc:VGNC:86406]	2.55	0.000004
ssc-miR-181b	CD4	CD4 molecule [Source:VGNC Symbol;Acc:VGNC:86417]	2.55	0.000004
ssc-miR-181b	CD47	CD47 molecule [Source:VGNC Symbol;Acc:VGNC:108637]	2.55	0.000004
ssc-miR-181b	CD69	CD69 molecule [Source:VGNC Symbol;Acc:VGNC:103223]	2.55	0.000004
ssc-miR-181b	CDC27	cell division cycle 27 [Source:VGNC Symbol;Acc:VGNC:86448]	2.55	0.000004
ssc-miR-181b	CDC42BPA	CDC42 binding protein kinase alpha [Source:VGNC Symbol;Acc:VGNC:95847]	2.55	0.000004
ssc-miR-181b	CDC5L	cell division cycle 5 like [Source:VGNC Symbol;Acc:VGNC:86463]	2.55	0.000004
ssc-miR-181b	CDC73	cell division cycle 73 [Source:VGNC Symbol;Acc:VGNC:95817]	2.55	0.000004
ssc-miR-181b	CDH8	cadherin 8 [Source:VGNC Symbol;Acc:VGNC:86487]	2.55	0.000004
ssc-miR-181b	CDK17	cyclin dependent kinase 17 [Source:VGNC Symbol;Acc:VGNC:86500]	2.55	0.000004
ssc-miR-181b	CDK8	cyclin dependent kinase 8 [Source:VGNC Symbol;Acc:VGNC:86507]	2.55	0.000004
ssc-miR-181b	CDKL2	cyclin dependent kinase like 2 [Source:VGNC Symbol;Acc:VGNC:86511]	2.55	0.000004
ssc-miR-181b	CDON	cell adhesion associated, onco regulated [Source:VGNC Symbol;Acc:VGNC:86519]	2.55	0.000004
ssc-miR-181b	CDS1	CDP-diacylglycerol synthase 1 [Source:VGNC Symbol;Acc:VGNC:86523]	2.55	0.000004
ssc-miR-181b	CDS2	CDP-diacylglycerol synthase 2 [Source:VGNC Symbol;Acc:VGNC:95869]	2.55	0.000004
ssc-miR-181b	CDX2	caudal type homeobox 2 [Source:VGNC Symbol;Acc:VGNC:86527]	2.55	0.000004
ssc-miR-181b	CDYL	chromodomain Y like [Source:HGNC Symbol;Acc:HGNC:1811]	2.55	0.000004
ssc-miR-181b	CECR2	CECR2 histone acetyl-lysine reader [Source:VGNC Symbol;Acc:VGNC:96576]	2.55	0.000004
ssc-miR-181b	CELSR3	cadherin EGF LAG seven-pass G-type receptor 3 [Source:VGNC Symbol;Acc:VGNC:86541]	2.55	0.000004
ssc-miR-181b	CENPO	centromere protein O [Source:HGNC Symbol;Acc:HGNC:28152]	2.55	0.000004
ssc-miR-181b	CEP135	centrosomal protein 135 [Source:VGNC Symbol;Acc:VGNC:86561]	2.55	0.000004
ssc-miR-181b	CEP350	centrosomal protein 350 [Source:HGNC Symbol;Acc:HGNC:24238]	2.55	0.000004
ssc-miR-181b	CEP85L	centrosomal protein 85 like [Source:VGNC Symbol;Acc:VGNC:86580]	2.55	0.000004
ssc-miR-181b	CEP97	centrosomal protein 97 [Source:VGNC Symbol;Acc:VGNC:108638]	2.55	0.000004
ssc-miR-181b	CGRRF1	cell growth regulator with ring finger domain 1 [Source:VGNC Symbol;Acc:VGNC:86617]	2.55	0.000004
ssc-miR-181b	CHCHD4	coiled-coil-helix-coiled-coil-helix domain containing 4 [Source:NCBI gene (formerly Entrezgene);Acc:100154663]	2.55	0.000004
ssc-miR-181b	CHD1	chromodomain helicase DNA binding protein 1 [Source:VGNC Symbol;Acc:VGNC:86629]	2.55	0.000004
ssc-miR-181b	CHD7	chromodomain helicase DNA binding protein 7 [Source:VGNC Symbol;Acc:VGNC:86633]	2.55	0.000004
ssc-miR-181b	CHD9	chromodomain helicase DNA binding protein 9 [Source:VGNC Symbol;Acc:VGNC:86635]	2.55	0.000004
ssc-miR-181b	CHIC1	cysteine rich hydrophobic domain 1 [Source:VGNC Symbol;Acc:VGNC:86642]	2.55	0.000004
ssc-miR-181b	CHL1	cell adhesion molecule L1 like [Source:VGNC Symbol;Acc:VGNC:108639]	2.55	0.000004
ssc-miR-181b	CHMP1B	hypothetical gene	2.55	0.000004
ssc-miR-181b	CHMP3	charged multivesicular body protein 3 [Source:VGNC Symbol;Acc:VGNC:106570]	2.55	0.000004

ssc-miR-181b	CHP1	calcineurin like EF-hand protein 1 [Source:VGNC Symbol;Acc:VGNC:86657]	2.55	0.000004
ssc-miR-181b	CHRM3	cholinergic receptor muscarinic 3 [Source:VGNC Symbol;Acc:VGNC:86664]	2.55	0.000004
ssc-miR-181b	CHST1	carbohydrate sulfotransferase 1 [Source:VGNC Symbol;Acc:VGNC:86673]	2.55	0.000004
ssc-miR-181b	CHURC1	churchill domain containing 1 [Source:VGNC Symbol;Acc:VGNC:86689]	2.55	0.000004
ssc-miR-181b	CISD1	hypothetical gene	2.55	0.000004
ssc-miR-181b	CKAP4	cytoskeleton associated protein 4 [Source:VGNC Symbol;Acc:VGNC:86716]	2.55	0.000004
ssc-miR-181b	CLASP1	cytoplasmic linker associated protein 1 [Source:VGNC Symbol;Acc:VGNC:95829]	2.55	0.000004
ssc-miR-181b	CLCC1	chloride channel CLIC like 1 [Source:VGNC Symbol;Acc:VGNC:86724]	2.55	0.000004
ssc-miR-181b	CLIP1	CAP-Gly domain containing linker protein 1 [Source:VGNC Symbol;Acc:VGNC:86766]	2.55	0.000004
ssc-miR-181b	CLMN	calmin [Source:VGNC Symbol;Acc:VGNC:86769]	2.55	0.000004
ssc-miR-181b	CLOCK	clock circadian regulator [Source:VGNC Symbol;Acc:VGNC:86774]	2.55	0.000004
ssc-miR-181b	CLUH	clustered mitochondria homolog [Source:VGNC Symbol;Acc:VGNC:86792]	2.55	0.000004
ssc-miR-181b	CLVS1	clavesin 1 [Source:VGNC Symbol;Acc:VGNC:86794]	2.55	0.000004
ssc-miR-181b	CMPK2	cytidine/uridine monophosphate kinase 2 [Source:VGNC Symbol;Acc:VGNC:86801]	2.55	0.000004
ssc-miR-181b	CNKSR2	connector enhancer of kinase suppressor of Ras 2 [Source:VGNC Symbol;Acc:VGNC:86822]	2.55	0.000004
ssc-miR-181b	CNKSR3	CNKSR family member 3 [Source:VGNC Symbol;Acc:VGNC:86823]	2.55	0.000004
ssc-miR-181b	CNNM2	cyclin and CBS domain divalent metal cation transport mediator 2 [Source:VGNC Symbol;Acc:VGNC:86828]	2.55	0.000004
ssc-miR-181b	CNOT1	CCR4-NOT transcription complex subunit 1 [Source:VGNC Symbol;Acc:VGNC:86831]	2.55	0.000004
ssc-miR-181b	CNOT2	CCR4-NOT transcription complex subunit 2 [Source:VGNC Symbol;Acc:VGNC:86834]	2.55	0.000004
ssc-miR-181b	CNOT6L	CCR4-NOT transcription complex subunit 6 like [Source:VGNC Symbol;Acc:VGNC:86838]	2.55	0.000004
ssc-miR-181b	CNR1	cannabinoid receptor 1 [Source:VGNC Symbol;Acc:VGNC:86843]	2.55	0.000004
ssc-miR-181b	CNTN4	contactin 4 [Source:VGNC Symbol;Acc:VGNC:97934]	2.55	0.000004
ssc-miR-181b	CNTNAP2	hypothetical gene	2.55	0.000004
ssc-miR-181b	COL16A1	collagen type XVI alpha 1 chain [Source:VGNC Symbol;Acc:VGNC:86867]	2.55	0.000004
ssc-miR-181b	COL5A1	collagen type V alpha 1 chain [Source:VGNC Symbol;Acc:VGNC:86877]	2.55	0.000004
ssc-miR-181b	COLGALT1	collagen beta(1-O)galactosyltransferase 1 [Source:HGNC Symbol;Acc:HGNC:26182]	2.55	0.000004
ssc-miR-181b	COLQ	collagen like tail subunit of asymmetric acetylcholinesterase [Source:VGNC Symbol;Acc:VGNC:86884]	2.55	0.000004
ssc-miR-181b	COMMD6	hypothetical gene	2.55	0.000004
ssc-miR-181b	COPS2	COP9 signalosome subunit 2 [Source:VGNC Symbol;Acc:VGNC:86899]	2.55	0.000004
ssc-miR-181b	CORO1C	coronin 1C [Source:VGNC Symbol;Acc:VGNC:86914]	2.55	0.000004
ssc-miR-181b	CPD	carboxypeptidase D [Source:VGNC Symbol;Acc:VGNC:86935]	2.55	0.000004
ssc-miR-181b	CPEB4	cytoplasmic polyadenylation element binding protein 4 [Source:VGNC Symbol;Acc:VGNC:86939]	2.55	0.000004
ssc-miR-181b	CPNE2	copine 2 [Source:VGNC Symbol;Acc:VGNC:86950]	2.55	0.000004
ssc-miR-181b	CPOX	coproporphyrinogen oxidase [Source:VGNC Symbol;Acc:VGNC:86956]	2.55	0.000004
ssc-miR-181b	CPSF6	cleavage and polyadenylation specific factor 6 [Source:VGNC Symbol;Acc:VGNC:86962]	2.55	0.000004
ssc-miR-181b	CPT1A	carnitine palmitoyltransferase 1A [Source:VGNC Symbol;Acc:VGNC:86964]	2.55	0.000004
ssc-miR-181b	CREB1	cAMP responsive element binding protein 1 [Source:VGNC Symbol;Acc:VGNC:96004]	2.55	0.000004
ssc-miR-181b	CREBL2	cAMP responsive element binding protein like 2 [Source:VGNC Symbol;Acc:VGNC:86986]	2.55	0.000004
ssc-miR-181b	CREBRF	CREB3 regulatory factor [Source:VGNC Symbol;Acc:VGNC:86987]	2.55	0.000004
ssc-miR-181b	CREBZF	CREB/ATF bZIP transcription factor [Source:VGNC Symbol;Acc:VGNC:86988]	2.55	0.000004
ssc-miR-181b	CRIM1	cysteine rich transmembrane BMP regulator 1 [Source:VGNC Symbol;Acc:VGNC:86995]	2.55	0.000004
ssc-miR-181b	CRISPLD1	cysteine rich secretory protein LCCL domain containing 1 [Source:VGNC Symbol;Acc:VGNC:97945]	2.55	0.000004
ssc-miR-181b	CRYBG3	crystallin beta-gamma domain containing 3 [Source:VGNC Symbol;Acc:VGNC:87020]	2.55	0.000004
ssc-miR-181b	CSNK1A1	casein kinase 1 alpha 1 [Source:VGNC Symbol;Acc:VGNC:99630]	2.55	0.000004
ssc-miR-181b	CSNK1G1	casein kinase 1 gamma 1 [Source:VGNC Symbol;Acc:VGNC:97949]	2.55	0.000004
ssc-miR-181b	CSNK1G3	casein kinase 1 gamma 3 [Source:VGNC Symbol;Acc:VGNC:97951]	2.55	0.000004
ssc-miR-181b	CSRNP3	cysteine and serine rich nuclear protein 3 [Source:VGNC Symbol;Acc:VGNC:96249]	2.55	0.000004
ssc-miR-181b	CSTF3	cleavage stimulation factor subunit 3 [Source:VGNC Symbol;Acc:VGNC:87052]	2.55	0.000004
ssc-miR-181b	CTBP1	C-terminal binding protein 1 [Source:VGNC Symbol;Acc:VGNC:87053]	2.55	0.000004

ssc-miR-181b	CTC-432M15.3	hypothetical gene	2.55	0.000004
ssc-miR-181b	CTD-2140B24.4	hypothetical gene	2.55	0.000004
ssc-miR-181b	CTD-2228K2.5	hypothetical gene	2.55	0.000004
ssc-miR-181b	CTDSPL	CTD small phosphatase like [Source:VGNC Symbol;Acc:VGNC:107131]	2.55	0.000004
ssc-miR-181b	CTH	cystathionine gamma-lyase [Source:VGNC Symbol;Acc:VGNC:96961]	2.55	0.000004
ssc-miR-181b	CTIF	cap binding complex dependent translation initiation factor [Source:VGNC Symbol;Acc:VGNC:87062]	2.55	0.000004
ssc-miR-181b	CTNND1	catenin delta 1 [Source:VGNC Symbol;Acc:VGNC:87067]	2.55	0.000004
ssc-miR-181b	CTTNBP2NL	CTTNBP2 N-terminal like [Source:VGNC Symbol;Acc:VGNC:87082]	2.55	0.000004
ssc-miR-181b	CUL3	cullin 3 [Source:VGNC Symbol;Acc:VGNC:96091]	2.55	0.000004
ssc-miR-181b	CUL4A	cullin 4A [Source:VGNC Symbol;Acc:VGNC:87088]	2.55	0.000004
ssc-miR-181b	CUL5	cullin 5 [Source:VGNC Symbol;Acc:VGNC:87089]	2.55	0.000004
ssc-miR-181b	CXADR	CXADR Ig-like cell adhesion molecule [Source:VGNC Symbol;Acc:VGNC:87100]	2.55	0.000004
ssc-miR-181b	CXXC5	CXXC finger protein 5 [Source:VGNC Symbol;Acc:VGNC:87118]	2.55	0.000004
ssc-miR-181b	CYB5B	cytochrome b5 type B [Source:VGNC Symbol;Acc:VGNC:96728]	2.55	0.000004
ssc-miR-181b	CYLD	CYLD lysine 63 deubiquitinase [Source:VGNC Symbol;Acc:VGNC:87130]	2.55	0.000004
ssc-miR-181b	CYP26B1	cytochrome P450 family 26 subfamily B member 1 [Source:VGNC Symbol;Acc:VGNC:103374]	2.55	0.000004
ssc-miR-181b	CYP4X1	hypothetical gene	2.55	0.000004
ssc-miR-181b	CYR61	hypothetical gene	2.55	0.000004
ssc-miR-181b	DAZAP2	DAZ associated protein 2 [Source:VGNC Symbol;Acc:VGNC:87162]	2.55	0.000004
ssc-miR-181b	DCAF12L1	hypothetical gene	2.55	0.000004
ssc-miR-181b	DCAF16	DDB1 and CUL4 associated factor 16 [Source:HGNC Symbol;Acc:HGNC:25987]	2.55	0.000004
ssc-miR-181b	DCBLD2	discoidin, CUB and LCCL domain containing 2 [Source:VGNC Symbol;Acc:VGNC:87180]	2.55	0.000004
ssc-miR-181b	DCLK1	doublecortin like kinase 1 [Source:HGNC Symbol;Acc:HGNC:2700]	2.55	0.000004
ssc-miR-181b	DCLK3	doublecortin like kinase 3 [Source:VGNC Symbol;Acc:VGNC:87184]	2.55	0.000004
ssc-miR-181b	DCUN1D1	hypothetical gene	2.55	0.000004
ssc-miR-181b	DDIT4	DNA damage inducible transcript 4 [Source:VGNC Symbol;Acc:VGNC:87208]	2.55	0.000004
ssc-miR-181b	DDR2	discoidin domain receptor tyrosine kinase 2 [Source:VGNC Symbol;Acc:VGNC:87212]	2.55	0.000004
ssc-miR-181b	DDX3X	DEAD-box helicase 3 X-linked [Source:NCBI gene (formerly Entrezgene);Acc:100515940]	2.55	0.000004
ssc-miR-181b	DDX3Y	hypothetical gene	2.55	0.000004
ssc-miR-181b	DDX5	DEAD-box helicase 5 [Source:VGNC Symbol;Acc:VGNC:87226]	2.55	0.000004
ssc-miR-181b	DDX52	DExD-box helicase 52 [Source:VGNC Symbol;Acc:VGNC:87229]	2.55	0.000004
ssc-miR-181b	DEK	hypothetical gene	2.55	0.000004
ssc-miR-181b	DENND5B	DENN domain containing 5B [Source:VGNC Symbol;Acc:VGNC:87255]	2.55	0.000004
ssc-miR-181b	DEPTOR	DEP domain containing MTOR interacting protein [Source:HGNC Symbol;Acc:HGNC:22953]	2.55	0.000004
ssc-miR-181b	DERL1	derlin 1 [Source:NCBI gene (formerly Entrezgene);Acc:100626802]	2.55	0.000004
ssc-miR-181b	DERL2	derlin 2 [Source:VGNC Symbol;Acc:VGNC:98986]	2.55	0.000004
ssc-miR-181b	DESI2	desumoylating isopeptidase 2 [Source:VGNC Symbol;Acc:VGNC:96037]	2.55	0.000004
ssc-miR-181b	DGCR2	DiGeorge syndrome critical region gene 2 [Source:HGNC Symbol;Acc:HGNC:2845]	2.55	0.000004
ssc-miR-181b	DHX57	DExH-box helicase 57 [Source:VGNC Symbol;Acc:VGNC:87296]	2.55	0.000004
ssc-miR-181b	DIP2A	disco interacting A [Source:VGNC Symbol;Acc:VGNC:95836]	2.55	0.000004
ssc-miR-181b	DIP2B	disco interacting B [Source:VGNC Symbol;Acc:VGNC:87306]	2.55	0.000004
ssc-miR-181b	DIP2C	disco interacting C [Source:VGNC Symbol;Acc:VGNC:96201]	2.55	0.000004
ssc-miR-181b	DIXDC1	hypothetical gene	2.55	0.000004
ssc-miR-181b	DLG2	discs large MAGUK scaffold protein 2 [Source:VGNC Symbol;Acc:VGNC:108581]	2.55	0.000004
ssc-miR-181b	DLGAP2	DLG associated protein 2 [Source:VGNC Symbol;Acc:VGNC:99711]	2.55	0.000004
ssc-miR-181b	DMTN	dematin actin binding protein [Source:VGNC Symbol;Acc:VGNC:87358]	2.55	0.000004
ssc-miR-181b	DMXL2	Dmx like 2 [Source:VGNC Symbol;Acc:VGNC:87360]	2.55	0.000004
ssc-miR-181b	DNAJA4	DnaJ heat shock protein family (Hsp40) member A4 [Source:NCBI gene (formerly Entrezgene);Acc:397613]	2.55	0.000004
ssc-miR-181b	DNAJB1	DnaJ heat shock protein family (Hsp40) member B1 [Source:VGNC Symbol;Acc:VGNC:96614]	2.55	0.000004

ssc-miR-181b	DNAJB14	DnaJ heat shock protein family (Hsp40) member B14 [Source:VGNC Symbol;Acc:VGNC:98919]	2.55	0.000004
ssc-miR-181b	DNAJB5	DnaJ heat shock protein family (Hsp40) member B5 [Source:VGNC Symbol;Acc:VGNC:103081]	2.55	0.000004
ssc-miR-181b	DNAJC13	DnaJ heat shock protein family (Hsp40) member C13 [Source:VGNC Symbol;Acc:VGNC:108650]	2.55	0.000004
ssc-miR-181b	DNAJC21	hypothetical gene	2.55	0.000004
ssc-miR-181b	DNAJC3	DnaJ heat shock protein family (Hsp40) member C3 [Source:NCBI gene (formerly Entrezgene);Acc:100154166]	2.55	0.000004
ssc-miR-181b	DNAJC5	DnaJ heat shock protein family (Hsp40) member C5 [Source:VGNC Symbol;Acc:VGNC:108714]	2.55	0.000004
ssc-miR-181b	DNAL1	dynein axonemal light chain 1 [Source:VGNC Symbol;Acc:VGNC:87373]	2.55	0.000004
ssc-miR-181b	DOCK10	dedicator of cytokinesis 10 [Source:VGNC Symbol;Acc:VGNC:96007]	2.55	0.000004
ssc-miR-181b	DOCK4	dedicator of cytokinesis 4 [Source:VGNC Symbol;Acc:VGNC:87394]	2.55	0.000004
ssc-miR-181b	DOCK7	dedicator of cytokinesis 7 [Source:VGNC Symbol;Acc:VGNC:87397]	2.55	0.000004
ssc-miR-181b	DPP6	dipeptidyl peptidase like 6 [Source:VGNC Symbol;Acc:VGNC:87421]	2.55	0.000004
ssc-miR-181b	DPYSL2	dihydropyrimidinase like 2 [Source:VGNC Symbol;Acc:VGNC:87430]	2.55	0.000004
ssc-miR-181b	DR1	down-regulator of transcription 1 [Source:VGNC Symbol;Acc:VGNC:87435]	2.55	0.000004
ssc-miR-181b	DRAM1	DNA damage regulated autophagy modulator 1 [Source:VGNC Symbol;Acc:VGNC:87436]	2.55	0.000004
ssc-miR-181b	DRD1	dopamine receptor D1 [Source:VGNC Symbol;Acc:VGNC:87443]	2.55	0.000004
ssc-miR-181b	DSC1	desmocollin 1 [Source:VGNC Symbol;Acc:VGNC:87452]	2.55	0.000004
ssc-miR-181b	DTNA	dystrobrevin alpha [Source:VGNC Symbol;Acc:VGNC:87462]	2.55	0.000004
ssc-miR-181b	DUSP10	dual specificity phosphatase 10 [Source:VGNC Symbol;Acc:VGNC:96246]	2.55	0.000004
ssc-miR-181b	DUSP5	dual specificity phosphatase 5 [Source:VGNC Symbol;Acc:VGNC:87487]	2.55	0.000004
ssc-miR-181b	DUSP6	dual specificity phosphatase 6 [Source:VGNC Symbol;Acc:VGNC:87488]	2.55	0.000004
ssc-miR-181b	DYNC1LI2	dynein cytoplasmic 1 light intermediate chain 2 [Source:VGNC Symbol;Acc:VGNC:87498]	2.55	0.000004
ssc-miR-181b	DYNLL2	dynein light chain LC8-type 2 [Source:VGNC Symbol;Acc:VGNC:87501]	2.55	0.000004
ssc-miR-181b	DYRK2	dual specificity tyrosine phosphorylation regulated kinase 2 [Source:VGNC Symbol;Acc:VGNC:87507]	2.55	0.000004
ssc-miR-181b	E2F5	E2F transcription factor 5 [Source:VGNC Symbol;Acc:VGNC:87516]	2.55	0.000004
ssc-miR-181b	E2F7	E2F transcription factor 7 [Source:VGNC Symbol;Acc:VGNC:87518]	2.55	0.000004
ssc-miR-181b	ECT2L	epithelial cell transforming 2 like [Source:VGNC Symbol;Acc:VGNC:87539]	2.55	0.000004
ssc-miR-181b	EDA2R	hypothetical gene	2.55	0.000004
ssc-miR-181b	EDAR	ectodysplasin A receptor [Source:VGNC Symbol;Acc:VGNC:87541]	2.55	0.000004
ssc-miR-181b	EED	embryonic ectoderm development [Source:VGNC Symbol;Acc:VGNC:87552]	2.55	0.000004
ssc-miR-181b	EGR1	early growth response 1 [Source:VGNC Symbol;Acc:VGNC:87590]	2.55	0.000004
ssc-miR-181b	EGR3	early growth response 3 [Source:VGNC Symbol;Acc:VGNC:87591]	2.55	0.000004
ssc-miR-181b	EHF	ETS homologous factor [Source:VGNC Symbol;Acc:VGNC:87599]	2.55	0.000004
ssc-miR-181b	EIF2S2	eukaryotic translation initiation factor 2 subunit beta [Source:VGNC Symbol;Acc:VGNC:96265]	2.55	0.000004
ssc-miR-181b	EIF4A2	eukaryotic translation initiation factor 4A2 [Source:VGNC Symbol;Acc:VGNC:87624]	2.55	0.000004
ssc-miR-181b	EIF4E3	eukaryotic translation initiation factor 4E family member 3 [Source:VGNC Symbol;Acc:VGNC:87628]	2.55	0.000004
ssc-miR-181b	ELAVL2	ELAV like RNA binding protein 2 [Source:VGNC Symbol;Acc:VGNC:87639]	2.55	0.000004
ssc-miR-181b	ELAVL4	ELAV like RNA binding protein 4 [Source:VGNC Symbol;Acc:VGNC:97047]	2.55	0.000004
ssc-miR-181b	ELMSAN1	hypothetical gene	2.55	0.000004
ssc-miR-181b	ELN	elastin [Source:VGNC Symbol;Acc:VGNC:87655]	2.55	0.000004
ssc-miR-181b	ELP4	elongator acetyltransferase complex subunit 4 [Source:VGNC Symbol;Acc:VGNC:87665]	2.55	0.000004
ssc-miR-181b	EMC1	ER membrane protein complex subunit 1 [Source:VGNC Symbol;Acc:VGNC:97050]	2.55	0.000004
ssc-miR-181b	EMC3	ER membrane protein complex subunit 3 [Source:VGNC Symbol;Acc:VGNC:87671]	2.55	0.000004
ssc-miR-181b	EML1	EMAP like 1 [Source:VGNC Symbol;Acc:VGNC:87682]	2.55	0.000004
ssc-miR-181b	EMX2	empty spiracles homeobox 2 [Source:VGNC Symbol;Acc:VGNC:87693]	2.55	0.000004
ssc-miR-181b	EN2	engrailed homeobox 2 [Source:VGNC Symbol;Acc:VGNC:87694]	2.55	0.000004
ssc-miR-181b	ENAH	ENAH actin regulator [Source:VGNC Symbol;Acc:VGNC:108271]	2.55	0.000004
ssc-miR-181b	ENOX2	ecto-NOX disulfide-thiol exchanger 2 [Source:VGNC Symbol;Acc:VGNC:87707]	2.55	0.000004
ssc-miR-181b	ENPP1	ectonucleotide pyrophosphatase/phosphodiesterase 1 [Source:VGNC Symbol;Acc:VGNC:87708]	2.55	0.000004
ssc-miR-181b	ENTPD6	ectonucleoside triphosphate diphosphohydrolase 6 [Source:VGNC Symbol;Acc:VGNC:96731]	2.55	0.000004

ssc-miR-181b	EPB41	erythrocyte membrane protein band 4.1 [Source:VGNC Symbol;Acc:VGNC:87722]	2.55	0.000004
ssc-miR-181b	EPB41L3	erythrocyte membrane protein band 4.1 like 3 [Source:VGNC Symbol;Acc:VGNC:87724]	2.55	0.000004
ssc-miR-181b	EPC1	enhancer of polycomb homolog 1 [Source:VGNC Symbol;Acc:VGNC:95923]	2.55	0.000004
ssc-miR-181b	EPC2	enhancer of polycomb homolog 2 [Source:VGNC Symbol;Acc:VGNC:96045]	2.55	0.000004
ssc-miR-181b	EPHA4	EPH receptor A4 [Source:VGNC Symbol;Acc:VGNC:96280]	2.55	0.000004
ssc-miR-181b	EPHA7	EPH receptor A7 [Source:VGNC Symbol;Acc:VGNC:87734]	2.55	0.000004
ssc-miR-181b	EPM2AIP1	EPM2A interacting protein 1 [Source:VGNC Symbol;Acc:VGNC:87741]	2.55	0.000004
ssc-miR-181b	EPN2	epsin 2 [Source:VGNC Symbol;Acc:VGNC:97982]	2.55	0.000004
ssc-miR-181b	EPT1	hypothetical gene	2.55	0.000004
ssc-miR-181b	ERF	ETS2 repressor factor [Source:VGNC Symbol;Acc:VGNC:87767]	2.55	0.000004
ssc-miR-181b	ERG	ETS transcription factor ERG [Source:VGNC Symbol;Acc:VGNC:87768]	2.55	0.000004
ssc-miR-181b	ERGIC2	ERGIC and golgi 2 [Source:VGNC Symbol;Acc:VGNC:87771]	2.55	0.000004
ssc-miR-181b	ERH	ERH mRNA splicing and mitosis factor [Source:VGNC Symbol;Acc:VGNC:87772]	2.55	0.000004
ssc-miR-181b	ERI1	exoribonuclease 1 [Source:VGNC Symbol;Acc:VGNC:97985]	2.55	0.000004
ssc-miR-181b	ERLIN2	ER lipid raft associated 2 [Source:VGNC Symbol;Acc:VGNC:95586]	2.55	0.000004
ssc-miR-181b	ERMN	ermin [Source:VGNC Symbol;Acc:VGNC:96207]	2.55	0.000004
ssc-miR-181b	ERO1L	hypothetical gene	2.55	0.000004
ssc-miR-181b	ESD	esterase D [Source:VGNC Symbol;Acc:VGNC:87788]	2.55	0.000004
ssc-miR-181b	ESM1	endothelial cell specific molecule 1 [Source:VGNC Symbol;Acc:VGNC:87789]	2.55	0.000004
ssc-miR-181b	ESR1	estrogen receptor 1 [Source:VGNC Symbol;Acc:VGNC:103089]	2.55	0.000004
ssc-miR-181b	ETNK1	ethanolamine kinase 1 [Source:VGNC Symbol;Acc:VGNC:87806]	2.55	0.000004
ssc-miR-181b	ETS1	ETS proto-onco 1, transcription factor [Source:VGNC Symbol;Acc:VGNC:87808]	2.55	0.000004
ssc-miR-181b	ETV6	ETS variant transcription factor 6 [Source:VGNC Symbol;Acc:VGNC:87815]	2.55	0.000004
ssc-miR-181b	EVI2A	ecotropic viral integration site 2A [Source:HGNC Symbol;Acc:HGNC:3499]	2.55	0.000004
ssc-miR-181b	EVI5	ecotropic viral integration site 5 [Source:VGNC Symbol;Acc:VGNC:98793]	2.55	0.000004
ssc-miR-181b	EVX1	even-skipped homeobox 1 [Source:VGNC Symbol;Acc:VGNC:87821]	2.55	0.000004
ssc-miR-181b	EVX2	even-skipped homeobox 2 [Source:VGNC Symbol;Acc:VGNC:96290]	2.55	0.000004
ssc-miR-181b	EXO1	exonuclease 1 [Source:VGNC Symbol;Acc:VGNC:96291]	2.55	0.000004
ssc-miR-181b	EXOSC2	exosome component 2 [Source:VGNC Symbol;Acc:VGNC:87838]	2.55	0.000004
ssc-miR-181b	EXOSC3	exosome component 3 [Source:VGNC Symbol;Acc:VGNC:87839]	2.55	0.000004
ssc-miR-181b	EXT1	exostosin glycosyltransferase 1 [Source:VGNC Symbol;Acc:VGNC:87846]	2.55	0.000004
ssc-miR-181b	EYA3	EYA transcriptional coactivator and phosphatase 3 [Source:VGNC Symbol;Acc:VGNC:87852]	2.55	0.000004
ssc-miR-181b	F2R	coagulation factor II thrombin receptor [Source:VGNC Symbol;Acc:VGNC:87860]	2.55	0.000004
ssc-miR-181b	F3	coagulation factor III, tissue factor [Source:VGNC Symbol;Acc:VGNC:87864]	2.55	0.000004
ssc-miR-181b	FAF1	Fas associated factor 1 [Source:VGNC Symbol;Acc:VGNC:87879]	2.55	0.000004
ssc-miR-181b	FAM102A	family with sequence similarity 102 member A [Source:HGNC Symbol;Acc:HGNC:31419]	2.55	0.000004
ssc-miR-181b	FAM105B	hypothetical gene	2.55	0.000004
ssc-miR-181b	FAM118A	family with sequence similarity 118 member A [Source:VGNC Symbol;Acc:VGNC:87893]	2.55	0.000004
ssc-miR-181b	FAM126B	family with sequence similarity 126 member B [Source:HGNC Symbol;Acc:HGNC:28593]	2.55	0.000004
ssc-miR-181b	FAM135A	family with sequence similarity 135 member A [Source:VGNC Symbol;Acc:VGNC:87904]	2.55	0.000004
ssc-miR-181b	FAM13B	family with sequence similarity 13 member B [Source:VGNC Symbol;Acc:VGNC:87906]	2.55	0.000004
ssc-miR-181b	FAM160A1	hypothetical gene	2.55	0.000004
ssc-miR-181b	FAM160A2	hypothetical gene	2.55	0.000004
ssc-miR-181b	FAM163A	family with sequence similarity 163 member A [Source:HGNC Symbol;Acc:HGNC:28274]	2.55	0.000004
ssc-miR-181b	FAM171A1	family with sequence similarity 171 member A1 [Source:VGNC Symbol;Acc:VGNC:96011]	2.55	0.000004
ssc-miR-181b	FAM178A	hypothetical gene	2.55	0.000004
ssc-miR-181b	FAM179B	hypothetical gene	2.55	0.000004
ssc-miR-181b	FAM19A2	hypothetical gene	2.55	0.000004
ssc-miR-181b	FAM216B	family with sequence similarity 216 member B [Source:VGNC Symbol;Acc:VGNC:87955]	2.55	0.000004

ssc-miR-181b	FAM222B	family with sequence similarity 222 member B [Source:VGNC Symbol;Acc:VGNC:87960]	2.55	0.000004
ssc-miR-181b	FAM3C	FAM3 metabolism regulating signaling molecule C [Source:VGNC Symbol;Acc:VGNC:87969]	2.55	0.000004
ssc-miR-181b	FAM46C	hypothetical gene	2.55	0.000004
ssc-miR-181b	FAM49A	hypothetical gene	2.55	0.000004
ssc-miR-181b	FAM58A	hypothetical gene	2.55	0.000004
ssc-miR-181b	FAM73B	hypothetical gene	2.55	0.000004
ssc-miR-181b	FAM89A	family with sequence similarity 89 member A [Source:VGNC Symbol;Acc:VGNC:87994]	2.55	0.000004
ssc-miR-181b	FAXC	hypothetical gene	2.55	0.000004
ssc-miR-181b	FBXL17	F-box and leucine rich repeat protein 17 [Source:VGNC Symbol;Acc:VGNC:99650]	2.55	0.000004
ssc-miR-181b	FBXL3	F-box and leucine rich repeat protein 3 [Source:VGNC Symbol;Acc:VGNC:88030]	2.55	0.000004
ssc-miR-181b	FBXO10	F-box protein 10 [Source:VGNC Symbol;Acc:VGNC:103092]	2.55	0.000004
ssc-miR-181b	FBXO11	F-box protein 11 [Source:VGNC Symbol;Acc:VGNC:88032]	2.55	0.000004
ssc-miR-181b	FBXO33	F-box protein 33 [Source:VGNC Symbol;Acc:VGNC:88040]	2.55	0.000004
ssc-miR-181b	FBXO34	F-box protein 34 [Source:VGNC Symbol;Acc:VGNC:88041]	2.55	0.000004
ssc-miR-181b	FBXO41	F-box protein 41 [Source:VGNC Symbol;Acc:VGNC:88046]	2.55	0.000004
ssc-miR-181b	FBXO45	F-box protein 45 [Source:VGNC Symbol;Acc:VGNC:88048]	2.55	0.000004
ssc-miR-181b	FBXO48	F-box protein 48 [Source:VGNC Symbol;Acc:VGNC:88051]	2.55	0.000004
ssc-miR-181b	FGD4	FYVE, RhoGEF and PH domain containing 4 [Source:VGNC Symbol;Acc:VGNC:88097]	2.55	0.000004
ssc-miR-181b	FGFR3	fibroblast growth factor receptor 3 [Source:VGNC Symbol;Acc:VGNC:98929]	2.55	0.000004
ssc-miR-181b	FHAD1	forkhead associated phosphopeptide binding domain 1 [Source:VGNC Symbol;Acc:VGNC:88125]	2.55	0.000004
ssc-miR-181b	FHDC1	FH2 domain containing 1 [Source:VGNC Symbol;Acc:VGNC:88126]	2.55	0.000004
ssc-miR-181b	FIGN	fidgetin, microtubule severing factor [Source:VGNC Symbol;Acc:VGNC:95580]	2.55	0.000004
ssc-miR-181b	FKBP1A	hypothetical gene	2.55	0.000004
ssc-miR-181b	FLJ00104	hypothetical gene	2.55	0.000004
ssc-miR-181b	FLT1	fms related receptor tyrosine kinase 1 [Source:VGNC Symbol;Acc:VGNC:88162]	2.55	0.000004
ssc-miR-181b	FMNL2	formin like 2 [Source:VGNC Symbol;Acc:VGNC:95879]	2.55	0.000004
ssc-miR-181b	FMR1	FMRP translational regulator 1 [Source:VGNC Symbol;Acc:VGNC:88175]	2.55	0.000004
ssc-miR-181b	FNBP4	formin binding protein 4 [Source:VGNC Symbol;Acc:VGNC:88180]	2.55	0.000004
ssc-miR-181b	FNDC3A	fibronectin type III domain containing 3A [Source:VGNC Symbol;Acc:VGNC:88182]	2.55	0.000004
ssc-miR-181b	FNDC3B	fibronectin type III domain containing 3B [Source:VGNC Symbol;Acc:VGNC:88183]	2.55	0.000004
ssc-miR-181b	FNIP2	folliculin interacting protein 2 [Source:VGNC Symbol;Acc:VGNC:88188]	2.55	0.000004
ssc-miR-181b	FOS	Fos proto-onco, AP-1 transcription factor subunit [Source:NCBI gene (formerly Entrezgene);Acc:100144486]	2.55	0.000004
ssc-miR-181b	FOXB1	forkhead box B1 [Source:VGNC Symbol;Acc:VGNC:88195]	2.55	0.000004
ssc-miR-181b	FOXK1	forkhead box K1 [Source:VGNC Symbol;Acc:VGNC:88214]	2.55	0.000004
ssc-miR-181b	FOXP1	forkhead box P1 [Source:VGNC Symbol;Acc:VGNC:88222]	2.55	0.000004
ssc-miR-181b	FOXP2	forkhead box P2 [Source:VGNC Symbol;Acc:VGNC:98014]	2.55	0.000004
ssc-miR-181b	FRS2	fibroblast growth factor receptor substrate 2 [Source:VGNC Symbol;Acc:VGNC:88246]	2.55	0.000004
ssc-miR-181b	FRYL	FRY like transcription coactivator [Source:VGNC Symbol;Acc:VGNC:98015]	2.55	0.000004
ssc-miR-181b	FSD1L	fibronectin type III and SPRY domain containing 1 like [Source:HGNC Symbol;Acc:HGNC:13753]	2.55	0.000004
ssc-miR-181b	FSTL1	follistatin like 1 [Source:VGNC Symbol;Acc:VGNC:88255]	2.55	0.000004
ssc-miR-181b	FTO	FTO alpha-ketoglutarate dependent dioxygenase [Source:VGNC Symbol;Acc:VGNC:88259]	2.55	0.000004
ssc-miR-181b	G3BP2	G3BP stress granule assembly factor 2 [Source:VGNC Symbol;Acc:VGNC:88289]	2.55	0.000004
ssc-miR-181b	G6PC3	glucose-6-phosphatase catalytic subunit 3 [Source:VGNC Symbol;Acc:VGNC:88291]	2.55	0.000004
ssc-miR-181b	GABRA1	gamma-aminobutyric acid type A receptor subunit alpha1 [Source:VGNC Symbol;Acc:VGNC:88300]	2.55	0.000004
ssc-miR-181b	GABRA4	gamma-aminobutyric acid type A receptor subunit alpha4 [Source:VGNC Symbol;Acc:VGNC:88303]	2.55	0.000004
ssc-miR-181b	GABRB1	gamma-aminobutyric acid type A receptor subunit beta1 [Source:VGNC Symbol;Acc:VGNC:88306]	2.55	0.000004
ssc-miR-181b	GABRQ	gamma-aminobutyric acid type A receptor subunit theta [Source:VGNC Symbol;Acc:VGNC:88315]	2.55	0.000004
ssc-miR-181b	GAD2	glutamate decarboxylase 2 [Source:VGNC Symbol;Acc:VGNC:108273]	2.55	0.000004
ssc-miR-181b	GALNT16	polypeptide N-acetylgalactosaminyltransferase 16 [Source:VGNC Symbol;Acc:VGNC:88332]	2.55	0.000004

ssc-miR-181b	GALNT4	polypeptide N-acetylgalactosaminyltransferase 4 [Source:HGNC Symbol;Acc:HGNC:4126]	2.55	0.000004
ssc-miR-181b	GAPVD1	GTPase activating protein and VPS9 domains 1 [Source:VGNC Symbol;Acc:VGNC:88347]	2.55	0.000004
ssc-miR-181b	GAS7	growth arrest specific 7 [Source:VGNC Symbol;Acc:VGNC:88359]	2.55	0.000004
ssc-miR-181b	GATA6	GATA binding protein 6 [Source:VGNC Symbol;Acc:VGNC:88366]	2.55	0.000004
ssc-miR-181b	GATC	glutamyl-tRNA amidotransferase subunit C [Source:VGNC Symbol;Acc:VGNC:103948]	2.55	0.000004
ssc-miR-181b	GCC2	GRIP and coiled-coil domain containing 2 [Source:VGNC Symbol;Acc:VGNC:88380]	2.55	0.000004
ssc-miR-181b	GCNT2	glucosaminyl (N-acetyl) transferase 2 (I blood group) [Source:HGNC Symbol;Acc:HGNC:4204]	2.55	0.000004
ssc-miR-181b	GCNT4	glucosaminyl (N-acetyl) transferase 4 [Source:VGNC Symbol;Acc:VGNC:96616]	2.55	0.000004
ssc-miR-181b	GDAP1	ganglioside induced differentiation associated protein 1 [Source:VGNC Symbol;Acc:VGNC:88394]	2.55	0.000004
ssc-miR-181b	GDI1	GDP dissociation inhibitor 1 [Source:VGNC Symbol;Acc:VGNC:88405]	2.55	0.000004
ssc-miR-181b	GDPD1	hypothetical gene	2.55	0.000004
ssc-miR-181b	GEMIN6	gem nuclear organelle associated protein 6 [Source:VGNC Symbol;Acc:VGNC:88414]	2.55	0.000004
ssc-miR-181b	GFPT1	glutamine--fructose-6-phosphate transaminase 1 [Source:VGNC Symbol;Acc:VGNC:88426]	2.55	0.000004
ssc-miR-181b	GHITM	growth hormone inducible transmembrane protein [Source:VGNC Symbol;Acc:VGNC:88442]	2.55	0.000004
ssc-miR-181b	GHR	growth hormone receptor [Source:NCBI gene (formerly Entrezgene);Acc:397488]	2.55	0.000004
ssc-miR-181b	GID4	GID complex subunit 4 homolog [Source:VGNC Symbol;Acc:VGNC:98996]	2.55	0.000004
ssc-miR-181b	GIGYF1	GRB10 interacting GYF protein 1 [Source:VGNC Symbol;Acc:VGNC:88444]	2.55	0.000004
ssc-miR-181b	GJA9	gap junction protein alpha 9 [Source:VGNC Symbol;Acc:VGNC:97065]	2.55	0.000004
ssc-miR-181b	GLB1L	galactosidase beta 1 like [Source:VGNC Symbol;Acc:VGNC:96165]	2.55	0.000004
ssc-miR-181b	GLCCI1	glucocorticoid induced 1 [Source:VGNC Symbol;Acc:VGNC:88476]	2.55	0.000004
ssc-miR-181b	GLIS3	GLIS family zinc finger 3 [Source:VGNC Symbol;Acc:VGNC:88485]	2.55	0.000004
ssc-miR-181b	GLO1	glyoxalase I [Source:HGNC Symbol;Acc:HGNC:4323]	2.55	0.000004
ssc-miR-181b	GMFB	glia maturation factor beta [Source:VGNC Symbol;Acc:VGNC:88509]	2.55	0.000004
ssc-miR-181b	GMPS	guanine monophosphate synthase [Source:VGNC Symbol;Acc:VGNC:88516]	2.55	0.000004
ssc-miR-181b	GNA13	G protein subunit alpha 13 [Source:VGNC Symbol;Acc:VGNC:98997]	2.55	0.000004
ssc-miR-181b	GNAO1	hypothetical gene	2.55	0.000004
ssc-miR-181b	GNAQ	G protein subunit alpha q [Source:VGNC Symbol;Acc:VGNC:103100]	2.55	0.000004
ssc-miR-181b	GNB4	G protein subunit beta 4 [Source:VGNC Symbol;Acc:VGNC:88533]	2.55	0.000004
ssc-miR-181b	GNG2	hypothetical gene	2.55	0.000004
ssc-miR-181b	GOLGA1	golgin A1 [Source:VGNC Symbol;Acc:VGNC:88549]	2.55	0.000004
ssc-miR-181b	GOLIM4	golgi integral membrane protein 4 [Source:VGNC Symbol;Acc:VGNC:88555]	2.55	0.000004
ssc-miR-181b	GOT2	glutamic-oxaloacetic transaminase 2 [Source:VGNC Symbol;Acc:VGNC:88562]	2.55	0.000004
ssc-miR-181b	GP5	glycoprotein V platelet [Source:VGNC Symbol;Acc:VGNC:88566]	2.55	0.000004
ssc-miR-181b	GPBP1	GC-rich promoter binding protein 1 [Source:VGNC Symbol;Acc:VGNC:88578]	2.55	0.000004
ssc-miR-181b	GPCPD1	glycerophosphocholine phosphodiesterase 1 [Source:VGNC Symbol;Acc:VGNC:96148]	2.55	0.000004
ssc-miR-181b	GPD1L	glycerol-3-phosphate dehydrogenase 1 like [Source:VGNC Symbol;Acc:VGNC:108661]	2.55	0.000004
ssc-miR-181b	GPD2	glycerol-3-phosphate dehydrogenase 2 [Source:VGNC Symbol;Acc:VGNC:96330]	2.55	0.000004
ssc-miR-181b	GPR137C	G protein-coupled receptor 137C [Source:VGNC Symbol;Acc:VGNC:88602]	2.55	0.000004
ssc-miR-181b	GPR180	G protein-coupled receptor 180 [Source:VGNC Symbol;Acc:VGNC:88621]	2.55	0.000004
ssc-miR-181b	GPSM1	G protein signaling modulator 1 [Source:VGNC Symbol;Acc:VGNC:88648]	2.55	0.000004
ssc-miR-181b	GRAMD4	GRAM domain containing 4 [Source:VGNC Symbol;Acc:VGNC:88659]	2.55	0.000004
ssc-miR-181b	GRB10	growth factor receptor bound protein 10 [Source:VGNC Symbol;Acc:VGNC:88662]	2.55	0.000004
ssc-miR-181b	GREB1L	GREB1 like retinoic acid receptor coactivator [Source:VGNC Symbol;Acc:VGNC:96584]	2.55	0.000004
ssc-miR-181b	GRIA2	glutamate ionotropic receptor AMPA type subunit 2 [Source:VGNC Symbol;Acc:VGNC:88671]	2.55	0.000004
ssc-miR-181b	GRID1	glutamate ionotropic receptor delta type subunit 1 [Source:VGNC Symbol;Acc:VGNC:88674]	2.55	0.000004
ssc-miR-181b	GRIK2	glutamate ionotropic receptor kainate type subunit 2 [Source:VGNC Symbol;Acc:VGNC:88678]	2.55	0.000004
ssc-miR-181b	GRIK3	glutamate ionotropic receptor kainate type subunit 3 [Source:VGNC Symbol;Acc:VGNC:88679]	2.55	0.000004
ssc-miR-181b	GRIK4	glutamate ionotropic receptor kainate type subunit 4 [Source:VGNC Symbol;Acc:VGNC:88680]	2.55	0.000004
ssc-miR-181b	GRM1	glutamate metabotropic receptor 1 [Source:VGNC Symbol;Acc:VGNC:88700]	2.55	0.000004

ssc-miR-181b	GRM5	glutamate metabotropic receptor 5 [Source:VGNC Symbol;Acc:VGNC:88703]	2.55	0.000004
ssc-miR-181b	GRM7	glutamate metabotropic receptor 7 [Source:VGNC Symbol;Acc:VGNC:98028]	2.55	0.000004
ssc-miR-181b	GSE1	Gse1 coiled-coil protein [Source:VGNC Symbol;Acc:VGNC:88720]	2.55	0.000004
ssc-miR-181b	GSKIP	GSK3B interacting protein [Source:VGNC Symbol;Acc:VGNC:103956]	2.55	0.000004
ssc-miR-181b	GSPT1	G1 to S phase transition 1 [Source:VGNC Symbol;Acc:VGNC:88724]	2.55	0.000004
ssc-miR-181b	GUCY1A2	guanylate cyclase 1 soluble subunit alpha 2 [Source:VGNC Symbol;Acc:VGNC:88751]	2.55	0.000004
ssc-miR-181b	HAND2	heart and neural crest derivatives expressed 2 [Source:VGNC Symbol;Acc:VGNC:88778]	2.55	0.000004
ssc-miR-181b	HAPLN1	hyaluronan and proteoglycan link protein 1 [Source:VGNC Symbol;Acc:VGNC:88780]	2.55	0.000004
ssc-miR-181b	HAUS3	hypothetical gene	2.55	0.000004
ssc-miR-181b	HCN1	hyperpolarization activated cyclic nucleotide gated potassium channel 1 [Source:VGNC Symbol;Acc:VGNC:88802]	2.55	0.000004
ssc-miR-181b	HCN2	hyperpolarization activated cyclic nucleotide gated potassium and sodium channel 2 [Source:VGNC Symbol;Acc:VGNC:88803]	2.55	0.000004
ssc-miR-181b	HDAC9	histone deacetylase 9 [Source:HGNC Symbol;Acc:HGNC:14065]	2.55	0.000004
ssc-miR-181b	HECA	hdc homolog, cell cycle regulator [Source:VGNC Symbol;Acc:VGNC:88831]	2.55	0.000004
ssc-miR-181b	HECW2	HECT, C2 and WW domain containing E3 ubiquitin protein ligase 2 [Source:NCBI gene (formerly Entrezgene);Acc:100155879]	2.55	0.000004
ssc-miR-181b	HEPHL1	hephaestin like 1 [Source:VGNC Symbol;Acc:VGNC:88848]	2.55	0.000004
ssc-miR-181b	HEXIM1	HEXIM P-TEFb complex subunit 1 [Source:HGNC Symbol;Acc:HGNC:24953]	2.55	0.000004
ssc-miR-181b	HEY2	hes related family bHLH transcription factor with YRPW motif 2 [Source:VGNC Symbol;Acc:VGNC:88864]	2.55	0.000004
ssc-miR-181b	HGF	hepatocyte growth factor [Source:VGNC Symbol;Acc:VGNC:88869]	2.55	0.000004
ssc-miR-181b	HIC2	HIC ZBTB transcriptional repressor 2 [Source:HGNC Symbol;Acc:HGNC:18595]	2.55	0.000004
ssc-miR-181b	HINT3	histidine triad nucleotide binding protein 3 [Source:VGNC Symbol;Acc:VGNC:88884]	2.55	0.000004
ssc-miR-181b	HIPK1	homeodomain interacting protein kinase 1 [Source:VGNC Symbol;Acc:VGNC:88887]	2.55	0.000004
ssc-miR-181b	HIPK2	homeodomain interacting protein kinase 2 [Source:VGNC Symbol;Acc:VGNC:88888]	2.55	0.000004
ssc-miR-181b	HIPK3	homeodomain interacting protein kinase 3 [Source:VGNC Symbol;Acc:VGNC:88889]	2.55	0.000004
ssc-miR-181b	HLF	HLF transcription factor, PAR bZIP family member [Source:VGNC Symbol;Acc:VGNC:88896]	2.55	0.000004
ssc-miR-181b	HLTF	helicase like transcription factor [Source:VGNC Symbol;Acc:VGNC:88897]	2.55	0.000004
ssc-miR-181b	HMBS	hydroxymethylbilane synthase [Source:VGNC Symbol;Acc:VGNC:88899]	2.55	0.000004
ssc-miR-181b	HMGB1	hypothetical gene	2.55	0.000004
ssc-miR-181b	HMGB2	high mobility group box 2 [Source:VGNC Symbol;Acc:VGNC:88902]	2.55	0.000004
ssc-miR-181b	HMX1	H6 family homeobox 1 [Source:VGNC Symbol;Acc:VGNC:88913]	2.55	0.000004
ssc-miR-181b	HNRNPA0	hypothetical gene	2.55	0.000004
ssc-miR-181b	HNRNPH1	heterous nuclear ribonucleoprotein H1 [Source:VGNC Symbol;Acc:VGNC:88920]	2.55	0.000004
ssc-miR-181b	HNRNPK	heterous nuclear ribonucleoprotein K [Source:VGNC Symbol;Acc:VGNC:103963]	2.55	0.000004
ssc-miR-181b	HNRNPR	heterous nuclear ribonucleoprotein R [Source:VGNC Symbol;Acc:VGNC:88925]	2.55	0.000004
ssc-miR-181b	HOMER1	homer scaffold protein 1 [Source:VGNC Symbol;Acc:VGNC:88928]	2.55	0.000004
ssc-miR-181b	HOXA1	homeobox A1 [Source:VGNC Symbol;Acc:VGNC:88933]	2.55	0.000004
ssc-miR-181b	HOXA11	homeobox A11 [Source:VGNC Symbol;Acc:VGNC:88935]	2.55	0.000004
ssc-miR-181b	HOXB4	homeobox B4 [Source:HGNC Symbol;Acc:HGNC:5115]	2.55	0.000004
ssc-miR-181b	HOXB5	homeobox B5 [Source:VGNC Symbol;Acc:VGNC:88945]	2.55	0.000004
ssc-miR-181b	HOXB8	homeobox B8 [Source:VGNC Symbol;Acc:VGNC:88948]	2.55	0.000004
ssc-miR-181b	HOXC8	homeobox C8 [Source:VGNC Symbol;Acc:VGNC:88954]	2.55	0.000004
ssc-miR-181b	HOXD1	homeobox D1 [Source:VGNC Symbol;Acc:VGNC:96350]	2.55	0.000004
ssc-miR-181b	HS2ST1	heparan sulfate 2-O-sulfotransferase 1 [Source:HGNC Symbol;Acc:HGNC:5193]	2.55	0.000004
ssc-miR-181b	HS3ST3A1	heparan sulfate-glucosamine 3-sulfotransferase 3A1 [Source:VGNC Symbol;Acc:VGNC:99002]	2.55	0.000004
ssc-miR-181b	HS6ST1	heparan sulfate 6-O-sulfotransferase 1 [Source:VGNC Symbol;Acc:VGNC:96362]	2.55	0.000004
ssc-miR-181b	HSP90B1	heat shock protein 90 beta family member 1 [Source:VGNC Symbol;Acc:VGNC:103290]	2.55	0.000004
ssc-miR-181b	HSPA5	heat shock protein family A (Hsp70) member 5 [Source:VGNC Symbol;Acc:VGNC:103107]	2.55	0.000004
ssc-miR-181b	HTR2C	5-hydroxytryptamine receptor 2C [Source:VGNC Symbol;Acc:VGNC:96737]	2.55	0.000004
ssc-miR-181b	HTT	huntingtin [Source:VGNC Symbol;Acc:VGNC:89006]	2.55	0.000004
ssc-miR-181b	HYOU1	hypoxia up-regulated 1 [Source:VGNC Symbol;Acc:VGNC:89014]	2.55	0.000004

ssc-miR-181b	IARS2	isoleucyl-tRNA synthetase 2, mitochondrial [Source:VGNC Symbol;Acc:VGNC:96226]	2.55	0.000004
ssc-miR-181b	IFNE	interferon epsilon [Source:VGNC Symbol;Acc:VGNC:89039]	2.55	0.000004
ssc-miR-181b	IFRG15	hypothetical gene	2.55	0.000004
ssc-miR-181b	IGDCC3	immunoglobulin superfamily DCC subclass member 3 [Source:HGNC Symbol;Acc:HGNC:9700]	2.55	0.000004
ssc-miR-181b	IGF2BP2	insulin like growth factor 2 mRNA binding protein 2 [Source:VGNC Symbol;Acc:VGNC:89055]	2.55	0.000004
ssc-miR-181b	IGF2BP3	insulin like growth factor 2 mRNA binding protein 3 [Source:VGNC Symbol;Acc:VGNC:89056]	2.55	0.000004
ssc-miR-181b	IGSF11	immunoglobulin superfamily member 11 [Source:VGNC Symbol;Acc:VGNC:89064]	2.55	0.000004
ssc-miR-181b	IK	IK cytokine [Source:VGNC Symbol;Acc:VGNC:89070]	2.55	0.000004
ssc-miR-181b	IL1A	interleukin 1 alpha [Source:VGNC Symbol;Acc:VGNC:89091]	2.55	0.000004
ssc-miR-181b	IL1RAPL1	interleukin 1 receptor accessory protein like 1 [Source:VGNC Symbol;Acc:VGNC:103967]	2.55	0.000004
ssc-miR-181b	IL6ST	interleukin 6 cytokine family signal transducer [Source:VGNC Symbol;Acc:VGNC:89113]	2.55	0.000004
ssc-miR-181b	ILF3	interleukin enhancer binding factor 3 [Source:VGNC Symbol;Acc:VGNC:89120]	2.55	0.000004
ssc-miR-181b	IMPG2	interphotoreceptor matrix proteoglycan 2 [Source:VGNC Symbol;Acc:VGNC:98048]	2.55	0.000004
ssc-miR-181b	ING5	inhibitor of growth family member 5 [Source:VGNC Symbol;Acc:VGNC:95912]	2.55	0.000004
ssc-miR-181b	INHBA	inhibin subunit beta A [Source:VGNC Symbol;Acc:VGNC:89133]	2.55	0.000004
ssc-miR-181b	INO80	INO80 complex ATPase subunit [Source:VGNC Symbol;Acc:VGNC:89138]	2.55	0.000004
ssc-miR-181b	INO80D	INO80 complex subunit D [Source:VGNC Symbol;Acc:VGNC:96113]	2.55	0.000004
ssc-miR-181b	INPP5A	inositol polyphosphate-5-phosphatase A [Source:VGNC Symbol;Acc:VGNC:89142]	2.55	0.000004
ssc-miR-181b	INPP5E	inositol polyphosphate-5-phosphatase E [Source:VGNC Symbol;Acc:VGNC:89144]	2.55	0.000004
ssc-miR-181b	IPMK	inositol polyphosphate multikinase [Source:VGNC Symbol;Acc:VGNC:89175]	2.55	0.000004
ssc-miR-181b	IPO5	importin 5 [Source:VGNC Symbol;Acc:VGNC:89179]	2.55	0.000004
ssc-miR-181b	IPO8	importin 8 [Source:VGNC Symbol;Acc:VGNC:89181]	2.55	0.000004
ssc-miR-181b	IPPK	inositol-pentakisphosphate 2-kinase [Source:VGNC Symbol;Acc:VGNC:89183]	2.55	0.000004
ssc-miR-181b	IQCJ-SCHIP1	hypothetical gene	2.55	0.000004
ssc-miR-181b	IQGAP2	IQ motif containing GTPase activating protein 2 [Source:VGNC Symbol;Acc:VGNC:89192]	2.55	0.000004
ssc-miR-181b	IQSEC2	IQ motif and Sec7 domain ArfGEF 2 [Source:VGNC Symbol;Acc:VGNC:89195]	2.55	0.000004
ssc-miR-181b	IRAK1BP1	interleukin 1 receptor associated kinase 1 binding protein 1 [Source:VGNC Symbol;Acc:VGNC:89199]	2.55	0.000004
ssc-miR-181b	IRS2	insulin receptor substrate 2 [Source:VGNC Symbol;Acc:VGNC:89214]	2.55	0.000004
ssc-miR-181b	ISPD	hypothetical gene	2.55	0.000004
ssc-miR-181b	ITFG1	integrin alpha FG-GAP repeat containing 1 [Source:VGNC Symbol;Acc:VGNC:89229]	2.55	0.000004
ssc-miR-181b	ITGA1	integrin subunit alpha 1 [Source:VGNC Symbol;Acc:VGNC:89231]	2.55	0.000004
ssc-miR-181b	ITGA2	integrin subunit alpha 2 [Source:VGNC Symbol;Acc:VGNC:89234]	2.55	0.000004
ssc-miR-181b	ITGA3	integrin subunit alpha 3 [Source:VGNC Symbol;Acc:VGNC:89235]	2.55	0.000004
ssc-miR-181b	ITGA6	integrin subunit alpha 6 [Source:VGNC Symbol;Acc:VGNC:96378]	2.55	0.000004
ssc-miR-181b	ITGB8	integrin subunit beta 8 [Source:VGNC Symbol;Acc:VGNC:89246]	2.55	0.000004
ssc-miR-181b	ITPK1	inositol-tetrakisphosphate 1-kinase [Source:VGNC Symbol;Acc:VGNC:89251]	2.55	0.000004
ssc-miR-181b	ITSN1	intersectin 1 [Source:VGNC Symbol;Acc:VGNC:108669]	2.55	0.000004
ssc-miR-181b	JAK2	Janus kinase 2 [Source:VGNC Symbol;Acc:VGNC:89271]	2.55	0.000004
ssc-miR-181b	JAKMIP3	Janus kinase and microtubule interacting protein 3 [Source:VGNC Symbol;Acc:VGNC:89275]	2.55	0.000004
ssc-miR-181b	JARID2	jumonji and AT-rich interaction domain containing 2 [Source:VGNC Symbol;Acc:VGNC:89279]	2.55	0.000004
ssc-miR-181b	JAZF1	JAZF zinc finger 1 [Source:VGNC Symbol;Acc:VGNC:89280]	2.55	0.000004
ssc-miR-181b	JDP2	Jun dimerization protein 2 [Source:VGNC Symbol;Acc:VGNC:89282]	2.55	0.000004
ssc-miR-181b	KANK1	KN motif and ankyrin repeat domains 1 [Source:VGNC Symbol;Acc:VGNC:103112]	2.55	0.000004
ssc-miR-181b	KARS	hypothetical gene	2.55	0.000004
ssc-miR-181b	KAT2B	lysine acetyltransferase 2B [Source:VGNC Symbol;Acc:VGNC:89304]	2.55	0.000004
ssc-miR-181b	KAT7	lysine acetyltransferase 7 [Source:VGNC Symbol;Acc:VGNC:89307]	2.55	0.000004
ssc-miR-181b	KATNBL1	katanin regulatory subunit B1 like 1 [Source:VGNC Symbol;Acc:VGNC:89313]	2.55	0.000004
ssc-miR-181b	KCMF1	potassium channel modulatory factor 1 [Source:VGNC Symbol;Acc:VGNC:89322]	2.55	0.000004
ssc-miR-181b	KCNA4	potassium voltage-gated channel subfamily A member 4 [Source:VGNC Symbol;Acc:VGNC:89326]	2.55	0.000004

ssc-miR-181b	KCNC2	potassium voltage-gated channel subfamily C member 2 [Source:VGNC Symbol;Acc:VGNC:89333]	2.55	0.000004
ssc-miR-181b	KCNH1	potassium voltage-gated channel subfamily H member 1 [Source:VGNC Symbol;Acc:VGNC:108599]	2.55	0.000004
ssc-miR-181b	KCNJ1	potassium inwardly rectifying channel subfamily J member 1 [Source:VGNC Symbol;Acc:VGNC:89352]	2.55	0.000004
ssc-miR-181b	KCNJ10	potassium inwardly rectifying channel subfamily J member 10 [Source:VGNC Symbol;Acc:VGNC:89353]	2.55	0.000004
ssc-miR-181b	KCNJ15	potassium inwardly rectifying channel subfamily J member 15 [Source:VGNC Symbol;Acc:VGNC:89355]	2.55	0.000004
ssc-miR-181b	KCNK10	potassium two pore domain channel subfamily K member 10 [Source:VGNC Symbol;Acc:VGNC:89364]	2.55	0.000004
ssc-miR-181b	KCNK12	potassium two pore domain channel subfamily K member 12 [Source:HGNC Symbol;Acc:HGNC:6274]	2.55	0.000004
ssc-miR-181b	KCNN3	potassium calcium-activated channel subfamily N member 3 [Source:VGNC Symbol;Acc:VGNC:98056]	2.55	0.000004
ssc-miR-181b	KCNQ5	potassium voltage-gated channel subfamily Q member 5 [Source:VGNC Symbol;Acc:VGNC:89384]	2.55	0.000004
ssc-miR-181b	KCTD10	potassium channel tetramerization domain containing 10 [Source:VGNC Symbol;Acc:VGNC:103972]	2.55	0.000004
ssc-miR-181b	KCTD16	potassium channel tetramerization domain containing 16 [Source:VGNC Symbol;Acc:VGNC:89395]	2.55	0.000004
ssc-miR-181b	KDM5A	lysine demethylase 5A [Source:VGNC Symbol;Acc:VGNC:89415]	2.55	0.000004
ssc-miR-181b	KIAA0195	hypothetical gene	2.55	0.000004
ssc-miR-181b	KIAA0226	hypothetical gene	2.55	0.000004
ssc-miR-181b	KIAA0247	hypothetical gene	2.55	0.000004
ssc-miR-181b	KIAA1024	hypothetical gene	2.55	0.000004
ssc-miR-181b	KIAA1239	hypothetical gene	2.55	0.000004
ssc-miR-181b	KIAA1244	hypothetical gene	2.55	0.000004
ssc-miR-181b	KIAA1324L	hypothetical gene	2.55	0.000004
ssc-miR-181b	KIAA1462	hypothetical gene	2.55	0.000004
ssc-miR-181b	KIAA1549L	KIAA1549 like [Source:VGNC Symbol;Acc:VGNC:89445]	2.55	0.000004
ssc-miR-181b	KIAA1551	hypothetical gene	2.55	0.000004
ssc-miR-181b	KIAA1644	hypothetical gene	2.55	0.000004
ssc-miR-181b	KIAA2018	hypothetical gene	2.55	0.000004
ssc-miR-181b	KIAA2022	hypothetical gene	2.55	0.000004
ssc-miR-181b	KIF1B	kinesin family member 1B [Source:VGNC Symbol;Acc:VGNC:89460]	2.55	0.000004
ssc-miR-181b	KIF3A	kinesin family member 3A [Source:VGNC Symbol;Acc:VGNC:89470]	2.55	0.000004
ssc-miR-181b	KIF3B	kinesin family member 3B [Source:VGNC Symbol;Acc:VGNC:96392]	2.55	0.000004
ssc-miR-181b	KITLG	KIT ligand [Source:VGNC Symbol;Acc:VGNC:98061]	2.55	0.000004
ssc-miR-181b	KLF15	Kruppel like factor 15 [Source:VGNC Symbol;Acc:VGNC:89495]	2.55	0.000004
ssc-miR-181b	KLF3	Kruppel like factor 3 [Source:VGNC Symbol;Acc:VGNC:89498]	2.55	0.000004
ssc-miR-181b	KLF6	Kruppel like factor 6 [Source:VGNC Symbol;Acc:VGNC:98063]	2.55	0.000004
ssc-miR-181b	KLF7	Kruppel like factor 7 [Source:VGNC Symbol;Acc:VGNC:96396]	2.55	0.000004
ssc-miR-181b	KLHL14	kelch like family member 14 [Source:VGNC Symbol;Acc:VGNC:89515]	2.55	0.000004
ssc-miR-181b	KLHL15	kelch like family member 15 [Source:VGNC Symbol;Acc:VGNC:89516]	2.55	0.000004
ssc-miR-181b	KLHL2	kelch like family member 2 [Source:VGNC Symbol;Acc:VGNC:89518]	2.55	0.000004
ssc-miR-181b	KLHL24	kelch like family member 24 [Source:VGNC Symbol;Acc:VGNC:89521]	2.55	0.000004
ssc-miR-181b	KLHL29	kelch like family member 29 [Source:VGNC Symbol;Acc:VGNC:89524]	2.55	0.000004
ssc-miR-181b	KLHL3	kelch like family member 3 [Source:VGNC Symbol;Acc:VGNC:89525]	2.55	0.000004
ssc-miR-181b	KLHL36	kelch like family member 36 [Source:VGNC Symbol;Acc:VGNC:89530]	2.55	0.000004
ssc-miR-181b	KLHL42	kelch like family member 42 [Source:VGNC Symbol;Acc:VGNC:89534]	2.55	0.000004
ssc-miR-181b	KLHL5	kelch like family member 5 [Source:VGNC Symbol;Acc:VGNC:89535]	2.55	0.000004
ssc-miR-181b	KMT2A	lysine methyltransferase 2A [Source:VGNC Symbol;Acc:VGNC:108600]	2.55	0.000004
ssc-miR-181b	KMT2C	lysine methyltransferase 2C [Source:VGNC Symbol;Acc:VGNC:89550]	2.55	0.000004
ssc-miR-181b	KMT2E	lysine methyltransferase 2E (inactive) [Source:VGNC Symbol;Acc:VGNC:89551]	2.55	0.000004
ssc-miR-181b	KPNA1	karyopherin subunit alpha 1 [Source:VGNC Symbol;Acc:VGNC:89560]	2.55	0.000004
ssc-miR-181b	KPNA4	karyopherin subunit alpha 4 [Source:VGNC Symbol;Acc:VGNC:89563]	2.55	0.000004
ssc-miR-181b	KPNB1	karyopherin subunit beta 1 [Source:VGNC Symbol;Acc:VGNC:89567]	2.55	0.000004
ssc-miR-181b	KRAS	KRAS proto-onco, GTPase [Source:VGNC Symbol;Acc:VGNC:89569]	2.55	0.000004

ssc-miR-181b	KRBOX4	hypothetical gene	2.55	0.000004
ssc-miR-181b	KREMEN1	kringle containing transmembrane protein 1 [Source:VGNC Symbol;Acc:VGNC:89572]	2.55	0.000004
ssc-miR-181b	KRT222	keratin 222 [Source:HGNC Symbol;Acc:HGNC:28695]	2.55	0.000004
ssc-miR-181b	KSR1	kinase suppressor of ras 1 [Source:VGNC Symbol;Acc:VGNC:89606]	2.55	0.000004
ssc-miR-181b	L1CAM	L1 cell adhesion molecule [Source:HGNC Symbol;Acc:HGNC:6470]	2.55	0.000004
ssc-miR-181b	LAMC1	laminin subunit gamma 1 [Source:VGNC Symbol;Acc:VGNC:89624]	2.55	0.000004
ssc-miR-181b	LAMP3	hypothetical gene	2.55	0.000004
ssc-miR-181b	LARP4	La ribonucleoprotein 4 [Source:VGNC Symbol;Acc:VGNC:89641]	2.55	0.000004
ssc-miR-181b	LBR	lamin B receptor [Source:VGNC Symbol;Acc:VGNC:98068]	2.55	0.000004
ssc-miR-181b	LCLAT1	lysocardiolipin acyltransferase 1 [Source:VGNC Symbol;Acc:VGNC:89658]	2.55	0.000004
ssc-miR-181b	LCOR	ligand dependent nuclear receptor corepressor [Source:HGNC Symbol;Acc:HGNC:29503]	2.55	0.000004
ssc-miR-181b	LEMD3	LEM domain containing 3 [Source:VGNC Symbol;Acc:VGNC:89679]	2.55	0.000004
ssc-miR-181b	LGALSL	galectin like [Source:VGNC Symbol;Acc:VGNC:89698]	2.55	0.000004
ssc-miR-181b	LGI2	leucine rich repeat LGI family member 2 [Source:VGNC Symbol;Acc:VGNC:89699]	2.55	0.000004
ssc-miR-181b	LHFPL4	LHFPL tetraspan subfamily member 4 [Source:VGNC Symbol;Acc:VGNC:89707]	2.55	0.000004
ssc-miR-181b	LHX9	LIM homeobox 9 [Source:VGNC Symbol;Acc:VGNC:95608]	2.55	0.000004
ssc-miR-181b	LIF	LIF interleukin 6 family cytokine [Source:VGNC Symbol;Acc:VGNC:89719]	2.55	0.000004
ssc-miR-181b	LIMCH1	LIM and calponin homology domains 1 [Source:VGNC Symbol;Acc:VGNC:89724]	2.55	0.000004
ssc-miR-181b	LIMS1	hypothetical gene	2.55	0.000004
ssc-miR-181b	LIN28A	lin-28 homolog A [Source:VGNC Symbol;Acc:VGNC:98492]	2.55	0.000004
ssc-miR-181b	LIN28B	lin-28 homolog B [Source:VGNC Symbol;Acc:VGNC:89729]	2.55	0.000004
ssc-miR-181b	LIN7C	lin-7 homolog C, crumbs cell polarity complex component [Source:VGNC Symbol;Acc:VGNC:89732]	2.55	0.000004
ssc-miR-181b	LLPH	LLP homolog, long-term synaptic facilitation factor [Source:VGNC Symbol;Acc:VGNC:103297]	2.55	0.000004
ssc-miR-181b	LMBRD2	LMBR1 domain containing 2 [Source:VGNC Symbol;Acc:VGNC:89755]	2.55	0.000004
ssc-miR-181b	LMO1	LIM domain only 1 [Source:VGNC Symbol;Acc:VGNC:89764]	2.55	0.000004
ssc-miR-181b	LMO3	hypothetical gene	2.55	0.000004
ssc-miR-181b	LMTK2	lemur tyrosine kinase 2 [Source:HGNC Symbol;Acc:HGNC:17880]	2.55	0.000004
ssc-miR-181b	LNX2	ligand of numb-protein X 2 [Source:VGNC Symbol;Acc:VGNC:89774]	2.55	0.000004
ssc-miR-181b	LONRF2	LON peptidase N-terminal domain and ring finger 2 [Source:VGNC Symbol;Acc:VGNC:89777]	2.55	0.000004
ssc-miR-181b	LOX	lysyl oxidase [Source:VGNC Symbol;Acc:VGNC:99785]	2.55	0.000004
ssc-miR-181b	LPCAT1	lysophosphatidylcholine acyltransferase 1 [Source:VGNC Symbol;Acc:VGNC:89788]	2.55	0.000004
ssc-miR-181b	LPCAT2	lysophosphatidylcholine acyltransferase 2 [Source:VGNC Symbol;Acc:VGNC:89789]	2.55	0.000004
ssc-miR-181b	LPGAT1	lysophosphatidylglycerol acyltransferase 1 [Source:VGNC Symbol;Acc:VGNC:89792]	2.55	0.000004
ssc-miR-181b	LPP	LIM domain containing preferred translocation partner in lipoma [Source:HGNC Symbol;Acc:HGNC:6679]	2.55	0.000004
ssc-miR-181b	LPPR4	hypothetical gene	2.55	0.000004
ssc-miR-181b	LRBA	LPS responsive beige-like anchor protein [Source:VGNC Symbol;Acc:VGNC:98074]	2.55	0.000004
ssc-miR-181b	LRIG2	leucine rich repeats and immunoglobulin like domains 2 [Source:VGNC Symbol;Acc:VGNC:89811]	2.55	0.000004
ssc-miR-181b	LRP12	LDL receptor related protein 12 [Source:VGNC Symbol;Acc:VGNC:89818]	2.55	0.000004
ssc-miR-181b	LRP4	LDL receptor related protein 4 [Source:VGNC Symbol;Acc:VGNC:89820]	2.55	0.000004
ssc-miR-181b	LRP6	LDL receptor related protein 6 [Source:VGNC Symbol;Acc:VGNC:89821]	2.55	0.000004
ssc-miR-181b	LRRC32	leucine rich repeat containing 32 [Source:VGNC Symbol;Acc:VGNC:108602]	2.55	0.000004
ssc-miR-181b	LRRC8D	leucine rich repeat containing 8 VRAC subunit D [Source:VGNC Symbol;Acc:VGNC:98088]	2.55	0.000004
ssc-miR-181b	LRRFIP1	hypothetical gene	2.55	0.000004
ssc-miR-181b	LRRN1	leucine rich repeat neuronal 1 [Source:VGNC Symbol;Acc:VGNC:89860]	2.55	0.000004
ssc-miR-181b	LUZP1	leucine zipper protein 1 [Source:VGNC Symbol;Acc:VGNC:89897]	2.55	0.000004
ssc-miR-181b	LYRM1	LYR motif containing 1 [Source:VGNC Symbol;Acc:VGNC:89915]	2.55	0.000004
ssc-miR-181b	MAB21L2	mab-21 like 2 [Source:HGNC Symbol;Acc:HGNC:6758]	2.55	0.000004
ssc-miR-181b	MADD	MAP kinase activating death domain [Source:VGNC Symbol;Acc:VGNC:89942]	2.55	0.000004
ssc-miR-181b	MAEA	macrophage erythroblast attacher, E3 ubiquitin ligase [Source:VGNC Symbol;Acc:VGNC:89943]	2.55	0.000004

ssc-miR-181b	MAGOHB	hypothetical gene	2.55	0.000004
ssc-miR-181b	MAMDC2	MAM domain containing 2 [Source:VGNC Symbol;Acc:VGNC:89962]	2.55	0.000004
ssc-miR-181b	MAN2A1	mannosidase alpha class 2A member 1 [Source:VGNC Symbol;Acc:VGNC:98098]	2.55	0.000004
ssc-miR-181b	MAP1A	microtubule associated protein 1A [Source:VGNC Symbol;Acc:VGNC:89978]	2.55	0.000004
ssc-miR-181b	MAP1B	microtubule associated protein 1B [Source:VGNC Symbol;Acc:VGNC:89979]	2.55	0.000004
ssc-miR-181b	MAP2K1	mitogen-activated protein kinase kinase 1 [Source:VGNC Symbol;Acc:VGNC:103121]	2.55	0.000004
ssc-miR-181b	MAP3K1	mitogen-activated protein kinase kinase kinase 1 [Source:VGNC Symbol;Acc:VGNC:98104]	2.55	0.000004
ssc-miR-181b	MAP3K10	mitogen-activated protein kinase kinase kinase 10 [Source:VGNC Symbol;Acc:VGNC:89981]	2.55	0.000004
ssc-miR-181b	MAP3K2	mitogen-activated protein kinase kinase kinase 2 [Source:VGNC Symbol;Acc:VGNC:98107]	2.55	0.000004
ssc-miR-181b	MAP3K3	mitogen-activated protein kinase kinase kinase 3 [Source:VGNC Symbol;Acc:VGNC:98108]	2.55	0.000004
ssc-miR-181b	MAP3K8	mitogen-activated protein kinase kinase kinase 8 [Source:VGNC Symbol;Acc:VGNC:98110]	2.55	0.000004
ssc-miR-181b	MAP3K9	mitogen-activated protein kinase kinase kinase 9 [Source:VGNC Symbol;Acc:VGNC:89989]	2.55	0.000004
ssc-miR-181b	MAP4K4	mitogen-activated protein kinase kinase kinase kinase 4 [Source:VGNC Symbol;Acc:VGNC:98114]	2.55	0.000004
ssc-miR-181b	MAPK1	mitogen-activated protein kinase 1 [Source:VGNC Symbol;Acc:VGNC:89996]	2.55	0.000004
ssc-miR-181b	MAPK1IP1L	mitogen-activated protein kinase 1 interacting protein 1 like [Source:VGNC Symbol;Acc:VGNC:90002]	2.55	0.000004
ssc-miR-181b	MAPK8	mitogen-activated protein kinase 8 [Source:VGNC Symbol;Acc:VGNC:90006]	2.55	0.000004
ssc-miR-181b	MAPRE2	microtubule associated protein RP/EB family member 2 [Source:VGNC Symbol;Acc:VGNC:98117]	2.55	0.000004
ssc-miR-181b	MAPT	microtubule associated protein tau [Source:VGNC Symbol;Acc:VGNC:90016]	2.55	0.000004
ssc-miR-181b	MARCH11	hypothetical gene	2.55	0.000004
ssc-miR-181b	MARCH6	hypothetical gene	2.55	0.000004
ssc-miR-181b	MARCKS	myristoylated alanine rich protein kinase C substrate [Source:VGNC Symbol;Acc:VGNC:90024]	2.55	0.000004
ssc-miR-181b	MARK1	microtubule affinity regulating kinase 1 [Source:VGNC Symbol;Acc:VGNC:96411]	2.55	0.000004
ssc-miR-181b	MB21D2	Mab-21 domain containing 2 [Source:VGNC Symbol;Acc:VGNC:90047]	2.55	0.000004
ssc-miR-181b	MBD2	methyl-CpG binding domain protein 2 [Source:VGNC Symbol;Acc:VGNC:90048]	2.55	0.000004
ssc-miR-181b	MBLAC2	metallo-beta-lactamase domain containing 2 [Source:VGNC Symbol;Acc:VGNC:90053]	2.55	0.000004
ssc-miR-181b	MBNL1	muscleblind like splicing regulator 1 [Source:VGNC Symbol;Acc:VGNC:90054]	2.55	0.000004
ssc-miR-181b	MBNL2	muscleblind like splicing regulator 2 [Source:VGNC Symbol;Acc:VGNC:90055]	2.55	0.000004
ssc-miR-181b	MBOAT1	membrane bound O-acyltransferase domain containing 1 [Source:VGNC Symbol;Acc:VGNC:90057]	2.55	0.000004
ssc-miR-181b	MBOAT2	membrane bound O-acyltransferase domain containing 2 [Source:VGNC Symbol;Acc:VGNC:90058]	2.55	0.000004
ssc-miR-181b	MBTPS2	hypothetical gene	2.55	0.000004
ssc-miR-181b	MCC	MCC regulator of WNT signaling pathway [Source:VGNC Symbol;Acc:VGNC:96588]	2.55	0.000004
ssc-miR-181b	MCL1	MCL1 apoptosis regulator, BCL2 family member [Source:VGNC Symbol;Acc:VGNC:90072]	2.55	0.000004
ssc-miR-181b	MCUR1	mitochondrial calcium uniporter regulator 1 [Source:HGNC Symbol;Acc:HGNC:21097]	2.55	0.000004
ssc-miR-181b	MECP2	methyl-CpG binding protein 2 [Source:VGNC Symbol;Acc:VGNC:90101]	2.55	0.000004
ssc-miR-181b	MED12L	mediator complex subunit 12L [Source:VGNC Symbol;Acc:VGNC:90105]	2.55	0.000004
ssc-miR-181b	MED26	mediator complex subunit 26 [Source:VGNC Symbol;Acc:VGNC:100168]	2.55	0.000004
ssc-miR-181b	MED4	mediator complex subunit 4 [Source:VGNC Symbol;Acc:VGNC:90121]	2.55	0.000004
ssc-miR-181b	MED8	mediator complex subunit 8 [Source:VGNC Symbol;Acc:VGNC:90124]	2.55	0.000004
ssc-miR-181b	MEF2A	myocyte enhancer factor 2A [Source:VGNC Symbol;Acc:VGNC:98123]	2.55	0.000004
ssc-miR-181b	MEGF9	multiple EGF like domains 9 [Source:VGNC Symbol;Acc:VGNC:90130]	2.55	0.000004
ssc-miR-181b	MELK	maternal embryonic leucine zipper kinase [Source:VGNC Symbol;Acc:VGNC:90137]	2.55	0.000004
ssc-miR-181b	METAP1	methionyl aminopeptidase 1 [Source:VGNC Symbol;Acc:VGNC:90151]	2.55	0.000004
ssc-miR-181b	METAP2	methionyl aminopeptidase 2 [Source:VGNC Symbol;Acc:VGNC:90152]	2.55	0.000004
ssc-miR-181b	MEX3A	mex-3 RNA binding family member A [Source:VGNC Symbol;Acc:VGNC:90168]	2.55	0.000004
ssc-miR-181b	MEX3B	mex-3 RNA binding family member B [Source:VGNC Symbol;Acc:VGNC:90169]	2.55	0.000004
ssc-miR-181b	MFAP3	microfibril associated protein 3 [Source:VGNC Symbol;Acc:VGNC:90174]	2.55	0.000004
ssc-miR-181b	MFAP3L	microfibril associated protein 3 like [Source:VGNC Symbol;Acc:VGNC:90175]	2.55	0.000004
ssc-miR-181b	MFSD6	major facilitator superfamily domain containing 6 [Source:VGNC Symbol;Acc:VGNC:96052]	2.55	0.000004
ssc-miR-181b	MGA	MAX dimerization protein MGA [Source:VGNC Symbol;Acc:VGNC:90195]	2.55	0.000004

ssc-miR-181b	MGAT2	alpha-1,6-mannosyl-glycoprotein 2-beta-N-acetylglucosaminyltransferase [Source:HGNC Symbol;Acc:HGNC:7045]	2.55	0.000004
ssc-miR-181b	MID2	midline 2 [Source:VGNC Symbol;Acc:VGNC:98127]	2.55	0.000004
ssc-miR-181b	MIER3	MIER family member 3 [Source:VGNC Symbol;Acc:VGNC:90221]	2.55	0.000004
ssc-miR-181b	MINK1	misshapen like kinase 1 [Source:VGNC Symbol;Acc:VGNC:98128]	2.55	0.000004
ssc-miR-181b	MIP	major intrinsic protein of lens fiber [Source:VGNC Symbol;Acc:VGNC:90235]	2.55	0.000004
ssc-miR-181b	MITF	melanocyte inducing transcription factor [Source:VGNC Symbol;Acc:VGNC:90243]	2.55	0.000004
ssc-miR-181b	MKNK2	MAPK interacting serine/threonine kinase 2 [Source:VGNC Symbol;Acc:VGNC:90247]	2.55	0.000004
ssc-miR-181b	MLEC	malectin [Source:HGNC Symbol;Acc:HGNC:28973]	2.55	0.000004
ssc-miR-181b	MLF1	myeloid leukemia factor 1 [Source:HGNC Symbol;Acc:HGNC:7125]	2.55	0.000004
ssc-miR-181b	MLK4	hypothetical gene	2.55	0.000004
ssc-miR-181b	MLXIP	MLX interacting protein [Source:VGNC Symbol;Acc:VGNC:90262]	2.55	0.000004
ssc-miR-181b	MME	membrane metalloendopeptidase [Source:VGNC Symbol;Acc:VGNC:90265]	2.55	0.000004
ssc-miR-181b	MMP14	matrix metallopeptidase 14 [Source:NCBI gene (formerly Entrezgene);Acc:397471]	2.55	0.000004
ssc-miR-181b	MNX1	motor neuron and pancreas homeobox 1 [Source:VGNC Symbol;Acc:VGNC:107152]	2.55	0.000004
ssc-miR-181b	MOB3B	MOB kinase activator 3B [Source:VGNC Symbol;Acc:VGNC:96024]	2.55	0.000004
ssc-miR-181b	MON2	MON2 homolog, regulator of endosome-to-Golgi trafficking [Source:VGNC Symbol;Acc:VGNC:90301]	2.55	0.000004
ssc-miR-181b	MOSPD1	motile sperm domain containing 1 [Source:VGNC Symbol;Acc:VGNC:103990]	2.55	0.000004
ssc-miR-181b	MPP5	hypothetical gene	2.55	0.000004
ssc-miR-181b	MPP7	MAGUK p55 scaffold protein 7 [Source:VGNC Symbol;Acc:VGNC:98138]	2.55	0.000004
ssc-miR-181b	MPZL3	myelin protein zero like 3 [Source:VGNC Symbol;Acc:VGNC:90337]	2.55	0.000004
ssc-miR-181b	MRPL17	mitochondrial ribosomal protein L17 [Source:VGNC Symbol;Acc:VGNC:103992]	2.55	0.000004
ssc-miR-181b	MSANTD3	Myb/SANT DNA binding domain containing 3 [Source:HGNC Symbol;Acc:HGNC:23370]	2.55	0.000004
ssc-miR-181b	MSANTD3-TMEFF1	hypothetical gene	2.55	0.000004
ssc-miR-181b	MSI1	musashi RNA binding protein 1 [Source:VGNC Symbol;Acc:VGNC:90421]	2.55	0.000004
ssc-miR-181b	MSI2	musashi RNA binding protein 2 [Source:VGNC Symbol;Acc:VGNC:90422]	2.55	0.000004
ssc-miR-181b	MSL2	MSL complex subunit 2 [Source:VGNC Symbol;Acc:VGNC:90424]	2.55	0.000004
ssc-miR-181b	MTF1	metal regulatory transcription factor 1 [Source:VGNC Symbol;Acc:VGNC:90443]	2.55	0.000004
ssc-miR-181b	MTF2	metal response element binding transcription factor 2 [Source:VGNC Symbol;Acc:VGNC:90444]	2.55	0.000004
ssc-miR-181b	MTMR1	myotubularin related protein 1 [Source:VGNC Symbol;Acc:VGNC:90456]	2.55	0.000004
ssc-miR-181b	MTMR10	myotubularin related protein 10 [Source:VGNC Symbol;Acc:VGNC:90457]	2.55	0.000004
ssc-miR-181b	MTMR12	myotubularin related protein 12 [Source:VGNC Symbol;Acc:VGNC:90459]	2.55	0.000004
ssc-miR-181b	MTMR9	myotubularin related protein 9 [Source:VGNC Symbol;Acc:VGNC:90464]	2.55	0.000004
ssc-miR-181b	MTPN	myotrophin [Source:VGNC Symbol;Acc:VGNC:90467]	2.55	0.000004
ssc-miR-181b	MTX3	metaxin 3 [Source:VGNC Symbol;Acc:VGNC:90479]	2.55	0.000004
ssc-miR-181b	MUC19	hypothetical gene	2.55	0.000004
ssc-miR-181b	MYBL1	MYB proto-onco like 1 [Source:VGNC Symbol;Acc:VGNC:90498]	2.55	0.000004
ssc-miR-181b	MYCBP	MYC binding protein [Source:NCBI gene (formerly Entrezgene);Acc:100513990]	2.55	0.000004
ssc-miR-181b	MYCBP2	MYC binding protein 2 [Source:VGNC Symbol;Acc:VGNC:90502]	2.55	0.000004
ssc-miR-181b	MYLK	myosin light chain kinase [Source:VGNC Symbol;Acc:VGNC:108676]	2.55	0.000004
ssc-miR-181b	MYLK3	myosin light chain kinase 3 [Source:VGNC Symbol;Acc:VGNC:90518]	2.55	0.000004
ssc-miR-181b	MYO1E	myosin IE [Source:VGNC Symbol;Acc:VGNC:90528]	2.55	0.000004
ssc-miR-181b	NAA15	N-alpha-acetyltransferase 15, NatA auxiliary subunit [Source:VGNC Symbol;Acc:VGNC:96747]	2.55	0.000004
ssc-miR-181b	NAA20	N-alpha-acetyltransferase 20, NatB catalytic subunit [Source:VGNC Symbol;Acc:VGNC:96749]	2.55	0.000004
ssc-miR-181b	NAA38	hypothetical gene	2.55	0.000004
ssc-miR-181b	NAA50	N-alpha-acetyltransferase 50, NatE catalytic subunit [Source:VGNC Symbol;Acc:VGNC:104002]	2.55	0.000004
ssc-miR-181b	NAALADL2	N-acetylated alpha-linked acidic dipeptidase like 2 [Source:VGNC Symbol;Acc:VGNC:90559]	2.55	0.000004
ssc-miR-181b	NAB1	NGFI-A binding protein 1 [Source:VGNC Symbol;Acc:VGNC:96431]	2.55	0.000004
ssc-miR-181b	NACC2	NACC family member 2 [Source:VGNC Symbol;Acc:VGNC:90564]	2.55	0.000004
ssc-miR-181b	NAIF1	hypothetical gene	2.55	0.000004

ssc-miR-181b	NBEA	neurobeachin [Source:VGNC Symbol;Acc:VGNC:90590]	2.55	0.000004
ssc-miR-181b	NBEAL1	cytochrome P450 family 20 subfamily A member 1 [Source:VGNC Symbol;Acc:VGNC:103366]	2.55	0.000004
ssc-miR-181b	NCALD	neurocalcin delta [Source:VGNC Symbol;Acc:VGNC:90594]	2.55	0.000004
ssc-miR-181b	NCOA2	nuclear receptor coactivator 2 [Source:VGNC Symbol;Acc:VGNC:90616]	2.55	0.000004
ssc-miR-181b	NDRG2	NDRG family member 2 [Source:VGNC Symbol;Acc:VGNC:90632]	2.55	0.000004
ssc-miR-181b	NDRG4	NDRG family member 4 [Source:VGNC Symbol;Acc:VGNC:90633]	2.55	0.000004
ssc-miR-181b	NEGR1	neuronal growth regulator 1 [Source:VGNC Symbol;Acc:VGNC:90671]	2.55	0.000004
ssc-miR-181b	NEK7	NIMA related kinase 7 [Source:VGNC Symbol;Acc:VGNC:98152]	2.55	0.000004
ssc-miR-181b	NELFA	negative elongation factor complex member A [Source:VGNC Symbol;Acc:VGNC:90680]	2.55	0.000004
ssc-miR-181b	NETO2	neuropilin and tolloid like 2 [Source:VGNC Symbol;Acc:VGNC:90691]	2.55	0.000004
ssc-miR-181b	NEURL1B	neuralized E3 ubiquitin protein ligase 1B [Source:VGNC Symbol;Acc:VGNC:90695]	2.55	0.000004
ssc-miR-181b	NFAT5	nuclear factor of activated T cells 5 [Source:VGNC Symbol;Acc:VGNC:90708]	2.55	0.000004
ssc-miR-181b	NFATC2	nuclear factor of activated T cells 2 [Source:VGNC Symbol;Acc:VGNC:96440]	2.55	0.000004
ssc-miR-181b	NFATC2IP	nuclear factor of activated T cells 2 interacting protein [Source:VGNC Symbol;Acc:VGNC:90709]	2.55	0.000004
ssc-miR-181b	NFIB	nuclear factor I B [Source:VGNC Symbol;Acc:VGNC:90716]	2.55	0.000004
ssc-miR-181b	NHSL2	NHS like 2 [Source:VGNC Symbol;Acc:VGNC:98155]	2.55	0.000004
ssc-miR-181b	NIPAL4	NIPA like domain containing 4 [Source:VGNC Symbol;Acc:VGNC:90751]	2.55	0.000004
ssc-miR-181b	NIPBL	NIPBL cohesin loading factor [Source:VGNC Symbol;Acc:VGNC:90752]	2.55	0.000004
ssc-miR-181b	NKAIN2	sodium/potassium transporting ATPase interacting 2 [Source:VGNC Symbol;Acc:VGNC:103141]	2.55	0.000004
ssc-miR-181b	NKAP	NFKB activating protein [Source:VGNC Symbol;Acc:VGNC:90756]	2.55	0.000004
ssc-miR-181b	NKRF	NFKB repressing factor [Source:VGNC Symbol;Acc:VGNC:90764]	2.55	0.000004
ssc-miR-181b	NKX3-2	NK3 homeobox 2 [Source:VGNC Symbol;Acc:VGNC:90773]	2.55	0.000004
ssc-miR-181b	NLK	nemo like kinase [Source:VGNC Symbol;Acc:VGNC:90779]	2.55	0.000004
ssc-miR-181b	NLN	neurolysin [Source:VGNC Symbol;Acc:VGNC:90780]	2.55	0.000004
ssc-miR-181b	NME1	NME/NM23 nucleoside diphosphate kinase 1 [Source:VGNC Symbol;Acc:VGNC:99023]	2.55	0.000004
ssc-miR-181b	NMNAT2	nicotinamide nucleotide adenylyltransferase 2 [Source:VGNC Symbol;Acc:VGNC:90798]	2.55	0.000004
ssc-miR-181b	NMNAT3	nicotinamide nucleotide adenylyltransferase 3 [Source:VGNC Symbol;Acc:VGNC:108679]	2.55	0.000004
ssc-miR-181b	NMT1	N-myristoyltransferase 1 [Source:VGNC Symbol;Acc:VGNC:90802]	2.55	0.000004
ssc-miR-181b	NMT2	N-myristoyltransferase 2 [Source:VGNC Symbol;Acc:VGNC:96448]	2.55	0.000004
ssc-miR-181b	NOG	noggin [Source:VGNC Symbol;Acc:VGNC:90812]	2.55	0.000004
ssc-miR-181b	NOL4	nucleolar protein 4 [Source:VGNC Symbol;Acc:VGNC:90814]	2.55	0.000004
ssc-miR-181b	NOTCH2	notch receptor 2 [Source:HGNC Symbol;Acc:HGNC:7882]	2.55	0.000004
ssc-miR-181b	NOVA1	NOVA alternative splicing regulator 1 [Source:VGNC Symbol;Acc:VGNC:90827]	2.55	0.000004
ssc-miR-181b	NPEPPS	aminopeptidase puromycin sensitive [Source:VGNC Symbol;Acc:VGNC:98157]	2.55	0.000004
ssc-miR-181b	NPTN	neuroplastin [Source:VGNC Symbol;Acc:VGNC:90861]	2.55	0.000004
ssc-miR-181b	NPTXR	neuronal pentraxin receptor [Source:VGNC Symbol;Acc:VGNC:98158]	2.55	0.000004
ssc-miR-181b	NR1D2	nuclear receptor subfamily 1 group D member 2 [Source:VGNC Symbol;Acc:VGNC:99726]	2.55	0.000004
ssc-miR-181b	NR2C2	nuclear receptor subfamily 2 group C member 2 [Source:VGNC Symbol;Acc:VGNC:90876]	2.55	0.000004
ssc-miR-181b	NR3C1	nuclear receptor subfamily 3 group C member 1 [Source:VGNC Symbol;Acc:VGNC:90883]	2.55	0.000004
ssc-miR-181b	NR4A3	nuclear receptor subfamily 4 group A member 3 [Source:VGNC Symbol;Acc:VGNC:90885]	2.55	0.000004
ssc-miR-181b	NR6A1	nuclear receptor subfamily 6 group A member 1 [Source:VGNC Symbol;Acc:VGNC:90887]	2.55	0.000004
ssc-miR-181b	NRAS	NRAS proto-onco, GTPase [Source:VGNC Symbol;Acc:VGNC:98827]	2.55	0.000004
ssc-miR-181b	NREP	neuronal regeneration related protein [Source:HGNC Symbol;Acc:HGNC:16834]	2.55	0.000004
ssc-miR-181b	NRP1	neuropilin 1 [Source:VGNC Symbol;Acc:VGNC:104012]	2.55	0.000004
ssc-miR-181b	NRXN1	neurexin 1 [Source:HGNC Symbol;Acc:HGNC:8008]	2.55	0.000004
ssc-miR-181b	NSG1	neuronal vesicle trafficking associated 1 [Source:VGNC Symbol;Acc:VGNC:90907]	2.55	0.000004
ssc-miR-181b	NSMAF	neutral sphingomyelinase activation associated factor [Source:VGNC Symbol;Acc:VGNC:90909]	2.55	0.000004
ssc-miR-181b	NSUN3	NOP2/Sun RNA methyltransferase 3 [Source:VGNC Symbol;Acc:VGNC:90915]	2.55	0.000004
ssc-miR-181b	NT5DC3	5'-nucleotidase domain containing 3 [Source:VGNC Symbol;Acc:VGNC:90924]	2.55	0.000004

ssc-miR-181b	NUCKS1	nuclear casein kinase and cyclin dependent kinase substrate 1 [Source:VGNC Symbol;Acc:VGNC:90947]	2.55	0.000004
ssc-miR-181b	NUDT21	nudix hydrolase 21 [Source:VGNC Symbol;Acc:VGNC:98523]	2.55	0.000004
ssc-miR-181b	NUFIP2	nuclear FMR1 interacting protein 2 [Source:VGNC Symbol;Acc:VGNC:90967]	2.55	0.000004
ssc-miR-181b	NUP155	nucleoporin 155 [Source:VGNC Symbol;Acc:VGNC:90974]	2.55	0.000004
ssc-miR-181b	NUS1	NUS1 dehydrodolichyl diphosphate synthase subunit [Source:VGNC Symbol;Acc:VGNC:90990]	2.55	0.000004
ssc-miR-181b	OGT	O-linked N-acetylglucosamine (GlcNAc) transferase [Source:NCBI gene (formerly Entrezgene);Acc:664652]	2.55	0.000004
ssc-miR-181b	OLFM3	olfactomedin 3 [Source:VGNC Symbol;Acc:VGNC:91033]	2.55	0.000004
ssc-miR-181b	ONECUT2	one cut homeobox 2 [Source:VGNC Symbol;Acc:VGNC:91043]	2.55	0.000004
ssc-miR-181b	ONECUT3	one cut homeobox 3 [Source:VGNC Symbol;Acc:VGNC:91044]	2.55	0.000004
ssc-miR-181b	OSBPL3	oxysterol binding protein like 3 [Source:VGNC Symbol;Acc:VGNC:91071]	2.55	0.000004
ssc-miR-181b	OSBPL8	oxysterol binding protein like 8 [Source:VGNC Symbol;Acc:VGNC:91074]	2.55	0.000004
ssc-miR-181b	OTOGL	otogelin like [Source:VGNC Symbol;Acc:VGNC:91091]	2.55	0.000004
ssc-miR-181b	OTUD4	OTU deubiquitinase 4 [Source:VGNC Symbol;Acc:VGNC:91100]	2.55	0.000004
ssc-miR-181b	PACS2	phosphofurin acidic cluster sorting protein 2 [Source:VGNC Symbol;Acc:VGNC:91141]	2.55	0.000004
ssc-miR-181b	PAFAH1B1	platelet activating factor acetylhydrolase 1b regulatory subunit 1 [Source:VGNC Symbol;Acc:VGNC:99026]	2.55	0.000004
ssc-miR-181b	PAFAH1B2	platelet activating factor acetylhydrolase 1b catalytic subunit 2 [Source:VGNC Symbol;Acc:VGNC:91151]	2.55	0.000004
ssc-miR-181b	PAK4	p21 (RAC1) activated kinase 4 [Source:VGNC Symbol;Acc:VGNC:98526]	2.55	0.000004
ssc-miR-181b	PAK6	p21 (RAC1) activated kinase 6 [Source:VGNC Symbol;Acc:VGNC:103144]	2.55	0.000004
ssc-miR-181b	PAK7	hypothetical gene	2.55	0.000004
ssc-miR-181b	PALM2	hypothetical gene	2.55	0.000004
ssc-miR-181b	PAM	peptidylglycine alpha-amidating monooxygenase [Source:VGNC Symbol;Acc:VGNC:91163]	2.55	0.000004
ssc-miR-181b	PAN3	poly(A) specific ribonuclease subunit PAN3 [Source:HGNC Symbol;Acc:HGNC:29991]	2.55	0.000004
ssc-miR-181b	PANK3	pantothenate kinase 3 [Source:VGNC Symbol;Acc:VGNC:91165]	2.55	0.000004
ssc-miR-181b	PANK4	pantothenate kinase 4 (inactive) [Source:VGNC Symbol;Acc:VGNC:98527]	2.55	0.000004
ssc-miR-181b	PAPD5	hypothetical gene	2.55	0.000004
ssc-miR-181b	PAPOLG	poly(A) polymerase gamma [Source:HGNC Symbol;Acc:HGNC:14982]	2.55	0.000004
ssc-miR-181b	PAQR3	progestin and adipoQ receptor family member 3 [Source:VGNC Symbol;Acc:VGNC:91174]	2.55	0.000004
ssc-miR-181b	PARK2	hypothetical gene	2.55	0.000004
ssc-miR-181b	PARM1	prostate androgen-regulated mucin-like protein 1 [Source:VGNC Symbol;Acc:VGNC:91182]	2.55	0.000004
ssc-miR-181b	PARP2	poly(ADP-ribose) polymerase 2 [Source:HGNC Symbol;Acc:HGNC:272]	2.55	0.000004
ssc-miR-181b	PAWR	pro-apoptotic WT1 regulator [Source:VGNC Symbol;Acc:VGNC:91191]	2.55	0.000004
ssc-miR-181b	PAX5	paired box 5 [Source:VGNC Symbol;Acc:VGNC:91194]	2.55	0.000004
ssc-miR-181b	PAX9	paired box 9 [Source:HGNC Symbol;Acc:HGNC:8623]	2.55	0.000004
ssc-miR-181b	PBX1	hypothetical gene	2.55	0.000004
ssc-miR-181b	PBX3	PBX homeobox 3 [Source:VGNC Symbol;Acc:VGNC:91205]	2.55	0.000004
ssc-miR-181b	PCDH11Y	hypothetical gene	2.55	0.000004
ssc-miR-181b	PCDHA1	hypothetical gene	2.55	0.000004
ssc-miR-181b	PCDHA10	hypothetical gene	2.55	0.000004
ssc-miR-181b	PCDHA11	hypothetical gene	2.55	0.000004
ssc-miR-181b	PCDHA12	hypothetical gene	2.55	0.000004
ssc-miR-181b	PCDHA13	protocadherin alpha 13 [Source:HGNC Symbol;Acc:HGNC:8667]	2.55	0.000004
ssc-miR-181b	PCDHA2	hypothetical gene	2.55	0.000004
ssc-miR-181b	PCDHA3	protocadherin alpha 3 [Source:HGNC Symbol;Acc:HGNC:8669]	2.55	0.000004
ssc-miR-181b	PCDHA4	hypothetical gene	2.55	0.000004
ssc-miR-181b	PCDHA5	hypothetical gene	2.55	0.000004
ssc-miR-181b	PCDHA6	hypothetical gene	2.55	0.000004
ssc-miR-181b	PCDHA7	hypothetical gene	2.55	0.000004
ssc-miR-181b	PCDHA8	hypothetical gene	2.55	0.000004
ssc-miR-181b	PCDHA9	hypothetical gene	2.55	0.000004

ssc-miR-181b	PCDHAC1	hypothetical gene	2.55	0.000004
ssc-miR-181b	PCDHAC2	protocadherin alpha subfamily C, 2 [Source:HGNC Symbol;Acc:HGNC:8677]	2.55	0.000004
ssc-miR-181b	PCNP	PEST proteolytic signal containing nuclear protein [Source:VGNC Symbol;Acc:VGNC:104018]	2.55	0.000004
ssc-miR-181b	PCSK1	proprotein convertase subtilisin/kexin type 1 [Source:VGNC Symbol;Acc:VGNC:91230]	2.55	0.000004
ssc-miR-181b	PDAP1	PDGFA associated protein 1 [Source:VGNC Symbol;Acc:VGNC:91239]	2.55	0.000004
ssc-miR-181b	PDCD4	programmed cell death 4 [Source:VGNC Symbol;Acc:VGNC:91244]	2.55	0.000004
ssc-miR-181b	PDCD6IP	hypothetical gene	2.55	0.000004
ssc-miR-181b	PDE10A	phosphodiesterase 10A [Source:VGNC Symbol;Acc:VGNC:91248]	2.55	0.000004
ssc-miR-181b	PDE3A	phosphodiesterase 3A [Source:VGNC Symbol;Acc:VGNC:91252]	2.55	0.000004
ssc-miR-181b	PDE5A	phosphodiesterase 5A [Source:VGNC Symbol;Acc:VGNC:91257]	2.55	0.000004
ssc-miR-181b	PDGFA	platelet derived growth factor subunit A [Source:VGNC Symbol;Acc:VGNC:91265]	2.55	0.000004
ssc-miR-181b	PDGFRA	platelet derived growth factor receptor alpha [Source:VGNC Symbol;Acc:VGNC:98179]	2.55	0.000004
ssc-miR-181b	PDHX	pyruvate dehydrogenase complex component X [Source:VGNC Symbol;Acc:VGNC:91270]	2.55	0.000004
ssc-miR-181b	PDIA6	protein disulfide isomerase family A member 6 [Source:VGNC Symbol;Acc:VGNC:91275]	2.55	0.000004
ssc-miR-181b	PDIK1L	PDLIM1 interacting kinase 1 like [Source:VGNC Symbol;Acc:VGNC:91276]	2.55	0.000004
ssc-miR-181b	PDK3	pyruvate dehydrogenase kinase 3 [Source:VGNC Symbol;Acc:VGNC:91279]	2.55	0.000004
ssc-miR-181b	PDK4	pyruvate dehydrogenase kinase 4 [Source:VGNC Symbol;Acc:VGNC:91280]	2.55	0.000004
ssc-miR-181b	PDP1	pyruvate dehyrogenase phosphatase catalytic subunit 1 [Source:VGNC Symbol;Acc:VGNC:91286]	2.55	0.000004
ssc-miR-181b	PDPK1	3-phosphoinositide dependent protein kinase 1 [Source:HGNC Symbol;Acc:HGNC:8816]	2.55	0.000004
ssc-miR-181b	PDXDC1	pyridoxal dependent decarboxylase domain containing 1 [Source:HGNC Symbol;Acc:HGNC:28995]	2.55	0.000004
ssc-miR-181b	PEAK1	pseudopodium enriched atypical kinase 1 [Source:VGNC Symbol;Acc:VGNC:91300]	2.55	0.000004
ssc-miR-181b	PET117	PET117 cytochrome c oxidase chaperone [Source:HGNC Symbol;Acc:HGNC:40045]	2.55	0.000004
ssc-miR-181b	PEX5L	peroxisomal biosis factor 5 like [Source:VGNC Symbol;Acc:VGNC:91329]	2.55	0.000004
ssc-miR-181b	PGM2L1	phosphoglucomutase 2 like 1 [Source:VGNC Symbol;Acc:VGNC:91357]	2.55	0.000004
ssc-miR-181b	PGR	progesterone receptor [Source:VGNC Symbol;Acc:VGNC:91362]	2.55	0.000004
ssc-miR-181b	PGRMC2	progesterone receptor membrane component 2 [Source:VGNC Symbol;Acc:VGNC:98952]	2.55	0.000004
ssc-miR-181b	PHACTR2	phosphatase and actin regulator 2 [Source:VGNC Symbol;Acc:VGNC:91366]	2.55	0.000004
ssc-miR-181b	PHACTR4	phosphatase and actin regulator 4 [Source:VGNC Symbol;Acc:VGNC:98531]	2.55	0.000004
ssc-miR-181b	PHC3	polyhomeotic homolog 3 [Source:VGNC Symbol;Acc:VGNC:91372]	2.55	0.000004
ssc-miR-181b	PHF15	hypothetical gene	2.55	0.000004
ssc-miR-181b	PHF2	PHD finger protein 2 [Source:VGNC Symbol;Acc:VGNC:91382]	2.55	0.000004
ssc-miR-181b	PHF3	PHD finger protein 3 [Source:VGNC Symbol;Acc:VGNC:91387]	2.55	0.000004
ssc-miR-181b	PHIP	pleckstrin homology domain interacting protein [Source:VGNC Symbol;Acc:VGNC:91392]	2.55	0.000004
ssc-miR-181b	PHLDA1	pleckstrin homology like domain family A member 1 [Source:HGNC Symbol;Acc:HGNC:8933]	2.55	0.000004
ssc-miR-181b	PHLDB2	pleckstrin homology like domain family B member 2 [Source:VGNC Symbol;Acc:VGNC:91399]	2.55	0.000004
ssc-miR-181b	PHLPP2	PH domain and leucine rich repeat protein phosphatase 2 [Source:VGNC Symbol;Acc:VGNC:98186]	2.55	0.000004
ssc-miR-181b	PHOX2B	paired like homeobox 2B [Source:VGNC Symbol;Acc:VGNC:91402]	2.55	0.000004
ssc-miR-181b	PHTF2	putative homeodomain transcription factor 2 [Source:VGNC Symbol;Acc:VGNC:91405]	2.55	0.000004
ssc-miR-181b	PI4K2A	phosphatidylinositol 4-kinase type 2 alpha [Source:HGNC Symbol;Acc:HGNC:30031]	2.55	0.000004
ssc-miR-181b	PI4K2B	phosphatidylinositol 4-kinase type 2 beta [Source:VGNC Symbol;Acc:VGNC:98190]	2.55	0.000004
ssc-miR-181b	PIAS1	protein inhibitor of activated STAT 1 [Source:VGNC Symbol;Acc:VGNC:91410]	2.55	0.000004
ssc-miR-181b	PICALM	phosphatidylinositol binding clathrin assembly protein [Source:VGNC Symbol;Acc:VGNC:91415]	2.55	0.000004
ssc-miR-181b	PIK3C2A	phosphatidylinositol-4-phosphate 3-kinase catalytic subunit type 2 alpha [Source:VGNC Symbol;Acc:VGNC:91436]	2.55	0.000004
ssc-miR-181b	PIK3CB	phosphatidylinositol-4,5-bisphosphate 3-kinase catalytic subunit beta [Source:VGNC Symbol;Acc:VGNC:91441]	2.55	0.000004
ssc-miR-181b	PIK3R1	phosphoinositide-3-kinase regulatory subunit 1 [Source:VGNC Symbol;Acc:VGNC:91445]	2.55	0.000004
ssc-miR-181b	PIK3R3	phosphoinositide-3-kinase regulatory subunit 3 [Source:HGNC Symbol;Acc:HGNC:8981]	2.55	0.000004
ssc-miR-181b	PIP4K2B	phosphatidylinositol-5-phosphate 4-kinase type 2 beta [Source:VGNC Symbol;Acc:VGNC:91454]	2.55	0.000004
ssc-miR-181b	PITPNB	phosphatidylinositol transfer protein beta [Source:VGNC Symbol;Acc:VGNC:104026]	2.55	0.000004
ssc-miR-181b	PITX2	paired like homeodomain 2 [Source:VGNC Symbol;Acc:VGNC:91467]	2.55	0.000004

ssc-miR-181b	PJA2	praja ring finger ubiquitin ligase 2 [Source:VGNC Symbol;Acc:VGNC:91470]	2.55	0.000004
ssc-miR-181b	PKDCC	protein kinase domain containing, cytoplasmic [Source:VGNC Symbol;Acc:VGNC:91475]	2.55	0.000004
ssc-miR-181b	PKHD1	PKHD1 ciliary IPT domain containing fibrocystin/polyductin [Source:VGNC Symbol;Acc:VGNC:91476]	2.55	0.000004
ssc-miR-181b	PKN2	protein kinase N2 [Source:VGNC Symbol;Acc:VGNC:91482]	2.55	0.000004
ssc-miR-181b	PKNOX1	PBX/knotted 1 homeobox 1 [Source:VGNC Symbol;Acc:VGNC:91484]	2.55	0.000004
ssc-miR-181b	PKNOX2	PBX/knotted 1 homeobox 2 [Source:VGNC Symbol;Acc:VGNC:91485]	2.55	0.000004
ssc-miR-181b	PKP2	plakophilin 2 [Source:VGNC Symbol;Acc:VGNC:91486]	2.55	0.000004
ssc-miR-181b	PLA2G4A	phospholipase A2 group IVA [Source:VGNC Symbol;Acc:VGNC:91495]	2.55	0.000004
ssc-miR-181b	PLAG1	PLAG1 zinc finger [Source:VGNC Symbol;Acc:VGNC:91509]	2.55	0.000004
ssc-miR-181b	PLAU	plasminogen activator, urokinase [Source:VGNC Symbol;Acc:VGNC:91511]	2.55	0.000004
ssc-miR-181b	PLCB1	phospholipase C beta 1 [Source:VGNC Symbol;Acc:VGNC:95706]	2.55	0.000004
ssc-miR-181b	PLCL2	phospholipase C like 2 [Source:VGNC Symbol;Acc:VGNC:91523]	2.55	0.000004
ssc-miR-181b	PLCXD3	phosphatidylinositol specific phospholipase C X domain containing 3 [Source:VGNC Symbol;Acc:VGNC:91524]	2.55	0.000004
ssc-miR-181b	PLEK	pleckstrin [Source:VGNC Symbol;Acc:VGNC:91531]	2.55	0.000004
ssc-miR-181b	PLEKHA3	pleckstrin homology domain containing A3 [Source:NCBI gene (formerly Entrezgene);Acc:100154010]	2.55	0.000004
ssc-miR-181b	PLEKHG3	pleckstrin homology and RhoGEF domain containing G3 [Source:VGNC Symbol;Acc:VGNC:91544]	2.55	0.000004
ssc-miR-181b	PLOD3	procollagen-lysine,2-oxoglutarate 5-dioxygenase 3 [Source:VGNC Symbol;Acc:VGNC:91568]	2.55	0.000004
ssc-miR-181b	PLXDC2	plexin domain containing 2 [Source:VGNC Symbol;Acc:VGNC:95958]	2.55	0.000004
ssc-miR-181b	PLXNC1	plexin C1 [Source:VGNC Symbol;Acc:VGNC:91584]	2.55	0.000004
ssc-miR-181b	PMAIP1	phorbol-12-myristate-13-acetate-induced protein 1 [Source:NCBI gene (formerly Entrezgene);Acc:397278]	2.55	0.000004
ssc-miR-181b	PNISR	PNN interacting serine and arginine rich protein [Source:VGNC Symbol;Acc:VGNC:91594]	2.55	0.000004
ssc-miR-181b	PNRC1	proline rich nuclear receptor coactivator 1 [Source:VGNC Symbol;Acc:VGNC:91613]	2.55	0.000004
ssc-miR-181b	PNRC2	proline rich nuclear receptor coactivator 2 [Source:VGNC Symbol;Acc:VGNC:91614]	2.55	0.000004
ssc-miR-181b	POC1B-GALNT4	hypothetical gene	2.55	0.000004
ssc-miR-181b	PODXL	hypothetical gene	2.55	0.000004
ssc-miR-181b	POLA1	DNA polymerase alpha 1, catalytic subunit [Source:VGNC Symbol;Acc:VGNC:91626]	2.55	0.000004
ssc-miR-181b	POLQ	DNA polymerase theta [Source:VGNC Symbol;Acc:VGNC:91641]	2.55	0.000004
ssc-miR-181b	POM121C	hypothetical gene	2.55	0.000004
ssc-miR-181b	POU2F1	POU class 2 homeobox 1 [Source:VGNC Symbol;Acc:VGNC:91672]	2.55	0.000004
ssc-miR-181b	POU3F3	POU class 3 homeobox 3 [Source:VGNC Symbol;Acc:VGNC:91676]	2.55	0.000004
ssc-miR-181b	PPAP2B	hypothetical gene	2.55	0.000004
ssc-miR-181b	PPARA	peroxisome proliferator activated receptor alpha [Source:VGNC Symbol;Acc:VGNC:91682]	2.55	0.000004
ssc-miR-181b	PPFIA1	PTPRF interacting protein alpha 1 [Source:VGNC Symbol;Acc:VGNC:91691]	2.55	0.000004
ssc-miR-181b	PPIP5K2	diphosphoinositol pentakisphosphate kinase 2 [Source:VGNC Symbol;Acc:VGNC:91701]	2.55	0.000004
ssc-miR-181b	PPP1CB	protein phosphatase 1 catalytic subunit beta [Source:NCBI gene (formerly Entrezgene);Acc:397378]	2.55	0.000004
ssc-miR-181b	PPP1R11	protein phosphatase 1 regulatory inhibitor subunit 11 [Source:VGNC Symbol;Acc:VGNC:91718]	2.55	0.000004
ssc-miR-181b	PPP1R12B	protein phosphatase 1 regulatory subunit 12B [Source:VGNC Symbol;Acc:VGNC:96430]	2.55	0.000004
ssc-miR-181b	PPP1R2	hypothetical gene	2.55	0.000004
ssc-miR-181b	PPP1R3B	protein phosphatase 1 regulatory subunit 3B [Source:VGNC Symbol;Acc:VGNC:95636]	2.55	0.000004
ssc-miR-181b	PPP1R3E	protein phosphatase 1 regulatory subunit 3E [Source:HGNC Symbol;Acc:HGNC:14943]	2.55	0.000004
ssc-miR-181b	PPP2R2C	protein phosphatase 2 regulatory subunit Bgamma [Source:VGNC Symbol;Acc:VGNC:91749]	2.55	0.000004
ssc-miR-181b	PPP2R5E	protein phosphatase 2 regulatory subunit B'epsilon [Source:VGNC Symbol;Acc:VGNC:91754]	2.55	0.000004
ssc-miR-181b	PPP3R1	protein phosphatase 3 regulatory subunit B, alpha [Source:VGNC Symbol;Acc:VGNC:106453]	2.55	0.000004
ssc-miR-181b	PPP4R2	protein phosphatase 4 regulatory subunit 2 [Source:VGNC Symbol;Acc:VGNC:91756]	2.55	0.000004
ssc-miR-181b	PPP6C	protein phosphatase 6 catalytic subunit [Source:VGNC Symbol;Acc:VGNC:98219]	2.55	0.000004
ssc-miR-181b	PRDM1	PR/SET domain 1 [Source:VGNC Symbol;Acc:VGNC:91772]	2.55	0.000004
ssc-miR-181b	PRDM16	PR/SET domain 16 [Source:VGNC Symbol;Acc:VGNC:91777]	2.55	0.000004
ssc-miR-181b	PRDM4	PR/SET domain 4 [Source:VGNC Symbol;Acc:VGNC:91779]	2.55	0.000004
ssc-miR-181b	PRICKLE2	prickle planar cell polarity protein 2 [Source:VGNC Symbol;Acc:VGNC:91794]	2.55	0.000004

ssc-miR-181b	PRIMA1	proline rich membrane anchor 1 [Source:HGNC Symbol;Acc:HGNC:18319]	2.55	0.000004
ssc-miR-181b	PRKAA1	protein kinase AMP-activated catalytic subunit alpha 1 [Source:VGNC Symbol;Acc:VGNC:91797]	2.55	0.000004
ssc-miR-181b	PRKACB	protein kinase cAMP-activated catalytic subunit beta [Source:VGNC Symbol;Acc:VGNC:91800]	2.55	0.000004
ssc-miR-181b	PRKAR2A	protein kinase cAMP-dependent type II regulatory subunit alpha [Source:VGNC Symbol;Acc:VGNC:91804]	2.55	0.000004
ssc-miR-181b	PRKCD	protein kinase C delta [Source:VGNC Symbol;Acc:VGNC:91806]	2.55	0.000004
ssc-miR-181b	PRKCE	protein kinase C epsilon [Source:VGNC Symbol;Acc:VGNC:91807]	2.55	0.000004
ssc-miR-181b	PRKG1	protein kinase cGMP-dependent 1 [Source:VGNC Symbol;Acc:VGNC:91816]	2.55	0.000004
ssc-miR-181b	PRLR	prolactin receptor [Source:VGNC Symbol;Acc:VGNC:91819]	2.55	0.000004
ssc-miR-181b	PROSC	hypothetical gene	2.55	0.000004
ssc-miR-181b	PROSER1	proline and serine rich 1 [Source:VGNC Symbol;Acc:VGNC:91836]	2.55	0.000004
ssc-miR-181b	PROX1	prospero homeobox 1 [Source:VGNC Symbol;Acc:VGNC:91837]	2.55	0.000004
ssc-miR-181b	PROX2	prospero homeobox 2 [Source:VGNC Symbol;Acc:VGNC:91838]	2.55	0.000004
ssc-miR-181b	PRPF40A	pre-mRNA processing factor 40 homolog A [Source:VGNC Symbol;Acc:VGNC:98222]	2.55	0.000004
ssc-miR-181b	PRRC2C	proline rich coiled-coil 2C [Source:VGNC Symbol;Acc:VGNC:91867]	2.55	0.000004
ssc-miR-181b	PRRG1	proline rich and Gla domain 1 [Source:VGNC Symbol;Acc:VGNC:101494]	2.55	0.000004
ssc-miR-181b	PSAP	prosaposin [Source:NCBI gene (formerly Entrezgene);Acc:100153167]	2.55	0.000004
ssc-miR-181b	PSD3	pleckstrin and Sec7 domain containing 3 [Source:VGNC Symbol;Acc:VGNC:107166]	2.55	0.000004
ssc-miR-181b	PSPC1	paraspeckle component 1 [Source:VGNC Symbol;Acc:VGNC:91930]	2.55	0.000004
ssc-miR-181b	PTBP2	polypyrimidine tract binding protein 2 [Source:VGNC Symbol;Acc:VGNC:91937]	2.55	0.000004
ssc-miR-181b	PTBP3	polypyrimidine tract binding protein 3 [Source:VGNC Symbol;Acc:VGNC:91938]	2.55	0.000004
ssc-miR-181b	PTEN	hypothetical gene	2.55	0.000004
ssc-miR-181b	PTGER3	prostaglandin E receptor 3 [Source:VGNC Symbol;Acc:VGNC:98225]	2.55	0.000004
ssc-miR-181b	PTGR2	prostaglandin reductase 2 [Source:HGNC Symbol;Acc:HGNC:20149]	2.55	0.000004
ssc-miR-181b	PTGS2	prostaglandin-endoperoxide synthase 2 [Source:VGNC Symbol;Acc:VGNC:91959]	2.55	0.000004
ssc-miR-181b	PTPN4	protein tyrosine phosphatase non-receptor type 4 [Source:VGNC Symbol;Acc:VGNC:96521]	2.55	0.000004
ssc-miR-181b	PTPN9	protein tyrosine phosphatase non-receptor type 9 [Source:VGNC Symbol;Acc:VGNC:91983]	2.55	0.000004
ssc-miR-181b	PTPRE	protein tyrosine phosphatase receptor type E [Source:VGNC Symbol;Acc:VGNC:91986]	2.55	0.000004
ssc-miR-181b	PTPRR	protein tyrosine phosphatase receptor type R [Source:HGNC Symbol;Acc:HGNC:9680]	2.55	0.000004
ssc-miR-181b	PUM1	pumilio RNA binding family member 1 [Source:VGNC Symbol;Acc:VGNC:92001]	2.55	0.000004
ssc-miR-181b	PURB	purine rich element binding protein B [Source:VGNC Symbol;Acc:VGNC:92004]	2.55	0.000004
ssc-miR-181b	QDPR	quinoid dihydropteridine reductase [Source:VGNC Symbol;Acc:VGNC:98232]	2.55	0.000004
ssc-miR-181b	QKI	QKI, KH domain containing RNA binding [Source:VGNC Symbol;Acc:VGNC:92025]	2.55	0.000004
ssc-miR-181b	QSER1	glutamine and serine rich 1 [Source:VGNC Symbol;Acc:VGNC:92032]	2.55	0.000004
ssc-miR-181b	RAB11A	RAB11A, member RAS onco family [Source:VGNC Symbol;Acc:VGNC:98236]	2.55	0.000004
ssc-miR-181b	RAB11FIP2	RAB11 family interacting protein 2 [Source:VGNC Symbol;Acc:VGNC:92039]	2.55	0.000004
ssc-miR-181b	RAB22A	RAB22A, member RAS onco family [Source:VGNC Symbol;Acc:VGNC:98244]	2.55	0.000004
ssc-miR-181b	RAB27B	RAB27B, member RAS onco family [Source:VGNC Symbol;Acc:VGNC:98250]	2.55	0.000004
ssc-miR-181b	RAB30	RAB30, member RAS onco family [Source:VGNC Symbol;Acc:VGNC:98254]	2.55	0.000004
ssc-miR-181b	RAB3C	RAB3C, member RAS onco family [Source:VGNC Symbol;Acc:VGNC:104043]	2.55	0.000004
ssc-miR-181b	RAB3GAP2	RAB3 GTPase activating non-catalytic protein subunit 2 [Source:VGNC Symbol;Acc:VGNC:95833]	2.55	0.000004
ssc-miR-181b	RAB3IL1	RAB3A interacting protein like 1 [Source:VGNC Symbol;Acc:VGNC:92042]	2.55	0.000004
ssc-miR-181b	RAB3IP	RAB3A interacting protein [Source:HGNC Symbol;Acc:HGNC:16508]	2.55	0.000004
ssc-miR-181b	RAB6B	RAB6B, member RAS onco family [Source:VGNC Symbol;Acc:VGNC:98272]	2.55	0.000004
ssc-miR-181b	RAB8B	RAB8B, member RAS onco family [Source:VGNC Symbol;Acc:VGNC:98276]	2.55	0.000004
ssc-miR-181b	RABGEF1	RAB guanine nucleotide exchange factor 1 [Source:VGNC Symbol;Acc:VGNC:108510]	2.55	0.000004
ssc-miR-181b	RAD21	RAD21 cohesin complex component [Source:VGNC Symbol;Acc:VGNC:92054]	2.55	0.000004
ssc-miR-181b	RAD23B	RAD23 homolog B, nucleotide excision repair protein [Source:VGNC Symbol;Acc:VGNC:103160]	2.55	0.000004
ssc-miR-181b	RAD51L3-RFFL	hypothetical gene	2.55	0.000004
ssc-miR-181b	RALA	RAS like proto-oncogene A [Source:HGNC Symbol;Acc:HGNC:9839]	2.55	0.000004

ssc-miR-181b	RALBP1	ralA binding protein 1 [Source:VGNC Symbol;Acc:VGNC:92070]	2.55	0.000004
ssc-miR-181b	RALGAPA2	Ral GTPase activating protein catalytic subunit alpha 2 [Source:VGNC Symbol;Acc:VGNC:95454]	2.55	0.000004
ssc-miR-181b	RALGAPB	Ral GTPase activating protein non-catalytic subunit beta [Source:VGNC Symbol;Acc:VGNC:95463]	2.55	0.000004
ssc-miR-181b	RAN	hypothetical gene	2.55	0.000004
ssc-miR-181b	RANBP3	RAN binding protein 3 [Source:VGNC Symbol;Acc:VGNC:92079]	2.55	0.000004
ssc-miR-181b	RAP1A	RAP1A, member of RAS onco family [Source:VGNC Symbol;Acc:VGNC:92084]	2.55	0.000004
ssc-miR-181b	RAP1B	RAP1B, member of RAS oncogene family [Source:HGNC Symbol;Acc:HGNC:9857]	2.55	0.000004
ssc-miR-181b	RAPGEF2	Rap guanine nucleotide exchange factor 2 [Source:VGNC Symbol;Acc:VGNC:92092]	2.55	0.000004
ssc-miR-181b	RASA2	RAS p21 protein activator 2 [Source:VGNC Symbol;Acc:VGNC:92102]	2.55	0.000004
ssc-miR-181b	RASAL2	RAS protein activator like 2 [Source:VGNC Symbol;Acc:VGNC:92105]	2.55	0.000004
ssc-miR-181b	RASL10B	RAS like family 10 member B [Source:VGNC Symbol;Acc:VGNC:92118]	2.55	0.000004
ssc-miR-181b	RASSF1	Ras association domain family member 1 [Source:VGNC Symbol;Acc:VGNC:92122]	2.55	0.000004
ssc-miR-181b	RASSF2	Ras association domain family member 2 [Source:VGNC Symbol;Acc:VGNC:96531]	2.55	0.000004
ssc-miR-181b	RASSF6	Ras association domain family member 6 [Source:VGNC Symbol;Acc:VGNC:92126]	2.55	0.000004
ssc-miR-181b	RASSF8	Ras association domain family member 8 [Source:VGNC Symbol;Acc:VGNC:92128]	2.55	0.000004
ssc-miR-181b	RBAK	RB associated KRAB zinc finger [Source:VGNC Symbol;Acc:VGNC:92134]	2.55	0.000004
ssc-miR-181b	RBBP7	RB binding protein 7, chromatin remodeling factor [Source:VGNC Symbol;Acc:VGNC:92137]	2.55	0.000004
ssc-miR-181b	RBM22	RNA binding motif protein 22 [Source:VGNC Symbol;Acc:VGNC:92151]	2.55	0.000004
ssc-miR-181b	RBM25	RNA binding motif protein 25 [Source:NCBI gene (formerly Entrezgene);Acc:100511390]	2.55	0.000004
ssc-miR-181b	RBM26	RNA binding motif protein 26 [Source:VGNC Symbol;Acc:VGNC:92153]	2.55	0.000004
ssc-miR-181b	RBM27	RNA binding motif protein 27 [Source:HGNC Symbol;Acc:HGNC:29243]	2.55	0.000004
ssc-miR-181b	RBM47	RNA binding motif protein 47 [Source:VGNC Symbol;Acc:VGNC:92158]	2.55	0.000004
ssc-miR-181b	RBPMS	RNA binding protein, mRNA processing factor [Source:VGNC Symbol;Acc:VGNC:95904]	2.55	0.000004
ssc-miR-181b	RCAN1	regulator of calcineurin 1 [Source:VGNC Symbol;Acc:VGNC:92170]	2.55	0.000004
ssc-miR-181b	RCAN3	RCAN family member 3 [Source:VGNC Symbol;Acc:VGNC:92172]	2.55	0.000004
ssc-miR-181b	RCN2	reticulocalbin 2 [Source:VGNC Symbol;Acc:VGNC:92181]	2.55	0.000004
ssc-miR-181b	RCOR1	REST corepressor 1 [Source:VGNC Symbol;Acc:VGNC:92183]	2.55	0.000004
ssc-miR-181b	RECK	reversion inducing cysteine rich protein with kazal motifs [Source:VGNC Symbol;Acc:VGNC:92194]	2.55	0.000004
ssc-miR-181b	RFFL	ring finger and FYVE like domain containing E3 ubiquitin protein ligase [Source:VGNC Symbol;Acc:VGNC:98289]	2.55	0.000004
ssc-miR-181b	RFTN2	raftlin family member 2 [Source:VGNC Symbol;Acc:VGNC:96128]	2.55	0.000004
ssc-miR-181b	RFX2	regulatory factor X2 [Source:VGNC Symbol;Acc:VGNC:92244]	2.55	0.000004
ssc-miR-181b	RFX3	regulatory factor X3 [Source:VGNC Symbol;Acc:VGNC:92245]	2.55	0.000004
ssc-miR-181b	RFX5	regulatory factor X5 [Source:VGNC Symbol;Acc:VGNC:92246]	2.55	0.000004
ssc-miR-181b	RGMA	repulsive guidance molecule BMP co-receptor a [Source:VGNC Symbol;Acc:VGNC:92254]	2.55	0.000004
ssc-miR-181b	RGPD4	hypothetical gene	2.55	0.000004
ssc-miR-181b	RGPD6	hypothetical gene	2.55	0.000004
ssc-miR-181b	RIMKLB	ribosomal modification protein rimK like family member B [Source:VGNC Symbol;Acc:VGNC:92308]	2.55	0.000004
ssc-miR-181b	RIMS3	regulating synaptic membrane exocytosis 3 [Source:VGNC Symbol;Acc:VGNC:92310]	2.55	0.000004
ssc-miR-181b	RIN2	hypothetical gene	2.55	0.000004
ssc-miR-181b	RLF	RLF zinc finger [Source:VGNC Symbol;Acc:VGNC:92328]	2.55	0.000004
ssc-miR-181b	RLIM	ring finger protein, LIM domain interacting [Source:HGNC Symbol;Acc:HGNC:13429]	2.55	0.000004
ssc-miR-181b	RMND5A	required for meiotic nuclear division 5 homolog A [Source:VGNC Symbol;Acc:VGNC:92336]	2.55	0.000004
ssc-miR-181b	RNF103-CHMP3	hypothetical gene	2.55	0.000004
ssc-miR-181b	RNF125	ring finger protein 125 [Source:VGNC Symbol;Acc:VGNC:92352]	2.55	0.000004
ssc-miR-181b	RNF141	ring finger protein 141 [Source:VGNC Symbol;Acc:VGNC:98292]	2.55	0.000004
ssc-miR-181b	RNF144B	ring finger protein 144B [Source:VGNC Symbol;Acc:VGNC:92359]	2.55	0.000004
ssc-miR-181b	RNF145	ring finger protein 145 [Source:VGNC Symbol;Acc:VGNC:92360]	2.55	0.000004
ssc-miR-181b	RNF146	ring finger protein 146 [Source:VGNC Symbol;Acc:VGNC:92361]	2.55	0.000004
ssc-miR-181b	RNF150	ring finger protein 150 [Source:VGNC Symbol;Acc:VGNC:98293]	2.55	0.000004

ssc-miR-181b	RNF152	ring finger protein 152 [Source:VGNC Symbol;Acc:VGNC:92364]	2.55	0.000004
ssc-miR-181b	RNF169	ring finger protein 169 [Source:VGNC Symbol;Acc:VGNC:92368]	2.55	0.000004
ssc-miR-181b	RNF182	ring finger protein 182 [Source:VGNC Symbol;Acc:VGNC:92372]	2.55	0.000004
ssc-miR-181b	RNF217	ring finger protein 217 [Source:VGNC Symbol;Acc:VGNC:103162]	2.55	0.000004
ssc-miR-181b	RNF32	hypothetical gene	2.55	0.000004
ssc-miR-181b	RNF34	ring finger protein 34 [Source:VGNC Symbol;Acc:VGNC:92389]	2.55	0.000004
ssc-miR-181b	RNF38	ring finger protein 38 [Source:VGNC Symbol;Acc:VGNC:92390]	2.55	0.000004
ssc-miR-181b	RNF43	ring finger protein 43 [Source:VGNC Symbol;Acc:VGNC:99034]	2.55	0.000004
ssc-miR-181b	RNF8	ring finger protein 8 [Source:HGNC Symbol;Acc:HGNC:10071]	2.55	0.000004
ssc-miR-181b	ROBO2	roundabout guidance receptor 2 [Source:HGNC Symbol;Acc:HGNC:10250]	2.55	0.000004
ssc-miR-181b	RORA	RAR related orphan receptor A [Source:VGNC Symbol;Acc:VGNC:92408]	2.55	0.000004
ssc-miR-181b	RORB	RAR related orphan receptor B [Source:VGNC Symbol;Acc:VGNC:92409]	2.55	0.000004
ssc-miR-181b	RP11-644F5.10	hypothetical gene	2.55	0.000004
ssc-miR-181b	RPL34	ribosomal protein L34 [Source:VGNC Symbol;Acc:VGNC:98957]	2.55	0.000004
ssc-miR-181b	RPS6KA3	ribosomal protein S6 kinase A3 [Source:VGNC Symbol;Acc:VGNC:92442]	2.55	0.000004
ssc-miR-181b	RPS6KA6	ribosomal protein S6 kinase A6 [Source:VGNC Symbol;Acc:VGNC:92445]	2.55	0.000004
ssc-miR-181b	RPS6KB1	ribosomal protein S6 kinase B1 [Source:VGNC Symbol;Acc:VGNC:99037]	2.55	0.000004
ssc-miR-181b	RRAS2	hypothetical gene	2.55	0.000004
ssc-miR-181b	RSF1	remodeling and spacing factor 1 [Source:VGNC Symbol;Acc:VGNC:92476]	2.55	0.000004
ssc-miR-181b	RSPO2	R-spondin 2 [Source:VGNC Symbol;Acc:VGNC:92484]	2.55	0.000004
ssc-miR-181b	RTKN2	rhotekin 2 [Source:VGNC Symbol;Acc:VGNC:92495]	2.55	0.000004
ssc-miR-181b	RUFY3	RUN and FYVE domain containing 3 [Source:VGNC Symbol;Acc:VGNC:98959]	2.55	0.000004
ssc-miR-181b	RUNX1	RUNX family transcription factor 1 [Source:VGNC Symbol;Acc:VGNC:92516]	2.55	0.000004
ssc-miR-181b	RYR3	ryanodine receptor 3 [Source:HGNC Symbol;Acc:HGNC:10485]	2.55	0.000004
ssc-miR-181b	S1PR1	sphingosine-1-phosphate receptor 1 [Source:VGNC Symbol;Acc:VGNC:92551]	2.55	0.000004
ssc-miR-181b	SACM1L	SAC1 like phosphatidylinositide phosphatase [Source:VGNC Symbol;Acc:VGNC:92557]	2.55	0.000004
ssc-miR-181b	SALL3	spalt like transcription factor 3 [Source:VGNC Symbol;Acc:VGNC:92563]	2.55	0.000004
ssc-miR-181b	SALL4	spalt like transcription factor 4 [Source:NCBI gene (formerly Entrezgene);Acc:100136902]	2.55	0.000004
ssc-miR-181b	SAMD12	sterile alpha motif domain containing 12 [Source:VGNC Symbol;Acc:VGNC:92565]	2.55	0.000004
ssc-miR-181b	SAMD4A	sterile alpha motif domain containing 4A [Source:VGNC Symbol;Acc:VGNC:92569]	2.55	0.000004
ssc-miR-181b	SAMHD1	SAM and HD domain containing deoxynucleoside triphosphate triphosphohydrolase 1 [Source:VGNC Symbol;Acc:VGNC:95712]	2.55	0.000004
ssc-miR-181b	SAP30L	SAP30 like [Source:VGNC Symbol;Acc:VGNC:92577]	2.55	0.000004
ssc-miR-181b	SAR1B	secretion associated Ras related GTPase 1B [Source:VGNC Symbol;Acc:VGNC:92580]	2.55	0.000004
ssc-miR-181b	SBNO1	strawberry notch homolog 1 [Source:VGNC Symbol;Acc:VGNC:92596]	2.55	0.000004
ssc-miR-181b	SCAI	suppressor of cancer cell invasion [Source:VGNC Symbol;Acc:VGNC:103170]	2.55	0.000004
ssc-miR-181b	SCAMP2	secretory carrier membrane protein 2 [Source:VGNC Symbol;Acc:VGNC:92606]	2.55	0.000004
ssc-miR-181b	SCD	stearoyl-CoA desaturase [Source:NCBI gene (formerly Entrezgene);Acc:396670]	2.55	0.000004
ssc-miR-181b	SCHIP1	hypothetical gene	2.55	0.000004
ssc-miR-181b	SCML2	Scm polycomb group protein like 2 [Source:VGNC Symbol;Acc:VGNC:92628]	2.55	0.000004
ssc-miR-181b	SCOC	short coiled-coil protein [Source:VGNC Symbol;Acc:VGNC:98960]	2.55	0.000004
ssc-miR-181b	SDC2	syndecan 2 [Source:VGNC Symbol;Acc:VGNC:92655]	2.55	0.000004
ssc-miR-181b	SEC24C	SEC24 homolog C, COPII coat complex component [Source:VGNC Symbol;Acc:VGNC:92678]	2.55	0.000004
ssc-miR-181b	SECISBP2	SECIS binding protein 2 [Source:VGNC Symbol;Acc:VGNC:92685]	2.55	0.000004
ssc-miR-181b	SEL1L	SEL1L adaptor subunit of ERAD E3 ubiquitin ligase [Source:VGNC Symbol;Acc:VGNC:92687]	2.55	0.000004
ssc-miR-181b	SELK	hypothetical gene	2.55	0.000004
ssc-miR-181b	SEMA3C	semaphorin 3C [Source:VGNC Symbol;Acc:VGNC:92695]	2.55	0.000004
ssc-miR-181b	SEMA4C	semaphorin 4C [Source:VGNC Symbol;Acc:VGNC:92702]	2.55	0.000004
ssc-miR-181b	SEMA4F	ssemaphorin 4F [Source:VGNC Symbol;Acc:VGNC:92704]	2.55	0.000004
ssc-miR-181b	SEMA4G	semaphorin 4G [Source:VGNC Symbol;Acc:VGNC:92705]	2.55	0.000004

ssc-miR-181b	SENP1	SUMO specific peptidase 1 [Source:VGNC Symbol;Acc:VGNC:92712]	2.55	0.000004
ssc-miR-181b	SENP2	SUMO specific peptidase 2 [Source:VGNC Symbol;Acc:VGNC:92713]	2.55	0.000004
ssc-miR-181b	SENP5	SUMO specific peptidase 5 [Source:VGNC Symbol;Acc:VGNC:92714]	2.55	0.000004
ssc-miR-181b	SEPT11	hypothetical gene	2.55	0.000004
ssc-miR-181b	SEPT3	hypothetical gene	2.55	0.000004
ssc-miR-181b	SEPT8	hypothetical gene	2.55	0.000004
ssc-miR-181b	SERPINE1	serpin family E member 1 [Source:VGNC Symbol;Acc:VGNC:98310]	2.55	0.000004
ssc-miR-181b	SERTAD2	SERTA domain containing 2 [Source:VGNC Symbol;Acc:VGNC:92750]	2.55	0.000004
ssc-miR-181b	SERTAD4	SERTA domain containing 4 [Source:VGNC Symbol;Acc:VGNC:92752]	2.55	0.000004
ssc-miR-181b	SESN3	sestrin 3 [Source:VGNC Symbol;Acc:VGNC:92755]	2.55	0.000004
ssc-miR-181b	SF1	splicing factor 1 [Source:HGNC Symbol;Acc:HGNC:12950]	2.55	0.000004
ssc-miR-181b	SFMBT1	Scm like with four mbt domains 1 [Source:VGNC Symbol;Acc:VGNC:92773]	2.55	0.000004
ssc-miR-181b	SFRP4	secreted frizzled related protein 4 [Source:VGNC Symbol;Acc:VGNC:92776]	2.55	0.000004
ssc-miR-181b	SGIP1	SH3GL interacting endocytic adaptor 1 [Source:VGNC Symbol;Acc:VGNC:92792]	2.55	0.000004
ssc-miR-181b	SGK3	serum/glucocorticoid regulated kinase family member 3 [Source:HGNC Symbol;Acc:HGNC:10812]	2.55	0.000004
ssc-miR-181b	SGPP1	sphingosine-1-phosphate phosphatase 1 [Source:VGNC Symbol;Acc:VGNC:92798]	2.55	0.000004
ssc-miR-181b	SGTB	small glutamine rich tetratricopeptide repeat co-chaperone beta [Source:VGNC Symbol;Acc:VGNC:92804]	2.55	0.000004
ssc-miR-181b	SH2B3	SH2B adaptor protein 3 [Source:VGNC Symbol;Acc:VGNC:92807]	2.55	0.000004
ssc-miR-181b	SHC3	SHC adaptor protein 3 [Source:VGNC Symbol;Acc:VGNC:92838]	2.55	0.000004
ssc-miR-181b	SHE	Src homology 2 domain containing E [Source:VGNC Symbol;Acc:VGNC:92842]	2.55	0.000004
ssc-miR-181b	SHH	sonic hedgehog signaling molecule [Source:VGNC Symbol;Acc:VGNC:92844]	2.55	0.000004
ssc-miR-181b	SHOC2	SHOC2 leucine rich repeat scaffold protein [Source:VGNC Symbol;Acc:VGNC:98318]	2.55	0.000004
ssc-miR-181b	SHROOM3	hypothetical gene	2.55	0.000004
ssc-miR-181b	SIAE	sialic acid acetylesterase [Source:VGNC Symbol;Acc:VGNC:92864]	2.55	0.000004
ssc-miR-181b	SIK3	SIK family kinase 3 [Source:VGNC Symbol;Acc:VGNC:92873]	2.55	0.000004
ssc-miR-181b	SIM1	SIM bHLH transcription factor 1 [Source:VGNC Symbol;Acc:VGNC:92876]	2.55	0.000004
ssc-miR-181b	SIN3B	SIN3 transcription regulator family member B [Source:VGNC Symbol;Acc:VGNC:92880]	2.55	0.000004
ssc-miR-181b	SIPA1L1	signal induced proliferation associated 1 like 1 [Source:VGNC Symbol;Acc:VGNC:92882]	2.55	0.000004
ssc-miR-181b	SIPA1L2	signal induced proliferation associated 1 like 2 [Source:VGNC Symbol;Acc:VGNC:92883]	2.55	0.000004
ssc-miR-181b	SIRT1	sirtuin 1 [Source:VGNC Symbol;Acc:VGNC:92884]	2.55	0.000004
ssc-miR-181b	SIX2	SIX homeobox 2 [Source:VGNC Symbol;Acc:VGNC:92893]	2.55	0.000004
ssc-miR-181b	SIX4	SIX homeobox 4 [Source:VGNC Symbol;Acc:VGNC:92895]	2.55	0.000004
ssc-miR-181b	SLA	Src like adaptor [Source:VGNC Symbol;Acc:VGNC:92908]	2.55	0.000004
ssc-miR-181b	SLAIN2	SLAIN motif family member 2 [Source:VGNC Symbol;Acc:VGNC:92910]	2.55	0.000004
ssc-miR-181b	SLC10A7	solute carrier family 10 member 7 [Source:VGNC Symbol;Acc:VGNC:92919]	2.55	0.000004
ssc-miR-181b	SLC12A5	solute carrier family 12 member 5 [Source:VGNC Symbol;Acc:VGNC:95594]	2.55	0.000004
ssc-miR-181b	SLC16A12	solute carrier family 16 member 12 [Source:VGNC Symbol;Acc:VGNC:92940]	2.55	0.000004
ssc-miR-181b	SLC16A6	solute carrier family 16 member 6 [Source:VGNC Symbol;Acc:VGNC:92946]	2.55	0.000004
ssc-miR-181b	SLC16A7	solute carrier family 16 member 7 [Source:VGNC Symbol;Acc:VGNC:92947]	2.55	0.000004
ssc-miR-181b	SLC19A2	solute carrier family 19 member 2 [Source:VGNC Symbol;Acc:VGNC:92962]	2.55	0.000004
ssc-miR-181b	SLC25A12	solute carrier family 25 member 12 [Source:VGNC Symbol;Acc:VGNC:95502]	2.55	0.000004
ssc-miR-181b	SLC25A16	solute carrier family 25 member 16 [Source:VGNC Symbol;Acc:VGNC:92995]	2.55	0.000004
ssc-miR-181b	SLC25A24	solute carrier family 25 member 24 [Source:VGNC Symbol;Acc:VGNC:93001]	2.55	0.000004
ssc-miR-181b	SLC25A25	solute carrier family 25 member 25 [Source:VGNC Symbol;Acc:VGNC:93002]	2.55	0.000004
ssc-miR-181b	SLC25A36	solute carrier family 25 member 36 [Source:VGNC Symbol;Acc:VGNC:93012]	2.55	0.000004
ssc-miR-181b	SLC25A37	hypothetical gene	2.55	0.000004
ssc-miR-181b	SLC25A4	solute carrier family 25 member 4 [Source:VGNC Symbol;Acc:VGNC:95503]	2.55	0.000004
ssc-miR-181b	SLC25A53	solute carrier family 25 member 53 [Source:VGNC Symbol;Acc:VGNC:93023]	2.55	0.000004
ssc-miR-181b	SLC26A4	solute carrier family 26 member 4 [Source:VGNC Symbol;Acc:VGNC:93029]	2.55	0.000004

ssc-miR-181b	SLC26A9	solute carrier family 26 member 9 [Source:VGNC Symbol;Acc:VGNC:93034]	2.55	0.000004
ssc-miR-181b	SLC2A14	hypothetical gene	2.55	0.000004
ssc-miR-181b	SLC2A3	hypothetical gene	2.55	0.000004
ssc-miR-181b	SLC30A1	solute carrier family 30 member 1 [Source:VGNC Symbol;Acc:VGNC:93055]	2.55	0.000004
ssc-miR-181b	SLC35A3	solute carrier family 35 member A3 [Source:VGNC Symbol;Acc:VGNC:98860]	2.55	0.000004
ssc-miR-181b	SLC35E1	solute carrier family 35 member E1 [Source:VGNC Symbol;Acc:VGNC:93077]	2.55	0.000004
ssc-miR-181b	SLC35F1	solute carrier family 35 member F1 [Source:VGNC Symbol;Acc:VGNC:93081]	2.55	0.000004
ssc-miR-181b	SLC35F3	solute carrier family 35 member F3 [Source:VGNC Symbol;Acc:VGNC:93083]	2.55	0.000004
ssc-miR-181b	SLC35G1	solute carrier family 35 member G1 [Source:VGNC Symbol;Acc:VGNC:93086]	2.55	0.000004
ssc-miR-181b	SLC37A3	solute carrier family 37 member 3 [Source:VGNC Symbol;Acc:VGNC:98324]	2.55	0.000004
ssc-miR-181b	SLC38A2	solute carrier family 38 member 2 [Source:VGNC Symbol;Acc:VGNC:93094]	2.55	0.000004
ssc-miR-181b	SLC44A1	solute carrier family 44 member 1 [Source:VGNC Symbol;Acc:VGNC:93118]	2.55	0.000004
ssc-miR-181b	SLC45A4	solute carrier family 45 member 4 [Source:VGNC Symbol;Acc:VGNC:93125]	2.55	0.000004
ssc-miR-181b	SLC4A10	solute carrier family 4 member 10 [Source:VGNC Symbol;Acc:VGNC:95592]	2.55	0.000004
ssc-miR-181b	SLC4A8	solute carrier family 4 member 8 [Source:VGNC Symbol;Acc:VGNC:98326]	2.55	0.000004
ssc-miR-181b	SLC5A7	solute carrier family 5 member 7 [Source:VGNC Symbol;Acc:VGNC:93147]	2.55	0.000004
ssc-miR-181b	SLC6A2	solute carrier family 6 member 2 [Source:VGNC Symbol;Acc:VGNC:93161]	2.55	0.000004
ssc-miR-181b	SLC7A11	solute carrier family 7 member 11 [Source:VGNC Symbol;Acc:VGNC:93171]	2.55	0.000004
ssc-miR-181b	SLC8A1	solute carrier family 8 member A1 [Source:VGNC Symbol;Acc:VGNC:93178]	2.55	0.000004
ssc-miR-181b	SLC9A6	solute carrier family 9 member A6 [Source:VGNC Symbol;Acc:VGNC:93188]	2.55	0.000004
ssc-miR-181b	SLC9A8	solute carrier family 9 member A8 [Source:VGNC Symbol;Acc:VGNC:95943]	2.55	0.000004
ssc-miR-181b	SLIT1	slit guidance ligand 1 [Source:VGNC Symbol;Acc:VGNC:107171]	2.55	0.000004
ssc-miR-181b	SLITRK1	SLIT and NTRK like family member 1 [Source:VGNC Symbol;Acc:VGNC:93206]	2.55	0.000004
ssc-miR-181b	SLITRK3	SLIT and NTRK like family member 3 [Source:VGNC Symbol;Acc:VGNC:93208]	2.55	0.000004
ssc-miR-181b	SLITRK4	SLIT and NTRK like family member 4 [Source:VGNC Symbol;Acc:VGNC:93209]	2.55	0.000004
ssc-miR-181b	SLMAP	sarcolemma associated protein [Source:VGNC Symbol;Acc:VGNC:93212]	2.55	0.000004
ssc-miR-181b	SLX4	SLX4 structure-specific endonuclease subunit [Source:VGNC Symbol;Acc:VGNC:93215]	2.55	0.000004
ssc-miR-181b	SMAD2	SMAD family member 2 [Source:VGNC Symbol;Acc:VGNC:98329]	2.55	0.000004
ssc-miR-181b	SMAD7	SMAD family member 7 [Source:VGNC Symbol;Acc:VGNC:93221]	2.55	0.000004
ssc-miR-181b	SMAP1	small ArfGAP 1 [Source:VGNC Symbol;Acc:VGNC:93223]	2.55	0.000004
ssc-miR-181b	SMCO1	single-pass membrane protein with coiled-coil domains 1 [Source:VGNC Symbol;Acc:VGNC:93243]	2.55	0.000004
ssc-miR-181b	SMG7	SMG7 nonsense mediated mRNA decay factor [Source:VGNC Symbol;Acc:VGNC:93250]	2.55	0.000004
ssc-miR-181b	SMNDC1	survival motor neuron domain containing 1 [Source:VGNC Symbol;Acc:VGNC:93257]	2.55	0.000004
ssc-miR-181b	SNAI2	snail family transcriptional repressor 2 [Source:VGNC Symbol;Acc:VGNC:98330]	2.55	0.000004
ssc-miR-181b	SNAP25	synaptosome associated protein 25 [Source:VGNC Symbol;Acc:VGNC:95509]	2.55	0.000004
ssc-miR-181b	SNN	stannin [Source:HGNC Symbol;Acc:HGNC:11149]	2.55	0.000004
ssc-miR-181b	SNTB1	syntrophin beta 1 [Source:VGNC Symbol;Acc:VGNC:93297]	2.55	0.000004
ssc-miR-181b	SNTB2	syntrophin beta 2 [Source:VGNC Symbol;Acc:VGNC:93298]	2.55	0.000004
ssc-miR-181b	SNX30	sorting nexin family member 30 [Source:VGNC Symbol;Acc:VGNC:93318]	2.55	0.000004
ssc-miR-181b	SOCS2	suppressor of cytokine signaling 2 [Source:NCBI gene (formerly Entrezgene);Acc:100037966]	2.55	0.000004
ssc-miR-181b	SOCS3	suppressor of cytokine signaling 3 [Source:VGNC Symbol;Acc:VGNC:99052]	2.55	0.000004
ssc-miR-181b	SOCS4	suppressor of cytokine signaling 4 [Source:VGNC Symbol;Acc:VGNC:93331]	2.55	0.000004
ssc-miR-181b	SOGA2	hypothetical gene	2.55	0.000004
ssc-miR-181b	SORBS1	sorbin and SH3 domain containing 1 [Source:VGNC Symbol;Acc:VGNC:93337]	2.55	0.000004
ssc-miR-181b	SOS1	SOS Ras/Rac guanine nucleotide exchange factor 1 [Source:HGNC Symbol;Acc:HGNC:11187]	2.55	0.000004
ssc-miR-181b	SOWAHA	sosondowah ankyrin repeat domain family member A [Source:VGNC Symbol;Acc:VGNC:93347]	2.55	0.000004
ssc-miR-181b	SOWAHC	hypothetical gene	2.55	0.000004
ssc-miR-181b	SOX5	SRY-box transcription factor 5 [Source:VGNC Symbol;Acc:VGNC:93357]	2.55	0.000004
ssc-miR-181b	SOX6	SRY-box transcription factor 6 [Source:VGNC Symbol;Acc:VGNC:93358]	2.55	0.000004

ssc-miR-181b	SP1	Sp1 transcription factor [Source:VGNC Symbol;Acc:VGNC:93360]	2.55	0.000004
ssc-miR-181b	SPAG9	sperm associated antigen 9 [Source:VGNC Symbol;Acc:VGNC:93374]	2.55	0.000004
ssc-miR-181b	SPATS2	spermatogenesis associated serine rich 2 [Source:HGNC Symbol;Acc:HGNC:18650]	2.55	0.000004
ssc-miR-181b	SPECC1L	sperm antigen with calponin homology and coiled-coil domains 1 like [Source:HGNC Symbol;Acc:HGNC:29022]	2.55	0.000004
ssc-miR-181b	SPICE1	spindle and centriole associated protein 1 [Source:HGNC Symbol;Acc:HGNC:25083]	2.55	0.000004
ssc-miR-181b	SPIRE1	spire type actin nucleation factor 1 [Source:VGNC Symbol;Acc:VGNC:98336]	2.55	0.000004
ssc-miR-181b	SPP1	secreted phosphoprotein 1 [Source:VGNC Symbol;Acc:VGNC:93419]	2.55	0.000004
ssc-miR-181b	SPRY4	sprouty RTK signaling antagonist 4 [Source:VGNC Symbol;Acc:VGNC:93427]	2.55	0.000004
ssc-miR-181b	SPTY2D1	SPT2 chromatin protein domain containing 1 [Source:VGNC Symbol;Acc:VGNC:100867]	2.55	0.000004
ssc-miR-181b	SRCIN1	SRC kinase signaling inhibitor 1 [Source:VGNC Symbol;Acc:VGNC:93448]	2.55	0.000004
ssc-miR-181b	SRGAP1	SLIT-ROBO Rho GTPase activating protein 1 [Source:VGNC Symbol;Acc:VGNC:93454]	2.55	0.000004
ssc-miR-181b	SRGAP2	hypothetical gene	2.55	0.000004
ssc-miR-181b	SRPK2	SRSF protein kinase 2 [Source:VGNC Symbol;Acc:VGNC:93463]	2.55	0.000004
ssc-miR-181b	SRSF10	serine and arginine rich splicing factor 10 [Source:VGNC Symbol;Acc:VGNC:93472]	2.55	0.000004
ssc-miR-181b	SRSF11	serine and arginine rich splicing factor 11 [Source:VGNC Symbol;Acc:VGNC:93473]	2.55	0.000004
ssc-miR-181b	SRSF5	serine and arginine rich splicing factor 5 [Source:VGNC Symbol;Acc:VGNC:93476]	2.55	0.000004
ssc-miR-181b	SRSF7	serine and arginine rich splicing factor 7 [Source:VGNC Symbol;Acc:VGNC:93477]	2.55	0.000004
ssc-miR-181b	SS18L1	SS18L1 subunit of BAF chromatin remodeling complex [Source:VGNC Symbol;Acc:VGNC:98342]	2.55	0.000004
ssc-miR-181b	SSBP2	single stranded DNA binding protein 2 [Source:VGNC Symbol;Acc:VGNC:93480]	2.55	0.000004
ssc-miR-181b	SSX2IP	SSX family member 2 interacting protein [Source:VGNC Symbol;Acc:VGNC:93499]	2.55	0.000004
ssc-miR-181b	ST8SIA3	ST8 alpha-N-acetyl-neuraminide alpha-2,8-sialyltransferase 3 [Source:VGNC Symbol;Acc:VGNC:93518]	2.55	0.000004
ssc-miR-181b	ST8SIA4	ST8 alpha-N-acetyl-neuraminide alpha-2,8-sialyltransferase 4 [Source:VGNC Symbol;Acc:VGNC:93519]	2.55	0.000004
ssc-miR-181b	STAT3	signal transducer and activator of transcription 3 [Source:VGNC Symbol;Acc:VGNC:93540]	2.55	0.000004
ssc-miR-181b	STAU1	staufen double-stranded RNA binding protein 1 [Source:VGNC Symbol;Acc:VGNC:95525]	2.55	0.000004
ssc-miR-181b	STC1	stanniocalcin 1 [Source:VGNC Symbol;Acc:VGNC:93542]	2.55	0.000004
ssc-miR-181b	STIM2	stromal interaction molecule 2 [Source:VGNC Symbol;Acc:VGNC:98967]	2.55	0.000004
ssc-miR-181b	STK10	serine/threonine kinase 10 [Source:HGNC Symbol;Acc:HGNC:11388]	2.55	0.000004
ssc-miR-181b	STOX2	storkhead box 2 [Source:VGNC Symbol;Acc:VGNC:96082]	2.55	0.000004
ssc-miR-181b	STRN	striatin [Source:VGNC Symbol;Acc:VGNC:93578]	2.55	0.000004
ssc-miR-181b	STXBP5	syntaxin binding protein 5 [Source:VGNC Symbol;Acc:VGNC:93599]	2.55	0.000004
ssc-miR-181b	STXBP6	syntaxin binding protein 6 [Source:NCBI gene (formerly Entrezgene);Acc:100514852]	2.55	0.000004
ssc-miR-181b	SUDS3	SDS3 homolog, SIN3A corepressor complex component [Source:VGNC Symbol;Acc:VGNC:93607]	2.55	0.000004
ssc-miR-181b	SUGT1	SGT1 homolog, MIS12 kinetochore complex assembly cochaperone [Source:VGNC Symbol;Acc:VGNC:93611]	2.55	0.000004
ssc-miR-181b	SUMF1	sulfatase modifying factor 1 [Source:VGNC Symbol;Acc:VGNC:93617]	2.55	0.000004
ssc-miR-181b	SUN1	Sad1 and UNC84 domain containing 1 [Source:VGNC Symbol;Acc:VGNC:93618]	2.55	0.000004
ssc-miR-181b	SVIP	small VCP interacting protein [Source:HGNC Symbol;Acc:HGNC:25238]	2.55	0.000004
ssc-miR-181b	SYNE1	hypothetical gene	2.55	0.000004
ssc-miR-181b	SYNGR2	synaptogyrin 2 [Source:HGNC Symbol;Acc:HGNC:11499]	2.55	0.000004
ssc-miR-181b	SYNPR	synaptoporin [Source:VGNC Symbol;Acc:VGNC:93674]	2.55	0.000004
ssc-miR-181b	SYPL2	synaptophysin like 2 [Source:VGNC Symbol;Acc:VGNC:93677]	2.55	0.000004
ssc-miR-181b	SYT11	synaptotagmin 11 [Source:VGNC Symbol;Acc:VGNC:93680]	2.55	0.000004
ssc-miR-181b	SYT3	synaptotagmin 3 [Source:VGNC Symbol;Acc:VGNC:93684]	2.55	0.000004
ssc-miR-181b	TAB3	TGF-beta activated kinase 1 (MAP3K7) binding protein 3 [Source:HGNC Symbol;Acc:HGNC:30681]	2.55	0.000004
ssc-miR-181b	TADA2B	transcriptional adaptor 2B [Source:VGNC Symbol;Acc:VGNC:93707]	2.55	0.000004
ssc-miR-181b	TAF13	TATA-box binding protein associated factor 13 [Source:VGNC Symbol;Acc:VGNC:93710]	2.55	0.000004
ssc-miR-181b	TAF4	TATA-box binding protein associated factor 4 [Source:VGNC Symbol;Acc:VGNC:95532]	2.55	0.000004
ssc-miR-181b	TAF9B	TATA-box binding protein associated factor 9b [Source:VGNC Symbol;Acc:VGNC:93725]	2.55	0.000004
ssc-miR-181b	TANC2	tetratricopeptide repeat, ankyrin repeat and coiled-coil containing 2 [Source:VGNC Symbol;Acc:VGNC:93733]	2.55	0.000004
ssc-miR-181b	TAOK1	TAO kinase 1 [Source:VGNC Symbol;Acc:VGNC:98355]	2.55	0.000004

ssc-miR-181b	TARDBP	TAR DNA binding protein [Source:VGNC Symbol;Acc:VGNC:98624]	2.55	0.000004
ssc-miR-181b	TBC1D1	TBC1 domain family member 1 [Source:VGNC Symbol;Acc:VGNC:93758]	2.55	0.000004
ssc-miR-181b	TBC1D14	hypothetical gene	2.55	0.000004
ssc-miR-181b	TBC1D15	TBC1 domain family member 15 [Source:VGNC Symbol;Acc:VGNC:93764]	2.55	0.000004
ssc-miR-181b	TBC1D24	TBC1 domain family member 24 [Source:HGNC Symbol;Acc:HGNC:29203]	2.55	0.000004
ssc-miR-181b	TBC1D25	TBC1 domain family member 25 [Source:VGNC Symbol;Acc:VGNC:93773]	2.55	0.000004
ssc-miR-181b	TBC1D2B	TBC1 domain family member 2B [Source:VGNC Symbol;Acc:VGNC:93774]	2.55	0.000004
ssc-miR-181b	TBC1D4	TBC1 domain family member 4 [Source:HGNC Symbol;Acc:HGNC:19165]	2.55	0.000004
ssc-miR-181b	TBC1D9	TBC1 domain family member 9 [Source:VGNC Symbol;Acc:VGNC:93781]	2.55	0.000004
ssc-miR-181b	TBCEL	tubulin folding cofactor E like [Source:HGNC Symbol;Acc:HGNC:28115]	2.55	0.000004
ssc-miR-181b	TBL1X	hypothetical gene	2.55	0.000004
ssc-miR-181b	TBL1XR1	TBL1X receptor 1 [Source:VGNC Symbol;Acc:VGNC:96600]	2.55	0.000004
ssc-miR-181b	TBL1Y	hypothetical gene	2.55	0.000004
ssc-miR-181b	TBPL1	TATA-box binding protein like 1 [Source:VGNC Symbol;Acc:VGNC:93792]	2.55	0.000004
ssc-miR-181b	TBX18	T-box transcription factor 18 [Source:VGNC Symbol;Acc:VGNC:103187]	2.55	0.000004
ssc-miR-181b	TCERG1	transcription elongation regulator 1 [Source:VGNC Symbol;Acc:VGNC:93815]	2.55	0.000004
ssc-miR-181b	TCF7L2	transcription factor 7 like 2 [Source:VGNC Symbol;Acc:VGNC:93825]	2.55	0.000004
ssc-miR-181b	TDRD6	tudor domain containing 6 [Source:VGNC Symbol;Acc:VGNC:93850]	2.55	0.000004
ssc-miR-181b	TEAD1	TEA domain transcription factor 1 [Source:VGNC Symbol;Acc:VGNC:93853]	2.55	0.000004
ssc-miR-181b	TFAM	transcription factor A, mitochondrial [Source:NCBI gene (formerly Entrezgene);Acc:397279]	2.55	0.000004
ssc-miR-181b	TGFBR1	transforming growth factor beta receptor 1 [Source:VGNC Symbol;Acc:VGNC:98368]	2.55	0.000004
ssc-miR-181b	TGFBR2	transforming growth factor beta receptor 2 [Source:VGNC Symbol;Acc:VGNC:93931]	2.55	0.000004
ssc-miR-181b	TGFBR3	transforming growth factor beta receptor 3 [Source:VGNC Symbol;Acc:VGNC:93932]	2.55	0.000004
ssc-miR-181b	TGIF2	TGFB induced factor homeobox 2 [Source:VGNC Symbol;Acc:VGNC:95656]	2.55	0.000004
ssc-miR-181b	THAP1	THAP domain containing 1 [Source:VGNC Symbol;Acc:VGNC:95950]	2.55	0.000004
ssc-miR-181b	THBS2	thrombospondin 2 [Source:VGNC Symbol;Acc:VGNC:93947]	2.55	0.000004
ssc-miR-181b	THBS4	thrombospondin 4 [Source:VGNC Symbol;Acc:VGNC:93949]	2.55	0.000004
ssc-miR-181b	THRA	thyroid hormone receptor alpha [Source:VGNC Symbol;Acc:VGNC:93963]	2.55	0.000004
ssc-miR-181b	THRB	thyroid hormone receptor beta [Source:VGNC Symbol;Acc:VGNC:93965]	2.55	0.000004
ssc-miR-181b	TIAL1	TIA1 cytotoxic granule associated RNA binding protein like 1 [Source:VGNC Symbol;Acc:VGNC:104073]	2.55	0.000004
ssc-miR-181b	TIMP3	TIMP metallopeptidase inhibitor 3 [Source:VGNC Symbol;Acc:VGNC:93996]	2.55	0.000004
ssc-miR-181b	TLK1	tousled like kinase 1 [Source:VGNC Symbol;Acc:VGNC:95543]	2.55	0.000004
ssc-miR-181b	TLK2	tousled like kinase 2 [Source:VGNC Symbol;Acc:VGNC:94017]	2.55	0.000004
ssc-miR-181b	TLL1	tolloid like 1 [Source:VGNC Symbol;Acc:VGNC:94018]	2.55	0.000004
ssc-miR-181b	TM9SF3	transmembrane 9 superfamily member 3 [Source:VGNC Symbol;Acc:VGNC:94037]	2.55	0.000004
ssc-miR-181b	TM9SF4	transmembrane 9 superfamily member 4 [Source:VGNC Symbol;Acc:VGNC:96250]	2.55	0.000004
ssc-miR-181b	TMC7	transmembrane channel like 7 [Source:VGNC Symbol;Acc:VGNC:94044]	2.55	0.000004
ssc-miR-181b	TMCC1	transmembrane and coiled-coil domain family 1 [Source:VGNC Symbol;Acc:VGNC:94046]	2.55	0.000004
ssc-miR-181b	TMED4	transmembrane p24 trafficking protein 4 [Source:VGNC Symbol;Acc:VGNC:94056]	2.55	0.000004
ssc-miR-181b	TMED8	transmembrane p24 trafficking protein family member 8 [Source:VGNC Symbol;Acc:VGNC:94058]	2.55	0.000004
ssc-miR-181b	TMEFF1	transmembrane protein with EGF like and two follistatin like domains 1 [Source:HGNC Symbol;Acc:HGNC:11866]	2.55	0.000004
ssc-miR-181b	TMEM106B	transmembrane protein 106B [Source:VGNC Symbol;Acc:VGNC:104075]	2.55	0.000004
ssc-miR-181b	TMEM108	transmembrane protein 108 [Source:VGNC Symbol;Acc:VGNC:94065]	2.55	0.000004
ssc-miR-181b	TMEM127	transmembrane protein 127 [Source:HGNC Symbol;Acc:HGNC:26038]	2.55	0.000004
ssc-miR-181b	TMEM131	transmembrane protein 131 [Source:VGNC Symbol;Acc:VGNC:94078]	2.55	0.000004
ssc-miR-181b	TMEM132B	transmembrane protein 132B [Source:HGNC Symbol;Acc:HGNC:29397]	2.55	0.000004
ssc-miR-181b	TMEM132D	transmembrane protein 132D [Source:VGNC Symbol;Acc:VGNC:94082]	2.55	0.000004
ssc-miR-181b	TMEM134	transmembrane protein 134 [Source:VGNC Symbol;Acc:VGNC:94084]	2.55	0.000004
ssc-miR-181b	TMEM135	transmembrane protein 135 [Source:VGNC Symbol;Acc:VGNC:94085]	2.55	0.000004

ssc-miR-181b	TMEM151B	transmembrane protein 151B [Source:VGNC Symbol;Acc:VGNC:98376]	2.55	0.000004
ssc-miR-181b	TMEM161B	transmembrane protein 161B [Source:VGNC Symbol;Acc:VGNC:94102]	2.55	0.000004
ssc-miR-181b	TMEM165	transmembrane protein 165 [Source:VGNC Symbol;Acc:VGNC:94104]	2.55	0.000004
ssc-miR-181b	TMEM170A	transmembrane protein 170A [Source:VGNC Symbol;Acc:VGNC:98659]	2.55	0.000004
ssc-miR-181b	TMEM178B	transmembrane protein 178B [Source:HGNC Symbol;Acc:HGNC:44112]	2.55	0.000004
ssc-miR-181b	TMEM181	transmembrane protein 181 [Source:VGNC Symbol;Acc:VGNC:94117]	2.55	0.000004
ssc-miR-181b	TMEM242	transmembrane protein 242 [Source:HGNC Symbol;Acc:HGNC:17206]	2.55	0.000004
ssc-miR-181b	TMEM30A	transmembrane protein 30A [Source:VGNC Symbol;Acc:VGNC:94170]	2.55	0.000004
ssc-miR-181b	TMEM47	transmembrane protein 47 [Source:VGNC Symbol;Acc:VGNC:94184]	2.55	0.000004
ssc-miR-181b	TMEM64	transmembrane protein 64 [Source:VGNC Symbol;Acc:VGNC:94199]	2.55	0.000004
ssc-miR-181b	TMEM71	transmembrane protein 71 [Source:VGNC Symbol;Acc:VGNC:94205]	2.55	0.000004
ssc-miR-181b	TMEM87B	transmembrane protein 87B [Source:VGNC Symbol;Acc:VGNC:94213]	2.55	0.000004
ssc-miR-181b	TMF1	TATA element modulatory factor 1 [Source:VGNC Symbol;Acc:VGNC:94223]	2.55	0.000004
ssc-miR-181b	TMX3	thioredoxin related transmembrane protein 3 [Source:VGNC Symbol;Acc:VGNC:94248]	2.55	0.000004
ssc-miR-181b	TNF	tumor necrosis factor [Source:NCBI gene (formerly Entrezgene);Acc:397086]	2.55	0.000004
ssc-miR-181b	TNFAIP1	TNF alpha induced protein 1 [Source:VGNC Symbol;Acc:VGNC:94250]	2.55	0.000004
ssc-miR-181b	TNFRSF11B	TNF receptor superfamily member 11b [Source:HGNC Symbol;Acc:HGNC:11909]	2.55	0.000004
ssc-miR-181b	TNIK	TRAF2 and NCK interacting kinase [Source:VGNC Symbol;Acc:VGNC:98381]	2.55	0.000004
ssc-miR-181b	TNPO1	transportin 1 [Source:VGNC Symbol;Acc:VGNC:94287]	2.55	0.000004
ssc-miR-181b	TNRC6B	trinucleotide repeat containing adaptor 6B [Source:VGNC Symbol;Acc:VGNC:94293]	2.55	0.000004
ssc-miR-181b	TNS1	tensin 1 [Source:VGNC Symbol;Acc:VGNC:104082]	2.55	0.000004
ssc-miR-181b	TNS3	tensin 3 [Source:VGNC Symbol;Acc:VGNC:94296]	2.55	0.000004
ssc-miR-181b	TOPBP1	DNA topoisomerase II binding protein 1 [Source:VGNC Symbol;Acc:VGNC:94315]	2.55	0.000004
ssc-miR-181b	TOR1AIP2	torsin 1A interacting protein 2 [Source:VGNC Symbol;Acc:VGNC:94318]	2.55	0.000004
ssc-miR-181b	TOX	thymocyte selection associated high mobility group box [Source:VGNC Symbol;Acc:VGNC:94322]	2.55	0.000004
ssc-miR-181b	TPST2	tyrosylprotein sulfotransferase 2 [Source:NCBI gene (formerly Entrezgene);Acc:100154448]	2.55	0.000004
ssc-miR-181b	TRAF3IP2	TRAF3 interacting protein 2 [Source:VGNC Symbol;Acc:VGNC:94361]	2.55	0.000004
ssc-miR-181b	TRAF6	TNF receptor associated factor 6 [Source:VGNC Symbol;Acc:VGNC:94365]	2.55	0.000004
ssc-miR-181b	TRAK2	trafficking kinesin protein 2 [Source:VGNC Symbol;Acc:VGNC:95577]	2.55	0.000004
ssc-miR-181b	TRANK1	tetratricopeptide repeat and ankyrin repeat containing 1 [Source:VGNC Symbol;Acc:VGNC:94370]	2.55	0.000004
ssc-miR-181b	TRERF1	transcriptional regulating factor 1 [Source:VGNC Symbol;Acc:VGNC:94387]	2.55	0.000004
ssc-miR-181b	TRIM13	tripartite motif containing 13 [Source:VGNC Symbol;Acc:VGNC:94394]	2.55	0.000004
ssc-miR-181b	TRIM2	tripartite motif containing 2 [Source:VGNC Symbol;Acc:VGNC:94397]	2.55	0.000004
ssc-miR-181b	TRIM3	tripartite motif containing 3 [Source:HGNC Symbol;Acc:HGNC:10064]	2.55	0.000004
ssc-miR-181b	TRIM71	tripartite motif containing 71 [Source:VGNC Symbol;Acc:VGNC:94431]	2.55	0.000004
ssc-miR-181b	TRIP4	thyroid hormone receptor interactor 4 [Source:VGNC Symbol;Acc:VGNC:94438]	2.55	0.000004
ssc-miR-181b	TRNP1	TMF1 regulated nuclear protein 1 [Source:VGNC Symbol;Acc:VGNC:94456]	2.55	0.000004
ssc-miR-181b	TRPC1	transient receptor potential cation channel subfamily C member 1 [Source:VGNC Symbol;Acc:VGNC:94460]	2.55	0.000004
ssc-miR-181b	TRPM3	transient receptor potential cation channel subfamily M member 3 [Source:VGNC Symbol;Acc:VGNC:98384]	2.55	0.000004
ssc-miR-181b	TRPM7	transient receptor potential cation channel subfamily M member 7 [Source:VGNC Symbol;Acc:VGNC:94470]	2.55	0.000004
ssc-miR-181b	TRPV3	transient receptor potential cation channel subfamily V member 3 [Source:VGNC Symbol;Acc:VGNC:94475]	2.55	0.000004
ssc-miR-181b	TRRAP	transformation/transcription domain associated protein [Source:VGNC Symbol;Acc:VGNC:94477]	2.55	0.000004
ssc-miR-181b	TSC22D2	TSC22 domain family member 2 [Source:VGNC Symbol;Acc:VGNC:94482]	2.55	0.000004
ssc-miR-181b	TSFM	Ts translation elongation factor, mitochondrial [Source:VGNC Symbol;Acc:VGNC:94486]	2.55	0.000004
ssc-miR-181b	TSPAN13	tetraspanin 13 [Source:VGNC Symbol;Acc:VGNC:94502]	2.55	0.000004
ssc-miR-181b	TTBK1	tau tubulin kinase 1 [Source:VGNC Symbol;Acc:VGNC:98386]	2.55	0.000004
ssc-miR-181b	TTC30A	hypothetical gene	2.55	0.000004
ssc-miR-181b	TTC39A	tetratricopeptide repeat domain 39A [Source:VGNC Symbol;Acc:VGNC:94551]	2.55	0.000004
ssc-miR-181b	TTC39B	tetratricopeptide repeat domain 39B [Source:VGNC Symbol;Acc:VGNC:94552]	2.55	0.000004

ssc-miR-181b	TTL	hypothetical gene	2.55	0.000004
ssc-miR-181b	TTLL7	tubulin tyrosine ligase like 7 [Source:VGNC Symbol;Acc:VGNC:94571]	2.55	0.000004
ssc-miR-181b	TTYH3	tweety family member 3 [Source:VGNC Symbol;Acc:VGNC:94576]	2.55	0.000004
ssc-miR-181b	TUB	TUB bipartite transcription factor [Source:VGNC Symbol;Acc:VGNC:94577]	2.55	0.000004
ssc-miR-181b	TUBB	tubulin beta class I [Source:VGNC Symbol;Acc:VGNC:94578]	2.55	0.000004
ssc-miR-181b	TUBD1	tubulin delta 1 [Source:VGNC Symbol;Acc:VGNC:94582]	2.55	0.000004
ssc-miR-181b	TULP4	hypothetical gene	2.55	0.000004
ssc-miR-181b	TUSC3	tumor suppressor candidate 3 [Source:VGNC Symbol;Acc:VGNC:108742]	2.55	0.000004
ssc-miR-181b	TXK	TXK tyrosine kinase [Source:VGNC Symbol;Acc:VGNC:94599]	2.55	0.000004
ssc-miR-181b	TXNDC12	thioredoxin domain containing 12 [Source:VGNC Symbol;Acc:VGNC:94604]	2.55	0.000004
ssc-miR-181b	TXNDC15	thioredoxin domain containing 15 [Source:VGNC Symbol;Acc:VGNC:94605]	2.55	0.000004
ssc-miR-181b	TXNDC5	thioredoxin domain containing 5 [Source:VGNC Symbol;Acc:VGNC:94608]	2.55	0.000004
ssc-miR-181b	TXNL1	thioredoxin like 1 [Source:VGNC Symbol;Acc:VGNC:94611]	2.55	0.000004
ssc-miR-181b	UBA6	ubiquitin like modifier activating enzyme 6 [Source:VGNC Symbol;Acc:VGNC:94630]	2.55	0.000004
ssc-miR-181b	UBE2B	ubiquitin conjugating enzyme E2 B [Source:VGNC Symbol;Acc:VGNC:94640]	2.55	0.000004
ssc-miR-181b	UBE2D3	ubiquitin conjugating enzyme E2 D3 [Source:NCBI gene (formerly Entrezgene);Acc:780418]	2.55	0.000004
ssc-miR-181b	UBE2H	ubiquitin conjugating enzyme E2 H [Source:VGNC Symbol;Acc:VGNC:94643]	2.55	0.000004
ssc-miR-181b	UBE2J1	ubiquitin conjugating enzyme E2 J1 [Source:VGNC Symbol;Acc:VGNC:94644]	2.55	0.000004
ssc-miR-181b	UBE2L3	hypothetical gene	2.55	0.000004
ssc-miR-181b	UBE2N	hypothetical gene	2.55	0.000004
ssc-miR-181b	UBE2T	ubiquitin conjugating enzyme E2 T [Source:VGNC Symbol;Acc:VGNC:98392]	2.55	0.000004
ssc-miR-181b	UBE2W	ubiquitin conjugating enzyme E2 W [Source:VGNC Symbol;Acc:VGNC:98890]	2.55	0.000004
ssc-miR-181b	UBE3C	ubiquitin protein ligase E3C [Source:VGNC Symbol;Acc:VGNC:94654]	2.55	0.000004
ssc-miR-181b	UBN2	ubinuclein 2 [Source:VGNC Symbol;Acc:VGNC:94664]	2.55	0.000004
ssc-miR-181b	UBP1	upstream binding protein 1 [Source:VGNC Symbol;Acc:VGNC:108701]	2.55	0.000004
ssc-miR-181b	UBR2	ubiquitin protein ligase E3 component n-recognin 2 [Source:VGNC Symbol;Acc:VGNC:94669]	2.55	0.000004
ssc-miR-181b	UGCG	UDP-glucose ceramide glucosyltransferase [Source:VGNC Symbol;Acc:VGNC:94685]	2.55	0.000004
ssc-miR-181b	UNC5A	unc-5 netrin receptor A [Source:VGNC Symbol;Acc:VGNC:94707]	2.55	0.000004
ssc-miR-181b	UNC5D	unc-5 netrin receptor D [Source:VGNC Symbol;Acc:VGNC:95892]	2.55	0.000004
ssc-miR-181b	UNC80	unc-80 homolog, NALCN channel complex subunit [Source:VGNC Symbol;Acc:VGNC:96134]	2.55	0.000004
ssc-miR-181b	UNKL	unk like zinc finger [Source:VGNC Symbol;Acc:VGNC:94716]	2.55	0.000004
ssc-miR-181b	USP13	ubiquitin specific peptidase 13 [Source:VGNC Symbol;Acc:VGNC:94746]	2.55	0.000004
ssc-miR-181b	USP15	ubiquitin specific peptidase 15 [Source:VGNC Symbol;Acc:VGNC:94748]	2.55	0.000004
ssc-miR-181b	USP24	ubiquitin specific peptidase 24 [Source:VGNC Symbol;Acc:VGNC:94754]	2.55	0.000004
ssc-miR-181b	USP31	ubiquitin specific peptidase 31 [Source:HGNC Symbol;Acc:HGNC:20060]	2.55	0.000004
ssc-miR-181b	USP33	ubiquitin specific peptidase 33 [Source:HGNC Symbol;Acc:HGNC:20059]	2.55	0.000004
ssc-miR-181b	USP42	ubiquitin specific peptidase 42 [Source:HGNC Symbol;Acc:HGNC:20068]	2.55	0.000004
ssc-miR-181b	USP47	ubiquitin specific peptidase 47 [Source:VGNC Symbol;Acc:VGNC:94769]	2.55	0.000004
ssc-miR-181b	USP6NL	USP6 N-terminal like [Source:VGNC Symbol;Acc:VGNC:95820]	2.55	0.000004
ssc-miR-181b	USP8	ubiquitin specific peptidase 8 [Source:VGNC Symbol;Acc:VGNC:94778]	2.55	0.000004
ssc-miR-181b	USP9X	ubiquitin specific peptidase 9 X-linked [Source:HGNC Symbol;Acc:HGNC:12632]	2.55	0.000004
ssc-miR-181b	USP9Y	hypothetical gene	2.55	0.000004
ssc-miR-181b	VANGL1	VANGL planar cell polarity protein 1 [Source:VGNC Symbol;Acc:VGNC:94797]	2.55	0.000004
ssc-miR-181b	VCAM1	vascular cell adhesion molecule 1 [Source:VGNC Symbol;Acc:VGNC:94811]	2.55	0.000004
ssc-miR-181b	VCAN	versican [Source:VGNC Symbol;Acc:VGNC:108163]	2.55	0.000004
ssc-miR-181b	VCL	vinculin [Source:VGNC Symbol;Acc:VGNC:94812]	2.55	0.000004
ssc-miR-181b	VGLL4	vestigial like family member 4 [Source:VGNC Symbol;Acc:VGNC:94824]	2.55	0.000004
ssc-miR-181b	VHL	hypothetical gene	2.55	0.000004
ssc-miR-181b	VIP	vasoactive intestinal peptide [Source:VGNC Symbol;Acc:VGNC:103201]	2.55	0.000004

ssc-miR-181b	VKORC1L1	vitamin K epoxide reductase complex subunit 1 like 1 [Source:VGNC Symbol;Acc:VGNC:94831]	2.55	0.000004
ssc-miR-181b	VMP1	vacuole membrane protein 1 [Source:HGNC Symbol;Acc:HGNC:29559]	2.55	0.000004
ssc-miR-181b	VPS4A	vacuolar protein sorting 4 homolog A [Source:NCBI gene (formerly Entrezgene);Acc:100627841]	2.55	0.000004
ssc-miR-181b	VSX1	visual system homeobox 1 [Source:VGNC Symbol;Acc:VGNC:95566]	2.55	0.000004
ssc-miR-181b	WASF1	hypothetical gene	2.55	0.000004
ssc-miR-181b	WASL	WASP like actin nucleation promoting factor [Source:VGNC Symbol;Acc:VGNC:96604]	2.55	0.000004
ssc-miR-181b	WDFY2	WD repeat and FYVE domain containing 2 [Source:VGNC Symbol;Acc:VGNC:94902]	2.55	0.000004
ssc-miR-181b	WDFY3	WD repeat and FYVE domain containing 3 [Source:VGNC Symbol;Acc:VGNC:94903]	2.55	0.000004
ssc-miR-181b	WDR37	WD repeat domain 37 [Source:VGNC Symbol;Acc:VGNC:96258]	2.55	0.000004
ssc-miR-181b	WDR7	WD repeat domain 7 [Source:VGNC Symbol;Acc:VGNC:94936]	2.55	0.000004
ssc-miR-181b	WDR82	WD repeat domain 82 [Source:VGNC Symbol;Acc:VGNC:94944]	2.55	0.000004
ssc-miR-181b	WHAMM	WASP homolog associated with actin, golgi membranes and microtubules [Source:VGNC Symbol;Acc:VGNC:98414]	2.55	0.000004
ssc-miR-181b	WIF1	WNT inhibitory factor 1 [Source:VGNC Symbol;Acc:VGNC:94957]	2.55	0.000004
ssc-miR-181b	WNK1	WNK lysine deficient protein kinase 1 [Source:VGNC Symbol;Acc:VGNC:94962]	2.55	0.000004
ssc-miR-181b	WNK3	WNK lysine deficient protein kinase 3 [Source:VGNC Symbol;Acc:VGNC:94964]	2.55	0.000004
ssc-miR-181b	WNT9A	Wnt family member 9A [Source:VGNC Symbol;Acc:VGNC:94978]	2.55	0.000004
ssc-miR-181b	WSB2	WD repeat and SOCS box containing 2 [Source:VGNC Symbol;Acc:VGNC:94982]	2.55	0.000004
ssc-miR-181b	WSCD2	WSC domain containing 2 [Source:HGNC Symbol;Acc:HGNC:29117]	2.55	0.000004
ssc-miR-181b	WWC1	WW and C2 domain containing 1 [Source:VGNC Symbol;Acc:VGNC:94986]	2.55	0.000004
ssc-miR-181b	WWC2	WW and C2 domain containing 2 [Source:VGNC Symbol;Acc:VGNC:96033]	2.55	0.000004
ssc-miR-181b	XIAP	X-linked inhibitor of apoptosis [Source:NCBI gene (formerly Entrezgene);Acc:100037300]	2.55	0.000004
ssc-miR-181b	XK	X-linked Kx blood group [Source:HGNC Symbol;Acc:HGNC:12811]	2.55	0.000004
ssc-miR-181b	XPO7	exportin 7 [Source:VGNC Symbol;Acc:VGNC:95007]	2.55	0.000004
ssc-miR-181b	XRN1	5'-3' exoribonuclease 1 [Source:VGNC Symbol;Acc:VGNC:108705]	2.55	0.000004
ssc-miR-181b	XYLT1	xylosyltransferase 1 [Source:VGNC Symbol;Acc:VGNC:95015]	2.55	0.000004
ssc-miR-181b	YAP1	Yes1 associated transcriptional regulator [Source:VGNC Symbol;Acc:VGNC:95019]	2.55	0.000004
ssc-miR-181b	YIPF4	Yip1 domain family member 4 [Source:VGNC Symbol;Acc:VGNC:95030]	2.55	0.000004
ssc-miR-181b	YIPF6	Yip1 domain family member 6 [Source:VGNC Symbol;Acc:VGNC:95032]	2.55	0.000004
ssc-miR-181b	YLPM1	YLP motif containing 1 [Source:VGNC Symbol;Acc:VGNC:95034]	2.55	0.000004
ssc-miR-181b	YOD1	YOD1 deubiquitinase [Source:VGNC Symbol;Acc:VGNC:95035]	2.55	0.000004
ssc-miR-181b	YTHDC1	YTH domain containing 1 [Source:VGNC Symbol;Acc:VGNC:95041]	2.55	0.000004
ssc-miR-181b	YTHDC2	YTH domain containing 2 [Source:VGNC Symbol;Acc:VGNC:95042]	2.55	0.000004
ssc-miR-181b	YTHDF2	YTH N6-methyladenosine RNA binding protein 2 [Source:VGNC Symbol;Acc:VGNC:95043]	2.55	0.000004
ssc-miR-181b	YTHDF3	YTH N6-methyladenosine RNA binding protein 3 [Source:VGNC Symbol;Acc:VGNC:95044]	2.55	0.000004
ssc-miR-181b	YWHAB	tyrosine 3-monooxygenase/tryptophan 5-monooxygenase activation protein beta [Source:VGNC Symbol;Acc:VGNC:98417]	2.55	0.000004
ssc-miR-181b	YWHAG	hypothetical gene	2.55	0.000004
ssc-miR-181b	YWHAZ	tyrosine 3-monooxygenase/tryptophan 5-monooxygenase activation protein zeta [Source:VGNC Symbol;Acc:VGNC:95047]	2.55	0.000004
ssc-miR-181b	YY1	YY1 transcription factor [Source:HGNC Symbol;Acc:HGNC:12856]	2.55	0.000004
ssc-miR-181b	YY2	hypothetical gene	2.55	0.000004
ssc-miR-181b	ZADH2	prostaglandin reductase 3 [Source:VGNC Symbol;Acc:VGNC:95049]	2.55	0.000004
ssc-miR-181b	ZBTB20	zinc finger and BTB domain containing 20 [Source:VGNC Symbol;Acc:VGNC:95063]	2.55	0.000004
ssc-miR-181b	ZBTB34	zinc finger and BTB domain containing 34 [Source:VGNC Symbol;Acc:VGNC:95070]	2.55	0.000004
ssc-miR-181b	ZBTB4	hypothetical gene	2.55	0.000004
ssc-miR-181b	ZBTB41	zinc finger and BTB domain containing 41 [Source:VGNC Symbol;Acc:VGNC:108284]	2.55	0.000004
ssc-miR-181b	ZBTB43	zinc finger and BTB domain containing 43 [Source:VGNC Symbol;Acc:VGNC:95075]	2.55	0.000004
ssc-miR-181b	ZBTB7A	zinc finger and BTB domain containing 7A [Source:VGNC Symbol;Acc:VGNC:95081]	2.55	0.000004
ssc-miR-181b	ZBTB8A	zinc finger and BTB domain containing 8A [Source:VGNC Symbol;Acc:VGNC:95084]	2.55	0.000004
ssc-miR-181b	ZBTB8B	zinc finger and BTB domain containing 8B [Source:HGNC Symbol;Acc:HGNC:37057]	2.55	0.000004
ssc-miR-181b	ZC3H6	zinc finger CCCH-type containing 6 [Source:VGNC Symbol;Acc:VGNC:95098]	2.55	0.000004

ssc-miR-181b	ZCCHC14	zinc finger CCHC-type containing 14 [Source:VGNC Symbol;Acc:VGNC:95107]	2.55	0.000004
ssc-miR-181b	ZDHHC21	zinc finger DHHC-type palmitoyltransferase 21 [Source:VGNC Symbol;Acc:VGNC:95123]	2.55	0.000004
ssc-miR-181b	ZDHHC3	zinc finger DHHC-type palmitoyltransferase 3 [Source:VGNC Symbol;Acc:VGNC:95126]	2.55	0.000004
ssc-miR-181b	ZDHHC7	zinc finger DHHC-type palmitoyltransferase 7 [Source:VGNC Symbol;Acc:VGNC:95129]	2.55	0.000004
ssc-miR-181b	ZEB1	zinc finger E-box binding homeobox 1 [Source:VGNC Symbol;Acc:VGNC:95536]	2.55	0.000004
ssc-miR-181b	ZEB2	hypothetical gene	2.55	0.000004
ssc-miR-181b	ZFAND5	zinc finger AN1-type containing 5 [Source:VGNC Symbol;Acc:VGNC:95137]	2.55	0.000004
ssc-miR-181b	ZFAND6	zinc finger AN1-type containing 6 [Source:VGNC Symbol;Acc:VGNC:104102]	2.55	0.000004
ssc-miR-181b	ZFHX3	zinc finger homeobox 3 [Source:VGNC Symbol;Acc:VGNC:95141]	2.55	0.000004
ssc-miR-181b	ZFHX4	zinc finger homeobox 4 [Source:VGNC Symbol;Acc:VGNC:95142]	2.55	0.000004
ssc-miR-181b	ZFP1	ZFP1 zinc finger protein [Source:VGNC Symbol;Acc:VGNC:95143]	2.55	0.000004
ssc-miR-181b	ZFP14	ZFP14 zinc finger protein [Source:VGNC Symbol;Acc:VGNC:95144]	2.55	0.000004
ssc-miR-181b	ZFP30	ZFP30 zinc finger protein [Source:VGNC Symbol;Acc:VGNC:98741]	2.55	0.000004
ssc-miR-181b	ZFP36L1	ZFP36 ring finger protein like 1 [Source:VGNC Symbol;Acc:VGNC:95146]	2.55	0.000004
ssc-miR-181b	ZFP36L2	ZFP36 ring finger protein like 2 [Source:VGNC Symbol;Acc:VGNC:95147]	2.55	0.000004
ssc-miR-181b	ZFP62	ZFP62 zinc finger protein [Source:VGNC Symbol;Acc:VGNC:95148]	2.55	0.000004
ssc-miR-181b	ZFP69B	ZFP69 zinc finger protein B [Source:HGNC Symbol;Acc:HGNC:28053]	2.55	0.000004
ssc-miR-181b	ZFP82	ZFP82 zinc finger protein [Source:HGNC Symbol;Acc:HGNC:28682]	2.55	0.000004
ssc-miR-181b	ZFX	hypothetical gene	2.55	0.000004
ssc-miR-181b	ZIC2	Zic family member 2 [Source:VGNC Symbol;Acc:VGNC:95167]	2.55	0.000004
ssc-miR-181b	ZIC3	Zic family member 3 [Source:VGNC Symbol;Acc:VGNC:95168]	2.55	0.000004
ssc-miR-181b	ZIK1	hypothetical gene	2.55	0.000004
ssc-miR-181b	ZKSCAN1	zinc finger with KRAB and SCAN domains 1 [Source:VGNC Symbol;Acc:VGNC:95171]	2.55	0.000004
ssc-miR-181b	ZKSCAN5	zinc finger with KRAB and SCAN domains 5 [Source:VGNC Symbol;Acc:VGNC:95174]	2.55	0.000004
ssc-miR-181b	ZMYND11	zinc finger MYND-type containing 11 [Source:VGNC Symbol;Acc:VGNC:95827]	2.55	0.000004
ssc-miR-181b	ZNF136	hypothetical gene	2.55	0.000004
ssc-miR-181b	ZNF200	zinc finger protein 200 [Source:VGNC Symbol;Acc:VGNC:95203]	2.55	0.000004
ssc-miR-181b	ZNF207	zinc finger protein 207 [Source:VGNC Symbol;Acc:VGNC:95205]	2.55	0.000004
ssc-miR-181b	ZNF23	zinc finger protein 23 [Source:HGNC Symbol;Acc:HGNC:13023]	2.55	0.000004
ssc-miR-181b	ZNF236	zinc finger protein 236 [Source:VGNC Symbol;Acc:VGNC:95209]	2.55	0.000004
ssc-miR-181b	ZNF24	zinc finger protein 24 [Source:VGNC Symbol;Acc:VGNC:98422]	2.55	0.000004
ssc-miR-181b	ZNF256	hypothetical gene	2.55	0.000004
ssc-miR-181b	ZNF266	hypothetical gene	2.55	0.000004
ssc-miR-181b	ZNF268	zinc finger protein 268 [Source:HGNC Symbol;Acc:HGNC:13061]	2.55	0.000004
ssc-miR-181b	ZNF280B	zinc finger protein 280B [Source:HGNC Symbol;Acc:HGNC:23022]	2.55	0.000004
ssc-miR-181b	ZNF283	hypothetical gene	2.55	0.000004
ssc-miR-181b	ZNF286A	zinc finger protein 286A [Source:HGNC Symbol;Acc:HGNC:13501]	2.55	0.000004
ssc-miR-181b	ZNF286B	hypothetical gene	2.55	0.000004
ssc-miR-181b	ZNF292	zinc finger protein 292 [Source:VGNC Symbol;Acc:VGNC:95218]	2.55	0.000004
ssc-miR-181b	ZNF300	zinc finger protein 300 [Source:VGNC Symbol;Acc:VGNC:98424]	2.55	0.000004
ssc-miR-181b	ZNF302	hypothetical gene	2.55	0.000004
ssc-miR-181b	ZNF33B	hypothetical gene	2.55	0.000004
ssc-miR-181b	ZNF394	zinc finger protein 394 [Source:HGNC Symbol;Acc:HGNC:18832]	2.55	0.000004
ssc-miR-181b	ZNF397	zinc finger protein 397 [Source:VGNC Symbol;Acc:VGNC:95236]	2.55	0.000004
ssc-miR-181b	ZNF426	hypothetical gene	2.55	0.000004
ssc-miR-181b	ZNF449	zinc finger protein 449 [Source:VGNC Symbol;Acc:VGNC:95242]	2.55	0.000004
ssc-miR-181b	ZNF454	zinc finger protein 454 [Source:HGNC Symbol;Acc:HGNC:21200]	2.55	0.000004
ssc-miR-181b	ZNF470	zinc finger protein 470 [Source:HGNC Symbol;Acc:HGNC:22220]	2.55	0.000004
ssc-miR-181b	ZNF471	zinc finger protein 471 [Source:VGNC Symbol;Acc:VGNC:95246]	2.55	0.000004

ssc-miR-181b	ZNF487	hypothetical gene	2.55	0.000004
ssc-miR-181b	ZNF514	zinc finger protein 514 [Source:VGNC Symbol;Acc:VGNC:95255]	2.55	0.000004
ssc-miR-181b	ZNF536	zinc finger protein 536 [Source:VGNC Symbol;Acc:VGNC:95262]	2.55	0.000004
ssc-miR-181b	ZNF562	hypothetical gene	2.55	0.000004
ssc-miR-181b	ZNF594	hypothetical gene	2.55	0.000004
ssc-miR-181b	ZNF597	hypothetical gene	2.55	0.000004
ssc-miR-181b	ZNF613	zinc finger protein 613 [Source:HGNC Symbol;Acc:HGNC:25827]	2.55	0.000004
ssc-miR-181b	ZNF618	zinc finger protein 618 [Source:VGNC Symbol;Acc:VGNC:95276]	2.55	0.000004
ssc-miR-181b	ZNF625	hypothetical gene	2.55	0.000004
ssc-miR-181b	ZNF652	zinc finger protein 652 [Source:VGNC Symbol;Acc:VGNC:99108]	2.55	0.000004
ssc-miR-181b	ZNF654	zinc finger protein 654 [Source:VGNC Symbol;Acc:VGNC:95285]	2.55	0.000004
ssc-miR-181b	ZNF655	zinc finger protein 655 [Source:VGNC Symbol;Acc:VGNC:95286]	2.55	0.000004
ssc-miR-181b	ZNF664	zinc finger protein 664 [Source:VGNC Symbol;Acc:VGNC:95288]	2.55	0.000004
ssc-miR-181b	ZNF667	zinc finger protein 667 [Source:VGNC Symbol;Acc:VGNC:95289]	2.55	0.000004
ssc-miR-181b	ZNF697	zinc finger protein 697 [Source:HGNC Symbol;Acc:HGNC:32034]	2.55	0.000004
ssc-miR-181b	ZNF699	hypothetical gene	2.55	0.000004
ssc-miR-181b	ZNF704	zinc finger protein 704 [Source:VGNC Symbol;Acc:VGNC:95296]	2.55	0.000004
ssc-miR-181b	ZNF763	hypothetical gene	2.55	0.000004
ssc-miR-181b	ZNF772	zinc finger protein 772 [Source:HGNC Symbol;Acc:HGNC:33106]	2.55	0.000004
ssc-miR-181b	ZNF780A	hypothetical gene	2.55	0.000004
ssc-miR-181b	ZNF780B	hypothetical gene	2.55	0.000004
ssc-miR-181b	ZNF781	hypothetical gene	2.55	0.000004
ssc-miR-181b	ZNF788	hypothetical gene	2.55	0.000004
ssc-miR-181b	ZNF791	hypothetical gene	2.55	0.000004
ssc-miR-181b	ZNF800	zinc finger protein 800 [Source:VGNC Symbol;Acc:VGNC:95306]	2.55	0.000004
ssc-miR-181b	ZNRF2	zinc and ring finger 2 [Source:VGNC Symbol;Acc:VGNC:95317]	2.55	0.000004
ssc-miR-181b	ZSCAN12	zinc finger and SCAN domain containing 12 [Source:VGNC Symbol;Acc:VGNC:95325]	2.55	0.000004
ssc-miR-181b	ZSWIM1	zinc finger SWIM-type containing 1 [Source:VGNC Symbol;Acc:VGNC:95759]	2.55	0.000004
ssc-miR-181b	ZZZ3	zinc finger ZZ-type containing 3 [Source:VGNC Symbol;Acc:VGNC:95348]	2.55	0.000004
ssc-miR-181a	AAK1	AP2 associated kinase 1 [Source:VGNC Symbol;Acc:VGNC:100379]	2.42	0.041
ssc-miR-181a	AASDHPPT	aminoadipate-semialdehyde dehydrogenase-phosphopantetheinyl transferase [Source:VGNC Symbol;Acc:VGNC:84943]	2.42	0.041
ssc-miR-181a	ABHD13	abhydrolase domain containing 13 [Source:VGNC Symbol;Acc:VGNC:84972]	2.42	0.041
ssc-miR-181a	ABI1	abl interactor 1 [Source:VGNC Symbol;Acc:VGNC:95519]	2.42	0.041
ssc-miR-181a	ABI3BP	ABI family member 3 binding protein [Source:VGNC Symbol;Acc:VGNC:108628]	2.42	0.041
ssc-miR-181a	ABTB2	ankyrin repeat and BTB domain containing 2 [Source:VGNC Symbol;Acc:VGNC:84994]	2.42	0.041
ssc-miR-181a	AC012215.1	hypothetical gene	2.42	0.041
ssc-miR-181a	AC068987.1	hypothetical gene	2.42	0.041
ssc-miR-181a	ACAN	aggrecan [Source:NCBI gene (formerly Entrezgene);Acc:397255]	2.42	0.041
ssc-miR-181a	ACAP2	ArfGAP with coiled-coil, ankyrin repeat and PH domains 2 [Source:VGNC Symbol;Acc:VGNC:85002]	2.42	0.041
ssc-miR-181a	ACER3	alkaline ceramidase 3 [Source:VGNC Symbol;Acc:VGNC:103893]	2.42	0.041
ssc-miR-181a	ACSL6	acyl-CoA synthetase long chain family member 6 [Source:VGNC Symbol;Acc:VGNC:99581]	2.42	0.041
ssc-miR-181a	ACTA2	actin alpha 2, smooth muscle [Source:VGNC Symbol;Acc:VGNC:85038]	2.42	0.041
ssc-miR-181a	ACTR3	actin related protein 3 [Source:VGNC Symbol;Acc:VGNC:103894]	2.42	0.041
ssc-miR-181a	ACTRT3	actin related protein T3 [Source:VGNC Symbol;Acc:VGNC:85055]	2.42	0.041
ssc-miR-181a	ACVR1C	activin A receptor type 1C [Source:VGNC Symbol;Acc:VGNC:95873]	2.42	0.041
ssc-miR-181a	ACVR2A	activin A receptor type 2A [Source:VGNC Symbol;Acc:VGNC:95843]	2.42	0.041
ssc-miR-181a	ACVR2B	activin A receptor type 2B [Source:VGNC Symbol;Acc:VGNC:108629]	2.42	0.041
ssc-miR-181a	ADAM11	ADAM metallopeptidase domain 11 [Source:VGNC Symbol;Acc:VGNC:85062]	2.42	0.041
ssc-miR-181a	ADAMTS1	ADAM metallopeptidase with thrombospondin type 1 motif 1 [Source:VGNC Symbol;Acc:VGNC:85072]	2.42	0.041

ssc-miR-181a	ADAMTS18	ADAM metallopeptidase with thrombospondin type 1 motif 18 [Source:VGNC Symbol;Acc:VGNC:85080]	2.42	0.041
ssc-miR-181a	ADAMTS5	ADAM metallopeptidase with thrombospondin type 1 motif 5 [Source:VGNC Symbol;Acc:VGNC:85085]	2.42	0.041
ssc-miR-181a	ADAMTSL1	ADAMTS like 1 [Source:VGNC Symbol;Acc:VGNC:85090]	2.42	0.041
ssc-miR-181a	ADARB1	adenosine deaminase RNA specific B1 [Source:VGNC Symbol;Acc:VGNC:85098]	2.42	0.041
ssc-miR-181a	ADARB2	adenosine deaminase RNA specific B2 (inactive) [Source:HGNC Symbol;Acc:HGNC:227]	2.42	0.041
ssc-miR-181a	ADAT2	adenosine deaminase tRNA specific 2 [Source:VGNC Symbol;Acc:VGNC:85100]	2.42	0.041
ssc-miR-181a	ADCY9	adenylate cyclase 9 [Source:VGNC Symbol;Acc:VGNC:85113]	2.42	0.041
ssc-miR-181a	ADHFE1	alcohol dehydrogenase iron containing 1 [Source:VGNC Symbol;Acc:VGNC:98730]	2.42	0.041
ssc-miR-181a	ADO	2-aminoethanethiol dioxygenase [Source:VGNC Symbol;Acc:VGNC:85146]	2.42	0.041
ssc-miR-181a	ADRBK1	hypothetical gene	2.42	0.041
ssc-miR-181a	AFAP1	actin filament associated protein 1 [Source:VGNC Symbol;Acc:VGNC:85165]	2.42	0.041
ssc-miR-181a	AFF1	AF4/FMR2 family member 1 [Source:VGNC Symbol;Acc:VGNC:85167]	2.42	0.041
ssc-miR-181a	AFG3L2	AFG3 like matrix AAA peptidase subunit 2 [Source:HGNC Symbol;Acc:HGNC:315]	2.42	0.041
ssc-miR-181a	AFTPH	aftiphilin [Source:VGNC Symbol;Acc:VGNC:85173]	2.42	0.041
ssc-miR-181a	AGFG1	ArfGAP with FG repeats 1 [Source:VGNC Symbol;Acc:VGNC:96359]	2.42	0.041
ssc-miR-181a	AGO4	hypothetical gene	2.42	0.041
ssc-miR-181a	AHNAK	hypothetical gene	2.42	0.041
ssc-miR-181a	AHSA2	hypothetical gene	2.42	0.041
ssc-miR-181a	AKAP5	A-kinase anchoring protein 5 [Source:VGNC Symbol;Acc:VGNC:85220]	2.42	0.041
ssc-miR-181a	AKIRIN1	akirin 1 [Source:VGNC Symbol;Acc:VGNC:85227]	2.42	0.041
ssc-miR-181a	AKT3	AKT serine/threonine kinase 3 [Source:VGNC Symbol;Acc:VGNC:96306]	2.42	0.041
ssc-miR-181a	AL021546.6	hypothetical gene	2.42	0.041
ssc-miR-181a	AL590483.1	hypothetical gene	2.42	0.041
ssc-miR-181a	ALDH1A3	aldehyde dehydrogenase 1 family member A3 [Source:VGNC Symbol;Acc:VGNC:97879]	2.42	0.041
ssc-miR-181a	AMER2	APC membrane recruitment protein 2 [Source:VGNC Symbol;Acc:VGNC:85277]	2.42	0.041
ssc-miR-181a	ANAPC16	anaphase promoting complex subunit 16 [Source:NCBI gene (formerly Entrezgene);Acc:100155019]	2.42	0.041
ssc-miR-181a	ANGPTL3	angiopoietin like 3 [Source:VGNC Symbol;Acc:VGNC:85305]	2.42	0.041
ssc-miR-181a	ANKFY1	ankyrin repeat and FYVE domain containing 1 [Source:VGNC Symbol;Acc:VGNC:85314]	2.42	0.041
ssc-miR-181a	ANKRD13C	ankyrin repeat domain 13C [Source:VGNC Symbol;Acc:VGNC:85326]	2.42	0.041
ssc-miR-181a	ANKRD33B	ankyrin repeat domain 33B [Source:VGNC Symbol;Acc:VGNC:85334]	2.42	0.041
ssc-miR-181a	ANKRD44	ankyrin repeat domain 44 [Source:VGNC Symbol;Acc:VGNC:107123]	2.42	0.041
ssc-miR-181a	ANKRD50	ankyrin repeat domain containing 50 [Source:VGNC Symbol;Acc:VGNC:85341]	2.42	0.041
ssc-miR-181a	ANKRD52	ankyrin repeat domain 52 [Source:VGNC Symbol;Acc:VGNC:85342]	2.42	0.041
ssc-miR-181a	ANP32A	hypothetical gene	2.42	0.041
ssc-miR-181a	ANXA11	annexin A11 [Source:VGNC Symbol;Acc:VGNC:85367]	2.42	0.041
ssc-miR-181a	AP1AR	adaptor related protein complex 1 associated regulatory protein [Source:HGNC Symbol;Acc:HGNC:28808]	2.42	0.041
ssc-miR-181a	AP1G1	adaptor related protein complex 1 subunit gamma 1 [Source:VGNC Symbol;Acc:VGNC:85378]	2.42	0.041
ssc-miR-181a	AP1S3	adaptor related protein complex 1 subunit sigma 3 [Source:VGNC Symbol;Acc:VGNC:95901]	2.42	0.041
ssc-miR-181a	AP5M1	adaptor related protein complex 5 subunit mu 1 [Source:VGNC Symbol;Acc:VGNC:85396]	2.42	0.041
ssc-miR-181a	APBA1	amyloid beta protein binding family A member 1 [Source:VGNC Symbol;Acc:VGNC:85399]	2.42	0.041
ssc-miR-181a	APLP2	amyloid beta like protein 2 [Source:VGNC Symbol;Acc:VGNC:85414]	2.42	0.041
ssc-miR-181a	APOO	apolipoprotein O [Source:VGNC Symbol;Acc:VGNC:103900]	2.42	0.041
ssc-miR-181a	ARF3	ADP ribosylation factor 3 [Source:NCBI gene (formerly Entrezgene);Acc:100301570]	2.42	0.041
ssc-miR-181a	ARF6	ADP ribosylation factor 6 [Source:VGNC Symbol;Acc:VGNC:85448]	2.42	0.041
ssc-miR-181a	ARHGEF3	Rho guanine nucleotide exchange factor 3 [Source:VGNC Symbol;Acc:VGNC:85496]	2.42	0.041
ssc-miR-181a	ARHGEF37	Rho guanine nucleotide exchange factor 37 [Source:VGNC Symbol;Acc:VGNC:85498]	2.42	0.041
ssc-miR-181a	ARID2	AT-rich interaction domain 2 [Source:HGNC Symbol;Acc:HGNC:18037]	2.42	0.041
ssc-miR-181a	ARIH1	ariadne RBR E3 ubiquitin protein ligase 1 [Source:HGNC Symbol;Acc:HGNC:689]	2.42	0.041
ssc-miR-181a	ARL1	ADP ribosylation factor like GTPase 1 [Source:VGNC Symbol;Acc:VGNC:85513]	2.42	0.041

ssc-miR-181a	ARL3	ADP ribosylation factor like GTPase 3 [Source:VGNC Symbol;Acc:VGNC:107366]	2.42	0.041
ssc-miR-181a	ARL5A	ADP ribosylation factor like GTPase 5A [Source:VGNC Symbol;Acc:VGNC:96418]	2.42	0.041
ssc-miR-181a	ARNT2	aryl hydrocarbon receptor nuclear translocator 2 [Source:HGNC Symbol;Acc:HGNC:16876]	2.42	0.041
ssc-miR-181a	ARRDC3	arrestin domain containing 3 [Source:VGNC Symbol;Acc:VGNC:85546]	2.42	0.041
ssc-miR-181a	ARSJ	arylsulfatase family member J [Source:VGNC Symbol;Acc:VGNC:85549]	2.42	0.041
ssc-miR-181a	ASAH2	hypothetical gene	2.42	0.041
ssc-miR-181a	ASAH2B	hypothetical gene	2.42	0.041
ssc-miR-181a	ASIC1	acid sensing ion channel subunit 1 [Source:VGNC Symbol;Acc:VGNC:85578]	2.42	0.041
ssc-miR-181a	ASPHD2	aspartate beta-hydroxylase domain containing 2 [Source:VGNC Symbol;Acc:VGNC:85585]	2.42	0.041
ssc-miR-181a	ASXL2	ASXL transcriptional regulator 2 [Source:VGNC Symbol;Acc:VGNC:85595]	2.42	0.041
ssc-miR-181a	ASXL3	ASXL transcriptional regulator 3 [Source:VGNC Symbol;Acc:VGNC:85596]	2.42	0.041
ssc-miR-181a	ATF2	activating transcription factor 2 [Source:VGNC Symbol;Acc:VGNC:96446]	2.42	0.041
ssc-miR-181a	ATG2B	autophagy related 2B [Source:VGNC Symbol;Acc:VGNC:85620]	2.42	0.041
ssc-miR-181a	ATG7	autophagy related 7 [Source:VGNC Symbol;Acc:VGNC:85625]	2.42	0.041
ssc-miR-181a	ATL3	atlastin GTPase 3 [Source:VGNC Symbol;Acc:VGNC:85629]	2.42	0.041
ssc-miR-181a	ATM	ATM serine/threonine kinase [Source:VGNC Symbol;Acc:VGNC:108572]	2.42	0.041
ssc-miR-181a	ATMIN	hypothetical gene	2.42	0.041
ssc-miR-181a	ATP11A	ATPase phospholipid transporting 11A [Source:VGNC Symbol;Acc:VGNC:85634]	2.42	0.041
ssc-miR-181a	ATP11C	ATPase phospholipid transporting 11C [Source:VGNC Symbol;Acc:VGNC:85635]	2.42	0.041
ssc-miR-181a	ATP1B1	ATPase Na+/K+ transporting subunit beta 1 [Source:VGNC Symbol;Acc:VGNC:85642]	2.42	0.041
ssc-miR-181a	ATP2A2	ATPase sarcoplasmic/endoplasmic reticulum Ca2+ transporting 2 [Source:VGNC Symbol;Acc:VGNC:85647]	2.42	0.041
ssc-miR-181a	ATP2B1	ATPase plasma membrane Ca2+ transporting 1 [Source:VGNC Symbol;Acc:VGNC:103219]	2.42	0.041
ssc-miR-181a	ATP2B2	ATPase plasma membrane Ca2+ transporting 2 [Source:VGNC Symbol;Acc:VGNC:85649]	2.42	0.041
ssc-miR-181a	ATP6V1A	ATPase H+ transporting V1 subunit A [Source:VGNC Symbol;Acc:VGNC:85669]	2.42	0.041
ssc-miR-181a	ATP6V1C1	ATPase H+ transporting V1 subunit C1 [Source:VGNC Symbol;Acc:VGNC:85672]	2.42	0.041
ssc-miR-181a	ATP8A1	ATPase phospholipid transporting 8A1 [Source:VGNC Symbol;Acc:VGNC:97894]	2.42	0.041
ssc-miR-181a	ATP8B1	ATPase phospholipid transporting 8B1 [Source:VGNC Symbol;Acc:VGNC:85679]	2.42	0.041
ssc-miR-181a	ATP8B2	ATPase phospholipid transporting 8B2 [Source:VGNC Symbol;Acc:VGNC:85680]	2.42	0.041
ssc-miR-181a	ATP9A	ATPase phospholipid transporting 9A (putative) [Source:NCBI gene (formerly Entrezgene);Acc:100136901]	2.42	0.041
ssc-miR-181a	ATRNL1	attractin like 1 [Source:VGNC Symbol;Acc:VGNC:85685]	2.42	0.041
ssc-miR-181a	ATXN1	ataxin 1 [Source:VGNC Symbol;Acc:VGNC:85687]	2.42	0.041
ssc-miR-181a	ATXN3	hypothetical gene	2.42	0.041
ssc-miR-181a	ATXN7	ataxin 7 [Source:VGNC Symbol;Acc:VGNC:99704]	2.42	0.041
ssc-miR-181a	AVL9	AVL9 cell migration associated [Source:VGNC Symbol;Acc:VGNC:85701]	2.42	0.041
ssc-miR-181a	B3GALT1	beta-1,3-galactosyltransferase 1 [Source:VGNC Symbol;Acc:VGNC:96494]	2.42	0.041
ssc-miR-181a	B4GALT1	beta-1,4-galactosyltransferase 1 [Source:VGNC Symbol;Acc:VGNC:96497]	2.42	0.041
ssc-miR-181a	BACH2	BTB domain and CNC homolog 2 [Source:VGNC Symbol;Acc:VGNC:85742]	2.42	0.041
ssc-miR-181a	BAG4	BAG cochaperone 4 [Source:VGNC Symbol;Acc:VGNC:96503]	2.42	0.041
ssc-miR-181a	BAI3	hypothetical gene	2.42	0.041
ssc-miR-181a	BAZ1A	bromodomain adjacent to zinc finger domain 1A [Source:VGNC Symbol;Acc:VGNC:85761]	2.42	0.041
ssc-miR-181a	BAZ2A	bromodomain adjacent to zinc finger domain 2A [Source:VGNC Symbol;Acc:VGNC:85763]	2.42	0.041
ssc-miR-181a	BAZ2B	bromodomain adjacent to zinc finger domain 2B [Source:HGNC Symbol;Acc:HGNC:963]	2.42	0.041
ssc-miR-181a	BCL2	BCL2 apoptosis regulator [Source:HGNC Symbol;Acc:HGNC:990]	2.42	0.041
ssc-miR-181a	BCL2L11	BCL2 like 11 [Source:NCBI gene (formerly Entrezgene);Acc:396632]	2.42	0.041
ssc-miR-181a	BCL2L13	BCL2 like 13 [Source:VGNC Symbol;Acc:VGNC:85781]	2.42	0.041
ssc-miR-181a	BCL6	BCL6 transcription repressor [Source:VGNC Symbol;Acc:VGNC:96565]	2.42	0.041
ssc-miR-181a	BCL9	BCL9 transcription coactivator [Source:VGNC Symbol;Acc:VGNC:96567]	2.42	0.041
ssc-miR-181a	BCLAF1	BCL2 associated transcription factor 1 [Source:VGNC Symbol;Acc:VGNC:85787]	2.42	0.041
ssc-miR-181a	BCR	BCR activator of RhoGEF and GTPase [Source:HGNC Symbol;Acc:HGNC:1014]	2.42	0.041

ssc-miR-181a	BEND3	BEN domain containing 3 [Source:VGNC Symbol;Acc:VGNC:85800]	2.42	0.041
ssc-miR-181a	BEND4	BEN domain containing 4 [Source:VGNC Symbol;Acc:VGNC:85801]	2.42	0.041
ssc-miR-181a	BHLHE40	basic helix-loop-helix family member e40 [Source:VGNC Symbol;Acc:VGNC:85814]	2.42	0.041
ssc-miR-181a	BHLHE41	basic helix-loop-helix family member e41 [Source:VGNC Symbol;Acc:VGNC:85815]	2.42	0.041
ssc-miR-181a	BIRC6	baculoviral IAP repeat containing 6 [Source:VGNC Symbol;Acc:VGNC:97038]	2.42	0.041
ssc-miR-181a	BLOC1S6	biosis of lysosomal organelles complex 1 subunit 6 [Source:VGNC Symbol;Acc:VGNC:85832]	2.42	0.041
ssc-miR-181a	BMF	Bcl2 modifying factor [Source:VGNC Symbol;Acc:VGNC:85837]	2.42	0.041
ssc-miR-181a	BMP3	bone morphotic protein 3 [Source:VGNC Symbol;Acc:VGNC:85842]	2.42	0.041
ssc-miR-181a	BMPR2	bone morphotic protein receptor type 2 [Source:VGNC Symbol;Acc:VGNC:95494]	2.42	0.041
ssc-miR-181a	BNC2	basonuclin 2 [Source:VGNC Symbol;Acc:VGNC:85852]	2.42	0.041
ssc-miR-181a	BPTF	bromodomain PHD finger transcription factor [Source:VGNC Symbol;Acc:VGNC:97904]	2.42	0.041
ssc-miR-181a	BRAP	BRCA1 associated protein [Source:VGNC Symbol;Acc:VGNC:85867]	2.42	0.041
ssc-miR-181a	BRD1	bromodomain containing 1 [Source:VGNC Symbol;Acc:VGNC:85871]	2.42	0.041
ssc-miR-181a	BRWD1	bromodomain and WD repeat domain containing 1 [Source:VGNC Symbol;Acc:VGNC:108153]	2.42	0.041
ssc-miR-181a	BTBD3	BTB domain containing 3 [Source:VGNC Symbol;Acc:VGNC:95673]	2.42	0.041
ssc-miR-181a	BTG1	BTG anti-proliferation factor 1 [Source:VGNC Symbol;Acc:VGNC:103907]	2.42	0.041
ssc-miR-181a	BTG2	BTG anti-proliferation factor 2 [Source:NCBI gene (formerly Entrezgene);Acc:100048932]	2.42	0.041
ssc-miR-181a	BTLA	hypothetical gene	2.42	0.041
ssc-miR-181a	C11orf30	hypothetical gene	2.42	0.041
ssc-miR-181a	C11orf73	hypothetical gene	2.42	0.041
ssc-miR-181a	C14orf28	chromosome 1 C14orf28 homolog [Source:VGNC Symbol;Acc:VGNC:85958]	2.42	0.041
ssc-miR-181a	C15ORF37	hypothetical gene	2.42	0.041
ssc-miR-181a	C16orf87	chromosome 6 C16orf87 homolog [Source:VGNC Symbol;Acc:VGNC:96927]	2.42	0.041
ssc-miR-181a	C1QTNF3	C1q and TNF related 3 [Source:NCBI gene (formerly Entrezgene);Acc:100270814]	2.42	0.041
ssc-miR-181a	C2CD5	C2 calcium dependent domain containing 5 [Source:VGNC Symbol;Acc:VGNC:85995]	2.42	0.041
ssc-miR-181a	C2orf69	chromosome 2 open reading frame 69 [Source:HGNC Symbol;Acc:HGNC:26799]	2.42	0.041
ssc-miR-181a	C4orf29	hypothetical gene	2.42	0.041
ssc-miR-181a	C6orf62	chromosome 7 C6orf62 homolog [Source:VGNC Symbol;Acc:VGNC:86074]	2.42	0.041
ssc-miR-181a	C6orf89	chromosome 7 C6orf89 homolog [Source:VGNC Symbol;Acc:VGNC:86075]	2.42	0.041
ssc-miR-181a	C7orf41	hypothetical gene	2.42	0.041
ssc-miR-181a	C8orf4	hypothetical gene	2.42	0.041
ssc-miR-181a	C8orf44-SGK3	hypothetical gene	2.42	0.041
ssc-miR-181a	CACNA2D1	calcium voltage-gated channel auxiliary subunit alpha2delta 1 [Source:VGNC Symbol;Acc:VGNC:86120]	2.42	0.041
ssc-miR-181a	CACNA2D2	calcium voltage-gated channel auxiliary subunit alpha2delta 2 [Source:VGNC Symbol;Acc:VGNC:86121]	2.42	0.041
ssc-miR-181a	CALCR	calcitonin receptor [Source:VGNC Symbol;Acc:VGNC:86142]	2.42	0.041
ssc-miR-181a	CALM1	calmodulin 1 [Source:NCBI gene (formerly Entrezgene);Acc:100154056]	2.42	0.041
ssc-miR-181a	CALR	calreticulin [Source:VGNC Symbol;Acc:VGNC:86149]	2.42	0.041
ssc-miR-181a	CAMK2D	calcium/calmodulin dependent protein kinase II delta [Source:VGNC Symbol;Acc:VGNC:97911]	2.42	0.041
ssc-miR-181a	CAMK2G	calcium/calmodulin dependent protein kinase II gamma [Source:VGNC Symbol;Acc:VGNC:86157]	2.42	0.041
ssc-miR-181a	CAMKK1	calcium/calmodulin dependent protein kinase kinase 1 [Source:VGNC Symbol;Acc:VGNC:98977]	2.42	0.041
ssc-miR-181a	CAMSAP1	calmodulin regulated spectrin associated protein 1 [Source:VGNC Symbol;Acc:VGNC:86162]	2.42	0.041
ssc-miR-181a	CAMSAP2	calmodulin regulated spectrin associated protein family member 2 [Source:VGNC Symbol;Acc:VGNC:96205]	2.42	0.041
ssc-miR-181a	CAMTA2	calmodulin binding transcription activator 2 [Source:VGNC Symbol;Acc:VGNC:86165]	2.42	0.041
ssc-miR-181a	CAND1	cullin associated and neddylation dissociated 1 [Source:VGNC Symbol;Acc:VGNC:97912]	2.42	0.041
ssc-miR-181a	CAPRIN2	caprin family member 2 [Source:VGNC Symbol;Acc:VGNC:86179]	2.42	0.041
ssc-miR-181a	CARD10	caspase recruitment domain family member 10 [Source:VGNC Symbol;Acc:VGNC:86185]	2.42	0.041
ssc-miR-181a	CARD11	caspase recruitment domain family member 11 [Source:VGNC Symbol;Acc:VGNC:86186]	2.42	0.041
ssc-miR-181a	CARM1	coactivator associated arginine methyltransferase 1 [Source:VGNC Symbol;Acc:VGNC:86191]	2.42	0.041
ssc-miR-181a	CASP10	caspase 10 [Source:VGNC Symbol;Acc:VGNC:95638]	2.42	0.041

ssc-miR-181a	CBFA2T2	CBFA2/RUNX1 partner transcriptional co-repressor 2 [Source:VGNC Symbol;Acc:VGNC:96573]	2.42	0.041
ssc-miR-181a	CBFA2T3	CBFA2/RUNX1 partner transcriptional co-repressor 3 [Source:VGNC Symbol;Acc:VGNC:96574]	2.42	0.041
ssc-miR-181a	CBLB	Cbl proto-onco B [Source:VGNC Symbol;Acc:VGNC:86223]	2.42	0.041
ssc-miR-181a	CBX4	chromobox 4 [Source:VGNC Symbol;Acc:VGNC:86231]	2.42	0.041
ssc-miR-181a	CBX5	chromobox 5 [Source:VGNC Symbol;Acc:VGNC:86232]	2.42	0.041
ssc-miR-181a	CBX7	chromobox 7 [Source:HGNC Symbol;Acc:HGNC:1557]	2.42	0.041
ssc-miR-181a	CBX8	chromobox 8 [Source:VGNC Symbol;Acc:VGNC:86233]	2.42	0.041
ssc-miR-181a	CCAR1	cell division cycle and apoptosis regulator 1 [Source:VGNC Symbol;Acc:VGNC:86238]	2.42	0.041
ssc-miR-181a	CCDC117	coiled-coil domain containing 117 [Source:VGNC Symbol;Acc:VGNC:86248]	2.42	0.041
ssc-miR-181a	CCDC14	coiled-coil domain containing 14 [Source:VGNC Symbol;Acc:VGNC:86260]	2.42	0.041
ssc-miR-181a	CCDC6	coiled-coil domain containing 6 [Source:VGNC Symbol;Acc:VGNC:86304]	2.42	0.041
ssc-miR-181a	CCDC64	hypothetical gene	2.42	0.041
ssc-miR-181a	CCDC85A	coiled-coil domain containing 85A [Source:VGNC Symbol;Acc:VGNC:86320]	2.42	0.041
ssc-miR-181a	CCDC92	coiled-coil domain containing 92 [Source:VGNC Symbol;Acc:VGNC:86330]	2.42	0.041
ssc-miR-181a	CCL8	chemokine (C-C motif) ligand 8 [Source:NCBI gene (formerly Entrezgene);Acc:100302703]	2.42	0.041
ssc-miR-181a	CCNB1	cyclin B1 [Source:VGNC Symbol;Acc:VGNC:86349]	2.42	0.041
ssc-miR-181a	CCNJ	cyclin J [Source:VGNC Symbol;Acc:VGNC:86360]	2.42	0.041
ssc-miR-181a	CCNK	cyclin K [Source:VGNC Symbol;Acc:VGNC:86362]	2.42	0.041
ssc-miR-181a	CCNT1	cyclin T1 [Source:VGNC Symbol;Acc:VGNC:86366]	2.42	0.041
ssc-miR-181a	CCP110	centriolar coiled-coil protein 110 [Source:VGNC Symbol;Acc:VGNC:86367]	2.42	0.041
ssc-miR-181a	CD163	CD163 molecule [Source:NCBI gene (formerly Entrezgene);Acc:397031]	2.42	0.041
ssc-miR-181a	CD4	CD4 molecule [Source:VGNC Symbol;Acc:VGNC:86417]	2.42	0.041
ssc-miR-181a	CD47	CD47 molecule [Source:VGNC Symbol;Acc:VGNC:108637]	2.42	0.041
ssc-miR-181a	CD69	CD69 molecule [Source:VGNC Symbol;Acc:VGNC:103223]	2.42	0.041
ssc-miR-181a	CDC27	cell division cycle 27 [Source:VGNC Symbol;Acc:VGNC:86448]	2.42	0.041
ssc-miR-181a	CDC42BPA	CDC42 binding protein kinase alpha [Source:VGNC Symbol;Acc:VGNC:95847]	2.42	0.041
ssc-miR-181a	CDC5L	cell division cycle 5 like [Source:VGNC Symbol;Acc:VGNC:86463]	2.42	0.041
ssc-miR-181a	CDC73	cell division cycle 73 [Source:VGNC Symbol;Acc:VGNC:95817]	2.42	0.041
ssc-miR-181a	CDH8	cadherin 8 [Source:VGNC Symbol;Acc:VGNC:86487]	2.42	0.041
ssc-miR-181a	CDK17	cyclin dependent kinase 17 [Source:VGNC Symbol;Acc:VGNC:86500]	2.42	0.041
ssc-miR-181a	CDK8	cyclin dependent kinase 8 [Source:VGNC Symbol;Acc:VGNC:86507]	2.42	0.041
ssc-miR-181a	CDKL2	cyclin dependent kinase like 2 [Source:VGNC Symbol;Acc:VGNC:86511]	2.42	0.041
ssc-miR-181a	CDON	cell adhesion associated, onco regulated [Source:VGNC Symbol;Acc:VGNC:86519]	2.42	0.041
ssc-miR-181a	CDS1	CDP-diacylglycerol synthase 1 [Source:VGNC Symbol;Acc:VGNC:86523]	2.42	0.041
ssc-miR-181a	CDS2	CDP-diacylglycerol synthase 2 [Source:VGNC Symbol;Acc:VGNC:95869]	2.42	0.041
ssc-miR-181a	CDX2	caudal type homeobox 2 [Source:VGNC Symbol;Acc:VGNC:86527]	2.42	0.041
ssc-miR-181a	CDYL	chromodomain Y like [Source:HGNC Symbol;Acc:HGNC:1811]	2.42	0.041
ssc-miR-181a	CECR2	CECR2 histone acetyl-lysine reader [Source:VGNC Symbol;Acc:VGNC:96576]	2.42	0.041
ssc-miR-181a	CELSR3	cadherin EGF LAG seven-pass G-type receptor 3 [Source:VGNC Symbol;Acc:VGNC:86541]	2.42	0.041
ssc-miR-181a	CENPO	centromere protein O [Source:HGNC Symbol;Acc:HGNC:28152]	2.42	0.041
ssc-miR-181a	CEP350	centrosomal protein 350 [Source:HGNC Symbol;Acc:HGNC:24238]	2.42	0.041
ssc-miR-181a	CEP85L	centrosomal protein 85 like [Source:VGNC Symbol;Acc:VGNC:86580]	2.42	0.041
ssc-miR-181a	CEP97	centrosomal protein 97 [Source:VGNC Symbol;Acc:VGNC:108638]	2.42	0.041
ssc-miR-181a	CGRRF1	cell growth regulator with ring finger domain 1 [Source:VGNC Symbol;Acc:VGNC:86617]	2.42	0.041
ssc-miR-181a	CHCHD4	coiled-coil-helix-coiled-coil-helix domain containing 4 [Source:NCBI gene (formerly Entrezgene);Acc:100154663]	2.42	0.041
ssc-miR-181a	CHD1	chromodomain helicase DNA binding protein 1 [Source:VGNC Symbol;Acc:VGNC:86629]	2.42	0.041
ssc-miR-181a	CHD7	chromodomain helicase DNA binding protein 7 [Source:VGNC Symbol;Acc:VGNC:86633]	2.42	0.041
ssc-miR-181a	CHD9	chromodomain helicase DNA binding protein 9 [Source:VGNC Symbol;Acc:VGNC:86635]	2.42	0.041
ssc-miR-181a	CHIC1	cysteine rich hydrophobic domain 1 [Source:VGNC Symbol;Acc:VGNC:86642]	2.42	0.041

ssc-miR-181a	CHL1	cell adhesion molecule L1 like [Source:VGNC Symbol;Acc:VGNC:108639]	2.42	0.041
ssc-miR-181a	CHMP1B	hypothetical gene	2.42	0.041
ssc-miR-181a	CHMP3	charged multivesicular body protein 3 [Source:VGNC Symbol;Acc:VGNC:106570]	2.42	0.041
ssc-miR-181a	CHP1	calcineurin like EF-hand protein 1 [Source:VGNC Symbol;Acc:VGNC:86657]	2.42	0.041
ssc-miR-181a	CHRM3	cholinergic receptor muscarinic 3 [Source:VGNC Symbol;Acc:VGNC:86664]	2.42	0.041
ssc-miR-181a	CHST1	carbohydrate sulfotransferase 1 [Source:VGNC Symbol;Acc:VGNC:86673]	2.42	0.041
ssc-miR-181a	CHURC1	churchill domain containing 1 [Source:VGNC Symbol;Acc:VGNC:86689]	2.42	0.041
ssc-miR-181a	CISD1	hypothetical gene	2.42	0.041
ssc-miR-181a	CKAP4	cytoskeleton associated protein 4 [Source:VGNC Symbol;Acc:VGNC:86716]	2.42	0.041
ssc-miR-181a	CLASP1	cytoplasmic linker associated protein 1 [Source:VGNC Symbol;Acc:VGNC:95829]	2.42	0.041
ssc-miR-181a	CLCC1	chloride channel CLIC like 1 [Source:VGNC Symbol;Acc:VGNC:86724]	2.42	0.041
ssc-miR-181a	CLIP1	CAP-Gly domain containing linker protein 1 [Source:VGNC Symbol;Acc:VGNC:86766]	2.42	0.041
ssc-miR-181a	CLOCK	clock circadian regulator [Source:VGNC Symbol;Acc:VGNC:86774]	2.42	0.041
ssc-miR-181a	CLUH	clustered mitochondria homolog [Source:VGNC Symbol;Acc:VGNC:86792]	2.42	0.041
ssc-miR-181a	CLVS1	clavesin 1 [Source:VGNC Symbol;Acc:VGNC:86794]	2.42	0.041
ssc-miR-181a	CMPK2	cytidine/uridine monophosphate kinase 2 [Source:VGNC Symbol;Acc:VGNC:86801]	2.42	0.041
ssc-miR-181a	CNKSR2	connector enhancer of kinase suppressor of Ras 2 [Source:VGNC Symbol;Acc:VGNC:86822]	2.42	0.041
ssc-miR-181a	CNKSR3	CNKSR family member 3 [Source:VGNC Symbol;Acc:VGNC:86823]	2.42	0.041
ssc-miR-181a	CNNM2	cyclin and CBS domain divalent metal cation transport mediator 2 [Source:VGNC Symbol;Acc:VGNC:86828]	2.42	0.041
ssc-miR-181a	CNOT1	CCR4-NOT transcription complex subunit 1 [Source:VGNC Symbol;Acc:VGNC:86831]	2.42	0.041
ssc-miR-181a	CNOT2	CCR4-NOT transcription complex subunit 2 [Source:VGNC Symbol;Acc:VGNC:86834]	2.42	0.041
ssc-miR-181a	CNOT6L	CCR4-NOT transcription complex subunit 6 like [Source:VGNC Symbol;Acc:VGNC:86838]	2.42	0.041
ssc-miR-181a	CNR1	cannabinoid receptor 1 [Source:VGNC Symbol;Acc:VGNC:86843]	2.42	0.041
ssc-miR-181a	CNTN4	contactin 4 [Source:VGNC Symbol;Acc:VGNC:97934]	2.42	0.041
ssc-miR-181a	CNTNAP2	hypothetical gene	2.42	0.041
ssc-miR-181a	COL16A1	collagen type XVI alpha 1 chain [Source:VGNC Symbol;Acc:VGNC:86867]	2.42	0.041
ssc-miR-181a	COLGALT1	collagen beta(1-O)galactosyltransferase 1 [Source:HGNC Symbol;Acc:HGNC:26182]	2.42	0.041
ssc-miR-181a	COLQ	collagen like tail subunit of asymmetric acetylcholinesterase [Source:VGNC Symbol;Acc:VGNC:86884]	2.42	0.041
ssc-miR-181a	COMMD6	hypothetical gene	2.42	0.041
ssc-miR-181a	COPS2	COP9 signalosome subunit 2 [Source:VGNC Symbol;Acc:VGNC:86899]	2.42	0.041
ssc-miR-181a	CORO1C	coronin 1C [Source:VGNC Symbol;Acc:VGNC:86914]	2.42	0.041
ssc-miR-181a	CPD	carboxypeptidase D [Source:VGNC Symbol;Acc:VGNC:86935]	2.42	0.041
ssc-miR-181a	CPEB4	cytoplasmic polyadenylation element binding protein 4 [Source:VGNC Symbol;Acc:VGNC:86939]	2.42	0.041
ssc-miR-181a	CPNE2	copine 2 [Source:VGNC Symbol;Acc:VGNC:86950]	2.42	0.041
ssc-miR-181a	CPOX	coproporphyrinogen oxidase [Source:VGNC Symbol;Acc:VGNC:86956]	2.42	0.041
ssc-miR-181a	CPSF6	cleavage and polyadenylation specific factor 6 [Source:VGNC Symbol;Acc:VGNC:86962]	2.42	0.041
ssc-miR-181a	CPT1A	carnitine palmitoyltransferase 1A [Source:VGNC Symbol;Acc:VGNC:86964]	2.42	0.041
ssc-miR-181a	CREB1	cAMP responsive element binding protein 1 [Source:VGNC Symbol;Acc:VGNC:96004]	2.42	0.041
ssc-miR-181a	CREBL2	cAMP responsive element binding protein like 2 [Source:VGNC Symbol;Acc:VGNC:86986]	2.42	0.041
ssc-miR-181a	CREBRF	CREB3 regulatory factor [Source:VGNC Symbol;Acc:VGNC:86987]	2.42	0.041
ssc-miR-181a	CREBZF	CREB/ATF bZIP transcription factor [Source:VGNC Symbol;Acc:VGNC:86988]	2.42	0.041
ssc-miR-181a	CRIM1	cysteine rich transmembrane BMP regulator 1 [Source:VGNC Symbol;Acc:VGNC:86995]	2.42	0.041
ssc-miR-181a	CRISPLD1	cysteine rich secretory protein LCCL domain containing 1 [Source:VGNC Symbol;Acc:VGNC:97945]	2.42	0.041
ssc-miR-181a	CRYBG3	crystallin beta-gamma domain containing 3 [Source:VGNC Symbol;Acc:VGNC:87020]	2.42	0.041
ssc-miR-181a	CSNK1A1	casein kinase 1 alpha 1 [Source:VGNC Symbol;Acc:VGNC:99630]	2.42	0.041
ssc-miR-181a	CSNK1G1	casein kinase 1 gamma 1 [Source:VGNC Symbol;Acc:VGNC:97949]	2.42	0.041
ssc-miR-181a	CSNK1G3	casein kinase 1 gamma 3 [Source:VGNC Symbol;Acc:VGNC:97951]	2.42	0.041
ssc-miR-181a	CSRNP3	cysteine and serine rich nuclear protein 3 [Source:VGNC Symbol;Acc:VGNC:96249]	2.42	0.041
ssc-miR-181a	CSTF3	cleavage stimulation factor subunit 3 [Source:VGNC Symbol;Acc:VGNC:87052]	2.42	0.041

ssc-miR-181a	CTBP1	C-terminal binding protein 1 [Source:VGNC Symbol;Acc:VGNC:87053]	2.42	0.041
ssc-miR-181a	CTC-432M15.3	hypothetical gene	2.42	0.041
ssc-miR-181a	CTD-2140B24.4	hypothetical gene	2.42	0.041
ssc-miR-181a	CTD-2228K2.5	hypothetical gene	2.42	0.041
ssc-miR-181a	CTDSPL	CTD small phosphatase like [Source:VGNC Symbol;Acc:VGNC:107131]	2.42	0.041
ssc-miR-181a	CTH	cystathionine gamma-lyase [Source:VGNC Symbol;Acc:VGNC:96961]	2.42	0.041
ssc-miR-181a	CTIF	cap binding complex dependent translation initiation factor [Source:VGNC Symbol;Acc:VGNC:87062]	2.42	0.041
ssc-miR-181a	CTNND1	catenin delta 1 [Source:VGNC Symbol;Acc:VGNC:87067]	2.42	0.041
ssc-miR-181a	CTTNBP2NL	CTTNBP2 N-terminal like [Source:VGNC Symbol;Acc:VGNC:87082]	2.42	0.041
ssc-miR-181a	CUL3	cullin 3 [Source:VGNC Symbol;Acc:VGNC:96091]	2.42	0.041
ssc-miR-181a	CUL4A	cullin 4A [Source:VGNC Symbol;Acc:VGNC:87088]	2.42	0.041
ssc-miR-181a	CUL5	cullin 5 [Source:VGNC Symbol;Acc:VGNC:87089]	2.42	0.041
ssc-miR-181a	CXADR	CXADR Ig-like cell adhesion molecule [Source:VGNC Symbol;Acc:VGNC:87100]	2.42	0.041
ssc-miR-181a	CXXC5	CXXC finger protein 5 [Source:VGNC Symbol;Acc:VGNC:87118]	2.42	0.041
ssc-miR-181a	CYB5B	cytochrome b5 type B [Source:VGNC Symbol;Acc:VGNC:96728]	2.42	0.041
ssc-miR-181a	CYLD	CYLD lysine 63 deubiquitinase [Source:VGNC Symbol;Acc:VGNC:87130]	2.42	0.041
ssc-miR-181a	CYP26B1	cytochrome P450 family 26 subfamily B member 1 [Source:VGNC Symbol;Acc:VGNC:103374]	2.42	0.041
ssc-miR-181a	CYP4X1	hypothetical gene	2.42	0.041
ssc-miR-181a	CYR61	hypothetical gene	2.42	0.041
ssc-miR-181a	DAZAP2	DAZ associated protein 2 [Source:VGNC Symbol;Acc:VGNC:87162]	2.42	0.041
ssc-miR-181a	DCAF12L1	hypothetical gene	2.42	0.041
ssc-miR-181a	DCAF16	DDB1 and CUL4 associated factor 16 [Source:HGNC Symbol;Acc:HGNC:25987]	2.42	0.041
ssc-miR-181a	DCBLD2	discoidin, CUB and LCCL domain containing 2 [Source:VGNC Symbol;Acc:VGNC:87180]	2.42	0.041
ssc-miR-181a	DCLK1	doublecortin like kinase 1 [Source:HGNC Symbol;Acc:HGNC:2700]	2.42	0.041
ssc-miR-181a	DCLK3	doublecortin like kinase 3 [Source:VGNC Symbol;Acc:VGNC:87184]	2.42	0.041
ssc-miR-181a	DCUN1D1	hypothetical gene	2.42	0.041
ssc-miR-181a	DDIT4	DNA damage inducible transcript 4 [Source:VGNC Symbol;Acc:VGNC:87208]	2.42	0.041
ssc-miR-181a	DDR2	discoidin domain receptor tyrosine kinase 2 [Source:VGNC Symbol;Acc:VGNC:87212]	2.42	0.041
ssc-miR-181a	DDX3X	DEAD-box helicase 3 X-linked [Source:NCBI gene (formerly Entrezgene);Acc:100515940]	2.42	0.041
ssc-miR-181a	DDX3Y	hypothetical gene	2.42	0.041
ssc-miR-181a	DDX5	DEAD-box helicase 5 [Source:VGNC Symbol;Acc:VGNC:87226]	2.42	0.041
ssc-miR-181a	DDX52	DExD-box helicase 52 [Source:VGNC Symbol;Acc:VGNC:87229]	2.42	0.041
ssc-miR-181a	DEK	hypothetical gene	2.42	0.041
ssc-miR-181a	DENND5B	DENN domain containing 5B [Source:VGNC Symbol;Acc:VGNC:87255]	2.42	0.041
ssc-miR-181a	DEPTOR	DEP domain containing MTOR interacting protein [Source:HGNC Symbol;Acc:HGNC:22953]	2.42	0.041
ssc-miR-181a	DERL1	derlin 1 [Source:NCBI gene (formerly Entrezgene);Acc:100626802]	2.42	0.041
ssc-miR-181a	DERL2	derlin 2 [Source:VGNC Symbol;Acc:VGNC:98986]	2.42	0.041
ssc-miR-181a	DESI2	desumoylating isopeptidase 2 [Source:VGNC Symbol;Acc:VGNC:96037]	2.42	0.041
ssc-miR-181a	DHX57	DExH-box helicase 57 [Source:VGNC Symbol;Acc:VGNC:87296]	2.42	0.041
ssc-miR-181a	DIP2A	disco interacting A [Source:VGNC Symbol;Acc:VGNC:95836]	2.42	0.041
ssc-miR-181a	DIP2B	disco interacting B [Source:VGNC Symbol;Acc:VGNC:87306]	2.42	0.041
ssc-miR-181a	DIP2C	disco interacting C [Source:VGNC Symbol;Acc:VGNC:96201]	2.42	0.041
ssc-miR-181a	DLG2	discs large MAGUK scaffold protein 2 [Source:VGNC Symbol;Acc:VGNC:108581]	2.42	0.041
ssc-miR-181a	DLGAP2	DLG associated protein 2 [Source:VGNC Symbol;Acc:VGNC:99711]	2.42	0.041
ssc-miR-181a	DMTN	dematin actin binding protein [Source:VGNC Symbol;Acc:VGNC:87358]	2.42	0.041
ssc-miR-181a	DMXL2	Dmx like 2 [Source:VGNC Symbol;Acc:VGNC:87360]	2.42	0.041
ssc-miR-181a	DNAJA4	DnaJ heat shock protein family (Hsp40) member A4 [Source:NCBI gene (formerly Entrezgene);Acc:397613]	2.42	0.041
ssc-miR-181a	DNAJB1	DnaJ heat shock protein family (Hsp40) member B1 [Source:VGNC Symbol;Acc:VGNC:96614]	2.42	0.041
ssc-miR-181a	DNAJB14	DnaJ heat shock protein family (Hsp40) member B14 [Source:VGNC Symbol;Acc:VGNC:98919]	2.42	0.041

ssc-miR-181a	DNAJB5	DnaJ heat shock protein family (Hsp40) member B5 [Source:VGNC Symbol;Acc:VGNC:103081]	2.42	0.041
ssc-miR-181a	DNAJC13	DnaJ heat shock protein family (Hsp40) member C13 [Source:VGNC Symbol;Acc:VGNC:108650]	2.42	0.041
ssc-miR-181a	DNAJC21	hypothetical gene	2.42	0.041
ssc-miR-181a	DNAJC3	DnaJ heat shock protein family (Hsp40) member C3 [Source:NCBI gene (formerly Entrezgene);Acc:100154166]	2.42	0.041
ssc-miR-181a	DNAJC5	DnaJ heat shock protein family (Hsp40) member C5 [Source:VGNC Symbol;Acc:VGNC:108714]	2.42	0.041
ssc-miR-181a	DNAL1	dynein axonemal light chain 1 [Source:VGNC Symbol;Acc:VGNC:87373]	2.42	0.041
ssc-miR-181a	DOCK10	dedicator of cytokinesis 10 [Source:VGNC Symbol;Acc:VGNC:96007]	2.42	0.041
ssc-miR-181a	DOCK4	dedicator of cytokinesis 4 [Source:VGNC Symbol;Acc:VGNC:87394]	2.42	0.041
ssc-miR-181a	DOCK7	dedicator of cytokinesis 7 [Source:VGNC Symbol;Acc:VGNC:87397]	2.42	0.041
ssc-miR-181a	DPP6	dipeptidyl peptidase like 6 [Source:VGNC Symbol;Acc:VGNC:87421]	2.42	0.041
ssc-miR-181a	DPYSL2	dihydropyrimidinase like 2 [Source:VGNC Symbol;Acc:VGNC:87430]	2.42	0.041
ssc-miR-181a	DR1	down-regulator of transcription 1 [Source:VGNC Symbol;Acc:VGNC:87435]	2.42	0.041
ssc-miR-181a	DRAM1	DNA damage regulated autophagy modulator 1 [Source:VGNC Symbol;Acc:VGNC:87436]	2.42	0.041
ssc-miR-181a	DRD1	dopamine receptor D1 [Source:VGNC Symbol;Acc:VGNC:87443]	2.42	0.041
ssc-miR-181a	DSC1	desmocollin 1 [Source:VGNC Symbol;Acc:VGNC:87452]	2.42	0.041
ssc-miR-181a	DTNA	dystrobrevin alpha [Source:VGNC Symbol;Acc:VGNC:87462]	2.42	0.041
ssc-miR-181a	DUSP10	dual specificity phosphatase 10 [Source:VGNC Symbol;Acc:VGNC:96246]	2.42	0.041
ssc-miR-181a	DUSP5	dual specificity phosphatase 5 [Source:VGNC Symbol;Acc:VGNC:87487]	2.42	0.041
ssc-miR-181a	DUSP6	dual specificity phosphatase 6 [Source:VGNC Symbol;Acc:VGNC:87488]	2.42	0.041
ssc-miR-181a	DYNC1LI2	dynein cytoplasmic 1 light intermediate chain 2 [Source:VGNC Symbol;Acc:VGNC:87498]	2.42	0.041
ssc-miR-181a	DYNLL2	dynein light chain LC8-type 2 [Source:VGNC Symbol;Acc:VGNC:87501]	2.42	0.041
ssc-miR-181a	DYRK2	dual specificity tyrosine phosphorylation regulated kinase 2 [Source:VGNC Symbol;Acc:VGNC:87507]	2.42	0.041
ssc-miR-181a	E2F5	E2F transcription factor 5 [Source:VGNC Symbol;Acc:VGNC:87516]	2.42	0.041
ssc-miR-181a	E2F7	E2F transcription factor 7 [Source:VGNC Symbol;Acc:VGNC:87518]	2.42	0.041
ssc-miR-181a	ECT2L	epithelial cell transforming 2 like [Source:VGNC Symbol;Acc:VGNC:87539]	2.42	0.041
ssc-miR-181a	EDA2R	hypothetical gene	2.42	0.041
ssc-miR-181a	EDAR	ectodysplasin A receptor [Source:VGNC Symbol;Acc:VGNC:87541]	2.42	0.041
ssc-miR-181a	EED	embryonic ectoderm development [Source:VGNC Symbol;Acc:VGNC:87552]	2.42	0.041
ssc-miR-181a	EGR1	early growth response 1 [Source:VGNC Symbol;Acc:VGNC:87590]	2.42	0.041
ssc-miR-181a	EGR3	early growth response 3 [Source:VGNC Symbol;Acc:VGNC:87591]	2.42	0.041
ssc-miR-181a	EHF	ETS homologous factor [Source:VGNC Symbol;Acc:VGNC:87599]	2.42	0.041
ssc-miR-181a	EIF2S2	eukaryotic translation initiation factor 2 subunit beta [Source:VGNC Symbol;Acc:VGNC:96265]	2.42	0.041
ssc-miR-181a	EIF4A2	eukaryotic translation initiation factor 4A2 [Source:VGNC Symbol;Acc:VGNC:87624]	2.42	0.041
ssc-miR-181a	EIF4E3	eukaryotic translation initiation factor 4E family member 3 [Source:VGNC Symbol;Acc:VGNC:87628]	2.42	0.041
ssc-miR-181a	ELAVL2	ELAV like RNA binding protein 2 [Source:VGNC Symbol;Acc:VGNC:87639]	2.42	0.041
ssc-miR-181a	ELAVL4	ELAV like RNA binding protein 4 [Source:VGNC Symbol;Acc:VGNC:97047]	2.42	0.041
ssc-miR-181a	ELMSAN1	hypothetical gene	2.42	0.041
ssc-miR-181a	ELN	elastin [Source:VGNC Symbol;Acc:VGNC:87655]	2.42	0.041
ssc-miR-181a	ELP4	elongator acetyltransferase complex subunit 4 [Source:VGNC Symbol;Acc:VGNC:87665]	2.42	0.041
ssc-miR-181a	EMC3	ER membrane protein complex subunit 3 [Source:VGNC Symbol;Acc:VGNC:87671]	2.42	0.041
ssc-miR-181a	EML1	EMAP like 1 [Source:VGNC Symbol;Acc:VGNC:87682]	2.42	0.041
ssc-miR-181a	EMX2	empty spiracles homeobox 2 [Source:VGNC Symbol;Acc:VGNC:87693]	2.42	0.041
ssc-miR-181a	EN2	engrailed homeobox 2 [Source:VGNC Symbol;Acc:VGNC:87694]	2.42	0.041
ssc-miR-181a	ENAH	ENAH actin regulator [Source:VGNC Symbol;Acc:VGNC:108271]	2.42	0.041
ssc-miR-181a	ENOX2	ecto-NOX disulfide-thiol exchanger 2 [Source:VGNC Symbol;Acc:VGNC:87707]	2.42	0.041
ssc-miR-181a	ENPP1	ectonucleotide pyrophosphatase/phosphodiesterase 1 [Source:VGNC Symbol;Acc:VGNC:87708]	2.42	0.041
ssc-miR-181a	ENTPD6	ectonucleoside triphosphate diphosphohydrolase 6 [Source:VGNC Symbol;Acc:VGNC:96731]	2.42	0.041
ssc-miR-181a	EPB41	erythrocyte membrane protein band 4.1 [Source:VGNC Symbol;Acc:VGNC:87722]	2.42	0.041
ssc-miR-181a	EPB41L3	erythrocyte membrane protein band 4.1 like 3 [Source:VGNC Symbol;Acc:VGNC:87724]	2.42	0.041

ssc-miR-181a	EPC1	enhancer of polycomb homolog 1 [Source:VGNC Symbol;Acc:VGNC:95923]	2.42	0.041
ssc-miR-181a	EPC2	enhancer of polycomb homolog 2 [Source:VGNC Symbol;Acc:VGNC:96045]	2.42	0.041
ssc-miR-181a	EPHA4	EPH receptor A4 [Source:VGNC Symbol;Acc:VGNC:96280]	2.42	0.041
ssc-miR-181a	EPHA7	EPH receptor A7 [Source:VGNC Symbol;Acc:VGNC:87734]	2.42	0.041
ssc-miR-181a	EPM2AIP1	EPM2A interacting protein 1 [Source:VGNC Symbol;Acc:VGNC:87741]	2.42	0.041
ssc-miR-181a	EPN2	epsin 2 [Source:VGNC Symbol;Acc:VGNC:97982]	2.42	0.041
ssc-miR-181a	EPT1	hypothetical gene	2.42	0.041
ssc-miR-181a	ERF	ETS2 repressor factor [Source:VGNC Symbol;Acc:VGNC:87767]	2.42	0.041
ssc-miR-181a	ERG	ETS transcription factor ERG [Source:VGNC Symbol;Acc:VGNC:87768]	2.42	0.041
ssc-miR-181a	ERGIC2	ERGIC and golgi 2 [Source:VGNC Symbol;Acc:VGNC:87771]	2.42	0.041
ssc-miR-181a	ERH	ERH mRNA splicing and mitosis factor [Source:VGNC Symbol;Acc:VGNC:87772]	2.42	0.041
ssc-miR-181a	ERI1	exoribonuclease 1 [Source:VGNC Symbol;Acc:VGNC:97985]	2.42	0.041
ssc-miR-181a	ERLIN2	ER lipid raft associated 2 [Source:VGNC Symbol;Acc:VGNC:95586]	2.42	0.041
ssc-miR-181a	ERMN	ermin [Source:VGNC Symbol;Acc:VGNC:96207]	2.42	0.041
ssc-miR-181a	ERO1L	hypothetical gene	2.42	0.041
ssc-miR-181a	ESM1	endothelial cell specific molecule 1 [Source:VGNC Symbol;Acc:VGNC:87789]	2.42	0.041
ssc-miR-181a	ESR1	estrogen receptor 1 [Source:VGNC Symbol;Acc:VGNC:103089]	2.42	0.041
ssc-miR-181a	ETNK1	ethanolamine kinase 1 [Source:VGNC Symbol;Acc:VGNC:87806]	2.42	0.041
ssc-miR-181a	ETS1	ETS proto-onco 1, transcription factor [Source:VGNC Symbol;Acc:VGNC:87808]	2.42	0.041
ssc-miR-181a	ETV6	ETS variant transcription factor 6 [Source:VGNC Symbol;Acc:VGNC:87815]	2.42	0.041
ssc-miR-181a	EVI5	ecotropic viral integration site 5 [Source:VGNC Symbol;Acc:VGNC:98793]	2.42	0.041
ssc-miR-181a	EVX1	even-skipped homeobox 1 [Source:VGNC Symbol;Acc:VGNC:87821]	2.42	0.041
ssc-miR-181a	EVX2	even-skipped homeobox 2 [Source:VGNC Symbol;Acc:VGNC:96290]	2.42	0.041
ssc-miR-181a	EXOSC2	exosome component 2 [Source:VGNC Symbol;Acc:VGNC:87838]	2.42	0.041
ssc-miR-181a	EXOSC3	exosome component 3 [Source:VGNC Symbol;Acc:VGNC:87839]	2.42	0.041
ssc-miR-181a	EYA3	EYA transcriptional coactivator and phosphatase 3 [Source:VGNC Symbol;Acc:VGNC:87852]	2.42	0.041
ssc-miR-181a	F3	coagulation factor III, tissue factor [Source:VGNC Symbol;Acc:VGNC:87864]	2.42	0.041
ssc-miR-181a	FAF1	Fas associated factor 1 [Source:VGNC Symbol;Acc:VGNC:87879]	2.42	0.041
ssc-miR-181a	FAM102A	family with sequence similarity 102 member A [Source:HGNC Symbol;Acc:HGNC:31419]	2.42	0.041
ssc-miR-181a	FAM105B	hypothetical gene	2.42	0.041
ssc-miR-181a	FAM118A	family with sequence similarity 118 member A [Source:VGNC Symbol;Acc:VGNC:87893]	2.42	0.041
ssc-miR-181a	FAM126B	family with sequence similarity 126 member B [Source:HGNC Symbol;Acc:HGNC:28593]	2.42	0.041
ssc-miR-181a	FAM135A	family with sequence similarity 135 member A [Source:VGNC Symbol;Acc:VGNC:87904]	2.42	0.041
ssc-miR-181a	FAM13B	family with sequence similarity 13 member B [Source:VGNC Symbol;Acc:VGNC:87906]	2.42	0.041
ssc-miR-181a	FAM160A1	hypothetical gene	2.42	0.041
ssc-miR-181a	FAM160A2	hypothetical gene	2.42	0.041
ssc-miR-181a	FAM163A	family with sequence similarity 163 member A [Source:HGNC Symbol;Acc:HGNC:28274]	2.42	0.041
ssc-miR-181a	FAM171A1	family with sequence similarity 171 member A1 [Source:VGNC Symbol;Acc:VGNC:96011]	2.42	0.041
ssc-miR-181a	FAM178A	hypothetical gene	2.42	0.041
ssc-miR-181a	FAM179B	hypothetical gene	2.42	0.041
ssc-miR-181a	FAM19A2	hypothetical gene	2.42	0.041
ssc-miR-181a	FAM222B	family with sequence similarity 222 member B [Source:VGNC Symbol;Acc:VGNC:87960]	2.42	0.041
ssc-miR-181a	FAM3C	FAM3 metabolism regulating signaling molecule C [Source:VGNC Symbol;Acc:VGNC:87969]	2.42	0.041
ssc-miR-181a	FAM46C	hypothetical gene	2.42	0.041
ssc-miR-181a	FAM49A	hypothetical gene	2.42	0.041
ssc-miR-181a	FAM58A	hypothetical gene	2.42	0.041
ssc-miR-181a	FAM73B	hypothetical gene	2.42	0.041
ssc-miR-181a	FAXC	hypothetical gene	2.42	0.041
ssc-miR-181a	FBXL17	F-box and leucine rich repeat protein 17 [Source:VGNC Symbol;Acc:VGNC:99650]	2.42	0.041

ssc-miR-181a	FBXL3	F-box and leucine rich repeat protein 3 [Source:VGNC Symbol;Acc:VGNC:88030]	2.42	0.041
ssc-miR-181a	FBXO10	F-box protein 10 [Source:VGNC Symbol;Acc:VGNC:103092]	2.42	0.041
ssc-miR-181a	FBXO11	F-box protein 11 [Source:VGNC Symbol;Acc:VGNC:88032]	2.42	0.041
ssc-miR-181a	FBXO33	F-box protein 33 [Source:VGNC Symbol;Acc:VGNC:88040]	2.42	0.041
ssc-miR-181a	FBXO34	F-box protein 34 [Source:VGNC Symbol;Acc:VGNC:88041]	2.42	0.041
ssc-miR-181a	FBXO41	F-box protein 41 [Source:VGNC Symbol;Acc:VGNC:88046]	2.42	0.041
ssc-miR-181a	FBXO45	F-box protein 45 [Source:VGNC Symbol;Acc:VGNC:88048]	2.42	0.041
ssc-miR-181a	FBXO48	F-box protein 48 [Source:VGNC Symbol;Acc:VGNC:88051]	2.42	0.041
ssc-miR-181a	FGD4	FYVE, RhoGEF and PH domain containing 4 [Source:VGNC Symbol;Acc:VGNC:88097]	2.42	0.041
ssc-miR-181a	FGFR3	fibroblast growth factor receptor 3 [Source:VGNC Symbol;Acc:VGNC:98929]	2.42	0.041
ssc-miR-181a	FHDC1	FH2 domain containing 1 [Source:VGNC Symbol;Acc:VGNC:88126]	2.42	0.041
ssc-miR-181a	FIGN	fidgetin, microtubule severing factor [Source:VGNC Symbol;Acc:VGNC:95580]	2.42	0.041
ssc-miR-181a	FKBP1A	hypothetical gene	2.42	0.041
ssc-miR-181a	FLJ00104	hypothetical gene	2.42	0.041
ssc-miR-181a	FLT1	fms related receptor tyrosine kinase 1 [Source:VGNC Symbol;Acc:VGNC:88162]	2.42	0.041
ssc-miR-181a	FMNL2	formin like 2 [Source:VGNC Symbol;Acc:VGNC:95879]	2.42	0.041
ssc-miR-181a	FMR1	FMRP translational regulator 1 [Source:VGNC Symbol;Acc:VGNC:88175]	2.42	0.041
ssc-miR-181a	FNBP4	formin binding protein 4 [Source:VGNC Symbol;Acc:VGNC:88180]	2.42	0.041
ssc-miR-181a	FNDC3A	fibronectin type III domain containing 3A [Source:VGNC Symbol;Acc:VGNC:88182]	2.42	0.041
ssc-miR-181a	FNDC3B	fibronectin type III domain containing 3B [Source:VGNC Symbol;Acc:VGNC:88183]	2.42	0.041
ssc-miR-181a	FNIP2	folliculin interacting protein 2 [Source:VGNC Symbol;Acc:VGNC:88188]	2.42	0.041
ssc-miR-181a	FOS	Fos proto-onco, AP-1 transcription factor subunit [Source:NCBI gene (formerly Entrezgene);Acc:100144486]	2.42	0.041
ssc-miR-181a	FOXB1	forkhead box B1 [Source:VGNC Symbol;Acc:VGNC:88195]	2.42	0.041
ssc-miR-181a	FOXK1	forkhead box K1 [Source:VGNC Symbol;Acc:VGNC:88214]	2.42	0.041
ssc-miR-181a	FOXP1	forkhead box P1 [Source:VGNC Symbol;Acc:VGNC:88222]	2.42	0.041
ssc-miR-181a	FOXP2	forkhead box P2 [Source:VGNC Symbol;Acc:VGNC:98014]	2.42	0.041
ssc-miR-181a	FRS2	fibroblast growth factor receptor substrate 2 [Source:VGNC Symbol;Acc:VGNC:88246]	2.42	0.041
ssc-miR-181a	FRYL	FRY like transcription coactivator [Source:VGNC Symbol;Acc:VGNC:98015]	2.42	0.041
ssc-miR-181a	FSD1L	fibronectin type III and SPRY domain containing 1 like [Source:HGNC Symbol;Acc:HGNC:13753]	2.42	0.041
ssc-miR-181a	FSTL1	follistatin like 1 [Source:VGNC Symbol;Acc:VGNC:88255]	2.42	0.041
ssc-miR-181a	FTO	FTO alpha-ketoglutarate dependent dioxygenase [Source:VGNC Symbol;Acc:VGNC:88259]	2.42	0.041
ssc-miR-181a	G3BP2	G3BP stress granule assembly factor 2 [Source:VGNC Symbol;Acc:VGNC:88289]	2.42	0.041
ssc-miR-181a	G6PC3	glucose-6-phosphatase catalytic subunit 3 [Source:VGNC Symbol;Acc:VGNC:88291]	2.42	0.041
ssc-miR-181a	GABRA1	gamma-aminobutyric acid type A receptor subunit alpha1 [Source:VGNC Symbol;Acc:VGNC:88300]	2.42	0.041
ssc-miR-181a	GABRA4	gamma-aminobutyric acid type A receptor subunit alpha4 [Source:VGNC Symbol;Acc:VGNC:88303]	2.42	0.041
ssc-miR-181a	GABRB1	gamma-aminobutyric acid type A receptor subunit beta1 [Source:VGNC Symbol;Acc:VGNC:88306]	2.42	0.041
ssc-miR-181a	GABRQ	gamma-aminobutyric acid type A receptor subunit theta [Source:VGNC Symbol;Acc:VGNC:88315]	2.42	0.041
ssc-miR-181a	GAD2	glutamate decarboxylase 2 [Source:VGNC Symbol;Acc:VGNC:108273]	2.42	0.041
ssc-miR-181a	GALNT16	polypeptide N-acetylgalactosaminyltransferase 16 [Source:VGNC Symbol;Acc:VGNC:88332]	2.42	0.041
ssc-miR-181a	GALNT4	polypeptide N-acetylgalactosaminyltransferase 4 [Source:HGNC Symbol;Acc:HGNC:4126]	2.42	0.041
ssc-miR-181a	GAPVD1	GTPase activating protein and VPS9 domains 1 [Source:VGNC Symbol;Acc:VGNC:88347]	2.42	0.041
ssc-miR-181a	GAS7	growth arrest specific 7 [Source:VGNC Symbol;Acc:VGNC:88359]	2.42	0.041
ssc-miR-181a	GATA6	GATA binding protein 6 [Source:VGNC Symbol;Acc:VGNC:88366]	2.42	0.041
ssc-miR-181a	GATC	glutamyl-tRNA amidotransferase subunit C [Source:VGNC Symbol;Acc:VGNC:103948]	2.42	0.041
ssc-miR-181a	GCC2	GRIP and coiled-coil domain containing 2 [Source:VGNC Symbol;Acc:VGNC:88380]	2.42	0.041
ssc-miR-181a	GCNT2	glucosaminyl (N-acetyl) transferase 2 (I blood group) [Source:HGNC Symbol;Acc:HGNC:4204]	2.42	0.041
ssc-miR-181a	GCNT4	glucosaminyl (N-acetyl) transferase 4 [Source:VGNC Symbol;Acc:VGNC:96616]	2.42	0.041
ssc-miR-181a	GDAP1	ganglioside induced differentiation associated protein 1 [Source:VGNC Symbol;Acc:VGNC:88394]	2.42	0.041
ssc-miR-181a	GDI1	GDP dissociation inhibitor 1 [Source:VGNC Symbol;Acc:VGNC:88405]	2.42	0.041

ssc-miR-181a	GDPD1	hypothetical gene	2.42	0.041
ssc-miR-181a	GEMIN6	gem nuclear organelle associated protein 6 [Source:VGNC Symbol;Acc:VGNC:88414]	2.42	0.041
ssc-miR-181a	GFPT1	glutamine--fructose-6-phosphate transaminase 1 [Source:VGNC Symbol;Acc:VGNC:88426]	2.42	0.041
ssc-miR-181a	GHITM	growth hormone inducible transmembrane protein [Source:VGNC Symbol;Acc:VGNC:88442]	2.42	0.041
ssc-miR-181a	GHR	growth hormone receptor [Source:NCBI gene (formerly Entrezgene);Acc:397488]	2.42	0.041
ssc-miR-181a	GID4	GID complex subunit 4 homolog [Source:VGNC Symbol;Acc:VGNC:98996]	2.42	0.041
ssc-miR-181a	GIGYF1	GRB10 interacting GYF protein 1 [Source:VGNC Symbol;Acc:VGNC:88444]	2.42	0.041
ssc-miR-181a	GJA9	gap junction protein alpha 9 [Source:VGNC Symbol;Acc:VGNC:97065]	2.42	0.041
ssc-miR-181a	GLCCI1	glucocorticoid induced 1 [Source:VGNC Symbol;Acc:VGNC:88476]	2.42	0.041
ssc-miR-181a	GLIS3	GLIS family zinc finger 3 [Source:VGNC Symbol;Acc:VGNC:88485]	2.42	0.041
ssc-miR-181a	GLO1	glyoxalase I [Source:HGNC Symbol;Acc:HGNC:4323]	2.42	0.041
ssc-miR-181a	GMFB	glia maturation factor beta [Source:VGNC Symbol;Acc:VGNC:88509]	2.42	0.041
ssc-miR-181a	GMPS	guanine monophosphate synthase [Source:VGNC Symbol;Acc:VGNC:88516]	2.42	0.041
ssc-miR-181a	GNA13	G protein subunit alpha 13 [Source:VGNC Symbol;Acc:VGNC:98997]	2.42	0.041
ssc-miR-181a	GNAO1	hypothetical gene	2.42	0.041
ssc-miR-181a	GNAQ	G protein subunit alpha q [Source:VGNC Symbol;Acc:VGNC:103100]	2.42	0.041
ssc-miR-181a	GNB4	G protein subunit beta 4 [Source:VGNC Symbol;Acc:VGNC:88533]	2.42	0.041
ssc-miR-181a	GNG2	hypothetical gene	2.42	0.041
ssc-miR-181a	GOLGA1	golgin A1 [Source:VGNC Symbol;Acc:VGNC:88549]	2.42	0.041
ssc-miR-181a	GOLIM4	golgi integral membrane protein 4 [Source:VGNC Symbol;Acc:VGNC:88555]	2.42	0.041
ssc-miR-181a	GOT2	glutamic-oxaloacetic transaminase 2 [Source:VGNC Symbol;Acc:VGNC:88562]	2.42	0.041
ssc-miR-181a	GP5	glycoprotein V platelet [Source:VGNC Symbol;Acc:VGNC:88566]	2.42	0.041
ssc-miR-181a	GPBP1	GC-rich promoter binding protein 1 [Source:VGNC Symbol;Acc:VGNC:88578]	2.42	0.041
ssc-miR-181a	GPCPD1	glycerophosphocholine phosphodiesterase 1 [Source:VGNC Symbol;Acc:VGNC:96148]	2.42	0.041
ssc-miR-181a	GPD1L	glycerol-3-phosphate dehydrogenase 1 like [Source:VGNC Symbol;Acc:VGNC:108661]	2.42	0.041
ssc-miR-181a	GPD2	glycerol-3-phosphate dehydrogenase 2 [Source:VGNC Symbol;Acc:VGNC:96330]	2.42	0.041
ssc-miR-181a	GPR137C	G protein-coupled receptor 137C [Source:VGNC Symbol;Acc:VGNC:88602]	2.42	0.041
ssc-miR-181a	GPR180	G protein-coupled receptor 180 [Source:VGNC Symbol;Acc:VGNC:88621]	2.42	0.041
ssc-miR-181a	GPSM1	G protein signaling modulator 1 [Source:VGNC Symbol;Acc:VGNC:88648]	2.42	0.041
ssc-miR-181a	GRAMD4	GRAM domain containing 4 [Source:VGNC Symbol;Acc:VGNC:88659]	2.42	0.041
ssc-miR-181a	GRB10	growth factor receptor bound protein 10 [Source:VGNC Symbol;Acc:VGNC:88662]	2.42	0.041
ssc-miR-181a	GREB1L	GREB1 like retinoic acid receptor coactivator [Source:VGNC Symbol;Acc:VGNC:96584]	2.42	0.041
ssc-miR-181a	GRIA2	glutamate ionotropic receptor AMPA type subunit 2 [Source:VGNC Symbol;Acc:VGNC:88671]	2.42	0.041
ssc-miR-181a	GRID1	glutamate ionotropic receptor delta type subunit 1 [Source:VGNC Symbol;Acc:VGNC:88674]	2.42	0.041
ssc-miR-181a	GRIK2	glutamate ionotropic receptor kainate type subunit 2 [Source:VGNC Symbol;Acc:VGNC:88678]	2.42	0.041
ssc-miR-181a	GRIK3	glutamate ionotropic receptor kainate type subunit 3 [Source:VGNC Symbol;Acc:VGNC:88679]	2.42	0.041
ssc-miR-181a	GRIK4	glutamate ionotropic receptor kainate type subunit 4 [Source:VGNC Symbol;Acc:VGNC:88680]	2.42	0.041
ssc-miR-181a	GRM1	glutamate metabotropic receptor 1 [Source:VGNC Symbol;Acc:VGNC:88700]	2.42	0.041
ssc-miR-181a	GRM5	glutamate metabotropic receptor 5 [Source:VGNC Symbol;Acc:VGNC:88703]	2.42	0.041
ssc-miR-181a	GRM7	glutamate metabotropic receptor 7 [Source:VGNC Symbol;Acc:VGNC:98028]	2.42	0.041
ssc-miR-181a	GSE1	Gse1 coiled-coil protein [Source:VGNC Symbol;Acc:VGNC:88720]	2.42	0.041
ssc-miR-181a	GSKIP	GSK3B interacting protein [Source:VGNC Symbol;Acc:VGNC:103956]	2.42	0.041
ssc-miR-181a	GSPT1	G1 to S phase transition 1 [Source:VGNC Symbol;Acc:VGNC:88724]	2.42	0.041
ssc-miR-181a	GUCY1A2	guanylate cyclase 1 soluble subunit alpha 2 [Source:VGNC Symbol;Acc:VGNC:88751]	2.42	0.041
ssc-miR-181a	HAND2	heart and neural crest derivatives expressed 2 [Source:VGNC Symbol;Acc:VGNC:88778]	2.42	0.041
ssc-miR-181a	HAPLN1	hyaluronan and proteoglycan link protein 1 [Source:VGNC Symbol;Acc:VGNC:88780]	2.42	0.041
ssc-miR-181a	HAUS3	hypothetical gene	2.42	0.041
ssc-miR-181a	HCN1	hyperpolarization activated cyclic nucleotide gated potassium channel 1 [Source:VGNC Symbol;Acc:VGNC:88802]	2.42	0.041
ssc-miR-181a	HCN2	hyperpolarization activated cyclic nucleotide gated potassium and sodium channel 2 [Source:VGNC Symbol;Acc:VGNC:88803]	2.42	0.041

ssc-miR-181a	HDAC9	histone deacetylase 9 [Source:HGNC Symbol;Acc:HGNC:14065]	2.42	0.041
ssc-miR-181a	HECA	hdc homolog, cell cycle regulator [Source:VGNC Symbol;Acc:VGNC:88831]	2.42	0.041
ssc-miR-181a	HECW2	HECT, C2 and WW domain containing E3 ubiquitin protein ligase 2 [Source:NCBI gene (formerly Entrezgene);Acc:100155879]	2.42	0.041
ssc-miR-181a	HEPHL1	hephaestin like 1 [Source:VGNC Symbol;Acc:VGNC:88848]	2.42	0.041
ssc-miR-181a	HEXIM1	HEXIM P-TEFb complex subunit 1 [Source:HGNC Symbol;Acc:HGNC:24953]	2.42	0.041
ssc-miR-181a	HEY2	hes related family bHLH transcription factor with YRPW motif 2 [Source:VGNC Symbol;Acc:VGNC:88864]	2.42	0.041
ssc-miR-181a	HGF	hepatocyte growth factor [Source:VGNC Symbol;Acc:VGNC:88869]	2.42	0.041
ssc-miR-181a	HIC2	HIC ZBTB transcriptional repressor 2 [Source:HGNC Symbol;Acc:HGNC:18595]	2.42	0.041
ssc-miR-181a	HINT3	histidine triad nucleotide binding protein 3 [Source:VGNC Symbol;Acc:VGNC:88884]	2.42	0.041
ssc-miR-181a	HIPK1	homeodomain interacting protein kinase 1 [Source:VGNC Symbol;Acc:VGNC:88887]	2.42	0.041
ssc-miR-181a	HIPK2	homeodomain interacting protein kinase 2 [Source:VGNC Symbol;Acc:VGNC:88888]	2.42	0.041
ssc-miR-181a	HIPK3	homeodomain interacting protein kinase 3 [Source:VGNC Symbol;Acc:VGNC:88889]	2.42	0.041
ssc-miR-181a	HLF	HLF transcription factor, PAR bZIP family member [Source:VGNC Symbol;Acc:VGNC:88896]	2.42	0.041
ssc-miR-181a	HMBS	hydroxymethylbilane synthase [Source:VGNC Symbol;Acc:VGNC:88899]	2.42	0.041
ssc-miR-181a	HMGB1	hypothetical gene	2.42	0.041
ssc-miR-181a	HMGB2	high mobility group box 2 [Source:VGNC Symbol;Acc:VGNC:88902]	2.42	0.041
ssc-miR-181a	HMX1	H6 family homeobox 1 [Source:VGNC Symbol;Acc:VGNC:88913]	2.42	0.041
ssc-miR-181a	HNRNPA0	hypothetical gene	2.42	0.041
ssc-miR-181a	HNRNPH1	heterous nuclear ribonucleoprotein H1 [Source:VGNC Symbol;Acc:VGNC:88920]	2.42	0.041
ssc-miR-181a	HNRNPK	heterous nuclear ribonucleoprotein K [Source:VGNC Symbol;Acc:VGNC:103963]	2.42	0.041
ssc-miR-181a	HNRNPR	heterous nuclear ribonucleoprotein R [Source:VGNC Symbol;Acc:VGNC:88925]	2.42	0.041
ssc-miR-181a	HOMER1	homer scaffold protein 1 [Source:VGNC Symbol;Acc:VGNC:88928]	2.42	0.041
ssc-miR-181a	HOXA1	homeobox A1 [Source:VGNC Symbol;Acc:VGNC:88933]	2.42	0.041
ssc-miR-181a	HOXA11	homeobox A11 [Source:VGNC Symbol;Acc:VGNC:88935]	2.42	0.041
ssc-miR-181a	HOXB4	homeobox B4 [Source:HGNC Symbol;Acc:HGNC:5115]	2.42	0.041
ssc-miR-181a	HOXB5	homeobox B5 [Source:VGNC Symbol;Acc:VGNC:88945]	2.42	0.041
ssc-miR-181a	HOXB8	homeobox B8 [Source:VGNC Symbol;Acc:VGNC:88948]	2.42	0.041
ssc-miR-181a	HOXC8	homeobox C8 [Source:VGNC Symbol;Acc:VGNC:88954]	2.42	0.041
ssc-miR-181a	HOXD1	homeobox D1 [Source:VGNC Symbol;Acc:VGNC:96350]	2.42	0.041
ssc-miR-181a	HS2ST1	heparan sulfate 2-O-sulfotransferase 1 [Source:HGNC Symbol;Acc:HGNC:5193]	2.42	0.041
ssc-miR-181a	HS3ST3A1	heparan sulfate-glucosamine 3-sulfotransferase 3A1 [Source:VGNC Symbol;Acc:VGNC:99002]	2.42	0.041
ssc-miR-181a	HS6ST1	heparan sulfate 6-O-sulfotransferase 1 [Source:VGNC Symbol;Acc:VGNC:96362]	2.42	0.041
ssc-miR-181a	HSP90B1	heat shock protein 90 beta family member 1 [Source:VGNC Symbol;Acc:VGNC:103290]	2.42	0.041
ssc-miR-181a	HSPA5	heat shock protein family A (Hsp70) member 5 [Source:VGNC Symbol;Acc:VGNC:103107]	2.42	0.041
ssc-miR-181a	HTR2C	5-hydroxytryptamine receptor 2C [Source:VGNC Symbol;Acc:VGNC:96737]	2.42	0.041
ssc-miR-181a	HTT	huntingtin [Source:VGNC Symbol;Acc:VGNC:89006]	2.42	0.041
ssc-miR-181a	HYOU1	hypoxia up-regulated 1 [Source:VGNC Symbol;Acc:VGNC:89014]	2.42	0.041
ssc-miR-181a	IARS2	isoleucyl-tRNA synthetase 2, mitochondrial [Source:VGNC Symbol;Acc:VGNC:96226]	2.42	0.041
ssc-miR-181a	IFNE	interferon epsilon [Source:VGNC Symbol;Acc:VGNC:89039]	2.42	0.041
ssc-miR-181a	IFRG15	hypothetical gene	2.42	0.041
ssc-miR-181a	IGDCC3	immunoglobulin superfamily DCC subclass member 3 [Source:HGNC Symbol;Acc:HGNC:9700]	2.42	0.041
ssc-miR-181a	IGF2BP2	insulin like growth factor 2 mRNA binding protein 2 [Source:VGNC Symbol;Acc:VGNC:89055]	2.42	0.041
ssc-miR-181a	IGF2BP3	insulin like growth factor 2 mRNA binding protein 3 [Source:VGNC Symbol;Acc:VGNC:89056]	2.42	0.041
ssc-miR-181a	IGSF11	immunoglobulin superfamily member 11 [Source:VGNC Symbol;Acc:VGNC:89064]	2.42	0.041
ssc-miR-181a	IK	IK cytokine [Source:VGNC Symbol;Acc:VGNC:89070]	2.42	0.041
ssc-miR-181a	IL1A	interleukin 1 alpha [Source:VGNC Symbol;Acc:VGNC:89091]	2.42	0.041
ssc-miR-181a	IL1RAPL1	interleukin 1 receptor accessory protein like 1 [Source:VGNC Symbol;Acc:VGNC:103967]	2.42	0.041
ssc-miR-181a	IL6ST	interleukin 6 cytokine family signal transducer [Source:VGNC Symbol;Acc:VGNC:89113]	2.42	0.041
ssc-miR-181a	ILF3	interleukin enhancer binding factor 3 [Source:VGNC Symbol;Acc:VGNC:89120]	2.42	0.041

ssc-miR-181a	IMPG2	interphotoreceptor matrix proteoglycan 2 [Source:VGNC Symbol;Acc:VGNC:98048]	2.42	0.041
ssc-miR-181a	INHBA	inhibin subunit beta A [Source:VGNC Symbol;Acc:VGNC:89133]	2.42	0.041
ssc-miR-181a	INO80	INO80 complex ATPase subunit [Source:VGNC Symbol;Acc:VGNC:89138]	2.42	0.041
ssc-miR-181a	INO80D	INO80 complex subunit D [Source:VGNC Symbol;Acc:VGNC:96113]	2.42	0.041
ssc-miR-181a	INPP5A	inositol polyphosphate-5-phosphatase A [Source:VGNC Symbol;Acc:VGNC:89142]	2.42	0.041
ssc-miR-181a	INPP5E	inositol polyphosphate-5-phosphatase E [Source:VGNC Symbol;Acc:VGNC:89144]	2.42	0.041
ssc-miR-181a	IPMK	inositol polyphosphate multikinase [Source:VGNC Symbol;Acc:VGNC:89175]	2.42	0.041
ssc-miR-181a	IPO5	importin 5 [Source:VGNC Symbol;Acc:VGNC:89179]	2.42	0.041
ssc-miR-181a	IPO8	importin 8 [Source:VGNC Symbol;Acc:VGNC:89181]	2.42	0.041
ssc-miR-181a	IPPK	inositol-pentakisphosphate 2-kinase [Source:VGNC Symbol;Acc:VGNC:89183]	2.42	0.041
ssc-miR-181a	IQCJ-SCHIP1	hypothetical gene	2.42	0.041
ssc-miR-181a	IQGAP2	IQ motif containing GTPase activating protein 2 [Source:VGNC Symbol;Acc:VGNC:89192]	2.42	0.041
ssc-miR-181a	IQSEC2	IQ motif and Sec7 domain ArfGEF 2 [Source:VGNC Symbol;Acc:VGNC:89195]	2.42	0.041
ssc-miR-181a	IRAK1BP1	interleukin 1 receptor associated kinase 1 binding protein 1 [Source:VGNC Symbol;Acc:VGNC:89199]	2.42	0.041
ssc-miR-181a	IRS2	insulin receptor substrate 2 [Source:VGNC Symbol;Acc:VGNC:89214]	2.42	0.041
ssc-miR-181a	ISPD	hypothetical gene	2.42	0.041
ssc-miR-181a	ITFG1	integrin alpha FG-GAP repeat containing 1 [Source:VGNC Symbol;Acc:VGNC:89229]	2.42	0.041
ssc-miR-181a	ITGA1	integrin subunit alpha 1 [Source:VGNC Symbol;Acc:VGNC:89231]	2.42	0.041
ssc-miR-181a	ITGA2	integrin subunit alpha 2 [Source:VGNC Symbol;Acc:VGNC:89234]	2.42	0.041
ssc-miR-181a	ITGA3	integrin subunit alpha 3 [Source:VGNC Symbol;Acc:VGNC:89235]	2.42	0.041
ssc-miR-181a	ITGA6	integrin subunit alpha 6 [Source:VGNC Symbol;Acc:VGNC:96378]	2.42	0.041
ssc-miR-181a	ITGB8	integrin subunit beta 8 [Source:VGNC Symbol;Acc:VGNC:89246]	2.42	0.041
ssc-miR-181a	ITPK1	inositol-tetrakisphosphate 1-kinase [Source:VGNC Symbol;Acc:VGNC:89251]	2.42	0.041
ssc-miR-181a	ITSN1	intersectin 1 [Source:VGNC Symbol;Acc:VGNC:108669]	2.42	0.041
ssc-miR-181a	JAK2	Janus kinase 2 [Source:VGNC Symbol;Acc:VGNC:89271]	2.42	0.041
ssc-miR-181a	JAKMIP3	Janus kinase and microtubule interacting protein 3 [Source:VGNC Symbol;Acc:VGNC:89275]	2.42	0.041
ssc-miR-181a	JARID2	jumonji and AT-rich interaction domain containing 2 [Source:VGNC Symbol;Acc:VGNC:89279]	2.42	0.041
ssc-miR-181a	JAZF1	JAZF zinc finger 1 [Source:VGNC Symbol;Acc:VGNC:89280]	2.42	0.041
ssc-miR-181a	JDP2	Jun dimerization protein 2 [Source:VGNC Symbol;Acc:VGNC:89282]	2.42	0.041
ssc-miR-181a	KANK1	KN motif and ankyrin repeat domains 1 [Source:VGNC Symbol;Acc:VGNC:103112]	2.42	0.041
ssc-miR-181a	KARS	hypothetical gene	2.42	0.041
ssc-miR-181a	KAT2B	lysine acetyltransferase 2B [Source:VGNC Symbol;Acc:VGNC:89304]	2.42	0.041
ssc-miR-181a	KAT7	lysine acetyltransferase 7 [Source:VGNC Symbol;Acc:VGNC:89307]	2.42	0.041
ssc-miR-181a	KATNBL1	katanin regulatory subunit B1 like 1 [Source:VGNC Symbol;Acc:VGNC:89313]	2.42	0.041
ssc-miR-181a	KCMF1	potassium channel modulatory factor 1 [Source:VGNC Symbol;Acc:VGNC:89322]	2.42	0.041
ssc-miR-181a	KCNA4	potassium voltage-gated channel subfamily A member 4 [Source:VGNC Symbol;Acc:VGNC:89326]	2.42	0.041
ssc-miR-181a	KCNC2	potassium voltage-gated channel subfamily C member 2 [Source:VGNC Symbol;Acc:VGNC:89333]	2.42	0.041
ssc-miR-181a	KCNH1	potassium voltage-gated channel subfamily H member 1 [Source:VGNC Symbol;Acc:VGNC:108599]	2.42	0.041
ssc-miR-181a	KCNJ1	potassium inwardly rectifying channel subfamily J member 1 [Source:VGNC Symbol;Acc:VGNC:89352]	2.42	0.041
ssc-miR-181a	KCNJ10	potassium inwardly rectifying channel subfamily J member 10 [Source:VGNC Symbol;Acc:VGNC:89353]	2.42	0.041
ssc-miR-181a	KCNJ15	potassium inwardly rectifying channel subfamily J member 15 [Source:VGNC Symbol;Acc:VGNC:89355]	2.42	0.041
ssc-miR-181a	KCNK10	potassium two pore domain channel subfamily K member 10 [Source:VGNC Symbol;Acc:VGNC:89364]	2.42	0.041
ssc-miR-181a	KCNK12	potassium two pore domain channel subfamily K member 12 [Source:HGNC Symbol;Acc:HGNC:6274]	2.42	0.041
ssc-miR-181a	KCNN3	potassium calcium-activated channel subfamily N member 3 [Source:VGNC Symbol;Acc:VGNC:98056]	2.42	0.041
ssc-miR-181a	KCNQ5	potassium voltage-gated channel subfamily Q member 5 [Source:VGNC Symbol;Acc:VGNC:89384]	2.42	0.041
ssc-miR-181a	KCTD10	potassium channel tetramerization domain containing 10 [Source:VGNC Symbol;Acc:VGNC:103972]	2.42	0.041
ssc-miR-181a	KCTD16	potassium channel tetramerization domain containing 16 [Source:VGNC Symbol;Acc:VGNC:89395]	2.42	0.041
ssc-miR-181a	KDM5A	lysine demethylase 5A [Source:VGNC Symbol;Acc:VGNC:89415]	2.42	0.041
ssc-miR-181a	KIAA0195	hypothetical gene	2.42	0.041

ssc-miR-181a	KIAA0226	hypothetical gene	2.42	0.041
ssc-miR-181a	KIAA0247	hypothetical gene	2.42	0.041
ssc-miR-181a	KIAA1024	hypothetical gene	2.42	0.041
ssc-miR-181a	KIAA1239	hypothetical gene	2.42	0.041
ssc-miR-181a	KIAA1244	hypothetical gene	2.42	0.041
ssc-miR-181a	KIAA1324L	hypothetical gene	2.42	0.041
ssc-miR-181a	KIAA1462	hypothetical gene	2.42	0.041
ssc-miR-181a	KIAA1549L	KIAA1549 like [Source:VGNC Symbol;Acc:VGNC:89445]	2.42	0.041
ssc-miR-181a	KIAA1551	hypothetical gene	2.42	0.041
ssc-miR-181a	KIAA1644	hypothetical gene	2.42	0.041
ssc-miR-181a	KIAA2018	hypothetical gene	2.42	0.041
ssc-miR-181a	KIAA2022	hypothetical gene	2.42	0.041
ssc-miR-181a	KIF1B	kinesin family member 1B [Source:VGNC Symbol;Acc:VGNC:89460]	2.42	0.041
ssc-miR-181a	KIF3A	kinesin family member 3A [Source:VGNC Symbol;Acc:VGNC:89470]	2.42	0.041
ssc-miR-181a	KIF3B	kinesin family member 3B [Source:VGNC Symbol;Acc:VGNC:96392]	2.42	0.041
ssc-miR-181a	KITLG	KIT ligand [Source:VGNC Symbol;Acc:VGNC:98061]	2.42	0.041
ssc-miR-181a	KLF15	Kruppel like factor 15 [Source:VGNC Symbol;Acc:VGNC:89495]	2.42	0.041
ssc-miR-181a	KLF3	Kruppel like factor 3 [Source:VGNC Symbol;Acc:VGNC:89498]	2.42	0.041
ssc-miR-181a	KLF6	Kruppel like factor 6 [Source:VGNC Symbol;Acc:VGNC:98063]	2.42	0.041
ssc-miR-181a	KLF7	Kruppel like factor 7 [Source:VGNC Symbol;Acc:VGNC:96396]	2.42	0.041
ssc-miR-181a	KLHL14	kelch like family member 14 [Source:VGNC Symbol;Acc:VGNC:89515]	2.42	0.041
ssc-miR-181a	KLHL15	kelch like family member 15 [Source:VGNC Symbol;Acc:VGNC:89516]	2.42	0.041
ssc-miR-181a	KLHL2	kelch like family member 2 [Source:VGNC Symbol;Acc:VGNC:89518]	2.42	0.041
ssc-miR-181a	KLHL24	kelch like family member 24 [Source:VGNC Symbol;Acc:VGNC:89521]	2.42	0.041
ssc-miR-181a	KLHL29	kelch like family member 29 [Source:VGNC Symbol;Acc:VGNC:89524]	2.42	0.041
ssc-miR-181a	KLHL3	kelch like family member 3 [Source:VGNC Symbol;Acc:VGNC:89525]	2.42	0.041
ssc-miR-181a	KLHL42	kelch like family member 42 [Source:VGNC Symbol;Acc:VGNC:89534]	2.42	0.041
ssc-miR-181a	KLHL5	kelch like family member 5 [Source:VGNC Symbol;Acc:VGNC:89535]	2.42	0.041
ssc-miR-181a	KMT2A	lysine methyltransferase 2A [Source:VGNC Symbol;Acc:VGNC:108600]	2.42	0.041
ssc-miR-181a	KMT2C	lysine methyltransferase 2C [Source:VGNC Symbol;Acc:VGNC:89550]	2.42	0.041
ssc-miR-181a	KMT2E	lysine methyltransferase 2E (inactive) [Source:VGNC Symbol;Acc:VGNC:89551]	2.42	0.041
ssc-miR-181a	KPNA1	karyopherin subunit alpha 1 [Source:VGNC Symbol;Acc:VGNC:89560]	2.42	0.041
ssc-miR-181a	KPNA4	karyopherin subunit alpha 4 [Source:VGNC Symbol;Acc:VGNC:89563]	2.42	0.041
ssc-miR-181a	KPNB1	karyopherin subunit beta 1 [Source:VGNC Symbol;Acc:VGNC:89567]	2.42	0.041
ssc-miR-181a	KRAS	KRAS proto-onco, GTPase [Source:VGNC Symbol;Acc:VGNC:89569]	2.42	0.041
ssc-miR-181a	KRBOX4	hypothetical gene	2.42	0.041
ssc-miR-181a	KREMEN1	kringle containing transmembrane protein 1 [Source:VGNC Symbol;Acc:VGNC:89572]	2.42	0.041
ssc-miR-181a	KRT222	keratin 222 [Source:HGNC Symbol;Acc:HGNC:28695]	2.42	0.041
ssc-miR-181a	KSR1	kinase suppressor of ras 1 [Source:VGNC Symbol;Acc:VGNC:89606]	2.42	0.041
ssc-miR-181a	L1CAM	L1 cell adhesion molecule [Source:HGNC Symbol;Acc:HGNC:6470]	2.42	0.041
ssc-miR-181a	LAMC1	laminin subunit gamma 1 [Source:VGNC Symbol;Acc:VGNC:89624]	2.42	0.041
ssc-miR-181a	LAMP3	hypothetical gene	2.42	0.041
ssc-miR-181a	LARP4	La ribonucleoprotein 4 [Source:VGNC Symbol;Acc:VGNC:89641]	2.42	0.041
ssc-miR-181a	LBR	lamin B receptor [Source:VGNC Symbol;Acc:VGNC:98068]	2.42	0.041
ssc-miR-181a	LCLAT1	lysocardiolipin acyltransferase 1 [Source:VGNC Symbol;Acc:VGNC:89658]	2.42	0.041
ssc-miR-181a	LCOR	ligand dependent nuclear receptor corepressor [Source:HGNC Symbol;Acc:HGNC:29503]	2.42	0.041
ssc-miR-181a	LEMD3	LEM domain containing 3 [Source:VGNC Symbol;Acc:VGNC:89679]	2.42	0.041
ssc-miR-181a	LGALSL	galectin like [Source:VGNC Symbol;Acc:VGNC:89698]	2.42	0.041
ssc-miR-181a	LGI2	leucine rich repeat LGI family member 2 [Source:VGNC Symbol;Acc:VGNC:89699]	2.42	0.041

ssc-miR-181a	LHFPL4	LHFPL tetraspan subfamily member 4 [Source:VGNC Symbol;Acc:VGNC:89707]	2.42	0.041
ssc-miR-181a	LHX9	LIM homeobox 9 [Source:VGNC Symbol;Acc:VGNC:95608]	2.42	0.041
ssc-miR-181a	LIF	LIF interleukin 6 family cytokine [Source:VGNC Symbol;Acc:VGNC:89719]	2.42	0.041
ssc-miR-181a	LIMCH1	LIM and calponin homology domains 1 [Source:VGNC Symbol;Acc:VGNC:89724]	2.42	0.041
ssc-miR-181a	LIMS1	hypothetical gene	2.42	0.041
ssc-miR-181a	LIN28A	lin-28 homolog A [Source:VGNC Symbol;Acc:VGNC:98492]	2.42	0.041
ssc-miR-181a	LIN28B	lin-28 homolog B [Source:VGNC Symbol;Acc:VGNC:89729]	2.42	0.041
ssc-miR-181a	LIN7C	lin-7 homolog C, crumbs cell polarity complex component [Source:VGNC Symbol;Acc:VGNC:89732]	2.42	0.041
ssc-miR-181a	LLPH	LLP homolog, long-term synaptic facilitation factor [Source:VGNC Symbol;Acc:VGNC:103297]	2.42	0.041
ssc-miR-181a	LMBRD2	LMBR1 domain containing 2 [Source:VGNC Symbol;Acc:VGNC:89755]	2.42	0.041
ssc-miR-181a	LMO1	LIM domain only 1 [Source:VGNC Symbol;Acc:VGNC:89764]	2.42	0.041
ssc-miR-181a	LMO3	hypothetical gene	2.42	0.041
ssc-miR-181a	LNX2	ligand of numb-protein X 2 [Source:VGNC Symbol;Acc:VGNC:89774]	2.42	0.041
ssc-miR-181a	LOX	lysyl oxidase [Source:VGNC Symbol;Acc:VGNC:99785]	2.42	0.041
ssc-miR-181a	LPCAT1	lysophosphatidylcholine acyltransferase 1 [Source:VGNC Symbol;Acc:VGNC:89788]	2.42	0.041
ssc-miR-181a	LPGAT1	lysophosphatidylglycerol acyltransferase 1 [Source:VGNC Symbol;Acc:VGNC:89792]	2.42	0.041
ssc-miR-181a	LPP	LIM domain containing preferred translocation partner in lipoma [Source:HGNC Symbol;Acc:HGNC:6679]	2.42	0.041
ssc-miR-181a	LPPR4	hypothetical gene	2.42	0.041
ssc-miR-181a	LRBA	LPS responsive beige-like anchor protein [Source:VGNC Symbol;Acc:VGNC:98074]	2.42	0.041
ssc-miR-181a	LRIG2	leucine rich repeats and immunoglobulin like domains 2 [Source:VGNC Symbol;Acc:VGNC:89811]	2.42	0.041
ssc-miR-181a	LRP12	LDL receptor related protein 12 [Source:VGNC Symbol;Acc:VGNC:89818]	2.42	0.041
ssc-miR-181a	LRP4	LDL receptor related protein 4 [Source:VGNC Symbol;Acc:VGNC:89820]	2.42	0.041
ssc-miR-181a	LRRC32	leucine rich repeat containing 32 [Source:VGNC Symbol;Acc:VGNC:108602]	2.42	0.041
ssc-miR-181a	LRRC8D	leucine rich repeat containing 8 VRAC subunit D [Source:VGNC Symbol;Acc:VGNC:98088]	2.42	0.041
ssc-miR-181a	LRRFIP1	hypothetical gene	2.42	0.041
ssc-miR-181a	LRRN1	leucine rich repeat neuronal 1 [Source:VGNC Symbol;Acc:VGNC:89860]	2.42	0.041
ssc-miR-181a	LUZP1	leucine zipper protein 1 [Source:VGNC Symbol;Acc:VGNC:89897]	2.42	0.041
ssc-miR-181a	LYRM1	LYR motif containing 1 [Source:VGNC Symbol;Acc:VGNC:89915]	2.42	0.041
ssc-miR-181a	MAB21L2	mab-21 like 2 [Source:HGNC Symbol;Acc:HGNC:6758]	2.42	0.041
ssc-miR-181a	MADD	MAP kinase activating death domain [Source:VGNC Symbol;Acc:VGNC:89942]	2.42	0.041
ssc-miR-181a	MAEA	macrophage erythroblast attacher, E3 ubiquitin ligase [Source:VGNC Symbol;Acc:VGNC:89943]	2.42	0.041
ssc-miR-181a	MAGOHB	hypothetical gene	2.42	0.041
ssc-miR-181a	MAMDC2	MAM domain containing 2 [Source:VGNC Symbol;Acc:VGNC:89962]	2.42	0.041
ssc-miR-181a	MAN2A1	mannosidase alpha class 2A member 1 [Source:VGNC Symbol;Acc:VGNC:98098]	2.42	0.041
ssc-miR-181a	MAP1A	microtubule associated protein 1A [Source:VGNC Symbol;Acc:VGNC:89978]	2.42	0.041
ssc-miR-181a	MAP1B	microtubule associated protein 1B [Source:VGNC Symbol;Acc:VGNC:89979]	2.42	0.041
ssc-miR-181a	MAP2K1	mitogen-activated protein kinase kinase 1 [Source:VGNC Symbol;Acc:VGNC:103121]	2.42	0.041
ssc-miR-181a	MAP3K1	mitogen-activated protein kinase kinase kinase 1 [Source:VGNC Symbol;Acc:VGNC:98104]	2.42	0.041
ssc-miR-181a	MAP3K10	mitogen-activated protein kinase kinase kinase 10 [Source:VGNC Symbol;Acc:VGNC:89981]	2.42	0.041
ssc-miR-181a	MAP3K2	mitogen-activated protein kinase kinase kinase 2 [Source:VGNC Symbol;Acc:VGNC:98107]	2.42	0.041
ssc-miR-181a	MAP3K3	mitogen-activated protein kinase kinase kinase 3 [Source:VGNC Symbol;Acc:VGNC:98108]	2.42	0.041
ssc-miR-181a	MAP3K8	mitogen-activated protein kinase kinase kinase 8 [Source:VGNC Symbol;Acc:VGNC:98110]	2.42	0.041
ssc-miR-181a	MAP3K9	mitogen-activated protein kinase kinase kinase 9 [Source:VGNC Symbol;Acc:VGNC:89989]	2.42	0.041
ssc-miR-181a	MAP4K4	mitogen-activated protein kinase kinase kinase kinase 4 [Source:VGNC Symbol;Acc:VGNC:98114]	2.42	0.041
ssc-miR-181a	MAPK1	mitogen-activated protein kinase 1 [Source:VGNC Symbol;Acc:VGNC:89996]	2.42	0.041
ssc-miR-181a	MAPK1IP1L	mitogen-activated protein kinase 1 interacting protein 1 like [Source:VGNC Symbol;Acc:VGNC:90002]	2.42	0.041
ssc-miR-181a	MAPK8	mitogen-activated protein kinase 8 [Source:VGNC Symbol;Acc:VGNC:90006]	2.42	0.041
ssc-miR-181a	MAPRE2	microtubule associated protein RP/EB family member 2 [Source:VGNC Symbol;Acc:VGNC:98117]	2.42	0.041
ssc-miR-181a	MAPT	microtubule associated protein tau [Source:VGNC Symbol;Acc:VGNC:90016]	2.42	0.041

ssc-miR-181a	MARCH11	hypothetical gene	2.42	0.041
ssc-miR-181a	MARCKS	myristoylated alanine rich protein kinase C substrate [Source:VGNC Symbol;Acc:VGNC:90024]	2.42	0.041
ssc-miR-181a	MARK1	microtubule affinity regulating kinase 1 [Source:VGNC Symbol;Acc:VGNC:96411]	2.42	0.041
ssc-miR-181a	MB21D2	Mab-21 domain containing 2 [Source:VGNC Symbol;Acc:VGNC:90047]	2.42	0.041
ssc-miR-181a	MBD2	methyl-CpG binding domain protein 2 [Source:VGNC Symbol;Acc:VGNC:90048]	2.42	0.041
ssc-miR-181a	MBLAC2	metallo-beta-lactamase domain containing 2 [Source:VGNC Symbol;Acc:VGNC:90053]	2.42	0.041
ssc-miR-181a	MBNL1	muscleblind like splicing regulator 1 [Source:VGNC Symbol;Acc:VGNC:90054]	2.42	0.041
ssc-miR-181a	MBNL2	muscleblind like splicing regulator 2 [Source:VGNC Symbol;Acc:VGNC:90055]	2.42	0.041
ssc-miR-181a	MBOAT1	membrane bound O-acyltransferase domain containing 1 [Source:VGNC Symbol;Acc:VGNC:90057]	2.42	0.041
ssc-miR-181a	MBOAT2	membrane bound O-acyltransferase domain containing 2 [Source:VGNC Symbol;Acc:VGNC:90058]	2.42	0.041
ssc-miR-181a	MBTPS2	hypothetical gene	2.42	0.041
ssc-miR-181a	MCC	MCC regulator of WNT signaling pathway [Source:VGNC Symbol;Acc:VGNC:96588]	2.42	0.041
ssc-miR-181a	MCL1	MCL1 apoptosis regulator, BCL2 family member [Source:VGNC Symbol;Acc:VGNC:90072]	2.42	0.041
ssc-miR-181a	MECP2	methyl-CpG binding protein 2 [Source:VGNC Symbol;Acc:VGNC:90101]	2.42	0.041
ssc-miR-181a	MED12L	mediator complex subunit 12L [Source:VGNC Symbol;Acc:VGNC:90105]	2.42	0.041
ssc-miR-181a	MED26	mediator complex subunit 26 [Source:VGNC Symbol;Acc:VGNC:100168]	2.42	0.041
ssc-miR-181a	MED4	mediator complex subunit 4 [Source:VGNC Symbol;Acc:VGNC:90121]	2.42	0.041
ssc-miR-181a	MED8	mediator complex subunit 8 [Source:VGNC Symbol;Acc:VGNC:90124]	2.42	0.041
ssc-miR-181a	MEF2A	myocyte enhancer factor 2A [Source:VGNC Symbol;Acc:VGNC:98123]	2.42	0.041
ssc-miR-181a	METAP1	methionyl aminopeptidase 1 [Source:VGNC Symbol;Acc:VGNC:90151]	2.42	0.041
ssc-miR-181a	METAP2	methionyl aminopeptidase 2 [Source:VGNC Symbol;Acc:VGNC:90152]	2.42	0.041
ssc-miR-181a	MEX3A	mex-3 RNA binding family member A [Source:VGNC Symbol;Acc:VGNC:90168]	2.42	0.041
ssc-miR-181a	MEX3B	mex-3 RNA binding family member B [Source:VGNC Symbol;Acc:VGNC:90169]	2.42	0.041
ssc-miR-181a	MFAP3	microfibril associated protein 3 [Source:VGNC Symbol;Acc:VGNC:90174]	2.42	0.041
ssc-miR-181a	MFAP3L	microfibril associated protein 3 like [Source:VGNC Symbol;Acc:VGNC:90175]	2.42	0.041
ssc-miR-181a	MFSD6	major facilitator superfamily domain containing 6 [Source:VGNC Symbol;Acc:VGNC:96052]	2.42	0.041
ssc-miR-181a	MGA	MAX dimerization protein MGA [Source:VGNC Symbol;Acc:VGNC:90195]	2.42	0.041
ssc-miR-181a	MGAT2	alpha-1,6-mannosyl-glycoprotein 2-beta-N-acetylglucosaminyltransferase [Source:HGNC Symbol;Acc:HGNC:7045]	2.42	0.041
ssc-miR-181a	MID2	midline 2 [Source:VGNC Symbol;Acc:VGNC:98127]	2.42	0.041
ssc-miR-181a	MIER3	MIER family member 3 [Source:VGNC Symbol;Acc:VGNC:90221]	2.42	0.041
ssc-miR-181a	MINK1	misshapen like kinase 1 [Source:VGNC Symbol;Acc:VGNC:98128]	2.42	0.041
ssc-miR-181a	MIP	major intrinsic protein of lens fiber [Source:VGNC Symbol;Acc:VGNC:90235]	2.42	0.041
ssc-miR-181a	MITF	melanocyte inducing transcription factor [Source:VGNC Symbol;Acc:VGNC:90243]	2.42	0.041
ssc-miR-181a	MKNK2	MAPK interacting serine/threonine kinase 2 [Source:VGNC Symbol;Acc:VGNC:90247]	2.42	0.041
ssc-miR-181a	MLEC	malectin [Source:HGNC Symbol;Acc:HGNC:28973]	2.42	0.041
ssc-miR-181a	MLF1	myeloid leukemia factor 1 [Source:HGNC Symbol;Acc:HGNC:7125]	2.42	0.041
ssc-miR-181a	MLK4	hypothetical gene	2.42	0.041
ssc-miR-181a	MME	membrane metalloendopeptidase [Source:VGNC Symbol;Acc:VGNC:90265]	2.42	0.041
ssc-miR-181a	MMP14	matrix metallopeptidase 14 [Source:NCBI gene (formerly Entrezgene);Acc:397471]	2.42	0.041
ssc-miR-181a	MNX1	motor neuron and pancreas homeobox 1 [Source:VGNC Symbol;Acc:VGNC:107152]	2.42	0.041
ssc-miR-181a	MOB3B	MOB kinase activator 3B [Source:VGNC Symbol;Acc:VGNC:96024]	2.42	0.041
ssc-miR-181a	MON2	MON2 homolog, regulator of endosome-to-Golgi trafficking [Source:VGNC Symbol;Acc:VGNC:90301]	2.42	0.041
ssc-miR-181a	MOSPD1	motile sperm domain containing 1 [Source:VGNC Symbol;Acc:VGNC:103990]	2.42	0.041
ssc-miR-181a	MPP5	hypothetical gene	2.42	0.041
ssc-miR-181a	MPP7	MAGUK p55 scaffold protein 7 [Source:VGNC Symbol;Acc:VGNC:98138]	2.42	0.041
ssc-miR-181a	MPZL3	myelin protein zero like 3 [Source:VGNC Symbol;Acc:VGNC:90337]	2.42	0.041
ssc-miR-181a	MRPL17	mitochondrial ribosomal protein L17 [Source:VGNC Symbol;Acc:VGNC:103992]	2.42	0.041
ssc-miR-181a	MSANTD3	Myb/SANT DNA binding domain containing 3 [Source:HGNC Symbol;Acc:HGNC:23370]	2.42	0.041
ssc-miR-181a	MSANTD3-TMEFF1	hypothetical gene	2.42	0.041

ssc-miR-181a	MSI1	musashi RNA binding protein 1 [Source:VGNC Symbol;Acc:VGNC:90421]	2.42	0.041
ssc-miR-181a	MSI2	musashi RNA binding protein 2 [Source:VGNC Symbol;Acc:VGNC:90422]	2.42	0.041
ssc-miR-181a	MSL2	MSL complex subunit 2 [Source:VGNC Symbol;Acc:VGNC:90424]	2.42	0.041
ssc-miR-181a	MTF1	metal regulatory transcription factor 1 [Source:VGNC Symbol;Acc:VGNC:90443]	2.42	0.041
ssc-miR-181a	MTF2	metal response element binding transcription factor 2 [Source:VGNC Symbol;Acc:VGNC:90444]	2.42	0.041
ssc-miR-181a	MTMR1	myotubularin related protein 1 [Source:VGNC Symbol;Acc:VGNC:90456]	2.42	0.041
ssc-miR-181a	MTMR10	myotubularin related protein 10 [Source:VGNC Symbol;Acc:VGNC:90457]	2.42	0.041
ssc-miR-181a	MTMR12	myotubularin related protein 12 [Source:VGNC Symbol;Acc:VGNC:90459]	2.42	0.041
ssc-miR-181a	MTMR9	myotubularin related protein 9 [Source:VGNC Symbol;Acc:VGNC:90464]	2.42	0.041
ssc-miR-181a	MTPN	myotrophin [Source:VGNC Symbol;Acc:VGNC:90467]	2.42	0.041
ssc-miR-181a	MTX3	metaxin 3 [Source:VGNC Symbol;Acc:VGNC:90479]	2.42	0.041
ssc-miR-181a	MUC19	hypothetical gene	2.42	0.041
ssc-miR-181a	MYBL1	MYB proto-onco like 1 [Source:VGNC Symbol;Acc:VGNC:90498]	2.42	0.041
ssc-miR-181a	MYCBP	MYC binding protein [Source:NCBI gene (formerly Entrezgene);Acc:100513990]	2.42	0.041
ssc-miR-181a	MYCBP2	MYC binding protein 2 [Source:VGNC Symbol;Acc:VGNC:90502]	2.42	0.041
ssc-miR-181a	MYLK	myosin light chain kinase [Source:VGNC Symbol;Acc:VGNC:108676]	2.42	0.041
ssc-miR-181a	MYLK3	myosin light chain kinase 3 [Source:VGNC Symbol;Acc:VGNC:90518]	2.42	0.041
ssc-miR-181a	MYO1E	myosin IE [Source:VGNC Symbol;Acc:VGNC:90528]	2.42	0.041
ssc-miR-181a	NAA15	N-alpha-acetyltransferase 15, NatA auxiliary subunit [Source:VGNC Symbol;Acc:VGNC:96747]	2.42	0.041
ssc-miR-181a	NAA20	N-alpha-acetyltransferase 20, NatB catalytic subunit [Source:VGNC Symbol;Acc:VGNC:96749]	2.42	0.041
ssc-miR-181a	NAA50	N-alpha-acetyltransferase 50, NatE catalytic subunit [Source:VGNC Symbol;Acc:VGNC:104002]	2.42	0.041
ssc-miR-181a	NAALADL2	N-acetylated alpha-linked acidic dipeptidase like 2 [Source:VGNC Symbol;Acc:VGNC:90559]	2.42	0.041
ssc-miR-181a	NAB1	NGFI-A binding protein 1 [Source:VGNC Symbol;Acc:VGNC:96431]	2.42	0.041
ssc-miR-181a	NACC2	NACC family member 2 [Source:VGNC Symbol;Acc:VGNC:90564]	2.42	0.041
ssc-miR-181a	NBEA	neurobeachin [Source:VGNC Symbol;Acc:VGNC:90590]	2.42	0.041
ssc-miR-181a	NBEAL1	cytochrome P450 family 20 subfamily A member 1 [Source:VGNC Symbol;Acc:VGNC:103366]	2.42	0.041
ssc-miR-181a	NCALD	neurocalcin delta [Source:VGNC Symbol;Acc:VGNC:90594]	2.42	0.041
ssc-miR-181a	NCOA2	nuclear receptor coactivator 2 [Source:VGNC Symbol;Acc:VGNC:90616]	2.42	0.041
ssc-miR-181a	NDRG2	NDRG family member 2 [Source:VGNC Symbol;Acc:VGNC:90632]	2.42	0.041
ssc-miR-181a	NEGR1	neuronal growth regulator 1 [Source:VGNC Symbol;Acc:VGNC:90671]	2.42	0.041
ssc-miR-181a	NEK7	NIMA related kinase 7 [Source:VGNC Symbol;Acc:VGNC:98152]	2.42	0.041
ssc-miR-181a	NELFA	negative elongation factor complex member A [Source:VGNC Symbol;Acc:VGNC:90680]	2.42	0.041
ssc-miR-181a	NETO2	neuropilin and tolloid like 2 [Source:VGNC Symbol;Acc:VGNC:90691]	2.42	0.041
ssc-miR-181a	NEURL1B	neuralized E3 ubiquitin protein ligase 1B [Source:VGNC Symbol;Acc:VGNC:90695]	2.42	0.041
ssc-miR-181a	NFAT5	nuclear factor of activated T cells 5 [Source:VGNC Symbol;Acc:VGNC:90708]	2.42	0.041
ssc-miR-181a	NFATC2	nuclear factor of activated T cells 2 [Source:VGNC Symbol;Acc:VGNC:96440]	2.42	0.041
ssc-miR-181a	NFATC2IP	nuclear factor of activated T cells 2 interacting protein [Source:VGNC Symbol;Acc:VGNC:90709]	2.42	0.041
ssc-miR-181a	NFIB	nuclear factor I B [Source:VGNC Symbol;Acc:VGNC:90716]	2.42	0.041
ssc-miR-181a	NHSL2	NHS like 2 [Source:VGNC Symbol;Acc:VGNC:98155]	2.42	0.041
ssc-miR-181a	NIPAL4	NIPA like domain containing 4 [Source:VGNC Symbol;Acc:VGNC:90751]	2.42	0.041
ssc-miR-181a	NIPBL	NIPBL cohesin loading factor [Source:VGNC Symbol;Acc:VGNC:90752]	2.42	0.041
ssc-miR-181a	NKAIN2	sodium/potassium transporting ATPase interacting 2 [Source:VGNC Symbol;Acc:VGNC:103141]	2.42	0.041
ssc-miR-181a	NKAP	NFKB activating protein [Source:VGNC Symbol;Acc:VGNC:90756]	2.42	0.041
ssc-miR-181a	NKRF	NFKB repressing factor [Source:VGNC Symbol;Acc:VGNC:90764]	2.42	0.041
ssc-miR-181a	NKX3-2	NK3 homeobox 2 [Source:VGNC Symbol;Acc:VGNC:90773]	2.42	0.041
ssc-miR-181a	NLK	nemo like kinase [Source:VGNC Symbol;Acc:VGNC:90779]	2.42	0.041
ssc-miR-181a	NLN	neurolysin [Source:VGNC Symbol;Acc:VGNC:90780]	2.42	0.041
ssc-miR-181a	NME1	NME/NM23 nucleoside diphosphate kinase 1 [Source:VGNC Symbol;Acc:VGNC:99023]	2.42	0.041
ssc-miR-181a	NMNAT2	nicotinamide nucleotide adenylyltransferase 2 [Source:VGNC Symbol;Acc:VGNC:90798]	2.42	0.041

ssc-miR-181a	NMNAT3	nicotinamide nucleotide adenylyltransferase 3 [Source:VGNC Symbol;Acc:VGNC:108679]	2.42	0.041
ssc-miR-181a	NMT1	N-myristoyltransferase 1 [Source:VGNC Symbol;Acc:VGNC:90802]	2.42	0.041
ssc-miR-181a	NMT2	N-myristoyltransferase 2 [Source:VGNC Symbol;Acc:VGNC:96448]	2.42	0.041
ssc-miR-181a	NOG	noggin [Source:VGNC Symbol;Acc:VGNC:90812]	2.42	0.041
ssc-miR-181a	NOL4	nucleolar protein 4 [Source:VGNC Symbol;Acc:VGNC:90814]	2.42	0.041
ssc-miR-181a	NOTCH2	notch receptor 2 [Source:HGNC Symbol;Acc:HGNC:7882]	2.42	0.041
ssc-miR-181a	NOVA1	NOVA alternative splicing regulator 1 [Source:VGNC Symbol;Acc:VGNC:90827]	2.42	0.041
ssc-miR-181a	NPEPPS	aminopeptidase puromycin sensitive [Source:VGNC Symbol;Acc:VGNC:98157]	2.42	0.041
ssc-miR-181a	NPTN	neuroplastin [Source:VGNC Symbol;Acc:VGNC:90861]	2.42	0.041
ssc-miR-181a	NPTXR	neuronal pentraxin receptor [Source:VGNC Symbol;Acc:VGNC:98158]	2.42	0.041
ssc-miR-181a	NR1D2	nuclear receptor subfamily 1 group D member 2 [Source:VGNC Symbol;Acc:VGNC:99726]	2.42	0.041
ssc-miR-181a	NR2C2	nuclear receptor subfamily 2 group C member 2 [Source:VGNC Symbol;Acc:VGNC:90876]	2.42	0.041
ssc-miR-181a	NR3C1	nuclear receptor subfamily 3 group C member 1 [Source:VGNC Symbol;Acc:VGNC:90883]	2.42	0.041
ssc-miR-181a	NR4A3	nuclear receptor subfamily 4 group A member 3 [Source:VGNC Symbol;Acc:VGNC:90885]	2.42	0.041
ssc-miR-181a	NR6A1	nuclear receptor subfamily 6 group A member 1 [Source:VGNC Symbol;Acc:VGNC:90887]	2.42	0.041
ssc-miR-181a	NRAS	NRAS proto-onco, GTPase [Source:VGNC Symbol;Acc:VGNC:98827]	2.42	0.041
ssc-miR-181a	NREP	neuronal regeneration related protein [Source:HGNC Symbol;Acc:HGNC:16834]	2.42	0.041
ssc-miR-181a	NRP1	neuropilin 1 [Source:VGNC Symbol;Acc:VGNC:104012]	2.42	0.041
ssc-miR-181a	NRXN1	neurexin 1 [Source:HGNC Symbol;Acc:HGNC:8008]	2.42	0.041
ssc-miR-181a	NSG1	neuronal vesicle trafficking associated 1 [Source:VGNC Symbol;Acc:VGNC:90907]	2.42	0.041
ssc-miR-181a	NSMAF	neutral sphingomyelinase activation associated factor [Source:VGNC Symbol;Acc:VGNC:90909]	2.42	0.041
ssc-miR-181a	NSUN3	NOP2/Sun RNA methyltransferase 3 [Source:VGNC Symbol;Acc:VGNC:90915]	2.42	0.041
ssc-miR-181a	NT5DC3	5'-nucleotidase domain containing 3 [Source:VGNC Symbol;Acc:VGNC:90924]	2.42	0.041
ssc-miR-181a	NUCKS1	nuclear casein kinase and cyclin dependent kinase substrate 1 [Source:VGNC Symbol;Acc:VGNC:90947]	2.42	0.041
ssc-miR-181a	NUDT21	nudix hydrolase 21 [Source:VGNC Symbol;Acc:VGNC:98523]	2.42	0.041
ssc-miR-181a	NUFIP2	nuclear FMR1 interacting protein 2 [Source:VGNC Symbol;Acc:VGNC:90967]	2.42	0.041
ssc-miR-181a	NUP155	nucleoporin 155 [Source:VGNC Symbol;Acc:VGNC:90974]	2.42	0.041
ssc-miR-181a	NUS1	NUS1 dehydrodolichyl diphosphate synthase subunit [Source:VGNC Symbol;Acc:VGNC:90990]	2.42	0.041
ssc-miR-181a	OGT	O-linked N-acetylglucosamine (GlcNAc) transferase [Source:NCBI gene (formerly Entrezgene);Acc:664652]	2.42	0.041
ssc-miR-181a	OLFM3	olfactomedin 3 [Source:VGNC Symbol;Acc:VGNC:91033]	2.42	0.041
ssc-miR-181a	ONECUT2	one cut homeobox 2 [Source:VGNC Symbol;Acc:VGNC:91043]	2.42	0.041
ssc-miR-181a	ONECUT3	one cut homeobox 3 [Source:VGNC Symbol;Acc:VGNC:91044]	2.42	0.041
ssc-miR-181a	OSBPL3	oxysterol binding protein like 3 [Source:VGNC Symbol;Acc:VGNC:91071]	2.42	0.041
ssc-miR-181a	OSBPL8	oxysterol binding protein like 8 [Source:VGNC Symbol;Acc:VGNC:91074]	2.42	0.041
ssc-miR-181a	OTOGL	otogelin like [Source:VGNC Symbol;Acc:VGNC:91091]	2.42	0.041
ssc-miR-181a	OTUD4	OTU deubiquitinase 4 [Source:VGNC Symbol;Acc:VGNC:91100]	2.42	0.041
ssc-miR-181a	PACS2	phosphofurin acidic cluster sorting protein 2 [Source:VGNC Symbol;Acc:VGNC:91141]	2.42	0.041
ssc-miR-181a	PAFAH1B1	platelet activating factor acetylhydrolase 1b regulatory subunit 1 [Source:VGNC Symbol;Acc:VGNC:99026]	2.42	0.041
ssc-miR-181a	PAFAH1B2	platelet activating factor acetylhydrolase 1b catalytic subunit 2 [Source:VGNC Symbol;Acc:VGNC:91151]	2.42	0.041
ssc-miR-181a	PAK4	p21 (RAC1) activated kinase 4 [Source:VGNC Symbol;Acc:VGNC:98526]	2.42	0.041
ssc-miR-181a	PAK6	p21 (RAC1) activated kinase 6 [Source:VGNC Symbol;Acc:VGNC:103144]	2.42	0.041
ssc-miR-181a	PAK7	hypothetical gene	2.42	0.041
ssc-miR-181a	PALM2	hypothetical gene	2.42	0.041
ssc-miR-181a	PAM	peptidylglycine alpha-amidating monooxygenase [Source:VGNC Symbol;Acc:VGNC:91163]	2.42	0.041
ssc-miR-181a	PAN3	poly(A) specific ribonuclease subunit PAN3 [Source:HGNC Symbol;Acc:HGNC:29991]	2.42	0.041
ssc-miR-181a	PANK4	pantothenate kinase 4 (inactive) [Source:VGNC Symbol;Acc:VGNC:98527]	2.42	0.041
ssc-miR-181a	PAPD5	hypothetical gene	2.42	0.041
ssc-miR-181a	PAPOLG	poly(A) polymerase gamma [Source:HGNC Symbol;Acc:HGNC:14982]	2.42	0.041
ssc-miR-181a	PAQR3	progestin and adipoQ receptor family member 3 [Source:VGNC Symbol;Acc:VGNC:91174]	2.42	0.041

ssc-miR-181a	PARK2	hypothetical gene	2.42	0.041
ssc-miR-181a	PARM1	prostate androgen-regulated mucin-like protein 1 [Source:VGNC Symbol;Acc:VGNC:91182]	2.42	0.041
ssc-miR-181a	PAWR	pro-apoptotic WT1 regulator [Source:VGNC Symbol;Acc:VGNC:91191]	2.42	0.041
ssc-miR-181a	PAX5	paired box 5 [Source:VGNC Symbol;Acc:VGNC:91194]	2.42	0.041
ssc-miR-181a	PAX9	paired box 9 [Source:HGNC Symbol;Acc:HGNC:8623]	2.42	0.041
ssc-miR-181a	PBX1	hypothetical gene	2.42	0.041
ssc-miR-181a	PBX3	PBX homeobox 3 [Source:VGNC Symbol;Acc:VGNC:91205]	2.42	0.041
ssc-miR-181a	PCDH11Y	hypothetical gene	2.42	0.041
ssc-miR-181a	PCDHA1	hypothetical gene	2.42	0.041
ssc-miR-181a	PCDHA10	hypothetical gene	2.42	0.041
ssc-miR-181a	PCDHA11	hypothetical gene	2.42	0.041
ssc-miR-181a	PCDHA12	hypothetical gene	2.42	0.041
ssc-miR-181a	PCDHA13	protocadherin alpha 13 [Source:HGNC Symbol;Acc:HGNC:8667]	2.42	0.041
ssc-miR-181a	PCDHA2	hypothetical gene	2.42	0.041
ssc-miR-181a	PCDHA3	protocadherin alpha 3 [Source:HGNC Symbol;Acc:HGNC:8669]	2.42	0.041
ssc-miR-181a	PCDHA4	hypothetical gene	2.42	0.041
ssc-miR-181a	PCDHA5	hypothetical gene	2.42	0.041
ssc-miR-181a	PCDHA6	hypothetical gene	2.42	0.041
ssc-miR-181a	PCDHA7	hypothetical gene	2.42	0.041
ssc-miR-181a	PCDHA8	hypothetical gene	2.42	0.041
ssc-miR-181a	PCDHA9	hypothetical gene	2.42	0.041
ssc-miR-181a	PCDHAC1	hypothetical gene	2.42	0.041
ssc-miR-181a	PCDHAC2	protocadherin alpha subfamily C, 2 [Source:HGNC Symbol;Acc:HGNC:8677]	2.42	0.041
ssc-miR-181a	PCNP	PEST proteolytic signal containing nuclear protein [Source:VGNC Symbol;Acc:VGNC:104018]	2.42	0.041
ssc-miR-181a	PCSK1	proprotein convertase subtilisin/kexin type 1 [Source:VGNC Symbol;Acc:VGNC:91230]	2.42	0.041
ssc-miR-181a	PDCD4	programmed cell death 4 [Source:VGNC Symbol;Acc:VGNC:91244]	2.42	0.041
ssc-miR-181a	PDCD6IP	hypothetical gene	2.42	0.041
ssc-miR-181a	PDE10A	phosphodiesterase 10A [Source:VGNC Symbol;Acc:VGNC:91248]	2.42	0.041
ssc-miR-181a	PDE3A	phosphodiesterase 3A [Source:VGNC Symbol;Acc:VGNC:91252]	2.42	0.041
ssc-miR-181a	PDE5A	phosphodiesterase 5A [Source:VGNC Symbol;Acc:VGNC:91257]	2.42	0.041
ssc-miR-181a	PDGFRA	platelet derived growth factor receptor alpha [Source:VGNC Symbol;Acc:VGNC:98179]	2.42	0.041
ssc-miR-181a	PDHX	pyruvate dehydrogenase complex component X [Source:VGNC Symbol;Acc:VGNC:91270]	2.42	0.041
ssc-miR-181a	PDIA6	protein disulfide isomerase family A member 6 [Source:VGNC Symbol;Acc:VGNC:91275]	2.42	0.041
ssc-miR-181a	PDIK1L	PDLIM1 interacting kinase 1 like [Source:VGNC Symbol;Acc:VGNC:91276]	2.42	0.041
ssc-miR-181a	PDK3	pyruvate dehydrogenase kinase 3 [Source:VGNC Symbol;Acc:VGNC:91279]	2.42	0.041
ssc-miR-181a	PDK4	pyruvate dehydrogenase kinase 4 [Source:VGNC Symbol;Acc:VGNC:91280]	2.42	0.041
ssc-miR-181a	PDP1	pyruvate dehydrogenase phosphatase catalytic subunit 1 [Source:VGNC Symbol;Acc:VGNC:91286]	2.42	0.041
ssc-miR-181a	PDPK1	3-phosphoinositide dependent protein kinase 1 [Source:HGNC Symbol;Acc:HGNC:8816]	2.42	0.041
ssc-miR-181a	PDXDC1	pyridoxal dependent decarboxylase domain containing 1 [Source:HGNC Symbol;Acc:HGNC:28995]	2.42	0.041
ssc-miR-181a	PEAK1	pseudopodium enriched atypical kinase 1 [Source:VGNC Symbol;Acc:VGNC:91300]	2.42	0.041
ssc-miR-181a	PET117	PET117 cytochrome c oxidase chaperone [Source:HGNC Symbol;Acc:HGNC:40045]	2.42	0.041
ssc-miR-181a	PEX5L	peroxisomal biosis factor 5 like [Source:VGNC Symbol;Acc:VGNC:91329]	2.42	0.041
ssc-miR-181a	PGR	progesterone receptor [Source:VGNC Symbol;Acc:VGNC:91362]	2.42	0.041
ssc-miR-181a	PGRMC2	progesterone receptor membrane component 2 [Source:VGNC Symbol;Acc:VGNC:98952]	2.42	0.041
ssc-miR-181a	PHACTR2	phosphatase and actin regulator 2 [Source:VGNC Symbol;Acc:VGNC:91366]	2.42	0.041
ssc-miR-181a	PHACTR4	phosphatase and actin regulator 4 [Source:VGNC Symbol;Acc:VGNC:98531]	2.42	0.041
ssc-miR-181a	PHC3	polyhomeotic homolog 3 [Source:VGNC Symbol;Acc:VGNC:91372]	2.42	0.041
ssc-miR-181a	PHF15	hypothetical gene	2.42	0.041
ssc-miR-181a	PHF2	PHD finger protein 2 [Source:VGNC Symbol;Acc:VGNC:91382]	2.42	0.041

ssc-miR-181a	PHF3	PHD finger protein 3 [Source:VGNC Symbol;Acc:VGNC:91387]	2.42	0.041
ssc-miR-181a	PHIP	pleckstrin homology domain interacting protein [Source:VGNC Symbol;Acc:VGNC:91392]	2.42	0.041
ssc-miR-181a	PHLDA1	pleckstrin homology like domain family A member 1 [Source:HGNC Symbol;Acc:HGNC:8933]	2.42	0.041
ssc-miR-181a	PHLDB2	pleckstrin homology like domain family B member 2 [Source:VGNC Symbol;Acc:VGNC:91399]	2.42	0.041
ssc-miR-181a	PHLPP2	PH domain and leucine rich repeat protein phosphatase 2 [Source:VGNC Symbol;Acc:VGNC:98186]	2.42	0.041
ssc-miR-181a	PHOX2B	paired like homeobox 2B [Source:VGNC Symbol;Acc:VGNC:91402]	2.42	0.041
ssc-miR-181a	PHTF2	putative homeodomain transcription factor 2 [Source:VGNC Symbol;Acc:VGNC:91405]	2.42	0.041
ssc-miR-181a	PI4K2A	phosphatidylinositol 4-kinase type 2 alpha [Source:HGNC Symbol;Acc:HGNC:30031]	2.42	0.041
ssc-miR-181a	PI4K2B	phosphatidylinositol 4-kinase type 2 beta [Source:VGNC Symbol;Acc:VGNC:98190]	2.42	0.041
ssc-miR-181a	PIAS1	protein inhibitor of activated STAT 1 [Source:VGNC Symbol;Acc:VGNC:91410]	2.42	0.041
ssc-miR-181a	PICALM	phosphatidylinositol binding clathrin assembly protein [Source:VGNC Symbol;Acc:VGNC:91415]	2.42	0.041
ssc-miR-181a	PIK3C2A	phosphatidylinositol-4-phosphate 3-kinase catalytic subunit type 2 alpha [Source:VGNC Symbol;Acc:VGNC:91436]	2.42	0.041
ssc-miR-181a	PIK3CB	phosphatidylinositol-4,5-bisphosphate 3-kinase catalytic subunit beta [Source:VGNC Symbol;Acc:VGNC:91441]	2.42	0.041
ssc-miR-181a	PIK3R3	phosphoinositide-3-kinase regulatory subunit 3 [Source:HGNC Symbol;Acc:HGNC:8981]	2.42	0.041
ssc-miR-181a	PIP4K2B	phosphatidylinositol-5-phosphate 4-kinase type 2 beta [Source:VGNC Symbol;Acc:VGNC:91454]	2.42	0.041
ssc-miR-181a	PITPNB	phosphatidylinositol transfer protein beta [Source:VGNC Symbol;Acc:VGNC:104026]	2.42	0.041
ssc-miR-181a	PITX2	paired like homeodomain 2 [Source:VGNC Symbol;Acc:VGNC:91467]	2.42	0.041
ssc-miR-181a	PJA2	praja ring finger ubiquitin ligase 2 [Source:VGNC Symbol;Acc:VGNC:91470]	2.42	0.041
ssc-miR-181a	PKDCC	protein kinase domain containing, cytoplasmic [Source:VGNC Symbol;Acc:VGNC:91475]	2.42	0.041
ssc-miR-181a	PKHD1	PKHD1 ciliary IPT domain containing fibrocystin/polyductin [Source:VGNC Symbol;Acc:VGNC:91476]	2.42	0.041
ssc-miR-181a	PKN2	protein kinase N2 [Source:VGNC Symbol;Acc:VGNC:91482]	2.42	0.041
ssc-miR-181a	PKNOX2	PBX/knotted 1 homeobox 2 [Source:VGNC Symbol;Acc:VGNC:91485]	2.42	0.041
ssc-miR-181a	PKP2	plakophilin 2 [Source:VGNC Symbol;Acc:VGNC:91486]	2.42	0.041
ssc-miR-181a	PLAG1	PLAG1 zinc finger [Source:VGNC Symbol;Acc:VGNC:91509]	2.42	0.041
ssc-miR-181a	PLAU	plasminogen activator, urokinase [Source:VGNC Symbol;Acc:VGNC:91511]	2.42	0.041
ssc-miR-181a	PLCB1	phospholipase C beta 1 [Source:VGNC Symbol;Acc:VGNC:95706]	2.42	0.041
ssc-miR-181a	PLCL2	phospholipase C like 2 [Source:VGNC Symbol;Acc:VGNC:91523]	2.42	0.041
ssc-miR-181a	PLCXD3	phosphatidylinositol specific phospholipase C X domain containing 3 [Source:VGNC Symbol;Acc:VGNC:91524]	2.42	0.041
ssc-miR-181a	PLEK	pleckstrin [Source:VGNC Symbol;Acc:VGNC:91531]	2.42	0.041
ssc-miR-181a	PLEKHA3	pleckstrin homology domain containing A3 [Source:NCBI gene (formerly Entrezgene);Acc:100154010]	2.42	0.041
ssc-miR-181a	PLEKHG3	pleckstrin homology and RhoGEF domain containing G3 [Source:VGNC Symbol;Acc:VGNC:91544]	2.42	0.041
ssc-miR-181a	PLOD3	procollagen-lysine,2-oxoglutarate 5-dioxygenase 3 [Source:VGNC Symbol;Acc:VGNC:91568]	2.42	0.041
ssc-miR-181a	PLXDC2	plexin domain containing 2 [Source:VGNC Symbol;Acc:VGNC:95958]	2.42	0.041
ssc-miR-181a	PLXNC1	plexin C1 [Source:VGNC Symbol;Acc:VGNC:91584]	2.42	0.041
ssc-miR-181a	PMAIP1	phorbol-12-myristate-13-acetate-induced protein 1 [Source:NCBI gene (formerly Entrezgene);Acc:397278]	2.42	0.041
ssc-miR-181a	PNISR	PNN interacting serine and arginine rich protein [Source:VGNC Symbol;Acc:VGNC:91594]	2.42	0.041
ssc-miR-181a	PNRC1	proline rich nuclear receptor coactivator 1 [Source:VGNC Symbol;Acc:VGNC:91613]	2.42	0.041
ssc-miR-181a	PNRC2	proline rich nuclear receptor coactivator 2 [Source:VGNC Symbol;Acc:VGNC:91614]	2.42	0.041
ssc-miR-181a	POC1B-GALNT4	hypothetical gene	2.42	0.041
ssc-miR-181a	PODXL	hypothetical gene	2.42	0.041
ssc-miR-181a	POLQ	DNA polymerase theta [Source:VGNC Symbol;Acc:VGNC:91641]	2.42	0.041
ssc-miR-181a	POM121C	hypothetical gene	2.42	0.041
ssc-miR-181a	POU2F1	POU class 2 homeobox 1 [Source:VGNC Symbol;Acc:VGNC:91672]	2.42	0.041
ssc-miR-181a	POU3F3	POU class 3 homeobox 3 [Source:VGNC Symbol;Acc:VGNC:91676]	2.42	0.041
ssc-miR-181a	PPAP2B	hypothetical gene	2.42	0.041
ssc-miR-181a	PPARA	peroxisome proliferator activated receptor alpha [Source:VGNC Symbol;Acc:VGNC:91682]	2.42	0.041
ssc-miR-181a	PPFIA1	PTPRF interacting protein alpha 1 [Source:VGNC Symbol;Acc:VGNC:91691]	2.42	0.041
ssc-miR-181a	PPIP5K2	diphosphoinositol pentakisphosphate kinase 2 [Source:VGNC Symbol;Acc:VGNC:91701]	2.42	0.041
ssc-miR-181a	PPP1CB	protein phosphatase 1 catalytic subunit beta [Source:NCBI gene (formerly Entrezgene);Acc:397378]	2.42	0.041

ssc-miR-181a	PPP1R11	protein phosphatase 1 regulatory inhibitor subunit 11 [Source:VGNC Symbol;Acc:VGNC:91718]	2.42	0.041
ssc-miR-181a	PPP1R12B	protein phosphatase 1 regulatory subunit 12B [Source:VGNC Symbol;Acc:VGNC:96430]	2.42	0.041
ssc-miR-181a	PPP1R2	hypothetical gene	2.42	0.041
ssc-miR-181a	PPP1R3B	protein phosphatase 1 regulatory subunit 3B [Source:VGNC Symbol;Acc:VGNC:95636]	2.42	0.041
ssc-miR-181a	PPP1R3E	protein phosphatase 1 regulatory subunit 3E [Source:HGNC Symbol;Acc:HGNC:14943]	2.42	0.041
ssc-miR-181a	PPP2R2C	protein phosphatase 2 regulatory subunit Bgamma [Source:VGNC Symbol;Acc:VGNC:91749]	2.42	0.041
ssc-miR-181a	PPP2R5E	protein phosphatase 2 regulatory subunit B'epsilon [Source:VGNC Symbol;Acc:VGNC:91754]	2.42	0.041
ssc-miR-181a	PPP3R1	protein phosphatase 3 regulatory subunit B, alpha [Source:VGNC Symbol;Acc:VGNC:106453]	2.42	0.041
ssc-miR-181a	PPP4R2	protein phosphatase 4 regulatory subunit 2 [Source:VGNC Symbol;Acc:VGNC:91756]	2.42	0.041
ssc-miR-181a	PPP6C	protein phosphatase 6 catalytic subunit [Source:VGNC Symbol;Acc:VGNC:98219]	2.42	0.041
ssc-miR-181a	PRDM1	PR/SET domain 1 [Source:VGNC Symbol;Acc:VGNC:91772]	2.42	0.041
ssc-miR-181a	PRDM4	PR/SET domain 4 [Source:VGNC Symbol;Acc:VGNC:91779]	2.42	0.041
ssc-miR-181a	PRICKLE2	prickle planar cell polarity protein 2 [Source:VGNC Symbol;Acc:VGNC:91794]	2.42	0.041
ssc-miR-181a	PRIMA1	proline rich membrane anchor 1 [Source:HGNC Symbol;Acc:HGNC:18319]	2.42	0.041
ssc-miR-181a	PRKAA1	protein kinase AMP-activated catalytic subunit alpha 1 [Source:VGNC Symbol;Acc:VGNC:91797]	2.42	0.041
ssc-miR-181a	PRKAR2A	protein kinase cAMP-dependent type II regulatory subunit alpha [Source:VGNC Symbol;Acc:VGNC:91804]	2.42	0.041
ssc-miR-181a	PRKCD	protein kinase C delta [Source:VGNC Symbol;Acc:VGNC:91806]	2.42	0.041
ssc-miR-181a	PRKCE	protein kinase C epsilon [Source:VGNC Symbol;Acc:VGNC:91807]	2.42	0.041
ssc-miR-181a	PRKG1	protein kinase cGMP-dependent 1 [Source:VGNC Symbol;Acc:VGNC:91816]	2.42	0.041
ssc-miR-181a	PROSC	hypothetical gene	2.42	0.041
ssc-miR-181a	PROSER1	proline and serine rich 1 [Source:VGNC Symbol;Acc:VGNC:91836]	2.42	0.041
ssc-miR-181a	PROX1	prospero homeobox 1 [Source:VGNC Symbol;Acc:VGNC:91837]	2.42	0.041
ssc-miR-181a	PROX2	prospero homeobox 2 [Source:VGNC Symbol;Acc:VGNC:91838]	2.42	0.041
ssc-miR-181a	PRPF40A	pre-mRNA processing factor 40 homolog A [Source:VGNC Symbol;Acc:VGNC:98222]	2.42	0.041
ssc-miR-181a	PRRC2C	proline rich coiled-coil 2C [Source:VGNC Symbol;Acc:VGNC:91867]	2.42	0.041
ssc-miR-181a	PRRG1	proline rich and Gla domain 1 [Source:VGNC Symbol;Acc:VGNC:101494]	2.42	0.041
ssc-miR-181a	PSAP	prosaposin [Source:NCBI gene (formerly Entrezgene);Acc:100153167]	2.42	0.041
ssc-miR-181a	PSD3	pleckstrin and Sec7 domain containing 3 [Source:VGNC Symbol;Acc:VGNC:107166]	2.42	0.041
ssc-miR-181a	PSPC1	paraspeckle component 1 [Source:VGNC Symbol;Acc:VGNC:91930]	2.42	0.041
ssc-miR-181a	PTBP2	polypyrimidine tract binding protein 2 [Source:VGNC Symbol;Acc:VGNC:91937]	2.42	0.041
ssc-miR-181a	PTBP3	polypyrimidine tract binding protein 3 [Source:VGNC Symbol;Acc:VGNC:91938]	2.42	0.041
ssc-miR-181a	PTEN	hypothetical gene	2.42	0.041
ssc-miR-181a	PTGER3	prostaglandin E receptor 3 [Source:VGNC Symbol;Acc:VGNC:98225]	2.42	0.041
ssc-miR-181a	PTGS2	prostaglandin-endoperoxide synthase 2 [Source:VGNC Symbol;Acc:VGNC:91959]	2.42	0.041
ssc-miR-181a	PTPN4	protein tyrosine phosphatase non-receptor type 4 [Source:VGNC Symbol;Acc:VGNC:96521]	2.42	0.041
ssc-miR-181a	PTPRE	protein tyrosine phosphatase receptor type E [Source:VGNC Symbol;Acc:VGNC:91986]	2.42	0.041
ssc-miR-181a	PUM1	pumilio RNA binding family member 1 [Source:VGNC Symbol;Acc:VGNC:92001]	2.42	0.041
ssc-miR-181a	PURB	purine rich element binding protein B [Source:VGNC Symbol;Acc:VGNC:92004]	2.42	0.041
ssc-miR-181a	QDPR	quinoid dihydropteridine reductase [Source:VGNC Symbol;Acc:VGNC:98232]	2.42	0.041
ssc-miR-181a	QKI	QKI, KH domain containing RNA binding [Source:VGNC Symbol;Acc:VGNC:92025]	2.42	0.041
ssc-miR-181a	QSER1	glutamine and serine rich 1 [Source:VGNC Symbol;Acc:VGNC:92032]	2.42	0.041
ssc-miR-181a	RAB11A	RAB11A, member RAS onco family [Source:VGNC Symbol;Acc:VGNC:98236]	2.42	0.041
ssc-miR-181a	RAB11FIP2	RAB11 family interacting protein 2 [Source:VGNC Symbol;Acc:VGNC:92039]	2.42	0.041
ssc-miR-181a	RAB22A	RAB22A, member RAS onco family [Source:VGNC Symbol;Acc:VGNC:98244]	2.42	0.041
ssc-miR-181a	RAB27B	RAB27B, member RAS onco family [Source:VGNC Symbol;Acc:VGNC:98250]	2.42	0.041
ssc-miR-181a	RAB30	RAB30, member RAS onco family [Source:VGNC Symbol;Acc:VGNC:98254]	2.42	0.041
ssc-miR-181a	RAB3C	RAB3C, member RAS onco family [Source:VGNC Symbol;Acc:VGNC:104043]	2.42	0.041
ssc-miR-181a	RAB3GAP2	RAB3 GTPase activating non-catalytic protein subunit 2 [Source:VGNC Symbol;Acc:VGNC:95833]	2.42	0.041
ssc-miR-181a	RAB3IL1	RAB3A interacting protein like 1 [Source:VGNC Symbol;Acc:VGNC:92042]	2.42	0.041

ssc-miR-181a	RAB6B	RAB6B, member RAS onco family [Source:VGNC Symbol;Acc:VGNC:98272]	2.42	0.041
ssc-miR-181a	RAB8B	RAB8B, member RAS onco family [Source:VGNC Symbol;Acc:VGNC:98276]	2.42	0.041
ssc-miR-181a	RABGEF1	RAB guanine nucleotide exchange factor 1 [Source:VGNC Symbol;Acc:VGNC:108510]	2.42	0.041
ssc-miR-181a	RAD21	RAD21 cohesin complex component [Source:VGNC Symbol;Acc:VGNC:92054]	2.42	0.041
ssc-miR-181a	RAD23B	RAD23 homolog B, nucleotide excision repair protein [Source:VGNC Symbol;Acc:VGNC:103160]	2.42	0.041
ssc-miR-181a	RAD51L3-RFFL	hypothetical gene	2.42	0.041
ssc-miR-181a	RALA	RAS like proto-oncogene A [Source:HGNC Symbol;Acc:HGNC:9839]	2.42	0.041
ssc-miR-181a	RALBP1	ralA binding protein 1 [Source:VGNC Symbol;Acc:VGNC:92070]	2.42	0.041
ssc-miR-181a	RALGAPA2	Ral GTPase activating protein catalytic subunit alpha 2 [Source:VGNC Symbol;Acc:VGNC:95454]	2.42	0.041
ssc-miR-181a	RALGAPB	Ral GTPase activating protein non-catalytic subunit beta [Source:VGNC Symbol;Acc:VGNC:95463]	2.42	0.041
ssc-miR-181a	RAN	hypothetical gene	2.42	0.041
ssc-miR-181a	RANBP3	RAN binding protein 3 [Source:VGNC Symbol;Acc:VGNC:92079]	2.42	0.041
ssc-miR-181a	RAP1A	RAP1A, member of RAS onco family [Source:VGNC Symbol;Acc:VGNC:92084]	2.42	0.041
ssc-miR-181a	RAP1B	RAP1B, member of RAS oncogene family [Source:HGNC Symbol;Acc:HGNC:9857]	2.42	0.041
ssc-miR-181a	RAPGEF2	Rap guanine nucleotide exchange factor 2 [Source:VGNC Symbol;Acc:VGNC:92092]	2.42	0.041
ssc-miR-181a	RASA2	RAS p21 protein activator 2 [Source:VGNC Symbol;Acc:VGNC:92102]	2.42	0.041
ssc-miR-181a	RASAL2	RAS protein activator like 2 [Source:VGNC Symbol;Acc:VGNC:92105]	2.42	0.041
ssc-miR-181a	RASL10B	RAS like family 10 member B [Source:VGNC Symbol;Acc:VGNC:92118]	2.42	0.041
ssc-miR-181a	RASSF8	Ras association domain family member 8 [Source:VGNC Symbol;Acc:VGNC:92128]	2.42	0.041
ssc-miR-181a	RBAK	RB associated KRAB zinc finger [Source:VGNC Symbol;Acc:VGNC:92134]	2.42	0.041
ssc-miR-181a	RBBP7	RB binding protein 7, chromatin remodeling factor [Source:VGNC Symbol;Acc:VGNC:92137]	2.42	0.041
ssc-miR-181a	RBM22	RNA binding motif protein 22 [Source:VGNC Symbol;Acc:VGNC:92151]	2.42	0.041
ssc-miR-181a	RBM26	RNA binding motif protein 26 [Source:VGNC Symbol;Acc:VGNC:92153]	2.42	0.041
ssc-miR-181a	RBM27	RNA binding motif protein 27 [Source:HGNC Symbol;Acc:HGNC:29243]	2.42	0.041
ssc-miR-181a	RBM47	RNA binding motif protein 47 [Source:VGNC Symbol;Acc:VGNC:92158]	2.42	0.041
ssc-miR-181a	RBPMS	RNA binding protein, mRNA processing factor [Source:VGNC Symbol;Acc:VGNC:95904]	2.42	0.041
ssc-miR-181a	RCAN1	regulator of calcineurin 1 [Source:VGNC Symbol;Acc:VGNC:92170]	2.42	0.041
ssc-miR-181a	RCAN3	RCAN family member 3 [Source:VGNC Symbol;Acc:VGNC:92172]	2.42	0.041
ssc-miR-181a	RCN2	reticulocalbin 2 [Source:VGNC Symbol;Acc:VGNC:92181]	2.42	0.041
ssc-miR-181a	RCOR1	REST corepressor 1 [Source:VGNC Symbol;Acc:VGNC:92183]	2.42	0.041
ssc-miR-181a	RECK	reversion inducing cysteine rich protein with kazal motifs [Source:VGNC Symbol;Acc:VGNC:92194]	2.42	0.041
ssc-miR-181a	RFFL	ring finger and FYVE like domain containing E3 ubiquitin protein ligase [Source:VGNC Symbol;Acc:VGNC:98289]	2.42	0.041
ssc-miR-181a	RFX2	regulatory factor X2 [Source:VGNC Symbol;Acc:VGNC:92244]	2.42	0.041
ssc-miR-181a	RFX3	regulatory factor X3 [Source:VGNC Symbol;Acc:VGNC:92245]	2.42	0.041
ssc-miR-181a	RFX5	regulatory factor X5 [Source:VGNC Symbol;Acc:VGNC:92246]	2.42	0.041
ssc-miR-181a	RGMA	repulsive guidance molecule BMP co-receptor a [Source:VGNC Symbol;Acc:VGNC:92254]	2.42	0.041
ssc-miR-181a	RGPD4	hypothetical gene	2.42	0.041
ssc-miR-181a	RGPD6	hypothetical gene	2.42	0.041
ssc-miR-181a	RIMKLB	ribosomal modification protein rimK like family member B [Source:VGNC Symbol;Acc:VGNC:92308]	2.42	0.041
ssc-miR-181a	RIMS3	regulating synaptic membrane exocytosis 3 [Source:VGNC Symbol;Acc:VGNC:92310]	2.42	0.041
ssc-miR-181a	RIN2	hypothetical gene	2.42	0.041
ssc-miR-181a	RLF	RLF zinc finger [Source:VGNC Symbol;Acc:VGNC:92328]	2.42	0.041
ssc-miR-181a	RLIM	ring finger protein, LIM domain interacting [Source:HGNC Symbol;Acc:HGNC:13429]	2.42	0.041
ssc-miR-181a	RMND5A	required for meiotic nuclear division 5 homolog A [Source:VGNC Symbol;Acc:VGNC:92336]	2.42	0.041
ssc-miR-181a	RNF103-CHMP3	hypothetical gene	2.42	0.041
ssc-miR-181a	RNF141	ring finger protein 141 [Source:VGNC Symbol;Acc:VGNC:98292]	2.42	0.041
ssc-miR-181a	RNF144B	ring finger protein 144B [Source:VGNC Symbol;Acc:VGNC:92359]	2.42	0.041
ssc-miR-181a	RNF145	ring finger protein 145 [Source:VGNC Symbol;Acc:VGNC:92360]	2.42	0.041
ssc-miR-181a	RNF146	ring finger protein 146 [Source:VGNC Symbol;Acc:VGNC:92361]	2.42	0.041

ssc-miR-181a	RNF150	ring finger protein 150 [Source:VGNC Symbol;Acc:VGNC:98293]	2.42	0.041
ssc-miR-181a	RNF152	ring finger protein 152 [Source:VGNC Symbol;Acc:VGNC:92364]	2.42	0.041
ssc-miR-181a	RNF169	ring finger protein 169 [Source:VGNC Symbol;Acc:VGNC:92368]	2.42	0.041
ssc-miR-181a	RNF182	ring finger protein 182 [Source:VGNC Symbol;Acc:VGNC:92372]	2.42	0.041
ssc-miR-181a	RNF217	ring finger protein 217 [Source:VGNC Symbol;Acc:VGNC:103162]	2.42	0.041
ssc-miR-181a	RNF34	ring finger protein 34 [Source:VGNC Symbol;Acc:VGNC:92389]	2.42	0.041
ssc-miR-181a	RNF38	ring finger protein 38 [Source:VGNC Symbol;Acc:VGNC:92390]	2.42	0.041
ssc-miR-181a	RNF43	ring finger protein 43 [Source:VGNC Symbol;Acc:VGNC:99034]	2.42	0.041
ssc-miR-181a	RNF8	ring finger protein 8 [Source:HGNC Symbol;Acc:HGNC:10071]	2.42	0.041
ssc-miR-181a	ROBO2	roundabout guidance receptor 2 [Source:HGNC Symbol;Acc:HGNC:10250]	2.42	0.041
ssc-miR-181a	RORA	RAR related orphan receptor A [Source:VGNC Symbol;Acc:VGNC:92408]	2.42	0.041
ssc-miR-181a	RORB	RAR related orphan receptor B [Source:VGNC Symbol;Acc:VGNC:92409]	2.42	0.041
ssc-miR-181a	RP11-644F5.10	hypothetical gene	2.42	0.041
ssc-miR-181a	RPL34	ribosomal protein L34 [Source:VGNC Symbol;Acc:VGNC:98957]	2.42	0.041
ssc-miR-181a	RPS6KA3	ribosomal protein S6 kinase A3 [Source:VGNC Symbol;Acc:VGNC:92442]	2.42	0.041
ssc-miR-181a	RPS6KA6	ribosomal protein S6 kinase A6 [Source:VGNC Symbol;Acc:VGNC:92445]	2.42	0.041
ssc-miR-181a	RPS6KB1	ribosomal protein S6 kinase B1 [Source:VGNC Symbol;Acc:VGNC:99037]	2.42	0.041
ssc-miR-181a	RRAS2	hypothetical gene	2.42	0.041
ssc-miR-181a	RSF1	remodeling and spacing factor 1 [Source:VGNC Symbol;Acc:VGNC:92476]	2.42	0.041
ssc-miR-181a	RSPO2	R-spondin 2 [Source:VGNC Symbol;Acc:VGNC:92484]	2.42	0.041
ssc-miR-181a	RTKN2	rhotekin 2 [Source:VGNC Symbol;Acc:VGNC:92495]	2.42	0.041
ssc-miR-181a	RUFY3	RUN and FYVE domain containing 3 [Source:VGNC Symbol;Acc:VGNC:98959]	2.42	0.041
ssc-miR-181a	RUNX1	RUNX family transcription factor 1 [Source:VGNC Symbol;Acc:VGNC:92516]	2.42	0.041
ssc-miR-181a	RYR3	ryanodine receptor 3 [Source:HGNC Symbol;Acc:HGNC:10485]	2.42	0.041
ssc-miR-181a	S1PR1	sphingosine-1-phosphate receptor 1 [Source:VGNC Symbol;Acc:VGNC:92551]	2.42	0.041
ssc-miR-181a	SACM1L	SAC1 like phosphatidylinositide phosphatase [Source:VGNC Symbol;Acc:VGNC:92557]	2.42	0.041
ssc-miR-181a	SALL3	spalt like transcription factor 3 [Source:VGNC Symbol;Acc:VGNC:92563]	2.42	0.041
ssc-miR-181a	SALL4	spalt like transcription factor 4 [Source:NCBI gene (formerly Entrezgene);Acc:100136902]	2.42	0.041
ssc-miR-181a	SAMD12	sterile alpha motif domain containing 12 [Source:VGNC Symbol;Acc:VGNC:92565]	2.42	0.041
ssc-miR-181a	SAMD4A	sterile alpha motif domain containing 4A [Source:VGNC Symbol;Acc:VGNC:92569]	2.42	0.041
ssc-miR-181a	SAMHD1	SAM and HD domain containing deoxynucleoside triphosphate triphosphohydrolase 1 [Source:VGNC Symbol;Acc:VGNC:95712]	2.42	0.041
ssc-miR-181a	SAP30L	SAP30 like [Source:VGNC Symbol;Acc:VGNC:92577]	2.42	0.041
ssc-miR-181a	SAR1B	secretion associated Ras related GTPase 1B [Source:VGNC Symbol;Acc:VGNC:92580]	2.42	0.041
ssc-miR-181a	SBNO1	strawberry notch homolog 1 [Source:VGNC Symbol;Acc:VGNC:92596]	2.42	0.041
ssc-miR-181a	SCAI	suppressor of cancer cell invasion [Source:VGNC Symbol;Acc:VGNC:103170]	2.42	0.041
ssc-miR-181a	SCAMP2	secretory carrier membrane protein 2 [Source:VGNC Symbol;Acc:VGNC:92606]	2.42	0.041
ssc-miR-181a	SCD	stearoyl-CoA desaturase [Source:NCBI gene (formerly Entrezgene);Acc:396670]	2.42	0.041
ssc-miR-181a	SCHIP1	hypothetical gene	2.42	0.041
ssc-miR-181a	SCML2	Scm polycomb group protein like 2 [Source:VGNC Symbol;Acc:VGNC:92628]	2.42	0.041
ssc-miR-181a	SCOC	short coiled-coil protein [Source:VGNC Symbol;Acc:VGNC:98960]	2.42	0.041
ssc-miR-181a	SDC2	syndecan 2 [Source:VGNC Symbol;Acc:VGNC:92655]	2.42	0.041
ssc-miR-181a	SEC24C	SEC24 homolog C, COPII coat complex component [Source:VGNC Symbol;Acc:VGNC:92678]	2.42	0.041
ssc-miR-181a	SECISBP2	SECIS binding protein 2 [Source:VGNC Symbol;Acc:VGNC:92685]	2.42	0.041
ssc-miR-181a	SEL1L	SEL1L adaptor subunit of ERAD E3 ubiquitin ligase [Source:VGNC Symbol;Acc:VGNC:92687]	2.42	0.041
ssc-miR-181a	SELK	hypothetical gene	2.42	0.041
ssc-miR-181a	SEMA3C	semaphorin 3C [Source:VGNC Symbol;Acc:VGNC:92695]	2.42	0.041
ssc-miR-181a	SEMA4C	semaphorin 4C [Source:VGNC Symbol;Acc:VGNC:92702]	2.42	0.041
ssc-miR-181a	SEMA4F	ssemaphorin 4F [Source:VGNC Symbol;Acc:VGNC:92704]	2.42	0.041
ssc-miR-181a	SEMA4G	semaphorin 4G [Source:VGNC Symbol;Acc:VGNC:92705]	2.42	0.041

ssc-miR-181a	SENP1	SUMO specific peptidase 1 [Source:VGNC Symbol;Acc:VGNC:92712]	2.42	0.041
ssc-miR-181a	SENP2	SUMO specific peptidase 2 [Source:VGNC Symbol;Acc:VGNC:92713]	2.42	0.041
ssc-miR-181a	SENP5	SUMO specific peptidase 5 [Source:VGNC Symbol;Acc:VGNC:92714]	2.42	0.041
ssc-miR-181a	SEPT11	hypothetical gene	2.42	0.041
ssc-miR-181a	SEPT3	hypothetical gene	2.42	0.041
ssc-miR-181a	SEPT8	hypothetical gene	2.42	0.041
ssc-miR-181a	SERPINE1	serpin family E member 1 [Source:VGNC Symbol;Acc:VGNC:98310]	2.42	0.041
ssc-miR-181a	SERTAD2	SERTA domain containing 2 [Source:VGNC Symbol;Acc:VGNC:92750]	2.42	0.041
ssc-miR-181a	SERTAD4	SERTA domain containing 4 [Source:VGNC Symbol;Acc:VGNC:92752]	2.42	0.041
ssc-miR-181a	SESN3	sestrin 3 [Source:VGNC Symbol;Acc:VGNC:92755]	2.42	0.041
ssc-miR-181a	SF1	splicing factor 1 [Source:HGNC Symbol;Acc:HGNC:12950]	2.42	0.041
ssc-miR-181a	SFMBT1	Scm like with four mbt domains 1 [Source:VGNC Symbol;Acc:VGNC:92773]	2.42	0.041
ssc-miR-181a	SFRP4	secreted frizzled related protein 4 [Source:VGNC Symbol;Acc:VGNC:92776]	2.42	0.041
ssc-miR-181a	SGIP1	SH3GL interacting endocytic adaptor 1 [Source:VGNC Symbol;Acc:VGNC:92792]	2.42	0.041
ssc-miR-181a	SGK3	serum/glucocorticoid regulated kinase family member 3 [Source:HGNC Symbol;Acc:HGNC:10812]	2.42	0.041
ssc-miR-181a	SGPP1	sphingosine-1-phosphate phosphatase 1 [Source:VGNC Symbol;Acc:VGNC:92798]	2.42	0.041
ssc-miR-181a	SGTB	small glutamine rich tetratricopeptide repeat co-chaperone beta [Source:VGNC Symbol;Acc:VGNC:92804]	2.42	0.041
ssc-miR-181a	SH2B3	SH2B adaptor protein 3 [Source:VGNC Symbol;Acc:VGNC:92807]	2.42	0.041
ssc-miR-181a	SHC3	SHC adaptor protein 3 [Source:VGNC Symbol;Acc:VGNC:92838]	2.42	0.041
ssc-miR-181a	SHE	Src homology 2 domain containing E [Source:VGNC Symbol;Acc:VGNC:92842]	2.42	0.041
ssc-miR-181a	SHH	sonic hedgehog signaling molecule [Source:VGNC Symbol;Acc:VGNC:92844]	2.42	0.041
ssc-miR-181a	SHOC2	SHOC2 leucine rich repeat scaffold protein [Source:VGNC Symbol;Acc:VGNC:98318]	2.42	0.041
ssc-miR-181a	SHROOM3	hypothetical gene	2.42	0.041
ssc-miR-181a	SIAE	sialic acid acetylesterase [Source:VGNC Symbol;Acc:VGNC:92864]	2.42	0.041
ssc-miR-181a	SIK3	SIK family kinase 3 [Source:VGNC Symbol;Acc:VGNC:92873]	2.42	0.041
ssc-miR-181a	SIM1	SIM bHLH transcription factor 1 [Source:VGNC Symbol;Acc:VGNC:92876]	2.42	0.041
ssc-miR-181a	SIN3B	SIN3 transcription regulator family member B [Source:VGNC Symbol;Acc:VGNC:92880]	2.42	0.041
ssc-miR-181a	SIPA1L1	signal induced proliferation associated 1 like 1 [Source:VGNC Symbol;Acc:VGNC:92882]	2.42	0.041
ssc-miR-181a	SIPA1L2	signal induced proliferation associated 1 like 2 [Source:VGNC Symbol;Acc:VGNC:92883]	2.42	0.041
ssc-miR-181a	SIRT1	sirtuin 1 [Source:VGNC Symbol;Acc:VGNC:92884]	2.42	0.041
ssc-miR-181a	SIX2	SIX homeobox 2 [Source:VGNC Symbol;Acc:VGNC:92893]	2.42	0.041
ssc-miR-181a	SIX4	SIX homeobox 4 [Source:VGNC Symbol;Acc:VGNC:92895]	2.42	0.041
ssc-miR-181a	SLA	Src like adaptor [Source:VGNC Symbol;Acc:VGNC:92908]	2.42	0.041
ssc-miR-181a	SLAIN2	SLAIN motif family member 2 [Source:VGNC Symbol;Acc:VGNC:92910]	2.42	0.041
ssc-miR-181a	SLC10A7	solute carrier family 10 member 7 [Source:VGNC Symbol;Acc:VGNC:92919]	2.42	0.041
ssc-miR-181a	SLC12A5	solute carrier family 12 member 5 [Source:VGNC Symbol;Acc:VGNC:95594]	2.42	0.041
ssc-miR-181a	SLC16A12	solute carrier family 16 member 12 [Source:VGNC Symbol;Acc:VGNC:92940]	2.42	0.041
ssc-miR-181a	SLC16A6	solute carrier family 16 member 6 [Source:VGNC Symbol;Acc:VGNC:92946]	2.42	0.041
ssc-miR-181a	SLC16A7	solute carrier family 16 member 7 [Source:VGNC Symbol;Acc:VGNC:92947]	2.42	0.041
ssc-miR-181a	SLC25A12	solute carrier family 25 member 12 [Source:VGNC Symbol;Acc:VGNC:95502]	2.42	0.041
ssc-miR-181a	SLC25A16	solute carrier family 25 member 16 [Source:VGNC Symbol;Acc:VGNC:92995]	2.42	0.041
ssc-miR-181a	SLC25A24	solute carrier family 25 member 24 [Source:VGNC Symbol;Acc:VGNC:93001]	2.42	0.041
ssc-miR-181a	SLC25A25	solute carrier family 25 member 25 [Source:VGNC Symbol;Acc:VGNC:93002]	2.42	0.041
ssc-miR-181a	SLC25A36	solute carrier family 25 member 36 [Source:VGNC Symbol;Acc:VGNC:93012]	2.42	0.041
ssc-miR-181a	SLC25A37	hypothetical gene	2.42	0.041
ssc-miR-181a	SLC25A53	solute carrier family 25 member 53 [Source:VGNC Symbol;Acc:VGNC:93023]	2.42	0.041
ssc-miR-181a	SLC26A4	solute carrier family 26 member 4 [Source:VGNC Symbol;Acc:VGNC:93029]	2.42	0.041
ssc-miR-181a	SLC26A9	solute carrier family 26 member 9 [Source:VGNC Symbol;Acc:VGNC:93034]	2.42	0.041
ssc-miR-181a	SLC2A14	hypothetical gene	2.42	0.041

ssc-miR-181a	SLC2A3	hypothetical gene	2.42	0.041
ssc-miR-181a	SLC30A1	solute carrier family 30 member 1 [Source:VGNC Symbol;Acc:VGNC:93055]	2.42	0.041
ssc-miR-181a	SLC35A3	solute carrier family 35 member A3 [Source:VGNC Symbol;Acc:VGNC:98860]	2.42	0.041
ssc-miR-181a	SLC35E1	solute carrier family 35 member E1 [Source:VGNC Symbol;Acc:VGNC:93077]	2.42	0.041
ssc-miR-181a	SLC35F1	solute carrier family 35 member F1 [Source:VGNC Symbol;Acc:VGNC:93081]	2.42	0.041
ssc-miR-181a	SLC35F3	solute carrier family 35 member F3 [Source:VGNC Symbol;Acc:VGNC:93083]	2.42	0.041
ssc-miR-181a	SLC35G1	solute carrier family 35 member G1 [Source:VGNC Symbol;Acc:VGNC:93086]	2.42	0.041
ssc-miR-181a	SLC37A3	solute carrier family 37 member 3 [Source:VGNC Symbol;Acc:VGNC:98324]	2.42	0.041
ssc-miR-181a	SLC38A2	solute carrier family 38 member 2 [Source:VGNC Symbol;Acc:VGNC:93094]	2.42	0.041
ssc-miR-181a	SLC4A10	solute carrier family 4 member 10 [Source:VGNC Symbol;Acc:VGNC:95592]	2.42	0.041
ssc-miR-181a	SLC4A8	solute carrier family 4 member 8 [Source:VGNC Symbol;Acc:VGNC:98326]	2.42	0.041
ssc-miR-181a	SLC5A7	solute carrier family 5 member 7 [Source:VGNC Symbol;Acc:VGNC:93147]	2.42	0.041
ssc-miR-181a	SLC6A2	solute carrier family 6 member 2 [Source:VGNC Symbol;Acc:VGNC:93161]	2.42	0.041
ssc-miR-181a	SLC7A11	solute carrier family 7 member 11 [Source:VGNC Symbol;Acc:VGNC:93171]	2.42	0.041
ssc-miR-181a	SLC8A1	solute carrier family 8 member A1 [Source:VGNC Symbol;Acc:VGNC:93178]	2.42	0.041
ssc-miR-181a	SLC9A6	solute carrier family 9 member A6 [Source:VGNC Symbol;Acc:VGNC:93188]	2.42	0.041
ssc-miR-181a	SLC9A8	solute carrier family 9 member A8 [Source:VGNC Symbol;Acc:VGNC:95943]	2.42	0.041
ssc-miR-181a	SLIT1	slit guidance ligand 1 [Source:VGNC Symbol;Acc:VGNC:107171]	2.42	0.041
ssc-miR-181a	SLITRK1	SLIT and NTRK like family member 1 [Source:VGNC Symbol;Acc:VGNC:93206]	2.42	0.041
ssc-miR-181a	SLITRK3	SLIT and NTRK like family member 3 [Source:VGNC Symbol;Acc:VGNC:93208]	2.42	0.041
ssc-miR-181a	SLITRK4	SLIT and NTRK like family member 4 [Source:VGNC Symbol;Acc:VGNC:93209]	2.42	0.041
ssc-miR-181a	SLMAP	sarcolemma associated protein [Source:VGNC Symbol;Acc:VGNC:93212]	2.42	0.041
ssc-miR-181a	SLX4	SLX4 structure-specific endonuclease subunit [Source:VGNC Symbol;Acc:VGNC:93215]	2.42	0.041
ssc-miR-181a	SMAD2	SMAD family member 2 [Source:VGNC Symbol;Acc:VGNC:98329]	2.42	0.041
ssc-miR-181a	SMAD7	SMAD family member 7 [Source:VGNC Symbol;Acc:VGNC:93221]	2.42	0.041
ssc-miR-181a	SMAP1	small ArfGAP 1 [Source:VGNC Symbol;Acc:VGNC:93223]	2.42	0.041
ssc-miR-181a	SMCO1	single-pass membrane protein with coiled-coil domains 1 [Source:VGNC Symbol;Acc:VGNC:93243]	2.42	0.041
ssc-miR-181a	SMG7	SMG7 nonsense mediated mRNA decay factor [Source:VGNC Symbol;Acc:VGNC:93250]	2.42	0.041
ssc-miR-181a	SMNDC1	survival motor neuron domain containing 1 [Source:VGNC Symbol;Acc:VGNC:93257]	2.42	0.041
ssc-miR-181a	SNAI2	snail family transcriptional repressor 2 [Source:VGNC Symbol;Acc:VGNC:98330]	2.42	0.041
ssc-miR-181a	SNAP25	synaptosome associated protein 25 [Source:VGNC Symbol;Acc:VGNC:95509]	2.42	0.041
ssc-miR-181a	SNN	stannin [Source:HGNC Symbol;Acc:HGNC:11149]	2.42	0.041
ssc-miR-181a	SNTB1	syntrophin beta 1 [Source:VGNC Symbol;Acc:VGNC:93297]	2.42	0.041
ssc-miR-181a	SNTB2	syntrophin beta 2 [Source:VGNC Symbol;Acc:VGNC:93298]	2.42	0.041
ssc-miR-181a	SNX30	sorting nexin family member 30 [Source:VGNC Symbol;Acc:VGNC:93318]	2.42	0.041
ssc-miR-181a	SOCS2	suppressor of cytokine signaling 2 [Source:NCBI gene (formerly Entrezgene);Acc:100037966]	2.42	0.041
ssc-miR-181a	SOCS3	suppressor of cytokine signaling 3 [Source:VGNC Symbol;Acc:VGNC:99052]	2.42	0.041
ssc-miR-181a	SOCS4	suppressor of cytokine signaling 4 [Source:VGNC Symbol;Acc:VGNC:93331]	2.42	0.041
ssc-miR-181a	SOGA2	hypothetical gene	2.42	0.041
ssc-miR-181a	SOS1	SOS Ras/Rac guanine nucleotide exchange factor 1 [Source:HGNC Symbol;Acc:HGNC:11187]	2.42	0.041
ssc-miR-181a	SOWAHA	sosondowah ankyrin repeat domain family member A [Source:VGNC Symbol;Acc:VGNC:93347]	2.42	0.041
ssc-miR-181a	SOWAHC	hypothetical gene	2.42	0.041
ssc-miR-181a	SOX5	SRY-box transcription factor 5 [Source:VGNC Symbol;Acc:VGNC:93357]	2.42	0.041
ssc-miR-181a	SOX6	SRY-box transcription factor 6 [Source:VGNC Symbol;Acc:VGNC:93358]	2.42	0.041
ssc-miR-181a	SP1	Sp1 transcription factor [Source:VGNC Symbol;Acc:VGNC:93360]	2.42	0.041
ssc-miR-181a	SPAG9	sperm associated antigen 9 [Source:VGNC Symbol;Acc:VGNC:93374]	2.42	0.041
ssc-miR-181a	SPATS2	spermatogenesis associated serine rich 2 [Source:HGNC Symbol;Acc:HGNC:18650]	2.42	0.041
ssc-miR-181a	SPECC1L	sperm antigen with calponin homology and coiled-coil domains 1 like [Source:HGNC Symbol;Acc:HGNC:29022]	2.42	0.041
ssc-miR-181a	SPICE1	spindle and centriole associated protein 1 [Source:HGNC Symbol;Acc:HGNC:25083]	2.42	0.041

ssc-miR-181a	SPIRE1	spire type actin nucleation factor 1 [Source:VGNC Symbol;Acc:VGNC:98336]	2.42	0.041
ssc-miR-181a	SPP1	secreted phosphoprotein 1 [Source:VGNC Symbol;Acc:VGNC:93419]	2.42	0.041
ssc-miR-181a	SPRY4	sprouty RTK signaling antagonist 4 [Source:VGNC Symbol;Acc:VGNC:93427]	2.42	0.041
ssc-miR-181a	SPTY2D1	SPT2 chromatin protein domain containing 1 [Source:VGNC Symbol;Acc:VGNC:100867]	2.42	0.041
ssc-miR-181a	SRCIN1	SRC kinase signaling inhibitor 1 [Source:VGNC Symbol;Acc:VGNC:93448]	2.42	0.041
ssc-miR-181a	SRGAP1	SLIT-ROBO Rho GTPase activating protein 1 [Source:VGNC Symbol;Acc:VGNC:93454]	2.42	0.041
ssc-miR-181a	SRGAP2	hypothetical gene	2.42	0.041
ssc-miR-181a	SRPK2	SRSF protein kinase 2 [Source:VGNC Symbol;Acc:VGNC:93463]	2.42	0.041
ssc-miR-181a	SRSF10	serine and arginine rich splicing factor 10 [Source:VGNC Symbol;Acc:VGNC:93472]	2.42	0.041
ssc-miR-181a	SRSF11	serine and arginine rich splicing factor 11 [Source:VGNC Symbol;Acc:VGNC:93473]	2.42	0.041
ssc-miR-181a	SRSF5	serine and arginine rich splicing factor 5 [Source:VGNC Symbol;Acc:VGNC:93476]	2.42	0.041
ssc-miR-181a	SRSF7	serine and arginine rich splicing factor 7 [Source:VGNC Symbol;Acc:VGNC:93477]	2.42	0.041
ssc-miR-181a	SS18L1	SS18L1 subunit of BAF chromatin remodeling complex [Source:VGNC Symbol;Acc:VGNC:98342]	2.42	0.041
ssc-miR-181a	SSBP2	single stranded DNA binding protein 2 [Source:VGNC Symbol;Acc:VGNC:93480]	2.42	0.041
ssc-miR-181a	SSX2IP	SSX family member 2 interacting protein [Source:VGNC Symbol;Acc:VGNC:93499]	2.42	0.041
ssc-miR-181a	ST8SIA3	ST8 alpha-N-acetyl-neuraminide alpha-2,8-sialyltransferase 3 [Source:VGNC Symbol;Acc:VGNC:93518]	2.42	0.041
ssc-miR-181a	ST8SIA4	ST8 alpha-N-acetyl-neuraminide alpha-2,8-sialyltransferase 4 [Source:VGNC Symbol;Acc:VGNC:93519]	2.42	0.041
ssc-miR-181a	STAT3	signal transducer and activator of transcription 3 [Source:VGNC Symbol;Acc:VGNC:93540]	2.42	0.041
ssc-miR-181a	STAU1	staufen double-stranded RNA binding protein 1 [Source:VGNC Symbol;Acc:VGNC:95525]	2.42	0.041
ssc-miR-181a	STC1	stanniocalcin 1 [Source:VGNC Symbol;Acc:VGNC:93542]	2.42	0.041
ssc-miR-181a	STIM2	stromal interaction molecule 2 [Source:VGNC Symbol;Acc:VGNC:98967]	2.42	0.041
ssc-miR-181a	STK10	serine/threonine kinase 10 [Source:HGNC Symbol;Acc:HGNC:11388]	2.42	0.041
ssc-miR-181a	STOX2	storkhead box 2 [Source:VGNC Symbol;Acc:VGNC:96082]	2.42	0.041
ssc-miR-181a	STRN	striatin [Source:VGNC Symbol;Acc:VGNC:93578]	2.42	0.041
ssc-miR-181a	STXBP5	syntaxin binding protein 5 [Source:VGNC Symbol;Acc:VGNC:93599]	2.42	0.041
ssc-miR-181a	STXBP6	syntaxin binding protein 6 [Source:NCBI gene (formerly Entrezgene);Acc:100514852]	2.42	0.041
ssc-miR-181a	SUDS3	SDS3 homolog, SIN3A corepressor complex component [Source:VGNC Symbol;Acc:VGNC:93607]	2.42	0.041
ssc-miR-181a	SUGT1	SGT1 homolog, MIS12 kinetochore complex assembly cochaperone [Source:VGNC Symbol;Acc:VGNC:93611]	2.42	0.041
ssc-miR-181a	SUMF1	sulfatase modifying factor 1 [Source:VGNC Symbol;Acc:VGNC:93617]	2.42	0.041
ssc-miR-181a	SUN1	Sad1 and UNC84 domain containing 1 [Source:VGNC Symbol;Acc:VGNC:93618]	2.42	0.041
ssc-miR-181a	SYNE1	hypothetical gene	2.42	0.041
ssc-miR-181a	SYNGR2	synaptogyrin 2 [Source:HGNC Symbol;Acc:HGNC:11499]	2.42	0.041
ssc-miR-181a	SYNPR	synaptoporin [Source:VGNC Symbol;Acc:VGNC:93674]	2.42	0.041
ssc-miR-181a	SYPL2	synaptophysin like 2 [Source:VGNC Symbol;Acc:VGNC:93677]	2.42	0.041
ssc-miR-181a	SYT11	synaptotagmin 11 [Source:VGNC Symbol;Acc:VGNC:93680]	2.42	0.041
ssc-miR-181a	SYT3	synaptotagmin 3 [Source:VGNC Symbol;Acc:VGNC:93684]	2.42	0.041
ssc-miR-181a	TAB3	TGF-beta activated kinase 1 (MAP3K7) binding protein 3 [Source:HGNC Symbol;Acc:HGNC:30681]	2.42	0.041
ssc-miR-181a	TADA2B	transcriptional adaptor 2B [Source:VGNC Symbol;Acc:VGNC:93707]	2.42	0.041
ssc-miR-181a	TAF4	TATA-box binding protein associated factor 4 [Source:VGNC Symbol;Acc:VGNC:95532]	2.42	0.041
ssc-miR-181a	TAF9B	TATA-box binding protein associated factor 9b [Source:VGNC Symbol;Acc:VGNC:93725]	2.42	0.041
ssc-miR-181a	TANC2	tetratricopeptide repeat, ankyrin repeat and coiled-coil containing 2 [Source:VGNC Symbol;Acc:VGNC:93733]	2.42	0.041
ssc-miR-181a	TAOK1	TAO kinase 1 [Source:VGNC Symbol;Acc:VGNC:98355]	2.42	0.041
ssc-miR-181a	TARDBP	TAR DNA binding protein [Source:VGNC Symbol;Acc:VGNC:98624]	2.42	0.041
ssc-miR-181a	TBC1D1	TBC1 domain family member 1 [Source:VGNC Symbol;Acc:VGNC:93758]	2.42	0.041
ssc-miR-181a	TBC1D15	TBC1 domain family member 15 [Source:VGNC Symbol;Acc:VGNC:93764]	2.42	0.041
ssc-miR-181a	TBC1D24	TBC1 domain family member 24 [Source:HGNC Symbol;Acc:HGNC:29203]	2.42	0.041
ssc-miR-181a	TBC1D25	TBC1 domain family member 25 [Source:VGNC Symbol;Acc:VGNC:93773]	2.42	0.041
ssc-miR-181a	TBC1D2B	TBC1 domain family member 2B [Source:VGNC Symbol;Acc:VGNC:93774]	2.42	0.041
ssc-miR-181a	TBC1D4	TBC1 domain family member 4 [Source:HGNC Symbol;Acc:HGNC:19165]	2.42	0.041

ssc-miR-181a	TBC1D9	TBC1 domain family member 9 [Source:VGNC Symbol;Acc:VGNC:93781]	2.42	0.041
ssc-miR-181a	TBCEL	tubulin folding cofactor E like [Source:HGNC Symbol;Acc:HGNC:28115]	2.42	0.041
ssc-miR-181a	TBL1X	hypothetical gene	2.42	0.041
ssc-miR-181a	TBL1XR1	TBL1X receptor 1 [Source:VGNC Symbol;Acc:VGNC:96600]	2.42	0.041
ssc-miR-181a	TBL1Y	hypothetical gene	2.42	0.041
ssc-miR-181a	TBPL1	TATA-box binding protein like 1 [Source:VGNC Symbol;Acc:VGNC:93792]	2.42	0.041
ssc-miR-181a	TBX18	T-box transcription factor 18 [Source:VGNC Symbol;Acc:VGNC:103187]	2.42	0.041
ssc-miR-181a	TCERG1	transcription elongation regulator 1 [Source:VGNC Symbol;Acc:VGNC:93815]	2.42	0.041
ssc-miR-181a	TCF7L2	transcription factor 7 like 2 [Source:VGNC Symbol;Acc:VGNC:93825]	2.42	0.041
ssc-miR-181a	TDRD6	tudor domain containing 6 [Source:VGNC Symbol;Acc:VGNC:93850]	2.42	0.041
ssc-miR-181a	TEAD1	TEA domain transcription factor 1 [Source:VGNC Symbol;Acc:VGNC:93853]	2.42	0.041
ssc-miR-181a	TFAM	transcription factor A, mitochondrial [Source:NCBI gene (formerly Entrezgene);Acc:397279]	2.42	0.041
ssc-miR-181a	TGFBR1	transforming growth factor beta receptor 1 [Source:VGNC Symbol;Acc:VGNC:98368]	2.42	0.041
ssc-miR-181a	TGFBR2	transforming growth factor beta receptor 2 [Source:VGNC Symbol;Acc:VGNC:93931]	2.42	0.041
ssc-miR-181a	TGFBR3	transforming growth factor beta receptor 3 [Source:VGNC Symbol;Acc:VGNC:93932]	2.42	0.041
ssc-miR-181a	THBS2	thrombospondin 2 [Source:VGNC Symbol;Acc:VGNC:93947]	2.42	0.041
ssc-miR-181a	THBS4	thrombospondin 4 [Source:VGNC Symbol;Acc:VGNC:93949]	2.42	0.041
ssc-miR-181a	THRA	thyroid hormone receptor alpha [Source:VGNC Symbol;Acc:VGNC:93963]	2.42	0.041
ssc-miR-181a	THRB	thyroid hormone receptor beta [Source:VGNC Symbol;Acc:VGNC:93965]	2.42	0.041
ssc-miR-181a	TIAL1	TIA1 cytotoxic granule associated RNA binding protein like 1 [Source:VGNC Symbol;Acc:VGNC:104073]	2.42	0.041
ssc-miR-181a	TIMP3	TIMP metallopeptidase inhibitor 3 [Source:VGNC Symbol;Acc:VGNC:93996]	2.42	0.041
ssc-miR-181a	TLK1	tousled like kinase 1 [Source:VGNC Symbol;Acc:VGNC:95543]	2.42	0.041
ssc-miR-181a	TLK2	tousled like kinase 2 [Source:VGNC Symbol;Acc:VGNC:94017]	2.42	0.041
ssc-miR-181a	TLL1	tolloid like 1 [Source:VGNC Symbol;Acc:VGNC:94018]	2.42	0.041
ssc-miR-181a	TM9SF3	transmembrane 9 superfamily member 3 [Source:VGNC Symbol;Acc:VGNC:94037]	2.42	0.041
ssc-miR-181a	TM9SF4	transmembrane 9 superfamily member 4 [Source:VGNC Symbol;Acc:VGNC:96250]	2.42	0.041
ssc-miR-181a	TMC7	transmembrane channel like 7 [Source:VGNC Symbol;Acc:VGNC:94044]	2.42	0.041
ssc-miR-181a	TMCC1	transmembrane and coiled-coil domain family 1 [Source:VGNC Symbol;Acc:VGNC:94046]	2.42	0.041
ssc-miR-181a	TMED4	transmembrane p24 trafficking protein 4 [Source:VGNC Symbol;Acc:VGNC:94056]	2.42	0.041
ssc-miR-181a	TMED8	transmembrane p24 trafficking protein family member 8 [Source:VGNC Symbol;Acc:VGNC:94058]	2.42	0.041
ssc-miR-181a	TMEFF1	transmembrane protein with EGF like and two follistatin like domains 1 [Source:HGNC Symbol;Acc:HGNC:11866]	2.42	0.041
ssc-miR-181a	TMEM106B	transmembrane protein 106B [Source:VGNC Symbol;Acc:VGNC:104075]	2.42	0.041
ssc-miR-181a	TMEM108	transmembrane protein 108 [Source:VGNC Symbol;Acc:VGNC:94065]	2.42	0.041
ssc-miR-181a	TMEM127	transmembrane protein 127 [Source:HGNC Symbol;Acc:HGNC:26038]	2.42	0.041
ssc-miR-181a	TMEM131	transmembrane protein 131 [Source:VGNC Symbol;Acc:VGNC:94078]	2.42	0.041
ssc-miR-181a	TMEM132B	transmembrane protein 132B [Source:HGNC Symbol;Acc:HGNC:29397]	2.42	0.041
ssc-miR-181a	TMEM132D	transmembrane protein 132D [Source:VGNC Symbol;Acc:VGNC:94082]	2.42	0.041
ssc-miR-181a	TMEM135	transmembrane protein 135 [Source:VGNC Symbol;Acc:VGNC:94085]	2.42	0.041
ssc-miR-181a	TMEM151B	transmembrane protein 151B [Source:VGNC Symbol;Acc:VGNC:98376]	2.42	0.041
ssc-miR-181a	TMEM161B	transmembrane protein 161B [Source:VGNC Symbol;Acc:VGNC:94102]	2.42	0.041
ssc-miR-181a	TMEM170A	transmembrane protein 170A [Source:VGNC Symbol;Acc:VGNC:98659]	2.42	0.041
ssc-miR-181a	TMEM178B	transmembrane protein 178B [Source:HGNC Symbol;Acc:HGNC:44112]	2.42	0.041
ssc-miR-181a	TMEM242	transmembrane protein 242 [Source:HGNC Symbol;Acc:HGNC:17206]	2.42	0.041
ssc-miR-181a	TMEM30A	transmembrane protein 30A [Source:VGNC Symbol;Acc:VGNC:94170]	2.42	0.041
ssc-miR-181a	TMEM47	transmembrane protein 47 [Source:VGNC Symbol;Acc:VGNC:94184]	2.42	0.041
ssc-miR-181a	TMEM64	transmembrane protein 64 [Source:VGNC Symbol;Acc:VGNC:94199]	2.42	0.041
ssc-miR-181a	TMEM71	transmembrane protein 71 [Source:VGNC Symbol;Acc:VGNC:94205]	2.42	0.041
ssc-miR-181a	TMEM87B	transmembrane protein 87B [Source:VGNC Symbol;Acc:VGNC:94213]	2.42	0.041
ssc-miR-181a	TMF1	TATA element modulatory factor 1 [Source:VGNC Symbol;Acc:VGNC:94223]	2.42	0.041

ssc-miR-181a	TMX3	thioredoxin related transmembrane protein 3 [Source:VGNC Symbol;Acc:VGNC:94248]	2.42	0.041
ssc-miR-181a	TNF	tumor necrosis factor [Source:NCBI gene (formerly Entrezgene);Acc:397086]	2.42	0.041
ssc-miR-181a	TNFAIP1	TNF alpha induced protein 1 [Source:VGNC Symbol;Acc:VGNC:94250]	2.42	0.041
ssc-miR-181a	TNFRSF11B	TNF receptor superfamily member 11b [Source:HGNC Symbol;Acc:HGNC:11909]	2.42	0.041
ssc-miR-181a	TNIK	TRAF2 and NCK interacting kinase [Source:VGNC Symbol;Acc:VGNC:98381]	2.42	0.041
ssc-miR-181a	TNPO1	transportin 1 [Source:VGNC Symbol;Acc:VGNC:94287]	2.42	0.041
ssc-miR-181a	TNRC6B	trinucleotide repeat containing adaptor 6B [Source:VGNC Symbol;Acc:VGNC:94293]	2.42	0.041
ssc-miR-181a	TNS1	tensin 1 [Source:VGNC Symbol;Acc:VGNC:104082]	2.42	0.041
ssc-miR-181a	TNS3	tensin 3 [Source:VGNC Symbol;Acc:VGNC:94296]	2.42	0.041
ssc-miR-181a	TOPBP1	DNA topoisomerase II binding protein 1 [Source:VGNC Symbol;Acc:VGNC:94315]	2.42	0.041
ssc-miR-181a	TOR1AIP2	torsin 1A interacting protein 2 [Source:VGNC Symbol;Acc:VGNC:94318]	2.42	0.041
ssc-miR-181a	TOX	thymocyte selection associated high mobility group box [Source:VGNC Symbol;Acc:VGNC:94322]	2.42	0.041
ssc-miR-181a	TPST2	tyrosylprotein sulfotransferase 2 [Source:NCBI gene (formerly Entrezgene);Acc:100154448]	2.42	0.041
ssc-miR-181a	TRAF3IP2	TRAF3 interacting protein 2 [Source:VGNC Symbol;Acc:VGNC:94361]	2.42	0.041
ssc-miR-181a	TRAF6	TNF receptor associated factor 6 [Source:VGNC Symbol;Acc:VGNC:94365]	2.42	0.041
ssc-miR-181a	TRAK2	trafficking kinesin protein 2 [Source:VGNC Symbol;Acc:VGNC:95577]	2.42	0.041
ssc-miR-181a	TRANK1	tetratricopeptide repeat and ankyrin repeat containing 1 [Source:VGNC Symbol;Acc:VGNC:94370]	2.42	0.041
ssc-miR-181a	TRERF1	transcriptional regulating factor 1 [Source:VGNC Symbol;Acc:VGNC:94387]	2.42	0.041
ssc-miR-181a	TRIM13	tripartite motif containing 13 [Source:VGNC Symbol;Acc:VGNC:94394]	2.42	0.041
ssc-miR-181a	TRIM2	tripartite motif containing 2 [Source:VGNC Symbol;Acc:VGNC:94397]	2.42	0.041
ssc-miR-181a	TRIM3	tripartite motif containing 3 [Source:HGNC Symbol;Acc:HGNC:10064]	2.42	0.041
ssc-miR-181a	TRIM71	tripartite motif containing 71 [Source:VGNC Symbol;Acc:VGNC:94431]	2.42	0.041
ssc-miR-181a	TRIP4	thyroid hormone receptor interactor 4 [Source:VGNC Symbol;Acc:VGNC:94438]	2.42	0.041
ssc-miR-181a	TRNP1	TMF1 regulated nuclear protein 1 [Source:VGNC Symbol;Acc:VGNC:94456]	2.42	0.041
ssc-miR-181a	TRPC1	transient receptor potential cation channel subfamily C member 1 [Source:VGNC Symbol;Acc:VGNC:94460]	2.42	0.041
ssc-miR-181a	TRPM3	transient receptor potential cation channel subfamily M member 3 [Source:VGNC Symbol;Acc:VGNC:98384]	2.42	0.041
ssc-miR-181a	TRPM7	transient receptor potential cation channel subfamily M member 7 [Source:VGNC Symbol;Acc:VGNC:94470]	2.42	0.041
ssc-miR-181a	TRPV3	transient receptor potential cation channel subfamily V member 3 [Source:VGNC Symbol;Acc:VGNC:94475]	2.42	0.041
ssc-miR-181a	TRRAP	transformation/transcription domain associated protein [Source:VGNC Symbol;Acc:VGNC:94477]	2.42	0.041
ssc-miR-181a	TSC22D2	TSC22 domain family member 2 [Source:VGNC Symbol;Acc:VGNC:94482]	2.42	0.041
ssc-miR-181a	TSFM	Ts translation elongation factor, mitochondrial [Source:VGNC Symbol;Acc:VGNC:94486]	2.42	0.041
ssc-miR-181a	TSPAN13	tetraspanin 13 [Source:VGNC Symbol;Acc:VGNC:94502]	2.42	0.041
ssc-miR-181a	TTBK1	tau tubulin kinase 1 [Source:VGNC Symbol;Acc:VGNC:98386]	2.42	0.041
ssc-miR-181a	TTC39A	tetratricopeptide repeat domain 39A [Source:VGNC Symbol;Acc:VGNC:94551]	2.42	0.041
ssc-miR-181a	TTC39B	tetratricopeptide repeat domain 39B [Source:VGNC Symbol;Acc:VGNC:94552]	2.42	0.041
ssc-miR-181a	TTL	hypothetical gene	2.42	0.041
ssc-miR-181a	TTLL7	tubulin tyrosine ligase like 7 [Source:VGNC Symbol;Acc:VGNC:94571]	2.42	0.041
ssc-miR-181a	TTYH3	tweety family member 3 [Source:VGNC Symbol;Acc:VGNC:94576]	2.42	0.041
ssc-miR-181a	TUB	TUB bipartite transcription factor [Source:VGNC Symbol;Acc:VGNC:94577]	2.42	0.041
ssc-miR-181a	TUBB	tubulin beta class I [Source:VGNC Symbol;Acc:VGNC:94578]	2.42	0.041
ssc-miR-181a	TUBD1	tubulin delta 1 [Source:VGNC Symbol;Acc:VGNC:94582]	2.42	0.041
ssc-miR-181a	TULP4	hypothetical gene	2.42	0.041
ssc-miR-181a	TUSC3	tumor suppressor candidate 3 [Source:VGNC Symbol;Acc:VGNC:108742]	2.42	0.041
ssc-miR-181a	TXK	TXK tyrosine kinase [Source:VGNC Symbol;Acc:VGNC:94599]	2.42	0.041
ssc-miR-181a	TXNDC15	thioredoxin domain containing 15 [Source:VGNC Symbol;Acc:VGNC:94605]	2.42	0.041
ssc-miR-181a	TXNDC5	thioredoxin domain containing 5 [Source:VGNC Symbol;Acc:VGNC:94608]	2.42	0.041
ssc-miR-181a	TXNL1	thioredoxin like 1 [Source:VGNC Symbol;Acc:VGNC:94611]	2.42	0.041
ssc-miR-181a	UBA6	ubiquitin like modifier activating enzyme 6 [Source:VGNC Symbol;Acc:VGNC:94630]	2.42	0.041
ssc-miR-181a	UBE2B	ubiquitin conjugating enzyme E2 B [Source:VGNC Symbol;Acc:VGNC:94640]	2.42	0.041

ssc-miR-181a	UBE2D3	ubiquitin conjugating enzyme E2 D3 [Source:NCBI gene (formerly Entrezgene);Acc:780418]	2.42	0.041
ssc-miR-181a	UBE2H	ubiquitin conjugating enzyme E2 H [Source:VGNC Symbol;Acc:VGNC:94643]	2.42	0.041
ssc-miR-181a	UBE2J1	ubiquitin conjugating enzyme E2 J1 [Source:VGNC Symbol;Acc:VGNC:94644]	2.42	0.041
ssc-miR-181a	UBE2L3	hypothetical gene	2.42	0.041
ssc-miR-181a	UBE2N	hypothetical gene	2.42	0.041
ssc-miR-181a	UBE2T	ubiquitin conjugating enzyme E2 T [Source:VGNC Symbol;Acc:VGNC:98392]	2.42	0.041
ssc-miR-181a	UBE2W	ubiquitin conjugating enzyme E2 W [Source:VGNC Symbol;Acc:VGNC:98890]	2.42	0.041
ssc-miR-181a	UBE3C	ubiquitin protein ligase E3C [Source:VGNC Symbol;Acc:VGNC:94654]	2.42	0.041
ssc-miR-181a	UBN2	ubinuclein 2 [Source:VGNC Symbol;Acc:VGNC:94664]	2.42	0.041
ssc-miR-181a	UBP1	upstream binding protein 1 [Source:VGNC Symbol;Acc:VGNC:108701]	2.42	0.041
ssc-miR-181a	UBR2	ubiquitin protein ligase E3 component n-recognin 2 [Source:VGNC Symbol;Acc:VGNC:94669]	2.42	0.041
ssc-miR-181a	UGCG	UDP-glucose ceramide glucosyltransferase [Source:VGNC Symbol;Acc:VGNC:94685]	2.42	0.041
ssc-miR-181a	UNC5A	unc-5 netrin receptor A [Source:VGNC Symbol;Acc:VGNC:94707]	2.42	0.041
ssc-miR-181a	UNC5D	unc-5 netrin receptor D [Source:VGNC Symbol;Acc:VGNC:95892]	2.42	0.041
ssc-miR-181a	UNC80	unc-80 homolog, NALCN channel complex subunit [Source:VGNC Symbol;Acc:VGNC:96134]	2.42	0.041
ssc-miR-181a	UNKL	unk like zinc finger [Source:VGNC Symbol;Acc:VGNC:94716]	2.42	0.041
ssc-miR-181a	USP13	ubiquitin specific peptidase 13 [Source:VGNC Symbol;Acc:VGNC:94746]	2.42	0.041
ssc-miR-181a	USP15	ubiquitin specific peptidase 15 [Source:VGNC Symbol;Acc:VGNC:94748]	2.42	0.041
ssc-miR-181a	USP31	ubiquitin specific peptidase 31 [Source:HGNC Symbol;Acc:HGNC:20060]	2.42	0.041
ssc-miR-181a	USP33	ubiquitin specific peptidase 33 [Source:HGNC Symbol;Acc:HGNC:20059]	2.42	0.041
ssc-miR-181a	USP42	ubiquitin specific peptidase 42 [Source:HGNC Symbol;Acc:HGNC:20068]	2.42	0.041
ssc-miR-181a	USP47	ubiquitin specific peptidase 47 [Source:VGNC Symbol;Acc:VGNC:94769]	2.42	0.041
ssc-miR-181a	USP6NL	USP6 N-terminal like [Source:VGNC Symbol;Acc:VGNC:95820]	2.42	0.041
ssc-miR-181a	USP8	ubiquitin specific peptidase 8 [Source:VGNC Symbol;Acc:VGNC:94778]	2.42	0.041
ssc-miR-181a	USP9X	ubiquitin specific peptidase 9 X-linked [Source:HGNC Symbol;Acc:HGNC:12632]	2.42	0.041
ssc-miR-181a	USP9Y	hypothetical gene	2.42	0.041
ssc-miR-181a	VANGL1	VANGL planar cell polarity protein 1 [Source:VGNC Symbol;Acc:VGNC:94797]	2.42	0.041
ssc-miR-181a	VCAM1	vascular cell adhesion molecule 1 [Source:VGNC Symbol;Acc:VGNC:94811]	2.42	0.041
ssc-miR-181a	VCAN	versican [Source:VGNC Symbol;Acc:VGNC:108163]	2.42	0.041
ssc-miR-181a	VCL	vinculin [Source:VGNC Symbol;Acc:VGNC:94812]	2.42	0.041
ssc-miR-181a	VGLL4	vestigial like family member 4 [Source:VGNC Symbol;Acc:VGNC:94824]	2.42	0.041
ssc-miR-181a	VHL	hypothetical gene	2.42	0.041
ssc-miR-181a	VKORC1L1	vitamin K epoxide reductase complex subunit 1 like 1 [Source:VGNC Symbol;Acc:VGNC:94831]	2.42	0.041
ssc-miR-181a	VMP1	vacuole membrane protein 1 [Source:HGNC Symbol;Acc:HGNC:29559]	2.42	0.041
ssc-miR-181a	VPS4A	vacuolar protein sorting 4 homolog A [Source:NCBI gene (formerly Entrezgene);Acc:100627841]	2.42	0.041
ssc-miR-181a	VSX1	visual system homeobox 1 [Source:VGNC Symbol;Acc:VGNC:95566]	2.42	0.041
ssc-miR-181a	WASF1	hypothetical gene	2.42	0.041
ssc-miR-181a	WASL	WASP like actin nucleation promoting factor [Source:VGNC Symbol;Acc:VGNC:96604]	2.42	0.041
ssc-miR-181a	WDFY2	WD repeat and FYVE domain containing 2 [Source:VGNC Symbol;Acc:VGNC:94902]	2.42	0.041
ssc-miR-181a	WDFY3	WD repeat and FYVE domain containing 3 [Source:VGNC Symbol;Acc:VGNC:94903]	2.42	0.041
ssc-miR-181a	WDR37	WD repeat domain 37 [Source:VGNC Symbol;Acc:VGNC:96258]	2.42	0.041
ssc-miR-181a	WDR7	WD repeat domain 7 [Source:VGNC Symbol;Acc:VGNC:94936]	2.42	0.041
ssc-miR-181a	WDR82	WD repeat domain 82 [Source:VGNC Symbol;Acc:VGNC:94944]	2.42	0.041
ssc-miR-181a	WHAMM	WASP homolog associated with actin, golgi membranes and microtubules [Source:VGNC Symbol;Acc:VGNC:98414]	2.42	0.041
ssc-miR-181a	WIF1	WNT inhibitory factor 1 [Source:VGNC Symbol;Acc:VGNC:94957]	2.42	0.041
ssc-miR-181a	WNK1	WNK lysine deficient protein kinase 1 [Source:VGNC Symbol;Acc:VGNC:94962]	2.42	0.041
ssc-miR-181a	WNK3	WNK lysine deficient protein kinase 3 [Source:VGNC Symbol;Acc:VGNC:94964]	2.42	0.041
ssc-miR-181a	WNT9A	Wnt family member 9A [Source:VGNC Symbol;Acc:VGNC:94978]	2.42	0.041
ssc-miR-181a	WSB2	WD repeat and SOCS box containing 2 [Source:VGNC Symbol;Acc:VGNC:94982]	2.42	0.041

ssc-miR-181a	WSCD2	WSC domain containing 2 [Source:HGNC Symbol;Acc:HGNC:29117]	2.42	0.041
ssc-miR-181a	WWC1	WW and C2 domain containing 1 [Source:VGNC Symbol;Acc:VGNC:94986]	2.42	0.041
ssc-miR-181a	WWC2	WW and C2 domain containing 2 [Source:VGNC Symbol;Acc:VGNC:96033]	2.42	0.041
ssc-miR-181a	XIAP	X-linked inhibitor of apoptosis [Source:NCBI gene (formerly Entrezgene);Acc:100037300]	2.42	0.041
ssc-miR-181a	XK	X-linked Kx blood group [Source:HGNC Symbol;Acc:HGNC:12811]	2.42	0.041
ssc-miR-181a	XPO7	exportin 7 [Source:VGNC Symbol;Acc:VGNC:95007]	2.42	0.041
ssc-miR-181a	XRN1	5'-3' exoribonuclease 1 [Source:VGNC Symbol;Acc:VGNC:108705]	2.42	0.041
ssc-miR-181a	XYLT1	xylosyltransferase 1 [Source:VGNC Symbol;Acc:VGNC:95015]	2.42	0.041
ssc-miR-181a	YAP1	Yes1 associated transcriptional regulator [Source:VGNC Symbol;Acc:VGNC:95019]	2.42	0.041
ssc-miR-181a	YIPF4	Yip1 domain family member 4 [Source:VGNC Symbol;Acc:VGNC:95030]	2.42	0.041
ssc-miR-181a	YIPF6	Yip1 domain family member 6 [Source:VGNC Symbol;Acc:VGNC:95032]	2.42	0.041
ssc-miR-181a	YLPM1	YLP motif containing 1 [Source:VGNC Symbol;Acc:VGNC:95034]	2.42	0.041
ssc-miR-181a	YOD1	YOD1 deubiquitinase [Source:VGNC Symbol;Acc:VGNC:95035]	2.42	0.041
ssc-miR-181a	YTHDC1	YTH domain containing 1 [Source:VGNC Symbol;Acc:VGNC:95041]	2.42	0.041
ssc-miR-181a	YTHDC2	YTH domain containing 2 [Source:VGNC Symbol;Acc:VGNC:95042]	2.42	0.041
ssc-miR-181a	YTHDF2	YTH N6-methyladenosine RNA binding protein 2 [Source:VGNC Symbol;Acc:VGNC:95043]	2.42	0.041
ssc-miR-181a	YTHDF3	YTH N6-methyladenosine RNA binding protein 3 [Source:VGNC Symbol;Acc:VGNC:95044]	2.42	0.041
ssc-miR-181a	YWHAB	tyrosine 3-monooxygenase/tryptophan 5-monooxygenase activation protein beta [Source:VGNC Symbol;Acc:VGNC:98417]	2.42	0.041
ssc-miR-181a	YWHAG	hypothetical gene	2.42	0.041
ssc-miR-181a	YWHAZ	tyrosine 3-monooxygenase/tryptophan 5-monooxygenase activation protein zeta [Source:VGNC Symbol;Acc:VGNC:95047]	2.42	0.041
ssc-miR-181a	YY1	YY1 transcription factor [Source:HGNC Symbol;Acc:HGNC:12856]	2.42	0.041
ssc-miR-181a	ZADH2	prostaglandin reductase 3 [Source:VGNC Symbol;Acc:VGNC:95049]	2.42	0.041
ssc-miR-181a	ZBTB20	zinc finger and BTB domain containing 20 [Source:VGNC Symbol;Acc:VGNC:95063]	2.42	0.041
ssc-miR-181a	ZBTB34	zinc finger and BTB domain containing 34 [Source:VGNC Symbol;Acc:VGNC:95070]	2.42	0.041
ssc-miR-181a	ZBTB4	hypothetical gene	2.42	0.041
ssc-miR-181a	ZBTB41	zinc finger and BTB domain containing 41 [Source:VGNC Symbol;Acc:VGNC:108284]	2.42	0.041
ssc-miR-181a	ZBTB43	zinc finger and BTB domain containing 43 [Source:VGNC Symbol;Acc:VGNC:95075]	2.42	0.041
ssc-miR-181a	ZBTB7A	zinc finger and BTB domain containing 7A [Source:VGNC Symbol;Acc:VGNC:95081]	2.42	0.041
ssc-miR-181a	ZBTB8A	zinc finger and BTB domain containing 8A [Source:VGNC Symbol;Acc:VGNC:95084]	2.42	0.041
ssc-miR-181a	ZBTB8B	zinc finger and BTB domain containing 8B [Source:HGNC Symbol;Acc:HGNC:37057]	2.42	0.041
ssc-miR-181a	ZC3H6	zinc finger CCCH-type containing 6 [Source:VGNC Symbol;Acc:VGNC:95098]	2.42	0.041
ssc-miR-181a	ZCCHC14	zinc finger CCHC-type containing 14 [Source:VGNC Symbol;Acc:VGNC:95107]	2.42	0.041
ssc-miR-181a	ZDHHC21	zinc finger DHHC-type palmitoyltransferase 21 [Source:VGNC Symbol;Acc:VGNC:95123]	2.42	0.041
ssc-miR-181a	ZDHHC3	zinc finger DHHC-type palmitoyltransferase 3 [Source:VGNC Symbol;Acc:VGNC:95126]	2.42	0.041
ssc-miR-181a	ZDHHC7	zinc finger DHHC-type palmitoyltransferase 7 [Source:VGNC Symbol;Acc:VGNC:95129]	2.42	0.041
ssc-miR-181a	ZEB1	zinc finger E-box binding homeobox 1 [Source:VGNC Symbol;Acc:VGNC:95536]	2.42	0.041
ssc-miR-181a	ZEB2	hypothetical gene	2.42	0.041
ssc-miR-181a	ZFAND5	zinc finger AN1-type containing 5 [Source:VGNC Symbol;Acc:VGNC:95137]	2.42	0.041
ssc-miR-181a	ZFAND6	zinc finger AN1-type containing 6 [Source:VGNC Symbol;Acc:VGNC:104102]	2.42	0.041
ssc-miR-181a	ZFHX3	zinc finger homeobox 3 [Source:VGNC Symbol;Acc:VGNC:95141]	2.42	0.041
ssc-miR-181a	ZFHX4	zinc finger homeobox 4 [Source:VGNC Symbol;Acc:VGNC:95142]	2.42	0.041
ssc-miR-181a	ZFP1	ZFP1 zinc finger protein [Source:VGNC Symbol;Acc:VGNC:95143]	2.42	0.041
ssc-miR-181a	ZFP14	ZFP14 zinc finger protein [Source:VGNC Symbol;Acc:VGNC:95144]	2.42	0.041
ssc-miR-181a	ZFP30	ZFP30 zinc finger protein [Source:VGNC Symbol;Acc:VGNC:98741]	2.42	0.041
ssc-miR-181a	ZFP36L1	ZFP36 ring finger protein like 1 [Source:VGNC Symbol;Acc:VGNC:95146]	2.42	0.041
ssc-miR-181a	ZFP36L2	ZFP36 ring finger protein like 2 [Source:VGNC Symbol;Acc:VGNC:95147]	2.42	0.041
ssc-miR-181a	ZFP69B	ZFP69 zinc finger protein B [Source:HGNC Symbol;Acc:HGNC:28053]	2.42	0.041
ssc-miR-181a	ZFP82	ZFP82 zinc finger protein [Source:HGNC Symbol;Acc:HGNC:28682]	2.42	0.041
ssc-miR-181a	ZIC2	Zic family member 2 [Source:VGNC Symbol;Acc:VGNC:95167]	2.42	0.041

ssc-miR-181a	ZIC3	Zic family member 3 [Source:VGNC Symbol;Acc:VGNC:95168]	2.42	0.041
ssc-miR-181a	ZIK1	hypothetical gene	2.42	0.041
ssc-miR-181a	ZKSCAN5	zinc finger with KRAB and SCAN domains 5 [Source:VGNC Symbol;Acc:VGNC:95174]	2.42	0.041
ssc-miR-181a	ZMYND11	zinc finger MYND-type containing 11 [Source:VGNC Symbol;Acc:VGNC:95827]	2.42	0.041
ssc-miR-181a	ZNF136	hypothetical gene	2.42	0.041
ssc-miR-181a	ZNF200	zinc finger protein 200 [Source:VGNC Symbol;Acc:VGNC:95203]	2.42	0.041
ssc-miR-181a	ZNF207	zinc finger protein 207 [Source:VGNC Symbol;Acc:VGNC:95205]	2.42	0.041
ssc-miR-181a	ZNF23	zinc finger protein 23 [Source:HGNC Symbol;Acc:HGNC:13023]	2.42	0.041
ssc-miR-181a	ZNF236	zinc finger protein 236 [Source:VGNC Symbol;Acc:VGNC:95209]	2.42	0.041
ssc-miR-181a	ZNF24	zinc finger protein 24 [Source:VGNC Symbol;Acc:VGNC:98422]	2.42	0.041
ssc-miR-181a	ZNF256	hypothetical gene	2.42	0.041
ssc-miR-181a	ZNF266	hypothetical gene	2.42	0.041
ssc-miR-181a	ZNF268	zinc finger protein 268 [Source:HGNC Symbol;Acc:HGNC:13061]	2.42	0.041
ssc-miR-181a	ZNF280B	zinc finger protein 280B [Source:HGNC Symbol;Acc:HGNC:23022]	2.42	0.041
ssc-miR-181a	ZNF283	hypothetical gene	2.42	0.041
ssc-miR-181a	ZNF286A	zinc finger protein 286A [Source:HGNC Symbol;Acc:HGNC:13501]	2.42	0.041
ssc-miR-181a	ZNF286B	hypothetical gene	2.42	0.041
ssc-miR-181a	ZNF292	zinc finger protein 292 [Source:VGNC Symbol;Acc:VGNC:95218]	2.42	0.041
ssc-miR-181a	ZNF300	zinc finger protein 300 [Source:VGNC Symbol;Acc:VGNC:98424]	2.42	0.041
ssc-miR-181a	ZNF302	hypothetical gene	2.42	0.041
ssc-miR-181a	ZNF33B	hypothetical gene	2.42	0.041
ssc-miR-181a	ZNF394	zinc finger protein 394 [Source:HGNC Symbol;Acc:HGNC:18832]	2.42	0.041
ssc-miR-181a	ZNF397	zinc finger protein 397 [Source:VGNC Symbol;Acc:VGNC:95236]	2.42	0.041
ssc-miR-181a	ZNF449	zinc finger protein 449 [Source:VGNC Symbol;Acc:VGNC:95242]	2.42	0.041
ssc-miR-181a	ZNF454	zinc finger protein 454 [Source:HGNC Symbol;Acc:HGNC:21200]	2.42	0.041
ssc-miR-181a	ZNF470	zinc finger protein 470 [Source:HGNC Symbol;Acc:HGNC:22220]	2.42	0.041
ssc-miR-181a	ZNF471	zinc finger protein 471 [Source:VGNC Symbol;Acc:VGNC:95246]	2.42	0.041
ssc-miR-181a	ZNF487	hypothetical gene	2.42	0.041
ssc-miR-181a	ZNF514	zinc finger protein 514 [Source:VGNC Symbol;Acc:VGNC:95255]	2.42	0.041
ssc-miR-181a	ZNF536	zinc finger protein 536 [Source:VGNC Symbol;Acc:VGNC:95262]	2.42	0.041
ssc-miR-181a	ZNF594	hypothetical gene	2.42	0.041
ssc-miR-181a	ZNF613	zinc finger protein 613 [Source:HGNC Symbol;Acc:HGNC:25827]	2.42	0.041
ssc-miR-181a	ZNF618	zinc finger protein 618 [Source:VGNC Symbol;Acc:VGNC:95276]	2.42	0.041
ssc-miR-181a	ZNF625	hypothetical gene	2.42	0.041
ssc-miR-181a	ZNF652	zinc finger protein 652 [Source:VGNC Symbol;Acc:VGNC:99108]	2.42	0.041
ssc-miR-181a	ZNF664	zinc finger protein 664 [Source:VGNC Symbol;Acc:VGNC:95288]	2.42	0.041
ssc-miR-181a	ZNF667	zinc finger protein 667 [Source:VGNC Symbol;Acc:VGNC:95289]	2.42	0.041
ssc-miR-181a	ZNF697	zinc finger protein 697 [Source:HGNC Symbol;Acc:HGNC:32034]	2.42	0.041
ssc-miR-181a	ZNF699	hypothetical gene	2.42	0.041
ssc-miR-181a	ZNF704	zinc finger protein 704 [Source:VGNC Symbol;Acc:VGNC:95296]	2.42	0.041
ssc-miR-181a	ZNF763	hypothetical gene	2.42	0.041
ssc-miR-181a	ZNF772	zinc finger protein 772 [Source:HGNC Symbol;Acc:HGNC:33106]	2.42	0.041
ssc-miR-181a	ZNF780A	hypothetical gene	2.42	0.041
ssc-miR-181a	ZNF780B	hypothetical gene	2.42	0.041
ssc-miR-181a	ZNF781	hypothetical gene	2.42	0.041
ssc-miR-181a	ZNF788	hypothetical gene	2.42	0.041
ssc-miR-181a	ZNF791	hypothetical gene	2.42	0.041
ssc-miR-181a	ZNF800	zinc finger protein 800 [Source:VGNC Symbol;Acc:VGNC:95306]	2.42	0.041
ssc-miR-181a	ZNRF2	zinc and ring finger 2 [Source:VGNC Symbol;Acc:VGNC:95317]	2.42	0.041

ssc-miR-181a	ZSCAN12	zinc finger and SCAN domain containing 12 [Source:VGNC Symbol;Acc:VGNC:95325]	2.42	0.041
ssc-miR-181a	ZSWIM1	zinc finger SWIM-type containing 1 [Source:VGNC Symbol;Acc:VGNC:95759]	2.42	0.041
ssc-miR-181a	ZZZ3	zinc finger ZZ-type containing 3 [Source:VGNC Symbol;Acc:VGNC:95348]	2.42	0.041
ssc-miR-185	AAK1	AP2 associated kinase 1 [Source:VGNC Symbol;Acc:VGNC:100379]	2.08	0.058
ssc-miR-185	ABCA9	ATP binding cassette subfamily A member 9 [Source:HGNC Symbol;Acc:HGNC:39]	2.08	0.058
ssc-miR-185	ABCC1	ATP binding cassette subfamily C member 1 [Source:VGNC Symbol;Acc:VGNC:84952]	2.08	0.058
ssc-miR-185	ABCG1	ATP binding cassette subfamily G member 1 [Source:VGNC Symbol;Acc:VGNC:97858]	2.08	0.058
ssc-miR-185	ABCG4	ATP binding cassette subfamily G member 4 [Source:HGNC Symbol;Acc:HGNC:13884]	2.08	0.058
ssc-miR-185	ABHD15	abhydrolase domain containing 15 [Source:VGNC Symbol;Acc:VGNC:84974]	2.08	0.058
ssc-miR-185	ABHD4	abhydrolase domain containing 4, N-acyl phospholipase B [Source:VGNC Symbol;Acc:VGNC:97859]	2.08	0.058
ssc-miR-185	ABL1	ABL proto-onco 1, non-receptor tyrosine kinase [Source:VGNC Symbol;Acc:VGNC:84984]	2.08	0.058
ssc-miR-185	AC005003.1	hypothetical gene	2.08	0.058
ssc-miR-185	AC138655.1	hypothetical gene	2.08	0.058
ssc-miR-185	ACTN4	actinin alpha 4 [Source:VGNC Symbol;Acc:VGNC:85047]	2.08	0.058
ssc-miR-185	ADIPOQ	adiponectin, C1Q and collagen domain containing [Source:VGNC Symbol;Acc:VGNC:85140]	2.08	0.058
ssc-miR-185	AGFG1	ArfGAP with FG repeats 1 [Source:VGNC Symbol;Acc:VGNC:96359]	2.08	0.058
ssc-miR-185	AGO1	hypothetical gene	2.08	0.058
ssc-miR-185	AGPAT6	hypothetical gene	2.08	0.058
ssc-miR-185	AMIGO2	adhesion molecule with Ig like domain 2 [Source:VGNC Symbol;Acc:VGNC:85281]	2.08	0.058
ssc-miR-185	ANKFY1	ankyrin repeat and FYVE domain containing 1 [Source:VGNC Symbol;Acc:VGNC:85314]	2.08	0.058
ssc-miR-185	ANKRD34A	ankyrin repeat domain 34A [Source:VGNC Symbol;Acc:VGNC:85335]	2.08	0.058
ssc-miR-185	APBA1	amyloid beta protein binding family A member 1 [Source:VGNC Symbol;Acc:VGNC:85399]	2.08	0.058
ssc-miR-185	APP	amyloid beta protein [Source:VGNC Symbol;Acc:VGNC:103902]	2.08	0.058
ssc-miR-185	AQPEP	hypothetical gene	2.08	0.058
ssc-miR-185	ARAP1	ArfGAP with RhoGAP domain, ankyrin repeat and PH domain 1 [Source:VGNC Symbol;Acc:VGNC:85439]	2.08	0.058
ssc-miR-185	ARHGAP19-SLIT1	hypothetical gene	2.08	0.058
ssc-miR-185	ARHGAP32	Rho GTPase activating protein 32 [Source:VGNC Symbol;Acc:VGNC:85471]	2.08	0.058
ssc-miR-185	ARPC4-TTLL3	hypothetical gene	2.08	0.058
ssc-miR-185	ASB16	ankyrin repeat and SOCS box containing 16 [Source:VGNC Symbol;Acc:VGNC:98974]	2.08	0.058
ssc-miR-185	ASIC1	acid sensing ion channel subunit 1 [Source:VGNC Symbol;Acc:VGNC:85578]	2.08	0.058
ssc-miR-185	ATP1A3	ATPase Na+/K+ transporting subunit alpha 3 [Source:VGNC Symbol;Acc:VGNC:96923]	2.08	0.058
ssc-miR-185	ATP1B4	ATPase Na+/K+ transporting family member beta 4 [Source:VGNC Symbol;Acc:VGNC:85644]	2.08	0.058
ssc-miR-185	ATP6V1F	ATPase H+ transporting V1 subunit F [Source:VGNC Symbol;Acc:VGNC:85676]	2.08	0.058
ssc-miR-185	ATP8B1	ATPase phospholipid transporting 8B1 [Source:VGNC Symbol;Acc:VGNC:85679]	2.08	0.058
ssc-miR-185	BAI1	hypothetical gene	2.08	0.058
ssc-miR-185	BAZ2A	bromodomain adjacent to zinc finger domain 2A [Source:VGNC Symbol;Acc:VGNC:85763]	2.08	0.058
ssc-miR-185	BCL2L2	BCL2 like 2 [Source:HGNC Symbol;Acc:HGNC:995]	2.08	0.058
ssc-miR-185	BEND3	BEN domain containing 3 [Source:VGNC Symbol;Acc:VGNC:85800]	2.08	0.058
ssc-miR-185	BICD1	BICD cargo adaptor 1 [Source:VGNC Symbol;Acc:VGNC:85818]	2.08	0.058
ssc-miR-185	BMF	Bcl2 modifying factor [Source:VGNC Symbol;Acc:VGNC:85837]	2.08	0.058
ssc-miR-185	BSN	bassoon presynaptic cytomatrix protein [Source:VGNC Symbol;Acc:VGNC:85893]	2.08	0.058
ssc-miR-185	BTN1A1	butyrophilin subfamily 1 member A1 [Source:VGNC Symbol;Acc:VGNC:85915]	2.08	0.058
ssc-miR-185	C11orf87	chromosome 11 open reading frame 87 [Source:HGNC Symbol;Acc:HGNC:33788]	2.08	0.058
ssc-miR-185	C16orf89	chromosome 3 C16orf89 homolog [Source:VGNC Symbol;Acc:VGNC:86017]	2.08	0.058
ssc-miR-185	C19orf54	chromosome 6 C19orf54 homolog [Source:VGNC Symbol;Acc:VGNC:86057]	2.08	0.058
ssc-miR-185	C1orf95	hypothetical gene	2.08	0.058
ssc-miR-185	C2CD2L	C2CD2 like [Source:VGNC Symbol;Acc:VGNC:85991]	2.08	0.058
ssc-miR-185	C5AR2	complement C5a receptor 2 [Source:VGNC Symbol;Acc:VGNC:86039]	2.08	0.058
ssc-miR-185	CA10	carbonic anhydrase 10 [Source:VGNC Symbol;Acc:VGNC:86096]	2.08	0.058

ssc-miR-185	CABP4	calcium binding protein 4 [Source:VGNC Symbol;Acc:VGNC:86111]	2.08	0.058
ssc-miR-185	CADM1	cell adhesion molecule 1 [Source:VGNC Symbol;Acc:VGNC:86134]	2.08	0.058
ssc-miR-185	CADM4	cell adhesion molecule 4 [Source:VGNC Symbol;Acc:VGNC:86136]	2.08	0.058
ssc-miR-185	CALCOCO1	calcium binding and coiled-coil domain 1 [Source:VGNC Symbol;Acc:VGNC:86140]	2.08	0.058
ssc-miR-185	CALN1	hypothetical gene	2.08	0.058
ssc-miR-185	CAMK1G	calcium/calmodulin dependent protein kinase IG [Source:VGNC Symbol;Acc:VGNC:86154]	2.08	0.058
ssc-miR-185	CAMK2D	calcium/calmodulin dependent protein kinase II delta [Source:VGNC Symbol;Acc:VGNC:97911]	2.08	0.058
ssc-miR-185	CAPZA1	capping actin protein of muscle Z-line subunit alpha 1 [Source:VGNC Symbol;Acc:VGNC:86183]	2.08	0.058
ssc-miR-185	CAPZB	capping actin protein of muscle Z-line subunit beta [Source:VGNC Symbol;Acc:VGNC:86184]	2.08	0.058
ssc-miR-185	CASC10	hypothetical gene	2.08	0.058
ssc-miR-185	CBFA2T3	CBFA2/RUNX1 partner transcriptional co-repressor 3 [Source:VGNC Symbol;Acc:VGNC:96574]	2.08	0.058
ssc-miR-185	CBX5	chromobox 5 [Source:VGNC Symbol;Acc:VGNC:86232]	2.08	0.058
ssc-miR-185	CCBE1	collagen and calcium binding EGF domains 1 [Source:VGNC Symbol;Acc:VGNC:86240]	2.08	0.058
ssc-miR-185	CCDC171	coiled-coil domain containing 171 [Source:VGNC Symbol;Acc:VGNC:103044]	2.08	0.058
ssc-miR-185	CCDC9	coiled-coil domain containing 9 [Source:VGNC Symbol;Acc:VGNC:86327]	2.08	0.058
ssc-miR-185	CCND2	cyclin D2 [Source:VGNC Symbol;Acc:VGNC:103222]	2.08	0.058
ssc-miR-185	CCNK	cyclin K [Source:VGNC Symbol;Acc:VGNC:86362]	2.08	0.058
ssc-miR-185	CD300LF	hypothetical gene	2.08	0.058
ssc-miR-185	CDC42	cell division cycle 42 [Source:NCBI gene (formerly Entrezgene);Acc:780428]	2.08	0.058
ssc-miR-185	CDC42EP3	CDC42 effector protein 3 [Source:VGNC Symbol;Acc:VGNC:86457]	2.08	0.058
ssc-miR-185	CDH24	cadherin 24 [Source:VGNC Symbol;Acc:VGNC:86485]	2.08	0.058
ssc-miR-185	CDH4	cadherin 4 [Source:VGNC Symbol;Acc:VGNC:95853]	2.08	0.058
ssc-miR-185	CEACAM1	hypothetical gene	2.08	0.058
ssc-miR-185	CECR6	hypothetical gene	2.08	0.058
ssc-miR-185	CHMP7	charged multivesicular body protein 7 [Source:VGNC Symbol;Acc:VGNC:86654]	2.08	0.058
ssc-miR-185	CHST11	carbohydrate sulfotransferase 11 [Source:VGNC Symbol;Acc:VGNC:86675]	2.08	0.058
ssc-miR-185	CLDN18	claudin 18 [Source:VGNC Symbol;Acc:VGNC:86735]	2.08	0.058
ssc-miR-185	CLLU1	hypothetical gene	2.08	0.058
ssc-miR-185	CLTC	clathrin heavy chain [Source:VGNC Symbol;Acc:VGNC:86789]	2.08	0.058
ssc-miR-185	CNKSR3	CNKSR family member 3 [Source:VGNC Symbol;Acc:VGNC:86823]	2.08	0.058
ssc-miR-185	CNNM3	cyclin and CBS domain divalent metal cation transport mediator 3 [Source:VGNC Symbol;Acc:VGNC:86829]	2.08	0.058
ssc-miR-185	CNTD1	cyclin N-terminal domain containing 1 [Source:VGNC Symbol;Acc:VGNC:86846]	2.08	0.058
ssc-miR-185	CNTN2	contactin 2 [Source:VGNC Symbol;Acc:VGNC:108577]	2.08	0.058
ssc-miR-185	COL17A1	collagen type XVII alpha 1 chain [Source:VGNC Symbol;Acc:VGNC:86868]	2.08	0.058
ssc-miR-185	CORO2B	coronin 2B [Source:VGNC Symbol;Acc:VGNC:86916]	2.08	0.058
ssc-miR-185	CREBRF	CREB3 regulatory factor [Source:VGNC Symbol;Acc:VGNC:86987]	2.08	0.058
ssc-miR-185	CSF1	colony stimulating factor 1 [Source:VGNC Symbol;Acc:VGNC:87030]	2.08	0.058
ssc-miR-185	CSMD2	hypothetical gene	2.08	0.058
ssc-miR-185	CSNK1D	casein kinase 1 delta [Source:VGNC Symbol;Acc:VGNC:97948]	2.08	0.058
ssc-miR-185	CSTF2	cleavage stimulation factor subunit 2 [Source:VGNC Symbol;Acc:VGNC:87050]	2.08	0.058
ssc-miR-185	CTA-299D3.8	hypothetical gene	2.08	0.058
ssc-miR-185	CTSE	hypothetical gene	2.08	0.058
ssc-miR-185	CTSK	cathepsin K [Source:NCBI gene (formerly Entrezgene);Acc:397569]	2.08	0.058
ssc-miR-185	CXorf36	hypothetical gene	2.08	0.058
ssc-miR-185	CYB561	cytochrome b561 [Source:VGNC Symbol;Acc:VGNC:87119]	2.08	0.058
ssc-miR-185	CYP20A1	cytochrome P450 family 20 subfamily A member 1 [Source:NCBI gene (formerly Entrezgene);Acc:100124408]	2.08	0.058
ssc-miR-185	CYP26B1	cytochrome P450 family 26 subfamily B member 1 [Source:VGNC Symbol;Acc:VGNC:103374]	2.08	0.058
ssc-miR-185	CYP4V2	cytochrome P450 family 4 subfamily V member 2 [Source:VGNC Symbol;Acc:VGNC:103361]	2.08	0.058
ssc-miR-185	DAB2IP	DAB2 interacting protein [Source:VGNC Symbol;Acc:VGNC:87145]	2.08	0.058

ssc-miR-185	DCLRE1C	DNA cross-link repair 1C [Source:VGNC Symbol;Acc:VGNC:108268]	2.08	0.058
ssc-miR-185	DCTN3	dynactin subunit 3 [Source:VGNC Symbol;Acc:VGNC:96152]	2.08	0.058
ssc-miR-185	DGCR14	hypothetical gene	2.08	0.058
ssc-miR-185	DGKB	diacylglycerol kinase beta [Source:VGNC Symbol;Acc:VGNC:87270]	2.08	0.058
ssc-miR-185	DGKI	diacylglycerol kinase iota [Source:VGNC Symbol;Acc:VGNC:87274]	2.08	0.058
ssc-miR-185	DHDDS	dehydrodolichyl diphosphate synthase subunit [Source:VGNC Symbol;Acc:VGNC:87279]	2.08	0.058
ssc-miR-185	DLG2	discs large MAGUK scaffold protein 2 [Source:VGNC Symbol;Acc:VGNC:108581]	2.08	0.058
ssc-miR-185	DLGAP3	DLG associated protein 3 [Source:VGNC Symbol;Acc:VGNC:87331]	2.08	0.058
ssc-miR-185	DLGAP4	DLG associated protein 4 [Source:VGNC Symbol;Acc:VGNC:96043]	2.08	0.058
ssc-miR-185	DLST	dihydrolipoamide S-succinyltransferase [Source:VGNC Symbol;Acc:VGNC:87338]	2.08	0.058
ssc-miR-185	DNAH10OS	hypothetical gene	2.08	0.058
ssc-miR-185	DNAH17	dynein axonemal heavy chain 17 [Source:VGNC Symbol;Acc:VGNC:87369]	2.08	0.058
ssc-miR-185	DNAH17-AS1	hypothetical gene	2.08	0.058
ssc-miR-185	DNAJB2	DnaJ heat shock protein family (Hsp40) member B2 [Source:HGNC Symbol;Acc:HGNC:5228]	2.08	0.058
ssc-miR-185	DNM1	dynamin 1 [Source:VGNC Symbol;Acc:VGNC:87380]	2.08	0.058
ssc-miR-185	DPCR1	hypothetical gene	2.08	0.058
ssc-miR-185	DPF2	double PHD fingers 2 [Source:VGNC Symbol;Acc:VGNC:87413]	2.08	0.058
ssc-miR-185	DRP2	dystrophin related protein 2 [Source:VGNC Symbol;Acc:VGNC:87451]	2.08	0.058
ssc-miR-185	DTX3L	deltex E3 ubiquitin ligase 3L [Source:VGNC Symbol;Acc:VGNC:87469]	2.08	0.058
ssc-miR-185	DTX4	deltex E3 ubiquitin ligase 4 [Source:VGNC Symbol;Acc:VGNC:87470]	2.08	0.058
ssc-miR-185	DUSP3	dual specificity phosphatase 3 [Source:VGNC Symbol;Acc:VGNC:87486]	2.08	0.058
ssc-miR-185	DYNLL2	dynein light chain LC8-type 2 [Source:VGNC Symbol;Acc:VGNC:87501]	2.08	0.058
ssc-miR-185	DYRK1B	dual specificity tyrosine phosphorylation regulated kinase 1B [Source:VGNC Symbol;Acc:VGNC:87506]	2.08	0.058
ssc-miR-185	E2F6	E2F transcription factor 6 [Source:VGNC Symbol;Acc:VGNC:87517]	2.08	0.058
ssc-miR-185	EBF4	EBF family member 4 [Source:VGNC Symbol;Acc:VGNC:96211]	2.08	0.058
ssc-miR-185	EDA	ectodysplasin A [Source:VGNC Symbol;Acc:VGNC:87540]	2.08	0.058
ssc-miR-185	EHD4	EH domain containing 4 [Source:VGNC Symbol;Acc:VGNC:87598]	2.08	0.058
ssc-miR-185	EIF4B	eukaryotic translation initiation factor 4B [Source:HGNC Symbol;Acc:HGNC:3285]	2.08	0.058
ssc-miR-185	EIF5A	hypothetical gene	2.08	0.058
ssc-miR-185	ELAVL3	ELAV like RNA binding protein 3 [Source:VGNC Symbol;Acc:VGNC:87640]	2.08	0.058
ssc-miR-185	ELF4	E74 like ETS transcription factor 4 [Source:VGNC Symbol;Acc:VGNC:87643]	2.08	0.058
ssc-miR-185	ELK1	ETS transcription factor ELK1 [Source:HGNC Symbol;Acc:HGNC:3321]	2.08	0.058
ssc-miR-185	ELOVL4	ELOVL fatty acid elongase 4 [Source:VGNC Symbol;Acc:VGNC:87660]	2.08	0.058
ssc-miR-185	ELP4	elongator acetyltransferase complex subunit 4 [Source:VGNC Symbol;Acc:VGNC:87665]	2.08	0.058
ssc-miR-185	EMC1	ER membrane protein complex subunit 1 [Source:VGNC Symbol;Acc:VGNC:97050]	2.08	0.058
ssc-miR-185	EMC10	ER membrane protein complex subunit 10 [Source:VGNC Symbol;Acc:VGNC:87669]	2.08	0.058
ssc-miR-185	ENAH	ENAH actin regulator [Source:VGNC Symbol;Acc:VGNC:108271]	2.08	0.058
ssc-miR-185	EPHB2	EPH receptor B2 [Source:VGNC Symbol;Acc:VGNC:87737]	2.08	0.058
ssc-miR-185	ERBB2	erb-b2 receptor tyrosine kinase 2 [Source:VGNC Symbol;Acc:VGNC:87759]	2.08	0.058
ssc-miR-185	EVI5	ecotropic viral integration site 5 [Source:VGNC Symbol;Acc:VGNC:98793]	2.08	0.058
ssc-miR-185	F9	coagulation factor IX [Source:NCBI gene (formerly Entrezgene);Acc:397518]	2.08	0.058
ssc-miR-185	FAIM2	Fas apoptotic inhibitory molecule 2 [Source:VGNC Symbol;Acc:VGNC:87883]	2.08	0.058
ssc-miR-185	FAM101A	hypothetical gene	2.08	0.058
ssc-miR-185	FAM107A	hypothetical gene	2.08	0.058
ssc-miR-185	FAM114A1	family with sequence similarity 114 member A1 [Source:VGNC Symbol;Acc:VGNC:87890]	2.08	0.058
ssc-miR-185	FAM134C	hypothetical gene	2.08	0.058
ssc-miR-185	FAM168A	family with sequence similarity 168 member A [Source:VGNC Symbol;Acc:VGNC:87926]	2.08	0.058
ssc-miR-185	FAM222A	family with sequence similarity 222 member A [Source:VGNC Symbol;Acc:VGNC:87959]	2.08	0.058
ssc-miR-185	FAM53B	family with sequence similarity 53 member B [Source:VGNC Symbol;Acc:VGNC:87974]	2.08	0.058

ssc-miR-185	FAM53C	family with sequence similarity 53 member C [Source:VGNC Symbol;Acc:VGNC:87975]	2.08	0.058
ssc-miR-185	FAM73B	hypothetical gene	2.08	0.058
ssc-miR-185	FAM76A	family with sequence similarity 76 member A [Source:VGNC Symbol;Acc:VGNC:87982]	2.08	0.058
ssc-miR-185	FAXC	hypothetical gene	2.08	0.058
ssc-miR-185	FBLN7	fibulin 7 [Source:VGNC Symbol;Acc:VGNC:97999]	2.08	0.058
ssc-miR-185	FBXL20	F-box and leucine rich repeat protein 20 [Source:VGNC Symbol;Acc:VGNC:98006]	2.08	0.058
ssc-miR-185	FCHSD2	FCH and double SH3 domains 2 [Source:VGNC Symbol;Acc:VGNC:88068]	2.08	0.058
ssc-miR-185	FNDC5	fibronectin type III domain containing 5 [Source:VGNC Symbol;Acc:VGNC:88185]	2.08	0.058
ssc-miR-185	FOSL2	FOS like 2, AP-1 transcription factor subunit [Source:VGNC Symbol;Acc:VGNC:88192]	2.08	0.058
ssc-miR-185	FRK	fyn related Src family tyrosine kinase [Source:VGNC Symbol;Acc:VGNC:88235]	2.08	0.058
ssc-miR-185	FSTL4	follistatin like 4 [Source:VGNC Symbol;Acc:VGNC:88256]	2.08	0.058
ssc-miR-185	G6PC	hypothetical gene	2.08	0.058
ssc-miR-185	GABPB2	GA binding protein transcription factor subunit beta 2 [Source:VGNC Symbol;Acc:VGNC:98796]	2.08	0.058
ssc-miR-185	GADD45G	growth arrest and DNA damage inducible gamma [Source:VGNC Symbol;Acc:VGNC:88318]	2.08	0.058
ssc-miR-185	GALM	galactose mutarotase [Source:NCBI gene (formerly Entrezgene);Acc:399536]	2.08	0.058
ssc-miR-185	GALNT8	polypeptide N-acetylgalactosaminyltransferase 8 [Source:HGNC Symbol;Acc:HGNC:4130]	2.08	0.058
ssc-miR-185	GATAD2B	GATA zinc finger domain containing 2B [Source:VGNC Symbol;Acc:VGNC:88369]	2.08	0.058
ssc-miR-185	GATSL2	hypothetical gene	2.08	0.058
ssc-miR-185	GFRA2	GDNF family receptor alpha 2 [Source:VGNC Symbol;Acc:VGNC:88429]	2.08	0.058
ssc-miR-185	GK	hypothetical gene	2.08	0.058
ssc-miR-185	GNA13	G protein subunit alpha 13 [Source:VGNC Symbol;Acc:VGNC:98997]	2.08	0.058
ssc-miR-185	GPR107	G protein-coupled receptor 107 [Source:VGNC Symbol;Acc:VGNC:88595]	2.08	0.058
ssc-miR-185	GPR12	G protein-coupled receptor 12 [Source:VGNC Symbol;Acc:VGNC:88598]	2.08	0.058
ssc-miR-185	GPR156	G protein-coupled receptor 156 [Source:VGNC Symbol;Acc:VGNC:88610]	2.08	0.058
ssc-miR-185	GPR26	G protein-coupled receptor 26 [Source:VGNC Symbol;Acc:VGNC:88626]	2.08	0.058
ssc-miR-185	GPR37L1	G protein-coupled receptor 37 like 1 [Source:VGNC Symbol;Acc:VGNC:95633]	2.08	0.058
ssc-miR-185	GPR61	G protein-coupled receptor 61 [Source:HGNC Symbol;Acc:HGNC:13300]	2.08	0.058
ssc-miR-185	GPR75	G protein-coupled receptor 75 [Source:VGNC Symbol;Acc:VGNC:88636]	2.08	0.058
ssc-miR-185	GPRIN3	hypothetical gene	2.08	0.058
ssc-miR-185	GPX8	glutathione peroxidase 8 (putative) [Source:HGNC Symbol;Acc:HGNC:33100]	2.08	0.058
ssc-miR-185	GRM6	glutamate metabotropic receptor 6 [Source:VGNC Symbol;Acc:VGNC:88704]	2.08	0.058
ssc-miR-185	GTPBP1	GTP binding protein 1 [Source:VGNC Symbol;Acc:VGNC:88742]	2.08	0.058
ssc-miR-185	HAUS3	hypothetical gene	2.08	0.058
ssc-miR-185	HIC2	HIC ZBTB transcriptional repressor 2 [Source:HGNC Symbol;Acc:HGNC:18595]	2.08	0.058
ssc-miR-185	HIF3A	hypoxia inducible factor 3 subunit alpha [Source:VGNC Symbol;Acc:VGNC:88881]	2.08	0.058
ssc-miR-185	HNRNPA0	hypothetical gene	2.08	0.058
ssc-miR-185	HOMEZ	homeobox and leucine zipper encoding [Source:VGNC Symbol;Acc:VGNC:88930]	2.08	0.058
ssc-miR-185	HOOK3	hook microtubule tethering protein 3 [Source:VGNC Symbol;Acc:VGNC:98037]	2.08	0.058
ssc-miR-185	HOXA13	homeobox A13 [Source:NCBI gene (formerly Entrezgene);Acc:100359352]	2.08	0.058
ssc-miR-185	HOXB6	homeobox B6 [Source:VGNC Symbol;Acc:VGNC:88946]	2.08	0.058
ssc-miR-185	HP1BP3	heterochromatin protein 1 binding protein 3 [Source:VGNC Symbol;Acc:VGNC:88956]	2.08	0.058
ssc-miR-185	HS3ST4	heparan sulfate-glucosamine 3-sulfotransferase 4 [Source:VGNC Symbol;Acc:VGNC:106584]	2.08	0.058
ssc-miR-185	HSD17B8	hydroxysteroid 17-beta dehydrogenase 8 [Source:VGNC Symbol;Acc:VGNC:88989]	2.08	0.058
ssc-miR-185	HSPH1	heat shock protein family H (Hsp110) member 1 [Source:NCBI gene (formerly Entrezgene);Acc:100048931]	2.08	0.058
ssc-miR-185	HTR2A	5-hydroxytryptamine receptor 2A [Source:VGNC Symbol;Acc:VGNC:103964]	2.08	0.058
ssc-miR-185	IFRG15	hypothetical gene	2.08	0.058
ssc-miR-185	IGF2R	insulin like growth factor 2 receptor [Source:VGNC Symbol;Acc:VGNC:89057]	2.08	0.058
ssc-miR-185	IGJ	hypothetical gene	2.08	0.058
ssc-miR-185	IKZF3	IKAROS family zinc finger 3 [Source:VGNC Symbol;Acc:VGNC:89074]	2.08	0.058

ssc-miR-185	IKZF4	IKAROS family zinc finger 4 [Source:VGNC Symbol;Acc:VGNC:89075]	2.08	0.058
ssc-miR-185	INHBE	inhibin subunit beta E [Source:VGNC Symbol;Acc:VGNC:89135]	2.08	0.058
ssc-miR-185	INPP5K	inositol polyphosphate-5-phosphatase K [Source:VGNC Symbol;Acc:VGNC:89147]	2.08	0.058
ssc-miR-185	IRAK3	interleukin 1 receptor associated kinase 3 [Source:VGNC Symbol;Acc:VGNC:89201]	2.08	0.058
ssc-miR-185	JOSD1	Josephin domain containing 1 [Source:VGNC Symbol;Acc:VGNC:89290]	2.08	0.058
ssc-miR-185	KAT7	lysine acetyltransferase 7 [Source:VGNC Symbol;Acc:VGNC:89307]	2.08	0.058
ssc-miR-185	KCNAB3	potassium voltage-gated channel subfamily A regulatory beta subunit 3 [Source:VGNC Symbol;Acc:VGNC:89330]	2.08	0.058
ssc-miR-185	KCNC2	potassium voltage-gated channel subfamily C member 2 [Source:VGNC Symbol;Acc:VGNC:89333]	2.08	0.058
ssc-miR-185	KCND3	potassium voltage-gated channel subfamily D member 3 [Source:VGNC Symbol;Acc:VGNC:98814]	2.08	0.058
ssc-miR-185	KCNJ6	potassium inwardly rectifying channel subfamily J member 6 [Source:VGNC Symbol;Acc:VGNC:89360]	2.08	0.058
ssc-miR-185	KCNN3	potassium calcium-activated channel subfamily N member 3 [Source:VGNC Symbol;Acc:VGNC:98056]	2.08	0.058
ssc-miR-185	KDM2A	lysine demethylase 2A [Source:VGNC Symbol;Acc:VGNC:89410]	2.08	0.058
ssc-miR-185	KIAA0141	hypothetical gene	2.08	0.058
ssc-miR-185	KIAA1024	hypothetical gene	2.08	0.058
ssc-miR-185	KIAA1199	hypothetical gene	2.08	0.058
ssc-miR-185	KIAA1429	hypothetical gene	2.08	0.058
ssc-miR-185	KIF1B	kinesin family member 1B [Source:VGNC Symbol;Acc:VGNC:89460]	2.08	0.058
ssc-miR-185	KIF21B	kinesin family member 21B [Source:VGNC Symbol;Acc:VGNC:96219]	2.08	0.058
ssc-miR-185	KIF3C	kinesin family member 3C [Source:VGNC Symbol;Acc:VGNC:89471]	2.08	0.058
ssc-miR-185	KLF7	Kruppel like factor 7 [Source:VGNC Symbol;Acc:VGNC:96396]	2.08	0.058
ssc-miR-185	KLHL3	kelch like family member 3 [Source:VGNC Symbol;Acc:VGNC:89525]	2.08	0.058
ssc-miR-185	KLK5	kallikrein related peptidase 5 [Source:VGNC Symbol;Acc:VGNC:89544]	2.08	0.058
ssc-miR-185	KRT78	keratin 78 [Source:VGNC Symbol;Acc:VGNC:89594]	2.08	0.058
ssc-miR-185	LCOR	ligand dependent nuclear receptor corepressor [Source:HGNC Symbol;Acc:HGNC:29503]	2.08	0.058
ssc-miR-185	LDLRAD3	low density lipoprotein receptor class A domain containing 3 [Source:VGNC Symbol;Acc:VGNC:89672]	2.08	0.058
ssc-miR-185	LIF	LIF interleukin 6 family cytokine [Source:VGNC Symbol;Acc:VGNC:89719]	2.08	0.058
ssc-miR-185	LITAF	hypothetical gene	2.08	0.058
ssc-miR-185	LPCAT3	lysophosphatidylcholine acyltransferase 3 [Source:VGNC Symbol;Acc:VGNC:89790]	2.08	0.058
ssc-miR-185	LPHN1	hypothetical gene	2.08	0.058
ssc-miR-185	LRCH3	leucine rich repeats and calponin homology domain containing 3 [Source:VGNC Symbol;Acc:VGNC:89803]	2.08	0.058
ssc-miR-185	LRRC10B	leucine rich repeat containing 10B [Source:VGNC Symbol;Acc:VGNC:99787]	2.08	0.058
ssc-miR-185	LRRC15	leucine rich repeat containing 15 [Source:VGNC Symbol;Acc:VGNC:98077]	2.08	0.058
ssc-miR-185	LRRC16B	hypothetical gene	2.08	0.058
ssc-miR-185	LRRC55	leucine rich repeat containing 55 [Source:VGNC Symbol;Acc:VGNC:89850]	2.08	0.058
ssc-miR-185	LRRC8E	leucine rich repeat containing 8 VRAC subunit E [Source:VGNC Symbol;Acc:VGNC:98089]	2.08	0.058
ssc-miR-185	LSAMP	limbic system associated membrane protein [Source:VGNC Symbol;Acc:VGNC:89868]	2.08	0.058
ssc-miR-185	LZIC	leucine zipper and CTNNBIP1 domain containing [Source:VGNC Symbol;Acc:VGNC:89925]	2.08	0.058
ssc-miR-185	MAPK14	mitogen-activated protein kinase 14 [Source:VGNC Symbol;Acc:VGNC:90000]	2.08	0.058
ssc-miR-185	MAPK8IP3	mitogen-activated protein kinase 8 interacting protein 3 [Source:VGNC Symbol;Acc:VGNC:90008]	2.08	0.058
ssc-miR-185	MAPRE2	microtubule associated protein RP/EB family member 2 [Source:VGNC Symbol;Acc:VGNC:98117]	2.08	0.058
ssc-miR-185	MAT2A	methionine adenosyltransferase 2A [Source:VGNC Symbol;Acc:VGNC:90039]	2.08	0.058
ssc-miR-185	MBNL3	muscleblind like splicing regulator 3 [Source:VGNC Symbol;Acc:VGNC:90056]	2.08	0.058
ssc-miR-185	MDM4	MDM4 regulator of p53 [Source:VGNC Symbol;Acc:VGNC:90094]	2.08	0.058
ssc-miR-185	MEF2B	myocyte enhancer factor 2B [Source:VGNC Symbol;Acc:VGNC:100169]	2.08	0.058
ssc-miR-185	MEOX1	mesenchyme homeobox 1 [Source:VGNC Symbol;Acc:VGNC:90141]	2.08	0.058
ssc-miR-185	MESDC1	hypothetical gene	2.08	0.058
ssc-miR-185	MEX3A	mex-3 RNA binding family member A [Source:VGNC Symbol;Acc:VGNC:90168]	2.08	0.058
ssc-miR-185	MID2	midline 2 [Source:VGNC Symbol;Acc:VGNC:98127]	2.08	0.058
ssc-miR-185	MIEF1	hypothetical gene	2.08	0.058

ssc-miR-185	MIER1	MIER1 transcriptional regulator [Source:VGNC Symbol;Acc:VGNC:90219]	2.08	0.058
ssc-miR-185	MLLT3	MLLT3 super elongation complex subunit [Source:VGNC Symbol;Acc:VGNC:90256]	2.08	0.058
ssc-miR-185	MORN4	MORN repeat containing 4 [Source:VGNC Symbol;Acc:VGNC:90310]	2.08	0.058
ssc-miR-185	MPP2	MAGUK p55 scaffold protein 2 [Source:VGNC Symbol;Acc:VGNC:98133]	2.08	0.058
ssc-miR-185	MRPL17	mitochondrial ribosomal protein L17 [Source:VGNC Symbol;Acc:VGNC:103992]	2.08	0.058
ssc-miR-185	MRRF	mitochondrial ribosome recycling factor [Source:VGNC Symbol;Acc:VGNC:90402]	2.08	0.058
ssc-miR-185	MSI1	musashi RNA binding protein 1 [Source:VGNC Symbol;Acc:VGNC:90421]	2.08	0.058
ssc-miR-185	MTMR9	myotubularin related protein 9 [Source:VGNC Symbol;Acc:VGNC:90464]	2.08	0.058
ssc-miR-185	MTSS1L	hypothetical gene	2.08	0.058
ssc-miR-185	MXI1	hypothetical gene	2.08	0.058
ssc-miR-185	MYBL1	MYB proto-onco like 1 [Source:VGNC Symbol;Acc:VGNC:90498]	2.08	0.058
ssc-miR-185	MYLK	myosin light chain kinase [Source:VGNC Symbol;Acc:VGNC:108676]	2.08	0.058
ssc-miR-185	MYO18A	myosin XVIIIA [Source:HGNC Symbol;Acc:HGNC:31104]	2.08	0.058
ssc-miR-185	MYPN	myopalladin [Source:VGNC Symbol;Acc:VGNC:107412]	2.08	0.058
ssc-miR-185	NAA50	N-alpha-acetyltransferase 50, NatE catalytic subunit [Source:VGNC Symbol;Acc:VGNC:104002]	2.08	0.058
ssc-miR-185	NAGS	N-acetylglutamate synthase [Source:VGNC Symbol;Acc:VGNC:90571]	2.08	0.058
ssc-miR-185	NCOA2	nuclear receptor coactivator 2 [Source:VGNC Symbol;Acc:VGNC:90616]	2.08	0.058
ssc-miR-185	NDST1	N-deacetylase and N-sulfotransferase 1 [Source:VGNC Symbol;Acc:VGNC:90634]	2.08	0.058
ssc-miR-185	NECAB3	N-terminal EF-hand calcium binding protein 3 [Source:VGNC Symbol;Acc:VGNC:95670]	2.08	0.058
ssc-miR-185	NFATC3	nuclear factor of activated T cells 3 [Source:VGNC Symbol;Acc:VGNC:90710]	2.08	0.058
ssc-miR-185	NFIX	nuclear factor I X [Source:VGNC Symbol;Acc:VGNC:90718]	2.08	0.058
ssc-miR-185	NLRP1	hypothetical gene	2.08	0.058
ssc-miR-185	NMNAT2	nicotinamide nucleotide adenylyltransferase 2 [Source:VGNC Symbol;Acc:VGNC:90798]	2.08	0.058
ssc-miR-185	NOP9	NOP9 nucleolar protein [Source:VGNC Symbol;Acc:VGNC:90822]	2.08	0.058
ssc-miR-185	NOX5	NADPH oxidase 5 [Source:VGNC Symbol;Acc:VGNC:90832]	2.08	0.058
ssc-miR-185	NPFFR1	neuropeptide FF receptor 1 [Source:VGNC Symbol;Acc:VGNC:90846]	2.08	0.058
ssc-miR-185	NPR2	natriuretic peptide receptor 2 [Source:VGNC Symbol;Acc:VGNC:90857]	2.08	0.058
ssc-miR-185	NR1D1	nuclear receptor subfamily 1 group D member 1 [Source:VGNC Symbol;Acc:VGNC:90869]	2.08	0.058
ssc-miR-185	NR6A1	nuclear receptor subfamily 6 group A member 1 [Source:VGNC Symbol;Acc:VGNC:90887]	2.08	0.058
ssc-miR-185	NRSN1	neurensin 1 [Source:VGNC Symbol;Acc:VGNC:90902]	2.08	0.058
ssc-miR-185	NSDHL	NAD(P) dependent steroid dehydrogenase-like [Source:NCBI gene (formerly Entrezgene);Acc:100157894]	2.08	0.058
ssc-miR-185	NT5C1A	5'-nucleotidase, cytosolic IA [Source:VGNC Symbol;Acc:VGNC:90919]	2.08	0.058
ssc-miR-185	NTPCR	nucleoside-triphosphatase, cancer-related [Source:HGNC Symbol;Acc:HGNC:28204]	2.08	0.058
ssc-miR-185	NUP37	nucleoporin 37 [Source:VGNC Symbol;Acc:VGNC:90980]	2.08	0.058
ssc-miR-185	ODF2L	outer dense fiber of sperm tails 2 like [Source:VGNC Symbol;Acc:VGNC:91018]	2.08	0.058
ssc-miR-185	OLFM2	olfactomedin 2 [Source:VGNC Symbol;Acc:VGNC:91032]	2.08	0.058
ssc-miR-185	OSBP2	oxysterol binding protein 2 [Source:VGNC Symbol;Acc:VGNC:91067]	2.08	0.058
ssc-miR-185	OTOP3	otopetrin 3 [Source:VGNC Symbol;Acc:VGNC:91095]	2.08	0.058
ssc-miR-185	PAK6	p21 (RAC1) activated kinase 6 [Source:VGNC Symbol;Acc:VGNC:103144]	2.08	0.058
ssc-miR-185	PAK7	hypothetical gene	2.08	0.058
ssc-miR-185	PARPBP	PARP1 binding protein [Source:VGNC Symbol;Acc:VGNC:91183]	2.08	0.058
ssc-miR-185	PARS2	prolyl-tRNA synthetase 2, mitochondrial [Source:VGNC Symbol;Acc:VGNC:91184]	2.08	0.058
ssc-miR-185	PAX2	paired box 2 [Source:VGNC Symbol;Acc:VGNC:91192]	2.08	0.058
ssc-miR-185	PAX5	paired box 5 [Source:VGNC Symbol;Acc:VGNC:91194]	2.08	0.058
ssc-miR-185	PAX7	paired box 7 [Source:VGNC Symbol;Acc:VGNC:91196]	2.08	0.058
ssc-miR-185	PAX8	hypothetical gene	2.08	0.058
ssc-miR-185	PBX1	hypothetical gene	2.08	0.058
ssc-miR-185	PBX2	PBX homeobox 2 [Source:VGNC Symbol;Acc:VGNC:91204]	2.08	0.058
ssc-miR-185	PCDH1	protocadherin 1 [Source:VGNC Symbol;Acc:VGNC:91211]	2.08	0.058

ssc-miR-185	PCDH10	protocadherin 10 [Source:HGNC Symbol;Acc:HGNC:13404]	2.08	0.058
ssc-miR-185	PCDHA1	hypothetical gene	2.08	0.058
ssc-miR-185	PCDHA10	hypothetical gene	2.08	0.058
ssc-miR-185	PCDHA11	hypothetical gene	2.08	0.058
ssc-miR-185	PCDHA12	hypothetical gene	2.08	0.058
ssc-miR-185	PCDHA13	protocadherin alpha 13 [Source:HGNC Symbol;Acc:HGNC:8667]	2.08	0.058
ssc-miR-185	PCDHA2	hypothetical gene	2.08	0.058
ssc-miR-185	PCDHA3	protocadherin alpha 3 [Source:HGNC Symbol;Acc:HGNC:8669]	2.08	0.058
ssc-miR-185	PCDHA4	hypothetical gene	2.08	0.058
ssc-miR-185	PCDHA5	hypothetical gene	2.08	0.058
ssc-miR-185	PCDHA6	hypothetical gene	2.08	0.058
ssc-miR-185	PCDHA7	hypothetical gene	2.08	0.058
ssc-miR-185	PCDHA8	hypothetical gene	2.08	0.058
ssc-miR-185	PCDHA9	hypothetical gene	2.08	0.058
ssc-miR-185	PCDHAC1	hypothetical gene	2.08	0.058
ssc-miR-185	PCDHAC2	protocadherin alpha subfamily C, 2 [Source:HGNC Symbol;Acc:HGNC:8677]	2.08	0.058
ssc-miR-185	PCP4L1	Purkinje cell protein 4 like 1 [Source:VGNC Symbol;Acc:VGNC:98831]	2.08	0.058
ssc-miR-185	PDE7A	phosphodiesterase 7A [Source:VGNC Symbol;Acc:VGNC:91261]	2.08	0.058
ssc-miR-185	PER1	period circadian regulator 1 [Source:VGNC Symbol;Acc:VGNC:91314]	2.08	0.058
ssc-miR-185	PGM1	hypothetical gene	2.08	0.058
ssc-miR-185	PHF7	PHD finger protein 7 [Source:VGNC Symbol;Acc:VGNC:91390]	2.08	0.058
ssc-miR-185	PHLDB1	pleckstrin homology like domain family B member 1 [Source:VGNC Symbol;Acc:VGNC:91398]	2.08	0.058
ssc-miR-185	PHLPP2	PH domain and leucine rich repeat protein phosphatase 2 [Source:VGNC Symbol;Acc:VGNC:98186]	2.08	0.058
ssc-miR-185	PHYHIP	phytanoyl-CoA 2-hydroxylase interacting protein [Source:VGNC Symbol;Acc:VGNC:91406]	2.08	0.058
ssc-miR-185	PILRA	hypothetical gene	2.08	0.058
ssc-miR-185	PITPNA	phosphatidylinositol transfer protein alpha [Source:VGNC Symbol;Acc:VGNC:91464]	2.08	0.058
ssc-miR-185	PITPNC1	phosphatidylinositol transfer protein cytoplasmic 1 [Source:NCBI gene (formerly Entrezgene);Acc:100233199]	2.08	0.058
ssc-miR-185	PKIA	cAMP-dependent protein kinase inhibitor alpha [Source:VGNC Symbol;Acc:VGNC:91477]	2.08	0.058
ssc-miR-185	PLA2G2F	phospholipase A2 group IIF [Source:VGNC Symbol;Acc:VGNC:91493]	2.08	0.058
ssc-miR-185	PLAC8	hypothetical gene	2.08	0.058
ssc-miR-185	PLEKHA1	pleckstrin homology domain containing A1 [Source:VGNC Symbol;Acc:VGNC:91533]	2.08	0.058
ssc-miR-185	PLEKHG3	pleckstrin homology and RhoGEF domain containing G3 [Source:VGNC Symbol;Acc:VGNC:91544]	2.08	0.058
ssc-miR-185	PLXNA4	plexin A4 [Source:VGNC Symbol;Acc:VGNC:98204]	2.08	0.058
ssc-miR-185	POC1A	POC1 centriolar protein A [Source:VGNC Symbol;Acc:VGNC:91615]	2.08	0.058
ssc-miR-185	PPAP2B	hypothetical gene	2.08	0.058
ssc-miR-185	PPARGC1B	PPARG coactivator 1 beta [Source:VGNC Symbol;Acc:VGNC:91686]	2.08	0.058
ssc-miR-185	PPIP5K2	diphosphoinositol pentakisphosphate kinase 2 [Source:VGNC Symbol;Acc:VGNC:91701]	2.08	0.058
ssc-miR-185	PPM1L	protein phosphatase, Mg2+/Mn2+ dependent 1L [Source:VGNC Symbol;Acc:VGNC:91711]	2.08	0.058
ssc-miR-185	PPP1R3E	protein phosphatase 1 regulatory subunit 3E [Source:HGNC Symbol;Acc:HGNC:14943]	2.08	0.058
ssc-miR-185	PRIM2	DNA primase subunit 2 [Source:VGNC Symbol;Acc:VGNC:91796]	2.08	0.058
ssc-miR-185	PRKAR2A	protein kinase cAMP-dependent type II regulatory subunit alpha [Source:VGNC Symbol;Acc:VGNC:91804]	2.08	0.058
ssc-miR-185	PRRT2	proline rich transmembrane protein 2 [Source:HGNC Symbol;Acc:HGNC:30500]	2.08	0.058
ssc-miR-185	PRX	periaxin [Source:VGNC Symbol;Acc:VGNC:91891]	2.08	0.058
ssc-miR-185	PSMD11	proteasome 26S subunit, non-ATPase 11 [Source:VGNC Symbol;Acc:VGNC:91919]	2.08	0.058
ssc-miR-185	PTCHD1	patched domain containing 1 [Source:VGNC Symbol;Acc:VGNC:91942]	2.08	0.058
ssc-miR-185	PTPRA	protein tyrosine phosphatase receptor type A [Source:VGNC Symbol;Acc:VGNC:96522]	2.08	0.058
ssc-miR-185	PTPRG	protein tyrosine phosphatase receptor type G [Source:VGNC Symbol;Acc:VGNC:91988]	2.08	0.058
ssc-miR-185	RAB14	RAB14, member RAS onco family [Source:VGNC Symbol;Acc:VGNC:98238]	2.08	0.058
ssc-miR-185	RAB35	RAB35, member RAS onco family [Source:VGNC Symbol;Acc:VGNC:98259]	2.08	0.058

ssc-miR-185	RAB3B	RAB3B, member RAS onco family [Source:VGNC Symbol;Acc:VGNC:98265]	2.08	0.058
ssc-miR-185	RAD54L2	RAD54 like 2 [Source:VGNC Symbol;Acc:VGNC:92061]	2.08	0.058
ssc-miR-185	RALGPS2	Ral GEF with PH domain and SH3 binding motif 2 [Source:VGNC Symbol;Acc:VGNC:92073]	2.08	0.058
ssc-miR-185	RBM8A	RNA binding motif protein 8A [Source:VGNC Symbol;Acc:VGNC:92162]	2.08	0.058
ssc-miR-185	RERE	hypothetical gene	2.08	0.058
ssc-miR-185	RIC8B	RIC8 guanine nucleotide exchange factor B [Source:VGNC Symbol;Acc:VGNC:92302]	2.08	0.058
ssc-miR-185	RIMS4	regulating synaptic membrane exocytosis 4 [Source:VGNC Symbol;Acc:VGNC:95775]	2.08	0.058
ssc-miR-185	RMND5A	required for meiotic nuclear division 5 homolog A [Source:VGNC Symbol;Acc:VGNC:92336]	2.08	0.058
ssc-miR-185	RNF20	ring finger protein 20 [Source:VGNC Symbol;Acc:VGNC:92378]	2.08	0.058
ssc-miR-185	RNF26	ring finger protein 26 [Source:VGNC Symbol;Acc:VGNC:92387]	2.08	0.058
ssc-miR-185	ROBO2	roundabout guidance receptor 2 [Source:HGNC Symbol;Acc:HGNC:10250]	2.08	0.058
ssc-miR-185	ROCK2	Rho associated coiled-coil containing protein kinase 2 [Source:HGNC Symbol;Acc:HGNC:10252]	2.08	0.058
ssc-miR-185	RORB	RAR related orphan receptor B [Source:VGNC Symbol;Acc:VGNC:92409]	2.08	0.058
ssc-miR-185	RP11-73M18.2	hypothetical gene	2.08	0.058
ssc-miR-185	RSBN1	round spermatid basic protein 1 [Source:VGNC Symbol;Acc:VGNC:92473]	2.08	0.058
ssc-miR-185	RSU1	Ras suppressor protein 1 [Source:VGNC Symbol;Acc:VGNC:98298]	2.08	0.058
ssc-miR-185	S1PR1	sphingosine-1-phosphate receptor 1 [Source:VGNC Symbol;Acc:VGNC:92551]	2.08	0.058
ssc-miR-185	SAMD12	sterile alpha motif domain containing 12 [Source:VGNC Symbol;Acc:VGNC:92565]	2.08	0.058
ssc-miR-185	SAP30L	SAP30 like [Source:VGNC Symbol;Acc:VGNC:92577]	2.08	0.058
ssc-miR-185	SAR1A	secretion associated Ras related GTPase 1A [Source:VGNC Symbol;Acc:VGNC:92579]	2.08	0.058
ssc-miR-185	SCARB1	scavenger receptor class B member 1 [Source:VGNC Symbol;Acc:VGNC:92613]	2.08	0.058
ssc-miR-185	SDHC	succinate dehydrogenase complex subunit C [Source:VGNC Symbol;Acc:VGNC:98852]	2.08	0.058
ssc-miR-185	SEC22A	SEC22 homolog A, vesicle trafficking protein [Source:VGNC Symbol;Acc:VGNC:92673]	2.08	0.058
ssc-miR-185	SEL1L	SEL1L adaptor subunit of ERAD E3 ubiquitin ligase [Source:VGNC Symbol;Acc:VGNC:92687]	2.08	0.058
ssc-miR-185	SEL1L3	SEL1L family member 3 [Source:VGNC Symbol;Acc:VGNC:92688]	2.08	0.058
ssc-miR-185	SELO	hypothetical gene	2.08	0.058
ssc-miR-185	SELRC1	hypothetical gene	2.08	0.058
ssc-miR-185	SETD5	SET domain containing 5 [Source:VGNC Symbol;Acc:VGNC:92760]	2.08	0.058
ssc-miR-185	SF1	splicing factor 1 [Source:HGNC Symbol;Acc:HGNC:12950]	2.08	0.058
ssc-miR-185	SFXN3	sideroflexin 3 [Source:VGNC Symbol;Acc:VGNC:92785]	2.08	0.058
ssc-miR-185	SGMS1	sphingomyelin synthase 1 [Source:VGNC Symbol;Acc:VGNC:92794]	2.08	0.058
ssc-miR-185	SGPP2	sphingosine-1-phosphate phosphatase 2 [Source:VGNC Symbol;Acc:VGNC:95931]	2.08	0.058
ssc-miR-185	SH3BGRL2	SH3 domain binding glutamate rich protein like 2 [Source:VGNC Symbol;Acc:VGNC:92816]	2.08	0.058
ssc-miR-185	SH3BP5	SH3 domain binding protein 5 [Source:VGNC Symbol;Acc:VGNC:92819]	2.08	0.058
ssc-miR-185	SHC2	hypothetical gene	2.08	0.058
ssc-miR-185	SHOC2	SHOC2 leucine rich repeat scaffold protein [Source:VGNC Symbol;Acc:VGNC:98318]	2.08	0.058
ssc-miR-185	SIX3	SIX homeobox 3 [Source:VGNC Symbol;Acc:VGNC:92894]	2.08	0.058
ssc-miR-185	SLA2	Src like adaptor 2 [Source:HGNC Symbol;Acc:HGNC:17329]	2.08	0.058
ssc-miR-185	SLAMF6	SLAM family member 6 [Source:VGNC Symbol;Acc:VGNC:92912]	2.08	0.058
ssc-miR-185	SLC16A2	solute carrier family 16 member 2 [Source:VGNC Symbol;Acc:VGNC:92942]	2.08	0.058
ssc-miR-185	SLC22A23	solute carrier family 22 member 23 [Source:HGNC Symbol;Acc:HGNC:21106]	2.08	0.058
ssc-miR-185	SLC24A2	solute carrier family 24 member 2 [Source:VGNC Symbol;Acc:VGNC:92987]	2.08	0.058
ssc-miR-185	SLC24A4	solute carrier family 24 member 4 [Source:VGNC Symbol;Acc:VGNC:92988]	2.08	0.058
ssc-miR-185	SLC25A29	hypothetical gene	2.08	0.058
ssc-miR-185	SLC25A53	solute carrier family 25 member 53 [Source:VGNC Symbol;Acc:VGNC:93023]	2.08	0.058
ssc-miR-185	SLC2A13	solute carrier family 2 member 13 [Source:VGNC Symbol;Acc:VGNC:93047]	2.08	0.058
ssc-miR-185	SLC35C2	solute carrier family 35 member C2 [Source:VGNC Symbol;Acc:VGNC:95831]	2.08	0.058
ssc-miR-185	SLC37A2	solute carrier family 37 member 2 [Source:VGNC Symbol;Acc:VGNC:98323]	2.08	0.058
ssc-miR-185	SLC38A1	solute carrier family 38 member 1 [Source:HGNC Symbol;Acc:HGNC:13447]	2.08	0.058

ssc-miR-185	SLC39A14	solute carrier family 39 member 14 [Source:HGNC Symbol;Acc:HGNC:20858]	2.08	0.058
ssc-miR-185	SLC44A2	solute carrier family 44 member 2 [Source:VGNC Symbol;Acc:VGNC:100862]	2.08	0.058
ssc-miR-185	SLC6A16	hypothetical gene	2.08	0.058
ssc-miR-185	SLC8A1	solute carrier family 8 member A1 [Source:VGNC Symbol;Acc:VGNC:93178]	2.08	0.058
ssc-miR-185	SMG5	SMG5 nonsense mediated mRNA decay factor [Source:VGNC Symbol;Acc:VGNC:98861]	2.08	0.058
ssc-miR-185	SMG7	SMG7 nonsense mediated mRNA decay factor [Source:VGNC Symbol;Acc:VGNC:93250]	2.08	0.058
ssc-miR-185	SMUG1	single-strand-selective monofunctional uracil-DNA glycosylase 1 [Source:VGNC Symbol;Acc:VGNC:93269]	2.08	0.058
ssc-miR-185	SND1	staphylococcal nuclease and tudor domain containing 1 [Source:VGNC Symbol;Acc:VGNC:93287]	2.08	0.058
ssc-miR-185	SNTB2	syntrophin beta 2 [Source:VGNC Symbol;Acc:VGNC:93298]	2.08	0.058
ssc-miR-185	SNX22	sorting nexin 22 [Source:VGNC Symbol;Acc:VGNC:93314]	2.08	0.058
ssc-miR-185	SNX30	sorting nexin family member 30 [Source:VGNC Symbol;Acc:VGNC:93318]	2.08	0.058
ssc-miR-185	SORCS1	sortilin related VPS10 domain containing receptor 1 [Source:VGNC Symbol;Acc:VGNC:93339]	2.08	0.058
ssc-miR-185	SORL1	sortilin related receptor 1 [Source:VGNC Symbol;Acc:VGNC:108617]	2.08	0.058
ssc-miR-185	SOX13	SRY-box transcription factor 13 [Source:VGNC Symbol;Acc:VGNC:93352]	2.08	0.058
ssc-miR-185	SPATA2	spermatosis associated 2 [Source:VGNC Symbol;Acc:VGNC:95628]	2.08	0.058
ssc-miR-185	SPATS2	spermatogenesis associated serine rich 2 [Source:HGNC Symbol;Acc:HGNC:18650]	2.08	0.058
ssc-miR-185	SPECC1L	sperm antigen with calponin homology and coiled-coil domains 1 like [Source:HGNC Symbol;Acc:HGNC:29022]	2.08	0.058
ssc-miR-185	SPIN2A	hypothetical gene	2.08	0.058
ssc-miR-185	SPIN3	spindlin family member 3 [Source:HGNC Symbol;Acc:HGNC:27272]	2.08	0.058
ssc-miR-185	SPRYD4	SPRY domain containing 4 [Source:HGNC Symbol;Acc:HGNC:27468]	2.08	0.058
ssc-miR-185	SRM	spermidine synthase [Source:HGNC Symbol;Acc:HGNC:11296]	2.08	0.058
ssc-miR-185	SRRM4	serine/arginine repetitive matrix 4 [Source:VGNC Symbol;Acc:VGNC:93470]	2.08	0.058
ssc-miR-185	SSBP2	single stranded DNA binding protein 2 [Source:VGNC Symbol;Acc:VGNC:93480]	2.08	0.058
ssc-miR-185	ST6GALNAC3	ST6 N-acetylgalactosaminide alpha-2,6-sialyltransferase 3 [Source:VGNC Symbol;Acc:VGNC:93511]	2.08	0.058
ssc-miR-185	ST6GALNAC5	ST6 N-acetylgalactosaminide alpha-2,6-sialyltransferase 5 [Source:VGNC Symbol;Acc:VGNC:93513]	2.08	0.058
ssc-miR-185	ST7L	suppression of tumorigenicity 7 like [Source:VGNC Symbol;Acc:VGNC:93515]	2.08	0.058
ssc-miR-185	ST8SIA2	ST8 alpha-N-acetyl-neuraminide alpha-2,8-sialyltransferase 2 [Source:VGNC Symbol;Acc:VGNC:93517]	2.08	0.058
ssc-miR-185	STAC2	SH3 and cysteine rich domain 2 [Source:VGNC Symbol;Acc:VGNC:93522]	2.08	0.058
ssc-miR-185	STX4	syntaxin 4 [Source:VGNC Symbol;Acc:VGNC:93592]	2.08	0.058
ssc-miR-185	STXBP4	syntaxin binding protein 4 [Source:VGNC Symbol;Acc:VGNC:93598]	2.08	0.058
ssc-miR-185	SUPT6H	SPT6 homolog, histone chaperone and transcription elongation factor [Source:VGNC Symbol;Acc:VGNC:93626]	2.08	0.058
ssc-miR-185	SYNGAP1	synaptic Ras GTPase activating protein 1 [Source:VGNC Symbol;Acc:VGNC:93664]	2.08	0.058
ssc-miR-185	SYNM	hypothetical gene	2.08	0.058
ssc-miR-185	TAF3	TATA-box binding protein associated factor 3 [Source:VGNC Symbol;Acc:VGNC:95844]	2.08	0.058
ssc-miR-185	TANC2	tetratricopeptide repeat, ankyrin repeat and coiled-coil containing 2 [Source:VGNC Symbol;Acc:VGNC:93733]	2.08	0.058
ssc-miR-185	TAPBP	TAP binding protein [Source:HGNC Symbol;Acc:HGNC:11566]	2.08	0.058
ssc-miR-185	TBC1D20	TBC1 domain family member 20 [Source:VGNC Symbol;Acc:VGNC:95752]	2.08	0.058
ssc-miR-185	TBL2	transducin beta like 2 [Source:VGNC Symbol;Acc:VGNC:93789]	2.08	0.058
ssc-miR-185	TBX19	T-box transcription factor 19 [Source:VGNC Symbol;Acc:VGNC:93797]	2.08	0.058
ssc-miR-185	TCF12	transcription factor 12 [Source:VGNC Symbol;Acc:VGNC:93817]	2.08	0.058
ssc-miR-185	TCF23	transcription factor 23 [Source:VGNC Symbol;Acc:VGNC:93820]	2.08	0.058
ssc-miR-185	TEAD1	TEA domain transcription factor 1 [Source:VGNC Symbol;Acc:VGNC:93853]	2.08	0.058
ssc-miR-185	TENM4	teneurin transmembrane protein 4 [Source:HGNC Symbol;Acc:HGNC:29945]	2.08	0.058
ssc-miR-185	THBS2	thrombospondin 2 [Source:VGNC Symbol;Acc:VGNC:93947]	2.08	0.058
ssc-miR-185	THRA	thyroid hormone receptor alpha [Source:VGNC Symbol;Acc:VGNC:93963]	2.08	0.058
ssc-miR-185	THSD7A	thrombospondin type 1 domain containing 7A [Source:VGNC Symbol;Acc:VGNC:93968]	2.08	0.058
ssc-miR-185	TIAF1	hypothetical gene	2.08	0.058
ssc-miR-185	TJP1	tight junction protein 1 [Source:VGNC Symbol;Acc:VGNC:94005]	2.08	0.058
ssc-miR-185	TMC5	transmembrane channel like 5 [Source:VGNC Symbol;Acc:VGNC:94042]	2.08	0.058

ssc-miR-185	TMEM233	transmembrane protein 233 [Source:HGNC Symbol;Acc:HGNC:37219]	2.08	0.058
ssc-miR-185	TMEM254	transmembrane protein 254 [Source:VGNC Symbol;Acc:VGNC:94160]	2.08	0.058
ssc-miR-185	TMEM39A	transmembrane protein 39A [Source:VGNC Symbol;Acc:VGNC:94176]	2.08	0.058
ssc-miR-185	TMEM51	transmembrane protein 51 [Source:VGNC Symbol;Acc:VGNC:94187]	2.08	0.058
ssc-miR-185	TMEM98	transmembrane protein 98 [Source:VGNC Symbol;Acc:VGNC:94221]	2.08	0.058
ssc-miR-185	TMOD2	tropomodulin 2 [Source:VGNC Symbol;Acc:VGNC:94227]	2.08	0.058
ssc-miR-185	TNFSF8	TNF superfamily member 8 [Source:VGNC Symbol;Acc:VGNC:94273]	2.08	0.058
ssc-miR-185	TNPO3	transportin 3 [Source:VGNC Symbol;Acc:VGNC:94289]	2.08	0.058
ssc-miR-185	TNS4	tensin 4 [Source:VGNC Symbol;Acc:VGNC:94297]	2.08	0.058
ssc-miR-185	TOM1L2	target of myb1 like 2 membrane trafficking protein [Source:VGNC Symbol;Acc:VGNC:94303]	2.08	0.058
ssc-miR-185	TOX3	TOX high mobility group box family member 3 [Source:VGNC Symbol;Acc:VGNC:94323]	2.08	0.058
ssc-miR-185	TPD52	tumor protein D52 [Source:VGNC Symbol;Acc:VGNC:98383]	2.08	0.058
ssc-miR-185	TPM3	tropomyosin 3 [Source:VGNC Symbol;Acc:VGNC:98877]	2.08	0.058
ssc-miR-185	TPR	translocated promoter region, nuclear basket protein [Source:VGNC Symbol;Acc:VGNC:108623]	2.08	0.058
ssc-miR-185	TRIM24	tripartite motif containing 24 [Source:VGNC Symbol;Acc:VGNC:94400]	2.08	0.058
ssc-miR-185	TRIM44	tripartite motif containing 44 [Source:VGNC Symbol;Acc:VGNC:94416]	2.08	0.058
ssc-miR-185	TRIM5	hypothetical gene	2.08	0.058
ssc-miR-185	TRIM67	tripartite motif containing 67 [Source:VGNC Symbol;Acc:VGNC:94427]	2.08	0.058
ssc-miR-185	TRIOBP	hypothetical gene	2.08	0.058
ssc-miR-185	TSN	translin [Source:VGNC Symbol;Acc:VGNC:95556]	2.08	0.058
ssc-miR-185	TSPAN6	tetraspanin 6 [Source:NCBI gene (formerly Entrezgene);Acc:100157741]	2.08	0.058
ssc-miR-185	TTC5	tetratricopeptide repeat domain 5 [Source:VGNC Symbol;Acc:VGNC:94555]	2.08	0.058
ssc-miR-185	TTLL3	actin related protein 2/3 complex subunit 4 [Source:NCBI gene (formerly Entrezgene);Acc:100155875]	2.08	0.058
ssc-miR-185	TTYH3	tweety family member 3 [Source:VGNC Symbol;Acc:VGNC:94576]	2.08	0.058
ssc-miR-185	UBE2B	ubiquitin conjugating enzyme E2 B [Source:VGNC Symbol;Acc:VGNC:94640]	2.08	0.058
ssc-miR-185	UHMK1	U2AF homology motif kinase 1 [Source:VGNC Symbol;Acc:VGNC:94688]	2.08	0.058
ssc-miR-185	ULK2	unc-51 like autophagy activating kinase 2 [Source:VGNC Symbol;Acc:VGNC:94695]	2.08	0.058
ssc-miR-185	UPF3B	UPF3B regulator of nonsense mediated mRNA decay [Source:VGNC Symbol;Acc:VGNC:94720]	2.08	0.058
ssc-miR-185	URM1	ubiquitin related modifier 1 [Source:VGNC Symbol;Acc:VGNC:94732]	2.08	0.058
ssc-miR-185	USH1C	USH1 protein network component harmonin [Source:VGNC Symbol;Acc:VGNC:94738]	2.08	0.058
ssc-miR-185	USP3	ubiquitin specific peptidase 3 [Source:VGNC Symbol;Acc:VGNC:94757]	2.08	0.058
ssc-miR-185	VAMP2	vesicle associated membrane protein 2 [Source:HGNC Symbol;Acc:HGNC:12643]	2.08	0.058
ssc-miR-185	VIPAS39	VPS33B interacting protein, apical-basolateral polarity regulator, spe-39 homolog [Source:VGNC Symbol;Acc:VGNC:94826]	2.08	0.058
ssc-miR-185	VSTM5	V-set and transmembrane domain containing 5 [Source:VGNC Symbol;Acc:VGNC:94875]	2.08	0.058
ssc-miR-185	WDR52	hypothetical gene	2.08	0.058
ssc-miR-185	WDR55	WD repeat domain 55 [Source:VGNC Symbol;Acc:VGNC:94929]	2.08	0.058
ssc-miR-185	WISP2	hypothetical gene	2.08	0.058
ssc-miR-185	WNT7A	Wnt family member 7A [Source:VGNC Symbol;Acc:VGNC:94974]	2.08	0.058
ssc-miR-185	WWC2	WW and C2 domain containing 2 [Source:VGNC Symbol;Acc:VGNC:96033]	2.08	0.058
ssc-miR-185	XPR1	xenotropic and polytropic retrovirus receptor 1 [Source:VGNC Symbol;Acc:VGNC:108625]	2.08	0.058
ssc-miR-185	XYLT1	xylosyltransferase 1 [Source:VGNC Symbol;Acc:VGNC:95015]	2.08	0.058
ssc-miR-185	YARS	hypothetical gene	2.08	0.058
ssc-miR-185	YWHAZ	tyrosine 3-monooxygenase/tryptophan 5-monooxygenase activation protein zeta [Source:VGNC Symbol;Acc:VGNC:95047]	2.08	0.058
ssc-miR-185	ZBTB16	zinc finger and BTB domain containing 16 [Source:VGNC Symbol;Acc:VGNC:108626]	2.08	0.058
ssc-miR-185	ZBTB20	zinc finger and BTB domain containing 20 [Source:VGNC Symbol;Acc:VGNC:95063]	2.08	0.058
ssc-miR-185	ZC3H11A	hypothetical gene	2.08	0.058
ssc-miR-185	ZCCHC3	zinc finger CCHC-type containing 3 [Source:HGNC Symbol;Acc:HGNC:16230]	2.08	0.058
ssc-miR-185	ZFYVE9	zinc finger FYVE-type containing 9 [Source:VGNC Symbol;Acc:VGNC:95163]	2.08	0.058
ssc-miR-185	ZNF185	zinc finger protein 185 with LIM domain [Source:VGNC Symbol;Acc:VGNC:95201]	2.08	0.058

ssc-miR-185	ZNF226	zinc finger protein 226 [Source:HGNC Symbol;Acc:HGNC:13019]	2.08	0.058
ssc-miR-185	ZNF280C	zinc finger protein 280C [Source:VGNC Symbol;Acc:VGNC:95216]	2.08	0.058
ssc-miR-185	ZNF385A	zinc finger protein 385A [Source:VGNC Symbol;Acc:VGNC:95233]	2.08	0.058
ssc-miR-185	ZNF423	zinc finger protein 423 [Source:VGNC Symbol;Acc:VGNC:98752]	2.08	0.058
ssc-miR-185	ZNF609	zinc finger protein 609 [Source:VGNC Symbol;Acc:VGNC:95274]	2.08	0.058
ssc-miR-185	ZNF646	hypothetical gene	2.08	0.058
ssc-miR-185	ZNF703	zinc finger protein 703 [Source:VGNC Symbol;Acc:VGNC:96112]	2.08	0.058
ssc-miR-185	ZNF704	zinc finger protein 704 [Source:VGNC Symbol;Acc:VGNC:95296]	2.08	0.058
ssc-miR-185	ZNF81	zinc finger protein 81 [Source:VGNC Symbol;Acc:VGNC:95308]	2.08	0.058
ssc-miR-132	ABCG4	ATP binding cassette subfamily G member 4 [Source:HGNC Symbol;Acc:HGNC:13884]	1.98	0.0542
ssc-miR-132	ABR	ABR activator of RhoGEF and GTPase [Source:VGNC Symbol;Acc:VGNC:84989]	1.98	0.0542
ssc-miR-132	ACSL3	acyl-CoA synthetase long chain family member 3 [Source:VGNC Symbol;Acc:VGNC:96295]	1.98	0.0542
ssc-miR-132	ACTR3	actin related protein 3 [Source:VGNC Symbol;Acc:VGNC:103894]	1.98	0.0542
ssc-miR-132	ACVR1	activin A receptor type 1 [Source:VGNC Symbol;Acc:VGNC:95830]	1.98	0.0542
ssc-miR-132	ACVR2B	activin A receptor type 2B [Source:VGNC Symbol;Acc:VGNC:108629]	1.98	0.0542
ssc-miR-132	ADAMTS5	ADAM metallopeptidase with thrombospondin type 1 motif 5 [Source:VGNC Symbol;Acc:VGNC:85085]	1.98	0.0542
ssc-miR-132	ADCY3	adenylate cyclase 3 [Source:VGNC Symbol;Acc:VGNC:85107]	1.98	0.0542
ssc-miR-132	ADCYAP1	adenylate cyclase activating polypeptide 1 [Source:VGNC Symbol;Acc:VGNC:85114]	1.98	0.0542
ssc-miR-132	AEBP2	AE binding protein 2 [Source:VGNC Symbol;Acc:VGNC:85163]	1.98	0.0542
ssc-miR-132	AFF2	AF4/FMR2 family member 2 [Source:VGNC Symbol;Acc:VGNC:85168]	1.98	0.0542
ssc-miR-132	AFF4	AF4/FMR2 family member 4 [Source:VGNC Symbol;Acc:VGNC:85169]	1.98	0.0542
ssc-miR-132	AGO1	hypothetical gene	1.98	0.0542
ssc-miR-132	AGO2	argonaute RISC catalytic component 2 [Source:VGNC Symbol;Acc:VGNC:97871]	1.98	0.0542
ssc-miR-132	AHSA2	hypothetical gene	1.98	0.0542
ssc-miR-132	AKIRIN1	akirin 1 [Source:VGNC Symbol;Acc:VGNC:85227]	1.98	0.0542
ssc-miR-132	AKT3	AKT serine/threonine kinase 3 [Source:VGNC Symbol;Acc:VGNC:96306]	1.98	0.0542
ssc-miR-132	AMD1	hypothetical gene	1.98	0.0542
ssc-miR-132	AMHR2	anti-Mullerian hormone receptor type 2 [Source:VGNC Symbol;Acc:VGNC:85279]	1.98	0.0542
ssc-miR-132	AMMECR1	AMMECR nuclear protein 1 [Source:VGNC Symbol;Acc:VGNC:96559]	1.98	0.0542
ssc-miR-132	AMOT	angiomotin [Source:VGNC Symbol;Acc:VGNC:85283]	1.98	0.0542
ssc-miR-132	ANGEL2	angel homolog 2 [Source:VGNC Symbol;Acc:VGNC:85302]	1.98	0.0542
ssc-miR-132	ANKRD29	ankyrin repeat domain 29 [Source:VGNC Symbol;Acc:VGNC:85332]	1.98	0.0542
ssc-miR-132	ANKRD34C	ankyrin repeat domain 34C [Source:HGNC Symbol;Acc:HGNC:33888]	1.98	0.0542
ssc-miR-132	ANKRD40	ankyrin repeat domain 40 [Source:VGNC Symbol;Acc:VGNC:85338]	1.98	0.0542
ssc-miR-132	ANP32A	hypothetical gene	1.98	0.0542
ssc-miR-132	APAF1	apoptotic peptidase activating factor 1 [Source:VGNC Symbol;Acc:VGNC:85398]	1.98	0.0542
ssc-miR-132	ARF3	ADP ribosylation factor 3 [Source:NCBI gene (formerly Entrezgene);Acc:100301570]	1.98	0.0542
ssc-miR-132	ARFGAP2	ADP ribosylation factor GTPase activating protein 2 [Source:VGNC Symbol;Acc:VGNC:85449]	1.98	0.0542
ssc-miR-132	ARFGEF2	ADP ribosylation factor guanine nucleotide exchange factor 2 [Source:VGNC Symbol;Acc:VGNC:95672]	1.98	0.0542
ssc-miR-132	ARHGAP17	Rho GTPase activating protein 17 [Source:VGNC Symbol;Acc:VGNC:85459]	1.98	0.0542
ssc-miR-132	ARHGAP32	Rho GTPase activating protein 32 [Source:VGNC Symbol;Acc:VGNC:85471]	1.98	0.0542
ssc-miR-132	ARHGEF11	Rho guanine nucleotide exchange factor 11 [Source:VGNC Symbol;Acc:VGNC:85487]	1.98	0.0542
ssc-miR-132	ARID1A	AT-rich interaction domain 1A [Source:VGNC Symbol;Acc:VGNC:85505]	1.98	0.0542
ssc-miR-132	ARID1B	AT-rich interaction domain 1B [Source:VGNC Symbol;Acc:VGNC:85506]	1.98	0.0542
ssc-miR-132	ARID2	AT-rich interaction domain 2 [Source:HGNC Symbol;Acc:HGNC:18037]	1.98	0.0542
ssc-miR-132	ARID4B	AT-rich interaction domain 4B [Source:HGNC Symbol;Acc:HGNC:15550]	1.98	0.0542
ssc-miR-132	ARIH1	ariadne RBR E3 ubiquitin protein ligase 1 [Source:HGNC Symbol;Acc:HGNC:689]	1.98	0.0542
ssc-miR-132	ARPP19	cAMP regulated phosphoprotein 19 [Source:NCBI gene (formerly Entrezgene);Acc:397362]	1.98	0.0542
ssc-miR-132	ARX	aristaless related homeobox [Source:VGNC Symbol;Acc:VGNC:85556]	1.98	0.0542

ssc-miR-132	ASXL2	ASXL transcriptional regulator 2 [Source:VGNC Symbol;Acc:VGNC:85595]	1.98	0.0542
ssc-miR-132	ATL3	atlastin GTPase 3 [Source:VGNC Symbol;Acc:VGNC:85629]	1.98	0.0542
ssc-miR-132	ATXN1	ataxin 1 [Source:VGNC Symbol;Acc:VGNC:85687]	1.98	0.0542
ssc-miR-132	ATXN7	ataxin 7 [Source:VGNC Symbol;Acc:VGNC:99704]	1.98	0.0542
ssc-miR-132	AZIN1	antizyme inhibitor 1 [Source:VGNC Symbol;Acc:VGNC:85712]	1.98	0.0542
ssc-miR-132	B3GAT2	beta-1,3-glucuronyltransferase 2 [Source:VGNC Symbol;Acc:VGNC:85720]	1.98	0.0542
ssc-miR-132	B4GALT5	beta-1,4-galactosyltransferase 5 [Source:VGNC Symbol;Acc:VGNC:96499]	1.98	0.0542
ssc-miR-132	B4GALT6	beta-1,4-galactosyltransferase 6 [Source:VGNC Symbol;Acc:VGNC:85734]	1.98	0.0542
ssc-miR-132	BAALC	BAALC binder of MAP3K1 and KLF4 [Source:NCBI gene (formerly Entrezgene);Acc:100170128]	1.98	0.0542
ssc-miR-132	BAMBI	BMP and activin membrane bound inhibitor [Source:VGNC Symbol;Acc:VGNC:96238]	1.98	0.0542
ssc-miR-132	BBX	BBX high mobility group box domain containing [Source:VGNC Symbol;Acc:VGNC:85766]	1.98	0.0542
ssc-miR-132	BICD1	BICD cargo adaptor 1 [Source:VGNC Symbol;Acc:VGNC:85818]	1.98	0.0542
ssc-miR-132	BMP2	bone morphotic protein 2 [Source:VGNC Symbol;Acc:VGNC:95488]	1.98	0.0542
ssc-miR-132	BMP3	bone morphotic protein 3 [Source:VGNC Symbol;Acc:VGNC:85842]	1.98	0.0542
ssc-miR-132	BMPR1A	bone morphotic protein receptor type 1A [Source:VGNC Symbol;Acc:VGNC:85846]	1.98	0.0542
ssc-miR-132	BNC2	basonuclin 2 [Source:VGNC Symbol;Acc:VGNC:85852]	1.98	0.0542
ssc-miR-132	BNIP2	BCL2 interacting protein 2 [Source:VGNC Symbol;Acc:VGNC:85854]	1.98	0.0542
ssc-miR-132	BOLL	boule homolog, RNA binding protein [Source:VGNC Symbol;Acc:VGNC:95610]	1.98	0.0542
ssc-miR-132	BRCA1	BRCA1 DNA repair associated [Source:VGNC Symbol;Acc:VGNC:85869]	1.98	0.0542
ssc-miR-132	BRI3	brain protein I3 [Source:VGNC Symbol;Acc:VGNC:103906]	1.98	0.0542
ssc-miR-132	BRWD1	bromodomain and WD repeat domain containing 1 [Source:VGNC Symbol;Acc:VGNC:108153]	1.98	0.0542
ssc-miR-132	BSN	bassoon presynaptic cytomatrix protein [Source:VGNC Symbol;Acc:VGNC:85893]	1.98	0.0542
ssc-miR-132	BTAF1	B-TFIID TATA-box binding protein associated factor 1 [Source:VGNC Symbol;Acc:VGNC:85899]	1.98	0.0542
ssc-miR-132	BTBD11	BTB domain containing 11 [Source:VGNC Symbol;Acc:VGNC:85901]	1.98	0.0542
ssc-miR-132	BTBD7	BTB domain containing 7 [Source:VGNC Symbol;Acc:VGNC:85908]	1.98	0.0542
ssc-miR-132	BTG2	BTG anti-proliferation factor 2 [Source:NCBI gene (formerly Entrezgene);Acc:100048932]	1.98	0.0542
ssc-miR-132	C11orf87	chromosome 11 open reading frame 87 [Source:HGNC Symbol;Acc:HGNC:33788]	1.98	0.0542
ssc-miR-132	C16orf87	chromosome 6 C16orf87 homolog [Source:VGNC Symbol;Acc:VGNC:96927]	1.98	0.0542
ssc-miR-132	C19orf47	chromosome 6 C19orf47 homolog [Source:VGNC Symbol;Acc:VGNC:86056]	1.98	0.0542
ssc-miR-132	C1orf115	chromosome 10 C1orf115 homolog [Source:VGNC Symbol;Acc:VGNC:96111]	1.98	0.0542
ssc-miR-132	C3orf14	chromosome 13 C3orf14 homolog [Source:VGNC Symbol;Acc:VGNC:85931]	1.98	0.0542
ssc-miR-132	C8orf44-SGK3	hypothetical gene	1.98	0.0542
ssc-miR-132	C9orf9	hypothetical gene	1.98	0.0542
ssc-miR-132	CAAP1	caspase activity and apoptosis inhibitor 1 [Source:HGNC Symbol;Acc:HGNC:25834]	1.98	0.0542
ssc-miR-132	CACNG2	calcium voltage-gated channel auxiliary subunit gamma 2 [Source:VGNC Symbol;Acc:VGNC:86125]	1.98	0.0542
ssc-miR-132	CALU	calumenin [Source:VGNC Symbol;Acc:VGNC:86151]	1.98	0.0542
ssc-miR-132	CAMSAP2	calmodulin regulated spectrin associated protein family member 2 [Source:VGNC Symbol;Acc:VGNC:96205]	1.98	0.0542
ssc-miR-132	CAPRIN1	cell cycle associated protein 1 [Source:VGNC Symbol;Acc:VGNC:86178]	1.98	0.0542
ssc-miR-132	CASD1	CAS1 domain containing 1 [Source:VGNC Symbol;Acc:VGNC:86199]	1.98	0.0542
ssc-miR-132	CBFA2T2	CBFA2/RUNX1 partner transcriptional co-repressor 2 [Source:VGNC Symbol;Acc:VGNC:96573]	1.98	0.0542
ssc-miR-132	CBLL1	Cbl proto-onco like 1 [Source:VGNC Symbol;Acc:VGNC:108573]	1.98	0.0542
ssc-miR-132	CC2D1B	coiled-coil and C2 domain containing 1B [Source:VGNC Symbol;Acc:VGNC:86236]	1.98	0.0542
ssc-miR-132	CCDC117	coiled-coil domain containing 117 [Source:VGNC Symbol;Acc:VGNC:86248]	1.98	0.0542
ssc-miR-132	CCDC144A	hypothetical gene	1.98	0.0542
ssc-miR-132	CCDC171	coiled-coil domain containing 171 [Source:VGNC Symbol;Acc:VGNC:103044]	1.98	0.0542
ssc-miR-132	CCDC71L	coiled-coil domain containing 71 like [Source:HGNC Symbol;Acc:HGNC:26685]	1.98	0.0542
ssc-miR-132	CCDC88A	coiled-coil domain containing 88A [Source:VGNC Symbol;Acc:VGNC:86325]	1.98	0.0542
ssc-miR-132	CCR10	C-C motif chemokine receptor 10 [Source:VGNC Symbol;Acc:VGNC:86369]	1.98	0.0542
ssc-miR-132	CDC42BPA	CDC42 binding protein kinase alpha [Source:VGNC Symbol;Acc:VGNC:95847]	1.98	0.0542

ssc-miR-132	CDH8	cadherin 8 [Source:VGNC Symbol;Acc:VGNC:86487]	1.98	0.0542
ssc-miR-132	CDK19	cyclin dependent kinase 19 [Source:VGNC Symbol;Acc:VGNC:86502]	1.98	0.0542
ssc-miR-132	CELF2	hypothetical gene	1.98	0.0542
ssc-miR-132	CELSR3	cadherin EGF LAG seven-pass G-type receptor 3 [Source:VGNC Symbol;Acc:VGNC:86541]	1.98	0.0542
ssc-miR-132	CFL2	cofilin 2 [Source:VGNC Symbol;Acc:VGNC:86611]	1.98	0.0542
ssc-miR-132	CHD1	chromodomain helicase DNA binding protein 1 [Source:VGNC Symbol;Acc:VGNC:86629]	1.98	0.0542
ssc-miR-132	CHD8	chromodomain helicase DNA binding protein 8 [Source:VGNC Symbol;Acc:VGNC:86634]	1.98	0.0542
ssc-miR-132	CHSY1	chondroitin sulfate synthase 1 [Source:VGNC Symbol;Acc:VGNC:86684]	1.98	0.0542
ssc-miR-132	CITED2	Cbp/p300 interacting transactivator with Glu/Asp rich carboxy-terminal domain 2 [Source:VGNC Symbol;Acc:VGNC:86712]	1.98	0.0542
ssc-miR-132	CLMN	calmin [Source:VGNC Symbol;Acc:VGNC:86769]	1.98	0.0542
ssc-miR-132	CLOCK	clock circadian regulator [Source:VGNC Symbol;Acc:VGNC:86774]	1.98	0.0542
ssc-miR-132	CLSTN2	calsyntenin 2 [Source:VGNC Symbol;Acc:VGNC:86785]	1.98	0.0542
ssc-miR-132	CLYBL	citramalyl-CoA lyase [Source:VGNC Symbol;Acc:VGNC:86796]	1.98	0.0542
ssc-miR-132	CNIH1	cornichon family AMPA receptor auxiliary protein 1 [Source:VGNC Symbol;Acc:VGNC:86820]	1.98	0.0542
ssc-miR-132	CNOT2	CCR4-NOT transcription complex subunit 2 [Source:VGNC Symbol;Acc:VGNC:86834]	1.98	0.0542
ssc-miR-132	CNTNAP3B	hypothetical gene	1.98	0.0542
ssc-miR-132	COL4A4	collagen type IV alpha 4 chain [Source:VGNC Symbol;Acc:VGNC:95980]	1.98	0.0542
ssc-miR-132	COLCA1	hypothetical gene	1.98	0.0542
ssc-miR-132	COLEC10	collectin subfamily member 10 [Source:VGNC Symbol;Acc:VGNC:86882]	1.98	0.0542
ssc-miR-132	COLQ	collagen like tail subunit of asymmetric acetylcholinesterase [Source:VGNC Symbol;Acc:VGNC:86884]	1.98	0.0542
ssc-miR-132	CPSF6	cleavage and polyadenylation specific factor 6 [Source:VGNC Symbol;Acc:VGNC:86962]	1.98	0.0542
ssc-miR-132	CREB5	cAMP responsive element binding protein 5 [Source:VGNC Symbol;Acc:VGNC:86984]	1.98	0.0542
ssc-miR-132	CRK	CRK proto-onco, adaptor protein [Source:NCBI gene (formerly Entrezgene);Acc:100192444]	1.98	0.0542
ssc-miR-132	CRTC1	CREB regulated transcription coactivator 1 [Source:VGNC Symbol;Acc:VGNC:87007]	1.98	0.0542
ssc-miR-132	CSDE1	cold shock domain containing E1 [Source:VGNC Symbol;Acc:VGNC:87029]	1.98	0.0542
ssc-miR-132	CSRNP3	cysteine and serine rich nuclear protein 3 [Source:VGNC Symbol;Acc:VGNC:96249]	1.98	0.0542
ssc-miR-132	CTDSPL2	CTD small phosphatase like 2 [Source:VGNC Symbol;Acc:VGNC:87060]	1.98	0.0542
ssc-miR-132	CTGF	hypothetical gene	1.98	0.0542
ssc-miR-132	CTSV	cathepsin V [Source:NCBI gene (formerly Entrezgene);Acc:396926]	1.98	0.0542
ssc-miR-132	CWF19L1	CWF19 like cell cycle control factor 1 [Source:VGNC Symbol;Acc:VGNC:96725]	1.98	0.0542
ssc-miR-132	CYB5R4	cytochrome b5 reductase 4 [Source:HGNC Symbol;Acc:HGNC:20147]	1.98	0.0542
ssc-miR-132	DAAM1	dishevelled associated activator of morphosis 1 [Source:VGNC Symbol;Acc:VGNC:87141]	1.98	0.0542
ssc-miR-132	DAB1	DAB adaptor protein 1 [Source:VGNC Symbol;Acc:VGNC:87143]	1.98	0.0542
ssc-miR-132	DACH1	dachshund family transcription factor 1 [Source:VGNC Symbol;Acc:VGNC:87146]	1.98	0.0542
ssc-miR-132	DAZAP2	DAZ associated protein 2 [Source:VGNC Symbol;Acc:VGNC:87162]	1.98	0.0542
ssc-miR-132	DCAF8	DDB1 and CUL4 associated factor 8 [Source:NCBI gene (formerly Entrezgene);Acc:100153655]	1.98	0.0542
ssc-miR-132	DCC	DCC netrin 1 receptor [Source:VGNC Symbol;Acc:VGNC:103078]	1.98	0.0542
ssc-miR-132	DCUN1D3	defective in cullin neddylation 1 domain containing 3 [Source:VGNC Symbol;Acc:VGNC:87196]	1.98	0.0542
ssc-miR-132	DCUN1D4	defective in cullin neddylation 1 domain containing 4 [Source:VGNC Symbol;Acc:VGNC:87197]	1.98	0.0542
ssc-miR-132	DESI2	desumoylating isopeptidase 2 [Source:VGNC Symbol;Acc:VGNC:96037]	1.98	0.0542
ssc-miR-132	DGKH	diacylglycerol kinase eta [Source:VGNC Symbol;Acc:VGNC:87273]	1.98	0.0542
ssc-miR-132	DIAPH1	diaphanous related formin 1 [Source:VGNC Symbol;Acc:VGNC:99632]	1.98	0.0542
ssc-miR-132	DKK3	dickkopf WNT signaling pathway inhibitor 3 [Source:VGNC Symbol;Acc:VGNC:87323]	1.98	0.0542
ssc-miR-132	DNAJA2	DnaJ heat shock protein family (Hsp40) member A2 [Source:VGNC Symbol;Acc:VGNC:96621]	1.98	0.0542
ssc-miR-132	DNMT3A	DNA methyltransferase 3 alpha [Source:VGNC Symbol;Acc:VGNC:87384]	1.98	0.0542
ssc-miR-132	DOCK4	dedicator of cytokinesis 4 [Source:VGNC Symbol;Acc:VGNC:87394]	1.98	0.0542
ssc-miR-132	DPYSL3	dihydropyrimidinase like 3 [Source:VGNC Symbol;Acc:VGNC:87431]	1.98	0.0542
ssc-miR-132	DSC2	desmocollin 2 [Source:HGNC Symbol;Acc:HGNC:3036]	1.98	0.0542
ssc-miR-132	DUSP9	dual specificity phosphatase 9 [Source:VGNC Symbol;Acc:VGNC:87490]	1.98	0.0542

ssc-miR-132	DYNC1LI2	dynein cytoplasmic 1 light intermediate chain 2 [Source:VGNC Symbol;Acc:VGNC:87498]	1.98	0.0542
ssc-miR-132	DYNLL2	dynein light chain LC8-type 2 [Source:VGNC Symbol;Acc:VGNC:87501]	1.98	0.0542
ssc-miR-132	DYRK2	dual specificity tyrosine phosphorylation regulated kinase 2 [Source:VGNC Symbol;Acc:VGNC:87507]	1.98	0.0542
ssc-miR-132	E2F5	E2F transcription factor 5 [Source:VGNC Symbol;Acc:VGNC:87516]	1.98	0.0542
ssc-miR-132	EBF3	EBF transcription factor 3 [Source:VGNC Symbol;Acc:VGNC:87527]	1.98	0.0542
ssc-miR-132	EBPL	EBP like [Source:VGNC Symbol;Acc:VGNC:87529]	1.98	0.0542
ssc-miR-132	EDIL3	EGF like repeats and discoidin domains 3 [Source:VGNC Symbol;Acc:VGNC:99642]	1.98	0.0542
ssc-miR-132	EGR1	early growth response 1 [Source:VGNC Symbol;Acc:VGNC:87590]	1.98	0.0542
ssc-miR-132	EIF4A2	eukaryotic translation initiation factor 4A2 [Source:VGNC Symbol;Acc:VGNC:87624]	1.98	0.0542
ssc-miR-132	EIF5A2	eukaryotic translation initiation factor 5A2 [Source:VGNC Symbol;Acc:VGNC:87633]	1.98	0.0542
ssc-miR-132	ELF2	E74 like ETS transcription factor 2 [Source:VGNC Symbol;Acc:VGNC:87642]	1.98	0.0542
ssc-miR-132	ELFN2	extracellular leucine rich repeat and fibronectin type III domain containing 2 [Source:VGNC Symbol;Acc:VGNC:103232]	1.98	0.0542
ssc-miR-132	ELK4	ETS transcription factor ELK4 [Source:VGNC Symbol;Acc:VGNC:87647]	1.98	0.0542
ssc-miR-132	ELMSAN1	hypothetical gene	1.98	0.0542
ssc-miR-132	EP300	E1A binding protein p300 [Source:VGNC Symbol;Acc:VGNC:87720]	1.98	0.0542
ssc-miR-132	EPC1	enhancer of polycomb homolog 1 [Source:VGNC Symbol;Acc:VGNC:95923]	1.98	0.0542
ssc-miR-132	ESRRG	estrogen related receptor gamma [Source:VGNC Symbol;Acc:VGNC:96289]	1.98	0.0542
ssc-miR-132	ETNK1	ethanolamine kinase 1 [Source:VGNC Symbol;Acc:VGNC:87806]	1.98	0.0542
ssc-miR-132	ETV1	ETS variant transcription factor 1 [Source:VGNC Symbol;Acc:VGNC:87810]	1.98	0.0542
ssc-miR-132	EXOC5	exocyst complex component 5 [Source:VGNC Symbol;Acc:VGNC:87831]	1.98	0.0542
ssc-miR-132	EXTL2	exostosin like glycosyltransferase 2 [Source:VGNC Symbol;Acc:VGNC:87849]	1.98	0.0542
ssc-miR-132	FAM105B	hypothetical gene	1.98	0.0542
ssc-miR-132	FAM167A	family with sequence similarity 167 member A [Source:VGNC Symbol;Acc:VGNC:87924]	1.98	0.0542
ssc-miR-132	FAM91A1	family with sequence similarity 91 member A1 [Source:VGNC Symbol;Acc:VGNC:97998]	1.98	0.0542
ssc-miR-132	FAT2	FAT atypical cadherin 2 [Source:VGNC Symbol;Acc:VGNC:88019]	1.98	0.0542
ssc-miR-132	FBXL20	F-box and leucine rich repeat protein 20 [Source:VGNC Symbol;Acc:VGNC:98006]	1.98	0.0542
ssc-miR-132	FBXO21	F-box protein 21 [Source:VGNC Symbol;Acc:VGNC:88035]	1.98	0.0542
ssc-miR-132	FBXO28	F-box protein 28 [Source:HGNC Symbol;Acc:HGNC:29046]	1.98	0.0542
ssc-miR-132	FBXO42	F-box protein 42 [Source:VGNC Symbol;Acc:VGNC:88047]	1.98	0.0542
ssc-miR-132	FBXW7	F-box and WD repeat domain containing 7 [Source:VGNC Symbol;Acc:VGNC:98925]	1.98	0.0542
ssc-miR-132	FEM1C	fem-1 homolog C [Source:VGNC Symbol;Acc:VGNC:88084]	1.98	0.0542
ssc-miR-132	FGD4	FYVE, RhoGEF and PH domain containing 4 [Source:VGNC Symbol;Acc:VGNC:88097]	1.98	0.0542
ssc-miR-132	FGF7	fibroblast growth factor 7 [Source:HGNC Symbol;Acc:HGNC:3685]	1.98	0.0542
ssc-miR-132	FKBP2	FKBP prolyl isomerase 2 [Source:VGNC Symbol;Acc:VGNC:99652]	1.98	0.0542
ssc-miR-132	FLNB	filamin B [Source:VGNC Symbol;Acc:VGNC:88156]	1.98	0.0542
ssc-miR-132	FMNL3	formin like 3 [Source:VGNC Symbol;Acc:VGNC:88169]	1.98	0.0542
ssc-miR-132	FNBP1L	formin binding protein 1 like [Source:VGNC Symbol;Acc:VGNC:88179]	1.98	0.0542
ssc-miR-132	FOXA1	forkhead box A1 [Source:VGNC Symbol;Acc:VGNC:88193]	1.98	0.0542
ssc-miR-132	FOXN3	forkhead box N3 [Source:VGNC Symbol;Acc:VGNC:88220]	1.98	0.0542
ssc-miR-132	FOXO1	forkhead box O1 [Source:VGNC Symbol;Acc:VGNC:98013]	1.98	0.0542
ssc-miR-132	FOXO3	forkhead box O3 [Source:VGNC Symbol;Acc:VGNC:99715]	1.98	0.0542
ssc-miR-132	FOXP1	forkhead box P1 [Source:VGNC Symbol;Acc:VGNC:88222]	1.98	0.0542
ssc-miR-132	FOXP2	forkhead box P2 [Source:VGNC Symbol;Acc:VGNC:98014]	1.98	0.0542
ssc-miR-132	FRK	fyn related Src family tyrosine kinase [Source:VGNC Symbol;Acc:VGNC:88235]	1.98	0.0542
ssc-miR-132	FRYL	FRY like transcription coactivator [Source:VGNC Symbol;Acc:VGNC:98015]	1.98	0.0542
ssc-miR-132	FUBP3	far upstream element binding protein 3 [Source:VGNC Symbol;Acc:VGNC:103093]	1.98	0.0542
ssc-miR-132	FXR1	FMR1 autosomal homolog 1 [Source:VGNC Symbol;Acc:VGNC:108659]	1.98	0.0542
ssc-miR-132	GAB1	GRB2 associated binding protein 1 [Source:VGNC Symbol;Acc:VGNC:88294]	1.98	0.0542
ssc-miR-132	GABPB2	GA binding protein transcription factor subunit beta 2 [Source:VGNC Symbol;Acc:VGNC:98796]	1.98	0.0542

ssc-miR-132	GALNT13	polypeptide N-acetylgalactosaminyltransferase 13 [Source:VGNC Symbol;Acc:VGNC:95992]	1.98	0.0542
ssc-miR-132	GAPVD1	GTPase activating protein and VPS9 domains 1 [Source:VGNC Symbol;Acc:VGNC:88347]	1.98	0.0542
ssc-miR-132	GDF5	growth differentiation factor 5 [Source:VGNC Symbol;Acc:VGNC:96319]	1.98	0.0542
ssc-miR-132	GEMIN6	gem nuclear organelle associated protein 6 [Source:VGNC Symbol;Acc:VGNC:88414]	1.98	0.0542
ssc-miR-132	GHR	growth hormone receptor [Source:NCBI gene (formerly Entrezgene);Acc:397488]	1.98	0.0542
ssc-miR-132	GIGYF1	GRB10 interacting GYF protein 1 [Source:VGNC Symbol;Acc:VGNC:88444]	1.98	0.0542
ssc-miR-132	GLCCI1	glucocorticoid induced 1 [Source:VGNC Symbol;Acc:VGNC:88476]	1.98	0.0542
ssc-miR-132	GLIS3	GLIS family zinc finger 3 [Source:VGNC Symbol;Acc:VGNC:88485]	1.98	0.0542
ssc-miR-132	GLRB	glycine receptor beta [Source:VGNC Symbol;Acc:VGNC:88494]	1.98	0.0542
ssc-miR-132	GLTSCR1L	hypothetical gene	1.98	0.0542
ssc-miR-132	GMFB	glia maturation factor beta [Source:VGNC Symbol;Acc:VGNC:88509]	1.98	0.0542
ssc-miR-132	GMPR	guanosine monophosphate reductase [Source:VGNC Symbol;Acc:VGNC:88514]	1.98	0.0542
ssc-miR-132	GNA12	G protein subunit alpha 12 [Source:HGNC Symbol;Acc:HGNC:4380]	1.98	0.0542
ssc-miR-132	GNB1L	G protein subunit beta 1 like [Source:VGNC Symbol;Acc:VGNC:88530]	1.98	0.0542
ssc-miR-132	GOLGA4	hypothetical gene	1.98	0.0542
ssc-miR-132	GOLM1	golgi membrane protein 1 [Source:VGNC Symbol;Acc:VGNC:95640]	1.98	0.0542
ssc-miR-132	GPD2	glycerol-3-phosphate dehydrogenase 2 [Source:VGNC Symbol;Acc:VGNC:96330]	1.98	0.0542
ssc-miR-132	GPR137C	G protein-coupled receptor 137C [Source:VGNC Symbol;Acc:VGNC:88602]	1.98	0.0542
ssc-miR-132	GPR37L1	G protein-coupled receptor 37 like 1 [Source:VGNC Symbol;Acc:VGNC:95633]	1.98	0.0542
ssc-miR-132	GPRIN3	hypothetical gene	1.98	0.0542
ssc-miR-132	GRM3	glutamate metabotropic receptor 3 [Source:VGNC Symbol;Acc:VGNC:88702]	1.98	0.0542
ssc-miR-132	GRSF1	G-rich RNA sequence binding factor 1 [Source:VGNC Symbol;Acc:VGNC:88710]	1.98	0.0542
ssc-miR-132	GSK3B	glycogen synthase kinase 3 beta [Source:VGNC Symbol;Acc:VGNC:88723]	1.98	0.0542
ssc-miR-132	GTDC1	glycosyltransferase like domain containing 1 [Source:VGNC Symbol;Acc:VGNC:95955]	1.98	0.0542
ssc-miR-132	GTF2H1	ral transcription factor IIH subunit 1 [Source:VGNC Symbol;Acc:VGNC:88734]	1.98	0.0542
ssc-miR-132	H2AFZ	H2A histone family, member Z [Source:NCBI gene (formerly Entrezgene);Acc:100141311]	1.98	0.0542
ssc-miR-132	H3F3B	hypothetical gene	1.98	0.0542
ssc-miR-132	HAO1	hydroxyacid oxidase 1 [Source:VGNC Symbol;Acc:VGNC:98029]	1.98	0.0542
ssc-miR-132	HAPLN1	hyaluronan and proteoglycan link protein 1 [Source:VGNC Symbol;Acc:VGNC:88780]	1.98	0.0542
ssc-miR-132	HBEGF	heparin binding EGF like growth factor [Source:VGNC Symbol;Acc:VGNC:88792]	1.98	0.0542
ssc-miR-132	HECTD1	HECT domain E3 ubiquitin protein ligase 1 [Source:VGNC Symbol;Acc:VGNC:88832]	1.98	0.0542
ssc-miR-132	HHIP	hedgehog interacting protein [Source:VGNC Symbol;Acc:VGNC:88875]	1.98	0.0542
ssc-miR-132	HHIPL1	hypothetical gene	1.98	0.0542
ssc-miR-132	HIC2	HIC ZBTB transcriptional repressor 2 [Source:HGNC Symbol;Acc:HGNC:18595]	1.98	0.0542
ssc-miR-132	HIVEP1	HIVEP zinc finger 1 [Source:VGNC Symbol;Acc:VGNC:96585]	1.98	0.0542
ssc-miR-132	HMGA2	hypothetical gene	1.98	0.0542
ssc-miR-132	HMGN2	high mobility group nucleosomal binding domain 2 [Source:NCBI gene (formerly Entrezgene);Acc:100524500]	1.98	0.0542
ssc-miR-132	HMGXB4	HMG-box containing 4 [Source:VGNC Symbol;Acc:VGNC:96346]	1.98	0.0542
ssc-miR-132	HNRNPR	heterous nuclear ribonucleoprotein R [Source:VGNC Symbol;Acc:VGNC:88925]	1.98	0.0542
ssc-miR-132	HNRNPU	heterogeneous nuclear ribonucleoprotein U [Source:HGNC Symbol;Acc:HGNC:5048]	1.98	0.0542
ssc-miR-132	HOXD12	homeobox D12 [Source:VGNC Symbol;Acc:VGNC:96353]	1.98	0.0542
ssc-miR-132	HSD11B1	hydroxysteroid 11-beta dehydrogenase 1 [Source:VGNC Symbol;Acc:VGNC:88983]	1.98	0.0542
ssc-miR-132	IQSEC1	IQ motif and Sec7 domain ArfGEF 1 [Source:VGNC Symbol;Acc:VGNC:89194]	1.98	0.0542
ssc-miR-132	IRF1	interferon regulatory factor 1 [Source:VGNC Symbol;Acc:VGNC:89203]	1.98	0.0542
ssc-miR-132	ISL1	ISL LIM homeobox 1 [Source:VGNC Symbol;Acc:VGNC:89225]	1.98	0.0542
ssc-miR-132	ITCH	itchy E3 ubiquitin protein ligase [Source:HGNC Symbol;Acc:HGNC:13890]	1.98	0.0542
ssc-miR-132	ITGA9	integrin subunit alpha 9 [Source:VGNC Symbol;Acc:VGNC:89238]	1.98	0.0542
ssc-miR-132	JHDM1D	hypothetical gene	1.98	0.0542
ssc-miR-132	JMY	junction mediating and regulatory protein, p53 cofactor [Source:VGNC Symbol;Acc:VGNC:89289]	1.98	0.0542

ssc-miR-132	KAT6A	hypothetical gene	1.98	0.0542
ssc-miR-132	KAT7	lysine acetyltransferase 7 [Source:VGNC Symbol;Acc:VGNC:89307]	1.98	0.0542
ssc-miR-132	KCMF1	potassium channel modulatory factor 1 [Source:VGNC Symbol;Acc:VGNC:89322]	1.98	0.0542
ssc-miR-132	KCNA6	potassium voltage-gated channel subfamily A member 6 [Source:HGNC Symbol;Acc:HGNC:6225]	1.98	0.0542
ssc-miR-132	KCNJ12	hypothetical gene	1.98	0.0542
ssc-miR-132	KCNJ6	potassium inwardly rectifying channel subfamily J member 6 [Source:VGNC Symbol;Acc:VGNC:89360]	1.98	0.0542
ssc-miR-132	KCNK2	potassium two pore domain channel subfamily K member 2 [Source:VGNC Symbol;Acc:VGNC:89369]	1.98	0.0542
ssc-miR-132	KCNN3	potassium calcium-activated channel subfamily N member 3 [Source:VGNC Symbol;Acc:VGNC:98056]	1.98	0.0542
ssc-miR-132	KDM5A	lysine demethylase 5A [Source:VGNC Symbol;Acc:VGNC:89415]	1.98	0.0542
ssc-miR-132	KDM5B	lysine demethylase 5B [Source:VGNC Symbol;Acc:VGNC:95870]	1.98	0.0542
ssc-miR-132	KHDRBS2	KH RNA binding domain containing, signal transduction associated 2 [Source:VGNC Symbol;Acc:VGNC:89423]	1.98	0.0542
ssc-miR-132	KIAA1211	hypothetical gene	1.98	0.0542
ssc-miR-132	KIAA1211L	hypothetical gene	1.98	0.0542
ssc-miR-132	KIAA1456	hypothetical gene	1.98	0.0542
ssc-miR-132	KIAA1549	KIAA1549 [Source:VGNC Symbol;Acc:VGNC:99719]	1.98	0.0542
ssc-miR-132	KIAA1958	KIAA1958 [Source:VGNC Symbol;Acc:VGNC:89448]	1.98	0.0542
ssc-miR-132	KIF21B	kinesin family member 21B [Source:VGNC Symbol;Acc:VGNC:96219]	1.98	0.0542
ssc-miR-132	KITLG	KIT ligand [Source:VGNC Symbol;Acc:VGNC:98061]	1.98	0.0542
ssc-miR-132	KLF7	Kruppel like factor 7 [Source:VGNC Symbol;Acc:VGNC:96396]	1.98	0.0542
ssc-miR-132	KLF8	Kruppel like factor 8 [Source:VGNC Symbol;Acc:VGNC:89500]	1.98	0.0542
ssc-miR-132	KLHL11	kelch like family member 11 [Source:VGNC Symbol;Acc:VGNC:89513]	1.98	0.0542
ssc-miR-132	KPNA1	karyopherin subunit alpha 1 [Source:VGNC Symbol;Acc:VGNC:89560]	1.98	0.0542
ssc-miR-132	L3MBTL3	L3MBTL histone methyl-lysine binding protein 3 [Source:VGNC Symbol;Acc:VGNC:98066]	1.98	0.0542
ssc-miR-132	LARGE	hypothetical gene	1.98	0.0542
ssc-miR-132	LCMT2	hypothetical gene	1.98	0.0542
ssc-miR-132	LEMD3	LEM domain containing 3 [Source:VGNC Symbol;Acc:VGNC:89679]	1.98	0.0542
ssc-miR-132	LIMD2	LIM domain containing 2 [Source:VGNC Symbol;Acc:VGNC:89726]	1.98	0.0542
ssc-miR-132	LIN28B	lin-28 homolog B [Source:VGNC Symbol;Acc:VGNC:89729]	1.98	0.0542
ssc-miR-132	LMLN	leishmanolysin like peptidase [Source:VGNC Symbol;Acc:VGNC:89759]	1.98	0.0542
ssc-miR-132	LRRC28	hypothetical gene	1.98	0.0542
ssc-miR-132	LRRTM3	leucine rich repeat transmembrane neuronal 3 [Source:VGNC Symbol;Acc:VGNC:107409]	1.98	0.0542
ssc-miR-132	LSAMP	limbic system associated membrane protein [Source:VGNC Symbol;Acc:VGNC:89868]	1.98	0.0542
ssc-miR-132	LSM11	LSM11, U7 small nuclear RNA associated [Source:VGNC Symbol;Acc:VGNC:89871]	1.98	0.0542
ssc-miR-132	LZTS3	leucine zipper tumor suppressor family member 3 [Source:VGNC Symbol;Acc:VGNC:96237]	1.98	0.0542
ssc-miR-132	MAF	MAF bZIP transcription factor [Source:VGNC Symbol;Acc:VGNC:89945]	1.98	0.0542
ssc-miR-132	MAP3K3	mitogen-activated protein kinase kinase kinase 3 [Source:VGNC Symbol;Acc:VGNC:98108]	1.98	0.0542
ssc-miR-132	MAP3K9	mitogen-activated protein kinase kinase kinase 9 [Source:VGNC Symbol;Acc:VGNC:89989]	1.98	0.0542
ssc-miR-132	MAPK1	mitogen-activated protein kinase 1 [Source:VGNC Symbol;Acc:VGNC:89996]	1.98	0.0542
ssc-miR-132	MAPK3	mitogen-activated protein kinase 3 [Source:VGNC Symbol;Acc:VGNC:90003]	1.98	0.0542
ssc-miR-132	MAPKBP1	mitogen-activated protein kinase binding protein 1 [Source:VGNC Symbol;Acc:VGNC:90014]	1.98	0.0542
ssc-miR-132	MAPT	microtubule associated protein tau [Source:VGNC Symbol;Acc:VGNC:90016]	1.98	0.0542
ssc-miR-132	MBNL3	muscleblind like splicing regulator 3 [Source:VGNC Symbol;Acc:VGNC:90056]	1.98	0.0542
ssc-miR-132	MCIDAS	multiciliate differentiation and DNA synthesis associated cell cycle protein [Source:VGNC Symbol;Acc:VGNC:90071]	1.98	0.0542
ssc-miR-132	MDFIC	MyoD family inhibitor domain containing [Source:VGNC Symbol;Acc:VGNC:90089]	1.98	0.0542
ssc-miR-132	MECOM	MDS1 and EVI1 complex locus [Source:VGNC Symbol;Acc:VGNC:90100]	1.98	0.0542
ssc-miR-132	MECP2	methyl-CpG binding protein 2 [Source:VGNC Symbol;Acc:VGNC:90101]	1.98	0.0542
ssc-miR-132	MED28	mediator complex subunit 28 [Source:VGNC Symbol;Acc:VGNC:90118]	1.98	0.0542
ssc-miR-132	MED9	mediator complex subunit 9 [Source:VGNC Symbol;Acc:VGNC:90125]	1.98	0.0542
ssc-miR-132	MEF2A	myocyte enhancer factor 2A [Source:VGNC Symbol;Acc:VGNC:98123]	1.98	0.0542

ssc-miR-132	MEIS1	Meis homeobox 1 [Source:VGNC Symbol;Acc:VGNC:90135]	1.98	0.0542
ssc-miR-132	MEIS2	Meis homeobox 2 [Source:VGNC Symbol;Acc:VGNC:90136]	1.98	0.0542
ssc-miR-132	MEPCE	methylphosphate capping enzyme [Source:VGNC Symbol;Acc:VGNC:90144]	1.98	0.0542
ssc-miR-132	METTL24	methyltransferase like 24 [Source:VGNC Symbol;Acc:VGNC:90161]	1.98	0.0542
ssc-miR-132	MEX3A	mex-3 RNA binding family member A [Source:VGNC Symbol;Acc:VGNC:90168]	1.98	0.0542
ssc-miR-132	MIA3	hypothetical gene	1.98	0.0542
ssc-miR-132	MINA	hypothetical gene	1.98	0.0542
ssc-miR-132	MITF	melanocyte inducing transcription factor [Source:VGNC Symbol;Acc:VGNC:90243]	1.98	0.0542
ssc-miR-132	MLLT6	MLLT6, PHD finger containing [Source:VGNC Symbol;Acc:VGNC:90257]	1.98	0.0542
ssc-miR-132	MMGT1	membrane magnesium transporter 1 [Source:VGNC Symbol;Acc:VGNC:98130]	1.98	0.0542
ssc-miR-132	MMP16	matrix metallopeptidase 16 [Source:VGNC Symbol;Acc:VGNC:90271]	1.98	0.0542
ssc-miR-132	MSANTD2	Myb/SANT DNA binding domain containing 2 [Source:VGNC Symbol;Acc:VGNC:90414]	1.98	0.0542
ssc-miR-132	MSANTD3-TMEFF1	hypothetical gene	1.98	0.0542
ssc-miR-132	MSH6	mutS homolog 6 [Source:VGNC Symbol;Acc:VGNC:90420]	1.98	0.0542
ssc-miR-132	MTF1	metal regulatory transcription factor 1 [Source:VGNC Symbol;Acc:VGNC:90443]	1.98	0.0542
ssc-miR-132	MTMR10	myotubularin related protein 10 [Source:VGNC Symbol;Acc:VGNC:90457]	1.98	0.0542
ssc-miR-132	MUC19	hypothetical gene	1.98	0.0542
ssc-miR-132	MYCBP2	MYC binding protein 2 [Source:VGNC Symbol;Acc:VGNC:90502]	1.98	0.0542
ssc-miR-132	MYPN	myopalladin [Source:VGNC Symbol;Acc:VGNC:107412]	1.98	0.0542
ssc-miR-132	MYRF	hypothetical gene	1.98	0.0542
ssc-miR-132	NACC2	NACC family member 2 [Source:VGNC Symbol;Acc:VGNC:90564]	1.98	0.0542
ssc-miR-132	NCALD	neurocalcin delta [Source:VGNC Symbol;Acc:VGNC:90594]	1.98	0.0542
ssc-miR-132	NCOR1	nuclear receptor corepressor 1 [Source:VGNC Symbol;Acc:VGNC:99723]	1.98	0.0542
ssc-miR-132	NDRG4	NDRG family member 4 [Source:VGNC Symbol;Acc:VGNC:90633]	1.98	0.0542
ssc-miR-132	NET1	neuroepithelial cell transforming 1 [Source:VGNC Symbol;Acc:VGNC:95622]	1.98	0.0542
ssc-miR-132	NFAT5	nuclear factor of activated T cells 5 [Source:VGNC Symbol;Acc:VGNC:90708]	1.98	0.0542
ssc-miR-132	NFATC2	nuclear factor of activated T cells 2 [Source:VGNC Symbol;Acc:VGNC:96440]	1.98	0.0542
ssc-miR-132	NFIA	nuclear factor I A [Source:VGNC Symbol;Acc:VGNC:90715]	1.98	0.0542
ssc-miR-132	NFIB	nuclear factor I B [Source:VGNC Symbol;Acc:VGNC:90716]	1.98	0.0542
ssc-miR-132	NFYA	nuclear transcription factor Y subunit alpha [Source:VGNC Symbol;Acc:VGNC:90729]	1.98	0.0542
ssc-miR-132	NLK	nemo like kinase [Source:VGNC Symbol;Acc:VGNC:90779]	1.98	0.0542
ssc-miR-132	NMNAT2	nicotinamide nucleotide adenylyltransferase 2 [Source:VGNC Symbol;Acc:VGNC:90798]	1.98	0.0542
ssc-miR-132	NMT2	N-myristoyltransferase 2 [Source:VGNC Symbol;Acc:VGNC:96448]	1.98	0.0542
ssc-miR-132	NOL7	nucleolar protein 7 [Source:VGNC Symbol;Acc:VGNC:90815]	1.98	0.0542
ssc-miR-132	NOVA1	NOVA alternative splicing regulator 1 [Source:VGNC Symbol;Acc:VGNC:90827]	1.98	0.0542
ssc-miR-132	NR4A2	nuclear receptor subfamily 4 group A member 2 [Source:VGNC Symbol;Acc:VGNC:96451]	1.98	0.0542
ssc-miR-132	NRCAM	neuronal cell adhesion molecule [Source:HGNC Symbol;Acc:HGNC:7994]	1.98	0.0542
ssc-miR-132	NREP	neuronal regeneration related protein [Source:HGNC Symbol;Acc:HGNC:16834]	1.98	0.0542
ssc-miR-132	NRG2	neuregulin 2 [Source:VGNC Symbol;Acc:VGNC:98160]	1.98	0.0542
ssc-miR-132	NRXN1	neurexin 1 [Source:HGNC Symbol;Acc:HGNC:8008]	1.98	0.0542
ssc-miR-132	NTNG1	netrin G1 [Source:VGNC Symbol;Acc:VGNC:90935]	1.98	0.0542
ssc-miR-132	NTNG2	netrin G2 [Source:VGNC Symbol;Acc:VGNC:90936]	1.98	0.0542
ssc-miR-132	NUDCD3	NudC domain containing 3 [Source:VGNC Symbol;Acc:VGNC:90951]	1.98	0.0542
ssc-miR-132	NUP37	nucleoporin 37 [Source:VGNC Symbol;Acc:VGNC:90980]	1.98	0.0542
ssc-miR-132	NUPL1	hypothetical gene	1.98	0.0542
ssc-miR-132	OLFM1	olfactomedin 1 [Source:VGNC Symbol;Acc:VGNC:91031]	1.98	0.0542
ssc-miR-132	OSBPL8	oxysterol binding protein like 8 [Source:VGNC Symbol;Acc:VGNC:91074]	1.98	0.0542
ssc-miR-132	OTUD3	OTU deubiquitinase 3 [Source:VGNC Symbol;Acc:VGNC:91099]	1.98	0.0542
ssc-miR-132	OTUD7B	OTU deubiquitinase 7B [Source:VGNC Symbol;Acc:VGNC:91105]	1.98	0.0542

ssc-miR-132	PAIP2	poly(A) binding protein interacting protein 2 [Source:VGNC Symbol;Acc:VGNC:96618]	1.98	0.0542
ssc-miR-132	PALM2	hypothetical gene	1.98	0.0542
ssc-miR-132	PAM	peptidylglycine alpha-amidating monooxygenase [Source:VGNC Symbol;Acc:VGNC:91163]	1.98	0.0542
ssc-miR-132	PAN3	poly(A) specific ribonuclease subunit PAN3 [Source:HGNC Symbol;Acc:HGNC:29991]	1.98	0.0542
ssc-miR-132	PANK4	pantothenate kinase 4 (inactive) [Source:VGNC Symbol;Acc:VGNC:98527]	1.98	0.0542
ssc-miR-132	PAX5	paired box 5 [Source:VGNC Symbol;Acc:VGNC:91194]	1.98	0.0542
ssc-miR-132	PAX7	paired box 7 [Source:VGNC Symbol;Acc:VGNC:91196]	1.98	0.0542
ssc-miR-132	PCDH10	protocadherin 10 [Source:HGNC Symbol;Acc:HGNC:13404]	1.98	0.0542
ssc-miR-132	PCDHB15	hypothetical gene	1.98	0.0542
ssc-miR-132	PCGF3	polycomb group ring finger 3 [Source:VGNC Symbol;Acc:VGNC:91220]	1.98	0.0542
ssc-miR-132	PDE5A	phosphodiesterase 5A [Source:VGNC Symbol;Acc:VGNC:91257]	1.98	0.0542
ssc-miR-132	PDE7A	phosphodiesterase 7A [Source:VGNC Symbol;Acc:VGNC:91261]	1.98	0.0542
ssc-miR-132	PDE7B	phosphodiesterase 7B [Source:VGNC Symbol;Acc:VGNC:91262]	1.98	0.0542
ssc-miR-132	PDGFRA	platelet derived growth factor receptor alpha [Source:VGNC Symbol;Acc:VGNC:98179]	1.98	0.0542
ssc-miR-132	PDZD8	hypothetical gene	1.98	0.0542
ssc-miR-132	PEA15	proliferation and apoptosis adaptor protein 15 [Source:VGNC Symbol;Acc:VGNC:98181]	1.98	0.0542
ssc-miR-132	PGPEP1	pyroglutamyl-peptidase I [Source:VGNC Symbol;Acc:VGNC:91360]	1.98	0.0542
ssc-miR-132	PHF20L1	PHD finger protein 20 like 1 [Source:VGNC Symbol;Acc:VGNC:91383]	1.98	0.0542
ssc-miR-132	PIK3CA	phosphatidylinositol-4,5-bisphosphate 3-kinase catalytic subunit alpha [Source:VGNC Symbol;Acc:VGNC:91440]	1.98	0.0542
ssc-miR-132	PIK3IP1	phosphoinositide-3-kinase interacting protein 1 [Source:VGNC Symbol;Acc:VGNC:91444]	1.98	0.0542
ssc-miR-132	PLK5	hypothetical gene	1.98	0.0542
ssc-miR-132	PLXNA4	plexin A4 [Source:VGNC Symbol;Acc:VGNC:98204]	1.98	0.0542
ssc-miR-132	PNN	pinin, desmosome associated protein [Source:VGNC Symbol;Acc:VGNC:91603]	1.98	0.0542
ssc-miR-132	POC1B	POC1 centriolar protein B [Source:VGNC Symbol;Acc:VGNC:91616]	1.98	0.0542
ssc-miR-132	POC1B-GALNT4	hypothetical gene	1.98	0.0542
ssc-miR-132	POLE3	DNA polymerase epsilon 3, accessory subunit [Source:VGNC Symbol;Acc:VGNC:103154]	1.98	0.0542
ssc-miR-132	POM121C	hypothetical gene	1.98	0.0542
ssc-miR-132	PPFIBP1	PPFIA binding protein 1 [Source:VGNC Symbol;Acc:VGNC:91695]	1.98	0.0542
ssc-miR-132	PPM1G	protein phosphatase, Mg2+/Mn2+ dependent 1G [Source:VGNC Symbol;Acc:VGNC:91707]	1.98	0.0542
ssc-miR-132	PPP2CB	protein phosphatase 2 catalytic subunit beta [Source:VGNC Symbol;Acc:VGNC:96501]	1.98	0.0542
ssc-miR-132	PPP2R5C	protein phosphatase 2 regulatory subunit B'gamma [Source:VGNC Symbol;Acc:VGNC:91753]	1.98	0.0542
ssc-miR-132	PPP2R5E	protein phosphatase 2 regulatory subunit B'epsilon [Source:VGNC Symbol;Acc:VGNC:91754]	1.98	0.0542
ssc-miR-132	PPP3CA	protein phosphatase 3 catalytic subunit alpha [Source:VGNC Symbol;Acc:VGNC:98218]	1.98	0.0542
ssc-miR-132	PRDM6	PR/SET domain 6 [Source:VGNC Symbol;Acc:VGNC:91780]	1.98	0.0542
ssc-miR-132	PRICKLE2	prickle planar cell polarity protein 2 [Source:VGNC Symbol;Acc:VGNC:91794]	1.98	0.0542
ssc-miR-132	PRKAA2	protein kinase AMP-activated catalytic subunit alpha 2 [Source:VGNC Symbol;Acc:VGNC:91798]	1.98	0.0542
ssc-miR-132	PRKD1	protein kinase D1 [Source:VGNC Symbol;Acc:VGNC:91812]	1.98	0.0542
ssc-miR-132	PRPF38B	hypothetical gene	1.98	0.0542
ssc-miR-132	PRR14L	proline rich 14 like [Source:VGNC Symbol;Acc:VGNC:91853]	1.98	0.0542
ssc-miR-132	PRRT2	proline rich transmembrane protein 2 [Source:HGNC Symbol;Acc:HGNC:30500]	1.98	0.0542
ssc-miR-132	PSMA2	proteasome 20S subunit alpha 2 [Source:HGNC Symbol;Acc:HGNC:9531]	1.98	0.0542
ssc-miR-132	PSMD12	proteasome 26S subunit, non-ATPase 12 [Source:VGNC Symbol;Acc:VGNC:91920]	1.98	0.0542
ssc-miR-132	PTBP2	polypyrimidine tract binding protein 2 [Source:VGNC Symbol;Acc:VGNC:91937]	1.98	0.0542
ssc-miR-132	PTCH1	patched 1 [Source:VGNC Symbol;Acc:VGNC:96513]	1.98	0.0542
ssc-miR-132	PTEN	hypothetical gene	1.98	0.0542
ssc-miR-132	PTPMT1	protein tyrosine phosphatase mitochondrial 1 [Source:VGNC Symbol;Acc:VGNC:91971]	1.98	0.0542
ssc-miR-132	PTPRD	protein tyrosine phosphatase receptor type D [Source:HGNC Symbol;Acc:HGNC:9668]	1.98	0.0542
ssc-miR-132	PXN	paxillin [Source:HGNC Symbol;Acc:HGNC:9718]	1.98	0.0542
ssc-miR-132	PYGO1	pygopus family PHD finger 1 [Source:VGNC Symbol;Acc:VGNC:98231]	1.98	0.0542

ssc-miR-132	PYURF	hypothetical gene	1.98	0.0542
ssc-miR-132	QKI	QKI, KH domain containing RNA binding [Source:VGNC Symbol;Acc:VGNC:92025]	1.98	0.0542
ssc-miR-132	RAB15	RAB15, member RAS onco family [Source:VGNC Symbol;Acc:VGNC:98239]	1.98	0.0542
ssc-miR-132	RAB1A	RAB1A, member RAS onco family [Source:VGNC Symbol;Acc:VGNC:104042]	1.98	0.0542
ssc-miR-132	RAB6B	RAB6B, member RAS onco family [Source:VGNC Symbol;Acc:VGNC:98272]	1.98	0.0542
ssc-miR-132	RAP2B	RAP2B, member of RAS onco family [Source:VGNC Symbol;Acc:VGNC:92089]	1.98	0.0542
ssc-miR-132	RASA1	RAS p21 protein activator 1 [Source:VGNC Symbol;Acc:VGNC:92101]	1.98	0.0542
ssc-miR-132	RASGRP1	RAS guanyl releasing protein 1 [Source:VGNC Symbol;Acc:VGNC:92113]	1.98	0.0542
ssc-miR-132	RB1	RB transcriptional corepressor 1 [Source:VGNC Symbol;Acc:VGNC:92132]	1.98	0.0542
ssc-miR-132	RBFOX1	RNA binding fox-1 homolog 1 [Source:VGNC Symbol;Acc:VGNC:92139]	1.98	0.0542
ssc-miR-132	RBM12	RNA binding motif protein 12 [Source:HGNC Symbol;Acc:HGNC:9898]	1.98	0.0542
ssc-miR-132	RDX	radixin [Source:NCBI gene (formerly Entrezgene);Acc:494457]	1.98	0.0542
ssc-miR-132	RFX3	regulatory factor X3 [Source:VGNC Symbol;Acc:VGNC:92245]	1.98	0.0542
ssc-miR-132	RGS7BP	regulator of G protein signaling 7 binding protein [Source:VGNC Symbol;Acc:VGNC:92268]	1.98	0.0542
ssc-miR-132	RND3	Rho family GTPase 3 [Source:VGNC Symbol;Acc:VGNC:96407]	1.98	0.0542
ssc-miR-132	RNF152	ring finger protein 152 [Source:VGNC Symbol;Acc:VGNC:92364]	1.98	0.0542
ssc-miR-132	ROCK1	Rho associated coiled-coil containing protein kinase 1 [Source:VGNC Symbol;Acc:VGNC:98294]	1.98	0.0542
ssc-miR-132	RORB	RAR related orphan receptor B [Source:VGNC Symbol;Acc:VGNC:92409]	1.98	0.0542
ssc-miR-132	RPH3A	rabphilin 3A [Source:VGNC Symbol;Acc:VGNC:92425]	1.98	0.0542
ssc-miR-132	RRAGC	Ras related GTP binding C [Source:HGNC Symbol;Acc:HGNC:19902]	1.98	0.0542
ssc-miR-132	RSU1	Ras suppressor protein 1 [Source:VGNC Symbol;Acc:VGNC:98298]	1.98	0.0542
ssc-miR-132	RTF1	RTF1 homolog, Paf1/RNA polymerase II complex component [Source:VGNC Symbol;Acc:VGNC:92493]	1.98	0.0542
ssc-miR-132	RUNX1T1	RUNX1 partner transcriptional co-repressor 1 [Source:VGNC Symbol;Acc:VGNC:96594]	1.98	0.0542
ssc-miR-132	SALL1	spalt like transcription factor 1 [Source:VGNC Symbol;Acc:VGNC:92561]	1.98	0.0542
ssc-miR-132	SALL3	spalt like transcription factor 3 [Source:VGNC Symbol;Acc:VGNC:92563]	1.98	0.0542
ssc-miR-132	SAMD12	sterile alpha motif domain containing 12 [Source:VGNC Symbol;Acc:VGNC:92565]	1.98	0.0542
ssc-miR-132	SAP30	Sin3A associated protein 30 [Source:VGNC Symbol;Acc:VGNC:92575]	1.98	0.0542
ssc-miR-132	SAP30L	SAP30 like [Source:VGNC Symbol;Acc:VGNC:92577]	1.98	0.0542
ssc-miR-132	SATB2	SATB homeobox 2 [Source:VGNC Symbol;Acc:VGNC:95972]	1.98	0.0542
ssc-miR-132	SCN1A	sodium voltage-gated channel alpha subunit 1 [Source:VGNC Symbol;Acc:VGNC:95478]	1.98	0.0542
ssc-miR-132	SCN2A	hypothetical gene	1.98	0.0542
ssc-miR-132	SCN3A	sodium voltage-gated channel alpha subunit 3 [Source:VGNC Symbol;Acc:VGNC:95479]	1.98	0.0542
ssc-miR-132	SCYL3	SCY1 like pseudokinase 3 [Source:VGNC Symbol;Acc:VGNC:92653]	1.98	0.0542
ssc-miR-132	SDC2	syndecan 2 [Source:VGNC Symbol;Acc:VGNC:92655]	1.98	0.0542
ssc-miR-132	SDHAF1	succinate dehydrogenase complex assembly factor 1 [Source:HGNC Symbol;Acc:HGNC:33867]	1.98	0.0542
ssc-miR-132	SEC16A	SEC16 homolog A, endoplasmic reticulum export factor [Source:VGNC Symbol;Acc:VGNC:92671]	1.98	0.0542
ssc-miR-132	SEC23IP	SEC23 interacting protein [Source:VGNC Symbol;Acc:VGNC:92675]	1.98	0.0542
ssc-miR-132	SEC24A	SEC24 homolog A, COPII coat complex component [Source:VGNC Symbol;Acc:VGNC:92676]	1.98	0.0542
ssc-miR-132	SEC62	SEC62 homolog, preprotein translocation factor [Source:VGNC Symbol;Acc:VGNC:108691]	1.98	0.0542
ssc-miR-132	SEMA4G	semaphorin 4G [Source:VGNC Symbol;Acc:VGNC:92705]	1.98	0.0542
ssc-miR-132	SEMA6A	semaphorin 6A [Source:VGNC Symbol;Acc:VGNC:92708]	1.98	0.0542
ssc-miR-132	SEPHS1	selenophosphate synthetase 1 [Source:VGNC Symbol;Acc:VGNC:104053]	1.98	0.0542
ssc-miR-132	SERP1	stress associated endoplasmic reticulum protein 1 [Source:NCBI gene (formerly Entrezgene);Acc:100156392]	1.98	0.0542
ssc-miR-132	SESN3	sestrin 3 [Source:VGNC Symbol;Acc:VGNC:92755]	1.98	0.0542
ssc-miR-132	SETD5	SET domain containing 5 [Source:VGNC Symbol;Acc:VGNC:92760]	1.98	0.0542
ssc-miR-132	SGK3	serum/glucocorticoid regulated kinase family member 3 [Source:HGNC Symbol;Acc:HGNC:10812]	1.98	0.0542
ssc-miR-132	SHANK2	SH3 and multiple ankyrin repeat domains 2 [Source:VGNC Symbol;Acc:VGNC:92835]	1.98	0.0542
ssc-miR-132	SHH	sonic hedgehog signaling molecule [Source:VGNC Symbol;Acc:VGNC:92844]	1.98	0.0542
ssc-miR-132	SHISA9	shisa family member 9 [Source:HGNC Symbol;Acc:HGNC:37231]	1.98	0.0542

ssc-miR-132	SIRT1	sirtuin 1 [Source:VGNC Symbol;Acc:VGNC:92884]	1.98	0.0542
ssc-miR-132	SIX4	SIX homeobox 4 [Source:VGNC Symbol;Acc:VGNC:92895]	1.98	0.0542
ssc-miR-132	SKAP2	src kinase associated phosphoprotein 2 [Source:VGNC Symbol;Acc:VGNC:92901]	1.98	0.0542
ssc-miR-132	SLAIN2	SLAIN motif family member 2 [Source:VGNC Symbol;Acc:VGNC:92910]	1.98	0.0542
ssc-miR-132	SLBP	stem-loop binding protein [Source:NCBI gene (formerly Entrezgene);Acc:100513605]	1.98	0.0542
ssc-miR-132	SLC10A7	solute carrier family 10 member 7 [Source:VGNC Symbol;Acc:VGNC:92919]	1.98	0.0542
ssc-miR-132	SLC12A6	solute carrier family 12 member 6 [Source:VGNC Symbol;Acc:VGNC:92925]	1.98	0.0542
ssc-miR-132	SLC23A2	solute carrier family 23 member 2 [Source:VGNC Symbol;Acc:VGNC:108737]	1.98	0.0542
ssc-miR-132	SLC25A28	solute carrier family 25 member 28 [Source:VGNC Symbol;Acc:VGNC:93005]	1.98	0.0542
ssc-miR-132	SLC2A1	solute carrier family 2 member 1 [Source:HGNC Symbol;Acc:HGNC:11005]	1.98	0.0542
ssc-miR-132	SLC2A3	hypothetical gene	1.98	0.0542
ssc-miR-132	SLC30A6	solute carrier family 30 member 6 [Source:VGNC Symbol;Acc:VGNC:93060]	1.98	0.0542
ssc-miR-132	SLC6A1	solute carrier family 6 member 1 [Source:VGNC Symbol;Acc:VGNC:93153]	1.98	0.0542
ssc-miR-132	SLC8A1	solute carrier family 8 member A1 [Source:VGNC Symbol;Acc:VGNC:93178]	1.98	0.0542
ssc-miR-132	SLTM	SAFB like transcription modulator [Source:VGNC Symbol;Acc:VGNC:93213]	1.98	0.0542
ssc-miR-132	SMAD2	SMAD family member 2 [Source:VGNC Symbol;Acc:VGNC:98329]	1.98	0.0542
ssc-miR-132	SMAD5	SMAD family member 5 [Source:VGNC Symbol;Acc:VGNC:93219]	1.98	0.0542
ssc-miR-132	SMARCA5	SWI/SNF related, matrix associated, actin dependent regulator of chromatin, subfamily a, member 5 [Source:VGNC Symbol;Acc:VGNC:93228]	1.98	0.0542
ssc-miR-132	SNIP1	Smad nuclear interacting protein 1 [Source:VGNC Symbol;Acc:VGNC:93289]	1.98	0.0542
ssc-miR-132	SNX11	sorting nexin 11 [Source:VGNC Symbol;Acc:VGNC:93304]	1.98	0.0542
ssc-miR-132	SNX18	sorting nexin 18 [Source:VGNC Symbol;Acc:VGNC:93310]	1.98	0.0542
ssc-miR-132	SNX29	sorting nexin 29 [Source:VGNC Symbol;Acc:VGNC:98331]	1.98	0.0542
ssc-miR-132	SNX30	sorting nexin family member 30 [Source:VGNC Symbol;Acc:VGNC:93318]	1.98	0.0542
ssc-miR-132	SOCS2	suppressor of cytokine signaling 2 [Source:NCBI gene (formerly Entrezgene);Acc:100037966]	1.98	0.0542
ssc-miR-132	SOS1	SOS Ras/Rac guanine nucleotide exchange factor 1 [Source:HGNC Symbol;Acc:HGNC:11187]	1.98	0.0542
ssc-miR-132	SOX4	SRY-box transcription factor 4 [Source:HGNC Symbol;Acc:HGNC:11200]	1.98	0.0542
ssc-miR-132	SOX5	SRY-box transcription factor 5 [Source:VGNC Symbol;Acc:VGNC:93357]	1.98	0.0542
ssc-miR-132	SOX6	SRY-box transcription factor 6 [Source:VGNC Symbol;Acc:VGNC:93358]	1.98	0.0542
ssc-miR-132	SPAST	spastin [Source:VGNC Symbol;Acc:VGNC:98333]	1.98	0.0542
ssc-miR-132	SPPL3	signal peptide peptidase like 3 [Source:VGNC Symbol;Acc:VGNC:98340]	1.98	0.0542
ssc-miR-132	SPRED1	sprouty related EVH1 domain containing 1 [Source:VGNC Symbol;Acc:VGNC:93420]	1.98	0.0542
ssc-miR-132	SPRY1	sprouty RTK signaling antagonist 1 [Source:VGNC Symbol;Acc:VGNC:93424]	1.98	0.0542
ssc-miR-132	SPRYD7	SPRY domain containing 7 [Source:VGNC Symbol;Acc:VGNC:93429]	1.98	0.0542
ssc-miR-132	SPTSSA	serine palmitoyltransferase small subunit A [Source:HGNC Symbol;Acc:HGNC:20361]	1.98	0.0542
ssc-miR-132	SPTY2D1	SPT2 chromatin protein domain containing 1 [Source:VGNC Symbol;Acc:VGNC:100867]	1.98	0.0542
ssc-miR-132	SREBF1	sterol regulatory element binding transcription factor 1 [Source:VGNC Symbol;Acc:VGNC:99083]	1.98	0.0542
ssc-miR-132	SRGAP1	SLIT-ROBO Rho GTPase activating protein 1 [Source:VGNC Symbol;Acc:VGNC:93454]	1.98	0.0542
ssc-miR-132	SRGAP2	hypothetical gene	1.98	0.0542
ssc-miR-132	SRGAP3	SLIT-ROBO Rho GTPase activating protein 3 [Source:VGNC Symbol;Acc:VGNC:93455]	1.98	0.0542
ssc-miR-132	SRSF1	serine and arginine rich splicing factor 1 [Source:VGNC Symbol;Acc:VGNC:99084]	1.98	0.0542
ssc-miR-132	SRSF10	serine and arginine rich splicing factor 10 [Source:VGNC Symbol;Acc:VGNC:93472]	1.98	0.0542
ssc-miR-132	SS18	SS18 subunit of BAF chromatin remodeling complex [Source:VGNC Symbol;Acc:VGNC:93478]	1.98	0.0542
ssc-miR-132	STIM2	stromal interaction molecule 2 [Source:VGNC Symbol;Acc:VGNC:98967]	1.98	0.0542
ssc-miR-132	STX16	syntaxin 16 [Source:NCBI gene (formerly Entrezgene);Acc:100144526]	1.98	0.0542
ssc-miR-132	SV2A	synaptic vesicle glycoprotein 2A [Source:VGNC Symbol;Acc:VGNC:93639]	1.98	0.0542
ssc-miR-132	TACC1	hypothetical gene	1.98	0.0542
ssc-miR-132	TADA2B	transcriptional adaptor 2B [Source:VGNC Symbol;Acc:VGNC:93707]	1.98	0.0542
ssc-miR-132	TAF4	TATA-box binding protein associated factor 4 [Source:VGNC Symbol;Acc:VGNC:95532]	1.98	0.0542
ssc-miR-132	TAF4B	TATA-box binding protein associated factor 4b [Source:VGNC Symbol;Acc:VGNC:93716]	1.98	0.0542

ssc-miR-132	TBC1D30	TBC1 domain family member 30 [Source:VGNC Symbol;Acc:VGNC:93775]	1.98	0.0542
ssc-miR-132	TCEB1	hypothetical gene	1.98	0.0542
ssc-miR-132	TCF15	transcription factor 15 [Source:VGNC Symbol;Acc:VGNC:95535]	1.98	0.0542
ssc-miR-132	TCF21	transcription factor 21 [Source:VGNC Symbol;Acc:VGNC:93819]	1.98	0.0542
ssc-miR-132	TCF7L1	hypothetical gene	1.98	0.0542
ssc-miR-132	TCF7L2	transcription factor 7 like 2 [Source:VGNC Symbol;Acc:VGNC:93825]	1.98	0.0542
ssc-miR-132	TEAD1	TEA domain transcription factor 1 [Source:VGNC Symbol;Acc:VGNC:93853]	1.98	0.0542
ssc-miR-132	TENM1	teneurin transmembrane protein 1 [Source:VGNC Symbol;Acc:VGNC:98363]	1.98	0.0542
ssc-miR-132	TFDP1	transcription factor Dp-1 [Source:HGNC Symbol;Acc:HGNC:11749]	1.98	0.0542
ssc-miR-132	TFDP2	transcription factor Dp-2 [Source:VGNC Symbol;Acc:VGNC:93915]	1.98	0.0542
ssc-miR-132	THBS1	thrombospondin 1 [Source:VGNC Symbol;Acc:VGNC:93946]	1.98	0.0542
ssc-miR-132	TIMM9	hypothetical gene	1.98	0.0542
ssc-miR-132	TJAP1	tight junction associated protein 1 [Source:VGNC Symbol;Acc:VGNC:94004]	1.98	0.0542
ssc-miR-132	TLK2	tousled like kinase 2 [Source:VGNC Symbol;Acc:VGNC:94017]	1.98	0.0542
ssc-miR-132	TLN2	talin 2 [Source:VGNC Symbol;Acc:VGNC:103190]	1.98	0.0542
ssc-miR-132	TMED5	transmembrane p24 trafficking protein 5 [Source:VGNC Symbol;Acc:VGNC:94057]	1.98	0.0542
ssc-miR-132	TMED7	transmembrane p24 trafficking protein 7 [Source:NCBI gene (formerly Entrezgene);Acc:100522183]	1.98	0.0542
ssc-miR-132	TMEFF1	transmembrane protein with EGF like and two follistatin like domains 1 [Source:HGNC Symbol;Acc:HGNC:11866]	1.98	0.0542
ssc-miR-132	TMEM106B	transmembrane protein 106B [Source:VGNC Symbol;Acc:VGNC:104075]	1.98	0.0542
ssc-miR-132	TMEM178B	transmembrane protein 178B [Source:HGNC Symbol;Acc:HGNC:44112]	1.98	0.0542
ssc-miR-132	TMEM194B	hypothetical gene	1.98	0.0542
ssc-miR-132	TMEM2	hypothetical gene	1.98	0.0542
ssc-miR-132	TMEM215	transmembrane protein 215 [Source:VGNC Symbol;Acc:VGNC:96279]	1.98	0.0542
ssc-miR-132	TMEM257	hypothetical gene	1.98	0.0542
ssc-miR-132	TMEM43	transmembrane protein 43 [Source:VGNC Symbol;Acc:VGNC:99732]	1.98	0.0542
ssc-miR-132	TRIB2	tribbles pseudokinase 2 [Source:VGNC Symbol;Acc:VGNC:94392]	1.98	0.0542
ssc-miR-132	TSNAX	translin associated factor X [Source:VGNC Symbol;Acc:VGNC:103075]	1.98	0.0542
ssc-miR-132	TSPAN18	tetraspanin 18 [Source:VGNC Symbol;Acc:VGNC:94507]	1.98	0.0542
ssc-miR-132	TTI1	TELO2 interacting protein 1 [Source:VGNC Symbol;Acc:VGNC:96194]	1.98	0.0542
ssc-miR-132	TUSC1	tumor suppressor candidate 1 [Source:HGNC Symbol;Acc:HGNC:31010]	1.98	0.0542
ssc-miR-132	UBE2D2	ubiquitin conjugating enzyme E2 D2 [Source:VGNC Symbol;Acc:VGNC:98391]	1.98	0.0542
ssc-miR-132	UNKL	unk like zinc finger [Source:VGNC Symbol;Acc:VGNC:94716]	1.98	0.0542
ssc-miR-132	UQCRFS1	ubiquinol-cytochrome c reductase, Rieske iron-sulfur polypeptide 1 [Source:HGNC Symbol;Acc:HGNC:12587]	1.98	0.0542
ssc-miR-132	USP14	ubiquitin specific peptidase 14 [Source:VGNC Symbol;Acc:VGNC:94747]	1.98	0.0542
ssc-miR-132	USP15	ubiquitin specific peptidase 15 [Source:VGNC Symbol;Acc:VGNC:94748]	1.98	0.0542
ssc-miR-132	USP38	ubiquitin specific peptidase 38 [Source:VGNC Symbol;Acc:VGNC:94762]	1.98	0.0542
ssc-miR-132	USP8	ubiquitin specific peptidase 8 [Source:VGNC Symbol;Acc:VGNC:94778]	1.98	0.0542
ssc-miR-132	USP9X	ubiquitin specific peptidase 9 X-linked [Source:HGNC Symbol;Acc:HGNC:12632]	1.98	0.0542
ssc-miR-132	USP9Y	hypothetical gene	1.98	0.0542
ssc-miR-132	VAPA	VAMP associated protein A [Source:VGNC Symbol;Acc:VGNC:94799]	1.98	0.0542
ssc-miR-132	VMP1	vacuole membrane protein 1 [Source:HGNC Symbol;Acc:HGNC:29559]	1.98	0.0542
ssc-miR-132	VTI1A	vesicle transport through interaction with t-SNAREs 1A [Source:VGNC Symbol;Acc:VGNC:94879]	1.98	0.0542
ssc-miR-132	WDFY2	WD repeat and FYVE domain containing 2 [Source:VGNC Symbol;Acc:VGNC:94902]	1.98	0.0542
ssc-miR-132	WSB2	WD repeat and SOCS box containing 2 [Source:VGNC Symbol;Acc:VGNC:94982]	1.98	0.0542
ssc-miR-132	WT1	WT1 transcription factor [Source:VGNC Symbol;Acc:VGNC:100884]	1.98	0.0542
ssc-miR-132	WTAP	WT1 associated protein [Source:VGNC Symbol;Acc:VGNC:94984]	1.98	0.0542
ssc-miR-132	YIPF6	Yip1 domain family member 6 [Source:VGNC Symbol;Acc:VGNC:95032]	1.98	0.0542
ssc-miR-132	YWHAG	hypothetical gene	1.98	0.0542
ssc-miR-132	ZBTB18	zinc finger and BTB domain containing 18 [Source:HGNC Symbol;Acc:HGNC:13030]	1.98	0.0542

ssc-miR-132	ZBTB20	zinc finger and BTB domain containing 20 [Source:VGNC Symbol;Acc:VGNC:95063]	1.98	0.0542
ssc-miR-132	ZBTB34	zinc finger and BTB domain containing 34 [Source:VGNC Symbol;Acc:VGNC:95070]	1.98	0.0542
ssc-miR-132	ZBTB5	zinc finger and BTB domain containing 5 [Source:VGNC Symbol;Acc:VGNC:103204]	1.98	0.0542
ssc-miR-132	ZCCHC11	hypothetical gene	1.98	0.0542
ssc-miR-132	ZDHHC13	zinc finger DHHC-type palmitoyltransferase 13 [Source:VGNC Symbol;Acc:VGNC:95116]	1.98	0.0542
ssc-miR-132	ZDHHC18	zinc finger DHHC-type palmitoyltransferase 18 [Source:VGNC Symbol;Acc:VGNC:95120]	1.98	0.0542
ssc-miR-132	ZEB2	hypothetical gene	1.98	0.0542
ssc-miR-132	ZFC3H1	zinc finger C3H1-type containing [Source:VGNC Symbol;Acc:VGNC:95139]	1.98	0.0542
ssc-miR-132	ZFHX4	zinc finger homeobox 4 [Source:VGNC Symbol;Acc:VGNC:95142]	1.98	0.0542
ssc-miR-132	ZFYVE1	zinc finger FYVE-type containing 1 [Source:VGNC Symbol;Acc:VGNC:95156]	1.98	0.0542
ssc-miR-132	ZNF207	zinc finger protein 207 [Source:VGNC Symbol;Acc:VGNC:95205]	1.98	0.0542
ssc-miR-132	ZNF292	zinc finger protein 292 [Source:VGNC Symbol;Acc:VGNC:95218]	1.98	0.0542
ssc-miR-132	ZNF395	zinc finger protein 395 [Source:VGNC Symbol;Acc:VGNC:95235]	1.98	0.0542
ssc-miR-132	ZNF474	hypothetical gene	1.98	0.0542
ssc-miR-132	ZNF516	zinc finger protein 516 [Source:VGNC Symbol;Acc:VGNC:95256]	1.98	0.0542
ssc-miR-132	ZNF521	zinc finger protein 521 [Source:VGNC Symbol;Acc:VGNC:98715]	1.98	0.0542
ssc-miR-132	ZNF644	zinc finger protein 644 [Source:VGNC Symbol;Acc:VGNC:95283]	1.98	0.0542
ssc-miR-132	ZNF652	zinc finger protein 652 [Source:VGNC Symbol;Acc:VGNC:99108]	1.98	0.0542
ssc-miR-132	ZNF704	zinc finger protein 704 [Source:VGNC Symbol;Acc:VGNC:95296]	1.98	0.0542
ssc-miR-132	ZSWIM6	zinc finger SWIM-type containing 6 [Source:VGNC Symbol;Acc:VGNC:95335]	1.98	0.0542
ssc-miR-191	AJAP1	adherens junctions associated protein 1 [Source:VGNC Symbol;Acc:VGNC:85207]	1.85	0.0115
ssc-miR-191	AMMECR1	AMMECR nuclear protein 1 [Source:VGNC Symbol;Acc:VGNC:96559]	1.85	0.0115
ssc-miR-191	AMMECR1L	AMMECR1 like [Source:VGNC Symbol;Acc:VGNC:103895]	1.85	0.0115
ssc-miR-191	ANKRD27	ankyrin repeat domain 27 [Source:VGNC Symbol;Acc:VGNC:96907]	1.85	0.0115
ssc-miR-191	AP1G1	adaptor related protein complex 1 subunit gamma 1 [Source:VGNC Symbol;Acc:VGNC:85378]	1.85	0.0115
ssc-miR-191	ATP2B2	ATPase plasma membrane Ca2+ transporting 2 [Source:VGNC Symbol;Acc:VGNC:85649]	1.85	0.0115
ssc-miR-191	BDNF	brain derived neurotrophic factor [Source:VGNC Symbol;Acc:VGNC:85795]	1.85	0.0115
ssc-miR-191	BNC2	basonuclin 2 [Source:VGNC Symbol;Acc:VGNC:85852]	1.85	0.0115
ssc-miR-191	BRMS1L	BRMS1 like transcriptional repressor [Source:VGNC Symbol;Acc:VGNC:96571]	1.85	0.0115
ssc-miR-191	C7	complement C7 [Source:VGNC Symbol;Acc:VGNC:86067]	1.85	0.0115
ssc-miR-191	CALN1	hypothetical gene	1.85	0.0115
ssc-miR-191	CASK	calcium/calmodulin dependent serine protein kinase [Source:HGNC Symbol;Acc:HGNC:1497]	1.85	0.0115
ssc-miR-191	CDK6	cyclin dependent kinase 6 [Source:HGNC Symbol;Acc:HGNC:1777]	1.85	0.0115
ssc-miR-191	CDKN1B	cyclin dependent kinase inhibitor 1B [Source:VGNC Symbol;Acc:VGNC:86515]	1.85	0.0115
ssc-miR-191	CHMP5	charged multivesicular body protein 5 [Source:VGNC Symbol;Acc:VGNC:103920]	1.85	0.0115
ssc-miR-191	CTDSP2	CTD small phosphatase 2 [Source:VGNC Symbol;Acc:VGNC:87059]	1.85	0.0115
ssc-miR-191	DCAF7	DDB1 and CUL4 associated factor 7 [Source:VGNC Symbol;Acc:VGNC:87178]	1.85	0.0115
ssc-miR-191	DLX1	distal-less homeobox 1 [Source:VGNC Symbol;Acc:VGNC:96199]	1.85	0.0115
ssc-miR-191	DRGX	dorsal root ganglia homeobox [Source:VGNC Symbol;Acc:VGNC:97968]	1.85	0.0115
ssc-miR-191	DSE	dermatan sulfate epimerase [Source:VGNC Symbol;Acc:VGNC:103084]	1.85	0.0115
ssc-miR-191	DUSP7	dual specificity phosphatase 7 [Source:HGNC Symbol;Acc:HGNC:3073]	1.85	0.0115
ssc-miR-191	EGR1	early growth response 1 [Source:VGNC Symbol;Acc:VGNC:87590]	1.85	0.0115
ssc-miR-191	EMX2	empty spiracles homeobox 2 [Source:VGNC Symbol;Acc:VGNC:87693]	1.85	0.0115
ssc-miR-191	FUBP3	far upstream element binding protein 3 [Source:VGNC Symbol;Acc:VGNC:103093]	1.85	0.0115
ssc-miR-191	FZD5	frizzled class receptor 5 [Source:VGNC Symbol;Acc:VGNC:96309]	1.85	0.0115
ssc-miR-191	GAP43	growth associated protein 43 [Source:VGNC Symbol;Acc:VGNC:98018]	1.85	0.0115
ssc-miR-191	GATSL2	hypothetical gene	1.85	0.0115
ssc-miR-191	GLYR1	glyoxylate reductase 1 homolog [Source:VGNC Symbol;Acc:VGNC:88505]	1.85	0.0115
ssc-miR-191	HIP1	huntingtin interacting protein 1 [Source:VGNC Symbol;Acc:VGNC:88885]	1.85	0.0115

ssc-miR-191	HOXB2	homeobox B2 [Source:VGNC Symbol;Acc:VGNC:88943]	1.85	0.0115
ssc-miR-191	HRK	harakiri, BCL2 interacting protein [Source:HGNC Symbol;Acc:HGNC:5185]	1.85	0.0115
ssc-miR-191	IFFO2	intermediate filament family orphan 2 [Source:VGNC Symbol;Acc:VGNC:98475]	1.85	0.0115
ssc-miR-191	LBH	LBH regulator of WNT signaling pathway [Source:HGNC Symbol;Acc:HGNC:29532]	1.85	0.0115
ssc-miR-191	LIN54	lin-54 DREAM MuvB core complex component [Source:VGNC Symbol;Acc:VGNC:98072]	1.85	0.0115
ssc-miR-191	LRRC8A	leucine rich repeat containing 8 VRAC subunit A [Source:VGNC Symbol;Acc:VGNC:98085]	1.85	0.0115
ssc-miR-191	MAPRE2	microtubule associated protein RP/EB family member 2 [Source:VGNC Symbol;Acc:VGNC:98117]	1.85	0.0115
ssc-miR-191	MAPRE3	microtubule associated protein RP/EB family member 3 [Source:VGNC Symbol;Acc:VGNC:90015]	1.85	0.0115
ssc-miR-191	MEIS2	Meis homeobox 2 [Source:VGNC Symbol;Acc:VGNC:90136]	1.85	0.0115
ssc-miR-191	MYEOV	hypothetical gene	1.85	0.0115
ssc-miR-191	NDST1	N-deacetylase and N-sulfotransferase 1 [Source:VGNC Symbol;Acc:VGNC:90634]	1.85	0.0115
ssc-miR-191	NEURL4	neuralized E3 ubiquitin protein ligase 4 [Source:VGNC Symbol;Acc:VGNC:99022]	1.85	0.0115
ssc-miR-191	NFIA	nuclear factor I A [Source:VGNC Symbol;Acc:VGNC:90715]	1.85	0.0115
ssc-miR-191	NFIC	nuclear factor I C [Source:VGNC Symbol;Acc:VGNC:100313]	1.85	0.0115
ssc-miR-191	NRCAM	neuronal cell adhesion molecule [Source:HGNC Symbol;Acc:HGNC:7994]	1.85	0.0115
ssc-miR-191	OXSR1	hypothetical gene	1.85	0.0115
ssc-miR-191	PEG10	paternally expressed 10 [Source:NCBI gene (formerly Entrezgene);Acc:654416]	1.85	0.0115
ssc-miR-191	PHC2	polyhomeotic homolog 2 [Source:VGNC Symbol;Acc:VGNC:91371]	1.85	0.0115
ssc-miR-191	PITPNM2	phosphatidylinositol transfer protein membrane associated 2 [Source:VGNC Symbol;Acc:VGNC:91466]	1.85	0.0115
ssc-miR-191	PITPNM3	PITPNM family member 3 [Source:HGNC Symbol;Acc:HGNC:21043]	1.85	0.0115
ssc-miR-191	PLCD1	phospholipase C delta 1 [Source:VGNC Symbol;Acc:VGNC:91517]	1.85	0.0115
ssc-miR-191	PLXNA4	plexin A4 [Source:VGNC Symbol;Acc:VGNC:98204]	1.85	0.0115
ssc-miR-191	RCC2	regulator of chromosome condensation 2 [Source:VGNC Symbol;Acc:VGNC:98547]	1.85	0.0115
ssc-miR-191	RFX3	regulatory factor X3 [Source:VGNC Symbol;Acc:VGNC:92245]	1.85	0.0115
ssc-miR-191	SALL1	spalt like transcription factor 1 [Source:VGNC Symbol;Acc:VGNC:92561]	1.85	0.0115
ssc-miR-191	SATB1	SATB homeobox 1 [Source:VGNC Symbol;Acc:VGNC:92587]	1.85	0.0115
ssc-miR-191	SATB2	SATB homeobox 2 [Source:VGNC Symbol;Acc:VGNC:95972]	1.85	0.0115
ssc-miR-191	SETBP1	SET binding protein 1 [Source:VGNC Symbol;Acc:VGNC:92756]	1.85	0.0115
ssc-miR-191	SETD1B	SET domain containing 1B, histone lysine methyltransferase [Source:HGNC Symbol;Acc:HGNC:29187]	1.85	0.0115
ssc-miR-191	SHANK3	hypothetical gene	1.85	0.0115
ssc-miR-191	SLA	Src like adaptor [Source:VGNC Symbol;Acc:VGNC:92908]	1.85	0.0115
ssc-miR-191	SLC16A2	solute carrier family 16 member 2 [Source:VGNC Symbol;Acc:VGNC:92942]	1.85	0.0115
ssc-miR-191	SMAD2	SMAD family member 2 [Source:VGNC Symbol;Acc:VGNC:98329]	1.85	0.0115
ssc-miR-191	SMARCD1	SWI/SNF related, matrix associated, actin dependent regulator of chromatin, subfamily d, member 1 [Source:VGNC Symbol;Acc:VGNC:93233]	1.85	0.0115
ssc-miR-191	SMUG1	single-strand-selective monofunctional uracil-DNA glycosylase 1 [Source:VGNC Symbol;Acc:VGNC:93269]	1.85	0.0115
ssc-miR-191	SOX4	SRY-box transcription factor 4 [Source:HGNC Symbol;Acc:HGNC:11200]	1.85	0.0115
ssc-miR-191	SRGAP2	hypothetical gene	1.85	0.0115
ssc-miR-191	SUV420H1	hypothetical gene	1.85	0.0115
ssc-miR-191	TAF5	TATA-box binding protein associated factor 5 [Source:VGNC Symbol;Acc:VGNC:93717]	1.85	0.0115
ssc-miR-191	TBC1D15	TBC1 domain family member 15 [Source:VGNC Symbol;Acc:VGNC:93764]	1.85	0.0115
ssc-miR-191	TCF7L2	transcription factor 7 like 2 [Source:VGNC Symbol;Acc:VGNC:93825]	1.85	0.0115
ssc-miR-191	TJP1	tight junction protein 1 [Source:VGNC Symbol;Acc:VGNC:94005]	1.85	0.0115
ssc-miR-191	TMC7	transmembrane channel like 7 [Source:VGNC Symbol;Acc:VGNC:94044]	1.85	0.0115
ssc-miR-191	TMOD2	tropomodulin 2 [Source:VGNC Symbol;Acc:VGNC:94227]	1.85	0.0115
ssc-miR-191	TOR1AIP2	torsin 1A interacting protein 2 [Source:VGNC Symbol;Acc:VGNC:94318]	1.85	0.0115
ssc-miR-191	VEZF1	vascular endothelial zinc finger 1 [Source:VGNC Symbol;Acc:VGNC:94818]	1.85	0.0115
ssc-miR-191	WIZ	WIZ zinc finger [Source:VGNC Symbol;Acc:VGNC:100883]	1.85	0.0115
ssc-miR-191	WSB1	WD repeat and SOCS box containing 1 [Source:VGNC Symbol;Acc:VGNC:94981]	1.85	0.0115
ssc-miR-191	ZBTB34	zinc finger and BTB domain containing 34 [Source:VGNC Symbol;Acc:VGNC:95070]	1.85	0.0115

ssc-miR-191	ZCCHC14	zinc finger CCHC-type containing 14 [Source:VGNC Symbol;Acc:VGNC:95107]	1.85	0.0115
ssc-miR-191	ZCCHC24	zinc finger CCHC-type containing 24 [Source:HGNC Symbol;Acc:HGNC:26911]	1.85	0.0115
ssc-miR-191	ZNF362	zinc finger protein 362 [Source:VGNC Symbol;Acc:VGNC:95229]	1.85	0.0115
ssc-miR-425-5p	AC004466.1	hypothetical gene	1.8	0.0188
ssc-miR-425-5p	ACAN	aggrecan [Source:NCBI gene (formerly Entrezgene);Acc:397255]	1.8	0.0188
ssc-miR-425-5p	ACTR3	actin related protein 3 [Source:VGNC Symbol;Acc:VGNC:103894]	1.8	0.0188
ssc-miR-425-5p	ACVR1C	activin A receptor type 1C [Source:VGNC Symbol;Acc:VGNC:95873]	1.8	0.0188
ssc-miR-425-5p	ADCYAP1	adenylate cyclase activating polypeptide 1 [Source:VGNC Symbol;Acc:VGNC:85114]	1.8	0.0188
ssc-miR-425-5p	ADK	adenosine kinase [Source:VGNC Symbol;Acc:VGNC:85143]	1.8	0.0188
ssc-miR-425-5p	AFF4	AF4/FMR2 family member 4 [Source:VGNC Symbol;Acc:VGNC:85169]	1.8	0.0188
ssc-miR-425-5p	AGAP1	ArfGAP with GTPase domain, ankyrin repeat and PH domain 1 [Source:HGNC Symbol;Acc:HGNC:16922]	1.8	0.0188
ssc-miR-425-5p	AGFG1	ArfGAP with FG repeats 1 [Source:VGNC Symbol;Acc:VGNC:96359]	1.8	0.0188
ssc-miR-425-5p	AGFG2	ArfGAP with FG repeats 2 [Source:VGNC Symbol;Acc:VGNC:85179]	1.8	0.0188
ssc-miR-425-5p	AHSA2	hypothetical gene	1.8	0.0188
ssc-miR-425-5p	AIFM1	apoptosis inducing factor mitochondria associated 1 [Source:VGNC Symbol;Acc:VGNC:85198]	1.8	0.0188
ssc-miR-425-5p	ALG9	ALG9 alpha-1,2-mannosyltransferase [Source:NCBI gene (formerly Entrezgene);Acc:100519965]	1.8	0.0188
ssc-miR-425-5p	AMPH	amphiphysin [Source:VGNC Symbol;Acc:VGNC:85289]	1.8	0.0188
ssc-miR-425-5p	ANK3	hypothetical gene	1.8	0.0188
ssc-miR-425-5p	ANKHD1	ankyrin repeat and KH domain containing 1 [Source:NCBI gene (formerly Entrezgene);Acc:100512270]	1.8	0.0188
ssc-miR-425-5p	ANO8	anoctamin 8 [Source:VGNC Symbol;Acc:VGNC:85360]	1.8	0.0188
ssc-miR-425-5p	ANXA11	annexin A11 [Source:VGNC Symbol;Acc:VGNC:85367]	1.8	0.0188
ssc-miR-425-5p	AP1AR	adaptor related protein complex 1 associated regulatory protein [Source:HGNC Symbol;Acc:HGNC:28808]	1.8	0.0188
ssc-miR-425-5p	AP3M1	adaptor related protein complex 3 subunit mu 1 [Source:VGNC Symbol;Acc:VGNC:85390]	1.8	0.0188
ssc-miR-425-5p	AQR	aquarius intron-binding spliceosomal factor [Source:VGNC Symbol;Acc:VGNC:85437]	1.8	0.0188
ssc-miR-425-5p	ARHGAP36	hypothetical gene	1.8	0.0188
ssc-miR-425-5p	ARIH1	ariadne RBR E3 ubiquitin protein ligase 1 [Source:HGNC Symbol;Acc:HGNC:689]	1.8	0.0188
ssc-miR-425-5p	ARL3	ADP ribosylation factor like GTPase 3 [Source:VGNC Symbol;Acc:VGNC:107366]	1.8	0.0188
ssc-miR-425-5p	ARL5B	ADP ribosylation factor like GTPase 5B [Source:VGNC Symbol;Acc:VGNC:95985]	1.8	0.0188
ssc-miR-425-5p	ARMCX3	armadillo repeat containing X-linked 3 [Source:HGNC Symbol;Acc:HGNC:24065]	1.8	0.0188
ssc-miR-425-5p	ATP1B1	ATPase Na+/K+ transporting subunit beta 1 [Source:VGNC Symbol;Acc:VGNC:85642]	1.8	0.0188
ssc-miR-425-5p	ATP1B3	hypothetical gene	1.8	0.0188
ssc-miR-425-5p	ATP5E	hypothetical gene	1.8	0.0188
ssc-miR-425-5p	ATP5F1	hypothetical gene	1.8	0.0188
ssc-miR-425-5p	ATP5G3	hypothetical gene	1.8	0.0188
ssc-miR-425-5p	ATP8A2	ATPase phospholipid transporting 8A2 [Source:HGNC Symbol;Acc:HGNC:13533]	1.8	0.0188
ssc-miR-425-5p	B3GALT5	hypothetical gene	1.8	0.0188
ssc-miR-425-5p	B3GAT2	beta-1,3-glucuronyltransferase 2 [Source:VGNC Symbol;Acc:VGNC:85720]	1.8	0.0188
ssc-miR-425-5p	BCL2L2-PABPN1	hypothetical gene	1.8	0.0188
ssc-miR-425-5p	BCLAF1	BCL2 associated transcription factor 1 [Source:VGNC Symbol;Acc:VGNC:85787]	1.8	0.0188
ssc-miR-425-5p	BCOR	BCL6 corepressor [Source:HGNC Symbol;Acc:HGNC:20893]	1.8	0.0188
ssc-miR-425-5p	BFAR	bifunctional apoptosis regulator [Source:VGNC Symbol;Acc:VGNC:85809]	1.8	0.0188
ssc-miR-425-5p	BLOC1S1	biogenesis of lysosomal organelles complex 1 subunit 1 [Source:HGNC Symbol;Acc:HGNC:4200]	1.8	0.0188
ssc-miR-425-5p	BMP8A	hypothetical gene	1.8	0.0188
ssc-miR-425-5p	BNC2	basonuclin 2 [Source:VGNC Symbol;Acc:VGNC:85852]	1.8	0.0188
ssc-miR-425-5p	C3orf17	hypothetical gene	1.8	0.0188
ssc-miR-425-5p	C3orf70	chromosome 13 C3orf70 homolog [Source:VGNC Symbol;Acc:VGNC:85937]	1.8	0.0188
ssc-miR-425-5p	CAB39	calcium binding protein 39 [Source:VGNC Symbol;Acc:VGNC:95936]	1.8	0.0188
ssc-miR-425-5p	CAB39L	calcium binding protein 39 like [Source:VGNC Symbol;Acc:VGNC:86106]	1.8	0.0188
ssc-miR-425-5p	CACNA1D	calcium voltage-gated channel subunit alpha1 D [Source:VGNC Symbol;Acc:VGNC:86117]	1.8	0.0188

ssc-miR-425-5p	CADM4	cell adhesion molecule 4 [Source:VGNC Symbol;Acc:VGNC:86136]	1.8	0.0188
ssc-miR-425-5p	CALM1	calmodulin 1 [Source:NCBI gene (formerly Entrezgene);Acc:100154056]	1.8	0.0188
ssc-miR-425-5p	CBL	Cbl proto-onco [Source:VGNC Symbol;Acc:VGNC:86222]	1.8	0.0188
ssc-miR-425-5p	CBX6	chromobox 6 [Source:VGNC Symbol;Acc:VGNC:97915]	1.8	0.0188
ssc-miR-425-5p	CCDC73	coiled-coil domain containing 73 [Source:VGNC Symbol;Acc:VGNC:86312]	1.8	0.0188
ssc-miR-425-5p	CD209	CD209 molecule [Source:NCBI gene (formerly Entrezgene);Acc:100170134]	1.8	0.0188
ssc-miR-425-5p	CDC5L	cell division cycle 5 like [Source:VGNC Symbol;Acc:VGNC:86463]	1.8	0.0188
ssc-miR-425-5p	CDK8	cyclin dependent kinase 8 [Source:VGNC Symbol;Acc:VGNC:86507]	1.8	0.0188
ssc-miR-425-5p	CDON	cell adhesion associated, onco regulated [Source:VGNC Symbol;Acc:VGNC:86519]	1.8	0.0188
ssc-miR-425-5p	CELF2	hypothetical gene	1.8	0.0188
ssc-miR-425-5p	CENPO	centromere protein O [Source:HGNC Symbol;Acc:HGNC:28152]	1.8	0.0188
ssc-miR-425-5p	CEP57	hypothetical gene	1.8	0.0188
ssc-miR-425-5p	CFL2	cofilin 2 [Source:VGNC Symbol;Acc:VGNC:86611]	1.8	0.0188
ssc-miR-425-5p	CLCN4	chloride voltage-gated channel 4 [Source:VGNC Symbol;Acc:VGNC:86728]	1.8	0.0188
ssc-miR-425-5p	CLVS2	clavesin 2 [Source:VGNC Symbol;Acc:VGNC:86795]	1.8	0.0188
ssc-miR-425-5p	CNNM2	cyclin and CBS domain divalent metal cation transport mediator 2 [Source:VGNC Symbol;Acc:VGNC:86828]	1.8	0.0188
ssc-miR-425-5p	CNR1	cannabinoid receptor 1 [Source:VGNC Symbol;Acc:VGNC:86843]	1.8	0.0188
ssc-miR-425-5p	CPEB1	cytoplasmic polyadenylation element binding protein 1 [Source:NCBI gene (formerly Entrezgene);Acc:100048944]	1.8	0.0188
ssc-miR-425-5p	CPEB2	cytoplasmic polyadenylation element binding protein 2 [Source:VGNC Symbol;Acc:VGNC:86937]	1.8	0.0188
ssc-miR-425-5p	CREB1	cAMP responsive element binding protein 1 [Source:VGNC Symbol;Acc:VGNC:96004]	1.8	0.0188
ssc-miR-425-5p	CREBZF	CREB/ATF bZIP transcription factor [Source:VGNC Symbol;Acc:VGNC:86988]	1.8	0.0188
ssc-miR-425-5p	CSNK1G1	casein kinase 1 gamma 1 [Source:VGNC Symbol;Acc:VGNC:97949]	1.8	0.0188
ssc-miR-425-5p	CTNND1	catenin delta 1 [Source:VGNC Symbol;Acc:VGNC:87067]	1.8	0.0188
ssc-miR-425-5p	CTSS	cathepsin S [Source:VGNC Symbol;Acc:VGNC:87079]	1.8	0.0188
ssc-miR-425-5p	CUL4B	cullin 4B [Source:HGNC Symbol;Acc:HGNC:2555]	1.8	0.0188
ssc-miR-425-5p	CXorf56	hypothetical gene	1.8	0.0188
ssc-miR-425-5p	DACH1	dachshund family transcription factor 1 [Source:VGNC Symbol;Acc:VGNC:87146]	1.8	0.0188
ssc-miR-425-5p	DCUN1D1	hypothetical gene	1.8	0.0188
ssc-miR-425-5p	DDX18	DEAD-box helicase 18 [Source:VGNC Symbol;Acc:VGNC:99709]	1.8	0.0188
ssc-miR-425-5p	DEK	hypothetical gene	1.8	0.0188
ssc-miR-425-5p	DENND6A	DENN domain containing 6A [Source:VGNC Symbol;Acc:VGNC:87256]	1.8	0.0188
ssc-miR-425-5p	DGCR14	hypothetical gene	1.8	0.0188
ssc-miR-425-5p	DGKG	diacylglycerol kinase gamma [Source:VGNC Symbol;Acc:VGNC:87272]	1.8	0.0188
ssc-miR-425-5p	DGKI	diacylglycerol kinase iota [Source:VGNC Symbol;Acc:VGNC:87274]	1.8	0.0188
ssc-miR-425-5p	DHX40	DEAH-box helicase 40 [Source:VGNC Symbol;Acc:VGNC:87295]	1.8	0.0188
ssc-miR-425-5p	DICER1	dicer 1, ribonuclease III [Source:VGNC Symbol;Acc:VGNC:87302]	1.8	0.0188
ssc-miR-425-5p	DIP2C	disco interacting C [Source:VGNC Symbol;Acc:VGNC:96201]	1.8	0.0188
ssc-miR-425-5p	DIS3L2	DIS3 like 3'-5' exoribonuclease 2 [Source:VGNC Symbol;Acc:VGNC:96183]	1.8	0.0188
ssc-miR-425-5p	DNAJB6	DnaJ heat shock protein family (Hsp40) member B6 [Source:HGNC Symbol;Acc:HGNC:14888]	1.8	0.0188
ssc-miR-425-5p	DNAJC27	DnaJ heat shock protein family (Hsp40) member C27 [Source:VGNC Symbol;Acc:VGNC:102563]	1.8	0.0188
ssc-miR-425-5p	DPYSL2	dihydropyrimidinase like 2 [Source:VGNC Symbol;Acc:VGNC:87430]	1.8	0.0188
ssc-miR-425-5p	DR1	down-regulator of transcription 1 [Source:VGNC Symbol;Acc:VGNC:87435]	1.8	0.0188
ssc-miR-425-5p	DUSP16	dual specificity phosphatase 16 [Source:VGNC Symbol;Acc:VGNC:87480]	1.8	0.0188
ssc-miR-425-5p	DUSP19	dual specificity phosphatase 19 [Source:VGNC Symbol;Acc:VGNC:95899]	1.8	0.0188
ssc-miR-425-5p	DYNC1I2	dynein cytoplasmic 1 intermediate chain 2 [Source:VGNC Symbol;Acc:VGNC:96224]	1.8	0.0188
ssc-miR-425-5p	DYRK1A	dual specificity tyrosine phosphorylation regulated kinase 1A [Source:VGNC Symbol;Acc:VGNC:87505]	1.8	0.0188
ssc-miR-425-5p	ECE2	endothelin converting enzyme 2 [Source:HGNC Symbol;Acc:HGNC:13275]	1.8	0.0188
ssc-miR-425-5p	EFNB2	ephrin B2 [Source:VGNC Symbol;Acc:VGNC:87577]	1.8	0.0188
ssc-miR-425-5p	EIF4E3	eukaryotic translation initiation factor 4E family member 3 [Source:VGNC Symbol;Acc:VGNC:87628]	1.8	0.0188

ssc-miR-425-5p	ELAVL4	ELAV like RNA binding protein 4 [Source:VGNC Symbol;Acc:VGNC:97047]	1.8	0.0188
ssc-miR-425-5p	ELK3	ETS transcription factor ELK3 [Source:VGNC Symbol;Acc:VGNC:87646]	1.8	0.0188
ssc-miR-425-5p	ELMSAN1	hypothetical gene	1.8	0.0188
ssc-miR-425-5p	ENAH	ENAH actin regulator [Source:VGNC Symbol;Acc:VGNC:108271]	1.8	0.0188
ssc-miR-425-5p	EPC1	enhancer of polycomb homolog 1 [Source:VGNC Symbol;Acc:VGNC:95923]	1.8	0.0188
ssc-miR-425-5p	EPN2	epsin 2 [Source:VGNC Symbol;Acc:VGNC:97982]	1.8	0.0188
ssc-miR-425-5p	ERP44	endoplasmic reticulum protein 44 [Source:VGNC Symbol;Acc:VGNC:87783]	1.8	0.0188
ssc-miR-425-5p	FAM133B	family with sequence similarity 133 member B [Source:HGNC Symbol;Acc:HGNC:28629]	1.8	0.0188
ssc-miR-425-5p	FAM135B	family with sequence similarity 135 member B [Source:VGNC Symbol;Acc:VGNC:87905]	1.8	0.0188
ssc-miR-425-5p	FAM63B	hypothetical gene	1.8	0.0188
ssc-miR-425-5p	FBN1	fibrillin 1 [Source:VGNC Symbol;Acc:VGNC:103090]	1.8	0.0188
ssc-miR-425-5p	FBXL17	F-box and leucine rich repeat protein 17 [Source:VGNC Symbol;Acc:VGNC:99650]	1.8	0.0188
ssc-miR-425-5p	FGF10	fibroblast growth factor 10 [Source:VGNC Symbol;Acc:VGNC:88101]	1.8	0.0188
ssc-miR-425-5p	FGF9	fibroblast growth factor 9 [Source:VGNC Symbol;Acc:VGNC:103943]	1.8	0.0188
ssc-miR-425-5p	FLJ00104	hypothetical gene	1.8	0.0188
ssc-miR-425-5p	FOXD1	forkhead box D1 [Source:VGNC Symbol;Acc:VGNC:88199]	1.8	0.0188
ssc-miR-425-5p	FOXD3	forkhead box D3 [Source:VGNC Symbol;Acc:VGNC:88201]	1.8	0.0188
ssc-miR-425-5p	FOXJ3	forkhead box J3 [Source:VGNC Symbol;Acc:VGNC:88213]	1.8	0.0188
ssc-miR-425-5p	FRK	fyn related Src family tyrosine kinase [Source:VGNC Symbol;Acc:VGNC:88235]	1.8	0.0188
ssc-miR-425-5p	FSCN1	fascin actin-bundling protein 1 [Source:VGNC Symbol;Acc:VGNC:88248]	1.8	0.0188
ssc-miR-425-5p	FST	follistatin [Source:NCBI gene (formerly Entrezgene);Acc:445002]	1.8	0.0188
ssc-miR-425-5p	FZD4	frizzled class receptor 4 [Source:VGNC Symbol;Acc:VGNC:88282]	1.8	0.0188
ssc-miR-425-5p	GABRB3	gamma-aminobutyric acid type A receptor subunit beta3 [Source:VGNC Symbol;Acc:VGNC:88308]	1.8	0.0188
ssc-miR-425-5p	GIT1	GIT ArfGAP 1 [Source:VGNC Symbol;Acc:VGNC:88457]	1.8	0.0188
ssc-miR-425-5p	GJA3	gap junction protein alpha 3 [Source:HGNC Symbol;Acc:HGNC:4277]	1.8	0.0188
ssc-miR-425-5p	GLIS3	GLIS family zinc finger 3 [Source:VGNC Symbol;Acc:VGNC:88485]	1.8	0.0188
ssc-miR-425-5p	GNS	glucosamine (N-acetyl)-6-sulfatase [Source:VGNC Symbol;Acc:VGNC:103284]	1.8	0.0188
ssc-miR-425-5p	GOLGA4	hypothetical gene	1.8	0.0188
ssc-miR-425-5p	GPATCH2L	G-patch domain containing 2 like [Source:VGNC Symbol;Acc:VGNC:88575]	1.8	0.0188
ssc-miR-425-5p	GRIN2A	glutamate ionotropic receptor NMDA type subunit 2A [Source:VGNC Symbol;Acc:VGNC:88683]	1.8	0.0188
ssc-miR-425-5p	GRM1	glutamate metabotropic receptor 1 [Source:VGNC Symbol;Acc:VGNC:88700]	1.8	0.0188
ssc-miR-425-5p	GTDC1	glycosyltransferase like domain containing 1 [Source:VGNC Symbol;Acc:VGNC:95955]	1.8	0.0188
ssc-miR-425-5p	HDLBP	high density lipoprotein binding protein [Source:HGNC Symbol;Acc:HGNC:4857]	1.8	0.0188
ssc-miR-425-5p	HECTD1	HECT domain E3 ubiquitin protein ligase 1 [Source:VGNC Symbol;Acc:VGNC:88832]	1.8	0.0188
ssc-miR-425-5p	HIC2	HIC ZBTB transcriptional repressor 2 [Source:HGNC Symbol;Acc:HGNC:18595]	1.8	0.0188
ssc-miR-425-5p	HIPK3	homeodomain interacting protein kinase 3 [Source:VGNC Symbol;Acc:VGNC:88889]	1.8	0.0188
ssc-miR-425-5p	HNRNPA3	hypothetical gene	1.8	0.0188
ssc-miR-425-5p	HNRNPAB	hypothetical gene	1.8	0.0188
ssc-miR-425-5p	HNRNPD	heterous nuclear ribonucleoprotein D [Source:VGNC Symbol;Acc:VGNC:88919]	1.8	0.0188
ssc-miR-425-5p	HNRNPUL2	heterous nuclear ribonucleoprotein U like 2 [Source:VGNC Symbol;Acc:VGNC:99758]	1.8	0.0188
ssc-miR-425-5p	HOXC5	homeobox C5 [Source:VGNC Symbol;Acc:VGNC:103288]	1.8	0.0188
ssc-miR-425-5p	HOXD9	homeobox D9 [Source:VGNC Symbol;Acc:VGNC:96357]	1.8	0.0188
ssc-miR-425-5p	HSPB8	heat shock protein family B (small) member 8 [Source:VGNC Symbol;Acc:VGNC:96685]	1.8	0.0188
ssc-miR-425-5p	HSPH1	heat shock protein family H (Hsp110) member 1 [Source:NCBI gene (formerly Entrezgene);Acc:100048931]	1.8	0.0188
ssc-miR-425-5p	ID1	inhibitor of DNA binding 1, HLH protein [Source:VGNC Symbol;Acc:VGNC:96366]	1.8	0.0188
ssc-miR-425-5p	IFFO2	intermediate filament family orphan 2 [Source:VGNC Symbol;Acc:VGNC:98475]	1.8	0.0188
ssc-miR-425-5p	IGF1	insulin like growth factor 1 [Source:VGNC Symbol;Acc:VGNC:98044]	1.8	0.0188
ssc-miR-425-5p	IL1RAPL1	interleukin 1 receptor accessory protein like 1 [Source:VGNC Symbol;Acc:VGNC:103967]	1.8	0.0188
ssc-miR-425-5p	IMPAD1	hypothetical gene	1.8	0.0188

ssc-miR-425-5p	INTS8	integrator complex subunit 8 [Source:VGNC Symbol;Acc:VGNC:89168]	1.8	0.0188
ssc-miR-425-5p	ITM2B	integral membrane protein 2B [Source:VGNC Symbol;Acc:VGNC:103971]	1.8	0.0188
ssc-miR-425-5p	ITPKB	hypothetical gene	1.8	0.0188
ssc-miR-425-5p	JPH1	junctophilin 1 [Source:VGNC Symbol;Acc:VGNC:89291]	1.8	0.0188
ssc-miR-425-5p	KAT6B	lysine acetyltransferase 6B [Source:VGNC Symbol;Acc:VGNC:89306]	1.8	0.0188
ssc-miR-425-5p	KCNC3	potassium voltage-gated channel subfamily C member 3 [Source:VGNC Symbol;Acc:VGNC:89334]	1.8	0.0188
ssc-miR-425-5p	KCNJ15	potassium inwardly rectifying channel subfamily J member 15 [Source:VGNC Symbol;Acc:VGNC:89355]	1.8	0.0188
ssc-miR-425-5p	KIAA1244	hypothetical gene	1.8	0.0188
ssc-miR-425-5p	KIAA2022	hypothetical gene	1.8	0.0188
ssc-miR-425-5p	KIF21B	kinesin family member 21B [Source:VGNC Symbol;Acc:VGNC:96219]	1.8	0.0188
ssc-miR-425-5p	KLF3	Kruppel like factor 3 [Source:VGNC Symbol;Acc:VGNC:89498]	1.8	0.0188
ssc-miR-425-5p	KPNA6	karyopherin subunit alpha 6 [Source:VGNC Symbol;Acc:VGNC:89565]	1.8	0.0188
ssc-miR-425-5p	LARP4B	hypothetical gene	1.8	0.0188
ssc-miR-425-5p	LCOR	ligand dependent nuclear receptor corepressor [Source:HGNC Symbol;Acc:HGNC:29503]	1.8	0.0188
ssc-miR-425-5p	LDHA	hypothetical gene	1.8	0.0188
ssc-miR-425-5p	LGSN	lengsin, lens protein with glutamine synthetase domain [Source:HGNC Symbol;Acc:HGNC:21016]	1.8	0.0188
ssc-miR-425-5p	LHX9	LIM homeobox 9 [Source:VGNC Symbol;Acc:VGNC:95608]	1.8	0.0188
ssc-miR-425-5p	LIF	LIF interleukin 6 family cytokine [Source:VGNC Symbol;Acc:VGNC:89719]	1.8	0.0188
ssc-miR-425-5p	LIN7C	lin-7 homolog C, crumbs cell polarity complex component [Source:VGNC Symbol;Acc:VGNC:89732]	1.8	0.0188
ssc-miR-425-5p	LOXL2	lysyl oxidase like 2 [Source:VGNC Symbol;Acc:VGNC:89781]	1.8	0.0188
ssc-miR-425-5p	LPP	LIM domain containing preferred translocation partner in lipoma [Source:HGNC Symbol;Acc:HGNC:6679]	1.8	0.0188
ssc-miR-425-5p	LRG1	hypothetical gene	1.8	0.0188
ssc-miR-425-5p	LRRC40	hypothetical gene	1.8	0.0188
ssc-miR-425-5p	MAML1	mastermind like transcriptional coactivator 1 [Source:VGNC Symbol;Acc:VGNC:89963]	1.8	0.0188
ssc-miR-425-5p	MAP2K6	mitogen-activated protein kinase kinase 6 [Source:VGNC Symbol;Acc:VGNC:98102]	1.8	0.0188
ssc-miR-425-5p	MAP3K5	mitogen-activated protein kinase kinase kinase 5 [Source:VGNC Symbol;Acc:VGNC:89985]	1.8	0.0188
ssc-miR-425-5p	MAPK6	mitogen-activated protein kinase 6 [Source:VGNC Symbol;Acc:VGNC:103123]	1.8	0.0188
ssc-miR-425-5p	MARC1	hypothetical gene	1.8	0.0188
ssc-miR-425-5p	MATR3	matrin 3 [Source:HGNC Symbol;Acc:HGNC:6912]	1.8	0.0188
ssc-miR-425-5p	MBNL1	muscleblind like splicing regulator 1 [Source:VGNC Symbol;Acc:VGNC:90054]	1.8	0.0188
ssc-miR-425-5p	MEF2C	myocyte enhancer factor 2C [Source:VGNC Symbol;Acc:VGNC:90127]	1.8	0.0188
ssc-miR-425-5p	MEGF9	multiple EGF like domains 9 [Source:VGNC Symbol;Acc:VGNC:90130]	1.8	0.0188
ssc-miR-425-5p	MEIS1	Meis homeobox 1 [Source:VGNC Symbol;Acc:VGNC:90135]	1.8	0.0188
ssc-miR-425-5p	MIEN1	migration and invasion enhancer 1 [Source:VGNC Symbol;Acc:VGNC:90218]	1.8	0.0188
ssc-miR-425-5p	MINA	hypothetical gene	1.8	0.0188
ssc-miR-425-5p	MLLT3	MLLT3 super elongation complex subunit [Source:VGNC Symbol;Acc:VGNC:90256]	1.8	0.0188
ssc-miR-425-5p	MR1	major histocompatibility complex, class I-related [Source:VGNC Symbol;Acc:VGNC:90338]	1.8	0.0188
ssc-miR-425-5p	MRC2	mannose receptor C type 2 [Source:VGNC Symbol;Acc:VGNC:90341]	1.8	0.0188
ssc-miR-425-5p	MSL2	MSL complex subunit 2 [Source:VGNC Symbol;Acc:VGNC:90424]	1.8	0.0188
ssc-miR-425-5p	N4BP2L1	NEDD4 binding protein 2 like 1 [Source:HGNC Symbol;Acc:HGNC:25037]	1.8	0.0188
ssc-miR-425-5p	NCAM1	neural cell adhesion molecule 1 [Source:VGNC Symbol;Acc:VGNC:108603]	1.8	0.0188
ssc-miR-425-5p	NEK10	NIMA related kinase 10 [Source:VGNC Symbol;Acc:VGNC:90674]	1.8	0.0188
ssc-miR-425-5p	NETO1	neuropilin and tolloid like 1 [Source:VGNC Symbol;Acc:VGNC:90690]	1.8	0.0188
ssc-miR-425-5p	NOP9	NOP9 nucleolar protein [Source:VGNC Symbol;Acc:VGNC:90822]	1.8	0.0188
ssc-miR-425-5p	NOS1AP	hypothetical gene	1.8	0.0188
ssc-miR-425-5p	NRARP	NOTCH regulated ankyrin repeat protein [Source:VGNC Symbol;Acc:VGNC:90889]	1.8	0.0188
ssc-miR-425-5p	NT5C1B	5'-nucleotidase, cytosolic IB [Source:HGNC Symbol;Acc:HGNC:17818]	1.8	0.0188
ssc-miR-425-5p	NT5C1B-RDH14	hypothetical gene	1.8	0.0188
ssc-miR-425-5p	NUFIP2	nuclear FMR1 interacting protein 2 [Source:VGNC Symbol;Acc:VGNC:90967]	1.8	0.0188

ssc-miR-425-5p	NUP62CL	nucleoporin 62 C-terminal like [Source:VGNC Symbol;Acc:VGNC:104013]	1.8	0.0188
ssc-miR-425-5p	PABPN1	poly(A) binding protein nuclear 1 [Source:NCBI gene (formerly Entrezgene);Acc:100155260]	1.8	0.0188
ssc-miR-425-5p	PAK2	p21 (RAC1) activated kinase 2 [Source:HGNC Symbol;Acc:HGNC:8591]	1.8	0.0188
ssc-miR-425-5p	PAK3	p21 (RAC1) activated kinase 3 [Source:HGNC Symbol;Acc:HGNC:8592]	1.8	0.0188
ssc-miR-425-5p	PAPD5	hypothetical gene	1.8	0.0188
ssc-miR-425-5p	PCMT1	protein-L-isoaspartate (D-aspartate) O-methyltransferase [Source:VGNC Symbol;Acc:VGNC:103146]	1.8	0.0188
ssc-miR-425-5p	PDCD10	programmed cell death 10 [Source:VGNC Symbol;Acc:VGNC:91241]	1.8	0.0188
ssc-miR-425-5p	PDE3B	phosphodiesterase 3B [Source:VGNC Symbol;Acc:VGNC:91253]	1.8	0.0188
ssc-miR-425-5p	PER2	period circadian regulator 2 [Source:VGNC Symbol;Acc:VGNC:96478]	1.8	0.0188
ssc-miR-425-5p	PEX13	hypothetical gene	1.8	0.0188
ssc-miR-425-5p	PGK1	phosphoglycerate kinase 1 [Source:VGNC Symbol;Acc:VGNC:104025]	1.8	0.0188
ssc-miR-425-5p	PGPEP1	pyroglutamyl-peptidase I [Source:VGNC Symbol;Acc:VGNC:91360]	1.8	0.0188
ssc-miR-425-5p	PGR	progesterone receptor [Source:VGNC Symbol;Acc:VGNC:91362]	1.8	0.0188
ssc-miR-425-5p	PHF12	PHD finger protein 12 [Source:VGNC Symbol;Acc:VGNC:91378]	1.8	0.0188
ssc-miR-425-5p	PHF21A	PHD finger protein 21A [Source:VGNC Symbol;Acc:VGNC:91384]	1.8	0.0188
ssc-miR-425-5p	PHIP	pleckstrin homology domain interacting protein [Source:VGNC Symbol;Acc:VGNC:91392]	1.8	0.0188
ssc-miR-425-5p	PITPNC1	phosphatidylinositol transfer protein cytoplasmic 1 [Source:NCBI gene (formerly Entrezgene);Acc:100233199]	1.8	0.0188
ssc-miR-425-5p	PKP4	plakophilin 4 [Source:VGNC Symbol;Acc:VGNC:96488]	1.8	0.0188
ssc-miR-425-5p	PLN	phospholamban [Source:NCBI gene (formerly Entrezgene);Acc:397421]	1.8	0.0188
ssc-miR-425-5p	POMT2	protein O-mannosyltransferase 2 [Source:VGNC Symbol;Acc:VGNC:91660]	1.8	0.0188
ssc-miR-425-5p	POU2F2	POU class 2 homeobox 2 [Source:VGNC Symbol;Acc:VGNC:91673]	1.8	0.0188
ssc-miR-425-5p	PPM1F	protein phosphatase, Mg2+/Mn2+ dependent 1F [Source:VGNC Symbol;Acc:VGNC:91706]	1.8	0.0188
ssc-miR-425-5p	PPP2CB	protein phosphatase 2 catalytic subunit beta [Source:VGNC Symbol;Acc:VGNC:96501]	1.8	0.0188
ssc-miR-425-5p	PPP3CA	protein phosphatase 3 catalytic subunit alpha [Source:VGNC Symbol;Acc:VGNC:98218]	1.8	0.0188
ssc-miR-425-5p	PRDM8	PR/SET domain 8 [Source:VGNC Symbol;Acc:VGNC:91781]	1.8	0.0188
ssc-miR-425-5p	PSMD12	proteasome 26S subunit, non-ATPase 12 [Source:VGNC Symbol;Acc:VGNC:91920]	1.8	0.0188
ssc-miR-425-5p	PTCH1	patched 1 [Source:VGNC Symbol;Acc:VGNC:96513]	1.8	0.0188
ssc-miR-425-5p	PTEN	hypothetical gene	1.8	0.0188
ssc-miR-425-5p	PTPRN2	protein tyrosine phosphatase receptor type N2 [Source:VGNC Symbol;Acc:VGNC:91993]	1.8	0.0188
ssc-miR-425-5p	RAB2B	RAB2B, member RAS onco family [Source:VGNC Symbol;Acc:VGNC:98253]	1.8	0.0188
ssc-miR-425-5p	RAB3C	RAB3C, member RAS onco family [Source:VGNC Symbol;Acc:VGNC:104043]	1.8	0.0188
ssc-miR-425-5p	RALGAPA2	Ral GTPase activating protein catalytic subunit alpha 2 [Source:VGNC Symbol;Acc:VGNC:95454]	1.8	0.0188
ssc-miR-425-5p	RANBP3	RAN binding protein 3 [Source:VGNC Symbol;Acc:VGNC:92079]	1.8	0.0188
ssc-miR-425-5p	RBM48	RNA binding motif protein 48 [Source:VGNC Symbol;Acc:VGNC:92159]	1.8	0.0188
ssc-miR-425-5p	RBM7	RNA binding motif protein 7 [Source:VGNC Symbol;Acc:VGNC:108612]	1.8	0.0188
ssc-miR-425-5p	RCOR1	REST corepressor 1 [Source:VGNC Symbol;Acc:VGNC:92183]	1.8	0.0188
ssc-miR-425-5p	RD3	RD3 regulator of GUCY2D [Source:VGNC Symbol;Acc:VGNC:92188]	1.8	0.0188
ssc-miR-425-5p	RGMB	repulsive guidance molecule BMP co-receptor b [Source:VGNC Symbol;Acc:VGNC:92255]	1.8	0.0188
ssc-miR-425-5p	RGS17	regulator of G protein signaling 17 [Source:VGNC Symbol;Acc:VGNC:92263]	1.8	0.0188
ssc-miR-425-5p	RHNO1	RAD9-HUS1-RAD1 interacting nuclear orphan 1 [Source:VGNC Symbol;Acc:VGNC:92282]	1.8	0.0188
ssc-miR-425-5p	RLIM	ring finger protein, LIM domain interacting [Source:HGNC Symbol;Acc:HGNC:13429]	1.8	0.0188
ssc-miR-425-5p	RNF11	ring finger protein 11 [Source:VGNC Symbol;Acc:VGNC:98601]	1.8	0.0188
ssc-miR-425-5p	RNF14	ring finger protein 14 [Source:VGNC Symbol;Acc:VGNC:92357]	1.8	0.0188
ssc-miR-425-5p	RNF145	ring finger protein 145 [Source:VGNC Symbol;Acc:VGNC:92360]	1.8	0.0188
ssc-miR-425-5p	RNF150	ring finger protein 150 [Source:VGNC Symbol;Acc:VGNC:98293]	1.8	0.0188
ssc-miR-425-5p	RNF219	hypothetical gene	1.8	0.0188
ssc-miR-425-5p	RNF222	ring finger protein 222 [Source:VGNC Symbol;Acc:VGNC:92384]	1.8	0.0188
ssc-miR-425-5p	RP11-758M4.1	hypothetical gene	1.8	0.0188
ssc-miR-425-5p	RP11-834C11.12	hypothetical gene	1.8	0.0188

ssc-miR-425-5p	RPP14	hypothetical gene	1.8	0.0188
ssc-miR-425-5p	RSF1	remodeling and spacing factor 1 [Source:VGNC Symbol;Acc:VGNC:92476]	1.8	0.0188
ssc-miR-425-5p	RUNX1T1	RUNX1 partner transcriptional co-repressor 1 [Source:VGNC Symbol;Acc:VGNC:96594]	1.8	0.0188
ssc-miR-425-5p	RYR2	ryanodine receptor 2 [Source:VGNC Symbol;Acc:VGNC:92534]	1.8	0.0188
ssc-miR-425-5p	S1PR1	sphingosine-1-phosphate receptor 1 [Source:VGNC Symbol;Acc:VGNC:92551]	1.8	0.0188
ssc-miR-425-5p	SAMD4A	sterile alpha motif domain containing 4A [Source:VGNC Symbol;Acc:VGNC:92569]	1.8	0.0188
ssc-miR-425-5p	SCAMP1	secretory carrier membrane protein 1 [Source:VGNC Symbol;Acc:VGNC:92605]	1.8	0.0188
ssc-miR-425-5p	SDK2	hypothetical gene	1.8	0.0188
ssc-miR-425-5p	SENP1	SUMO specific peptidase 1 [Source:VGNC Symbol;Acc:VGNC:92712]	1.8	0.0188
ssc-miR-425-5p	SENP6	SUMO specific peptidase 6 [Source:VGNC Symbol;Acc:VGNC:92715]	1.8	0.0188
ssc-miR-425-5p	SERP1	stress associated endoplasmic reticulum protein 1 [Source:NCBI gene (formerly Entrezgene);Acc:100156392]	1.8	0.0188
ssc-miR-425-5p	SERPINE1	serpin family E member 1 [Source:VGNC Symbol;Acc:VGNC:98310]	1.8	0.0188
ssc-miR-425-5p	SH3RF1	SH3 domain containing ring finger 1 [Source:VGNC Symbol;Acc:VGNC:98316]	1.8	0.0188
ssc-miR-425-5p	SIAH2	siah E3 ubiquitin protein ligase 2 [Source:VGNC Symbol;Acc:VGNC:92866]	1.8	0.0188
ssc-miR-425-5p	SLC16A1	hypothetical gene	1.8	0.0188
ssc-miR-425-5p	SLC16A3	solute carrier family 16 member 3 [Source:VGNC Symbol;Acc:VGNC:92943]	1.8	0.0188
ssc-miR-425-5p	SLC25A16	solute carrier family 25 member 16 [Source:VGNC Symbol;Acc:VGNC:92995]	1.8	0.0188
ssc-miR-425-5p	SLC35F1	solute carrier family 35 member F1 [Source:VGNC Symbol;Acc:VGNC:93081]	1.8	0.0188
ssc-miR-425-5p	SLC6A1	solute carrier family 6 member 1 [Source:VGNC Symbol;Acc:VGNC:93153]	1.8	0.0188
ssc-miR-425-5p	SLC7A6	hypothetical gene	1.8	0.0188
ssc-miR-425-5p	SLC8A1	solute carrier family 8 member A1 [Source:VGNC Symbol;Acc:VGNC:93178]	1.8	0.0188
ssc-miR-425-5p	SLIT3	slit guidance ligand 3 [Source:VGNC Symbol;Acc:VGNC:93205]	1.8	0.0188
ssc-miR-425-5p	SMAD2	SMAD family member 2 [Source:VGNC Symbol;Acc:VGNC:98329]	1.8	0.0188
ssc-miR-425-5p	SMAD5	SMAD family member 5 [Source:VGNC Symbol;Acc:VGNC:93219]	1.8	0.0188
ssc-miR-425-5p	SMARCA5	SWI/SNF related, matrix associated, actin dependent regulator of chromatin, subfamily a, member 5 [Source:VGNC Symbol;Acc:VGNC:93228]	1.8	0.0188
ssc-miR-425-5p	SMEK1	hypothetical gene	1.8	0.0188
ssc-miR-425-5p	SNAP25	synaptosome associated protein 25 [Source:VGNC Symbol;Acc:VGNC:95509]	1.8	0.0188
ssc-miR-425-5p	SNX12	sorting nexin 12 [Source:VGNC Symbol;Acc:VGNC:93305]	1.8	0.0188
ssc-miR-425-5p	SOGA1	suppressor of glucose, autophagy associated 1 [Source:VGNC Symbol;Acc:VGNC:95740]	1.8	0.0188
ssc-miR-425-5p	SPATS2L	spermatosis associated serine rich 2 like [Source:VGNC Symbol;Acc:VGNC:96047]	1.8	0.0188
ssc-miR-425-5p	SPRY3	sprouty RTK signaling antagonist 3 [Source:VGNC Symbol;Acc:VGNC:93426]	1.8	0.0188
ssc-miR-425-5p	SRSF1	serine and arginine rich splicing factor 1 [Source:VGNC Symbol;Acc:VGNC:99084]	1.8	0.0188
ssc-miR-425-5p	SRSF11	serine and arginine rich splicing factor 11 [Source:VGNC Symbol;Acc:VGNC:93473]	1.8	0.0188
ssc-miR-425-5p	SSX2IP	SSX family member 2 interacting protein [Source:VGNC Symbol;Acc:VGNC:93499]	1.8	0.0188
ssc-miR-425-5p	ST6GALNAC5	ST6 N-acetylgalactosaminide alpha-2,6-sialyltransferase 5 [Source:VGNC Symbol;Acc:VGNC:93513]	1.8	0.0188
ssc-miR-425-5p	STIM2	stromal interaction molecule 2 [Source:VGNC Symbol;Acc:VGNC:98967]	1.8	0.0188
ssc-miR-425-5p	STK24	serine/threonine kinase 24 [Source:VGNC Symbol;Acc:VGNC:98344]	1.8	0.0188
ssc-miR-425-5p	STK39	serine/threonine kinase 39 [Source:VGNC Symbol;Acc:VGNC:98348]	1.8	0.0188
ssc-miR-425-5p	STMN1	stathmin 1 [Source:VGNC Symbol;Acc:VGNC:93560]	1.8	0.0188
ssc-miR-425-5p	STRIP2	striatin interacting protein 2 [Source:VGNC Symbol;Acc:VGNC:93577]	1.8	0.0188
ssc-miR-425-5p	STX2	syntaxin 2 [Source:VGNC Symbol;Acc:VGNC:93590]	1.8	0.0188
ssc-miR-425-5p	SUV420H1	hypothetical gene	1.8	0.0188
ssc-miR-425-5p	SYNCRIP	synaptotagmin binding cytoplasmic RNA interacting protein [Source:VGNC Symbol;Acc:VGNC:103185]	1.8	0.0188
ssc-miR-425-5p	SYNPO	synaptopodin [Source:VGNC Symbol;Acc:VGNC:93671]	1.8	0.0188
ssc-miR-425-5p	TANC2	tetratricopeptide repeat, ankyrin repeat and coiled-coil containing 2 [Source:VGNC Symbol;Acc:VGNC:93733]	1.8	0.0188
ssc-miR-425-5p	TAOK1	TAO kinase 1 [Source:VGNC Symbol;Acc:VGNC:98355]	1.8	0.0188
ssc-miR-425-5p	TET2	hypothetical gene	1.8	0.0188
ssc-miR-425-5p	TFIP11	tuftelin interacting protein 11 [Source:NCBI gene (formerly Entrezgene);Acc:768099]	1.8	0.0188
ssc-miR-425-5p	TGFB1	transforming growth factor beta 1 [Source:VGNC Symbol;Acc:VGNC:98656]	1.8	0.0188

ssc-miR-425-5p	TGFBR2	transforming growth factor beta receptor 2 [Source:VGNC Symbol;Acc:VGNC:93931]	1.8	0.0188
ssc-miR-425-5p	THRB	thyroid hormone receptor beta [Source:VGNC Symbol;Acc:VGNC:93965]	1.8	0.0188
ssc-miR-425-5p	TIMM10	translocase of inner mitochondrial membrane 10 [Source:HGNC Symbol;Acc:HGNC:11814]	1.8	0.0188
ssc-miR-425-5p	TM9SF3	transmembrane 9 superfamily member 3 [Source:VGNC Symbol;Acc:VGNC:94037]	1.8	0.0188
ssc-miR-425-5p	TMED8	transmembrane p24 trafficking protein family member 8 [Source:VGNC Symbol;Acc:VGNC:94058]	1.8	0.0188
ssc-miR-425-5p	TMEM120B	transmembrane protein 120B [Source:VGNC Symbol;Acc:VGNC:94071]	1.8	0.0188
ssc-miR-425-5p	TMEM127	transmembrane protein 127 [Source:HGNC Symbol;Acc:HGNC:26038]	1.8	0.0188
ssc-miR-425-5p	TMEM170B	transmembrane protein 170B [Source:VGNC Symbol;Acc:VGNC:94108]	1.8	0.0188
ssc-miR-425-5p	TMEM237	transmembrane protein 237 [Source:VGNC Symbol;Acc:VGNC:95613]	1.8	0.0188
ssc-miR-425-5p	TMEM260	transmembrane protein 260 [Source:VGNC Symbol;Acc:VGNC:94164]	1.8	0.0188
ssc-miR-425-5p	TNC	tenascin C [Source:VGNC Symbol;Acc:VGNC:103192]	1.8	0.0188
ssc-miR-425-5p	TNFSF13B	TNF superfamily member 13b [Source:VGNC Symbol;Acc:VGNC:94269]	1.8	0.0188
ssc-miR-425-5p	TOMM22	translocase of outer mitochondrial membrane 22 [Source:HGNC Symbol;Acc:HGNC:18002]	1.8	0.0188
ssc-miR-425-5p	TXNL1	thioredoxin like 1 [Source:VGNC Symbol;Acc:VGNC:94611]	1.8	0.0188
ssc-miR-425-5p	UBE2N	hypothetical gene	1.8	0.0188
ssc-miR-425-5p	UBQLN1	ubiquilin 1 [Source:VGNC Symbol;Acc:VGNC:95560]	1.8	0.0188
ssc-miR-425-5p	UBQLN2	ubiquilin 2 [Source:VGNC Symbol;Acc:VGNC:94665]	1.8	0.0188
ssc-miR-425-5p	USP1	ubiquitin specific peptidase 1 [Source:VGNC Symbol;Acc:VGNC:94742]	1.8	0.0188
ssc-miR-425-5p	USP22	ubiquitin specific peptidase 22 [Source:HGNC Symbol;Acc:HGNC:12621]	1.8	0.0188
ssc-miR-425-5p	USP8	ubiquitin specific peptidase 8 [Source:VGNC Symbol;Acc:VGNC:94778]	1.8	0.0188
ssc-miR-425-5p	UXS1	UDP-glucuronate decarboxylase 1 [Source:VGNC Symbol;Acc:VGNC:94791]	1.8	0.0188
ssc-miR-425-5p	VEZF1	vascular endothelial zinc finger 1 [Source:VGNC Symbol;Acc:VGNC:94818]	1.8	0.0188
ssc-miR-425-5p	WASL	WASP like actin nucleation promoting factor [Source:VGNC Symbol;Acc:VGNC:96604]	1.8	0.0188
ssc-miR-425-5p	WDR13	WD repeat domain 13 [Source:VGNC Symbol;Acc:VGNC:94907]	1.8	0.0188
ssc-miR-425-5p	WDR41	hypothetical gene	1.8	0.0188
ssc-miR-425-5p	WRNIP1	WRN helicase interacting protein 1 [Source:VGNC Symbol;Acc:VGNC:104096]	1.8	0.0188
ssc-miR-425-5p	WTAP	WT1 associated protein [Source:VGNC Symbol;Acc:VGNC:94984]	1.8	0.0188
ssc-miR-425-5p	WWC3	WWC family member 3 [Source:VGNC Symbol;Acc:VGNC:94987]	1.8	0.0188
ssc-miR-425-5p	XPO4	exportin 4 [Source:VGNC Symbol;Acc:VGNC:95004]	1.8	0.0188
ssc-miR-425-5p	XYLT1	xylosyltransferase 1 [Source:VGNC Symbol;Acc:VGNC:95015]	1.8	0.0188
ssc-miR-425-5p	YPEL2	yippee like 2 [Source:VGNC Symbol;Acc:VGNC:95036]	1.8	0.0188
ssc-miR-425-5p	ZBTB34	zinc finger and BTB domain containing 34 [Source:VGNC Symbol;Acc:VGNC:95070]	1.8	0.0188
ssc-miR-425-5p	ZBTB43	zinc finger and BTB domain containing 43 [Source:VGNC Symbol;Acc:VGNC:95075]	1.8	0.0188
ssc-miR-425-5p	ZC3H6	zinc finger CCCH-type containing 6 [Source:VGNC Symbol;Acc:VGNC:95098]	1.8	0.0188
ssc-miR-425-5p	ZFHX3	zinc finger homeobox 3 [Source:VGNC Symbol;Acc:VGNC:95141]	1.8	0.0188
ssc-miR-425-5p	ZFHX4	zinc finger homeobox 4 [Source:VGNC Symbol;Acc:VGNC:95142]	1.8	0.0188
ssc-miR-425-5p	ZNF148	zinc finger protein 148 [Source:VGNC Symbol;Acc:VGNC:95192]	1.8	0.0188
ssc-miR-425-5p	ZNF24	zinc finger protein 24 [Source:VGNC Symbol;Acc:VGNC:98422]	1.8	0.0188
ssc-miR-425-5p	ZNF423	zinc finger protein 423 [Source:VGNC Symbol;Acc:VGNC:98752]	1.8	0.0188
ssc-miR-425-5p	ZNF449	zinc finger protein 449 [Source:VGNC Symbol;Acc:VGNC:95242]	1.8	0.0188
ssc-miR-425-5p	ZNF695	hypothetical gene	1.8	0.0188
ssc-miR-425-5p	ZNF831	zinc finger protein 831 [Source:VGNC Symbol;Acc:VGNC:95765]	1.8	0.0188
ssc-miR-425-5p	ZSCAN29	zinc finger and SCAN domain containing 29 [Source:HGNC Symbol;Acc:HGNC:26673]	1.8	0.0188
ssc-miR-628	NA	hypothetical gene	1.73	0.0598
ssc-miR-425-3p	NA	hypothetical gene	2.15	0.0073
ssc-miR-22-3p	A4GNT	alpha-1,4-N-acetylglucosaminyltransferase [Source:VGNC Symbol;Acc:VGNC:84935]	2.1	0.0644
ssc-miR-22-3p	ABCG8	ATP binding cassette subfamily G member 8 [Source:VGNC Symbol;Acc:VGNC:96913]	2.1	0.0644
ssc-miR-22-3p	ABHD15	abhydrolase domain containing 15 [Source:VGNC Symbol;Acc:VGNC:84974]	2.1	0.0644
ssc-miR-22-3p	AC006486.1	hypothetical gene	2.1	0.0644

ssc-miR-22-3p	AC007375.1	hypothetical gene	2.1	0.0644
ssc-miR-22-3p	AC012215.1	hypothetical gene	2.1	0.0644
ssc-miR-22-3p	AC074091.13	hypothetical gene	2.1	0.0644
ssc-miR-22-3p	ACER3	alkaline ceramidase 3 [Source:VGNC Symbol;Acc:VGNC:103893]	2.1	0.0644
ssc-miR-22-3p	ACLY	ATP citrate lyase [Source:VGNC Symbol;Acc:VGNC:85014]	2.1	0.0644
ssc-miR-22-3p	ACVR1B	activin A receptor type 1B [Source:VGNC Symbol;Acc:VGNC:85056]	2.1	0.0644
ssc-miR-22-3p	ACVR2B	activin A receptor type 2B [Source:VGNC Symbol;Acc:VGNC:108629]	2.1	0.0644
ssc-miR-22-3p	ACVRL1	activin A receptor like type 1 [Source:VGNC Symbol;Acc:VGNC:85057]	2.1	0.0644
ssc-miR-22-3p	ADAM11	ADAM metallopeptidase domain 11 [Source:VGNC Symbol;Acc:VGNC:85062]	2.1	0.0644
ssc-miR-22-3p	ADAMTS5	ADAM metallopeptidase with thrombospondin type 1 motif 5 [Source:VGNC Symbol;Acc:VGNC:85085]	2.1	0.0644
ssc-miR-22-3p	ADAT2	adenosine deaminase tRNA specific 2 [Source:VGNC Symbol;Acc:VGNC:85100]	2.1	0.0644
ssc-miR-22-3p	ADNP	activity dependent neuroprotector homeobox [Source:VGNC Symbol;Acc:VGNC:95657]	2.1	0.0644
ssc-miR-22-3p	AGMAT	agmatinase [Source:VGNC Symbol;Acc:VGNC:85181]	2.1	0.0644
ssc-miR-22-3p	AGO1	hypothetical gene	2.1	0.0644
ssc-miR-22-3p	AGPAT6	hypothetical gene	2.1	0.0644
ssc-miR-22-3p	AIDA	axin interactor, dorsalization associated [Source:VGNC Symbol;Acc:VGNC:96103]	2.1	0.0644
ssc-miR-22-3p	AIFM1	apoptosis inducing factor mitochondria associated 1 [Source:VGNC Symbol;Acc:VGNC:85198]	2.1	0.0644
ssc-miR-22-3p	AK2	hypothetical gene	2.1	0.0644
ssc-miR-22-3p	AKT3	AKT serine/threonine kinase 3 [Source:VGNC Symbol;Acc:VGNC:96306]	2.1	0.0644
ssc-miR-22-3p	AL589765.1	hypothetical gene	2.1	0.0644
ssc-miR-22-3p	ALDH5A1	aldehyde dehydrogenase 5 family member A1 [Source:VGNC Symbol;Acc:VGNC:85241]	2.1	0.0644
ssc-miR-22-3p	ALG9	ALG9 alpha-1,2-mannosyltransferase [Source:NCBI gene (formerly Entrezgene);Acc:100519965]	2.1	0.0644
ssc-miR-22-3p	ALX1	ALX homeobox 1 [Source:VGNC Symbol;Acc:VGNC:85271]	2.1	0.0644
ssc-miR-22-3p	AMMECR1L	AMMECR1 like [Source:VGNC Symbol;Acc:VGNC:103895]	2.1	0.0644
ssc-miR-22-3p	AMOT	angiomotin [Source:VGNC Symbol;Acc:VGNC:85283]	2.1	0.0644
ssc-miR-22-3p	ANGEL2	angel homolog 2 [Source:VGNC Symbol;Acc:VGNC:85302]	2.1	0.0644
ssc-miR-22-3p	ANKFY1	ankyrin repeat and FYVE domain containing 1 [Source:VGNC Symbol;Acc:VGNC:85314]	2.1	0.0644
ssc-miR-22-3p	ANKHD1-EIF4EBP3	hypothetical gene	2.1	0.0644
ssc-miR-22-3p	ANKRD13A	ankyrin repeat domain 13A [Source:VGNC Symbol;Acc:VGNC:85324]	2.1	0.0644
ssc-miR-22-3p	ANKRD52	ankyrin repeat domain 52 [Source:VGNC Symbol;Acc:VGNC:85342]	2.1	0.0644
ssc-miR-22-3p	AP1S1	adaptor related protein complex 1 subunit sigma 1 [Source:VGNC Symbol;Acc:VGNC:97032]	2.1	0.0644
ssc-miR-22-3p	APBB2	amyloid beta protein binding family B member 2 [Source:VGNC Symbol;Acc:VGNC:85403]	2.1	0.0644
ssc-miR-22-3p	ARHGAP19-SLIT1	hypothetical gene	2.1	0.0644
ssc-miR-22-3p	ARHGAP26	Rho GTPase activating protein 26 [Source:VGNC Symbol;Acc:VGNC:85465]	2.1	0.0644
ssc-miR-22-3p	ARHGAP27	Rho GTPase activating protein 27 [Source:VGNC Symbol;Acc:VGNC:85466]	2.1	0.0644
ssc-miR-22-3p	ARHGAP9	Rho GTPase activating protein 9 [Source:VGNC Symbol;Acc:VGNC:85482]	2.1	0.0644
ssc-miR-22-3p	ARHGEF12	Rho guanine nucleotide exchange factor 12 [Source:VGNC Symbol;Acc:VGNC:85488]	2.1	0.0644
ssc-miR-22-3p	ARHGEF26	Rho guanine nucleotide exchange factor 26 [Source:VGNC Symbol;Acc:VGNC:85494]	2.1	0.0644
ssc-miR-22-3p	ARMC5	armadillo repeat containing 5 [Source:VGNC Symbol;Acc:VGNC:85529]	2.1	0.0644
ssc-miR-22-3p	ARPC5	actin related protein 2/3 complex subunit 5 [Source:VGNC Symbol;Acc:VGNC:85539]	2.1	0.0644
ssc-miR-22-3p	ARRB1	arrestin beta 1 [Source:VGNC Symbol;Acc:VGNC:85542]	2.1	0.0644
ssc-miR-22-3p	ASB14	ankyrin repeat and SOCS box containing 14 [Source:VGNC Symbol;Acc:VGNC:85562]	2.1	0.0644
ssc-miR-22-3p	ASB6	ankyrin repeat and SOCS box containing 6 [Source:VGNC Symbol;Acc:VGNC:85565]	2.1	0.0644
ssc-miR-22-3p	ASCL1	achaete-scute family bHLH transcription factor 1 [Source:HGNC Symbol;Acc:HGNC:738]	2.1	0.0644
ssc-miR-22-3p	ATG2B	autophagy related 2B [Source:VGNC Symbol;Acc:VGNC:85620]	2.1	0.0644
ssc-miR-22-3p	ATOH1	atonal bHLH transcription factor 1 [Source:VGNC Symbol;Acc:VGNC:85631]	2.1	0.0644
ssc-miR-22-3p	ATP8A1	ATPase phospholipid transporting 8A1 [Source:VGNC Symbol;Acc:VGNC:97894]	2.1	0.0644
ssc-miR-22-3p	ATP8A2	ATPase phospholipid transporting 8A2 [Source:HGNC Symbol;Acc:HGNC:13533]	2.1	0.0644
ssc-miR-22-3p	B3GAT2	beta-1,3-glucuronyltransferase 2 [Source:VGNC Symbol;Acc:VGNC:85720]	2.1	0.0644

ssc-miR-22-3p	B4GALT5	beta-1,4-galactosyltransferase 5 [Source:VGNC Symbol;Acc:VGNC:96499]	2.1	0.0644
ssc-miR-22-3p	BAG5	BAG cochaperone 5 [Source:VGNC Symbol;Acc:VGNC:85746]	2.1	0.0644
ssc-miR-22-3p	BATF3	basic leucine zipper ATF-like transcription factor 3 [Source:VGNC Symbol;Acc:VGNC:85760]	2.1	0.0644
ssc-miR-22-3p	BBS1	Bardet-Biedl syndrome 1 [Source:HGNC Symbol;Acc:HGNC:966]	2.1	0.0644
ssc-miR-22-3p	BCL9	BCL9 transcription coactivator [Source:VGNC Symbol;Acc:VGNC:96567]	2.1	0.0644
ssc-miR-22-3p	BCL9L	BCL9 like [Source:VGNC Symbol;Acc:VGNC:96568]	2.1	0.0644
ssc-miR-22-3p	BDH1	3-hydroxybutyrate dehydrogenase 1 [Source:VGNC Symbol;Acc:VGNC:85792]	2.1	0.0644
ssc-miR-22-3p	BIN1	bridging integrator 1 [Source:VGNC Symbol;Acc:VGNC:95475]	2.1	0.0644
ssc-miR-22-3p	BLOC1S1	biogenesis of lysosomal organelles complex 1 subunit 1 [Source:HGNC Symbol;Acc:HGNC:4200]	2.1	0.0644
ssc-miR-22-3p	BRI3BP	BRI3 binding protein [Source:HGNC Symbol;Acc:HGNC:14251]	2.1	0.0644
ssc-miR-22-3p	BRSK2	BR serine/threonine kinase 2 [Source:VGNC Symbol;Acc:VGNC:85889]	2.1	0.0644
ssc-miR-22-3p	BRWD3	bromodomain and WD repeat domain containing 3 [Source:VGNC Symbol;Acc:VGNC:85890]	2.1	0.0644
ssc-miR-22-3p	BTG1	BTG anti-proliferation factor 1 [Source:VGNC Symbol;Acc:VGNC:103907]	2.1	0.0644
ssc-miR-22-3p	C10orf11	hypothetical gene	2.1	0.0644
ssc-miR-22-3p	C17orf58	chromosome 12 C17orf58 homolog [Source:VGNC Symbol;Acc:VGNC:107125]	2.1	0.0644
ssc-miR-22-3p	C17orf85	hypothetical gene	2.1	0.0644
ssc-miR-22-3p	C17orf96	hypothetical gene	2.1	0.0644
ssc-miR-22-3p	C19orf70	hypothetical gene	2.1	0.0644
ssc-miR-22-3p	C1orf21	hypothetical gene	2.1	0.0644
ssc-miR-22-3p	C1orf95	hypothetical gene	2.1	0.0644
ssc-miR-22-3p	C20orf112	hypothetical gene	2.1	0.0644
ssc-miR-22-3p	C5orf24	chromosome 2 C5orf24 homolog [Source:VGNC Symbol;Acc:VGNC:86010]	2.1	0.0644
ssc-miR-22-3p	C6orf62	chromosome 7 C6orf62 homolog [Source:VGNC Symbol;Acc:VGNC:86074]	2.1	0.0644
ssc-miR-22-3p	C7orf73	hypothetical gene	2.1	0.0644
ssc-miR-22-3p	C8orf46	hypothetical gene	2.1	0.0644
ssc-miR-22-3p	CA7	carbonic anhydrase 7 [Source:VGNC Symbol;Acc:VGNC:86104]	2.1	0.0644
ssc-miR-22-3p	CABP7	calcium binding protein 7 [Source:VGNC Symbol;Acc:VGNC:107372]	2.1	0.0644
ssc-miR-22-3p	CACNG3	calcium voltage-gated channel auxiliary subunit gamma 3 [Source:VGNC Symbol;Acc:VGNC:86126]	2.1	0.0644
ssc-miR-22-3p	CACUL1	CDK2 associated cullin domain 1 [Source:VGNC Symbol;Acc:VGNC:86132]	2.1	0.0644
ssc-miR-22-3p	CADM1	cell adhesion molecule 1 [Source:VGNC Symbol;Acc:VGNC:86134]	2.1	0.0644
ssc-miR-22-3p	CADM3	cell adhesion molecule 3 [Source:VGNC Symbol;Acc:VGNC:86135]	2.1	0.0644
ssc-miR-22-3p	CALCR	calcitonin receptor [Source:VGNC Symbol;Acc:VGNC:86142]	2.1	0.0644
ssc-miR-22-3p	CAMK1G	calcium/calmodulin dependent protein kinase IG [Source:VGNC Symbol;Acc:VGNC:86154]	2.1	0.0644
ssc-miR-22-3p	CAMK2G	calcium/calmodulin dependent protein kinase II gamma [Source:VGNC Symbol;Acc:VGNC:86157]	2.1	0.0644
ssc-miR-22-3p	CAMK2N1	calcium/calmodulin dependent protein kinase II inhibitor 1 [Source:VGNC Symbol;Acc:VGNC:96935]	2.1	0.0644
ssc-miR-22-3p	CAMTA2	calmodulin binding transcription activator 2 [Source:VGNC Symbol;Acc:VGNC:86165]	2.1	0.0644
ssc-miR-22-3p	CAPN1	calpain 1 [Source:VGNC Symbol;Acc:VGNC:86170]	2.1	0.0644
ssc-miR-22-3p	CASP10	caspase 10 [Source:VGNC Symbol;Acc:VGNC:95638]	2.1	0.0644
ssc-miR-22-3p	CATSPERG	cation channel sperm associated auxiliary subunit gamma [Source:VGNC Symbol;Acc:VGNC:86215]	2.1	0.0644
ssc-miR-22-3p	CAV3	caveolin 3 [Source:NCBI gene (formerly Entrezgene);Acc:641358]	2.1	0.0644
ssc-miR-22-3p	CBL	Cbl proto-onco [Source:VGNC Symbol;Acc:VGNC:86222]	2.1	0.0644
ssc-miR-22-3p	CBX6	chromobox 6 [Source:VGNC Symbol;Acc:VGNC:97915]	2.1	0.0644
ssc-miR-22-3p	CCDC104	hypothetical gene	2.1	0.0644
ssc-miR-22-3p	CCDC149	coiled-coil domain containing 149 [Source:VGNC Symbol;Acc:VGNC:86262]	2.1	0.0644
ssc-miR-22-3p	CCDC152	coiled-coil domain containing 152 [Source:VGNC Symbol;Acc:VGNC:86264]	2.1	0.0644
ssc-miR-22-3p	CCDC47	coiled-coil domain containing 47 [Source:VGNC Symbol;Acc:VGNC:97916]	2.1	0.0644
ssc-miR-22-3p	CCDC67	hypothetical gene	2.1	0.0644
ssc-miR-22-3p	CCDC71L	coiled-coil domain containing 71 like [Source:HGNC Symbol;Acc:HGNC:26685]	2.1	0.0644
ssc-miR-22-3p	CCM2L	CCM2 like scaffold protein [Source:VGNC Symbol;Acc:VGNC:95757]	2.1	0.0644

ssc-miR-22-3p	CCNJL	cyclin J like [Source:VGNC Symbol;Acc:VGNC:86361]	2.1	0.0644
ssc-miR-22-3p	CCNT2	cyclin T2 [Source:VGNC Symbol;Acc:VGNC:103916]	2.1	0.0644
ssc-miR-22-3p	CD207	CD207 molecule [Source:VGNC Symbol;Acc:VGNC:86397]	2.1	0.0644
ssc-miR-22-3p	CD72	hypothetical gene	2.1	0.0644
ssc-miR-22-3p	CDC14A	cell division cycle 14A [Source:VGNC Symbol;Acc:VGNC:86442]	2.1	0.0644
ssc-miR-22-3p	CDC25A	cell division cycle 25A [Source:VGNC Symbol;Acc:VGNC:86446]	2.1	0.0644
ssc-miR-22-3p	CDC6	cell division cycle 6 [Source:VGNC Symbol;Acc:VGNC:86464]	2.1	0.0644
ssc-miR-22-3p	CDH4	cadherin 4 [Source:VGNC Symbol;Acc:VGNC:95853]	2.1	0.0644
ssc-miR-22-3p	CDH5	cadherin 5 [Source:VGNC Symbol;Acc:VGNC:86486]	2.1	0.0644
ssc-miR-22-3p	CDK13	cyclin dependent kinase 13 [Source:VGNC Symbol;Acc:VGNC:86497]	2.1	0.0644
ssc-miR-22-3p	CDX2	caudal type homeobox 2 [Source:VGNC Symbol;Acc:VGNC:86527]	2.1	0.0644
ssc-miR-22-3p	CEBPD	CCAAT enhancer binding protein delta [Source:VGNC Symbol;Acc:VGNC:86532]	2.1	0.0644
ssc-miR-22-3p	CECR2	CECR2 histone acetyl-lysine reader [Source:VGNC Symbol;Acc:VGNC:96576]	2.1	0.0644
ssc-miR-22-3p	CELF1	CUGBP Elav-like family member 1 [Source:VGNC Symbol;Acc:VGNC:86537]	2.1	0.0644
ssc-miR-22-3p	CENPB	centromere protein B [Source:HGNC Symbol;Acc:HGNC:1852]	2.1	0.0644
ssc-miR-22-3p	CENPV	hypothetical gene	2.1	0.0644
ssc-miR-22-3p	CERK	ceramide kinase [Source:VGNC Symbol;Acc:VGNC:86586]	2.1	0.0644
ssc-miR-22-3p	CGNL1	cingulin like 1 [Source:VGNC Symbol;Acc:VGNC:86616]	2.1	0.0644
ssc-miR-22-3p	CHD7	chromodomain helicase DNA binding protein 7 [Source:VGNC Symbol;Acc:VGNC:86633]	2.1	0.0644
ssc-miR-22-3p	CHD9	chromodomain helicase DNA binding protein 9 [Source:VGNC Symbol;Acc:VGNC:86635]	2.1	0.0644
ssc-miR-22-3p	CHDH	choline dehydrogenase [Source:VGNC Symbol;Acc:VGNC:86636]	2.1	0.0644
ssc-miR-22-3p	CHKA	choline kinase alpha [Source:VGNC Symbol;Acc:VGNC:86646]	2.1	0.0644
ssc-miR-22-3p	CIITA	class II major histocompatibility complex transactivator [Source:VGNC Symbol;Acc:VGNC:86701]	2.1	0.0644
ssc-miR-22-3p	CISH	cytokine inducible SH2 containing protein [Source:VGNC Symbol;Acc:VGNC:86709]	2.1	0.0644
ssc-miR-22-3p	CLIC4	chloride intracellular channel 4 [Source:VGNC Symbol;Acc:VGNC:86763]	2.1	0.0644
ssc-miR-22-3p	CLIP2	hypothetical gene	2.1	0.0644
ssc-miR-22-3p	CLPB	caseinolytic mitochondrial matrix peptidase chaperone subunit B [Source:VGNC Symbol;Acc:VGNC:86776]	2.1	0.0644
ssc-miR-22-3p	CLVS2	clavesin 2 [Source:VGNC Symbol;Acc:VGNC:86795]	2.1	0.0644
ssc-miR-22-3p	CMTR1	cap methyltransferase 1 [Source:HGNC Symbol;Acc:HGNC:21077]	2.1	0.0644
ssc-miR-22-3p	CNIH4	cornichon family AMPA receptor auxiliary protein 4 [Source:VGNC Symbol;Acc:VGNC:96060]	2.1	0.0644
ssc-miR-22-3p	CNKSR2	connector enhancer of kinase suppressor of Ras 2 [Source:VGNC Symbol;Acc:VGNC:86822]	2.1	0.0644
ssc-miR-22-3p	CNOT1	CCR4-NOT transcription complex subunit 1 [Source:VGNC Symbol;Acc:VGNC:86831]	2.1	0.0644
ssc-miR-22-3p	CNOT6	CCR4-NOT transcription complex subunit 6 [Source:VGNC Symbol;Acc:VGNC:86837]	2.1	0.0644
ssc-miR-22-3p	CNOT6L	CCR4-NOT transcription complex subunit 6 like [Source:VGNC Symbol;Acc:VGNC:86838]	2.1	0.0644
ssc-miR-22-3p	CNP	2',3'-cyclic nucleotide 3' phosphodiesterase [Source:VGNC Symbol;Acc:VGNC:86840]	2.1	0.0644
ssc-miR-22-3p	CNTN2	contactin 2 [Source:VGNC Symbol;Acc:VGNC:108577]	2.1	0.0644
ssc-miR-22-3p	CNTNAP5	contactin associated protein family member 5 [Source:VGNC Symbol;Acc:VGNC:95459]	2.1	0.0644
ssc-miR-22-3p	CNTROB	centrobin, centriole duplication and spindle assembly protein [Source:VGNC Symbol;Acc:VGNC:86850]	2.1	0.0644
ssc-miR-22-3p	COPS7B	COP9 signalosome subunit 7B [Source:HGNC Symbol;Acc:HGNC:16760]	2.1	0.0644
ssc-miR-22-3p	CPEB1	cytoplasmic polyadenylation element binding protein 1 [Source:NCBI gene (formerly Entrezgene);Acc:100048944]	2.1	0.0644
ssc-miR-22-3p	CREB1	cAMP responsive element binding protein 1 [Source:VGNC Symbol;Acc:VGNC:96004]	2.1	0.0644
ssc-miR-22-3p	CSF1R	colony stimulating factor 1 receptor [Source:VGNC Symbol;Acc:VGNC:87031]	2.1	0.0644
ssc-miR-22-3p	CSNK1A1	casein kinase 1 alpha 1 [Source:VGNC Symbol;Acc:VGNC:99630]	2.1	0.0644
ssc-miR-22-3p	CSRNP3	cysteine and serine rich nuclear protein 3 [Source:VGNC Symbol;Acc:VGNC:96249]	2.1	0.0644
ssc-miR-22-3p	CTDSPL2	CTD small phosphatase like 2 [Source:VGNC Symbol;Acc:VGNC:87060]	2.1	0.0644
ssc-miR-22-3p	CTIF	cap binding complex dependent translation initiation factor [Source:VGNC Symbol;Acc:VGNC:87062]	2.1	0.0644
ssc-miR-22-3p	CUL3	cullin 3 [Source:VGNC Symbol;Acc:VGNC:96091]	2.1	0.0644
ssc-miR-22-3p	CYR61	hypothetical gene	2.1	0.0644
ssc-miR-22-3p	CYTH1	cytohesin 1 [Source:VGNC Symbol;Acc:VGNC:87135]	2.1	0.0644

ssc-miR-22-3p	CYTH3	cytohesin 3 [Source:VGNC Symbol;Acc:VGNC:87136]	2.1	0.0644
ssc-miR-22-3p	DAPK2	death associated protein kinase 2 [Source:VGNC Symbol;Acc:VGNC:87157]	2.1	0.0644
ssc-miR-22-3p	DAW1	dynein assembly factor with WD repeats 1 [Source:HGNC Symbol;Acc:HGNC:26383]	2.1	0.0644
ssc-miR-22-3p	DBNDD2	dysbindin domain containing 2 [Source:VGNC Symbol;Acc:VGNC:107134]	2.1	0.0644
ssc-miR-22-3p	DBNL	drebrin like [Source:VGNC Symbol;Acc:VGNC:87166]	2.1	0.0644
ssc-miR-22-3p	DDIT4	DNA damage inducible transcript 4 [Source:VGNC Symbol;Acc:VGNC:87208]	2.1	0.0644
ssc-miR-22-3p	DDRGK1	DDRGK domain containing 1 [Source:VGNC Symbol;Acc:VGNC:103933]	2.1	0.0644
ssc-miR-22-3p	DENND2C	DENN domain containing 2C [Source:HGNC Symbol;Acc:HGNC:24748]	2.1	0.0644
ssc-miR-22-3p	DGKE	diacylglycerol kinase epsilon [Source:VGNC Symbol;Acc:VGNC:87271]	2.1	0.0644
ssc-miR-22-3p	DGKI	diacylglycerol kinase iota [Source:VGNC Symbol;Acc:VGNC:87274]	2.1	0.0644
ssc-miR-22-3p	DLGAP2	DLG associated protein 2 [Source:VGNC Symbol;Acc:VGNC:99711]	2.1	0.0644
ssc-miR-22-3p	DLK1	delta like non-canonical Notch ligand 1 [Source:VGNC Symbol;Acc:VGNC:87333]	2.1	0.0644
ssc-miR-22-3p	DNAJB5	DnaJ heat shock protein family (Hsp40) member B5 [Source:VGNC Symbol;Acc:VGNC:103081]	2.1	0.0644
ssc-miR-22-3p	DNAJC16	DnaJ heat shock protein family (Hsp40) member C16 [Source:VGNC Symbol;Acc:VGNC:96624]	2.1	0.0644
ssc-miR-22-3p	DNAJC27	DnaJ heat shock protein family (Hsp40) member C27 [Source:VGNC Symbol;Acc:VGNC:102563]	2.1	0.0644
ssc-miR-22-3p	DNAJC5	DnaJ heat shock protein family (Hsp40) member C5 [Source:VGNC Symbol;Acc:VGNC:108714]	2.1	0.0644
ssc-miR-22-3p	DNM3	dynamin 3 [Source:VGNC Symbol;Acc:VGNC:87382]	2.1	0.0644
ssc-miR-22-3p	DNMT3A	DNA methyltransferase 3 alpha [Source:VGNC Symbol;Acc:VGNC:87384]	2.1	0.0644
ssc-miR-22-3p	DOK7	hypothetical gene	2.1	0.0644
ssc-miR-22-3p	DPF2	double PHD fingers 2 [Source:VGNC Symbol;Acc:VGNC:87413]	2.1	0.0644
ssc-miR-22-3p	DPM2	dolichyl-phosphate mannosyltransferase subunit 2, regulatory [Source:VGNC Symbol;Acc:VGNC:87419]	2.1	0.0644
ssc-miR-22-3p	DPP10	dipeptidyl peptidase like 10 [Source:VGNC Symbol;Acc:VGNC:95949]	2.1	0.0644
ssc-miR-22-3p	DPY30	dpy-30 histone methyltransferase complex regulatory subunit [Source:VGNC Symbol;Acc:VGNC:87428]	2.1	0.0644
ssc-miR-22-3p	DPYSL3	dihydropyrimidinase like 3 [Source:VGNC Symbol;Acc:VGNC:87431]	2.1	0.0644
ssc-miR-22-3p	DST	dystonin [Source:HGNC Symbol;Acc:HGNC:1090]	2.1	0.0644
ssc-miR-22-3p	DUSP13	hypothetical gene	2.1	0.0644
ssc-miR-22-3p	DUSP16	dual specificity phosphatase 16 [Source:VGNC Symbol;Acc:VGNC:87480]	2.1	0.0644
ssc-miR-22-3p	E2F2	E2F transcription factor 2 [Source:VGNC Symbol;Acc:VGNC:87513]	2.1	0.0644
ssc-miR-22-3p	EDC3	enhancer of mRNA decapping 3 [Source:VGNC Symbol;Acc:VGNC:87543]	2.1	0.0644
ssc-miR-22-3p	EDNRA	endothelin receptor type A [Source:VGNC Symbol;Acc:VGNC:87549]	2.1	0.0644
ssc-miR-22-3p	EHD1	EH domain containing 1 [Source:VGNC Symbol;Acc:VGNC:87595]	2.1	0.0644
ssc-miR-22-3p	EIF4EBP2	eukaryotic translation initiation factor 4E binding protein 2 [Source:VGNC Symbol;Acc:VGNC:87629]	2.1	0.0644
ssc-miR-22-3p	EIF4EBP3	eukaryotic translation initiation factor 4E binding protein 3 [Source:NCBI gene (formerly Entrezgene);Acc:100529254]	2.1	0.0644
ssc-miR-22-3p	ELOVL6	ELOVL fatty acid elongase 6 [Source:VGNC Symbol;Acc:VGNC:87662]	2.1	0.0644
ssc-miR-22-3p	EMILIN3	elastin microfibril interfacer 3 [Source:VGNC Symbol;Acc:VGNC:95748]	2.1	0.0644
ssc-miR-22-3p	EMX2	empty spiracles homeobox 2 [Source:VGNC Symbol;Acc:VGNC:87693]	2.1	0.0644
ssc-miR-22-3p	ENO1	enolase 1 [Source:VGNC Symbol;Acc:VGNC:87701]	2.1	0.0644
ssc-miR-22-3p	EP300	E1A binding protein p300 [Source:VGNC Symbol;Acc:VGNC:87720]	2.1	0.0644
ssc-miR-22-3p	EPB41L2	erythrocyte membrane protein band 4.1 like 2 [Source:VGNC Symbol;Acc:VGNC:87723]	2.1	0.0644
ssc-miR-22-3p	EPC1	enhancer of polycomb homolog 1 [Source:VGNC Symbol;Acc:VGNC:95923]	2.1	0.0644
ssc-miR-22-3p	ERBB3	erb-b2 receptor tyrosine kinase 3 [Source:VGNC Symbol;Acc:VGNC:87760]	2.1	0.0644
ssc-miR-22-3p	ERBB4	erb-b2 receptor tyrosine kinase 4 [Source:VGNC Symbol;Acc:VGNC:96284]	2.1	0.0644
ssc-miR-22-3p	ERCC8	ERCC excision repair 8, CSA ubiquitin ligase complex subunit [Source:VGNC Symbol;Acc:VGNC:87765]	2.1	0.0644
ssc-miR-22-3p	ERI2	ERI1 exoribonuclease family member 2 [Source:VGNC Symbol;Acc:VGNC:106577]	2.1	0.0644
ssc-miR-22-3p	ESR1	estrogen receptor 1 [Source:VGNC Symbol;Acc:VGNC:103089]	2.1	0.0644
ssc-miR-22-3p	ETS2	ETS proto-onco 2, transcription factor [Source:VGNC Symbol;Acc:VGNC:87809]	2.1	0.0644
ssc-miR-22-3p	ETV1	ETS variant transcription factor 1 [Source:VGNC Symbol;Acc:VGNC:87810]	2.1	0.0644
ssc-miR-22-3p	EXOC5	exocyst complex component 5 [Source:VGNC Symbol;Acc:VGNC:87831]	2.1	0.0644
ssc-miR-22-3p	EYA3	EYA transcriptional coactivator and phosphatase 3 [Source:VGNC Symbol;Acc:VGNC:87852]	2.1	0.0644

ssc-miR-22-3p	EZH1	enhancer of zeste 1 polycomb repressive complex 2 subunit [Source:VGNC Symbol;Acc:VGNC:87854]	2.1	0.0644
ssc-miR-22-3p	F2	coagulation factor II, thrombin [Source:VGNC Symbol;Acc:VGNC:97993]	2.1	0.0644
ssc-miR-22-3p	FAM117A	family with sequence similarity 117 member A [Source:VGNC Symbol;Acc:VGNC:87892]	2.1	0.0644
ssc-miR-22-3p	FAM136A	hypothetical gene	2.1	0.0644
ssc-miR-22-3p	FAM160B2	hypothetical gene	2.1	0.0644
ssc-miR-22-3p	FAM168B	family with sequence similarity 168 member B [Source:VGNC Symbol;Acc:VGNC:95462]	2.1	0.0644
ssc-miR-22-3p	FAM49B	hypothetical gene	2.1	0.0644
ssc-miR-22-3p	FAM53C	family with sequence similarity 53 member C [Source:VGNC Symbol;Acc:VGNC:87975]	2.1	0.0644
ssc-miR-22-3p	FAM83F	family with sequence similarity 83 member F [Source:VGNC Symbol;Acc:VGNC:87991]	2.1	0.0644
ssc-miR-22-3p	FAM89B	family with sequence similarity 89 member B [Source:VGNC Symbol;Acc:VGNC:87995]	2.1	0.0644
ssc-miR-22-3p	FAM96A	hypothetical gene	2.1	0.0644
ssc-miR-22-3p	FASLG	Fas ligand [Source:VGNC Symbol;Acc:VGNC:103942]	2.1	0.0644
ssc-miR-22-3p	FBLN7	fibulin 7 [Source:VGNC Symbol;Acc:VGNC:97999]	2.1	0.0644
ssc-miR-22-3p	FBN2	fibrillin 2 [Source:VGNC Symbol;Acc:VGNC:88025]	2.1	0.0644
ssc-miR-22-3p	FBRS	fibrosin [Source:VGNC Symbol;Acc:VGNC:88027]	2.1	0.0644
ssc-miR-22-3p	FBXL13	F-box and leucine rich repeat protein 13 [Source:VGNC Symbol;Acc:VGNC:98000]	2.1	0.0644
ssc-miR-22-3p	FBXL19	F-box and leucine rich repeat protein 19 [Source:VGNC Symbol;Acc:VGNC:98004]	2.1	0.0644
ssc-miR-22-3p	FBXO34	F-box protein 34 [Source:VGNC Symbol;Acc:VGNC:88041]	2.1	0.0644
ssc-miR-22-3p	FBXO41	F-box protein 41 [Source:VGNC Symbol;Acc:VGNC:88046]	2.1	0.0644
ssc-miR-22-3p	FBXO45	F-box protein 45 [Source:VGNC Symbol;Acc:VGNC:88048]	2.1	0.0644
ssc-miR-22-3p	FBXO46	F-box protein 46 [Source:VGNC Symbol;Acc:VGNC:88049]	2.1	0.0644
ssc-miR-22-3p	FBXW7	F-box and WD repeat domain containing 7 [Source:VGNC Symbol;Acc:VGNC:98925]	2.1	0.0644
ssc-miR-22-3p	FEM1B	fem-1 homolog B [Source:VGNC Symbol;Acc:VGNC:88083]	2.1	0.0644
ssc-miR-22-3p	FKBP10	FKBP prolyl isomerase 10 [Source:VGNC Symbol;Acc:VGNC:88143]	2.1	0.0644
ssc-miR-22-3p	FMNL2	formin like 2 [Source:VGNC Symbol;Acc:VGNC:95879]	2.1	0.0644
ssc-miR-22-3p	FMNL3	formin like 3 [Source:VGNC Symbol;Acc:VGNC:88169]	2.1	0.0644
ssc-miR-22-3p	FNBP4	formin binding protein 4 [Source:VGNC Symbol;Acc:VGNC:88180]	2.1	0.0644
ssc-miR-22-3p	FOSL2	FOS like 2, AP-1 transcription factor subunit [Source:VGNC Symbol;Acc:VGNC:88192]	2.1	0.0644
ssc-miR-22-3p	FOXD3	forkhead box D3 [Source:VGNC Symbol;Acc:VGNC:88201]	2.1	0.0644
ssc-miR-22-3p	FOXK2	forkhead box K2 [Source:VGNC Symbol;Acc:VGNC:88215]	2.1	0.0644
ssc-miR-22-3p	FOXP1	forkhead box P1 [Source:VGNC Symbol;Acc:VGNC:88222]	2.1	0.0644
ssc-miR-22-3p	FRAT2	FRAT regulator of WNT signaling pathway 2 [Source:VGNC Symbol;Acc:VGNC:107394]	2.1	0.0644
ssc-miR-22-3p	FSTL1	follistatin like 1 [Source:VGNC Symbol;Acc:VGNC:88255]	2.1	0.0644
ssc-miR-22-3p	FTL	ferritin light chain [Source:NCBI gene (formerly Entrezgene);Acc:397035]	2.1	0.0644
ssc-miR-22-3p	FTO	FTO alpha-ketoglutarate dependent dioxygenase [Source:VGNC Symbol;Acc:VGNC:88259]	2.1	0.0644
ssc-miR-22-3p	FUT9	fucosyltransferase 9 [Source:VGNC Symbol;Acc:VGNC:88271]	2.1	0.0644
ssc-miR-22-3p	FZD8	frizzled class receptor 8 [Source:VGNC Symbol;Acc:VGNC:96311]	2.1	0.0644
ssc-miR-22-3p	GABBR2	gamma-aminobutyric acid type B receptor subunit 2 [Source:VGNC Symbol;Acc:VGNC:88298]	2.1	0.0644
ssc-miR-22-3p	GABRE	gamma-aminobutyric acid type A receptor subunit epsilon [Source:VGNC Symbol;Acc:VGNC:88310]	2.1	0.0644
ssc-miR-22-3p	GALNT3	polypeptide N-acetylgalactosaminyltransferase 3 [Source:VGNC Symbol;Acc:VGNC:96313]	2.1	0.0644
ssc-miR-22-3p	GAN	gigaxonin [Source:VGNC Symbol;Acc:VGNC:88343]	2.1	0.0644
ssc-miR-22-3p	GATAD2B	GATA zinc finger domain containing 2B [Source:VGNC Symbol;Acc:VGNC:88369]	2.1	0.0644
ssc-miR-22-3p	GATM	glycine amidinotransferase [Source:VGNC Symbol;Acc:VGNC:88372]	2.1	0.0644
ssc-miR-22-3p	GATS	hypothetical gene	2.1	0.0644
ssc-miR-22-3p	GATSL2	hypothetical gene	2.1	0.0644
ssc-miR-22-3p	GBA	glucosylceramidase beta [Source:VGNC Symbol;Acc:VGNC:98802]	2.1	0.0644
ssc-miR-22-3p	GDAP1	ganglioside induced differentiation associated protein 1 [Source:VGNC Symbol;Acc:VGNC:88394]	2.1	0.0644
ssc-miR-22-3p	GFRA1	GDNF family receptor alpha 1 [Source:VGNC Symbol;Acc:VGNC:88428]	2.1	0.0644
ssc-miR-22-3p	GHRHR	growth hormone releasing hormone receptor [Source:VGNC Symbol;Acc:VGNC:88443]	2.1	0.0644

ssc-miR-22-3p	GINS4	GINS complex subunit 4 [Source:VGNC Symbol;Acc:VGNC:96171]	2.1	0.0644
ssc-miR-22-3p	GJA9	gap junction protein alpha 9 [Source:VGNC Symbol;Acc:VGNC:97065]	2.1	0.0644
ssc-miR-22-3p	GLDN	gliomedin [Source:VGNC Symbol;Acc:VGNC:88479]	2.1	0.0644
ssc-miR-22-3p	GLIPR2	GLI pathosis related 2 [Source:VGNC Symbol;Acc:VGNC:98023]	2.1	0.0644
ssc-miR-22-3p	GMPPB	GDP-mannose pyrophosphorylase B [Source:NCBI gene (formerly Entrezgene);Acc:100513376]	2.1	0.0644
ssc-miR-22-3p	GPBP1	GC-rich promoter binding protein 1 [Source:VGNC Symbol;Acc:VGNC:88578]	2.1	0.0644
ssc-miR-22-3p	GPR107	G protein-coupled receptor 107 [Source:VGNC Symbol;Acc:VGNC:88595]	2.1	0.0644
ssc-miR-22-3p	GPR161	G protein-coupled receptor 161 [Source:VGNC Symbol;Acc:VGNC:88613]	2.1	0.0644
ssc-miR-22-3p	GPR37L1	G protein-coupled receptor 37 like 1 [Source:VGNC Symbol;Acc:VGNC:95633]	2.1	0.0644
ssc-miR-22-3p	GRM1	glutamate metabotropic receptor 1 [Source:VGNC Symbol;Acc:VGNC:88700]	2.1	0.0644
ssc-miR-22-3p	GRM5	glutamate metabotropic receptor 5 [Source:VGNC Symbol;Acc:VGNC:88703]	2.1	0.0644
ssc-miR-22-3p	GTDC1	glycosyltransferase like domain containing 1 [Source:VGNC Symbol;Acc:VGNC:95955]	2.1	0.0644
ssc-miR-22-3p	GTF2B	ral transcription factor IIB [Source:VGNC Symbol;Acc:VGNC:88731]	2.1	0.0644
ssc-miR-22-3p	H2AFX	hypothetical gene	2.1	0.0644
ssc-miR-22-3p	H3F3B	hypothetical gene	2.1	0.0644
ssc-miR-22-3p	H3F3C	hypothetical gene	2.1	0.0644
ssc-miR-22-3p	HAUS5	HAUS augmin like complex subunit 5 [Source:VGNC Symbol;Acc:VGNC:88788]	2.1	0.0644
ssc-miR-22-3p	HCN4	hyperpolarization activated cyclic nucleotide gated potassium channel 4 [Source:VGNC Symbol;Acc:VGNC:88805]	2.1	0.0644
ssc-miR-22-3p	HDAC4	histone deacetylase 4 [Source:VGNC Symbol;Acc:VGNC:95602]	2.1	0.0644
ssc-miR-22-3p	HELZ	helicase with zinc finger [Source:VGNC Symbol;Acc:VGNC:88840]	2.1	0.0644
ssc-miR-22-3p	HEMK1	HemK methyltransferase family member 1 [Source:VGNC Symbol;Acc:VGNC:88843]	2.1	0.0644
ssc-miR-22-3p	HERPUD2	HERPUD family member 2 [Source:VGNC Symbol;Acc:VGNC:88853]	2.1	0.0644
ssc-miR-22-3p	HIF1AN	hypoxia inducible factor 1 subunit alpha inhibitor [Source:VGNC Symbol;Acc:VGNC:98033]	2.1	0.0644
ssc-miR-22-3p	HIPK1	homeodomain interacting protein kinase 1 [Source:VGNC Symbol;Acc:VGNC:88887]	2.1	0.0644
ssc-miR-22-3p	HNRNPA3	hypothetical gene	2.1	0.0644
ssc-miR-22-3p	HNRNPH1	heterous nuclear ribonucleoprotein H1 [Source:VGNC Symbol;Acc:VGNC:88920]	2.1	0.0644
ssc-miR-22-3p	HNRNPUL2	heterous nuclear ribonucleoprotein U like 2 [Source:VGNC Symbol;Acc:VGNC:99758]	2.1	0.0644
ssc-miR-22-3p	HOMER1	homer scaffold protein 1 [Source:VGNC Symbol;Acc:VGNC:88928]	2.1	0.0644
ssc-miR-22-3p	HOXA4	hypothetical gene	2.1	0.0644
ssc-miR-22-3p	HOXC11	homeobox C11 [Source:VGNC Symbol;Acc:VGNC:103287]	2.1	0.0644
ssc-miR-22-3p	HUNK	hormonally up-regulated Neu-associated kinase [Source:VGNC Symbol;Acc:VGNC:89007]	2.1	0.0644
ssc-miR-22-3p	ICOSLG	hypothetical gene	2.1	0.0644
ssc-miR-22-3p	IFFO2	intermediate filament family orphan 2 [Source:VGNC Symbol;Acc:VGNC:98475]	2.1	0.0644
ssc-miR-22-3p	IGF2BP1	insulin like growth factor 2 mRNA binding protein 1 [Source:VGNC Symbol;Acc:VGNC:99006]	2.1	0.0644
ssc-miR-22-3p	IKZF4	IKAROS family zinc finger 4 [Source:VGNC Symbol;Acc:VGNC:89075]	2.1	0.0644
ssc-miR-22-3p	IL13RA1	interleukin 13 receptor subunit alpha 1 [Source:VGNC Symbol;Acc:VGNC:89081]	2.1	0.0644
ssc-miR-22-3p	IL17RD	interleukin 17 receptor D [Source:VGNC Symbol;Acc:VGNC:89087]	2.1	0.0644
ssc-miR-22-3p	IL31	hypothetical gene	2.1	0.0644
ssc-miR-22-3p	IL6R	interleukin 6 receptor [Source:VGNC Symbol;Acc:VGNC:89112]	2.1	0.0644
ssc-miR-22-3p	INO80E	INO80 complex subunit E [Source:VGNC Symbol;Acc:VGNC:89139]	2.1	0.0644
ssc-miR-22-3p	IP6K1	inositol hexakisphosphate kinase 1 [Source:VGNC Symbol;Acc:VGNC:89172]	2.1	0.0644
ssc-miR-22-3p	IPO7	importin 7 [Source:VGNC Symbol;Acc:VGNC:89180]	2.1	0.0644
ssc-miR-22-3p	IRF5	interferon regulatory factor 5 [Source:VGNC Symbol;Acc:VGNC:89208]	2.1	0.0644
ssc-miR-22-3p	ITGA10	integrin subunit alpha 10 [Source:VGNC Symbol;Acc:VGNC:89232]	2.1	0.0644
ssc-miR-22-3p	ITGB3BP	hypothetical gene	2.1	0.0644
ssc-miR-22-3p	ITGB8	integrin subunit beta 8 [Source:VGNC Symbol;Acc:VGNC:89246]	2.1	0.0644
ssc-miR-22-3p	JARID2	jumonji and AT-rich interaction domain containing 2 [Source:VGNC Symbol;Acc:VGNC:89279]	2.1	0.0644
ssc-miR-22-3p	KAT5	lysine acetyltransferase 5 [Source:VGNC Symbol;Acc:VGNC:89305]	2.1	0.0644
ssc-miR-22-3p	KAT6A	hypothetical gene	2.1	0.0644

ssc-miR-22-3p	KAT6B	lysine acetyltransferase 6B [Source:VGNC Symbol;Acc:VGNC:89306]	2.1	0.0644
ssc-miR-22-3p	KAT7	lysine acetyltransferase 7 [Source:VGNC Symbol;Acc:VGNC:89307]	2.1	0.0644
ssc-miR-22-3p	KATNBL1	katanin regulatory subunit B1 like 1 [Source:VGNC Symbol;Acc:VGNC:89313]	2.1	0.0644
ssc-miR-22-3p	KB-1507C5.2	hypothetical gene	2.1	0.0644
ssc-miR-22-3p	KBTBD11	kelch repeat and BTB domain containing 11 [Source:VGNC Symbol;Acc:VGNC:96196]	2.1	0.0644
ssc-miR-22-3p	KCNE1	potassium voltage-gated channel subfamily E regulatory subunit 1 [Source:NCBI gene (formerly Entrezgene);Acc:100621502]	2.1	0.0644
ssc-miR-22-3p	KCNE1L	hypothetical gene	2.1	0.0644
ssc-miR-22-3p	KCNH1	potassium voltage-gated channel subfamily H member 1 [Source:VGNC Symbol;Acc:VGNC:108599]	2.1	0.0644
ssc-miR-22-3p	KCNJ6	potassium inwardly rectifying channel subfamily J member 6 [Source:VGNC Symbol;Acc:VGNC:89360]	2.1	0.0644
ssc-miR-22-3p	KCNK10	potassium two pore domain channel subfamily K member 10 [Source:VGNC Symbol;Acc:VGNC:89364]	2.1	0.0644
ssc-miR-22-3p	KCNK12	potassium two pore domain channel subfamily K member 12 [Source:HGNC Symbol;Acc:HGNC:6274]	2.1	0.0644
ssc-miR-22-3p	KCNMB4	potassium calcium-activated channel subfamily M regulatory beta subunit 4 [Source:VGNC Symbol;Acc:VGNC:89378]	2.1	0.0644
ssc-miR-22-3p	KCTD10	potassium channel tetramerization domain containing 10 [Source:VGNC Symbol;Acc:VGNC:103972]	2.1	0.0644
ssc-miR-22-3p	KDM3A	lysine demethylase 3A [Source:VGNC Symbol;Acc:VGNC:89411]	2.1	0.0644
ssc-miR-22-3p	KDM6B	lysine demethylase 6B [Source:VGNC Symbol;Acc:VGNC:89416]	2.1	0.0644
ssc-miR-22-3p	KHDRBS2	KH RNA binding domain containing, signal transduction associated 2 [Source:VGNC Symbol;Acc:VGNC:89423]	2.1	0.0644
ssc-miR-22-3p	KHNYN	KH and NYN domain containing [Source:VGNC Symbol;Acc:VGNC:89426]	2.1	0.0644
ssc-miR-22-3p	KIAA0040	KIAA0040 [Source:VGNC Symbol;Acc:VGNC:98059]	2.1	0.0644
ssc-miR-22-3p	KIAA0226	hypothetical gene	2.1	0.0644
ssc-miR-22-3p	KIAA0247	hypothetical gene	2.1	0.0644
ssc-miR-22-3p	KIAA0319L	KIAA0319 like [Source:VGNC Symbol;Acc:VGNC:89430]	2.1	0.0644
ssc-miR-22-3p	KIAA0355	hypothetical gene	2.1	0.0644
ssc-miR-22-3p	KIAA0556	hypothetical gene	2.1	0.0644
ssc-miR-22-3p	KIAA1211L	hypothetical gene	2.1	0.0644
ssc-miR-22-3p	KIRREL3	kirre like nephrin family adhesion molecule 3 [Source:VGNC Symbol;Acc:VGNC:89482]	2.1	0.0644
ssc-miR-22-3p	KLF6	Kruppel like factor 6 [Source:VGNC Symbol;Acc:VGNC:98063]	2.1	0.0644
ssc-miR-22-3p	KLF7	Kruppel like factor 7 [Source:VGNC Symbol;Acc:VGNC:96396]	2.1	0.0644
ssc-miR-22-3p	KMT2A	lysine methyltransferase 2A [Source:VGNC Symbol;Acc:VGNC:108600]	2.1	0.0644
ssc-miR-22-3p	L3MBTL2	L3MBTL histone methyl-lysine binding protein 2 [Source:VGNC Symbol;Acc:VGNC:89613]	2.1	0.0644
ssc-miR-22-3p	LAMC1	laminin subunit gamma 1 [Source:VGNC Symbol;Acc:VGNC:89624]	2.1	0.0644
ssc-miR-22-3p	LAS1L	LAS1 like ribosome biosis factor [Source:VGNC Symbol;Acc:VGNC:89645]	2.1	0.0644
ssc-miR-22-3p	LGALS1	galectin 1 [Source:VGNC Symbol;Acc:VGNC:89691]	2.1	0.0644
ssc-miR-22-3p	LIF	LIF interleukin 6 family cytokine [Source:VGNC Symbol;Acc:VGNC:89719]	2.1	0.0644
ssc-miR-22-3p	LIN7C	lin-7 homolog C, crumbs cell polarity complex component [Source:VGNC Symbol;Acc:VGNC:89732]	2.1	0.0644
ssc-miR-22-3p	LINGO2	leucine rich repeat and Ig domain containing 2 [Source:HGNC Symbol;Acc:HGNC:21207]	2.1	0.0644
ssc-miR-22-3p	LMTK2	lemur tyrosine kinase 2 [Source:HGNC Symbol;Acc:HGNC:17880]	2.1	0.0644
ssc-miR-22-3p	LPP	LIM domain containing preferred translocation partner in lipoma [Source:HGNC Symbol;Acc:HGNC:6679]	2.1	0.0644
ssc-miR-22-3p	LRCH1	leucine rich repeats and calponin homology domain containing 1 [Source:VGNC Symbol;Acc:VGNC:89801]	2.1	0.0644
ssc-miR-22-3p	LRCH4	leucine rich repeats and calponin homology domain containing 4 [Source:VGNC Symbol;Acc:VGNC:89804]	2.1	0.0644
ssc-miR-22-3p	LRRC1	leucine rich repeat containing 1 [Source:VGNC Symbol;Acc:VGNC:103977]	2.1	0.0644
ssc-miR-22-3p	LRRC16A	hypothetical gene	2.1	0.0644
ssc-miR-22-3p	LRRC40	hypothetical gene	2.1	0.0644
ssc-miR-22-3p	LRRC73	leucine rich repeat containing 73 [Source:VGNC Symbol;Acc:VGNC:89854]	2.1	0.0644
ssc-miR-22-3p	LRRN2	leucine rich repeat neuronal 2 [Source:VGNC Symbol;Acc:VGNC:89861]	2.1	0.0644
ssc-miR-22-3p	LSG1	large 60S subunit nuclear export GTPase 1 [Source:VGNC Symbol;Acc:VGNC:89869]	2.1	0.0644
ssc-miR-22-3p	LTB4R	leukotriene B4 receptor [Source:HGNC Symbol;Acc:HGNC:6713]	2.1	0.0644
ssc-miR-22-3p	LTBP2	latent transforming growth factor beta binding protein 2 [Source:VGNC Symbol;Acc:VGNC:89886]	2.1	0.0644
ssc-miR-22-3p	LZIC	leucine zipper and CTNNBIP1 domain containing [Source:VGNC Symbol;Acc:VGNC:89925]	2.1	0.0644
ssc-miR-22-3p	MACROD2	mono-ADP ribosylhydrolase 2 [Source:VGNC Symbol;Acc:VGNC:108725]	2.1	0.0644

ssc-miR-22-3p	MAGI2	membrane associated guanylate kinase, WW and PDZ domain containing 2 [Source:VGNC Symbol;Acc:VGNC:89955]	2.1	0.0644
ssc-miR-22-3p	MALL	mal, T cell differentiation protein like [Source:NCBI gene (formerly Entrezgene);Acc:100626835]	2.1	0.0644
ssc-miR-22-3p	MANEAL	mannosidase endo-alpha like [Source:VGNC Symbol;Acc:VGNC:89972]	2.1	0.0644
ssc-miR-22-3p	MAP3K1	mitogen-activated protein kinase kinase kinase 1 [Source:VGNC Symbol;Acc:VGNC:98104]	2.1	0.0644
ssc-miR-22-3p	MAP3K12	mitogen-activated protein kinase kinase kinase 12 [Source:VGNC Symbol;Acc:VGNC:89983]	2.1	0.0644
ssc-miR-22-3p	MAP3K3	mitogen-activated protein kinase kinase kinase 3 [Source:VGNC Symbol;Acc:VGNC:98108]	2.1	0.0644
ssc-miR-22-3p	MAPK1	mitogen-activated protein kinase 1 [Source:VGNC Symbol;Acc:VGNC:89996]	2.1	0.0644
ssc-miR-22-3p	MAPK10	mitogen-activated protein kinase 10 [Source:VGNC Symbol;Acc:VGNC:98116]	2.1	0.0644
ssc-miR-22-3p	MAPK14	mitogen-activated protein kinase 14 [Source:VGNC Symbol;Acc:VGNC:90000]	2.1	0.0644
ssc-miR-22-3p	MAPK8IP3	mitogen-activated protein kinase 8 interacting protein 3 [Source:VGNC Symbol;Acc:VGNC:90008]	2.1	0.0644
ssc-miR-22-3p	MARCH8	hypothetical gene	2.1	0.0644
ssc-miR-22-3p	MAT2A	methionine adenosyltransferase 2A [Source:VGNC Symbol;Acc:VGNC:90039]	2.1	0.0644
ssc-miR-22-3p	MAX	MYC associated factor X [Source:VGNC Symbol;Acc:VGNC:90046]	2.1	0.0644
ssc-miR-22-3p	MCAT	malonyl-CoA-acyl carrier protein transacylase [Source:VGNC Symbol;Acc:VGNC:90062]	2.1	0.0644
ssc-miR-22-3p	MECOM	MDS1 and EVI1 complex locus [Source:VGNC Symbol;Acc:VGNC:90100]	2.1	0.0644
ssc-miR-22-3p	MECP2	methyl-CpG binding protein 2 [Source:VGNC Symbol;Acc:VGNC:90101]	2.1	0.0644
ssc-miR-22-3p	MED19	mediator complex subunit 19 [Source:VGNC Symbol;Acc:VGNC:90111]	2.1	0.0644
ssc-miR-22-3p	MED20	mediator complex subunit 20 [Source:VGNC Symbol;Acc:VGNC:90112]	2.1	0.0644
ssc-miR-22-3p	MED28	mediator complex subunit 28 [Source:VGNC Symbol;Acc:VGNC:90118]	2.1	0.0644
ssc-miR-22-3p	MEGF11	multiple EGF like domains 11 [Source:VGNC Symbol;Acc:VGNC:103126]	2.1	0.0644
ssc-miR-22-3p	MEIS2	Meis homeobox 2 [Source:VGNC Symbol;Acc:VGNC:90136]	2.1	0.0644
ssc-miR-22-3p	METTL15	methyltransferase like 15 [Source:VGNC Symbol;Acc:VGNC:98124]	2.1	0.0644
ssc-miR-22-3p	MEX3A	mex-3 RNA binding family member A [Source:VGNC Symbol;Acc:VGNC:90168]	2.1	0.0644
ssc-miR-22-3p	MFGE8	milk fat globule EGF and factor V/VIII domain containing [Source:NCBI gene (formerly Entrezgene);Acc:397545]	2.1	0.0644
ssc-miR-22-3p	MGAT5	alpha-1,6-mannosylglycoprotein 6-beta-N-acetylglucosaminyltransferase [Source:VGNC Symbol;Acc:VGNC:96741]	2.1	0.0644
ssc-miR-22-3p	MICALCL	hypothetical gene	2.1	0.0644
ssc-miR-22-3p	MIER2	MIER family member 2 [Source:VGNC Symbol;Acc:VGNC:90220]	2.1	0.0644
ssc-miR-22-3p	MMACHC	metabolism of cobalamin associated C [Source:VGNC Symbol;Acc:VGNC:96744]	2.1	0.0644
ssc-miR-22-3p	MMP24	matrix metallopeptidase 24 [Source:VGNC Symbol;Acc:VGNC:96421]	2.1	0.0644
ssc-miR-22-3p	MMS19	MMS19 homolog, cytosolic iron-sulfur assembly component [Source:VGNC Symbol;Acc:VGNC:90283]	2.1	0.0644
ssc-miR-22-3p	MOCS1	molybdenum cofactor synthesis 1 [Source:VGNC Symbol;Acc:VGNC:107153]	2.1	0.0644
ssc-miR-22-3p	MON2	MON2 homolog, regulator of endosome-to-Golgi trafficking [Source:VGNC Symbol;Acc:VGNC:90301]	2.1	0.0644
ssc-miR-22-3p	MPPED1	metallophosphoesterase domain containing 1 [Source:VGNC Symbol;Acc:VGNC:90330]	2.1	0.0644
ssc-miR-22-3p	MPV17L	MPV17 mitochondrial inner membrane protein like [Source:VGNC Symbol;Acc:VGNC:98139]	2.1	0.0644
ssc-miR-22-3p	MPZL3	myelin protein zero like 3 [Source:VGNC Symbol;Acc:VGNC:90337]	2.1	0.0644
ssc-miR-22-3p	MR1	major histocompatibility complex, class I-related [Source:VGNC Symbol;Acc:VGNC:90338]	2.1	0.0644
ssc-miR-22-3p	MRPL11	mitochondrial ribosomal protein L11 [Source:VGNC Symbol;Acc:VGNC:90350]	2.1	0.0644
ssc-miR-22-3p	MSL2	MSL complex subunit 2 [Source:VGNC Symbol;Acc:VGNC:90424]	2.1	0.0644
ssc-miR-22-3p	MST4	hypothetical gene	2.1	0.0644
ssc-miR-22-3p	MTDH	metadherin [Source:VGNC Symbol;Acc:VGNC:90439]	2.1	0.0644
ssc-miR-22-3p	MTF2	metal response element binding transcription factor 2 [Source:VGNC Symbol;Acc:VGNC:90444]	2.1	0.0644
ssc-miR-22-3p	MTHFD2	methylenetetrahydrofolate dehydrogenase (NADP+ dependent) 2, methenyltetrahydrofolate cyclohydrolase [Source:HGNC Symbol;Acc:HGNC:7434]	2.1	0.0644
ssc-miR-22-3p	MTHFR	methylenetetrahydrofolate reductase [Source:VGNC Symbol;Acc:VGNC:90451]	2.1	0.0644
ssc-miR-22-3p	MTMR2	myotubularin related protein 2 [Source:VGNC Symbol;Acc:VGNC:90460]	2.1	0.0644
ssc-miR-22-3p	MUM1L1	hypothetical gene	2.1	0.0644
ssc-miR-22-3p	MURC	hypothetical gene	2.1	0.0644
ssc-miR-22-3p	MVB12B	multivesicular body subunit 12B [Source:VGNC Symbol;Acc:VGNC:90488]	2.1	0.0644
ssc-miR-22-3p	MXD1	MAX dimerization protein 1 [Source:VGNC Symbol;Acc:VGNC:90492]	2.1	0.0644
ssc-miR-22-3p	MXD4	MAX dimerization protein 4 [Source:VGNC Symbol;Acc:VGNC:90493]	2.1	0.0644

ssc-miR-22-3p	MYCBP	MYC binding protein [Source:NCBI gene (formerly Entrezgene);Acc:100513990]	2.1	0.0644
ssc-miR-22-3p	MYCL	hypothetical gene	2.1	0.0644
ssc-miR-22-3p	MYO18A	myosin XVIIIA [Source:HGNC Symbol;Acc:HGNC:31104]	2.1	0.0644
ssc-miR-22-3p	MYO1A	myosin IA [Source:VGNC Symbol;Acc:VGNC:90525]	2.1	0.0644
ssc-miR-22-3p	MYO9A	myosin IXA [Source:VGNC Symbol;Acc:VGNC:103138]	2.1	0.0644
ssc-miR-22-3p	N4BP2	NEDD4 binding protein 2 [Source:VGNC Symbol;Acc:VGNC:90554]	2.1	0.0644
ssc-miR-22-3p	N4BP3	NEDD4 binding protein 3 [Source:VGNC Symbol;Acc:VGNC:90555]	2.1	0.0644
ssc-miR-22-3p	NAA10	N-alpha-acetyltransferase 10, NatA catalytic subunit [Source:VGNC Symbol;Acc:VGNC:96746]	2.1	0.0644
ssc-miR-22-3p	NAA20	N-alpha-acetyltransferase 20, NatB catalytic subunit [Source:VGNC Symbol;Acc:VGNC:96749]	2.1	0.0644
ssc-miR-22-3p	NAALADL1	N-acetylated alpha-linked acidic dipeptidase like 1 [Source:VGNC Symbol;Acc:VGNC:90558]	2.1	0.0644
ssc-miR-22-3p	NBR1	NBR1 autophagy cargo receptor [Source:VGNC Symbol;Acc:VGNC:90593]	2.1	0.0644
ssc-miR-22-3p	NCKAP1	NCK associated protein 1 [Source:VGNC Symbol;Acc:VGNC:96432]	2.1	0.0644
ssc-miR-22-3p	NCMAP	hypothetical gene	2.1	0.0644
ssc-miR-22-3p	NCOA1	nuclear receptor coactivator 1 [Source:VGNC Symbol;Acc:VGNC:90615]	2.1	0.0644
ssc-miR-22-3p	NDUFC2-KCTD14	hypothetical gene	2.1	0.0644
ssc-miR-22-3p	NECAP1	NECAP endocytosis associated 1 [Source:VGNC Symbol;Acc:VGNC:90660]	2.1	0.0644
ssc-miR-22-3p	NET1	neuroepithelial cell transforming 1 [Source:VGNC Symbol;Acc:VGNC:95622]	2.1	0.0644
ssc-miR-22-3p	NFAT5	nuclear factor of activated T cells 5 [Source:VGNC Symbol;Acc:VGNC:90708]	2.1	0.0644
ssc-miR-22-3p	NFIB	nuclear factor I B [Source:VGNC Symbol;Acc:VGNC:90716]	2.1	0.0644
ssc-miR-22-3p	NFYA	nuclear transcription factor Y subunit alpha [Source:VGNC Symbol;Acc:VGNC:90729]	2.1	0.0644
ssc-miR-22-3p	NHP2	hypothetical gene	2.1	0.0644
ssc-miR-22-3p	NKIRAS2	NFKB inhibitor interacting Ras like 2 [Source:VGNC Symbol;Acc:VGNC:90763]	2.1	0.0644
ssc-miR-22-3p	NLGN2	hypothetical gene	2.1	0.0644
ssc-miR-22-3p	NOP9	NOP9 nucleolar protein [Source:VGNC Symbol;Acc:VGNC:90822]	2.1	0.0644
ssc-miR-22-3p	NPAS3	neuronal PAS domain protein 3 [Source:VGNC Symbol;Acc:VGNC:90838]	2.1	0.0644
ssc-miR-22-3p	NPNT	nephronectin [Source:VGNC Symbol;Acc:VGNC:90855]	2.1	0.0644
ssc-miR-22-3p	NR3C1	nuclear receptor subfamily 3 group C member 1 [Source:VGNC Symbol;Acc:VGNC:90883]	2.1	0.0644
ssc-miR-22-3p	NRAS	NRAS proto-onco, GTPase [Source:VGNC Symbol;Acc:VGNC:98827]	2.1	0.0644
ssc-miR-22-3p	NTNG1	netrin G1 [Source:VGNC Symbol;Acc:VGNC:90935]	2.1	0.0644
ssc-miR-22-3p	NTRK2	neurotrophic receptor tyrosine kinase 2 [Source:VGNC Symbol;Acc:VGNC:96455]	2.1	0.0644
ssc-miR-22-3p	NUDT4	hypothetical gene	2.1	0.0644
ssc-miR-22-3p	NUP210	nucleoporin 210 [Source:VGNC Symbol;Acc:VGNC:90977]	2.1	0.0644
ssc-miR-22-3p	NUS1	NUS1 dehydrodolichyl diphosphate synthase subunit [Source:VGNC Symbol;Acc:VGNC:90990]	2.1	0.0644
ssc-miR-22-3p	NXF1	nuclear RNA export factor 1 [Source:VGNC Symbol;Acc:VGNC:90993]	2.1	0.0644
ssc-miR-22-3p	NXPH3	neurexophilin 3 [Source:VGNC Symbol;Acc:VGNC:90999]	2.1	0.0644
ssc-miR-22-3p	NXT2	nuclear transport factor 2 like export factor 2 [Source:VGNC Symbol;Acc:VGNC:91001]	2.1	0.0644
ssc-miR-22-3p	NYAP2	neuronal tyrosine-phosphorylated phosphoinositide-3-kinase adaptor 2 [Source:VGNC Symbol;Acc:VGNC:96213]	2.1	0.0644
ssc-miR-22-3p	ODF1	outer dense fiber of sperm tails 1 [Source:VGNC Symbol;Acc:VGNC:91016]	2.1	0.0644
ssc-miR-22-3p	OGN	osteoglycin [Source:VGNC Symbol;Acc:VGNC:91028]	2.1	0.0644
ssc-miR-22-3p	OLA1	Obg like ATPase 1 [Source:VGNC Symbol;Acc:VGNC:96188]	2.1	0.0644
ssc-miR-22-3p	OLFML2A	olfactomedin like 2A [Source:VGNC Symbol;Acc:VGNC:98170]	2.1	0.0644
ssc-miR-22-3p	ONECUT1	one cut homeobox 1 [Source:VGNC Symbol;Acc:VGNC:91042]	2.1	0.0644
ssc-miR-22-3p	ORMDL2	ORMDL sphingolipid biosynthesis regulator 2 [Source:VGNC Symbol;Acc:VGNC:103307]	2.1	0.0644
ssc-miR-22-3p	OSM	oncostatin M [Source:HGNC Symbol;Acc:HGNC:8506]	2.1	0.0644
ssc-miR-22-3p	OTUD7A	OTU deubiquitinase 7A [Source:VGNC Symbol;Acc:VGNC:91104]	2.1	0.0644
ssc-miR-22-3p	PACRGL	parkin coregulated like [Source:VGNC Symbol;Acc:VGNC:91139]	2.1	0.0644
ssc-miR-22-3p	PADI2	peptidyl arginine deiminase 2 [Source:VGNC Symbol;Acc:VGNC:91146]	2.1	0.0644
ssc-miR-22-3p	PAFAH1B2	platelet activating factor acetylhydrolase 1b catalytic subunit 2 [Source:VGNC Symbol;Acc:VGNC:91151]	2.1	0.0644
ssc-miR-22-3p	PAIP2B	poly(A) binding protein interacting protein 2B [Source:HGNC Symbol;Acc:HGNC:29200]	2.1	0.0644

ssc-miR-22-3p	PALD1	phosphatase domain containing paladin 1 [Source:VGNC Symbol;Acc:VGNC:91159]	2.1	0.0644
ssc-miR-22-3p	PALM	paralemmin [Source:VGNC Symbol;Acc:VGNC:91160]	2.1	0.0644
ssc-miR-22-3p	PAOX	polyamine oxidase [Source:HGNC Symbol;Acc:HGNC:20837]	2.1	0.0644
ssc-miR-22-3p	PAWR	pro-apoptotic WT1 regulator [Source:VGNC Symbol;Acc:VGNC:91191]	2.1	0.0644
ssc-miR-22-3p	PAX6	paired box 6 [Source:VGNC Symbol;Acc:VGNC:91195]	2.1	0.0644
ssc-miR-22-3p	PAX7	paired box 7 [Source:VGNC Symbol;Acc:VGNC:91196]	2.1	0.0644
ssc-miR-22-3p	PCDH1	protocadherin 1 [Source:VGNC Symbol;Acc:VGNC:91211]	2.1	0.0644
ssc-miR-22-3p	PCGF5	hypothetical gene	2.1	0.0644
ssc-miR-22-3p	PCNP	PEST proteolytic signal containing nuclear protein [Source:VGNC Symbol;Acc:VGNC:104018]	2.1	0.0644
ssc-miR-22-3p	PCYT2	phosphate cytidylyltransferase 2, ethanolamine [Source:VGNC Symbol;Acc:VGNC:91238]	2.1	0.0644
ssc-miR-22-3p	PDAP1	PDGFA associated protein 1 [Source:VGNC Symbol;Acc:VGNC:91239]	2.1	0.0644
ssc-miR-22-3p	PDE5A	phosphodiesterase 5A [Source:VGNC Symbol;Acc:VGNC:91257]	2.1	0.0644
ssc-miR-22-3p	PDIK1L	PDLIM1 interacting kinase 1 like [Source:VGNC Symbol;Acc:VGNC:91276]	2.1	0.0644
ssc-miR-22-3p	PDK3	pyruvate dehydrogenase kinase 3 [Source:VGNC Symbol;Acc:VGNC:91279]	2.1	0.0644
ssc-miR-22-3p	PDS5A	PDS5 cohesin associated factor A [Source:HGNC Symbol;Acc:HGNC:29088]	2.1	0.0644
ssc-miR-22-3p	PDSS1	decaprenyl diphosphate synthase subunit 1 [Source:VGNC Symbol;Acc:VGNC:95858]	2.1	0.0644
ssc-miR-22-3p	PDZD4	PDZ domain containing 4 [Source:VGNC Symbol;Acc:VGNC:91296]	2.1	0.0644
ssc-miR-22-3p	PER1	period circadian regulator 1 [Source:VGNC Symbol;Acc:VGNC:91314]	2.1	0.0644
ssc-miR-22-3p	PER2	period circadian regulator 2 [Source:VGNC Symbol;Acc:VGNC:96478]	2.1	0.0644
ssc-miR-22-3p	PEX7	peroxisomal biosis factor 7 [Source:VGNC Symbol;Acc:VGNC:91331]	2.1	0.0644
ssc-miR-22-3p	PFDN6	prefoldin subunit 6 [Source:VGNC Symbol;Acc:VGNC:104024]	2.1	0.0644
ssc-miR-22-3p	PHACTR4	phosphatase and actin regulator 4 [Source:VGNC Symbol;Acc:VGNC:98531]	2.1	0.0644
ssc-miR-22-3p	PHC1	polyhomeotic homolog 1 [Source:VGNC Symbol;Acc:VGNC:91370]	2.1	0.0644
ssc-miR-22-3p	PHEX	phosphate regulating endopeptidase homolog X-linked [Source:NCBI gene (formerly Entrezgene);Acc:100154258]	2.1	0.0644
ssc-miR-22-3p	PHF12	PHD finger protein 12 [Source:VGNC Symbol;Acc:VGNC:91378]	2.1	0.0644
ssc-miR-22-3p	PHF13	PHD finger protein 13 [Source:VGNC Symbol;Acc:VGNC:91379]	2.1	0.0644
ssc-miR-22-3p	PHF15	hypothetical gene	2.1	0.0644
ssc-miR-22-3p	PHF7	PHD finger protein 7 [Source:VGNC Symbol;Acc:VGNC:91390]	2.1	0.0644
ssc-miR-22-3p	PHF8	PHD finger protein 8 [Source:VGNC Symbol;Acc:VGNC:91391]	2.1	0.0644
ssc-miR-22-3p	PIANP	PILR alpha associated neural protein [Source:VGNC Symbol;Acc:VGNC:91409]	2.1	0.0644
ssc-miR-22-3p	PIP4K2B	phosphatidylinositol-5-phosphate 4-kinase type 2 beta [Source:VGNC Symbol;Acc:VGNC:91454]	2.1	0.0644
ssc-miR-22-3p	PLAGL2	PLAG1 like zinc finger 2 [Source:VGNC Symbol;Acc:VGNC:96490]	2.1	0.0644
ssc-miR-22-3p	PLBD2	phospholipase B domain containing 2 [Source:VGNC Symbol;Acc:VGNC:91514]	2.1	0.0644
ssc-miR-22-3p	PLCXD3	phosphatidylinositol specific phospholipase C X domain containing 3 [Source:VGNC Symbol;Acc:VGNC:91524]	2.1	0.0644
ssc-miR-22-3p	PLEKHA6	pleckstrin homology domain containing A6 [Source:VGNC Symbol;Acc:VGNC:91536]	2.1	0.0644
ssc-miR-22-3p	PNISR	PNN interacting serine and arginine rich protein [Source:VGNC Symbol;Acc:VGNC:91594]	2.1	0.0644
ssc-miR-22-3p	POGK	pogo transposable element derived with KRAB domain [Source:VGNC Symbol;Acc:VGNC:91622]	2.1	0.0644
ssc-miR-22-3p	POM121	hypothetical gene	2.1	0.0644
ssc-miR-22-3p	POM121C	hypothetical gene	2.1	0.0644
ssc-miR-22-3p	PPARGC1B	PPARG coactivator 1 beta [Source:VGNC Symbol;Acc:VGNC:91686]	2.1	0.0644
ssc-miR-22-3p	PPM1K	protein phosphatase, Mg2+/Mn2+ dependent 1K [Source:VGNC Symbol;Acc:VGNC:91710]	2.1	0.0644
ssc-miR-22-3p	PPM1L	protein phosphatase, Mg2+/Mn2+ dependent 1L [Source:VGNC Symbol;Acc:VGNC:91711]	2.1	0.0644
ssc-miR-22-3p	PPP1R15B	protein phosphatase 1 regulatory subunit 15B [Source:VGNC Symbol;Acc:VGNC:91726]	2.1	0.0644
ssc-miR-22-3p	PPP1R9A	protein phosphatase 1 regulatory subunit 9A [Source:VGNC Symbol;Acc:VGNC:91743]	2.1	0.0644
ssc-miR-22-3p	PRCD	photoreceptor disc component [Source:VGNC Symbol;Acc:VGNC:91770]	2.1	0.0644
ssc-miR-22-3p	PRCP	prolylcarboxypeptidase [Source:VGNC Symbol;Acc:VGNC:91771]	2.1	0.0644
ssc-miR-22-3p	PRDM16	PR/SET domain 16 [Source:VGNC Symbol;Acc:VGNC:91777]	2.1	0.0644
ssc-miR-22-3p	PRELP	proline and arginine rich end leucine rich repeat protein [Source:VGNC Symbol;Acc:VGNC:91789]	2.1	0.0644
ssc-miR-22-3p	PRKAB1	protein kinase AMP-activated non-catalytic subunit beta 1 [Source:NCBI gene (formerly Entrezgene);Acc:100156537]	2.1	0.0644

ssc-miR-22-3p	PRKAR2A	protein kinase cAMP-dependent type II regulatory subunit alpha [Source:VGNC Symbol;Acc:VGNC:91804]	2.1	0.0644
ssc-miR-22-3p	PROX1	prospero homeobox 1 [Source:VGNC Symbol;Acc:VGNC:91837]	2.1	0.0644
ssc-miR-22-3p	PRPF38A	pre-mRNA processing factor 38A [Source:HGNC Symbol;Acc:HGNC:25930]	2.1	0.0644
ssc-miR-22-3p	PRR14L	proline rich 14 like [Source:VGNC Symbol;Acc:VGNC:91853]	2.1	0.0644
ssc-miR-22-3p	PTEN	hypothetical gene	2.1	0.0644
ssc-miR-22-3p	PTGIS	prostaglandin I2 synthase [Source:VGNC Symbol;Acc:VGNC:96515]	2.1	0.0644
ssc-miR-22-3p	PTGS1	prostaglandin-endoperoxide synthase 1 [Source:VGNC Symbol;Acc:VGNC:91958]	2.1	0.0644
ssc-miR-22-3p	PTPN1	protein tyrosine phosphatase non-receptor type 1 [Source:VGNC Symbol;Acc:VGNC:96519]	2.1	0.0644
ssc-miR-22-3p	PTPN9	protein tyrosine phosphatase non-receptor type 9 [Source:VGNC Symbol;Acc:VGNC:91983]	2.1	0.0644
ssc-miR-22-3p	PURB	purine rich element binding protein B [Source:VGNC Symbol;Acc:VGNC:92004]	2.1	0.0644
ssc-miR-22-3p	QSOX1	quiescin sulfhydryl oxidase 1 [Source:VGNC Symbol;Acc:VGNC:92033]	2.1	0.0644
ssc-miR-22-3p	RAB3C	RAB3C, member RAS onco family [Source:VGNC Symbol;Acc:VGNC:104043]	2.1	0.0644
ssc-miR-22-3p	RAB5B	RAB5B, member RAS oncogene family [Source:HGNC Symbol;Acc:HGNC:9784]	2.1	0.0644
ssc-miR-22-3p	RAP2B	RAP2B, member of RAS onco family [Source:VGNC Symbol;Acc:VGNC:92089]	2.1	0.0644
ssc-miR-22-3p	RAPGEF1	Rap guanine nucleotide exchange factor 1 [Source:VGNC Symbol;Acc:VGNC:92091]	2.1	0.0644
ssc-miR-22-3p	RAPGEFL1	Rap guanine nucleotide exchange factor like 1 [Source:VGNC Symbol;Acc:VGNC:92095]	2.1	0.0644
ssc-miR-22-3p	RASL10B	RAS like family 10 member B [Source:VGNC Symbol;Acc:VGNC:92118]	2.1	0.0644
ssc-miR-22-3p	RASSF9	Ras association domain family member 9 [Source:VGNC Symbol;Acc:VGNC:92129]	2.1	0.0644
ssc-miR-22-3p	RBFOX2	RNA binding fox-1 homolog 2 [Source:VGNC Symbol;Acc:VGNC:92140]	2.1	0.0644
ssc-miR-22-3p	RBM15	RNA binding motif protein 15 [Source:VGNC Symbol;Acc:VGNC:92148]	2.1	0.0644
ssc-miR-22-3p	RCC2	regulator of chromosome condensation 2 [Source:VGNC Symbol;Acc:VGNC:98547]	2.1	0.0644
ssc-miR-22-3p	RCOR1	REST corepressor 1 [Source:VGNC Symbol;Acc:VGNC:92183]	2.1	0.0644
ssc-miR-22-3p	RFXANK	regulatory factor X associated ankyrin containing protein [Source:VGNC Symbol;Acc:VGNC:92250]	2.1	0.0644
ssc-miR-22-3p	RGP1	RGP1 homolog, RAB6A GEF complex partner 1 [Source:VGNC Symbol;Acc:VGNC:92257]	2.1	0.0644
ssc-miR-22-3p	RGS14	regulator of G protein signaling 14 [Source:VGNC Symbol;Acc:VGNC:92261]	2.1	0.0644
ssc-miR-22-3p	RGS2	regulator of G protein signaling 2 [Source:VGNC Symbol;Acc:VGNC:96538]	2.1	0.0644
ssc-miR-22-3p	RGS3	hypothetical gene	2.1	0.0644
ssc-miR-22-3p	RHOC	ras homolog family member C [Source:VGNC Symbol;Acc:VGNC:98846]	2.1	0.0644
ssc-miR-22-3p	RIC8B	RIC8 guanine nucleotide exchange factor B [Source:VGNC Symbol;Acc:VGNC:92302]	2.1	0.0644
ssc-miR-22-3p	RILPL1	Rab interacting lysosomal protein like 1 [Source:VGNC Symbol;Acc:VGNC:92304]	2.1	0.0644
ssc-miR-22-3p	RIMS4	regulating synaptic membrane exocytosis 4 [Source:VGNC Symbol;Acc:VGNC:95775]	2.1	0.0644
ssc-miR-22-3p	RMDN3	regulator of microtubule dynamics 3 [Source:VGNC Symbol;Acc:VGNC:92333]	2.1	0.0644
ssc-miR-22-3p	RMND5B	required for meiotic nuclear division 5 homolog B [Source:VGNC Symbol;Acc:VGNC:92337]	2.1	0.0644
ssc-miR-22-3p	RNF121	interleukin 18 binding protein [Source:VGNC Symbol;Acc:VGNC:89089]	2.1	0.0644
ssc-miR-22-3p	RNF150	ring finger protein 150 [Source:VGNC Symbol;Acc:VGNC:98293]	2.1	0.0644
ssc-miR-22-3p	RNF151	ring finger protein 151 [Source:VGNC Symbol;Acc:VGNC:92363]	2.1	0.0644
ssc-miR-22-3p	RNF19B	ring finger protein 19B [Source:VGNC Symbol;Acc:VGNC:92376]	2.1	0.0644
ssc-miR-22-3p	RNF215	ring finger protein 215 [Source:HGNC Symbol;Acc:HGNC:33434]	2.1	0.0644
ssc-miR-22-3p	RNF44	ring finger protein 44 [Source:VGNC Symbol;Acc:VGNC:92395]	2.1	0.0644
ssc-miR-22-3p	RNF8	ring finger protein 8 [Source:HGNC Symbol;Acc:HGNC:10071]	2.1	0.0644
ssc-miR-22-3p	RPH3AL	rabphilin 3A like (without C2 domains) [Source:HGNC Symbol;Acc:HGNC:10296]	2.1	0.0644
ssc-miR-22-3p	RPL14	hypothetical gene	2.1	0.0644
ssc-miR-22-3p	RPL36A-HNRNPH2	hypothetical gene	2.1	0.0644
ssc-miR-22-3p	RPRD2	regulation of nuclear pre-mRNA domain containing 2 [Source:VGNC Symbol;Acc:VGNC:92436]	2.1	0.0644
ssc-miR-22-3p	RPS3	ribosomal protein S3 [Source:VGNC Symbol;Acc:VGNC:104049]	2.1	0.0644
ssc-miR-22-3p	RPS9	ribosomal protein S9 [Source:VGNC Symbol;Acc:VGNC:92449]	2.1	0.0644
ssc-miR-22-3p	RSBN1	round spermatid basic protein 1 [Source:VGNC Symbol;Acc:VGNC:92473]	2.1	0.0644
ssc-miR-22-3p	RSU1	Ras suppressor protein 1 [Source:VGNC Symbol;Acc:VGNC:98298]	2.1	0.0644
ssc-miR-22-3p	RUNX1	RUNX family transcription factor 1 [Source:VGNC Symbol;Acc:VGNC:92516]	2.1	0.0644

ssc-miR-22-3p	RUNX1T1	RUNX1 partner transcriptional co-repressor 1 [Source:VGNC Symbol;Acc:VGNC:96594]	2.1	0.0644
ssc-miR-22-3p	SAMD12	sterile alpha motif domain containing 12 [Source:VGNC Symbol;Acc:VGNC:92565]	2.1	0.0644
ssc-miR-22-3p	SATB2	SATB homeobox 2 [Source:VGNC Symbol;Acc:VGNC:95972]	2.1	0.0644
ssc-miR-22-3p	SAV1	salvador family WW domain containing protein 1 [Source:VGNC Symbol;Acc:VGNC:92588]	2.1	0.0644
ssc-miR-22-3p	SCAMP1	secretory carrier membrane protein 1 [Source:VGNC Symbol;Acc:VGNC:92605]	2.1	0.0644
ssc-miR-22-3p	SCARF1	scavenger receptor class F member 1 [Source:VGNC Symbol;Acc:VGNC:92615]	2.1	0.0644
ssc-miR-22-3p	SCYL3	SCY1 like pseudokinase 3 [Source:VGNC Symbol;Acc:VGNC:92653]	2.1	0.0644
ssc-miR-22-3p	SEC14L4	SEC14 like lipid binding 4 [Source:HGNC Symbol;Acc:HGNC:20627]	2.1	0.0644
ssc-miR-22-3p	SEC22A	SEC22 homolog A, vesicle trafficking protein [Source:VGNC Symbol;Acc:VGNC:92673]	2.1	0.0644
ssc-miR-22-3p	SEC23B	SEC23 homolog B, COPII coat complex component [Source:VGNC Symbol;Acc:VGNC:95490]	2.1	0.0644
ssc-miR-22-3p	SEMA4F	ssemaphorin 4F [Source:VGNC Symbol;Acc:VGNC:92704]	2.1	0.0644
ssc-miR-22-3p	SEPHS1	selenophosphate synthetase 1 [Source:VGNC Symbol;Acc:VGNC:104053]	2.1	0.0644
ssc-miR-22-3p	SESN3	sestrin 3 [Source:VGNC Symbol;Acc:VGNC:92755]	2.1	0.0644
ssc-miR-22-3p	SFMBT2	Scm like with four mbt domains 2 [Source:VGNC Symbol;Acc:VGNC:95935]	2.1	0.0644
ssc-miR-22-3p	SGSM2	small G protein signaling modulator 2 [Source:VGNC Symbol;Acc:VGNC:92801]	2.1	0.0644
ssc-miR-22-3p	SH2D1B	hypothetical gene	2.1	0.0644
ssc-miR-22-3p	SH3BP5L	SH3 binding domain protein 5 like [Source:VGNC Symbol;Acc:VGNC:92820]	2.1	0.0644
ssc-miR-22-3p	SH3KBP1	SH3 domain containing kinase binding protein 1 [Source:VGNC Symbol;Acc:VGNC:92828]	2.1	0.0644
ssc-miR-22-3p	SH3PXD2A	SH3 and PX domains 2A [Source:VGNC Symbol;Acc:VGNC:92829]	2.1	0.0644
ssc-miR-22-3p	SHISA9	shisa family member 9 [Source:HGNC Symbol;Acc:HGNC:37231]	2.1	0.0644
ssc-miR-22-3p	SIRT1	sirtuin 1 [Source:VGNC Symbol;Acc:VGNC:92884]	2.1	0.0644
ssc-miR-22-3p	SKI	SKI proto-onco [Source:VGNC Symbol;Acc:VGNC:92902]	2.1	0.0644
ssc-miR-22-3p	SLC16A14	solute carrier family 16 member 14 [Source:VGNC Symbol;Acc:VGNC:96129]	2.1	0.0644
ssc-miR-22-3p	SLC16A2	solute carrier family 16 member 2 [Source:VGNC Symbol;Acc:VGNC:92942]	2.1	0.0644
ssc-miR-22-3p	SLC22A3	solute carrier family 22 member 3 [Source:VGNC Symbol;Acc:VGNC:92980]	2.1	0.0644
ssc-miR-22-3p	SLC24A2	solute carrier family 24 member 2 [Source:VGNC Symbol;Acc:VGNC:92987]	2.1	0.0644
ssc-miR-22-3p	SLC24A4	solute carrier family 24 member 4 [Source:VGNC Symbol;Acc:VGNC:92988]	2.1	0.0644
ssc-miR-22-3p	SLC26A9	solute carrier family 26 member 9 [Source:VGNC Symbol;Acc:VGNC:93034]	2.1	0.0644
ssc-miR-22-3p	SLC2A1	solute carrier family 2 member 1 [Source:HGNC Symbol;Acc:HGNC:11005]	2.1	0.0644
ssc-miR-22-3p	SLC2A12	solute carrier family 2 member 12 [Source:VGNC Symbol;Acc:VGNC:93046]	2.1	0.0644
ssc-miR-22-3p	SLC35E4	solute carrier family 35 member E4 [Source:VGNC Symbol;Acc:VGNC:93080]	2.1	0.0644
ssc-miR-22-3p	SLC35F6	solute carrier family 35 member F6 [Source:VGNC Symbol;Acc:VGNC:93085]	2.1	0.0644
ssc-miR-22-3p	SLC39A2	solute carrier family 39 member 2 [Source:VGNC Symbol;Acc:VGNC:93104]	2.1	0.0644
ssc-miR-22-3p	SLC48A1	solute carrier family 48 member 1 [Source:VGNC Symbol;Acc:VGNC:93129]	2.1	0.0644
ssc-miR-22-3p	SLC6A1	solute carrier family 6 member 1 [Source:VGNC Symbol;Acc:VGNC:93153]	2.1	0.0644
ssc-miR-22-3p	SLC6A17	solute carrier family 6 member 17 [Source:VGNC Symbol;Acc:VGNC:93158]	2.1	0.0644
ssc-miR-22-3p	SLC7A2	solute carrier family 7 member 2 [Source:VGNC Symbol;Acc:VGNC:95506]	2.1	0.0644
ssc-miR-22-3p	SLC7A7	solute carrier family 7 member 7 [Source:VGNC Symbol;Acc:VGNC:93175]	2.1	0.0644
ssc-miR-22-3p	SLC9A1	solute carrier family 9 member A1 [Source:NCBI gene (formerly Entrezgene);Acc:397458]	2.1	0.0644
ssc-miR-22-3p	SMAP1	small ArfGAP 1 [Source:VGNC Symbol;Acc:VGNC:93223]	2.1	0.0644
ssc-miR-22-3p	SMG7	SMG7 nonsense mediated mRNA decay factor [Source:VGNC Symbol;Acc:VGNC:93250]	2.1	0.0644
ssc-miR-22-3p	SMIM12	small integral membrane protein 12 [Source:HGNC Symbol;Acc:HGNC:25154]	2.1	0.0644
ssc-miR-22-3p	SMLR1	small leucine rich protein 1 [Source:HGNC Symbol;Acc:HGNC:44670]	2.1	0.0644
ssc-miR-22-3p	SNAI1	snail family transcriptional repressor 1 [Source:VGNC Symbol;Acc:VGNC:95508]	2.1	0.0644
ssc-miR-22-3p	SNAP91	synaptosome associated protein 91 [Source:VGNC Symbol;Acc:VGNC:93279]	2.1	0.0644
ssc-miR-22-3p	SNRK	SNF related kinase [Source:VGNC Symbol;Acc:VGNC:93290]	2.1	0.0644
ssc-miR-22-3p	SNX18	sorting nexin 18 [Source:VGNC Symbol;Acc:VGNC:93310]	2.1	0.0644
ssc-miR-22-3p	SNX21	sorting nexin family member 21 [Source:VGNC Symbol;Acc:VGNC:95758]	2.1	0.0644
ssc-miR-22-3p	SNX33	sorting nexin 33 [Source:VGNC Symbol;Acc:VGNC:93321]	2.1	0.0644

ssc-miR-22-3p	SOCS2	suppressor of cytokine signaling 2 [Source:NCBI gene (formerly Entrezgene);Acc:100037966]	2.1	0.0644
ssc-miR-22-3p	SOCS3	suppressor of cytokine signaling 3 [Source:VGNC Symbol;Acc:VGNC:99052]	2.1	0.0644
ssc-miR-22-3p	SOCS7	suppressor of cytokine signaling 7 [Source:VGNC Symbol;Acc:VGNC:93334]	2.1	0.0644
ssc-miR-22-3p	SORCS1	sortilin related VPS10 domain containing receptor 1 [Source:VGNC Symbol;Acc:VGNC:93339]	2.1	0.0644
ssc-miR-22-3p	SOSTDC1	sclerostin domain containing 1 [Source:VGNC Symbol;Acc:VGNC:93346]	2.1	0.0644
ssc-miR-22-3p	SP1	Sp1 transcription factor [Source:VGNC Symbol;Acc:VGNC:93360]	2.1	0.0644
ssc-miR-22-3p	SPOCK1	SPARC (osteonectin), cwcv and kazal like domains proteoglycan 1 [Source:VGNC Symbol;Acc:VGNC:96619]	2.1	0.0644
ssc-miR-22-3p	SPTLC2	serine palmitoyltransferase long chain base subunit 2 [Source:VGNC Symbol;Acc:VGNC:93440]	2.1	0.0644
ssc-miR-22-3p	SRCAP	Snf2 related CREBBP activator protein [Source:HGNC Symbol;Acc:HGNC:16974]	2.1	0.0644
ssc-miR-22-3p	SRL	sarcalumenin [Source:HGNC Symbol;Acc:HGNC:11295]	2.1	0.0644
ssc-miR-22-3p	SRRD	SRR1 domain containing [Source:HGNC Symbol;Acc:HGNC:33910]	2.1	0.0644
ssc-miR-22-3p	SRSF10	serine and arginine rich splicing factor 10 [Source:VGNC Symbol;Acc:VGNC:93472]	2.1	0.0644
ssc-miR-22-3p	SSPN	sarcospan [Source:VGNC Symbol;Acc:VGNC:93489]	2.1	0.0644
ssc-miR-22-3p	ST6GAL2	ST6 beta-galactoside alpha-2,6-sialyltransferase 2 [Source:VGNC Symbol;Acc:VGNC:93508]	2.1	0.0644
ssc-miR-22-3p	ST8SIA4	ST8 alpha-N-acetyl-neuraminide alpha-2,8-sialyltransferase 4 [Source:VGNC Symbol;Acc:VGNC:93519]	2.1	0.0644
ssc-miR-22-3p	STAG2	stromal antigen 2 [Source:VGNC Symbol;Acc:VGNC:93525]	2.1	0.0644
ssc-miR-22-3p	STEAP3	STEAP3 metalloreductase [Source:VGNC Symbol;Acc:VGNC:96048]	2.1	0.0644
ssc-miR-22-3p	STK39	serine/threonine kinase 39 [Source:VGNC Symbol;Acc:VGNC:98348]	2.1	0.0644
ssc-miR-22-3p	STK4	serine/threonine kinase 4 [Source:VGNC Symbol;Acc:VGNC:98349]	2.1	0.0644
ssc-miR-22-3p	STOX2	storkhead box 2 [Source:VGNC Symbol;Acc:VGNC:96082]	2.1	0.0644
ssc-miR-22-3p	STRN	striatin [Source:VGNC Symbol;Acc:VGNC:93578]	2.1	0.0644
ssc-miR-22-3p	STRN3	striatin 3 [Source:VGNC Symbol;Acc:VGNC:93579]	2.1	0.0644
ssc-miR-22-3p	STX4	syntaxin 4 [Source:VGNC Symbol;Acc:VGNC:93592]	2.1	0.0644
ssc-miR-22-3p	STYX	serine/threonine/tyrosine interacting protein [Source:VGNC Symbol;Acc:VGNC:93602]	2.1	0.0644
ssc-miR-22-3p	SV2A	synaptic vesicle glycoprotein 2A [Source:VGNC Symbol;Acc:VGNC:93639]	2.1	0.0644
ssc-miR-22-3p	SYNE1	hypothetical gene	2.1	0.0644
ssc-miR-22-3p	SYNRG	synergin gamma [Source:VGNC Symbol;Acc:VGNC:93675]	2.1	0.0644
ssc-miR-22-3p	SZRD1	hypothetical gene	2.1	0.0644
ssc-miR-22-3p	TACR3	tachykinin receptor 3 [Source:VGNC Symbol;Acc:VGNC:93703]	2.1	0.0644
ssc-miR-22-3p	TADA2A	transcriptional adaptor 2A [Source:VGNC Symbol;Acc:VGNC:93706]	2.1	0.0644
ssc-miR-22-3p	TAGLN	transgelin [Source:VGNC Symbol;Acc:VGNC:93730]	2.1	0.0644
ssc-miR-22-3p	TBC1D1	TBC1 domain family member 1 [Source:VGNC Symbol;Acc:VGNC:93758]	2.1	0.0644
ssc-miR-22-3p	TBC1D12	TBC1 domain family member 12 [Source:VGNC Symbol;Acc:VGNC:93762]	2.1	0.0644
ssc-miR-22-3p	TBC1D16	hypothetical gene	2.1	0.0644
ssc-miR-22-3p	TBC1D2	TBC1 domain family member 2 [Source:VGNC Symbol;Acc:VGNC:93768]	2.1	0.0644
ssc-miR-22-3p	TBL2	transducin beta like 2 [Source:VGNC Symbol;Acc:VGNC:93789]	2.1	0.0644
ssc-miR-22-3p	TCF23	transcription factor 23 [Source:VGNC Symbol;Acc:VGNC:93820]	2.1	0.0644
ssc-miR-22-3p	TCF7L2	transcription factor 7 like 2 [Source:VGNC Symbol;Acc:VGNC:93825]	2.1	0.0644
ssc-miR-22-3p	TEF	TEF transcription factor, PAR bZIP family member [Source:VGNC Symbol;Acc:VGNC:93864]	2.1	0.0644
ssc-miR-22-3p	TESK2	testis associated actin remodelling kinase 2 [Source:VGNC Symbol;Acc:VGNC:93886]	2.1	0.0644
ssc-miR-22-3p	TET2	hypothetical gene	2.1	0.0644
ssc-miR-22-3p	TFCP2	transcription factor CP2 [Source:VGNC Symbol;Acc:VGNC:93914]	2.1	0.0644
ssc-miR-22-3p	TGFBR1	transforming growth factor beta receptor 1 [Source:VGNC Symbol;Acc:VGNC:98368]	2.1	0.0644
ssc-miR-22-3p	TGIF2	TGFB induced factor homeobox 2 [Source:VGNC Symbol;Acc:VGNC:95656]	2.1	0.0644
ssc-miR-22-3p	TGM6	transglutaminase 6 [Source:VGNC Symbol;Acc:VGNC:95803]	2.1	0.0644
ssc-miR-22-3p	THAP1	THAP domain containing 1 [Source:VGNC Symbol;Acc:VGNC:95950]	2.1	0.0644
ssc-miR-22-3p	THPO	thrombopoietin [Source:VGNC Symbol;Acc:VGNC:93962]	2.1	0.0644
ssc-miR-22-3p	THRA	thyroid hormone receptor alpha [Source:VGNC Symbol;Acc:VGNC:93963]	2.1	0.0644
ssc-miR-22-3p	THSD7A	thrombospondin type 1 domain containing 7A [Source:VGNC Symbol;Acc:VGNC:93968]	2.1	0.0644

ssc-miR-22-3p	TIAM1	TIAM Rac1 associated GEF 1 [Source:VGNC Symbol;Acc:VGNC:96601]	2.1	0.0644
ssc-miR-22-3p	TLK2	tousled like kinase 2 [Source:VGNC Symbol;Acc:VGNC:94017]	2.1	0.0644
ssc-miR-22-3p	TMED4	transmembrane p24 trafficking protein 4 [Source:VGNC Symbol;Acc:VGNC:94056]	2.1	0.0644
ssc-miR-22-3p	TMEM164	transmembrane protein 164 [Source:VGNC Symbol;Acc:VGNC:94103]	2.1	0.0644
ssc-miR-22-3p	TMEM183A	transmembrane protein 183A [Source:NCBI gene (formerly Entrezgene);Acc:100512006]	2.1	0.0644
ssc-miR-22-3p	TMEM184A	transmembrane protein 184A [Source:VGNC Symbol;Acc:VGNC:94119]	2.1	0.0644
ssc-miR-22-3p	TMEM229B	hypothetical gene	2.1	0.0644
ssc-miR-22-3p	TMEM26	transmembrane protein 26 [Source:VGNC Symbol;Acc:VGNC:94163]	2.1	0.0644
ssc-miR-22-3p	TMEM33	transmembrane protein 33 [Source:VGNC Symbol;Acc:VGNC:94171]	2.1	0.0644
ssc-miR-22-3p	TMEM50B	transmembrane protein 50B [Source:VGNC Symbol;Acc:VGNC:94186]	2.1	0.0644
ssc-miR-22-3p	TMOD3	tropomodulin 3 [Source:VGNC Symbol;Acc:VGNC:94228]	2.1	0.0644
ssc-miR-22-3p	TMPRSS11A	transmembrane serine protease 11A [Source:HGNC Symbol;Acc:HGNC:27954]	2.1	0.0644
ssc-miR-22-3p	TNFRSF13C	TNF receptor superfamily member 13C [Source:VGNC Symbol;Acc:VGNC:94260]	2.1	0.0644
ssc-miR-22-3p	TNFRSF1B	TNF receptor superfamily member 1B [Source:VGNC Symbol;Acc:VGNC:94264]	2.1	0.0644
ssc-miR-22-3p	TNFSF15	TNF superfamily member 15 [Source:NCBI gene (formerly Entrezgene);Acc:100624969]	2.1	0.0644
ssc-miR-22-3p	TNIP1	TNFAIP3 interacting protein 1 [Source:VGNC Symbol;Acc:VGNC:94275]	2.1	0.0644
ssc-miR-22-3p	TNKS2	tankyrase 2 [Source:VGNC Symbol;Acc:VGNC:94280]	2.1	0.0644
ssc-miR-22-3p	TNRC6B	trinucleotide repeat containing adaptor 6B [Source:VGNC Symbol;Acc:VGNC:94293]	2.1	0.0644
ssc-miR-22-3p	TOM1	target of myb1 membrane trafficking protein [Source:VGNC Symbol;Acc:VGNC:95552]	2.1	0.0644
ssc-miR-22-3p	TP53	tumor protein p53 [Source:VGNC Symbol;Acc:VGNC:94324]	2.1	0.0644
ssc-miR-22-3p	TP53INP1	tumor protein p53 inducible nuclear protein 1 [Source:VGNC Symbol;Acc:VGNC:94329]	2.1	0.0644
ssc-miR-22-3p	TP63	tumor protein p63 [Source:VGNC Symbol;Acc:VGNC:94330]	2.1	0.0644
ssc-miR-22-3p	TP73	tumor protein p73 [Source:VGNC Symbol;Acc:VGNC:94331]	2.1	0.0644
ssc-miR-22-3p	TPM3	tropomyosin 3 [Source:VGNC Symbol;Acc:VGNC:98877]	2.1	0.0644
ssc-miR-22-3p	TRIB2	tribbles pseudokinase 2 [Source:VGNC Symbol;Acc:VGNC:94392]	2.1	0.0644
ssc-miR-22-3p	TRIM67	tripartite motif containing 67 [Source:VGNC Symbol;Acc:VGNC:94427]	2.1	0.0644
ssc-miR-22-3p	TRPM7	transient receptor potential cation channel subfamily M member 7 [Source:VGNC Symbol;Acc:VGNC:94470]	2.1	0.0644
ssc-miR-22-3p	TRUB1	TruB pseudouridine synthase family member 1 [Source:VGNC Symbol;Acc:VGNC:94478]	2.1	0.0644
ssc-miR-22-3p	TTC31	tetratricopeptide repeat domain 31 [Source:VGNC Symbol;Acc:VGNC:94546]	2.1	0.0644
ssc-miR-22-3p	TTC9	tetratricopeptide repeat domain 9 [Source:VGNC Symbol;Acc:VGNC:94560]	2.1	0.0644
ssc-miR-22-3p	TTYH3	tweety family member 3 [Source:VGNC Symbol;Acc:VGNC:94576]	2.1	0.0644
ssc-miR-22-3p	TUB	TUB bipartite transcription factor [Source:VGNC Symbol;Acc:VGNC:94577]	2.1	0.0644
ssc-miR-22-3p	TUBB6	tubulin beta 6 class V [Source:VGNC Symbol;Acc:VGNC:94581]	2.1	0.0644
ssc-miR-22-3p	TYRO3	TYRO3 protein tyrosine kinase [Source:VGNC Symbol;Acc:VGNC:94618]	2.1	0.0644
ssc-miR-22-3p	UBALD1	hypothetical gene	2.1	0.0644
ssc-miR-22-3p	UBD	ubiquitin D [Source:NCBI gene (formerly Entrezgene);Acc:100294688]	2.1	0.0644
ssc-miR-22-3p	UBE2N	hypothetical gene	2.1	0.0644
ssc-miR-22-3p	UBE2O	ubiquitin conjugating enzyme E2 O [Source:VGNC Symbol;Acc:VGNC:94649]	2.1	0.0644
ssc-miR-22-3p	UGCG	UDP-glucose ceramide glucosyltransferase [Source:VGNC Symbol;Acc:VGNC:94685]	2.1	0.0644
ssc-miR-22-3p	ULK2	unc-51 like autophagy activating kinase 2 [Source:VGNC Symbol;Acc:VGNC:94695]	2.1	0.0644
ssc-miR-22-3p	UNC5D	unc-5 netrin receptor D [Source:VGNC Symbol;Acc:VGNC:95892]	2.1	0.0644
ssc-miR-22-3p	UNC79	unc-79 homolog, NALCN channel complex subunit [Source:HGNC Symbol;Acc:HGNC:19966]	2.1	0.0644
ssc-miR-22-3p	UNK	unk zinc finger [Source:VGNC Symbol;Acc:VGNC:94715]	2.1	0.0644
ssc-miR-22-3p	UQCC1	ubiquinol-cytochrome c reductase complex assembly factor 1 [Source:VGNC Symbol;Acc:VGNC:95694]	2.1	0.0644
ssc-miR-22-3p	USP37	ubiquitin specific peptidase 37 [Source:VGNC Symbol;Acc:VGNC:98404]	2.1	0.0644
ssc-miR-22-3p	USP46	ubiquitin specific peptidase 46 [Source:VGNC Symbol;Acc:VGNC:94768]	2.1	0.0644
ssc-miR-22-3p	USP47	ubiquitin specific peptidase 47 [Source:VGNC Symbol;Acc:VGNC:94769]	2.1	0.0644
ssc-miR-22-3p	USP8	ubiquitin specific peptidase 8 [Source:VGNC Symbol;Acc:VGNC:94778]	2.1	0.0644
ssc-miR-22-3p	VASH1	vasohibin 1 [Source:VGNC Symbol;Acc:VGNC:94801]	2.1	0.0644

ssc-miR-22-3p	VASP	vasodilator stimulated phosphoprotein [Source:VGNC Symbol;Acc:VGNC:94804]	2.1	0.0644
ssc-miR-22-3p	VCAM1	vascular cell adhesion molecule 1 [Source:VGNC Symbol;Acc:VGNC:94811]	2.1	0.0644
ssc-miR-22-3p	VEZF1	vascular endothelial zinc finger 1 [Source:VGNC Symbol;Acc:VGNC:94818]	2.1	0.0644
ssc-miR-22-3p	VPS13B	vacuolar protein sorting 13 homolog B [Source:VGNC Symbol;Acc:VGNC:94839]	2.1	0.0644
ssc-miR-22-3p	VPS53	VPS53 subunit of GARP complex [Source:VGNC Symbol;Acc:VGNC:94861]	2.1	0.0644
ssc-miR-22-3p	VSNL1	visinin like 1 [Source:VGNC Symbol;Acc:VGNC:94871]	2.1	0.0644
ssc-miR-22-3p	WAPAL	hypothetical gene	2.1	0.0644
ssc-miR-22-3p	WARS2	tryptophanyl tRNA synthetase 2, mitochondrial [Source:VGNC Symbol;Acc:VGNC:94892]	2.1	0.0644
ssc-miR-22-3p	WASF1	hypothetical gene	2.1	0.0644
ssc-miR-22-3p	WDFY3	WD repeat and FYVE domain containing 3 [Source:VGNC Symbol;Acc:VGNC:94903]	2.1	0.0644
ssc-miR-22-3p	WDR82	WD repeat domain 82 [Source:VGNC Symbol;Acc:VGNC:94944]	2.1	0.0644
ssc-miR-22-3p	WDTC1	WD and tetratricopeptide repeats 1 [Source:VGNC Symbol;Acc:VGNC:94952]	2.1	0.0644
ssc-miR-22-3p	WFIKKN2	WAP, follistatin/kazal, immunoglobulin, kunitz and netrin domain containing 2 [Source:VGNC Symbol;Acc:VGNC:99105]	2.1	0.0644
ssc-miR-22-3p	WNK3	WNK lysine deficient protein kinase 3 [Source:VGNC Symbol;Acc:VGNC:94964]	2.1	0.0644
ssc-miR-22-3p	WNT1	Wnt family member 1 [Source:HGNC Symbol;Acc:HGNC:12774]	2.1	0.0644
ssc-miR-22-3p	WNT10B	Wnt family member 10B [Source:VGNC Symbol;Acc:VGNC:94965]	2.1	0.0644
ssc-miR-22-3p	WRNIP1	WRN helicase interacting protein 1 [Source:VGNC Symbol;Acc:VGNC:104096]	2.1	0.0644
ssc-miR-22-3p	WSCD1	WSC domain containing 1 [Source:VGNC Symbol;Acc:VGNC:94983]	2.1	0.0644
ssc-miR-22-3p	WWC2	WW and C2 domain containing 2 [Source:VGNC Symbol;Acc:VGNC:96033]	2.1	0.0644
ssc-miR-22-3p	XXbac-BPG32J3.20	hypothetical gene	2.1	0.0644
ssc-miR-22-3p	YWHAZ	tyrosine 3-monooxygenase/tryptophan 5-monooxygenase activation protein zeta [Source:VGNC Symbol;Acc:VGNC:95047]	2.1	0.0644
ssc-miR-22-3p	ZBTB16	zinc finger and BTB domain containing 16 [Source:VGNC Symbol;Acc:VGNC:108626]	2.1	0.0644
ssc-miR-22-3p	ZBTB20	zinc finger and BTB domain containing 20 [Source:VGNC Symbol;Acc:VGNC:95063]	2.1	0.0644
ssc-miR-22-3p	ZBTB39	zinc finger and BTB domain containing 39 [Source:VGNC Symbol;Acc:VGNC:95073]	2.1	0.0644
ssc-miR-22-3p	ZBTB47	zinc finger and BTB domain containing 47 [Source:VGNC Symbol;Acc:VGNC:95078]	2.1	0.0644
ssc-miR-22-3p	ZCCHC14	zinc finger CCHC-type containing 14 [Source:VGNC Symbol;Acc:VGNC:95107]	2.1	0.0644
ssc-miR-22-3p	ZCCHC8	zinc finger CCHC-type containing 8 [Source:VGNC Symbol;Acc:VGNC:95112]	2.1	0.0644
ssc-miR-22-3p	ZDHHC16	zinc finger DHHC-type palmitoyltransferase 16 [Source:VGNC Symbol;Acc:VGNC:104101]	2.1	0.0644
ssc-miR-22-3p	ZDHHC3	zinc finger DHHC-type palmitoyltransferase 3 [Source:VGNC Symbol;Acc:VGNC:95126]	2.1	0.0644
ssc-miR-22-3p	ZFP36L2	ZFP36 ring finger protein like 2 [Source:VGNC Symbol;Acc:VGNC:95147]	2.1	0.0644
ssc-miR-22-3p	ZFP91	ZFP91 zinc finger protein, atypical E3 ubiquitin ligase [Source:HGNC Symbol;Acc:HGNC:14983]	2.1	0.0644
ssc-miR-22-3p	ZFYVE9	zinc finger FYVE-type containing 9 [Source:VGNC Symbol;Acc:VGNC:95163]	2.1	0.0644
ssc-miR-22-3p	ZKSCAN1	zinc finger with KRAB and SCAN domains 1 [Source:VGNC Symbol;Acc:VGNC:95171]	2.1	0.0644
ssc-miR-22-3p	ZMYM4	zinc finger MYM-type containing 4 [Source:VGNC Symbol;Acc:VGNC:95183]	2.1	0.0644
ssc-miR-22-3p	ZNF106	zinc finger protein 106 [Source:VGNC Symbol;Acc:VGNC:95190]	2.1	0.0644
ssc-miR-22-3p	ZNF500	hypothetical gene	2.1	0.0644
ssc-miR-22-3p	ZNF593	zinc finger protein 593 [Source:HGNC Symbol;Acc:HGNC:30943]	2.1	0.0644
ssc-miR-22-3p	ZNF609	zinc finger protein 609 [Source:VGNC Symbol;Acc:VGNC:95274]	2.1	0.0644
ssc-miR-22-3p	ZNF618	zinc finger protein 618 [Source:VGNC Symbol;Acc:VGNC:95276]	2.1	0.0644
ssc-miR-22-3p	ZNF638	zinc finger protein 638 [Source:VGNC Symbol;Acc:VGNC:95281]	2.1	0.0644
ssc-miR-22-3p	ZNF652	zinc finger protein 652 [Source:VGNC Symbol;Acc:VGNC:99108]	2.1	0.0644
ssc-miR-22-3p	ZNF668	zinc finger protein 668 [Source:VGNC Symbol;Acc:VGNC:95290]	2.1	0.0644
ssc-miR-22-3p	ZNF704	zinc finger protein 704 [Source:VGNC Symbol;Acc:VGNC:95296]	2.1	0.0644
ssc-miR-22-3p	ZNF706	hypothetical gene	2.1	0.0644
ssc-miR-22-3p	ZNF740	zinc finger protein 740 [Source:VGNC Symbol;Acc:VGNC:95298]	2.1	0.0644
ssc-miR-22-3p	ZNF827	zinc finger protein 827 [Source:VGNC Symbol;Acc:VGNC:98970]	2.1	0.0644
ssc-miR-22-3p	ZNHIT6	zinc finger HIT-type containing 6 [Source:VGNC Symbol;Acc:VGNC:98909]	2.1	0.0644
ssc-miR-22-3p	ZNRF2	zinc and ring finger 2 [Source:VGNC Symbol;Acc:VGNC:95317]	2.1	0.0644
ssc-miR-125a	ABAT	4-aminobutyrate aminotransferase [Source:VGNC Symbol;Acc:VGNC:96910]	2.08	0.0653

ssc-miR-125a	ABCC5	ATP binding cassette subfamily C member 5 [Source:VGNC Symbol;Acc:VGNC:84958]	2.08	0.0653
ssc-miR-125a	ABHD3	abhydrolase domain containing 3, phospholipase [Source:VGNC Symbol;Acc:VGNC:84981]	2.08	0.0653
ssc-miR-125a	ABHD6	abhydrolase domain containing 6, acylglycerol lipase [Source:VGNC Symbol;Acc:VGNC:84982]	2.08	0.0653
ssc-miR-125a	ABI2	abl interactor 2 [Source:VGNC Symbol;Acc:VGNC:96027]	2.08	0.0653
ssc-miR-125a	ABL2	ABL proto-onco 2, non-receptor tyrosine kinase [Source:VGNC Symbol;Acc:VGNC:84985]	2.08	0.0653
ssc-miR-125a	ABTB1	ankyrin repeat and BTB domain containing 1 [Source:VGNC Symbol;Acc:VGNC:84993]	2.08	0.0653
ssc-miR-125a	AC140061.12	hypothetical gene	2.08	0.0653
ssc-miR-125a	ACACB	acetyl-CoA carboxylase beta [Source:NCBI gene (formerly Entrezgene);Acc:100154993]	2.08	0.0653
ssc-miR-125a	ACER2	alkaline ceramidase 2 [Source:VGNC Symbol;Acc:VGNC:85010]	2.08	0.0653
ssc-miR-125a	ACHE	acetylcholinesterase [Source:VGNC Symbol;Acc:VGNC:97029]	2.08	0.0653
ssc-miR-125a	ACOT13	acyl-CoA thioesterase 13 [Source:VGNC Symbol;Acc:VGNC:85018]	2.08	0.0653
ssc-miR-125a	ACVR1C	activin A receptor type 1C [Source:VGNC Symbol;Acc:VGNC:95873]	2.08	0.0653
ssc-miR-125a	ACVR2A	activin A receptor type 2A [Source:VGNC Symbol;Acc:VGNC:95843]	2.08	0.0653
ssc-miR-125a	ACVR2B	activin A receptor type 2B [Source:VGNC Symbol;Acc:VGNC:108629]	2.08	0.0653
ssc-miR-125a	ADAM11	ADAM metallopeptidase domain 11 [Source:VGNC Symbol;Acc:VGNC:85062]	2.08	0.0653
ssc-miR-125a	ADAM9	ADAM metallopeptidase domain 9 [Source:VGNC Symbol;Acc:VGNC:95969]	2.08	0.0653
ssc-miR-125a	ADAMTS14	ADAM metallopeptidase with thrombospondin type 1 motif 14 [Source:VGNC Symbol;Acc:VGNC:85076]	2.08	0.0653
ssc-miR-125a	ADAMTS15	ADAM metallopeptidase with thrombospondin type 1 motif 15 [Source:VGNC Symbol;Acc:VGNC:85077]	2.08	0.0653
ssc-miR-125a	ADCY5	adenylate cyclase 5 [Source:VGNC Symbol;Acc:VGNC:85109]	2.08	0.0653
ssc-miR-125a	ADCY6	adenylate cyclase 6 [Source:VGNC Symbol;Acc:VGNC:85110]	2.08	0.0653
ssc-miR-125a	ADD2	adducin 2 [Source:VGNC Symbol;Acc:VGNC:85117]	2.08	0.0653
ssc-miR-125a	AGGF1	angiogenic factor with G-patch and FHA domains 1 [Source:VGNC Symbol;Acc:VGNC:96789]	2.08	0.0653
ssc-miR-125a	AGO2	argonaute RISC catalytic component 2 [Source:VGNC Symbol;Acc:VGNC:97871]	2.08	0.0653
ssc-miR-125a	AGO4	hypothetical gene	2.08	0.0653
ssc-miR-125a	AHCY	adenosylhomocysteinase [Source:VGNC Symbol;Acc:VGNC:108708]	2.08	0.0653
ssc-miR-125a	AHSA2	hypothetical gene	2.08	0.0653
ssc-miR-125a	AIFM1	apoptosis inducing factor mitochondria associated 1 [Source:VGNC Symbol;Acc:VGNC:85198]	2.08	0.0653
ssc-miR-125a	AJUBA	ajuba LIM protein [Source:VGNC Symbol;Acc:VGNC:85208]	2.08	0.0653
ssc-miR-125a	AKAP1	A-kinase anchoring protein 1 [Source:VGNC Symbol;Acc:VGNC:85214]	2.08	0.0653
ssc-miR-125a	AKAP13	hypothetical gene	2.08	0.0653
ssc-miR-125a	AKT1S1	AKT1 substrate 1 [Source:VGNC Symbol;Acc:VGNC:85231]	2.08	0.0653
ssc-miR-125a	AKT3	AKT serine/threonine kinase 3 [Source:VGNC Symbol;Acc:VGNC:96306]	2.08	0.0653
ssc-miR-125a	AL626787.1	hypothetical gene	2.08	0.0653
ssc-miR-125a	ALDH1A3	aldehyde dehydrogenase 1 family member A3 [Source:VGNC Symbol;Acc:VGNC:97879]	2.08	0.0653
ssc-miR-125a	ALG6	ALG6 alpha-1,3-glucosyltransferase [Source:VGNC Symbol;Acc:VGNC:85253]	2.08	0.0653
ssc-miR-125a	ALPK3	alpha kinase 3 [Source:HGNC Symbol;Acc:HGNC:17574]	2.08	0.0653
ssc-miR-125a	AMER2	APC membrane recruitment protein 2 [Source:VGNC Symbol;Acc:VGNC:85277]	2.08	0.0653
ssc-miR-125a	ANAPC16	anaphase promoting complex subunit 16 [Source:NCBI gene (formerly Entrezgene);Acc:100155019]	2.08	0.0653
ssc-miR-125a	ANGPT2	angiopoietin 2 [Source:NCBI gene (formerly Entrezgene);Acc:396730]	2.08	0.0653
ssc-miR-125a	ANKRD13B	ankyrin repeat domain 13B [Source:VGNC Symbol;Acc:VGNC:85325]	2.08	0.0653
ssc-miR-125a	ANKRD33B	ankyrin repeat domain 33B [Source:VGNC Symbol;Acc:VGNC:85334]	2.08	0.0653
ssc-miR-125a	ANKRD44	ankyrin repeat domain 44 [Source:VGNC Symbol;Acc:VGNC:107123]	2.08	0.0653
ssc-miR-125a	ANKRD50	ankyrin repeat domain containing 50 [Source:VGNC Symbol;Acc:VGNC:85341]	2.08	0.0653
ssc-miR-125a	ANO3	anoctamin 3 [Source:VGNC Symbol;Acc:VGNC:85356]	2.08	0.0653
ssc-miR-125a	ANPEP	alanyl aminopeptidase, membrane [Source:VGNC Symbol;Acc:VGNC:85362]	2.08	0.0653
ssc-miR-125a	ANTXR2	ANTXR cell adhesion molecule 2 [Source:VGNC Symbol;Acc:VGNC:85365]	2.08	0.0653
ssc-miR-125a	AP5Z1	adaptor related protein complex 5 subunit zeta 1 [Source:VGNC Symbol;Acc:VGNC:85397]	2.08	0.0653
ssc-miR-125a	APC	APC regulator of WNT signaling pathway [Source:VGNC Symbol;Acc:VGNC:99584]	2.08	0.0653
ssc-miR-125a	APMAP	adipocyte plasma membrane associated protein [Source:VGNC Symbol;Acc:VGNC:95578]	2.08	0.0653

ssc-miR-125a	APOC4	apolipoprotein C4 [Source:NCBI gene (formerly Entrezgene);Acc:100381253]	2.08	0.0653
ssc-miR-125a	AQPEP	hypothetical gene	2.08	0.0653
ssc-miR-125a	ARCN1	archain 1 [Source:VGNC Symbol;Acc:VGNC:85443]	2.08	0.0653
ssc-miR-125a	AREL1	apoptosis resistant E3 ubiquitin protein ligase 1 [Source:VGNC Symbol;Acc:VGNC:85445]	2.08	0.0653
ssc-miR-125a	ARF3	ADP ribosylation factor 3 [Source:NCBI gene (formerly Entrezgene);Acc:100301570]	2.08	0.0653
ssc-miR-125a	ARHGEF1	Rho guanine nucleotide exchange factor 1 [Source:VGNC Symbol;Acc:VGNC:96922]	2.08	0.0653
ssc-miR-125a	ARID3A	AT-rich interaction domain 3A [Source:VGNC Symbol;Acc:VGNC:85507]	2.08	0.0653
ssc-miR-125a	ARID3B	AT-rich interaction domain 3B [Source:VGNC Symbol;Acc:VGNC:85508]	2.08	0.0653
ssc-miR-125a	ASB13	ankyrin repeat and SOCS box containing 13 [Source:VGNC Symbol;Acc:VGNC:95921]	2.08	0.0653
ssc-miR-125a	ASB4	ankyrin repeat and SOCS box containing 4 [Source:VGNC Symbol;Acc:VGNC:97891]	2.08	0.0653
ssc-miR-125a	ASB6	ankyrin repeat and SOCS box containing 6 [Source:VGNC Symbol;Acc:VGNC:85565]	2.08	0.0653
ssc-miR-125a	ASCC1	activating signal cointegrator 1 complex subunit 1 [Source:VGNC Symbol;Acc:VGNC:85569]	2.08	0.0653
ssc-miR-125a	ASIC1	acid sensing ion channel subunit 1 [Source:VGNC Symbol;Acc:VGNC:85578]	2.08	0.0653
ssc-miR-125a	ASXL2	ASXL transcriptional regulator 2 [Source:VGNC Symbol;Acc:VGNC:85595]	2.08	0.0653
ssc-miR-125a	ASXL3	ASXL transcriptional regulator 3 [Source:VGNC Symbol;Acc:VGNC:85596]	2.08	0.0653
ssc-miR-125a	ATG4D	autophagy related 4D cysteine peptidase [Source:VGNC Symbol;Acc:VGNC:85624]	2.08	0.0653
ssc-miR-125a	ATL2	atlastin GTPase 2 [Source:VGNC Symbol;Acc:VGNC:85628]	2.08	0.0653
ssc-miR-125a	ATOH8	atonal bHLH transcription factor 8 [Source:VGNC Symbol;Acc:VGNC:85633]	2.08	0.0653
ssc-miR-125a	ATP10D	ATPase phospholipid transporting 10D (putative) [Source:VGNC Symbol;Acc:VGNC:98913]	2.08	0.0653
ssc-miR-125a	ATP13A3	ATPase 13A3 [Source:VGNC Symbol;Acc:VGNC:85639]	2.08	0.0653
ssc-miR-125a	ATXN1	ataxin 1 [Source:VGNC Symbol;Acc:VGNC:85687]	2.08	0.0653
ssc-miR-125a	ATXN1L	ataxin 1 like [Source:VGNC Symbol;Acc:VGNC:85689]	2.08	0.0653
ssc-miR-125a	ATXN3	hypothetical gene	2.08	0.0653
ssc-miR-125a	ATXN7	ataxin 7 [Source:VGNC Symbol;Acc:VGNC:99704]	2.08	0.0653
ssc-miR-125a	ATXN7L3	ataxin 7 like 3 [Source:VGNC Symbol;Acc:VGNC:85694]	2.08	0.0653
ssc-miR-125a	AVL9	AVL9 cell migration associated [Source:VGNC Symbol;Acc:VGNC:85701]	2.08	0.0653
ssc-miR-125a	B3GALNT2	beta-1,3-N-acetylgalactosaminyltransferase 2 [Source:VGNC Symbol;Acc:VGNC:85715]	2.08	0.0653
ssc-miR-125a	B4GALNT3	beta-1,4-N-acetyl-galactosaminyltransferase 3 [Source:VGNC Symbol;Acc:VGNC:85731]	2.08	0.0653
ssc-miR-125a	B4GALT1	beta-1,4-galactosyltransferase 1 [Source:VGNC Symbol;Acc:VGNC:96497]	2.08	0.0653
ssc-miR-125a	B4GALT6	beta-1,4-galactosyltransferase 6 [Source:VGNC Symbol;Acc:VGNC:85734]	2.08	0.0653
ssc-miR-125a	BAG4	BAG cochaperone 4 [Source:VGNC Symbol;Acc:VGNC:96503]	2.08	0.0653
ssc-miR-125a	BAK1	hypothetical gene	2.08	0.0653
ssc-miR-125a	BAP1	BRCA1 associated protein 1 [Source:VGNC Symbol;Acc:VGNC:85754]	2.08	0.0653
ssc-miR-125a	BAZ2A	bromodomain adjacent to zinc finger domain 2A [Source:VGNC Symbol;Acc:VGNC:85763]	2.08	0.0653
ssc-miR-125a	BCAS3	BCAS3 microtubule associated cell migration factor [Source:VGNC Symbol;Acc:VGNC:85772]	2.08	0.0653
ssc-miR-125a	BCAT1	branched chain amino acid transaminase 1 [Source:VGNC Symbol;Acc:VGNC:85773]	2.08	0.0653
ssc-miR-125a	BCL2	BCL2 apoptosis regulator [Source:HGNC Symbol;Acc:HGNC:990]	2.08	0.0653
ssc-miR-125a	BCL2L12	hypothetical gene	2.08	0.0653
ssc-miR-125a	BCL2L2	BCL2 like 2 [Source:HGNC Symbol;Acc:HGNC:995]	2.08	0.0653
ssc-miR-125a	BDH1	3-hydroxybutyrate dehydrogenase 1 [Source:VGNC Symbol;Acc:VGNC:85792]	2.08	0.0653
ssc-miR-125a	BLZF1	basic leucine zipper nuclear factor 1 [Source:VGNC Symbol;Acc:VGNC:85835]	2.08	0.0653
ssc-miR-125a	BMF	Bcl2 modifying factor [Source:VGNC Symbol;Acc:VGNC:85837]	2.08	0.0653
ssc-miR-125a	BMPR1B	bone morphotic protein receptor type 1B [Source:VGNC Symbol;Acc:VGNC:85847]	2.08	0.0653
ssc-miR-125a	BMPR2	bone morphotic protein receptor type 2 [Source:VGNC Symbol;Acc:VGNC:95494]	2.08	0.0653
ssc-miR-125a	BNIP2	BCL2 interacting protein 2 [Source:VGNC Symbol;Acc:VGNC:85854]	2.08	0.0653
ssc-miR-125a	BRPF1	bromodomain and PHD finger containing 1 [Source:VGNC Symbol;Acc:VGNC:85885]	2.08	0.0653
ssc-miR-125a	BRSK2	BR serine/threonine kinase 2 [Source:VGNC Symbol;Acc:VGNC:85889]	2.08	0.0653
ssc-miR-125a	BRWD1	bromodomain and WD repeat domain containing 1 [Source:VGNC Symbol;Acc:VGNC:108153]	2.08	0.0653
ssc-miR-125a	BSN	bassoon presynaptic cytomatrix protein [Source:VGNC Symbol;Acc:VGNC:85893]	2.08	0.0653

ssc-miR-125a	BTG2	BTG anti-proliferation factor 2 [Source:NCBI gene (formerly Entrezgene);Acc:100048932]	2.08	0.0653
ssc-miR-125a	BTN2A2	hypothetical gene	2.08	0.0653
ssc-miR-125a	BTN3A1	hypothetical gene	2.08	0.0653
ssc-miR-125a	BTN3A2	hypothetical gene	2.08	0.0653
ssc-miR-125a	C10orf105	chromosome 14 C10orf105 homolog [Source:VGNC Symbol;Acc:VGNC:85940]	2.08	0.0653
ssc-miR-125a	C10orf54	hypothetical gene	2.08	0.0653
ssc-miR-125a	C11orf57	hypothetical gene	2.08	0.0653
ssc-miR-125a	C15orf39	chromosome 7 C15orf39 homolog [Source:VGNC Symbol;Acc:VGNC:86070]	2.08	0.0653
ssc-miR-125a	C17orf59	hypothetical gene	2.08	0.0653
ssc-miR-125a	C17orf96	hypothetical gene	2.08	0.0653
ssc-miR-125a	C19orf38	chromosome 2 C19orf38 homolog [Source:VGNC Symbol;Acc:VGNC:86003]	2.08	0.0653
ssc-miR-125a	C19orf54	chromosome 6 C19orf54 homolog [Source:VGNC Symbol;Acc:VGNC:86057]	2.08	0.0653
ssc-miR-125a	C1orf210	chromosome 6 C1orf210 homolog [Source:VGNC Symbol;Acc:VGNC:86063]	2.08	0.0653
ssc-miR-125a	C1orf226	hypothetical gene	2.08	0.0653
ssc-miR-125a	C1QTNF3	C1q and TNF related 3 [Source:NCBI gene (formerly Entrezgene);Acc:100270814]	2.08	0.0653
ssc-miR-125a	C20orf112	hypothetical gene	2.08	0.0653
ssc-miR-125a	C4orf19	chromosome 8 C4orf19 homolog [Source:VGNC Symbol;Acc:VGNC:86080]	2.08	0.0653
ssc-miR-125a	C6orf47	hypothetical gene	2.08	0.0653
ssc-miR-125a	CA12	carbonic anhydrase 12 [Source:VGNC Symbol;Acc:VGNC:86097]	2.08	0.0653
ssc-miR-125a	CACNB1	calcium voltage-gated channel auxiliary subunit beta 1 [Source:VGNC Symbol;Acc:VGNC:97909]	2.08	0.0653
ssc-miR-125a	CACNB3	calcium voltage-gated channel auxiliary subunit beta 3 [Source:VGNC Symbol;Acc:VGNC:103221]	2.08	0.0653
ssc-miR-125a	CACNG2	calcium voltage-gated channel auxiliary subunit gamma 2 [Source:VGNC Symbol;Acc:VGNC:86125]	2.08	0.0653
ssc-miR-125a	CALHM1	calcium homeostasis modulator 1 [Source:HGNC Symbol;Acc:HGNC:23494]	2.08	0.0653
ssc-miR-125a	CALN1	hypothetical gene	2.08	0.0653
ssc-miR-125a	CAMK4	calcium/calmodulin dependent protein kinase IV [Source:VGNC Symbol;Acc:VGNC:99602]	2.08	0.0653
ssc-miR-125a	CAMKV	CaM kinase like vesicle associated [Source:VGNC Symbol;Acc:VGNC:86160]	2.08	0.0653
ssc-miR-125a	CAMSAP3	calmodulin regulated spectrin associated protein family member 3 [Source:VGNC Symbol;Acc:VGNC:86163]	2.08	0.0653
ssc-miR-125a	CASP2	caspase 2 [Source:HGNC Symbol;Acc:HGNC:1503]	2.08	0.0653
ssc-miR-125a	CBFB	core-binding factor subunit beta [Source:VGNC Symbol;Acc:VGNC:86221]	2.08	0.0653
ssc-miR-125a	CBX7	chromobox 7 [Source:HGNC Symbol;Acc:HGNC:1557]	2.08	0.0653
ssc-miR-125a	CCBE1	collagen and calcium binding EGF domains 1 [Source:VGNC Symbol;Acc:VGNC:86240]	2.08	0.0653
ssc-miR-125a	CCDC71L	coiled-coil domain containing 71 like [Source:HGNC Symbol;Acc:HGNC:26685]	2.08	0.0653
ssc-miR-125a	CCNJ	cyclin J [Source:VGNC Symbol;Acc:VGNC:86360]	2.08	0.0653
ssc-miR-125a	CCNJL	cyclin J like [Source:VGNC Symbol;Acc:VGNC:86361]	2.08	0.0653
ssc-miR-125a	CD248	CD248 molecule [Source:VGNC Symbol;Acc:VGNC:86402]	2.08	0.0653
ssc-miR-125a	CD300LF	hypothetical gene	2.08	0.0653
ssc-miR-125a	CD34	CD34 molecule [Source:VGNC Symbol;Acc:VGNC:86410]	2.08	0.0653
ssc-miR-125a	CDC37L1	cell division cycle 37 like 1 [Source:VGNC Symbol;Acc:VGNC:86451]	2.08	0.0653
ssc-miR-125a	CDC42BPG	CDC42 binding protein kinase gamma [Source:VGNC Symbol;Acc:VGNC:86454]	2.08	0.0653
ssc-miR-125a	CDC42SE1	CDC42 small effector 1 [Source:VGNC Symbol;Acc:VGNC:86460]	2.08	0.0653
ssc-miR-125a	CDH5	cadherin 5 [Source:VGNC Symbol;Acc:VGNC:86486]	2.08	0.0653
ssc-miR-125a	CDK16	cyclin dependent kinase 16 [Source:VGNC Symbol;Acc:VGNC:86499]	2.08	0.0653
ssc-miR-125a	CDK19	cyclin dependent kinase 19 [Source:VGNC Symbol;Acc:VGNC:86502]	2.08	0.0653
ssc-miR-125a	CDR2L	cerebellar deration related protein 2 like [Source:VGNC Symbol;Acc:VGNC:86522]	2.08	0.0653
ssc-miR-125a	CDS2	CDP-diacylglycerol synthase 2 [Source:VGNC Symbol;Acc:VGNC:95869]	2.08	0.0653
ssc-miR-125a	CECR2	CECR2 histone acetyl-lysine reader [Source:VGNC Symbol;Acc:VGNC:96576]	2.08	0.0653
ssc-miR-125a	CELSR2	cadherin EGF LAG seven-pass G-type receptor 2 [Source:VGNC Symbol;Acc:VGNC:98748]	2.08	0.0653
ssc-miR-125a	CEP85	centrosomal protein 85 [Source:VGNC Symbol;Acc:VGNC:86579]	2.08	0.0653
ssc-miR-125a	CGN	hypothetical gene	2.08	0.0653

ssc-miR-125a	CHAMP1	hypothetical gene	2.08	0.0653
ssc-miR-125a	CHTF8	hypothetical gene	2.08	0.0653
ssc-miR-125a	CLCC1	chloride channel CLIC like 1 [Source:VGNC Symbol;Acc:VGNC:86724]	2.08	0.0653
ssc-miR-125a	CLDN12	hypothetical gene	2.08	0.0653
ssc-miR-125a	CLIC4	chloride intracellular channel 4 [Source:VGNC Symbol;Acc:VGNC:86763]	2.08	0.0653
ssc-miR-125a	CLIP2	hypothetical gene	2.08	0.0653
ssc-miR-125a	CMTM7	CKLF like MARVEL transmembrane domain containing 7 [Source:VGNC Symbol;Acc:VGNC:103928]	2.08	0.0653
ssc-miR-125a	CNNM1	cyclin and CBS domain divalent metal cation transport mediator 1 [Source:VGNC Symbol;Acc:VGNC:86827]	2.08	0.0653
ssc-miR-125a	CNNM2	cyclin and CBS domain divalent metal cation transport mediator 2 [Source:VGNC Symbol;Acc:VGNC:86828]	2.08	0.0653
ssc-miR-125a	CNNM4	cyclin and CBS domain divalent metal cation transport mediator 4 [Source:VGNC Symbol;Acc:VGNC:86830]	2.08	0.0653
ssc-miR-125a	CNTNAP5	contactin associated protein family member 5 [Source:VGNC Symbol;Acc:VGNC:95459]	2.08	0.0653
ssc-miR-125a	COL11A2	collagen type XI alpha 2 chain [Source:VGNC Symbol;Acc:VGNC:86863]	2.08	0.0653
ssc-miR-125a	COL4A3	collagen type IV alpha 3 chain [Source:HGNC Symbol;Acc:HGNC:2204]	2.08	0.0653
ssc-miR-125a	COPS7B	COP9 signalosome subunit 7B [Source:HGNC Symbol;Acc:HGNC:16760]	2.08	0.0653
ssc-miR-125a	COPZ1	COPI coat complex subunit zeta 1 [Source:VGNC Symbol;Acc:VGNC:86903]	2.08	0.0653
ssc-miR-125a	CORO1C	coronin 1C [Source:VGNC Symbol;Acc:VGNC:86914]	2.08	0.0653
ssc-miR-125a	CORO2A	coronin 2A [Source:VGNC Symbol;Acc:VGNC:86915]	2.08	0.0653
ssc-miR-125a	CORO2B	coronin 2B [Source:VGNC Symbol;Acc:VGNC:86916]	2.08	0.0653
ssc-miR-125a	COX20	hypothetical gene	2.08	0.0653
ssc-miR-125a	CPEB3	cytoplasmic polyadenylation element binding protein 3 [Source:VGNC Symbol;Acc:VGNC:86938]	2.08	0.0653
ssc-miR-125a	CPEB4	cytoplasmic polyadenylation element binding protein 4 [Source:VGNC Symbol;Acc:VGNC:86939]	2.08	0.0653
ssc-miR-125a	CPM	carboxypeptidase M [Source:VGNC Symbol;Acc:VGNC:86947]	2.08	0.0653
ssc-miR-125a	CPNE2	copine 2 [Source:VGNC Symbol;Acc:VGNC:86950]	2.08	0.0653
ssc-miR-125a	CPSF6	cleavage and polyadenylation specific factor 6 [Source:VGNC Symbol;Acc:VGNC:86962]	2.08	0.0653
ssc-miR-125a	CRAMP1L	hypothetical gene	2.08	0.0653
ssc-miR-125a	CRB2	crumbs cell polarity complex component 2 [Source:VGNC Symbol;Acc:VGNC:86977]	2.08	0.0653
ssc-miR-125a	CREB1	cAMP responsive element binding protein 1 [Source:VGNC Symbol;Acc:VGNC:96004]	2.08	0.0653
ssc-miR-125a	CRTC1	CREB regulated transcription coactivator 1 [Source:VGNC Symbol;Acc:VGNC:87007]	2.08	0.0653
ssc-miR-125a	CSNK2A1	hypothetical gene	2.08	0.0653
ssc-miR-125a	CSRNP1	cysteine and serine rich nuclear protein 1 [Source:VGNC Symbol;Acc:VGNC:87046]	2.08	0.0653
ssc-miR-125a	CSRNP3	cysteine and serine rich nuclear protein 3 [Source:VGNC Symbol;Acc:VGNC:96249]	2.08	0.0653
ssc-miR-125a	CTDSP2	CTD small phosphatase 2 [Source:VGNC Symbol;Acc:VGNC:87059]	2.08	0.0653
ssc-miR-125a	CTNNBL1	catenin beta like 1 [Source:VGNC Symbol;Acc:VGNC:95688]	2.08	0.0653
ssc-miR-125a	CXorf23	hypothetical gene	2.08	0.0653
ssc-miR-125a	CYP24A1	cytochrome P450 family 24 subfamily A member 1 [Source:VGNC Symbol;Acc:VGNC:103371]	2.08	0.0653
ssc-miR-125a	CYTH1	cytohesin 1 [Source:VGNC Symbol;Acc:VGNC:87135]	2.08	0.0653
ssc-miR-125a	DAAM1	dishevelled associated activator of morphosis 1 [Source:VGNC Symbol;Acc:VGNC:87141]	2.08	0.0653
ssc-miR-125a	DAAM2	dishevelled associated activator of morphosis 2 [Source:VGNC Symbol;Acc:VGNC:87142]	2.08	0.0653
ssc-miR-125a	DAGLA	diacylglycerol lipase alpha [Source:VGNC Symbol;Acc:VGNC:87152]	2.08	0.0653
ssc-miR-125a	DAZAP2	DAZ associated protein 2 [Source:VGNC Symbol;Acc:VGNC:87162]	2.08	0.0653
ssc-miR-125a	DCAF10	DDB1 and CUL4 associated factor 10 [Source:VGNC Symbol;Acc:VGNC:87173]	2.08	0.0653
ssc-miR-125a	DCP1A	decapping mRNA 1A [Source:VGNC Symbol;Acc:VGNC:97960]	2.08	0.0653
ssc-miR-125a	DCTN1	dynactin subunit 1 [Source:VGNC Symbol;Acc:VGNC:101486]	2.08	0.0653
ssc-miR-125a	DDB2	damage specific DNA binding protein 2 [Source:VGNC Symbol;Acc:VGNC:87203]	2.08	0.0653
ssc-miR-125a	DDX42	DEAD-box helicase 42 [Source:VGNC Symbol;Acc:VGNC:87222]	2.08	0.0653
ssc-miR-125a	DENND6A	DENN domain containing 6A [Source:VGNC Symbol;Acc:VGNC:87256]	2.08	0.0653
ssc-miR-125a	DGAT1	diacylglycerol O-acyltransferase 1 [Source:VGNC Symbol;Acc:VGNC:87267]	2.08	0.0653
ssc-miR-125a	DHX33	DEAH-box helicase 33 [Source:VGNC Symbol;Acc:VGNC:87290]	2.08	0.0653
ssc-miR-125a	DICER1	dicer 1, ribonuclease III [Source:VGNC Symbol;Acc:VGNC:87302]	2.08	0.0653

ssc-miR-125a	DIRAS1	DIRAS family GTPase 1 [Source:VGNC Symbol;Acc:VGNC:87312]	2.08	0.0653
ssc-miR-125a	DIRAS2	DIRAS family GTPase 2 [Source:VGNC Symbol;Acc:VGNC:87313]	2.08	0.0653
ssc-miR-125a	DIS3L2	DIS3 like 3'-5' exoribonuclease 2 [Source:VGNC Symbol;Acc:VGNC:96183]	2.08	0.0653
ssc-miR-125a	DKFZP779J2370	hypothetical gene	2.08	0.0653
ssc-miR-125a	DNAJB1	DnaJ heat shock protein family (Hsp40) member B1 [Source:VGNC Symbol;Acc:VGNC:96614]	2.08	0.0653
ssc-miR-125a	DNAJB5	DnaJ heat shock protein family (Hsp40) member B5 [Source:VGNC Symbol;Acc:VGNC:103081]	2.08	0.0653
ssc-miR-125a	DNAJC10	DnaJ heat shock protein family (Hsp40) member C10 [Source:HGNC Symbol;Acc:HGNC:24637]	2.08	0.0653
ssc-miR-125a	DNAJC14	DnaJ heat shock protein family (Hsp40) member C14 [Source:NCBI gene (formerly Entrezgene);Acc:100151876]	2.08	0.0653
ssc-miR-125a	DNAL1	dynein axonemal light chain 1 [Source:VGNC Symbol;Acc:VGNC:87373]	2.08	0.0653
ssc-miR-125a	DNAL4	dynein axonemal light chain 4 [Source:VGNC Symbol;Acc:VGNC:87374]	2.08	0.0653
ssc-miR-125a	DOCK3	dedicator of cytokinesis 3 [Source:VGNC Symbol;Acc:VGNC:87393]	2.08	0.0653
ssc-miR-125a	DPF2	double PHD fingers 2 [Source:VGNC Symbol;Acc:VGNC:87413]	2.08	0.0653
ssc-miR-125a	DPH2	diphthamide biosynthesis 2 [Source:VGNC Symbol;Acc:VGNC:87415]	2.08	0.0653
ssc-miR-125a	DPP9	dipeptidyl peptidase 9 [Source:VGNC Symbol;Acc:VGNC:87424]	2.08	0.0653
ssc-miR-125a	DPY19L1	dpy-19 like C-mannosyltransferase 1 [Source:VGNC Symbol;Acc:VGNC:99712]	2.08	0.0653
ssc-miR-125a	DPYSL4	dihydropyrimidinase like 4 [Source:VGNC Symbol;Acc:VGNC:87432]	2.08	0.0653
ssc-miR-125a	DRAM2	DNA damage regulated autophagy modulator 2 [Source:VGNC Symbol;Acc:VGNC:87437]	2.08	0.0653
ssc-miR-125a	DRD2	dopamine receptor D2 [Source:VGNC Symbol;Acc:VGNC:87444]	2.08	0.0653
ssc-miR-125a	DTX4	deltex E3 ubiquitin ligase 4 [Source:VGNC Symbol;Acc:VGNC:87470]	2.08	0.0653
ssc-miR-125a	DUS1L	dihydrouridine synthase 1 like [Source:VGNC Symbol;Acc:VGNC:87474]	2.08	0.0653
ssc-miR-125a	DUSP3	dual specificity phosphatase 3 [Source:VGNC Symbol;Acc:VGNC:87486]	2.08	0.0653
ssc-miR-125a	DUSP6	dual specificity phosphatase 6 [Source:VGNC Symbol;Acc:VGNC:87488]	2.08	0.0653
ssc-miR-125a	DUSP7	dual specificity phosphatase 7 [Source:HGNC Symbol;Acc:HGNC:3073]	2.08	0.0653
ssc-miR-125a	DVL1	dishevelled segment polarity protein 1 [Source:VGNC Symbol;Acc:VGNC:96985]	2.08	0.0653
ssc-miR-125a	DVL3	dishevelled segment polarity protein 3 [Source:VGNC Symbol;Acc:VGNC:87492]	2.08	0.0653
ssc-miR-125a	DYRK2	dual specificity tyrosine phosphorylation regulated kinase 2 [Source:VGNC Symbol;Acc:VGNC:87507]	2.08	0.0653
ssc-miR-125a	E2F2	E2F transcription factor 2 [Source:VGNC Symbol;Acc:VGNC:87513]	2.08	0.0653
ssc-miR-125a	E2F3	E2F transcription factor 3 [Source:VGNC Symbol;Acc:VGNC:87514]	2.08	0.0653
ssc-miR-125a	EAF1	ELL associated factor 1 [Source:VGNC Symbol;Acc:VGNC:87521]	2.08	0.0653
ssc-miR-125a	EBF4	EBF family member 4 [Source:VGNC Symbol;Acc:VGNC:96211]	2.08	0.0653
ssc-miR-125a	ECE1	endothelin converting enzyme 1 [Source:VGNC Symbol;Acc:VGNC:87531]	2.08	0.0653
ssc-miR-125a	ECHDC3	enoyl-CoA hydratase domain containing 3 [Source:VGNC Symbol;Acc:VGNC:96009]	2.08	0.0653
ssc-miR-125a	EDC3	enhancer of mRNA decapping 3 [Source:VGNC Symbol;Acc:VGNC:87543]	2.08	0.0653
ssc-miR-125a	EDEM1	ER degradation enhancing alpha-mannosidase like protein 1 [Source:VGNC Symbol;Acc:VGNC:87545]	2.08	0.0653
ssc-miR-125a	EEF1G	hypothetical gene	2.08	0.0653
ssc-miR-125a	EIF1AD	eukaryotic translation initiation factor 1A domain containing [Source:VGNC Symbol;Acc:VGNC:87603]	2.08	0.0653
ssc-miR-125a	EIF2B5	eukaryotic translation initiation factor 2B subunit epsilon [Source:VGNC Symbol;Acc:VGNC:87612]	2.08	0.0653
ssc-miR-125a	EIF4EBP1	eukaryotic translation initiation factor 4E binding protein 1 [Source:VGNC Symbol;Acc:VGNC:96268]	2.08	0.0653
ssc-miR-125a	EIF5A2	eukaryotic translation initiation factor 5A2 [Source:VGNC Symbol;Acc:VGNC:87633]	2.08	0.0653
ssc-miR-125a	ELAC2	elaC ribonuclease Z 2 [Source:VGNC Symbol;Acc:VGNC:87636]	2.08	0.0653
ssc-miR-125a	ELAVL4	ELAV like RNA binding protein 4 [Source:VGNC Symbol;Acc:VGNC:97047]	2.08	0.0653
ssc-miR-125a	ELL	elongation factor for RNA polymerase II [Source:VGNC Symbol;Acc:VGNC:87648]	2.08	0.0653
ssc-miR-125a	ELMSAN1	hypothetical gene	2.08	0.0653
ssc-miR-125a	ELOVL1	ELOVL fatty acid elongase 1 [Source:VGNC Symbol;Acc:VGNC:87657]	2.08	0.0653
ssc-miR-125a	ELOVL2	ELOVL fatty acid elongase 2 [Source:VGNC Symbol;Acc:VGNC:87658]	2.08	0.0653
ssc-miR-125a	ELOVL4	ELOVL fatty acid elongase 4 [Source:VGNC Symbol;Acc:VGNC:87660]	2.08	0.0653
ssc-miR-125a	ELOVL6	ELOVL fatty acid elongase 6 [Source:VGNC Symbol;Acc:VGNC:87662]	2.08	0.0653
ssc-miR-125a	EMID1	EMI domain containing 1 [Source:VGNC Symbol;Acc:VGNC:87679]	2.08	0.0653
ssc-miR-125a	ENPEP	glutamyl aminopeptidase [Source:VGNC Symbol;Acc:VGNC:98923]	2.08	0.0653

ssc-miR-125a	ENPP1	ectonucleotide pyrophosphatase/phosphodiesterase 1 [Source:VGNC Symbol;Acc:VGNC:87708]	2.08	0.0653
ssc-miR-125a	ENTPD4	ectonucleoside triphosphate diphosphohydrolase 4 [Source:VGNC Symbol;Acc:VGNC:107386]	2.08	0.0653
ssc-miR-125a	EPHA1	EPH receptor A1 [Source:VGNC Symbol;Acc:VGNC:87729]	2.08	0.0653
ssc-miR-125a	EPHA7	EPH receptor A7 [Source:VGNC Symbol;Acc:VGNC:87734]	2.08	0.0653
ssc-miR-125a	EPOR	erythropoietin receptor [Source:VGNC Symbol;Acc:VGNC:87746]	2.08	0.0653
ssc-miR-125a	ERBB3	erb-b2 receptor tyrosine kinase 3 [Source:VGNC Symbol;Acc:VGNC:87760]	2.08	0.0653
ssc-miR-125a	ERBB4	erb-b2 receptor tyrosine kinase 4 [Source:VGNC Symbol;Acc:VGNC:96284]	2.08	0.0653
ssc-miR-125a	ERCC6	hypothetical gene	2.08	0.0653
ssc-miR-125a	ERN2	endoplasmic reticulum to nucleus signaling 2 [Source:VGNC Symbol;Acc:VGNC:87778]	2.08	0.0653
ssc-miR-125a	ESRRA	estrogen related receptor alpha [Source:VGNC Symbol;Acc:VGNC:87793]	2.08	0.0653
ssc-miR-125a	ESRRG	estrogen related receptor gamma [Source:VGNC Symbol;Acc:VGNC:96289]	2.08	0.0653
ssc-miR-125a	ESYT1	extended synaptotagmin 1 [Source:VGNC Symbol;Acc:VGNC:87796]	2.08	0.0653
ssc-miR-125a	ETS1	ETS proto-onco 1, transcription factor [Source:VGNC Symbol;Acc:VGNC:87808]	2.08	0.0653
ssc-miR-125a	ETV3	ETS variant transcription factor 3 [Source:VGNC Symbol;Acc:VGNC:87812]	2.08	0.0653
ssc-miR-125a	ETV6	ETS variant transcription factor 6 [Source:VGNC Symbol;Acc:VGNC:87815]	2.08	0.0653
ssc-miR-125a	EVA1A	eva-1 homolog A, regulator of programmed cell death [Source:VGNC Symbol;Acc:VGNC:97988]	2.08	0.0653
ssc-miR-125a	FAM107B	hypothetical gene	2.08	0.0653
ssc-miR-125a	FAM118A	family with sequence similarity 118 member A [Source:VGNC Symbol;Acc:VGNC:87893]	2.08	0.0653
ssc-miR-125a	FAM126B	family with sequence similarity 126 member B [Source:HGNC Symbol;Acc:HGNC:28593]	2.08	0.0653
ssc-miR-125a	FAM129B	hypothetical gene	2.08	0.0653
ssc-miR-125a	FAM131B	family with sequence similarity 131 member B [Source:VGNC Symbol;Acc:VGNC:87901]	2.08	0.0653
ssc-miR-125a	FAM134A	hypothetical gene	2.08	0.0653
ssc-miR-125a	FAM134C	hypothetical gene	2.08	0.0653
ssc-miR-125a	FAM135B	family with sequence similarity 135 member B [Source:VGNC Symbol;Acc:VGNC:87905]	2.08	0.0653
ssc-miR-125a	FAM169B	hypothetical gene	2.08	0.0653
ssc-miR-125a	FAM174B	family with sequence similarity 174 member B [Source:VGNC Symbol;Acc:VGNC:87932]	2.08	0.0653
ssc-miR-125a	FAM213A	hypothetical gene	2.08	0.0653
ssc-miR-125a	FAM222B	family with sequence similarity 222 member B [Source:VGNC Symbol;Acc:VGNC:87960]	2.08	0.0653
ssc-miR-125a	FAM46A	hypothetical gene	2.08	0.0653
ssc-miR-125a	FAM53C	family with sequence similarity 53 member C [Source:VGNC Symbol;Acc:VGNC:87975]	2.08	0.0653
ssc-miR-125a	FAM65B	hypothetical gene	2.08	0.0653
ssc-miR-125a	FAM73A	hypothetical gene	2.08	0.0653
ssc-miR-125a	FAM78A	family with sequence similarity 78 member A [Source:VGNC Symbol;Acc:VGNC:87984]	2.08	0.0653
ssc-miR-125a	FAM83F	family with sequence similarity 83 member F [Source:VGNC Symbol;Acc:VGNC:87991]	2.08	0.0653
ssc-miR-125a	FAM89B	family with sequence similarity 89 member B [Source:VGNC Symbol;Acc:VGNC:87995]	2.08	0.0653
ssc-miR-125a	FAM92B	hypothetical gene	2.08	0.0653
ssc-miR-125a	FAT4	FAT atypical cadherin 4 [Source:HGNC Symbol;Acc:HGNC:23109]	2.08	0.0653
ssc-miR-125a	FBXO10	F-box protein 10 [Source:VGNC Symbol;Acc:VGNC:103092]	2.08	0.0653
ssc-miR-125a	FBXO42	F-box protein 42 [Source:VGNC Symbol;Acc:VGNC:88047]	2.08	0.0653
ssc-miR-125a	FBXO45	F-box protein 45 [Source:VGNC Symbol;Acc:VGNC:88048]	2.08	0.0653
ssc-miR-125a	FBXW4	F-box and WD repeat domain containing 4 [Source:VGNC Symbol;Acc:VGNC:88055]	2.08	0.0653
ssc-miR-125a	FGF14	fibroblast growth factor 14 [Source:VGNC Symbol;Acc:VGNC:88104]	2.08	0.0653
ssc-miR-125a	FGFR1	fibroblast growth factor receptor 1 [Source:HGNC Symbol;Acc:HGNC:3688]	2.08	0.0653
ssc-miR-125a	FGFR2	fibroblast growth factor receptor 2 [Source:NCBI gene (formerly Entrezgene);Acc:396762]	2.08	0.0653
ssc-miR-125a	FKBP15	FKBP prolyl isomerase family member 15 [Source:VGNC Symbol;Acc:VGNC:88145]	2.08	0.0653
ssc-miR-125a	FLOT2	flotillin 2 [Source:VGNC Symbol;Acc:VGNC:88159]	2.08	0.0653
ssc-miR-125a	FLVCR1	FLVCR heme transporter 1 [Source:VGNC Symbol;Acc:VGNC:96580]	2.08	0.0653
ssc-miR-125a	FMN1	formin 1 [Source:HGNC Symbol;Acc:HGNC:3768]	2.08	0.0653
ssc-miR-125a	FMR1	FMRP translational regulator 1 [Source:VGNC Symbol;Acc:VGNC:88175]	2.08	0.0653

ssc-miR-125a	FNDC3B	fibronectin type III domain containing 3B [Source:VGNC Symbol;Acc:VGNC:88183]	2.08	0.0653
ssc-miR-125a	FOSL2	FOS like 2, AP-1 transcription factor subunit [Source:VGNC Symbol;Acc:VGNC:88192]	2.08	0.0653
ssc-miR-125a	FOXD2	forkhead box D2 [Source:VGNC Symbol;Acc:VGNC:88200]	2.08	0.0653
ssc-miR-125a	FOXK2	forkhead box K2 [Source:VGNC Symbol;Acc:VGNC:88215]	2.08	0.0653
ssc-miR-125a	FOXN3	forkhead box N3 [Source:VGNC Symbol;Acc:VGNC:88220]	2.08	0.0653
ssc-miR-125a	FOXQ1	forkhead box Q1 [Source:VGNC Symbol;Acc:VGNC:88225]	2.08	0.0653
ssc-miR-125a	FRAS1	Fraser extracellular matrix complex subunit 1 [Source:VGNC Symbol;Acc:VGNC:88232]	2.08	0.0653
ssc-miR-125a	FRMD5	FERM domain containing 5 [Source:VGNC Symbol;Acc:VGNC:88238]	2.08	0.0653
ssc-miR-125a	FUT4	fucosyltransferase 4 [Source:HGNC Symbol;Acc:HGNC:4015]	2.08	0.0653
ssc-miR-125a	G3BP1	G3BP stress granule assembly factor 1 [Source:VGNC Symbol;Acc:VGNC:88288]	2.08	0.0653
ssc-miR-125a	GAB2	GRB2 associated binding protein 2 [Source:VGNC Symbol;Acc:VGNC:108586]	2.08	0.0653
ssc-miR-125a	GAB4	hypothetical gene	2.08	0.0653
ssc-miR-125a	GALNT14	polypeptide N-acetylgalactosaminyltransferase 14 [Source:VGNC Symbol;Acc:VGNC:88330]	2.08	0.0653
ssc-miR-125a	GALNT5	polypeptide N-acetylgalactosaminyltransferase 5 [Source:VGNC Symbol;Acc:VGNC:96314]	2.08	0.0653
ssc-miR-125a	GALNT7	polypeptide N-acetylgalactosaminyltransferase 7 [Source:VGNC Symbol;Acc:VGNC:103946]	2.08	0.0653
ssc-miR-125a	GCNT1	glucosaminyl (N-acetyl) transferase 1 [Source:VGNC Symbol;Acc:VGNC:103096]	2.08	0.0653
ssc-miR-125a	GDF11	growth differentiation factor 11 [Source:VGNC Symbol;Acc:VGNC:88398]	2.08	0.0653
ssc-miR-125a	GDNF	glial cell derived neurotrophic factor [Source:VGNC Symbol;Acc:VGNC:88406]	2.08	0.0653
ssc-miR-125a	GDPD5	glycerophosphodiester phosphodiesterase domain containing 5 [Source:VGNC Symbol;Acc:VGNC:88409]	2.08	0.0653
ssc-miR-125a	GEMIN8	gem nuclear organelle associated protein 8 [Source:VGNC Symbol;Acc:VGNC:88416]	2.08	0.0653
ssc-miR-125a	GFOD2	glucose-fructose oxidoreductase domain containing 2 [Source:VGNC Symbol;Acc:VGNC:88425]	2.08	0.0653
ssc-miR-125a	GGA2	golgi associated, gamma adaptin ear containing, ARF binding protein 2 [Source:VGNC Symbol;Acc:VGNC:88432]	2.08	0.0653
ssc-miR-125a	GGT7	gamma-glutamyltransferase 7 [Source:VGNC Symbol;Acc:VGNC:96321]	2.08	0.0653
ssc-miR-125a	GINS4	GINS complex subunit 4 [Source:VGNC Symbol;Acc:VGNC:96171]	2.08	0.0653
ssc-miR-125a	GJC1	gap junction protein gamma 1 [Source:VGNC Symbol;Acc:VGNC:88468]	2.08	0.0653
ssc-miR-125a	GK5	glycerol kinase 5 [Source:VGNC Symbol;Acc:VGNC:96734]	2.08	0.0653
ssc-miR-125a	GLIS2	GLIS family zinc finger 2 [Source:VGNC Symbol;Acc:VGNC:88484]	2.08	0.0653
ssc-miR-125a	GLTP	glycolipid transfer protein [Source:VGNC Symbol;Acc:VGNC:107395]	2.08	0.0653
ssc-miR-125a	GMIP	GEM interacting protein [Source:VGNC Symbol;Acc:VGNC:88511]	2.08	0.0653
ssc-miR-125a	GOLGA5	golgin A5 [Source:VGNC Symbol;Acc:VGNC:88552]	2.08	0.0653
ssc-miR-125a	GOPC	golgi associated PDZ and coiled-coil motif containing [Source:VGNC Symbol;Acc:VGNC:98024]	2.08	0.0653
ssc-miR-125a	GPC4	glypican 4 [Source:VGNC Symbol;Acc:VGNC:88581]	2.08	0.0653
ssc-miR-125a	GPC6	glypican 6 [Source:HGNC Symbol;Acc:HGNC:4454]	2.08	0.0653
ssc-miR-125a	GPR107	G protein-coupled receptor 107 [Source:VGNC Symbol;Acc:VGNC:88595]	2.08	0.0653
ssc-miR-125a	GPR157	G protein-coupled receptor 157 [Source:VGNC Symbol;Acc:VGNC:88611]	2.08	0.0653
ssc-miR-125a	GPRC5A	G protein-coupled receptor class C group 5 member A [Source:VGNC Symbol;Acc:VGNC:88642]	2.08	0.0653
ssc-miR-125a	GRAMD1B	GRAM domain containing 1B [Source:VGNC Symbol;Acc:VGNC:88656]	2.08	0.0653
ssc-miR-125a	GRB10	growth factor receptor bound protein 10 [Source:VGNC Symbol;Acc:VGNC:88662]	2.08	0.0653
ssc-miR-125a	GREB1L	GREB1 like retinoic acid receptor coactivator [Source:VGNC Symbol;Acc:VGNC:96584]	2.08	0.0653
ssc-miR-125a	GRHL1	grainyhead like transcription factor 1 [Source:VGNC Symbol;Acc:VGNC:88666]	2.08	0.0653
ssc-miR-125a	GRIN2A	glutamate ionotropic receptor NMDA type subunit 2A [Source:VGNC Symbol;Acc:VGNC:88683]	2.08	0.0653
ssc-miR-125a	GRK5	G protein-coupled receptor kinase 5 [Source:VGNC Symbol;Acc:VGNC:88697]	2.08	0.0653
ssc-miR-125a	GRSF1	G-rich RNA sequence binding factor 1 [Source:VGNC Symbol;Acc:VGNC:88710]	2.08	0.0653
ssc-miR-125a	GSTM5	hypothetical gene	2.08	0.0653
ssc-miR-125a	GTPBP2	GTP binding protein 2 [Source:VGNC Symbol;Acc:VGNC:88743]	2.08	0.0653
ssc-miR-125a	GXYLT1	glucoside xylosyltransferase 1 [Source:VGNC Symbol;Acc:VGNC:88756]	2.08	0.0653
ssc-miR-125a	H6PD	hexose-6-phosphate dehydrogenase/glucose 1-dehydrogenase [Source:VGNC Symbol;Acc:VGNC:88764]	2.08	0.0653
ssc-miR-125a	HAP1	huntingtin associated protein 1 [Source:VGNC Symbol;Acc:VGNC:88779]	2.08	0.0653
ssc-miR-125a	HAPLN1	hyaluronan and proteoglycan link protein 1 [Source:VGNC Symbol;Acc:VGNC:88780]	2.08	0.0653

ssc-miR-125a	HCN1	hyperpolarization activated cyclic nucleotide gated potassium channel 1 [Source:VGNC Symbol;Acc:VGNC:88802]	2.08	0.0653
ssc-miR-125a	HCN3	hyperpolarization activated cyclic nucleotide gated potassium channel 3 [Source:VGNC Symbol;Acc:VGNC:88804]	2.08	0.0653
ssc-miR-125a	HCN4	hyperpolarization activated cyclic nucleotide gated potassium channel 4 [Source:VGNC Symbol;Acc:VGNC:88805]	2.08	0.0653
ssc-miR-125a	HDDC3	HD domain containing 3 [Source:VGNC Symbol;Acc:VGNC:88818]	2.08	0.0653
ssc-miR-125a	HIC2	HIC ZBTB transcriptional repressor 2 [Source:HGNC Symbol;Acc:HGNC:18595]	2.08	0.0653
ssc-miR-125a	HIF1AN	hypoxia inducible factor 1 subunit alpha inhibitor [Source:VGNC Symbol;Acc:VGNC:98033]	2.08	0.0653
ssc-miR-125a	HINFP	histone H4 transcription factor [Source:HGNC Symbol;Acc:HGNC:17850]	2.08	0.0653
ssc-miR-125a	HIPK3	homeodomain interacting protein kinase 3 [Source:VGNC Symbol;Acc:VGNC:88889]	2.08	0.0653
ssc-miR-125a	HK2	hexokinase 2 [Source:VGNC Symbol;Acc:VGNC:98034]	2.08	0.0653
ssc-miR-125a	HMGB3	hypothetical gene	2.08	0.0653
ssc-miR-125a	HMGCR	3-hydroxy-3-methylglutaryl-CoA reductase [Source:VGNC Symbol;Acc:VGNC:88906]	2.08	0.0653
ssc-miR-125a	HNF4G	hepatocyte nuclear factor 4 gamma [Source:VGNC Symbol;Acc:VGNC:88917]	2.08	0.0653
ssc-miR-125a	HNRNPR	heterous nuclear ribonucleoprotein R [Source:VGNC Symbol;Acc:VGNC:88925]	2.08	0.0653
ssc-miR-125a	HNRNPUL2	heterous nuclear ribonucleoprotein U like 2 [Source:VGNC Symbol;Acc:VGNC:99758]	2.08	0.0653
ssc-miR-125a	HOMEZ	homeobox and leucine zipper encoding [Source:VGNC Symbol;Acc:VGNC:88930]	2.08	0.0653
ssc-miR-125a	HOXB3	homeobox B3 [Source:VGNC Symbol;Acc:VGNC:88944]	2.08	0.0653
ssc-miR-125a	HOXD1	homeobox D1 [Source:VGNC Symbol;Acc:VGNC:96350]	2.08	0.0653
ssc-miR-125a	HOXD9	homeobox D9 [Source:VGNC Symbol;Acc:VGNC:96357]	2.08	0.0653
ssc-miR-125a	HRK	harakiri, BCL2 interacting protein [Source:HGNC Symbol;Acc:HGNC:5185]	2.08	0.0653
ssc-miR-125a	HS3ST3A1	heparan sulfate-glucosamine 3-sulfotransferase 3A1 [Source:VGNC Symbol;Acc:VGNC:99002]	2.08	0.0653
ssc-miR-125a	IER2	immediate early response 2 [Source:HGNC Symbol;Acc:HGNC:28871]	2.08	0.0653
ssc-miR-125a	IER3IP1	immediate early response 3 interacting protein 1 [Source:HGNC Symbol;Acc:HGNC:18550]	2.08	0.0653
ssc-miR-125a	IFFO1	intermediate filament family orphan 1 [Source:VGNC Symbol;Acc:VGNC:89030]	2.08	0.0653
ssc-miR-125a	IFNG	interferon gamma [Source:VGNC Symbol;Acc:VGNC:89040]	2.08	0.0653
ssc-miR-125a	IGF2BP1	insulin like growth factor 2 mRNA binding protein 1 [Source:VGNC Symbol;Acc:VGNC:99006]	2.08	0.0653
ssc-miR-125a	IGSF11	immunoglobulin superfamily member 11 [Source:VGNC Symbol;Acc:VGNC:89064]	2.08	0.0653
ssc-miR-125a	IKBKE	inhibitor of nuclear factor kappa B kinase subunit epsilon [Source:VGNC Symbol;Acc:VGNC:89071]	2.08	0.0653
ssc-miR-125a	IKZF3	IKAROS family zinc finger 3 [Source:VGNC Symbol;Acc:VGNC:89074]	2.08	0.0653
ssc-miR-125a	IKZF4	IKAROS family zinc finger 4 [Source:VGNC Symbol;Acc:VGNC:89075]	2.08	0.0653
ssc-miR-125a	IL10RB	interleukin 10 receptor subunit beta [Source:NCBI gene (formerly Entrezgene);Acc:396657]	2.08	0.0653
ssc-miR-125a	IL16	interleukin 16 [Source:VGNC Symbol;Acc:VGNC:89084]	2.08	0.0653
ssc-miR-125a	IL1RAPL1	interleukin 1 receptor accessory protein like 1 [Source:VGNC Symbol;Acc:VGNC:103967]	2.08	0.0653
ssc-miR-125a	IL6R	interleukin 6 receptor [Source:VGNC Symbol;Acc:VGNC:89112]	2.08	0.0653
ssc-miR-125a	IMPAD1	hypothetical gene	2.08	0.0653
ssc-miR-125a	INO80D	INO80 complex subunit D [Source:VGNC Symbol;Acc:VGNC:96113]	2.08	0.0653
ssc-miR-125a	INPP5K	inositol polyphosphate-5-phosphatase K [Source:VGNC Symbol;Acc:VGNC:89147]	2.08	0.0653
ssc-miR-125a	INTS7	integrator complex subunit 7 [Source:VGNC Symbol;Acc:VGNC:89167]	2.08	0.0653
ssc-miR-125a	IP6K1	inositol hexakisphosphate kinase 1 [Source:VGNC Symbol;Acc:VGNC:89172]	2.08	0.0653
ssc-miR-125a	IPMK	inositol polyphosphate multikinase [Source:VGNC Symbol;Acc:VGNC:89175]	2.08	0.0653
ssc-miR-125a	IRF4	interferon regulatory factor 4 [Source:VGNC Symbol;Acc:VGNC:89207]	2.08	0.0653
ssc-miR-125a	IST1	IST1 factor associated with ESCRT-III [Source:VGNC Symbol;Acc:VGNC:98480]	2.08	0.0653
ssc-miR-125a	ITGA1	integrin subunit alpha 1 [Source:VGNC Symbol;Acc:VGNC:89231]	2.08	0.0653
ssc-miR-125a	ITGA7	integrin subunit alpha 7 [Source:VGNC Symbol;Acc:VGNC:89237]	2.08	0.0653
ssc-miR-125a	ITGA8	integrin subunit alpha 8 [Source:VGNC Symbol;Acc:VGNC:96379]	2.08	0.0653
ssc-miR-125a	ITGA9	integrin subunit alpha 9 [Source:VGNC Symbol;Acc:VGNC:89238]	2.08	0.0653
ssc-miR-125a	ITGB3	integrin subunit beta 3 [Source:NCBI gene (formerly Entrezgene);Acc:397063]	2.08	0.0653
ssc-miR-125a	JHDM1D	hypothetical gene	2.08	0.0653
ssc-miR-125a	JMY	junction mediating and regulatory protein, p53 cofactor [Source:VGNC Symbol;Acc:VGNC:89289]	2.08	0.0653
ssc-miR-125a	KCNA1	potassium voltage-gated channel subfamily A member 1 [Source:VGNC Symbol;Acc:VGNC:89323]	2.08	0.0653

ssc-miR-125a	KCNB1	potassium voltage-gated channel subfamily B member 1 [Source:VGNC Symbol;Acc:VGNC:96386]	2.08	0.0653
ssc-miR-125a	KCNC2	potassium voltage-gated channel subfamily C member 2 [Source:VGNC Symbol;Acc:VGNC:89333]	2.08	0.0653
ssc-miR-125a	KCNC3	potassium voltage-gated channel subfamily C member 3 [Source:VGNC Symbol;Acc:VGNC:89334]	2.08	0.0653
ssc-miR-125a	KCNC4	potassium voltage-gated channel subfamily C member 4 [Source:VGNC Symbol;Acc:VGNC:89335]	2.08	0.0653
ssc-miR-125a	KCNH1	potassium voltage-gated channel subfamily H member 1 [Source:VGNC Symbol;Acc:VGNC:108599]	2.08	0.0653
ssc-miR-125a	KCNH3	potassium voltage-gated channel subfamily H member 3 [Source:VGNC Symbol;Acc:VGNC:89344]	2.08	0.0653
ssc-miR-125a	KCNH4	potassium voltage-gated channel subfamily H member 4 [Source:HGNC Symbol;Acc:HGNC:6253]	2.08	0.0653
ssc-miR-125a	KCNH7	potassium voltage-gated channel subfamily H member 7 [Source:VGNC Symbol;Acc:VGNC:95897]	2.08	0.0653
ssc-miR-125a	KCNIP3	potassium voltage-gated channel interacting protein 3 [Source:VGNC Symbol;Acc:VGNC:89350]	2.08	0.0653
ssc-miR-125a	KCNJ12	hypothetical gene	2.08	0.0653
ssc-miR-125a	KCNJ6	potassium inwardly rectifying channel subfamily J member 6 [Source:VGNC Symbol;Acc:VGNC:89360]	2.08	0.0653
ssc-miR-125a	KCNJ9	potassium inwardly rectifying channel subfamily J member 9 [Source:VGNC Symbol;Acc:VGNC:89362]	2.08	0.0653
ssc-miR-125a	KCNK10	potassium two pore domain channel subfamily K member 10 [Source:VGNC Symbol;Acc:VGNC:89364]	2.08	0.0653
ssc-miR-125a	KCNS3	potassium voltage-gated channel modifier subfamily S member 3 [Source:VGNC Symbol;Acc:VGNC:89387]	2.08	0.0653
ssc-miR-125a	KCTD15	potassium channel tetramerization domain containing 15 [Source:VGNC Symbol;Acc:VGNC:89394]	2.08	0.0653
ssc-miR-125a	KCTD2	potassium channel tetramerization domain containing 2 [Source:VGNC Symbol;Acc:VGNC:89398]	2.08	0.0653
ssc-miR-125a	KCTD21	potassium channel tetramerization domain containing 21 [Source:VGNC Symbol;Acc:VGNC:89400]	2.08	0.0653
ssc-miR-125a	KDM4B	lysine demethylase 4B [Source:VGNC Symbol;Acc:VGNC:89413]	2.08	0.0653
ssc-miR-125a	KHNYN	KH and NYN domain containing [Source:VGNC Symbol;Acc:VGNC:89426]	2.08	0.0653
ssc-miR-125a	KIAA0247	hypothetical gene	2.08	0.0653
ssc-miR-125a	KIAA0319L	KIAA0319 like [Source:VGNC Symbol;Acc:VGNC:89430]	2.08	0.0653
ssc-miR-125a	KIAA0556	hypothetical gene	2.08	0.0653
ssc-miR-125a	KIAA1244	hypothetical gene	2.08	0.0653
ssc-miR-125a	KIAA1467	hypothetical gene	2.08	0.0653
ssc-miR-125a	KIAA1522	KIAA1522 [Source:VGNC Symbol;Acc:VGNC:89444]	2.08	0.0653
ssc-miR-125a	KIAA1598	hypothetical gene	2.08	0.0653
ssc-miR-125a	KIAA1644	hypothetical gene	2.08	0.0653
ssc-miR-125a	KIAA1841	hypothetical gene	2.08	0.0653
ssc-miR-125a	KIAA1919	hypothetical gene	2.08	0.0653
ssc-miR-125a	KIF18B	kinesin family member 18B [Source:VGNC Symbol;Acc:VGNC:89458]	2.08	0.0653
ssc-miR-125a	KIF1C	kinesin family member 1C [Source:VGNC Symbol;Acc:VGNC:89461]	2.08	0.0653
ssc-miR-125a	KLC2	kinesin light chain 2 [Source:VGNC Symbol;Acc:VGNC:89486]	2.08	0.0653
ssc-miR-125a	KLF13	Kruppel like factor 13 [Source:VGNC Symbol;Acc:VGNC:89493]	2.08	0.0653
ssc-miR-125a	KLF16	Kruppel like factor 16 [Source:VGNC Symbol;Acc:VGNC:89496]	2.08	0.0653
ssc-miR-125a	KLF3	Kruppel like factor 3 [Source:VGNC Symbol;Acc:VGNC:89498]	2.08	0.0653
ssc-miR-125a	KLHL24	kelch like family member 24 [Source:VGNC Symbol;Acc:VGNC:89521]	2.08	0.0653
ssc-miR-125a	KLHL31	kelch like family member 31 [Source:VGNC Symbol;Acc:VGNC:89526]	2.08	0.0653
ssc-miR-125a	KLHL6	hypothetical gene	2.08	0.0653
ssc-miR-125a	KNCN	kinocilin [Source:HGNC Symbol;Acc:HGNC:26488]	2.08	0.0653
ssc-miR-125a	KNOP1	lysine rich nucleolar protein 1 [Source:VGNC Symbol;Acc:VGNC:89558]	2.08	0.0653
ssc-miR-125a	KPNA6	karyopherin subunit alpha 6 [Source:VGNC Symbol;Acc:VGNC:89565]	2.08	0.0653
ssc-miR-125a	KRBA1	KRAB-A domain containing 1 [Source:VGNC Symbol;Acc:VGNC:89570]	2.08	0.0653
ssc-miR-125a	LAS1L	LAS1 like ribosome biosis factor [Source:VGNC Symbol;Acc:VGNC:89645]	2.08	0.0653
ssc-miR-125a	LBH	LBH regulator of WNT signaling pathway [Source:HGNC Symbol;Acc:HGNC:29532]	2.08	0.0653
ssc-miR-125a	LCLAT1	lysocardiolipin acyltransferase 1 [Source:VGNC Symbol;Acc:VGNC:89658]	2.08	0.0653
ssc-miR-125a	LCOR	ligand dependent nuclear receptor corepressor [Source:HGNC Symbol;Acc:HGNC:29503]	2.08	0.0653
ssc-miR-125a	LEP	leptin [Source:VGNC Symbol;Acc:VGNC:89684]	2.08	0.0653
ssc-miR-125a	LFNG	LFNG O-fucosylpeptide 3-beta-N-acetylglucosaminyltransferase [Source:VGNC Symbol;Acc:VGNC:89690]	2.08	0.0653
ssc-miR-125a	LGI2	leucine rich repeat LGI family member 2 [Source:VGNC Symbol;Acc:VGNC:89699]	2.08	0.0653

ssc-miR-125a	LHFPL4	LHFPL tetraspan subfamily member 4 [Source:VGNC Symbol;Acc:VGNC:89707]	2.08	0.0653
ssc-miR-125a	LHX9	LIM homeobox 9 [Source:VGNC Symbol;Acc:VGNC:95608]	2.08	0.0653
ssc-miR-125a	LIFR	LIF receptor subunit alpha [Source:HGNC Symbol;Acc:HGNC:6597]	2.08	0.0653
ssc-miR-125a	LIMD2	LIM domain containing 2 [Source:VGNC Symbol;Acc:VGNC:89726]	2.08	0.0653
ssc-miR-125a	LIMK1	LIM domain kinase 1 [Source:VGNC Symbol;Acc:VGNC:89727]	2.08	0.0653
ssc-miR-125a	LIN28A	lin-28 homolog A [Source:VGNC Symbol;Acc:VGNC:98492]	2.08	0.0653
ssc-miR-125a	LIN28B	lin-28 homolog B [Source:VGNC Symbol;Acc:VGNC:89729]	2.08	0.0653
ssc-miR-125a	LIPA	lipase A, lysosomal acid type [Source:VGNC Symbol;Acc:VGNC:89737]	2.08	0.0653
ssc-miR-125a	LNPEP	leucyl and cystinyl aminopeptidase [Source:VGNC Symbol;Acc:VGNC:89772]	2.08	0.0653
ssc-miR-125a	LOXL1	lysyl oxidase like 1 [Source:VGNC Symbol;Acc:VGNC:89780]	2.08	0.0653
ssc-miR-125a	LPAR4	lysophosphatidic acid receptor 4 [Source:VGNC Symbol;Acc:VGNC:89786]	2.08	0.0653
ssc-miR-125a	LPCAT4	lysophosphatidylcholine acyltransferase 4 [Source:VGNC Symbol;Acc:VGNC:89791]	2.08	0.0653
ssc-miR-125a	LPP	LIM domain containing preferred translocation partner in lipoma [Source:HGNC Symbol;Acc:HGNC:6679]	2.08	0.0653
ssc-miR-125a	LRFN2	leucine rich repeat and fibronectin type III domain containing 2 [Source:VGNC Symbol;Acc:VGNC:89805]	2.08	0.0653
ssc-miR-125a	LRP4	LDL receptor related protein 4 [Source:VGNC Symbol;Acc:VGNC:89820]	2.08	0.0653
ssc-miR-125a	LRRC10B	leucine rich repeat containing 10B [Source:VGNC Symbol;Acc:VGNC:99787]	2.08	0.0653
ssc-miR-125a	LRRC8B	leucine rich repeat containing 8 VRAC subunit B [Source:VGNC Symbol;Acc:VGNC:98086]	2.08	0.0653
ssc-miR-125a	LURAP1	leucine rich adaptor protein 1 [Source:VGNC Symbol;Acc:VGNC:89895]	2.08	0.0653
ssc-miR-125a	LURAP1L	leucine rich adaptor protein 1 like [Source:VGNC Symbol;Acc:VGNC:89896]	2.08	0.0653
ssc-miR-125a	LUZP1	leucine zipper protein 1 [Source:VGNC Symbol;Acc:VGNC:89897]	2.08	0.0653
ssc-miR-125a	LYPD6	LY6/PLAUR domain containing 6 [Source:HGNC Symbol;Acc:HGNC:28751]	2.08	0.0653
ssc-miR-125a	M6PR	mannose-6-phosphate receptor, cation dependent [Source:VGNC Symbol;Acc:VGNC:89931]	2.08	0.0653
ssc-miR-125a	MAF	MAF bZIP transcription factor [Source:VGNC Symbol;Acc:VGNC:89945]	2.08	0.0653
ssc-miR-125a	MAMDC2	MAM domain containing 2 [Source:VGNC Symbol;Acc:VGNC:89962]	2.08	0.0653
ssc-miR-125a	MAN1B1	hypothetical gene	2.08	0.0653
ssc-miR-125a	MAP2K7	mitogen-activated protein kinase kinase 7 [Source:VGNC Symbol;Acc:VGNC:98103]	2.08	0.0653
ssc-miR-125a	MAP3K1	mitogen-activated protein kinase kinase kinase 1 [Source:VGNC Symbol;Acc:VGNC:98104]	2.08	0.0653
ssc-miR-125a	MAP3K10	mitogen-activated protein kinase kinase kinase 10 [Source:VGNC Symbol;Acc:VGNC:89981]	2.08	0.0653
ssc-miR-125a	MAP3K11	mitogen-activated protein kinase kinase kinase 11 [Source:VGNC Symbol;Acc:VGNC:89982]	2.08	0.0653
ssc-miR-125a	MAP3K3	mitogen-activated protein kinase kinase kinase 3 [Source:VGNC Symbol;Acc:VGNC:98108]	2.08	0.0653
ssc-miR-125a	MAP3K9	mitogen-activated protein kinase kinase kinase 9 [Source:VGNC Symbol;Acc:VGNC:89989]	2.08	0.0653
ssc-miR-125a	MAPK10	mitogen-activated protein kinase 10 [Source:VGNC Symbol;Acc:VGNC:98116]	2.08	0.0653
ssc-miR-125a	MAPK12	mitogen-activated protein kinase 12 [Source:VGNC Symbol;Acc:VGNC:89998]	2.08	0.0653
ssc-miR-125a	MAPK14	mitogen-activated protein kinase 14 [Source:VGNC Symbol;Acc:VGNC:90000]	2.08	0.0653
ssc-miR-125a	MAPK1IP1L	mitogen-activated protein kinase 1 interacting protein 1 like [Source:VGNC Symbol;Acc:VGNC:90002]	2.08	0.0653
ssc-miR-125a	MAPRE2	microtubule associated protein RP/EB family member 2 [Source:VGNC Symbol;Acc:VGNC:98117]	2.08	0.0653
ssc-miR-125a	MARK2	microtubule affinity regulating kinase 2 [Source:VGNC Symbol;Acc:VGNC:98118]	2.08	0.0653
ssc-miR-125a	MBD1	methyl-CpG binding domain protein 1 [Source:HGNC Symbol;Acc:HGNC:6916]	2.08	0.0653
ssc-miR-125a	MBNL3	muscleblind like splicing regulator 3 [Source:VGNC Symbol;Acc:VGNC:90056]	2.08	0.0653
ssc-miR-125a	MBOAT2	membrane bound O-acyltransferase domain containing 2 [Source:VGNC Symbol;Acc:VGNC:90058]	2.08	0.0653
ssc-miR-125a	MCL1	MCL1 apoptosis regulator, BCL2 family member [Source:VGNC Symbol;Acc:VGNC:90072]	2.08	0.0653
ssc-miR-125a	ME2	malic enzyme 2 [Source:VGNC Symbol;Acc:VGNC:90096]	2.08	0.0653
ssc-miR-125a	MEF2D	myocyte enhancer factor 2D [Source:VGNC Symbol;Acc:VGNC:90128]	2.08	0.0653
ssc-miR-125a	MEGF8	hypothetical gene	2.08	0.0653
ssc-miR-125a	MEIS2	Meis homeobox 2 [Source:VGNC Symbol;Acc:VGNC:90136]	2.08	0.0653
ssc-miR-125a	METTL24	methyltransferase like 24 [Source:VGNC Symbol;Acc:VGNC:90161]	2.08	0.0653
ssc-miR-125a	MFHAS1	multifunctional ROCO family signaling regulator 1 [Source:VGNC Symbol;Acc:VGNC:107361]	2.08	0.0653
ssc-miR-125a	MFN1	mitofusin 1 [Source:VGNC Symbol;Acc:VGNC:90178]	2.08	0.0653
ssc-miR-125a	MFSD9	major facilitator superfamily domain containing 9 [Source:VGNC Symbol;Acc:VGNC:90194]	2.08	0.0653

ssc-miR-125a	MGAT4A	alpha-1,3-mannosyl-glycoprotein 4-beta-N-acetylglucosaminyltransferase A [Source:VGNC Symbol;Acc:VGNC:108158]	2.08	0.0653
ssc-miR-125a	MGAT5	alpha-1,6-mannosylglycoprotein 6-beta-N-acetylglucosaminyltransferase [Source:VGNC Symbol;Acc:VGNC:96741]	2.08	0.0653
ssc-miR-125a	MIB1	MIB E3 ubiquitin protein ligase 1 [Source:VGNC Symbol;Acc:VGNC:90205]	2.08	0.0653
ssc-miR-125a	MICALL1	MICAL like 1 [Source:VGNC Symbol;Acc:VGNC:90209]	2.08	0.0653
ssc-miR-125a	MIP	major intrinsic protein of lens fiber [Source:VGNC Symbol;Acc:VGNC:90235]	2.08	0.0653
ssc-miR-125a	MKNK2	MAPK interacting serine/threonine kinase 2 [Source:VGNC Symbol;Acc:VGNC:90247]	2.08	0.0653
ssc-miR-125a	MLF2	myeloid leukemia factor 2 [Source:VGNC Symbol;Acc:VGNC:103302]	2.08	0.0653
ssc-miR-125a	MMP11	matrix metallopeptidase 11 [Source:VGNC Symbol;Acc:VGNC:90267]	2.08	0.0653
ssc-miR-125a	MOCS1	molybdenum cofactor synthesis 1 [Source:VGNC Symbol;Acc:VGNC:107153]	2.08	0.0653
ssc-miR-125a	MORC2	MORC family CW-type zinc finger 2 [Source:VGNC Symbol;Acc:VGNC:90303]	2.08	0.0653
ssc-miR-125a	MRPL17	mitochondrial ribosomal protein L17 [Source:VGNC Symbol;Acc:VGNC:103992]	2.08	0.0653
ssc-miR-125a	MSI1	musashi RNA binding protein 1 [Source:VGNC Symbol;Acc:VGNC:90421]	2.08	0.0653
ssc-miR-125a	MSL1	MSL complex subunit 1 [Source:VGNC Symbol;Acc:VGNC:90423]	2.08	0.0653
ssc-miR-125a	MSRB3	methionine sulfoxide reductase B3 [Source:VGNC Symbol;Acc:VGNC:90427]	2.08	0.0653
ssc-miR-125a	MT1A	metallothionein 1A [Source:NCBI gene (formerly Entrezgene);Acc:397417]	2.08	0.0653
ssc-miR-125a	MTF1	metal regulatory transcription factor 1 [Source:VGNC Symbol;Acc:VGNC:90443]	2.08	0.0653
ssc-miR-125a	MTMR3	myotubularin related protein 3 [Source:VGNC Symbol;Acc:VGNC:90461]	2.08	0.0653
ssc-miR-125a	MTUS1	microtubule associated scaffold protein 1 [Source:VGNC Symbol;Acc:VGNC:107157]	2.08	0.0653
ssc-miR-125a	MTUS2	microtubule associated scaffold protein 2 [Source:VGNC Symbol;Acc:VGNC:90477]	2.08	0.0653
ssc-miR-125a	MXD4	MAX dimerization protein 4 [Source:VGNC Symbol;Acc:VGNC:90493]	2.08	0.0653
ssc-miR-125a	MYL3	myosin light chain 3 [Source:VGNC Symbol;Acc:VGNC:90515]	2.08	0.0653
ssc-miR-125a	MYO18A	myosin XVIIIA [Source:HGNC Symbol;Acc:HGNC:31104]	2.08	0.0653
ssc-miR-125a	MYO1E	myosin IE [Source:VGNC Symbol;Acc:VGNC:90528]	2.08	0.0653
ssc-miR-125a	MYO6	myosin VI [Source:VGNC Symbol;Acc:VGNC:103137]	2.08	0.0653
ssc-miR-125a	MYO9A	myosin IXA [Source:VGNC Symbol;Acc:VGNC:103138]	2.08	0.0653
ssc-miR-125a	MYT1	myelin transcription factor 1 [Source:VGNC Symbol;Acc:VGNC:104000]	2.08	0.0653
ssc-miR-125a	NAA25	N-alpha-acetyltransferase 25, NatB auxiliary subunit [Source:VGNC Symbol;Acc:VGNC:96750]	2.08	0.0653
ssc-miR-125a	NAA40	N-alpha-acetyltransferase 40, NatD catalytic subunit [Source:VGNC Symbol;Acc:VGNC:96753]	2.08	0.0653
ssc-miR-125a	NAIF1	hypothetical gene	2.08	0.0653
ssc-miR-125a	NBEAL2	neurobeachin like 2 [Source:VGNC Symbol;Acc:VGNC:90591]	2.08	0.0653
ssc-miR-125a	NCAN	neurocan [Source:VGNC Symbol;Acc:VGNC:90596]	2.08	0.0653
ssc-miR-125a	NCKAP5L	NCK associated protein 5 like [Source:VGNC Symbol;Acc:VGNC:90611]	2.08	0.0653
ssc-miR-125a	NCLN	nicalin [Source:VGNC Symbol;Acc:VGNC:90613]	2.08	0.0653
ssc-miR-125a	NCOR2	nuclear receptor corepressor 2 [Source:VGNC Symbol;Acc:VGNC:98148]	2.08	0.0653
ssc-miR-125a	NDRG3	NDRG family member 3 [Source:HGNC Symbol;Acc:HGNC:14462]	2.08	0.0653
ssc-miR-125a	NECAB3	N-terminal EF-hand calcium binding protein 3 [Source:VGNC Symbol;Acc:VGNC:95670]	2.08	0.0653
ssc-miR-125a	NEDD9	neural cell expressed, developmentally down-regulated 9 [Source:VGNC Symbol;Acc:VGNC:90668]	2.08	0.0653
ssc-miR-125a	NEK10	NIMA related kinase 10 [Source:VGNC Symbol;Acc:VGNC:90674]	2.08	0.0653
ssc-miR-125a	NEO1	neogenin 1 [Source:HGNC Symbol;Acc:HGNC:7754]	2.08	0.0653
ssc-miR-125a	NEU1	neuraminidase 1 [Source:VGNC Symbol;Acc:VGNC:90692]	2.08	0.0653
ssc-miR-125a	NFASC	neurofascin [Source:VGNC Symbol;Acc:VGNC:90707]	2.08	0.0653
ssc-miR-125a	NFIB	nuclear factor I B [Source:VGNC Symbol;Acc:VGNC:90716]	2.08	0.0653
ssc-miR-125a	NFIC	nuclear factor I C [Source:VGNC Symbol;Acc:VGNC:100313]	2.08	0.0653
ssc-miR-125a	NHSL2	NHS like 2 [Source:VGNC Symbol;Acc:VGNC:98155]	2.08	0.0653
ssc-miR-125a	NICN1	nicolin 1, tubulin polyglutamylase complex subunit [Source:VGNC Symbol;Acc:VGNC:107158]	2.08	0.0653
ssc-miR-125a	NIN	ninein [Source:VGNC Symbol;Acc:VGNC:90745]	2.08	0.0653
ssc-miR-125a	NINL	hypothetical gene	2.08	0.0653
ssc-miR-125a	NIPA1	NIPA magnesium transporter 1 [Source:VGNC Symbol;Acc:VGNC:95455]	2.08	0.0653
ssc-miR-125a	NIPAL4	NIPA like domain containing 4 [Source:VGNC Symbol;Acc:VGNC:90751]	2.08	0.0653

ssc-miR-125a	NKIRAS2	NFKB inhibitor interacting Ras like 2 [Source:VGNC Symbol;Acc:VGNC:90763]	2.08	0.0653
ssc-miR-125a	NKPD1	NTPase KAP family P-loop domain containing 1 [Source:VGNC Symbol;Acc:VGNC:98518]	2.08	0.0653
ssc-miR-125a	NLE1	notchless homolog 1 [Source:VGNC Symbol;Acc:VGNC:90776]	2.08	0.0653
ssc-miR-125a	NLRC5	NLR family CARD domain containing 5 [Source:VGNC Symbol;Acc:VGNC:90782]	2.08	0.0653
ssc-miR-125a	NOL12	nucleolar protein 12 [Source:HGNC Symbol;Acc:HGNC:28585]	2.08	0.0653
ssc-miR-125a	NR5A2	nuclear receptor subfamily 5 group A member 2 [Source:VGNC Symbol;Acc:VGNC:96452]	2.08	0.0653
ssc-miR-125a	NR6A1	nuclear receptor subfamily 6 group A member 1 [Source:VGNC Symbol;Acc:VGNC:90887]	2.08	0.0653
ssc-miR-125a	NRG3	neuregulin 3 [Source:VGNC Symbol;Acc:VGNC:104011]	2.08	0.0653
ssc-miR-125a	NRM	nurim [Source:VGNC Symbol;Acc:VGNC:90899]	2.08	0.0653
ssc-miR-125a	NRXN1	neurexin 1 [Source:HGNC Symbol;Acc:HGNC:8008]	2.08	0.0653
ssc-miR-125a	NT5DC1	5'-nucleotidase domain containing 1 [Source:VGNC Symbol;Acc:VGNC:90922]	2.08	0.0653
ssc-miR-125a	NUDCD2	NudC domain containing 2 [Source:VGNC Symbol;Acc:VGNC:90950]	2.08	0.0653
ssc-miR-125a	NUDT12	nudix hydrolase 12 [Source:VGNC Symbol;Acc:VGNC:90953]	2.08	0.0653
ssc-miR-125a	NUMBL	NUMB like endocytic adaptor protein [Source:HGNC Symbol;Acc:HGNC:8061]	2.08	0.0653
ssc-miR-125a	NUP210	nucleoporin 210 [Source:VGNC Symbol;Acc:VGNC:90977]	2.08	0.0653
ssc-miR-125a	NUP50	nucleoporin 50 [Source:VGNC Symbol;Acc:VGNC:90983]	2.08	0.0653
ssc-miR-125a	NXF1	nuclear RNA export factor 1 [Source:VGNC Symbol;Acc:VGNC:90993]	2.08	0.0653
ssc-miR-125a	OAZ2	ornithine decarboxylase antizyme 2 [Source:VGNC Symbol;Acc:VGNC:91009]	2.08	0.0653
ssc-miR-125a	OLFML2A	olfactomedin like 2A [Source:VGNC Symbol;Acc:VGNC:98170]	2.08	0.0653
ssc-miR-125a	ONECUT2	one cut homeobox 2 [Source:VGNC Symbol;Acc:VGNC:91043]	2.08	0.0653
ssc-miR-125a	OPALIN	oligodendrocytic myelin paranodal and inner loop protein [Source:VGNC Symbol;Acc:VGNC:91046]	2.08	0.0653
ssc-miR-125a	ORC2	origin recognition complex subunit 2 [Source:VGNC Symbol;Acc:VGNC:96464]	2.08	0.0653
ssc-miR-125a	OSBPL9	oxysterol binding protein like 9 [Source:VGNC Symbol;Acc:VGNC:91075]	2.08	0.0653
ssc-miR-125a	OTUB2	OTU deubiquitinase, ubiquitin aldehyde binding 2 [Source:VGNC Symbol;Acc:VGNC:91098]	2.08	0.0653
ssc-miR-125a	OVOL1	ovo like transcriptional repressor 1 [Source:VGNC Symbol;Acc:VGNC:91111]	2.08	0.0653
ssc-miR-125a	PABPC4L	poly(A) binding protein cytoplasmic 4 like [Source:VGNC Symbol;Acc:VGNC:108517]	2.08	0.0653
ssc-miR-125a	PACS2	phosphofurin acidic cluster sorting protein 2 [Source:VGNC Symbol;Acc:VGNC:91141]	2.08	0.0653
ssc-miR-125a	PAFAH1B1	platelet activating factor acetylhydrolase 1b regulatory subunit 1 [Source:VGNC Symbol;Acc:VGNC:99026]	2.08	0.0653
ssc-miR-125a	PAK3	p21 (RAC1) activated kinase 3 [Source:HGNC Symbol;Acc:HGNC:8592]	2.08	0.0653
ssc-miR-125a	PAN2	poly(A) specific ribonuclease subunit PAN2 [Source:HGNC Symbol;Acc:HGNC:20074]	2.08	0.0653
ssc-miR-125a	PARM1	prostate androgen-regulated mucin-like protein 1 [Source:VGNC Symbol;Acc:VGNC:91182]	2.08	0.0653
ssc-miR-125a	PARP11	poly(ADP-ribose) polymerase family member 11 [Source:VGNC Symbol;Acc:VGNC:103308]	2.08	0.0653
ssc-miR-125a	PAX9	paired box 9 [Source:HGNC Symbol;Acc:HGNC:8623]	2.08	0.0653
ssc-miR-125a	PCDH1	protocadherin 1 [Source:VGNC Symbol;Acc:VGNC:91211]	2.08	0.0653
ssc-miR-125a	PCGF6	polycomb group ring finger 6 [Source:VGNC Symbol;Acc:VGNC:91221]	2.08	0.0653
ssc-miR-125a	PCNX	hypothetical gene	2.08	0.0653
ssc-miR-125a	PCSK7	proprotein convertase subtilisin/kexin type 7 [Source:VGNC Symbol;Acc:VGNC:91233]	2.08	0.0653
ssc-miR-125a	PCTP	hypothetical gene	2.08	0.0653
ssc-miR-125a	PDE7A	phosphodiesterase 7A [Source:VGNC Symbol;Acc:VGNC:91261]	2.08	0.0653
ssc-miR-125a	PDGFRA	platelet derived growth factor receptor alpha [Source:VGNC Symbol;Acc:VGNC:98179]	2.08	0.0653
ssc-miR-125a	PDK3	pyruvate dehydrogenase kinase 3 [Source:VGNC Symbol;Acc:VGNC:91279]	2.08	0.0653
ssc-miR-125a	PEAK1	pseudopodium enriched atypical kinase 1 [Source:VGNC Symbol;Acc:VGNC:91300]	2.08	0.0653
ssc-miR-125a	PELI2	pellino E3 ubiquitin protein ligase family member 2 [Source:VGNC Symbol;Acc:VGNC:91307]	2.08	0.0653
ssc-miR-125a	PET100	PET100 cytochrome c oxidase chaperone [Source:VGNC Symbol;Acc:VGNC:91318]	2.08	0.0653
ssc-miR-125a	PGAP3	post-GPI attachment to proteins phospholipase 3 [Source:VGNC Symbol;Acc:VGNC:91343]	2.08	0.0653
ssc-miR-125a	PGP	phosphoglycolate phosphatase [Source:VGNC Symbol;Acc:VGNC:91359]	2.08	0.0653
ssc-miR-125a	PHACTR3	phosphatase and actin regulator 3 [Source:VGNC Symbol;Acc:VGNC:95667]	2.08	0.0653
ssc-miR-125a	PHC2	polyhomeotic homolog 2 [Source:VGNC Symbol;Acc:VGNC:91371]	2.08	0.0653
ssc-miR-125a	PHF15	hypothetical gene	2.08	0.0653

ssc-miR-125a	PHF20	PHD finger protein 20 [Source:HGNC Symbol;Acc:HGNC:16098]	2.08	0.0653
ssc-miR-125a	PHOX2B	paired like homeobox 2B [Source:VGNC Symbol;Acc:VGNC:91402]	2.08	0.0653
ssc-miR-125a	PHYHIP	phytanoyl-CoA 2-hydroxylase interacting protein [Source:VGNC Symbol;Acc:VGNC:91406]	2.08	0.0653
ssc-miR-125a	PI4K2B	phosphatidylinositol 4-kinase type 2 beta [Source:VGNC Symbol;Acc:VGNC:98190]	2.08	0.0653
ssc-miR-125a	PIK3C2B	phosphatidylinositol-4-phosphate 3-kinase catalytic subunit type 2 beta [Source:VGNC Symbol;Acc:VGNC:91437]	2.08	0.0653
ssc-miR-125a	PIP4K2B	phosphatidylinositol-5-phosphate 4-kinase type 2 beta [Source:VGNC Symbol;Acc:VGNC:91454]	2.08	0.0653
ssc-miR-125a	PKP4	plakophilin 4 [Source:VGNC Symbol;Acc:VGNC:96488]	2.08	0.0653
ssc-miR-125a	PLAGL2	PLAG1 like zinc finger 2 [Source:VGNC Symbol;Acc:VGNC:96490]	2.08	0.0653
ssc-miR-125a	PLEKHA8	pleckstrin homology domain containing A8 [Source:HGNC Symbol;Acc:HGNC:30037]	2.08	0.0653
ssc-miR-125a	PLEKHH1	pleckstrin homology, MyTH4 and FERM domain containing H1 [Source:HGNC Symbol;Acc:HGNC:17733]	2.08	0.0653
ssc-miR-125a	PLEKHM3	pleckstrin homology domain containing M3 [Source:VGNC Symbol;Acc:VGNC:96281]	2.08	0.0653
ssc-miR-125a	PLXNA1	plexin A1 [Source:VGNC Symbol;Acc:VGNC:91579]	2.08	0.0653
ssc-miR-125a	PMM2	hypothetical gene	2.08	0.0653
ssc-miR-125a	PODXL	hypothetical gene	2.08	0.0653
ssc-miR-125a	POLR3H	RNA polymerase III subunit H [Source:VGNC Symbol;Acc:VGNC:91658]	2.08	0.0653
ssc-miR-125a	POM121	hypothetical gene	2.08	0.0653
ssc-miR-125a	PPAT	phosphoribosyl pyrophosphate amidotransferase [Source:VGNC Symbol;Acc:VGNC:91687]	2.08	0.0653
ssc-miR-125a	PPM1A	protein phosphatase, Mg2+/Mn2+ dependent 1A [Source:VGNC Symbol;Acc:VGNC:91703]	2.08	0.0653
ssc-miR-125a	PPM1H	protein phosphatase, Mg2+/Mn2+ dependent 1H [Source:VGNC Symbol;Acc:VGNC:91708]	2.08	0.0653
ssc-miR-125a	PPME1	protein phosphatase methylesterase 1 [Source:VGNC Symbol;Acc:VGNC:91713]	2.08	0.0653
ssc-miR-125a	PPP1CA	protein phosphatase 1 catalytic subunit alpha [Source:VGNC Symbol;Acc:VGNC:91715]	2.08	0.0653
ssc-miR-125a	PPP1R37	protein phosphatase 1 regulatory subunit 37 [Source:VGNC Symbol;Acc:VGNC:91735]	2.08	0.0653
ssc-miR-125a	PPP1R9B	protein phosphatase 1 regulatory subunit 9B [Source:VGNC Symbol;Acc:VGNC:91744]	2.08	0.0653
ssc-miR-125a	PPP2CA	protein phosphatase 2 catalytic subunit alpha [Source:VGNC Symbol;Acc:VGNC:91745]	2.08	0.0653
ssc-miR-125a	PPP2R1B	protein phosphatase 2 scaffold subunit Abeta [Source:VGNC Symbol;Acc:VGNC:91747]	2.08	0.0653
ssc-miR-125a	PPP2R5C	protein phosphatase 2 regulatory subunit B'gamma [Source:VGNC Symbol;Acc:VGNC:91753]	2.08	0.0653
ssc-miR-125a	PPP5C	protein phosphatase 5 catalytic subunit [Source:VGNC Symbol;Acc:VGNC:98541]	2.08	0.0653
ssc-miR-125a	PPT1	palmitoyl-protein thioesterase 1 [Source:VGNC Symbol;Acc:VGNC:91764]	2.08	0.0653
ssc-miR-125a	PQLC1	hypothetical gene	2.08	0.0653
ssc-miR-125a	PRDM1	PR/SET domain 1 [Source:VGNC Symbol;Acc:VGNC:91772]	2.08	0.0653
ssc-miR-125a	PRKAA2	protein kinase AMP-activated catalytic subunit alpha 2 [Source:VGNC Symbol;Acc:VGNC:91798]	2.08	0.0653
ssc-miR-125a	PROX1	prospero homeobox 1 [Source:VGNC Symbol;Acc:VGNC:91837]	2.08	0.0653
ssc-miR-125a	PRRC1	proline rich coiled-coil 1 [Source:VGNC Symbol;Acc:VGNC:91864]	2.08	0.0653
ssc-miR-125a	PRSS35	serine protease 35 [Source:VGNC Symbol;Acc:VGNC:91883]	2.08	0.0653
ssc-miR-125a	PSMB5	hypothetical gene	2.08	0.0653
ssc-miR-125a	PSMB8	proteasome 20S subunit beta 8 [Source:VGNC Symbol;Acc:VGNC:91910]	2.08	0.0653
ssc-miR-125a	PSME3	proteasome activator subunit 3 [Source:VGNC Symbol;Acc:VGNC:91926]	2.08	0.0653
ssc-miR-125a	PSTPIP2	proline-serine-threonine phosphatase interacting protein 2 [Source:HGNC Symbol;Acc:HGNC:9581]	2.08	0.0653
ssc-miR-125a	PTAR1	protein prenyltransferase alpha subunit repeat containing 1 [Source:VGNC Symbol;Acc:VGNC:91936]	2.08	0.0653
ssc-miR-125a	PTH1R	parathyroid hormone 1 receptor [Source:VGNC Symbol;Acc:VGNC:91961]	2.08	0.0653
ssc-miR-125a	PTPN1	protein tyrosine phosphatase non-receptor type 1 [Source:VGNC Symbol;Acc:VGNC:96519]	2.08	0.0653
ssc-miR-125a	PTPN18	protein tyrosine phosphatase non-receptor type 18 [Source:VGNC Symbol;Acc:VGNC:96520]	2.08	0.0653
ssc-miR-125a	PTPRF	protein tyrosine phosphatase receptor type F [Source:VGNC Symbol;Acc:VGNC:91987]	2.08	0.0653
ssc-miR-125a	PTPRU	protein tyrosine phosphatase receptor type U [Source:VGNC Symbol;Acc:VGNC:91995]	2.08	0.0653
ssc-miR-125a	PTRH2	peptidyl-tRNA hydrolase 2 [Source:VGNC Symbol;Acc:VGNC:91998]	2.08	0.0653
ssc-miR-125a	QKI	QKI, KH domain containing RNA binding [Source:VGNC Symbol;Acc:VGNC:92025]	2.08	0.0653
ssc-miR-125a	QSOX2	quiescin sulfhydryl oxidase 2 [Source:VGNC Symbol;Acc:VGNC:92034]	2.08	0.0653
ssc-miR-125a	RAB22A	RAB22A, member RAS onco family [Source:VGNC Symbol;Acc:VGNC:98244]	2.08	0.0653
ssc-miR-125a	RAB3C	RAB3C, member RAS onco family [Source:VGNC Symbol;Acc:VGNC:104043]	2.08	0.0653

ssc-miR-125a	RAB3D	RAB3D, member RAS onco family [Source:VGNC Symbol;Acc:VGNC:98266]	2.08	0.0653
ssc-miR-125a	RAB3IP	RAB3A interacting protein [Source:HGNC Symbol;Acc:HGNC:16508]	2.08	0.0653
ssc-miR-125a	RAB6B	RAB6B, member RAS onco family [Source:VGNC Symbol;Acc:VGNC:98272]	2.08	0.0653
ssc-miR-125a	RAB8B	RAB8B, member RAS onco family [Source:VGNC Symbol;Acc:VGNC:98276]	2.08	0.0653
ssc-miR-125a	RABL2B	hypothetical gene	2.08	0.0653
ssc-miR-125a	RAD54L2	RAD54 like 2 [Source:VGNC Symbol;Acc:VGNC:92061]	2.08	0.0653
ssc-miR-125a	RAF1	Raf-1 proto-onco, serine/threonine kinase [Source:VGNC Symbol;Acc:VGNC:108688]	2.08	0.0653
ssc-miR-125a	RALGPS2	Ral GEF with PH domain and SH3 binding motif 2 [Source:VGNC Symbol;Acc:VGNC:92073]	2.08	0.0653
ssc-miR-125a	RAP1A	RAP1A, member of RAS onco family [Source:VGNC Symbol;Acc:VGNC:92084]	2.08	0.0653
ssc-miR-125a	RAPGEF4	Rap guanine nucleotide exchange factor 4 [Source:VGNC Symbol;Acc:VGNC:95814]	2.08	0.0653
ssc-miR-125a	RAPGEF5	hypothetical gene	2.08	0.0653
ssc-miR-125a	RAPGEFL1	Rap guanine nucleotide exchange factor like 1 [Source:VGNC Symbol;Acc:VGNC:92095]	2.08	0.0653
ssc-miR-125a	RASA2	RAS p21 protein activator 2 [Source:VGNC Symbol;Acc:VGNC:92102]	2.08	0.0653
ssc-miR-125a	RASAL2	RAS protein activator like 2 [Source:VGNC Symbol;Acc:VGNC:92105]	2.08	0.0653
ssc-miR-125a	RASGEF1A	RasGEF domain family member 1A [Source:VGNC Symbol;Acc:VGNC:92108]	2.08	0.0653
ssc-miR-125a	RASGRF1	Ras protein specific guanine nucleotide releasing factor 1 [Source:VGNC Symbol;Acc:VGNC:92111]	2.08	0.0653
ssc-miR-125a	RASGRF2	Ras protein specific guanine nucleotide releasing factor 2 [Source:VGNC Symbol;Acc:VGNC:92112]	2.08	0.0653
ssc-miR-125a	RASL10B	RAS like family 10 member B [Source:VGNC Symbol;Acc:VGNC:92118]	2.08	0.0653
ssc-miR-125a	RASSF3	Ras association domain family member 3 [Source:VGNC Symbol;Acc:VGNC:92124]	2.08	0.0653
ssc-miR-125a	RBAK	RB associated KRAB zinc finger [Source:VGNC Symbol;Acc:VGNC:92134]	2.08	0.0653
ssc-miR-125a	RBFOX2	RNA binding fox-1 homolog 2 [Source:VGNC Symbol;Acc:VGNC:92140]	2.08	0.0653
ssc-miR-125a	RBM18	RNA binding motif protein 18 [Source:VGNC Symbol;Acc:VGNC:92149]	2.08	0.0653
ssc-miR-125a	RBM20	RNA binding motif protein 20 [Source:HGNC Symbol;Acc:HGNC:27424]	2.08	0.0653
ssc-miR-125a	RBM24	hypothetical gene	2.08	0.0653
ssc-miR-125a	RBM38	RNA binding motif protein 38 [Source:VGNC Symbol;Acc:VGNC:95666]	2.08	0.0653
ssc-miR-125a	RBM7	RNA binding motif protein 7 [Source:VGNC Symbol;Acc:VGNC:108612]	2.08	0.0653
ssc-miR-125a	RCN3	reticulocalbin 3 [Source:VGNC Symbol;Acc:VGNC:92182]	2.08	0.0653
ssc-miR-125a	REEP3	receptor accessory protein 3 [Source:VGNC Symbol;Acc:VGNC:92199]	2.08	0.0653
ssc-miR-125a	REST	hypothetical gene	2.08	0.0653
ssc-miR-125a	RFX3	regulatory factor X3 [Source:VGNC Symbol;Acc:VGNC:92245]	2.08	0.0653
ssc-miR-125a	RFX5	regulatory factor X5 [Source:VGNC Symbol;Acc:VGNC:92246]	2.08	0.0653
ssc-miR-125a	RFXANK	regulatory factor X associated ankyrin containing protein [Source:VGNC Symbol;Acc:VGNC:92250]	2.08	0.0653
ssc-miR-125a	RGS7BP	regulator of G protein signaling 7 binding protein [Source:VGNC Symbol;Acc:VGNC:92268]	2.08	0.0653
ssc-miR-125a	RHOQ	ras homolog family member Q [Source:VGNC Symbol;Acc:VGNC:92292]	2.08	0.0653
ssc-miR-125a	RHOT2	ras homolog family member T2 [Source:NCBI gene (formerly Entrezgene);Acc:397596]	2.08	0.0653
ssc-miR-125a	RIMKLA	ribosomal modification protein rimK like family member A [Source:HGNC Symbol;Acc:HGNC:28725]	2.08	0.0653
ssc-miR-125a	RIT1	Ras like without CAAX 1 [Source:VGNC Symbol;Acc:VGNC:92325]	2.08	0.0653
ssc-miR-125a	RNASEH2C	ribonuclease H2 subunit C [Source:HGNC Symbol;Acc:HGNC:24116]	2.08	0.0653
ssc-miR-125a	RND2	Rho family GTPase 2 [Source:VGNC Symbol;Acc:VGNC:92344]	2.08	0.0653
ssc-miR-125a	RNF121	interleukin 18 binding protein [Source:VGNC Symbol;Acc:VGNC:89089]	2.08	0.0653
ssc-miR-125a	RNF144A	ring finger protein 144A [Source:VGNC Symbol;Acc:VGNC:92358]	2.08	0.0653
ssc-miR-125a	RNF144B	ring finger protein 144B [Source:VGNC Symbol;Acc:VGNC:92359]	2.08	0.0653
ssc-miR-125a	RNF168	hypothetical gene	2.08	0.0653
ssc-miR-125a	RNF217	ring finger protein 217 [Source:VGNC Symbol;Acc:VGNC:103162]	2.08	0.0653
ssc-miR-125a	RNF44	ring finger protein 44 [Source:VGNC Symbol;Acc:VGNC:92395]	2.08	0.0653
ssc-miR-125a	RNPEPL1	arginyl aminopeptidase like 1 [Source:VGNC Symbol;Acc:VGNC:95470]	2.08	0.0653
ssc-miR-125a	RORA	RAR related orphan receptor A [Source:VGNC Symbol;Acc:VGNC:92408]	2.08	0.0653
ssc-miR-125a	RORB	RAR related orphan receptor B [Source:VGNC Symbol;Acc:VGNC:92409]	2.08	0.0653
ssc-miR-125a	RORC	RAR related orphan receptor C [Source:VGNC Symbol;Acc:VGNC:92410]	2.08	0.0653

ssc-miR-125a	RP11-17M16.1	hypothetical gene	2.08	0.0653
ssc-miR-125a	RP11-766F14.2	hypothetical gene	2.08	0.0653
ssc-miR-125a	RPS6KA1	ribosomal protein S6 kinase A1 [Source:VGNC Symbol;Acc:VGNC:92440]	2.08	0.0653
ssc-miR-125a	RPUSD2	RNA pseudouridine synthase domain containing 2 [Source:HGNC Symbol;Acc:HGNC:24180]	2.08	0.0653
ssc-miR-125a	RREB1	hypothetical gene	2.08	0.0653
ssc-miR-125a	RTN2	reticulon 2 [Source:VGNC Symbol;Acc:VGNC:98608]	2.08	0.0653
ssc-miR-125a	RUFY3	RUN and FYVE domain containing 3 [Source:VGNC Symbol;Acc:VGNC:98959]	2.08	0.0653
ssc-miR-125a	RUSC2	RUN and SH3 domain containing 2 [Source:VGNC Symbol;Acc:VGNC:92520]	2.08	0.0653
ssc-miR-125a	RYBP	RING1 and YY1 binding protein [Source:VGNC Symbol;Acc:VGNC:92532]	2.08	0.0653
ssc-miR-125a	RYR2	ryanodine receptor 2 [Source:VGNC Symbol;Acc:VGNC:92534]	2.08	0.0653
ssc-miR-125a	SAMD10	sterile alpha motif domain containing 10 [Source:VGNC Symbol;Acc:VGNC:95750]	2.08	0.0653
ssc-miR-125a	SAMD14	sterile alpha motif domain containing 14 [Source:VGNC Symbol;Acc:VGNC:92566]	2.08	0.0653
ssc-miR-125a	SARM1	hypothetical gene	2.08	0.0653
ssc-miR-125a	SATB2	SATB homeobox 2 [Source:VGNC Symbol;Acc:VGNC:95972]	2.08	0.0653
ssc-miR-125a	SAV1	salvador family WW domain containing protein 1 [Source:VGNC Symbol;Acc:VGNC:92588]	2.08	0.0653
ssc-miR-125a	SBK1	SH3 domain binding kinase 1 [Source:HGNC Symbol;Acc:HGNC:17699]	2.08	0.0653
ssc-miR-125a	SBNO1	strawberry notch homolog 1 [Source:VGNC Symbol;Acc:VGNC:92596]	2.08	0.0653
ssc-miR-125a	SCARB1	scavenger receptor class B member 1 [Source:VGNC Symbol;Acc:VGNC:92613]	2.08	0.0653
ssc-miR-125a	SCARF1	scavenger receptor class F member 1 [Source:VGNC Symbol;Acc:VGNC:92615]	2.08	0.0653
ssc-miR-125a	SCARF2	scavenger receptor class F member 2 [Source:VGNC Symbol;Acc:VGNC:92616]	2.08	0.0653
ssc-miR-125a	SCD	stearoyl-CoA desaturase [Source:NCBI gene (formerly Entrezgene);Acc:396670]	2.08	0.0653
ssc-miR-125a	SCLY	selenocysteine lyase [Source:VGNC Symbol;Acc:VGNC:98302]	2.08	0.0653
ssc-miR-125a	SCN2B	sodium voltage-gated channel beta subunit 2 [Source:VGNC Symbol;Acc:VGNC:92633]	2.08	0.0653
ssc-miR-125a	SCN4B	hypothetical gene	2.08	0.0653
ssc-miR-125a	SCN5A	sodium voltage-gated channel alpha subunit 5 [Source:VGNC Symbol;Acc:VGNC:92637]	2.08	0.0653
ssc-miR-125a	SCRT1	scratch family transcriptional repressor 1 [Source:VGNC Symbol;Acc:VGNC:92649]	2.08	0.0653
ssc-miR-125a	SCRT2	scratch family transcriptional repressor 2 [Source:VGNC Symbol;Acc:VGNC:95721]	2.08	0.0653
ssc-miR-125a	SCUBE3	signal peptide, CUB domain and EGF like domain containing 3 [Source:VGNC Symbol;Acc:VGNC:92651]	2.08	0.0653
ssc-miR-125a	SEL1L	SEL1L adaptor subunit of ERAD E3 ubiquitin ligase [Source:VGNC Symbol;Acc:VGNC:92687]	2.08	0.0653
ssc-miR-125a	SEMA4B	semaphorin 4B [Source:VGNC Symbol;Acc:VGNC:92701]	2.08	0.0653
ssc-miR-125a	SEMA4C	semaphorin 4C [Source:VGNC Symbol;Acc:VGNC:92702]	2.08	0.0653
ssc-miR-125a	SEMA4D	semaphorin 4D [Source:VGNC Symbol;Acc:VGNC:92703]	2.08	0.0653
ssc-miR-125a	SEMA4F	ssemaphorin 4F [Source:VGNC Symbol;Acc:VGNC:92704]	2.08	0.0653
ssc-miR-125a	SEPN1	hypothetical gene	2.08	0.0653
ssc-miR-125a	SEPT11	hypothetical gene	2.08	0.0653
ssc-miR-125a	SERINC5	serine incorporator 5 [Source:VGNC Symbol;Acc:VGNC:92730]	2.08	0.0653
ssc-miR-125a	SERTAD3	SERTA domain containing 3 [Source:VGNC Symbol;Acc:VGNC:92751]	2.08	0.0653
ssc-miR-125a	SESTD1	SEC14 and spectrin domain containing 1 [Source:VGNC Symbol;Acc:VGNC:95884]	2.08	0.0653
ssc-miR-125a	SFMBT1	Scm like with four mbt domains 1 [Source:VGNC Symbol;Acc:VGNC:92773]	2.08	0.0653
ssc-miR-125a	SGIP1	SH3GL interacting endocytic adaptor 1 [Source:VGNC Symbol;Acc:VGNC:92792]	2.08	0.0653
ssc-miR-125a	SGOL1	hypothetical gene	2.08	0.0653
ssc-miR-125a	SGPL1	sphingosine-1-phosphate lyase 1 [Source:VGNC Symbol;Acc:VGNC:92797]	2.08	0.0653
ssc-miR-125a	SGSM2	small G protein signaling modulator 2 [Source:VGNC Symbol;Acc:VGNC:92801]	2.08	0.0653
ssc-miR-125a	SH2B3	SH2B adaptor protein 3 [Source:VGNC Symbol;Acc:VGNC:92807]	2.08	0.0653
ssc-miR-125a	SH2D4B	SH2 domain containing 4B [Source:VGNC Symbol;Acc:VGNC:92811]	2.08	0.0653
ssc-miR-125a	SH3BP4	SH3 domain binding protein 4 [Source:VGNC Symbol;Acc:VGNC:95495]	2.08	0.0653
ssc-miR-125a	SH3BP5L	SH3 binding domain protein 5 like [Source:VGNC Symbol;Acc:VGNC:92820]	2.08	0.0653
ssc-miR-125a	SH3TC2	SH3 domain and tetratricopeptide repeats 2 [Source:VGNC Symbol;Acc:VGNC:92833]	2.08	0.0653
ssc-miR-125a	SHISA6	hypothetical gene	2.08	0.0653

ssc-miR-125a	SIGIRR	single Ig and TIR domain containing [Source:VGNC Symbol;Acc:VGNC:92870]	2.08	0.0653
ssc-miR-125a	SIM1	SIM bHLH transcription factor 1 [Source:VGNC Symbol;Acc:VGNC:92876]	2.08	0.0653
ssc-miR-125a	SIPA1L3	signal induced proliferation associated 1 like 3 [Source:VGNC Symbol;Acc:VGNC:98613]	2.08	0.0653
ssc-miR-125a	SIRT7	sirtuin 7 [Source:VGNC Symbol;Acc:VGNC:92890]	2.08	0.0653
ssc-miR-125a	SIT1	signaling threshold regulating transmembrane adaptor 1 [Source:VGNC Symbol;Acc:VGNC:92891]	2.08	0.0653
ssc-miR-125a	SLC16A6	solute carrier family 16 member 6 [Source:VGNC Symbol;Acc:VGNC:92946]	2.08	0.0653
ssc-miR-125a	SLC17A7	solute carrier family 17 member 7 [Source:VGNC Symbol;Acc:VGNC:92955]	2.08	0.0653
ssc-miR-125a	SLC1A5	solute carrier family 1 member 5 [Source:VGNC Symbol;Acc:VGNC:92967]	2.08	0.0653
ssc-miR-125a	SLC23A2	solute carrier family 23 member 2 [Source:VGNC Symbol;Acc:VGNC:108737]	2.08	0.0653
ssc-miR-125a	SLC24A2	solute carrier family 24 member 2 [Source:VGNC Symbol;Acc:VGNC:92987]	2.08	0.0653
ssc-miR-125a	SLC25A15	solute carrier family 25 member 15 [Source:VGNC Symbol;Acc:VGNC:92994]	2.08	0.0653
ssc-miR-125a	SLC25A35	solute carrier family 25 member 35 [Source:VGNC Symbol;Acc:VGNC:93011]	2.08	0.0653
ssc-miR-125a	SLC25A53	solute carrier family 25 member 53 [Source:VGNC Symbol;Acc:VGNC:93023]	2.08	0.0653
ssc-miR-125a	SLC26A6	solute carrier family 26 member 6 [Source:VGNC Symbol;Acc:VGNC:93031]	2.08	0.0653
ssc-miR-125a	SLC27A4	solute carrier family 27 member 4 [Source:VGNC Symbol;Acc:VGNC:93038]	2.08	0.0653
ssc-miR-125a	SLC31A1	solute carrier family 31 member 1 [Source:VGNC Symbol;Acc:VGNC:103180]	2.08	0.0653
ssc-miR-125a	SLC35A4	solute carrier family 35 member A4 [Source:VGNC Symbol;Acc:VGNC:93069]	2.08	0.0653
ssc-miR-125a	SLC35C1	solute carrier family 35 member C1 [Source:VGNC Symbol;Acc:VGNC:93075]	2.08	0.0653
ssc-miR-125a	SLC35G1	solute carrier family 35 member G1 [Source:VGNC Symbol;Acc:VGNC:93086]	2.08	0.0653
ssc-miR-125a	SLC38A9	solute carrier family 38 member 9 [Source:VGNC Symbol;Acc:VGNC:93100]	2.08	0.0653
ssc-miR-125a	SLC39A9	solute carrier family 39 member 9 [Source:VGNC Symbol;Acc:VGNC:93110]	2.08	0.0653
ssc-miR-125a	SLC4A10	solute carrier family 4 member 10 [Source:VGNC Symbol;Acc:VGNC:95592]	2.08	0.0653
ssc-miR-125a	SLC4A4	solute carrier family 4 member 4 [Source:VGNC Symbol;Acc:VGNC:93133]	2.08	0.0653
ssc-miR-125a	SLC4A8	solute carrier family 4 member 8 [Source:VGNC Symbol;Acc:VGNC:98326]	2.08	0.0653
ssc-miR-125a	SLC6A17	solute carrier family 6 member 17 [Source:VGNC Symbol;Acc:VGNC:93158]	2.08	0.0653
ssc-miR-125a	SLC7A1	solute carrier family 7 member 1 [Source:VGNC Symbol;Acc:VGNC:93169]	2.08	0.0653
ssc-miR-125a	SLC7A6	hypothetical gene	2.08	0.0653
ssc-miR-125a	SLC8A2	solute carrier family 8 member A2 [Source:VGNC Symbol;Acc:VGNC:93179]	2.08	0.0653
ssc-miR-125a	SLC8B1	solute carrier family 8 member B1 [Source:VGNC Symbol;Acc:VGNC:93181]	2.08	0.0653
ssc-miR-125a	SLCO2A1	solute carrier organic anion transporter family member 2A1 [Source:VGNC Symbol;Acc:VGNC:93195]	2.08	0.0653
ssc-miR-125a	SLITRK6	SLIT and NTRK like family member 6 [Source:VGNC Symbol;Acc:VGNC:93211]	2.08	0.0653
ssc-miR-125a	SMAD2	SMAD family member 2 [Source:VGNC Symbol;Acc:VGNC:98329]	2.08	0.0653
ssc-miR-125a	SMCR8	SMCR8-C9orf72 complex subunit [Source:VGNC Symbol;Acc:VGNC:96598]	2.08	0.0653
ssc-miR-125a	SMEK1	hypothetical gene	2.08	0.0653
ssc-miR-125a	SMG1	SMG1 nonsense mediated mRNA decay associated PI3K related kinase [Source:VGNC Symbol;Acc:VGNC:93248]	2.08	0.0653
ssc-miR-125a	SMG5	SMG5 nonsense mediated mRNA decay factor [Source:VGNC Symbol;Acc:VGNC:98861]	2.08	0.0653
ssc-miR-125a	SMURF1	SMAD specific E3 ubiquitin protein ligase 1 [Source:VGNC Symbol;Acc:VGNC:93270]	2.08	0.0653
ssc-miR-125a	SNAP25	synaptosome associated protein 25 [Source:VGNC Symbol;Acc:VGNC:95509]	2.08	0.0653
ssc-miR-125a	SNTB2	syntrophin beta 2 [Source:VGNC Symbol;Acc:VGNC:93298]	2.08	0.0653
ssc-miR-125a	SNX18	sorting nexin 18 [Source:VGNC Symbol;Acc:VGNC:93310]	2.08	0.0653
ssc-miR-125a	SNX22	sorting nexin 22 [Source:VGNC Symbol;Acc:VGNC:93314]	2.08	0.0653
ssc-miR-125a	SNX27	sorting nexin 27 [Source:VGNC Symbol;Acc:VGNC:93316]	2.08	0.0653
ssc-miR-125a	SOCS4	suppressor of cytokine signaling 4 [Source:VGNC Symbol;Acc:VGNC:93331]	2.08	0.0653
ssc-miR-125a	SORCS2	sortilin related VPS10 domain containing receptor 2 [Source:VGNC Symbol;Acc:VGNC:93340]	2.08	0.0653
ssc-miR-125a	SORT1	sortilin 1 [Source:VGNC Symbol;Acc:VGNC:93343]	2.08	0.0653
ssc-miR-125a	SOX11	SRY-box transcription factor 11 [Source:VGNC Symbol;Acc:VGNC:93351]	2.08	0.0653
ssc-miR-125a	SOX6	SRY-box transcription factor 6 [Source:VGNC Symbol;Acc:VGNC:93358]	2.08	0.0653
ssc-miR-125a	SP1	Sp1 transcription factor [Source:VGNC Symbol;Acc:VGNC:93360]	2.08	0.0653
ssc-miR-125a	SP9	Sp9 transcription factor [Source:VGNC Symbol;Acc:VGNC:96247]	2.08	0.0653

ssc-miR-125a	SPATS2	spermatogenesis associated serine rich 2 [Source:HGNC Symbol;Acc:HGNC:18650]	2.08	0.0653
ssc-miR-125a	SPSB4	splA/ryanodine receptor domain and SOCS box containing 4 [Source:VGNC Symbol;Acc:VGNC:93433]	2.08	0.0653
ssc-miR-125a	SPTBN2	spectrin beta, non-erythrocytic 2 [Source:VGNC Symbol;Acc:VGNC:93437]	2.08	0.0653
ssc-miR-125a	SREBF2	sterol regulatory element binding transcription factor 2 [Source:VGNC Symbol;Acc:VGNC:93450]	2.08	0.0653
ssc-miR-125a	SRF	serum response factor [Source:VGNC Symbol;Acc:VGNC:93452]	2.08	0.0653
ssc-miR-125a	SRGAP2	hypothetical gene	2.08	0.0653
ssc-miR-125a	SRRM3	serine/arginine repetitive matrix 3 [Source:VGNC Symbol;Acc:VGNC:93469]	2.08	0.0653
ssc-miR-125a	SSTR3	somatostatin receptor 3 [Source:VGNC Symbol;Acc:VGNC:93497]	2.08	0.0653
ssc-miR-125a	ST18	ST18 C2H2C-type zinc finger transcription factor [Source:VGNC Symbol;Acc:VGNC:93501]	2.08	0.0653
ssc-miR-125a	ST3GAL6	ST3 beta-galactoside alpha-2,3-sialyltransferase 6 [Source:VGNC Symbol;Acc:VGNC:93506]	2.08	0.0653
ssc-miR-125a	ST6GAL1	ST6 beta-galactoside alpha-2,6-sialyltransferase 1 [Source:VGNC Symbol;Acc:VGNC:93507]	2.08	0.0653
ssc-miR-125a	ST6GALNAC6	hypothetical gene	2.08	0.0653
ssc-miR-125a	ST8SIA4	ST8 alpha-N-acetyl-neuraminide alpha-2,8-sialyltransferase 4 [Source:VGNC Symbol;Acc:VGNC:93519]	2.08	0.0653
ssc-miR-125a	STARD13	hypothetical gene	2.08	0.0653
ssc-miR-125a	STAT3	signal transducer and activator of transcription 3 [Source:VGNC Symbol;Acc:VGNC:93540]	2.08	0.0653
ssc-miR-125a	STC1	stanniocalcin 1 [Source:VGNC Symbol;Acc:VGNC:93542]	2.08	0.0653
ssc-miR-125a	STOX2	storkhead box 2 [Source:VGNC Symbol;Acc:VGNC:96082]	2.08	0.0653
ssc-miR-125a	STX6	syntaxin 6 [Source:VGNC Symbol;Acc:VGNC:93594]	2.08	0.0653
ssc-miR-125a	STXBP5L	syntaxin binding protein 5L [Source:VGNC Symbol;Acc:VGNC:93600]	2.08	0.0653
ssc-miR-125a	SUFU	SUFU negative regulator of hedgehog signaling [Source:VGNC Symbol;Acc:VGNC:93608]	2.08	0.0653
ssc-miR-125a	SULT4A1	sulfotransferase family 4A member 1 [Source:VGNC Symbol;Acc:VGNC:93615]	2.08	0.0653
ssc-miR-125a	SUN1	Sad1 and UNC84 domain containing 1 [Source:VGNC Symbol;Acc:VGNC:93618]	2.08	0.0653
ssc-miR-125a	SUV39H1	SUV39H1 histone lysine methyltransferase [Source:VGNC Symbol;Acc:VGNC:93637]	2.08	0.0653
ssc-miR-125a	SUV39H2	SUV39H2 histone lysine methyltransferase [Source:VGNC Symbol;Acc:VGNC:95841]	2.08	0.0653
ssc-miR-125a	SUV420H2	hypothetical gene	2.08	0.0653
ssc-miR-125a	SVIP	small VCP interacting protein [Source:HGNC Symbol;Acc:HGNC:25238]	2.08	0.0653
ssc-miR-125a	SYT7	synaptotagmin 7 [Source:VGNC Symbol;Acc:VGNC:100868]	2.08	0.0653
ssc-miR-125a	SYVN1	synoviolin 1 [Source:HGNC Symbol;Acc:HGNC:20738]	2.08	0.0653
ssc-miR-125a	SZRD1	hypothetical gene	2.08	0.0653
ssc-miR-125a	TACC2	transforming acidic coiled-coil containing protein 2 [Source:NCBI gene (formerly Entrezgene);Acc:100152804]	2.08	0.0653
ssc-miR-125a	TADA2B	transcriptional adaptor 2B [Source:VGNC Symbol;Acc:VGNC:93707]	2.08	0.0653
ssc-miR-125a	TAF5L	TATA-box binding protein associated factor 5 like [Source:VGNC Symbol;Acc:VGNC:93718]	2.08	0.0653
ssc-miR-125a	TAF7L	TATA-box binding protein associated factor 7 like [Source:VGNC Symbol;Acc:VGNC:93722]	2.08	0.0653
ssc-miR-125a	TAF9B	TATA-box binding protein associated factor 9b [Source:VGNC Symbol;Acc:VGNC:93725]	2.08	0.0653
ssc-miR-125a	TAL2	hypothetical gene	2.08	0.0653
ssc-miR-125a	TANC2	tetratricopeptide repeat, ankyrin repeat and coiled-coil containing 2 [Source:VGNC Symbol;Acc:VGNC:93733]	2.08	0.0653
ssc-miR-125a	TANGO2	transport and golgi organization 2 homolog [Source:VGNC Symbol;Acc:VGNC:93734]	2.08	0.0653
ssc-miR-125a	TAOK1	TAO kinase 1 [Source:VGNC Symbol;Acc:VGNC:98355]	2.08	0.0653
ssc-miR-125a	TAZ	hypothetical gene	2.08	0.0653
ssc-miR-125a	TBC1D1	TBC1 domain family member 1 [Source:VGNC Symbol;Acc:VGNC:93758]	2.08	0.0653
ssc-miR-125a	TBC1D16	hypothetical gene	2.08	0.0653
ssc-miR-125a	TBX4	T-box transcription factor 4 [Source:VGNC Symbol;Acc:VGNC:93803]	2.08	0.0653
ssc-miR-125a	TDG	thymine DNA glycosylase [Source:VGNC Symbol;Acc:VGNC:93842]	2.08	0.0653
ssc-miR-125a	TEF	TEF transcription factor, PAR bZIP family member [Source:VGNC Symbol;Acc:VGNC:93864]	2.08	0.0653
ssc-miR-125a	TET2	hypothetical gene	2.08	0.0653
ssc-miR-125a	TET3	tet methylcytosine dioxygenase 3 [Source:VGNC Symbol;Acc:VGNC:93890]	2.08	0.0653
ssc-miR-125a	TEX261	testis expressed 261 [Source:HGNC Symbol;Acc:HGNC:30712]	2.08	0.0653
ssc-miR-125a	TGFBR1	transforming growth factor beta receptor 1 [Source:VGNC Symbol;Acc:VGNC:98368]	2.08	0.0653
ssc-miR-125a	TGOLN2	hypothetical gene	2.08	0.0653

ssc-miR-125a	THEMIS2	thymocyte selection associated family member 2 [Source:VGNC Symbol;Acc:VGNC:93954]	2.08	0.0653
ssc-miR-125a	THRA	thyroid hormone receptor alpha [Source:VGNC Symbol;Acc:VGNC:93963]	2.08	0.0653
ssc-miR-125a	THY1	hypothetical gene	2.08	0.0653
ssc-miR-125a	TIFAB	TIFA inhibitor [Source:VGNC Symbol;Acc:VGNC:93979]	2.08	0.0653
ssc-miR-125a	TIMM17B	translocase of inner mitochondrial membrane 17B [Source:VGNC Symbol;Acc:VGNC:93986]	2.08	0.0653
ssc-miR-125a	TJAP1	tight junction associated protein 1 [Source:VGNC Symbol;Acc:VGNC:94004]	2.08	0.0653
ssc-miR-125a	TLE3	TLE family member 3, transcriptional corepressor [Source:VGNC Symbol;Acc:VGNC:94016]	2.08	0.0653
ssc-miR-125a	TLK2	tousled like kinase 2 [Source:VGNC Symbol;Acc:VGNC:94017]	2.08	0.0653
ssc-miR-125a	TMBIM6	transmembrane BAX inhibitor motif containing 6 [Source:VGNC Symbol;Acc:VGNC:98374]	2.08	0.0653
ssc-miR-125a	TMCC2	transmembrane and coiled-coil domain family 2 [Source:VGNC Symbol;Acc:VGNC:94047]	2.08	0.0653
ssc-miR-125a	TMED1	transmembrane p24 trafficking protein 1 [Source:VGNC Symbol;Acc:VGNC:94053]	2.08	0.0653
ssc-miR-125a	TMED9	transmembrane p24 trafficking protein 9 [Source:VGNC Symbol;Acc:VGNC:100875]	2.08	0.0653
ssc-miR-125a	TMEM120B	transmembrane protein 120B [Source:VGNC Symbol;Acc:VGNC:94071]	2.08	0.0653
ssc-miR-125a	TMEM123	hypothetical gene	2.08	0.0653
ssc-miR-125a	TMEM132E	transmembrane protein 132E [Source:VGNC Symbol;Acc:VGNC:94083]	2.08	0.0653
ssc-miR-125a	TMEM135	transmembrane protein 135 [Source:VGNC Symbol;Acc:VGNC:94085]	2.08	0.0653
ssc-miR-125a	TMEM136	hypothetical gene	2.08	0.0653
ssc-miR-125a	TMEM161B	transmembrane protein 161B [Source:VGNC Symbol;Acc:VGNC:94102]	2.08	0.0653
ssc-miR-125a	TMEM168	transmembrane protein 168 [Source:VGNC Symbol;Acc:VGNC:94106]	2.08	0.0653
ssc-miR-125a	TMEM170B	transmembrane protein 170B [Source:VGNC Symbol;Acc:VGNC:94108]	2.08	0.0653
ssc-miR-125a	TMEM180	hypothetical gene	2.08	0.0653
ssc-miR-125a	TMEM19	transmembrane protein 19 [Source:VGNC Symbol;Acc:VGNC:94124]	2.08	0.0653
ssc-miR-125a	TMEM194A	hypothetical gene	2.08	0.0653
ssc-miR-125a	TMEM198	transmembrane protein 198 [Source:VGNC Symbol;Acc:VGNC:96274]	2.08	0.0653
ssc-miR-125a	TMEM25	transmembrane protein 25 [Source:VGNC Symbol;Acc:VGNC:94157]	2.08	0.0653
ssc-miR-125a	TMEM72	transmembrane protein 72 [Source:VGNC Symbol;Acc:VGNC:94206]	2.08	0.0653
ssc-miR-125a	TMEM86A	transmembrane protein 86A [Source:VGNC Symbol;Acc:VGNC:94210]	2.08	0.0653
ssc-miR-125a	TMEM87B	transmembrane protein 87B [Source:VGNC Symbol;Acc:VGNC:94213]	2.08	0.0653
ssc-miR-125a	TMPRSS2	transmembrane serine protease 2 [Source:VGNC Symbol;Acc:VGNC:94234]	2.08	0.0653
ssc-miR-125a	TMTC2	transmembrane O-mannosyltransferase targeting cadherins 2 [Source:VGNC Symbol;Acc:VGNC:94242]	2.08	0.0653
ssc-miR-125a	TNFAIP3	TNF alpha induced protein 3 [Source:VGNC Symbol;Acc:VGNC:94252]	2.08	0.0653
ssc-miR-125a	TNFRSF1B	TNF receptor superfamily member 1B [Source:VGNC Symbol;Acc:VGNC:94264]	2.08	0.0653
ssc-miR-125a	TNFSF4	TNF superfamily member 4 [Source:VGNC Symbol;Acc:VGNC:104081]	2.08	0.0653
ssc-miR-125a	TOMM40	translocase of outer mitochondrial membrane 40 [Source:VGNC Symbol;Acc:VGNC:94305]	2.08	0.0653
ssc-miR-125a	TOR2A	torsin family 2 member A [Source:VGNC Symbol;Acc:VGNC:94319]	2.08	0.0653
ssc-miR-125a	TP53INP1	tumor protein p53 inducible nuclear protein 1 [Source:VGNC Symbol;Acc:VGNC:94329]	2.08	0.0653
ssc-miR-125a	TRAF6	TNF receptor associated factor 6 [Source:VGNC Symbol;Acc:VGNC:94365]	2.08	0.0653
ssc-miR-125a	TRAPPC6B	trafficking protein particle complex subunit 6B [Source:VGNC Symbol;Acc:VGNC:94378]	2.08	0.0653
ssc-miR-125a	TRIAP1	hypothetical gene	2.08	0.0653
ssc-miR-125a	TRIB2	tribbles pseudokinase 2 [Source:VGNC Symbol;Acc:VGNC:94392]	2.08	0.0653
ssc-miR-125a	TRIM5	hypothetical gene	2.08	0.0653
ssc-miR-125a	TRIM68	tripartite motif containing 68 [Source:VGNC Symbol;Acc:VGNC:94428]	2.08	0.0653
ssc-miR-125a	TRIM71	tripartite motif containing 71 [Source:VGNC Symbol;Acc:VGNC:94431]	2.08	0.0653
ssc-miR-125a	TRPS1	transcriptional repressor GATA binding 1 [Source:VGNC Symbol;Acc:VGNC:94471]	2.08	0.0653
ssc-miR-125a	TSEN54	tRNA splicing endonuclease subunit 54 [Source:VGNC Symbol;Acc:VGNC:94485]	2.08	0.0653
ssc-miR-125a	TSHZ2	teashirt zinc finger homeobox 2 [Source:NCBI gene (formerly Entrezgene);Acc:100136903]	2.08	0.0653
ssc-miR-125a	TSNARE1	hypothetical gene	2.08	0.0653
ssc-miR-125a	TSPAN12	tetraspanin 12 [Source:VGNC Symbol;Acc:VGNC:94501]	2.08	0.0653
ssc-miR-125a	TSPAN14	tetraspanin 14 [Source:VGNC Symbol;Acc:VGNC:94503]	2.08	0.0653

ssc-miR-125a	TSTA3	hypothetical gene	2.08	0.0653
ssc-miR-125a	TTC7A	tetratricopeptide repeat domain 7A [Source:VGNC Symbol;Acc:VGNC:94557]	2.08	0.0653
ssc-miR-125a	TTPA	alpha tocopherol transfer protein [Source:HGNC Symbol;Acc:HGNC:12404]	2.08	0.0653
ssc-miR-125a	TXNDC16	thioredoxin domain containing 16 [Source:VGNC Symbol;Acc:VGNC:94606]	2.08	0.0653
ssc-miR-125a	TXNRD1	thioredoxin reductase 1 [Source:NCBI gene (formerly Entrezgene);Acc:396681]	2.08	0.0653
ssc-miR-125a	TYSND1	trypsin like peroxisomal matrix peptidase 1 [Source:VGNC Symbol;Acc:VGNC:94621]	2.08	0.0653
ssc-miR-125a	UBASH3B	ubiquitin associated and SH3 domain containing B [Source:VGNC Symbol;Acc:VGNC:94637]	2.08	0.0653
ssc-miR-125a	UBE2G1	ubiquitin conjugating enzyme E2 G1 [Source:HGNC Symbol;Acc:HGNC:12482]	2.08	0.0653
ssc-miR-125a	UBE2J1	ubiquitin conjugating enzyme E2 J1 [Source:VGNC Symbol;Acc:VGNC:94644]	2.08	0.0653
ssc-miR-125a	UBE2L3	hypothetical gene	2.08	0.0653
ssc-miR-125a	UBE2R2	ubiquitin conjugating enzyme E2 R2 [Source:VGNC Symbol;Acc:VGNC:95924]	2.08	0.0653
ssc-miR-125a	UBE2W	ubiquitin conjugating enzyme E2 W [Source:VGNC Symbol;Acc:VGNC:98890]	2.08	0.0653
ssc-miR-125a	UBN1	ubinuclein 1 [Source:VGNC Symbol;Acc:VGNC:94663]	2.08	0.0653
ssc-miR-125a	UBR7	ubiquitin protein ligase E3 component n-recognin 7 [Source:HGNC Symbol;Acc:HGNC:20344]	2.08	0.0653
ssc-miR-125a	UBTD1	ubiquitin domain containing 1 [Source:VGNC Symbol;Acc:VGNC:94672]	2.08	0.0653
ssc-miR-125a	UCK2	uridine-cytidine kinase 2 [Source:VGNC Symbol;Acc:VGNC:98399]	2.08	0.0653
ssc-miR-125a	UHRF1BP1L	UHRF1 binding protein 1 like [Source:VGNC Symbol;Acc:VGNC:94691]	2.08	0.0653
ssc-miR-125a	ULK3	unc-51 like kinase 3 [Source:VGNC Symbol;Acc:VGNC:94696]	2.08	0.0653
ssc-miR-125a	UQCC1	ubiquinol-cytochrome c reductase complex assembly factor 1 [Source:VGNC Symbol;Acc:VGNC:95694]	2.08	0.0653
ssc-miR-125a	USP12	ubiquitin specific peptidase 12 [Source:VGNC Symbol;Acc:VGNC:94745]	2.08	0.0653
ssc-miR-125a	USP2	ubiquitin specific peptidase 2 [Source:VGNC Symbol;Acc:VGNC:94751]	2.08	0.0653
ssc-miR-125a	USP37	ubiquitin specific peptidase 37 [Source:VGNC Symbol;Acc:VGNC:98404]	2.08	0.0653
ssc-miR-125a	USP38	ubiquitin specific peptidase 38 [Source:VGNC Symbol;Acc:VGNC:94762]	2.08	0.0653
ssc-miR-125a	USP45	ubiquitin specific peptidase 45 [Source:VGNC Symbol;Acc:VGNC:94767]	2.08	0.0653
ssc-miR-125a	USP46	ubiquitin specific peptidase 46 [Source:VGNC Symbol;Acc:VGNC:94768]	2.08	0.0653
ssc-miR-125a	UVRAG	UV radiation resistance associated [Source:VGNC Symbol;Acc:VGNC:94789]	2.08	0.0653
ssc-miR-125a	VANGL1	VANGL planar cell polarity protein 1 [Source:VGNC Symbol;Acc:VGNC:94797]	2.08	0.0653
ssc-miR-125a	VANGL2	VANGL planar cell polarity protein 2 [Source:VGNC Symbol;Acc:VGNC:94798]	2.08	0.0653
ssc-miR-125a	VAV3	hypothetical gene	2.08	0.0653
ssc-miR-125a	VCPIP1	valosin containing protein interacting protein 1 [Source:VGNC Symbol;Acc:VGNC:94813]	2.08	0.0653
ssc-miR-125a	VDR	vitamin D receptor [Source:VGNC Symbol;Acc:VGNC:94814]	2.08	0.0653
ssc-miR-125a	VMAC	vimentin type intermediate filament associated coiled-coil protein [Source:HGNC Symbol;Acc:HGNC:33803]	2.08	0.0653
ssc-miR-125a	VPS36	vacuolar protein sorting 36 homolog [Source:VGNC Symbol;Acc:VGNC:94850]	2.08	0.0653
ssc-miR-125a	VPS37B	VPS37B subunit of ESCRT-I [Source:VGNC Symbol;Acc:VGNC:94851]	2.08	0.0653
ssc-miR-125a	VPS4B	vacuolar protein sorting 4 homolog B [Source:VGNC Symbol;Acc:VGNC:94857]	2.08	0.0653
ssc-miR-125a	VSTM2A	V-set and transmembrane domain containing 2A [Source:VGNC Symbol;Acc:VGNC:94872]	2.08	0.0653
ssc-miR-125a	VTCN1	V-set domain containing T cell activation inhibitor 1 [Source:VGNC Symbol;Acc:VGNC:94878]	2.08	0.0653
ssc-miR-125a	VWC2	von Willebrand factor C domain containing 2 [Source:VGNC Symbol;Acc:VGNC:94887]	2.08	0.0653
ssc-miR-125a	WARS	hypothetical gene	2.08	0.0653
ssc-miR-125a	WDR1	WD repeat domain 1 [Source:VGNC Symbol;Acc:VGNC:94905]	2.08	0.0653
ssc-miR-125a	WDR25	WD repeat domain 25 [Source:VGNC Symbol;Acc:VGNC:94912]	2.08	0.0653
ssc-miR-125a	WDTC1	WD and tetratricopeptide repeats 1 [Source:VGNC Symbol;Acc:VGNC:94952]	2.08	0.0653
ssc-miR-125a	WIPF2	WAS/WASL interacting protein family member 2 [Source:VGNC Symbol;Acc:VGNC:99106]	2.08	0.0653
ssc-miR-125a	WIZ	WIZ zinc finger [Source:VGNC Symbol;Acc:VGNC:100883]	2.08	0.0653
ssc-miR-125a	WNT7A	Wnt family member 7A [Source:VGNC Symbol;Acc:VGNC:94974]	2.08	0.0653
ssc-miR-125a	XKRX	XK related X-linked [Source:HGNC Symbol;Acc:HGNC:29845]	2.08	0.0653
ssc-miR-125a	YES1	YES proto-onco 1, Src family tyrosine kinase [Source:VGNC Symbol;Acc:VGNC:95024]	2.08	0.0653
ssc-miR-125a	YIPF2	Yip1 domain family member 2 [Source:VGNC Symbol;Acc:VGNC:95028]	2.08	0.0653
ssc-miR-125a	YOD1	YOD1 deubiquitinase [Source:VGNC Symbol;Acc:VGNC:95035]	2.08	0.0653

ssc-miR-125a	YPEL2	yippee like 2 [Source:VGNC Symbol;Acc:VGNC:95036]	2.08	0.0653
ssc-miR-125a	YWHAG	hypothetical gene	2.08	0.0653
ssc-miR-125a	ZBTB34	zinc finger and BTB domain containing 34 [Source:VGNC Symbol;Acc:VGNC:95070]	2.08	0.0653
ssc-miR-125a	ZBTB37	zinc finger and BTB domain containing 37 [Source:VGNC Symbol;Acc:VGNC:95071]	2.08	0.0653
ssc-miR-125a	ZBTB38	zinc finger and BTB domain containing 38 [Source:VGNC Symbol;Acc:VGNC:95072]	2.08	0.0653
ssc-miR-125a	ZBTB47	zinc finger and BTB domain containing 47 [Source:VGNC Symbol;Acc:VGNC:95078]	2.08	0.0653
ssc-miR-125a	ZBTB7A	zinc finger and BTB domain containing 7A [Source:VGNC Symbol;Acc:VGNC:95081]	2.08	0.0653
ssc-miR-125a	ZBTB9	zinc finger and BTB domain containing 9 [Source:HGNC Symbol;Acc:HGNC:28323]	2.08	0.0653
ssc-miR-125a	ZC3H11A	hypothetical gene	2.08	0.0653
ssc-miR-125a	ZC3H12B	zinc finger CCCH-type containing 12B [Source:VGNC Symbol;Acc:VGNC:95090]	2.08	0.0653
ssc-miR-125a	ZC3H7B	zinc finger CCCH-type containing 7B [Source:VGNC Symbol;Acc:VGNC:95100]	2.08	0.0653
ssc-miR-125a	ZCCHC8	zinc finger CCHC-type containing 8 [Source:VGNC Symbol;Acc:VGNC:95112]	2.08	0.0653
ssc-miR-125a	ZDHHC7	zinc finger DHHC-type palmitoyltransferase 7 [Source:VGNC Symbol;Acc:VGNC:95129]	2.08	0.0653
ssc-miR-125a	ZDHHC9	zinc finger DHHC-type palmitoyltransferase 9 [Source:VGNC Symbol;Acc:VGNC:95131]	2.08	0.0653
ssc-miR-125a	ZFHX2	zinc finger homeobox 2 [Source:VGNC Symbol;Acc:VGNC:95140]	2.08	0.0653
ssc-miR-125a	ZFP62	ZFP62 zinc finger protein [Source:VGNC Symbol;Acc:VGNC:95148]	2.08	0.0653
ssc-miR-125a	ZFYVE1	zinc finger FYVE-type containing 1 [Source:VGNC Symbol;Acc:VGNC:95156]	2.08	0.0653
ssc-miR-125a	ZKSCAN5	zinc finger with KRAB and SCAN domains 5 [Source:VGNC Symbol;Acc:VGNC:95174]	2.08	0.0653
ssc-miR-125a	ZNF12	zinc finger protein 12 [Source:HGNC Symbol;Acc:HGNC:12902]	2.08	0.0653
ssc-miR-125a	ZNF148	zinc finger protein 148 [Source:VGNC Symbol;Acc:VGNC:95192]	2.08	0.0653
ssc-miR-125a	ZNF236	zinc finger protein 236 [Source:VGNC Symbol;Acc:VGNC:95209]	2.08	0.0653
ssc-miR-125a	ZNF260	zinc finger protein 260 [Source:VGNC Symbol;Acc:VGNC:98750]	2.08	0.0653
ssc-miR-125a	ZNF281	zinc finger protein 281 [Source:VGNC Symbol;Acc:VGNC:108285]	2.08	0.0653
ssc-miR-125a	ZNF385A	zinc finger protein 385A [Source:VGNC Symbol;Acc:VGNC:95233]	2.08	0.0653
ssc-miR-125a	ZNF395	zinc finger protein 395 [Source:VGNC Symbol;Acc:VGNC:95235]	2.08	0.0653
ssc-miR-125a	ZNF460	hypothetical gene	2.08	0.0653
ssc-miR-125a	ZNF512B	zinc finger protein 512B [Source:HGNC Symbol;Acc:HGNC:29212]	2.08	0.0653
ssc-miR-125a	ZNF570	zinc finger protein 570 [Source:VGNC Symbol;Acc:VGNC:95267]	2.08	0.0653
ssc-miR-125a	ZNF618	zinc finger protein 618 [Source:VGNC Symbol;Acc:VGNC:95276]	2.08	0.0653
ssc-miR-125a	ZNF624	zinc finger protein 624 [Source:VGNC Symbol;Acc:VGNC:95278]	2.08	0.0653
ssc-miR-125a	ZNF652	zinc finger protein 652 [Source:VGNC Symbol;Acc:VGNC:99108]	2.08	0.0653
ssc-miR-125a	ZNF691	zinc finger protein 691 [Source:HGNC Symbol;Acc:HGNC:28028]	2.08	0.0653
ssc-miR-125a	ZNF704	zinc finger protein 704 [Source:VGNC Symbol;Acc:VGNC:95296]	2.08	0.0653
ssc-miR-125a	ZNF710	zinc finger protein 710 [Source:VGNC Symbol;Acc:VGNC:107180]	2.08	0.0653
ssc-miR-125a	ZNF76	zinc finger protein 76 [Source:HGNC Symbol;Acc:HGNC:13149]	2.08	0.0653
ssc-miR-125a	ZNF792	zinc finger protein 792 [Source:VGNC Symbol;Acc:VGNC:95304]	2.08	0.0653
ssc-miR-125a	ZNF8	zinc finger protein 8 [Source:VGNC Symbol;Acc:VGNC:95305]	2.08	0.0653
ssc-miR-125a	ZNF827	zinc finger protein 827 [Source:VGNC Symbol;Acc:VGNC:98970]	2.08	0.0653
ssc-miR-125a	ZNRF3	zinc and ring finger 3 [Source:VGNC Symbol;Acc:VGNC:95318]	2.08	0.0653
ssc-miR-125a	ZSCAN29	zinc finger and SCAN domain containing 29 [Source:HGNC Symbol;Acc:HGNC:26673]	2.08	0.0653
ssc-miR-125a	ZSWIM4	zinc finger SWIM-type containing 4 [Source:VGNC Symbol;Acc:VGNC:95333]	2.08	0.0653
ssc-miR-125a	ZSWIM5	zinc finger SWIM-type containing 5 [Source:VGNC Symbol;Acc:VGNC:95334]	2.08	0.0653
ssc-miR-125a	ZSWIM6	zinc finger SWIM-type containing 6 [Source:VGNC Symbol;Acc:VGNC:95335]	2.08	0.0653
ssc-miR-30d	A1CF	APOBEC1 complementation factor [Source:VGNC Symbol;Acc:VGNC:84933]	2.07	0.0098
ssc-miR-30d	ABAT	4-aminobutyrate aminotransferase [Source:VGNC Symbol;Acc:VGNC:96910]	2.07	0.0098
ssc-miR-30d	ABCB7	ATP binding cassette subfamily B member 7 [Source:VGNC Symbol;Acc:VGNC:97855]	2.07	0.0098
ssc-miR-30d	ABCC9	ATP binding cassette subfamily C member 9 [Source:VGNC Symbol;Acc:VGNC:84960]	2.07	0.0098
ssc-miR-30d	ABCD2	ATP binding cassette subfamily D member 2 [Source:VGNC Symbol;Acc:VGNC:84962]	2.07	0.0098
ssc-miR-30d	ABHD6	abhydrolase domain containing 6, acylglycerol lipase [Source:VGNC Symbol;Acc:VGNC:84982]	2.07	0.0098

ssc-miR-30d	ABI2	abl interactor 2 [Source:VGNC Symbol;Acc:VGNC:96027]	2.07	0.0098
ssc-miR-30d	ABI3BP	ABI family member 3 binding protein [Source:VGNC Symbol;Acc:VGNC:108628]	2.07	0.0098
ssc-miR-30d	ABL1	ABL proto-onco 1, non-receptor tyrosine kinase [Source:VGNC Symbol;Acc:VGNC:84984]	2.07	0.0098
ssc-miR-30d	ABL2	ABL proto-onco 2, non-receptor tyrosine kinase [Source:VGNC Symbol;Acc:VGNC:84985]	2.07	0.0098
ssc-miR-30d	AC002451.1	hypothetical gene	2.07	0.0098
ssc-miR-30d	AC012215.1	hypothetical gene	2.07	0.0098
ssc-miR-30d	AC104472.1	hypothetical gene	2.07	0.0098
ssc-miR-30d	ACAP2	ArfGAP with coiled-coil, ankyrin repeat and PH domains 2 [Source:VGNC Symbol;Acc:VGNC:85002]	2.07	0.0098
ssc-miR-30d	ACSBG1	acyl-CoA synthetase bubblegum family member 1 [Source:VGNC Symbol;Acc:VGNC:85030]	2.07	0.0098
ssc-miR-30d	ACTC1	actin alpha cardiac muscle 1 [Source:VGNC Symbol;Acc:VGNC:85040]	2.07	0.0098
ssc-miR-30d	ACTN1	actinin alpha 1 [Source:NCBI gene (formerly Entrezgene);Acc:100513412]	2.07	0.0098
ssc-miR-30d	ACTR1A	actin related protein 1A [Source:VGNC Symbol;Acc:VGNC:85049]	2.07	0.0098
ssc-miR-30d	ACTR3	actin related protein 3 [Source:VGNC Symbol;Acc:VGNC:103894]	2.07	0.0098
ssc-miR-30d	ACVR1	activin A receptor type 1 [Source:VGNC Symbol;Acc:VGNC:95830]	2.07	0.0098
ssc-miR-30d	ADAM10	ADAM metallopeptidase domain 10 [Source:VGNC Symbol;Acc:VGNC:85061]	2.07	0.0098
ssc-miR-30d	ADAM11	ADAM metallopeptidase domain 11 [Source:VGNC Symbol;Acc:VGNC:85062]	2.07	0.0098
ssc-miR-30d	ADAM12	ADAM metallopeptidase domain 12 [Source:VGNC Symbol;Acc:VGNC:85063]	2.07	0.0098
ssc-miR-30d	ADAM19	ADAM metallopeptidase domain 19 [Source:VGNC Symbol;Acc:VGNC:85066]	2.07	0.0098
ssc-miR-30d	ADAM22	ADAM metallopeptidase domain 22 [Source:VGNC Symbol;Acc:VGNC:85067]	2.07	0.0098
ssc-miR-30d	ADAM9	ADAM metallopeptidase domain 9 [Source:VGNC Symbol;Acc:VGNC:95969]	2.07	0.0098
ssc-miR-30d	ADAMTS14	ADAM metallopeptidase with thrombospondin type 1 motif 14 [Source:VGNC Symbol;Acc:VGNC:85076]	2.07	0.0098
ssc-miR-30d	ADAMTS3	ADAM metallopeptidase with thrombospondin type 1 motif 3 [Source:VGNC Symbol;Acc:VGNC:85083]	2.07	0.0098
ssc-miR-30d	ADAMTS5	ADAM metallopeptidase with thrombospondin type 1 motif 5 [Source:VGNC Symbol;Acc:VGNC:85085]	2.07	0.0098
ssc-miR-30d	ADAMTS9	ADAM metallopeptidase with thrombospondin type 1 motif 9 [Source:VGNC Symbol;Acc:VGNC:85089]	2.07	0.0098
ssc-miR-30d	ADAMTSL2	ADAMTS like 2 [Source:VGNC Symbol;Acc:VGNC:85091]	2.07	0.0098
ssc-miR-30d	ADARB2	adenosine deaminase RNA specific B2 (inactive) [Source:HGNC Symbol;Acc:HGNC:227]	2.07	0.0098
ssc-miR-30d	ADO	2-aminoethanethiol dioxygenase [Source:VGNC Symbol;Acc:VGNC:85146]	2.07	0.0098
ssc-miR-30d	ADRA1D	adrenoceptor alpha 1D [Source:NCBI gene (formerly Entrezgene);Acc:552898]	2.07	0.0098
ssc-miR-30d	ADRA2A	adrenoceptor alpha 2A [Source:VGNC Symbol;Acc:VGNC:85156]	2.07	0.0098
ssc-miR-30d	ADRA2B	adrenoceptor alpha 2B [Source:VGNC Symbol;Acc:VGNC:85157]	2.07	0.0098
ssc-miR-30d	ADRB1	adrenoceptor beta 1 [Source:VGNC Symbol;Acc:VGNC:107363]	2.07	0.0098
ssc-miR-30d	AFAP1L2	actin filament associated protein 1 like 2 [Source:HGNC Symbol;Acc:HGNC:25901]	2.07	0.0098
ssc-miR-30d	AFF3	AF4/FMR2 family member 3 [Source:HGNC Symbol;Acc:HGNC:6473]	2.07	0.0098
ssc-miR-30d	AFF4	AF4/FMR2 family member 4 [Source:VGNC Symbol;Acc:VGNC:85169]	2.07	0.0098
ssc-miR-30d	AGO1	hypothetical gene	2.07	0.0098
ssc-miR-30d	AGO2	argonaute RISC catalytic component 2 [Source:VGNC Symbol;Acc:VGNC:97871]	2.07	0.0098
ssc-miR-30d	AGO3	argonaute RISC component 1 [Source:NCBI gene (formerly Entrezgene);Acc:100499510]	2.07	0.0098
ssc-miR-30d	AGO4	hypothetical gene	2.07	0.0098
ssc-miR-30d	AGPAT4	1-acylglycerol-3-phosphate O-acyltransferase 4 [Source:VGNC Symbol;Acc:VGNC:85186]	2.07	0.0098
ssc-miR-30d	AHNAK	hypothetical gene	2.07	0.0098
ssc-miR-30d	AIDA	axin interactor, dorsalization associated [Source:VGNC Symbol;Acc:VGNC:96103]	2.07	0.0098
ssc-miR-30d	AK4	hypothetical gene	2.07	0.0098
ssc-miR-30d	AKAP10	A-kinase anchoring protein 10 [Source:VGNC Symbol;Acc:VGNC:85215]	2.07	0.0098
ssc-miR-30d	AKIRIN1	akirin 1 [Source:VGNC Symbol;Acc:VGNC:85227]	2.07	0.0098
ssc-miR-30d	AL139099.1	hypothetical gene	2.07	0.0098
ssc-miR-30d	ALG10	hypothetical gene	2.07	0.0098
ssc-miR-30d	ALG10B	hypothetical gene	2.07	0.0098
ssc-miR-30d	ALG9	ALG9 alpha-1,2-mannosyltransferase [Source:NCBI gene (formerly Entrezgene);Acc:100519965]	2.07	0.0098
ssc-miR-30d	ALPK3	alpha kinase 3 [Source:HGNC Symbol;Acc:HGNC:17574]	2.07	0.0098

ssc-miR-30d	AMER1	APC membrane recruitment protein 1 [Source:VGNC Symbol;Acc:VGNC:85276]	2.07	0.0098
ssc-miR-30d	AMOT	angiomotin [Source:VGNC Symbol;Acc:VGNC:85283]	2.07	0.0098
ssc-miR-30d	AMOTL1	angiomotin like 1 [Source:VGNC Symbol;Acc:VGNC:85284]	2.07	0.0098
ssc-miR-30d	AMOTL2	angiomotin like 2 [Source:VGNC Symbol;Acc:VGNC:85285]	2.07	0.0098
ssc-miR-30d	ANGEL2	angel homolog 2 [Source:VGNC Symbol;Acc:VGNC:85302]	2.07	0.0098
ssc-miR-30d	ANKHD1	ankyrin repeat and KH domain containing 1 [Source:NCBI gene (formerly Entrezgene);Acc:100512270]	2.07	0.0098
ssc-miR-30d	ANKRA2	ankyrin repeat family A member 2 [Source:VGNC Symbol;Acc:VGNC:85320]	2.07	0.0098
ssc-miR-30d	ANKRD17	ankyrin repeat domain 17 [Source:VGNC Symbol;Acc:VGNC:85328]	2.07	0.0098
ssc-miR-30d	ANKRD26	hypothetical gene	2.07	0.0098
ssc-miR-30d	ANKRD52	ankyrin repeat domain 52 [Source:VGNC Symbol;Acc:VGNC:85342]	2.07	0.0098
ssc-miR-30d	ANKS4B	ankyrin repeat and sterile alpha motif domain containing 4B [Source:VGNC Symbol;Acc:VGNC:85350]	2.07	0.0098
ssc-miR-30d	ANO4	anoctamin 4 [Source:VGNC Symbol;Acc:VGNC:85357]	2.07	0.0098
ssc-miR-30d	AP1B1	adaptor related protein complex 1 subunit beta 1 [Source:VGNC Symbol;Acc:VGNC:85377]	2.07	0.0098
ssc-miR-30d	AP1S2	adaptor related protein complex 1 subunit sigma 2 [Source:VGNC Symbol;Acc:VGNC:85382]	2.07	0.0098
ssc-miR-30d	AP2A1	adaptor related protein complex 2 subunit alpha 1 [Source:VGNC Symbol;Acc:VGNC:85383]	2.07	0.0098
ssc-miR-30d	AP3M1	adaptor related protein complex 3 subunit mu 1 [Source:VGNC Symbol;Acc:VGNC:85390]	2.07	0.0098
ssc-miR-30d	AP3S1	hypothetical gene	2.07	0.0098
ssc-miR-30d	AP4E1	adaptor related protein complex 4 subunit epsilon 1 [Source:VGNC Symbol;Acc:VGNC:85392]	2.07	0.0098
ssc-miR-30d	AP5M1	adaptor related protein complex 5 subunit mu 1 [Source:VGNC Symbol;Acc:VGNC:85396]	2.07	0.0098
ssc-miR-30d	APBA1	amyloid beta protein binding family A member 1 [Source:VGNC Symbol;Acc:VGNC:85399]	2.07	0.0098
ssc-miR-30d	AQR	aquarius intron-binding spliceosomal factor [Source:VGNC Symbol;Acc:VGNC:85437]	2.07	0.0098
ssc-miR-30d	ARAP2	ArfGAP with RhoGAP domain, ankyrin repeat and PH domain 2 [Source:VGNC Symbol;Acc:VGNC:85440]	2.07	0.0098
ssc-miR-30d	ARF4	ADP ribosylation factor 4 [Source:VGNC Symbol;Acc:VGNC:85446]	2.07	0.0098
ssc-miR-30d	ARHGAP26	Rho GTPase activating protein 26 [Source:VGNC Symbol;Acc:VGNC:85465]	2.07	0.0098
ssc-miR-30d	ARHGEF3	Rho guanine nucleotide exchange factor 3 [Source:VGNC Symbol;Acc:VGNC:85496]	2.07	0.0098
ssc-miR-30d	ARHGEF6	Rac/Cdc42 guanine nucleotide exchange factor 6 [Source:VGNC Symbol;Acc:VGNC:85502]	2.07	0.0098
ssc-miR-30d	ARID1A	AT-rich interaction domain 1A [Source:VGNC Symbol;Acc:VGNC:85505]	2.07	0.0098
ssc-miR-30d	ARID3A	AT-rich interaction domain 3A [Source:VGNC Symbol;Acc:VGNC:85507]	2.07	0.0098
ssc-miR-30d	ARID4A	AT-rich interaction domain 4A [Source:VGNC Symbol;Acc:VGNC:85509]	2.07	0.0098
ssc-miR-30d	ARID4B	AT-rich interaction domain 4B [Source:HGNC Symbol;Acc:HGNC:15550]	2.07	0.0098
ssc-miR-30d	ARID5B	AT-rich interaction domain 5B [Source:VGNC Symbol;Acc:VGNC:85511]	2.07	0.0098
ssc-miR-30d	ARIH1	ariadne RBR E3 ubiquitin protein ligase 1 [Source:HGNC Symbol;Acc:HGNC:689]	2.07	0.0098
ssc-miR-30d	ARL10	ADP ribosylation factor like GTPase 10 [Source:VGNC Symbol;Acc:VGNC:85514]	2.07	0.0098
ssc-miR-30d	ARL15	ADP ribosylation factor like GTPase 15 [Source:VGNC Symbol;Acc:VGNC:85520]	2.07	0.0098
ssc-miR-30d	ARL4A	ADP ribosylation factor like GTPase 4A [Source:VGNC Symbol;Acc:VGNC:85522]	2.07	0.0098
ssc-miR-30d	ARL4C	ADP ribosylation factor like GTPase 4C [Source:VGNC Symbol;Acc:VGNC:96419]	2.07	0.0098
ssc-miR-30d	ARL6IP5	ADP ribosylation factor like GTPase 6 interacting protein 5 [Source:VGNC Symbol;Acc:VGNC:103904]	2.07	0.0098
ssc-miR-30d	ARL6IP6	ADP ribosylation factor like GTPase 6 interacting protein 6 [Source:VGNC Symbol;Acc:VGNC:96029]	2.07	0.0098
ssc-miR-30d	ASAH1	N-acylsphingosine amidohydrolase 1 [Source:VGNC Symbol;Acc:VGNC:96424]	2.07	0.0098
ssc-miR-30d	ASB14	ankyrin repeat and SOCS box containing 14 [Source:VGNC Symbol;Acc:VGNC:85562]	2.07	0.0098
ssc-miR-30d	ASB3	ankyrin repeat and SOCS box containing 3 [Source:VGNC Symbol;Acc:VGNC:97034]	2.07	0.0098
ssc-miR-30d	ASB4	ankyrin repeat and SOCS box containing 4 [Source:VGNC Symbol;Acc:VGNC:97891]	2.07	0.0098
ssc-miR-30d	ASCC1	activating signal cointegrator 1 complex subunit 1 [Source:VGNC Symbol;Acc:VGNC:85569]	2.07	0.0098
ssc-miR-30d	ASCC3	activating signal cointegrator 1 complex subunit 3 [Source:VGNC Symbol;Acc:VGNC:85571]	2.07	0.0098
ssc-miR-30d	ASXL3	ASXL transcriptional regulator 3 [Source:VGNC Symbol;Acc:VGNC:85596]	2.07	0.0098
ssc-miR-30d	ATAD2B	ATPase family AAA domain containing 2B [Source:VGNC Symbol;Acc:VGNC:85600]	2.07	0.0098
ssc-miR-30d	ATAD5	ATPase family AAA domain containing 5 [Source:VGNC Symbol;Acc:VGNC:85601]	2.07	0.0098
ssc-miR-30d	ATF1	activating transcription factor 1 [Source:VGNC Symbol;Acc:VGNC:85605]	2.07	0.0098
ssc-miR-30d	ATF7IP2	activating transcription factor 7 interacting protein 2 [Source:VGNC Symbol;Acc:VGNC:85612]	2.07	0.0098

ssc-miR-30d	ATG12	autophagy related 12 [Source:VGNC Symbol;Acc:VGNC:85615]	2.07	0.0098
ssc-miR-30d	ATG5	autophagy related 5 [Source:VGNC Symbol;Acc:VGNC:103029]	2.07	0.0098
ssc-miR-30d	ATL2	atlastin GTPase 2 [Source:VGNC Symbol;Acc:VGNC:85628]	2.07	0.0098
ssc-miR-30d	ATP2A2	ATPase sarcoplasmic/endoplasmic reticulum Ca2+ transporting 2 [Source:VGNC Symbol;Acc:VGNC:85647]	2.07	0.0098
ssc-miR-30d	ATP2B1	ATPase plasma membrane Ca2+ transporting 1 [Source:VGNC Symbol;Acc:VGNC:103219]	2.07	0.0098
ssc-miR-30d	ATP2B2	ATPase plasma membrane Ca2+ transporting 2 [Source:VGNC Symbol;Acc:VGNC:85649]	2.07	0.0098
ssc-miR-30d	ATP6V0A1	ATPase H+ transporting V0 subunit a1 [Source:VGNC Symbol;Acc:VGNC:85663]	2.07	0.0098
ssc-miR-30d	ATP6V0D1	ATPase H+ transporting V0 subunit d1 [Source:VGNC Symbol;Acc:VGNC:85667]	2.07	0.0098
ssc-miR-30d	ATP6V1C1	ATPase H+ transporting V1 subunit C1 [Source:VGNC Symbol;Acc:VGNC:85672]	2.07	0.0098
ssc-miR-30d	ATP8A1	ATPase phospholipid transporting 8A1 [Source:VGNC Symbol;Acc:VGNC:97894]	2.07	0.0098
ssc-miR-30d	ATP8B1	ATPase phospholipid transporting 8B1 [Source:VGNC Symbol;Acc:VGNC:85679]	2.07	0.0098
ssc-miR-30d	ATP8B2	ATPase phospholipid transporting 8B2 [Source:VGNC Symbol;Acc:VGNC:85680]	2.07	0.0098
ssc-miR-30d	ATRN	attractin [Source:VGNC Symbol;Acc:VGNC:96479]	2.07	0.0098
ssc-miR-30d	ATRNL1	attractin like 1 [Source:VGNC Symbol;Acc:VGNC:85685]	2.07	0.0098
ssc-miR-30d	ATXN1	ataxin 1 [Source:VGNC Symbol;Acc:VGNC:85687]	2.07	0.0098
ssc-miR-30d	ATXN3	hypothetical gene	2.07	0.0098
ssc-miR-30d	AVEN	apoptosis and caspase activation inhibitor [Source:HGNC Symbol;Acc:HGNC:13509]	2.07	0.0098
ssc-miR-30d	AVL9	AVL9 cell migration associated [Source:VGNC Symbol;Acc:VGNC:85701]	2.07	0.0098
ssc-miR-30d	AZIN1	antizyme inhibitor 1 [Source:VGNC Symbol;Acc:VGNC:85712]	2.07	0.0098
ssc-miR-30d	B3GALT5	hypothetical gene	2.07	0.0098
ssc-miR-30d	B3GALT6	beta-1,3-galactosyltransferase 6 [Source:VGNC Symbol;Acc:VGNC:85718]	2.07	0.0098
ssc-miR-30d	B3GALTL	hypothetical gene	2.07	0.0098
ssc-miR-30d	B3GAT1	beta-1,3-glucuronyltransferase 1 [Source:VGNC Symbol;Acc:VGNC:85719]	2.07	0.0098
ssc-miR-30d	B3GNT5	UDP-GlcNAc:betaGal beta-1,3-N-acetylglucosaminyltransferase 5 [Source:VGNC Symbol;Acc:VGNC:85726]	2.07	0.0098
ssc-miR-30d	B4GALT5	beta-1,4-galactosyltransferase 5 [Source:VGNC Symbol;Acc:VGNC:96499]	2.07	0.0098
ssc-miR-30d	B4GALT6	beta-1,4-galactosyltransferase 6 [Source:VGNC Symbol;Acc:VGNC:85734]	2.07	0.0098
ssc-miR-30d	BACH1	BTB domain and CNC homolog 1 [Source:VGNC Symbol;Acc:VGNC:85741]	2.07	0.0098
ssc-miR-30d	BAHD1	bromo adjacent homology domain containing 1 [Source:VGNC Symbol;Acc:VGNC:85747]	2.07	0.0098
ssc-miR-30d	BAZ1A	bromodomain adjacent to zinc finger domain 1A [Source:VGNC Symbol;Acc:VGNC:85761]	2.07	0.0098
ssc-miR-30d	BAZ2B	bromodomain adjacent to zinc finger domain 2B [Source:HGNC Symbol;Acc:HGNC:963]	2.07	0.0098
ssc-miR-30d	BBX	BBX high mobility group box domain containing [Source:VGNC Symbol;Acc:VGNC:85766]	2.07	0.0098
ssc-miR-30d	BCL11B	BAF chromatin remodeling complex subunit BCL11B [Source:VGNC Symbol;Acc:VGNC:96563]	2.07	0.0098
ssc-miR-30d	BCL2	BCL2 apoptosis regulator [Source:HGNC Symbol;Acc:HGNC:990]	2.07	0.0098
ssc-miR-30d	BCL2L11	BCL2 like 11 [Source:NCBI gene (formerly Entrezgene);Acc:396632]	2.07	0.0098
ssc-miR-30d	BCL6	BCL6 transcription repressor [Source:VGNC Symbol;Acc:VGNC:96565]	2.07	0.0098
ssc-miR-30d	BCL9	BCL9 transcription coactivator [Source:VGNC Symbol;Acc:VGNC:96567]	2.07	0.0098
ssc-miR-30d	BCLAF1	BCL2 associated transcription factor 1 [Source:VGNC Symbol;Acc:VGNC:85787]	2.07	0.0098
ssc-miR-30d	BCOR	BCL6 corepressor [Source:HGNC Symbol;Acc:HGNC:20893]	2.07	0.0098
ssc-miR-30d	BDNF	brain derived neurotrophic factor [Source:VGNC Symbol;Acc:VGNC:85795]	2.07	0.0098
ssc-miR-30d	BDP1	B double prime 1, subunit of RNA polymerase III transcription initiation factor IIIB [Source:VGNC Symbol;Acc:VGNC:85796]	2.07	0.0098
ssc-miR-30d	BEAN1	brain expressed associated with NEDD4 1 [Source:VGNC Symbol;Acc:VGNC:85797]	2.07	0.0098
ssc-miR-30d	BECN1	beclin 1 [Source:VGNC Symbol;Acc:VGNC:85798]	2.07	0.0098
ssc-miR-30d	BEND3	BEN domain containing 3 [Source:VGNC Symbol;Acc:VGNC:85800]	2.07	0.0098
ssc-miR-30d	BEND4	BEN domain containing 4 [Source:VGNC Symbol;Acc:VGNC:85801]	2.07	0.0098
ssc-miR-30d	BEND7	BEN domain containing 7 [Source:VGNC Symbol;Acc:VGNC:106445]	2.07	0.0098
ssc-miR-30d	BIRC6	baculoviral IAP repeat containing 6 [Source:VGNC Symbol;Acc:VGNC:97038]	2.07	0.0098
ssc-miR-30d	BMP7	bone morphotic protein 7 [Source:VGNC Symbol;Acc:VGNC:95491]	2.07	0.0098
ssc-miR-30d	BMPR2	bone morphotic protein receptor type 2 [Source:VGNC Symbol;Acc:VGNC:95494]	2.07	0.0098
ssc-miR-30d	BNC1	basonuclin 1 [Source:VGNC Symbol;Acc:VGNC:85851]	2.07	0.0098

ssc-miR-30d	BNC2	basonuclin 2 [Source:VGNC Symbol;Acc:VGNC:85852]	2.07	0.0098
ssc-miR-30d	BNIP3L	BCL2 interacting protein 3 like [Source:VGNC Symbol;Acc:VGNC:85856]	2.07	0.0098
ssc-miR-30d	BRAP	BRCA1 associated protein [Source:VGNC Symbol;Acc:VGNC:85867]	2.07	0.0098
ssc-miR-30d	BRD1	bromodomain containing 1 [Source:VGNC Symbol;Acc:VGNC:85871]	2.07	0.0098
ssc-miR-30d	BRWD1	bromodomain and WD repeat domain containing 1 [Source:VGNC Symbol;Acc:VGNC:108153]	2.07	0.0098
ssc-miR-30d	BRWD3	bromodomain and WD repeat domain containing 3 [Source:VGNC Symbol;Acc:VGNC:85890]	2.07	0.0098
ssc-miR-30d	BSN	bassoon presynaptic cytomatrix protein [Source:VGNC Symbol;Acc:VGNC:85893]	2.07	0.0098
ssc-miR-30d	BTAF1	B-TFIID TATA-box binding protein associated factor 1 [Source:VGNC Symbol;Acc:VGNC:85899]	2.07	0.0098
ssc-miR-30d	BTBD10	hypothetical gene	2.07	0.0098
ssc-miR-30d	BTBD7	BTB domain containing 7 [Source:VGNC Symbol;Acc:VGNC:85908]	2.07	0.0098
ssc-miR-30d	BZW1	basic leucine zipper and W2 domains 1 [Source:VGNC Symbol;Acc:VGNC:95885]	2.07	0.0098
ssc-miR-30d	C10orf105	chromosome 14 C10orf105 homolog [Source:VGNC Symbol;Acc:VGNC:85940]	2.07	0.0098
ssc-miR-30d	C10orf11	hypothetical gene	2.07	0.0098
ssc-miR-30d	C10orf118	hypothetical gene	2.07	0.0098
ssc-miR-30d	C10orf25	hypothetical gene	2.07	0.0098
ssc-miR-30d	C10orf76	hypothetical gene	2.07	0.0098
ssc-miR-30d	C11orf84	hypothetical gene	2.07	0.0098
ssc-miR-30d	C14orf28	chromosome 1 C14orf28 homolog [Source:VGNC Symbol;Acc:VGNC:85958]	2.07	0.0098
ssc-miR-30d	C16orf52	hypothetical gene	2.07	0.0098
ssc-miR-30d	C16orf87	chromosome 6 C16orf87 homolog [Source:VGNC Symbol;Acc:VGNC:96927]	2.07	0.0098
ssc-miR-30d	C20orf112	hypothetical gene	2.07	0.0098
ssc-miR-30d	C21orf91	chromosome 13 C21orf91 homolog [Source:VGNC Symbol;Acc:VGNC:85930]	2.07	0.0098
ssc-miR-30d	C3orf18	chromosome 13 C3orf18 homolog [Source:VGNC Symbol;Acc:VGNC:85932]	2.07	0.0098
ssc-miR-30d	C3orf58	hypothetical gene	2.07	0.0098
ssc-miR-30d	C4orf19	chromosome 8 C4orf19 homolog [Source:VGNC Symbol;Acc:VGNC:86080]	2.07	0.0098
ssc-miR-30d	C7orf43	hypothetical gene	2.07	0.0098
ssc-miR-30d	C7orf55-LUC7L2	hypothetical gene	2.07	0.0098
ssc-miR-30d	C7orf60	hypothetical gene	2.07	0.0098
ssc-miR-30d	C8orf4	hypothetical gene	2.07	0.0098
ssc-miR-30d	C8orf44-SGK3	hypothetical gene	2.07	0.0098
ssc-miR-30d	C9orf170	hypothetical gene	2.07	0.0098
ssc-miR-30d	C9orf41	hypothetical gene	2.07	0.0098
ssc-miR-30d	C9orf72	chromosome 10 C9orf72 homolog [Source:VGNC Symbol;Acc:VGNC:96173]	2.07	0.0098
ssc-miR-30d	CA10	carbonic anhydrase 10 [Source:VGNC Symbol;Acc:VGNC:86096]	2.07	0.0098
ssc-miR-30d	CABLES2	Cdk5 and Abl enzyme substrate 2 [Source:VGNC Symbol;Acc:VGNC:95753]	2.07	0.0098
ssc-miR-30d	CACHD1	cache domain containing 1 [Source:VGNC Symbol;Acc:VGNC:86116]	2.07	0.0098
ssc-miR-30d	CACNA1C	calcium voltage-gated channel subunit alpha1 C [Source:HGNC Symbol;Acc:HGNC:1390]	2.07	0.0098
ssc-miR-30d	CACNA1D	calcium voltage-gated channel subunit alpha1 D [Source:VGNC Symbol;Acc:VGNC:86117]	2.07	0.0098
ssc-miR-30d	CACNB2	calcium voltage-gated channel auxiliary subunit beta 2 [Source:VGNC Symbol;Acc:VGNC:95599]	2.07	0.0098
ssc-miR-30d	CACUL1	CDK2 associated cullin domain 1 [Source:VGNC Symbol;Acc:VGNC:86132]	2.07	0.0098
ssc-miR-30d	CACYBP	calcyclin binding protein [Source:VGNC Symbol;Acc:VGNC:86133]	2.07	0.0098
ssc-miR-30d	CADM1	cell adhesion molecule 1 [Source:VGNC Symbol;Acc:VGNC:86134]	2.07	0.0098
ssc-miR-30d	CADM2	cell adhesion molecule 2 [Source:VGNC Symbol;Acc:VGNC:97910]	2.07	0.0098
ssc-miR-30d	CADPS	hypothetical gene	2.07	0.0098
ssc-miR-30d	CALB2	calbindin 2 [Source:VGNC Symbol;Acc:VGNC:86139]	2.07	0.0098
ssc-miR-30d	CALCOCO1	calcium binding and coiled-coil domain 1 [Source:VGNC Symbol;Acc:VGNC:86140]	2.07	0.0098
ssc-miR-30d	CALCR	calcitonin receptor [Source:VGNC Symbol;Acc:VGNC:86142]	2.07	0.0098
ssc-miR-30d	CALD1	caldesmon 1 [Source:VGNC Symbol;Acc:VGNC:86143]	2.07	0.0098
ssc-miR-30d	CALU	calumenin [Source:VGNC Symbol;Acc:VGNC:86151]	2.07	0.0098

ssc-miR-30d	CAMK2D	calcium/calmodulin dependent protein kinase II delta [Source:VGNC Symbol;Acc:VGNC:97911]	2.07	0.0098
ssc-miR-30d	CAMK2N1	calcium/calmodulin dependent protein kinase II inhibitor 1 [Source:VGNC Symbol;Acc:VGNC:96935]	2.07	0.0098
ssc-miR-30d	CAMK2N2	calcium/calmodulin dependent protein kinase II inhibitor 2 [Source:HGNC Symbol;Acc:HGNC:24197]	2.07	0.0098
ssc-miR-30d	CAMK4	calcium/calmodulin dependent protein kinase IV [Source:VGNC Symbol;Acc:VGNC:99602]	2.07	0.0098
ssc-miR-30d	CAMKK2	calcium/calmodulin dependent protein kinase kinase 2 [Source:VGNC Symbol;Acc:VGNC:86158]	2.07	0.0098
ssc-miR-30d	CAND1	cullin associated and neddylation dissociated 1 [Source:VGNC Symbol;Acc:VGNC:97912]	2.07	0.0098
ssc-miR-30d	CAPN5	calpain 5 [Source:VGNC Symbol;Acc:VGNC:86175]	2.07	0.0098
ssc-miR-30d	CAPN7	calpain 7 [Source:NCBI gene (formerly Entrezgene);Acc:100037936]	2.07	0.0098
ssc-miR-30d	CAPRIN1	cell cycle associated protein 1 [Source:VGNC Symbol;Acc:VGNC:86178]	2.07	0.0098
ssc-miR-30d	CAPZA1	capping actin protein of muscle Z-line subunit alpha 1 [Source:VGNC Symbol;Acc:VGNC:86183]	2.07	0.0098
ssc-miR-30d	CARF	calcium responsive transcription factor [Source:VGNC Symbol;Acc:VGNC:95615]	2.07	0.0098
ssc-miR-30d	CARS	hypothetical gene	2.07	0.0098
ssc-miR-30d	CASD1	CAS1 domain containing 1 [Source:VGNC Symbol;Acc:VGNC:86199]	2.07	0.0098
ssc-miR-30d	CASP10	caspase 10 [Source:VGNC Symbol;Acc:VGNC:95638]	2.07	0.0098
ssc-miR-30d	CAT	catalase [Source:VGNC Symbol;Acc:VGNC:86208]	2.07	0.0098
ssc-miR-30d	CBFB	core-binding factor subunit beta [Source:VGNC Symbol;Acc:VGNC:86221]	2.07	0.0098
ssc-miR-30d	CBLB	Cbl proto-onco B [Source:VGNC Symbol;Acc:VGNC:86223]	2.07	0.0098
ssc-miR-30d	CBX2	chromobox 2 [Source:VGNC Symbol;Acc:VGNC:86230]	2.07	0.0098
ssc-miR-30d	CBX3	chromobox 3 [Source:HGNC Symbol;Acc:HGNC:1553]	2.07	0.0098
ssc-miR-30d	CBX7	chromobox 7 [Source:HGNC Symbol;Acc:HGNC:1557]	2.07	0.0098
ssc-miR-30d	CBX8	chromobox 8 [Source:VGNC Symbol;Acc:VGNC:86233]	2.07	0.0098
ssc-miR-30d	CCDC117	coiled-coil domain containing 117 [Source:VGNC Symbol;Acc:VGNC:86248]	2.07	0.0098
ssc-miR-30d	CCDC120	coiled-coil domain containing 120 [Source:VGNC Symbol;Acc:VGNC:86249]	2.07	0.0098
ssc-miR-30d	CCDC148	hypothetical gene	2.07	0.0098
ssc-miR-30d	CCDC160	coiled-coil domain containing 160 [Source:VGNC Symbol;Acc:VGNC:86269]	2.07	0.0098
ssc-miR-30d	CCDC178	coiled-coil domain containing 178 [Source:VGNC Symbol;Acc:VGNC:86276]	2.07	0.0098
ssc-miR-30d	CCDC43	coiled-coil domain containing 43 [Source:VGNC Symbol;Acc:VGNC:98979]	2.07	0.0098
ssc-miR-30d	CCDC6	coiled-coil domain containing 6 [Source:VGNC Symbol;Acc:VGNC:86304]	2.07	0.0098
ssc-miR-30d	CCDC71L	coiled-coil domain containing 71 like [Source:HGNC Symbol;Acc:HGNC:26685]	2.07	0.0098
ssc-miR-30d	CCDC97	coiled-coil domain containing 97 [Source:VGNC Symbol;Acc:VGNC:86331]	2.07	0.0098
ssc-miR-30d	CCNE2	cyclin E2 [Source:VGNC Symbol;Acc:VGNC:86355]	2.07	0.0098
ssc-miR-30d	CCNJL	cyclin J like [Source:VGNC Symbol;Acc:VGNC:86361]	2.07	0.0098
ssc-miR-30d	CCNK	cyclin K [Source:VGNC Symbol;Acc:VGNC:86362]	2.07	0.0098
ssc-miR-30d	CCNT2	cyclin T2 [Source:VGNC Symbol;Acc:VGNC:103916]	2.07	0.0098
ssc-miR-30d	CCNY	cyclin Y [Source:VGNC Symbol;Acc:VGNC:95998]	2.07	0.0098
ssc-miR-30d	CCP110	centriolar coiled-coil protein 110 [Source:VGNC Symbol;Acc:VGNC:86367]	2.07	0.0098
ssc-miR-30d	CCPG1	cell cycle progression 1 [Source:VGNC Symbol;Acc:VGNC:86368]	2.07	0.0098
ssc-miR-30d	CCSER1	coiled-coil serine rich protein 1 [Source:VGNC Symbol;Acc:VGNC:86378]	2.07	0.0098
ssc-miR-30d	CCZ1B	hypothetical gene	2.07	0.0098
ssc-miR-30d	CD226	CD226 molecule [Source:VGNC Symbol;Acc:VGNC:86399]	2.07	0.0098
ssc-miR-30d	CD2AP	CD2 associated protein [Source:VGNC Symbol;Acc:VGNC:86406]	2.07	0.0098
ssc-miR-30d	CD302	CD302 molecule [Source:NCBI gene (formerly Entrezgene);Acc:100126280]	2.07	0.0098
ssc-miR-30d	CDC37L1	cell division cycle 37 like 1 [Source:VGNC Symbol;Acc:VGNC:86451]	2.07	0.0098
ssc-miR-30d	CDC42BPA	CDC42 binding protein kinase alpha [Source:VGNC Symbol;Acc:VGNC:95847]	2.07	0.0098
ssc-miR-30d	CDC7	cell division cycle 7 [Source:VGNC Symbol;Acc:VGNC:97922]	2.07	0.0098
ssc-miR-30d	CDCA7	cell division cycle associated 7 [Source:VGNC Symbol;Acc:VGNC:95623]	2.07	0.0098
ssc-miR-30d	CDH13	cadherin 13 [Source:VGNC Symbol;Acc:VGNC:86477]	2.07	0.0098
ssc-miR-30d	CDH20	cadherin 20 [Source:VGNC Symbol;Acc:VGNC:86484]	2.07	0.0098
ssc-miR-30d	CDK12	cyclin dependent kinase 12 [Source:VGNC Symbol;Acc:VGNC:86496]	2.07	0.0098

ssc-miR-30d	CDYL2	chromodomain Y like 2 [Source:VGNC Symbol;Acc:VGNC:86529]	2.07	0.0098
ssc-miR-30d	CEACAM1	hypothetical gene	2.07	0.0098
ssc-miR-30d	CECR2	CECR2 histone acetyl-lysine reader [Source:VGNC Symbol;Acc:VGNC:96576]	2.07	0.0098
ssc-miR-30d	CECR6	hypothetical gene	2.07	0.0098
ssc-miR-30d	CELF1	CUGBP Elav-like family member 1 [Source:VGNC Symbol;Acc:VGNC:86537]	2.07	0.0098
ssc-miR-30d	CELF2	hypothetical gene	2.07	0.0098
ssc-miR-30d	CELF3	CUGBP Elav-like family member 3 [Source:VGNC Symbol;Acc:VGNC:86538]	2.07	0.0098
ssc-miR-30d	CELF5	CUGBP Elav-like family member 5 [Source:VGNC Symbol;Acc:VGNC:86540]	2.07	0.0098
ssc-miR-30d	CELSR3	cadherin EGF LAG seven-pass G-type receptor 3 [Source:VGNC Symbol;Acc:VGNC:86541]	2.07	0.0098
ssc-miR-30d	CEP170	centrosomal protein 170 [Source:VGNC Symbol;Acc:VGNC:97925]	2.07	0.0098
ssc-miR-30d	CEP170B	centrosomal protein 170B [Source:VGNC Symbol;Acc:VGNC:86564]	2.07	0.0098
ssc-miR-30d	CEP350	centrosomal protein 350 [Source:HGNC Symbol;Acc:HGNC:24238]	2.07	0.0098
ssc-miR-30d	CEP41	centrosomal protein 41 [Source:VGNC Symbol;Acc:VGNC:86569]	2.07	0.0098
ssc-miR-30d	CEP44	centrosomal protein 44 [Source:VGNC Symbol;Acc:VGNC:86571]	2.07	0.0098
ssc-miR-30d	CEP76	centrosomal protein 76 [Source:VGNC Symbol;Acc:VGNC:96947]	2.07	0.0098
ssc-miR-30d	CEP85L	centrosomal protein 85 like [Source:VGNC Symbol;Acc:VGNC:86580]	2.07	0.0098
ssc-miR-30d	CERS5	ceramide synthase 5 [Source:VGNC Symbol;Acc:VGNC:97926]	2.07	0.0098
ssc-miR-30d	CERS6	ceramide synthase 6 [Source:VGNC Symbol;Acc:VGNC:96025]	2.07	0.0098
ssc-miR-30d	CFDP1	craniofacial development protein 1 [Source:VGNC Symbol;Acc:VGNC:86610]	2.07	0.0098
ssc-miR-30d	CFL2	cofilin 2 [Source:VGNC Symbol;Acc:VGNC:86611]	2.07	0.0098
ssc-miR-30d	CHD1	chromodomain helicase DNA binding protein 1 [Source:VGNC Symbol;Acc:VGNC:86629]	2.07	0.0098
ssc-miR-30d	CHD5	chromodomain helicase DNA binding protein 5 [Source:VGNC Symbol;Acc:VGNC:86632]	2.07	0.0098
ssc-miR-30d	CHD7	chromodomain helicase DNA binding protein 7 [Source:VGNC Symbol;Acc:VGNC:86633]	2.07	0.0098
ssc-miR-30d	CHD9	chromodomain helicase DNA binding protein 9 [Source:VGNC Symbol;Acc:VGNC:86635]	2.07	0.0098
ssc-miR-30d	CHFR	checkpoint with forkhead and ring finger domains [Source:HGNC Symbol;Acc:HGNC:20455]	2.07	0.0098
ssc-miR-30d	CHIC1	cysteine rich hydrophobic domain 1 [Source:VGNC Symbol;Acc:VGNC:86642]	2.07	0.0098
ssc-miR-30d	CHIC2	cysteine rich hydrophobic domain 2 [Source:VGNC Symbol;Acc:VGNC:86643]	2.07	0.0098
ssc-miR-30d	CHKA	choline kinase alpha [Source:VGNC Symbol;Acc:VGNC:86646]	2.07	0.0098
ssc-miR-30d	CHL1	cell adhesion molecule L1 like [Source:VGNC Symbol;Acc:VGNC:108639]	2.07	0.0098
ssc-miR-30d	CHMP2B	charged multivesicular body protein 2B [Source:VGNC Symbol;Acc:VGNC:86650]	2.07	0.0098
ssc-miR-30d	CHRM3	cholinergic receptor muscarinic 3 [Source:VGNC Symbol;Acc:VGNC:86664]	2.07	0.0098
ssc-miR-30d	CHST1	carbohydrate sulfotransferase 1 [Source:VGNC Symbol;Acc:VGNC:86673]	2.07	0.0098
ssc-miR-30d	CHST12	carbohydrate sulfotransferase 12 [Source:HGNC Symbol;Acc:HGNC:17423]	2.07	0.0098
ssc-miR-30d	CHST2	carbohydrate sulfotransferase 2 [Source:VGNC Symbol;Acc:VGNC:86678]	2.07	0.0098
ssc-miR-30d	CLCC1	chloride channel CLIC like 1 [Source:VGNC Symbol;Acc:VGNC:86724]	2.07	0.0098
ssc-miR-30d	CLCF1	cardiotrophin like cytokine factor 1 [Source:VGNC Symbol;Acc:VGNC:86725]	2.07	0.0098
ssc-miR-30d	CLCN3	chloride voltage-gated channel 3 [Source:VGNC Symbol;Acc:VGNC:86727]	2.07	0.0098
ssc-miR-30d	CLDN19	claudin 19 [Source:VGNC Symbol;Acc:VGNC:86736]	2.07	0.0098
ssc-miR-30d	CLEC14A	C-type lectin domain containing 14A [Source:VGNC Symbol;Acc:VGNC:86747]	2.07	0.0098
ssc-miR-30d	CLN8	CLN8 transmembrane ER and ERGIC protein [Source:VGNC Symbol;Acc:VGNC:103067]	2.07	0.0098
ssc-miR-30d	CLOCK	clock circadian regulator [Source:VGNC Symbol;Acc:VGNC:86774]	2.07	0.0098
ssc-miR-30d	CLRN1	clarin 1 [Source:VGNC Symbol;Acc:VGNC:97933]	2.07	0.0098
ssc-miR-30d	CLSPN	claspin [Source:VGNC Symbol;Acc:VGNC:86783]	2.07	0.0098
ssc-miR-30d	CMTM4	CKLF like MARVEL transmembrane domain containing 4 [Source:VGNC Symbol;Acc:VGNC:86804]	2.07	0.0098
ssc-miR-30d	CNGB3	cyclic nucleotide gated channel subunit beta 3 [Source:VGNC Symbol;Acc:VGNC:86819]	2.07	0.0098
ssc-miR-30d	CNKSR2	connector enhancer of kinase suppressor of Ras 2 [Source:VGNC Symbol;Acc:VGNC:86822]	2.07	0.0098
ssc-miR-30d	CNOT6	CCR4-NOT transcription complex subunit 6 [Source:VGNC Symbol;Acc:VGNC:86837]	2.07	0.0098
ssc-miR-30d	CNTN2	contactin 2 [Source:VGNC Symbol;Acc:VGNC:108577]	2.07	0.0098
ssc-miR-30d	CNTN4	contactin 4 [Source:VGNC Symbol;Acc:VGNC:97934]	2.07	0.0098

ssc-miR-30d	COG3	component of oligomeric golgi complex 3 [Source:HGNC Symbol;Acc:HGNC:18619]	2.07	0.0098
ssc-miR-30d	COG5	component of oligomeric golgi complex 5 [Source:VGNC Symbol;Acc:VGNC:86857]	2.07	0.0098
ssc-miR-30d	COL13A1	collagen type XIII alpha 1 chain [Source:VGNC Symbol;Acc:VGNC:86865]	2.07	0.0098
ssc-miR-30d	COL25A1	collagen type XXV alpha 1 chain [Source:HGNC Symbol;Acc:HGNC:18603]	2.07	0.0098
ssc-miR-30d	COL4A3BP	hypothetical gene	2.07	0.0098
ssc-miR-30d	COL9A3	collagen type IX alpha 3 chain [Source:VGNC Symbol;Acc:VGNC:108713]	2.07	0.0098
ssc-miR-30d	COMT	catechol-O-methyltransferase [Source:VGNC Symbol;Acc:VGNC:107378]	2.07	0.0098
ssc-miR-30d	COPS7B	COP9 signalosome subunit 7B [Source:HGNC Symbol;Acc:HGNC:16760]	2.07	0.0098
ssc-miR-30d	COQ3	coenzyme Q3, methyltransferase [Source:HGNC Symbol;Acc:HGNC:18175]	2.07	0.0098
ssc-miR-30d	CORO2A	coronin 2A [Source:VGNC Symbol;Acc:VGNC:86915]	2.07	0.0098
ssc-miR-30d	COTL1	coactosin like F-actin binding protein 1 [Source:HGNC Symbol;Acc:HGNC:18304]	2.07	0.0098
ssc-miR-30d	CPEB2	cytoplasmic polyadenylation element binding protein 2 [Source:VGNC Symbol;Acc:VGNC:86937]	2.07	0.0098
ssc-miR-30d	CPEB3	cytoplasmic polyadenylation element binding protein 3 [Source:VGNC Symbol;Acc:VGNC:86938]	2.07	0.0098
ssc-miR-30d	CPEB4	cytoplasmic polyadenylation element binding protein 4 [Source:VGNC Symbol;Acc:VGNC:86939]	2.07	0.0098
ssc-miR-30d	CPNE8	copine 8 [Source:VGNC Symbol;Acc:VGNC:86955]	2.07	0.0098
ssc-miR-30d	CPOX	coproporphyrinogen oxidase [Source:VGNC Symbol;Acc:VGNC:86956]	2.07	0.0098
ssc-miR-30d	CPSF6	cleavage and polyadenylation specific factor 6 [Source:VGNC Symbol;Acc:VGNC:86962]	2.07	0.0098
ssc-miR-30d	CREB1	cAMP responsive element binding protein 1 [Source:VGNC Symbol;Acc:VGNC:96004]	2.07	0.0098
ssc-miR-30d	CRKL	CRK like proto-onco, adaptor protein [Source:VGNC Symbol;Acc:VGNC:86997]	2.07	0.0098
ssc-miR-30d	CRLF3	cytokine receptor like factor 3 [Source:VGNC Symbol;Acc:VGNC:86999]	2.07	0.0098
ssc-miR-30d	CRMP1	collapsin response mediator protein 1 [Source:VGNC Symbol;Acc:VGNC:87000]	2.07	0.0098
ssc-miR-30d	CRY2	cryptochrome circadian regulator 2 [Source:VGNC Symbol;Acc:VGNC:87012]	2.07	0.0098
ssc-miR-30d	CSAD	cysteine sulfinic acid decarboxylase [Source:VGNC Symbol;Acc:VGNC:87027]	2.07	0.0098
ssc-miR-30d	CSGALNACT1	chondroitin sulfate N-acetylgalactosaminyltransferase 1 [Source:HGNC Symbol;Acc:HGNC:24290]	2.07	0.0098
ssc-miR-30d	CSNK1A1	casein kinase 1 alpha 1 [Source:VGNC Symbol;Acc:VGNC:99630]	2.07	0.0098
ssc-miR-30d	CSNK1A1L	hypothetical gene	2.07	0.0098
ssc-miR-30d	CSNK1G1	casein kinase 1 gamma 1 [Source:VGNC Symbol;Acc:VGNC:97949]	2.07	0.0098
ssc-miR-30d	CSNK2A1	hypothetical gene	2.07	0.0098
ssc-miR-30d	CTDSPL2	CTD small phosphatase like 2 [Source:VGNC Symbol;Acc:VGNC:87060]	2.07	0.0098
ssc-miR-30d	CTH	cystathionine gamma-lyase [Source:VGNC Symbol;Acc:VGNC:96961]	2.07	0.0098
ssc-miR-30d	CTHRC1	collagen triple helix repeat containing 1 [Source:VGNC Symbol;Acc:VGNC:87061]	2.07	0.0098
ssc-miR-30d	CTPS1	CTP synthase 1 [Source:VGNC Symbol;Acc:VGNC:87070]	2.07	0.0098
ssc-miR-30d	CTTNBP2NL	CTTNBP2 N-terminal like [Source:VGNC Symbol;Acc:VGNC:87082]	2.07	0.0098
ssc-miR-30d	CUL2	cullin 2 [Source:VGNC Symbol;Acc:VGNC:96090]	2.07	0.0098
ssc-miR-30d	CUL9	cullin 9 [Source:VGNC Symbol;Acc:VGNC:87090]	2.07	0.0098
ssc-miR-30d	CYB561	cytochrome b561 [Source:VGNC Symbol;Acc:VGNC:87119]	2.07	0.0098
ssc-miR-30d	CYB5B	cytochrome b5 type B [Source:VGNC Symbol;Acc:VGNC:96728]	2.07	0.0098
ssc-miR-30d	CYLD	CYLD lysine 63 deubiquitinase [Source:VGNC Symbol;Acc:VGNC:87130]	2.07	0.0098
ssc-miR-30d	CYP24A1	cytochrome P450 family 24 subfamily A member 1 [Source:VGNC Symbol;Acc:VGNC:103371]	2.07	0.0098
ssc-miR-30d	CYSLTR1	cysteinyl leukotriene receptor 1 [Source:VGNC Symbol;Acc:VGNC:87132]	2.07	0.0098
ssc-miR-30d	CYYR1	cysteine and tyrosine rich 1 [Source:VGNC Symbol;Acc:VGNC:87139]	2.07	0.0098
ssc-miR-30d	DAAM2	dishevelled associated activator of morphosis 2 [Source:VGNC Symbol;Acc:VGNC:87142]	2.07	0.0098
ssc-miR-30d	DAB1	DAB adaptor protein 1 [Source:VGNC Symbol;Acc:VGNC:87143]	2.07	0.0098
ssc-miR-30d	DACH2	dachshund family transcription factor 2 [Source:HGNC Symbol;Acc:HGNC:16814]	2.07	0.0098
ssc-miR-30d	DACT1	dishevelled binding antagonist of beta catenin 1 [Source:VGNC Symbol;Acc:VGNC:87147]	2.07	0.0098
ssc-miR-30d	DAG1	dystroglycan 1 [Source:VGNC Symbol;Acc:VGNC:87151]	2.07	0.0098
ssc-miR-30d	DAGLA	diacylglycerol lipase alpha [Source:VGNC Symbol;Acc:VGNC:87152]	2.07	0.0098
ssc-miR-30d	DBF4	hypothetical gene	2.07	0.0098
ssc-miR-30d	DCAF5	DDB1 and CUL4 associated factor 5 [Source:VGNC Symbol;Acc:VGNC:87176]	2.07	0.0098

ssc-miR-30d	DCBLD1	discoidin, CUB and LCCL domain containing 1 [Source:VGNC Symbol;Acc:VGNC:87179]	2.07	0.0098
ssc-miR-30d	DCP1A	decapping mRNA 1A [Source:VGNC Symbol;Acc:VGNC:97960]	2.07	0.0098
ssc-miR-30d	DCP2	decapping mRNA 2 [Source:VGNC Symbol;Acc:VGNC:87188]	2.07	0.0098
ssc-miR-30d	DCTN4	dynactin subunit 4 [Source:VGNC Symbol;Acc:VGNC:99635]	2.07	0.0098
ssc-miR-30d	DCUN1D1	hypothetical gene	2.07	0.0098
ssc-miR-30d	DCUN1D3	defective in cullin neddylation 1 domain containing 3 [Source:VGNC Symbol;Acc:VGNC:87196]	2.07	0.0098
ssc-miR-30d	DCX	doublecortin [Source:HGNC Symbol;Acc:HGNC:2714]	2.07	0.0098
ssc-miR-30d	DDAH1	dimethylarginine dimethylaminohydrolase 1 [Source:VGNC Symbol;Acc:VGNC:87200]	2.07	0.0098
ssc-miR-30d	DDI2	hypothetical gene	2.07	0.0098
ssc-miR-30d	DDIT4	DNA damage inducible transcript 4 [Source:VGNC Symbol;Acc:VGNC:87208]	2.07	0.0098
ssc-miR-30d	DDX19A	hypothetical gene	2.07	0.0098
ssc-miR-30d	DDX19B	hypothetical gene	2.07	0.0098
ssc-miR-30d	DDX46	DEAD-box helicase 46 [Source:VGNC Symbol;Acc:VGNC:87223]	2.07	0.0098
ssc-miR-30d	DENND2C	DENN domain containing 2C [Source:HGNC Symbol;Acc:HGNC:24748]	2.07	0.0098
ssc-miR-30d	DENND4A	DENN domain containing 4A [Source:VGNC Symbol;Acc:VGNC:87251]	2.07	0.0098
ssc-miR-30d	DENND5B	DENN domain containing 5B [Source:VGNC Symbol;Acc:VGNC:87255]	2.07	0.0098
ssc-miR-30d	DESI2	desumoylating isopeptidase 2 [Source:VGNC Symbol;Acc:VGNC:96037]	2.07	0.0098
ssc-miR-30d	DEXI	Dexi homolog [Source:HGNC Symbol;Acc:HGNC:13267]	2.07	0.0098
ssc-miR-30d	DGKB	diacylglycerol kinase beta [Source:VGNC Symbol;Acc:VGNC:87270]	2.07	0.0098
ssc-miR-30d	DGKD	diacylglycerol kinase delta [Source:VGNC Symbol;Acc:VGNC:96180]	2.07	0.0098
ssc-miR-30d	DGKE	diacylglycerol kinase epsilon [Source:VGNC Symbol;Acc:VGNC:87271]	2.07	0.0098
ssc-miR-30d	DGKH	diacylglycerol kinase eta [Source:VGNC Symbol;Acc:VGNC:87273]	2.07	0.0098
ssc-miR-30d	DGKI	diacylglycerol kinase iota [Source:VGNC Symbol;Acc:VGNC:87274]	2.07	0.0098
ssc-miR-30d	DGKQ	diacylglycerol kinase theta [Source:VGNC Symbol;Acc:VGNC:87276]	2.07	0.0098
ssc-miR-30d	DGKZ	diacylglycerol kinase zeta [Source:VGNC Symbol;Acc:VGNC:87277]	2.07	0.0098
ssc-miR-30d	DHX36	DEAH-box helicase 36 [Source:VGNC Symbol;Acc:VGNC:87292]	2.07	0.0098
ssc-miR-30d	DHX40	DEAH-box helicase 40 [Source:VGNC Symbol;Acc:VGNC:87295]	2.07	0.0098
ssc-miR-30d	DIO2	iodothyronine deiodinase 2 [Source:VGNC Symbol;Acc:VGNC:103935]	2.07	0.0098
ssc-miR-30d	DIP2B	disco interacting B [Source:VGNC Symbol;Acc:VGNC:87306]	2.07	0.0098
ssc-miR-30d	DIP2C	disco interacting C [Source:VGNC Symbol;Acc:VGNC:96201]	2.07	0.0098
ssc-miR-30d	DLD	dihydrolipoamide dehydrogenase [Source:VGNC Symbol;Acc:VGNC:108580]	2.07	0.0098
ssc-miR-30d	DLG5	discs large MAGUK scaffold protein 5 [Source:VGNC Symbol;Acc:VGNC:87329]	2.07	0.0098
ssc-miR-30d	DLGAP1	DLG associated protein 1 [Source:VGNC Symbol;Acc:VGNC:87330]	2.07	0.0098
ssc-miR-30d	DLGAP2	DLG associated protein 2 [Source:VGNC Symbol;Acc:VGNC:99711]	2.07	0.0098
ssc-miR-30d	DLGAP4	DLG associated protein 4 [Source:VGNC Symbol;Acc:VGNC:96043]	2.07	0.0098
ssc-miR-30d	DLK1	delta like non-canonical Notch ligand 1 [Source:VGNC Symbol;Acc:VGNC:87333]	2.07	0.0098
ssc-miR-30d	DLL4	delta like canonical Notch ligand 4 [Source:VGNC Symbol;Acc:VGNC:87337]	2.07	0.0098
ssc-miR-30d	DMD	dystrophin [Source:NCBI gene (formerly Entrezgene);Acc:497636]	2.07	0.0098
ssc-miR-30d	DNAH17	dynein axonemal heavy chain 17 [Source:VGNC Symbol;Acc:VGNC:87369]	2.07	0.0098
ssc-miR-30d	DNAJC13	DnaJ heat shock protein family (Hsp40) member C13 [Source:VGNC Symbol;Acc:VGNC:108650]	2.07	0.0098
ssc-miR-30d	DNAJC15	DnaJ heat shock protein family (Hsp40) member C15 [Source:HGNC Symbol;Acc:HGNC:20325]	2.07	0.0098
ssc-miR-30d	DNAJC25	DnaJ heat shock protein family (Hsp40) member C25 [Source:VGNC Symbol;Acc:VGNC:103083]	2.07	0.0098
ssc-miR-30d	DNAJC25-GNG10	hypothetical gene	2.07	0.0098
ssc-miR-30d	DNMT3A	DNA methyltransferase 3 alpha [Source:VGNC Symbol;Acc:VGNC:87384]	2.07	0.0098
ssc-miR-30d	DOC2A	double C2 domain alpha [Source:VGNC Symbol;Acc:VGNC:87388]	2.07	0.0098
ssc-miR-30d	DOCK7	dedicator of cytokinesis 7 [Source:VGNC Symbol;Acc:VGNC:87397]	2.07	0.0098
ssc-miR-30d	DOLPP1	dolichyldiphosphatase 1 [Source:VGNC Symbol;Acc:VGNC:87407]	2.07	0.0098
ssc-miR-30d	DPY19L1	dpy-19 like C-mannosyltransferase 1 [Source:VGNC Symbol;Acc:VGNC:99712]	2.07	0.0098
ssc-miR-30d	DPY19L3	dpy-19 like C-mannosyltransferase 3 [Source:VGNC Symbol;Acc:VGNC:87426]	2.07	0.0098

ssc-miR-30d	DPYSL2	dihydropyrimidinase like 2 [Source:VGNC Symbol;Acc:VGNC:87430]	2.07	0.0098
ssc-miR-30d	DRAXIN	dorsal inhibitory axon guidance protein [Source:VGNC Symbol;Acc:VGNC:87439]	2.07	0.0098
ssc-miR-30d	DRD1	dopamine receptor D1 [Source:VGNC Symbol;Acc:VGNC:87443]	2.07	0.0098
ssc-miR-30d	DRP2	dystrophin related protein 2 [Source:VGNC Symbol;Acc:VGNC:87451]	2.07	0.0098
ssc-miR-30d	DSC2	desmocollin 2 [Source:HGNC Symbol;Acc:HGNC:3036]	2.07	0.0098
ssc-miR-30d	DSTYK	dual serine/threonine and tyrosine protein kinase [Source:VGNC Symbol;Acc:VGNC:87460]	2.07	0.0098
ssc-miR-30d	DTNA	dystrobrevin alpha [Source:VGNC Symbol;Acc:VGNC:87462]	2.07	0.0098
ssc-miR-30d	DUSP18	dual specificity phosphatase 18 [Source:VGNC Symbol;Acc:VGNC:103937]	2.07	0.0098
ssc-miR-30d	E2F3	E2F transcription factor 3 [Source:VGNC Symbol;Acc:VGNC:87514]	2.07	0.0098
ssc-miR-30d	E2F7	E2F transcription factor 7 [Source:VGNC Symbol;Acc:VGNC:87518]	2.07	0.0098
ssc-miR-30d	EAF1	ELL associated factor 1 [Source:VGNC Symbol;Acc:VGNC:87521]	2.07	0.0098
ssc-miR-30d	EBF2	EBF transcription factor 2 [Source:VGNC Symbol;Acc:VGNC:87526]	2.07	0.0098
ssc-miR-30d	EBF3	EBF transcription factor 3 [Source:VGNC Symbol;Acc:VGNC:87527]	2.07	0.0098
ssc-miR-30d	EDA	ectodysplasin A [Source:VGNC Symbol;Acc:VGNC:87540]	2.07	0.0098
ssc-miR-30d	EDC3	enhancer of mRNA decapping 3 [Source:VGNC Symbol;Acc:VGNC:87543]	2.07	0.0098
ssc-miR-30d	EDEM3	ER degradation enhancing alpha-mannosidase like protein 3 [Source:VGNC Symbol;Acc:VGNC:87546]	2.07	0.0098
ssc-miR-30d	EDNRA	endothelin receptor type A [Source:VGNC Symbol;Acc:VGNC:87549]	2.07	0.0098
ssc-miR-30d	EDNRB	endothelin receptor type B [Source:VGNC Symbol;Acc:VGNC:87550]	2.07	0.0098
ssc-miR-30d	EEA1	early endosome antigen 1 [Source:HGNC Symbol;Acc:HGNC:3185]	2.07	0.0098
ssc-miR-30d	EED	embryonic ectoderm development [Source:VGNC Symbol;Acc:VGNC:87552]	2.07	0.0098
ssc-miR-30d	EEF2K	eukaryotic elongation factor 2 kinase [Source:VGNC Symbol;Acc:VGNC:87557]	2.07	0.0098
ssc-miR-30d	EFNA3	ephrin A3 [Source:VGNC Symbol;Acc:VGNC:98790]	2.07	0.0098
ssc-miR-30d	EFR3A	EFR3 homolog A [Source:VGNC Symbol;Acc:VGNC:87579]	2.07	0.0098
ssc-miR-30d	EIF3A	eukaryotic translation initiation factor 3 subunit A [Source:VGNC Symbol;Acc:VGNC:87615]	2.07	0.0098
ssc-miR-30d	EIF5A2	eukaryotic translation initiation factor 5A2 [Source:VGNC Symbol;Acc:VGNC:87633]	2.07	0.0098
ssc-miR-30d	ELAVL2	ELAV like RNA binding protein 2 [Source:VGNC Symbol;Acc:VGNC:87639]	2.07	0.0098
ssc-miR-30d	ELAVL3	ELAV like RNA binding protein 3 [Source:VGNC Symbol;Acc:VGNC:87640]	2.07	0.0098
ssc-miR-30d	ELAVL4	ELAV like RNA binding protein 4 [Source:VGNC Symbol;Acc:VGNC:97047]	2.07	0.0098
ssc-miR-30d	ELFN2	extracellular leucine rich repeat and fibronectin type III domain containing 2 [Source:VGNC Symbol;Acc:VGNC:103232]	2.07	0.0098
ssc-miR-30d	ELK3	ETS transcription factor ELK3 [Source:VGNC Symbol;Acc:VGNC:87646]	2.07	0.0098
ssc-miR-30d	ELL	elongation factor for RNA polymerase II [Source:VGNC Symbol;Acc:VGNC:87648]	2.07	0.0098
ssc-miR-30d	ELL2	elongation factor for RNA polymerase II 2 [Source:VGNC Symbol;Acc:VGNC:87649]	2.07	0.0098
ssc-miR-30d	ELMO1	engulfment and cell motility 1 [Source:HGNC Symbol;Acc:HGNC:16286]	2.07	0.0098
ssc-miR-30d	ELMOD2	ELMO domain containing 2 [Source:VGNC Symbol;Acc:VGNC:87653]	2.07	0.0098
ssc-miR-30d	ELMSAN1	hypothetical gene	2.07	0.0098
ssc-miR-30d	ELOVL5	ELOVL fatty acid elongase 5 [Source:VGNC Symbol;Acc:VGNC:87661]	2.07	0.0098
ssc-miR-30d	ELOVL6	ELOVL fatty acid elongase 6 [Source:VGNC Symbol;Acc:VGNC:87662]	2.07	0.0098
ssc-miR-30d	EML1	EMAP like 1 [Source:VGNC Symbol;Acc:VGNC:87682]	2.07	0.0098
ssc-miR-30d	EML4	EMAP like 4 [Source:VGNC Symbol;Acc:VGNC:87685]	2.07	0.0098
ssc-miR-30d	ENAH	ENAH actin regulator [Source:VGNC Symbol;Acc:VGNC:108271]	2.07	0.0098
ssc-miR-30d	ENOX2	ecto-NOX disulfide-thiol exchanger 2 [Source:VGNC Symbol;Acc:VGNC:87707]	2.07	0.0098
ssc-miR-30d	ENTHD2	hypothetical gene	2.07	0.0098
ssc-miR-30d	EOGT	EGF domain specific O-linked N-acetylglucosamine transferase [Source:VGNC Symbol;Acc:VGNC:87718]	2.07	0.0098
ssc-miR-30d	EPB41	erythrocyte membrane protein band 4.1 [Source:VGNC Symbol;Acc:VGNC:87722]	2.07	0.0098
ssc-miR-30d	EPB41L3	erythrocyte membrane protein band 4.1 like 3 [Source:VGNC Symbol;Acc:VGNC:87724]	2.07	0.0098
ssc-miR-30d	EPB41L4B	erythrocyte membrane protein band 4.1 like 4B [Source:VGNC Symbol;Acc:VGNC:103088]	2.07	0.0098
ssc-miR-30d	EPB41L5	erythrocyte membrane protein band 4.1 like 5 [Source:VGNC Symbol;Acc:VGNC:97979]	2.07	0.0098
ssc-miR-30d	EPC1	enhancer of polycomb homolog 1 [Source:VGNC Symbol;Acc:VGNC:95923]	2.07	0.0098
ssc-miR-30d	EPC2	enhancer of polycomb homolog 2 [Source:VGNC Symbol;Acc:VGNC:96045]	2.07	0.0098

ssc-miR-30d	EPDR1	ependymin related 1 [Source:VGNC Symbol;Acc:VGNC:87727]	2.07	0.0098
ssc-miR-30d	EPG5	ectopic P-granules 5 autophagy tethering factor [Source:VGNC Symbol;Acc:VGNC:87728]	2.07	0.0098
ssc-miR-30d	EPHB2	EPH receptor B2 [Source:VGNC Symbol;Acc:VGNC:87737]	2.07	0.0098
ssc-miR-30d	EPN2	epsin 2 [Source:VGNC Symbol;Acc:VGNC:97982]	2.07	0.0098
ssc-miR-30d	ERC2	ELKS/RAB6-interacting/CAST family member 2 [Source:VGNC Symbol;Acc:VGNC:87762]	2.07	0.0098
ssc-miR-30d	ERCC6	hypothetical gene	2.07	0.0098
ssc-miR-30d	ERG	ETS transcription factor ERG [Source:VGNC Symbol;Acc:VGNC:87768]	2.07	0.0098
ssc-miR-30d	ERLIN1	hypothetical gene	2.07	0.0098
ssc-miR-30d	ERMAP	erythroblast membrane associated protein [Source:VGNC Symbol;Acc:VGNC:97053]	2.07	0.0098
ssc-miR-30d	ERRFI1	ERBB receptor feedback inhibitor 1 [Source:VGNC Symbol;Acc:VGNC:87784]	2.07	0.0098
ssc-miR-30d	ESCO1	establishment of sister chromatid cohesion N-acetyltransferase 1 [Source:VGNC Symbol;Acc:VGNC:87786]	2.07	0.0098
ssc-miR-30d	ESPN	espin [Source:VGNC Symbol;Acc:VGNC:97054]	2.07	0.0098
ssc-miR-30d	ESRRG	estrogen related receptor gamma [Source:VGNC Symbol;Acc:VGNC:96289]	2.07	0.0098
ssc-miR-30d	EVA1C	eva-1 homolog C [Source:VGNC Symbol;Acc:VGNC:87817]	2.07	0.0098
ssc-miR-30d	EVI5	ecotropic viral integration site 5 [Source:VGNC Symbol;Acc:VGNC:98793]	2.07	0.0098
ssc-miR-30d	EVI5L	ecotropic viral integration site 5 like [Source:VGNC Symbol;Acc:VGNC:99646]	2.07	0.0098
ssc-miR-30d	EVX2	even-skipped homeobox 2 [Source:VGNC Symbol;Acc:VGNC:96290]	2.07	0.0098
ssc-miR-30d	EXTL2	exostosin like glycosyltransferase 2 [Source:VGNC Symbol;Acc:VGNC:87849]	2.07	0.0098
ssc-miR-30d	EYA2	EYA transcriptional coactivator and phosphatase 2 [Source:VGNC Symbol;Acc:VGNC:96292]	2.07	0.0098
ssc-miR-30d	FA2H	fatty acid 2-hydroxylase [Source:VGNC Symbol;Acc:VGNC:87868]	2.07	0.0098
ssc-miR-30d	FAF2	Fas associated factor family member 2 [Source:VGNC Symbol;Acc:VGNC:87880]	2.07	0.0098
ssc-miR-30d	FAM104A	family with sequence similarity 104 member A [Source:VGNC Symbol;Acc:VGNC:98990]	2.07	0.0098
ssc-miR-30d	FAM104B	hypothetical gene	2.07	0.0098
ssc-miR-30d	FAM105B	hypothetical gene	2.07	0.0098
ssc-miR-30d	FAM109A	hypothetical gene	2.07	0.0098
ssc-miR-30d	FAM109B	hypothetical gene	2.07	0.0098
ssc-miR-30d	FAM110B	family with sequence similarity 110 member B [Source:VGNC Symbol;Acc:VGNC:87885]	2.07	0.0098
ssc-miR-30d	FAM124A	family with sequence similarity 124 member A [Source:VGNC Symbol;Acc:VGNC:87898]	2.07	0.0098
ssc-miR-30d	FAM126A	family with sequence similarity 126 member A [Source:VGNC Symbol;Acc:VGNC:87899]	2.07	0.0098
ssc-miR-30d	FAM126B	family with sequence similarity 126 member B [Source:HGNC Symbol;Acc:HGNC:28593]	2.07	0.0098
ssc-miR-30d	FAM129A	hypothetical gene	2.07	0.0098
ssc-miR-30d	FAM131B	family with sequence similarity 131 member B [Source:VGNC Symbol;Acc:VGNC:87901]	2.07	0.0098
ssc-miR-30d	FAM133A	family with sequence similarity 133 member A [Source:VGNC Symbol;Acc:VGNC:87903]	2.07	0.0098
ssc-miR-30d	FAM13A	family with sequence similarity 13 member A [Source:VGNC Symbol;Acc:VGNC:98924]	2.07	0.0098
ssc-miR-30d	FAM13C	family with sequence similarity 13 member C [Source:VGNC Symbol;Acc:VGNC:87907]	2.07	0.0098
ssc-miR-30d	FAM155A	hypothetical gene	2.07	0.0098
ssc-miR-30d	FAM160B1	hypothetical gene	2.07	0.0098
ssc-miR-30d	FAM168B	family with sequence similarity 168 member B [Source:VGNC Symbol;Acc:VGNC:95462]	2.07	0.0098
ssc-miR-30d	FAM175B	hypothetical gene	2.07	0.0098
ssc-miR-30d	FAM178A	hypothetical gene	2.07	0.0098
ssc-miR-30d	FAM179B	hypothetical gene	2.07	0.0098
ssc-miR-30d	FAM199X	family with sequence similarity 199, X-linked [Source:HGNC Symbol;Acc:HGNC:25195]	2.07	0.0098
ssc-miR-30d	FAM210B	family with sequence similarity 210 member B [Source:VGNC Symbol;Acc:VGNC:95736]	2.07	0.0098
ssc-miR-30d	FAM214A	family with sequence similarity 214 member A [Source:VGNC Symbol;Acc:VGNC:87952]	2.07	0.0098
ssc-miR-30d	FAM216A	family with sequence similarity 216 member A [Source:VGNC Symbol;Acc:VGNC:87954]	2.07	0.0098
ssc-miR-30d	FAM217B	family with sequence similarity 217 member B [Source:VGNC Symbol;Acc:VGNC:95767]	2.07	0.0098
ssc-miR-30d	FAM219B	family with sequence similarity 219 member B [Source:VGNC Symbol;Acc:VGNC:87956]	2.07	0.0098
ssc-miR-30d	FAM43A	family with sequence similarity 43 member A [Source:VGNC Symbol;Acc:VGNC:87970]	2.07	0.0098
ssc-miR-30d	FAM46A	hypothetical gene	2.07	0.0098

ssc-miR-30d	FAM46C	hypothetical gene	2.07	0.0098
ssc-miR-30d	FAM49A	hypothetical gene	2.07	0.0098
ssc-miR-30d	FAM53B	family with sequence similarity 53 member B [Source:VGNC Symbol;Acc:VGNC:87974]	2.07	0.0098
ssc-miR-30d	FAM72B	hypothetical gene	2.07	0.0098
ssc-miR-30d	FAM72D	hypothetical gene	2.07	0.0098
ssc-miR-30d	FAM73B	hypothetical gene	2.07	0.0098
ssc-miR-30d	FAM81A	family with sequence similarity 81 member A [Source:VGNC Symbol;Acc:VGNC:87986]	2.07	0.0098
ssc-miR-30d	FAM83F	family with sequence similarity 83 member F [Source:VGNC Symbol;Acc:VGNC:87991]	2.07	0.0098
ssc-miR-30d	FAM83G	family with sequence similarity 83 member G [Source:VGNC Symbol;Acc:VGNC:87992]	2.07	0.0098
ssc-miR-30d	FAM91A1	family with sequence similarity 91 member A1 [Source:VGNC Symbol;Acc:VGNC:97998]	2.07	0.0098
ssc-miR-30d	FANCF	FA complementation group F [Source:VGNC Symbol;Acc:VGNC:88004]	2.07	0.0098
ssc-miR-30d	FAP	fibroblast activation protein alpha [Source:VGNC Symbol;Acc:VGNC:99714]	2.07	0.0098
ssc-miR-30d	FBXL14	hypothetical gene	2.07	0.0098
ssc-miR-30d	FBXL17	F-box and leucine rich repeat protein 17 [Source:VGNC Symbol;Acc:VGNC:99650]	2.07	0.0098
ssc-miR-30d	FBXL20	F-box and leucine rich repeat protein 20 [Source:VGNC Symbol;Acc:VGNC:98006]	2.07	0.0098
ssc-miR-30d	FBXO28	F-box protein 28 [Source:HGNC Symbol;Acc:HGNC:29046]	2.07	0.0098
ssc-miR-30d	FBXO32	F-box protein 32 [Source:VGNC Symbol;Acc:VGNC:88039]	2.07	0.0098
ssc-miR-30d	FBXO34	F-box protein 34 [Source:VGNC Symbol;Acc:VGNC:88041]	2.07	0.0098
ssc-miR-30d	FBXO42	F-box protein 42 [Source:VGNC Symbol;Acc:VGNC:88047]	2.07	0.0098
ssc-miR-30d	FBXO45	F-box protein 45 [Source:VGNC Symbol;Acc:VGNC:88048]	2.07	0.0098
ssc-miR-30d	FECH	ferrochelatase [Source:VGNC Symbol;Acc:VGNC:88081]	2.07	0.0098
ssc-miR-30d	FGD6	FYVE, RhoGEF and PH domain containing 6 [Source:VGNC Symbol;Acc:VGNC:88099]	2.07	0.0098
ssc-miR-30d	FGF20	fibroblast growth factor 20 [Source:VGNC Symbol;Acc:VGNC:96298]	2.07	0.0098
ssc-miR-30d	FIGN	fidgetin, microtubule severing factor [Source:VGNC Symbol;Acc:VGNC:95580]	2.07	0.0098
ssc-miR-30d	FKBP14	FKBP prolyl isomerase 14 [Source:VGNC Symbol;Acc:VGNC:88144]	2.07	0.0098
ssc-miR-30d	FKBP3	FKBP prolyl isomerase 3 [Source:VGNC Symbol;Acc:VGNC:88146]	2.07	0.0098
ssc-miR-30d	FLJ20373	hypothetical gene	2.07	0.0098
ssc-miR-30d	FLVCR1	FLVCR heme transporter 1 [Source:VGNC Symbol;Acc:VGNC:96580]	2.07	0.0098
ssc-miR-30d	FNDC3A	fibronectin type III domain containing 3A [Source:VGNC Symbol;Acc:VGNC:88182]	2.07	0.0098
ssc-miR-30d	FNDC3B	fibronectin type III domain containing 3B [Source:VGNC Symbol;Acc:VGNC:88183]	2.07	0.0098
ssc-miR-30d	FNIP2	folliculin interacting protein 2 [Source:VGNC Symbol;Acc:VGNC:88188]	2.07	0.0098
ssc-miR-30d	FOSL2	FOS like 2, AP-1 transcription factor subunit [Source:VGNC Symbol;Acc:VGNC:88192]	2.07	0.0098
ssc-miR-30d	FOXA1	forkhead box A1 [Source:VGNC Symbol;Acc:VGNC:88193]	2.07	0.0098
ssc-miR-30d	FOXB1	forkhead box B1 [Source:VGNC Symbol;Acc:VGNC:88195]	2.07	0.0098
ssc-miR-30d	FOXD1	forkhead box D1 [Source:VGNC Symbol;Acc:VGNC:88199]	2.07	0.0098
ssc-miR-30d	FOXG1	forkhead box G1 [Source:VGNC Symbol;Acc:VGNC:88206]	2.07	0.0098
ssc-miR-30d	FOXN2	forkhead box N2 [Source:VGNC Symbol;Acc:VGNC:88219]	2.07	0.0098
ssc-miR-30d	FOXO3	forkhead box O3 [Source:VGNC Symbol;Acc:VGNC:99715]	2.07	0.0098
ssc-miR-30d	FOXP4	forkhead box P4 [Source:VGNC Symbol;Acc:VGNC:88224]	2.07	0.0098
ssc-miR-30d	FRK	fyn related Src family tyrosine kinase [Source:VGNC Symbol;Acc:VGNC:88235]	2.07	0.0098
ssc-miR-30d	FRMD4A	FERM domain containing 4A [Source:VGNC Symbol;Acc:VGNC:96088]	2.07	0.0098
ssc-miR-30d	FRMD6	FERM domain containing 6 [Source:VGNC Symbol;Acc:VGNC:88239]	2.07	0.0098
ssc-miR-30d	FRMPD1	FERM and PDZ domain containing 1 [Source:VGNC Symbol;Acc:VGNC:88240]	2.07	0.0098
ssc-miR-30d	FRMPD4	FERM and PDZ domain containing 4 [Source:VGNC Symbol;Acc:VGNC:88243]	2.07	0.0098
ssc-miR-30d	FRS2	fibroblast growth factor receptor substrate 2 [Source:VGNC Symbol;Acc:VGNC:88246]	2.07	0.0098
ssc-miR-30d	FRZB	frizzled related protein [Source:VGNC Symbol;Acc:VGNC:96308]	2.07	0.0098
ssc-miR-30d	FST	follistatin [Source:NCBI gene (formerly Entrezgene);Acc:445002]	2.07	0.0098
ssc-miR-30d	FSTL4	follistatin like 4 [Source:VGNC Symbol;Acc:VGNC:88256]	2.07	0.0098
ssc-miR-30d	FTO	FTO alpha-ketoglutarate dependent dioxygenase [Source:VGNC Symbol;Acc:VGNC:88259]	2.07	0.0098

ssc-miR-30d	FUBP1	far upstream element binding protein 1 [Source:VGNC Symbol;Acc:VGNC:96781]	2.07	0.0098
ssc-miR-30d	FUBP3	far upstream element binding protein 3 [Source:VGNC Symbol;Acc:VGNC:103093]	2.07	0.0098
ssc-miR-30d	FUCA1	alpha-L-fucosidase 1 [Source:VGNC Symbol;Acc:VGNC:88262]	2.07	0.0098
ssc-miR-30d	FUT4	fucosyltransferase 4 [Source:HGNC Symbol;Acc:HGNC:4015]	2.07	0.0098
ssc-miR-30d	FXR1	FMR1 autosomal homolog 1 [Source:VGNC Symbol;Acc:VGNC:108659]	2.07	0.0098
ssc-miR-30d	FYCO1	hypothetical gene	2.07	0.0098
ssc-miR-30d	FYN	FYN proto-onco, Src family tyrosine kinase [Source:VGNC Symbol;Acc:VGNC:88276]	2.07	0.0098
ssc-miR-30d	FYTTD1	forty-two-three domain containing 1 [Source:VGNC Symbol;Acc:VGNC:88277]	2.07	0.0098
ssc-miR-30d	FZD2	frizzled class receptor 2 [Source:VGNC Symbol;Acc:VGNC:88280]	2.07	0.0098
ssc-miR-30d	FZD3	frizzled class receptor 3 [Source:VGNC Symbol;Acc:VGNC:88281]	2.07	0.0098
ssc-miR-30d	G3BP1	G3BP stress granule assembly factor 1 [Source:VGNC Symbol;Acc:VGNC:88288]	2.07	0.0098
ssc-miR-30d	G3BP2	G3BP stress granule assembly factor 2 [Source:VGNC Symbol;Acc:VGNC:88289]	2.07	0.0098
ssc-miR-30d	G6PC3	glucose-6-phosphatase catalytic subunit 3 [Source:VGNC Symbol;Acc:VGNC:88291]	2.07	0.0098
ssc-miR-30d	GABRA1	gamma-aminobutyric acid type A receptor subunit alpha1 [Source:VGNC Symbol;Acc:VGNC:88300]	2.07	0.0098
ssc-miR-30d	GABRA5	gamma-aminobutyric acid type A receptor subunit alpha5 [Source:VGNC Symbol;Acc:VGNC:88304]	2.07	0.0098
ssc-miR-30d	GABRB1	gamma-aminobutyric acid type A receptor subunit beta1 [Source:VGNC Symbol;Acc:VGNC:88306]	2.07	0.0098
ssc-miR-30d	GADD45GIP1	GADD45G interacting protein 1 [Source:VGNC Symbol;Acc:VGNC:99751]	2.07	0.0098
ssc-miR-30d	GALNT1	polypeptide N-acetylgalactosaminyltransferase 1 [Source:VGNC Symbol;Acc:VGNC:97062]	2.07	0.0098
ssc-miR-30d	GALNT2	polypeptide N-acetylgalactosaminyltransferase 2 [Source:VGNC Symbol;Acc:VGNC:88334]	2.07	0.0098
ssc-miR-30d	GALNT3	polypeptide N-acetylgalactosaminyltransferase 3 [Source:VGNC Symbol;Acc:VGNC:96313]	2.07	0.0098
ssc-miR-30d	GALNT7	polypeptide N-acetylgalactosaminyltransferase 7 [Source:VGNC Symbol;Acc:VGNC:103946]	2.07	0.0098
ssc-miR-30d	GALR1	galanin receptor 1 [Source:VGNC Symbol;Acc:VGNC:88339]	2.07	0.0098
ssc-miR-30d	GAN	gigaxonin [Source:VGNC Symbol;Acc:VGNC:88343]	2.07	0.0098
ssc-miR-30d	GAREM	hypothetical gene	2.07	0.0098
ssc-miR-30d	GAS2	growth arrest specific 2 [Source:VGNC Symbol;Acc:VGNC:88354]	2.07	0.0098
ssc-miR-30d	GAS7	growth arrest specific 7 [Source:VGNC Symbol;Acc:VGNC:88359]	2.07	0.0098
ssc-miR-30d	GATA5	GATA binding protein 5 [Source:VGNC Symbol;Acc:VGNC:95661]	2.07	0.0098
ssc-miR-30d	GATA6	GATA binding protein 6 [Source:VGNC Symbol;Acc:VGNC:88366]	2.07	0.0098
ssc-miR-30d	GATM	glycine amidinotransferase [Source:VGNC Symbol;Acc:VGNC:88372]	2.07	0.0098
ssc-miR-30d	GBP2	guanylate binding protein 2, interferon-inducible [Source:NCBI gene (formerly Entrezgene);Acc:100153137]	2.07	0.0098
ssc-miR-30d	GCH1	GTP cyclohydrolase 1 [Source:VGNC Symbol;Acc:VGNC:88384]	2.07	0.0098
ssc-miR-30d	GCLC	glutamate-cysteine ligase catalytic subunit [Source:VGNC Symbol;Acc:VGNC:88386]	2.07	0.0098
ssc-miR-30d	GCNT2	glucosaminyl (N-acetyl) transferase 2 (I blood group) [Source:HGNC Symbol;Acc:HGNC:4204]	2.07	0.0098
ssc-miR-30d	GCNT3	glucosaminyl (N-acetyl) transferase 3, mucin type [Source:VGNC Symbol;Acc:VGNC:103097]	2.07	0.0098
ssc-miR-30d	GDA	guanine deaminase [Source:VGNC Symbol;Acc:VGNC:88393]	2.07	0.0098
ssc-miR-30d	GDF2	growth differentiation factor 2 [Source:VGNC Symbol;Acc:VGNC:88400]	2.07	0.0098
ssc-miR-30d	GDI2	GDP dissociation inhibitor 2 [Source:VGNC Symbol;Acc:VGNC:98019]	2.07	0.0098
ssc-miR-30d	GDNF	glial cell derived neurotrophic factor [Source:VGNC Symbol;Acc:VGNC:88406]	2.07	0.0098
ssc-miR-30d	GFPT2	glutamine-fructose-6-phosphate transaminase 2 [Source:VGNC Symbol;Acc:VGNC:88427]	2.07	0.0098
ssc-miR-30d	GID4	GID complex subunit 4 homolog [Source:VGNC Symbol;Acc:VGNC:98996]	2.07	0.0098
ssc-miR-30d	GIGYF1	GRB10 interacting GYF protein 1 [Source:VGNC Symbol;Acc:VGNC:88444]	2.07	0.0098
ssc-miR-30d	GIGYF2	GRB10 interacting GYF protein 2 [Source:VGNC Symbol;Acc:VGNC:95551]	2.07	0.0098
ssc-miR-30d	GJA1	gap junction protein alpha 1 [Source:VGNC Symbol;Acc:VGNC:103098]	2.07	0.0098
ssc-miR-30d	GJA3	gap junction protein alpha 3 [Source:HGNC Symbol;Acc:HGNC:4277]	2.07	0.0098
ssc-miR-30d	GJC2	gap junction protein gamma 2 [Source:VGNC Symbol;Acc:VGNC:88469]	2.07	0.0098
ssc-miR-30d	GLCCI1	glucocorticoid induced 1 [Source:VGNC Symbol;Acc:VGNC:88476]	2.07	0.0098
ssc-miR-30d	GLCE	glucuronic acid epimerase [Source:VGNC Symbol;Acc:VGNC:88477]	2.07	0.0098
ssc-miR-30d	GLDC	glycine decarboxylase [Source:VGNC Symbol;Acc:VGNC:88478]	2.07	0.0098
ssc-miR-30d	GLI2	GLI family zinc finger 2 [Source:VGNC Symbol;Acc:VGNC:96324]	2.07	0.0098

ssc-miR-30d	GLUD1	glutamate dehydrogenase 1 [Source:NCBI gene (formerly Entrezgene);Acc:100157162]	2.07	0.0098
ssc-miR-30d	GLUD2	hypothetical gene	2.07	0.0098
ssc-miR-30d	GMEB1	glucocorticoid modulatory element binding protein 1 [Source:VGNC Symbol;Acc:VGNC:88508]	2.07	0.0098
ssc-miR-30d	GMEB2	glucocorticoid modulatory element binding protein 2 [Source:VGNC Symbol;Acc:VGNC:96326]	2.07	0.0098
ssc-miR-30d	GMNC	geminin coiled-coil domain containing [Source:VGNC Symbol;Acc:VGNC:88512]	2.07	0.0098
ssc-miR-30d	GNA13	G protein subunit alpha 13 [Source:VGNC Symbol;Acc:VGNC:98997]	2.07	0.0098
ssc-miR-30d	GNAI2	G protein subunit alpha i2 [Source:VGNC Symbol;Acc:VGNC:88522]	2.07	0.0098
ssc-miR-30d	GNAO1	hypothetical gene	2.07	0.0098
ssc-miR-30d	GNAQ	G protein subunit alpha q [Source:VGNC Symbol;Acc:VGNC:103100]	2.07	0.0098
ssc-miR-30d	GNG10	G protein subunit gamma 10 [Source:HGNC Symbol;Acc:HGNC:4402]	2.07	0.0098
ssc-miR-30d	GNPDA1	glucosamine-6-phosphate deaminase 1 [Source:VGNC Symbol;Acc:VGNC:88544]	2.07	0.0098
ssc-miR-30d	GOLGA1	golgin A1 [Source:VGNC Symbol;Acc:VGNC:88549]	2.07	0.0098
ssc-miR-30d	GOLGA4	hypothetical gene	2.07	0.0098
ssc-miR-30d	GOLGA6A	hypothetical gene	2.07	0.0098
ssc-miR-30d	GOLGA6B	hypothetical gene	2.07	0.0098
ssc-miR-30d	GOLGA6C	hypothetical gene	2.07	0.0098
ssc-miR-30d	GOLGA6D	hypothetical gene	2.07	0.0098
ssc-miR-30d	GOLGA6L10	hypothetical gene	2.07	0.0098
ssc-miR-30d	GOLGA6L4	hypothetical gene	2.07	0.0098
ssc-miR-30d	GOLGA6L9	hypothetical gene	2.07	0.0098
ssc-miR-30d	GOLGA8B	hypothetical gene	2.07	0.0098
ssc-miR-30d	GOT2	glutamic-oxaloacetic transaminase 2 [Source:VGNC Symbol;Acc:VGNC:88562]	2.07	0.0098
ssc-miR-30d	GPCPD1	glycerophosphocholine phosphodiesterase 1 [Source:VGNC Symbol;Acc:VGNC:96148]	2.07	0.0098
ssc-miR-30d	GPD1L	glycerol-3-phosphate dehydrogenase 1 like [Source:VGNC Symbol;Acc:VGNC:108661]	2.07	0.0098
ssc-miR-30d	GPR108	G protein-coupled receptor 108 [Source:VGNC Symbol;Acc:VGNC:88596]	2.07	0.0098
ssc-miR-30d	GPR124	hypothetical gene	2.07	0.0098
ssc-miR-30d	GPR125	hypothetical gene	2.07	0.0098
ssc-miR-30d	GPR137C	G protein-coupled receptor 137C [Source:VGNC Symbol;Acc:VGNC:88602]	2.07	0.0098
ssc-miR-30d	GPR150	G protein-coupled receptor 150 [Source:VGNC Symbol;Acc:VGNC:88607]	2.07	0.0098
ssc-miR-30d	GPR156	G protein-coupled receptor 156 [Source:VGNC Symbol;Acc:VGNC:88610]	2.07	0.0098
ssc-miR-30d	GPR157	G protein-coupled receptor 157 [Source:VGNC Symbol;Acc:VGNC:88611]	2.07	0.0098
ssc-miR-30d	GPR161	G protein-coupled receptor 161 [Source:VGNC Symbol;Acc:VGNC:88613]	2.07	0.0098
ssc-miR-30d	GPR180	G protein-coupled receptor 180 [Source:VGNC Symbol;Acc:VGNC:88621]	2.07	0.0098
ssc-miR-30d	GPR26	G protein-coupled receptor 26 [Source:VGNC Symbol;Acc:VGNC:88626]	2.07	0.0098
ssc-miR-30d	GPR63	G protein-coupled receptor 63 [Source:VGNC Symbol;Acc:VGNC:88634]	2.07	0.0098
ssc-miR-30d	GPR75-ASB3	hypothetical gene	2.07	0.0098
ssc-miR-30d	GPRIN3	hypothetical gene	2.07	0.0098
ssc-miR-30d	GPT2	glutamic--pyruvic transaminase 2 [Source:VGNC Symbol;Acc:VGNC:88651]	2.07	0.0098
ssc-miR-30d	GRAMD2	hypothetical gene	2.07	0.0098
ssc-miR-30d	GRB10	growth factor receptor bound protein 10 [Source:VGNC Symbol;Acc:VGNC:88662]	2.07	0.0098
ssc-miR-30d	GREB1L	GREB1 like retinoic acid receptor coactivator [Source:VGNC Symbol;Acc:VGNC:96584]	2.07	0.0098
ssc-miR-30d	GRHL1	grainyhead like transcription factor 1 [Source:VGNC Symbol;Acc:VGNC:88666]	2.07	0.0098
ssc-miR-30d	GRHL2	grainyhead like transcription factor 2 [Source:VGNC Symbol;Acc:VGNC:88667]	2.07	0.0098
ssc-miR-30d	GRIA2	glutamate ionotropic receptor AMPA type subunit 2 [Source:VGNC Symbol;Acc:VGNC:88671]	2.07	0.0098
ssc-miR-30d	GRIN2A	glutamate ionotropic receptor NMDA type subunit 2A [Source:VGNC Symbol;Acc:VGNC:88683]	2.07	0.0098
ssc-miR-30d	GRIN3A	glutamate ionotropic receptor NMDA type subunit 3A [Source:VGNC Symbol;Acc:VGNC:88687]	2.07	0.0098
ssc-miR-30d	GRK5	G protein-coupled receptor kinase 5 [Source:VGNC Symbol;Acc:VGNC:88697]	2.07	0.0098
ssc-miR-30d	GRK6	G protein-coupled receptor kinase 6 [Source:VGNC Symbol;Acc:VGNC:88698]	2.07	0.0098
ssc-miR-30d	GRM3	glutamate metabotropic receptor 3 [Source:VGNC Symbol;Acc:VGNC:88702]	2.07	0.0098

ssc-miR-30d	GRM5	glutamate metabotropic receptor 5 [Source:VGNC Symbol;Acc:VGNC:88703]	2.07	0.0098
ssc-miR-30d	GSKIP	GSK3B interacting protein [Source:VGNC Symbol;Acc:VGNC:103956]	2.07	0.0098
ssc-miR-30d	GTDC1	glycosyltransferase like domain containing 1 [Source:VGNC Symbol;Acc:VGNC:95955]	2.07	0.0098
ssc-miR-30d	GTF2H1	ral transcription factor IIH subunit 1 [Source:VGNC Symbol;Acc:VGNC:88734]	2.07	0.0098
ssc-miR-30d	GUF1	GTP binding elongation factor GUF1 [Source:VGNC Symbol;Acc:VGNC:88754]	2.07	0.0098
ssc-miR-30d	GXYLT1	glucoside xylosyltransferase 1 [Source:VGNC Symbol;Acc:VGNC:88756]	2.07	0.0098
ssc-miR-30d	GZF1	GDNF inducible zinc finger protein 1 [Source:VGNC Symbol;Acc:VGNC:108722]	2.07	0.0098
ssc-miR-30d	H2AFY	hypothetical gene	2.07	0.0098
ssc-miR-30d	H6PD	hexose-6-phosphate dehydrogenase/glucose 1-dehydrogenase [Source:VGNC Symbol;Acc:VGNC:88764]	2.07	0.0098
ssc-miR-30d	HACE1	HECT domain and ankyrin repeat containing E3 ubiquitin protein ligase 1 [Source:VGNC Symbol;Acc:VGNC:88769]	2.07	0.0098
ssc-miR-30d	HAS2	hyaluronan synthase 2 [Source:HGNC Symbol;Acc:HGNC:4819]	2.07	0.0098
ssc-miR-30d	HBS1L	HBS1 like translational GTPase [Source:VGNC Symbol;Acc:VGNC:88796]	2.07	0.0098
ssc-miR-30d	HCAR2	hypothetical gene	2.07	0.0098
ssc-miR-30d	HCAR3	hypothetical gene	2.07	0.0098
ssc-miR-30d	HCFC2	host cell factor C2 [Source:VGNC Symbol;Acc:VGNC:88800]	2.07	0.0098
ssc-miR-30d	HCN1	hyperpolarization activated cyclic nucleotide gated potassium channel 1 [Source:VGNC Symbol;Acc:VGNC:88802]	2.07	0.0098
ssc-miR-30d	HDAC5	histone deacetylase 5 [Source:VGNC Symbol;Acc:VGNC:88815]	2.07	0.0098
ssc-miR-30d	HDAC9	histone deacetylase 9 [Source:HGNC Symbol;Acc:HGNC:14065]	2.07	0.0098
ssc-miR-30d	HECTD4	HECT domain E3 ubiquitin protein ligase 4 [Source:VGNC Symbol;Acc:VGNC:88834]	2.07	0.0098
ssc-miR-30d	HECW1	HECT, C2 and WW domain containing E3 ubiquitin protein ligase 1 [Source:VGNC Symbol;Acc:VGNC:88835]	2.07	0.0098
ssc-miR-30d	HELZ	helicase with zinc finger [Source:VGNC Symbol;Acc:VGNC:88840]	2.07	0.0098
ssc-miR-30d	HEPHL1	hephaestin like 1 [Source:VGNC Symbol;Acc:VGNC:88848]	2.07	0.0098
ssc-miR-30d	HERC2	HECT and RLD domain containing E3 ubiquitin protein ligase 2 [Source:VGNC Symbol;Acc:VGNC:99716]	2.07	0.0098
ssc-miR-30d	HERC3	HECT and RLD domain containing E3 ubiquitin protein ligase 3 [Source:VGNC Symbol;Acc:VGNC:98934]	2.07	0.0098
ssc-miR-30d	HHIPL1	hypothetical gene	2.07	0.0098
ssc-miR-30d	HIAT1	hypothetical gene	2.07	0.0098
ssc-miR-30d	HIC2	HIC ZBTB transcriptional repressor 2 [Source:HGNC Symbol;Acc:HGNC:18595]	2.07	0.0098
ssc-miR-30d	HIF1AN	hypoxia inducible factor 1 subunit alpha inhibitor [Source:VGNC Symbol;Acc:VGNC:98033]	2.07	0.0098
ssc-miR-30d	HIPK1	homeodomain interacting protein kinase 1 [Source:VGNC Symbol;Acc:VGNC:88887]	2.07	0.0098
ssc-miR-30d	HIPK2	homeodomain interacting protein kinase 2 [Source:VGNC Symbol;Acc:VGNC:88888]	2.07	0.0098
ssc-miR-30d	HIRIP3	HIRA interacting protein 3 [Source:HGNC Symbol;Acc:HGNC:4917]	2.07	0.0098
ssc-miR-30d	HIVEP1	HIVEP zinc finger 1 [Source:VGNC Symbol;Acc:VGNC:96585]	2.07	0.0098
ssc-miR-30d	HLF	HLF transcription factor, PAR bZIP family member [Source:VGNC Symbol;Acc:VGNC:88896]	2.07	0.0098
ssc-miR-30d	HLX	H2.0 like homeobox [Source:VGNC Symbol;Acc:VGNC:96345]	2.07	0.0098
ssc-miR-30d	HMGB3	hypothetical gene	2.07	0.0098
ssc-miR-30d	HNRNPA1	heterous nuclear ribonucleoprotein A1 [Source:VGNC Symbol;Acc:VGNC:88918]	2.07	0.0098
ssc-miR-30d	HNRNPA2B1	heterogeneous nuclear ribonucleoprotein A2/B1 [Source:HGNC Symbol;Acc:HGNC:5033]	2.07	0.0098
ssc-miR-30d	HNRNPA3	hypothetical gene	2.07	0.0098
ssc-miR-30d	HNRNPC	hypothetical gene	2.07	0.0098
ssc-miR-30d	HNRNPUL2	heterous nuclear ribonucleoprotein U like 2 [Source:VGNC Symbol;Acc:VGNC:99758]	2.07	0.0098
ssc-miR-30d	HOOK3	hook microtubule tethering protein 3 [Source:VGNC Symbol;Acc:VGNC:98037]	2.07	0.0098
ssc-miR-30d	HOXA1	homeobox A1 [Source:VGNC Symbol;Acc:VGNC:88933]	2.07	0.0098
ssc-miR-30d	HOXA11	homeobox A11 [Source:VGNC Symbol;Acc:VGNC:88935]	2.07	0.0098
ssc-miR-30d	HOXA2	homeobox A2 [Source:VGNC Symbol;Acc:VGNC:88937]	2.07	0.0098
ssc-miR-30d	HOXA9	homeobox A9 [Source:HGNC Symbol;Acc:HGNC:5109]	2.07	0.0098
ssc-miR-30d	HOXB3	homeobox B3 [Source:VGNC Symbol;Acc:VGNC:88944]	2.07	0.0098
ssc-miR-30d	HOXB4	homeobox B4 [Source:HGNC Symbol;Acc:HGNC:5115]	2.07	0.0098
ssc-miR-30d	HOXB8	homeobox B8 [Source:VGNC Symbol;Acc:VGNC:88948]	2.07	0.0098
ssc-miR-30d	HOXD11	homeobox D11 [Source:VGNC Symbol;Acc:VGNC:96352]	2.07	0.0098

ssc-miR-30d	HPCAL1	hippocalcin like 1 [Source:VGNC Symbol;Acc:VGNC:88958]	2.07	0.0098
ssc-miR-30d	HPS4	HPS4 biosis of lysosomal organelles complex 3 subunit 2 [Source:VGNC Symbol;Acc:VGNC:88966]	2.07	0.0098
ssc-miR-30d	HSPA2	hypothetical gene	2.07	0.0098
ssc-miR-30d	HSPA4L	heat shock protein family A (Hsp70) member 4 like [Source:HGNC Symbol;Acc:HGNC:17041]	2.07	0.0098
ssc-miR-30d	HSPA5	heat shock protein family A (Hsp70) member 5 [Source:VGNC Symbol;Acc:VGNC:103107]	2.07	0.0098
ssc-miR-30d	HSPD1	heat shock protein family D (Hsp60) member 1 [Source:NCBI gene (formerly Entrezgene);Acc:492279]	2.07	0.0098
ssc-miR-30d	HSPE1-MOB4	hypothetical gene	2.07	0.0098
ssc-miR-30d	HTRA3	HtrA serine peptidase 3 [Source:VGNC Symbol;Acc:VGNC:89005]	2.07	0.0098
ssc-miR-30d	ICK	hypothetical gene	2.07	0.0098
ssc-miR-30d	IDE	insulin degrading enzyme [Source:VGNC Symbol;Acc:VGNC:89025]	2.07	0.0098
ssc-miR-30d	IDH1	isocitrate dehydrogenase (NADP(+)) 1 [Source:HGNC Symbol;Acc:HGNC:5382]	2.07	0.0098
ssc-miR-30d	IER2	immediate early response 2 [Source:HGNC Symbol;Acc:HGNC:28871]	2.07	0.0098
ssc-miR-30d	IER5	immediate early response 5 [Source:VGNC Symbol;Acc:VGNC:89028]	2.07	0.0098
ssc-miR-30d	IFFO2	intermediate filament family orphan 2 [Source:VGNC Symbol;Acc:VGNC:98475]	2.07	0.0098
ssc-miR-30d	IFNAR2	interferon alpha and beta receptor subunit 2 [Source:NCBI gene (formerly Entrezgene);Acc:100533555]	2.07	0.0098
ssc-miR-30d	IFNLR1	interferon lambda receptor 1 [Source:VGNC Symbol;Acc:VGNC:89043]	2.07	0.0098
ssc-miR-30d	IGF1R	insulin like growth factor 1 receptor [Source:NCBI gene (formerly Entrezgene);Acc:397350]	2.07	0.0098
ssc-miR-30d	IGF2R	insulin like growth factor 2 receptor [Source:VGNC Symbol;Acc:VGNC:89057]	2.07	0.0098
ssc-miR-30d	IGSF3	immunoglobulin superfamily member 3 [Source:VGNC Symbol;Acc:VGNC:89067]	2.07	0.0098
ssc-miR-30d	IKZF2	IKAROS family zinc finger 2 [Source:VGNC Symbol;Acc:VGNC:95576]	2.07	0.0098
ssc-miR-30d	IL1A	interleukin 1 alpha [Source:VGNC Symbol;Acc:VGNC:89091]	2.07	0.0098
ssc-miR-30d	IL1RAPL2	interleukin 1 receptor accessory protein like 2 [Source:VGNC Symbol;Acc:VGNC:103968]	2.07	0.0098
ssc-miR-30d	IL2RA	interleukin 2 receptor subunit alpha [Source:VGNC Symbol;Acc:VGNC:108275]	2.07	0.0098
ssc-miR-30d	ILDR2	immunoglobulin like domain containing receptor 2 [Source:VGNC Symbol;Acc:VGNC:89118]	2.07	0.0098
ssc-miR-30d	ING5	inhibitor of growth family member 5 [Source:VGNC Symbol;Acc:VGNC:95912]	2.07	0.0098
ssc-miR-30d	INHBA	inhibin subunit beta A [Source:VGNC Symbol;Acc:VGNC:89133]	2.07	0.0098
ssc-miR-30d	INO80D	INO80 complex subunit D [Source:VGNC Symbol;Acc:VGNC:96113]	2.07	0.0098
ssc-miR-30d	INPP4A	inositol polyphosphate-4-phosphatase type I A [Source:VGNC Symbol;Acc:VGNC:89140]	2.07	0.0098
ssc-miR-30d	INSIG2	insulin induced 2 [Source:VGNC Symbol;Acc:VGNC:103970]	2.07	0.0098
ssc-miR-30d	INTS2	integrator complex subunit 2 [Source:VGNC Symbol;Acc:VGNC:89162]	2.07	0.0098
ssc-miR-30d	IP6K3	inositol hexakisphosphate kinase 3 [Source:VGNC Symbol;Acc:VGNC:89174]	2.07	0.0098
ssc-miR-30d	IPMK	inositol polyphosphate multikinase [Source:VGNC Symbol;Acc:VGNC:89175]	2.07	0.0098
ssc-miR-30d	IQCG	IQ motif containing G [Source:VGNC Symbol;Acc:VGNC:89189]	2.07	0.0098
ssc-miR-30d	IRF2BP2	interferon regulatory factor 2 binding protein 2 [Source:VGNC Symbol;Acc:VGNC:89205]	2.07	0.0098
ssc-miR-30d	IRF4	interferon regulatory factor 4 [Source:VGNC Symbol;Acc:VGNC:89207]	2.07	0.0098
ssc-miR-30d	IRS1	insulin receptor substrate 1 [Source:VGNC Symbol;Acc:VGNC:96376]	2.07	0.0098
ssc-miR-30d	IRS2	insulin receptor substrate 2 [Source:VGNC Symbol;Acc:VGNC:89214]	2.07	0.0098
ssc-miR-30d	IRX4	iroquois homeobox 4 [Source:VGNC Symbol;Acc:VGNC:89218]	2.07	0.0098
ssc-miR-30d	ISG20L2	interferon stimulated exonuclease 20 like 2 [Source:VGNC Symbol;Acc:VGNC:89224]	2.07	0.0098
ssc-miR-30d	ITGA2	integrin subunit alpha 2 [Source:VGNC Symbol;Acc:VGNC:89234]	2.07	0.0098
ssc-miR-30d	ITGA4	integrin subunit alpha 4 [Source:VGNC Symbol;Acc:VGNC:96377]	2.07	0.0098
ssc-miR-30d	ITGA6	integrin subunit alpha 6 [Source:VGNC Symbol;Acc:VGNC:96378]	2.07	0.0098
ssc-miR-30d	ITGA8	integrin subunit alpha 8 [Source:VGNC Symbol;Acc:VGNC:96379]	2.07	0.0098
ssc-miR-30d	ITGA9	integrin subunit alpha 9 [Source:VGNC Symbol;Acc:VGNC:89238]	2.07	0.0098
ssc-miR-30d	ITGB3	integrin subunit beta 3 [Source:NCBI gene (formerly Entrezgene);Acc:397063]	2.07	0.0098
ssc-miR-30d	ITPK1	inositol-tetrakisphosphate 1-kinase [Source:VGNC Symbol;Acc:VGNC:89251]	2.07	0.0098
ssc-miR-30d	ITSN1	intersectin 1 [Source:VGNC Symbol;Acc:VGNC:108669]	2.07	0.0098
ssc-miR-30d	JAG2	jagged canonical Notch ligand 2 [Source:VGNC Symbol;Acc:VGNC:89269]	2.07	0.0098
ssc-miR-30d	JAK1	Janus kinase 1 [Source:VGNC Symbol;Acc:VGNC:89270]	2.07	0.0098

ssc-miR-30d	JAKMIP2	janus kinase and microtubule interacting protein 2 [Source:VGNC Symbol;Acc:VGNC:89274]	2.07	0.0098
ssc-miR-30d	JAKMIP3	Janus kinase and microtubule interacting protein 3 [Source:VGNC Symbol;Acc:VGNC:89275]	2.07	0.0098
ssc-miR-30d	JAM2	junctional adhesion molecule 2 [Source:VGNC Symbol;Acc:VGNC:89276]	2.07	0.0098
ssc-miR-30d	JARID2	jumonji and AT-rich interaction domain containing 2 [Source:VGNC Symbol;Acc:VGNC:89279]	2.07	0.0098
ssc-miR-30d	JDP2	Jun dimerization protein 2 [Source:VGNC Symbol;Acc:VGNC:89282]	2.07	0.0098
ssc-miR-30d	JMY	junction mediating and regulatory protein, p53 cofactor [Source:VGNC Symbol;Acc:VGNC:89289]	2.07	0.0098
ssc-miR-30d	JOSD1	Josephin domain containing 1 [Source:VGNC Symbol;Acc:VGNC:89290]	2.07	0.0098
ssc-miR-30d	JPH4	junctophilin 4 [Source:VGNC Symbol;Acc:VGNC:89292]	2.07	0.0098
ssc-miR-30d	JUNB	JunB proto-onco, AP-1 transcription factor subunit [Source:VGNC Symbol;Acc:VGNC:89295]	2.07	0.0098
ssc-miR-30d	KATNBL1	katanin regulatory subunit B1 like 1 [Source:VGNC Symbol;Acc:VGNC:89313]	2.07	0.0098
ssc-miR-30d	KCNA4	potassium voltage-gated channel subfamily A member 4 [Source:VGNC Symbol;Acc:VGNC:89326]	2.07	0.0098
ssc-miR-30d	KCNJ12	hypothetical gene	2.07	0.0098
ssc-miR-30d	KCNJ15	potassium inwardly rectifying channel subfamily J member 15 [Source:VGNC Symbol;Acc:VGNC:89355]	2.07	0.0098
ssc-miR-30d	KCNJ3	potassium inwardly rectifying channel subfamily J member 3 [Source:HGNC Symbol;Acc:HGNC:6264]	2.07	0.0098
ssc-miR-30d	KCNJ6	potassium inwardly rectifying channel subfamily J member 6 [Source:VGNC Symbol;Acc:VGNC:89360]	2.07	0.0098
ssc-miR-30d	KCNN3	potassium calcium-activated channel subfamily N member 3 [Source:VGNC Symbol;Acc:VGNC:98056]	2.07	0.0098
ssc-miR-30d	KCTD16	potassium channel tetramerization domain containing 16 [Source:VGNC Symbol;Acc:VGNC:89395]	2.07	0.0098
ssc-miR-30d	KCTD20	potassium channel tetramerization domain containing 20 [Source:VGNC Symbol;Acc:VGNC:89399]	2.07	0.0098
ssc-miR-30d	KCTD3	potassium channel tetramerization domain containing 3 [Source:VGNC Symbol;Acc:VGNC:95963]	2.07	0.0098
ssc-miR-30d	KCTD5	hypothetical gene	2.07	0.0098
ssc-miR-30d	KCTD7	potassium channel tetramerization domain containing 7 [Source:VGNC Symbol;Acc:VGNC:89403]	2.07	0.0098
ssc-miR-30d	KCTD8	potassium channel tetramerization domain containing 8 [Source:HGNC Symbol;Acc:HGNC:22394]	2.07	0.0098
ssc-miR-30d	KDM3A	lysine demethylase 3A [Source:VGNC Symbol;Acc:VGNC:89411]	2.07	0.0098
ssc-miR-30d	KDM5B	lysine demethylase 5B [Source:VGNC Symbol;Acc:VGNC:95870]	2.07	0.0098
ssc-miR-30d	KDSR	3-ketodihydrosphingosine reductase [Source:VGNC Symbol;Acc:VGNC:108157]	2.07	0.0098
ssc-miR-30d	KHNYN	KH and NYN domain containing [Source:VGNC Symbol;Acc:VGNC:89426]	2.07	0.0098
ssc-miR-30d	KIAA0087	hypothetical gene	2.07	0.0098
ssc-miR-30d	KIAA0101	hypothetical gene	2.07	0.0098
ssc-miR-30d	KIAA0226L	hypothetical gene	2.07	0.0098
ssc-miR-30d	KIAA0247	hypothetical gene	2.07	0.0098
ssc-miR-30d	KIAA0355	hypothetical gene	2.07	0.0098
ssc-miR-30d	KIAA0408	KIAA0408 [Source:VGNC Symbol;Acc:VGNC:89432]	2.07	0.0098
ssc-miR-30d	KIAA0930	KIAA0930 [Source:VGNC Symbol;Acc:VGNC:89438]	2.07	0.0098
ssc-miR-30d	KIAA1024	hypothetical gene	2.07	0.0098
ssc-miR-30d	KIAA1033	hypothetical gene	2.07	0.0098
ssc-miR-30d	KIAA1147	hypothetical gene	2.07	0.0098
ssc-miR-30d	KIAA1211	hypothetical gene	2.07	0.0098
ssc-miR-30d	KIAA1211L	hypothetical gene	2.07	0.0098
ssc-miR-30d	KIAA1244	hypothetical gene	2.07	0.0098
ssc-miR-30d	KIAA1522	KIAA1522 [Source:VGNC Symbol;Acc:VGNC:89444]	2.07	0.0098
ssc-miR-30d	KIAA1549	KIAA1549 [Source:VGNC Symbol;Acc:VGNC:99719]	2.07	0.0098
ssc-miR-30d	KIAA1715	hypothetical gene	2.07	0.0098
ssc-miR-30d	KIAA2026	KIAA2026 [Source:VGNC Symbol;Acc:VGNC:89450]	2.07	0.0098
ssc-miR-30d	KIF16B	kinesin family member 16B [Source:VGNC Symbol;Acc:VGNC:106450]	2.07	0.0098
ssc-miR-30d	KIF1C	kinesin family member 1C [Source:VGNC Symbol;Acc:VGNC:89461]	2.07	0.0098
ssc-miR-30d	KIF21B	kinesin family member 21B [Source:VGNC Symbol;Acc:VGNC:96219]	2.07	0.0098
ssc-miR-30d	KIF3A	kinesin family member 3A [Source:VGNC Symbol;Acc:VGNC:89470]	2.07	0.0098
ssc-miR-30d	KIF3C	kinesin family member 3C [Source:VGNC Symbol;Acc:VGNC:89471]	2.07	0.0098
ssc-miR-30d	KLF10	Kruppel like factor 10 [Source:VGNC Symbol;Acc:VGNC:89490]	2.07	0.0098

ssc-miR-30d	KLF11	Kruppel like factor 11 [Source:VGNC Symbol;Acc:VGNC:89491]	2.07	0.0098
ssc-miR-30d	KLF12	Kruppel like factor 12 [Source:VGNC Symbol;Acc:VGNC:89492]	2.07	0.0098
ssc-miR-30d	KLF13	Kruppel like factor 13 [Source:VGNC Symbol;Acc:VGNC:89493]	2.07	0.0098
ssc-miR-30d	KLF14	Kruppel like factor 14 [Source:VGNC Symbol;Acc:VGNC:89494]	2.07	0.0098
ssc-miR-30d	KLF8	Kruppel like factor 8 [Source:VGNC Symbol;Acc:VGNC:89500]	2.07	0.0098
ssc-miR-30d	KLF9	Kruppel like factor 9 [Source:VGNC Symbol;Acc:VGNC:103114]	2.07	0.0098
ssc-miR-30d	KLHL20	kelch like family member 20 [Source:VGNC Symbol;Acc:VGNC:89519]	2.07	0.0098
ssc-miR-30d	KLHL23	kelch like family member 23 [Source:VGNC Symbol;Acc:VGNC:96158]	2.07	0.0098
ssc-miR-30d	KLHL24	kelch like family member 24 [Source:VGNC Symbol;Acc:VGNC:89521]	2.07	0.0098
ssc-miR-30d	KLHL28	kelch like family member 28 [Source:VGNC Symbol;Acc:VGNC:89523]	2.07	0.0098
ssc-miR-30d	KMT2A	lysine methyltransferase 2A [Source:VGNC Symbol;Acc:VGNC:108600]	2.07	0.0098
ssc-miR-30d	KMT2C	lysine methyltransferase 2C [Source:VGNC Symbol;Acc:VGNC:89550]	2.07	0.0098
ssc-miR-30d	KMT2D	hypothetical gene	2.07	0.0098
ssc-miR-30d	KPNA3	karyopherin subunit alpha 3 [Source:VGNC Symbol;Acc:VGNC:89562]	2.07	0.0098
ssc-miR-30d	KPNA6	karyopherin subunit alpha 6 [Source:VGNC Symbol;Acc:VGNC:89565]	2.07	0.0098
ssc-miR-30d	KRAS	KRAS proto-onco, GTPase [Source:VGNC Symbol;Acc:VGNC:89569]	2.07	0.0098
ssc-miR-30d	KREMEN1	kringle containing transmembrane protein 1 [Source:VGNC Symbol;Acc:VGNC:89572]	2.07	0.0098
ssc-miR-30d	KSR1	kinase suppressor of ras 1 [Source:VGNC Symbol;Acc:VGNC:89606]	2.07	0.0098
ssc-miR-30d	KXD1	KxDL motif containing 1 [Source:HGNC Symbol;Acc:HGNC:28420]	2.07	0.0098
ssc-miR-30d	LARGE	hypothetical gene	2.07	0.0098
ssc-miR-30d	LARP1	La ribonucleoprotein 1, translational regulator [Source:VGNC Symbol;Acc:VGNC:98067]	2.07	0.0098
ssc-miR-30d	LARP1B	La ribonucleoprotein 1B [Source:VGNC Symbol;Acc:VGNC:89640]	2.07	0.0098
ssc-miR-30d	LARP4	La ribonucleoprotein 4 [Source:VGNC Symbol;Acc:VGNC:89641]	2.07	0.0098
ssc-miR-30d	LATS2	large tumor suppressor kinase 2 [Source:VGNC Symbol;Acc:VGNC:89649]	2.07	0.0098
ssc-miR-30d	LAYN	layilin [Source:VGNC Symbol;Acc:VGNC:89651]	2.07	0.0098
ssc-miR-30d	LCLAT1	lysocardiolipin acyltransferase 1 [Source:VGNC Symbol;Acc:VGNC:89658]	2.07	0.0098
ssc-miR-30d	LCOR	ligand dependent nuclear receptor corepressor [Source:HGNC Symbol;Acc:HGNC:29503]	2.07	0.0098
ssc-miR-30d	LDLR	low density lipoprotein receptor [Source:VGNC Symbol;Acc:VGNC:89671]	2.07	0.0098
ssc-miR-30d	LEPR	leptin receptor [Source:VGNC Symbol;Acc:VGNC:89685]	2.07	0.0098
ssc-miR-30d	LETMD1	LETM1 domain containing 1 [Source:VGNC Symbol;Acc:VGNC:89688]	2.07	0.0098
ssc-miR-30d	LGI1	leucine rich glioma inactivated 1 [Source:HGNC Symbol;Acc:HGNC:6572]	2.07	0.0098
ssc-miR-30d	LHFPL2	LHFPL tetraspan subfamily member 2 [Source:VGNC Symbol;Acc:VGNC:89705]	2.07	0.0098
ssc-miR-30d	LHX1	LIM homeobox 1 [Source:VGNC Symbol;Acc:VGNC:89711]	2.07	0.0098
ssc-miR-30d	LHX5	LIM homeobox 5 [Source:VGNC Symbol;Acc:VGNC:89715]	2.07	0.0098
ssc-miR-30d	LHX8	LIM homeobox 8 [Source:VGNC Symbol;Acc:VGNC:89717]	2.07	0.0098
ssc-miR-30d	LHX9	LIM homeobox 9 [Source:VGNC Symbol;Acc:VGNC:95608]	2.07	0.0098
ssc-miR-30d	LIFR	LIF receptor subunit alpha [Source:HGNC Symbol;Acc:HGNC:6597]	2.07	0.0098
ssc-miR-30d	LIMCH1	LIM and calponin homology domains 1 [Source:VGNC Symbol;Acc:VGNC:89724]	2.07	0.0098
ssc-miR-30d	LIN28A	lin-28 homolog A [Source:VGNC Symbol;Acc:VGNC:98492]	2.07	0.0098
ssc-miR-30d	LIN28B	lin-28 homolog B [Source:VGNC Symbol;Acc:VGNC:89729]	2.07	0.0098
ssc-miR-30d	LIN7C	lin-7 homolog C, crumbs cell polarity complex component [Source:VGNC Symbol;Acc:VGNC:89732]	2.07	0.0098
ssc-miR-30d	LMBR1	limb development membrane protein 1 [Source:VGNC Symbol;Acc:VGNC:89752]	2.07	0.0098
ssc-miR-30d	LMBR1L	limb development membrane protein 1 like [Source:VGNC Symbol;Acc:VGNC:89753]	2.07	0.0098
ssc-miR-30d	LMLN	leishmanolysin like peptidase [Source:VGNC Symbol;Acc:VGNC:89759]	2.07	0.0098
ssc-miR-30d	LONRF1	LON peptidase N-terminal domain and ring finger 1 [Source:VGNC Symbol;Acc:VGNC:96123]	2.07	0.0098
ssc-miR-30d	LONRF3	LON peptidase N-terminal domain and ring finger 3 [Source:VGNC Symbol;Acc:VGNC:89778]	2.07	0.0098
ssc-miR-30d	LOX	lysyl oxidase [Source:VGNC Symbol;Acc:VGNC:99785]	2.07	0.0098
ssc-miR-30d	LPAR3	lysophosphatidic acid receptor 3 [Source:VGNC Symbol;Acc:VGNC:89785]	2.07	0.0098
ssc-miR-30d	LPGAT1	lysophosphatidylglycerol acyltransferase 1 [Source:VGNC Symbol;Acc:VGNC:89792]	2.07	0.0098

ssc-miR-30d	LPHN3	hypothetical gene	2.07	0.0098
ssc-miR-30d	LPP	LIM domain containing preferred translocation partner in lipoma [Source:HGNC Symbol;Acc:HGNC:6679]	2.07	0.0098
ssc-miR-30d	LPPR4	hypothetical gene	2.07	0.0098
ssc-miR-30d	LRCH2	leucine rich repeats and calponin homology domain containing 2 [Source:VGNC Symbol;Acc:VGNC:89802]	2.07	0.0098
ssc-miR-30d	LRFN2	leucine rich repeat and fibronectin type III domain containing 2 [Source:VGNC Symbol;Acc:VGNC:89805]	2.07	0.0098
ssc-miR-30d	LRIG2	leucine rich repeats and immunoglobulin like domains 2 [Source:VGNC Symbol;Acc:VGNC:89811]	2.07	0.0098
ssc-miR-30d	LRP6	LDL receptor related protein 6 [Source:VGNC Symbol;Acc:VGNC:89821]	2.07	0.0098
ssc-miR-30d	LRRC17	leucine rich repeat containing 17 [Source:VGNC Symbol;Acc:VGNC:89828]	2.07	0.0098
ssc-miR-30d	LRRC40	hypothetical gene	2.07	0.0098
ssc-miR-30d	LRRC58	leucine rich repeat containing 58 [Source:VGNC Symbol;Acc:VGNC:98081]	2.07	0.0098
ssc-miR-30d	LRRC8C	leucine rich repeat containing 8 VRAC subunit C [Source:VGNC Symbol;Acc:VGNC:98087]	2.07	0.0098
ssc-miR-30d	LRRC8D	leucine rich repeat containing 8 VRAC subunit D [Source:VGNC Symbol;Acc:VGNC:98088]	2.07	0.0098
ssc-miR-30d	LRRK2	leucine rich repeat kinase 2 [Source:VGNC Symbol;Acc:VGNC:98094]	2.07	0.0098
ssc-miR-30d	LSM14B	LSM family member 14B [Source:VGNC Symbol;Acc:VGNC:95691]	2.07	0.0098
ssc-miR-30d	LUC7L2	LUC7 like 2, pre-mRNA splicing factor [Source:HGNC Symbol;Acc:HGNC:21608]	2.07	0.0098
ssc-miR-30d	LY75-CD302	hypothetical gene	2.07	0.0098
ssc-miR-30d	LYN	LYN proto-onco, Src family tyrosine kinase [Source:VGNC Symbol;Acc:VGNC:89910]	2.07	0.0098
ssc-miR-30d	LYSMD3	LysM domain containing 3 [Source:VGNC Symbol;Acc:VGNC:89919]	2.07	0.0098
ssc-miR-30d	LYST	lysosomal trafficking regulator [Source:VGNC Symbol;Acc:VGNC:89921]	2.07	0.0098
ssc-miR-30d	MAB21L1	mab-21 like 1 [Source:VGNC Symbol;Acc:VGNC:89932]	2.07	0.0098
ssc-miR-30d	MAF	MAF bZIP transcription factor [Source:VGNC Symbol;Acc:VGNC:89945]	2.07	0.0098
ssc-miR-30d	MAFG	MAF bZIP transcription factor G [Source:VGNC Symbol;Acc:VGNC:89948]	2.07	0.0098
ssc-miR-30d	MAGI2	membrane associated guanylate kinase, WW and PDZ domain containing 2 [Source:VGNC Symbol;Acc:VGNC:89955]	2.07	0.0098
ssc-miR-30d	MAGI3	membrane associated guanylate kinase, WW and PDZ domain containing 3 [Source:VGNC Symbol;Acc:VGNC:89956]	2.07	0.0098
ssc-miR-30d	MAL	hypothetical gene	2.07	0.0098
ssc-miR-30d	MAML1	mastermind like transcriptional coactivator 1 [Source:VGNC Symbol;Acc:VGNC:89963]	2.07	0.0098
ssc-miR-30d	MAN1A2	mannosidase alpha class 1A member 2 [Source:VGNC Symbol;Acc:VGNC:89967]	2.07	0.0098
ssc-miR-30d	MAN1B1	hypothetical gene	2.07	0.0098
ssc-miR-30d	MAP2K6	mitogen-activated protein kinase kinase 6 [Source:VGNC Symbol;Acc:VGNC:98102]	2.07	0.0098
ssc-miR-30d	MAP3K1	mitogen-activated protein kinase kinase kinase 1 [Source:VGNC Symbol;Acc:VGNC:98104]	2.07	0.0098
ssc-miR-30d	MAP3K12	mitogen-activated protein kinase kinase kinase 12 [Source:VGNC Symbol;Acc:VGNC:89983]	2.07	0.0098
ssc-miR-30d	MAP3K19	mitogen-activated protein kinase kinase kinase 19 [Source:VGNC Symbol;Acc:VGNC:98106]	2.07	0.0098
ssc-miR-30d	MAP3K2	mitogen-activated protein kinase kinase kinase 2 [Source:VGNC Symbol;Acc:VGNC:98107]	2.07	0.0098
ssc-miR-30d	MAP3K5	mitogen-activated protein kinase kinase kinase 5 [Source:VGNC Symbol;Acc:VGNC:89985]	2.07	0.0098
ssc-miR-30d	MAP3K7	mitogen-activated protein kinase kinase kinase 7 [Source:VGNC Symbol;Acc:VGNC:89987]	2.07	0.0098
ssc-miR-30d	MAP4K4	mitogen-activated protein kinase kinase kinase kinase 4 [Source:VGNC Symbol;Acc:VGNC:98114]	2.07	0.0098
ssc-miR-30d	MAPK8	mitogen-activated protein kinase 8 [Source:VGNC Symbol;Acc:VGNC:90006]	2.07	0.0098
ssc-miR-30d	MAPKBP1	mitogen-activated protein kinase binding protein 1 [Source:VGNC Symbol;Acc:VGNC:90014]	2.07	0.0098
ssc-miR-30d	MARCH3	hypothetical gene	2.07	0.0098
ssc-miR-30d	MARCH4	hypothetical gene	2.07	0.0098
ssc-miR-30d	MARCH6	hypothetical gene	2.07	0.0098
ssc-miR-30d	MARCH8	hypothetical gene	2.07	0.0098
ssc-miR-30d	MARCKS	myristoylated alanine rich protein kinase C substrate [Source:VGNC Symbol;Acc:VGNC:90024]	2.07	0.0098
ssc-miR-30d	MARK1	microtubule affinity regulating kinase 1 [Source:VGNC Symbol;Acc:VGNC:96411]	2.07	0.0098
ssc-miR-30d	MARK3	microtubule affinity regulating kinase 3 [Source:VGNC Symbol;Acc:VGNC:90026]	2.07	0.0098
ssc-miR-30d	MAST3	microtubule associated serine/threonine kinase 3 [Source:VGNC Symbol;Acc:VGNC:90036]	2.07	0.0098
ssc-miR-30d	MAT2A	methionine adenosyltransferase 2A [Source:VGNC Symbol;Acc:VGNC:90039]	2.07	0.0098
ssc-miR-30d	MATR3	matrin 3 [Source:HGNC Symbol;Acc:HGNC:6912]	2.07	0.0098
ssc-miR-30d	MAU2	MAU2 sister chromatid cohesion factor [Source:VGNC Symbol;Acc:VGNC:90045]	2.07	0.0098

ssc-miR-30d	MBD6	methyl-CpG binding domain protein 6 [Source:VGNC Symbol;Acc:VGNC:90051]	2.07	0.0098
ssc-miR-30d	MBNL1	muscleblind like splicing regulator 1 [Source:VGNC Symbol;Acc:VGNC:90054]	2.07	0.0098
ssc-miR-30d	MBNL2	muscleblind like splicing regulator 2 [Source:VGNC Symbol;Acc:VGNC:90055]	2.07	0.0098
ssc-miR-30d	MBNL3	muscleblind like splicing regulator 3 [Source:VGNC Symbol;Acc:VGNC:90056]	2.07	0.0098
ssc-miR-30d	MBOAT1	membrane bound O-acyltransferase domain containing 1 [Source:VGNC Symbol;Acc:VGNC:90057]	2.07	0.0098
ssc-miR-30d	MBTPS2	hypothetical gene	2.07	0.0098
ssc-miR-30d	MCF2L	MCF.2 cell line derived transforming sequence like [Source:VGNC Symbol;Acc:VGNC:90068]	2.07	0.0098
ssc-miR-30d	MCTS1	MCTS1 re-initiation and release factor [Source:HGNC Symbol;Acc:HGNC:23357]	2.07	0.0098
ssc-miR-30d	MDM4	MDM4 regulator of p53 [Source:VGNC Symbol;Acc:VGNC:90094]	2.07	0.0098
ssc-miR-30d	ME1	malic enzyme 1 [Source:VGNC Symbol;Acc:VGNC:90095]	2.07	0.0098
ssc-miR-30d	MECP2	methyl-CpG binding protein 2 [Source:VGNC Symbol;Acc:VGNC:90101]	2.07	0.0098
ssc-miR-30d	MED12L	mediator complex subunit 12L [Source:VGNC Symbol;Acc:VGNC:90105]	2.07	0.0098
ssc-miR-30d	MEF2D	myocyte enhancer factor 2D [Source:VGNC Symbol;Acc:VGNC:90128]	2.07	0.0098
ssc-miR-30d	MEIS2	Meis homeobox 2 [Source:VGNC Symbol;Acc:VGNC:90136]	2.07	0.0098
ssc-miR-30d	METAP2	methionyl aminopeptidase 2 [Source:VGNC Symbol;Acc:VGNC:90152]	2.07	0.0098
ssc-miR-30d	METTL1	methyltransferase 1, tRNA methylguanosine [Source:HGNC Symbol;Acc:HGNC:7030]	2.07	0.0098
ssc-miR-30d	MEX3B	mex-3 RNA binding family member B [Source:VGNC Symbol;Acc:VGNC:90169]	2.07	0.0098
ssc-miR-30d	MEX3D	mex-3 RNA binding family member D [Source:VGNC Symbol;Acc:VGNC:90171]	2.07	0.0098
ssc-miR-30d	MFAP3L	microfibril associated protein 3 like [Source:VGNC Symbol;Acc:VGNC:90175]	2.07	0.0098
ssc-miR-30d	MFHAS1	multifunctional ROCO family signaling regulator 1 [Source:VGNC Symbol;Acc:VGNC:107361]	2.07	0.0098
ssc-miR-30d	MFSD11	major facilitator superfamily domain containing 11 [Source:VGNC Symbol;Acc:VGNC:90184]	2.07	0.0098
ssc-miR-30d	MFSD6	major facilitator superfamily domain containing 6 [Source:VGNC Symbol;Acc:VGNC:96052]	2.07	0.0098
ssc-miR-30d	MIA3	hypothetical gene	2.07	0.0098
ssc-miR-30d	MIB1	MIB E3 ubiquitin protein ligase 1 [Source:VGNC Symbol;Acc:VGNC:90205]	2.07	0.0098
ssc-miR-30d	MICAL1	microtubule associated monooxygenase, calponin and LIM domain containing 1 [Source:VGNC Symbol;Acc:VGNC:90207]	2.07	0.0098
ssc-miR-30d	MICU3	mitochondrial calcium uptake family member 3 [Source:VGNC Symbol;Acc:VGNC:96160]	2.07	0.0098
ssc-miR-30d	MID2	midline 2 [Source:VGNC Symbol;Acc:VGNC:98127]	2.07	0.0098
ssc-miR-30d	MIER2	MIER family member 2 [Source:VGNC Symbol;Acc:VGNC:90220]	2.07	0.0098
ssc-miR-30d	MIER3	MIER family member 3 [Source:VGNC Symbol;Acc:VGNC:90221]	2.07	0.0098
ssc-miR-30d	MINOS1	hypothetical gene	2.07	0.0098
ssc-miR-30d	MINPP1	multiple inositol-polyphosphate phosphatase 1 [Source:VGNC Symbol;Acc:VGNC:90233]	2.07	0.0098
ssc-miR-30d	MIS12	MIS12 kinetochore complex component [Source:VGNC Symbol;Acc:VGNC:90238]	2.07	0.0098
ssc-miR-30d	MIS18BP1	MIS18 binding protein 1 [Source:VGNC Symbol;Acc:VGNC:90240]	2.07	0.0098
ssc-miR-30d	MKL2	hypothetical gene	2.07	0.0098
ssc-miR-30d	MKRN3	hypothetical gene	2.07	0.0098
ssc-miR-30d	MLK4	hypothetical gene	2.07	0.0098
ssc-miR-30d	MLXIP	MLX interacting protein [Source:VGNC Symbol;Acc:VGNC:90262]	2.07	0.0098
ssc-miR-30d	MMD	monocyte to macrophage differentiation associated [Source:VGNC Symbol;Acc:VGNC:90264]	2.07	0.0098
ssc-miR-30d	MMP16	matrix metallopeptidase 16 [Source:VGNC Symbol;Acc:VGNC:90271]	2.07	0.0098
ssc-miR-30d	MMP19	matrix metallopeptidase 19 [Source:VGNC Symbol;Acc:VGNC:90273]	2.07	0.0098
ssc-miR-30d	MNT	MAX network transcriptional repressor [Source:VGNC Symbol;Acc:VGNC:90289]	2.07	0.0098
ssc-miR-30d	MOB4	hypothetical gene	2.07	0.0098
ssc-miR-30d	MOV10	Mov10 RISC complex RNA helicase [Source:VGNC Symbol;Acc:VGNC:90316]	2.07	0.0098
ssc-miR-30d	MPPED1	metallophosphoesterase domain containing 1 [Source:VGNC Symbol;Acc:VGNC:90330]	2.07	0.0098
ssc-miR-30d	MSANTD3-TMEFF1	hypothetical gene	2.07	0.0098
ssc-miR-30d	MSANTD4	Myb/SANT DNA binding domain containing 4 with coiled-coils [Source:VGNC Symbol;Acc:VGNC:90415]	2.07	0.0098
ssc-miR-30d	MSI2	musashi RNA binding protein 2 [Source:VGNC Symbol;Acc:VGNC:90422]	2.07	0.0098
ssc-miR-30d	MTA1	hypothetical gene	2.07	0.0098
ssc-miR-30d	MTDH	metadherin [Source:VGNC Symbol;Acc:VGNC:90439]	2.07	0.0098

ssc-miR-30d	MTF1	metal regulatory transcription factor 1 [Source:VGNC Symbol;Acc:VGNC:90443]	2.07	0.0098
ssc-miR-30d	MTF2	metal response element binding transcription factor 2 [Source:VGNC Symbol;Acc:VGNC:90444]	2.07	0.0098
ssc-miR-30d	MTHFD1L	methylenetetrahydrofolate dehydrogenase (NADP+ dependent) 1 like [Source:VGNC Symbol;Acc:VGNC:103129]	2.07	0.0098
ssc-miR-30d	MTTP	microsomal triglyceride transfer protein [Source:VGNC Symbol;Acc:VGNC:98942]	2.07	0.0098
ssc-miR-30d	MXRA5	hypothetical gene	2.07	0.0098
ssc-miR-30d	MYBL2	MYB proto-onco like 2 [Source:VGNC Symbol;Acc:VGNC:103997]	2.07	0.0098
ssc-miR-30d	MYLK	myosin light chain kinase [Source:VGNC Symbol;Acc:VGNC:108676]	2.07	0.0098
ssc-miR-30d	MYO5A	myosin VA [Source:HGNC Symbol;Acc:HGNC:7602]	2.07	0.0098
ssc-miR-30d	MYO9B	myosin IXB [Source:VGNC Symbol;Acc:VGNC:90534]	2.07	0.0098
ssc-miR-30d	MYOM2	myomesin 2 [Source:HGNC Symbol;Acc:HGNC:7614]	2.07	0.0098
ssc-miR-30d	MYSM1	Myb like, SWIRM and MPN domains 1 [Source:VGNC Symbol;Acc:VGNC:90548]	2.07	0.0098
ssc-miR-30d	MZT1	mitotic spindle organizing protein 1 [Source:VGNC Symbol;Acc:VGNC:90552]	2.07	0.0098
ssc-miR-30d	N4BP2	NEDD4 binding protein 2 [Source:VGNC Symbol;Acc:VGNC:90554]	2.07	0.0098
ssc-miR-30d	NAA25	N-alpha-acetyltransferase 25, NatB auxiliary subunit [Source:VGNC Symbol;Acc:VGNC:96750]	2.07	0.0098
ssc-miR-30d	NAALADL2	N-acetylated alpha-linked acidic dipeptidase like 2 [Source:VGNC Symbol;Acc:VGNC:90559]	2.07	0.0098
ssc-miR-30d	NACC2	NACC family member 2 [Source:VGNC Symbol;Acc:VGNC:90564]	2.07	0.0098
ssc-miR-30d	NADK	NAD kinase [Source:VGNC Symbol;Acc:VGNC:90565]	2.07	0.0098
ssc-miR-30d	NAGPA	N-acetylglucosamine-1-phosphodiester alpha-N-acetylglucosaminidase [Source:NCBI gene (formerly Entrezgene);Acc:100512596]	2.07	0.0098
ssc-miR-30d	NAP1L1	nucleosome assembly protein 1 like 1 [Source:VGNC Symbol;Acc:VGNC:90576]	2.07	0.0098
ssc-miR-30d	NAP1L5	nucleosome assembly protein 1 like 5 [Source:NCBI gene (formerly Entrezgene);Acc:100616076]	2.07	0.0098
ssc-miR-30d	NAPG	NSF attachment protein gamma [Source:VGNC Symbol;Acc:VGNC:90580]	2.07	0.0098
ssc-miR-30d	NAV1	neuron navigator 1 [Source:VGNC Symbol;Acc:VGNC:95725]	2.07	0.0098
ssc-miR-30d	NAV3	neuron navigator 3 [Source:VGNC Symbol;Acc:VGNC:90589]	2.07	0.0098
ssc-miR-30d	NCALD	neurocalcin delta [Source:VGNC Symbol;Acc:VGNC:90594]	2.07	0.0098
ssc-miR-30d	NCAM1	neural cell adhesion molecule 1 [Source:VGNC Symbol;Acc:VGNC:108603]	2.07	0.0098
ssc-miR-30d	NCOA3	nuclear receptor coactivator 3 [Source:VGNC Symbol;Acc:VGNC:96434]	2.07	0.0098
ssc-miR-30d	NCS1	neuronal calcium sensor 1 [Source:VGNC Symbol;Acc:VGNC:90620]	2.07	0.0098
ssc-miR-30d	NDEL1	nudE neurodevelopment protein 1 like 1 [Source:VGNC Symbol;Acc:VGNC:90625]	2.07	0.0098
ssc-miR-30d	NDNF	neuron derived neurotrophic factor [Source:VGNC Symbol;Acc:VGNC:90629]	2.07	0.0098
ssc-miR-30d	NECAB1	N-terminal EF-hand calcium binding protein 1 [Source:VGNC Symbol;Acc:VGNC:90659]	2.07	0.0098
ssc-miR-30d	NECAP1	NECAP endocytosis associated 1 [Source:VGNC Symbol;Acc:VGNC:90660]	2.07	0.0098
ssc-miR-30d	NEDD4	NEDD4 E3 ubiquitin protein ligase [Source:VGNC Symbol;Acc:VGNC:90666]	2.07	0.0098
ssc-miR-30d	NEDD4L	NEDD4 like E3 ubiquitin protein ligase [Source:VGNC Symbol;Acc:VGNC:90667]	2.07	0.0098
ssc-miR-30d	NEFM	hypothetical gene	2.07	0.0098
ssc-miR-30d	NEGR1	neuronal growth regulator 1 [Source:VGNC Symbol;Acc:VGNC:90671]	2.07	0.0098
ssc-miR-30d	NEURL1B	neuralized E3 ubiquitin protein ligase 1B [Source:VGNC Symbol;Acc:VGNC:90695]	2.07	0.0098
ssc-miR-30d	NEUROD1	neuronal differentiation 1 [Source:VGNC Symbol;Acc:VGNC:96439]	2.07	0.0098
ssc-miR-30d	NEUROD6	neuronal differentiation 6 [Source:VGNC Symbol;Acc:VGNC:90699]	2.07	0.0098
ssc-miR-30d	NF1	neurofibromin 1 [Source:VGNC Symbol;Acc:VGNC:90704]	2.07	0.0098
ssc-miR-30d	NFAT5	nuclear factor of activated T cells 5 [Source:VGNC Symbol;Acc:VGNC:90708]	2.07	0.0098
ssc-miR-30d	NFATC2	nuclear factor of activated T cells 2 [Source:VGNC Symbol;Acc:VGNC:96440]	2.07	0.0098
ssc-miR-30d	NFATC3	nuclear factor of activated T cells 3 [Source:VGNC Symbol;Acc:VGNC:90710]	2.07	0.0098
ssc-miR-30d	NFIA	nuclear factor I A [Source:VGNC Symbol;Acc:VGNC:90715]	2.07	0.0098
ssc-miR-30d	NFIB	nuclear factor I B [Source:VGNC Symbol;Acc:VGNC:90716]	2.07	0.0098
ssc-miR-30d	NFYA	nuclear transcription factor Y subunit alpha [Source:VGNC Symbol;Acc:VGNC:90729]	2.07	0.0098
ssc-miR-30d	NFYB	nuclear transcription factor Y subunit beta [Source:VGNC Symbol;Acc:VGNC:90730]	2.07	0.0098
ssc-miR-30d	NHLH2	nescient helix-loop-helix 2 [Source:VGNC Symbol;Acc:VGNC:90736]	2.07	0.0098
ssc-miR-30d	NHS	NHS actin remodeling regulator [Source:VGNC Symbol;Acc:VGNC:90738]	2.07	0.0098
ssc-miR-30d	NID1	nidogen 1 [Source:VGNC Symbol;Acc:VGNC:90742]	2.07	0.0098

ssc-miR-30d	NIPAL1	NIPA like domain containing 1 [Source:VGNC Symbol;Acc:VGNC:90748]	2.07	0.0098
ssc-miR-30d	NKAIN2	sodium/potassium transporting ATPase interacting 2 [Source:VGNC Symbol;Acc:VGNC:103141]	2.07	0.0098
ssc-miR-30d	NKX2-2	NK2 homeobox 2 [Source:VGNC Symbol;Acc:VGNC:96444]	2.07	0.0098
ssc-miR-30d	NLGN1	neuroligin 1 [Source:VGNC Symbol;Acc:VGNC:90777]	2.07	0.0098
ssc-miR-30d	NLK	nemo like kinase [Source:VGNC Symbol;Acc:VGNC:90779]	2.07	0.0098
ssc-miR-30d	NMT2	N-myristoyltransferase 2 [Source:VGNC Symbol;Acc:VGNC:96448]	2.07	0.0098
ssc-miR-30d	NOTCH1	notch receptor 1 [Source:HGNC Symbol;Acc:HGNC:7881]	2.07	0.0098
ssc-miR-30d	NOVA1	NOVA alternative splicing regulator 1 [Source:VGNC Symbol;Acc:VGNC:90827]	2.07	0.0098
ssc-miR-30d	NPTXR	neuronal pentraxin receptor [Source:VGNC Symbol;Acc:VGNC:98158]	2.07	0.0098
ssc-miR-30d	NR2F2	nuclear receptor subfamily 2 group F member 2 [Source:VGNC Symbol;Acc:VGNC:90881]	2.07	0.0098
ssc-miR-30d	NR3C1	nuclear receptor subfamily 3 group C member 1 [Source:VGNC Symbol;Acc:VGNC:90883]	2.07	0.0098
ssc-miR-30d	NR3C2	nuclear receptor subfamily 3 group C member 2 [Source:VGNC Symbol;Acc:VGNC:90884]	2.07	0.0098
ssc-miR-30d	NR4A2	nuclear receptor subfamily 4 group A member 2 [Source:VGNC Symbol;Acc:VGNC:96451]	2.07	0.0098
ssc-miR-30d	NR5A2	nuclear receptor subfamily 5 group A member 2 [Source:VGNC Symbol;Acc:VGNC:96452]	2.07	0.0098
ssc-miR-30d	NR6A1	nuclear receptor subfamily 6 group A member 1 [Source:VGNC Symbol;Acc:VGNC:90887]	2.07	0.0098
ssc-miR-30d	NRBF2	nuclear receptor binding factor 2 [Source:VGNC Symbol;Acc:VGNC:90890]	2.07	0.0098
ssc-miR-30d	NRBP1	nuclear receptor binding protein 1 [Source:VGNC Symbol;Acc:VGNC:90891]	2.07	0.0098
ssc-miR-30d	NREP	neuronal regeneration related protein [Source:HGNC Symbol;Acc:HGNC:16834]	2.07	0.0098
ssc-miR-30d	NRG2	neuregulin 2 [Source:VGNC Symbol;Acc:VGNC:98160]	2.07	0.0098
ssc-miR-30d	NRG3	neuregulin 3 [Source:VGNC Symbol;Acc:VGNC:104011]	2.07	0.0098
ssc-miR-30d	NRIP1	nuclear receptor interacting protein 1 [Source:VGNC Symbol;Acc:VGNC:90896]	2.07	0.0098
ssc-miR-30d	NRK	Nik related kinase [Source:VGNC Symbol;Acc:VGNC:98161]	2.07	0.0098
ssc-miR-30d	NRP1	neuropilin 1 [Source:VGNC Symbol;Acc:VGNC:104012]	2.07	0.0098
ssc-miR-30d	NRXN3	neurexin 3 [Source:HGNC Symbol;Acc:HGNC:8010]	2.07	0.0098
ssc-miR-30d	NSD1	nuclear receptor binding SET domain protein 1 [Source:VGNC Symbol;Acc:VGNC:90904]	2.07	0.0098
ssc-miR-30d	NSG1	neuronal vesicle trafficking associated 1 [Source:VGNC Symbol;Acc:VGNC:90907]	2.07	0.0098
ssc-miR-30d	NSUN3	NOP2/Sun RNA methyltransferase 3 [Source:VGNC Symbol;Acc:VGNC:90915]	2.07	0.0098
ssc-miR-30d	NT5DC1	5'-nucleotidase domain containing 1 [Source:VGNC Symbol;Acc:VGNC:90922]	2.07	0.0098
ssc-miR-30d	NT5DC3	5'-nucleotidase domain containing 3 [Source:VGNC Symbol;Acc:VGNC:90924]	2.07	0.0098
ssc-miR-30d	NT5E	5'-nucleotidase ecto [Source:VGNC Symbol;Acc:VGNC:90925]	2.07	0.0098
ssc-miR-30d	NTNG1	netrin G1 [Source:VGNC Symbol;Acc:VGNC:90935]	2.07	0.0098
ssc-miR-30d	NUAK1	NUAK family kinase 1 [Source:VGNC Symbol;Acc:VGNC:90940]	2.07	0.0098
ssc-miR-30d	NUCKS1	nuclear casein kinase and cyclin dependent kinase substrate 1 [Source:VGNC Symbol;Acc:VGNC:90947]	2.07	0.0098
ssc-miR-30d	NUDT5	nudix hydrolase 5 [Source:VGNC Symbol;Acc:VGNC:96458]	2.07	0.0098
ssc-miR-30d	NUFIP2	nuclear FMR1 interacting protein 2 [Source:VGNC Symbol;Acc:VGNC:90967]	2.07	0.0098
ssc-miR-30d	NUP43	nucleoporin 43 [Source:VGNC Symbol;Acc:VGNC:90982]	2.07	0.0098
ssc-miR-30d	NUS1	NUS1 dehydrodolichyl diphosphate synthase subunit [Source:VGNC Symbol;Acc:VGNC:90990]	2.07	0.0098
ssc-miR-30d	OCLN	occludin [Source:VGNC Symbol;Acc:VGNC:91013]	2.07	0.0098
ssc-miR-30d	OMG	oligodendrocyte myelin glycoprotein [Source:VGNC Symbol;Acc:VGNC:91041]	2.07	0.0098
ssc-miR-30d	ONECUT2	one cut homeobox 2 [Source:VGNC Symbol;Acc:VGNC:91043]	2.07	0.0098
ssc-miR-30d	ONECUT3	one cut homeobox 3 [Source:VGNC Symbol;Acc:VGNC:91044]	2.07	0.0098
ssc-miR-30d	ORC2	origin recognition complex subunit 2 [Source:VGNC Symbol;Acc:VGNC:96464]	2.07	0.0098
ssc-miR-30d	OSBPL8	oxysterol binding protein like 8 [Source:VGNC Symbol;Acc:VGNC:91074]	2.07	0.0098
ssc-miR-30d	OSTM1	osteoclastosis associated transmembrane protein 1 [Source:VGNC Symbol;Acc:VGNC:91086]	2.07	0.0098
ssc-miR-30d	OTOR	hypothetical gene	2.07	0.0098
ssc-miR-30d	OTUD4	OTU deubiquitinase 4 [Source:VGNC Symbol;Acc:VGNC:91100]	2.07	0.0098
ssc-miR-30d	OTUD6B	OTU deubiquitinase 6B [Source:VGNC Symbol;Acc:VGNC:91103]	2.07	0.0098
ssc-miR-30d	OVOL1	ovo like transcriptional repressor 1 [Source:VGNC Symbol;Acc:VGNC:91111]	2.07	0.0098
ssc-miR-30d	OVOL2	ovo like zinc finger 2 [Source:VGNC Symbol;Acc:VGNC:95663]	2.07	0.0098

ssc-miR-30d	OXR1	oxidation resistance 1 [Source:VGNC Symbol;Acc:VGNC:91116]	2.07	0.0098
ssc-miR-30d	OXTR	oxytocin receptor [Source:VGNC Symbol;Acc:VGNC:108052]	2.07	0.0098
ssc-miR-30d	P2RY2	purinergic receptor P2Y2 [Source:VGNC Symbol;Acc:VGNC:98173]	2.07	0.0098
ssc-miR-30d	P4HA1	prolyl 4-hydroxylase subunit alpha 1 [Source:VGNC Symbol;Acc:VGNC:91132]	2.07	0.0098
ssc-miR-30d	P4HA2	prolyl 4-hydroxylase subunit alpha 2 [Source:VGNC Symbol;Acc:VGNC:91133]	2.07	0.0098
ssc-miR-30d	P4HA3	prolyl 4-hydroxylase subunit alpha 3 [Source:VGNC Symbol;Acc:VGNC:91134]	2.07	0.0098
ssc-miR-30d	PAAF1	proteasomal ATPase associated factor 1 [Source:VGNC Symbol;Acc:VGNC:108605]	2.07	0.0098
ssc-miR-30d	PABPC1L2A	hypothetical gene	2.07	0.0098
ssc-miR-30d	PABPC1L2B	hypothetical gene	2.07	0.0098
ssc-miR-30d	PACRGL	parkin coregulated like [Source:VGNC Symbol;Acc:VGNC:91139]	2.07	0.0098
ssc-miR-30d	PAFAH1B2	platelet activating factor acetylhydrolase 1b catalytic subunit 2 [Source:VGNC Symbol;Acc:VGNC:91151]	2.07	0.0098
ssc-miR-30d	PAG1	phosphoprotein membrane anchor with glycosphingolipid microdomains 1 [Source:VGNC Symbol;Acc:VGNC:91154]	2.07	0.0098
ssc-miR-30d	PAK4	p21 (RAC1) activated kinase 4 [Source:VGNC Symbol;Acc:VGNC:98526]	2.07	0.0098
ssc-miR-30d	PALM2	hypothetical gene	2.07	0.0098
ssc-miR-30d	PANK3	pantothenate kinase 3 [Source:VGNC Symbol;Acc:VGNC:91165]	2.07	0.0098
ssc-miR-30d	PANX1	pannexin 1 [Source:VGNC Symbol;Acc:VGNC:91166]	2.07	0.0098
ssc-miR-30d	PAPD4	hypothetical gene	2.07	0.0098
ssc-miR-30d	PAPD5	hypothetical gene	2.07	0.0098
ssc-miR-30d	PAPOLB	hypothetical gene	2.07	0.0098
ssc-miR-30d	PAQR5	progestin and adipoQ receptor family member 5 [Source:VGNC Symbol;Acc:VGNC:91175]	2.07	0.0098
ssc-miR-30d	PARP16	poly(ADP-ribose) polymerase family member 16 [Source:VGNC Symbol;Acc:VGNC:103145]	2.07	0.0098
ssc-miR-30d	PARP8	poly(ADP-ribose) polymerase family member 8 [Source:HGNC Symbol;Acc:HGNC:26124]	2.07	0.0098
ssc-miR-30d	PAWR	pro-apoptotic WT1 regulator [Source:VGNC Symbol;Acc:VGNC:91191]	2.07	0.0098
ssc-miR-30d	PAX3	paired box 3 [Source:VGNC Symbol;Acc:VGNC:96468]	2.07	0.0098
ssc-miR-30d	PAX9	paired box 9 [Source:HGNC Symbol;Acc:HGNC:8623]	2.07	0.0098
ssc-miR-30d	PAXBP1	PAX3 and PAX7 binding protein 1 [Source:VGNC Symbol;Acc:VGNC:91197]	2.07	0.0098
ssc-miR-30d	PBRM1	polybromo 1 [Source:VGNC Symbol;Acc:VGNC:91202]	2.07	0.0098
ssc-miR-30d	PCDH10	protocadherin 10 [Source:HGNC Symbol;Acc:HGNC:13404]	2.07	0.0098
ssc-miR-30d	PCDH17	protocadherin 17 [Source:VGNC Symbol;Acc:VGNC:91214]	2.07	0.0098
ssc-miR-30d	PCDH19	protocadherin 19 [Source:VGNC Symbol;Acc:VGNC:91216]	2.07	0.0098
ssc-miR-30d	PCDH20	protocadherin 20 [Source:HGNC Symbol;Acc:HGNC:14257]	2.07	0.0098
ssc-miR-30d	PCGF3	polycomb group ring finger 3 [Source:VGNC Symbol;Acc:VGNC:91220]	2.07	0.0098
ssc-miR-30d	PCGF5	hypothetical gene	2.07	0.0098
ssc-miR-30d	PCNXL2	hypothetical gene	2.07	0.0098
ssc-miR-30d	PDAP1	PDGFA associated protein 1 [Source:VGNC Symbol;Acc:VGNC:91239]	2.07	0.0098
ssc-miR-30d	PDCD10	programmed cell death 10 [Source:VGNC Symbol;Acc:VGNC:91241]	2.07	0.0098
ssc-miR-30d	PDCL	phosducin like [Source:VGNC Symbol;Acc:VGNC:91245]	2.07	0.0098
ssc-miR-30d	PDE3A	phosphodiesterase 3A [Source:VGNC Symbol;Acc:VGNC:91252]	2.07	0.0098
ssc-miR-30d	PDE4D	phosphodiesterase 4D [Source:VGNC Symbol;Acc:VGNC:91256]	2.07	0.0098
ssc-miR-30d	PDE5A	phosphodiesterase 5A [Source:VGNC Symbol;Acc:VGNC:91257]	2.07	0.0098
ssc-miR-30d	PDE7A	phosphodiesterase 7A [Source:VGNC Symbol;Acc:VGNC:91261]	2.07	0.0098
ssc-miR-30d	PDGFRB	platelet derived growth factor receptor beta [Source:VGNC Symbol;Acc:VGNC:91268]	2.07	0.0098
ssc-miR-30d	PDP2	pyruvate dehyrogenase phosphatase catalytic subunit 2 [Source:VGNC Symbol;Acc:VGNC:91287]	2.07	0.0098
ssc-miR-30d	PDS5B	PDS5 cohesin associated factor B [Source:VGNC Symbol;Acc:VGNC:91290]	2.07	0.0098
ssc-miR-30d	PDSS1	decaprenyl diphosphate synthase subunit 1 [Source:VGNC Symbol;Acc:VGNC:95858]	2.07	0.0098
ssc-miR-30d	PDSS2	decaprenyl diphosphate synthase subunit 2 [Source:VGNC Symbol;Acc:VGNC:91291]	2.07	0.0098
ssc-miR-30d	PEAK1	pseudopodium enriched atypical kinase 1 [Source:VGNC Symbol;Acc:VGNC:91300]	2.07	0.0098
ssc-miR-30d	PELI1	pellino E3 ubiquitin protein ligase 1 [Source:VGNC Symbol;Acc:VGNC:91306]	2.07	0.0098
ssc-miR-30d	PELI2	pellino E3 ubiquitin protein ligase family member 2 [Source:VGNC Symbol;Acc:VGNC:91307]	2.07	0.0098

ssc-miR-30d	PER2	period circadian regulator 2 [Source:VGNC Symbol;Acc:VGNC:96478]	2.07	0.0098
ssc-miR-30d	PER3	hypothetical gene	2.07	0.0098
ssc-miR-30d	PEX26	peroxisomal biogenesis factor 26 [Source:HGNC Symbol;Acc:HGNC:22965]	2.07	0.0098
ssc-miR-30d	PEX5L	peroxisomal biosis factor 5 like [Source:VGNC Symbol;Acc:VGNC:91329]	2.07	0.0098
ssc-miR-30d	PFN2	hypothetical gene	2.07	0.0098
ssc-miR-30d	PGGT1B	protein geranylgeranyltransferase type I subunit beta [Source:VGNC Symbol;Acc:VGNC:91350]	2.07	0.0098
ssc-miR-30d	PGM1	hypothetical gene	2.07	0.0098
ssc-miR-30d	PGM2L1	phosphoglucomutase 2 like 1 [Source:VGNC Symbol;Acc:VGNC:91357]	2.07	0.0098
ssc-miR-30d	PGM3	phosphoglucomutase 3 [Source:VGNC Symbol;Acc:VGNC:103150]	2.07	0.0098
ssc-miR-30d	PGP	phosphoglycolate phosphatase [Source:VGNC Symbol;Acc:VGNC:91359]	2.07	0.0098
ssc-miR-30d	PGR	progesterone receptor [Source:VGNC Symbol;Acc:VGNC:91362]	2.07	0.0098
ssc-miR-30d	PHACTR2	phosphatase and actin regulator 2 [Source:VGNC Symbol;Acc:VGNC:91366]	2.07	0.0098
ssc-miR-30d	PHF13	PHD finger protein 13 [Source:VGNC Symbol;Acc:VGNC:91379]	2.07	0.0098
ssc-miR-30d	PHF16	hypothetical gene	2.07	0.0098
ssc-miR-30d	PHF6	PHD finger protein 6 [Source:VGNC Symbol;Acc:VGNC:91389]	2.07	0.0098
ssc-miR-30d	PHIP	pleckstrin homology domain interacting protein [Source:VGNC Symbol;Acc:VGNC:91392]	2.07	0.0098
ssc-miR-30d	PHKA2	phosphorylase kinase regulatory subunit alpha 2 [Source:VGNC Symbol;Acc:VGNC:91394]	2.07	0.0098
ssc-miR-30d	PHLDB2	pleckstrin homology like domain family B member 2 [Source:VGNC Symbol;Acc:VGNC:91399]	2.07	0.0098
ssc-miR-30d	PHTF2	putative homeodomain transcription factor 2 [Source:VGNC Symbol;Acc:VGNC:91405]	2.07	0.0098
ssc-miR-30d	PI4K2B	phosphatidylinositol 4-kinase type 2 beta [Source:VGNC Symbol;Acc:VGNC:98190]	2.07	0.0098
ssc-miR-30d	PIAS1	protein inhibitor of activated STAT 1 [Source:VGNC Symbol;Acc:VGNC:91410]	2.07	0.0098
ssc-miR-30d	PICALM	phosphatidylinositol binding clathrin assembly protein [Source:VGNC Symbol;Acc:VGNC:91415]	2.07	0.0098
ssc-miR-30d	PIEZO2	piezo type mechanosensitive ion channel component 2 [Source:HGNC Symbol;Acc:HGNC:26270]	2.07	0.0098
ssc-miR-30d	PIGA	phosphatidylinositol glycan anchor biosynthesis class A [Source:VGNC Symbol;Acc:VGNC:91419]	2.07	0.0098
ssc-miR-30d	PIGV	phosphatidylinositol glycan anchor biosynthesis class V [Source:VGNC Symbol;Acc:VGNC:91430]	2.07	0.0098
ssc-miR-30d	PIGX	phosphatidylinositol glycan anchor biosynthesis class X [Source:VGNC Symbol;Acc:VGNC:91432]	2.07	0.0098
ssc-miR-30d	PIK3CD	phosphatidylinositol-4,5-bisphosphate 3-kinase catalytic subunit delta [Source:VGNC Symbol;Acc:VGNC:91442]	2.07	0.0098
ssc-miR-30d	PIK3R2	phosphoinositide-3-kinase regulatory subunit 2 [Source:VGNC Symbol;Acc:VGNC:100324]	2.07	0.0098
ssc-miR-30d	PIN4	peptidylprolyl cis/trans isomerase, NIMA-interacting 4 [Source:VGNC Symbol;Acc:VGNC:91452]	2.07	0.0098
ssc-miR-30d	PIP4K2A	phosphatidylinositol-5-phosphate 4-kinase type 2 alpha [Source:VGNC Symbol;Acc:VGNC:96485]	2.07	0.0098
ssc-miR-30d	PIP4K2B	phosphatidylinositol-5-phosphate 4-kinase type 2 beta [Source:VGNC Symbol;Acc:VGNC:91454]	2.07	0.0098
ssc-miR-30d	PIRT	phosphoinositide interacting regulator of transient receptor potential channels [Source:VGNC Symbol;Acc:VGNC:91461]	2.07	0.0098
ssc-miR-30d	PITPNB	phosphatidylinositol transfer protein beta [Source:VGNC Symbol;Acc:VGNC:104026]	2.07	0.0098
ssc-miR-30d	PITPNC1	phosphatidylinositol transfer protein cytoplasmic 1 [Source:NCBI gene (formerly Entrezgene);Acc:100233199]	2.07	0.0098
ssc-miR-30d	PITPNM2	phosphatidylinositol transfer protein membrane associated 2 [Source:VGNC Symbol;Acc:VGNC:91466]	2.07	0.0098
ssc-miR-30d	PITPNM3	PITPNM family member 3 [Source:HGNC Symbol;Acc:HGNC:21043]	2.07	0.0098
ssc-miR-30d	PITX1	paired like homeodomain 1 [Source:NCBI gene (formerly Entrezgene);Acc:100689266]	2.07	0.0098
ssc-miR-30d	PKHD1	PKHD1 ciliary IPT domain containing fibrocystin/polyductin [Source:VGNC Symbol;Acc:VGNC:91476]	2.07	0.0098
ssc-miR-30d	PKNOX2	PBX/knotted 1 homeobox 2 [Source:VGNC Symbol;Acc:VGNC:91485]	2.07	0.0098
ssc-miR-30d	PLA2G12A	phospholipase A2 group XIIA [Source:HGNC Symbol;Acc:HGNC:18554]	2.07	0.0098
ssc-miR-30d	PLAG1	PLAG1 zinc finger [Source:VGNC Symbol;Acc:VGNC:91509]	2.07	0.0098
ssc-miR-30d	PLAGL2	PLAG1 like zinc finger 2 [Source:VGNC Symbol;Acc:VGNC:96490]	2.07	0.0098
ssc-miR-30d	PLCG1	phospholipase C gamma 1 [Source:VGNC Symbol;Acc:VGNC:96492]	2.07	0.0098
ssc-miR-30d	PLCXD3	phosphatidylinositol specific phospholipase C X domain containing 3 [Source:VGNC Symbol;Acc:VGNC:91524]	2.07	0.0098
ssc-miR-30d	PLEKHA6	pleckstrin homology domain containing A6 [Source:VGNC Symbol;Acc:VGNC:91536]	2.07	0.0098
ssc-miR-30d	PLEKHM3	pleckstrin homology domain containing M3 [Source:VGNC Symbol;Acc:VGNC:96281]	2.07	0.0098
ssc-miR-30d	PLEKHO2	pleckstrin homology domain containing O2 [Source:HGNC Symbol;Acc:HGNC:30026]	2.07	0.0098
ssc-miR-30d	PLS1	plastin 1 [Source:VGNC Symbol;Acc:VGNC:91572]	2.07	0.0098
ssc-miR-30d	PLXNA1	plexin A1 [Source:VGNC Symbol;Acc:VGNC:91579]	2.07	0.0098

ssc-miR-30d	PLXNA2	plexin A2 [Source:VGNC Symbol;Acc:VGNC:91580]	2.07	0.0098
ssc-miR-30d	PLXNC1	plexin C1 [Source:VGNC Symbol;Acc:VGNC:91584]	2.07	0.0098
ssc-miR-30d	PMEPA1	prostate transmembrane protein, androgen induced 1 [Source:VGNC Symbol;Acc:VGNC:95606]	2.07	0.0098
ssc-miR-30d	PNN	pinin, desmosome associated protein [Source:VGNC Symbol;Acc:VGNC:91603]	2.07	0.0098
ssc-miR-30d	PNPLA2	patatin like phospholipase domain containing 2 [Source:VGNC Symbol;Acc:VGNC:91607]	2.07	0.0098
ssc-miR-30d	POC1B	POC1 centriolar protein B [Source:VGNC Symbol;Acc:VGNC:91616]	2.07	0.0098
ssc-miR-30d	POGLUT1	protein O-glucosyltransferase 1 [Source:VGNC Symbol;Acc:VGNC:91623]	2.07	0.0098
ssc-miR-30d	POLE3	DNA polymerase epsilon 3, accessory subunit [Source:VGNC Symbol;Acc:VGNC:103154]	2.07	0.0098
ssc-miR-30d	POLR1D	RNA polymerase I and III subunit D [Source:VGNC Symbol;Acc:VGNC:104031]	2.07	0.0098
ssc-miR-30d	POLR3E	RNA polymerase III subunit E [Source:VGNC Symbol;Acc:VGNC:91657]	2.07	0.0098
ssc-miR-30d	POLR3G	hypothetical gene	2.07	0.0098
ssc-miR-30d	PON2	paraoxonase 2 [Source:VGNC Symbol;Acc:VGNC:91662]	2.07	0.0098
ssc-miR-30d	POP1	POP1 homolog, ribonuclease P/MRP subunit [Source:VGNC Symbol;Acc:VGNC:91663]	2.07	0.0098
ssc-miR-30d	POU3F2	POU class 3 homeobox 2 [Source:HGNC Symbol;Acc:HGNC:9215]	2.07	0.0098
ssc-miR-30d	POU4F2	POU class 4 homeobox 2 [Source:VGNC Symbol;Acc:VGNC:91679]	2.07	0.0098
ssc-miR-30d	PPA1	inorganic pyrophosphatase 1 [Source:VGNC Symbol;Acc:VGNC:96757]	2.07	0.0098
ssc-miR-30d	PPAPDC2	hypothetical gene	2.07	0.0098
ssc-miR-30d	PPARGC1A	PPARG coactivator 1 alpha [Source:VGNC Symbol;Acc:VGNC:91685]	2.07	0.0098
ssc-miR-30d	PPARGC1B	PPARG coactivator 1 beta [Source:VGNC Symbol;Acc:VGNC:91686]	2.07	0.0098
ssc-miR-30d	PPFIA2	PTPRF interacting protein alpha 2 [Source:VGNC Symbol;Acc:VGNC:91692]	2.07	0.0098
ssc-miR-30d	PPID	peptidylprolyl isomerase D [Source:VGNC Symbol;Acc:VGNC:98212]	2.07	0.0098
ssc-miR-30d	PPIL3	peptidylprolyl isomerase like 3 [Source:VGNC Symbol;Acc:VGNC:103073]	2.07	0.0098
ssc-miR-30d	PPIL4	peptidylprolyl isomerase like 4 [Source:VGNC Symbol;Acc:VGNC:91699]	2.07	0.0098
ssc-miR-30d	PPM1A	protein phosphatase, Mg2+/Mn2+ dependent 1A [Source:VGNC Symbol;Acc:VGNC:91703]	2.07	0.0098
ssc-miR-30d	PPM1E	protein phosphatase, Mg2+/Mn2+ dependent 1E [Source:VGNC Symbol;Acc:VGNC:91705]	2.07	0.0098
ssc-miR-30d	PPP1R12A	protein phosphatase 1 regulatory subunit 12A [Source:VGNC Symbol;Acc:VGNC:91719]	2.07	0.0098
ssc-miR-30d	PPP1R14C	protein phosphatase 1 regulatory inhibitor subunit 14C [Source:VGNC Symbol;Acc:VGNC:91724]	2.07	0.0098
ssc-miR-30d	PPP1R18	protein phosphatase 1 regulatory subunit 18 [Source:VGNC Symbol;Acc:VGNC:104034]	2.07	0.0098
ssc-miR-30d	PPP1R2	hypothetical gene	2.07	0.0098
ssc-miR-30d	PPP1R9A	protein phosphatase 1 regulatory subunit 9A [Source:VGNC Symbol;Acc:VGNC:91743]	2.07	0.0098
ssc-miR-30d	PPP2R1B	protein phosphatase 2 scaffold subunit Abeta [Source:VGNC Symbol;Acc:VGNC:91747]	2.07	0.0098
ssc-miR-30d	PPP2R4	hypothetical gene	2.07	0.0098
ssc-miR-30d	PPP2R5E	protein phosphatase 2 regulatory subunit B'epsilon [Source:VGNC Symbol;Acc:VGNC:91754]	2.07	0.0098
ssc-miR-30d	PPP3CA	protein phosphatase 3 catalytic subunit alpha [Source:VGNC Symbol;Acc:VGNC:98218]	2.07	0.0098
ssc-miR-30d	PPP3CB	protein phosphatase 3 catalytic subunit beta [Source:VGNC Symbol;Acc:VGNC:104035]	2.07	0.0098
ssc-miR-30d	PPP3R1	protein phosphatase 3 regulatory subunit B, alpha [Source:VGNC Symbol;Acc:VGNC:106453]	2.07	0.0098
ssc-miR-30d	PPP6C	protein phosphatase 6 catalytic subunit [Source:VGNC Symbol;Acc:VGNC:98219]	2.07	0.0098
ssc-miR-30d	PPTC7	protein phosphatase targeting COQ7 [Source:VGNC Symbol;Acc:VGNC:91765]	2.07	0.0098
ssc-miR-30d	PRCD	photoreceptor disc component [Source:VGNC Symbol;Acc:VGNC:91770]	2.07	0.0098
ssc-miR-30d	PRDM1	PR/SET domain 1 [Source:VGNC Symbol;Acc:VGNC:91772]	2.07	0.0098
ssc-miR-30d	PRDM5	PR/SET domain 5 [Source:VGNC Symbol;Acc:VGNC:98953]	2.07	0.0098
ssc-miR-30d	PRICKLE1	prickle planar cell polarity protein 1 [Source:VGNC Symbol;Acc:VGNC:91793]	2.07	0.0098
ssc-miR-30d	PRKAA2	protein kinase AMP-activated catalytic subunit alpha 2 [Source:VGNC Symbol;Acc:VGNC:91798]	2.07	0.0098
ssc-miR-30d	PRKAR1A	protein kinase cAMP-dependent type I regulatory subunit alpha [Source:VGNC Symbol;Acc:VGNC:91802]	2.07	0.0098
ssc-miR-30d	PRKRIR	hypothetical gene	2.07	0.0098
ssc-miR-30d	PRLR	prolactin receptor [Source:VGNC Symbol;Acc:VGNC:91819]	2.07	0.0098
ssc-miR-30d	PROM1	prominin 1 [Source:VGNC Symbol;Acc:VGNC:91832]	2.07	0.0098
ssc-miR-30d	PROSER1	proline and serine rich 1 [Source:VGNC Symbol;Acc:VGNC:91836]	2.07	0.0098
ssc-miR-30d	PRPF40A	pre-mRNA processing factor 40 homolog A [Source:VGNC Symbol;Acc:VGNC:98222]	2.07	0.0098

ssc-miR-30d	PRR14L	proline rich 14 like [Source:VGNC Symbol;Acc:VGNC:91853]	2.07	0.0098
ssc-miR-30d	PRRG1	proline rich and Gla domain 1 [Source:VGNC Symbol;Acc:VGNC:101494]	2.07	0.0098
ssc-miR-30d	PRRT2	proline rich transmembrane protein 2 [Source:HGNC Symbol;Acc:HGNC:30500]	2.07	0.0098
ssc-miR-30d	PRRX1	paired related homeobox 1 [Source:VGNC Symbol;Acc:VGNC:91875]	2.07	0.0098
ssc-miR-30d	PRUNE2	hypothetical gene	2.07	0.0098
ssc-miR-30d	PSD3	pleckstrin and Sec7 domain containing 3 [Source:VGNC Symbol;Acc:VGNC:107166]	2.07	0.0098
ssc-miR-30d	PSEN2	presenilin 2 [Source:VGNC Symbol;Acc:VGNC:96508]	2.07	0.0098
ssc-miR-30d	PSMD7	proteasome 26S subunit, non-ATPase 7 [Source:VGNC Symbol;Acc:VGNC:91925]	2.07	0.0098
ssc-miR-30d	PSMD9	proteasome 26S subunit, non-ATPase 9 [Source:HGNC Symbol;Acc:HGNC:9567]	2.07	0.0098
ssc-miR-30d	PSME3	proteasome activator subunit 3 [Source:VGNC Symbol;Acc:VGNC:91926]	2.07	0.0098
ssc-miR-30d	PTAFR	platelet activating factor receptor [Source:VGNC Symbol;Acc:VGNC:98224]	2.07	0.0098
ssc-miR-30d	PTBP3	polypyrimidine tract binding protein 3 [Source:VGNC Symbol;Acc:VGNC:91938]	2.07	0.0098
ssc-miR-30d	PTCHD1	patched domain containing 1 [Source:VGNC Symbol;Acc:VGNC:91942]	2.07	0.0098
ssc-miR-30d	PTGDR	prostaglandin D2 receptor [Source:HGNC Symbol;Acc:HGNC:9591]	2.07	0.0098
ssc-miR-30d	PTGER3	prostaglandin E receptor 3 [Source:VGNC Symbol;Acc:VGNC:98225]	2.07	0.0098
ssc-miR-30d	PTGFRN	prostaglandin F2 receptor inhibitor [Source:VGNC Symbol;Acc:VGNC:91955]	2.07	0.0098
ssc-miR-30d	PTP4A1	protein tyrosine phosphatase 4A1 [Source:HGNC Symbol;Acc:HGNC:9634]	2.07	0.0098
ssc-miR-30d	PTPDC1	protein tyrosine phosphatase domain containing 1 [Source:VGNC Symbol;Acc:VGNC:91970]	2.07	0.0098
ssc-miR-30d	PTPN13	protein tyrosine phosphatase non-receptor type 13 [Source:VGNC Symbol;Acc:VGNC:91974]	2.07	0.0098
ssc-miR-30d	PTPN21	protein tyrosine phosphatase non-receptor type 21 [Source:VGNC Symbol;Acc:VGNC:91977]	2.07	0.0098
ssc-miR-30d	PTPN4	protein tyrosine phosphatase non-receptor type 4 [Source:VGNC Symbol;Acc:VGNC:96521]	2.07	0.0098
ssc-miR-30d	PTPN9	protein tyrosine phosphatase non-receptor type 9 [Source:VGNC Symbol;Acc:VGNC:91983]	2.07	0.0098
ssc-miR-30d	PTPRK	protein tyrosine phosphatase receptor type K [Source:VGNC Symbol;Acc:VGNC:91991]	2.07	0.0098
ssc-miR-30d	PUM2	pumilio RNA binding family member 2 [Source:VGNC Symbol;Acc:VGNC:92002]	2.07	0.0098
ssc-miR-30d	PVRL3	hypothetical gene	2.07	0.0098
ssc-miR-30d	PXK	PX domain containing serine/threonine kinase like [Source:VGNC Symbol;Acc:VGNC:92015]	2.07	0.0098
ssc-miR-30d	PXN	paxillin [Source:HGNC Symbol;Acc:HGNC:9718]	2.07	0.0098
ssc-miR-30d	PYGO1	pygopus family PHD finger 1 [Source:VGNC Symbol;Acc:VGNC:98231]	2.07	0.0098
ssc-miR-30d	QKI	QKI, KH domain containing RNA binding [Source:VGNC Symbol;Acc:VGNC:92025]	2.07	0.0098
ssc-miR-30d	R3HDM1	R3H domain containing 1 [Source:VGNC Symbol;Acc:VGNC:96526]	2.07	0.0098
ssc-miR-30d	RAB10	RAB10, member RAS onco family [Source:VGNC Symbol;Acc:VGNC:98235]	2.07	0.0098
ssc-miR-30d	RAB11A	RAB11A, member RAS onco family [Source:VGNC Symbol;Acc:VGNC:98236]	2.07	0.0098
ssc-miR-30d	RAB15	RAB15, member RAS onco family [Source:VGNC Symbol;Acc:VGNC:98239]	2.07	0.0098
ssc-miR-30d	RAB22A	RAB22A, member RAS onco family [Source:VGNC Symbol;Acc:VGNC:98244]	2.07	0.0098
ssc-miR-30d	RAB23	RAB23, member RAS onco family [Source:VGNC Symbol;Acc:VGNC:98245]	2.07	0.0098
ssc-miR-30d	RAB27B	RAB27B, member RAS onco family [Source:VGNC Symbol;Acc:VGNC:98250]	2.07	0.0098
ssc-miR-30d	RAB32	RAB32, member RAS onco family [Source:NCBI gene (formerly Entrezgene);Acc:100144496]	2.07	0.0098
ssc-miR-30d	RAB33B	RAB33B, member RAS onco family [Source:VGNC Symbol;Acc:VGNC:98257]	2.07	0.0098
ssc-miR-30d	RAB38	RAB38, member RAS onco family [Source:VGNC Symbol;Acc:VGNC:98261]	2.07	0.0098
ssc-miR-30d	RAB3D	RAB3D, member RAS onco family [Source:VGNC Symbol;Acc:VGNC:98266]	2.07	0.0098
ssc-miR-30d	RAB4A	RAB4A, member RAS onco family [Source:VGNC Symbol;Acc:VGNC:98270]	2.07	0.0098
ssc-miR-30d	RAB4B	RAB4B, member RAS onco family [Source:VGNC Symbol;Acc:VGNC:98597]	2.07	0.0098
ssc-miR-30d	RAB7A	RAB7A, member RAS onco family [Source:VGNC Symbol;Acc:VGNC:98273]	2.07	0.0098
ssc-miR-30d	RAB8A	RAB8A, member RAS onco family [Source:VGNC Symbol;Acc:VGNC:98275]	2.07	0.0098
ssc-miR-30d	RABGAP1L	RAB GTPase activating protein 1 like [Source:VGNC Symbol;Acc:VGNC:108611]	2.07	0.0098
ssc-miR-30d	RAD23B	RAD23 homolog B, nucleotide excision repair protein [Source:VGNC Symbol;Acc:VGNC:103160]	2.07	0.0098
ssc-miR-30d	RAD51L3-RFFL	hypothetical gene	2.07	0.0098
ssc-miR-30d	RAD9B	RAD9 checkpoint clamp component B [Source:VGNC Symbol;Acc:VGNC:92063]	2.07	0.0098
ssc-miR-30d	RAI14	retinoic acid induced 14 [Source:VGNC Symbol;Acc:VGNC:92068]	2.07	0.0098

ssc-miR-30d	RALGDS	ral guanine nucleotide dissociation stimulator [Source:HGNC Symbol;Acc:HGNC:9842]	2.07	0.0098
ssc-miR-30d	RALGPS1	Ral GEF with PH domain and SH3 binding motif 1 [Source:VGNC Symbol;Acc:VGNC:92072]	2.07	0.0098
ssc-miR-30d	RALGPS2	Ral GEF with PH domain and SH3 binding motif 2 [Source:VGNC Symbol;Acc:VGNC:92073]	2.07	0.0098
ssc-miR-30d	RANBP10	RAN binding protein 10 [Source:VGNC Symbol;Acc:VGNC:92077]	2.07	0.0098
ssc-miR-30d	RANBP9	RAN binding protein 9 [Source:VGNC Symbol;Acc:VGNC:92082]	2.07	0.0098
ssc-miR-30d	RAP1B	RAP1B, member of RAS oncogene family [Source:HGNC Symbol;Acc:HGNC:9857]	2.07	0.0098
ssc-miR-30d	RAP2A	RAP2A, member of RAS onco family [Source:VGNC Symbol;Acc:VGNC:92088]	2.07	0.0098
ssc-miR-30d	RAP2B	RAP2B, member of RAS onco family [Source:VGNC Symbol;Acc:VGNC:92089]	2.07	0.0098
ssc-miR-30d	RAP2C	RAP2C, member of RAS onco family [Source:VGNC Symbol;Acc:VGNC:92090]	2.07	0.0098
ssc-miR-30d	RAPGEF2	Rap guanine nucleotide exchange factor 2 [Source:VGNC Symbol;Acc:VGNC:92092]	2.07	0.0098
ssc-miR-30d	RAPGEF4	Rap guanine nucleotide exchange factor 4 [Source:VGNC Symbol;Acc:VGNC:95814]	2.07	0.0098
ssc-miR-30d	RAPH1	hypothetical gene	2.07	0.0098
ssc-miR-30d	RARB	retinoic acid receptor beta [Source:HGNC Symbol;Acc:HGNC:9865]	2.07	0.0098
ssc-miR-30d	RARG	retinoic acid receptor gamma [Source:HGNC Symbol;Acc:HGNC:9866]	2.07	0.0098
ssc-miR-30d	RASA1	RAS p21 protein activator 1 [Source:VGNC Symbol;Acc:VGNC:92101]	2.07	0.0098
ssc-miR-30d	RASA2	RAS p21 protein activator 2 [Source:VGNC Symbol;Acc:VGNC:92102]	2.07	0.0098
ssc-miR-30d	RASAL2	RAS protein activator like 2 [Source:VGNC Symbol;Acc:VGNC:92105]	2.07	0.0098
ssc-miR-30d	RASD1	ras related dexamethasone induced 1 [Source:VGNC Symbol;Acc:VGNC:92107]	2.07	0.0098
ssc-miR-30d	RASEF	hypothetical gene	2.07	0.0098
ssc-miR-30d	RASGEF1A	RasGEF domain family member 1A [Source:VGNC Symbol;Acc:VGNC:92108]	2.07	0.0098
ssc-miR-30d	RASGEF1B	RasGEF domain family member 1B [Source:VGNC Symbol;Acc:VGNC:92109]	2.07	0.0098
ssc-miR-30d	RASGRF2	Ras protein specific guanine nucleotide releasing factor 2 [Source:VGNC Symbol;Acc:VGNC:92112]	2.07	0.0098
ssc-miR-30d	RASGRP3	RAS guanyl releasing protein 3 [Source:VGNC Symbol;Acc:VGNC:92115]	2.07	0.0098
ssc-miR-30d	RASL12	RAS like family 12 [Source:VGNC Symbol;Acc:VGNC:92121]	2.07	0.0098
ssc-miR-30d	RASSF4	Ras association domain family member 4 [Source:NCBI gene (formerly Entrezgene);Acc:100152580]	2.07	0.0098
ssc-miR-30d	RASSF8	Ras association domain family member 8 [Source:VGNC Symbol;Acc:VGNC:92128]	2.07	0.0098
ssc-miR-30d	RAVER2	ribonucleoprotein, PTB binding 2 [Source:VGNC Symbol;Acc:VGNC:98283]	2.07	0.0098
ssc-miR-30d	RBFOX1	RNA binding fox-1 homolog 1 [Source:VGNC Symbol;Acc:VGNC:92139]	2.07	0.0098
ssc-miR-30d	RBM12	RNA binding motif protein 12 [Source:HGNC Symbol;Acc:HGNC:9898]	2.07	0.0098
ssc-miR-30d	RBM15B	RNA binding motif protein 15B [Source:VGNC Symbol;Acc:VGNC:98285]	2.07	0.0098
ssc-miR-30d	RCAN1	regulator of calcineurin 1 [Source:VGNC Symbol;Acc:VGNC:92170]	2.07	0.0098
ssc-miR-30d	RCBTB1	RCC1 and BTB domain containing protein 1 [Source:VGNC Symbol;Acc:VGNC:92173]	2.07	0.0098
ssc-miR-30d	RCC2	regulator of chromosome condensation 2 [Source:VGNC Symbol;Acc:VGNC:98547]	2.07	0.0098
ssc-miR-30d	RCOR1	REST corepressor 1 [Source:VGNC Symbol;Acc:VGNC:92183]	2.07	0.0098
ssc-miR-30d	RECK	reversion inducing cysteine rich protein with kazal motifs [Source:VGNC Symbol;Acc:VGNC:92194]	2.07	0.0098
ssc-miR-30d	REEP1	receptor accessory protein 1 [Source:VGNC Symbol;Acc:VGNC:92197]	2.07	0.0098
ssc-miR-30d	REEP3	receptor accessory protein 3 [Source:VGNC Symbol;Acc:VGNC:92199]	2.07	0.0098
ssc-miR-30d	REV1	REV1 DNA directed polymerase [Source:HGNC Symbol;Acc:HGNC:14060]	2.07	0.0098
ssc-miR-30d	REV3L	REV3 like, DNA directed polymerase zeta catalytic subunit [Source:VGNC Symbol;Acc:VGNC:92226]	2.07	0.0098
ssc-miR-30d	RFFL	ring finger and FYVE like domain containing E3 ubiquitin protein ligase [Source:VGNC Symbol;Acc:VGNC:98289]	2.07	0.0098
ssc-miR-30d	RFTN2	raftlin family member 2 [Source:VGNC Symbol;Acc:VGNC:96128]	2.07	0.0098
ssc-miR-30d	RFX2	regulatory factor X2 [Source:VGNC Symbol;Acc:VGNC:92244]	2.07	0.0098
ssc-miR-30d	RFX3	regulatory factor X3 [Source:VGNC Symbol;Acc:VGNC:92245]	2.07	0.0098
ssc-miR-30d	RFX6	regulatory factor X6 [Source:VGNC Symbol;Acc:VGNC:92247]	2.07	0.0098
ssc-miR-30d	RFX7	regulatory factor X7 [Source:VGNC Symbol;Acc:VGNC:92248]	2.07	0.0098
ssc-miR-30d	RGL1	ral guanine nucleotide dissociation stimulator like 1 [Source:VGNC Symbol;Acc:VGNC:92252]	2.07	0.0098
ssc-miR-30d	RGS17	regulator of G protein signaling 17 [Source:VGNC Symbol;Acc:VGNC:92263]	2.07	0.0098
ssc-miR-30d	RGS9BP	regulator of G protein signaling 9 binding protein [Source:HGNC Symbol;Acc:HGNC:30304]	2.07	0.0098
ssc-miR-30d	RHEBL1	RHEB like 1 [Source:VGNC Symbol;Acc:VGNC:92280]	2.07	0.0098

ssc-miR-30d	RHOB	ras homolog family member B [Source:NCBI gene (formerly Entrezgene);Acc:100144503]	2.07	0.0098
ssc-miR-30d	RHOH	ras homolog family member H [Source:VGNC Symbol;Acc:VGNC:92290]	2.07	0.0098
ssc-miR-30d	RIMBP2	RIMS binding protein 2 [Source:VGNC Symbol;Acc:VGNC:92306]	2.07	0.0098
ssc-miR-30d	RIMKLB	ribosomal modification protein rimK like family member B [Source:VGNC Symbol;Acc:VGNC:92308]	2.07	0.0098
ssc-miR-30d	RIMS1	regulating synaptic membrane exocytosis 1 [Source:VGNC Symbol;Acc:VGNC:92309]	2.07	0.0098
ssc-miR-30d	RIOK3	RIO kinase 3 [Source:VGNC Symbol;Acc:VGNC:92316]	2.07	0.0098
ssc-miR-30d	RLIM	ring finger protein, LIM domain interacting [Source:HGNC Symbol;Acc:HGNC:13429]	2.07	0.0098
ssc-miR-30d	RNF115	ring finger protein 115 [Source:VGNC Symbol;Acc:VGNC:92348]	2.07	0.0098
ssc-miR-30d	RNF122	ring finger protein 122 [Source:VGNC Symbol;Acc:VGNC:92350]	2.07	0.0098
ssc-miR-30d	RNF157	ring finger protein 157 [Source:VGNC Symbol;Acc:VGNC:92365]	2.07	0.0098
ssc-miR-30d	RNF165	ring finger protein 165 [Source:VGNC Symbol;Acc:VGNC:92366]	2.07	0.0098
ssc-miR-30d	RNF169	ring finger protein 169 [Source:VGNC Symbol;Acc:VGNC:92368]	2.07	0.0098
ssc-miR-30d	RNF213	hypothetical gene	2.07	0.0098
ssc-miR-30d	RNF217	ring finger protein 217 [Source:VGNC Symbol;Acc:VGNC:103162]	2.07	0.0098
ssc-miR-30d	RNF220	ring finger protein 220 [Source:VGNC Symbol;Acc:VGNC:98602]	2.07	0.0098
ssc-miR-30d	RNF24	ring finger protein 24 [Source:HGNC Symbol;Acc:HGNC:13779]	2.07	0.0098
ssc-miR-30d	RNF34	ring finger protein 34 [Source:VGNC Symbol;Acc:VGNC:92389]	2.07	0.0098
ssc-miR-30d	RNF44	ring finger protein 44 [Source:VGNC Symbol;Acc:VGNC:92395]	2.07	0.0098
ssc-miR-30d	RNF7	ring finger protein 7 [Source:VGNC Symbol;Acc:VGNC:92398]	2.07	0.0098
ssc-miR-30d	RNMT	RNA guanine-7 methyltransferase [Source:VGNC Symbol;Acc:VGNC:92402]	2.07	0.0098
ssc-miR-30d	ROCK2	Rho associated coiled-coil containing protein kinase 2 [Source:HGNC Symbol;Acc:HGNC:10252]	2.07	0.0098
ssc-miR-30d	ROR1	receptor tyrosine kinase like orphan receptor 1 [Source:VGNC Symbol;Acc:VGNC:98295]	2.07	0.0098
ssc-miR-30d	ROR2	receptor tyrosine kinase like orphan receptor 2 [Source:VGNC Symbol;Acc:VGNC:92407]	2.07	0.0098
ssc-miR-30d	RORA	RAR related orphan receptor A [Source:VGNC Symbol;Acc:VGNC:92408]	2.07	0.0098
ssc-miR-30d	RP1-170O19.20	hypothetical gene	2.07	0.0098
ssc-miR-30d	RP11-160N1.10	hypothetical gene	2.07	0.0098
ssc-miR-30d	RP11-497E19.2	hypothetical gene	2.07	0.0098
ssc-miR-30d	RP11-766F14.2	hypothetical gene	2.07	0.0098
ssc-miR-30d	RP13-996F3.5	hypothetical gene	2.07	0.0098
ssc-miR-30d	RP6-24A23.6	hypothetical gene	2.07	0.0098
ssc-miR-30d	RPA1	replication protein A1 [Source:VGNC Symbol;Acc:VGNC:92413]	2.07	0.0098
ssc-miR-30d	RPP14	hypothetical gene	2.07	0.0098
ssc-miR-30d	RPRD1A	regulation of nuclear pre-mRNA domain containing 1A [Source:VGNC Symbol;Acc:VGNC:92435]	2.07	0.0098
ssc-miR-30d	RPRD2	regulation of nuclear pre-mRNA domain containing 2 [Source:VGNC Symbol;Acc:VGNC:92436]	2.07	0.0098
ssc-miR-30d	RPS6KA2	ribosomal protein S6 kinase A2 [Source:VGNC Symbol;Acc:VGNC:92441]	2.07	0.0098
ssc-miR-30d	RPS6KA5	ribosomal protein S6 kinase A5 [Source:VGNC Symbol;Acc:VGNC:92444]	2.07	0.0098
ssc-miR-30d	RRAD	RRAD, Ras related glycolysis inhibitor and calcium channel regulator [Source:VGNC Symbol;Acc:VGNC:92454]	2.07	0.0098
ssc-miR-30d	RSF1	remodeling and spacing factor 1 [Source:VGNC Symbol;Acc:VGNC:92476]	2.07	0.0098
ssc-miR-30d	RTCB	RNA 2',3'-cyclic phosphate and 5'-OH ligase [Source:VGNC Symbol;Acc:VGNC:92492]	2.07	0.0098
ssc-miR-30d	RTKN2	rhotekin 2 [Source:VGNC Symbol;Acc:VGNC:92495]	2.07	0.0098
ssc-miR-30d	RTN4R	reticulon 4 receptor [Source:HGNC Symbol;Acc:HGNC:18601]	2.07	0.0098
ssc-miR-30d	RUNDC3B	RUN domain containing 3B [Source:VGNC Symbol;Acc:VGNC:92515]	2.07	0.0098
ssc-miR-30d	RUNX1	RUNX family transcription factor 1 [Source:VGNC Symbol;Acc:VGNC:92516]	2.07	0.0098
ssc-miR-30d	RUNX2	RUNX family transcription factor 2 [Source:VGNC Symbol;Acc:VGNC:92517]	2.07	0.0098
ssc-miR-30d	RWDD4	RWD domain containing 4 [Source:VGNC Symbol;Acc:VGNC:96022]	2.07	0.0098
ssc-miR-30d	S100PBP	S100P binding protein [Source:VGNC Symbol;Acc:VGNC:92549]	2.07	0.0098
ssc-miR-30d	SACS	sacsin molecular chaperone [Source:HGNC Symbol;Acc:HGNC:10519]	2.07	0.0098
ssc-miR-30d	SALL4	spalt like transcription factor 4 [Source:NCBI gene (formerly Entrezgene);Acc:100136902]	2.07	0.0098
ssc-miR-30d	SAMD4A	sterile alpha motif domain containing 4A [Source:VGNC Symbol;Acc:VGNC:92569]	2.07	0.0098

ssc-miR-30d	SAMD8	sterile alpha motif domain containing 8 [Source:VGNC Symbol;Acc:VGNC:92571]	2.07	0.0098
ssc-miR-30d	SAP30	Sin3A associated protein 30 [Source:VGNC Symbol;Acc:VGNC:92575]	2.07	0.0098
ssc-miR-30d	SAP30BP	SAP30 binding protein [Source:VGNC Symbol;Acc:VGNC:92576]	2.07	0.0098
ssc-miR-30d	SAR1B	secretion associated Ras related GTPase 1B [Source:VGNC Symbol;Acc:VGNC:92580]	2.07	0.0098
ssc-miR-30d	SATB1	SATB homeobox 1 [Source:VGNC Symbol;Acc:VGNC:92587]	2.07	0.0098
ssc-miR-30d	SATB2	SATB homeobox 2 [Source:VGNC Symbol;Acc:VGNC:95972]	2.07	0.0098
ssc-miR-30d	SBF1	SET binding factor 1 [Source:VGNC Symbol;Acc:VGNC:92593]	2.07	0.0098
ssc-miR-30d	SBK1	SH3 domain binding kinase 1 [Source:HGNC Symbol;Acc:HGNC:17699]	2.07	0.0098
ssc-miR-30d	SCAF4	SR-related CTD associated factor 4 [Source:VGNC Symbol;Acc:VGNC:92603]	2.07	0.0098
ssc-miR-30d	SCAMP1	secretory carrier membrane protein 1 [Source:VGNC Symbol;Acc:VGNC:92605]	2.07	0.0098
ssc-miR-30d	SCN1A	sodium voltage-gated channel alpha subunit 1 [Source:VGNC Symbol;Acc:VGNC:95478]	2.07	0.0098
ssc-miR-30d	SCN2A	hypothetical gene	2.07	0.0098
ssc-miR-30d	SCN3A	sodium voltage-gated channel alpha subunit 3 [Source:VGNC Symbol;Acc:VGNC:95479]	2.07	0.0098
ssc-miR-30d	SCN8A	sodium voltage-gated channel alpha subunit 8 [Source:VGNC Symbol;Acc:VGNC:92638]	2.07	0.0098
ssc-miR-30d	SCN9A	sodium voltage-gated channel alpha subunit 9 [Source:VGNC Symbol;Acc:VGNC:95481]	2.07	0.0098
ssc-miR-30d	SCYL3	SCY1 like pseudokinase 3 [Source:VGNC Symbol;Acc:VGNC:92653]	2.07	0.0098
ssc-miR-30d	SDAD1	SDA1 domain containing 1 [Source:VGNC Symbol;Acc:VGNC:98961]	2.07	0.0098
ssc-miR-30d	SDC2	syndecan 2 [Source:VGNC Symbol;Acc:VGNC:92655]	2.07	0.0098
ssc-miR-30d	SDCBP	syndecan binding protein [Source:VGNC Symbol;Acc:VGNC:92657]	2.07	0.0098
ssc-miR-30d	SDCCAG3	hypothetical gene	2.07	0.0098
ssc-miR-30d	SDK2	hypothetical gene	2.07	0.0098
ssc-miR-30d	SEC14L2	SEC14 like lipid binding 2 [Source:NCBI gene (formerly Entrezgene);Acc:100152451]	2.07	0.0098
ssc-miR-30d	SEC22A	SEC22 homolog A, vesicle trafficking protein [Source:VGNC Symbol;Acc:VGNC:92673]	2.07	0.0098
ssc-miR-30d	SEC22C	SEC22 homolog C, vesicle trafficking protein [Source:VGNC Symbol;Acc:VGNC:92674]	2.07	0.0098
ssc-miR-30d	SEC23A	hypothetical gene	2.07	0.0098
ssc-miR-30d	SEC23IP	SEC23 interacting protein [Source:VGNC Symbol;Acc:VGNC:92675]	2.07	0.0098
ssc-miR-30d	SEC24A	SEC24 homolog A, COPII coat complex component [Source:VGNC Symbol;Acc:VGNC:92676]	2.07	0.0098
ssc-miR-30d	SEC61A2	SEC61 translocon subunit alpha 2 [Source:VGNC Symbol;Acc:VGNC:95457]	2.07	0.0098
ssc-miR-30d	SEC62	SEC62 homolog, preprotein translocation factor [Source:VGNC Symbol;Acc:VGNC:108691]	2.07	0.0098
ssc-miR-30d	SEL1L3	SEL1L family member 3 [Source:VGNC Symbol;Acc:VGNC:92688]	2.07	0.0098
ssc-miR-30d	SEMA3A	semaphorin 3A [Source:VGNC Symbol;Acc:VGNC:92693]	2.07	0.0098
ssc-miR-30d	SEMA6B	semaphorin 6B [Source:VGNC Symbol;Acc:VGNC:92709]	2.07	0.0098
ssc-miR-30d	SEMA6D	semaphorin 6D [Source:VGNC Symbol;Acc:VGNC:92711]	2.07	0.0098
ssc-miR-30d	SENP5	SUMO specific peptidase 5 [Source:VGNC Symbol;Acc:VGNC:92714]	2.07	0.0098
ssc-miR-30d	SEPHS1	selenophosphate synthetase 1 [Source:VGNC Symbol;Acc:VGNC:104053]	2.07	0.0098
ssc-miR-30d	SEPT3	hypothetical gene	2.07	0.0098
ssc-miR-30d	SEPT7	hypothetical gene	2.07	0.0098
ssc-miR-30d	SEPT8	hypothetical gene	2.07	0.0098
ssc-miR-30d	SERPINE1	serpin family E member 1 [Source:VGNC Symbol;Acc:VGNC:98310]	2.07	0.0098
ssc-miR-30d	SERPINF2	serpin family F member 2 [Source:VGNC Symbol;Acc:VGNC:92744]	2.07	0.0098
ssc-miR-30d	SETD1B	SET domain containing 1B, histone lysine methyltransferase [Source:HGNC Symbol;Acc:HGNC:29187]	2.07	0.0098
ssc-miR-30d	SETD3	SET domain containing 3, actin histidine methyltransferase [Source:VGNC Symbol;Acc:VGNC:92758]	2.07	0.0098
ssc-miR-30d	SETD5	SET domain containing 5 [Source:VGNC Symbol;Acc:VGNC:92760]	2.07	0.0098
ssc-miR-30d	SETD7	SET domain containing 7, histone lysine methyltransferase [Source:VGNC Symbol;Acc:VGNC:92762]	2.07	0.0098
ssc-miR-30d	SETD9	SET domain containing 9 [Source:VGNC Symbol;Acc:VGNC:92763]	2.07	0.0098
ssc-miR-30d	SFMBT1	Scm like with four mbt domains 1 [Source:VGNC Symbol;Acc:VGNC:92773]	2.07	0.0098
ssc-miR-30d	SFXN1	sideroflexin 1 [Source:VGNC Symbol;Acc:VGNC:92783]	2.07	0.0098
ssc-miR-30d	SGCB	sarcoglycan beta [Source:NCBI gene (formerly Entrezgene);Acc:100135674]	2.07	0.0098
ssc-miR-30d	SGK3	serum/glucocorticoid regulated kinase family member 3 [Source:HGNC Symbol;Acc:HGNC:10812]	2.07	0.0098

ssc-miR-30d	SGMS2	sphingomyelin synthase 2 [Source:VGNC Symbol;Acc:VGNC:92795]	2.07	0.0098
ssc-miR-30d	SGSM1	small G protein signaling modulator 1 [Source:VGNC Symbol;Acc:VGNC:92800]	2.07	0.0098
ssc-miR-30d	SH2B3	SH2B adaptor protein 3 [Source:VGNC Symbol;Acc:VGNC:92807]	2.07	0.0098
ssc-miR-30d	SH3GLB1	SH3 domain containing GRB2 like, endophilin B1 [Source:VGNC Symbol;Acc:VGNC:92826]	2.07	0.0098
ssc-miR-30d	SH3KBP1	SH3 domain containing kinase binding protein 1 [Source:VGNC Symbol;Acc:VGNC:92828]	2.07	0.0098
ssc-miR-30d	SH3PXD2A	SH3 and PX domains 2A [Source:VGNC Symbol;Acc:VGNC:92829]	2.07	0.0098
ssc-miR-30d	SH3RF1	SH3 domain containing ring finger 1 [Source:VGNC Symbol;Acc:VGNC:98316]	2.07	0.0098
ssc-miR-30d	SHISA2	shisa family member 2 [Source:VGNC Symbol;Acc:VGNC:92845]	2.07	0.0098
ssc-miR-30d	SHOC2	SHOC2 leucine rich repeat scaffold protein [Source:VGNC Symbol;Acc:VGNC:98318]	2.07	0.0098
ssc-miR-30d	SHROOM1	shroom family member 1 [Source:VGNC Symbol;Acc:VGNC:92860]	2.07	0.0098
ssc-miR-30d	SIAH2	siah E3 ubiquitin protein ligase 2 [Source:VGNC Symbol;Acc:VGNC:92866]	2.07	0.0098
ssc-miR-30d	SIDT2	SID1 transmembrane family member 2 [Source:VGNC Symbol;Acc:VGNC:92869]	2.07	0.0098
ssc-miR-30d	SIK3	SIK family kinase 3 [Source:VGNC Symbol;Acc:VGNC:92873]	2.07	0.0098
ssc-miR-30d	SIM1	SIM bHLH transcription factor 1 [Source:VGNC Symbol;Acc:VGNC:92876]	2.07	0.0098
ssc-miR-30d	SIRT1	sirtuin 1 [Source:VGNC Symbol;Acc:VGNC:92884]	2.07	0.0098
ssc-miR-30d	SIX1	SIX homeobox 1 [Source:VGNC Symbol;Acc:VGNC:92892]	2.07	0.0098
ssc-miR-30d	SIX4	SIX homeobox 4 [Source:VGNC Symbol;Acc:VGNC:92895]	2.07	0.0098
ssc-miR-30d	SKIL	SKI like proto-onco [Source:VGNC Symbol;Acc:VGNC:92903]	2.07	0.0098
ssc-miR-30d	SKP2	S-phase kinase associated protein 2 [Source:VGNC Symbol;Acc:VGNC:92907]	2.07	0.0098
ssc-miR-30d	SLAIN2	SLAIN motif family member 2 [Source:VGNC Symbol;Acc:VGNC:92910]	2.07	0.0098
ssc-miR-30d	SLC12A6	solute carrier family 12 member 6 [Source:VGNC Symbol;Acc:VGNC:92925]	2.07	0.0098
ssc-miR-30d	SLC16A14	solute carrier family 16 member 14 [Source:VGNC Symbol;Acc:VGNC:96129]	2.07	0.0098
ssc-miR-30d	SLC18A2	solute carrier family 18 member A2 [Source:VGNC Symbol;Acc:VGNC:92958]	2.07	0.0098
ssc-miR-30d	SLC22A23	solute carrier family 22 member 23 [Source:HGNC Symbol;Acc:HGNC:21106]	2.07	0.0098
ssc-miR-30d	SLC22A5	solute carrier family 22 member 5 [Source:HGNC Symbol;Acc:HGNC:10969]	2.07	0.0098
ssc-miR-30d	SLC25A34	solute carrier family 25 member 34 [Source:VGNC Symbol;Acc:VGNC:93010]	2.07	0.0098
ssc-miR-30d	SLC25A36	solute carrier family 25 member 36 [Source:VGNC Symbol;Acc:VGNC:93012]	2.07	0.0098
ssc-miR-30d	SLC29A3	solute carrier family 29 member 3 [Source:VGNC Symbol;Acc:VGNC:93044]	2.07	0.0098
ssc-miR-30d	SLC30A10	hypothetical gene	2.07	0.0098
ssc-miR-30d	SLC30A2	solute carrier family 30 member 2 [Source:VGNC Symbol;Acc:VGNC:93056]	2.07	0.0098
ssc-miR-30d	SLC30A4	solute carrier family 30 member 4 [Source:VGNC Symbol;Acc:VGNC:93058]	2.07	0.0098
ssc-miR-30d	SLC30A5	solute carrier family 30 member 5 [Source:VGNC Symbol;Acc:VGNC:93059]	2.07	0.0098
ssc-miR-30d	SLC35A3	solute carrier family 35 member A3 [Source:VGNC Symbol;Acc:VGNC:98860]	2.07	0.0098
ssc-miR-30d	SLC35A5	solute carrier family 35 member A5 [Source:VGNC Symbol;Acc:VGNC:93070]	2.07	0.0098
ssc-miR-30d	SLC35B4	solute carrier family 35 member B4 [Source:VGNC Symbol;Acc:VGNC:93074]	2.07	0.0098
ssc-miR-30d	SLC35C1	solute carrier family 35 member C1 [Source:VGNC Symbol;Acc:VGNC:93075]	2.07	0.0098
ssc-miR-30d	SLC35D3	solute carrier family 35 member D3 [Source:VGNC Symbol;Acc:VGNC:93076]	2.07	0.0098
ssc-miR-30d	SLC35F1	solute carrier family 35 member F1 [Source:VGNC Symbol;Acc:VGNC:93081]	2.07	0.0098
ssc-miR-30d	SLC35F3	solute carrier family 35 member F3 [Source:VGNC Symbol;Acc:VGNC:93083]	2.07	0.0098
ssc-miR-30d	SLC35F4	solute carrier family 35 member F4 [Source:VGNC Symbol;Acc:VGNC:93084]	2.07	0.0098
ssc-miR-30d	SLC36A1	solute carrier family 36 member 1 [Source:VGNC Symbol;Acc:VGNC:93088]	2.07	0.0098
ssc-miR-30d	SLC38A1	solute carrier family 38 member 1 [Source:HGNC Symbol;Acc:HGNC:13447]	2.07	0.0098
ssc-miR-30d	SLC38A2	solute carrier family 38 member 2 [Source:VGNC Symbol;Acc:VGNC:93094]	2.07	0.0098
ssc-miR-30d	SLC38A4	solute carrier family 38 member 4 [Source:VGNC Symbol;Acc:VGNC:93096]	2.07	0.0098
ssc-miR-30d	SLC38A7	solute carrier family 38 member 7 [Source:HGNC Symbol;Acc:HGNC:25582]	2.07	0.0098
ssc-miR-30d	SLC39A11	solute carrier family 39 member 11 [Source:VGNC Symbol;Acc:VGNC:93102]	2.07	0.0098
ssc-miR-30d	SLC41A2	solute carrier family 41 member 2 [Source:VGNC Symbol;Acc:VGNC:93114]	2.07	0.0098
ssc-miR-30d	SLC4A7	solute carrier family 4 member 7 [Source:VGNC Symbol;Acc:VGNC:93135]	2.07	0.0098
ssc-miR-30d	SLC5A11	solute carrier family 5 member 11 [Source:VGNC Symbol;Acc:VGNC:93141]	2.07	0.0098

ssc-miR-30d	SLC5A3	solute carrier family 5 member 3 [Source:VGNC Symbol;Acc:VGNC:93144]	2.07	0.0098
ssc-miR-30d	SLC6A15	solute carrier family 6 member 15 [Source:VGNC Symbol;Acc:VGNC:93157]	2.07	0.0098
ssc-miR-30d	SLC6A3	solute carrier family 6 member 3 [Source:VGNC Symbol;Acc:VGNC:93163]	2.07	0.0098
ssc-miR-30d	SLC6A6	solute carrier family 6 member 6 [Source:VGNC Symbol;Acc:VGNC:93166]	2.07	0.0098
ssc-miR-30d	SLC6A9	solute carrier family 6 member 9 [Source:VGNC Symbol;Acc:VGNC:93168]	2.07	0.0098
ssc-miR-30d	SLC7A10	solute carrier family 7 member 10 [Source:VGNC Symbol;Acc:VGNC:93170]	2.07	0.0098
ssc-miR-30d	SLC7A11	solute carrier family 7 member 11 [Source:VGNC Symbol;Acc:VGNC:93171]	2.07	0.0098
ssc-miR-30d	SLC7A6	hypothetical gene	2.07	0.0098
ssc-miR-30d	SLC9A4	solute carrier family 9 member A4 [Source:VGNC Symbol;Acc:VGNC:93186]	2.07	0.0098
ssc-miR-30d	SLC9A8	solute carrier family 9 member A8 [Source:VGNC Symbol;Acc:VGNC:95943]	2.07	0.0098
ssc-miR-30d	SMAD1	SMAD family member 1 [Source:VGNC Symbol;Acc:VGNC:93216]	2.07	0.0098
ssc-miR-30d	SMAD2	SMAD family member 2 [Source:VGNC Symbol;Acc:VGNC:98329]	2.07	0.0098
ssc-miR-30d	SMAD5	SMAD family member 5 [Source:VGNC Symbol;Acc:VGNC:93219]	2.07	0.0098
ssc-miR-30d	SMAP1	small ArfGAP 1 [Source:VGNC Symbol;Acc:VGNC:93223]	2.07	0.0098
ssc-miR-30d	SMARCA5	SWI/SNF related, matrix associated, actin dependent regulator of chromatin, subfamily a, member 5 [Source:VGNC Symbol;Acc:VGNC:93228]	2.07	0.0098
ssc-miR-30d	SMARCD2	SWI/SNF related, matrix associated, actin dependent regulator of chromatin, subfamily d, member 2 [Source:NCBI gene (formerly Entrezgene);Acc:100512367]	2.07	0.0098
ssc-miR-30d	SMIM14	small integral membrane protein 14 [Source:VGNC Symbol;Acc:VGNC:93252]	2.07	0.0098
ssc-miR-30d	SNAI1	snail family transcriptional repressor 1 [Source:VGNC Symbol;Acc:VGNC:95508]	2.07	0.0098
ssc-miR-30d	SNAPC3	small nuclear RNA activating complex polypeptide 3 [Source:VGNC Symbol;Acc:VGNC:103182]	2.07	0.0098
ssc-miR-30d	SNAPIN	SNAP associated protein [Source:VGNC Symbol;Acc:VGNC:93284]	2.07	0.0098
ssc-miR-30d	SNTB2	syntrophin beta 2 [Source:VGNC Symbol;Acc:VGNC:93298]	2.07	0.0098
ssc-miR-30d	SNX1	sorting nexin 1 [Source:HGNC Symbol;Acc:HGNC:11172]	2.07	0.0098
ssc-miR-30d	SNX10	sorting nexin 10 [Source:VGNC Symbol;Acc:VGNC:93303]	2.07	0.0098
ssc-miR-30d	SNX16	sorting nexin 16 [Source:VGNC Symbol;Acc:VGNC:93308]	2.07	0.0098
ssc-miR-30d	SNX18	sorting nexin 18 [Source:VGNC Symbol;Acc:VGNC:93310]	2.07	0.0098
ssc-miR-30d	SNX2	sorting nexin 2 [Source:VGNC Symbol;Acc:VGNC:93312]	2.07	0.0098
ssc-miR-30d	SNX27	sorting nexin 27 [Source:VGNC Symbol;Acc:VGNC:93316]	2.07	0.0098
ssc-miR-30d	SNX29	sorting nexin 29 [Source:VGNC Symbol;Acc:VGNC:98331]	2.07	0.0098
ssc-miR-30d	SNX30	sorting nexin family member 30 [Source:VGNC Symbol;Acc:VGNC:93318]	2.07	0.0098
ssc-miR-30d	SNX33	sorting nexin 33 [Source:VGNC Symbol;Acc:VGNC:93321]	2.07	0.0098
ssc-miR-30d	SNX6	sorting nexin 6 [Source:VGNC Symbol;Acc:VGNC:93323]	2.07	0.0098
ssc-miR-30d	SNX8	sorting nexin 8 [Source:VGNC Symbol;Acc:VGNC:93325]	2.07	0.0098
ssc-miR-30d	SOBP	sine oculis binding protein homolog [Source:VGNC Symbol;Acc:VGNC:93329]	2.07	0.0098
ssc-miR-30d	SOCS1	suppressor of cytokine signaling 1 [Source:VGNC Symbol;Acc:VGNC:93330]	2.07	0.0098
ssc-miR-30d	SOCS3	suppressor of cytokine signaling 3 [Source:VGNC Symbol;Acc:VGNC:99052]	2.07	0.0098
ssc-miR-30d	SOCS6	suppressor of cytokine signaling 6 [Source:VGNC Symbol;Acc:VGNC:93333]	2.07	0.0098
ssc-miR-30d	SOGA3	SOGA family member 3 [Source:HGNC Symbol;Acc:HGNC:21494]	2.07	0.0098
ssc-miR-30d	SON	SON DNA and RNA binding protein [Source:VGNC Symbol;Acc:VGNC:93336]	2.07	0.0098
ssc-miR-30d	SORCS3	sortilin related VPS10 domain containing receptor 3 [Source:VGNC Symbol;Acc:VGNC:93341]	2.07	0.0098
ssc-miR-30d	SOS1	SOS Ras/Rac guanine nucleotide exchange factor 1 [Source:HGNC Symbol;Acc:HGNC:11187]	2.07	0.0098
ssc-miR-30d	SOX12	SRY-box transcription factor 12 [Source:HGNC Symbol;Acc:HGNC:11198]	2.07	0.0098
ssc-miR-30d	SOX13	SRY-box transcription factor 13 [Source:VGNC Symbol;Acc:VGNC:93352]	2.07	0.0098
ssc-miR-30d	SOX4	SRY-box transcription factor 4 [Source:HGNC Symbol;Acc:HGNC:11200]	2.07	0.0098
ssc-miR-30d	SOX9	SRY-box transcription factor 9 [Source:VGNC Symbol;Acc:VGNC:99053]	2.07	0.0098
ssc-miR-30d	SP4	Sp4 transcription factor [Source:VGNC Symbol;Acc:VGNC:93362]	2.07	0.0098
ssc-miR-30d	SPAST	spastin [Source:VGNC Symbol;Acc:VGNC:98333]	2.07	0.0098
ssc-miR-30d	SPATS2L	spermatosis associated serine rich 2 like [Source:VGNC Symbol;Acc:VGNC:96047]	2.07	0.0098
ssc-miR-30d	SPCS3	signal peptidase complex subunit 3 [Source:VGNC Symbol;Acc:VGNC:96119]	2.07	0.0098
ssc-miR-30d	SPEN	hypothetical gene	2.07	0.0098

ssc-miR-30d	SPHKAP	SPHK1 interactor, AKAP domain containing [Source:VGNC Symbol;Acc:VGNC:96245]	2.07	0.0098
ssc-miR-30d	SPIN1	spindlin 1 [Source:VGNC Symbol;Acc:VGNC:101495]	2.07	0.0098
ssc-miR-30d	SPOPL	speckle type BTB/POZ protein like [Source:VGNC Symbol;Acc:VGNC:96161]	2.07	0.0098
ssc-miR-30d	SPRYD7	SPRY domain containing 7 [Source:VGNC Symbol;Acc:VGNC:93429]	2.07	0.0098
ssc-miR-30d	SPTLC2	serine palmitoyltransferase long chain base subunit 2 [Source:VGNC Symbol;Acc:VGNC:93440]	2.07	0.0098
ssc-miR-30d	SPTSSB	serine palmitoyltransferase small subunit B [Source:VGNC Symbol;Acc:VGNC:93441]	2.07	0.0098
ssc-miR-30d	SRGAP3	SLIT-ROBO Rho GTPase activating protein 3 [Source:VGNC Symbol;Acc:VGNC:93455]	2.07	0.0098
ssc-miR-30d	SRI	sorcin [Source:VGNC Symbol;Acc:VGNC:93457]	2.07	0.0098
ssc-miR-30d	SRP14	signal recognition particle 14 [Source:VGNC Symbol;Acc:VGNC:93458]	2.07	0.0098
ssc-miR-30d	SRSF10	serine and arginine rich splicing factor 10 [Source:VGNC Symbol;Acc:VGNC:93472]	2.07	0.0098
ssc-miR-30d	SRSF12	serine and arginine rich splicing factor 12 [Source:VGNC Symbol;Acc:VGNC:93474]	2.07	0.0098
ssc-miR-30d	SRSF7	serine and arginine rich splicing factor 7 [Source:VGNC Symbol;Acc:VGNC:93477]	2.07	0.0098
ssc-miR-30d	SSBP2	single stranded DNA binding protein 2 [Source:VGNC Symbol;Acc:VGNC:93480]	2.07	0.0098
ssc-miR-30d	SSBP3	single stranded DNA binding protein 3 [Source:VGNC Symbol;Acc:VGNC:93481]	2.07	0.0098
ssc-miR-30d	SSH1	slingshot protein phosphatase 1 [Source:VGNC Symbol;Acc:VGNC:93485]	2.07	0.0098
ssc-miR-30d	SSR1	signal sequence receptor subunit 1 [Source:VGNC Symbol;Acc:VGNC:93490]	2.07	0.0098
ssc-miR-30d	SSR3	signal sequence receptor subunit 3 [Source:VGNC Symbol;Acc:VGNC:93492]	2.07	0.0098
ssc-miR-30d	SSX2IP	SSX family member 2 interacting protein [Source:VGNC Symbol;Acc:VGNC:93499]	2.07	0.0098
ssc-miR-30d	ST6GALNAC5	ST6 N-acetylgalactosaminide alpha-2,6-sialyltransferase 5 [Source:VGNC Symbol;Acc:VGNC:93513]	2.07	0.0098
ssc-miR-30d	ST8SIA1	ST8 alpha-N-acetyl-neuraminide alpha-2,8-sialyltransferase 1 [Source:VGNC Symbol;Acc:VGNC:93516]	2.07	0.0098
ssc-miR-30d	ST8SIA4	ST8 alpha-N-acetyl-neuraminide alpha-2,8-sialyltransferase 4 [Source:VGNC Symbol;Acc:VGNC:93519]	2.07	0.0098
ssc-miR-30d	STAC	SH3 and cysteine rich domain [Source:HGNC Symbol;Acc:HGNC:11353]	2.07	0.0098
ssc-miR-30d	STAG2	stromal antigen 2 [Source:VGNC Symbol;Acc:VGNC:93525]	2.07	0.0098
ssc-miR-30d	STAU1	staufen double-stranded RNA binding protein 1 [Source:VGNC Symbol;Acc:VGNC:95525]	2.07	0.0098
ssc-miR-30d	STC1	stanniocalcin 1 [Source:VGNC Symbol;Acc:VGNC:93542]	2.07	0.0098
ssc-miR-30d	STIM2	stromal interaction molecule 2 [Source:VGNC Symbol;Acc:VGNC:98967]	2.07	0.0098
ssc-miR-30d	STK10	serine/threonine kinase 10 [Source:HGNC Symbol;Acc:HGNC:11388]	2.07	0.0098
ssc-miR-30d	STK17B	serine/threonine kinase 17b [Source:VGNC Symbol;Acc:VGNC:95527]	2.07	0.0098
ssc-miR-30d	STK35	serine/threonine kinase 35 [Source:VGNC Symbol;Acc:VGNC:95802]	2.07	0.0098
ssc-miR-30d	STK38L	serine/threonine kinase 38 like [Source:VGNC Symbol;Acc:VGNC:93557]	2.07	0.0098
ssc-miR-30d	STK39	serine/threonine kinase 39 [Source:VGNC Symbol;Acc:VGNC:98348]	2.07	0.0098
ssc-miR-30d	STK4	serine/threonine kinase 4 [Source:VGNC Symbol;Acc:VGNC:98349]	2.07	0.0098
ssc-miR-30d	STOML1	stomatin like 1 [Source:VGNC Symbol;Acc:VGNC:93566]	2.07	0.0098
ssc-miR-30d	STOX2	storkhead box 2 [Source:VGNC Symbol;Acc:VGNC:96082]	2.07	0.0098
ssc-miR-30d	STRIP1	striatin interacting protein 1 [Source:VGNC Symbol;Acc:VGNC:93576]	2.07	0.0098
ssc-miR-30d	STT3B	STT3 oligosaccharyltransferase complex catalytic subunit B [Source:VGNC Symbol;Acc:VGNC:93582]	2.07	0.0098
ssc-miR-30d	STX16	syntaxin 16 [Source:NCBI gene (formerly Entrezgene);Acc:100144526]	2.07	0.0098
ssc-miR-30d	STX17	syntaxin 17 [Source:VGNC Symbol;Acc:VGNC:93587]	2.07	0.0098
ssc-miR-30d	STX2	syntaxin 2 [Source:VGNC Symbol;Acc:VGNC:93590]	2.07	0.0098
ssc-miR-30d	STX6	syntaxin 6 [Source:VGNC Symbol;Acc:VGNC:93594]	2.07	0.0098
ssc-miR-30d	STX7	syntaxin 7 [Source:VGNC Symbol;Acc:VGNC:93595]	2.07	0.0098
ssc-miR-30d	STXBP1	syntaxin binding protein 1 [Source:VGNC Symbol;Acc:VGNC:93596]	2.07	0.0098
ssc-miR-30d	STXBP5	syntaxin binding protein 5 [Source:VGNC Symbol;Acc:VGNC:93599]	2.07	0.0098
ssc-miR-30d	STYX	serine/threonine/tyrosine interacting protein [Source:VGNC Symbol;Acc:VGNC:93602]	2.07	0.0098
ssc-miR-30d	SUCLG2	succinate-CoA ligase GDP-forming subunit beta [Source:VGNC Symbol;Acc:VGNC:93604]	2.07	0.0098
ssc-miR-30d	SUPT3H	SPT3 homolog, SAGA and STAGA complex component [Source:VGNC Symbol;Acc:VGNC:93623]	2.07	0.0098
ssc-miR-30d	SURF4	surfeit 4 [Source:VGNC Symbol;Acc:VGNC:93631]	2.07	0.0098
ssc-miR-30d	SUV39H2	SUV39H2 histone lysine methyltransferase [Source:VGNC Symbol;Acc:VGNC:95841]	2.07	0.0098
ssc-miR-30d	SV2C	synaptic vesicle glycoprotein 2C [Source:VGNC Symbol;Acc:VGNC:93641]	2.07	0.0098

ssc-miR-30d	SYNGR3	synaptogyrin 3 [Source:VGNC Symbol;Acc:VGNC:93666]	2.07	0.0098
ssc-miR-30d	SYPL1	synaptophysin like 1 [Source:HGNC Symbol;Acc:HGNC:11507]	2.07	0.0098
ssc-miR-30d	TAB3	TGF-beta activated kinase 1 (MAP3K7) binding protein 3 [Source:HGNC Symbol;Acc:HGNC:30681]	2.07	0.0098
ssc-miR-30d	TACC1	hypothetical gene	2.07	0.0098
ssc-miR-30d	TADA2B	transcriptional adaptor 2B [Source:VGNC Symbol;Acc:VGNC:93707]	2.07	0.0098
ssc-miR-30d	TAF4B	TATA-box binding protein associated factor 4b [Source:VGNC Symbol;Acc:VGNC:93716]	2.07	0.0098
ssc-miR-30d	TAOK1	TAO kinase 1 [Source:VGNC Symbol;Acc:VGNC:98355]	2.07	0.0098
ssc-miR-30d	TASP1	taspase 1 [Source:VGNC Symbol;Acc:VGNC:95676]	2.07	0.0098
ssc-miR-30d	TBC1D10B	TBC1 domain family member 10B [Source:VGNC Symbol;Acc:VGNC:93760]	2.07	0.0098
ssc-miR-30d	TBC1D15	TBC1 domain family member 15 [Source:VGNC Symbol;Acc:VGNC:93764]	2.07	0.0098
ssc-miR-30d	TBC1D2B	TBC1 domain family member 2B [Source:VGNC Symbol;Acc:VGNC:93774]	2.07	0.0098
ssc-miR-30d	TBC1D9	TBC1 domain family member 9 [Source:VGNC Symbol;Acc:VGNC:93781]	2.07	0.0098
ssc-miR-30d	TBL1X	hypothetical gene	2.07	0.0098
ssc-miR-30d	TBL1XR1	TBL1X receptor 1 [Source:VGNC Symbol;Acc:VGNC:96600]	2.07	0.0098
ssc-miR-30d	TBPL1	TATA-box binding protein like 1 [Source:VGNC Symbol;Acc:VGNC:93792]	2.07	0.0098
ssc-miR-30d	TCERG1	transcription elongation regulator 1 [Source:VGNC Symbol;Acc:VGNC:93815]	2.07	0.0098
ssc-miR-30d	TCF21	transcription factor 21 [Source:VGNC Symbol;Acc:VGNC:93819]	2.07	0.0098
ssc-miR-30d	TCF7	transcription factor 7 [Source:VGNC Symbol;Acc:VGNC:96767]	2.07	0.0098
ssc-miR-30d	TDG	thymine DNA glycosylase [Source:VGNC Symbol;Acc:VGNC:93842]	2.07	0.0098
ssc-miR-30d	TEAD1	TEA domain transcription factor 1 [Source:VGNC Symbol;Acc:VGNC:93853]	2.07	0.0098
ssc-miR-30d	TECPR1	tectonin beta-propeller repeat containing 1 [Source:VGNC Symbol;Acc:VGNC:93858]	2.07	0.0098
ssc-miR-30d	TENM1	teneurin transmembrane protein 1 [Source:VGNC Symbol;Acc:VGNC:98363]	2.07	0.0098
ssc-miR-30d	TENM3	teneurin transmembrane protein 3 [Source:VGNC Symbol;Acc:VGNC:96233]	2.07	0.0098
ssc-miR-30d	TET1	tet methylcytosine dioxygenase 1 [Source:VGNC Symbol;Acc:VGNC:93888]	2.07	0.0098
ssc-miR-30d	TET3	tet methylcytosine dioxygenase 3 [Source:VGNC Symbol;Acc:VGNC:93890]	2.07	0.0098
ssc-miR-30d	TEX2	testis expressed 2 [Source:VGNC Symbol;Acc:VGNC:93894]	2.07	0.0098
ssc-miR-30d	TFCP2L1	transcription factor CP2 like 1 [Source:HGNC Symbol;Acc:HGNC:17925]	2.07	0.0098
ssc-miR-30d	TFDP1	transcription factor Dp-1 [Source:HGNC Symbol;Acc:HGNC:11749]	2.07	0.0098
ssc-miR-30d	THBS2	thrombospondin 2 [Source:VGNC Symbol;Acc:VGNC:93947]	2.07	0.0098
ssc-miR-30d	TIA1	TIA1 cytotoxic granule associated RNA binding protein [Source:VGNC Symbol;Acc:VGNC:93975]	2.07	0.0098
ssc-miR-30d	TICAM2	hypothetical gene	2.07	0.0098
ssc-miR-30d	TIMM22	translocase of inner mitochondrial membrane 22 [Source:VGNC Symbol;Acc:VGNC:93988]	2.07	0.0098
ssc-miR-30d	TIMP2	hypothetical gene	2.07	0.0098
ssc-miR-30d	TIMP3	TIMP metallopeptidase inhibitor 3 [Source:VGNC Symbol;Acc:VGNC:93996]	2.07	0.0098
ssc-miR-30d	TLL2	tolloid like 2 [Source:VGNC Symbol;Acc:VGNC:94019]	2.07	0.0098
ssc-miR-30d	TMCC1	transmembrane and coiled-coil domain family 1 [Source:VGNC Symbol;Acc:VGNC:94046]	2.07	0.0098
ssc-miR-30d	TMED2	hypothetical gene	2.07	0.0098
ssc-miR-30d	TMEFF1	transmembrane protein with EGF like and two follistatin like domains 1 [Source:HGNC Symbol;Acc:HGNC:11866]	2.07	0.0098
ssc-miR-30d	TMEM106B	transmembrane protein 106B [Source:VGNC Symbol;Acc:VGNC:104075]	2.07	0.0098
ssc-miR-30d	TMEM110	hypothetical gene	2.07	0.0098
ssc-miR-30d	TMEM121	transmembrane protein 121 [Source:HGNC Symbol;Acc:HGNC:20511]	2.07	0.0098
ssc-miR-30d	TMEM133	hypothetical gene	2.07	0.0098
ssc-miR-30d	TMEM135	transmembrane protein 135 [Source:VGNC Symbol;Acc:VGNC:94085]	2.07	0.0098
ssc-miR-30d	TMEM154	transmembrane protein 154 [Source:VGNC Symbol;Acc:VGNC:94098]	2.07	0.0098
ssc-miR-30d	TMEM168	transmembrane protein 168 [Source:VGNC Symbol;Acc:VGNC:94106]	2.07	0.0098
ssc-miR-30d	TMEM170B	transmembrane protein 170B [Source:VGNC Symbol;Acc:VGNC:94108]	2.07	0.0098
ssc-miR-30d	TMEM181	transmembrane protein 181 [Source:VGNC Symbol;Acc:VGNC:94117]	2.07	0.0098
ssc-miR-30d	TMEM220	transmembrane protein 220 [Source:VGNC Symbol;Acc:VGNC:94142]	2.07	0.0098
ssc-miR-30d	TMEM229A	transmembrane protein 229A [Source:VGNC Symbol;Acc:VGNC:94146]	2.07	0.0098

ssc-miR-30d	TMEM245	transmembrane protein 245 [Source:HGNC Symbol;Acc:HGNC:1363]	2.07	0.0098
ssc-miR-30d	TMEM252	transmembrane protein 252 [Source:VGNC Symbol;Acc:VGNC:94158]	2.07	0.0098
ssc-miR-30d	TMEM41A	transmembrane protein 41A [Source:VGNC Symbol;Acc:VGNC:104080]	2.07	0.0098
ssc-miR-30d	TMEM55A	hypothetical gene	2.07	0.0098
ssc-miR-30d	TMEM56	hypothetical gene	2.07	0.0098
ssc-miR-30d	TMEM70	transmembrane protein 70 [Source:VGNC Symbol;Acc:VGNC:94204]	2.07	0.0098
ssc-miR-30d	TMEM86A	transmembrane protein 86A [Source:VGNC Symbol;Acc:VGNC:94210]	2.07	0.0098
ssc-miR-30d	TMEM87A	transmembrane protein 87A [Source:VGNC Symbol;Acc:VGNC:94212]	2.07	0.0098
ssc-miR-30d	TMOD2	tropomodulin 2 [Source:VGNC Symbol;Acc:VGNC:94227]	2.07	0.0098
ssc-miR-30d	TMOD3	tropomodulin 3 [Source:VGNC Symbol;Acc:VGNC:94228]	2.07	0.0098
ssc-miR-30d	TMTC3	transmembrane O-mannosyltransferase targeting cadherins 3 [Source:VGNC Symbol;Acc:VGNC:94243]	2.07	0.0098
ssc-miR-30d	TNFRSF8	hypothetical gene	2.07	0.0098
ssc-miR-30d	TNIK	TRAF2 and NCK interacting kinase [Source:VGNC Symbol;Acc:VGNC:98381]	2.07	0.0098
ssc-miR-30d	TNIP1	TNFAIP3 interacting protein 1 [Source:VGNC Symbol;Acc:VGNC:94275]	2.07	0.0098
ssc-miR-30d	TNKS	tankyrase [Source:VGNC Symbol;Acc:VGNC:95546]	2.07	0.0098
ssc-miR-30d	TNPO1	transportin 1 [Source:VGNC Symbol;Acc:VGNC:94287]	2.07	0.0098
ssc-miR-30d	TNPO3	transportin 3 [Source:VGNC Symbol;Acc:VGNC:94289]	2.07	0.0098
ssc-miR-30d	TNRC6A	trinucleotide repeat containing adaptor 6A [Source:VGNC Symbol;Acc:VGNC:94292]	2.07	0.0098
ssc-miR-30d	TNRC6B	trinucleotide repeat containing adaptor 6B [Source:VGNC Symbol;Acc:VGNC:94293]	2.07	0.0098
ssc-miR-30d	TNRC6C	trinucleotide repeat containing adaptor 6C [Source:VGNC Symbol;Acc:VGNC:94294]	2.07	0.0098
ssc-miR-30d	TNXB	tenascin XB [Source:NCBI gene (formerly Entrezgene);Acc:445520]	2.07	0.0098
ssc-miR-30d	TOR1B	torsin family 1 member B [Source:HGNC Symbol;Acc:HGNC:11995]	2.07	0.0098
ssc-miR-30d	TOX	thymocyte selection associated high mobility group box [Source:VGNC Symbol;Acc:VGNC:94322]	2.07	0.0098
ssc-miR-30d	TP53INP1	tumor protein p53 inducible nuclear protein 1 [Source:VGNC Symbol;Acc:VGNC:94329]	2.07	0.0098
ssc-miR-30d	TPM4	hypothetical gene	2.07	0.0098
ssc-miR-30d	TPRG1	tumor protein p63 regulated 1 [Source:VGNC Symbol;Acc:VGNC:94348]	2.07	0.0098
ssc-miR-30d	TPRG1L	tumor protein p63 regulated 1 like [Source:VGNC Symbol;Acc:VGNC:94349]	2.07	0.0098
ssc-miR-30d	TRAF3	TNF receptor associated factor 3 [Source:VGNC Symbol;Acc:VGNC:94360]	2.07	0.0098
ssc-miR-30d	TRAF3IP2	TRAF3 interacting protein 2 [Source:VGNC Symbol;Acc:VGNC:94361]	2.07	0.0098
ssc-miR-30d	TRAM2	translocation associated membrane protein 2 [Source:HGNC Symbol;Acc:HGNC:16855]	2.07	0.0098
ssc-miR-30d	TRDN	triadin [Source:VGNC Symbol;Acc:VGNC:103195]	2.07	0.0098
ssc-miR-30d	TRERF1	transcriptional regulating factor 1 [Source:VGNC Symbol;Acc:VGNC:94387]	2.07	0.0098
ssc-miR-30d	TRIM13	tripartite motif containing 13 [Source:VGNC Symbol;Acc:VGNC:94394]	2.07	0.0098
ssc-miR-30d	TRIM23	tripartite motif containing 23 [Source:VGNC Symbol;Acc:VGNC:94399]	2.07	0.0098
ssc-miR-30d	TRIM24	tripartite motif containing 24 [Source:VGNC Symbol;Acc:VGNC:94400]	2.07	0.0098
ssc-miR-30d	TRIM27	tripartite motif containing 27 [Source:VGNC Symbol;Acc:VGNC:94402]	2.07	0.0098
ssc-miR-30d	TRIM33	tripartite motif containing 33 [Source:VGNC Symbol;Acc:VGNC:98881]	2.07	0.0098
ssc-miR-30d	TRIM36	tripartite motif containing 36 [Source:VGNC Symbol;Acc:VGNC:94409]	2.07	0.0098
ssc-miR-30d	TRIM71	tripartite motif containing 71 [Source:VGNC Symbol;Acc:VGNC:94431]	2.07	0.0098
ssc-miR-30d	TRIM9	tripartite motif containing 9 [Source:VGNC Symbol;Acc:VGNC:94434]	2.07	0.0098
ssc-miR-30d	TRIO	trio Rho guanine nucleotide exchange factor [Source:VGNC Symbol;Acc:VGNC:94435]	2.07	0.0098
ssc-miR-30d	TRIP12	thyroid hormone receptor interactor 12 [Source:VGNC Symbol;Acc:VGNC:95555]	2.07	0.0098
ssc-miR-30d	TRMT5	tRNA methyltransferase 5 [Source:VGNC Symbol;Acc:VGNC:94451]	2.07	0.0098
ssc-miR-30d	TRO	trophinin [Source:NCBI gene (formerly Entrezgene);Acc:396561]	2.07	0.0098
ssc-miR-30d	TRPM3	transient receptor potential cation channel subfamily M member 3 [Source:VGNC Symbol;Acc:VGNC:98384]	2.07	0.0098
ssc-miR-30d	TRPM7	transient receptor potential cation channel subfamily M member 7 [Source:VGNC Symbol;Acc:VGNC:94470]	2.07	0.0098
ssc-miR-30d	TRPS1	transcriptional repressor GATA binding 1 [Source:VGNC Symbol;Acc:VGNC:94471]	2.07	0.0098
ssc-miR-30d	TRRAP	transformation/transcription domain associated protein [Source:VGNC Symbol;Acc:VGNC:94477]	2.07	0.0098
ssc-miR-30d	TSC1	TSC complex subunit 1 [Source:HGNC Symbol;Acc:HGNC:12362]	2.07	0.0098

ssc-miR-30d	TSC22D2	TSC22 domain family member 2 [Source:VGNC Symbol;Acc:VGNC:94482]	2.07	0.0098
ssc-miR-30d	TSPAN14	tetraspanin 14 [Source:VGNC Symbol;Acc:VGNC:94503]	2.07	0.0098
ssc-miR-30d	TSPAN2	tetraspanin 2 [Source:VGNC Symbol;Acc:VGNC:94509]	2.07	0.0098
ssc-miR-30d	TSPAN33	tetraspanin 33 [Source:VGNC Symbol;Acc:VGNC:94512]	2.07	0.0098
ssc-miR-30d	TSPYL4	TSPY like 4 [Source:HGNC Symbol;Acc:HGNC:21559]	2.07	0.0098
ssc-miR-30d	TTBK1	tau tubulin kinase 1 [Source:VGNC Symbol;Acc:VGNC:98386]	2.07	0.0098
ssc-miR-30d	TTC28	tetratricopeptide repeat domain 28 [Source:VGNC Symbol;Acc:VGNC:94543]	2.07	0.0098
ssc-miR-30d	TTC30A	hypothetical gene	2.07	0.0098
ssc-miR-30d	TTC39A	tetratricopeptide repeat domain 39A [Source:VGNC Symbol;Acc:VGNC:94551]	2.07	0.0098
ssc-miR-30d	TTLL7	tubulin tyrosine ligase like 7 [Source:VGNC Symbol;Acc:VGNC:94571]	2.07	0.0098
ssc-miR-30d	TTPA	alpha tocopherol transfer protein [Source:HGNC Symbol;Acc:HGNC:12404]	2.07	0.0098
ssc-miR-30d	TUBGCP3	tubulin gamma complex associated protein 3 [Source:VGNC Symbol;Acc:VGNC:94585]	2.07	0.0098
ssc-miR-30d	TULP4	hypothetical gene	2.07	0.0098
ssc-miR-30d	TUSC3	tumor suppressor candidate 3 [Source:VGNC Symbol;Acc:VGNC:108742]	2.07	0.0098
ssc-miR-30d	TVP23B	hypothetical gene	2.07	0.0098
ssc-miR-30d	TWF1	twinfilin actin binding protein 1 [Source:VGNC Symbol;Acc:VGNC:94595]	2.07	0.0098
ssc-miR-30d	TWSG1	twisted gastrulation BMP signaling modulator 1 [Source:VGNC Symbol;Acc:VGNC:94598]	2.07	0.0098
ssc-miR-30d	TXNDC5	thioredoxin domain containing 5 [Source:VGNC Symbol;Acc:VGNC:94608]	2.07	0.0098
ssc-miR-30d	TYRO3	TYRO3 protein tyrosine kinase [Source:VGNC Symbol;Acc:VGNC:94618]	2.07	0.0098
ssc-miR-30d	UACA	uveal autoantigen with coiled-coil domains and ankyrin repeats [Source:VGNC Symbol;Acc:VGNC:94625]	2.07	0.0098
ssc-miR-30d	UBAC1	UBA domain containing 1 [Source:VGNC Symbol;Acc:VGNC:94632]	2.07	0.0098
ssc-miR-30d	UBE2D2	ubiquitin conjugating enzyme E2 D2 [Source:VGNC Symbol;Acc:VGNC:98391]	2.07	0.0098
ssc-miR-30d	UBE2D3	ubiquitin conjugating enzyme E2 D3 [Source:NCBI gene (formerly Entrezgene);Acc:780418]	2.07	0.0098
ssc-miR-30d	UBE2F	hypothetical gene	2.07	0.0098
ssc-miR-30d	UBE2G1	ubiquitin conjugating enzyme E2 G1 [Source:HGNC Symbol;Acc:HGNC:12482]	2.07	0.0098
ssc-miR-30d	UBE2I	hypothetical gene	2.07	0.0098
ssc-miR-30d	UBE2J1	ubiquitin conjugating enzyme E2 J1 [Source:VGNC Symbol;Acc:VGNC:94644]	2.07	0.0098
ssc-miR-30d	UBE2K	ubiquitin conjugating enzyme E2 K [Source:VGNC Symbol;Acc:VGNC:94646]	2.07	0.0098
ssc-miR-30d	UBE2O	ubiquitin conjugating enzyme E2 O [Source:VGNC Symbol;Acc:VGNC:94649]	2.07	0.0098
ssc-miR-30d	UBE2QL1	ubiquitin conjugating enzyme E2 Q family like 1 [Source:VGNC Symbol;Acc:VGNC:94650]	2.07	0.0098
ssc-miR-30d	UBE2R2	ubiquitin conjugating enzyme E2 R2 [Source:VGNC Symbol;Acc:VGNC:95924]	2.07	0.0098
ssc-miR-30d	UBE2V2	ubiquitin conjugating enzyme E2 V2 [Source:VGNC Symbol;Acc:VGNC:98394]	2.07	0.0098
ssc-miR-30d	UBE3C	ubiquitin protein ligase E3C [Source:VGNC Symbol;Acc:VGNC:94654]	2.07	0.0098
ssc-miR-30d	UBN1	ubinuclein 1 [Source:VGNC Symbol;Acc:VGNC:94663]	2.07	0.0098
ssc-miR-30d	UBN2	ubinuclein 2 [Source:VGNC Symbol;Acc:VGNC:94664]	2.07	0.0098
ssc-miR-30d	UBR7	ubiquitin protein ligase E3 component n-recognin 7 [Source:HGNC Symbol;Acc:HGNC:20344]	2.07	0.0098
ssc-miR-30d	UBXN2A	UBX domain protein 2A [Source:VGNC Symbol;Acc:VGNC:94677]	2.07	0.0098
ssc-miR-30d	UBXN7	hypothetical gene	2.07	0.0098
ssc-miR-30d	UCP3	uncoupling protein 3 [Source:VGNC Symbol;Acc:VGNC:108624]	2.07	0.0098
ssc-miR-30d	UGT8	UDP glycosyltransferase 8 [Source:VGNC Symbol;Acc:VGNC:98401]	2.07	0.0098
ssc-miR-30d	UHRF1BP1	UHRF1 binding protein 1 [Source:VGNC Symbol;Acc:VGNC:94690]	2.07	0.0098
ssc-miR-30d	UNC5C	unc-5 netrin receptor C [Source:VGNC Symbol;Acc:VGNC:94709]	2.07	0.0098
ssc-miR-30d	UNC5D	unc-5 netrin receptor D [Source:VGNC Symbol;Acc:VGNC:95892]	2.07	0.0098
ssc-miR-30d	UNKL	unk like zinc finger [Source:VGNC Symbol;Acc:VGNC:94716]	2.07	0.0098
ssc-miR-30d	URM1	ubiquitin related modifier 1 [Source:VGNC Symbol;Acc:VGNC:94732]	2.07	0.0098
ssc-miR-30d	USP14	ubiquitin specific peptidase 14 [Source:VGNC Symbol;Acc:VGNC:94747]	2.07	0.0098
ssc-miR-30d	USP15	ubiquitin specific peptidase 15 [Source:VGNC Symbol;Acc:VGNC:94748]	2.07	0.0098
ssc-miR-30d	USP2	ubiquitin specific peptidase 2 [Source:VGNC Symbol;Acc:VGNC:94751]	2.07	0.0098
ssc-miR-30d	USP22	ubiquitin specific peptidase 22 [Source:HGNC Symbol;Acc:HGNC:12621]	2.07	0.0098

ssc-miR-30d	USP24	ubiquitin specific peptidase 24 [Source:VGNC Symbol;Acc:VGNC:94754]	2.07	0.0098
ssc-miR-30d	USP37	ubiquitin specific peptidase 37 [Source:VGNC Symbol;Acc:VGNC:98404]	2.07	0.0098
ssc-miR-30d	USP44	ubiquitin specific peptidase 44 [Source:VGNC Symbol;Acc:VGNC:94766]	2.07	0.0098
ssc-miR-30d	USP45	ubiquitin specific peptidase 45 [Source:VGNC Symbol;Acc:VGNC:94767]	2.07	0.0098
ssc-miR-30d	USP47	ubiquitin specific peptidase 47 [Source:VGNC Symbol;Acc:VGNC:94769]	2.07	0.0098
ssc-miR-30d	USP48	ubiquitin specific peptidase 48 [Source:VGNC Symbol;Acc:VGNC:94770]	2.07	0.0098
ssc-miR-30d	USP49	ubiquitin specific peptidase 49 [Source:VGNC Symbol;Acc:VGNC:94771]	2.07	0.0098
ssc-miR-30d	UST	uronyl 2-sulfotransferase [Source:VGNC Symbol;Acc:VGNC:94780]	2.07	0.0098
ssc-miR-30d	VAMP3	hypothetical gene	2.07	0.0098
ssc-miR-30d	VAPA	VAMP associated protein A [Source:VGNC Symbol;Acc:VGNC:94799]	2.07	0.0098
ssc-miR-30d	VASH1	vasohibin 1 [Source:VGNC Symbol;Acc:VGNC:94801]	2.07	0.0098
ssc-miR-30d	VAT1	vesicle amine transport 1 [Source:VGNC Symbol;Acc:VGNC:94805]	2.07	0.0098
ssc-miR-30d	VAT1L	vesicle amine transport 1 like [Source:VGNC Symbol;Acc:VGNC:94806]	2.07	0.0098
ssc-miR-30d	VAV2	vav guanine nucleotide exchange factor 2 [Source:VGNC Symbol;Acc:VGNC:94808]	2.07	0.0098
ssc-miR-30d	VAV3	hypothetical gene	2.07	0.0098
ssc-miR-30d	VCAN	versican [Source:VGNC Symbol;Acc:VGNC:108163]	2.07	0.0098
ssc-miR-30d	VIM	vimentin [Source:VGNC Symbol;Acc:VGNC:95565]	2.07	0.0098
ssc-miR-30d	VIP	vasoactive intestinal peptide [Source:VGNC Symbol;Acc:VGNC:103201]	2.07	0.0098
ssc-miR-30d	VKORC1L1	vitamin K epoxide reductase complex subunit 1 like 1 [Source:VGNC Symbol;Acc:VGNC:94831]	2.07	0.0098
ssc-miR-30d	VOPP1	VOPP1 WW domain binding protein [Source:VGNC Symbol;Acc:VGNC:94835]	2.07	0.0098
ssc-miR-30d	VPS13C	vacuolar protein sorting 13 homolog C [Source:VGNC Symbol;Acc:VGNC:94840]	2.07	0.0098
ssc-miR-30d	VPS26B	VPS26, retromer complex component B [Source:NCBI gene (formerly Entrezgene);Acc:100192441]	2.07	0.0098
ssc-miR-30d	VPS33A	VPS33A core subunit of CORVET and HOPS complexes [Source:NCBI gene (formerly Entrezgene);Acc:100049702]	2.07	0.0098
ssc-miR-30d	VPS53	VPS53 subunit of GARP complex [Source:VGNC Symbol;Acc:VGNC:94861]	2.07	0.0098
ssc-miR-30d	VSTM4	V-set and transmembrane domain containing 4 [Source:VGNC Symbol;Acc:VGNC:94874]	2.07	0.0098
ssc-miR-30d	VSTM5	V-set and transmembrane domain containing 5 [Source:VGNC Symbol;Acc:VGNC:94875]	2.07	0.0098
ssc-miR-30d	VWDE	von Willebrand factor D and EGF domains [Source:VGNC Symbol;Acc:VGNC:104093]	2.07	0.0098
ssc-miR-30d	WASL	WASP like actin nucleation promoting factor [Source:VGNC Symbol;Acc:VGNC:96604]	2.07	0.0098
ssc-miR-30d	WBP1L	WW domain binding protein 1 like [Source:VGNC Symbol;Acc:VGNC:94898]	2.07	0.0098
ssc-miR-30d	WDFY3	WD repeat and FYVE domain containing 3 [Source:VGNC Symbol;Acc:VGNC:94903]	2.07	0.0098
ssc-miR-30d	WDPCP	WD repeat containing planar cell polarity effector [Source:HGNC Symbol;Acc:HGNC:28027]	2.07	0.0098
ssc-miR-30d	WDR1	WD repeat domain 1 [Source:VGNC Symbol;Acc:VGNC:94905]	2.07	0.0098
ssc-miR-30d	WDR26	WD repeat domain 26 [Source:VGNC Symbol;Acc:VGNC:95960]	2.07	0.0098
ssc-miR-30d	WDR37	WD repeat domain 37 [Source:VGNC Symbol;Acc:VGNC:96258]	2.07	0.0098
ssc-miR-30d	WDR44	WD repeat domain 44 [Source:VGNC Symbol;Acc:VGNC:94921]	2.07	0.0098
ssc-miR-30d	WDR47	WD repeat domain 47 [Source:VGNC Symbol;Acc:VGNC:94924]	2.07	0.0098
ssc-miR-30d	WDR7	WD repeat domain 7 [Source:VGNC Symbol;Acc:VGNC:94936]	2.07	0.0098
ssc-miR-30d	WDR70	WD repeat domain 70 [Source:VGNC Symbol;Acc:VGNC:94937]	2.07	0.0098
ssc-miR-30d	WDR82	WD repeat domain 82 [Source:VGNC Symbol;Acc:VGNC:94944]	2.07	0.0098
ssc-miR-30d	WIPF1	hypothetical gene	2.07	0.0098
ssc-miR-30d	WIPF3	WAS/WASL interacting protein family member 3 [Source:VGNC Symbol;Acc:VGNC:94958]	2.07	0.0098
ssc-miR-30d	WNK3	WNK lysine deficient protein kinase 3 [Source:VGNC Symbol;Acc:VGNC:94964]	2.07	0.0098
ssc-miR-30d	WNT7B	Wnt family member 7B [Source:VGNC Symbol;Acc:VGNC:94975]	2.07	0.0098
ssc-miR-30d	WWP1	WW domain containing E3 ubiquitin protein ligase 1 [Source:VGNC Symbol;Acc:VGNC:94989]	2.07	0.0098
ssc-miR-30d	XKR4	XK related 4 [Source:VGNC Symbol;Acc:VGNC:98900]	2.07	0.0098
ssc-miR-30d	XPO1	exportin 1 [Source:VGNC Symbol;Acc:VGNC:95003]	2.07	0.0098
ssc-miR-30d	XPR1	xenotropic and polytropic retrovirus receptor 1 [Source:VGNC Symbol;Acc:VGNC:108625]	2.07	0.0098
ssc-miR-30d	YAF2	YY1 associated factor 2 [Source:VGNC Symbol;Acc:VGNC:95018]	2.07	0.0098
ssc-miR-30d	YBX1	hypothetical gene	2.07	0.0098

ssc-miR-30d	YES1	YES proto-onco 1, Src family tyrosine kinase [Source:VGNC Symbol;Acc:VGNC:95024]	2.07	0.0098
ssc-miR-30d	YOD1	YOD1 deubiquitinase [Source:VGNC Symbol;Acc:VGNC:95035]	2.07	0.0098
ssc-miR-30d	YPEL2	yippee like 2 [Source:VGNC Symbol;Acc:VGNC:95036]	2.07	0.0098
ssc-miR-30d	YPEL5	yippee like 5 [Source:VGNC Symbol;Acc:VGNC:95039]	2.07	0.0098
ssc-miR-30d	YTHDC1	YTH domain containing 1 [Source:VGNC Symbol;Acc:VGNC:95041]	2.07	0.0098
ssc-miR-30d	YTHDF2	YTH N6-methyladenosine RNA binding protein 2 [Source:VGNC Symbol;Acc:VGNC:95043]	2.07	0.0098
ssc-miR-30d	YTHDF3	YTH N6-methyladenosine RNA binding protein 3 [Source:VGNC Symbol;Acc:VGNC:95044]	2.07	0.0098
ssc-miR-30d	YWHAG	hypothetical gene	2.07	0.0098
ssc-miR-30d	YWHAZ	tyrosine 3-monooxygenase/tryptophan 5-monooxygenase activation protein zeta [Source:VGNC Symbol;Acc:VGNC:95047]	2.07	0.0098
ssc-miR-30d	YY1	YY1 transcription factor [Source:HGNC Symbol;Acc:HGNC:12856]	2.07	0.0098
ssc-miR-30d	ZBED4	hypothetical gene	2.07	0.0098
ssc-miR-30d	ZBTB10	zinc finger and BTB domain containing 10 [Source:VGNC Symbol;Acc:VGNC:95058]	2.07	0.0098
ssc-miR-30d	ZBTB11	zinc finger and BTB domain containing 11 [Source:VGNC Symbol;Acc:VGNC:95059]	2.07	0.0098
ssc-miR-30d	ZBTB18	zinc finger and BTB domain containing 18 [Source:HGNC Symbol;Acc:HGNC:13030]	2.07	0.0098
ssc-miR-30d	ZBTB20	zinc finger and BTB domain containing 20 [Source:VGNC Symbol;Acc:VGNC:95063]	2.07	0.0098
ssc-miR-30d	ZBTB34	zinc finger and BTB domain containing 34 [Source:VGNC Symbol;Acc:VGNC:95070]	2.07	0.0098
ssc-miR-30d	ZBTB39	zinc finger and BTB domain containing 39 [Source:VGNC Symbol;Acc:VGNC:95073]	2.07	0.0098
ssc-miR-30d	ZBTB40	zinc finger and BTB domain containing 40 [Source:VGNC Symbol;Acc:VGNC:95074]	2.07	0.0098
ssc-miR-30d	ZBTB41	zinc finger and BTB domain containing 41 [Source:VGNC Symbol;Acc:VGNC:108284]	2.07	0.0098
ssc-miR-30d	ZBTB44	zinc finger and BTB domain containing 44 [Source:VGNC Symbol;Acc:VGNC:95076]	2.07	0.0098
ssc-miR-30d	ZBTB6	zinc finger and BTB domain containing 6 [Source:VGNC Symbol;Acc:VGNC:95080]	2.07	0.0098
ssc-miR-30d	ZBTB7A	zinc finger and BTB domain containing 7A [Source:VGNC Symbol;Acc:VGNC:95081]	2.07	0.0098
ssc-miR-30d	ZCCHC14	zinc finger CCHC-type containing 14 [Source:VGNC Symbol;Acc:VGNC:95107]	2.07	0.0098
ssc-miR-30d	ZCCHC2	zinc finger CCHC-type containing 2 [Source:VGNC Symbol;Acc:VGNC:95109]	2.07	0.0098
ssc-miR-30d	ZCCHC24	zinc finger CCHC-type containing 24 [Source:HGNC Symbol;Acc:HGNC:26911]	2.07	0.0098
ssc-miR-30d	ZCCHC4	zinc finger CCHC-type containing 4 [Source:VGNC Symbol;Acc:VGNC:95110]	2.07	0.0098
ssc-miR-30d	ZDHHC14	zinc finger DHHC-type palmitoyltransferase 14 [Source:VGNC Symbol;Acc:VGNC:95117]	2.07	0.0098
ssc-miR-30d	ZDHHC17	zinc finger DHHC-type palmitoyltransferase 17 [Source:VGNC Symbol;Acc:VGNC:95119]	2.07	0.0098
ssc-miR-30d	ZDHHC20	zinc finger DHHC-type palmitoyltransferase 20 [Source:VGNC Symbol;Acc:VGNC:95122]	2.07	0.0098
ssc-miR-30d	ZDHHC21	zinc finger DHHC-type palmitoyltransferase 21 [Source:VGNC Symbol;Acc:VGNC:95123]	2.07	0.0098
ssc-miR-30d	ZEB2	hypothetical gene	2.07	0.0098
ssc-miR-30d	ZFAND5	zinc finger AN1-type containing 5 [Source:VGNC Symbol;Acc:VGNC:95137]	2.07	0.0098
ssc-miR-30d	ZFC3H1	zinc finger C3H1-type containing [Source:VGNC Symbol;Acc:VGNC:95139]	2.07	0.0098
ssc-miR-30d	ZFHX3	zinc finger homeobox 3 [Source:VGNC Symbol;Acc:VGNC:95141]	2.07	0.0098
ssc-miR-30d	ZFP36L1	ZFP36 ring finger protein like 1 [Source:VGNC Symbol;Acc:VGNC:95146]	2.07	0.0098
ssc-miR-30d	ZFP36L2	ZFP36 ring finger protein like 2 [Source:VGNC Symbol;Acc:VGNC:95147]	2.07	0.0098
ssc-miR-30d	ZFP90	ZFP90 zinc finger protein [Source:VGNC Symbol;Acc:VGNC:95149]	2.07	0.0098
ssc-miR-30d	ZFYVE26	zinc finger FYVE-type containing 26 [Source:VGNC Symbol;Acc:VGNC:95160]	2.07	0.0098
ssc-miR-30d	ZG16	hypothetical gene	2.07	0.0098
ssc-miR-30d	ZIC2	Zic family member 2 [Source:VGNC Symbol;Acc:VGNC:95167]	2.07	0.0098
ssc-miR-30d	ZMYND8	zinc finger MYND-type containing 8 [Source:VGNC Symbol;Acc:VGNC:108744]	2.07	0.0098
ssc-miR-30d	ZNF148	zinc finger protein 148 [Source:VGNC Symbol;Acc:VGNC:95192]	2.07	0.0098
ssc-miR-30d	ZNF197	zinc finger protein 197 [Source:VGNC Symbol;Acc:VGNC:95202]	2.07	0.0098
ssc-miR-30d	ZNF200	zinc finger protein 200 [Source:VGNC Symbol;Acc:VGNC:95203]	2.07	0.0098
ssc-miR-30d	ZNF264	hypothetical gene	2.07	0.0098
ssc-miR-30d	ZNF275	hypothetical gene	2.07	0.0098
ssc-miR-30d	ZNF280B	zinc finger protein 280B [Source:HGNC Symbol;Acc:HGNC:23022]	2.07	0.0098
ssc-miR-30d	ZNF382	zinc finger protein 382 [Source:VGNC Symbol;Acc:VGNC:95232]	2.07	0.0098
ssc-miR-30d	ZNF507	zinc finger protein 507 [Source:VGNC Symbol;Acc:VGNC:95253]	2.07	0.0098

ssc-miR-30d	ZNF521	zinc finger protein 521 [Source:VGNC Symbol;Acc:VGNC:98715]	2.07	0.0098
ssc-miR-30d	ZNF608	hypothetical gene	2.07	0.0098
ssc-miR-30d	ZNF644	zinc finger protein 644 [Source:VGNC Symbol;Acc:VGNC:95283]	2.07	0.0098
ssc-miR-30d	ZNF652	zinc finger protein 652 [Source:VGNC Symbol;Acc:VGNC:99108]	2.07	0.0098
ssc-miR-30d	ZNF704	zinc finger protein 704 [Source:VGNC Symbol;Acc:VGNC:95296]	2.07	0.0098
ssc-miR-30d	ZNF711	zinc finger protein 711 [Source:HGNC Symbol;Acc:HGNC:13128]	2.07	0.0098
ssc-miR-30d	ZNF746	hypothetical gene	2.07	0.0098
ssc-miR-30d	ZNF770	zinc finger protein 770 [Source:VGNC Symbol;Acc:VGNC:95300]	2.07	0.0098
ssc-miR-30d	ZNF827	zinc finger protein 827 [Source:VGNC Symbol;Acc:VGNC:98970]	2.07	0.0098
ssc-miR-30d	ZNF831	zinc finger protein 831 [Source:VGNC Symbol;Acc:VGNC:95765]	2.07	0.0098
ssc-miR-30d	ZNRF1	zinc and ring finger 1 [Source:HGNC Symbol;Acc:HGNC:18452]	2.07	0.0098
ssc-miR-30d	ZPBP2	zona pellucida binding protein 2 [Source:VGNC Symbol;Acc:VGNC:95320]	2.07	0.0098
ssc-miR-30d	ZRANB3	zinc finger RANBP2-type containing 3 [Source:VGNC Symbol;Acc:VGNC:96073]	2.07	0.0098
ssc-miR-30d	ZSCAN29	zinc finger and SCAN domain containing 29 [Source:HGNC Symbol;Acc:HGNC:26673]	2.07	0.0098
ssc-miR-30d	ZSWIM5	zinc finger SWIM-type containing 5 [Source:VGNC Symbol;Acc:VGNC:95334]	2.07	0.0098
ssc-miR-30d	ZSWIM6	zinc finger SWIM-type containing 6 [Source:VGNC Symbol;Acc:VGNC:95335]	2.07	0.0098
ssc-miR-99b	AGO2	argonaute RISC catalytic component 2 [Source:VGNC Symbol;Acc:VGNC:97871]	1.9	0.028
ssc-miR-99b	AP1AR	adaptor related protein complex 1 associated regulatory protein [Source:HGNC Symbol;Acc:HGNC:28808]	1.9	0.028
ssc-miR-99b	ARHGEF12	Rho guanine nucleotide exchange factor 12 [Source:VGNC Symbol;Acc:VGNC:85488]	1.9	0.028
ssc-miR-99b	BAZ2A	bromodomain adjacent to zinc finger domain 2A [Source:VGNC Symbol;Acc:VGNC:85763]	1.9	0.028
ssc-miR-99b	BMPR2	bone morphotic protein receptor type 2 [Source:VGNC Symbol;Acc:VGNC:95494]	1.9	0.028
ssc-miR-99b	CDYL2	chromodomain Y like 2 [Source:VGNC Symbol;Acc:VGNC:86529]	1.9	0.028
ssc-miR-99b	CEP85	centrosomal protein 85 [Source:VGNC Symbol;Acc:VGNC:86579]	1.9	0.028
ssc-miR-99b	CLDN11	claudin 11 [Source:NCBI gene (formerly Entrezgene);Acc:100302016]	1.9	0.028
ssc-miR-99b	CTDSPL	CTD small phosphatase like [Source:VGNC Symbol;Acc:VGNC:107131]	1.9	0.028
ssc-miR-99b	CYP26B1	cytochrome P450 family 26 subfamily B member 1 [Source:VGNC Symbol;Acc:VGNC:103374]	1.9	0.028
ssc-miR-99b	DESI2	desumoylating isopeptidase 2 [Source:VGNC Symbol;Acc:VGNC:96037]	1.9	0.028
ssc-miR-99b	EPDR1	ependymin related 1 [Source:VGNC Symbol;Acc:VGNC:87727]	1.9	0.028
ssc-miR-99b	ETV3	ETS variant transcription factor 3 [Source:VGNC Symbol;Acc:VGNC:87812]	1.9	0.028
ssc-miR-99b	FGFR3	fibroblast growth factor receptor 3 [Source:VGNC Symbol;Acc:VGNC:98929]	1.9	0.028
ssc-miR-99b	FKBP5	FKBP prolyl isomerase 5 [Source:VGNC Symbol;Acc:VGNC:88148]	1.9	0.028
ssc-miR-99b	FZD5	frizzled class receptor 5 [Source:VGNC Symbol;Acc:VGNC:96309]	1.9	0.028
ssc-miR-99b	FZD8	frizzled class receptor 8 [Source:VGNC Symbol;Acc:VGNC:96311]	1.9	0.028
ssc-miR-99b	GMPS	guanine monophosphate synthase [Source:VGNC Symbol;Acc:VGNC:88516]	1.9	0.028
ssc-miR-99b	GRHL1	grainyhead like transcription factor 1 [Source:VGNC Symbol;Acc:VGNC:88666]	1.9	0.028
ssc-miR-99b	HES7	hes family bHLH transcription factor 7 [Source:HGNC Symbol;Acc:HGNC:15977]	1.9	0.028
ssc-miR-99b	HOXA1	homeobox A1 [Source:VGNC Symbol;Acc:VGNC:88933]	1.9	0.028
ssc-miR-99b	HS3ST2	heparan sulfate-glucosamine 3-sulfotransferase 2 [Source:VGNC Symbol;Acc:VGNC:88978]	1.9	0.028
ssc-miR-99b	HS3ST3B1	heparan sulfate-glucosamine 3-sulfotransferase 3B1 [Source:VGNC Symbol;Acc:VGNC:99003]	1.9	0.028
ssc-miR-99b	ICMT	isoprenylcysteine carboxyl methyltransferase [Source:VGNC Symbol;Acc:VGNC:89022]	1.9	0.028
ssc-miR-99b	IGF1R	insulin like growth factor 1 receptor [Source:NCBI gene (formerly Entrezgene);Acc:397350]	1.9	0.028
ssc-miR-99b	INSM1	INSM transcriptional repressor 1 [Source:HGNC Symbol;Acc:HGNC:6090]	1.9	0.028
ssc-miR-99b	KBTBD8	kelch repeat and BTB domain containing 8 [Source:VGNC Symbol;Acc:VGNC:89321]	1.9	0.028
ssc-miR-99b	KDM6B	lysine demethylase 6B [Source:VGNC Symbol;Acc:VGNC:89416]	1.9	0.028
ssc-miR-99b	LRRC8B	leucine rich repeat containing 8 VRAC subunit B [Source:VGNC Symbol;Acc:VGNC:98086]	1.9	0.028
ssc-miR-99b	MBNL1	muscleblind like splicing regulator 1 [Source:VGNC Symbol;Acc:VGNC:90054]	1.9	0.028
ssc-miR-99b	MTMR3	myotubularin related protein 3 [Source:VGNC Symbol;Acc:VGNC:90461]	1.9	0.028
ssc-miR-99b	MTOR	mechanistic target of rapamycin kinase [Source:VGNC Symbol;Acc:VGNC:90466]	1.9	0.028
ssc-miR-99b	NIPBL	NIPBL cohesin loading factor [Source:VGNC Symbol;Acc:VGNC:90752]	1.9	0.028

ssc-miR-99b	NR6A1	nuclear receptor subfamily 6 group A member 1 [Source:VGNC Symbol;Acc:VGNC:90887]	1.9	0.028
ssc-miR-99b	NTRK3	neurotrophic receptor tyrosine kinase 3 [Source:VGNC Symbol;Acc:VGNC:103142]	1.9	0.028
ssc-miR-99b	NXF1	nuclear RNA export factor 1 [Source:VGNC Symbol;Acc:VGNC:90993]	1.9	0.028
ssc-miR-99b	PI15	peptidase inhibitor 15 [Source:VGNC Symbol;Acc:VGNC:98188]	1.9	0.028
ssc-miR-99b	PPP1CB	protein phosphatase 1 catalytic subunit beta [Source:NCBI gene (formerly Entrezgene);Acc:397378]	1.9	0.028
ssc-miR-99b	PPP3CA	protein phosphatase 3 catalytic subunit alpha [Source:VGNC Symbol;Acc:VGNC:98218]	1.9	0.028
ssc-miR-99b	RASGRP3	RAS guanyl releasing protein 3 [Source:VGNC Symbol;Acc:VGNC:92115]	1.9	0.028
ssc-miR-99b	RAVER2	ribonucleoprotein, PTB binding 2 [Source:VGNC Symbol;Acc:VGNC:98283]	1.9	0.028
ssc-miR-99b	RMND5A	required for meiotic nuclear division 5 homolog A [Source:VGNC Symbol;Acc:VGNC:92336]	1.9	0.028
ssc-miR-99b	RRAGD	Ras related GTP binding D [Source:VGNC Symbol;Acc:VGNC:92457]	1.9	0.028
ssc-miR-99b	SATB1	SATB homeobox 1 [Source:VGNC Symbol;Acc:VGNC:92587]	1.9	0.028
ssc-miR-99b	SEH1L	centrosomal protein 192 [Source:VGNC Symbol;Acc:VGNC:96946]	1.9	0.028
ssc-miR-99b	SIM1	SIM bHLH transcription factor 1 [Source:VGNC Symbol;Acc:VGNC:92876]	1.9	0.028
ssc-miR-99b	SLC44A1	solute carrier family 44 member 1 [Source:VGNC Symbol;Acc:VGNC:93118]	1.9	0.028
ssc-miR-99b	SMARCA5	SWI/SNF related, matrix associated, actin dependent regulator of chromatin, subfamily a, member 5 [Source:VGNC Symbol;Acc:VGNC:93228]	1.9	0.028
ssc-miR-99b	SMARCD1	SWI/SNF related, matrix associated, actin dependent regulator of chromatin, subfamily d, member 1 [Source:VGNC Symbol;Acc:VGNC:93233]	1.9	0.028
ssc-miR-99b	ST5	hypothetical gene	1.9	0.028
ssc-miR-99b	ST6GALNAC4	ST6 N-acetylgalactosaminide alpha-2,6-sialyltransferase 4 [Source:VGNC Symbol;Acc:VGNC:93512]	1.9	0.028
ssc-miR-99b	TAOK1	TAO kinase 1 [Source:VGNC Symbol;Acc:VGNC:98355]	1.9	0.028
ssc-miR-99b	THAP2	THAP domain containing 2 [Source:VGNC Symbol;Acc:VGNC:104071]	1.9	0.028
ssc-miR-99b	TMEM135	transmembrane protein 135 [Source:VGNC Symbol;Acc:VGNC:94085]	1.9	0.028
ssc-miR-99b	TMEM136	hypothetical gene	1.9	0.028
ssc-miR-99b	TMEM30A	transmembrane protein 30A [Source:VGNC Symbol;Acc:VGNC:94170]	1.9	0.028
ssc-miR-99b	TRIB1	tribbles pseudokinase 1 [Source:VGNC Symbol;Acc:VGNC:94391]	1.9	0.028
ssc-miR-99b	TRIB2	tribbles pseudokinase 2 [Source:VGNC Symbol;Acc:VGNC:94392]	1.9	0.028
ssc-miR-99b	TRIM71	tripartite motif containing 71 [Source:VGNC Symbol;Acc:VGNC:94431]	1.9	0.028
ssc-miR-99b	TTC39A	tetratricopeptide repeat domain 39A [Source:VGNC Symbol;Acc:VGNC:94551]	1.9	0.028
ssc-miR-99b	ZBTB7A	zinc finger and BTB domain containing 7A [Source:VGNC Symbol;Acc:VGNC:95081]	1.9	0.028
ssc-miR-99b	ZNRF2	zinc and ring finger 2 [Source:VGNC Symbol;Acc:VGNC:95317]	1.9	0.028
ssc-miR-99b	ZZEF1	zinc finger ZZ-type and EF-hand domain containing 1 [Source:VGNC Symbol;Acc:VGNC:95347]	1.9	0.028
ssc-miR-103	ABCF1	ATP binding cassette subfamily F member 1 [Source:VGNC Symbol;Acc:VGNC:84966]	1.89	0.0903
ssc-miR-103	ABHD2	abhydrolase domain containing 2, acylglycerol lipase [Source:VGNC Symbol;Acc:VGNC:84980]	1.89	0.0903
ssc-miR-103	ABI2	abl interactor 2 [Source:VGNC Symbol;Acc:VGNC:96027]	1.89	0.0903
ssc-miR-103	ABL2	ABL proto-onco 2, non-receptor tyrosine kinase [Source:VGNC Symbol;Acc:VGNC:84985]	1.89	0.0903
ssc-miR-103	AC011897.1	hypothetical gene	1.89	0.0903
ssc-miR-103	AC068987.1	hypothetical gene	1.89	0.0903
ssc-miR-103	AC137932.1	hypothetical gene	1.89	0.0903
ssc-miR-103	ACACB	acetyl-CoA carboxylase beta [Source:NCBI gene (formerly Entrezgene);Acc:100154993]	1.89	0.0903
ssc-miR-103	ACER3	alkaline ceramidase 3 [Source:VGNC Symbol;Acc:VGNC:103893]	1.89	0.0903
ssc-miR-103	ACOX1	acyl-CoA oxidase 1 [Source:VGNC Symbol;Acc:VGNC:85020]	1.89	0.0903
ssc-miR-103	ACTG1	actin gamma 1 [Source:HGNC Symbol;Acc:HGNC:144]	1.89	0.0903
ssc-miR-103	ACVR2B	activin A receptor type 2B [Source:VGNC Symbol;Acc:VGNC:108629]	1.89	0.0903
ssc-miR-103	ADAMTSL3	ADAMTS like 3 [Source:VGNC Symbol;Acc:VGNC:85092]	1.89	0.0903
ssc-miR-103	ADAP1	ArfGAP with dual PH domains 1 [Source:VGNC Symbol;Acc:VGNC:85095]	1.89	0.0903
ssc-miR-103	ADCYAP1	adenylate cyclase activating polypeptide 1 [Source:VGNC Symbol;Acc:VGNC:85114]	1.89	0.0903
ssc-miR-103	ADD1	adducin 1 [Source:VGNC Symbol;Acc:VGNC:85116]	1.89	0.0903
ssc-miR-103	ADNP	activity dependent neuroprotector homeobox [Source:VGNC Symbol;Acc:VGNC:95657]	1.89	0.0903
ssc-miR-103	ADNP2	ADNP homeobox 2 [Source:VGNC Symbol;Acc:VGNC:85145]	1.89	0.0903
ssc-miR-103	AFF4	AF4/FMR2 family member 4 [Source:VGNC Symbol;Acc:VGNC:85169]	1.89	0.0903

ssc-miR-103	AGFG1	ArfGAP with FG repeats 1 [Source:VGNC Symbol;Acc:VGNC:96359]	1.89	0.0903
ssc-miR-103	AGO1	hypothetical gene	1.89	0.0903
ssc-miR-103	AGO3	argonaute RISC component 1 [Source:NCBI gene (formerly Entrezgene);Acc:100499510]	1.89	0.0903
ssc-miR-103	AGO4	hypothetical gene	1.89	0.0903
ssc-miR-103	AGPAT3	1-acylglycerol-3-phosphate O-acyltransferase 3 [Source:VGNC Symbol;Acc:VGNC:85185]	1.89	0.0903
ssc-miR-103	AHSA2	hypothetical gene	1.89	0.0903
ssc-miR-103	AIFM1	apoptosis inducing factor mitochondria associated 1 [Source:VGNC Symbol;Acc:VGNC:85198]	1.89	0.0903
ssc-miR-103	AJUBA	ajuba LIM protein [Source:VGNC Symbol;Acc:VGNC:85208]	1.89	0.0903
ssc-miR-103	AK2	hypothetical gene	1.89	0.0903
ssc-miR-103	AK4	hypothetical gene	1.89	0.0903
ssc-miR-103	AKAP12	A-kinase anchoring protein 12 [Source:VGNC Symbol;Acc:VGNC:85216]	1.89	0.0903
ssc-miR-103	AKAP13	hypothetical gene	1.89	0.0903
ssc-miR-103	AKAP2	hypothetical gene	1.89	0.0903
ssc-miR-103	AKIRIN2	hypothetical gene	1.89	0.0903
ssc-miR-103	ALAD	aminolevulinate dehydratase [Source:VGNC Symbol;Acc:VGNC:85234]	1.89	0.0903
ssc-miR-103	ALG9	ALG9 alpha-1,2-mannosyltransferase [Source:NCBI gene (formerly Entrezgene);Acc:100519965]	1.89	0.0903
ssc-miR-103	AMMECR1	AMMECR nuclear protein 1 [Source:VGNC Symbol;Acc:VGNC:96559]	1.89	0.0903
ssc-miR-103	AMOT	angiomotin [Source:VGNC Symbol;Acc:VGNC:85283]	1.89	0.0903
ssc-miR-103	AMOTL2	angiomotin like 2 [Source:VGNC Symbol;Acc:VGNC:85285]	1.89	0.0903
ssc-miR-103	ANK1	ankyrin 1 [Source:VGNC Symbol;Acc:VGNC:96344]	1.89	0.0903
ssc-miR-103	ANK3	hypothetical gene	1.89	0.0903
ssc-miR-103	ANKFY1	ankyrin repeat and FYVE domain containing 1 [Source:VGNC Symbol;Acc:VGNC:85314]	1.89	0.0903
ssc-miR-103	ANKIB1	ankyrin repeat and IBR domain containing 1 [Source:VGNC Symbol;Acc:VGNC:85316]	1.89	0.0903
ssc-miR-103	ANKRD42	ankyrin repeat domain 42 [Source:VGNC Symbol;Acc:VGNC:108569]	1.89	0.0903
ssc-miR-103	ANKRD52	ankyrin repeat domain 52 [Source:VGNC Symbol;Acc:VGNC:85342]	1.89	0.0903
ssc-miR-103	ANO3	anoctamin 3 [Source:VGNC Symbol;Acc:VGNC:85356]	1.89	0.0903
ssc-miR-103	AP3M1	adaptor related protein complex 3 subunit mu 1 [Source:VGNC Symbol;Acc:VGNC:85390]	1.89	0.0903
ssc-miR-103	APBA1	amyloid beta protein binding family A member 1 [Source:VGNC Symbol;Acc:VGNC:85399]	1.89	0.0903
ssc-miR-103	APPBP2	amyloid beta protein binding protein 2 [Source:VGNC Symbol;Acc:VGNC:85425]	1.89	0.0903
ssc-miR-103	APPL1	adaptor protein, phosphotyrosine interacting with PH domain and leucine zipper 1 [Source:VGNC Symbol;Acc:VGNC:85426]	1.89	0.0903
ssc-miR-103	AQP11	aquaporin 11 [Source:VGNC Symbol;Acc:VGNC:85430]	1.89	0.0903
ssc-miR-103	ARHGAP17	Rho GTPase activating protein 17 [Source:VGNC Symbol;Acc:VGNC:85459]	1.89	0.0903
ssc-miR-103	ARHGAP19	Rho GTPase activating protein 19 [Source:HGNC Symbol;Acc:HGNC:23724]	1.89	0.0903
ssc-miR-103	ARHGAP5	Rho GTPase activating protein 5 [Source:VGNC Symbol;Acc:VGNC:85480]	1.89	0.0903
ssc-miR-103	ARID2	AT-rich interaction domain 2 [Source:HGNC Symbol;Acc:HGNC:18037]	1.89	0.0903
ssc-miR-103	ARID4A	AT-rich interaction domain 4A [Source:VGNC Symbol;Acc:VGNC:85509]	1.89	0.0903
ssc-miR-103	ARIH1	ariadne RBR E3 ubiquitin protein ligase 1 [Source:HGNC Symbol;Acc:HGNC:689]	1.89	0.0903
ssc-miR-103	ARIH2	ariadne RBR E3 ubiquitin protein ligase 2 [Source:VGNC Symbol;Acc:VGNC:85512]	1.89	0.0903
ssc-miR-103	ARL8A	ADP ribosylation factor like GTPase 8A [Source:VGNC Symbol;Acc:VGNC:96067]	1.89	0.0903
ssc-miR-103	ARMC1	armadillo repeat containing 1 [Source:VGNC Symbol;Acc:VGNC:85526]	1.89	0.0903
ssc-miR-103	ARMC5	armadillo repeat containing 5 [Source:VGNC Symbol;Acc:VGNC:85529]	1.89	0.0903
ssc-miR-103	ARMC7	hypothetical gene	1.89	0.0903
ssc-miR-103	ARMC8	armadillo repeat containing 8 [Source:VGNC Symbol;Acc:VGNC:85531]	1.89	0.0903
ssc-miR-103	ARNT	aryl hydrocarbon receptor nuclear translocator [Source:VGNC Symbol;Acc:VGNC:98733]	1.89	0.0903
ssc-miR-103	ARPC5L	actin related protein 2/3 complex subunit 5 like [Source:VGNC Symbol;Acc:VGNC:85540]	1.89	0.0903
ssc-miR-103	ASH1L	ASH1 like histone lysine methyltransferase [Source:VGNC Symbol;Acc:VGNC:85577]	1.89	0.0903
ssc-miR-103	ASIC1	acid sensing ion channel subunit 1 [Source:VGNC Symbol;Acc:VGNC:85578]	1.89	0.0903
ssc-miR-103	ATAD2B	ATPase family AAA domain containing 2B [Source:VGNC Symbol;Acc:VGNC:85600]	1.89	0.0903
ssc-miR-103	ATL2	atlastin GTPase 2 [Source:VGNC Symbol;Acc:VGNC:85628]	1.89	0.0903

ssc-miR-103	ATP13A3	ATPase 13A3 [Source:VGNC Symbol;Acc:VGNC:85639]	1.89	0.0903
ssc-miR-103	ATP1B2	ATPase Na+/K+ transporting subunit beta 2 [Source:VGNC Symbol;Acc:VGNC:85643]	1.89	0.0903
ssc-miR-103	ATXN1	ataxin 1 [Source:VGNC Symbol;Acc:VGNC:85687]	1.89	0.0903
ssc-miR-103	ATXN1L	ataxin 1 like [Source:VGNC Symbol;Acc:VGNC:85689]	1.89	0.0903
ssc-miR-103	ATXN7	ataxin 7 [Source:VGNC Symbol;Acc:VGNC:99704]	1.89	0.0903
ssc-miR-103	ATXN7L1	ataxin 7 like 1 [Source:VGNC Symbol;Acc:VGNC:85692]	1.89	0.0903
ssc-miR-103	AVL9	AVL9 cell migration associated [Source:VGNC Symbol;Acc:VGNC:85701]	1.89	0.0903
ssc-miR-103	AVPR2	vasopressin receptor 2 [Source:VGNC Symbol;Acc:VGNC:97898]	1.89	0.0903
ssc-miR-103	AXIN2	axin 2 [Source:VGNC Symbol;Acc:VGNC:85708]	1.89	0.0903
ssc-miR-103	BACH2	BTB domain and CNC homolog 2 [Source:VGNC Symbol;Acc:VGNC:85742]	1.89	0.0903
ssc-miR-103	BAI3	hypothetical gene	1.89	0.0903
ssc-miR-103	BAK1	hypothetical gene	1.89	0.0903
ssc-miR-103	BAZ2A	bromodomain adjacent to zinc finger domain 2A [Source:VGNC Symbol;Acc:VGNC:85763]	1.89	0.0903
ssc-miR-103	BAZ2B	bromodomain adjacent to zinc finger domain 2B [Source:HGNC Symbol;Acc:HGNC:963]	1.89	0.0903
ssc-miR-103	BCAT2	branched chain amino acid transaminase 2 [Source:VGNC Symbol;Acc:VGNC:85774]	1.89	0.0903
ssc-miR-103	BCKDK	branched chain keto acid dehydrogenase kinase [Source:VGNC Symbol;Acc:VGNC:85778]	1.89	0.0903
ssc-miR-103	BCL2L13	BCL2 like 13 [Source:VGNC Symbol;Acc:VGNC:85781]	1.89	0.0903
ssc-miR-103	BCL2L2	BCL2 like 2 [Source:HGNC Symbol;Acc:HGNC:995]	1.89	0.0903
ssc-miR-103	BCL9L	BCL9 like [Source:VGNC Symbol;Acc:VGNC:96568]	1.89	0.0903
ssc-miR-103	BDNF	brain derived neurotrophic factor [Source:VGNC Symbol;Acc:VGNC:85795]	1.89	0.0903
ssc-miR-103	BLMH	bleomycin hydrolase [Source:HGNC Symbol;Acc:HGNC:1059]	1.89	0.0903
ssc-miR-103	BOLA3	hypothetical gene	1.89	0.0903
ssc-miR-103	BTBD7	BTB domain containing 7 [Source:VGNC Symbol;Acc:VGNC:85908]	1.89	0.0903
ssc-miR-103	BTG2	BTG anti-proliferation factor 2 [Source:NCBI gene (formerly Entrezgene);Acc:100048932]	1.89	0.0903
ssc-miR-103	BTLA	hypothetical gene	1.89	0.0903
ssc-miR-103	BTRC	beta-transducin repeat containing E3 ubiquitin protein ligase [Source:VGNC Symbol;Acc:VGNC:107368]	1.89	0.0903
ssc-miR-103	C16orf72	chromosome 3 C16orf72 homolog [Source:VGNC Symbol;Acc:VGNC:86016]	1.89	0.0903
ssc-miR-103	C17orf85	hypothetical gene	1.89	0.0903
ssc-miR-103	C19orf26	hypothetical gene	1.89	0.0903
ssc-miR-103	C1orf226	hypothetical gene	1.89	0.0903
ssc-miR-103	C20orf112	hypothetical gene	1.89	0.0903
ssc-miR-103	C2CD5	C2 calcium dependent domain containing 5 [Source:VGNC Symbol;Acc:VGNC:85995]	1.89	0.0903
ssc-miR-103	C6orf211	hypothetical gene	1.89	0.0903
ssc-miR-103	C6orf57	hypothetical gene	1.89	0.0903
ssc-miR-103	C6orf62	chromosome 7 C6orf62 homolog [Source:VGNC Symbol;Acc:VGNC:86074]	1.89	0.0903
ssc-miR-103	C7orf55	hypothetical gene	1.89	0.0903
ssc-miR-103	C9orf114	hypothetical gene	1.89	0.0903
ssc-miR-103	C9orf69	hypothetical gene	1.89	0.0903
ssc-miR-103	CAB39	calcium binding protein 39 [Source:VGNC Symbol;Acc:VGNC:95936]	1.89	0.0903
ssc-miR-103	CACNA1C	calcium voltage-gated channel subunit alpha1 C [Source:HGNC Symbol;Acc:HGNC:1390]	1.89	0.0903
ssc-miR-103	CACNA2D1	calcium voltage-gated channel auxiliary subunit alpha2delta 1 [Source:VGNC Symbol;Acc:VGNC:86120]	1.89	0.0903
ssc-miR-103	CALU	calumenin [Source:VGNC Symbol;Acc:VGNC:86151]	1.89	0.0903
ssc-miR-103	CAMK2G	calcium/calmodulin dependent protein kinase II gamma [Source:VGNC Symbol;Acc:VGNC:86157]	1.89	0.0903
ssc-miR-103	CAMK2N1	calcium/calmodulin dependent protein kinase II inhibitor 1 [Source:VGNC Symbol;Acc:VGNC:96935]	1.89	0.0903
ssc-miR-103	CAMKK1	calcium/calmodulin dependent protein kinase kinase 1 [Source:VGNC Symbol;Acc:VGNC:98977]	1.89	0.0903
ssc-miR-103	CAMKV	CaM kinase like vesicle associated [Source:VGNC Symbol;Acc:VGNC:86160]	1.89	0.0903
ssc-miR-103	CAPZA1	capping actin protein of muscle Z-line subunit alpha 1 [Source:VGNC Symbol;Acc:VGNC:86183]	1.89	0.0903
ssc-miR-103	CAPZA2	capping actin protein of muscle Z-line subunit alpha 2 [Source:VGNC Symbol;Acc:VGNC:103910]	1.89	0.0903
ssc-miR-103	CARM1	coactivator associated arginine methyltransferase 1 [Source:VGNC Symbol;Acc:VGNC:86191]	1.89	0.0903

ssc-miR-103	CASC4	hypothetical gene	1.89	0.0903
ssc-miR-103	CASK	calcium/calmodulin dependent serine protein kinase [Source:HGNC Symbol;Acc:HGNC:1497]	1.89	0.0903
ssc-miR-103	CASP10	caspase 10 [Source:VGNC Symbol;Acc:VGNC:95638]	1.89	0.0903
ssc-miR-103	CASP8	caspase 8 [Source:VGNC Symbol;Acc:VGNC:95639]	1.89	0.0903
ssc-miR-103	CC2D1B	coiled-coil and C2 domain containing 1B [Source:VGNC Symbol;Acc:VGNC:86236]	1.89	0.0903
ssc-miR-103	CCAR1	cell division cycle and apoptosis regulator 1 [Source:VGNC Symbol;Acc:VGNC:86238]	1.89	0.0903
ssc-miR-103	CCBL2	hypothetical gene	1.89	0.0903
ssc-miR-103	CCDC171	coiled-coil domain containing 171 [Source:VGNC Symbol;Acc:VGNC:103044]	1.89	0.0903
ssc-miR-103	CCDC178	coiled-coil domain containing 178 [Source:VGNC Symbol;Acc:VGNC:86276]	1.89	0.0903
ssc-miR-103	CCDC50	coiled-coil domain containing 50 [Source:VGNC Symbol;Acc:VGNC:86299]	1.89	0.0903
ssc-miR-103	CCDC6	coiled-coil domain containing 6 [Source:VGNC Symbol;Acc:VGNC:86304]	1.89	0.0903
ssc-miR-103	CCNE1	hypothetical gene	1.89	0.0903
ssc-miR-103	CCNG2	cyclin G2 [Source:VGNC Symbol;Acc:VGNC:86358]	1.89	0.0903
ssc-miR-103	CCNJ	cyclin J [Source:VGNC Symbol;Acc:VGNC:86360]	1.89	0.0903
ssc-miR-103	CCNJL	cyclin J like [Source:VGNC Symbol;Acc:VGNC:86361]	1.89	0.0903
ssc-miR-103	CCNYL1	hypothetical gene	1.89	0.0903
ssc-miR-103	CD164L2	CD164 molecule like 2 [Source:HGNC Symbol;Acc:HGNC:32043]	1.89	0.0903
ssc-miR-103	CD69	CD69 molecule [Source:VGNC Symbol;Acc:VGNC:103223]	1.89	0.0903
ssc-miR-103	CDC20B	cell division cycle 20B [Source:HGNC Symbol;Acc:HGNC:24222]	1.89	0.0903
ssc-miR-103	CDC27	cell division cycle 27 [Source:VGNC Symbol;Acc:VGNC:86448]	1.89	0.0903
ssc-miR-103	CDC37L1	cell division cycle 37 like 1 [Source:VGNC Symbol;Acc:VGNC:86451]	1.89	0.0903
ssc-miR-103	CDC42SE1	CDC42 small effector 1 [Source:VGNC Symbol;Acc:VGNC:86460]	1.89	0.0903
ssc-miR-103	CDC6	cell division cycle 6 [Source:VGNC Symbol;Acc:VGNC:86464]	1.89	0.0903
ssc-miR-103	CDK12	cyclin dependent kinase 12 [Source:VGNC Symbol;Acc:VGNC:86496]	1.89	0.0903
ssc-miR-103	CDK14	cyclin dependent kinase 14 [Source:VGNC Symbol;Acc:VGNC:86498]	1.89	0.0903
ssc-miR-103	CDK5R1	cyclin dependent kinase 5 regulatory subunit 1 [Source:VGNC Symbol;Acc:VGNC:98980]	1.89	0.0903
ssc-miR-103	CDK6	cyclin dependent kinase 6 [Source:HGNC Symbol;Acc:HGNC:1777]	1.89	0.0903
ssc-miR-103	CDK8	cyclin dependent kinase 8 [Source:VGNC Symbol;Acc:VGNC:86507]	1.89	0.0903
ssc-miR-103	CDKL2	cyclin dependent kinase like 2 [Source:VGNC Symbol;Acc:VGNC:86511]	1.89	0.0903
ssc-miR-103	CDON	cell adhesion associated, onco regulated [Source:VGNC Symbol;Acc:VGNC:86519]	1.89	0.0903
ssc-miR-103	CDV3	CDV3 homolog [Source:VGNC Symbol;Acc:VGNC:86525]	1.89	0.0903
ssc-miR-103	CEACAM1	hypothetical gene	1.89	0.0903
ssc-miR-103	CECR2	CECR2 histone acetyl-lysine reader [Source:VGNC Symbol;Acc:VGNC:96576]	1.89	0.0903
ssc-miR-103	CECR6	hypothetical gene	1.89	0.0903
ssc-miR-103	CELF2	hypothetical gene	1.89	0.0903
ssc-miR-103	CELSR2	cadherin EGF LAG seven-pass G-type receptor 2 [Source:VGNC Symbol;Acc:VGNC:98748]	1.89	0.0903
ssc-miR-103	CENPBD1	hypothetical gene	1.89	0.0903
ssc-miR-103	CENPM	centromere protein M [Source:VGNC Symbol;Acc:VGNC:86553]	1.89	0.0903
ssc-miR-103	CENPP	centromere protein P [Source:VGNC Symbol;Acc:VGNC:98865]	1.89	0.0903
ssc-miR-103	CEP72	hypothetical gene	1.89	0.0903
ssc-miR-103	CEP85L	centrosomal protein 85 like [Source:VGNC Symbol;Acc:VGNC:86580]	1.89	0.0903
ssc-miR-103	CGGBP1	CGG triplet repeat binding protein 1 [Source:VGNC Symbol;Acc:VGNC:86615]	1.89	0.0903
ssc-miR-103	CHD1	chromodomain helicase DNA binding protein 1 [Source:VGNC Symbol;Acc:VGNC:86629]	1.89	0.0903
ssc-miR-103	CHD2	chromodomain helicase DNA binding protein 2 [Source:HGNC Symbol;Acc:HGNC:1917]	1.89	0.0903
ssc-miR-103	CHD6	chromodomain helicase DNA binding protein 6 [Source:VGNC Symbol;Acc:VGNC:95903]	1.89	0.0903
ssc-miR-103	CHIC1	cysteine rich hydrophobic domain 1 [Source:VGNC Symbol;Acc:VGNC:86642]	1.89	0.0903
ssc-miR-103	CHRM1	hypothetical gene	1.89	0.0903
ssc-miR-103	CHST11	carbohydrate sulfotransferase 11 [Source:VGNC Symbol;Acc:VGNC:86675]	1.89	0.0903
ssc-miR-103	CINP	cyclin dependent kinase 2 interacting protein [Source:VGNC Symbol;Acc:VGNC:86705]	1.89	0.0903

ssc-miR-103	CLCN5	chloride voltage-gated channel 5 [Source:VGNC Symbol;Acc:VGNC:103925]	1.89	0.0903
ssc-miR-103	CLIP1	CAP-Gly domain containing linker protein 1 [Source:VGNC Symbol;Acc:VGNC:86766]	1.89	0.0903
ssc-miR-103	CLOCK	clock circadian regulator [Source:VGNC Symbol;Acc:VGNC:86774]	1.89	0.0903
ssc-miR-103	CLSPN	claspin [Source:VGNC Symbol;Acc:VGNC:86783]	1.89	0.0903
ssc-miR-103	CNDP1	carnosine dipeptidase 1 [Source:VGNC Symbol;Acc:VGNC:86811]	1.89	0.0903
ssc-miR-103	CNNM2	cyclin and CBS domain divalent metal cation transport mediator 2 [Source:VGNC Symbol;Acc:VGNC:86828]	1.89	0.0903
ssc-miR-103	CNOT6L	CCR4-NOT transcription complex subunit 6 like [Source:VGNC Symbol;Acc:VGNC:86838]	1.89	0.0903
ssc-miR-103	CNTNAP1	contactin associated protein 1 [Source:VGNC Symbol;Acc:VGNC:86849]	1.89	0.0903
ssc-miR-103	COBLL1	cordon-bleu WH2 repeat protein like 1 [Source:VGNC Symbol;Acc:VGNC:96013]	1.89	0.0903
ssc-miR-103	COL22A1	collagen type XXII alpha 1 chain [Source:VGNC Symbol;Acc:VGNC:97937]	1.89	0.0903
ssc-miR-103	COPS2	COP9 signalosome subunit 2 [Source:VGNC Symbol;Acc:VGNC:86899]	1.89	0.0903
ssc-miR-103	COPZ1	COPI coat complex subunit zeta 1 [Source:VGNC Symbol;Acc:VGNC:86903]	1.89	0.0903
ssc-miR-103	CORO2B	coronin 2B [Source:VGNC Symbol;Acc:VGNC:86916]	1.89	0.0903
ssc-miR-103	CPD	carboxypeptidase D [Source:VGNC Symbol;Acc:VGNC:86935]	1.89	0.0903
ssc-miR-103	CPEB3	cytoplasmic polyadenylation element binding protein 3 [Source:VGNC Symbol;Acc:VGNC:86938]	1.89	0.0903
ssc-miR-103	CPSF2	cleavage and polyadenylation specific factor 2 [Source:VGNC Symbol;Acc:VGNC:86959]	1.89	0.0903
ssc-miR-103	CREBRF	CREB3 regulatory factor [Source:VGNC Symbol;Acc:VGNC:86987]	1.89	0.0903
ssc-miR-103	CRELD1	cysteine rich with EGF like domains 1 [Source:VGNC Symbol;Acc:VGNC:86991]	1.89	0.0903
ssc-miR-103	CSMD1	CUB and Sushi multiple domains 1 [Source:VGNC Symbol;Acc:VGNC:95600]	1.89	0.0903
ssc-miR-103	CSNK1A1	casein kinase 1 alpha 1 [Source:VGNC Symbol;Acc:VGNC:99630]	1.89	0.0903
ssc-miR-103	CSNK1G2	casein kinase 1 gamma 2 [Source:VGNC Symbol;Acc:VGNC:97950]	1.89	0.0903
ssc-miR-103	CSNK1G3	casein kinase 1 gamma 3 [Source:VGNC Symbol;Acc:VGNC:97951]	1.89	0.0903
ssc-miR-103	CTBS	chitobiase [Source:VGNC Symbol;Acc:VGNC:87055]	1.89	0.0903
ssc-miR-103	CTNND1	catenin delta 1 [Source:VGNC Symbol;Acc:VGNC:87067]	1.89	0.0903
ssc-miR-103	CUL4A	cullin 4A [Source:VGNC Symbol;Acc:VGNC:87088]	1.89	0.0903
ssc-miR-103	CXorf23	hypothetical gene	1.89	0.0903
ssc-miR-103	CXXC1	CXXC finger protein 1 [Source:VGNC Symbol;Acc:VGNC:87116]	1.89	0.0903
ssc-miR-103	DCAF10	DDB1 and CUL4 associated factor 10 [Source:VGNC Symbol;Acc:VGNC:87173]	1.89	0.0903
ssc-miR-103	DCAF5	DDB1 and CUL4 associated factor 5 [Source:VGNC Symbol;Acc:VGNC:87176]	1.89	0.0903
ssc-miR-103	DCBLD2	discoidin, CUB and LCCL domain containing 2 [Source:VGNC Symbol;Acc:VGNC:87180]	1.89	0.0903
ssc-miR-103	DCP1A	decapping mRNA 1A [Source:VGNC Symbol;Acc:VGNC:97960]	1.89	0.0903
ssc-miR-103	DCTN3	dynactin subunit 3 [Source:VGNC Symbol;Acc:VGNC:96152]	1.89	0.0903
ssc-miR-103	DCTN4	dynactin subunit 4 [Source:VGNC Symbol;Acc:VGNC:99635]	1.89	0.0903
ssc-miR-103	DCUN1D3	defective in cullin neddylation 1 domain containing 3 [Source:VGNC Symbol;Acc:VGNC:87196]	1.89	0.0903
ssc-miR-103	DCUN1D4	defective in cullin neddylation 1 domain containing 4 [Source:VGNC Symbol;Acc:VGNC:87197]	1.89	0.0903
ssc-miR-103	DCUN1D5	defective in cullin neddylation 1 domain containing 5 [Source:VGNC Symbol;Acc:VGNC:87198]	1.89	0.0903
ssc-miR-103	DDX49	DEAD-box helicase 49 [Source:VGNC Symbol;Acc:VGNC:87225]	1.89	0.0903
ssc-miR-103	DENND5A	DENN domain containing 5A [Source:VGNC Symbol;Acc:VGNC:87254]	1.89	0.0903
ssc-miR-103	DEPTOR	DEP domain containing MTOR interacting protein [Source:HGNC Symbol;Acc:HGNC:22953]	1.89	0.0903
ssc-miR-103	DESI1	desumoylating isopeptidase 1 [Source:VGNC Symbol;Acc:VGNC:87263]	1.89	0.0903
ssc-miR-103	DGCR2	DiGeorge syndrome critical region gene 2 [Source:HGNC Symbol;Acc:HGNC:2845]	1.89	0.0903
ssc-miR-103	DGKH	diacylglycerol kinase eta [Source:VGNC Symbol;Acc:VGNC:87273]	1.89	0.0903
ssc-miR-103	DIAPH2	diaphanous related formin 2 [Source:VGNC Symbol;Acc:VGNC:87300]	1.89	0.0903
ssc-miR-103	DICER1	dicer 1, ribonuclease III [Source:VGNC Symbol;Acc:VGNC:87302]	1.89	0.0903
ssc-miR-103	DISC1	DISC1 scaffold protein [Source:VGNC Symbol;Acc:VGNC:87317]	1.89	0.0903
ssc-miR-103	DKK1	dickkopf WNT signaling pathway inhibitor 1 [Source:VGNC Symbol;Acc:VGNC:87321]	1.89	0.0903
ssc-miR-103	DLG4	discs large MAGUK scaffold protein 4 [Source:VGNC Symbol;Acc:VGNC:87328]	1.89	0.0903
ssc-miR-103	DLG5	discs large MAGUK scaffold protein 5 [Source:VGNC Symbol;Acc:VGNC:87329]	1.89	0.0903
ssc-miR-103	DLL1	delta like canonical Notch ligand 1 [Source:VGNC Symbol;Acc:VGNC:87335]	1.89	0.0903

ssc-miR-103	DNAJB4	DnaJ heat shock protein family (Hsp40) member B4 [Source:VGNC Symbol;Acc:VGNC:96623]	1.89	0.0903
ssc-miR-103	DNAJC3	DnaJ heat shock protein family (Hsp40) member C3 [Source:NCBI gene (formerly Entrezgene);Acc:100154166]	1.89	0.0903
ssc-miR-103	DPP8	dipeptidyl peptidase 8 [Source:VGNC Symbol;Acc:VGNC:87423]	1.89	0.0903
ssc-miR-103	DPYSL3	dihydropyrimidinase like 3 [Source:VGNC Symbol;Acc:VGNC:87431]	1.89	0.0903
ssc-miR-103	DPYSL5	dihydropyrimidinase like 5 [Source:VGNC Symbol;Acc:VGNC:87433]	1.89	0.0903
ssc-miR-103	DSC1	desmocollin 1 [Source:VGNC Symbol;Acc:VGNC:87452]	1.89	0.0903
ssc-miR-103	DSC3	desmocollin 3 [Source:HGNC Symbol;Acc:HGNC:3037]	1.89	0.0903
ssc-miR-103	DST	dystonin [Source:HGNC Symbol;Acc:HGNC:1090]	1.89	0.0903
ssc-miR-103	DUS1L	dihydrouridine synthase 1 like [Source:VGNC Symbol;Acc:VGNC:87474]	1.89	0.0903
ssc-miR-103	DYNC1I1	dynein cytoplasmic 1 intermediate chain 1 [Source:VGNC Symbol;Acc:VGNC:87496]	1.89	0.0903
ssc-miR-103	DYNC1LI2	dynein cytoplasmic 1 light intermediate chain 2 [Source:VGNC Symbol;Acc:VGNC:87498]	1.89	0.0903
ssc-miR-103	DYNLL2	dynein light chain LC8-type 2 [Source:VGNC Symbol;Acc:VGNC:87501]	1.89	0.0903
ssc-miR-103	DYRK1A	dual specificity tyrosine phosphorylation regulated kinase 1A [Source:VGNC Symbol;Acc:VGNC:87505]	1.89	0.0903
ssc-miR-103	DYRK2	dual specificity tyrosine phosphorylation regulated kinase 2 [Source:VGNC Symbol;Acc:VGNC:87507]	1.89	0.0903
ssc-miR-103	EBF1	EBF transcription factor 1 [Source:VGNC Symbol;Acc:VGNC:87525]	1.89	0.0903
ssc-miR-103	EDA	ectodysplasin A [Source:VGNC Symbol;Acc:VGNC:87540]	1.89	0.0903
ssc-miR-103	EDIL3	EGF like repeats and discoidin domains 3 [Source:VGNC Symbol;Acc:VGNC:99642]	1.89	0.0903
ssc-miR-103	EFNB2	ephrin B2 [Source:VGNC Symbol;Acc:VGNC:87577]	1.89	0.0903
ssc-miR-103	EFR3B	EFR3 homolog B [Source:VGNC Symbol;Acc:VGNC:87580]	1.89	0.0903
ssc-miR-103	EIF1AX	eukaryotic translation initiation factor 1A X-linked [Source:NCBI gene (formerly Entrezgene);Acc:100522912]	1.89	0.0903
ssc-miR-103	EIF2S1	eukaryotic translation initiation factor 2 subunit alpha [Source:VGNC Symbol;Acc:VGNC:87614]	1.89	0.0903
ssc-miR-103	EIF4B	eukaryotic translation initiation factor 4B [Source:HGNC Symbol;Acc:HGNC:3285]	1.89	0.0903
ssc-miR-103	EIF4EBP2	eukaryotic translation initiation factor 4E binding protein 2 [Source:VGNC Symbol;Acc:VGNC:87629]	1.89	0.0903
ssc-miR-103	EIF4G1	eukaryotic translation initiation factor 4 gamma 1 [Source:VGNC Symbol;Acc:VGNC:87631]	1.89	0.0903
ssc-miR-103	EIF5	hypothetical gene	1.89	0.0903
ssc-miR-103	EIF5A2	eukaryotic translation initiation factor 5A2 [Source:VGNC Symbol;Acc:VGNC:87633]	1.89	0.0903
ssc-miR-103	ELAVL3	ELAV like RNA binding protein 3 [Source:VGNC Symbol;Acc:VGNC:87640]	1.89	0.0903
ssc-miR-103	ELK4	ETS transcription factor ELK4 [Source:VGNC Symbol;Acc:VGNC:87647]	1.89	0.0903
ssc-miR-103	ELOVL6	ELOVL fatty acid elongase 6 [Source:VGNC Symbol;Acc:VGNC:87662]	1.89	0.0903
ssc-miR-103	EML5	hypothetical gene	1.89	0.0903
ssc-miR-103	EN2	engrailed homeobox 2 [Source:VGNC Symbol;Acc:VGNC:87694]	1.89	0.0903
ssc-miR-103	ENPP2	ectonucleotide pyrophosphatase/phosphodiesterase 2 [Source:VGNC Symbol;Acc:VGNC:87709]	1.89	0.0903
ssc-miR-103	ENTPD4	ectonucleoside triphosphate diphosphohydrolase 4 [Source:VGNC Symbol;Acc:VGNC:107386]	1.89	0.0903
ssc-miR-103	ENTPD7	ectonucleoside triphosphate diphosphohydrolase 7 [Source:VGNC Symbol;Acc:VGNC:87716]	1.89	0.0903
ssc-miR-103	EPC2	enhancer of polycomb homolog 2 [Source:VGNC Symbol;Acc:VGNC:96045]	1.89	0.0903
ssc-miR-103	EPT1	hypothetical gene	1.89	0.0903
ssc-miR-103	ERLIN1	hypothetical gene	1.89	0.0903
ssc-miR-103	ESF1	ESF1 nucleolar pre-rRNA processing protein homolog [Source:VGNC Symbol;Acc:VGNC:95697]	1.89	0.0903
ssc-miR-103	ESR1	estrogen receptor 1 [Source:VGNC Symbol;Acc:VGNC:103089]	1.89	0.0903
ssc-miR-103	ESRRA	estrogen related receptor alpha [Source:VGNC Symbol;Acc:VGNC:87793]	1.89	0.0903
ssc-miR-103	ETF1	eukaryotic translation termination factor 1 [Source:VGNC Symbol;Acc:VGNC:87800]	1.89	0.0903
ssc-miR-103	ETNK1	ethanolamine kinase 1 [Source:VGNC Symbol;Acc:VGNC:87806]	1.89	0.0903
ssc-miR-103	ETS1	ETS proto-onco 1, transcription factor [Source:VGNC Symbol;Acc:VGNC:87808]	1.89	0.0903
ssc-miR-103	ETV3	ETS variant transcription factor 3 [Source:VGNC Symbol;Acc:VGNC:87812]	1.89	0.0903
ssc-miR-103	EVA1A	eva-1 homolog A, regulator of programmed cell death [Source:VGNC Symbol;Acc:VGNC:97988]	1.89	0.0903
ssc-miR-103	EXOC5	exocyst complex component 5 [Source:VGNC Symbol;Acc:VGNC:87831]	1.89	0.0903
ssc-miR-103	FAF2	Fas associated factor family member 2 [Source:VGNC Symbol;Acc:VGNC:87880]	1.89	0.0903
ssc-miR-103	FAM104A	family with sequence similarity 104 member A [Source:VGNC Symbol;Acc:VGNC:98990]	1.89	0.0903
ssc-miR-103	FAM104B	hypothetical gene	1.89	0.0903

ssc-miR-103	FAM110C	family with sequence similarity 110 member C [Source:HGNC Symbol;Acc:HGNC:33340]	1.89	0.0903
ssc-miR-103	FAM117B	family with sequence similarity 117 member B [Source:VGNC Symbol;Acc:VGNC:95616]	1.89	0.0903
ssc-miR-103	FAM126B	family with sequence similarity 126 member B [Source:HGNC Symbol;Acc:HGNC:28593]	1.89	0.0903
ssc-miR-103	FAM134C	hypothetical gene	1.89	0.0903
ssc-miR-103	FAM13B	family with sequence similarity 13 member B [Source:VGNC Symbol;Acc:VGNC:87906]	1.89	0.0903
ssc-miR-103	FAM168B	family with sequence similarity 168 member B [Source:VGNC Symbol;Acc:VGNC:95462]	1.89	0.0903
ssc-miR-103	FAM179B	hypothetical gene	1.89	0.0903
ssc-miR-103	FAM189A1	family with sequence similarity 189 member A1 [Source:VGNC Symbol;Acc:VGNC:87943]	1.89	0.0903
ssc-miR-103	FAM189B	family with sequence similarity 189 member B [Source:VGNC Symbol;Acc:VGNC:87945]	1.89	0.0903
ssc-miR-103	FAM49A	hypothetical gene	1.89	0.0903
ssc-miR-103	FAM57B	hypothetical gene	1.89	0.0903
ssc-miR-103	FAM60A	hypothetical gene	1.89	0.0903
ssc-miR-103	FAM73A	hypothetical gene	1.89	0.0903
ssc-miR-103	FAM81A	family with sequence similarity 81 member A [Source:VGNC Symbol;Acc:VGNC:87986]	1.89	0.0903
ssc-miR-103	FAM98A	family with sequence similarity 98 member A [Source:VGNC Symbol;Acc:VGNC:87997]	1.89	0.0903
ssc-miR-103	FAT4	FAT atypical cadherin 4 [Source:HGNC Symbol;Acc:HGNC:23109]	1.89	0.0903
ssc-miR-103	FBXL18	F-box and leucine rich repeat protein 18 [Source:VGNC Symbol;Acc:VGNC:88029]	1.89	0.0903
ssc-miR-103	FBXL20	F-box and leucine rich repeat protein 20 [Source:VGNC Symbol;Acc:VGNC:98006]	1.89	0.0903
ssc-miR-103	FBXO10	F-box protein 10 [Source:VGNC Symbol;Acc:VGNC:103092]	1.89	0.0903
ssc-miR-103	FBXO24	F-box protein 24 [Source:VGNC Symbol;Acc:VGNC:88036]	1.89	0.0903
ssc-miR-103	FBXW7	F-box and WD repeat domain containing 7 [Source:VGNC Symbol;Acc:VGNC:98925]	1.89	0.0903
ssc-miR-103	FDXR	ferredoxin reductase [Source:VGNC Symbol;Acc:VGNC:88080]	1.89	0.0903
ssc-miR-103	FERMT2	FERM domain containing kindlin 2 [Source:VGNC Symbol;Acc:VGNC:88088]	1.89	0.0903
ssc-miR-103	FEZF1	FEZ family zinc finger 1 [Source:HGNC Symbol;Acc:HGNC:22788]	1.89	0.0903
ssc-miR-103	FGF10	fibroblast growth factor 10 [Source:VGNC Symbol;Acc:VGNC:88101]	1.89	0.0903
ssc-miR-103	FGF18	fibroblast growth factor 18 [Source:VGNC Symbol;Acc:VGNC:88107]	1.89	0.0903
ssc-miR-103	FGF2	hypothetical gene	1.89	0.0903
ssc-miR-103	FGF7	fibroblast growth factor 7 [Source:HGNC Symbol;Acc:HGNC:3685]	1.89	0.0903
ssc-miR-103	FLOT2	flotillin 2 [Source:VGNC Symbol;Acc:VGNC:88159]	1.89	0.0903
ssc-miR-103	FLT1	fms related receptor tyrosine kinase 1 [Source:VGNC Symbol;Acc:VGNC:88162]	1.89	0.0903
ssc-miR-103	FMNL3	formin like 3 [Source:VGNC Symbol;Acc:VGNC:88169]	1.89	0.0903
ssc-miR-103	FNBP1L	formin binding protein 1 like [Source:VGNC Symbol;Acc:VGNC:88179]	1.89	0.0903
ssc-miR-103	FOXG1	forkhead box G1 [Source:VGNC Symbol;Acc:VGNC:88206]	1.89	0.0903
ssc-miR-103	FOXJ2	forkhead box J2 [Source:VGNC Symbol;Acc:VGNC:88212]	1.89	0.0903
ssc-miR-103	FOXJ3	forkhead box J3 [Source:VGNC Symbol;Acc:VGNC:88213]	1.89	0.0903
ssc-miR-103	FOXP1	forkhead box P1 [Source:VGNC Symbol;Acc:VGNC:88222]	1.89	0.0903
ssc-miR-103	FP15737	hypothetical gene	1.89	0.0903
ssc-miR-103	FRK	fyn related Src family tyrosine kinase [Source:VGNC Symbol;Acc:VGNC:88235]	1.89	0.0903
ssc-miR-103	FRYL	FRY like transcription coactivator [Source:VGNC Symbol;Acc:VGNC:98015]	1.89	0.0903
ssc-miR-103	FSTL4	follistatin like 4 [Source:VGNC Symbol;Acc:VGNC:88256]	1.89	0.0903
ssc-miR-103	FUBP1	far upstream element binding protein 1 [Source:VGNC Symbol;Acc:VGNC:96781]	1.89	0.0903
ssc-miR-103	FUCA2	alpha-L-fucosidase 2 [Source:VGNC Symbol;Acc:VGNC:88263]	1.89	0.0903
ssc-miR-103	FURIN	furin, paired basic amino acid cleaving enzyme [Source:VGNC Symbol;Acc:VGNC:88266]	1.89	0.0903
ssc-miR-103	FZD4	frizzled class receptor 4 [Source:VGNC Symbol;Acc:VGNC:88282]	1.89	0.0903
ssc-miR-103	G3BP2	G3BP stress granule assembly factor 2 [Source:VGNC Symbol;Acc:VGNC:88289]	1.89	0.0903
ssc-miR-103	GABRB1	gamma-aminobutyric acid type A receptor subunit beta1 [Source:VGNC Symbol;Acc:VGNC:88306]	1.89	0.0903
ssc-miR-103	GABRG2	gamma-aminobutyric acid type A receptor subunit gamma2 [Source:VGNC Symbol;Acc:VGNC:88312]	1.89	0.0903
ssc-miR-103	GABRQ	gamma-aminobutyric acid type A receptor subunit theta [Source:VGNC Symbol;Acc:VGNC:88315]	1.89	0.0903
ssc-miR-103	GADD45G	growth arrest and DNA damage inducible gamma [Source:VGNC Symbol;Acc:VGNC:88318]	1.89	0.0903

ssc-miR-103	GALNT13	polypeptide N-acetylgalactosaminyltransferase 13 [Source:VGNC Symbol;Acc:VGNC:95992]	1.89	0.0903
ssc-miR-103	GALNT7	polypeptide N-acetylgalactosaminyltransferase 7 [Source:VGNC Symbol;Acc:VGNC:103946]	1.89	0.0903
ssc-miR-103	GALNTL6	polypeptide N-acetylgalactosaminyltransferase like 6 [Source:VGNC Symbol;Acc:VGNC:103947]	1.89	0.0903
ssc-miR-103	GAN	gigaxonin [Source:VGNC Symbol;Acc:VGNC:88343]	1.89	0.0903
ssc-miR-103	GATAD2A	GATA zinc finger domain containing 2A [Source:VGNC Symbol;Acc:VGNC:88368]	1.89	0.0903
ssc-miR-103	GATAD2B	GATA zinc finger domain containing 2B [Source:VGNC Symbol;Acc:VGNC:88369]	1.89	0.0903
ssc-miR-103	GGA2	golgi associated, gamma adaptin ear containing, ARF binding protein 2 [Source:VGNC Symbol;Acc:VGNC:88432]	1.89	0.0903
ssc-miR-103	GGA3	golgi associated, gamma adaptin ear containing, ARF binding protein 3 [Source:VGNC Symbol;Acc:VGNC:88433]	1.89	0.0903
ssc-miR-103	GGACT	hypothetical gene	1.89	0.0903
ssc-miR-103	GIT2	GIT ArfGAP 2 [Source:VGNC Symbol;Acc:VGNC:88458]	1.89	0.0903
ssc-miR-103	GK	hypothetical gene	1.89	0.0903
ssc-miR-103	GLRA2	glycine receptor alpha 2 [Source:VGNC Symbol;Acc:VGNC:88492]	1.89	0.0903
ssc-miR-103	GLUD1	glutamate dehydrogenase 1 [Source:NCBI gene (formerly Entrezgene);Acc:100157162]	1.89	0.0903
ssc-miR-103	GNAI3	G protein subunit alpha i3 [Source:VGNC Symbol;Acc:VGNC:88523]	1.89	0.0903
ssc-miR-103	GNAL	G protein subunit alpha L [Source:VGNC Symbol;Acc:VGNC:88524]	1.89	0.0903
ssc-miR-103	GNAO1	hypothetical gene	1.89	0.0903
ssc-miR-103	GNB4	G protein subunit beta 4 [Source:VGNC Symbol;Acc:VGNC:88533]	1.89	0.0903
ssc-miR-103	GNPNAT1	glucosamine-phosphate N-acetyltransferase 1 [Source:VGNC Symbol;Acc:VGNC:103102]	1.89	0.0903
ssc-miR-103	GNS	glucosamine (N-acetyl)-6-sulfatase [Source:VGNC Symbol;Acc:VGNC:103284]	1.89	0.0903
ssc-miR-103	GOLGA7B	golgin A7 family member B [Source:VGNC Symbol;Acc:VGNC:88553]	1.89	0.0903
ssc-miR-103	GOPC	golgi associated PDZ and coiled-coil motif containing [Source:VGNC Symbol;Acc:VGNC:98024]	1.89	0.0903
ssc-miR-103	GPATCH8	G-patch domain containing 8 [Source:HGNC Symbol;Acc:HGNC:29066]	1.89	0.0903
ssc-miR-103	GPC6	glypican 6 [Source:HGNC Symbol;Acc:HGNC:4454]	1.89	0.0903
ssc-miR-103	GPCPD1	glycerophosphocholine phosphodiesterase 1 [Source:VGNC Symbol;Acc:VGNC:96148]	1.89	0.0903
ssc-miR-103	GPR12	G protein-coupled receptor 12 [Source:VGNC Symbol;Acc:VGNC:88598]	1.89	0.0903
ssc-miR-103	GPR124	hypothetical gene	1.89	0.0903
ssc-miR-103	GPR6	hypothetical gene	1.89	0.0903
ssc-miR-103	GPR63	G protein-coupled receptor 63 [Source:VGNC Symbol;Acc:VGNC:88634]	1.89	0.0903
ssc-miR-103	GPRIN3	hypothetical gene	1.89	0.0903
ssc-miR-103	GRAMD2	hypothetical gene	1.89	0.0903
ssc-miR-103	GREM2	gremlin 2, DAN family BMP antagonist [Source:VGNC Symbol;Acc:VGNC:95854]	1.89	0.0903
ssc-miR-103	GRID1	glutamate ionotropic receptor delta type subunit 1 [Source:VGNC Symbol;Acc:VGNC:88674]	1.89	0.0903
ssc-miR-103	GRIN2A	glutamate ionotropic receptor NMDA type subunit 2A [Source:VGNC Symbol;Acc:VGNC:88683]	1.89	0.0903
ssc-miR-103	GSKIP	GSK3B interacting protein [Source:VGNC Symbol;Acc:VGNC:103956]	1.89	0.0903
ssc-miR-103	GSTCD	glutathione S-transferase C-terminal domain containing [Source:VGNC Symbol;Acc:VGNC:88725]	1.89	0.0903
ssc-miR-103	GTPBP2	GTP binding protein 2 [Source:VGNC Symbol;Acc:VGNC:88743]	1.89	0.0903
ssc-miR-103	HAUS5	HAUS augmin like complex subunit 5 [Source:VGNC Symbol;Acc:VGNC:88788]	1.89	0.0903
ssc-miR-103	HCFC2	host cell factor C2 [Source:VGNC Symbol;Acc:VGNC:88800]	1.89	0.0903
ssc-miR-103	HCN1	hyperpolarization activated cyclic nucleotide gated potassium channel 1 [Source:VGNC Symbol;Acc:VGNC:88802]	1.89	0.0903
ssc-miR-103	HEG1	heart development protein with EGF like domains 1 [Source:VGNC Symbol;Acc:VGNC:88836]	1.89	0.0903
ssc-miR-103	HELLS	helicase, lymphoid specific [Source:VGNC Symbol;Acc:VGNC:88838]	1.89	0.0903
ssc-miR-103	HELZ	helicase with zinc finger [Source:VGNC Symbol;Acc:VGNC:88840]	1.89	0.0903
ssc-miR-103	HERC2	HECT and RLD domain containing E3 ubiquitin protein ligase 2 [Source:VGNC Symbol;Acc:VGNC:99716]	1.89	0.0903
ssc-miR-103	HIATL1	hypothetical gene	1.89	0.0903
ssc-miR-103	HIC2	HIC ZBTB transcriptional repressor 2 [Source:HGNC Symbol;Acc:HGNC:18595]	1.89	0.0903
ssc-miR-103	HIPK2	homeodomain interacting protein kinase 2 [Source:VGNC Symbol;Acc:VGNC:88888]	1.89	0.0903
ssc-miR-103	HLF	HLF transcription factor, PAR bZIP family member [Source:VGNC Symbol;Acc:VGNC:88896]	1.89	0.0903
ssc-miR-103	HMGA1	high mobility group AT-hook 1 [Source:VGNC Symbol;Acc:VGNC:103070]	1.89	0.0903
ssc-miR-103	HMGA2	hypothetical gene	1.89	0.0903

ssc-miR-103	HMGB1	hypothetical gene	1.89	0.0903
ssc-miR-103	HMGN2	high mobility group nucleosomal binding domain 2 [Source:NCBI gene (formerly Entrezgene);Acc:100524500]	1.89	0.0903
ssc-miR-103	HNMT	histamine N-methyltransferase [Source:NCBI gene (formerly Entrezgene);Acc:100624677]	1.89	0.0903
ssc-miR-103	HNRNPA2B1	heterogeneous nuclear ribonucleoprotein A2/B1 [Source:HGNC Symbol;Acc:HGNC:5033]	1.89	0.0903
ssc-miR-103	HOXA10	homeobox A10 [Source:VGNC Symbol;Acc:VGNC:88934]	1.89	0.0903
ssc-miR-103	HOXA7	homeobox A7 [Source:VGNC Symbol;Acc:VGNC:88940]	1.89	0.0903
ssc-miR-103	HOXB2	homeobox B2 [Source:VGNC Symbol;Acc:VGNC:88943]	1.89	0.0903
ssc-miR-103	HOXD10	homeobox D10 [Source:VGNC Symbol;Acc:VGNC:96351]	1.89	0.0903
ssc-miR-103	HPSE2	heparanase 2 (inactive) [Source:HGNC Symbol;Acc:HGNC:18374]	1.89	0.0903
ssc-miR-103	HSD3B7	hydroxy-delta-5-steroid dehydrogenase, 3 beta- and steroid delta-isomerase 7 [Source:VGNC Symbol;Acc:VGNC:88990]	1.89	0.0903
ssc-miR-103	HSDL1	hydroxysteroid dehydrogenase like 1 [Source:VGNC Symbol;Acc:VGNC:88991]	1.89	0.0903
ssc-miR-103	HSPA12A	heat shock protein family A (Hsp70) member 12A [Source:VGNC Symbol;Acc:VGNC:96684]	1.89	0.0903
ssc-miR-103	HTT	huntingtin [Source:VGNC Symbol;Acc:VGNC:89006]	1.89	0.0903
ssc-miR-103	IARS	hypothetical gene	1.89	0.0903
ssc-miR-103	ICMT	isoprenylcysteine carboxyl methyltransferase [Source:VGNC Symbol;Acc:VGNC:89022]	1.89	0.0903
ssc-miR-103	IFFO1	intermediate filament family orphan 1 [Source:VGNC Symbol;Acc:VGNC:89030]	1.89	0.0903
ssc-miR-103	IGSF3	immunoglobulin superfamily member 3 [Source:VGNC Symbol;Acc:VGNC:89067]	1.89	0.0903
ssc-miR-103	IHH	Indian hedgehog signaling molecule [Source:VGNC Symbol;Acc:VGNC:96370]	1.89	0.0903
ssc-miR-103	IKBKAP	hypothetical gene	1.89	0.0903
ssc-miR-103	IL7	interleukin 7 [Source:VGNC Symbol;Acc:VGNC:98812]	1.89	0.0903
ssc-miR-103	IMPA1	inositol monophosphatase 1 [Source:VGNC Symbol;Acc:VGNC:89125]	1.89	0.0903
ssc-miR-103	IMPG1	interphotoreceptor matrix proteoglycan 1 [Source:VGNC Symbol;Acc:VGNC:89127]	1.89	0.0903
ssc-miR-103	INADL	hypothetical gene	1.89	0.0903
ssc-miR-103	INSIG1	insulin induced 1 [Source:VGNC Symbol;Acc:VGNC:89150]	1.89	0.0903
ssc-miR-103	IRF2BP2	interferon regulatory factor 2 binding protein 2 [Source:VGNC Symbol;Acc:VGNC:89205]	1.89	0.0903
ssc-miR-103	ISL2	ISL LIM homeobox 2 [Source:VGNC Symbol;Acc:VGNC:89226]	1.89	0.0903
ssc-miR-103	ITPRIP	inositol 1,4,5-trisphosphate receptor interacting protein [Source:VGNC Symbol;Acc:VGNC:89256]	1.89	0.0903
ssc-miR-103	ITSN1	intersectin 1 [Source:VGNC Symbol;Acc:VGNC:108669]	1.89	0.0903
ssc-miR-103	JAKMIP2	janus kinase and microtubule interacting protein 2 [Source:VGNC Symbol;Acc:VGNC:89274]	1.89	0.0903
ssc-miR-103	JHDM1D	hypothetical gene	1.89	0.0903
ssc-miR-103	JOSD1	Josephin domain containing 1 [Source:VGNC Symbol;Acc:VGNC:89290]	1.89	0.0903
ssc-miR-103	JPH3	hypothetical gene	1.89	0.0903
ssc-miR-103	KANK4	KN motif and ankyrin repeat domains 4 [Source:VGNC Symbol;Acc:VGNC:89298]	1.89	0.0903
ssc-miR-103	KAT7	lysine acetyltransferase 7 [Source:VGNC Symbol;Acc:VGNC:89307]	1.89	0.0903
ssc-miR-103	KATNBL1	katanin regulatory subunit B1 like 1 [Source:VGNC Symbol;Acc:VGNC:89313]	1.89	0.0903
ssc-miR-103	KAZN	kazrin, periplakin interacting protein [Source:HGNC Symbol;Acc:HGNC:29173]	1.89	0.0903
ssc-miR-103	KB-1507C5.2	hypothetical gene	1.89	0.0903
ssc-miR-103	KCMF1	potassium channel modulatory factor 1 [Source:VGNC Symbol;Acc:VGNC:89322]	1.89	0.0903
ssc-miR-103	KCNA2	potassium voltage-gated channel subfamily A member 2 [Source:VGNC Symbol;Acc:VGNC:89325]	1.89	0.0903
ssc-miR-103	KCNC4	potassium voltage-gated channel subfamily C member 4 [Source:VGNC Symbol;Acc:VGNC:89335]	1.89	0.0903
ssc-miR-103	KCND1	potassium voltage-gated channel subfamily D member 1 [Source:VGNC Symbol;Acc:VGNC:89336]	1.89	0.0903
ssc-miR-103	KCNH1	potassium voltage-gated channel subfamily H member 1 [Source:VGNC Symbol;Acc:VGNC:108599]	1.89	0.0903
ssc-miR-103	KCNIP2	potassium voltage-gated channel interacting protein 2 [Source:VGNC Symbol;Acc:VGNC:89349]	1.89	0.0903
ssc-miR-103	KCNK12	potassium two pore domain channel subfamily K member 12 [Source:HGNC Symbol;Acc:HGNC:6274]	1.89	0.0903
ssc-miR-103	KCNK7	potassium two pore domain channel subfamily K member 7 [Source:VGNC Symbol;Acc:VGNC:89374]	1.89	0.0903
ssc-miR-103	KCNN3	potassium calcium-activated channel subfamily N member 3 [Source:VGNC Symbol;Acc:VGNC:98056]	1.89	0.0903
ssc-miR-103	KIAA0232	hypothetical gene	1.89	0.0903
ssc-miR-103	KIAA0247	hypothetical gene	1.89	0.0903
ssc-miR-103	KIAA0907	hypothetical gene	1.89	0.0903

ssc-miR-103	KIAA0947	hypothetical gene	1.89	0.0903
ssc-miR-103	KIAA1033	hypothetical gene	1.89	0.0903
ssc-miR-103	KIAA1429	hypothetical gene	1.89	0.0903
ssc-miR-103	KIAA1549L	KIAA1549 like [Source:VGNC Symbol;Acc:VGNC:89445]	1.89	0.0903
ssc-miR-103	KIAA1958	KIAA1958 [Source:VGNC Symbol;Acc:VGNC:89448]	1.89	0.0903
ssc-miR-103	KIAA2018	hypothetical gene	1.89	0.0903
ssc-miR-103	KIF1B	kinesin family member 1B [Source:VGNC Symbol;Acc:VGNC:89460]	1.89	0.0903
ssc-miR-103	KIF21A	kinesin family member 21A [Source:VGNC Symbol;Acc:VGNC:89464]	1.89	0.0903
ssc-miR-103	KIF23	kinesin family member 23 [Source:VGNC Symbol;Acc:VGNC:103113]	1.89	0.0903
ssc-miR-103	KIF3B	kinesin family member 3B [Source:VGNC Symbol;Acc:VGNC:96392]	1.89	0.0903
ssc-miR-103	KIF5A	kinesin family member 5A [Source:VGNC Symbol;Acc:VGNC:89472]	1.89	0.0903
ssc-miR-103	KIF5C	kinesin family member 5C [Source:VGNC Symbol;Acc:VGNC:96394]	1.89	0.0903
ssc-miR-103	KIFC2	kinesin family member C2 [Source:VGNC Symbol;Acc:VGNC:89478]	1.89	0.0903
ssc-miR-103	KLF4	Kruppel like factor 4 [Source:VGNC Symbol;Acc:VGNC:98062]	1.89	0.0903
ssc-miR-103	KLF7	Kruppel like factor 7 [Source:VGNC Symbol;Acc:VGNC:96396]	1.89	0.0903
ssc-miR-103	KLHL18	kelch like family member 18 [Source:VGNC Symbol;Acc:VGNC:89517]	1.89	0.0903
ssc-miR-103	KMT2D	hypothetical gene	1.89	0.0903
ssc-miR-103	KPNA1	karyopherin subunit alpha 1 [Source:VGNC Symbol;Acc:VGNC:89560]	1.89	0.0903
ssc-miR-103	KPNA3	karyopherin subunit alpha 3 [Source:VGNC Symbol;Acc:VGNC:89562]	1.89	0.0903
ssc-miR-103	KPNA4	karyopherin subunit alpha 4 [Source:VGNC Symbol;Acc:VGNC:89563]	1.89	0.0903
ssc-miR-103	KPNB1	karyopherin subunit beta 1 [Source:VGNC Symbol;Acc:VGNC:89567]	1.89	0.0903
ssc-miR-103	KRT85	keratin 85 [Source:HGNC Symbol;Acc:HGNC:6462]	1.89	0.0903
ssc-miR-103	LANCL3	LanC like 3 [Source:VGNC Symbol;Acc:VGNC:89634]	1.89	0.0903
ssc-miR-103	LARP4	La ribonucleoprotein 4 [Source:VGNC Symbol;Acc:VGNC:89641]	1.89	0.0903
ssc-miR-103	LARP4B	hypothetical gene	1.89	0.0903
ssc-miR-103	LATS2	large tumor suppressor kinase 2 [Source:VGNC Symbol;Acc:VGNC:89649]	1.89	0.0903
ssc-miR-103	LCOR	ligand dependent nuclear receptor corepressor [Source:HGNC Symbol;Acc:HGNC:29503]	1.89	0.0903
ssc-miR-103	LDHA	hypothetical gene	1.89	0.0903
ssc-miR-103	LDLRAD2	low density lipoprotein receptor class A domain containing 2 [Source:HGNC Symbol;Acc:HGNC:32071]	1.89	0.0903
ssc-miR-103	LETMD1	LETM1 domain containing 1 [Source:VGNC Symbol;Acc:VGNC:89688]	1.89	0.0903
ssc-miR-103	LGSN	lengsin, lens protein with glutamine synthetase domain [Source:HGNC Symbol;Acc:HGNC:21016]	1.89	0.0903
ssc-miR-103	LHPP	phospholysine phosphohistidine inorganic pyrophosphate phosphatase [Source:VGNC Symbol;Acc:VGNC:89710]	1.89	0.0903
ssc-miR-103	LHX4	LIM homeobox 4 [Source:VGNC Symbol;Acc:VGNC:89714]	1.89	0.0903
ssc-miR-103	LIN28A	lin-28 homolog A [Source:VGNC Symbol;Acc:VGNC:98492]	1.89	0.0903
ssc-miR-103	LIN28B	lin-28 homolog B [Source:VGNC Symbol;Acc:VGNC:89729]	1.89	0.0903
ssc-miR-103	LIN7A	lin-7 homolog A, crumbs cell polarity complex component [Source:VGNC Symbol;Acc:VGNC:89730]	1.89	0.0903
ssc-miR-103	LONRF1	LON peptidase N-terminal domain and ring finger 1 [Source:VGNC Symbol;Acc:VGNC:96123]	1.89	0.0903
ssc-miR-103	LONRF3	LON peptidase N-terminal domain and ring finger 3 [Source:VGNC Symbol;Acc:VGNC:89778]	1.89	0.0903
ssc-miR-103	LRIG2	leucine rich repeats and immunoglobulin like domains 2 [Source:VGNC Symbol;Acc:VGNC:89811]	1.89	0.0903
ssc-miR-103	LRP1	hypothetical gene	1.89	0.0903
ssc-miR-103	LRP1B	LDL receptor related protein 1B [Source:HGNC Symbol;Acc:HGNC:6693]	1.89	0.0903
ssc-miR-103	LRP2	LDL receptor related protein 2 [Source:HGNC Symbol;Acc:HGNC:6694]	1.89	0.0903
ssc-miR-103	LRRC14	leucine rich repeat containing 14 [Source:VGNC Symbol;Acc:VGNC:89826]	1.89	0.0903
ssc-miR-103	LRRC15	leucine rich repeat containing 15 [Source:VGNC Symbol;Acc:VGNC:98077]	1.89	0.0903
ssc-miR-103	LRRC32	leucine rich repeat containing 32 [Source:VGNC Symbol;Acc:VGNC:108602]	1.89	0.0903
ssc-miR-103	LRRC36	leucine rich repeat containing 36 [Source:VGNC Symbol;Acc:VGNC:89836]	1.89	0.0903
ssc-miR-103	LRRC40	hypothetical gene	1.89	0.0903
ssc-miR-103	LRRC4C	leucine rich repeat containing 4C [Source:VGNC Symbol;Acc:VGNC:89848]	1.89	0.0903
ssc-miR-103	LRRC55	leucine rich repeat containing 55 [Source:VGNC Symbol;Acc:VGNC:89850]	1.89	0.0903

ssc-miR-103	LRRC8D	leucine rich repeat containing 8 VRAC subunit D [Source:VGNC Symbol;Acc:VGNC:98088]	1.89	0.0903
ssc-miR-103	LUZP1	leucine zipper protein 1 [Source:VGNC Symbol;Acc:VGNC:89897]	1.89	0.0903
ssc-miR-103	MAF1	MAF1 homolog, negative regulator of RNA polymerase III [Source:HGNC Symbol;Acc:HGNC:24966]	1.89	0.0903
ssc-miR-103	MAK16	MAK16 homolog [Source:VGNC Symbol;Acc:VGNC:103979]	1.89	0.0903
ssc-miR-103	MAML1	mastermind like transcriptional coactivator 1 [Source:VGNC Symbol;Acc:VGNC:89963]	1.89	0.0903
ssc-miR-103	MAP2K6	mitogen-activated protein kinase kinase 6 [Source:VGNC Symbol;Acc:VGNC:98102]	1.89	0.0903
ssc-miR-103	MAP3K3	mitogen-activated protein kinase kinase kinase 3 [Source:VGNC Symbol;Acc:VGNC:98108]	1.89	0.0903
ssc-miR-103	MAP4	microtubule associated protein 4 [Source:VGNC Symbol;Acc:VGNC:103071]	1.89	0.0903
ssc-miR-103	MAPK1IP1L	mitogen-activated protein kinase 1 interacting protein 1 like [Source:VGNC Symbol;Acc:VGNC:90002]	1.89	0.0903
ssc-miR-103	MAPK8	mitogen-activated protein kinase 8 [Source:VGNC Symbol;Acc:VGNC:90006]	1.89	0.0903
ssc-miR-103	MAPKBP1	mitogen-activated protein kinase binding protein 1 [Source:VGNC Symbol;Acc:VGNC:90014]	1.89	0.0903
ssc-miR-103	MARCH3	hypothetical gene	1.89	0.0903
ssc-miR-103	MBNL1	muscleblind like splicing regulator 1 [Source:VGNC Symbol;Acc:VGNC:90054]	1.89	0.0903
ssc-miR-103	MBNL2	muscleblind like splicing regulator 2 [Source:VGNC Symbol;Acc:VGNC:90055]	1.89	0.0903
ssc-miR-103	MBNL3	muscleblind like splicing regulator 3 [Source:VGNC Symbol;Acc:VGNC:90056]	1.89	0.0903
ssc-miR-103	MCAT	malonyl-CoA-acyl carrier protein transacylase [Source:VGNC Symbol;Acc:VGNC:90062]	1.89	0.0903
ssc-miR-103	MCFD2	multiple coagulation factor deficiency 2, ER cargo receptor complex subunit [Source:VGNC Symbol;Acc:VGNC:103980]	1.89	0.0903
ssc-miR-103	MED26	mediator complex subunit 26 [Source:VGNC Symbol;Acc:VGNC:100168]	1.89	0.0903
ssc-miR-103	MEF2D	myocyte enhancer factor 2D [Source:VGNC Symbol;Acc:VGNC:90128]	1.89	0.0903
ssc-miR-103	MEGF11	multiple EGF like domains 11 [Source:VGNC Symbol;Acc:VGNC:103126]	1.89	0.0903
ssc-miR-103	MFAP3	microfibril associated protein 3 [Source:VGNC Symbol;Acc:VGNC:90174]	1.89	0.0903
ssc-miR-103	MFN1	mitofusin 1 [Source:VGNC Symbol;Acc:VGNC:90178]	1.89	0.0903
ssc-miR-103	MFN2	mitofusin 2 [Source:VGNC Symbol;Acc:VGNC:90179]	1.89	0.0903
ssc-miR-103	MGA	MAX dimerization protein MGA [Source:VGNC Symbol;Acc:VGNC:90195]	1.89	0.0903
ssc-miR-103	MIB1	MIB E3 ubiquitin protein ligase 1 [Source:VGNC Symbol;Acc:VGNC:90205]	1.89	0.0903
ssc-miR-103	MIF4GD	MIF4G domain containing [Source:VGNC Symbol;Acc:VGNC:90223]	1.89	0.0903
ssc-miR-103	MLK4	hypothetical gene	1.89	0.0903
ssc-miR-103	MMP24	matrix metallopeptidase 24 [Source:VGNC Symbol;Acc:VGNC:96421]	1.89	0.0903
ssc-miR-103	MMS19	MMS19 homolog, cytosolic iron-sulfur assembly component [Source:VGNC Symbol;Acc:VGNC:90283]	1.89	0.0903
ssc-miR-103	MOB3B	MOB kinase activator 3B [Source:VGNC Symbol;Acc:VGNC:96024]	1.89	0.0903
ssc-miR-103	MRPS23	mitochondrial ribosomal protein S23 [Source:VGNC Symbol;Acc:VGNC:90389]	1.89	0.0903
ssc-miR-103	MSH6	mutS homolog 6 [Source:VGNC Symbol;Acc:VGNC:90420]	1.89	0.0903
ssc-miR-103	MSI2	musashi RNA binding protein 2 [Source:VGNC Symbol;Acc:VGNC:90422]	1.89	0.0903
ssc-miR-103	MTF1	metal regulatory transcription factor 1 [Source:VGNC Symbol;Acc:VGNC:90443]	1.89	0.0903
ssc-miR-103	MTFR1L	mitochondrial fission regulator 1 like [Source:VGNC Symbol;Acc:VGNC:90448]	1.89	0.0903
ssc-miR-103	MTHFD1L	methylenetetrahydrofolate dehydrogenase (NADP+ dependent) 1 like [Source:VGNC Symbol;Acc:VGNC:103129]	1.89	0.0903
ssc-miR-103	MTMR12	myotubularin related protein 12 [Source:VGNC Symbol;Acc:VGNC:90459]	1.89	0.0903
ssc-miR-103	MTMR3	myotubularin related protein 3 [Source:VGNC Symbol;Acc:VGNC:90461]	1.89	0.0903
ssc-miR-103	MTMR4	myotubularin related protein 4 [Source:VGNC Symbol;Acc:VGNC:90462]	1.89	0.0903
ssc-miR-103	MTUS1	microtubule associated scaffold protein 1 [Source:VGNC Symbol;Acc:VGNC:107157]	1.89	0.0903
ssc-miR-103	MXD4	MAX dimerization protein 4 [Source:VGNC Symbol;Acc:VGNC:90493]	1.89	0.0903
ssc-miR-103	MYB	MYB proto-onco, transcription factor [Source:VGNC Symbol;Acc:VGNC:90496]	1.89	0.0903
ssc-miR-103	MYBL1	MYB proto-onco like 1 [Source:VGNC Symbol;Acc:VGNC:90498]	1.89	0.0903
ssc-miR-103	MYCN	MYCN proto-onco, bHLH transcription factor [Source:VGNC Symbol;Acc:VGNC:90504]	1.89	0.0903
ssc-miR-103	MYH9	myosin heavy chain 9 [Source:VGNC Symbol;Acc:VGNC:90512]	1.89	0.0903
ssc-miR-103	MYNN	myoneurin [Source:VGNC Symbol;Acc:VGNC:90521]	1.89	0.0903
ssc-miR-103	N4BP1	NEDD4 binding protein 1 [Source:VGNC Symbol;Acc:VGNC:90553]	1.89	0.0903
ssc-miR-103	NAA15	N-alpha-acetyltransferase 15, NatA auxiliary subunit [Source:VGNC Symbol;Acc:VGNC:96747]	1.89	0.0903
ssc-miR-103	NAA20	N-alpha-acetyltransferase 20, NatB catalytic subunit [Source:VGNC Symbol;Acc:VGNC:96749]	1.89	0.0903

ssc-miR-103	NAT8L	N-acetyltransferase 8 like [Source:VGNC Symbol;Acc:VGNC:90586]	1.89	0.0903
ssc-miR-103	NAV1	neuron navigator 1 [Source:VGNC Symbol;Acc:VGNC:95725]	1.89	0.0903
ssc-miR-103	NAV2	neuron navigator 2 [Source:VGNC Symbol;Acc:VGNC:90588]	1.89	0.0903
ssc-miR-103	NCOA2	nuclear receptor coactivator 2 [Source:VGNC Symbol;Acc:VGNC:90616]	1.89	0.0903
ssc-miR-103	NDEL1	nudE neurodevelopment protein 1 like 1 [Source:VGNC Symbol;Acc:VGNC:90625]	1.89	0.0903
ssc-miR-103	NEDD9	neural cell expressed, developmentally down-regulated 9 [Source:VGNC Symbol;Acc:VGNC:90668]	1.89	0.0903
ssc-miR-103	NEK10	NIMA related kinase 10 [Source:VGNC Symbol;Acc:VGNC:90674]	1.89	0.0903
ssc-miR-103	NF1	neurofibromin 1 [Source:VGNC Symbol;Acc:VGNC:90704]	1.89	0.0903
ssc-miR-103	NFAM1	NFAT activating protein with ITAM motif 1 [Source:VGNC Symbol;Acc:VGNC:90706]	1.89	0.0903
ssc-miR-103	NFAT5	nuclear factor of activated T cells 5 [Source:VGNC Symbol;Acc:VGNC:90708]	1.89	0.0903
ssc-miR-103	NFATC2	nuclear factor of activated T cells 2 [Source:VGNC Symbol;Acc:VGNC:96440]	1.89	0.0903
ssc-miR-103	NFIA	nuclear factor I A [Source:VGNC Symbol;Acc:VGNC:90715]	1.89	0.0903
ssc-miR-103	NFIB	nuclear factor I B [Source:VGNC Symbol;Acc:VGNC:90716]	1.89	0.0903
ssc-miR-103	NHSL2	NHS like 2 [Source:VGNC Symbol;Acc:VGNC:98155]	1.89	0.0903
ssc-miR-103	NMNAT1	nicotinamide nucleotide adenylyltransferase 1 [Source:VGNC Symbol;Acc:VGNC:90797]	1.89	0.0903
ssc-miR-103	NMT2	N-myristoyltransferase 2 [Source:VGNC Symbol;Acc:VGNC:96448]	1.89	0.0903
ssc-miR-103	NOS1	nitric oxide synthase 1 [Source:VGNC Symbol;Acc:VGNC:90823]	1.89	0.0903
ssc-miR-103	NOTCH2	notch receptor 2 [Source:HGNC Symbol;Acc:HGNC:7882]	1.89	0.0903
ssc-miR-103	NOVA1	NOVA alternative splicing regulator 1 [Source:VGNC Symbol;Acc:VGNC:90827]	1.89	0.0903
ssc-miR-103	NOVA2	NOVA alternative splicing regulator 2 [Source:VGNC Symbol;Acc:VGNC:90828]	1.89	0.0903
ssc-miR-103	NPAS2	neuronal PAS domain protein 2 [Source:VGNC Symbol;Acc:VGNC:90837]	1.89	0.0903
ssc-miR-103	NPAS3	neuronal PAS domain protein 3 [Source:VGNC Symbol;Acc:VGNC:90838]	1.89	0.0903
ssc-miR-103	NPFFR1	neuropeptide FF receptor 1 [Source:VGNC Symbol;Acc:VGNC:90846]	1.89	0.0903
ssc-miR-103	NR1D2	nuclear receptor subfamily 1 group D member 2 [Source:VGNC Symbol;Acc:VGNC:99726]	1.89	0.0903
ssc-miR-103	NR2C2	nuclear receptor subfamily 2 group C member 2 [Source:VGNC Symbol;Acc:VGNC:90876]	1.89	0.0903
ssc-miR-103	NR4A3	nuclear receptor subfamily 4 group A member 3 [Source:VGNC Symbol;Acc:VGNC:90885]	1.89	0.0903
ssc-miR-103	NREP	neuronal regeneration related protein [Source:HGNC Symbol;Acc:HGNC:16834]	1.89	0.0903
ssc-miR-103	NRIP1	nuclear receptor interacting protein 1 [Source:VGNC Symbol;Acc:VGNC:90896]	1.89	0.0903
ssc-miR-103	NSG1	neuronal vesicle trafficking associated 1 [Source:VGNC Symbol;Acc:VGNC:90907]	1.89	0.0903
ssc-miR-103	NUAK1	NUAK family kinase 1 [Source:VGNC Symbol;Acc:VGNC:90940]	1.89	0.0903
ssc-miR-103	NUDCD2	NudC domain containing 2 [Source:VGNC Symbol;Acc:VGNC:90950]	1.89	0.0903
ssc-miR-103	NUP37	nucleoporin 37 [Source:VGNC Symbol;Acc:VGNC:90980]	1.89	0.0903
ssc-miR-103	NUP50	nucleoporin 50 [Source:VGNC Symbol;Acc:VGNC:90983]	1.89	0.0903
ssc-miR-103	NUP62CL	nucleoporin 62 C-terminal like [Source:VGNC Symbol;Acc:VGNC:104013]	1.89	0.0903
ssc-miR-103	NXF1	nuclear RNA export factor 1 [Source:VGNC Symbol;Acc:VGNC:90993]	1.89	0.0903
ssc-miR-103	NXPH3	neurexophilin 3 [Source:VGNC Symbol;Acc:VGNC:90999]	1.89	0.0903
ssc-miR-103	OAF	out at first homolog [Source:VGNC Symbol;Acc:VGNC:91005]	1.89	0.0903
ssc-miR-103	OGT	O-linked N-acetylglucosamine (GlcNAc) transferase [Source:NCBI gene (formerly Entrezgene);Acc:664652]	1.89	0.0903
ssc-miR-103	OIP5	Opa interacting protein 5 [Source:VGNC Symbol;Acc:VGNC:91029]	1.89	0.0903
ssc-miR-103	ORC6	origin recognition complex subunit 6 [Source:VGNC Symbol;Acc:VGNC:91063]	1.89	0.0903
ssc-miR-103	OSBPL6	oxysterol binding protein like 6 [Source:VGNC Symbol;Acc:VGNC:95807]	1.89	0.0903
ssc-miR-103	OTUD4	OTU deubiquitinase 4 [Source:VGNC Symbol;Acc:VGNC:91100]	1.89	0.0903
ssc-miR-103	OXLD1	oxidoreductase like domain containing 1 [Source:VGNC Symbol;Acc:VGNC:91114]	1.89	0.0903
ssc-miR-103	PABPC1L	poly(A) binding protein cytoplasmic 1 like [Source:VGNC Symbol;Acc:VGNC:108728]	1.89	0.0903
ssc-miR-103	PACSIN1	protein kinase C and casein kinase substrate in neurons 1 [Source:VGNC Symbol;Acc:VGNC:91142]	1.89	0.0903
ssc-miR-103	PAFAH1B2	platelet activating factor acetylhydrolase 1b catalytic subunit 2 [Source:VGNC Symbol;Acc:VGNC:91151]	1.89	0.0903
ssc-miR-103	PAG1	phosphoprotein membrane anchor with glycosphingolipid microdomains 1 [Source:VGNC Symbol;Acc:VGNC:91154]	1.89	0.0903
ssc-miR-103	PAIP2B	poly(A) binding protein interacting protein 2B [Source:HGNC Symbol;Acc:HGNC:29200]	1.89	0.0903
ssc-miR-103	PAK3	p21 (RAC1) activated kinase 3 [Source:HGNC Symbol;Acc:HGNC:8592]	1.89	0.0903

ssc-miR-103	PALM2-AKAP2	hypothetical gene	1.89	0.0903
ssc-miR-103	PAPPA2	pappalysin 2 [Source:VGNC Symbol;Acc:VGNC:91171]	1.89	0.0903
ssc-miR-103	PARP12	poly(ADP-ribose) polymerase family member 12 [Source:VGNC Symbol;Acc:VGNC:96688]	1.89	0.0903
ssc-miR-103	PAWR	pro-apoptotic WT1 regulator [Source:VGNC Symbol;Acc:VGNC:91191]	1.89	0.0903
ssc-miR-103	PCDH17	protocadherin 17 [Source:VGNC Symbol;Acc:VGNC:91214]	1.89	0.0903
ssc-miR-103	PCGF2	polycomb group ring finger 2 [Source:VGNC Symbol;Acc:VGNC:91219]	1.89	0.0903
ssc-miR-103	PCGF5	hypothetical gene	1.89	0.0903
ssc-miR-103	PDCD10	programmed cell death 10 [Source:VGNC Symbol;Acc:VGNC:91241]	1.89	0.0903
ssc-miR-103	PDE11A	phosphodiesterase 11A [Source:HGNC Symbol;Acc:HGNC:8773]	1.89	0.0903
ssc-miR-103	PDE3A	phosphodiesterase 3A [Source:VGNC Symbol;Acc:VGNC:91252]	1.89	0.0903
ssc-miR-103	PDE3B	phosphodiesterase 3B [Source:VGNC Symbol;Acc:VGNC:91253]	1.89	0.0903
ssc-miR-103	PDE4D	phosphodiesterase 4D [Source:VGNC Symbol;Acc:VGNC:91256]	1.89	0.0903
ssc-miR-103	PDE8B	phosphodiesterase 8B [Source:VGNC Symbol;Acc:VGNC:100321]	1.89	0.0903
ssc-miR-103	PDHB	pyruvate dehydrogenase E1 subunit beta [Source:VGNC Symbol;Acc:VGNC:91269]	1.89	0.0903
ssc-miR-103	PDIK1L	PDLIM1 interacting kinase 1 like [Source:VGNC Symbol;Acc:VGNC:91276]	1.89	0.0903
ssc-miR-103	PDK3	pyruvate dehydrogenase kinase 3 [Source:VGNC Symbol;Acc:VGNC:91279]	1.89	0.0903
ssc-miR-103	PDK4	pyruvate dehydrogenase kinase 4 [Source:VGNC Symbol;Acc:VGNC:91280]	1.89	0.0903
ssc-miR-103	PDPK1	3-phosphoinositide dependent protein kinase 1 [Source:HGNC Symbol;Acc:HGNC:8816]	1.89	0.0903
ssc-miR-103	PDS5A	PDS5 cohesin associated factor A [Source:HGNC Symbol;Acc:HGNC:29088]	1.89	0.0903
ssc-miR-103	PDZD8	hypothetical gene	1.89	0.0903
ssc-miR-103	PEG10	paternally expressed 10 [Source:NCBI gene (formerly Entrezgene);Acc:654416]	1.89	0.0903
ssc-miR-103	PELI2	pellino E3 ubiquitin protein ligase family member 2 [Source:VGNC Symbol;Acc:VGNC:91307]	1.89	0.0903
ssc-miR-103	PER2	period circadian regulator 2 [Source:VGNC Symbol;Acc:VGNC:96478]	1.89	0.0903
ssc-miR-103	PGRMC2	progesterone receptor membrane component 2 [Source:VGNC Symbol;Acc:VGNC:98952]	1.89	0.0903
ssc-miR-103	PHF15	hypothetical gene	1.89	0.0903
ssc-miR-103	PHF19	PHD finger protein 19 [Source:VGNC Symbol;Acc:VGNC:91381]	1.89	0.0903
ssc-miR-103	PHF20	PHD finger protein 20 [Source:HGNC Symbol;Acc:HGNC:16098]	1.89	0.0903
ssc-miR-103	PHF5A	PHD finger protein 5A [Source:VGNC Symbol;Acc:VGNC:91388]	1.89	0.0903
ssc-miR-103	PHYHIPL	phytanoyl-CoA 2-hydroxylase interacting protein like [Source:VGNC Symbol;Acc:VGNC:91407]	1.89	0.0903
ssc-miR-103	PI4KB	phosphatidylinositol 4-kinase beta [Source:VGNC Symbol;Acc:VGNC:91408]	1.89	0.0903
ssc-miR-103	PIANP	PILR alpha associated neural protein [Source:VGNC Symbol;Acc:VGNC:91409]	1.89	0.0903
ssc-miR-103	PIEZO1	piezo type mechanosensitive ion channel component 1 [Source:VGNC Symbol;Acc:VGNC:91417]	1.89	0.0903
ssc-miR-103	PIGV	phosphatidylinositol glycan anchor biosynthesis class V [Source:VGNC Symbol;Acc:VGNC:91430]	1.89	0.0903
ssc-miR-103	PIK3CB	phosphatidylinositol-4,5-bisphosphate 3-kinase catalytic subunit beta [Source:VGNC Symbol;Acc:VGNC:91441]	1.89	0.0903
ssc-miR-103	PIK3R1	phosphoinositide-3-kinase regulatory subunit 1 [Source:VGNC Symbol;Acc:VGNC:91445]	1.89	0.0903
ssc-miR-103	PITPNA	phosphatidylinositol transfer protein alpha [Source:VGNC Symbol;Acc:VGNC:91464]	1.89	0.0903
ssc-miR-103	PKIA	cAMP-dependent protein kinase inhibitor alpha [Source:VGNC Symbol;Acc:VGNC:91477]	1.89	0.0903
ssc-miR-103	PLA2G2F	phospholipase A2 group IIF [Source:VGNC Symbol;Acc:VGNC:91493]	1.89	0.0903
ssc-miR-103	PLAG1	PLAG1 zinc finger [Source:VGNC Symbol;Acc:VGNC:91509]	1.89	0.0903
ssc-miR-103	PLCB1	phospholipase C beta 1 [Source:VGNC Symbol;Acc:VGNC:95706]	1.89	0.0903
ssc-miR-103	PLEKHA1	pleckstrin homology domain containing A1 [Source:VGNC Symbol;Acc:VGNC:91533]	1.89	0.0903
ssc-miR-103	PLEKHA7	pleckstrin homology domain containing A7 [Source:VGNC Symbol;Acc:VGNC:91537]	1.89	0.0903
ssc-miR-103	PLEKHF2	pleckstrin homology and FYVE domain containing 2 [Source:VGNC Symbol;Acc:VGNC:91541]	1.89	0.0903
ssc-miR-103	PNISR	PNN interacting serine and arginine rich protein [Source:VGNC Symbol;Acc:VGNC:91594]	1.89	0.0903
ssc-miR-103	PNN	pinin, desmosome associated protein [Source:VGNC Symbol;Acc:VGNC:91603]	1.89	0.0903
ssc-miR-103	POFUT2	hypothetical gene	1.89	0.0903
ssc-miR-103	POGLUT1	protein O-glucosyltransferase 1 [Source:VGNC Symbol;Acc:VGNC:91623]	1.89	0.0903
ssc-miR-103	POLDIP2	DNA polymerase delta interacting protein 2 [Source:VGNC Symbol;Acc:VGNC:91629]	1.89	0.0903
ssc-miR-103	POLR3H	RNA polymerase III subunit H [Source:VGNC Symbol;Acc:VGNC:91658]	1.89	0.0903

ssc-miR-103	POMGNT1	protein O-linked mannose N-acetylglucosaminyltransferase 1 (beta 1,2-) [Source:VGNC Symbol;Acc:VGNC:98536]	1.89	0.0903
ssc-miR-103	POU3F2	POU class 3 homeobox 2 [Source:HGNC Symbol;Acc:HGNC:9215]	1.89	0.0903
ssc-miR-103	POU3F3	POU class 3 homeobox 3 [Source:VGNC Symbol;Acc:VGNC:91676]	1.89	0.0903
ssc-miR-103	PPARG	peroxisome proliferator activated receptor gamma [Source:VGNC Symbol;Acc:VGNC:91684]	1.89	0.0903
ssc-miR-103	PPIG	peptidylprolyl isomerase G [Source:VGNC Symbol;Acc:VGNC:98214]	1.89	0.0903
ssc-miR-103	PPIL4	peptidylprolyl isomerase like 4 [Source:VGNC Symbol;Acc:VGNC:91699]	1.89	0.0903
ssc-miR-103	PPIP5K2	diphosphoinositol pentakisphosphate kinase 2 [Source:VGNC Symbol;Acc:VGNC:91701]	1.89	0.0903
ssc-miR-103	PPM1E	protein phosphatase, Mg2+/Mn2+ dependent 1E [Source:VGNC Symbol;Acc:VGNC:91705]	1.89	0.0903
ssc-miR-103	PPP2R3C	protein phosphatase 2 regulatory subunit B''gamma [Source:VGNC Symbol;Acc:VGNC:91750]	1.89	0.0903
ssc-miR-103	PPP2R5C	protein phosphatase 2 regulatory subunit B'gamma [Source:VGNC Symbol;Acc:VGNC:91753]	1.89	0.0903
ssc-miR-103	PPP3R1	protein phosphatase 3 regulatory subunit B, alpha [Source:VGNC Symbol;Acc:VGNC:106453]	1.89	0.0903
ssc-miR-103	PPP6C	protein phosphatase 6 catalytic subunit [Source:VGNC Symbol;Acc:VGNC:98219]	1.89	0.0903
ssc-miR-103	PPP6R2	protein phosphatase 6 regulatory subunit 2 [Source:VGNC Symbol;Acc:VGNC:91761]	1.89	0.0903
ssc-miR-103	PQLC1	hypothetical gene	1.89	0.0903
ssc-miR-103	PRKAB2	protein kinase AMP-activated non-catalytic subunit beta 2 [Source:VGNC Symbol;Acc:VGNC:91799]	1.89	0.0903
ssc-miR-103	PRKAR1B	protein kinase cAMP-dependent type I regulatory subunit beta [Source:VGNC Symbol;Acc:VGNC:91803]	1.89	0.0903
ssc-miR-103	PRKCE	protein kinase C epsilon [Source:VGNC Symbol;Acc:VGNC:91807]	1.89	0.0903
ssc-miR-103	PRKG1	protein kinase cGMP-dependent 1 [Source:VGNC Symbol;Acc:VGNC:91816]	1.89	0.0903
ssc-miR-103	PRMT8	protein arginine methyltransferase 8 [Source:VGNC Symbol;Acc:VGNC:91825]	1.89	0.0903
ssc-miR-103	PRPF38B	hypothetical gene	1.89	0.0903
ssc-miR-103	PRPF4B	pre-mRNA processing factor 4B [Source:VGNC Symbol;Acc:VGNC:91845]	1.89	0.0903
ssc-miR-103	PRR12	hypothetical gene	1.89	0.0903
ssc-miR-103	PRR14L	proline rich 14 like [Source:VGNC Symbol;Acc:VGNC:91853]	1.89	0.0903
ssc-miR-103	PRR15L	proline rich 15 like [Source:HGNC Symbol;Acc:HGNC:28149]	1.89	0.0903
ssc-miR-103	PRRT2	proline rich transmembrane protein 2 [Source:HGNC Symbol;Acc:HGNC:30500]	1.89	0.0903
ssc-miR-103	PSMB5	hypothetical gene	1.89	0.0903
ssc-miR-103	PSMF1	proteasome inhibitor subunit 1 [Source:VGNC Symbol;Acc:VGNC:96512]	1.89	0.0903
ssc-miR-103	PTEN	hypothetical gene	1.89	0.0903
ssc-miR-103	PTGS2	prostaglandin-endoperoxide synthase 2 [Source:VGNC Symbol;Acc:VGNC:91959]	1.89	0.0903
ssc-miR-103	PTH	parathyroid hormone [Source:VGNC Symbol;Acc:VGNC:91960]	1.89	0.0903
ssc-miR-103	PTPLAD1	hypothetical gene	1.89	0.0903
ssc-miR-103	PTPLB	hypothetical gene	1.89	0.0903
ssc-miR-103	PTPN4	protein tyrosine phosphatase non-receptor type 4 [Source:VGNC Symbol;Acc:VGNC:96521]	1.89	0.0903
ssc-miR-103	PURA	purine rich element binding protein A [Source:HGNC Symbol;Acc:HGNC:9701]	1.89	0.0903
ssc-miR-103	PURB	purine rich element binding protein B [Source:VGNC Symbol;Acc:VGNC:92004]	1.89	0.0903
ssc-miR-103	PVRL1	hypothetical gene	1.89	0.0903
ssc-miR-103	PWWP2B	PWWP domain containing 2B [Source:VGNC Symbol;Acc:VGNC:92010]	1.89	0.0903
ssc-miR-103	RAB10	RAB10, member RAS onco family [Source:VGNC Symbol;Acc:VGNC:98235]	1.89	0.0903
ssc-miR-103	RAB11FIP2	RAB11 family interacting protein 2 [Source:VGNC Symbol;Acc:VGNC:92039]	1.89	0.0903
ssc-miR-103	RAB11FIP4	RAB11 family interacting protein 4 [Source:VGNC Symbol;Acc:VGNC:92041]	1.89	0.0903
ssc-miR-103	RAB15	RAB15, member RAS onco family [Source:VGNC Symbol;Acc:VGNC:98239]	1.89	0.0903
ssc-miR-103	RAB1B	RAB1B, member RAS onco family [Source:NCBI gene (formerly Entrezgene);Acc:100144502]	1.89	0.0903
ssc-miR-103	RAB30	RAB30, member RAS onco family [Source:VGNC Symbol;Acc:VGNC:98254]	1.89	0.0903
ssc-miR-103	RAB40A	hypothetical gene	1.89	0.0903
ssc-miR-103	RAB40AL	hypothetical gene	1.89	0.0903
ssc-miR-103	RAI14	retinoic acid induced 14 [Source:VGNC Symbol;Acc:VGNC:92068]	1.89	0.0903
ssc-miR-103	RAN	hypothetical gene	1.89	0.0903
ssc-miR-103	RAP2B	RAP2B, member of RAS onco family [Source:VGNC Symbol;Acc:VGNC:92089]	1.89	0.0903
ssc-miR-103	RAP2C	RAP2C, member of RAS onco family [Source:VGNC Symbol;Acc:VGNC:92090]	1.89	0.0903

ssc-miR-103	RASAL2	RAS protein activator like 2 [Source:VGNC Symbol;Acc:VGNC:92105]	1.89	0.0903
ssc-miR-103	RASSF4	Ras association domain family member 4 [Source:NCBI gene (formerly Entrezgene);Acc:100152580]	1.89	0.0903
ssc-miR-103	RASSF5	Ras association domain family member 5 [Source:VGNC Symbol;Acc:VGNC:92125]	1.89	0.0903
ssc-miR-103	RASSF8	Ras association domain family member 8 [Source:VGNC Symbol;Acc:VGNC:92128]	1.89	0.0903
ssc-miR-103	RBAK	RB associated KRAB zinc finger [Source:VGNC Symbol;Acc:VGNC:92134]	1.89	0.0903
ssc-miR-103	RBBP6	RB binding protein 6, ubiquitin ligase [Source:VGNC Symbol;Acc:VGNC:92136]	1.89	0.0903
ssc-miR-103	RBM24	hypothetical gene	1.89	0.0903
ssc-miR-103	RBM8A	RNA binding motif protein 8A [Source:VGNC Symbol;Acc:VGNC:92162]	1.89	0.0903
ssc-miR-103	RC3H1	ring finger and CCCH-type domains 1 [Source:VGNC Symbol;Acc:VGNC:92168]	1.89	0.0903
ssc-miR-103	RCAN1	regulator of calcineurin 1 [Source:VGNC Symbol;Acc:VGNC:92170]	1.89	0.0903
ssc-miR-103	RDH10	retinol dehydrogenase 10 [Source:VGNC Symbol;Acc:VGNC:92190]	1.89	0.0903
ssc-miR-103	REST	hypothetical gene	1.89	0.0903
ssc-miR-103	RFWD3	ring finger and WD repeat domain 3 [Source:VGNC Symbol;Acc:VGNC:92242]	1.89	0.0903
ssc-miR-103	RGMA	repulsive guidance molecule BMP co-receptor a [Source:VGNC Symbol;Acc:VGNC:92254]	1.89	0.0903
ssc-miR-103	RGS17	regulator of G protein signaling 17 [Source:VGNC Symbol;Acc:VGNC:92263]	1.89	0.0903
ssc-miR-103	RGS4	regulator of G protein signaling 4 [Source:VGNC Symbol;Acc:VGNC:92266]	1.89	0.0903
ssc-miR-103	RIMS3	regulating synaptic membrane exocytosis 3 [Source:VGNC Symbol;Acc:VGNC:92310]	1.89	0.0903
ssc-miR-103	RNF125	ring finger protein 125 [Source:VGNC Symbol;Acc:VGNC:92352]	1.89	0.0903
ssc-miR-103	RNF152	ring finger protein 152 [Source:VGNC Symbol;Acc:VGNC:92364]	1.89	0.0903
ssc-miR-103	RNF157	ring finger protein 157 [Source:VGNC Symbol;Acc:VGNC:92365]	1.89	0.0903
ssc-miR-103	RNF24	ring finger protein 24 [Source:HGNC Symbol;Acc:HGNC:13779]	1.89	0.0903
ssc-miR-103	RNF38	ring finger protein 38 [Source:VGNC Symbol;Acc:VGNC:92390]	1.89	0.0903
ssc-miR-103	ROR1	receptor tyrosine kinase like orphan receptor 1 [Source:VGNC Symbol;Acc:VGNC:98295]	1.89	0.0903
ssc-miR-103	RORA	RAR related orphan receptor A [Source:VGNC Symbol;Acc:VGNC:92408]	1.89	0.0903
ssc-miR-103	RORB	RAR related orphan receptor B [Source:VGNC Symbol;Acc:VGNC:92409]	1.89	0.0903
ssc-miR-103	RP11-192H23.4	hypothetical gene	1.89	0.0903
ssc-miR-103	RP11-302B13.5	hypothetical gene	1.89	0.0903
ssc-miR-103	RP6-24A23.6	hypothetical gene	1.89	0.0903
ssc-miR-103	RPGRIP1L	RPGRIP1 like [Source:VGNC Symbol;Acc:VGNC:92424]	1.89	0.0903
ssc-miR-103	RPRD2	regulation of nuclear pre-mRNA domain containing 2 [Source:VGNC Symbol;Acc:VGNC:92436]	1.89	0.0903
ssc-miR-103	RPS6KA3	ribosomal protein S6 kinase A3 [Source:VGNC Symbol;Acc:VGNC:92442]	1.89	0.0903
ssc-miR-103	RPS6KA5	ribosomal protein S6 kinase A5 [Source:VGNC Symbol;Acc:VGNC:92444]	1.89	0.0903
ssc-miR-103	RPUSD1	RNA pseudouridine synthase domain containing 1 [Source:VGNC Symbol;Acc:VGNC:92451]	1.89	0.0903
ssc-miR-103	RRAGC	Ras related GTP binding C [Source:HGNC Symbol;Acc:HGNC:19902]	1.89	0.0903
ssc-miR-103	RSPO3	R-spondin 3 [Source:VGNC Symbol;Acc:VGNC:92485]	1.89	0.0903
ssc-miR-103	RTKN2	rhotekin 2 [Source:VGNC Symbol;Acc:VGNC:92495]	1.89	0.0903
ssc-miR-103	RUNX1T1	RUNX1 partner transcriptional co-repressor 1 [Source:VGNC Symbol;Acc:VGNC:96594]	1.89	0.0903
ssc-miR-103	RYBP	RING1 and YY1 binding protein [Source:VGNC Symbol;Acc:VGNC:92532]	1.89	0.0903
ssc-miR-103	SALL1	spalt like transcription factor 1 [Source:VGNC Symbol;Acc:VGNC:92561]	1.89	0.0903
ssc-miR-103	SALL4	spalt like transcription factor 4 [Source:NCBI gene (formerly Entrezgene);Acc:100136902]	1.89	0.0903
ssc-miR-103	SATB2	SATB homeobox 2 [Source:VGNC Symbol;Acc:VGNC:95972]	1.89	0.0903
ssc-miR-103	SAYSD1	SAYSVFN motif domain containing 1 [Source:HGNC Symbol;Acc:HGNC:21025]	1.89	0.0903
ssc-miR-103	SBDS	SBDS ribosome maturation factor [Source:VGNC Symbol;Acc:VGNC:92592]	1.89	0.0903
ssc-miR-103	SBNO1	strawberry notch homolog 1 [Source:VGNC Symbol;Acc:VGNC:92596]	1.89	0.0903
ssc-miR-103	SCAF8	SR-related CTD associated factor 8 [Source:VGNC Symbol;Acc:VGNC:92604]	1.89	0.0903
ssc-miR-103	SCAI	suppressor of cancer cell invasion [Source:VGNC Symbol;Acc:VGNC:103170]	1.89	0.0903
ssc-miR-103	SCAMP5	secretory carrier membrane protein 5 [Source:VGNC Symbol;Acc:VGNC:92609]	1.89	0.0903
ssc-miR-103	SCML4	Scm polycomb group protein like 4 [Source:VGNC Symbol;Acc:VGNC:92629]	1.89	0.0903
ssc-miR-103	SCN1A	sodium voltage-gated channel alpha subunit 1 [Source:VGNC Symbol;Acc:VGNC:95478]	1.89	0.0903

ssc-miR-103	SCN2A	hypothetical gene	1.89	0.0903
ssc-miR-103	SCN8A	sodium voltage-gated channel alpha subunit 8 [Source:VGNC Symbol;Acc:VGNC:92638]	1.89	0.0903
ssc-miR-103	SDC2	syndecan 2 [Source:VGNC Symbol;Acc:VGNC:92655]	1.89	0.0903
ssc-miR-103	SDCBP	syndecan binding protein [Source:VGNC Symbol;Acc:VGNC:92657]	1.89	0.0903
ssc-miR-103	SEC22A	SEC22 homolog A, vesicle trafficking protein [Source:VGNC Symbol;Acc:VGNC:92673]	1.89	0.0903
ssc-miR-103	SEC22C	SEC22 homolog C, vesicle trafficking protein [Source:VGNC Symbol;Acc:VGNC:92674]	1.89	0.0903
ssc-miR-103	SEL1L3	SEL1L family member 3 [Source:VGNC Symbol;Acc:VGNC:92688]	1.89	0.0903
ssc-miR-103	SEMA4F	ssemaphorin 4F [Source:VGNC Symbol;Acc:VGNC:92704]	1.89	0.0903
ssc-miR-103	SEMA6A	semaphorin 6A [Source:VGNC Symbol;Acc:VGNC:92708]	1.89	0.0903
ssc-miR-103	SENP5	SUMO specific peptidase 5 [Source:VGNC Symbol;Acc:VGNC:92714]	1.89	0.0903
ssc-miR-103	SEPT4	hypothetical gene	1.89	0.0903
ssc-miR-103	SEPT5	hypothetical gene	1.89	0.0903
ssc-miR-103	SEPT6	hypothetical gene	1.89	0.0903
ssc-miR-103	SERINC3	serine incorporator 3 [Source:VGNC Symbol;Acc:VGNC:95538]	1.89	0.0903
ssc-miR-103	SERPINB1	serpin family B member 1 [Source:HGNC Symbol;Acc:HGNC:3311]	1.89	0.0903
ssc-miR-103	SERPINH1	serpin family H member 1 [Source:VGNC Symbol;Acc:VGNC:92746]	1.89	0.0903
ssc-miR-103	SETD1B	SET domain containing 1B, histone lysine methyltransferase [Source:HGNC Symbol;Acc:HGNC:29187]	1.89	0.0903
ssc-miR-103	SETD5	SET domain containing 5 [Source:VGNC Symbol;Acc:VGNC:92760]	1.89	0.0903
ssc-miR-103	SF1	splicing factor 1 [Source:HGNC Symbol;Acc:HGNC:12950]	1.89	0.0903
ssc-miR-103	SF3A1	splicing factor 3a subunit 1 [Source:VGNC Symbol;Acc:VGNC:92769]	1.89	0.0903
ssc-miR-103	SGMS2	sphingomyelin synthase 2 [Source:VGNC Symbol;Acc:VGNC:92795]	1.89	0.0903
ssc-miR-103	SGSM2	small G protein signaling modulator 2 [Source:VGNC Symbol;Acc:VGNC:92801]	1.89	0.0903
ssc-miR-103	SGTB	small glutamine rich tetratricopeptide repeat co-chaperone beta [Source:VGNC Symbol;Acc:VGNC:92804]	1.89	0.0903
ssc-miR-103	SH2D2A	SH2 domain containing 2A [Source:VGNC Symbol;Acc:VGNC:92808]	1.89	0.0903
ssc-miR-103	SH3BP4	SH3 domain binding protein 4 [Source:VGNC Symbol;Acc:VGNC:95495]	1.89	0.0903
ssc-miR-103	SH3BP5	SH3 domain binding protein 5 [Source:VGNC Symbol;Acc:VGNC:92819]	1.89	0.0903
ssc-miR-103	SH3BP5L	SH3 binding domain protein 5 like [Source:VGNC Symbol;Acc:VGNC:92820]	1.89	0.0903
ssc-miR-103	SH3GL2	SH3 domain containing GRB2 like 2, endophilin A1 [Source:VGNC Symbol;Acc:VGNC:92824]	1.89	0.0903
ssc-miR-103	SH3PXD2A	SH3 and PX domains 2A [Source:VGNC Symbol;Acc:VGNC:92829]	1.89	0.0903
ssc-miR-103	SHC3	SHC adaptor protein 3 [Source:VGNC Symbol;Acc:VGNC:92838]	1.89	0.0903
ssc-miR-103	SHROOM3	hypothetical gene	1.89	0.0903
ssc-miR-103	SIK2	salt inducible kinase 2 [Source:VGNC Symbol;Acc:VGNC:92872]	1.89	0.0903
ssc-miR-103	SIM1	SIM bHLH transcription factor 1 [Source:VGNC Symbol;Acc:VGNC:92876]	1.89	0.0903
ssc-miR-103	SIPA1L2	signal induced proliferation associated 1 like 2 [Source:VGNC Symbol;Acc:VGNC:92883]	1.89	0.0903
ssc-miR-103	SIX4	SIX homeobox 4 [Source:VGNC Symbol;Acc:VGNC:92895]	1.89	0.0903
ssc-miR-103	SKI	SKI proto-onco [Source:VGNC Symbol;Acc:VGNC:92902]	1.89	0.0903
ssc-miR-103	SKOR1	SKI family transcriptional corepressor 1 [Source:VGNC Symbol;Acc:VGNC:92905]	1.89	0.0903
ssc-miR-103	SLA2	Src like adaptor 2 [Source:HGNC Symbol;Acc:HGNC:17329]	1.89	0.0903
ssc-miR-103	SLAIN2	SLAIN motif family member 2 [Source:VGNC Symbol;Acc:VGNC:92910]	1.89	0.0903
ssc-miR-103	SLC12A6	solute carrier family 12 member 6 [Source:VGNC Symbol;Acc:VGNC:92925]	1.89	0.0903
ssc-miR-103	SLC20A2	solute carrier family 20 member 2 [Source:VGNC Symbol;Acc:VGNC:95498]	1.89	0.0903
ssc-miR-103	SLC25A37	hypothetical gene	1.89	0.0903
ssc-miR-103	SLC26A10	hypothetical gene	1.89	0.0903
ssc-miR-103	SLC2A13	solute carrier family 2 member 13 [Source:VGNC Symbol;Acc:VGNC:93047]	1.89	0.0903
ssc-miR-103	SLC2A14	hypothetical gene	1.89	0.0903
ssc-miR-103	SLC2A3	hypothetical gene	1.89	0.0903
ssc-miR-103	SLC30A10	hypothetical gene	1.89	0.0903
ssc-miR-103	SLC35A3	solute carrier family 35 member A3 [Source:VGNC Symbol;Acc:VGNC:98860]	1.89	0.0903
ssc-miR-103	SLC35G1	solute carrier family 35 member G1 [Source:VGNC Symbol;Acc:VGNC:93086]	1.89	0.0903

ssc-miR-103	SLC44A1	solute carrier family 44 member 1 [Source:VGNC Symbol;Acc:VGNC:93118]	1.89	0.0903
ssc-miR-103	SLC45A4	solute carrier family 45 member 4 [Source:VGNC Symbol;Acc:VGNC:93125]	1.89	0.0903
ssc-miR-103	SLC5A3	solute carrier family 5 member 3 [Source:VGNC Symbol;Acc:VGNC:93144]	1.89	0.0903
ssc-miR-103	SLC8A1	solute carrier family 8 member A1 [Source:VGNC Symbol;Acc:VGNC:93178]	1.89	0.0903
ssc-miR-103	SLC8A2	solute carrier family 8 member A2 [Source:VGNC Symbol;Acc:VGNC:93179]	1.89	0.0903
ssc-miR-103	SLC9A7	solute carrier family 9 member A7 [Source:VGNC Symbol;Acc:VGNC:93189]	1.89	0.0903
ssc-miR-103	SLITRK1	SLIT and NTRK like family member 1 [Source:VGNC Symbol;Acc:VGNC:93206]	1.89	0.0903
ssc-miR-103	SLITRK3	SLIT and NTRK like family member 3 [Source:VGNC Symbol;Acc:VGNC:93208]	1.89	0.0903
ssc-miR-103	SLITRK6	SLIT and NTRK like family member 6 [Source:VGNC Symbol;Acc:VGNC:93211]	1.89	0.0903
ssc-miR-103	SMARCE1	SWI/SNF related, matrix associated, actin dependent regulator of chromatin, subfamily e, member 1 [Source:VGNC Symbol;Acc:VGNC:93235]	1.89	0.0903
ssc-miR-103	SMCHD1	structural maintenance of chromosomes flexible hinge domain containing 1 [Source:VGNC Symbol;Acc:VGNC:93242]	1.89	0.0903
ssc-miR-103	SMEK2	hypothetical gene	1.89	0.0903
ssc-miR-103	SMS	spermine synthase [Source:VGNC Symbol;Acc:VGNC:104059]	1.89	0.0903
ssc-miR-103	SMTNL2	smoothelin like 2 [Source:VGNC Symbol;Acc:VGNC:93268]	1.89	0.0903
ssc-miR-103	SNCG	synuclein gamma [Source:NCBI gene (formerly Entrezgene);Acc:100125343]	1.89	0.0903
ssc-miR-103	SNRK	SNF related kinase [Source:VGNC Symbol;Acc:VGNC:93290]	1.89	0.0903
ssc-miR-103	SNURF	SNRPN upstream open reading frame [Source:HGNC Symbol;Acc:HGNC:11171]	1.89	0.0903
ssc-miR-103	SNX11	sorting nexin 11 [Source:VGNC Symbol;Acc:VGNC:93304]	1.89	0.0903
ssc-miR-103	SNX12	sorting nexin 12 [Source:VGNC Symbol;Acc:VGNC:93305]	1.89	0.0903
ssc-miR-103	SNX18	sorting nexin 18 [Source:VGNC Symbol;Acc:VGNC:93310]	1.89	0.0903
ssc-miR-103	SNX24	sorting nexin 24 [Source:VGNC Symbol;Acc:VGNC:93315]	1.89	0.0903
ssc-miR-103	SNX3	sorting nexin 3 [Source:VGNC Symbol;Acc:VGNC:93317]	1.89	0.0903
ssc-miR-103	SNX30	sorting nexin family member 30 [Source:VGNC Symbol;Acc:VGNC:93318]	1.89	0.0903
ssc-miR-103	SOCS7	suppressor of cytokine signaling 7 [Source:VGNC Symbol;Acc:VGNC:93334]	1.89	0.0903
ssc-miR-103	SOWAHC	hypothetical gene	1.89	0.0903
ssc-miR-103	SOX6	SRY-box transcription factor 6 [Source:VGNC Symbol;Acc:VGNC:93358]	1.89	0.0903
ssc-miR-103	SP3	Sp3 transcription factor [Source:VGNC Symbol;Acc:VGNC:95511]	1.89	0.0903
ssc-miR-103	SPATS2L	spermatosis associated serine rich 2 like [Source:VGNC Symbol;Acc:VGNC:96047]	1.89	0.0903
ssc-miR-103	SPIN1	spindlin 1 [Source:VGNC Symbol;Acc:VGNC:101495]	1.89	0.0903
ssc-miR-103	SPRY1	sprouty RTK signaling antagonist 1 [Source:VGNC Symbol;Acc:VGNC:93424]	1.89	0.0903
ssc-miR-103	SPRY2	sprouty RTK signaling antagonist 2 [Source:VGNC Symbol;Acc:VGNC:93425]	1.89	0.0903
ssc-miR-103	SPRY3	sprouty RTK signaling antagonist 3 [Source:VGNC Symbol;Acc:VGNC:93426]	1.89	0.0903
ssc-miR-103	SPSB4	splA/ryanodine receptor domain and SOCS box containing 4 [Source:VGNC Symbol;Acc:VGNC:93433]	1.89	0.0903
ssc-miR-103	SPTBN4	spectrin beta, non-erythrocytic 4 [Source:VGNC Symbol;Acc:VGNC:93438]	1.89	0.0903
ssc-miR-103	SREK1	splicing regulatory glutamic acid and lysine rich protein 1 [Source:VGNC Symbol;Acc:VGNC:93451]	1.89	0.0903
ssc-miR-103	SRGAP1	SLIT-ROBO Rho GTPase activating protein 1 [Source:VGNC Symbol;Acc:VGNC:93454]	1.89	0.0903
ssc-miR-103	SRGAP2	hypothetical gene	1.89	0.0903
ssc-miR-103	SRM	spermidine synthase [Source:HGNC Symbol;Acc:HGNC:11296]	1.89	0.0903
ssc-miR-103	SRP19	signal recognition particle 19 [Source:VGNC Symbol;Acc:VGNC:98341]	1.89	0.0903
ssc-miR-103	SRSF10	serine and arginine rich splicing factor 10 [Source:VGNC Symbol;Acc:VGNC:93472]	1.89	0.0903
ssc-miR-103	SRSF6	serine and arginine rich splicing factor 6 [Source:NCBI gene (formerly Entrezgene);Acc:768102]	1.89	0.0903
ssc-miR-103	ST13	ST13 Hsp70 interacting protein [Source:HGNC Symbol;Acc:HGNC:11343]	1.89	0.0903
ssc-miR-103	ST3GAL2	ST3 beta-galactoside alpha-2,3-sialyltransferase 2 [Source:HGNC Symbol;Acc:HGNC:10863]	1.89	0.0903
ssc-miR-103	ST6GALNAC5	ST6 N-acetylgalactosaminide alpha-2,6-sialyltransferase 5 [Source:VGNC Symbol;Acc:VGNC:93513]	1.89	0.0903
ssc-miR-103	ST8SIA3	ST8 alpha-N-acetyl-neuraminide alpha-2,8-sialyltransferase 3 [Source:VGNC Symbol;Acc:VGNC:93518]	1.89	0.0903
ssc-miR-103	ST8SIA4	ST8 alpha-N-acetyl-neuraminide alpha-2,8-sialyltransferase 4 [Source:VGNC Symbol;Acc:VGNC:93519]	1.89	0.0903
ssc-miR-103	STAG1	stromal antigen 1 [Source:VGNC Symbol;Acc:VGNC:93524]	1.89	0.0903
ssc-miR-103	STAMBPL1	STAM binding protein like 1 [Source:VGNC Symbol;Acc:VGNC:93527]	1.89	0.0903
ssc-miR-103	STRBP	spermatid perinuclear RNA binding protein [Source:VGNC Symbol;Acc:VGNC:93575]	1.89	0.0903

ssc-miR-103	STRIP1	striatin interacting protein 1 [Source:VGNC Symbol;Acc:VGNC:93576]	1.89	0.0903
ssc-miR-103	STRN	striatin [Source:VGNC Symbol;Acc:VGNC:93578]	1.89	0.0903
ssc-miR-103	STX3	syntaxin 3 [Source:VGNC Symbol;Acc:VGNC:93591]	1.89	0.0903
ssc-miR-103	STXBP6	syntaxin binding protein 6 [Source:NCBI gene (formerly Entrezgene);Acc:100514852]	1.89	0.0903
ssc-miR-103	STYX	serine/threonine/tyrosine interacting protein [Source:VGNC Symbol;Acc:VGNC:93602]	1.89	0.0903
ssc-miR-103	SUFU	SUFU negative regulator of hedgehog signaling [Source:VGNC Symbol;Acc:VGNC:93608]	1.89	0.0903
ssc-miR-103	SUGT1	SGT1 homolog, MIS12 kinetochore complex assembly cochaperone [Source:VGNC Symbol;Acc:VGNC:93611]	1.89	0.0903
ssc-miR-103	SUN2	Sad1 and UNC84 domain containing 2 [Source:VGNC Symbol;Acc:VGNC:93619]	1.89	0.0903
ssc-miR-103	SUZ12	SUZ12 polycomb repressive complex 2 subunit [Source:VGNC Symbol;Acc:VGNC:93638]	1.89	0.0903
ssc-miR-103	SV2C	synaptic vesicle glycoprotein 2C [Source:VGNC Symbol;Acc:VGNC:93641]	1.89	0.0903
ssc-miR-103	SVEP1	sushi, von Willebrand factor type A, EGF and pentraxin domain containing 1 [Source:VGNC Symbol;Acc:VGNC:93642]	1.89	0.0903
ssc-miR-103	SYDE2	synapse defective Rho GTPase homolog 2 [Source:VGNC Symbol;Acc:VGNC:98874]	1.89	0.0903
ssc-miR-103	SYNDIG1	synapse differentiation inducing 1 [Source:VGNC Symbol;Acc:VGNC:95689]	1.89	0.0903
ssc-miR-103	SYNGR1	synaptogyrin 1 [Source:VGNC Symbol;Acc:VGNC:93665]	1.89	0.0903
ssc-miR-103	SYNRG	synergin gamma [Source:VGNC Symbol;Acc:VGNC:93675]	1.89	0.0903
ssc-miR-103	SYT2	synaptotagmin 2 [Source:VGNC Symbol;Acc:VGNC:95530]	1.89	0.0903
ssc-miR-103	SYT6	synaptotagmin 6 [Source:VGNC Symbol;Acc:VGNC:93687]	1.89	0.0903
ssc-miR-103	TACC1	hypothetical gene	1.89	0.0903
ssc-miR-103	TAOK1	TAO kinase 1 [Source:VGNC Symbol;Acc:VGNC:98355]	1.89	0.0903
ssc-miR-103	TAOK3	TAO kinase 3 [Source:VGNC Symbol;Acc:VGNC:98357]	1.89	0.0903
ssc-miR-103	TARDBP	TAR DNA binding protein [Source:VGNC Symbol;Acc:VGNC:98624]	1.89	0.0903
ssc-miR-103	TBC1D19	TBC1 domain family member 19 [Source:VGNC Symbol;Acc:VGNC:93767]	1.89	0.0903
ssc-miR-103	TBC1D4	TBC1 domain family member 4 [Source:HGNC Symbol;Acc:HGNC:19165]	1.89	0.0903
ssc-miR-103	TBKBP1	hypothetical gene	1.89	0.0903
ssc-miR-103	TBL1XR1	TBL1X receptor 1 [Source:VGNC Symbol;Acc:VGNC:96600]	1.89	0.0903
ssc-miR-103	TBPL1	TATA-box binding protein like 1 [Source:VGNC Symbol;Acc:VGNC:93792]	1.89	0.0903
ssc-miR-103	TDG	thymine DNA glycosylase [Source:VGNC Symbol;Acc:VGNC:93842]	1.89	0.0903
ssc-miR-103	TERT	telomerase reverse transcriptase [Source:VGNC Symbol;Acc:VGNC:93883]	1.89	0.0903
ssc-miR-103	TFCP2L1	transcription factor CP2 like 1 [Source:HGNC Symbol;Acc:HGNC:17925]	1.89	0.0903
ssc-miR-103	TFRC	transferrin receptor [Source:VGNC Symbol;Acc:VGNC:93925]	1.89	0.0903
ssc-miR-103	TGFBR2	transforming growth factor beta receptor 2 [Source:VGNC Symbol;Acc:VGNC:93931]	1.89	0.0903
ssc-miR-103	TGFBR3	transforming growth factor beta receptor 3 [Source:VGNC Symbol;Acc:VGNC:93932]	1.89	0.0903
ssc-miR-103	TGIF2	TGFB induced factor homeobox 2 [Source:VGNC Symbol;Acc:VGNC:95656]	1.89	0.0903
ssc-miR-103	TGM3	transglutaminase 3 [Source:HGNC Symbol;Acc:HGNC:11779]	1.89	0.0903
ssc-miR-103	THOC2	THO complex 2 [Source:VGNC Symbol;Acc:VGNC:93957]	1.89	0.0903
ssc-miR-103	THRA	thyroid hormone receptor alpha [Source:VGNC Symbol;Acc:VGNC:93963]	1.89	0.0903
ssc-miR-103	THRB	thyroid hormone receptor beta [Source:VGNC Symbol;Acc:VGNC:93965]	1.89	0.0903
ssc-miR-103	THSD7A	thrombospondin type 1 domain containing 7A [Source:VGNC Symbol;Acc:VGNC:93968]	1.89	0.0903
ssc-miR-103	THY1	hypothetical gene	1.89	0.0903
ssc-miR-103	TIGD5	tigger transposable element derived 5 [Source:VGNC Symbol;Acc:VGNC:98876]	1.89	0.0903
ssc-miR-103	TIMP4	TIMP metallopeptidase inhibitor 4 [Source:VGNC Symbol;Acc:VGNC:93997]	1.89	0.0903
ssc-miR-103	TJP1	tight junction protein 1 [Source:VGNC Symbol;Acc:VGNC:94005]	1.89	0.0903
ssc-miR-103	TLE4	TLE family member 4, transcriptional corepressor [Source:VGNC Symbol;Acc:VGNC:98372]	1.89	0.0903
ssc-miR-103	TLK1	tousled like kinase 1 [Source:VGNC Symbol;Acc:VGNC:95543]	1.89	0.0903
ssc-miR-103	TM7SF3	transmembrane 7 superfamily member 3 [Source:VGNC Symbol;Acc:VGNC:94035]	1.89	0.0903
ssc-miR-103	TMC7	transmembrane channel like 7 [Source:VGNC Symbol;Acc:VGNC:94044]	1.89	0.0903
ssc-miR-103	TMED7-TICAM2	hypothetical gene	1.89	0.0903
ssc-miR-103	TMEM127	transmembrane protein 127 [Source:HGNC Symbol;Acc:HGNC:26038]	1.89	0.0903
ssc-miR-103	TMEM135	transmembrane protein 135 [Source:VGNC Symbol;Acc:VGNC:94085]	1.89	0.0903

ssc-miR-103	TMEM150A	transmembrane protein 150A [Source:VGNC Symbol;Acc:VGNC:94095]	1.89	0.0903
ssc-miR-103	TMEM184B	transmembrane protein 184B [Source:VGNC Symbol;Acc:VGNC:94120]	1.89	0.0903
ssc-miR-103	TMEM184C	transmembrane protein 184C [Source:VGNC Symbol;Acc:VGNC:94121]	1.89	0.0903
ssc-miR-103	TMEM239	transmembrane protein 239 [Source:VGNC Symbol;Acc:VGNC:108739]	1.89	0.0903
ssc-miR-103	TMEM251	transmembrane protein 251 [Source:VGNC Symbol;Acc:VGNC:98378]	1.89	0.0903
ssc-miR-103	TMEM259	transmembrane protein 259 [Source:VGNC Symbol;Acc:VGNC:94162]	1.89	0.0903
ssc-miR-103	TMEM260	transmembrane protein 260 [Source:VGNC Symbol;Acc:VGNC:94164]	1.89	0.0903
ssc-miR-103	TMEM47	transmembrane protein 47 [Source:VGNC Symbol;Acc:VGNC:94184]	1.89	0.0903
ssc-miR-103	TMEM52	transmembrane protein 52 [Source:VGNC Symbol;Acc:VGNC:94188]	1.89	0.0903
ssc-miR-103	TMUB2	transmembrane and ubiquitin like domain containing 2 [Source:VGNC Symbol;Acc:VGNC:94246]	1.89	0.0903
ssc-miR-103	TNFAIP3	TNF alpha induced protein 3 [Source:VGNC Symbol;Acc:VGNC:94252]	1.89	0.0903
ssc-miR-103	TNIK	TRAF2 and NCK interacting kinase [Source:VGNC Symbol;Acc:VGNC:98381]	1.89	0.0903
ssc-miR-103	TNPO1	transportin 1 [Source:VGNC Symbol;Acc:VGNC:94287]	1.89	0.0903
ssc-miR-103	TNRC6B	trinucleotide repeat containing adaptor 6B [Source:VGNC Symbol;Acc:VGNC:94293]	1.89	0.0903
ssc-miR-103	TOB2	transducer of ERBB2, 2 [Source:VGNC Symbol;Acc:VGNC:94298]	1.89	0.0903
ssc-miR-103	TPD52	tumor protein D52 [Source:VGNC Symbol;Acc:VGNC:98383]	1.89	0.0903
ssc-miR-103	TRABD2B	TraB domain containing 2B [Source:VGNC Symbol;Acc:VGNC:94356]	1.89	0.0903
ssc-miR-103	TRAF3	TNF receptor associated factor 3 [Source:VGNC Symbol;Acc:VGNC:94360]	1.89	0.0903
ssc-miR-103	TRIAP1	hypothetical gene	1.89	0.0903
ssc-miR-103	TRIM26	tripartite motif containing 26 [Source:NCBI gene (formerly Entrezgene);Acc:100144460]	1.89	0.0903
ssc-miR-103	TRIM71	tripartite motif containing 71 [Source:VGNC Symbol;Acc:VGNC:94431]	1.89	0.0903
ssc-miR-103	TRIM9	tripartite motif containing 9 [Source:VGNC Symbol;Acc:VGNC:94434]	1.89	0.0903
ssc-miR-103	TRMT112	tRNA methyltransferase activator subunit 11-2 [Source:VGNC Symbol;Acc:VGNC:94445]	1.89	0.0903
ssc-miR-103	TRPV3	transient receptor potential cation channel subfamily V member 3 [Source:VGNC Symbol;Acc:VGNC:94475]	1.89	0.0903
ssc-miR-103	TSHZ1	teashirt zinc finger homeobox 1 [Source:VGNC Symbol;Acc:VGNC:94492]	1.89	0.0903
ssc-miR-103	TSHZ2	teashirt zinc finger homeobox 2 [Source:NCBI gene (formerly Entrezgene);Acc:100136903]	1.89	0.0903
ssc-miR-103	TSPAN31	tetraspanin 31 [Source:VGNC Symbol;Acc:VGNC:94511]	1.89	0.0903
ssc-miR-103	TSPAN5	tetraspanin 5 [Source:VGNC Symbol;Acc:VGNC:94514]	1.89	0.0903
ssc-miR-103	TSPAN9	tetraspanin 9 [Source:VGNC Symbol;Acc:VGNC:94516]	1.89	0.0903
ssc-miR-103	TSPYL1	TSPY like 1 [Source:VGNC Symbol;Acc:VGNC:103197]	1.89	0.0903
ssc-miR-103	TTBK2	tau tubulin kinase 2 [Source:VGNC Symbol;Acc:VGNC:98387]	1.89	0.0903
ssc-miR-103	TTC9	tetratricopeptide repeat domain 9 [Source:VGNC Symbol;Acc:VGNC:94560]	1.89	0.0903
ssc-miR-103	TTL	hypothetical gene	1.89	0.0903
ssc-miR-103	TTLL7	tubulin tyrosine ligase like 7 [Source:VGNC Symbol;Acc:VGNC:94571]	1.89	0.0903
ssc-miR-103	TULP4	hypothetical gene	1.89	0.0903
ssc-miR-103	TWF1	twinfilin actin binding protein 1 [Source:VGNC Symbol;Acc:VGNC:94595]	1.89	0.0903
ssc-miR-103	UBE2E3	ubiquitin conjugating enzyme E2 E3 [Source:VGNC Symbol;Acc:VGNC:106461]	1.89	0.0903
ssc-miR-103	UBE2J1	ubiquitin conjugating enzyme E2 J1 [Source:VGNC Symbol;Acc:VGNC:94644]	1.89	0.0903
ssc-miR-103	UBE2L3	hypothetical gene	1.89	0.0903
ssc-miR-103	UBE2N	hypothetical gene	1.89	0.0903
ssc-miR-103	UBE2Q1	ubiquitin conjugating enzyme E2 Q1 [Source:VGNC Symbol;Acc:VGNC:98888]	1.89	0.0903
ssc-miR-103	UBE2R2	ubiquitin conjugating enzyme E2 R2 [Source:VGNC Symbol;Acc:VGNC:95924]	1.89	0.0903
ssc-miR-103	UBE2W	ubiquitin conjugating enzyme E2 W [Source:VGNC Symbol;Acc:VGNC:98890]	1.89	0.0903
ssc-miR-103	UBE4A	ubiquitination factor E4A [Source:VGNC Symbol;Acc:VGNC:94656]	1.89	0.0903
ssc-miR-103	UBL3	hypothetical gene	1.89	0.0903
ssc-miR-103	UBR3	ubiquitin protein ligase E3 component n-recognin 3 [Source:VGNC Symbol;Acc:VGNC:96771]	1.89	0.0903
ssc-miR-103	UBR4	ubiquitin protein ligase E3 component n-recognin 4 [Source:VGNC Symbol;Acc:VGNC:94670]	1.89	0.0903
ssc-miR-103	UBXN2B	UBX domain protein 2B [Source:VGNC Symbol;Acc:VGNC:94678]	1.89	0.0903
ssc-miR-103	UBXN7	hypothetical gene	1.89	0.0903

ssc-miR-103	UGGT1	UDP-glucose glycoprotein glucosyltransferase 1 [Source:VGNC Symbol;Acc:VGNC:95646]	1.89	0.0903
ssc-miR-103	UHMK1	U2AF homology motif kinase 1 [Source:VGNC Symbol;Acc:VGNC:94688]	1.89	0.0903
ssc-miR-103	UMOD	uromodulin [Source:VGNC Symbol;Acc:VGNC:94698]	1.89	0.0903
ssc-miR-103	UNC80	unc-80 homolog, NALCN channel complex subunit [Source:VGNC Symbol;Acc:VGNC:96134]	1.89	0.0903
ssc-miR-103	UPF2	UPF2 regulator of nonsense mediated mRNA decay [Source:VGNC Symbol;Acc:VGNC:98402]	1.89	0.0903
ssc-miR-103	USP14	ubiquitin specific peptidase 14 [Source:VGNC Symbol;Acc:VGNC:94747]	1.89	0.0903
ssc-miR-103	USP24	ubiquitin specific peptidase 24 [Source:VGNC Symbol;Acc:VGNC:94754]	1.89	0.0903
ssc-miR-103	USP42	ubiquitin specific peptidase 42 [Source:HGNC Symbol;Acc:HGNC:20068]	1.89	0.0903
ssc-miR-103	USP45	ubiquitin specific peptidase 45 [Source:VGNC Symbol;Acc:VGNC:94767]	1.89	0.0903
ssc-miR-103	USP47	ubiquitin specific peptidase 47 [Source:VGNC Symbol;Acc:VGNC:94769]	1.89	0.0903
ssc-miR-103	USP6NL	USP6 N-terminal like [Source:VGNC Symbol;Acc:VGNC:95820]	1.89	0.0903
ssc-miR-103	VAMP1	hypothetical gene	1.89	0.0903
ssc-miR-103	VAMP4	vesicle associated membrane protein 4 [Source:VGNC Symbol;Acc:VGNC:94794]	1.89	0.0903
ssc-miR-103	VAMP8	vesicle associated membrane protein 8 [Source:HGNC Symbol;Acc:HGNC:12647]	1.89	0.0903
ssc-miR-103	VAV3	hypothetical gene	1.89	0.0903
ssc-miR-103	VCAN	versican [Source:VGNC Symbol;Acc:VGNC:108163]	1.89	0.0903
ssc-miR-103	VCL	vinculin [Source:VGNC Symbol;Acc:VGNC:94812]	1.89	0.0903
ssc-miR-103	VCP	valosin containing protein [Source:NCBI gene (formerly Entrezgene);Acc:397524]	1.89	0.0903
ssc-miR-103	VOPP1	VOPP1 WW domain binding protein [Source:VGNC Symbol;Acc:VGNC:94835]	1.89	0.0903
ssc-miR-103	VPS4A	vacuolar protein sorting 4 homolog A [Source:NCBI gene (formerly Entrezgene);Acc:100627841]	1.89	0.0903
ssc-miR-103	VSNL1	visinin like 1 [Source:VGNC Symbol;Acc:VGNC:94871]	1.89	0.0903
ssc-miR-103	VWC2L	von Willebrand factor C domain containing 2 like [Source:VGNC Symbol;Acc:VGNC:96301]	1.89	0.0903
ssc-miR-103	WASF3	WASP family member 3 [Source:VGNC Symbol;Acc:VGNC:94894]	1.89	0.0903
ssc-miR-103	WDFY2	WD repeat and FYVE domain containing 2 [Source:VGNC Symbol;Acc:VGNC:94902]	1.89	0.0903
ssc-miR-103	WDFY4	WDFY family member 4 [Source:HGNC Symbol;Acc:HGNC:29323]	1.89	0.0903
ssc-miR-103	WDR20	WD repeat domain 20 [Source:VGNC Symbol;Acc:VGNC:94910]	1.89	0.0903
ssc-miR-103	WIPI2	WD repeat domain, phosphoinositide interacting 2 [Source:VGNC Symbol;Acc:VGNC:94960]	1.89	0.0903
ssc-miR-103	WNK1	WNK lysine deficient protein kinase 1 [Source:VGNC Symbol;Acc:VGNC:94962]	1.89	0.0903
ssc-miR-103	WNT16	Wnt family member 16 [Source:VGNC Symbol;Acc:VGNC:94967]	1.89	0.0903
ssc-miR-103	WNT3A	Wnt family member 3A [Source:VGNC Symbol;Acc:VGNC:94971]	1.89	0.0903
ssc-miR-103	WNT4	hypothetical gene	1.89	0.0903
ssc-miR-103	WWP1	WW domain containing E3 ubiquitin protein ligase 1 [Source:VGNC Symbol;Acc:VGNC:94989]	1.89	0.0903
ssc-miR-103	WWP2	WW domain containing E3 ubiquitin protein ligase 2 [Source:VGNC Symbol;Acc:VGNC:94990]	1.89	0.0903
ssc-miR-103	XPO4	exportin 4 [Source:VGNC Symbol;Acc:VGNC:95004]	1.89	0.0903
ssc-miR-103	XPO7	exportin 7 [Source:VGNC Symbol;Acc:VGNC:95007]	1.89	0.0903
ssc-miR-103	YAF2	YY1 associated factor 2 [Source:VGNC Symbol;Acc:VGNC:95018]	1.89	0.0903
ssc-miR-103	YTHDC1	YTH domain containing 1 [Source:VGNC Symbol;Acc:VGNC:95041]	1.89	0.0903
ssc-miR-103	YWHAH	tyrosine 3-monooxygenase/tryptophan 5-monooxygenase activation protein eta [Source:VGNC Symbol;Acc:VGNC:95045]	1.89	0.0903
ssc-miR-103	ZBTB10	zinc finger and BTB domain containing 10 [Source:VGNC Symbol;Acc:VGNC:95058]	1.89	0.0903
ssc-miR-103	ZBTB16	zinc finger and BTB domain containing 16 [Source:VGNC Symbol;Acc:VGNC:108626]	1.89	0.0903
ssc-miR-103	ZBTB20	zinc finger and BTB domain containing 20 [Source:VGNC Symbol;Acc:VGNC:95063]	1.89	0.0903
ssc-miR-103	ZBTB34	zinc finger and BTB domain containing 34 [Source:VGNC Symbol;Acc:VGNC:95070]	1.89	0.0903
ssc-miR-103	ZBTB37	zinc finger and BTB domain containing 37 [Source:VGNC Symbol;Acc:VGNC:95071]	1.89	0.0903
ssc-miR-103	ZBTB39	zinc finger and BTB domain containing 39 [Source:VGNC Symbol;Acc:VGNC:95073]	1.89	0.0903
ssc-miR-103	ZBTB8A	zinc finger and BTB domain containing 8A [Source:VGNC Symbol;Acc:VGNC:95084]	1.89	0.0903
ssc-miR-103	ZC3H12B	zinc finger CCCH-type containing 12B [Source:VGNC Symbol;Acc:VGNC:95090]	1.89	0.0903
ssc-miR-103	ZC3H12C	zinc finger CCCH-type containing 12C [Source:VGNC Symbol;Acc:VGNC:95091]	1.89	0.0903
ssc-miR-103	ZC3H7B	zinc finger CCCH-type containing 7B [Source:VGNC Symbol;Acc:VGNC:95100]	1.89	0.0903
ssc-miR-103	ZCCHC2	zinc finger CCHC-type containing 2 [Source:VGNC Symbol;Acc:VGNC:95109]	1.89	0.0903

ssc-miR-103	ZDHHC3	zinc finger DHHC-type palmitoyltransferase 3 [Source:VGNC Symbol;Acc:VGNC:95126]	1.89	0.0903
ssc-miR-103	ZFPM2	zinc finger protein, FOG family member 2 [Source:VGNC Symbol;Acc:VGNC:95153]	1.89	0.0903
ssc-miR-103	ZHX1	zinc fingers and homeoboxes 1 [Source:VGNC Symbol;Acc:VGNC:95164]	1.89	0.0903
ssc-miR-103	ZHX3	zinc fingers and homeoboxes 3 [Source:VGNC Symbol;Acc:VGNC:95717]	1.89	0.0903
ssc-miR-103	ZKSCAN1	zinc finger with KRAB and SCAN domains 1 [Source:VGNC Symbol;Acc:VGNC:95171]	1.89	0.0903
ssc-miR-103	ZMYM2	zinc finger MYM-type containing 2 [Source:VGNC Symbol;Acc:VGNC:95182]	1.89	0.0903
ssc-miR-103	ZNF449	zinc finger protein 449 [Source:VGNC Symbol;Acc:VGNC:95242]	1.89	0.0903
ssc-miR-103	ZNF652	zinc finger protein 652 [Source:VGNC Symbol;Acc:VGNC:99108]	1.89	0.0903
ssc-miR-103	ZNF654	zinc finger protein 654 [Source:VGNC Symbol;Acc:VGNC:95285]	1.89	0.0903
ssc-miR-103	ZNF711	zinc finger protein 711 [Source:HGNC Symbol;Acc:HGNC:13128]	1.89	0.0903
ssc-miR-103	ZNF770	zinc finger protein 770 [Source:VGNC Symbol;Acc:VGNC:95300]	1.89	0.0903
ssc-miR-103	ZNF827	zinc finger protein 827 [Source:VGNC Symbol;Acc:VGNC:98970]	1.89	0.0903
ssc-miR-103	ZNRF2	zinc and ring finger 2 [Source:VGNC Symbol;Acc:VGNC:95317]	1.89	0.0903
ssc-miR-103	ZNRF3	zinc and ring finger 3 [Source:VGNC Symbol;Acc:VGNC:95318]	1.89	0.0903
ssc-miR-323	AAK1	AP2 associated kinase 1 [Source:VGNC Symbol;Acc:VGNC:100379]	1.78	0.0994
ssc-miR-323	ABHD13	abhydrolase domain containing 13 [Source:VGNC Symbol;Acc:VGNC:84972]	1.78	0.0994
ssc-miR-323	ABI2	abl interactor 2 [Source:VGNC Symbol;Acc:VGNC:96027]	1.78	0.0994
ssc-miR-323	AC007405.2	hypothetical gene	1.78	0.0994
ssc-miR-323	AC026703.1	hypothetical gene	1.78	0.0994
ssc-miR-323	ACVR2B	activin A receptor type 2B [Source:VGNC Symbol;Acc:VGNC:108629]	1.78	0.0994
ssc-miR-323	ADAM10	ADAM metallopeptidase domain 10 [Source:VGNC Symbol;Acc:VGNC:85061]	1.78	0.0994
ssc-miR-323	ADCY6	adenylate cyclase 6 [Source:VGNC Symbol;Acc:VGNC:85110]	1.78	0.0994
ssc-miR-323	ADIPOR1	adiponectin receptor 1 [Source:NCBI gene (formerly Entrezgene);Acc:404695]	1.78	0.0994
ssc-miR-323	ADIPOR2	adiponectin receptor 2 [Source:VGNC Symbol;Acc:VGNC:85141]	1.78	0.0994
ssc-miR-323	ADNP	activity dependent neuroprotector homeobox [Source:VGNC Symbol;Acc:VGNC:95657]	1.78	0.0994
ssc-miR-323	AFTPH	aftiphilin [Source:VGNC Symbol;Acc:VGNC:85173]	1.78	0.0994
ssc-miR-323	AGO1	hypothetical gene	1.78	0.0994
ssc-miR-323	AGO2	argonaute RISC catalytic component 2 [Source:VGNC Symbol;Acc:VGNC:97871]	1.78	0.0994
ssc-miR-323	AKAP11	A-kinase anchoring protein 11 [Source:HGNC Symbol;Acc:HGNC:369]	1.78	0.0994
ssc-miR-323	ALS2	alsin Rho guanine nucleotide exchange factor ALS2 [Source:VGNC Symbol;Acc:VGNC:96327]	1.78	0.0994
ssc-miR-323	ANKRD28	ankyrin repeat domain 28 [Source:VGNC Symbol;Acc:VGNC:85331]	1.78	0.0994
ssc-miR-323	ANKRD34B	ankyrin repeat domain 34B [Source:VGNC Symbol;Acc:VGNC:85336]	1.78	0.0994
ssc-miR-323	APP	amyloid beta protein [Source:VGNC Symbol;Acc:VGNC:103902]	1.78	0.0994
ssc-miR-323	APPL1	adaptor protein, phosphotyrosine interacting with PH domain and leucine zipper 1 [Source:VGNC Symbol;Acc:VGNC:85426]	1.78	0.0994
ssc-miR-323	ARF6	ADP ribosylation factor 6 [Source:VGNC Symbol;Acc:VGNC:85448]	1.78	0.0994
ssc-miR-323	ARHGAP44	Rho GTPase activating protein 44 [Source:VGNC Symbol;Acc:VGNC:85478]	1.78	0.0994
ssc-miR-323	ARHGEF11	Rho guanine nucleotide exchange factor 11 [Source:VGNC Symbol;Acc:VGNC:85487]	1.78	0.0994
ssc-miR-323	ARHGEF37	Rho guanine nucleotide exchange factor 37 [Source:VGNC Symbol;Acc:VGNC:85498]	1.78	0.0994
ssc-miR-323	ARID1B	AT-rich interaction domain 1B [Source:VGNC Symbol;Acc:VGNC:85506]	1.78	0.0994
ssc-miR-323	ARL1	ADP ribosylation factor like GTPase 1 [Source:VGNC Symbol;Acc:VGNC:85513]	1.78	0.0994
ssc-miR-323	ARL5A	ADP ribosylation factor like GTPase 5A [Source:VGNC Symbol;Acc:VGNC:96418]	1.78	0.0994
ssc-miR-323	ARNT2	aryl hydrocarbon receptor nuclear translocator 2 [Source:HGNC Symbol;Acc:HGNC:16876]	1.78	0.0994
ssc-miR-323	ARX	aristaless related homeobox [Source:VGNC Symbol;Acc:VGNC:85556]	1.78	0.0994
ssc-miR-323	ASB7	ankyrin repeat and SOCS box containing 7 [Source:VGNC Symbol;Acc:VGNC:85566]	1.78	0.0994
ssc-miR-323	ATAD5	ATPase family AAA domain containing 5 [Source:VGNC Symbol;Acc:VGNC:85601]	1.78	0.0994
ssc-miR-323	ATP11A	ATPase phospholipid transporting 11A [Source:VGNC Symbol;Acc:VGNC:85634]	1.78	0.0994
ssc-miR-323	ATP13A3	ATPase 13A3 [Source:VGNC Symbol;Acc:VGNC:85639]	1.78	0.0994
ssc-miR-323	ATP1B1	ATPase Na+/K+ transporting subunit beta 1 [Source:VGNC Symbol;Acc:VGNC:85642]	1.78	0.0994
ssc-miR-323	ATXN3	hypothetical gene	1.78	0.0994

ssc-miR-323	AUTS2	activator of transcription and developmental regulator AUTS2 [Source:VGNC Symbol;Acc:VGNC:97897]	1.78	0.0994
ssc-miR-323	AZI2	5-azacytidine induced 2 [Source:VGNC Symbol;Acc:VGNC:85711]	1.78	0.0994
ssc-miR-323	AZIN1	antizyme inhibitor 1 [Source:VGNC Symbol;Acc:VGNC:85712]	1.78	0.0994
ssc-miR-323	BAZ2A	bromodomain adjacent to zinc finger domain 2A [Source:VGNC Symbol;Acc:VGNC:85763]	1.78	0.0994
ssc-miR-323	BCL2	BCL2 apoptosis regulator [Source:HGNC Symbol;Acc:HGNC:990]	1.78	0.0994
ssc-miR-323	BCL2L11	BCL2 like 11 [Source:NCBI gene (formerly Entrezgene);Acc:396632]	1.78	0.0994
ssc-miR-323	BEND4	BEN domain containing 4 [Source:VGNC Symbol;Acc:VGNC:85801]	1.78	0.0994
ssc-miR-323	BMP3	bone morphotic protein 3 [Source:VGNC Symbol;Acc:VGNC:85842]	1.78	0.0994
ssc-miR-323	BNC2	basonuclin 2 [Source:VGNC Symbol;Acc:VGNC:85852]	1.78	0.0994
ssc-miR-323	BPTF	bromodomain PHD finger transcription factor [Source:VGNC Symbol;Acc:VGNC:97904]	1.78	0.0994
ssc-miR-323	BRI3	brain protein I3 [Source:VGNC Symbol;Acc:VGNC:103906]	1.78	0.0994
ssc-miR-323	BRINP1	BMP/retinoic acid inducible neural specific 1 [Source:VGNC Symbol;Acc:VGNC:85881]	1.78	0.0994
ssc-miR-323	BTBD3	BTB domain containing 3 [Source:VGNC Symbol;Acc:VGNC:95673]	1.78	0.0994
ssc-miR-323	BZW1	basic leucine zipper and W2 domains 1 [Source:VGNC Symbol;Acc:VGNC:95885]	1.78	0.0994
ssc-miR-323	C11orf30	hypothetical gene	1.78	0.0994
ssc-miR-323	C16orf87	chromosome 6 C16orf87 homolog [Source:VGNC Symbol;Acc:VGNC:96927]	1.78	0.0994
ssc-miR-323	C17orf75	chromosome 12 C17orf75 homolog [Source:VGNC Symbol;Acc:VGNC:85925]	1.78	0.0994
ssc-miR-323	C1orf52	chromosome 4 C1orf52 homolog [Source:VGNC Symbol;Acc:VGNC:98742]	1.78	0.0994
ssc-miR-323	C20orf194	hypothetical gene	1.78	0.0994
ssc-miR-323	C2orf49	chromosome 3 C2orf49 homolog [Source:VGNC Symbol;Acc:VGNC:86022]	1.78	0.0994
ssc-miR-323	C6orf89	chromosome 7 C6orf89 homolog [Source:VGNC Symbol;Acc:VGNC:86075]	1.78	0.0994
ssc-miR-323	C8orf44-SGK3	hypothetical gene	1.78	0.0994
ssc-miR-323	CABP7	calcium binding protein 7 [Source:VGNC Symbol;Acc:VGNC:107372]	1.78	0.0994
ssc-miR-323	CACNA1C	calcium voltage-gated channel subunit alpha1 C [Source:HGNC Symbol;Acc:HGNC:1390]	1.78	0.0994
ssc-miR-323	CACNA1G	calcium voltage-gated channel subunit alpha1 G [Source:VGNC Symbol;Acc:VGNC:86119]	1.78	0.0994
ssc-miR-323	CALB1	calbindin 1 [Source:VGNC Symbol;Acc:VGNC:86138]	1.78	0.0994
ssc-miR-323	CAND1	cullin associated and neddylation dissociated 1 [Source:VGNC Symbol;Acc:VGNC:97912]	1.78	0.0994
ssc-miR-323	CAPS	calcyphosine [Source:VGNC Symbol;Acc:VGNC:86180]	1.78	0.0994
ssc-miR-323	CASQ1	calsequestrin 1 [Source:VGNC Symbol;Acc:VGNC:86205]	1.78	0.0994
ssc-miR-323	CBFA2T2	CBFA2/RUNX1 partner transcriptional co-repressor 2 [Source:VGNC Symbol;Acc:VGNC:96573]	1.78	0.0994
ssc-miR-323	CBX5	chromobox 5 [Source:VGNC Symbol;Acc:VGNC:86232]	1.78	0.0994
ssc-miR-323	CCDC68	coiled-coil domain containing 68 [Source:VGNC Symbol;Acc:VGNC:86308]	1.78	0.0994
ssc-miR-323	CCND2	cyclin D2 [Source:VGNC Symbol;Acc:VGNC:103222]	1.78	0.0994
ssc-miR-323	CCNK	cyclin K [Source:VGNC Symbol;Acc:VGNC:86362]	1.78	0.0994
ssc-miR-323	CCNYL1	hypothetical gene	1.78	0.0994
ssc-miR-323	CDC27	cell division cycle 27 [Source:VGNC Symbol;Acc:VGNC:86448]	1.78	0.0994
ssc-miR-323	CDC42BPB	CDC42 binding protein kinase beta [Source:VGNC Symbol;Acc:VGNC:86453]	1.78	0.0994
ssc-miR-323	CDC73	cell division cycle 73 [Source:VGNC Symbol;Acc:VGNC:95817]	1.78	0.0994
ssc-miR-323	CDCA2	cell division cycle associated 2 [Source:VGNC Symbol;Acc:VGNC:86465]	1.78	0.0994
ssc-miR-323	CDH11	cadherin 11 [Source:VGNC Symbol;Acc:VGNC:86475]	1.78	0.0994
ssc-miR-323	CDH6	cadherin 6 [Source:HGNC Symbol;Acc:HGNC:1765]	1.78	0.0994
ssc-miR-323	CDK14	cyclin dependent kinase 14 [Source:VGNC Symbol;Acc:VGNC:86498]	1.78	0.0994
ssc-miR-323	CDKN1B	cyclin dependent kinase inhibitor 1B [Source:VGNC Symbol;Acc:VGNC:86515]	1.78	0.0994
ssc-miR-323	CERS5	ceramide synthase 5 [Source:VGNC Symbol;Acc:VGNC:97926]	1.78	0.0994
ssc-miR-323	CGGBP1	CGG triplet repeat binding protein 1 [Source:VGNC Symbol;Acc:VGNC:86615]	1.78	0.0994
ssc-miR-323	CHCHD4	coiled-coil-helix-coiled-coil-helix domain containing 4 [Source:NCBI gene (formerly Entrezgene);Acc:100154663]	1.78	0.0994
ssc-miR-323	CHMP5	charged multivesicular body protein 5 [Source:VGNC Symbol;Acc:VGNC:103920]	1.78	0.0994
ssc-miR-323	CHODL	chondrolectin [Source:VGNC Symbol;Acc:VGNC:86655]	1.78	0.0994
ssc-miR-323	CHRM2	cholinergic receptor muscarinic 2 [Source:VGNC Symbol;Acc:VGNC:103922]	1.78	0.0994

ssc-miR-323	CHUK	component of inhibitor of nuclear factor kappa B kinase complex [Source:VGNC Symbol;Acc:VGNC:86688]	1.78	0.0994
ssc-miR-323	CLLU1	hypothetical gene	1.78	0.0994
ssc-miR-323	CLOCK	clock circadian regulator [Source:VGNC Symbol;Acc:VGNC:86774]	1.78	0.0994
ssc-miR-323	CLPTM1L	CLPTM1 like [Source:VGNC Symbol;Acc:VGNC:86779]	1.78	0.0994
ssc-miR-323	CLSTN3	calsyntenin 3 [Source:VGNC Symbol;Acc:VGNC:86786]	1.78	0.0994
ssc-miR-323	CLVS2	clavesin 2 [Source:VGNC Symbol;Acc:VGNC:86795]	1.78	0.0994
ssc-miR-323	CLYBL	citramalyl-CoA lyase [Source:VGNC Symbol;Acc:VGNC:86796]	1.78	0.0994
ssc-miR-323	CMTM8	CKLF like MARVEL transmembrane domain containing 8 [Source:VGNC Symbol;Acc:VGNC:86807]	1.78	0.0994
ssc-miR-323	CNBP	hypothetical gene	1.78	0.0994
ssc-miR-323	CNTNAP5	contactin associated protein family member 5 [Source:VGNC Symbol;Acc:VGNC:95459]	1.78	0.0994
ssc-miR-323	COL4A3	collagen type IV alpha 3 chain [Source:HGNC Symbol;Acc:HGNC:2204]	1.78	0.0994
ssc-miR-323	COMMD8	COMM domain containing 8 [Source:VGNC Symbol;Acc:VGNC:86890]	1.78	0.0994
ssc-miR-323	CPE	carboxypeptidase E [Source:VGNC Symbol;Acc:VGNC:86936]	1.78	0.0994
ssc-miR-323	CPEB3	cytoplasmic polyadenylation element binding protein 3 [Source:VGNC Symbol;Acc:VGNC:86938]	1.78	0.0994
ssc-miR-323	CPEB4	cytoplasmic polyadenylation element binding protein 4 [Source:VGNC Symbol;Acc:VGNC:86939]	1.78	0.0994
ssc-miR-323	CREBL2	cAMP responsive element binding protein like 2 [Source:VGNC Symbol;Acc:VGNC:86986]	1.78	0.0994
ssc-miR-323	CRLS1	cardiolipin synthase 1 [Source:VGNC Symbol;Acc:VGNC:95756]	1.78	0.0994
ssc-miR-323	CSMD1	CUB and Sushi multiple domains 1 [Source:VGNC Symbol;Acc:VGNC:95600]	1.78	0.0994
ssc-miR-323	CSRNP3	cysteine and serine rich nuclear protein 3 [Source:VGNC Symbol;Acc:VGNC:96249]	1.78	0.0994
ssc-miR-323	CTDSPL2	CTD small phosphatase like 2 [Source:VGNC Symbol;Acc:VGNC:87060]	1.78	0.0994
ssc-miR-323	CTTNBP2NL	CTTNBP2 N-terminal like [Source:VGNC Symbol;Acc:VGNC:87082]	1.78	0.0994
ssc-miR-323	CUL2	cullin 2 [Source:VGNC Symbol;Acc:VGNC:96090]	1.78	0.0994
ssc-miR-323	CUL4A	cullin 4A [Source:VGNC Symbol;Acc:VGNC:87088]	1.78	0.0994
ssc-miR-323	CYTH3	cytohesin 3 [Source:VGNC Symbol;Acc:VGNC:87136]	1.78	0.0994
ssc-miR-323	DAB2IP	DAB2 interacting protein [Source:VGNC Symbol;Acc:VGNC:87145]	1.78	0.0994
ssc-miR-323	DAGLA	diacylglycerol lipase alpha [Source:VGNC Symbol;Acc:VGNC:87152]	1.78	0.0994
ssc-miR-323	DCTN4	dynactin subunit 4 [Source:VGNC Symbol;Acc:VGNC:99635]	1.78	0.0994
ssc-miR-323	DCUN1D3	defective in cullin neddylation 1 domain containing 3 [Source:VGNC Symbol;Acc:VGNC:87196]	1.78	0.0994
ssc-miR-323	DCX	doublecortin [Source:HGNC Symbol;Acc:HGNC:2714]	1.78	0.0994
ssc-miR-323	DDI2	hypothetical gene	1.78	0.0994
ssc-miR-323	DDN	dendrin [Source:VGNC Symbol;Acc:VGNC:87210]	1.78	0.0994
ssc-miR-323	DFNB59	hypothetical gene	1.78	0.0994
ssc-miR-323	DGKH	diacylglycerol kinase eta [Source:VGNC Symbol;Acc:VGNC:87273]	1.78	0.0994
ssc-miR-323	DIP2C	disco interacting C [Source:VGNC Symbol;Acc:VGNC:96201]	1.78	0.0994
ssc-miR-323	DKK2	dickkopf WNT signaling pathway inhibitor 2 [Source:VGNC Symbol;Acc:VGNC:87322]	1.78	0.0994
ssc-miR-323	DNAJC6	DnaJ heat shock protein family (Hsp40) member C6 [Source:VGNC Symbol;Acc:VGNC:96625]	1.78	0.0994
ssc-miR-323	DNMT3A	DNA methyltransferase 3 alpha [Source:VGNC Symbol;Acc:VGNC:87384]	1.78	0.0994
ssc-miR-323	DOCK4	dedicator of cytokinesis 4 [Source:VGNC Symbol;Acc:VGNC:87394]	1.78	0.0994
ssc-miR-323	DR1	down-regulator of transcription 1 [Source:VGNC Symbol;Acc:VGNC:87435]	1.78	0.0994
ssc-miR-323	DSTYK	dual serine/threonine and tyrosine protein kinase [Source:VGNC Symbol;Acc:VGNC:87460]	1.78	0.0994
ssc-miR-323	DUSP22	dual specificity phosphatase 22 [Source:VGNC Symbol;Acc:VGNC:87482]	1.78	0.0994
ssc-miR-323	E2F3	E2F transcription factor 3 [Source:VGNC Symbol;Acc:VGNC:87514]	1.78	0.0994
ssc-miR-323	EDA	ectodysplasin A [Source:VGNC Symbol;Acc:VGNC:87540]	1.78	0.0994
ssc-miR-323	EDEM1	ER degradation enhancing alpha-mannosidase like protein 1 [Source:VGNC Symbol;Acc:VGNC:87545]	1.78	0.0994
ssc-miR-323	EDN2	endothelin 2 [Source:VGNC Symbol;Acc:VGNC:87548]	1.78	0.0994
ssc-miR-323	EEA1	early endosome antigen 1 [Source:HGNC Symbol;Acc:HGNC:3185]	1.78	0.0994
ssc-miR-323	EED	embryonic ectoderm development [Source:VGNC Symbol;Acc:VGNC:87552]	1.78	0.0994
ssc-miR-323	EFCAB14	EF-hand calcium binding domain 14 [Source:VGNC Symbol;Acc:VGNC:87563]	1.78	0.0994
ssc-miR-323	EFNA3	ephrin A3 [Source:VGNC Symbol;Acc:VGNC:98790]	1.78	0.0994

ssc-miR-323	EGR3	early growth response 3 [Source:VGNC Symbol;Acc:VGNC:87591]	1.78	0.0994
ssc-miR-323	EIF1	eukaryotic translation initiation factor 1 [Source:VGNC Symbol;Acc:VGNC:98988]	1.78	0.0994
ssc-miR-323	EIF4E	eukaryotic translation initiation factor 4E [Source:VGNC Symbol;Acc:VGNC:87626]	1.78	0.0994
ssc-miR-323	ELAVL2	ELAV like RNA binding protein 2 [Source:VGNC Symbol;Acc:VGNC:87639]	1.78	0.0994
ssc-miR-323	ELF1	E74 like ETS transcription factor 1 [Source:VGNC Symbol;Acc:VGNC:87641]	1.78	0.0994
ssc-miR-323	ELMSAN1	hypothetical gene	1.78	0.0994
ssc-miR-323	EMC4	ER membrane protein complex subunit 4 [Source:VGNC Symbol;Acc:VGNC:103941]	1.78	0.0994
ssc-miR-323	EML5	hypothetical gene	1.78	0.0994
ssc-miR-323	ENAH	ENAH actin regulator [Source:VGNC Symbol;Acc:VGNC:108271]	1.78	0.0994
ssc-miR-323	ENOX1	ecto-NOX disulfide-thiol exchanger 1 [Source:VGNC Symbol;Acc:VGNC:87706]	1.78	0.0994
ssc-miR-323	ENPP5	ectonucleotide pyrophosphatase/phosphodiesterase family member 5 [Source:VGNC Symbol;Acc:VGNC:96730]	1.78	0.0994
ssc-miR-323	ENPP6	ectonucleotide pyrophosphatase/phosphodiesterase 6 [Source:VGNC Symbol;Acc:VGNC:95999]	1.78	0.0994
ssc-miR-323	EPHA5	EPH receptor A5 [Source:VGNC Symbol;Acc:VGNC:87733]	1.78	0.0994
ssc-miR-323	ERBB4	erb-b2 receptor tyrosine kinase 4 [Source:VGNC Symbol;Acc:VGNC:96284]	1.78	0.0994
ssc-miR-323	ERN2	endoplasmic reticulum to nucleus signaling 2 [Source:VGNC Symbol;Acc:VGNC:87778]	1.78	0.0994
ssc-miR-323	ESCO1	establishment of sister chromatid cohesion N-acetyltransferase 1 [Source:VGNC Symbol;Acc:VGNC:87786]	1.78	0.0994
ssc-miR-323	ESRRA	estrogen related receptor alpha [Source:VGNC Symbol;Acc:VGNC:87793]	1.78	0.0994
ssc-miR-323	ETV3	ETS variant transcription factor 3 [Source:VGNC Symbol;Acc:VGNC:87812]	1.78	0.0994
ssc-miR-323	EXPH5	exophilin 5 [Source:HGNC Symbol;Acc:HGNC:30578]	1.78	0.0994
ssc-miR-323	FAM102B	family with sequence similarity 102 member B [Source:VGNC Symbol;Acc:VGNC:87884]	1.78	0.0994
ssc-miR-323	FAM114A1	family with sequence similarity 114 member A1 [Source:VGNC Symbol;Acc:VGNC:87890]	1.78	0.0994
ssc-miR-323	FAM135A	family with sequence similarity 135 member A [Source:VGNC Symbol;Acc:VGNC:87904]	1.78	0.0994
ssc-miR-323	FAM160B1	hypothetical gene	1.78	0.0994
ssc-miR-323	FAM168B	family with sequence similarity 168 member B [Source:VGNC Symbol;Acc:VGNC:95462]	1.78	0.0994
ssc-miR-323	FAM177A1	family with sequence similarity 177 member A1 [Source:VGNC Symbol;Acc:VGNC:87933]	1.78	0.0994
ssc-miR-323	FAM189A1	family with sequence similarity 189 member A1 [Source:VGNC Symbol;Acc:VGNC:87943]	1.78	0.0994
ssc-miR-323	FAM196B	hypothetical gene	1.78	0.0994
ssc-miR-323	FAM199X	family with sequence similarity 199, X-linked [Source:HGNC Symbol;Acc:HGNC:25195]	1.78	0.0994
ssc-miR-323	FAM204A	family with sequence similarity 204 member A [Source:VGNC Symbol;Acc:VGNC:107389]	1.78	0.0994
ssc-miR-323	FAXC	hypothetical gene	1.78	0.0994
ssc-miR-323	FBRSL1	hypothetical gene	1.78	0.0994
ssc-miR-323	FBXO3	F-box protein 3 [Source:VGNC Symbol;Acc:VGNC:88037]	1.78	0.0994
ssc-miR-323	FBXO30	F-box protein 30 [Source:VGNC Symbol;Acc:VGNC:108155]	1.78	0.0994
ssc-miR-323	FHL5	four and a half LIM domains 5 [Source:VGNC Symbol;Acc:VGNC:88130]	1.78	0.0994
ssc-miR-323	FLRT3	fibronectin leucine rich transmembrane protein 3 [Source:VGNC Symbol;Acc:VGNC:96304]	1.78	0.0994
ssc-miR-323	FMR1	FMRP translational regulator 1 [Source:VGNC Symbol;Acc:VGNC:88175]	1.78	0.0994
ssc-miR-323	FNIP1	folliculin interacting protein 1 [Source:VGNC Symbol;Acc:VGNC:88187]	1.78	0.0994
ssc-miR-323	FOCAD	focadhesin [Source:VGNC Symbol;Acc:VGNC:88189]	1.78	0.0994
ssc-miR-323	FOXB1	forkhead box B1 [Source:VGNC Symbol;Acc:VGNC:88195]	1.78	0.0994
ssc-miR-323	FOXD1	forkhead box D1 [Source:VGNC Symbol;Acc:VGNC:88199]	1.78	0.0994
ssc-miR-323	FOXP2	forkhead box P2 [Source:VGNC Symbol;Acc:VGNC:98014]	1.78	0.0994
ssc-miR-323	FRK	fyn related Src family tyrosine kinase [Source:VGNC Symbol;Acc:VGNC:88235]	1.78	0.0994
ssc-miR-323	FUBP1	far upstream element binding protein 1 [Source:VGNC Symbol;Acc:VGNC:96781]	1.78	0.0994
ssc-miR-323	FUT9	fucosyltransferase 9 [Source:VGNC Symbol;Acc:VGNC:88271]	1.78	0.0994
ssc-miR-323	FZD5	frizzled class receptor 5 [Source:VGNC Symbol;Acc:VGNC:96309]	1.78	0.0994
ssc-miR-323	G3BP2	G3BP stress granule assembly factor 2 [Source:VGNC Symbol;Acc:VGNC:88289]	1.78	0.0994
ssc-miR-323	GABBR2	gamma-aminobutyric acid type B receptor subunit 2 [Source:VGNC Symbol;Acc:VGNC:88298]	1.78	0.0994
ssc-miR-323	GABRP	gamma-aminobutyric acid type A receptor subunit pi [Source:VGNC Symbol;Acc:VGNC:88314]	1.78	0.0994
ssc-miR-323	GAD1	glutamate decarboxylase 1 [Source:VGNC Symbol;Acc:VGNC:96312]	1.78	0.0994

ssc-miR-323	GALNT1	polypeptide N-acetylgalactosaminyltransferase 1 [Source:VGNC Symbol;Acc:VGNC:97062]	1.78	0.0994
ssc-miR-323	GAS2L3	growth arrest specific 2 like 3 [Source:VGNC Symbol;Acc:VGNC:88357]	1.78	0.0994
ssc-miR-323	GATAD2B	GATA zinc finger domain containing 2B [Source:VGNC Symbol;Acc:VGNC:88369]	1.78	0.0994
ssc-miR-323	GCA	grancalcin [Source:VGNC Symbol;Acc:VGNC:95726]	1.78	0.0994
ssc-miR-323	GCLC	glutamate-cysteine ligase catalytic subunit [Source:VGNC Symbol;Acc:VGNC:88386]	1.78	0.0994
ssc-miR-323	GCLM	glutamate-cysteine ligase modifier subunit [Source:VGNC Symbol;Acc:VGNC:88387]	1.78	0.0994
ssc-miR-323	GDF6	growth differentiation factor 6 [Source:VGNC Symbol;Acc:VGNC:88402]	1.78	0.0994
ssc-miR-323	GGNBP2	gametotin binding protein 2 [Source:VGNC Symbol;Acc:VGNC:88437]	1.78	0.0994
ssc-miR-323	GHR	growth hormone receptor [Source:NCBI gene (formerly Entrezgene);Acc:397488]	1.78	0.0994
ssc-miR-323	GLCE	glucuronic acid epimerase [Source:VGNC Symbol;Acc:VGNC:88477]	1.78	0.0994
ssc-miR-323	GLIS3	GLIS family zinc finger 3 [Source:VGNC Symbol;Acc:VGNC:88485]	1.78	0.0994
ssc-miR-323	GMCL1	hypothetical gene	1.78	0.0994
ssc-miR-323	GNAQ	G protein subunit alpha q [Source:VGNC Symbol;Acc:VGNC:103100]	1.78	0.0994
ssc-miR-323	GNE	glucosamine (UDP-N-acetyl)-2-epimerase/N-acetylmannosamine kinase [Source:VGNC Symbol;Acc:VGNC:103101]	1.78	0.0994
ssc-miR-323	GOLIM4	golgi integral membrane protein 4 [Source:VGNC Symbol;Acc:VGNC:88555]	1.78	0.0994
ssc-miR-323	GPBP1	GC-rich promoter binding protein 1 [Source:VGNC Symbol;Acc:VGNC:88578]	1.78	0.0994
ssc-miR-323	GPCPD1	glycerophosphocholine phosphodiesterase 1 [Source:VGNC Symbol;Acc:VGNC:96148]	1.78	0.0994
ssc-miR-323	GPR22	G protein-coupled receptor 22 [Source:VGNC Symbol;Acc:VGNC:88625]	1.78	0.0994
ssc-miR-323	GPSM2	G protein signaling modulator 2 [Source:VGNC Symbol;Acc:VGNC:88649]	1.78	0.0994
ssc-miR-323	GRAMD1B	GRAM domain containing 1B [Source:VGNC Symbol;Acc:VGNC:88656]	1.78	0.0994
ssc-miR-323	GREM1	gremlin 1, DAN family BMP antagonist [Source:VGNC Symbol;Acc:VGNC:88665]	1.78	0.0994
ssc-miR-323	GREM2	gremlin 2, DAN family BMP antagonist [Source:VGNC Symbol;Acc:VGNC:95854]	1.78	0.0994
ssc-miR-323	GRIN2B	glutamate ionotropic receptor NMDA type subunit 2B [Source:VGNC Symbol;Acc:VGNC:88684]	1.78	0.0994
ssc-miR-323	GRM7	glutamate metabotropic receptor 7 [Source:VGNC Symbol;Acc:VGNC:98028]	1.78	0.0994
ssc-miR-323	GRSF1	G-rich RNA sequence binding factor 1 [Source:VGNC Symbol;Acc:VGNC:88710]	1.78	0.0994
ssc-miR-323	HDX	highly divergent homeobox [Source:VGNC Symbol;Acc:VGNC:88824]	1.78	0.0994
ssc-miR-323	HIC2	HIC ZBTB transcriptional repressor 2 [Source:HGNC Symbol;Acc:HGNC:18595]	1.78	0.0994
ssc-miR-323	HIPK3	homeodomain interacting protein kinase 3 [Source:VGNC Symbol;Acc:VGNC:88889]	1.78	0.0994
ssc-miR-323	HK2	hexokinase 2 [Source:VGNC Symbol;Acc:VGNC:98034]	1.78	0.0994
ssc-miR-323	HMGCR	3-hydroxy-3-methylglutaryl-CoA reductase [Source:VGNC Symbol;Acc:VGNC:88906]	1.78	0.0994
ssc-miR-323	HMGCS1	3-hydroxy-3-methylglutaryl-CoA synthase 1 [Source:VGNC Symbol;Acc:VGNC:88907]	1.78	0.0994
ssc-miR-323	HMGN5	hypothetical gene	1.78	0.0994
ssc-miR-323	HNRNPK	heterous nuclear ribonucleoprotein K [Source:VGNC Symbol;Acc:VGNC:103963]	1.78	0.0994
ssc-miR-323	HP1BP3	heterochromatin protein 1 binding protein 3 [Source:VGNC Symbol;Acc:VGNC:88956]	1.78	0.0994
ssc-miR-323	HSDL2	hydroxysteroid dehydrogenase like 2 [Source:VGNC Symbol;Acc:VGNC:88992]	1.78	0.0994
ssc-miR-323	HSPA13	heat shock protein family A (Hsp70) member 13 [Source:VGNC Symbol;Acc:VGNC:108663]	1.78	0.0994
ssc-miR-323	HSPA4	heat shock protein family A (Hsp70) member 4 [Source:HGNC Symbol;Acc:HGNC:5237]	1.78	0.0994
ssc-miR-323	HTRA1	HtrA serine peptidase 1 [Source:VGNC Symbol;Acc:VGNC:107400]	1.78	0.0994
ssc-miR-323	IFRD1	interferon related developmental regulator 1 [Source:NCBI gene (formerly Entrezgene);Acc:493185]	1.78	0.0994
ssc-miR-323	IGF1	insulin like growth factor 1 [Source:VGNC Symbol;Acc:VGNC:98044]	1.78	0.0994
ssc-miR-323	IGSF3	immunoglobulin superfamily member 3 [Source:VGNC Symbol;Acc:VGNC:89067]	1.78	0.0994
ssc-miR-323	IKZF2	IKAROS family zinc finger 2 [Source:VGNC Symbol;Acc:VGNC:95576]	1.78	0.0994
ssc-miR-323	ING5	inhibitor of growth family member 5 [Source:VGNC Symbol;Acc:VGNC:95912]	1.78	0.0994
ssc-miR-323	IPO8	importin 8 [Source:VGNC Symbol;Acc:VGNC:89181]	1.78	0.0994
ssc-miR-323	IRF2BPL	interferon regulatory factor 2 binding protein like [Source:HGNC Symbol;Acc:HGNC:14282]	1.78	0.0994
ssc-miR-323	JAKMIP2	janus kinase and microtubule interacting protein 2 [Source:VGNC Symbol;Acc:VGNC:89274]	1.78	0.0994
ssc-miR-323	JHDM1D	hypothetical gene	1.78	0.0994
ssc-miR-323	JPH3	hypothetical gene	1.78	0.0994
ssc-miR-323	KALRN	hypothetical gene	1.78	0.0994

ssc-miR-323	KCMF1	potassium channel modulatory factor 1 [Source:VGNC Symbol;Acc:VGNC:89322]	1.78	0.0994
ssc-miR-323	KCNB1	potassium voltage-gated channel subfamily B member 1 [Source:VGNC Symbol;Acc:VGNC:96386]	1.78	0.0994
ssc-miR-323	KCND3	potassium voltage-gated channel subfamily D member 3 [Source:VGNC Symbol;Acc:VGNC:98814]	1.78	0.0994
ssc-miR-323	KCNJ6	potassium inwardly rectifying channel subfamily J member 6 [Source:VGNC Symbol;Acc:VGNC:89360]	1.78	0.0994
ssc-miR-323	KCNK10	potassium two pore domain channel subfamily K member 10 [Source:VGNC Symbol;Acc:VGNC:89364]	1.78	0.0994
ssc-miR-323	KCNMB2	potassium calcium-activated channel subfamily M regulatory beta subunit 2 [Source:HGNC Symbol;Acc:HGNC:6286]	1.78	0.0994
ssc-miR-323	KCNQ3	potassium voltage-gated channel subfamily Q member 3 [Source:VGNC Symbol;Acc:VGNC:89382]	1.78	0.0994
ssc-miR-323	KCTD12	potassium channel tetramerization domain containing 12 [Source:HGNC Symbol;Acc:HGNC:14678]	1.78	0.0994
ssc-miR-323	KCTD16	potassium channel tetramerization domain containing 16 [Source:VGNC Symbol;Acc:VGNC:89395]	1.78	0.0994
ssc-miR-323	KCTD3	potassium channel tetramerization domain containing 3 [Source:VGNC Symbol;Acc:VGNC:95963]	1.78	0.0994
ssc-miR-323	KDM3A	lysine demethylase 3A [Source:VGNC Symbol;Acc:VGNC:89411]	1.78	0.0994
ssc-miR-323	KIAA1211	hypothetical gene	1.78	0.0994
ssc-miR-323	KIAA1432	hypothetical gene	1.78	0.0994
ssc-miR-323	KIAA1468	hypothetical gene	1.78	0.0994
ssc-miR-323	KIAA1598	hypothetical gene	1.78	0.0994
ssc-miR-323	KIF26B	kinesin family member 26B [Source:VGNC Symbol;Acc:VGNC:96087]	1.78	0.0994
ssc-miR-323	KITLG	KIT ligand [Source:VGNC Symbol;Acc:VGNC:98061]	1.78	0.0994
ssc-miR-323	KLF11	Kruppel like factor 11 [Source:VGNC Symbol;Acc:VGNC:89491]	1.78	0.0994
ssc-miR-323	KLF3	Kruppel like factor 3 [Source:VGNC Symbol;Acc:VGNC:89498]	1.78	0.0994
ssc-miR-323	KLHL3	kelch like family member 3 [Source:VGNC Symbol;Acc:VGNC:89525]	1.78	0.0994
ssc-miR-323	KMT2C	lysine methyltransferase 2C [Source:VGNC Symbol;Acc:VGNC:89550]	1.78	0.0994
ssc-miR-323	KMT2E	lysine methyltransferase 2E (inactive) [Source:VGNC Symbol;Acc:VGNC:89551]	1.78	0.0994
ssc-miR-323	KPNA4	karyopherin subunit alpha 4 [Source:VGNC Symbol;Acc:VGNC:89563]	1.78	0.0994
ssc-miR-323	KRAS	KRAS proto-onco, GTPase [Source:VGNC Symbol;Acc:VGNC:89569]	1.78	0.0994
ssc-miR-323	LCA5	lebercilin LCA5 [Source:VGNC Symbol;Acc:VGNC:89654]	1.78	0.0994
ssc-miR-323	LCOR	ligand dependent nuclear receptor corepressor [Source:HGNC Symbol;Acc:HGNC:29503]	1.78	0.0994
ssc-miR-323	LDHA	hypothetical gene	1.78	0.0994
ssc-miR-323	LDLRAD4	low density lipoprotein receptor class A domain containing 4 [Source:VGNC Symbol;Acc:VGNC:89673]	1.78	0.0994
ssc-miR-323	LIPG	lipase G, endothelial type [Source:VGNC Symbol;Acc:VGNC:89739]	1.78	0.0994
ssc-miR-323	LMO3	hypothetical gene	1.78	0.0994
ssc-miR-323	LMO7	LIM domain 7 [Source:VGNC Symbol;Acc:VGNC:89767]	1.78	0.0994
ssc-miR-323	LPHN3	hypothetical gene	1.78	0.0994
ssc-miR-323	LPPR5	hypothetical gene	1.78	0.0994
ssc-miR-323	LRP12	LDL receptor related protein 12 [Source:VGNC Symbol;Acc:VGNC:89818]	1.78	0.0994
ssc-miR-323	LRRC4	leucine rich repeat containing 4 [Source:VGNC Symbol;Acc:VGNC:89839]	1.78	0.0994
ssc-miR-323	LRRTM3	leucine rich repeat transmembrane neuronal 3 [Source:VGNC Symbol;Acc:VGNC:107409]	1.78	0.0994
ssc-miR-323	LSM14B	LSM family member 14B [Source:VGNC Symbol;Acc:VGNC:95691]	1.78	0.0994
ssc-miR-323	MAB21L1	mab-21 like 1 [Source:VGNC Symbol;Acc:VGNC:89932]	1.78	0.0994
ssc-miR-323	MACC1	MET transcriptional regulator MACC1 [Source:VGNC Symbol;Acc:VGNC:89935]	1.78	0.0994
ssc-miR-323	MAML3	mastermind like transcriptional coactivator 3 [Source:VGNC Symbol;Acc:VGNC:98938]	1.78	0.0994
ssc-miR-323	MANSC1	hypothetical gene	1.78	0.0994
ssc-miR-323	MAP3K2	mitogen-activated protein kinase kinase kinase 2 [Source:VGNC Symbol;Acc:VGNC:98107]	1.78	0.0994
ssc-miR-323	MAP3K3	mitogen-activated protein kinase kinase kinase 3 [Source:VGNC Symbol;Acc:VGNC:98108]	1.78	0.0994
ssc-miR-323	MAP4K4	mitogen-activated protein kinase kinase kinase kinase 4 [Source:VGNC Symbol;Acc:VGNC:98114]	1.78	0.0994
ssc-miR-323	MAP4K5	mitogen-activated protein kinase kinase kinase kinase 5 [Source:VGNC Symbol;Acc:VGNC:98115]	1.78	0.0994
ssc-miR-323	MAP7	microtubule associated protein 7 [Source:VGNC Symbol;Acc:VGNC:89992]	1.78	0.0994
ssc-miR-323	MAPK1IP1L	mitogen-activated protein kinase 1 interacting protein 1 like [Source:VGNC Symbol;Acc:VGNC:90002]	1.78	0.0994
ssc-miR-323	MAPK6	mitogen-activated protein kinase 6 [Source:VGNC Symbol;Acc:VGNC:103123]	1.78	0.0994
ssc-miR-323	MAPKAPK5	MAPK activated protein kinase 5 [Source:VGNC Symbol;Acc:VGNC:90013]	1.78	0.0994

ssc-miR-323	MBD5	methyl-CpG binding domain protein 5 [Source:VGNC Symbol;Acc:VGNC:95940]	1.78	0.0994
ssc-miR-323	MCMBP	minichromosome maintenance complex binding protein [Source:VGNC Symbol;Acc:VGNC:90077]	1.78	0.0994
ssc-miR-323	MCTP1	multiple C2 and transmembrane domain containing 1 [Source:VGNC Symbol;Acc:VGNC:90083]	1.78	0.0994
ssc-miR-323	MED12L	mediator complex subunit 12L [Source:VGNC Symbol;Acc:VGNC:90105]	1.78	0.0994
ssc-miR-323	MEGF9	multiple EGF like domains 9 [Source:VGNC Symbol;Acc:VGNC:90130]	1.78	0.0994
ssc-miR-323	METAP2	methionyl aminopeptidase 2 [Source:VGNC Symbol;Acc:VGNC:90152]	1.78	0.0994
ssc-miR-323	MEX3A	mex-3 RNA binding family member A [Source:VGNC Symbol;Acc:VGNC:90168]	1.78	0.0994
ssc-miR-323	MFSD2A	major facilitator superfamily domain containing 2A [Source:VGNC Symbol;Acc:VGNC:90188]	1.78	0.0994
ssc-miR-323	MITF	melanocyte inducing transcription factor [Source:VGNC Symbol;Acc:VGNC:90243]	1.78	0.0994
ssc-miR-323	MKLN1	muskelin 1 [Source:VGNC Symbol;Acc:VGNC:90245]	1.78	0.0994
ssc-miR-323	MLLT3	MLLT3 super elongation complex subunit [Source:VGNC Symbol;Acc:VGNC:90256]	1.78	0.0994
ssc-miR-323	MME	membrane metalloendopeptidase [Source:VGNC Symbol;Acc:VGNC:90265]	1.78	0.0994
ssc-miR-323	MMP14	matrix metallopeptidase 14 [Source:NCBI gene (formerly Entrezgene);Acc:397471]	1.78	0.0994
ssc-miR-323	MOB1B	hypothetical gene	1.78	0.0994
ssc-miR-323	MPP6	hypothetical gene	1.78	0.0994
ssc-miR-323	MSANTD3	Myb/SANT DNA binding domain containing 3 [Source:HGNC Symbol;Acc:HGNC:23370]	1.78	0.0994
ssc-miR-323	MSL3	MSL complex subunit 3 [Source:NCBI gene (formerly Entrezgene);Acc:100526025]	1.78	0.0994
ssc-miR-323	MTCH2	hypothetical gene	1.78	0.0994
ssc-miR-323	MTMR2	myotubularin related protein 2 [Source:VGNC Symbol;Acc:VGNC:90460]	1.78	0.0994
ssc-miR-323	MXD1	MAX dimerization protein 1 [Source:VGNC Symbol;Acc:VGNC:90492]	1.78	0.0994
ssc-miR-323	MYSM1	Myb like, SWIRM and MPN domains 1 [Source:VGNC Symbol;Acc:VGNC:90548]	1.78	0.0994
ssc-miR-323	MYT1L	myelin transcription factor 1 like [Source:VGNC Symbol;Acc:VGNC:90549]	1.78	0.0994
ssc-miR-323	NASP	nuclear autoantigenic sperm protein [Source:VGNC Symbol;Acc:VGNC:90584]	1.78	0.0994
ssc-miR-323	NCAN	neurocan [Source:VGNC Symbol;Acc:VGNC:90596]	1.78	0.0994
ssc-miR-323	NDST1	N-deacetylase and N-sulfotransferase 1 [Source:VGNC Symbol;Acc:VGNC:90634]	1.78	0.0994
ssc-miR-323	NDUFB9	NADH:ubiquinone oxidoreductase subunit B9 [Source:VGNC Symbol;Acc:VGNC:90649]	1.78	0.0994
ssc-miR-323	NEK6	NIMA related kinase 6 [Source:VGNC Symbol;Acc:VGNC:90678]	1.78	0.0994
ssc-miR-323	NFATC2	nuclear factor of activated T cells 2 [Source:VGNC Symbol;Acc:VGNC:96440]	1.78	0.0994
ssc-miR-323	NFIA	nuclear factor I A [Source:VGNC Symbol;Acc:VGNC:90715]	1.78	0.0994
ssc-miR-323	NFIX	nuclear factor I X [Source:VGNC Symbol;Acc:VGNC:90718]	1.78	0.0994
ssc-miR-323	NFKBID	NFKB inhibitor delta [Source:VGNC Symbol;Acc:VGNC:90723]	1.78	0.0994
ssc-miR-323	NFYB	nuclear transcription factor Y subunit beta [Source:VGNC Symbol;Acc:VGNC:90730]	1.78	0.0994
ssc-miR-323	NIPBL	NIPBL cohesin loading factor [Source:VGNC Symbol;Acc:VGNC:90752]	1.78	0.0994
ssc-miR-323	NLK	nemo like kinase [Source:VGNC Symbol;Acc:VGNC:90779]	1.78	0.0994
ssc-miR-323	NLN	neurolysin [Source:VGNC Symbol;Acc:VGNC:90780]	1.78	0.0994
ssc-miR-323	NOL4	nucleolar protein 4 [Source:VGNC Symbol;Acc:VGNC:90814]	1.78	0.0994
ssc-miR-323	NPPC	natriuretic peptide C [Source:VGNC Symbol;Acc:VGNC:96450]	1.78	0.0994
ssc-miR-323	NPR3	natriuretic peptide receptor 3 [Source:VGNC Symbol;Acc:VGNC:90858]	1.78	0.0994
ssc-miR-323	NPVF	neuropeptide VF [Source:VGNC Symbol;Acc:VGNC:90864]	1.78	0.0994
ssc-miR-323	NR4A2	nuclear receptor subfamily 4 group A member 2 [Source:VGNC Symbol;Acc:VGNC:96451]	1.78	0.0994
ssc-miR-323	NRG2	neuregulin 2 [Source:VGNC Symbol;Acc:VGNC:98160]	1.78	0.0994
ssc-miR-323	NRK	Nik related kinase [Source:VGNC Symbol;Acc:VGNC:98161]	1.78	0.0994
ssc-miR-323	NRP2	neuropilin 2 [Source:VGNC Symbol;Acc:VGNC:96454]	1.78	0.0994
ssc-miR-323	NSL1	NSL1 component of MIS12 kinetochore complex [Source:VGNC Symbol;Acc:VGNC:90908]	1.78	0.0994
ssc-miR-323	NUDT5	nudix hydrolase 5 [Source:VGNC Symbol;Acc:VGNC:96458]	1.78	0.0994
ssc-miR-323	NUFIP2	nuclear FMR1 interacting protein 2 [Source:VGNC Symbol;Acc:VGNC:90967]	1.78	0.0994
ssc-miR-323	NUP62CL	nucleoporin 62 C-terminal like [Source:VGNC Symbol;Acc:VGNC:104013]	1.78	0.0994
ssc-miR-323	ONECUT2	one cut homeobox 2 [Source:VGNC Symbol;Acc:VGNC:91043]	1.78	0.0994
ssc-miR-323	ONECUT3	one cut homeobox 3 [Source:VGNC Symbol;Acc:VGNC:91044]	1.78	0.0994

ssc-miR-323	OSBPL8	oxysterol binding protein like 8 [Source:VGNC Symbol;Acc:VGNC:91074]	1.78	0.0994
ssc-miR-323	OTUD5	OTU deubiquitinase 5 [Source:VGNC Symbol;Acc:VGNC:91101]	1.78	0.0994
ssc-miR-323	PACS2	phosphofurin acidic cluster sorting protein 2 [Source:VGNC Symbol;Acc:VGNC:91141]	1.78	0.0994
ssc-miR-323	PAK7	hypothetical gene	1.78	0.0994
ssc-miR-323	PALM2	hypothetical gene	1.78	0.0994
ssc-miR-323	PAPPA2	pappalysin 2 [Source:VGNC Symbol;Acc:VGNC:91171]	1.78	0.0994
ssc-miR-323	PARP8	poly(ADP-ribose) polymerase family member 8 [Source:HGNC Symbol;Acc:HGNC:26124]	1.78	0.0994
ssc-miR-323	PAWR	pro-apoptotic WT1 regulator [Source:VGNC Symbol;Acc:VGNC:91191]	1.78	0.0994
ssc-miR-323	PBX3	PBX homeobox 3 [Source:VGNC Symbol;Acc:VGNC:91205]	1.78	0.0994
ssc-miR-323	PCBP1	poly(rC) binding protein 1 [Source:HGNC Symbol;Acc:HGNC:8647]	1.78	0.0994
ssc-miR-323	PCBP2	poly(rC) binding protein 2 [Source:VGNC Symbol;Acc:VGNC:103309]	1.78	0.0994
ssc-miR-323	PCDH11Y	hypothetical gene	1.78	0.0994
ssc-miR-323	PCDH17	protocadherin 17 [Source:VGNC Symbol;Acc:VGNC:91214]	1.78	0.0994
ssc-miR-323	PCDH8	protocadherin 8 [Source:HGNC Symbol;Acc:HGNC:8660]	1.78	0.0994
ssc-miR-323	PCNP	PEST proteolytic signal containing nuclear protein [Source:VGNC Symbol;Acc:VGNC:104018]	1.78	0.0994
ssc-miR-323	PCSK1	proprotein convertase subtilisin/kexin type 1 [Source:VGNC Symbol;Acc:VGNC:91230]	1.78	0.0994
ssc-miR-323	PDE4B	phosphodiesterase 4B [Source:VGNC Symbol;Acc:VGNC:91255]	1.78	0.0994
ssc-miR-323	PDE4D	phosphodiesterase 4D [Source:VGNC Symbol;Acc:VGNC:91256]	1.78	0.0994
ssc-miR-323	PHACTR4	phosphatase and actin regulator 4 [Source:VGNC Symbol;Acc:VGNC:98531]	1.78	0.0994
ssc-miR-323	PHF12	PHD finger protein 12 [Source:VGNC Symbol;Acc:VGNC:91378]	1.78	0.0994
ssc-miR-323	PHF8	PHD finger protein 8 [Source:VGNC Symbol;Acc:VGNC:91391]	1.78	0.0994
ssc-miR-323	PHLDA1	pleckstrin homology like domain family A member 1 [Source:HGNC Symbol;Acc:HGNC:8933]	1.78	0.0994
ssc-miR-323	PHLPP2	PH domain and leucine rich repeat protein phosphatase 2 [Source:VGNC Symbol;Acc:VGNC:98186]	1.78	0.0994
ssc-miR-323	PI4K2A	phosphatidylinositol 4-kinase type 2 alpha [Source:HGNC Symbol;Acc:HGNC:30031]	1.78	0.0994
ssc-miR-323	PIK3R3	phosphoinositide-3-kinase regulatory subunit 3 [Source:HGNC Symbol;Acc:HGNC:8981]	1.78	0.0994
ssc-miR-323	PIP5K1A	phosphatidylinositol-4-phosphate 5-kinase type 1 alpha [Source:VGNC Symbol;Acc:VGNC:98834]	1.78	0.0994
ssc-miR-323	PJA2	praja ring finger ubiquitin ligase 2 [Source:VGNC Symbol;Acc:VGNC:91470]	1.78	0.0994
ssc-miR-323	PKN2	protein kinase N2 [Source:VGNC Symbol;Acc:VGNC:91482]	1.78	0.0994
ssc-miR-323	PLD5	phospholipase D family member 5 [Source:VGNC Symbol;Acc:VGNC:96145]	1.78	0.0994
ssc-miR-323	PLEKHA5	pleckstrin homology domain containing A5 [Source:VGNC Symbol;Acc:VGNC:91535]	1.78	0.0994
ssc-miR-323	PNISR	PNN interacting serine and arginine rich protein [Source:VGNC Symbol;Acc:VGNC:91594]	1.78	0.0994
ssc-miR-323	POLI	DNA polymerase iota [Source:VGNC Symbol;Acc:VGNC:91636]	1.78	0.0994
ssc-miR-323	POU4F2	POU class 4 homeobox 2 [Source:VGNC Symbol;Acc:VGNC:91679]	1.78	0.0994
ssc-miR-323	PPAP2B	hypothetical gene	1.78	0.0994
ssc-miR-323	PPARGC1B	PPARG coactivator 1 beta [Source:VGNC Symbol;Acc:VGNC:91686]	1.78	0.0994
ssc-miR-323	PPFIBP1	PPFIA binding protein 1 [Source:VGNC Symbol;Acc:VGNC:91695]	1.78	0.0994
ssc-miR-323	PPP1CB	protein phosphatase 1 catalytic subunit beta [Source:NCBI gene (formerly Entrezgene);Acc:397378]	1.78	0.0994
ssc-miR-323	PPP4R2	protein phosphatase 4 regulatory subunit 2 [Source:VGNC Symbol;Acc:VGNC:91756]	1.78	0.0994
ssc-miR-323	PPP6C	protein phosphatase 6 catalytic subunit [Source:VGNC Symbol;Acc:VGNC:98219]	1.78	0.0994
ssc-miR-323	PPTC7	protein phosphatase targeting COQ7 [Source:VGNC Symbol;Acc:VGNC:91765]	1.78	0.0994
ssc-miR-323	PRDM4	PR/SET domain 4 [Source:VGNC Symbol;Acc:VGNC:91779]	1.78	0.0994
ssc-miR-323	PRICKLE1	prickle planar cell polarity protein 1 [Source:VGNC Symbol;Acc:VGNC:91793]	1.78	0.0994
ssc-miR-323	PRKAA2	protein kinase AMP-activated catalytic subunit alpha 2 [Source:VGNC Symbol;Acc:VGNC:91798]	1.78	0.0994
ssc-miR-323	PRKD3	protein kinase D3 [Source:VGNC Symbol;Acc:VGNC:91814]	1.78	0.0994
ssc-miR-323	PRPF40A	pre-mRNA processing factor 40 homolog A [Source:VGNC Symbol;Acc:VGNC:98222]	1.78	0.0994
ssc-miR-323	PRR12	hypothetical gene	1.78	0.0994
ssc-miR-323	PSMC6	proteasome 26S subunit, ATPase 6 [Source:VGNC Symbol;Acc:VGNC:91917]	1.78	0.0994
ssc-miR-323	PSME3	proteasome activator subunit 3 [Source:VGNC Symbol;Acc:VGNC:91926]	1.78	0.0994
ssc-miR-323	PSPC1	paraspeckle component 1 [Source:VGNC Symbol;Acc:VGNC:91930]	1.78	0.0994

ssc-miR-323	PTPLA	hypothetical gene	1.78	0.0994
ssc-miR-323	PTPRF	protein tyrosine phosphatase receptor type F [Source:VGNC Symbol;Acc:VGNC:91987]	1.78	0.0994
ssc-miR-323	PUM1	pumilio RNA binding family member 1 [Source:VGNC Symbol;Acc:VGNC:92001]	1.78	0.0994
ssc-miR-323	PXDC1	PX domain containing 1 [Source:VGNC Symbol;Acc:VGNC:92013]	1.78	0.0994
ssc-miR-323	RAB11FIP4	RAB11 family interacting protein 4 [Source:VGNC Symbol;Acc:VGNC:92041]	1.78	0.0994
ssc-miR-323	RAB2A	RAB2A, member RAS onco family [Source:VGNC Symbol;Acc:VGNC:98252]	1.78	0.0994
ssc-miR-323	RAB3GAP2	RAB3 GTPase activating non-catalytic protein subunit 2 [Source:VGNC Symbol;Acc:VGNC:95833]	1.78	0.0994
ssc-miR-323	RAB5B	RAB5B, member RAS oncogene family [Source:HGNC Symbol;Acc:HGNC:9784]	1.78	0.0994
ssc-miR-323	RALGPS1	Ral GEF with PH domain and SH3 binding motif 1 [Source:VGNC Symbol;Acc:VGNC:92072]	1.78	0.0994
ssc-miR-323	RAP1GAP2	RAP1 GTPase activating protein 2 [Source:VGNC Symbol;Acc:VGNC:92086]	1.78	0.0994
ssc-miR-323	RAP1GDS1	Rap1 GTPase-GDP dissociation stimulator 1 [Source:VGNC Symbol;Acc:VGNC:92087]	1.78	0.0994
ssc-miR-323	RASAL2	RAS protein activator like 2 [Source:VGNC Symbol;Acc:VGNC:92105]	1.78	0.0994
ssc-miR-323	RBM24	hypothetical gene	1.78	0.0994
ssc-miR-323	RBM27	RNA binding motif protein 27 [Source:HGNC Symbol;Acc:HGNC:29243]	1.78	0.0994
ssc-miR-323	RBM5	RNA binding motif protein 5 [Source:VGNC Symbol;Acc:VGNC:92160]	1.78	0.0994
ssc-miR-323	RCAN1	regulator of calcineurin 1 [Source:VGNC Symbol;Acc:VGNC:92170]	1.78	0.0994
ssc-miR-323	RCN2	reticulocalbin 2 [Source:VGNC Symbol;Acc:VGNC:92181]	1.78	0.0994
ssc-miR-323	REEP5	receptor accessory protein 5 [Source:VGNC Symbol;Acc:VGNC:92201]	1.78	0.0994
ssc-miR-323	REL	REL proto-onco, NF-kB subunit [Source:VGNC Symbol;Acc:VGNC:92202]	1.78	0.0994
ssc-miR-323	RFX3	regulatory factor X3 [Source:VGNC Symbol;Acc:VGNC:92245]	1.78	0.0994
ssc-miR-323	RHEB	Ras homolog, mTORC1 binding [Source:NCBI gene (formerly Entrezgene);Acc:100516050]	1.78	0.0994
ssc-miR-323	RIPK4	receptor interacting serine/threonine kinase 4 [Source:VGNC Symbol;Acc:VGNC:92321]	1.78	0.0994
ssc-miR-323	RNF11	ring finger protein 11 [Source:VGNC Symbol;Acc:VGNC:98601]	1.78	0.0994
ssc-miR-323	RNF149	ring finger protein 149 [Source:VGNC Symbol;Acc:VGNC:92362]	1.78	0.0994
ssc-miR-323	RNF152	ring finger protein 152 [Source:VGNC Symbol;Acc:VGNC:92364]	1.78	0.0994
ssc-miR-323	RNF2	ring finger protein 2 [Source:VGNC Symbol;Acc:VGNC:92377]	1.78	0.0994
ssc-miR-323	RNF20	ring finger protein 20 [Source:VGNC Symbol;Acc:VGNC:92378]	1.78	0.0994
ssc-miR-323	RNF214	ring finger protein 214 [Source:VGNC Symbol;Acc:VGNC:92382]	1.78	0.0994
ssc-miR-323	ROCK1	Rho associated coiled-coil containing protein kinase 1 [Source:VGNC Symbol;Acc:VGNC:98294]	1.78	0.0994
ssc-miR-323	ROCK2	Rho associated coiled-coil containing protein kinase 2 [Source:HGNC Symbol;Acc:HGNC:10252]	1.78	0.0994
ssc-miR-323	RORA	RAR related orphan receptor A [Source:VGNC Symbol;Acc:VGNC:92408]	1.78	0.0994
ssc-miR-323	RPP14	hypothetical gene	1.78	0.0994
ssc-miR-323	RPS27L	ribosomal protein S27 like [Source:HGNC Symbol;Acc:HGNC:18476]	1.78	0.0994
ssc-miR-323	RPS6KA3	ribosomal protein S6 kinase A3 [Source:VGNC Symbol;Acc:VGNC:92442]	1.78	0.0994
ssc-miR-323	RSBN1	round spermatid basic protein 1 [Source:VGNC Symbol;Acc:VGNC:92473]	1.78	0.0994
ssc-miR-323	RSBN1L	round spermatid basic protein 1 like [Source:VGNC Symbol;Acc:VGNC:92474]	1.78	0.0994
ssc-miR-323	RUNX1T1	RUNX1 partner transcriptional co-repressor 1 [Source:VGNC Symbol;Acc:VGNC:96594]	1.78	0.0994
ssc-miR-323	RYK	receptor like tyrosine kinase [Source:VGNC Symbol;Acc:VGNC:92533]	1.78	0.0994
ssc-miR-323	SALL3	spalt like transcription factor 3 [Source:VGNC Symbol;Acc:VGNC:92563]	1.78	0.0994
ssc-miR-323	SBNO1	strawberry notch homolog 1 [Source:VGNC Symbol;Acc:VGNC:92596]	1.78	0.0994
ssc-miR-323	SCN1A	sodium voltage-gated channel alpha subunit 1 [Source:VGNC Symbol;Acc:VGNC:95478]	1.78	0.0994
ssc-miR-323	SCYL2	SCY1 like pseudokinase 2 [Source:VGNC Symbol;Acc:VGNC:98305]	1.78	0.0994
ssc-miR-323	SEC16B	SEC16 homolog B, endoplasmic reticulum export factor [Source:VGNC Symbol;Acc:VGNC:92672]	1.78	0.0994
ssc-miR-323	SEC23B	SEC23 homolog B, COPII coat complex component [Source:VGNC Symbol;Acc:VGNC:95490]	1.78	0.0994
ssc-miR-323	SEC63	SEC63 homolog, protein translocation regulator [Source:VGNC Symbol;Acc:VGNC:103172]	1.78	0.0994
ssc-miR-323	SEMA7A	semaphorin 7A (John Milton Hagen blood group) [Source:HGNC Symbol;Acc:HGNC:10741]	1.78	0.0994
ssc-miR-323	SEPT7	hypothetical gene	1.78	0.0994
ssc-miR-323	SERTAD4	SERTA domain containing 4 [Source:VGNC Symbol;Acc:VGNC:92752]	1.78	0.0994
ssc-miR-323	SESTD1	SEC14 and spectrin domain containing 1 [Source:VGNC Symbol;Acc:VGNC:95884]	1.78	0.0994

ssc-miR-323	SETBP1	SET binding protein 1 [Source:VGNC Symbol;Acc:VGNC:92756]	1.78	0.0994
ssc-miR-323	SETD9	SET domain containing 9 [Source:VGNC Symbol;Acc:VGNC:92763]	1.78	0.0994
ssc-miR-323	SEZ6	seizure related 6 homolog [Source:VGNC Symbol;Acc:VGNC:92767]	1.78	0.0994
ssc-miR-323	SGK1	serum/glucocorticoid regulated kinase 1 [Source:VGNC Symbol;Acc:VGNC:92793]	1.78	0.0994
ssc-miR-323	SGK3	serum/glucocorticoid regulated kinase family member 3 [Source:HGNC Symbol;Acc:HGNC:10812]	1.78	0.0994
ssc-miR-323	SGTB	small glutamine rich tetratricopeptide repeat co-chaperone beta [Source:VGNC Symbol;Acc:VGNC:92804]	1.78	0.0994
ssc-miR-323	SH2B3	SH2B adaptor protein 3 [Source:VGNC Symbol;Acc:VGNC:92807]	1.78	0.0994
ssc-miR-323	SIKE1	suppressor of IKBKE 1 [Source:VGNC Symbol;Acc:VGNC:92874]	1.78	0.0994
ssc-miR-323	SIN3A	SIN3 transcription regulator family member A [Source:VGNC Symbol;Acc:VGNC:92879]	1.78	0.0994
ssc-miR-323	SKI	SKI proto-onco [Source:VGNC Symbol;Acc:VGNC:92902]	1.78	0.0994
ssc-miR-323	SLAIN1	SLAIN motif family member 1 [Source:VGNC Symbol;Acc:VGNC:92909]	1.78	0.0994
ssc-miR-323	SLC16A10	solute carrier family 16 member 10 [Source:VGNC Symbol;Acc:VGNC:103178]	1.78	0.0994
ssc-miR-323	SLC22A23	solute carrier family 22 member 23 [Source:HGNC Symbol;Acc:HGNC:21106]	1.78	0.0994
ssc-miR-323	SLC25A12	solute carrier family 25 member 12 [Source:VGNC Symbol;Acc:VGNC:95502]	1.78	0.0994
ssc-miR-323	SLC30A7	solute carrier family 30 member 7 [Source:VGNC Symbol;Acc:VGNC:93061]	1.78	0.0994
ssc-miR-323	SLC44A1	solute carrier family 44 member 1 [Source:VGNC Symbol;Acc:VGNC:93118]	1.78	0.0994
ssc-miR-323	SLC7A14	solute carrier family 7 member 14 [Source:VGNC Symbol;Acc:VGNC:93173]	1.78	0.0994
ssc-miR-323	SLC7A8	solute carrier family 7 member 8 [Source:VGNC Symbol;Acc:VGNC:93176]	1.78	0.0994
ssc-miR-323	SLITRK3	SLIT and NTRK like family member 3 [Source:VGNC Symbol;Acc:VGNC:93208]	1.78	0.0994
ssc-miR-323	SMAD2	SMAD family member 2 [Source:VGNC Symbol;Acc:VGNC:98329]	1.78	0.0994
ssc-miR-323	SMAD3	SMAD family member 3 [Source:VGNC Symbol;Acc:VGNC:93217]	1.78	0.0994
ssc-miR-323	SMAD5	SMAD family member 5 [Source:VGNC Symbol;Acc:VGNC:93219]	1.78	0.0994
ssc-miR-323	SMC3	structural maintenance of chromosomes 3 [Source:VGNC Symbol;Acc:VGNC:93239]	1.78	0.0994
ssc-miR-323	SMCHD1	structural maintenance of chromosomes flexible hinge domain containing 1 [Source:VGNC Symbol;Acc:VGNC:93242]	1.78	0.0994
ssc-miR-323	SMNDC1	survival motor neuron domain containing 1 [Source:VGNC Symbol;Acc:VGNC:93257]	1.78	0.0994
ssc-miR-323	SMURF1	SMAD specific E3 ubiquitin protein ligase 1 [Source:VGNC Symbol;Acc:VGNC:93270]	1.78	0.0994
ssc-miR-323	SNX27	sorting nexin 27 [Source:VGNC Symbol;Acc:VGNC:93316]	1.78	0.0994
ssc-miR-323	SOCS5	suppressor of cytokine signaling 5 [Source:VGNC Symbol;Acc:VGNC:93332]	1.78	0.0994
ssc-miR-323	SORCS1	sortilin related VPS10 domain containing receptor 1 [Source:VGNC Symbol;Acc:VGNC:93339]	1.78	0.0994
ssc-miR-323	SP8	Sp8 transcription factor [Source:VGNC Symbol;Acc:VGNC:93365]	1.78	0.0994
ssc-miR-323	SP9	Sp9 transcription factor [Source:VGNC Symbol;Acc:VGNC:96247]	1.78	0.0994
ssc-miR-323	SPPL2A	signal peptide peptidase like 2A [Source:VGNC Symbol;Acc:VGNC:98337]	1.78	0.0994
ssc-miR-323	SPPL3	signal peptide peptidase like 3 [Source:VGNC Symbol;Acc:VGNC:98340]	1.78	0.0994
ssc-miR-323	SPRY3	sprouty RTK signaling antagonist 3 [Source:VGNC Symbol;Acc:VGNC:93426]	1.78	0.0994
ssc-miR-323	SPTBN1	spectrin beta, non-erythrocytic 1 [Source:VGNC Symbol;Acc:VGNC:93436]	1.78	0.0994
ssc-miR-323	SPTSSA	serine palmitoyltransferase small subunit A [Source:HGNC Symbol;Acc:HGNC:20361]	1.78	0.0994
ssc-miR-323	SREK1IP1	SREK1 interacting protein 1 [Source:HGNC Symbol;Acc:HGNC:26716]	1.78	0.0994
ssc-miR-323	SRGAP1	SLIT-ROBO Rho GTPase activating protein 1 [Source:VGNC Symbol;Acc:VGNC:93454]	1.78	0.0994
ssc-miR-323	ST7L	suppression of tumorigenicity 7 like [Source:VGNC Symbol;Acc:VGNC:93515]	1.78	0.0994
ssc-miR-323	STAG2	stromal antigen 2 [Source:VGNC Symbol;Acc:VGNC:93525]	1.78	0.0994
ssc-miR-323	STOX2	storkhead box 2 [Source:VGNC Symbol;Acc:VGNC:96082]	1.78	0.0994
ssc-miR-323	STRN	striatin [Source:VGNC Symbol;Acc:VGNC:93578]	1.78	0.0994
ssc-miR-323	STRN3	striatin 3 [Source:VGNC Symbol;Acc:VGNC:93579]	1.78	0.0994
ssc-miR-323	SUCO	SUN domain containing ossification factor [Source:VGNC Symbol;Acc:VGNC:93606]	1.78	0.0994
ssc-miR-323	SUMO1	small ubiquitin like modifier 1 [Source:NCBI gene (formerly Entrezgene);Acc:100127139]	1.78	0.0994
ssc-miR-323	SV2C	synaptic vesicle glycoprotein 2C [Source:VGNC Symbol;Acc:VGNC:93641]	1.78	0.0994
ssc-miR-323	SYDE2	synapse defective Rho GTPase homolog 2 [Source:VGNC Symbol;Acc:VGNC:98874]	1.78	0.0994
ssc-miR-323	TBC1D19	TBC1 domain family member 19 [Source:VGNC Symbol;Acc:VGNC:93767]	1.78	0.0994
ssc-miR-323	TBC1D4	TBC1 domain family member 4 [Source:HGNC Symbol;Acc:HGNC:19165]	1.78	0.0994

ssc-miR-323	TBL1XR1	TBL1X receptor 1 [Source:VGNC Symbol;Acc:VGNC:96600]	1.78	0.0994
ssc-miR-323	TBX20	T-box transcription factor 20 [Source:VGNC Symbol;Acc:VGNC:93799]	1.78	0.0994
ssc-miR-323	TEAD3	TEA domain transcription factor 3 [Source:VGNC Symbol;Acc:VGNC:93855]	1.78	0.0994
ssc-miR-323	TET2	hypothetical gene	1.78	0.0994
ssc-miR-323	TGFA	transforming growth factor alpha [Source:VGNC Symbol;Acc:VGNC:93928]	1.78	0.0994
ssc-miR-323	TGFB2	transforming growth factor beta 2 [Source:VGNC Symbol;Acc:VGNC:95541]	1.78	0.0994
ssc-miR-323	THSD7A	thrombospondin type 1 domain containing 7A [Source:VGNC Symbol;Acc:VGNC:93968]	1.78	0.0994
ssc-miR-323	TIMM23	hypothetical gene	1.78	0.0994
ssc-miR-323	TIMP3	TIMP metallopeptidase inhibitor 3 [Source:VGNC Symbol;Acc:VGNC:93996]	1.78	0.0994
ssc-miR-323	TM2D3	TM2 domain containing 3 [Source:VGNC Symbol;Acc:VGNC:94029]	1.78	0.0994
ssc-miR-323	TM9SF3	transmembrane 9 superfamily member 3 [Source:VGNC Symbol;Acc:VGNC:94037]	1.78	0.0994
ssc-miR-323	TMEFF2	transmembrane protein with EGF like and two follistatin like domains 2 [Source:HGNC Symbol;Acc:HGNC:11867]	1.78	0.0994
ssc-miR-323	TMEM106B	transmembrane protein 106B [Source:VGNC Symbol;Acc:VGNC:104075]	1.78	0.0994
ssc-miR-323	TMEM164	transmembrane protein 164 [Source:VGNC Symbol;Acc:VGNC:94103]	1.78	0.0994
ssc-miR-323	TMEM168	transmembrane protein 168 [Source:VGNC Symbol;Acc:VGNC:94106]	1.78	0.0994
ssc-miR-323	TMEM242	transmembrane protein 242 [Source:HGNC Symbol;Acc:HGNC:17206]	1.78	0.0994
ssc-miR-323	TMEM30A	transmembrane protein 30A [Source:VGNC Symbol;Acc:VGNC:94170]	1.78	0.0994
ssc-miR-323	TMEM33	transmembrane protein 33 [Source:VGNC Symbol;Acc:VGNC:94171]	1.78	0.0994
ssc-miR-323	TMEM65	transmembrane protein 65 [Source:VGNC Symbol;Acc:VGNC:94200]	1.78	0.0994
ssc-miR-323	TMX1	thioredoxin related transmembrane protein 1 [Source:VGNC Symbol;Acc:VGNC:94247]	1.78	0.0994
ssc-miR-323	TNFSF11	TNF superfamily member 11 [Source:HGNC Symbol;Acc:HGNC:11926]	1.78	0.0994
ssc-miR-323	TNKS2	tankyrase 2 [Source:VGNC Symbol;Acc:VGNC:94280]	1.78	0.0994
ssc-miR-323	TP73	tumor protein p73 [Source:VGNC Symbol;Acc:VGNC:94331]	1.78	0.0994
ssc-miR-323	TRAPPC10	trafficking protein particle complex subunit 10 [Source:VGNC Symbol;Acc:VGNC:101497]	1.78	0.0994
ssc-miR-323	TRERF1	transcriptional regulating factor 1 [Source:VGNC Symbol;Acc:VGNC:94387]	1.78	0.0994
ssc-miR-323	TRPS1	transcriptional repressor GATA binding 1 [Source:VGNC Symbol;Acc:VGNC:94471]	1.78	0.0994
ssc-miR-323	TSC22D2	TSC22 domain family member 2 [Source:VGNC Symbol;Acc:VGNC:94482]	1.78	0.0994
ssc-miR-323	TSPAN3	tetraspanin 3 [Source:VGNC Symbol;Acc:VGNC:94510]	1.78	0.0994
ssc-miR-323	UBA2	hypothetical gene	1.78	0.0994
ssc-miR-323	UBALD2	UBA like domain containing 2 [Source:VGNC Symbol;Acc:VGNC:94633]	1.78	0.0994
ssc-miR-323	UBAP2	ubiquitin associated protein 2 [Source:VGNC Symbol;Acc:VGNC:95607]	1.78	0.0994
ssc-miR-323	UBE2B	ubiquitin conjugating enzyme E2 B [Source:VGNC Symbol;Acc:VGNC:94640]	1.78	0.0994
ssc-miR-323	UBE2F	hypothetical gene	1.78	0.0994
ssc-miR-323	UBE2K	ubiquitin conjugating enzyme E2 K [Source:VGNC Symbol;Acc:VGNC:94646]	1.78	0.0994
ssc-miR-323	UBE2Q1	ubiquitin conjugating enzyme E2 Q1 [Source:VGNC Symbol;Acc:VGNC:98888]	1.78	0.0994
ssc-miR-323	UBE2W	ubiquitin conjugating enzyme E2 W [Source:VGNC Symbol;Acc:VGNC:98890]	1.78	0.0994
ssc-miR-323	UBQLN2	ubiquilin 2 [Source:VGNC Symbol;Acc:VGNC:94665]	1.78	0.0994
ssc-miR-323	UHRF1BP1L	UHRF1 binding protein 1 like [Source:VGNC Symbol;Acc:VGNC:94691]	1.78	0.0994
ssc-miR-323	ULK2	unc-51 like autophagy activating kinase 2 [Source:VGNC Symbol;Acc:VGNC:94695]	1.78	0.0994
ssc-miR-323	UNC80	unc-80 homolog, NALCN channel complex subunit [Source:VGNC Symbol;Acc:VGNC:96134]	1.78	0.0994
ssc-miR-323	UQCRFS1	ubiquinol-cytochrome c reductase, Rieske iron-sulfur polypeptide 1 [Source:HGNC Symbol;Acc:HGNC:12587]	1.78	0.0994
ssc-miR-323	USP15	ubiquitin specific peptidase 15 [Source:VGNC Symbol;Acc:VGNC:94748]	1.78	0.0994
ssc-miR-323	USP6NL	USP6 N-terminal like [Source:VGNC Symbol;Acc:VGNC:95820]	1.78	0.0994
ssc-miR-323	VAPA	VAMP associated protein A [Source:VGNC Symbol;Acc:VGNC:94799]	1.78	0.0994
ssc-miR-323	VAPB	VAMP associated protein B and C [Source:VGNC Symbol;Acc:VGNC:108743]	1.78	0.0994
ssc-miR-323	VIL1	villin 1 [Source:VGNC Symbol;Acc:VGNC:95564]	1.78	0.0994
ssc-miR-323	VSNL1	visinin like 1 [Source:VGNC Symbol;Acc:VGNC:94871]	1.78	0.0994
ssc-miR-323	VSTM4	V-set and transmembrane domain containing 4 [Source:VGNC Symbol;Acc:VGNC:94874]	1.78	0.0994
ssc-miR-323	VWDE	von Willebrand factor D and EGF domains [Source:VGNC Symbol;Acc:VGNC:104093]	1.78	0.0994

ssc-miR-323	WEE1	WEE1 G2 checkpoint kinase [Source:VGNC Symbol;Acc:VGNC:104095]	1.78	0.0994
ssc-miR-323	WIPF3	WAS/WASL interacting protein family member 3 [Source:VGNC Symbol;Acc:VGNC:94958]	1.78	0.0994
ssc-miR-323	WT1	WT1 transcription factor [Source:VGNC Symbol;Acc:VGNC:100884]	1.78	0.0994
ssc-miR-323	XYLT1	xylosyltransferase 1 [Source:VGNC Symbol;Acc:VGNC:95015]	1.78	0.0994
ssc-miR-323	YAF2	YY1 associated factor 2 [Source:VGNC Symbol;Acc:VGNC:95018]	1.78	0.0994
ssc-miR-323	ZADH2	prostaglandin reductase 3 [Source:VGNC Symbol;Acc:VGNC:95049]	1.78	0.0994
ssc-miR-323	ZBTB10	zinc finger and BTB domain containing 10 [Source:VGNC Symbol;Acc:VGNC:95058]	1.78	0.0994
ssc-miR-323	ZBTB16	zinc finger and BTB domain containing 16 [Source:VGNC Symbol;Acc:VGNC:108626]	1.78	0.0994
ssc-miR-323	ZBTB20	zinc finger and BTB domain containing 20 [Source:VGNC Symbol;Acc:VGNC:95063]	1.78	0.0994
ssc-miR-323	ZC3H12B	zinc finger CCCH-type containing 12B [Source:VGNC Symbol;Acc:VGNC:95090]	1.78	0.0994
ssc-miR-323	ZC3H7B	zinc finger CCCH-type containing 7B [Source:VGNC Symbol;Acc:VGNC:95100]	1.78	0.0994
ssc-miR-323	ZCCHC14	zinc finger CCHC-type containing 14 [Source:VGNC Symbol;Acc:VGNC:95107]	1.78	0.0994
ssc-miR-323	ZFAND5	zinc finger AN1-type containing 5 [Source:VGNC Symbol;Acc:VGNC:95137]	1.78	0.0994
ssc-miR-323	ZFHX3	zinc finger homeobox 3 [Source:VGNC Symbol;Acc:VGNC:95141]	1.78	0.0994
ssc-miR-323	ZFP69B	ZFP69 zinc finger protein B [Source:HGNC Symbol;Acc:HGNC:28053]	1.78	0.0994
ssc-miR-323	ZFP91	ZFP91 zinc finger protein, atypical E3 ubiquitin ligase [Source:HGNC Symbol;Acc:HGNC:14983]	1.78	0.0994
ssc-miR-323	ZFPM2	zinc finger protein, FOG family member 2 [Source:VGNC Symbol;Acc:VGNC:95153]	1.78	0.0994
ssc-miR-323	ZMYM2	zinc finger MYM-type containing 2 [Source:VGNC Symbol;Acc:VGNC:95182]	1.78	0.0994
ssc-miR-323	ZNF148	zinc finger protein 148 [Source:VGNC Symbol;Acc:VGNC:95192]	1.78	0.0994
ssc-miR-323	ZNF207	zinc finger protein 207 [Source:VGNC Symbol;Acc:VGNC:95205]	1.78	0.0994
ssc-miR-323	ZNF24	zinc finger protein 24 [Source:VGNC Symbol;Acc:VGNC:98422]	1.78	0.0994
ssc-miR-323	ZNF280C	zinc finger protein 280C [Source:VGNC Symbol;Acc:VGNC:95216]	1.78	0.0994
ssc-miR-323	ZNF281	zinc finger protein 281 [Source:VGNC Symbol;Acc:VGNC:108285]	1.78	0.0994
ssc-miR-323	ZNF365	zinc finger protein 365 [Source:VGNC Symbol;Acc:VGNC:95230]	1.78	0.0994
ssc-miR-323	ZNF395	zinc finger protein 395 [Source:VGNC Symbol;Acc:VGNC:95235]	1.78	0.0994
ssc-miR-323	ZNF469	hypothetical gene	1.78	0.0994
ssc-miR-323	ZNF706	hypothetical gene	1.78	0.0994
ssc-miR-323	ZZZ3	zinc finger ZZ-type containing 3 [Source:VGNC Symbol;Acc:VGNC:95348]	1.78	0.0994
ssc-miR-133a-5p	NA	hypothetical gene	1.76	0.0941
ssc-miR-374b-3p	NA	hypothetical gene	1.75	0.0706
ssc-miR-345-5p	NA	hypothetical gene	1.72	0.0043
ssc-miR-376a-5p	NA	hypothetical gene	1.71	0.0444
ssc-miR-146a-3p	NA	hypothetical gene	-1.91	0.0282
ssc-miR-144	ABCA1	ATP binding cassette subfamily A member 1 [Source:VGNC Symbol;Acc:VGNC:84947]	-1.94	0.0167
ssc-miR-144	ABHD17B	abhydrolase domain containing 17B, depalmitoylase [Source:VGNC Symbol;Acc:VGNC:84977]	-1.94	0.0167
ssc-miR-144	ABHD17C	abhydrolase domain containing 17C, depalmitoylase [Source:VGNC Symbol;Acc:VGNC:84978]	-1.94	0.0167
ssc-miR-144	ABHD5	abhydrolase domain containing 5, lysophosphatidic acid acyltransferase [Source:VGNC Symbol;Acc:VGNC:97860]	-1.94	0.0167
ssc-miR-144	ABI2	abl interactor 2 [Source:VGNC Symbol;Acc:VGNC:96027]	-1.94	0.0167
ssc-miR-144	ABTB2	ankyrin repeat and BTB domain containing 2 [Source:VGNC Symbol;Acc:VGNC:84994]	-1.94	0.0167
ssc-miR-144	AC068987.1	hypothetical gene	-1.94	0.0167
ssc-miR-144	ACBD3	acyl-CoA binding domain containing 3 [Source:VGNC Symbol;Acc:VGNC:95641]	-1.94	0.0167
ssc-miR-144	ACBD5	acyl-CoA binding domain containing 5 [Source:VGNC Symbol;Acc:VGNC:95997]	-1.94	0.0167
ssc-miR-144	ACSL4	acyl-CoA synthetase long chain family member 4 [Source:VGNC Symbol;Acc:VGNC:85033]	-1.94	0.0167
ssc-miR-144	ACVR2B	activin A receptor type 2B [Source:VGNC Symbol;Acc:VGNC:108629]	-1.94	0.0167
ssc-miR-144	ADAMTS15	ADAM metallopeptidase with thrombospondin type 1 motif 15 [Source:VGNC Symbol;Acc:VGNC:85077]	-1.94	0.0167
ssc-miR-144	ADAMTS17	ADAM metallopeptidase with thrombospondin type 1 motif 17 [Source:VGNC Symbol;Acc:VGNC:85079]	-1.94	0.0167
ssc-miR-144	ADAMTS3	ADAM metallopeptidase with thrombospondin type 1 motif 3 [Source:VGNC Symbol;Acc:VGNC:85083]	-1.94	0.0167
ssc-miR-144	ADAMTS5	ADAM metallopeptidase with thrombospondin type 1 motif 5 [Source:VGNC Symbol;Acc:VGNC:85085]	-1.94	0.0167
ssc-miR-144	ADAMTSL3	ADAMTS like 3 [Source:VGNC Symbol;Acc:VGNC:85092]	-1.94	0.0167

ssc-miR-144	ADARB2	adenosine deaminase RNA specific B2 (inactive) [Source:HGNC Symbol;Acc:HGNC:227]	-1.94	0.0167
ssc-miR-144	ADCYAP1	adenylate cyclase activating polypeptide 1 [Source:VGNC Symbol;Acc:VGNC:85114]	-1.94	0.0167
ssc-miR-144	ADRB1	adrenoceptor beta 1 [Source:VGNC Symbol;Acc:VGNC:107363]	-1.94	0.0167
ssc-miR-144	AEBP2	AE binding protein 2 [Source:VGNC Symbol;Acc:VGNC:85163]	-1.94	0.0167
ssc-miR-144	AFAP1	actin filament associated protein 1 [Source:VGNC Symbol;Acc:VGNC:85165]	-1.94	0.0167
ssc-miR-144	AFF4	AF4/FMR2 family member 4 [Source:VGNC Symbol;Acc:VGNC:85169]	-1.94	0.0167
ssc-miR-144	AGFG1	ArfGAP with FG repeats 1 [Source:VGNC Symbol;Acc:VGNC:96359]	-1.94	0.0167
ssc-miR-144	AGO3	argonaute RISC component 1 [Source:NCBI gene (formerly Entrezgene);Acc:100499510]	-1.94	0.0167
ssc-miR-144	AGPAT3	1-acylglycerol-3-phosphate O-acyltransferase 3 [Source:VGNC Symbol;Acc:VGNC:85185]	-1.94	0.0167
ssc-miR-144	AGRN	agrin [Source:VGNC Symbol;Acc:VGNC:85188]	-1.94	0.0167
ssc-miR-144	AHCY	adenosylhomocysteinase [Source:VGNC Symbol;Acc:VGNC:108708]	-1.94	0.0167
ssc-miR-144	AHDC1	hypothetical gene	-1.94	0.0167
ssc-miR-144	AHRR	aryl-hydrocarbon receptor repressor [Source:VGNC Symbol;Acc:VGNC:103065]	-1.94	0.0167
ssc-miR-144	AJAP1	adherens junctions associated protein 1 [Source:VGNC Symbol;Acc:VGNC:85207]	-1.94	0.0167
ssc-miR-144	AKAP2	hypothetical gene	-1.94	0.0167
ssc-miR-144	AKIRIN1	akirin 1 [Source:VGNC Symbol;Acc:VGNC:85227]	-1.94	0.0167
ssc-miR-144	AL162407.1	hypothetical gene	-1.94	0.0167
ssc-miR-144	ALCAM	activated leukocyte cell adhesion molecule [Source:VGNC Symbol;Acc:VGNC:85236]	-1.94	0.0167
ssc-miR-144	ALDH1A3	aldehyde dehydrogenase 1 family member A3 [Source:VGNC Symbol;Acc:VGNC:97879]	-1.94	0.0167
ssc-miR-144	ALS2	alsin Rho guanine nucleotide exchange factor ALS2 [Source:VGNC Symbol;Acc:VGNC:96327]	-1.94	0.0167
ssc-miR-144	AMMECR1	AMMECR nuclear protein 1 [Source:VGNC Symbol;Acc:VGNC:96559]	-1.94	0.0167
ssc-miR-144	ANGPTL3	angiopoietin like 3 [Source:VGNC Symbol;Acc:VGNC:85305]	-1.94	0.0167
ssc-miR-144	ANK2	hypothetical gene	-1.94	0.0167
ssc-miR-144	ANK3	hypothetical gene	-1.94	0.0167
ssc-miR-144	ANKH	ANKH inorganic pyrophosphate transport regulator [Source:VGNC Symbol;Acc:VGNC:85315]	-1.94	0.0167
ssc-miR-144	ANKRD11	ankyrin repeat domain containing 11 [Source:VGNC Symbol;Acc:VGNC:85323]	-1.94	0.0167
ssc-miR-144	ANKRD12	ankyrin repeat domain 12 [Source:VGNC Symbol;Acc:VGNC:96906]	-1.94	0.0167
ssc-miR-144	ANKRD17	ankyrin repeat domain 17 [Source:VGNC Symbol;Acc:VGNC:85328]	-1.94	0.0167
ssc-miR-144	ANKRD28	ankyrin repeat domain 28 [Source:VGNC Symbol;Acc:VGNC:85331]	-1.94	0.0167
ssc-miR-144	ANKRD50	ankyrin repeat domain containing 50 [Source:VGNC Symbol;Acc:VGNC:85341]	-1.94	0.0167
ssc-miR-144	ANKRD52	ankyrin repeat domain 52 [Source:VGNC Symbol;Acc:VGNC:85342]	-1.94	0.0167
ssc-miR-144	ANKS1A	ankyrin repeat and sterile alpha motif domain containing 1A [Source:VGNC Symbol;Acc:VGNC:85348]	-1.94	0.0167
ssc-miR-144	ANO1	anoctamin 1 [Source:VGNC Symbol;Acc:VGNC:85354]	-1.94	0.0167
ssc-miR-144	ANTXR2	ANTXR cell adhesion molecule 2 [Source:VGNC Symbol;Acc:VGNC:85365]	-1.94	0.0167
ssc-miR-144	APAF1	apoptotic peptidase activating factor 1 [Source:VGNC Symbol;Acc:VGNC:85398]	-1.94	0.0167
ssc-miR-144	APBA2	amyloid beta protein binding family A member 2 [Source:VGNC Symbol;Acc:VGNC:85400]	-1.94	0.0167
ssc-miR-144	APC	APC regulator of WNT signaling pathway [Source:VGNC Symbol;Acc:VGNC:99584]	-1.94	0.0167
ssc-miR-144	APLP2	amyloid beta like protein 2 [Source:VGNC Symbol;Acc:VGNC:85414]	-1.94	0.0167
ssc-miR-144	APP	amyloid beta protein [Source:VGNC Symbol;Acc:VGNC:103902]	-1.94	0.0167
ssc-miR-144	APPBP2	amyloid beta protein binding protein 2 [Source:VGNC Symbol;Acc:VGNC:85425]	-1.94	0.0167
ssc-miR-144	APPL1	adaptor protein, phosphotyrosine interacting with PH domain and leucine zipper 1 [Source:VGNC Symbol;Acc:VGNC:85426]	-1.94	0.0167
ssc-miR-144	ARAP2	ArfGAP with RhoGAP domain, ankyrin repeat and PH domain 2 [Source:VGNC Symbol;Acc:VGNC:85440]	-1.94	0.0167
ssc-miR-144	ARFGEF1	ADP ribosylation factor guanine nucleotide exchange factor 1 [Source:VGNC Symbol;Acc:VGNC:85451]	-1.94	0.0167
ssc-miR-144	ARFGEF2	ADP ribosylation factor guanine nucleotide exchange factor 2 [Source:VGNC Symbol;Acc:VGNC:95672]	-1.94	0.0167
ssc-miR-144	ARHGAP18	Rho GTPase activating protein 18 [Source:VGNC Symbol;Acc:VGNC:85460]	-1.94	0.0167
ssc-miR-144	ARHGAP26	Rho GTPase activating protein 26 [Source:VGNC Symbol;Acc:VGNC:85465]	-1.94	0.0167
ssc-miR-144	ARHGAP5	Rho GTPase activating protein 5 [Source:VGNC Symbol;Acc:VGNC:85480]	-1.94	0.0167
ssc-miR-144	ARHGEF3	Rho guanine nucleotide exchange factor 3 [Source:VGNC Symbol;Acc:VGNC:85496]	-1.94	0.0167
ssc-miR-144	ARID1A	AT-rich interaction domain 1A [Source:VGNC Symbol;Acc:VGNC:85505]	-1.94	0.0167

ssc-miR-144	ARID2	AT-rich interaction domain 2 [Source:HGNC Symbol;Acc:HGNC:18037]	-1.94	0.0167
ssc-miR-144	ARID4B	AT-rich interaction domain 4B [Source:HGNC Symbol;Acc:HGNC:15550]	-1.94	0.0167
ssc-miR-144	ARID5B	AT-rich interaction domain 5B [Source:VGNC Symbol;Acc:VGNC:85511]	-1.94	0.0167
ssc-miR-144	ARL4C	ADP ribosylation factor like GTPase 4C [Source:VGNC Symbol;Acc:VGNC:96419]	-1.94	0.0167
ssc-miR-144	ARNTL2	aryl hydrocarbon receptor nuclear translocator like 2 [Source:VGNC Symbol;Acc:VGNC:103217]	-1.94	0.0167
ssc-miR-144	ARPP21	cAMP regulated phosphoprotein 21 [Source:VGNC Symbol;Acc:VGNC:85541]	-1.94	0.0167
ssc-miR-144	ARRDC3	arrestin domain containing 3 [Source:VGNC Symbol;Acc:VGNC:85546]	-1.94	0.0167
ssc-miR-144	ARSJ	arylsulfatase family member J [Source:VGNC Symbol;Acc:VGNC:85549]	-1.94	0.0167
ssc-miR-144	ASAP1	ArfGAP with SH3 domain, ankyrin repeat and PH domain 1 [Source:VGNC Symbol;Acc:VGNC:98734]	-1.94	0.0167
ssc-miR-144	ASH1L	ASH1 like histone lysine methyltransferase [Source:VGNC Symbol;Acc:VGNC:85577]	-1.94	0.0167
ssc-miR-144	ATAD2B	ATPase family AAA domain containing 2B [Source:VGNC Symbol;Acc:VGNC:85600]	-1.94	0.0167
ssc-miR-144	ATF7IP	activating transcription factor 7 interacting protein [Source:VGNC Symbol;Acc:VGNC:85611]	-1.94	0.0167
ssc-miR-144	ATG4D	autophagy related 4D cysteine peptidase [Source:VGNC Symbol;Acc:VGNC:85624]	-1.94	0.0167
ssc-miR-144	ATP1B1	ATPase Na+/K+ transporting subunit beta 1 [Source:VGNC Symbol;Acc:VGNC:85642]	-1.94	0.0167
ssc-miR-144	ATP1B2	ATPase Na+/K+ transporting subunit beta 2 [Source:VGNC Symbol;Acc:VGNC:85643]	-1.94	0.0167
ssc-miR-144	ATP2B1	ATPase plasma membrane Ca2+ transporting 1 [Source:VGNC Symbol;Acc:VGNC:103219]	-1.94	0.0167
ssc-miR-144	ATP2B2	ATPase plasma membrane Ca2+ transporting 2 [Source:VGNC Symbol;Acc:VGNC:85649]	-1.94	0.0167
ssc-miR-144	ATP2C1	ATPase secretory pathway Ca2+ transporting 1 [Source:VGNC Symbol;Acc:VGNC:85652]	-1.94	0.0167
ssc-miR-144	ATP5G2	hypothetical gene	-1.94	0.0167
ssc-miR-144	ATP8A1	ATPase phospholipid transporting 8A1 [Source:VGNC Symbol;Acc:VGNC:97894]	-1.94	0.0167
ssc-miR-144	ATRX	ATRX chromatin remodeler [Source:VGNC Symbol;Acc:VGNC:85686]	-1.94	0.0167
ssc-miR-144	ATXN1	ataxin 1 [Source:VGNC Symbol;Acc:VGNC:85687]	-1.94	0.0167
ssc-miR-144	ATXN1L	ataxin 1 like [Source:VGNC Symbol;Acc:VGNC:85689]	-1.94	0.0167
ssc-miR-144	AXIN2	axin 2 [Source:VGNC Symbol;Acc:VGNC:85708]	-1.94	0.0167
ssc-miR-144	B3GALNT2	beta-1,3-N-acetylgalactosaminyltransferase 2 [Source:VGNC Symbol;Acc:VGNC:85715]	-1.94	0.0167
ssc-miR-144	B3GALT6	beta-1,3-galactosyltransferase 6 [Source:VGNC Symbol;Acc:VGNC:85718]	-1.94	0.0167
ssc-miR-144	B3GAT2	beta-1,3-glucuronyltransferase 2 [Source:VGNC Symbol;Acc:VGNC:85720]	-1.94	0.0167
ssc-miR-144	BACH2	BTB domain and CNC homolog 2 [Source:VGNC Symbol;Acc:VGNC:85742]	-1.94	0.0167
ssc-miR-144	BAZ2B	bromodomain adjacent to zinc finger domain 2B [Source:HGNC Symbol;Acc:HGNC:963]	-1.94	0.0167
ssc-miR-144	BBC3	BCL2 binding component 3 [Source:HGNC Symbol;Acc:HGNC:17868]	-1.94	0.0167
ssc-miR-144	BBX	BBX high mobility group box domain containing [Source:VGNC Symbol;Acc:VGNC:85766]	-1.94	0.0167
ssc-miR-144	BCL11B	BAF chromatin remodeling complex subunit BCL11B [Source:VGNC Symbol;Acc:VGNC:96563]	-1.94	0.0167
ssc-miR-144	BCL2L11	BCL2 like 11 [Source:NCBI gene (formerly Entrezgene);Acc:396632]	-1.94	0.0167
ssc-miR-144	BCL2L2-PABPN1	hypothetical gene	-1.94	0.0167
ssc-miR-144	BCL6	BCL6 transcription repressor [Source:VGNC Symbol;Acc:VGNC:96565]	-1.94	0.0167
ssc-miR-144	BCL9	BCL9 transcription coactivator [Source:VGNC Symbol;Acc:VGNC:96567]	-1.94	0.0167
ssc-miR-144	BCL9L	BCL9 like [Source:VGNC Symbol;Acc:VGNC:96568]	-1.94	0.0167
ssc-miR-144	BEAN1	brain expressed associated with NEDD4 1 [Source:VGNC Symbol;Acc:VGNC:85797]	-1.94	0.0167
ssc-miR-144	BEGAIN	brain enriched guanylate kinase associated [Source:VGNC Symbol;Acc:VGNC:85799]	-1.94	0.0167
ssc-miR-144	BEND3	BEN domain containing 3 [Source:VGNC Symbol;Acc:VGNC:85800]	-1.94	0.0167
ssc-miR-144	BET1	Bet1 golgi vesicular membrane trafficking protein [Source:VGNC Symbol;Acc:VGNC:85807]	-1.94	0.0167
ssc-miR-144	BHLHE41	basic helix-loop-helix family member e41 [Source:VGNC Symbol;Acc:VGNC:85815]	-1.94	0.0167
ssc-miR-144	BICC1	BicC family RNA binding protein 1 [Source:VGNC Symbol;Acc:VGNC:85817]	-1.94	0.0167
ssc-miR-144	BICD2	BICD cargo adaptor 2 [Source:VGNC Symbol;Acc:VGNC:85819]	-1.94	0.0167
ssc-miR-144	BLOC1S5	biosis of lysosomal organelles complex 1 subunit 5 [Source:NCBI gene (formerly Entrezgene);Acc:100049677]	-1.94	0.0167
ssc-miR-144	BMPR1A	bone morphotic protein receptor type 1A [Source:VGNC Symbol;Acc:VGNC:85846]	-1.94	0.0167
ssc-miR-144	BMPR1B	bone morphotic protein receptor type 1B [Source:VGNC Symbol;Acc:VGNC:85847]	-1.94	0.0167
ssc-miR-144	BNC2	basonuclin 2 [Source:VGNC Symbol;Acc:VGNC:85852]	-1.94	0.0167
ssc-miR-144	BRCC3	BRCA1/BRCA2-containing complex subunit 3 [Source:VGNC Symbol;Acc:VGNC:85870]	-1.94	0.0167

ssc-miR-144	BRPF1	bromodomain and PHD finger containing 1 [Source:VGNC Symbol;Acc:VGNC:85885]	-1.94	0.0167
ssc-miR-144	BRPF3	bromodomain and PHD finger containing 3 [Source:VGNC Symbol;Acc:VGNC:85886]	-1.94	0.0167
ssc-miR-144	BRSK2	BR serine/threonine kinase 2 [Source:VGNC Symbol;Acc:VGNC:85889]	-1.94	0.0167
ssc-miR-144	BRWD3	bromodomain and WD repeat domain containing 3 [Source:VGNC Symbol;Acc:VGNC:85890]	-1.94	0.0167
ssc-miR-144	BTAF1	B-TFIID TATA-box binding protein associated factor 1 [Source:VGNC Symbol;Acc:VGNC:85899]	-1.94	0.0167
ssc-miR-144	BTBD3	BTB domain containing 3 [Source:VGNC Symbol;Acc:VGNC:95673]	-1.94	0.0167
ssc-miR-144	BZW1	basic leucine zipper and W2 domains 1 [Source:VGNC Symbol;Acc:VGNC:95885]	-1.94	0.0167
ssc-miR-144	C10orf118	hypothetical gene	-1.94	0.0167
ssc-miR-144	C10orf12	hypothetical gene	-1.94	0.0167
ssc-miR-144	C11orf30	hypothetical gene	-1.94	0.0167
ssc-miR-144	C12orf5	hypothetical gene	-1.94	0.0167
ssc-miR-144	C19orf73	hypothetical gene	-1.94	0.0167
ssc-miR-144	C19orf81	chromosome 6 C19orf81 homolog [Source:VGNC Symbol;Acc:VGNC:86058]	-1.94	0.0167
ssc-miR-144	C20orf194	hypothetical gene	-1.94	0.0167
ssc-miR-144	C2CD2	C2 calcium dependent domain containing 2 [Source:VGNC Symbol;Acc:VGNC:85990]	-1.94	0.0167
ssc-miR-144	C3orf58	hypothetical gene	-1.94	0.0167
ssc-miR-144	C5orf24	chromosome 2 C5orf24 homolog [Source:VGNC Symbol;Acc:VGNC:86010]	-1.94	0.0167
ssc-miR-144	C6orf62	chromosome 7 C6orf62 homolog [Source:VGNC Symbol;Acc:VGNC:86074]	-1.94	0.0167
ssc-miR-144	C8orf44-SGK3	hypothetical gene	-1.94	0.0167
ssc-miR-144	CACNA2D1	calcium voltage-gated channel auxiliary subunit alpha2delta 1 [Source:VGNC Symbol;Acc:VGNC:86120]	-1.94	0.0167
ssc-miR-144	CACNB2	calcium voltage-gated channel auxiliary subunit beta 2 [Source:VGNC Symbol;Acc:VGNC:95599]	-1.94	0.0167
ssc-miR-144	CADM1	cell adhesion molecule 1 [Source:VGNC Symbol;Acc:VGNC:86134]	-1.94	0.0167
ssc-miR-144	CALB1	calbindin 1 [Source:VGNC Symbol;Acc:VGNC:86138]	-1.94	0.0167
ssc-miR-144	CALCRL	calcitonin receptor like receptor [Source:NCBI gene (formerly Entrezgene);Acc:397172]	-1.94	0.0167
ssc-miR-144	CAMK2D	calcium/calmodulin dependent protein kinase II delta [Source:VGNC Symbol;Acc:VGNC:97911]	-1.94	0.0167
ssc-miR-144	CAMKK2	calcium/calmodulin dependent protein kinase kinase 2 [Source:VGNC Symbol;Acc:VGNC:86158]	-1.94	0.0167
ssc-miR-144	CAMSAP2	calmodulin regulated spectrin associated protein family member 2 [Source:VGNC Symbol;Acc:VGNC:96205]	-1.94	0.0167
ssc-miR-144	CAND1	cullin associated and neddylation dissociated 1 [Source:VGNC Symbol;Acc:VGNC:97912]	-1.94	0.0167
ssc-miR-144	CAPN7	calpain 7 [Source:NCBI gene (formerly Entrezgene);Acc:100037936]	-1.94	0.0167
ssc-miR-144	CAPZA1	capping actin protein of muscle Z-line subunit alpha 1 [Source:VGNC Symbol;Acc:VGNC:86183]	-1.94	0.0167
ssc-miR-144	CASC4	hypothetical gene	-1.94	0.0167
ssc-miR-144	CASK	calcium/calmodulin dependent serine protein kinase [Source:HGNC Symbol;Acc:HGNC:1497]	-1.94	0.0167
ssc-miR-144	CAV2	caveolin 2 [Source:VGNC Symbol;Acc:VGNC:103914]	-1.94	0.0167
ssc-miR-144	CAV3	caveolin 3 [Source:NCBI gene (formerly Entrezgene);Acc:641358]	-1.94	0.0167
ssc-miR-144	CBFA2T2	CBFA2/RUNX1 partner transcriptional co-repressor 2 [Source:VGNC Symbol;Acc:VGNC:96573]	-1.94	0.0167
ssc-miR-144	CBLL1	Cbl proto-onco like 1 [Source:VGNC Symbol;Acc:VGNC:108573]	-1.94	0.0167
ssc-miR-144	CBX4	chromobox 4 [Source:VGNC Symbol;Acc:VGNC:86231]	-1.94	0.0167
ssc-miR-144	CBX5	chromobox 5 [Source:VGNC Symbol;Acc:VGNC:86232]	-1.94	0.0167
ssc-miR-144	CCDC144A	hypothetical gene	-1.94	0.0167
ssc-miR-144	CCDC85A	coiled-coil domain containing 85A [Source:VGNC Symbol;Acc:VGNC:86320]	-1.94	0.0167
ssc-miR-144	CCDC88A	coiled-coil domain containing 88A [Source:VGNC Symbol;Acc:VGNC:86325]	-1.94	0.0167
ssc-miR-144	CCNE2	cyclin E2 [Source:VGNC Symbol;Acc:VGNC:86355]	-1.94	0.0167
ssc-miR-144	CCNG2	cyclin G2 [Source:VGNC Symbol;Acc:VGNC:86358]	-1.94	0.0167
ssc-miR-144	CCNK	cyclin K [Source:VGNC Symbol;Acc:VGNC:86362]	-1.94	0.0167
ssc-miR-144	CCNT2	cyclin T2 [Source:VGNC Symbol;Acc:VGNC:103916]	-1.94	0.0167
ssc-miR-144	CCRN4L	hypothetical gene	-1.94	0.0167
ssc-miR-144	CCSER2	coiled-coil serine rich protein 2 [Source:VGNC Symbol;Acc:VGNC:86379]	-1.94	0.0167
ssc-miR-144	CD34	CD34 molecule [Source:VGNC Symbol;Acc:VGNC:86410]	-1.94	0.0167
ssc-miR-144	CDC42EP3	CDC42 effector protein 3 [Source:VGNC Symbol;Acc:VGNC:86457]	-1.94	0.0167

ssc-miR-144	CDC73	cell division cycle 73 [Source:VGNC Symbol;Acc:VGNC:95817]	-1.94	0.0167
ssc-miR-144	CDH11	cadherin 11 [Source:VGNC Symbol;Acc:VGNC:86475]	-1.94	0.0167
ssc-miR-144	CDH2	cadherin 2 [Source:VGNC Symbol;Acc:VGNC:86483]	-1.94	0.0167
ssc-miR-144	CDH20	cadherin 20 [Source:VGNC Symbol;Acc:VGNC:86484]	-1.94	0.0167
ssc-miR-144	CDH5	cadherin 5 [Source:VGNC Symbol;Acc:VGNC:86486]	-1.94	0.0167
ssc-miR-144	CDH6	cadherin 6 [Source:HGNC Symbol;Acc:HGNC:1765]	-1.94	0.0167
ssc-miR-144	CDH8	cadherin 8 [Source:VGNC Symbol;Acc:VGNC:86487]	-1.94	0.0167
ssc-miR-144	CDK17	cyclin dependent kinase 17 [Source:VGNC Symbol;Acc:VGNC:86500]	-1.94	0.0167
ssc-miR-144	CDK19	cyclin dependent kinase 19 [Source:VGNC Symbol;Acc:VGNC:86502]	-1.94	0.0167
ssc-miR-144	CDK8	cyclin dependent kinase 8 [Source:VGNC Symbol;Acc:VGNC:86507]	-1.94	0.0167
ssc-miR-144	CDR2	cerebellar deration related protein 2 [Source:VGNC Symbol;Acc:VGNC:86521]	-1.94	0.0167
ssc-miR-144	CDS2	CDP-diacylglycerol synthase 2 [Source:VGNC Symbol;Acc:VGNC:95869]	-1.94	0.0167
ssc-miR-144	CDV3	CDV3 homolog [Source:VGNC Symbol;Acc:VGNC:86525]	-1.94	0.0167
ssc-miR-144	CDYL	chromodomain Y like [Source:HGNC Symbol;Acc:HGNC:1811]	-1.94	0.0167
ssc-miR-144	CEBPA	CCAAT enhancer binding protein alpha [Source:VGNC Symbol;Acc:VGNC:86531]	-1.94	0.0167
ssc-miR-144	CECR2	CECR2 histone acetyl-lysine reader [Source:VGNC Symbol;Acc:VGNC:96576]	-1.94	0.0167
ssc-miR-144	CELF2	hypothetical gene	-1.94	0.0167
ssc-miR-144	CEP135	centrosomal protein 135 [Source:VGNC Symbol;Acc:VGNC:86561]	-1.94	0.0167
ssc-miR-144	CEP170	centrosomal protein 170 [Source:VGNC Symbol;Acc:VGNC:97925]	-1.94	0.0167
ssc-miR-144	CEP55	centrosomal protein 55 [Source:VGNC Symbol;Acc:VGNC:86572]	-1.94	0.0167
ssc-miR-144	CFL2	cofilin 2 [Source:VGNC Symbol;Acc:VGNC:86611]	-1.94	0.0167
ssc-miR-144	CGGBP1	CGG triplet repeat binding protein 1 [Source:VGNC Symbol;Acc:VGNC:86615]	-1.94	0.0167
ssc-miR-144	CHIC2	cysteine rich hydrophobic domain 2 [Source:VGNC Symbol;Acc:VGNC:86643]	-1.94	0.0167
ssc-miR-144	CHN2	chimerin 2 [Source:HGNC Symbol;Acc:HGNC:1944]	-1.94	0.0167
ssc-miR-144	CHST11	carbohydrate sulfotransferase 11 [Source:VGNC Symbol;Acc:VGNC:86675]	-1.94	0.0167
ssc-miR-144	CHTOP	chromatin target of PRMT1 [Source:VGNC Symbol;Acc:VGNC:86687]	-1.94	0.0167
ssc-miR-144	CITED2	Cbp/p300 interacting transactivator with Glu/Asp rich carboxy-terminal domain 2 [Source:VGNC Symbol;Acc:VGNC:86712]	-1.94	0.0167
ssc-miR-144	CLK4	CDC like kinase 4 [Source:VGNC Symbol;Acc:VGNC:86768]	-1.94	0.0167
ssc-miR-144	CLMP	CXADR like membrane protein [Source:VGNC Symbol;Acc:VGNC:86770]	-1.94	0.0167
ssc-miR-144	CNN3	calponin 3 [Source:VGNC Symbol;Acc:VGNC:86826]	-1.94	0.0167
ssc-miR-144	CNNM2	cyclin and CBS domain divalent metal cation transport mediator 2 [Source:VGNC Symbol;Acc:VGNC:86828]	-1.94	0.0167
ssc-miR-144	CNOT1	CCR4-NOT transcription complex subunit 1 [Source:VGNC Symbol;Acc:VGNC:86831]	-1.94	0.0167
ssc-miR-144	CNTN3	contactin 3 [Source:VGNC Symbol;Acc:VGNC:108642]	-1.94	0.0167
ssc-miR-144	COL11A1	collagen type XI alpha 1 chain [Source:VGNC Symbol;Acc:VGNC:86862]	-1.94	0.0167
ssc-miR-144	COL5A2	collagen type V alpha 2 chain [Source:VGNC Symbol;Acc:VGNC:95981]	-1.94	0.0167
ssc-miR-144	COL8A1	collagen type VIII alpha 1 chain [Source:VGNC Symbol;Acc:VGNC:86880]	-1.94	0.0167
ssc-miR-144	COPS2	COP9 signalosome subunit 2 [Source:VGNC Symbol;Acc:VGNC:86899]	-1.94	0.0167
ssc-miR-144	COX7C	cytochrome c oxidase subunit 7C [Source:VGNC Symbol;Acc:VGNC:99628]	-1.94	0.0167
ssc-miR-144	CPEB1	cytoplasmic polyadenylation element binding protein 1 [Source:NCBI gene (formerly Entrezgene);Acc:100048944]	-1.94	0.0167
ssc-miR-144	CPEB2	cytoplasmic polyadenylation element binding protein 2 [Source:VGNC Symbol;Acc:VGNC:86937]	-1.94	0.0167
ssc-miR-144	CPEB3	cytoplasmic polyadenylation element binding protein 3 [Source:VGNC Symbol;Acc:VGNC:86938]	-1.94	0.0167
ssc-miR-144	CPNE2	copine 2 [Source:VGNC Symbol;Acc:VGNC:86950]	-1.94	0.0167
ssc-miR-144	CREB1	cAMP responsive element binding protein 1 [Source:VGNC Symbol;Acc:VGNC:96004]	-1.94	0.0167
ssc-miR-144	CREBRF	CREB3 regulatory factor [Source:VGNC Symbol;Acc:VGNC:86987]	-1.94	0.0167
ssc-miR-144	CREBZF	CREB/ATF bZIP transcription factor [Source:VGNC Symbol;Acc:VGNC:86988]	-1.94	0.0167
ssc-miR-144	CRISPLD1	cysteine rich secretory protein LCCL domain containing 1 [Source:VGNC Symbol;Acc:VGNC:97945]	-1.94	0.0167
ssc-miR-144	CRK	CRK proto-onco, adaptor protein [Source:NCBI gene (formerly Entrezgene);Acc:100192444]	-1.94	0.0167
ssc-miR-144	CRTC1	CREB regulated transcription coactivator 1 [Source:VGNC Symbol;Acc:VGNC:87007]	-1.94	0.0167
ssc-miR-144	CSNK1A1	casein kinase 1 alpha 1 [Source:VGNC Symbol;Acc:VGNC:99630]	-1.94	0.0167

ssc-miR-144	CSNK1D	casein kinase 1 delta [Source:VGNC Symbol;Acc:VGNC:97948]	-1.94	0.0167
ssc-miR-144	CSRNP2	cysteine and serine rich nuclear protein 2 [Source:VGNC Symbol;Acc:VGNC:87047]	-1.94	0.0167
ssc-miR-144	CTBP2	C-terminal binding protein 2 [Source:VGNC Symbol;Acc:VGNC:87054]	-1.94	0.0167
ssc-miR-144	CTDSPL2	CTD small phosphatase like 2 [Source:VGNC Symbol;Acc:VGNC:87060]	-1.94	0.0167
ssc-miR-144	CUL3	cullin 3 [Source:VGNC Symbol;Acc:VGNC:96091]	-1.94	0.0167
ssc-miR-144	CUL4A	cullin 4A [Source:VGNC Symbol;Acc:VGNC:87088]	-1.94	0.0167
ssc-miR-144	CUL4B	cullin 4B [Source:HGNC Symbol;Acc:HGNC:2555]	-1.94	0.0167
ssc-miR-144	CUL5	cullin 5 [Source:VGNC Symbol;Acc:VGNC:87089]	-1.94	0.0167
ssc-miR-144	CXorf23	hypothetical gene	-1.94	0.0167
ssc-miR-144	CXXC4	CXXC finger protein 4 [Source:HGNC Symbol;Acc:HGNC:24593]	-1.94	0.0167
ssc-miR-144	CXXC5	CXXC finger protein 5 [Source:VGNC Symbol;Acc:VGNC:87118]	-1.94	0.0167
ssc-miR-144	CYB5B	cytochrome b5 type B [Source:VGNC Symbol;Acc:VGNC:96728]	-1.94	0.0167
ssc-miR-144	CYP24A1	cytochrome P450 family 24 subfamily A member 1 [Source:VGNC Symbol;Acc:VGNC:103371]	-1.94	0.0167
ssc-miR-144	CYP27C1	hypothetical gene	-1.94	0.0167
ssc-miR-144	CYTH3	cytohesin 3 [Source:VGNC Symbol;Acc:VGNC:87136]	-1.94	0.0167
ssc-miR-144	DACT1	dishevelled binding antagonist of beta catenin 1 [Source:VGNC Symbol;Acc:VGNC:87147]	-1.94	0.0167
ssc-miR-144	DACT3	dishevelled binding antagonist of beta catenin 3 [Source:VGNC Symbol;Acc:VGNC:87149]	-1.94	0.0167
ssc-miR-144	DAZ1	hypothetical gene	-1.94	0.0167
ssc-miR-144	DAZ2	hypothetical gene	-1.94	0.0167
ssc-miR-144	DAZ3	hypothetical gene	-1.94	0.0167
ssc-miR-144	DAZ4	hypothetical gene	-1.94	0.0167
ssc-miR-144	DAZAP2	DAZ associated protein 2 [Source:VGNC Symbol;Acc:VGNC:87162]	-1.94	0.0167
ssc-miR-144	DAZL	hypothetical gene	-1.94	0.0167
ssc-miR-144	DCAF7	DDB1 and CUL4 associated factor 7 [Source:VGNC Symbol;Acc:VGNC:87178]	-1.94	0.0167
ssc-miR-144	DCBLD2	discoidin, CUB and LCCL domain containing 2 [Source:VGNC Symbol;Acc:VGNC:87180]	-1.94	0.0167
ssc-miR-144	DCLRE1C	DNA cross-link repair 1C [Source:VGNC Symbol;Acc:VGNC:108268]	-1.94	0.0167
ssc-miR-144	DCTN2	dynactin subunit 2 [Source:VGNC Symbol;Acc:VGNC:87193]	-1.94	0.0167
ssc-miR-144	DDHD1	DDHD domain containing 1 [Source:VGNC Symbol;Acc:VGNC:87205]	-1.94	0.0167
ssc-miR-144	DENND5B	DENN domain containing 5B [Source:VGNC Symbol;Acc:VGNC:87255]	-1.94	0.0167
ssc-miR-144	DGCR2	DiGeorge syndrome critical region gene 2 [Source:HGNC Symbol;Acc:HGNC:2845]	-1.94	0.0167
ssc-miR-144	DICER1	dicer 1, ribonuclease III [Source:VGNC Symbol;Acc:VGNC:87302]	-1.94	0.0167
ssc-miR-144	DIP2B	disco interacting B [Source:VGNC Symbol;Acc:VGNC:87306]	-1.94	0.0167
ssc-miR-144	DIP2C	disco interacting C [Source:VGNC Symbol;Acc:VGNC:96201]	-1.94	0.0167
ssc-miR-144	DLC1	DLC1 Rho GTPase activating protein [Source:VGNC Symbol;Acc:VGNC:96191]	-1.94	0.0167
ssc-miR-144	DLG5	discs large MAGUK scaffold protein 5 [Source:VGNC Symbol;Acc:VGNC:87329]	-1.94	0.0167
ssc-miR-144	DLGAP3	DLG associated protein 3 [Source:VGNC Symbol;Acc:VGNC:87331]	-1.94	0.0167
ssc-miR-144	DMD	dystrophin [Source:NCBI gene (formerly Entrezgene);Acc:497636]	-1.94	0.0167
ssc-miR-144	DNAJC16	DnaJ heat shock protein family (Hsp40) member C16 [Source:VGNC Symbol;Acc:VGNC:96624]	-1.94	0.0167
ssc-miR-144	DNAJC2	DnaJ heat shock protein family (Hsp40) member C2 [Source:VGNC Symbol;Acc:VGNC:108583]	-1.94	0.0167
ssc-miR-144	DNAJC6	DnaJ heat shock protein family (Hsp40) member C6 [Source:VGNC Symbol;Acc:VGNC:96625]	-1.94	0.0167
ssc-miR-144	DNMT3A	DNA methyltransferase 3 alpha [Source:VGNC Symbol;Acc:VGNC:87384]	-1.94	0.0167
ssc-miR-144	DOCK3	dedicator of cytokinesis 3 [Source:VGNC Symbol;Acc:VGNC:87393]	-1.94	0.0167
ssc-miR-144	DOK4	docking protein 4 [Source:VGNC Symbol;Acc:VGNC:87403]	-1.94	0.0167
ssc-miR-144	DOK6	docking protein 6 [Source:VGNC Symbol;Acc:VGNC:87404]	-1.94	0.0167
ssc-miR-144	DPP10	dipeptidyl peptidase like 10 [Source:VGNC Symbol;Acc:VGNC:95949]	-1.94	0.0167
ssc-miR-144	DPP8	dipeptidyl peptidase 8 [Source:VGNC Symbol;Acc:VGNC:87423]	-1.94	0.0167
ssc-miR-144	DPYSL5	dihydropyrimidinase like 5 [Source:VGNC Symbol;Acc:VGNC:87433]	-1.94	0.0167
ssc-miR-144	DR1	down-regulator of transcription 1 [Source:VGNC Symbol;Acc:VGNC:87435]	-1.94	0.0167
ssc-miR-144	DRP2	dystrophin related protein 2 [Source:VGNC Symbol;Acc:VGNC:87451]	-1.94	0.0167

ssc-miR-144	DST	dystonin [Source:HGNC Symbol;Acc:HGNC:1090]	-1.94	0.0167
ssc-miR-144	DSTYK	dual serine/threonine and tyrosine protein kinase [Source:VGNC Symbol;Acc:VGNC:87460]	-1.94	0.0167
ssc-miR-144	DTNA	dystrobrevin alpha [Source:VGNC Symbol;Acc:VGNC:87462]	-1.94	0.0167
ssc-miR-144	DUSP1	dual specificity phosphatase 1 [Source:VGNC Symbol;Acc:VGNC:87476]	-1.94	0.0167
ssc-miR-144	DUSP16	dual specificity phosphatase 16 [Source:VGNC Symbol;Acc:VGNC:87480]	-1.94	0.0167
ssc-miR-144	DVL1	dishevelled segment polarity protein 1 [Source:VGNC Symbol;Acc:VGNC:96985]	-1.94	0.0167
ssc-miR-144	DYRK1A	dual specificity tyrosine phosphorylation regulated kinase 1A [Source:VGNC Symbol;Acc:VGNC:87505]	-1.94	0.0167
ssc-miR-144	DYRK2	dual specificity tyrosine phosphorylation regulated kinase 2 [Source:VGNC Symbol;Acc:VGNC:87507]	-1.94	0.0167
ssc-miR-144	E2F3	E2F transcription factor 3 [Source:VGNC Symbol;Acc:VGNC:87514]	-1.94	0.0167
ssc-miR-144	E2F8	E2F transcription factor 8 [Source:VGNC Symbol;Acc:VGNC:87519]	-1.94	0.0167
ssc-miR-144	EBF1	EBF transcription factor 1 [Source:VGNC Symbol;Acc:VGNC:87525]	-1.94	0.0167
ssc-miR-144	EBF2	EBF transcription factor 2 [Source:VGNC Symbol;Acc:VGNC:87526]	-1.94	0.0167
ssc-miR-144	ECE1	endothelin converting enzyme 1 [Source:VGNC Symbol;Acc:VGNC:87531]	-1.94	0.0167
ssc-miR-144	EDA	ectodysplasin A [Source:VGNC Symbol;Acc:VGNC:87540]	-1.94	0.0167
ssc-miR-144	EDAR	ectodysplasin A receptor [Source:VGNC Symbol;Acc:VGNC:87541]	-1.94	0.0167
ssc-miR-144	EFCAB14	EF-hand calcium binding domain 14 [Source:VGNC Symbol;Acc:VGNC:87563]	-1.94	0.0167
ssc-miR-144	EFNA2	ephrin A2 [Source:VGNC Symbol;Acc:VGNC:87573]	-1.94	0.0167
ssc-miR-144	EFNA5	ephrin A5 [Source:VGNC Symbol;Acc:VGNC:87575]	-1.94	0.0167
ssc-miR-144	EFNB2	ephrin B2 [Source:VGNC Symbol;Acc:VGNC:87577]	-1.94	0.0167
ssc-miR-144	EFR3A	EFR3 homolog A [Source:VGNC Symbol;Acc:VGNC:87579]	-1.94	0.0167
ssc-miR-144	EGFR	epidermal growth factor receptor [Source:VGNC Symbol;Acc:VGNC:87587]	-1.94	0.0167
ssc-miR-144	EHMT1	euchromatic histone lysine methyltransferase 1 [Source:VGNC Symbol;Acc:VGNC:107138]	-1.94	0.0167
ssc-miR-144	EIF4G2	eukaryotic translation initiation factor 4 gamma 2 [Source:VGNC Symbol;Acc:VGNC:99645]	-1.94	0.0167
ssc-miR-144	EIF5	hypothetical gene	-1.94	0.0167
ssc-miR-144	EIF5A2	eukaryotic translation initiation factor 5A2 [Source:VGNC Symbol;Acc:VGNC:87633]	-1.94	0.0167
ssc-miR-144	ELAVL2	ELAV like RNA binding protein 2 [Source:VGNC Symbol;Acc:VGNC:87639]	-1.94	0.0167
ssc-miR-144	ELF5	E74 like ETS transcription factor 5 [Source:VGNC Symbol;Acc:VGNC:87644]	-1.94	0.0167
ssc-miR-144	ELFN2	extracellular leucine rich repeat and fibronectin type III domain containing 2 [Source:VGNC Symbol;Acc:VGNC:103232]	-1.94	0.0167
ssc-miR-144	ELL	elongation factor for RNA polymerase II [Source:VGNC Symbol;Acc:VGNC:87648]	-1.94	0.0167
ssc-miR-144	ELL2	elongation factor for RNA polymerase II 2 [Source:VGNC Symbol;Acc:VGNC:87649]	-1.94	0.0167
ssc-miR-144	ELN	elastin [Source:VGNC Symbol;Acc:VGNC:87655]	-1.94	0.0167
ssc-miR-144	ELP4	elongator acetyltransferase complex subunit 4 [Source:VGNC Symbol;Acc:VGNC:87665]	-1.94	0.0167
ssc-miR-144	EMC7	ER membrane protein complex subunit 7 [Source:VGNC Symbol;Acc:VGNC:87672]	-1.94	0.0167
ssc-miR-144	EMP1	epithelial membrane protein 1 [Source:VGNC Symbol;Acc:VGNC:87688]	-1.94	0.0167
ssc-miR-144	EMP2	epithelial membrane protein 2 [Source:VGNC Symbol;Acc:VGNC:87689]	-1.94	0.0167
ssc-miR-144	ENAH	ENAH actin regulator [Source:VGNC Symbol;Acc:VGNC:108271]	-1.94	0.0167
ssc-miR-144	EPB41	erythrocyte membrane protein band 4.1 [Source:VGNC Symbol;Acc:VGNC:87722]	-1.94	0.0167
ssc-miR-144	EPB41L2	erythrocyte membrane protein band 4.1 like 2 [Source:VGNC Symbol;Acc:VGNC:87723]	-1.94	0.0167
ssc-miR-144	EPB41L4A	erythrocyte membrane protein band 4.1 like 4A [Source:HGNC Symbol;Acc:HGNC:13278]	-1.94	0.0167
ssc-miR-144	EPHA5	EPH receptor A5 [Source:VGNC Symbol;Acc:VGNC:87733]	-1.94	0.0167
ssc-miR-144	EPHA7	EPH receptor A7 [Source:VGNC Symbol;Acc:VGNC:87734]	-1.94	0.0167
ssc-miR-144	EPN2	epsin 2 [Source:VGNC Symbol;Acc:VGNC:97982]	-1.94	0.0167
ssc-miR-144	EPS8	epidermal growth factor receptor pathway substrate 8 [Source:VGNC Symbol;Acc:VGNC:87749]	-1.94	0.0167
ssc-miR-144	ERBB4	erb-b2 receptor tyrosine kinase 4 [Source:VGNC Symbol;Acc:VGNC:96284]	-1.94	0.0167
ssc-miR-144	ERN1	endoplasmic reticulum to nucleus signaling 1 [Source:HGNC Symbol;Acc:HGNC:3449]	-1.94	0.0167
ssc-miR-144	ERO1L	hypothetical gene	-1.94	0.0167
ssc-miR-144	ESCO1	establishment of sister chromatid cohesion N-acetyltransferase 1 [Source:VGNC Symbol;Acc:VGNC:87786]	-1.94	0.0167
ssc-miR-144	ESRRG	estrogen related receptor gamma [Source:VGNC Symbol;Acc:VGNC:96289]	-1.94	0.0167
ssc-miR-144	ETNK1	ethanolamine kinase 1 [Source:VGNC Symbol;Acc:VGNC:87806]	-1.94	0.0167

ssc-miR-144	ETS1	ETS proto-onco 1, transcription factor [Source:VGNC Symbol;Acc:VGNC:87808]	-1.94	0.0167
ssc-miR-144	ETV1	ETS variant transcription factor 1 [Source:VGNC Symbol;Acc:VGNC:87810]	-1.94	0.0167
ssc-miR-144	EZH2	enhancer of zeste 2 polycomb repressive complex 2 subunit [Source:VGNC Symbol;Acc:VGNC:87855]	-1.94	0.0167
ssc-miR-144	FAM126B	family with sequence similarity 126 member B [Source:HGNC Symbol;Acc:HGNC:28593]	-1.94	0.0167
ssc-miR-144	FAM150B	hypothetical gene	-1.94	0.0167
ssc-miR-144	FAM168A	family with sequence similarity 168 member A [Source:VGNC Symbol;Acc:VGNC:87926]	-1.94	0.0167
ssc-miR-144	FAM168B	family with sequence similarity 168 member B [Source:VGNC Symbol;Acc:VGNC:95462]	-1.94	0.0167
ssc-miR-144	FAM174A	family with sequence similarity 174 member A [Source:VGNC Symbol;Acc:VGNC:87931]	-1.94	0.0167
ssc-miR-144	FAM179B	hypothetical gene	-1.94	0.0167
ssc-miR-144	FAM193B	family with sequence similarity 193 member B [Source:VGNC Symbol;Acc:VGNC:87947]	-1.94	0.0167
ssc-miR-144	FAM196A	hypothetical gene	-1.94	0.0167
ssc-miR-144	FAM199X	family with sequence similarity 199, X-linked [Source:HGNC Symbol;Acc:HGNC:25195]	-1.94	0.0167
ssc-miR-144	FAM222A	family with sequence similarity 222 member A [Source:VGNC Symbol;Acc:VGNC:87959]	-1.94	0.0167
ssc-miR-144	FAM222B	family with sequence similarity 222 member B [Source:VGNC Symbol;Acc:VGNC:87960]	-1.94	0.0167
ssc-miR-144	FAM46A	hypothetical gene	-1.94	0.0167
ssc-miR-144	FAM60A	hypothetical gene	-1.94	0.0167
ssc-miR-144	FAM73A	hypothetical gene	-1.94	0.0167
ssc-miR-144	FAM76B	family with sequence similarity 76 member B [Source:VGNC Symbol;Acc:VGNC:87983]	-1.94	0.0167
ssc-miR-144	FAM84A	hypothetical gene	-1.94	0.0167
ssc-miR-144	FAT3	hypothetical gene	-1.94	0.0167
ssc-miR-144	FAT4	FAT atypical cadherin 4 [Source:HGNC Symbol;Acc:HGNC:23109]	-1.94	0.0167
ssc-miR-144	FAXC	hypothetical gene	-1.94	0.0167
ssc-miR-144	FBN1	fibrillin 1 [Source:VGNC Symbol;Acc:VGNC:103090]	-1.94	0.0167
ssc-miR-144	FBN2	fibrillin 2 [Source:VGNC Symbol;Acc:VGNC:88025]	-1.94	0.0167
ssc-miR-144	FBXL3	F-box and leucine rich repeat protein 3 [Source:VGNC Symbol;Acc:VGNC:88030]	-1.94	0.0167
ssc-miR-144	FBXO30	F-box protein 30 [Source:VGNC Symbol;Acc:VGNC:108155]	-1.94	0.0167
ssc-miR-144	FBXO32	F-box protein 32 [Source:VGNC Symbol;Acc:VGNC:88039]	-1.94	0.0167
ssc-miR-144	FBXW11	F-box and WD repeat domain containing 11 [Source:HGNC Symbol;Acc:HGNC:13607]	-1.94	0.0167
ssc-miR-144	FBXW7	F-box and WD repeat domain containing 7 [Source:VGNC Symbol;Acc:VGNC:98925]	-1.94	0.0167
ssc-miR-144	FGD6	FYVE, RhoGEF and PH domain containing 6 [Source:VGNC Symbol;Acc:VGNC:88099]	-1.94	0.0167
ssc-miR-144	FGF16	fibroblast growth factor 16 [Source:VGNC Symbol;Acc:VGNC:88105]	-1.94	0.0167
ssc-miR-144	FGF7	fibroblast growth factor 7 [Source:HGNC Symbol;Acc:HGNC:3685]	-1.94	0.0167
ssc-miR-144	FGFR1OP	hypothetical gene	-1.94	0.0167
ssc-miR-144	FIGN	fidgetin, microtubule severing factor [Source:VGNC Symbol;Acc:VGNC:95580]	-1.94	0.0167
ssc-miR-144	FILIP1L	filamin A interacting protein 1 like [Source:VGNC Symbol;Acc:VGNC:88140]	-1.94	0.0167
ssc-miR-144	FKBP7	FKBP prolyl isomerase 7 [Source:VGNC Symbol;Acc:VGNC:96302]	-1.94	0.0167
ssc-miR-144	FLI1	Fli-1 proto-onco, ETS transcription factor [Source:VGNC Symbol;Acc:VGNC:88153]	-1.94	0.0167
ssc-miR-144	FLRT2	fibronectin leucine rich transmembrane protein 2 [Source:VGNC Symbol;Acc:VGNC:88161]	-1.94	0.0167
ssc-miR-144	FLRT3	fibronectin leucine rich transmembrane protein 3 [Source:VGNC Symbol;Acc:VGNC:96304]	-1.94	0.0167
ssc-miR-144	FMN2	hypothetical gene	-1.94	0.0167
ssc-miR-144	FMR1	FMRP translational regulator 1 [Source:VGNC Symbol;Acc:VGNC:88175]	-1.94	0.0167
ssc-miR-144	FN1	fibronectin 1 [Source:VGNC Symbol;Acc:VGNC:96305]	-1.94	0.0167
ssc-miR-144	FNDC3A	fibronectin type III domain containing 3A [Source:VGNC Symbol;Acc:VGNC:88182]	-1.94	0.0167
ssc-miR-144	FOS	Fos proto-onco, AP-1 transcription factor subunit [Source:NCBI gene (formerly Entrezgene);Acc:100144486]	-1.94	0.0167
ssc-miR-144	FOSB	FosB proto-onco, AP-1 transcription factor subunit [Source:VGNC Symbol;Acc:VGNC:88190]	-1.94	0.0167
ssc-miR-144	FOSL2	FOS like 2, AP-1 transcription factor subunit [Source:VGNC Symbol;Acc:VGNC:88192]	-1.94	0.0167
ssc-miR-144	FOXF1	forkhead box F1 [Source:VGNC Symbol;Acc:VGNC:88204]	-1.94	0.0167
ssc-miR-144	FOXG1	forkhead box G1 [Source:VGNC Symbol;Acc:VGNC:88206]	-1.94	0.0167
ssc-miR-144	FOXN4	hypothetical gene	-1.94	0.0167

ssc-miR-144	FOXO1	forkhead box O1 [Source:VGNC Symbol;Acc:VGNC:98013]	-1.94	0.0167
ssc-miR-144	FOXP1	forkhead box P1 [Source:VGNC Symbol;Acc:VGNC:88222]	-1.94	0.0167
ssc-miR-144	FRS2	fibroblast growth factor receptor substrate 2 [Source:VGNC Symbol;Acc:VGNC:88246]	-1.94	0.0167
ssc-miR-144	FRY	hypothetical gene	-1.94	0.0167
ssc-miR-144	FRYL	FRY like transcription coactivator [Source:VGNC Symbol;Acc:VGNC:98015]	-1.94	0.0167
ssc-miR-144	FST	follistatin [Source:NCBI gene (formerly Entrezgene);Acc:445002]	-1.94	0.0167
ssc-miR-144	FTO	FTO alpha-ketoglutarate dependent dioxygenase [Source:VGNC Symbol;Acc:VGNC:88259]	-1.94	0.0167
ssc-miR-144	FUBP3	far upstream element binding protein 3 [Source:VGNC Symbol;Acc:VGNC:103093]	-1.94	0.0167
ssc-miR-144	FZD4	frizzled class receptor 4 [Source:VGNC Symbol;Acc:VGNC:88282]	-1.94	0.0167
ssc-miR-144	GAB1	GRB2 associated binding protein 1 [Source:VGNC Symbol;Acc:VGNC:88294]	-1.94	0.0167
ssc-miR-144	GABBR2	gamma-aminobutyric acid type B receptor subunit 2 [Source:VGNC Symbol;Acc:VGNC:88298]	-1.94	0.0167
ssc-miR-144	GABPA	GA binding protein transcription factor subunit alpha [Source:VGNC Symbol;Acc:VGNC:103945]	-1.94	0.0167
ssc-miR-144	GABRA1	gamma-aminobutyric acid type A receptor subunit alpha1 [Source:VGNC Symbol;Acc:VGNC:88300]	-1.94	0.0167
ssc-miR-144	GABRB2	gamma-aminobutyric acid type A receptor subunit beta2 [Source:VGNC Symbol;Acc:VGNC:88307]	-1.94	0.0167
ssc-miR-144	GALNT1	polypeptide N-acetylgalactosaminyltransferase 1 [Source:VGNC Symbol;Acc:VGNC:97062]	-1.94	0.0167
ssc-miR-144	GATA2	GATA binding protein 2 [Source:VGNC Symbol;Acc:VGNC:88364]	-1.94	0.0167
ssc-miR-144	GATA3	GATA binding protein 3 [Source:VGNC Symbol;Acc:VGNC:96315]	-1.94	0.0167
ssc-miR-144	GATSL2	hypothetical gene	-1.94	0.0167
ssc-miR-144	GBE1	1,4-alpha-glucan branching enzyme 1 [Source:VGNC Symbol;Acc:VGNC:88374]	-1.94	0.0167
ssc-miR-144	GCA	grancalcin [Source:VGNC Symbol;Acc:VGNC:95726]	-1.94	0.0167
ssc-miR-144	GCLC	glutamate-cysteine ligase catalytic subunit [Source:VGNC Symbol;Acc:VGNC:88386]	-1.94	0.0167
ssc-miR-144	GDE1	glycerophosphodiester phosphodiesterase 1 [Source:VGNC Symbol;Acc:VGNC:88396]	-1.94	0.0167
ssc-miR-144	GDF10	growth differentiation factor 10 [Source:VGNC Symbol;Acc:VGNC:88397]	-1.94	0.0167
ssc-miR-144	GET4	hypothetical gene	-1.94	0.0167
ssc-miR-144	GHR	growth hormone receptor [Source:NCBI gene (formerly Entrezgene);Acc:397488]	-1.94	0.0167
ssc-miR-144	GID4	GID complex subunit 4 homolog [Source:VGNC Symbol;Acc:VGNC:98996]	-1.94	0.0167
ssc-miR-144	GJA1	gap junction protein alpha 1 [Source:VGNC Symbol;Acc:VGNC:103098]	-1.94	0.0167
ssc-miR-144	GK5	glycerol kinase 5 [Source:VGNC Symbol;Acc:VGNC:96734]	-1.94	0.0167
ssc-miR-144	GLCCI1	glucocorticoid induced 1 [Source:VGNC Symbol;Acc:VGNC:88476]	-1.94	0.0167
ssc-miR-144	GLG1	golgi glycoprotein 1 [Source:VGNC Symbol;Acc:VGNC:88481]	-1.94	0.0167
ssc-miR-144	GLI2	GLI family zinc finger 2 [Source:VGNC Symbol;Acc:VGNC:96324]	-1.94	0.0167
ssc-miR-144	GLTSCR1	hypothetical gene	-1.94	0.0167
ssc-miR-144	GLTSCR1L	hypothetical gene	-1.94	0.0167
ssc-miR-144	GMEB2	glucocorticoid modulatory element binding protein 2 [Source:VGNC Symbol;Acc:VGNC:96326]	-1.94	0.0167
ssc-miR-144	GMFB	glia maturation factor beta [Source:VGNC Symbol;Acc:VGNC:88509]	-1.94	0.0167
ssc-miR-144	GNA13	G protein subunit alpha 13 [Source:VGNC Symbol;Acc:VGNC:98997]	-1.94	0.0167
ssc-miR-144	GNAI3	G protein subunit alpha i3 [Source:VGNC Symbol;Acc:VGNC:88523]	-1.94	0.0167
ssc-miR-144	GNAL	G protein subunit alpha L [Source:VGNC Symbol;Acc:VGNC:88524]	-1.94	0.0167
ssc-miR-144	GNPTAB	N-acetylglucosamine-1-phosphate transferase subunits alpha and beta [Source:VGNC Symbol;Acc:VGNC:88546]	-1.94	0.0167
ssc-miR-144	GOLGA2	hypothetical gene	-1.94	0.0167
ssc-miR-144	GOLGA4	hypothetical gene	-1.94	0.0167
ssc-miR-144	GOLGA7	golgin A7 [Source:VGNC Symbol;Acc:VGNC:96059]	-1.94	0.0167
ssc-miR-144	GPM6B	hypothetical gene	-1.94	0.0167
ssc-miR-144	GPR158	G protein-coupled receptor 158 [Source:VGNC Symbol;Acc:VGNC:96016]	-1.94	0.0167
ssc-miR-144	GPR174	G protein-coupled receptor 174 [Source:VGNC Symbol;Acc:VGNC:88617]	-1.94	0.0167
ssc-miR-144	GPR183	G protein-coupled receptor 183 [Source:VGNC Symbol;Acc:VGNC:88623]	-1.94	0.0167
ssc-miR-144	GPR85	G protein-coupled receptor 85 [Source:VGNC Symbol;Acc:VGNC:88638]	-1.94	0.0167
ssc-miR-144	GPRIN3	hypothetical gene	-1.94	0.0167
ssc-miR-144	GRAMD4	GRAM domain containing 4 [Source:VGNC Symbol;Acc:VGNC:88659]	-1.94	0.0167

ssc-miR-144	GRID2	glutamate ionotropic receptor delta type subunit 2 [Source:VGNC Symbol;Acc:VGNC:98932]	-1.94	0.0167
ssc-miR-144	GRIK2	glutamate ionotropic receptor kainate type subunit 2 [Source:VGNC Symbol;Acc:VGNC:88678]	-1.94	0.0167
ssc-miR-144	GRIK3	glutamate ionotropic receptor kainate type subunit 3 [Source:VGNC Symbol;Acc:VGNC:88679]	-1.94	0.0167
ssc-miR-144	GRIN2A	glutamate ionotropic receptor NMDA type subunit 2A [Source:VGNC Symbol;Acc:VGNC:88683]	-1.94	0.0167
ssc-miR-144	GRM5	glutamate metabotropic receptor 5 [Source:VGNC Symbol;Acc:VGNC:88703]	-1.94	0.0167
ssc-miR-144	GRSF1	G-rich RNA sequence binding factor 1 [Source:VGNC Symbol;Acc:VGNC:88710]	-1.94	0.0167
ssc-miR-144	GSK3B	glycogen synthase kinase 3 beta [Source:VGNC Symbol;Acc:VGNC:88723]	-1.94	0.0167
ssc-miR-144	GSPT1	G1 to S phase transition 1 [Source:VGNC Symbol;Acc:VGNC:88724]	-1.94	0.0167
ssc-miR-144	GSPT2	G1 to S phase transition 2 [Source:HGNC Symbol;Acc:HGNC:4622]	-1.94	0.0167
ssc-miR-144	GTF2A1	ral transcription factor IIA subunit 1 [Source:VGNC Symbol;Acc:VGNC:88730]	-1.94	0.0167
ssc-miR-144	GUCY1A2	guanylate cyclase 1 soluble subunit alpha 2 [Source:VGNC Symbol;Acc:VGNC:88751]	-1.94	0.0167
ssc-miR-144	H2AFV	hypothetical gene	-1.94	0.0167
ssc-miR-144	HAPLN1	hyaluronan and proteoglycan link protein 1 [Source:VGNC Symbol;Acc:VGNC:88780]	-1.94	0.0167
ssc-miR-144	HAT1	histone acetyltransferase 1 [Source:VGNC Symbol;Acc:VGNC:96340]	-1.94	0.0167
ssc-miR-144	HDAC4	histone deacetylase 4 [Source:VGNC Symbol;Acc:VGNC:95602]	-1.94	0.0167
ssc-miR-144	HDAC9	histone deacetylase 9 [Source:HGNC Symbol;Acc:HGNC:14065]	-1.94	0.0167
ssc-miR-144	HDGFRP3	hypothetical gene	-1.94	0.0167
ssc-miR-144	HEG1	heart development protein with EGF like domains 1 [Source:VGNC Symbol;Acc:VGNC:88836]	-1.94	0.0167
ssc-miR-144	HELZ	helicase with zinc finger [Source:VGNC Symbol;Acc:VGNC:88840]	-1.94	0.0167
ssc-miR-144	HERC2	HECT and RLD domain containing E3 ubiquitin protein ligase 2 [Source:VGNC Symbol;Acc:VGNC:99716]	-1.94	0.0167
ssc-miR-144	HERPUD1	homocysteine inducible ER protein with ubiquitin like domain 1 [Source:VGNC Symbol;Acc:VGNC:88852]	-1.94	0.0167
ssc-miR-144	HEY2	hes related family bHLH transcription factor with YRPW motif 2 [Source:VGNC Symbol;Acc:VGNC:88864]	-1.94	0.0167
ssc-miR-144	HGF	hepatocyte growth factor [Source:VGNC Symbol;Acc:VGNC:88869]	-1.94	0.0167
ssc-miR-144	HIPK2	homeodomain interacting protein kinase 2 [Source:VGNC Symbol;Acc:VGNC:88888]	-1.94	0.0167
ssc-miR-144	HIPK3	homeodomain interacting protein kinase 3 [Source:VGNC Symbol;Acc:VGNC:88889]	-1.94	0.0167
ssc-miR-144	HIVEP3	hypothetical gene	-1.94	0.0167
ssc-miR-144	HLTF	helicase like transcription factor [Source:VGNC Symbol;Acc:VGNC:88897]	-1.94	0.0167
ssc-miR-144	HMGCR	3-hydroxy-3-methylglutaryl-CoA reductase [Source:VGNC Symbol;Acc:VGNC:88906]	-1.94	0.0167
ssc-miR-144	HMGN2	high mobility group nucleosomal binding domain 2 [Source:NCBI gene (formerly Entrezgene);Acc:100524500]	-1.94	0.0167
ssc-miR-144	HMGN3	high mobility group nucleosomal binding domain 3 [Source:VGNC Symbol;Acc:VGNC:88908]	-1.94	0.0167
ssc-miR-144	HNRNPAB	hypothetical gene	-1.94	0.0167
ssc-miR-144	HNRNPC	hypothetical gene	-1.94	0.0167
ssc-miR-144	HNRNPF	hypothetical gene	-1.94	0.0167
ssc-miR-144	HNRNPU	heterogeneous nuclear ribonucleoprotein U [Source:HGNC Symbol;Acc:HGNC:5048]	-1.94	0.0167
ssc-miR-144	HOXA10	homeobox A10 [Source:VGNC Symbol;Acc:VGNC:88934]	-1.94	0.0167
ssc-miR-144	HOXA7	homeobox A7 [Source:VGNC Symbol;Acc:VGNC:88940]	-1.94	0.0167
ssc-miR-144	HS2ST1	heparan sulfate 2-O-sulfotransferase 1 [Source:HGNC Symbol;Acc:HGNC:5193]	-1.94	0.0167
ssc-miR-144	HS3ST3B1	heparan sulfate-glucosamine 3-sulfotransferase 3B1 [Source:VGNC Symbol;Acc:VGNC:99003]	-1.94	0.0167
ssc-miR-144	HSF2	heat shock transcription factor 2 [Source:VGNC Symbol;Acc:VGNC:88994]	-1.94	0.0167
ssc-miR-144	HSP90AA1	heat shock protein 90 alpha family class A member 1 [Source:NCBI gene (formerly Entrezgene);Acc:397028]	-1.94	0.0167
ssc-miR-144	HSPA4L	heat shock protein family A (Hsp70) member 4 like [Source:HGNC Symbol;Acc:HGNC:17041]	-1.94	0.0167
ssc-miR-144	HSPE1-MOB4	hypothetical gene	-1.94	0.0167
ssc-miR-144	HTRA3	HtrA serine peptidase 3 [Source:VGNC Symbol;Acc:VGNC:89005]	-1.94	0.0167
ssc-miR-144	ICK	hypothetical gene	-1.94	0.0167
ssc-miR-144	ID4	inhibitor of DNA binding 4, HLH protein [Source:VGNC Symbol;Acc:VGNC:89024]	-1.94	0.0167
ssc-miR-144	IDH2	isocitrate dehydrogenase (NADP(+)) 2 [Source:NCBI gene (formerly Entrezgene);Acc:397603]	-1.94	0.0167
ssc-miR-144	IFFO2	intermediate filament family orphan 2 [Source:VGNC Symbol;Acc:VGNC:98475]	-1.94	0.0167
ssc-miR-144	IGF2R	insulin like growth factor 2 receptor [Source:VGNC Symbol;Acc:VGNC:89057]	-1.94	0.0167
ssc-miR-144	IGIP	hypothetical gene	-1.94	0.0167

ssc-miR-144	IKZF4	IKAROS family zinc finger 4 [Source:VGNC Symbol;Acc:VGNC:89075]	-1.94	0.0167
ssc-miR-144	IMPA1	inositol monophosphatase 1 [Source:VGNC Symbol;Acc:VGNC:89125]	-1.94	0.0167
ssc-miR-144	IMPACT	impact RWD domain protein [Source:HGNC Symbol;Acc:HGNC:20387]	-1.94	0.0167
ssc-miR-144	INO80D	INO80 complex subunit D [Source:VGNC Symbol;Acc:VGNC:96113]	-1.94	0.0167
ssc-miR-144	IPO7	importin 7 [Source:VGNC Symbol;Acc:VGNC:89180]	-1.94	0.0167
ssc-miR-144	IPO8	importin 8 [Source:VGNC Symbol;Acc:VGNC:89181]	-1.94	0.0167
ssc-miR-144	IQGAP1	IQ motif containing GTPase activating protein 1 [Source:HGNC Symbol;Acc:HGNC:6110]	-1.94	0.0167
ssc-miR-144	IRS1	insulin receptor substrate 1 [Source:VGNC Symbol;Acc:VGNC:96376]	-1.94	0.0167
ssc-miR-144	ISCA1	iron-sulfur cluster assembly 1 [Source:HGNC Symbol;Acc:HGNC:28660]	-1.94	0.0167
ssc-miR-144	ITCH	itchy E3 ubiquitin protein ligase [Source:HGNC Symbol;Acc:HGNC:13890]	-1.94	0.0167
ssc-miR-144	ITGA6	integrin subunit alpha 6 [Source:VGNC Symbol;Acc:VGNC:96378]	-1.94	0.0167
ssc-miR-144	ITGA8	integrin subunit alpha 8 [Source:VGNC Symbol;Acc:VGNC:96379]	-1.94	0.0167
ssc-miR-144	ITGB6	integrin subunit beta 6 [Source:VGNC Symbol;Acc:VGNC:98054]	-1.94	0.0167
ssc-miR-144	ITGB8	integrin subunit beta 8 [Source:VGNC Symbol;Acc:VGNC:89246]	-1.94	0.0167
ssc-miR-144	ITPR1	inositol 1,4,5-trisphosphate receptor type 1 [Source:VGNC Symbol;Acc:VGNC:89253]	-1.94	0.0167
ssc-miR-144	ITPRIPL1	ITPRIP like 1 [Source:VGNC Symbol;Acc:VGNC:89257]	-1.94	0.0167
ssc-miR-144	ITSN2	intersectin 2 [Source:VGNC Symbol;Acc:VGNC:89259]	-1.94	0.0167
ssc-miR-144	JAK2	Janus kinase 2 [Source:VGNC Symbol;Acc:VGNC:89271]	-1.94	0.0167
ssc-miR-144	JAKMIP2	janus kinase and microtubule interacting protein 2 [Source:VGNC Symbol;Acc:VGNC:89274]	-1.94	0.0167
ssc-miR-144	JAKMIP3	Janus kinase and microtubule interacting protein 3 [Source:VGNC Symbol;Acc:VGNC:89275]	-1.94	0.0167
ssc-miR-144	JDP2	Jun dimerization protein 2 [Source:VGNC Symbol;Acc:VGNC:89282]	-1.94	0.0167
ssc-miR-144	JHDM1D	hypothetical gene	-1.94	0.0167
ssc-miR-144	JPH1	junctophilin 1 [Source:VGNC Symbol;Acc:VGNC:89291]	-1.94	0.0167
ssc-miR-144	KAT6A	hypothetical gene	-1.94	0.0167
ssc-miR-144	KAT6B	lysine acetyltransferase 6B [Source:VGNC Symbol;Acc:VGNC:89306]	-1.94	0.0167
ssc-miR-144	KAT7	lysine acetyltransferase 7 [Source:VGNC Symbol;Acc:VGNC:89307]	-1.94	0.0167
ssc-miR-144	KATNBL1	katanin regulatory subunit B1 like 1 [Source:VGNC Symbol;Acc:VGNC:89313]	-1.94	0.0167
ssc-miR-144	KBTBD2	kelch repeat and BTB domain containing 2 [Source:VGNC Symbol;Acc:VGNC:89317]	-1.94	0.0167
ssc-miR-144	KBTBD8	kelch repeat and BTB domain containing 8 [Source:VGNC Symbol;Acc:VGNC:89321]	-1.94	0.0167
ssc-miR-144	KCMF1	potassium channel modulatory factor 1 [Source:VGNC Symbol;Acc:VGNC:89322]	-1.94	0.0167
ssc-miR-144	KCNA1	potassium voltage-gated channel subfamily A member 1 [Source:VGNC Symbol;Acc:VGNC:89323]	-1.94	0.0167
ssc-miR-144	KCNA4	potassium voltage-gated channel subfamily A member 4 [Source:VGNC Symbol;Acc:VGNC:89326]	-1.94	0.0167
ssc-miR-144	KCNC4	potassium voltage-gated channel subfamily C member 4 [Source:VGNC Symbol;Acc:VGNC:89335]	-1.94	0.0167
ssc-miR-144	KCND2	potassium voltage-gated channel subfamily D member 2 [Source:HGNC Symbol;Acc:HGNC:6238]	-1.94	0.0167
ssc-miR-144	KCND3	potassium voltage-gated channel subfamily D member 3 [Source:VGNC Symbol;Acc:VGNC:98814]	-1.94	0.0167
ssc-miR-144	KCNH7	potassium voltage-gated channel subfamily H member 7 [Source:VGNC Symbol;Acc:VGNC:95897]	-1.94	0.0167
ssc-miR-144	KCNJ6	potassium inwardly rectifying channel subfamily J member 6 [Source:VGNC Symbol;Acc:VGNC:89360]	-1.94	0.0167
ssc-miR-144	KCNJ8	potassium inwardly rectifying channel subfamily J member 8 [Source:VGNC Symbol;Acc:VGNC:89361]	-1.94	0.0167
ssc-miR-144	KCNK12	potassium two pore domain channel subfamily K member 12 [Source:HGNC Symbol;Acc:HGNC:6274]	-1.94	0.0167
ssc-miR-144	KCNMB2	potassium calcium-activated channel subfamily M regulatory beta subunit 2 [Source:HGNC Symbol;Acc:HGNC:6286]	-1.94	0.0167
ssc-miR-144	KCNN3	potassium calcium-activated channel subfamily N member 3 [Source:VGNC Symbol;Acc:VGNC:98056]	-1.94	0.0167
ssc-miR-144	KCTD10	potassium channel tetramerization domain containing 10 [Source:VGNC Symbol;Acc:VGNC:103972]	-1.94	0.0167
ssc-miR-144	KCTD12	potassium channel tetramerization domain containing 12 [Source:HGNC Symbol;Acc:HGNC:14678]	-1.94	0.0167
ssc-miR-144	KDM3A	lysine demethylase 3A [Source:VGNC Symbol;Acc:VGNC:89411]	-1.94	0.0167
ssc-miR-144	KDM3B	lysine demethylase 3B [Source:VGNC Symbol;Acc:VGNC:89412]	-1.94	0.0167
ssc-miR-144	KDM6A	lysine demethylase 6A [Source:HGNC Symbol;Acc:HGNC:12637]	-1.94	0.0167
ssc-miR-144	KDM6B	lysine demethylase 6B [Source:VGNC Symbol;Acc:VGNC:89416]	-1.94	0.0167
ssc-miR-144	KHDRBS2	KH RNA binding domain containing, signal transduction associated 2 [Source:VGNC Symbol;Acc:VGNC:89423]	-1.94	0.0167
ssc-miR-144	KHDRBS3	KH RNA binding domain containing, signal transduction associated 3 [Source:VGNC Symbol;Acc:VGNC:89424]	-1.94	0.0167

ssc-miR-144	KIAA0232	hypothetical gene	-1.94	0.0167
ssc-miR-144	KIAA0556	hypothetical gene	-1.94	0.0167
ssc-miR-144	KIAA1024	hypothetical gene	-1.94	0.0167
ssc-miR-144	KIAA1033	hypothetical gene	-1.94	0.0167
ssc-miR-144	KIAA1217	KIAA1217 [Source:VGNC Symbol;Acc:VGNC:96079]	-1.94	0.0167
ssc-miR-144	KIAA1522	KIAA1522 [Source:VGNC Symbol;Acc:VGNC:89444]	-1.94	0.0167
ssc-miR-144	KIAA1549L	KIAA1549 like [Source:VGNC Symbol;Acc:VGNC:89445]	-1.94	0.0167
ssc-miR-144	KIF14	kinesin family member 14 [Source:VGNC Symbol;Acc:VGNC:108276]	-1.94	0.0167
ssc-miR-144	KIF2A	kinesin family member 2A [Source:VGNC Symbol;Acc:VGNC:89467]	-1.94	0.0167
ssc-miR-144	KIF3A	kinesin family member 3A [Source:VGNC Symbol;Acc:VGNC:89470]	-1.94	0.0167
ssc-miR-144	KIN	Kin17 DNA and RNA binding protein [Source:VGNC Symbol;Acc:VGNC:96395]	-1.94	0.0167
ssc-miR-144	KIT	KIT proto-onco, receptor tyrosine kinase [Source:VGNC Symbol;Acc:VGNC:98060]	-1.94	0.0167
ssc-miR-144	KITLG	KIT ligand [Source:VGNC Symbol;Acc:VGNC:98061]	-1.94	0.0167
ssc-miR-144	KLF11	Kruppel like factor 11 [Source:VGNC Symbol;Acc:VGNC:89491]	-1.94	0.0167
ssc-miR-144	KLF12	Kruppel like factor 12 [Source:VGNC Symbol;Acc:VGNC:89492]	-1.94	0.0167
ssc-miR-144	KLF3	Kruppel like factor 3 [Source:VGNC Symbol;Acc:VGNC:89498]	-1.94	0.0167
ssc-miR-144	KLF6	Kruppel like factor 6 [Source:VGNC Symbol;Acc:VGNC:98063]	-1.94	0.0167
ssc-miR-144	KLF8	Kruppel like factor 8 [Source:VGNC Symbol;Acc:VGNC:89500]	-1.94	0.0167
ssc-miR-144	KLF9	Kruppel like factor 9 [Source:VGNC Symbol;Acc:VGNC:103114]	-1.94	0.0167
ssc-miR-144	KLHL23	kelch like family member 23 [Source:VGNC Symbol;Acc:VGNC:96158]	-1.94	0.0167
ssc-miR-144	KLLN	hypothetical gene	-1.94	0.0167
ssc-miR-144	KMT2E	lysine methyltransferase 2E (inactive) [Source:VGNC Symbol;Acc:VGNC:89551]	-1.94	0.0167
ssc-miR-144	KPNA1	karyopherin subunit alpha 1 [Source:VGNC Symbol;Acc:VGNC:89560]	-1.94	0.0167
ssc-miR-144	KPNA2	karyopherin subunit alpha 2 [Source:VGNC Symbol;Acc:VGNC:89561]	-1.94	0.0167
ssc-miR-144	KPNA3	karyopherin subunit alpha 3 [Source:VGNC Symbol;Acc:VGNC:89562]	-1.94	0.0167
ssc-miR-144	KPNA6	karyopherin subunit alpha 6 [Source:VGNC Symbol;Acc:VGNC:89565]	-1.94	0.0167
ssc-miR-144	KRT222	keratin 222 [Source:HGNC Symbol;Acc:HGNC:28695]	-1.94	0.0167
ssc-miR-144	KSR1	kinase suppressor of ras 1 [Source:VGNC Symbol;Acc:VGNC:89606]	-1.94	0.0167
ssc-miR-144	LANCL3	LanC like 3 [Source:VGNC Symbol;Acc:VGNC:89634]	-1.94	0.0167
ssc-miR-144	LARP4B	hypothetical gene	-1.94	0.0167
ssc-miR-144	LBR	lamin B receptor [Source:VGNC Symbol;Acc:VGNC:98068]	-1.94	0.0167
ssc-miR-144	LCOR	ligand dependent nuclear receptor corepressor [Source:HGNC Symbol;Acc:HGNC:29503]	-1.94	0.0167
ssc-miR-144	LDLRAD4	low density lipoprotein receptor class A domain containing 4 [Source:VGNC Symbol;Acc:VGNC:89673]	-1.94	0.0167
ssc-miR-144	LGR4	leucine rich repeat containing G protein-coupled receptor 4 [Source:VGNC Symbol;Acc:VGNC:89703]	-1.94	0.0167
ssc-miR-144	LHX1	LIM homeobox 1 [Source:VGNC Symbol;Acc:VGNC:89711]	-1.94	0.0167
ssc-miR-144	LHX2	LIM homeobox 2 [Source:VGNC Symbol;Acc:VGNC:89712]	-1.94	0.0167
ssc-miR-144	LIFR	LIF receptor subunit alpha [Source:HGNC Symbol;Acc:HGNC:6597]	-1.94	0.0167
ssc-miR-144	LIMCH1	LIM and calponin homology domains 1 [Source:VGNC Symbol;Acc:VGNC:89724]	-1.94	0.0167
ssc-miR-144	LIMK2	LIM domain kinase 2 [Source:VGNC Symbol;Acc:VGNC:89728]	-1.94	0.0167
ssc-miR-144	LMO3	hypothetical gene	-1.94	0.0167
ssc-miR-144	LMTK2	lemur tyrosine kinase 2 [Source:HGNC Symbol;Acc:HGNC:17880]	-1.94	0.0167
ssc-miR-144	LNPEP	leucyl and cystinyl aminopeptidase [Source:VGNC Symbol;Acc:VGNC:89772]	-1.94	0.0167
ssc-miR-144	LONRF3	LON peptidase N-terminal domain and ring finger 3 [Source:VGNC Symbol;Acc:VGNC:89778]	-1.94	0.0167
ssc-miR-144	LPHN2	hypothetical gene	-1.94	0.0167
ssc-miR-144	LPPR5	hypothetical gene	-1.94	0.0167
ssc-miR-144	LRIG2	leucine rich repeats and immunoglobulin like domains 2 [Source:VGNC Symbol;Acc:VGNC:89811]	-1.94	0.0167
ssc-miR-144	LRP12	LDL receptor related protein 12 [Source:VGNC Symbol;Acc:VGNC:89818]	-1.94	0.0167
ssc-miR-144	LRRC1	leucine rich repeat containing 1 [Source:VGNC Symbol;Acc:VGNC:103977]	-1.94	0.0167
ssc-miR-144	LRRN1	leucine rich repeat neuronal 1 [Source:VGNC Symbol;Acc:VGNC:89860]	-1.94	0.0167

ssc-miR-144	LRRTM2	leucine rich repeat transmembrane neuronal 2 [Source:VGNC Symbol;Acc:VGNC:99788]	-1.94	0.0167
ssc-miR-144	LSAMP	limbic system associated membrane protein [Source:VGNC Symbol;Acc:VGNC:89868]	-1.94	0.0167
ssc-miR-144	LSM14A	LSM14A mRNA processing body assembly factor [Source:VGNC Symbol;Acc:VGNC:89872]	-1.94	0.0167
ssc-miR-144	LSM14B	LSM family member 14B [Source:VGNC Symbol;Acc:VGNC:95691]	-1.94	0.0167
ssc-miR-144	LUZP6	hypothetical gene	-1.94	0.0167
ssc-miR-144	LYPD6	LY6/PLAUR domain containing 6 [Source:HGNC Symbol;Acc:HGNC:28751]	-1.94	0.0167
ssc-miR-144	LYSMD3	LysM domain containing 3 [Source:VGNC Symbol;Acc:VGNC:89919]	-1.94	0.0167
ssc-miR-144	LZIC	leucine zipper and CTNNBIP1 domain containing [Source:VGNC Symbol;Acc:VGNC:89925]	-1.94	0.0167
ssc-miR-144	MAFK	MAF bZIP transcription factor K [Source:HGNC Symbol;Acc:HGNC:6782]	-1.94	0.0167
ssc-miR-144	MAGI2	membrane associated guanylate kinase, WW and PDZ domain containing 2 [Source:VGNC Symbol;Acc:VGNC:89955]	-1.94	0.0167
ssc-miR-144	MAGI3	membrane associated guanylate kinase, WW and PDZ domain containing 3 [Source:VGNC Symbol;Acc:VGNC:89956]	-1.94	0.0167
ssc-miR-144	MAML3	mastermind like transcriptional coactivator 3 [Source:VGNC Symbol;Acc:VGNC:98938]	-1.94	0.0167
ssc-miR-144	MAP1B	microtubule associated protein 1B [Source:VGNC Symbol;Acc:VGNC:89979]	-1.94	0.0167
ssc-miR-144	MAP3K2	mitogen-activated protein kinase kinase kinase 2 [Source:VGNC Symbol;Acc:VGNC:98107]	-1.94	0.0167
ssc-miR-144	MAP3K4	mitogen-activated protein kinase kinase kinase 4 [Source:VGNC Symbol;Acc:VGNC:98109]	-1.94	0.0167
ssc-miR-144	MAP3K8	mitogen-activated protein kinase kinase kinase 8 [Source:VGNC Symbol;Acc:VGNC:98110]	-1.94	0.0167
ssc-miR-144	MAP3K9	mitogen-activated protein kinase kinase kinase 9 [Source:VGNC Symbol;Acc:VGNC:89989]	-1.94	0.0167
ssc-miR-144	MAP7D1	MAP7 domain containing 1 [Source:VGNC Symbol;Acc:VGNC:89993]	-1.94	0.0167
ssc-miR-144	MAPK1	mitogen-activated protein kinase 1 [Source:VGNC Symbol;Acc:VGNC:89996]	-1.94	0.0167
ssc-miR-144	MAPK6	mitogen-activated protein kinase 6 [Source:VGNC Symbol;Acc:VGNC:103123]	-1.94	0.0167
ssc-miR-144	MARCH3	hypothetical gene	-1.94	0.0167
ssc-miR-144	MARCH7	hypothetical gene	-1.94	0.0167
ssc-miR-144	MARCKS	myristoylated alanine rich protein kinase C substrate [Source:VGNC Symbol;Acc:VGNC:90024]	-1.94	0.0167
ssc-miR-144	MARK1	microtubule affinity regulating kinase 1 [Source:VGNC Symbol;Acc:VGNC:96411]	-1.94	0.0167
ssc-miR-144	MATN2	matrilin 2 [Source:VGNC Symbol;Acc:VGNC:90043]	-1.94	0.0167
ssc-miR-144	MBNL1	muscleblind like splicing regulator 1 [Source:VGNC Symbol;Acc:VGNC:90054]	-1.94	0.0167
ssc-miR-144	MBNL2	muscleblind like splicing regulator 2 [Source:VGNC Symbol;Acc:VGNC:90055]	-1.94	0.0167
ssc-miR-144	MBNL3	muscleblind like splicing regulator 3 [Source:VGNC Symbol;Acc:VGNC:90056]	-1.94	0.0167
ssc-miR-144	MBTD1	mbt domain containing 1 [Source:VGNC Symbol;Acc:VGNC:90060]	-1.94	0.0167
ssc-miR-144	MBTPS2	hypothetical gene	-1.94	0.0167
ssc-miR-144	MCF2L	MCF.2 cell line derived transforming sequence like [Source:VGNC Symbol;Acc:VGNC:90068]	-1.94	0.0167
ssc-miR-144	MDM4	MDM4 regulator of p53 [Source:VGNC Symbol;Acc:VGNC:90094]	-1.94	0.0167
ssc-miR-144	MED12L	mediator complex subunit 12L [Source:VGNC Symbol;Acc:VGNC:90105]	-1.94	0.0167
ssc-miR-144	MED13	mediator complex subunit 13 [Source:VGNC Symbol;Acc:VGNC:90106]	-1.94	0.0167
ssc-miR-144	MED14	mediator complex subunit 14 [Source:VGNC Symbol;Acc:VGNC:90108]	-1.94	0.0167
ssc-miR-144	MEF2A	myocyte enhancer factor 2A [Source:VGNC Symbol;Acc:VGNC:98123]	-1.94	0.0167
ssc-miR-144	MEF2D	myocyte enhancer factor 2D [Source:VGNC Symbol;Acc:VGNC:90128]	-1.94	0.0167
ssc-miR-144	MEGF9	multiple EGF like domains 9 [Source:VGNC Symbol;Acc:VGNC:90130]	-1.94	0.0167
ssc-miR-144	MEIS1	Meis homeobox 1 [Source:VGNC Symbol;Acc:VGNC:90135]	-1.94	0.0167
ssc-miR-144	MEIS2	Meis homeobox 2 [Source:VGNC Symbol;Acc:VGNC:90136]	-1.94	0.0167
ssc-miR-144	MET	MET proto-onco, receptor tyrosine kinase [Source:NCBI gene (formerly Entrezgene);Acc:654328]	-1.94	0.0167
ssc-miR-144	METTL23	methyltransferase like 23 [Source:VGNC Symbol;Acc:VGNC:98125]	-1.94	0.0167
ssc-miR-144	MFN1	mitofusin 1 [Source:VGNC Symbol;Acc:VGNC:90178]	-1.94	0.0167
ssc-miR-144	MFSD6	major facilitator superfamily domain containing 6 [Source:VGNC Symbol;Acc:VGNC:96052]	-1.94	0.0167
ssc-miR-144	MGAT4A	alpha-1,3-mannosyl-glycoprotein 4-beta-N-acetylglucosaminyltransferase A [Source:VGNC Symbol;Acc:VGNC:108158]	-1.94	0.0167
ssc-miR-144	MGAT5	alpha-1,6-mannosylglycoprotein 6-beta-N-acetylglucosaminyltransferase [Source:VGNC Symbol;Acc:VGNC:96741]	-1.94	0.0167
ssc-miR-144	MGRN1	mahogunin ring finger 1 [Source:VGNC Symbol;Acc:VGNC:90202]	-1.94	0.0167
ssc-miR-144	MIER3	MIER family member 3 [Source:VGNC Symbol;Acc:VGNC:90221]	-1.94	0.0167
ssc-miR-144	MITF	melanocyte inducing transcription factor [Source:VGNC Symbol;Acc:VGNC:90243]	-1.94	0.0167

ssc-miR-144	MKL2	hypothetical gene	-1.94	0.0167
ssc-miR-144	MLEC	malectin [Source:HGNC Symbol;Acc:HGNC:28973]	-1.94	0.0167
ssc-miR-144	MLLT4	hypothetical gene	-1.94	0.0167
ssc-miR-144	MMGT1	membrane magnesium transporter 1 [Source:VGNC Symbol;Acc:VGNC:98130]	-1.94	0.0167
ssc-miR-144	MMP15	matrix metallopeptidase 15 [Source:VGNC Symbol;Acc:VGNC:90270]	-1.94	0.0167
ssc-miR-144	MOB4	hypothetical gene	-1.94	0.0167
ssc-miR-144	MON2	MON2 homolog, regulator of endosome-to-Golgi trafficking [Source:VGNC Symbol;Acc:VGNC:90301]	-1.94	0.0167
ssc-miR-144	MORC3	MORC family CW-type zinc finger 3 [Source:VGNC Symbol;Acc:VGNC:90304]	-1.94	0.0167
ssc-miR-144	MORN4	MORN repeat containing 4 [Source:VGNC Symbol;Acc:VGNC:90310]	-1.94	0.0167
ssc-miR-144	MRGBP	MRG domain binding protein [Source:HGNC Symbol;Acc:HGNC:15866]	-1.94	0.0167
ssc-miR-144	MRPS14	mitochondrial ribosomal protein S14 [Source:VGNC Symbol;Acc:VGNC:90383]	-1.94	0.0167
ssc-miR-144	MSI1	musashi RNA binding protein 1 [Source:VGNC Symbol;Acc:VGNC:90421]	-1.94	0.0167
ssc-miR-144	MSI2	musashi RNA binding protein 2 [Source:VGNC Symbol;Acc:VGNC:90422]	-1.94	0.0167
ssc-miR-144	MSX1	msh homeobox 1 [Source:VGNC Symbol;Acc:VGNC:90431]	-1.94	0.0167
ssc-miR-144	MTFR1L	mitochondrial fission regulator 1 like [Source:VGNC Symbol;Acc:VGNC:90448]	-1.94	0.0167
ssc-miR-144	MTMR12	myotubularin related protein 12 [Source:VGNC Symbol;Acc:VGNC:90459]	-1.94	0.0167
ssc-miR-144	MTMR2	myotubularin related protein 2 [Source:VGNC Symbol;Acc:VGNC:90460]	-1.94	0.0167
ssc-miR-144	MTPN	myotrophin [Source:VGNC Symbol;Acc:VGNC:90467]	-1.94	0.0167
ssc-miR-144	MTSS1L	hypothetical gene	-1.94	0.0167
ssc-miR-144	MTX3	metaxin 3 [Source:VGNC Symbol;Acc:VGNC:90479]	-1.94	0.0167
ssc-miR-144	MUC19	hypothetical gene	-1.94	0.0167
ssc-miR-144	MYB	MYB proto-onco, transcription factor [Source:VGNC Symbol;Acc:VGNC:90496]	-1.94	0.0167
ssc-miR-144	MYBL1	MYB proto-onco like 1 [Source:VGNC Symbol;Acc:VGNC:90498]	-1.94	0.0167
ssc-miR-144	MYCL	hypothetical gene	-1.94	0.0167
ssc-miR-144	MYCN	MYCN proto-onco, bHLH transcription factor [Source:VGNC Symbol;Acc:VGNC:90504]	-1.94	0.0167
ssc-miR-144	MYEF2	myelin expression factor 2 [Source:VGNC Symbol;Acc:VGNC:90507]	-1.94	0.0167
ssc-miR-144	MYH10	hypothetical gene	-1.94	0.0167
ssc-miR-144	MYO1E	myosin IE [Source:VGNC Symbol;Acc:VGNC:90528]	-1.94	0.0167
ssc-miR-144	MYO9A	myosin IXA [Source:VGNC Symbol;Acc:VGNC:103138]	-1.94	0.0167
ssc-miR-144	MYT1	myelin transcription factor 1 [Source:VGNC Symbol;Acc:VGNC:104000]	-1.94	0.0167
ssc-miR-144	NAA30	N-alpha-acetyltransferase 30, NatC catalytic subunit [Source:VGNC Symbol;Acc:VGNC:96751]	-1.94	0.0167
ssc-miR-144	NAA38	hypothetical gene	-1.94	0.0167
ssc-miR-144	NACC1	nucleus accumbens associated 1 [Source:VGNC Symbol;Acc:VGNC:90563]	-1.94	0.0167
ssc-miR-144	NACC2	NACC family member 2 [Source:VGNC Symbol;Acc:VGNC:90564]	-1.94	0.0167
ssc-miR-144	NANOS1	nanos C2HC-type zinc finger 1 [Source:VGNC Symbol;Acc:VGNC:90574]	-1.94	0.0167
ssc-miR-144	NAV3	neuron navigator 3 [Source:VGNC Symbol;Acc:VGNC:90589]	-1.94	0.0167
ssc-miR-144	NCKAP1	NCK associated protein 1 [Source:VGNC Symbol;Acc:VGNC:96432]	-1.94	0.0167
ssc-miR-144	NCOA2	nuclear receptor coactivator 2 [Source:VGNC Symbol;Acc:VGNC:90616]	-1.94	0.0167
ssc-miR-144	NCOA7	nuclear receptor coactivator 7 [Source:VGNC Symbol;Acc:VGNC:90618]	-1.94	0.0167
ssc-miR-144	NCS1	neuronal calcium sensor 1 [Source:VGNC Symbol;Acc:VGNC:90620]	-1.94	0.0167
ssc-miR-144	NDFIP1	Nedd4 family interacting protein 1 [Source:VGNC Symbol;Acc:VGNC:90626]	-1.94	0.0167
ssc-miR-144	NDNF	neuron derived neurotrophic factor [Source:VGNC Symbol;Acc:VGNC:90629]	-1.94	0.0167
ssc-miR-144	NDRG4	NDRG family member 4 [Source:VGNC Symbol;Acc:VGNC:90633]	-1.94	0.0167
ssc-miR-144	NEDD4	NEDD4 E3 ubiquitin protein ligase [Source:VGNC Symbol;Acc:VGNC:90666]	-1.94	0.0167
ssc-miR-144	NEK7	NIMA related kinase 7 [Source:VGNC Symbol;Acc:VGNC:98152]	-1.94	0.0167
ssc-miR-144	NET1	neuroepithelial cell transforming 1 [Source:VGNC Symbol;Acc:VGNC:95622]	-1.94	0.0167
ssc-miR-144	NETO2	neuropilin and tolloid like 2 [Source:VGNC Symbol;Acc:VGNC:90691]	-1.94	0.0167
ssc-miR-144	NEURL1B	neuralized E3 ubiquitin protein ligase 1B [Source:VGNC Symbol;Acc:VGNC:90695]	-1.94	0.0167
ssc-miR-144	NEUROD1	neuronal differentiation 1 [Source:VGNC Symbol;Acc:VGNC:96439]	-1.94	0.0167

ssc-miR-144	NFE2L1	NFE2 like bZIP transcription factor 1 [Source:VGNC Symbol;Acc:VGNC:90713]	-1.94	0.0167
ssc-miR-144	NFE2L2	NFE2 like bZIP transcription factor 2 [Source:VGNC Symbol;Acc:VGNC:96441]	-1.94	0.0167
ssc-miR-144	NFIA	nuclear factor I A [Source:VGNC Symbol;Acc:VGNC:90715]	-1.94	0.0167
ssc-miR-144	NFIB	nuclear factor I B [Source:VGNC Symbol;Acc:VGNC:90716]	-1.94	0.0167
ssc-miR-144	NFYB	nuclear transcription factor Y subunit beta [Source:VGNC Symbol;Acc:VGNC:90730]	-1.94	0.0167
ssc-miR-144	NGFRAP1	hypothetical gene	-1.94	0.0167
ssc-miR-144	NHLRC2	NHL repeat containing 2 [Source:VGNC Symbol;Acc:VGNC:90737]	-1.94	0.0167
ssc-miR-144	NID2	nidogen 2 [Source:VGNC Symbol;Acc:VGNC:90743]	-1.94	0.0167
ssc-miR-144	NKRF	NFKB repressing factor [Source:VGNC Symbol;Acc:VGNC:90764]	-1.94	0.0167
ssc-miR-144	NLK	nemo like kinase [Source:VGNC Symbol;Acc:VGNC:90779]	-1.94	0.0167
ssc-miR-144	NLN	neurolysin [Source:VGNC Symbol;Acc:VGNC:90780]	-1.94	0.0167
ssc-miR-144	NOG	noggin [Source:VGNC Symbol;Acc:VGNC:90812]	-1.94	0.0167
ssc-miR-144	NOL4	nucleolar protein 4 [Source:VGNC Symbol;Acc:VGNC:90814]	-1.94	0.0167
ssc-miR-144	NOL7	nucleolar protein 7 [Source:VGNC Symbol;Acc:VGNC:90815]	-1.94	0.0167
ssc-miR-144	NOVA1	NOVA alternative splicing regulator 1 [Source:VGNC Symbol;Acc:VGNC:90827]	-1.94	0.0167
ssc-miR-144	NPAT	nuclear protein, coactivator of histone transcription [Source:VGNC Symbol;Acc:VGNC:90840]	-1.94	0.0167
ssc-miR-144	NPNT	nephronectin [Source:VGNC Symbol;Acc:VGNC:90855]	-1.94	0.0167
ssc-miR-144	NPTX1	neuronal pentraxin 1 [Source:VGNC Symbol;Acc:VGNC:90862]	-1.94	0.0167
ssc-miR-144	NR1D2	nuclear receptor subfamily 1 group D member 2 [Source:VGNC Symbol;Acc:VGNC:99726]	-1.94	0.0167
ssc-miR-144	NR2F2	nuclear receptor subfamily 2 group F member 2 [Source:VGNC Symbol;Acc:VGNC:90881]	-1.94	0.0167
ssc-miR-144	NR3C1	nuclear receptor subfamily 3 group C member 1 [Source:VGNC Symbol;Acc:VGNC:90883]	-1.94	0.0167
ssc-miR-144	NR4A3	nuclear receptor subfamily 4 group A member 3 [Source:VGNC Symbol;Acc:VGNC:90885]	-1.94	0.0167
ssc-miR-144	NR5A2	nuclear receptor subfamily 5 group A member 2 [Source:VGNC Symbol;Acc:VGNC:96452]	-1.94	0.0167
ssc-miR-144	NR6A1	nuclear receptor subfamily 6 group A member 1 [Source:VGNC Symbol;Acc:VGNC:90887]	-1.94	0.0167
ssc-miR-144	NRK	Nik related kinase [Source:VGNC Symbol;Acc:VGNC:98161]	-1.94	0.0167
ssc-miR-144	NSD1	nuclear receptor binding SET domain protein 1 [Source:VGNC Symbol;Acc:VGNC:90904]	-1.94	0.0167
ssc-miR-144	NT5DC1	5'-nucleotidase domain containing 1 [Source:VGNC Symbol;Acc:VGNC:90922]	-1.94	0.0167
ssc-miR-144	NTNG1	netrin G1 [Source:VGNC Symbol;Acc:VGNC:90935]	-1.94	0.0167
ssc-miR-144	NUDCD1	NudC domain containing 1 [Source:VGNC Symbol;Acc:VGNC:90949]	-1.94	0.0167
ssc-miR-144	NUDT11	hypothetical gene	-1.94	0.0167
ssc-miR-144	NUFIP2	nuclear FMR1 interacting protein 2 [Source:VGNC Symbol;Acc:VGNC:90967]	-1.94	0.0167
ssc-miR-144	NUP188	nucleoporin 188 [Source:VGNC Symbol;Acc:VGNC:98165]	-1.94	0.0167
ssc-miR-144	NUP37	nucleoporin 37 [Source:VGNC Symbol;Acc:VGNC:90980]	-1.94	0.0167
ssc-miR-144	NUP62CL	nucleoporin 62 C-terminal like [Source:VGNC Symbol;Acc:VGNC:104013]	-1.94	0.0167
ssc-miR-144	NYAP2	neuronal tyrosine-phosphorylated phosphoinositide-3-kinase adaptor 2 [Source:VGNC Symbol;Acc:VGNC:96213]	-1.94	0.0167
ssc-miR-144	OGFRL1	opioid growth factor receptor like 1 [Source:VGNC Symbol;Acc:VGNC:91026]	-1.94	0.0167
ssc-miR-144	ONECUT2	one cut homeobox 2 [Source:VGNC Symbol;Acc:VGNC:91043]	-1.94	0.0167
ssc-miR-144	OSBPL10	oxysterol binding protein like 10 [Source:VGNC Symbol;Acc:VGNC:91068]	-1.94	0.0167
ssc-miR-144	OSBPL1A	oxysterol binding protein like 1A [Source:VGNC Symbol;Acc:VGNC:91070]	-1.94	0.0167
ssc-miR-144	OTUD1	OTU deubiquitinase 1 [Source:VGNC Symbol;Acc:VGNC:96156]	-1.94	0.0167
ssc-miR-144	OTUD3	OTU deubiquitinase 3 [Source:VGNC Symbol;Acc:VGNC:91099]	-1.94	0.0167
ssc-miR-144	OTUD4	OTU deubiquitinase 4 [Source:VGNC Symbol;Acc:VGNC:91100]	-1.94	0.0167
ssc-miR-144	OTUD7A	OTU deubiquitinase 7A [Source:VGNC Symbol;Acc:VGNC:91104]	-1.94	0.0167
ssc-miR-144	PABPC1L2A	hypothetical gene	-1.94	0.0167
ssc-miR-144	PABPC1L2B	hypothetical gene	-1.94	0.0167
ssc-miR-144	PABPN1	poly(A) binding protein nuclear 1 [Source:NCBI gene (formerly Entrezgene);Acc:100155260]	-1.94	0.0167
ssc-miR-144	PAFAH1B1	platelet activating factor acetylhydrolase 1b regulatory subunit 1 [Source:VGNC Symbol;Acc:VGNC:99026]	-1.94	0.0167
ssc-miR-144	PAG1	phosphoprotein membrane anchor with glycosphingolipid microdomains 1 [Source:VGNC Symbol;Acc:VGNC:91154]	-1.94	0.0167
ssc-miR-144	PAK7	hypothetical gene	-1.94	0.0167

ssc-miR-144	PALD1	phosphatase domain containing paladin 1 [Source:VGNC Symbol;Acc:VGNC:91159]	-1.94	0.0167
ssc-miR-144	PALM2	hypothetical gene	-1.94	0.0167
ssc-miR-144	PALM2-AKAP2	hypothetical gene	-1.94	0.0167
ssc-miR-144	PANK1	pantothenate kinase 1 [Source:VGNC Symbol;Acc:VGNC:107164]	-1.94	0.0167
ssc-miR-144	PANK3	pantothenate kinase 3 [Source:VGNC Symbol;Acc:VGNC:91165]	-1.94	0.0167
ssc-miR-144	PANX2	pannexin 2 [Source:VGNC Symbol;Acc:VGNC:91167]	-1.94	0.0167
ssc-miR-144	PAPD5	hypothetical gene	-1.94	0.0167
ssc-miR-144	PAPOLG	poly(A) polymerase gamma [Source:HGNC Symbol;Acc:HGNC:14982]	-1.94	0.0167
ssc-miR-144	PAPPA	pappalysin 1 [Source:VGNC Symbol;Acc:VGNC:91170]	-1.94	0.0167
ssc-miR-144	PAPPA2	pappalysin 2 [Source:VGNC Symbol;Acc:VGNC:91171]	-1.94	0.0167
ssc-miR-144	PARN	poly(A)-specific ribonuclease [Source:HGNC Symbol;Acc:HGNC:8609]	-1.94	0.0167
ssc-miR-144	PAX3	paired box 3 [Source:VGNC Symbol;Acc:VGNC:96468]	-1.94	0.0167
ssc-miR-144	PAX5	paired box 5 [Source:VGNC Symbol;Acc:VGNC:91194]	-1.94	0.0167
ssc-miR-144	PAX8	hypothetical gene	-1.94	0.0167
ssc-miR-144	PBRM1	polybromo 1 [Source:VGNC Symbol;Acc:VGNC:91202]	-1.94	0.0167
ssc-miR-144	PBX3	PBX homeobox 3 [Source:VGNC Symbol;Acc:VGNC:91205]	-1.94	0.0167
ssc-miR-144	PCDH10	protocadherin 10 [Source:HGNC Symbol;Acc:HGNC:13404]	-1.94	0.0167
ssc-miR-144	PCDH18	protocadherin 18 [Source:VGNC Symbol;Acc:VGNC:91215]	-1.94	0.0167
ssc-miR-144	PCDH7	protocadherin 7 [Source:NCBI gene (formerly Entrezgene);Acc:100520035]	-1.94	0.0167
ssc-miR-144	PCGF5	hypothetical gene	-1.94	0.0167
ssc-miR-144	PCSK5	proprotein convertase subtilisin/kexin type 5 [Source:VGNC Symbol;Acc:VGNC:103147]	-1.94	0.0167
ssc-miR-144	PDCD4	programmed cell death 4 [Source:VGNC Symbol;Acc:VGNC:91244]	-1.94	0.0167
ssc-miR-144	PDCL	phosducin like [Source:VGNC Symbol;Acc:VGNC:91245]	-1.94	0.0167
ssc-miR-144	PDE3B	phosphodiesterase 3B [Source:VGNC Symbol;Acc:VGNC:91253]	-1.94	0.0167
ssc-miR-144	PDE4A	phosphodiesterase 4A [Source:VGNC Symbol;Acc:VGNC:91254]	-1.94	0.0167
ssc-miR-144	PDE4D	phosphodiesterase 4D [Source:VGNC Symbol;Acc:VGNC:91256]	-1.94	0.0167
ssc-miR-144	PDE7A	phosphodiesterase 7A [Source:VGNC Symbol;Acc:VGNC:91261]	-1.94	0.0167
ssc-miR-144	PDE7B	phosphodiesterase 7B [Source:VGNC Symbol;Acc:VGNC:91262]	-1.94	0.0167
ssc-miR-144	PDS5B	PDS5 cohesin associated factor B [Source:VGNC Symbol;Acc:VGNC:91290]	-1.94	0.0167
ssc-miR-144	PEX7	peroxisomal biosis factor 7 [Source:VGNC Symbol;Acc:VGNC:91331]	-1.94	0.0167
ssc-miR-144	PFDN6	prefoldin subunit 6 [Source:VGNC Symbol;Acc:VGNC:104024]	-1.94	0.0167
ssc-miR-144	PGGT1B	protein geranylgeranyltransferase type I subunit beta [Source:VGNC Symbol;Acc:VGNC:91350]	-1.94	0.0167
ssc-miR-144	PGRMC2	progesterone receptor membrane component 2 [Source:VGNC Symbol;Acc:VGNC:98952]	-1.94	0.0167
ssc-miR-144	PHACTR2	phosphatase and actin regulator 2 [Source:VGNC Symbol;Acc:VGNC:91366]	-1.94	0.0167
ssc-miR-144	PHF20L1	PHD finger protein 20 like 1 [Source:VGNC Symbol;Acc:VGNC:91383]	-1.94	0.0167
ssc-miR-144	PHF3	PHD finger protein 3 [Source:VGNC Symbol;Acc:VGNC:91387]	-1.94	0.0167
ssc-miR-144	PHF6	PHD finger protein 6 [Source:VGNC Symbol;Acc:VGNC:91389]	-1.94	0.0167
ssc-miR-144	PHLDA1	pleckstrin homology like domain family A member 1 [Source:HGNC Symbol;Acc:HGNC:8933]	-1.94	0.0167
ssc-miR-144	PHTF2	putative homeodomain transcription factor 2 [Source:VGNC Symbol;Acc:VGNC:91405]	-1.94	0.0167
ssc-miR-144	PIAS4	protein inhibitor of activated STAT 4 [Source:VGNC Symbol;Acc:VGNC:91413]	-1.94	0.0167
ssc-miR-144	PIEZO1	piezo type mechanosensitive ion channel component 1 [Source:VGNC Symbol;Acc:VGNC:91417]	-1.94	0.0167
ssc-miR-144	PIK3C2A	phosphatidylinositol-4-phosphate 3-kinase catalytic subunit type 2 alpha [Source:VGNC Symbol;Acc:VGNC:91436]	-1.94	0.0167
ssc-miR-144	PIK3C2B	phosphatidylinositol-4-phosphate 3-kinase catalytic subunit type 2 beta [Source:VGNC Symbol;Acc:VGNC:91437]	-1.94	0.0167
ssc-miR-144	PIK3CB	phosphatidylinositol-4,5-bisphosphate 3-kinase catalytic subunit beta [Source:VGNC Symbol;Acc:VGNC:91441]	-1.94	0.0167
ssc-miR-144	PIM1	Pim-1 proto-oncogene, serine/threonine kinase [Source:HGNC Symbol;Acc:HGNC:8986]	-1.94	0.0167
ssc-miR-144	PIP5K1C	phosphatidylinositol-4-phosphate 5-kinase type 1 gamma [Source:VGNC Symbol;Acc:VGNC:91459]	-1.94	0.0167
ssc-miR-144	PJA2	praja ring finger ubiquitin ligase 2 [Source:VGNC Symbol;Acc:VGNC:91470]	-1.94	0.0167
ssc-miR-144	PKIA	cAMP-dependent protein kinase inhibitor alpha [Source:VGNC Symbol;Acc:VGNC:91477]	-1.94	0.0167
ssc-miR-144	PKIB	cAMP-dependent protein kinase inhibitor beta [Source:VGNC Symbol;Acc:VGNC:91478]	-1.94	0.0167

ssc-miR-144	PKNOX1	PBX/knotted 1 homeobox 1 [Source:VGNC Symbol;Acc:VGNC:91484]	-1.94	0.0167
ssc-miR-144	PLA2G4A	phospholipase A2 group IVA [Source:VGNC Symbol;Acc:VGNC:91495]	-1.94	0.0167
ssc-miR-144	PLAA	phospholipase A2 activating protein [Source:VGNC Symbol;Acc:VGNC:91502]	-1.94	0.0167
ssc-miR-144	PLAG1	PLAG1 zinc finger [Source:VGNC Symbol;Acc:VGNC:91509]	-1.94	0.0167
ssc-miR-144	PLAT	plasminogen activator, tissue type [Source:VGNC Symbol;Acc:VGNC:98201]	-1.94	0.0167
ssc-miR-144	PLD5	phospholipase D family member 5 [Source:VGNC Symbol;Acc:VGNC:96145]	-1.94	0.0167
ssc-miR-144	PLEKHG1	pleckstrin homology and RhoGEF domain containing G1 [Source:VGNC Symbol;Acc:VGNC:91542]	-1.94	0.0167
ssc-miR-144	PLEKHG3	pleckstrin homology and RhoGEF domain containing G3 [Source:VGNC Symbol;Acc:VGNC:91544]	-1.94	0.0167
ssc-miR-144	PLXNC1	plexin C1 [Source:VGNC Symbol;Acc:VGNC:91584]	-1.94	0.0167
ssc-miR-144	PMAIP1	phorbol-12-myristate-13-acetate-induced protein 1 [Source:NCBI gene (formerly Entrezgene);Acc:397278]	-1.94	0.0167
ssc-miR-144	PMM2	hypothetical gene	-1.94	0.0167
ssc-miR-144	PNRC1	proline rich nuclear receptor coactivator 1 [Source:VGNC Symbol;Acc:VGNC:91613]	-1.94	0.0167
ssc-miR-144	PODXL	hypothetical gene	-1.94	0.0167
ssc-miR-144	POU2F1	POU class 2 homeobox 1 [Source:VGNC Symbol;Acc:VGNC:91672]	-1.94	0.0167
ssc-miR-144	POU3F1	POU class 3 homeobox 1 [Source:VGNC Symbol;Acc:VGNC:91675]	-1.94	0.0167
ssc-miR-144	PPAP2B	hypothetical gene	-1.94	0.0167
ssc-miR-144	PPARGC1B	PPARG coactivator 1 beta [Source:VGNC Symbol;Acc:VGNC:91686]	-1.94	0.0167
ssc-miR-144	PPFIA1	PTPRF interacting protein alpha 1 [Source:VGNC Symbol;Acc:VGNC:91691]	-1.94	0.0167
ssc-miR-144	PPFIA4	PTPRF interacting protein alpha 4 [Source:VGNC Symbol;Acc:VGNC:91694]	-1.94	0.0167
ssc-miR-144	PPIG	peptidylprolyl isomerase G [Source:VGNC Symbol;Acc:VGNC:98214]	-1.94	0.0167
ssc-miR-144	PPP1CC	protein phosphatase 1 catalytic subunit gamma [Source:VGNC Symbol;Acc:VGNC:91716]	-1.94	0.0167
ssc-miR-144	PPP1R13B	protein phosphatase 1 regulatory subunit 13B [Source:VGNC Symbol;Acc:VGNC:91721]	-1.94	0.0167
ssc-miR-144	PPP1R16B	protein phosphatase 1 regulatory subunit 16B [Source:VGNC Symbol;Acc:VGNC:95669]	-1.94	0.0167
ssc-miR-144	PPP2R2A	protein phosphatase 2 regulatory subunit Balpha [Source:VGNC Symbol;Acc:VGNC:91748]	-1.94	0.0167
ssc-miR-144	PPP2R2D	hypothetical gene	-1.94	0.0167
ssc-miR-144	PPP2R5E	protein phosphatase 2 regulatory subunit B'epsilon [Source:VGNC Symbol;Acc:VGNC:91754]	-1.94	0.0167
ssc-miR-144	PPP3R1	protein phosphatase 3 regulatory subunit B, alpha [Source:VGNC Symbol;Acc:VGNC:106453]	-1.94	0.0167
ssc-miR-144	PPP4R1	protein phosphatase 4 regulatory subunit 1 [Source:VGNC Symbol;Acc:VGNC:98540]	-1.94	0.0167
ssc-miR-144	PPP4R1L	hypothetical gene	-1.94	0.0167
ssc-miR-144	PPTC7	protein phosphatase targeting COQ7 [Source:VGNC Symbol;Acc:VGNC:91765]	-1.94	0.0167
ssc-miR-144	PRDM16	PR/SET domain 16 [Source:VGNC Symbol;Acc:VGNC:91777]	-1.94	0.0167
ssc-miR-144	PRICKLE1	prickle planar cell polarity protein 1 [Source:VGNC Symbol;Acc:VGNC:91793]	-1.94	0.0167
ssc-miR-144	PRKAA1	protein kinase AMP-activated catalytic subunit alpha 1 [Source:VGNC Symbol;Acc:VGNC:91797]	-1.94	0.0167
ssc-miR-144	PRKAR1A	protein kinase cAMP-dependent type I regulatory subunit alpha [Source:VGNC Symbol;Acc:VGNC:91802]	-1.94	0.0167
ssc-miR-144	PRKCE	protein kinase C epsilon [Source:VGNC Symbol;Acc:VGNC:91807]	-1.94	0.0167
ssc-miR-144	PRKD3	protein kinase D3 [Source:VGNC Symbol;Acc:VGNC:91814]	-1.94	0.0167
ssc-miR-144	PROX1	prospero homeobox 1 [Source:VGNC Symbol;Acc:VGNC:91837]	-1.94	0.0167
ssc-miR-144	PRR11	proline rich 11 [Source:VGNC Symbol;Acc:VGNC:91850]	-1.94	0.0167
ssc-miR-144	PRRC2C	proline rich coiled-coil 2C [Source:VGNC Symbol;Acc:VGNC:91867]	-1.94	0.0167
ssc-miR-144	PRRX1	paired related homeobox 1 [Source:VGNC Symbol;Acc:VGNC:91875]	-1.94	0.0167
ssc-miR-144	PSD2	pleckstrin and Sec7 domain containing 2 [Source:VGNC Symbol;Acc:VGNC:91896]	-1.94	0.0167
ssc-miR-144	PSKH1	protein serine kinase H1 [Source:VGNC Symbol;Acc:VGNC:91899]	-1.94	0.0167
ssc-miR-144	PTBP1	polypyrimidine tract binding protein 1 [Source:VGNC Symbol;Acc:VGNC:100330]	-1.94	0.0167
ssc-miR-144	PTBP3	polypyrimidine tract binding protein 3 [Source:VGNC Symbol;Acc:VGNC:91938]	-1.94	0.0167
ssc-miR-144	PTCH1	patched 1 [Source:VGNC Symbol;Acc:VGNC:96513]	-1.94	0.0167
ssc-miR-144	PTEN	hypothetical gene	-1.94	0.0167
ssc-miR-144	PTGFRN	prostaglandin F2 receptor inhibitor [Source:VGNC Symbol;Acc:VGNC:91955]	-1.94	0.0167
ssc-miR-144	PTGS2	prostaglandin-endoperoxide synthase 2 [Source:VGNC Symbol;Acc:VGNC:91959]	-1.94	0.0167
ssc-miR-144	PTHLH	parathyroid hormone like hormone [Source:VGNC Symbol;Acc:VGNC:91963]	-1.94	0.0167

ssc-miR-144	PTP4A1	protein tyrosine phosphatase 4A1 [Source:HGNC Symbol;Acc:HGNC:9634]	-1.94	0.0167
ssc-miR-144	PTPN12	protein tyrosine phosphatase non-receptor type 12 [Source:VGNC Symbol;Acc:VGNC:91973]	-1.94	0.0167
ssc-miR-144	PTPN3	protein tyrosine phosphatase non-receptor type 3 [Source:VGNC Symbol;Acc:VGNC:91980]	-1.94	0.0167
ssc-miR-144	PTPN4	protein tyrosine phosphatase non-receptor type 4 [Source:VGNC Symbol;Acc:VGNC:96521]	-1.94	0.0167
ssc-miR-144	PTPN5	protein tyrosine phosphatase non-receptor type 5 [Source:VGNC Symbol;Acc:VGNC:91981]	-1.94	0.0167
ssc-miR-144	PTPN9	protein tyrosine phosphatase non-receptor type 9 [Source:VGNC Symbol;Acc:VGNC:91983]	-1.94	0.0167
ssc-miR-144	PTPRJ	protein tyrosine phosphatase receptor type J [Source:VGNC Symbol;Acc:VGNC:91990]	-1.94	0.0167
ssc-miR-144	PTPRZ1	protein tyrosine phosphatase receptor type Z1 [Source:VGNC Symbol;Acc:VGNC:91996]	-1.94	0.0167
ssc-miR-144	PUM1	pumilio RNA binding family member 1 [Source:VGNC Symbol;Acc:VGNC:92001]	-1.94	0.0167
ssc-miR-144	PUM2	pumilio RNA binding family member 2 [Source:VGNC Symbol;Acc:VGNC:92002]	-1.94	0.0167
ssc-miR-144	PURA	purine rich element binding protein A [Source:HGNC Symbol;Acc:HGNC:9701]	-1.94	0.0167
ssc-miR-144	PURB	purine rich element binding protein B [Source:VGNC Symbol;Acc:VGNC:92004]	-1.94	0.0167
ssc-miR-144	PYGO1	pygopus family PHD finger 1 [Source:VGNC Symbol;Acc:VGNC:98231]	-1.94	0.0167
ssc-miR-144	QKI	QKI, KH domain containing RNA binding [Source:VGNC Symbol;Acc:VGNC:92025]	-1.94	0.0167
ssc-miR-144	QSER1	glutamine and serine rich 1 [Source:VGNC Symbol;Acc:VGNC:92032]	-1.94	0.0167
ssc-miR-144	RAB11FIP1	hypothetical gene	-1.94	0.0167
ssc-miR-144	RAB18	RAB18, member RAS onco family [Source:VGNC Symbol;Acc:VGNC:98241]	-1.94	0.0167
ssc-miR-144	RAB1A	RAB1A, member RAS onco family [Source:VGNC Symbol;Acc:VGNC:104042]	-1.94	0.0167
ssc-miR-144	RAB2A	RAB2A, member RAS onco family [Source:VGNC Symbol;Acc:VGNC:98252]	-1.94	0.0167
ssc-miR-144	RAB30	RAB30, member RAS onco family [Source:VGNC Symbol;Acc:VGNC:98254]	-1.94	0.0167
ssc-miR-144	RAB39B	RAB39B, member RAS onco family [Source:VGNC Symbol;Acc:VGNC:98263]	-1.94	0.0167
ssc-miR-144	RAB4A	RAB4A, member RAS onco family [Source:VGNC Symbol;Acc:VGNC:98270]	-1.94	0.0167
ssc-miR-144	RAB5A	RAB5A, member RAS onco family [Source:VGNC Symbol;Acc:VGNC:108687]	-1.94	0.0167
ssc-miR-144	RAD54L2	RAD54 like 2 [Source:VGNC Symbol;Acc:VGNC:92061]	-1.94	0.0167
ssc-miR-144	RALBP1	ralA binding protein 1 [Source:VGNC Symbol;Acc:VGNC:92070]	-1.94	0.0167
ssc-miR-144	RALGPS1	Ral GEF with PH domain and SH3 binding motif 1 [Source:VGNC Symbol;Acc:VGNC:92072]	-1.94	0.0167
ssc-miR-144	RALGPS2	Ral GEF with PH domain and SH3 binding motif 2 [Source:VGNC Symbol;Acc:VGNC:92073]	-1.94	0.0167
ssc-miR-144	RANBP9	RAN binding protein 9 [Source:VGNC Symbol;Acc:VGNC:92082]	-1.94	0.0167
ssc-miR-144	RAP1A	RAP1A, member of RAS onco family [Source:VGNC Symbol;Acc:VGNC:92084]	-1.94	0.0167
ssc-miR-144	RAP1B	RAP1B, member of RAS oncogene family [Source:HGNC Symbol;Acc:HGNC:9857]	-1.94	0.0167
ssc-miR-144	RAP1GAP2	RAP1 GTPase activating protein 2 [Source:VGNC Symbol;Acc:VGNC:92086]	-1.94	0.0167
ssc-miR-144	RAP2B	RAP2B, member of RAS onco family [Source:VGNC Symbol;Acc:VGNC:92089]	-1.94	0.0167
ssc-miR-144	RAP2C	RAP2C, member of RAS onco family [Source:VGNC Symbol;Acc:VGNC:92090]	-1.94	0.0167
ssc-miR-144	RAPGEF2	Rap guanine nucleotide exchange factor 2 [Source:VGNC Symbol;Acc:VGNC:92092]	-1.94	0.0167
ssc-miR-144	RARB	retinoic acid receptor beta [Source:HGNC Symbol;Acc:HGNC:9865]	-1.94	0.0167
ssc-miR-144	RASA1	RAS p21 protein activator 1 [Source:VGNC Symbol;Acc:VGNC:92101]	-1.94	0.0167
ssc-miR-144	RASD2	hypothetical gene	-1.94	0.0167
ssc-miR-144	RASEF	hypothetical gene	-1.94	0.0167
ssc-miR-144	RASGRF1	Ras protein specific guanine nucleotide releasing factor 1 [Source:VGNC Symbol;Acc:VGNC:92111]	-1.94	0.0167
ssc-miR-144	RASSF6	Ras association domain family member 6 [Source:VGNC Symbol;Acc:VGNC:92126]	-1.94	0.0167
ssc-miR-144	RBM12	RNA binding motif protein 12 [Source:HGNC Symbol;Acc:HGNC:9898]	-1.94	0.0167
ssc-miR-144	RBM15B	RNA binding motif protein 15B [Source:VGNC Symbol;Acc:VGNC:98285]	-1.94	0.0167
ssc-miR-144	RBM24	hypothetical gene	-1.94	0.0167
ssc-miR-144	RBM25	RNA binding motif protein 25 [Source:NCBI gene (formerly Entrezgene);Acc:100511390]	-1.94	0.0167
ssc-miR-144	RC3H1	ring finger and CCCH-type domains 1 [Source:VGNC Symbol;Acc:VGNC:92168]	-1.94	0.0167
ssc-miR-144	RCN2	reticulocalbin 2 [Source:VGNC Symbol;Acc:VGNC:92181]	-1.94	0.0167
ssc-miR-144	REV1	REV1 DNA directed polymerase [Source:HGNC Symbol;Acc:HGNC:14060]	-1.94	0.0167
ssc-miR-144	REV3L	REV3 like, DNA directed polymerase zeta catalytic subunit [Source:VGNC Symbol;Acc:VGNC:92226]	-1.94	0.0167
ssc-miR-144	RFX3	regulatory factor X3 [Source:VGNC Symbol;Acc:VGNC:92245]	-1.94	0.0167

ssc-miR-144	RGL1	ral guanine nucleotide dissociation stimulator like 1 [Source:VGNC Symbol;Acc:VGNC:92252]	-1.94	0.0167
ssc-miR-144	RGMA	repulsive guidance molecule BMP co-receptor a [Source:VGNC Symbol;Acc:VGNC:92254]	-1.94	0.0167
ssc-miR-144	RGMB	repulsive guidance molecule BMP co-receptor b [Source:VGNC Symbol;Acc:VGNC:92255]	-1.94	0.0167
ssc-miR-144	RGS16	regulator of G protein signaling 16 [Source:VGNC Symbol;Acc:VGNC:92262]	-1.94	0.0167
ssc-miR-144	RGS17	regulator of G protein signaling 17 [Source:VGNC Symbol;Acc:VGNC:92263]	-1.94	0.0167
ssc-miR-144	RHPN2	rhophilin Rho GTPase binding protein 2 [Source:VGNC Symbol;Acc:VGNC:98548]	-1.94	0.0167
ssc-miR-144	RIN2	hypothetical gene	-1.94	0.0167
ssc-miR-144	RNF111	ring finger protein 111 [Source:VGNC Symbol;Acc:VGNC:92346]	-1.94	0.0167
ssc-miR-144	RNF125	ring finger protein 125 [Source:VGNC Symbol;Acc:VGNC:92352]	-1.94	0.0167
ssc-miR-144	RNF139	ring finger protein 139 [Source:HGNC Symbol;Acc:HGNC:17023]	-1.94	0.0167
ssc-miR-144	RNF219	hypothetical gene	-1.94	0.0167
ssc-miR-144	RNF38	ring finger protein 38 [Source:VGNC Symbol;Acc:VGNC:92390]	-1.94	0.0167
ssc-miR-144	RNF44	ring finger protein 44 [Source:VGNC Symbol;Acc:VGNC:92395]	-1.94	0.0167
ssc-miR-144	RNPC3	RNA binding region (RNP1, RRM) containing 3 [Source:HGNC Symbol;Acc:HGNC:18666]	-1.94	0.0167
ssc-miR-144	ROBO2	roundabout guidance receptor 2 [Source:HGNC Symbol;Acc:HGNC:10250]	-1.94	0.0167
ssc-miR-144	ROCK1	Rho associated coiled-coil containing protein kinase 1 [Source:VGNC Symbol;Acc:VGNC:98294]	-1.94	0.0167
ssc-miR-144	ROCK2	Rho associated coiled-coil containing protein kinase 2 [Source:HGNC Symbol;Acc:HGNC:10252]	-1.94	0.0167
ssc-miR-144	RORA	RAR related orphan receptor A [Source:VGNC Symbol;Acc:VGNC:92408]	-1.94	0.0167
ssc-miR-144	RORB	RAR related orphan receptor B [Source:VGNC Symbol;Acc:VGNC:92409]	-1.94	0.0167
ssc-miR-144	RPA1	replication protein A1 [Source:VGNC Symbol;Acc:VGNC:92413]	-1.94	0.0167
ssc-miR-144	RPF1	ribosome production factor 1 homolog [Source:VGNC Symbol;Acc:VGNC:92421]	-1.94	0.0167
ssc-miR-144	RPGRIP1L	RPGRIP1 like [Source:VGNC Symbol;Acc:VGNC:92424]	-1.94	0.0167
ssc-miR-144	RPP40	ribonuclease P/MRP subunit p40 [Source:VGNC Symbol;Acc:VGNC:92434]	-1.94	0.0167
ssc-miR-144	RRAGD	Ras related GTP binding D [Source:VGNC Symbol;Acc:VGNC:92457]	-1.94	0.0167
ssc-miR-144	RREB1	hypothetical gene	-1.94	0.0167
ssc-miR-144	RSPO2	R-spondin 2 [Source:VGNC Symbol;Acc:VGNC:92484]	-1.94	0.0167
ssc-miR-144	RSPO3	R-spondin 3 [Source:VGNC Symbol;Acc:VGNC:92485]	-1.94	0.0167
ssc-miR-144	RSRC2	arginine and serine rich coiled-coil 2 [Source:VGNC Symbol;Acc:VGNC:92488]	-1.94	0.0167
ssc-miR-144	RUNX1	RUNX family transcription factor 1 [Source:VGNC Symbol;Acc:VGNC:92516]	-1.94	0.0167
ssc-miR-144	RXRB	retinoid X receptor beta [Source:VGNC Symbol;Acc:VGNC:92529]	-1.94	0.0167
ssc-miR-144	S1PR3	sphingosine-1-phosphate receptor 3 [Source:VGNC Symbol;Acc:VGNC:92553]	-1.94	0.0167
ssc-miR-144	SALL1	spalt like transcription factor 1 [Source:VGNC Symbol;Acc:VGNC:92561]	-1.94	0.0167
ssc-miR-144	SAMD12	sterile alpha motif domain containing 12 [Source:VGNC Symbol;Acc:VGNC:92565]	-1.94	0.0167
ssc-miR-144	SAP30L	SAP30 like [Source:VGNC Symbol;Acc:VGNC:92577]	-1.94	0.0167
ssc-miR-144	SASH1	SAM and SH3 domain containing 1 [Source:VGNC Symbol;Acc:VGNC:92583]	-1.94	0.0167
ssc-miR-144	SCAMP1	secretory carrier membrane protein 1 [Source:VGNC Symbol;Acc:VGNC:92605]	-1.94	0.0167
ssc-miR-144	SCN1A	sodium voltage-gated channel alpha subunit 1 [Source:VGNC Symbol;Acc:VGNC:95478]	-1.94	0.0167
ssc-miR-144	SCN2A	hypothetical gene	-1.94	0.0167
ssc-miR-144	SCN8A	sodium voltage-gated channel alpha subunit 8 [Source:VGNC Symbol;Acc:VGNC:92638]	-1.94	0.0167
ssc-miR-144	SEC24A	SEC24 homolog A, COPII coat complex component [Source:VGNC Symbol;Acc:VGNC:92676]	-1.94	0.0167
ssc-miR-144	SEL1L	SEL1L adaptor subunit of ERAD E3 ubiquitin ligase [Source:VGNC Symbol;Acc:VGNC:92687]	-1.94	0.0167
ssc-miR-144	SELT	hypothetical gene	-1.94	0.0167
ssc-miR-144	SEMA6A	semaphorin 6A [Source:VGNC Symbol;Acc:VGNC:92708]	-1.94	0.0167
ssc-miR-144	SEMA6D	semaphorin 6D [Source:VGNC Symbol;Acc:VGNC:92711]	-1.94	0.0167
ssc-miR-144	SENP7	SUMO specific peptidase 7 [Source:VGNC Symbol;Acc:VGNC:92716]	-1.94	0.0167
ssc-miR-144	SEPHS1	selenophosphate synthetase 1 [Source:VGNC Symbol;Acc:VGNC:104053]	-1.94	0.0167
ssc-miR-144	SESN3	sestrin 3 [Source:VGNC Symbol;Acc:VGNC:92755]	-1.94	0.0167
ssc-miR-144	SESTD1	SEC14 and spectrin domain containing 1 [Source:VGNC Symbol;Acc:VGNC:95884]	-1.94	0.0167
ssc-miR-144	SETD3	SET domain containing 3, actin histidine methyltransferase [Source:VGNC Symbol;Acc:VGNC:92758]	-1.94	0.0167

ssc-miR-144	SETD5	SET domain containing 5 [Source:VGNC Symbol;Acc:VGNC:92760]	-1.94	0.0167
ssc-miR-144	SFRP1	secreted frizzled related protein 1 [Source:VGNC Symbol;Acc:VGNC:95493]	-1.94	0.0167
ssc-miR-144	SGK3	serum/glucocorticoid regulated kinase family member 3 [Source:HGNC Symbol;Acc:HGNC:10812]	-1.94	0.0167
ssc-miR-144	SGMS2	sphingomyelin synthase 2 [Source:VGNC Symbol;Acc:VGNC:92795]	-1.94	0.0167
ssc-miR-144	SGPP1	sphingosine-1-phosphate phosphatase 1 [Source:VGNC Symbol;Acc:VGNC:92798]	-1.94	0.0167
ssc-miR-144	SH2B3	SH2B adaptor protein 3 [Source:VGNC Symbol;Acc:VGNC:92807]	-1.94	0.0167
ssc-miR-144	SH3BP5	SH3 domain binding protein 5 [Source:VGNC Symbol;Acc:VGNC:92819]	-1.94	0.0167
ssc-miR-144	SH3GLB1	SH3 domain containing GRB2 like, endophilin B1 [Source:VGNC Symbol;Acc:VGNC:92826]	-1.94	0.0167
ssc-miR-144	SH3PXD2A	SH3 and PX domains 2A [Source:VGNC Symbol;Acc:VGNC:92829]	-1.94	0.0167
ssc-miR-144	SHANK2	SH3 and multiple ankyrin repeat domains 2 [Source:VGNC Symbol;Acc:VGNC:92835]	-1.94	0.0167
ssc-miR-144	SHH	sonic hedgehog signaling molecule [Source:VGNC Symbol;Acc:VGNC:92844]	-1.94	0.0167
ssc-miR-144	SHISA6	hypothetical gene	-1.94	0.0167
ssc-miR-144	SHOX	hypothetical gene	-1.94	0.0167
ssc-miR-144	SIK1	salt inducible kinase 1 [Source:HGNC Symbol;Acc:HGNC:11142]	-1.94	0.0167
ssc-miR-144	SIK2	salt inducible kinase 2 [Source:VGNC Symbol;Acc:VGNC:92872]	-1.94	0.0167
ssc-miR-144	SIM1	SIM bHLH transcription factor 1 [Source:VGNC Symbol;Acc:VGNC:92876]	-1.94	0.0167
ssc-miR-144	SIN3A	SIN3 transcription regulator family member A [Source:VGNC Symbol;Acc:VGNC:92879]	-1.94	0.0167
ssc-miR-144	SIX4	SIX homeobox 4 [Source:VGNC Symbol;Acc:VGNC:92895]	-1.94	0.0167
ssc-miR-144	SKIL	SKI like proto-onco [Source:VGNC Symbol;Acc:VGNC:92903]	-1.94	0.0167
ssc-miR-144	SLA2	Src like adaptor 2 [Source:HGNC Symbol;Acc:HGNC:17329]	-1.94	0.0167
ssc-miR-144	SLAIN2	SLAIN motif family member 2 [Source:VGNC Symbol;Acc:VGNC:92910]	-1.94	0.0167
ssc-miR-144	SLC10A7	solute carrier family 10 member 7 [Source:VGNC Symbol;Acc:VGNC:92919]	-1.94	0.0167
ssc-miR-144	SLC12A2	solute carrier family 12 member 2 [Source:VGNC Symbol;Acc:VGNC:92922]	-1.94	0.0167
ssc-miR-144	SLC19A2	solute carrier family 19 member 2 [Source:VGNC Symbol;Acc:VGNC:92962]	-1.94	0.0167
ssc-miR-144	SLC1A1	solute carrier family 1 member 1 [Source:VGNC Symbol;Acc:VGNC:92963]	-1.94	0.0167
ssc-miR-144	SLC20A2	solute carrier family 20 member 2 [Source:VGNC Symbol;Acc:VGNC:95498]	-1.94	0.0167
ssc-miR-144	SLC22A23	solute carrier family 22 member 23 [Source:HGNC Symbol;Acc:HGNC:21106]	-1.94	0.0167
ssc-miR-144	SLC23A2	solute carrier family 23 member 2 [Source:VGNC Symbol;Acc:VGNC:108737]	-1.94	0.0167
ssc-miR-144	SLC25A21	solute carrier family 25 member 21 [Source:VGNC Symbol;Acc:VGNC:98320]	-1.94	0.0167
ssc-miR-144	SLC30A4	solute carrier family 30 member 4 [Source:VGNC Symbol;Acc:VGNC:93058]	-1.94	0.0167
ssc-miR-144	SLC30A7	solute carrier family 30 member 7 [Source:VGNC Symbol;Acc:VGNC:93061]	-1.94	0.0167
ssc-miR-144	SLC35A3	solute carrier family 35 member A3 [Source:VGNC Symbol;Acc:VGNC:98860]	-1.94	0.0167
ssc-miR-144	SLC45A3	solute carrier family 45 member 3 [Source:VGNC Symbol;Acc:VGNC:93124]	-1.94	0.0167
ssc-miR-144	SLC4A10	solute carrier family 4 member 10 [Source:VGNC Symbol;Acc:VGNC:95592]	-1.94	0.0167
ssc-miR-144	SLC4A4	solute carrier family 4 member 4 [Source:VGNC Symbol;Acc:VGNC:93133]	-1.94	0.0167
ssc-miR-144	SLC6A17	solute carrier family 6 member 17 [Source:VGNC Symbol;Acc:VGNC:93158]	-1.94	0.0167
ssc-miR-144	SLC7A11	solute carrier family 7 member 11 [Source:VGNC Symbol;Acc:VGNC:93171]	-1.94	0.0167
ssc-miR-144	SLC8A1	solute carrier family 8 member A1 [Source:VGNC Symbol;Acc:VGNC:93178]	-1.94	0.0167
ssc-miR-144	SLITRK4	SLIT and NTRK like family member 4 [Source:VGNC Symbol;Acc:VGNC:93209]	-1.94	0.0167
ssc-miR-144	SMAD1	SMAD family member 1 [Source:VGNC Symbol;Acc:VGNC:93216]	-1.94	0.0167
ssc-miR-144	SMAD5	SMAD family member 5 [Source:VGNC Symbol;Acc:VGNC:93219]	-1.94	0.0167
ssc-miR-144	SMARCA4	SWI/SNF related, matrix associated, actin dependent regulator of chromatin, subfamily a, member 4 [Source:VGNC Symbol;Acc:VGNC:93227]	-1.94	0.0167
ssc-miR-144	SMARCA5	SWI/SNF related, matrix associated, actin dependent regulator of chromatin, subfamily a, member 5 [Source:VGNC Symbol;Acc:VGNC:93228]	-1.94	0.0167
ssc-miR-144	SMOC1	SPARC related modular calcium binding 1 [Source:VGNC Symbol;Acc:VGNC:93259]	-1.94	0.0167
ssc-miR-144	SMPD3	sphingomyelin phosphodiesterase 3 [Source:VGNC Symbol;Acc:VGNC:93263]	-1.94	0.0167
ssc-miR-144	SNAP91	synaptosome associated protein 91 [Source:VGNC Symbol;Acc:VGNC:93279]	-1.94	0.0167
ssc-miR-144	SNN	stannin [Source:HGNC Symbol;Acc:HGNC:11149]	-1.94	0.0167
ssc-miR-144	SNTB2	syntrophin beta 2 [Source:VGNC Symbol;Acc:VGNC:93298]	-1.94	0.0167
ssc-miR-144	SOAT1	sterol O-acyltransferase 1 [Source:VGNC Symbol;Acc:VGNC:93327]	-1.94	0.0167

ssc-miR-144	SOBP	sine oculis binding protein homolog [Source:VGNC Symbol;Acc:VGNC:93329]	-1.94	0.0167
ssc-miR-144	SOCS2	suppressor of cytokine signaling 2 [Source:NCBI gene (formerly Entrezgene);Acc:100037966]	-1.94	0.0167
ssc-miR-144	SOCS5	suppressor of cytokine signaling 5 [Source:VGNC Symbol;Acc:VGNC:93332]	-1.94	0.0167
ssc-miR-144	SOCS6	suppressor of cytokine signaling 6 [Source:VGNC Symbol;Acc:VGNC:93333]	-1.94	0.0167
ssc-miR-144	SOCS7	suppressor of cytokine signaling 7 [Source:VGNC Symbol;Acc:VGNC:93334]	-1.94	0.0167
ssc-miR-144	SOGA2	hypothetical gene	-1.94	0.0167
ssc-miR-144	SON	SON DNA and RNA binding protein [Source:VGNC Symbol;Acc:VGNC:93336]	-1.94	0.0167
ssc-miR-144	SORCS3	sortilin related VPS10 domain containing receptor 3 [Source:VGNC Symbol;Acc:VGNC:93341]	-1.94	0.0167
ssc-miR-144	SOS1	SOS Ras/Rac guanine nucleotide exchange factor 1 [Source:HGNC Symbol;Acc:HGNC:11187]	-1.94	0.0167
ssc-miR-144	SOX1	SRY-box transcription factor 1 [Source:HGNC Symbol;Acc:HGNC:11189]	-1.94	0.0167
ssc-miR-144	SOX11	SRY-box transcription factor 11 [Source:VGNC Symbol;Acc:VGNC:93351]	-1.94	0.0167
ssc-miR-144	SOX6	SRY-box transcription factor 6 [Source:VGNC Symbol;Acc:VGNC:93358]	-1.94	0.0167
ssc-miR-144	SP4	Sp4 transcription factor [Source:VGNC Symbol;Acc:VGNC:93362]	-1.94	0.0167
ssc-miR-144	SPATA2	spermatosis associated 2 [Source:VGNC Symbol;Acc:VGNC:95628]	-1.94	0.0167
ssc-miR-144	SPIRE1	spire type actin nucleation factor 1 [Source:VGNC Symbol;Acc:VGNC:98336]	-1.94	0.0167
ssc-miR-144	SPOPL	speckle type BTB/POZ protein like [Source:VGNC Symbol;Acc:VGNC:96161]	-1.94	0.0167
ssc-miR-144	SPRED1	sprouty related EVH1 domain containing 1 [Source:VGNC Symbol;Acc:VGNC:93420]	-1.94	0.0167
ssc-miR-144	SPRY3	sprouty RTK signaling antagonist 3 [Source:VGNC Symbol;Acc:VGNC:93426]	-1.94	0.0167
ssc-miR-144	SRC	SRC proto-onco, non-receptor tyrosine kinase [Source:VGNC Symbol;Acc:VGNC:95516]	-1.94	0.0167
ssc-miR-144	SREK1	splicing regulatory glutamic acid and lysine rich protein 1 [Source:VGNC Symbol;Acc:VGNC:93451]	-1.94	0.0167
ssc-miR-144	SRGAP1	SLIT-ROBO Rho GTPase activating protein 1 [Source:VGNC Symbol;Acc:VGNC:93454]	-1.94	0.0167
ssc-miR-144	SRSF10	serine and arginine rich splicing factor 10 [Source:VGNC Symbol;Acc:VGNC:93472]	-1.94	0.0167
ssc-miR-144	SS18	SS18 subunit of BAF chromatin remodeling complex [Source:VGNC Symbol;Acc:VGNC:93478]	-1.94	0.0167
ssc-miR-144	SSH1	slingshot protein phosphatase 1 [Source:VGNC Symbol;Acc:VGNC:93485]	-1.94	0.0167
ssc-miR-144	SSX2IP	SSX family member 2 interacting protein [Source:VGNC Symbol;Acc:VGNC:93499]	-1.94	0.0167
ssc-miR-144	ST18	ST18 C2H2C-type zinc finger transcription factor [Source:VGNC Symbol;Acc:VGNC:93501]	-1.94	0.0167
ssc-miR-144	ST5	hypothetical gene	-1.94	0.0167
ssc-miR-144	ST6GALNAC3	ST6 N-acetylgalactosaminide alpha-2,6-sialyltransferase 3 [Source:VGNC Symbol;Acc:VGNC:93511]	-1.94	0.0167
ssc-miR-144	STAC	SH3 and cysteine rich domain [Source:HGNC Symbol;Acc:HGNC:11353]	-1.94	0.0167
ssc-miR-144	STAG1	stromal antigen 1 [Source:VGNC Symbol;Acc:VGNC:93524]	-1.94	0.0167
ssc-miR-144	STAM	signal transducing adaptor molecule [Source:VGNC Symbol;Acc:VGNC:95520]	-1.94	0.0167
ssc-miR-144	STARD8	StAR related lipid transfer domain containing 8 [Source:VGNC Symbol;Acc:VGNC:93538]	-1.94	0.0167
ssc-miR-144	STAT6	signal transducer and activator of transcription 6 [Source:VGNC Symbol;Acc:VGNC:93541]	-1.94	0.0167
ssc-miR-144	STAU1	staufen double-stranded RNA binding protein 1 [Source:VGNC Symbol;Acc:VGNC:95525]	-1.94	0.0167
ssc-miR-144	STC1	stanniocalcin 1 [Source:VGNC Symbol;Acc:VGNC:93542]	-1.94	0.0167
ssc-miR-144	STIL	STIL centriolar assembly protein [Source:VGNC Symbol;Acc:VGNC:93545]	-1.94	0.0167
ssc-miR-144	STK38L	serine/threonine kinase 38 like [Source:VGNC Symbol;Acc:VGNC:93557]	-1.94	0.0167
ssc-miR-144	STMN1	stathmin 1 [Source:VGNC Symbol;Acc:VGNC:93560]	-1.94	0.0167
ssc-miR-144	STOX2	storkhead box 2 [Source:VGNC Symbol;Acc:VGNC:96082]	-1.94	0.0167
ssc-miR-144	STRN	striatin [Source:VGNC Symbol;Acc:VGNC:93578]	-1.94	0.0167
ssc-miR-144	STRN3	striatin 3 [Source:VGNC Symbol;Acc:VGNC:93579]	-1.94	0.0167
ssc-miR-144	STX7	syntaxin 7 [Source:VGNC Symbol;Acc:VGNC:93595]	-1.94	0.0167
ssc-miR-144	STXBP4	syntaxin binding protein 4 [Source:VGNC Symbol;Acc:VGNC:93598]	-1.94	0.0167
ssc-miR-144	SUCLA2	succinate-CoA ligase ADP-forming subunit beta [Source:HGNC Symbol;Acc:HGNC:11448]	-1.94	0.0167
ssc-miR-144	SULF2	sulfatase 2 [Source:VGNC Symbol;Acc:VGNC:95938]	-1.94	0.0167
ssc-miR-144	SULT4A1	sulfotransferase family 4A member 1 [Source:VGNC Symbol;Acc:VGNC:93615]	-1.94	0.0167
ssc-miR-144	SUMO3	small ubiquitin like modifier 3 [Source:HGNC Symbol;Acc:HGNC:11124]	-1.94	0.0167
ssc-miR-144	SV2C	synaptic vesicle glycoprotein 2C [Source:VGNC Symbol;Acc:VGNC:93641]	-1.94	0.0167
ssc-miR-144	SWAP70	switching B cell complex subunit SWAP70 [Source:VGNC Symbol;Acc:VGNC:93645]	-1.94	0.0167

ssc-miR-144	SYAP1	synapse associated protein 1 [Source:VGNC Symbol;Acc:VGNC:93648]	-1.94	0.0167
ssc-miR-144	SYNCRIP	synaptotagmin binding cytoplasmic RNA interacting protein [Source:VGNC Symbol;Acc:VGNC:103185]	-1.94	0.0167
ssc-miR-144	SYNGAP1	synaptic Ras GTPase activating protein 1 [Source:VGNC Symbol;Acc:VGNC:93664]	-1.94	0.0167
ssc-miR-144	SYNRG	synergin gamma [Source:VGNC Symbol;Acc:VGNC:93675]	-1.94	0.0167
ssc-miR-144	SYT11	synaptotagmin 11 [Source:VGNC Symbol;Acc:VGNC:93680]	-1.94	0.0167
ssc-miR-144	SYT14	synaptotagmin 14 [Source:VGNC Symbol;Acc:VGNC:108619]	-1.94	0.0167
ssc-miR-144	TAB2	TGF-beta activated kinase 1 (MAP3K7) binding protein 2 [Source:VGNC Symbol;Acc:VGNC:103186]	-1.94	0.0167
ssc-miR-144	TAL1	TAL bHLH transcription factor 1, erythroid differentiation factor [Source:HGNC Symbol;Acc:HGNC:11556]	-1.94	0.0167
ssc-miR-144	TANC1	tetratricopeptide repeat, ankyrin repeat and coiled-coil containing 1 [Source:VGNC Symbol;Acc:VGNC:99730]	-1.94	0.0167
ssc-miR-144	TANC2	tetratricopeptide repeat, ankyrin repeat and coiled-coil containing 2 [Source:VGNC Symbol;Acc:VGNC:93733]	-1.94	0.0167
ssc-miR-144	TAOK1	TAO kinase 1 [Source:VGNC Symbol;Acc:VGNC:98355]	-1.94	0.0167
ssc-miR-144	TBC1D12	TBC1 domain family member 12 [Source:VGNC Symbol;Acc:VGNC:93762]	-1.94	0.0167
ssc-miR-144	TBC1D14	hypothetical gene	-1.94	0.0167
ssc-miR-144	TBC1D19	TBC1 domain family member 19 [Source:VGNC Symbol;Acc:VGNC:93767]	-1.94	0.0167
ssc-miR-144	TBL1XR1	TBL1X receptor 1 [Source:VGNC Symbol;Acc:VGNC:96600]	-1.94	0.0167
ssc-miR-144	TBX1	T-box transcription factor 1 [Source:VGNC Symbol;Acc:VGNC:93795]	-1.94	0.0167
ssc-miR-144	TBX18	T-box transcription factor 18 [Source:VGNC Symbol;Acc:VGNC:103187]	-1.94	0.0167
ssc-miR-144	TCEB1	hypothetical gene	-1.94	0.0167
ssc-miR-144	TCF21	transcription factor 21 [Source:VGNC Symbol;Acc:VGNC:93819]	-1.94	0.0167
ssc-miR-144	TCF4	transcription factor 4 [Source:VGNC Symbol;Acc:VGNC:93823]	-1.94	0.0167
ssc-miR-144	TCFL5	transcription factor like 5 [Source:VGNC Symbol;Acc:VGNC:95537]	-1.94	0.0167
ssc-miR-144	TDG	thymine DNA glycosylase [Source:VGNC Symbol;Acc:VGNC:93842]	-1.94	0.0167
ssc-miR-144	TECTA	tectorin alpha [Source:HGNC Symbol;Acc:HGNC:11720]	-1.94	0.0167
ssc-miR-144	TEK	TEK receptor tyrosine kinase [Source:VGNC Symbol;Acc:VGNC:93866]	-1.94	0.0167
ssc-miR-144	TENM1	teneurin transmembrane protein 1 [Source:VGNC Symbol;Acc:VGNC:98363]	-1.94	0.0167
ssc-miR-144	TENM3	teneurin transmembrane protein 3 [Source:VGNC Symbol;Acc:VGNC:96233]	-1.94	0.0167
ssc-miR-144	TES	testin LIM domain protein [Source:VGNC Symbol;Acc:VGNC:93884]	-1.94	0.0167
ssc-miR-144	TET2	hypothetical gene	-1.94	0.0167
ssc-miR-144	TET3	tet methylcytosine dioxygenase 3 [Source:VGNC Symbol;Acc:VGNC:93890]	-1.94	0.0167
ssc-miR-144	TFAP4	transcription factor AP-4 [Source:VGNC Symbol;Acc:VGNC:93912]	-1.94	0.0167
ssc-miR-144	TFRC	transferrin receptor [Source:VGNC Symbol;Acc:VGNC:93925]	-1.94	0.0167
ssc-miR-144	TGFA	transforming growth factor alpha [Source:VGNC Symbol;Acc:VGNC:93928]	-1.94	0.0167
ssc-miR-144	TGFBR2	transforming growth factor beta receptor 2 [Source:VGNC Symbol;Acc:VGNC:93931]	-1.94	0.0167
ssc-miR-144	TGIF1	TGFB induced factor homeobox 1 [Source:VGNC Symbol;Acc:VGNC:93935]	-1.94	0.0167
ssc-miR-144	TGIF2	TGFB induced factor homeobox 2 [Source:VGNC Symbol;Acc:VGNC:95656]	-1.94	0.0167
ssc-miR-144	THAP1	THAP domain containing 1 [Source:VGNC Symbol;Acc:VGNC:95950]	-1.94	0.0167
ssc-miR-144	THBS1	thrombospondin 1 [Source:VGNC Symbol;Acc:VGNC:93946]	-1.94	0.0167
ssc-miR-144	THBS2	thrombospondin 2 [Source:VGNC Symbol;Acc:VGNC:93947]	-1.94	0.0167
ssc-miR-144	THOC2	THO complex 2 [Source:VGNC Symbol;Acc:VGNC:93957]	-1.94	0.0167
ssc-miR-144	TIMM8A	translocase of inner mitochondrial membrane 8A [Source:VGNC Symbol;Acc:VGNC:93992]	-1.94	0.0167
ssc-miR-144	TJP1	tight junction protein 1 [Source:VGNC Symbol;Acc:VGNC:94005]	-1.94	0.0167
ssc-miR-144	TM9SF3	transmembrane 9 superfamily member 3 [Source:VGNC Symbol;Acc:VGNC:94037]	-1.94	0.0167
ssc-miR-144	TMBIM6	transmembrane BAX inhibitor motif containing 6 [Source:VGNC Symbol;Acc:VGNC:98374]	-1.94	0.0167
ssc-miR-144	TMCC1	transmembrane and coiled-coil domain family 1 [Source:VGNC Symbol;Acc:VGNC:94046]	-1.94	0.0167
ssc-miR-144	TMED5	transmembrane p24 trafficking protein 5 [Source:VGNC Symbol;Acc:VGNC:94057]	-1.94	0.0167
ssc-miR-144	TMEM106B	transmembrane protein 106B [Source:VGNC Symbol;Acc:VGNC:104075]	-1.94	0.0167
ssc-miR-144	TMEM126B	transmembrane protein 126B [Source:VGNC Symbol;Acc:VGNC:104078]	-1.94	0.0167
ssc-miR-144	TMEM161B	transmembrane protein 161B [Source:VGNC Symbol;Acc:VGNC:94102]	-1.94	0.0167
ssc-miR-144	TMEM163	transmembrane protein 163 [Source:VGNC Symbol;Acc:VGNC:96078]	-1.94	0.0167

ssc-miR-144	TMEM165	transmembrane protein 165 [Source:VGNC Symbol;Acc:VGNC:94104]	-1.94	0.0167
ssc-miR-144	TMEM178B	transmembrane protein 178B [Source:HGNC Symbol;Acc:HGNC:44112]	-1.94	0.0167
ssc-miR-144	TMEM184B	transmembrane protein 184B [Source:VGNC Symbol;Acc:VGNC:94120]	-1.94	0.0167
ssc-miR-144	TMEM189	hypothetical gene	-1.94	0.0167
ssc-miR-144	TMEM189-UBE2V1	hypothetical gene	-1.94	0.0167
ssc-miR-144	TMEM257	hypothetical gene	-1.94	0.0167
ssc-miR-144	TMEM55A	hypothetical gene	-1.94	0.0167
ssc-miR-144	TMEM65	transmembrane protein 65 [Source:VGNC Symbol;Acc:VGNC:94200]	-1.94	0.0167
ssc-miR-144	TMF1	TATA element modulatory factor 1 [Source:VGNC Symbol;Acc:VGNC:94223]	-1.94	0.0167
ssc-miR-144	TMTC3	transmembrane O-mannosyltransferase targeting cadherins 3 [Source:VGNC Symbol;Acc:VGNC:94243]	-1.94	0.0167
ssc-miR-144	TNC	tenascin C [Source:VGNC Symbol;Acc:VGNC:103192]	-1.94	0.0167
ssc-miR-144	TNFSF11	TNF superfamily member 11 [Source:HGNC Symbol;Acc:HGNC:11926]	-1.94	0.0167
ssc-miR-144	TNIK	TRAF2 and NCK interacting kinase [Source:VGNC Symbol;Acc:VGNC:98381]	-1.94	0.0167
ssc-miR-144	TNKS2	tankyrase 2 [Source:VGNC Symbol;Acc:VGNC:94280]	-1.94	0.0167
ssc-miR-144	TNPO1	transportin 1 [Source:VGNC Symbol;Acc:VGNC:94287]	-1.94	0.0167
ssc-miR-144	TNRC18	trinucleotide repeat containing 18 [Source:VGNC Symbol;Acc:VGNC:94291]	-1.94	0.0167
ssc-miR-144	TNRC6A	trinucleotide repeat containing adaptor 6A [Source:VGNC Symbol;Acc:VGNC:94292]	-1.94	0.0167
ssc-miR-144	TNRC6B	trinucleotide repeat containing adaptor 6B [Source:VGNC Symbol;Acc:VGNC:94293]	-1.94	0.0167
ssc-miR-144	TNS3	tensin 3 [Source:VGNC Symbol;Acc:VGNC:94296]	-1.94	0.0167
ssc-miR-144	TOP1	hypothetical gene	-1.94	0.0167
ssc-miR-144	TOP2A	DNA topoisomerase II alpha [Source:VGNC Symbol;Acc:VGNC:94310]	-1.94	0.0167
ssc-miR-144	TOX	thymocyte selection associated high mobility group box [Source:VGNC Symbol;Acc:VGNC:94322]	-1.94	0.0167
ssc-miR-144	TP53INP1	tumor protein p53 inducible nuclear protein 1 [Source:VGNC Symbol;Acc:VGNC:94329]	-1.94	0.0167
ssc-miR-144	TP53INP2	tumor protein p53 inducible nuclear protein 2 [Source:VGNC Symbol;Acc:VGNC:95737]	-1.94	0.0167
ssc-miR-144	TPD52	tumor protein D52 [Source:VGNC Symbol;Acc:VGNC:98383]	-1.94	0.0167
ssc-miR-144	TRAF3	TNF receptor associated factor 3 [Source:VGNC Symbol;Acc:VGNC:94360]	-1.94	0.0167
ssc-miR-144	TRAPPC8	trafficking protein particle complex subunit 8 [Source:VGNC Symbol;Acc:VGNC:94379]	-1.94	0.0167
ssc-miR-144	TRDMT1	tRNA aspartic acid methyltransferase 1 [Source:VGNC Symbol;Acc:VGNC:96227]	-1.94	0.0167
ssc-miR-144	TRIB1	tribbles pseudokinase 1 [Source:VGNC Symbol;Acc:VGNC:94391]	-1.94	0.0167
ssc-miR-144	TRIM24	tripartite motif containing 24 [Source:VGNC Symbol;Acc:VGNC:94400]	-1.94	0.0167
[truncated: 101,279 more chars]
